# Mediating Effects of Lipid Biomarkers and Sex‐Specific Moderation on the Associations Between Anthropometric and Dietary Factors With Insulin Resistance

**DOI:** 10.1155/jobe/7216950

**Published:** 2026-02-27

**Authors:** Brice Ulrich Foudjo Saha, Lifoter Kenneth Navti, Ismael Teta, Fanuel Yibume Bey, Edouard Akono Nantia, Tume Christopher Bonglavnyuy, Eugene Sobngwi

**Affiliations:** ^1^ Department of Biochemistry, Faculty of Science, The University of Bamenda, P.O. Box 39 Bambili, Bamenda, Cameroon, unibda.net; ^2^ UNICEF Kenya Country Office, P.O. Box 44145—00100, Nairobi, Kenya; ^3^ Department of Internal Medicine and Specialties, Faculty of Medicine and Biomedical Sciences, University of Yaoundé 1, Yaoundé, Cameroon, uy1.uninet.cm; ^4^ National Obesity Center and Endocrinology and Metabolic Disease Unit, Yaoundé Central Hospital, Yaoundé, Cameroon, hopitalcentral.cm; ^5^ Laboratory of Molecular Medicine and Metabolism, Biotechnology Center, University of Yaoundé 1, Yaoundé, Cameroon, uy1.uninet.cm

**Keywords:** anthropometry, diet, dyslipidemia, insulin resistance, sex

## Abstract

**Introduction:**

Various anthropometric and dietary indicators influence insulin resistance (IR), and lipid biomarkers may play a pivotal mediating role in these relationships. To investigate the mediating effects of lipid biomarkers on the relationships between anthropometric, dietary indicators, and IR, and the moderating role of sex in these associations.

**Methods:**

A hospital‐based cross‐sectional study was conducted with 169 participants. Parallel mediation models assessed the mediating effects of lipid biomarkers (triglycerides, HDL, LDL, and total cholesterol) on the relationships between anthropometric (BMI, waist circumference, waist‐to‐hip ratio, and waist‐to‐height ratio) and dietary indicators (at least one starchy staple, at least one fruit, consumed all five recommended food groups (ALL‐5), global dietary recommendations (GDR) score, and noncommunicable disease risk/protect scores) and IR. Moderated mediation analyses evaluated the moderating effect of sex. Statistical analyses were bootstrapped.

**Results:**

The cumulative effect of lipid biomarkers fully mediated the relationship between WHtR and IR (Indirect Coef. = 0.65; 95% CI [0.02, 1.70]). TG significantly mediated the associations between WHR and IR (indirect Coef. = 0.55; 95% CI [0.11, 2.63]), BMI and IR (indirect Coef. = 0.76; 95% CI [0.17, 2.27]), and WC and IR (indirect Coef. = 0.72; 95% CI [0.04, 2.63]). Furthermore, LDL‐C (Indirect Coef. = 1.99; 95% CI [0.24, 9.61]) and HDL‐C (Indirect Coef. = 0.55; 95% CI [0.03, 2.57]) mediated the relationships of starchy staple and fruit intake with IR, respectively. Sex moderated the direct effect of WC on IR (interaction Coef. = 5.11, *p* = 0.002) but did not moderate the indirect pathways involving lipid biomarkers.

**Conclusions:**

These findings elucidate the intricate interplay between body composition, diet, lipid biomarkers, sex, and IR, providing insights for developing targeted prevention and intervention strategies to combat IR.

## 1. Introduction

Insulin resistance (IR) is a major metabolic disorder where the body fails to respond to insulin, leading to elevated blood glucose and increasing the risk of Type 2 diabetes and cardiovascular diseases [1, 2]. Globally, IR prevalence is rising, driven by obesity and sedentary lifestyles [3]. In sub‐Saharan Africa, a rapid epidemiological transition has doubled the prevalence of metabolic disorders in just 6 years, yet the specific metabolic drivers in these populations remain under‐characterized compared to Western cohorts [4]. Key contributors to IR include anthropometric indicators like BMI and waist circumference (WC), which are linked to obesity and metabolic dysfunction [5].

The diet also plays a pivotal role in metabolic health and IR. In the Cameroonian context, dietary patterns are characterized by a high consumption of starchy staples (such as cassava, plantains, and tubers) and a growing intake of processed foods. This dietary profile has been linked to distinct glycemic responses and lipid profiles that may not align with Western metabolic models [6, 7]. Mechanistically, these dietary and anthropometric factors influence IR through the modulation of lipid biomarkers. Triglycerides and low‐density lipoproteins (LDL) act as key signaling molecules that bridge the gap between central adiposity and impaired insulin sensitivity [8]. However, most studies in African hospital‐based populations have treated these factors in isolation, primarily focusing on bivariate associations or prevalence without exploring the complex mediating pathways that explain how specific diets and body compositions lead to IR in this population [9–11].

Sex differences further complicate these relationships, as men and women exhibit distinct fat distribution and hormonal regulation [12]. In sub‐Saharan Africa, women often present with higher rates of obesity, yet the sex‐specific moderation of the lipid‐IR pathway remains poorly understood [13].

This research aims to fill these gaps by using an innovative moderated mediation approach to examine how lipid biomarkers mediate the relationships between anthropometric/dietary indicators and IR in a Cameroonian clinical cohort. This comprehensive approach seeks to enhance the precision of metabolic health interventions by identifying whether sex influences these indirect pathways. The following research hypotheses were formulated:

 H1: Lipid biomarkers significantly mediate the relationship between anthropometric indicators and IR.
 H2: Lipid biomarkers significantly mediate the influence of dietary indicators on IR.
 H3: Sex significantly moderates these associations, leading to sex‐specific metabolic profiles in the development of IR.

## 2. Materials and Methods

### 2.1. Study Area

The research was conducted at the National Obesity and Diabetic Centre (NODC) within the Yaoundé Central Hospital, situated in Cameroon’s Center Region.

### 2.2. Study Design

A cross‐sectional hospital‐based study design aimed to explore the relationships between diet quality indices, anthropometric parameters, lipid biomarkers, sex, and IR among individuals visiting the NODC. It is important to emphasize that this hospital‐based, cross‐sectional design limits the external validity of the findings to the general population and does not allow for the establishment of temporal causality.

### 2.3. Study Population

The study focused on individuals attending the NODC.

### 2.4. Sample Size Calculation

The sample size was determined using the Hsieh et al. [14] methodology, considering an alpha level of 0.05, a beta of 0.8, a coefficient of determination of 0.7, a prevalence rate of 53.9% [9], an odds ratio of 3 [15], and a 5% nonresponse rate, resulting in a required sample size of 153.

### 2.5. Sampling Procedure

Eligible patients at NODC during the study period were recruited via convenience sampling. The recruited patients primarily consisted of individuals seeking specialized care for metabolic screening, obesity management, or glycemic follow‐up. While this clinical profile is highly relevant for investigating IR, the use of convenience sampling in a tertiary care setting may introduce selection bias, as these participants may present higher metabolic risk than the general community.

#### 2.5.1. Inclusion Criteria

Inclusion encompassed adults aged 20 and older who sought care at the NODC and consented to participate in this study. Eligible participants were available for IR screening and other study‐related assessments. Both males and females were included, regardless of their presenting medical condition or comorbidities, unless these conditions or their treatments are known to directly affect insulin sensitivity.

#### 2.5.2. Exclusion Criteria

Exclusions were made for individuals on medications impacting insulin sensitivity, pregnant women, those with cognitive impairment or communication barriers, and critically ill patients.

### 2.6. Ethical Considerations

Ethical approval was obtained from the Center Regional Ethics Committee for Human Health Research (approval number 0119/CRERSHC/2024). Participants freely consented to the study by signing an informed consent form. The study was conducted under the ethical standards of the 1964 Declaration of Helsinki. Blood samples were collected following WHO guidelines [16].

### 2.7. Data Collection

Data were gathered through a structured questionnaire covering sociodemographics, anthropometric measurements, dietary indicators, and biochemical parameters.

#### 2.7.1. Outcome Variable

Serum glucose concentration was analyzed using the glucose oxidase 4‐aminoantipyrine peroxidase (GOD‐PAP) method [17]. In brief, serum was incubated with the GOD‐PAP reagent according to standard protocols, and absorbance was measured at 500 nm against the blank for the test samples and the standard. Insulin was determined by enzyme immunoassay using a commercial kit (AccuDiag^TM^—Insulin ELISA, Woodland Hills, USA) [18]. The assay followed the manufacturer’s instructions, utilizing a horseradish peroxidase‐conjugated enzyme reagent and tetramethylbenzidine (TMB) substrate. Optical densities were read at 450 nm using an ELISA plate reader (BMG LABTECH, Ortenberg, Germany).

IR was evaluated by the homeostasis model assessment (HOMA‐IR) using fasting blood glucose (mmol/L) and fasting insulin (mU/L), calculated as follows [18]:

(1) HOMA−IR=insulinmU/L×Glucosemmol/L22.5.

IR was used as a continuous variable.

#### 2.7.2. Independent Variables

Independent variables comprised anthropometric measures and dietary indices. Anthropometric assessments were conducted three times, and mean values were used for analysis. Weight (kg) and height (m) were measured following the techniques recommended by Lohman et al. [19]. Body mass index (BMI) was computed as weight (kg) divided by height squared (m^2^). WC was measured using a nonflexible tape with an accuracy of 0.1 cm, placed directly on the skin at the narrowest point between the lower rib and the iliac crest during expiration [19]. Hip circumference was measured at the point of maximum circumference over the buttocks. These measurements allowed the calculation of the waist‐to‐hip ratio (WHR) (WC divided by hip circumference) and the waist‐to‐height ratio (WHtR) (WC divided by height).

Dietary indices were derived from a 24‐h dietary recall using the Cameroon‐adapted Diet Quality Questionnaire (DQQ) [20]. Multiple indicators were computed: the Food Group Dietary Score (FGDS), a diversity score ranging from 0 to 10; the minimum dietary diversity (MDD‐W) reflecting the consumption of at least five food groups, the ALL‐5 score, a binary indicator reflecting consumption of all five recommended food groups; and specific indicators for starchy staples, animal‐source foods, fruits, vegetables, and pulses, nuts, or seeds. Furthermore, adherence to global dietary recommendations (GDR) was assessed through the NCD Protect score (healthy components, 0–9), NCD Risk score (components to limit, 0–9), and GDR score (0–18), calculated as NCD Protect minus NCD Risk plus 9 [20].

#### 2.7.3. Mediating Factors

The lipid biomarkers analyzed were total cholesterol, high‐density lipoprotein (HDL)‐cholesterol, and triglycerides, determined using standardized enzymatic methods [17]. LDL concentrations were calculated using the Friedewald equation (LDL = total cholesterol−HDL−triglycerides/5) [21, 22]. Abnormal lipid profiles were defined according to the US NCEP‐ATP III criteria [23]. Mediating factors were treated as continuous variables.

#### 2.7.4. Moderation Factor

Sex was self‐reported by participants based on the question “What is your sex?” Sex was treated as a binary variable (Male/Female).

#### 2.7.5. Other Demographics

The study documented key demographic variables, including age (in years), educational attainment (categorized as none, primary, secondary, or post‐secondary), marital status (categorized as divorced, married, single, or widowed), and employment status (dichotomized as employed or unemployed).

### 2.8. Data Quality Control

To ensure data integrity, investigators participated in a training session, conducting a presurvey to reduce variability and maintain supervision throughout the study.

### 2.9. Statistical Analysis

Data were processed and analyzed using IBM‐SPSS 27.0 for Windows (IBM Corporation, Armonk, NY, USA) with the PROCESS macro version 4.3 [24]. Initial data screening verified data entry accuracy, identified missing data and outliers, and basic assumptions of linear regression analyses. Descriptive statistics were presented as frequencies and percentages for categorical variables, and means and standard errors for continuous variables.

To test the mediation hypothesis, PROCESS model 4 examined the parallel mediation effects of lipid biomarkers on the associations between anthropometric/dietary indicators and IR. Anthropometric and dietary indicators served as independent variables, with IR as the dependent variable. Unstandardized coefficients for total, direct, and indirect effects were reported, with significance set at *p* < 0.05 for total and direct effects, and 95% confidence intervals excluding zero for indirect effects.

To test the moderation and moderated mediation hypotheses, PROCESS model 5 examined the interaction between sex and direct effects, while PROCESS model 14 assessed the interaction between sex and indirect effects from the initial mediation analysis. Sex was entered as a moderator, with significant mediators from the first hypothesis (BMI, WC, WHR, WHtR, fruit intake, and starchy staple intake) as independent variables. Standardized coefficients for direct effects and the index of moderated mediation for conditional indirect effects were reported, with significance set at *p* < 0.05 for direct effects and 95% confidence intervals excluding zero for the index of moderated mediation.

Mediation and moderated mediation analyses used a bootstrap of 5000 resamples to estimate indirect effects and 95% confidence intervals.

## 3. Results

### 3.1. General Characteristics of the Population

In this study, 169 participants were enrolled. Table 1 presents the sociodemographic characteristics of the study participants. More than two‐thirds of participants were aged 20–59 years, with over half being female. Most had secondary education (49.1%), and the majority were married, while 51.5% were unemployed.

**TABLE 1:** Sociodemographic characteristics of the study population visiting the National Obesity and Diabetic Centre, Yaoundé.

Variable	Statistics
Age (years)—*N* (%)	
20–39	48 (28.4)
40–59	67 (39.7)
60–79	50 (29.6)
80–99	4 (2.4)
Sex—*N* (%)	
Female	99 (58.6)
Male	70 (41.4)
Education—*N* (%)	
No education	9 (5.3)
Primary	26 (15.4)
Secondary	83 (49.1)
Post‐secondary	51 (30.2)
Marital status—*N* (%)	
Divorced	3 (1.8)
Married	109 (64.5)
Single	44 (26.0)
Widow	13 (7.7)
Occupation—*N* (%)	
Employed	82 (48.5)
Unemployed	87 (51.5)

Anthropometric data indicated that two‐thirds of the participants were either overweight or obese, with central obesity affecting 50.3% (Table 2). Additionally, nearly two‐thirds had an abnormal WHR, and 35.5% exhibited an abnormal WHtR.

**TABLE 2:** Anthropometric and biochemical characteristics of the study population visiting the National Obesity and Diabetic Centre, Yaoundé.

Variables	Statistics
*Anthropometric indicators*	
Body mass index (BMI)—*N* (%)	
Undernourished	4 (2.4)
Normal	53 (31.4)
Overweight	55 (32.5)
Obese	57 (33.7)
Waist circumference—*N* (%)	
Normal	84 (49.7)
Abnormal	85 (50.3)
Waist‐to‐hip ratio—*N* (%)	
Normal	59 (34.9)
Abnormal	110 (65.1)
Waist‐to‐height ratio—*N* (%)	
Normal	109 (64.5)
Abnormal	60 (35.5)

*Lipid biomarkers*	
Total cholesterol (g/L)—mean (SE)	135.76 (2.90)
LDL‐cholesterol (g/L)—mean (SE)	76.17 (2.67)
HDL‐cholesterol (g/L)—mean (SE)	41.16 (1.02)
Triglycerides (g/L)—mean (SE)	88.11 (4.88)
Fasting blood glucose (g/dL)—mean (SE)	1.46 (0.06)
Insulin (μUI/mL)—mean (SE)	11.07 (0.78)

The mean values for total cholesterol, LDL‐cholesterol, HDL‐cholesterol, and triglycerides were 135.76, 76.17, 41.16, and 88.11 g/L, respectively. Fasting blood glucose averaged 1.46 g/dL, and insulin levels were recorded at 11.07 µUI/mL.

Regarding dietary quality (Table 3), 45.5% of participants met the MDD, while only 15.1% achieved the ALL‐5 indicator. Food consumption statistics showed that 79.9%, 59.8%, 37.3%, 87.0%, and 91.1% of participants consumed at least one vegetable, one fruit, one pulse/nut/seed, one animal‐source food, and starchy food, respectively. On average, participants consumed about four food groups, with NCD Protect Score, NCD Risk Score, and GDR Score being 2.8, 1.1, and 10.7, respectively.

**TABLE 3:** Dietary quality characteristics of the study population visiting the National Obesity and Diabetic Centre, Yaoundé.

Variable	Statistics
Minimum dietary diversity (MDD‐W)—*N* (%)	
No	54 (54.5)
Yes	45 (45.5)
ALL‐5—*N* (%)	
No	144 (85.2)
Yes	25 (15.1)
At least one vegetable—*N* (%)	
No	34 (20.1)
Yes	135 (79.9)
At least one fruit—*N* (%)	
No	68 (40.2)
Yes	101 (59.8)
At least one pulse, nut, or seed—*N* (%)	
No	106 (62.7)
Yes	63 (37.3)
At least one animal‐source food (ASF)—*N* (%)	
No	22 (13.0)
Yes	147 (87.0)
At least starchy staple—*N* (%)	
No	15 (8.9)
Yes	154 (91.1)
Food Group Dietary Score (FGDS)—mean (SE)	4.2 (1.0)
NCD Protect Score—mean (SE)	2.8 (1.0)
NCD Risk Score—mean (SE)	1.1 (0.1)
GDR Score—mean (SE)	10.7 (0.1)

Abbreviations: GDR, global dietary recommendations; NCD, noncommunicable disease.

### 3.2. Mediation Effect of Lipid Biomarkers

Table 4 illustrates the mediating effect of lipid biomarkers on the relationship between anthropometric and dietary quality indicators, as independent variables, and IR, as the dependent variable, using parallel mediation models.

**TABLE 4:** Parallel mediating effect of lipid biomarkers on the relationship between the independent variables (anthropometric measurements and diet quality indicators) and insulin resistance as the dependent variable.

Independent variable	Total effect‐coef. (*p* value)	Direct effect‐coef. (*p* value)	Indirect effect‐Coef. (95% CI)				
			HDL	LDL	Triglyceride	Total cholesterol	Combined
*Anthropometric indicators*							
BMI	1.36 (0.004)^∗^	1.07 (0.038)^∗^	−0.08 (−0.53, 0.35)	0.60 (0.00, 3.04)	0.76 (0.17, 2.27)^∗^	−0.98 (−4.22, −0.17)	0.29 (−0.21, 0.94)
WC	3.15 (< 0.001)^∗^	2.50 (< 0.001)^∗^	0.29 (−0.14, 1.66)	0.20 (−1.05, 1.45)	0.72 (0.04, 2.63)^∗^	−0.56 (−4.30, 0.45)	0.65 (0.09, 1.45)^∗^
WHR	1.56 (0.053)	1.14 (0.186)	0.16 (−0.49, 1.40)	0.27 (−0.99, 3.24)	0.55 (0.11, 2.63)^∗^	−0.57 (−5.45, 0.37)	0.42 (−0.01, 1.05)
WHtR	1.59 (0.053)	0.94 (0.240)	−0.04 (−0.80, 0.69)	1.01 (−0.01, 5.71)	0.84 (−0.04, 2.97)	−1.14 (−6.64, −0.05)	0.65 (0.02, 1.70)^∗^

*Diet quality indicators*							
FGDS	0.29 (0.351)	0.36 (0.234)	0.16 (−0.07, 0.81)	−0.04 (−1.14, 0.61)	−0.17 (−0.63, 0.15)	−0.01 (−0.86, 0.77)	−0.06 (−0.44, 0.18)
ALL‐5	0.28 (0.799)	0.42 (0.692)	0.45 (−0.19, 2.36)	0.31 (−1.44, 2.83)	−0.57 (−2.23, 0.38)	−0.33 (−3.16, 1.22)	−0.13 (−0.91, 0.73)
At least SS	−2.40 (0.082)	−2.59 (0.049)^∗^	0.02 (−1.09, 1.24)	1.99 (0.24, 9.61)^∗^	−0.36 (−2.40, 1.13)	−1.46 (−9.57, −0.02)	0.19 (−1.34, 1.63)
At least veg	0.46 (0.640)	0.65 (0.484)	0.00 (−1.17, 1.29)	0.02 (−3.11, 2.85)	0.06 (−1.05, 1.29)	−0.28 (−3.59, 2.28)	−0.19 (−1.15, 0.50)
At least one fruit	1.23 (0.125)	1.57 (0.042)^∗^	0.55 (0.03, 2.57)^∗^	−0.57 (−3.67, 0.62)	−0.81 (−3.25, 0.05)	0.49 (−0.93, 3.73)	−0.34 (−1.35, 0.36)
At least PNS	−0.97 (0.232)	−0.35 (0.649)	−0.03 (−0.72, 0.82)	−0.18 (−2.53, 1.09)	−0.76 (−2.53, −0.03)	0.35 (−0.72, 3.49)	−0.62 (−1.31, −0.05)
At least ASF	−0.27 (0.820)	−0.38 (0.737)	0.66 (−0.17, 2.70)	−0.89 (−7.87, 1.17)	−0.73 (−3.47, 1.07)	1.08 (−1.56, 6.87)	0.11 (−1.74, 1.33)
NCD protect score	0.42 (0.187)	0.45 (0.139)	0.22 (−0.02, 1.06)	0.01 (−0.79, 0.80)	−0.05 (−0.47, 0.31)	−0.21 (−1.44, 0.34)	−0.03 (−0.29, 0.20)
NCD risk score	0.04 (0.920)	−0.29 (0.427)	0.05 (−0.32, 0.47)	−0.17 (−1.46, 0.43)	0.44 (−0.13, 2.00)	0.00 (−1.21, 0.83)	0.32 (−0.14, 0.98)
GDR score	0.22 (0.360)	0.36 (0.109)	0.11 (−0.06, 0.59)	0.07 (−0.39, 0.77)	−0.21 (−0.92, 0.09)	−0.12 (−0.85, 0.43)	−0.15 (−0.47, 0.06)

*Note:* WHR: waist‐to‐hip ratio, WHtR: waist‐to‐height ratio, FDGS: food group dietary score, At least veg: at least one vegetable, At least PNS: at least one pulse, nut or seed, At least ASF: at least one animal‐source food, At least SS: at least starchy staple, Coef.: coefficient, Combined: cumulative effect of HDL‐C, LDL‐C, triglycerides, and total cholesterol.

Abbreviations: 95% CI, 95% confidence interval; BMI, body mass index; HDL, high‐density lipoprotein; LDL, low‐density lipoprotein; WC, waist circumference.

^∗^ *p* values carrying the superscript are statistically significant (*p* < 0.05).

Regarding anthropometric indicators, the results showed that lipid biomarkers fully or partially mediated all anthropometric indicators. The parallel mediation analysis elucidated that BMI significantly affects IR (Coef = 1.36, *p* = 0.004). The direct effect of BMI on IR was also significant (Coef = 1.07, *p* = 0.038), indicating a strong independent association. Although the cumulative indirect effect of all lipid biomarkers was not significant (*p* ≥ 0.05), a notable finding was the significant indirect effect of triglycerides (Coef = 0.76, 95% CI 0.17–2.27). This suggests that triglycerides partially mediate the relationship between BMI and IR, with a substantial portion of the total effect being attributable to this pathway. In contrast, HDL, LDL, and total cholesterol did not show significant mediation effects. These results underscore the specific and prominent role of triglycerides in the BMI–IR association.

The parallel mediation analysis demonstrated that WC has a significant total effect on IR (Coef = 3.15, *p* < 0.001). The direct effect of WC on IR was also significant (Coef = 2.50, *p* < 0.001), indicating a strong independent association. Notably, the cumulative indirect effect of lipid biomarkers was significant (Coef = 0.65, *p* < 0.05), with triglycerides emerging as a significant mediator (Coef = 0.72, 95% CI 0.04–2.63). This suggests that a substantial portion of the effect of WC on IR is mediated through triglycerides, highlighting the importance of this lipid biomarker in the WC‐IR pathway. In contrast, HDL, LDL, and total cholesterol did not show significant mediation effects. These findings emphasize the role of triglycerides in linking central adiposity to IR.

The parallel mediation analysis examining the relationship between WHR and IR revealed nonsignificant total and direct effects, indicating that WHR alone does not significantly predict IR in this model. Furthermore, the cumulative indirect effect of lipid biomarkers was not significant. However, a notable finding was the significant indirect effect of triglycerides (Coef = 0.55, 95% CI 0.11–2.63), suggesting that triglycerides may still play a role in the pathway between WHR and IR, despite the nonsignificant total effect. HDL, LDL, and total cholesterol did not show significant mediation effects. These results highlight the complex nature of the relationship between body fat distribution, lipid biomarkers, and IR, with triglycerides emerging as a potential mediator independent of the overall effect of WHR.

The parallel mediation analysis investigating the relationship between WHtR and IR yielded a nonsignificant total effect and direct effect, suggesting that WHtR alone does not strongly predict IR. Interestingly, the cumulative indirect effect of lipid biomarkers was significant (Coef = 0.65, 95% CI 0.02–1.70), indicating that lipid biomarkers collectively mediate the WHtR–IR relationship, even in the absence of a significant direct effect. However, none of the individual lipid biomarkers— HDL, LDL, triglycerides, and total cholesterol—showed significant mediation effects independently. These findings suggest a potential synergistic effect of lipid biomarkers in the pathway between WHtR and IR.

Regarding dietary quality indicators, out of 10 indicators studied, only the relationship between “at least starchy staple” or “at least one fruit” and IR was found to be mediated by lipid biomarkers. The parallel mediation analysis investigating the relationship between the intake of at least one starchy staple and IR revealed a nonsignificant total effect. Notably, the direct effect of starchy staple intake on IR was significant and negative (Coef = −2.59, *p* = 0.049), suggesting that consuming at least one starchy staple is independently associated with lower IR. The cumulative indirect effect of lipid biomarkers was not significant. However, LDL emerged as a significant mediator (Coef = 1.99, 95% CI 0.24–9.61), indicating that LDL levels partially mediate the relationship between starchy staple intake and IR, despite the nonsignificant total effect. HDL, triglycerides, and total cholesterol did not show significant mediation effects. These findings suggest a complex interplay where starchy staple intake may have a direct beneficial effect on IR, while also influencing IR indirectly through LDL levels.

The parallel mediation analysis examining the relationship between the intake of at least one fruit and IR revealed a nonsignificant total effect. However, the direct effect of fruit intake on IR was significant and positive (Coef = 1.57, *p* = 0.042), indicating that consuming at least one fruit is independently associated with higher IR, contrary to expectations. The cumulative indirect effect of lipid biomarkers was not significant. Notably, HDL emerged as a significant mediator (Coef = 0.55, 95% CI 0.03–2.57), suggesting that HDL levels partially mediate the relationship between fruit intake and IR, despite the nonsignificant total effect. LDL, triglycerides, and total cholesterol did not show significant mediation effects. These findings present a complex picture, where fruit intake may have an unexpected direct effect on IR, while also influencing IR indirectly through HDL levels.

### 3.3. Moderation Effect of Sex

Table 5 illustrates the moderating effect of sex on the direct and indirect relationships between specific anthropometrics (BMI, WC, WHR, and WHtR) and dietary indicators (consumption of at least one starchy staple and at least one fruit) with IR as the outcome variable.

**TABLE 5:** Moderation effect of sex on the direct and indirect relationships between some anthropometric and diet quality indicators on insulin resistance.

Independent variable	Direct effect	Conditional indirect effect			
	Coef. (*p* value)	HDL	LDL	TG	TC
		Index of moderated mediation (95% CI)			
BMI	−1.17 (0.182)	0.28 (−1.23, 2.08)	−2.82 (−9.44, 1.25)	−2.09 (−6.75, 0.28)	4.62 (−1.67, 15.14)
WC	5.11 (0.002)^∗^	−1.22 (−6.26, 0.72)	−1.09 (−7.62, 5.91)	−2.03 (−7.38, 0.30)	3.13 (−3.05, 13.35)
WHR	−0.64 (0.711)	2.15 (−6.47, 2.45)	−2.22 (−13.98, 8.04)	−2.94 (−8.72, 1.20)	5.19 (−5.56, 20.25)
WHtR	−2.05 (0.197)	0.18 (−2.65, 2.95)	−5.31 (−18.31, 2.05)	−2.30 (−8.50, 0.56)	6.19 (−2.39, 21.97)
At least SS	1.89 (0.474)	−0.08 (−3.79, 4.20)	−9.05 (−33.95, 3.85)	0.92 (−3.06, 5.79)	7.20 (−3.05, 31.14)
At least one fruit	−2.36 (0.123)	−2.18 (−8.62, 1.12)	2.73 (−3.05, 15.21)	2.04 (−0.82, 7.71)	−2.51 (−14.46, 3.61)

*Note:* WHtR: waist‐to‐height ratio, At least SS: at least starchy staple, TG: triglycerides.

Abbreviations: 95% CI, 95% confidence interval; BMI, body mass index; HDL, high‐density lipoprotein; LDL, low‐density lipoprotein; TC, total cholesterol; WC, waist circumference; WHR, waist‐to‐hip ratio.

^∗^values carrying the superscript are statistically significant (*p* < 0.05).

The analysis demonstrated that the interaction term for BMI and sex was not statistically significant (*p* = 0.182), indicating that sex did not moderate the direct effect of BMI on IR. Furthermore, the indices of moderated mediation for specific lipid biomarkers included zero within the 95% bootstrap confidence interval, suggesting that sex did not moderate the mediating effects of these biomarkers.

However, the interaction term for WC and sex was statistically significant (*p* = 0.002), implying that sex moderated the direct effect of WC on IR. No significant moderated mediation was observed for the index of moderated mediation.

The interaction terms for WHR and sex, WHtR and sex, consumption of at least one starchy staple and sex, and consumption of at least one fruit and sex were not statistically significant (*p* ≥ 0.05). This suggests that sex did not moderate the direct effects of these variables on IR. Additionally, no significant moderated mediation was observed for WHR, WHtR, or the dietary indicators, as indicated by their respective indices of moderated mediation.

## 4. Discussion

The findings of this research provide a detailed elucidation of the intricate pathways linking body composition, dietary quality, and lipid biomarkers to IR within a Cameroonian clinical cohort. This study is among the first in the Central African region to utilize a moderated mediation framework to explore these interconnections, filling a significant gap in the understanding of metabolic health in populations undergoing rapid epidemiological transition. Our results underscore that anthropometric indicators, particularly BMI and WC, exert profound effects on IR, with triglycerides emerging as a central and consistent mediator across different models.

The prominent role of triglycerides as a bridge between adiposity and IR aligns with the widely recognized lipotoxicity hypothesis. According to this framework, when the storage capacity of subcutaneous adipose tissue (SAT) is exceeded, lipids spill over and accumulate as ectopic fat in nonadipose tissues such as the liver and skeletal muscle. This accumulation of intracellular lipid metabolites, such as diacylglycerols and ceramides, interferes with the insulin receptor substrate (IRS‐1) signaling pathway, subsequently impairing glucose transporter translocation and promoting systemic IR [25, 26]. In sub‐Saharan African populations, where metabolic profiles often differ from Western norms due to both genetic and environmental factors, hypertriglyceridemia has been increasingly identified as a critical metabolic driver. Recent evidence from Central African cohorts suggests that elevated circulating triglycerides may be a more sensitive predictor of IR than total body mass, potentially reflecting ethnic‐specific variations in adipose tissue distribution and lipid metabolism efficiency [27, 28].

Regarding dietary indicators, the analysis revealed complex and multifaceted relationships that challenge some conventional nutritional paradigms. A particularly striking finding was that the intake of at least one starchy staple was independently associated with lower IR. In the Cameroonian context, this observation may be potentially explained by the nature of the staples consumed. Traditional diets in the region rely heavily on green plantains, cassava, and various tubers, which are known to be rich in resistant starch when prepared through traditional boiling methods. Resistant starch acts similarly to dietary fiber; it escapes digestion in the small intestine and undergoes fermentation by the gut microbiota in the colon. This process produces short‐chain fatty acids (SCFAs), such as butyrate and propionate, which have been shown in several studies to enhance systemic insulin sensitivity and modulate the secretion of incretin hormones like glucagon‐like peptide‐1 (GLP‐1) [29, 30]. However, this interpretation must remain tentative. As highlighted by dietary studies in Africa, the glycemic response to these staples is highly sensitive to processing and cooking techniques, such as frying or overprocessing, which significantly increase the glycemic load. Since our 24‐h recall did not capture the granular details of food preparation, this independent beneficial effect of staples warrants further investigation through longitudinal and interventional designs [31–33].

The unexpected positive direct association between fruit intake and IR presents a significant paradox that requires a highly cautious and nuanced interpretation. While fruit consumption is generally promoted as a protective factor against metabolic diseases, our findings suggest an association with higher IR in this specific cohort. This potentially counterintuitive result may be attributed to several factors specific to the local context. First, the varieties of fruits commonly consumed in tropical regions, such as ripe mangoes, pineapples, and bananas, often possess a higher glycemic index and higher fructose content compared to the temperate fruits typically studied in the Western literature. Excessive fructose intake, particularly when fiber intake is insufficient, can accelerate hepatic de novo lipogenesis, leading to increased intrahepatic fat and worsened insulin signaling [34, 35]. Second, this finding might be partially influenced by methodological factors such as recall bias or social desirability bias. Individuals who are already aware of their metabolic risk or have received prior health counseling might over‐report healthy behaviors like fruit intake, even while their metabolic status continues to deteriorate. Furthermore, residual confounding from other lifestyle factors, such as the consumption of sugar‐sweetened beverages or specific fruit‐based preparations, cannot be entirely excluded [36, 37]. The emergence of HDL as a significant partial mediator in the fruit–IR relationship suggests that the metabolic impact of fruits may be complex and dependent on their ability to maintain or improve the lipid profile, reinforcing the idea that nutritional impact is rarely isolated but depends on the overall dietary matrix [38].

The moderated mediation analysis provides further depth into the sex‐specific nature of these associations. Sex significantly moderated the direct effect of WC on IR, but did not affect the mediating role of lipid biomarkers. This finding suggests a dual‐track mechanism: while the initial impact of central adiposity on insulin sensitivity is influenced by sex‐specific factors, the subsequent downstream effects of established dyslipidemia (hypertriglyceridemia and elevated LDL) on insulin signaling appear to follow a uniform physiological pathway across both men and women. The stronger direct impact of WC in one sex may be elucidated by the distinct patterns of fat distribution described in the recent Central African literature. Men in these regions tend to accumulate more visceral adipose tissue (VAT), which is highly metabolic and secretes a higher concentration of pro‐inflammatory cytokines (such as IL‐6 and TNF‐α) directly into the portal circulation. In contrast, women, particularly those in the premenopausal stage, tend to store more SAT in the gluteofemoral region, which may act as a “metabolic sink”, temporarily protecting against the lipotoxic effects seen with central obesity [39, 40]. These observations are consistent with the findings of Rodrigues et al. [41], who demonstrated that for the same average BMI, sex‐specific differences in VAT and SAT accumulation lead to vastly different metabolic risk profiles. Our study reinforces their conclusion that clinical assessments must look beyond BMI and adopt sex‐sensitive thresholds for WC to accurately capture metabolic vulnerability.

Furthermore, the lack of significant moderated mediation for lipid biomarkers implies that the mediating role of triglycerides in the BMI‐IR and WC‐IR pathways is robust across genders. Once excess adiposity leads to the failure of adipose tissue expansion and the subsequent rise in circulating triglycerides, the biochemical interference with the glucose transporter (GLUT4) translocation process seems to be a sex‐neutral biological event [42, 43]. This underscores the critical importance of targeting dyslipidemia as a universal clinical priority in both Cameroonian men and women to mitigate the rising burden of Type 2 diabetes. The intricate interplay identified here suggests that interventions should not only focus on weight loss but also on specific lipid management and the promotion of dietary patterns that include high‐fiber, resistant‐starch staples to potentially buffer the insulin‐resistant effects of central obesity.

## 5. Strengths and Limitations

A primary strength of this study is the use of a well‐characterized clinical cohort and a validated, locally adapted DQQ, which accurately captures the cultural nuances of the Cameroonian diet [20]. Furthermore, the application of moderated mediation models provides a more granular understanding of metabolic interactions than standard bivariate analyses.

However, several limitations warrant consideration. The cross‐sectional design precludes the establishment of temporal causality, and the hospital‐based recruitment may limit the generalizability of the findings to asymptomatic community populations. While the DQQ is culturally adapted, the 24‐h dietary recall remains subject to social desirability and recall biases. Most importantly, the absence of physical activity data represents a notable limitation. As a major determinant of both lipid metabolism and insulin sensitivity, the lack of this variable may result in residual confounding. Future longitudinal research incorporating objective measures of physical activity, such as accelerometry, is necessary to confirm these mediating pathways and support the development of multifactorial, sex‐specific metabolic interventions.

## 6. Conclusion

In summary, these findings reveal that anthropometric indicators such as BMI and WC exert significant effects on IR, with triglycerides emerging as a critical mediator in their associations. While triglycerides partially mediate the relationship between BMI and IR, they also significantly mediate the impact of WC, underscoring their role in linking body fat distribution to IR. In contrast, WHR and WHtR do not show significant direct effects on IR, although triglycerides may still play a mediating role for WHR. Dietary quality indicators demonstrate a complex interplay, where the intake of starchy staples appears to independently lower IR, with LDL levels mediating this effect, whereas fruit intake may be linked to increased IR, with HDL acting as a mediator. Additionally, the influence of sex on these relationships varies, significantly moderating the impact of WC on IR, while having no notable effects on BMI or other indicators. Overall, these results highlight the multifactorial nature of IR, emphasizing the need for further exploration of lipid biomarkers and dietary influences in managing this condition. Future research should aim to explore underlying mechanisms to inform targeted prevention and intervention strategies for IR.

## Author Contributions

Brice Ulrich Foudjo Saha and Lifoter Kenneth Navti conceived and designed the study and analyses. Brice Ulrich Foudjo Saha drafted the manuscript. Ismael Teta supported the data curation and analysis. Material preparation and data collection were performed by Fanuel Yibume Bey. Edouard Akono Nantia, Tume Christopher Bonglavnyuy, Eugene Sobngwi, and Ismael Teta provided guidance and oversight in the development of the research idea, supervised the research project, and provided mentorship.

## Funding

This research did not receive any specific grant from funding agencies in the public, commercial, or not‐for‐profit sectors.

## Disclosure

All the authors made a substantial, direct, and intellectual contribution to the work and approved the final version of the manuscript for publication.

## Conflicts of Interest

The authors declare no conflicts of interest.

## Supporting Information

Additional supporting information can be found online in the Supporting Information section.

## Supporting information


**Supporting Information 1** *Supporting information 1 for mediation statistics*. This supporting file presents the statistical outputs generated by the PROCESS Macro, including descriptive statistics (means, standard deviations, and correlations) for all variables. It also reports regression coefficients for the direct, indirect, and total effects of the independent variable on the dependent variable, accompanied by standard errors and confidence intervals for assessing statistical significance and precision. Indirect effects are estimated, and bootstrapped confidence intervals are provided to evaluate their significance. Additionally, the file includes model summary statistics, such as R‐squared values and F‐statistics, to assess overall model fit.

**Supporting Information 2** *Supporting information 2 for moderation statistics*. This supporting file includes descriptive statistics, encompassing means, standard deviations, and correlations for the variables involved. It presents regression coefficients for the direct, indirect, and total effects of the independent variable on the dependent variable, along with standard errors and confidence intervals to assess statistical significance and precision. The file also contains conditional effects of the independent variable on the dependent variable at different levels of the moderator. Additionally, model summary statistics, such as R‐squared values and F‐statistics, are provided to evaluate overall model fit.

## Data Availability

The dataset supporting the findings of this study is openly available in Mendeley Data at https://data.mendeley.com/preview/8wd3gndzc9?a=259ad994-cdbf-4f69-9adc-843ee53d63fd.
