# Supplementary material for: Mediating Effects of Lipid Biomarkers and Sex‐Specific Moderation on the Associations Between Anthropometric and Dietary Factors With Insulin Resistance
Source: J Obes. 2026 Feb 27;2026:7216950. doi: 10.1155/jobe/7216950 (PMC12949073; doi:10.1155/jobe/7216950)
Supplement: Supplementary file 1 — Supporting Information 1 Supporting information 1 for mediation statistics. This supporting file presents the statistical outputs generated by the PROCESS Macro, including descriptive statistics (means, standard deviations, and correlations) for all variables. It also reports regression coefficients for the direct, indirect, and total effects of the independent variable on the dependent variable, accompanied by standard errors and confidence intervals for assessing statistical significance and precision. Indirect effects are estimated, and bootstrapped confidence intervals are provided to evaluate their significance. Additionally, the file includes model summary statistics, such as R‐squared values and F‐statistics, to assess overall model fit. [file JOBE-2026-7216950-s001.doc]

GET
  FILE='C:\Users\Brice SAHA\Documents\UBa\Master Thesis 2021\Fanuel\Database fanuel.sav'.
DATASET NAME Jeu_de_données1 WINDOW=FRONT.
* Encoding: UTF-8.
preserve.
set printback=off.


Matrice


Remarques	
Sortie obtenue	10-NOV-2024 16:55:08	
Commentaires		
Entrée	Données	C:\Users\Brice SAHA\Documents\UBa\Master Thesis 2021\Fanuel\Database fanuel.sav	
	Jeu de données actif	Jeu_de_données1	
	Filtre	<sans>	
	Pondération	<sans>	
	Scinder un fichier	<sans>	
	N de lignes dans le fichier de travail	169	
Syntaxe	MATRIX.
compute wnames='xxxxx'.
compute znames='xxxxx'.
compute mcerpt=0.
compute wiscov=0.
compute ziscov=0.
compute tooman=0.
compute errcode=make(100,1,0).
compute notecode=make(100,1,0).
compute model = trunc( 4 ).
compute iterate = abs(trunc( 100 )).
compute converge = abs( 0.00001 ).
compute itprobtg=0.
compute v2tag=0.
compute ydich=0.
compute maxwwarn=0.
compute minwwarn=0.
compute maxzwarn=0.
compute minzwarn=0.
compute toomany=0.
compute wdich=0.
compute zdich=0.
compute wnotev=0.
compute znotev=0.
compute nxpval=1.
compute nwpval=1.
compute nzpval=1.
compute errs=1.
compute notes=1.
compute criterr=0.
compute novar=0.
compute adjust=0.
compute ncs=0.
compute serial=0.
compute sobelok=0.
compute hasw=0.
compute hasz=0.
compute printw=0.
compute printz=0.
compute xmint=( 0 =1).
compute wmodcust=0.
compute zmodcust=0.
compute booting=0.
compute bootiter=0.
compute iterrmod=0.
compute cov = 'xxxxx'.
compute varorder=( 0 <> 0).
compute nws=0.
compute w= 'xxxxx'.
compute nzs=0.
compute z = 'xxxxx'.
compute nms=0.
compute m = 'TG HDL LDL TC'.
compute nys=0.
compute y = 'HOMAIR'.
compute nxs=0.
compute x = 'BMIStat'.
compute effsize=( 0 =1).
compute stand=( 1 =1).
compute intprobe = .1.
compute xrefvals={ 999 }.
compute center=trunc( 0 ).
compute xcontcf=0.
compute xscaling=1.
compute cdeval={ -999 }.
compute cuscoval=0.
do if (model=74).
compute errcode(errs,1)=7.
compute errs=errs+1.
compute criterr=1.
end if.
do if (xmint=1 and model <> 4).
compute errcode(errs,1)=63.
compute errs=errs+1.
compute criterr=1.
end if.
do if (xmint=1 and model=4).
compute w=x.
compute model=74.
compute intprobe=1.
compute notecode(notes,1) = 32.
compute notes = notes + 1.
do if (effsize=1 or stand=1).
compute notecode(notes,1) = 34.
compute notes = notes + 1.
compute stand=0.
compute effsize=0.
end if.
do if (center <> 0).
compute center=0.
compute errcode(errs,1)=71.
compute errs=errs+1.
compute criterr=1.
end if.
end if.
compute v = 'xxxxx'.
compute q = 'xxxxx'.
compute linsum={ -999 }.
compute nlinsum=ncol(linsum).
do if (linsum(1,1) = -999).
compute nlinsum=0.
end if.
compute oldvars= 'xxxxx'.
compute mcxok=0.
compute mcwok=0.
compute mczok=0.
compute xprod=0.
compute zprod=0.
compute wprod=0.
compute modcok=0.
compute alttotal=0.
compute hc3=trunc( 0 ).
compute jn=( 0 = 1).
compute listmiss=( 0 =1).
compute modelres=( 0 =1).
compute outscree=( 1 =1).
compute activate=( 0 =1).
compute booterr=0.
compute normal=( 0 =1).
compute xmtest=( 0 =1).
compute describe=( 0 =1).
compute longname=( 0 =1).
do if (stand=1).
compute effsize=1.
end if.
compute pstog=0.
compute sobelok=0.
compute normal=( 0 =1).
compute mdichok=( 0 =1).
compute contrast={ 999 }.
compute ncontr=ncol(contrast).
compute ncontrow=nrow(contrast).
do if (contrast(1,1) = 999).
compute ncontr=1.
compute contrast=0.
end if.
do if (ncontr = 1).
compute contrast=trunc(contrast).
do if (contrast > 3 or contrast < 0)).
compute ncontr=1.
compute contrast = 0.
end if.
end if.
do if (ncontr > 1).
compute contvec=contrast.
compute contrast=4.
do if (ncontrow > 1).
compute contrast=0.
compute modcok=1.
compute wcontval=contvec(:,1).
compute zcontval=contvec(:,2).
do if ((ncontr <> 2) or (ncontrow <> 2)).
compute notecode(notes,1) = 19.
compute notes = notes + 1.
compute modcok=0.
end if.
end if.
end if.
do if (xmint = 1 and contrast(1,1) <> 0).
compute contrast=0.
compute notecode(notes,1) = 37.
compute notes = notes + 1.
end if.
do if (varorder = 1).
compute notecode(notes,1) = 21.
compute notes = notes + 1.
end if.
do if ( 999 <> 999 or 999 <> 999).
compute notecode(notes,1) = 22.
compute notes = notes + 1.
end if.
compute modelbt=( 0 =1).
compute cluster= 'xxxxx'.
compute matrices=( 0 =1).
compute covcoeff=( 0 =1).
compute covmy=trunc( 0 ).
do if (covmy < 0 or covmy > 2).
compute covmy = 0.
end if.
compute boot = abs(trunc( 5000 )).
compute bc=( 0 =1).
compute mc=abs(trunc( 0 )).
compute hc=trunc( 5 ).
do if (intprobe < 0 or intprobe > 1).
compute intprobe = .10.
end if.
compute plot=trunc( 0 ).
do if (plot < 0 or plot > 2).
compute plot=0.
end if.
compute total=( 1 =1).
compute dototal=0.
compute saveboot = ( 0 = 1).
compute saveest=( 0 = 2).
do if (saveest=1).
compute intprobe=1.
end if.
do if (hc >= 0 and hc < 5).
compute notecode(notes,1) = 4.
compute notes = notes + 1.
end if.
do if (hc > 5 or hc < 0).
compute hc=5.
end if.
compute mcw=trunc( 0 ).
compute mcz=trunc( 0 ).
compute mcx=trunc( 0 ).
do if (mcx > 0 and mcx < 3 and model = 74).
compute mcw=mcx.
compute xscaling=1.
end if.
do if (mcx > 2 and model = 74).
compute errcode(errs,1)=65.
compute errs=errs+1.
compute criterr=1.
end if.
do if (model = 74 and normal=1).
compute notecode(notes,1) = 33.
compute normal=0.
compute notes = notes + 1.
end if.
do if (mcx > 0 and contrast > 0).
compute notecode(notes,1) = 28.
compute notes = notes + 1.
compute contrast=0.
end if.
compute nxvls=1.
compute nmvls=1.
compute nwvls=1.
compute nzvls=1.
compute paths=999.
compute pathsw=999.
compute pathsz=999.
compute pathswz=999.
compute pathsmod=999.
compute pathtype=999.
compute obscoeff=999.
compute pathsdv={' '}.
compute quantile=1.
do if ( 999 <>999).
compute notecode(notes,1) = 23.
compute notes = notes + 1.
end if.
compute moments=( 0 =1).
do if (moments=1).
compute quantile=0.
end if.
compute bmatrix={ -999 }.
compute wmatrix={ -999 }.
compute zmatrix={ -999 }.
compute wzmatrix={ -999 }.
compute cmatrix={ -999 }.
compute xcatcode={ -999 }.
compute wcatcode={ -999 }.
compute zcatcode={ -999 }.
compute needed=0.
compute conf= 95.
do if (trunc( 95 ) >= 100 or (trunc( 95 ) <= 50)).
compute conf = 95.
compute notecode(notes,1)=2.
compute notes=notes+1.
end if.
do if (model >= 0 and model < 4 and modelbt=0).
compute boot=0.
compute mc=0.
compute bc=0.
end if.
do if (mc > 0 and boot > 0).
compute boot=0.
compute bc=0.
end if.
do if ((boot < 1000) and (mc = 0) and (boot > 0)).
compute boot=5000.
end if.
do if ((mc < 1000) and (boot = 0) and (mc > 0)).
compute mc=5000.
end if.
compute p0=-.322232431088.
compute p1 = -1.
compute p2 = -.342242088547.
compute p3 = -.0204231210245.
compute p4 = -.0000453642210148.
compute q0 = .0993484626060.
compute q1 = .588581570495.
compute q2 = .531103462366.
compute q3 = .103537752850.
compute q4 = .0038560700634.
compute badend=0.
compute priorlo = -9999999.
compute priorhi = 9999999.
compute alpha2 = (1-(conf/100))/2.
compute cilm=alpha2*2.
compute y5=sqrt(-2*ln(alpha2)).
compute xp2=(y5+((((y5*p4+p3)*y5+p2)*y5+p1)*y5+p0)/((((y5*q4+q3)*y5+q2)*y5+q1)*y5+q0)).
compute medlb={'   M1  :';'   M2  :';'   M3  :';'   M4  :';'   M5  :';'   M6  :';'   M7  :';'   M8  :';'   M9  :';'   M10 :'}.
compute medlb2={'(M1)','(M2)','(M3)','(M4)','(M5)','(M6)','(M7)','(M8)','(M9)','(M10)'}.
compute xlb={'   X1  :';'   X2  :';'   X3  :';'   X4  :';'   X5  :';'   X6  :';'   X7  :';'   X8  :';'   X9  :'}.
compute highlbw={'M1*W'; 'M2*W'; 'M3*W'; 'M4*W'; 'M5*W'; 'M6*W'; 'M7*W'; 'M8*W'; 'M9*W'; 'M10*W'}.
do if (xmint=1).
compute highlbw={'M1*X'; 'M2*X'; 'M3*X'; 'M4*X'; 'M5*X'; 'M6*X'; 'M7*X'; 'M8*X'; 'M9*X'; 'M10*X'}.
end if.
compute highlbz={'M1*Z'; 'M2*Z'; 'M3*Z'; 'M4*Z'; 'M5*Z'; 'M6*Z'; 'M7*Z'; 'M8*Z'; 'M9*Z';'M10*Z'}.
compute highlbwz={'M1*W*Z'; 'M2*W*Z'; 'M3*W*Z'; 'M4*W*Z'; 'M5*W*Z'; 'M6*W*Z'; 'M7*W*Z'; 'M8*W*Z'; 'M9*W*Z';'M10*W*Z'}.
compute highlbbt={'BOTH(M1)'; 'BOTH(M2)'; 'BOTH(M3)'; 'BOTH(M4)'; 'BOTH(M5)'; 'BOTH(M6)'; 'BOTH(M7)'; 'BOTH(M8)'; 'BOTH(M9)';'BTH(M10)'}.
compute highlbx={'M1*X'; 'M2*X'; 'M3*X'; 'M4*X'; 'M5*X'; 'M6*X'; 'M7*X'; 'M8*X'; 'M9*X'; 'M10*X'}.
compute skipwz=0.
compute validm={1,1,1,1,1,1,1,1,1,1,1,1,1,1,1,1,1,1,1,1,1,1,0,0,0,0,0,1,1,0,0,0,0, 0,0,0,0,0,0,0,0,0,0,0,0,0,0,0,0,0,0,0,0,0,0,0,0,1,1,1,1,1,1,1,1,1,1,1,1,1,1,1,1,1,1,1,0,0,0,1,1, 1,1,1,1,1,1,1,1,1,1,1}.
do if (activate=1).
compute errcode(errs,1)=60.
compute errs=errs+1.
compute criterr=1.
end if.
do if (criterr=0).
do if (( 0 =1)=1).
compute errcode(errs,1)=42.
compute errs=errs+1.
compute criterr=1.
end if.
do if (model > 0 and model < 93).
do if (validm(1,model)=0).
compute errcode(errs,1)=6.
compute errs=errs+1.
compute criterr=1.
end if.
release validm.
end if.
do if ((model > 92 or model < 0) and model <> 999)).
compute errcode(errs,1)=7.
compute errs=errs+1.
compute criterr=1.
end if.
do if (model = 999 and bmatrix(1,1)=-999).
compute errcode(errs,1)=24.
compute errs=errs+1.
compute criterr=1.
end if.
do if (model <> 999 and bmatrix(1,1) <> -999).
compute errcode(errs,1)=25.
compute errs=errs+1.
compute criterr=1.
end if.
do if ((model = 74 or (model > 0 and model < 4)) and ((wmatrix(1,1) <> -999) or (zmatrix(1,1)<>-999) or (wzmatrix(1,1)<>-999)))).
compute errcode(errs,1)=41.
compute errs=errs+1.
compute criterr=1.
end if.
do if (hc3 <> 0).
compute notecode(notes,1) = 5.
compute notes = notes + 1.
do if (hc3 = 1).
compute hc=3.
end if.
end if.
do if ((v <> 'xxxxx') or (q <> 'xxxxx')).
compute errcode(errs,1)=14.
compute errs=errs+1.
compute errcode(errs,1)=48.
compute errs=errs+1.
compute criterr=1.
end if.
do if (oldvars <> 'xxxxx').
compute errcode(errs,1)=48.
compute errs=errs+1.
compute criterr=1.
end if.
do if (cluster <> 'xxxxx').
compute errcode(errs,1)=27.
compute errs=errs+1.
compute criterr=1.
end if.
do if ((y = 'xxxxx') or (x = 'xxxxx')).
compute errcode(errs,1)=1.
compute errs=errs+1.
compute criterr=1.
end if.
do if ((m = 'xxxxx') and model > 3).
compute errcode(errs,1)=8.
compute errs=errs+1.
compute criterr=1.
end if.
end if.
do if (criterr=0).
get ytmp/variables = HOMAIR /names = ynames/MISSING = 99999.
compute nys=ncol(ytmp).
compute needed=nys.
compute n=nrow(ytmp).
compute varnames={ynames}.
compute dat=ytmp.

.


do if (toomany=1 and longname=0).
compute criterr=1.
do if (tooman=0).
compute tooman=1.
compute errcode(errs,1) = 61.
compute errs = errs + 1.
end if.
end if
.
compute modelvar={ '4' ;t(ynames)}.
do if ( 4 =999).
compute modelvar(1,1)='CUSTOM'.
end if.
get xtmp/variables = BMIStat /names = xnames/MISSING = 99999.
compute nxs=ncol(xtmp).
compute n=nrow(xtmp).
compute needed=needed+nxs.
compute varnames={varnames,xnames}.
compute xcatlab=t(xnames).
compute dat={dat,xtmp}.

.


do if (toomany=1 and longname=0).
compute criterr=1.
do if (tooman=0).
compute tooman=1.
compute errcode(errs,1) = 61.
compute errs = errs + 1.
end if.
end if
.
compute modelvar={modelvar;t(xnames)}.
do if (nxs = 1).
compute modelvlb={'Model  :';'    Y  :';'    X  :'}.
else.
compute modelvlb={'Model  :';'    Y  :';xlb(1:nxs,1)}.
end if.
do if (m <> 'xxxxx').
get mtmp/variables = TG HDL LDL TC /names = mnames/MISSING = 99999.
compute nms=ncol(mtmp).
compute mprod=make(1,nms,0).
compute n=nrow(mtmp).
compute needed=needed+nms.
compute varnames={varnames,mnames}.
compute dat={dat,mtmp}.
compute modelvar={modelvar;t(mnames)}.
compute x2m=make(99,nms,0).
compute m2y=make(99,nms,0).
compute onem=make(nms,1,1).

.


do if (toomany=1 and longname=0).
compute criterr=1.
do if (tooman=0).
compute tooman=1.
compute errcode(errs,1) = 61.
compute errs = errs + 1.
end if.
end if
.
do if (nms > 1 and nms < 11).
compute modelvlb={modelvlb;medlb(1:nms,1)}.
else.
compute modelvlb={modelvlb;'    M  :'}.
end if.
do if (nms > 0 and model < 4).
compute errcode(errs,1)=9.
compute errs=errs+1.
do if (model <> 0).
compute errcode(errs,1)=48.
compute errs=errs+1.
end if.
compute criterr=1.
end if.
end if.
compute wlocatet=0.
compute wlocate=0.
do if (w <> 'xxxxx').
do if (xmint=0).
get wtmp/variables = xxxxx /names = wnames/MISSING = 99999.

.


do if (toomany=1 and longname=0).
compute criterr=1.
do if (tooman=0).
compute tooman=1.
compute errcode(errs,1) = 61.
compute errs = errs + 1.
end if.
end if
.
end if.
do if (xmint=1).
get wtmp/variables = BMIStat /names = wnames/MISSING = 99999.
end if.
compute nws=ncol(wtmp).
compute n=nrow(wtmp).
compute varnames={varnames,wnames}.
compute wlocate=ncol(varnames).
do if (model=74).
compute wlocatet=1.
do if (xnames <> wnames).
compute errcode(errs,1)=45.
compute errs=errs+1.
compute criterr=1.
end if.
end if.
compute wcatlab=t(wnames).
compute dat={dat,wtmp}.
do if (xmint <> 1).
compute modelvar={modelvar;t(wnames)}.
compute modelvlb={modelvlb;'    W  :'}.
end if.
end if.
do if (z <> 'xxxxx').
get ztmp/variables = xxxxx /names = znames/MISSING = 99999.
compute nzs=ncol(ztmp).
compute n=nrow(ztmp).

.


do if (toomany=1 and longname=0).
compute criterr=1.
do if (tooman=0).
compute tooman=1.
compute errcode(errs,1) = 61.
compute errs = errs + 1.
end if.
end if
.
compute varnames={varnames,znames}.
compute zcatlab=t(znames).
compute dat={dat,ztmp}.
compute modelvar={modelvar;t(znames)}.
compute modelvlb={modelvlb;'    Z  :'}.
end if.
do if (cov <> 'xxxxx').
get ctmp/variables = xxxxx /names = covnames/MISSING = 99999.
compute ncs=ncol(ctmp).
compute n=nrow(ctmp).

.


do if (toomany=1 and longname=0).
compute criterr=1.
do if (tooman=0).
compute tooman=1.
compute errcode(errs,1) = 61.
compute errs = errs + 1.
end if.
end if
.
compute varnames={varnames,covnames}.
compute dat={dat,ctmp}.
end if.
do if (nws > 1 or nzs > 1 or nys > 1 or nxs > 1).
compute errcode(errs,1)=3.
compute errs=errs+1.
compute criterr=1.
end if.
do if ((model = 80 or model = 81) and (nms < 3 or nms > 6)).
compute errcode(errs,1)=32.
compute errs=errs+1.
compute criterr=1.
end if.
do if (model = 82 and nms <> 4).
compute errcode(errs,1)=33.
compute errs=errs+1.
compute criterr=1.
end if.
do if (nms > 10).
compute errcode(errs,1)=37.
compute errs=errs+1.
compute criterr=1.
end if.
do if ((model = 6 or (model > 82 and model < 999)) and (nms < 2 or nms > 6)).
compute errcode(errs,1)=34.
compute errs=errs+1.
compute criterr=1.
end if.
compute match=0.
compute match2=0.
compute mcwzcov=0.
loop i = 1 to (ncol(varnames)-1).
loop j = (i+1) to ncol(varnames).
do if (varnames(i)=varnames(j)).
do if (i < (nxs+nms+nys+1)).
compute match2=match2+1.
end if.
do if (wlocatet=1 and i=2 and j=wlocate).
compute match2=match2-1.
end if.
do if ((wnames=znames) and (nws > 0 or nzs > 0))).
compute match2=match2+1.
end if.
do if (i < (ncol(varnames)-ncs+1)) and j > (ncol(varnames)-ncs)).
do if ((varnames(j)=wnames) and mcw=0)).
compute match=0.
compute wiscov=(j-(ncol(varnames)-ncs)).
end if.
do if ((varnames(j)=wnames) and mcw <>0)).
compute mcwzcov=1.
end if.
do if ((varnames(j)=znames) and mcz=0).
compute match=0.
compute ziscov=(j-(ncol(varnames)-ncs)).
end if.
do if ((varnames(j)=znames) and mcz<>0)).
compute mcwzcov=1.
end if.
end if.
end if.
end loop.
end loop.
do if (match2>0 or match=1).
compute errcode(errs,1)=2.
compute errs=errs+1.
compute criterr=1.
end if.
do if (mcwzcov=1).
compute errcode(errs,1)=50.
compute errs=errs+1.
compute criterr=1.
end if.
compute ninit=nrow(dat).
compute rownum=make(ninit,1,0).
loop i = 1 to ninit.
compute rownum(i,1)=i.
end loop.
compute dat={rownum,dat}.
compute j=1.
compute missrow=0.
loop i = 1 to n.
do if (rsum(dat(i,2:ncol(dat))=99999)=0).
compute dat(j,:)=dat(i,:).
compute j=j+1.
else.
compute missrow={missrow;dat(i,1)}.
end if.
end loop.
do if (j < 5).
compute errcode(errs,1)=62.
compute errs=errs+1.
compute criterr=1.
end if.
do if (criterr=0).
compute rownum=dat(1:(j-1),1).
do if (nrow(missrow) > 1).
compute missrow=t(missrow(2:nrow(missrow),1)).
compute notecode(notes,1) = 29.
compute notes = notes + 1.
end if.
compute dat=dat(1:(j-1),2:ncol(dat)).
compute n=nrow(dat).
compute nmiss=ninit-n.
compute ytmp=dat(:,1:nys).

.
compute desctmp=make((8-(4* 0 )),ncol( ytmp ),-999).
loop jd=1 to ncol( ytmp ).
compute descdat= ytmp (:,jd).
compute desctmp(1,jd) = csum(descdat)/nrow(descdat).
compute desctmp(2,jd) = (nrow(descdat)*sscp(descdat))-(t(csum(descdat))*(csum(descdat))).
compute desctmp(2,jd) = sqrt(desctmp(2,jd)/(nrow(descdat)*(nrow(descdat)-1))).
compute desctmp(3,jd)=cmin(descdat).
compute desctmp(4,jd)=cmax(descdat).
do if ( 0 =0).
compute minwarn=0.
compute maxwarn=0.
do if ((desctmp(3,jd)=desctmp(4,jd)) and novar=0).
compute errcode(errs,1)=15.
compute errs=errs+1.
compute criterr=1.
compute novar=1.
end if.
compute tmp=((descdat(:,1)=desctmp(3,jd))+(descdat(:,1)=desctmp(4,jd))).
compute desctmp(8,jd)=(csum(tmp)=nrow(tmp)).
compute tmp = descdat.
compute tmp(GRADE(descdat),:) = descdat.
compute descdat = tmp.
release tmp.
compute decval={.16;.5;.84}.
loop kd=1 to 3.
compute low=trunc(decval(kd,1)*(nrow(descdat)+1)).
compute lowdec=decval(kd,1)*(nrow(descdat)+1)-low.
compute value=descdat(low,1)+(descdat((low+1),1)-descdat(low,1))*lowdec.
compute desctmp((4+kd),jd)=value.
end loop.
compute mnotev=1.
compute modvals=desctmp(5:7,:).
do if (quantile <> 1).
compute desctmp(5,jd)=desctmp(1,jd)-desctmp(2,jd).
compute desctmp(6,jd)=desctmp(1,jd).
compute desctmp(7,jd)=desctmp(1,jd)+desctmp(2,jd).
compute modvals=desctmp(5:7,:).
compute mnotev=2.
do if (modvals(1,1) < desctmp(3,1)).
compute modvals(1,1)=desctmp(3,1).
compute minwarn=1.
end if.
do if (modvals(3,1) > desctmp(4,1)).
compute modvals(3,1)=desctmp(4,1).
compute maxwarn=1.
end if.
end if.
do if (desctmp(8,1)=1).
compute modvals={desctmp(3,1);desctmp(4,1)}.
compute mnotev=0.
compute minwarn=0.
compute maxwarn=0.
end if.
end if.
end loop
.
compute ysd=desctmp(2,:).
compute ovsd=ysd.
do if (desctmp(8,1)=1).
compute ydich=1.
do if (total=1).
compute total=0.
compute notecode(notes,1) = 24.
compute notes = notes + 1.
end if.
do if (effsize=1).
compute effsize=0.
compute notecode(notes,1) = 25.
compute notes = notes + 1.
end if.
do if (model=74).
compute errcode(errs,1) = 72.
compute errs=errs+1.
compute criterr=1.
end if.
compute omx = cmax(ytmp).
compute omn = cmin(ytmp).
compute ytmp = (ytmp = omx).
compute dat(:,1:nys)=(dat(:,1:nys)=omx).
compute rcd = {omn, 0; omx, 1}.
end if.
compute xtmp=dat(:,(nys+1):(nys+nxs)).

.
compute desctmp=make((8-(4* 0 )),ncol( xtmp ),-999).
loop jd=1 to ncol( xtmp ).
compute descdat= xtmp (:,jd).
compute desctmp(1,jd) = csum(descdat)/nrow(descdat).
compute desctmp(2,jd) = (nrow(descdat)*sscp(descdat))-(t(csum(descdat))*(csum(descdat))).
compute desctmp(2,jd) = sqrt(desctmp(2,jd)/(nrow(descdat)*(nrow(descdat)-1))).
compute desctmp(3,jd)=cmin(descdat).
compute desctmp(4,jd)=cmax(descdat).
do if ( 0 =0).
compute minwarn=0.
compute maxwarn=0.
do if ((desctmp(3,jd)=desctmp(4,jd)) and novar=0).
compute errcode(errs,1)=15.
compute errs=errs+1.
compute criterr=1.
compute novar=1.
end if.
compute tmp=((descdat(:,1)=desctmp(3,jd))+(descdat(:,1)=desctmp(4,jd))).
compute desctmp(8,jd)=(csum(tmp)=nrow(tmp)).
compute tmp = descdat.
compute tmp(GRADE(descdat),:) = descdat.
compute descdat = tmp.
release tmp.
compute decval={.16;.5;.84}.
loop kd=1 to 3.
compute low=trunc(decval(kd,1)*(nrow(descdat)+1)).
compute lowdec=decval(kd,1)*(nrow(descdat)+1)-low.
compute value=descdat(low,1)+(descdat((low+1),1)-descdat(low,1))*lowdec.
compute desctmp((4+kd),jd)=value.
end loop.
compute mnotev=1.
compute modvals=desctmp(5:7,:).
do if (quantile <> 1).
compute desctmp(5,jd)=desctmp(1,jd)-desctmp(2,jd).
compute desctmp(6,jd)=desctmp(1,jd).
compute desctmp(7,jd)=desctmp(1,jd)+desctmp(2,jd).
compute modvals=desctmp(5:7,:).
compute mnotev=2.
do if (modvals(1,1) < desctmp(3,1)).
compute modvals(1,1)=desctmp(3,1).
compute minwarn=1.
end if.
do if (modvals(3,1) > desctmp(4,1)).
compute modvals(3,1)=desctmp(4,1).
compute maxwarn=1.
end if.
end if.
do if (desctmp(8,1)=1).
compute modvals={desctmp(3,1);desctmp(4,1)}.
compute mnotev=0.
compute minwarn=0.
compute maxwarn=0.
end if.
end if.
end loop
.
compute xsd=desctmp(2,:).
compute xmodvals=modvals.
compute xdich=desctmp(8,1).
compute xmx=cmax(xtmp).
compute xmn=cmin(xtmp).
do if ((mcx > 0) and (xrefvals(1,1) <> 999) and (xmint=1) and (model= 74)).
compute notecode(notes,1) = 36.
compute notes = notes + 1.
end if.
do if (mcx=0).
do if (ncol(xrefvals)>2 and model=74 and xmint=1).
compute errcode(errs,1)=67.
compute errs=errs+1.
compute criterr=1.
end if.
do if ((model=74) and (xmint=1)).
do if ((xrefvals(1,1)=999) and nxvls=1 and xdich=0).
compute errcode(errs,1)=66.
compute errs=errs+1.
compute criterr=1.
end if.
do if ((xrefvals(1,1)=999) and (xdich=1)).
compute xrefvals={xmn,xmx}.
compute xscaling=xrefvals(1,2)-xrefvals(1,1).
end if.
do if (ncol(xrefvals)=1 and xrefvals(1,1) <> 999).
do if (xdich=0).
compute xrefvals={xrefvals,(xrefvals(1,1)+1)}.
compute xscaling=xrefvals(1,2)-xrefvals(1,1).
end if.
do if (xdich=1).
do if ((xrefvals(1,1) <> xmx) and (xrefvals(1,1) <> xmn)).
compute errcode(errs,1)=70.
compute errs=errs+1.
compute criterr=1.
end if.
do if (xrefvals(1,1) = xmx).
compute xrefvals={xrefvals,xmn}.
compute xscaling=xrefvals(1,2)-xrefvals(1,1).
end if.
do if (xrefvals(1,1) = xmn).
compute xrefvals={xrefvals,xmx}.
compute xscaling=xrefvals(1,2)-xrefvals(1,1).
end if.
end if.
end if.
do if (ncol(xrefvals)=2).
compute xscaling=xrefvals(1,2)-xrefvals(1,1).
do if (xdich=1).
compute xreferr=1.
do if (((xrefvals(1,1) = xmx) and (xrefvals(1,2)=xmn)) or ((xrefvals(1,1) = xmn) and (xrefvals(1,2)=xmx))).
compute xreferr=0.
end if.
do if (xreferr=1).
compute errcode(errs,1)=70.
compute errs=errs+1.
compute criterr=1.
end if.
end if.
end if.
end if.
end if.
do if (xmint=1 and model=74 and mcx=0).
compute xmodvals=t(xrefvals).
compute xcontcf=1.
end if.
compute nxpval=nrow(xmodvals).
compute xprobval=xmodvals.
do if (xdich =1 and mcx > 0).
compute mcx=0.
compute errcode(errs,1) = 52.
compute errs = errs + 1.
compute criterr = 1.
end if.
do if (nms > 0).
compute mtmp=dat(:,(nys+nxs+1):(nys+nxs+nms)).

.
compute desctmp=make((8-(4* 0 )),ncol( mtmp ),-999).
loop jd=1 to ncol( mtmp ).
compute descdat= mtmp (:,jd).
compute desctmp(1,jd) = csum(descdat)/nrow(descdat).
compute desctmp(2,jd) = (nrow(descdat)*sscp(descdat))-(t(csum(descdat))*(csum(descdat))).
compute desctmp(2,jd) = sqrt(desctmp(2,jd)/(nrow(descdat)*(nrow(descdat)-1))).
compute desctmp(3,jd)=cmin(descdat).
compute desctmp(4,jd)=cmax(descdat).
do if ( 0 =0).
compute minwarn=0.
compute maxwarn=0.
do if ((desctmp(3,jd)=desctmp(4,jd)) and novar=0).
compute errcode(errs,1)=15.
compute errs=errs+1.
compute criterr=1.
compute novar=1.
end if.
compute tmp=((descdat(:,1)=desctmp(3,jd))+(descdat(:,1)=desctmp(4,jd))).
compute desctmp(8,jd)=(csum(tmp)=nrow(tmp)).
compute tmp = descdat.
compute tmp(GRADE(descdat),:) = descdat.
compute descdat = tmp.
release tmp.
compute decval={.16;.5;.84}.
loop kd=1 to 3.
compute low=trunc(decval(kd,1)*(nrow(descdat)+1)).
compute lowdec=decval(kd,1)*(nrow(descdat)+1)-low.
compute value=descdat(low,1)+(descdat((low+1),1)-descdat(low,1))*lowdec.
compute desctmp((4+kd),jd)=value.
end loop.
compute mnotev=1.
compute modvals=desctmp(5:7,:).
do if (quantile <> 1).
compute desctmp(5,jd)=desctmp(1,jd)-desctmp(2,jd).
compute desctmp(6,jd)=desctmp(1,jd).
compute desctmp(7,jd)=desctmp(1,jd)+desctmp(2,jd).
compute modvals=desctmp(5:7,:).
compute mnotev=2.
do if (modvals(1,1) < desctmp(3,1)).
compute modvals(1,1)=desctmp(3,1).
compute minwarn=1.
end if.
do if (modvals(3,1) > desctmp(4,1)).
compute modvals(3,1)=desctmp(4,1).
compute maxwarn=1.
end if.
end if.
do if (desctmp(8,1)=1).
compute modvals={desctmp(3,1);desctmp(4,1)}.
compute mnotev=0.
compute minwarn=0.
compute maxwarn=0.
end if.
end if.
end loop
.
compute ovsd={desctmp(2,:),ysd}.
compute medmeans=cdeval.
do if ((cdeval(1,1) <> -999) and (ncol(medmeans) <> nms) and (model=74)).
compute errcode(errs,1)=64.
compute errs=errs+1.
compute criterr=1.
end if.
do if ((cdeval(1,1)=-999) and (model=74)).
compute medmeans=desctmp(1,:).
end if.
do if ((cdeval(1,1)<> -999) and (model=74) and (criterr=0)).
compute notecode(notes,1) = 31.
compute notes = notes + 1.
end if.
do if ((rsum(desctmp(8,:))>0) and (mdichok <> 1)).
compute errcode(errs,1)=43.
compute errs=errs+1.
compute criterr=1.
end if.
compute mmodvals=modvals.
compute mprobval=mmodvals.
end if.
do if (nws > 0).
compute wtmp=dat(:,(nys+nxs+nms+1):(nys+nxs+nms+nws)).

.
compute desctmp=make((8-(4* 0 )),ncol( wtmp ),-999).
loop jd=1 to ncol( wtmp ).
compute descdat= wtmp (:,jd).
compute desctmp(1,jd) = csum(descdat)/nrow(descdat).
compute desctmp(2,jd) = (nrow(descdat)*sscp(descdat))-(t(csum(descdat))*(csum(descdat))).
compute desctmp(2,jd) = sqrt(desctmp(2,jd)/(nrow(descdat)*(nrow(descdat)-1))).
compute desctmp(3,jd)=cmin(descdat).
compute desctmp(4,jd)=cmax(descdat).
do if ( 0 =0).
compute minwarn=0.
compute maxwarn=0.
do if ((desctmp(3,jd)=desctmp(4,jd)) and novar=0).
compute errcode(errs,1)=15.
compute errs=errs+1.
compute criterr=1.
compute novar=1.
end if.
compute tmp=((descdat(:,1)=desctmp(3,jd))+(descdat(:,1)=desctmp(4,jd))).
compute desctmp(8,jd)=(csum(tmp)=nrow(tmp)).
compute tmp = descdat.
compute tmp(GRADE(descdat),:) = descdat.
compute descdat = tmp.
release tmp.
compute decval={.16;.5;.84}.
loop kd=1 to 3.
compute low=trunc(decval(kd,1)*(nrow(descdat)+1)).
compute lowdec=decval(kd,1)*(nrow(descdat)+1)-low.
compute value=descdat(low,1)+(descdat((low+1),1)-descdat(low,1))*lowdec.
compute desctmp((4+kd),jd)=value.
end loop.
compute mnotev=1.
compute modvals=desctmp(5:7,:).
do if (quantile <> 1).
compute desctmp(5,jd)=desctmp(1,jd)-desctmp(2,jd).
compute desctmp(6,jd)=desctmp(1,jd).
compute desctmp(7,jd)=desctmp(1,jd)+desctmp(2,jd).
compute modvals=desctmp(5:7,:).
compute mnotev=2.
do if (modvals(1,1) < desctmp(3,1)).
compute modvals(1,1)=desctmp(3,1).
compute minwarn=1.
end if.
do if (modvals(3,1) > desctmp(4,1)).
compute modvals(3,1)=desctmp(4,1).
compute maxwarn=1.
end if.
end if.
do if (desctmp(8,1)=1).
compute modvals={desctmp(3,1);desctmp(4,1)}.
compute mnotev=0.
compute minwarn=0.
compute maxwarn=0.
end if.
end if.
end loop
.
compute wmodvals=modvals.
compute wdich=desctmp(8,1).
do if (wdich =1 and mcw > 0).
compute mcw=0.
compute errcode(errs,1) = 52.
compute errs = errs + 1.
compute criterr = 1.
end if.
compute wmin=desctmp(3,1).
compute wmax=desctmp(4,1).
compute minwwarn=minwarn.
compute maxwwarn=maxwarn.
compute wnotev=mnotev.
compute wmodval={ 999 }.
do if (xmint=1 and model=74 and mcx=0).
compute wmodval=xrefvals.
end if.
compute nwcontr=ncol(wmodval).
do if (wmodval(1,1) <> 999).
compute wmodvals=wmodval(1,1).
compute wmodcust=1.
do if (nwcontr > 1).
compute wmodvals=t(wmodval).
end if.
compute minwwarn=0.
compute maxwwarn=0.
compute wnotev=0.
end if.
compute wprobval=wmodvals.
compute nwpval=nrow(wmodvals).
end if.
do if (nzs > 0).
compute ztmp=dat(:,(nys+nxs+nms+nws+1):(nys+nxs+nms+nws+nzs)).

.
compute desctmp=make((8-(4* 0 )),ncol( ztmp ),-999).
loop jd=1 to ncol( ztmp ).
compute descdat= ztmp (:,jd).
compute desctmp(1,jd) = csum(descdat)/nrow(descdat).
compute desctmp(2,jd) = (nrow(descdat)*sscp(descdat))-(t(csum(descdat))*(csum(descdat))).
compute desctmp(2,jd) = sqrt(desctmp(2,jd)/(nrow(descdat)*(nrow(descdat)-1))).
compute desctmp(3,jd)=cmin(descdat).
compute desctmp(4,jd)=cmax(descdat).
do if ( 0 =0).
compute minwarn=0.
compute maxwarn=0.
do if ((desctmp(3,jd)=desctmp(4,jd)) and novar=0).
compute errcode(errs,1)=15.
compute errs=errs+1.
compute criterr=1.
compute novar=1.
end if.
compute tmp=((descdat(:,1)=desctmp(3,jd))+(descdat(:,1)=desctmp(4,jd))).
compute desctmp(8,jd)=(csum(tmp)=nrow(tmp)).
compute tmp = descdat.
compute tmp(GRADE(descdat),:) = descdat.
compute descdat = tmp.
release tmp.
compute decval={.16;.5;.84}.
loop kd=1 to 3.
compute low=trunc(decval(kd,1)*(nrow(descdat)+1)).
compute lowdec=decval(kd,1)*(nrow(descdat)+1)-low.
compute value=descdat(low,1)+(descdat((low+1),1)-descdat(low,1))*lowdec.
compute desctmp((4+kd),jd)=value.
end loop.
compute mnotev=1.
compute modvals=desctmp(5:7,:).
do if (quantile <> 1).
compute desctmp(5,jd)=desctmp(1,jd)-desctmp(2,jd).
compute desctmp(6,jd)=desctmp(1,jd).
compute desctmp(7,jd)=desctmp(1,jd)+desctmp(2,jd).
compute modvals=desctmp(5:7,:).
compute mnotev=2.
do if (modvals(1,1) < desctmp(3,1)).
compute modvals(1,1)=desctmp(3,1).
compute minwarn=1.
end if.
do if (modvals(3,1) > desctmp(4,1)).
compute modvals(3,1)=desctmp(4,1).
compute maxwarn=1.
end if.
end if.
do if (desctmp(8,1)=1).
compute modvals={desctmp(3,1);desctmp(4,1)}.
compute mnotev=0.
compute minwarn=0.
compute maxwarn=0.
end if.
end if.
end loop
.
compute zmodvals=modvals.
compute zdich=desctmp(8,1).
do if (zdich =1 and mcz > 0).
compute mcz=0.
compute errcode(errs,1) = 52.
compute errs = errs + 1.
compute criterr = 1.
end if.
compute zmin=desctmp(3,1).
compute zmax=desctmp(4,1).
compute minzwarn=minwarn.
compute maxzwarn=maxwarn.
compute znotev=mnotev.
compute zmodval={ 999 }.
compute nzcontr=ncol(zmodval).
do if (zmodval(1,1) <> 999).
compute zmodvals=zmodval(1,1).
compute zmodcust=1.
do if (nzcontr > 1).
compute zmodvals=t(zmodval).
end if.
compute minzwarn=0.
compute maxzwarn=0.
compute znotev=0.
end if.
compute zprobval=zmodvals.
compute nzpval=nrow(zmodvals).
end if.
do if (ncs > 0).
compute ctmp=dat(:,(nys+nxs+nms+nws+nzs+1):(nys+nxs+nms+nws+nzs+ncs)).

.
compute desctmp=make((8-(4* 0 )),ncol( ctmp ),-999).
loop jd=1 to ncol( ctmp ).
compute descdat= ctmp (:,jd).
compute desctmp(1,jd) = csum(descdat)/nrow(descdat).
compute desctmp(2,jd) = (nrow(descdat)*sscp(descdat))-(t(csum(descdat))*(csum(descdat))).
compute desctmp(2,jd) = sqrt(desctmp(2,jd)/(nrow(descdat)*(nrow(descdat)-1))).
compute desctmp(3,jd)=cmin(descdat).
compute desctmp(4,jd)=cmax(descdat).
do if ( 0 =0).
compute minwarn=0.
compute maxwarn=0.
do if ((desctmp(3,jd)=desctmp(4,jd)) and novar=0).
compute errcode(errs,1)=15.
compute errs=errs+1.
compute criterr=1.
compute novar=1.
end if.
compute tmp=((descdat(:,1)=desctmp(3,jd))+(descdat(:,1)=desctmp(4,jd))).
compute desctmp(8,jd)=(csum(tmp)=nrow(tmp)).
compute tmp = descdat.
compute tmp(GRADE(descdat),:) = descdat.
compute descdat = tmp.
release tmp.
compute decval={.16;.5;.84}.
loop kd=1 to 3.
compute low=trunc(decval(kd,1)*(nrow(descdat)+1)).
compute lowdec=decval(kd,1)*(nrow(descdat)+1)-low.
compute value=descdat(low,1)+(descdat((low+1),1)-descdat(low,1))*lowdec.
compute desctmp((4+kd),jd)=value.
end loop.
compute mnotev=1.
compute modvals=desctmp(5:7,:).
do if (quantile <> 1).
compute desctmp(5,jd)=desctmp(1,jd)-desctmp(2,jd).
compute desctmp(6,jd)=desctmp(1,jd).
compute desctmp(7,jd)=desctmp(1,jd)+desctmp(2,jd).
compute modvals=desctmp(5:7,:).
compute mnotev=2.
do if (modvals(1,1) < desctmp(3,1)).
compute modvals(1,1)=desctmp(3,1).
compute minwarn=1.
end if.
do if (modvals(3,1) > desctmp(4,1)).
compute modvals(3,1)=desctmp(4,1).
compute maxwarn=1.
end if.
end if.
do if (desctmp(8,1)=1).
compute modvals={desctmp(3,1);desctmp(4,1)}.
compute mnotev=0.
compute minwarn=0.
compute maxwarn=0.
end if.
end if.
end loop
.
compute covmeans=desctmp(1,:).
compute coval={ -999 }.
do if ((coval(1,1) <> -999) and (ncol(coval) <> ncs) and (model=74)).
compute errcode(errs,1)=69.
compute errs=errs+1.
compute criterr=1.
end if.
do if ((coval(1,1)<> -999) and (criterr=0) and (model=74)).
compute notecode(notes,1) = 35.
compute notes = notes + 1.
compute cuscoval=1.
end if.
end if.
compute n=nrow(ytmp).
compute ones=make(n,1,1).
do if (nws > 0 and mcw > 0).
compute tmp={rownum,wtmp(:,1)}.

.
compute dd= tmp.
compute temp = dd.
compute temp(GRADE(dd(:,2)),:) = dd.
compute dd = temp.
compute dummy = design(dd(:,2)).
compute nvls = ncol(dummy).
compute nnvls = csum(dummy).
compute mnvls = cmin(t(nnvls)).
compute conmat1=1.
do if (mnvls < 2).
compute errcode(errs,1) = 5.
compute errs = errs + 1.
compute criterr = 1.
end if.
do if (nvls > 9).
compute errcode(errs,1) = 4.
compute errs = errs+1.
compute criterr = 1.
end if.
do if (criterr = 0).
compute dumok = 1.
compute nnvls=make(nvls,1,0).
compute nnvls(1,1)=dd(1,2).
compute temp = 2.
loop i = 2 to n.
do if (dd(i,2) <> nnvls((temp-1),1)).
compute nnvls(temp,1)=dd(i,2).
compute temp = temp+1.
end if.
end loop.
do if ( mcw > 0).
compute x = dummy(:,2:ncol(dummy)).
compute nx = ncol(x).
compute minus1 = make(1,ncol(x),-1).
compute xdes=make((nx+1),3,0).
compute xdes(1,1)=dd(1,2).
compute xdes(1,2)=1.
compute temp = 2.
loop k = 2 to n.
do if (dd(k,2) <> dd((k-1),2)).
compute xdes(temp,2) = k.
compute xdes(temp,1) = dd(k,2).
compute xdes((temp-1),3) = k-1.
compute temp=temp+1.
end if.
end loop.
compute xdes((temp-1),3)=n.
compute xdes = {xdes, (xdes(:,3)-xdes(:,2)+1)}.
do if ( mcw = 4).
loop k = 1 to n.
do if (rsum(x(k,:)) = 0).
compute x(k,:) = minus1.
end if.
end loop.
end if.
do if ( mcw = 2 or mcw = 3 or mcw =5).
loop k = 1 to n.
do if (rsum(x(k,:)) > 0).
loop i = 1 to ncol(x).
do if (x(k,i) = 0).
compute x(k,i) = 1.
else.
break.
end if.
end loop.
end if.
end loop.
do if ( mcw = 3).
compute conmat1={-8,1,1,1,1,1,1,1,1; 0,-7,1,1,1,1,1,1,1; 0,0,-6,1,1,1,1,1,1; 0,0,0,-5,1,1,1,1,1; 0,0,0,0,-4,1,1,1,1; 0,0,0,0,0,-3,1,1,1; 0,0,0,0,0,0,-2,1,1; 0,0,0,0,0,0,0,-1,1}.
loop i = 1 to 8.
compute conmat1(i,:)=conmat1(i,:)/(10-i).
end loop.
compute conmat1=t(conmat1((10-nvls):8,(10-nvls):9)).
loop k=1 to n.
compute x(k,:)=conmat1((rsum(x(k,:))+1),:).
end loop.
end if.
end if.
do if ( mcw = 5).
compute custcode={ -999 }.
do if (ncol(custcode) <> (nvls*(nvls-1))).
compute errcode(errs,1) = (37+ 2 ).
compute errs = errs + 1.
compute criterr = 1.
end if.
do if (ncol(custcode) = (nvls*(nvls-1))).
compute conmat1=make(nvls,(nvls-1),0).
compute cnt=1.
loop i = 1 to nvls.
loop k = 1 to (nvls-1).
compute conmat1(i,k)=custcode(1,cnt).
compute cnt=cnt+1.
end loop.
end loop.
loop k=1 to n.
compute x(k,:)=conmat1((rsum(x(k,:))+1),:).
end loop.
end if.
end if.
compute xskip = 1.
compute dummat = make((nx+1),nx,0).
compute dummat((2:nrow(dummat)),:)=ident(nx).
do if ( mcw = 4).
compute dummat(1,:) = minus1.
end if.
do if ( mcw = 2).
loop i = 2 to nrow(dummat).
loop j = 1 to (i-1).
compute dummat(i,j) = 1.
end loop.
end loop.
end if.
do if ( mcw = 3).
compute dummat=conmat1.
end if.
do if ( mcw = 5 and criterr=0).
compute dummat=conmat1.
end if.
compute dummat={nnvls, dummat}.
compute x={dd(:,1),x}.
compute temp = x.
compute temp(GRADE(x(:,1)),:) = x.
compute x = temp.
release conmat1,temp,dd,xskip,xdes,dummy.
end if.
end if
.
compute wmodvals=nnvls.
compute nwpval=nrow(wmodvals).
do if (criterr=0).
compute minwwarn=0.
compute maxwwarn=0.
compute wnotev=0.
compute wtmp=x(:,2:ncol(x)).
compute wcatlab={'W1';'W2';'W3';'W4';'W5';'W6';'W7';'W8';'W9'}.
do if (xmint=1).
compute wcatlab={'X1';'X2';'X3';'X4';'X5';'X6';'X7';'X8';'X9'}.
end if.
compute nwvls=nvls-1.
compute mcwok=1.
compute dummatw=dummat.
compute wprobval=dummatw(:,2:ncol(dummatw)).
do if (modcok=1).
compute wcontval=make(2,ncol(wprobval),-999).
compute temp=0.
loop i = 1 to 2.
loop j = 1 to nrow(dummatw).
do if (contvec(i,1)=dummatw(j,1)).
compute wcontval(i,:)=wprobval(j,:).
compute temp=temp+1.
end if.
end loop.
end loop.
do if (temp < 2).
compute notecode(notes,1) = 20.
compute notes = notes + 1.
compute modcok=0.
end if.
end if.
do if ((wmodval(1,1) <> 999) and (xmint <> 1)).
compute notecode(notes,1) = 9.
compute notes = notes + 1.
end if.
release tmp, dummat.
end if.
end if.
do if (nzs > 0 and mcz > 0).
compute tmp={rownum,ztmp(:,1)}.

.
compute dd= tmp.
compute temp = dd.
compute temp(GRADE(dd(:,2)),:) = dd.
compute dd = temp.
compute dummy = design(dd(:,2)).
compute nvls = ncol(dummy).
compute nnvls = csum(dummy).
compute mnvls = cmin(t(nnvls)).
compute conmat1=1.
do if (mnvls < 2).
compute errcode(errs,1) = 5.
compute errs = errs + 1.
compute criterr = 1.
end if.
do if (nvls > 9).
compute errcode(errs,1) = 4.
compute errs = errs+1.
compute criterr = 1.
end if.
do if (criterr = 0).
compute dumok = 1.
compute nnvls=make(nvls,1,0).
compute nnvls(1,1)=dd(1,2).
compute temp = 2.
loop i = 2 to n.
do if (dd(i,2) <> nnvls((temp-1),1)).
compute nnvls(temp,1)=dd(i,2).
compute temp = temp+1.
end if.
end loop.
do if ( mcz > 0).
compute x = dummy(:,2:ncol(dummy)).
compute nx = ncol(x).
compute minus1 = make(1,ncol(x),-1).
compute xdes=make((nx+1),3,0).
compute xdes(1,1)=dd(1,2).
compute xdes(1,2)=1.
compute temp = 2.
loop k = 2 to n.
do if (dd(k,2) <> dd((k-1),2)).
compute xdes(temp,2) = k.
compute xdes(temp,1) = dd(k,2).
compute xdes((temp-1),3) = k-1.
compute temp=temp+1.
end if.
end loop.
compute xdes((temp-1),3)=n.
compute xdes = {xdes, (xdes(:,3)-xdes(:,2)+1)}.
do if ( mcz = 4).
loop k = 1 to n.
do if (rsum(x(k,:)) = 0).
compute x(k,:) = minus1.
end if.
end loop.
end if.
do if ( mcz = 2 or mcz = 3 or mcz =5).
loop k = 1 to n.
do if (rsum(x(k,:)) > 0).
loop i = 1 to ncol(x).
do if (x(k,i) = 0).
compute x(k,i) = 1.
else.
break.
end if.
end loop.
end if.
end loop.
do if ( mcz = 3).
compute conmat1={-8,1,1,1,1,1,1,1,1; 0,-7,1,1,1,1,1,1,1; 0,0,-6,1,1,1,1,1,1; 0,0,0,-5,1,1,1,1,1; 0,0,0,0,-4,1,1,1,1; 0,0,0,0,0,-3,1,1,1; 0,0,0,0,0,0,-2,1,1; 0,0,0,0,0,0,0,-1,1}.
loop i = 1 to 8.
compute conmat1(i,:)=conmat1(i,:)/(10-i).
end loop.
compute conmat1=t(conmat1((10-nvls):8,(10-nvls):9)).
loop k=1 to n.
compute x(k,:)=conmat1((rsum(x(k,:))+1),:).
end loop.
end if.
end if.
do if ( mcz = 5).
compute custcode={ -999 }.
do if (ncol(custcode) <> (nvls*(nvls-1))).
compute errcode(errs,1) = (37+ 3 ).
compute errs = errs + 1.
compute criterr = 1.
end if.
do if (ncol(custcode) = (nvls*(nvls-1))).
compute conmat1=make(nvls,(nvls-1),0).
compute cnt=1.
loop i = 1 to nvls.
loop k = 1 to (nvls-1).
compute conmat1(i,k)=custcode(1,cnt).
compute cnt=cnt+1.
end loop.
end loop.
loop k=1 to n.
compute x(k,:)=conmat1((rsum(x(k,:))+1),:).
end loop.
end if.
end if.
compute xskip = 1.
compute dummat = make((nx+1),nx,0).
compute dummat((2:nrow(dummat)),:)=ident(nx).
do if ( mcz = 4).
compute dummat(1,:) = minus1.
end if.
do if ( mcz = 2).
loop i = 2 to nrow(dummat).
loop j = 1 to (i-1).
compute dummat(i,j) = 1.
end loop.
end loop.
end if.
do if ( mcz = 3).
compute dummat=conmat1.
end if.
do if ( mcz = 5 and criterr=0).
compute dummat=conmat1.
end if.
compute dummat={nnvls, dummat}.
compute x={dd(:,1),x}.
compute temp = x.
compute temp(GRADE(x(:,1)),:) = x.
compute x = temp.
release conmat1,temp,dd,xskip,xdes,dummy.
end if.
end if
.
compute zmodvals=nnvls.
compute nzpval=nrow(zmodvals).
do if (criterr=0).
compute minzwarn=0.
compute maxzwarn=0.
compute znotev=0.
compute ztmp=x(:,2:ncol(x)).
compute zcatlab={'Z1';'Z2';'Z3';'Z4';'Z5';'Z6';'Z7';'Z8';'Z9'}.
compute nzvls=nvls-1.
compute mczok=1.
compute dummatz=dummat.
compute zprobval=dummatz(:,2:ncol(dummatz)).
do if (modcok=1).
compute zcontval=make(2,ncol(zprobval),-999).
compute temp=0.
loop i = 1 to 2.
loop j = 1 to nrow(dummatz).
do if (contvec(i,2)=dummatz(j,1)).
compute zcontval(i,:)=zprobval(j,:).
compute temp=temp+1.
end if.
end loop.
end loop.
do if (temp < 2).
compute notecode(notes,1) = 20.
compute notes = notes + 1.
compute modcok=0.
end if.
end if.
do if (zmodval(1,1) <> 999).
compute notecode(notes,1) = 10.
compute notes = notes + 1.
end if.
release tmp, dummat.
end if.
end if.
do if (nxs > 0 and mcx > 0).
compute tmp={rownum,xtmp(:,1)}.

.
compute dd= tmp.
compute temp = dd.
compute temp(GRADE(dd(:,2)),:) = dd.
compute dd = temp.
compute dummy = design(dd(:,2)).
compute nvls = ncol(dummy).
compute nnvls = csum(dummy).
compute mnvls = cmin(t(nnvls)).
compute conmat1=1.
do if (mnvls < 2).
compute errcode(errs,1) = 5.
compute errs = errs + 1.
compute criterr = 1.
end if.
do if (nvls > 9).
compute errcode(errs,1) = 4.
compute errs = errs+1.
compute criterr = 1.
end if.
do if (criterr = 0).
compute dumok = 1.
compute nnvls=make(nvls,1,0).
compute nnvls(1,1)=dd(1,2).
compute temp = 2.
loop i = 2 to n.
do if (dd(i,2) <> nnvls((temp-1),1)).
compute nnvls(temp,1)=dd(i,2).
compute temp = temp+1.
end if.
end loop.
do if ( mcx > 0).
compute x = dummy(:,2:ncol(dummy)).
compute nx = ncol(x).
compute minus1 = make(1,ncol(x),-1).
compute xdes=make((nx+1),3,0).
compute xdes(1,1)=dd(1,2).
compute xdes(1,2)=1.
compute temp = 2.
loop k = 2 to n.
do if (dd(k,2) <> dd((k-1),2)).
compute xdes(temp,2) = k.
compute xdes(temp,1) = dd(k,2).
compute xdes((temp-1),3) = k-1.
compute temp=temp+1.
end if.
end loop.
compute xdes((temp-1),3)=n.
compute xdes = {xdes, (xdes(:,3)-xdes(:,2)+1)}.
do if ( mcx = 4).
loop k = 1 to n.
do if (rsum(x(k,:)) = 0).
compute x(k,:) = minus1.
end if.
end loop.
end if.
do if ( mcx = 2 or mcx = 3 or mcx =5).
loop k = 1 to n.
do if (rsum(x(k,:)) > 0).
loop i = 1 to ncol(x).
do if (x(k,i) = 0).
compute x(k,i) = 1.
else.
break.
end if.
end loop.
end if.
end loop.
do if ( mcx = 3).
compute conmat1={-8,1,1,1,1,1,1,1,1; 0,-7,1,1,1,1,1,1,1; 0,0,-6,1,1,1,1,1,1; 0,0,0,-5,1,1,1,1,1; 0,0,0,0,-4,1,1,1,1; 0,0,0,0,0,-3,1,1,1; 0,0,0,0,0,0,-2,1,1; 0,0,0,0,0,0,0,-1,1}.
loop i = 1 to 8.
compute conmat1(i,:)=conmat1(i,:)/(10-i).
end loop.
compute conmat1=t(conmat1((10-nvls):8,(10-nvls):9)).
loop k=1 to n.
compute x(k,:)=conmat1((rsum(x(k,:))+1),:).
end loop.
end if.
end if.
do if ( mcx = 5).
compute custcode={ -999 }.
do if (ncol(custcode) <> (nvls*(nvls-1))).
compute errcode(errs,1) = (37+ 1 ).
compute errs = errs + 1.
compute criterr = 1.
end if.
do if (ncol(custcode) = (nvls*(nvls-1))).
compute conmat1=make(nvls,(nvls-1),0).
compute cnt=1.
loop i = 1 to nvls.
loop k = 1 to (nvls-1).
compute conmat1(i,k)=custcode(1,cnt).
compute cnt=cnt+1.
end loop.
end loop.
loop k=1 to n.
compute x(k,:)=conmat1((rsum(x(k,:))+1),:).
end loop.
end if.
end if.
compute xskip = 1.
compute dummat = make((nx+1),nx,0).
compute dummat((2:nrow(dummat)),:)=ident(nx).
do if ( mcx = 4).
compute dummat(1,:) = minus1.
end if.
do if ( mcx = 2).
loop i = 2 to nrow(dummat).
loop j = 1 to (i-1).
compute dummat(i,j) = 1.
end loop.
end loop.
end if.
do if ( mcx = 3).
compute dummat=conmat1.
end if.
do if ( mcx = 5 and criterr=0).
compute dummat=conmat1.
end if.
compute dummat={nnvls, dummat}.
compute x={dd(:,1),x}.
compute temp = x.
compute temp(GRADE(x(:,1)),:) = x.
compute x = temp.
release conmat1,temp,dd,xskip,xdes,dummy.
end if.
end if
.
do if (criterr=0).
compute xtmp=x(:,2:ncol(x)).
compute xcatlab={'X1';'X2';'X3';'X4';'X5';'X6';'X7';'X8';'X9'}.
compute nxvls=nvls-1.
compute xdich=(nvls=2).
compute mcxok=1.
compute dummatx=dummat.
compute xmodvals=dummatx(:,1).
compute nxpval=nrow(xmodvals).
release tmp, dummat.
end if.
end if.
compute intlab=make(100,1,' ').


compute intlab( 1 ,1)= 'Int_1'.


compute intlab( 2 ,1)= 'Int_2'.


compute intlab( 3 ,1)= 'Int_3'.


compute intlab( 4 ,1)= 'Int_4'.


compute intlab( 5 ,1)= 'Int_5'.


compute intlab( 6 ,1)= 'Int_6'.


compute intlab( 7 ,1)= 'Int_7'.


compute intlab( 8 ,1)= 'Int_8'.


compute intlab( 9 ,1)= 'Int_9'.


compute intlab( 10 ,1)= 'Int_10'.


compute intlab( 11 ,1)= 'Int_11'.


compute intlab( 12 ,1)= 'Int_12'.


compute intlab( 13 ,1)= 'Int_13'.


compute intlab( 14 ,1)= 'Int_14'.


compute intlab( 15 ,1)= 'Int_15'.


compute intlab( 16 ,1)= 'Int_16'.


compute intlab( 17 ,1)= 'Int_17'.


compute intlab( 18 ,1)= 'Int_18'.


compute intlab( 19 ,1)= 'Int_19'.


compute intlab( 20 ,1)= 'Int_20'.


compute intlab( 21 ,1)= 'Int_21'.


compute intlab( 22 ,1)= 'Int_22'.


compute intlab( 23 ,1)= 'Int_23'.


compute intlab( 24 ,1)= 'Int_24'.


compute intlab( 25 ,1)= 'Int_25'.


compute intlab( 26 ,1)= 'Int_26'.


compute intlab( 27 ,1)= 'Int_27'.


compute intlab( 28 ,1)= 'Int_28'.


compute intlab( 29 ,1)= 'Int_29'.


compute intlab( 30 ,1)= 'Int_30'.


compute intlab( 31 ,1)= 'Int_31'.


compute intlab( 32 ,1)= 'Int_32'.


compute intlab( 33 ,1)= 'Int_33'.


compute intlab( 34 ,1)= 'Int_34'.


compute intlab( 35 ,1)= 'Int_35'.


compute intlab( 36 ,1)= 'Int_36'.


compute intlab( 37 ,1)= 'Int_37'.


compute intlab( 38 ,1)= 'Int_38'.


compute intlab( 39 ,1)= 'Int_39'.


compute intlab( 40 ,1)= 'Int_40'.


compute intlab( 41 ,1)= 'Int_41'.


compute intlab( 42 ,1)= 'Int_42'.


compute intlab( 43 ,1)= 'Int_43'.


compute intlab( 44 ,1)= 'Int_44'.


compute intlab( 45 ,1)= 'Int_45'.


compute intlab( 46 ,1)= 'Int_46'.


compute intlab( 47 ,1)= 'Int_47'.


compute intlab( 48 ,1)= 'Int_48'.


compute intlab( 49 ,1)= 'Int_49'.


compute intlab( 50 ,1)= 'Int_50'.


compute intlab( 51 ,1)= 'Int_51'.


compute intlab( 52 ,1)= 'Int_52'.


compute intlab( 53 ,1)= 'Int_53'.


compute intlab( 54 ,1)= 'Int_54'.


compute intlab( 55 ,1)= 'Int_55'.


compute intlab( 56 ,1)= 'Int_56'.


compute intlab( 57 ,1)= 'Int_57'.


compute intlab( 58 ,1)= 'Int_58'.


compute intlab( 59 ,1)= 'Int_59'.


compute intlab( 60 ,1)= 'Int_60'.


compute intlab( 61 ,1)= 'Int_61'.


compute intlab( 62 ,1)= 'Int_62'.


compute intlab( 63 ,1)= 'Int_63'.


compute intlab( 64 ,1)= 'Int_64'.


compute intlab( 65 ,1)= 'Int_65'.


compute intlab( 66 ,1)= 'Int_66'.


compute intlab( 67 ,1)= 'Int_67'.


compute intlab( 68 ,1)= 'Int_68'.


compute intlab( 69 ,1)= 'Int_69'.


compute intlab( 70 ,1)= 'Int_70'.


compute intlab( 71 ,1)= 'Int_71'.


compute intlab( 72 ,1)= 'Int_72'.


compute intlab( 73 ,1)= 'Int_73'.


compute intlab( 74 ,1)= 'Int_74'.


compute intlab( 75 ,1)= 'Int_75'.


compute intlab( 76 ,1)= 'Int_76'.


compute intlab( 77 ,1)= 'Int_77'.


compute intlab( 78 ,1)= 'Int_78'.


compute intlab( 79 ,1)= 'Int_79'.


compute intlab( 80 ,1)= 'Int_80'.


compute intlab( 81 ,1)= 'Int_81'.


compute intlab( 82 ,1)= 'Int_82'.


compute intlab( 83 ,1)= 'Int_83'.


compute intlab( 84 ,1)= 'Int_84'.


compute intlab( 85 ,1)= 'Int_85'.


compute intlab( 86 ,1)= 'Int_86'.


compute intlab( 87 ,1)= 'Int_87'.


compute intlab( 88 ,1)= 'Int_88'.


compute intlab( 89 ,1)= 'Int_89'.


compute intlab( 90 ,1)= 'Int_90'.


compute intlab( 91 ,1)= 'Int_91'.


compute intlab( 92 ,1)= 'Int_92'.


compute intlab( 93 ,1)= 'Int_93'.


compute intlab( 94 ,1)= 'Int_94'.


compute intlab( 95 ,1)= 'Int_95'.


compute intlab( 96 ,1)= 'Int_96'.


compute intlab( 97 ,1)= 'Int_97'.


compute intlab( 98 ,1)= 'Int_98'.


compute intlab( 99 ,1)= 'Int_99'.


compute intlab( 100 ,1)= 'Int_100'.

compute bcmat=make(needed,needed,0).
compute wcmat=make(needed,needed,0).
compute zcmat=make(needed,needed,0).
compute wzcmat=make(needed,needed,0).
compute wsum=0.
compute zsum=0.
compute wzsum=0.
end if.
end if.
do if (criterr = 0 and model <> 999).
compute modelmat= {1,0,0,0,0,0,0,1,0,0;2,0,0,0,0,0,0,1,1,0;3,0,0,0,0,0,0,1,1,1;4,0,0,0,0,0,0,0,0,0; 5,0,0,0,0,0,0,1,0,0;6,0,0,0,0,0,0,0,0,0;7,1,0,0,0,0,0,0,0,0;8,1,0,0,0,0,0,1,0,0; 9,1,1,0,0,0,0,0,0,0;10,1,1,0,0,0,0,1,1,0;11,1,1,1,0,0,0,0,0,0;12,1,1,1,0,0,0,1,1,1; 13,1,1,1,0,0,0,1,0,0;14,0,0,0,1,0,0,0,0,0;15,0,0,0,1,0,0,1,0,0;16,0,0,0,1,1,0,0,0,0; 17,0,0,0,1,1,0,1,1,0;18,0,0,0,1,1,1,0,0,0;19,0,0,0,1,1,1,1,1,1;20,0,0,0,1,1,1,1,0,0; 21,1,0,0,0,1,0,0,0,0;22,1,0,0,0,1,0,1,0,0;23,0,0,0,0,0,0,0,0,0;24,0,0,0,0,0,0,0,0,0; 25,0,0,0,0,0,0,0,0,0;26,0,0,0,0,0,0,0,0,0;27,0,0,0,0,0,0,0,0,0;28,1,0,0,0,1,0,0,1,0; 29,1,0,0,0,1,0,1,1,0;30,0,0,0,0,0,0,0,0,0;31,0,0,0,0,0,0,0,0,0;32,0,0,0,0,0,0,0,0,0; 33,0,0,0,0,0,0,0,0,0;34,0,0,0,0,0,0,0,0,0;35,0,0,0,0,0,0,0,0,0;36,0,0,0,0,0,0,0,0,0; 37,0,0,0,0,0,0,0,0,0;38,0,0,0,0,0,0,0,0,0;39,0,0,0,0,0,0,0,0,0;40,0,0,0,0,0,0,0,0,0; 41,0,0,0,0,0,0,0,0,0;42,0,0,0,0,0,0,0,0,0;43,0,0,0,0,0,0,0,0,0;44,0,0,0,0,0,0,0,0,0; 45,0,0,0,0,0,0,0,0,0;46,0,0,0,0,0,0,0,0,0;47,0,0,0,0,0,0,0,0,0;48,0,0,0,0,0,0,0

 ,0,0; 49,0,0,0,0,0,0,0,0,0;50,0,0,0,0,0,0,0,0,0;51,0,0,0,0,0,0,0,0,0;52,0,0,0,0,0,0,0,0,0; 53,0,0,0,0,0,0,0,0,0;54,0,0,0,0,0,0,0,0,0;55,0,0,0,0,0,0,0,0,0;56,0,0,0,0,0,0,0,0,0; 57,0,0,0,0,0,0,0,0,0;58,1,0,0,1,0,0,0,0,0;59,1,0,0,1,0,0,1,0,0;60,1,1,0,1,0,0,0,0,0; 61,1,1,0,1,0,0,1,0,0;62,1,1,0,1,0,0,0,1,0;63,1,1,0,1,0,0,1,1,0;64,1,0,0,1,1,0,0,0,0; 65,1,0,0,1,1,0,1,0,0;66,1,0,0,1,1,0,0,1,0;67,1,0,0,1,1,0,1,1,0;68,1,1,1,1,0,0,0,0,0; 69,1,1,1,1,0,0,1,1,1;70,1,0,0,1,1,1,0,0,0;71,1,0,0,1,1,1,1,1,1;72,1,1,1,1,1,1,0,0,0; 73,1,1,1,1,1,1,1,1,1;74,0,0,0,1,0,0,0,0,0;75,1,1,0,1,1,0,0,0,0;76,1,1,0,1,1,0,1,1,0; 77,0,0,0,0,0,0,0,0,0;78,0,0,0,0,0,0,0,0,0;79,0,0,0,0,0,0,0,0,0;80,0,0,0,0,0,0,0,0,0; 81,0,0,0,0,0,0,0,0,0;82,0,0,0,0,0,0,0,0,0;83,1,0,0,0,0,0,0,0,0;84,1,0,0,0,0,0,0,0,0; 85,1,0,0,0,0,0,1,0,0;86,1,0,0,0,0,0,1,0,0;87,0,0,0,1,0,0,0,0,0;88,0,0,0,1,0,0,0,0,0; 89,0,0,0,1,0,0,1,0,0;90,0,0,0,1,0,0,1,0,0;91,0,0,0,0,0,0,0,0,0;92,1,0,0,1,0,0,1,0,0}.
do if (model > 0).
compute tmp=modelmat(model,2:ncol(modelmat)).
end if.
do if (model=0).
compute tmp={0,0,0,0,0,0,0,0,0}.
end if.
do if (model < 4).
compute bcmat((nxs+1),1)=1.
end if.
do if ((model > 3) and (model <> 6)).
compute bcmat((nxs+1):(nxs+nms),1)=onem.
compute bcmat(nrow(bcmat),(nxs+1):(nxs+nms))=t(onem).
compute bcmat(nrow(bcmat),1)=1.
end if.
do if ((model = 6) or (model > 82 and model < 93)).
loop j = 2 to nrow(bcmat).
loop i = 1 to (j-1).
compute bcmat(j,i)=1.
end loop.
end loop.
end if.
do if (model = 80).
loop i = 1 to nms.
compute bcmat((nrow(bcmat)-1),i)=1.
end loop.
end if.
do if (model = 81).
loop j = 3 to nrow(bcmat).
compute bcmat(j,2)=1.
end loop.
end if.
do if (model = 82).
compute bcmat(3,2)=1.
compute bcmat(5,4)=1.
end if.
do if (tmp(1,1)=1).
compute wcmat((nxs+1):(nxs+nms),1)=onem.
compute wprod=1.
compute xprod=1.
do if (model = 83 or model = 86).
compute onemsx=onem.
loop i = 1 to (nms-1).
compute onemsx(i+1,1)=0.
end loop.
compute wcmat((nxs+1):(nxs+nms),1)=onemsx.
end if.
end if.
do if (tmp(1,4)=1).
compute wcmat(nrow(wcmat),(nxs+1):(nxs+nms))=t(onem).
compute wprod=1.
do if (model = 87 or model = 90).
compute onemsx=onem.
loop i = 1 to (nms-1).
compute onemsx(i,1)=0.
end loop.
compute wcmat(nrow(wcmat),(nxs+1):(nxs+nms))=t(onemsx).
end if.
end if.
do if (tmp(1,7)=1).
compute wcmat(nrow(wcmat),1)=1.
compute wprod=1.
compute xprod=1.
end if.
do if (tmp(1,2)=1).
compute zcmat((nxs+1):(nxs+nms),1)=onem.
compute zprod=1.
compute xprod=1.
end if.
do if (tmp(1,5)=1).
compute zcmat(nrow(zcmat),(nxs+1):(nxs+nms))=t(onem).
compute zprod=1.
end if.
do if (tmp(1,8)=1).
compute zcmat(nrow(zcmat),1)=1.
compute zprod=1.
compute xprod=1.
end if.
do if (tmp(1,3)=1).
compute wzcmat((nxs+1):(nxs+nms),1)=onem.
compute xprod=1.
compute wprod=1.
compute zprod=1.
end if.
do if (tmp(1,6)=1).
compute wzcmat(nrow(wzcmat),(nxs+1):(nxs+nms))=t(onem).
compute zprod=1.
compute wprod=1.
end if.
do if (tmp(1,9)=1).
compute wzcmat(nrow(wzcmat),1)=1.
compute xprod=1.
compute wprod=1.
compute zprod=1.
end if.
do if (model = 91 or model = 92).
loop j = 1 to (nms-1).
loop i = 1 to j.
compute wcmat((nxs+1+j),(nxs+i))=1.
end loop.
end loop.
end if.
do if (nms < 0).
loop i = 1 to nms.
compute tmp=csum(wcmat(:,(1+i)))+csum(zcmat(:,(1+i)))+csum(wzcmat(:,(1+i))).
compute mprod(1,i)=(tmp>0).
end loop.
end if.
end if.
do if (ncs > 0).
compute ccmat=make((nms+nys),ncs,1).
compute ccmatoff=ccmat.
do if (covmy=1).
compute ccmat(nrow(ccmat),:)=make(1,ncs,0).
end if.
do if (covmy=2).
compute ccmat(1:nms,:)=make(nms,ncs,0).
end if.
do if (cmatrix(1,1) <> -999).
do if (ncol(cmatrix) <> ((nms+nys)*ncs)).
compute errcode(errs,1)=29.
compute errs=errs+1.
compute criterr=1.
end if.
do if (criterr = 0).
compute tmp=1.
loop i = 1 to (nms+nys).
loop j = 1 to ncs.
compute ccmat(i,j)=1-(cmatrix(1,tmp) = 0).
compute tmp=tmp+1.
end loop.
end loop.
do if (rsum((csum(ccmat)=0)) <> 0).
compute errcode(errs,1)=30.
compute errs=errs+1.
compute criterr=1.
end if.
end if.
do if (covmy <> 0).
compute notecode(notes,1)=1.
compute notes=notes+1.
end if.
end if.
do if (xmint=1 and (covmy > 0 or (csum(rsum(ccmat)) <> ((nms+nys)*ncs)))).
compute errcode(errs,1)=68.
compute errs=errs+1.
compute criterr=1.
end if.
end if.
do if (criterr=0).
compute needed=needed*(needed-1)/2.
compute nopath=0.
do if (bmatrix(1,1) <> -999).
compute tmp=1.
do if ((ncol(bmatrix) <> needed) or (csum(rsum(bmatrix))=0)).
compute errcode(errs,1)=16.
compute errs=errs+1.
compute criterr=1.
else.
loop i = 2 to nrow(bcmat).
loop j = 1 to (i-1).
compute bcmat(i,j)=1-(bmatrix(1,tmp) = 0).
compute tmp=tmp+1.
end loop.
end loop.
end if.
do if ((csum(bcmat(:,1))=0) and criterr=0).
compute errcode(errs,1)=22.
compute errs=errs+1.
compute criterr=1.
end if.
do if ((rsum(bcmat(nrow(bcmat),:))=0) and criterr=0).
compute errcode(errs,1)=23.
compute errs=errs+1.
compute criterr=1.
end if.
compute dm=0.
do if (nms > 0).
loop i = 1 to nms.
do if (((rsum(bcmat((nxs+i),:)) = 0) or (csum(bcmat(:,(nxs+i))) = 0)) and (dm=0) and (criterr=0)).
compute errcode(errs,1)=26.
compute errs=errs+1.
compute criterr=1.
compute dm=1.
end if.
end loop.
end if.
release dm.
end if.
end if.
do if (criterr=0).
do if (wmatrix(1,1) <> -999).
compute tmp=1.
do if (ncol(wmatrix) <> needed).
compute errcode(errs,1)=17.
compute errs=errs+1.
compute criterr=1.
else.
compute modelvar(1,1)='CUSTOM'.
loop i = 2 to nrow(wcmat).
loop j = 1 to (i-1).
compute wcmat(i,j)=1-(wmatrix(1,tmp) = 0).
do if ((wcmat(i,j)=1) and (bcmat(i,j)=0) and (nopath=0)).
compute errcode(errs,1)=20.
compute errs=errs+1.
compute criterr=1.
compute nopath=1.
end if.
compute tmp=tmp+1.
end loop.
end loop.
end if.
end if.
do if (zmatrix(1,1) <> -999).
compute tmp=1.
do if (ncol(zmatrix) <> needed).
compute errcode(errs,1)=18.
compute errs=errs+1.
compute criterr=1.
else.
compute modelvar(1,1)='CUSTOM'.
do if (csum(rsum(wcmat))=0 and model=999).
compute errcode(errs,1)=21.
compute errs=errs+1.
compute criterr=1.
end if.
loop i = 2 to nrow(zcmat).
loop j = 1 to (i-1).
compute zcmat(i,j)=1-(zmatrix(1,tmp) = 0).
do if ((zcmat(i,j)=1) and (bcmat(i,j)=0) and (nopath=0)).
compute errcode(errs,1)=20.
compute errs=errs+1.
compute criterr=1.
compute nopath=1.
end if.
compute tmp=tmp+1.
end loop.
end loop.
end if.
end if.
compute tmp=1.
do if (wzmatrix(1,1) <> -999).
do if (ncol(wzmatrix) <> needed).
compute errcode(errs,1)=19.
compute errs=errs+1.
compute criterr=1.
end if.
compute modelvar(1,1)='CUSTOM'.
end if.
do if (criterr=0).
loop i = 2 to nrow(wzcmat).
loop j = 1 to (i-1).
do if (wzmatrix(1,1) <> -999).
compute wzcmat(i,j)=1-(wzmatrix(1,tmp) = 0).
end if.
do if (wzcmat(i,j)=1).
compute wcmat(i,j)=1.
compute zcmat(i,j)=1.
end if.
do if ((wzcmat(i,j)=1) and (bcmat(i,j)=0) and (nopath=0)).
compute errcode(errs,1)=20.
compute errs=errs+1.
compute criterr=1.
compute nopath=1.
end if.
compute tmp=tmp+1.
end loop.
end loop.
end if.
end if.
do if (criterr=0).
compute xprod=csum(wcmat(:,1))+csum(zcmat(:,1))+csum(wzcmat(:,1)).
compute xprod=(xprod > 0).
compute wsum=csum(rsum(wcmat)).
compute wprod=(wsum > 0).
do if (nms > 0).
loop i = 1 to nms.
compute tmp=csum(wcmat(:,(1+i)))+csum(zcmat(:,(1+i)))+csum(wzcmat(:,(1+i))).
compute mprod(1,i)=(tmp>0).
end loop.
end if.
do if ((wsum > 0) and (w = 'xxxxx')).
compute errcode(errs,1)=11.
compute errs=errs+1.
compute criterr=1.
end if.
do if ((wsum = 0) and (w <> 'xxxxx')).
compute errcode(errs,1)=10.
compute errs=errs+1.
compute criterr=1.
end if.
compute zsum=csum(rsum(zcmat)).
compute zprod=(zsum > 0).
do if ((zsum > 0) and (z = 'xxxxx')).
compute errcode(errs,1)=13.
compute errs=errs+1.
compute criterr=1.
end if.
do if ((zsum = 0) and (z <> 'xxxxx')).
compute errcode(errs,1)=12.
compute errs=errs+1.
compute criterr=1.
end if.
do if ((zsum > 0) and (wsum = 0)).
compute errcode(errs,1)=35.
compute errs=errs+1.
compute criterr=1.
end if.
end if.
do if (criterr=0 and nms > 1).
compute serchk=bcmat(2:(nrow(bcmat)-1),2:ncol(bcmat)).
do if (csum(rsum(serchk))) > 0.
compute serial=1.
do if (nms > 6).
compute errcode(errs,1)=36.
compute errs=errs+1.
compute criterr=1.
end if.
end if.
end if.
do if (center > 0 and criterr=0).
compute centvar={' '}.
do if (criterr=0).
do if ((center = 1) or (center = 2 and wdich = 0)).
do if (wprod=1 and mcwok=0 and nwpval > 0).
loop i = 1 to nws.
compute wtmp(:,i)=wtmp(:,i)-(csum(wtmp(:,i))/n).
compute centvar={centvar,wnames(1,i)}.
end loop.

.
compute desctmp=make((8-(4* wmodcust )),ncol( wtmp ),-999).
loop jd=1 to ncol( wtmp ).
compute descdat= wtmp (:,jd).
compute desctmp(1,jd) = csum(descdat)/nrow(descdat).
compute desctmp(2,jd) = (nrow(descdat)*sscp(descdat))-(t(csum(descdat))*(csum(descdat))).
compute desctmp(2,jd) = sqrt(desctmp(2,jd)/(nrow(descdat)*(nrow(descdat)-1))).
compute desctmp(3,jd)=cmin(descdat).
compute desctmp(4,jd)=cmax(descdat).
do if ( wmodcust =0).
compute minwarn=0.
compute maxwarn=0.
do if ((desctmp(3,jd)=desctmp(4,jd)) and novar=0).
compute errcode(errs,1)=15.
compute errs=errs+1.
compute criterr=1.
compute novar=1.
end if.
compute tmp=((descdat(:,1)=desctmp(3,jd))+(descdat(:,1)=desctmp(4,jd))).
compute desctmp(8,jd)=(csum(tmp)=nrow(tmp)).
compute tmp = descdat.
compute tmp(GRADE(descdat),:) = descdat.
compute descdat = tmp.
release tmp.
compute decval={.16;.5;.84}.
loop kd=1 to 3.
compute low=trunc(decval(kd,1)*(nrow(descdat)+1)).
compute lowdec=decval(kd,1)*(nrow(descdat)+1)-low.
compute value=descdat(low,1)+(descdat((low+1),1)-descdat(low,1))*lowdec.
compute desctmp((4+kd),jd)=value.
end loop.
compute mnotev=1.
compute modvals=desctmp(5:7,:).
do if (quantile <> 1).
compute desctmp(5,jd)=desctmp(1,jd)-desctmp(2,jd).
compute desctmp(6,jd)=desctmp(1,jd).
compute desctmp(7,jd)=desctmp(1,jd)+desctmp(2,jd).
compute modvals=desctmp(5:7,:).
compute mnotev=2.
do if (modvals(1,1) < desctmp(3,1)).
compute modvals(1,1)=desctmp(3,1).
compute minwarn=1.
end if.
do if (modvals(3,1) > desctmp(4,1)).
compute modvals(3,1)=desctmp(4,1).
compute maxwarn=1.
end if.
end if.
do if (desctmp(8,1)=1).
compute modvals={desctmp(3,1);desctmp(4,1)}.
compute mnotev=0.
compute minwarn=0.
compute maxwarn=0.
end if.
end if.
end loop
.
compute wmin=desctmp(3,1).
compute wmax=desctmp(4,1).
do if (wmodcust=0).
compute wmodvals=modvals.
compute wprobval=wmodvals.
end if.
end if.
end if.
do if ((center = 1) or (center = 2 and zdich = 0)).
do if (zprod=1 and mczok=0 and nzpval > 0).
loop i = 1 to nzs.
compute ztmp(:,i)=ztmp(:,i)-(csum(ztmp(:,i))/n).
compute centvar={centvar,znames(1,i)}.
end loop.

.
compute desctmp=make((8-(4* zmodcust )),ncol( ztmp ),-999).
loop jd=1 to ncol( ztmp ).
compute descdat= ztmp (:,jd).
compute desctmp(1,jd) = csum(descdat)/nrow(descdat).
compute desctmp(2,jd) = (nrow(descdat)*sscp(descdat))-(t(csum(descdat))*(csum(descdat))).
compute desctmp(2,jd) = sqrt(desctmp(2,jd)/(nrow(descdat)*(nrow(descdat)-1))).
compute desctmp(3,jd)=cmin(descdat).
compute desctmp(4,jd)=cmax(descdat).
do if ( zmodcust =0).
compute minwarn=0.
compute maxwarn=0.
do if ((desctmp(3,jd)=desctmp(4,jd)) and novar=0).
compute errcode(errs,1)=15.
compute errs=errs+1.
compute criterr=1.
compute novar=1.
end if.
compute tmp=((descdat(:,1)=desctmp(3,jd))+(descdat(:,1)=desctmp(4,jd))).
compute desctmp(8,jd)=(csum(tmp)=nrow(tmp)).
compute tmp = descdat.
compute tmp(GRADE(descdat),:) = descdat.
compute descdat = tmp.
release tmp.
compute decval={.16;.5;.84}.
loop kd=1 to 3.
compute low=trunc(decval(kd,1)*(nrow(descdat)+1)).
compute lowdec=decval(kd,1)*(nrow(descdat)+1)-low.
compute value=descdat(low,1)+(descdat((low+1),1)-descdat(low,1))*lowdec.
compute desctmp((4+kd),jd)=value.
end loop.
compute mnotev=1.
compute modvals=desctmp(5:7,:).
do if (quantile <> 1).
compute desctmp(5,jd)=desctmp(1,jd)-desctmp(2,jd).
compute desctmp(6,jd)=desctmp(1,jd).
compute desctmp(7,jd)=desctmp(1,jd)+desctmp(2,jd).
compute modvals=desctmp(5:7,:).
compute mnotev=2.
do if (modvals(1,1) < desctmp(3,1)).
compute modvals(1,1)=desctmp(3,1).
compute minwarn=1.
end if.
do if (modvals(3,1) > desctmp(4,1)).
compute modvals(3,1)=desctmp(4,1).
compute maxwarn=1.
end if.
end if.
do if (desctmp(8,1)=1).
compute modvals={desctmp(3,1);desctmp(4,1)}.
compute mnotev=0.
compute minwarn=0.
compute maxwarn=0.
end if.
end if.
end loop
.
compute zmin=desctmp(3,1).
compute zmax=desctmp(4,1).
do if (zmodcust=0).
compute zmodvals=modvals.
compute zprobval=zmodvals.
end if.
end if.
end if.
do if ((center = 1) or (center = 2 and xdich = 0)).
do if (xprod=1 and mcxok=0).
loop i = 1 to nxs.
compute xtmp(:,i)=xtmp(:,i)-(csum(xtmp(:,i))/n).
compute centvar={centvar,xnames(1,i)}.
end loop.

.
compute desctmp=make((8-(4* 0 )),ncol( xtmp ),-999).
loop jd=1 to ncol( xtmp ).
compute descdat= xtmp (:,jd).
compute desctmp(1,jd) = csum(descdat)/nrow(descdat).
compute desctmp(2,jd) = (nrow(descdat)*sscp(descdat))-(t(csum(descdat))*(csum(descdat))).
compute desctmp(2,jd) = sqrt(desctmp(2,jd)/(nrow(descdat)*(nrow(descdat)-1))).
compute desctmp(3,jd)=cmin(descdat).
compute desctmp(4,jd)=cmax(descdat).
do if ( 0 =0).
compute minwarn=0.
compute maxwarn=0.
do if ((desctmp(3,jd)=desctmp(4,jd)) and novar=0).
compute errcode(errs,1)=15.
compute errs=errs+1.
compute criterr=1.
compute novar=1.
end if.
compute tmp=((descdat(:,1)=desctmp(3,jd))+(descdat(:,1)=desctmp(4,jd))).
compute desctmp(8,jd)=(csum(tmp)=nrow(tmp)).
compute tmp = descdat.
compute tmp(GRADE(descdat),:) = descdat.
compute descdat = tmp.
release tmp.
compute decval={.16;.5;.84}.
loop kd=1 to 3.
compute low=trunc(decval(kd,1)*(nrow(descdat)+1)).
compute lowdec=decval(kd,1)*(nrow(descdat)+1)-low.
compute value=descdat(low,1)+(descdat((low+1),1)-descdat(low,1))*lowdec.
compute desctmp((4+kd),jd)=value.
end loop.
compute mnotev=1.
compute modvals=desctmp(5:7,:).
do if (quantile <> 1).
compute desctmp(5,jd)=desctmp(1,jd)-desctmp(2,jd).
compute desctmp(6,jd)=desctmp(1,jd).
compute desctmp(7,jd)=desctmp(1,jd)+desctmp(2,jd).
compute modvals=desctmp(5:7,:).
compute mnotev=2.
do if (modvals(1,1) < desctmp(3,1)).
compute modvals(1,1)=desctmp(3,1).
compute minwarn=1.
end if.
do if (modvals(3,1) > desctmp(4,1)).
compute modvals(3,1)=desctmp(4,1).
compute maxwarn=1.
end if.
end if.
do if (desctmp(8,1)=1).
compute modvals={desctmp(3,1);desctmp(4,1)}.
compute mnotev=0.
compute minwarn=0.
compute maxwarn=0.
end if.
end if.
end loop
.
compute xmodvals=modvals.
compute xprobval=xmodvals.
end if.
end if.
do if (nms > 0).
loop i = 1 to nms.
do if (mprod(1,i)=1).
compute mtmp(:,i)=mtmp(:,i)-(csum(mtmp(:,i))/n).
compute centvar={centvar,mnames(1,i)}.
end if.
end loop.

.
compute desctmp=make((8-(4* 0 )),ncol( mtmp ),-999).
loop jd=1 to ncol( mtmp ).
compute descdat= mtmp (:,jd).
compute desctmp(1,jd) = csum(descdat)/nrow(descdat).
compute desctmp(2,jd) = (nrow(descdat)*sscp(descdat))-(t(csum(descdat))*(csum(descdat))).
compute desctmp(2,jd) = sqrt(desctmp(2,jd)/(nrow(descdat)*(nrow(descdat)-1))).
compute desctmp(3,jd)=cmin(descdat).
compute desctmp(4,jd)=cmax(descdat).
do if ( 0 =0).
compute minwarn=0.
compute maxwarn=0.
do if ((desctmp(3,jd)=desctmp(4,jd)) and novar=0).
compute errcode(errs,1)=15.
compute errs=errs+1.
compute criterr=1.
compute novar=1.
end if.
compute tmp=((descdat(:,1)=desctmp(3,jd))+(descdat(:,1)=desctmp(4,jd))).
compute desctmp(8,jd)=(csum(tmp)=nrow(tmp)).
compute tmp = descdat.
compute tmp(GRADE(descdat),:) = descdat.
compute descdat = tmp.
release tmp.
compute decval={.16;.5;.84}.
loop kd=1 to 3.
compute low=trunc(decval(kd,1)*(nrow(descdat)+1)).
compute lowdec=decval(kd,1)*(nrow(descdat)+1)-low.
compute value=descdat(low,1)+(descdat((low+1),1)-descdat(low,1))*lowdec.
compute desctmp((4+kd),jd)=value.
end loop.
compute mnotev=1.
compute modvals=desctmp(5:7,:).
do if (quantile <> 1).
compute desctmp(5,jd)=desctmp(1,jd)-desctmp(2,jd).
compute desctmp(6,jd)=desctmp(1,jd).
compute desctmp(7,jd)=desctmp(1,jd)+desctmp(2,jd).
compute modvals=desctmp(5:7,:).
compute mnotev=2.
do if (modvals(1,1) < desctmp(3,1)).
compute modvals(1,1)=desctmp(3,1).
compute minwarn=1.
end if.
do if (modvals(3,1) > desctmp(4,1)).
compute modvals(3,1)=desctmp(4,1).
compute maxwarn=1.
end if.
end if.
do if (desctmp(8,1)=1).
compute modvals={desctmp(3,1);desctmp(4,1)}.
compute mnotev=0.
compute minwarn=0.
compute maxwarn=0.
end if.
end if.
end loop
.
do if ((cdeval(1,1)=-999) and (model=74)).
compute medmeans=desctmp(1,:).
end if.
compute mmodvals=modvals.
compute mprobval=mmodvals.
end if.
end if.
do if (ncol(centvar) > 1).
compute notecode(notes,1)=3.
compute notes=notes+1.
end if.
end if.
do if (criterr=0).
compute wsum=rsum(csum(wcmat)).
compute zsum=rsum(csum(zcmat)).
compute wzsum=rsum(csum(wzcmat)).
compute nump=make(1,(nys+nms),-999).
compue numint=make(1,(nys+nms),0).
compute datcount=1.
compute xtmpuse=0.
compute wtmpuse=0.
compute ztmpuse=0.
compute xwtmpus=0.
compute xztmpus=0.
compute wztmpus=0.
compute xwztmpu=0.
compute xtmploc=-999.
compute wtmploc=-999.
compute xwtmplo=-999.
compute ztmploc=-999.
compute xztmplo=-999.
compute wztmplo=-999.
compute xwztmplo=-999.
compute vlabs={' '}.
do if (ncs > 0).
compute ctmpuse=make(1,ncs,0).
end if.
do if (nms > 0).
compute mtmpuse=make(1,nms,0).
compute mwtmpus=make(1,nms,0).
compute mztmpus=make(1,nms,0).
compute mwztmpu=make(1,nms,0).
compute mtmploc=make(1,nms,0).
compute mwtmplo=make(nwvls,nms,-999).
compute mztmplo=make(nzvls,nms,-999).
compute mwztmplo=make((nwvls*nzvls),nms,-999).
end if.
do if (ncs > 0).
compute ctmploc=make(1,ncs,0).
end if.
compute fulldat=make(n,1,1).
compute datindx=make(1000,(nms+nys),-999).
compute wherew=make(2,(nms+nys),-999).
compute wherex=make(2,(nms+nys),-999).
compute wherez=make(2,(nms+nys),-999).
compute wherexw=make(2,(nms+nys),-999).
compute wherexz=make(2,(nms+nys),-999).
compute wherewz=make(2,(nms+nys),-999).
compute wherexwz=make(2,(nms+nys),-999).
do if (nms > 0).
compute wherem=make(nms,(nms+nys),-999).
compute wheremw = make(nms*2,(nms+nys),-999).
compute wheremz = make(nms*2,(nms+nys),-999).
compute wheremwz = make(nms*2,(nms+nys),-999).
end if.
compute wzhigh=make(1000,(((nms+1)*(nms+2))/2),0).
compute whigh=make(1000,(((nms+1)*(nms+2))/2),0).
compute zhigh=make(1000,(((nms+1)*(nms+2))/2),0).
compute fochigh=make(1000,(((nms+1)*(nms+2))/2),0).
compute xcoefloc={1;2;3;4;5;6;7;8;9}.
compute intkey = {' ', ' ', ' ', ' ', ' ', ' ', ' '}.
compute wzhighct=0.
compute whighct=0.
compute zhighct=0.
compute foccnt=0.
loop i = 2 to nrow(bcmat).
compute wdid=0.
compute zdid=0.
compute wzdid=0.
compute cntmp=1.
compute start=1.
do if (i < nrow(bcmat)).
compute outv=mtmp(:,(i-1)).
compute modlabel={mnames(1,(i-1));'constant'}.
end if.
do if (i = nrow(bcmat)).
compute outv=ytmp.
compute modlabel={ynames;'constant'}.
end if.
loop j = 1 to (i-1).
compute foccnt=foccnt+1.
do if (j = 1 and bcmat(i,j)=1).
compute outv={outv,xtmp}.
compute modlabel={modlabel;xcatlab(1:nxvls,1)}.
do if (xtmpuse=0).
compute fulldat={fulldat,xtmp}.
compute xtmpuse=1.
loop k4=datcount to (datcount+(nxvls-1)).
compute xtmploc={xtmploc;k4}.
end loop.
compute xtmploc=xtmploc(2:nrow(xtmploc),1).
compute datcount=datcount+nxvls.
end if.
compute datindx(start:(start+nrow(xtmploc)-1),(i-1))=xtmploc.
compute wherex(1,(i-1))=start+1.
compute wherex(2,(i-1))=start+nrow(xtmploc)-1+1.
do if (model = 74).
end if.
compute onebl=make(nrow(xtmploc),1,1).
compute fochigh((start+1):(start+nrow(xtmploc)),foccnt)=onebl.
compute start=start+nrow(xtmploc).
end if.
do if (j > 1 and bcmat(i,j)=1).
compute outv={outv,mtmp(:,(j-1))}.
compute modlabel={modlabel;mnames(1,(j-1))}.
do if (mtmpuse(1,(j-1))=0).
compute fulldat={fulldat,mtmp(:,(j-1))}.
compute mtmpuse(1,(j-1))=1.
compute mtmploc(1,(j-1))=datcount.
compute datcount=datcount+1.
end if.
compute datindx(start:(start+nrow(mtmploc)-1),(i-1))=mtmploc(1,(j-1)).
compute wherem((j-1),(i-1))=start+1.
compute onebl=make(nrow(mtmploc(1,j-1)),1,1).
compute ttt=nrow(mtmploc(1,(j-1)))+start-1.
compute fochigh((start+1):(start+nrow(mtmploc(1,(j-1)))),foccnt)=onebl.
compute start=start+nrow(mtmploc(1,(j-1))).
end if.
end loop.
do if (wsum > 0).
loop j = 1 to (i-1).
compute whighct=whighct+1.
do if (j = 1 and wcmat(i,j)=1).
do if (wdid=0).
compute outv={outv,wtmp}.
do if (ncs > 0 and wiscov > 0).
compute ccmatoff((i-1),wiscov)=0.
end if.
compute modlabel={modlabel;wcatlab(1:nwvls,1)}.
compute wdid=1.
do if (wtmpuse=0).
compute fulldat={fulldat,wtmp}.
do if (ncs > 0 and wiscov > 0).
compute ccmatoff((i-1),wiscov)=0.
end if.
compute wtmpuse=1.
loop k4=datcount to (datcount+(nwvls-1)).
compute wtmploc={wtmploc;k4}.
end loop.
compute wtmploc=wtmploc(2:nrow(wtmploc),1).
compute datcount=datcount+nwvls.
end if.
end if.
compute datindx(start:(start+nrow(wtmploc)-1),(i-1))=wtmploc.
compute wherew(1,(i-1))=start+1.
compute wherew(2,(i-1))=start+nrow(wtmploc)-1+1.
compute start=start+nrow(wtmploc).
loop k1=1 to nxvls.
loop k2 = 1 to nwvls.
compute outv={outv,(xtmp(:,k1)&*wtmp(:,k2))}.
do if (ncs > 0 and wiscov > 0).
compute ccmatoff((i-1),wiscov)=0.
end if.
compute modlabel={modlabel;intlab(cntmp,1)}.
compute intkey={intkey;intlab(cntmp,1),':',xcatlab(k1,1),'x',wcatlab(k2,1),' ',' '}.
compute cntmp=cntmp+1.
end loop.
end loop.
do if (xwtmpus=0).
compute fulldat={fulldat,outv(:,(ncol(outv)-(nxvls*nwvls)+1):ncol(outv))}.
compute xwtmpus=1.
do if (ncs > 0 and wiscov > 0).
compute ccmatoff((i-1),wiscov)=0.
end if.
loop k4=datcount to (datcount+((nwvls*nxvls)-1)).
compute xwtmplo={xwtmplo;k4}.
end loop.
compute xwtmplo=xwtmplo(2:nrow(xwtmplo),1).
compute datcount=datcount+(nxvls*nwvls).
end if.
compute datindx(start:(start+nrow(xwtmplo)-1),(i-1))=xwtmplo.
compute wherexw(1,(i-1))=start+1.
compute wherexw(2,(i-1))=start+nrow(xwtmplo)-1+1.
compute onebl=make(nrow(xwtmplo),1,1).
compute whigh((start+1):(start+nrow(xwtmplo)),whighct)=onebl.
compute start=start+nrow(xwtmplo).
end if.
do if (j > 1 and wcmat(i,j)=1).
do if (wdid=0 and model <> 74).
compute outv={outv,wtmp}.
do if (ncs > 0 and wiscov > 0).
compute ccmatoff((i-1),wiscov)=0.
end if.
compute modlabel={modlabel;wcatlab(1:nwvls,1)}.
compute wdid=1.
do if (wtmpuse=0).
compute fulldat={fulldat,wtmp}.
do if (ncs > 0 and wiscov > 0).
compute ccmatoff((i-1),wiscov)=0.
end if.
compute wtmpuse=1.
loop k4=datcount to (datcount+(nwvls-1)).
compute wtmploc={wtmploc;k4}.
end loop.
compute wtmploc=wtmploc(2:nrow(wtmploc),1).
compute datcount=datcount+nwvls.
end if.
compute datindx(start:(start+nrow(wtmploc)-1),(i-1))=wtmploc.
compute wherew(1,(i-1))=start+1.
compute wherew(2,(i-1))=start+nrow(wtmploc)-1+1.
compute start=start+nrow(wtmploc).
end if.
loop k2 = 1 to nwvls.
compute outv={outv,(mtmp(:,(j-1))&*wtmp(:,k2))}.
do if (ncs > 0 and wiscov > 0).
compute ccmatoff((i-1),wiscov)=0.
end if.
compute modlabel={modlabel;intlab(cntmp,1)}.
compute intkey={intkey;intlab(cntmp,1),':', mnames(1,(j-1)),'x',wcatlab(k2,1),' ',' '}.
compute cntmp=cntmp+1.
end loop.
do if (mwtmpus(1,(j-1))=0).
compute fulldat={fulldat,outv(:,(ncol(outv)-nwvls+1):ncol(outv))}.
do if (ncs > 0 and wiscov > 0).
compute ccmatoff((i-1),wiscov)=0.
end if.
compute mwtmpus(1,(j-1))=1.
compute mw22=-999.
loop k4=datcount to (datcount+(nwvls-1)).
compute mw22={mw22;k4}.
end loop.
compute mwtmplo(:,(j-1))=mw22(2:nrow(mw22),1).
compute datcount=datcount+nwvls.
end if.
compute datindx(start:(start+nrow(mwtmplo)-1),(i-1))=mwtmplo(:,(j-1)).
compute wheremw(((2*j)-3),(i-1))=start+1.
compute wheremw(((2*j)-2),(i-1))=start+nrow(mwtmplo)-1+1.
compute onebl=make(nrow(mwtmplo),1,1).
compute whigh((start+1):(start+nrow(mwtmplo)),whighct)=onebl.
compute start=start+nrow(mwtmplo).
end if.
end loop.
end if.
do if (zsum > 0).
loop j = 1 to (i-1).
compute zhighct=zhighct+1.
do if (j = 1 and zcmat(i,j)=1).
do if (zdid=0).
compute outv={outv,ztmp}.
do if (ncs > 0 and ziscov > 0).
compute ccmatoff((i-1),ziscov)=0.
end if.
compute modlabel={modlabel;zcatlab(1:nzvls,1)}.
compute zdid=1.
do if (ztmpuse=0).
compute fulldat={fulldat,ztmp}.
do if (ncs > 0 and ziscov > 0).
compute ccmatoff((i-1),ziscov)=0.
end if.
compute ztmpuse=1.
loop k4=datcount to (datcount+(nzvls-1)).
compute ztmploc={ztmploc;k4}.
end loop.
compute ztmploc=ztmploc(2:nrow(ztmploc),1).
compute datcount=datcount+nzvls.
end if.
end if.
compute datindx(start:(start+nrow(ztmploc)-1),(i-1))=ztmploc.
compute wherez(1,(i-1))=start+1.
compute wherez(2,(i-1))=start+nrow(ztmploc)-1+1.
compute start=start+nrow(ztmploc).
loop k1=1 to nxvls.
loop k2 = 1 to nzvls.
compute outv={outv,(xtmp(:,k1)&*ztmp(:,k2))}.
do if (ncs > 0 and ziscov > 0).
compute ccmatoff((i-1),ziscov)=0.
end if.
compute modlabel={modlabel;intlab(cntmp,1)}.
compute intkey={intkey;intlab(cntmp,1),':',xcatlab(k1,1),'x',zcatlab(k2,1),' ',' '}.
compute cntmp=cntmp+1.
end loop.
end loop.
do if (xztmpus=0).
compute fulldat={fulldat,outv(:,(ncol(outv)-(nxvls*nzvls)+1):ncol(outv))}.
do if (ncs > 0 and ziscov > 0).
compute ccmatoff((i-1),ziscov)=0.
end if.
compute xztmpus=1.
loop k4=datcount to (datcount+((nzvls*nxvls)-1)).
compute xztmplo={xztmplo;k4}.
end loop.
compute xztmplo=xztmplo(2:nrow(xztmplo),1).
compute datcount=datcount+(nxvls*nzvls).
end if.
compute datindx(start:(start+nrow(xztmplo)-1),(i-1))=xztmplo.
compute wherexz(1,(i-1))=start+1.
compute wherexz(2,(i-1))=start+nrow(xztmplo)-1+1.
compute onebl=make(nrow(xztmplo),1,1).
compute zhigh((start+1):(start+nrow(xztmplo)),zhighct)=onebl.
compute start=start+nrow(xztmplo).
end if.
do if (j > 1 and zcmat(i,j)=1).
do if (zdid=0).
compute outv={outv,ztmp}.
do if (ncs > 0 and ziscov > 0).
compute ccmatoff((i-1),ziscov)=0.
end if.
compute modlabel={modlabel;zcatlab(1:nzvls,1)}.
compute zdid=1.
do if (ztmpuse=0).
compute fulldat={fulldat,ztmp}.
do if (ncs > 0 and ziscov > 0).
compute ccmatoff((i-1),ziscov)=0.
end if.
compute ztmpuse=1.
loop k4=datcount to (datcount+(nzvls-1)).
compute ztmploc={ztmploc;k4}.
end loop.
compute ztmploc=ztmploc(2:nrow(ztmploc),1).
compute datcount=datcount+nzvls.
end if.
compute datindx(start:(start+nrow(ztmploc)-1),(i-1))=ztmploc.
compute wherez(1,(i-1))=start+1.
compute wherez(2,(i-1))=start+nrow(ztmploc)-1+1.
compute start=start+nrow(ztmploc).
end if.
loop k2 = 1 to nzvls.
compute outv={outv,(mtmp(:,(j-1))&*ztmp(:,k2))}.
do if (ncs > 0 and ziscov > 0).
compute ccmatoff((i-1),ziscov)=0.
end if.
compute modlabel={modlabel;intlab(cntmp,1)}.
compute intkey={intkey;intlab(cntmp,1),':', mnames(1,(j-1)),'x',zcatlab(k2,1),' ',' '}.
compute cntmp=cntmp+1.
end loop.
do if (mztmpus(1,(j-1))=0).
compute fulldat={fulldat,outv(:,(ncol(outv)-nzvls+1):ncol(outv))}.
do if (ncs > 0 and ziscov > 0).
compute ccmatoff((i-1),ziscov)=0.
end if.
compute mztmpus(1,(j-1))=1.
compute mz22=-999.
loop k4=datcount to (datcount+(nzvls-1)).
compute mz22={mz22;k4}.
end loop.
compute mztmplo(:,(j-1))=mz22(2:nrow(mz22),1).
compute datcount=datcount+nzvls.
end if.
compute datindx(start:(start+nrow(mztmplo)-1),(i-1))=mztmplo(:,(j-1)).
compute wheremz(((2*j)-3),(i-1))=start+1.
compute wheremz(((2*j)-2),(i-1))=start+nrow(mztmplo)-1+1.
compute onebl=make(nrow(mztmplo),1,1).
compute zhigh((start+1):(start+nrow(mztmplo)),zhighct)=onebl.
compute start=start+nrow(mztmplo).
end if.
end loop.
end if.
do if (wzsum > 0).
loop j = 1 to (i-1).
compute wzhighct=wzhighct+1.
do if (j = 1 and wzcmat(i,j)=1).
do if (wzdid=0).
loop k1=1 to nwvls.
loop k2 = 1 to nzvls.
compute outv={outv,(wtmp(:,k1)&*ztmp(:,k2))}.
do if (ncs > 0 and (ziscov > 0)).
compute ccmatoff((i-1),ziscov)=0.
end if.
do if (ncs > 0 and (wiscov > 0)).
compute ccmatoff((i-1),wiscov)=0.
end if.
compute modlabel={modlabel;intlab(cntmp,1)}.
compute intkey={intkey;intlab(cntmp,1),':',wcatlab(k1,1),'x',zcatlab(k2,1),' ',' '}.
compute cntmp=cntmp+1.
end loop.
end loop.
do if (wztmpus=0).
compute fulldat={fulldat,outv(:,(ncol(outv)-(nwvls*nzvls)+1):ncol(outv))}.
do if (ncs > 0 and (ziscov > 0)).
compute ccmatoff((i-1),ziscov)=0.
end if.
do if (ncs > 0 and (wiscov > 0)).
compute ccmatoff((i-1),wiscov)=0.
end if.
compute wztmpus=1.
loop k4=datcount to (datcount+((nwvls*nzvls)-1)).
compute wztmplo={wztmplo;k4}.
end loop.
compute wztmplo=wztmplo(2:nrow(wztmplo),1).
compute datcount=datcount+(nzvls*nwvls).
end if.
compute wzdid=1.
end if.
compute datindx(start:(start+nrow(wztmplo)-1),(i-1))=wztmplo.
compute wherewz(1,(i-1))=start+1.
compute wherewz(2,(i-1))=start+nrow(wztmplo)-1+1.
compute start=start+nrow(wztmplo).
loop k1=1 to nxvls.
loop k2=1 to nwvls.
loop k3=1 to nzvls.
compute outv={outv,(xtmp(:,k1)&*wtmp(:,k2)&*ztmp(:,k3))}.
do if (ncs > 0 and (ziscov > 0)).
compute ccmatoff((i-1),ziscov)=0.
end if.
do if (ncs > 0 and (wiscov > 0)).
compute ccmatoff((i-1),wiscov)=0.
end if.
compute modlabel={modlabel;intlab(cntmp,1)}.
compute intkey={intkey;intlab(cntmp,1),':',xcatlab(k1,1),'x',wcatlab(k2,1),'x', zcatlab(k3,1)}.
compute cntmp=cntmp+1.
end loop.
end loop.
end loop.
do if (xwztmpu=0).
compute fulldat={fulldat,outv(:,(ncol(outv)-(nxvls*nwvls*nzvls)+1):ncol(outv))}.
do if (ncs > 0 and (ziscov > 0)).
compute ccmatoff((i-1),ziscov)=0.
end if.
do if (ncs > 0 and (wiscov > 0)).
compute ccmatoff((i-1),wiscov)=0.
end if.
compute xwztmpu=1.
loop k4=datcount to (datcount+((nzvls*nxvls*nwvls)-1)).
compute xwztmplo={xwztmplo;k4}.
end loop.
compute xwztmplo=xwztmplo(2:nrow(xwztmplo),1).
compute datcount=datcount+(nxvls*nzvls*nwvls).
end if.
compute datindx(start:(start+nrow(xwztmplo)-1),(i-1))=xwztmplo.
compute wherexwz(1,(i-1))=start+1.
compute wherexwz(2,(i-1))=start+nrow(xwztmplo)-1+1.
compute onebl=make(nrow(xwztmplo),1,1).
compute wzhigh((start+1):(start+nrow(xwztmplo)),wzhighct)=onebl.
compute start=start+nrow(xwztmplo).
end if.
do if (j > 1 and wzcmat(i,j)=1).
do if (wzdid=0).
loop k1=1 to nwvls.
loop k2 = 1 to nzvls.
compute outv={outv,(wtmp(:,k1)&*ztmp(:,k2))}.
do if (ncs > 0 and (ziscov > 0)).
compute ccmatoff((i-1),ziscov)=0.
end if.
do if (ncs > 0 and (wiscov > 0)).
compute ccmatoff((i-1),wiscov)=0.
end if.
compute modlabel={modlabel;intlab(cntmp,1)}.
compute intkey={intkey;intlab(cntmp,1),':',wcatlab(k1,1),'x',zcatlab(k2,1),' ',' '}.
compute cntmp=cntmp+1.
end loop.
end loop.
do if (wztmpus=0).
compute fulldat={fulldat,outv(:,(ncol(outv)-(nwvls*nzvls)+1):ncol(outv))}.
do if (ncs > 0 and (ziscov > 0)).
compute ccmatoff((i-1),ziscov)=0.
end if.
do if (ncs > 0 and (wiscov > 0)).
compute ccmatoff((i-1),wiscov)=0.
end if.
compute wztmpus=1.
loop k4=datcount to (datcount+((nwvls*nzvls)-1)).
compute wztmplo={wztmplo;k4}.
end loop.
compute wztmplo=wztmplo(2:nrow(wztmplo),1).
compute datcount=datcount+(nzvls*nwvls).
end if.
compute wzdid=1.
compute datindx(start:(start+nrow(wztmplo)-1),(i-1))=wztmplo.
compute wherewz(1,(i-1))=start+1.
compute wherewz(2,(i-1))=start+nrow(wztmplo)-1+1.
compute start=start+nrow(wztmplo).
end if.
loop k1 = 1 to nwvls.
loop k2 = 1 to nzvls.
compute outv={outv,(mtmp(:,(j-1))&*wtmp(:,k1)&*ztmp(:,k2))}.
do if (ncs > 0 and (ziscov > 0)).
compute ccmatoff((i-1),ziscov)=0.
end if.
do if (ncs > 0 and (wiscov > 0)).
compute ccmatoff((i-1),wiscov)=0.
end if.
compute modlabel={modlabel;intlab(cntmp,1)}.
compute intkey={intkey;intlab(cntmp,1),':',mnames(1,(j-1)),'x',wcatlab(k1,1),'x', zcatlab(k2,1)}.
compute cntmp=cntmp+1.
end loop.
end loop.
do if (mwztmpu(1,(j-1))=0).
compute fulldat={fulldat,outv(:,(ncol(outv)-(nwvls*nzvls)+1):ncol(outv))}.
do if (ncs > 0 and (ziscov > 0)).
compute ccmatoff((i-1),ziscov)=0.
end if.
do if (ncs > 0 and (wiscov > 0)).
compute ccmatoff((i-1),wiscov)=0.
end if.
compute mwztmpu(1,(j-1))=1.
compute mz22=-999.
loop k4=datcount to (datcount+(nwvls*nzvls)-1).
compute mz22={mz22;k4}.
end loop.
compute mwztmplo(:,(j-1))=mz22(2:nrow(mz22),1).
compute datcount=datcount+(nwvls*nzvls).
end if.
compute datindx(start:(start+nrow(mwztmplo)-1),(i-1))=mwztmplo(:,(j-1)).
compute wheremwz(((2*j)-3),(i-1))=start+1.
compute wheremwz(((2*j)-2),(i-1))=start+nrow(mwztmplo)-1+1.
compute onebl=make(nrow(mwztmplo),1,1).
compute wzhigh((start+1):(start+nrow(mwztmplo)),wzhighct)=onebl.
compute start=start+nrow(mwztmplo).
end if.
end loop.
end if.
do if (ncs > 0).
compute ccmat=ccmat&*ccmatoff.
loop j = 1 to ncs.
do if (ccmat((i-1),j))=1.
do if (j=wiscov).
compute ctmp(:,j)=wtmp.
end if.
do if (j=ziscov).
compute ctmp(:,j)=ztmp.
end if.
compute outv={outv,ctmp(:,j)}.
compute modlabel={modlabel;covnames(1,j)}.
do if (ctmpuse(1,j)=0).
compute fulldat={fulldat,ctmp(:,j)}.
compute ctmpuse(1,j)=1.
compute ctmploc(1,j)=datcount.
compute datcount=datcount+1.
end if.
compute datindx(start:(start+nrow(ctmploc)-1),(i-1))=ctmploc(1,j).
compute start=start+nrow(ctmploc(1,j)).
end if.
end loop.
end if.
compute wdid=0.
compute zdid=0.
compute wzdid=0.
compute vlabs={vlabs;modlabel(2:nrow(modlabel),1)}.
compute numint(1,(i-1))=cntmp-1.
compute nump(1,(i-1))=nrow(modlabel)-1.
end loop.
release datcount, xtmpuse, wtmpuse, ztmpuse, xwtmpus, xztmpus, wztmpus, xwztmpu.
release xtmploc, wtmploc, xwtmplo, ztmploc, xztmplo, wztmplo, xwztmplo, foccnt.
do if (modcok=1 and ((nms > 0) or (zcmat(2,1) <> 1) or (mcx <> 0))).
compute notecode(notes,1) = 19.
compute notes = notes + 1.
compute modcok=0.
end if.
do if ((serial = 1 or (rsum(numint)>0) or nms=0) and mc > 0).
compute notecode(notes,1) = 15.
compute notes = notes + 1.
compute boot=mc.
compute mc=0.
end if.
do if (boot <> 0 or mc <> 0).
compute bootsz=boot.
do if (mc > 0).
compute bootsz=mc.
compute saveboot=0.
end if.
loop.
compute cilow = rnd(bootsz*(1-(conf/100))/2).
compute cihigh = trunc((bootsz*(conf/100)+(bootsz*(1-(conf/100))/2)))+1.
do if (cilow < 1 or cihigh > bootsz).
compute bootsz=trunc((bootsz+1000)/1000)*1000.
compute adjust = 1.
end if.
end loop if (cilow gt 0 and cihigh le bootsz).
do if (boot > 0).
compute boot=bootsz.
end if.
do if (mc > 0).
compute mc=bootsz.
end if.
do if (adjust = 1 and boot > 0).
compute notecode(notes,1) = 8.
compute notes = notes + 1.
end if.
do if (adjust = 1 and mc > 0).
compute notecode(notes,1) = 16.
compute notes = notes + 1.
end if.
end if.
compute maxboot = trunc(2*boot).
do if ( 0 > maxboot).
compute maxboot=trunc( 0 ).
end if.
do if (nms > 0).
release mtmpuse, mwtmpus, mwztmpu, mtmploc, mwtmplo, mztmplo, mwztmplo.
end if.
release wdid, zdid, wzdid, start,modlabel.
compute vlabs=vlabs(2:nrow(vlabs),1).
do if (rsum(numint) > 0).
compute intkey=intkey(2:nrow(intkey),:).
end if.
compute fulldat=fulldat(:,2:ncol(fulldat)).
compute fochigh=fochigh(1:rmax(nump),:).
compute whigh=whigh(1:rmax(nump),:).
compute zhigh=zhigh(1:rmax(nump),:).
compute wzhigh=wzhigh(1:rmax(nump),:).
compute coeffs=fochigh+whigh+zhigh+wzhigh.
compute bootloc=make(rmax(nump),ncol(nump),0).
do if (nms > 0).
compute cntmp=1.
loop i = 1 to ncol(nump).
loop j = 1 to nump(1,i).
compute bootloc(j,i)=cntmp.
compute cntmp=cntmp+1.
end loop.
end loop.
compute fochighb=make(nrow(fochigh),ncol(fochigh),0).
compute whighb=fochighb.
compute zhighb=fochighb.
compute wzhighb=fochighb.
compute thetaxmb=make(nrow(fochighb),nms,0).
compute thetaxyb=make(nrow(fochighb),1,0).
compute pathsfoc=make(nxvls,1,0).
compute cntmp=1.
loop i = 1 to (nms+nys).
loop j = 1 to i.
compute fochighb(:,cntmp)=fochigh(:,cntmp)&*bootloc(:,i).
compute whighb(:,cntmp)=whigh(:,cntmp)&*bootloc(:,i).
compute zhighb(:,cntmp)=zhigh(:,cntmp)&*bootloc(:,i).
compute wzhighb(:,cntmp)=wzhigh(:,cntmp)&*bootloc(:,i).
compute coeffsb=fochighb+whighb+zhighb+wzhighb.
do if ((i < (nms+nys)) and (j = 1)).
compute thetaxmb(:,i)=coeffsb(:,cntmp).
end if.
do if ((i = (nms+nys)) and (j = 1)).
compute thetaxyb(:,1)=coeffsb(:,cntmp).
end if.
compute cntmp=cntmp+1.
end loop.
end loop.
compute thetamyb=coeffsb(:,(ncol(coeffsb)-nms+1):ncol(coeffsb)).
do if (serial = 1).
compute thetammb=make(nrow(coeffsb),((nms*(nms-1))/2),0).
end if.
compute cntmp=1.
do if (nms > 1 and serial = 1).
loop i = 1 to (nms-1).
compute start=((i+2)*(i+1))/2.
loop j = 2 to (nms-i+1).
compute thetammb(:,cntmp)=coeffsb(:,start).
compute start=start+j+i-1.
compute cntmp=cntmp+1.
end loop.
end loop.
end if.
end if.
do if ((total = 1) and (rsum(numint)=0 or (xmint=1))).
compute dototal=1.
do if ((csum(bcmat(:,1)) <> (nms+nys)) or (rsum(bcmat(nrow(bcmat),:)) <> (nms+nys))).
compute dototal=0.
compute alttotal=1.
compute notecode(notes,1) = 12.
compute notes = notes + 1.
end if.
do if (ncs > 0).
do if ((csum(rsum(ccmat))) < (nrow(ccmat)*ncol(ccmat))).
compute dototal=0.
compute alttotal=1.
compute notecode(notes,1) = 11.
compute notes = notes + 1.
end if.
end if.
do if (model=74).
do if (xdich=0 and nxvls=1).
compute dototal=0.
compute alttotal=1.
end if.
do if ((xdich=1 or nxvls > 1) and (ncs > 0) and model=74).
compute dototal=0.
compute alttotal=1.
end if.
end if.
end if.
end if.
do if (criterr=0 and ncs > 0).
do if (rsum((csum(ccmat)=0)) <> 0).
compute errcode(errs,1)=51.
compute errs=errs+1.
compute criterr=1.
end if.
end if.
compute debug= 0.
do if (outscree=1).
print/title = '***************** PROCESS Procedure for SPSS Version 4.2 *****************'.
print/title = '          Written by Andrew F. Hayes, Ph.D.       www.afhayes.com'.
print/title = '    Documentation available in Hayes (2022). www.guilford.com/p/hayes3'/space=0.
end if.
do if (criterr=0).
compute modresid=make(n,1,99999).
do if (stand=1 and ydich=1).
compute stand=0.
end if.
compute anymod2=csum(rsum(wcmat+zcmat+wzcmat)).
do if (anymod2 > 0 and stand = 1).
compute notecode(notes,1) = 27.
compute notes = notes + 1.
compute stand=0.
end if.
compute funny=1.
do if (outscree=1).
print modelvar/title = '**************************************************************************'/format = A8/rnames=modelvlb.
do if (ncs > 0).
print covnames/title='Covariates:'/format=A8.
end if.
print n/title='Sample'/rlabel='Size:'.
do if (( 'random' <> 'random')).
compute seedt= 'random'.
print seedt/title='Custom'/format=A12/rlabel = 'Seed:'.
end if.
end if.
compute maxresm=9.
compute resultm=make(1,maxresm,99999).
do if (describe=1).
compute means=csum(dat)/n.
compute sigmatal = (t(dat)*(ident(n)-(1/n)*ones*t(ones))*dat)*(1/(n-1)).
compute sdvec=sqrt(diag(sigmatal)).
compute sdall = mdiag(1/sdvec).
compute corall=sdall*sigmatal*t(sdall).
compute means={means;t(sdvec)}.
do if (ncol(means) > 9).
compute resultm=make(1,ncol(means),99999).
compute maxresm=ncol(means).
end if.
compute resultm2=make(2,maxresm,99999).
compute resultm2(1:2,1:ncol(means))=means.
compute resultm={resultm;resultm2}.
compute resultm2=make(ncol(corall),maxresm,99999).
compute resultm2(1:ncol(corall),1:ncol(corall))=corall.
compute resultm={resultm;resultm2}.
do if (outscree=1).
print means/title='Variable means and standard deviations'/cnames=varnames/rlabels='Mean','SD'/format= F10.4.
print corall/title='Variable intercorrelations (Pearson r)'/cnames=varnames/rnames=varnames/format= F10.4.
end if.
end if.
do if (outscree=1).
do if (mcxok=1).
compute labtmp={xnames,t(xcatlab(1:nxvls,1))}.
print dummatx/title = 'Coding of categorical X variable for analysis:'/cnames = labtmp/format = F6.3.
end if.
do if (mcwok=1 and xmint=0).
compute labtmp={wnames,t(wcatlab(1:nwvls,1))}.
print dummatw/title = 'Coding of categorical W variable for analysis:'/cnames = labtmp/format = F6.3.
end if.
do if (mczok=1).
compute labtmp={znames,t(zcatlab(1:nzvls,1))}.
print dummatz/title = 'Coding of categorical Z variable for analysis:'/cnames = labtmp/format = F6.3.
end if.
end if.
end if.
do if (criterr = 0).
compute outnames=ynames.
compute outvars=ytmp.
do if (nms > 0).
compute outnames={mnames,ynames}.
compute outvars={mtmp,ytmp}.
compute indcov=make(((nms*2)+(nms*(nxvls-1))),((nms*2)+(nms*(nxvls-1))),0).
compute mcsopath=make(((nms*2)+(nms*(nxvls-1))),1,0).
end if.
compute labstart=1.
compute intstart=1.
compute start=1.
compute coeffmat=make(1,6,0).
compute conseq={'        '}.
compute dfmat=0.
compute coeffcol=0.
compute pathscnt=1.
compute pathscn2=1.
loop i = 1 to (nms+nys).
do if (outscree=1).
print/title = '**************************************************************************'.
end if.
compute highf=make(1,5,0).
compute highf2=highf.
do if ((i = (nms+nys)) and (ydich=1)).
compute highf=make(1,3,0).
compute highf2=highf.
end if.
compute flabel={' '}.
compute y=outvars(:,i).
compute xindx=datindx(1:(nump(1,i)-1),i).
compute x = fulldat(:,xindx).
compute x={ones,x}.
compute xsq=t(x)*x.
compute exsq=eval(xsq).
release xsq.
compute zeroeig=csum(exsq <= 0.000000000002).
do if (outscree=1).
print outnames(1,i)/title = 'OUTCOME VARIABLE:'/format = A8/space=0.
do if (ydich=1 and (i = (nms+nys))).
compute nmsd = {outnames(1,i), 'Analysis'}.
print rcd/title = 'Coding of binary Y for logistic regression analysis:'/cnames = nmsd/format = F9.2.
end if.
end if.
do if (zeroeig > 0).
print / title = 'SINGULAR OR NEAR SINGULAR DATA MATRIX.'.
compute criterr=1.
compute errcode(errs,1)=31.
compute errs=errs+1.
end if.
compute means=csum(x)/n.
compute vlabsm=vlabs(labstart:(labstart+(nump(1,i)-1)),1).
do if (criterr=0).
do if (ydich=0 or (i < (nms+nys))).

.
do if ( 1 =1).
compute hatmat=inv(t( x )* x )*t( x ).
compute b = hatmat* y.
compute modres=b.
do if ( 1 =1).
compute n1=nrow( x ).
compute dfres=n1-(ncol( x )).
compute sstotal = t( y -(csum( y )/n1))*( y -(csum( y )/n1)).
compute resid= y - x *b.
compute ssresid = csum((resid)&**2).
compute r2 = (sstotal-ssresid)/sstotal.
do if (r2 < 0).
compute r2=0.
end if.
compute adjr2 = 1-((1-r2)*(n1-1)/(dfres)).
compute mse=ssresid/(n1-ncol( x )).

.
compute n1=nrow( x ).
compute invXtX = inv(t( x )* x ).
compute varb = mse *invXtX.
compute k3 = ncol( x ).
compute xhc=0.
do if ( hc <> 5).
compute xhc= x.
compute hat = xhc(:,1).
loop i3=1 to nrow(xhc).
compute hat(i3,1)= xhc(i3,:)*invXtX*t(xhc(i3,:)).
end loop.
do if ( hc = 0 or hc =1).
loop i3 = 1 to k3.
compute xhc(:,i3)=xhc(:,i3)&* resid.
end loop.
end if.
do if ( hc =3 or hc =2).
loop i3=1 to k3.
compute xhc(:,i3) = ( resid &/(1-hat)&**(1/(4- hc )))&*xhc(:,i3).
end loop.
end if.
do if ( hc = 4).
compute hcmn=make(n,2,4).
compute hcmn(:,2)=(n1*hat)/k3.
loop i3= 1 to k3.
compute xhc(:,i3) = ( resid &/(1-hat)&**(rmin(hcmn)/2))&*xhc(:,i3).
end loop.
end if.
compute varb=(invXtX*t(xhc)*xhc*invXtX).
do if ( hc =1).
compute varb=(n1/(n1-ncol( x )))&*varb.
end if.
end if.
compute hclab={'se(HC0)','se(HC1)','se(HC2)','se(HC3)','se(HC4)','se'}.
compute hclab=hclab(1,( hc +1)).
compute hcflab={'F(HC0)','F(HC1)','F(HC2)','F(HC3)','F(HC4)','F'}.
compute hcflab=hcflab(1,( hc +1)).
release xhc
.
compute seb=sqrt(diag(varb)).
compute trat = b&/seb.
compute p = 2*(1-tcdf(abs(trat), (dfres))).
compute tval = sqrt(dfres* (exp((dfres-(5/6))*((xp2/(dfres-(2/3)+(.11/dfres)))* (xp2/(dfres-(2/3)+(.11/dfres)))))-1)).
compute modres={modres,seb,trat,p}.
compute modres={modres,(b-tval&*seb),(b+tval&*seb)}.
compute modresl={'coeff',hclab,'t','p','LLCI','ULCI'}.
compute lmat = ident(ncol( x )).
compute lmat = lmat(:,2:ncol(lmat)).
compute fratio = (t(t(lmat)*b)*inv(t(lmat)*varb*lmat)*((t(lmat)*b)))/(ncol( x )-1).
compute pfr = 1-fcdf(fratio,(ncol( x )-1),dfres).
compute modsum={sqrt(r2),r2,mse,fratio,(ncol( x )-1),dfres,pfr}.
compute modsuml={'R','R-sq','MSE',hcflab,'df1','df2', 'p'}.
end if.
end if.
do if ( 1 = 2 or 1 =3).
compute xlp= x.
compute ylp= y.
compute pt2 = make(nrow(ylp),1,(csum(ylp)/nrow(ylp))).
do if ( 1 =2).
compute LL3 = ylp&*ln(pt2)+(1-ylp)&*ln(1-pt2).
end if.
compute LL3 = -2*csum(LL3).
compute bt1 = make(ncol(xlp),1,0).
compute LL1 = 0.
compute pt1 = make(nrow(ylp),1,0.5).
compute pt1lp=pt1.
loop jjj = 1 to iterate.
compute xlptmp=t(xlp).
compute vecprb=pt1lp&*(1-pt1lp).
loop kkk=1 to ncol(xlp).
compute xlptmp(kkk,:)=xlptmp(kkk,:)&*t(vecprb).
end loop.
compute b = bt1+inv(xlptmp*xlp)*t(xlp)*(ylp-pt1lp).
do if ( 1 =2).
compute xlpb=xlp*b.
compute xlpbt=(xlpb > -709.7).
compute xlpb709=(1-xlpbt)*(-709.7).
compute xlpb=(xlpb&*xlpbt)+xlpb709.
compute pt1lp = 1/(1+exp(-(xlpb))).
end if.
compute itprob = csum((pt1lp < .00000001) or (pt1lp > .9999999)).
do if (itprob > 0).
loop kkk = 1 to nrow(pt1lp).
do if (pt1lp(kkk,1) > .9999999).
compute pt1lp(kkk,1) = .9999999.
end if.
do if (pt1lp(kkk,1) < .00000001).
compute pt1lp(kkk,1) = .00000001.
end if.
end loop.
compute itprob = 0.
end if.
do if (itprob = 0).
do if ( 1 =2).
compute LL = ylp&*ln(pt1lp)+(1-ylp)&*ln(1-pt1lp).
end if.
compute LL2 = -2*csum(ll).
end if.
do if (abs(LL1-LL2) < converge).
do if ( 1 =1).
compute xlptmp=t(xlp).
compute vecprb=pt1lp&*(1-pt1lp).
loop kkk=1 to ncol(xlp).
compute xlptmp(kkk,:)=xlptmp(kkk,:)&*t(vecprb).
end loop.
compute varb = inv(xlptmp*xlp).
compute seb = sqrt(diag(varb)).
release xlptmp.
end if.
break.
end if.
compute bt1 = b.
compute LL1 = LL2.
end loop.
compute modres=b.
do if (jjj > iterate).
compute itprob = 2.
do if (booting=0).
compute iterrmod=1.
end if.
do if (booting=1).
compute bootiter=1.
end if.
do if (itprobtg=0).
compute itprobtg=1.
compute errcode(errs,1) = 47.
compute errs = errs + 1.
do if (booting = 0 and 1 =1).
compute vt1 = mdiag(pt1lp&*(1-pt1lp)).
compute varb = inv(t(xlp)*vt1*xlp).
compute seb = sqrt(diag(varb)).
end if.
end if.
end if.
do if ( 1 =1).
compute trat = b&/seb.
compute dfres=nrow(xlp).
compute p = 2*(1-cdfnorm(abs(trat))).
compute modres={modres,seb,trat,p}.
compute modres={modres,(b-xp2&*seb),(b+xp2&*seb)}.
compute pvchi=1-chicdf((LL3-LL2),(nrow(modres)-1)).
compute mcF = (LL3-LL2)/LL3.
compute cox = 1-exp(-(LL3-LL2)/nrow(xlp)).
compute nagel = cox/(1-exp(-(LL3)/nrow(xlp))).
compute modsum={LL2,(LL3-LL2),(nrow(modres)-1),pvchi, mcF,cox,nagel}.
compute modsuml={'-2LL','ModelLL', 'df', 'p', 'McFadden', 'CoxSnell', 'Nagelkrk'}.
compute modresl={'coeff','se','Z','p','LLCI','ULCI'}.
end if.
end if
.
compute dfmatt=make(nrow(modres),1,modsum(1,6)).
compute modresid={modresid,resid}.
end if.
do if (ydich=1 and (i = (nms+nys))).

.
do if ( 2 =1).
compute hatmat=inv(t( x )* x )*t( x ).
compute b = hatmat* y.
compute modres=b.
do if ( 1 =1).
compute n1=nrow( x ).
compute dfres=n1-(ncol( x )).
compute sstotal = t( y -(csum( y )/n1))*( y -(csum( y )/n1)).
compute resid= y - x *b.
compute ssresid = csum((resid)&**2).
compute r2 = (sstotal-ssresid)/sstotal.
do if (r2 < 0).
compute r2=0.
end if.
compute adjr2 = 1-((1-r2)*(n1-1)/(dfres)).
compute mse=ssresid/(n1-ncol( x )).

.
compute n1=nrow( x ).
compute invXtX = inv(t( x )* x ).
compute varb = mse *invXtX.
compute k3 = ncol( x ).
compute xhc=0.
do if ( hc <> 5).
compute xhc= x.
compute hat = xhc(:,1).
loop i3=1 to nrow(xhc).
compute hat(i3,1)= xhc(i3,:)*invXtX*t(xhc(i3,:)).
end loop.
do if ( hc = 0 or hc =1).
loop i3 = 1 to k3.
compute xhc(:,i3)=xhc(:,i3)&* resid.
end loop.
end if.
do if ( hc =3 or hc =2).
loop i3=1 to k3.
compute xhc(:,i3) = ( resid &/(1-hat)&**(1/(4- hc )))&*xhc(:,i3).
end loop.
end if.
do if ( hc = 4).
compute hcmn=make(n,2,4).
compute hcmn(:,2)=(n1*hat)/k3.
loop i3= 1 to k3.
compute xhc(:,i3) = ( resid &/(1-hat)&**(rmin(hcmn)/2))&*xhc(:,i3).
end loop.
end if.
compute varb=(invXtX*t(xhc)*xhc*invXtX).
do if ( hc =1).
compute varb=(n1/(n1-ncol( x )))&*varb.
end if.
end if.
compute hclab={'se(HC0)','se(HC1)','se(HC2)','se(HC3)','se(HC4)','se'}.
compute hclab=hclab(1,( hc +1)).
compute hcflab={'F(HC0)','F(HC1)','F(HC2)','F(HC3)','F(HC4)','F'}.
compute hcflab=hcflab(1,( hc +1)).
release xhc
.
compute seb=sqrt(diag(varb)).
compute trat = b&/seb.
compute p = 2*(1-tcdf(abs(trat), (dfres))).
compute tval = sqrt(dfres* (exp((dfres-(5/6))*((xp2/(dfres-(2/3)+(.11/dfres)))* (xp2/(dfres-(2/3)+(.11/dfres)))))-1)).
compute modres={modres,seb,trat,p}.
compute modres={modres,(b-tval&*seb),(b+tval&*seb)}.
compute modresl={'coeff',hclab,'t','p','LLCI','ULCI'}.
compute lmat = ident(ncol( x )).
compute lmat = lmat(:,2:ncol(lmat)).
compute fratio = (t(t(lmat)*b)*inv(t(lmat)*varb*lmat)*((t(lmat)*b)))/(ncol( x )-1).
compute pfr = 1-fcdf(fratio,(ncol( x )-1),dfres).
compute modsum={sqrt(r2),r2,mse,fratio,(ncol( x )-1),dfres,pfr}.
compute modsuml={'R','R-sq','MSE',hcflab,'df1','df2', 'p'}.
end if.
end if.
do if ( 2 = 2 or 2 =3).
compute xlp= x.
compute ylp= y.
compute pt2 = make(nrow(ylp),1,(csum(ylp)/nrow(ylp))).
do if ( 2 =2).
compute LL3 = ylp&*ln(pt2)+(1-ylp)&*ln(1-pt2).
end if.
compute LL3 = -2*csum(LL3).
compute bt1 = make(ncol(xlp),1,0).
compute LL1 = 0.
compute pt1 = make(nrow(ylp),1,0.5).
compute pt1lp=pt1.
loop jjj = 1 to iterate.
compute xlptmp=t(xlp).
compute vecprb=pt1lp&*(1-pt1lp).
loop kkk=1 to ncol(xlp).
compute xlptmp(kkk,:)=xlptmp(kkk,:)&*t(vecprb).
end loop.
compute b = bt1+inv(xlptmp*xlp)*t(xlp)*(ylp-pt1lp).
do if ( 2 =2).
compute xlpb=xlp*b.
compute xlpbt=(xlpb > -709.7).
compute xlpb709=(1-xlpbt)*(-709.7).
compute xlpb=(xlpb&*xlpbt)+xlpb709.
compute pt1lp = 1/(1+exp(-(xlpb))).
end if.
compute itprob = csum((pt1lp < .00000001) or (pt1lp > .9999999)).
do if (itprob > 0).
loop kkk = 1 to nrow(pt1lp).
do if (pt1lp(kkk,1) > .9999999).
compute pt1lp(kkk,1) = .9999999.
end if.
do if (pt1lp(kkk,1) < .00000001).
compute pt1lp(kkk,1) = .00000001.
end if.
end loop.
compute itprob = 0.
end if.
do if (itprob = 0).
do if ( 2 =2).
compute LL = ylp&*ln(pt1lp)+(1-ylp)&*ln(1-pt1lp).
end if.
compute LL2 = -2*csum(ll).
end if.
do if (abs(LL1-LL2) < converge).
do if ( 1 =1).
compute xlptmp=t(xlp).
compute vecprb=pt1lp&*(1-pt1lp).
loop kkk=1 to ncol(xlp).
compute xlptmp(kkk,:)=xlptmp(kkk,:)&*t(vecprb).
end loop.
compute varb = inv(xlptmp*xlp).
compute seb = sqrt(diag(varb)).
release xlptmp.
end if.
break.
end if.
compute bt1 = b.
compute LL1 = LL2.
end loop.
compute modres=b.
do if (jjj > iterate).
compute itprob = 2.
do if (booting=0).
compute iterrmod=1.
end if.
do if (booting=1).
compute bootiter=1.
end if.
do if (itprobtg=0).
compute itprobtg=1.
compute errcode(errs,1) = 47.
compute errs = errs + 1.
do if (booting = 0 and 1 =1).
compute vt1 = mdiag(pt1lp&*(1-pt1lp)).
compute varb = inv(t(xlp)*vt1*xlp).
compute seb = sqrt(diag(varb)).
end if.
end if.
end if.
do if ( 1 =1).
compute trat = b&/seb.
compute dfres=nrow(xlp).
compute p = 2*(1-cdfnorm(abs(trat))).
compute modres={modres,seb,trat,p}.
compute modres={modres,(b-xp2&*seb),(b+xp2&*seb)}.
compute pvchi=1-chicdf((LL3-LL2),(nrow(modres)-1)).
compute mcF = (LL3-LL2)/LL3.
compute cox = 1-exp(-(LL3-LL2)/nrow(xlp)).
compute nagel = cox/(1-exp(-(LL3)/nrow(xlp))).
compute modsum={LL2,(LL3-LL2),(nrow(modres)-1),pvchi, mcF,cox,nagel}.
compute modsuml={'-2LL','ModelLL', 'df', 'p', 'McFadden', 'CoxSnell', 'Nagelkrk'}.
compute modresl={'coeff','se','Z','p','LLCI','ULCI'}.
end if.
end if
.
compute dfmatt=make(nrow(modres),1,-999).
end if.
compute resultm2=make(1,maxresm,99999).
compute resultm2(1,1:ncol(modsum))=modsum.
compute resultm={resultm;resultm2}.
compute resultm2=make(nrow(modres),maxresm,99999).
compute resultm2(1:nrow(modres),1:ncol(modres))=modres.
compute resultm={resultm;resultm2}.
compute obscoeff={obscoeff,t(b)}.
do if (outscree=1).
print modsum/title = 'Model Summary'/cnames = modsuml/format= F10.4.
print modres/title='Model'/rnames=vlabsm/cnames=modresl/format= F10.4.
end if.
compute basemod=modsum(1,1).
compute basemodx=basemod.
do if (ydich=1 and (i = (nms+nys))).
do if (outscree=1).
print/title='These results are expressed in a log-odds metric.'.
end if.
compute notecode(notes,1) = 26.
compute notes = notes + 1.
end if.
compute coeffmat={coeffmat;modres}.
compute conseqt=make(nrow(modres),1,outnames(1,i)).
compute conseq={conseq;conseqt}.
compute dfmat={dfmat;dfmatt}.
compute labstart=labstart+nump(1,i).
do if (stand=1).
compute predsd=make(nrow(modres),1,0).
compute stdmod=modres(:,1)&/ovsd(1,i).
loop jd=1 to ncol(x).
compute descdat=x(:,jd).
compute predsd(jd,1) = (nrow(descdat)*sscp(descdat))-(t(csum(descdat))*(csum(descdat))).
compute predsd(jd,1) = sqrt(predsd(jd,1)/(nrow(descdat)*(nrow(descdat)-1))).
end loop.
do if (wherex(1,i) <> -999 and ((nxvls > 1) or (xdich=1))).
compute sdmsone=make(nxvls,1,1).
compute predsd(wherex(1,i):wherex(2,i),1)=sdmsone.
compute pstog=1.
end if.
compute predsd(1,1)=1.
compute stdmod=stdmod&*predsd.
compute stdmod=stdmod(2:nrow(stdmod),1).
compute sdvlabs=vlabsm(2:nrow(vlabsm),1).
compute resultm2=make(nrow(stdmod),maxresm,99999).
compute resultm2(1:nrow(stdmod),1:ncol(stdmod))=stdmod.
compute resultm={resultm;resultm2}.
do if (outscree=1).
print stdmod/title='Standardized coefficients'/clabels='coeff'/rnames=sdvlabs/format= F10.4.
end if.
end if.
do if (nms > 0 and serial = 0 and (rsum(numint) = 0) and (normal=1 or mc > 0)).
do if (i < (nms+nys)).
compute indcov((((i-1)*nxvls)+1):(i*nxvls),(((i-1)*nxvls)+1):(i*nxvls))=varb(2:(1+nxvls), 2:(1+nxvls)).
compute mcsopath((((i-1)*nxvls)+1):(i*nxvls) ,1)=modres(2:(1+nxvls),1).
end if.
do if (i = (nms+nys)).
compute atm=ncol(wherem).
compute indcov(((nms*nxvls)+1):nrow(mcsopath),((nms*nxvls)+1):nrow(mcsopath))= varb(wherem(1,atm):(wherem(1,atm)+nms-1),wherem(1,atm):(wherem(1,atm)+nms-1)).
compute mcsopath(((nms*nxvls)+1):nrow(mcsopath),1)=modres(wherem(1,atm):(wherem(1,atm)+ nms-1),1).
compute sobelok=1.
end if.
end if.
compute obsdirfx=make(1,nxvls,0).
compute dirzes=make(1,nxvls,0).
do if ((i = (nms+nys)) and (bcmat(nrow(bcmat),1)=1)).
compute direff=modres(2:(1+nxvls),:).
compute obsdirfx=t(direff(:,1)).
compute direfflb=modresl.
compute direffl2=vlabsm(2:(1+nxvls),:).
compute lmat=make(nrow(b),1,0).
compute lmat2=make(nxvls,1,1).
compute lmat(2:(1+nxvls),1)=lmat2.
do if (ydich <> 1).

.
compute lmat2= lmat.
do if ( 0 =0).
compute lmat2 = mdiag( lmat ).
compute lmat3=make(nrow(lmat2),1,0).
loop flp=1 to ncol(lmat2).
do if (csum(lmat2(:,flp))=1).
compute lmat3={lmat3,lmat2(:,flp)}.
end if.
end loop.
compute lmat2=lmat3(:,2:ncol(lmat3)).
end if.
compute fratio = (t(t(lmat2)* b )*inv(t(lmat2)* varb *lmat2)*((t(lmat2)* b )))/ncol(lmat2).
compute pfr = 1-fcdf(fratio,ncol(lmat2),(n-nrow( b ))).
compute fresult={fratio,ncol(lmat2),(n-nrow( b )),pfr}.
do if (i = (nms+nys) and (ydich=1)).
compute fratio=fratio*ncol(lmat2).
compute pfr=1-chicdf(fratio,ncol(lmat2)).
compute fresult={fratio,ncol(lmat2),pfr}.
end if.
do if ( 1 =1).
compute lmat3=1-rsum(lmat2).
compute xfm=make(n,csum(lmat3),0).
compute flpc=1.
loop flp=1 to nrow(lmat3).
do if (lmat3(flp,1)=1).
compute xfm(:,flpc)=x(:,flp).
compute flpc=flpc+1.
end if.
end loop.
compute bfm=inv(t(xfm)*xfm)*t(xfm)*y.
compute resid=y-(xfm*bfm).
compute sstotal=(y-(csum(y)/n)).
compute sstotal=csum(sstotal&*sstotal).
compute ssresid=csum(resid&*resid).
compute rsqch= r2 -((sstotal-ssresid)/sstotal).
compute fresult={rsqch,fresult}.
release xfm,flpc, resid, ssresid, bfm.
end if
.
compute diromni=fresult.
end if.
do if (ydich = 1 ).

.
compute btemphld=b.
compute llrdat=make(nrow(x),nrow( lmat )-csum( lmat ),-999).
compute llrdf=ncol(x)-ncol(llrdat).
compute llrcnt=0.
loop llri=1 to nrow( lmat ).
do if ( lmat (llri,1)=0).
compute llrcnt=llrcnt+1.
compute llrdat(:,llrcnt)=x(:,llri).
end if.
end loop.

.
do if ( 2 =1).
compute hatmat=inv(t( llrdat )* llrdat )*t( llrdat ).
compute b = hatmat* y.
compute modres=b.
do if ( 0 =1).
compute n1=nrow( llrdat ).
compute dfres=n1-(ncol( llrdat )).
compute sstotal = t( y -(csum( y )/n1))*( y -(csum( y )/n1)).
compute resid= y - llrdat *b.
compute ssresid = csum((resid)&**2).
compute r2 = (sstotal-ssresid)/sstotal.
do if (r2 < 0).
compute r2=0.
end if.
compute adjr2 = 1-((1-r2)*(n1-1)/(dfres)).
compute mse=ssresid/(n1-ncol( llrdat )).

.
compute n1=nrow( x ).
compute invXtX = inv(t( x )* x ).
compute varb = mse *invXtX.
compute k3 = ncol( x ).
compute xhc=0.
do if ( hc <> 5).
compute xhc= x.
compute hat = xhc(:,1).
loop i3=1 to nrow(xhc).
compute hat(i3,1)= xhc(i3,:)*invXtX*t(xhc(i3,:)).
end loop.
do if ( hc = 0 or hc =1).
loop i3 = 1 to k3.
compute xhc(:,i3)=xhc(:,i3)&* resid.
end loop.
end if.
do if ( hc =3 or hc =2).
loop i3=1 to k3.
compute xhc(:,i3) = ( resid &/(1-hat)&**(1/(4- hc )))&*xhc(:,i3).
end loop.
end if.
do if ( hc = 4).
compute hcmn=make(n,2,4).
compute hcmn(:,2)=(n1*hat)/k3.
loop i3= 1 to k3.
compute xhc(:,i3) = ( resid &/(1-hat)&**(rmin(hcmn)/2))&*xhc(:,i3).
end loop.
end if.
compute varb=(invXtX*t(xhc)*xhc*invXtX).
do if ( hc =1).
compute varb=(n1/(n1-ncol( x )))&*varb.
end if.
end if.
compute hclab={'se(HC0)','se(HC1)','se(HC2)','se(HC3)','se(HC4)','se'}.
compute hclab=hclab(1,( hc +1)).
compute hcflab={'F(HC0)','F(HC1)','F(HC2)','F(HC3)','F(HC4)','F'}.
compute hcflab=hcflab(1,( hc +1)).
release xhc
.
compute seb=sqrt(diag(varb)).
compute trat = b&/seb.
compute p = 2*(1-tcdf(abs(trat), (dfres))).
compute tval = sqrt(dfres* (exp((dfres-(5/6))*((xp2/(dfres-(2/3)+(.11/dfres)))* (xp2/(dfres-(2/3)+(.11/dfres)))))-1)).
compute modres={modres,seb,trat,p}.
compute modres={modres,(b-tval&*seb),(b+tval&*seb)}.
compute modresl={'coeff',hclab,'t','p','LLCI','ULCI'}.
compute lmat = ident(ncol( llrdat )).
compute lmat = lmat(:,2:ncol(lmat)).
compute fratio = (t(t(lmat)*b)*inv(t(lmat)*varb*lmat)*((t(lmat)*b)))/(ncol( llrdat )-1).
compute pfr = 1-fcdf(fratio,(ncol( llrdat )-1),dfres).
compute modsum={sqrt(r2),r2,mse,fratio,(ncol( llrdat )-1),dfres,pfr}.
compute modsuml={'R','R-sq','MSE',hcflab,'df1','df2', 'p'}.
end if.
end if.
do if ( 2 = 2 or 2 =3).
compute xlp= llrdat.
compute ylp= y.
compute pt2 = make(nrow(ylp),1,(csum(ylp)/nrow(ylp))).
do if ( 2 =2).
compute LL3 = ylp&*ln(pt2)+(1-ylp)&*ln(1-pt2).
end if.
compute LL3 = -2*csum(LL3).
compute bt1 = make(ncol(xlp),1,0).
compute LL1 = 0.
compute pt1 = make(nrow(ylp),1,0.5).
compute pt1lp=pt1.
loop jjj = 1 to iterate.
compute xlptmp=t(xlp).
compute vecprb=pt1lp&*(1-pt1lp).
loop kkk=1 to ncol(xlp).
compute xlptmp(kkk,:)=xlptmp(kkk,:)&*t(vecprb).
end loop.
compute b = bt1+inv(xlptmp*xlp)*t(xlp)*(ylp-pt1lp).
do if ( 2 =2).
compute xlpb=xlp*b.
compute xlpbt=(xlpb > -709.7).
compute xlpb709=(1-xlpbt)*(-709.7).
compute xlpb=(xlpb&*xlpbt)+xlpb709.
compute pt1lp = 1/(1+exp(-(xlpb))).
end if.
compute itprob = csum((pt1lp < .00000001) or (pt1lp > .9999999)).
do if (itprob > 0).
loop kkk = 1 to nrow(pt1lp).
do if (pt1lp(kkk,1) > .9999999).
compute pt1lp(kkk,1) = .9999999.
end if.
do if (pt1lp(kkk,1) < .00000001).
compute pt1lp(kkk,1) = .00000001.
end if.
end loop.
compute itprob = 0.
end if.
do if (itprob = 0).
do if ( 2 =2).
compute LL = ylp&*ln(pt1lp)+(1-ylp)&*ln(1-pt1lp).
end if.
compute LL2 = -2*csum(ll).
end if.
do if (abs(LL1-LL2) < converge).
do if ( 0 =1).
compute xlptmp=t(xlp).
compute vecprb=pt1lp&*(1-pt1lp).
loop kkk=1 to ncol(xlp).
compute xlptmp(kkk,:)=xlptmp(kkk,:)&*t(vecprb).
end loop.
compute varb = inv(xlptmp*xlp).
compute seb = sqrt(diag(varb)).
release xlptmp.
end if.
break.
end if.
compute bt1 = b.
compute LL1 = LL2.
end loop.
compute modres=b.
do if (jjj > iterate).
compute itprob = 2.
do if (booting=0).
compute iterrmod=1.
end if.
do if (booting=1).
compute bootiter=1.
end if.
do if (itprobtg=0).
compute itprobtg=1.
compute errcode(errs,1) = 47.
compute errs = errs + 1.
do if (booting = 0 and 0 =1).
compute vt1 = mdiag(pt1lp&*(1-pt1lp)).
compute varb = inv(t(xlp)*vt1*xlp).
compute seb = sqrt(diag(varb)).
end if.
end if.
end if.
do if ( 0 =1).
compute trat = b&/seb.
compute dfres=nrow(xlp).
compute p = 2*(1-cdfnorm(abs(trat))).
compute modres={modres,seb,trat,p}.
compute modres={modres,(b-xp2&*seb),(b+xp2&*seb)}.
compute pvchi=1-chicdf((LL3-LL2),(nrow(modres)-1)).
compute mcF = (LL3-LL2)/LL3.
compute cox = 1-exp(-(LL3-LL2)/nrow(xlp)).
compute nagel = cox/(1-exp(-(LL3)/nrow(xlp))).
compute modsum={LL2,(LL3-LL2),(nrow(modres)-1),pvchi, mcF,cox,nagel}.
compute modsuml={'-2LL','ModelLL', 'df', 'p', 'McFadden', 'CoxSnell', 'Nagelkrk'}.
compute modresl={'coeff','se','Z','p','LLCI','ULCI'}.
end if.
end if
.
compute b=btemphld.
compute fresult={(LL2-basemod),llrdf,1-chicdf((LL2-basemod),llrdf)}
.
compute diromni=fresult.
end if.
end if.
do if (numint(1,i) > 0).
compute intkeym=intkey(intstart:(intstart+numint(1,i)-1),:).
do if (outscree=1).
print intkeym/title='Product terms key:'/format = A8.
end if.
end if.
do if (covcoeff=1).
do if (outscree=1).
print varb/title='Covariance matrix of regression parameter estimates:'/rnames=vlabsm /cnames=vlabsm/format= F10.4.
end if.
compute resultm2=make(nrow(varb),maxresm,99999).
do if (ncol(varb) <= maxresm).
compute resultm2(1:nrow(varb),1:ncol(varb))=varb.
compute resultm={resultm;resultm2}.
end if.
do if (ncol(varb) > maxresm).
compute resultmt=make(nrow(resultm),ncol(varb),99999).
compute resultmt(1:nrow(resultm),1:ncol(resultm))=resultm.
compute resultm=resultmt.
compute resultm2=make(nrow(varb),ncol(resultm),99999).
compute resultm2(1:nrow(varb),1:ncol(varb))=varb.
compute resultm={resultm;resultm2}.
compute maxresm=ncol(resultm).
end if.
end if.
do if (model <> 74 and xmtest=1 and nms > 0).
compute r2tmp=r2.
compute btmp=b.
compute varbtmp=varb.
compute dfrestmp=dfres.
compute tvaltmp=tval.
compute xmtst=make(nms,4,0).
compute xmtstlbc={hcflab,'df1','df2','p'}.
do if ((i=(nms+nys)) and (ydich=1)).
compute xmtst=make(nms,3,0).
compute xmtstlbc={'Chi-sq','df','p'}.
end if.
compute xmtstlb={' '}.
compute xmtmat=x.
compute numxint=0.
loop xmints=2 to i.
compute x=xmtmat.
do if ((bcmat((i+1),xmints)=1) and (wzcmat((i+1),xmints) <> 1)).
do if (bcmat((i+1),1)=0).
compute x={xmtmat,xtmp}.
do if ((ydich=1) and (i=(nms+nys))).

.
do if ( 2 =1).
compute hatmat=inv(t( x )* x )*t( x ).
compute b = hatmat* y.
compute modres=b.
do if ( 1 =1).
compute n1=nrow( x ).
compute dfres=n1-(ncol( x )).
compute sstotal = t( y -(csum( y )/n1))*( y -(csum( y )/n1)).
compute resid= y - x *b.
compute ssresid = csum((resid)&**2).
compute r2 = (sstotal-ssresid)/sstotal.
do if (r2 < 0).
compute r2=0.
end if.
compute adjr2 = 1-((1-r2)*(n1-1)/(dfres)).
compute mse=ssresid/(n1-ncol( x )).

.
compute n1=nrow( x ).
compute invXtX = inv(t( x )* x ).
compute varb = mse *invXtX.
compute k3 = ncol( x ).
compute xhc=0.
do if ( hc <> 5).
compute xhc= x.
compute hat = xhc(:,1).
loop i3=1 to nrow(xhc).
compute hat(i3,1)= xhc(i3,:)*invXtX*t(xhc(i3,:)).
end loop.
do if ( hc = 0 or hc =1).
loop i3 = 1 to k3.
compute xhc(:,i3)=xhc(:,i3)&* resid.
end loop.
end if.
do if ( hc =3 or hc =2).
loop i3=1 to k3.
compute xhc(:,i3) = ( resid &/(1-hat)&**(1/(4- hc )))&*xhc(:,i3).
end loop.
end if.
do if ( hc = 4).
compute hcmn=make(n,2,4).
compute hcmn(:,2)=(n1*hat)/k3.
loop i3= 1 to k3.
compute xhc(:,i3) = ( resid &/(1-hat)&**(rmin(hcmn)/2))&*xhc(:,i3).
end loop.
end if.
compute varb=(invXtX*t(xhc)*xhc*invXtX).
do if ( hc =1).
compute varb=(n1/(n1-ncol( x )))&*varb.
end if.
end if.
compute hclab={'se(HC0)','se(HC1)','se(HC2)','se(HC3)','se(HC4)','se'}.
compute hclab=hclab(1,( hc +1)).
compute hcflab={'F(HC0)','F(HC1)','F(HC2)','F(HC3)','F(HC4)','F'}.
compute hcflab=hcflab(1,( hc +1)).
release xhc
.
compute seb=sqrt(diag(varb)).
compute trat = b&/seb.
compute p = 2*(1-tcdf(abs(trat), (dfres))).
compute tval = sqrt(dfres* (exp((dfres-(5/6))*((xp2/(dfres-(2/3)+(.11/dfres)))* (xp2/(dfres-(2/3)+(.11/dfres)))))-1)).
compute modres={modres,seb,trat,p}.
compute modres={modres,(b-tval&*seb),(b+tval&*seb)}.
compute modresl={'coeff',hclab,'t','p','LLCI','ULCI'}.
compute lmat = ident(ncol( x )).
compute lmat = lmat(:,2:ncol(lmat)).
compute fratio = (t(t(lmat)*b)*inv(t(lmat)*varb*lmat)*((t(lmat)*b)))/(ncol( x )-1).
compute pfr = 1-fcdf(fratio,(ncol( x )-1),dfres).
compute modsum={sqrt(r2),r2,mse,fratio,(ncol( x )-1),dfres,pfr}.
compute modsuml={'R','R-sq','MSE',hcflab,'df1','df2', 'p'}.
end if.
end if.
do if ( 2 = 2 or 2 =3).
compute xlp= x.
compute ylp= y.
compute pt2 = make(nrow(ylp),1,(csum(ylp)/nrow(ylp))).
do if ( 2 =2).
compute LL3 = ylp&*ln(pt2)+(1-ylp)&*ln(1-pt2).
end if.
compute LL3 = -2*csum(LL3).
compute bt1 = make(ncol(xlp),1,0).
compute LL1 = 0.
compute pt1 = make(nrow(ylp),1,0.5).
compute pt1lp=pt1.
loop jjj = 1 to iterate.
compute xlptmp=t(xlp).
compute vecprb=pt1lp&*(1-pt1lp).
loop kkk=1 to ncol(xlp).
compute xlptmp(kkk,:)=xlptmp(kkk,:)&*t(vecprb).
end loop.
compute b = bt1+inv(xlptmp*xlp)*t(xlp)*(ylp-pt1lp).
do if ( 2 =2).
compute xlpb=xlp*b.
compute xlpbt=(xlpb > -709.7).
compute xlpb709=(1-xlpbt)*(-709.7).
compute xlpb=(xlpb&*xlpbt)+xlpb709.
compute pt1lp = 1/(1+exp(-(xlpb))).
end if.
compute itprob = csum((pt1lp < .00000001) or (pt1lp > .9999999)).
do if (itprob > 0).
loop kkk = 1 to nrow(pt1lp).
do if (pt1lp(kkk,1) > .9999999).
compute pt1lp(kkk,1) = .9999999.
end if.
do if (pt1lp(kkk,1) < .00000001).
compute pt1lp(kkk,1) = .00000001.
end if.
end loop.
compute itprob = 0.
end if.
do if (itprob = 0).
do if ( 2 =2).
compute LL = ylp&*ln(pt1lp)+(1-ylp)&*ln(1-pt1lp).
end if.
compute LL2 = -2*csum(ll).
end if.
do if (abs(LL1-LL2) < converge).
do if ( 1 =1).
compute xlptmp=t(xlp).
compute vecprb=pt1lp&*(1-pt1lp).
loop kkk=1 to ncol(xlp).
compute xlptmp(kkk,:)=xlptmp(kkk,:)&*t(vecprb).
end loop.
compute varb = inv(xlptmp*xlp).
compute seb = sqrt(diag(varb)).
release xlptmp.
end if.
break.
end if.
compute bt1 = b.
compute LL1 = LL2.
end loop.
compute modres=b.
do if (jjj > iterate).
compute itprob = 2.
do if (booting=0).
compute iterrmod=1.
end if.
do if (booting=1).
compute bootiter=1.
end if.
do if (itprobtg=0).
compute itprobtg=1.
compute errcode(errs,1) = 47.
compute errs = errs + 1.
do if (booting = 0 and 1 =1).
compute vt1 = mdiag(pt1lp&*(1-pt1lp)).
compute varb = inv(t(xlp)*vt1*xlp).
compute seb = sqrt(diag(varb)).
end if.
end if.
end if.
do if ( 1 =1).
compute trat = b&/seb.
compute dfres=nrow(xlp).
compute p = 2*(1-cdfnorm(abs(trat))).
compute modres={modres,seb,trat,p}.
compute modres={modres,(b-xp2&*seb),(b+xp2&*seb)}.
compute pvchi=1-chicdf((LL3-LL2),(nrow(modres)-1)).
compute mcF = (LL3-LL2)/LL3.
compute cox = 1-exp(-(LL3-LL2)/nrow(xlp)).
compute nagel = cox/(1-exp(-(LL3)/nrow(xlp))).
compute modsum={LL2,(LL3-LL2),(nrow(modres)-1),pvchi, mcF,cox,nagel}.
compute modsuml={'-2LL','ModelLL', 'df', 'p', 'McFadden', 'CoxSnell', 'Nagelkrk'}.
compute modresl={'coeff','se','Z','p','LLCI','ULCI'}.
end if.
end if
.
compute basemodx=LL2.
end if.
end if.
loop xmtlp1=1 to nxvls.
compute x={x,xtmp(:,xmtlp1)&*(mtmp(:,(xmints-1))-(csum(mtmp(:,(xmints-1)))/nrow(mtmp)) )}.
end loop.
do if ((i < (nms+nys)) or (ydich=0)).

.
do if ( 1 =1).
compute hatmat=inv(t( x )* x )*t( x ).
compute b = hatmat* y.
compute modres=b.
do if ( 1 =1).
compute n1=nrow( x ).
compute dfres=n1-(ncol( x )).
compute sstotal = t( y -(csum( y )/n1))*( y -(csum( y )/n1)).
compute resid= y - x *b.
compute ssresid = csum((resid)&**2).
compute r2 = (sstotal-ssresid)/sstotal.
do if (r2 < 0).
compute r2=0.
end if.
compute adjr2 = 1-((1-r2)*(n1-1)/(dfres)).
compute mse=ssresid/(n1-ncol( x )).

.
compute n1=nrow( x ).
compute invXtX = inv(t( x )* x ).
compute varb = mse *invXtX.
compute k3 = ncol( x ).
compute xhc=0.
do if ( hc <> 5).
compute xhc= x.
compute hat = xhc(:,1).
loop i3=1 to nrow(xhc).
compute hat(i3,1)= xhc(i3,:)*invXtX*t(xhc(i3,:)).
end loop.
do if ( hc = 0 or hc =1).
loop i3 = 1 to k3.
compute xhc(:,i3)=xhc(:,i3)&* resid.
end loop.
end if.
do if ( hc =3 or hc =2).
loop i3=1 to k3.
compute xhc(:,i3) = ( resid &/(1-hat)&**(1/(4- hc )))&*xhc(:,i3).
end loop.
end if.
do if ( hc = 4).
compute hcmn=make(n,2,4).
compute hcmn(:,2)=(n1*hat)/k3.
loop i3= 1 to k3.
compute xhc(:,i3) = ( resid &/(1-hat)&**(rmin(hcmn)/2))&*xhc(:,i3).
end loop.
end if.
compute varb=(invXtX*t(xhc)*xhc*invXtX).
do if ( hc =1).
compute varb=(n1/(n1-ncol( x )))&*varb.
end if.
end if.
compute hclab={'se(HC0)','se(HC1)','se(HC2)','se(HC3)','se(HC4)','se'}.
compute hclab=hclab(1,( hc +1)).
compute hcflab={'F(HC0)','F(HC1)','F(HC2)','F(HC3)','F(HC4)','F'}.
compute hcflab=hcflab(1,( hc +1)).
release xhc
.
compute seb=sqrt(diag(varb)).
compute trat = b&/seb.
compute p = 2*(1-tcdf(abs(trat), (dfres))).
compute tval = sqrt(dfres* (exp((dfres-(5/6))*((xp2/(dfres-(2/3)+(.11/dfres)))* (xp2/(dfres-(2/3)+(.11/dfres)))))-1)).
compute modres={modres,seb,trat,p}.
compute modres={modres,(b-tval&*seb),(b+tval&*seb)}.
compute modresl={'coeff',hclab,'t','p','LLCI','ULCI'}.
compute lmat = ident(ncol( x )).
compute lmat = lmat(:,2:ncol(lmat)).
compute fratio = (t(t(lmat)*b)*inv(t(lmat)*varb*lmat)*((t(lmat)*b)))/(ncol( x )-1).
compute pfr = 1-fcdf(fratio,(ncol( x )-1),dfres).
compute modsum={sqrt(r2),r2,mse,fratio,(ncol( x )-1),dfres,pfr}.
compute modsuml={'R','R-sq','MSE',hcflab,'df1','df2', 'p'}.
end if.
end if.
do if ( 1 = 2 or 1 =3).
compute xlp= x.
compute ylp= y.
compute pt2 = make(nrow(ylp),1,(csum(ylp)/nrow(ylp))).
do if ( 1 =2).
compute LL3 = ylp&*ln(pt2)+(1-ylp)&*ln(1-pt2).
end if.
compute LL3 = -2*csum(LL3).
compute bt1 = make(ncol(xlp),1,0).
compute LL1 = 0.
compute pt1 = make(nrow(ylp),1,0.5).
compute pt1lp=pt1.
loop jjj = 1 to iterate.
compute xlptmp=t(xlp).
compute vecprb=pt1lp&*(1-pt1lp).
loop kkk=1 to ncol(xlp).
compute xlptmp(kkk,:)=xlptmp(kkk,:)&*t(vecprb).
end loop.
compute b = bt1+inv(xlptmp*xlp)*t(xlp)*(ylp-pt1lp).
do if ( 1 =2).
compute xlpb=xlp*b.
compute xlpbt=(xlpb > -709.7).
compute xlpb709=(1-xlpbt)*(-709.7).
compute xlpb=(xlpb&*xlpbt)+xlpb709.
compute pt1lp = 1/(1+exp(-(xlpb))).
end if.
compute itprob = csum((pt1lp < .00000001) or (pt1lp > .9999999)).
do if (itprob > 0).
loop kkk = 1 to nrow(pt1lp).
do if (pt1lp(kkk,1) > .9999999).
compute pt1lp(kkk,1) = .9999999.
end if.
do if (pt1lp(kkk,1) < .00000001).
compute pt1lp(kkk,1) = .00000001.
end if.
end loop.
compute itprob = 0.
end if.
do if (itprob = 0).
do if ( 1 =2).
compute LL = ylp&*ln(pt1lp)+(1-ylp)&*ln(1-pt1lp).
end if.
compute LL2 = -2*csum(ll).
end if.
do if (abs(LL1-LL2) < converge).
do if ( 1 =1).
compute xlptmp=t(xlp).
compute vecprb=pt1lp&*(1-pt1lp).
loop kkk=1 to ncol(xlp).
compute xlptmp(kkk,:)=xlptmp(kkk,:)&*t(vecprb).
end loop.
compute varb = inv(xlptmp*xlp).
compute seb = sqrt(diag(varb)).
release xlptmp.
end if.
break.
end if.
compute bt1 = b.
compute LL1 = LL2.
end loop.
compute modres=b.
do if (jjj > iterate).
compute itprob = 2.
do if (booting=0).
compute iterrmod=1.
end if.
do if (booting=1).
compute bootiter=1.
end if.
do if (itprobtg=0).
compute itprobtg=1.
compute errcode(errs,1) = 47.
compute errs = errs + 1.
do if (booting = 0 and 1 =1).
compute vt1 = mdiag(pt1lp&*(1-pt1lp)).
compute varb = inv(t(xlp)*vt1*xlp).
compute seb = sqrt(diag(varb)).
end if.
end if.
end if.
do if ( 1 =1).
compute trat = b&/seb.
compute dfres=nrow(xlp).
compute p = 2*(1-cdfnorm(abs(trat))).
compute modres={modres,seb,trat,p}.
compute modres={modres,(b-xp2&*seb),(b+xp2&*seb)}.
compute pvchi=1-chicdf((LL3-LL2),(nrow(modres)-1)).
compute mcF = (LL3-LL2)/LL3.
compute cox = 1-exp(-(LL3-LL2)/nrow(xlp)).
compute nagel = cox/(1-exp(-(LL3)/nrow(xlp))).
compute modsum={LL2,(LL3-LL2),(nrow(modres)-1),pvchi, mcF,cox,nagel}.
compute modsuml={'-2LL','ModelLL', 'df', 'p', 'McFadden', 'CoxSnell', 'Nagelkrk'}.
compute modresl={'coeff','se','Z','p','LLCI','ULCI'}.
end if.
end if
.
end if.
do if ((i = (nms+nys)) and (ydich=1)).

.
do if ( 2 =1).
compute hatmat=inv(t( x )* x )*t( x ).
compute b = hatmat* y.
compute modres=b.
do if ( 1 =1).
compute n1=nrow( x ).
compute dfres=n1-(ncol( x )).
compute sstotal = t( y -(csum( y )/n1))*( y -(csum( y )/n1)).
compute resid= y - x *b.
compute ssresid = csum((resid)&**2).
compute r2 = (sstotal-ssresid)/sstotal.
do if (r2 < 0).
compute r2=0.
end if.
compute adjr2 = 1-((1-r2)*(n1-1)/(dfres)).
compute mse=ssresid/(n1-ncol( x )).

.
compute n1=nrow( x ).
compute invXtX = inv(t( x )* x ).
compute varb = mse *invXtX.
compute k3 = ncol( x ).
compute xhc=0.
do if ( hc <> 5).
compute xhc= x.
compute hat = xhc(:,1).
loop i3=1 to nrow(xhc).
compute hat(i3,1)= xhc(i3,:)*invXtX*t(xhc(i3,:)).
end loop.
do if ( hc = 0 or hc =1).
loop i3 = 1 to k3.
compute xhc(:,i3)=xhc(:,i3)&* resid.
end loop.
end if.
do if ( hc =3 or hc =2).
loop i3=1 to k3.
compute xhc(:,i3) = ( resid &/(1-hat)&**(1/(4- hc )))&*xhc(:,i3).
end loop.
end if.
do if ( hc = 4).
compute hcmn=make(n,2,4).
compute hcmn(:,2)=(n1*hat)/k3.
loop i3= 1 to k3.
compute xhc(:,i3) = ( resid &/(1-hat)&**(rmin(hcmn)/2))&*xhc(:,i3).
end loop.
end if.
compute varb=(invXtX*t(xhc)*xhc*invXtX).
do if ( hc =1).
compute varb=(n1/(n1-ncol( x )))&*varb.
end if.
end if.
compute hclab={'se(HC0)','se(HC1)','se(HC2)','se(HC3)','se(HC4)','se'}.
compute hclab=hclab(1,( hc +1)).
compute hcflab={'F(HC0)','F(HC1)','F(HC2)','F(HC3)','F(HC4)','F'}.
compute hcflab=hcflab(1,( hc +1)).
release xhc
.
compute seb=sqrt(diag(varb)).
compute trat = b&/seb.
compute p = 2*(1-tcdf(abs(trat), (dfres))).
compute tval = sqrt(dfres* (exp((dfres-(5/6))*((xp2/(dfres-(2/3)+(.11/dfres)))* (xp2/(dfres-(2/3)+(.11/dfres)))))-1)).
compute modres={modres,seb,trat,p}.
compute modres={modres,(b-tval&*seb),(b+tval&*seb)}.
compute modresl={'coeff',hclab,'t','p','LLCI','ULCI'}.
compute lmat = ident(ncol( x )).
compute lmat = lmat(:,2:ncol(lmat)).
compute fratio = (t(t(lmat)*b)*inv(t(lmat)*varb*lmat)*((t(lmat)*b)))/(ncol( x )-1).
compute pfr = 1-fcdf(fratio,(ncol( x )-1),dfres).
compute modsum={sqrt(r2),r2,mse,fratio,(ncol( x )-1),dfres,pfr}.
compute modsuml={'R','R-sq','MSE',hcflab,'df1','df2', 'p'}.
end if.
end if.
do if ( 2 = 2 or 2 =3).
compute xlp= x.
compute ylp= y.
compute pt2 = make(nrow(ylp),1,(csum(ylp)/nrow(ylp))).
do if ( 2 =2).
compute LL3 = ylp&*ln(pt2)+(1-ylp)&*ln(1-pt2).
end if.
compute LL3 = -2*csum(LL3).
compute bt1 = make(ncol(xlp),1,0).
compute LL1 = 0.
compute pt1 = make(nrow(ylp),1,0.5).
compute pt1lp=pt1.
loop jjj = 1 to iterate.
compute xlptmp=t(xlp).
compute vecprb=pt1lp&*(1-pt1lp).
loop kkk=1 to ncol(xlp).
compute xlptmp(kkk,:)=xlptmp(kkk,:)&*t(vecprb).
end loop.
compute b = bt1+inv(xlptmp*xlp)*t(xlp)*(ylp-pt1lp).
do if ( 2 =2).
compute xlpb=xlp*b.
compute xlpbt=(xlpb > -709.7).
compute xlpb709=(1-xlpbt)*(-709.7).
compute xlpb=(xlpb&*xlpbt)+xlpb709.
compute pt1lp = 1/(1+exp(-(xlpb))).
end if.
compute itprob = csum((pt1lp < .00000001) or (pt1lp > .9999999)).
do if (itprob > 0).
loop kkk = 1 to nrow(pt1lp).
do if (pt1lp(kkk,1) > .9999999).
compute pt1lp(kkk,1) = .9999999.
end if.
do if (pt1lp(kkk,1) < .00000001).
compute pt1lp(kkk,1) = .00000001.
end if.
end loop.
compute itprob = 0.
end if.
do if (itprob = 0).
do if ( 2 =2).
compute LL = ylp&*ln(pt1lp)+(1-ylp)&*ln(1-pt1lp).
end if.
compute LL2 = -2*csum(ll).
end if.
do if (abs(LL1-LL2) < converge).
do if ( 1 =1).
compute xlptmp=t(xlp).
compute vecprb=pt1lp&*(1-pt1lp).
loop kkk=1 to ncol(xlp).
compute xlptmp(kkk,:)=xlptmp(kkk,:)&*t(vecprb).
end loop.
compute varb = inv(xlptmp*xlp).
compute seb = sqrt(diag(varb)).
release xlptmp.
end if.
break.
end if.
compute bt1 = b.
compute LL1 = LL2.
end loop.
compute modres=b.
do if (jjj > iterate).
compute itprob = 2.
do if (booting=0).
compute iterrmod=1.
end if.
do if (booting=1).
compute bootiter=1.
end if.
do if (itprobtg=0).
compute itprobtg=1.
compute errcode(errs,1) = 47.
compute errs = errs + 1.
do if (booting = 0 and 1 =1).
compute vt1 = mdiag(pt1lp&*(1-pt1lp)).
compute varb = inv(t(xlp)*vt1*xlp).
compute seb = sqrt(diag(varb)).
end if.
end if.
end if.
do if ( 1 =1).
compute trat = b&/seb.
compute dfres=nrow(xlp).
compute p = 2*(1-cdfnorm(abs(trat))).
compute modres={modres,seb,trat,p}.
compute modres={modres,(b-xp2&*seb),(b+xp2&*seb)}.
compute pvchi=1-chicdf((LL3-LL2),(nrow(modres)-1)).
compute mcF = (LL3-LL2)/LL3.
compute cox = 1-exp(-(LL3-LL2)/nrow(xlp)).
compute nagel = cox/(1-exp(-(LL3)/nrow(xlp))).
compute modsum={LL2,(LL3-LL2),(nrow(modres)-1),pvchi, mcF,cox,nagel}.
compute modsuml={'-2LL','ModelLL', 'df', 'p', 'McFadden', 'CoxSnell', 'Nagelkrk'}.
compute modresl={'coeff','se','Z','p','LLCI','ULCI'}.
end if.
end if
.
compute chidfxm=basemodx-LL2.
end if.
compute lmat=make(nrow(b),nxvls,0).
compute lmattmp=ident(nxvls).
compute lmat((nrow(lmat)-nxvls+1):nrow(lmat),:)=lmattmp.

.
compute lmat2= lmat.
do if ( 1 =0).
compute lmat2 = mdiag( lmat ).
compute lmat3=make(nrow(lmat2),1,0).
loop flp=1 to ncol(lmat2).
do if (csum(lmat2(:,flp))=1).
compute lmat3={lmat3,lmat2(:,flp)}.
end if.
end loop.
compute lmat2=lmat3(:,2:ncol(lmat3)).
end if.
compute fratio = (t(t(lmat2)* b )*inv(t(lmat2)* varb *lmat2)*((t(lmat2)* b )))/ncol(lmat2).
compute pfr = 1-fcdf(fratio,ncol(lmat2),(n-nrow( b ))).
compute fresult={fratio,ncol(lmat2),(n-nrow( b )),pfr}.
do if (i = (nms+nys) and (ydich=1)).
compute fratio=fratio*ncol(lmat2).
compute pfr=1-chicdf(fratio,ncol(lmat2)).
compute fresult={fratio,ncol(lmat2),pfr}.
end if.
do if ( 0 =1).
compute lmat3=1-rsum(lmat2).
compute xfm=make(n,csum(lmat3),0).
compute flpc=1.
loop flp=1 to nrow(lmat3).
do if (lmat3(flp,1)=1).
compute xfm(:,flpc)=x(:,flp).
compute flpc=flpc+1.
end if.
end loop.
compute bfm=inv(t(xfm)*xfm)*t(xfm)*y.
compute resid=y-(xfm*bfm).
compute sstotal=(y-(csum(y)/n)).
compute sstotal=csum(sstotal&*sstotal).
compute ssresid=csum(resid&*resid).
compute rsqch= 0 -((sstotal-ssresid)/sstotal).
compute fresult={rsqch,fresult}.
release xfm,flpc, resid, ssresid, bfm.
end if
.
compute numxint=numxint+1.
compute xmtst(numxint,:)=fresult.
do if ((i = (nms+nys)) and (ydich=1)).
compute xmtst(numxint,1)=chidfxm.
compute xmtst(numxint,3)=1-chicdf(chidfxm,nxvls).
end if.
compute xmtstlb={xmtstlb;highlbx((xmints-1),1)}.
end if.
end loop.
compute x=xmtmat.
release xmtmat.
do if (numxint>0).
compute xmtstlb=xmtstlb((2:(numxint+1)),:).
compute xmtst=xmtst(1:numxint,:).
compute resultm2=make(nrow(xmtst),maxresm,99999).
compute resultm2(1:nrow(xmtst),1:ncol(xmtst))=xmtst.
compute resultm={resultm;resultm2}.
do if (nms=1).
compute xmtstlb=' '.
end if.
do if (outscree=1).
do if ((i < (nms+nys)) or (ydich=0)).
print xmtst/title='Test(s) of X by M interaction:'/rnames=xmtstlb/cnames=xmtstlbc/format= F10.4.
end if.
do if ((i = (nms+nys)) and (ydich=1)).
print xmtst/title='Likelihood ratio test(s) of X by M interaction:'/rnames=xmtstlb/cnames=xmtstlbc/format= F10.4.
end if.
end if.
end if.
compute r2=r2tmp.
compute b=btmp.
compute varb=varbtmp.
compute dfres=dfrestmp.
compute tval=tvaltmp.
end if.
do if (criterr = 0).
compute jj=0.
loop j = start to ((start+i)-1).
compute dbint=0.
compute lmat=whigh(1:nump(1,i),j).
compute lmat2=wzhigh(1:nump(1,i),j).
do if ((csum(lmat) > 0) and (csum(lmat2) = 0)).
do if ((i < (nms+nys)) or (ydich <> 1)).

.
compute lmat2= lmat.
do if ( 0 =0).
compute lmat2 = mdiag( lmat ).
compute lmat3=make(nrow(lmat2),1,0).
loop flp=1 to ncol(lmat2).
do if (csum(lmat2(:,flp))=1).
compute lmat3={lmat3,lmat2(:,flp)}.
end if.
end loop.
compute lmat2=lmat3(:,2:ncol(lmat3)).
end if.
compute fratio = (t(t(lmat2)* b )*inv(t(lmat2)* varb *lmat2)*((t(lmat2)* b )))/ncol(lmat2).
compute pfr = 1-fcdf(fratio,ncol(lmat2),(n-nrow( b ))).
compute fresult={fratio,ncol(lmat2),(n-nrow( b )),pfr}.
do if (i = (nms+nys) and (ydich=1)).
compute fratio=fratio*ncol(lmat2).
compute pfr=1-chicdf(fratio,ncol(lmat2)).
compute fresult={fratio,ncol(lmat2),pfr}.
end if.
do if ( 1 =1).
compute lmat3=1-rsum(lmat2).
compute xfm=make(n,csum(lmat3),0).
compute flpc=1.
loop flp=1 to nrow(lmat3).
do if (lmat3(flp,1)=1).
compute xfm(:,flpc)=x(:,flp).
compute flpc=flpc+1.
end if.
end loop.
compute bfm=inv(t(xfm)*xfm)*t(xfm)*y.
compute resid=y-(xfm*bfm).
compute sstotal=(y-(csum(y)/n)).
compute sstotal=csum(sstotal&*sstotal).
compute ssresid=csum(resid&*resid).
compute rsqch= r2 -((sstotal-ssresid)/sstotal).
compute fresult={rsqch,fresult}.
release xfm,flpc, resid, ssresid, bfm.
end if
.
compute lmatdb=lmat.
compute dbint=dbint+1.
end if.
do if ((ydich = 1) and (i = (nms+nys))).

.
compute btemphld=b.
compute llrdat=make(nrow(x),nrow( lmat )-csum( lmat ),-999).
compute llrdf=ncol(x)-ncol(llrdat).
compute llrcnt=0.
loop llri=1 to nrow( lmat ).
do if ( lmat (llri,1)=0).
compute llrcnt=llrcnt+1.
compute llrdat(:,llrcnt)=x(:,llri).
end if.
end loop.

.
do if ( 2 =1).
compute hatmat=inv(t( llrdat )* llrdat )*t( llrdat ).
compute b = hatmat* y.
compute modres=b.
do if ( 0 =1).
compute n1=nrow( llrdat ).
compute dfres=n1-(ncol( llrdat )).
compute sstotal = t( y -(csum( y )/n1))*( y -(csum( y )/n1)).
compute resid= y - llrdat *b.
compute ssresid = csum((resid)&**2).
compute r2 = (sstotal-ssresid)/sstotal.
do if (r2 < 0).
compute r2=0.
end if.
compute adjr2 = 1-((1-r2)*(n1-1)/(dfres)).
compute mse=ssresid/(n1-ncol( llrdat )).

.
compute n1=nrow( x ).
compute invXtX = inv(t( x )* x ).
compute varb = mse *invXtX.
compute k3 = ncol( x ).
compute xhc=0.
do if ( hc <> 5).
compute xhc= x.
compute hat = xhc(:,1).
loop i3=1 to nrow(xhc).
compute hat(i3,1)= xhc(i3,:)*invXtX*t(xhc(i3,:)).
end loop.
do if ( hc = 0 or hc =1).
loop i3 = 1 to k3.
compute xhc(:,i3)=xhc(:,i3)&* resid.
end loop.
end if.
do if ( hc =3 or hc =2).
loop i3=1 to k3.
compute xhc(:,i3) = ( resid &/(1-hat)&**(1/(4- hc )))&*xhc(:,i3).
end loop.
end if.
do if ( hc = 4).
compute hcmn=make(n,2,4).
compute hcmn(:,2)=(n1*hat)/k3.
loop i3= 1 to k3.
compute xhc(:,i3) = ( resid &/(1-hat)&**(rmin(hcmn)/2))&*xhc(:,i3).
end loop.
end if.
compute varb=(invXtX*t(xhc)*xhc*invXtX).
do if ( hc =1).
compute varb=(n1/(n1-ncol( x )))&*varb.
end if.
end if.
compute hclab={'se(HC0)','se(HC1)','se(HC2)','se(HC3)','se(HC4)','se'}.
compute hclab=hclab(1,( hc +1)).
compute hcflab={'F(HC0)','F(HC1)','F(HC2)','F(HC3)','F(HC4)','F'}.
compute hcflab=hcflab(1,( hc +1)).
release xhc
.
compute seb=sqrt(diag(varb)).
compute trat = b&/seb.
compute p = 2*(1-tcdf(abs(trat), (dfres))).
compute tval = sqrt(dfres* (exp((dfres-(5/6))*((xp2/(dfres-(2/3)+(.11/dfres)))* (xp2/(dfres-(2/3)+(.11/dfres)))))-1)).
compute modres={modres,seb,trat,p}.
compute modres={modres,(b-tval&*seb),(b+tval&*seb)}.
compute modresl={'coeff',hclab,'t','p','LLCI','ULCI'}.
compute lmat = ident(ncol( llrdat )).
compute lmat = lmat(:,2:ncol(lmat)).
compute fratio = (t(t(lmat)*b)*inv(t(lmat)*varb*lmat)*((t(lmat)*b)))/(ncol( llrdat )-1).
compute pfr = 1-fcdf(fratio,(ncol( llrdat )-1),dfres).
compute modsum={sqrt(r2),r2,mse,fratio,(ncol( llrdat )-1),dfres,pfr}.
compute modsuml={'R','R-sq','MSE',hcflab,'df1','df2', 'p'}.
end if.
end if.
do if ( 2 = 2 or 2 =3).
compute xlp= llrdat.
compute ylp= y.
compute pt2 = make(nrow(ylp),1,(csum(ylp)/nrow(ylp))).
do if ( 2 =2).
compute LL3 = ylp&*ln(pt2)+(1-ylp)&*ln(1-pt2).
end if.
compute LL3 = -2*csum(LL3).
compute bt1 = make(ncol(xlp),1,0).
compute LL1 = 0.
compute pt1 = make(nrow(ylp),1,0.5).
compute pt1lp=pt1.
loop jjj = 1 to iterate.
compute xlptmp=t(xlp).
compute vecprb=pt1lp&*(1-pt1lp).
loop kkk=1 to ncol(xlp).
compute xlptmp(kkk,:)=xlptmp(kkk,:)&*t(vecprb).
end loop.
compute b = bt1+inv(xlptmp*xlp)*t(xlp)*(ylp-pt1lp).
do if ( 2 =2).
compute xlpb=xlp*b.
compute xlpbt=(xlpb > -709.7).
compute xlpb709=(1-xlpbt)*(-709.7).
compute xlpb=(xlpb&*xlpbt)+xlpb709.
compute pt1lp = 1/(1+exp(-(xlpb))).
end if.
compute itprob = csum((pt1lp < .00000001) or (pt1lp > .9999999)).
do if (itprob > 0).
loop kkk = 1 to nrow(pt1lp).
do if (pt1lp(kkk,1) > .9999999).
compute pt1lp(kkk,1) = .9999999.
end if.
do if (pt1lp(kkk,1) < .00000001).
compute pt1lp(kkk,1) = .00000001.
end if.
end loop.
compute itprob = 0.
end if.
do if (itprob = 0).
do if ( 2 =2).
compute LL = ylp&*ln(pt1lp)+(1-ylp)&*ln(1-pt1lp).
end if.
compute LL2 = -2*csum(ll).
end if.
do if (abs(LL1-LL2) < converge).
do if ( 0 =1).
compute xlptmp=t(xlp).
compute vecprb=pt1lp&*(1-pt1lp).
loop kkk=1 to ncol(xlp).
compute xlptmp(kkk,:)=xlptmp(kkk,:)&*t(vecprb).
end loop.
compute varb = inv(xlptmp*xlp).
compute seb = sqrt(diag(varb)).
release xlptmp.
end if.
break.
end if.
compute bt1 = b.
compute LL1 = LL2.
end loop.
compute modres=b.
do if (jjj > iterate).
compute itprob = 2.
do if (booting=0).
compute iterrmod=1.
end if.
do if (booting=1).
compute bootiter=1.
end if.
do if (itprobtg=0).
compute itprobtg=1.
compute errcode(errs,1) = 47.
compute errs = errs + 1.
do if (booting = 0 and 0 =1).
compute vt1 = mdiag(pt1lp&*(1-pt1lp)).
compute varb = inv(t(xlp)*vt1*xlp).
compute seb = sqrt(diag(varb)).
end if.
end if.
end if.
do if ( 0 =1).
compute trat = b&/seb.
compute dfres=nrow(xlp).
compute p = 2*(1-cdfnorm(abs(trat))).
compute modres={modres,seb,trat,p}.
compute modres={modres,(b-xp2&*seb),(b+xp2&*seb)}.
compute pvchi=1-chicdf((LL3-LL2),(nrow(modres)-1)).
compute mcF = (LL3-LL2)/LL3.
compute cox = 1-exp(-(LL3-LL2)/nrow(xlp)).
compute nagel = cox/(1-exp(-(LL3)/nrow(xlp))).
compute modsum={LL2,(LL3-LL2),(nrow(modres)-1),pvchi, mcF,cox,nagel}.
compute modsuml={'-2LL','ModelLL', 'df', 'p', 'McFadden', 'CoxSnell', 'Nagelkrk'}.
compute modresl={'coeff','se','Z','p','LLCI','ULCI'}.
end if.
end if
.
compute b=btemphld.
compute fresult={(LL2-basemod),llrdf,1-chicdf((LL2-basemod),llrdf)}
.
compute lmatdb=lmat.
compute dbint=dbint+1.
end if.
compute highf={highf;fresult}.
compute highf2={highf2;fresult}.
do if (j = start).
compute flabel={flabel;'X*W'}.
end if.
do if (j > start).
do if (nms > 1).
compute flabel={flabel;highlbw(jj,1)}.
else if (nms = 1).
do if (xmint=0).
compute flabel={flabel;'M*W'}.
end if.
do if (xmint=1).
compute flabel={flabel;'X*M'}.
end if.
end if.
end if.
end if.
compute lmat=zhigh(1:nump(1,i),j).
compute lmat2=wzhigh(1:nump(1,i),j).
do if ((csum(lmat) > 0) and (csum(lmat2) = 0)).
do if ((i < (nms+nys)) or (ydich <> 1)).

.
compute lmat2= lmat.
do if ( 0 =0).
compute lmat2 = mdiag( lmat ).
compute lmat3=make(nrow(lmat2),1,0).
loop flp=1 to ncol(lmat2).
do if (csum(lmat2(:,flp))=1).
compute lmat3={lmat3,lmat2(:,flp)}.
end if.
end loop.
compute lmat2=lmat3(:,2:ncol(lmat3)).
end if.
compute fratio = (t(t(lmat2)* b )*inv(t(lmat2)* varb *lmat2)*((t(lmat2)* b )))/ncol(lmat2).
compute pfr = 1-fcdf(fratio,ncol(lmat2),(n-nrow( b ))).
compute fresult={fratio,ncol(lmat2),(n-nrow( b )),pfr}.
do if (i = (nms+nys) and (ydich=1)).
compute fratio=fratio*ncol(lmat2).
compute pfr=1-chicdf(fratio,ncol(lmat2)).
compute fresult={fratio,ncol(lmat2),pfr}.
end if.
do if ( 1 =1).
compute lmat3=1-rsum(lmat2).
compute xfm=make(n,csum(lmat3),0).
compute flpc=1.
loop flp=1 to nrow(lmat3).
do if (lmat3(flp,1)=1).
compute xfm(:,flpc)=x(:,flp).
compute flpc=flpc+1.
end if.
end loop.
compute bfm=inv(t(xfm)*xfm)*t(xfm)*y.
compute resid=y-(xfm*bfm).
compute sstotal=(y-(csum(y)/n)).
compute sstotal=csum(sstotal&*sstotal).
compute ssresid=csum(resid&*resid).
compute rsqch= r2 -((sstotal-ssresid)/sstotal).
compute fresult={rsqch,fresult}.
release xfm,flpc, resid, ssresid, bfm.
end if
.
compute dbint=dbint+1.
end if.
do if ((ydich = 1) and (i = (nms+nys))).

.
compute btemphld=b.
compute llrdat=make(nrow(x),nrow( lmat )-csum( lmat ),-999).
compute llrdf=ncol(x)-ncol(llrdat).
compute llrcnt=0.
loop llri=1 to nrow( lmat ).
do if ( lmat (llri,1)=0).
compute llrcnt=llrcnt+1.
compute llrdat(:,llrcnt)=x(:,llri).
end if.
end loop.

.
do if ( 2 =1).
compute hatmat=inv(t( llrdat )* llrdat )*t( llrdat ).
compute b = hatmat* y.
compute modres=b.
do if ( 0 =1).
compute n1=nrow( llrdat ).
compute dfres=n1-(ncol( llrdat )).
compute sstotal = t( y -(csum( y )/n1))*( y -(csum( y )/n1)).
compute resid= y - llrdat *b.
compute ssresid = csum((resid)&**2).
compute r2 = (sstotal-ssresid)/sstotal.
do if (r2 < 0).
compute r2=0.
end if.
compute adjr2 = 1-((1-r2)*(n1-1)/(dfres)).
compute mse=ssresid/(n1-ncol( llrdat )).

.
compute n1=nrow( x ).
compute invXtX = inv(t( x )* x ).
compute varb = mse *invXtX.
compute k3 = ncol( x ).
compute xhc=0.
do if ( hc <> 5).
compute xhc= x.
compute hat = xhc(:,1).
loop i3=1 to nrow(xhc).
compute hat(i3,1)= xhc(i3,:)*invXtX*t(xhc(i3,:)).
end loop.
do if ( hc = 0 or hc =1).
loop i3 = 1 to k3.
compute xhc(:,i3)=xhc(:,i3)&* resid.
end loop.
end if.
do if ( hc =3 or hc =2).
loop i3=1 to k3.
compute xhc(:,i3) = ( resid &/(1-hat)&**(1/(4- hc )))&*xhc(:,i3).
end loop.
end if.
do if ( hc = 4).
compute hcmn=make(n,2,4).
compute hcmn(:,2)=(n1*hat)/k3.
loop i3= 1 to k3.
compute xhc(:,i3) = ( resid &/(1-hat)&**(rmin(hcmn)/2))&*xhc(:,i3).
end loop.
end if.
compute varb=(invXtX*t(xhc)*xhc*invXtX).
do if ( hc =1).
compute varb=(n1/(n1-ncol( x )))&*varb.
end if.
end if.
compute hclab={'se(HC0)','se(HC1)','se(HC2)','se(HC3)','se(HC4)','se'}.
compute hclab=hclab(1,( hc +1)).
compute hcflab={'F(HC0)','F(HC1)','F(HC2)','F(HC3)','F(HC4)','F'}.
compute hcflab=hcflab(1,( hc +1)).
release xhc
.
compute seb=sqrt(diag(varb)).
compute trat = b&/seb.
compute p = 2*(1-tcdf(abs(trat), (dfres))).
compute tval = sqrt(dfres* (exp((dfres-(5/6))*((xp2/(dfres-(2/3)+(.11/dfres)))* (xp2/(dfres-(2/3)+(.11/dfres)))))-1)).
compute modres={modres,seb,trat,p}.
compute modres={modres,(b-tval&*seb),(b+tval&*seb)}.
compute modresl={'coeff',hclab,'t','p','LLCI','ULCI'}.
compute lmat = ident(ncol( llrdat )).
compute lmat = lmat(:,2:ncol(lmat)).
compute fratio = (t(t(lmat)*b)*inv(t(lmat)*varb*lmat)*((t(lmat)*b)))/(ncol( llrdat )-1).
compute pfr = 1-fcdf(fratio,(ncol( llrdat )-1),dfres).
compute modsum={sqrt(r2),r2,mse,fratio,(ncol( llrdat )-1),dfres,pfr}.
compute modsuml={'R','R-sq','MSE',hcflab,'df1','df2', 'p'}.
end if.
end if.
do if ( 2 = 2 or 2 =3).
compute xlp= llrdat.
compute ylp= y.
compute pt2 = make(nrow(ylp),1,(csum(ylp)/nrow(ylp))).
do if ( 2 =2).
compute LL3 = ylp&*ln(pt2)+(1-ylp)&*ln(1-pt2).
end if.
compute LL3 = -2*csum(LL3).
compute bt1 = make(ncol(xlp),1,0).
compute LL1 = 0.
compute pt1 = make(nrow(ylp),1,0.5).
compute pt1lp=pt1.
loop jjj = 1 to iterate.
compute xlptmp=t(xlp).
compute vecprb=pt1lp&*(1-pt1lp).
loop kkk=1 to ncol(xlp).
compute xlptmp(kkk,:)=xlptmp(kkk,:)&*t(vecprb).
end loop.
compute b = bt1+inv(xlptmp*xlp)*t(xlp)*(ylp-pt1lp).
do if ( 2 =2).
compute xlpb=xlp*b.
compute xlpbt=(xlpb > -709.7).
compute xlpb709=(1-xlpbt)*(-709.7).
compute xlpb=(xlpb&*xlpbt)+xlpb709.
compute pt1lp = 1/(1+exp(-(xlpb))).
end if.
compute itprob = csum((pt1lp < .00000001) or (pt1lp > .9999999)).
do if (itprob > 0).
loop kkk = 1 to nrow(pt1lp).
do if (pt1lp(kkk,1) > .9999999).
compute pt1lp(kkk,1) = .9999999.
end if.
do if (pt1lp(kkk,1) < .00000001).
compute pt1lp(kkk,1) = .00000001.
end if.
end loop.
compute itprob = 0.
end if.
do if (itprob = 0).
do if ( 2 =2).
compute LL = ylp&*ln(pt1lp)+(1-ylp)&*ln(1-pt1lp).
end if.
compute LL2 = -2*csum(ll).
end if.
do if (abs(LL1-LL2) < converge).
do if ( 0 =1).
compute xlptmp=t(xlp).
compute vecprb=pt1lp&*(1-pt1lp).
loop kkk=1 to ncol(xlp).
compute xlptmp(kkk,:)=xlptmp(kkk,:)&*t(vecprb).
end loop.
compute varb = inv(xlptmp*xlp).
compute seb = sqrt(diag(varb)).
release xlptmp.
end if.
break.
end if.
compute bt1 = b.
compute LL1 = LL2.
end loop.
compute modres=b.
do if (jjj > iterate).
compute itprob = 2.
do if (booting=0).
compute iterrmod=1.
end if.
do if (booting=1).
compute bootiter=1.
end if.
do if (itprobtg=0).
compute itprobtg=1.
compute errcode(errs,1) = 47.
compute errs = errs + 1.
do if (booting = 0 and 0 =1).
compute vt1 = mdiag(pt1lp&*(1-pt1lp)).
compute varb = inv(t(xlp)*vt1*xlp).
compute seb = sqrt(diag(varb)).
end if.
end if.
end if.
do if ( 0 =1).
compute trat = b&/seb.
compute dfres=nrow(xlp).
compute p = 2*(1-cdfnorm(abs(trat))).
compute modres={modres,seb,trat,p}.
compute modres={modres,(b-xp2&*seb),(b+xp2&*seb)}.
compute pvchi=1-chicdf((LL3-LL2),(nrow(modres)-1)).
compute mcF = (LL3-LL2)/LL3.
compute cox = 1-exp(-(LL3-LL2)/nrow(xlp)).
compute nagel = cox/(1-exp(-(LL3)/nrow(xlp))).
compute modsum={LL2,(LL3-LL2),(nrow(modres)-1),pvchi, mcF,cox,nagel}.
compute modsuml={'-2LL','ModelLL', 'df', 'p', 'McFadden', 'CoxSnell', 'Nagelkrk'}.
compute modresl={'coeff','se','Z','p','LLCI','ULCI'}.
end if.
end if
.
compute b=btemphld.
compute fresult={(LL2-basemod),llrdf,1-chicdf((LL2-basemod),llrdf)}
.
compute dbint=dbint+1.
end if.
compute highf={highf;fresult}.
compute highf2={highf2;fresult}.
do if (j = start).
compute flabel={flabel;'X*Z'}.
end if.
do if (j > start).
do if (nms > 1).
compute flabel={flabel;highlbz(jj,1)}.
else if (nms = 1).
compute flabel={flabel;'M*Z'}.
end if.
end if.
end if.
do if (dbint=2).
compute lmatdb=lmatdb+lmat.
do if ((ydich = 1) and (i = (nms+nys))).

.
compute btemphld=b.
compute llrdat=make(nrow(x),nrow( lmatdb )-csum( lmatdb ),-999).
compute llrdf=ncol(x)-ncol(llrdat).
compute llrcnt=0.
loop llri=1 to nrow( lmatdb ).
do if ( lmatdb (llri,1)=0).
compute llrcnt=llrcnt+1.
compute llrdat(:,llrcnt)=x(:,llri).
end if.
end loop.

.
do if ( 2 =1).
compute hatmat=inv(t( llrdat )* llrdat )*t( llrdat ).
compute b = hatmat* y.
compute modres=b.
do if ( 0 =1).
compute n1=nrow( llrdat ).
compute dfres=n1-(ncol( llrdat )).
compute sstotal = t( y -(csum( y )/n1))*( y -(csum( y )/n1)).
compute resid= y - llrdat *b.
compute ssresid = csum((resid)&**2).
compute r2 = (sstotal-ssresid)/sstotal.
do if (r2 < 0).
compute r2=0.
end if.
compute adjr2 = 1-((1-r2)*(n1-1)/(dfres)).
compute mse=ssresid/(n1-ncol( llrdat )).

.
compute n1=nrow( x ).
compute invXtX = inv(t( x )* x ).
compute varb = mse *invXtX.
compute k3 = ncol( x ).
compute xhc=0.
do if ( hc <> 5).
compute xhc= x.
compute hat = xhc(:,1).
loop i3=1 to nrow(xhc).
compute hat(i3,1)= xhc(i3,:)*invXtX*t(xhc(i3,:)).
end loop.
do if ( hc = 0 or hc =1).
loop i3 = 1 to k3.
compute xhc(:,i3)=xhc(:,i3)&* resid.
end loop.
end if.
do if ( hc =3 or hc =2).
loop i3=1 to k3.
compute xhc(:,i3) = ( resid &/(1-hat)&**(1/(4- hc )))&*xhc(:,i3).
end loop.
end if.
do if ( hc = 4).
compute hcmn=make(n,2,4).
compute hcmn(:,2)=(n1*hat)/k3.
loop i3= 1 to k3.
compute xhc(:,i3) = ( resid &/(1-hat)&**(rmin(hcmn)/2))&*xhc(:,i3).
end loop.
end if.
compute varb=(invXtX*t(xhc)*xhc*invXtX).
do if ( hc =1).
compute varb=(n1/(n1-ncol( x )))&*varb.
end if.
end if.
compute hclab={'se(HC0)','se(HC1)','se(HC2)','se(HC3)','se(HC4)','se'}.
compute hclab=hclab(1,( hc +1)).
compute hcflab={'F(HC0)','F(HC1)','F(HC2)','F(HC3)','F(HC4)','F'}.
compute hcflab=hcflab(1,( hc +1)).
release xhc
.
compute seb=sqrt(diag(varb)).
compute trat = b&/seb.
compute p = 2*(1-tcdf(abs(trat), (dfres))).
compute tval = sqrt(dfres* (exp((dfres-(5/6))*((xp2/(dfres-(2/3)+(.11/dfres)))* (xp2/(dfres-(2/3)+(.11/dfres)))))-1)).
compute modres={modres,seb,trat,p}.
compute modres={modres,(b-tval&*seb),(b+tval&*seb)}.
compute modresl={'coeff',hclab,'t','p','LLCI','ULCI'}.
compute lmat = ident(ncol( llrdat )).
compute lmat = lmat(:,2:ncol(lmat)).
compute fratio = (t(t(lmat)*b)*inv(t(lmat)*varb*lmat)*((t(lmat)*b)))/(ncol( llrdat )-1).
compute pfr = 1-fcdf(fratio,(ncol( llrdat )-1),dfres).
compute modsum={sqrt(r2),r2,mse,fratio,(ncol( llrdat )-1),dfres,pfr}.
compute modsuml={'R','R-sq','MSE',hcflab,'df1','df2', 'p'}.
end if.
end if.
do if ( 2 = 2 or 2 =3).
compute xlp= llrdat.
compute ylp= y.
compute pt2 = make(nrow(ylp),1,(csum(ylp)/nrow(ylp))).
do if ( 2 =2).
compute LL3 = ylp&*ln(pt2)+(1-ylp)&*ln(1-pt2).
end if.
compute LL3 = -2*csum(LL3).
compute bt1 = make(ncol(xlp),1,0).
compute LL1 = 0.
compute pt1 = make(nrow(ylp),1,0.5).
compute pt1lp=pt1.
loop jjj = 1 to iterate.
compute xlptmp=t(xlp).
compute vecprb=pt1lp&*(1-pt1lp).
loop kkk=1 to ncol(xlp).
compute xlptmp(kkk,:)=xlptmp(kkk,:)&*t(vecprb).
end loop.
compute b = bt1+inv(xlptmp*xlp)*t(xlp)*(ylp-pt1lp).
do if ( 2 =2).
compute xlpb=xlp*b.
compute xlpbt=(xlpb > -709.7).
compute xlpb709=(1-xlpbt)*(-709.7).
compute xlpb=(xlpb&*xlpbt)+xlpb709.
compute pt1lp = 1/(1+exp(-(xlpb))).
end if.
compute itprob = csum((pt1lp < .00000001) or (pt1lp > .9999999)).
do if (itprob > 0).
loop kkk = 1 to nrow(pt1lp).
do if (pt1lp(kkk,1) > .9999999).
compute pt1lp(kkk,1) = .9999999.
end if.
do if (pt1lp(kkk,1) < .00000001).
compute pt1lp(kkk,1) = .00000001.
end if.
end loop.
compute itprob = 0.
end if.
do if (itprob = 0).
do if ( 2 =2).
compute LL = ylp&*ln(pt1lp)+(1-ylp)&*ln(1-pt1lp).
end if.
compute LL2 = -2*csum(ll).
end if.
do if (abs(LL1-LL2) < converge).
do if ( 0 =1).
compute xlptmp=t(xlp).
compute vecprb=pt1lp&*(1-pt1lp).
loop kkk=1 to ncol(xlp).
compute xlptmp(kkk,:)=xlptmp(kkk,:)&*t(vecprb).
end loop.
compute varb = inv(xlptmp*xlp).
compute seb = sqrt(diag(varb)).
release xlptmp.
end if.
break.
end if.
compute bt1 = b.
compute LL1 = LL2.
end loop.
compute modres=b.
do if (jjj > iterate).
compute itprob = 2.
do if (booting=0).
compute iterrmod=1.
end if.
do if (booting=1).
compute bootiter=1.
end if.
do if (itprobtg=0).
compute itprobtg=1.
compute errcode(errs,1) = 47.
compute errs = errs + 1.
do if (booting = 0 and 0 =1).
compute vt1 = mdiag(pt1lp&*(1-pt1lp)).
compute varb = inv(t(xlp)*vt1*xlp).
compute seb = sqrt(diag(varb)).
end if.
end if.
end if.
do if ( 0 =1).
compute trat = b&/seb.
compute dfres=nrow(xlp).
compute p = 2*(1-cdfnorm(abs(trat))).
compute modres={modres,seb,trat,p}.
compute modres={modres,(b-xp2&*seb),(b+xp2&*seb)}.
compute pvchi=1-chicdf((LL3-LL2),(nrow(modres)-1)).
compute mcF = (LL3-LL2)/LL3.
compute cox = 1-exp(-(LL3-LL2)/nrow(xlp)).
compute nagel = cox/(1-exp(-(LL3)/nrow(xlp))).
compute modsum={LL2,(LL3-LL2),(nrow(modres)-1),pvchi, mcF,cox,nagel}.
compute modsuml={'-2LL','ModelLL', 'df', 'p', 'McFadden', 'CoxSnell', 'Nagelkrk'}.
compute modresl={'coeff','se','Z','p','LLCI','ULCI'}.
end if.
end if
.
compute b=btemphld.
compute fresult={(LL2-basemod),llrdf,1-chicdf((LL2-basemod),llrdf)}
.
end if.
do if (ydich <> 1 or i < (nms+nys)).

.
compute lmat2= lmatdb.
do if ( 0 =0).
compute lmat2 = mdiag( lmatdb ).
compute lmat3=make(nrow(lmat2),1,0).
loop flp=1 to ncol(lmat2).
do if (csum(lmat2(:,flp))=1).
compute lmat3={lmat3,lmat2(:,flp)}.
end if.
end loop.
compute lmat2=lmat3(:,2:ncol(lmat3)).
end if.
compute fratio = (t(t(lmat2)* b )*inv(t(lmat2)* varb *lmat2)*((t(lmat2)* b )))/ncol(lmat2).
compute pfr = 1-fcdf(fratio,ncol(lmat2),(n-nrow( b ))).
compute fresult={fratio,ncol(lmat2),(n-nrow( b )),pfr}.
do if (i = (nms+nys) and (ydich=1)).
compute fratio=fratio*ncol(lmat2).
compute pfr=1-chicdf(fratio,ncol(lmat2)).
compute fresult={fratio,ncol(lmat2),pfr}.
end if.
do if ( 1 =1).
compute lmat3=1-rsum(lmat2).
compute xfm=make(n,csum(lmat3),0).
compute flpc=1.
loop flp=1 to nrow(lmat3).
do if (lmat3(flp,1)=1).
compute xfm(:,flpc)=x(:,flp).
compute flpc=flpc+1.
end if.
end loop.
compute bfm=inv(t(xfm)*xfm)*t(xfm)*y.
compute resid=y-(xfm*bfm).
compute sstotal=(y-(csum(y)/n)).
compute sstotal=csum(sstotal&*sstotal).
compute ssresid=csum(resid&*resid).
compute rsqch= r2 -((sstotal-ssresid)/sstotal).
compute fresult={rsqch,fresult}.
release xfm,flpc, resid, ssresid, bfm.
end if
.
end if.
compute dbint=0.
compute highf={highf;fresult}.
do if (jj=0 and nms > 0).
compute flabel={flabel;'BOTH(X)'}.
end if.
do if (jj=0 and nms = 0).
compute flabel={flabel;'BOTH'}.
end if.
do if (jj>0 and nms = 1).
compute flabel={flabel;'BOTH(M)'}.
end if.
do if (nms > 1 and jj > 0).
compute flabel={flabel;highlbbt(jj,1)}.
end if.
end if.
compute lmat2=wzhigh(1:nump(1,i),j).
do if (csum(lmat2) > 0).
do if ((i < (nms+nys)) or (ydich <> 1)).

.
compute lmat2= lmat2.
do if ( 0 =0).
compute lmat2 = mdiag( lmat2 ).
compute lmat3=make(nrow(lmat2),1,0).
loop flp=1 to ncol(lmat2).
do if (csum(lmat2(:,flp))=1).
compute lmat3={lmat3,lmat2(:,flp)}.
end if.
end loop.
compute lmat2=lmat3(:,2:ncol(lmat3)).
end if.
compute fratio = (t(t(lmat2)* b )*inv(t(lmat2)* varb *lmat2)*((t(lmat2)* b )))/ncol(lmat2).
compute pfr = 1-fcdf(fratio,ncol(lmat2),(n-nrow( b ))).
compute fresult={fratio,ncol(lmat2),(n-nrow( b )),pfr}.
do if (i = (nms+nys) and (ydich=1)).
compute fratio=fratio*ncol(lmat2).
compute pfr=1-chicdf(fratio,ncol(lmat2)).
compute fresult={fratio,ncol(lmat2),pfr}.
end if.
do if ( 1 =1).
compute lmat3=1-rsum(lmat2).
compute xfm=make(n,csum(lmat3),0).
compute flpc=1.
loop flp=1 to nrow(lmat3).
do if (lmat3(flp,1)=1).
compute xfm(:,flpc)=x(:,flp).
compute flpc=flpc+1.
end if.
end loop.
compute bfm=inv(t(xfm)*xfm)*t(xfm)*y.
compute resid=y-(xfm*bfm).
compute sstotal=(y-(csum(y)/n)).
compute sstotal=csum(sstotal&*sstotal).
compute ssresid=csum(resid&*resid).
compute rsqch= r2 -((sstotal-ssresid)/sstotal).
compute fresult={rsqch,fresult}.
release xfm,flpc, resid, ssresid, bfm.
end if
.
end if.
do if ((ydich = 1) and (i = (nms+nys))).

.
compute btemphld=b.
compute llrdat=make(nrow(x),nrow( lmat2 )-csum( lmat2 ),-999).
compute llrdf=ncol(x)-ncol(llrdat).
compute llrcnt=0.
loop llri=1 to nrow( lmat2 ).
do if ( lmat2 (llri,1)=0).
compute llrcnt=llrcnt+1.
compute llrdat(:,llrcnt)=x(:,llri).
end if.
end loop.

.
do if ( 2 =1).
compute hatmat=inv(t( llrdat )* llrdat )*t( llrdat ).
compute b = hatmat* y.
compute modres=b.
do if ( 0 =1).
compute n1=nrow( llrdat ).
compute dfres=n1-(ncol( llrdat )).
compute sstotal = t( y -(csum( y )/n1))*( y -(csum( y )/n1)).
compute resid= y - llrdat *b.
compute ssresid = csum((resid)&**2).
compute r2 = (sstotal-ssresid)/sstotal.
do if (r2 < 0).
compute r2=0.
end if.
compute adjr2 = 1-((1-r2)*(n1-1)/(dfres)).
compute mse=ssresid/(n1-ncol( llrdat )).

.
compute n1=nrow( x ).
compute invXtX = inv(t( x )* x ).
compute varb = mse *invXtX.
compute k3 = ncol( x ).
compute xhc=0.
do if ( hc <> 5).
compute xhc= x.
compute hat = xhc(:,1).
loop i3=1 to nrow(xhc).
compute hat(i3,1)= xhc(i3,:)*invXtX*t(xhc(i3,:)).
end loop.
do if ( hc = 0 or hc =1).
loop i3 = 1 to k3.
compute xhc(:,i3)=xhc(:,i3)&* resid.
end loop.
end if.
do if ( hc =3 or hc =2).
loop i3=1 to k3.
compute xhc(:,i3) = ( resid &/(1-hat)&**(1/(4- hc )))&*xhc(:,i3).
end loop.
end if.
do if ( hc = 4).
compute hcmn=make(n,2,4).
compute hcmn(:,2)=(n1*hat)/k3.
loop i3= 1 to k3.
compute xhc(:,i3) = ( resid &/(1-hat)&**(rmin(hcmn)/2))&*xhc(:,i3).
end loop.
end if.
compute varb=(invXtX*t(xhc)*xhc*invXtX).
do if ( hc =1).
compute varb=(n1/(n1-ncol( x )))&*varb.
end if.
end if.
compute hclab={'se(HC0)','se(HC1)','se(HC2)','se(HC3)','se(HC4)','se'}.
compute hclab=hclab(1,( hc +1)).
compute hcflab={'F(HC0)','F(HC1)','F(HC2)','F(HC3)','F(HC4)','F'}.
compute hcflab=hcflab(1,( hc +1)).
release xhc
.
compute seb=sqrt(diag(varb)).
compute trat = b&/seb.
compute p = 2*(1-tcdf(abs(trat), (dfres))).
compute tval = sqrt(dfres* (exp((dfres-(5/6))*((xp2/(dfres-(2/3)+(.11/dfres)))* (xp2/(dfres-(2/3)+(.11/dfres)))))-1)).
compute modres={modres,seb,trat,p}.
compute modres={modres,(b-tval&*seb),(b+tval&*seb)}.
compute modresl={'coeff',hclab,'t','p','LLCI','ULCI'}.
compute lmat = ident(ncol( llrdat )).
compute lmat = lmat(:,2:ncol(lmat)).
compute fratio = (t(t(lmat)*b)*inv(t(lmat)*varb*lmat)*((t(lmat)*b)))/(ncol( llrdat )-1).
compute pfr = 1-fcdf(fratio,(ncol( llrdat )-1),dfres).
compute modsum={sqrt(r2),r2,mse,fratio,(ncol( llrdat )-1),dfres,pfr}.
compute modsuml={'R','R-sq','MSE',hcflab,'df1','df2', 'p'}.
end if.
end if.
do if ( 2 = 2 or 2 =3).
compute xlp= llrdat.
compute ylp= y.
compute pt2 = make(nrow(ylp),1,(csum(ylp)/nrow(ylp))).
do if ( 2 =2).
compute LL3 = ylp&*ln(pt2)+(1-ylp)&*ln(1-pt2).
end if.
compute LL3 = -2*csum(LL3).
compute bt1 = make(ncol(xlp),1,0).
compute LL1 = 0.
compute pt1 = make(nrow(ylp),1,0.5).
compute pt1lp=pt1.
loop jjj = 1 to iterate.
compute xlptmp=t(xlp).
compute vecprb=pt1lp&*(1-pt1lp).
loop kkk=1 to ncol(xlp).
compute xlptmp(kkk,:)=xlptmp(kkk,:)&*t(vecprb).
end loop.
compute b = bt1+inv(xlptmp*xlp)*t(xlp)*(ylp-pt1lp).
do if ( 2 =2).
compute xlpb=xlp*b.
compute xlpbt=(xlpb > -709.7).
compute xlpb709=(1-xlpbt)*(-709.7).
compute xlpb=(xlpb&*xlpbt)+xlpb709.
compute pt1lp = 1/(1+exp(-(xlpb))).
end if.
compute itprob = csum((pt1lp < .00000001) or (pt1lp > .9999999)).
do if (itprob > 0).
loop kkk = 1 to nrow(pt1lp).
do if (pt1lp(kkk,1) > .9999999).
compute pt1lp(kkk,1) = .9999999.
end if.
do if (pt1lp(kkk,1) < .00000001).
compute pt1lp(kkk,1) = .00000001.
end if.
end loop.
compute itprob = 0.
end if.
do if (itprob = 0).
do if ( 2 =2).
compute LL = ylp&*ln(pt1lp)+(1-ylp)&*ln(1-pt1lp).
end if.
compute LL2 = -2*csum(ll).
end if.
do if (abs(LL1-LL2) < converge).
do if ( 0 =1).
compute xlptmp=t(xlp).
compute vecprb=pt1lp&*(1-pt1lp).
loop kkk=1 to ncol(xlp).
compute xlptmp(kkk,:)=xlptmp(kkk,:)&*t(vecprb).
end loop.
compute varb = inv(xlptmp*xlp).
compute seb = sqrt(diag(varb)).
release xlptmp.
end if.
break.
end if.
compute bt1 = b.
compute LL1 = LL2.
end loop.
compute modres=b.
do if (jjj > iterate).
compute itprob = 2.
do if (booting=0).
compute iterrmod=1.
end if.
do if (booting=1).
compute bootiter=1.
end if.
do if (itprobtg=0).
compute itprobtg=1.
compute errcode(errs,1) = 47.
compute errs = errs + 1.
do if (booting = 0 and 0 =1).
compute vt1 = mdiag(pt1lp&*(1-pt1lp)).
compute varb = inv(t(xlp)*vt1*xlp).
compute seb = sqrt(diag(varb)).
end if.
end if.
end if.
do if ( 0 =1).
compute trat = b&/seb.
compute dfres=nrow(xlp).
compute p = 2*(1-cdfnorm(abs(trat))).
compute modres={modres,seb,trat,p}.
compute modres={modres,(b-xp2&*seb),(b+xp2&*seb)}.
compute pvchi=1-chicdf((LL3-LL2),(nrow(modres)-1)).
compute mcF = (LL3-LL2)/LL3.
compute cox = 1-exp(-(LL3-LL2)/nrow(xlp)).
compute nagel = cox/(1-exp(-(LL3)/nrow(xlp))).
compute modsum={LL2,(LL3-LL2),(nrow(modres)-1),pvchi, mcF,cox,nagel}.
compute modsuml={'-2LL','ModelLL', 'df', 'p', 'McFadden', 'CoxSnell', 'Nagelkrk'}.
compute modresl={'coeff','se','Z','p','LLCI','ULCI'}.
end if.
end if
.
compute b=btemphld.
compute fresult={(LL2-basemod),llrdf,1-chicdf((LL2-basemod),llrdf)}
.
end if.
compute highf={highf;fresult}.
compute highf2={highf2;fresult}.
do if (j = start).
compute flabel={flabel;'X*W*Z'}.
end if.
do if (j > start).
do if (nms > 1).
compute flabel={flabel;highlbwz(jj,1)}.
else if (nms = 1).
compute flabel={flabel;'M*W*Z'}.
end if.
end if.
end if.
compute jj=jj+1.
end loop.
release jj.
compute start=start+i.
end if.
do if (nrow(highf) > 1).
compute highf=highf(2:nrow(highf),:).
compute highf2=highf2(2:nrow(highf2),:).
compute flabel=flabel(2:nrow(flabel),1).
compute resultm2=make(nrow(highf),maxresm,99999).
compute resultm2(1:nrow(highf),1:ncol(highf))=highf.
compute resultm={resultm;resultm2}.
do if (outscree=1).
do if ((i < nms+nys) or (ydich=0)).
compute clabtmp={'R2-chng', hcflab,'df1','df2','p'}.
print highf/format = F10.4 /rnames=flabel/cnames=clabtmp/ title = 'Test(s) of highest order unconditional interaction(s):'.
end if.
do if (ydich=1 and i=(nms+nys)).
compute clabtmp={'Chi-sq', 'df','p'}.
print/title='Likelihood ratio test(s) of highest order'.
print highf/format = F10.4 /rnames=flabel/cnames=clabtmp/ title = 'unconditional interactions(s):'/space=0.
end if.
end if.
compute intpb=highf2(:,ncol(highf2)).
end if.
compute intstart=intstart+numint(1,i).
end if.

.
do if (criterr=0).
compute threeway=0.
compute didprint=0.
compute didsome=0.
compute sigintct=0.
loop jmed =1 to (nms+1).
compute hasw=0.
compute hasz=0.
compute jnok=0.
compute nm1vls=0.
compute nm2vls=0.
compute panelgrp=0.
compute graphixs={'WITH', outnames(1,i), 'BY'}.
compute focpred4={' '}.
compute intprint=0.
compute modcat=0.
do if (jmed <= i).
do if ((jmed = 1) and ((i+1) = nrow(bcmat))).
compute pathscnt=pathscnt+1.
else.
compute paths={paths,bcmat((i+1),jmed)}.
compute pathsw={pathsw,wcmat((i+1),jmed)}.
compute pathsz={pathsz,zcmat((i+1),jmed)}.
compute pathswz={pathswz,wzcmat((i+1),jmed)}.
compute temp=fochigh(:,pathscnt)&*bootloc(:,i).
compute pathsfoc={pathsfoc,pathsfoc(:,1)}.
do if (jmed=1).
compute pathtype={pathtype,1}.
end if.
do if ((i+1)=nrow(bcmat)).
compute pathtype={pathtype,3}.
end if.
do if (jmed > 1) and ((i+1) < nrow(bcmat)).
compute pathtype={pathtype,2}.
end if.
do if (jmed=1 and nxvls > 1 and (bcmat((i+1),jmed)=1)).
compute pathsfoc(:,(pathscn2+1))=temp(2:(nxvls+1),1).
end if.
do if ((jmed > 1) or (jmed=1 and nxvls=1)).
compute temp=cmax(temp).
compute pathsfoc(1,(pathscn2+1))=temp.
end if.
compute pathscnt=pathscnt+1.
compute pathscn2=pathscn2+1.
do if (i <= nms).
compute pathsdv={pathsdv,mnames(1,i)}.
end if.
do if (i > nms).
compute pathsdv={pathsdv,ynames}.
end if.
end if.
compute coeffcol=coeffcol+1.
compute probettt=coeffs(1:nrow(b),coeffcol).
do if (jmed=1 and (bcmat((i+1),jmed)=1)).
compute omni=make(nrow(probettt),nxvls,0).
compute omnitmp=ident(nxvls).
compute omni(2:(1+nxvls),:)=omnitmp.
end if.
do if (csum(probettt)>0).
compute probvarb=make(csum(probettt),csum(probettt),999).
compute probcoef=make(csum(probettt),1,999).
compute coefflp2=1.
loop coefflp=1 to nrow(probettt).
do if (probettt(coefflp,1)=1).
compute probcoef(coefflp2,1)=b(coefflp,1).
compute coefflp2=coefflp2+1.
end if.
end loop.
compute coefflp=0.
compute coefflp2=0.
loop iclp=1 to nrow(probettt).
do if probettt(iclp,1)=1.
compute coefflp=coefflp+1.
compute coefflp2=coefflp.
compute probvarb(coefflp,coefflp) = varb(iclp,iclp).
do if (iclp < nrow(probettt)).
loop jclp=(iclp+1) to nrow(probettt).
do if (probettt(jclp,1)=1).
compute coefflp2=coefflp2+1.
compute probvarb(coefflp,coefflp2)=varb(iclp, jclp).
compute probvarb(coefflp2,coefflp)=varb(iclp, jclp).
end if.
end loop.
end if.
end if.
end loop.
end if.
end if.
compute xprobval=xmodvals.
do if (nxvls > 1 or mcx > 0).
compute xprobval=dummatx(:,2:ncol(dummatx)).
end if.
do if ((wcmat((i+1),jmed)=1) and (zcmat((i+1),jmed)=0)).
compute numplps=1.
compute modvals=wmodvals.
compute probeval=wmodvals.
compute wheremv1=wherexw.
compute nm1vls=nwvls.
compute lpstsp={1,1}.
compute modcat=0.
compute jnmod=wtmp.
compute jnmodlab=wnames.
compute jnok=1.
compute jnmin=wmin.
compute jnmax=wmax.
compute wherejn1=2.
do if (jmed=1).
compute wherejn3=wherexw(1,i).
do if (nxvls > 1).
compute jnok=0.
end if.
end if.
do if (jmed > 1).
compute wherejn1=wherem((jmed-1),i).
compute wherejn3=wheremw(((2*jmed)-3),i).
end if.
do if (nwvls > 1).
compute probeval=wprobval.
compute lpstsp(1,2)=ncol(probeval).
compute modcat=1.
compute jnok=0.
end if.
do if (wdich = 1).
compute modcat=1.
compute jnok=0.
end if.
compute problabs=wnames.
compute focpred3={wnames,'(W)'}.
do if (xmint=1).
compute focpred3={wnames,'(X)'}.
end if.
compute hasw=1.
compute modgrph=wnames.
compute intprint=1.
compute sigintct=sigintct+1.
compute printpbe=intpb(sigintct,1).
end if.
do if ((wcmat((i+1),jmed)=0) and (zcmat((i+1),jmed)=1)).
compute numplps=1.
compute modvals=zmodvals.
compute probeval=zmodvals.
compute wheremv1=wherexz.
compute nm1vls=nzvls.
compute lpstsp={1,1}.
compute jnok=1.
compute jnmod=ztmp.
compute jnmin=zmin.
compute jnmax=zmax.
compute jnmodlab=znames.
compute wherejn1=2.
do if (jmed=1).
compute wherejn3=wherexz(1,i).
do if (nxvls > 1).
compute jnok=0.
end if.
end if.
do if (jmed > 1).
compute wherejn1=wherem((jmed-1),i).
compute wherejn3=wheremz(((2*jmed)-3),i).
end if.
do if (nzvls > 1).
compute probeval=zprobval.
compute lpstsp(1,2)=ncol(probeval).
compute modcat=1.
compute jnok=0.
end if.
do if (zdich = 1).
compute modcat=1.
compute jnok=0.
end if.
compute problabs=znames.
compute focpred3={znames,'(Z)'}.
compute modgrph=znames.
compute hasz=1.
compute intprint=1.
compute sigintct=sigintct+1.
compute printpbe=intpb(sigintct,1).
end if.
do if ((wzcmat((i+1),jmed)=1) or ((wcmat((i+1),jmed)=1) and (zcmat((i+1),jmed)=1))).
compute numplps=2.
compute probecnt=1.
compute intprint=1.
do if (wzcmat((i+1),jmed)=1).
compute sigintct=sigintct+1.
compute printpbe=intpb(sigintct,1).
else.
compute sigintct=sigintct+2.
compute printpbe=cmin(intpb((sigintct-1):sigintct,1)).
end if.
compute panelgrp=1.
compute hasw=1.
compute hasz=1.
compute panelcde={'/PANEL','ROWVAR=',znames,'.'}.
compute modgrph=wnames.
compute lpstsp={1,1;1,1}.
compute wheremv1=wherexw.
compute nm1vls=nwvls.
compute wheremv2=wherexz.
compute nm2vls=nzvls.
compute jnok=0.
do if (wzcmat((i+1),jmed)=1).
compute jnok=1.
end if.
do if (jmed > 1).
compute mprobval=mmodvals.
end if.
do if (jmed=1).
do if (nxvls > 1).
compute jnok=0.
end if.
end if.
do if (nwvls > 1).
compute lpstsp(1,2)=ncol(wprobval).
compute modcat=1.
compute jnok=0.
end if.
do if (zdich=1).
compute modcat=1.
compute jnok=0.
end if.
compute lpstsp(2,1)=lpstsp(1,2)+1.
compute lpstsp(2,2)=lpstsp(1,2)+1.
do if (nzvls > 1).
compute lpstsp(2,1)=lpstsp(1,2)+1.
compute lpstsp(2,2)=lpstsp(1,2)+ncol(zprobval).
compute jnok=0.
end if.
do if (zdich=1).
compute jnok=0.
end if.
compute omni3=make(nrow(b),(nxvls*nwvls),0).
do if (jmed > 1).
compute omni3=make(nrow(b),nwvls,0).
end if.
compute focpred3={wnames,'(W)'}.
do if (xmint=1).
compute focpred3={wnames,'(X)'}.
end if.
compute focpred4={'    ', 'Mod var:', znames, '(Z)'}.
compute modvals=make((nrow(wmodvals)*nrow(zmodvals)),2,0).
compute probeval=make((nrow(wmodvals)*nrow(zmodvals)),(ncol(wprobval)+ncol(zprobval)),0).
loop probei= 1 to nrow(wmodvals).
loop probej =1 to nrow(zmodvals).
compute modvals(probecnt,1)=wmodvals(probei,1).
compute probeval(probecnt,1:nwvls)=wprobval(probei,:).
compute modvals(probecnt,2)=zmodvals(probej,1).
compute probeval(probecnt,(nwvls+1):(nwvls+nzvls))=zprobval(probej,:).
compute probecnt=probecnt+1.
end loop.
end loop.
do if (wzcmat((i+1),jmed)=1).
compute numplps=numplps+1.
compute probprod=make(1,(ncol(wprobval)*ncol(zprobval)),0).
compute lpstsp2={1,1}.
compute lpstsp={lpstsp;lpstsp2}.
compute lpstsp(3,1)=lpstsp(2,2)+1.
compute lpstsp(3,2)=lpstsp(2,2)+ncol(probprod).
compute jnmod=ztmp.
compute jnmin=zmin.
compute jnmax=zmax.
compute jnmodlab=znames.
do if (jmed = 1).
compute wherejn1=wherexw(1,i).
compute wherejn3=wherexwz(1,i).
end if.
do if (jmed > 1).
compute wherejn1=wheremw(((2*jmed)-3),i).
compute wherejn3=wheremwz(((2*jmed)-3),i).
end if.
loop probei = 1 to nrow(wmodvals).
loop probej = 1 to nrow(zmodvals).
compute probtemp=1.
loop probek = 1 to ncol(wprobval).
compute probtemp={probtemp,(wprobval(probei,probek)&*zprobval(probej,:))}.
end loop.
compute probprod={probprod;probtemp(1,2:ncol(probtemp))}.
end loop.
end loop.
compute probprod=probprod(2:nrow(probprod),:).
compute probeval={probeval,probprod}.
end if.
compute problabs={wnames,znames}.
release probecnt, probei, probej.
end if.
do if (intprint=1).
compute focpred={'   Focal', 'predict:'}.
do if (jmed=1).
compute focpred={focpred,xnames,'(X)'}.
compute focplotv=xmodvals.
end if.
do if (jmed >1).
do if (nms > 1).
compute focpred={focpred,mnames(1,(jmed-1)), medlb2(1,(jmed-1))}.
end if.
do if (nms = 1).
compute focpred={focpred,mnames(1,(jmed-1)), '(M)'}.
end if.
compute focplotv=mmodvals(:,(jmed-1)).
end if.
compute focpred2={'    ', 'Mod var:',focpred3}.
compute focpred={focpred;focpred2}.
do if (ncol(focpred4) > 1).
compute focpred={focpred;focpred4}.
compute focpred4={' '}.
end if.
release focpred2,focpred3.
do if (outscree=1).
do if ((plot = 1 or plot = 2) or (printpbe <= intprobe)).
print focpred/title='----------'/format=A8/space=0.
end if.
end if.
compute foctmp=make(nrow(modvals),1,1).
compute probexpl=1.
compute probeva2={foctmp,probeval}.
do if (jmed=1 and nxs > 0 and mcx > 0).
compute probexpl=nxvls.
end if.
compute foctmp=make(nrow(modvals),1,1).
compute modvals3=make(1,(6+ncol(problabs)),0).
compute probrown=make(nrow(probeval),1,0).
compute jtmp=1.
loop probei = 1 to nrow(probeval).
compute probrown(probei,1)=jtmp.
compute jtmp=jtmp+nxvls.
end loop.
release jtmp.
compute probrow=999.
compute modvarl=problabs.
do if (plot = 1 or plot = 2 or nxvls > 1).
compute plotvals=make((nrow(modvals)*nrow(focplotv)),(ncol(modvals)+1),999).
loop ploti=1 to nrow(modvals).
loop plotj=1 to nrow(focplotv).
compute plotvals((((ploti-1)*nrow(focplotv))+plotj),2:ncol(plotvals))=modvals(ploti,:).
compute plotvals((((ploti-1)*nrow(focplotv))+plotj),1)=focplotv(plotj,1).
end loop.
end loop.
compute focpredn=3.
do if (jmed=1).
do if (nxvls > 1).
compute focpredn=(nxvls+1).
end if.
do if (nxvls=1 and xdich=1).
compute focpredn=2.
end if.
end if.
compute meanmat=mdiag(means).
compute onesmat=make(nrow(meanmat),(nrow(probeval)*focpredn),1).
compute probeplt=t(mdiag(means)*onesmat).
do if (jmed=1).
do if ((wcmat((i+1),1)=1) or (zcmat((i+1),1)=1)).
compute plotcnt=1.
compute iloops=nwpval*nzpval.
compute plotmx=nxpval*nzpval.
do if ((wcmat((i+1),1)=1) and (zcmat((i+1),1)=0)).
compute iloops=nwpval.
compute plotmx=nxpval.
end if.
do if ((wcmat((i+1),1)=0) and (zcmat((i+1),1)=1)).
compute iloops=nzpval.
compute plotmx=nxpval.
end if.
compute xestvals=make((nxpval*iloops),ncol(xprobval),-999).
do if (wcmat((i+1),1)=1).
compute westvals=make(nrow(xestvals),ncol(wprobval),-999).
end if.
do if (zcmat((i+1),1)=1).
compute zestvals=make(nrow(xestvals),ncol(zprobval),-999).
end if.
loop ploti=1 to iloops.
loop plotj=1 to nxpval.
compute xestvals(plotcnt,:)=xprobval(plotj,:).
compute plotcnt=plotcnt+1.
end loop.
end loop.
compute plotcnt=1.
compute plotcnt1=1.
compute plotcnt2=1.
compute plotcntz=1.
loop ploti = 1 to (iloops*nxpval).
do if (wcmat((i+1),1)=1).
compute westvals(ploti,:)=wprobval(plotcnt1,:).
end if.
do if ((wcmat((i+1),1)=0) and (zcmat((i+1),1)=1)).
compute zestvals(ploti,:)=zprobval(plotcnt1,:).
end if.
do if ((wcmat((i+1),1)=1) and (zcmat((i+1),1)=1)).
compute zestvals(ploti,:)=zprobval(plotcnt2,:).
compute plotcntz=plotcntz+1.
end if.
compute plotcnt=plotcnt+1.
do if (plotcnt > plotmx).
compute plotcnt=1.
compute plotcnt1=plotcnt1+1.
end if.
do if (plotcntz > nxpval).
compute plotcnt2=plotcnt2+1.
compute plotcntz=1.
do if (plotcnt2 > nzpval).
compute plotcnt2=1.
end if.
end if.
end loop.
compute probeplt(:,2:(1+(ncol(xestvals))))=xestvals.
do if (wcmat((i+1),1)=1).
compute probeplt(:,(wherew(1,i)):(wherew(2,i)))=westvals.
end if.
do if (zcmat((i+1),1)=1).
compute probeplt(:,(wherez(1,i)):(wherez(2,i)))=zestvals.
end if.
end if.
end if.
do if (jmed > 1).
do if ((wcmat((i+1),jmed)=1) or (zcmat((i+1),jmed)=1)).
compute plotcnt=1.
compute iloops=nwpval*nzpval.
compute plotmx=3*nzpval.
do if ((wcmat((i+1),jmed)=1) and (zcmat((i+1),jmed)=0)).
compute iloops=nwpval.
compute plotmx=3.
end if.
do if ((wcmat((i+1),jmed)=0) and (zcmat((i+1),jmed)=1)).
compute iloops=nzpval.
compute plotmx=3.
end if.
compute mestvals=make((3*iloops),1,-999).
do if (wcmat((i+1),jmed)=1).
compute westvals=make(nrow(mestvals),ncol(wprobval),-999).
end if.
do if (zcmat((i+1),jmed)=1).
compute zestvals=make(nrow(mestvals),ncol(zprobval),-999).
end if.
loop ploti=1 to iloops.
loop plotj=1 to 3.
compute mestvals(plotcnt,:)=mprobval(plotj,(jmed-1)).
compute plotcnt=plotcnt+1.
end loop.
end loop.
compute plotcnt=1.
compute plotcnt1=1.
compute plotcnt2=1.
compute plotcntz=1.
loop ploti = 1 to (iloops*3).
do if (wcmat((i+1),jmed)=1).
compute westvals(ploti,:)=wprobval(plotcnt1,:).
end if.
do if ((wcmat((i+1),jmed)=0) and (zcmat((i+1),jmed)=1)).
compute zestvals(ploti,:)=zprobval(plotcnt1,:).
end if.
do if ((wcmat((i+1),jmed)=1) and (zcmat((i+1),jmed)=1)).
compute zestvals(ploti,:)=zprobval(plotcnt2,:).
compute plotcntz=plotcntz+1.
end if.
compute plotcnt=plotcnt+1.
do if (plotcnt > plotmx).
compute plotcnt=1.
compute plotcnt1=plotcnt1+1.
end if.
do if (plotcntz > 3).
compute plotcnt2=plotcnt2+1.
compute plotcntz=1.
do if (plotcnt2 > nzpval).
compute plotcnt2=1.
end if.
end if.
end loop.
compute probeplt(:,wherem((jmed-1),i))=mestvals.
do if (wcmat((i+1),jmed)=1).
do if (model <> 74).
compute probeplt(:,(wherew(1,i)):(wherew(2,i)))=westvals.
end if.
do if (model = 74).
compute probeplt(:,(wherex(1,i)):(wherex(2,i)))=westvals.
end if.
end if.
do if (zcmat((i+1),jmed)=1).
compute probeplt(:,(wherez(1,i)):(wherez(2,i)))=zestvals.
end if.
end if.
end if.
compute prodloop = 1.
do if (jmed=1).
compute prodloop=ncol(xestvals).
end if.
do if (wcmat((i+1),jmed))=1.
compute plotcnt=0.
loop ploti = 1 to prodloop.
loop plotj = 1 to ncol(westvals).
do if (jmed=1).
compute probeplt(:,(wherexw(1,i)+plotcnt))=xestvals(:,ploti)&*westvals(:,plotj).
end if.
do if (jmed > 1).
compute probeplt(:,(wheremw(((jmed*2)-3) ,i)+plotcnt))=mestvals(:,ploti)&*westvals(:,plotj).
end if.
compute plotcnt=plotcnt+1.
end loop.
end loop.
end if.
do if (zcmat((i+1),jmed))=1.
compute plotcnt=0.
loop ploti = 1 to prodloop.
loop plotj = 1 to ncol(zestvals).
do if (jmed = 1).
compute probeplt(:,(wherexz(1,i)+plotcnt))=xestvals(:,ploti)&*zestvals(:,plotj).
end if.
do if (jmed > 1).
compute probeplt(:,(wheremz(((jmed*2)-3),i)+plotcnt))=mestvals(:,ploti)&*zestvals(:, plotj).
end if.
compute plotcnt=plotcnt+1.
end loop.
end loop.
end if.
do if (wzcmat((i+1),jmed))=1.
compute plotcnt=0.
compute threeway=1.
loop ploti = 1 to ncol(westvals).
loop plotj = 1 to ncol(zestvals).
compute probeplt(:,(wherewz(1,i)+plotcnt))=westvals(:,ploti)&*zestvals(:,plotj).
compute plotcnt=plotcnt+1.
end loop.
end loop.
compute plotcnt=0.
loop plotk = 1 to prodloop.
loop ploti = 1 to ncol(westvals).
loop plotj = 1 to ncol(zestvals).
do if (jmed = 1).
compute probeplt(:,(wherexwz(1,i)+plotcnt))=xestvals(:,plotk)&*westvals(:,ploti)&* zestvals(:,plotj).
end if.
do if (jmed > 1).
compute probeplt(:,(wheremwz(((jmed*2)-3),i)+plotcnt))=mestvals(:,plotk)&* westvals(:,ploti)&*zestvals(:,plotj).
end if.
compute plotcnt=plotcnt+1.
end loop.
end loop.
end loop.
end if.
loop newplp=1 to i.
do if (newplp <> jmed).
do if (wcmat((i+1),newplp))=1.
compute prodloop=1.
do if (newplp=1).
compute prodloop=nxvls.
end if.
compute plotcnt=0.
loop ploti = 1 to prodloop.
loop plotj = 1 to nwvls.
do if (newplp = 1).
compute probeplt(:,(wherexw(1,i)+plotcnt))=probeplt(:,(1+ploti))&*probeplt(:, (wherew(1,i)+plotj-1)).
end if.
do if (newplp > 1).
do if (model <> 74).
compute probeplt(:,(wheremw(((newplp*2)-3) ,i)+plotcnt))=probeplt(:,wherem((newplp-1),i))&*probeplt(:,(wherew(1,i)+plotj-1)).
end if.
do if (model = 74).
compute probeplt(:,(wheremw(((newplp*2)-3) ,i)+plotcnt))=probeplt(:,wherem((newplp-1),i))&*probeplt(:,(wherex(1,i)+plotj-1)).
end if.
end if.
compute plotcnt=plotcnt+1.
end loop.
end loop.
end if.
do if (zcmat((i+1),newplp))=1.
compute prodloop=1.
do if (newplp=1).
compute prodloop=nxvls.
end if.
compute plotcnt=0.
loop ploti = 1 to prodloop.
loop plotj = 1 to nzvls.
do if (newplp = 1).
compute probeplt(:,(wherexz(1,i)+plotcnt))=probeplt(:,(1+ploti))&*probeplt(:, (wherez(1,i)+plotj-1)).
end if.
do if (newplp > 1).
compute probeplt(:,(wheremz(((newplp*2)-3),i)+plotcnt))=probeplt(:, wherem((newplp-1),i))&*probeplt(:,(wherez(1,i)+plotj-1)).
end if.
compute plotcnt=plotcnt+1.
end loop.
end loop.
end if.
do if (wzcmat((i+1),newplp))=1.
compute plotcnt=0.
do if (threeway=0).
loop ploti = 1 to nwvls.
loop plotj = 1 to nzvls.
compute probeplt(:,(wherewz(1,i)+plotcnt))=probeplt(:,(wherew(1,i)+ploti-1))&* probeplt(:,(wherez(1,i)+plotj-1)).
compute plotcnt=plotcnt+1.
end loop.
end loop.
end if.
compute prodloop=1.
do if (newplp=1).
compute prodloop=nxvls.
end if.
compute plotcnt=0.
loop plotk = 1 to prodloop.
loop ploti = 1 to nwvls.
loop plotj = 1 to nzvls.
do if (newplp = 1).
compute probeplt(:,(wherexwz(1,i)+plotcnt))=probeplt(:,(1+plotk))&*probeplt(:, (wherew(1,i)+ploti-1))&*probeplt(:,(wherez(1,i)+plotj-1)).
end if.
do if (newplp > 1).
compute probeplt(:,(wheremwz(((newplp*2)-3),i)+plotcnt))=probeplt(:, wherem((newplp-1),i))&*probeplt(:,(wherew(1,i)+ploti-1))&*probeplt(:,(wherez(1,i)+plotj-1)).
end if.
compute plotcnt=plotcnt+1.
end loop.
end loop.
end loop.
end if.
end if.
end loop.
compute predvals=probeplt*b.
do if (debug <> 0).
print probeplt.
print b.
end if.
do if (i = nms+nys) and (ydich=1).
compute predvalt=(predvals < 709.7).
compute prevalt7=(1-predvalt)*(709.7).
compute predvals=(predvals&*predvalt)+prevalt7.
compute expyhat=exp(predvals)&/(1+exp(predvals)).
end if.
compute sepred=make(nrow(plotvals),3,999).
loop sei=1 to nrow(plotvals).
compute ask=probeplt(sei,:).
compute sepred(sei,1)=sqrt(ask*varb*t(ask)).
do if ((i < nms+nys) or (ydich=0)).
compute sepred(sei,2)=predvals(sei,1)-tval*sepred(sei,1).
compute sepred(sei,3)=predvals(sei,1)+tval*sepred(sei,1).
end if.
do if ((i = nms+nys) and (ydich=1)).
compute sepred(sei,2)=predvals(sei,1)-xp2*sepred(sei,1).
compute sepred(sei,3)=predvals(sei,1)+xp2*sepred(sei,1).
end if.
end loop.
compute prevloc=ncol(plotvals)+1.
compute probeplt={plotvals,predvals}.
do if (plot = 2).
compute probeplt={probeplt,sepred}.
end if.
do if ((i = nms+nys) and (ydich=1)).
compute probeplt={probeplt,expyhat}.
end if.
compute didsome=0.
end if.
do if ((wzcmat((i+1),jmed)=1) and (printpbe <= intprobe)).
do if (jmed=1).
compute omnilp2=nxvls*nwvls.
compute omnitmp=ident(omnilp2).
compute omni3(wherexw(1,i):wherexw(2,i),:)=omnitmp.
end if.
do if (jmed>1).
compute omnilp2=nwvls.
compute omnitmp=ident(omnilp2).
compute omni3(wheremw(((jmed*2)-3),i):wheremw(((jmed*2)-2),i),:)=omnitmp.
end if.
compute omnif=make(1,4,0).
do if ((i = nms+nys) and (ydich=1)).
compute omnif=make(1,3,0).
end if.
compute condeff3=0.
loop omnilp1=1 to nrow(zprobval).
loop omnilp=1 to (omnilp2).
do if (jmed=1).
compute omni3((wherexwz(1,i)+((omnilp-1)*nzvls)):(wherexwz(1,i)+((omnilp-1)*nzvls)+ (nzvls-1)),omnilp)=t(zprobval(omnilp1,:)).
end if.
do if (jmed > 1).
compute omni3((wheremwz(((jmed*2)-3),i)+((omnilp-1)*nzvls)):(wheremwz(((jmed*2)-3),i)+ ((omnilp-1)*nzvls)+(nzvls-1)),omnilp)=t(zprobval(omnilp1,:)).
end if.
end loop.
compute condeff=t(omni3)*b.
compute condeff3={condeff3;condeff}.

.
compute lmat2= omni3.
do if ( 1 =0).
compute lmat2 = mdiag( omni3 ).
compute lmat3=make(nrow(lmat2),1,0).
loop flp=1 to ncol(lmat2).
do if (csum(lmat2(:,flp))=1).
compute lmat3={lmat3,lmat2(:,flp)}.
end if.
end loop.
compute lmat2=lmat3(:,2:ncol(lmat3)).
end if.
compute fratio = (t(t(lmat2)* b )*inv(t(lmat2)* varb *lmat2)*((t(lmat2)* b )))/ncol(lmat2).
compute pfr = 1-fcdf(fratio,ncol(lmat2),(n-nrow( b ))).
compute fresult={fratio,ncol(lmat2),(n-nrow( b )),pfr}.
do if (i = (nms+nys) and (ydich=1)).
compute fratio=fratio*ncol(lmat2).
compute pfr=1-chicdf(fratio,ncol(lmat2)).
compute fresult={fratio,ncol(lmat2),pfr}.
end if.
do if ( 0 =1).
compute lmat3=1-rsum(lmat2).
compute xfm=make(n,csum(lmat3),0).
compute flpc=1.
loop flp=1 to nrow(lmat3).
do if (lmat3(flp,1)=1).
compute xfm(:,flpc)=x(:,flp).
compute flpc=flpc+1.
end if.
end loop.
compute bfm=inv(t(xfm)*xfm)*t(xfm)*y.
compute resid=y-(xfm*bfm).
compute sstotal=(y-(csum(y)/n)).
compute sstotal=csum(sstotal&*sstotal).
compute ssresid=csum(resid&*resid).
compute rsqch= 0 -((sstotal-ssresid)/sstotal).
compute fresult={rsqch,fresult}.
release xfm,flpc, resid, ssresid, bfm.
end if
.
compute omnif={omnif;fresult}.
end loop.
compute omnif=omnif(2:nrow(omnif),:).
compute clabtmp=znames.
compute condeff3=condeff3(2:nrow(condeff3),:).
do if ((nxvls*nwvls)=1).
compute omnif={condeff3,omnif}.
compute clabtmp={clabtmp,'Effect'}.
end if.
compute omnif={zmodvals,omnif}.
do if ((i < nms+nys) or (ydich=0)).
compute clabtmp={clabtmp,hcflab,'df1','df2','p'}.
end if.
do if ((i = (nms+nys)) and (ydich=1)).
compute clabtmp={clabtmp,'Chi-sq','df','p'}.
end if.
compute resultm2=make(nrow(omnif),maxresm,99999).
compute resultm2(1:nrow(omnif),1:ncol(omnif))=omnif.
compute resultm={resultm;resultm2}.
do if (outscree=1).
do if (jmed=1).
print omnif/title='Test of conditional X*W interaction at value(s) of Z:'/cnames=clabtmp/format= F10.4.
end if.
do if (jmed>1).
print omnif/title='Test of conditional M*W interaction at value(s) of Z:'/cnames=clabtmp/format= F10.4.
end if.
end if.
release omni3.
end if.
loop probei = 1 to probexpl.
do if (probexpl > 1).
compute foctmp=make(nrow(modvals),probexpl,0).
compute foctmp(:,probei)=foctmp(:,probei)+1.
compute probtemp=make(nrow(modvals),1,0).
loop probem = 1 to numplps.
loop probek = 1 to nxvls.
loop probej=lpstsp(probem,1) to lpstsp(probem,2).
compute probtemp={probtemp,foctmp(:,probek)&*probeval(:,probej)}.
end loop.
end loop.
end loop.
compute probeva2=probtemp(:,2:ncol(probtemp)).
compute probeva2={foctmp,probeva2}.
end if.
compute probres=probeva2*probcoef.
compute probrese=sqrt(diag(probeva2*probvarb*t(probeva2))).
compute tratio = probres&/probrese.
compute p = 2*(1-tcdf(abs(tratio), dfres)).
do if (ydich=1 and i = (nms+nys)).
compute p = 2*(1-cdfnorm(abs(tratio))).
end if.
compute modvals2={modvals,probres,probrese,tratio, p}.
do if ((i < nms+nys) or (ydich=0)).
compute modvals2={modvals2,(probres-tval&*probrese),(probres+tval&*probrese)}.
compute problabs={problabs,'Effect',hclab,'t', 'p', 'LLCI', 'ULCI'}.
end if.
do if (ydich=1 and i = (nms+nys)).
compute modvals2={modvals2,(probres-xp2&*probrese),(probres+xp2&*probrese)}.
compute problabs={problabs,'Effect','se','Z', 'p', 'LLCI', 'ULCI'}.
end if.
do if (probexpl > 1 and (printpbe <= intprobe)).
do if (hasz = 1).
compute printz=1.
end if.
do if (hasw=1).
compute printw=1.
end if.
compute probrlab=make(nrow(modvals),1,xcatlab(probei,1)).
compute modvals3={modvals3; modvals2}.
compute probrow={probrow;probrown}.
compute probrown=probrown+1.
do if (probei=probexpl).
compute xproblab=xcatlab(1:nxvls,1).
compute probrow=probrow(2:nrow(probrow),1).
compute modvals3=modvals3(2:nrow(modvals3),:).
compute temp=modvals3.
compute temp(GRADE(probrow(:,1)),:)=modvals3.
compute modvals3=temp.
compute start2=1.
compute problabs=problabs(1,(1+(ncol(modvarl))):ncol(problabs)).
compute pstart=1.
loop probek= 1 to nrow(probeval).
compute endstart=start2+(nxvls-1).
compute temp=modvals3(start2:endstart,(1+ncol(modvarl)):ncol(modvals3)).
compute temp2=t(modvals3(start2:start2,1:ncol(modvarl))).
compute trnames=t(modvarl).
do if (outscree=1).
do if (probek > 1).
print/title='----------'/space=0.
else.
print/title = 'Conditional effects of the focal predictor at values of the moderator(s):'.
do if ((jmed=1) and (i = (nms+nys)) and (nms > 0)).
do if (nxvls = 1).
print/title = '(These are also the conditional direct effects of X on Y)'/space=0.
else.
print/title = '(These are also the relative conditional direct effects of X on Y)'/space=0.
end if.
end if.
print.
end if.
print temp2/title = 'Moderator value(s):'/rnames=trnames/format= F10.4 /space=0.
print temp/title = ' '/cnames=problabs/rnames=xproblab/format= F10.4 /space=0.
end if.
compute resultm2=make(nrow(temp2),maxresm,99999).
compute resultm2(1:nrow(temp2),1:ncol(temp2))=temp2.
compute resultm={resultm;resultm2}.
compute resultm2=make(nrow(temp),maxresm,99999).
compute resultm2(1:nrow(temp),1:ncol(temp))=temp.
compute resultm={resultm;resultm2}.
compute start2=start2+nxvls.
compute didsome=1.
do if (jmed=1).
compute mod1val=probeval(probek,1:nm1vls).
loop omnilp=1 to nxvls.
compute omni((wheremv1(1,i)+((omnilp-1)*nm1vls)):(wheremv1(1,i)+((omnilp-1)* nm1vls)+(nm1vls-1)),omnilp)=t(mod1val).
do if (nm1vls < ncol(probeval)).
compute mod2val=probeval(probek,(nm1vls+1):(nm1vls+nm2vls)).
compute omni((wheremv2(1,i)+((omnilp-1)*nm2vls)):(wheremv2(1,i)+((omnilp-1)* nm2vls)+(nm2vls-1)),omnilp)=t(mod2val).
do if ((nm1vls+nm2vls) < ncol(probeval)).
compute intlen=nm1vls*nm2vls.
compute modintvl=probeval(probek,(nm1vls+nm2vls+1):ncol(probeval)).
compute omni((wherexwz(1,i)+((omnilp-1)*intlen)):(wherexwz(1,i)+((omnilp-1)* intlen)+(intlen-1)),omnilp)=t(modintvl).
end if.
end if.
end loop.

.
compute lmat2= omni.
do if ( 1 =0).
compute lmat2 = mdiag( omni ).
compute lmat3=make(nrow(lmat2),1,0).
loop flp=1 to ncol(lmat2).
do if (csum(lmat2(:,flp))=1).
compute lmat3={lmat3,lmat2(:,flp)}.
end if.
end loop.
compute lmat2=lmat3(:,2:ncol(lmat3)).
end if.
compute fratio = (t(t(lmat2)* b )*inv(t(lmat2)* varb *lmat2)*((t(lmat2)* b )))/ncol(lmat2).
compute pfr = 1-fcdf(fratio,ncol(lmat2),(n-nrow( b ))).
compute fresult={fratio,ncol(lmat2),(n-nrow( b )),pfr}.
do if (i = (nms+nys) and (ydich=1)).
compute fratio=fratio*ncol(lmat2).
compute pfr=1-chicdf(fratio,ncol(lmat2)).
compute fresult={fratio,ncol(lmat2),pfr}.
end if.
do if ( 0 =1).
compute lmat3=1-rsum(lmat2).
compute xfm=make(n,csum(lmat3),0).
compute flpc=1.
loop flp=1 to nrow(lmat3).
do if (lmat3(flp,1)=1).
compute xfm(:,flpc)=x(:,flp).
compute flpc=flpc+1.
end if.
end loop.
compute bfm=inv(t(xfm)*xfm)*t(xfm)*y.
compute resid=y-(xfm*bfm).
compute sstotal=(y-(csum(y)/n)).
compute sstotal=csum(sstotal&*sstotal).
compute ssresid=csum(resid&*resid).
compute rsqch= 0 -((sstotal-ssresid)/sstotal).
compute fresult={rsqch,fresult}.
release xfm,flpc, resid, ssresid, bfm.
end if
.
compute resultm2=make(nrow(fresult),maxresm,99999).
compute resultm2(1:nrow(fresult),1:ncol(fresult))=fresult.
compute resultm={resultm;resultm2}.
do if (outscree=1).
do if (i < (nms + nys) or (ydich = 0)).
compute clabtmp={hcflab,'df1','df2','p'}.
print fresult/title='Test of equality of conditional means'/cnames=clabtmp/format= F10.4.
end if.
do if (i = (nms + nys) and (ydich = 1)).
compute clabtmp={'Chi-sq','df','p'}.
print fresult/title='Test of equality of conditional logits or probabilities'/cnames=clabtmp/format= F10.4.
end if.
end if.
compute probetmp=probeplt(pstart:(pstart+nxvls),1).
compute probetmp={probetmp,probeplt(pstart:(pstart+nxvls),prevloc:ncol(probeplt))}.
compute pstart=pstart+(nxvls+1).
compute resultm2=make(nrow(probetmp),maxresm,99999).
compute resultm2(1:nrow(probetmp),1:ncol(probetmp))=probetmp.
compute resultm={resultm;resultm2}.
do if (i < ((nms + nys)) or (ydich = 0)).
compute clabtmp={xnames, outnames(1,i), hclab, 'LLCI', 'ULCI'}.
do if (outscree=1).
print probetmp/title = 'Estimated conditional means being compared:'/cnames=clabtmp/format= F10.4.
end if.
end if.
do if ((i = (nms+nys)) and (ydich = 1)).
compute clabtmp={xnames, outnames(1,i), 'prob'}.
compute probetm2=probetmp(:,1:2).
compute probetm2={probetm2,probetmp(:,ncol(probetmp))}.
do if (outscree=1).
print probetm2/title = 'Estimated conditional logits and probabilities:'/cnames=clabtmp/format= F10.4.
end if.
end if.
end if.
end loop.
release probrow, start2, endstart, temp, temp2.
end if.
end if.
do if (probexpl = 1 and (printpbe <= intprobe)).
compute resultm2=make(nrow(modvals2),maxresm,99999).
compute resultm2(1:nrow(modvals2),1:ncol(modvals2))=modvals2.
compute resultm={resultm;resultm2}.
do if (outscree=1).
print/title = 'Conditional effects of the focal predictor at values of the moderator(s):'.
print modvals2/cnames=problabs/title = ' '/space=0/format= F10.4.
end if.
compute didsome=1.
do if (hasz = 1).
compute printz=1.
end if.
do if (hasw=1).
compute printw=1.
end if.
do if (jn = 1 and jnok=1).
do if (criterr = 0).
compute dfres=n-nrow(b).
compute roots=99999.
compute jncrit =(dfres* (exp((dfres-(5/6))*((xp2/(dfres-(2/3)+(.11/dfres)))* (xp2/(dfres-(2/3)+(.11/dfres)))))-1)).
do if (i = (nms+nys) and (ydich=1)).
compute jncrit=xp2*xp2.
end if.
compute jnb1=b(wherejn1,1).
compute jnb3=b(wherejn3,1).
compute jnsb1=varb(wherejn1,wherejn1).
compute jnsb3=varb(wherejn3,wherejn3).
compute jnsb1b3=varb(wherejn1,wherejn3).
compute ajn =(jncrit*jnsb3)-(jnb3*jnb3).
compute bjn = 2*((jncrit*jnsb1b3)-(jnb1*jnb3)).
compute cjn = (jncrit*jnsb1)-(jnb1*jnb1).
compute radarg = (bjn*bjn)-(4*ajn*cjn).
compute den = 2*ajn.
compute nrts = 0.
do if (radarg >= 0 and den <> 0).
compute x21 = (-bjn+sqrt(radarg))/den.
compute x22 = (-bjn-sqrt(radarg))/den.
compute roots = 0.
do if (x21 >= jnmin and x21 <= jnmax).
compute nrts = 1.
compute roots = {roots; x21}.
end if.
do if (x22 >= jnmin and x22 <= jnmax).
compute nrts = nrts + 1.
compute roots = {roots; x22}.
end if.
compute roots={roots,make(nrow(roots),2,0)}.
end if.
do if (nrts > 0).
compute roots = roots(2:nrow(roots),1:3).
compute roots(1,2)=(csum(jnmod < roots(1,1))/n)*100.
compute roots(1,3)=(csum(jnmod > roots(1,1))/n)*100.
do if (nrow(roots)=2).
compute roots(2,2)=(csum(jnmod < roots(2,1))/n)*100.
compute roots(2,3)=(csum(jnmod > roots(2,1))/n)*100.
end if.
do if (outscree=1).
print roots/title = 'Moderator value(s) defining Johnson-Neyman significance region(s):'/clabels = 'Value', '% below', '% above'/format F10.4.
end if.
do if (nrts=1).
compute tmprts=make(1,3,99999).
compute roots={roots;tmprts}.
end if.
end if.
do if (nrts = 0).
compute roots=make(2,1,99999).
do if (outscree=1).
print/title = 'There are no statistical significance transition points within the observed'.
print/title = 'range of the moderator found using the Johnson-Neyman method.'/space=0.
end if.
end if.
compute resultm2=make(nrow(roots),maxresm,99999).
compute resultm2(1:nrow(roots),1:ncol(roots))=roots.
compute resultm={resultm;resultm2}.
compute jnvals=make(23,7,0).
loop jni= 0 to (21-(nrts)).
compute jnvals((jni+1),1)=jnmin+(jni*((jnmax-jnmin)/(21-nrts))).
end loop.
do if (nrts > 0).
loop jni = 1 to nrts.
loop jnj = 2 to (nrow(jnvals)-1).
do if ((roots(jni,1) > jnvals((jnj-1),1)) and (roots(jni,1) < jnvals(jnj,1))).
compute jnvals((jnj+1):(21+jni),1)=jnvals(jnj:(20+jni),1).
compute jnvals(jnj,1)=roots(jni,1).
end if.
end loop.
end loop.
end if.
compute jnvals=jnvals(1:22,:).
loop jni = 1 to nrow(jnvals).
compute jnvals(jni,2)=jnb1+jnb3*jnvals(jni,1).
compute jnvals(jni,3)=sqrt(jnsb1+2*jnvals(jni,1)*jnsb1b3+(jnvals(jni,1)*jnvals(jni, 1))*jnsb3).
compute jnvals(jni,4)=jnvals(jni,2)/jnvals(jni,3).
compute jnvals(jni,5)=2*(1-tcdf(abs(jnvals(jni,4)), dfres)).
compute jnvals(jni,6)=jnvals(jni,2)-sqrt(jncrit)*jnvals(jni,3).
compute jnvals(jni,7)=jnvals(jni,2)+sqrt(jncrit)*jnvals(jni,3).
do if ((i = nms + nys) and (ydich=1)).
compute jnvals(jni,5)=2*(1-cdfnorm(abs(jnvals(jni,4)))).
compute jnvals(jni,6)=jnvals(jni,2)-xp2*jnvals(jni,3).
compute jnvals(jni,7)=jnvals(jni,2)+xp2*jnvals(jni,3).
end if.
end loop.
compute resultm2=make(nrow(jnvals),maxresm,99999).
compute resultm2(1:nrow(jnvals),1:ncol(jnvals))=jnvals.
compute resultm={resultm;resultm2}.
do if ((i < nms+nys) or (ydich=0)).
compute jnclbs={jnmodlab,'Effect',hclab,'t', 'p', 'LLCI', 'ULCI'}.
end if.
do if ((i = nms + nys) and (ydich=1)).
compute jnclbs={jnmodlab,'Effect','se','Z', 'p', 'LLCI', 'ULCI'}.
end if.
do if (((wcmat((i+1),jmed)=1) or (zcmat((i+1),jmed)=1)) and (wzcmat((i+1),jmed)=0)).
do if (outscree=1).
print jnvals/title = 'Conditional effect of focal predictor at values of the moderator:'/cnames =jnclbs/format = F10.4.
end if.
end if.
do if (outscree=1).
do if ((jmed = 1) and (wzcmat((i+1),jmed)=1)).
print jnvals/title = 'Conditional X*W interaction at values of the moderator Z:'/cnames =jnclbs/format = F10.4.
end if.
do if ((jmed > 1) and (wzcmat((i+1),jmed)=1)).
print jnvals/title = 'Conditional M*W interaction at values of the moderator Z:'/cnames =jnclbs/format = F10.4.
end if.
end if.
end if.
end if.
end if.
do if ((i = (nms+nys)) and (jmed=1) and (bcmat(nrow(bcmat),1)=1)).
do if (probei=1).
compute direfflb=problabs.
compute direff=modvals2.
end if.
do if (probei>1).
compute direff={direff;modvals2}.
end if.
end if.
compute intprint=0.
do if ((jmed=1) and (i=1) and nms=0) and modcok=1).
compute contvec2=make(2,1,1).
compute contvec2={contvec2,wcontval,zcontval}.
do if (wzcmat((i+1),jmed)=1).
loop conti= 1 to ncol(wcontval).
loop contj = 1 to ncol(zcontval).
compute contvec2={contvec2,wcontval(:,conti)&*zcontval(:,contj)}.
end loop.
end loop.
end if.
compute conteff=contvec2*probcoef.
compute contdiff=contvec2(1,:)-contvec2(2,:).
compute contse=sqrt(contdiff*probvarb*t(contdiff)).
compute conteffd=conteff(1,1)-conteff(2,1).
compute contvec={contvec,conteff}.
compute contvecm=contvec.
compute resultm2=make(nrow(contvecm),maxresm,99999).
compute resultm2(1:nrow(contvecm),1:ncol(contvecm))=contvecm.
compute resultm={resultm;resultm2}.
do if (outscree=1).
print/title='Contrast between conditional effects of X:'.
print contvec/title=' '/rlabels='Effect1:','Effect2:'/cnames=problabs/format = F10.4 /space=0.
end if.
do if (ydich=0).
compute p=2*(1-tcdf(abs(conteffd/contse), dfres)).
compute contvec={conteffd,contse,conteffd/contse, p}.
compute contvec={contvec,(conteffd-(tval*contse))}.
compute contvec={contvec,(conteffd+(tval*contse))}.
compute contlabs={'Contrast', hclab, 't', 'p', 'LLCI', 'ULCI'}.
end if.
do if (ydich=1).
compute p=2*(1-cdfnorm(abs(conteffd/contse))).
compute contvec={conteffd,contse,conteffd/contse, p}.
compute contvec={contvec,(conteffd-(xp2*contse))}.
compute contvec={contvec,(conteffd+(xp2*contse))}.
compute contlabs={'Contrast', 'se', 'Z', 'p', 'LLCI', 'ULCI'}.
end if.
compute resultm2=make(nrow(contvec),maxresm,99999).
compute resultm2(1:nrow(contvec),1:ncol(contvec))=contvec.
compute resultm={resultm;resultm2}.
do if (outscree=1).
print contvec/title='Test of Effect1 minus Effect2'/format= F10.4 /cnames=contlabs.
end if.
end if.
end loop.
do if (plot = 1 or plot = 2).
compute datalabs={t(focpred(:,3)),outnames(1,i)}.
do if (plot = 1).
compute datalabs={datalabs}.
end if.
do if (plot = 2).
compute datalabs={datalabs,'se', 'LLCI', 'ULCI'}.
end if.
do if ((i = nms+nys) and (ydich=1)).
compute datalabs={datalabs,'prob'}.
end if.
compute resultm2=make(nrow(probeplt),maxresm,99999).
compute resultm2(1:nrow(probeplt),1:ncol(probeplt))=probeplt.
compute resultm={resultm;resultm2}.
compute datalabs={datalabs,'.'}.
do if (outscree=1).
print/title = 'Data for visualizing the conditional effect of the focal predictor:'.
print/title = 'Paste text below into a SPSS syntax window and execute to produce plot.'/space=0.
compute dumb = {' ', ' ', ' ', ' ', ' ', ' ', ' '}.
print datalabs/title = 'DATA LIST FREE/'/format=A10.
print probeplt/title = 'BEGIN DATA.'/format= F10.4 /space=0.
print/title = 'END DATA.'/space=0.
end if.
compute focgrph=datalabs(1,1).
compute graphix={focgrph,graphixs,modgrph}.
do if (((xdich=1) or (nxvls > 1)) and ((modcat=0) and (focgrph = xnames))).
compute graphix={modgrph,graphixs,focgrph}.
end if.
do if (panelgrp = 0).
compute graphix={graphix,'.'}.
else.
compute graphix={graphix,panelcde}.
end if.
do if (outscree=1).
print graphix/title = 'GRAPH/SCATTERPLOT='/format=A8/space=0.
end if.
do if (i = (nms + nys) and ydich = 1).
compute graphixd=graphix.
compute graphixd(1,3)='prob'.
do if (outscree=1).
print graphixd/title = 'GRAPH/SCATTERPLOT='/format=A8/space=0.
end if.
end if.
end if.
end if.
end loop.
release jmed, intprint,didprint.
end if
.
do if (model = 74 and i <= nms).
compute onetemp=make(nrow(xprobval),1,1).
compute mestmt74={onetemp,xprobval}.
do if (ncs > 0).
compute ncovmdl=rsum(ccmat(i,:)).
do if (ncovmdl > 0).
compute cvmnc=make(nrow(mestmt74),ncovmdl,1).
compute cvmnctmp=csum(x(:,(ncol(x)-ncovmdl+1):ncol(x)))/nrow(x).
do if (cuscoval > 0).
compute cvmnctmp=coval.
end if.
loop mestlp=1 to ncovmdl.
compute cvmnc(:,mestlp)=cvmnc(:,mestlp)*cvmnctmp(:,mestlp).
end loop.
compute mestmt74={mestmt74,cvmnc}.
end if.
end if.
compute mest74t=mestmt74*b.
do if (i = 1).
compute mest74=mest74t.
end if.
do if (i > 1).
compute mest74={mest74,mest74t}.
end if.
end if.
do if ((i = (nms+nys)) and (model >= 0 and model < 4) and (linsum(1,1) <> -999)).
compute lhyprob=1.
compute meansub=0.
do if (((nlinsum=nrow(b)) or (nlinsum = (nrow(b)-ncs)))).
do if (nlinsum = (nrow(b)-ncs) and ncs > 0).
compute linsum={linsum,covmeans}.
compute meansub=1.
end if.
compute lhyprob=0.
compute hypest=linsum*b.
compute sehypest=sqrt(linsum*varb*t(linsum)).
compute phypest=2*(1-tcdf(abs(hypest/sehypest), (dfres))).
compute hypest={hypest,sehypest,(hypest/sehypest),phypest,(hypest-tval*sehypest),(hypest+ tval*sehypest)}.
compute resultm2=make(ncol(linsum),maxresm,99999).
compute resultm2(:,1)=t(linsum).
compute resultm={resultm;resultm2}.
compute resultm2=make(1,maxresm,99999).
compute resultm2(1,1:ncol(hypest))=hypest.
compute resultm={resultm;resultm2}.
do if (outscree=1).
compute hyplabs={'Estimate',hclab,'t','p','LLCI','ULCI'}.
print/title='----------'.
print/title='Linear Combination Estimate and Hypothesis Test'/space=0.
print t(linsum)/title= 'Weight vector:'/clabels='weight'/rnames=vlabsm/format F10.4.
print hypest/title=' '/cnames=hyplabs/format= F10.4 /space=0.
do if (meansub=1).
print/title='Covariate weight(s) set to the sample mean.'.
end if.
end if.
end if.
do if (lhyprob=1).
compute notecode(notes,1) = 30.
compute notes = notes + 1.
end if.
end if.
end loop if criterr=1.
compute lastb=b.
compute lastcov=varb.
do if (criterr=0 and dototal = 1).
compute x=xtmp.
compute vlabsm={'constant';xcatlab(1:nxvls,1)}.
do if (ncs > 0).
compute x = {x,ctmp}.
compute vlabsm={vlabsm;t(covnames)}.
end if.
compute x = {ones,x}.

.
do if ( 1 =1).
compute hatmat=inv(t( x )* x )*t( x ).
compute b = hatmat* y.
compute modres=b.
do if ( 1 =1).
compute n1=nrow( x ).
compute dfres=n1-(ncol( x )).
compute sstotal = t( y -(csum( y )/n1))*( y -(csum( y )/n1)).
compute resid= y - x *b.
compute ssresid = csum((resid)&**2).
compute r2 = (sstotal-ssresid)/sstotal.
do if (r2 < 0).
compute r2=0.
end if.
compute adjr2 = 1-((1-r2)*(n1-1)/(dfres)).
compute mse=ssresid/(n1-ncol( x )).

.
compute n1=nrow( x ).
compute invXtX = inv(t( x )* x ).
compute varb = mse *invXtX.
compute k3 = ncol( x ).
compute xhc=0.
do if ( hc <> 5).
compute xhc= x.
compute hat = xhc(:,1).
loop i3=1 to nrow(xhc).
compute hat(i3,1)= xhc(i3,:)*invXtX*t(xhc(i3,:)).
end loop.
do if ( hc = 0 or hc =1).
loop i3 = 1 to k3.
compute xhc(:,i3)=xhc(:,i3)&* resid.
end loop.
end if.
do if ( hc =3 or hc =2).
loop i3=1 to k3.
compute xhc(:,i3) = ( resid &/(1-hat)&**(1/(4- hc )))&*xhc(:,i3).
end loop.
end if.
do if ( hc = 4).
compute hcmn=make(n,2,4).
compute hcmn(:,2)=(n1*hat)/k3.
loop i3= 1 to k3.
compute xhc(:,i3) = ( resid &/(1-hat)&**(rmin(hcmn)/2))&*xhc(:,i3).
end loop.
end if.
compute varb=(invXtX*t(xhc)*xhc*invXtX).
do if ( hc =1).
compute varb=(n1/(n1-ncol( x )))&*varb.
end if.
end if.
compute hclab={'se(HC0)','se(HC1)','se(HC2)','se(HC3)','se(HC4)','se'}.
compute hclab=hclab(1,( hc +1)).
compute hcflab={'F(HC0)','F(HC1)','F(HC2)','F(HC3)','F(HC4)','F'}.
compute hcflab=hcflab(1,( hc +1)).
release xhc
.
compute seb=sqrt(diag(varb)).
compute trat = b&/seb.
compute p = 2*(1-tcdf(abs(trat), (dfres))).
compute tval = sqrt(dfres* (exp((dfres-(5/6))*((xp2/(dfres-(2/3)+(.11/dfres)))* (xp2/(dfres-(2/3)+(.11/dfres)))))-1)).
compute modres={modres,seb,trat,p}.
compute modres={modres,(b-tval&*seb),(b+tval&*seb)}.
compute modresl={'coeff',hclab,'t','p','LLCI','ULCI'}.
compute lmat = ident(ncol( x )).
compute lmat = lmat(:,2:ncol(lmat)).
compute fratio = (t(t(lmat)*b)*inv(t(lmat)*varb*lmat)*((t(lmat)*b)))/(ncol( x )-1).
compute pfr = 1-fcdf(fratio,(ncol( x )-1),dfres).
compute modsum={sqrt(r2),r2,mse,fratio,(ncol( x )-1),dfres,pfr}.
compute modsuml={'R','R-sq','MSE',hcflab,'df1','df2', 'p'}.
end if.
end if.
do if ( 1 = 2 or 1 =3).
compute xlp= x.
compute ylp= y.
compute pt2 = make(nrow(ylp),1,(csum(ylp)/nrow(ylp))).
do if ( 1 =2).
compute LL3 = ylp&*ln(pt2)+(1-ylp)&*ln(1-pt2).
end if.
compute LL3 = -2*csum(LL3).
compute bt1 = make(ncol(xlp),1,0).
compute LL1 = 0.
compute pt1 = make(nrow(ylp),1,0.5).
compute pt1lp=pt1.
loop jjj = 1 to iterate.
compute xlptmp=t(xlp).
compute vecprb=pt1lp&*(1-pt1lp).
loop kkk=1 to ncol(xlp).
compute xlptmp(kkk,:)=xlptmp(kkk,:)&*t(vecprb).
end loop.
compute b = bt1+inv(xlptmp*xlp)*t(xlp)*(ylp-pt1lp).
do if ( 1 =2).
compute xlpb=xlp*b.
compute xlpbt=(xlpb > -709.7).
compute xlpb709=(1-xlpbt)*(-709.7).
compute xlpb=(xlpb&*xlpbt)+xlpb709.
compute pt1lp = 1/(1+exp(-(xlpb))).
end if.
compute itprob = csum((pt1lp < .00000001) or (pt1lp > .9999999)).
do if (itprob > 0).
loop kkk = 1 to nrow(pt1lp).
do if (pt1lp(kkk,1) > .9999999).
compute pt1lp(kkk,1) = .9999999.
end if.
do if (pt1lp(kkk,1) < .00000001).
compute pt1lp(kkk,1) = .00000001.
end if.
end loop.
compute itprob = 0.
end if.
do if (itprob = 0).
do if ( 1 =2).
compute LL = ylp&*ln(pt1lp)+(1-ylp)&*ln(1-pt1lp).
end if.
compute LL2 = -2*csum(ll).
end if.
do if (abs(LL1-LL2) < converge).
do if ( 1 =1).
compute xlptmp=t(xlp).
compute vecprb=pt1lp&*(1-pt1lp).
loop kkk=1 to ncol(xlp).
compute xlptmp(kkk,:)=xlptmp(kkk,:)&*t(vecprb).
end loop.
compute varb = inv(xlptmp*xlp).
compute seb = sqrt(diag(varb)).
release xlptmp.
end if.
break.
end if.
compute bt1 = b.
compute LL1 = LL2.
end loop.
compute modres=b.
do if (jjj > iterate).
compute itprob = 2.
do if (booting=0).
compute iterrmod=1.
end if.
do if (booting=1).
compute bootiter=1.
end if.
do if (itprobtg=0).
compute itprobtg=1.
compute errcode(errs,1) = 47.
compute errs = errs + 1.
do if (booting = 0 and 1 =1).
compute vt1 = mdiag(pt1lp&*(1-pt1lp)).
compute varb = inv(t(xlp)*vt1*xlp).
compute seb = sqrt(diag(varb)).
end if.
end if.
end if.
do if ( 1 =1).
compute trat = b&/seb.
compute dfres=nrow(xlp).
compute p = 2*(1-cdfnorm(abs(trat))).
compute modres={modres,seb,trat,p}.
compute modres={modres,(b-xp2&*seb),(b+xp2&*seb)}.
compute pvchi=1-chicdf((LL3-LL2),(nrow(modres)-1)).
compute mcF = (LL3-LL2)/LL3.
compute cox = 1-exp(-(LL3-LL2)/nrow(xlp)).
compute nagel = cox/(1-exp(-(LL3)/nrow(xlp))).
compute modsum={LL2,(LL3-LL2),(nrow(modres)-1),pvchi, mcF,cox,nagel}.
compute modsuml={'-2LL','ModelLL', 'df', 'p', 'McFadden', 'CoxSnell', 'Nagelkrk'}.
compute modresl={'coeff','se','Z','p','LLCI','ULCI'}.
end if.
end if
.
compute toteff=modres(2:(1+nxvls),:).
compute nodotot=0.
do if (xdich=1 and xmint=1 and model=74).
compute toteff(:,1)=toteff(:,1)*xscaling.
compute toteff(:,2)=toteff(:,2)*abs(xscaling).
compute toteff(:,3)=toteff(:,3)*xscaling.
compute toteff(:,5)=toteff(:,5)*xscaling.
compute toteff(:,6)=toteff(:,6)*xscaling.
compute citmp=toteff(:,5:6).
do if (xscaling < 0).
compute toteff(:,5)=citmp(:,2).
compute toteff(:,6)=citmp(:,1).
end if.
compute nodotot=1.
end if.
compute totefflb=modresl.
do if (outscree=1).
do if (nodotot=0).
print/title = '************************** TOTAL EFFECT MODEL ****************************'.
end if.
do if (nodotot=1).
print/title = '**************************************************************************'.
end if.
print outnames(1,ncol(outnames))/title = 'OUTCOME VARIABLE:'/format = A8/space=0.
end if.
compute toteffl2=vlabsm(2:(1+nxvls),:).
compute resultm2=make(1,maxresm,99999).
compute resultm2(1,1:ncol(modsum))=modsum.
compute resultm={resultm;resultm2}.
compute resultm2=make(nrow(modres),maxresm,99999).
compute resultm2(1:nrow(modres),1:ncol(modres))=modres.
compute resultm={resultm;resultm2}.
do if (outscree=1).
print modsum/title = 'Model Summary'/cnames = modsuml/format= F10.4.
print modres/title='Model'/rnames=vlabsm/cnames=modresl/format= F10.4.
end if.
compute lmat=make(nrow(b),1,0).
compute lmat2=make(nxvls,1,1).
compute lmat(2:(1+nxvls),1)=lmat2.
do if (ydich <> 1).

.
compute lmat2= lmat.
do if ( 0 =0).
compute lmat2 = mdiag( lmat ).
compute lmat3=make(nrow(lmat2),1,0).
loop flp=1 to ncol(lmat2).
do if (csum(lmat2(:,flp))=1).
compute lmat3={lmat3,lmat2(:,flp)}.
end if.
end loop.
compute lmat2=lmat3(:,2:ncol(lmat3)).
end if.
compute fratio = (t(t(lmat2)* b )*inv(t(lmat2)* varb *lmat2)*((t(lmat2)* b )))/ncol(lmat2).
compute pfr = 1-fcdf(fratio,ncol(lmat2),(n-nrow( b ))).
compute fresult={fratio,ncol(lmat2),(n-nrow( b )),pfr}.
do if (i = (nms+nys) and (ydich=1)).
compute fratio=fratio*ncol(lmat2).
compute pfr=1-chicdf(fratio,ncol(lmat2)).
compute fresult={fratio,ncol(lmat2),pfr}.
end if.
do if ( 1 =1).
compute lmat3=1-rsum(lmat2).
compute xfm=make(n,csum(lmat3),0).
compute flpc=1.
loop flp=1 to nrow(lmat3).
do if (lmat3(flp,1)=1).
compute xfm(:,flpc)=x(:,flp).
compute flpc=flpc+1.
end if.
end loop.
compute bfm=inv(t(xfm)*xfm)*t(xfm)*y.
compute resid=y-(xfm*bfm).
compute sstotal=(y-(csum(y)/n)).
compute sstotal=csum(sstotal&*sstotal).
compute ssresid=csum(resid&*resid).
compute rsqch= r2 -((sstotal-ssresid)/sstotal).
compute fresult={rsqch,fresult}.
release xfm,flpc, resid, ssresid, bfm.
end if
.
compute totomni=fresult.
end if.
do if (stand=1).
compute predsd=make(nrow(modres),1,0).
compute stdmod=modres(:,1)&/ovsd(1,ncol(ovsd)).
loop jd=1 to ncol(x).
compute descdat=x(:,jd).
compute predsd(jd,1) = (nrow(descdat)*sscp(descdat))-(t(csum(descdat))*(csum(descdat))).
compute predsd(jd,1) = sqrt(predsd(jd,1)/(nrow(descdat)*(nrow(descdat)-1))).
end loop.
do if (wherex(1,ncol(wherex)) <> -999 and ((nxvls > 1) or (xdich=1))).
compute sdmsone=make(nxvls,1,1).
compute predsd(wherex(1,ncol(wherex)):wherex(2,ncol(wherex)),1)=sdmsone.
compute pstog=1.
end if.
compute predsd(1,1)=1.
compute stdmod=stdmod&*predsd.
compute stdmod=stdmod(2:nrow(stdmod),1).
compute sdvlabs=vlabsm(2:nrow(vlabsm),1).
compute resultm2=make(nrow(stdmod),maxresm,99999).
compute resultm2(1:nrow(stdmod),1:ncol(stdmod))=stdmod.
compute resultm={resultm;resultm2}.
do if (outscree=1).
print stdmod/title='Standardized coefficients'/clabels='coeff'/rnames=sdvlabs/format= F10.4.
end if.
end if.
do if (covcoeff=1).
do if (outscree=1).
print varb/title='Covariance matrix of regression parameter estimates:'/rnames=vlabsm/cnames=vlabsm/format= F10.4.
end if.
compute resultm2=make(nrow(varb),maxresm,99999).
do if (ncol(varb) <= maxresm).
compute resultm2(1:nrow(varb),1:ncol(varb))=varb.
compute resultm={resultm;resultm2}.
end if.
do if (ncol(varb) > maxresm).
compute resultmt=make(nrow(resultm),ncol(varb),99999).
compute resultmt(1:nrow(resultm),1:ncol(resultm))=resultm.
compute resultm=resultmt.
compute resultm2=make(nrow(varb),ncol(resultm),99999).
compute resultm2(1:nrow(varb),1:ncol(varb))=varb.
compute resultm={resultm;resultm2}.
compute maxresm=ncol(resultm).
end if.
end if.
end if.
end if.
do if (criterr=0 and nms > 0 and ydich=0 and modelres=1).
compute modresid=modresid(:,2:ncol(modresid)).
compute sigmatal = (t(modresid)*(ident(n)-(1/n)*ones*t(ones))*modresid)*(1/(n-1)).
compute sdall = mdiag(1/sqrt(diag(sigmatal))).
compute corall=sdall*sigmatal*t(sdall).
compute resultm2=make(nrow(corall),maxresm,99999).
compute resultm2(1:nrow(corall),1:ncol(corall))=corall.
compute resultm={resultm;resultm2}.
do if (outscree=1).
print/title = '****************** CORRELATIONS BETWEEN MODEL RESIDUALS ******************'.
print corall/title=' '/format= F10.4 / cnames=outnames/rnames=outnames/space=0.
end if.
end if.
do if (criterr=0 and boot > 0).
compute bootres=make(1,rsum(nump),-999).
compute bootdir=obsdirfx.
compute natdirbt=make(1,nxvls,-999).
do if (effsize=1).
compute bootysd=make(1,1,-999).
compute bootxsd=make(1,1,-999).
end if.
compute badboot=0.
compute goodboot=0.
compute smallest=1.
compute booting=1.
loop j = 1 to maxboot.
compute nobootx=1.
compute modres2=999.
compute v=trunc(uniform(n,1)*n)+1.
compute bad=0.
loop i = 1 to (nms+nys).
compute y=outvars(v,i).
compute ynovar= (nrow(y)*sscp(y))-(t(csum(y))*(csum(y))).
do if (ynovar = 0).
compute bad=1.
end if.
compute xindx=datindx(1:(nump(1,i)-1),i).
compute hello=0.
compute x = fulldat(v,xindx).
compute x={ones,x}.
compute xsq=t(x)*x.
compute exsq=eval(xsq).
release xsq.
compute holymoly=cmin(exsq).
compute zeroeig=csum(exsq <= 0.000000000002).
compute bad=bad + (zeroeig > 0).

.
compute desctmp=make((8-(4* 1 )),ncol( y ),-999).
loop jd=1 to ncol( y ).
compute descdat= y (:,jd).
compute desctmp(1,jd) = csum(descdat)/nrow(descdat).
compute desctmp(2,jd) = (nrow(descdat)*sscp(descdat))-(t(csum(descdat))*(csum(descdat))).
compute desctmp(2,jd) = sqrt(desctmp(2,jd)/(nrow(descdat)*(nrow(descdat)-1))).
compute desctmp(3,jd)=cmin(descdat).
compute desctmp(4,jd)=cmax(descdat).
do if ( 1 =0).
compute minwarn=0.
compute maxwarn=0.
do if ((desctmp(3,jd)=desctmp(4,jd)) and novar=0).
compute errcode(errs,1)=15.
compute errs=errs+1.
compute criterr=1.
compute novar=1.
end if.
compute tmp=((descdat(:,1)=desctmp(3,jd))+(descdat(:,1)=desctmp(4,jd))).
compute desctmp(8,jd)=(csum(tmp)=nrow(tmp)).
compute tmp = descdat.
compute tmp(GRADE(descdat),:) = descdat.
compute descdat = tmp.
release tmp.
compute decval={.16;.5;.84}.
loop kd=1 to 3.
compute low=trunc(decval(kd,1)*(nrow(descdat)+1)).
compute lowdec=decval(kd,1)*(nrow(descdat)+1)-low.
compute value=descdat(low,1)+(descdat((low+1),1)-descdat(low,1))*lowdec.
compute desctmp((4+kd),jd)=value.
end loop.
compute mnotev=1.
compute modvals=desctmp(5:7,:).
do if (quantile <> 1).
compute desctmp(5,jd)=desctmp(1,jd)-desctmp(2,jd).
compute desctmp(6,jd)=desctmp(1,jd).
compute desctmp(7,jd)=desctmp(1,jd)+desctmp(2,jd).
compute modvals=desctmp(5:7,:).
compute mnotev=2.
do if (modvals(1,1) < desctmp(3,1)).
compute modvals(1,1)=desctmp(3,1).
compute minwarn=1.
end if.
do if (modvals(3,1) > desctmp(4,1)).
compute modvals(3,1)=desctmp(4,1).
compute maxwarn=1.
end if.
end if.
do if (desctmp(8,1)=1).
compute modvals={desctmp(3,1);desctmp(4,1)}.
compute mnotev=0.
compute minwarn=0.
compute maxwarn=0.
end if.
end if.
end loop
.
compute bad=bad+ (desctmp(2,1) <= 0.00000000001).
do if (bad = 0).
do if (holymoly < smallest).
compute smallest=holymoly.
end if.
do if (ydich=0 or (i < (nms+nys))).

.
do if ( 1 =1).
compute hatmat=inv(t( x )* x )*t( x ).
compute b = hatmat* y.
compute modres=b.
do if ( 0 =1).
compute n1=nrow( x ).
compute dfres=n1-(ncol( x )).
compute sstotal = t( y -(csum( y )/n1))*( y -(csum( y )/n1)).
compute resid= y - x *b.
compute ssresid = csum((resid)&**2).
compute r2 = (sstotal-ssresid)/sstotal.
do if (r2 < 0).
compute r2=0.
end if.
compute adjr2 = 1-((1-r2)*(n1-1)/(dfres)).
compute mse=ssresid/(n1-ncol( x )).

.
compute n1=nrow( x ).
compute invXtX = inv(t( x )* x ).
compute varb = mse *invXtX.
compute k3 = ncol( x ).
compute xhc=0.
do if ( hc <> 5).
compute xhc= x.
compute hat = xhc(:,1).
loop i3=1 to nrow(xhc).
compute hat(i3,1)= xhc(i3,:)*invXtX*t(xhc(i3,:)).
end loop.
do if ( hc = 0 or hc =1).
loop i3 = 1 to k3.
compute xhc(:,i3)=xhc(:,i3)&* resid.
end loop.
end if.
do if ( hc =3 or hc =2).
loop i3=1 to k3.
compute xhc(:,i3) = ( resid &/(1-hat)&**(1/(4- hc )))&*xhc(:,i3).
end loop.
end if.
do if ( hc = 4).
compute hcmn=make(n,2,4).
compute hcmn(:,2)=(n1*hat)/k3.
loop i3= 1 to k3.
compute xhc(:,i3) = ( resid &/(1-hat)&**(rmin(hcmn)/2))&*xhc(:,i3).
end loop.
end if.
compute varb=(invXtX*t(xhc)*xhc*invXtX).
do if ( hc =1).
compute varb=(n1/(n1-ncol( x )))&*varb.
end if.
end if.
compute hclab={'se(HC0)','se(HC1)','se(HC2)','se(HC3)','se(HC4)','se'}.
compute hclab=hclab(1,( hc +1)).
compute hcflab={'F(HC0)','F(HC1)','F(HC2)','F(HC3)','F(HC4)','F'}.
compute hcflab=hcflab(1,( hc +1)).
release xhc
.
compute seb=sqrt(diag(varb)).
compute trat = b&/seb.
compute p = 2*(1-tcdf(abs(trat), (dfres))).
compute tval = sqrt(dfres* (exp((dfres-(5/6))*((xp2/(dfres-(2/3)+(.11/dfres)))* (xp2/(dfres-(2/3)+(.11/dfres)))))-1)).
compute modres={modres,seb,trat,p}.
compute modres={modres,(b-tval&*seb),(b+tval&*seb)}.
compute modresl={'coeff',hclab,'t','p','LLCI','ULCI'}.
compute lmat = ident(ncol( x )).
compute lmat = lmat(:,2:ncol(lmat)).
compute fratio = (t(t(lmat)*b)*inv(t(lmat)*varb*lmat)*((t(lmat)*b)))/(ncol( x )-1).
compute pfr = 1-fcdf(fratio,(ncol( x )-1),dfres).
compute modsum={sqrt(r2),r2,mse,fratio,(ncol( x )-1),dfres,pfr}.
compute modsuml={'R','R-sq','MSE',hcflab,'df1','df2', 'p'}.
end if.
end if.
do if ( 1 = 2 or 1 =3).
compute xlp= x.
compute ylp= y.
compute pt2 = make(nrow(ylp),1,(csum(ylp)/nrow(ylp))).
do if ( 1 =2).
compute LL3 = ylp&*ln(pt2)+(1-ylp)&*ln(1-pt2).
end if.
compute LL3 = -2*csum(LL3).
compute bt1 = make(ncol(xlp),1,0).
compute LL1 = 0.
compute pt1 = make(nrow(ylp),1,0.5).
compute pt1lp=pt1.
loop jjj = 1 to iterate.
compute xlptmp=t(xlp).
compute vecprb=pt1lp&*(1-pt1lp).
loop kkk=1 to ncol(xlp).
compute xlptmp(kkk,:)=xlptmp(kkk,:)&*t(vecprb).
end loop.
compute b = bt1+inv(xlptmp*xlp)*t(xlp)*(ylp-pt1lp).
do if ( 1 =2).
compute xlpb=xlp*b.
compute xlpbt=(xlpb > -709.7).
compute xlpb709=(1-xlpbt)*(-709.7).
compute xlpb=(xlpb&*xlpbt)+xlpb709.
compute pt1lp = 1/(1+exp(-(xlpb))).
end if.
compute itprob = csum((pt1lp < .00000001) or (pt1lp > .9999999)).
do if (itprob > 0).
loop kkk = 1 to nrow(pt1lp).
do if (pt1lp(kkk,1) > .9999999).
compute pt1lp(kkk,1) = .9999999.
end if.
do if (pt1lp(kkk,1) < .00000001).
compute pt1lp(kkk,1) = .00000001.
end if.
end loop.
compute itprob = 0.
end if.
do if (itprob = 0).
do if ( 1 =2).
compute LL = ylp&*ln(pt1lp)+(1-ylp)&*ln(1-pt1lp).
end if.
compute LL2 = -2*csum(ll).
end if.
do if (abs(LL1-LL2) < converge).
do if ( 0 =1).
compute xlptmp=t(xlp).
compute vecprb=pt1lp&*(1-pt1lp).
loop kkk=1 to ncol(xlp).
compute xlptmp(kkk,:)=xlptmp(kkk,:)&*t(vecprb).
end loop.
compute varb = inv(xlptmp*xlp).
compute seb = sqrt(diag(varb)).
release xlptmp.
end if.
break.
end if.
compute bt1 = b.
compute LL1 = LL2.
end loop.
compute modres=b.
do if (jjj > iterate).
compute itprob = 2.
do if (booting=0).
compute iterrmod=1.
end if.
do if (booting=1).
compute bootiter=1.
end if.
do if (itprobtg=0).
compute itprobtg=1.
compute errcode(errs,1) = 47.
compute errs = errs + 1.
do if (booting = 0 and 0 =1).
compute vt1 = mdiag(pt1lp&*(1-pt1lp)).
compute varb = inv(t(xlp)*vt1*xlp).
compute seb = sqrt(diag(varb)).
end if.
end if.
end if.
do if ( 0 =1).
compute trat = b&/seb.
compute dfres=nrow(xlp).
compute p = 2*(1-cdfnorm(abs(trat))).
compute modres={modres,seb,trat,p}.
compute modres={modres,(b-xp2&*seb),(b+xp2&*seb)}.
compute pvchi=1-chicdf((LL3-LL2),(nrow(modres)-1)).
compute mcF = (LL3-LL2)/LL3.
compute cox = 1-exp(-(LL3-LL2)/nrow(xlp)).
compute nagel = cox/(1-exp(-(LL3)/nrow(xlp))).
compute modsum={LL2,(LL3-LL2),(nrow(modres)-1),pvchi, mcF,cox,nagel}.
compute modsuml={'-2LL','ModelLL', 'df', 'p', 'McFadden', 'CoxSnell', 'Nagelkrk'}.
compute modresl={'coeff','se','Z','p','LLCI','ULCI'}.
end if.
end if
.
end if.
do if (ydich=1 and (i = (nms+nys))).

.
do if ( 2 =1).
compute hatmat=inv(t( x )* x )*t( x ).
compute b = hatmat* y.
compute modres=b.
do if ( 0 =1).
compute n1=nrow( x ).
compute dfres=n1-(ncol( x )).
compute sstotal = t( y -(csum( y )/n1))*( y -(csum( y )/n1)).
compute resid= y - x *b.
compute ssresid = csum((resid)&**2).
compute r2 = (sstotal-ssresid)/sstotal.
do if (r2 < 0).
compute r2=0.
end if.
compute adjr2 = 1-((1-r2)*(n1-1)/(dfres)).
compute mse=ssresid/(n1-ncol( x )).

.
compute n1=nrow( x ).
compute invXtX = inv(t( x )* x ).
compute varb = mse *invXtX.
compute k3 = ncol( x ).
compute xhc=0.
do if ( hc <> 5).
compute xhc= x.
compute hat = xhc(:,1).
loop i3=1 to nrow(xhc).
compute hat(i3,1)= xhc(i3,:)*invXtX*t(xhc(i3,:)).
end loop.
do if ( hc = 0 or hc =1).
loop i3 = 1 to k3.
compute xhc(:,i3)=xhc(:,i3)&* resid.
end loop.
end if.
do if ( hc =3 or hc =2).
loop i3=1 to k3.
compute xhc(:,i3) = ( resid &/(1-hat)&**(1/(4- hc )))&*xhc(:,i3).
end loop.
end if.
do if ( hc = 4).
compute hcmn=make(n,2,4).
compute hcmn(:,2)=(n1*hat)/k3.
loop i3= 1 to k3.
compute xhc(:,i3) = ( resid &/(1-hat)&**(rmin(hcmn)/2))&*xhc(:,i3).
end loop.
end if.
compute varb=(invXtX*t(xhc)*xhc*invXtX).
do if ( hc =1).
compute varb=(n1/(n1-ncol( x )))&*varb.
end if.
end if.
compute hclab={'se(HC0)','se(HC1)','se(HC2)','se(HC3)','se(HC4)','se'}.
compute hclab=hclab(1,( hc +1)).
compute hcflab={'F(HC0)','F(HC1)','F(HC2)','F(HC3)','F(HC4)','F'}.
compute hcflab=hcflab(1,( hc +1)).
release xhc
.
compute seb=sqrt(diag(varb)).
compute trat = b&/seb.
compute p = 2*(1-tcdf(abs(trat), (dfres))).
compute tval = sqrt(dfres* (exp((dfres-(5/6))*((xp2/(dfres-(2/3)+(.11/dfres)))* (xp2/(dfres-(2/3)+(.11/dfres)))))-1)).
compute modres={modres,seb,trat,p}.
compute modres={modres,(b-tval&*seb),(b+tval&*seb)}.
compute modresl={'coeff',hclab,'t','p','LLCI','ULCI'}.
compute lmat = ident(ncol( x )).
compute lmat = lmat(:,2:ncol(lmat)).
compute fratio = (t(t(lmat)*b)*inv(t(lmat)*varb*lmat)*((t(lmat)*b)))/(ncol( x )-1).
compute pfr = 1-fcdf(fratio,(ncol( x )-1),dfres).
compute modsum={sqrt(r2),r2,mse,fratio,(ncol( x )-1),dfres,pfr}.
compute modsuml={'R','R-sq','MSE',hcflab,'df1','df2', 'p'}.
end if.
end if.
do if ( 2 = 2 or 2 =3).
compute xlp= x.
compute ylp= y.
compute pt2 = make(nrow(ylp),1,(csum(ylp)/nrow(ylp))).
do if ( 2 =2).
compute LL3 = ylp&*ln(pt2)+(1-ylp)&*ln(1-pt2).
end if.
compute LL3 = -2*csum(LL3).
compute bt1 = make(ncol(xlp),1,0).
compute LL1 = 0.
compute pt1 = make(nrow(ylp),1,0.5).
compute pt1lp=pt1.
loop jjj = 1 to iterate.
compute xlptmp=t(xlp).
compute vecprb=pt1lp&*(1-pt1lp).
loop kkk=1 to ncol(xlp).
compute xlptmp(kkk,:)=xlptmp(kkk,:)&*t(vecprb).
end loop.
compute b = bt1+inv(xlptmp*xlp)*t(xlp)*(ylp-pt1lp).
do if ( 2 =2).
compute xlpb=xlp*b.
compute xlpbt=(xlpb > -709.7).
compute xlpb709=(1-xlpbt)*(-709.7).
compute xlpb=(xlpb&*xlpbt)+xlpb709.
compute pt1lp = 1/(1+exp(-(xlpb))).
end if.
compute itprob = csum((pt1lp < .00000001) or (pt1lp > .9999999)).
do if (itprob > 0).
loop kkk = 1 to nrow(pt1lp).
do if (pt1lp(kkk,1) > .9999999).
compute pt1lp(kkk,1) = .9999999.
end if.
do if (pt1lp(kkk,1) < .00000001).
compute pt1lp(kkk,1) = .00000001.
end if.
end loop.
compute itprob = 0.
end if.
do if (itprob = 0).
do if ( 2 =2).
compute LL = ylp&*ln(pt1lp)+(1-ylp)&*ln(1-pt1lp).
end if.
compute LL2 = -2*csum(ll).
end if.
do if (abs(LL1-LL2) < converge).
do if ( 0 =1).
compute xlptmp=t(xlp).
compute vecprb=pt1lp&*(1-pt1lp).
loop kkk=1 to ncol(xlp).
compute xlptmp(kkk,:)=xlptmp(kkk,:)&*t(vecprb).
end loop.
compute varb = inv(xlptmp*xlp).
compute seb = sqrt(diag(varb)).
release xlptmp.
end if.
break.
end if.
compute bt1 = b.
compute LL1 = LL2.
end loop.
compute modres=b.
do if (jjj > iterate).
compute itprob = 2.
do if (booting=0).
compute iterrmod=1.
end if.
do if (booting=1).
compute bootiter=1.
end if.
do if (itprobtg=0).
compute itprobtg=1.
compute errcode(errs,1) = 47.
compute errs = errs + 1.
do if (booting = 0 and 0 =1).
compute vt1 = mdiag(pt1lp&*(1-pt1lp)).
compute varb = inv(t(xlp)*vt1*xlp).
compute seb = sqrt(diag(varb)).
end if.
end if.
end if.
do if ( 0 =1).
compute trat = b&/seb.
compute dfres=nrow(xlp).
compute p = 2*(1-cdfnorm(abs(trat))).
compute modres={modres,seb,trat,p}.
compute modres={modres,(b-xp2&*seb),(b+xp2&*seb)}.
compute pvchi=1-chicdf((LL3-LL2),(nrow(modres)-1)).
compute mcF = (LL3-LL2)/LL3.
compute cox = 1-exp(-(LL3-LL2)/nrow(xlp)).
compute nagel = cox/(1-exp(-(LL3)/nrow(xlp))).
compute modsum={LL2,(LL3-LL2),(nrow(modres)-1),pvchi, mcF,cox,nagel}.
compute modsuml={'-2LL','ModelLL', 'df', 'p', 'McFadden', 'CoxSnell', 'Nagelkrk'}.
compute modresl={'coeff','se','Z','p','LLCI','ULCI'}.
end if.
end if
.
end if.
compute modres2={modres2,t(modres)}.
do if (i = (nms+nys)).
do if (bcmat((i+1),1) = 1).
compute bootdir={bootdir;t(modres(wherex(1,i):wherex(2,i),1))}.
end if.
do if (bcmat((i+1),1) = 0).
compute bootdir={bootdir;dirzes}.
end if.
end if.
do if (model = 74 and i <= nms).
compute onetemp=make(nrow(xprobval),1,1).
compute mestmtb={onetemp,xprobval}.
do if (ncs > 0).
compute ncovmdlb=rsum(ccmat(i,:)).
do if (ncovmdl > 0).
compute cvmncb=make(nrow(mestmtb),ncovmdlb,1).
compute cvmnctmp=csum(x(:,(ncol(x)-ncovmdlb+1):ncol(x)))/nrow(x).
do if (cuscoval > 0).
compute cvmnctmp=coval.
end if.
loop mestlp=1 to ncovmdlb.
compute cvmncb(:,mestlp)=cvmncb(:,mestlp)*cvmnctmp(:,mestlp).
end loop.
compute mestmtb={mestmtb,cvmncb}.
end if.
end if.
compute mestbt=mestmtb*modres.
do if (i = 1).
compute mestb=mestbt.
end if.
do if (i > 1).
compute mestb={mestb,mestbt}.
end if.
end if.
do if (model = 74 and i = (nms+nys)).
compute xvalptmp=make(1,nxvls,0).
compute mest74sp=make(1,nms,0).
compute mest74sp={mest74sp;mestb}.
compute xvalptmp={xvalptmp;ident(nxvls)}.
compute dirfxcf=make(nrow(mestb),1,0).
loop cfloop1=1 to nrow(mestb).
compute ndirfx=make(nrow(modres),1,0).
compute ndirfx(2:(nxvls+1),1)=t(xvalptmp(cfloop1,:)).
loop cfloop3=1 to nms.
do if (mcx=1 or mcx=0).
compute ndirfx(wheremw((1+((cfloop3-1)*2)),ncol(wheremw)):wheremw((2+((cfloop3-1)*2) ),ncol(wheremw)))=t(xvalptmp(cfloop1,:)*mestb(1,cfloop3)).
end if.
do if (mcx=2).
compute ndirfx(wheremw((1+((cfloop3-1)*2)),ncol(wheremw)):wheremw((2+((cfloop3-1)*2) ),ncol(wheremw)))=t(xvalptmp(cfloop1,:)*mest74sp(cfloop1,cfloop3)).
end if.
end loop.
do if (cfloop1 > 1).
compute dirfxcf(cfloop1,1)=t(ndirfx)*modres*xscaling.
end if.
end loop.
compute natdirbt={natdirbt;t(dirfxcf(2:nrow(dirfxcf),:))}.
end if.
do if ( (bcmat((i+1),1) = 1) and (nobootx=1) and (effsize=1)).
compute nobootx=0.
compute xsdtemp= (nrow(x)*sscp(x(:,2)))-(t(csum(x(:,2)))*(csum(x(:,2)))).
compute xsdtemp= sqrt(xsdtemp/(nrow(x)*(nrow(x)-1))).
end if.
end if.
end loop.
do if (bad = 0).
compute bootres={bootres;modres2(:,2:ncol(modres2))}.
do if (effsize=1).
compute ysdtemp= (nrow(y)*sscp(y))-(t(csum(y))*(csum(y))).
compute ysdtemp= sqrt(ysdtemp/(nrow(y)*(nrow(y)-1))).
compute bootysd={bootysd;ysdtemp}.
compute bootxsd={bootxsd;xsdtemp}.
end if.
compute goodboot=goodboot+1.
end if.
do if (bad <> 0).
compute badboot=badboot+1.
end if.
end loop if (goodboot = boot).
compute bootres=bootres(2:nrow(bootres),:).
do if (effsize=1).
compute bootysd=bootysd(2:nrow(bootysd),:).
do if (nrow(bootxsd) > 1).
compute bootxsd=bootxsd(2:nrow(bootxsd),:).
end if.
end if.
do if (goodboot < (boot)).
compute boot=0.
compute modelbt=0.
compute notecode(notes,1) = 7.
compute notes = notes + 1.
end if.
do if (boot > 0).
do if (effsize=1).
compute bootysd={ysd;bootysd}.
compute bootxsd={xsd;bootxsd}.
end if.
do if (saveboot = 1).
save bootres/outfile = *.
end if.
do if (modelbt=1).
compute bootcim=make(ncol(bootres),5,-99999).
compute bootcim(:,2) = t(csum(bootres)/nrow(bootres)).
compute bootcim(:,1) = coeffmat(2:nrow(coeffmat),1).
loop i = 1 to ncol(bootres).

.
compute temp = bootres(:,i).
compute temp(GRADE( bootres(:,i) )) = bootres(:,i).
compute badlo = 0.
compute badhi = 0.
do if ( (bootcim(i,1)*bc)+(9999*(1-bc)) <> 9999).
compute pv=csum(temp < (bootcim(i,1)*bc)+(9999*(1-bc)) )/nrow(temp).
compute ppv = pv.
do if (pv > .5).
compute ppv = 1-pv.
end if.
compute y5=sqrt(-2*ln(ppv)).
compute xp=y5+((((y5*p4+p3)*y5+p2)*y5+p1)*y5+p0)/((((y5*q4+q3)*y5+q2)*y5+q1)*y5+q0).
do if (pv <= .5).
compute xp = -xp.
end if.
compute cilow=rnd(nrow(temp)*(cdfnorm(2*xp-xp2))).
compute cihigh=trunc(nrow(temp)*(cdfnorm(2*xp+(xp2))))+1.
do if (cilow < 1).
compute cilow = 1.
compute booterr=1.
compute badlo = 1.
end if.
do if (cihigh > nrow(temp)).
compute cihigh = boot.
compute booterr=1.
compute badhi = 1.
end if.
compute llcit=temp(cilow,1).
compute ulcit=temp(cihigh,1).
do if (badlo = 1 and llcit <> priorlo).
compute badend={badend, llcit}.
compute priorlo = llcit.
end if.
do if (badhi = 1 and ulcit <> priorhi).
compute badend={badend, ulcit}.
compute priorhi = ulcit.
end if.
end if.
do if ( (bootcim(i,1)*bc)+(9999*(1-bc)) = 9999).
compute llcit=temp(cilow,1).
compute ulcit=temp(cihigh,1).
end if.
compute bootse=t(sqrt((cssq(temp)-((csum(temp)&**2)/nrow(temp)))/(nrow(temp)-1)))
.
compute bootcim(i,4:5)={llcit,ulcit}.
compute bootcim(i,3)=bootse.
end loop.
end if.
end if.
do if (badboot > 0).
compute notecode(notes,1) = 6.
compute notes = notes + 1.
end if.
end if.
do if (criterr=0).
release fulldat,x,y.
end if.
do if (xmint=1 and criterr=0).
compute xvalptmp=make(1,nxvls,0).
compute mest74sp=make(1,nms,0).
compute mest74sp={mest74sp;mest74}.
compute xvalptmp={xvalptmp;ident(nxvls)}.
loop kcfuhd=1 to 2.
compute dirfxcf=make(nrow(mest74),6,0).
loop cfloop1=1 to nrow(mest74).
compute ndirfx=make(nrow(lastb),1,0).
compute ndirfx(2:(nxvls+1),1)=t(xvalptmp(cfloop1,:)).
loop cfloop3=1 to nms.
do if (kcfuhd=1).
do if (mcx=1 or mcx=0).
compute ndirfx(wheremw((1+((cfloop3-1)*2)),ncol(wheremw)):wheremw((2+((cfloop3-1)*2)), ncol(wheremw)))=t(xvalptmp(cfloop1,:)*mest74(1,cfloop3)).
end if.
do if (mcx=2).
compute ndirfx(wheremw((1+((cfloop3-1)*2)),ncol(wheremw)):wheremw((2+((cfloop3-1)*2)), ncol(wheremw)))=t(xvalptmp(cfloop1,:)*mest74sp(cfloop1,cfloop3)).
end if.
end if.
do if (kcfuhd=2).
compute ndirfx(wheremw((1+((cfloop3-1)*2)),ncol(wheremw)):wheremw((2+((cfloop3-1)*2)), ncol(wheremw)))=t(xvalptmp(cfloop1,:)*medmeans(1,cfloop3)).
end if.
end loop.
do if (cfloop1 > 1).
compute dirfxcf(cfloop1,1)=t(ndirfx)*lastb*xscaling.
compute dirfxcf(cfloop1,2)=sqrt(diag(t(ndirfx)*lastcov*ndirfx))*abs(xscaling).
compute dirfxcf(cfloop1,3)=dirfxcf(cfloop1,1)/dirfxcf(cfloop1,2).
compute dirfxcf(cfloop1,4)=2*(1-tcdf(abs(dirfxcf(cfloop1,3)), dfres)).
compute dirfxcf(cfloop1,5)=dirfxcf(cfloop1,1)-tval*dirfxcf(cfloop1,2).
compute dirfxcf(cfloop1,6)=dirfxcf(cfloop1,1)+tval*dirfxcf(cfloop1,2).
end if.
end loop.
compute codireff=dirfxcf(2:nrow(dirfxcf),:).
do if (kcfuhd=1).
compute direff=codireff.
end if.
end loop.
end if.

.
do if (criterr = 0 and nms > 0).
compute paths=paths(:,2:ncol(paths)).
compute pathsw=pathsw(:,2:ncol(pathsw)).
compute pathsz=pathsz(:,2:ncol(pathsz)).
compute pathswz=pathswz(:,2:ncol(pathswz)).
compute pathsmod=pathsw+pathsz+pathswz.
compute pathsdv=pathsdv(:,2:ncol(pathsdv)).
compute pathsfoc=pathsfoc(:,2:ncol(pathsfoc)).
compute pathtype=pathtype(:,2:ncol(pathtype)).
compute anymod=(rsum(pathsmod) > 0).
compute obscoeff=obscoeff(1,2:ncol(obscoeff)).
do if (outscree=1).
do if (xmint=1).
print/title = '************************* COUNTERFACTUALLY DEFINED ************************'.
else.
print/title = ' '.
end if.
do if (dototal = 0 and alttotal=0).
print/title = '****************** DIRECT AND INDIRECT EFFECTS OF X ON Y *****************'/space=0.
end if.
do if (alttotal=1).
print/title = '************** TOTAL, DIRECT, AND INDIRECT EFFECTS OF X ON Y **************'/space=0.
end if.
end if.
do if (dototal = 1).
do if (outscree=1).
print/title = '************** TOTAL, DIRECT, AND INDIRECT EFFECTS OF X ON Y **************'/space=0.
end if.
compute totefflb(1,1)='Effect'.
do if (effsize=1).
compute toteffsz=toteff(:,1)/ysd.
do if (xdich=1 or mcx > 0).
compute totefflb={totefflb,'c_ps'}.
end if.
do if (xdich = 0 and mcx = 0).
compute toteffsz=toteffsz*xsd.
compute totefflb={totefflb,'c_cs'}.
end if.
compute toteff={toteff,toteffsz}.
end if.
compute resultm2=make(nrow(toteff),maxresm,99999).
compute resultm2(1:nrow(toteff),1:ncol(toteff))=toteff.
compute resultm={resultm;resultm2}.
do if (nxvls > 1).
compute resultm2=make(nrow(totomni),maxresm,99999).
compute resultm2(1:nrow(totomni),1:ncol(totomni))=totomni.
compute resultm={resultm;resultm2}.
compute clabtmp={'R2-chng', hcflab, 'df1','df2','p'}.
do if (outscree=1).
print toteff/title='Relative total effects of X on Y'/rnames=toteffl2/cnames=totefflb/format= F10.4.
print totomni/title='Omnibus test of total effect of X on Y'/cnames=clabtmp/format= F10.4.
print/title= '----------'/space=0.
end if.
else.
do if (outscree=1).
print toteff/title='Total effect of X on Y'/cnames=totefflb/format= F10.4.
end if.
end if.
end if.
compute moddir=wcmat(nrow(bcmat),1)+zcmat(nrow(bcmat),1).
do if (xmint=1).
compute moddir=1.
end if.
do if (bcmat(nrow(bcmat),1)=1).
do if (ydich=1).
compute direfflb(:,(ncol(direfflb)-5):ncol(direfflb))={'Effect','se','Z','p','LLCI','ULCI'}.
end if.
do if (moddir=0 or xmint=1).
compute direfflb(1,1)='Effect'.
end if.
do if (effsize=1 and moddir=0 and anymod = 0).
compute direffsz=direff(:,1)/ysd.
do if (xdich=1 or mcx > 0).
compute direfflb={direfflb,'c''_ps'}.
end if.
do if (xdich = 0 and mcx = 0).
compute direffsz=direffsz*xsd.
compute direfflb={direfflb,'c''_cs'}.
end if.
compute direff={direff,direffsz}.
end if.
compute resultm2=make(nrow(direff),maxresm,99999).
compute resultm2(1:nrow(direff),1:ncol(direff))=direff.
compute resultm={resultm;resultm2}.
do if (xmint=1).
compute resultm2=make(nrow(codireff),maxresm,99999).
compute resultm2(1:nrow(codireff),1:ncol(codireff))=codireff.
compute resultm={resultm;resultm2}.
end if.
do if (moddir = 0 and nxvls=1).
do if (outscree=1).
print direff/title='Direct effect of X on Y'/cnames=direfflb/format= F10.4.
end if.
end if.
do if (moddir = 0 and nxvls>1).
compute resultm2=make(nrow(diromni),maxresm,99999).
compute resultm2(1:nrow(diromni),1:ncol(diromni))=diromni.
compute resultm={resultm;resultm2}.
do if (outscree=1).
print direff/title='Relative direct effects of X on Y'/rnames=direffl2/cnames=direfflb/format= F10.4.
do if (ydich = 0).
compute clabtmp={'R2-chng', hcflab, 'df1','df2','p'}.
print diromni/title='Omnibus test of direct effect of X on Y:'/cnames=clabtmp/format= F10.4.
end if.
do if (ydich = 1).
compute clabtmp={'Chi-sq', 'df', 'p'}.
print diromni/title='Omnibus likelihood ratio test of direct effect of X on Y'/cnames=clabtmp/format= F10.4.
end if.
print/title= '----------'/space=0.
end if.
end if.
do if (moddir > 0 and nxvls=1).
do if (xmint=0 and outscree=1).
print direff/title='Conditional direct effects of X on Y'/cnames=direfflb/format= F10.4.
end if.
do if (xmint=1).
do if (outscree=1).
print direff/title='(Pure) Natural direct effect of X on Y'/cnames=direfflb/format= F10.4.
print codireff/title='Controlled direct effect of X on Y'/cnames=direfflb/format= F10.4.
print/title= '----------'/space=0.
end if.
compute obnatdfx=direff(:,1).
end if.
end if.
compute direffl4=direffl2.
do if (moddir > 0 and nxvls>1).
compute direffl2={' '}.
loop i = 1 to nxvls.
loop j = 1 to (nrow(direff)/nxvls).
compute direffl2={direffl2;xcatlab(i,1)}.
end loop.
end loop.
compute direffl2=direffl2(2:nrow(direffl2),1).
do if (xmint=0 and outscree=1).
print direff/title='Relative conditional direct effects of X on Y'/cnames=direfflb/rnames=direffl2/format= F10.4.
end if.
do if (xmint=1).
do if (outscree=1).
print direff/title='Relative (pure) natural direct effects of X on Y'/cnames=direfflb/rnames=direffl2/format= F10.4.
print codireff/title='Relative controlled direct effects of X on Y'/cnames=direfflb/rnames=direffl2/format= F10.4.
print/title= '----------'/space=0.
end if.
compute obnatdfx=direff(:,1).
end if.
end if.
compute direffl2=direffl4.
end if.
do if (bcmat(nrow(bcmat),1)=0 and xmint <> 1 and outscree=1).
print/title='The direct effect of X on Y is fixed to zero.'.
end if.
do if (nms = 1).
compute indmark={2}.
compute indsets={1,2}.
end if.
do if (nms = 2).
compute indmark={2,2,3}.
compute indsets={1,4,2,5,1,3,5}.
compute thetam=1.
end if.
do if (nms = 3).
compute indmark={2,2,2,3,3,3,4}.
compute indsets={1,7,2,8,4,9,1,3,8,1,5,9,2,6,9,1,3,6,9}.
compute thetam={1,2,3}.
end if.
do if (nms = 4).
compute indmark={2,2,2,2,3,3,3,3,3,3,4,4,4,4,5}.
compute indsets={1,11,2,12,4,13,7,14,1,3,12,1,5,13,1,8,14,2,6,13,2,9,14,4,10,14,1,3,6,13,1,3,9, 14,1,5,10,14,2,6,10,14,1,3,6,10,14}.
compute thetam={1,2,4,3,5,6}.
end if.
do if (nms = 5).
compute indmark={2,2,2,2,2,3,3,3,3,3,3,3,3,3,3,4,4,4,4,4,4,4,4,4,4,5,5,5,5,5,6}.
compute indsets={1,16,2,17,4,18,7,19,11,20,1,3,17,1,5,18,1,8,19,1,12,20,2,6,18,2,9,19,2,13,20,4, 10,19,4,14,20,7,15,20,1,3,6,18, 1,3,9,19,1,3,13,20,1,5,10,19,1,5,14,20,1,8,15,20,2,6,10,19,2,6, 14,20,2,9,15,20,4,10,15,20,1,3,6,10,19, 1,3,6,14,20,1,3,9,15,20,1,5,10,15,20,2,6,10,15,20,1,3,6,10,15, 20}.
compute thetam={1,2,5,3,6,8,4,7,9,10}.
end if.
do if (nms = 6).
compute indmark={2,2,2,2,2,2,3,3,3,3,3,3,3,3,3,3,3,3,3,3,3,4,4,4,4,4,4,4,4,4,4,4,4,4,4,4,4,4,4, 4,4,5,5,5,5,5,5,5,5,5,5,5,5,5,5,5,6,6,6,6,6,6,7}.
compute indsets={1,22,2,23,4,24,7,25,11,26,16,27,1,3,23,1,5,24,1,8,25,1,12,26,1,17,27,2,6,24,2, 9,25,2,13,26,2,18,27,4,10,25,4,14,26, 4,19,27,7,15,26,7,20,27,11,21,27,1,3,6,24,1,3,9,25,1,3,13,26,1, 3,18,27,1,5,10,25,1,5,14,26,1,5,19,27,1,8,15,26,1,8,20,27, 1,12,21,27,2,6,10,25,2,6,14,26,2,6,19,27,2,9,15,26,2,9,20,27,2, 13,21,27,4,10,15,26,4,10,20,27,4,14,21,27,7,15,21,27, 1,3,6,10,25,1,3,6,14,26,1,3,6,19,27,1,3,9,15,26,1,3,9,20,27,1,3, 13,21,27,1,5,10,15,26,1,5,10,20,27,1,5,14,21,27, 1,8,15,21,27,2,6,10,15,26,2,6,10,20,27,2,6,14,21,27,2,9,15,21, 27,4,10,15,21,27,1,3,6,10,15,26,1,3,6,10,20,27, 1,3,6,14,21,27,1,3,9,15,21,27,1,5,10,15,21,27,2,6,10,15,21,27, 1,3,6,10,15,21,27}.
compute thetam={1,2,6,3,7,10,4,8,11,13,5,9,12,14,15}.
end if.
do if (nms = 7).
compute indmark={2,2,2,2,2,2,2}.
compute indsets={1,29,2,30,4,31,7,32,11,33,16,34,22,35}.
end if.
do if (nms = 8).
compute indmark={2,2,2,2,2,2,2,2}.
compute indsets={1,37,2,38,4,39,7,40,11,41,16,42,22,43,29,44}.
end if.
do if (nms = 9).
compute indmark={2,2,2,2,2,2,2,2,2}.
compute indsets={1,46,2,47,4,48,7,49,11,50,16,51,22,52,29,53,37,54}.
end if.
do if (nms=10).
compute indmark={2,2,2,2,2,2,2,2,2,2}.
compute indsets={1,56,2,57,4,58,7,59,11,60,16,61,22,62,29,63,37,64,46,65}.
end if.
compute indlbl = {'Ind1'; 'Ind2'; 'Ind3'; 'Ind4'; 'Ind5'; 'Ind6'; 'Ind7'; 'Ind8'; 'Ind9'; 'Ind10'; 'Ind11'; 'Ind12'; 'Ind13'; 'Ind14'; 'Ind15'}.
compute indlbl = {indlbl; 'Ind16';'Ind17';'Ind18';'Ind19';'Ind20';'Ind21';'Ind22';'Ind23';'Ind24';'Ind25';'Ind26';'Ind27';'Ind28';'Ind29';'Ind30'}.
compute indlbl = {indlbl; 'Ind31';'Ind32';'Ind33';'Ind34';'Ind35';'Ind36';'Ind37';'Ind38';'Ind39';'Ind40';'Ind41';'Ind42';'Ind43';'Ind44';'Ind45'}.
compute indlbl = {indlbl; 'Ind46';'Ind47';'Ind48';'Ind49';'Ind50';'Ind51';'Ind52';'Ind53';'Ind54';'Ind55';'Ind56';'Ind57';'Ind58';'Ind59';'Ind60'}.
compute indlbl = {indlbl; 'Ind61';'Ind62';'Ind63';'Ind64';'Ind65';'Ind66';'Ind67';'Ind68';'Ind69';'Ind70';'Ind71';'Ind72';'Ind73';'Ind74';'Ind75'}.
compute indlbl = {indlbl; 'Ind76';'Ind77';'Ind78';'Ind79';'Ind80';'Ind81';'Ind82';'Ind83';'Ind84';'Ind85';'Ind86';'Ind87';'Ind88';'Ind89';'Ind90'}.
compute cntname={'(C1)';'(C2)';'(C3)';'(C4)';'(C5)';'(C6)';'(C7)';'(C8)';'(C9)';'(C10)';'(C11)';'(C12)';'(C13)';'(C14)';'(C15)';'(C16)';'(C17)'}.
compute cntname={cntname;'(C18)';'(C19)';'(C20)';'(C21)';'(C22)';'(C23)';'(C24)';'(C25)';'(C26)';'(C27)';'(C28)';'(C29)';'(C30)';'(C31)'}.
compute cntname={cntname;'(C32)';'(C33)';'(C34)';'(C35)';'(C36)';'(C37)';'(C38)';'(C39)';'(C40)';'(C41)';'(C42)';'(C43)';'(C44)';'(C45)'}.
compute cntname={cntname;'(C46)';'(C47)';'(C48)';'(C49)';'(C50)';'(C51)';'(C52)';'(C53)';'(C54)';'(C55)';'(C56)';'(C57)';'(C58)';'(C59)'}.
compute cntname={cntname;'(C60)';'(C61)';'(C62)';'(C63)';'(C64)';'(C65)';'(C66)';'(C67)';'(C68)';'(C69)';'(C70)';'(C71)';'(C72)';'(C73)'}.
compute cntname={cntname;'(C74)';'(C75)';'(C76)';'(C77)';'(C78)';'(C79)';'(C80)';'(C81)';'(C82)';'(C83)';'(C84)';'(C85)';'(C86)';'(C87)'}.
compute cntname={cntname;'(C88)';'(C89)';'(C90)';'(C91)';'(C92)';'(C93)';'(C94)';'(C95)';'(C96)';'(C97)';'(C98)';'(C99)';'(C100)';'(C101)'}.
compute cntname={cntname;'(C102)';'(C103)';'(C104)';'(C105)'}.
compute indmake=make(ncol(indmark),(nms+2),0).
compute indmod=make(ncol(indmark),1,999).
compute indmmm=make(ncol(indmark),1,0).
compute indmmmt=make(ncol(indmark),1,0).
compute start=1.
compute end=0.
compute nindfx=0.
compute indlocs=make(nrow(thetaxmb),ncol(paths),999).
compute indkey=make(ncol(indmark),1+((rmax(indmark)*2)+1),'     ').
compute c1=1.
compute c2=1.
compute c3=1.
loop i = 1 to ncol(paths).
do if (pathtype(1,i)=1).
compute indlocs(:,i)=thetaxmb(:,c1).
compute c1=c1+1.
end if.
do if (pathtype(1,i)=3).
compute indlocs(:,i)=thetamyb(:,c2).
compute c2=c2+1.
end if.
do if (pathtype(1,i)=2 and nms < 7 and serial=1).
compute indlocs(:,i)=thetammb(:,thetam(1,c3)).
compute c3=c3+1.
end if.
end loop.
loop i = 1 to ncol(indlocs).
compute c1=2.
loop j = 2 to nrow(indlocs).
do if (indlocs(j,i) <> 0).
compute indlocs(c1,i)=indlocs(j,i).
compute c1=c1+1.
end if.
end loop.
compute indlocs(1,i)=c1-2.
end loop.
compute indlocs=indlocs(1:rmax((indlocs(1,:))+1),:).
loop i = 1 to ncol(indmark).
compute numget=indmark(1,i).
compute end=end+numget.
compute gotcha=indsets(1,start:end).
compute start=end+1.
compute ok=1.
compute temp=0.
compute repoman=make(4,1,0).
loop j = 1 to ncol(gotcha).
do if paths(1,gotcha(1,j))=0.
compute ok=0.
end if.
do if (pathsmod(1,gotcha(1,j)) > 0).
compute temp=1.
compute temp2={pathsw(1,gotcha(1,j));pathsz(1,gotcha(1,j));pathswz(1,gotcha(1,j));0}.
do if (temp2(1,1)=1 and temp2(2,1)=1 and temp2(3,1)=0).
compute temp2(4,1)=1.
end if.
compute repoman=repoman+temp2.
end if.
end loop.
compute temp=0.
compute tempmmm=0.
compute typemmm=0.
do if ((repoman(1,1) > 0) and (repoman(2,1) = 0)).
compute temp=1.
do if (repoman(1,1)=1).
compute tempmmm=1.
end if.
do if (repoman(1,1) > 1 and (wdich=1 or mcw > 0)).
compute tempmmm=12.
compute typemmm=mcw.
do if (wdich=1).
compute typemmm=1.
end if.
end if.
do if (repoman(1,1) > 1 and (wdich=0 and mcw = 0)).
compute tempmmm=101.
end if.
end if.
do if ((repoman(1,1) = 0) and (repoman(2,1) > 0)).
compute temp=2.
do if (repoman(2,1)=1).
compute tempmmm=2.
end if.
do if (repoman(2,1) > 1 and (zdich = 1 or mcz > 0)).
compute tempmmm=22.
compute typemmm=mcz.
do if (zdich=1).
compute typemmm=1.
end if.
end if.
do if (repoman(2,1) > 1 and (zdich = 0 and mcw = 0)).
compute tempmmm=102.
end if.
end if.
do if (repoman(1,1)>0 and repoman(2,1)>0).
compute temp=3.
do if (repoman(1,1)=1 and repoman(2,1)=1).
do if (repoman(4,1)=1).
compute tempmmm=31.
end if.
do if (repoman(3,1)=1).
compute tempmmm=41.
end if.
end if.
end if.
do if (repoman(1,1)=1 and repoman(2,1)=1 and repoman(3,1)=0 and repoman(4,1)=0).
compute tempmmm=51.
end if.
do if (ok = 1).
compute nindfx=nindfx+1.
compute indmake(nindfx,1)=numget.
compute indmod(nindfx,1)=temp.
compute indmmm(nindfx,1)=tempmmm.
compute indmmmt(nindfx,1)=typemmm.
compute indmake(nindfx,2:(1+numget))=gotcha.
compute indkey(nindfx,1)=xnames.
loop j = 1 to numget.
compute indkey(nindfx,(j*2+1))=pathsdv(1,gotcha(1,j)).
compute indkey(nindfx,(j*2))='   ->   '.
end loop.
end if.
end loop.
compute indkey=indkey(1:nindfx,1:((cmax(indmake(:,1))*2)+1)).
compute indmake=indmake(1:nindfx,1:(cmax(indmake(:,1))+1)).
compute indmod=indmod(1:nrow(indmake),1).
compute indmmm=indmmm(1:nrow(indmake),1).
compute indmmmt=indmmmt(1:nrow(indmake),1).
compute ncpairs = (((nindfx)*(nindfx-1))/2).
do if ((contrast = 1 or contrast = 2 or contrast=3) and (ncpairs > 105)).
compute contrast=0.
compute notecode(notes,1) = 13.
compute notes = notes + 1.
end if.
do if (contrast = 4).
do if (ncol(contvec) <> nindfx).
compute contrast=0.
compute notecode(notes,1) = 14.
compute notes = notes + 1.
end if.
end if.
do if (anymod=0).
do if (nms = 1 and contrast > 0).
compute contrast=0.
end if.
compute efloop=(((1-(effsize=0))*2)+1)-(((mcx>0 or xdich=1))*(1-(effsize=0))).
loop kk=1 to efloop.
do if (boot = 0).
compute bootres=obscoeff.
do if (kk=1).
compute totbtvec=make(1,nxvls,0).
end if.
compute bootdir=obsdirfx.
compute indtab=999.
compute inddiff=999.
compute bootysd=ysd.
compute bootxsd=xsd.
end if.
do if (boot > 0).
compute bootres={obscoeff;bootres}.
do if (kk=1).
compute totbtvec=make(nrow(bootres),nxvls,0).
end if.
compute indtab=make(1,4,999).
compute inddiff=make(nrow(bootres),1,999).
end if.
compute indtotal=make(nrow(bootres),1,0).
loop i = 1 to nrow(indmake).
loop j = 1 to nxvls.
compute indtemp=make(nrow(bootres),1,1).
loop k = 1 to indmake(i,1).
compute jtemp=1.
do if (j > 1 and k=1).
compute jtemp=j.
end if.
compute indtemp=indtemp&*bootres(:,pathsfoc(jtemp,indmake(i,(k+1)))).
end loop.
do if (kk = 2).
compute indtemp=indtemp/bootysd.
end if.
do if (kk = 3).
compute indtemp=(bootxsd&*indtemp)/bootysd.
end if.
do if (contrast <> 0).
compute inddiff={inddiff,indtemp}.
end if.
do if (nxvls=1).
compute indtotal=indtotal+indtemp.
end if.
compute indeff=indtemp(1,1).
do if (kk=1).
compute totbtvec(:,j)=totbtvec(:,j)+indtemp.
end if.
do if (boot > 0).

.
compute temp = indtemp(2:nrow(indtemp),1).
compute temp(GRADE( indtemp(2:nrow(indtemp),1) )) = indtemp(2:nrow(indtemp),1).
compute badlo = 0.
compute badhi = 0.
do if ( (indtemp(1,1)*bc)+(9999*(1-bc)) <> 9999).
compute pv=csum(temp < (indtemp(1,1)*bc)+(9999*(1-bc)) )/nrow(temp).
compute ppv = pv.
do if (pv > .5).
compute ppv = 1-pv.
end if.
compute y5=sqrt(-2*ln(ppv)).
compute xp=y5+((((y5*p4+p3)*y5+p2)*y5+p1)*y5+p0)/((((y5*q4+q3)*y5+q2)*y5+q1)*y5+q0).
do if (pv <= .5).
compute xp = -xp.
end if.
compute cilow=rnd(nrow(temp)*(cdfnorm(2*xp-xp2))).
compute cihigh=trunc(nrow(temp)*(cdfnorm(2*xp+(xp2))))+1.
do if (cilow < 1).
compute cilow = 1.
compute booterr=1.
compute badlo = 1.
end if.
do if (cihigh > nrow(temp)).
compute cihigh = boot.
compute booterr=1.
compute badhi = 1.
end if.
compute llcit=temp(cilow,1).
compute ulcit=temp(cihigh,1).
do if (badlo = 1 and llcit <> priorlo).
compute badend={badend, llcit}.
compute priorlo = llcit.
end if.
do if (badhi = 1 and ulcit <> priorhi).
compute badend={badend, ulcit}.
compute priorhi = ulcit.
end if.
end if.
do if ( (indtemp(1,1)*bc)+(9999*(1-bc)) = 9999).
compute llcit=temp(cilow,1).
compute ulcit=temp(cihigh,1).
end if.
compute bootse=t(sqrt((cssq(temp)-((csum(temp)&**2)/nrow(temp)))/(nrow(temp)-1)))
.
compute indeff={indeff,bootse,llcit,ulcit}.
end if.
do if (kk=1).
compute indtabn={indtab;indeff}.
end if.
compute indtab={indtab;indeff}.
end loop.
end loop.
compute indtab=indtab(2:nrow(indtab),:).
do if (kk=1).
compute indtabn=indtabn(2:nrow(indtabn),:).
end if.
compute rowlbs=indlbl(1:nrow(indtab),1).
do if (mc > 0).
compute inddiff=make(mc,1,-999).
compute indtab2=make(nrow(indtab),4,-999).
compute indtab2(:,1)=indtab.
compute indtab=indtab2.
release indtab2.
compute mcct=0.
compute indtotal=make(mc,1,0).
do if (kk = 1).
compute x1 = sqrt(-2*ln(uniform(mc,nrow(mcsopath))))&*cos((2*3.14159265358979)*uniform(mc, nrow(mcsopath))).
compute x1=x1*chol(indcov).
loop ii=1 to nrow(x1).
compute x1(ii,:)=x1(ii,:)+t(mcsopath).
end loop.
end if.
loop ii=1 to nms.
compute tmpb=x1(:,((nms*nxvls)+ii)).
compute tmpb2=tmpb.
do if (nxvls > 1).
loop jj=1 to (nxvls-1).
compute tmpb2={tmpb2,tmpb}.
end loop.
end if.
compute indtemp=x1(:,(((ii-1)*nxvls)+1):(ii*nxvls))&*tmpb2.
loop jj=1 to ncol(indtemp).
do if (kk = 2).
compute indtemp(:,jj)=indtemp(:,jj)/ysd.
end if.
do if (kk = 3).
compute indtemp(:,jj)=(xsd*indtemp(:,jj))/ysd.
end if.

.
compute temp = indtemp(:,jj).
compute temp(GRADE( indtemp(:,jj) )) = indtemp(:,jj).
compute badlo = 0.
compute badhi = 0.
do if ( 9999 <> 9999).
compute pv=csum(temp < 9999 )/nrow(temp).
compute ppv = pv.
do if (pv > .5).
compute ppv = 1-pv.
end if.
compute y5=sqrt(-2*ln(ppv)).
compute xp=y5+((((y5*p4+p3)*y5+p2)*y5+p1)*y5+p0)/((((y5*q4+q3)*y5+q2)*y5+q1)*y5+q0).
do if (pv <= .5).
compute xp = -xp.
end if.
compute cilow=rnd(nrow(temp)*(cdfnorm(2*xp-xp2))).
compute cihigh=trunc(nrow(temp)*(cdfnorm(2*xp+(xp2))))+1.
do if (cilow < 1).
compute cilow = 1.
compute booterr=1.
compute badlo = 1.
end if.
do if (cihigh > nrow(temp)).
compute cihigh = boot.
compute booterr=1.
compute badhi = 1.
end if.
compute llcit=temp(cilow,1).
compute ulcit=temp(cihigh,1).
do if (badlo = 1 and llcit <> priorlo).
compute badend={badend, llcit}.
compute priorlo = llcit.
end if.
do if (badhi = 1 and ulcit <> priorhi).
compute badend={badend, ulcit}.
compute priorhi = ulcit.
end if.
end if.
do if ( 9999 = 9999).
compute llcit=temp(cilow,1).
compute ulcit=temp(cihigh,1).
end if.
compute bootse=t(sqrt((cssq(temp)-((csum(temp)&**2)/nrow(temp)))/(nrow(temp)-1)))
.
compute mcct=mcct+1.
compute indtab(mcct,2:4)={bootse,llcit,ulcit}.
end loop.
do if (nxvls=1).
compute indtotal=indtotal+indtemp.
do if (contrast <> 0).
compute inddiff={inddiff,indtemp}.
end if.
end if.
end loop.
release indtemp,tmpb.
end if.
do if (normal = 1 and sobelok=1).
compute sobelmat=indtab(:,1).
compute sobelmat={sobelmat,(sobelmat/2),sobelmat,sobelmat}.
loop ii=1 to nms.
compute se2b=indcov(((nms*nxvls)+ii),((nms*nxvls)+ii)).
compute bpath2=mcsopath(((nms*nxvls)+ii),1)&**2.
compute se2a=diag(indcov((((ii-1)*nxvls)+1):(ii*nxvls),(((ii-1)*nxvls)+1):(ii*nxvls))).
compute apath2=mcsopath((((ii-1)*nxvls)+1):(ii*nxvls) ,1)&**2.
compute sesobel=sqrt(apath2*se2b+bpath2*se2a+se2a*se2b).
compute sobelmat((((ii-1)*nxvls)+1):(ii*nxvls),2)=sesobel.
end loop.
release se2b,bpath2,se2a,apath2,sesobel,ii.
compute sobelmat(:,3)=sobelmat(:,1)&/sobelmat(:,2).
compute sobelmat(:,4) = 2*(1-cdfnorm(abs(sobelmat(:,3)))).
end if.
do if (serial = 0).
compute rowlbs=t(mnames).
end if.
do if (nxvls=1 and nms > 1).
compute rowlbs={'TOTAL';rowlbs}.
compute indtemp=indtotal(1,1).
do if (boot > 0 and nxvls=1).

.
compute temp = indtotal(2:nrow(indtotal),1).
compute temp(GRADE( indtotal(2:nrow(indtotal),1) )) = indtotal(2:nrow(indtotal),1).
compute badlo = 0.
compute badhi = 0.
do if ( (indtotal(1,1)*bc)+(9999*(1-bc)) <> 9999).
compute pv=csum(temp < (indtotal(1,1)*bc)+(9999*(1-bc)) )/nrow(temp).
compute ppv = pv.
do if (pv > .5).
compute ppv = 1-pv.
end if.
compute y5=sqrt(-2*ln(ppv)).
compute xp=y5+((((y5*p4+p3)*y5+p2)*y5+p1)*y5+p0)/((((y5*q4+q3)*y5+q2)*y5+q1)*y5+q0).
do if (pv <= .5).
compute xp = -xp.
end if.
compute cilow=rnd(nrow(temp)*(cdfnorm(2*xp-xp2))).
compute cihigh=trunc(nrow(temp)*(cdfnorm(2*xp+(xp2))))+1.
do if (cilow < 1).
compute cilow = 1.
compute booterr=1.
compute badlo = 1.
end if.
do if (cihigh > nrow(temp)).
compute cihigh = boot.
compute booterr=1.
compute badhi = 1.
end if.
compute llcit=temp(cilow,1).
compute ulcit=temp(cihigh,1).
do if (badlo = 1 and llcit <> priorlo).
compute badend={badend, llcit}.
compute priorlo = llcit.
end if.
do if (badhi = 1 and ulcit <> priorhi).
compute badend={badend, ulcit}.
compute priorhi = ulcit.
end if.
end if.
do if ( (indtotal(1,1)*bc)+(9999*(1-bc)) = 9999).
compute llcit=temp(cilow,1).
compute ulcit=temp(cihigh,1).
end if.
compute bootse=t(sqrt((cssq(temp)-((csum(temp)&**2)/nrow(temp)))/(nrow(temp)-1)))
.
compute indtemp={indtemp, bootse,llcit,ulcit}.
end if.
do if (mc > 0).
compute obtmc=indtab(:,1).
compute indtemp=csum(obtmc).

.
compute temp = indtotal(:,1).
compute temp(GRADE( indtotal(:,1) )) = indtotal(:,1).
compute badlo = 0.
compute badhi = 0.
do if ( 9999 <> 9999).
compute pv=csum(temp < 9999 )/nrow(temp).
compute ppv = pv.
do if (pv > .5).
compute ppv = 1-pv.
end if.
compute y5=sqrt(-2*ln(ppv)).
compute xp=y5+((((y5*p4+p3)*y5+p2)*y5+p1)*y5+p0)/((((y5*q4+q3)*y5+q2)*y5+q1)*y5+q0).
do if (pv <= .5).
compute xp = -xp.
end if.
compute cilow=rnd(nrow(temp)*(cdfnorm(2*xp-xp2))).
compute cihigh=trunc(nrow(temp)*(cdfnorm(2*xp+(xp2))))+1.
do if (cilow < 1).
compute cilow = 1.
compute booterr=1.
compute badlo = 1.
end if.
do if (cihigh > nrow(temp)).
compute cihigh = boot.
compute booterr=1.
compute badhi = 1.
end if.
compute llcit=temp(cilow,1).
compute ulcit=temp(cihigh,1).
do if (badlo = 1 and llcit <> priorlo).
compute badend={badend, llcit}.
compute priorlo = llcit.
end if.
do if (badhi = 1 and ulcit <> priorhi).
compute badend={badend, ulcit}.
compute priorhi = ulcit.
end if.
end if.
do if ( 9999 = 9999).
compute llcit=temp(cilow,1).
compute ulcit=temp(cihigh,1).
end if.
compute bootse=t(sqrt((cssq(temp)-((csum(temp)&**2)/nrow(temp)))/(nrow(temp)-1)))
.
compute indtemp={indtemp, bootse,llcit,ulcit}.
end if.
compute indtab={indtemp;indtab}.
end if.
compute bootlbs={'Effect', 'BootSE','BootLLCI','BootULCI'}.
do if (mc > 0).
compute bootlbs={'Effect', 'MC SE','MC LLCI','MC ULCI'}.
end if.
do if (nxvls = 1).
do if (contrast <> 0).
compute inddiff=inddiff(:,2:ncol(inddiff)).
do if (mc > 0).
compute inddiff={t(obtmc);inddiff}.
end if.
do if (contrast = 4).
compute inddifft=inddiff*t(contvec).
compute indtemp=inddifft(1,1).
do if (boot > 0 or mc > 0).
do if (mc > 0).

.
compute temp = inddifft(2:nrow(inddifft),1).
compute temp(GRADE( inddifft(2:nrow(inddifft),1) )) = inddifft(2:nrow(inddifft),1).
compute badlo = 0.
compute badhi = 0.
do if ( 9999 <> 9999).
compute pv=csum(temp < 9999 )/nrow(temp).
compute ppv = pv.
do if (pv > .5).
compute ppv = 1-pv.
end if.
compute y5=sqrt(-2*ln(ppv)).
compute xp=y5+((((y5*p4+p3)*y5+p2)*y5+p1)*y5+p0)/((((y5*q4+q3)*y5+q2)*y5+q1)*y5+q0).
do if (pv <= .5).
compute xp = -xp.
end if.
compute cilow=rnd(nrow(temp)*(cdfnorm(2*xp-xp2))).
compute cihigh=trunc(nrow(temp)*(cdfnorm(2*xp+(xp2))))+1.
do if (cilow < 1).
compute cilow = 1.
compute booterr=1.
compute badlo = 1.
end if.
do if (cihigh > nrow(temp)).
compute cihigh = boot.
compute booterr=1.
compute badhi = 1.
end if.
compute llcit=temp(cilow,1).
compute ulcit=temp(cihigh,1).
do if (badlo = 1 and llcit <> priorlo).
compute badend={badend, llcit}.
compute priorlo = llcit.
end if.
do if (badhi = 1 and ulcit <> priorhi).
compute badend={badend, ulcit}.
compute priorhi = ulcit.
end if.
end if.
do if ( 9999 = 9999).
compute llcit=temp(cilow,1).
compute ulcit=temp(cihigh,1).
end if.
compute bootse=t(sqrt((cssq(temp)-((csum(temp)&**2)/nrow(temp)))/(nrow(temp)-1)))
.
end if.
do if (boot > 0).

.
compute temp = inddifft(2:nrow(inddifft),1).
compute temp(GRADE( inddifft(2:nrow(inddifft),1) )) = inddifft(2:nrow(inddifft),1).
compute badlo = 0.
compute badhi = 0.
do if ( (inddifft(1,1)*bc)+(9999* (1-bc)) <> 9999).
compute pv=csum(temp < (inddifft(1,1)*bc)+(9999* (1-bc)) )/nrow(temp).
compute ppv = pv.
do if (pv > .5).
compute ppv = 1-pv.
end if.
compute y5=sqrt(-2*ln(ppv)).
compute xp=y5+((((y5*p4+p3)*y5+p2)*y5+p1)*y5+p0)/((((y5*q4+q3)*y5+q2)*y5+q1)*y5+q0).
do if (pv <= .5).
compute xp = -xp.
end if.
compute cilow=rnd(nrow(temp)*(cdfnorm(2*xp-xp2))).
compute cihigh=trunc(nrow(temp)*(cdfnorm(2*xp+(xp2))))+1.
do if (cilow < 1).
compute cilow = 1.
compute booterr=1.
compute badlo = 1.
end if.
do if (cihigh > nrow(temp)).
compute cihigh = boot.
compute booterr=1.
compute badhi = 1.
end if.
compute llcit=temp(cilow,1).
compute ulcit=temp(cihigh,1).
do if (badlo = 1 and llcit <> priorlo).
compute badend={badend, llcit}.
compute priorlo = llcit.
end if.
do if (badhi = 1 and ulcit <> priorhi).
compute badend={badend, ulcit}.
compute priorhi = ulcit.
end if.
end if.
do if ( (inddifft(1,1)*bc)+(9999* (1-bc)) = 9999).
compute llcit=temp(cilow,1).
compute ulcit=temp(cihigh,1).
end if.
compute bootse=t(sqrt((cssq(temp)-((csum(temp)&**2)/nrow(temp)))/(nrow(temp)-1)))
.
end if.
compute indtemp={indtemp, bootse,llcit,ulcit}.
end if.
compute indtab={indtab;indtemp}.
end if.
do if (contrast = 1 or contrast = 2 or contrast=3).
compute conkey=make(1,4,' ').
loop i = 1 to ncol(inddiff)-1.
loop j = (i+1) to ncol(inddiff).
compute inddifft=inddiff(:,i)-inddiff(:,j).
do if (contrast=2).
compute inddifft=abs(inddiff(:,i))-abs(inddiff(:,j)).
end if.
do if (contrast=3).
compute inddifft=inddiff(:,i)+inddiff(:,j).
end if.
compute indtemp=inddifft(1,1).
do if (contrast=1 or contrast=2).
compute conkeyt={' ', rowlbs((i+1),1),' minus  ',rowlbs((j+1),1)}.
end if.
do if (contrast=3).
compute conkeyt={' ', rowlbs((i+1),1),'  plus  ',rowlbs((j+1),1)}.
end if.
compute conkey={conkey;conkeyt}.
do if (boot > 0 or mc > 0).
do if (mc > 0).

.
compute temp = inddifft(2:nrow(inddifft),1).
compute temp(GRADE( inddifft(2:nrow(inddifft),1) )) = inddifft(2:nrow(inddifft),1).
compute badlo = 0.
compute badhi = 0.
do if ( 9999 <> 9999).
compute pv=csum(temp < 9999 )/nrow(temp).
compute ppv = pv.
do if (pv > .5).
compute ppv = 1-pv.
end if.
compute y5=sqrt(-2*ln(ppv)).
compute xp=y5+((((y5*p4+p3)*y5+p2)*y5+p1)*y5+p0)/((((y5*q4+q3)*y5+q2)*y5+q1)*y5+q0).
do if (pv <= .5).
compute xp = -xp.
end if.
compute cilow=rnd(nrow(temp)*(cdfnorm(2*xp-xp2))).
compute cihigh=trunc(nrow(temp)*(cdfnorm(2*xp+(xp2))))+1.
do if (cilow < 1).
compute cilow = 1.
compute booterr=1.
compute badlo = 1.
end if.
do if (cihigh > nrow(temp)).
compute cihigh = boot.
compute booterr=1.
compute badhi = 1.
end if.
compute llcit=temp(cilow,1).
compute ulcit=temp(cihigh,1).
do if (badlo = 1 and llcit <> priorlo).
compute badend={badend, llcit}.
compute priorlo = llcit.
end if.
do if (badhi = 1 and ulcit <> priorhi).
compute badend={badend, ulcit}.
compute priorhi = ulcit.
end if.
end if.
do if ( 9999 = 9999).
compute llcit=temp(cilow,1).
compute ulcit=temp(cihigh,1).
end if.
compute bootse=t(sqrt((cssq(temp)-((csum(temp)&**2)/nrow(temp)))/(nrow(temp)-1)))
.
end if.
do if (boot > 0).

.
compute temp = inddifft(2:nrow(inddifft),1).
compute temp(GRADE( inddifft(2:nrow(inddifft),1) )) = inddifft(2:nrow(inddifft),1).
compute badlo = 0.
compute badhi = 0.
do if ( (inddifft(1,1)*bc)+(9999* (1-bc)) <> 9999).
compute pv=csum(temp < (inddifft(1,1)*bc)+(9999* (1-bc)) )/nrow(temp).
compute ppv = pv.
do if (pv > .5).
compute ppv = 1-pv.
end if.
compute y5=sqrt(-2*ln(ppv)).
compute xp=y5+((((y5*p4+p3)*y5+p2)*y5+p1)*y5+p0)/((((y5*q4+q3)*y5+q2)*y5+q1)*y5+q0).
do if (pv <= .5).
compute xp = -xp.
end if.
compute cilow=rnd(nrow(temp)*(cdfnorm(2*xp-xp2))).
compute cihigh=trunc(nrow(temp)*(cdfnorm(2*xp+(xp2))))+1.
do if (cilow < 1).
compute cilow = 1.
compute booterr=1.
compute badlo = 1.
end if.
do if (cihigh > nrow(temp)).
compute cihigh = boot.
compute booterr=1.
compute badhi = 1.
end if.
compute llcit=temp(cilow,1).
compute ulcit=temp(cihigh,1).
do if (badlo = 1 and llcit <> priorlo).
compute badend={badend, llcit}.
compute priorlo = llcit.
end if.
do if (badhi = 1 and ulcit <> priorhi).
compute badend={badend, ulcit}.
compute priorhi = ulcit.
end if.
end if.
do if ( (inddifft(1,1)*bc)+(9999* (1-bc)) = 9999).
compute llcit=temp(cilow,1).
compute ulcit=temp(cihigh,1).
end if.
compute bootse=t(sqrt((cssq(temp)-((csum(temp)&**2)/nrow(temp)))/(nrow(temp)-1)))
.
end if.
compute indtemp={indtemp, bootse,llcit,ulcit}.
end if.
compute indtab={indtab;indtemp}.
end loop.
end loop.
end if.
release inddiff.
compute contlbs=cntname(1:(((nindfx)*(nindfx-1))/2),1).
compute rowlbs={rowlbs;contlbs}.
end if.
compute resultm2=make(nrow(indtab),maxresm,99999).
compute resultm2(1:nrow(indtab),1:ncol(indtab))=indtab.
compute resultm={resultm;resultm2}.
do if (outscree=1).
do if (kk=1).
print indtab/title = 'Indirect effect(s) of X on Y:'/rnames=rowlbs/cnames=bootlbs/format= F10.4.
end if.
do if (kk = 2 and (xdich=1 or mcx > 0)).
print indtab/title = 'Partially standardized indirect effect(s) of X on Y:'/rnames=rowlbs/cnames=bootlbs/format= F10.4.
end if.
do if (kk = 3).
print indtab/title = 'Completely standardized indirect effect(s) of X on Y:'/rnames=rowlbs/cnames=bootlbs/format= F10.4.
end if.
end if.
do if (normal=1 and sobelok=1 and kk=1).
compute resultm2=make(nrow(sobelmat),maxresm,99999).
compute resultm2(1:nrow(sobelmat),1:ncol(sobelmat))=sobelmat.
compute resultm={resultm;resultm2}.
compute sobellab={'Effect',hclab,'Z','p'}.
compute sobelrlb=rowlbs.
do if (nms > 1).
compute sobelrlb=rowlbs(2:(1+nms),1).
end if.
do if (outscree=1).
print sobelmat/title='   Normal theory test for indirect effect(s):'/cnames=sobellab/rnames=sobelrlb/format= F10.4.
end if.
end if.
do if (contrast <> 0).
do if ((contrast=1 or contrast = 2 or contrast=3) and kk=efloop ).
compute conkey=conkey(2:nrow(conkey),:).
do if (outscree=1).
print conkey/title = 'Specific indirect effect contrast definition(s):'/rnames=contlbs/format=A8.
end if.
end if.
do if (outscree=1).
do if (contrast = 4 and kk=efloop).
compute crowlbs=rowlbs(2:(nindfx+1),1).
print contvec/title = 'Specific indirect effect contrast weights:'/cnames=crowlbs/rlabels='(C1)'/format= F10.4.
end if.
do if (contrast = 2 and kk=efloop).
print/title = 'Contrasts are differences between absolute values of indirect effects'.
end if.
end if.
end if.
do if (serial = 1 and kk=efloop).
compute rowlbst=rowlbs(2:nrow(rowlbs),1).
do if (outscree=1).
print indkey/title = 'Indirect effect key:'/rnames=rowlbst/format = A8.
end if.
end if.
else.
do if (outscree=1).
do if (kk = 1).
print/title = 'Relative indirect effects of X on Y'.
end if.
do if (kk = 2).
print/title = 'Partially standardized relative indirect effect(s) of X on Y:'.
end if.
do if (kk = 3).
print/title = 'Completely standardized relative indirect effect(s) of X on Y:'.
end if.
end if.
loop i = 1 to nrow(indmake).
compute indtabsm=indtab((((i-1)*nxvls)+1):(nxvls*i),:).
compute indkeyt=indkey(i,:).
do if (outscree=1).
print indkeyt/title=' '/space=0/format=A8.
end if.
do if (bcmat(nrow(bcmat),1)=0)).
compute direffl2=xcatlab(1:nxvls,1).
end if.
compute resultm2=make(nrow(indtabsm),maxresm,99999).
compute resultm2(1:nrow(indtabsm),1:ncol(indtabsm))=indtabsm.
compute resultm={resultm;resultm2}.
do if (outscree=1).
print indtabsm/title = ' '/cnames=bootlbs/rnames=direffl2/format= F10.4 /space=0.
end if.
do if (normal=1 and sobelok=1 and kk=1).
compute sobelsm=sobelmat((((i-1)*nxvls)+1):(nxvls*i),:).
compute sobellab={'Effect',hclab,'Z','p'}.
compute resultm2=make(nrow(sobelsm),maxresm,99999).
compute resultm2(1:nrow(sobelsm),1:ncol(sobelsm))=sobelsm.
compute resultm={resultm;resultm2}.
do if (outscree=1).
print sobelsm/title='      Normal theory test for relative indirect effects:'/cnames=sobellab/rnames=direffl2/format= F10.4.
end if.
end if.
end loop.
end if.
do if (effsize = 1 and boot > 0).
compute bootres=bootres(2:nrow(bootres),:).
end if.
end loop.
do if (alttotal=1).
compute altcnms='Effect'.
compute totbtvec=totbtvec+bootdir.
compute alttotfx=t(totbtvec(1,:)).
do if (boot > 0).
compute alttotfx=make(ncol(totbtvec),4,0).
compute alttotfx(:,1)=t(totbtvec(1,:)).
loop cec=1 to ncol(totbtvec).

.
compute temp = totbtvec(2:nrow(totbtvec),cec).
compute temp(GRADE( totbtvec(2:nrow(totbtvec),cec) )) = totbtvec(2:nrow(totbtvec),cec).
compute badlo = 0.
compute badhi = 0.
do if ( (totbtvec(1,cec)*bc)+(9999* (1-bc)) <> 9999).
compute pv=csum(temp < (totbtvec(1,cec)*bc)+(9999* (1-bc)) )/nrow(temp).
compute ppv = pv.
do if (pv > .5).
compute ppv = 1-pv.
end if.
compute y5=sqrt(-2*ln(ppv)).
compute xp=y5+((((y5*p4+p3)*y5+p2)*y5+p1)*y5+p0)/((((y5*q4+q3)*y5+q2)*y5+q1)*y5+q0).
do if (pv <= .5).
compute xp = -xp.
end if.
compute cilow=rnd(nrow(temp)*(cdfnorm(2*xp-xp2))).
compute cihigh=trunc(nrow(temp)*(cdfnorm(2*xp+(xp2))))+1.
do if (cilow < 1).
compute cilow = 1.
compute booterr=1.
compute badlo = 1.
end if.
do if (cihigh > nrow(temp)).
compute cihigh = boot.
compute booterr=1.
compute badhi = 1.
end if.
compute llcit=temp(cilow,1).
compute ulcit=temp(cihigh,1).
do if (badlo = 1 and llcit <> priorlo).
compute badend={badend, llcit}.
compute priorlo = llcit.
end if.
do if (badhi = 1 and ulcit <> priorhi).
compute badend={badend, ulcit}.
compute priorhi = ulcit.
end if.
end if.
do if ( (totbtvec(1,cec)*bc)+(9999* (1-bc)) = 9999).
compute llcit=temp(cilow,1).
compute ulcit=temp(cihigh,1).
end if.
compute bootse=t(sqrt((cssq(temp)-((csum(temp)&**2)/nrow(temp)))/(nrow(temp)-1)))
.
compute alttotfx(cec,2:4)={bootse,llcit,ulcit}.
end loop.
compute altcnms={altcnms,'BootSE', 'BootLLCI','BootULCI'}.
end if.
compute resultm2=make(nrow(alttotfx),maxresm,99999).
compute resultm2(1:nrow(alttotfx),1:ncol(alttotfx))=alttotfx.
compute resultm={resultm;resultm2}.
do if (outscree=1).
do if (nxvls > 1).
print/title= '----------'/space=0.
print alttotfx/title='Relative total effects of X on Y (sum of direct and indirect effects)'/rnames=direffl2/cnames=altcnms/format= F10.4.
end if.
do if (nxvls = 1).
print alttotfx/title='Total effect of X on Y (sum of direct and indirect effects)'/cnames=altcnms/format= F10.4.
end if.
end if.
end if.
end if.
do if (anymod > 0).
do if (boot = 0).
compute bootres=obscoeff.
compute indtab=999.
end if.
do if (boot > 0).
compute bootres={obscoeff;bootres}.
compute indtab=make(1,4,999).
end if.
do if (csum((indmod > 0))=nrow(indmod)).
do if (outscree=1).
do if (nxvls > 1).
do if (xmint=0).
print/title= 'Relative conditional indirect effects of X on Y:'.
end if.
do if (xmint=1).
print/title= 'Relative (total) natural indirect effects of X on Y:'.
end if.
end if.
do if (nxvls = 1).
do if (xmint=0).
print/title= 'Conditional indirect effects of X on Y:'.
end if.
do if (xmint=1).
print/title= '(Total) Natural indirect effect(s) of X on Y:'.
end if.
end if.
end if.
end if.
do if (csum((indmod > 0)) < nrow(indmod)).
do if (outscree=1).
do if (nxvls > 1).
print/title= 'Relative conditional and unconditional indirect effects of X on Y:'.
end if.
do if (nxvls = 1).
print/title= 'Conditional and unconditional indirect effects of X on Y:'.
end if.
end if.
end if.
compute cftotfx=make(nrow(bootres),nxvls,0).
loop i = 1 to nrow(indmake).
compute indtab=indtab(1,:)*0.
do if (outscree=1).
do if (xmint=0).
print indkey(i,:)/title = 'INDIRECT EFFECT:'/format=A8.
end if.
do if (xmint=1).
print indkey(i,:)/title = ' '/format=A8/space=0.
end if.
end if.
do if (indmod(i,1)=0).
loop j = 1 to nxvls.
compute indtemp=make(nrow(bootres),1,1).
loop k = 1 to indmake(i,1).
compute jtemp=1.
do if (j > 1 and k=1).
compute jtemp=j.
end if.
compute indtemp=indtemp&*bootres(:,pathsfoc(jtemp,indmake(i,(k+1)))).
end loop.
compute indeff=indtemp(1,1).
do if (boot > 0).

.
compute temp = indtemp(2:nrow(indtemp),1).
compute temp(GRADE( indtemp(2:nrow(indtemp),1) )) = indtemp(2:nrow(indtemp),1).
compute badlo = 0.
compute badhi = 0.
do if ( (indtemp(1,1)*bc)+(9999*(1-bc)) <> 9999).
compute pv=csum(temp < (indtemp(1,1)*bc)+(9999*(1-bc)) )/nrow(temp).
compute ppv = pv.
do if (pv > .5).
compute ppv = 1-pv.
end if.
compute y5=sqrt(-2*ln(ppv)).
compute xp=y5+((((y5*p4+p3)*y5+p2)*y5+p1)*y5+p0)/((((y5*q4+q3)*y5+q2)*y5+q1)*y5+q0).
do if (pv <= .5).
compute xp = -xp.
end if.
compute cilow=rnd(nrow(temp)*(cdfnorm(2*xp-xp2))).
compute cihigh=trunc(nrow(temp)*(cdfnorm(2*xp+(xp2))))+1.
do if (cilow < 1).
compute cilow = 1.
compute booterr=1.
compute badlo = 1.
end if.
do if (cihigh > nrow(temp)).
compute cihigh = boot.
compute booterr=1.
compute badhi = 1.
end if.
compute llcit=temp(cilow,1).
compute ulcit=temp(cihigh,1).
do if (badlo = 1 and llcit <> priorlo).
compute badend={badend, llcit}.
compute priorlo = llcit.
end if.
do if (badhi = 1 and ulcit <> priorhi).
compute badend={badend, ulcit}.
compute priorhi = ulcit.
end if.
end if.
do if ( (indtemp(1,1)*bc)+(9999*(1-bc)) = 9999).
compute llcit=temp(cilow,1).
compute ulcit=temp(cihigh,1).
end if.
compute bootse=t(sqrt((cssq(temp)-((csum(temp)&**2)/nrow(temp)))/(nrow(temp)-1)))
.
compute indeff={indeff,bootse,llcit,ulcit}.
end if.
compute indtab={indtab;indeff}.
end loop.
compute resultm2=make(nrow(indtab),maxresm,99999).
compute resultm2(1:nrow(indtab),1:ncol(indtab))=indtab.
compute resultm={resultm;resultm2}.
do if (outscree=1).
do if (nxvls > 1).
compute indefflb=xcatlab(1:nxvls,1).
print indtab(2:nrow(indtab),:)/title = ' '/clabels='Effect', 'BootSE', 'BootLLCI', 'BootULCI'/rnames=indefflb/format= F10.4 /space=0.
end if.
do if (nxvls = 1).
print indtab(2:nrow(indtab),:)/title = ' '/clabels='Effect', 'BootSE', 'BootLLCI', 'BootULCI'/format= F10.4 /space=0.
end if.
end if.
end if.
do if (indmod(i,1)>0).
do if (indmod(i,1)=1).
compute indmodva=wmodvals.
compute indprova=wprobval.
compute condlbs={wnames}.
compute printw=1.
else if (indmod(i,1)=2).
compute indmodva=zmodvals.
compute indprova=zprobval.
compute condlbs={znames}.
compute printz=1.
else if (indmod(i,1)=3).
compute cntmp=1.
compute printz=1.
compute printw=1.
compute indmodva=make((nrow(wmodvals)*nrow(zmodvals)),2,999).
loop k7 = 1 to nrow(wmodvals).
loop k8 = 1 to nrow(zmodvals).
compute indmodva(cntmp,:)={wmodvals(k7,1),zmodvals(k8,1)}.
compute cntmp=cntmp+1.
end loop.
end loop.
compute condlbs={wnames,znames}.
end if.
compute condres=make(nrow(indmodva),1,999).
do if (boot > 0).
compute condres=make(nrow(indmodva),4,999).
end if.
compute condres={indmodva,condres}.
loop k4 = 1 to nxvls.
compute imm3=make(nrow(bootres),1,1).
compute imm4=make(nrow(bootres),1,1).
compute indcontr=0.
do if (indmod(i,1)=3).
compute tihsw=wprobval.
compute tihsz=zprobval.
end if.
loop k1=1 to nrow(indmodva).
compute tucker2=make(nrow(bootres),1,1).
compute imm2=make(nrow(bootres),1,1).
compute wfirst=0.
compute zfirst=0.
compute immset=0.
loop k2=1 to indmake(i,1).
compute colnumb=indmake(i,(k2+1)).
do if (k2=1).
compute wbb=make(nrow(bootres),(nwvls*nxvls),0).
compute zbb=make(nrow(bootres),(nzvls*nxvls),0).
compute wzbb=make(nrow(bootres),(nwvls*nzvls*nxvls),0).
end if.
do if (k2<>1).
compute wbb=make(nrow(bootres),nwvls,0).
compute zbb=make(nrow(bootres),nzvls,0).
compute wzbb=make(nrow(bootres),(nwvls*nzvls),0).
end if.
compute cnt=1.
compute tihs=indlocs(2:((indlocs(1,colnumb))+1),colnumb).
do if (k2 = 1).
compute focbb=tihs(1:nxvls,1).
compute focbb=bootres(:,focbb).
do if (indmmm(i,1)>0).
compute imm=focbb(:,k4).
compute condbb=imm.
end if.
compute focaddon=make(1,nxvls,0).
compute focaddon(1,k4)=1.
compute cnt=cnt+nxvls.
compute placeh=nxvls.
do if (indmod(i,1)=1).
compute tihsz=make(nrow(wprobval),(nzvls*nxvls),0).
compute tihswz=make(nrow(wprobval),(nwvls*nzvls*nxvls),0).
do if (pathsw(1,colnumb)=1).
compute temp=make(nrow(wprobval),(nxvls*nwvls),0).
loop k5 = 1 to nrow(wprobval).
loop k6=1 to nwvls.
compute temp(k5, (((k4-1)*nwvls)+k6))=wprobval(k5,k6).
end loop.
end loop.
compute indprova={temp,tihsz,tihswz}.
else.
compute indprova={wprobval,tihsz,tihswz}.
end if.
end if.
do if (indmod(i,1)=2).
compute tihsw=make(nrow(zprobval),(nwvls*nxvls),0).
compute tihswz=make(nrow(zprobval),(nwvls*nzvls*nxvls),0).
do if (pathsz(1,colnumb)=1).
compute temp=make(nrow(zprobval),(nxvls*nzvls),0).
loop k5 = 1 to nrow(zprobval).
loop k6 =1 to nzvls.
compute temp(k5,(((k4-1)*nzvls)+k6))=zprobval(k5,k6).
end loop.
end loop.
compute indprova={tihsw,temp,tihswz}.
else.
compute indprova={tihsw,zprobval,tihswz}.
end if.
end if.
do if (indmod(i,1)=3).
compute indprova=make((nrow(wprobval)*nrow(zprobval)),((ncol(wprobval)*nxvls)+ (ncol(zprobval)*nxvls)+(nwvls*nzvls*nxvls)),0).
compute cntemp=1.
loop k7=1 to nrow(wprobval).
loop k8 =1 to nrow(zprobval).
compute temp=wprobval(k7,:)*focaddon(1,k4).
compute indprova(cntemp,(((k4-1)*nwvls)+1):(k4*(nwvls)))=temp.
compute temp=zprobval(k8,:)*focaddon(1,k4).
compute indprova(cntemp, ((((k4-1)*nzvls)+1)+(nxvls*nwvls)) : ((((k4-1)*nzvls)+1)+(nxvls*nwvls)+(nzvls-1)))=temp.
compute cntemp=cntemp+1.
end loop.
end loop.
do if (pathsz(1,colnumb)=0).
compute temp=make(nrow(indprova),(ncol(zprobval)*nxvls),0).
compute indprova(:,((ncol(wprobval)*nxvls)+1):((ncol(wprobval)+ncol(zprobval))* nxvls))=temp.
end if.
do if (pathsw(1,colnumb)=0).
compute temp=make(nrow(indprova),(ncol(wprobval)*nxvls),0).
compute indprova(:,1:(ncol(wprobval)*nxvls))=temp.
end if.
do if (pathswz(1,colnumb)=1).
compute cntemp=(ncol(wprobval)*nxvls)+(ncol(zprobval)*nxvls)+((k4-1)* ncol(wprobval)*ncol(zprobval))+1.
loop k7=1 to ncol(wprobval).
loop k8=1 to ncol(zprobval).
compute indprova(:,cntemp)=indprova(:,((ncol(wprobval)*(k4-1))+k7))&* indprova(:,((((k4-1)*ncol(zprobval))+k8)+(nxvls*ncol(wprobval)))).
compute cntemp=cntemp+1.
end loop.
end loop.
end if.
end if.
end if.
do if (k2 > 1).
compute focbb=tihs(1,1).
compute focbb=bootres(:,focbb).
do if (indmmm(i,1)>0).
compute imm=focbb(:,1).
compute condbb=imm.
end if.
compute focaddon=1.
compute cnt=cnt+1.
compute placeh=1.
do if (indmod(i,1)=1).
compute tihsz=make(nrow(wprobval),nzvls,0).
compute tihswz=make(nrow(wprobval),(nwvls*nzvls),0).
compute indprova={wprobval,tihsz,tihswz}.
end if.
do if (indmod(i,1)=2).
compute tihsw=make(nrow(zprobval),nwvls,0).
compute tihswz=make(nrow(zprobval),(nwvls*nzvls),0).
compute indprova={tihsw,zprobval,tihswz}.
end if.
do if (indmod(i,1)=3).
compute indprova=make((nrow(wprobval)*nrow(zprobval)),((ncol(wprobval)+ ncol(zprobval))+(nwvls*nzvls)),0).
compute cntemp=1.
loop k7=1 to nrow(wprobval).
loop k8 =1 to nrow(zprobval).
compute indprova(cntemp,1:(ncol(wprobval)+ncol(zprobval)))={wprobval(k7,:), zprobval(k8,:)}.
compute cntemp=cntemp+1.
end loop.
end loop.
do if (pathsz(1,colnumb)=0).
compute temp=make(nrow(indprova),ncol(zprobval),0).
compute indprova(:,(ncol(wprobval)+1):(ncol(wprobval)+ncol(zprobval)))=temp.
end if.
do if (pathsw(1,colnumb)=0).
compute temp=make(nrow(indprova),ncol(wprobval),0).
compute indprova(:,1:ncol(wprobval))=temp.
end if.
do if (pathswz(1,colnumb)=1).
compute cntemp=ncol(wprobval)+ncol(zprobval)+1.
loop k7=1 to ncol(wprobval).
loop k8=1 to ncol(zprobval).
compute indprova(:,cntemp)=indprova(:,k7)&*indprova(:,(ncol(wprobval)+k8)).
compute cntemp=cntemp+1.
end loop.
end loop.
end if.
end if.
end if.
do if (pathsw(1,colnumb)) = 1.
compute wbb=tihs(cnt:(cnt+(placeh*nwvls)-1),1).
compute wbb=bootres(:,wbb).
compute immlbs2=wcatlab(1:nwvls,1).
do if (zfirst=0).
compute wfirst=1.
end if.
do if (indmmm(i,1)=1 or indmmm(i,1)=31 or indmmm(i,1)=51).
compute imm=wbb(:,1).
loop k7 = 1 to nwvls.
compute imm={imm,wbb(:,(((k4-1)*nwvls*(k2=1))+k7))}.
end loop.
compute imm=imm(:,2:ncol(imm)).
end if.
do if (indmmm(i,1)=41 or indmmm(i,1)=51).
compute condbb=make(nrow(bootres),1,0).
loop k7 = 1 to nwvls.
compute condbb={condbb,wbb(:,(((k4-1)*nwvls*(k2=1))+k7))}.
end loop.
compute condbb=condbb(:,2:ncol(condbb)).
end if.
compute cnt=cnt+(placeh*nwvls).
end if.
do if (pathsz(1,colnumb)) = 1.
compute zbb=tihs(cnt:(cnt+(placeh*nzvls)-1),1).
compute zbb=bootres(:,zbb).
do if (wfirst=0).
compute zfirst=1.
end if.
do if (indmmm(i,1) <> 31).
compute immlbs2=zcatlab(1:nzvls,1).
end if.
do if (indmmm(i,1)=2 or indmmm(i,1)=31 or indmmm(i,1)=51).
do if (indmmm(i,1)=2).
compute imm=zbb(:,1).
end if.
loop k7 = 1 to nzvls.
compute imm={imm,zbb(:,(((k4-1)*nzvls*(k2=1))+k7))}.
end loop.
do if (indmmm(i,1)=2 or indmmm(i,1)=51).
compute imm=imm(:,2:ncol(imm)).
do if (indmmm(i,1)=51).
compute condbb={condbb,imm}.
end if.
end if.
end if.
compute cnt=cnt+(placeh*nzvls).
end if.
do if (pathswz(1,colnumb)) = 1.
compute wzbb=tihs(cnt:(cnt+(placeh*nwvls*nzvls)-1),1).
compute wzbb=bootres(:,wzbb).
do if (indmmm(i,1)=41).
compute imm=wzbb(:,1).
loop k7=1 to nwvls*nzvls.
compute imm={imm,wzbb(:,(((k4-1)*nzvls*nwvls*(k2=1))+k7))}.
end loop.
end if.
do if (indmmm(i,1)=41).
compute imm=imm(:,2:ncol(imm)).
compute condbb={condbb,imm(:,(ncol(imm)-(nwvls*nzvls)+1):ncol(imm))}.
end if.
compute cnt=cnt+(placeh*nzvls*nwvls).
end if.
compute indprobe={focaddon,indprova(k1,:)}.
compute tucker={focbb,wbb,zbb,wzbb}.
loop k3=1 to ncol(indprobe).
compute tucker(:,k3)=tucker(:,k3)*indprobe(1,k3).
end loop.
compute tucker2=tucker2&*rsum(tucker).
do if (indmmm(i,1) = 1 or indmmm(i,1)=2 or indmmm(i,1)=31 or indmmm(i,1)=41 or indmmm(i,1)=51).
do if (immset=1).
do if (ncol(imm2)=1 and ncol(imm) = 1).
compute imm2=imm2&*imm.
end if.
do if (indmmm(i,1)=41 or indmmm(i,1)=51).
do if ((ncol(condbb2) > 1) and (ncol(condbb)>1)).
compute condbb2t=make(nrow(condbb2),(ncol(condbb2)*ncol(condbb)),-999999).
compute k9=1.
do if (wfirst=1).
loop k7=1 to ncol(condbb2).
loop k8 = 1 to ncol(condbb).
compute condbb2t(:,k9)=condbb2(:,k7)&*condbb(:,k8).
compute k9=k9+1.
end loop.
end loop.
end if.
do if (zfirst=1).
loop k7=1 to ncol(condbb).
loop k8 = 1 to ncol(condbb2).
compute condbb2t(:,k9)=condbb(:,k7)&*condbb2(:,k8).
compute k9=k9+1.
end loop.
end loop.
end if.
compute condbb2=condbb2t.
release condbb2t.
end if.
do if ((ncol(condbb2) > 1) and (ncol(condbb)=1)).
loop k7 = 1 to ncol(condbb2).
compute condbb2(:,k7)=condbb2(:,k7)&*condbb.
end loop.
end if.
do if ((ncol(condbb2) = 1) and (ncol(condbb)>1)).
loop k7 = 1 to ncol(condbb).
compute condbb(:,k7)=condbb2&*condbb(:,k7).
end loop.
compute condbb2=condbb.
end if.
end if.
do if (ncol(imm2) <> 1 and ncol(imm) <> 1).
compute imm2t=make(nrow(imm2),(ncol(imm2)*ncol(imm)),-999999).
compute k9=1.
do if (wfirst=1).
loop k7=1 to ncol(imm2).
loop k8 = 1 to ncol(imm).
compute imm2t(:,k9)=imm2(:,k7)&*imm(:,k8).
compute k9=k9+1.
end loop.
end loop.
end if.
do if (zfirst=1).
loop k7=1 to ncol(imm).
loop k8 = 1 to ncol(imm2).
compute imm2t(:,k9)=imm(:,k7)&*imm2(:,k8).
compute k9=k9+1.
end loop.
end loop.
end if.
compute imm2=imm2t.
release imm2t.
end if.
do if ((ncol(imm2) > 1) and (ncol(imm)=1)).
loop k7=1 to ncol(imm2).
compute imm2(:,k7)=imm2(:,k7)&*imm.
end loop.
end if.
do if ((ncol(imm2) = 1) and (ncol(imm) > 1)).
loop k7=1 to ncol(imm).
compute imm(:,k7)=imm2&*imm(:,k7).
end loop.
compute imm2=imm.
end if.
end if.
do if (immset=0).
compute imm2=imm.
do if (indmmm(i,1)=41 or indmmm(i,1)=51).
compute condbb2=condbb.
end if.
compute immset=1.
end if.
end if.
end loop.
compute indtemp=tucker2(1,1).
do if (indmmm(i,1)=12 or indmmm(i,1)=22).
compute imm3={imm3,tucker2}.
do if (k1=nrow(indmodva)).
compute imm3=imm3(:,2:ncol(imm3)).
compute immstop=ncol(imm3).
loop k8=2 to immstop.
do if (indmmmt(i,1)=1).
compute imm3={imm3,(imm3(:,k8)-imm3(:,1))}.
end if.
do if (indmmmt(i,1)=2).
compute imm3={imm3,(imm3(:,k8)-imm3(:,(k8-1)))}.
end if.
do if (indmmmt(i,1)=3).
compute imm3={imm3,((rsum(imm3(:,(k8:immstop)))/(immstop-k8+1))-imm3(:,(k8-1))) }.
end if.
do if (indmmmt(i,1)=4).
compute imm3={imm3,(imm3(:,k8)-(rsum(imm3(:,1:immstop))/immstop))}.
end if.
end loop.
do if (indmmmt(i,1)<5).
compute imm2=imm3(:,(immstop+1):ncol(imm3)).
release imm3.
end if.
end if.
end if.
do if (indmmm(i,1)>-1 and (contrast = 1 or contrast = 2 or contrast=3)).
compute imm4={imm4,tucker2}.
do if (k1=nrow(indmodva) and k1 > 1).
compute imm4=imm4(:,2:ncol(imm4)).
compute immstop=ncol(imm4).
compute condcont=make((immstop*(immstop-1)/2),6,-999).
loop k8 = 1 to (immstop-1).
loop k9 = (k8+1) to immstop.
do if (contrast=1).
compute imm4={imm4,(imm4(:,k9)-imm4(:,k8))}.
end if.
do if (contrast=2).
compute imm4={imm4,(abs(imm4(:,k9))-abs(imm4(:,k8)))}.
end if.
do if (contrast=3).
compute imm4={imm4,(imm4(:,k9)+imm4(:,k8))}.
end if.
compute condcont((ncol(imm4)-immstop),1)=imm4(1,k9).
compute condcont((ncol(imm4)-immstop),2)=imm4(1,k8).
end loop.
end loop.
compute imm4=imm4(:,(immstop+1):ncol(imm4)).
loop k8=1 to ncol(imm4).
compute condcont(k8,3)=imm4(1,k8).
do if (boot > 0).

.
compute temp = imm4(2:nrow(imm4),k8).
compute temp(GRADE( imm4(2:nrow(imm4),k8) )) = imm4(2:nrow(imm4),k8).
compute badlo = 0.
compute badhi = 0.
do if ( (imm4(1,k8)*bc)+(9999*(1-bc)) <> 9999).
compute pv=csum(temp < (imm4(1,k8)*bc)+(9999*(1-bc)) )/nrow(temp).
compute ppv = pv.
do if (pv > .5).
compute ppv = 1-pv.
end if.
compute y5=sqrt(-2*ln(ppv)).
compute xp=y5+((((y5*p4+p3)*y5+p2)*y5+p1)*y5+p0)/((((y5*q4+q3)*y5+q2)*y5+q1)*y5+q0).
do if (pv <= .5).
compute xp = -xp.
end if.
compute cilow=rnd(nrow(temp)*(cdfnorm(2*xp-xp2))).
compute cihigh=trunc(nrow(temp)*(cdfnorm(2*xp+(xp2))))+1.
do if (cilow < 1).
compute cilow = 1.
compute booterr=1.
compute badlo = 1.
end if.
do if (cihigh > nrow(temp)).
compute cihigh = boot.
compute booterr=1.
compute badhi = 1.
end if.
compute llcit=temp(cilow,1).
compute ulcit=temp(cihigh,1).
do if (badlo = 1 and llcit <> priorlo).
compute badend={badend, llcit}.
compute priorlo = llcit.
end if.
do if (badhi = 1 and ulcit <> priorhi).
compute badend={badend, ulcit}.
compute priorhi = ulcit.
end if.
end if.
do if ( (imm4(1,k8)*bc)+(9999*(1-bc)) = 9999).
compute llcit=temp(cilow,1).
compute ulcit=temp(cihigh,1).
end if.
compute bootse=t(sqrt((cssq(temp)-((csum(temp)&**2)/nrow(temp)))/(nrow(temp)-1)))
.
compute condcont(k8,4:6)={bootse,llcit,ulcit}.
end if.
end loop.
do if (boot=0).
compute condcont=condcont(:,1:3).
end if.
compute indcontr=1.
end if.
end if.
do if (xmint=1).
do if (k1=(k4+1)).
compute cftotfx(:,k4)=cftotfx(:,k4)+(tucker2*xscaling).
end if.
end if.
do if (boot > 0).
compute tucker2=tucker2*xscaling.

.
compute temp = tucker2(2:nrow(tucker2),1).
compute temp(GRADE( tucker2(2:nrow(tucker2),1) )) = tucker2(2:nrow(tucker2),1).
compute badlo = 0.
compute badhi = 0.
do if ( (tucker2(1,1)*bc)+(9999*(1-bc)) <> 9999).
compute pv=csum(temp < (tucker2(1,1)*bc)+(9999*(1-bc)) )/nrow(temp).
compute ppv = pv.
do if (pv > .5).
compute ppv = 1-pv.
end if.
compute y5=sqrt(-2*ln(ppv)).
compute xp=y5+((((y5*p4+p3)*y5+p2)*y5+p1)*y5+p0)/((((y5*q4+q3)*y5+q2)*y5+q1)*y5+q0).
do if (pv <= .5).
compute xp = -xp.
end if.
compute cilow=rnd(nrow(temp)*(cdfnorm(2*xp-xp2))).
compute cihigh=trunc(nrow(temp)*(cdfnorm(2*xp+(xp2))))+1.
do if (cilow < 1).
compute cilow = 1.
compute booterr=1.
compute badlo = 1.
end if.
do if (cihigh > nrow(temp)).
compute cihigh = boot.
compute booterr=1.
compute badhi = 1.
end if.
compute llcit=temp(cilow,1).
compute ulcit=temp(cihigh,1).
do if (badlo = 1 and llcit <> priorlo).
compute badend={badend, llcit}.
compute priorlo = llcit.
end if.
do if (badhi = 1 and ulcit <> priorhi).
compute badend={badend, ulcit}.
compute priorhi = ulcit.
end if.
end if.
do if ( (tucker2(1,1)*bc)+(9999*(1-bc)) = 9999).
compute llcit=temp(cilow,1).
compute ulcit=temp(cihigh,1).
end if.
compute bootse=t(sqrt((cssq(temp)-((csum(temp)&**2)/nrow(temp)))/(nrow(temp)-1)))
.
compute indtemp={indtemp*xscaling, bootse,llcit,ulcit}.
end if.
do if (boot=0).
compute indtemp=indtemp*xscaling.
end if.
compute condres(k1,(ncol(indmodva)+1):ncol(condres))=indtemp.
end loop.
do if (xmint=0).
compute resultm2=make(nrow(condres),maxresm,99999).
compute resultm2(1:nrow(condres),1:ncol(condres))=condres.
compute resultm={resultm;resultm2}.
end if.
compute condlbs={condlbs,'Effect'}.
do if (boot > 0).
compute condlbs={condlbs,'BootSE', 'BootLLCI', 'BootULCI'}.
end if.
do if (xmint = 1).
do if (k4 = 1).
compute natindfx=condres((2+(k4-1)),2:ncol(condres)).
end if.
do if (k4 > 1).
compute natindfx={natindfx;condres((2+(k4-1)),2:ncol(condres))}.
end if.
do if (k4=nxvls).
compute resultm2=make(nrow(natindfx),maxresm,99999).
compute resultm2(1:nrow(natindfx),1:ncol(natindfx))=natindfx.
compute resultm={resultm;resultm2}.
end if.
end if.
do if (outscree=1 and xmint=0).
do if (nxvls=1).
print condres/title=' '/cnames=condlbs/format= F10.4 /space=0.
else.
compute condrlb=make(nrow(condres),1,xcatlab(k4,1)).
print condres/title=' '/cnames=condlbs/rnames=condrlb/format= F10.4 /space=0.
end if.
end if.
do if (outscree=1 and xmint=1 and k4=nxvls).
do if (nxvls=1).
print natindfx/title=' '/clabels='Effect', 'BootSE', 'BootLLCI', 'BootULCI'/format= F10.4 /space=0.
else.
compute condrlb=make(nrow(condres),1,xcatlab(k4,1)).
print natindfx/title=' '/clabels='Effect', 'BootSE', 'BootLLCI', 'BootULCI'/rnames=direffl2/format= F10.4 /space=0.
end if.
end if.
compute dichadj=0.
compute immcat=0.
do if ((indmmm(i,1)>0) and (xmint=0)).
do if (indmmm(i,1)=1 or indmmm(i,1)=12 or indmmm(i,1)=31).
do if (wdich=1 and mcw=0).
do if (indmmm(i,1) <> 12).
compute imm2(:,1)=imm2(:,1)*(wmax-wmin).
end if.
do if (indmmm(i,1) <> 31).
compute dichadj=1.
end if.
end if.
do if ((mcw = 1 or mcw = 2) and indmmm(i,1) <> 31)).
compute immcat=1.
end if.
end if.
do if (indmmm(i,1)=2 or indmmm(i,1)=22 or indmmm(i,1)=31).
do if (zdich=1 and mcz=0).
do if (indmmm(i,1) = 31).
compute imm2(:,(nwvls+1):ncol(imm2))=imm2(:,(nwvls+1):ncol(imm2))*(zmax-zmin).
end if.
do if (indmmm(i,1) = 2).
compute imm2(:,1)=imm2(:,1)*(zmax-zmin).
end if.
do if (indmmm(i,1) <> 31).
compute dichadj=1.
end if.
end if.
do if ((mcz = 1 or mcz = 2) and indmmm(i,1) <> 31)).
compute immcat=1.
end if.
end if.
compute immtemp2=t(imm2(1,:)).
compute immtemp=immtemp2.
compute immlbs={'Index'}.
do if (boot > 0).
compute immtemp=make(1,3,0).
loop k7=1 to ncol(imm2).

.
compute temp = imm2(2:nrow(imm2),k7).
compute temp(GRADE( imm2(2:nrow(imm2),k7) )) = imm2(2:nrow(imm2),k7).
compute badlo = 0.
compute badhi = 0.
do if ( (imm2(1,k7)*bc)+(9999*(1-bc)) <> 9999).
compute pv=csum(temp < (imm2(1,k7)*bc)+(9999*(1-bc)) )/nrow(temp).
compute ppv = pv.
do if (pv > .5).
compute ppv = 1-pv.
end if.
compute y5=sqrt(-2*ln(ppv)).
compute xp=y5+((((y5*p4+p3)*y5+p2)*y5+p1)*y5+p0)/((((y5*q4+q3)*y5+q2)*y5+q1)*y5+q0).
do if (pv <= .5).
compute xp = -xp.
end if.
compute cilow=rnd(nrow(temp)*(cdfnorm(2*xp-xp2))).
compute cihigh=trunc(nrow(temp)*(cdfnorm(2*xp+(xp2))))+1.
do if (cilow < 1).
compute cilow = 1.
compute booterr=1.
compute badlo = 1.
end if.
do if (cihigh > nrow(temp)).
compute cihigh = boot.
compute booterr=1.
compute badhi = 1.
end if.
compute llcit=temp(cilow,1).
compute ulcit=temp(cihigh,1).
do if (badlo = 1 and llcit <> priorlo).
compute badend={badend, llcit}.
compute priorlo = llcit.
end if.
do if (badhi = 1 and ulcit <> priorhi).
compute badend={badend, ulcit}.
compute priorhi = ulcit.
end if.
end if.
do if ( (imm2(1,k7)*bc)+(9999*(1-bc)) = 9999).
compute llcit=temp(cilow,1).
compute ulcit=temp(cihigh,1).
end if.
compute bootse=t(sqrt((cssq(temp)-((csum(temp)&**2)/nrow(temp)))/(nrow(temp)-1)))
.
compute temp={bootse,llcit,ulcit}.
compute immtemp={immtemp;temp}.
end loop.
compute immtemp=immtemp(2:nrow(immtemp),:).
compute immtemp={immtemp2,immtemp}.
compute immlbs={immlbs,'BootSE', 'BootLLCI', 'BootULCI'}.
end if.
compute resultm2=make(nrow(immtemp),maxresm,99999).
compute resultm2(1:nrow(immtemp),1:ncol(immtemp))=immtemp.
compute resultm={resultm;resultm2}.
do if (dichadj=0 and immcat=0 and indmmmt(i,1)<>5 and indmmm(i,1) < 100 ).
do if (outscree=1).
do if (indmmm(i,1) < 30).
print immtemp/title='      Index of moderated mediation:'/cnames=immlbs/rnames=immlbs2/format= F10.4.
end if.
do if (indmmm(i,1) = 31).
compute immlbs2={immlbs2;zcatlab(1:nzvls,1)}.
print immtemp/title='      Indices of partial moderated mediation:'/cnames=immlbs/rnames=immlbs2/format= F10.4.
end if.
end if.
do if ((nzvls = 1) and (nwvls = 1)).
do if (indmmm(i,1) = 41 or indmmm(i,1)=51).
loop k7=1 to nwvls.
compute immlbs2=zcatlab(1:nzvls,1).
compute immtemp2=immtemp((((k7-1)*nzvls)+1):(((k7-1)*nzvls)+nzvls),:).
do if (outscree=1).
do if (nwvls > 1).
compute primodv={'        ', wcatlab(k7,1)}.
print primodv/title='      Primary moderator:'/format=A8.
end if.
do if (nzvls=1).
print immtemp2/title='      Index of moderated moderated mediation'/cnames=immlbs/format= F10.4.
else.
print immtemp2/title='      Indices of moderated moderated mediation'/cnames=immlbs/rnames=immlbs2/format= F10.4.
end if.
end if.
compute cmmtemp=make(nrow(zprobval),4,0).
loop k8=1 to nrow(zprobval).
compute condbb3=condbb2(:,((nwvls+1)+((k7-1)*nzvls)):((nwvls+1)+((k7-1)*nzvls)+ (nzvls-1))).
do if (ncol(zprobval) > 1).
compute condbb3=condbb3*mdiag(zprobval(k8,:)).
else.
compute condbb3=condbb3&*zprobval(k8,:).
end if.
compute condbb3={condbb2(:,k7),condbb3}.
compute icmm=rsum(condbb3).
compute cmmtemp(k8,1)=icmm(1,1).
do if (boot > 0).

.
compute temp = icmm(2:nrow(icmm),1).
compute temp(GRADE( icmm(2:nrow(icmm),1) )) = icmm(2:nrow(icmm),1).
compute badlo = 0.
compute badhi = 0.
do if ( (icmm(1,1)*bc)+(9999*(1-bc)) <> 9999).
compute pv=csum(temp < (icmm(1,1)*bc)+(9999*(1-bc)) )/nrow(temp).
compute ppv = pv.
do if (pv > .5).
compute ppv = 1-pv.
end if.
compute y5=sqrt(-2*ln(ppv)).
compute xp=y5+((((y5*p4+p3)*y5+p2)*y5+p1)*y5+p0)/((((y5*q4+q3)*y5+q2)*y5+q1)*y5+q0).
do if (pv <= .5).
compute xp = -xp.
end if.
compute cilow=rnd(nrow(temp)*(cdfnorm(2*xp-xp2))).
compute cihigh=trunc(nrow(temp)*(cdfnorm(2*xp+(xp2))))+1.
do if (cilow < 1).
compute cilow = 1.
compute booterr=1.
compute badlo = 1.
end if.
do if (cihigh > nrow(temp)).
compute cihigh = boot.
compute booterr=1.
compute badhi = 1.
end if.
compute llcit=temp(cilow,1).
compute ulcit=temp(cihigh,1).
do if (badlo = 1 and llcit <> priorlo).
compute badend={badend, llcit}.
compute priorlo = llcit.
end if.
do if (badhi = 1 and ulcit <> priorhi).
compute badend={badend, ulcit}.
compute priorhi = ulcit.
end if.
end if.
do if ( (icmm(1,1)*bc)+(9999*(1-bc)) = 9999).
compute llcit=temp(cilow,1).
compute ulcit=temp(cihigh,1).
end if.
compute bootse=t(sqrt((cssq(temp)-((csum(temp)&**2)/nrow(temp)))/(nrow(temp)-1)))
.
compute cmmtemp(k8,2:4)={bootse,llcit,ulcit}.
end if.
end loop.
compute cmmtemp={zmodvals,cmmtemp}.
do if (boot=0).
compute cmmtemp=cmmtemp(:,1:2).
end if.
compute cmmlbs={znames,immlbs}.
compute resultm2=make(nrow(cmmtemp),maxresm,99999).
compute resultm2(1:nrow(cmmtemp),1:ncol(cmmtemp))=cmmtemp.
compute resultm={resultm;resultm2}.
do if (outscree=1).
print cmmtemp/title='      Indices of conditional moderated mediation by W'/cnames=cmmlbs/format= F10.4.
end if.
end loop.
end if.
end if.
end if.
do if (dichadj=1 or immcat=1 and indmmm(i,1) < 30).
do if (outscree=1).
print immtemp/title='Index of moderated mediation (difference between conditional indirect effects):'/cnames=immlbs/rnames=immlbs2/format= F10.4.
end if.
end if.
end if.
do if (indcontr=1).
compute resultm2=make(nrow(condcont),maxresm,99999).
compute resultm2(1:nrow(condcont),1:ncol(condcont))=condcont.
compute resultm={resultm;resultm2}.
compute condctlb={'Effect1','Effect2','Contrast','BootSE', 'BootLLCI','BootULCI'}.
do if (outscree=1).
print condcont/title=' Pairwise contrasts between conditional indirect effects (Effect1 minus Effect2)'/cnames=condctlb/format= F10.4.
end if.
end if.
end loop.
end if.
end loop.
do if (alttotal=1).
compute altcnms='Effect'.
compute alttotfx=t(cftotfx(1,:))+obnatdfx.
do if (boot > 0).
compute alttotfx=make(ncol(cftotfx),4,0).
compute alttotfx(:,1)=t(cftotfx(1,:))+obnatdfx.
compute natdirbt(1,:)=t(obnatdfx).
compute cftotfx=cftotfx+natdirbt.
loop cec=1 to ncol(cftotfx).

.
compute temp = cftotfx(2:nrow(cftotfx),cec).
compute temp(GRADE( cftotfx(2:nrow(cftotfx),cec) )) = cftotfx(2:nrow(cftotfx),cec).
compute badlo = 0.
compute badhi = 0.
do if ( (cftotfx(1,cec)*bc)+(9999*(1-bc)) <> 9999).
compute pv=csum(temp < (cftotfx(1,cec)*bc)+(9999*(1-bc)) )/nrow(temp).
compute ppv = pv.
do if (pv > .5).
compute ppv = 1-pv.
end if.
compute y5=sqrt(-2*ln(ppv)).
compute xp=y5+((((y5*p4+p3)*y5+p2)*y5+p1)*y5+p0)/((((y5*q4+q3)*y5+q2)*y5+q1)*y5+q0).
do if (pv <= .5).
compute xp = -xp.
end if.
compute cilow=rnd(nrow(temp)*(cdfnorm(2*xp-xp2))).
compute cihigh=trunc(nrow(temp)*(cdfnorm(2*xp+(xp2))))+1.
do if (cilow < 1).
compute cilow = 1.
compute booterr=1.
compute badlo = 1.
end if.
do if (cihigh > nrow(temp)).
compute cihigh = boot.
compute booterr=1.
compute badhi = 1.
end if.
compute llcit=temp(cilow,1).
compute ulcit=temp(cihigh,1).
do if (badlo = 1 and llcit <> priorlo).
compute badend={badend, llcit}.
compute priorlo = llcit.
end if.
do if (badhi = 1 and ulcit <> priorhi).
compute badend={badend, ulcit}.
compute priorhi = ulcit.
end if.
end if.
do if ( (cftotfx(1,cec)*bc)+(9999*(1-bc)) = 9999).
compute llcit=temp(cilow,1).
compute ulcit=temp(cihigh,1).
end if.
compute bootse=t(sqrt((cssq(temp)-((csum(temp)&**2)/nrow(temp)))/(nrow(temp)-1)))
.
compute alttotfx(cec,2:4)={bootse,llcit,ulcit}.
end loop.
compute altcnms={altcnms,'BootSE', 'BootLLCI','BootULCI'}.
end if.
compute resultm2=make(nrow(alttotfx),maxresm,99999).
compute resultm2(1:nrow(alttotfx),1:ncol(alttotfx))=alttotfx.
compute resultm={resultm;resultm2}.
do if (outscree=1).
print/title='----------'.
do if (nxvls > 1).
print alttotfx/title='Relative total effects of X on Y (sum of direct and indirect effects)'/cnames=altcnms/rnames=direffl2/format= F10.4.
end if.
do if (nxvls = 1).
print alttotfx/title='Total effect of X on Y (sum of direct and indirect effects)'/cnames=altcnms/format= F10.4.
end if.
end if.
end if.
end if.
end if
.
do if (criterr=0 and (saveest = 1 or saveboot=1)).
compute coeffsav=coeffmat(2:nrow(coeffmat),:).
compute conseq=conseq(2:nrow(conseq),1).
compute dfmat=dfmat(2:nrow(dfmat),1).
compute dfmat=dfmat.
compute saven={'conseqnt','antecdnt','coeff','se','t','p','LLCI','ULCI','df'}.
do if (ydich=1).
compute saven={'conseqnt','antecdnt','coeff','se','t_or_Z','p','LLCI','ULCI','df'}.
end if.
do if (saveboot=1 and boot > 0 ).
compute colslab=make(300,1,' ').


compute colslab( 1 ,1)= 'COL1'.


compute colslab( 2 ,1)= 'COL2'.


compute colslab( 3 ,1)= 'COL3'.


compute colslab( 4 ,1)= 'COL4'.


compute colslab( 5 ,1)= 'COL5'.


compute colslab( 6 ,1)= 'COL6'.


compute colslab( 7 ,1)= 'COL7'.


compute colslab( 8 ,1)= 'COL8'.


compute colslab( 9 ,1)= 'COL9'.


compute colslab( 10 ,1)= 'COL10'.


compute colslab( 11 ,1)= 'COL11'.


compute colslab( 12 ,1)= 'COL12'.


compute colslab( 13 ,1)= 'COL13'.


compute colslab( 14 ,1)= 'COL14'.


compute colslab( 15 ,1)= 'COL15'.


compute colslab( 16 ,1)= 'COL16'.


compute colslab( 17 ,1)= 'COL17'.


compute colslab( 18 ,1)= 'COL18'.


compute colslab( 19 ,1)= 'COL19'.


compute colslab( 20 ,1)= 'COL20'.


compute colslab( 21 ,1)= 'COL21'.


compute colslab( 22 ,1)= 'COL22'.


compute colslab( 23 ,1)= 'COL23'.


compute colslab( 24 ,1)= 'COL24'.


compute colslab( 25 ,1)= 'COL25'.


compute colslab( 26 ,1)= 'COL26'.


compute colslab( 27 ,1)= 'COL27'.


compute colslab( 28 ,1)= 'COL28'.


compute colslab( 29 ,1)= 'COL29'.


compute colslab( 30 ,1)= 'COL30'.


compute colslab( 31 ,1)= 'COL31'.


compute colslab( 32 ,1)= 'COL32'.


compute colslab( 33 ,1)= 'COL33'.


compute colslab( 34 ,1)= 'COL34'.


compute colslab( 35 ,1)= 'COL35'.


compute colslab( 36 ,1)= 'COL36'.


compute colslab( 37 ,1)= 'COL37'.


compute colslab( 38 ,1)= 'COL38'.


compute colslab( 39 ,1)= 'COL39'.


compute colslab( 40 ,1)= 'COL40'.


compute colslab( 41 ,1)= 'COL41'.


compute colslab( 42 ,1)= 'COL42'.


compute colslab( 43 ,1)= 'COL43'.


compute colslab( 44 ,1)= 'COL44'.


compute colslab( 45 ,1)= 'COL45'.


compute colslab( 46 ,1)= 'COL46'.


compute colslab( 47 ,1)= 'COL47'.


compute colslab( 48 ,1)= 'COL48'.


compute colslab( 49 ,1)= 'COL49'.


compute colslab( 50 ,1)= 'COL50'.


compute colslab( 51 ,1)= 'COL51'.


compute colslab( 52 ,1)= 'COL52'.


compute colslab( 53 ,1)= 'COL53'.


compute colslab( 54 ,1)= 'COL54'.


compute colslab( 55 ,1)= 'COL55'.


compute colslab( 56 ,1)= 'COL56'.


compute colslab( 57 ,1)= 'COL57'.


compute colslab( 58 ,1)= 'COL58'.


compute colslab( 59 ,1)= 'COL59'.


compute colslab( 60 ,1)= 'COL60'.


compute colslab( 61 ,1)= 'COL61'.


compute colslab( 62 ,1)= 'COL62'.


compute colslab( 63 ,1)= 'COL63'.


compute colslab( 64 ,1)= 'COL64'.


compute colslab( 65 ,1)= 'COL65'.


compute colslab( 66 ,1)= 'COL66'.


compute colslab( 67 ,1)= 'COL67'.


compute colslab( 68 ,1)= 'COL68'.


compute colslab( 69 ,1)= 'COL69'.


compute colslab( 70 ,1)= 'COL70'.


compute colslab( 71 ,1)= 'COL71'.


compute colslab( 72 ,1)= 'COL72'.


compute colslab( 73 ,1)= 'COL73'.


compute colslab( 74 ,1)= 'COL74'.


compute colslab( 75 ,1)= 'COL75'.


compute colslab( 76 ,1)= 'COL76'.


compute colslab( 77 ,1)= 'COL77'.


compute colslab( 78 ,1)= 'COL78'.


compute colslab( 79 ,1)= 'COL79'.


compute colslab( 80 ,1)= 'COL80'.


compute colslab( 81 ,1)= 'COL81'.


compute colslab( 82 ,1)= 'COL82'.


compute colslab( 83 ,1)= 'COL83'.


compute colslab( 84 ,1)= 'COL84'.


compute colslab( 85 ,1)= 'COL85'.


compute colslab( 86 ,1)= 'COL86'.


compute colslab( 87 ,1)= 'COL87'.


compute colslab( 88 ,1)= 'COL88'.


compute colslab( 89 ,1)= 'COL89'.


compute colslab( 90 ,1)= 'COL90'.


compute colslab( 91 ,1)= 'COL91'.


compute colslab( 92 ,1)= 'COL92'.


compute colslab( 93 ,1)= 'COL93'.


compute colslab( 94 ,1)= 'COL94'.


compute colslab( 95 ,1)= 'COL95'.


compute colslab( 96 ,1)= 'COL96'.


compute colslab( 97 ,1)= 'COL97'.


compute colslab( 98 ,1)= 'COL98'.


compute colslab( 99 ,1)= 'COL99'.


compute colslab( 100 ,1)= 'COL100'.


compute colslab( 101 ,1)= 'COL101'.


compute colslab( 102 ,1)= 'COL102'.


compute colslab( 103 ,1)= 'COL103'.


compute colslab( 104 ,1)= 'COL104'.


compute colslab( 105 ,1)= 'COL105'.


compute colslab( 106 ,1)= 'COL106'.


compute colslab( 107 ,1)= 'COL107'.


compute colslab( 108 ,1)= 'COL108'.


compute colslab( 109 ,1)= 'COL109'.


compute colslab( 110 ,1)= 'COL110'.


compute colslab( 111 ,1)= 'COL111'.


compute colslab( 112 ,1)= 'COL112'.


compute colslab( 113 ,1)= 'COL113'.


compute colslab( 114 ,1)= 'COL114'.


compute colslab( 115 ,1)= 'COL115'.


compute colslab( 116 ,1)= 'COL116'.


compute colslab( 117 ,1)= 'COL117'.


compute colslab( 118 ,1)= 'COL118'.


compute colslab( 119 ,1)= 'COL119'.


compute colslab( 120 ,1)= 'COL120'.


compute colslab( 121 ,1)= 'COL121'.


compute colslab( 122 ,1)= 'COL122'.


compute colslab( 123 ,1)= 'COL123'.


compute colslab( 124 ,1)= 'COL124'.


compute colslab( 125 ,1)= 'COL125'.


compute colslab( 126 ,1)= 'COL126'.


compute colslab( 127 ,1)= 'COL127'.


compute colslab( 128 ,1)= 'COL128'.


compute colslab( 129 ,1)= 'COL129'.


compute colslab( 130 ,1)= 'COL130'.


compute colslab( 131 ,1)= 'COL131'.


compute colslab( 132 ,1)= 'COL132'.


compute colslab( 133 ,1)= 'COL133'.


compute colslab( 134 ,1)= 'COL134'.


compute colslab( 135 ,1)= 'COL135'.


compute colslab( 136 ,1)= 'COL136'.


compute colslab( 137 ,1)= 'COL137'.


compute colslab( 138 ,1)= 'COL138'.


compute colslab( 139 ,1)= 'COL139'.


compute colslab( 140 ,1)= 'COL140'.


compute colslab( 141 ,1)= 'COL141'.


compute colslab( 142 ,1)= 'COL142'.


compute colslab( 143 ,1)= 'COL143'.


compute colslab( 144 ,1)= 'COL144'.


compute colslab( 145 ,1)= 'COL145'.


compute colslab( 146 ,1)= 'COL146'.


compute colslab( 147 ,1)= 'COL147'.


compute colslab( 148 ,1)= 'COL148'.


compute colslab( 149 ,1)= 'COL149'.


compute colslab( 150 ,1)= 'COL150'.


compute colslab( 151 ,1)= 'COL151'.


compute colslab( 152 ,1)= 'COL152'.


compute colslab( 153 ,1)= 'COL153'.


compute colslab( 154 ,1)= 'COL154'.


compute colslab( 155 ,1)= 'COL155'.


compute colslab( 156 ,1)= 'COL156'.


compute colslab( 157 ,1)= 'COL157'.


compute colslab( 158 ,1)= 'COL158'.


compute colslab( 159 ,1)= 'COL159'.


compute colslab( 160 ,1)= 'COL160'.


compute colslab( 161 ,1)= 'COL161'.


compute colslab( 162 ,1)= 'COL162'.


compute colslab( 163 ,1)= 'COL163'.


compute colslab( 164 ,1)= 'COL164'.


compute colslab( 165 ,1)= 'COL165'.


compute colslab( 166 ,1)= 'COL166'.


compute colslab( 167 ,1)= 'COL167'.


compute colslab( 168 ,1)= 'COL168'.


compute colslab( 169 ,1)= 'COL169'.


compute colslab( 170 ,1)= 'COL170'.


compute colslab( 171 ,1)= 'COL171'.


compute colslab( 172 ,1)= 'COL172'.


compute colslab( 173 ,1)= 'COL173'.


compute colslab( 174 ,1)= 'COL174'.


compute colslab( 175 ,1)= 'COL175'.


compute colslab( 176 ,1)= 'COL176'.


compute colslab( 177 ,1)= 'COL177'.


compute colslab( 178 ,1)= 'COL178'.


compute colslab( 179 ,1)= 'COL179'.


compute colslab( 180 ,1)= 'COL180'.


compute colslab( 181 ,1)= 'COL181'.


compute colslab( 182 ,1)= 'COL182'.


compute colslab( 183 ,1)= 'COL183'.


compute colslab( 184 ,1)= 'COL184'.


compute colslab( 185 ,1)= 'COL185'.


compute colslab( 186 ,1)= 'COL186'.


compute colslab( 187 ,1)= 'COL187'.


compute colslab( 188 ,1)= 'COL188'.


compute colslab( 189 ,1)= 'COL189'.


compute colslab( 190 ,1)= 'COL190'.


compute colslab( 191 ,1)= 'COL191'.


compute colslab( 192 ,1)= 'COL192'.


compute colslab( 193 ,1)= 'COL193'.


compute colslab( 194 ,1)= 'COL194'.


compute colslab( 195 ,1)= 'COL195'.


compute colslab( 196 ,1)= 'COL196'.


compute colslab( 197 ,1)= 'COL197'.


compute colslab( 198 ,1)= 'COL198'.


compute colslab( 199 ,1)= 'COL199'.


compute colslab( 200 ,1)= 'COL200'.


compute colslab( 201 ,1)= 'COL201'.


compute colslab( 202 ,1)= 'COL202'.


compute colslab( 203 ,1)= 'COL203'.


compute colslab( 204 ,1)= 'COL204'.


compute colslab( 205 ,1)= 'COL205'.


compute colslab( 206 ,1)= 'COL206'.


compute colslab( 207 ,1)= 'COL207'.


compute colslab( 208 ,1)= 'COL208'.


compute colslab( 209 ,1)= 'COL209'.


compute colslab( 210 ,1)= 'COL210'.


compute colslab( 211 ,1)= 'COL211'.


compute colslab( 212 ,1)= 'COL212'.


compute colslab( 213 ,1)= 'COL213'.


compute colslab( 214 ,1)= 'COL214'.


compute colslab( 215 ,1)= 'COL215'.


compute colslab( 216 ,1)= 'COL216'.


compute colslab( 217 ,1)= 'COL217'.


compute colslab( 218 ,1)= 'COL218'.


compute colslab( 219 ,1)= 'COL219'.


compute colslab( 220 ,1)= 'COL220'.


compute colslab( 221 ,1)= 'COL221'.


compute colslab( 222 ,1)= 'COL222'.


compute colslab( 223 ,1)= 'COL223'.


compute colslab( 224 ,1)= 'COL224'.


compute colslab( 225 ,1)= 'COL225'.


compute colslab( 226 ,1)= 'COL226'.


compute colslab( 227 ,1)= 'COL227'.


compute colslab( 228 ,1)= 'COL228'.


compute colslab( 229 ,1)= 'COL229'.


compute colslab( 230 ,1)= 'COL230'.


compute colslab( 231 ,1)= 'COL231'.


compute colslab( 232 ,1)= 'COL232'.


compute colslab( 233 ,1)= 'COL233'.


compute colslab( 234 ,1)= 'COL234'.


compute colslab( 235 ,1)= 'COL235'.


compute colslab( 236 ,1)= 'COL236'.


compute colslab( 237 ,1)= 'COL237'.


compute colslab( 238 ,1)= 'COL238'.


compute colslab( 239 ,1)= 'COL239'.


compute colslab( 240 ,1)= 'COL240'.


compute colslab( 241 ,1)= 'COL241'.


compute colslab( 242 ,1)= 'COL242'.


compute colslab( 243 ,1)= 'COL243'.


compute colslab( 244 ,1)= 'COL244'.


compute colslab( 245 ,1)= 'COL245'.


compute colslab( 246 ,1)= 'COL246'.


compute colslab( 247 ,1)= 'COL247'.


compute colslab( 248 ,1)= 'COL248'.


compute colslab( 249 ,1)= 'COL249'.


compute colslab( 250 ,1)= 'COL250'.


compute colslab( 251 ,1)= 'COL251'.


compute colslab( 252 ,1)= 'COL252'.


compute colslab( 253 ,1)= 'COL253'.


compute colslab( 254 ,1)= 'COL254'.


compute colslab( 255 ,1)= 'COL255'.


compute colslab( 256 ,1)= 'COL256'.


compute colslab( 257 ,1)= 'COL257'.


compute colslab( 258 ,1)= 'COL258'.


compute colslab( 259 ,1)= 'COL259'.


compute colslab( 260 ,1)= 'COL260'.


compute colslab( 261 ,1)= 'COL261'.


compute colslab( 262 ,1)= 'COL262'.


compute colslab( 263 ,1)= 'COL263'.


compute colslab( 264 ,1)= 'COL264'.


compute colslab( 265 ,1)= 'COL265'.


compute colslab( 266 ,1)= 'COL266'.


compute colslab( 267 ,1)= 'COL267'.


compute colslab( 268 ,1)= 'COL268'.


compute colslab( 269 ,1)= 'COL269'.


compute colslab( 270 ,1)= 'COL270'.


compute colslab( 271 ,1)= 'COL271'.


compute colslab( 272 ,1)= 'COL272'.


compute colslab( 273 ,1)= 'COL273'.


compute colslab( 274 ,1)= 'COL274'.


compute colslab( 275 ,1)= 'COL275'.


compute colslab( 276 ,1)= 'COL276'.


compute colslab( 277 ,1)= 'COL277'.


compute colslab( 278 ,1)= 'COL278'.


compute colslab( 279 ,1)= 'COL279'.


compute colslab( 280 ,1)= 'COL280'.


compute colslab( 281 ,1)= 'COL281'.


compute colslab( 282 ,1)= 'COL282'.


compute colslab( 283 ,1)= 'COL283'.


compute colslab( 284 ,1)= 'COL284'.


compute colslab( 285 ,1)= 'COL285'.


compute colslab( 286 ,1)= 'COL286'.


compute colslab( 287 ,1)= 'COL287'.


compute colslab( 288 ,1)= 'COL288'.


compute colslab( 289 ,1)= 'COL289'.


compute colslab( 290 ,1)= 'COL290'.


compute colslab( 291 ,1)= 'COL291'.


compute colslab( 292 ,1)= 'COL292'.


compute colslab( 293 ,1)= 'COL293'.


compute colslab( 294 ,1)= 'COL294'.


compute colslab( 295 ,1)= 'COL295'.


compute colslab( 296 ,1)= 'COL296'.


compute colslab( 297 ,1)= 'COL297'.


compute colslab( 298 ,1)= 'COL298'.


compute colslab( 299 ,1)= 'COL299'.


compute colslab( 300 ,1)= 'COL300'.

compute colslab=colslab(1:ncol(bootres),1).
compute colslab={colslab,conseq,vlabs}.
do if (outscree=1).
print/title = '**************************************************************************'.
print/title='Bootstrap estimates were saved to a file'/space=0.
print colslab/title='Map of column names to model coefficients:'/clabels=' ','Conseqnt','Antecdnt'/format=a8.
end if.
end if.
end if.
do if (criterr=0 and boot > 0 and modelbt=1).
compute labstart=1.
do if (outscree=1).
print/title = '*********** BOOTSTRAP RESULTS FOR REGRESSION MODEL PARAMETERS ************'.
end if.
loop iboot = 1 to (nms+nys).
do if (outscree=1).
print outnames(1,iboot)/title = 'OUTCOME VARIABLE:'/format = A8.
end if.
compute vlabsm=vlabs(labstart:(labstart+(nump(1,iboot)-1)),1).
compute outnmtmp=bootcim(labstart:(labstart+(nump(1,iboot)-1)),:).
compute resultm2=make(nrow(outnmtmp),maxresm,99999).
compute resultm2(1:nrow(outnmtmp),1:ncol(outnmtmp))=outnmtmp.
compute resultm={resultm;resultm2}.
do if (outscree=1).
print bootcim(labstart:(labstart+(nump(1,iboot)-1)),:)/title=' '/rnames=vlabsm/ clabels='Coeff' 'BootMean' 'BootSE' 'BootLLCI' 'BootULCI'/format= F10.4 /space=0.
end if.
compute labstart=labstart+nump(1,iboot).
do if (iboot < (nms+nys)).
do if (outscree=1).
print/title= '----------'.
end if.
end if.
end loop.
end if.
do if (criterr=0 and saveest=1).
compute resultm=resultm(2:nrow(resultm),:).
compute bocaj=(resultm=99999).
compute bocaj=csum(bocaj).
compute bocaj=1-(bocaj=nrow(resultm)).
compute j=1.
loop i = 1 to ncol(resultm).
do if (bocaj(1,i)=1).
compute j=j+1.
end if.
end loop.
compute resultm=resultm(:,1:(j-1)).
save resultm/outfile = *.
end if.
do if (criterr = 0 and matrices=1 and outscree=1).
print/title = '************************ MODEL DEFINITION MATRICES ************************'.
print/title = 'FROM variables are columns, TO variables are rows.'.
compute temp2=make(nrow(bcmat),ncol(bcmat),'0').
loop i = 2 to nrow(bcmat).
loop j = 1 to (ncol(bcmat)-1).
do if (bcmat(i,j)=1).
compute temp2(i,j)='1'.
end if.
do if (j >= i).
compute temp2(i,j)=' '.
end if.
end loop.
end loop.
compute temp2=temp2(2:nrow(bcmat),(1:(ncol(bcmat)-1))).
do if (nms > 0).
compute cmatlabs={xnames,mnames}.
compute rmatlabs={mnames,ynames}.
end if.
do if (nms = 0).
compute cmatlabs={xnames}.
compute rmatlabs={ynames}.
end if.
print temp2/title='BMATRIX: Paths freely estimated (1) and fixed to zero (0):'/cnames=cmatlabs/rnames=rmatlabs/format A3.
compute z=0.
do if (rsum(csum(wcmat))<>0).
compute temp2=make(nrow(wcmat),ncol(wcmat),'0').
loop i = 2 to nrow(wcmat).
loop j = 1 to (ncol(wcmat)-1).
do if (wcmat(i,j)=1).
compute temp2(i,j)='1'.
end if.
do if (j >= i).
compute temp2(i,j)=' '.
end if.
end loop.
end loop.
compute temp2=temp2(2:nrow(wcmat),(1:(ncol(wcmat)-1))).
print temp2/title='WMATRIX: Paths moderated (1) and not moderated (0) by W:'/cnames=cmatlabs/rnames=rmatlabs/format A3.
end if.
do if (rsum(csum(zcmat))<>0).
compute temp2=make(nrow(zcmat),ncol(zcmat),'0').
loop i = 2 to nrow(zcmat).
loop j = 1 to (ncol(zcmat)-1).
do if (zcmat(i,j)=1).
compute temp2(i,j)='1'.
end if.
do if (j >= i).
compute temp2(i,j)=' '.
end if.
end loop.
end loop.
compute temp2=temp2(2:nrow(zcmat),(1:(ncol(zcmat)-1))).
print temp2/title='ZMATRIX: Paths moderated (1) and not moderated (0) by Z:'/cnames=cmatlabs/rnames=rmatlabs/format a3.
end if.
do if (rsum(csum(wzcmat))<>0).
compute temp2=make(nrow(wzcmat),ncol(wzcmat),'0').
loop i = 2 to nrow(wzcmat).
loop j = 1 to (ncol(wzcmat)-1).
do if (wzcmat(i,j)=1).
compute temp2(i,j)='1'.
end if.
do if (j >= i).
compute temp2(i,j)=' '.
end if.
end loop.
end loop.
compute temp2=temp2(2:nrow(wzcmat),(1:(ncol(wzcmat)-1))).
print temp2/title='WZMATRIX: W moderated paths moderated (1) and not moderated (0) by Z:'/cnames=cmatlabs/rnames=rmatlabs/format a3.
end if.
do if (ncs > 0).
print ccmat/title='CMATRIX: Covariates (columns) in (1) and not in (0) the models of M and Y (rows):'/rnames=rmatlabs/cnames=covnames.
end if.
end if.
do if (outscree=1).
do if (activate=0).
print/title = '*********************** ANALYSIS NOTES AND ERRORS ************************'.
end if.
end if.
do if (activate=1).
print/title = '**************************************************************************'.
end if.
do if (criterr=0).
do if (outscree=1).
print conf/title = 'Level of confidence for all confidence intervals in output:'/format = F8.4.
do if (boot > 0).
do if (goodboot = boot and bc=0).
print boot/title='Number of bootstrap samples for percentile bootstrap confidence intervals:'.
end if.
do if (goodboot = boot and bc=1).
print boot/title='Number of bootstrap samples for bias-corrected bootstrap confidence intervals:'.
end if.
do if (booterr = 1).
compute badend = badend(1,2:ncol(badend)).
print/title = 'WARNING: Bootstrap CI endpoints below not trustworthy. Decrease confidence'.
print badend/title='or increase the number of bootstrap samples.'/space=0/format = F10.4.
end if.
end if.
do if (mc > 0).
print mc/title='Number of samples for Monte Carlo confidence intervals:'.
end if.
do if (wnotev > 0 and printw=1).
do if (wnotev=1).
print/title = 'W values in conditional tables are the 16th, 50th, and 84th percentiles.'.
else if (wnotev=2).
do if (minwwarn=0 and maxwwarn=0).
print/title = 'W values in conditional tables are the mean and +/- SD from the mean.'.
end if.
do if (minwwarn=1).
print/title = 'W values in conditional tables are the minimum, the mean, and 1 SD above the mean.'.
end if.
do if (maxwwarn=1).
print/title = 'W values in conditional tables are 1 SD below the mean, the mean, and the maximum.'.
end if.
end if.
end if.
do if (znotev > 0 and printz=1).
do if (znotev=1).
print/title = 'Z values in conditional tables are the 16th, 50th, and 84th percentiles.'.
else if (znotev=2).
do if (minzwarn=0 and maxzwarn=0).
print/title = 'Z values in conditional tables are the mean and +/- SD from the mean.'.
end if.
do if (minzwarn=1).
print/title = 'Z values in conditional tables are the minimum, the mean, and 1 SD above the mean.'.
end if.
do if (maxzwarn=1).
print/title = 'Z values in conditional tables are 1 SD below the mean, the mean, and the maximum.'.
end if.
end if.
end if.
do if (minwwarn > 0).
print/title = 'NOTE: One SD below the mean is below the minimum observed in the data for W,'.
print/title = '      so the minimum measurement on W is used for conditioning instead.'/space=0.
end if.
do if (maxwwarn > 0).
print/title = 'NOTE: One SD above the mean is above the maximum observed in the data for W,'.
print/title = '      so the maximum measurement for W is used for conditioning instead.'/space=0.
end if.
do if (minzwarn > 0).
print/title = 'NOTE: One SD below the mean is below the minimum observed in the data for Z,'.
print/title = '      so the minimum measurement for Z is used for conditioning instead.'/space=0.
end if.
do if (maxzwarn > 0).
print/title = 'NOTE: One SD above the mean is above the maximum observed in the data for Z,'.
print/title = '      so the maximum measurement for Z is used for conditioning instead.'/space=0.
end if.
do if (pstog=1).
print/title= 'NOTE: Standardized coefficients for dichotomous or multicategorical X are in'.
print/title= '      partially standardized form.'/space=0.
end if.
loop i = 1 to 100.
do if (notecode(i,1) = 32).
print/title = 'Direct, indirect, and total effects are counterfactually defined'.
do if (xcontcf=0).
print/title = 'assuming X by M interaction.'/space=0.
end if.
do if (xcontcf=1).
print/title = 'assuming X by M interaction and with the following reference (x_ref)'/space=0.
compute states=t(xrefvals).
print states/title = 'and counterfactual (x_cf) states for X:'/rlabels='x_ref' 'x_cf'/format= F10.4 /space=0.
end if.
end if.
do if (notecode(i,1)=1).
print/title = 'NOTE: COVMY is ignored when using CMATRIX option.'.
end if.
do if (notecode(i,1)=2).
print/title = 'NOTE: Confidence level restricted to between 50 and 99.9999%.  95% confidence is provided in output'.
end if.
do if (notecode(i,1)=3).
print centvar/title = 'NOTE: The following variables were mean centered prior to analysis:'/format = a8.
end if.
do if (notecode(i,1) = 4).
print/title = 'NOTE: A heteroscedasticity consistent standard error and covariance matrix estimator was used.'.
end if.
do if (notecode(i,1) = 5).
print/title = 'NOTE: The HC3 option has been replaced with HC.  See the documentation.'.
end if.
do if (notecode(i,1) = 6).
print/title = 'NOTE: Due to estimation problems, some bootstrap samples had to be replaced.'.
print badboot/title='      The number of times this happened was:'/space=0/format=F8.0.
end if.
do if (notecode(i,1) = 7).
print/title = 'NOTE: The bootstrapping was not completed due to problematic bootstrap samples.'.
print/title = '      Bootstrap confidence intervals are therefore suppressed.'/space=0.
end if.
do if (notecode(i,1) = 8).
print/title = 'NOTE: The number of bootstrap samples was adjusted upward given your desired confidence.'.
end if.
do if (notecode(i,1) = 9).
print/title = 'NOTE: WMODVAL is ignored when W is specified as multicategorical.'.
end if.
do if (notecode(i,1) = 10).
print/title = 'NOTE: ZMODVAL is ignored when Z is specified as multicategorical.'.
end if.
do if (notecode(i,1) = 11).
print/title = 'NOTE: Total effect model generated only when all covariates are specified in all'.
print/title = '      models of M and Y.'/space=0.
end if.
do if (notecode(i,1) = 30).
print/title = 'NOTE: Your vector of linear hypothesis weights is of the wrong length for this model.'.
end if.
do if (notecode(i,1) = 12).
print/title = 'NOTE: Total effect model generated only when X is freely estimated to affect each M'.
print/title = '      and both X and M are freely estimated to affect Y'/space=0.
end if.
do if (notecode(i,1) = 13).
print/title = 'NOTE: There are too many pairwise contrasts to conduct with this model.'.
end if.
do if (notecode(i,1) = 14).
print/title = 'NOTE: The number of contrast weights must equal the number of indirect effects.'.
end if.
do if (notecode(i,1) = 15).
print/title = 'NOTE: Monte Carlo confidence intervals not available for this model.'.
print/title = '      Bootstrapping is used instead.'/space=0.
end if.
do if (notecode(i,1) = 16).
print/title = 'NOTE: The number of Monte Carlo samples was adjusted upward given your desired confidence.'.
end if.
do if (notecode(i,1) = 19).
print/title = 'NOTE: Your contrast matrix is invalid or not applicable to this model.'.
end if.
do if (notecode(i,1) = 20).
print/title = 'NOTE: One of the groups specified by your contrast matrix does not exist in the data.'.
end if.
do if (notecode(i,1) = 21).
print/title = 'NOTE: The VARORDER option is not available in this release.'.
end if.
do if (notecode(i,1) = 22).
print/title = 'NOTE: The VMODVAL and QMODVAL options are not available in this release.'.
end if.
do if (notecode(i,1) = 23).
print/title = 'NOTE: The QUANTILE option is not available in this release.'.
end if.
do if (notecode(i,1) = 24).
print/title = 'NOTE: Total effect model not available with dichotomous Y.'.
end if.
do if (notecode(i,1) = 25).
print/title = 'NOTE: STAND/EFFSIZE options not available with dichotomous Y.'.
end if.
do if ((notecode(i,1) = 26) and nms > 0).
print/title = 'NOTE: Direct and indirect effects of X on Y are on a log-odds metric.'.
end if.
do if (notecode(i,1) = 27).
print/title = 'NOTE: Standardized coefficients are not available for models with moderators.'.
end if.
do if (notecode(i,1) = 28).
print/title = 'NOTE: The contrast option is not available with a multicategorical X.'.
end if.
do if (notecode(i,1) = 31).
do if (nms > 1).
print medmeans/title = 'NOTE: Controlled direct effect(s) estimated at the following mediator values:'/cnames=mnames/format= F10.4.
end if.
do if (nms=1).
print medmeans/title = 'NOTE: Controlled direct effect(s) estimated at the following mediator value:'/format= F10.4.
end if.
end if.
do if (notecode(i,1) = 33).
print/title = 'NOTE: Sobel test is not available with the XMINT option.'.
end if.
do if (notecode(i,1) = 37).
print/title = 'NOTE: The CONTRAST option is not available when using the XMINT option.'.
end if.
do if (notecode(i,1) = 34).
print/title = 'NOTE: Standardized effects are not available when using the XMINT option.'.
end if.
do if (notecode(i,1) = 36).
print/title = 'NOTE: The XREFVAL option is ignored when X is declared as multicategorical.'.
end if.
do if (notecode(i,1) = 35).
print coval/title = 'NOTE: Counterfactual effects estimated at the following covariate values:'/cnames=covnames/format= F10.4.
end if.
do if (notecode(i,1) = 29 and (listmiss=1)).
compute a=missrow.
compute conum=ncol(a).
compute allgood=0.
compute smremain=12.
compute largesti=1.
compute smallrow=0.
do if (conum > 12).
loop ii = 1 to 12.
compute check=(conum/ii).
do if (check = trunc(check)).
compute check2=conum/ii.
compute aok=ii.
do if (aok > 2).
compute allgood=1.
end if.
else.
compute remain=conum-(ii*trunc(check)).
do if (remain <= smremain).
compute smremain=remain.
compute largesti=ii.
compute smallrow=trunc(conum/largesti).
end if.
end if.
end loop.
compute atemp=a(1,1:(aok*check2)).
compute atemp=reshape(atemp,check2,aok).
do if (ncol(atemp) > 2).
print/title='NOTE: Missing data resulted in the deletion of the following row(s) of'.
print atemp/title='      data from the analysis:'/format=F6.0/space=0.
end if.
do if (allgood=0).
compute atemp=a(1,1:(smallrow*largesti)).
compute atemp=reshape(atemp,smallrow,largesti).
compute btemp=a(1,((largesti*smallrow)+1):conum).
print/title='NOTE: Missing data resulted in the deletion of the following row(s) of'.
print atemp/title='      data from the analysis:'/format=F6.0/space=0.
print btemp/title=' '/space=0/format=f6.0.
end if.
else if (conum <= 12).
print/title='NOTE: Missing data resulted in the deletion of the following row(s) of'.
print a/title='      data from the analysis:'/format=F6.0/space=0.
end if.
end if.
end loop.
do if (toomany=1).
print/title='WARNING: Variables names longer than eight characters can produce incorrect output'.
print/title='when some variables in the data file have the same first eight characters. Shorter'/space=0.
print/title='variable names are recommended. By using this output, you are accepting all risk'/space=0.
print/title='and consequences of interpreting or reporting results that may be incorrect.'/space=0.
end if.
end if.
end if.
loop i = 1 to 100.
do if (errcode(i,1)=1).
print/title = 'ERROR: You must specify a Y and an X variable.'.
end if.
do if (errcode(i,1)=2).
print/title = 'ERROR: X, M, or Y variable used more than once or W and Z are the same variable.'.
do if (toomany = 1).
print/title = '       This could be caused by the use of variables names longer'/space=0.
print varnames/title = '       than eight characters. Here are the variables I see:'/space=0/format=A8.
end if.
end if.
do if (errcode(i,1)=3).
print/title = 'ERROR: You have specified more than one variable for W, Y, X, or Z'.
end if.
do if (errcode(i,1)=4).
print/title = 'ERROR: A variable specified as multicategorical has more than nine categories.'.
end if.
do if (errcode(i,1)=5).
print/title = 'ERROR: One of the categories contains only a single case.'.
end if.
do if (errcode(i,1)=6).
print/title = 'ERROR: Invalid model number in this version of PROCESS.'.
end if.
do if (errcode(i,1)=7).
print/title = 'ERROR: Invalid model number.'.
end if.
do if (errcode(i,1)=8).
print/title = 'ERROR: You must specify an M variable for this model.'.
end if.
do if (errcode(i,1)=9).
print/title = 'ERROR: You have specified an M variable in a model that does not use it.'.
end if.
do if (errcode(i,1)=10).
print/title = 'ERROR: You have specified a W variable in a model that does not use it.'.
end if.
do if (errcode(i,1)=11).
print/title = 'ERROR: You have not specified a W variable in a model that requires it.'.
end if.
do if (errcode(i,1)=12).
print/title = 'ERROR: You have specified a Z variable in a model that does not use it.'.
end if.
do if (errcode(i,1)=13).
print/title = 'ERROR: You have not specified a Z variable in a model that requires it.'.
end if.
do if (errcode(i,1)=14).
print/title = 'ERROR: V and Q are not proper specifications in this release of PROCESS.'.
print/title = '       Moderators must be specified as W and/or Z.'/space=0.
end if.
do if (errcode(i,1)=15).
print/title = 'ERROR: One of your model variables exhibits no variation (it is a constant).'.
end if.
do if (errcode(i,1)=16).
print/title = 'ERROR: BMATRIX is not the correct length or is otherwise invalid.'.
end if.
do if (errcode(i,1)=17).
print/title = 'ERROR: WMATRIX is not the correct length or is otherwise invalid.'.
end if.
do if (errcode(i,1)=18).
print/title = 'ERROR: ZMATRIX is not the correct length or is otherwise invalid.'.
end if.
do if (errcode(i,1)=19).
print/title = 'ERROR: WZMATRIX is not the correct length or is otherwise invalid.'.
end if.
do if (errcode(i,1)=20).
print/title = 'ERROR: A path fixed at zero cannot be moderated.'.
end if.
do if (errcode(i,1)=21).
print/title = 'ERROR: If only one moderator is specified, it must be specified as W.'.
end if.
do if (errcode(i,1)=22).
print/title = 'ERROR: In BMATRIX, X must be specified to affect at least one variable.'.
end if.
do if (errcode(i,1)=23).
print/title = 'ERROR: In BMATRIX, at least one variable must be specified to affect Y.'.
end if.
do if (errcode(i,1)=24).
print/title = 'ERROR: You must specify a model number or a custom BMATRIX specification.'.
end if.
do if (errcode(i,1)=25).
print/title = 'ERROR: BMATRIX cannot be used in conjunction with a model number.'.
end if.
do if (errcode(i,1)=26).
print/title = 'ERROR: Your model has a dangling mediator (all Ms must affect and be affected).'.
end if.
do if (errcode(i,1)=27).
print/title = 'ERROR: CLUSTER is not available on this release of PROCESS.'.
end if.
do if (errcode(i,1)=29).
print/title = 'ERROR: CMATRIX is not the correct length or is otherwise invalid.'.
end if.
do if (errcode(i,1)=30).
print/title = 'ERROR: In CMATRIX, all covariates must be assigned to an M or a Y.'.
end if.
do if (errcode(i,1)=31).
print/title = 'ERROR: A linear or near linear dependency (singularity) exists in the data.'.
end if.
do if (errcode(i,1)=32).
print/title = 'ERROR: Models 80 and 81 require between 3 and 6 mediators.'.
end if.
do if (errcode(i,1)=33).
print/title = 'ERROR: Model 82 requires 4 mediators.'.
end if.
do if (errcode(i,1)=34).
print/title = 'ERROR: This model number requires between 2 and 6 mediators.'.
end if.
do if (errcode(i,1)=35).
print/title = 'ERROR: In a model with only one moderator, that moderator must be W.'.
end if.
do if (errcode(i,1)=36).
print/title = 'ERROR: A serial mediation model cannot have more than 6 mediators.'.
end if.
do if (errcode(i,1)=37).
print/title = 'ERROR: No more than 10 mediators are allowed in a PROCESS command.'.
end if.
do if (errcode(i,1)=38).
print/title = 'ERROR: XCATCODE is not provided, not the correct length, or is otherwise invalid.'.
end if.
do if (errcode(i,1)=39).
print/title = 'ERROR: WCATCODE is not provided, not the correct length, or is otherwise invalid.'.
end if.
do if (errcode(i,1)=40).
print/title = 'ERROR: ZCATCODE is not provided, not the correct length, or is otherwise invalid.'.
end if.
do if (errcode(i,1)=41).
print/title = 'ERROR: Models 1, 2, and 3 cannot be customized.'.
end if.
do if (errcode(i,1)=42).
print/title = 'ERROR: WS option available only in PROCESS v2. Or use the MEMORE macro instead.'.
print/title = '       MEMORE can be downloaded from www.akmontoya.com.'/space=0.
end if.
do if (errcode(i,1)=43).
print/title = 'ERROR: PROCESS does not allow dichotomous mediators.'.
end if.
do if (errcode(i,1)=60).
print/title = 'PROCESS is now ready for use.'.
print/title='Copyright 2017-2022 by Andrew F. Hayes. ALL RIGHTS RESERVED.'/space=0.
print/title='Workshop schedule available at http://haskayne.ucalgary.ca/CCRAM'/space=0.
end if.
do if (errcode(i,1)=50).
print/title = 'ERROR: A multicategorical moderator cannot be specified as a covariate.'.
end if.
do if (errcode(i,1)=51).
print/title = 'ERROR: A variable you specified as a covariate is a moderator in all equations.'.
end if.
do if (errcode(i,1)=52 and mcerpt=0).
compute mcerpt=1.
print/title = 'ERROR: A variable specified as multicategorical must have at least three categories'.
end if.
do if (errcode(i,1) = 47).
do if (iterrmod=1).
print/title = 'ERROR: Iteration for Y model didn''t converge to a solution. Interpret results with caution.'.
print/title = '       Try increasing the number of iterations, though this will slow down computation.'/space=0.
end if.
do if (bootiter=1).
print/title = 'ERROR: Nonconvergence during bootstrapping. Interpet bootstrap results with caution.'.
end if.
end if.
do if (errcode(i,1) = 61).
print/title = 'ERROR: Variable names should be no more than eight characters in length.'.
end if.
do if (errcode(i,1) = 62).
print/title = 'ERROR: After listwise deletion of cases with missing data, too few cases remain.'.
end if.
do if (errcode(i,1) = 63).
print/title = 'ERROR: The XMINT option is available only for model 4.'.
end if.
do if (errcode(i,1) = 64).
print/title = 'ERROR: Incorrect number of values specified in CDEVAL option.'.
end if.
do if (errcode(i,1) = 65).
print/title = 'ERROR: Only indicator or sequential coding of X is allowed with the XMINT option.'.
end if.
do if (errcode(i,1) = 66).
print/title = 'ERROR: XMINT option not available with continuous X using PROCESS GUI. Use syntax.'.
end if.
do if (errcode(i,1) = 67).
print/title = 'ERROR: Too many elements provided in XREFVAL option.'.
end if.
do if (errcode(i,1)=68).
print/title = 'ERROR: Covariate assignment is not allowed with XMINT option.'.
end if.
do if (errcode(i,1) = 69).
print/title = 'ERROR: Incorrect number of values specified in COVAL option.'.
end if.
do if (errcode(i,1) = 70).
print/title = 'ERROR: Incorrect value(s) in XREFVAL for this dichotomous X variable.'.
end if.
do if (errcode(i,1) = 71).
print/title = 'ERROR: The CENTER option is not available when using the XMINT option.'.
end if.
do if (errcode(i,1) = 72).
print/title = 'ERROR: The XMINT option is not available for models with a dichotomous Y.'.
end if.
end loop.
END MATRIX.	
Ressources	Temps de processeur	00:00:06,53	
	Temps écoulé	00:00:06,52	


[Jeu_de_données1] C:\Users\Brice SAHA\Documents\UBa\Master Thesis 2021\Fanuel\Database fanuel.sav


Run MATRIX procedure:

***************** PROCESS Procedure for SPSS Version 4.2 *****************

          Written by Andrew F. Hayes, Ph.D.       www.afhayes.com
    Documentation available in Hayes (2022). www.guilford.com/p/hayes3

**************************************************************************
Model  : 4
    Y  : HOMAIR
    X  : BMIStat
   M1  : TG
   M2  : HDL
   M3  : LDL
   M4  : TC

Sample
Size:  169

**************************************************************************
OUTCOME VARIABLE:
 TG

Model Summary
          R       R-sq        MSE          F        df1        df2          p
      ,2482      ,0616  3802,1097    10,9607     1,0000   167,0000      ,0011

Model
              coeff         se          t          p       LLCI       ULCI
constant    68,5626     7,5727     9,0539      ,0000    53,6120    83,5132
BMIStat     18,2482     5,5119     3,3107      ,0011     7,3662    29,1302

Standardized coefficients
             coeff
BMIStat      ,2482

**************************************************************************
OUTCOME VARIABLE:
 HDL

Model Summary
          R       R-sq        MSE          F        df1        df2          p
      ,0399      ,0016   177,8528      ,2658     1,0000   167,0000      ,6068

Model
              coeff         se          t          p       LLCI       ULCI
constant    41,8180     1,6378    25,5325      ,0000    38,5845    45,0515
BMIStat      -,6146     1,1921     -,5156      ,6068    -2,9682     1,7390

Standardized coefficients
             coeff
BMIStat     -,0399

**************************************************************************
OUTCOME VARIABLE:
 LDL

Model Summary
          R       R-sq        MSE          F        df1        df2          p
      ,1564      ,0245  1160,9220     4,1901     1,0000   167,0000      ,0422

Model
              coeff         se          t          p       LLCI       ULCI
constant    69,6364     4,1845    16,6416      ,0000    61,3751    77,8977
BMIStat      6,2345     3,0457     2,0470      ,0422      ,2214    12,2476

Standardized coefficients
             coeff
BMIStat      ,1564

**************************************************************************
OUTCOME VARIABLE:
 TC

Model Summary
          R       R-sq        MSE          F        df1        df2          p
      ,2354      ,0554  1371,4616     9,7986     1,0000   167,0000      ,0021

Model
              coeff         se          t          p       LLCI       ULCI
constant   124,6651     4,5481    27,4102      ,0000   115,6858   133,6443
BMIStat     10,3625     3,3104     3,1303      ,0021     3,8268    16,8981

Standardized coefficients
             coeff
BMIStat      ,2354

**************************************************************************
OUTCOME VARIABLE:
 HOMAIR

Model Summary
          R       R-sq        MSE          F        df1        df2          p
      ,4036      ,1629    22,4450     6,3426     5,0000   163,0000      ,0000

Model
              coeff         se          t          p       LLCI       ULCI
constant     -,4218     1,6457     -,2563      ,7981    -3,6714     2,8279
BMIStat      1,0705      ,4445     2,4082      ,0171      ,1927     1,9482
TG            ,0416      ,0096     4,3298      ,0000      ,0226      ,0606
HDL           ,1312      ,0425     3,0833      ,0024      ,0472      ,2152
LDL           ,0955      ,0352     2,7167      ,0073      ,0261      ,1649
TC           -,0949      ,0350    -2,7093      ,0075     -,1641     -,0257

Standardized coefficients
             coeff
BMIStat      ,1811
TG           ,5179
HDL          ,3422
LDL          ,6440
TC          -,7068

************************** TOTAL EFFECT MODEL ****************************
OUTCOME VARIABLE:
 HOMAIR

Model Summary
          R       R-sq        MSE          F        df1        df2          p
      ,2304      ,0531    24,7807     9,3609     1,0000   167,0000      ,0026

Model
              coeff         se          t          p       LLCI       ULCI
constant     2,7377      ,6114     4,4780      ,0000     1,5307     3,9446
BMIStat      1,3615      ,4450     3,0596      ,0026      ,4829     2,2400

Standardized coefficients
             coeff
BMIStat      ,2304


************** TOTAL, DIRECT, AND INDIRECT EFFECTS OF X ON Y **************

Total effect of X on Y
     Effect         se          t          p       LLCI       ULCI       c_cs
     1,3615      ,4450     3,0596      ,0026      ,4829     2,2400      ,2304

Direct effect of X on Y
     Effect         se          t          p       LLCI       ULCI      c'_cs
     1,0705      ,4445     2,4082      ,0171      ,1927     1,9482      ,1811

Indirect effect(s) of X on Y:
          Effect     BootSE   BootLLCI   BootULCI
TOTAL      ,2910      ,2941     -,1955      ,9584
TG         ,7596      ,5741      ,1793     2,2850
HDL       -,0806      ,2301     -,5136      ,3590
LDL        ,5954      ,8545     -,0017     3,1871
TC        -,9834     1,1672    -4,5962     -,1633

Completely standardized indirect effect(s) of X on Y:
          Effect     BootSE   BootLLCI   BootULCI
TOTAL      ,0492      ,0485     -,0331      ,1565
TG         ,1285      ,0892      ,0336      ,3659
HDL       -,0136      ,0371     -,0819      ,0570
LDL        ,1008      ,1388     -,0003      ,5262
TC        -,1664      ,1870     -,7397     -,0309

*********************** ANALYSIS NOTES AND ERRORS ************************

Level of confidence for all confidence intervals in output:
  95,0000

Number of bootstrap samples for percentile bootstrap confidence intervals:
  5000

------ END MATRIX -----


* Encoding: UTF-8.
preserve.
set printback=off.


Matrice


Remarques	
Sortie obtenue	10-NOV-2024 16:57:37	
Commentaires		
Entrée	Données	C:\Users\Brice SAHA\Documents\UBa\Master Thesis 2021\Fanuel\Database fanuel.sav	
	Jeu de données actif	Jeu_de_données1	
	Filtre	<sans>	
	Pondération	<sans>	
	Scinder un fichier	<sans>	
	N de lignes dans le fichier de travail	169	
Syntaxe	MATRIX.
compute wnames='xxxxx'.
compute znames='xxxxx'.
compute mcerpt=0.
compute wiscov=0.
compute ziscov=0.
compute tooman=0.
compute errcode=make(100,1,0).
compute notecode=make(100,1,0).
compute model = trunc( 4 ).
compute iterate = abs(trunc( 100 )).
compute converge = abs( 0.00001 ).
compute itprobtg=0.
compute v2tag=0.
compute ydich=0.
compute maxwwarn=0.
compute minwwarn=0.
compute maxzwarn=0.
compute minzwarn=0.
compute toomany=0.
compute wdich=0.
compute zdich=0.
compute wnotev=0.
compute znotev=0.
compute nxpval=1.
compute nwpval=1.
compute nzpval=1.
compute errs=1.
compute notes=1.
compute criterr=0.
compute novar=0.
compute adjust=0.
compute ncs=0.
compute serial=0.
compute sobelok=0.
compute hasw=0.
compute hasz=0.
compute printw=0.
compute printz=0.
compute xmint=( 0 =1).
compute wmodcust=0.
compute zmodcust=0.
compute booting=0.
compute bootiter=0.
compute iterrmod=0.
compute cov = 'xxxxx'.
compute varorder=( 0 <> 0).
compute nws=0.
compute w= 'xxxxx'.
compute nzs=0.
compute z = 'xxxxx'.
compute nms=0.
compute m = 'TG HDL LDL TC'.
compute nys=0.
compute y = 'HOMAIR'.
compute nxs=0.
compute x = 'WCStatus'.
compute effsize=( 0 =1).
compute stand=( 1 =1).
compute intprobe = .1.
compute xrefvals={ 999 }.
compute center=trunc( 0 ).
compute xcontcf=0.
compute xscaling=1.
compute cdeval={ -999 }.
compute cuscoval=0.
do if (model=74).
compute errcode(errs,1)=7.
compute errs=errs+1.
compute criterr=1.
end if.
do if (xmint=1 and model <> 4).
compute errcode(errs,1)=63.
compute errs=errs+1.
compute criterr=1.
end if.
do if (xmint=1 and model=4).
compute w=x.
compute model=74.
compute intprobe=1.
compute notecode(notes,1) = 32.
compute notes = notes + 1.
do if (effsize=1 or stand=1).
compute notecode(notes,1) = 34.
compute notes = notes + 1.
compute stand=0.
compute effsize=0.
end if.
do if (center <> 0).
compute center=0.
compute errcode(errs,1)=71.
compute errs=errs+1.
compute criterr=1.
end if.
end if.
compute v = 'xxxxx'.
compute q = 'xxxxx'.
compute linsum={ -999 }.
compute nlinsum=ncol(linsum).
do if (linsum(1,1) = -999).
compute nlinsum=0.
end if.
compute oldvars= 'xxxxx'.
compute mcxok=0.
compute mcwok=0.
compute mczok=0.
compute xprod=0.
compute zprod=0.
compute wprod=0.
compute modcok=0.
compute alttotal=0.
compute hc3=trunc( 0 ).
compute jn=( 0 = 1).
compute listmiss=( 0 =1).
compute modelres=( 0 =1).
compute outscree=( 1 =1).
compute activate=( 0 =1).
compute booterr=0.
compute normal=( 0 =1).
compute xmtest=( 0 =1).
compute describe=( 0 =1).
compute longname=( 0 =1).
do if (stand=1).
compute effsize=1.
end if.
compute pstog=0.
compute sobelok=0.
compute normal=( 0 =1).
compute mdichok=( 0 =1).
compute contrast={ 999 }.
compute ncontr=ncol(contrast).
compute ncontrow=nrow(contrast).
do if (contrast(1,1) = 999).
compute ncontr=1.
compute contrast=0.
end if.
do if (ncontr = 1).
compute contrast=trunc(contrast).
do if (contrast > 3 or contrast < 0)).
compute ncontr=1.
compute contrast = 0.
end if.
end if.
do if (ncontr > 1).
compute contvec=contrast.
compute contrast=4.
do if (ncontrow > 1).
compute contrast=0.
compute modcok=1.
compute wcontval=contvec(:,1).
compute zcontval=contvec(:,2).
do if ((ncontr <> 2) or (ncontrow <> 2)).
compute notecode(notes,1) = 19.
compute notes = notes + 1.
compute modcok=0.
end if.
end if.
end if.
do if (xmint = 1 and contrast(1,1) <> 0).
compute contrast=0.
compute notecode(notes,1) = 37.
compute notes = notes + 1.
end if.
do if (varorder = 1).
compute notecode(notes,1) = 21.
compute notes = notes + 1.
end if.
do if ( 999 <> 999 or 999 <> 999).
compute notecode(notes,1) = 22.
compute notes = notes + 1.
end if.
compute modelbt=( 0 =1).
compute cluster= 'xxxxx'.
compute matrices=( 0 =1).
compute covcoeff=( 0 =1).
compute covmy=trunc( 0 ).
do if (covmy < 0 or covmy > 2).
compute covmy = 0.
end if.
compute boot = abs(trunc( 5000 )).
compute bc=( 0 =1).
compute mc=abs(trunc( 0 )).
compute hc=trunc( 5 ).
do if (intprobe < 0 or intprobe > 1).
compute intprobe = .10.
end if.
compute plot=trunc( 0 ).
do if (plot < 0 or plot > 2).
compute plot=0.
end if.
compute total=( 1 =1).
compute dototal=0.
compute saveboot = ( 0 = 1).
compute saveest=( 0 = 2).
do if (saveest=1).
compute intprobe=1.
end if.
do if (hc >= 0 and hc < 5).
compute notecode(notes,1) = 4.
compute notes = notes + 1.
end if.
do if (hc > 5 or hc < 0).
compute hc=5.
end if.
compute mcw=trunc( 0 ).
compute mcz=trunc( 0 ).
compute mcx=trunc( 0 ).
do if (mcx > 0 and mcx < 3 and model = 74).
compute mcw=mcx.
compute xscaling=1.
end if.
do if (mcx > 2 and model = 74).
compute errcode(errs,1)=65.
compute errs=errs+1.
compute criterr=1.
end if.
do if (model = 74 and normal=1).
compute notecode(notes,1) = 33.
compute normal=0.
compute notes = notes + 1.
end if.
do if (mcx > 0 and contrast > 0).
compute notecode(notes,1) = 28.
compute notes = notes + 1.
compute contrast=0.
end if.
compute nxvls=1.
compute nmvls=1.
compute nwvls=1.
compute nzvls=1.
compute paths=999.
compute pathsw=999.
compute pathsz=999.
compute pathswz=999.
compute pathsmod=999.
compute pathtype=999.
compute obscoeff=999.
compute pathsdv={' '}.
compute quantile=1.
do if ( 999 <>999).
compute notecode(notes,1) = 23.
compute notes = notes + 1.
end if.
compute moments=( 0 =1).
do if (moments=1).
compute quantile=0.
end if.
compute bmatrix={ -999 }.
compute wmatrix={ -999 }.
compute zmatrix={ -999 }.
compute wzmatrix={ -999 }.
compute cmatrix={ -999 }.
compute xcatcode={ -999 }.
compute wcatcode={ -999 }.
compute zcatcode={ -999 }.
compute needed=0.
compute conf= 95.
do if (trunc( 95 ) >= 100 or (trunc( 95 ) <= 50)).
compute conf = 95.
compute notecode(notes,1)=2.
compute notes=notes+1.
end if.
do if (model >= 0 and model < 4 and modelbt=0).
compute boot=0.
compute mc=0.
compute bc=0.
end if.
do if (mc > 0 and boot > 0).
compute boot=0.
compute bc=0.
end if.
do if ((boot < 1000) and (mc = 0) and (boot > 0)).
compute boot=5000.
end if.
do if ((mc < 1000) and (boot = 0) and (mc > 0)).
compute mc=5000.
end if.
compute p0=-.322232431088.
compute p1 = -1.
compute p2 = -.342242088547.
compute p3 = -.0204231210245.
compute p4 = -.0000453642210148.
compute q0 = .0993484626060.
compute q1 = .588581570495.
compute q2 = .531103462366.
compute q3 = .103537752850.
compute q4 = .0038560700634.
compute badend=0.
compute priorlo = -9999999.
compute priorhi = 9999999.
compute alpha2 = (1-(conf/100))/2.
compute cilm=alpha2*2.
compute y5=sqrt(-2*ln(alpha2)).
compute xp2=(y5+((((y5*p4+p3)*y5+p2)*y5+p1)*y5+p0)/((((y5*q4+q3)*y5+q2)*y5+q1)*y5+q0)).
compute medlb={'   M1  :';'   M2  :';'   M3  :';'   M4  :';'   M5  :';'   M6  :';'   M7  :';'   M8  :';'   M9  :';'   M10 :'}.
compute medlb2={'(M1)','(M2)','(M3)','(M4)','(M5)','(M6)','(M7)','(M8)','(M9)','(M10)'}.
compute xlb={'   X1  :';'   X2  :';'   X3  :';'   X4  :';'   X5  :';'   X6  :';'   X7  :';'   X8  :';'   X9  :'}.
compute highlbw={'M1*W'; 'M2*W'; 'M3*W'; 'M4*W'; 'M5*W'; 'M6*W'; 'M7*W'; 'M8*W'; 'M9*W'; 'M10*W'}.
do if (xmint=1).
compute highlbw={'M1*X'; 'M2*X'; 'M3*X'; 'M4*X'; 'M5*X'; 'M6*X'; 'M7*X'; 'M8*X'; 'M9*X'; 'M10*X'}.
end if.
compute highlbz={'M1*Z'; 'M2*Z'; 'M3*Z'; 'M4*Z'; 'M5*Z'; 'M6*Z'; 'M7*Z'; 'M8*Z'; 'M9*Z';'M10*Z'}.
compute highlbwz={'M1*W*Z'; 'M2*W*Z'; 'M3*W*Z'; 'M4*W*Z'; 'M5*W*Z'; 'M6*W*Z'; 'M7*W*Z'; 'M8*W*Z'; 'M9*W*Z';'M10*W*Z'}.
compute highlbbt={'BOTH(M1)'; 'BOTH(M2)'; 'BOTH(M3)'; 'BOTH(M4)'; 'BOTH(M5)'; 'BOTH(M6)'; 'BOTH(M7)'; 'BOTH(M8)'; 'BOTH(M9)';'BTH(M10)'}.
compute highlbx={'M1*X'; 'M2*X'; 'M3*X'; 'M4*X'; 'M5*X'; 'M6*X'; 'M7*X'; 'M8*X'; 'M9*X'; 'M10*X'}.
compute skipwz=0.
compute validm={1,1,1,1,1,1,1,1,1,1,1,1,1,1,1,1,1,1,1,1,1,1,0,0,0,0,0,1,1,0,0,0,0, 0,0,0,0,0,0,0,0,0,0,0,0,0,0,0,0,0,0,0,0,0,0,0,0,1,1,1,1,1,1,1,1,1,1,1,1,1,1,1,1,1,1,1,0,0,0,1,1, 1,1,1,1,1,1,1,1,1,1,1}.
do if (activate=1).
compute errcode(errs,1)=60.
compute errs=errs+1.
compute criterr=1.
end if.
do if (criterr=0).
do if (( 0 =1)=1).
compute errcode(errs,1)=42.
compute errs=errs+1.
compute criterr=1.
end if.
do if (model > 0 and model < 93).
do if (validm(1,model)=0).
compute errcode(errs,1)=6.
compute errs=errs+1.
compute criterr=1.
end if.
release validm.
end if.
do if ((model > 92 or model < 0) and model <> 999)).
compute errcode(errs,1)=7.
compute errs=errs+1.
compute criterr=1.
end if.
do if (model = 999 and bmatrix(1,1)=-999).
compute errcode(errs,1)=24.
compute errs=errs+1.
compute criterr=1.
end if.
do if (model <> 999 and bmatrix(1,1) <> -999).
compute errcode(errs,1)=25.
compute errs=errs+1.
compute criterr=1.
end if.
do if ((model = 74 or (model > 0 and model < 4)) and ((wmatrix(1,1) <> -999) or (zmatrix(1,1)<>-999) or (wzmatrix(1,1)<>-999)))).
compute errcode(errs,1)=41.
compute errs=errs+1.
compute criterr=1.
end if.
do if (hc3 <> 0).
compute notecode(notes,1) = 5.
compute notes = notes + 1.
do if (hc3 = 1).
compute hc=3.
end if.
end if.
do if ((v <> 'xxxxx') or (q <> 'xxxxx')).
compute errcode(errs,1)=14.
compute errs=errs+1.
compute errcode(errs,1)=48.
compute errs=errs+1.
compute criterr=1.
end if.
do if (oldvars <> 'xxxxx').
compute errcode(errs,1)=48.
compute errs=errs+1.
compute criterr=1.
end if.
do if (cluster <> 'xxxxx').
compute errcode(errs,1)=27.
compute errs=errs+1.
compute criterr=1.
end if.
do if ((y = 'xxxxx') or (x = 'xxxxx')).
compute errcode(errs,1)=1.
compute errs=errs+1.
compute criterr=1.
end if.
do if ((m = 'xxxxx') and model > 3).
compute errcode(errs,1)=8.
compute errs=errs+1.
compute criterr=1.
end if.
end if.
do if (criterr=0).
get ytmp/variables = HOMAIR /names = ynames/MISSING = 99999.
compute nys=ncol(ytmp).
compute needed=nys.
compute n=nrow(ytmp).
compute varnames={ynames}.
compute dat=ytmp.

.


do if (toomany=1 and longname=0).
compute criterr=1.
do if (tooman=0).
compute tooman=1.
compute errcode(errs,1) = 61.
compute errs = errs + 1.
end if.
end if
.
compute modelvar={ '4' ;t(ynames)}.
do if ( 4 =999).
compute modelvar(1,1)='CUSTOM'.
end if.
get xtmp/variables = WCStatus /names = xnames/MISSING = 99999.
compute nxs=ncol(xtmp).
compute n=nrow(xtmp).
compute needed=needed+nxs.
compute varnames={varnames,xnames}.
compute xcatlab=t(xnames).
compute dat={dat,xtmp}.

.


do if (toomany=1 and longname=0).
compute criterr=1.
do if (tooman=0).
compute tooman=1.
compute errcode(errs,1) = 61.
compute errs = errs + 1.
end if.
end if
.
compute modelvar={modelvar;t(xnames)}.
do if (nxs = 1).
compute modelvlb={'Model  :';'    Y  :';'    X  :'}.
else.
compute modelvlb={'Model  :';'    Y  :';xlb(1:nxs,1)}.
end if.
do if (m <> 'xxxxx').
get mtmp/variables = TG HDL LDL TC /names = mnames/MISSING = 99999.
compute nms=ncol(mtmp).
compute mprod=make(1,nms,0).
compute n=nrow(mtmp).
compute needed=needed+nms.
compute varnames={varnames,mnames}.
compute dat={dat,mtmp}.
compute modelvar={modelvar;t(mnames)}.
compute x2m=make(99,nms,0).
compute m2y=make(99,nms,0).
compute onem=make(nms,1,1).

.


do if (toomany=1 and longname=0).
compute criterr=1.
do if (tooman=0).
compute tooman=1.
compute errcode(errs,1) = 61.
compute errs = errs + 1.
end if.
end if
.
do if (nms > 1 and nms < 11).
compute modelvlb={modelvlb;medlb(1:nms,1)}.
else.
compute modelvlb={modelvlb;'    M  :'}.
end if.
do if (nms > 0 and model < 4).
compute errcode(errs,1)=9.
compute errs=errs+1.
do if (model <> 0).
compute errcode(errs,1)=48.
compute errs=errs+1.
end if.
compute criterr=1.
end if.
end if.
compute wlocatet=0.
compute wlocate=0.
do if (w <> 'xxxxx').
do if (xmint=0).
get wtmp/variables = xxxxx /names = wnames/MISSING = 99999.

.


do if (toomany=1 and longname=0).
compute criterr=1.
do if (tooman=0).
compute tooman=1.
compute errcode(errs,1) = 61.
compute errs = errs + 1.
end if.
end if
.
end if.
do if (xmint=1).
get wtmp/variables = WCStatus /names = wnames/MISSING = 99999.
end if.
compute nws=ncol(wtmp).
compute n=nrow(wtmp).
compute varnames={varnames,wnames}.
compute wlocate=ncol(varnames).
do if (model=74).
compute wlocatet=1.
do if (xnames <> wnames).
compute errcode(errs,1)=45.
compute errs=errs+1.
compute criterr=1.
end if.
end if.
compute wcatlab=t(wnames).
compute dat={dat,wtmp}.
do if (xmint <> 1).
compute modelvar={modelvar;t(wnames)}.
compute modelvlb={modelvlb;'    W  :'}.
end if.
end if.
do if (z <> 'xxxxx').
get ztmp/variables = xxxxx /names = znames/MISSING = 99999.
compute nzs=ncol(ztmp).
compute n=nrow(ztmp).

.


do if (toomany=1 and longname=0).
compute criterr=1.
do if (tooman=0).
compute tooman=1.
compute errcode(errs,1) = 61.
compute errs = errs + 1.
end if.
end if
.
compute varnames={varnames,znames}.
compute zcatlab=t(znames).
compute dat={dat,ztmp}.
compute modelvar={modelvar;t(znames)}.
compute modelvlb={modelvlb;'    Z  :'}.
end if.
do if (cov <> 'xxxxx').
get ctmp/variables = xxxxx /names = covnames/MISSING = 99999.
compute ncs=ncol(ctmp).
compute n=nrow(ctmp).

.


do if (toomany=1 and longname=0).
compute criterr=1.
do if (tooman=0).
compute tooman=1.
compute errcode(errs,1) = 61.
compute errs = errs + 1.
end if.
end if
.
compute varnames={varnames,covnames}.
compute dat={dat,ctmp}.
end if.
do if (nws > 1 or nzs > 1 or nys > 1 or nxs > 1).
compute errcode(errs,1)=3.
compute errs=errs+1.
compute criterr=1.
end if.
do if ((model = 80 or model = 81) and (nms < 3 or nms > 6)).
compute errcode(errs,1)=32.
compute errs=errs+1.
compute criterr=1.
end if.
do if (model = 82 and nms <> 4).
compute errcode(errs,1)=33.
compute errs=errs+1.
compute criterr=1.
end if.
do if (nms > 10).
compute errcode(errs,1)=37.
compute errs=errs+1.
compute criterr=1.
end if.
do if ((model = 6 or (model > 82 and model < 999)) and (nms < 2 or nms > 6)).
compute errcode(errs,1)=34.
compute errs=errs+1.
compute criterr=1.
end if.
compute match=0.
compute match2=0.
compute mcwzcov=0.
loop i = 1 to (ncol(varnames)-1).
loop j = (i+1) to ncol(varnames).
do if (varnames(i)=varnames(j)).
do if (i < (nxs+nms+nys+1)).
compute match2=match2+1.
end if.
do if (wlocatet=1 and i=2 and j=wlocate).
compute match2=match2-1.
end if.
do if ((wnames=znames) and (nws > 0 or nzs > 0))).
compute match2=match2+1.
end if.
do if (i < (ncol(varnames)-ncs+1)) and j > (ncol(varnames)-ncs)).
do if ((varnames(j)=wnames) and mcw=0)).
compute match=0.
compute wiscov=(j-(ncol(varnames)-ncs)).
end if.
do if ((varnames(j)=wnames) and mcw <>0)).
compute mcwzcov=1.
end if.
do if ((varnames(j)=znames) and mcz=0).
compute match=0.
compute ziscov=(j-(ncol(varnames)-ncs)).
end if.
do if ((varnames(j)=znames) and mcz<>0)).
compute mcwzcov=1.
end if.
end if.
end if.
end loop.
end loop.
do if (match2>0 or match=1).
compute errcode(errs,1)=2.
compute errs=errs+1.
compute criterr=1.
end if.
do if (mcwzcov=1).
compute errcode(errs,1)=50.
compute errs=errs+1.
compute criterr=1.
end if.
compute ninit=nrow(dat).
compute rownum=make(ninit,1,0).
loop i = 1 to ninit.
compute rownum(i,1)=i.
end loop.
compute dat={rownum,dat}.
compute j=1.
compute missrow=0.
loop i = 1 to n.
do if (rsum(dat(i,2:ncol(dat))=99999)=0).
compute dat(j,:)=dat(i,:).
compute j=j+1.
else.
compute missrow={missrow;dat(i,1)}.
end if.
end loop.
do if (j < 5).
compute errcode(errs,1)=62.
compute errs=errs+1.
compute criterr=1.
end if.
do if (criterr=0).
compute rownum=dat(1:(j-1),1).
do if (nrow(missrow) > 1).
compute missrow=t(missrow(2:nrow(missrow),1)).
compute notecode(notes,1) = 29.
compute notes = notes + 1.
end if.
compute dat=dat(1:(j-1),2:ncol(dat)).
compute n=nrow(dat).
compute nmiss=ninit-n.
compute ytmp=dat(:,1:nys).

.
compute desctmp=make((8-(4* 0 )),ncol( ytmp ),-999).
loop jd=1 to ncol( ytmp ).
compute descdat= ytmp (:,jd).
compute desctmp(1,jd) = csum(descdat)/nrow(descdat).
compute desctmp(2,jd) = (nrow(descdat)*sscp(descdat))-(t(csum(descdat))*(csum(descdat))).
compute desctmp(2,jd) = sqrt(desctmp(2,jd)/(nrow(descdat)*(nrow(descdat)-1))).
compute desctmp(3,jd)=cmin(descdat).
compute desctmp(4,jd)=cmax(descdat).
do if ( 0 =0).
compute minwarn=0.
compute maxwarn=0.
do if ((desctmp(3,jd)=desctmp(4,jd)) and novar=0).
compute errcode(errs,1)=15.
compute errs=errs+1.
compute criterr=1.
compute novar=1.
end if.
compute tmp=((descdat(:,1)=desctmp(3,jd))+(descdat(:,1)=desctmp(4,jd))).
compute desctmp(8,jd)=(csum(tmp)=nrow(tmp)).
compute tmp = descdat.
compute tmp(GRADE(descdat),:) = descdat.
compute descdat = tmp.
release tmp.
compute decval={.16;.5;.84}.
loop kd=1 to 3.
compute low=trunc(decval(kd,1)*(nrow(descdat)+1)).
compute lowdec=decval(kd,1)*(nrow(descdat)+1)-low.
compute value=descdat(low,1)+(descdat((low+1),1)-descdat(low,1))*lowdec.
compute desctmp((4+kd),jd)=value.
end loop.
compute mnotev=1.
compute modvals=desctmp(5:7,:).
do if (quantile <> 1).
compute desctmp(5,jd)=desctmp(1,jd)-desctmp(2,jd).
compute desctmp(6,jd)=desctmp(1,jd).
compute desctmp(7,jd)=desctmp(1,jd)+desctmp(2,jd).
compute modvals=desctmp(5:7,:).
compute mnotev=2.
do if (modvals(1,1) < desctmp(3,1)).
compute modvals(1,1)=desctmp(3,1).
compute minwarn=1.
end if.
do if (modvals(3,1) > desctmp(4,1)).
compute modvals(3,1)=desctmp(4,1).
compute maxwarn=1.
end if.
end if.
do if (desctmp(8,1)=1).
compute modvals={desctmp(3,1);desctmp(4,1)}.
compute mnotev=0.
compute minwarn=0.
compute maxwarn=0.
end if.
end if.
end loop
.
compute ysd=desctmp(2,:).
compute ovsd=ysd.
do if (desctmp(8,1)=1).
compute ydich=1.
do if (total=1).
compute total=0.
compute notecode(notes,1) = 24.
compute notes = notes + 1.
end if.
do if (effsize=1).
compute effsize=0.
compute notecode(notes,1) = 25.
compute notes = notes + 1.
end if.
do if (model=74).
compute errcode(errs,1) = 72.
compute errs=errs+1.
compute criterr=1.
end if.
compute omx = cmax(ytmp).
compute omn = cmin(ytmp).
compute ytmp = (ytmp = omx).
compute dat(:,1:nys)=(dat(:,1:nys)=omx).
compute rcd = {omn, 0; omx, 1}.
end if.
compute xtmp=dat(:,(nys+1):(nys+nxs)).

.
compute desctmp=make((8-(4* 0 )),ncol( xtmp ),-999).
loop jd=1 to ncol( xtmp ).
compute descdat= xtmp (:,jd).
compute desctmp(1,jd) = csum(descdat)/nrow(descdat).
compute desctmp(2,jd) = (nrow(descdat)*sscp(descdat))-(t(csum(descdat))*(csum(descdat))).
compute desctmp(2,jd) = sqrt(desctmp(2,jd)/(nrow(descdat)*(nrow(descdat)-1))).
compute desctmp(3,jd)=cmin(descdat).
compute desctmp(4,jd)=cmax(descdat).
do if ( 0 =0).
compute minwarn=0.
compute maxwarn=0.
do if ((desctmp(3,jd)=desctmp(4,jd)) and novar=0).
compute errcode(errs,1)=15.
compute errs=errs+1.
compute criterr=1.
compute novar=1.
end if.
compute tmp=((descdat(:,1)=desctmp(3,jd))+(descdat(:,1)=desctmp(4,jd))).
compute desctmp(8,jd)=(csum(tmp)=nrow(tmp)).
compute tmp = descdat.
compute tmp(GRADE(descdat),:) = descdat.
compute descdat = tmp.
release tmp.
compute decval={.16;.5;.84}.
loop kd=1 to 3.
compute low=trunc(decval(kd,1)*(nrow(descdat)+1)).
compute lowdec=decval(kd,1)*(nrow(descdat)+1)-low.
compute value=descdat(low,1)+(descdat((low+1),1)-descdat(low,1))*lowdec.
compute desctmp((4+kd),jd)=value.
end loop.
compute mnotev=1.
compute modvals=desctmp(5:7,:).
do if (quantile <> 1).
compute desctmp(5,jd)=desctmp(1,jd)-desctmp(2,jd).
compute desctmp(6,jd)=desctmp(1,jd).
compute desctmp(7,jd)=desctmp(1,jd)+desctmp(2,jd).
compute modvals=desctmp(5:7,:).
compute mnotev=2.
do if (modvals(1,1) < desctmp(3,1)).
compute modvals(1,1)=desctmp(3,1).
compute minwarn=1.
end if.
do if (modvals(3,1) > desctmp(4,1)).
compute modvals(3,1)=desctmp(4,1).
compute maxwarn=1.
end if.
end if.
do if (desctmp(8,1)=1).
compute modvals={desctmp(3,1);desctmp(4,1)}.
compute mnotev=0.
compute minwarn=0.
compute maxwarn=0.
end if.
end if.
end loop
.
compute xsd=desctmp(2,:).
compute xmodvals=modvals.
compute xdich=desctmp(8,1).
compute xmx=cmax(xtmp).
compute xmn=cmin(xtmp).
do if ((mcx > 0) and (xrefvals(1,1) <> 999) and (xmint=1) and (model= 74)).
compute notecode(notes,1) = 36.
compute notes = notes + 1.
end if.
do if (mcx=0).
do if (ncol(xrefvals)>2 and model=74 and xmint=1).
compute errcode(errs,1)=67.
compute errs=errs+1.
compute criterr=1.
end if.
do if ((model=74) and (xmint=1)).
do if ((xrefvals(1,1)=999) and nxvls=1 and xdich=0).
compute errcode(errs,1)=66.
compute errs=errs+1.
compute criterr=1.
end if.
do if ((xrefvals(1,1)=999) and (xdich=1)).
compute xrefvals={xmn,xmx}.
compute xscaling=xrefvals(1,2)-xrefvals(1,1).
end if.
do if (ncol(xrefvals)=1 and xrefvals(1,1) <> 999).
do if (xdich=0).
compute xrefvals={xrefvals,(xrefvals(1,1)+1)}.
compute xscaling=xrefvals(1,2)-xrefvals(1,1).
end if.
do if (xdich=1).
do if ((xrefvals(1,1) <> xmx) and (xrefvals(1,1) <> xmn)).
compute errcode(errs,1)=70.
compute errs=errs+1.
compute criterr=1.
end if.
do if (xrefvals(1,1) = xmx).
compute xrefvals={xrefvals,xmn}.
compute xscaling=xrefvals(1,2)-xrefvals(1,1).
end if.
do if (xrefvals(1,1) = xmn).
compute xrefvals={xrefvals,xmx}.
compute xscaling=xrefvals(1,2)-xrefvals(1,1).
end if.
end if.
end if.
do if (ncol(xrefvals)=2).
compute xscaling=xrefvals(1,2)-xrefvals(1,1).
do if (xdich=1).
compute xreferr=1.
do if (((xrefvals(1,1) = xmx) and (xrefvals(1,2)=xmn)) or ((xrefvals(1,1) = xmn) and (xrefvals(1,2)=xmx))).
compute xreferr=0.
end if.
do if (xreferr=1).
compute errcode(errs,1)=70.
compute errs=errs+1.
compute criterr=1.
end if.
end if.
end if.
end if.
end if.
do if (xmint=1 and model=74 and mcx=0).
compute xmodvals=t(xrefvals).
compute xcontcf=1.
end if.
compute nxpval=nrow(xmodvals).
compute xprobval=xmodvals.
do if (xdich =1 and mcx > 0).
compute mcx=0.
compute errcode(errs,1) = 52.
compute errs = errs + 1.
compute criterr = 1.
end if.
do if (nms > 0).
compute mtmp=dat(:,(nys+nxs+1):(nys+nxs+nms)).

.
compute desctmp=make((8-(4* 0 )),ncol( mtmp ),-999).
loop jd=1 to ncol( mtmp ).
compute descdat= mtmp (:,jd).
compute desctmp(1,jd) = csum(descdat)/nrow(descdat).
compute desctmp(2,jd) = (nrow(descdat)*sscp(descdat))-(t(csum(descdat))*(csum(descdat))).
compute desctmp(2,jd) = sqrt(desctmp(2,jd)/(nrow(descdat)*(nrow(descdat)-1))).
compute desctmp(3,jd)=cmin(descdat).
compute desctmp(4,jd)=cmax(descdat).
do if ( 0 =0).
compute minwarn=0.
compute maxwarn=0.
do if ((desctmp(3,jd)=desctmp(4,jd)) and novar=0).
compute errcode(errs,1)=15.
compute errs=errs+1.
compute criterr=1.
compute novar=1.
end if.
compute tmp=((descdat(:,1)=desctmp(3,jd))+(descdat(:,1)=desctmp(4,jd))).
compute desctmp(8,jd)=(csum(tmp)=nrow(tmp)).
compute tmp = descdat.
compute tmp(GRADE(descdat),:) = descdat.
compute descdat = tmp.
release tmp.
compute decval={.16;.5;.84}.
loop kd=1 to 3.
compute low=trunc(decval(kd,1)*(nrow(descdat)+1)).
compute lowdec=decval(kd,1)*(nrow(descdat)+1)-low.
compute value=descdat(low,1)+(descdat((low+1),1)-descdat(low,1))*lowdec.
compute desctmp((4+kd),jd)=value.
end loop.
compute mnotev=1.
compute modvals=desctmp(5:7,:).
do if (quantile <> 1).
compute desctmp(5,jd)=desctmp(1,jd)-desctmp(2,jd).
compute desctmp(6,jd)=desctmp(1,jd).
compute desctmp(7,jd)=desctmp(1,jd)+desctmp(2,jd).
compute modvals=desctmp(5:7,:).
compute mnotev=2.
do if (modvals(1,1) < desctmp(3,1)).
compute modvals(1,1)=desctmp(3,1).
compute minwarn=1.
end if.
do if (modvals(3,1) > desctmp(4,1)).
compute modvals(3,1)=desctmp(4,1).
compute maxwarn=1.
end if.
end if.
do if (desctmp(8,1)=1).
compute modvals={desctmp(3,1);desctmp(4,1)}.
compute mnotev=0.
compute minwarn=0.
compute maxwarn=0.
end if.
end if.
end loop
.
compute ovsd={desctmp(2,:),ysd}.
compute medmeans=cdeval.
do if ((cdeval(1,1) <> -999) and (ncol(medmeans) <> nms) and (model=74)).
compute errcode(errs,1)=64.
compute errs=errs+1.
compute criterr=1.
end if.
do if ((cdeval(1,1)=-999) and (model=74)).
compute medmeans=desctmp(1,:).
end if.
do if ((cdeval(1,1)<> -999) and (model=74) and (criterr=0)).
compute notecode(notes,1) = 31.
compute notes = notes + 1.
end if.
do if ((rsum(desctmp(8,:))>0) and (mdichok <> 1)).
compute errcode(errs,1)=43.
compute errs=errs+1.
compute criterr=1.
end if.
compute mmodvals=modvals.
compute mprobval=mmodvals.
end if.
do if (nws > 0).
compute wtmp=dat(:,(nys+nxs+nms+1):(nys+nxs+nms+nws)).

.
compute desctmp=make((8-(4* 0 )),ncol( wtmp ),-999).
loop jd=1 to ncol( wtmp ).
compute descdat= wtmp (:,jd).
compute desctmp(1,jd) = csum(descdat)/nrow(descdat).
compute desctmp(2,jd) = (nrow(descdat)*sscp(descdat))-(t(csum(descdat))*(csum(descdat))).
compute desctmp(2,jd) = sqrt(desctmp(2,jd)/(nrow(descdat)*(nrow(descdat)-1))).
compute desctmp(3,jd)=cmin(descdat).
compute desctmp(4,jd)=cmax(descdat).
do if ( 0 =0).
compute minwarn=0.
compute maxwarn=0.
do if ((desctmp(3,jd)=desctmp(4,jd)) and novar=0).
compute errcode(errs,1)=15.
compute errs=errs+1.
compute criterr=1.
compute novar=1.
end if.
compute tmp=((descdat(:,1)=desctmp(3,jd))+(descdat(:,1)=desctmp(4,jd))).
compute desctmp(8,jd)=(csum(tmp)=nrow(tmp)).
compute tmp = descdat.
compute tmp(GRADE(descdat),:) = descdat.
compute descdat = tmp.
release tmp.
compute decval={.16;.5;.84}.
loop kd=1 to 3.
compute low=trunc(decval(kd,1)*(nrow(descdat)+1)).
compute lowdec=decval(kd,1)*(nrow(descdat)+1)-low.
compute value=descdat(low,1)+(descdat((low+1),1)-descdat(low,1))*lowdec.
compute desctmp((4+kd),jd)=value.
end loop.
compute mnotev=1.
compute modvals=desctmp(5:7,:).
do if (quantile <> 1).
compute desctmp(5,jd)=desctmp(1,jd)-desctmp(2,jd).
compute desctmp(6,jd)=desctmp(1,jd).
compute desctmp(7,jd)=desctmp(1,jd)+desctmp(2,jd).
compute modvals=desctmp(5:7,:).
compute mnotev=2.
do if (modvals(1,1) < desctmp(3,1)).
compute modvals(1,1)=desctmp(3,1).
compute minwarn=1.
end if.
do if (modvals(3,1) > desctmp(4,1)).
compute modvals(3,1)=desctmp(4,1).
compute maxwarn=1.
end if.
end if.
do if (desctmp(8,1)=1).
compute modvals={desctmp(3,1);desctmp(4,1)}.
compute mnotev=0.
compute minwarn=0.
compute maxwarn=0.
end if.
end if.
end loop
.
compute wmodvals=modvals.
compute wdich=desctmp(8,1).
do if (wdich =1 and mcw > 0).
compute mcw=0.
compute errcode(errs,1) = 52.
compute errs = errs + 1.
compute criterr = 1.
end if.
compute wmin=desctmp(3,1).
compute wmax=desctmp(4,1).
compute minwwarn=minwarn.
compute maxwwarn=maxwarn.
compute wnotev=mnotev.
compute wmodval={ 999 }.
do if (xmint=1 and model=74 and mcx=0).
compute wmodval=xrefvals.
end if.
compute nwcontr=ncol(wmodval).
do if (wmodval(1,1) <> 999).
compute wmodvals=wmodval(1,1).
compute wmodcust=1.
do if (nwcontr > 1).
compute wmodvals=t(wmodval).
end if.
compute minwwarn=0.
compute maxwwarn=0.
compute wnotev=0.
end if.
compute wprobval=wmodvals.
compute nwpval=nrow(wmodvals).
end if.
do if (nzs > 0).
compute ztmp=dat(:,(nys+nxs+nms+nws+1):(nys+nxs+nms+nws+nzs)).

.
compute desctmp=make((8-(4* 0 )),ncol( ztmp ),-999).
loop jd=1 to ncol( ztmp ).
compute descdat= ztmp (:,jd).
compute desctmp(1,jd) = csum(descdat)/nrow(descdat).
compute desctmp(2,jd) = (nrow(descdat)*sscp(descdat))-(t(csum(descdat))*(csum(descdat))).
compute desctmp(2,jd) = sqrt(desctmp(2,jd)/(nrow(descdat)*(nrow(descdat)-1))).
compute desctmp(3,jd)=cmin(descdat).
compute desctmp(4,jd)=cmax(descdat).
do if ( 0 =0).
compute minwarn=0.
compute maxwarn=0.
do if ((desctmp(3,jd)=desctmp(4,jd)) and novar=0).
compute errcode(errs,1)=15.
compute errs=errs+1.
compute criterr=1.
compute novar=1.
end if.
compute tmp=((descdat(:,1)=desctmp(3,jd))+(descdat(:,1)=desctmp(4,jd))).
compute desctmp(8,jd)=(csum(tmp)=nrow(tmp)).
compute tmp = descdat.
compute tmp(GRADE(descdat),:) = descdat.
compute descdat = tmp.
release tmp.
compute decval={.16;.5;.84}.
loop kd=1 to 3.
compute low=trunc(decval(kd,1)*(nrow(descdat)+1)).
compute lowdec=decval(kd,1)*(nrow(descdat)+1)-low.
compute value=descdat(low,1)+(descdat((low+1),1)-descdat(low,1))*lowdec.
compute desctmp((4+kd),jd)=value.
end loop.
compute mnotev=1.
compute modvals=desctmp(5:7,:).
do if (quantile <> 1).
compute desctmp(5,jd)=desctmp(1,jd)-desctmp(2,jd).
compute desctmp(6,jd)=desctmp(1,jd).
compute desctmp(7,jd)=desctmp(1,jd)+desctmp(2,jd).
compute modvals=desctmp(5:7,:).
compute mnotev=2.
do if (modvals(1,1) < desctmp(3,1)).
compute modvals(1,1)=desctmp(3,1).
compute minwarn=1.
end if.
do if (modvals(3,1) > desctmp(4,1)).
compute modvals(3,1)=desctmp(4,1).
compute maxwarn=1.
end if.
end if.
do if (desctmp(8,1)=1).
compute modvals={desctmp(3,1);desctmp(4,1)}.
compute mnotev=0.
compute minwarn=0.
compute maxwarn=0.
end if.
end if.
end loop
.
compute zmodvals=modvals.
compute zdich=desctmp(8,1).
do if (zdich =1 and mcz > 0).
compute mcz=0.
compute errcode(errs,1) = 52.
compute errs = errs + 1.
compute criterr = 1.
end if.
compute zmin=desctmp(3,1).
compute zmax=desctmp(4,1).
compute minzwarn=minwarn.
compute maxzwarn=maxwarn.
compute znotev=mnotev.
compute zmodval={ 999 }.
compute nzcontr=ncol(zmodval).
do if (zmodval(1,1) <> 999).
compute zmodvals=zmodval(1,1).
compute zmodcust=1.
do if (nzcontr > 1).
compute zmodvals=t(zmodval).
end if.
compute minzwarn=0.
compute maxzwarn=0.
compute znotev=0.
end if.
compute zprobval=zmodvals.
compute nzpval=nrow(zmodvals).
end if.
do if (ncs > 0).
compute ctmp=dat(:,(nys+nxs+nms+nws+nzs+1):(nys+nxs+nms+nws+nzs+ncs)).

.
compute desctmp=make((8-(4* 0 )),ncol( ctmp ),-999).
loop jd=1 to ncol( ctmp ).
compute descdat= ctmp (:,jd).
compute desctmp(1,jd) = csum(descdat)/nrow(descdat).
compute desctmp(2,jd) = (nrow(descdat)*sscp(descdat))-(t(csum(descdat))*(csum(descdat))).
compute desctmp(2,jd) = sqrt(desctmp(2,jd)/(nrow(descdat)*(nrow(descdat)-1))).
compute desctmp(3,jd)=cmin(descdat).
compute desctmp(4,jd)=cmax(descdat).
do if ( 0 =0).
compute minwarn=0.
compute maxwarn=0.
do if ((desctmp(3,jd)=desctmp(4,jd)) and novar=0).
compute errcode(errs,1)=15.
compute errs=errs+1.
compute criterr=1.
compute novar=1.
end if.
compute tmp=((descdat(:,1)=desctmp(3,jd))+(descdat(:,1)=desctmp(4,jd))).
compute desctmp(8,jd)=(csum(tmp)=nrow(tmp)).
compute tmp = descdat.
compute tmp(GRADE(descdat),:) = descdat.
compute descdat = tmp.
release tmp.
compute decval={.16;.5;.84}.
loop kd=1 to 3.
compute low=trunc(decval(kd,1)*(nrow(descdat)+1)).
compute lowdec=decval(kd,1)*(nrow(descdat)+1)-low.
compute value=descdat(low,1)+(descdat((low+1),1)-descdat(low,1))*lowdec.
compute desctmp((4+kd),jd)=value.
end loop.
compute mnotev=1.
compute modvals=desctmp(5:7,:).
do if (quantile <> 1).
compute desctmp(5,jd)=desctmp(1,jd)-desctmp(2,jd).
compute desctmp(6,jd)=desctmp(1,jd).
compute desctmp(7,jd)=desctmp(1,jd)+desctmp(2,jd).
compute modvals=desctmp(5:7,:).
compute mnotev=2.
do if (modvals(1,1) < desctmp(3,1)).
compute modvals(1,1)=desctmp(3,1).
compute minwarn=1.
end if.
do if (modvals(3,1) > desctmp(4,1)).
compute modvals(3,1)=desctmp(4,1).
compute maxwarn=1.
end if.
end if.
do if (desctmp(8,1)=1).
compute modvals={desctmp(3,1);desctmp(4,1)}.
compute mnotev=0.
compute minwarn=0.
compute maxwarn=0.
end if.
end if.
end loop
.
compute covmeans=desctmp(1,:).
compute coval={ -999 }.
do if ((coval(1,1) <> -999) and (ncol(coval) <> ncs) and (model=74)).
compute errcode(errs,1)=69.
compute errs=errs+1.
compute criterr=1.
end if.
do if ((coval(1,1)<> -999) and (criterr=0) and (model=74)).
compute notecode(notes,1) = 35.
compute notes = notes + 1.
compute cuscoval=1.
end if.
end if.
compute n=nrow(ytmp).
compute ones=make(n,1,1).
do if (nws > 0 and mcw > 0).
compute tmp={rownum,wtmp(:,1)}.

.
compute dd= tmp.
compute temp = dd.
compute temp(GRADE(dd(:,2)),:) = dd.
compute dd = temp.
compute dummy = design(dd(:,2)).
compute nvls = ncol(dummy).
compute nnvls = csum(dummy).
compute mnvls = cmin(t(nnvls)).
compute conmat1=1.
do if (mnvls < 2).
compute errcode(errs,1) = 5.
compute errs = errs + 1.
compute criterr = 1.
end if.
do if (nvls > 9).
compute errcode(errs,1) = 4.
compute errs = errs+1.
compute criterr = 1.
end if.
do if (criterr = 0).
compute dumok = 1.
compute nnvls=make(nvls,1,0).
compute nnvls(1,1)=dd(1,2).
compute temp = 2.
loop i = 2 to n.
do if (dd(i,2) <> nnvls((temp-1),1)).
compute nnvls(temp,1)=dd(i,2).
compute temp = temp+1.
end if.
end loop.
do if ( mcw > 0).
compute x = dummy(:,2:ncol(dummy)).
compute nx = ncol(x).
compute minus1 = make(1,ncol(x),-1).
compute xdes=make((nx+1),3,0).
compute xdes(1,1)=dd(1,2).
compute xdes(1,2)=1.
compute temp = 2.
loop k = 2 to n.
do if (dd(k,2) <> dd((k-1),2)).
compute xdes(temp,2) = k.
compute xdes(temp,1) = dd(k,2).
compute xdes((temp-1),3) = k-1.
compute temp=temp+1.
end if.
end loop.
compute xdes((temp-1),3)=n.
compute xdes = {xdes, (xdes(:,3)-xdes(:,2)+1)}.
do if ( mcw = 4).
loop k = 1 to n.
do if (rsum(x(k,:)) = 0).
compute x(k,:) = minus1.
end if.
end loop.
end if.
do if ( mcw = 2 or mcw = 3 or mcw =5).
loop k = 1 to n.
do if (rsum(x(k,:)) > 0).
loop i = 1 to ncol(x).
do if (x(k,i) = 0).
compute x(k,i) = 1.
else.
break.
end if.
end loop.
end if.
end loop.
do if ( mcw = 3).
compute conmat1={-8,1,1,1,1,1,1,1,1; 0,-7,1,1,1,1,1,1,1; 0,0,-6,1,1,1,1,1,1; 0,0,0,-5,1,1,1,1,1; 0,0,0,0,-4,1,1,1,1; 0,0,0,0,0,-3,1,1,1; 0,0,0,0,0,0,-2,1,1; 0,0,0,0,0,0,0,-1,1}.
loop i = 1 to 8.
compute conmat1(i,:)=conmat1(i,:)/(10-i).
end loop.
compute conmat1=t(conmat1((10-nvls):8,(10-nvls):9)).
loop k=1 to n.
compute x(k,:)=conmat1((rsum(x(k,:))+1),:).
end loop.
end if.
end if.
do if ( mcw = 5).
compute custcode={ -999 }.
do if (ncol(custcode) <> (nvls*(nvls-1))).
compute errcode(errs,1) = (37+ 2 ).
compute errs = errs + 1.
compute criterr = 1.
end if.
do if (ncol(custcode) = (nvls*(nvls-1))).
compute conmat1=make(nvls,(nvls-1),0).
compute cnt=1.
loop i = 1 to nvls.
loop k = 1 to (nvls-1).
compute conmat1(i,k)=custcode(1,cnt).
compute cnt=cnt+1.
end loop.
end loop.
loop k=1 to n.
compute x(k,:)=conmat1((rsum(x(k,:))+1),:).
end loop.
end if.
end if.
compute xskip = 1.
compute dummat = make((nx+1),nx,0).
compute dummat((2:nrow(dummat)),:)=ident(nx).
do if ( mcw = 4).
compute dummat(1,:) = minus1.
end if.
do if ( mcw = 2).
loop i = 2 to nrow(dummat).
loop j = 1 to (i-1).
compute dummat(i,j) = 1.
end loop.
end loop.
end if.
do if ( mcw = 3).
compute dummat=conmat1.
end if.
do if ( mcw = 5 and criterr=0).
compute dummat=conmat1.
end if.
compute dummat={nnvls, dummat}.
compute x={dd(:,1),x}.
compute temp = x.
compute temp(GRADE(x(:,1)),:) = x.
compute x = temp.
release conmat1,temp,dd,xskip,xdes,dummy.
end if.
end if
.
compute wmodvals=nnvls.
compute nwpval=nrow(wmodvals).
do if (criterr=0).
compute minwwarn=0.
compute maxwwarn=0.
compute wnotev=0.
compute wtmp=x(:,2:ncol(x)).
compute wcatlab={'W1';'W2';'W3';'W4';'W5';'W6';'W7';'W8';'W9'}.
do if (xmint=1).
compute wcatlab={'X1';'X2';'X3';'X4';'X5';'X6';'X7';'X8';'X9'}.
end if.
compute nwvls=nvls-1.
compute mcwok=1.
compute dummatw=dummat.
compute wprobval=dummatw(:,2:ncol(dummatw)).
do if (modcok=1).
compute wcontval=make(2,ncol(wprobval),-999).
compute temp=0.
loop i = 1 to 2.
loop j = 1 to nrow(dummatw).
do if (contvec(i,1)=dummatw(j,1)).
compute wcontval(i,:)=wprobval(j,:).
compute temp=temp+1.
end if.
end loop.
end loop.
do if (temp < 2).
compute notecode(notes,1) = 20.
compute notes = notes + 1.
compute modcok=0.
end if.
end if.
do if ((wmodval(1,1) <> 999) and (xmint <> 1)).
compute notecode(notes,1) = 9.
compute notes = notes + 1.
end if.
release tmp, dummat.
end if.
end if.
do if (nzs > 0 and mcz > 0).
compute tmp={rownum,ztmp(:,1)}.

.
compute dd= tmp.
compute temp = dd.
compute temp(GRADE(dd(:,2)),:) = dd.
compute dd = temp.
compute dummy = design(dd(:,2)).
compute nvls = ncol(dummy).
compute nnvls = csum(dummy).
compute mnvls = cmin(t(nnvls)).
compute conmat1=1.
do if (mnvls < 2).
compute errcode(errs,1) = 5.
compute errs = errs + 1.
compute criterr = 1.
end if.
do if (nvls > 9).
compute errcode(errs,1) = 4.
compute errs = errs+1.
compute criterr = 1.
end if.
do if (criterr = 0).
compute dumok = 1.
compute nnvls=make(nvls,1,0).
compute nnvls(1,1)=dd(1,2).
compute temp = 2.
loop i = 2 to n.
do if (dd(i,2) <> nnvls((temp-1),1)).
compute nnvls(temp,1)=dd(i,2).
compute temp = temp+1.
end if.
end loop.
do if ( mcz > 0).
compute x = dummy(:,2:ncol(dummy)).
compute nx = ncol(x).
compute minus1 = make(1,ncol(x),-1).
compute xdes=make((nx+1),3,0).
compute xdes(1,1)=dd(1,2).
compute xdes(1,2)=1.
compute temp = 2.
loop k = 2 to n.
do if (dd(k,2) <> dd((k-1),2)).
compute xdes(temp,2) = k.
compute xdes(temp,1) = dd(k,2).
compute xdes((temp-1),3) = k-1.
compute temp=temp+1.
end if.
end loop.
compute xdes((temp-1),3)=n.
compute xdes = {xdes, (xdes(:,3)-xdes(:,2)+1)}.
do if ( mcz = 4).
loop k = 1 to n.
do if (rsum(x(k,:)) = 0).
compute x(k,:) = minus1.
end if.
end loop.
end if.
do if ( mcz = 2 or mcz = 3 or mcz =5).
loop k = 1 to n.
do if (rsum(x(k,:)) > 0).
loop i = 1 to ncol(x).
do if (x(k,i) = 0).
compute x(k,i) = 1.
else.
break.
end if.
end loop.
end if.
end loop.
do if ( mcz = 3).
compute conmat1={-8,1,1,1,1,1,1,1,1; 0,-7,1,1,1,1,1,1,1; 0,0,-6,1,1,1,1,1,1; 0,0,0,-5,1,1,1,1,1; 0,0,0,0,-4,1,1,1,1; 0,0,0,0,0,-3,1,1,1; 0,0,0,0,0,0,-2,1,1; 0,0,0,0,0,0,0,-1,1}.
loop i = 1 to 8.
compute conmat1(i,:)=conmat1(i,:)/(10-i).
end loop.
compute conmat1=t(conmat1((10-nvls):8,(10-nvls):9)).
loop k=1 to n.
compute x(k,:)=conmat1((rsum(x(k,:))+1),:).
end loop.
end if.
end if.
do if ( mcz = 5).
compute custcode={ -999 }.
do if (ncol(custcode) <> (nvls*(nvls-1))).
compute errcode(errs,1) = (37+ 3 ).
compute errs = errs + 1.
compute criterr = 1.
end if.
do if (ncol(custcode) = (nvls*(nvls-1))).
compute conmat1=make(nvls,(nvls-1),0).
compute cnt=1.
loop i = 1 to nvls.
loop k = 1 to (nvls-1).
compute conmat1(i,k)=custcode(1,cnt).
compute cnt=cnt+1.
end loop.
end loop.
loop k=1 to n.
compute x(k,:)=conmat1((rsum(x(k,:))+1),:).
end loop.
end if.
end if.
compute xskip = 1.
compute dummat = make((nx+1),nx,0).
compute dummat((2:nrow(dummat)),:)=ident(nx).
do if ( mcz = 4).
compute dummat(1,:) = minus1.
end if.
do if ( mcz = 2).
loop i = 2 to nrow(dummat).
loop j = 1 to (i-1).
compute dummat(i,j) = 1.
end loop.
end loop.
end if.
do if ( mcz = 3).
compute dummat=conmat1.
end if.
do if ( mcz = 5 and criterr=0).
compute dummat=conmat1.
end if.
compute dummat={nnvls, dummat}.
compute x={dd(:,1),x}.
compute temp = x.
compute temp(GRADE(x(:,1)),:) = x.
compute x = temp.
release conmat1,temp,dd,xskip,xdes,dummy.
end if.
end if
.
compute zmodvals=nnvls.
compute nzpval=nrow(zmodvals).
do if (criterr=0).
compute minzwarn=0.
compute maxzwarn=0.
compute znotev=0.
compute ztmp=x(:,2:ncol(x)).
compute zcatlab={'Z1';'Z2';'Z3';'Z4';'Z5';'Z6';'Z7';'Z8';'Z9'}.
compute nzvls=nvls-1.
compute mczok=1.
compute dummatz=dummat.
compute zprobval=dummatz(:,2:ncol(dummatz)).
do if (modcok=1).
compute zcontval=make(2,ncol(zprobval),-999).
compute temp=0.
loop i = 1 to 2.
loop j = 1 to nrow(dummatz).
do if (contvec(i,2)=dummatz(j,1)).
compute zcontval(i,:)=zprobval(j,:).
compute temp=temp+1.
end if.
end loop.
end loop.
do if (temp < 2).
compute notecode(notes,1) = 20.
compute notes = notes + 1.
compute modcok=0.
end if.
end if.
do if (zmodval(1,1) <> 999).
compute notecode(notes,1) = 10.
compute notes = notes + 1.
end if.
release tmp, dummat.
end if.
end if.
do if (nxs > 0 and mcx > 0).
compute tmp={rownum,xtmp(:,1)}.

.
compute dd= tmp.
compute temp = dd.
compute temp(GRADE(dd(:,2)),:) = dd.
compute dd = temp.
compute dummy = design(dd(:,2)).
compute nvls = ncol(dummy).
compute nnvls = csum(dummy).
compute mnvls = cmin(t(nnvls)).
compute conmat1=1.
do if (mnvls < 2).
compute errcode(errs,1) = 5.
compute errs = errs + 1.
compute criterr = 1.
end if.
do if (nvls > 9).
compute errcode(errs,1) = 4.
compute errs = errs+1.
compute criterr = 1.
end if.
do if (criterr = 0).
compute dumok = 1.
compute nnvls=make(nvls,1,0).
compute nnvls(1,1)=dd(1,2).
compute temp = 2.
loop i = 2 to n.
do if (dd(i,2) <> nnvls((temp-1),1)).
compute nnvls(temp,1)=dd(i,2).
compute temp = temp+1.
end if.
end loop.
do if ( mcx > 0).
compute x = dummy(:,2:ncol(dummy)).
compute nx = ncol(x).
compute minus1 = make(1,ncol(x),-1).
compute xdes=make((nx+1),3,0).
compute xdes(1,1)=dd(1,2).
compute xdes(1,2)=1.
compute temp = 2.
loop k = 2 to n.
do if (dd(k,2) <> dd((k-1),2)).
compute xdes(temp,2) = k.
compute xdes(temp,1) = dd(k,2).
compute xdes((temp-1),3) = k-1.
compute temp=temp+1.
end if.
end loop.
compute xdes((temp-1),3)=n.
compute xdes = {xdes, (xdes(:,3)-xdes(:,2)+1)}.
do if ( mcx = 4).
loop k = 1 to n.
do if (rsum(x(k,:)) = 0).
compute x(k,:) = minus1.
end if.
end loop.
end if.
do if ( mcx = 2 or mcx = 3 or mcx =5).
loop k = 1 to n.
do if (rsum(x(k,:)) > 0).
loop i = 1 to ncol(x).
do if (x(k,i) = 0).
compute x(k,i) = 1.
else.
break.
end if.
end loop.
end if.
end loop.
do if ( mcx = 3).
compute conmat1={-8,1,1,1,1,1,1,1,1; 0,-7,1,1,1,1,1,1,1; 0,0,-6,1,1,1,1,1,1; 0,0,0,-5,1,1,1,1,1; 0,0,0,0,-4,1,1,1,1; 0,0,0,0,0,-3,1,1,1; 0,0,0,0,0,0,-2,1,1; 0,0,0,0,0,0,0,-1,1}.
loop i = 1 to 8.
compute conmat1(i,:)=conmat1(i,:)/(10-i).
end loop.
compute conmat1=t(conmat1((10-nvls):8,(10-nvls):9)).
loop k=1 to n.
compute x(k,:)=conmat1((rsum(x(k,:))+1),:).
end loop.
end if.
end if.
do if ( mcx = 5).
compute custcode={ -999 }.
do if (ncol(custcode) <> (nvls*(nvls-1))).
compute errcode(errs,1) = (37+ 1 ).
compute errs = errs + 1.
compute criterr = 1.
end if.
do if (ncol(custcode) = (nvls*(nvls-1))).
compute conmat1=make(nvls,(nvls-1),0).
compute cnt=1.
loop i = 1 to nvls.
loop k = 1 to (nvls-1).
compute conmat1(i,k)=custcode(1,cnt).
compute cnt=cnt+1.
end loop.
end loop.
loop k=1 to n.
compute x(k,:)=conmat1((rsum(x(k,:))+1),:).
end loop.
end if.
end if.
compute xskip = 1.
compute dummat = make((nx+1),nx,0).
compute dummat((2:nrow(dummat)),:)=ident(nx).
do if ( mcx = 4).
compute dummat(1,:) = minus1.
end if.
do if ( mcx = 2).
loop i = 2 to nrow(dummat).
loop j = 1 to (i-1).
compute dummat(i,j) = 1.
end loop.
end loop.
end if.
do if ( mcx = 3).
compute dummat=conmat1.
end if.
do if ( mcx = 5 and criterr=0).
compute dummat=conmat1.
end if.
compute dummat={nnvls, dummat}.
compute x={dd(:,1),x}.
compute temp = x.
compute temp(GRADE(x(:,1)),:) = x.
compute x = temp.
release conmat1,temp,dd,xskip,xdes,dummy.
end if.
end if
.
do if (criterr=0).
compute xtmp=x(:,2:ncol(x)).
compute xcatlab={'X1';'X2';'X3';'X4';'X5';'X6';'X7';'X8';'X9'}.
compute nxvls=nvls-1.
compute xdich=(nvls=2).
compute mcxok=1.
compute dummatx=dummat.
compute xmodvals=dummatx(:,1).
compute nxpval=nrow(xmodvals).
release tmp, dummat.
end if.
end if.
compute intlab=make(100,1,' ').


compute intlab( 1 ,1)= 'Int_1'.


compute intlab( 2 ,1)= 'Int_2'.


compute intlab( 3 ,1)= 'Int_3'.


compute intlab( 4 ,1)= 'Int_4'.


compute intlab( 5 ,1)= 'Int_5'.


compute intlab( 6 ,1)= 'Int_6'.


compute intlab( 7 ,1)= 'Int_7'.


compute intlab( 8 ,1)= 'Int_8'.


compute intlab( 9 ,1)= 'Int_9'.


compute intlab( 10 ,1)= 'Int_10'.


compute intlab( 11 ,1)= 'Int_11'.


compute intlab( 12 ,1)= 'Int_12'.


compute intlab( 13 ,1)= 'Int_13'.


compute intlab( 14 ,1)= 'Int_14'.


compute intlab( 15 ,1)= 'Int_15'.


compute intlab( 16 ,1)= 'Int_16'.


compute intlab( 17 ,1)= 'Int_17'.


compute intlab( 18 ,1)= 'Int_18'.


compute intlab( 19 ,1)= 'Int_19'.


compute intlab( 20 ,1)= 'Int_20'.


compute intlab( 21 ,1)= 'Int_21'.


compute intlab( 22 ,1)= 'Int_22'.


compute intlab( 23 ,1)= 'Int_23'.


compute intlab( 24 ,1)= 'Int_24'.


compute intlab( 25 ,1)= 'Int_25'.


compute intlab( 26 ,1)= 'Int_26'.


compute intlab( 27 ,1)= 'Int_27'.


compute intlab( 28 ,1)= 'Int_28'.


compute intlab( 29 ,1)= 'Int_29'.


compute intlab( 30 ,1)= 'Int_30'.


compute intlab( 31 ,1)= 'Int_31'.


compute intlab( 32 ,1)= 'Int_32'.


compute intlab( 33 ,1)= 'Int_33'.


compute intlab( 34 ,1)= 'Int_34'.


compute intlab( 35 ,1)= 'Int_35'.


compute intlab( 36 ,1)= 'Int_36'.


compute intlab( 37 ,1)= 'Int_37'.


compute intlab( 38 ,1)= 'Int_38'.


compute intlab( 39 ,1)= 'Int_39'.


compute intlab( 40 ,1)= 'Int_40'.


compute intlab( 41 ,1)= 'Int_41'.


compute intlab( 42 ,1)= 'Int_42'.


compute intlab( 43 ,1)= 'Int_43'.


compute intlab( 44 ,1)= 'Int_44'.


compute intlab( 45 ,1)= 'Int_45'.


compute intlab( 46 ,1)= 'Int_46'.


compute intlab( 47 ,1)= 'Int_47'.


compute intlab( 48 ,1)= 'Int_48'.


compute intlab( 49 ,1)= 'Int_49'.


compute intlab( 50 ,1)= 'Int_50'.


compute intlab( 51 ,1)= 'Int_51'.


compute intlab( 52 ,1)= 'Int_52'.


compute intlab( 53 ,1)= 'Int_53'.


compute intlab( 54 ,1)= 'Int_54'.


compute intlab( 55 ,1)= 'Int_55'.


compute intlab( 56 ,1)= 'Int_56'.


compute intlab( 57 ,1)= 'Int_57'.


compute intlab( 58 ,1)= 'Int_58'.


compute intlab( 59 ,1)= 'Int_59'.


compute intlab( 60 ,1)= 'Int_60'.


compute intlab( 61 ,1)= 'Int_61'.


compute intlab( 62 ,1)= 'Int_62'.


compute intlab( 63 ,1)= 'Int_63'.


compute intlab( 64 ,1)= 'Int_64'.


compute intlab( 65 ,1)= 'Int_65'.


compute intlab( 66 ,1)= 'Int_66'.


compute intlab( 67 ,1)= 'Int_67'.


compute intlab( 68 ,1)= 'Int_68'.


compute intlab( 69 ,1)= 'Int_69'.


compute intlab( 70 ,1)= 'Int_70'.


compute intlab( 71 ,1)= 'Int_71'.


compute intlab( 72 ,1)= 'Int_72'.


compute intlab( 73 ,1)= 'Int_73'.


compute intlab( 74 ,1)= 'Int_74'.


compute intlab( 75 ,1)= 'Int_75'.


compute intlab( 76 ,1)= 'Int_76'.


compute intlab( 77 ,1)= 'Int_77'.


compute intlab( 78 ,1)= 'Int_78'.


compute intlab( 79 ,1)= 'Int_79'.


compute intlab( 80 ,1)= 'Int_80'.


compute intlab( 81 ,1)= 'Int_81'.


compute intlab( 82 ,1)= 'Int_82'.


compute intlab( 83 ,1)= 'Int_83'.


compute intlab( 84 ,1)= 'Int_84'.


compute intlab( 85 ,1)= 'Int_85'.


compute intlab( 86 ,1)= 'Int_86'.


compute intlab( 87 ,1)= 'Int_87'.


compute intlab( 88 ,1)= 'Int_88'.


compute intlab( 89 ,1)= 'Int_89'.


compute intlab( 90 ,1)= 'Int_90'.


compute intlab( 91 ,1)= 'Int_91'.


compute intlab( 92 ,1)= 'Int_92'.


compute intlab( 93 ,1)= 'Int_93'.


compute intlab( 94 ,1)= 'Int_94'.


compute intlab( 95 ,1)= 'Int_95'.


compute intlab( 96 ,1)= 'Int_96'.


compute intlab( 97 ,1)= 'Int_97'.


compute intlab( 98 ,1)= 'Int_98'.


compute intlab( 99 ,1)= 'Int_99'.


compute intlab( 100 ,1)= 'Int_100'.

compute bcmat=make(needed,needed,0).
compute wcmat=make(needed,needed,0).
compute zcmat=make(needed,needed,0).
compute wzcmat=make(needed,needed,0).
compute wsum=0.
compute zsum=0.
compute wzsum=0.
end if.
end if.
do if (criterr = 0 and model <> 999).
compute modelmat= {1,0,0,0,0,0,0,1,0,0;2,0,0,0,0,0,0,1,1,0;3,0,0,0,0,0,0,1,1,1;4,0,0,0,0,0,0,0,0,0; 5,0,0,0,0,0,0,1,0,0;6,0,0,0,0,0,0,0,0,0;7,1,0,0,0,0,0,0,0,0;8,1,0,0,0,0,0,1,0,0; 9,1,1,0,0,0,0,0,0,0;10,1,1,0,0,0,0,1,1,0;11,1,1,1,0,0,0,0,0,0;12,1,1,1,0,0,0,1,1,1; 13,1,1,1,0,0,0,1,0,0;14,0,0,0,1,0,0,0,0,0;15,0,0,0,1,0,0,1,0,0;16,0,0,0,1,1,0,0,0,0; 17,0,0,0,1,1,0,1,1,0;18,0,0,0,1,1,1,0,0,0;19,0,0,0,1,1,1,1,1,1;20,0,0,0,1,1,1,1,0,0; 21,1,0,0,0,1,0,0,0,0;22,1,0,0,0,1,0,1,0,0;23,0,0,0,0,0,0,0,0,0;24,0,0,0,0,0,0,0,0,0; 25,0,0,0,0,0,0,0,0,0;26,0,0,0,0,0,0,0,0,0;27,0,0,0,0,0,0,0,0,0;28,1,0,0,0,1,0,0,1,0; 29,1,0,0,0,1,0,1,1,0;30,0,0,0,0,0,0,0,0,0;31,0,0,0,0,0,0,0,0,0;32,0,0,0,0,0,0,0,0,0; 33,0,0,0,0,0,0,0,0,0;34,0,0,0,0,0,0,0,0,0;35,0,0,0,0,0,0,0,0,0;36,0,0,0,0,0,0,0,0,0; 37,0,0,0,0,0,0,0,0,0;38,0,0,0,0,0,0,0,0,0;39,0,0,0,0,0,0,0,0,0;40,0,0,0,0,0,0,0,0,0; 41,0,0,0,0,0,0,0,0,0;42,0,0,0,0,0,0,0,0,0;43,0,0,0,0,0,0,0,0,0;44,0,0,0,0,0,0,0,0,0; 45,0,0,0,0,0,0,0,0,0;46,0,0,0,0,0,0,0,0,0;47,0,0,0,0,0,0,0,0,0;48,0,0,0,0,0,0,0

 ,0,0; 49,0,0,0,0,0,0,0,0,0;50,0,0,0,0,0,0,0,0,0;51,0,0,0,0,0,0,0,0,0;52,0,0,0,0,0,0,0,0,0; 53,0,0,0,0,0,0,0,0,0;54,0,0,0,0,0,0,0,0,0;55,0,0,0,0,0,0,0,0,0;56,0,0,0,0,0,0,0,0,0; 57,0,0,0,0,0,0,0,0,0;58,1,0,0,1,0,0,0,0,0;59,1,0,0,1,0,0,1,0,0;60,1,1,0,1,0,0,0,0,0; 61,1,1,0,1,0,0,1,0,0;62,1,1,0,1,0,0,0,1,0;63,1,1,0,1,0,0,1,1,0;64,1,0,0,1,1,0,0,0,0; 65,1,0,0,1,1,0,1,0,0;66,1,0,0,1,1,0,0,1,0;67,1,0,0,1,1,0,1,1,0;68,1,1,1,1,0,0,0,0,0; 69,1,1,1,1,0,0,1,1,1;70,1,0,0,1,1,1,0,0,0;71,1,0,0,1,1,1,1,1,1;72,1,1,1,1,1,1,0,0,0; 73,1,1,1,1,1,1,1,1,1;74,0,0,0,1,0,0,0,0,0;75,1,1,0,1,1,0,0,0,0;76,1,1,0,1,1,0,1,1,0; 77,0,0,0,0,0,0,0,0,0;78,0,0,0,0,0,0,0,0,0;79,0,0,0,0,0,0,0,0,0;80,0,0,0,0,0,0,0,0,0; 81,0,0,0,0,0,0,0,0,0;82,0,0,0,0,0,0,0,0,0;83,1,0,0,0,0,0,0,0,0;84,1,0,0,0,0,0,0,0,0; 85,1,0,0,0,0,0,1,0,0;86,1,0,0,0,0,0,1,0,0;87,0,0,0,1,0,0,0,0,0;88,0,0,0,1,0,0,0,0,0; 89,0,0,0,1,0,0,1,0,0;90,0,0,0,1,0,0,1,0,0;91,0,0,0,0,0,0,0,0,0;92,1,0,0,1,0,0,1,0,0}.
do if (model > 0).
compute tmp=modelmat(model,2:ncol(modelmat)).
end if.
do if (model=0).
compute tmp={0,0,0,0,0,0,0,0,0}.
end if.
do if (model < 4).
compute bcmat((nxs+1),1)=1.
end if.
do if ((model > 3) and (model <> 6)).
compute bcmat((nxs+1):(nxs+nms),1)=onem.
compute bcmat(nrow(bcmat),(nxs+1):(nxs+nms))=t(onem).
compute bcmat(nrow(bcmat),1)=1.
end if.
do if ((model = 6) or (model > 82 and model < 93)).
loop j = 2 to nrow(bcmat).
loop i = 1 to (j-1).
compute bcmat(j,i)=1.
end loop.
end loop.
end if.
do if (model = 80).
loop i = 1 to nms.
compute bcmat((nrow(bcmat)-1),i)=1.
end loop.
end if.
do if (model = 81).
loop j = 3 to nrow(bcmat).
compute bcmat(j,2)=1.
end loop.
end if.
do if (model = 82).
compute bcmat(3,2)=1.
compute bcmat(5,4)=1.
end if.
do if (tmp(1,1)=1).
compute wcmat((nxs+1):(nxs+nms),1)=onem.
compute wprod=1.
compute xprod=1.
do if (model = 83 or model = 86).
compute onemsx=onem.
loop i = 1 to (nms-1).
compute onemsx(i+1,1)=0.
end loop.
compute wcmat((nxs+1):(nxs+nms),1)=onemsx.
end if.
end if.
do if (tmp(1,4)=1).
compute wcmat(nrow(wcmat),(nxs+1):(nxs+nms))=t(onem).
compute wprod=1.
do if (model = 87 or model = 90).
compute onemsx=onem.
loop i = 1 to (nms-1).
compute onemsx(i,1)=0.
end loop.
compute wcmat(nrow(wcmat),(nxs+1):(nxs+nms))=t(onemsx).
end if.
end if.
do if (tmp(1,7)=1).
compute wcmat(nrow(wcmat),1)=1.
compute wprod=1.
compute xprod=1.
end if.
do if (tmp(1,2)=1).
compute zcmat((nxs+1):(nxs+nms),1)=onem.
compute zprod=1.
compute xprod=1.
end if.
do if (tmp(1,5)=1).
compute zcmat(nrow(zcmat),(nxs+1):(nxs+nms))=t(onem).
compute zprod=1.
end if.
do if (tmp(1,8)=1).
compute zcmat(nrow(zcmat),1)=1.
compute zprod=1.
compute xprod=1.
end if.
do if (tmp(1,3)=1).
compute wzcmat((nxs+1):(nxs+nms),1)=onem.
compute xprod=1.
compute wprod=1.
compute zprod=1.
end if.
do if (tmp(1,6)=1).
compute wzcmat(nrow(wzcmat),(nxs+1):(nxs+nms))=t(onem).
compute zprod=1.
compute wprod=1.
end if.
do if (tmp(1,9)=1).
compute wzcmat(nrow(wzcmat),1)=1.
compute xprod=1.
compute wprod=1.
compute zprod=1.
end if.
do if (model = 91 or model = 92).
loop j = 1 to (nms-1).
loop i = 1 to j.
compute wcmat((nxs+1+j),(nxs+i))=1.
end loop.
end loop.
end if.
do if (nms < 0).
loop i = 1 to nms.
compute tmp=csum(wcmat(:,(1+i)))+csum(zcmat(:,(1+i)))+csum(wzcmat(:,(1+i))).
compute mprod(1,i)=(tmp>0).
end loop.
end if.
end if.
do if (ncs > 0).
compute ccmat=make((nms+nys),ncs,1).
compute ccmatoff=ccmat.
do if (covmy=1).
compute ccmat(nrow(ccmat),:)=make(1,ncs,0).
end if.
do if (covmy=2).
compute ccmat(1:nms,:)=make(nms,ncs,0).
end if.
do if (cmatrix(1,1) <> -999).
do if (ncol(cmatrix) <> ((nms+nys)*ncs)).
compute errcode(errs,1)=29.
compute errs=errs+1.
compute criterr=1.
end if.
do if (criterr = 0).
compute tmp=1.
loop i = 1 to (nms+nys).
loop j = 1 to ncs.
compute ccmat(i,j)=1-(cmatrix(1,tmp) = 0).
compute tmp=tmp+1.
end loop.
end loop.
do if (rsum((csum(ccmat)=0)) <> 0).
compute errcode(errs,1)=30.
compute errs=errs+1.
compute criterr=1.
end if.
end if.
do if (covmy <> 0).
compute notecode(notes,1)=1.
compute notes=notes+1.
end if.
end if.
do if (xmint=1 and (covmy > 0 or (csum(rsum(ccmat)) <> ((nms+nys)*ncs)))).
compute errcode(errs,1)=68.
compute errs=errs+1.
compute criterr=1.
end if.
end if.
do if (criterr=0).
compute needed=needed*(needed-1)/2.
compute nopath=0.
do if (bmatrix(1,1) <> -999).
compute tmp=1.
do if ((ncol(bmatrix) <> needed) or (csum(rsum(bmatrix))=0)).
compute errcode(errs,1)=16.
compute errs=errs+1.
compute criterr=1.
else.
loop i = 2 to nrow(bcmat).
loop j = 1 to (i-1).
compute bcmat(i,j)=1-(bmatrix(1,tmp) = 0).
compute tmp=tmp+1.
end loop.
end loop.
end if.
do if ((csum(bcmat(:,1))=0) and criterr=0).
compute errcode(errs,1)=22.
compute errs=errs+1.
compute criterr=1.
end if.
do if ((rsum(bcmat(nrow(bcmat),:))=0) and criterr=0).
compute errcode(errs,1)=23.
compute errs=errs+1.
compute criterr=1.
end if.
compute dm=0.
do if (nms > 0).
loop i = 1 to nms.
do if (((rsum(bcmat((nxs+i),:)) = 0) or (csum(bcmat(:,(nxs+i))) = 0)) and (dm=0) and (criterr=0)).
compute errcode(errs,1)=26.
compute errs=errs+1.
compute criterr=1.
compute dm=1.
end if.
end loop.
end if.
release dm.
end if.
end if.
do if (criterr=0).
do if (wmatrix(1,1) <> -999).
compute tmp=1.
do if (ncol(wmatrix) <> needed).
compute errcode(errs,1)=17.
compute errs=errs+1.
compute criterr=1.
else.
compute modelvar(1,1)='CUSTOM'.
loop i = 2 to nrow(wcmat).
loop j = 1 to (i-1).
compute wcmat(i,j)=1-(wmatrix(1,tmp) = 0).
do if ((wcmat(i,j)=1) and (bcmat(i,j)=0) and (nopath=0)).
compute errcode(errs,1)=20.
compute errs=errs+1.
compute criterr=1.
compute nopath=1.
end if.
compute tmp=tmp+1.
end loop.
end loop.
end if.
end if.
do if (zmatrix(1,1) <> -999).
compute tmp=1.
do if (ncol(zmatrix) <> needed).
compute errcode(errs,1)=18.
compute errs=errs+1.
compute criterr=1.
else.
compute modelvar(1,1)='CUSTOM'.
do if (csum(rsum(wcmat))=0 and model=999).
compute errcode(errs,1)=21.
compute errs=errs+1.
compute criterr=1.
end if.
loop i = 2 to nrow(zcmat).
loop j = 1 to (i-1).
compute zcmat(i,j)=1-(zmatrix(1,tmp) = 0).
do if ((zcmat(i,j)=1) and (bcmat(i,j)=0) and (nopath=0)).
compute errcode(errs,1)=20.
compute errs=errs+1.
compute criterr=1.
compute nopath=1.
end if.
compute tmp=tmp+1.
end loop.
end loop.
end if.
end if.
compute tmp=1.
do if (wzmatrix(1,1) <> -999).
do if (ncol(wzmatrix) <> needed).
compute errcode(errs,1)=19.
compute errs=errs+1.
compute criterr=1.
end if.
compute modelvar(1,1)='CUSTOM'.
end if.
do if (criterr=0).
loop i = 2 to nrow(wzcmat).
loop j = 1 to (i-1).
do if (wzmatrix(1,1) <> -999).
compute wzcmat(i,j)=1-(wzmatrix(1,tmp) = 0).
end if.
do if (wzcmat(i,j)=1).
compute wcmat(i,j)=1.
compute zcmat(i,j)=1.
end if.
do if ((wzcmat(i,j)=1) and (bcmat(i,j)=0) and (nopath=0)).
compute errcode(errs,1)=20.
compute errs=errs+1.
compute criterr=1.
compute nopath=1.
end if.
compute tmp=tmp+1.
end loop.
end loop.
end if.
end if.
do if (criterr=0).
compute xprod=csum(wcmat(:,1))+csum(zcmat(:,1))+csum(wzcmat(:,1)).
compute xprod=(xprod > 0).
compute wsum=csum(rsum(wcmat)).
compute wprod=(wsum > 0).
do if (nms > 0).
loop i = 1 to nms.
compute tmp=csum(wcmat(:,(1+i)))+csum(zcmat(:,(1+i)))+csum(wzcmat(:,(1+i))).
compute mprod(1,i)=(tmp>0).
end loop.
end if.
do if ((wsum > 0) and (w = 'xxxxx')).
compute errcode(errs,1)=11.
compute errs=errs+1.
compute criterr=1.
end if.
do if ((wsum = 0) and (w <> 'xxxxx')).
compute errcode(errs,1)=10.
compute errs=errs+1.
compute criterr=1.
end if.
compute zsum=csum(rsum(zcmat)).
compute zprod=(zsum > 0).
do if ((zsum > 0) and (z = 'xxxxx')).
compute errcode(errs,1)=13.
compute errs=errs+1.
compute criterr=1.
end if.
do if ((zsum = 0) and (z <> 'xxxxx')).
compute errcode(errs,1)=12.
compute errs=errs+1.
compute criterr=1.
end if.
do if ((zsum > 0) and (wsum = 0)).
compute errcode(errs,1)=35.
compute errs=errs+1.
compute criterr=1.
end if.
end if.
do if (criterr=0 and nms > 1).
compute serchk=bcmat(2:(nrow(bcmat)-1),2:ncol(bcmat)).
do if (csum(rsum(serchk))) > 0.
compute serial=1.
do if (nms > 6).
compute errcode(errs,1)=36.
compute errs=errs+1.
compute criterr=1.
end if.
end if.
end if.
do if (center > 0 and criterr=0).
compute centvar={' '}.
do if (criterr=0).
do if ((center = 1) or (center = 2 and wdich = 0)).
do if (wprod=1 and mcwok=0 and nwpval > 0).
loop i = 1 to nws.
compute wtmp(:,i)=wtmp(:,i)-(csum(wtmp(:,i))/n).
compute centvar={centvar,wnames(1,i)}.
end loop.

.
compute desctmp=make((8-(4* wmodcust )),ncol( wtmp ),-999).
loop jd=1 to ncol( wtmp ).
compute descdat= wtmp (:,jd).
compute desctmp(1,jd) = csum(descdat)/nrow(descdat).
compute desctmp(2,jd) = (nrow(descdat)*sscp(descdat))-(t(csum(descdat))*(csum(descdat))).
compute desctmp(2,jd) = sqrt(desctmp(2,jd)/(nrow(descdat)*(nrow(descdat)-1))).
compute desctmp(3,jd)=cmin(descdat).
compute desctmp(4,jd)=cmax(descdat).
do if ( wmodcust =0).
compute minwarn=0.
compute maxwarn=0.
do if ((desctmp(3,jd)=desctmp(4,jd)) and novar=0).
compute errcode(errs,1)=15.
compute errs=errs+1.
compute criterr=1.
compute novar=1.
end if.
compute tmp=((descdat(:,1)=desctmp(3,jd))+(descdat(:,1)=desctmp(4,jd))).
compute desctmp(8,jd)=(csum(tmp)=nrow(tmp)).
compute tmp = descdat.
compute tmp(GRADE(descdat),:) = descdat.
compute descdat = tmp.
release tmp.
compute decval={.16;.5;.84}.
loop kd=1 to 3.
compute low=trunc(decval(kd,1)*(nrow(descdat)+1)).
compute lowdec=decval(kd,1)*(nrow(descdat)+1)-low.
compute value=descdat(low,1)+(descdat((low+1),1)-descdat(low,1))*lowdec.
compute desctmp((4+kd),jd)=value.
end loop.
compute mnotev=1.
compute modvals=desctmp(5:7,:).
do if (quantile <> 1).
compute desctmp(5,jd)=desctmp(1,jd)-desctmp(2,jd).
compute desctmp(6,jd)=desctmp(1,jd).
compute desctmp(7,jd)=desctmp(1,jd)+desctmp(2,jd).
compute modvals=desctmp(5:7,:).
compute mnotev=2.
do if (modvals(1,1) < desctmp(3,1)).
compute modvals(1,1)=desctmp(3,1).
compute minwarn=1.
end if.
do if (modvals(3,1) > desctmp(4,1)).
compute modvals(3,1)=desctmp(4,1).
compute maxwarn=1.
end if.
end if.
do if (desctmp(8,1)=1).
compute modvals={desctmp(3,1);desctmp(4,1)}.
compute mnotev=0.
compute minwarn=0.
compute maxwarn=0.
end if.
end if.
end loop
.
compute wmin=desctmp(3,1).
compute wmax=desctmp(4,1).
do if (wmodcust=0).
compute wmodvals=modvals.
compute wprobval=wmodvals.
end if.
end if.
end if.
do if ((center = 1) or (center = 2 and zdich = 0)).
do if (zprod=1 and mczok=0 and nzpval > 0).
loop i = 1 to nzs.
compute ztmp(:,i)=ztmp(:,i)-(csum(ztmp(:,i))/n).
compute centvar={centvar,znames(1,i)}.
end loop.

.
compute desctmp=make((8-(4* zmodcust )),ncol( ztmp ),-999).
loop jd=1 to ncol( ztmp ).
compute descdat= ztmp (:,jd).
compute desctmp(1,jd) = csum(descdat)/nrow(descdat).
compute desctmp(2,jd) = (nrow(descdat)*sscp(descdat))-(t(csum(descdat))*(csum(descdat))).
compute desctmp(2,jd) = sqrt(desctmp(2,jd)/(nrow(descdat)*(nrow(descdat)-1))).
compute desctmp(3,jd)=cmin(descdat).
compute desctmp(4,jd)=cmax(descdat).
do if ( zmodcust =0).
compute minwarn=0.
compute maxwarn=0.
do if ((desctmp(3,jd)=desctmp(4,jd)) and novar=0).
compute errcode(errs,1)=15.
compute errs=errs+1.
compute criterr=1.
compute novar=1.
end if.
compute tmp=((descdat(:,1)=desctmp(3,jd))+(descdat(:,1)=desctmp(4,jd))).
compute desctmp(8,jd)=(csum(tmp)=nrow(tmp)).
compute tmp = descdat.
compute tmp(GRADE(descdat),:) = descdat.
compute descdat = tmp.
release tmp.
compute decval={.16;.5;.84}.
loop kd=1 to 3.
compute low=trunc(decval(kd,1)*(nrow(descdat)+1)).
compute lowdec=decval(kd,1)*(nrow(descdat)+1)-low.
compute value=descdat(low,1)+(descdat((low+1),1)-descdat(low,1))*lowdec.
compute desctmp((4+kd),jd)=value.
end loop.
compute mnotev=1.
compute modvals=desctmp(5:7,:).
do if (quantile <> 1).
compute desctmp(5,jd)=desctmp(1,jd)-desctmp(2,jd).
compute desctmp(6,jd)=desctmp(1,jd).
compute desctmp(7,jd)=desctmp(1,jd)+desctmp(2,jd).
compute modvals=desctmp(5:7,:).
compute mnotev=2.
do if (modvals(1,1) < desctmp(3,1)).
compute modvals(1,1)=desctmp(3,1).
compute minwarn=1.
end if.
do if (modvals(3,1) > desctmp(4,1)).
compute modvals(3,1)=desctmp(4,1).
compute maxwarn=1.
end if.
end if.
do if (desctmp(8,1)=1).
compute modvals={desctmp(3,1);desctmp(4,1)}.
compute mnotev=0.
compute minwarn=0.
compute maxwarn=0.
end if.
end if.
end loop
.
compute zmin=desctmp(3,1).
compute zmax=desctmp(4,1).
do if (zmodcust=0).
compute zmodvals=modvals.
compute zprobval=zmodvals.
end if.
end if.
end if.
do if ((center = 1) or (center = 2 and xdich = 0)).
do if (xprod=1 and mcxok=0).
loop i = 1 to nxs.
compute xtmp(:,i)=xtmp(:,i)-(csum(xtmp(:,i))/n).
compute centvar={centvar,xnames(1,i)}.
end loop.

.
compute desctmp=make((8-(4* 0 )),ncol( xtmp ),-999).
loop jd=1 to ncol( xtmp ).
compute descdat= xtmp (:,jd).
compute desctmp(1,jd) = csum(descdat)/nrow(descdat).
compute desctmp(2,jd) = (nrow(descdat)*sscp(descdat))-(t(csum(descdat))*(csum(descdat))).
compute desctmp(2,jd) = sqrt(desctmp(2,jd)/(nrow(descdat)*(nrow(descdat)-1))).
compute desctmp(3,jd)=cmin(descdat).
compute desctmp(4,jd)=cmax(descdat).
do if ( 0 =0).
compute minwarn=0.
compute maxwarn=0.
do if ((desctmp(3,jd)=desctmp(4,jd)) and novar=0).
compute errcode(errs,1)=15.
compute errs=errs+1.
compute criterr=1.
compute novar=1.
end if.
compute tmp=((descdat(:,1)=desctmp(3,jd))+(descdat(:,1)=desctmp(4,jd))).
compute desctmp(8,jd)=(csum(tmp)=nrow(tmp)).
compute tmp = descdat.
compute tmp(GRADE(descdat),:) = descdat.
compute descdat = tmp.
release tmp.
compute decval={.16;.5;.84}.
loop kd=1 to 3.
compute low=trunc(decval(kd,1)*(nrow(descdat)+1)).
compute lowdec=decval(kd,1)*(nrow(descdat)+1)-low.
compute value=descdat(low,1)+(descdat((low+1),1)-descdat(low,1))*lowdec.
compute desctmp((4+kd),jd)=value.
end loop.
compute mnotev=1.
compute modvals=desctmp(5:7,:).
do if (quantile <> 1).
compute desctmp(5,jd)=desctmp(1,jd)-desctmp(2,jd).
compute desctmp(6,jd)=desctmp(1,jd).
compute desctmp(7,jd)=desctmp(1,jd)+desctmp(2,jd).
compute modvals=desctmp(5:7,:).
compute mnotev=2.
do if (modvals(1,1) < desctmp(3,1)).
compute modvals(1,1)=desctmp(3,1).
compute minwarn=1.
end if.
do if (modvals(3,1) > desctmp(4,1)).
compute modvals(3,1)=desctmp(4,1).
compute maxwarn=1.
end if.
end if.
do if (desctmp(8,1)=1).
compute modvals={desctmp(3,1);desctmp(4,1)}.
compute mnotev=0.
compute minwarn=0.
compute maxwarn=0.
end if.
end if.
end loop
.
compute xmodvals=modvals.
compute xprobval=xmodvals.
end if.
end if.
do if (nms > 0).
loop i = 1 to nms.
do if (mprod(1,i)=1).
compute mtmp(:,i)=mtmp(:,i)-(csum(mtmp(:,i))/n).
compute centvar={centvar,mnames(1,i)}.
end if.
end loop.

.
compute desctmp=make((8-(4* 0 )),ncol( mtmp ),-999).
loop jd=1 to ncol( mtmp ).
compute descdat= mtmp (:,jd).
compute desctmp(1,jd) = csum(descdat)/nrow(descdat).
compute desctmp(2,jd) = (nrow(descdat)*sscp(descdat))-(t(csum(descdat))*(csum(descdat))).
compute desctmp(2,jd) = sqrt(desctmp(2,jd)/(nrow(descdat)*(nrow(descdat)-1))).
compute desctmp(3,jd)=cmin(descdat).
compute desctmp(4,jd)=cmax(descdat).
do if ( 0 =0).
compute minwarn=0.
compute maxwarn=0.
do if ((desctmp(3,jd)=desctmp(4,jd)) and novar=0).
compute errcode(errs,1)=15.
compute errs=errs+1.
compute criterr=1.
compute novar=1.
end if.
compute tmp=((descdat(:,1)=desctmp(3,jd))+(descdat(:,1)=desctmp(4,jd))).
compute desctmp(8,jd)=(csum(tmp)=nrow(tmp)).
compute tmp = descdat.
compute tmp(GRADE(descdat),:) = descdat.
compute descdat = tmp.
release tmp.
compute decval={.16;.5;.84}.
loop kd=1 to 3.
compute low=trunc(decval(kd,1)*(nrow(descdat)+1)).
compute lowdec=decval(kd,1)*(nrow(descdat)+1)-low.
compute value=descdat(low,1)+(descdat((low+1),1)-descdat(low,1))*lowdec.
compute desctmp((4+kd),jd)=value.
end loop.
compute mnotev=1.
compute modvals=desctmp(5:7,:).
do if (quantile <> 1).
compute desctmp(5,jd)=desctmp(1,jd)-desctmp(2,jd).
compute desctmp(6,jd)=desctmp(1,jd).
compute desctmp(7,jd)=desctmp(1,jd)+desctmp(2,jd).
compute modvals=desctmp(5:7,:).
compute mnotev=2.
do if (modvals(1,1) < desctmp(3,1)).
compute modvals(1,1)=desctmp(3,1).
compute minwarn=1.
end if.
do if (modvals(3,1) > desctmp(4,1)).
compute modvals(3,1)=desctmp(4,1).
compute maxwarn=1.
end if.
end if.
do if (desctmp(8,1)=1).
compute modvals={desctmp(3,1);desctmp(4,1)}.
compute mnotev=0.
compute minwarn=0.
compute maxwarn=0.
end if.
end if.
end loop
.
do if ((cdeval(1,1)=-999) and (model=74)).
compute medmeans=desctmp(1,:).
end if.
compute mmodvals=modvals.
compute mprobval=mmodvals.
end if.
end if.
do if (ncol(centvar) > 1).
compute notecode(notes,1)=3.
compute notes=notes+1.
end if.
end if.
do if (criterr=0).
compute wsum=rsum(csum(wcmat)).
compute zsum=rsum(csum(zcmat)).
compute wzsum=rsum(csum(wzcmat)).
compute nump=make(1,(nys+nms),-999).
compue numint=make(1,(nys+nms),0).
compute datcount=1.
compute xtmpuse=0.
compute wtmpuse=0.
compute ztmpuse=0.
compute xwtmpus=0.
compute xztmpus=0.
compute wztmpus=0.
compute xwztmpu=0.
compute xtmploc=-999.
compute wtmploc=-999.
compute xwtmplo=-999.
compute ztmploc=-999.
compute xztmplo=-999.
compute wztmplo=-999.
compute xwztmplo=-999.
compute vlabs={' '}.
do if (ncs > 0).
compute ctmpuse=make(1,ncs,0).
end if.
do if (nms > 0).
compute mtmpuse=make(1,nms,0).
compute mwtmpus=make(1,nms,0).
compute mztmpus=make(1,nms,0).
compute mwztmpu=make(1,nms,0).
compute mtmploc=make(1,nms,0).
compute mwtmplo=make(nwvls,nms,-999).
compute mztmplo=make(nzvls,nms,-999).
compute mwztmplo=make((nwvls*nzvls),nms,-999).
end if.
do if (ncs > 0).
compute ctmploc=make(1,ncs,0).
end if.
compute fulldat=make(n,1,1).
compute datindx=make(1000,(nms+nys),-999).
compute wherew=make(2,(nms+nys),-999).
compute wherex=make(2,(nms+nys),-999).
compute wherez=make(2,(nms+nys),-999).
compute wherexw=make(2,(nms+nys),-999).
compute wherexz=make(2,(nms+nys),-999).
compute wherewz=make(2,(nms+nys),-999).
compute wherexwz=make(2,(nms+nys),-999).
do if (nms > 0).
compute wherem=make(nms,(nms+nys),-999).
compute wheremw = make(nms*2,(nms+nys),-999).
compute wheremz = make(nms*2,(nms+nys),-999).
compute wheremwz = make(nms*2,(nms+nys),-999).
end if.
compute wzhigh=make(1000,(((nms+1)*(nms+2))/2),0).
compute whigh=make(1000,(((nms+1)*(nms+2))/2),0).
compute zhigh=make(1000,(((nms+1)*(nms+2))/2),0).
compute fochigh=make(1000,(((nms+1)*(nms+2))/2),0).
compute xcoefloc={1;2;3;4;5;6;7;8;9}.
compute intkey = {' ', ' ', ' ', ' ', ' ', ' ', ' '}.
compute wzhighct=0.
compute whighct=0.
compute zhighct=0.
compute foccnt=0.
loop i = 2 to nrow(bcmat).
compute wdid=0.
compute zdid=0.
compute wzdid=0.
compute cntmp=1.
compute start=1.
do if (i < nrow(bcmat)).
compute outv=mtmp(:,(i-1)).
compute modlabel={mnames(1,(i-1));'constant'}.
end if.
do if (i = nrow(bcmat)).
compute outv=ytmp.
compute modlabel={ynames;'constant'}.
end if.
loop j = 1 to (i-1).
compute foccnt=foccnt+1.
do if (j = 1 and bcmat(i,j)=1).
compute outv={outv,xtmp}.
compute modlabel={modlabel;xcatlab(1:nxvls,1)}.
do if (xtmpuse=0).
compute fulldat={fulldat,xtmp}.
compute xtmpuse=1.
loop k4=datcount to (datcount+(nxvls-1)).
compute xtmploc={xtmploc;k4}.
end loop.
compute xtmploc=xtmploc(2:nrow(xtmploc),1).
compute datcount=datcount+nxvls.
end if.
compute datindx(start:(start+nrow(xtmploc)-1),(i-1))=xtmploc.
compute wherex(1,(i-1))=start+1.
compute wherex(2,(i-1))=start+nrow(xtmploc)-1+1.
do if (model = 74).
end if.
compute onebl=make(nrow(xtmploc),1,1).
compute fochigh((start+1):(start+nrow(xtmploc)),foccnt)=onebl.
compute start=start+nrow(xtmploc).
end if.
do if (j > 1 and bcmat(i,j)=1).
compute outv={outv,mtmp(:,(j-1))}.
compute modlabel={modlabel;mnames(1,(j-1))}.
do if (mtmpuse(1,(j-1))=0).
compute fulldat={fulldat,mtmp(:,(j-1))}.
compute mtmpuse(1,(j-1))=1.
compute mtmploc(1,(j-1))=datcount.
compute datcount=datcount+1.
end if.
compute datindx(start:(start+nrow(mtmploc)-1),(i-1))=mtmploc(1,(j-1)).
compute wherem((j-1),(i-1))=start+1.
compute onebl=make(nrow(mtmploc(1,j-1)),1,1).
compute ttt=nrow(mtmploc(1,(j-1)))+start-1.
compute fochigh((start+1):(start+nrow(mtmploc(1,(j-1)))),foccnt)=onebl.
compute start=start+nrow(mtmploc(1,(j-1))).
end if.
end loop.
do if (wsum > 0).
loop j = 1 to (i-1).
compute whighct=whighct+1.
do if (j = 1 and wcmat(i,j)=1).
do if (wdid=0).
compute outv={outv,wtmp}.
do if (ncs > 0 and wiscov > 0).
compute ccmatoff((i-1),wiscov)=0.
end if.
compute modlabel={modlabel;wcatlab(1:nwvls,1)}.
compute wdid=1.
do if (wtmpuse=0).
compute fulldat={fulldat,wtmp}.
do if (ncs > 0 and wiscov > 0).
compute ccmatoff((i-1),wiscov)=0.
end if.
compute wtmpuse=1.
loop k4=datcount to (datcount+(nwvls-1)).
compute wtmploc={wtmploc;k4}.
end loop.
compute wtmploc=wtmploc(2:nrow(wtmploc),1).
compute datcount=datcount+nwvls.
end if.
end if.
compute datindx(start:(start+nrow(wtmploc)-1),(i-1))=wtmploc.
compute wherew(1,(i-1))=start+1.
compute wherew(2,(i-1))=start+nrow(wtmploc)-1+1.
compute start=start+nrow(wtmploc).
loop k1=1 to nxvls.
loop k2 = 1 to nwvls.
compute outv={outv,(xtmp(:,k1)&*wtmp(:,k2))}.
do if (ncs > 0 and wiscov > 0).
compute ccmatoff((i-1),wiscov)=0.
end if.
compute modlabel={modlabel;intlab(cntmp,1)}.
compute intkey={intkey;intlab(cntmp,1),':',xcatlab(k1,1),'x',wcatlab(k2,1),' ',' '}.
compute cntmp=cntmp+1.
end loop.
end loop.
do if (xwtmpus=0).
compute fulldat={fulldat,outv(:,(ncol(outv)-(nxvls*nwvls)+1):ncol(outv))}.
compute xwtmpus=1.
do if (ncs > 0 and wiscov > 0).
compute ccmatoff((i-1),wiscov)=0.
end if.
loop k4=datcount to (datcount+((nwvls*nxvls)-1)).
compute xwtmplo={xwtmplo;k4}.
end loop.
compute xwtmplo=xwtmplo(2:nrow(xwtmplo),1).
compute datcount=datcount+(nxvls*nwvls).
end if.
compute datindx(start:(start+nrow(xwtmplo)-1),(i-1))=xwtmplo.
compute wherexw(1,(i-1))=start+1.
compute wherexw(2,(i-1))=start+nrow(xwtmplo)-1+1.
compute onebl=make(nrow(xwtmplo),1,1).
compute whigh((start+1):(start+nrow(xwtmplo)),whighct)=onebl.
compute start=start+nrow(xwtmplo).
end if.
do if (j > 1 and wcmat(i,j)=1).
do if (wdid=0 and model <> 74).
compute outv={outv,wtmp}.
do if (ncs > 0 and wiscov > 0).
compute ccmatoff((i-1),wiscov)=0.
end if.
compute modlabel={modlabel;wcatlab(1:nwvls,1)}.
compute wdid=1.
do if (wtmpuse=0).
compute fulldat={fulldat,wtmp}.
do if (ncs > 0 and wiscov > 0).
compute ccmatoff((i-1),wiscov)=0.
end if.
compute wtmpuse=1.
loop k4=datcount to (datcount+(nwvls-1)).
compute wtmploc={wtmploc;k4}.
end loop.
compute wtmploc=wtmploc(2:nrow(wtmploc),1).
compute datcount=datcount+nwvls.
end if.
compute datindx(start:(start+nrow(wtmploc)-1),(i-1))=wtmploc.
compute wherew(1,(i-1))=start+1.
compute wherew(2,(i-1))=start+nrow(wtmploc)-1+1.
compute start=start+nrow(wtmploc).
end if.
loop k2 = 1 to nwvls.
compute outv={outv,(mtmp(:,(j-1))&*wtmp(:,k2))}.
do if (ncs > 0 and wiscov > 0).
compute ccmatoff((i-1),wiscov)=0.
end if.
compute modlabel={modlabel;intlab(cntmp,1)}.
compute intkey={intkey;intlab(cntmp,1),':', mnames(1,(j-1)),'x',wcatlab(k2,1),' ',' '}.
compute cntmp=cntmp+1.
end loop.
do if (mwtmpus(1,(j-1))=0).
compute fulldat={fulldat,outv(:,(ncol(outv)-nwvls+1):ncol(outv))}.
do if (ncs > 0 and wiscov > 0).
compute ccmatoff((i-1),wiscov)=0.
end if.
compute mwtmpus(1,(j-1))=1.
compute mw22=-999.
loop k4=datcount to (datcount+(nwvls-1)).
compute mw22={mw22;k4}.
end loop.
compute mwtmplo(:,(j-1))=mw22(2:nrow(mw22),1).
compute datcount=datcount+nwvls.
end if.
compute datindx(start:(start+nrow(mwtmplo)-1),(i-1))=mwtmplo(:,(j-1)).
compute wheremw(((2*j)-3),(i-1))=start+1.
compute wheremw(((2*j)-2),(i-1))=start+nrow(mwtmplo)-1+1.
compute onebl=make(nrow(mwtmplo),1,1).
compute whigh((start+1):(start+nrow(mwtmplo)),whighct)=onebl.
compute start=start+nrow(mwtmplo).
end if.
end loop.
end if.
do if (zsum > 0).
loop j = 1 to (i-1).
compute zhighct=zhighct+1.
do if (j = 1 and zcmat(i,j)=1).
do if (zdid=0).
compute outv={outv,ztmp}.
do if (ncs > 0 and ziscov > 0).
compute ccmatoff((i-1),ziscov)=0.
end if.
compute modlabel={modlabel;zcatlab(1:nzvls,1)}.
compute zdid=1.
do if (ztmpuse=0).
compute fulldat={fulldat,ztmp}.
do if (ncs > 0 and ziscov > 0).
compute ccmatoff((i-1),ziscov)=0.
end if.
compute ztmpuse=1.
loop k4=datcount to (datcount+(nzvls-1)).
compute ztmploc={ztmploc;k4}.
end loop.
compute ztmploc=ztmploc(2:nrow(ztmploc),1).
compute datcount=datcount+nzvls.
end if.
end if.
compute datindx(start:(start+nrow(ztmploc)-1),(i-1))=ztmploc.
compute wherez(1,(i-1))=start+1.
compute wherez(2,(i-1))=start+nrow(ztmploc)-1+1.
compute start=start+nrow(ztmploc).
loop k1=1 to nxvls.
loop k2 = 1 to nzvls.
compute outv={outv,(xtmp(:,k1)&*ztmp(:,k2))}.
do if (ncs > 0 and ziscov > 0).
compute ccmatoff((i-1),ziscov)=0.
end if.
compute modlabel={modlabel;intlab(cntmp,1)}.
compute intkey={intkey;intlab(cntmp,1),':',xcatlab(k1,1),'x',zcatlab(k2,1),' ',' '}.
compute cntmp=cntmp+1.
end loop.
end loop.
do if (xztmpus=0).
compute fulldat={fulldat,outv(:,(ncol(outv)-(nxvls*nzvls)+1):ncol(outv))}.
do if (ncs > 0 and ziscov > 0).
compute ccmatoff((i-1),ziscov)=0.
end if.
compute xztmpus=1.
loop k4=datcount to (datcount+((nzvls*nxvls)-1)).
compute xztmplo={xztmplo;k4}.
end loop.
compute xztmplo=xztmplo(2:nrow(xztmplo),1).
compute datcount=datcount+(nxvls*nzvls).
end if.
compute datindx(start:(start+nrow(xztmplo)-1),(i-1))=xztmplo.
compute wherexz(1,(i-1))=start+1.
compute wherexz(2,(i-1))=start+nrow(xztmplo)-1+1.
compute onebl=make(nrow(xztmplo),1,1).
compute zhigh((start+1):(start+nrow(xztmplo)),zhighct)=onebl.
compute start=start+nrow(xztmplo).
end if.
do if (j > 1 and zcmat(i,j)=1).
do if (zdid=0).
compute outv={outv,ztmp}.
do if (ncs > 0 and ziscov > 0).
compute ccmatoff((i-1),ziscov)=0.
end if.
compute modlabel={modlabel;zcatlab(1:nzvls,1)}.
compute zdid=1.
do if (ztmpuse=0).
compute fulldat={fulldat,ztmp}.
do if (ncs > 0 and ziscov > 0).
compute ccmatoff((i-1),ziscov)=0.
end if.
compute ztmpuse=1.
loop k4=datcount to (datcount+(nzvls-1)).
compute ztmploc={ztmploc;k4}.
end loop.
compute ztmploc=ztmploc(2:nrow(ztmploc),1).
compute datcount=datcount+nzvls.
end if.
compute datindx(start:(start+nrow(ztmploc)-1),(i-1))=ztmploc.
compute wherez(1,(i-1))=start+1.
compute wherez(2,(i-1))=start+nrow(ztmploc)-1+1.
compute start=start+nrow(ztmploc).
end if.
loop k2 = 1 to nzvls.
compute outv={outv,(mtmp(:,(j-1))&*ztmp(:,k2))}.
do if (ncs > 0 and ziscov > 0).
compute ccmatoff((i-1),ziscov)=0.
end if.
compute modlabel={modlabel;intlab(cntmp,1)}.
compute intkey={intkey;intlab(cntmp,1),':', mnames(1,(j-1)),'x',zcatlab(k2,1),' ',' '}.
compute cntmp=cntmp+1.
end loop.
do if (mztmpus(1,(j-1))=0).
compute fulldat={fulldat,outv(:,(ncol(outv)-nzvls+1):ncol(outv))}.
do if (ncs > 0 and ziscov > 0).
compute ccmatoff((i-1),ziscov)=0.
end if.
compute mztmpus(1,(j-1))=1.
compute mz22=-999.
loop k4=datcount to (datcount+(nzvls-1)).
compute mz22={mz22;k4}.
end loop.
compute mztmplo(:,(j-1))=mz22(2:nrow(mz22),1).
compute datcount=datcount+nzvls.
end if.
compute datindx(start:(start+nrow(mztmplo)-1),(i-1))=mztmplo(:,(j-1)).
compute wheremz(((2*j)-3),(i-1))=start+1.
compute wheremz(((2*j)-2),(i-1))=start+nrow(mztmplo)-1+1.
compute onebl=make(nrow(mztmplo),1,1).
compute zhigh((start+1):(start+nrow(mztmplo)),zhighct)=onebl.
compute start=start+nrow(mztmplo).
end if.
end loop.
end if.
do if (wzsum > 0).
loop j = 1 to (i-1).
compute wzhighct=wzhighct+1.
do if (j = 1 and wzcmat(i,j)=1).
do if (wzdid=0).
loop k1=1 to nwvls.
loop k2 = 1 to nzvls.
compute outv={outv,(wtmp(:,k1)&*ztmp(:,k2))}.
do if (ncs > 0 and (ziscov > 0)).
compute ccmatoff((i-1),ziscov)=0.
end if.
do if (ncs > 0 and (wiscov > 0)).
compute ccmatoff((i-1),wiscov)=0.
end if.
compute modlabel={modlabel;intlab(cntmp,1)}.
compute intkey={intkey;intlab(cntmp,1),':',wcatlab(k1,1),'x',zcatlab(k2,1),' ',' '}.
compute cntmp=cntmp+1.
end loop.
end loop.
do if (wztmpus=0).
compute fulldat={fulldat,outv(:,(ncol(outv)-(nwvls*nzvls)+1):ncol(outv))}.
do if (ncs > 0 and (ziscov > 0)).
compute ccmatoff((i-1),ziscov)=0.
end if.
do if (ncs > 0 and (wiscov > 0)).
compute ccmatoff((i-1),wiscov)=0.
end if.
compute wztmpus=1.
loop k4=datcount to (datcount+((nwvls*nzvls)-1)).
compute wztmplo={wztmplo;k4}.
end loop.
compute wztmplo=wztmplo(2:nrow(wztmplo),1).
compute datcount=datcount+(nzvls*nwvls).
end if.
compute wzdid=1.
end if.
compute datindx(start:(start+nrow(wztmplo)-1),(i-1))=wztmplo.
compute wherewz(1,(i-1))=start+1.
compute wherewz(2,(i-1))=start+nrow(wztmplo)-1+1.
compute start=start+nrow(wztmplo).
loop k1=1 to nxvls.
loop k2=1 to nwvls.
loop k3=1 to nzvls.
compute outv={outv,(xtmp(:,k1)&*wtmp(:,k2)&*ztmp(:,k3))}.
do if (ncs > 0 and (ziscov > 0)).
compute ccmatoff((i-1),ziscov)=0.
end if.
do if (ncs > 0 and (wiscov > 0)).
compute ccmatoff((i-1),wiscov)=0.
end if.
compute modlabel={modlabel;intlab(cntmp,1)}.
compute intkey={intkey;intlab(cntmp,1),':',xcatlab(k1,1),'x',wcatlab(k2,1),'x', zcatlab(k3,1)}.
compute cntmp=cntmp+1.
end loop.
end loop.
end loop.
do if (xwztmpu=0).
compute fulldat={fulldat,outv(:,(ncol(outv)-(nxvls*nwvls*nzvls)+1):ncol(outv))}.
do if (ncs > 0 and (ziscov > 0)).
compute ccmatoff((i-1),ziscov)=0.
end if.
do if (ncs > 0 and (wiscov > 0)).
compute ccmatoff((i-1),wiscov)=0.
end if.
compute xwztmpu=1.
loop k4=datcount to (datcount+((nzvls*nxvls*nwvls)-1)).
compute xwztmplo={xwztmplo;k4}.
end loop.
compute xwztmplo=xwztmplo(2:nrow(xwztmplo),1).
compute datcount=datcount+(nxvls*nzvls*nwvls).
end if.
compute datindx(start:(start+nrow(xwztmplo)-1),(i-1))=xwztmplo.
compute wherexwz(1,(i-1))=start+1.
compute wherexwz(2,(i-1))=start+nrow(xwztmplo)-1+1.
compute onebl=make(nrow(xwztmplo),1,1).
compute wzhigh((start+1):(start+nrow(xwztmplo)),wzhighct)=onebl.
compute start=start+nrow(xwztmplo).
end if.
do if (j > 1 and wzcmat(i,j)=1).
do if (wzdid=0).
loop k1=1 to nwvls.
loop k2 = 1 to nzvls.
compute outv={outv,(wtmp(:,k1)&*ztmp(:,k2))}.
do if (ncs > 0 and (ziscov > 0)).
compute ccmatoff((i-1),ziscov)=0.
end if.
do if (ncs > 0 and (wiscov > 0)).
compute ccmatoff((i-1),wiscov)=0.
end if.
compute modlabel={modlabel;intlab(cntmp,1)}.
compute intkey={intkey;intlab(cntmp,1),':',wcatlab(k1,1),'x',zcatlab(k2,1),' ',' '}.
compute cntmp=cntmp+1.
end loop.
end loop.
do if (wztmpus=0).
compute fulldat={fulldat,outv(:,(ncol(outv)-(nwvls*nzvls)+1):ncol(outv))}.
do if (ncs > 0 and (ziscov > 0)).
compute ccmatoff((i-1),ziscov)=0.
end if.
do if (ncs > 0 and (wiscov > 0)).
compute ccmatoff((i-1),wiscov)=0.
end if.
compute wztmpus=1.
loop k4=datcount to (datcount+((nwvls*nzvls)-1)).
compute wztmplo={wztmplo;k4}.
end loop.
compute wztmplo=wztmplo(2:nrow(wztmplo),1).
compute datcount=datcount+(nzvls*nwvls).
end if.
compute wzdid=1.
compute datindx(start:(start+nrow(wztmplo)-1),(i-1))=wztmplo.
compute wherewz(1,(i-1))=start+1.
compute wherewz(2,(i-1))=start+nrow(wztmplo)-1+1.
compute start=start+nrow(wztmplo).
end if.
loop k1 = 1 to nwvls.
loop k2 = 1 to nzvls.
compute outv={outv,(mtmp(:,(j-1))&*wtmp(:,k1)&*ztmp(:,k2))}.
do if (ncs > 0 and (ziscov > 0)).
compute ccmatoff((i-1),ziscov)=0.
end if.
do if (ncs > 0 and (wiscov > 0)).
compute ccmatoff((i-1),wiscov)=0.
end if.
compute modlabel={modlabel;intlab(cntmp,1)}.
compute intkey={intkey;intlab(cntmp,1),':',mnames(1,(j-1)),'x',wcatlab(k1,1),'x', zcatlab(k2,1)}.
compute cntmp=cntmp+1.
end loop.
end loop.
do if (mwztmpu(1,(j-1))=0).
compute fulldat={fulldat,outv(:,(ncol(outv)-(nwvls*nzvls)+1):ncol(outv))}.
do if (ncs > 0 and (ziscov > 0)).
compute ccmatoff((i-1),ziscov)=0.
end if.
do if (ncs > 0 and (wiscov > 0)).
compute ccmatoff((i-1),wiscov)=0.
end if.
compute mwztmpu(1,(j-1))=1.
compute mz22=-999.
loop k4=datcount to (datcount+(nwvls*nzvls)-1).
compute mz22={mz22;k4}.
end loop.
compute mwztmplo(:,(j-1))=mz22(2:nrow(mz22),1).
compute datcount=datcount+(nwvls*nzvls).
end if.
compute datindx(start:(start+nrow(mwztmplo)-1),(i-1))=mwztmplo(:,(j-1)).
compute wheremwz(((2*j)-3),(i-1))=start+1.
compute wheremwz(((2*j)-2),(i-1))=start+nrow(mwztmplo)-1+1.
compute onebl=make(nrow(mwztmplo),1,1).
compute wzhigh((start+1):(start+nrow(mwztmplo)),wzhighct)=onebl.
compute start=start+nrow(mwztmplo).
end if.
end loop.
end if.
do if (ncs > 0).
compute ccmat=ccmat&*ccmatoff.
loop j = 1 to ncs.
do if (ccmat((i-1),j))=1.
do if (j=wiscov).
compute ctmp(:,j)=wtmp.
end if.
do if (j=ziscov).
compute ctmp(:,j)=ztmp.
end if.
compute outv={outv,ctmp(:,j)}.
compute modlabel={modlabel;covnames(1,j)}.
do if (ctmpuse(1,j)=0).
compute fulldat={fulldat,ctmp(:,j)}.
compute ctmpuse(1,j)=1.
compute ctmploc(1,j)=datcount.
compute datcount=datcount+1.
end if.
compute datindx(start:(start+nrow(ctmploc)-1),(i-1))=ctmploc(1,j).
compute start=start+nrow(ctmploc(1,j)).
end if.
end loop.
end if.
compute wdid=0.
compute zdid=0.
compute wzdid=0.
compute vlabs={vlabs;modlabel(2:nrow(modlabel),1)}.
compute numint(1,(i-1))=cntmp-1.
compute nump(1,(i-1))=nrow(modlabel)-1.
end loop.
release datcount, xtmpuse, wtmpuse, ztmpuse, xwtmpus, xztmpus, wztmpus, xwztmpu.
release xtmploc, wtmploc, xwtmplo, ztmploc, xztmplo, wztmplo, xwztmplo, foccnt.
do if (modcok=1 and ((nms > 0) or (zcmat(2,1) <> 1) or (mcx <> 0))).
compute notecode(notes,1) = 19.
compute notes = notes + 1.
compute modcok=0.
end if.
do if ((serial = 1 or (rsum(numint)>0) or nms=0) and mc > 0).
compute notecode(notes,1) = 15.
compute notes = notes + 1.
compute boot=mc.
compute mc=0.
end if.
do if (boot <> 0 or mc <> 0).
compute bootsz=boot.
do if (mc > 0).
compute bootsz=mc.
compute saveboot=0.
end if.
loop.
compute cilow = rnd(bootsz*(1-(conf/100))/2).
compute cihigh = trunc((bootsz*(conf/100)+(bootsz*(1-(conf/100))/2)))+1.
do if (cilow < 1 or cihigh > bootsz).
compute bootsz=trunc((bootsz+1000)/1000)*1000.
compute adjust = 1.
end if.
end loop if (cilow gt 0 and cihigh le bootsz).
do if (boot > 0).
compute boot=bootsz.
end if.
do if (mc > 0).
compute mc=bootsz.
end if.
do if (adjust = 1 and boot > 0).
compute notecode(notes,1) = 8.
compute notes = notes + 1.
end if.
do if (adjust = 1 and mc > 0).
compute notecode(notes,1) = 16.
compute notes = notes + 1.
end if.
end if.
compute maxboot = trunc(2*boot).
do if ( 0 > maxboot).
compute maxboot=trunc( 0 ).
end if.
do if (nms > 0).
release mtmpuse, mwtmpus, mwztmpu, mtmploc, mwtmplo, mztmplo, mwztmplo.
end if.
release wdid, zdid, wzdid, start,modlabel.
compute vlabs=vlabs(2:nrow(vlabs),1).
do if (rsum(numint) > 0).
compute intkey=intkey(2:nrow(intkey),:).
end if.
compute fulldat=fulldat(:,2:ncol(fulldat)).
compute fochigh=fochigh(1:rmax(nump),:).
compute whigh=whigh(1:rmax(nump),:).
compute zhigh=zhigh(1:rmax(nump),:).
compute wzhigh=wzhigh(1:rmax(nump),:).
compute coeffs=fochigh+whigh+zhigh+wzhigh.
compute bootloc=make(rmax(nump),ncol(nump),0).
do if (nms > 0).
compute cntmp=1.
loop i = 1 to ncol(nump).
loop j = 1 to nump(1,i).
compute bootloc(j,i)=cntmp.
compute cntmp=cntmp+1.
end loop.
end loop.
compute fochighb=make(nrow(fochigh),ncol(fochigh),0).
compute whighb=fochighb.
compute zhighb=fochighb.
compute wzhighb=fochighb.
compute thetaxmb=make(nrow(fochighb),nms,0).
compute thetaxyb=make(nrow(fochighb),1,0).
compute pathsfoc=make(nxvls,1,0).
compute cntmp=1.
loop i = 1 to (nms+nys).
loop j = 1 to i.
compute fochighb(:,cntmp)=fochigh(:,cntmp)&*bootloc(:,i).
compute whighb(:,cntmp)=whigh(:,cntmp)&*bootloc(:,i).
compute zhighb(:,cntmp)=zhigh(:,cntmp)&*bootloc(:,i).
compute wzhighb(:,cntmp)=wzhigh(:,cntmp)&*bootloc(:,i).
compute coeffsb=fochighb+whighb+zhighb+wzhighb.
do if ((i < (nms+nys)) and (j = 1)).
compute thetaxmb(:,i)=coeffsb(:,cntmp).
end if.
do if ((i = (nms+nys)) and (j = 1)).
compute thetaxyb(:,1)=coeffsb(:,cntmp).
end if.
compute cntmp=cntmp+1.
end loop.
end loop.
compute thetamyb=coeffsb(:,(ncol(coeffsb)-nms+1):ncol(coeffsb)).
do if (serial = 1).
compute thetammb=make(nrow(coeffsb),((nms*(nms-1))/2),0).
end if.
compute cntmp=1.
do if (nms > 1 and serial = 1).
loop i = 1 to (nms-1).
compute start=((i+2)*(i+1))/2.
loop j = 2 to (nms-i+1).
compute thetammb(:,cntmp)=coeffsb(:,start).
compute start=start+j+i-1.
compute cntmp=cntmp+1.
end loop.
end loop.
end if.
end if.
do if ((total = 1) and (rsum(numint)=0 or (xmint=1))).
compute dototal=1.
do if ((csum(bcmat(:,1)) <> (nms+nys)) or (rsum(bcmat(nrow(bcmat),:)) <> (nms+nys))).
compute dototal=0.
compute alttotal=1.
compute notecode(notes,1) = 12.
compute notes = notes + 1.
end if.
do if (ncs > 0).
do if ((csum(rsum(ccmat))) < (nrow(ccmat)*ncol(ccmat))).
compute dototal=0.
compute alttotal=1.
compute notecode(notes,1) = 11.
compute notes = notes + 1.
end if.
end if.
do if (model=74).
do if (xdich=0 and nxvls=1).
compute dototal=0.
compute alttotal=1.
end if.
do if ((xdich=1 or nxvls > 1) and (ncs > 0) and model=74).
compute dototal=0.
compute alttotal=1.
end if.
end if.
end if.
end if.
do if (criterr=0 and ncs > 0).
do if (rsum((csum(ccmat)=0)) <> 0).
compute errcode(errs,1)=51.
compute errs=errs+1.
compute criterr=1.
end if.
end if.
compute debug= 0.
do if (outscree=1).
print/title = '***************** PROCESS Procedure for SPSS Version 4.2 *****************'.
print/title = '          Written by Andrew F. Hayes, Ph.D.       www.afhayes.com'.
print/title = '    Documentation available in Hayes (2022). www.guilford.com/p/hayes3'/space=0.
end if.
do if (criterr=0).
compute modresid=make(n,1,99999).
do if (stand=1 and ydich=1).
compute stand=0.
end if.
compute anymod2=csum(rsum(wcmat+zcmat+wzcmat)).
do if (anymod2 > 0 and stand = 1).
compute notecode(notes,1) = 27.
compute notes = notes + 1.
compute stand=0.
end if.
compute funny=1.
do if (outscree=1).
print modelvar/title = '**************************************************************************'/format = A8/rnames=modelvlb.
do if (ncs > 0).
print covnames/title='Covariates:'/format=A8.
end if.
print n/title='Sample'/rlabel='Size:'.
do if (( 'random' <> 'random')).
compute seedt= 'random'.
print seedt/title='Custom'/format=A12/rlabel = 'Seed:'.
end if.
end if.
compute maxresm=9.
compute resultm=make(1,maxresm,99999).
do if (describe=1).
compute means=csum(dat)/n.
compute sigmatal = (t(dat)*(ident(n)-(1/n)*ones*t(ones))*dat)*(1/(n-1)).
compute sdvec=sqrt(diag(sigmatal)).
compute sdall = mdiag(1/sdvec).
compute corall=sdall*sigmatal*t(sdall).
compute means={means;t(sdvec)}.
do if (ncol(means) > 9).
compute resultm=make(1,ncol(means),99999).
compute maxresm=ncol(means).
end if.
compute resultm2=make(2,maxresm,99999).
compute resultm2(1:2,1:ncol(means))=means.
compute resultm={resultm;resultm2}.
compute resultm2=make(ncol(corall),maxresm,99999).
compute resultm2(1:ncol(corall),1:ncol(corall))=corall.
compute resultm={resultm;resultm2}.
do if (outscree=1).
print means/title='Variable means and standard deviations'/cnames=varnames/rlabels='Mean','SD'/format= F10.4.
print corall/title='Variable intercorrelations (Pearson r)'/cnames=varnames/rnames=varnames/format= F10.4.
end if.
end if.
do if (outscree=1).
do if (mcxok=1).
compute labtmp={xnames,t(xcatlab(1:nxvls,1))}.
print dummatx/title = 'Coding of categorical X variable for analysis:'/cnames = labtmp/format = F6.3.
end if.
do if (mcwok=1 and xmint=0).
compute labtmp={wnames,t(wcatlab(1:nwvls,1))}.
print dummatw/title = 'Coding of categorical W variable for analysis:'/cnames = labtmp/format = F6.3.
end if.
do if (mczok=1).
compute labtmp={znames,t(zcatlab(1:nzvls,1))}.
print dummatz/title = 'Coding of categorical Z variable for analysis:'/cnames = labtmp/format = F6.3.
end if.
end if.
end if.
do if (criterr = 0).
compute outnames=ynames.
compute outvars=ytmp.
do if (nms > 0).
compute outnames={mnames,ynames}.
compute outvars={mtmp,ytmp}.
compute indcov=make(((nms*2)+(nms*(nxvls-1))),((nms*2)+(nms*(nxvls-1))),0).
compute mcsopath=make(((nms*2)+(nms*(nxvls-1))),1,0).
end if.
compute labstart=1.
compute intstart=1.
compute start=1.
compute coeffmat=make(1,6,0).
compute conseq={'        '}.
compute dfmat=0.
compute coeffcol=0.
compute pathscnt=1.
compute pathscn2=1.
loop i = 1 to (nms+nys).
do if (outscree=1).
print/title = '**************************************************************************'.
end if.
compute highf=make(1,5,0).
compute highf2=highf.
do if ((i = (nms+nys)) and (ydich=1)).
compute highf=make(1,3,0).
compute highf2=highf.
end if.
compute flabel={' '}.
compute y=outvars(:,i).
compute xindx=datindx(1:(nump(1,i)-1),i).
compute x = fulldat(:,xindx).
compute x={ones,x}.
compute xsq=t(x)*x.
compute exsq=eval(xsq).
release xsq.
compute zeroeig=csum(exsq <= 0.000000000002).
do if (outscree=1).
print outnames(1,i)/title = 'OUTCOME VARIABLE:'/format = A8/space=0.
do if (ydich=1 and (i = (nms+nys))).
compute nmsd = {outnames(1,i), 'Analysis'}.
print rcd/title = 'Coding of binary Y for logistic regression analysis:'/cnames = nmsd/format = F9.2.
end if.
end if.
do if (zeroeig > 0).
print / title = 'SINGULAR OR NEAR SINGULAR DATA MATRIX.'.
compute criterr=1.
compute errcode(errs,1)=31.
compute errs=errs+1.
end if.
compute means=csum(x)/n.
compute vlabsm=vlabs(labstart:(labstart+(nump(1,i)-1)),1).
do if (criterr=0).
do if (ydich=0 or (i < (nms+nys))).

.
do if ( 1 =1).
compute hatmat=inv(t( x )* x )*t( x ).
compute b = hatmat* y.
compute modres=b.
do if ( 1 =1).
compute n1=nrow( x ).
compute dfres=n1-(ncol( x )).
compute sstotal = t( y -(csum( y )/n1))*( y -(csum( y )/n1)).
compute resid= y - x *b.
compute ssresid = csum((resid)&**2).
compute r2 = (sstotal-ssresid)/sstotal.
do if (r2 < 0).
compute r2=0.
end if.
compute adjr2 = 1-((1-r2)*(n1-1)/(dfres)).
compute mse=ssresid/(n1-ncol( x )).

.
compute n1=nrow( x ).
compute invXtX = inv(t( x )* x ).
compute varb = mse *invXtX.
compute k3 = ncol( x ).
compute xhc=0.
do if ( hc <> 5).
compute xhc= x.
compute hat = xhc(:,1).
loop i3=1 to nrow(xhc).
compute hat(i3,1)= xhc(i3,:)*invXtX*t(xhc(i3,:)).
end loop.
do if ( hc = 0 or hc =1).
loop i3 = 1 to k3.
compute xhc(:,i3)=xhc(:,i3)&* resid.
end loop.
end if.
do if ( hc =3 or hc =2).
loop i3=1 to k3.
compute xhc(:,i3) = ( resid &/(1-hat)&**(1/(4- hc )))&*xhc(:,i3).
end loop.
end if.
do if ( hc = 4).
compute hcmn=make(n,2,4).
compute hcmn(:,2)=(n1*hat)/k3.
loop i3= 1 to k3.
compute xhc(:,i3) = ( resid &/(1-hat)&**(rmin(hcmn)/2))&*xhc(:,i3).
end loop.
end if.
compute varb=(invXtX*t(xhc)*xhc*invXtX).
do if ( hc =1).
compute varb=(n1/(n1-ncol( x )))&*varb.
end if.
end if.
compute hclab={'se(HC0)','se(HC1)','se(HC2)','se(HC3)','se(HC4)','se'}.
compute hclab=hclab(1,( hc +1)).
compute hcflab={'F(HC0)','F(HC1)','F(HC2)','F(HC3)','F(HC4)','F'}.
compute hcflab=hcflab(1,( hc +1)).
release xhc
.
compute seb=sqrt(diag(varb)).
compute trat = b&/seb.
compute p = 2*(1-tcdf(abs(trat), (dfres))).
compute tval = sqrt(dfres* (exp((dfres-(5/6))*((xp2/(dfres-(2/3)+(.11/dfres)))* (xp2/(dfres-(2/3)+(.11/dfres)))))-1)).
compute modres={modres,seb,trat,p}.
compute modres={modres,(b-tval&*seb),(b+tval&*seb)}.
compute modresl={'coeff',hclab,'t','p','LLCI','ULCI'}.
compute lmat = ident(ncol( x )).
compute lmat = lmat(:,2:ncol(lmat)).
compute fratio = (t(t(lmat)*b)*inv(t(lmat)*varb*lmat)*((t(lmat)*b)))/(ncol( x )-1).
compute pfr = 1-fcdf(fratio,(ncol( x )-1),dfres).
compute modsum={sqrt(r2),r2,mse,fratio,(ncol( x )-1),dfres,pfr}.
compute modsuml={'R','R-sq','MSE',hcflab,'df1','df2', 'p'}.
end if.
end if.
do if ( 1 = 2 or 1 =3).
compute xlp= x.
compute ylp= y.
compute pt2 = make(nrow(ylp),1,(csum(ylp)/nrow(ylp))).
do if ( 1 =2).
compute LL3 = ylp&*ln(pt2)+(1-ylp)&*ln(1-pt2).
end if.
compute LL3 = -2*csum(LL3).
compute bt1 = make(ncol(xlp),1,0).
compute LL1 = 0.
compute pt1 = make(nrow(ylp),1,0.5).
compute pt1lp=pt1.
loop jjj = 1 to iterate.
compute xlptmp=t(xlp).
compute vecprb=pt1lp&*(1-pt1lp).
loop kkk=1 to ncol(xlp).
compute xlptmp(kkk,:)=xlptmp(kkk,:)&*t(vecprb).
end loop.
compute b = bt1+inv(xlptmp*xlp)*t(xlp)*(ylp-pt1lp).
do if ( 1 =2).
compute xlpb=xlp*b.
compute xlpbt=(xlpb > -709.7).
compute xlpb709=(1-xlpbt)*(-709.7).
compute xlpb=(xlpb&*xlpbt)+xlpb709.
compute pt1lp = 1/(1+exp(-(xlpb))).
end if.
compute itprob = csum((pt1lp < .00000001) or (pt1lp > .9999999)).
do if (itprob > 0).
loop kkk = 1 to nrow(pt1lp).
do if (pt1lp(kkk,1) > .9999999).
compute pt1lp(kkk,1) = .9999999.
end if.
do if (pt1lp(kkk,1) < .00000001).
compute pt1lp(kkk,1) = .00000001.
end if.
end loop.
compute itprob = 0.
end if.
do if (itprob = 0).
do if ( 1 =2).
compute LL = ylp&*ln(pt1lp)+(1-ylp)&*ln(1-pt1lp).
end if.
compute LL2 = -2*csum(ll).
end if.
do if (abs(LL1-LL2) < converge).
do if ( 1 =1).
compute xlptmp=t(xlp).
compute vecprb=pt1lp&*(1-pt1lp).
loop kkk=1 to ncol(xlp).
compute xlptmp(kkk,:)=xlptmp(kkk,:)&*t(vecprb).
end loop.
compute varb = inv(xlptmp*xlp).
compute seb = sqrt(diag(varb)).
release xlptmp.
end if.
break.
end if.
compute bt1 = b.
compute LL1 = LL2.
end loop.
compute modres=b.
do if (jjj > iterate).
compute itprob = 2.
do if (booting=0).
compute iterrmod=1.
end if.
do if (booting=1).
compute bootiter=1.
end if.
do if (itprobtg=0).
compute itprobtg=1.
compute errcode(errs,1) = 47.
compute errs = errs + 1.
do if (booting = 0 and 1 =1).
compute vt1 = mdiag(pt1lp&*(1-pt1lp)).
compute varb = inv(t(xlp)*vt1*xlp).
compute seb = sqrt(diag(varb)).
end if.
end if.
end if.
do if ( 1 =1).
compute trat = b&/seb.
compute dfres=nrow(xlp).
compute p = 2*(1-cdfnorm(abs(trat))).
compute modres={modres,seb,trat,p}.
compute modres={modres,(b-xp2&*seb),(b+xp2&*seb)}.
compute pvchi=1-chicdf((LL3-LL2),(nrow(modres)-1)).
compute mcF = (LL3-LL2)/LL3.
compute cox = 1-exp(-(LL3-LL2)/nrow(xlp)).
compute nagel = cox/(1-exp(-(LL3)/nrow(xlp))).
compute modsum={LL2,(LL3-LL2),(nrow(modres)-1),pvchi, mcF,cox,nagel}.
compute modsuml={'-2LL','ModelLL', 'df', 'p', 'McFadden', 'CoxSnell', 'Nagelkrk'}.
compute modresl={'coeff','se','Z','p','LLCI','ULCI'}.
end if.
end if
.
compute dfmatt=make(nrow(modres),1,modsum(1,6)).
compute modresid={modresid,resid}.
end if.
do if (ydich=1 and (i = (nms+nys))).

.
do if ( 2 =1).
compute hatmat=inv(t( x )* x )*t( x ).
compute b = hatmat* y.
compute modres=b.
do if ( 1 =1).
compute n1=nrow( x ).
compute dfres=n1-(ncol( x )).
compute sstotal = t( y -(csum( y )/n1))*( y -(csum( y )/n1)).
compute resid= y - x *b.
compute ssresid = csum((resid)&**2).
compute r2 = (sstotal-ssresid)/sstotal.
do if (r2 < 0).
compute r2=0.
end if.
compute adjr2 = 1-((1-r2)*(n1-1)/(dfres)).
compute mse=ssresid/(n1-ncol( x )).

.
compute n1=nrow( x ).
compute invXtX = inv(t( x )* x ).
compute varb = mse *invXtX.
compute k3 = ncol( x ).
compute xhc=0.
do if ( hc <> 5).
compute xhc= x.
compute hat = xhc(:,1).
loop i3=1 to nrow(xhc).
compute hat(i3,1)= xhc(i3,:)*invXtX*t(xhc(i3,:)).
end loop.
do if ( hc = 0 or hc =1).
loop i3 = 1 to k3.
compute xhc(:,i3)=xhc(:,i3)&* resid.
end loop.
end if.
do if ( hc =3 or hc =2).
loop i3=1 to k3.
compute xhc(:,i3) = ( resid &/(1-hat)&**(1/(4- hc )))&*xhc(:,i3).
end loop.
end if.
do if ( hc = 4).
compute hcmn=make(n,2,4).
compute hcmn(:,2)=(n1*hat)/k3.
loop i3= 1 to k3.
compute xhc(:,i3) = ( resid &/(1-hat)&**(rmin(hcmn)/2))&*xhc(:,i3).
end loop.
end if.
compute varb=(invXtX*t(xhc)*xhc*invXtX).
do if ( hc =1).
compute varb=(n1/(n1-ncol( x )))&*varb.
end if.
end if.
compute hclab={'se(HC0)','se(HC1)','se(HC2)','se(HC3)','se(HC4)','se'}.
compute hclab=hclab(1,( hc +1)).
compute hcflab={'F(HC0)','F(HC1)','F(HC2)','F(HC3)','F(HC4)','F'}.
compute hcflab=hcflab(1,( hc +1)).
release xhc
.
compute seb=sqrt(diag(varb)).
compute trat = b&/seb.
compute p = 2*(1-tcdf(abs(trat), (dfres))).
compute tval = sqrt(dfres* (exp((dfres-(5/6))*((xp2/(dfres-(2/3)+(.11/dfres)))* (xp2/(dfres-(2/3)+(.11/dfres)))))-1)).
compute modres={modres,seb,trat,p}.
compute modres={modres,(b-tval&*seb),(b+tval&*seb)}.
compute modresl={'coeff',hclab,'t','p','LLCI','ULCI'}.
compute lmat = ident(ncol( x )).
compute lmat = lmat(:,2:ncol(lmat)).
compute fratio = (t(t(lmat)*b)*inv(t(lmat)*varb*lmat)*((t(lmat)*b)))/(ncol( x )-1).
compute pfr = 1-fcdf(fratio,(ncol( x )-1),dfres).
compute modsum={sqrt(r2),r2,mse,fratio,(ncol( x )-1),dfres,pfr}.
compute modsuml={'R','R-sq','MSE',hcflab,'df1','df2', 'p'}.
end if.
end if.
do if ( 2 = 2 or 2 =3).
compute xlp= x.
compute ylp= y.
compute pt2 = make(nrow(ylp),1,(csum(ylp)/nrow(ylp))).
do if ( 2 =2).
compute LL3 = ylp&*ln(pt2)+(1-ylp)&*ln(1-pt2).
end if.
compute LL3 = -2*csum(LL3).
compute bt1 = make(ncol(xlp),1,0).
compute LL1 = 0.
compute pt1 = make(nrow(ylp),1,0.5).
compute pt1lp=pt1.
loop jjj = 1 to iterate.
compute xlptmp=t(xlp).
compute vecprb=pt1lp&*(1-pt1lp).
loop kkk=1 to ncol(xlp).
compute xlptmp(kkk,:)=xlptmp(kkk,:)&*t(vecprb).
end loop.
compute b = bt1+inv(xlptmp*xlp)*t(xlp)*(ylp-pt1lp).
do if ( 2 =2).
compute xlpb=xlp*b.
compute xlpbt=(xlpb > -709.7).
compute xlpb709=(1-xlpbt)*(-709.7).
compute xlpb=(xlpb&*xlpbt)+xlpb709.
compute pt1lp = 1/(1+exp(-(xlpb))).
end if.
compute itprob = csum((pt1lp < .00000001) or (pt1lp > .9999999)).
do if (itprob > 0).
loop kkk = 1 to nrow(pt1lp).
do if (pt1lp(kkk,1) > .9999999).
compute pt1lp(kkk,1) = .9999999.
end if.
do if (pt1lp(kkk,1) < .00000001).
compute pt1lp(kkk,1) = .00000001.
end if.
end loop.
compute itprob = 0.
end if.
do if (itprob = 0).
do if ( 2 =2).
compute LL = ylp&*ln(pt1lp)+(1-ylp)&*ln(1-pt1lp).
end if.
compute LL2 = -2*csum(ll).
end if.
do if (abs(LL1-LL2) < converge).
do if ( 1 =1).
compute xlptmp=t(xlp).
compute vecprb=pt1lp&*(1-pt1lp).
loop kkk=1 to ncol(xlp).
compute xlptmp(kkk,:)=xlptmp(kkk,:)&*t(vecprb).
end loop.
compute varb = inv(xlptmp*xlp).
compute seb = sqrt(diag(varb)).
release xlptmp.
end if.
break.
end if.
compute bt1 = b.
compute LL1 = LL2.
end loop.
compute modres=b.
do if (jjj > iterate).
compute itprob = 2.
do if (booting=0).
compute iterrmod=1.
end if.
do if (booting=1).
compute bootiter=1.
end if.
do if (itprobtg=0).
compute itprobtg=1.
compute errcode(errs,1) = 47.
compute errs = errs + 1.
do if (booting = 0 and 1 =1).
compute vt1 = mdiag(pt1lp&*(1-pt1lp)).
compute varb = inv(t(xlp)*vt1*xlp).
compute seb = sqrt(diag(varb)).
end if.
end if.
end if.
do if ( 1 =1).
compute trat = b&/seb.
compute dfres=nrow(xlp).
compute p = 2*(1-cdfnorm(abs(trat))).
compute modres={modres,seb,trat,p}.
compute modres={modres,(b-xp2&*seb),(b+xp2&*seb)}.
compute pvchi=1-chicdf((LL3-LL2),(nrow(modres)-1)).
compute mcF = (LL3-LL2)/LL3.
compute cox = 1-exp(-(LL3-LL2)/nrow(xlp)).
compute nagel = cox/(1-exp(-(LL3)/nrow(xlp))).
compute modsum={LL2,(LL3-LL2),(nrow(modres)-1),pvchi, mcF,cox,nagel}.
compute modsuml={'-2LL','ModelLL', 'df', 'p', 'McFadden', 'CoxSnell', 'Nagelkrk'}.
compute modresl={'coeff','se','Z','p','LLCI','ULCI'}.
end if.
end if
.
compute dfmatt=make(nrow(modres),1,-999).
end if.
compute resultm2=make(1,maxresm,99999).
compute resultm2(1,1:ncol(modsum))=modsum.
compute resultm={resultm;resultm2}.
compute resultm2=make(nrow(modres),maxresm,99999).
compute resultm2(1:nrow(modres),1:ncol(modres))=modres.
compute resultm={resultm;resultm2}.
compute obscoeff={obscoeff,t(b)}.
do if (outscree=1).
print modsum/title = 'Model Summary'/cnames = modsuml/format= F10.4.
print modres/title='Model'/rnames=vlabsm/cnames=modresl/format= F10.4.
end if.
compute basemod=modsum(1,1).
compute basemodx=basemod.
do if (ydich=1 and (i = (nms+nys))).
do if (outscree=1).
print/title='These results are expressed in a log-odds metric.'.
end if.
compute notecode(notes,1) = 26.
compute notes = notes + 1.
end if.
compute coeffmat={coeffmat;modres}.
compute conseqt=make(nrow(modres),1,outnames(1,i)).
compute conseq={conseq;conseqt}.
compute dfmat={dfmat;dfmatt}.
compute labstart=labstart+nump(1,i).
do if (stand=1).
compute predsd=make(nrow(modres),1,0).
compute stdmod=modres(:,1)&/ovsd(1,i).
loop jd=1 to ncol(x).
compute descdat=x(:,jd).
compute predsd(jd,1) = (nrow(descdat)*sscp(descdat))-(t(csum(descdat))*(csum(descdat))).
compute predsd(jd,1) = sqrt(predsd(jd,1)/(nrow(descdat)*(nrow(descdat)-1))).
end loop.
do if (wherex(1,i) <> -999 and ((nxvls > 1) or (xdich=1))).
compute sdmsone=make(nxvls,1,1).
compute predsd(wherex(1,i):wherex(2,i),1)=sdmsone.
compute pstog=1.
end if.
compute predsd(1,1)=1.
compute stdmod=stdmod&*predsd.
compute stdmod=stdmod(2:nrow(stdmod),1).
compute sdvlabs=vlabsm(2:nrow(vlabsm),1).
compute resultm2=make(nrow(stdmod),maxresm,99999).
compute resultm2(1:nrow(stdmod),1:ncol(stdmod))=stdmod.
compute resultm={resultm;resultm2}.
do if (outscree=1).
print stdmod/title='Standardized coefficients'/clabels='coeff'/rnames=sdvlabs/format= F10.4.
end if.
end if.
do if (nms > 0 and serial = 0 and (rsum(numint) = 0) and (normal=1 or mc > 0)).
do if (i < (nms+nys)).
compute indcov((((i-1)*nxvls)+1):(i*nxvls),(((i-1)*nxvls)+1):(i*nxvls))=varb(2:(1+nxvls), 2:(1+nxvls)).
compute mcsopath((((i-1)*nxvls)+1):(i*nxvls) ,1)=modres(2:(1+nxvls),1).
end if.
do if (i = (nms+nys)).
compute atm=ncol(wherem).
compute indcov(((nms*nxvls)+1):nrow(mcsopath),((nms*nxvls)+1):nrow(mcsopath))= varb(wherem(1,atm):(wherem(1,atm)+nms-1),wherem(1,atm):(wherem(1,atm)+nms-1)).
compute mcsopath(((nms*nxvls)+1):nrow(mcsopath),1)=modres(wherem(1,atm):(wherem(1,atm)+ nms-1),1).
compute sobelok=1.
end if.
end if.
compute obsdirfx=make(1,nxvls,0).
compute dirzes=make(1,nxvls,0).
do if ((i = (nms+nys)) and (bcmat(nrow(bcmat),1)=1)).
compute direff=modres(2:(1+nxvls),:).
compute obsdirfx=t(direff(:,1)).
compute direfflb=modresl.
compute direffl2=vlabsm(2:(1+nxvls),:).
compute lmat=make(nrow(b),1,0).
compute lmat2=make(nxvls,1,1).
compute lmat(2:(1+nxvls),1)=lmat2.
do if (ydich <> 1).

.
compute lmat2= lmat.
do if ( 0 =0).
compute lmat2 = mdiag( lmat ).
compute lmat3=make(nrow(lmat2),1,0).
loop flp=1 to ncol(lmat2).
do if (csum(lmat2(:,flp))=1).
compute lmat3={lmat3,lmat2(:,flp)}.
end if.
end loop.
compute lmat2=lmat3(:,2:ncol(lmat3)).
end if.
compute fratio = (t(t(lmat2)* b )*inv(t(lmat2)* varb *lmat2)*((t(lmat2)* b )))/ncol(lmat2).
compute pfr = 1-fcdf(fratio,ncol(lmat2),(n-nrow( b ))).
compute fresult={fratio,ncol(lmat2),(n-nrow( b )),pfr}.
do if (i = (nms+nys) and (ydich=1)).
compute fratio=fratio*ncol(lmat2).
compute pfr=1-chicdf(fratio,ncol(lmat2)).
compute fresult={fratio,ncol(lmat2),pfr}.
end if.
do if ( 1 =1).
compute lmat3=1-rsum(lmat2).
compute xfm=make(n,csum(lmat3),0).
compute flpc=1.
loop flp=1 to nrow(lmat3).
do if (lmat3(flp,1)=1).
compute xfm(:,flpc)=x(:,flp).
compute flpc=flpc+1.
end if.
end loop.
compute bfm=inv(t(xfm)*xfm)*t(xfm)*y.
compute resid=y-(xfm*bfm).
compute sstotal=(y-(csum(y)/n)).
compute sstotal=csum(sstotal&*sstotal).
compute ssresid=csum(resid&*resid).
compute rsqch= r2 -((sstotal-ssresid)/sstotal).
compute fresult={rsqch,fresult}.
release xfm,flpc, resid, ssresid, bfm.
end if
.
compute diromni=fresult.
end if.
do if (ydich = 1 ).

.
compute btemphld=b.
compute llrdat=make(nrow(x),nrow( lmat )-csum( lmat ),-999).
compute llrdf=ncol(x)-ncol(llrdat).
compute llrcnt=0.
loop llri=1 to nrow( lmat ).
do if ( lmat (llri,1)=0).
compute llrcnt=llrcnt+1.
compute llrdat(:,llrcnt)=x(:,llri).
end if.
end loop.

.
do if ( 2 =1).
compute hatmat=inv(t( llrdat )* llrdat )*t( llrdat ).
compute b = hatmat* y.
compute modres=b.
do if ( 0 =1).
compute n1=nrow( llrdat ).
compute dfres=n1-(ncol( llrdat )).
compute sstotal = t( y -(csum( y )/n1))*( y -(csum( y )/n1)).
compute resid= y - llrdat *b.
compute ssresid = csum((resid)&**2).
compute r2 = (sstotal-ssresid)/sstotal.
do if (r2 < 0).
compute r2=0.
end if.
compute adjr2 = 1-((1-r2)*(n1-1)/(dfres)).
compute mse=ssresid/(n1-ncol( llrdat )).

.
compute n1=nrow( x ).
compute invXtX = inv(t( x )* x ).
compute varb = mse *invXtX.
compute k3 = ncol( x ).
compute xhc=0.
do if ( hc <> 5).
compute xhc= x.
compute hat = xhc(:,1).
loop i3=1 to nrow(xhc).
compute hat(i3,1)= xhc(i3,:)*invXtX*t(xhc(i3,:)).
end loop.
do if ( hc = 0 or hc =1).
loop i3 = 1 to k3.
compute xhc(:,i3)=xhc(:,i3)&* resid.
end loop.
end if.
do if ( hc =3 or hc =2).
loop i3=1 to k3.
compute xhc(:,i3) = ( resid &/(1-hat)&**(1/(4- hc )))&*xhc(:,i3).
end loop.
end if.
do if ( hc = 4).
compute hcmn=make(n,2,4).
compute hcmn(:,2)=(n1*hat)/k3.
loop i3= 1 to k3.
compute xhc(:,i3) = ( resid &/(1-hat)&**(rmin(hcmn)/2))&*xhc(:,i3).
end loop.
end if.
compute varb=(invXtX*t(xhc)*xhc*invXtX).
do if ( hc =1).
compute varb=(n1/(n1-ncol( x )))&*varb.
end if.
end if.
compute hclab={'se(HC0)','se(HC1)','se(HC2)','se(HC3)','se(HC4)','se'}.
compute hclab=hclab(1,( hc +1)).
compute hcflab={'F(HC0)','F(HC1)','F(HC2)','F(HC3)','F(HC4)','F'}.
compute hcflab=hcflab(1,( hc +1)).
release xhc
.
compute seb=sqrt(diag(varb)).
compute trat = b&/seb.
compute p = 2*(1-tcdf(abs(trat), (dfres))).
compute tval = sqrt(dfres* (exp((dfres-(5/6))*((xp2/(dfres-(2/3)+(.11/dfres)))* (xp2/(dfres-(2/3)+(.11/dfres)))))-1)).
compute modres={modres,seb,trat,p}.
compute modres={modres,(b-tval&*seb),(b+tval&*seb)}.
compute modresl={'coeff',hclab,'t','p','LLCI','ULCI'}.
compute lmat = ident(ncol( llrdat )).
compute lmat = lmat(:,2:ncol(lmat)).
compute fratio = (t(t(lmat)*b)*inv(t(lmat)*varb*lmat)*((t(lmat)*b)))/(ncol( llrdat )-1).
compute pfr = 1-fcdf(fratio,(ncol( llrdat )-1),dfres).
compute modsum={sqrt(r2),r2,mse,fratio,(ncol( llrdat )-1),dfres,pfr}.
compute modsuml={'R','R-sq','MSE',hcflab,'df1','df2', 'p'}.
end if.
end if.
do if ( 2 = 2 or 2 =3).
compute xlp= llrdat.
compute ylp= y.
compute pt2 = make(nrow(ylp),1,(csum(ylp)/nrow(ylp))).
do if ( 2 =2).
compute LL3 = ylp&*ln(pt2)+(1-ylp)&*ln(1-pt2).
end if.
compute LL3 = -2*csum(LL3).
compute bt1 = make(ncol(xlp),1,0).
compute LL1 = 0.
compute pt1 = make(nrow(ylp),1,0.5).
compute pt1lp=pt1.
loop jjj = 1 to iterate.
compute xlptmp=t(xlp).
compute vecprb=pt1lp&*(1-pt1lp).
loop kkk=1 to ncol(xlp).
compute xlptmp(kkk,:)=xlptmp(kkk,:)&*t(vecprb).
end loop.
compute b = bt1+inv(xlptmp*xlp)*t(xlp)*(ylp-pt1lp).
do if ( 2 =2).
compute xlpb=xlp*b.
compute xlpbt=(xlpb > -709.7).
compute xlpb709=(1-xlpbt)*(-709.7).
compute xlpb=(xlpb&*xlpbt)+xlpb709.
compute pt1lp = 1/(1+exp(-(xlpb))).
end if.
compute itprob = csum((pt1lp < .00000001) or (pt1lp > .9999999)).
do if (itprob > 0).
loop kkk = 1 to nrow(pt1lp).
do if (pt1lp(kkk,1) > .9999999).
compute pt1lp(kkk,1) = .9999999.
end if.
do if (pt1lp(kkk,1) < .00000001).
compute pt1lp(kkk,1) = .00000001.
end if.
end loop.
compute itprob = 0.
end if.
do if (itprob = 0).
do if ( 2 =2).
compute LL = ylp&*ln(pt1lp)+(1-ylp)&*ln(1-pt1lp).
end if.
compute LL2 = -2*csum(ll).
end if.
do if (abs(LL1-LL2) < converge).
do if ( 0 =1).
compute xlptmp=t(xlp).
compute vecprb=pt1lp&*(1-pt1lp).
loop kkk=1 to ncol(xlp).
compute xlptmp(kkk,:)=xlptmp(kkk,:)&*t(vecprb).
end loop.
compute varb = inv(xlptmp*xlp).
compute seb = sqrt(diag(varb)).
release xlptmp.
end if.
break.
end if.
compute bt1 = b.
compute LL1 = LL2.
end loop.
compute modres=b.
do if (jjj > iterate).
compute itprob = 2.
do if (booting=0).
compute iterrmod=1.
end if.
do if (booting=1).
compute bootiter=1.
end if.
do if (itprobtg=0).
compute itprobtg=1.
compute errcode(errs,1) = 47.
compute errs = errs + 1.
do if (booting = 0 and 0 =1).
compute vt1 = mdiag(pt1lp&*(1-pt1lp)).
compute varb = inv(t(xlp)*vt1*xlp).
compute seb = sqrt(diag(varb)).
end if.
end if.
end if.
do if ( 0 =1).
compute trat = b&/seb.
compute dfres=nrow(xlp).
compute p = 2*(1-cdfnorm(abs(trat))).
compute modres={modres,seb,trat,p}.
compute modres={modres,(b-xp2&*seb),(b+xp2&*seb)}.
compute pvchi=1-chicdf((LL3-LL2),(nrow(modres)-1)).
compute mcF = (LL3-LL2)/LL3.
compute cox = 1-exp(-(LL3-LL2)/nrow(xlp)).
compute nagel = cox/(1-exp(-(LL3)/nrow(xlp))).
compute modsum={LL2,(LL3-LL2),(nrow(modres)-1),pvchi, mcF,cox,nagel}.
compute modsuml={'-2LL','ModelLL', 'df', 'p', 'McFadden', 'CoxSnell', 'Nagelkrk'}.
compute modresl={'coeff','se','Z','p','LLCI','ULCI'}.
end if.
end if
.
compute b=btemphld.
compute fresult={(LL2-basemod),llrdf,1-chicdf((LL2-basemod),llrdf)}
.
compute diromni=fresult.
end if.
end if.
do if (numint(1,i) > 0).
compute intkeym=intkey(intstart:(intstart+numint(1,i)-1),:).
do if (outscree=1).
print intkeym/title='Product terms key:'/format = A8.
end if.
end if.
do if (covcoeff=1).
do if (outscree=1).
print varb/title='Covariance matrix of regression parameter estimates:'/rnames=vlabsm /cnames=vlabsm/format= F10.4.
end if.
compute resultm2=make(nrow(varb),maxresm,99999).
do if (ncol(varb) <= maxresm).
compute resultm2(1:nrow(varb),1:ncol(varb))=varb.
compute resultm={resultm;resultm2}.
end if.
do if (ncol(varb) > maxresm).
compute resultmt=make(nrow(resultm),ncol(varb),99999).
compute resultmt(1:nrow(resultm),1:ncol(resultm))=resultm.
compute resultm=resultmt.
compute resultm2=make(nrow(varb),ncol(resultm),99999).
compute resultm2(1:nrow(varb),1:ncol(varb))=varb.
compute resultm={resultm;resultm2}.
compute maxresm=ncol(resultm).
end if.
end if.
do if (model <> 74 and xmtest=1 and nms > 0).
compute r2tmp=r2.
compute btmp=b.
compute varbtmp=varb.
compute dfrestmp=dfres.
compute tvaltmp=tval.
compute xmtst=make(nms,4,0).
compute xmtstlbc={hcflab,'df1','df2','p'}.
do if ((i=(nms+nys)) and (ydich=1)).
compute xmtst=make(nms,3,0).
compute xmtstlbc={'Chi-sq','df','p'}.
end if.
compute xmtstlb={' '}.
compute xmtmat=x.
compute numxint=0.
loop xmints=2 to i.
compute x=xmtmat.
do if ((bcmat((i+1),xmints)=1) and (wzcmat((i+1),xmints) <> 1)).
do if (bcmat((i+1),1)=0).
compute x={xmtmat,xtmp}.
do if ((ydich=1) and (i=(nms+nys))).

.
do if ( 2 =1).
compute hatmat=inv(t( x )* x )*t( x ).
compute b = hatmat* y.
compute modres=b.
do if ( 1 =1).
compute n1=nrow( x ).
compute dfres=n1-(ncol( x )).
compute sstotal = t( y -(csum( y )/n1))*( y -(csum( y )/n1)).
compute resid= y - x *b.
compute ssresid = csum((resid)&**2).
compute r2 = (sstotal-ssresid)/sstotal.
do if (r2 < 0).
compute r2=0.
end if.
compute adjr2 = 1-((1-r2)*(n1-1)/(dfres)).
compute mse=ssresid/(n1-ncol( x )).

.
compute n1=nrow( x ).
compute invXtX = inv(t( x )* x ).
compute varb = mse *invXtX.
compute k3 = ncol( x ).
compute xhc=0.
do if ( hc <> 5).
compute xhc= x.
compute hat = xhc(:,1).
loop i3=1 to nrow(xhc).
compute hat(i3,1)= xhc(i3,:)*invXtX*t(xhc(i3,:)).
end loop.
do if ( hc = 0 or hc =1).
loop i3 = 1 to k3.
compute xhc(:,i3)=xhc(:,i3)&* resid.
end loop.
end if.
do if ( hc =3 or hc =2).
loop i3=1 to k3.
compute xhc(:,i3) = ( resid &/(1-hat)&**(1/(4- hc )))&*xhc(:,i3).
end loop.
end if.
do if ( hc = 4).
compute hcmn=make(n,2,4).
compute hcmn(:,2)=(n1*hat)/k3.
loop i3= 1 to k3.
compute xhc(:,i3) = ( resid &/(1-hat)&**(rmin(hcmn)/2))&*xhc(:,i3).
end loop.
end if.
compute varb=(invXtX*t(xhc)*xhc*invXtX).
do if ( hc =1).
compute varb=(n1/(n1-ncol( x )))&*varb.
end if.
end if.
compute hclab={'se(HC0)','se(HC1)','se(HC2)','se(HC3)','se(HC4)','se'}.
compute hclab=hclab(1,( hc +1)).
compute hcflab={'F(HC0)','F(HC1)','F(HC2)','F(HC3)','F(HC4)','F'}.
compute hcflab=hcflab(1,( hc +1)).
release xhc
.
compute seb=sqrt(diag(varb)).
compute trat = b&/seb.
compute p = 2*(1-tcdf(abs(trat), (dfres))).
compute tval = sqrt(dfres* (exp((dfres-(5/6))*((xp2/(dfres-(2/3)+(.11/dfres)))* (xp2/(dfres-(2/3)+(.11/dfres)))))-1)).
compute modres={modres,seb,trat,p}.
compute modres={modres,(b-tval&*seb),(b+tval&*seb)}.
compute modresl={'coeff',hclab,'t','p','LLCI','ULCI'}.
compute lmat = ident(ncol( x )).
compute lmat = lmat(:,2:ncol(lmat)).
compute fratio = (t(t(lmat)*b)*inv(t(lmat)*varb*lmat)*((t(lmat)*b)))/(ncol( x )-1).
compute pfr = 1-fcdf(fratio,(ncol( x )-1),dfres).
compute modsum={sqrt(r2),r2,mse,fratio,(ncol( x )-1),dfres,pfr}.
compute modsuml={'R','R-sq','MSE',hcflab,'df1','df2', 'p'}.
end if.
end if.
do if ( 2 = 2 or 2 =3).
compute xlp= x.
compute ylp= y.
compute pt2 = make(nrow(ylp),1,(csum(ylp)/nrow(ylp))).
do if ( 2 =2).
compute LL3 = ylp&*ln(pt2)+(1-ylp)&*ln(1-pt2).
end if.
compute LL3 = -2*csum(LL3).
compute bt1 = make(ncol(xlp),1,0).
compute LL1 = 0.
compute pt1 = make(nrow(ylp),1,0.5).
compute pt1lp=pt1.
loop jjj = 1 to iterate.
compute xlptmp=t(xlp).
compute vecprb=pt1lp&*(1-pt1lp).
loop kkk=1 to ncol(xlp).
compute xlptmp(kkk,:)=xlptmp(kkk,:)&*t(vecprb).
end loop.
compute b = bt1+inv(xlptmp*xlp)*t(xlp)*(ylp-pt1lp).
do if ( 2 =2).
compute xlpb=xlp*b.
compute xlpbt=(xlpb > -709.7).
compute xlpb709=(1-xlpbt)*(-709.7).
compute xlpb=(xlpb&*xlpbt)+xlpb709.
compute pt1lp = 1/(1+exp(-(xlpb))).
end if.
compute itprob = csum((pt1lp < .00000001) or (pt1lp > .9999999)).
do if (itprob > 0).
loop kkk = 1 to nrow(pt1lp).
do if (pt1lp(kkk,1) > .9999999).
compute pt1lp(kkk,1) = .9999999.
end if.
do if (pt1lp(kkk,1) < .00000001).
compute pt1lp(kkk,1) = .00000001.
end if.
end loop.
compute itprob = 0.
end if.
do if (itprob = 0).
do if ( 2 =2).
compute LL = ylp&*ln(pt1lp)+(1-ylp)&*ln(1-pt1lp).
end if.
compute LL2 = -2*csum(ll).
end if.
do if (abs(LL1-LL2) < converge).
do if ( 1 =1).
compute xlptmp=t(xlp).
compute vecprb=pt1lp&*(1-pt1lp).
loop kkk=1 to ncol(xlp).
compute xlptmp(kkk,:)=xlptmp(kkk,:)&*t(vecprb).
end loop.
compute varb = inv(xlptmp*xlp).
compute seb = sqrt(diag(varb)).
release xlptmp.
end if.
break.
end if.
compute bt1 = b.
compute LL1 = LL2.
end loop.
compute modres=b.
do if (jjj > iterate).
compute itprob = 2.
do if (booting=0).
compute iterrmod=1.
end if.
do if (booting=1).
compute bootiter=1.
end if.
do if (itprobtg=0).
compute itprobtg=1.
compute errcode(errs,1) = 47.
compute errs = errs + 1.
do if (booting = 0 and 1 =1).
compute vt1 = mdiag(pt1lp&*(1-pt1lp)).
compute varb = inv(t(xlp)*vt1*xlp).
compute seb = sqrt(diag(varb)).
end if.
end if.
end if.
do if ( 1 =1).
compute trat = b&/seb.
compute dfres=nrow(xlp).
compute p = 2*(1-cdfnorm(abs(trat))).
compute modres={modres,seb,trat,p}.
compute modres={modres,(b-xp2&*seb),(b+xp2&*seb)}.
compute pvchi=1-chicdf((LL3-LL2),(nrow(modres)-1)).
compute mcF = (LL3-LL2)/LL3.
compute cox = 1-exp(-(LL3-LL2)/nrow(xlp)).
compute nagel = cox/(1-exp(-(LL3)/nrow(xlp))).
compute modsum={LL2,(LL3-LL2),(nrow(modres)-1),pvchi, mcF,cox,nagel}.
compute modsuml={'-2LL','ModelLL', 'df', 'p', 'McFadden', 'CoxSnell', 'Nagelkrk'}.
compute modresl={'coeff','se','Z','p','LLCI','ULCI'}.
end if.
end if
.
compute basemodx=LL2.
end if.
end if.
loop xmtlp1=1 to nxvls.
compute x={x,xtmp(:,xmtlp1)&*(mtmp(:,(xmints-1))-(csum(mtmp(:,(xmints-1)))/nrow(mtmp)) )}.
end loop.
do if ((i < (nms+nys)) or (ydich=0)).

.
do if ( 1 =1).
compute hatmat=inv(t( x )* x )*t( x ).
compute b = hatmat* y.
compute modres=b.
do if ( 1 =1).
compute n1=nrow( x ).
compute dfres=n1-(ncol( x )).
compute sstotal = t( y -(csum( y )/n1))*( y -(csum( y )/n1)).
compute resid= y - x *b.
compute ssresid = csum((resid)&**2).
compute r2 = (sstotal-ssresid)/sstotal.
do if (r2 < 0).
compute r2=0.
end if.
compute adjr2 = 1-((1-r2)*(n1-1)/(dfres)).
compute mse=ssresid/(n1-ncol( x )).

.
compute n1=nrow( x ).
compute invXtX = inv(t( x )* x ).
compute varb = mse *invXtX.
compute k3 = ncol( x ).
compute xhc=0.
do if ( hc <> 5).
compute xhc= x.
compute hat = xhc(:,1).
loop i3=1 to nrow(xhc).
compute hat(i3,1)= xhc(i3,:)*invXtX*t(xhc(i3,:)).
end loop.
do if ( hc = 0 or hc =1).
loop i3 = 1 to k3.
compute xhc(:,i3)=xhc(:,i3)&* resid.
end loop.
end if.
do if ( hc =3 or hc =2).
loop i3=1 to k3.
compute xhc(:,i3) = ( resid &/(1-hat)&**(1/(4- hc )))&*xhc(:,i3).
end loop.
end if.
do if ( hc = 4).
compute hcmn=make(n,2,4).
compute hcmn(:,2)=(n1*hat)/k3.
loop i3= 1 to k3.
compute xhc(:,i3) = ( resid &/(1-hat)&**(rmin(hcmn)/2))&*xhc(:,i3).
end loop.
end if.
compute varb=(invXtX*t(xhc)*xhc*invXtX).
do if ( hc =1).
compute varb=(n1/(n1-ncol( x )))&*varb.
end if.
end if.
compute hclab={'se(HC0)','se(HC1)','se(HC2)','se(HC3)','se(HC4)','se'}.
compute hclab=hclab(1,( hc +1)).
compute hcflab={'F(HC0)','F(HC1)','F(HC2)','F(HC3)','F(HC4)','F'}.
compute hcflab=hcflab(1,( hc +1)).
release xhc
.
compute seb=sqrt(diag(varb)).
compute trat = b&/seb.
compute p = 2*(1-tcdf(abs(trat), (dfres))).
compute tval = sqrt(dfres* (exp((dfres-(5/6))*((xp2/(dfres-(2/3)+(.11/dfres)))* (xp2/(dfres-(2/3)+(.11/dfres)))))-1)).
compute modres={modres,seb,trat,p}.
compute modres={modres,(b-tval&*seb),(b+tval&*seb)}.
compute modresl={'coeff',hclab,'t','p','LLCI','ULCI'}.
compute lmat = ident(ncol( x )).
compute lmat = lmat(:,2:ncol(lmat)).
compute fratio = (t(t(lmat)*b)*inv(t(lmat)*varb*lmat)*((t(lmat)*b)))/(ncol( x )-1).
compute pfr = 1-fcdf(fratio,(ncol( x )-1),dfres).
compute modsum={sqrt(r2),r2,mse,fratio,(ncol( x )-1),dfres,pfr}.
compute modsuml={'R','R-sq','MSE',hcflab,'df1','df2', 'p'}.
end if.
end if.
do if ( 1 = 2 or 1 =3).
compute xlp= x.
compute ylp= y.
compute pt2 = make(nrow(ylp),1,(csum(ylp)/nrow(ylp))).
do if ( 1 =2).
compute LL3 = ylp&*ln(pt2)+(1-ylp)&*ln(1-pt2).
end if.
compute LL3 = -2*csum(LL3).
compute bt1 = make(ncol(xlp),1,0).
compute LL1 = 0.
compute pt1 = make(nrow(ylp),1,0.5).
compute pt1lp=pt1.
loop jjj = 1 to iterate.
compute xlptmp=t(xlp).
compute vecprb=pt1lp&*(1-pt1lp).
loop kkk=1 to ncol(xlp).
compute xlptmp(kkk,:)=xlptmp(kkk,:)&*t(vecprb).
end loop.
compute b = bt1+inv(xlptmp*xlp)*t(xlp)*(ylp-pt1lp).
do if ( 1 =2).
compute xlpb=xlp*b.
compute xlpbt=(xlpb > -709.7).
compute xlpb709=(1-xlpbt)*(-709.7).
compute xlpb=(xlpb&*xlpbt)+xlpb709.
compute pt1lp = 1/(1+exp(-(xlpb))).
end if.
compute itprob = csum((pt1lp < .00000001) or (pt1lp > .9999999)).
do if (itprob > 0).
loop kkk = 1 to nrow(pt1lp).
do if (pt1lp(kkk,1) > .9999999).
compute pt1lp(kkk,1) = .9999999.
end if.
do if (pt1lp(kkk,1) < .00000001).
compute pt1lp(kkk,1) = .00000001.
end if.
end loop.
compute itprob = 0.
end if.
do if (itprob = 0).
do if ( 1 =2).
compute LL = ylp&*ln(pt1lp)+(1-ylp)&*ln(1-pt1lp).
end if.
compute LL2 = -2*csum(ll).
end if.
do if (abs(LL1-LL2) < converge).
do if ( 1 =1).
compute xlptmp=t(xlp).
compute vecprb=pt1lp&*(1-pt1lp).
loop kkk=1 to ncol(xlp).
compute xlptmp(kkk,:)=xlptmp(kkk,:)&*t(vecprb).
end loop.
compute varb = inv(xlptmp*xlp).
compute seb = sqrt(diag(varb)).
release xlptmp.
end if.
break.
end if.
compute bt1 = b.
compute LL1 = LL2.
end loop.
compute modres=b.
do if (jjj > iterate).
compute itprob = 2.
do if (booting=0).
compute iterrmod=1.
end if.
do if (booting=1).
compute bootiter=1.
end if.
do if (itprobtg=0).
compute itprobtg=1.
compute errcode(errs,1) = 47.
compute errs = errs + 1.
do if (booting = 0 and 1 =1).
compute vt1 = mdiag(pt1lp&*(1-pt1lp)).
compute varb = inv(t(xlp)*vt1*xlp).
compute seb = sqrt(diag(varb)).
end if.
end if.
end if.
do if ( 1 =1).
compute trat = b&/seb.
compute dfres=nrow(xlp).
compute p = 2*(1-cdfnorm(abs(trat))).
compute modres={modres,seb,trat,p}.
compute modres={modres,(b-xp2&*seb),(b+xp2&*seb)}.
compute pvchi=1-chicdf((LL3-LL2),(nrow(modres)-1)).
compute mcF = (LL3-LL2)/LL3.
compute cox = 1-exp(-(LL3-LL2)/nrow(xlp)).
compute nagel = cox/(1-exp(-(LL3)/nrow(xlp))).
compute modsum={LL2,(LL3-LL2),(nrow(modres)-1),pvchi, mcF,cox,nagel}.
compute modsuml={'-2LL','ModelLL', 'df', 'p', 'McFadden', 'CoxSnell', 'Nagelkrk'}.
compute modresl={'coeff','se','Z','p','LLCI','ULCI'}.
end if.
end if
.
end if.
do if ((i = (nms+nys)) and (ydich=1)).

.
do if ( 2 =1).
compute hatmat=inv(t( x )* x )*t( x ).
compute b = hatmat* y.
compute modres=b.
do if ( 1 =1).
compute n1=nrow( x ).
compute dfres=n1-(ncol( x )).
compute sstotal = t( y -(csum( y )/n1))*( y -(csum( y )/n1)).
compute resid= y - x *b.
compute ssresid = csum((resid)&**2).
compute r2 = (sstotal-ssresid)/sstotal.
do if (r2 < 0).
compute r2=0.
end if.
compute adjr2 = 1-((1-r2)*(n1-1)/(dfres)).
compute mse=ssresid/(n1-ncol( x )).

.
compute n1=nrow( x ).
compute invXtX = inv(t( x )* x ).
compute varb = mse *invXtX.
compute k3 = ncol( x ).
compute xhc=0.
do if ( hc <> 5).
compute xhc= x.
compute hat = xhc(:,1).
loop i3=1 to nrow(xhc).
compute hat(i3,1)= xhc(i3,:)*invXtX*t(xhc(i3,:)).
end loop.
do if ( hc = 0 or hc =1).
loop i3 = 1 to k3.
compute xhc(:,i3)=xhc(:,i3)&* resid.
end loop.
end if.
do if ( hc =3 or hc =2).
loop i3=1 to k3.
compute xhc(:,i3) = ( resid &/(1-hat)&**(1/(4- hc )))&*xhc(:,i3).
end loop.
end if.
do if ( hc = 4).
compute hcmn=make(n,2,4).
compute hcmn(:,2)=(n1*hat)/k3.
loop i3= 1 to k3.
compute xhc(:,i3) = ( resid &/(1-hat)&**(rmin(hcmn)/2))&*xhc(:,i3).
end loop.
end if.
compute varb=(invXtX*t(xhc)*xhc*invXtX).
do if ( hc =1).
compute varb=(n1/(n1-ncol( x )))&*varb.
end if.
end if.
compute hclab={'se(HC0)','se(HC1)','se(HC2)','se(HC3)','se(HC4)','se'}.
compute hclab=hclab(1,( hc +1)).
compute hcflab={'F(HC0)','F(HC1)','F(HC2)','F(HC3)','F(HC4)','F'}.
compute hcflab=hcflab(1,( hc +1)).
release xhc
.
compute seb=sqrt(diag(varb)).
compute trat = b&/seb.
compute p = 2*(1-tcdf(abs(trat), (dfres))).
compute tval = sqrt(dfres* (exp((dfres-(5/6))*((xp2/(dfres-(2/3)+(.11/dfres)))* (xp2/(dfres-(2/3)+(.11/dfres)))))-1)).
compute modres={modres,seb,trat,p}.
compute modres={modres,(b-tval&*seb),(b+tval&*seb)}.
compute modresl={'coeff',hclab,'t','p','LLCI','ULCI'}.
compute lmat = ident(ncol( x )).
compute lmat = lmat(:,2:ncol(lmat)).
compute fratio = (t(t(lmat)*b)*inv(t(lmat)*varb*lmat)*((t(lmat)*b)))/(ncol( x )-1).
compute pfr = 1-fcdf(fratio,(ncol( x )-1),dfres).
compute modsum={sqrt(r2),r2,mse,fratio,(ncol( x )-1),dfres,pfr}.
compute modsuml={'R','R-sq','MSE',hcflab,'df1','df2', 'p'}.
end if.
end if.
do if ( 2 = 2 or 2 =3).
compute xlp= x.
compute ylp= y.
compute pt2 = make(nrow(ylp),1,(csum(ylp)/nrow(ylp))).
do if ( 2 =2).
compute LL3 = ylp&*ln(pt2)+(1-ylp)&*ln(1-pt2).
end if.
compute LL3 = -2*csum(LL3).
compute bt1 = make(ncol(xlp),1,0).
compute LL1 = 0.
compute pt1 = make(nrow(ylp),1,0.5).
compute pt1lp=pt1.
loop jjj = 1 to iterate.
compute xlptmp=t(xlp).
compute vecprb=pt1lp&*(1-pt1lp).
loop kkk=1 to ncol(xlp).
compute xlptmp(kkk,:)=xlptmp(kkk,:)&*t(vecprb).
end loop.
compute b = bt1+inv(xlptmp*xlp)*t(xlp)*(ylp-pt1lp).
do if ( 2 =2).
compute xlpb=xlp*b.
compute xlpbt=(xlpb > -709.7).
compute xlpb709=(1-xlpbt)*(-709.7).
compute xlpb=(xlpb&*xlpbt)+xlpb709.
compute pt1lp = 1/(1+exp(-(xlpb))).
end if.
compute itprob = csum((pt1lp < .00000001) or (pt1lp > .9999999)).
do if (itprob > 0).
loop kkk = 1 to nrow(pt1lp).
do if (pt1lp(kkk,1) > .9999999).
compute pt1lp(kkk,1) = .9999999.
end if.
do if (pt1lp(kkk,1) < .00000001).
compute pt1lp(kkk,1) = .00000001.
end if.
end loop.
compute itprob = 0.
end if.
do if (itprob = 0).
do if ( 2 =2).
compute LL = ylp&*ln(pt1lp)+(1-ylp)&*ln(1-pt1lp).
end if.
compute LL2 = -2*csum(ll).
end if.
do if (abs(LL1-LL2) < converge).
do if ( 1 =1).
compute xlptmp=t(xlp).
compute vecprb=pt1lp&*(1-pt1lp).
loop kkk=1 to ncol(xlp).
compute xlptmp(kkk,:)=xlptmp(kkk,:)&*t(vecprb).
end loop.
compute varb = inv(xlptmp*xlp).
compute seb = sqrt(diag(varb)).
release xlptmp.
end if.
break.
end if.
compute bt1 = b.
compute LL1 = LL2.
end loop.
compute modres=b.
do if (jjj > iterate).
compute itprob = 2.
do if (booting=0).
compute iterrmod=1.
end if.
do if (booting=1).
compute bootiter=1.
end if.
do if (itprobtg=0).
compute itprobtg=1.
compute errcode(errs,1) = 47.
compute errs = errs + 1.
do if (booting = 0 and 1 =1).
compute vt1 = mdiag(pt1lp&*(1-pt1lp)).
compute varb = inv(t(xlp)*vt1*xlp).
compute seb = sqrt(diag(varb)).
end if.
end if.
end if.
do if ( 1 =1).
compute trat = b&/seb.
compute dfres=nrow(xlp).
compute p = 2*(1-cdfnorm(abs(trat))).
compute modres={modres,seb,trat,p}.
compute modres={modres,(b-xp2&*seb),(b+xp2&*seb)}.
compute pvchi=1-chicdf((LL3-LL2),(nrow(modres)-1)).
compute mcF = (LL3-LL2)/LL3.
compute cox = 1-exp(-(LL3-LL2)/nrow(xlp)).
compute nagel = cox/(1-exp(-(LL3)/nrow(xlp))).
compute modsum={LL2,(LL3-LL2),(nrow(modres)-1),pvchi, mcF,cox,nagel}.
compute modsuml={'-2LL','ModelLL', 'df', 'p', 'McFadden', 'CoxSnell', 'Nagelkrk'}.
compute modresl={'coeff','se','Z','p','LLCI','ULCI'}.
end if.
end if
.
compute chidfxm=basemodx-LL2.
end if.
compute lmat=make(nrow(b),nxvls,0).
compute lmattmp=ident(nxvls).
compute lmat((nrow(lmat)-nxvls+1):nrow(lmat),:)=lmattmp.

.
compute lmat2= lmat.
do if ( 1 =0).
compute lmat2 = mdiag( lmat ).
compute lmat3=make(nrow(lmat2),1,0).
loop flp=1 to ncol(lmat2).
do if (csum(lmat2(:,flp))=1).
compute lmat3={lmat3,lmat2(:,flp)}.
end if.
end loop.
compute lmat2=lmat3(:,2:ncol(lmat3)).
end if.
compute fratio = (t(t(lmat2)* b )*inv(t(lmat2)* varb *lmat2)*((t(lmat2)* b )))/ncol(lmat2).
compute pfr = 1-fcdf(fratio,ncol(lmat2),(n-nrow( b ))).
compute fresult={fratio,ncol(lmat2),(n-nrow( b )),pfr}.
do if (i = (nms+nys) and (ydich=1)).
compute fratio=fratio*ncol(lmat2).
compute pfr=1-chicdf(fratio,ncol(lmat2)).
compute fresult={fratio,ncol(lmat2),pfr}.
end if.
do if ( 0 =1).
compute lmat3=1-rsum(lmat2).
compute xfm=make(n,csum(lmat3),0).
compute flpc=1.
loop flp=1 to nrow(lmat3).
do if (lmat3(flp,1)=1).
compute xfm(:,flpc)=x(:,flp).
compute flpc=flpc+1.
end if.
end loop.
compute bfm=inv(t(xfm)*xfm)*t(xfm)*y.
compute resid=y-(xfm*bfm).
compute sstotal=(y-(csum(y)/n)).
compute sstotal=csum(sstotal&*sstotal).
compute ssresid=csum(resid&*resid).
compute rsqch= 0 -((sstotal-ssresid)/sstotal).
compute fresult={rsqch,fresult}.
release xfm,flpc, resid, ssresid, bfm.
end if
.
compute numxint=numxint+1.
compute xmtst(numxint,:)=fresult.
do if ((i = (nms+nys)) and (ydich=1)).
compute xmtst(numxint,1)=chidfxm.
compute xmtst(numxint,3)=1-chicdf(chidfxm,nxvls).
end if.
compute xmtstlb={xmtstlb;highlbx((xmints-1),1)}.
end if.
end loop.
compute x=xmtmat.
release xmtmat.
do if (numxint>0).
compute xmtstlb=xmtstlb((2:(numxint+1)),:).
compute xmtst=xmtst(1:numxint,:).
compute resultm2=make(nrow(xmtst),maxresm,99999).
compute resultm2(1:nrow(xmtst),1:ncol(xmtst))=xmtst.
compute resultm={resultm;resultm2}.
do if (nms=1).
compute xmtstlb=' '.
end if.
do if (outscree=1).
do if ((i < (nms+nys)) or (ydich=0)).
print xmtst/title='Test(s) of X by M interaction:'/rnames=xmtstlb/cnames=xmtstlbc/format= F10.4.
end if.
do if ((i = (nms+nys)) and (ydich=1)).
print xmtst/title='Likelihood ratio test(s) of X by M interaction:'/rnames=xmtstlb/cnames=xmtstlbc/format= F10.4.
end if.
end if.
end if.
compute r2=r2tmp.
compute b=btmp.
compute varb=varbtmp.
compute dfres=dfrestmp.
compute tval=tvaltmp.
end if.
do if (criterr = 0).
compute jj=0.
loop j = start to ((start+i)-1).
compute dbint=0.
compute lmat=whigh(1:nump(1,i),j).
compute lmat2=wzhigh(1:nump(1,i),j).
do if ((csum(lmat) > 0) and (csum(lmat2) = 0)).
do if ((i < (nms+nys)) or (ydich <> 1)).

.
compute lmat2= lmat.
do if ( 0 =0).
compute lmat2 = mdiag( lmat ).
compute lmat3=make(nrow(lmat2),1,0).
loop flp=1 to ncol(lmat2).
do if (csum(lmat2(:,flp))=1).
compute lmat3={lmat3,lmat2(:,flp)}.
end if.
end loop.
compute lmat2=lmat3(:,2:ncol(lmat3)).
end if.
compute fratio = (t(t(lmat2)* b )*inv(t(lmat2)* varb *lmat2)*((t(lmat2)* b )))/ncol(lmat2).
compute pfr = 1-fcdf(fratio,ncol(lmat2),(n-nrow( b ))).
compute fresult={fratio,ncol(lmat2),(n-nrow( b )),pfr}.
do if (i = (nms+nys) and (ydich=1)).
compute fratio=fratio*ncol(lmat2).
compute pfr=1-chicdf(fratio,ncol(lmat2)).
compute fresult={fratio,ncol(lmat2),pfr}.
end if.
do if ( 1 =1).
compute lmat3=1-rsum(lmat2).
compute xfm=make(n,csum(lmat3),0).
compute flpc=1.
loop flp=1 to nrow(lmat3).
do if (lmat3(flp,1)=1).
compute xfm(:,flpc)=x(:,flp).
compute flpc=flpc+1.
end if.
end loop.
compute bfm=inv(t(xfm)*xfm)*t(xfm)*y.
compute resid=y-(xfm*bfm).
compute sstotal=(y-(csum(y)/n)).
compute sstotal=csum(sstotal&*sstotal).
compute ssresid=csum(resid&*resid).
compute rsqch= r2 -((sstotal-ssresid)/sstotal).
compute fresult={rsqch,fresult}.
release xfm,flpc, resid, ssresid, bfm.
end if
.
compute lmatdb=lmat.
compute dbint=dbint+1.
end if.
do if ((ydich = 1) and (i = (nms+nys))).

.
compute btemphld=b.
compute llrdat=make(nrow(x),nrow( lmat )-csum( lmat ),-999).
compute llrdf=ncol(x)-ncol(llrdat).
compute llrcnt=0.
loop llri=1 to nrow( lmat ).
do if ( lmat (llri,1)=0).
compute llrcnt=llrcnt+1.
compute llrdat(:,llrcnt)=x(:,llri).
end if.
end loop.

.
do if ( 2 =1).
compute hatmat=inv(t( llrdat )* llrdat )*t( llrdat ).
compute b = hatmat* y.
compute modres=b.
do if ( 0 =1).
compute n1=nrow( llrdat ).
compute dfres=n1-(ncol( llrdat )).
compute sstotal = t( y -(csum( y )/n1))*( y -(csum( y )/n1)).
compute resid= y - llrdat *b.
compute ssresid = csum((resid)&**2).
compute r2 = (sstotal-ssresid)/sstotal.
do if (r2 < 0).
compute r2=0.
end if.
compute adjr2 = 1-((1-r2)*(n1-1)/(dfres)).
compute mse=ssresid/(n1-ncol( llrdat )).

.
compute n1=nrow( x ).
compute invXtX = inv(t( x )* x ).
compute varb = mse *invXtX.
compute k3 = ncol( x ).
compute xhc=0.
do if ( hc <> 5).
compute xhc= x.
compute hat = xhc(:,1).
loop i3=1 to nrow(xhc).
compute hat(i3,1)= xhc(i3,:)*invXtX*t(xhc(i3,:)).
end loop.
do if ( hc = 0 or hc =1).
loop i3 = 1 to k3.
compute xhc(:,i3)=xhc(:,i3)&* resid.
end loop.
end if.
do if ( hc =3 or hc =2).
loop i3=1 to k3.
compute xhc(:,i3) = ( resid &/(1-hat)&**(1/(4- hc )))&*xhc(:,i3).
end loop.
end if.
do if ( hc = 4).
compute hcmn=make(n,2,4).
compute hcmn(:,2)=(n1*hat)/k3.
loop i3= 1 to k3.
compute xhc(:,i3) = ( resid &/(1-hat)&**(rmin(hcmn)/2))&*xhc(:,i3).
end loop.
end if.
compute varb=(invXtX*t(xhc)*xhc*invXtX).
do if ( hc =1).
compute varb=(n1/(n1-ncol( x )))&*varb.
end if.
end if.
compute hclab={'se(HC0)','se(HC1)','se(HC2)','se(HC3)','se(HC4)','se'}.
compute hclab=hclab(1,( hc +1)).
compute hcflab={'F(HC0)','F(HC1)','F(HC2)','F(HC3)','F(HC4)','F'}.
compute hcflab=hcflab(1,( hc +1)).
release xhc
.
compute seb=sqrt(diag(varb)).
compute trat = b&/seb.
compute p = 2*(1-tcdf(abs(trat), (dfres))).
compute tval = sqrt(dfres* (exp((dfres-(5/6))*((xp2/(dfres-(2/3)+(.11/dfres)))* (xp2/(dfres-(2/3)+(.11/dfres)))))-1)).
compute modres={modres,seb,trat,p}.
compute modres={modres,(b-tval&*seb),(b+tval&*seb)}.
compute modresl={'coeff',hclab,'t','p','LLCI','ULCI'}.
compute lmat = ident(ncol( llrdat )).
compute lmat = lmat(:,2:ncol(lmat)).
compute fratio = (t(t(lmat)*b)*inv(t(lmat)*varb*lmat)*((t(lmat)*b)))/(ncol( llrdat )-1).
compute pfr = 1-fcdf(fratio,(ncol( llrdat )-1),dfres).
compute modsum={sqrt(r2),r2,mse,fratio,(ncol( llrdat )-1),dfres,pfr}.
compute modsuml={'R','R-sq','MSE',hcflab,'df1','df2', 'p'}.
end if.
end if.
do if ( 2 = 2 or 2 =3).
compute xlp= llrdat.
compute ylp= y.
compute pt2 = make(nrow(ylp),1,(csum(ylp)/nrow(ylp))).
do if ( 2 =2).
compute LL3 = ylp&*ln(pt2)+(1-ylp)&*ln(1-pt2).
end if.
compute LL3 = -2*csum(LL3).
compute bt1 = make(ncol(xlp),1,0).
compute LL1 = 0.
compute pt1 = make(nrow(ylp),1,0.5).
compute pt1lp=pt1.
loop jjj = 1 to iterate.
compute xlptmp=t(xlp).
compute vecprb=pt1lp&*(1-pt1lp).
loop kkk=1 to ncol(xlp).
compute xlptmp(kkk,:)=xlptmp(kkk,:)&*t(vecprb).
end loop.
compute b = bt1+inv(xlptmp*xlp)*t(xlp)*(ylp-pt1lp).
do if ( 2 =2).
compute xlpb=xlp*b.
compute xlpbt=(xlpb > -709.7).
compute xlpb709=(1-xlpbt)*(-709.7).
compute xlpb=(xlpb&*xlpbt)+xlpb709.
compute pt1lp = 1/(1+exp(-(xlpb))).
end if.
compute itprob = csum((pt1lp < .00000001) or (pt1lp > .9999999)).
do if (itprob > 0).
loop kkk = 1 to nrow(pt1lp).
do if (pt1lp(kkk,1) > .9999999).
compute pt1lp(kkk,1) = .9999999.
end if.
do if (pt1lp(kkk,1) < .00000001).
compute pt1lp(kkk,1) = .00000001.
end if.
end loop.
compute itprob = 0.
end if.
do if (itprob = 0).
do if ( 2 =2).
compute LL = ylp&*ln(pt1lp)+(1-ylp)&*ln(1-pt1lp).
end if.
compute LL2 = -2*csum(ll).
end if.
do if (abs(LL1-LL2) < converge).
do if ( 0 =1).
compute xlptmp=t(xlp).
compute vecprb=pt1lp&*(1-pt1lp).
loop kkk=1 to ncol(xlp).
compute xlptmp(kkk,:)=xlptmp(kkk,:)&*t(vecprb).
end loop.
compute varb = inv(xlptmp*xlp).
compute seb = sqrt(diag(varb)).
release xlptmp.
end if.
break.
end if.
compute bt1 = b.
compute LL1 = LL2.
end loop.
compute modres=b.
do if (jjj > iterate).
compute itprob = 2.
do if (booting=0).
compute iterrmod=1.
end if.
do if (booting=1).
compute bootiter=1.
end if.
do if (itprobtg=0).
compute itprobtg=1.
compute errcode(errs,1) = 47.
compute errs = errs + 1.
do if (booting = 0 and 0 =1).
compute vt1 = mdiag(pt1lp&*(1-pt1lp)).
compute varb = inv(t(xlp)*vt1*xlp).
compute seb = sqrt(diag(varb)).
end if.
end if.
end if.
do if ( 0 =1).
compute trat = b&/seb.
compute dfres=nrow(xlp).
compute p = 2*(1-cdfnorm(abs(trat))).
compute modres={modres,seb,trat,p}.
compute modres={modres,(b-xp2&*seb),(b+xp2&*seb)}.
compute pvchi=1-chicdf((LL3-LL2),(nrow(modres)-1)).
compute mcF = (LL3-LL2)/LL3.
compute cox = 1-exp(-(LL3-LL2)/nrow(xlp)).
compute nagel = cox/(1-exp(-(LL3)/nrow(xlp))).
compute modsum={LL2,(LL3-LL2),(nrow(modres)-1),pvchi, mcF,cox,nagel}.
compute modsuml={'-2LL','ModelLL', 'df', 'p', 'McFadden', 'CoxSnell', 'Nagelkrk'}.
compute modresl={'coeff','se','Z','p','LLCI','ULCI'}.
end if.
end if
.
compute b=btemphld.
compute fresult={(LL2-basemod),llrdf,1-chicdf((LL2-basemod),llrdf)}
.
compute lmatdb=lmat.
compute dbint=dbint+1.
end if.
compute highf={highf;fresult}.
compute highf2={highf2;fresult}.
do if (j = start).
compute flabel={flabel;'X*W'}.
end if.
do if (j > start).
do if (nms > 1).
compute flabel={flabel;highlbw(jj,1)}.
else if (nms = 1).
do if (xmint=0).
compute flabel={flabel;'M*W'}.
end if.
do if (xmint=1).
compute flabel={flabel;'X*M'}.
end if.
end if.
end if.
end if.
compute lmat=zhigh(1:nump(1,i),j).
compute lmat2=wzhigh(1:nump(1,i),j).
do if ((csum(lmat) > 0) and (csum(lmat2) = 0)).
do if ((i < (nms+nys)) or (ydich <> 1)).

.
compute lmat2= lmat.
do if ( 0 =0).
compute lmat2 = mdiag( lmat ).
compute lmat3=make(nrow(lmat2),1,0).
loop flp=1 to ncol(lmat2).
do if (csum(lmat2(:,flp))=1).
compute lmat3={lmat3,lmat2(:,flp)}.
end if.
end loop.
compute lmat2=lmat3(:,2:ncol(lmat3)).
end if.
compute fratio = (t(t(lmat2)* b )*inv(t(lmat2)* varb *lmat2)*((t(lmat2)* b )))/ncol(lmat2).
compute pfr = 1-fcdf(fratio,ncol(lmat2),(n-nrow( b ))).
compute fresult={fratio,ncol(lmat2),(n-nrow( b )),pfr}.
do if (i = (nms+nys) and (ydich=1)).
compute fratio=fratio*ncol(lmat2).
compute pfr=1-chicdf(fratio,ncol(lmat2)).
compute fresult={fratio,ncol(lmat2),pfr}.
end if.
do if ( 1 =1).
compute lmat3=1-rsum(lmat2).
compute xfm=make(n,csum(lmat3),0).
compute flpc=1.
loop flp=1 to nrow(lmat3).
do if (lmat3(flp,1)=1).
compute xfm(:,flpc)=x(:,flp).
compute flpc=flpc+1.
end if.
end loop.
compute bfm=inv(t(xfm)*xfm)*t(xfm)*y.
compute resid=y-(xfm*bfm).
compute sstotal=(y-(csum(y)/n)).
compute sstotal=csum(sstotal&*sstotal).
compute ssresid=csum(resid&*resid).
compute rsqch= r2 -((sstotal-ssresid)/sstotal).
compute fresult={rsqch,fresult}.
release xfm,flpc, resid, ssresid, bfm.
end if
.
compute dbint=dbint+1.
end if.
do if ((ydich = 1) and (i = (nms+nys))).

.
compute btemphld=b.
compute llrdat=make(nrow(x),nrow( lmat )-csum( lmat ),-999).
compute llrdf=ncol(x)-ncol(llrdat).
compute llrcnt=0.
loop llri=1 to nrow( lmat ).
do if ( lmat (llri,1)=0).
compute llrcnt=llrcnt+1.
compute llrdat(:,llrcnt)=x(:,llri).
end if.
end loop.

.
do if ( 2 =1).
compute hatmat=inv(t( llrdat )* llrdat )*t( llrdat ).
compute b = hatmat* y.
compute modres=b.
do if ( 0 =1).
compute n1=nrow( llrdat ).
compute dfres=n1-(ncol( llrdat )).
compute sstotal = t( y -(csum( y )/n1))*( y -(csum( y )/n1)).
compute resid= y - llrdat *b.
compute ssresid = csum((resid)&**2).
compute r2 = (sstotal-ssresid)/sstotal.
do if (r2 < 0).
compute r2=0.
end if.
compute adjr2 = 1-((1-r2)*(n1-1)/(dfres)).
compute mse=ssresid/(n1-ncol( llrdat )).

.
compute n1=nrow( x ).
compute invXtX = inv(t( x )* x ).
compute varb = mse *invXtX.
compute k3 = ncol( x ).
compute xhc=0.
do if ( hc <> 5).
compute xhc= x.
compute hat = xhc(:,1).
loop i3=1 to nrow(xhc).
compute hat(i3,1)= xhc(i3,:)*invXtX*t(xhc(i3,:)).
end loop.
do if ( hc = 0 or hc =1).
loop i3 = 1 to k3.
compute xhc(:,i3)=xhc(:,i3)&* resid.
end loop.
end if.
do if ( hc =3 or hc =2).
loop i3=1 to k3.
compute xhc(:,i3) = ( resid &/(1-hat)&**(1/(4- hc )))&*xhc(:,i3).
end loop.
end if.
do if ( hc = 4).
compute hcmn=make(n,2,4).
compute hcmn(:,2)=(n1*hat)/k3.
loop i3= 1 to k3.
compute xhc(:,i3) = ( resid &/(1-hat)&**(rmin(hcmn)/2))&*xhc(:,i3).
end loop.
end if.
compute varb=(invXtX*t(xhc)*xhc*invXtX).
do if ( hc =1).
compute varb=(n1/(n1-ncol( x )))&*varb.
end if.
end if.
compute hclab={'se(HC0)','se(HC1)','se(HC2)','se(HC3)','se(HC4)','se'}.
compute hclab=hclab(1,( hc +1)).
compute hcflab={'F(HC0)','F(HC1)','F(HC2)','F(HC3)','F(HC4)','F'}.
compute hcflab=hcflab(1,( hc +1)).
release xhc
.
compute seb=sqrt(diag(varb)).
compute trat = b&/seb.
compute p = 2*(1-tcdf(abs(trat), (dfres))).
compute tval = sqrt(dfres* (exp((dfres-(5/6))*((xp2/(dfres-(2/3)+(.11/dfres)))* (xp2/(dfres-(2/3)+(.11/dfres)))))-1)).
compute modres={modres,seb,trat,p}.
compute modres={modres,(b-tval&*seb),(b+tval&*seb)}.
compute modresl={'coeff',hclab,'t','p','LLCI','ULCI'}.
compute lmat = ident(ncol( llrdat )).
compute lmat = lmat(:,2:ncol(lmat)).
compute fratio = (t(t(lmat)*b)*inv(t(lmat)*varb*lmat)*((t(lmat)*b)))/(ncol( llrdat )-1).
compute pfr = 1-fcdf(fratio,(ncol( llrdat )-1),dfres).
compute modsum={sqrt(r2),r2,mse,fratio,(ncol( llrdat )-1),dfres,pfr}.
compute modsuml={'R','R-sq','MSE',hcflab,'df1','df2', 'p'}.
end if.
end if.
do if ( 2 = 2 or 2 =3).
compute xlp= llrdat.
compute ylp= y.
compute pt2 = make(nrow(ylp),1,(csum(ylp)/nrow(ylp))).
do if ( 2 =2).
compute LL3 = ylp&*ln(pt2)+(1-ylp)&*ln(1-pt2).
end if.
compute LL3 = -2*csum(LL3).
compute bt1 = make(ncol(xlp),1,0).
compute LL1 = 0.
compute pt1 = make(nrow(ylp),1,0.5).
compute pt1lp=pt1.
loop jjj = 1 to iterate.
compute xlptmp=t(xlp).
compute vecprb=pt1lp&*(1-pt1lp).
loop kkk=1 to ncol(xlp).
compute xlptmp(kkk,:)=xlptmp(kkk,:)&*t(vecprb).
end loop.
compute b = bt1+inv(xlptmp*xlp)*t(xlp)*(ylp-pt1lp).
do if ( 2 =2).
compute xlpb=xlp*b.
compute xlpbt=(xlpb > -709.7).
compute xlpb709=(1-xlpbt)*(-709.7).
compute xlpb=(xlpb&*xlpbt)+xlpb709.
compute pt1lp = 1/(1+exp(-(xlpb))).
end if.
compute itprob = csum((pt1lp < .00000001) or (pt1lp > .9999999)).
do if (itprob > 0).
loop kkk = 1 to nrow(pt1lp).
do if (pt1lp(kkk,1) > .9999999).
compute pt1lp(kkk,1) = .9999999.
end if.
do if (pt1lp(kkk,1) < .00000001).
compute pt1lp(kkk,1) = .00000001.
end if.
end loop.
compute itprob = 0.
end if.
do if (itprob = 0).
do if ( 2 =2).
compute LL = ylp&*ln(pt1lp)+(1-ylp)&*ln(1-pt1lp).
end if.
compute LL2 = -2*csum(ll).
end if.
do if (abs(LL1-LL2) < converge).
do if ( 0 =1).
compute xlptmp=t(xlp).
compute vecprb=pt1lp&*(1-pt1lp).
loop kkk=1 to ncol(xlp).
compute xlptmp(kkk,:)=xlptmp(kkk,:)&*t(vecprb).
end loop.
compute varb = inv(xlptmp*xlp).
compute seb = sqrt(diag(varb)).
release xlptmp.
end if.
break.
end if.
compute bt1 = b.
compute LL1 = LL2.
end loop.
compute modres=b.
do if (jjj > iterate).
compute itprob = 2.
do if (booting=0).
compute iterrmod=1.
end if.
do if (booting=1).
compute bootiter=1.
end if.
do if (itprobtg=0).
compute itprobtg=1.
compute errcode(errs,1) = 47.
compute errs = errs + 1.
do if (booting = 0 and 0 =1).
compute vt1 = mdiag(pt1lp&*(1-pt1lp)).
compute varb = inv(t(xlp)*vt1*xlp).
compute seb = sqrt(diag(varb)).
end if.
end if.
end if.
do if ( 0 =1).
compute trat = b&/seb.
compute dfres=nrow(xlp).
compute p = 2*(1-cdfnorm(abs(trat))).
compute modres={modres,seb,trat,p}.
compute modres={modres,(b-xp2&*seb),(b+xp2&*seb)}.
compute pvchi=1-chicdf((LL3-LL2),(nrow(modres)-1)).
compute mcF = (LL3-LL2)/LL3.
compute cox = 1-exp(-(LL3-LL2)/nrow(xlp)).
compute nagel = cox/(1-exp(-(LL3)/nrow(xlp))).
compute modsum={LL2,(LL3-LL2),(nrow(modres)-1),pvchi, mcF,cox,nagel}.
compute modsuml={'-2LL','ModelLL', 'df', 'p', 'McFadden', 'CoxSnell', 'Nagelkrk'}.
compute modresl={'coeff','se','Z','p','LLCI','ULCI'}.
end if.
end if
.
compute b=btemphld.
compute fresult={(LL2-basemod),llrdf,1-chicdf((LL2-basemod),llrdf)}
.
compute dbint=dbint+1.
end if.
compute highf={highf;fresult}.
compute highf2={highf2;fresult}.
do if (j = start).
compute flabel={flabel;'X*Z'}.
end if.
do if (j > start).
do if (nms > 1).
compute flabel={flabel;highlbz(jj,1)}.
else if (nms = 1).
compute flabel={flabel;'M*Z'}.
end if.
end if.
end if.
do if (dbint=2).
compute lmatdb=lmatdb+lmat.
do if ((ydich = 1) and (i = (nms+nys))).

.
compute btemphld=b.
compute llrdat=make(nrow(x),nrow( lmatdb )-csum( lmatdb ),-999).
compute llrdf=ncol(x)-ncol(llrdat).
compute llrcnt=0.
loop llri=1 to nrow( lmatdb ).
do if ( lmatdb (llri,1)=0).
compute llrcnt=llrcnt+1.
compute llrdat(:,llrcnt)=x(:,llri).
end if.
end loop.

.
do if ( 2 =1).
compute hatmat=inv(t( llrdat )* llrdat )*t( llrdat ).
compute b = hatmat* y.
compute modres=b.
do if ( 0 =1).
compute n1=nrow( llrdat ).
compute dfres=n1-(ncol( llrdat )).
compute sstotal = t( y -(csum( y )/n1))*( y -(csum( y )/n1)).
compute resid= y - llrdat *b.
compute ssresid = csum((resid)&**2).
compute r2 = (sstotal-ssresid)/sstotal.
do if (r2 < 0).
compute r2=0.
end if.
compute adjr2 = 1-((1-r2)*(n1-1)/(dfres)).
compute mse=ssresid/(n1-ncol( llrdat )).

.
compute n1=nrow( x ).
compute invXtX = inv(t( x )* x ).
compute varb = mse *invXtX.
compute k3 = ncol( x ).
compute xhc=0.
do if ( hc <> 5).
compute xhc= x.
compute hat = xhc(:,1).
loop i3=1 to nrow(xhc).
compute hat(i3,1)= xhc(i3,:)*invXtX*t(xhc(i3,:)).
end loop.
do if ( hc = 0 or hc =1).
loop i3 = 1 to k3.
compute xhc(:,i3)=xhc(:,i3)&* resid.
end loop.
end if.
do if ( hc =3 or hc =2).
loop i3=1 to k3.
compute xhc(:,i3) = ( resid &/(1-hat)&**(1/(4- hc )))&*xhc(:,i3).
end loop.
end if.
do if ( hc = 4).
compute hcmn=make(n,2,4).
compute hcmn(:,2)=(n1*hat)/k3.
loop i3= 1 to k3.
compute xhc(:,i3) = ( resid &/(1-hat)&**(rmin(hcmn)/2))&*xhc(:,i3).
end loop.
end if.
compute varb=(invXtX*t(xhc)*xhc*invXtX).
do if ( hc =1).
compute varb=(n1/(n1-ncol( x )))&*varb.
end if.
end if.
compute hclab={'se(HC0)','se(HC1)','se(HC2)','se(HC3)','se(HC4)','se'}.
compute hclab=hclab(1,( hc +1)).
compute hcflab={'F(HC0)','F(HC1)','F(HC2)','F(HC3)','F(HC4)','F'}.
compute hcflab=hcflab(1,( hc +1)).
release xhc
.
compute seb=sqrt(diag(varb)).
compute trat = b&/seb.
compute p = 2*(1-tcdf(abs(trat), (dfres))).
compute tval = sqrt(dfres* (exp((dfres-(5/6))*((xp2/(dfres-(2/3)+(.11/dfres)))* (xp2/(dfres-(2/3)+(.11/dfres)))))-1)).
compute modres={modres,seb,trat,p}.
compute modres={modres,(b-tval&*seb),(b+tval&*seb)}.
compute modresl={'coeff',hclab,'t','p','LLCI','ULCI'}.
compute lmat = ident(ncol( llrdat )).
compute lmat = lmat(:,2:ncol(lmat)).
compute fratio = (t(t(lmat)*b)*inv(t(lmat)*varb*lmat)*((t(lmat)*b)))/(ncol( llrdat )-1).
compute pfr = 1-fcdf(fratio,(ncol( llrdat )-1),dfres).
compute modsum={sqrt(r2),r2,mse,fratio,(ncol( llrdat )-1),dfres,pfr}.
compute modsuml={'R','R-sq','MSE',hcflab,'df1','df2', 'p'}.
end if.
end if.
do if ( 2 = 2 or 2 =3).
compute xlp= llrdat.
compute ylp= y.
compute pt2 = make(nrow(ylp),1,(csum(ylp)/nrow(ylp))).
do if ( 2 =2).
compute LL3 = ylp&*ln(pt2)+(1-ylp)&*ln(1-pt2).
end if.
compute LL3 = -2*csum(LL3).
compute bt1 = make(ncol(xlp),1,0).
compute LL1 = 0.
compute pt1 = make(nrow(ylp),1,0.5).
compute pt1lp=pt1.
loop jjj = 1 to iterate.
compute xlptmp=t(xlp).
compute vecprb=pt1lp&*(1-pt1lp).
loop kkk=1 to ncol(xlp).
compute xlptmp(kkk,:)=xlptmp(kkk,:)&*t(vecprb).
end loop.
compute b = bt1+inv(xlptmp*xlp)*t(xlp)*(ylp-pt1lp).
do if ( 2 =2).
compute xlpb=xlp*b.
compute xlpbt=(xlpb > -709.7).
compute xlpb709=(1-xlpbt)*(-709.7).
compute xlpb=(xlpb&*xlpbt)+xlpb709.
compute pt1lp = 1/(1+exp(-(xlpb))).
end if.
compute itprob = csum((pt1lp < .00000001) or (pt1lp > .9999999)).
do if (itprob > 0).
loop kkk = 1 to nrow(pt1lp).
do if (pt1lp(kkk,1) > .9999999).
compute pt1lp(kkk,1) = .9999999.
end if.
do if (pt1lp(kkk,1) < .00000001).
compute pt1lp(kkk,1) = .00000001.
end if.
end loop.
compute itprob = 0.
end if.
do if (itprob = 0).
do if ( 2 =2).
compute LL = ylp&*ln(pt1lp)+(1-ylp)&*ln(1-pt1lp).
end if.
compute LL2 = -2*csum(ll).
end if.
do if (abs(LL1-LL2) < converge).
do if ( 0 =1).
compute xlptmp=t(xlp).
compute vecprb=pt1lp&*(1-pt1lp).
loop kkk=1 to ncol(xlp).
compute xlptmp(kkk,:)=xlptmp(kkk,:)&*t(vecprb).
end loop.
compute varb = inv(xlptmp*xlp).
compute seb = sqrt(diag(varb)).
release xlptmp.
end if.
break.
end if.
compute bt1 = b.
compute LL1 = LL2.
end loop.
compute modres=b.
do if (jjj > iterate).
compute itprob = 2.
do if (booting=0).
compute iterrmod=1.
end if.
do if (booting=1).
compute bootiter=1.
end if.
do if (itprobtg=0).
compute itprobtg=1.
compute errcode(errs,1) = 47.
compute errs = errs + 1.
do if (booting = 0 and 0 =1).
compute vt1 = mdiag(pt1lp&*(1-pt1lp)).
compute varb = inv(t(xlp)*vt1*xlp).
compute seb = sqrt(diag(varb)).
end if.
end if.
end if.
do if ( 0 =1).
compute trat = b&/seb.
compute dfres=nrow(xlp).
compute p = 2*(1-cdfnorm(abs(trat))).
compute modres={modres,seb,trat,p}.
compute modres={modres,(b-xp2&*seb),(b+xp2&*seb)}.
compute pvchi=1-chicdf((LL3-LL2),(nrow(modres)-1)).
compute mcF = (LL3-LL2)/LL3.
compute cox = 1-exp(-(LL3-LL2)/nrow(xlp)).
compute nagel = cox/(1-exp(-(LL3)/nrow(xlp))).
compute modsum={LL2,(LL3-LL2),(nrow(modres)-1),pvchi, mcF,cox,nagel}.
compute modsuml={'-2LL','ModelLL', 'df', 'p', 'McFadden', 'CoxSnell', 'Nagelkrk'}.
compute modresl={'coeff','se','Z','p','LLCI','ULCI'}.
end if.
end if
.
compute b=btemphld.
compute fresult={(LL2-basemod),llrdf,1-chicdf((LL2-basemod),llrdf)}
.
end if.
do if (ydich <> 1 or i < (nms+nys)).

.
compute lmat2= lmatdb.
do if ( 0 =0).
compute lmat2 = mdiag( lmatdb ).
compute lmat3=make(nrow(lmat2),1,0).
loop flp=1 to ncol(lmat2).
do if (csum(lmat2(:,flp))=1).
compute lmat3={lmat3,lmat2(:,flp)}.
end if.
end loop.
compute lmat2=lmat3(:,2:ncol(lmat3)).
end if.
compute fratio = (t(t(lmat2)* b )*inv(t(lmat2)* varb *lmat2)*((t(lmat2)* b )))/ncol(lmat2).
compute pfr = 1-fcdf(fratio,ncol(lmat2),(n-nrow( b ))).
compute fresult={fratio,ncol(lmat2),(n-nrow( b )),pfr}.
do if (i = (nms+nys) and (ydich=1)).
compute fratio=fratio*ncol(lmat2).
compute pfr=1-chicdf(fratio,ncol(lmat2)).
compute fresult={fratio,ncol(lmat2),pfr}.
end if.
do if ( 1 =1).
compute lmat3=1-rsum(lmat2).
compute xfm=make(n,csum(lmat3),0).
compute flpc=1.
loop flp=1 to nrow(lmat3).
do if (lmat3(flp,1)=1).
compute xfm(:,flpc)=x(:,flp).
compute flpc=flpc+1.
end if.
end loop.
compute bfm=inv(t(xfm)*xfm)*t(xfm)*y.
compute resid=y-(xfm*bfm).
compute sstotal=(y-(csum(y)/n)).
compute sstotal=csum(sstotal&*sstotal).
compute ssresid=csum(resid&*resid).
compute rsqch= r2 -((sstotal-ssresid)/sstotal).
compute fresult={rsqch,fresult}.
release xfm,flpc, resid, ssresid, bfm.
end if
.
end if.
compute dbint=0.
compute highf={highf;fresult}.
do if (jj=0 and nms > 0).
compute flabel={flabel;'BOTH(X)'}.
end if.
do if (jj=0 and nms = 0).
compute flabel={flabel;'BOTH'}.
end if.
do if (jj>0 and nms = 1).
compute flabel={flabel;'BOTH(M)'}.
end if.
do if (nms > 1 and jj > 0).
compute flabel={flabel;highlbbt(jj,1)}.
end if.
end if.
compute lmat2=wzhigh(1:nump(1,i),j).
do if (csum(lmat2) > 0).
do if ((i < (nms+nys)) or (ydich <> 1)).

.
compute lmat2= lmat2.
do if ( 0 =0).
compute lmat2 = mdiag( lmat2 ).
compute lmat3=make(nrow(lmat2),1,0).
loop flp=1 to ncol(lmat2).
do if (csum(lmat2(:,flp))=1).
compute lmat3={lmat3,lmat2(:,flp)}.
end if.
end loop.
compute lmat2=lmat3(:,2:ncol(lmat3)).
end if.
compute fratio = (t(t(lmat2)* b )*inv(t(lmat2)* varb *lmat2)*((t(lmat2)* b )))/ncol(lmat2).
compute pfr = 1-fcdf(fratio,ncol(lmat2),(n-nrow( b ))).
compute fresult={fratio,ncol(lmat2),(n-nrow( b )),pfr}.
do if (i = (nms+nys) and (ydich=1)).
compute fratio=fratio*ncol(lmat2).
compute pfr=1-chicdf(fratio,ncol(lmat2)).
compute fresult={fratio,ncol(lmat2),pfr}.
end if.
do if ( 1 =1).
compute lmat3=1-rsum(lmat2).
compute xfm=make(n,csum(lmat3),0).
compute flpc=1.
loop flp=1 to nrow(lmat3).
do if (lmat3(flp,1)=1).
compute xfm(:,flpc)=x(:,flp).
compute flpc=flpc+1.
end if.
end loop.
compute bfm=inv(t(xfm)*xfm)*t(xfm)*y.
compute resid=y-(xfm*bfm).
compute sstotal=(y-(csum(y)/n)).
compute sstotal=csum(sstotal&*sstotal).
compute ssresid=csum(resid&*resid).
compute rsqch= r2 -((sstotal-ssresid)/sstotal).
compute fresult={rsqch,fresult}.
release xfm,flpc, resid, ssresid, bfm.
end if
.
end if.
do if ((ydich = 1) and (i = (nms+nys))).

.
compute btemphld=b.
compute llrdat=make(nrow(x),nrow( lmat2 )-csum( lmat2 ),-999).
compute llrdf=ncol(x)-ncol(llrdat).
compute llrcnt=0.
loop llri=1 to nrow( lmat2 ).
do if ( lmat2 (llri,1)=0).
compute llrcnt=llrcnt+1.
compute llrdat(:,llrcnt)=x(:,llri).
end if.
end loop.

.
do if ( 2 =1).
compute hatmat=inv(t( llrdat )* llrdat )*t( llrdat ).
compute b = hatmat* y.
compute modres=b.
do if ( 0 =1).
compute n1=nrow( llrdat ).
compute dfres=n1-(ncol( llrdat )).
compute sstotal = t( y -(csum( y )/n1))*( y -(csum( y )/n1)).
compute resid= y - llrdat *b.
compute ssresid = csum((resid)&**2).
compute r2 = (sstotal-ssresid)/sstotal.
do if (r2 < 0).
compute r2=0.
end if.
compute adjr2 = 1-((1-r2)*(n1-1)/(dfres)).
compute mse=ssresid/(n1-ncol( llrdat )).

.
compute n1=nrow( x ).
compute invXtX = inv(t( x )* x ).
compute varb = mse *invXtX.
compute k3 = ncol( x ).
compute xhc=0.
do if ( hc <> 5).
compute xhc= x.
compute hat = xhc(:,1).
loop i3=1 to nrow(xhc).
compute hat(i3,1)= xhc(i3,:)*invXtX*t(xhc(i3,:)).
end loop.
do if ( hc = 0 or hc =1).
loop i3 = 1 to k3.
compute xhc(:,i3)=xhc(:,i3)&* resid.
end loop.
end if.
do if ( hc =3 or hc =2).
loop i3=1 to k3.
compute xhc(:,i3) = ( resid &/(1-hat)&**(1/(4- hc )))&*xhc(:,i3).
end loop.
end if.
do if ( hc = 4).
compute hcmn=make(n,2,4).
compute hcmn(:,2)=(n1*hat)/k3.
loop i3= 1 to k3.
compute xhc(:,i3) = ( resid &/(1-hat)&**(rmin(hcmn)/2))&*xhc(:,i3).
end loop.
end if.
compute varb=(invXtX*t(xhc)*xhc*invXtX).
do if ( hc =1).
compute varb=(n1/(n1-ncol( x )))&*varb.
end if.
end if.
compute hclab={'se(HC0)','se(HC1)','se(HC2)','se(HC3)','se(HC4)','se'}.
compute hclab=hclab(1,( hc +1)).
compute hcflab={'F(HC0)','F(HC1)','F(HC2)','F(HC3)','F(HC4)','F'}.
compute hcflab=hcflab(1,( hc +1)).
release xhc
.
compute seb=sqrt(diag(varb)).
compute trat = b&/seb.
compute p = 2*(1-tcdf(abs(trat), (dfres))).
compute tval = sqrt(dfres* (exp((dfres-(5/6))*((xp2/(dfres-(2/3)+(.11/dfres)))* (xp2/(dfres-(2/3)+(.11/dfres)))))-1)).
compute modres={modres,seb,trat,p}.
compute modres={modres,(b-tval&*seb),(b+tval&*seb)}.
compute modresl={'coeff',hclab,'t','p','LLCI','ULCI'}.
compute lmat = ident(ncol( llrdat )).
compute lmat = lmat(:,2:ncol(lmat)).
compute fratio = (t(t(lmat)*b)*inv(t(lmat)*varb*lmat)*((t(lmat)*b)))/(ncol( llrdat )-1).
compute pfr = 1-fcdf(fratio,(ncol( llrdat )-1),dfres).
compute modsum={sqrt(r2),r2,mse,fratio,(ncol( llrdat )-1),dfres,pfr}.
compute modsuml={'R','R-sq','MSE',hcflab,'df1','df2', 'p'}.
end if.
end if.
do if ( 2 = 2 or 2 =3).
compute xlp= llrdat.
compute ylp= y.
compute pt2 = make(nrow(ylp),1,(csum(ylp)/nrow(ylp))).
do if ( 2 =2).
compute LL3 = ylp&*ln(pt2)+(1-ylp)&*ln(1-pt2).
end if.
compute LL3 = -2*csum(LL3).
compute bt1 = make(ncol(xlp),1,0).
compute LL1 = 0.
compute pt1 = make(nrow(ylp),1,0.5).
compute pt1lp=pt1.
loop jjj = 1 to iterate.
compute xlptmp=t(xlp).
compute vecprb=pt1lp&*(1-pt1lp).
loop kkk=1 to ncol(xlp).
compute xlptmp(kkk,:)=xlptmp(kkk,:)&*t(vecprb).
end loop.
compute b = bt1+inv(xlptmp*xlp)*t(xlp)*(ylp-pt1lp).
do if ( 2 =2).
compute xlpb=xlp*b.
compute xlpbt=(xlpb > -709.7).
compute xlpb709=(1-xlpbt)*(-709.7).
compute xlpb=(xlpb&*xlpbt)+xlpb709.
compute pt1lp = 1/(1+exp(-(xlpb))).
end if.
compute itprob = csum((pt1lp < .00000001) or (pt1lp > .9999999)).
do if (itprob > 0).
loop kkk = 1 to nrow(pt1lp).
do if (pt1lp(kkk,1) > .9999999).
compute pt1lp(kkk,1) = .9999999.
end if.
do if (pt1lp(kkk,1) < .00000001).
compute pt1lp(kkk,1) = .00000001.
end if.
end loop.
compute itprob = 0.
end if.
do if (itprob = 0).
do if ( 2 =2).
compute LL = ylp&*ln(pt1lp)+(1-ylp)&*ln(1-pt1lp).
end if.
compute LL2 = -2*csum(ll).
end if.
do if (abs(LL1-LL2) < converge).
do if ( 0 =1).
compute xlptmp=t(xlp).
compute vecprb=pt1lp&*(1-pt1lp).
loop kkk=1 to ncol(xlp).
compute xlptmp(kkk,:)=xlptmp(kkk,:)&*t(vecprb).
end loop.
compute varb = inv(xlptmp*xlp).
compute seb = sqrt(diag(varb)).
release xlptmp.
end if.
break.
end if.
compute bt1 = b.
compute LL1 = LL2.
end loop.
compute modres=b.
do if (jjj > iterate).
compute itprob = 2.
do if (booting=0).
compute iterrmod=1.
end if.
do if (booting=1).
compute bootiter=1.
end if.
do if (itprobtg=0).
compute itprobtg=1.
compute errcode(errs,1) = 47.
compute errs = errs + 1.
do if (booting = 0 and 0 =1).
compute vt1 = mdiag(pt1lp&*(1-pt1lp)).
compute varb = inv(t(xlp)*vt1*xlp).
compute seb = sqrt(diag(varb)).
end if.
end if.
end if.
do if ( 0 =1).
compute trat = b&/seb.
compute dfres=nrow(xlp).
compute p = 2*(1-cdfnorm(abs(trat))).
compute modres={modres,seb,trat,p}.
compute modres={modres,(b-xp2&*seb),(b+xp2&*seb)}.
compute pvchi=1-chicdf((LL3-LL2),(nrow(modres)-1)).
compute mcF = (LL3-LL2)/LL3.
compute cox = 1-exp(-(LL3-LL2)/nrow(xlp)).
compute nagel = cox/(1-exp(-(LL3)/nrow(xlp))).
compute modsum={LL2,(LL3-LL2),(nrow(modres)-1),pvchi, mcF,cox,nagel}.
compute modsuml={'-2LL','ModelLL', 'df', 'p', 'McFadden', 'CoxSnell', 'Nagelkrk'}.
compute modresl={'coeff','se','Z','p','LLCI','ULCI'}.
end if.
end if
.
compute b=btemphld.
compute fresult={(LL2-basemod),llrdf,1-chicdf((LL2-basemod),llrdf)}
.
end if.
compute highf={highf;fresult}.
compute highf2={highf2;fresult}.
do if (j = start).
compute flabel={flabel;'X*W*Z'}.
end if.
do if (j > start).
do if (nms > 1).
compute flabel={flabel;highlbwz(jj,1)}.
else if (nms = 1).
compute flabel={flabel;'M*W*Z'}.
end if.
end if.
end if.
compute jj=jj+1.
end loop.
release jj.
compute start=start+i.
end if.
do if (nrow(highf) > 1).
compute highf=highf(2:nrow(highf),:).
compute highf2=highf2(2:nrow(highf2),:).
compute flabel=flabel(2:nrow(flabel),1).
compute resultm2=make(nrow(highf),maxresm,99999).
compute resultm2(1:nrow(highf),1:ncol(highf))=highf.
compute resultm={resultm;resultm2}.
do if (outscree=1).
do if ((i < nms+nys) or (ydich=0)).
compute clabtmp={'R2-chng', hcflab,'df1','df2','p'}.
print highf/format = F10.4 /rnames=flabel/cnames=clabtmp/ title = 'Test(s) of highest order unconditional interaction(s):'.
end if.
do if (ydich=1 and i=(nms+nys)).
compute clabtmp={'Chi-sq', 'df','p'}.
print/title='Likelihood ratio test(s) of highest order'.
print highf/format = F10.4 /rnames=flabel/cnames=clabtmp/ title = 'unconditional interactions(s):'/space=0.
end if.
end if.
compute intpb=highf2(:,ncol(highf2)).
end if.
compute intstart=intstart+numint(1,i).
end if.

.
do if (criterr=0).
compute threeway=0.
compute didprint=0.
compute didsome=0.
compute sigintct=0.
loop jmed =1 to (nms+1).
compute hasw=0.
compute hasz=0.
compute jnok=0.
compute nm1vls=0.
compute nm2vls=0.
compute panelgrp=0.
compute graphixs={'WITH', outnames(1,i), 'BY'}.
compute focpred4={' '}.
compute intprint=0.
compute modcat=0.
do if (jmed <= i).
do if ((jmed = 1) and ((i+1) = nrow(bcmat))).
compute pathscnt=pathscnt+1.
else.
compute paths={paths,bcmat((i+1),jmed)}.
compute pathsw={pathsw,wcmat((i+1),jmed)}.
compute pathsz={pathsz,zcmat((i+1),jmed)}.
compute pathswz={pathswz,wzcmat((i+1),jmed)}.
compute temp=fochigh(:,pathscnt)&*bootloc(:,i).
compute pathsfoc={pathsfoc,pathsfoc(:,1)}.
do if (jmed=1).
compute pathtype={pathtype,1}.
end if.
do if ((i+1)=nrow(bcmat)).
compute pathtype={pathtype,3}.
end if.
do if (jmed > 1) and ((i+1) < nrow(bcmat)).
compute pathtype={pathtype,2}.
end if.
do if (jmed=1 and nxvls > 1 and (bcmat((i+1),jmed)=1)).
compute pathsfoc(:,(pathscn2+1))=temp(2:(nxvls+1),1).
end if.
do if ((jmed > 1) or (jmed=1 and nxvls=1)).
compute temp=cmax(temp).
compute pathsfoc(1,(pathscn2+1))=temp.
end if.
compute pathscnt=pathscnt+1.
compute pathscn2=pathscn2+1.
do if (i <= nms).
compute pathsdv={pathsdv,mnames(1,i)}.
end if.
do if (i > nms).
compute pathsdv={pathsdv,ynames}.
end if.
end if.
compute coeffcol=coeffcol+1.
compute probettt=coeffs(1:nrow(b),coeffcol).
do if (jmed=1 and (bcmat((i+1),jmed)=1)).
compute omni=make(nrow(probettt),nxvls,0).
compute omnitmp=ident(nxvls).
compute omni(2:(1+nxvls),:)=omnitmp.
end if.
do if (csum(probettt)>0).
compute probvarb=make(csum(probettt),csum(probettt),999).
compute probcoef=make(csum(probettt),1,999).
compute coefflp2=1.
loop coefflp=1 to nrow(probettt).
do if (probettt(coefflp,1)=1).
compute probcoef(coefflp2,1)=b(coefflp,1).
compute coefflp2=coefflp2+1.
end if.
end loop.
compute coefflp=0.
compute coefflp2=0.
loop iclp=1 to nrow(probettt).
do if probettt(iclp,1)=1.
compute coefflp=coefflp+1.
compute coefflp2=coefflp.
compute probvarb(coefflp,coefflp) = varb(iclp,iclp).
do if (iclp < nrow(probettt)).
loop jclp=(iclp+1) to nrow(probettt).
do if (probettt(jclp,1)=1).
compute coefflp2=coefflp2+1.
compute probvarb(coefflp,coefflp2)=varb(iclp, jclp).
compute probvarb(coefflp2,coefflp)=varb(iclp, jclp).
end if.
end loop.
end if.
end if.
end loop.
end if.
end if.
compute xprobval=xmodvals.
do if (nxvls > 1 or mcx > 0).
compute xprobval=dummatx(:,2:ncol(dummatx)).
end if.
do if ((wcmat((i+1),jmed)=1) and (zcmat((i+1),jmed)=0)).
compute numplps=1.
compute modvals=wmodvals.
compute probeval=wmodvals.
compute wheremv1=wherexw.
compute nm1vls=nwvls.
compute lpstsp={1,1}.
compute modcat=0.
compute jnmod=wtmp.
compute jnmodlab=wnames.
compute jnok=1.
compute jnmin=wmin.
compute jnmax=wmax.
compute wherejn1=2.
do if (jmed=1).
compute wherejn3=wherexw(1,i).
do if (nxvls > 1).
compute jnok=0.
end if.
end if.
do if (jmed > 1).
compute wherejn1=wherem((jmed-1),i).
compute wherejn3=wheremw(((2*jmed)-3),i).
end if.
do if (nwvls > 1).
compute probeval=wprobval.
compute lpstsp(1,2)=ncol(probeval).
compute modcat=1.
compute jnok=0.
end if.
do if (wdich = 1).
compute modcat=1.
compute jnok=0.
end if.
compute problabs=wnames.
compute focpred3={wnames,'(W)'}.
do if (xmint=1).
compute focpred3={wnames,'(X)'}.
end if.
compute hasw=1.
compute modgrph=wnames.
compute intprint=1.
compute sigintct=sigintct+1.
compute printpbe=intpb(sigintct,1).
end if.
do if ((wcmat((i+1),jmed)=0) and (zcmat((i+1),jmed)=1)).
compute numplps=1.
compute modvals=zmodvals.
compute probeval=zmodvals.
compute wheremv1=wherexz.
compute nm1vls=nzvls.
compute lpstsp={1,1}.
compute jnok=1.
compute jnmod=ztmp.
compute jnmin=zmin.
compute jnmax=zmax.
compute jnmodlab=znames.
compute wherejn1=2.
do if (jmed=1).
compute wherejn3=wherexz(1,i).
do if (nxvls > 1).
compute jnok=0.
end if.
end if.
do if (jmed > 1).
compute wherejn1=wherem((jmed-1),i).
compute wherejn3=wheremz(((2*jmed)-3),i).
end if.
do if (nzvls > 1).
compute probeval=zprobval.
compute lpstsp(1,2)=ncol(probeval).
compute modcat=1.
compute jnok=0.
end if.
do if (zdich = 1).
compute modcat=1.
compute jnok=0.
end if.
compute problabs=znames.
compute focpred3={znames,'(Z)'}.
compute modgrph=znames.
compute hasz=1.
compute intprint=1.
compute sigintct=sigintct+1.
compute printpbe=intpb(sigintct,1).
end if.
do if ((wzcmat((i+1),jmed)=1) or ((wcmat((i+1),jmed)=1) and (zcmat((i+1),jmed)=1))).
compute numplps=2.
compute probecnt=1.
compute intprint=1.
do if (wzcmat((i+1),jmed)=1).
compute sigintct=sigintct+1.
compute printpbe=intpb(sigintct,1).
else.
compute sigintct=sigintct+2.
compute printpbe=cmin(intpb((sigintct-1):sigintct,1)).
end if.
compute panelgrp=1.
compute hasw=1.
compute hasz=1.
compute panelcde={'/PANEL','ROWVAR=',znames,'.'}.
compute modgrph=wnames.
compute lpstsp={1,1;1,1}.
compute wheremv1=wherexw.
compute nm1vls=nwvls.
compute wheremv2=wherexz.
compute nm2vls=nzvls.
compute jnok=0.
do if (wzcmat((i+1),jmed)=1).
compute jnok=1.
end if.
do if (jmed > 1).
compute mprobval=mmodvals.
end if.
do if (jmed=1).
do if (nxvls > 1).
compute jnok=0.
end if.
end if.
do if (nwvls > 1).
compute lpstsp(1,2)=ncol(wprobval).
compute modcat=1.
compute jnok=0.
end if.
do if (zdich=1).
compute modcat=1.
compute jnok=0.
end if.
compute lpstsp(2,1)=lpstsp(1,2)+1.
compute lpstsp(2,2)=lpstsp(1,2)+1.
do if (nzvls > 1).
compute lpstsp(2,1)=lpstsp(1,2)+1.
compute lpstsp(2,2)=lpstsp(1,2)+ncol(zprobval).
compute jnok=0.
end if.
do if (zdich=1).
compute jnok=0.
end if.
compute omni3=make(nrow(b),(nxvls*nwvls),0).
do if (jmed > 1).
compute omni3=make(nrow(b),nwvls,0).
end if.
compute focpred3={wnames,'(W)'}.
do if (xmint=1).
compute focpred3={wnames,'(X)'}.
end if.
compute focpred4={'    ', 'Mod var:', znames, '(Z)'}.
compute modvals=make((nrow(wmodvals)*nrow(zmodvals)),2,0).
compute probeval=make((nrow(wmodvals)*nrow(zmodvals)),(ncol(wprobval)+ncol(zprobval)),0).
loop probei= 1 to nrow(wmodvals).
loop probej =1 to nrow(zmodvals).
compute modvals(probecnt,1)=wmodvals(probei,1).
compute probeval(probecnt,1:nwvls)=wprobval(probei,:).
compute modvals(probecnt,2)=zmodvals(probej,1).
compute probeval(probecnt,(nwvls+1):(nwvls+nzvls))=zprobval(probej,:).
compute probecnt=probecnt+1.
end loop.
end loop.
do if (wzcmat((i+1),jmed)=1).
compute numplps=numplps+1.
compute probprod=make(1,(ncol(wprobval)*ncol(zprobval)),0).
compute lpstsp2={1,1}.
compute lpstsp={lpstsp;lpstsp2}.
compute lpstsp(3,1)=lpstsp(2,2)+1.
compute lpstsp(3,2)=lpstsp(2,2)+ncol(probprod).
compute jnmod=ztmp.
compute jnmin=zmin.
compute jnmax=zmax.
compute jnmodlab=znames.
do if (jmed = 1).
compute wherejn1=wherexw(1,i).
compute wherejn3=wherexwz(1,i).
end if.
do if (jmed > 1).
compute wherejn1=wheremw(((2*jmed)-3),i).
compute wherejn3=wheremwz(((2*jmed)-3),i).
end if.
loop probei = 1 to nrow(wmodvals).
loop probej = 1 to nrow(zmodvals).
compute probtemp=1.
loop probek = 1 to ncol(wprobval).
compute probtemp={probtemp,(wprobval(probei,probek)&*zprobval(probej,:))}.
end loop.
compute probprod={probprod;probtemp(1,2:ncol(probtemp))}.
end loop.
end loop.
compute probprod=probprod(2:nrow(probprod),:).
compute probeval={probeval,probprod}.
end if.
compute problabs={wnames,znames}.
release probecnt, probei, probej.
end if.
do if (intprint=1).
compute focpred={'   Focal', 'predict:'}.
do if (jmed=1).
compute focpred={focpred,xnames,'(X)'}.
compute focplotv=xmodvals.
end if.
do if (jmed >1).
do if (nms > 1).
compute focpred={focpred,mnames(1,(jmed-1)), medlb2(1,(jmed-1))}.
end if.
do if (nms = 1).
compute focpred={focpred,mnames(1,(jmed-1)), '(M)'}.
end if.
compute focplotv=mmodvals(:,(jmed-1)).
end if.
compute focpred2={'    ', 'Mod var:',focpred3}.
compute focpred={focpred;focpred2}.
do if (ncol(focpred4) > 1).
compute focpred={focpred;focpred4}.
compute focpred4={' '}.
end if.
release focpred2,focpred3.
do if (outscree=1).
do if ((plot = 1 or plot = 2) or (printpbe <= intprobe)).
print focpred/title='----------'/format=A8/space=0.
end if.
end if.
compute foctmp=make(nrow(modvals),1,1).
compute probexpl=1.
compute probeva2={foctmp,probeval}.
do if (jmed=1 and nxs > 0 and mcx > 0).
compute probexpl=nxvls.
end if.
compute foctmp=make(nrow(modvals),1,1).
compute modvals3=make(1,(6+ncol(problabs)),0).
compute probrown=make(nrow(probeval),1,0).
compute jtmp=1.
loop probei = 1 to nrow(probeval).
compute probrown(probei,1)=jtmp.
compute jtmp=jtmp+nxvls.
end loop.
release jtmp.
compute probrow=999.
compute modvarl=problabs.
do if (plot = 1 or plot = 2 or nxvls > 1).
compute plotvals=make((nrow(modvals)*nrow(focplotv)),(ncol(modvals)+1),999).
loop ploti=1 to nrow(modvals).
loop plotj=1 to nrow(focplotv).
compute plotvals((((ploti-1)*nrow(focplotv))+plotj),2:ncol(plotvals))=modvals(ploti,:).
compute plotvals((((ploti-1)*nrow(focplotv))+plotj),1)=focplotv(plotj,1).
end loop.
end loop.
compute focpredn=3.
do if (jmed=1).
do if (nxvls > 1).
compute focpredn=(nxvls+1).
end if.
do if (nxvls=1 and xdich=1).
compute focpredn=2.
end if.
end if.
compute meanmat=mdiag(means).
compute onesmat=make(nrow(meanmat),(nrow(probeval)*focpredn),1).
compute probeplt=t(mdiag(means)*onesmat).
do if (jmed=1).
do if ((wcmat((i+1),1)=1) or (zcmat((i+1),1)=1)).
compute plotcnt=1.
compute iloops=nwpval*nzpval.
compute plotmx=nxpval*nzpval.
do if ((wcmat((i+1),1)=1) and (zcmat((i+1),1)=0)).
compute iloops=nwpval.
compute plotmx=nxpval.
end if.
do if ((wcmat((i+1),1)=0) and (zcmat((i+1),1)=1)).
compute iloops=nzpval.
compute plotmx=nxpval.
end if.
compute xestvals=make((nxpval*iloops),ncol(xprobval),-999).
do if (wcmat((i+1),1)=1).
compute westvals=make(nrow(xestvals),ncol(wprobval),-999).
end if.
do if (zcmat((i+1),1)=1).
compute zestvals=make(nrow(xestvals),ncol(zprobval),-999).
end if.
loop ploti=1 to iloops.
loop plotj=1 to nxpval.
compute xestvals(plotcnt,:)=xprobval(plotj,:).
compute plotcnt=plotcnt+1.
end loop.
end loop.
compute plotcnt=1.
compute plotcnt1=1.
compute plotcnt2=1.
compute plotcntz=1.
loop ploti = 1 to (iloops*nxpval).
do if (wcmat((i+1),1)=1).
compute westvals(ploti,:)=wprobval(plotcnt1,:).
end if.
do if ((wcmat((i+1),1)=0) and (zcmat((i+1),1)=1)).
compute zestvals(ploti,:)=zprobval(plotcnt1,:).
end if.
do if ((wcmat((i+1),1)=1) and (zcmat((i+1),1)=1)).
compute zestvals(ploti,:)=zprobval(plotcnt2,:).
compute plotcntz=plotcntz+1.
end if.
compute plotcnt=plotcnt+1.
do if (plotcnt > plotmx).
compute plotcnt=1.
compute plotcnt1=plotcnt1+1.
end if.
do if (plotcntz > nxpval).
compute plotcnt2=plotcnt2+1.
compute plotcntz=1.
do if (plotcnt2 > nzpval).
compute plotcnt2=1.
end if.
end if.
end loop.
compute probeplt(:,2:(1+(ncol(xestvals))))=xestvals.
do if (wcmat((i+1),1)=1).
compute probeplt(:,(wherew(1,i)):(wherew(2,i)))=westvals.
end if.
do if (zcmat((i+1),1)=1).
compute probeplt(:,(wherez(1,i)):(wherez(2,i)))=zestvals.
end if.
end if.
end if.
do if (jmed > 1).
do if ((wcmat((i+1),jmed)=1) or (zcmat((i+1),jmed)=1)).
compute plotcnt=1.
compute iloops=nwpval*nzpval.
compute plotmx=3*nzpval.
do if ((wcmat((i+1),jmed)=1) and (zcmat((i+1),jmed)=0)).
compute iloops=nwpval.
compute plotmx=3.
end if.
do if ((wcmat((i+1),jmed)=0) and (zcmat((i+1),jmed)=1)).
compute iloops=nzpval.
compute plotmx=3.
end if.
compute mestvals=make((3*iloops),1,-999).
do if (wcmat((i+1),jmed)=1).
compute westvals=make(nrow(mestvals),ncol(wprobval),-999).
end if.
do if (zcmat((i+1),jmed)=1).
compute zestvals=make(nrow(mestvals),ncol(zprobval),-999).
end if.
loop ploti=1 to iloops.
loop plotj=1 to 3.
compute mestvals(plotcnt,:)=mprobval(plotj,(jmed-1)).
compute plotcnt=plotcnt+1.
end loop.
end loop.
compute plotcnt=1.
compute plotcnt1=1.
compute plotcnt2=1.
compute plotcntz=1.
loop ploti = 1 to (iloops*3).
do if (wcmat((i+1),jmed)=1).
compute westvals(ploti,:)=wprobval(plotcnt1,:).
end if.
do if ((wcmat((i+1),jmed)=0) and (zcmat((i+1),jmed)=1)).
compute zestvals(ploti,:)=zprobval(plotcnt1,:).
end if.
do if ((wcmat((i+1),jmed)=1) and (zcmat((i+1),jmed)=1)).
compute zestvals(ploti,:)=zprobval(plotcnt2,:).
compute plotcntz=plotcntz+1.
end if.
compute plotcnt=plotcnt+1.
do if (plotcnt > plotmx).
compute plotcnt=1.
compute plotcnt1=plotcnt1+1.
end if.
do if (plotcntz > 3).
compute plotcnt2=plotcnt2+1.
compute plotcntz=1.
do if (plotcnt2 > nzpval).
compute plotcnt2=1.
end if.
end if.
end loop.
compute probeplt(:,wherem((jmed-1),i))=mestvals.
do if (wcmat((i+1),jmed)=1).
do if (model <> 74).
compute probeplt(:,(wherew(1,i)):(wherew(2,i)))=westvals.
end if.
do if (model = 74).
compute probeplt(:,(wherex(1,i)):(wherex(2,i)))=westvals.
end if.
end if.
do if (zcmat((i+1),jmed)=1).
compute probeplt(:,(wherez(1,i)):(wherez(2,i)))=zestvals.
end if.
end if.
end if.
compute prodloop = 1.
do if (jmed=1).
compute prodloop=ncol(xestvals).
end if.
do if (wcmat((i+1),jmed))=1.
compute plotcnt=0.
loop ploti = 1 to prodloop.
loop plotj = 1 to ncol(westvals).
do if (jmed=1).
compute probeplt(:,(wherexw(1,i)+plotcnt))=xestvals(:,ploti)&*westvals(:,plotj).
end if.
do if (jmed > 1).
compute probeplt(:,(wheremw(((jmed*2)-3) ,i)+plotcnt))=mestvals(:,ploti)&*westvals(:,plotj).
end if.
compute plotcnt=plotcnt+1.
end loop.
end loop.
end if.
do if (zcmat((i+1),jmed))=1.
compute plotcnt=0.
loop ploti = 1 to prodloop.
loop plotj = 1 to ncol(zestvals).
do if (jmed = 1).
compute probeplt(:,(wherexz(1,i)+plotcnt))=xestvals(:,ploti)&*zestvals(:,plotj).
end if.
do if (jmed > 1).
compute probeplt(:,(wheremz(((jmed*2)-3),i)+plotcnt))=mestvals(:,ploti)&*zestvals(:, plotj).
end if.
compute plotcnt=plotcnt+1.
end loop.
end loop.
end if.
do if (wzcmat((i+1),jmed))=1.
compute plotcnt=0.
compute threeway=1.
loop ploti = 1 to ncol(westvals).
loop plotj = 1 to ncol(zestvals).
compute probeplt(:,(wherewz(1,i)+plotcnt))=westvals(:,ploti)&*zestvals(:,plotj).
compute plotcnt=plotcnt+1.
end loop.
end loop.
compute plotcnt=0.
loop plotk = 1 to prodloop.
loop ploti = 1 to ncol(westvals).
loop plotj = 1 to ncol(zestvals).
do if (jmed = 1).
compute probeplt(:,(wherexwz(1,i)+plotcnt))=xestvals(:,plotk)&*westvals(:,ploti)&* zestvals(:,plotj).
end if.
do if (jmed > 1).
compute probeplt(:,(wheremwz(((jmed*2)-3),i)+plotcnt))=mestvals(:,plotk)&* westvals(:,ploti)&*zestvals(:,plotj).
end if.
compute plotcnt=plotcnt+1.
end loop.
end loop.
end loop.
end if.
loop newplp=1 to i.
do if (newplp <> jmed).
do if (wcmat((i+1),newplp))=1.
compute prodloop=1.
do if (newplp=1).
compute prodloop=nxvls.
end if.
compute plotcnt=0.
loop ploti = 1 to prodloop.
loop plotj = 1 to nwvls.
do if (newplp = 1).
compute probeplt(:,(wherexw(1,i)+plotcnt))=probeplt(:,(1+ploti))&*probeplt(:, (wherew(1,i)+plotj-1)).
end if.
do if (newplp > 1).
do if (model <> 74).
compute probeplt(:,(wheremw(((newplp*2)-3) ,i)+plotcnt))=probeplt(:,wherem((newplp-1),i))&*probeplt(:,(wherew(1,i)+plotj-1)).
end if.
do if (model = 74).
compute probeplt(:,(wheremw(((newplp*2)-3) ,i)+plotcnt))=probeplt(:,wherem((newplp-1),i))&*probeplt(:,(wherex(1,i)+plotj-1)).
end if.
end if.
compute plotcnt=plotcnt+1.
end loop.
end loop.
end if.
do if (zcmat((i+1),newplp))=1.
compute prodloop=1.
do if (newplp=1).
compute prodloop=nxvls.
end if.
compute plotcnt=0.
loop ploti = 1 to prodloop.
loop plotj = 1 to nzvls.
do if (newplp = 1).
compute probeplt(:,(wherexz(1,i)+plotcnt))=probeplt(:,(1+ploti))&*probeplt(:, (wherez(1,i)+plotj-1)).
end if.
do if (newplp > 1).
compute probeplt(:,(wheremz(((newplp*2)-3),i)+plotcnt))=probeplt(:, wherem((newplp-1),i))&*probeplt(:,(wherez(1,i)+plotj-1)).
end if.
compute plotcnt=plotcnt+1.
end loop.
end loop.
end if.
do if (wzcmat((i+1),newplp))=1.
compute plotcnt=0.
do if (threeway=0).
loop ploti = 1 to nwvls.
loop plotj = 1 to nzvls.
compute probeplt(:,(wherewz(1,i)+plotcnt))=probeplt(:,(wherew(1,i)+ploti-1))&* probeplt(:,(wherez(1,i)+plotj-1)).
compute plotcnt=plotcnt+1.
end loop.
end loop.
end if.
compute prodloop=1.
do if (newplp=1).
compute prodloop=nxvls.
end if.
compute plotcnt=0.
loop plotk = 1 to prodloop.
loop ploti = 1 to nwvls.
loop plotj = 1 to nzvls.
do if (newplp = 1).
compute probeplt(:,(wherexwz(1,i)+plotcnt))=probeplt(:,(1+plotk))&*probeplt(:, (wherew(1,i)+ploti-1))&*probeplt(:,(wherez(1,i)+plotj-1)).
end if.
do if (newplp > 1).
compute probeplt(:,(wheremwz(((newplp*2)-3),i)+plotcnt))=probeplt(:, wherem((newplp-1),i))&*probeplt(:,(wherew(1,i)+ploti-1))&*probeplt(:,(wherez(1,i)+plotj-1)).
end if.
compute plotcnt=plotcnt+1.
end loop.
end loop.
end loop.
end if.
end if.
end loop.
compute predvals=probeplt*b.
do if (debug <> 0).
print probeplt.
print b.
end if.
do if (i = nms+nys) and (ydich=1).
compute predvalt=(predvals < 709.7).
compute prevalt7=(1-predvalt)*(709.7).
compute predvals=(predvals&*predvalt)+prevalt7.
compute expyhat=exp(predvals)&/(1+exp(predvals)).
end if.
compute sepred=make(nrow(plotvals),3,999).
loop sei=1 to nrow(plotvals).
compute ask=probeplt(sei,:).
compute sepred(sei,1)=sqrt(ask*varb*t(ask)).
do if ((i < nms+nys) or (ydich=0)).
compute sepred(sei,2)=predvals(sei,1)-tval*sepred(sei,1).
compute sepred(sei,3)=predvals(sei,1)+tval*sepred(sei,1).
end if.
do if ((i = nms+nys) and (ydich=1)).
compute sepred(sei,2)=predvals(sei,1)-xp2*sepred(sei,1).
compute sepred(sei,3)=predvals(sei,1)+xp2*sepred(sei,1).
end if.
end loop.
compute prevloc=ncol(plotvals)+1.
compute probeplt={plotvals,predvals}.
do if (plot = 2).
compute probeplt={probeplt,sepred}.
end if.
do if ((i = nms+nys) and (ydich=1)).
compute probeplt={probeplt,expyhat}.
end if.
compute didsome=0.
end if.
do if ((wzcmat((i+1),jmed)=1) and (printpbe <= intprobe)).
do if (jmed=1).
compute omnilp2=nxvls*nwvls.
compute omnitmp=ident(omnilp2).
compute omni3(wherexw(1,i):wherexw(2,i),:)=omnitmp.
end if.
do if (jmed>1).
compute omnilp2=nwvls.
compute omnitmp=ident(omnilp2).
compute omni3(wheremw(((jmed*2)-3),i):wheremw(((jmed*2)-2),i),:)=omnitmp.
end if.
compute omnif=make(1,4,0).
do if ((i = nms+nys) and (ydich=1)).
compute omnif=make(1,3,0).
end if.
compute condeff3=0.
loop omnilp1=1 to nrow(zprobval).
loop omnilp=1 to (omnilp2).
do if (jmed=1).
compute omni3((wherexwz(1,i)+((omnilp-1)*nzvls)):(wherexwz(1,i)+((omnilp-1)*nzvls)+ (nzvls-1)),omnilp)=t(zprobval(omnilp1,:)).
end if.
do if (jmed > 1).
compute omni3((wheremwz(((jmed*2)-3),i)+((omnilp-1)*nzvls)):(wheremwz(((jmed*2)-3),i)+ ((omnilp-1)*nzvls)+(nzvls-1)),omnilp)=t(zprobval(omnilp1,:)).
end if.
end loop.
compute condeff=t(omni3)*b.
compute condeff3={condeff3;condeff}.

.
compute lmat2= omni3.
do if ( 1 =0).
compute lmat2 = mdiag( omni3 ).
compute lmat3=make(nrow(lmat2),1,0).
loop flp=1 to ncol(lmat2).
do if (csum(lmat2(:,flp))=1).
compute lmat3={lmat3,lmat2(:,flp)}.
end if.
end loop.
compute lmat2=lmat3(:,2:ncol(lmat3)).
end if.
compute fratio = (t(t(lmat2)* b )*inv(t(lmat2)* varb *lmat2)*((t(lmat2)* b )))/ncol(lmat2).
compute pfr = 1-fcdf(fratio,ncol(lmat2),(n-nrow( b ))).
compute fresult={fratio,ncol(lmat2),(n-nrow( b )),pfr}.
do if (i = (nms+nys) and (ydich=1)).
compute fratio=fratio*ncol(lmat2).
compute pfr=1-chicdf(fratio,ncol(lmat2)).
compute fresult={fratio,ncol(lmat2),pfr}.
end if.
do if ( 0 =1).
compute lmat3=1-rsum(lmat2).
compute xfm=make(n,csum(lmat3),0).
compute flpc=1.
loop flp=1 to nrow(lmat3).
do if (lmat3(flp,1)=1).
compute xfm(:,flpc)=x(:,flp).
compute flpc=flpc+1.
end if.
end loop.
compute bfm=inv(t(xfm)*xfm)*t(xfm)*y.
compute resid=y-(xfm*bfm).
compute sstotal=(y-(csum(y)/n)).
compute sstotal=csum(sstotal&*sstotal).
compute ssresid=csum(resid&*resid).
compute rsqch= 0 -((sstotal-ssresid)/sstotal).
compute fresult={rsqch,fresult}.
release xfm,flpc, resid, ssresid, bfm.
end if
.
compute omnif={omnif;fresult}.
end loop.
compute omnif=omnif(2:nrow(omnif),:).
compute clabtmp=znames.
compute condeff3=condeff3(2:nrow(condeff3),:).
do if ((nxvls*nwvls)=1).
compute omnif={condeff3,omnif}.
compute clabtmp={clabtmp,'Effect'}.
end if.
compute omnif={zmodvals,omnif}.
do if ((i < nms+nys) or (ydich=0)).
compute clabtmp={clabtmp,hcflab,'df1','df2','p'}.
end if.
do if ((i = (nms+nys)) and (ydich=1)).
compute clabtmp={clabtmp,'Chi-sq','df','p'}.
end if.
compute resultm2=make(nrow(omnif),maxresm,99999).
compute resultm2(1:nrow(omnif),1:ncol(omnif))=omnif.
compute resultm={resultm;resultm2}.
do if (outscree=1).
do if (jmed=1).
print omnif/title='Test of conditional X*W interaction at value(s) of Z:'/cnames=clabtmp/format= F10.4.
end if.
do if (jmed>1).
print omnif/title='Test of conditional M*W interaction at value(s) of Z:'/cnames=clabtmp/format= F10.4.
end if.
end if.
release omni3.
end if.
loop probei = 1 to probexpl.
do if (probexpl > 1).
compute foctmp=make(nrow(modvals),probexpl,0).
compute foctmp(:,probei)=foctmp(:,probei)+1.
compute probtemp=make(nrow(modvals),1,0).
loop probem = 1 to numplps.
loop probek = 1 to nxvls.
loop probej=lpstsp(probem,1) to lpstsp(probem,2).
compute probtemp={probtemp,foctmp(:,probek)&*probeval(:,probej)}.
end loop.
end loop.
end loop.
compute probeva2=probtemp(:,2:ncol(probtemp)).
compute probeva2={foctmp,probeva2}.
end if.
compute probres=probeva2*probcoef.
compute probrese=sqrt(diag(probeva2*probvarb*t(probeva2))).
compute tratio = probres&/probrese.
compute p = 2*(1-tcdf(abs(tratio), dfres)).
do if (ydich=1 and i = (nms+nys)).
compute p = 2*(1-cdfnorm(abs(tratio))).
end if.
compute modvals2={modvals,probres,probrese,tratio, p}.
do if ((i < nms+nys) or (ydich=0)).
compute modvals2={modvals2,(probres-tval&*probrese),(probres+tval&*probrese)}.
compute problabs={problabs,'Effect',hclab,'t', 'p', 'LLCI', 'ULCI'}.
end if.
do if (ydich=1 and i = (nms+nys)).
compute modvals2={modvals2,(probres-xp2&*probrese),(probres+xp2&*probrese)}.
compute problabs={problabs,'Effect','se','Z', 'p', 'LLCI', 'ULCI'}.
end if.
do if (probexpl > 1 and (printpbe <= intprobe)).
do if (hasz = 1).
compute printz=1.
end if.
do if (hasw=1).
compute printw=1.
end if.
compute probrlab=make(nrow(modvals),1,xcatlab(probei,1)).
compute modvals3={modvals3; modvals2}.
compute probrow={probrow;probrown}.
compute probrown=probrown+1.
do if (probei=probexpl).
compute xproblab=xcatlab(1:nxvls,1).
compute probrow=probrow(2:nrow(probrow),1).
compute modvals3=modvals3(2:nrow(modvals3),:).
compute temp=modvals3.
compute temp(GRADE(probrow(:,1)),:)=modvals3.
compute modvals3=temp.
compute start2=1.
compute problabs=problabs(1,(1+(ncol(modvarl))):ncol(problabs)).
compute pstart=1.
loop probek= 1 to nrow(probeval).
compute endstart=start2+(nxvls-1).
compute temp=modvals3(start2:endstart,(1+ncol(modvarl)):ncol(modvals3)).
compute temp2=t(modvals3(start2:start2,1:ncol(modvarl))).
compute trnames=t(modvarl).
do if (outscree=1).
do if (probek > 1).
print/title='----------'/space=0.
else.
print/title = 'Conditional effects of the focal predictor at values of the moderator(s):'.
do if ((jmed=1) and (i = (nms+nys)) and (nms > 0)).
do if (nxvls = 1).
print/title = '(These are also the conditional direct effects of X on Y)'/space=0.
else.
print/title = '(These are also the relative conditional direct effects of X on Y)'/space=0.
end if.
end if.
print.
end if.
print temp2/title = 'Moderator value(s):'/rnames=trnames/format= F10.4 /space=0.
print temp/title = ' '/cnames=problabs/rnames=xproblab/format= F10.4 /space=0.
end if.
compute resultm2=make(nrow(temp2),maxresm,99999).
compute resultm2(1:nrow(temp2),1:ncol(temp2))=temp2.
compute resultm={resultm;resultm2}.
compute resultm2=make(nrow(temp),maxresm,99999).
compute resultm2(1:nrow(temp),1:ncol(temp))=temp.
compute resultm={resultm;resultm2}.
compute start2=start2+nxvls.
compute didsome=1.
do if (jmed=1).
compute mod1val=probeval(probek,1:nm1vls).
loop omnilp=1 to nxvls.
compute omni((wheremv1(1,i)+((omnilp-1)*nm1vls)):(wheremv1(1,i)+((omnilp-1)* nm1vls)+(nm1vls-1)),omnilp)=t(mod1val).
do if (nm1vls < ncol(probeval)).
compute mod2val=probeval(probek,(nm1vls+1):(nm1vls+nm2vls)).
compute omni((wheremv2(1,i)+((omnilp-1)*nm2vls)):(wheremv2(1,i)+((omnilp-1)* nm2vls)+(nm2vls-1)),omnilp)=t(mod2val).
do if ((nm1vls+nm2vls) < ncol(probeval)).
compute intlen=nm1vls*nm2vls.
compute modintvl=probeval(probek,(nm1vls+nm2vls+1):ncol(probeval)).
compute omni((wherexwz(1,i)+((omnilp-1)*intlen)):(wherexwz(1,i)+((omnilp-1)* intlen)+(intlen-1)),omnilp)=t(modintvl).
end if.
end if.
end loop.

.
compute lmat2= omni.
do if ( 1 =0).
compute lmat2 = mdiag( omni ).
compute lmat3=make(nrow(lmat2),1,0).
loop flp=1 to ncol(lmat2).
do if (csum(lmat2(:,flp))=1).
compute lmat3={lmat3,lmat2(:,flp)}.
end if.
end loop.
compute lmat2=lmat3(:,2:ncol(lmat3)).
end if.
compute fratio = (t(t(lmat2)* b )*inv(t(lmat2)* varb *lmat2)*((t(lmat2)* b )))/ncol(lmat2).
compute pfr = 1-fcdf(fratio,ncol(lmat2),(n-nrow( b ))).
compute fresult={fratio,ncol(lmat2),(n-nrow( b )),pfr}.
do if (i = (nms+nys) and (ydich=1)).
compute fratio=fratio*ncol(lmat2).
compute pfr=1-chicdf(fratio,ncol(lmat2)).
compute fresult={fratio,ncol(lmat2),pfr}.
end if.
do if ( 0 =1).
compute lmat3=1-rsum(lmat2).
compute xfm=make(n,csum(lmat3),0).
compute flpc=1.
loop flp=1 to nrow(lmat3).
do if (lmat3(flp,1)=1).
compute xfm(:,flpc)=x(:,flp).
compute flpc=flpc+1.
end if.
end loop.
compute bfm=inv(t(xfm)*xfm)*t(xfm)*y.
compute resid=y-(xfm*bfm).
compute sstotal=(y-(csum(y)/n)).
compute sstotal=csum(sstotal&*sstotal).
compute ssresid=csum(resid&*resid).
compute rsqch= 0 -((sstotal-ssresid)/sstotal).
compute fresult={rsqch,fresult}.
release xfm,flpc, resid, ssresid, bfm.
end if
.
compute resultm2=make(nrow(fresult),maxresm,99999).
compute resultm2(1:nrow(fresult),1:ncol(fresult))=fresult.
compute resultm={resultm;resultm2}.
do if (outscree=1).
do if (i < (nms + nys) or (ydich = 0)).
compute clabtmp={hcflab,'df1','df2','p'}.
print fresult/title='Test of equality of conditional means'/cnames=clabtmp/format= F10.4.
end if.
do if (i = (nms + nys) and (ydich = 1)).
compute clabtmp={'Chi-sq','df','p'}.
print fresult/title='Test of equality of conditional logits or probabilities'/cnames=clabtmp/format= F10.4.
end if.
end if.
compute probetmp=probeplt(pstart:(pstart+nxvls),1).
compute probetmp={probetmp,probeplt(pstart:(pstart+nxvls),prevloc:ncol(probeplt))}.
compute pstart=pstart+(nxvls+1).
compute resultm2=make(nrow(probetmp),maxresm,99999).
compute resultm2(1:nrow(probetmp),1:ncol(probetmp))=probetmp.
compute resultm={resultm;resultm2}.
do if (i < ((nms + nys)) or (ydich = 0)).
compute clabtmp={xnames, outnames(1,i), hclab, 'LLCI', 'ULCI'}.
do if (outscree=1).
print probetmp/title = 'Estimated conditional means being compared:'/cnames=clabtmp/format= F10.4.
end if.
end if.
do if ((i = (nms+nys)) and (ydich = 1)).
compute clabtmp={xnames, outnames(1,i), 'prob'}.
compute probetm2=probetmp(:,1:2).
compute probetm2={probetm2,probetmp(:,ncol(probetmp))}.
do if (outscree=1).
print probetm2/title = 'Estimated conditional logits and probabilities:'/cnames=clabtmp/format= F10.4.
end if.
end if.
end if.
end loop.
release probrow, start2, endstart, temp, temp2.
end if.
end if.
do if (probexpl = 1 and (printpbe <= intprobe)).
compute resultm2=make(nrow(modvals2),maxresm,99999).
compute resultm2(1:nrow(modvals2),1:ncol(modvals2))=modvals2.
compute resultm={resultm;resultm2}.
do if (outscree=1).
print/title = 'Conditional effects of the focal predictor at values of the moderator(s):'.
print modvals2/cnames=problabs/title = ' '/space=0/format= F10.4.
end if.
compute didsome=1.
do if (hasz = 1).
compute printz=1.
end if.
do if (hasw=1).
compute printw=1.
end if.
do if (jn = 1 and jnok=1).
do if (criterr = 0).
compute dfres=n-nrow(b).
compute roots=99999.
compute jncrit =(dfres* (exp((dfres-(5/6))*((xp2/(dfres-(2/3)+(.11/dfres)))* (xp2/(dfres-(2/3)+(.11/dfres)))))-1)).
do if (i = (nms+nys) and (ydich=1)).
compute jncrit=xp2*xp2.
end if.
compute jnb1=b(wherejn1,1).
compute jnb3=b(wherejn3,1).
compute jnsb1=varb(wherejn1,wherejn1).
compute jnsb3=varb(wherejn3,wherejn3).
compute jnsb1b3=varb(wherejn1,wherejn3).
compute ajn =(jncrit*jnsb3)-(jnb3*jnb3).
compute bjn = 2*((jncrit*jnsb1b3)-(jnb1*jnb3)).
compute cjn = (jncrit*jnsb1)-(jnb1*jnb1).
compute radarg = (bjn*bjn)-(4*ajn*cjn).
compute den = 2*ajn.
compute nrts = 0.
do if (radarg >= 0 and den <> 0).
compute x21 = (-bjn+sqrt(radarg))/den.
compute x22 = (-bjn-sqrt(radarg))/den.
compute roots = 0.
do if (x21 >= jnmin and x21 <= jnmax).
compute nrts = 1.
compute roots = {roots; x21}.
end if.
do if (x22 >= jnmin and x22 <= jnmax).
compute nrts = nrts + 1.
compute roots = {roots; x22}.
end if.
compute roots={roots,make(nrow(roots),2,0)}.
end if.
do if (nrts > 0).
compute roots = roots(2:nrow(roots),1:3).
compute roots(1,2)=(csum(jnmod < roots(1,1))/n)*100.
compute roots(1,3)=(csum(jnmod > roots(1,1))/n)*100.
do if (nrow(roots)=2).
compute roots(2,2)=(csum(jnmod < roots(2,1))/n)*100.
compute roots(2,3)=(csum(jnmod > roots(2,1))/n)*100.
end if.
do if (outscree=1).
print roots/title = 'Moderator value(s) defining Johnson-Neyman significance region(s):'/clabels = 'Value', '% below', '% above'/format F10.4.
end if.
do if (nrts=1).
compute tmprts=make(1,3,99999).
compute roots={roots;tmprts}.
end if.
end if.
do if (nrts = 0).
compute roots=make(2,1,99999).
do if (outscree=1).
print/title = 'There are no statistical significance transition points within the observed'.
print/title = 'range of the moderator found using the Johnson-Neyman method.'/space=0.
end if.
end if.
compute resultm2=make(nrow(roots),maxresm,99999).
compute resultm2(1:nrow(roots),1:ncol(roots))=roots.
compute resultm={resultm;resultm2}.
compute jnvals=make(23,7,0).
loop jni= 0 to (21-(nrts)).
compute jnvals((jni+1),1)=jnmin+(jni*((jnmax-jnmin)/(21-nrts))).
end loop.
do if (nrts > 0).
loop jni = 1 to nrts.
loop jnj = 2 to (nrow(jnvals)-1).
do if ((roots(jni,1) > jnvals((jnj-1),1)) and (roots(jni,1) < jnvals(jnj,1))).
compute jnvals((jnj+1):(21+jni),1)=jnvals(jnj:(20+jni),1).
compute jnvals(jnj,1)=roots(jni,1).
end if.
end loop.
end loop.
end if.
compute jnvals=jnvals(1:22,:).
loop jni = 1 to nrow(jnvals).
compute jnvals(jni,2)=jnb1+jnb3*jnvals(jni,1).
compute jnvals(jni,3)=sqrt(jnsb1+2*jnvals(jni,1)*jnsb1b3+(jnvals(jni,1)*jnvals(jni, 1))*jnsb3).
compute jnvals(jni,4)=jnvals(jni,2)/jnvals(jni,3).
compute jnvals(jni,5)=2*(1-tcdf(abs(jnvals(jni,4)), dfres)).
compute jnvals(jni,6)=jnvals(jni,2)-sqrt(jncrit)*jnvals(jni,3).
compute jnvals(jni,7)=jnvals(jni,2)+sqrt(jncrit)*jnvals(jni,3).
do if ((i = nms + nys) and (ydich=1)).
compute jnvals(jni,5)=2*(1-cdfnorm(abs(jnvals(jni,4)))).
compute jnvals(jni,6)=jnvals(jni,2)-xp2*jnvals(jni,3).
compute jnvals(jni,7)=jnvals(jni,2)+xp2*jnvals(jni,3).
end if.
end loop.
compute resultm2=make(nrow(jnvals),maxresm,99999).
compute resultm2(1:nrow(jnvals),1:ncol(jnvals))=jnvals.
compute resultm={resultm;resultm2}.
do if ((i < nms+nys) or (ydich=0)).
compute jnclbs={jnmodlab,'Effect',hclab,'t', 'p', 'LLCI', 'ULCI'}.
end if.
do if ((i = nms + nys) and (ydich=1)).
compute jnclbs={jnmodlab,'Effect','se','Z', 'p', 'LLCI', 'ULCI'}.
end if.
do if (((wcmat((i+1),jmed)=1) or (zcmat((i+1),jmed)=1)) and (wzcmat((i+1),jmed)=0)).
do if (outscree=1).
print jnvals/title = 'Conditional effect of focal predictor at values of the moderator:'/cnames =jnclbs/format = F10.4.
end if.
end if.
do if (outscree=1).
do if ((jmed = 1) and (wzcmat((i+1),jmed)=1)).
print jnvals/title = 'Conditional X*W interaction at values of the moderator Z:'/cnames =jnclbs/format = F10.4.
end if.
do if ((jmed > 1) and (wzcmat((i+1),jmed)=1)).
print jnvals/title = 'Conditional M*W interaction at values of the moderator Z:'/cnames =jnclbs/format = F10.4.
end if.
end if.
end if.
end if.
end if.
do if ((i = (nms+nys)) and (jmed=1) and (bcmat(nrow(bcmat),1)=1)).
do if (probei=1).
compute direfflb=problabs.
compute direff=modvals2.
end if.
do if (probei>1).
compute direff={direff;modvals2}.
end if.
end if.
compute intprint=0.
do if ((jmed=1) and (i=1) and nms=0) and modcok=1).
compute contvec2=make(2,1,1).
compute contvec2={contvec2,wcontval,zcontval}.
do if (wzcmat((i+1),jmed)=1).
loop conti= 1 to ncol(wcontval).
loop contj = 1 to ncol(zcontval).
compute contvec2={contvec2,wcontval(:,conti)&*zcontval(:,contj)}.
end loop.
end loop.
end if.
compute conteff=contvec2*probcoef.
compute contdiff=contvec2(1,:)-contvec2(2,:).
compute contse=sqrt(contdiff*probvarb*t(contdiff)).
compute conteffd=conteff(1,1)-conteff(2,1).
compute contvec={contvec,conteff}.
compute contvecm=contvec.
compute resultm2=make(nrow(contvecm),maxresm,99999).
compute resultm2(1:nrow(contvecm),1:ncol(contvecm))=contvecm.
compute resultm={resultm;resultm2}.
do if (outscree=1).
print/title='Contrast between conditional effects of X:'.
print contvec/title=' '/rlabels='Effect1:','Effect2:'/cnames=problabs/format = F10.4 /space=0.
end if.
do if (ydich=0).
compute p=2*(1-tcdf(abs(conteffd/contse), dfres)).
compute contvec={conteffd,contse,conteffd/contse, p}.
compute contvec={contvec,(conteffd-(tval*contse))}.
compute contvec={contvec,(conteffd+(tval*contse))}.
compute contlabs={'Contrast', hclab, 't', 'p', 'LLCI', 'ULCI'}.
end if.
do if (ydich=1).
compute p=2*(1-cdfnorm(abs(conteffd/contse))).
compute contvec={conteffd,contse,conteffd/contse, p}.
compute contvec={contvec,(conteffd-(xp2*contse))}.
compute contvec={contvec,(conteffd+(xp2*contse))}.
compute contlabs={'Contrast', 'se', 'Z', 'p', 'LLCI', 'ULCI'}.
end if.
compute resultm2=make(nrow(contvec),maxresm,99999).
compute resultm2(1:nrow(contvec),1:ncol(contvec))=contvec.
compute resultm={resultm;resultm2}.
do if (outscree=1).
print contvec/title='Test of Effect1 minus Effect2'/format= F10.4 /cnames=contlabs.
end if.
end if.
end loop.
do if (plot = 1 or plot = 2).
compute datalabs={t(focpred(:,3)),outnames(1,i)}.
do if (plot = 1).
compute datalabs={datalabs}.
end if.
do if (plot = 2).
compute datalabs={datalabs,'se', 'LLCI', 'ULCI'}.
end if.
do if ((i = nms+nys) and (ydich=1)).
compute datalabs={datalabs,'prob'}.
end if.
compute resultm2=make(nrow(probeplt),maxresm,99999).
compute resultm2(1:nrow(probeplt),1:ncol(probeplt))=probeplt.
compute resultm={resultm;resultm2}.
compute datalabs={datalabs,'.'}.
do if (outscree=1).
print/title = 'Data for visualizing the conditional effect of the focal predictor:'.
print/title = 'Paste text below into a SPSS syntax window and execute to produce plot.'/space=0.
compute dumb = {' ', ' ', ' ', ' ', ' ', ' ', ' '}.
print datalabs/title = 'DATA LIST FREE/'/format=A10.
print probeplt/title = 'BEGIN DATA.'/format= F10.4 /space=0.
print/title = 'END DATA.'/space=0.
end if.
compute focgrph=datalabs(1,1).
compute graphix={focgrph,graphixs,modgrph}.
do if (((xdich=1) or (nxvls > 1)) and ((modcat=0) and (focgrph = xnames))).
compute graphix={modgrph,graphixs,focgrph}.
end if.
do if (panelgrp = 0).
compute graphix={graphix,'.'}.
else.
compute graphix={graphix,panelcde}.
end if.
do if (outscree=1).
print graphix/title = 'GRAPH/SCATTERPLOT='/format=A8/space=0.
end if.
do if (i = (nms + nys) and ydich = 1).
compute graphixd=graphix.
compute graphixd(1,3)='prob'.
do if (outscree=1).
print graphixd/title = 'GRAPH/SCATTERPLOT='/format=A8/space=0.
end if.
end if.
end if.
end if.
end loop.
release jmed, intprint,didprint.
end if
.
do if (model = 74 and i <= nms).
compute onetemp=make(nrow(xprobval),1,1).
compute mestmt74={onetemp,xprobval}.
do if (ncs > 0).
compute ncovmdl=rsum(ccmat(i,:)).
do if (ncovmdl > 0).
compute cvmnc=make(nrow(mestmt74),ncovmdl,1).
compute cvmnctmp=csum(x(:,(ncol(x)-ncovmdl+1):ncol(x)))/nrow(x).
do if (cuscoval > 0).
compute cvmnctmp=coval.
end if.
loop mestlp=1 to ncovmdl.
compute cvmnc(:,mestlp)=cvmnc(:,mestlp)*cvmnctmp(:,mestlp).
end loop.
compute mestmt74={mestmt74,cvmnc}.
end if.
end if.
compute mest74t=mestmt74*b.
do if (i = 1).
compute mest74=mest74t.
end if.
do if (i > 1).
compute mest74={mest74,mest74t}.
end if.
end if.
do if ((i = (nms+nys)) and (model >= 0 and model < 4) and (linsum(1,1) <> -999)).
compute lhyprob=1.
compute meansub=0.
do if (((nlinsum=nrow(b)) or (nlinsum = (nrow(b)-ncs)))).
do if (nlinsum = (nrow(b)-ncs) and ncs > 0).
compute linsum={linsum,covmeans}.
compute meansub=1.
end if.
compute lhyprob=0.
compute hypest=linsum*b.
compute sehypest=sqrt(linsum*varb*t(linsum)).
compute phypest=2*(1-tcdf(abs(hypest/sehypest), (dfres))).
compute hypest={hypest,sehypest,(hypest/sehypest),phypest,(hypest-tval*sehypest),(hypest+ tval*sehypest)}.
compute resultm2=make(ncol(linsum),maxresm,99999).
compute resultm2(:,1)=t(linsum).
compute resultm={resultm;resultm2}.
compute resultm2=make(1,maxresm,99999).
compute resultm2(1,1:ncol(hypest))=hypest.
compute resultm={resultm;resultm2}.
do if (outscree=1).
compute hyplabs={'Estimate',hclab,'t','p','LLCI','ULCI'}.
print/title='----------'.
print/title='Linear Combination Estimate and Hypothesis Test'/space=0.
print t(linsum)/title= 'Weight vector:'/clabels='weight'/rnames=vlabsm/format F10.4.
print hypest/title=' '/cnames=hyplabs/format= F10.4 /space=0.
do if (meansub=1).
print/title='Covariate weight(s) set to the sample mean.'.
end if.
end if.
end if.
do if (lhyprob=1).
compute notecode(notes,1) = 30.
compute notes = notes + 1.
end if.
end if.
end loop if criterr=1.
compute lastb=b.
compute lastcov=varb.
do if (criterr=0 and dototal = 1).
compute x=xtmp.
compute vlabsm={'constant';xcatlab(1:nxvls,1)}.
do if (ncs > 0).
compute x = {x,ctmp}.
compute vlabsm={vlabsm;t(covnames)}.
end if.
compute x = {ones,x}.

.
do if ( 1 =1).
compute hatmat=inv(t( x )* x )*t( x ).
compute b = hatmat* y.
compute modres=b.
do if ( 1 =1).
compute n1=nrow( x ).
compute dfres=n1-(ncol( x )).
compute sstotal = t( y -(csum( y )/n1))*( y -(csum( y )/n1)).
compute resid= y - x *b.
compute ssresid = csum((resid)&**2).
compute r2 = (sstotal-ssresid)/sstotal.
do if (r2 < 0).
compute r2=0.
end if.
compute adjr2 = 1-((1-r2)*(n1-1)/(dfres)).
compute mse=ssresid/(n1-ncol( x )).

.
compute n1=nrow( x ).
compute invXtX = inv(t( x )* x ).
compute varb = mse *invXtX.
compute k3 = ncol( x ).
compute xhc=0.
do if ( hc <> 5).
compute xhc= x.
compute hat = xhc(:,1).
loop i3=1 to nrow(xhc).
compute hat(i3,1)= xhc(i3,:)*invXtX*t(xhc(i3,:)).
end loop.
do if ( hc = 0 or hc =1).
loop i3 = 1 to k3.
compute xhc(:,i3)=xhc(:,i3)&* resid.
end loop.
end if.
do if ( hc =3 or hc =2).
loop i3=1 to k3.
compute xhc(:,i3) = ( resid &/(1-hat)&**(1/(4- hc )))&*xhc(:,i3).
end loop.
end if.
do if ( hc = 4).
compute hcmn=make(n,2,4).
compute hcmn(:,2)=(n1*hat)/k3.
loop i3= 1 to k3.
compute xhc(:,i3) = ( resid &/(1-hat)&**(rmin(hcmn)/2))&*xhc(:,i3).
end loop.
end if.
compute varb=(invXtX*t(xhc)*xhc*invXtX).
do if ( hc =1).
compute varb=(n1/(n1-ncol( x )))&*varb.
end if.
end if.
compute hclab={'se(HC0)','se(HC1)','se(HC2)','se(HC3)','se(HC4)','se'}.
compute hclab=hclab(1,( hc +1)).
compute hcflab={'F(HC0)','F(HC1)','F(HC2)','F(HC3)','F(HC4)','F'}.
compute hcflab=hcflab(1,( hc +1)).
release xhc
.
compute seb=sqrt(diag(varb)).
compute trat = b&/seb.
compute p = 2*(1-tcdf(abs(trat), (dfres))).
compute tval = sqrt(dfres* (exp((dfres-(5/6))*((xp2/(dfres-(2/3)+(.11/dfres)))* (xp2/(dfres-(2/3)+(.11/dfres)))))-1)).
compute modres={modres,seb,trat,p}.
compute modres={modres,(b-tval&*seb),(b+tval&*seb)}.
compute modresl={'coeff',hclab,'t','p','LLCI','ULCI'}.
compute lmat = ident(ncol( x )).
compute lmat = lmat(:,2:ncol(lmat)).
compute fratio = (t(t(lmat)*b)*inv(t(lmat)*varb*lmat)*((t(lmat)*b)))/(ncol( x )-1).
compute pfr = 1-fcdf(fratio,(ncol( x )-1),dfres).
compute modsum={sqrt(r2),r2,mse,fratio,(ncol( x )-1),dfres,pfr}.
compute modsuml={'R','R-sq','MSE',hcflab,'df1','df2', 'p'}.
end if.
end if.
do if ( 1 = 2 or 1 =3).
compute xlp= x.
compute ylp= y.
compute pt2 = make(nrow(ylp),1,(csum(ylp)/nrow(ylp))).
do if ( 1 =2).
compute LL3 = ylp&*ln(pt2)+(1-ylp)&*ln(1-pt2).
end if.
compute LL3 = -2*csum(LL3).
compute bt1 = make(ncol(xlp),1,0).
compute LL1 = 0.
compute pt1 = make(nrow(ylp),1,0.5).
compute pt1lp=pt1.
loop jjj = 1 to iterate.
compute xlptmp=t(xlp).
compute vecprb=pt1lp&*(1-pt1lp).
loop kkk=1 to ncol(xlp).
compute xlptmp(kkk,:)=xlptmp(kkk,:)&*t(vecprb).
end loop.
compute b = bt1+inv(xlptmp*xlp)*t(xlp)*(ylp-pt1lp).
do if ( 1 =2).
compute xlpb=xlp*b.
compute xlpbt=(xlpb > -709.7).
compute xlpb709=(1-xlpbt)*(-709.7).
compute xlpb=(xlpb&*xlpbt)+xlpb709.
compute pt1lp = 1/(1+exp(-(xlpb))).
end if.
compute itprob = csum((pt1lp < .00000001) or (pt1lp > .9999999)).
do if (itprob > 0).
loop kkk = 1 to nrow(pt1lp).
do if (pt1lp(kkk,1) > .9999999).
compute pt1lp(kkk,1) = .9999999.
end if.
do if (pt1lp(kkk,1) < .00000001).
compute pt1lp(kkk,1) = .00000001.
end if.
end loop.
compute itprob = 0.
end if.
do if (itprob = 0).
do if ( 1 =2).
compute LL = ylp&*ln(pt1lp)+(1-ylp)&*ln(1-pt1lp).
end if.
compute LL2 = -2*csum(ll).
end if.
do if (abs(LL1-LL2) < converge).
do if ( 1 =1).
compute xlptmp=t(xlp).
compute vecprb=pt1lp&*(1-pt1lp).
loop kkk=1 to ncol(xlp).
compute xlptmp(kkk,:)=xlptmp(kkk,:)&*t(vecprb).
end loop.
compute varb = inv(xlptmp*xlp).
compute seb = sqrt(diag(varb)).
release xlptmp.
end if.
break.
end if.
compute bt1 = b.
compute LL1 = LL2.
end loop.
compute modres=b.
do if (jjj > iterate).
compute itprob = 2.
do if (booting=0).
compute iterrmod=1.
end if.
do if (booting=1).
compute bootiter=1.
end if.
do if (itprobtg=0).
compute itprobtg=1.
compute errcode(errs,1) = 47.
compute errs = errs + 1.
do if (booting = 0 and 1 =1).
compute vt1 = mdiag(pt1lp&*(1-pt1lp)).
compute varb = inv(t(xlp)*vt1*xlp).
compute seb = sqrt(diag(varb)).
end if.
end if.
end if.
do if ( 1 =1).
compute trat = b&/seb.
compute dfres=nrow(xlp).
compute p = 2*(1-cdfnorm(abs(trat))).
compute modres={modres,seb,trat,p}.
compute modres={modres,(b-xp2&*seb),(b+xp2&*seb)}.
compute pvchi=1-chicdf((LL3-LL2),(nrow(modres)-1)).
compute mcF = (LL3-LL2)/LL3.
compute cox = 1-exp(-(LL3-LL2)/nrow(xlp)).
compute nagel = cox/(1-exp(-(LL3)/nrow(xlp))).
compute modsum={LL2,(LL3-LL2),(nrow(modres)-1),pvchi, mcF,cox,nagel}.
compute modsuml={'-2LL','ModelLL', 'df', 'p', 'McFadden', 'CoxSnell', 'Nagelkrk'}.
compute modresl={'coeff','se','Z','p','LLCI','ULCI'}.
end if.
end if
.
compute toteff=modres(2:(1+nxvls),:).
compute nodotot=0.
do if (xdich=1 and xmint=1 and model=74).
compute toteff(:,1)=toteff(:,1)*xscaling.
compute toteff(:,2)=toteff(:,2)*abs(xscaling).
compute toteff(:,3)=toteff(:,3)*xscaling.
compute toteff(:,5)=toteff(:,5)*xscaling.
compute toteff(:,6)=toteff(:,6)*xscaling.
compute citmp=toteff(:,5:6).
do if (xscaling < 0).
compute toteff(:,5)=citmp(:,2).
compute toteff(:,6)=citmp(:,1).
end if.
compute nodotot=1.
end if.
compute totefflb=modresl.
do if (outscree=1).
do if (nodotot=0).
print/title = '************************** TOTAL EFFECT MODEL ****************************'.
end if.
do if (nodotot=1).
print/title = '**************************************************************************'.
end if.
print outnames(1,ncol(outnames))/title = 'OUTCOME VARIABLE:'/format = A8/space=0.
end if.
compute toteffl2=vlabsm(2:(1+nxvls),:).
compute resultm2=make(1,maxresm,99999).
compute resultm2(1,1:ncol(modsum))=modsum.
compute resultm={resultm;resultm2}.
compute resultm2=make(nrow(modres),maxresm,99999).
compute resultm2(1:nrow(modres),1:ncol(modres))=modres.
compute resultm={resultm;resultm2}.
do if (outscree=1).
print modsum/title = 'Model Summary'/cnames = modsuml/format= F10.4.
print modres/title='Model'/rnames=vlabsm/cnames=modresl/format= F10.4.
end if.
compute lmat=make(nrow(b),1,0).
compute lmat2=make(nxvls,1,1).
compute lmat(2:(1+nxvls),1)=lmat2.
do if (ydich <> 1).

.
compute lmat2= lmat.
do if ( 0 =0).
compute lmat2 = mdiag( lmat ).
compute lmat3=make(nrow(lmat2),1,0).
loop flp=1 to ncol(lmat2).
do if (csum(lmat2(:,flp))=1).
compute lmat3={lmat3,lmat2(:,flp)}.
end if.
end loop.
compute lmat2=lmat3(:,2:ncol(lmat3)).
end if.
compute fratio = (t(t(lmat2)* b )*inv(t(lmat2)* varb *lmat2)*((t(lmat2)* b )))/ncol(lmat2).
compute pfr = 1-fcdf(fratio,ncol(lmat2),(n-nrow( b ))).
compute fresult={fratio,ncol(lmat2),(n-nrow( b )),pfr}.
do if (i = (nms+nys) and (ydich=1)).
compute fratio=fratio*ncol(lmat2).
compute pfr=1-chicdf(fratio,ncol(lmat2)).
compute fresult={fratio,ncol(lmat2),pfr}.
end if.
do if ( 1 =1).
compute lmat3=1-rsum(lmat2).
compute xfm=make(n,csum(lmat3),0).
compute flpc=1.
loop flp=1 to nrow(lmat3).
do if (lmat3(flp,1)=1).
compute xfm(:,flpc)=x(:,flp).
compute flpc=flpc+1.
end if.
end loop.
compute bfm=inv(t(xfm)*xfm)*t(xfm)*y.
compute resid=y-(xfm*bfm).
compute sstotal=(y-(csum(y)/n)).
compute sstotal=csum(sstotal&*sstotal).
compute ssresid=csum(resid&*resid).
compute rsqch= r2 -((sstotal-ssresid)/sstotal).
compute fresult={rsqch,fresult}.
release xfm,flpc, resid, ssresid, bfm.
end if
.
compute totomni=fresult.
end if.
do if (stand=1).
compute predsd=make(nrow(modres),1,0).
compute stdmod=modres(:,1)&/ovsd(1,ncol(ovsd)).
loop jd=1 to ncol(x).
compute descdat=x(:,jd).
compute predsd(jd,1) = (nrow(descdat)*sscp(descdat))-(t(csum(descdat))*(csum(descdat))).
compute predsd(jd,1) = sqrt(predsd(jd,1)/(nrow(descdat)*(nrow(descdat)-1))).
end loop.
do if (wherex(1,ncol(wherex)) <> -999 and ((nxvls > 1) or (xdich=1))).
compute sdmsone=make(nxvls,1,1).
compute predsd(wherex(1,ncol(wherex)):wherex(2,ncol(wherex)),1)=sdmsone.
compute pstog=1.
end if.
compute predsd(1,1)=1.
compute stdmod=stdmod&*predsd.
compute stdmod=stdmod(2:nrow(stdmod),1).
compute sdvlabs=vlabsm(2:nrow(vlabsm),1).
compute resultm2=make(nrow(stdmod),maxresm,99999).
compute resultm2(1:nrow(stdmod),1:ncol(stdmod))=stdmod.
compute resultm={resultm;resultm2}.
do if (outscree=1).
print stdmod/title='Standardized coefficients'/clabels='coeff'/rnames=sdvlabs/format= F10.4.
end if.
end if.
do if (covcoeff=1).
do if (outscree=1).
print varb/title='Covariance matrix of regression parameter estimates:'/rnames=vlabsm/cnames=vlabsm/format= F10.4.
end if.
compute resultm2=make(nrow(varb),maxresm,99999).
do if (ncol(varb) <= maxresm).
compute resultm2(1:nrow(varb),1:ncol(varb))=varb.
compute resultm={resultm;resultm2}.
end if.
do if (ncol(varb) > maxresm).
compute resultmt=make(nrow(resultm),ncol(varb),99999).
compute resultmt(1:nrow(resultm),1:ncol(resultm))=resultm.
compute resultm=resultmt.
compute resultm2=make(nrow(varb),ncol(resultm),99999).
compute resultm2(1:nrow(varb),1:ncol(varb))=varb.
compute resultm={resultm;resultm2}.
compute maxresm=ncol(resultm).
end if.
end if.
end if.
end if.
do if (criterr=0 and nms > 0 and ydich=0 and modelres=1).
compute modresid=modresid(:,2:ncol(modresid)).
compute sigmatal = (t(modresid)*(ident(n)-(1/n)*ones*t(ones))*modresid)*(1/(n-1)).
compute sdall = mdiag(1/sqrt(diag(sigmatal))).
compute corall=sdall*sigmatal*t(sdall).
compute resultm2=make(nrow(corall),maxresm,99999).
compute resultm2(1:nrow(corall),1:ncol(corall))=corall.
compute resultm={resultm;resultm2}.
do if (outscree=1).
print/title = '****************** CORRELATIONS BETWEEN MODEL RESIDUALS ******************'.
print corall/title=' '/format= F10.4 / cnames=outnames/rnames=outnames/space=0.
end if.
end if.
do if (criterr=0 and boot > 0).
compute bootres=make(1,rsum(nump),-999).
compute bootdir=obsdirfx.
compute natdirbt=make(1,nxvls,-999).
do if (effsize=1).
compute bootysd=make(1,1,-999).
compute bootxsd=make(1,1,-999).
end if.
compute badboot=0.
compute goodboot=0.
compute smallest=1.
compute booting=1.
loop j = 1 to maxboot.
compute nobootx=1.
compute modres2=999.
compute v=trunc(uniform(n,1)*n)+1.
compute bad=0.
loop i = 1 to (nms+nys).
compute y=outvars(v,i).
compute ynovar= (nrow(y)*sscp(y))-(t(csum(y))*(csum(y))).
do if (ynovar = 0).
compute bad=1.
end if.
compute xindx=datindx(1:(nump(1,i)-1),i).
compute hello=0.
compute x = fulldat(v,xindx).
compute x={ones,x}.
compute xsq=t(x)*x.
compute exsq=eval(xsq).
release xsq.
compute holymoly=cmin(exsq).
compute zeroeig=csum(exsq <= 0.000000000002).
compute bad=bad + (zeroeig > 0).

.
compute desctmp=make((8-(4* 1 )),ncol( y ),-999).
loop jd=1 to ncol( y ).
compute descdat= y (:,jd).
compute desctmp(1,jd) = csum(descdat)/nrow(descdat).
compute desctmp(2,jd) = (nrow(descdat)*sscp(descdat))-(t(csum(descdat))*(csum(descdat))).
compute desctmp(2,jd) = sqrt(desctmp(2,jd)/(nrow(descdat)*(nrow(descdat)-1))).
compute desctmp(3,jd)=cmin(descdat).
compute desctmp(4,jd)=cmax(descdat).
do if ( 1 =0).
compute minwarn=0.
compute maxwarn=0.
do if ((desctmp(3,jd)=desctmp(4,jd)) and novar=0).
compute errcode(errs,1)=15.
compute errs=errs+1.
compute criterr=1.
compute novar=1.
end if.
compute tmp=((descdat(:,1)=desctmp(3,jd))+(descdat(:,1)=desctmp(4,jd))).
compute desctmp(8,jd)=(csum(tmp)=nrow(tmp)).
compute tmp = descdat.
compute tmp(GRADE(descdat),:) = descdat.
compute descdat = tmp.
release tmp.
compute decval={.16;.5;.84}.
loop kd=1 to 3.
compute low=trunc(decval(kd,1)*(nrow(descdat)+1)).
compute lowdec=decval(kd,1)*(nrow(descdat)+1)-low.
compute value=descdat(low,1)+(descdat((low+1),1)-descdat(low,1))*lowdec.
compute desctmp((4+kd),jd)=value.
end loop.
compute mnotev=1.
compute modvals=desctmp(5:7,:).
do if (quantile <> 1).
compute desctmp(5,jd)=desctmp(1,jd)-desctmp(2,jd).
compute desctmp(6,jd)=desctmp(1,jd).
compute desctmp(7,jd)=desctmp(1,jd)+desctmp(2,jd).
compute modvals=desctmp(5:7,:).
compute mnotev=2.
do if (modvals(1,1) < desctmp(3,1)).
compute modvals(1,1)=desctmp(3,1).
compute minwarn=1.
end if.
do if (modvals(3,1) > desctmp(4,1)).
compute modvals(3,1)=desctmp(4,1).
compute maxwarn=1.
end if.
end if.
do if (desctmp(8,1)=1).
compute modvals={desctmp(3,1);desctmp(4,1)}.
compute mnotev=0.
compute minwarn=0.
compute maxwarn=0.
end if.
end if.
end loop
.
compute bad=bad+ (desctmp(2,1) <= 0.00000000001).
do if (bad = 0).
do if (holymoly < smallest).
compute smallest=holymoly.
end if.
do if (ydich=0 or (i < (nms+nys))).

.
do if ( 1 =1).
compute hatmat=inv(t( x )* x )*t( x ).
compute b = hatmat* y.
compute modres=b.
do if ( 0 =1).
compute n1=nrow( x ).
compute dfres=n1-(ncol( x )).
compute sstotal = t( y -(csum( y )/n1))*( y -(csum( y )/n1)).
compute resid= y - x *b.
compute ssresid = csum((resid)&**2).
compute r2 = (sstotal-ssresid)/sstotal.
do if (r2 < 0).
compute r2=0.
end if.
compute adjr2 = 1-((1-r2)*(n1-1)/(dfres)).
compute mse=ssresid/(n1-ncol( x )).

.
compute n1=nrow( x ).
compute invXtX = inv(t( x )* x ).
compute varb = mse *invXtX.
compute k3 = ncol( x ).
compute xhc=0.
do if ( hc <> 5).
compute xhc= x.
compute hat = xhc(:,1).
loop i3=1 to nrow(xhc).
compute hat(i3,1)= xhc(i3,:)*invXtX*t(xhc(i3,:)).
end loop.
do if ( hc = 0 or hc =1).
loop i3 = 1 to k3.
compute xhc(:,i3)=xhc(:,i3)&* resid.
end loop.
end if.
do if ( hc =3 or hc =2).
loop i3=1 to k3.
compute xhc(:,i3) = ( resid &/(1-hat)&**(1/(4- hc )))&*xhc(:,i3).
end loop.
end if.
do if ( hc = 4).
compute hcmn=make(n,2,4).
compute hcmn(:,2)=(n1*hat)/k3.
loop i3= 1 to k3.
compute xhc(:,i3) = ( resid &/(1-hat)&**(rmin(hcmn)/2))&*xhc(:,i3).
end loop.
end if.
compute varb=(invXtX*t(xhc)*xhc*invXtX).
do if ( hc =1).
compute varb=(n1/(n1-ncol( x )))&*varb.
end if.
end if.
compute hclab={'se(HC0)','se(HC1)','se(HC2)','se(HC3)','se(HC4)','se'}.
compute hclab=hclab(1,( hc +1)).
compute hcflab={'F(HC0)','F(HC1)','F(HC2)','F(HC3)','F(HC4)','F'}.
compute hcflab=hcflab(1,( hc +1)).
release xhc
.
compute seb=sqrt(diag(varb)).
compute trat = b&/seb.
compute p = 2*(1-tcdf(abs(trat), (dfres))).
compute tval = sqrt(dfres* (exp((dfres-(5/6))*((xp2/(dfres-(2/3)+(.11/dfres)))* (xp2/(dfres-(2/3)+(.11/dfres)))))-1)).
compute modres={modres,seb,trat,p}.
compute modres={modres,(b-tval&*seb),(b+tval&*seb)}.
compute modresl={'coeff',hclab,'t','p','LLCI','ULCI'}.
compute lmat = ident(ncol( x )).
compute lmat = lmat(:,2:ncol(lmat)).
compute fratio = (t(t(lmat)*b)*inv(t(lmat)*varb*lmat)*((t(lmat)*b)))/(ncol( x )-1).
compute pfr = 1-fcdf(fratio,(ncol( x )-1),dfres).
compute modsum={sqrt(r2),r2,mse,fratio,(ncol( x )-1),dfres,pfr}.
compute modsuml={'R','R-sq','MSE',hcflab,'df1','df2', 'p'}.
end if.
end if.
do if ( 1 = 2 or 1 =3).
compute xlp= x.
compute ylp= y.
compute pt2 = make(nrow(ylp),1,(csum(ylp)/nrow(ylp))).
do if ( 1 =2).
compute LL3 = ylp&*ln(pt2)+(1-ylp)&*ln(1-pt2).
end if.
compute LL3 = -2*csum(LL3).
compute bt1 = make(ncol(xlp),1,0).
compute LL1 = 0.
compute pt1 = make(nrow(ylp),1,0.5).
compute pt1lp=pt1.
loop jjj = 1 to iterate.
compute xlptmp=t(xlp).
compute vecprb=pt1lp&*(1-pt1lp).
loop kkk=1 to ncol(xlp).
compute xlptmp(kkk,:)=xlptmp(kkk,:)&*t(vecprb).
end loop.
compute b = bt1+inv(xlptmp*xlp)*t(xlp)*(ylp-pt1lp).
do if ( 1 =2).
compute xlpb=xlp*b.
compute xlpbt=(xlpb > -709.7).
compute xlpb709=(1-xlpbt)*(-709.7).
compute xlpb=(xlpb&*xlpbt)+xlpb709.
compute pt1lp = 1/(1+exp(-(xlpb))).
end if.
compute itprob = csum((pt1lp < .00000001) or (pt1lp > .9999999)).
do if (itprob > 0).
loop kkk = 1 to nrow(pt1lp).
do if (pt1lp(kkk,1) > .9999999).
compute pt1lp(kkk,1) = .9999999.
end if.
do if (pt1lp(kkk,1) < .00000001).
compute pt1lp(kkk,1) = .00000001.
end if.
end loop.
compute itprob = 0.
end if.
do if (itprob = 0).
do if ( 1 =2).
compute LL = ylp&*ln(pt1lp)+(1-ylp)&*ln(1-pt1lp).
end if.
compute LL2 = -2*csum(ll).
end if.
do if (abs(LL1-LL2) < converge).
do if ( 0 =1).
compute xlptmp=t(xlp).
compute vecprb=pt1lp&*(1-pt1lp).
loop kkk=1 to ncol(xlp).
compute xlptmp(kkk,:)=xlptmp(kkk,:)&*t(vecprb).
end loop.
compute varb = inv(xlptmp*xlp).
compute seb = sqrt(diag(varb)).
release xlptmp.
end if.
break.
end if.
compute bt1 = b.
compute LL1 = LL2.
end loop.
compute modres=b.
do if (jjj > iterate).
compute itprob = 2.
do if (booting=0).
compute iterrmod=1.
end if.
do if (booting=1).
compute bootiter=1.
end if.
do if (itprobtg=0).
compute itprobtg=1.
compute errcode(errs,1) = 47.
compute errs = errs + 1.
do if (booting = 0 and 0 =1).
compute vt1 = mdiag(pt1lp&*(1-pt1lp)).
compute varb = inv(t(xlp)*vt1*xlp).
compute seb = sqrt(diag(varb)).
end if.
end if.
end if.
do if ( 0 =1).
compute trat = b&/seb.
compute dfres=nrow(xlp).
compute p = 2*(1-cdfnorm(abs(trat))).
compute modres={modres,seb,trat,p}.
compute modres={modres,(b-xp2&*seb),(b+xp2&*seb)}.
compute pvchi=1-chicdf((LL3-LL2),(nrow(modres)-1)).
compute mcF = (LL3-LL2)/LL3.
compute cox = 1-exp(-(LL3-LL2)/nrow(xlp)).
compute nagel = cox/(1-exp(-(LL3)/nrow(xlp))).
compute modsum={LL2,(LL3-LL2),(nrow(modres)-1),pvchi, mcF,cox,nagel}.
compute modsuml={'-2LL','ModelLL', 'df', 'p', 'McFadden', 'CoxSnell', 'Nagelkrk'}.
compute modresl={'coeff','se','Z','p','LLCI','ULCI'}.
end if.
end if
.
end if.
do if (ydich=1 and (i = (nms+nys))).

.
do if ( 2 =1).
compute hatmat=inv(t( x )* x )*t( x ).
compute b = hatmat* y.
compute modres=b.
do if ( 0 =1).
compute n1=nrow( x ).
compute dfres=n1-(ncol( x )).
compute sstotal = t( y -(csum( y )/n1))*( y -(csum( y )/n1)).
compute resid= y - x *b.
compute ssresid = csum((resid)&**2).
compute r2 = (sstotal-ssresid)/sstotal.
do if (r2 < 0).
compute r2=0.
end if.
compute adjr2 = 1-((1-r2)*(n1-1)/(dfres)).
compute mse=ssresid/(n1-ncol( x )).

.
compute n1=nrow( x ).
compute invXtX = inv(t( x )* x ).
compute varb = mse *invXtX.
compute k3 = ncol( x ).
compute xhc=0.
do if ( hc <> 5).
compute xhc= x.
compute hat = xhc(:,1).
loop i3=1 to nrow(xhc).
compute hat(i3,1)= xhc(i3,:)*invXtX*t(xhc(i3,:)).
end loop.
do if ( hc = 0 or hc =1).
loop i3 = 1 to k3.
compute xhc(:,i3)=xhc(:,i3)&* resid.
end loop.
end if.
do if ( hc =3 or hc =2).
loop i3=1 to k3.
compute xhc(:,i3) = ( resid &/(1-hat)&**(1/(4- hc )))&*xhc(:,i3).
end loop.
end if.
do if ( hc = 4).
compute hcmn=make(n,2,4).
compute hcmn(:,2)=(n1*hat)/k3.
loop i3= 1 to k3.
compute xhc(:,i3) = ( resid &/(1-hat)&**(rmin(hcmn)/2))&*xhc(:,i3).
end loop.
end if.
compute varb=(invXtX*t(xhc)*xhc*invXtX).
do if ( hc =1).
compute varb=(n1/(n1-ncol( x )))&*varb.
end if.
end if.
compute hclab={'se(HC0)','se(HC1)','se(HC2)','se(HC3)','se(HC4)','se'}.
compute hclab=hclab(1,( hc +1)).
compute hcflab={'F(HC0)','F(HC1)','F(HC2)','F(HC3)','F(HC4)','F'}.
compute hcflab=hcflab(1,( hc +1)).
release xhc
.
compute seb=sqrt(diag(varb)).
compute trat = b&/seb.
compute p = 2*(1-tcdf(abs(trat), (dfres))).
compute tval = sqrt(dfres* (exp((dfres-(5/6))*((xp2/(dfres-(2/3)+(.11/dfres)))* (xp2/(dfres-(2/3)+(.11/dfres)))))-1)).
compute modres={modres,seb,trat,p}.
compute modres={modres,(b-tval&*seb),(b+tval&*seb)}.
compute modresl={'coeff',hclab,'t','p','LLCI','ULCI'}.
compute lmat = ident(ncol( x )).
compute lmat = lmat(:,2:ncol(lmat)).
compute fratio = (t(t(lmat)*b)*inv(t(lmat)*varb*lmat)*((t(lmat)*b)))/(ncol( x )-1).
compute pfr = 1-fcdf(fratio,(ncol( x )-1),dfres).
compute modsum={sqrt(r2),r2,mse,fratio,(ncol( x )-1),dfres,pfr}.
compute modsuml={'R','R-sq','MSE',hcflab,'df1','df2', 'p'}.
end if.
end if.
do if ( 2 = 2 or 2 =3).
compute xlp= x.
compute ylp= y.
compute pt2 = make(nrow(ylp),1,(csum(ylp)/nrow(ylp))).
do if ( 2 =2).
compute LL3 = ylp&*ln(pt2)+(1-ylp)&*ln(1-pt2).
end if.
compute LL3 = -2*csum(LL3).
compute bt1 = make(ncol(xlp),1,0).
compute LL1 = 0.
compute pt1 = make(nrow(ylp),1,0.5).
compute pt1lp=pt1.
loop jjj = 1 to iterate.
compute xlptmp=t(xlp).
compute vecprb=pt1lp&*(1-pt1lp).
loop kkk=1 to ncol(xlp).
compute xlptmp(kkk,:)=xlptmp(kkk,:)&*t(vecprb).
end loop.
compute b = bt1+inv(xlptmp*xlp)*t(xlp)*(ylp-pt1lp).
do if ( 2 =2).
compute xlpb=xlp*b.
compute xlpbt=(xlpb > -709.7).
compute xlpb709=(1-xlpbt)*(-709.7).
compute xlpb=(xlpb&*xlpbt)+xlpb709.
compute pt1lp = 1/(1+exp(-(xlpb))).
end if.
compute itprob = csum((pt1lp < .00000001) or (pt1lp > .9999999)).
do if (itprob > 0).
loop kkk = 1 to nrow(pt1lp).
do if (pt1lp(kkk,1) > .9999999).
compute pt1lp(kkk,1) = .9999999.
end if.
do if (pt1lp(kkk,1) < .00000001).
compute pt1lp(kkk,1) = .00000001.
end if.
end loop.
compute itprob = 0.
end if.
do if (itprob = 0).
do if ( 2 =2).
compute LL = ylp&*ln(pt1lp)+(1-ylp)&*ln(1-pt1lp).
end if.
compute LL2 = -2*csum(ll).
end if.
do if (abs(LL1-LL2) < converge).
do if ( 0 =1).
compute xlptmp=t(xlp).
compute vecprb=pt1lp&*(1-pt1lp).
loop kkk=1 to ncol(xlp).
compute xlptmp(kkk,:)=xlptmp(kkk,:)&*t(vecprb).
end loop.
compute varb = inv(xlptmp*xlp).
compute seb = sqrt(diag(varb)).
release xlptmp.
end if.
break.
end if.
compute bt1 = b.
compute LL1 = LL2.
end loop.
compute modres=b.
do if (jjj > iterate).
compute itprob = 2.
do if (booting=0).
compute iterrmod=1.
end if.
do if (booting=1).
compute bootiter=1.
end if.
do if (itprobtg=0).
compute itprobtg=1.
compute errcode(errs,1) = 47.
compute errs = errs + 1.
do if (booting = 0 and 0 =1).
compute vt1 = mdiag(pt1lp&*(1-pt1lp)).
compute varb = inv(t(xlp)*vt1*xlp).
compute seb = sqrt(diag(varb)).
end if.
end if.
end if.
do if ( 0 =1).
compute trat = b&/seb.
compute dfres=nrow(xlp).
compute p = 2*(1-cdfnorm(abs(trat))).
compute modres={modres,seb,trat,p}.
compute modres={modres,(b-xp2&*seb),(b+xp2&*seb)}.
compute pvchi=1-chicdf((LL3-LL2),(nrow(modres)-1)).
compute mcF = (LL3-LL2)/LL3.
compute cox = 1-exp(-(LL3-LL2)/nrow(xlp)).
compute nagel = cox/(1-exp(-(LL3)/nrow(xlp))).
compute modsum={LL2,(LL3-LL2),(nrow(modres)-1),pvchi, mcF,cox,nagel}.
compute modsuml={'-2LL','ModelLL', 'df', 'p', 'McFadden', 'CoxSnell', 'Nagelkrk'}.
compute modresl={'coeff','se','Z','p','LLCI','ULCI'}.
end if.
end if
.
end if.
compute modres2={modres2,t(modres)}.
do if (i = (nms+nys)).
do if (bcmat((i+1),1) = 1).
compute bootdir={bootdir;t(modres(wherex(1,i):wherex(2,i),1))}.
end if.
do if (bcmat((i+1),1) = 0).
compute bootdir={bootdir;dirzes}.
end if.
end if.
do if (model = 74 and i <= nms).
compute onetemp=make(nrow(xprobval),1,1).
compute mestmtb={onetemp,xprobval}.
do if (ncs > 0).
compute ncovmdlb=rsum(ccmat(i,:)).
do if (ncovmdl > 0).
compute cvmncb=make(nrow(mestmtb),ncovmdlb,1).
compute cvmnctmp=csum(x(:,(ncol(x)-ncovmdlb+1):ncol(x)))/nrow(x).
do if (cuscoval > 0).
compute cvmnctmp=coval.
end if.
loop mestlp=1 to ncovmdlb.
compute cvmncb(:,mestlp)=cvmncb(:,mestlp)*cvmnctmp(:,mestlp).
end loop.
compute mestmtb={mestmtb,cvmncb}.
end if.
end if.
compute mestbt=mestmtb*modres.
do if (i = 1).
compute mestb=mestbt.
end if.
do if (i > 1).
compute mestb={mestb,mestbt}.
end if.
end if.
do if (model = 74 and i = (nms+nys)).
compute xvalptmp=make(1,nxvls,0).
compute mest74sp=make(1,nms,0).
compute mest74sp={mest74sp;mestb}.
compute xvalptmp={xvalptmp;ident(nxvls)}.
compute dirfxcf=make(nrow(mestb),1,0).
loop cfloop1=1 to nrow(mestb).
compute ndirfx=make(nrow(modres),1,0).
compute ndirfx(2:(nxvls+1),1)=t(xvalptmp(cfloop1,:)).
loop cfloop3=1 to nms.
do if (mcx=1 or mcx=0).
compute ndirfx(wheremw((1+((cfloop3-1)*2)),ncol(wheremw)):wheremw((2+((cfloop3-1)*2) ),ncol(wheremw)))=t(xvalptmp(cfloop1,:)*mestb(1,cfloop3)).
end if.
do if (mcx=2).
compute ndirfx(wheremw((1+((cfloop3-1)*2)),ncol(wheremw)):wheremw((2+((cfloop3-1)*2) ),ncol(wheremw)))=t(xvalptmp(cfloop1,:)*mest74sp(cfloop1,cfloop3)).
end if.
end loop.
do if (cfloop1 > 1).
compute dirfxcf(cfloop1,1)=t(ndirfx)*modres*xscaling.
end if.
end loop.
compute natdirbt={natdirbt;t(dirfxcf(2:nrow(dirfxcf),:))}.
end if.
do if ( (bcmat((i+1),1) = 1) and (nobootx=1) and (effsize=1)).
compute nobootx=0.
compute xsdtemp= (nrow(x)*sscp(x(:,2)))-(t(csum(x(:,2)))*(csum(x(:,2)))).
compute xsdtemp= sqrt(xsdtemp/(nrow(x)*(nrow(x)-1))).
end if.
end if.
end loop.
do if (bad = 0).
compute bootres={bootres;modres2(:,2:ncol(modres2))}.
do if (effsize=1).
compute ysdtemp= (nrow(y)*sscp(y))-(t(csum(y))*(csum(y))).
compute ysdtemp= sqrt(ysdtemp/(nrow(y)*(nrow(y)-1))).
compute bootysd={bootysd;ysdtemp}.
compute bootxsd={bootxsd;xsdtemp}.
end if.
compute goodboot=goodboot+1.
end if.
do if (bad <> 0).
compute badboot=badboot+1.
end if.
end loop if (goodboot = boot).
compute bootres=bootres(2:nrow(bootres),:).
do if (effsize=1).
compute bootysd=bootysd(2:nrow(bootysd),:).
do if (nrow(bootxsd) > 1).
compute bootxsd=bootxsd(2:nrow(bootxsd),:).
end if.
end if.
do if (goodboot < (boot)).
compute boot=0.
compute modelbt=0.
compute notecode(notes,1) = 7.
compute notes = notes + 1.
end if.
do if (boot > 0).
do if (effsize=1).
compute bootysd={ysd;bootysd}.
compute bootxsd={xsd;bootxsd}.
end if.
do if (saveboot = 1).
save bootres/outfile = *.
end if.
do if (modelbt=1).
compute bootcim=make(ncol(bootres),5,-99999).
compute bootcim(:,2) = t(csum(bootres)/nrow(bootres)).
compute bootcim(:,1) = coeffmat(2:nrow(coeffmat),1).
loop i = 1 to ncol(bootres).

.
compute temp = bootres(:,i).
compute temp(GRADE( bootres(:,i) )) = bootres(:,i).
compute badlo = 0.
compute badhi = 0.
do if ( (bootcim(i,1)*bc)+(9999*(1-bc)) <> 9999).
compute pv=csum(temp < (bootcim(i,1)*bc)+(9999*(1-bc)) )/nrow(temp).
compute ppv = pv.
do if (pv > .5).
compute ppv = 1-pv.
end if.
compute y5=sqrt(-2*ln(ppv)).
compute xp=y5+((((y5*p4+p3)*y5+p2)*y5+p1)*y5+p0)/((((y5*q4+q3)*y5+q2)*y5+q1)*y5+q0).
do if (pv <= .5).
compute xp = -xp.
end if.
compute cilow=rnd(nrow(temp)*(cdfnorm(2*xp-xp2))).
compute cihigh=trunc(nrow(temp)*(cdfnorm(2*xp+(xp2))))+1.
do if (cilow < 1).
compute cilow = 1.
compute booterr=1.
compute badlo = 1.
end if.
do if (cihigh > nrow(temp)).
compute cihigh = boot.
compute booterr=1.
compute badhi = 1.
end if.
compute llcit=temp(cilow,1).
compute ulcit=temp(cihigh,1).
do if (badlo = 1 and llcit <> priorlo).
compute badend={badend, llcit}.
compute priorlo = llcit.
end if.
do if (badhi = 1 and ulcit <> priorhi).
compute badend={badend, ulcit}.
compute priorhi = ulcit.
end if.
end if.
do if ( (bootcim(i,1)*bc)+(9999*(1-bc)) = 9999).
compute llcit=temp(cilow,1).
compute ulcit=temp(cihigh,1).
end if.
compute bootse=t(sqrt((cssq(temp)-((csum(temp)&**2)/nrow(temp)))/(nrow(temp)-1)))
.
compute bootcim(i,4:5)={llcit,ulcit}.
compute bootcim(i,3)=bootse.
end loop.
end if.
end if.
do if (badboot > 0).
compute notecode(notes,1) = 6.
compute notes = notes + 1.
end if.
end if.
do if (criterr=0).
release fulldat,x,y.
end if.
do if (xmint=1 and criterr=0).
compute xvalptmp=make(1,nxvls,0).
compute mest74sp=make(1,nms,0).
compute mest74sp={mest74sp;mest74}.
compute xvalptmp={xvalptmp;ident(nxvls)}.
loop kcfuhd=1 to 2.
compute dirfxcf=make(nrow(mest74),6,0).
loop cfloop1=1 to nrow(mest74).
compute ndirfx=make(nrow(lastb),1,0).
compute ndirfx(2:(nxvls+1),1)=t(xvalptmp(cfloop1,:)).
loop cfloop3=1 to nms.
do if (kcfuhd=1).
do if (mcx=1 or mcx=0).
compute ndirfx(wheremw((1+((cfloop3-1)*2)),ncol(wheremw)):wheremw((2+((cfloop3-1)*2)), ncol(wheremw)))=t(xvalptmp(cfloop1,:)*mest74(1,cfloop3)).
end if.
do if (mcx=2).
compute ndirfx(wheremw((1+((cfloop3-1)*2)),ncol(wheremw)):wheremw((2+((cfloop3-1)*2)), ncol(wheremw)))=t(xvalptmp(cfloop1,:)*mest74sp(cfloop1,cfloop3)).
end if.
end if.
do if (kcfuhd=2).
compute ndirfx(wheremw((1+((cfloop3-1)*2)),ncol(wheremw)):wheremw((2+((cfloop3-1)*2)), ncol(wheremw)))=t(xvalptmp(cfloop1,:)*medmeans(1,cfloop3)).
end if.
end loop.
do if (cfloop1 > 1).
compute dirfxcf(cfloop1,1)=t(ndirfx)*lastb*xscaling.
compute dirfxcf(cfloop1,2)=sqrt(diag(t(ndirfx)*lastcov*ndirfx))*abs(xscaling).
compute dirfxcf(cfloop1,3)=dirfxcf(cfloop1,1)/dirfxcf(cfloop1,2).
compute dirfxcf(cfloop1,4)=2*(1-tcdf(abs(dirfxcf(cfloop1,3)), dfres)).
compute dirfxcf(cfloop1,5)=dirfxcf(cfloop1,1)-tval*dirfxcf(cfloop1,2).
compute dirfxcf(cfloop1,6)=dirfxcf(cfloop1,1)+tval*dirfxcf(cfloop1,2).
end if.
end loop.
compute codireff=dirfxcf(2:nrow(dirfxcf),:).
do if (kcfuhd=1).
compute direff=codireff.
end if.
end loop.
end if.

.
do if (criterr = 0 and nms > 0).
compute paths=paths(:,2:ncol(paths)).
compute pathsw=pathsw(:,2:ncol(pathsw)).
compute pathsz=pathsz(:,2:ncol(pathsz)).
compute pathswz=pathswz(:,2:ncol(pathswz)).
compute pathsmod=pathsw+pathsz+pathswz.
compute pathsdv=pathsdv(:,2:ncol(pathsdv)).
compute pathsfoc=pathsfoc(:,2:ncol(pathsfoc)).
compute pathtype=pathtype(:,2:ncol(pathtype)).
compute anymod=(rsum(pathsmod) > 0).
compute obscoeff=obscoeff(1,2:ncol(obscoeff)).
do if (outscree=1).
do if (xmint=1).
print/title = '************************* COUNTERFACTUALLY DEFINED ************************'.
else.
print/title = ' '.
end if.
do if (dototal = 0 and alttotal=0).
print/title = '****************** DIRECT AND INDIRECT EFFECTS OF X ON Y *****************'/space=0.
end if.
do if (alttotal=1).
print/title = '************** TOTAL, DIRECT, AND INDIRECT EFFECTS OF X ON Y **************'/space=0.
end if.
end if.
do if (dototal = 1).
do if (outscree=1).
print/title = '************** TOTAL, DIRECT, AND INDIRECT EFFECTS OF X ON Y **************'/space=0.
end if.
compute totefflb(1,1)='Effect'.
do if (effsize=1).
compute toteffsz=toteff(:,1)/ysd.
do if (xdich=1 or mcx > 0).
compute totefflb={totefflb,'c_ps'}.
end if.
do if (xdich = 0 and mcx = 0).
compute toteffsz=toteffsz*xsd.
compute totefflb={totefflb,'c_cs'}.
end if.
compute toteff={toteff,toteffsz}.
end if.
compute resultm2=make(nrow(toteff),maxresm,99999).
compute resultm2(1:nrow(toteff),1:ncol(toteff))=toteff.
compute resultm={resultm;resultm2}.
do if (nxvls > 1).
compute resultm2=make(nrow(totomni),maxresm,99999).
compute resultm2(1:nrow(totomni),1:ncol(totomni))=totomni.
compute resultm={resultm;resultm2}.
compute clabtmp={'R2-chng', hcflab, 'df1','df2','p'}.
do if (outscree=1).
print toteff/title='Relative total effects of X on Y'/rnames=toteffl2/cnames=totefflb/format= F10.4.
print totomni/title='Omnibus test of total effect of X on Y'/cnames=clabtmp/format= F10.4.
print/title= '----------'/space=0.
end if.
else.
do if (outscree=1).
print toteff/title='Total effect of X on Y'/cnames=totefflb/format= F10.4.
end if.
end if.
end if.
compute moddir=wcmat(nrow(bcmat),1)+zcmat(nrow(bcmat),1).
do if (xmint=1).
compute moddir=1.
end if.
do if (bcmat(nrow(bcmat),1)=1).
do if (ydich=1).
compute direfflb(:,(ncol(direfflb)-5):ncol(direfflb))={'Effect','se','Z','p','LLCI','ULCI'}.
end if.
do if (moddir=0 or xmint=1).
compute direfflb(1,1)='Effect'.
end if.
do if (effsize=1 and moddir=0 and anymod = 0).
compute direffsz=direff(:,1)/ysd.
do if (xdich=1 or mcx > 0).
compute direfflb={direfflb,'c''_ps'}.
end if.
do if (xdich = 0 and mcx = 0).
compute direffsz=direffsz*xsd.
compute direfflb={direfflb,'c''_cs'}.
end if.
compute direff={direff,direffsz}.
end if.
compute resultm2=make(nrow(direff),maxresm,99999).
compute resultm2(1:nrow(direff),1:ncol(direff))=direff.
compute resultm={resultm;resultm2}.
do if (xmint=1).
compute resultm2=make(nrow(codireff),maxresm,99999).
compute resultm2(1:nrow(codireff),1:ncol(codireff))=codireff.
compute resultm={resultm;resultm2}.
end if.
do if (moddir = 0 and nxvls=1).
do if (outscree=1).
print direff/title='Direct effect of X on Y'/cnames=direfflb/format= F10.4.
end if.
end if.
do if (moddir = 0 and nxvls>1).
compute resultm2=make(nrow(diromni),maxresm,99999).
compute resultm2(1:nrow(diromni),1:ncol(diromni))=diromni.
compute resultm={resultm;resultm2}.
do if (outscree=1).
print direff/title='Relative direct effects of X on Y'/rnames=direffl2/cnames=direfflb/format= F10.4.
do if (ydich = 0).
compute clabtmp={'R2-chng', hcflab, 'df1','df2','p'}.
print diromni/title='Omnibus test of direct effect of X on Y:'/cnames=clabtmp/format= F10.4.
end if.
do if (ydich = 1).
compute clabtmp={'Chi-sq', 'df', 'p'}.
print diromni/title='Omnibus likelihood ratio test of direct effect of X on Y'/cnames=clabtmp/format= F10.4.
end if.
print/title= '----------'/space=0.
end if.
end if.
do if (moddir > 0 and nxvls=1).
do if (xmint=0 and outscree=1).
print direff/title='Conditional direct effects of X on Y'/cnames=direfflb/format= F10.4.
end if.
do if (xmint=1).
do if (outscree=1).
print direff/title='(Pure) Natural direct effect of X on Y'/cnames=direfflb/format= F10.4.
print codireff/title='Controlled direct effect of X on Y'/cnames=direfflb/format= F10.4.
print/title= '----------'/space=0.
end if.
compute obnatdfx=direff(:,1).
end if.
end if.
compute direffl4=direffl2.
do if (moddir > 0 and nxvls>1).
compute direffl2={' '}.
loop i = 1 to nxvls.
loop j = 1 to (nrow(direff)/nxvls).
compute direffl2={direffl2;xcatlab(i,1)}.
end loop.
end loop.
compute direffl2=direffl2(2:nrow(direffl2),1).
do if (xmint=0 and outscree=1).
print direff/title='Relative conditional direct effects of X on Y'/cnames=direfflb/rnames=direffl2/format= F10.4.
end if.
do if (xmint=1).
do if (outscree=1).
print direff/title='Relative (pure) natural direct effects of X on Y'/cnames=direfflb/rnames=direffl2/format= F10.4.
print codireff/title='Relative controlled direct effects of X on Y'/cnames=direfflb/rnames=direffl2/format= F10.4.
print/title= '----------'/space=0.
end if.
compute obnatdfx=direff(:,1).
end if.
end if.
compute direffl2=direffl4.
end if.
do if (bcmat(nrow(bcmat),1)=0 and xmint <> 1 and outscree=1).
print/title='The direct effect of X on Y is fixed to zero.'.
end if.
do if (nms = 1).
compute indmark={2}.
compute indsets={1,2}.
end if.
do if (nms = 2).
compute indmark={2,2,3}.
compute indsets={1,4,2,5,1,3,5}.
compute thetam=1.
end if.
do if (nms = 3).
compute indmark={2,2,2,3,3,3,4}.
compute indsets={1,7,2,8,4,9,1,3,8,1,5,9,2,6,9,1,3,6,9}.
compute thetam={1,2,3}.
end if.
do if (nms = 4).
compute indmark={2,2,2,2,3,3,3,3,3,3,4,4,4,4,5}.
compute indsets={1,11,2,12,4,13,7,14,1,3,12,1,5,13,1,8,14,2,6,13,2,9,14,4,10,14,1,3,6,13,1,3,9, 14,1,5,10,14,2,6,10,14,1,3,6,10,14}.
compute thetam={1,2,4,3,5,6}.
end if.
do if (nms = 5).
compute indmark={2,2,2,2,2,3,3,3,3,3,3,3,3,3,3,4,4,4,4,4,4,4,4,4,4,5,5,5,5,5,6}.
compute indsets={1,16,2,17,4,18,7,19,11,20,1,3,17,1,5,18,1,8,19,1,12,20,2,6,18,2,9,19,2,13,20,4, 10,19,4,14,20,7,15,20,1,3,6,18, 1,3,9,19,1,3,13,20,1,5,10,19,1,5,14,20,1,8,15,20,2,6,10,19,2,6, 14,20,2,9,15,20,4,10,15,20,1,3,6,10,19, 1,3,6,14,20,1,3,9,15,20,1,5,10,15,20,2,6,10,15,20,1,3,6,10,15, 20}.
compute thetam={1,2,5,3,6,8,4,7,9,10}.
end if.
do if (nms = 6).
compute indmark={2,2,2,2,2,2,3,3,3,3,3,3,3,3,3,3,3,3,3,3,3,4,4,4,4,4,4,4,4,4,4,4,4,4,4,4,4,4,4, 4,4,5,5,5,5,5,5,5,5,5,5,5,5,5,5,5,6,6,6,6,6,6,7}.
compute indsets={1,22,2,23,4,24,7,25,11,26,16,27,1,3,23,1,5,24,1,8,25,1,12,26,1,17,27,2,6,24,2, 9,25,2,13,26,2,18,27,4,10,25,4,14,26, 4,19,27,7,15,26,7,20,27,11,21,27,1,3,6,24,1,3,9,25,1,3,13,26,1, 3,18,27,1,5,10,25,1,5,14,26,1,5,19,27,1,8,15,26,1,8,20,27, 1,12,21,27,2,6,10,25,2,6,14,26,2,6,19,27,2,9,15,26,2,9,20,27,2, 13,21,27,4,10,15,26,4,10,20,27,4,14,21,27,7,15,21,27, 1,3,6,10,25,1,3,6,14,26,1,3,6,19,27,1,3,9,15,26,1,3,9,20,27,1,3, 13,21,27,1,5,10,15,26,1,5,10,20,27,1,5,14,21,27, 1,8,15,21,27,2,6,10,15,26,2,6,10,20,27,2,6,14,21,27,2,9,15,21, 27,4,10,15,21,27,1,3,6,10,15,26,1,3,6,10,20,27, 1,3,6,14,21,27,1,3,9,15,21,27,1,5,10,15,21,27,2,6,10,15,21,27, 1,3,6,10,15,21,27}.
compute thetam={1,2,6,3,7,10,4,8,11,13,5,9,12,14,15}.
end if.
do if (nms = 7).
compute indmark={2,2,2,2,2,2,2}.
compute indsets={1,29,2,30,4,31,7,32,11,33,16,34,22,35}.
end if.
do if (nms = 8).
compute indmark={2,2,2,2,2,2,2,2}.
compute indsets={1,37,2,38,4,39,7,40,11,41,16,42,22,43,29,44}.
end if.
do if (nms = 9).
compute indmark={2,2,2,2,2,2,2,2,2}.
compute indsets={1,46,2,47,4,48,7,49,11,50,16,51,22,52,29,53,37,54}.
end if.
do if (nms=10).
compute indmark={2,2,2,2,2,2,2,2,2,2}.
compute indsets={1,56,2,57,4,58,7,59,11,60,16,61,22,62,29,63,37,64,46,65}.
end if.
compute indlbl = {'Ind1'; 'Ind2'; 'Ind3'; 'Ind4'; 'Ind5'; 'Ind6'; 'Ind7'; 'Ind8'; 'Ind9'; 'Ind10'; 'Ind11'; 'Ind12'; 'Ind13'; 'Ind14'; 'Ind15'}.
compute indlbl = {indlbl; 'Ind16';'Ind17';'Ind18';'Ind19';'Ind20';'Ind21';'Ind22';'Ind23';'Ind24';'Ind25';'Ind26';'Ind27';'Ind28';'Ind29';'Ind30'}.
compute indlbl = {indlbl; 'Ind31';'Ind32';'Ind33';'Ind34';'Ind35';'Ind36';'Ind37';'Ind38';'Ind39';'Ind40';'Ind41';'Ind42';'Ind43';'Ind44';'Ind45'}.
compute indlbl = {indlbl; 'Ind46';'Ind47';'Ind48';'Ind49';'Ind50';'Ind51';'Ind52';'Ind53';'Ind54';'Ind55';'Ind56';'Ind57';'Ind58';'Ind59';'Ind60'}.
compute indlbl = {indlbl; 'Ind61';'Ind62';'Ind63';'Ind64';'Ind65';'Ind66';'Ind67';'Ind68';'Ind69';'Ind70';'Ind71';'Ind72';'Ind73';'Ind74';'Ind75'}.
compute indlbl = {indlbl; 'Ind76';'Ind77';'Ind78';'Ind79';'Ind80';'Ind81';'Ind82';'Ind83';'Ind84';'Ind85';'Ind86';'Ind87';'Ind88';'Ind89';'Ind90'}.
compute cntname={'(C1)';'(C2)';'(C3)';'(C4)';'(C5)';'(C6)';'(C7)';'(C8)';'(C9)';'(C10)';'(C11)';'(C12)';'(C13)';'(C14)';'(C15)';'(C16)';'(C17)'}.
compute cntname={cntname;'(C18)';'(C19)';'(C20)';'(C21)';'(C22)';'(C23)';'(C24)';'(C25)';'(C26)';'(C27)';'(C28)';'(C29)';'(C30)';'(C31)'}.
compute cntname={cntname;'(C32)';'(C33)';'(C34)';'(C35)';'(C36)';'(C37)';'(C38)';'(C39)';'(C40)';'(C41)';'(C42)';'(C43)';'(C44)';'(C45)'}.
compute cntname={cntname;'(C46)';'(C47)';'(C48)';'(C49)';'(C50)';'(C51)';'(C52)';'(C53)';'(C54)';'(C55)';'(C56)';'(C57)';'(C58)';'(C59)'}.
compute cntname={cntname;'(C60)';'(C61)';'(C62)';'(C63)';'(C64)';'(C65)';'(C66)';'(C67)';'(C68)';'(C69)';'(C70)';'(C71)';'(C72)';'(C73)'}.
compute cntname={cntname;'(C74)';'(C75)';'(C76)';'(C77)';'(C78)';'(C79)';'(C80)';'(C81)';'(C82)';'(C83)';'(C84)';'(C85)';'(C86)';'(C87)'}.
compute cntname={cntname;'(C88)';'(C89)';'(C90)';'(C91)';'(C92)';'(C93)';'(C94)';'(C95)';'(C96)';'(C97)';'(C98)';'(C99)';'(C100)';'(C101)'}.
compute cntname={cntname;'(C102)';'(C103)';'(C104)';'(C105)'}.
compute indmake=make(ncol(indmark),(nms+2),0).
compute indmod=make(ncol(indmark),1,999).
compute indmmm=make(ncol(indmark),1,0).
compute indmmmt=make(ncol(indmark),1,0).
compute start=1.
compute end=0.
compute nindfx=0.
compute indlocs=make(nrow(thetaxmb),ncol(paths),999).
compute indkey=make(ncol(indmark),1+((rmax(indmark)*2)+1),'     ').
compute c1=1.
compute c2=1.
compute c3=1.
loop i = 1 to ncol(paths).
do if (pathtype(1,i)=1).
compute indlocs(:,i)=thetaxmb(:,c1).
compute c1=c1+1.
end if.
do if (pathtype(1,i)=3).
compute indlocs(:,i)=thetamyb(:,c2).
compute c2=c2+1.
end if.
do if (pathtype(1,i)=2 and nms < 7 and serial=1).
compute indlocs(:,i)=thetammb(:,thetam(1,c3)).
compute c3=c3+1.
end if.
end loop.
loop i = 1 to ncol(indlocs).
compute c1=2.
loop j = 2 to nrow(indlocs).
do if (indlocs(j,i) <> 0).
compute indlocs(c1,i)=indlocs(j,i).
compute c1=c1+1.
end if.
end loop.
compute indlocs(1,i)=c1-2.
end loop.
compute indlocs=indlocs(1:rmax((indlocs(1,:))+1),:).
loop i = 1 to ncol(indmark).
compute numget=indmark(1,i).
compute end=end+numget.
compute gotcha=indsets(1,start:end).
compute start=end+1.
compute ok=1.
compute temp=0.
compute repoman=make(4,1,0).
loop j = 1 to ncol(gotcha).
do if paths(1,gotcha(1,j))=0.
compute ok=0.
end if.
do if (pathsmod(1,gotcha(1,j)) > 0).
compute temp=1.
compute temp2={pathsw(1,gotcha(1,j));pathsz(1,gotcha(1,j));pathswz(1,gotcha(1,j));0}.
do if (temp2(1,1)=1 and temp2(2,1)=1 and temp2(3,1)=0).
compute temp2(4,1)=1.
end if.
compute repoman=repoman+temp2.
end if.
end loop.
compute temp=0.
compute tempmmm=0.
compute typemmm=0.
do if ((repoman(1,1) > 0) and (repoman(2,1) = 0)).
compute temp=1.
do if (repoman(1,1)=1).
compute tempmmm=1.
end if.
do if (repoman(1,1) > 1 and (wdich=1 or mcw > 0)).
compute tempmmm=12.
compute typemmm=mcw.
do if (wdich=1).
compute typemmm=1.
end if.
end if.
do if (repoman(1,1) > 1 and (wdich=0 and mcw = 0)).
compute tempmmm=101.
end if.
end if.
do if ((repoman(1,1) = 0) and (repoman(2,1) > 0)).
compute temp=2.
do if (repoman(2,1)=1).
compute tempmmm=2.
end if.
do if (repoman(2,1) > 1 and (zdich = 1 or mcz > 0)).
compute tempmmm=22.
compute typemmm=mcz.
do if (zdich=1).
compute typemmm=1.
end if.
end if.
do if (repoman(2,1) > 1 and (zdich = 0 and mcw = 0)).
compute tempmmm=102.
end if.
end if.
do if (repoman(1,1)>0 and repoman(2,1)>0).
compute temp=3.
do if (repoman(1,1)=1 and repoman(2,1)=1).
do if (repoman(4,1)=1).
compute tempmmm=31.
end if.
do if (repoman(3,1)=1).
compute tempmmm=41.
end if.
end if.
end if.
do if (repoman(1,1)=1 and repoman(2,1)=1 and repoman(3,1)=0 and repoman(4,1)=0).
compute tempmmm=51.
end if.
do if (ok = 1).
compute nindfx=nindfx+1.
compute indmake(nindfx,1)=numget.
compute indmod(nindfx,1)=temp.
compute indmmm(nindfx,1)=tempmmm.
compute indmmmt(nindfx,1)=typemmm.
compute indmake(nindfx,2:(1+numget))=gotcha.
compute indkey(nindfx,1)=xnames.
loop j = 1 to numget.
compute indkey(nindfx,(j*2+1))=pathsdv(1,gotcha(1,j)).
compute indkey(nindfx,(j*2))='   ->   '.
end loop.
end if.
end loop.
compute indkey=indkey(1:nindfx,1:((cmax(indmake(:,1))*2)+1)).
compute indmake=indmake(1:nindfx,1:(cmax(indmake(:,1))+1)).
compute indmod=indmod(1:nrow(indmake),1).
compute indmmm=indmmm(1:nrow(indmake),1).
compute indmmmt=indmmmt(1:nrow(indmake),1).
compute ncpairs = (((nindfx)*(nindfx-1))/2).
do if ((contrast = 1 or contrast = 2 or contrast=3) and (ncpairs > 105)).
compute contrast=0.
compute notecode(notes,1) = 13.
compute notes = notes + 1.
end if.
do if (contrast = 4).
do if (ncol(contvec) <> nindfx).
compute contrast=0.
compute notecode(notes,1) = 14.
compute notes = notes + 1.
end if.
end if.
do if (anymod=0).
do if (nms = 1 and contrast > 0).
compute contrast=0.
end if.
compute efloop=(((1-(effsize=0))*2)+1)-(((mcx>0 or xdich=1))*(1-(effsize=0))).
loop kk=1 to efloop.
do if (boot = 0).
compute bootres=obscoeff.
do if (kk=1).
compute totbtvec=make(1,nxvls,0).
end if.
compute bootdir=obsdirfx.
compute indtab=999.
compute inddiff=999.
compute bootysd=ysd.
compute bootxsd=xsd.
end if.
do if (boot > 0).
compute bootres={obscoeff;bootres}.
do if (kk=1).
compute totbtvec=make(nrow(bootres),nxvls,0).
end if.
compute indtab=make(1,4,999).
compute inddiff=make(nrow(bootres),1,999).
end if.
compute indtotal=make(nrow(bootres),1,0).
loop i = 1 to nrow(indmake).
loop j = 1 to nxvls.
compute indtemp=make(nrow(bootres),1,1).
loop k = 1 to indmake(i,1).
compute jtemp=1.
do if (j > 1 and k=1).
compute jtemp=j.
end if.
compute indtemp=indtemp&*bootres(:,pathsfoc(jtemp,indmake(i,(k+1)))).
end loop.
do if (kk = 2).
compute indtemp=indtemp/bootysd.
end if.
do if (kk = 3).
compute indtemp=(bootxsd&*indtemp)/bootysd.
end if.
do if (contrast <> 0).
compute inddiff={inddiff,indtemp}.
end if.
do if (nxvls=1).
compute indtotal=indtotal+indtemp.
end if.
compute indeff=indtemp(1,1).
do if (kk=1).
compute totbtvec(:,j)=totbtvec(:,j)+indtemp.
end if.
do if (boot > 0).

.
compute temp = indtemp(2:nrow(indtemp),1).
compute temp(GRADE( indtemp(2:nrow(indtemp),1) )) = indtemp(2:nrow(indtemp),1).
compute badlo = 0.
compute badhi = 0.
do if ( (indtemp(1,1)*bc)+(9999*(1-bc)) <> 9999).
compute pv=csum(temp < (indtemp(1,1)*bc)+(9999*(1-bc)) )/nrow(temp).
compute ppv = pv.
do if (pv > .5).
compute ppv = 1-pv.
end if.
compute y5=sqrt(-2*ln(ppv)).
compute xp=y5+((((y5*p4+p3)*y5+p2)*y5+p1)*y5+p0)/((((y5*q4+q3)*y5+q2)*y5+q1)*y5+q0).
do if (pv <= .5).
compute xp = -xp.
end if.
compute cilow=rnd(nrow(temp)*(cdfnorm(2*xp-xp2))).
compute cihigh=trunc(nrow(temp)*(cdfnorm(2*xp+(xp2))))+1.
do if (cilow < 1).
compute cilow = 1.
compute booterr=1.
compute badlo = 1.
end if.
do if (cihigh > nrow(temp)).
compute cihigh = boot.
compute booterr=1.
compute badhi = 1.
end if.
compute llcit=temp(cilow,1).
compute ulcit=temp(cihigh,1).
do if (badlo = 1 and llcit <> priorlo).
compute badend={badend, llcit}.
compute priorlo = llcit.
end if.
do if (badhi = 1 and ulcit <> priorhi).
compute badend={badend, ulcit}.
compute priorhi = ulcit.
end if.
end if.
do if ( (indtemp(1,1)*bc)+(9999*(1-bc)) = 9999).
compute llcit=temp(cilow,1).
compute ulcit=temp(cihigh,1).
end if.
compute bootse=t(sqrt((cssq(temp)-((csum(temp)&**2)/nrow(temp)))/(nrow(temp)-1)))
.
compute indeff={indeff,bootse,llcit,ulcit}.
end if.
do if (kk=1).
compute indtabn={indtab;indeff}.
end if.
compute indtab={indtab;indeff}.
end loop.
end loop.
compute indtab=indtab(2:nrow(indtab),:).
do if (kk=1).
compute indtabn=indtabn(2:nrow(indtabn),:).
end if.
compute rowlbs=indlbl(1:nrow(indtab),1).
do if (mc > 0).
compute inddiff=make(mc,1,-999).
compute indtab2=make(nrow(indtab),4,-999).
compute indtab2(:,1)=indtab.
compute indtab=indtab2.
release indtab2.
compute mcct=0.
compute indtotal=make(mc,1,0).
do if (kk = 1).
compute x1 = sqrt(-2*ln(uniform(mc,nrow(mcsopath))))&*cos((2*3.14159265358979)*uniform(mc, nrow(mcsopath))).
compute x1=x1*chol(indcov).
loop ii=1 to nrow(x1).
compute x1(ii,:)=x1(ii,:)+t(mcsopath).
end loop.
end if.
loop ii=1 to nms.
compute tmpb=x1(:,((nms*nxvls)+ii)).
compute tmpb2=tmpb.
do if (nxvls > 1).
loop jj=1 to (nxvls-1).
compute tmpb2={tmpb2,tmpb}.
end loop.
end if.
compute indtemp=x1(:,(((ii-1)*nxvls)+1):(ii*nxvls))&*tmpb2.
loop jj=1 to ncol(indtemp).
do if (kk = 2).
compute indtemp(:,jj)=indtemp(:,jj)/ysd.
end if.
do if (kk = 3).
compute indtemp(:,jj)=(xsd*indtemp(:,jj))/ysd.
end if.

.
compute temp = indtemp(:,jj).
compute temp(GRADE( indtemp(:,jj) )) = indtemp(:,jj).
compute badlo = 0.
compute badhi = 0.
do if ( 9999 <> 9999).
compute pv=csum(temp < 9999 )/nrow(temp).
compute ppv = pv.
do if (pv > .5).
compute ppv = 1-pv.
end if.
compute y5=sqrt(-2*ln(ppv)).
compute xp=y5+((((y5*p4+p3)*y5+p2)*y5+p1)*y5+p0)/((((y5*q4+q3)*y5+q2)*y5+q1)*y5+q0).
do if (pv <= .5).
compute xp = -xp.
end if.
compute cilow=rnd(nrow(temp)*(cdfnorm(2*xp-xp2))).
compute cihigh=trunc(nrow(temp)*(cdfnorm(2*xp+(xp2))))+1.
do if (cilow < 1).
compute cilow = 1.
compute booterr=1.
compute badlo = 1.
end if.
do if (cihigh > nrow(temp)).
compute cihigh = boot.
compute booterr=1.
compute badhi = 1.
end if.
compute llcit=temp(cilow,1).
compute ulcit=temp(cihigh,1).
do if (badlo = 1 and llcit <> priorlo).
compute badend={badend, llcit}.
compute priorlo = llcit.
end if.
do if (badhi = 1 and ulcit <> priorhi).
compute badend={badend, ulcit}.
compute priorhi = ulcit.
end if.
end if.
do if ( 9999 = 9999).
compute llcit=temp(cilow,1).
compute ulcit=temp(cihigh,1).
end if.
compute bootse=t(sqrt((cssq(temp)-((csum(temp)&**2)/nrow(temp)))/(nrow(temp)-1)))
.
compute mcct=mcct+1.
compute indtab(mcct,2:4)={bootse,llcit,ulcit}.
end loop.
do if (nxvls=1).
compute indtotal=indtotal+indtemp.
do if (contrast <> 0).
compute inddiff={inddiff,indtemp}.
end if.
end if.
end loop.
release indtemp,tmpb.
end if.
do if (normal = 1 and sobelok=1).
compute sobelmat=indtab(:,1).
compute sobelmat={sobelmat,(sobelmat/2),sobelmat,sobelmat}.
loop ii=1 to nms.
compute se2b=indcov(((nms*nxvls)+ii),((nms*nxvls)+ii)).
compute bpath2=mcsopath(((nms*nxvls)+ii),1)&**2.
compute se2a=diag(indcov((((ii-1)*nxvls)+1):(ii*nxvls),(((ii-1)*nxvls)+1):(ii*nxvls))).
compute apath2=mcsopath((((ii-1)*nxvls)+1):(ii*nxvls) ,1)&**2.
compute sesobel=sqrt(apath2*se2b+bpath2*se2a+se2a*se2b).
compute sobelmat((((ii-1)*nxvls)+1):(ii*nxvls),2)=sesobel.
end loop.
release se2b,bpath2,se2a,apath2,sesobel,ii.
compute sobelmat(:,3)=sobelmat(:,1)&/sobelmat(:,2).
compute sobelmat(:,4) = 2*(1-cdfnorm(abs(sobelmat(:,3)))).
end if.
do if (serial = 0).
compute rowlbs=t(mnames).
end if.
do if (nxvls=1 and nms > 1).
compute rowlbs={'TOTAL';rowlbs}.
compute indtemp=indtotal(1,1).
do if (boot > 0 and nxvls=1).

.
compute temp = indtotal(2:nrow(indtotal),1).
compute temp(GRADE( indtotal(2:nrow(indtotal),1) )) = indtotal(2:nrow(indtotal),1).
compute badlo = 0.
compute badhi = 0.
do if ( (indtotal(1,1)*bc)+(9999*(1-bc)) <> 9999).
compute pv=csum(temp < (indtotal(1,1)*bc)+(9999*(1-bc)) )/nrow(temp).
compute ppv = pv.
do if (pv > .5).
compute ppv = 1-pv.
end if.
compute y5=sqrt(-2*ln(ppv)).
compute xp=y5+((((y5*p4+p3)*y5+p2)*y5+p1)*y5+p0)/((((y5*q4+q3)*y5+q2)*y5+q1)*y5+q0).
do if (pv <= .5).
compute xp = -xp.
end if.
compute cilow=rnd(nrow(temp)*(cdfnorm(2*xp-xp2))).
compute cihigh=trunc(nrow(temp)*(cdfnorm(2*xp+(xp2))))+1.
do if (cilow < 1).
compute cilow = 1.
compute booterr=1.
compute badlo = 1.
end if.
do if (cihigh > nrow(temp)).
compute cihigh = boot.
compute booterr=1.
compute badhi = 1.
end if.
compute llcit=temp(cilow,1).
compute ulcit=temp(cihigh,1).
do if (badlo = 1 and llcit <> priorlo).
compute badend={badend, llcit}.
compute priorlo = llcit.
end if.
do if (badhi = 1 and ulcit <> priorhi).
compute badend={badend, ulcit}.
compute priorhi = ulcit.
end if.
end if.
do if ( (indtotal(1,1)*bc)+(9999*(1-bc)) = 9999).
compute llcit=temp(cilow,1).
compute ulcit=temp(cihigh,1).
end if.
compute bootse=t(sqrt((cssq(temp)-((csum(temp)&**2)/nrow(temp)))/(nrow(temp)-1)))
.
compute indtemp={indtemp, bootse,llcit,ulcit}.
end if.
do if (mc > 0).
compute obtmc=indtab(:,1).
compute indtemp=csum(obtmc).

.
compute temp = indtotal(:,1).
compute temp(GRADE( indtotal(:,1) )) = indtotal(:,1).
compute badlo = 0.
compute badhi = 0.
do if ( 9999 <> 9999).
compute pv=csum(temp < 9999 )/nrow(temp).
compute ppv = pv.
do if (pv > .5).
compute ppv = 1-pv.
end if.
compute y5=sqrt(-2*ln(ppv)).
compute xp=y5+((((y5*p4+p3)*y5+p2)*y5+p1)*y5+p0)/((((y5*q4+q3)*y5+q2)*y5+q1)*y5+q0).
do if (pv <= .5).
compute xp = -xp.
end if.
compute cilow=rnd(nrow(temp)*(cdfnorm(2*xp-xp2))).
compute cihigh=trunc(nrow(temp)*(cdfnorm(2*xp+(xp2))))+1.
do if (cilow < 1).
compute cilow = 1.
compute booterr=1.
compute badlo = 1.
end if.
do if (cihigh > nrow(temp)).
compute cihigh = boot.
compute booterr=1.
compute badhi = 1.
end if.
compute llcit=temp(cilow,1).
compute ulcit=temp(cihigh,1).
do if (badlo = 1 and llcit <> priorlo).
compute badend={badend, llcit}.
compute priorlo = llcit.
end if.
do if (badhi = 1 and ulcit <> priorhi).
compute badend={badend, ulcit}.
compute priorhi = ulcit.
end if.
end if.
do if ( 9999 = 9999).
compute llcit=temp(cilow,1).
compute ulcit=temp(cihigh,1).
end if.
compute bootse=t(sqrt((cssq(temp)-((csum(temp)&**2)/nrow(temp)))/(nrow(temp)-1)))
.
compute indtemp={indtemp, bootse,llcit,ulcit}.
end if.
compute indtab={indtemp;indtab}.
end if.
compute bootlbs={'Effect', 'BootSE','BootLLCI','BootULCI'}.
do if (mc > 0).
compute bootlbs={'Effect', 'MC SE','MC LLCI','MC ULCI'}.
end if.
do if (nxvls = 1).
do if (contrast <> 0).
compute inddiff=inddiff(:,2:ncol(inddiff)).
do if (mc > 0).
compute inddiff={t(obtmc);inddiff}.
end if.
do if (contrast = 4).
compute inddifft=inddiff*t(contvec).
compute indtemp=inddifft(1,1).
do if (boot > 0 or mc > 0).
do if (mc > 0).

.
compute temp = inddifft(2:nrow(inddifft),1).
compute temp(GRADE( inddifft(2:nrow(inddifft),1) )) = inddifft(2:nrow(inddifft),1).
compute badlo = 0.
compute badhi = 0.
do if ( 9999 <> 9999).
compute pv=csum(temp < 9999 )/nrow(temp).
compute ppv = pv.
do if (pv > .5).
compute ppv = 1-pv.
end if.
compute y5=sqrt(-2*ln(ppv)).
compute xp=y5+((((y5*p4+p3)*y5+p2)*y5+p1)*y5+p0)/((((y5*q4+q3)*y5+q2)*y5+q1)*y5+q0).
do if (pv <= .5).
compute xp = -xp.
end if.
compute cilow=rnd(nrow(temp)*(cdfnorm(2*xp-xp2))).
compute cihigh=trunc(nrow(temp)*(cdfnorm(2*xp+(xp2))))+1.
do if (cilow < 1).
compute cilow = 1.
compute booterr=1.
compute badlo = 1.
end if.
do if (cihigh > nrow(temp)).
compute cihigh = boot.
compute booterr=1.
compute badhi = 1.
end if.
compute llcit=temp(cilow,1).
compute ulcit=temp(cihigh,1).
do if (badlo = 1 and llcit <> priorlo).
compute badend={badend, llcit}.
compute priorlo = llcit.
end if.
do if (badhi = 1 and ulcit <> priorhi).
compute badend={badend, ulcit}.
compute priorhi = ulcit.
end if.
end if.
do if ( 9999 = 9999).
compute llcit=temp(cilow,1).
compute ulcit=temp(cihigh,1).
end if.
compute bootse=t(sqrt((cssq(temp)-((csum(temp)&**2)/nrow(temp)))/(nrow(temp)-1)))
.
end if.
do if (boot > 0).

.
compute temp = inddifft(2:nrow(inddifft),1).
compute temp(GRADE( inddifft(2:nrow(inddifft),1) )) = inddifft(2:nrow(inddifft),1).
compute badlo = 0.
compute badhi = 0.
do if ( (inddifft(1,1)*bc)+(9999* (1-bc)) <> 9999).
compute pv=csum(temp < (inddifft(1,1)*bc)+(9999* (1-bc)) )/nrow(temp).
compute ppv = pv.
do if (pv > .5).
compute ppv = 1-pv.
end if.
compute y5=sqrt(-2*ln(ppv)).
compute xp=y5+((((y5*p4+p3)*y5+p2)*y5+p1)*y5+p0)/((((y5*q4+q3)*y5+q2)*y5+q1)*y5+q0).
do if (pv <= .5).
compute xp = -xp.
end if.
compute cilow=rnd(nrow(temp)*(cdfnorm(2*xp-xp2))).
compute cihigh=trunc(nrow(temp)*(cdfnorm(2*xp+(xp2))))+1.
do if (cilow < 1).
compute cilow = 1.
compute booterr=1.
compute badlo = 1.
end if.
do if (cihigh > nrow(temp)).
compute cihigh = boot.
compute booterr=1.
compute badhi = 1.
end if.
compute llcit=temp(cilow,1).
compute ulcit=temp(cihigh,1).
do if (badlo = 1 and llcit <> priorlo).
compute badend={badend, llcit}.
compute priorlo = llcit.
end if.
do if (badhi = 1 and ulcit <> priorhi).
compute badend={badend, ulcit}.
compute priorhi = ulcit.
end if.
end if.
do if ( (inddifft(1,1)*bc)+(9999* (1-bc)) = 9999).
compute llcit=temp(cilow,1).
compute ulcit=temp(cihigh,1).
end if.
compute bootse=t(sqrt((cssq(temp)-((csum(temp)&**2)/nrow(temp)))/(nrow(temp)-1)))
.
end if.
compute indtemp={indtemp, bootse,llcit,ulcit}.
end if.
compute indtab={indtab;indtemp}.
end if.
do if (contrast = 1 or contrast = 2 or contrast=3).
compute conkey=make(1,4,' ').
loop i = 1 to ncol(inddiff)-1.
loop j = (i+1) to ncol(inddiff).
compute inddifft=inddiff(:,i)-inddiff(:,j).
do if (contrast=2).
compute inddifft=abs(inddiff(:,i))-abs(inddiff(:,j)).
end if.
do if (contrast=3).
compute inddifft=inddiff(:,i)+inddiff(:,j).
end if.
compute indtemp=inddifft(1,1).
do if (contrast=1 or contrast=2).
compute conkeyt={' ', rowlbs((i+1),1),' minus  ',rowlbs((j+1),1)}.
end if.
do if (contrast=3).
compute conkeyt={' ', rowlbs((i+1),1),'  plus  ',rowlbs((j+1),1)}.
end if.
compute conkey={conkey;conkeyt}.
do if (boot > 0 or mc > 0).
do if (mc > 0).

.
compute temp = inddifft(2:nrow(inddifft),1).
compute temp(GRADE( inddifft(2:nrow(inddifft),1) )) = inddifft(2:nrow(inddifft),1).
compute badlo = 0.
compute badhi = 0.
do if ( 9999 <> 9999).
compute pv=csum(temp < 9999 )/nrow(temp).
compute ppv = pv.
do if (pv > .5).
compute ppv = 1-pv.
end if.
compute y5=sqrt(-2*ln(ppv)).
compute xp=y5+((((y5*p4+p3)*y5+p2)*y5+p1)*y5+p0)/((((y5*q4+q3)*y5+q2)*y5+q1)*y5+q0).
do if (pv <= .5).
compute xp = -xp.
end if.
compute cilow=rnd(nrow(temp)*(cdfnorm(2*xp-xp2))).
compute cihigh=trunc(nrow(temp)*(cdfnorm(2*xp+(xp2))))+1.
do if (cilow < 1).
compute cilow = 1.
compute booterr=1.
compute badlo = 1.
end if.
do if (cihigh > nrow(temp)).
compute cihigh = boot.
compute booterr=1.
compute badhi = 1.
end if.
compute llcit=temp(cilow,1).
compute ulcit=temp(cihigh,1).
do if (badlo = 1 and llcit <> priorlo).
compute badend={badend, llcit}.
compute priorlo = llcit.
end if.
do if (badhi = 1 and ulcit <> priorhi).
compute badend={badend, ulcit}.
compute priorhi = ulcit.
end if.
end if.
do if ( 9999 = 9999).
compute llcit=temp(cilow,1).
compute ulcit=temp(cihigh,1).
end if.
compute bootse=t(sqrt((cssq(temp)-((csum(temp)&**2)/nrow(temp)))/(nrow(temp)-1)))
.
end if.
do if (boot > 0).

.
compute temp = inddifft(2:nrow(inddifft),1).
compute temp(GRADE( inddifft(2:nrow(inddifft),1) )) = inddifft(2:nrow(inddifft),1).
compute badlo = 0.
compute badhi = 0.
do if ( (inddifft(1,1)*bc)+(9999* (1-bc)) <> 9999).
compute pv=csum(temp < (inddifft(1,1)*bc)+(9999* (1-bc)) )/nrow(temp).
compute ppv = pv.
do if (pv > .5).
compute ppv = 1-pv.
end if.
compute y5=sqrt(-2*ln(ppv)).
compute xp=y5+((((y5*p4+p3)*y5+p2)*y5+p1)*y5+p0)/((((y5*q4+q3)*y5+q2)*y5+q1)*y5+q0).
do if (pv <= .5).
compute xp = -xp.
end if.
compute cilow=rnd(nrow(temp)*(cdfnorm(2*xp-xp2))).
compute cihigh=trunc(nrow(temp)*(cdfnorm(2*xp+(xp2))))+1.
do if (cilow < 1).
compute cilow = 1.
compute booterr=1.
compute badlo = 1.
end if.
do if (cihigh > nrow(temp)).
compute cihigh = boot.
compute booterr=1.
compute badhi = 1.
end if.
compute llcit=temp(cilow,1).
compute ulcit=temp(cihigh,1).
do if (badlo = 1 and llcit <> priorlo).
compute badend={badend, llcit}.
compute priorlo = llcit.
end if.
do if (badhi = 1 and ulcit <> priorhi).
compute badend={badend, ulcit}.
compute priorhi = ulcit.
end if.
end if.
do if ( (inddifft(1,1)*bc)+(9999* (1-bc)) = 9999).
compute llcit=temp(cilow,1).
compute ulcit=temp(cihigh,1).
end if.
compute bootse=t(sqrt((cssq(temp)-((csum(temp)&**2)/nrow(temp)))/(nrow(temp)-1)))
.
end if.
compute indtemp={indtemp, bootse,llcit,ulcit}.
end if.
compute indtab={indtab;indtemp}.
end loop.
end loop.
end if.
release inddiff.
compute contlbs=cntname(1:(((nindfx)*(nindfx-1))/2),1).
compute rowlbs={rowlbs;contlbs}.
end if.
compute resultm2=make(nrow(indtab),maxresm,99999).
compute resultm2(1:nrow(indtab),1:ncol(indtab))=indtab.
compute resultm={resultm;resultm2}.
do if (outscree=1).
do if (kk=1).
print indtab/title = 'Indirect effect(s) of X on Y:'/rnames=rowlbs/cnames=bootlbs/format= F10.4.
end if.
do if (kk = 2 and (xdich=1 or mcx > 0)).
print indtab/title = 'Partially standardized indirect effect(s) of X on Y:'/rnames=rowlbs/cnames=bootlbs/format= F10.4.
end if.
do if (kk = 3).
print indtab/title = 'Completely standardized indirect effect(s) of X on Y:'/rnames=rowlbs/cnames=bootlbs/format= F10.4.
end if.
end if.
do if (normal=1 and sobelok=1 and kk=1).
compute resultm2=make(nrow(sobelmat),maxresm,99999).
compute resultm2(1:nrow(sobelmat),1:ncol(sobelmat))=sobelmat.
compute resultm={resultm;resultm2}.
compute sobellab={'Effect',hclab,'Z','p'}.
compute sobelrlb=rowlbs.
do if (nms > 1).
compute sobelrlb=rowlbs(2:(1+nms),1).
end if.
do if (outscree=1).
print sobelmat/title='   Normal theory test for indirect effect(s):'/cnames=sobellab/rnames=sobelrlb/format= F10.4.
end if.
end if.
do if (contrast <> 0).
do if ((contrast=1 or contrast = 2 or contrast=3) and kk=efloop ).
compute conkey=conkey(2:nrow(conkey),:).
do if (outscree=1).
print conkey/title = 'Specific indirect effect contrast definition(s):'/rnames=contlbs/format=A8.
end if.
end if.
do if (outscree=1).
do if (contrast = 4 and kk=efloop).
compute crowlbs=rowlbs(2:(nindfx+1),1).
print contvec/title = 'Specific indirect effect contrast weights:'/cnames=crowlbs/rlabels='(C1)'/format= F10.4.
end if.
do if (contrast = 2 and kk=efloop).
print/title = 'Contrasts are differences between absolute values of indirect effects'.
end if.
end if.
end if.
do if (serial = 1 and kk=efloop).
compute rowlbst=rowlbs(2:nrow(rowlbs),1).
do if (outscree=1).
print indkey/title = 'Indirect effect key:'/rnames=rowlbst/format = A8.
end if.
end if.
else.
do if (outscree=1).
do if (kk = 1).
print/title = 'Relative indirect effects of X on Y'.
end if.
do if (kk = 2).
print/title = 'Partially standardized relative indirect effect(s) of X on Y:'.
end if.
do if (kk = 3).
print/title = 'Completely standardized relative indirect effect(s) of X on Y:'.
end if.
end if.
loop i = 1 to nrow(indmake).
compute indtabsm=indtab((((i-1)*nxvls)+1):(nxvls*i),:).
compute indkeyt=indkey(i,:).
do if (outscree=1).
print indkeyt/title=' '/space=0/format=A8.
end if.
do if (bcmat(nrow(bcmat),1)=0)).
compute direffl2=xcatlab(1:nxvls,1).
end if.
compute resultm2=make(nrow(indtabsm),maxresm,99999).
compute resultm2(1:nrow(indtabsm),1:ncol(indtabsm))=indtabsm.
compute resultm={resultm;resultm2}.
do if (outscree=1).
print indtabsm/title = ' '/cnames=bootlbs/rnames=direffl2/format= F10.4 /space=0.
end if.
do if (normal=1 and sobelok=1 and kk=1).
compute sobelsm=sobelmat((((i-1)*nxvls)+1):(nxvls*i),:).
compute sobellab={'Effect',hclab,'Z','p'}.
compute resultm2=make(nrow(sobelsm),maxresm,99999).
compute resultm2(1:nrow(sobelsm),1:ncol(sobelsm))=sobelsm.
compute resultm={resultm;resultm2}.
do if (outscree=1).
print sobelsm/title='      Normal theory test for relative indirect effects:'/cnames=sobellab/rnames=direffl2/format= F10.4.
end if.
end if.
end loop.
end if.
do if (effsize = 1 and boot > 0).
compute bootres=bootres(2:nrow(bootres),:).
end if.
end loop.
do if (alttotal=1).
compute altcnms='Effect'.
compute totbtvec=totbtvec+bootdir.
compute alttotfx=t(totbtvec(1,:)).
do if (boot > 0).
compute alttotfx=make(ncol(totbtvec),4,0).
compute alttotfx(:,1)=t(totbtvec(1,:)).
loop cec=1 to ncol(totbtvec).

.
compute temp = totbtvec(2:nrow(totbtvec),cec).
compute temp(GRADE( totbtvec(2:nrow(totbtvec),cec) )) = totbtvec(2:nrow(totbtvec),cec).
compute badlo = 0.
compute badhi = 0.
do if ( (totbtvec(1,cec)*bc)+(9999* (1-bc)) <> 9999).
compute pv=csum(temp < (totbtvec(1,cec)*bc)+(9999* (1-bc)) )/nrow(temp).
compute ppv = pv.
do if (pv > .5).
compute ppv = 1-pv.
end if.
compute y5=sqrt(-2*ln(ppv)).
compute xp=y5+((((y5*p4+p3)*y5+p2)*y5+p1)*y5+p0)/((((y5*q4+q3)*y5+q2)*y5+q1)*y5+q0).
do if (pv <= .5).
compute xp = -xp.
end if.
compute cilow=rnd(nrow(temp)*(cdfnorm(2*xp-xp2))).
compute cihigh=trunc(nrow(temp)*(cdfnorm(2*xp+(xp2))))+1.
do if (cilow < 1).
compute cilow = 1.
compute booterr=1.
compute badlo = 1.
end if.
do if (cihigh > nrow(temp)).
compute cihigh = boot.
compute booterr=1.
compute badhi = 1.
end if.
compute llcit=temp(cilow,1).
compute ulcit=temp(cihigh,1).
do if (badlo = 1 and llcit <> priorlo).
compute badend={badend, llcit}.
compute priorlo = llcit.
end if.
do if (badhi = 1 and ulcit <> priorhi).
compute badend={badend, ulcit}.
compute priorhi = ulcit.
end if.
end if.
do if ( (totbtvec(1,cec)*bc)+(9999* (1-bc)) = 9999).
compute llcit=temp(cilow,1).
compute ulcit=temp(cihigh,1).
end if.
compute bootse=t(sqrt((cssq(temp)-((csum(temp)&**2)/nrow(temp)))/(nrow(temp)-1)))
.
compute alttotfx(cec,2:4)={bootse,llcit,ulcit}.
end loop.
compute altcnms={altcnms,'BootSE', 'BootLLCI','BootULCI'}.
end if.
compute resultm2=make(nrow(alttotfx),maxresm,99999).
compute resultm2(1:nrow(alttotfx),1:ncol(alttotfx))=alttotfx.
compute resultm={resultm;resultm2}.
do if (outscree=1).
do if (nxvls > 1).
print/title= '----------'/space=0.
print alttotfx/title='Relative total effects of X on Y (sum of direct and indirect effects)'/rnames=direffl2/cnames=altcnms/format= F10.4.
end if.
do if (nxvls = 1).
print alttotfx/title='Total effect of X on Y (sum of direct and indirect effects)'/cnames=altcnms/format= F10.4.
end if.
end if.
end if.
end if.
do if (anymod > 0).
do if (boot = 0).
compute bootres=obscoeff.
compute indtab=999.
end if.
do if (boot > 0).
compute bootres={obscoeff;bootres}.
compute indtab=make(1,4,999).
end if.
do if (csum((indmod > 0))=nrow(indmod)).
do if (outscree=1).
do if (nxvls > 1).
do if (xmint=0).
print/title= 'Relative conditional indirect effects of X on Y:'.
end if.
do if (xmint=1).
print/title= 'Relative (total) natural indirect effects of X on Y:'.
end if.
end if.
do if (nxvls = 1).
do if (xmint=0).
print/title= 'Conditional indirect effects of X on Y:'.
end if.
do if (xmint=1).
print/title= '(Total) Natural indirect effect(s) of X on Y:'.
end if.
end if.
end if.
end if.
do if (csum((indmod > 0)) < nrow(indmod)).
do if (outscree=1).
do if (nxvls > 1).
print/title= 'Relative conditional and unconditional indirect effects of X on Y:'.
end if.
do if (nxvls = 1).
print/title= 'Conditional and unconditional indirect effects of X on Y:'.
end if.
end if.
end if.
compute cftotfx=make(nrow(bootres),nxvls,0).
loop i = 1 to nrow(indmake).
compute indtab=indtab(1,:)*0.
do if (outscree=1).
do if (xmint=0).
print indkey(i,:)/title = 'INDIRECT EFFECT:'/format=A8.
end if.
do if (xmint=1).
print indkey(i,:)/title = ' '/format=A8/space=0.
end if.
end if.
do if (indmod(i,1)=0).
loop j = 1 to nxvls.
compute indtemp=make(nrow(bootres),1,1).
loop k = 1 to indmake(i,1).
compute jtemp=1.
do if (j > 1 and k=1).
compute jtemp=j.
end if.
compute indtemp=indtemp&*bootres(:,pathsfoc(jtemp,indmake(i,(k+1)))).
end loop.
compute indeff=indtemp(1,1).
do if (boot > 0).

.
compute temp = indtemp(2:nrow(indtemp),1).
compute temp(GRADE( indtemp(2:nrow(indtemp),1) )) = indtemp(2:nrow(indtemp),1).
compute badlo = 0.
compute badhi = 0.
do if ( (indtemp(1,1)*bc)+(9999*(1-bc)) <> 9999).
compute pv=csum(temp < (indtemp(1,1)*bc)+(9999*(1-bc)) )/nrow(temp).
compute ppv = pv.
do if (pv > .5).
compute ppv = 1-pv.
end if.
compute y5=sqrt(-2*ln(ppv)).
compute xp=y5+((((y5*p4+p3)*y5+p2)*y5+p1)*y5+p0)/((((y5*q4+q3)*y5+q2)*y5+q1)*y5+q0).
do if (pv <= .5).
compute xp = -xp.
end if.
compute cilow=rnd(nrow(temp)*(cdfnorm(2*xp-xp2))).
compute cihigh=trunc(nrow(temp)*(cdfnorm(2*xp+(xp2))))+1.
do if (cilow < 1).
compute cilow = 1.
compute booterr=1.
compute badlo = 1.
end if.
do if (cihigh > nrow(temp)).
compute cihigh = boot.
compute booterr=1.
compute badhi = 1.
end if.
compute llcit=temp(cilow,1).
compute ulcit=temp(cihigh,1).
do if (badlo = 1 and llcit <> priorlo).
compute badend={badend, llcit}.
compute priorlo = llcit.
end if.
do if (badhi = 1 and ulcit <> priorhi).
compute badend={badend, ulcit}.
compute priorhi = ulcit.
end if.
end if.
do if ( (indtemp(1,1)*bc)+(9999*(1-bc)) = 9999).
compute llcit=temp(cilow,1).
compute ulcit=temp(cihigh,1).
end if.
compute bootse=t(sqrt((cssq(temp)-((csum(temp)&**2)/nrow(temp)))/(nrow(temp)-1)))
.
compute indeff={indeff,bootse,llcit,ulcit}.
end if.
compute indtab={indtab;indeff}.
end loop.
compute resultm2=make(nrow(indtab),maxresm,99999).
compute resultm2(1:nrow(indtab),1:ncol(indtab))=indtab.
compute resultm={resultm;resultm2}.
do if (outscree=1).
do if (nxvls > 1).
compute indefflb=xcatlab(1:nxvls,1).
print indtab(2:nrow(indtab),:)/title = ' '/clabels='Effect', 'BootSE', 'BootLLCI', 'BootULCI'/rnames=indefflb/format= F10.4 /space=0.
end if.
do if (nxvls = 1).
print indtab(2:nrow(indtab),:)/title = ' '/clabels='Effect', 'BootSE', 'BootLLCI', 'BootULCI'/format= F10.4 /space=0.
end if.
end if.
end if.
do if (indmod(i,1)>0).
do if (indmod(i,1)=1).
compute indmodva=wmodvals.
compute indprova=wprobval.
compute condlbs={wnames}.
compute printw=1.
else if (indmod(i,1)=2).
compute indmodva=zmodvals.
compute indprova=zprobval.
compute condlbs={znames}.
compute printz=1.
else if (indmod(i,1)=3).
compute cntmp=1.
compute printz=1.
compute printw=1.
compute indmodva=make((nrow(wmodvals)*nrow(zmodvals)),2,999).
loop k7 = 1 to nrow(wmodvals).
loop k8 = 1 to nrow(zmodvals).
compute indmodva(cntmp,:)={wmodvals(k7,1),zmodvals(k8,1)}.
compute cntmp=cntmp+1.
end loop.
end loop.
compute condlbs={wnames,znames}.
end if.
compute condres=make(nrow(indmodva),1,999).
do if (boot > 0).
compute condres=make(nrow(indmodva),4,999).
end if.
compute condres={indmodva,condres}.
loop k4 = 1 to nxvls.
compute imm3=make(nrow(bootres),1,1).
compute imm4=make(nrow(bootres),1,1).
compute indcontr=0.
do if (indmod(i,1)=3).
compute tihsw=wprobval.
compute tihsz=zprobval.
end if.
loop k1=1 to nrow(indmodva).
compute tucker2=make(nrow(bootres),1,1).
compute imm2=make(nrow(bootres),1,1).
compute wfirst=0.
compute zfirst=0.
compute immset=0.
loop k2=1 to indmake(i,1).
compute colnumb=indmake(i,(k2+1)).
do if (k2=1).
compute wbb=make(nrow(bootres),(nwvls*nxvls),0).
compute zbb=make(nrow(bootres),(nzvls*nxvls),0).
compute wzbb=make(nrow(bootres),(nwvls*nzvls*nxvls),0).
end if.
do if (k2<>1).
compute wbb=make(nrow(bootres),nwvls,0).
compute zbb=make(nrow(bootres),nzvls,0).
compute wzbb=make(nrow(bootres),(nwvls*nzvls),0).
end if.
compute cnt=1.
compute tihs=indlocs(2:((indlocs(1,colnumb))+1),colnumb).
do if (k2 = 1).
compute focbb=tihs(1:nxvls,1).
compute focbb=bootres(:,focbb).
do if (indmmm(i,1)>0).
compute imm=focbb(:,k4).
compute condbb=imm.
end if.
compute focaddon=make(1,nxvls,0).
compute focaddon(1,k4)=1.
compute cnt=cnt+nxvls.
compute placeh=nxvls.
do if (indmod(i,1)=1).
compute tihsz=make(nrow(wprobval),(nzvls*nxvls),0).
compute tihswz=make(nrow(wprobval),(nwvls*nzvls*nxvls),0).
do if (pathsw(1,colnumb)=1).
compute temp=make(nrow(wprobval),(nxvls*nwvls),0).
loop k5 = 1 to nrow(wprobval).
loop k6=1 to nwvls.
compute temp(k5, (((k4-1)*nwvls)+k6))=wprobval(k5,k6).
end loop.
end loop.
compute indprova={temp,tihsz,tihswz}.
else.
compute indprova={wprobval,tihsz,tihswz}.
end if.
end if.
do if (indmod(i,1)=2).
compute tihsw=make(nrow(zprobval),(nwvls*nxvls),0).
compute tihswz=make(nrow(zprobval),(nwvls*nzvls*nxvls),0).
do if (pathsz(1,colnumb)=1).
compute temp=make(nrow(zprobval),(nxvls*nzvls),0).
loop k5 = 1 to nrow(zprobval).
loop k6 =1 to nzvls.
compute temp(k5,(((k4-1)*nzvls)+k6))=zprobval(k5,k6).
end loop.
end loop.
compute indprova={tihsw,temp,tihswz}.
else.
compute indprova={tihsw,zprobval,tihswz}.
end if.
end if.
do if (indmod(i,1)=3).
compute indprova=make((nrow(wprobval)*nrow(zprobval)),((ncol(wprobval)*nxvls)+ (ncol(zprobval)*nxvls)+(nwvls*nzvls*nxvls)),0).
compute cntemp=1.
loop k7=1 to nrow(wprobval).
loop k8 =1 to nrow(zprobval).
compute temp=wprobval(k7,:)*focaddon(1,k4).
compute indprova(cntemp,(((k4-1)*nwvls)+1):(k4*(nwvls)))=temp.
compute temp=zprobval(k8,:)*focaddon(1,k4).
compute indprova(cntemp, ((((k4-1)*nzvls)+1)+(nxvls*nwvls)) : ((((k4-1)*nzvls)+1)+(nxvls*nwvls)+(nzvls-1)))=temp.
compute cntemp=cntemp+1.
end loop.
end loop.
do if (pathsz(1,colnumb)=0).
compute temp=make(nrow(indprova),(ncol(zprobval)*nxvls),0).
compute indprova(:,((ncol(wprobval)*nxvls)+1):((ncol(wprobval)+ncol(zprobval))* nxvls))=temp.
end if.
do if (pathsw(1,colnumb)=0).
compute temp=make(nrow(indprova),(ncol(wprobval)*nxvls),0).
compute indprova(:,1:(ncol(wprobval)*nxvls))=temp.
end if.
do if (pathswz(1,colnumb)=1).
compute cntemp=(ncol(wprobval)*nxvls)+(ncol(zprobval)*nxvls)+((k4-1)* ncol(wprobval)*ncol(zprobval))+1.
loop k7=1 to ncol(wprobval).
loop k8=1 to ncol(zprobval).
compute indprova(:,cntemp)=indprova(:,((ncol(wprobval)*(k4-1))+k7))&* indprova(:,((((k4-1)*ncol(zprobval))+k8)+(nxvls*ncol(wprobval)))).
compute cntemp=cntemp+1.
end loop.
end loop.
end if.
end if.
end if.
do if (k2 > 1).
compute focbb=tihs(1,1).
compute focbb=bootres(:,focbb).
do if (indmmm(i,1)>0).
compute imm=focbb(:,1).
compute condbb=imm.
end if.
compute focaddon=1.
compute cnt=cnt+1.
compute placeh=1.
do if (indmod(i,1)=1).
compute tihsz=make(nrow(wprobval),nzvls,0).
compute tihswz=make(nrow(wprobval),(nwvls*nzvls),0).
compute indprova={wprobval,tihsz,tihswz}.
end if.
do if (indmod(i,1)=2).
compute tihsw=make(nrow(zprobval),nwvls,0).
compute tihswz=make(nrow(zprobval),(nwvls*nzvls),0).
compute indprova={tihsw,zprobval,tihswz}.
end if.
do if (indmod(i,1)=3).
compute indprova=make((nrow(wprobval)*nrow(zprobval)),((ncol(wprobval)+ ncol(zprobval))+(nwvls*nzvls)),0).
compute cntemp=1.
loop k7=1 to nrow(wprobval).
loop k8 =1 to nrow(zprobval).
compute indprova(cntemp,1:(ncol(wprobval)+ncol(zprobval)))={wprobval(k7,:), zprobval(k8,:)}.
compute cntemp=cntemp+1.
end loop.
end loop.
do if (pathsz(1,colnumb)=0).
compute temp=make(nrow(indprova),ncol(zprobval),0).
compute indprova(:,(ncol(wprobval)+1):(ncol(wprobval)+ncol(zprobval)))=temp.
end if.
do if (pathsw(1,colnumb)=0).
compute temp=make(nrow(indprova),ncol(wprobval),0).
compute indprova(:,1:ncol(wprobval))=temp.
end if.
do if (pathswz(1,colnumb)=1).
compute cntemp=ncol(wprobval)+ncol(zprobval)+1.
loop k7=1 to ncol(wprobval).
loop k8=1 to ncol(zprobval).
compute indprova(:,cntemp)=indprova(:,k7)&*indprova(:,(ncol(wprobval)+k8)).
compute cntemp=cntemp+1.
end loop.
end loop.
end if.
end if.
end if.
do if (pathsw(1,colnumb)) = 1.
compute wbb=tihs(cnt:(cnt+(placeh*nwvls)-1),1).
compute wbb=bootres(:,wbb).
compute immlbs2=wcatlab(1:nwvls,1).
do if (zfirst=0).
compute wfirst=1.
end if.
do if (indmmm(i,1)=1 or indmmm(i,1)=31 or indmmm(i,1)=51).
compute imm=wbb(:,1).
loop k7 = 1 to nwvls.
compute imm={imm,wbb(:,(((k4-1)*nwvls*(k2=1))+k7))}.
end loop.
compute imm=imm(:,2:ncol(imm)).
end if.
do if (indmmm(i,1)=41 or indmmm(i,1)=51).
compute condbb=make(nrow(bootres),1,0).
loop k7 = 1 to nwvls.
compute condbb={condbb,wbb(:,(((k4-1)*nwvls*(k2=1))+k7))}.
end loop.
compute condbb=condbb(:,2:ncol(condbb)).
end if.
compute cnt=cnt+(placeh*nwvls).
end if.
do if (pathsz(1,colnumb)) = 1.
compute zbb=tihs(cnt:(cnt+(placeh*nzvls)-1),1).
compute zbb=bootres(:,zbb).
do if (wfirst=0).
compute zfirst=1.
end if.
do if (indmmm(i,1) <> 31).
compute immlbs2=zcatlab(1:nzvls,1).
end if.
do if (indmmm(i,1)=2 or indmmm(i,1)=31 or indmmm(i,1)=51).
do if (indmmm(i,1)=2).
compute imm=zbb(:,1).
end if.
loop k7 = 1 to nzvls.
compute imm={imm,zbb(:,(((k4-1)*nzvls*(k2=1))+k7))}.
end loop.
do if (indmmm(i,1)=2 or indmmm(i,1)=51).
compute imm=imm(:,2:ncol(imm)).
do if (indmmm(i,1)=51).
compute condbb={condbb,imm}.
end if.
end if.
end if.
compute cnt=cnt+(placeh*nzvls).
end if.
do if (pathswz(1,colnumb)) = 1.
compute wzbb=tihs(cnt:(cnt+(placeh*nwvls*nzvls)-1),1).
compute wzbb=bootres(:,wzbb).
do if (indmmm(i,1)=41).
compute imm=wzbb(:,1).
loop k7=1 to nwvls*nzvls.
compute imm={imm,wzbb(:,(((k4-1)*nzvls*nwvls*(k2=1))+k7))}.
end loop.
end if.
do if (indmmm(i,1)=41).
compute imm=imm(:,2:ncol(imm)).
compute condbb={condbb,imm(:,(ncol(imm)-(nwvls*nzvls)+1):ncol(imm))}.
end if.
compute cnt=cnt+(placeh*nzvls*nwvls).
end if.
compute indprobe={focaddon,indprova(k1,:)}.
compute tucker={focbb,wbb,zbb,wzbb}.
loop k3=1 to ncol(indprobe).
compute tucker(:,k3)=tucker(:,k3)*indprobe(1,k3).
end loop.
compute tucker2=tucker2&*rsum(tucker).
do if (indmmm(i,1) = 1 or indmmm(i,1)=2 or indmmm(i,1)=31 or indmmm(i,1)=41 or indmmm(i,1)=51).
do if (immset=1).
do if (ncol(imm2)=1 and ncol(imm) = 1).
compute imm2=imm2&*imm.
end if.
do if (indmmm(i,1)=41 or indmmm(i,1)=51).
do if ((ncol(condbb2) > 1) and (ncol(condbb)>1)).
compute condbb2t=make(nrow(condbb2),(ncol(condbb2)*ncol(condbb)),-999999).
compute k9=1.
do if (wfirst=1).
loop k7=1 to ncol(condbb2).
loop k8 = 1 to ncol(condbb).
compute condbb2t(:,k9)=condbb2(:,k7)&*condbb(:,k8).
compute k9=k9+1.
end loop.
end loop.
end if.
do if (zfirst=1).
loop k7=1 to ncol(condbb).
loop k8 = 1 to ncol(condbb2).
compute condbb2t(:,k9)=condbb(:,k7)&*condbb2(:,k8).
compute k9=k9+1.
end loop.
end loop.
end if.
compute condbb2=condbb2t.
release condbb2t.
end if.
do if ((ncol(condbb2) > 1) and (ncol(condbb)=1)).
loop k7 = 1 to ncol(condbb2).
compute condbb2(:,k7)=condbb2(:,k7)&*condbb.
end loop.
end if.
do if ((ncol(condbb2) = 1) and (ncol(condbb)>1)).
loop k7 = 1 to ncol(condbb).
compute condbb(:,k7)=condbb2&*condbb(:,k7).
end loop.
compute condbb2=condbb.
end if.
end if.
do if (ncol(imm2) <> 1 and ncol(imm) <> 1).
compute imm2t=make(nrow(imm2),(ncol(imm2)*ncol(imm)),-999999).
compute k9=1.
do if (wfirst=1).
loop k7=1 to ncol(imm2).
loop k8 = 1 to ncol(imm).
compute imm2t(:,k9)=imm2(:,k7)&*imm(:,k8).
compute k9=k9+1.
end loop.
end loop.
end if.
do if (zfirst=1).
loop k7=1 to ncol(imm).
loop k8 = 1 to ncol(imm2).
compute imm2t(:,k9)=imm(:,k7)&*imm2(:,k8).
compute k9=k9+1.
end loop.
end loop.
end if.
compute imm2=imm2t.
release imm2t.
end if.
do if ((ncol(imm2) > 1) and (ncol(imm)=1)).
loop k7=1 to ncol(imm2).
compute imm2(:,k7)=imm2(:,k7)&*imm.
end loop.
end if.
do if ((ncol(imm2) = 1) and (ncol(imm) > 1)).
loop k7=1 to ncol(imm).
compute imm(:,k7)=imm2&*imm(:,k7).
end loop.
compute imm2=imm.
end if.
end if.
do if (immset=0).
compute imm2=imm.
do if (indmmm(i,1)=41 or indmmm(i,1)=51).
compute condbb2=condbb.
end if.
compute immset=1.
end if.
end if.
end loop.
compute indtemp=tucker2(1,1).
do if (indmmm(i,1)=12 or indmmm(i,1)=22).
compute imm3={imm3,tucker2}.
do if (k1=nrow(indmodva)).
compute imm3=imm3(:,2:ncol(imm3)).
compute immstop=ncol(imm3).
loop k8=2 to immstop.
do if (indmmmt(i,1)=1).
compute imm3={imm3,(imm3(:,k8)-imm3(:,1))}.
end if.
do if (indmmmt(i,1)=2).
compute imm3={imm3,(imm3(:,k8)-imm3(:,(k8-1)))}.
end if.
do if (indmmmt(i,1)=3).
compute imm3={imm3,((rsum(imm3(:,(k8:immstop)))/(immstop-k8+1))-imm3(:,(k8-1))) }.
end if.
do if (indmmmt(i,1)=4).
compute imm3={imm3,(imm3(:,k8)-(rsum(imm3(:,1:immstop))/immstop))}.
end if.
end loop.
do if (indmmmt(i,1)<5).
compute imm2=imm3(:,(immstop+1):ncol(imm3)).
release imm3.
end if.
end if.
end if.
do if (indmmm(i,1)>-1 and (contrast = 1 or contrast = 2 or contrast=3)).
compute imm4={imm4,tucker2}.
do if (k1=nrow(indmodva) and k1 > 1).
compute imm4=imm4(:,2:ncol(imm4)).
compute immstop=ncol(imm4).
compute condcont=make((immstop*(immstop-1)/2),6,-999).
loop k8 = 1 to (immstop-1).
loop k9 = (k8+1) to immstop.
do if (contrast=1).
compute imm4={imm4,(imm4(:,k9)-imm4(:,k8))}.
end if.
do if (contrast=2).
compute imm4={imm4,(abs(imm4(:,k9))-abs(imm4(:,k8)))}.
end if.
do if (contrast=3).
compute imm4={imm4,(imm4(:,k9)+imm4(:,k8))}.
end if.
compute condcont((ncol(imm4)-immstop),1)=imm4(1,k9).
compute condcont((ncol(imm4)-immstop),2)=imm4(1,k8).
end loop.
end loop.
compute imm4=imm4(:,(immstop+1):ncol(imm4)).
loop k8=1 to ncol(imm4).
compute condcont(k8,3)=imm4(1,k8).
do if (boot > 0).

.
compute temp = imm4(2:nrow(imm4),k8).
compute temp(GRADE( imm4(2:nrow(imm4),k8) )) = imm4(2:nrow(imm4),k8).
compute badlo = 0.
compute badhi = 0.
do if ( (imm4(1,k8)*bc)+(9999*(1-bc)) <> 9999).
compute pv=csum(temp < (imm4(1,k8)*bc)+(9999*(1-bc)) )/nrow(temp).
compute ppv = pv.
do if (pv > .5).
compute ppv = 1-pv.
end if.
compute y5=sqrt(-2*ln(ppv)).
compute xp=y5+((((y5*p4+p3)*y5+p2)*y5+p1)*y5+p0)/((((y5*q4+q3)*y5+q2)*y5+q1)*y5+q0).
do if (pv <= .5).
compute xp = -xp.
end if.
compute cilow=rnd(nrow(temp)*(cdfnorm(2*xp-xp2))).
compute cihigh=trunc(nrow(temp)*(cdfnorm(2*xp+(xp2))))+1.
do if (cilow < 1).
compute cilow = 1.
compute booterr=1.
compute badlo = 1.
end if.
do if (cihigh > nrow(temp)).
compute cihigh = boot.
compute booterr=1.
compute badhi = 1.
end if.
compute llcit=temp(cilow,1).
compute ulcit=temp(cihigh,1).
do if (badlo = 1 and llcit <> priorlo).
compute badend={badend, llcit}.
compute priorlo = llcit.
end if.
do if (badhi = 1 and ulcit <> priorhi).
compute badend={badend, ulcit}.
compute priorhi = ulcit.
end if.
end if.
do if ( (imm4(1,k8)*bc)+(9999*(1-bc)) = 9999).
compute llcit=temp(cilow,1).
compute ulcit=temp(cihigh,1).
end if.
compute bootse=t(sqrt((cssq(temp)-((csum(temp)&**2)/nrow(temp)))/(nrow(temp)-1)))
.
compute condcont(k8,4:6)={bootse,llcit,ulcit}.
end if.
end loop.
do if (boot=0).
compute condcont=condcont(:,1:3).
end if.
compute indcontr=1.
end if.
end if.
do if (xmint=1).
do if (k1=(k4+1)).
compute cftotfx(:,k4)=cftotfx(:,k4)+(tucker2*xscaling).
end if.
end if.
do if (boot > 0).
compute tucker2=tucker2*xscaling.

.
compute temp = tucker2(2:nrow(tucker2),1).
compute temp(GRADE( tucker2(2:nrow(tucker2),1) )) = tucker2(2:nrow(tucker2),1).
compute badlo = 0.
compute badhi = 0.
do if ( (tucker2(1,1)*bc)+(9999*(1-bc)) <> 9999).
compute pv=csum(temp < (tucker2(1,1)*bc)+(9999*(1-bc)) )/nrow(temp).
compute ppv = pv.
do if (pv > .5).
compute ppv = 1-pv.
end if.
compute y5=sqrt(-2*ln(ppv)).
compute xp=y5+((((y5*p4+p3)*y5+p2)*y5+p1)*y5+p0)/((((y5*q4+q3)*y5+q2)*y5+q1)*y5+q0).
do if (pv <= .5).
compute xp = -xp.
end if.
compute cilow=rnd(nrow(temp)*(cdfnorm(2*xp-xp2))).
compute cihigh=trunc(nrow(temp)*(cdfnorm(2*xp+(xp2))))+1.
do if (cilow < 1).
compute cilow = 1.
compute booterr=1.
compute badlo = 1.
end if.
do if (cihigh > nrow(temp)).
compute cihigh = boot.
compute booterr=1.
compute badhi = 1.
end if.
compute llcit=temp(cilow,1).
compute ulcit=temp(cihigh,1).
do if (badlo = 1 and llcit <> priorlo).
compute badend={badend, llcit}.
compute priorlo = llcit.
end if.
do if (badhi = 1 and ulcit <> priorhi).
compute badend={badend, ulcit}.
compute priorhi = ulcit.
end if.
end if.
do if ( (tucker2(1,1)*bc)+(9999*(1-bc)) = 9999).
compute llcit=temp(cilow,1).
compute ulcit=temp(cihigh,1).
end if.
compute bootse=t(sqrt((cssq(temp)-((csum(temp)&**2)/nrow(temp)))/(nrow(temp)-1)))
.
compute indtemp={indtemp*xscaling, bootse,llcit,ulcit}.
end if.
do if (boot=0).
compute indtemp=indtemp*xscaling.
end if.
compute condres(k1,(ncol(indmodva)+1):ncol(condres))=indtemp.
end loop.
do if (xmint=0).
compute resultm2=make(nrow(condres),maxresm,99999).
compute resultm2(1:nrow(condres),1:ncol(condres))=condres.
compute resultm={resultm;resultm2}.
end if.
compute condlbs={condlbs,'Effect'}.
do if (boot > 0).
compute condlbs={condlbs,'BootSE', 'BootLLCI', 'BootULCI'}.
end if.
do if (xmint = 1).
do if (k4 = 1).
compute natindfx=condres((2+(k4-1)),2:ncol(condres)).
end if.
do if (k4 > 1).
compute natindfx={natindfx;condres((2+(k4-1)),2:ncol(condres))}.
end if.
do if (k4=nxvls).
compute resultm2=make(nrow(natindfx),maxresm,99999).
compute resultm2(1:nrow(natindfx),1:ncol(natindfx))=natindfx.
compute resultm={resultm;resultm2}.
end if.
end if.
do if (outscree=1 and xmint=0).
do if (nxvls=1).
print condres/title=' '/cnames=condlbs/format= F10.4 /space=0.
else.
compute condrlb=make(nrow(condres),1,xcatlab(k4,1)).
print condres/title=' '/cnames=condlbs/rnames=condrlb/format= F10.4 /space=0.
end if.
end if.
do if (outscree=1 and xmint=1 and k4=nxvls).
do if (nxvls=1).
print natindfx/title=' '/clabels='Effect', 'BootSE', 'BootLLCI', 'BootULCI'/format= F10.4 /space=0.
else.
compute condrlb=make(nrow(condres),1,xcatlab(k4,1)).
print natindfx/title=' '/clabels='Effect', 'BootSE', 'BootLLCI', 'BootULCI'/rnames=direffl2/format= F10.4 /space=0.
end if.
end if.
compute dichadj=0.
compute immcat=0.
do if ((indmmm(i,1)>0) and (xmint=0)).
do if (indmmm(i,1)=1 or indmmm(i,1)=12 or indmmm(i,1)=31).
do if (wdich=1 and mcw=0).
do if (indmmm(i,1) <> 12).
compute imm2(:,1)=imm2(:,1)*(wmax-wmin).
end if.
do if (indmmm(i,1) <> 31).
compute dichadj=1.
end if.
end if.
do if ((mcw = 1 or mcw = 2) and indmmm(i,1) <> 31)).
compute immcat=1.
end if.
end if.
do if (indmmm(i,1)=2 or indmmm(i,1)=22 or indmmm(i,1)=31).
do if (zdich=1 and mcz=0).
do if (indmmm(i,1) = 31).
compute imm2(:,(nwvls+1):ncol(imm2))=imm2(:,(nwvls+1):ncol(imm2))*(zmax-zmin).
end if.
do if (indmmm(i,1) = 2).
compute imm2(:,1)=imm2(:,1)*(zmax-zmin).
end if.
do if (indmmm(i,1) <> 31).
compute dichadj=1.
end if.
end if.
do if ((mcz = 1 or mcz = 2) and indmmm(i,1) <> 31)).
compute immcat=1.
end if.
end if.
compute immtemp2=t(imm2(1,:)).
compute immtemp=immtemp2.
compute immlbs={'Index'}.
do if (boot > 0).
compute immtemp=make(1,3,0).
loop k7=1 to ncol(imm2).

.
compute temp = imm2(2:nrow(imm2),k7).
compute temp(GRADE( imm2(2:nrow(imm2),k7) )) = imm2(2:nrow(imm2),k7).
compute badlo = 0.
compute badhi = 0.
do if ( (imm2(1,k7)*bc)+(9999*(1-bc)) <> 9999).
compute pv=csum(temp < (imm2(1,k7)*bc)+(9999*(1-bc)) )/nrow(temp).
compute ppv = pv.
do if (pv > .5).
compute ppv = 1-pv.
end if.
compute y5=sqrt(-2*ln(ppv)).
compute xp=y5+((((y5*p4+p3)*y5+p2)*y5+p1)*y5+p0)/((((y5*q4+q3)*y5+q2)*y5+q1)*y5+q0).
do if (pv <= .5).
compute xp = -xp.
end if.
compute cilow=rnd(nrow(temp)*(cdfnorm(2*xp-xp2))).
compute cihigh=trunc(nrow(temp)*(cdfnorm(2*xp+(xp2))))+1.
do if (cilow < 1).
compute cilow = 1.
compute booterr=1.
compute badlo = 1.
end if.
do if (cihigh > nrow(temp)).
compute cihigh = boot.
compute booterr=1.
compute badhi = 1.
end if.
compute llcit=temp(cilow,1).
compute ulcit=temp(cihigh,1).
do if (badlo = 1 and llcit <> priorlo).
compute badend={badend, llcit}.
compute priorlo = llcit.
end if.
do if (badhi = 1 and ulcit <> priorhi).
compute badend={badend, ulcit}.
compute priorhi = ulcit.
end if.
end if.
do if ( (imm2(1,k7)*bc)+(9999*(1-bc)) = 9999).
compute llcit=temp(cilow,1).
compute ulcit=temp(cihigh,1).
end if.
compute bootse=t(sqrt((cssq(temp)-((csum(temp)&**2)/nrow(temp)))/(nrow(temp)-1)))
.
compute temp={bootse,llcit,ulcit}.
compute immtemp={immtemp;temp}.
end loop.
compute immtemp=immtemp(2:nrow(immtemp),:).
compute immtemp={immtemp2,immtemp}.
compute immlbs={immlbs,'BootSE', 'BootLLCI', 'BootULCI'}.
end if.
compute resultm2=make(nrow(immtemp),maxresm,99999).
compute resultm2(1:nrow(immtemp),1:ncol(immtemp))=immtemp.
compute resultm={resultm;resultm2}.
do if (dichadj=0 and immcat=0 and indmmmt(i,1)<>5 and indmmm(i,1) < 100 ).
do if (outscree=1).
do if (indmmm(i,1) < 30).
print immtemp/title='      Index of moderated mediation:'/cnames=immlbs/rnames=immlbs2/format= F10.4.
end if.
do if (indmmm(i,1) = 31).
compute immlbs2={immlbs2;zcatlab(1:nzvls,1)}.
print immtemp/title='      Indices of partial moderated mediation:'/cnames=immlbs/rnames=immlbs2/format= F10.4.
end if.
end if.
do if ((nzvls = 1) and (nwvls = 1)).
do if (indmmm(i,1) = 41 or indmmm(i,1)=51).
loop k7=1 to nwvls.
compute immlbs2=zcatlab(1:nzvls,1).
compute immtemp2=immtemp((((k7-1)*nzvls)+1):(((k7-1)*nzvls)+nzvls),:).
do if (outscree=1).
do if (nwvls > 1).
compute primodv={'        ', wcatlab(k7,1)}.
print primodv/title='      Primary moderator:'/format=A8.
end if.
do if (nzvls=1).
print immtemp2/title='      Index of moderated moderated mediation'/cnames=immlbs/format= F10.4.
else.
print immtemp2/title='      Indices of moderated moderated mediation'/cnames=immlbs/rnames=immlbs2/format= F10.4.
end if.
end if.
compute cmmtemp=make(nrow(zprobval),4,0).
loop k8=1 to nrow(zprobval).
compute condbb3=condbb2(:,((nwvls+1)+((k7-1)*nzvls)):((nwvls+1)+((k7-1)*nzvls)+ (nzvls-1))).
do if (ncol(zprobval) > 1).
compute condbb3=condbb3*mdiag(zprobval(k8,:)).
else.
compute condbb3=condbb3&*zprobval(k8,:).
end if.
compute condbb3={condbb2(:,k7),condbb3}.
compute icmm=rsum(condbb3).
compute cmmtemp(k8,1)=icmm(1,1).
do if (boot > 0).

.
compute temp = icmm(2:nrow(icmm),1).
compute temp(GRADE( icmm(2:nrow(icmm),1) )) = icmm(2:nrow(icmm),1).
compute badlo = 0.
compute badhi = 0.
do if ( (icmm(1,1)*bc)+(9999*(1-bc)) <> 9999).
compute pv=csum(temp < (icmm(1,1)*bc)+(9999*(1-bc)) )/nrow(temp).
compute ppv = pv.
do if (pv > .5).
compute ppv = 1-pv.
end if.
compute y5=sqrt(-2*ln(ppv)).
compute xp=y5+((((y5*p4+p3)*y5+p2)*y5+p1)*y5+p0)/((((y5*q4+q3)*y5+q2)*y5+q1)*y5+q0).
do if (pv <= .5).
compute xp = -xp.
end if.
compute cilow=rnd(nrow(temp)*(cdfnorm(2*xp-xp2))).
compute cihigh=trunc(nrow(temp)*(cdfnorm(2*xp+(xp2))))+1.
do if (cilow < 1).
compute cilow = 1.
compute booterr=1.
compute badlo = 1.
end if.
do if (cihigh > nrow(temp)).
compute cihigh = boot.
compute booterr=1.
compute badhi = 1.
end if.
compute llcit=temp(cilow,1).
compute ulcit=temp(cihigh,1).
do if (badlo = 1 and llcit <> priorlo).
compute badend={badend, llcit}.
compute priorlo = llcit.
end if.
do if (badhi = 1 and ulcit <> priorhi).
compute badend={badend, ulcit}.
compute priorhi = ulcit.
end if.
end if.
do if ( (icmm(1,1)*bc)+(9999*(1-bc)) = 9999).
compute llcit=temp(cilow,1).
compute ulcit=temp(cihigh,1).
end if.
compute bootse=t(sqrt((cssq(temp)-((csum(temp)&**2)/nrow(temp)))/(nrow(temp)-1)))
.
compute cmmtemp(k8,2:4)={bootse,llcit,ulcit}.
end if.
end loop.
compute cmmtemp={zmodvals,cmmtemp}.
do if (boot=0).
compute cmmtemp=cmmtemp(:,1:2).
end if.
compute cmmlbs={znames,immlbs}.
compute resultm2=make(nrow(cmmtemp),maxresm,99999).
compute resultm2(1:nrow(cmmtemp),1:ncol(cmmtemp))=cmmtemp.
compute resultm={resultm;resultm2}.
do if (outscree=1).
print cmmtemp/title='      Indices of conditional moderated mediation by W'/cnames=cmmlbs/format= F10.4.
end if.
end loop.
end if.
end if.
end if.
do if (dichadj=1 or immcat=1 and indmmm(i,1) < 30).
do if (outscree=1).
print immtemp/title='Index of moderated mediation (difference between conditional indirect effects):'/cnames=immlbs/rnames=immlbs2/format= F10.4.
end if.
end if.
end if.
do if (indcontr=1).
compute resultm2=make(nrow(condcont),maxresm,99999).
compute resultm2(1:nrow(condcont),1:ncol(condcont))=condcont.
compute resultm={resultm;resultm2}.
compute condctlb={'Effect1','Effect2','Contrast','BootSE', 'BootLLCI','BootULCI'}.
do if (outscree=1).
print condcont/title=' Pairwise contrasts between conditional indirect effects (Effect1 minus Effect2)'/cnames=condctlb/format= F10.4.
end if.
end if.
end loop.
end if.
end loop.
do if (alttotal=1).
compute altcnms='Effect'.
compute alttotfx=t(cftotfx(1,:))+obnatdfx.
do if (boot > 0).
compute alttotfx=make(ncol(cftotfx),4,0).
compute alttotfx(:,1)=t(cftotfx(1,:))+obnatdfx.
compute natdirbt(1,:)=t(obnatdfx).
compute cftotfx=cftotfx+natdirbt.
loop cec=1 to ncol(cftotfx).

.
compute temp = cftotfx(2:nrow(cftotfx),cec).
compute temp(GRADE( cftotfx(2:nrow(cftotfx),cec) )) = cftotfx(2:nrow(cftotfx),cec).
compute badlo = 0.
compute badhi = 0.
do if ( (cftotfx(1,cec)*bc)+(9999*(1-bc)) <> 9999).
compute pv=csum(temp < (cftotfx(1,cec)*bc)+(9999*(1-bc)) )/nrow(temp).
compute ppv = pv.
do if (pv > .5).
compute ppv = 1-pv.
end if.
compute y5=sqrt(-2*ln(ppv)).
compute xp=y5+((((y5*p4+p3)*y5+p2)*y5+p1)*y5+p0)/((((y5*q4+q3)*y5+q2)*y5+q1)*y5+q0).
do if (pv <= .5).
compute xp = -xp.
end if.
compute cilow=rnd(nrow(temp)*(cdfnorm(2*xp-xp2))).
compute cihigh=trunc(nrow(temp)*(cdfnorm(2*xp+(xp2))))+1.
do if (cilow < 1).
compute cilow = 1.
compute booterr=1.
compute badlo = 1.
end if.
do if (cihigh > nrow(temp)).
compute cihigh = boot.
compute booterr=1.
compute badhi = 1.
end if.
compute llcit=temp(cilow,1).
compute ulcit=temp(cihigh,1).
do if (badlo = 1 and llcit <> priorlo).
compute badend={badend, llcit}.
compute priorlo = llcit.
end if.
do if (badhi = 1 and ulcit <> priorhi).
compute badend={badend, ulcit}.
compute priorhi = ulcit.
end if.
end if.
do if ( (cftotfx(1,cec)*bc)+(9999*(1-bc)) = 9999).
compute llcit=temp(cilow,1).
compute ulcit=temp(cihigh,1).
end if.
compute bootse=t(sqrt((cssq(temp)-((csum(temp)&**2)/nrow(temp)))/(nrow(temp)-1)))
.
compute alttotfx(cec,2:4)={bootse,llcit,ulcit}.
end loop.
compute altcnms={altcnms,'BootSE', 'BootLLCI','BootULCI'}.
end if.
compute resultm2=make(nrow(alttotfx),maxresm,99999).
compute resultm2(1:nrow(alttotfx),1:ncol(alttotfx))=alttotfx.
compute resultm={resultm;resultm2}.
do if (outscree=1).
print/title='----------'.
do if (nxvls > 1).
print alttotfx/title='Relative total effects of X on Y (sum of direct and indirect effects)'/cnames=altcnms/rnames=direffl2/format= F10.4.
end if.
do if (nxvls = 1).
print alttotfx/title='Total effect of X on Y (sum of direct and indirect effects)'/cnames=altcnms/format= F10.4.
end if.
end if.
end if.
end if.
end if
.
do if (criterr=0 and (saveest = 1 or saveboot=1)).
compute coeffsav=coeffmat(2:nrow(coeffmat),:).
compute conseq=conseq(2:nrow(conseq),1).
compute dfmat=dfmat(2:nrow(dfmat),1).
compute dfmat=dfmat.
compute saven={'conseqnt','antecdnt','coeff','se','t','p','LLCI','ULCI','df'}.
do if (ydich=1).
compute saven={'conseqnt','antecdnt','coeff','se','t_or_Z','p','LLCI','ULCI','df'}.
end if.
do if (saveboot=1 and boot > 0 ).
compute colslab=make(300,1,' ').


compute colslab( 1 ,1)= 'COL1'.


compute colslab( 2 ,1)= 'COL2'.


compute colslab( 3 ,1)= 'COL3'.


compute colslab( 4 ,1)= 'COL4'.


compute colslab( 5 ,1)= 'COL5'.


compute colslab( 6 ,1)= 'COL6'.


compute colslab( 7 ,1)= 'COL7'.


compute colslab( 8 ,1)= 'COL8'.


compute colslab( 9 ,1)= 'COL9'.


compute colslab( 10 ,1)= 'COL10'.


compute colslab( 11 ,1)= 'COL11'.


compute colslab( 12 ,1)= 'COL12'.


compute colslab( 13 ,1)= 'COL13'.


compute colslab( 14 ,1)= 'COL14'.


compute colslab( 15 ,1)= 'COL15'.


compute colslab( 16 ,1)= 'COL16'.


compute colslab( 17 ,1)= 'COL17'.


compute colslab( 18 ,1)= 'COL18'.


compute colslab( 19 ,1)= 'COL19'.


compute colslab( 20 ,1)= 'COL20'.


compute colslab( 21 ,1)= 'COL21'.


compute colslab( 22 ,1)= 'COL22'.


compute colslab( 23 ,1)= 'COL23'.


compute colslab( 24 ,1)= 'COL24'.


compute colslab( 25 ,1)= 'COL25'.


compute colslab( 26 ,1)= 'COL26'.


compute colslab( 27 ,1)= 'COL27'.


compute colslab( 28 ,1)= 'COL28'.


compute colslab( 29 ,1)= 'COL29'.


compute colslab( 30 ,1)= 'COL30'.


compute colslab( 31 ,1)= 'COL31'.


compute colslab( 32 ,1)= 'COL32'.


compute colslab( 33 ,1)= 'COL33'.


compute colslab( 34 ,1)= 'COL34'.


compute colslab( 35 ,1)= 'COL35'.


compute colslab( 36 ,1)= 'COL36'.


compute colslab( 37 ,1)= 'COL37'.


compute colslab( 38 ,1)= 'COL38'.


compute colslab( 39 ,1)= 'COL39'.


compute colslab( 40 ,1)= 'COL40'.


compute colslab( 41 ,1)= 'COL41'.


compute colslab( 42 ,1)= 'COL42'.


compute colslab( 43 ,1)= 'COL43'.


compute colslab( 44 ,1)= 'COL44'.


compute colslab( 45 ,1)= 'COL45'.


compute colslab( 46 ,1)= 'COL46'.


compute colslab( 47 ,1)= 'COL47'.


compute colslab( 48 ,1)= 'COL48'.


compute colslab( 49 ,1)= 'COL49'.


compute colslab( 50 ,1)= 'COL50'.


compute colslab( 51 ,1)= 'COL51'.


compute colslab( 52 ,1)= 'COL52'.


compute colslab( 53 ,1)= 'COL53'.


compute colslab( 54 ,1)= 'COL54'.


compute colslab( 55 ,1)= 'COL55'.


compute colslab( 56 ,1)= 'COL56'.


compute colslab( 57 ,1)= 'COL57'.


compute colslab( 58 ,1)= 'COL58'.


compute colslab( 59 ,1)= 'COL59'.


compute colslab( 60 ,1)= 'COL60'.


compute colslab( 61 ,1)= 'COL61'.


compute colslab( 62 ,1)= 'COL62'.


compute colslab( 63 ,1)= 'COL63'.


compute colslab( 64 ,1)= 'COL64'.


compute colslab( 65 ,1)= 'COL65'.


compute colslab( 66 ,1)= 'COL66'.


compute colslab( 67 ,1)= 'COL67'.


compute colslab( 68 ,1)= 'COL68'.


compute colslab( 69 ,1)= 'COL69'.


compute colslab( 70 ,1)= 'COL70'.


compute colslab( 71 ,1)= 'COL71'.


compute colslab( 72 ,1)= 'COL72'.


compute colslab( 73 ,1)= 'COL73'.


compute colslab( 74 ,1)= 'COL74'.


compute colslab( 75 ,1)= 'COL75'.


compute colslab( 76 ,1)= 'COL76'.


compute colslab( 77 ,1)= 'COL77'.


compute colslab( 78 ,1)= 'COL78'.


compute colslab( 79 ,1)= 'COL79'.


compute colslab( 80 ,1)= 'COL80'.


compute colslab( 81 ,1)= 'COL81'.


compute colslab( 82 ,1)= 'COL82'.


compute colslab( 83 ,1)= 'COL83'.


compute colslab( 84 ,1)= 'COL84'.


compute colslab( 85 ,1)= 'COL85'.


compute colslab( 86 ,1)= 'COL86'.


compute colslab( 87 ,1)= 'COL87'.


compute colslab( 88 ,1)= 'COL88'.


compute colslab( 89 ,1)= 'COL89'.


compute colslab( 90 ,1)= 'COL90'.


compute colslab( 91 ,1)= 'COL91'.


compute colslab( 92 ,1)= 'COL92'.


compute colslab( 93 ,1)= 'COL93'.


compute colslab( 94 ,1)= 'COL94'.


compute colslab( 95 ,1)= 'COL95'.


compute colslab( 96 ,1)= 'COL96'.


compute colslab( 97 ,1)= 'COL97'.


compute colslab( 98 ,1)= 'COL98'.


compute colslab( 99 ,1)= 'COL99'.


compute colslab( 100 ,1)= 'COL100'.


compute colslab( 101 ,1)= 'COL101'.


compute colslab( 102 ,1)= 'COL102'.


compute colslab( 103 ,1)= 'COL103'.


compute colslab( 104 ,1)= 'COL104'.


compute colslab( 105 ,1)= 'COL105'.


compute colslab( 106 ,1)= 'COL106'.


compute colslab( 107 ,1)= 'COL107'.


compute colslab( 108 ,1)= 'COL108'.


compute colslab( 109 ,1)= 'COL109'.


compute colslab( 110 ,1)= 'COL110'.


compute colslab( 111 ,1)= 'COL111'.


compute colslab( 112 ,1)= 'COL112'.


compute colslab( 113 ,1)= 'COL113'.


compute colslab( 114 ,1)= 'COL114'.


compute colslab( 115 ,1)= 'COL115'.


compute colslab( 116 ,1)= 'COL116'.


compute colslab( 117 ,1)= 'COL117'.


compute colslab( 118 ,1)= 'COL118'.


compute colslab( 119 ,1)= 'COL119'.


compute colslab( 120 ,1)= 'COL120'.


compute colslab( 121 ,1)= 'COL121'.


compute colslab( 122 ,1)= 'COL122'.


compute colslab( 123 ,1)= 'COL123'.


compute colslab( 124 ,1)= 'COL124'.


compute colslab( 125 ,1)= 'COL125'.


compute colslab( 126 ,1)= 'COL126'.


compute colslab( 127 ,1)= 'COL127'.


compute colslab( 128 ,1)= 'COL128'.


compute colslab( 129 ,1)= 'COL129'.


compute colslab( 130 ,1)= 'COL130'.


compute colslab( 131 ,1)= 'COL131'.


compute colslab( 132 ,1)= 'COL132'.


compute colslab( 133 ,1)= 'COL133'.


compute colslab( 134 ,1)= 'COL134'.


compute colslab( 135 ,1)= 'COL135'.


compute colslab( 136 ,1)= 'COL136'.


compute colslab( 137 ,1)= 'COL137'.


compute colslab( 138 ,1)= 'COL138'.


compute colslab( 139 ,1)= 'COL139'.


compute colslab( 140 ,1)= 'COL140'.


compute colslab( 141 ,1)= 'COL141'.


compute colslab( 142 ,1)= 'COL142'.


compute colslab( 143 ,1)= 'COL143'.


compute colslab( 144 ,1)= 'COL144'.


compute colslab( 145 ,1)= 'COL145'.


compute colslab( 146 ,1)= 'COL146'.


compute colslab( 147 ,1)= 'COL147'.


compute colslab( 148 ,1)= 'COL148'.


compute colslab( 149 ,1)= 'COL149'.


compute colslab( 150 ,1)= 'COL150'.


compute colslab( 151 ,1)= 'COL151'.


compute colslab( 152 ,1)= 'COL152'.


compute colslab( 153 ,1)= 'COL153'.


compute colslab( 154 ,1)= 'COL154'.


compute colslab( 155 ,1)= 'COL155'.


compute colslab( 156 ,1)= 'COL156'.


compute colslab( 157 ,1)= 'COL157'.


compute colslab( 158 ,1)= 'COL158'.


compute colslab( 159 ,1)= 'COL159'.


compute colslab( 160 ,1)= 'COL160'.


compute colslab( 161 ,1)= 'COL161'.


compute colslab( 162 ,1)= 'COL162'.


compute colslab( 163 ,1)= 'COL163'.


compute colslab( 164 ,1)= 'COL164'.


compute colslab( 165 ,1)= 'COL165'.


compute colslab( 166 ,1)= 'COL166'.


compute colslab( 167 ,1)= 'COL167'.


compute colslab( 168 ,1)= 'COL168'.


compute colslab( 169 ,1)= 'COL169'.


compute colslab( 170 ,1)= 'COL170'.


compute colslab( 171 ,1)= 'COL171'.


compute colslab( 172 ,1)= 'COL172'.


compute colslab( 173 ,1)= 'COL173'.


compute colslab( 174 ,1)= 'COL174'.


compute colslab( 175 ,1)= 'COL175'.


compute colslab( 176 ,1)= 'COL176'.


compute colslab( 177 ,1)= 'COL177'.


compute colslab( 178 ,1)= 'COL178'.


compute colslab( 179 ,1)= 'COL179'.


compute colslab( 180 ,1)= 'COL180'.


compute colslab( 181 ,1)= 'COL181'.


compute colslab( 182 ,1)= 'COL182'.


compute colslab( 183 ,1)= 'COL183'.


compute colslab( 184 ,1)= 'COL184'.


compute colslab( 185 ,1)= 'COL185'.


compute colslab( 186 ,1)= 'COL186'.


compute colslab( 187 ,1)= 'COL187'.


compute colslab( 188 ,1)= 'COL188'.


compute colslab( 189 ,1)= 'COL189'.


compute colslab( 190 ,1)= 'COL190'.


compute colslab( 191 ,1)= 'COL191'.


compute colslab( 192 ,1)= 'COL192'.


compute colslab( 193 ,1)= 'COL193'.


compute colslab( 194 ,1)= 'COL194'.


compute colslab( 195 ,1)= 'COL195'.


compute colslab( 196 ,1)= 'COL196'.


compute colslab( 197 ,1)= 'COL197'.


compute colslab( 198 ,1)= 'COL198'.


compute colslab( 199 ,1)= 'COL199'.


compute colslab( 200 ,1)= 'COL200'.


compute colslab( 201 ,1)= 'COL201'.


compute colslab( 202 ,1)= 'COL202'.


compute colslab( 203 ,1)= 'COL203'.


compute colslab( 204 ,1)= 'COL204'.


compute colslab( 205 ,1)= 'COL205'.


compute colslab( 206 ,1)= 'COL206'.


compute colslab( 207 ,1)= 'COL207'.


compute colslab( 208 ,1)= 'COL208'.


compute colslab( 209 ,1)= 'COL209'.


compute colslab( 210 ,1)= 'COL210'.


compute colslab( 211 ,1)= 'COL211'.


compute colslab( 212 ,1)= 'COL212'.


compute colslab( 213 ,1)= 'COL213'.


compute colslab( 214 ,1)= 'COL214'.


compute colslab( 215 ,1)= 'COL215'.


compute colslab( 216 ,1)= 'COL216'.


compute colslab( 217 ,1)= 'COL217'.


compute colslab( 218 ,1)= 'COL218'.


compute colslab( 219 ,1)= 'COL219'.


compute colslab( 220 ,1)= 'COL220'.


compute colslab( 221 ,1)= 'COL221'.


compute colslab( 222 ,1)= 'COL222'.


compute colslab( 223 ,1)= 'COL223'.


compute colslab( 224 ,1)= 'COL224'.


compute colslab( 225 ,1)= 'COL225'.


compute colslab( 226 ,1)= 'COL226'.


compute colslab( 227 ,1)= 'COL227'.


compute colslab( 228 ,1)= 'COL228'.


compute colslab( 229 ,1)= 'COL229'.


compute colslab( 230 ,1)= 'COL230'.


compute colslab( 231 ,1)= 'COL231'.


compute colslab( 232 ,1)= 'COL232'.


compute colslab( 233 ,1)= 'COL233'.


compute colslab( 234 ,1)= 'COL234'.


compute colslab( 235 ,1)= 'COL235'.


compute colslab( 236 ,1)= 'COL236'.


compute colslab( 237 ,1)= 'COL237'.


compute colslab( 238 ,1)= 'COL238'.


compute colslab( 239 ,1)= 'COL239'.


compute colslab( 240 ,1)= 'COL240'.


compute colslab( 241 ,1)= 'COL241'.


compute colslab( 242 ,1)= 'COL242'.


compute colslab( 243 ,1)= 'COL243'.


compute colslab( 244 ,1)= 'COL244'.


compute colslab( 245 ,1)= 'COL245'.


compute colslab( 246 ,1)= 'COL246'.


compute colslab( 247 ,1)= 'COL247'.


compute colslab( 248 ,1)= 'COL248'.


compute colslab( 249 ,1)= 'COL249'.


compute colslab( 250 ,1)= 'COL250'.


compute colslab( 251 ,1)= 'COL251'.


compute colslab( 252 ,1)= 'COL252'.


compute colslab( 253 ,1)= 'COL253'.


compute colslab( 254 ,1)= 'COL254'.


compute colslab( 255 ,1)= 'COL255'.


compute colslab( 256 ,1)= 'COL256'.


compute colslab( 257 ,1)= 'COL257'.


compute colslab( 258 ,1)= 'COL258'.


compute colslab( 259 ,1)= 'COL259'.


compute colslab( 260 ,1)= 'COL260'.


compute colslab( 261 ,1)= 'COL261'.


compute colslab( 262 ,1)= 'COL262'.


compute colslab( 263 ,1)= 'COL263'.


compute colslab( 264 ,1)= 'COL264'.


compute colslab( 265 ,1)= 'COL265'.


compute colslab( 266 ,1)= 'COL266'.


compute colslab( 267 ,1)= 'COL267'.


compute colslab( 268 ,1)= 'COL268'.


compute colslab( 269 ,1)= 'COL269'.


compute colslab( 270 ,1)= 'COL270'.


compute colslab( 271 ,1)= 'COL271'.


compute colslab( 272 ,1)= 'COL272'.


compute colslab( 273 ,1)= 'COL273'.


compute colslab( 274 ,1)= 'COL274'.


compute colslab( 275 ,1)= 'COL275'.


compute colslab( 276 ,1)= 'COL276'.


compute colslab( 277 ,1)= 'COL277'.


compute colslab( 278 ,1)= 'COL278'.


compute colslab( 279 ,1)= 'COL279'.


compute colslab( 280 ,1)= 'COL280'.


compute colslab( 281 ,1)= 'COL281'.


compute colslab( 282 ,1)= 'COL282'.


compute colslab( 283 ,1)= 'COL283'.


compute colslab( 284 ,1)= 'COL284'.


compute colslab( 285 ,1)= 'COL285'.


compute colslab( 286 ,1)= 'COL286'.


compute colslab( 287 ,1)= 'COL287'.


compute colslab( 288 ,1)= 'COL288'.


compute colslab( 289 ,1)= 'COL289'.


compute colslab( 290 ,1)= 'COL290'.


compute colslab( 291 ,1)= 'COL291'.


compute colslab( 292 ,1)= 'COL292'.


compute colslab( 293 ,1)= 'COL293'.


compute colslab( 294 ,1)= 'COL294'.


compute colslab( 295 ,1)= 'COL295'.


compute colslab( 296 ,1)= 'COL296'.


compute colslab( 297 ,1)= 'COL297'.


compute colslab( 298 ,1)= 'COL298'.


compute colslab( 299 ,1)= 'COL299'.


compute colslab( 300 ,1)= 'COL300'.

compute colslab=colslab(1:ncol(bootres),1).
compute colslab={colslab,conseq,vlabs}.
do if (outscree=1).
print/title = '**************************************************************************'.
print/title='Bootstrap estimates were saved to a file'/space=0.
print colslab/title='Map of column names to model coefficients:'/clabels=' ','Conseqnt','Antecdnt'/format=a8.
end if.
end if.
end if.
do if (criterr=0 and boot > 0 and modelbt=1).
compute labstart=1.
do if (outscree=1).
print/title = '*********** BOOTSTRAP RESULTS FOR REGRESSION MODEL PARAMETERS ************'.
end if.
loop iboot = 1 to (nms+nys).
do if (outscree=1).
print outnames(1,iboot)/title = 'OUTCOME VARIABLE:'/format = A8.
end if.
compute vlabsm=vlabs(labstart:(labstart+(nump(1,iboot)-1)),1).
compute outnmtmp=bootcim(labstart:(labstart+(nump(1,iboot)-1)),:).
compute resultm2=make(nrow(outnmtmp),maxresm,99999).
compute resultm2(1:nrow(outnmtmp),1:ncol(outnmtmp))=outnmtmp.
compute resultm={resultm;resultm2}.
do if (outscree=1).
print bootcim(labstart:(labstart+(nump(1,iboot)-1)),:)/title=' '/rnames=vlabsm/ clabels='Coeff' 'BootMean' 'BootSE' 'BootLLCI' 'BootULCI'/format= F10.4 /space=0.
end if.
compute labstart=labstart+nump(1,iboot).
do if (iboot < (nms+nys)).
do if (outscree=1).
print/title= '----------'.
end if.
end if.
end loop.
end if.
do if (criterr=0 and saveest=1).
compute resultm=resultm(2:nrow(resultm),:).
compute bocaj=(resultm=99999).
compute bocaj=csum(bocaj).
compute bocaj=1-(bocaj=nrow(resultm)).
compute j=1.
loop i = 1 to ncol(resultm).
do if (bocaj(1,i)=1).
compute j=j+1.
end if.
end loop.
compute resultm=resultm(:,1:(j-1)).
save resultm/outfile = *.
end if.
do if (criterr = 0 and matrices=1 and outscree=1).
print/title = '************************ MODEL DEFINITION MATRICES ************************'.
print/title = 'FROM variables are columns, TO variables are rows.'.
compute temp2=make(nrow(bcmat),ncol(bcmat),'0').
loop i = 2 to nrow(bcmat).
loop j = 1 to (ncol(bcmat)-1).
do if (bcmat(i,j)=1).
compute temp2(i,j)='1'.
end if.
do if (j >= i).
compute temp2(i,j)=' '.
end if.
end loop.
end loop.
compute temp2=temp2(2:nrow(bcmat),(1:(ncol(bcmat)-1))).
do if (nms > 0).
compute cmatlabs={xnames,mnames}.
compute rmatlabs={mnames,ynames}.
end if.
do if (nms = 0).
compute cmatlabs={xnames}.
compute rmatlabs={ynames}.
end if.
print temp2/title='BMATRIX: Paths freely estimated (1) and fixed to zero (0):'/cnames=cmatlabs/rnames=rmatlabs/format A3.
compute z=0.
do if (rsum(csum(wcmat))<>0).
compute temp2=make(nrow(wcmat),ncol(wcmat),'0').
loop i = 2 to nrow(wcmat).
loop j = 1 to (ncol(wcmat)-1).
do if (wcmat(i,j)=1).
compute temp2(i,j)='1'.
end if.
do if (j >= i).
compute temp2(i,j)=' '.
end if.
end loop.
end loop.
compute temp2=temp2(2:nrow(wcmat),(1:(ncol(wcmat)-1))).
print temp2/title='WMATRIX: Paths moderated (1) and not moderated (0) by W:'/cnames=cmatlabs/rnames=rmatlabs/format A3.
end if.
do if (rsum(csum(zcmat))<>0).
compute temp2=make(nrow(zcmat),ncol(zcmat),'0').
loop i = 2 to nrow(zcmat).
loop j = 1 to (ncol(zcmat)-1).
do if (zcmat(i,j)=1).
compute temp2(i,j)='1'.
end if.
do if (j >= i).
compute temp2(i,j)=' '.
end if.
end loop.
end loop.
compute temp2=temp2(2:nrow(zcmat),(1:(ncol(zcmat)-1))).
print temp2/title='ZMATRIX: Paths moderated (1) and not moderated (0) by Z:'/cnames=cmatlabs/rnames=rmatlabs/format a3.
end if.
do if (rsum(csum(wzcmat))<>0).
compute temp2=make(nrow(wzcmat),ncol(wzcmat),'0').
loop i = 2 to nrow(wzcmat).
loop j = 1 to (ncol(wzcmat)-1).
do if (wzcmat(i,j)=1).
compute temp2(i,j)='1'.
end if.
do if (j >= i).
compute temp2(i,j)=' '.
end if.
end loop.
end loop.
compute temp2=temp2(2:nrow(wzcmat),(1:(ncol(wzcmat)-1))).
print temp2/title='WZMATRIX: W moderated paths moderated (1) and not moderated (0) by Z:'/cnames=cmatlabs/rnames=rmatlabs/format a3.
end if.
do if (ncs > 0).
print ccmat/title='CMATRIX: Covariates (columns) in (1) and not in (0) the models of M and Y (rows):'/rnames=rmatlabs/cnames=covnames.
end if.
end if.
do if (outscree=1).
do if (activate=0).
print/title = '*********************** ANALYSIS NOTES AND ERRORS ************************'.
end if.
end if.
do if (activate=1).
print/title = '**************************************************************************'.
end if.
do if (criterr=0).
do if (outscree=1).
print conf/title = 'Level of confidence for all confidence intervals in output:'/format = F8.4.
do if (boot > 0).
do if (goodboot = boot and bc=0).
print boot/title='Number of bootstrap samples for percentile bootstrap confidence intervals:'.
end if.
do if (goodboot = boot and bc=1).
print boot/title='Number of bootstrap samples for bias-corrected bootstrap confidence intervals:'.
end if.
do if (booterr = 1).
compute badend = badend(1,2:ncol(badend)).
print/title = 'WARNING: Bootstrap CI endpoints below not trustworthy. Decrease confidence'.
print badend/title='or increase the number of bootstrap samples.'/space=0/format = F10.4.
end if.
end if.
do if (mc > 0).
print mc/title='Number of samples for Monte Carlo confidence intervals:'.
end if.
do if (wnotev > 0 and printw=1).
do if (wnotev=1).
print/title = 'W values in conditional tables are the 16th, 50th, and 84th percentiles.'.
else if (wnotev=2).
do if (minwwarn=0 and maxwwarn=0).
print/title = 'W values in conditional tables are the mean and +/- SD from the mean.'.
end if.
do if (minwwarn=1).
print/title = 'W values in conditional tables are the minimum, the mean, and 1 SD above the mean.'.
end if.
do if (maxwwarn=1).
print/title = 'W values in conditional tables are 1 SD below the mean, the mean, and the maximum.'.
end if.
end if.
end if.
do if (znotev > 0 and printz=1).
do if (znotev=1).
print/title = 'Z values in conditional tables are the 16th, 50th, and 84th percentiles.'.
else if (znotev=2).
do if (minzwarn=0 and maxzwarn=0).
print/title = 'Z values in conditional tables are the mean and +/- SD from the mean.'.
end if.
do if (minzwarn=1).
print/title = 'Z values in conditional tables are the minimum, the mean, and 1 SD above the mean.'.
end if.
do if (maxzwarn=1).
print/title = 'Z values in conditional tables are 1 SD below the mean, the mean, and the maximum.'.
end if.
end if.
end if.
do if (minwwarn > 0).
print/title = 'NOTE: One SD below the mean is below the minimum observed in the data for W,'.
print/title = '      so the minimum measurement on W is used for conditioning instead.'/space=0.
end if.
do if (maxwwarn > 0).
print/title = 'NOTE: One SD above the mean is above the maximum observed in the data for W,'.
print/title = '      so the maximum measurement for W is used for conditioning instead.'/space=0.
end if.
do if (minzwarn > 0).
print/title = 'NOTE: One SD below the mean is below the minimum observed in the data for Z,'.
print/title = '      so the minimum measurement for Z is used for conditioning instead.'/space=0.
end if.
do if (maxzwarn > 0).
print/title = 'NOTE: One SD above the mean is above the maximum observed in the data for Z,'.
print/title = '      so the maximum measurement for Z is used for conditioning instead.'/space=0.
end if.
do if (pstog=1).
print/title= 'NOTE: Standardized coefficients for dichotomous or multicategorical X are in'.
print/title= '      partially standardized form.'/space=0.
end if.
loop i = 1 to 100.
do if (notecode(i,1) = 32).
print/title = 'Direct, indirect, and total effects are counterfactually defined'.
do if (xcontcf=0).
print/title = 'assuming X by M interaction.'/space=0.
end if.
do if (xcontcf=1).
print/title = 'assuming X by M interaction and with the following reference (x_ref)'/space=0.
compute states=t(xrefvals).
print states/title = 'and counterfactual (x_cf) states for X:'/rlabels='x_ref' 'x_cf'/format= F10.4 /space=0.
end if.
end if.
do if (notecode(i,1)=1).
print/title = 'NOTE: COVMY is ignored when using CMATRIX option.'.
end if.
do if (notecode(i,1)=2).
print/title = 'NOTE: Confidence level restricted to between 50 and 99.9999%.  95% confidence is provided in output'.
end if.
do if (notecode(i,1)=3).
print centvar/title = 'NOTE: The following variables were mean centered prior to analysis:'/format = a8.
end if.
do if (notecode(i,1) = 4).
print/title = 'NOTE: A heteroscedasticity consistent standard error and covariance matrix estimator was used.'.
end if.
do if (notecode(i,1) = 5).
print/title = 'NOTE: The HC3 option has been replaced with HC.  See the documentation.'.
end if.
do if (notecode(i,1) = 6).
print/title = 'NOTE: Due to estimation problems, some bootstrap samples had to be replaced.'.
print badboot/title='      The number of times this happened was:'/space=0/format=F8.0.
end if.
do if (notecode(i,1) = 7).
print/title = 'NOTE: The bootstrapping was not completed due to problematic bootstrap samples.'.
print/title = '      Bootstrap confidence intervals are therefore suppressed.'/space=0.
end if.
do if (notecode(i,1) = 8).
print/title = 'NOTE: The number of bootstrap samples was adjusted upward given your desired confidence.'.
end if.
do if (notecode(i,1) = 9).
print/title = 'NOTE: WMODVAL is ignored when W is specified as multicategorical.'.
end if.
do if (notecode(i,1) = 10).
print/title = 'NOTE: ZMODVAL is ignored when Z is specified as multicategorical.'.
end if.
do if (notecode(i,1) = 11).
print/title = 'NOTE: Total effect model generated only when all covariates are specified in all'.
print/title = '      models of M and Y.'/space=0.
end if.
do if (notecode(i,1) = 30).
print/title = 'NOTE: Your vector of linear hypothesis weights is of the wrong length for this model.'.
end if.
do if (notecode(i,1) = 12).
print/title = 'NOTE: Total effect model generated only when X is freely estimated to affect each M'.
print/title = '      and both X and M are freely estimated to affect Y'/space=0.
end if.
do if (notecode(i,1) = 13).
print/title = 'NOTE: There are too many pairwise contrasts to conduct with this model.'.
end if.
do if (notecode(i,1) = 14).
print/title = 'NOTE: The number of contrast weights must equal the number of indirect effects.'.
end if.
do if (notecode(i,1) = 15).
print/title = 'NOTE: Monte Carlo confidence intervals not available for this model.'.
print/title = '      Bootstrapping is used instead.'/space=0.
end if.
do if (notecode(i,1) = 16).
print/title = 'NOTE: The number of Monte Carlo samples was adjusted upward given your desired confidence.'.
end if.
do if (notecode(i,1) = 19).
print/title = 'NOTE: Your contrast matrix is invalid or not applicable to this model.'.
end if.
do if (notecode(i,1) = 20).
print/title = 'NOTE: One of the groups specified by your contrast matrix does not exist in the data.'.
end if.
do if (notecode(i,1) = 21).
print/title = 'NOTE: The VARORDER option is not available in this release.'.
end if.
do if (notecode(i,1) = 22).
print/title = 'NOTE: The VMODVAL and QMODVAL options are not available in this release.'.
end if.
do if (notecode(i,1) = 23).
print/title = 'NOTE: The QUANTILE option is not available in this release.'.
end if.
do if (notecode(i,1) = 24).
print/title = 'NOTE: Total effect model not available with dichotomous Y.'.
end if.
do if (notecode(i,1) = 25).
print/title = 'NOTE: STAND/EFFSIZE options not available with dichotomous Y.'.
end if.
do if ((notecode(i,1) = 26) and nms > 0).
print/title = 'NOTE: Direct and indirect effects of X on Y are on a log-odds metric.'.
end if.
do if (notecode(i,1) = 27).
print/title = 'NOTE: Standardized coefficients are not available for models with moderators.'.
end if.
do if (notecode(i,1) = 28).
print/title = 'NOTE: The contrast option is not available with a multicategorical X.'.
end if.
do if (notecode(i,1) = 31).
do if (nms > 1).
print medmeans/title = 'NOTE: Controlled direct effect(s) estimated at the following mediator values:'/cnames=mnames/format= F10.4.
end if.
do if (nms=1).
print medmeans/title = 'NOTE: Controlled direct effect(s) estimated at the following mediator value:'/format= F10.4.
end if.
end if.
do if (notecode(i,1) = 33).
print/title = 'NOTE: Sobel test is not available with the XMINT option.'.
end if.
do if (notecode(i,1) = 37).
print/title = 'NOTE: The CONTRAST option is not available when using the XMINT option.'.
end if.
do if (notecode(i,1) = 34).
print/title = 'NOTE: Standardized effects are not available when using the XMINT option.'.
end if.
do if (notecode(i,1) = 36).
print/title = 'NOTE: The XREFVAL option is ignored when X is declared as multicategorical.'.
end if.
do if (notecode(i,1) = 35).
print coval/title = 'NOTE: Counterfactual effects estimated at the following covariate values:'/cnames=covnames/format= F10.4.
end if.
do if (notecode(i,1) = 29 and (listmiss=1)).
compute a=missrow.
compute conum=ncol(a).
compute allgood=0.
compute smremain=12.
compute largesti=1.
compute smallrow=0.
do if (conum > 12).
loop ii = 1 to 12.
compute check=(conum/ii).
do if (check = trunc(check)).
compute check2=conum/ii.
compute aok=ii.
do if (aok > 2).
compute allgood=1.
end if.
else.
compute remain=conum-(ii*trunc(check)).
do if (remain <= smremain).
compute smremain=remain.
compute largesti=ii.
compute smallrow=trunc(conum/largesti).
end if.
end if.
end loop.
compute atemp=a(1,1:(aok*check2)).
compute atemp=reshape(atemp,check2,aok).
do if (ncol(atemp) > 2).
print/title='NOTE: Missing data resulted in the deletion of the following row(s) of'.
print atemp/title='      data from the analysis:'/format=F6.0/space=0.
end if.
do if (allgood=0).
compute atemp=a(1,1:(smallrow*largesti)).
compute atemp=reshape(atemp,smallrow,largesti).
compute btemp=a(1,((largesti*smallrow)+1):conum).
print/title='NOTE: Missing data resulted in the deletion of the following row(s) of'.
print atemp/title='      data from the analysis:'/format=F6.0/space=0.
print btemp/title=' '/space=0/format=f6.0.
end if.
else if (conum <= 12).
print/title='NOTE: Missing data resulted in the deletion of the following row(s) of'.
print a/title='      data from the analysis:'/format=F6.0/space=0.
end if.
end if.
end loop.
do if (toomany=1).
print/title='WARNING: Variables names longer than eight characters can produce incorrect output'.
print/title='when some variables in the data file have the same first eight characters. Shorter'/space=0.
print/title='variable names are recommended. By using this output, you are accepting all risk'/space=0.
print/title='and consequences of interpreting or reporting results that may be incorrect.'/space=0.
end if.
end if.
end if.
loop i = 1 to 100.
do if (errcode(i,1)=1).
print/title = 'ERROR: You must specify a Y and an X variable.'.
end if.
do if (errcode(i,1)=2).
print/title = 'ERROR: X, M, or Y variable used more than once or W and Z are the same variable.'.
do if (toomany = 1).
print/title = '       This could be caused by the use of variables names longer'/space=0.
print varnames/title = '       than eight characters. Here are the variables I see:'/space=0/format=A8.
end if.
end if.
do if (errcode(i,1)=3).
print/title = 'ERROR: You have specified more than one variable for W, Y, X, or Z'.
end if.
do if (errcode(i,1)=4).
print/title = 'ERROR: A variable specified as multicategorical has more than nine categories.'.
end if.
do if (errcode(i,1)=5).
print/title = 'ERROR: One of the categories contains only a single case.'.
end if.
do if (errcode(i,1)=6).
print/title = 'ERROR: Invalid model number in this version of PROCESS.'.
end if.
do if (errcode(i,1)=7).
print/title = 'ERROR: Invalid model number.'.
end if.
do if (errcode(i,1)=8).
print/title = 'ERROR: You must specify an M variable for this model.'.
end if.
do if (errcode(i,1)=9).
print/title = 'ERROR: You have specified an M variable in a model that does not use it.'.
end if.
do if (errcode(i,1)=10).
print/title = 'ERROR: You have specified a W variable in a model that does not use it.'.
end if.
do if (errcode(i,1)=11).
print/title = 'ERROR: You have not specified a W variable in a model that requires it.'.
end if.
do if (errcode(i,1)=12).
print/title = 'ERROR: You have specified a Z variable in a model that does not use it.'.
end if.
do if (errcode(i,1)=13).
print/title = 'ERROR: You have not specified a Z variable in a model that requires it.'.
end if.
do if (errcode(i,1)=14).
print/title = 'ERROR: V and Q are not proper specifications in this release of PROCESS.'.
print/title = '       Moderators must be specified as W and/or Z.'/space=0.
end if.
do if (errcode(i,1)=15).
print/title = 'ERROR: One of your model variables exhibits no variation (it is a constant).'.
end if.
do if (errcode(i,1)=16).
print/title = 'ERROR: BMATRIX is not the correct length or is otherwise invalid.'.
end if.
do if (errcode(i,1)=17).
print/title = 'ERROR: WMATRIX is not the correct length or is otherwise invalid.'.
end if.
do if (errcode(i,1)=18).
print/title = 'ERROR: ZMATRIX is not the correct length or is otherwise invalid.'.
end if.
do if (errcode(i,1)=19).
print/title = 'ERROR: WZMATRIX is not the correct length or is otherwise invalid.'.
end if.
do if (errcode(i,1)=20).
print/title = 'ERROR: A path fixed at zero cannot be moderated.'.
end if.
do if (errcode(i,1)=21).
print/title = 'ERROR: If only one moderator is specified, it must be specified as W.'.
end if.
do if (errcode(i,1)=22).
print/title = 'ERROR: In BMATRIX, X must be specified to affect at least one variable.'.
end if.
do if (errcode(i,1)=23).
print/title = 'ERROR: In BMATRIX, at least one variable must be specified to affect Y.'.
end if.
do if (errcode(i,1)=24).
print/title = 'ERROR: You must specify a model number or a custom BMATRIX specification.'.
end if.
do if (errcode(i,1)=25).
print/title = 'ERROR: BMATRIX cannot be used in conjunction with a model number.'.
end if.
do if (errcode(i,1)=26).
print/title = 'ERROR: Your model has a dangling mediator (all Ms must affect and be affected).'.
end if.
do if (errcode(i,1)=27).
print/title = 'ERROR: CLUSTER is not available on this release of PROCESS.'.
end if.
do if (errcode(i,1)=29).
print/title = 'ERROR: CMATRIX is not the correct length or is otherwise invalid.'.
end if.
do if (errcode(i,1)=30).
print/title = 'ERROR: In CMATRIX, all covariates must be assigned to an M or a Y.'.
end if.
do if (errcode(i,1)=31).
print/title = 'ERROR: A linear or near linear dependency (singularity) exists in the data.'.
end if.
do if (errcode(i,1)=32).
print/title = 'ERROR: Models 80 and 81 require between 3 and 6 mediators.'.
end if.
do if (errcode(i,1)=33).
print/title = 'ERROR: Model 82 requires 4 mediators.'.
end if.
do if (errcode(i,1)=34).
print/title = 'ERROR: This model number requires between 2 and 6 mediators.'.
end if.
do if (errcode(i,1)=35).
print/title = 'ERROR: In a model with only one moderator, that moderator must be W.'.
end if.
do if (errcode(i,1)=36).
print/title = 'ERROR: A serial mediation model cannot have more than 6 mediators.'.
end if.
do if (errcode(i,1)=37).
print/title = 'ERROR: No more than 10 mediators are allowed in a PROCESS command.'.
end if.
do if (errcode(i,1)=38).
print/title = 'ERROR: XCATCODE is not provided, not the correct length, or is otherwise invalid.'.
end if.
do if (errcode(i,1)=39).
print/title = 'ERROR: WCATCODE is not provided, not the correct length, or is otherwise invalid.'.
end if.
do if (errcode(i,1)=40).
print/title = 'ERROR: ZCATCODE is not provided, not the correct length, or is otherwise invalid.'.
end if.
do if (errcode(i,1)=41).
print/title = 'ERROR: Models 1, 2, and 3 cannot be customized.'.
end if.
do if (errcode(i,1)=42).
print/title = 'ERROR: WS option available only in PROCESS v2. Or use the MEMORE macro instead.'.
print/title = '       MEMORE can be downloaded from www.akmontoya.com.'/space=0.
end if.
do if (errcode(i,1)=43).
print/title = 'ERROR: PROCESS does not allow dichotomous mediators.'.
end if.
do if (errcode(i,1)=60).
print/title = 'PROCESS is now ready for use.'.
print/title='Copyright 2017-2022 by Andrew F. Hayes. ALL RIGHTS RESERVED.'/space=0.
print/title='Workshop schedule available at http://haskayne.ucalgary.ca/CCRAM'/space=0.
end if.
do if (errcode(i,1)=50).
print/title = 'ERROR: A multicategorical moderator cannot be specified as a covariate.'.
end if.
do if (errcode(i,1)=51).
print/title = 'ERROR: A variable you specified as a covariate is a moderator in all equations.'.
end if.
do if (errcode(i,1)=52 and mcerpt=0).
compute mcerpt=1.
print/title = 'ERROR: A variable specified as multicategorical must have at least three categories'.
end if.
do if (errcode(i,1) = 47).
do if (iterrmod=1).
print/title = 'ERROR: Iteration for Y model didn''t converge to a solution. Interpret results with caution.'.
print/title = '       Try increasing the number of iterations, though this will slow down computation.'/space=0.
end if.
do if (bootiter=1).
print/title = 'ERROR: Nonconvergence during bootstrapping. Interpet bootstrap results with caution.'.
end if.
end if.
do if (errcode(i,1) = 61).
print/title = 'ERROR: Variable names should be no more than eight characters in length.'.
end if.
do if (errcode(i,1) = 62).
print/title = 'ERROR: After listwise deletion of cases with missing data, too few cases remain.'.
end if.
do if (errcode(i,1) = 63).
print/title = 'ERROR: The XMINT option is available only for model 4.'.
end if.
do if (errcode(i,1) = 64).
print/title = 'ERROR: Incorrect number of values specified in CDEVAL option.'.
end if.
do if (errcode(i,1) = 65).
print/title = 'ERROR: Only indicator or sequential coding of X is allowed with the XMINT option.'.
end if.
do if (errcode(i,1) = 66).
print/title = 'ERROR: XMINT option not available with continuous X using PROCESS GUI. Use syntax.'.
end if.
do if (errcode(i,1) = 67).
print/title = 'ERROR: Too many elements provided in XREFVAL option.'.
end if.
do if (errcode(i,1)=68).
print/title = 'ERROR: Covariate assignment is not allowed with XMINT option.'.
end if.
do if (errcode(i,1) = 69).
print/title = 'ERROR: Incorrect number of values specified in COVAL option.'.
end if.
do if (errcode(i,1) = 70).
print/title = 'ERROR: Incorrect value(s) in XREFVAL for this dichotomous X variable.'.
end if.
do if (errcode(i,1) = 71).
print/title = 'ERROR: The CENTER option is not available when using the XMINT option.'.
end if.
do if (errcode(i,1) = 72).
print/title = 'ERROR: The XMINT option is not available for models with a dichotomous Y.'.
end if.
end loop.
END MATRIX.	
Ressources	Temps de processeur	00:00:06,41	
	Temps écoulé	00:00:06,38	


Run MATRIX procedure:

***************** PROCESS Procedure for SPSS Version 4.2 *****************

          Written by Andrew F. Hayes, Ph.D.       www.afhayes.com
    Documentation available in Hayes (2022). www.guilford.com/p/hayes3

**************************************************************************
Model  : 4
    Y  : HOMAIR
    X  : WCStatus
   M1  : TG
   M2  : HDL
   M3  : LDL
   M4  : TC

Sample
Size:  169

**************************************************************************
OUTCOME VARIABLE:
 TG

Model Summary
          R       R-sq        MSE          F        df1        df2          p
      ,1491      ,0222  3961,6349     3,7947     1,0000   167,0000      ,0531

Model
              coeff         se          t          p       LLCI       ULCI
constant    78,6190     6,8675    11,4480      ,0000    65,0608    92,1773
WCStatus    18,8633     9,6835     1,9480      ,0531     -,2545    37,9811

Standardized coefficients
              coeff
WCStatus      ,2972

**************************************************************************
OUTCOME VARIABLE:
 HDL

Model Summary
          R       R-sq        MSE          F        df1        df2          p
      ,1047      ,0110   176,1817     1,8523     1,0000   167,0000      ,1753

Model
              coeff         se          t          p       LLCI       ULCI
constant    39,7619     1,4482    27,4553      ,0000    36,9027    42,6211
WCStatus     2,7793     2,0421     1,3610      ,1753    -1,2524     6,8109

Standardized coefficients
              coeff
WCStatus      ,2089

**************************************************************************
OUTCOME VARIABLE:
 LDL

Model Summary
          R       R-sq        MSE          F        df1        df2          p
      ,0364      ,0013  1188,4773      ,2210     1,0000   167,0000      ,6389

Model
              coeff         se          t          p       LLCI       ULCI
constant    75,0595     3,7615    19,9549      ,0000    67,6334    82,4857
WCStatus     2,4934     5,3038      ,4701      ,6389    -7,9778    12,9646

Standardized coefficients
              coeff
WCStatus      ,0725

**************************************************************************
OUTCOME VARIABLE:
 TC

Model Summary
          R       R-sq        MSE          F        df1        df2          p
      ,0956      ,0091  1438,6497     1,5417     1,0000   167,0000      ,2161

Model
              coeff         se          t          p       LLCI       ULCI
constant   132,1190     4,1385    31,9248      ,0000   123,9486   140,2895
WCStatus     7,2457     5,8354     1,2417      ,2161    -4,2750    18,7664

Standardized coefficients
              coeff
WCStatus      ,1907

**************************************************************************
OUTCOME VARIABLE:
 HOMAIR

Model Summary
          R       R-sq        MSE          F        df1        df2          p
      ,4367      ,1907    21,6991     7,6813     5,0000   163,0000      ,0000

Model
              coeff         se          t          p       LLCI       ULCI
constant     -,3625     1,6112     -,2250      ,8223    -3,5441     2,8191
WCStatus     2,5005      ,7341     3,4062      ,0008     1,0509     3,9502
TG            ,0382      ,0096     3,9974      ,0001      ,0193      ,0570
HDL           ,1031      ,0422     2,4421      ,0157      ,0197      ,1865
LDL           ,0813      ,0347     2,3410      ,0204      ,0127      ,1498
TC           -,0774      ,0345    -2,2469      ,0260     -,1455     -,0094

Standardized coefficients
              coeff
WCStatus      ,4903
TG            ,4751
HDL           ,2690
LDL           ,5481
TC           -,5767

************************** TOTAL EFFECT MODEL ****************************
OUTCOME VARIABLE:
 HOMAIR

Model Summary
          R       R-sq        MSE          F        df1        df2          p
      ,3096      ,0959    23,6608    17,7078     1,0000   167,0000      ,0000

Model
              coeff         se          t          p       LLCI       ULCI
constant     2,6119      ,5307     4,9213      ,0000     1,5641     3,6597
WCStatus     3,1491      ,7484     4,2081      ,0000     1,6717     4,6266

Standardized coefficients
              coeff
WCStatus      ,6174


************** TOTAL, DIRECT, AND INDIRECT EFFECTS OF X ON Y **************

Total effect of X on Y
     Effect         se          t          p       LLCI       ULCI       c_ps
     3,1491      ,7484     4,2081      ,0000     1,6717     4,6266      ,6174

Direct effect of X on Y
     Effect         se          t          p       LLCI       ULCI      c'_ps
     2,5005      ,7341     3,4062      ,0008     1,0509     3,9502      ,4903

Indirect effect(s) of X on Y:
          Effect     BootSE   BootLLCI   BootULCI
TOTAL      ,6486      ,3553      ,0881     1,4761
TG         ,7203      ,6956      ,0140     2,6833
HDL        ,2866      ,5332     -,1643     1,7444
LDL        ,2027      ,8993    -1,1527     2,5823
TC        -,5610     1,3016    -4,6724      ,3492

Partially standardized indirect effect(s) of X on Y:
          Effect     BootSE   BootLLCI   BootULCI
TOTAL      ,1272      ,0636      ,0203      ,2674
TG         ,1412      ,1274      ,0029      ,4943
HDL        ,0562      ,1001     -,0318      ,3226
LDL        ,0397      ,1719     -,2214      ,4872
TC        -,1100      ,2451     -,8847      ,0722

*********************** ANALYSIS NOTES AND ERRORS ************************

Level of confidence for all confidence intervals in output:
  95,0000

Number of bootstrap samples for percentile bootstrap confidence intervals:
  5000

NOTE: Standardized coefficients for dichotomous or multicategorical X are in
      partially standardized form.

------ END MATRIX -----


* Encoding: UTF-8.
preserve.
set printback=off.


Matrice


Remarques	
Sortie obtenue	10-NOV-2024 16:59:25	
Commentaires		
Entrée	Données	C:\Users\Brice SAHA\Documents\UBa\Master Thesis 2021\Fanuel\Database fanuel.sav	
	Jeu de données actif	Jeu_de_données1	
	Filtre	<sans>	
	Pondération	<sans>	
	Scinder un fichier	<sans>	
	N de lignes dans le fichier de travail	169	
Syntaxe	MATRIX.
compute wnames='xxxxx'.
compute znames='xxxxx'.
compute mcerpt=0.
compute wiscov=0.
compute ziscov=0.
compute tooman=0.
compute errcode=make(100,1,0).
compute notecode=make(100,1,0).
compute model = trunc( 4 ).
compute iterate = abs(trunc( 100 )).
compute converge = abs( 0.00001 ).
compute itprobtg=0.
compute v2tag=0.
compute ydich=0.
compute maxwwarn=0.
compute minwwarn=0.
compute maxzwarn=0.
compute minzwarn=0.
compute toomany=0.
compute wdich=0.
compute zdich=0.
compute wnotev=0.
compute znotev=0.
compute nxpval=1.
compute nwpval=1.
compute nzpval=1.
compute errs=1.
compute notes=1.
compute criterr=0.
compute novar=0.
compute adjust=0.
compute ncs=0.
compute serial=0.
compute sobelok=0.
compute hasw=0.
compute hasz=0.
compute printw=0.
compute printz=0.
compute xmint=( 0 =1).
compute wmodcust=0.
compute zmodcust=0.
compute booting=0.
compute bootiter=0.
compute iterrmod=0.
compute cov = 'xxxxx'.
compute varorder=( 0 <> 0).
compute nws=0.
compute w= 'xxxxx'.
compute nzs=0.
compute z = 'xxxxx'.
compute nms=0.
compute m = 'TG HDL LDL TC'.
compute nys=0.
compute y = 'HOMAIR'.
compute nxs=0.
compute x = 'WHRSt'.
compute effsize=( 0 =1).
compute stand=( 1 =1).
compute intprobe = .1.
compute xrefvals={ 999 }.
compute center=trunc( 0 ).
compute xcontcf=0.
compute xscaling=1.
compute cdeval={ -999 }.
compute cuscoval=0.
do if (model=74).
compute errcode(errs,1)=7.
compute errs=errs+1.
compute criterr=1.
end if.
do if (xmint=1 and model <> 4).
compute errcode(errs,1)=63.
compute errs=errs+1.
compute criterr=1.
end if.
do if (xmint=1 and model=4).
compute w=x.
compute model=74.
compute intprobe=1.
compute notecode(notes,1) = 32.
compute notes = notes + 1.
do if (effsize=1 or stand=1).
compute notecode(notes,1) = 34.
compute notes = notes + 1.
compute stand=0.
compute effsize=0.
end if.
do if (center <> 0).
compute center=0.
compute errcode(errs,1)=71.
compute errs=errs+1.
compute criterr=1.
end if.
end if.
compute v = 'xxxxx'.
compute q = 'xxxxx'.
compute linsum={ -999 }.
compute nlinsum=ncol(linsum).
do if (linsum(1,1) = -999).
compute nlinsum=0.
end if.
compute oldvars= 'xxxxx'.
compute mcxok=0.
compute mcwok=0.
compute mczok=0.
compute xprod=0.
compute zprod=0.
compute wprod=0.
compute modcok=0.
compute alttotal=0.
compute hc3=trunc( 0 ).
compute jn=( 0 = 1).
compute listmiss=( 0 =1).
compute modelres=( 0 =1).
compute outscree=( 1 =1).
compute activate=( 0 =1).
compute booterr=0.
compute normal=( 0 =1).
compute xmtest=( 0 =1).
compute describe=( 0 =1).
compute longname=( 0 =1).
do if (stand=1).
compute effsize=1.
end if.
compute pstog=0.
compute sobelok=0.
compute normal=( 0 =1).
compute mdichok=( 0 =1).
compute contrast={ 999 }.
compute ncontr=ncol(contrast).
compute ncontrow=nrow(contrast).
do if (contrast(1,1) = 999).
compute ncontr=1.
compute contrast=0.
end if.
do if (ncontr = 1).
compute contrast=trunc(contrast).
do if (contrast > 3 or contrast < 0)).
compute ncontr=1.
compute contrast = 0.
end if.
end if.
do if (ncontr > 1).
compute contvec=contrast.
compute contrast=4.
do if (ncontrow > 1).
compute contrast=0.
compute modcok=1.
compute wcontval=contvec(:,1).
compute zcontval=contvec(:,2).
do if ((ncontr <> 2) or (ncontrow <> 2)).
compute notecode(notes,1) = 19.
compute notes = notes + 1.
compute modcok=0.
end if.
end if.
end if.
do if (xmint = 1 and contrast(1,1) <> 0).
compute contrast=0.
compute notecode(notes,1) = 37.
compute notes = notes + 1.
end if.
do if (varorder = 1).
compute notecode(notes,1) = 21.
compute notes = notes + 1.
end if.
do if ( 999 <> 999 or 999 <> 999).
compute notecode(notes,1) = 22.
compute notes = notes + 1.
end if.
compute modelbt=( 0 =1).
compute cluster= 'xxxxx'.
compute matrices=( 0 =1).
compute covcoeff=( 0 =1).
compute covmy=trunc( 0 ).
do if (covmy < 0 or covmy > 2).
compute covmy = 0.
end if.
compute boot = abs(trunc( 5000 )).
compute bc=( 0 =1).
compute mc=abs(trunc( 0 )).
compute hc=trunc( 5 ).
do if (intprobe < 0 or intprobe > 1).
compute intprobe = .10.
end if.
compute plot=trunc( 0 ).
do if (plot < 0 or plot > 2).
compute plot=0.
end if.
compute total=( 1 =1).
compute dototal=0.
compute saveboot = ( 0 = 1).
compute saveest=( 0 = 2).
do if (saveest=1).
compute intprobe=1.
end if.
do if (hc >= 0 and hc < 5).
compute notecode(notes,1) = 4.
compute notes = notes + 1.
end if.
do if (hc > 5 or hc < 0).
compute hc=5.
end if.
compute mcw=trunc( 0 ).
compute mcz=trunc( 0 ).
compute mcx=trunc( 0 ).
do if (mcx > 0 and mcx < 3 and model = 74).
compute mcw=mcx.
compute xscaling=1.
end if.
do if (mcx > 2 and model = 74).
compute errcode(errs,1)=65.
compute errs=errs+1.
compute criterr=1.
end if.
do if (model = 74 and normal=1).
compute notecode(notes,1) = 33.
compute normal=0.
compute notes = notes + 1.
end if.
do if (mcx > 0 and contrast > 0).
compute notecode(notes,1) = 28.
compute notes = notes + 1.
compute contrast=0.
end if.
compute nxvls=1.
compute nmvls=1.
compute nwvls=1.
compute nzvls=1.
compute paths=999.
compute pathsw=999.
compute pathsz=999.
compute pathswz=999.
compute pathsmod=999.
compute pathtype=999.
compute obscoeff=999.
compute pathsdv={' '}.
compute quantile=1.
do if ( 999 <>999).
compute notecode(notes,1) = 23.
compute notes = notes + 1.
end if.
compute moments=( 0 =1).
do if (moments=1).
compute quantile=0.
end if.
compute bmatrix={ -999 }.
compute wmatrix={ -999 }.
compute zmatrix={ -999 }.
compute wzmatrix={ -999 }.
compute cmatrix={ -999 }.
compute xcatcode={ -999 }.
compute wcatcode={ -999 }.
compute zcatcode={ -999 }.
compute needed=0.
compute conf= 95.
do if (trunc( 95 ) >= 100 or (trunc( 95 ) <= 50)).
compute conf = 95.
compute notecode(notes,1)=2.
compute notes=notes+1.
end if.
do if (model >= 0 and model < 4 and modelbt=0).
compute boot=0.
compute mc=0.
compute bc=0.
end if.
do if (mc > 0 and boot > 0).
compute boot=0.
compute bc=0.
end if.
do if ((boot < 1000) and (mc = 0) and (boot > 0)).
compute boot=5000.
end if.
do if ((mc < 1000) and (boot = 0) and (mc > 0)).
compute mc=5000.
end if.
compute p0=-.322232431088.
compute p1 = -1.
compute p2 = -.342242088547.
compute p3 = -.0204231210245.
compute p4 = -.0000453642210148.
compute q0 = .0993484626060.
compute q1 = .588581570495.
compute q2 = .531103462366.
compute q3 = .103537752850.
compute q4 = .0038560700634.
compute badend=0.
compute priorlo = -9999999.
compute priorhi = 9999999.
compute alpha2 = (1-(conf/100))/2.
compute cilm=alpha2*2.
compute y5=sqrt(-2*ln(alpha2)).
compute xp2=(y5+((((y5*p4+p3)*y5+p2)*y5+p1)*y5+p0)/((((y5*q4+q3)*y5+q2)*y5+q1)*y5+q0)).
compute medlb={'   M1  :';'   M2  :';'   M3  :';'   M4  :';'   M5  :';'   M6  :';'   M7  :';'   M8  :';'   M9  :';'   M10 :'}.
compute medlb2={'(M1)','(M2)','(M3)','(M4)','(M5)','(M6)','(M7)','(M8)','(M9)','(M10)'}.
compute xlb={'   X1  :';'   X2  :';'   X3  :';'   X4  :';'   X5  :';'   X6  :';'   X7  :';'   X8  :';'   X9  :'}.
compute highlbw={'M1*W'; 'M2*W'; 'M3*W'; 'M4*W'; 'M5*W'; 'M6*W'; 'M7*W'; 'M8*W'; 'M9*W'; 'M10*W'}.
do if (xmint=1).
compute highlbw={'M1*X'; 'M2*X'; 'M3*X'; 'M4*X'; 'M5*X'; 'M6*X'; 'M7*X'; 'M8*X'; 'M9*X'; 'M10*X'}.
end if.
compute highlbz={'M1*Z'; 'M2*Z'; 'M3*Z'; 'M4*Z'; 'M5*Z'; 'M6*Z'; 'M7*Z'; 'M8*Z'; 'M9*Z';'M10*Z'}.
compute highlbwz={'M1*W*Z'; 'M2*W*Z'; 'M3*W*Z'; 'M4*W*Z'; 'M5*W*Z'; 'M6*W*Z'; 'M7*W*Z'; 'M8*W*Z'; 'M9*W*Z';'M10*W*Z'}.
compute highlbbt={'BOTH(M1)'; 'BOTH(M2)'; 'BOTH(M3)'; 'BOTH(M4)'; 'BOTH(M5)'; 'BOTH(M6)'; 'BOTH(M7)'; 'BOTH(M8)'; 'BOTH(M9)';'BTH(M10)'}.
compute highlbx={'M1*X'; 'M2*X'; 'M3*X'; 'M4*X'; 'M5*X'; 'M6*X'; 'M7*X'; 'M8*X'; 'M9*X'; 'M10*X'}.
compute skipwz=0.
compute validm={1,1,1,1,1,1,1,1,1,1,1,1,1,1,1,1,1,1,1,1,1,1,0,0,0,0,0,1,1,0,0,0,0, 0,0,0,0,0,0,0,0,0,0,0,0,0,0,0,0,0,0,0,0,0,0,0,0,1,1,1,1,1,1,1,1,1,1,1,1,1,1,1,1,1,1,1,0,0,0,1,1, 1,1,1,1,1,1,1,1,1,1,1}.
do if (activate=1).
compute errcode(errs,1)=60.
compute errs=errs+1.
compute criterr=1.
end if.
do if (criterr=0).
do if (( 0 =1)=1).
compute errcode(errs,1)=42.
compute errs=errs+1.
compute criterr=1.
end if.
do if (model > 0 and model < 93).
do if (validm(1,model)=0).
compute errcode(errs,1)=6.
compute errs=errs+1.
compute criterr=1.
end if.
release validm.
end if.
do if ((model > 92 or model < 0) and model <> 999)).
compute errcode(errs,1)=7.
compute errs=errs+1.
compute criterr=1.
end if.
do if (model = 999 and bmatrix(1,1)=-999).
compute errcode(errs,1)=24.
compute errs=errs+1.
compute criterr=1.
end if.
do if (model <> 999 and bmatrix(1,1) <> -999).
compute errcode(errs,1)=25.
compute errs=errs+1.
compute criterr=1.
end if.
do if ((model = 74 or (model > 0 and model < 4)) and ((wmatrix(1,1) <> -999) or (zmatrix(1,1)<>-999) or (wzmatrix(1,1)<>-999)))).
compute errcode(errs,1)=41.
compute errs=errs+1.
compute criterr=1.
end if.
do if (hc3 <> 0).
compute notecode(notes,1) = 5.
compute notes = notes + 1.
do if (hc3 = 1).
compute hc=3.
end if.
end if.
do if ((v <> 'xxxxx') or (q <> 'xxxxx')).
compute errcode(errs,1)=14.
compute errs=errs+1.
compute errcode(errs,1)=48.
compute errs=errs+1.
compute criterr=1.
end if.
do if (oldvars <> 'xxxxx').
compute errcode(errs,1)=48.
compute errs=errs+1.
compute criterr=1.
end if.
do if (cluster <> 'xxxxx').
compute errcode(errs,1)=27.
compute errs=errs+1.
compute criterr=1.
end if.
do if ((y = 'xxxxx') or (x = 'xxxxx')).
compute errcode(errs,1)=1.
compute errs=errs+1.
compute criterr=1.
end if.
do if ((m = 'xxxxx') and model > 3).
compute errcode(errs,1)=8.
compute errs=errs+1.
compute criterr=1.
end if.
end if.
do if (criterr=0).
get ytmp/variables = HOMAIR /names = ynames/MISSING = 99999.
compute nys=ncol(ytmp).
compute needed=nys.
compute n=nrow(ytmp).
compute varnames={ynames}.
compute dat=ytmp.

.


do if (toomany=1 and longname=0).
compute criterr=1.
do if (tooman=0).
compute tooman=1.
compute errcode(errs,1) = 61.
compute errs = errs + 1.
end if.
end if
.
compute modelvar={ '4' ;t(ynames)}.
do if ( 4 =999).
compute modelvar(1,1)='CUSTOM'.
end if.
get xtmp/variables = WHRSt /names = xnames/MISSING = 99999.
compute nxs=ncol(xtmp).
compute n=nrow(xtmp).
compute needed=needed+nxs.
compute varnames={varnames,xnames}.
compute xcatlab=t(xnames).
compute dat={dat,xtmp}.

.


do if (toomany=1 and longname=0).
compute criterr=1.
do if (tooman=0).
compute tooman=1.
compute errcode(errs,1) = 61.
compute errs = errs + 1.
end if.
end if
.
compute modelvar={modelvar;t(xnames)}.
do if (nxs = 1).
compute modelvlb={'Model  :';'    Y  :';'    X  :'}.
else.
compute modelvlb={'Model  :';'    Y  :';xlb(1:nxs,1)}.
end if.
do if (m <> 'xxxxx').
get mtmp/variables = TG HDL LDL TC /names = mnames/MISSING = 99999.
compute nms=ncol(mtmp).
compute mprod=make(1,nms,0).
compute n=nrow(mtmp).
compute needed=needed+nms.
compute varnames={varnames,mnames}.
compute dat={dat,mtmp}.
compute modelvar={modelvar;t(mnames)}.
compute x2m=make(99,nms,0).
compute m2y=make(99,nms,0).
compute onem=make(nms,1,1).

.


do if (toomany=1 and longname=0).
compute criterr=1.
do if (tooman=0).
compute tooman=1.
compute errcode(errs,1) = 61.
compute errs = errs + 1.
end if.
end if
.
do if (nms > 1 and nms < 11).
compute modelvlb={modelvlb;medlb(1:nms,1)}.
else.
compute modelvlb={modelvlb;'    M  :'}.
end if.
do if (nms > 0 and model < 4).
compute errcode(errs,1)=9.
compute errs=errs+1.
do if (model <> 0).
compute errcode(errs,1)=48.
compute errs=errs+1.
end if.
compute criterr=1.
end if.
end if.
compute wlocatet=0.
compute wlocate=0.
do if (w <> 'xxxxx').
do if (xmint=0).
get wtmp/variables = xxxxx /names = wnames/MISSING = 99999.

.


do if (toomany=1 and longname=0).
compute criterr=1.
do if (tooman=0).
compute tooman=1.
compute errcode(errs,1) = 61.
compute errs = errs + 1.
end if.
end if
.
end if.
do if (xmint=1).
get wtmp/variables = WHRSt /names = wnames/MISSING = 99999.
end if.
compute nws=ncol(wtmp).
compute n=nrow(wtmp).
compute varnames={varnames,wnames}.
compute wlocate=ncol(varnames).
do if (model=74).
compute wlocatet=1.
do if (xnames <> wnames).
compute errcode(errs,1)=45.
compute errs=errs+1.
compute criterr=1.
end if.
end if.
compute wcatlab=t(wnames).
compute dat={dat,wtmp}.
do if (xmint <> 1).
compute modelvar={modelvar;t(wnames)}.
compute modelvlb={modelvlb;'    W  :'}.
end if.
end if.
do if (z <> 'xxxxx').
get ztmp/variables = xxxxx /names = znames/MISSING = 99999.
compute nzs=ncol(ztmp).
compute n=nrow(ztmp).

.


do if (toomany=1 and longname=0).
compute criterr=1.
do if (tooman=0).
compute tooman=1.
compute errcode(errs,1) = 61.
compute errs = errs + 1.
end if.
end if
.
compute varnames={varnames,znames}.
compute zcatlab=t(znames).
compute dat={dat,ztmp}.
compute modelvar={modelvar;t(znames)}.
compute modelvlb={modelvlb;'    Z  :'}.
end if.
do if (cov <> 'xxxxx').
get ctmp/variables = xxxxx /names = covnames/MISSING = 99999.
compute ncs=ncol(ctmp).
compute n=nrow(ctmp).

.


do if (toomany=1 and longname=0).
compute criterr=1.
do if (tooman=0).
compute tooman=1.
compute errcode(errs,1) = 61.
compute errs = errs + 1.
end if.
end if
.
compute varnames={varnames,covnames}.
compute dat={dat,ctmp}.
end if.
do if (nws > 1 or nzs > 1 or nys > 1 or nxs > 1).
compute errcode(errs,1)=3.
compute errs=errs+1.
compute criterr=1.
end if.
do if ((model = 80 or model = 81) and (nms < 3 or nms > 6)).
compute errcode(errs,1)=32.
compute errs=errs+1.
compute criterr=1.
end if.
do if (model = 82 and nms <> 4).
compute errcode(errs,1)=33.
compute errs=errs+1.
compute criterr=1.
end if.
do if (nms > 10).
compute errcode(errs,1)=37.
compute errs=errs+1.
compute criterr=1.
end if.
do if ((model = 6 or (model > 82 and model < 999)) and (nms < 2 or nms > 6)).
compute errcode(errs,1)=34.
compute errs=errs+1.
compute criterr=1.
end if.
compute match=0.
compute match2=0.
compute mcwzcov=0.
loop i = 1 to (ncol(varnames)-1).
loop j = (i+1) to ncol(varnames).
do if (varnames(i)=varnames(j)).
do if (i < (nxs+nms+nys+1)).
compute match2=match2+1.
end if.
do if (wlocatet=1 and i=2 and j=wlocate).
compute match2=match2-1.
end if.
do if ((wnames=znames) and (nws > 0 or nzs > 0))).
compute match2=match2+1.
end if.
do if (i < (ncol(varnames)-ncs+1)) and j > (ncol(varnames)-ncs)).
do if ((varnames(j)=wnames) and mcw=0)).
compute match=0.
compute wiscov=(j-(ncol(varnames)-ncs)).
end if.
do if ((varnames(j)=wnames) and mcw <>0)).
compute mcwzcov=1.
end if.
do if ((varnames(j)=znames) and mcz=0).
compute match=0.
compute ziscov=(j-(ncol(varnames)-ncs)).
end if.
do if ((varnames(j)=znames) and mcz<>0)).
compute mcwzcov=1.
end if.
end if.
end if.
end loop.
end loop.
do if (match2>0 or match=1).
compute errcode(errs,1)=2.
compute errs=errs+1.
compute criterr=1.
end if.
do if (mcwzcov=1).
compute errcode(errs,1)=50.
compute errs=errs+1.
compute criterr=1.
end if.
compute ninit=nrow(dat).
compute rownum=make(ninit,1,0).
loop i = 1 to ninit.
compute rownum(i,1)=i.
end loop.
compute dat={rownum,dat}.
compute j=1.
compute missrow=0.
loop i = 1 to n.
do if (rsum(dat(i,2:ncol(dat))=99999)=0).
compute dat(j,:)=dat(i,:).
compute j=j+1.
else.
compute missrow={missrow;dat(i,1)}.
end if.
end loop.
do if (j < 5).
compute errcode(errs,1)=62.
compute errs=errs+1.
compute criterr=1.
end if.
do if (criterr=0).
compute rownum=dat(1:(j-1),1).
do if (nrow(missrow) > 1).
compute missrow=t(missrow(2:nrow(missrow),1)).
compute notecode(notes,1) = 29.
compute notes = notes + 1.
end if.
compute dat=dat(1:(j-1),2:ncol(dat)).
compute n=nrow(dat).
compute nmiss=ninit-n.
compute ytmp=dat(:,1:nys).

.
compute desctmp=make((8-(4* 0 )),ncol( ytmp ),-999).
loop jd=1 to ncol( ytmp ).
compute descdat= ytmp (:,jd).
compute desctmp(1,jd) = csum(descdat)/nrow(descdat).
compute desctmp(2,jd) = (nrow(descdat)*sscp(descdat))-(t(csum(descdat))*(csum(descdat))).
compute desctmp(2,jd) = sqrt(desctmp(2,jd)/(nrow(descdat)*(nrow(descdat)-1))).
compute desctmp(3,jd)=cmin(descdat).
compute desctmp(4,jd)=cmax(descdat).
do if ( 0 =0).
compute minwarn=0.
compute maxwarn=0.
do if ((desctmp(3,jd)=desctmp(4,jd)) and novar=0).
compute errcode(errs,1)=15.
compute errs=errs+1.
compute criterr=1.
compute novar=1.
end if.
compute tmp=((descdat(:,1)=desctmp(3,jd))+(descdat(:,1)=desctmp(4,jd))).
compute desctmp(8,jd)=(csum(tmp)=nrow(tmp)).
compute tmp = descdat.
compute tmp(GRADE(descdat),:) = descdat.
compute descdat = tmp.
release tmp.
compute decval={.16;.5;.84}.
loop kd=1 to 3.
compute low=trunc(decval(kd,1)*(nrow(descdat)+1)).
compute lowdec=decval(kd,1)*(nrow(descdat)+1)-low.
compute value=descdat(low,1)+(descdat((low+1),1)-descdat(low,1))*lowdec.
compute desctmp((4+kd),jd)=value.
end loop.
compute mnotev=1.
compute modvals=desctmp(5:7,:).
do if (quantile <> 1).
compute desctmp(5,jd)=desctmp(1,jd)-desctmp(2,jd).
compute desctmp(6,jd)=desctmp(1,jd).
compute desctmp(7,jd)=desctmp(1,jd)+desctmp(2,jd).
compute modvals=desctmp(5:7,:).
compute mnotev=2.
do if (modvals(1,1) < desctmp(3,1)).
compute modvals(1,1)=desctmp(3,1).
compute minwarn=1.
end if.
do if (modvals(3,1) > desctmp(4,1)).
compute modvals(3,1)=desctmp(4,1).
compute maxwarn=1.
end if.
end if.
do if (desctmp(8,1)=1).
compute modvals={desctmp(3,1);desctmp(4,1)}.
compute mnotev=0.
compute minwarn=0.
compute maxwarn=0.
end if.
end if.
end loop
.
compute ysd=desctmp(2,:).
compute ovsd=ysd.
do if (desctmp(8,1)=1).
compute ydich=1.
do if (total=1).
compute total=0.
compute notecode(notes,1) = 24.
compute notes = notes + 1.
end if.
do if (effsize=1).
compute effsize=0.
compute notecode(notes,1) = 25.
compute notes = notes + 1.
end if.
do if (model=74).
compute errcode(errs,1) = 72.
compute errs=errs+1.
compute criterr=1.
end if.
compute omx = cmax(ytmp).
compute omn = cmin(ytmp).
compute ytmp = (ytmp = omx).
compute dat(:,1:nys)=(dat(:,1:nys)=omx).
compute rcd = {omn, 0; omx, 1}.
end if.
compute xtmp=dat(:,(nys+1):(nys+nxs)).

.
compute desctmp=make((8-(4* 0 )),ncol( xtmp ),-999).
loop jd=1 to ncol( xtmp ).
compute descdat= xtmp (:,jd).
compute desctmp(1,jd) = csum(descdat)/nrow(descdat).
compute desctmp(2,jd) = (nrow(descdat)*sscp(descdat))-(t(csum(descdat))*(csum(descdat))).
compute desctmp(2,jd) = sqrt(desctmp(2,jd)/(nrow(descdat)*(nrow(descdat)-1))).
compute desctmp(3,jd)=cmin(descdat).
compute desctmp(4,jd)=cmax(descdat).
do if ( 0 =0).
compute minwarn=0.
compute maxwarn=0.
do if ((desctmp(3,jd)=desctmp(4,jd)) and novar=0).
compute errcode(errs,1)=15.
compute errs=errs+1.
compute criterr=1.
compute novar=1.
end if.
compute tmp=((descdat(:,1)=desctmp(3,jd))+(descdat(:,1)=desctmp(4,jd))).
compute desctmp(8,jd)=(csum(tmp)=nrow(tmp)).
compute tmp = descdat.
compute tmp(GRADE(descdat),:) = descdat.
compute descdat = tmp.
release tmp.
compute decval={.16;.5;.84}.
loop kd=1 to 3.
compute low=trunc(decval(kd,1)*(nrow(descdat)+1)).
compute lowdec=decval(kd,1)*(nrow(descdat)+1)-low.
compute value=descdat(low,1)+(descdat((low+1),1)-descdat(low,1))*lowdec.
compute desctmp((4+kd),jd)=value.
end loop.
compute mnotev=1.
compute modvals=desctmp(5:7,:).
do if (quantile <> 1).
compute desctmp(5,jd)=desctmp(1,jd)-desctmp(2,jd).
compute desctmp(6,jd)=desctmp(1,jd).
compute desctmp(7,jd)=desctmp(1,jd)+desctmp(2,jd).
compute modvals=desctmp(5:7,:).
compute mnotev=2.
do if (modvals(1,1) < desctmp(3,1)).
compute modvals(1,1)=desctmp(3,1).
compute minwarn=1.
end if.
do if (modvals(3,1) > desctmp(4,1)).
compute modvals(3,1)=desctmp(4,1).
compute maxwarn=1.
end if.
end if.
do if (desctmp(8,1)=1).
compute modvals={desctmp(3,1);desctmp(4,1)}.
compute mnotev=0.
compute minwarn=0.
compute maxwarn=0.
end if.
end if.
end loop
.
compute xsd=desctmp(2,:).
compute xmodvals=modvals.
compute xdich=desctmp(8,1).
compute xmx=cmax(xtmp).
compute xmn=cmin(xtmp).
do if ((mcx > 0) and (xrefvals(1,1) <> 999) and (xmint=1) and (model= 74)).
compute notecode(notes,1) = 36.
compute notes = notes + 1.
end if.
do if (mcx=0).
do if (ncol(xrefvals)>2 and model=74 and xmint=1).
compute errcode(errs,1)=67.
compute errs=errs+1.
compute criterr=1.
end if.
do if ((model=74) and (xmint=1)).
do if ((xrefvals(1,1)=999) and nxvls=1 and xdich=0).
compute errcode(errs,1)=66.
compute errs=errs+1.
compute criterr=1.
end if.
do if ((xrefvals(1,1)=999) and (xdich=1)).
compute xrefvals={xmn,xmx}.
compute xscaling=xrefvals(1,2)-xrefvals(1,1).
end if.
do if (ncol(xrefvals)=1 and xrefvals(1,1) <> 999).
do if (xdich=0).
compute xrefvals={xrefvals,(xrefvals(1,1)+1)}.
compute xscaling=xrefvals(1,2)-xrefvals(1,1).
end if.
do if (xdich=1).
do if ((xrefvals(1,1) <> xmx) and (xrefvals(1,1) <> xmn)).
compute errcode(errs,1)=70.
compute errs=errs+1.
compute criterr=1.
end if.
do if (xrefvals(1,1) = xmx).
compute xrefvals={xrefvals,xmn}.
compute xscaling=xrefvals(1,2)-xrefvals(1,1).
end if.
do if (xrefvals(1,1) = xmn).
compute xrefvals={xrefvals,xmx}.
compute xscaling=xrefvals(1,2)-xrefvals(1,1).
end if.
end if.
end if.
do if (ncol(xrefvals)=2).
compute xscaling=xrefvals(1,2)-xrefvals(1,1).
do if (xdich=1).
compute xreferr=1.
do if (((xrefvals(1,1) = xmx) and (xrefvals(1,2)=xmn)) or ((xrefvals(1,1) = xmn) and (xrefvals(1,2)=xmx))).
compute xreferr=0.
end if.
do if (xreferr=1).
compute errcode(errs,1)=70.
compute errs=errs+1.
compute criterr=1.
end if.
end if.
end if.
end if.
end if.
do if (xmint=1 and model=74 and mcx=0).
compute xmodvals=t(xrefvals).
compute xcontcf=1.
end if.
compute nxpval=nrow(xmodvals).
compute xprobval=xmodvals.
do if (xdich =1 and mcx > 0).
compute mcx=0.
compute errcode(errs,1) = 52.
compute errs = errs + 1.
compute criterr = 1.
end if.
do if (nms > 0).
compute mtmp=dat(:,(nys+nxs+1):(nys+nxs+nms)).

.
compute desctmp=make((8-(4* 0 )),ncol( mtmp ),-999).
loop jd=1 to ncol( mtmp ).
compute descdat= mtmp (:,jd).
compute desctmp(1,jd) = csum(descdat)/nrow(descdat).
compute desctmp(2,jd) = (nrow(descdat)*sscp(descdat))-(t(csum(descdat))*(csum(descdat))).
compute desctmp(2,jd) = sqrt(desctmp(2,jd)/(nrow(descdat)*(nrow(descdat)-1))).
compute desctmp(3,jd)=cmin(descdat).
compute desctmp(4,jd)=cmax(descdat).
do if ( 0 =0).
compute minwarn=0.
compute maxwarn=0.
do if ((desctmp(3,jd)=desctmp(4,jd)) and novar=0).
compute errcode(errs,1)=15.
compute errs=errs+1.
compute criterr=1.
compute novar=1.
end if.
compute tmp=((descdat(:,1)=desctmp(3,jd))+(descdat(:,1)=desctmp(4,jd))).
compute desctmp(8,jd)=(csum(tmp)=nrow(tmp)).
compute tmp = descdat.
compute tmp(GRADE(descdat),:) = descdat.
compute descdat = tmp.
release tmp.
compute decval={.16;.5;.84}.
loop kd=1 to 3.
compute low=trunc(decval(kd,1)*(nrow(descdat)+1)).
compute lowdec=decval(kd,1)*(nrow(descdat)+1)-low.
compute value=descdat(low,1)+(descdat((low+1),1)-descdat(low,1))*lowdec.
compute desctmp((4+kd),jd)=value.
end loop.
compute mnotev=1.
compute modvals=desctmp(5:7,:).
do if (quantile <> 1).
compute desctmp(5,jd)=desctmp(1,jd)-desctmp(2,jd).
compute desctmp(6,jd)=desctmp(1,jd).
compute desctmp(7,jd)=desctmp(1,jd)+desctmp(2,jd).
compute modvals=desctmp(5:7,:).
compute mnotev=2.
do if (modvals(1,1) < desctmp(3,1)).
compute modvals(1,1)=desctmp(3,1).
compute minwarn=1.
end if.
do if (modvals(3,1) > desctmp(4,1)).
compute modvals(3,1)=desctmp(4,1).
compute maxwarn=1.
end if.
end if.
do if (desctmp(8,1)=1).
compute modvals={desctmp(3,1);desctmp(4,1)}.
compute mnotev=0.
compute minwarn=0.
compute maxwarn=0.
end if.
end if.
end loop
.
compute ovsd={desctmp(2,:),ysd}.
compute medmeans=cdeval.
do if ((cdeval(1,1) <> -999) and (ncol(medmeans) <> nms) and (model=74)).
compute errcode(errs,1)=64.
compute errs=errs+1.
compute criterr=1.
end if.
do if ((cdeval(1,1)=-999) and (model=74)).
compute medmeans=desctmp(1,:).
end if.
do if ((cdeval(1,1)<> -999) and (model=74) and (criterr=0)).
compute notecode(notes,1) = 31.
compute notes = notes + 1.
end if.
do if ((rsum(desctmp(8,:))>0) and (mdichok <> 1)).
compute errcode(errs,1)=43.
compute errs=errs+1.
compute criterr=1.
end if.
compute mmodvals=modvals.
compute mprobval=mmodvals.
end if.
do if (nws > 0).
compute wtmp=dat(:,(nys+nxs+nms+1):(nys+nxs+nms+nws)).

.
compute desctmp=make((8-(4* 0 )),ncol( wtmp ),-999).
loop jd=1 to ncol( wtmp ).
compute descdat= wtmp (:,jd).
compute desctmp(1,jd) = csum(descdat)/nrow(descdat).
compute desctmp(2,jd) = (nrow(descdat)*sscp(descdat))-(t(csum(descdat))*(csum(descdat))).
compute desctmp(2,jd) = sqrt(desctmp(2,jd)/(nrow(descdat)*(nrow(descdat)-1))).
compute desctmp(3,jd)=cmin(descdat).
compute desctmp(4,jd)=cmax(descdat).
do if ( 0 =0).
compute minwarn=0.
compute maxwarn=0.
do if ((desctmp(3,jd)=desctmp(4,jd)) and novar=0).
compute errcode(errs,1)=15.
compute errs=errs+1.
compute criterr=1.
compute novar=1.
end if.
compute tmp=((descdat(:,1)=desctmp(3,jd))+(descdat(:,1)=desctmp(4,jd))).
compute desctmp(8,jd)=(csum(tmp)=nrow(tmp)).
compute tmp = descdat.
compute tmp(GRADE(descdat),:) = descdat.
compute descdat = tmp.
release tmp.
compute decval={.16;.5;.84}.
loop kd=1 to 3.
compute low=trunc(decval(kd,1)*(nrow(descdat)+1)).
compute lowdec=decval(kd,1)*(nrow(descdat)+1)-low.
compute value=descdat(low,1)+(descdat((low+1),1)-descdat(low,1))*lowdec.
compute desctmp((4+kd),jd)=value.
end loop.
compute mnotev=1.
compute modvals=desctmp(5:7,:).
do if (quantile <> 1).
compute desctmp(5,jd)=desctmp(1,jd)-desctmp(2,jd).
compute desctmp(6,jd)=desctmp(1,jd).
compute desctmp(7,jd)=desctmp(1,jd)+desctmp(2,jd).
compute modvals=desctmp(5:7,:).
compute mnotev=2.
do if (modvals(1,1) < desctmp(3,1)).
compute modvals(1,1)=desctmp(3,1).
compute minwarn=1.
end if.
do if (modvals(3,1) > desctmp(4,1)).
compute modvals(3,1)=desctmp(4,1).
compute maxwarn=1.
end if.
end if.
do if (desctmp(8,1)=1).
compute modvals={desctmp(3,1);desctmp(4,1)}.
compute mnotev=0.
compute minwarn=0.
compute maxwarn=0.
end if.
end if.
end loop
.
compute wmodvals=modvals.
compute wdich=desctmp(8,1).
do if (wdich =1 and mcw > 0).
compute mcw=0.
compute errcode(errs,1) = 52.
compute errs = errs + 1.
compute criterr = 1.
end if.
compute wmin=desctmp(3,1).
compute wmax=desctmp(4,1).
compute minwwarn=minwarn.
compute maxwwarn=maxwarn.
compute wnotev=mnotev.
compute wmodval={ 999 }.
do if (xmint=1 and model=74 and mcx=0).
compute wmodval=xrefvals.
end if.
compute nwcontr=ncol(wmodval).
do if (wmodval(1,1) <> 999).
compute wmodvals=wmodval(1,1).
compute wmodcust=1.
do if (nwcontr > 1).
compute wmodvals=t(wmodval).
end if.
compute minwwarn=0.
compute maxwwarn=0.
compute wnotev=0.
end if.
compute wprobval=wmodvals.
compute nwpval=nrow(wmodvals).
end if.
do if (nzs > 0).
compute ztmp=dat(:,(nys+nxs+nms+nws+1):(nys+nxs+nms+nws+nzs)).

.
compute desctmp=make((8-(4* 0 )),ncol( ztmp ),-999).
loop jd=1 to ncol( ztmp ).
compute descdat= ztmp (:,jd).
compute desctmp(1,jd) = csum(descdat)/nrow(descdat).
compute desctmp(2,jd) = (nrow(descdat)*sscp(descdat))-(t(csum(descdat))*(csum(descdat))).
compute desctmp(2,jd) = sqrt(desctmp(2,jd)/(nrow(descdat)*(nrow(descdat)-1))).
compute desctmp(3,jd)=cmin(descdat).
compute desctmp(4,jd)=cmax(descdat).
do if ( 0 =0).
compute minwarn=0.
compute maxwarn=0.
do if ((desctmp(3,jd)=desctmp(4,jd)) and novar=0).
compute errcode(errs,1)=15.
compute errs=errs+1.
compute criterr=1.
compute novar=1.
end if.
compute tmp=((descdat(:,1)=desctmp(3,jd))+(descdat(:,1)=desctmp(4,jd))).
compute desctmp(8,jd)=(csum(tmp)=nrow(tmp)).
compute tmp = descdat.
compute tmp(GRADE(descdat),:) = descdat.
compute descdat = tmp.
release tmp.
compute decval={.16;.5;.84}.
loop kd=1 to 3.
compute low=trunc(decval(kd,1)*(nrow(descdat)+1)).
compute lowdec=decval(kd,1)*(nrow(descdat)+1)-low.
compute value=descdat(low,1)+(descdat((low+1),1)-descdat(low,1))*lowdec.
compute desctmp((4+kd),jd)=value.
end loop.
compute mnotev=1.
compute modvals=desctmp(5:7,:).
do if (quantile <> 1).
compute desctmp(5,jd)=desctmp(1,jd)-desctmp(2,jd).
compute desctmp(6,jd)=desctmp(1,jd).
compute desctmp(7,jd)=desctmp(1,jd)+desctmp(2,jd).
compute modvals=desctmp(5:7,:).
compute mnotev=2.
do if (modvals(1,1) < desctmp(3,1)).
compute modvals(1,1)=desctmp(3,1).
compute minwarn=1.
end if.
do if (modvals(3,1) > desctmp(4,1)).
compute modvals(3,1)=desctmp(4,1).
compute maxwarn=1.
end if.
end if.
do if (desctmp(8,1)=1).
compute modvals={desctmp(3,1);desctmp(4,1)}.
compute mnotev=0.
compute minwarn=0.
compute maxwarn=0.
end if.
end if.
end loop
.
compute zmodvals=modvals.
compute zdich=desctmp(8,1).
do if (zdich =1 and mcz > 0).
compute mcz=0.
compute errcode(errs,1) = 52.
compute errs = errs + 1.
compute criterr = 1.
end if.
compute zmin=desctmp(3,1).
compute zmax=desctmp(4,1).
compute minzwarn=minwarn.
compute maxzwarn=maxwarn.
compute znotev=mnotev.
compute zmodval={ 999 }.
compute nzcontr=ncol(zmodval).
do if (zmodval(1,1) <> 999).
compute zmodvals=zmodval(1,1).
compute zmodcust=1.
do if (nzcontr > 1).
compute zmodvals=t(zmodval).
end if.
compute minzwarn=0.
compute maxzwarn=0.
compute znotev=0.
end if.
compute zprobval=zmodvals.
compute nzpval=nrow(zmodvals).
end if.
do if (ncs > 0).
compute ctmp=dat(:,(nys+nxs+nms+nws+nzs+1):(nys+nxs+nms+nws+nzs+ncs)).

.
compute desctmp=make((8-(4* 0 )),ncol( ctmp ),-999).
loop jd=1 to ncol( ctmp ).
compute descdat= ctmp (:,jd).
compute desctmp(1,jd) = csum(descdat)/nrow(descdat).
compute desctmp(2,jd) = (nrow(descdat)*sscp(descdat))-(t(csum(descdat))*(csum(descdat))).
compute desctmp(2,jd) = sqrt(desctmp(2,jd)/(nrow(descdat)*(nrow(descdat)-1))).
compute desctmp(3,jd)=cmin(descdat).
compute desctmp(4,jd)=cmax(descdat).
do if ( 0 =0).
compute minwarn=0.
compute maxwarn=0.
do if ((desctmp(3,jd)=desctmp(4,jd)) and novar=0).
compute errcode(errs,1)=15.
compute errs=errs+1.
compute criterr=1.
compute novar=1.
end if.
compute tmp=((descdat(:,1)=desctmp(3,jd))+(descdat(:,1)=desctmp(4,jd))).
compute desctmp(8,jd)=(csum(tmp)=nrow(tmp)).
compute tmp = descdat.
compute tmp(GRADE(descdat),:) = descdat.
compute descdat = tmp.
release tmp.
compute decval={.16;.5;.84}.
loop kd=1 to 3.
compute low=trunc(decval(kd,1)*(nrow(descdat)+1)).
compute lowdec=decval(kd,1)*(nrow(descdat)+1)-low.
compute value=descdat(low,1)+(descdat((low+1),1)-descdat(low,1))*lowdec.
compute desctmp((4+kd),jd)=value.
end loop.
compute mnotev=1.
compute modvals=desctmp(5:7,:).
do if (quantile <> 1).
compute desctmp(5,jd)=desctmp(1,jd)-desctmp(2,jd).
compute desctmp(6,jd)=desctmp(1,jd).
compute desctmp(7,jd)=desctmp(1,jd)+desctmp(2,jd).
compute modvals=desctmp(5:7,:).
compute mnotev=2.
do if (modvals(1,1) < desctmp(3,1)).
compute modvals(1,1)=desctmp(3,1).
compute minwarn=1.
end if.
do if (modvals(3,1) > desctmp(4,1)).
compute modvals(3,1)=desctmp(4,1).
compute maxwarn=1.
end if.
end if.
do if (desctmp(8,1)=1).
compute modvals={desctmp(3,1);desctmp(4,1)}.
compute mnotev=0.
compute minwarn=0.
compute maxwarn=0.
end if.
end if.
end loop
.
compute covmeans=desctmp(1,:).
compute coval={ -999 }.
do if ((coval(1,1) <> -999) and (ncol(coval) <> ncs) and (model=74)).
compute errcode(errs,1)=69.
compute errs=errs+1.
compute criterr=1.
end if.
do if ((coval(1,1)<> -999) and (criterr=0) and (model=74)).
compute notecode(notes,1) = 35.
compute notes = notes + 1.
compute cuscoval=1.
end if.
end if.
compute n=nrow(ytmp).
compute ones=make(n,1,1).
do if (nws > 0 and mcw > 0).
compute tmp={rownum,wtmp(:,1)}.

.
compute dd= tmp.
compute temp = dd.
compute temp(GRADE(dd(:,2)),:) = dd.
compute dd = temp.
compute dummy = design(dd(:,2)).
compute nvls = ncol(dummy).
compute nnvls = csum(dummy).
compute mnvls = cmin(t(nnvls)).
compute conmat1=1.
do if (mnvls < 2).
compute errcode(errs,1) = 5.
compute errs = errs + 1.
compute criterr = 1.
end if.
do if (nvls > 9).
compute errcode(errs,1) = 4.
compute errs = errs+1.
compute criterr = 1.
end if.
do if (criterr = 0).
compute dumok = 1.
compute nnvls=make(nvls,1,0).
compute nnvls(1,1)=dd(1,2).
compute temp = 2.
loop i = 2 to n.
do if (dd(i,2) <> nnvls((temp-1),1)).
compute nnvls(temp,1)=dd(i,2).
compute temp = temp+1.
end if.
end loop.
do if ( mcw > 0).
compute x = dummy(:,2:ncol(dummy)).
compute nx = ncol(x).
compute minus1 = make(1,ncol(x),-1).
compute xdes=make((nx+1),3,0).
compute xdes(1,1)=dd(1,2).
compute xdes(1,2)=1.
compute temp = 2.
loop k = 2 to n.
do if (dd(k,2) <> dd((k-1),2)).
compute xdes(temp,2) = k.
compute xdes(temp,1) = dd(k,2).
compute xdes((temp-1),3) = k-1.
compute temp=temp+1.
end if.
end loop.
compute xdes((temp-1),3)=n.
compute xdes = {xdes, (xdes(:,3)-xdes(:,2)+1)}.
do if ( mcw = 4).
loop k = 1 to n.
do if (rsum(x(k,:)) = 0).
compute x(k,:) = minus1.
end if.
end loop.
end if.
do if ( mcw = 2 or mcw = 3 or mcw =5).
loop k = 1 to n.
do if (rsum(x(k,:)) > 0).
loop i = 1 to ncol(x).
do if (x(k,i) = 0).
compute x(k,i) = 1.
else.
break.
end if.
end loop.
end if.
end loop.
do if ( mcw = 3).
compute conmat1={-8,1,1,1,1,1,1,1,1; 0,-7,1,1,1,1,1,1,1; 0,0,-6,1,1,1,1,1,1; 0,0,0,-5,1,1,1,1,1; 0,0,0,0,-4,1,1,1,1; 0,0,0,0,0,-3,1,1,1; 0,0,0,0,0,0,-2,1,1; 0,0,0,0,0,0,0,-1,1}.
loop i = 1 to 8.
compute conmat1(i,:)=conmat1(i,:)/(10-i).
end loop.
compute conmat1=t(conmat1((10-nvls):8,(10-nvls):9)).
loop k=1 to n.
compute x(k,:)=conmat1((rsum(x(k,:))+1),:).
end loop.
end if.
end if.
do if ( mcw = 5).
compute custcode={ -999 }.
do if (ncol(custcode) <> (nvls*(nvls-1))).
compute errcode(errs,1) = (37+ 2 ).
compute errs = errs + 1.
compute criterr = 1.
end if.
do if (ncol(custcode) = (nvls*(nvls-1))).
compute conmat1=make(nvls,(nvls-1),0).
compute cnt=1.
loop i = 1 to nvls.
loop k = 1 to (nvls-1).
compute conmat1(i,k)=custcode(1,cnt).
compute cnt=cnt+1.
end loop.
end loop.
loop k=1 to n.
compute x(k,:)=conmat1((rsum(x(k,:))+1),:).
end loop.
end if.
end if.
compute xskip = 1.
compute dummat = make((nx+1),nx,0).
compute dummat((2:nrow(dummat)),:)=ident(nx).
do if ( mcw = 4).
compute dummat(1,:) = minus1.
end if.
do if ( mcw = 2).
loop i = 2 to nrow(dummat).
loop j = 1 to (i-1).
compute dummat(i,j) = 1.
end loop.
end loop.
end if.
do if ( mcw = 3).
compute dummat=conmat1.
end if.
do if ( mcw = 5 and criterr=0).
compute dummat=conmat1.
end if.
compute dummat={nnvls, dummat}.
compute x={dd(:,1),x}.
compute temp = x.
compute temp(GRADE(x(:,1)),:) = x.
compute x = temp.
release conmat1,temp,dd,xskip,xdes,dummy.
end if.
end if
.
compute wmodvals=nnvls.
compute nwpval=nrow(wmodvals).
do if (criterr=0).
compute minwwarn=0.
compute maxwwarn=0.
compute wnotev=0.
compute wtmp=x(:,2:ncol(x)).
compute wcatlab={'W1';'W2';'W3';'W4';'W5';'W6';'W7';'W8';'W9'}.
do if (xmint=1).
compute wcatlab={'X1';'X2';'X3';'X4';'X5';'X6';'X7';'X8';'X9'}.
end if.
compute nwvls=nvls-1.
compute mcwok=1.
compute dummatw=dummat.
compute wprobval=dummatw(:,2:ncol(dummatw)).
do if (modcok=1).
compute wcontval=make(2,ncol(wprobval),-999).
compute temp=0.
loop i = 1 to 2.
loop j = 1 to nrow(dummatw).
do if (contvec(i,1)=dummatw(j,1)).
compute wcontval(i,:)=wprobval(j,:).
compute temp=temp+1.
end if.
end loop.
end loop.
do if (temp < 2).
compute notecode(notes,1) = 20.
compute notes = notes + 1.
compute modcok=0.
end if.
end if.
do if ((wmodval(1,1) <> 999) and (xmint <> 1)).
compute notecode(notes,1) = 9.
compute notes = notes + 1.
end if.
release tmp, dummat.
end if.
end if.
do if (nzs > 0 and mcz > 0).
compute tmp={rownum,ztmp(:,1)}.

.
compute dd= tmp.
compute temp = dd.
compute temp(GRADE(dd(:,2)),:) = dd.
compute dd = temp.
compute dummy = design(dd(:,2)).
compute nvls = ncol(dummy).
compute nnvls = csum(dummy).
compute mnvls = cmin(t(nnvls)).
compute conmat1=1.
do if (mnvls < 2).
compute errcode(errs,1) = 5.
compute errs = errs + 1.
compute criterr = 1.
end if.
do if (nvls > 9).
compute errcode(errs,1) = 4.
compute errs = errs+1.
compute criterr = 1.
end if.
do if (criterr = 0).
compute dumok = 1.
compute nnvls=make(nvls,1,0).
compute nnvls(1,1)=dd(1,2).
compute temp = 2.
loop i = 2 to n.
do if (dd(i,2) <> nnvls((temp-1),1)).
compute nnvls(temp,1)=dd(i,2).
compute temp = temp+1.
end if.
end loop.
do if ( mcz > 0).
compute x = dummy(:,2:ncol(dummy)).
compute nx = ncol(x).
compute minus1 = make(1,ncol(x),-1).
compute xdes=make((nx+1),3,0).
compute xdes(1,1)=dd(1,2).
compute xdes(1,2)=1.
compute temp = 2.
loop k = 2 to n.
do if (dd(k,2) <> dd((k-1),2)).
compute xdes(temp,2) = k.
compute xdes(temp,1) = dd(k,2).
compute xdes((temp-1),3) = k-1.
compute temp=temp+1.
end if.
end loop.
compute xdes((temp-1),3)=n.
compute xdes = {xdes, (xdes(:,3)-xdes(:,2)+1)}.
do if ( mcz = 4).
loop k = 1 to n.
do if (rsum(x(k,:)) = 0).
compute x(k,:) = minus1.
end if.
end loop.
end if.
do if ( mcz = 2 or mcz = 3 or mcz =5).
loop k = 1 to n.
do if (rsum(x(k,:)) > 0).
loop i = 1 to ncol(x).
do if (x(k,i) = 0).
compute x(k,i) = 1.
else.
break.
end if.
end loop.
end if.
end loop.
do if ( mcz = 3).
compute conmat1={-8,1,1,1,1,1,1,1,1; 0,-7,1,1,1,1,1,1,1; 0,0,-6,1,1,1,1,1,1; 0,0,0,-5,1,1,1,1,1; 0,0,0,0,-4,1,1,1,1; 0,0,0,0,0,-3,1,1,1; 0,0,0,0,0,0,-2,1,1; 0,0,0,0,0,0,0,-1,1}.
loop i = 1 to 8.
compute conmat1(i,:)=conmat1(i,:)/(10-i).
end loop.
compute conmat1=t(conmat1((10-nvls):8,(10-nvls):9)).
loop k=1 to n.
compute x(k,:)=conmat1((rsum(x(k,:))+1),:).
end loop.
end if.
end if.
do if ( mcz = 5).
compute custcode={ -999 }.
do if (ncol(custcode) <> (nvls*(nvls-1))).
compute errcode(errs,1) = (37+ 3 ).
compute errs = errs + 1.
compute criterr = 1.
end if.
do if (ncol(custcode) = (nvls*(nvls-1))).
compute conmat1=make(nvls,(nvls-1),0).
compute cnt=1.
loop i = 1 to nvls.
loop k = 1 to (nvls-1).
compute conmat1(i,k)=custcode(1,cnt).
compute cnt=cnt+1.
end loop.
end loop.
loop k=1 to n.
compute x(k,:)=conmat1((rsum(x(k,:))+1),:).
end loop.
end if.
end if.
compute xskip = 1.
compute dummat = make((nx+1),nx,0).
compute dummat((2:nrow(dummat)),:)=ident(nx).
do if ( mcz = 4).
compute dummat(1,:) = minus1.
end if.
do if ( mcz = 2).
loop i = 2 to nrow(dummat).
loop j = 1 to (i-1).
compute dummat(i,j) = 1.
end loop.
end loop.
end if.
do if ( mcz = 3).
compute dummat=conmat1.
end if.
do if ( mcz = 5 and criterr=0).
compute dummat=conmat1.
end if.
compute dummat={nnvls, dummat}.
compute x={dd(:,1),x}.
compute temp = x.
compute temp(GRADE(x(:,1)),:) = x.
compute x = temp.
release conmat1,temp,dd,xskip,xdes,dummy.
end if.
end if
.
compute zmodvals=nnvls.
compute nzpval=nrow(zmodvals).
do if (criterr=0).
compute minzwarn=0.
compute maxzwarn=0.
compute znotev=0.
compute ztmp=x(:,2:ncol(x)).
compute zcatlab={'Z1';'Z2';'Z3';'Z4';'Z5';'Z6';'Z7';'Z8';'Z9'}.
compute nzvls=nvls-1.
compute mczok=1.
compute dummatz=dummat.
compute zprobval=dummatz(:,2:ncol(dummatz)).
do if (modcok=1).
compute zcontval=make(2,ncol(zprobval),-999).
compute temp=0.
loop i = 1 to 2.
loop j = 1 to nrow(dummatz).
do if (contvec(i,2)=dummatz(j,1)).
compute zcontval(i,:)=zprobval(j,:).
compute temp=temp+1.
end if.
end loop.
end loop.
do if (temp < 2).
compute notecode(notes,1) = 20.
compute notes = notes + 1.
compute modcok=0.
end if.
end if.
do if (zmodval(1,1) <> 999).
compute notecode(notes,1) = 10.
compute notes = notes + 1.
end if.
release tmp, dummat.
end if.
end if.
do if (nxs > 0 and mcx > 0).
compute tmp={rownum,xtmp(:,1)}.

.
compute dd= tmp.
compute temp = dd.
compute temp(GRADE(dd(:,2)),:) = dd.
compute dd = temp.
compute dummy = design(dd(:,2)).
compute nvls = ncol(dummy).
compute nnvls = csum(dummy).
compute mnvls = cmin(t(nnvls)).
compute conmat1=1.
do if (mnvls < 2).
compute errcode(errs,1) = 5.
compute errs = errs + 1.
compute criterr = 1.
end if.
do if (nvls > 9).
compute errcode(errs,1) = 4.
compute errs = errs+1.
compute criterr = 1.
end if.
do if (criterr = 0).
compute dumok = 1.
compute nnvls=make(nvls,1,0).
compute nnvls(1,1)=dd(1,2).
compute temp = 2.
loop i = 2 to n.
do if (dd(i,2) <> nnvls((temp-1),1)).
compute nnvls(temp,1)=dd(i,2).
compute temp = temp+1.
end if.
end loop.
do if ( mcx > 0).
compute x = dummy(:,2:ncol(dummy)).
compute nx = ncol(x).
compute minus1 = make(1,ncol(x),-1).
compute xdes=make((nx+1),3,0).
compute xdes(1,1)=dd(1,2).
compute xdes(1,2)=1.
compute temp = 2.
loop k = 2 to n.
do if (dd(k,2) <> dd((k-1),2)).
compute xdes(temp,2) = k.
compute xdes(temp,1) = dd(k,2).
compute xdes((temp-1),3) = k-1.
compute temp=temp+1.
end if.
end loop.
compute xdes((temp-1),3)=n.
compute xdes = {xdes, (xdes(:,3)-xdes(:,2)+1)}.
do if ( mcx = 4).
loop k = 1 to n.
do if (rsum(x(k,:)) = 0).
compute x(k,:) = minus1.
end if.
end loop.
end if.
do if ( mcx = 2 or mcx = 3 or mcx =5).
loop k = 1 to n.
do if (rsum(x(k,:)) > 0).
loop i = 1 to ncol(x).
do if (x(k,i) = 0).
compute x(k,i) = 1.
else.
break.
end if.
end loop.
end if.
end loop.
do if ( mcx = 3).
compute conmat1={-8,1,1,1,1,1,1,1,1; 0,-7,1,1,1,1,1,1,1; 0,0,-6,1,1,1,1,1,1; 0,0,0,-5,1,1,1,1,1; 0,0,0,0,-4,1,1,1,1; 0,0,0,0,0,-3,1,1,1; 0,0,0,0,0,0,-2,1,1; 0,0,0,0,0,0,0,-1,1}.
loop i = 1 to 8.
compute conmat1(i,:)=conmat1(i,:)/(10-i).
end loop.
compute conmat1=t(conmat1((10-nvls):8,(10-nvls):9)).
loop k=1 to n.
compute x(k,:)=conmat1((rsum(x(k,:))+1),:).
end loop.
end if.
end if.
do if ( mcx = 5).
compute custcode={ -999 }.
do if (ncol(custcode) <> (nvls*(nvls-1))).
compute errcode(errs,1) = (37+ 1 ).
compute errs = errs + 1.
compute criterr = 1.
end if.
do if (ncol(custcode) = (nvls*(nvls-1))).
compute conmat1=make(nvls,(nvls-1),0).
compute cnt=1.
loop i = 1 to nvls.
loop k = 1 to (nvls-1).
compute conmat1(i,k)=custcode(1,cnt).
compute cnt=cnt+1.
end loop.
end loop.
loop k=1 to n.
compute x(k,:)=conmat1((rsum(x(k,:))+1),:).
end loop.
end if.
end if.
compute xskip = 1.
compute dummat = make((nx+1),nx,0).
compute dummat((2:nrow(dummat)),:)=ident(nx).
do if ( mcx = 4).
compute dummat(1,:) = minus1.
end if.
do if ( mcx = 2).
loop i = 2 to nrow(dummat).
loop j = 1 to (i-1).
compute dummat(i,j) = 1.
end loop.
end loop.
end if.
do if ( mcx = 3).
compute dummat=conmat1.
end if.
do if ( mcx = 5 and criterr=0).
compute dummat=conmat1.
end if.
compute dummat={nnvls, dummat}.
compute x={dd(:,1),x}.
compute temp = x.
compute temp(GRADE(x(:,1)),:) = x.
compute x = temp.
release conmat1,temp,dd,xskip,xdes,dummy.
end if.
end if
.
do if (criterr=0).
compute xtmp=x(:,2:ncol(x)).
compute xcatlab={'X1';'X2';'X3';'X4';'X5';'X6';'X7';'X8';'X9'}.
compute nxvls=nvls-1.
compute xdich=(nvls=2).
compute mcxok=1.
compute dummatx=dummat.
compute xmodvals=dummatx(:,1).
compute nxpval=nrow(xmodvals).
release tmp, dummat.
end if.
end if.
compute intlab=make(100,1,' ').


compute intlab( 1 ,1)= 'Int_1'.


compute intlab( 2 ,1)= 'Int_2'.


compute intlab( 3 ,1)= 'Int_3'.


compute intlab( 4 ,1)= 'Int_4'.


compute intlab( 5 ,1)= 'Int_5'.


compute intlab( 6 ,1)= 'Int_6'.


compute intlab( 7 ,1)= 'Int_7'.


compute intlab( 8 ,1)= 'Int_8'.


compute intlab( 9 ,1)= 'Int_9'.


compute intlab( 10 ,1)= 'Int_10'.


compute intlab( 11 ,1)= 'Int_11'.


compute intlab( 12 ,1)= 'Int_12'.


compute intlab( 13 ,1)= 'Int_13'.


compute intlab( 14 ,1)= 'Int_14'.


compute intlab( 15 ,1)= 'Int_15'.


compute intlab( 16 ,1)= 'Int_16'.


compute intlab( 17 ,1)= 'Int_17'.


compute intlab( 18 ,1)= 'Int_18'.


compute intlab( 19 ,1)= 'Int_19'.


compute intlab( 20 ,1)= 'Int_20'.


compute intlab( 21 ,1)= 'Int_21'.


compute intlab( 22 ,1)= 'Int_22'.


compute intlab( 23 ,1)= 'Int_23'.


compute intlab( 24 ,1)= 'Int_24'.


compute intlab( 25 ,1)= 'Int_25'.


compute intlab( 26 ,1)= 'Int_26'.


compute intlab( 27 ,1)= 'Int_27'.


compute intlab( 28 ,1)= 'Int_28'.


compute intlab( 29 ,1)= 'Int_29'.


compute intlab( 30 ,1)= 'Int_30'.


compute intlab( 31 ,1)= 'Int_31'.


compute intlab( 32 ,1)= 'Int_32'.


compute intlab( 33 ,1)= 'Int_33'.


compute intlab( 34 ,1)= 'Int_34'.


compute intlab( 35 ,1)= 'Int_35'.


compute intlab( 36 ,1)= 'Int_36'.


compute intlab( 37 ,1)= 'Int_37'.


compute intlab( 38 ,1)= 'Int_38'.


compute intlab( 39 ,1)= 'Int_39'.


compute intlab( 40 ,1)= 'Int_40'.


compute intlab( 41 ,1)= 'Int_41'.


compute intlab( 42 ,1)= 'Int_42'.


compute intlab( 43 ,1)= 'Int_43'.


compute intlab( 44 ,1)= 'Int_44'.


compute intlab( 45 ,1)= 'Int_45'.


compute intlab( 46 ,1)= 'Int_46'.


compute intlab( 47 ,1)= 'Int_47'.


compute intlab( 48 ,1)= 'Int_48'.


compute intlab( 49 ,1)= 'Int_49'.


compute intlab( 50 ,1)= 'Int_50'.


compute intlab( 51 ,1)= 'Int_51'.


compute intlab( 52 ,1)= 'Int_52'.


compute intlab( 53 ,1)= 'Int_53'.


compute intlab( 54 ,1)= 'Int_54'.


compute intlab( 55 ,1)= 'Int_55'.


compute intlab( 56 ,1)= 'Int_56'.


compute intlab( 57 ,1)= 'Int_57'.


compute intlab( 58 ,1)= 'Int_58'.


compute intlab( 59 ,1)= 'Int_59'.


compute intlab( 60 ,1)= 'Int_60'.


compute intlab( 61 ,1)= 'Int_61'.


compute intlab( 62 ,1)= 'Int_62'.


compute intlab( 63 ,1)= 'Int_63'.


compute intlab( 64 ,1)= 'Int_64'.


compute intlab( 65 ,1)= 'Int_65'.


compute intlab( 66 ,1)= 'Int_66'.


compute intlab( 67 ,1)= 'Int_67'.


compute intlab( 68 ,1)= 'Int_68'.


compute intlab( 69 ,1)= 'Int_69'.


compute intlab( 70 ,1)= 'Int_70'.


compute intlab( 71 ,1)= 'Int_71'.


compute intlab( 72 ,1)= 'Int_72'.


compute intlab( 73 ,1)= 'Int_73'.


compute intlab( 74 ,1)= 'Int_74'.


compute intlab( 75 ,1)= 'Int_75'.


compute intlab( 76 ,1)= 'Int_76'.


compute intlab( 77 ,1)= 'Int_77'.


compute intlab( 78 ,1)= 'Int_78'.


compute intlab( 79 ,1)= 'Int_79'.


compute intlab( 80 ,1)= 'Int_80'.


compute intlab( 81 ,1)= 'Int_81'.


compute intlab( 82 ,1)= 'Int_82'.


compute intlab( 83 ,1)= 'Int_83'.


compute intlab( 84 ,1)= 'Int_84'.


compute intlab( 85 ,1)= 'Int_85'.


compute intlab( 86 ,1)= 'Int_86'.


compute intlab( 87 ,1)= 'Int_87'.


compute intlab( 88 ,1)= 'Int_88'.


compute intlab( 89 ,1)= 'Int_89'.


compute intlab( 90 ,1)= 'Int_90'.


compute intlab( 91 ,1)= 'Int_91'.


compute intlab( 92 ,1)= 'Int_92'.


compute intlab( 93 ,1)= 'Int_93'.


compute intlab( 94 ,1)= 'Int_94'.


compute intlab( 95 ,1)= 'Int_95'.


compute intlab( 96 ,1)= 'Int_96'.


compute intlab( 97 ,1)= 'Int_97'.


compute intlab( 98 ,1)= 'Int_98'.


compute intlab( 99 ,1)= 'Int_99'.


compute intlab( 100 ,1)= 'Int_100'.

compute bcmat=make(needed,needed,0).
compute wcmat=make(needed,needed,0).
compute zcmat=make(needed,needed,0).
compute wzcmat=make(needed,needed,0).
compute wsum=0.
compute zsum=0.
compute wzsum=0.
end if.
end if.
do if (criterr = 0 and model <> 999).
compute modelmat= {1,0,0,0,0,0,0,1,0,0;2,0,0,0,0,0,0,1,1,0;3,0,0,0,0,0,0,1,1,1;4,0,0,0,0,0,0,0,0,0; 5,0,0,0,0,0,0,1,0,0;6,0,0,0,0,0,0,0,0,0;7,1,0,0,0,0,0,0,0,0;8,1,0,0,0,0,0,1,0,0; 9,1,1,0,0,0,0,0,0,0;10,1,1,0,0,0,0,1,1,0;11,1,1,1,0,0,0,0,0,0;12,1,1,1,0,0,0,1,1,1; 13,1,1,1,0,0,0,1,0,0;14,0,0,0,1,0,0,0,0,0;15,0,0,0,1,0,0,1,0,0;16,0,0,0,1,1,0,0,0,0; 17,0,0,0,1,1,0,1,1,0;18,0,0,0,1,1,1,0,0,0;19,0,0,0,1,1,1,1,1,1;20,0,0,0,1,1,1,1,0,0; 21,1,0,0,0,1,0,0,0,0;22,1,0,0,0,1,0,1,0,0;23,0,0,0,0,0,0,0,0,0;24,0,0,0,0,0,0,0,0,0; 25,0,0,0,0,0,0,0,0,0;26,0,0,0,0,0,0,0,0,0;27,0,0,0,0,0,0,0,0,0;28,1,0,0,0,1,0,0,1,0; 29,1,0,0,0,1,0,1,1,0;30,0,0,0,0,0,0,0,0,0;31,0,0,0,0,0,0,0,0,0;32,0,0,0,0,0,0,0,0,0; 33,0,0,0,0,0,0,0,0,0;34,0,0,0,0,0,0,0,0,0;35,0,0,0,0,0,0,0,0,0;36,0,0,0,0,0,0,0,0,0; 37,0,0,0,0,0,0,0,0,0;38,0,0,0,0,0,0,0,0,0;39,0,0,0,0,0,0,0,0,0;40,0,0,0,0,0,0,0,0,0; 41,0,0,0,0,0,0,0,0,0;42,0,0,0,0,0,0,0,0,0;43,0,0,0,0,0,0,0,0,0;44,0,0,0,0,0,0,0,0,0; 45,0,0,0,0,0,0,0,0,0;46,0,0,0,0,0,0,0,0,0;47,0,0,0,0,0,0,0,0,0;48,0,0,0,0,0,0,0

 ,0,0; 49,0,0,0,0,0,0,0,0,0;50,0,0,0,0,0,0,0,0,0;51,0,0,0,0,0,0,0,0,0;52,0,0,0,0,0,0,0,0,0; 53,0,0,0,0,0,0,0,0,0;54,0,0,0,0,0,0,0,0,0;55,0,0,0,0,0,0,0,0,0;56,0,0,0,0,0,0,0,0,0; 57,0,0,0,0,0,0,0,0,0;58,1,0,0,1,0,0,0,0,0;59,1,0,0,1,0,0,1,0,0;60,1,1,0,1,0,0,0,0,0; 61,1,1,0,1,0,0,1,0,0;62,1,1,0,1,0,0,0,1,0;63,1,1,0,1,0,0,1,1,0;64,1,0,0,1,1,0,0,0,0; 65,1,0,0,1,1,0,1,0,0;66,1,0,0,1,1,0,0,1,0;67,1,0,0,1,1,0,1,1,0;68,1,1,1,1,0,0,0,0,0; 69,1,1,1,1,0,0,1,1,1;70,1,0,0,1,1,1,0,0,0;71,1,0,0,1,1,1,1,1,1;72,1,1,1,1,1,1,0,0,0; 73,1,1,1,1,1,1,1,1,1;74,0,0,0,1,0,0,0,0,0;75,1,1,0,1,1,0,0,0,0;76,1,1,0,1,1,0,1,1,0; 77,0,0,0,0,0,0,0,0,0;78,0,0,0,0,0,0,0,0,0;79,0,0,0,0,0,0,0,0,0;80,0,0,0,0,0,0,0,0,0; 81,0,0,0,0,0,0,0,0,0;82,0,0,0,0,0,0,0,0,0;83,1,0,0,0,0,0,0,0,0;84,1,0,0,0,0,0,0,0,0; 85,1,0,0,0,0,0,1,0,0;86,1,0,0,0,0,0,1,0,0;87,0,0,0,1,0,0,0,0,0;88,0,0,0,1,0,0,0,0,0; 89,0,0,0,1,0,0,1,0,0;90,0,0,0,1,0,0,1,0,0;91,0,0,0,0,0,0,0,0,0;92,1,0,0,1,0,0,1,0,0}.
do if (model > 0).
compute tmp=modelmat(model,2:ncol(modelmat)).
end if.
do if (model=0).
compute tmp={0,0,0,0,0,0,0,0,0}.
end if.
do if (model < 4).
compute bcmat((nxs+1),1)=1.
end if.
do if ((model > 3) and (model <> 6)).
compute bcmat((nxs+1):(nxs+nms),1)=onem.
compute bcmat(nrow(bcmat),(nxs+1):(nxs+nms))=t(onem).
compute bcmat(nrow(bcmat),1)=1.
end if.
do if ((model = 6) or (model > 82 and model < 93)).
loop j = 2 to nrow(bcmat).
loop i = 1 to (j-1).
compute bcmat(j,i)=1.
end loop.
end loop.
end if.
do if (model = 80).
loop i = 1 to nms.
compute bcmat((nrow(bcmat)-1),i)=1.
end loop.
end if.
do if (model = 81).
loop j = 3 to nrow(bcmat).
compute bcmat(j,2)=1.
end loop.
end if.
do if (model = 82).
compute bcmat(3,2)=1.
compute bcmat(5,4)=1.
end if.
do if (tmp(1,1)=1).
compute wcmat((nxs+1):(nxs+nms),1)=onem.
compute wprod=1.
compute xprod=1.
do if (model = 83 or model = 86).
compute onemsx=onem.
loop i = 1 to (nms-1).
compute onemsx(i+1,1)=0.
end loop.
compute wcmat((nxs+1):(nxs+nms),1)=onemsx.
end if.
end if.
do if (tmp(1,4)=1).
compute wcmat(nrow(wcmat),(nxs+1):(nxs+nms))=t(onem).
compute wprod=1.
do if (model = 87 or model = 90).
compute onemsx=onem.
loop i = 1 to (nms-1).
compute onemsx(i,1)=0.
end loop.
compute wcmat(nrow(wcmat),(nxs+1):(nxs+nms))=t(onemsx).
end if.
end if.
do if (tmp(1,7)=1).
compute wcmat(nrow(wcmat),1)=1.
compute wprod=1.
compute xprod=1.
end if.
do if (tmp(1,2)=1).
compute zcmat((nxs+1):(nxs+nms),1)=onem.
compute zprod=1.
compute xprod=1.
end if.
do if (tmp(1,5)=1).
compute zcmat(nrow(zcmat),(nxs+1):(nxs+nms))=t(onem).
compute zprod=1.
end if.
do if (tmp(1,8)=1).
compute zcmat(nrow(zcmat),1)=1.
compute zprod=1.
compute xprod=1.
end if.
do if (tmp(1,3)=1).
compute wzcmat((nxs+1):(nxs+nms),1)=onem.
compute xprod=1.
compute wprod=1.
compute zprod=1.
end if.
do if (tmp(1,6)=1).
compute wzcmat(nrow(wzcmat),(nxs+1):(nxs+nms))=t(onem).
compute zprod=1.
compute wprod=1.
end if.
do if (tmp(1,9)=1).
compute wzcmat(nrow(wzcmat),1)=1.
compute xprod=1.
compute wprod=1.
compute zprod=1.
end if.
do if (model = 91 or model = 92).
loop j = 1 to (nms-1).
loop i = 1 to j.
compute wcmat((nxs+1+j),(nxs+i))=1.
end loop.
end loop.
end if.
do if (nms < 0).
loop i = 1 to nms.
compute tmp=csum(wcmat(:,(1+i)))+csum(zcmat(:,(1+i)))+csum(wzcmat(:,(1+i))).
compute mprod(1,i)=(tmp>0).
end loop.
end if.
end if.
do if (ncs > 0).
compute ccmat=make((nms+nys),ncs,1).
compute ccmatoff=ccmat.
do if (covmy=1).
compute ccmat(nrow(ccmat),:)=make(1,ncs,0).
end if.
do if (covmy=2).
compute ccmat(1:nms,:)=make(nms,ncs,0).
end if.
do if (cmatrix(1,1) <> -999).
do if (ncol(cmatrix) <> ((nms+nys)*ncs)).
compute errcode(errs,1)=29.
compute errs=errs+1.
compute criterr=1.
end if.
do if (criterr = 0).
compute tmp=1.
loop i = 1 to (nms+nys).
loop j = 1 to ncs.
compute ccmat(i,j)=1-(cmatrix(1,tmp) = 0).
compute tmp=tmp+1.
end loop.
end loop.
do if (rsum((csum(ccmat)=0)) <> 0).
compute errcode(errs,1)=30.
compute errs=errs+1.
compute criterr=1.
end if.
end if.
do if (covmy <> 0).
compute notecode(notes,1)=1.
compute notes=notes+1.
end if.
end if.
do if (xmint=1 and (covmy > 0 or (csum(rsum(ccmat)) <> ((nms+nys)*ncs)))).
compute errcode(errs,1)=68.
compute errs=errs+1.
compute criterr=1.
end if.
end if.
do if (criterr=0).
compute needed=needed*(needed-1)/2.
compute nopath=0.
do if (bmatrix(1,1) <> -999).
compute tmp=1.
do if ((ncol(bmatrix) <> needed) or (csum(rsum(bmatrix))=0)).
compute errcode(errs,1)=16.
compute errs=errs+1.
compute criterr=1.
else.
loop i = 2 to nrow(bcmat).
loop j = 1 to (i-1).
compute bcmat(i,j)=1-(bmatrix(1,tmp) = 0).
compute tmp=tmp+1.
end loop.
end loop.
end if.
do if ((csum(bcmat(:,1))=0) and criterr=0).
compute errcode(errs,1)=22.
compute errs=errs+1.
compute criterr=1.
end if.
do if ((rsum(bcmat(nrow(bcmat),:))=0) and criterr=0).
compute errcode(errs,1)=23.
compute errs=errs+1.
compute criterr=1.
end if.
compute dm=0.
do if (nms > 0).
loop i = 1 to nms.
do if (((rsum(bcmat((nxs+i),:)) = 0) or (csum(bcmat(:,(nxs+i))) = 0)) and (dm=0) and (criterr=0)).
compute errcode(errs,1)=26.
compute errs=errs+1.
compute criterr=1.
compute dm=1.
end if.
end loop.
end if.
release dm.
end if.
end if.
do if (criterr=0).
do if (wmatrix(1,1) <> -999).
compute tmp=1.
do if (ncol(wmatrix) <> needed).
compute errcode(errs,1)=17.
compute errs=errs+1.
compute criterr=1.
else.
compute modelvar(1,1)='CUSTOM'.
loop i = 2 to nrow(wcmat).
loop j = 1 to (i-1).
compute wcmat(i,j)=1-(wmatrix(1,tmp) = 0).
do if ((wcmat(i,j)=1) and (bcmat(i,j)=0) and (nopath=0)).
compute errcode(errs,1)=20.
compute errs=errs+1.
compute criterr=1.
compute nopath=1.
end if.
compute tmp=tmp+1.
end loop.
end loop.
end if.
end if.
do if (zmatrix(1,1) <> -999).
compute tmp=1.
do if (ncol(zmatrix) <> needed).
compute errcode(errs,1)=18.
compute errs=errs+1.
compute criterr=1.
else.
compute modelvar(1,1)='CUSTOM'.
do if (csum(rsum(wcmat))=0 and model=999).
compute errcode(errs,1)=21.
compute errs=errs+1.
compute criterr=1.
end if.
loop i = 2 to nrow(zcmat).
loop j = 1 to (i-1).
compute zcmat(i,j)=1-(zmatrix(1,tmp) = 0).
do if ((zcmat(i,j)=1) and (bcmat(i,j)=0) and (nopath=0)).
compute errcode(errs,1)=20.
compute errs=errs+1.
compute criterr=1.
compute nopath=1.
end if.
compute tmp=tmp+1.
end loop.
end loop.
end if.
end if.
compute tmp=1.
do if (wzmatrix(1,1) <> -999).
do if (ncol(wzmatrix) <> needed).
compute errcode(errs,1)=19.
compute errs=errs+1.
compute criterr=1.
end if.
compute modelvar(1,1)='CUSTOM'.
end if.
do if (criterr=0).
loop i = 2 to nrow(wzcmat).
loop j = 1 to (i-1).
do if (wzmatrix(1,1) <> -999).
compute wzcmat(i,j)=1-(wzmatrix(1,tmp) = 0).
end if.
do if (wzcmat(i,j)=1).
compute wcmat(i,j)=1.
compute zcmat(i,j)=1.
end if.
do if ((wzcmat(i,j)=1) and (bcmat(i,j)=0) and (nopath=0)).
compute errcode(errs,1)=20.
compute errs=errs+1.
compute criterr=1.
compute nopath=1.
end if.
compute tmp=tmp+1.
end loop.
end loop.
end if.
end if.
do if (criterr=0).
compute xprod=csum(wcmat(:,1))+csum(zcmat(:,1))+csum(wzcmat(:,1)).
compute xprod=(xprod > 0).
compute wsum=csum(rsum(wcmat)).
compute wprod=(wsum > 0).
do if (nms > 0).
loop i = 1 to nms.
compute tmp=csum(wcmat(:,(1+i)))+csum(zcmat(:,(1+i)))+csum(wzcmat(:,(1+i))).
compute mprod(1,i)=(tmp>0).
end loop.
end if.
do if ((wsum > 0) and (w = 'xxxxx')).
compute errcode(errs,1)=11.
compute errs=errs+1.
compute criterr=1.
end if.
do if ((wsum = 0) and (w <> 'xxxxx')).
compute errcode(errs,1)=10.
compute errs=errs+1.
compute criterr=1.
end if.
compute zsum=csum(rsum(zcmat)).
compute zprod=(zsum > 0).
do if ((zsum > 0) and (z = 'xxxxx')).
compute errcode(errs,1)=13.
compute errs=errs+1.
compute criterr=1.
end if.
do if ((zsum = 0) and (z <> 'xxxxx')).
compute errcode(errs,1)=12.
compute errs=errs+1.
compute criterr=1.
end if.
do if ((zsum > 0) and (wsum = 0)).
compute errcode(errs,1)=35.
compute errs=errs+1.
compute criterr=1.
end if.
end if.
do if (criterr=0 and nms > 1).
compute serchk=bcmat(2:(nrow(bcmat)-1),2:ncol(bcmat)).
do if (csum(rsum(serchk))) > 0.
compute serial=1.
do if (nms > 6).
compute errcode(errs,1)=36.
compute errs=errs+1.
compute criterr=1.
end if.
end if.
end if.
do if (center > 0 and criterr=0).
compute centvar={' '}.
do if (criterr=0).
do if ((center = 1) or (center = 2 and wdich = 0)).
do if (wprod=1 and mcwok=0 and nwpval > 0).
loop i = 1 to nws.
compute wtmp(:,i)=wtmp(:,i)-(csum(wtmp(:,i))/n).
compute centvar={centvar,wnames(1,i)}.
end loop.

.
compute desctmp=make((8-(4* wmodcust )),ncol( wtmp ),-999).
loop jd=1 to ncol( wtmp ).
compute descdat= wtmp (:,jd).
compute desctmp(1,jd) = csum(descdat)/nrow(descdat).
compute desctmp(2,jd) = (nrow(descdat)*sscp(descdat))-(t(csum(descdat))*(csum(descdat))).
compute desctmp(2,jd) = sqrt(desctmp(2,jd)/(nrow(descdat)*(nrow(descdat)-1))).
compute desctmp(3,jd)=cmin(descdat).
compute desctmp(4,jd)=cmax(descdat).
do if ( wmodcust =0).
compute minwarn=0.
compute maxwarn=0.
do if ((desctmp(3,jd)=desctmp(4,jd)) and novar=0).
compute errcode(errs,1)=15.
compute errs=errs+1.
compute criterr=1.
compute novar=1.
end if.
compute tmp=((descdat(:,1)=desctmp(3,jd))+(descdat(:,1)=desctmp(4,jd))).
compute desctmp(8,jd)=(csum(tmp)=nrow(tmp)).
compute tmp = descdat.
compute tmp(GRADE(descdat),:) = descdat.
compute descdat = tmp.
release tmp.
compute decval={.16;.5;.84}.
loop kd=1 to 3.
compute low=trunc(decval(kd,1)*(nrow(descdat)+1)).
compute lowdec=decval(kd,1)*(nrow(descdat)+1)-low.
compute value=descdat(low,1)+(descdat((low+1),1)-descdat(low,1))*lowdec.
compute desctmp((4+kd),jd)=value.
end loop.
compute mnotev=1.
compute modvals=desctmp(5:7,:).
do if (quantile <> 1).
compute desctmp(5,jd)=desctmp(1,jd)-desctmp(2,jd).
compute desctmp(6,jd)=desctmp(1,jd).
compute desctmp(7,jd)=desctmp(1,jd)+desctmp(2,jd).
compute modvals=desctmp(5:7,:).
compute mnotev=2.
do if (modvals(1,1) < desctmp(3,1)).
compute modvals(1,1)=desctmp(3,1).
compute minwarn=1.
end if.
do if (modvals(3,1) > desctmp(4,1)).
compute modvals(3,1)=desctmp(4,1).
compute maxwarn=1.
end if.
end if.
do if (desctmp(8,1)=1).
compute modvals={desctmp(3,1);desctmp(4,1)}.
compute mnotev=0.
compute minwarn=0.
compute maxwarn=0.
end if.
end if.
end loop
.
compute wmin=desctmp(3,1).
compute wmax=desctmp(4,1).
do if (wmodcust=0).
compute wmodvals=modvals.
compute wprobval=wmodvals.
end if.
end if.
end if.
do if ((center = 1) or (center = 2 and zdich = 0)).
do if (zprod=1 and mczok=0 and nzpval > 0).
loop i = 1 to nzs.
compute ztmp(:,i)=ztmp(:,i)-(csum(ztmp(:,i))/n).
compute centvar={centvar,znames(1,i)}.
end loop.

.
compute desctmp=make((8-(4* zmodcust )),ncol( ztmp ),-999).
loop jd=1 to ncol( ztmp ).
compute descdat= ztmp (:,jd).
compute desctmp(1,jd) = csum(descdat)/nrow(descdat).
compute desctmp(2,jd) = (nrow(descdat)*sscp(descdat))-(t(csum(descdat))*(csum(descdat))).
compute desctmp(2,jd) = sqrt(desctmp(2,jd)/(nrow(descdat)*(nrow(descdat)-1))).
compute desctmp(3,jd)=cmin(descdat).
compute desctmp(4,jd)=cmax(descdat).
do if ( zmodcust =0).
compute minwarn=0.
compute maxwarn=0.
do if ((desctmp(3,jd)=desctmp(4,jd)) and novar=0).
compute errcode(errs,1)=15.
compute errs=errs+1.
compute criterr=1.
compute novar=1.
end if.
compute tmp=((descdat(:,1)=desctmp(3,jd))+(descdat(:,1)=desctmp(4,jd))).
compute desctmp(8,jd)=(csum(tmp)=nrow(tmp)).
compute tmp = descdat.
compute tmp(GRADE(descdat),:) = descdat.
compute descdat = tmp.
release tmp.
compute decval={.16;.5;.84}.
loop kd=1 to 3.
compute low=trunc(decval(kd,1)*(nrow(descdat)+1)).
compute lowdec=decval(kd,1)*(nrow(descdat)+1)-low.
compute value=descdat(low,1)+(descdat((low+1),1)-descdat(low,1))*lowdec.
compute desctmp((4+kd),jd)=value.
end loop.
compute mnotev=1.
compute modvals=desctmp(5:7,:).
do if (quantile <> 1).
compute desctmp(5,jd)=desctmp(1,jd)-desctmp(2,jd).
compute desctmp(6,jd)=desctmp(1,jd).
compute desctmp(7,jd)=desctmp(1,jd)+desctmp(2,jd).
compute modvals=desctmp(5:7,:).
compute mnotev=2.
do if (modvals(1,1) < desctmp(3,1)).
compute modvals(1,1)=desctmp(3,1).
compute minwarn=1.
end if.
do if (modvals(3,1) > desctmp(4,1)).
compute modvals(3,1)=desctmp(4,1).
compute maxwarn=1.
end if.
end if.
do if (desctmp(8,1)=1).
compute modvals={desctmp(3,1);desctmp(4,1)}.
compute mnotev=0.
compute minwarn=0.
compute maxwarn=0.
end if.
end if.
end loop
.
compute zmin=desctmp(3,1).
compute zmax=desctmp(4,1).
do if (zmodcust=0).
compute zmodvals=modvals.
compute zprobval=zmodvals.
end if.
end if.
end if.
do if ((center = 1) or (center = 2 and xdich = 0)).
do if (xprod=1 and mcxok=0).
loop i = 1 to nxs.
compute xtmp(:,i)=xtmp(:,i)-(csum(xtmp(:,i))/n).
compute centvar={centvar,xnames(1,i)}.
end loop.

.
compute desctmp=make((8-(4* 0 )),ncol( xtmp ),-999).
loop jd=1 to ncol( xtmp ).
compute descdat= xtmp (:,jd).
compute desctmp(1,jd) = csum(descdat)/nrow(descdat).
compute desctmp(2,jd) = (nrow(descdat)*sscp(descdat))-(t(csum(descdat))*(csum(descdat))).
compute desctmp(2,jd) = sqrt(desctmp(2,jd)/(nrow(descdat)*(nrow(descdat)-1))).
compute desctmp(3,jd)=cmin(descdat).
compute desctmp(4,jd)=cmax(descdat).
do if ( 0 =0).
compute minwarn=0.
compute maxwarn=0.
do if ((desctmp(3,jd)=desctmp(4,jd)) and novar=0).
compute errcode(errs,1)=15.
compute errs=errs+1.
compute criterr=1.
compute novar=1.
end if.
compute tmp=((descdat(:,1)=desctmp(3,jd))+(descdat(:,1)=desctmp(4,jd))).
compute desctmp(8,jd)=(csum(tmp)=nrow(tmp)).
compute tmp = descdat.
compute tmp(GRADE(descdat),:) = descdat.
compute descdat = tmp.
release tmp.
compute decval={.16;.5;.84}.
loop kd=1 to 3.
compute low=trunc(decval(kd,1)*(nrow(descdat)+1)).
compute lowdec=decval(kd,1)*(nrow(descdat)+1)-low.
compute value=descdat(low,1)+(descdat((low+1),1)-descdat(low,1))*lowdec.
compute desctmp((4+kd),jd)=value.
end loop.
compute mnotev=1.
compute modvals=desctmp(5:7,:).
do if (quantile <> 1).
compute desctmp(5,jd)=desctmp(1,jd)-desctmp(2,jd).
compute desctmp(6,jd)=desctmp(1,jd).
compute desctmp(7,jd)=desctmp(1,jd)+desctmp(2,jd).
compute modvals=desctmp(5:7,:).
compute mnotev=2.
do if (modvals(1,1) < desctmp(3,1)).
compute modvals(1,1)=desctmp(3,1).
compute minwarn=1.
end if.
do if (modvals(3,1) > desctmp(4,1)).
compute modvals(3,1)=desctmp(4,1).
compute maxwarn=1.
end if.
end if.
do if (desctmp(8,1)=1).
compute modvals={desctmp(3,1);desctmp(4,1)}.
compute mnotev=0.
compute minwarn=0.
compute maxwarn=0.
end if.
end if.
end loop
.
compute xmodvals=modvals.
compute xprobval=xmodvals.
end if.
end if.
do if (nms > 0).
loop i = 1 to nms.
do if (mprod(1,i)=1).
compute mtmp(:,i)=mtmp(:,i)-(csum(mtmp(:,i))/n).
compute centvar={centvar,mnames(1,i)}.
end if.
end loop.

.
compute desctmp=make((8-(4* 0 )),ncol( mtmp ),-999).
loop jd=1 to ncol( mtmp ).
compute descdat= mtmp (:,jd).
compute desctmp(1,jd) = csum(descdat)/nrow(descdat).
compute desctmp(2,jd) = (nrow(descdat)*sscp(descdat))-(t(csum(descdat))*(csum(descdat))).
compute desctmp(2,jd) = sqrt(desctmp(2,jd)/(nrow(descdat)*(nrow(descdat)-1))).
compute desctmp(3,jd)=cmin(descdat).
compute desctmp(4,jd)=cmax(descdat).
do if ( 0 =0).
compute minwarn=0.
compute maxwarn=0.
do if ((desctmp(3,jd)=desctmp(4,jd)) and novar=0).
compute errcode(errs,1)=15.
compute errs=errs+1.
compute criterr=1.
compute novar=1.
end if.
compute tmp=((descdat(:,1)=desctmp(3,jd))+(descdat(:,1)=desctmp(4,jd))).
compute desctmp(8,jd)=(csum(tmp)=nrow(tmp)).
compute tmp = descdat.
compute tmp(GRADE(descdat),:) = descdat.
compute descdat = tmp.
release tmp.
compute decval={.16;.5;.84}.
loop kd=1 to 3.
compute low=trunc(decval(kd,1)*(nrow(descdat)+1)).
compute lowdec=decval(kd,1)*(nrow(descdat)+1)-low.
compute value=descdat(low,1)+(descdat((low+1),1)-descdat(low,1))*lowdec.
compute desctmp((4+kd),jd)=value.
end loop.
compute mnotev=1.
compute modvals=desctmp(5:7,:).
do if (quantile <> 1).
compute desctmp(5,jd)=desctmp(1,jd)-desctmp(2,jd).
compute desctmp(6,jd)=desctmp(1,jd).
compute desctmp(7,jd)=desctmp(1,jd)+desctmp(2,jd).
compute modvals=desctmp(5:7,:).
compute mnotev=2.
do if (modvals(1,1) < desctmp(3,1)).
compute modvals(1,1)=desctmp(3,1).
compute minwarn=1.
end if.
do if (modvals(3,1) > desctmp(4,1)).
compute modvals(3,1)=desctmp(4,1).
compute maxwarn=1.
end if.
end if.
do if (desctmp(8,1)=1).
compute modvals={desctmp(3,1);desctmp(4,1)}.
compute mnotev=0.
compute minwarn=0.
compute maxwarn=0.
end if.
end if.
end loop
.
do if ((cdeval(1,1)=-999) and (model=74)).
compute medmeans=desctmp(1,:).
end if.
compute mmodvals=modvals.
compute mprobval=mmodvals.
end if.
end if.
do if (ncol(centvar) > 1).
compute notecode(notes,1)=3.
compute notes=notes+1.
end if.
end if.
do if (criterr=0).
compute wsum=rsum(csum(wcmat)).
compute zsum=rsum(csum(zcmat)).
compute wzsum=rsum(csum(wzcmat)).
compute nump=make(1,(nys+nms),-999).
compue numint=make(1,(nys+nms),0).
compute datcount=1.
compute xtmpuse=0.
compute wtmpuse=0.
compute ztmpuse=0.
compute xwtmpus=0.
compute xztmpus=0.
compute wztmpus=0.
compute xwztmpu=0.
compute xtmploc=-999.
compute wtmploc=-999.
compute xwtmplo=-999.
compute ztmploc=-999.
compute xztmplo=-999.
compute wztmplo=-999.
compute xwztmplo=-999.
compute vlabs={' '}.
do if (ncs > 0).
compute ctmpuse=make(1,ncs,0).
end if.
do if (nms > 0).
compute mtmpuse=make(1,nms,0).
compute mwtmpus=make(1,nms,0).
compute mztmpus=make(1,nms,0).
compute mwztmpu=make(1,nms,0).
compute mtmploc=make(1,nms,0).
compute mwtmplo=make(nwvls,nms,-999).
compute mztmplo=make(nzvls,nms,-999).
compute mwztmplo=make((nwvls*nzvls),nms,-999).
end if.
do if (ncs > 0).
compute ctmploc=make(1,ncs,0).
end if.
compute fulldat=make(n,1,1).
compute datindx=make(1000,(nms+nys),-999).
compute wherew=make(2,(nms+nys),-999).
compute wherex=make(2,(nms+nys),-999).
compute wherez=make(2,(nms+nys),-999).
compute wherexw=make(2,(nms+nys),-999).
compute wherexz=make(2,(nms+nys),-999).
compute wherewz=make(2,(nms+nys),-999).
compute wherexwz=make(2,(nms+nys),-999).
do if (nms > 0).
compute wherem=make(nms,(nms+nys),-999).
compute wheremw = make(nms*2,(nms+nys),-999).
compute wheremz = make(nms*2,(nms+nys),-999).
compute wheremwz = make(nms*2,(nms+nys),-999).
end if.
compute wzhigh=make(1000,(((nms+1)*(nms+2))/2),0).
compute whigh=make(1000,(((nms+1)*(nms+2))/2),0).
compute zhigh=make(1000,(((nms+1)*(nms+2))/2),0).
compute fochigh=make(1000,(((nms+1)*(nms+2))/2),0).
compute xcoefloc={1;2;3;4;5;6;7;8;9}.
compute intkey = {' ', ' ', ' ', ' ', ' ', ' ', ' '}.
compute wzhighct=0.
compute whighct=0.
compute zhighct=0.
compute foccnt=0.
loop i = 2 to nrow(bcmat).
compute wdid=0.
compute zdid=0.
compute wzdid=0.
compute cntmp=1.
compute start=1.
do if (i < nrow(bcmat)).
compute outv=mtmp(:,(i-1)).
compute modlabel={mnames(1,(i-1));'constant'}.
end if.
do if (i = nrow(bcmat)).
compute outv=ytmp.
compute modlabel={ynames;'constant'}.
end if.
loop j = 1 to (i-1).
compute foccnt=foccnt+1.
do if (j = 1 and bcmat(i,j)=1).
compute outv={outv,xtmp}.
compute modlabel={modlabel;xcatlab(1:nxvls,1)}.
do if (xtmpuse=0).
compute fulldat={fulldat,xtmp}.
compute xtmpuse=1.
loop k4=datcount to (datcount+(nxvls-1)).
compute xtmploc={xtmploc;k4}.
end loop.
compute xtmploc=xtmploc(2:nrow(xtmploc),1).
compute datcount=datcount+nxvls.
end if.
compute datindx(start:(start+nrow(xtmploc)-1),(i-1))=xtmploc.
compute wherex(1,(i-1))=start+1.
compute wherex(2,(i-1))=start+nrow(xtmploc)-1+1.
do if (model = 74).
end if.
compute onebl=make(nrow(xtmploc),1,1).
compute fochigh((start+1):(start+nrow(xtmploc)),foccnt)=onebl.
compute start=start+nrow(xtmploc).
end if.
do if (j > 1 and bcmat(i,j)=1).
compute outv={outv,mtmp(:,(j-1))}.
compute modlabel={modlabel;mnames(1,(j-1))}.
do if (mtmpuse(1,(j-1))=0).
compute fulldat={fulldat,mtmp(:,(j-1))}.
compute mtmpuse(1,(j-1))=1.
compute mtmploc(1,(j-1))=datcount.
compute datcount=datcount+1.
end if.
compute datindx(start:(start+nrow(mtmploc)-1),(i-1))=mtmploc(1,(j-1)).
compute wherem((j-1),(i-1))=start+1.
compute onebl=make(nrow(mtmploc(1,j-1)),1,1).
compute ttt=nrow(mtmploc(1,(j-1)))+start-1.
compute fochigh((start+1):(start+nrow(mtmploc(1,(j-1)))),foccnt)=onebl.
compute start=start+nrow(mtmploc(1,(j-1))).
end if.
end loop.
do if (wsum > 0).
loop j = 1 to (i-1).
compute whighct=whighct+1.
do if (j = 1 and wcmat(i,j)=1).
do if (wdid=0).
compute outv={outv,wtmp}.
do if (ncs > 0 and wiscov > 0).
compute ccmatoff((i-1),wiscov)=0.
end if.
compute modlabel={modlabel;wcatlab(1:nwvls,1)}.
compute wdid=1.
do if (wtmpuse=0).
compute fulldat={fulldat,wtmp}.
do if (ncs > 0 and wiscov > 0).
compute ccmatoff((i-1),wiscov)=0.
end if.
compute wtmpuse=1.
loop k4=datcount to (datcount+(nwvls-1)).
compute wtmploc={wtmploc;k4}.
end loop.
compute wtmploc=wtmploc(2:nrow(wtmploc),1).
compute datcount=datcount+nwvls.
end if.
end if.
compute datindx(start:(start+nrow(wtmploc)-1),(i-1))=wtmploc.
compute wherew(1,(i-1))=start+1.
compute wherew(2,(i-1))=start+nrow(wtmploc)-1+1.
compute start=start+nrow(wtmploc).
loop k1=1 to nxvls.
loop k2 = 1 to nwvls.
compute outv={outv,(xtmp(:,k1)&*wtmp(:,k2))}.
do if (ncs > 0 and wiscov > 0).
compute ccmatoff((i-1),wiscov)=0.
end if.
compute modlabel={modlabel;intlab(cntmp,1)}.
compute intkey={intkey;intlab(cntmp,1),':',xcatlab(k1,1),'x',wcatlab(k2,1),' ',' '}.
compute cntmp=cntmp+1.
end loop.
end loop.
do if (xwtmpus=0).
compute fulldat={fulldat,outv(:,(ncol(outv)-(nxvls*nwvls)+1):ncol(outv))}.
compute xwtmpus=1.
do if (ncs > 0 and wiscov > 0).
compute ccmatoff((i-1),wiscov)=0.
end if.
loop k4=datcount to (datcount+((nwvls*nxvls)-1)).
compute xwtmplo={xwtmplo;k4}.
end loop.
compute xwtmplo=xwtmplo(2:nrow(xwtmplo),1).
compute datcount=datcount+(nxvls*nwvls).
end if.
compute datindx(start:(start+nrow(xwtmplo)-1),(i-1))=xwtmplo.
compute wherexw(1,(i-1))=start+1.
compute wherexw(2,(i-1))=start+nrow(xwtmplo)-1+1.
compute onebl=make(nrow(xwtmplo),1,1).
compute whigh((start+1):(start+nrow(xwtmplo)),whighct)=onebl.
compute start=start+nrow(xwtmplo).
end if.
do if (j > 1 and wcmat(i,j)=1).
do if (wdid=0 and model <> 74).
compute outv={outv,wtmp}.
do if (ncs > 0 and wiscov > 0).
compute ccmatoff((i-1),wiscov)=0.
end if.
compute modlabel={modlabel;wcatlab(1:nwvls,1)}.
compute wdid=1.
do if (wtmpuse=0).
compute fulldat={fulldat,wtmp}.
do if (ncs > 0 and wiscov > 0).
compute ccmatoff((i-1),wiscov)=0.
end if.
compute wtmpuse=1.
loop k4=datcount to (datcount+(nwvls-1)).
compute wtmploc={wtmploc;k4}.
end loop.
compute wtmploc=wtmploc(2:nrow(wtmploc),1).
compute datcount=datcount+nwvls.
end if.
compute datindx(start:(start+nrow(wtmploc)-1),(i-1))=wtmploc.
compute wherew(1,(i-1))=start+1.
compute wherew(2,(i-1))=start+nrow(wtmploc)-1+1.
compute start=start+nrow(wtmploc).
end if.
loop k2 = 1 to nwvls.
compute outv={outv,(mtmp(:,(j-1))&*wtmp(:,k2))}.
do if (ncs > 0 and wiscov > 0).
compute ccmatoff((i-1),wiscov)=0.
end if.
compute modlabel={modlabel;intlab(cntmp,1)}.
compute intkey={intkey;intlab(cntmp,1),':', mnames(1,(j-1)),'x',wcatlab(k2,1),' ',' '}.
compute cntmp=cntmp+1.
end loop.
do if (mwtmpus(1,(j-1))=0).
compute fulldat={fulldat,outv(:,(ncol(outv)-nwvls+1):ncol(outv))}.
do if (ncs > 0 and wiscov > 0).
compute ccmatoff((i-1),wiscov)=0.
end if.
compute mwtmpus(1,(j-1))=1.
compute mw22=-999.
loop k4=datcount to (datcount+(nwvls-1)).
compute mw22={mw22;k4}.
end loop.
compute mwtmplo(:,(j-1))=mw22(2:nrow(mw22),1).
compute datcount=datcount+nwvls.
end if.
compute datindx(start:(start+nrow(mwtmplo)-1),(i-1))=mwtmplo(:,(j-1)).
compute wheremw(((2*j)-3),(i-1))=start+1.
compute wheremw(((2*j)-2),(i-1))=start+nrow(mwtmplo)-1+1.
compute onebl=make(nrow(mwtmplo),1,1).
compute whigh((start+1):(start+nrow(mwtmplo)),whighct)=onebl.
compute start=start+nrow(mwtmplo).
end if.
end loop.
end if.
do if (zsum > 0).
loop j = 1 to (i-1).
compute zhighct=zhighct+1.
do if (j = 1 and zcmat(i,j)=1).
do if (zdid=0).
compute outv={outv,ztmp}.
do if (ncs > 0 and ziscov > 0).
compute ccmatoff((i-1),ziscov)=0.
end if.
compute modlabel={modlabel;zcatlab(1:nzvls,1)}.
compute zdid=1.
do if (ztmpuse=0).
compute fulldat={fulldat,ztmp}.
do if (ncs > 0 and ziscov > 0).
compute ccmatoff((i-1),ziscov)=0.
end if.
compute ztmpuse=1.
loop k4=datcount to (datcount+(nzvls-1)).
compute ztmploc={ztmploc;k4}.
end loop.
compute ztmploc=ztmploc(2:nrow(ztmploc),1).
compute datcount=datcount+nzvls.
end if.
end if.
compute datindx(start:(start+nrow(ztmploc)-1),(i-1))=ztmploc.
compute wherez(1,(i-1))=start+1.
compute wherez(2,(i-1))=start+nrow(ztmploc)-1+1.
compute start=start+nrow(ztmploc).
loop k1=1 to nxvls.
loop k2 = 1 to nzvls.
compute outv={outv,(xtmp(:,k1)&*ztmp(:,k2))}.
do if (ncs > 0 and ziscov > 0).
compute ccmatoff((i-1),ziscov)=0.
end if.
compute modlabel={modlabel;intlab(cntmp,1)}.
compute intkey={intkey;intlab(cntmp,1),':',xcatlab(k1,1),'x',zcatlab(k2,1),' ',' '}.
compute cntmp=cntmp+1.
end loop.
end loop.
do if (xztmpus=0).
compute fulldat={fulldat,outv(:,(ncol(outv)-(nxvls*nzvls)+1):ncol(outv))}.
do if (ncs > 0 and ziscov > 0).
compute ccmatoff((i-1),ziscov)=0.
end if.
compute xztmpus=1.
loop k4=datcount to (datcount+((nzvls*nxvls)-1)).
compute xztmplo={xztmplo;k4}.
end loop.
compute xztmplo=xztmplo(2:nrow(xztmplo),1).
compute datcount=datcount+(nxvls*nzvls).
end if.
compute datindx(start:(start+nrow(xztmplo)-1),(i-1))=xztmplo.
compute wherexz(1,(i-1))=start+1.
compute wherexz(2,(i-1))=start+nrow(xztmplo)-1+1.
compute onebl=make(nrow(xztmplo),1,1).
compute zhigh((start+1):(start+nrow(xztmplo)),zhighct)=onebl.
compute start=start+nrow(xztmplo).
end if.
do if (j > 1 and zcmat(i,j)=1).
do if (zdid=0).
compute outv={outv,ztmp}.
do if (ncs > 0 and ziscov > 0).
compute ccmatoff((i-1),ziscov)=0.
end if.
compute modlabel={modlabel;zcatlab(1:nzvls,1)}.
compute zdid=1.
do if (ztmpuse=0).
compute fulldat={fulldat,ztmp}.
do if (ncs > 0 and ziscov > 0).
compute ccmatoff((i-1),ziscov)=0.
end if.
compute ztmpuse=1.
loop k4=datcount to (datcount+(nzvls-1)).
compute ztmploc={ztmploc;k4}.
end loop.
compute ztmploc=ztmploc(2:nrow(ztmploc),1).
compute datcount=datcount+nzvls.
end if.
compute datindx(start:(start+nrow(ztmploc)-1),(i-1))=ztmploc.
compute wherez(1,(i-1))=start+1.
compute wherez(2,(i-1))=start+nrow(ztmploc)-1+1.
compute start=start+nrow(ztmploc).
end if.
loop k2 = 1 to nzvls.
compute outv={outv,(mtmp(:,(j-1))&*ztmp(:,k2))}.
do if (ncs > 0 and ziscov > 0).
compute ccmatoff((i-1),ziscov)=0.
end if.
compute modlabel={modlabel;intlab(cntmp,1)}.
compute intkey={intkey;intlab(cntmp,1),':', mnames(1,(j-1)),'x',zcatlab(k2,1),' ',' '}.
compute cntmp=cntmp+1.
end loop.
do if (mztmpus(1,(j-1))=0).
compute fulldat={fulldat,outv(:,(ncol(outv)-nzvls+1):ncol(outv))}.
do if (ncs > 0 and ziscov > 0).
compute ccmatoff((i-1),ziscov)=0.
end if.
compute mztmpus(1,(j-1))=1.
compute mz22=-999.
loop k4=datcount to (datcount+(nzvls-1)).
compute mz22={mz22;k4}.
end loop.
compute mztmplo(:,(j-1))=mz22(2:nrow(mz22),1).
compute datcount=datcount+nzvls.
end if.
compute datindx(start:(start+nrow(mztmplo)-1),(i-1))=mztmplo(:,(j-1)).
compute wheremz(((2*j)-3),(i-1))=start+1.
compute wheremz(((2*j)-2),(i-1))=start+nrow(mztmplo)-1+1.
compute onebl=make(nrow(mztmplo),1,1).
compute zhigh((start+1):(start+nrow(mztmplo)),zhighct)=onebl.
compute start=start+nrow(mztmplo).
end if.
end loop.
end if.
do if (wzsum > 0).
loop j = 1 to (i-1).
compute wzhighct=wzhighct+1.
do if (j = 1 and wzcmat(i,j)=1).
do if (wzdid=0).
loop k1=1 to nwvls.
loop k2 = 1 to nzvls.
compute outv={outv,(wtmp(:,k1)&*ztmp(:,k2))}.
do if (ncs > 0 and (ziscov > 0)).
compute ccmatoff((i-1),ziscov)=0.
end if.
do if (ncs > 0 and (wiscov > 0)).
compute ccmatoff((i-1),wiscov)=0.
end if.
compute modlabel={modlabel;intlab(cntmp,1)}.
compute intkey={intkey;intlab(cntmp,1),':',wcatlab(k1,1),'x',zcatlab(k2,1),' ',' '}.
compute cntmp=cntmp+1.
end loop.
end loop.
do if (wztmpus=0).
compute fulldat={fulldat,outv(:,(ncol(outv)-(nwvls*nzvls)+1):ncol(outv))}.
do if (ncs > 0 and (ziscov > 0)).
compute ccmatoff((i-1),ziscov)=0.
end if.
do if (ncs > 0 and (wiscov > 0)).
compute ccmatoff((i-1),wiscov)=0.
end if.
compute wztmpus=1.
loop k4=datcount to (datcount+((nwvls*nzvls)-1)).
compute wztmplo={wztmplo;k4}.
end loop.
compute wztmplo=wztmplo(2:nrow(wztmplo),1).
compute datcount=datcount+(nzvls*nwvls).
end if.
compute wzdid=1.
end if.
compute datindx(start:(start+nrow(wztmplo)-1),(i-1))=wztmplo.
compute wherewz(1,(i-1))=start+1.
compute wherewz(2,(i-1))=start+nrow(wztmplo)-1+1.
compute start=start+nrow(wztmplo).
loop k1=1 to nxvls.
loop k2=1 to nwvls.
loop k3=1 to nzvls.
compute outv={outv,(xtmp(:,k1)&*wtmp(:,k2)&*ztmp(:,k3))}.
do if (ncs > 0 and (ziscov > 0)).
compute ccmatoff((i-1),ziscov)=0.
end if.
do if (ncs > 0 and (wiscov > 0)).
compute ccmatoff((i-1),wiscov)=0.
end if.
compute modlabel={modlabel;intlab(cntmp,1)}.
compute intkey={intkey;intlab(cntmp,1),':',xcatlab(k1,1),'x',wcatlab(k2,1),'x', zcatlab(k3,1)}.
compute cntmp=cntmp+1.
end loop.
end loop.
end loop.
do if (xwztmpu=0).
compute fulldat={fulldat,outv(:,(ncol(outv)-(nxvls*nwvls*nzvls)+1):ncol(outv))}.
do if (ncs > 0 and (ziscov > 0)).
compute ccmatoff((i-1),ziscov)=0.
end if.
do if (ncs > 0 and (wiscov > 0)).
compute ccmatoff((i-1),wiscov)=0.
end if.
compute xwztmpu=1.
loop k4=datcount to (datcount+((nzvls*nxvls*nwvls)-1)).
compute xwztmplo={xwztmplo;k4}.
end loop.
compute xwztmplo=xwztmplo(2:nrow(xwztmplo),1).
compute datcount=datcount+(nxvls*nzvls*nwvls).
end if.
compute datindx(start:(start+nrow(xwztmplo)-1),(i-1))=xwztmplo.
compute wherexwz(1,(i-1))=start+1.
compute wherexwz(2,(i-1))=start+nrow(xwztmplo)-1+1.
compute onebl=make(nrow(xwztmplo),1,1).
compute wzhigh((start+1):(start+nrow(xwztmplo)),wzhighct)=onebl.
compute start=start+nrow(xwztmplo).
end if.
do if (j > 1 and wzcmat(i,j)=1).
do if (wzdid=0).
loop k1=1 to nwvls.
loop k2 = 1 to nzvls.
compute outv={outv,(wtmp(:,k1)&*ztmp(:,k2))}.
do if (ncs > 0 and (ziscov > 0)).
compute ccmatoff((i-1),ziscov)=0.
end if.
do if (ncs > 0 and (wiscov > 0)).
compute ccmatoff((i-1),wiscov)=0.
end if.
compute modlabel={modlabel;intlab(cntmp,1)}.
compute intkey={intkey;intlab(cntmp,1),':',wcatlab(k1,1),'x',zcatlab(k2,1),' ',' '}.
compute cntmp=cntmp+1.
end loop.
end loop.
do if (wztmpus=0).
compute fulldat={fulldat,outv(:,(ncol(outv)-(nwvls*nzvls)+1):ncol(outv))}.
do if (ncs > 0 and (ziscov > 0)).
compute ccmatoff((i-1),ziscov)=0.
end if.
do if (ncs > 0 and (wiscov > 0)).
compute ccmatoff((i-1),wiscov)=0.
end if.
compute wztmpus=1.
loop k4=datcount to (datcount+((nwvls*nzvls)-1)).
compute wztmplo={wztmplo;k4}.
end loop.
compute wztmplo=wztmplo(2:nrow(wztmplo),1).
compute datcount=datcount+(nzvls*nwvls).
end if.
compute wzdid=1.
compute datindx(start:(start+nrow(wztmplo)-1),(i-1))=wztmplo.
compute wherewz(1,(i-1))=start+1.
compute wherewz(2,(i-1))=start+nrow(wztmplo)-1+1.
compute start=start+nrow(wztmplo).
end if.
loop k1 = 1 to nwvls.
loop k2 = 1 to nzvls.
compute outv={outv,(mtmp(:,(j-1))&*wtmp(:,k1)&*ztmp(:,k2))}.
do if (ncs > 0 and (ziscov > 0)).
compute ccmatoff((i-1),ziscov)=0.
end if.
do if (ncs > 0 and (wiscov > 0)).
compute ccmatoff((i-1),wiscov)=0.
end if.
compute modlabel={modlabel;intlab(cntmp,1)}.
compute intkey={intkey;intlab(cntmp,1),':',mnames(1,(j-1)),'x',wcatlab(k1,1),'x', zcatlab(k2,1)}.
compute cntmp=cntmp+1.
end loop.
end loop.
do if (mwztmpu(1,(j-1))=0).
compute fulldat={fulldat,outv(:,(ncol(outv)-(nwvls*nzvls)+1):ncol(outv))}.
do if (ncs > 0 and (ziscov > 0)).
compute ccmatoff((i-1),ziscov)=0.
end if.
do if (ncs > 0 and (wiscov > 0)).
compute ccmatoff((i-1),wiscov)=0.
end if.
compute mwztmpu(1,(j-1))=1.
compute mz22=-999.
loop k4=datcount to (datcount+(nwvls*nzvls)-1).
compute mz22={mz22;k4}.
end loop.
compute mwztmplo(:,(j-1))=mz22(2:nrow(mz22),1).
compute datcount=datcount+(nwvls*nzvls).
end if.
compute datindx(start:(start+nrow(mwztmplo)-1),(i-1))=mwztmplo(:,(j-1)).
compute wheremwz(((2*j)-3),(i-1))=start+1.
compute wheremwz(((2*j)-2),(i-1))=start+nrow(mwztmplo)-1+1.
compute onebl=make(nrow(mwztmplo),1,1).
compute wzhigh((start+1):(start+nrow(mwztmplo)),wzhighct)=onebl.
compute start=start+nrow(mwztmplo).
end if.
end loop.
end if.
do if (ncs > 0).
compute ccmat=ccmat&*ccmatoff.
loop j = 1 to ncs.
do if (ccmat((i-1),j))=1.
do if (j=wiscov).
compute ctmp(:,j)=wtmp.
end if.
do if (j=ziscov).
compute ctmp(:,j)=ztmp.
end if.
compute outv={outv,ctmp(:,j)}.
compute modlabel={modlabel;covnames(1,j)}.
do if (ctmpuse(1,j)=0).
compute fulldat={fulldat,ctmp(:,j)}.
compute ctmpuse(1,j)=1.
compute ctmploc(1,j)=datcount.
compute datcount=datcount+1.
end if.
compute datindx(start:(start+nrow(ctmploc)-1),(i-1))=ctmploc(1,j).
compute start=start+nrow(ctmploc(1,j)).
end if.
end loop.
end if.
compute wdid=0.
compute zdid=0.
compute wzdid=0.
compute vlabs={vlabs;modlabel(2:nrow(modlabel),1)}.
compute numint(1,(i-1))=cntmp-1.
compute nump(1,(i-1))=nrow(modlabel)-1.
end loop.
release datcount, xtmpuse, wtmpuse, ztmpuse, xwtmpus, xztmpus, wztmpus, xwztmpu.
release xtmploc, wtmploc, xwtmplo, ztmploc, xztmplo, wztmplo, xwztmplo, foccnt.
do if (modcok=1 and ((nms > 0) or (zcmat(2,1) <> 1) or (mcx <> 0))).
compute notecode(notes,1) = 19.
compute notes = notes + 1.
compute modcok=0.
end if.
do if ((serial = 1 or (rsum(numint)>0) or nms=0) and mc > 0).
compute notecode(notes,1) = 15.
compute notes = notes + 1.
compute boot=mc.
compute mc=0.
end if.
do if (boot <> 0 or mc <> 0).
compute bootsz=boot.
do if (mc > 0).
compute bootsz=mc.
compute saveboot=0.
end if.
loop.
compute cilow = rnd(bootsz*(1-(conf/100))/2).
compute cihigh = trunc((bootsz*(conf/100)+(bootsz*(1-(conf/100))/2)))+1.
do if (cilow < 1 or cihigh > bootsz).
compute bootsz=trunc((bootsz+1000)/1000)*1000.
compute adjust = 1.
end if.
end loop if (cilow gt 0 and cihigh le bootsz).
do if (boot > 0).
compute boot=bootsz.
end if.
do if (mc > 0).
compute mc=bootsz.
end if.
do if (adjust = 1 and boot > 0).
compute notecode(notes,1) = 8.
compute notes = notes + 1.
end if.
do if (adjust = 1 and mc > 0).
compute notecode(notes,1) = 16.
compute notes = notes + 1.
end if.
end if.
compute maxboot = trunc(2*boot).
do if ( 0 > maxboot).
compute maxboot=trunc( 0 ).
end if.
do if (nms > 0).
release mtmpuse, mwtmpus, mwztmpu, mtmploc, mwtmplo, mztmplo, mwztmplo.
end if.
release wdid, zdid, wzdid, start,modlabel.
compute vlabs=vlabs(2:nrow(vlabs),1).
do if (rsum(numint) > 0).
compute intkey=intkey(2:nrow(intkey),:).
end if.
compute fulldat=fulldat(:,2:ncol(fulldat)).
compute fochigh=fochigh(1:rmax(nump),:).
compute whigh=whigh(1:rmax(nump),:).
compute zhigh=zhigh(1:rmax(nump),:).
compute wzhigh=wzhigh(1:rmax(nump),:).
compute coeffs=fochigh+whigh+zhigh+wzhigh.
compute bootloc=make(rmax(nump),ncol(nump),0).
do if (nms > 0).
compute cntmp=1.
loop i = 1 to ncol(nump).
loop j = 1 to nump(1,i).
compute bootloc(j,i)=cntmp.
compute cntmp=cntmp+1.
end loop.
end loop.
compute fochighb=make(nrow(fochigh),ncol(fochigh),0).
compute whighb=fochighb.
compute zhighb=fochighb.
compute wzhighb=fochighb.
compute thetaxmb=make(nrow(fochighb),nms,0).
compute thetaxyb=make(nrow(fochighb),1,0).
compute pathsfoc=make(nxvls,1,0).
compute cntmp=1.
loop i = 1 to (nms+nys).
loop j = 1 to i.
compute fochighb(:,cntmp)=fochigh(:,cntmp)&*bootloc(:,i).
compute whighb(:,cntmp)=whigh(:,cntmp)&*bootloc(:,i).
compute zhighb(:,cntmp)=zhigh(:,cntmp)&*bootloc(:,i).
compute wzhighb(:,cntmp)=wzhigh(:,cntmp)&*bootloc(:,i).
compute coeffsb=fochighb+whighb+zhighb+wzhighb.
do if ((i < (nms+nys)) and (j = 1)).
compute thetaxmb(:,i)=coeffsb(:,cntmp).
end if.
do if ((i = (nms+nys)) and (j = 1)).
compute thetaxyb(:,1)=coeffsb(:,cntmp).
end if.
compute cntmp=cntmp+1.
end loop.
end loop.
compute thetamyb=coeffsb(:,(ncol(coeffsb)-nms+1):ncol(coeffsb)).
do if (serial = 1).
compute thetammb=make(nrow(coeffsb),((nms*(nms-1))/2),0).
end if.
compute cntmp=1.
do if (nms > 1 and serial = 1).
loop i = 1 to (nms-1).
compute start=((i+2)*(i+1))/2.
loop j = 2 to (nms-i+1).
compute thetammb(:,cntmp)=coeffsb(:,start).
compute start=start+j+i-1.
compute cntmp=cntmp+1.
end loop.
end loop.
end if.
end if.
do if ((total = 1) and (rsum(numint)=0 or (xmint=1))).
compute dototal=1.
do if ((csum(bcmat(:,1)) <> (nms+nys)) or (rsum(bcmat(nrow(bcmat),:)) <> (nms+nys))).
compute dototal=0.
compute alttotal=1.
compute notecode(notes,1) = 12.
compute notes = notes + 1.
end if.
do if (ncs > 0).
do if ((csum(rsum(ccmat))) < (nrow(ccmat)*ncol(ccmat))).
compute dototal=0.
compute alttotal=1.
compute notecode(notes,1) = 11.
compute notes = notes + 1.
end if.
end if.
do if (model=74).
do if (xdich=0 and nxvls=1).
compute dototal=0.
compute alttotal=1.
end if.
do if ((xdich=1 or nxvls > 1) and (ncs > 0) and model=74).
compute dototal=0.
compute alttotal=1.
end if.
end if.
end if.
end if.
do if (criterr=0 and ncs > 0).
do if (rsum((csum(ccmat)=0)) <> 0).
compute errcode(errs,1)=51.
compute errs=errs+1.
compute criterr=1.
end if.
end if.
compute debug= 0.
do if (outscree=1).
print/title = '***************** PROCESS Procedure for SPSS Version 4.2 *****************'.
print/title = '          Written by Andrew F. Hayes, Ph.D.       www.afhayes.com'.
print/title = '    Documentation available in Hayes (2022). www.guilford.com/p/hayes3'/space=0.
end if.
do if (criterr=0).
compute modresid=make(n,1,99999).
do if (stand=1 and ydich=1).
compute stand=0.
end if.
compute anymod2=csum(rsum(wcmat+zcmat+wzcmat)).
do if (anymod2 > 0 and stand = 1).
compute notecode(notes,1) = 27.
compute notes = notes + 1.
compute stand=0.
end if.
compute funny=1.
do if (outscree=1).
print modelvar/title = '**************************************************************************'/format = A8/rnames=modelvlb.
do if (ncs > 0).
print covnames/title='Covariates:'/format=A8.
end if.
print n/title='Sample'/rlabel='Size:'.
do if (( 'random' <> 'random')).
compute seedt= 'random'.
print seedt/title='Custom'/format=A12/rlabel = 'Seed:'.
end if.
end if.
compute maxresm=9.
compute resultm=make(1,maxresm,99999).
do if (describe=1).
compute means=csum(dat)/n.
compute sigmatal = (t(dat)*(ident(n)-(1/n)*ones*t(ones))*dat)*(1/(n-1)).
compute sdvec=sqrt(diag(sigmatal)).
compute sdall = mdiag(1/sdvec).
compute corall=sdall*sigmatal*t(sdall).
compute means={means;t(sdvec)}.
do if (ncol(means) > 9).
compute resultm=make(1,ncol(means),99999).
compute maxresm=ncol(means).
end if.
compute resultm2=make(2,maxresm,99999).
compute resultm2(1:2,1:ncol(means))=means.
compute resultm={resultm;resultm2}.
compute resultm2=make(ncol(corall),maxresm,99999).
compute resultm2(1:ncol(corall),1:ncol(corall))=corall.
compute resultm={resultm;resultm2}.
do if (outscree=1).
print means/title='Variable means and standard deviations'/cnames=varnames/rlabels='Mean','SD'/format= F10.4.
print corall/title='Variable intercorrelations (Pearson r)'/cnames=varnames/rnames=varnames/format= F10.4.
end if.
end if.
do if (outscree=1).
do if (mcxok=1).
compute labtmp={xnames,t(xcatlab(1:nxvls,1))}.
print dummatx/title = 'Coding of categorical X variable for analysis:'/cnames = labtmp/format = F6.3.
end if.
do if (mcwok=1 and xmint=0).
compute labtmp={wnames,t(wcatlab(1:nwvls,1))}.
print dummatw/title = 'Coding of categorical W variable for analysis:'/cnames = labtmp/format = F6.3.
end if.
do if (mczok=1).
compute labtmp={znames,t(zcatlab(1:nzvls,1))}.
print dummatz/title = 'Coding of categorical Z variable for analysis:'/cnames = labtmp/format = F6.3.
end if.
end if.
end if.
do if (criterr = 0).
compute outnames=ynames.
compute outvars=ytmp.
do if (nms > 0).
compute outnames={mnames,ynames}.
compute outvars={mtmp,ytmp}.
compute indcov=make(((nms*2)+(nms*(nxvls-1))),((nms*2)+(nms*(nxvls-1))),0).
compute mcsopath=make(((nms*2)+(nms*(nxvls-1))),1,0).
end if.
compute labstart=1.
compute intstart=1.
compute start=1.
compute coeffmat=make(1,6,0).
compute conseq={'        '}.
compute dfmat=0.
compute coeffcol=0.
compute pathscnt=1.
compute pathscn2=1.
loop i = 1 to (nms+nys).
do if (outscree=1).
print/title = '**************************************************************************'.
end if.
compute highf=make(1,5,0).
compute highf2=highf.
do if ((i = (nms+nys)) and (ydich=1)).
compute highf=make(1,3,0).
compute highf2=highf.
end if.
compute flabel={' '}.
compute y=outvars(:,i).
compute xindx=datindx(1:(nump(1,i)-1),i).
compute x = fulldat(:,xindx).
compute x={ones,x}.
compute xsq=t(x)*x.
compute exsq=eval(xsq).
release xsq.
compute zeroeig=csum(exsq <= 0.000000000002).
do if (outscree=1).
print outnames(1,i)/title = 'OUTCOME VARIABLE:'/format = A8/space=0.
do if (ydich=1 and (i = (nms+nys))).
compute nmsd = {outnames(1,i), 'Analysis'}.
print rcd/title = 'Coding of binary Y for logistic regression analysis:'/cnames = nmsd/format = F9.2.
end if.
end if.
do if (zeroeig > 0).
print / title = 'SINGULAR OR NEAR SINGULAR DATA MATRIX.'.
compute criterr=1.
compute errcode(errs,1)=31.
compute errs=errs+1.
end if.
compute means=csum(x)/n.
compute vlabsm=vlabs(labstart:(labstart+(nump(1,i)-1)),1).
do if (criterr=0).
do if (ydich=0 or (i < (nms+nys))).

.
do if ( 1 =1).
compute hatmat=inv(t( x )* x )*t( x ).
compute b = hatmat* y.
compute modres=b.
do if ( 1 =1).
compute n1=nrow( x ).
compute dfres=n1-(ncol( x )).
compute sstotal = t( y -(csum( y )/n1))*( y -(csum( y )/n1)).
compute resid= y - x *b.
compute ssresid = csum((resid)&**2).
compute r2 = (sstotal-ssresid)/sstotal.
do if (r2 < 0).
compute r2=0.
end if.
compute adjr2 = 1-((1-r2)*(n1-1)/(dfres)).
compute mse=ssresid/(n1-ncol( x )).

.
compute n1=nrow( x ).
compute invXtX = inv(t( x )* x ).
compute varb = mse *invXtX.
compute k3 = ncol( x ).
compute xhc=0.
do if ( hc <> 5).
compute xhc= x.
compute hat = xhc(:,1).
loop i3=1 to nrow(xhc).
compute hat(i3,1)= xhc(i3,:)*invXtX*t(xhc(i3,:)).
end loop.
do if ( hc = 0 or hc =1).
loop i3 = 1 to k3.
compute xhc(:,i3)=xhc(:,i3)&* resid.
end loop.
end if.
do if ( hc =3 or hc =2).
loop i3=1 to k3.
compute xhc(:,i3) = ( resid &/(1-hat)&**(1/(4- hc )))&*xhc(:,i3).
end loop.
end if.
do if ( hc = 4).
compute hcmn=make(n,2,4).
compute hcmn(:,2)=(n1*hat)/k3.
loop i3= 1 to k3.
compute xhc(:,i3) = ( resid &/(1-hat)&**(rmin(hcmn)/2))&*xhc(:,i3).
end loop.
end if.
compute varb=(invXtX*t(xhc)*xhc*invXtX).
do if ( hc =1).
compute varb=(n1/(n1-ncol( x )))&*varb.
end if.
end if.
compute hclab={'se(HC0)','se(HC1)','se(HC2)','se(HC3)','se(HC4)','se'}.
compute hclab=hclab(1,( hc +1)).
compute hcflab={'F(HC0)','F(HC1)','F(HC2)','F(HC3)','F(HC4)','F'}.
compute hcflab=hcflab(1,( hc +1)).
release xhc
.
compute seb=sqrt(diag(varb)).
compute trat = b&/seb.
compute p = 2*(1-tcdf(abs(trat), (dfres))).
compute tval = sqrt(dfres* (exp((dfres-(5/6))*((xp2/(dfres-(2/3)+(.11/dfres)))* (xp2/(dfres-(2/3)+(.11/dfres)))))-1)).
compute modres={modres,seb,trat,p}.
compute modres={modres,(b-tval&*seb),(b+tval&*seb)}.
compute modresl={'coeff',hclab,'t','p','LLCI','ULCI'}.
compute lmat = ident(ncol( x )).
compute lmat = lmat(:,2:ncol(lmat)).
compute fratio = (t(t(lmat)*b)*inv(t(lmat)*varb*lmat)*((t(lmat)*b)))/(ncol( x )-1).
compute pfr = 1-fcdf(fratio,(ncol( x )-1),dfres).
compute modsum={sqrt(r2),r2,mse,fratio,(ncol( x )-1),dfres,pfr}.
compute modsuml={'R','R-sq','MSE',hcflab,'df1','df2', 'p'}.
end if.
end if.
do if ( 1 = 2 or 1 =3).
compute xlp= x.
compute ylp= y.
compute pt2 = make(nrow(ylp),1,(csum(ylp)/nrow(ylp))).
do if ( 1 =2).
compute LL3 = ylp&*ln(pt2)+(1-ylp)&*ln(1-pt2).
end if.
compute LL3 = -2*csum(LL3).
compute bt1 = make(ncol(xlp),1,0).
compute LL1 = 0.
compute pt1 = make(nrow(ylp),1,0.5).
compute pt1lp=pt1.
loop jjj = 1 to iterate.
compute xlptmp=t(xlp).
compute vecprb=pt1lp&*(1-pt1lp).
loop kkk=1 to ncol(xlp).
compute xlptmp(kkk,:)=xlptmp(kkk,:)&*t(vecprb).
end loop.
compute b = bt1+inv(xlptmp*xlp)*t(xlp)*(ylp-pt1lp).
do if ( 1 =2).
compute xlpb=xlp*b.
compute xlpbt=(xlpb > -709.7).
compute xlpb709=(1-xlpbt)*(-709.7).
compute xlpb=(xlpb&*xlpbt)+xlpb709.
compute pt1lp = 1/(1+exp(-(xlpb))).
end if.
compute itprob = csum((pt1lp < .00000001) or (pt1lp > .9999999)).
do if (itprob > 0).
loop kkk = 1 to nrow(pt1lp).
do if (pt1lp(kkk,1) > .9999999).
compute pt1lp(kkk,1) = .9999999.
end if.
do if (pt1lp(kkk,1) < .00000001).
compute pt1lp(kkk,1) = .00000001.
end if.
end loop.
compute itprob = 0.
end if.
do if (itprob = 0).
do if ( 1 =2).
compute LL = ylp&*ln(pt1lp)+(1-ylp)&*ln(1-pt1lp).
end if.
compute LL2 = -2*csum(ll).
end if.
do if (abs(LL1-LL2) < converge).
do if ( 1 =1).
compute xlptmp=t(xlp).
compute vecprb=pt1lp&*(1-pt1lp).
loop kkk=1 to ncol(xlp).
compute xlptmp(kkk,:)=xlptmp(kkk,:)&*t(vecprb).
end loop.
compute varb = inv(xlptmp*xlp).
compute seb = sqrt(diag(varb)).
release xlptmp.
end if.
break.
end if.
compute bt1 = b.
compute LL1 = LL2.
end loop.
compute modres=b.
do if (jjj > iterate).
compute itprob = 2.
do if (booting=0).
compute iterrmod=1.
end if.
do if (booting=1).
compute bootiter=1.
end if.
do if (itprobtg=0).
compute itprobtg=1.
compute errcode(errs,1) = 47.
compute errs = errs + 1.
do if (booting = 0 and 1 =1).
compute vt1 = mdiag(pt1lp&*(1-pt1lp)).
compute varb = inv(t(xlp)*vt1*xlp).
compute seb = sqrt(diag(varb)).
end if.
end if.
end if.
do if ( 1 =1).
compute trat = b&/seb.
compute dfres=nrow(xlp).
compute p = 2*(1-cdfnorm(abs(trat))).
compute modres={modres,seb,trat,p}.
compute modres={modres,(b-xp2&*seb),(b+xp2&*seb)}.
compute pvchi=1-chicdf((LL3-LL2),(nrow(modres)-1)).
compute mcF = (LL3-LL2)/LL3.
compute cox = 1-exp(-(LL3-LL2)/nrow(xlp)).
compute nagel = cox/(1-exp(-(LL3)/nrow(xlp))).
compute modsum={LL2,(LL3-LL2),(nrow(modres)-1),pvchi, mcF,cox,nagel}.
compute modsuml={'-2LL','ModelLL', 'df', 'p', 'McFadden', 'CoxSnell', 'Nagelkrk'}.
compute modresl={'coeff','se','Z','p','LLCI','ULCI'}.
end if.
end if
.
compute dfmatt=make(nrow(modres),1,modsum(1,6)).
compute modresid={modresid,resid}.
end if.
do if (ydich=1 and (i = (nms+nys))).

.
do if ( 2 =1).
compute hatmat=inv(t( x )* x )*t( x ).
compute b = hatmat* y.
compute modres=b.
do if ( 1 =1).
compute n1=nrow( x ).
compute dfres=n1-(ncol( x )).
compute sstotal = t( y -(csum( y )/n1))*( y -(csum( y )/n1)).
compute resid= y - x *b.
compute ssresid = csum((resid)&**2).
compute r2 = (sstotal-ssresid)/sstotal.
do if (r2 < 0).
compute r2=0.
end if.
compute adjr2 = 1-((1-r2)*(n1-1)/(dfres)).
compute mse=ssresid/(n1-ncol( x )).

.
compute n1=nrow( x ).
compute invXtX = inv(t( x )* x ).
compute varb = mse *invXtX.
compute k3 = ncol( x ).
compute xhc=0.
do if ( hc <> 5).
compute xhc= x.
compute hat = xhc(:,1).
loop i3=1 to nrow(xhc).
compute hat(i3,1)= xhc(i3,:)*invXtX*t(xhc(i3,:)).
end loop.
do if ( hc = 0 or hc =1).
loop i3 = 1 to k3.
compute xhc(:,i3)=xhc(:,i3)&* resid.
end loop.
end if.
do if ( hc =3 or hc =2).
loop i3=1 to k3.
compute xhc(:,i3) = ( resid &/(1-hat)&**(1/(4- hc )))&*xhc(:,i3).
end loop.
end if.
do if ( hc = 4).
compute hcmn=make(n,2,4).
compute hcmn(:,2)=(n1*hat)/k3.
loop i3= 1 to k3.
compute xhc(:,i3) = ( resid &/(1-hat)&**(rmin(hcmn)/2))&*xhc(:,i3).
end loop.
end if.
compute varb=(invXtX*t(xhc)*xhc*invXtX).
do if ( hc =1).
compute varb=(n1/(n1-ncol( x )))&*varb.
end if.
end if.
compute hclab={'se(HC0)','se(HC1)','se(HC2)','se(HC3)','se(HC4)','se'}.
compute hclab=hclab(1,( hc +1)).
compute hcflab={'F(HC0)','F(HC1)','F(HC2)','F(HC3)','F(HC4)','F'}.
compute hcflab=hcflab(1,( hc +1)).
release xhc
.
compute seb=sqrt(diag(varb)).
compute trat = b&/seb.
compute p = 2*(1-tcdf(abs(trat), (dfres))).
compute tval = sqrt(dfres* (exp((dfres-(5/6))*((xp2/(dfres-(2/3)+(.11/dfres)))* (xp2/(dfres-(2/3)+(.11/dfres)))))-1)).
compute modres={modres,seb,trat,p}.
compute modres={modres,(b-tval&*seb),(b+tval&*seb)}.
compute modresl={'coeff',hclab,'t','p','LLCI','ULCI'}.
compute lmat = ident(ncol( x )).
compute lmat = lmat(:,2:ncol(lmat)).
compute fratio = (t(t(lmat)*b)*inv(t(lmat)*varb*lmat)*((t(lmat)*b)))/(ncol( x )-1).
compute pfr = 1-fcdf(fratio,(ncol( x )-1),dfres).
compute modsum={sqrt(r2),r2,mse,fratio,(ncol( x )-1),dfres,pfr}.
compute modsuml={'R','R-sq','MSE',hcflab,'df1','df2', 'p'}.
end if.
end if.
do if ( 2 = 2 or 2 =3).
compute xlp= x.
compute ylp= y.
compute pt2 = make(nrow(ylp),1,(csum(ylp)/nrow(ylp))).
do if ( 2 =2).
compute LL3 = ylp&*ln(pt2)+(1-ylp)&*ln(1-pt2).
end if.
compute LL3 = -2*csum(LL3).
compute bt1 = make(ncol(xlp),1,0).
compute LL1 = 0.
compute pt1 = make(nrow(ylp),1,0.5).
compute pt1lp=pt1.
loop jjj = 1 to iterate.
compute xlptmp=t(xlp).
compute vecprb=pt1lp&*(1-pt1lp).
loop kkk=1 to ncol(xlp).
compute xlptmp(kkk,:)=xlptmp(kkk,:)&*t(vecprb).
end loop.
compute b = bt1+inv(xlptmp*xlp)*t(xlp)*(ylp-pt1lp).
do if ( 2 =2).
compute xlpb=xlp*b.
compute xlpbt=(xlpb > -709.7).
compute xlpb709=(1-xlpbt)*(-709.7).
compute xlpb=(xlpb&*xlpbt)+xlpb709.
compute pt1lp = 1/(1+exp(-(xlpb))).
end if.
compute itprob = csum((pt1lp < .00000001) or (pt1lp > .9999999)).
do if (itprob > 0).
loop kkk = 1 to nrow(pt1lp).
do if (pt1lp(kkk,1) > .9999999).
compute pt1lp(kkk,1) = .9999999.
end if.
do if (pt1lp(kkk,1) < .00000001).
compute pt1lp(kkk,1) = .00000001.
end if.
end loop.
compute itprob = 0.
end if.
do if (itprob = 0).
do if ( 2 =2).
compute LL = ylp&*ln(pt1lp)+(1-ylp)&*ln(1-pt1lp).
end if.
compute LL2 = -2*csum(ll).
end if.
do if (abs(LL1-LL2) < converge).
do if ( 1 =1).
compute xlptmp=t(xlp).
compute vecprb=pt1lp&*(1-pt1lp).
loop kkk=1 to ncol(xlp).
compute xlptmp(kkk,:)=xlptmp(kkk,:)&*t(vecprb).
end loop.
compute varb = inv(xlptmp*xlp).
compute seb = sqrt(diag(varb)).
release xlptmp.
end if.
break.
end if.
compute bt1 = b.
compute LL1 = LL2.
end loop.
compute modres=b.
do if (jjj > iterate).
compute itprob = 2.
do if (booting=0).
compute iterrmod=1.
end if.
do if (booting=1).
compute bootiter=1.
end if.
do if (itprobtg=0).
compute itprobtg=1.
compute errcode(errs,1) = 47.
compute errs = errs + 1.
do if (booting = 0 and 1 =1).
compute vt1 = mdiag(pt1lp&*(1-pt1lp)).
compute varb = inv(t(xlp)*vt1*xlp).
compute seb = sqrt(diag(varb)).
end if.
end if.
end if.
do if ( 1 =1).
compute trat = b&/seb.
compute dfres=nrow(xlp).
compute p = 2*(1-cdfnorm(abs(trat))).
compute modres={modres,seb,trat,p}.
compute modres={modres,(b-xp2&*seb),(b+xp2&*seb)}.
compute pvchi=1-chicdf((LL3-LL2),(nrow(modres)-1)).
compute mcF = (LL3-LL2)/LL3.
compute cox = 1-exp(-(LL3-LL2)/nrow(xlp)).
compute nagel = cox/(1-exp(-(LL3)/nrow(xlp))).
compute modsum={LL2,(LL3-LL2),(nrow(modres)-1),pvchi, mcF,cox,nagel}.
compute modsuml={'-2LL','ModelLL', 'df', 'p', 'McFadden', 'CoxSnell', 'Nagelkrk'}.
compute modresl={'coeff','se','Z','p','LLCI','ULCI'}.
end if.
end if
.
compute dfmatt=make(nrow(modres),1,-999).
end if.
compute resultm2=make(1,maxresm,99999).
compute resultm2(1,1:ncol(modsum))=modsum.
compute resultm={resultm;resultm2}.
compute resultm2=make(nrow(modres),maxresm,99999).
compute resultm2(1:nrow(modres),1:ncol(modres))=modres.
compute resultm={resultm;resultm2}.
compute obscoeff={obscoeff,t(b)}.
do if (outscree=1).
print modsum/title = 'Model Summary'/cnames = modsuml/format= F10.4.
print modres/title='Model'/rnames=vlabsm/cnames=modresl/format= F10.4.
end if.
compute basemod=modsum(1,1).
compute basemodx=basemod.
do if (ydich=1 and (i = (nms+nys))).
do if (outscree=1).
print/title='These results are expressed in a log-odds metric.'.
end if.
compute notecode(notes,1) = 26.
compute notes = notes + 1.
end if.
compute coeffmat={coeffmat;modres}.
compute conseqt=make(nrow(modres),1,outnames(1,i)).
compute conseq={conseq;conseqt}.
compute dfmat={dfmat;dfmatt}.
compute labstart=labstart+nump(1,i).
do if (stand=1).
compute predsd=make(nrow(modres),1,0).
compute stdmod=modres(:,1)&/ovsd(1,i).
loop jd=1 to ncol(x).
compute descdat=x(:,jd).
compute predsd(jd,1) = (nrow(descdat)*sscp(descdat))-(t(csum(descdat))*(csum(descdat))).
compute predsd(jd,1) = sqrt(predsd(jd,1)/(nrow(descdat)*(nrow(descdat)-1))).
end loop.
do if (wherex(1,i) <> -999 and ((nxvls > 1) or (xdich=1))).
compute sdmsone=make(nxvls,1,1).
compute predsd(wherex(1,i):wherex(2,i),1)=sdmsone.
compute pstog=1.
end if.
compute predsd(1,1)=1.
compute stdmod=stdmod&*predsd.
compute stdmod=stdmod(2:nrow(stdmod),1).
compute sdvlabs=vlabsm(2:nrow(vlabsm),1).
compute resultm2=make(nrow(stdmod),maxresm,99999).
compute resultm2(1:nrow(stdmod),1:ncol(stdmod))=stdmod.
compute resultm={resultm;resultm2}.
do if (outscree=1).
print stdmod/title='Standardized coefficients'/clabels='coeff'/rnames=sdvlabs/format= F10.4.
end if.
end if.
do if (nms > 0 and serial = 0 and (rsum(numint) = 0) and (normal=1 or mc > 0)).
do if (i < (nms+nys)).
compute indcov((((i-1)*nxvls)+1):(i*nxvls),(((i-1)*nxvls)+1):(i*nxvls))=varb(2:(1+nxvls), 2:(1+nxvls)).
compute mcsopath((((i-1)*nxvls)+1):(i*nxvls) ,1)=modres(2:(1+nxvls),1).
end if.
do if (i = (nms+nys)).
compute atm=ncol(wherem).
compute indcov(((nms*nxvls)+1):nrow(mcsopath),((nms*nxvls)+1):nrow(mcsopath))= varb(wherem(1,atm):(wherem(1,atm)+nms-1),wherem(1,atm):(wherem(1,atm)+nms-1)).
compute mcsopath(((nms*nxvls)+1):nrow(mcsopath),1)=modres(wherem(1,atm):(wherem(1,atm)+ nms-1),1).
compute sobelok=1.
end if.
end if.
compute obsdirfx=make(1,nxvls,0).
compute dirzes=make(1,nxvls,0).
do if ((i = (nms+nys)) and (bcmat(nrow(bcmat),1)=1)).
compute direff=modres(2:(1+nxvls),:).
compute obsdirfx=t(direff(:,1)).
compute direfflb=modresl.
compute direffl2=vlabsm(2:(1+nxvls),:).
compute lmat=make(nrow(b),1,0).
compute lmat2=make(nxvls,1,1).
compute lmat(2:(1+nxvls),1)=lmat2.
do if (ydich <> 1).

.
compute lmat2= lmat.
do if ( 0 =0).
compute lmat2 = mdiag( lmat ).
compute lmat3=make(nrow(lmat2),1,0).
loop flp=1 to ncol(lmat2).
do if (csum(lmat2(:,flp))=1).
compute lmat3={lmat3,lmat2(:,flp)}.
end if.
end loop.
compute lmat2=lmat3(:,2:ncol(lmat3)).
end if.
compute fratio = (t(t(lmat2)* b )*inv(t(lmat2)* varb *lmat2)*((t(lmat2)* b )))/ncol(lmat2).
compute pfr = 1-fcdf(fratio,ncol(lmat2),(n-nrow( b ))).
compute fresult={fratio,ncol(lmat2),(n-nrow( b )),pfr}.
do if (i = (nms+nys) and (ydich=1)).
compute fratio=fratio*ncol(lmat2).
compute pfr=1-chicdf(fratio,ncol(lmat2)).
compute fresult={fratio,ncol(lmat2),pfr}.
end if.
do if ( 1 =1).
compute lmat3=1-rsum(lmat2).
compute xfm=make(n,csum(lmat3),0).
compute flpc=1.
loop flp=1 to nrow(lmat3).
do if (lmat3(flp,1)=1).
compute xfm(:,flpc)=x(:,flp).
compute flpc=flpc+1.
end if.
end loop.
compute bfm=inv(t(xfm)*xfm)*t(xfm)*y.
compute resid=y-(xfm*bfm).
compute sstotal=(y-(csum(y)/n)).
compute sstotal=csum(sstotal&*sstotal).
compute ssresid=csum(resid&*resid).
compute rsqch= r2 -((sstotal-ssresid)/sstotal).
compute fresult={rsqch,fresult}.
release xfm,flpc, resid, ssresid, bfm.
end if
.
compute diromni=fresult.
end if.
do if (ydich = 1 ).

.
compute btemphld=b.
compute llrdat=make(nrow(x),nrow( lmat )-csum( lmat ),-999).
compute llrdf=ncol(x)-ncol(llrdat).
compute llrcnt=0.
loop llri=1 to nrow( lmat ).
do if ( lmat (llri,1)=0).
compute llrcnt=llrcnt+1.
compute llrdat(:,llrcnt)=x(:,llri).
end if.
end loop.

.
do if ( 2 =1).
compute hatmat=inv(t( llrdat )* llrdat )*t( llrdat ).
compute b = hatmat* y.
compute modres=b.
do if ( 0 =1).
compute n1=nrow( llrdat ).
compute dfres=n1-(ncol( llrdat )).
compute sstotal = t( y -(csum( y )/n1))*( y -(csum( y )/n1)).
compute resid= y - llrdat *b.
compute ssresid = csum((resid)&**2).
compute r2 = (sstotal-ssresid)/sstotal.
do if (r2 < 0).
compute r2=0.
end if.
compute adjr2 = 1-((1-r2)*(n1-1)/(dfres)).
compute mse=ssresid/(n1-ncol( llrdat )).

.
compute n1=nrow( x ).
compute invXtX = inv(t( x )* x ).
compute varb = mse *invXtX.
compute k3 = ncol( x ).
compute xhc=0.
do if ( hc <> 5).
compute xhc= x.
compute hat = xhc(:,1).
loop i3=1 to nrow(xhc).
compute hat(i3,1)= xhc(i3,:)*invXtX*t(xhc(i3,:)).
end loop.
do if ( hc = 0 or hc =1).
loop i3 = 1 to k3.
compute xhc(:,i3)=xhc(:,i3)&* resid.
end loop.
end if.
do if ( hc =3 or hc =2).
loop i3=1 to k3.
compute xhc(:,i3) = ( resid &/(1-hat)&**(1/(4- hc )))&*xhc(:,i3).
end loop.
end if.
do if ( hc = 4).
compute hcmn=make(n,2,4).
compute hcmn(:,2)=(n1*hat)/k3.
loop i3= 1 to k3.
compute xhc(:,i3) = ( resid &/(1-hat)&**(rmin(hcmn)/2))&*xhc(:,i3).
end loop.
end if.
compute varb=(invXtX*t(xhc)*xhc*invXtX).
do if ( hc =1).
compute varb=(n1/(n1-ncol( x )))&*varb.
end if.
end if.
compute hclab={'se(HC0)','se(HC1)','se(HC2)','se(HC3)','se(HC4)','se'}.
compute hclab=hclab(1,( hc +1)).
compute hcflab={'F(HC0)','F(HC1)','F(HC2)','F(HC3)','F(HC4)','F'}.
compute hcflab=hcflab(1,( hc +1)).
release xhc
.
compute seb=sqrt(diag(varb)).
compute trat = b&/seb.
compute p = 2*(1-tcdf(abs(trat), (dfres))).
compute tval = sqrt(dfres* (exp((dfres-(5/6))*((xp2/(dfres-(2/3)+(.11/dfres)))* (xp2/(dfres-(2/3)+(.11/dfres)))))-1)).
compute modres={modres,seb,trat,p}.
compute modres={modres,(b-tval&*seb),(b+tval&*seb)}.
compute modresl={'coeff',hclab,'t','p','LLCI','ULCI'}.
compute lmat = ident(ncol( llrdat )).
compute lmat = lmat(:,2:ncol(lmat)).
compute fratio = (t(t(lmat)*b)*inv(t(lmat)*varb*lmat)*((t(lmat)*b)))/(ncol( llrdat )-1).
compute pfr = 1-fcdf(fratio,(ncol( llrdat )-1),dfres).
compute modsum={sqrt(r2),r2,mse,fratio,(ncol( llrdat )-1),dfres,pfr}.
compute modsuml={'R','R-sq','MSE',hcflab,'df1','df2', 'p'}.
end if.
end if.
do if ( 2 = 2 or 2 =3).
compute xlp= llrdat.
compute ylp= y.
compute pt2 = make(nrow(ylp),1,(csum(ylp)/nrow(ylp))).
do if ( 2 =2).
compute LL3 = ylp&*ln(pt2)+(1-ylp)&*ln(1-pt2).
end if.
compute LL3 = -2*csum(LL3).
compute bt1 = make(ncol(xlp),1,0).
compute LL1 = 0.
compute pt1 = make(nrow(ylp),1,0.5).
compute pt1lp=pt1.
loop jjj = 1 to iterate.
compute xlptmp=t(xlp).
compute vecprb=pt1lp&*(1-pt1lp).
loop kkk=1 to ncol(xlp).
compute xlptmp(kkk,:)=xlptmp(kkk,:)&*t(vecprb).
end loop.
compute b = bt1+inv(xlptmp*xlp)*t(xlp)*(ylp-pt1lp).
do if ( 2 =2).
compute xlpb=xlp*b.
compute xlpbt=(xlpb > -709.7).
compute xlpb709=(1-xlpbt)*(-709.7).
compute xlpb=(xlpb&*xlpbt)+xlpb709.
compute pt1lp = 1/(1+exp(-(xlpb))).
end if.
compute itprob = csum((pt1lp < .00000001) or (pt1lp > .9999999)).
do if (itprob > 0).
loop kkk = 1 to nrow(pt1lp).
do if (pt1lp(kkk,1) > .9999999).
compute pt1lp(kkk,1) = .9999999.
end if.
do if (pt1lp(kkk,1) < .00000001).
compute pt1lp(kkk,1) = .00000001.
end if.
end loop.
compute itprob = 0.
end if.
do if (itprob = 0).
do if ( 2 =2).
compute LL = ylp&*ln(pt1lp)+(1-ylp)&*ln(1-pt1lp).
end if.
compute LL2 = -2*csum(ll).
end if.
do if (abs(LL1-LL2) < converge).
do if ( 0 =1).
compute xlptmp=t(xlp).
compute vecprb=pt1lp&*(1-pt1lp).
loop kkk=1 to ncol(xlp).
compute xlptmp(kkk,:)=xlptmp(kkk,:)&*t(vecprb).
end loop.
compute varb = inv(xlptmp*xlp).
compute seb = sqrt(diag(varb)).
release xlptmp.
end if.
break.
end if.
compute bt1 = b.
compute LL1 = LL2.
end loop.
compute modres=b.
do if (jjj > iterate).
compute itprob = 2.
do if (booting=0).
compute iterrmod=1.
end if.
do if (booting=1).
compute bootiter=1.
end if.
do if (itprobtg=0).
compute itprobtg=1.
compute errcode(errs,1) = 47.
compute errs = errs + 1.
do if (booting = 0 and 0 =1).
compute vt1 = mdiag(pt1lp&*(1-pt1lp)).
compute varb = inv(t(xlp)*vt1*xlp).
compute seb = sqrt(diag(varb)).
end if.
end if.
end if.
do if ( 0 =1).
compute trat = b&/seb.
compute dfres=nrow(xlp).
compute p = 2*(1-cdfnorm(abs(trat))).
compute modres={modres,seb,trat,p}.
compute modres={modres,(b-xp2&*seb),(b+xp2&*seb)}.
compute pvchi=1-chicdf((LL3-LL2),(nrow(modres)-1)).
compute mcF = (LL3-LL2)/LL3.
compute cox = 1-exp(-(LL3-LL2)/nrow(xlp)).
compute nagel = cox/(1-exp(-(LL3)/nrow(xlp))).
compute modsum={LL2,(LL3-LL2),(nrow(modres)-1),pvchi, mcF,cox,nagel}.
compute modsuml={'-2LL','ModelLL', 'df', 'p', 'McFadden', 'CoxSnell', 'Nagelkrk'}.
compute modresl={'coeff','se','Z','p','LLCI','ULCI'}.
end if.
end if
.
compute b=btemphld.
compute fresult={(LL2-basemod),llrdf,1-chicdf((LL2-basemod),llrdf)}
.
compute diromni=fresult.
end if.
end if.
do if (numint(1,i) > 0).
compute intkeym=intkey(intstart:(intstart+numint(1,i)-1),:).
do if (outscree=1).
print intkeym/title='Product terms key:'/format = A8.
end if.
end if.
do if (covcoeff=1).
do if (outscree=1).
print varb/title='Covariance matrix of regression parameter estimates:'/rnames=vlabsm /cnames=vlabsm/format= F10.4.
end if.
compute resultm2=make(nrow(varb),maxresm,99999).
do if (ncol(varb) <= maxresm).
compute resultm2(1:nrow(varb),1:ncol(varb))=varb.
compute resultm={resultm;resultm2}.
end if.
do if (ncol(varb) > maxresm).
compute resultmt=make(nrow(resultm),ncol(varb),99999).
compute resultmt(1:nrow(resultm),1:ncol(resultm))=resultm.
compute resultm=resultmt.
compute resultm2=make(nrow(varb),ncol(resultm),99999).
compute resultm2(1:nrow(varb),1:ncol(varb))=varb.
compute resultm={resultm;resultm2}.
compute maxresm=ncol(resultm).
end if.
end if.
do if (model <> 74 and xmtest=1 and nms > 0).
compute r2tmp=r2.
compute btmp=b.
compute varbtmp=varb.
compute dfrestmp=dfres.
compute tvaltmp=tval.
compute xmtst=make(nms,4,0).
compute xmtstlbc={hcflab,'df1','df2','p'}.
do if ((i=(nms+nys)) and (ydich=1)).
compute xmtst=make(nms,3,0).
compute xmtstlbc={'Chi-sq','df','p'}.
end if.
compute xmtstlb={' '}.
compute xmtmat=x.
compute numxint=0.
loop xmints=2 to i.
compute x=xmtmat.
do if ((bcmat((i+1),xmints)=1) and (wzcmat((i+1),xmints) <> 1)).
do if (bcmat((i+1),1)=0).
compute x={xmtmat,xtmp}.
do if ((ydich=1) and (i=(nms+nys))).

.
do if ( 2 =1).
compute hatmat=inv(t( x )* x )*t( x ).
compute b = hatmat* y.
compute modres=b.
do if ( 1 =1).
compute n1=nrow( x ).
compute dfres=n1-(ncol( x )).
compute sstotal = t( y -(csum( y )/n1))*( y -(csum( y )/n1)).
compute resid= y - x *b.
compute ssresid = csum((resid)&**2).
compute r2 = (sstotal-ssresid)/sstotal.
do if (r2 < 0).
compute r2=0.
end if.
compute adjr2 = 1-((1-r2)*(n1-1)/(dfres)).
compute mse=ssresid/(n1-ncol( x )).

.
compute n1=nrow( x ).
compute invXtX = inv(t( x )* x ).
compute varb = mse *invXtX.
compute k3 = ncol( x ).
compute xhc=0.
do if ( hc <> 5).
compute xhc= x.
compute hat = xhc(:,1).
loop i3=1 to nrow(xhc).
compute hat(i3,1)= xhc(i3,:)*invXtX*t(xhc(i3,:)).
end loop.
do if ( hc = 0 or hc =1).
loop i3 = 1 to k3.
compute xhc(:,i3)=xhc(:,i3)&* resid.
end loop.
end if.
do if ( hc =3 or hc =2).
loop i3=1 to k3.
compute xhc(:,i3) = ( resid &/(1-hat)&**(1/(4- hc )))&*xhc(:,i3).
end loop.
end if.
do if ( hc = 4).
compute hcmn=make(n,2,4).
compute hcmn(:,2)=(n1*hat)/k3.
loop i3= 1 to k3.
compute xhc(:,i3) = ( resid &/(1-hat)&**(rmin(hcmn)/2))&*xhc(:,i3).
end loop.
end if.
compute varb=(invXtX*t(xhc)*xhc*invXtX).
do if ( hc =1).
compute varb=(n1/(n1-ncol( x )))&*varb.
end if.
end if.
compute hclab={'se(HC0)','se(HC1)','se(HC2)','se(HC3)','se(HC4)','se'}.
compute hclab=hclab(1,( hc +1)).
compute hcflab={'F(HC0)','F(HC1)','F(HC2)','F(HC3)','F(HC4)','F'}.
compute hcflab=hcflab(1,( hc +1)).
release xhc
.
compute seb=sqrt(diag(varb)).
compute trat = b&/seb.
compute p = 2*(1-tcdf(abs(trat), (dfres))).
compute tval = sqrt(dfres* (exp((dfres-(5/6))*((xp2/(dfres-(2/3)+(.11/dfres)))* (xp2/(dfres-(2/3)+(.11/dfres)))))-1)).
compute modres={modres,seb,trat,p}.
compute modres={modres,(b-tval&*seb),(b+tval&*seb)}.
compute modresl={'coeff',hclab,'t','p','LLCI','ULCI'}.
compute lmat = ident(ncol( x )).
compute lmat = lmat(:,2:ncol(lmat)).
compute fratio = (t(t(lmat)*b)*inv(t(lmat)*varb*lmat)*((t(lmat)*b)))/(ncol( x )-1).
compute pfr = 1-fcdf(fratio,(ncol( x )-1),dfres).
compute modsum={sqrt(r2),r2,mse,fratio,(ncol( x )-1),dfres,pfr}.
compute modsuml={'R','R-sq','MSE',hcflab,'df1','df2', 'p'}.
end if.
end if.
do if ( 2 = 2 or 2 =3).
compute xlp= x.
compute ylp= y.
compute pt2 = make(nrow(ylp),1,(csum(ylp)/nrow(ylp))).
do if ( 2 =2).
compute LL3 = ylp&*ln(pt2)+(1-ylp)&*ln(1-pt2).
end if.
compute LL3 = -2*csum(LL3).
compute bt1 = make(ncol(xlp),1,0).
compute LL1 = 0.
compute pt1 = make(nrow(ylp),1,0.5).
compute pt1lp=pt1.
loop jjj = 1 to iterate.
compute xlptmp=t(xlp).
compute vecprb=pt1lp&*(1-pt1lp).
loop kkk=1 to ncol(xlp).
compute xlptmp(kkk,:)=xlptmp(kkk,:)&*t(vecprb).
end loop.
compute b = bt1+inv(xlptmp*xlp)*t(xlp)*(ylp-pt1lp).
do if ( 2 =2).
compute xlpb=xlp*b.
compute xlpbt=(xlpb > -709.7).
compute xlpb709=(1-xlpbt)*(-709.7).
compute xlpb=(xlpb&*xlpbt)+xlpb709.
compute pt1lp = 1/(1+exp(-(xlpb))).
end if.
compute itprob = csum((pt1lp < .00000001) or (pt1lp > .9999999)).
do if (itprob > 0).
loop kkk = 1 to nrow(pt1lp).
do if (pt1lp(kkk,1) > .9999999).
compute pt1lp(kkk,1) = .9999999.
end if.
do if (pt1lp(kkk,1) < .00000001).
compute pt1lp(kkk,1) = .00000001.
end if.
end loop.
compute itprob = 0.
end if.
do if (itprob = 0).
do if ( 2 =2).
compute LL = ylp&*ln(pt1lp)+(1-ylp)&*ln(1-pt1lp).
end if.
compute LL2 = -2*csum(ll).
end if.
do if (abs(LL1-LL2) < converge).
do if ( 1 =1).
compute xlptmp=t(xlp).
compute vecprb=pt1lp&*(1-pt1lp).
loop kkk=1 to ncol(xlp).
compute xlptmp(kkk,:)=xlptmp(kkk,:)&*t(vecprb).
end loop.
compute varb = inv(xlptmp*xlp).
compute seb = sqrt(diag(varb)).
release xlptmp.
end if.
break.
end if.
compute bt1 = b.
compute LL1 = LL2.
end loop.
compute modres=b.
do if (jjj > iterate).
compute itprob = 2.
do if (booting=0).
compute iterrmod=1.
end if.
do if (booting=1).
compute bootiter=1.
end if.
do if (itprobtg=0).
compute itprobtg=1.
compute errcode(errs,1) = 47.
compute errs = errs + 1.
do if (booting = 0 and 1 =1).
compute vt1 = mdiag(pt1lp&*(1-pt1lp)).
compute varb = inv(t(xlp)*vt1*xlp).
compute seb = sqrt(diag(varb)).
end if.
end if.
end if.
do if ( 1 =1).
compute trat = b&/seb.
compute dfres=nrow(xlp).
compute p = 2*(1-cdfnorm(abs(trat))).
compute modres={modres,seb,trat,p}.
compute modres={modres,(b-xp2&*seb),(b+xp2&*seb)}.
compute pvchi=1-chicdf((LL3-LL2),(nrow(modres)-1)).
compute mcF = (LL3-LL2)/LL3.
compute cox = 1-exp(-(LL3-LL2)/nrow(xlp)).
compute nagel = cox/(1-exp(-(LL3)/nrow(xlp))).
compute modsum={LL2,(LL3-LL2),(nrow(modres)-1),pvchi, mcF,cox,nagel}.
compute modsuml={'-2LL','ModelLL', 'df', 'p', 'McFadden', 'CoxSnell', 'Nagelkrk'}.
compute modresl={'coeff','se','Z','p','LLCI','ULCI'}.
end if.
end if
.
compute basemodx=LL2.
end if.
end if.
loop xmtlp1=1 to nxvls.
compute x={x,xtmp(:,xmtlp1)&*(mtmp(:,(xmints-1))-(csum(mtmp(:,(xmints-1)))/nrow(mtmp)) )}.
end loop.
do if ((i < (nms+nys)) or (ydich=0)).

.
do if ( 1 =1).
compute hatmat=inv(t( x )* x )*t( x ).
compute b = hatmat* y.
compute modres=b.
do if ( 1 =1).
compute n1=nrow( x ).
compute dfres=n1-(ncol( x )).
compute sstotal = t( y -(csum( y )/n1))*( y -(csum( y )/n1)).
compute resid= y - x *b.
compute ssresid = csum((resid)&**2).
compute r2 = (sstotal-ssresid)/sstotal.
do if (r2 < 0).
compute r2=0.
end if.
compute adjr2 = 1-((1-r2)*(n1-1)/(dfres)).
compute mse=ssresid/(n1-ncol( x )).

.
compute n1=nrow( x ).
compute invXtX = inv(t( x )* x ).
compute varb = mse *invXtX.
compute k3 = ncol( x ).
compute xhc=0.
do if ( hc <> 5).
compute xhc= x.
compute hat = xhc(:,1).
loop i3=1 to nrow(xhc).
compute hat(i3,1)= xhc(i3,:)*invXtX*t(xhc(i3,:)).
end loop.
do if ( hc = 0 or hc =1).
loop i3 = 1 to k3.
compute xhc(:,i3)=xhc(:,i3)&* resid.
end loop.
end if.
do if ( hc =3 or hc =2).
loop i3=1 to k3.
compute xhc(:,i3) = ( resid &/(1-hat)&**(1/(4- hc )))&*xhc(:,i3).
end loop.
end if.
do if ( hc = 4).
compute hcmn=make(n,2,4).
compute hcmn(:,2)=(n1*hat)/k3.
loop i3= 1 to k3.
compute xhc(:,i3) = ( resid &/(1-hat)&**(rmin(hcmn)/2))&*xhc(:,i3).
end loop.
end if.
compute varb=(invXtX*t(xhc)*xhc*invXtX).
do if ( hc =1).
compute varb=(n1/(n1-ncol( x )))&*varb.
end if.
end if.
compute hclab={'se(HC0)','se(HC1)','se(HC2)','se(HC3)','se(HC4)','se'}.
compute hclab=hclab(1,( hc +1)).
compute hcflab={'F(HC0)','F(HC1)','F(HC2)','F(HC3)','F(HC4)','F'}.
compute hcflab=hcflab(1,( hc +1)).
release xhc
.
compute seb=sqrt(diag(varb)).
compute trat = b&/seb.
compute p = 2*(1-tcdf(abs(trat), (dfres))).
compute tval = sqrt(dfres* (exp((dfres-(5/6))*((xp2/(dfres-(2/3)+(.11/dfres)))* (xp2/(dfres-(2/3)+(.11/dfres)))))-1)).
compute modres={modres,seb,trat,p}.
compute modres={modres,(b-tval&*seb),(b+tval&*seb)}.
compute modresl={'coeff',hclab,'t','p','LLCI','ULCI'}.
compute lmat = ident(ncol( x )).
compute lmat = lmat(:,2:ncol(lmat)).
compute fratio = (t(t(lmat)*b)*inv(t(lmat)*varb*lmat)*((t(lmat)*b)))/(ncol( x )-1).
compute pfr = 1-fcdf(fratio,(ncol( x )-1),dfres).
compute modsum={sqrt(r2),r2,mse,fratio,(ncol( x )-1),dfres,pfr}.
compute modsuml={'R','R-sq','MSE',hcflab,'df1','df2', 'p'}.
end if.
end if.
do if ( 1 = 2 or 1 =3).
compute xlp= x.
compute ylp= y.
compute pt2 = make(nrow(ylp),1,(csum(ylp)/nrow(ylp))).
do if ( 1 =2).
compute LL3 = ylp&*ln(pt2)+(1-ylp)&*ln(1-pt2).
end if.
compute LL3 = -2*csum(LL3).
compute bt1 = make(ncol(xlp),1,0).
compute LL1 = 0.
compute pt1 = make(nrow(ylp),1,0.5).
compute pt1lp=pt1.
loop jjj = 1 to iterate.
compute xlptmp=t(xlp).
compute vecprb=pt1lp&*(1-pt1lp).
loop kkk=1 to ncol(xlp).
compute xlptmp(kkk,:)=xlptmp(kkk,:)&*t(vecprb).
end loop.
compute b = bt1+inv(xlptmp*xlp)*t(xlp)*(ylp-pt1lp).
do if ( 1 =2).
compute xlpb=xlp*b.
compute xlpbt=(xlpb > -709.7).
compute xlpb709=(1-xlpbt)*(-709.7).
compute xlpb=(xlpb&*xlpbt)+xlpb709.
compute pt1lp = 1/(1+exp(-(xlpb))).
end if.
compute itprob = csum((pt1lp < .00000001) or (pt1lp > .9999999)).
do if (itprob > 0).
loop kkk = 1 to nrow(pt1lp).
do if (pt1lp(kkk,1) > .9999999).
compute pt1lp(kkk,1) = .9999999.
end if.
do if (pt1lp(kkk,1) < .00000001).
compute pt1lp(kkk,1) = .00000001.
end if.
end loop.
compute itprob = 0.
end if.
do if (itprob = 0).
do if ( 1 =2).
compute LL = ylp&*ln(pt1lp)+(1-ylp)&*ln(1-pt1lp).
end if.
compute LL2 = -2*csum(ll).
end if.
do if (abs(LL1-LL2) < converge).
do if ( 1 =1).
compute xlptmp=t(xlp).
compute vecprb=pt1lp&*(1-pt1lp).
loop kkk=1 to ncol(xlp).
compute xlptmp(kkk,:)=xlptmp(kkk,:)&*t(vecprb).
end loop.
compute varb = inv(xlptmp*xlp).
compute seb = sqrt(diag(varb)).
release xlptmp.
end if.
break.
end if.
compute bt1 = b.
compute LL1 = LL2.
end loop.
compute modres=b.
do if (jjj > iterate).
compute itprob = 2.
do if (booting=0).
compute iterrmod=1.
end if.
do if (booting=1).
compute bootiter=1.
end if.
do if (itprobtg=0).
compute itprobtg=1.
compute errcode(errs,1) = 47.
compute errs = errs + 1.
do if (booting = 0 and 1 =1).
compute vt1 = mdiag(pt1lp&*(1-pt1lp)).
compute varb = inv(t(xlp)*vt1*xlp).
compute seb = sqrt(diag(varb)).
end if.
end if.
end if.
do if ( 1 =1).
compute trat = b&/seb.
compute dfres=nrow(xlp).
compute p = 2*(1-cdfnorm(abs(trat))).
compute modres={modres,seb,trat,p}.
compute modres={modres,(b-xp2&*seb),(b+xp2&*seb)}.
compute pvchi=1-chicdf((LL3-LL2),(nrow(modres)-1)).
compute mcF = (LL3-LL2)/LL3.
compute cox = 1-exp(-(LL3-LL2)/nrow(xlp)).
compute nagel = cox/(1-exp(-(LL3)/nrow(xlp))).
compute modsum={LL2,(LL3-LL2),(nrow(modres)-1),pvchi, mcF,cox,nagel}.
compute modsuml={'-2LL','ModelLL', 'df', 'p', 'McFadden', 'CoxSnell', 'Nagelkrk'}.
compute modresl={'coeff','se','Z','p','LLCI','ULCI'}.
end if.
end if
.
end if.
do if ((i = (nms+nys)) and (ydich=1)).

.
do if ( 2 =1).
compute hatmat=inv(t( x )* x )*t( x ).
compute b = hatmat* y.
compute modres=b.
do if ( 1 =1).
compute n1=nrow( x ).
compute dfres=n1-(ncol( x )).
compute sstotal = t( y -(csum( y )/n1))*( y -(csum( y )/n1)).
compute resid= y - x *b.
compute ssresid = csum((resid)&**2).
compute r2 = (sstotal-ssresid)/sstotal.
do if (r2 < 0).
compute r2=0.
end if.
compute adjr2 = 1-((1-r2)*(n1-1)/(dfres)).
compute mse=ssresid/(n1-ncol( x )).

.
compute n1=nrow( x ).
compute invXtX = inv(t( x )* x ).
compute varb = mse *invXtX.
compute k3 = ncol( x ).
compute xhc=0.
do if ( hc <> 5).
compute xhc= x.
compute hat = xhc(:,1).
loop i3=1 to nrow(xhc).
compute hat(i3,1)= xhc(i3,:)*invXtX*t(xhc(i3,:)).
end loop.
do if ( hc = 0 or hc =1).
loop i3 = 1 to k3.
compute xhc(:,i3)=xhc(:,i3)&* resid.
end loop.
end if.
do if ( hc =3 or hc =2).
loop i3=1 to k3.
compute xhc(:,i3) = ( resid &/(1-hat)&**(1/(4- hc )))&*xhc(:,i3).
end loop.
end if.
do if ( hc = 4).
compute hcmn=make(n,2,4).
compute hcmn(:,2)=(n1*hat)/k3.
loop i3= 1 to k3.
compute xhc(:,i3) = ( resid &/(1-hat)&**(rmin(hcmn)/2))&*xhc(:,i3).
end loop.
end if.
compute varb=(invXtX*t(xhc)*xhc*invXtX).
do if ( hc =1).
compute varb=(n1/(n1-ncol( x )))&*varb.
end if.
end if.
compute hclab={'se(HC0)','se(HC1)','se(HC2)','se(HC3)','se(HC4)','se'}.
compute hclab=hclab(1,( hc +1)).
compute hcflab={'F(HC0)','F(HC1)','F(HC2)','F(HC3)','F(HC4)','F'}.
compute hcflab=hcflab(1,( hc +1)).
release xhc
.
compute seb=sqrt(diag(varb)).
compute trat = b&/seb.
compute p = 2*(1-tcdf(abs(trat), (dfres))).
compute tval = sqrt(dfres* (exp((dfres-(5/6))*((xp2/(dfres-(2/3)+(.11/dfres)))* (xp2/(dfres-(2/3)+(.11/dfres)))))-1)).
compute modres={modres,seb,trat,p}.
compute modres={modres,(b-tval&*seb),(b+tval&*seb)}.
compute modresl={'coeff',hclab,'t','p','LLCI','ULCI'}.
compute lmat = ident(ncol( x )).
compute lmat = lmat(:,2:ncol(lmat)).
compute fratio = (t(t(lmat)*b)*inv(t(lmat)*varb*lmat)*((t(lmat)*b)))/(ncol( x )-1).
compute pfr = 1-fcdf(fratio,(ncol( x )-1),dfres).
compute modsum={sqrt(r2),r2,mse,fratio,(ncol( x )-1),dfres,pfr}.
compute modsuml={'R','R-sq','MSE',hcflab,'df1','df2', 'p'}.
end if.
end if.
do if ( 2 = 2 or 2 =3).
compute xlp= x.
compute ylp= y.
compute pt2 = make(nrow(ylp),1,(csum(ylp)/nrow(ylp))).
do if ( 2 =2).
compute LL3 = ylp&*ln(pt2)+(1-ylp)&*ln(1-pt2).
end if.
compute LL3 = -2*csum(LL3).
compute bt1 = make(ncol(xlp),1,0).
compute LL1 = 0.
compute pt1 = make(nrow(ylp),1,0.5).
compute pt1lp=pt1.
loop jjj = 1 to iterate.
compute xlptmp=t(xlp).
compute vecprb=pt1lp&*(1-pt1lp).
loop kkk=1 to ncol(xlp).
compute xlptmp(kkk,:)=xlptmp(kkk,:)&*t(vecprb).
end loop.
compute b = bt1+inv(xlptmp*xlp)*t(xlp)*(ylp-pt1lp).
do if ( 2 =2).
compute xlpb=xlp*b.
compute xlpbt=(xlpb > -709.7).
compute xlpb709=(1-xlpbt)*(-709.7).
compute xlpb=(xlpb&*xlpbt)+xlpb709.
compute pt1lp = 1/(1+exp(-(xlpb))).
end if.
compute itprob = csum((pt1lp < .00000001) or (pt1lp > .9999999)).
do if (itprob > 0).
loop kkk = 1 to nrow(pt1lp).
do if (pt1lp(kkk,1) > .9999999).
compute pt1lp(kkk,1) = .9999999.
end if.
do if (pt1lp(kkk,1) < .00000001).
compute pt1lp(kkk,1) = .00000001.
end if.
end loop.
compute itprob = 0.
end if.
do if (itprob = 0).
do if ( 2 =2).
compute LL = ylp&*ln(pt1lp)+(1-ylp)&*ln(1-pt1lp).
end if.
compute LL2 = -2*csum(ll).
end if.
do if (abs(LL1-LL2) < converge).
do if ( 1 =1).
compute xlptmp=t(xlp).
compute vecprb=pt1lp&*(1-pt1lp).
loop kkk=1 to ncol(xlp).
compute xlptmp(kkk,:)=xlptmp(kkk,:)&*t(vecprb).
end loop.
compute varb = inv(xlptmp*xlp).
compute seb = sqrt(diag(varb)).
release xlptmp.
end if.
break.
end if.
compute bt1 = b.
compute LL1 = LL2.
end loop.
compute modres=b.
do if (jjj > iterate).
compute itprob = 2.
do if (booting=0).
compute iterrmod=1.
end if.
do if (booting=1).
compute bootiter=1.
end if.
do if (itprobtg=0).
compute itprobtg=1.
compute errcode(errs,1) = 47.
compute errs = errs + 1.
do if (booting = 0 and 1 =1).
compute vt1 = mdiag(pt1lp&*(1-pt1lp)).
compute varb = inv(t(xlp)*vt1*xlp).
compute seb = sqrt(diag(varb)).
end if.
end if.
end if.
do if ( 1 =1).
compute trat = b&/seb.
compute dfres=nrow(xlp).
compute p = 2*(1-cdfnorm(abs(trat))).
compute modres={modres,seb,trat,p}.
compute modres={modres,(b-xp2&*seb),(b+xp2&*seb)}.
compute pvchi=1-chicdf((LL3-LL2),(nrow(modres)-1)).
compute mcF = (LL3-LL2)/LL3.
compute cox = 1-exp(-(LL3-LL2)/nrow(xlp)).
compute nagel = cox/(1-exp(-(LL3)/nrow(xlp))).
compute modsum={LL2,(LL3-LL2),(nrow(modres)-1),pvchi, mcF,cox,nagel}.
compute modsuml={'-2LL','ModelLL', 'df', 'p', 'McFadden', 'CoxSnell', 'Nagelkrk'}.
compute modresl={'coeff','se','Z','p','LLCI','ULCI'}.
end if.
end if
.
compute chidfxm=basemodx-LL2.
end if.
compute lmat=make(nrow(b),nxvls,0).
compute lmattmp=ident(nxvls).
compute lmat((nrow(lmat)-nxvls+1):nrow(lmat),:)=lmattmp.

.
compute lmat2= lmat.
do if ( 1 =0).
compute lmat2 = mdiag( lmat ).
compute lmat3=make(nrow(lmat2),1,0).
loop flp=1 to ncol(lmat2).
do if (csum(lmat2(:,flp))=1).
compute lmat3={lmat3,lmat2(:,flp)}.
end if.
end loop.
compute lmat2=lmat3(:,2:ncol(lmat3)).
end if.
compute fratio = (t(t(lmat2)* b )*inv(t(lmat2)* varb *lmat2)*((t(lmat2)* b )))/ncol(lmat2).
compute pfr = 1-fcdf(fratio,ncol(lmat2),(n-nrow( b ))).
compute fresult={fratio,ncol(lmat2),(n-nrow( b )),pfr}.
do if (i = (nms+nys) and (ydich=1)).
compute fratio=fratio*ncol(lmat2).
compute pfr=1-chicdf(fratio,ncol(lmat2)).
compute fresult={fratio,ncol(lmat2),pfr}.
end if.
do if ( 0 =1).
compute lmat3=1-rsum(lmat2).
compute xfm=make(n,csum(lmat3),0).
compute flpc=1.
loop flp=1 to nrow(lmat3).
do if (lmat3(flp,1)=1).
compute xfm(:,flpc)=x(:,flp).
compute flpc=flpc+1.
end if.
end loop.
compute bfm=inv(t(xfm)*xfm)*t(xfm)*y.
compute resid=y-(xfm*bfm).
compute sstotal=(y-(csum(y)/n)).
compute sstotal=csum(sstotal&*sstotal).
compute ssresid=csum(resid&*resid).
compute rsqch= 0 -((sstotal-ssresid)/sstotal).
compute fresult={rsqch,fresult}.
release xfm,flpc, resid, ssresid, bfm.
end if
.
compute numxint=numxint+1.
compute xmtst(numxint,:)=fresult.
do if ((i = (nms+nys)) and (ydich=1)).
compute xmtst(numxint,1)=chidfxm.
compute xmtst(numxint,3)=1-chicdf(chidfxm,nxvls).
end if.
compute xmtstlb={xmtstlb;highlbx((xmints-1),1)}.
end if.
end loop.
compute x=xmtmat.
release xmtmat.
do if (numxint>0).
compute xmtstlb=xmtstlb((2:(numxint+1)),:).
compute xmtst=xmtst(1:numxint,:).
compute resultm2=make(nrow(xmtst),maxresm,99999).
compute resultm2(1:nrow(xmtst),1:ncol(xmtst))=xmtst.
compute resultm={resultm;resultm2}.
do if (nms=1).
compute xmtstlb=' '.
end if.
do if (outscree=1).
do if ((i < (nms+nys)) or (ydich=0)).
print xmtst/title='Test(s) of X by M interaction:'/rnames=xmtstlb/cnames=xmtstlbc/format= F10.4.
end if.
do if ((i = (nms+nys)) and (ydich=1)).
print xmtst/title='Likelihood ratio test(s) of X by M interaction:'/rnames=xmtstlb/cnames=xmtstlbc/format= F10.4.
end if.
end if.
end if.
compute r2=r2tmp.
compute b=btmp.
compute varb=varbtmp.
compute dfres=dfrestmp.
compute tval=tvaltmp.
end if.
do if (criterr = 0).
compute jj=0.
loop j = start to ((start+i)-1).
compute dbint=0.
compute lmat=whigh(1:nump(1,i),j).
compute lmat2=wzhigh(1:nump(1,i),j).
do if ((csum(lmat) > 0) and (csum(lmat2) = 0)).
do if ((i < (nms+nys)) or (ydich <> 1)).

.
compute lmat2= lmat.
do if ( 0 =0).
compute lmat2 = mdiag( lmat ).
compute lmat3=make(nrow(lmat2),1,0).
loop flp=1 to ncol(lmat2).
do if (csum(lmat2(:,flp))=1).
compute lmat3={lmat3,lmat2(:,flp)}.
end if.
end loop.
compute lmat2=lmat3(:,2:ncol(lmat3)).
end if.
compute fratio = (t(t(lmat2)* b )*inv(t(lmat2)* varb *lmat2)*((t(lmat2)* b )))/ncol(lmat2).
compute pfr = 1-fcdf(fratio,ncol(lmat2),(n-nrow( b ))).
compute fresult={fratio,ncol(lmat2),(n-nrow( b )),pfr}.
do if (i = (nms+nys) and (ydich=1)).
compute fratio=fratio*ncol(lmat2).
compute pfr=1-chicdf(fratio,ncol(lmat2)).
compute fresult={fratio,ncol(lmat2),pfr}.
end if.
do if ( 1 =1).
compute lmat3=1-rsum(lmat2).
compute xfm=make(n,csum(lmat3),0).
compute flpc=1.
loop flp=1 to nrow(lmat3).
do if (lmat3(flp,1)=1).
compute xfm(:,flpc)=x(:,flp).
compute flpc=flpc+1.
end if.
end loop.
compute bfm=inv(t(xfm)*xfm)*t(xfm)*y.
compute resid=y-(xfm*bfm).
compute sstotal=(y-(csum(y)/n)).
compute sstotal=csum(sstotal&*sstotal).
compute ssresid=csum(resid&*resid).
compute rsqch= r2 -((sstotal-ssresid)/sstotal).
compute fresult={rsqch,fresult}.
release xfm,flpc, resid, ssresid, bfm.
end if
.
compute lmatdb=lmat.
compute dbint=dbint+1.
end if.
do if ((ydich = 1) and (i = (nms+nys))).

.
compute btemphld=b.
compute llrdat=make(nrow(x),nrow( lmat )-csum( lmat ),-999).
compute llrdf=ncol(x)-ncol(llrdat).
compute llrcnt=0.
loop llri=1 to nrow( lmat ).
do if ( lmat (llri,1)=0).
compute llrcnt=llrcnt+1.
compute llrdat(:,llrcnt)=x(:,llri).
end if.
end loop.

.
do if ( 2 =1).
compute hatmat=inv(t( llrdat )* llrdat )*t( llrdat ).
compute b = hatmat* y.
compute modres=b.
do if ( 0 =1).
compute n1=nrow( llrdat ).
compute dfres=n1-(ncol( llrdat )).
compute sstotal = t( y -(csum( y )/n1))*( y -(csum( y )/n1)).
compute resid= y - llrdat *b.
compute ssresid = csum((resid)&**2).
compute r2 = (sstotal-ssresid)/sstotal.
do if (r2 < 0).
compute r2=0.
end if.
compute adjr2 = 1-((1-r2)*(n1-1)/(dfres)).
compute mse=ssresid/(n1-ncol( llrdat )).

.
compute n1=nrow( x ).
compute invXtX = inv(t( x )* x ).
compute varb = mse *invXtX.
compute k3 = ncol( x ).
compute xhc=0.
do if ( hc <> 5).
compute xhc= x.
compute hat = xhc(:,1).
loop i3=1 to nrow(xhc).
compute hat(i3,1)= xhc(i3,:)*invXtX*t(xhc(i3,:)).
end loop.
do if ( hc = 0 or hc =1).
loop i3 = 1 to k3.
compute xhc(:,i3)=xhc(:,i3)&* resid.
end loop.
end if.
do if ( hc =3 or hc =2).
loop i3=1 to k3.
compute xhc(:,i3) = ( resid &/(1-hat)&**(1/(4- hc )))&*xhc(:,i3).
end loop.
end if.
do if ( hc = 4).
compute hcmn=make(n,2,4).
compute hcmn(:,2)=(n1*hat)/k3.
loop i3= 1 to k3.
compute xhc(:,i3) = ( resid &/(1-hat)&**(rmin(hcmn)/2))&*xhc(:,i3).
end loop.
end if.
compute varb=(invXtX*t(xhc)*xhc*invXtX).
do if ( hc =1).
compute varb=(n1/(n1-ncol( x )))&*varb.
end if.
end if.
compute hclab={'se(HC0)','se(HC1)','se(HC2)','se(HC3)','se(HC4)','se'}.
compute hclab=hclab(1,( hc +1)).
compute hcflab={'F(HC0)','F(HC1)','F(HC2)','F(HC3)','F(HC4)','F'}.
compute hcflab=hcflab(1,( hc +1)).
release xhc
.
compute seb=sqrt(diag(varb)).
compute trat = b&/seb.
compute p = 2*(1-tcdf(abs(trat), (dfres))).
compute tval = sqrt(dfres* (exp((dfres-(5/6))*((xp2/(dfres-(2/3)+(.11/dfres)))* (xp2/(dfres-(2/3)+(.11/dfres)))))-1)).
compute modres={modres,seb,trat,p}.
compute modres={modres,(b-tval&*seb),(b+tval&*seb)}.
compute modresl={'coeff',hclab,'t','p','LLCI','ULCI'}.
compute lmat = ident(ncol( llrdat )).
compute lmat = lmat(:,2:ncol(lmat)).
compute fratio = (t(t(lmat)*b)*inv(t(lmat)*varb*lmat)*((t(lmat)*b)))/(ncol( llrdat )-1).
compute pfr = 1-fcdf(fratio,(ncol( llrdat )-1),dfres).
compute modsum={sqrt(r2),r2,mse,fratio,(ncol( llrdat )-1),dfres,pfr}.
compute modsuml={'R','R-sq','MSE',hcflab,'df1','df2', 'p'}.
end if.
end if.
do if ( 2 = 2 or 2 =3).
compute xlp= llrdat.
compute ylp= y.
compute pt2 = make(nrow(ylp),1,(csum(ylp)/nrow(ylp))).
do if ( 2 =2).
compute LL3 = ylp&*ln(pt2)+(1-ylp)&*ln(1-pt2).
end if.
compute LL3 = -2*csum(LL3).
compute bt1 = make(ncol(xlp),1,0).
compute LL1 = 0.
compute pt1 = make(nrow(ylp),1,0.5).
compute pt1lp=pt1.
loop jjj = 1 to iterate.
compute xlptmp=t(xlp).
compute vecprb=pt1lp&*(1-pt1lp).
loop kkk=1 to ncol(xlp).
compute xlptmp(kkk,:)=xlptmp(kkk,:)&*t(vecprb).
end loop.
compute b = bt1+inv(xlptmp*xlp)*t(xlp)*(ylp-pt1lp).
do if ( 2 =2).
compute xlpb=xlp*b.
compute xlpbt=(xlpb > -709.7).
compute xlpb709=(1-xlpbt)*(-709.7).
compute xlpb=(xlpb&*xlpbt)+xlpb709.
compute pt1lp = 1/(1+exp(-(xlpb))).
end if.
compute itprob = csum((pt1lp < .00000001) or (pt1lp > .9999999)).
do if (itprob > 0).
loop kkk = 1 to nrow(pt1lp).
do if (pt1lp(kkk,1) > .9999999).
compute pt1lp(kkk,1) = .9999999.
end if.
do if (pt1lp(kkk,1) < .00000001).
compute pt1lp(kkk,1) = .00000001.
end if.
end loop.
compute itprob = 0.
end if.
do if (itprob = 0).
do if ( 2 =2).
compute LL = ylp&*ln(pt1lp)+(1-ylp)&*ln(1-pt1lp).
end if.
compute LL2 = -2*csum(ll).
end if.
do if (abs(LL1-LL2) < converge).
do if ( 0 =1).
compute xlptmp=t(xlp).
compute vecprb=pt1lp&*(1-pt1lp).
loop kkk=1 to ncol(xlp).
compute xlptmp(kkk,:)=xlptmp(kkk,:)&*t(vecprb).
end loop.
compute varb = inv(xlptmp*xlp).
compute seb = sqrt(diag(varb)).
release xlptmp.
end if.
break.
end if.
compute bt1 = b.
compute LL1 = LL2.
end loop.
compute modres=b.
do if (jjj > iterate).
compute itprob = 2.
do if (booting=0).
compute iterrmod=1.
end if.
do if (booting=1).
compute bootiter=1.
end if.
do if (itprobtg=0).
compute itprobtg=1.
compute errcode(errs,1) = 47.
compute errs = errs + 1.
do if (booting = 0 and 0 =1).
compute vt1 = mdiag(pt1lp&*(1-pt1lp)).
compute varb = inv(t(xlp)*vt1*xlp).
compute seb = sqrt(diag(varb)).
end if.
end if.
end if.
do if ( 0 =1).
compute trat = b&/seb.
compute dfres=nrow(xlp).
compute p = 2*(1-cdfnorm(abs(trat))).
compute modres={modres,seb,trat,p}.
compute modres={modres,(b-xp2&*seb),(b+xp2&*seb)}.
compute pvchi=1-chicdf((LL3-LL2),(nrow(modres)-1)).
compute mcF = (LL3-LL2)/LL3.
compute cox = 1-exp(-(LL3-LL2)/nrow(xlp)).
compute nagel = cox/(1-exp(-(LL3)/nrow(xlp))).
compute modsum={LL2,(LL3-LL2),(nrow(modres)-1),pvchi, mcF,cox,nagel}.
compute modsuml={'-2LL','ModelLL', 'df', 'p', 'McFadden', 'CoxSnell', 'Nagelkrk'}.
compute modresl={'coeff','se','Z','p','LLCI','ULCI'}.
end if.
end if
.
compute b=btemphld.
compute fresult={(LL2-basemod),llrdf,1-chicdf((LL2-basemod),llrdf)}
.
compute lmatdb=lmat.
compute dbint=dbint+1.
end if.
compute highf={highf;fresult}.
compute highf2={highf2;fresult}.
do if (j = start).
compute flabel={flabel;'X*W'}.
end if.
do if (j > start).
do if (nms > 1).
compute flabel={flabel;highlbw(jj,1)}.
else if (nms = 1).
do if (xmint=0).
compute flabel={flabel;'M*W'}.
end if.
do if (xmint=1).
compute flabel={flabel;'X*M'}.
end if.
end if.
end if.
end if.
compute lmat=zhigh(1:nump(1,i),j).
compute lmat2=wzhigh(1:nump(1,i),j).
do if ((csum(lmat) > 0) and (csum(lmat2) = 0)).
do if ((i < (nms+nys)) or (ydich <> 1)).

.
compute lmat2= lmat.
do if ( 0 =0).
compute lmat2 = mdiag( lmat ).
compute lmat3=make(nrow(lmat2),1,0).
loop flp=1 to ncol(lmat2).
do if (csum(lmat2(:,flp))=1).
compute lmat3={lmat3,lmat2(:,flp)}.
end if.
end loop.
compute lmat2=lmat3(:,2:ncol(lmat3)).
end if.
compute fratio = (t(t(lmat2)* b )*inv(t(lmat2)* varb *lmat2)*((t(lmat2)* b )))/ncol(lmat2).
compute pfr = 1-fcdf(fratio,ncol(lmat2),(n-nrow( b ))).
compute fresult={fratio,ncol(lmat2),(n-nrow( b )),pfr}.
do if (i = (nms+nys) and (ydich=1)).
compute fratio=fratio*ncol(lmat2).
compute pfr=1-chicdf(fratio,ncol(lmat2)).
compute fresult={fratio,ncol(lmat2),pfr}.
end if.
do if ( 1 =1).
compute lmat3=1-rsum(lmat2).
compute xfm=make(n,csum(lmat3),0).
compute flpc=1.
loop flp=1 to nrow(lmat3).
do if (lmat3(flp,1)=1).
compute xfm(:,flpc)=x(:,flp).
compute flpc=flpc+1.
end if.
end loop.
compute bfm=inv(t(xfm)*xfm)*t(xfm)*y.
compute resid=y-(xfm*bfm).
compute sstotal=(y-(csum(y)/n)).
compute sstotal=csum(sstotal&*sstotal).
compute ssresid=csum(resid&*resid).
compute rsqch= r2 -((sstotal-ssresid)/sstotal).
compute fresult={rsqch,fresult}.
release xfm,flpc, resid, ssresid, bfm.
end if
.
compute dbint=dbint+1.
end if.
do if ((ydich = 1) and (i = (nms+nys))).

.
compute btemphld=b.
compute llrdat=make(nrow(x),nrow( lmat )-csum( lmat ),-999).
compute llrdf=ncol(x)-ncol(llrdat).
compute llrcnt=0.
loop llri=1 to nrow( lmat ).
do if ( lmat (llri,1)=0).
compute llrcnt=llrcnt+1.
compute llrdat(:,llrcnt)=x(:,llri).
end if.
end loop.

.
do if ( 2 =1).
compute hatmat=inv(t( llrdat )* llrdat )*t( llrdat ).
compute b = hatmat* y.
compute modres=b.
do if ( 0 =1).
compute n1=nrow( llrdat ).
compute dfres=n1-(ncol( llrdat )).
compute sstotal = t( y -(csum( y )/n1))*( y -(csum( y )/n1)).
compute resid= y - llrdat *b.
compute ssresid = csum((resid)&**2).
compute r2 = (sstotal-ssresid)/sstotal.
do if (r2 < 0).
compute r2=0.
end if.
compute adjr2 = 1-((1-r2)*(n1-1)/(dfres)).
compute mse=ssresid/(n1-ncol( llrdat )).

.
compute n1=nrow( x ).
compute invXtX = inv(t( x )* x ).
compute varb = mse *invXtX.
compute k3 = ncol( x ).
compute xhc=0.
do if ( hc <> 5).
compute xhc= x.
compute hat = xhc(:,1).
loop i3=1 to nrow(xhc).
compute hat(i3,1)= xhc(i3,:)*invXtX*t(xhc(i3,:)).
end loop.
do if ( hc = 0 or hc =1).
loop i3 = 1 to k3.
compute xhc(:,i3)=xhc(:,i3)&* resid.
end loop.
end if.
do if ( hc =3 or hc =2).
loop i3=1 to k3.
compute xhc(:,i3) = ( resid &/(1-hat)&**(1/(4- hc )))&*xhc(:,i3).
end loop.
end if.
do if ( hc = 4).
compute hcmn=make(n,2,4).
compute hcmn(:,2)=(n1*hat)/k3.
loop i3= 1 to k3.
compute xhc(:,i3) = ( resid &/(1-hat)&**(rmin(hcmn)/2))&*xhc(:,i3).
end loop.
end if.
compute varb=(invXtX*t(xhc)*xhc*invXtX).
do if ( hc =1).
compute varb=(n1/(n1-ncol( x )))&*varb.
end if.
end if.
compute hclab={'se(HC0)','se(HC1)','se(HC2)','se(HC3)','se(HC4)','se'}.
compute hclab=hclab(1,( hc +1)).
compute hcflab={'F(HC0)','F(HC1)','F(HC2)','F(HC3)','F(HC4)','F'}.
compute hcflab=hcflab(1,( hc +1)).
release xhc
.
compute seb=sqrt(diag(varb)).
compute trat = b&/seb.
compute p = 2*(1-tcdf(abs(trat), (dfres))).
compute tval = sqrt(dfres* (exp((dfres-(5/6))*((xp2/(dfres-(2/3)+(.11/dfres)))* (xp2/(dfres-(2/3)+(.11/dfres)))))-1)).
compute modres={modres,seb,trat,p}.
compute modres={modres,(b-tval&*seb),(b+tval&*seb)}.
compute modresl={'coeff',hclab,'t','p','LLCI','ULCI'}.
compute lmat = ident(ncol( llrdat )).
compute lmat = lmat(:,2:ncol(lmat)).
compute fratio = (t(t(lmat)*b)*inv(t(lmat)*varb*lmat)*((t(lmat)*b)))/(ncol( llrdat )-1).
compute pfr = 1-fcdf(fratio,(ncol( llrdat )-1),dfres).
compute modsum={sqrt(r2),r2,mse,fratio,(ncol( llrdat )-1),dfres,pfr}.
compute modsuml={'R','R-sq','MSE',hcflab,'df1','df2', 'p'}.
end if.
end if.
do if ( 2 = 2 or 2 =3).
compute xlp= llrdat.
compute ylp= y.
compute pt2 = make(nrow(ylp),1,(csum(ylp)/nrow(ylp))).
do if ( 2 =2).
compute LL3 = ylp&*ln(pt2)+(1-ylp)&*ln(1-pt2).
end if.
compute LL3 = -2*csum(LL3).
compute bt1 = make(ncol(xlp),1,0).
compute LL1 = 0.
compute pt1 = make(nrow(ylp),1,0.5).
compute pt1lp=pt1.
loop jjj = 1 to iterate.
compute xlptmp=t(xlp).
compute vecprb=pt1lp&*(1-pt1lp).
loop kkk=1 to ncol(xlp).
compute xlptmp(kkk,:)=xlptmp(kkk,:)&*t(vecprb).
end loop.
compute b = bt1+inv(xlptmp*xlp)*t(xlp)*(ylp-pt1lp).
do if ( 2 =2).
compute xlpb=xlp*b.
compute xlpbt=(xlpb > -709.7).
compute xlpb709=(1-xlpbt)*(-709.7).
compute xlpb=(xlpb&*xlpbt)+xlpb709.
compute pt1lp = 1/(1+exp(-(xlpb))).
end if.
compute itprob = csum((pt1lp < .00000001) or (pt1lp > .9999999)).
do if (itprob > 0).
loop kkk = 1 to nrow(pt1lp).
do if (pt1lp(kkk,1) > .9999999).
compute pt1lp(kkk,1) = .9999999.
end if.
do if (pt1lp(kkk,1) < .00000001).
compute pt1lp(kkk,1) = .00000001.
end if.
end loop.
compute itprob = 0.
end if.
do if (itprob = 0).
do if ( 2 =2).
compute LL = ylp&*ln(pt1lp)+(1-ylp)&*ln(1-pt1lp).
end if.
compute LL2 = -2*csum(ll).
end if.
do if (abs(LL1-LL2) < converge).
do if ( 0 =1).
compute xlptmp=t(xlp).
compute vecprb=pt1lp&*(1-pt1lp).
loop kkk=1 to ncol(xlp).
compute xlptmp(kkk,:)=xlptmp(kkk,:)&*t(vecprb).
end loop.
compute varb = inv(xlptmp*xlp).
compute seb = sqrt(diag(varb)).
release xlptmp.
end if.
break.
end if.
compute bt1 = b.
compute LL1 = LL2.
end loop.
compute modres=b.
do if (jjj > iterate).
compute itprob = 2.
do if (booting=0).
compute iterrmod=1.
end if.
do if (booting=1).
compute bootiter=1.
end if.
do if (itprobtg=0).
compute itprobtg=1.
compute errcode(errs,1) = 47.
compute errs = errs + 1.
do if (booting = 0 and 0 =1).
compute vt1 = mdiag(pt1lp&*(1-pt1lp)).
compute varb = inv(t(xlp)*vt1*xlp).
compute seb = sqrt(diag(varb)).
end if.
end if.
end if.
do if ( 0 =1).
compute trat = b&/seb.
compute dfres=nrow(xlp).
compute p = 2*(1-cdfnorm(abs(trat))).
compute modres={modres,seb,trat,p}.
compute modres={modres,(b-xp2&*seb),(b+xp2&*seb)}.
compute pvchi=1-chicdf((LL3-LL2),(nrow(modres)-1)).
compute mcF = (LL3-LL2)/LL3.
compute cox = 1-exp(-(LL3-LL2)/nrow(xlp)).
compute nagel = cox/(1-exp(-(LL3)/nrow(xlp))).
compute modsum={LL2,(LL3-LL2),(nrow(modres)-1),pvchi, mcF,cox,nagel}.
compute modsuml={'-2LL','ModelLL', 'df', 'p', 'McFadden', 'CoxSnell', 'Nagelkrk'}.
compute modresl={'coeff','se','Z','p','LLCI','ULCI'}.
end if.
end if
.
compute b=btemphld.
compute fresult={(LL2-basemod),llrdf,1-chicdf((LL2-basemod),llrdf)}
.
compute dbint=dbint+1.
end if.
compute highf={highf;fresult}.
compute highf2={highf2;fresult}.
do if (j = start).
compute flabel={flabel;'X*Z'}.
end if.
do if (j > start).
do if (nms > 1).
compute flabel={flabel;highlbz(jj,1)}.
else if (nms = 1).
compute flabel={flabel;'M*Z'}.
end if.
end if.
end if.
do if (dbint=2).
compute lmatdb=lmatdb+lmat.
do if ((ydich = 1) and (i = (nms+nys))).

.
compute btemphld=b.
compute llrdat=make(nrow(x),nrow( lmatdb )-csum( lmatdb ),-999).
compute llrdf=ncol(x)-ncol(llrdat).
compute llrcnt=0.
loop llri=1 to nrow( lmatdb ).
do if ( lmatdb (llri,1)=0).
compute llrcnt=llrcnt+1.
compute llrdat(:,llrcnt)=x(:,llri).
end if.
end loop.

.
do if ( 2 =1).
compute hatmat=inv(t( llrdat )* llrdat )*t( llrdat ).
compute b = hatmat* y.
compute modres=b.
do if ( 0 =1).
compute n1=nrow( llrdat ).
compute dfres=n1-(ncol( llrdat )).
compute sstotal = t( y -(csum( y )/n1))*( y -(csum( y )/n1)).
compute resid= y - llrdat *b.
compute ssresid = csum((resid)&**2).
compute r2 = (sstotal-ssresid)/sstotal.
do if (r2 < 0).
compute r2=0.
end if.
compute adjr2 = 1-((1-r2)*(n1-1)/(dfres)).
compute mse=ssresid/(n1-ncol( llrdat )).

.
compute n1=nrow( x ).
compute invXtX = inv(t( x )* x ).
compute varb = mse *invXtX.
compute k3 = ncol( x ).
compute xhc=0.
do if ( hc <> 5).
compute xhc= x.
compute hat = xhc(:,1).
loop i3=1 to nrow(xhc).
compute hat(i3,1)= xhc(i3,:)*invXtX*t(xhc(i3,:)).
end loop.
do if ( hc = 0 or hc =1).
loop i3 = 1 to k3.
compute xhc(:,i3)=xhc(:,i3)&* resid.
end loop.
end if.
do if ( hc =3 or hc =2).
loop i3=1 to k3.
compute xhc(:,i3) = ( resid &/(1-hat)&**(1/(4- hc )))&*xhc(:,i3).
end loop.
end if.
do if ( hc = 4).
compute hcmn=make(n,2,4).
compute hcmn(:,2)=(n1*hat)/k3.
loop i3= 1 to k3.
compute xhc(:,i3) = ( resid &/(1-hat)&**(rmin(hcmn)/2))&*xhc(:,i3).
end loop.
end if.
compute varb=(invXtX*t(xhc)*xhc*invXtX).
do if ( hc =1).
compute varb=(n1/(n1-ncol( x )))&*varb.
end if.
end if.
compute hclab={'se(HC0)','se(HC1)','se(HC2)','se(HC3)','se(HC4)','se'}.
compute hclab=hclab(1,( hc +1)).
compute hcflab={'F(HC0)','F(HC1)','F(HC2)','F(HC3)','F(HC4)','F'}.
compute hcflab=hcflab(1,( hc +1)).
release xhc
.
compute seb=sqrt(diag(varb)).
compute trat = b&/seb.
compute p = 2*(1-tcdf(abs(trat), (dfres))).
compute tval = sqrt(dfres* (exp((dfres-(5/6))*((xp2/(dfres-(2/3)+(.11/dfres)))* (xp2/(dfres-(2/3)+(.11/dfres)))))-1)).
compute modres={modres,seb,trat,p}.
compute modres={modres,(b-tval&*seb),(b+tval&*seb)}.
compute modresl={'coeff',hclab,'t','p','LLCI','ULCI'}.
compute lmat = ident(ncol( llrdat )).
compute lmat = lmat(:,2:ncol(lmat)).
compute fratio = (t(t(lmat)*b)*inv(t(lmat)*varb*lmat)*((t(lmat)*b)))/(ncol( llrdat )-1).
compute pfr = 1-fcdf(fratio,(ncol( llrdat )-1),dfres).
compute modsum={sqrt(r2),r2,mse,fratio,(ncol( llrdat )-1),dfres,pfr}.
compute modsuml={'R','R-sq','MSE',hcflab,'df1','df2', 'p'}.
end if.
end if.
do if ( 2 = 2 or 2 =3).
compute xlp= llrdat.
compute ylp= y.
compute pt2 = make(nrow(ylp),1,(csum(ylp)/nrow(ylp))).
do if ( 2 =2).
compute LL3 = ylp&*ln(pt2)+(1-ylp)&*ln(1-pt2).
end if.
compute LL3 = -2*csum(LL3).
compute bt1 = make(ncol(xlp),1,0).
compute LL1 = 0.
compute pt1 = make(nrow(ylp),1,0.5).
compute pt1lp=pt1.
loop jjj = 1 to iterate.
compute xlptmp=t(xlp).
compute vecprb=pt1lp&*(1-pt1lp).
loop kkk=1 to ncol(xlp).
compute xlptmp(kkk,:)=xlptmp(kkk,:)&*t(vecprb).
end loop.
compute b = bt1+inv(xlptmp*xlp)*t(xlp)*(ylp-pt1lp).
do if ( 2 =2).
compute xlpb=xlp*b.
compute xlpbt=(xlpb > -709.7).
compute xlpb709=(1-xlpbt)*(-709.7).
compute xlpb=(xlpb&*xlpbt)+xlpb709.
compute pt1lp = 1/(1+exp(-(xlpb))).
end if.
compute itprob = csum((pt1lp < .00000001) or (pt1lp > .9999999)).
do if (itprob > 0).
loop kkk = 1 to nrow(pt1lp).
do if (pt1lp(kkk,1) > .9999999).
compute pt1lp(kkk,1) = .9999999.
end if.
do if (pt1lp(kkk,1) < .00000001).
compute pt1lp(kkk,1) = .00000001.
end if.
end loop.
compute itprob = 0.
end if.
do if (itprob = 0).
do if ( 2 =2).
compute LL = ylp&*ln(pt1lp)+(1-ylp)&*ln(1-pt1lp).
end if.
compute LL2 = -2*csum(ll).
end if.
do if (abs(LL1-LL2) < converge).
do if ( 0 =1).
compute xlptmp=t(xlp).
compute vecprb=pt1lp&*(1-pt1lp).
loop kkk=1 to ncol(xlp).
compute xlptmp(kkk,:)=xlptmp(kkk,:)&*t(vecprb).
end loop.
compute varb = inv(xlptmp*xlp).
compute seb = sqrt(diag(varb)).
release xlptmp.
end if.
break.
end if.
compute bt1 = b.
compute LL1 = LL2.
end loop.
compute modres=b.
do if (jjj > iterate).
compute itprob = 2.
do if (booting=0).
compute iterrmod=1.
end if.
do if (booting=1).
compute bootiter=1.
end if.
do if (itprobtg=0).
compute itprobtg=1.
compute errcode(errs,1) = 47.
compute errs = errs + 1.
do if (booting = 0 and 0 =1).
compute vt1 = mdiag(pt1lp&*(1-pt1lp)).
compute varb = inv(t(xlp)*vt1*xlp).
compute seb = sqrt(diag(varb)).
end if.
end if.
end if.
do if ( 0 =1).
compute trat = b&/seb.
compute dfres=nrow(xlp).
compute p = 2*(1-cdfnorm(abs(trat))).
compute modres={modres,seb,trat,p}.
compute modres={modres,(b-xp2&*seb),(b+xp2&*seb)}.
compute pvchi=1-chicdf((LL3-LL2),(nrow(modres)-1)).
compute mcF = (LL3-LL2)/LL3.
compute cox = 1-exp(-(LL3-LL2)/nrow(xlp)).
compute nagel = cox/(1-exp(-(LL3)/nrow(xlp))).
compute modsum={LL2,(LL3-LL2),(nrow(modres)-1),pvchi, mcF,cox,nagel}.
compute modsuml={'-2LL','ModelLL', 'df', 'p', 'McFadden', 'CoxSnell', 'Nagelkrk'}.
compute modresl={'coeff','se','Z','p','LLCI','ULCI'}.
end if.
end if
.
compute b=btemphld.
compute fresult={(LL2-basemod),llrdf,1-chicdf((LL2-basemod),llrdf)}
.
end if.
do if (ydich <> 1 or i < (nms+nys)).

.
compute lmat2= lmatdb.
do if ( 0 =0).
compute lmat2 = mdiag( lmatdb ).
compute lmat3=make(nrow(lmat2),1,0).
loop flp=1 to ncol(lmat2).
do if (csum(lmat2(:,flp))=1).
compute lmat3={lmat3,lmat2(:,flp)}.
end if.
end loop.
compute lmat2=lmat3(:,2:ncol(lmat3)).
end if.
compute fratio = (t(t(lmat2)* b )*inv(t(lmat2)* varb *lmat2)*((t(lmat2)* b )))/ncol(lmat2).
compute pfr = 1-fcdf(fratio,ncol(lmat2),(n-nrow( b ))).
compute fresult={fratio,ncol(lmat2),(n-nrow( b )),pfr}.
do if (i = (nms+nys) and (ydich=1)).
compute fratio=fratio*ncol(lmat2).
compute pfr=1-chicdf(fratio,ncol(lmat2)).
compute fresult={fratio,ncol(lmat2),pfr}.
end if.
do if ( 1 =1).
compute lmat3=1-rsum(lmat2).
compute xfm=make(n,csum(lmat3),0).
compute flpc=1.
loop flp=1 to nrow(lmat3).
do if (lmat3(flp,1)=1).
compute xfm(:,flpc)=x(:,flp).
compute flpc=flpc+1.
end if.
end loop.
compute bfm=inv(t(xfm)*xfm)*t(xfm)*y.
compute resid=y-(xfm*bfm).
compute sstotal=(y-(csum(y)/n)).
compute sstotal=csum(sstotal&*sstotal).
compute ssresid=csum(resid&*resid).
compute rsqch= r2 -((sstotal-ssresid)/sstotal).
compute fresult={rsqch,fresult}.
release xfm,flpc, resid, ssresid, bfm.
end if
.
end if.
compute dbint=0.
compute highf={highf;fresult}.
do if (jj=0 and nms > 0).
compute flabel={flabel;'BOTH(X)'}.
end if.
do if (jj=0 and nms = 0).
compute flabel={flabel;'BOTH'}.
end if.
do if (jj>0 and nms = 1).
compute flabel={flabel;'BOTH(M)'}.
end if.
do if (nms > 1 and jj > 0).
compute flabel={flabel;highlbbt(jj,1)}.
end if.
end if.
compute lmat2=wzhigh(1:nump(1,i),j).
do if (csum(lmat2) > 0).
do if ((i < (nms+nys)) or (ydich <> 1)).

.
compute lmat2= lmat2.
do if ( 0 =0).
compute lmat2 = mdiag( lmat2 ).
compute lmat3=make(nrow(lmat2),1,0).
loop flp=1 to ncol(lmat2).
do if (csum(lmat2(:,flp))=1).
compute lmat3={lmat3,lmat2(:,flp)}.
end if.
end loop.
compute lmat2=lmat3(:,2:ncol(lmat3)).
end if.
compute fratio = (t(t(lmat2)* b )*inv(t(lmat2)* varb *lmat2)*((t(lmat2)* b )))/ncol(lmat2).
compute pfr = 1-fcdf(fratio,ncol(lmat2),(n-nrow( b ))).
compute fresult={fratio,ncol(lmat2),(n-nrow( b )),pfr}.
do if (i = (nms+nys) and (ydich=1)).
compute fratio=fratio*ncol(lmat2).
compute pfr=1-chicdf(fratio,ncol(lmat2)).
compute fresult={fratio,ncol(lmat2),pfr}.
end if.
do if ( 1 =1).
compute lmat3=1-rsum(lmat2).
compute xfm=make(n,csum(lmat3),0).
compute flpc=1.
loop flp=1 to nrow(lmat3).
do if (lmat3(flp,1)=1).
compute xfm(:,flpc)=x(:,flp).
compute flpc=flpc+1.
end if.
end loop.
compute bfm=inv(t(xfm)*xfm)*t(xfm)*y.
compute resid=y-(xfm*bfm).
compute sstotal=(y-(csum(y)/n)).
compute sstotal=csum(sstotal&*sstotal).
compute ssresid=csum(resid&*resid).
compute rsqch= r2 -((sstotal-ssresid)/sstotal).
compute fresult={rsqch,fresult}.
release xfm,flpc, resid, ssresid, bfm.
end if
.
end if.
do if ((ydich = 1) and (i = (nms+nys))).

.
compute btemphld=b.
compute llrdat=make(nrow(x),nrow( lmat2 )-csum( lmat2 ),-999).
compute llrdf=ncol(x)-ncol(llrdat).
compute llrcnt=0.
loop llri=1 to nrow( lmat2 ).
do if ( lmat2 (llri,1)=0).
compute llrcnt=llrcnt+1.
compute llrdat(:,llrcnt)=x(:,llri).
end if.
end loop.

.
do if ( 2 =1).
compute hatmat=inv(t( llrdat )* llrdat )*t( llrdat ).
compute b = hatmat* y.
compute modres=b.
do if ( 0 =1).
compute n1=nrow( llrdat ).
compute dfres=n1-(ncol( llrdat )).
compute sstotal = t( y -(csum( y )/n1))*( y -(csum( y )/n1)).
compute resid= y - llrdat *b.
compute ssresid = csum((resid)&**2).
compute r2 = (sstotal-ssresid)/sstotal.
do if (r2 < 0).
compute r2=0.
end if.
compute adjr2 = 1-((1-r2)*(n1-1)/(dfres)).
compute mse=ssresid/(n1-ncol( llrdat )).

.
compute n1=nrow( x ).
compute invXtX = inv(t( x )* x ).
compute varb = mse *invXtX.
compute k3 = ncol( x ).
compute xhc=0.
do if ( hc <> 5).
compute xhc= x.
compute hat = xhc(:,1).
loop i3=1 to nrow(xhc).
compute hat(i3,1)= xhc(i3,:)*invXtX*t(xhc(i3,:)).
end loop.
do if ( hc = 0 or hc =1).
loop i3 = 1 to k3.
compute xhc(:,i3)=xhc(:,i3)&* resid.
end loop.
end if.
do if ( hc =3 or hc =2).
loop i3=1 to k3.
compute xhc(:,i3) = ( resid &/(1-hat)&**(1/(4- hc )))&*xhc(:,i3).
end loop.
end if.
do if ( hc = 4).
compute hcmn=make(n,2,4).
compute hcmn(:,2)=(n1*hat)/k3.
loop i3= 1 to k3.
compute xhc(:,i3) = ( resid &/(1-hat)&**(rmin(hcmn)/2))&*xhc(:,i3).
end loop.
end if.
compute varb=(invXtX*t(xhc)*xhc*invXtX).
do if ( hc =1).
compute varb=(n1/(n1-ncol( x )))&*varb.
end if.
end if.
compute hclab={'se(HC0)','se(HC1)','se(HC2)','se(HC3)','se(HC4)','se'}.
compute hclab=hclab(1,( hc +1)).
compute hcflab={'F(HC0)','F(HC1)','F(HC2)','F(HC3)','F(HC4)','F'}.
compute hcflab=hcflab(1,( hc +1)).
release xhc
.
compute seb=sqrt(diag(varb)).
compute trat = b&/seb.
compute p = 2*(1-tcdf(abs(trat), (dfres))).
compute tval = sqrt(dfres* (exp((dfres-(5/6))*((xp2/(dfres-(2/3)+(.11/dfres)))* (xp2/(dfres-(2/3)+(.11/dfres)))))-1)).
compute modres={modres,seb,trat,p}.
compute modres={modres,(b-tval&*seb),(b+tval&*seb)}.
compute modresl={'coeff',hclab,'t','p','LLCI','ULCI'}.
compute lmat = ident(ncol( llrdat )).
compute lmat = lmat(:,2:ncol(lmat)).
compute fratio = (t(t(lmat)*b)*inv(t(lmat)*varb*lmat)*((t(lmat)*b)))/(ncol( llrdat )-1).
compute pfr = 1-fcdf(fratio,(ncol( llrdat )-1),dfres).
compute modsum={sqrt(r2),r2,mse,fratio,(ncol( llrdat )-1),dfres,pfr}.
compute modsuml={'R','R-sq','MSE',hcflab,'df1','df2', 'p'}.
end if.
end if.
do if ( 2 = 2 or 2 =3).
compute xlp= llrdat.
compute ylp= y.
compute pt2 = make(nrow(ylp),1,(csum(ylp)/nrow(ylp))).
do if ( 2 =2).
compute LL3 = ylp&*ln(pt2)+(1-ylp)&*ln(1-pt2).
end if.
compute LL3 = -2*csum(LL3).
compute bt1 = make(ncol(xlp),1,0).
compute LL1 = 0.
compute pt1 = make(nrow(ylp),1,0.5).
compute pt1lp=pt1.
loop jjj = 1 to iterate.
compute xlptmp=t(xlp).
compute vecprb=pt1lp&*(1-pt1lp).
loop kkk=1 to ncol(xlp).
compute xlptmp(kkk,:)=xlptmp(kkk,:)&*t(vecprb).
end loop.
compute b = bt1+inv(xlptmp*xlp)*t(xlp)*(ylp-pt1lp).
do if ( 2 =2).
compute xlpb=xlp*b.
compute xlpbt=(xlpb > -709.7).
compute xlpb709=(1-xlpbt)*(-709.7).
compute xlpb=(xlpb&*xlpbt)+xlpb709.
compute pt1lp = 1/(1+exp(-(xlpb))).
end if.
compute itprob = csum((pt1lp < .00000001) or (pt1lp > .9999999)).
do if (itprob > 0).
loop kkk = 1 to nrow(pt1lp).
do if (pt1lp(kkk,1) > .9999999).
compute pt1lp(kkk,1) = .9999999.
end if.
do if (pt1lp(kkk,1) < .00000001).
compute pt1lp(kkk,1) = .00000001.
end if.
end loop.
compute itprob = 0.
end if.
do if (itprob = 0).
do if ( 2 =2).
compute LL = ylp&*ln(pt1lp)+(1-ylp)&*ln(1-pt1lp).
end if.
compute LL2 = -2*csum(ll).
end if.
do if (abs(LL1-LL2) < converge).
do if ( 0 =1).
compute xlptmp=t(xlp).
compute vecprb=pt1lp&*(1-pt1lp).
loop kkk=1 to ncol(xlp).
compute xlptmp(kkk,:)=xlptmp(kkk,:)&*t(vecprb).
end loop.
compute varb = inv(xlptmp*xlp).
compute seb = sqrt(diag(varb)).
release xlptmp.
end if.
break.
end if.
compute bt1 = b.
compute LL1 = LL2.
end loop.
compute modres=b.
do if (jjj > iterate).
compute itprob = 2.
do if (booting=0).
compute iterrmod=1.
end if.
do if (booting=1).
compute bootiter=1.
end if.
do if (itprobtg=0).
compute itprobtg=1.
compute errcode(errs,1) = 47.
compute errs = errs + 1.
do if (booting = 0 and 0 =1).
compute vt1 = mdiag(pt1lp&*(1-pt1lp)).
compute varb = inv(t(xlp)*vt1*xlp).
compute seb = sqrt(diag(varb)).
end if.
end if.
end if.
do if ( 0 =1).
compute trat = b&/seb.
compute dfres=nrow(xlp).
compute p = 2*(1-cdfnorm(abs(trat))).
compute modres={modres,seb,trat,p}.
compute modres={modres,(b-xp2&*seb),(b+xp2&*seb)}.
compute pvchi=1-chicdf((LL3-LL2),(nrow(modres)-1)).
compute mcF = (LL3-LL2)/LL3.
compute cox = 1-exp(-(LL3-LL2)/nrow(xlp)).
compute nagel = cox/(1-exp(-(LL3)/nrow(xlp))).
compute modsum={LL2,(LL3-LL2),(nrow(modres)-1),pvchi, mcF,cox,nagel}.
compute modsuml={'-2LL','ModelLL', 'df', 'p', 'McFadden', 'CoxSnell', 'Nagelkrk'}.
compute modresl={'coeff','se','Z','p','LLCI','ULCI'}.
end if.
end if
.
compute b=btemphld.
compute fresult={(LL2-basemod),llrdf,1-chicdf((LL2-basemod),llrdf)}
.
end if.
compute highf={highf;fresult}.
compute highf2={highf2;fresult}.
do if (j = start).
compute flabel={flabel;'X*W*Z'}.
end if.
do if (j > start).
do if (nms > 1).
compute flabel={flabel;highlbwz(jj,1)}.
else if (nms = 1).
compute flabel={flabel;'M*W*Z'}.
end if.
end if.
end if.
compute jj=jj+1.
end loop.
release jj.
compute start=start+i.
end if.
do if (nrow(highf) > 1).
compute highf=highf(2:nrow(highf),:).
compute highf2=highf2(2:nrow(highf2),:).
compute flabel=flabel(2:nrow(flabel),1).
compute resultm2=make(nrow(highf),maxresm,99999).
compute resultm2(1:nrow(highf),1:ncol(highf))=highf.
compute resultm={resultm;resultm2}.
do if (outscree=1).
do if ((i < nms+nys) or (ydich=0)).
compute clabtmp={'R2-chng', hcflab,'df1','df2','p'}.
print highf/format = F10.4 /rnames=flabel/cnames=clabtmp/ title = 'Test(s) of highest order unconditional interaction(s):'.
end if.
do if (ydich=1 and i=(nms+nys)).
compute clabtmp={'Chi-sq', 'df','p'}.
print/title='Likelihood ratio test(s) of highest order'.
print highf/format = F10.4 /rnames=flabel/cnames=clabtmp/ title = 'unconditional interactions(s):'/space=0.
end if.
end if.
compute intpb=highf2(:,ncol(highf2)).
end if.
compute intstart=intstart+numint(1,i).
end if.

.
do if (criterr=0).
compute threeway=0.
compute didprint=0.
compute didsome=0.
compute sigintct=0.
loop jmed =1 to (nms+1).
compute hasw=0.
compute hasz=0.
compute jnok=0.
compute nm1vls=0.
compute nm2vls=0.
compute panelgrp=0.
compute graphixs={'WITH', outnames(1,i), 'BY'}.
compute focpred4={' '}.
compute intprint=0.
compute modcat=0.
do if (jmed <= i).
do if ((jmed = 1) and ((i+1) = nrow(bcmat))).
compute pathscnt=pathscnt+1.
else.
compute paths={paths,bcmat((i+1),jmed)}.
compute pathsw={pathsw,wcmat((i+1),jmed)}.
compute pathsz={pathsz,zcmat((i+1),jmed)}.
compute pathswz={pathswz,wzcmat((i+1),jmed)}.
compute temp=fochigh(:,pathscnt)&*bootloc(:,i).
compute pathsfoc={pathsfoc,pathsfoc(:,1)}.
do if (jmed=1).
compute pathtype={pathtype,1}.
end if.
do if ((i+1)=nrow(bcmat)).
compute pathtype={pathtype,3}.
end if.
do if (jmed > 1) and ((i+1) < nrow(bcmat)).
compute pathtype={pathtype,2}.
end if.
do if (jmed=1 and nxvls > 1 and (bcmat((i+1),jmed)=1)).
compute pathsfoc(:,(pathscn2+1))=temp(2:(nxvls+1),1).
end if.
do if ((jmed > 1) or (jmed=1 and nxvls=1)).
compute temp=cmax(temp).
compute pathsfoc(1,(pathscn2+1))=temp.
end if.
compute pathscnt=pathscnt+1.
compute pathscn2=pathscn2+1.
do if (i <= nms).
compute pathsdv={pathsdv,mnames(1,i)}.
end if.
do if (i > nms).
compute pathsdv={pathsdv,ynames}.
end if.
end if.
compute coeffcol=coeffcol+1.
compute probettt=coeffs(1:nrow(b),coeffcol).
do if (jmed=1 and (bcmat((i+1),jmed)=1)).
compute omni=make(nrow(probettt),nxvls,0).
compute omnitmp=ident(nxvls).
compute omni(2:(1+nxvls),:)=omnitmp.
end if.
do if (csum(probettt)>0).
compute probvarb=make(csum(probettt),csum(probettt),999).
compute probcoef=make(csum(probettt),1,999).
compute coefflp2=1.
loop coefflp=1 to nrow(probettt).
do if (probettt(coefflp,1)=1).
compute probcoef(coefflp2,1)=b(coefflp,1).
compute coefflp2=coefflp2+1.
end if.
end loop.
compute coefflp=0.
compute coefflp2=0.
loop iclp=1 to nrow(probettt).
do if probettt(iclp,1)=1.
compute coefflp=coefflp+1.
compute coefflp2=coefflp.
compute probvarb(coefflp,coefflp) = varb(iclp,iclp).
do if (iclp < nrow(probettt)).
loop jclp=(iclp+1) to nrow(probettt).
do if (probettt(jclp,1)=1).
compute coefflp2=coefflp2+1.
compute probvarb(coefflp,coefflp2)=varb(iclp, jclp).
compute probvarb(coefflp2,coefflp)=varb(iclp, jclp).
end if.
end loop.
end if.
end if.
end loop.
end if.
end if.
compute xprobval=xmodvals.
do if (nxvls > 1 or mcx > 0).
compute xprobval=dummatx(:,2:ncol(dummatx)).
end if.
do if ((wcmat((i+1),jmed)=1) and (zcmat((i+1),jmed)=0)).
compute numplps=1.
compute modvals=wmodvals.
compute probeval=wmodvals.
compute wheremv1=wherexw.
compute nm1vls=nwvls.
compute lpstsp={1,1}.
compute modcat=0.
compute jnmod=wtmp.
compute jnmodlab=wnames.
compute jnok=1.
compute jnmin=wmin.
compute jnmax=wmax.
compute wherejn1=2.
do if (jmed=1).
compute wherejn3=wherexw(1,i).
do if (nxvls > 1).
compute jnok=0.
end if.
end if.
do if (jmed > 1).
compute wherejn1=wherem((jmed-1),i).
compute wherejn3=wheremw(((2*jmed)-3),i).
end if.
do if (nwvls > 1).
compute probeval=wprobval.
compute lpstsp(1,2)=ncol(probeval).
compute modcat=1.
compute jnok=0.
end if.
do if (wdich = 1).
compute modcat=1.
compute jnok=0.
end if.
compute problabs=wnames.
compute focpred3={wnames,'(W)'}.
do if (xmint=1).
compute focpred3={wnames,'(X)'}.
end if.
compute hasw=1.
compute modgrph=wnames.
compute intprint=1.
compute sigintct=sigintct+1.
compute printpbe=intpb(sigintct,1).
end if.
do if ((wcmat((i+1),jmed)=0) and (zcmat((i+1),jmed)=1)).
compute numplps=1.
compute modvals=zmodvals.
compute probeval=zmodvals.
compute wheremv1=wherexz.
compute nm1vls=nzvls.
compute lpstsp={1,1}.
compute jnok=1.
compute jnmod=ztmp.
compute jnmin=zmin.
compute jnmax=zmax.
compute jnmodlab=znames.
compute wherejn1=2.
do if (jmed=1).
compute wherejn3=wherexz(1,i).
do if (nxvls > 1).
compute jnok=0.
end if.
end if.
do if (jmed > 1).
compute wherejn1=wherem((jmed-1),i).
compute wherejn3=wheremz(((2*jmed)-3),i).
end if.
do if (nzvls > 1).
compute probeval=zprobval.
compute lpstsp(1,2)=ncol(probeval).
compute modcat=1.
compute jnok=0.
end if.
do if (zdich = 1).
compute modcat=1.
compute jnok=0.
end if.
compute problabs=znames.
compute focpred3={znames,'(Z)'}.
compute modgrph=znames.
compute hasz=1.
compute intprint=1.
compute sigintct=sigintct+1.
compute printpbe=intpb(sigintct,1).
end if.
do if ((wzcmat((i+1),jmed)=1) or ((wcmat((i+1),jmed)=1) and (zcmat((i+1),jmed)=1))).
compute numplps=2.
compute probecnt=1.
compute intprint=1.
do if (wzcmat((i+1),jmed)=1).
compute sigintct=sigintct+1.
compute printpbe=intpb(sigintct,1).
else.
compute sigintct=sigintct+2.
compute printpbe=cmin(intpb((sigintct-1):sigintct,1)).
end if.
compute panelgrp=1.
compute hasw=1.
compute hasz=1.
compute panelcde={'/PANEL','ROWVAR=',znames,'.'}.
compute modgrph=wnames.
compute lpstsp={1,1;1,1}.
compute wheremv1=wherexw.
compute nm1vls=nwvls.
compute wheremv2=wherexz.
compute nm2vls=nzvls.
compute jnok=0.
do if (wzcmat((i+1),jmed)=1).
compute jnok=1.
end if.
do if (jmed > 1).
compute mprobval=mmodvals.
end if.
do if (jmed=1).
do if (nxvls > 1).
compute jnok=0.
end if.
end if.
do if (nwvls > 1).
compute lpstsp(1,2)=ncol(wprobval).
compute modcat=1.
compute jnok=0.
end if.
do if (zdich=1).
compute modcat=1.
compute jnok=0.
end if.
compute lpstsp(2,1)=lpstsp(1,2)+1.
compute lpstsp(2,2)=lpstsp(1,2)+1.
do if (nzvls > 1).
compute lpstsp(2,1)=lpstsp(1,2)+1.
compute lpstsp(2,2)=lpstsp(1,2)+ncol(zprobval).
compute jnok=0.
end if.
do if (zdich=1).
compute jnok=0.
end if.
compute omni3=make(nrow(b),(nxvls*nwvls),0).
do if (jmed > 1).
compute omni3=make(nrow(b),nwvls,0).
end if.
compute focpred3={wnames,'(W)'}.
do if (xmint=1).
compute focpred3={wnames,'(X)'}.
end if.
compute focpred4={'    ', 'Mod var:', znames, '(Z)'}.
compute modvals=make((nrow(wmodvals)*nrow(zmodvals)),2,0).
compute probeval=make((nrow(wmodvals)*nrow(zmodvals)),(ncol(wprobval)+ncol(zprobval)),0).
loop probei= 1 to nrow(wmodvals).
loop probej =1 to nrow(zmodvals).
compute modvals(probecnt,1)=wmodvals(probei,1).
compute probeval(probecnt,1:nwvls)=wprobval(probei,:).
compute modvals(probecnt,2)=zmodvals(probej,1).
compute probeval(probecnt,(nwvls+1):(nwvls+nzvls))=zprobval(probej,:).
compute probecnt=probecnt+1.
end loop.
end loop.
do if (wzcmat((i+1),jmed)=1).
compute numplps=numplps+1.
compute probprod=make(1,(ncol(wprobval)*ncol(zprobval)),0).
compute lpstsp2={1,1}.
compute lpstsp={lpstsp;lpstsp2}.
compute lpstsp(3,1)=lpstsp(2,2)+1.
compute lpstsp(3,2)=lpstsp(2,2)+ncol(probprod).
compute jnmod=ztmp.
compute jnmin=zmin.
compute jnmax=zmax.
compute jnmodlab=znames.
do if (jmed = 1).
compute wherejn1=wherexw(1,i).
compute wherejn3=wherexwz(1,i).
end if.
do if (jmed > 1).
compute wherejn1=wheremw(((2*jmed)-3),i).
compute wherejn3=wheremwz(((2*jmed)-3),i).
end if.
loop probei = 1 to nrow(wmodvals).
loop probej = 1 to nrow(zmodvals).
compute probtemp=1.
loop probek = 1 to ncol(wprobval).
compute probtemp={probtemp,(wprobval(probei,probek)&*zprobval(probej,:))}.
end loop.
compute probprod={probprod;probtemp(1,2:ncol(probtemp))}.
end loop.
end loop.
compute probprod=probprod(2:nrow(probprod),:).
compute probeval={probeval,probprod}.
end if.
compute problabs={wnames,znames}.
release probecnt, probei, probej.
end if.
do if (intprint=1).
compute focpred={'   Focal', 'predict:'}.
do if (jmed=1).
compute focpred={focpred,xnames,'(X)'}.
compute focplotv=xmodvals.
end if.
do if (jmed >1).
do if (nms > 1).
compute focpred={focpred,mnames(1,(jmed-1)), medlb2(1,(jmed-1))}.
end if.
do if (nms = 1).
compute focpred={focpred,mnames(1,(jmed-1)), '(M)'}.
end if.
compute focplotv=mmodvals(:,(jmed-1)).
end if.
compute focpred2={'    ', 'Mod var:',focpred3}.
compute focpred={focpred;focpred2}.
do if (ncol(focpred4) > 1).
compute focpred={focpred;focpred4}.
compute focpred4={' '}.
end if.
release focpred2,focpred3.
do if (outscree=1).
do if ((plot = 1 or plot = 2) or (printpbe <= intprobe)).
print focpred/title='----------'/format=A8/space=0.
end if.
end if.
compute foctmp=make(nrow(modvals),1,1).
compute probexpl=1.
compute probeva2={foctmp,probeval}.
do if (jmed=1 and nxs > 0 and mcx > 0).
compute probexpl=nxvls.
end if.
compute foctmp=make(nrow(modvals),1,1).
compute modvals3=make(1,(6+ncol(problabs)),0).
compute probrown=make(nrow(probeval),1,0).
compute jtmp=1.
loop probei = 1 to nrow(probeval).
compute probrown(probei,1)=jtmp.
compute jtmp=jtmp+nxvls.
end loop.
release jtmp.
compute probrow=999.
compute modvarl=problabs.
do if (plot = 1 or plot = 2 or nxvls > 1).
compute plotvals=make((nrow(modvals)*nrow(focplotv)),(ncol(modvals)+1),999).
loop ploti=1 to nrow(modvals).
loop plotj=1 to nrow(focplotv).
compute plotvals((((ploti-1)*nrow(focplotv))+plotj),2:ncol(plotvals))=modvals(ploti,:).
compute plotvals((((ploti-1)*nrow(focplotv))+plotj),1)=focplotv(plotj,1).
end loop.
end loop.
compute focpredn=3.
do if (jmed=1).
do if (nxvls > 1).
compute focpredn=(nxvls+1).
end if.
do if (nxvls=1 and xdich=1).
compute focpredn=2.
end if.
end if.
compute meanmat=mdiag(means).
compute onesmat=make(nrow(meanmat),(nrow(probeval)*focpredn),1).
compute probeplt=t(mdiag(means)*onesmat).
do if (jmed=1).
do if ((wcmat((i+1),1)=1) or (zcmat((i+1),1)=1)).
compute plotcnt=1.
compute iloops=nwpval*nzpval.
compute plotmx=nxpval*nzpval.
do if ((wcmat((i+1),1)=1) and (zcmat((i+1),1)=0)).
compute iloops=nwpval.
compute plotmx=nxpval.
end if.
do if ((wcmat((i+1),1)=0) and (zcmat((i+1),1)=1)).
compute iloops=nzpval.
compute plotmx=nxpval.
end if.
compute xestvals=make((nxpval*iloops),ncol(xprobval),-999).
do if (wcmat((i+1),1)=1).
compute westvals=make(nrow(xestvals),ncol(wprobval),-999).
end if.
do if (zcmat((i+1),1)=1).
compute zestvals=make(nrow(xestvals),ncol(zprobval),-999).
end if.
loop ploti=1 to iloops.
loop plotj=1 to nxpval.
compute xestvals(plotcnt,:)=xprobval(plotj,:).
compute plotcnt=plotcnt+1.
end loop.
end loop.
compute plotcnt=1.
compute plotcnt1=1.
compute plotcnt2=1.
compute plotcntz=1.
loop ploti = 1 to (iloops*nxpval).
do if (wcmat((i+1),1)=1).
compute westvals(ploti,:)=wprobval(plotcnt1,:).
end if.
do if ((wcmat((i+1),1)=0) and (zcmat((i+1),1)=1)).
compute zestvals(ploti,:)=zprobval(plotcnt1,:).
end if.
do if ((wcmat((i+1),1)=1) and (zcmat((i+1),1)=1)).
compute zestvals(ploti,:)=zprobval(plotcnt2,:).
compute plotcntz=plotcntz+1.
end if.
compute plotcnt=plotcnt+1.
do if (plotcnt > plotmx).
compute plotcnt=1.
compute plotcnt1=plotcnt1+1.
end if.
do if (plotcntz > nxpval).
compute plotcnt2=plotcnt2+1.
compute plotcntz=1.
do if (plotcnt2 > nzpval).
compute plotcnt2=1.
end if.
end if.
end loop.
compute probeplt(:,2:(1+(ncol(xestvals))))=xestvals.
do if (wcmat((i+1),1)=1).
compute probeplt(:,(wherew(1,i)):(wherew(2,i)))=westvals.
end if.
do if (zcmat((i+1),1)=1).
compute probeplt(:,(wherez(1,i)):(wherez(2,i)))=zestvals.
end if.
end if.
end if.
do if (jmed > 1).
do if ((wcmat((i+1),jmed)=1) or (zcmat((i+1),jmed)=1)).
compute plotcnt=1.
compute iloops=nwpval*nzpval.
compute plotmx=3*nzpval.
do if ((wcmat((i+1),jmed)=1) and (zcmat((i+1),jmed)=0)).
compute iloops=nwpval.
compute plotmx=3.
end if.
do if ((wcmat((i+1),jmed)=0) and (zcmat((i+1),jmed)=1)).
compute iloops=nzpval.
compute plotmx=3.
end if.
compute mestvals=make((3*iloops),1,-999).
do if (wcmat((i+1),jmed)=1).
compute westvals=make(nrow(mestvals),ncol(wprobval),-999).
end if.
do if (zcmat((i+1),jmed)=1).
compute zestvals=make(nrow(mestvals),ncol(zprobval),-999).
end if.
loop ploti=1 to iloops.
loop plotj=1 to 3.
compute mestvals(plotcnt,:)=mprobval(plotj,(jmed-1)).
compute plotcnt=plotcnt+1.
end loop.
end loop.
compute plotcnt=1.
compute plotcnt1=1.
compute plotcnt2=1.
compute plotcntz=1.
loop ploti = 1 to (iloops*3).
do if (wcmat((i+1),jmed)=1).
compute westvals(ploti,:)=wprobval(plotcnt1,:).
end if.
do if ((wcmat((i+1),jmed)=0) and (zcmat((i+1),jmed)=1)).
compute zestvals(ploti,:)=zprobval(plotcnt1,:).
end if.
do if ((wcmat((i+1),jmed)=1) and (zcmat((i+1),jmed)=1)).
compute zestvals(ploti,:)=zprobval(plotcnt2,:).
compute plotcntz=plotcntz+1.
end if.
compute plotcnt=plotcnt+1.
do if (plotcnt > plotmx).
compute plotcnt=1.
compute plotcnt1=plotcnt1+1.
end if.
do if (plotcntz > 3).
compute plotcnt2=plotcnt2+1.
compute plotcntz=1.
do if (plotcnt2 > nzpval).
compute plotcnt2=1.
end if.
end if.
end loop.
compute probeplt(:,wherem((jmed-1),i))=mestvals.
do if (wcmat((i+1),jmed)=1).
do if (model <> 74).
compute probeplt(:,(wherew(1,i)):(wherew(2,i)))=westvals.
end if.
do if (model = 74).
compute probeplt(:,(wherex(1,i)):(wherex(2,i)))=westvals.
end if.
end if.
do if (zcmat((i+1),jmed)=1).
compute probeplt(:,(wherez(1,i)):(wherez(2,i)))=zestvals.
end if.
end if.
end if.
compute prodloop = 1.
do if (jmed=1).
compute prodloop=ncol(xestvals).
end if.
do if (wcmat((i+1),jmed))=1.
compute plotcnt=0.
loop ploti = 1 to prodloop.
loop plotj = 1 to ncol(westvals).
do if (jmed=1).
compute probeplt(:,(wherexw(1,i)+plotcnt))=xestvals(:,ploti)&*westvals(:,plotj).
end if.
do if (jmed > 1).
compute probeplt(:,(wheremw(((jmed*2)-3) ,i)+plotcnt))=mestvals(:,ploti)&*westvals(:,plotj).
end if.
compute plotcnt=plotcnt+1.
end loop.
end loop.
end if.
do if (zcmat((i+1),jmed))=1.
compute plotcnt=0.
loop ploti = 1 to prodloop.
loop plotj = 1 to ncol(zestvals).
do if (jmed = 1).
compute probeplt(:,(wherexz(1,i)+plotcnt))=xestvals(:,ploti)&*zestvals(:,plotj).
end if.
do if (jmed > 1).
compute probeplt(:,(wheremz(((jmed*2)-3),i)+plotcnt))=mestvals(:,ploti)&*zestvals(:, plotj).
end if.
compute plotcnt=plotcnt+1.
end loop.
end loop.
end if.
do if (wzcmat((i+1),jmed))=1.
compute plotcnt=0.
compute threeway=1.
loop ploti = 1 to ncol(westvals).
loop plotj = 1 to ncol(zestvals).
compute probeplt(:,(wherewz(1,i)+plotcnt))=westvals(:,ploti)&*zestvals(:,plotj).
compute plotcnt=plotcnt+1.
end loop.
end loop.
compute plotcnt=0.
loop plotk = 1 to prodloop.
loop ploti = 1 to ncol(westvals).
loop plotj = 1 to ncol(zestvals).
do if (jmed = 1).
compute probeplt(:,(wherexwz(1,i)+plotcnt))=xestvals(:,plotk)&*westvals(:,ploti)&* zestvals(:,plotj).
end if.
do if (jmed > 1).
compute probeplt(:,(wheremwz(((jmed*2)-3),i)+plotcnt))=mestvals(:,plotk)&* westvals(:,ploti)&*zestvals(:,plotj).
end if.
compute plotcnt=plotcnt+1.
end loop.
end loop.
end loop.
end if.
loop newplp=1 to i.
do if (newplp <> jmed).
do if (wcmat((i+1),newplp))=1.
compute prodloop=1.
do if (newplp=1).
compute prodloop=nxvls.
end if.
compute plotcnt=0.
loop ploti = 1 to prodloop.
loop plotj = 1 to nwvls.
do if (newplp = 1).
compute probeplt(:,(wherexw(1,i)+plotcnt))=probeplt(:,(1+ploti))&*probeplt(:, (wherew(1,i)+plotj-1)).
end if.
do if (newplp > 1).
do if (model <> 74).
compute probeplt(:,(wheremw(((newplp*2)-3) ,i)+plotcnt))=probeplt(:,wherem((newplp-1),i))&*probeplt(:,(wherew(1,i)+plotj-1)).
end if.
do if (model = 74).
compute probeplt(:,(wheremw(((newplp*2)-3) ,i)+plotcnt))=probeplt(:,wherem((newplp-1),i))&*probeplt(:,(wherex(1,i)+plotj-1)).
end if.
end if.
compute plotcnt=plotcnt+1.
end loop.
end loop.
end if.
do if (zcmat((i+1),newplp))=1.
compute prodloop=1.
do if (newplp=1).
compute prodloop=nxvls.
end if.
compute plotcnt=0.
loop ploti = 1 to prodloop.
loop plotj = 1 to nzvls.
do if (newplp = 1).
compute probeplt(:,(wherexz(1,i)+plotcnt))=probeplt(:,(1+ploti))&*probeplt(:, (wherez(1,i)+plotj-1)).
end if.
do if (newplp > 1).
compute probeplt(:,(wheremz(((newplp*2)-3),i)+plotcnt))=probeplt(:, wherem((newplp-1),i))&*probeplt(:,(wherez(1,i)+plotj-1)).
end if.
compute plotcnt=plotcnt+1.
end loop.
end loop.
end if.
do if (wzcmat((i+1),newplp))=1.
compute plotcnt=0.
do if (threeway=0).
loop ploti = 1 to nwvls.
loop plotj = 1 to nzvls.
compute probeplt(:,(wherewz(1,i)+plotcnt))=probeplt(:,(wherew(1,i)+ploti-1))&* probeplt(:,(wherez(1,i)+plotj-1)).
compute plotcnt=plotcnt+1.
end loop.
end loop.
end if.
compute prodloop=1.
do if (newplp=1).
compute prodloop=nxvls.
end if.
compute plotcnt=0.
loop plotk = 1 to prodloop.
loop ploti = 1 to nwvls.
loop plotj = 1 to nzvls.
do if (newplp = 1).
compute probeplt(:,(wherexwz(1,i)+plotcnt))=probeplt(:,(1+plotk))&*probeplt(:, (wherew(1,i)+ploti-1))&*probeplt(:,(wherez(1,i)+plotj-1)).
end if.
do if (newplp > 1).
compute probeplt(:,(wheremwz(((newplp*2)-3),i)+plotcnt))=probeplt(:, wherem((newplp-1),i))&*probeplt(:,(wherew(1,i)+ploti-1))&*probeplt(:,(wherez(1,i)+plotj-1)).
end if.
compute plotcnt=plotcnt+1.
end loop.
end loop.
end loop.
end if.
end if.
end loop.
compute predvals=probeplt*b.
do if (debug <> 0).
print probeplt.
print b.
end if.
do if (i = nms+nys) and (ydich=1).
compute predvalt=(predvals < 709.7).
compute prevalt7=(1-predvalt)*(709.7).
compute predvals=(predvals&*predvalt)+prevalt7.
compute expyhat=exp(predvals)&/(1+exp(predvals)).
end if.
compute sepred=make(nrow(plotvals),3,999).
loop sei=1 to nrow(plotvals).
compute ask=probeplt(sei,:).
compute sepred(sei,1)=sqrt(ask*varb*t(ask)).
do if ((i < nms+nys) or (ydich=0)).
compute sepred(sei,2)=predvals(sei,1)-tval*sepred(sei,1).
compute sepred(sei,3)=predvals(sei,1)+tval*sepred(sei,1).
end if.
do if ((i = nms+nys) and (ydich=1)).
compute sepred(sei,2)=predvals(sei,1)-xp2*sepred(sei,1).
compute sepred(sei,3)=predvals(sei,1)+xp2*sepred(sei,1).
end if.
end loop.
compute prevloc=ncol(plotvals)+1.
compute probeplt={plotvals,predvals}.
do if (plot = 2).
compute probeplt={probeplt,sepred}.
end if.
do if ((i = nms+nys) and (ydich=1)).
compute probeplt={probeplt,expyhat}.
end if.
compute didsome=0.
end if.
do if ((wzcmat((i+1),jmed)=1) and (printpbe <= intprobe)).
do if (jmed=1).
compute omnilp2=nxvls*nwvls.
compute omnitmp=ident(omnilp2).
compute omni3(wherexw(1,i):wherexw(2,i),:)=omnitmp.
end if.
do if (jmed>1).
compute omnilp2=nwvls.
compute omnitmp=ident(omnilp2).
compute omni3(wheremw(((jmed*2)-3),i):wheremw(((jmed*2)-2),i),:)=omnitmp.
end if.
compute omnif=make(1,4,0).
do if ((i = nms+nys) and (ydich=1)).
compute omnif=make(1,3,0).
end if.
compute condeff3=0.
loop omnilp1=1 to nrow(zprobval).
loop omnilp=1 to (omnilp2).
do if (jmed=1).
compute omni3((wherexwz(1,i)+((omnilp-1)*nzvls)):(wherexwz(1,i)+((omnilp-1)*nzvls)+ (nzvls-1)),omnilp)=t(zprobval(omnilp1,:)).
end if.
do if (jmed > 1).
compute omni3((wheremwz(((jmed*2)-3),i)+((omnilp-1)*nzvls)):(wheremwz(((jmed*2)-3),i)+ ((omnilp-1)*nzvls)+(nzvls-1)),omnilp)=t(zprobval(omnilp1,:)).
end if.
end loop.
compute condeff=t(omni3)*b.
compute condeff3={condeff3;condeff}.

.
compute lmat2= omni3.
do if ( 1 =0).
compute lmat2 = mdiag( omni3 ).
compute lmat3=make(nrow(lmat2),1,0).
loop flp=1 to ncol(lmat2).
do if (csum(lmat2(:,flp))=1).
compute lmat3={lmat3,lmat2(:,flp)}.
end if.
end loop.
compute lmat2=lmat3(:,2:ncol(lmat3)).
end if.
compute fratio = (t(t(lmat2)* b )*inv(t(lmat2)* varb *lmat2)*((t(lmat2)* b )))/ncol(lmat2).
compute pfr = 1-fcdf(fratio,ncol(lmat2),(n-nrow( b ))).
compute fresult={fratio,ncol(lmat2),(n-nrow( b )),pfr}.
do if (i = (nms+nys) and (ydich=1)).
compute fratio=fratio*ncol(lmat2).
compute pfr=1-chicdf(fratio,ncol(lmat2)).
compute fresult={fratio,ncol(lmat2),pfr}.
end if.
do if ( 0 =1).
compute lmat3=1-rsum(lmat2).
compute xfm=make(n,csum(lmat3),0).
compute flpc=1.
loop flp=1 to nrow(lmat3).
do if (lmat3(flp,1)=1).
compute xfm(:,flpc)=x(:,flp).
compute flpc=flpc+1.
end if.
end loop.
compute bfm=inv(t(xfm)*xfm)*t(xfm)*y.
compute resid=y-(xfm*bfm).
compute sstotal=(y-(csum(y)/n)).
compute sstotal=csum(sstotal&*sstotal).
compute ssresid=csum(resid&*resid).
compute rsqch= 0 -((sstotal-ssresid)/sstotal).
compute fresult={rsqch,fresult}.
release xfm,flpc, resid, ssresid, bfm.
end if
.
compute omnif={omnif;fresult}.
end loop.
compute omnif=omnif(2:nrow(omnif),:).
compute clabtmp=znames.
compute condeff3=condeff3(2:nrow(condeff3),:).
do if ((nxvls*nwvls)=1).
compute omnif={condeff3,omnif}.
compute clabtmp={clabtmp,'Effect'}.
end if.
compute omnif={zmodvals,omnif}.
do if ((i < nms+nys) or (ydich=0)).
compute clabtmp={clabtmp,hcflab,'df1','df2','p'}.
end if.
do if ((i = (nms+nys)) and (ydich=1)).
compute clabtmp={clabtmp,'Chi-sq','df','p'}.
end if.
compute resultm2=make(nrow(omnif),maxresm,99999).
compute resultm2(1:nrow(omnif),1:ncol(omnif))=omnif.
compute resultm={resultm;resultm2}.
do if (outscree=1).
do if (jmed=1).
print omnif/title='Test of conditional X*W interaction at value(s) of Z:'/cnames=clabtmp/format= F10.4.
end if.
do if (jmed>1).
print omnif/title='Test of conditional M*W interaction at value(s) of Z:'/cnames=clabtmp/format= F10.4.
end if.
end if.
release omni3.
end if.
loop probei = 1 to probexpl.
do if (probexpl > 1).
compute foctmp=make(nrow(modvals),probexpl,0).
compute foctmp(:,probei)=foctmp(:,probei)+1.
compute probtemp=make(nrow(modvals),1,0).
loop probem = 1 to numplps.
loop probek = 1 to nxvls.
loop probej=lpstsp(probem,1) to lpstsp(probem,2).
compute probtemp={probtemp,foctmp(:,probek)&*probeval(:,probej)}.
end loop.
end loop.
end loop.
compute probeva2=probtemp(:,2:ncol(probtemp)).
compute probeva2={foctmp,probeva2}.
end if.
compute probres=probeva2*probcoef.
compute probrese=sqrt(diag(probeva2*probvarb*t(probeva2))).
compute tratio = probres&/probrese.
compute p = 2*(1-tcdf(abs(tratio), dfres)).
do if (ydich=1 and i = (nms+nys)).
compute p = 2*(1-cdfnorm(abs(tratio))).
end if.
compute modvals2={modvals,probres,probrese,tratio, p}.
do if ((i < nms+nys) or (ydich=0)).
compute modvals2={modvals2,(probres-tval&*probrese),(probres+tval&*probrese)}.
compute problabs={problabs,'Effect',hclab,'t', 'p', 'LLCI', 'ULCI'}.
end if.
do if (ydich=1 and i = (nms+nys)).
compute modvals2={modvals2,(probres-xp2&*probrese),(probres+xp2&*probrese)}.
compute problabs={problabs,'Effect','se','Z', 'p', 'LLCI', 'ULCI'}.
end if.
do if (probexpl > 1 and (printpbe <= intprobe)).
do if (hasz = 1).
compute printz=1.
end if.
do if (hasw=1).
compute printw=1.
end if.
compute probrlab=make(nrow(modvals),1,xcatlab(probei,1)).
compute modvals3={modvals3; modvals2}.
compute probrow={probrow;probrown}.
compute probrown=probrown+1.
do if (probei=probexpl).
compute xproblab=xcatlab(1:nxvls,1).
compute probrow=probrow(2:nrow(probrow),1).
compute modvals3=modvals3(2:nrow(modvals3),:).
compute temp=modvals3.
compute temp(GRADE(probrow(:,1)),:)=modvals3.
compute modvals3=temp.
compute start2=1.
compute problabs=problabs(1,(1+(ncol(modvarl))):ncol(problabs)).
compute pstart=1.
loop probek= 1 to nrow(probeval).
compute endstart=start2+(nxvls-1).
compute temp=modvals3(start2:endstart,(1+ncol(modvarl)):ncol(modvals3)).
compute temp2=t(modvals3(start2:start2,1:ncol(modvarl))).
compute trnames=t(modvarl).
do if (outscree=1).
do if (probek > 1).
print/title='----------'/space=0.
else.
print/title = 'Conditional effects of the focal predictor at values of the moderator(s):'.
do if ((jmed=1) and (i = (nms+nys)) and (nms > 0)).
do if (nxvls = 1).
print/title = '(These are also the conditional direct effects of X on Y)'/space=0.
else.
print/title = '(These are also the relative conditional direct effects of X on Y)'/space=0.
end if.
end if.
print.
end if.
print temp2/title = 'Moderator value(s):'/rnames=trnames/format= F10.4 /space=0.
print temp/title = ' '/cnames=problabs/rnames=xproblab/format= F10.4 /space=0.
end if.
compute resultm2=make(nrow(temp2),maxresm,99999).
compute resultm2(1:nrow(temp2),1:ncol(temp2))=temp2.
compute resultm={resultm;resultm2}.
compute resultm2=make(nrow(temp),maxresm,99999).
compute resultm2(1:nrow(temp),1:ncol(temp))=temp.
compute resultm={resultm;resultm2}.
compute start2=start2+nxvls.
compute didsome=1.
do if (jmed=1).
compute mod1val=probeval(probek,1:nm1vls).
loop omnilp=1 to nxvls.
compute omni((wheremv1(1,i)+((omnilp-1)*nm1vls)):(wheremv1(1,i)+((omnilp-1)* nm1vls)+(nm1vls-1)),omnilp)=t(mod1val).
do if (nm1vls < ncol(probeval)).
compute mod2val=probeval(probek,(nm1vls+1):(nm1vls+nm2vls)).
compute omni((wheremv2(1,i)+((omnilp-1)*nm2vls)):(wheremv2(1,i)+((omnilp-1)* nm2vls)+(nm2vls-1)),omnilp)=t(mod2val).
do if ((nm1vls+nm2vls) < ncol(probeval)).
compute intlen=nm1vls*nm2vls.
compute modintvl=probeval(probek,(nm1vls+nm2vls+1):ncol(probeval)).
compute omni((wherexwz(1,i)+((omnilp-1)*intlen)):(wherexwz(1,i)+((omnilp-1)* intlen)+(intlen-1)),omnilp)=t(modintvl).
end if.
end if.
end loop.

.
compute lmat2= omni.
do if ( 1 =0).
compute lmat2 = mdiag( omni ).
compute lmat3=make(nrow(lmat2),1,0).
loop flp=1 to ncol(lmat2).
do if (csum(lmat2(:,flp))=1).
compute lmat3={lmat3,lmat2(:,flp)}.
end if.
end loop.
compute lmat2=lmat3(:,2:ncol(lmat3)).
end if.
compute fratio = (t(t(lmat2)* b )*inv(t(lmat2)* varb *lmat2)*((t(lmat2)* b )))/ncol(lmat2).
compute pfr = 1-fcdf(fratio,ncol(lmat2),(n-nrow( b ))).
compute fresult={fratio,ncol(lmat2),(n-nrow( b )),pfr}.
do if (i = (nms+nys) and (ydich=1)).
compute fratio=fratio*ncol(lmat2).
compute pfr=1-chicdf(fratio,ncol(lmat2)).
compute fresult={fratio,ncol(lmat2),pfr}.
end if.
do if ( 0 =1).
compute lmat3=1-rsum(lmat2).
compute xfm=make(n,csum(lmat3),0).
compute flpc=1.
loop flp=1 to nrow(lmat3).
do if (lmat3(flp,1)=1).
compute xfm(:,flpc)=x(:,flp).
compute flpc=flpc+1.
end if.
end loop.
compute bfm=inv(t(xfm)*xfm)*t(xfm)*y.
compute resid=y-(xfm*bfm).
compute sstotal=(y-(csum(y)/n)).
compute sstotal=csum(sstotal&*sstotal).
compute ssresid=csum(resid&*resid).
compute rsqch= 0 -((sstotal-ssresid)/sstotal).
compute fresult={rsqch,fresult}.
release xfm,flpc, resid, ssresid, bfm.
end if
.
compute resultm2=make(nrow(fresult),maxresm,99999).
compute resultm2(1:nrow(fresult),1:ncol(fresult))=fresult.
compute resultm={resultm;resultm2}.
do if (outscree=1).
do if (i < (nms + nys) or (ydich = 0)).
compute clabtmp={hcflab,'df1','df2','p'}.
print fresult/title='Test of equality of conditional means'/cnames=clabtmp/format= F10.4.
end if.
do if (i = (nms + nys) and (ydich = 1)).
compute clabtmp={'Chi-sq','df','p'}.
print fresult/title='Test of equality of conditional logits or probabilities'/cnames=clabtmp/format= F10.4.
end if.
end if.
compute probetmp=probeplt(pstart:(pstart+nxvls),1).
compute probetmp={probetmp,probeplt(pstart:(pstart+nxvls),prevloc:ncol(probeplt))}.
compute pstart=pstart+(nxvls+1).
compute resultm2=make(nrow(probetmp),maxresm,99999).
compute resultm2(1:nrow(probetmp),1:ncol(probetmp))=probetmp.
compute resultm={resultm;resultm2}.
do if (i < ((nms + nys)) or (ydich = 0)).
compute clabtmp={xnames, outnames(1,i), hclab, 'LLCI', 'ULCI'}.
do if (outscree=1).
print probetmp/title = 'Estimated conditional means being compared:'/cnames=clabtmp/format= F10.4.
end if.
end if.
do if ((i = (nms+nys)) and (ydich = 1)).
compute clabtmp={xnames, outnames(1,i), 'prob'}.
compute probetm2=probetmp(:,1:2).
compute probetm2={probetm2,probetmp(:,ncol(probetmp))}.
do if (outscree=1).
print probetm2/title = 'Estimated conditional logits and probabilities:'/cnames=clabtmp/format= F10.4.
end if.
end if.
end if.
end loop.
release probrow, start2, endstart, temp, temp2.
end if.
end if.
do if (probexpl = 1 and (printpbe <= intprobe)).
compute resultm2=make(nrow(modvals2),maxresm,99999).
compute resultm2(1:nrow(modvals2),1:ncol(modvals2))=modvals2.
compute resultm={resultm;resultm2}.
do if (outscree=1).
print/title = 'Conditional effects of the focal predictor at values of the moderator(s):'.
print modvals2/cnames=problabs/title = ' '/space=0/format= F10.4.
end if.
compute didsome=1.
do if (hasz = 1).
compute printz=1.
end if.
do if (hasw=1).
compute printw=1.
end if.
do if (jn = 1 and jnok=1).
do if (criterr = 0).
compute dfres=n-nrow(b).
compute roots=99999.
compute jncrit =(dfres* (exp((dfres-(5/6))*((xp2/(dfres-(2/3)+(.11/dfres)))* (xp2/(dfres-(2/3)+(.11/dfres)))))-1)).
do if (i = (nms+nys) and (ydich=1)).
compute jncrit=xp2*xp2.
end if.
compute jnb1=b(wherejn1,1).
compute jnb3=b(wherejn3,1).
compute jnsb1=varb(wherejn1,wherejn1).
compute jnsb3=varb(wherejn3,wherejn3).
compute jnsb1b3=varb(wherejn1,wherejn3).
compute ajn =(jncrit*jnsb3)-(jnb3*jnb3).
compute bjn = 2*((jncrit*jnsb1b3)-(jnb1*jnb3)).
compute cjn = (jncrit*jnsb1)-(jnb1*jnb1).
compute radarg = (bjn*bjn)-(4*ajn*cjn).
compute den = 2*ajn.
compute nrts = 0.
do if (radarg >= 0 and den <> 0).
compute x21 = (-bjn+sqrt(radarg))/den.
compute x22 = (-bjn-sqrt(radarg))/den.
compute roots = 0.
do if (x21 >= jnmin and x21 <= jnmax).
compute nrts = 1.
compute roots = {roots; x21}.
end if.
do if (x22 >= jnmin and x22 <= jnmax).
compute nrts = nrts + 1.
compute roots = {roots; x22}.
end if.
compute roots={roots,make(nrow(roots),2,0)}.
end if.
do if (nrts > 0).
compute roots = roots(2:nrow(roots),1:3).
compute roots(1,2)=(csum(jnmod < roots(1,1))/n)*100.
compute roots(1,3)=(csum(jnmod > roots(1,1))/n)*100.
do if (nrow(roots)=2).
compute roots(2,2)=(csum(jnmod < roots(2,1))/n)*100.
compute roots(2,3)=(csum(jnmod > roots(2,1))/n)*100.
end if.
do if (outscree=1).
print roots/title = 'Moderator value(s) defining Johnson-Neyman significance region(s):'/clabels = 'Value', '% below', '% above'/format F10.4.
end if.
do if (nrts=1).
compute tmprts=make(1,3,99999).
compute roots={roots;tmprts}.
end if.
end if.
do if (nrts = 0).
compute roots=make(2,1,99999).
do if (outscree=1).
print/title = 'There are no statistical significance transition points within the observed'.
print/title = 'range of the moderator found using the Johnson-Neyman method.'/space=0.
end if.
end if.
compute resultm2=make(nrow(roots),maxresm,99999).
compute resultm2(1:nrow(roots),1:ncol(roots))=roots.
compute resultm={resultm;resultm2}.
compute jnvals=make(23,7,0).
loop jni= 0 to (21-(nrts)).
compute jnvals((jni+1),1)=jnmin+(jni*((jnmax-jnmin)/(21-nrts))).
end loop.
do if (nrts > 0).
loop jni = 1 to nrts.
loop jnj = 2 to (nrow(jnvals)-1).
do if ((roots(jni,1) > jnvals((jnj-1),1)) and (roots(jni,1) < jnvals(jnj,1))).
compute jnvals((jnj+1):(21+jni),1)=jnvals(jnj:(20+jni),1).
compute jnvals(jnj,1)=roots(jni,1).
end if.
end loop.
end loop.
end if.
compute jnvals=jnvals(1:22,:).
loop jni = 1 to nrow(jnvals).
compute jnvals(jni,2)=jnb1+jnb3*jnvals(jni,1).
compute jnvals(jni,3)=sqrt(jnsb1+2*jnvals(jni,1)*jnsb1b3+(jnvals(jni,1)*jnvals(jni, 1))*jnsb3).
compute jnvals(jni,4)=jnvals(jni,2)/jnvals(jni,3).
compute jnvals(jni,5)=2*(1-tcdf(abs(jnvals(jni,4)), dfres)).
compute jnvals(jni,6)=jnvals(jni,2)-sqrt(jncrit)*jnvals(jni,3).
compute jnvals(jni,7)=jnvals(jni,2)+sqrt(jncrit)*jnvals(jni,3).
do if ((i = nms + nys) and (ydich=1)).
compute jnvals(jni,5)=2*(1-cdfnorm(abs(jnvals(jni,4)))).
compute jnvals(jni,6)=jnvals(jni,2)-xp2*jnvals(jni,3).
compute jnvals(jni,7)=jnvals(jni,2)+xp2*jnvals(jni,3).
end if.
end loop.
compute resultm2=make(nrow(jnvals),maxresm,99999).
compute resultm2(1:nrow(jnvals),1:ncol(jnvals))=jnvals.
compute resultm={resultm;resultm2}.
do if ((i < nms+nys) or (ydich=0)).
compute jnclbs={jnmodlab,'Effect',hclab,'t', 'p', 'LLCI', 'ULCI'}.
end if.
do if ((i = nms + nys) and (ydich=1)).
compute jnclbs={jnmodlab,'Effect','se','Z', 'p', 'LLCI', 'ULCI'}.
end if.
do if (((wcmat((i+1),jmed)=1) or (zcmat((i+1),jmed)=1)) and (wzcmat((i+1),jmed)=0)).
do if (outscree=1).
print jnvals/title = 'Conditional effect of focal predictor at values of the moderator:'/cnames =jnclbs/format = F10.4.
end if.
end if.
do if (outscree=1).
do if ((jmed = 1) and (wzcmat((i+1),jmed)=1)).
print jnvals/title = 'Conditional X*W interaction at values of the moderator Z:'/cnames =jnclbs/format = F10.4.
end if.
do if ((jmed > 1) and (wzcmat((i+1),jmed)=1)).
print jnvals/title = 'Conditional M*W interaction at values of the moderator Z:'/cnames =jnclbs/format = F10.4.
end if.
end if.
end if.
end if.
end if.
do if ((i = (nms+nys)) and (jmed=1) and (bcmat(nrow(bcmat),1)=1)).
do if (probei=1).
compute direfflb=problabs.
compute direff=modvals2.
end if.
do if (probei>1).
compute direff={direff;modvals2}.
end if.
end if.
compute intprint=0.
do if ((jmed=1) and (i=1) and nms=0) and modcok=1).
compute contvec2=make(2,1,1).
compute contvec2={contvec2,wcontval,zcontval}.
do if (wzcmat((i+1),jmed)=1).
loop conti= 1 to ncol(wcontval).
loop contj = 1 to ncol(zcontval).
compute contvec2={contvec2,wcontval(:,conti)&*zcontval(:,contj)}.
end loop.
end loop.
end if.
compute conteff=contvec2*probcoef.
compute contdiff=contvec2(1,:)-contvec2(2,:).
compute contse=sqrt(contdiff*probvarb*t(contdiff)).
compute conteffd=conteff(1,1)-conteff(2,1).
compute contvec={contvec,conteff}.
compute contvecm=contvec.
compute resultm2=make(nrow(contvecm),maxresm,99999).
compute resultm2(1:nrow(contvecm),1:ncol(contvecm))=contvecm.
compute resultm={resultm;resultm2}.
do if (outscree=1).
print/title='Contrast between conditional effects of X:'.
print contvec/title=' '/rlabels='Effect1:','Effect2:'/cnames=problabs/format = F10.4 /space=0.
end if.
do if (ydich=0).
compute p=2*(1-tcdf(abs(conteffd/contse), dfres)).
compute contvec={conteffd,contse,conteffd/contse, p}.
compute contvec={contvec,(conteffd-(tval*contse))}.
compute contvec={contvec,(conteffd+(tval*contse))}.
compute contlabs={'Contrast', hclab, 't', 'p', 'LLCI', 'ULCI'}.
end if.
do if (ydich=1).
compute p=2*(1-cdfnorm(abs(conteffd/contse))).
compute contvec={conteffd,contse,conteffd/contse, p}.
compute contvec={contvec,(conteffd-(xp2*contse))}.
compute contvec={contvec,(conteffd+(xp2*contse))}.
compute contlabs={'Contrast', 'se', 'Z', 'p', 'LLCI', 'ULCI'}.
end if.
compute resultm2=make(nrow(contvec),maxresm,99999).
compute resultm2(1:nrow(contvec),1:ncol(contvec))=contvec.
compute resultm={resultm;resultm2}.
do if (outscree=1).
print contvec/title='Test of Effect1 minus Effect2'/format= F10.4 /cnames=contlabs.
end if.
end if.
end loop.
do if (plot = 1 or plot = 2).
compute datalabs={t(focpred(:,3)),outnames(1,i)}.
do if (plot = 1).
compute datalabs={datalabs}.
end if.
do if (plot = 2).
compute datalabs={datalabs,'se', 'LLCI', 'ULCI'}.
end if.
do if ((i = nms+nys) and (ydich=1)).
compute datalabs={datalabs,'prob'}.
end if.
compute resultm2=make(nrow(probeplt),maxresm,99999).
compute resultm2(1:nrow(probeplt),1:ncol(probeplt))=probeplt.
compute resultm={resultm;resultm2}.
compute datalabs={datalabs,'.'}.
do if (outscree=1).
print/title = 'Data for visualizing the conditional effect of the focal predictor:'.
print/title = 'Paste text below into a SPSS syntax window and execute to produce plot.'/space=0.
compute dumb = {' ', ' ', ' ', ' ', ' ', ' ', ' '}.
print datalabs/title = 'DATA LIST FREE/'/format=A10.
print probeplt/title = 'BEGIN DATA.'/format= F10.4 /space=0.
print/title = 'END DATA.'/space=0.
end if.
compute focgrph=datalabs(1,1).
compute graphix={focgrph,graphixs,modgrph}.
do if (((xdich=1) or (nxvls > 1)) and ((modcat=0) and (focgrph = xnames))).
compute graphix={modgrph,graphixs,focgrph}.
end if.
do if (panelgrp = 0).
compute graphix={graphix,'.'}.
else.
compute graphix={graphix,panelcde}.
end if.
do if (outscree=1).
print graphix/title = 'GRAPH/SCATTERPLOT='/format=A8/space=0.
end if.
do if (i = (nms + nys) and ydich = 1).
compute graphixd=graphix.
compute graphixd(1,3)='prob'.
do if (outscree=1).
print graphixd/title = 'GRAPH/SCATTERPLOT='/format=A8/space=0.
end if.
end if.
end if.
end if.
end loop.
release jmed, intprint,didprint.
end if
.
do if (model = 74 and i <= nms).
compute onetemp=make(nrow(xprobval),1,1).
compute mestmt74={onetemp,xprobval}.
do if (ncs > 0).
compute ncovmdl=rsum(ccmat(i,:)).
do if (ncovmdl > 0).
compute cvmnc=make(nrow(mestmt74),ncovmdl,1).
compute cvmnctmp=csum(x(:,(ncol(x)-ncovmdl+1):ncol(x)))/nrow(x).
do if (cuscoval > 0).
compute cvmnctmp=coval.
end if.
loop mestlp=1 to ncovmdl.
compute cvmnc(:,mestlp)=cvmnc(:,mestlp)*cvmnctmp(:,mestlp).
end loop.
compute mestmt74={mestmt74,cvmnc}.
end if.
end if.
compute mest74t=mestmt74*b.
do if (i = 1).
compute mest74=mest74t.
end if.
do if (i > 1).
compute mest74={mest74,mest74t}.
end if.
end if.
do if ((i = (nms+nys)) and (model >= 0 and model < 4) and (linsum(1,1) <> -999)).
compute lhyprob=1.
compute meansub=0.
do if (((nlinsum=nrow(b)) or (nlinsum = (nrow(b)-ncs)))).
do if (nlinsum = (nrow(b)-ncs) and ncs > 0).
compute linsum={linsum,covmeans}.
compute meansub=1.
end if.
compute lhyprob=0.
compute hypest=linsum*b.
compute sehypest=sqrt(linsum*varb*t(linsum)).
compute phypest=2*(1-tcdf(abs(hypest/sehypest), (dfres))).
compute hypest={hypest,sehypest,(hypest/sehypest),phypest,(hypest-tval*sehypest),(hypest+ tval*sehypest)}.
compute resultm2=make(ncol(linsum),maxresm,99999).
compute resultm2(:,1)=t(linsum).
compute resultm={resultm;resultm2}.
compute resultm2=make(1,maxresm,99999).
compute resultm2(1,1:ncol(hypest))=hypest.
compute resultm={resultm;resultm2}.
do if (outscree=1).
compute hyplabs={'Estimate',hclab,'t','p','LLCI','ULCI'}.
print/title='----------'.
print/title='Linear Combination Estimate and Hypothesis Test'/space=0.
print t(linsum)/title= 'Weight vector:'/clabels='weight'/rnames=vlabsm/format F10.4.
print hypest/title=' '/cnames=hyplabs/format= F10.4 /space=0.
do if (meansub=1).
print/title='Covariate weight(s) set to the sample mean.'.
end if.
end if.
end if.
do if (lhyprob=1).
compute notecode(notes,1) = 30.
compute notes = notes + 1.
end if.
end if.
end loop if criterr=1.
compute lastb=b.
compute lastcov=varb.
do if (criterr=0 and dototal = 1).
compute x=xtmp.
compute vlabsm={'constant';xcatlab(1:nxvls,1)}.
do if (ncs > 0).
compute x = {x,ctmp}.
compute vlabsm={vlabsm;t(covnames)}.
end if.
compute x = {ones,x}.

.
do if ( 1 =1).
compute hatmat=inv(t( x )* x )*t( x ).
compute b = hatmat* y.
compute modres=b.
do if ( 1 =1).
compute n1=nrow( x ).
compute dfres=n1-(ncol( x )).
compute sstotal = t( y -(csum( y )/n1))*( y -(csum( y )/n1)).
compute resid= y - x *b.
compute ssresid = csum((resid)&**2).
compute r2 = (sstotal-ssresid)/sstotal.
do if (r2 < 0).
compute r2=0.
end if.
compute adjr2 = 1-((1-r2)*(n1-1)/(dfres)).
compute mse=ssresid/(n1-ncol( x )).

.
compute n1=nrow( x ).
compute invXtX = inv(t( x )* x ).
compute varb = mse *invXtX.
compute k3 = ncol( x ).
compute xhc=0.
do if ( hc <> 5).
compute xhc= x.
compute hat = xhc(:,1).
loop i3=1 to nrow(xhc).
compute hat(i3,1)= xhc(i3,:)*invXtX*t(xhc(i3,:)).
end loop.
do if ( hc = 0 or hc =1).
loop i3 = 1 to k3.
compute xhc(:,i3)=xhc(:,i3)&* resid.
end loop.
end if.
do if ( hc =3 or hc =2).
loop i3=1 to k3.
compute xhc(:,i3) = ( resid &/(1-hat)&**(1/(4- hc )))&*xhc(:,i3).
end loop.
end if.
do if ( hc = 4).
compute hcmn=make(n,2,4).
compute hcmn(:,2)=(n1*hat)/k3.
loop i3= 1 to k3.
compute xhc(:,i3) = ( resid &/(1-hat)&**(rmin(hcmn)/2))&*xhc(:,i3).
end loop.
end if.
compute varb=(invXtX*t(xhc)*xhc*invXtX).
do if ( hc =1).
compute varb=(n1/(n1-ncol( x )))&*varb.
end if.
end if.
compute hclab={'se(HC0)','se(HC1)','se(HC2)','se(HC3)','se(HC4)','se'}.
compute hclab=hclab(1,( hc +1)).
compute hcflab={'F(HC0)','F(HC1)','F(HC2)','F(HC3)','F(HC4)','F'}.
compute hcflab=hcflab(1,( hc +1)).
release xhc
.
compute seb=sqrt(diag(varb)).
compute trat = b&/seb.
compute p = 2*(1-tcdf(abs(trat), (dfres))).
compute tval = sqrt(dfres* (exp((dfres-(5/6))*((xp2/(dfres-(2/3)+(.11/dfres)))* (xp2/(dfres-(2/3)+(.11/dfres)))))-1)).
compute modres={modres,seb,trat,p}.
compute modres={modres,(b-tval&*seb),(b+tval&*seb)}.
compute modresl={'coeff',hclab,'t','p','LLCI','ULCI'}.
compute lmat = ident(ncol( x )).
compute lmat = lmat(:,2:ncol(lmat)).
compute fratio = (t(t(lmat)*b)*inv(t(lmat)*varb*lmat)*((t(lmat)*b)))/(ncol( x )-1).
compute pfr = 1-fcdf(fratio,(ncol( x )-1),dfres).
compute modsum={sqrt(r2),r2,mse,fratio,(ncol( x )-1),dfres,pfr}.
compute modsuml={'R','R-sq','MSE',hcflab,'df1','df2', 'p'}.
end if.
end if.
do if ( 1 = 2 or 1 =3).
compute xlp= x.
compute ylp= y.
compute pt2 = make(nrow(ylp),1,(csum(ylp)/nrow(ylp))).
do if ( 1 =2).
compute LL3 = ylp&*ln(pt2)+(1-ylp)&*ln(1-pt2).
end if.
compute LL3 = -2*csum(LL3).
compute bt1 = make(ncol(xlp),1,0).
compute LL1 = 0.
compute pt1 = make(nrow(ylp),1,0.5).
compute pt1lp=pt1.
loop jjj = 1 to iterate.
compute xlptmp=t(xlp).
compute vecprb=pt1lp&*(1-pt1lp).
loop kkk=1 to ncol(xlp).
compute xlptmp(kkk,:)=xlptmp(kkk,:)&*t(vecprb).
end loop.
compute b = bt1+inv(xlptmp*xlp)*t(xlp)*(ylp-pt1lp).
do if ( 1 =2).
compute xlpb=xlp*b.
compute xlpbt=(xlpb > -709.7).
compute xlpb709=(1-xlpbt)*(-709.7).
compute xlpb=(xlpb&*xlpbt)+xlpb709.
compute pt1lp = 1/(1+exp(-(xlpb))).
end if.
compute itprob = csum((pt1lp < .00000001) or (pt1lp > .9999999)).
do if (itprob > 0).
loop kkk = 1 to nrow(pt1lp).
do if (pt1lp(kkk,1) > .9999999).
compute pt1lp(kkk,1) = .9999999.
end if.
do if (pt1lp(kkk,1) < .00000001).
compute pt1lp(kkk,1) = .00000001.
end if.
end loop.
compute itprob = 0.
end if.
do if (itprob = 0).
do if ( 1 =2).
compute LL = ylp&*ln(pt1lp)+(1-ylp)&*ln(1-pt1lp).
end if.
compute LL2 = -2*csum(ll).
end if.
do if (abs(LL1-LL2) < converge).
do if ( 1 =1).
compute xlptmp=t(xlp).
compute vecprb=pt1lp&*(1-pt1lp).
loop kkk=1 to ncol(xlp).
compute xlptmp(kkk,:)=xlptmp(kkk,:)&*t(vecprb).
end loop.
compute varb = inv(xlptmp*xlp).
compute seb = sqrt(diag(varb)).
release xlptmp.
end if.
break.
end if.
compute bt1 = b.
compute LL1 = LL2.
end loop.
compute modres=b.
do if (jjj > iterate).
compute itprob = 2.
do if (booting=0).
compute iterrmod=1.
end if.
do if (booting=1).
compute bootiter=1.
end if.
do if (itprobtg=0).
compute itprobtg=1.
compute errcode(errs,1) = 47.
compute errs = errs + 1.
do if (booting = 0 and 1 =1).
compute vt1 = mdiag(pt1lp&*(1-pt1lp)).
compute varb = inv(t(xlp)*vt1*xlp).
compute seb = sqrt(diag(varb)).
end if.
end if.
end if.
do if ( 1 =1).
compute trat = b&/seb.
compute dfres=nrow(xlp).
compute p = 2*(1-cdfnorm(abs(trat))).
compute modres={modres,seb,trat,p}.
compute modres={modres,(b-xp2&*seb),(b+xp2&*seb)}.
compute pvchi=1-chicdf((LL3-LL2),(nrow(modres)-1)).
compute mcF = (LL3-LL2)/LL3.
compute cox = 1-exp(-(LL3-LL2)/nrow(xlp)).
compute nagel = cox/(1-exp(-(LL3)/nrow(xlp))).
compute modsum={LL2,(LL3-LL2),(nrow(modres)-1),pvchi, mcF,cox,nagel}.
compute modsuml={'-2LL','ModelLL', 'df', 'p', 'McFadden', 'CoxSnell', 'Nagelkrk'}.
compute modresl={'coeff','se','Z','p','LLCI','ULCI'}.
end if.
end if
.
compute toteff=modres(2:(1+nxvls),:).
compute nodotot=0.
do if (xdich=1 and xmint=1 and model=74).
compute toteff(:,1)=toteff(:,1)*xscaling.
compute toteff(:,2)=toteff(:,2)*abs(xscaling).
compute toteff(:,3)=toteff(:,3)*xscaling.
compute toteff(:,5)=toteff(:,5)*xscaling.
compute toteff(:,6)=toteff(:,6)*xscaling.
compute citmp=toteff(:,5:6).
do if (xscaling < 0).
compute toteff(:,5)=citmp(:,2).
compute toteff(:,6)=citmp(:,1).
end if.
compute nodotot=1.
end if.
compute totefflb=modresl.
do if (outscree=1).
do if (nodotot=0).
print/title = '************************** TOTAL EFFECT MODEL ****************************'.
end if.
do if (nodotot=1).
print/title = '**************************************************************************'.
end if.
print outnames(1,ncol(outnames))/title = 'OUTCOME VARIABLE:'/format = A8/space=0.
end if.
compute toteffl2=vlabsm(2:(1+nxvls),:).
compute resultm2=make(1,maxresm,99999).
compute resultm2(1,1:ncol(modsum))=modsum.
compute resultm={resultm;resultm2}.
compute resultm2=make(nrow(modres),maxresm,99999).
compute resultm2(1:nrow(modres),1:ncol(modres))=modres.
compute resultm={resultm;resultm2}.
do if (outscree=1).
print modsum/title = 'Model Summary'/cnames = modsuml/format= F10.4.
print modres/title='Model'/rnames=vlabsm/cnames=modresl/format= F10.4.
end if.
compute lmat=make(nrow(b),1,0).
compute lmat2=make(nxvls,1,1).
compute lmat(2:(1+nxvls),1)=lmat2.
do if (ydich <> 1).

.
compute lmat2= lmat.
do if ( 0 =0).
compute lmat2 = mdiag( lmat ).
compute lmat3=make(nrow(lmat2),1,0).
loop flp=1 to ncol(lmat2).
do if (csum(lmat2(:,flp))=1).
compute lmat3={lmat3,lmat2(:,flp)}.
end if.
end loop.
compute lmat2=lmat3(:,2:ncol(lmat3)).
end if.
compute fratio = (t(t(lmat2)* b )*inv(t(lmat2)* varb *lmat2)*((t(lmat2)* b )))/ncol(lmat2).
compute pfr = 1-fcdf(fratio,ncol(lmat2),(n-nrow( b ))).
compute fresult={fratio,ncol(lmat2),(n-nrow( b )),pfr}.
do if (i = (nms+nys) and (ydich=1)).
compute fratio=fratio*ncol(lmat2).
compute pfr=1-chicdf(fratio,ncol(lmat2)).
compute fresult={fratio,ncol(lmat2),pfr}.
end if.
do if ( 1 =1).
compute lmat3=1-rsum(lmat2).
compute xfm=make(n,csum(lmat3),0).
compute flpc=1.
loop flp=1 to nrow(lmat3).
do if (lmat3(flp,1)=1).
compute xfm(:,flpc)=x(:,flp).
compute flpc=flpc+1.
end if.
end loop.
compute bfm=inv(t(xfm)*xfm)*t(xfm)*y.
compute resid=y-(xfm*bfm).
compute sstotal=(y-(csum(y)/n)).
compute sstotal=csum(sstotal&*sstotal).
compute ssresid=csum(resid&*resid).
compute rsqch= r2 -((sstotal-ssresid)/sstotal).
compute fresult={rsqch,fresult}.
release xfm,flpc, resid, ssresid, bfm.
end if
.
compute totomni=fresult.
end if.
do if (stand=1).
compute predsd=make(nrow(modres),1,0).
compute stdmod=modres(:,1)&/ovsd(1,ncol(ovsd)).
loop jd=1 to ncol(x).
compute descdat=x(:,jd).
compute predsd(jd,1) = (nrow(descdat)*sscp(descdat))-(t(csum(descdat))*(csum(descdat))).
compute predsd(jd,1) = sqrt(predsd(jd,1)/(nrow(descdat)*(nrow(descdat)-1))).
end loop.
do if (wherex(1,ncol(wherex)) <> -999 and ((nxvls > 1) or (xdich=1))).
compute sdmsone=make(nxvls,1,1).
compute predsd(wherex(1,ncol(wherex)):wherex(2,ncol(wherex)),1)=sdmsone.
compute pstog=1.
end if.
compute predsd(1,1)=1.
compute stdmod=stdmod&*predsd.
compute stdmod=stdmod(2:nrow(stdmod),1).
compute sdvlabs=vlabsm(2:nrow(vlabsm),1).
compute resultm2=make(nrow(stdmod),maxresm,99999).
compute resultm2(1:nrow(stdmod),1:ncol(stdmod))=stdmod.
compute resultm={resultm;resultm2}.
do if (outscree=1).
print stdmod/title='Standardized coefficients'/clabels='coeff'/rnames=sdvlabs/format= F10.4.
end if.
end if.
do if (covcoeff=1).
do if (outscree=1).
print varb/title='Covariance matrix of regression parameter estimates:'/rnames=vlabsm/cnames=vlabsm/format= F10.4.
end if.
compute resultm2=make(nrow(varb),maxresm,99999).
do if (ncol(varb) <= maxresm).
compute resultm2(1:nrow(varb),1:ncol(varb))=varb.
compute resultm={resultm;resultm2}.
end if.
do if (ncol(varb) > maxresm).
compute resultmt=make(nrow(resultm),ncol(varb),99999).
compute resultmt(1:nrow(resultm),1:ncol(resultm))=resultm.
compute resultm=resultmt.
compute resultm2=make(nrow(varb),ncol(resultm),99999).
compute resultm2(1:nrow(varb),1:ncol(varb))=varb.
compute resultm={resultm;resultm2}.
compute maxresm=ncol(resultm).
end if.
end if.
end if.
end if.
do if (criterr=0 and nms > 0 and ydich=0 and modelres=1).
compute modresid=modresid(:,2:ncol(modresid)).
compute sigmatal = (t(modresid)*(ident(n)-(1/n)*ones*t(ones))*modresid)*(1/(n-1)).
compute sdall = mdiag(1/sqrt(diag(sigmatal))).
compute corall=sdall*sigmatal*t(sdall).
compute resultm2=make(nrow(corall),maxresm,99999).
compute resultm2(1:nrow(corall),1:ncol(corall))=corall.
compute resultm={resultm;resultm2}.
do if (outscree=1).
print/title = '****************** CORRELATIONS BETWEEN MODEL RESIDUALS ******************'.
print corall/title=' '/format= F10.4 / cnames=outnames/rnames=outnames/space=0.
end if.
end if.
do if (criterr=0 and boot > 0).
compute bootres=make(1,rsum(nump),-999).
compute bootdir=obsdirfx.
compute natdirbt=make(1,nxvls,-999).
do if (effsize=1).
compute bootysd=make(1,1,-999).
compute bootxsd=make(1,1,-999).
end if.
compute badboot=0.
compute goodboot=0.
compute smallest=1.
compute booting=1.
loop j = 1 to maxboot.
compute nobootx=1.
compute modres2=999.
compute v=trunc(uniform(n,1)*n)+1.
compute bad=0.
loop i = 1 to (nms+nys).
compute y=outvars(v,i).
compute ynovar= (nrow(y)*sscp(y))-(t(csum(y))*(csum(y))).
do if (ynovar = 0).
compute bad=1.
end if.
compute xindx=datindx(1:(nump(1,i)-1),i).
compute hello=0.
compute x = fulldat(v,xindx).
compute x={ones,x}.
compute xsq=t(x)*x.
compute exsq=eval(xsq).
release xsq.
compute holymoly=cmin(exsq).
compute zeroeig=csum(exsq <= 0.000000000002).
compute bad=bad + (zeroeig > 0).

.
compute desctmp=make((8-(4* 1 )),ncol( y ),-999).
loop jd=1 to ncol( y ).
compute descdat= y (:,jd).
compute desctmp(1,jd) = csum(descdat)/nrow(descdat).
compute desctmp(2,jd) = (nrow(descdat)*sscp(descdat))-(t(csum(descdat))*(csum(descdat))).
compute desctmp(2,jd) = sqrt(desctmp(2,jd)/(nrow(descdat)*(nrow(descdat)-1))).
compute desctmp(3,jd)=cmin(descdat).
compute desctmp(4,jd)=cmax(descdat).
do if ( 1 =0).
compute minwarn=0.
compute maxwarn=0.
do if ((desctmp(3,jd)=desctmp(4,jd)) and novar=0).
compute errcode(errs,1)=15.
compute errs=errs+1.
compute criterr=1.
compute novar=1.
end if.
compute tmp=((descdat(:,1)=desctmp(3,jd))+(descdat(:,1)=desctmp(4,jd))).
compute desctmp(8,jd)=(csum(tmp)=nrow(tmp)).
compute tmp = descdat.
compute tmp(GRADE(descdat),:) = descdat.
compute descdat = tmp.
release tmp.
compute decval={.16;.5;.84}.
loop kd=1 to 3.
compute low=trunc(decval(kd,1)*(nrow(descdat)+1)).
compute lowdec=decval(kd,1)*(nrow(descdat)+1)-low.
compute value=descdat(low,1)+(descdat((low+1),1)-descdat(low,1))*lowdec.
compute desctmp((4+kd),jd)=value.
end loop.
compute mnotev=1.
compute modvals=desctmp(5:7,:).
do if (quantile <> 1).
compute desctmp(5,jd)=desctmp(1,jd)-desctmp(2,jd).
compute desctmp(6,jd)=desctmp(1,jd).
compute desctmp(7,jd)=desctmp(1,jd)+desctmp(2,jd).
compute modvals=desctmp(5:7,:).
compute mnotev=2.
do if (modvals(1,1) < desctmp(3,1)).
compute modvals(1,1)=desctmp(3,1).
compute minwarn=1.
end if.
do if (modvals(3,1) > desctmp(4,1)).
compute modvals(3,1)=desctmp(4,1).
compute maxwarn=1.
end if.
end if.
do if (desctmp(8,1)=1).
compute modvals={desctmp(3,1);desctmp(4,1)}.
compute mnotev=0.
compute minwarn=0.
compute maxwarn=0.
end if.
end if.
end loop
.
compute bad=bad+ (desctmp(2,1) <= 0.00000000001).
do if (bad = 0).
do if (holymoly < smallest).
compute smallest=holymoly.
end if.
do if (ydich=0 or (i < (nms+nys))).

.
do if ( 1 =1).
compute hatmat=inv(t( x )* x )*t( x ).
compute b = hatmat* y.
compute modres=b.
do if ( 0 =1).
compute n1=nrow( x ).
compute dfres=n1-(ncol( x )).
compute sstotal = t( y -(csum( y )/n1))*( y -(csum( y )/n1)).
compute resid= y - x *b.
compute ssresid = csum((resid)&**2).
compute r2 = (sstotal-ssresid)/sstotal.
do if (r2 < 0).
compute r2=0.
end if.
compute adjr2 = 1-((1-r2)*(n1-1)/(dfres)).
compute mse=ssresid/(n1-ncol( x )).

.
compute n1=nrow( x ).
compute invXtX = inv(t( x )* x ).
compute varb = mse *invXtX.
compute k3 = ncol( x ).
compute xhc=0.
do if ( hc <> 5).
compute xhc= x.
compute hat = xhc(:,1).
loop i3=1 to nrow(xhc).
compute hat(i3,1)= xhc(i3,:)*invXtX*t(xhc(i3,:)).
end loop.
do if ( hc = 0 or hc =1).
loop i3 = 1 to k3.
compute xhc(:,i3)=xhc(:,i3)&* resid.
end loop.
end if.
do if ( hc =3 or hc =2).
loop i3=1 to k3.
compute xhc(:,i3) = ( resid &/(1-hat)&**(1/(4- hc )))&*xhc(:,i3).
end loop.
end if.
do if ( hc = 4).
compute hcmn=make(n,2,4).
compute hcmn(:,2)=(n1*hat)/k3.
loop i3= 1 to k3.
compute xhc(:,i3) = ( resid &/(1-hat)&**(rmin(hcmn)/2))&*xhc(:,i3).
end loop.
end if.
compute varb=(invXtX*t(xhc)*xhc*invXtX).
do if ( hc =1).
compute varb=(n1/(n1-ncol( x )))&*varb.
end if.
end if.
compute hclab={'se(HC0)','se(HC1)','se(HC2)','se(HC3)','se(HC4)','se'}.
compute hclab=hclab(1,( hc +1)).
compute hcflab={'F(HC0)','F(HC1)','F(HC2)','F(HC3)','F(HC4)','F'}.
compute hcflab=hcflab(1,( hc +1)).
release xhc
.
compute seb=sqrt(diag(varb)).
compute trat = b&/seb.
compute p = 2*(1-tcdf(abs(trat), (dfres))).
compute tval = sqrt(dfres* (exp((dfres-(5/6))*((xp2/(dfres-(2/3)+(.11/dfres)))* (xp2/(dfres-(2/3)+(.11/dfres)))))-1)).
compute modres={modres,seb,trat,p}.
compute modres={modres,(b-tval&*seb),(b+tval&*seb)}.
compute modresl={'coeff',hclab,'t','p','LLCI','ULCI'}.
compute lmat = ident(ncol( x )).
compute lmat = lmat(:,2:ncol(lmat)).
compute fratio = (t(t(lmat)*b)*inv(t(lmat)*varb*lmat)*((t(lmat)*b)))/(ncol( x )-1).
compute pfr = 1-fcdf(fratio,(ncol( x )-1),dfres).
compute modsum={sqrt(r2),r2,mse,fratio,(ncol( x )-1),dfres,pfr}.
compute modsuml={'R','R-sq','MSE',hcflab,'df1','df2', 'p'}.
end if.
end if.
do if ( 1 = 2 or 1 =3).
compute xlp= x.
compute ylp= y.
compute pt2 = make(nrow(ylp),1,(csum(ylp)/nrow(ylp))).
do if ( 1 =2).
compute LL3 = ylp&*ln(pt2)+(1-ylp)&*ln(1-pt2).
end if.
compute LL3 = -2*csum(LL3).
compute bt1 = make(ncol(xlp),1,0).
compute LL1 = 0.
compute pt1 = make(nrow(ylp),1,0.5).
compute pt1lp=pt1.
loop jjj = 1 to iterate.
compute xlptmp=t(xlp).
compute vecprb=pt1lp&*(1-pt1lp).
loop kkk=1 to ncol(xlp).
compute xlptmp(kkk,:)=xlptmp(kkk,:)&*t(vecprb).
end loop.
compute b = bt1+inv(xlptmp*xlp)*t(xlp)*(ylp-pt1lp).
do if ( 1 =2).
compute xlpb=xlp*b.
compute xlpbt=(xlpb > -709.7).
compute xlpb709=(1-xlpbt)*(-709.7).
compute xlpb=(xlpb&*xlpbt)+xlpb709.
compute pt1lp = 1/(1+exp(-(xlpb))).
end if.
compute itprob = csum((pt1lp < .00000001) or (pt1lp > .9999999)).
do if (itprob > 0).
loop kkk = 1 to nrow(pt1lp).
do if (pt1lp(kkk,1) > .9999999).
compute pt1lp(kkk,1) = .9999999.
end if.
do if (pt1lp(kkk,1) < .00000001).
compute pt1lp(kkk,1) = .00000001.
end if.
end loop.
compute itprob = 0.
end if.
do if (itprob = 0).
do if ( 1 =2).
compute LL = ylp&*ln(pt1lp)+(1-ylp)&*ln(1-pt1lp).
end if.
compute LL2 = -2*csum(ll).
end if.
do if (abs(LL1-LL2) < converge).
do if ( 0 =1).
compute xlptmp=t(xlp).
compute vecprb=pt1lp&*(1-pt1lp).
loop kkk=1 to ncol(xlp).
compute xlptmp(kkk,:)=xlptmp(kkk,:)&*t(vecprb).
end loop.
compute varb = inv(xlptmp*xlp).
compute seb = sqrt(diag(varb)).
release xlptmp.
end if.
break.
end if.
compute bt1 = b.
compute LL1 = LL2.
end loop.
compute modres=b.
do if (jjj > iterate).
compute itprob = 2.
do if (booting=0).
compute iterrmod=1.
end if.
do if (booting=1).
compute bootiter=1.
end if.
do if (itprobtg=0).
compute itprobtg=1.
compute errcode(errs,1) = 47.
compute errs = errs + 1.
do if (booting = 0 and 0 =1).
compute vt1 = mdiag(pt1lp&*(1-pt1lp)).
compute varb = inv(t(xlp)*vt1*xlp).
compute seb = sqrt(diag(varb)).
end if.
end if.
end if.
do if ( 0 =1).
compute trat = b&/seb.
compute dfres=nrow(xlp).
compute p = 2*(1-cdfnorm(abs(trat))).
compute modres={modres,seb,trat,p}.
compute modres={modres,(b-xp2&*seb),(b+xp2&*seb)}.
compute pvchi=1-chicdf((LL3-LL2),(nrow(modres)-1)).
compute mcF = (LL3-LL2)/LL3.
compute cox = 1-exp(-(LL3-LL2)/nrow(xlp)).
compute nagel = cox/(1-exp(-(LL3)/nrow(xlp))).
compute modsum={LL2,(LL3-LL2),(nrow(modres)-1),pvchi, mcF,cox,nagel}.
compute modsuml={'-2LL','ModelLL', 'df', 'p', 'McFadden', 'CoxSnell', 'Nagelkrk'}.
compute modresl={'coeff','se','Z','p','LLCI','ULCI'}.
end if.
end if
.
end if.
do if (ydich=1 and (i = (nms+nys))).

.
do if ( 2 =1).
compute hatmat=inv(t( x )* x )*t( x ).
compute b = hatmat* y.
compute modres=b.
do if ( 0 =1).
compute n1=nrow( x ).
compute dfres=n1-(ncol( x )).
compute sstotal = t( y -(csum( y )/n1))*( y -(csum( y )/n1)).
compute resid= y - x *b.
compute ssresid = csum((resid)&**2).
compute r2 = (sstotal-ssresid)/sstotal.
do if (r2 < 0).
compute r2=0.
end if.
compute adjr2 = 1-((1-r2)*(n1-1)/(dfres)).
compute mse=ssresid/(n1-ncol( x )).

.
compute n1=nrow( x ).
compute invXtX = inv(t( x )* x ).
compute varb = mse *invXtX.
compute k3 = ncol( x ).
compute xhc=0.
do if ( hc <> 5).
compute xhc= x.
compute hat = xhc(:,1).
loop i3=1 to nrow(xhc).
compute hat(i3,1)= xhc(i3,:)*invXtX*t(xhc(i3,:)).
end loop.
do if ( hc = 0 or hc =1).
loop i3 = 1 to k3.
compute xhc(:,i3)=xhc(:,i3)&* resid.
end loop.
end if.
do if ( hc =3 or hc =2).
loop i3=1 to k3.
compute xhc(:,i3) = ( resid &/(1-hat)&**(1/(4- hc )))&*xhc(:,i3).
end loop.
end if.
do if ( hc = 4).
compute hcmn=make(n,2,4).
compute hcmn(:,2)=(n1*hat)/k3.
loop i3= 1 to k3.
compute xhc(:,i3) = ( resid &/(1-hat)&**(rmin(hcmn)/2))&*xhc(:,i3).
end loop.
end if.
compute varb=(invXtX*t(xhc)*xhc*invXtX).
do if ( hc =1).
compute varb=(n1/(n1-ncol( x )))&*varb.
end if.
end if.
compute hclab={'se(HC0)','se(HC1)','se(HC2)','se(HC3)','se(HC4)','se'}.
compute hclab=hclab(1,( hc +1)).
compute hcflab={'F(HC0)','F(HC1)','F(HC2)','F(HC3)','F(HC4)','F'}.
compute hcflab=hcflab(1,( hc +1)).
release xhc
.
compute seb=sqrt(diag(varb)).
compute trat = b&/seb.
compute p = 2*(1-tcdf(abs(trat), (dfres))).
compute tval = sqrt(dfres* (exp((dfres-(5/6))*((xp2/(dfres-(2/3)+(.11/dfres)))* (xp2/(dfres-(2/3)+(.11/dfres)))))-1)).
compute modres={modres,seb,trat,p}.
compute modres={modres,(b-tval&*seb),(b+tval&*seb)}.
compute modresl={'coeff',hclab,'t','p','LLCI','ULCI'}.
compute lmat = ident(ncol( x )).
compute lmat = lmat(:,2:ncol(lmat)).
compute fratio = (t(t(lmat)*b)*inv(t(lmat)*varb*lmat)*((t(lmat)*b)))/(ncol( x )-1).
compute pfr = 1-fcdf(fratio,(ncol( x )-1),dfres).
compute modsum={sqrt(r2),r2,mse,fratio,(ncol( x )-1),dfres,pfr}.
compute modsuml={'R','R-sq','MSE',hcflab,'df1','df2', 'p'}.
end if.
end if.
do if ( 2 = 2 or 2 =3).
compute xlp= x.
compute ylp= y.
compute pt2 = make(nrow(ylp),1,(csum(ylp)/nrow(ylp))).
do if ( 2 =2).
compute LL3 = ylp&*ln(pt2)+(1-ylp)&*ln(1-pt2).
end if.
compute LL3 = -2*csum(LL3).
compute bt1 = make(ncol(xlp),1,0).
compute LL1 = 0.
compute pt1 = make(nrow(ylp),1,0.5).
compute pt1lp=pt1.
loop jjj = 1 to iterate.
compute xlptmp=t(xlp).
compute vecprb=pt1lp&*(1-pt1lp).
loop kkk=1 to ncol(xlp).
compute xlptmp(kkk,:)=xlptmp(kkk,:)&*t(vecprb).
end loop.
compute b = bt1+inv(xlptmp*xlp)*t(xlp)*(ylp-pt1lp).
do if ( 2 =2).
compute xlpb=xlp*b.
compute xlpbt=(xlpb > -709.7).
compute xlpb709=(1-xlpbt)*(-709.7).
compute xlpb=(xlpb&*xlpbt)+xlpb709.
compute pt1lp = 1/(1+exp(-(xlpb))).
end if.
compute itprob = csum((pt1lp < .00000001) or (pt1lp > .9999999)).
do if (itprob > 0).
loop kkk = 1 to nrow(pt1lp).
do if (pt1lp(kkk,1) > .9999999).
compute pt1lp(kkk,1) = .9999999.
end if.
do if (pt1lp(kkk,1) < .00000001).
compute pt1lp(kkk,1) = .00000001.
end if.
end loop.
compute itprob = 0.
end if.
do if (itprob = 0).
do if ( 2 =2).
compute LL = ylp&*ln(pt1lp)+(1-ylp)&*ln(1-pt1lp).
end if.
compute LL2 = -2*csum(ll).
end if.
do if (abs(LL1-LL2) < converge).
do if ( 0 =1).
compute xlptmp=t(xlp).
compute vecprb=pt1lp&*(1-pt1lp).
loop kkk=1 to ncol(xlp).
compute xlptmp(kkk,:)=xlptmp(kkk,:)&*t(vecprb).
end loop.
compute varb = inv(xlptmp*xlp).
compute seb = sqrt(diag(varb)).
release xlptmp.
end if.
break.
end if.
compute bt1 = b.
compute LL1 = LL2.
end loop.
compute modres=b.
do if (jjj > iterate).
compute itprob = 2.
do if (booting=0).
compute iterrmod=1.
end if.
do if (booting=1).
compute bootiter=1.
end if.
do if (itprobtg=0).
compute itprobtg=1.
compute errcode(errs,1) = 47.
compute errs = errs + 1.
do if (booting = 0 and 0 =1).
compute vt1 = mdiag(pt1lp&*(1-pt1lp)).
compute varb = inv(t(xlp)*vt1*xlp).
compute seb = sqrt(diag(varb)).
end if.
end if.
end if.
do if ( 0 =1).
compute trat = b&/seb.
compute dfres=nrow(xlp).
compute p = 2*(1-cdfnorm(abs(trat))).
compute modres={modres,seb,trat,p}.
compute modres={modres,(b-xp2&*seb),(b+xp2&*seb)}.
compute pvchi=1-chicdf((LL3-LL2),(nrow(modres)-1)).
compute mcF = (LL3-LL2)/LL3.
compute cox = 1-exp(-(LL3-LL2)/nrow(xlp)).
compute nagel = cox/(1-exp(-(LL3)/nrow(xlp))).
compute modsum={LL2,(LL3-LL2),(nrow(modres)-1),pvchi, mcF,cox,nagel}.
compute modsuml={'-2LL','ModelLL', 'df', 'p', 'McFadden', 'CoxSnell', 'Nagelkrk'}.
compute modresl={'coeff','se','Z','p','LLCI','ULCI'}.
end if.
end if
.
end if.
compute modres2={modres2,t(modres)}.
do if (i = (nms+nys)).
do if (bcmat((i+1),1) = 1).
compute bootdir={bootdir;t(modres(wherex(1,i):wherex(2,i),1))}.
end if.
do if (bcmat((i+1),1) = 0).
compute bootdir={bootdir;dirzes}.
end if.
end if.
do if (model = 74 and i <= nms).
compute onetemp=make(nrow(xprobval),1,1).
compute mestmtb={onetemp,xprobval}.
do if (ncs > 0).
compute ncovmdlb=rsum(ccmat(i,:)).
do if (ncovmdl > 0).
compute cvmncb=make(nrow(mestmtb),ncovmdlb,1).
compute cvmnctmp=csum(x(:,(ncol(x)-ncovmdlb+1):ncol(x)))/nrow(x).
do if (cuscoval > 0).
compute cvmnctmp=coval.
end if.
loop mestlp=1 to ncovmdlb.
compute cvmncb(:,mestlp)=cvmncb(:,mestlp)*cvmnctmp(:,mestlp).
end loop.
compute mestmtb={mestmtb,cvmncb}.
end if.
end if.
compute mestbt=mestmtb*modres.
do if (i = 1).
compute mestb=mestbt.
end if.
do if (i > 1).
compute mestb={mestb,mestbt}.
end if.
end if.
do if (model = 74 and i = (nms+nys)).
compute xvalptmp=make(1,nxvls,0).
compute mest74sp=make(1,nms,0).
compute mest74sp={mest74sp;mestb}.
compute xvalptmp={xvalptmp;ident(nxvls)}.
compute dirfxcf=make(nrow(mestb),1,0).
loop cfloop1=1 to nrow(mestb).
compute ndirfx=make(nrow(modres),1,0).
compute ndirfx(2:(nxvls+1),1)=t(xvalptmp(cfloop1,:)).
loop cfloop3=1 to nms.
do if (mcx=1 or mcx=0).
compute ndirfx(wheremw((1+((cfloop3-1)*2)),ncol(wheremw)):wheremw((2+((cfloop3-1)*2) ),ncol(wheremw)))=t(xvalptmp(cfloop1,:)*mestb(1,cfloop3)).
end if.
do if (mcx=2).
compute ndirfx(wheremw((1+((cfloop3-1)*2)),ncol(wheremw)):wheremw((2+((cfloop3-1)*2) ),ncol(wheremw)))=t(xvalptmp(cfloop1,:)*mest74sp(cfloop1,cfloop3)).
end if.
end loop.
do if (cfloop1 > 1).
compute dirfxcf(cfloop1,1)=t(ndirfx)*modres*xscaling.
end if.
end loop.
compute natdirbt={natdirbt;t(dirfxcf(2:nrow(dirfxcf),:))}.
end if.
do if ( (bcmat((i+1),1) = 1) and (nobootx=1) and (effsize=1)).
compute nobootx=0.
compute xsdtemp= (nrow(x)*sscp(x(:,2)))-(t(csum(x(:,2)))*(csum(x(:,2)))).
compute xsdtemp= sqrt(xsdtemp/(nrow(x)*(nrow(x)-1))).
end if.
end if.
end loop.
do if (bad = 0).
compute bootres={bootres;modres2(:,2:ncol(modres2))}.
do if (effsize=1).
compute ysdtemp= (nrow(y)*sscp(y))-(t(csum(y))*(csum(y))).
compute ysdtemp= sqrt(ysdtemp/(nrow(y)*(nrow(y)-1))).
compute bootysd={bootysd;ysdtemp}.
compute bootxsd={bootxsd;xsdtemp}.
end if.
compute goodboot=goodboot+1.
end if.
do if (bad <> 0).
compute badboot=badboot+1.
end if.
end loop if (goodboot = boot).
compute bootres=bootres(2:nrow(bootres),:).
do if (effsize=1).
compute bootysd=bootysd(2:nrow(bootysd),:).
do if (nrow(bootxsd) > 1).
compute bootxsd=bootxsd(2:nrow(bootxsd),:).
end if.
end if.
do if (goodboot < (boot)).
compute boot=0.
compute modelbt=0.
compute notecode(notes,1) = 7.
compute notes = notes + 1.
end if.
do if (boot > 0).
do if (effsize=1).
compute bootysd={ysd;bootysd}.
compute bootxsd={xsd;bootxsd}.
end if.
do if (saveboot = 1).
save bootres/outfile = *.
end if.
do if (modelbt=1).
compute bootcim=make(ncol(bootres),5,-99999).
compute bootcim(:,2) = t(csum(bootres)/nrow(bootres)).
compute bootcim(:,1) = coeffmat(2:nrow(coeffmat),1).
loop i = 1 to ncol(bootres).

.
compute temp = bootres(:,i).
compute temp(GRADE( bootres(:,i) )) = bootres(:,i).
compute badlo = 0.
compute badhi = 0.
do if ( (bootcim(i,1)*bc)+(9999*(1-bc)) <> 9999).
compute pv=csum(temp < (bootcim(i,1)*bc)+(9999*(1-bc)) )/nrow(temp).
compute ppv = pv.
do if (pv > .5).
compute ppv = 1-pv.
end if.
compute y5=sqrt(-2*ln(ppv)).
compute xp=y5+((((y5*p4+p3)*y5+p2)*y5+p1)*y5+p0)/((((y5*q4+q3)*y5+q2)*y5+q1)*y5+q0).
do if (pv <= .5).
compute xp = -xp.
end if.
compute cilow=rnd(nrow(temp)*(cdfnorm(2*xp-xp2))).
compute cihigh=trunc(nrow(temp)*(cdfnorm(2*xp+(xp2))))+1.
do if (cilow < 1).
compute cilow = 1.
compute booterr=1.
compute badlo = 1.
end if.
do if (cihigh > nrow(temp)).
compute cihigh = boot.
compute booterr=1.
compute badhi = 1.
end if.
compute llcit=temp(cilow,1).
compute ulcit=temp(cihigh,1).
do if (badlo = 1 and llcit <> priorlo).
compute badend={badend, llcit}.
compute priorlo = llcit.
end if.
do if (badhi = 1 and ulcit <> priorhi).
compute badend={badend, ulcit}.
compute priorhi = ulcit.
end if.
end if.
do if ( (bootcim(i,1)*bc)+(9999*(1-bc)) = 9999).
compute llcit=temp(cilow,1).
compute ulcit=temp(cihigh,1).
end if.
compute bootse=t(sqrt((cssq(temp)-((csum(temp)&**2)/nrow(temp)))/(nrow(temp)-1)))
.
compute bootcim(i,4:5)={llcit,ulcit}.
compute bootcim(i,3)=bootse.
end loop.
end if.
end if.
do if (badboot > 0).
compute notecode(notes,1) = 6.
compute notes = notes + 1.
end if.
end if.
do if (criterr=0).
release fulldat,x,y.
end if.
do if (xmint=1 and criterr=0).
compute xvalptmp=make(1,nxvls,0).
compute mest74sp=make(1,nms,0).
compute mest74sp={mest74sp;mest74}.
compute xvalptmp={xvalptmp;ident(nxvls)}.
loop kcfuhd=1 to 2.
compute dirfxcf=make(nrow(mest74),6,0).
loop cfloop1=1 to nrow(mest74).
compute ndirfx=make(nrow(lastb),1,0).
compute ndirfx(2:(nxvls+1),1)=t(xvalptmp(cfloop1,:)).
loop cfloop3=1 to nms.
do if (kcfuhd=1).
do if (mcx=1 or mcx=0).
compute ndirfx(wheremw((1+((cfloop3-1)*2)),ncol(wheremw)):wheremw((2+((cfloop3-1)*2)), ncol(wheremw)))=t(xvalptmp(cfloop1,:)*mest74(1,cfloop3)).
end if.
do if (mcx=2).
compute ndirfx(wheremw((1+((cfloop3-1)*2)),ncol(wheremw)):wheremw((2+((cfloop3-1)*2)), ncol(wheremw)))=t(xvalptmp(cfloop1,:)*mest74sp(cfloop1,cfloop3)).
end if.
end if.
do if (kcfuhd=2).
compute ndirfx(wheremw((1+((cfloop3-1)*2)),ncol(wheremw)):wheremw((2+((cfloop3-1)*2)), ncol(wheremw)))=t(xvalptmp(cfloop1,:)*medmeans(1,cfloop3)).
end if.
end loop.
do if (cfloop1 > 1).
compute dirfxcf(cfloop1,1)=t(ndirfx)*lastb*xscaling.
compute dirfxcf(cfloop1,2)=sqrt(diag(t(ndirfx)*lastcov*ndirfx))*abs(xscaling).
compute dirfxcf(cfloop1,3)=dirfxcf(cfloop1,1)/dirfxcf(cfloop1,2).
compute dirfxcf(cfloop1,4)=2*(1-tcdf(abs(dirfxcf(cfloop1,3)), dfres)).
compute dirfxcf(cfloop1,5)=dirfxcf(cfloop1,1)-tval*dirfxcf(cfloop1,2).
compute dirfxcf(cfloop1,6)=dirfxcf(cfloop1,1)+tval*dirfxcf(cfloop1,2).
end if.
end loop.
compute codireff=dirfxcf(2:nrow(dirfxcf),:).
do if (kcfuhd=1).
compute direff=codireff.
end if.
end loop.
end if.

.
do if (criterr = 0 and nms > 0).
compute paths=paths(:,2:ncol(paths)).
compute pathsw=pathsw(:,2:ncol(pathsw)).
compute pathsz=pathsz(:,2:ncol(pathsz)).
compute pathswz=pathswz(:,2:ncol(pathswz)).
compute pathsmod=pathsw+pathsz+pathswz.
compute pathsdv=pathsdv(:,2:ncol(pathsdv)).
compute pathsfoc=pathsfoc(:,2:ncol(pathsfoc)).
compute pathtype=pathtype(:,2:ncol(pathtype)).
compute anymod=(rsum(pathsmod) > 0).
compute obscoeff=obscoeff(1,2:ncol(obscoeff)).
do if (outscree=1).
do if (xmint=1).
print/title = '************************* COUNTERFACTUALLY DEFINED ************************'.
else.
print/title = ' '.
end if.
do if (dototal = 0 and alttotal=0).
print/title = '****************** DIRECT AND INDIRECT EFFECTS OF X ON Y *****************'/space=0.
end if.
do if (alttotal=1).
print/title = '************** TOTAL, DIRECT, AND INDIRECT EFFECTS OF X ON Y **************'/space=0.
end if.
end if.
do if (dototal = 1).
do if (outscree=1).
print/title = '************** TOTAL, DIRECT, AND INDIRECT EFFECTS OF X ON Y **************'/space=0.
end if.
compute totefflb(1,1)='Effect'.
do if (effsize=1).
compute toteffsz=toteff(:,1)/ysd.
do if (xdich=1 or mcx > 0).
compute totefflb={totefflb,'c_ps'}.
end if.
do if (xdich = 0 and mcx = 0).
compute toteffsz=toteffsz*xsd.
compute totefflb={totefflb,'c_cs'}.
end if.
compute toteff={toteff,toteffsz}.
end if.
compute resultm2=make(nrow(toteff),maxresm,99999).
compute resultm2(1:nrow(toteff),1:ncol(toteff))=toteff.
compute resultm={resultm;resultm2}.
do if (nxvls > 1).
compute resultm2=make(nrow(totomni),maxresm,99999).
compute resultm2(1:nrow(totomni),1:ncol(totomni))=totomni.
compute resultm={resultm;resultm2}.
compute clabtmp={'R2-chng', hcflab, 'df1','df2','p'}.
do if (outscree=1).
print toteff/title='Relative total effects of X on Y'/rnames=toteffl2/cnames=totefflb/format= F10.4.
print totomni/title='Omnibus test of total effect of X on Y'/cnames=clabtmp/format= F10.4.
print/title= '----------'/space=0.
end if.
else.
do if (outscree=1).
print toteff/title='Total effect of X on Y'/cnames=totefflb/format= F10.4.
end if.
end if.
end if.
compute moddir=wcmat(nrow(bcmat),1)+zcmat(nrow(bcmat),1).
do if (xmint=1).
compute moddir=1.
end if.
do if (bcmat(nrow(bcmat),1)=1).
do if (ydich=1).
compute direfflb(:,(ncol(direfflb)-5):ncol(direfflb))={'Effect','se','Z','p','LLCI','ULCI'}.
end if.
do if (moddir=0 or xmint=1).
compute direfflb(1,1)='Effect'.
end if.
do if (effsize=1 and moddir=0 and anymod = 0).
compute direffsz=direff(:,1)/ysd.
do if (xdich=1 or mcx > 0).
compute direfflb={direfflb,'c''_ps'}.
end if.
do if (xdich = 0 and mcx = 0).
compute direffsz=direffsz*xsd.
compute direfflb={direfflb,'c''_cs'}.
end if.
compute direff={direff,direffsz}.
end if.
compute resultm2=make(nrow(direff),maxresm,99999).
compute resultm2(1:nrow(direff),1:ncol(direff))=direff.
compute resultm={resultm;resultm2}.
do if (xmint=1).
compute resultm2=make(nrow(codireff),maxresm,99999).
compute resultm2(1:nrow(codireff),1:ncol(codireff))=codireff.
compute resultm={resultm;resultm2}.
end if.
do if (moddir = 0 and nxvls=1).
do if (outscree=1).
print direff/title='Direct effect of X on Y'/cnames=direfflb/format= F10.4.
end if.
end if.
do if (moddir = 0 and nxvls>1).
compute resultm2=make(nrow(diromni),maxresm,99999).
compute resultm2(1:nrow(diromni),1:ncol(diromni))=diromni.
compute resultm={resultm;resultm2}.
do if (outscree=1).
print direff/title='Relative direct effects of X on Y'/rnames=direffl2/cnames=direfflb/format= F10.4.
do if (ydich = 0).
compute clabtmp={'R2-chng', hcflab, 'df1','df2','p'}.
print diromni/title='Omnibus test of direct effect of X on Y:'/cnames=clabtmp/format= F10.4.
end if.
do if (ydich = 1).
compute clabtmp={'Chi-sq', 'df', 'p'}.
print diromni/title='Omnibus likelihood ratio test of direct effect of X on Y'/cnames=clabtmp/format= F10.4.
end if.
print/title= '----------'/space=0.
end if.
end if.
do if (moddir > 0 and nxvls=1).
do if (xmint=0 and outscree=1).
print direff/title='Conditional direct effects of X on Y'/cnames=direfflb/format= F10.4.
end if.
do if (xmint=1).
do if (outscree=1).
print direff/title='(Pure) Natural direct effect of X on Y'/cnames=direfflb/format= F10.4.
print codireff/title='Controlled direct effect of X on Y'/cnames=direfflb/format= F10.4.
print/title= '----------'/space=0.
end if.
compute obnatdfx=direff(:,1).
end if.
end if.
compute direffl4=direffl2.
do if (moddir > 0 and nxvls>1).
compute direffl2={' '}.
loop i = 1 to nxvls.
loop j = 1 to (nrow(direff)/nxvls).
compute direffl2={direffl2;xcatlab(i,1)}.
end loop.
end loop.
compute direffl2=direffl2(2:nrow(direffl2),1).
do if (xmint=0 and outscree=1).
print direff/title='Relative conditional direct effects of X on Y'/cnames=direfflb/rnames=direffl2/format= F10.4.
end if.
do if (xmint=1).
do if (outscree=1).
print direff/title='Relative (pure) natural direct effects of X on Y'/cnames=direfflb/rnames=direffl2/format= F10.4.
print codireff/title='Relative controlled direct effects of X on Y'/cnames=direfflb/rnames=direffl2/format= F10.4.
print/title= '----------'/space=0.
end if.
compute obnatdfx=direff(:,1).
end if.
end if.
compute direffl2=direffl4.
end if.
do if (bcmat(nrow(bcmat),1)=0 and xmint <> 1 and outscree=1).
print/title='The direct effect of X on Y is fixed to zero.'.
end if.
do if (nms = 1).
compute indmark={2}.
compute indsets={1,2}.
end if.
do if (nms = 2).
compute indmark={2,2,3}.
compute indsets={1,4,2,5,1,3,5}.
compute thetam=1.
end if.
do if (nms = 3).
compute indmark={2,2,2,3,3,3,4}.
compute indsets={1,7,2,8,4,9,1,3,8,1,5,9,2,6,9,1,3,6,9}.
compute thetam={1,2,3}.
end if.
do if (nms = 4).
compute indmark={2,2,2,2,3,3,3,3,3,3,4,4,4,4,5}.
compute indsets={1,11,2,12,4,13,7,14,1,3,12,1,5,13,1,8,14,2,6,13,2,9,14,4,10,14,1,3,6,13,1,3,9, 14,1,5,10,14,2,6,10,14,1,3,6,10,14}.
compute thetam={1,2,4,3,5,6}.
end if.
do if (nms = 5).
compute indmark={2,2,2,2,2,3,3,3,3,3,3,3,3,3,3,4,4,4,4,4,4,4,4,4,4,5,5,5,5,5,6}.
compute indsets={1,16,2,17,4,18,7,19,11,20,1,3,17,1,5,18,1,8,19,1,12,20,2,6,18,2,9,19,2,13,20,4, 10,19,4,14,20,7,15,20,1,3,6,18, 1,3,9,19,1,3,13,20,1,5,10,19,1,5,14,20,1,8,15,20,2,6,10,19,2,6, 14,20,2,9,15,20,4,10,15,20,1,3,6,10,19, 1,3,6,14,20,1,3,9,15,20,1,5,10,15,20,2,6,10,15,20,1,3,6,10,15, 20}.
compute thetam={1,2,5,3,6,8,4,7,9,10}.
end if.
do if (nms = 6).
compute indmark={2,2,2,2,2,2,3,3,3,3,3,3,3,3,3,3,3,3,3,3,3,4,4,4,4,4,4,4,4,4,4,4,4,4,4,4,4,4,4, 4,4,5,5,5,5,5,5,5,5,5,5,5,5,5,5,5,6,6,6,6,6,6,7}.
compute indsets={1,22,2,23,4,24,7,25,11,26,16,27,1,3,23,1,5,24,1,8,25,1,12,26,1,17,27,2,6,24,2, 9,25,2,13,26,2,18,27,4,10,25,4,14,26, 4,19,27,7,15,26,7,20,27,11,21,27,1,3,6,24,1,3,9,25,1,3,13,26,1, 3,18,27,1,5,10,25,1,5,14,26,1,5,19,27,1,8,15,26,1,8,20,27, 1,12,21,27,2,6,10,25,2,6,14,26,2,6,19,27,2,9,15,26,2,9,20,27,2, 13,21,27,4,10,15,26,4,10,20,27,4,14,21,27,7,15,21,27, 1,3,6,10,25,1,3,6,14,26,1,3,6,19,27,1,3,9,15,26,1,3,9,20,27,1,3, 13,21,27,1,5,10,15,26,1,5,10,20,27,1,5,14,21,27, 1,8,15,21,27,2,6,10,15,26,2,6,10,20,27,2,6,14,21,27,2,9,15,21, 27,4,10,15,21,27,1,3,6,10,15,26,1,3,6,10,20,27, 1,3,6,14,21,27,1,3,9,15,21,27,1,5,10,15,21,27,2,6,10,15,21,27, 1,3,6,10,15,21,27}.
compute thetam={1,2,6,3,7,10,4,8,11,13,5,9,12,14,15}.
end if.
do if (nms = 7).
compute indmark={2,2,2,2,2,2,2}.
compute indsets={1,29,2,30,4,31,7,32,11,33,16,34,22,35}.
end if.
do if (nms = 8).
compute indmark={2,2,2,2,2,2,2,2}.
compute indsets={1,37,2,38,4,39,7,40,11,41,16,42,22,43,29,44}.
end if.
do if (nms = 9).
compute indmark={2,2,2,2,2,2,2,2,2}.
compute indsets={1,46,2,47,4,48,7,49,11,50,16,51,22,52,29,53,37,54}.
end if.
do if (nms=10).
compute indmark={2,2,2,2,2,2,2,2,2,2}.
compute indsets={1,56,2,57,4,58,7,59,11,60,16,61,22,62,29,63,37,64,46,65}.
end if.
compute indlbl = {'Ind1'; 'Ind2'; 'Ind3'; 'Ind4'; 'Ind5'; 'Ind6'; 'Ind7'; 'Ind8'; 'Ind9'; 'Ind10'; 'Ind11'; 'Ind12'; 'Ind13'; 'Ind14'; 'Ind15'}.
compute indlbl = {indlbl; 'Ind16';'Ind17';'Ind18';'Ind19';'Ind20';'Ind21';'Ind22';'Ind23';'Ind24';'Ind25';'Ind26';'Ind27';'Ind28';'Ind29';'Ind30'}.
compute indlbl = {indlbl; 'Ind31';'Ind32';'Ind33';'Ind34';'Ind35';'Ind36';'Ind37';'Ind38';'Ind39';'Ind40';'Ind41';'Ind42';'Ind43';'Ind44';'Ind45'}.
compute indlbl = {indlbl; 'Ind46';'Ind47';'Ind48';'Ind49';'Ind50';'Ind51';'Ind52';'Ind53';'Ind54';'Ind55';'Ind56';'Ind57';'Ind58';'Ind59';'Ind60'}.
compute indlbl = {indlbl; 'Ind61';'Ind62';'Ind63';'Ind64';'Ind65';'Ind66';'Ind67';'Ind68';'Ind69';'Ind70';'Ind71';'Ind72';'Ind73';'Ind74';'Ind75'}.
compute indlbl = {indlbl; 'Ind76';'Ind77';'Ind78';'Ind79';'Ind80';'Ind81';'Ind82';'Ind83';'Ind84';'Ind85';'Ind86';'Ind87';'Ind88';'Ind89';'Ind90'}.
compute cntname={'(C1)';'(C2)';'(C3)';'(C4)';'(C5)';'(C6)';'(C7)';'(C8)';'(C9)';'(C10)';'(C11)';'(C12)';'(C13)';'(C14)';'(C15)';'(C16)';'(C17)'}.
compute cntname={cntname;'(C18)';'(C19)';'(C20)';'(C21)';'(C22)';'(C23)';'(C24)';'(C25)';'(C26)';'(C27)';'(C28)';'(C29)';'(C30)';'(C31)'}.
compute cntname={cntname;'(C32)';'(C33)';'(C34)';'(C35)';'(C36)';'(C37)';'(C38)';'(C39)';'(C40)';'(C41)';'(C42)';'(C43)';'(C44)';'(C45)'}.
compute cntname={cntname;'(C46)';'(C47)';'(C48)';'(C49)';'(C50)';'(C51)';'(C52)';'(C53)';'(C54)';'(C55)';'(C56)';'(C57)';'(C58)';'(C59)'}.
compute cntname={cntname;'(C60)';'(C61)';'(C62)';'(C63)';'(C64)';'(C65)';'(C66)';'(C67)';'(C68)';'(C69)';'(C70)';'(C71)';'(C72)';'(C73)'}.
compute cntname={cntname;'(C74)';'(C75)';'(C76)';'(C77)';'(C78)';'(C79)';'(C80)';'(C81)';'(C82)';'(C83)';'(C84)';'(C85)';'(C86)';'(C87)'}.
compute cntname={cntname;'(C88)';'(C89)';'(C90)';'(C91)';'(C92)';'(C93)';'(C94)';'(C95)';'(C96)';'(C97)';'(C98)';'(C99)';'(C100)';'(C101)'}.
compute cntname={cntname;'(C102)';'(C103)';'(C104)';'(C105)'}.
compute indmake=make(ncol(indmark),(nms+2),0).
compute indmod=make(ncol(indmark),1,999).
compute indmmm=make(ncol(indmark),1,0).
compute indmmmt=make(ncol(indmark),1,0).
compute start=1.
compute end=0.
compute nindfx=0.
compute indlocs=make(nrow(thetaxmb),ncol(paths),999).
compute indkey=make(ncol(indmark),1+((rmax(indmark)*2)+1),'     ').
compute c1=1.
compute c2=1.
compute c3=1.
loop i = 1 to ncol(paths).
do if (pathtype(1,i)=1).
compute indlocs(:,i)=thetaxmb(:,c1).
compute c1=c1+1.
end if.
do if (pathtype(1,i)=3).
compute indlocs(:,i)=thetamyb(:,c2).
compute c2=c2+1.
end if.
do if (pathtype(1,i)=2 and nms < 7 and serial=1).
compute indlocs(:,i)=thetammb(:,thetam(1,c3)).
compute c3=c3+1.
end if.
end loop.
loop i = 1 to ncol(indlocs).
compute c1=2.
loop j = 2 to nrow(indlocs).
do if (indlocs(j,i) <> 0).
compute indlocs(c1,i)=indlocs(j,i).
compute c1=c1+1.
end if.
end loop.
compute indlocs(1,i)=c1-2.
end loop.
compute indlocs=indlocs(1:rmax((indlocs(1,:))+1),:).
loop i = 1 to ncol(indmark).
compute numget=indmark(1,i).
compute end=end+numget.
compute gotcha=indsets(1,start:end).
compute start=end+1.
compute ok=1.
compute temp=0.
compute repoman=make(4,1,0).
loop j = 1 to ncol(gotcha).
do if paths(1,gotcha(1,j))=0.
compute ok=0.
end if.
do if (pathsmod(1,gotcha(1,j)) > 0).
compute temp=1.
compute temp2={pathsw(1,gotcha(1,j));pathsz(1,gotcha(1,j));pathswz(1,gotcha(1,j));0}.
do if (temp2(1,1)=1 and temp2(2,1)=1 and temp2(3,1)=0).
compute temp2(4,1)=1.
end if.
compute repoman=repoman+temp2.
end if.
end loop.
compute temp=0.
compute tempmmm=0.
compute typemmm=0.
do if ((repoman(1,1) > 0) and (repoman(2,1) = 0)).
compute temp=1.
do if (repoman(1,1)=1).
compute tempmmm=1.
end if.
do if (repoman(1,1) > 1 and (wdich=1 or mcw > 0)).
compute tempmmm=12.
compute typemmm=mcw.
do if (wdich=1).
compute typemmm=1.
end if.
end if.
do if (repoman(1,1) > 1 and (wdich=0 and mcw = 0)).
compute tempmmm=101.
end if.
end if.
do if ((repoman(1,1) = 0) and (repoman(2,1) > 0)).
compute temp=2.
do if (repoman(2,1)=1).
compute tempmmm=2.
end if.
do if (repoman(2,1) > 1 and (zdich = 1 or mcz > 0)).
compute tempmmm=22.
compute typemmm=mcz.
do if (zdich=1).
compute typemmm=1.
end if.
end if.
do if (repoman(2,1) > 1 and (zdich = 0 and mcw = 0)).
compute tempmmm=102.
end if.
end if.
do if (repoman(1,1)>0 and repoman(2,1)>0).
compute temp=3.
do if (repoman(1,1)=1 and repoman(2,1)=1).
do if (repoman(4,1)=1).
compute tempmmm=31.
end if.
do if (repoman(3,1)=1).
compute tempmmm=41.
end if.
end if.
end if.
do if (repoman(1,1)=1 and repoman(2,1)=1 and repoman(3,1)=0 and repoman(4,1)=0).
compute tempmmm=51.
end if.
do if (ok = 1).
compute nindfx=nindfx+1.
compute indmake(nindfx,1)=numget.
compute indmod(nindfx,1)=temp.
compute indmmm(nindfx,1)=tempmmm.
compute indmmmt(nindfx,1)=typemmm.
compute indmake(nindfx,2:(1+numget))=gotcha.
compute indkey(nindfx,1)=xnames.
loop j = 1 to numget.
compute indkey(nindfx,(j*2+1))=pathsdv(1,gotcha(1,j)).
compute indkey(nindfx,(j*2))='   ->   '.
end loop.
end if.
end loop.
compute indkey=indkey(1:nindfx,1:((cmax(indmake(:,1))*2)+1)).
compute indmake=indmake(1:nindfx,1:(cmax(indmake(:,1))+1)).
compute indmod=indmod(1:nrow(indmake),1).
compute indmmm=indmmm(1:nrow(indmake),1).
compute indmmmt=indmmmt(1:nrow(indmake),1).
compute ncpairs = (((nindfx)*(nindfx-1))/2).
do if ((contrast = 1 or contrast = 2 or contrast=3) and (ncpairs > 105)).
compute contrast=0.
compute notecode(notes,1) = 13.
compute notes = notes + 1.
end if.
do if (contrast = 4).
do if (ncol(contvec) <> nindfx).
compute contrast=0.
compute notecode(notes,1) = 14.
compute notes = notes + 1.
end if.
end if.
do if (anymod=0).
do if (nms = 1 and contrast > 0).
compute contrast=0.
end if.
compute efloop=(((1-(effsize=0))*2)+1)-(((mcx>0 or xdich=1))*(1-(effsize=0))).
loop kk=1 to efloop.
do if (boot = 0).
compute bootres=obscoeff.
do if (kk=1).
compute totbtvec=make(1,nxvls,0).
end if.
compute bootdir=obsdirfx.
compute indtab=999.
compute inddiff=999.
compute bootysd=ysd.
compute bootxsd=xsd.
end if.
do if (boot > 0).
compute bootres={obscoeff;bootres}.
do if (kk=1).
compute totbtvec=make(nrow(bootres),nxvls,0).
end if.
compute indtab=make(1,4,999).
compute inddiff=make(nrow(bootres),1,999).
end if.
compute indtotal=make(nrow(bootres),1,0).
loop i = 1 to nrow(indmake).
loop j = 1 to nxvls.
compute indtemp=make(nrow(bootres),1,1).
loop k = 1 to indmake(i,1).
compute jtemp=1.
do if (j > 1 and k=1).
compute jtemp=j.
end if.
compute indtemp=indtemp&*bootres(:,pathsfoc(jtemp,indmake(i,(k+1)))).
end loop.
do if (kk = 2).
compute indtemp=indtemp/bootysd.
end if.
do if (kk = 3).
compute indtemp=(bootxsd&*indtemp)/bootysd.
end if.
do if (contrast <> 0).
compute inddiff={inddiff,indtemp}.
end if.
do if (nxvls=1).
compute indtotal=indtotal+indtemp.
end if.
compute indeff=indtemp(1,1).
do if (kk=1).
compute totbtvec(:,j)=totbtvec(:,j)+indtemp.
end if.
do if (boot > 0).

.
compute temp = indtemp(2:nrow(indtemp),1).
compute temp(GRADE( indtemp(2:nrow(indtemp),1) )) = indtemp(2:nrow(indtemp),1).
compute badlo = 0.
compute badhi = 0.
do if ( (indtemp(1,1)*bc)+(9999*(1-bc)) <> 9999).
compute pv=csum(temp < (indtemp(1,1)*bc)+(9999*(1-bc)) )/nrow(temp).
compute ppv = pv.
do if (pv > .5).
compute ppv = 1-pv.
end if.
compute y5=sqrt(-2*ln(ppv)).
compute xp=y5+((((y5*p4+p3)*y5+p2)*y5+p1)*y5+p0)/((((y5*q4+q3)*y5+q2)*y5+q1)*y5+q0).
do if (pv <= .5).
compute xp = -xp.
end if.
compute cilow=rnd(nrow(temp)*(cdfnorm(2*xp-xp2))).
compute cihigh=trunc(nrow(temp)*(cdfnorm(2*xp+(xp2))))+1.
do if (cilow < 1).
compute cilow = 1.
compute booterr=1.
compute badlo = 1.
end if.
do if (cihigh > nrow(temp)).
compute cihigh = boot.
compute booterr=1.
compute badhi = 1.
end if.
compute llcit=temp(cilow,1).
compute ulcit=temp(cihigh,1).
do if (badlo = 1 and llcit <> priorlo).
compute badend={badend, llcit}.
compute priorlo = llcit.
end if.
do if (badhi = 1 and ulcit <> priorhi).
compute badend={badend, ulcit}.
compute priorhi = ulcit.
end if.
end if.
do if ( (indtemp(1,1)*bc)+(9999*(1-bc)) = 9999).
compute llcit=temp(cilow,1).
compute ulcit=temp(cihigh,1).
end if.
compute bootse=t(sqrt((cssq(temp)-((csum(temp)&**2)/nrow(temp)))/(nrow(temp)-1)))
.
compute indeff={indeff,bootse,llcit,ulcit}.
end if.
do if (kk=1).
compute indtabn={indtab;indeff}.
end if.
compute indtab={indtab;indeff}.
end loop.
end loop.
compute indtab=indtab(2:nrow(indtab),:).
do if (kk=1).
compute indtabn=indtabn(2:nrow(indtabn),:).
end if.
compute rowlbs=indlbl(1:nrow(indtab),1).
do if (mc > 0).
compute inddiff=make(mc,1,-999).
compute indtab2=make(nrow(indtab),4,-999).
compute indtab2(:,1)=indtab.
compute indtab=indtab2.
release indtab2.
compute mcct=0.
compute indtotal=make(mc,1,0).
do if (kk = 1).
compute x1 = sqrt(-2*ln(uniform(mc,nrow(mcsopath))))&*cos((2*3.14159265358979)*uniform(mc, nrow(mcsopath))).
compute x1=x1*chol(indcov).
loop ii=1 to nrow(x1).
compute x1(ii,:)=x1(ii,:)+t(mcsopath).
end loop.
end if.
loop ii=1 to nms.
compute tmpb=x1(:,((nms*nxvls)+ii)).
compute tmpb2=tmpb.
do if (nxvls > 1).
loop jj=1 to (nxvls-1).
compute tmpb2={tmpb2,tmpb}.
end loop.
end if.
compute indtemp=x1(:,(((ii-1)*nxvls)+1):(ii*nxvls))&*tmpb2.
loop jj=1 to ncol(indtemp).
do if (kk = 2).
compute indtemp(:,jj)=indtemp(:,jj)/ysd.
end if.
do if (kk = 3).
compute indtemp(:,jj)=(xsd*indtemp(:,jj))/ysd.
end if.

.
compute temp = indtemp(:,jj).
compute temp(GRADE( indtemp(:,jj) )) = indtemp(:,jj).
compute badlo = 0.
compute badhi = 0.
do if ( 9999 <> 9999).
compute pv=csum(temp < 9999 )/nrow(temp).
compute ppv = pv.
do if (pv > .5).
compute ppv = 1-pv.
end if.
compute y5=sqrt(-2*ln(ppv)).
compute xp=y5+((((y5*p4+p3)*y5+p2)*y5+p1)*y5+p0)/((((y5*q4+q3)*y5+q2)*y5+q1)*y5+q0).
do if (pv <= .5).
compute xp = -xp.
end if.
compute cilow=rnd(nrow(temp)*(cdfnorm(2*xp-xp2))).
compute cihigh=trunc(nrow(temp)*(cdfnorm(2*xp+(xp2))))+1.
do if (cilow < 1).
compute cilow = 1.
compute booterr=1.
compute badlo = 1.
end if.
do if (cihigh > nrow(temp)).
compute cihigh = boot.
compute booterr=1.
compute badhi = 1.
end if.
compute llcit=temp(cilow,1).
compute ulcit=temp(cihigh,1).
do if (badlo = 1 and llcit <> priorlo).
compute badend={badend, llcit}.
compute priorlo = llcit.
end if.
do if (badhi = 1 and ulcit <> priorhi).
compute badend={badend, ulcit}.
compute priorhi = ulcit.
end if.
end if.
do if ( 9999 = 9999).
compute llcit=temp(cilow,1).
compute ulcit=temp(cihigh,1).
end if.
compute bootse=t(sqrt((cssq(temp)-((csum(temp)&**2)/nrow(temp)))/(nrow(temp)-1)))
.
compute mcct=mcct+1.
compute indtab(mcct,2:4)={bootse,llcit,ulcit}.
end loop.
do if (nxvls=1).
compute indtotal=indtotal+indtemp.
do if (contrast <> 0).
compute inddiff={inddiff,indtemp}.
end if.
end if.
end loop.
release indtemp,tmpb.
end if.
do if (normal = 1 and sobelok=1).
compute sobelmat=indtab(:,1).
compute sobelmat={sobelmat,(sobelmat/2),sobelmat,sobelmat}.
loop ii=1 to nms.
compute se2b=indcov(((nms*nxvls)+ii),((nms*nxvls)+ii)).
compute bpath2=mcsopath(((nms*nxvls)+ii),1)&**2.
compute se2a=diag(indcov((((ii-1)*nxvls)+1):(ii*nxvls),(((ii-1)*nxvls)+1):(ii*nxvls))).
compute apath2=mcsopath((((ii-1)*nxvls)+1):(ii*nxvls) ,1)&**2.
compute sesobel=sqrt(apath2*se2b+bpath2*se2a+se2a*se2b).
compute sobelmat((((ii-1)*nxvls)+1):(ii*nxvls),2)=sesobel.
end loop.
release se2b,bpath2,se2a,apath2,sesobel,ii.
compute sobelmat(:,3)=sobelmat(:,1)&/sobelmat(:,2).
compute sobelmat(:,4) = 2*(1-cdfnorm(abs(sobelmat(:,3)))).
end if.
do if (serial = 0).
compute rowlbs=t(mnames).
end if.
do if (nxvls=1 and nms > 1).
compute rowlbs={'TOTAL';rowlbs}.
compute indtemp=indtotal(1,1).
do if (boot > 0 and nxvls=1).

.
compute temp = indtotal(2:nrow(indtotal),1).
compute temp(GRADE( indtotal(2:nrow(indtotal),1) )) = indtotal(2:nrow(indtotal),1).
compute badlo = 0.
compute badhi = 0.
do if ( (indtotal(1,1)*bc)+(9999*(1-bc)) <> 9999).
compute pv=csum(temp < (indtotal(1,1)*bc)+(9999*(1-bc)) )/nrow(temp).
compute ppv = pv.
do if (pv > .5).
compute ppv = 1-pv.
end if.
compute y5=sqrt(-2*ln(ppv)).
compute xp=y5+((((y5*p4+p3)*y5+p2)*y5+p1)*y5+p0)/((((y5*q4+q3)*y5+q2)*y5+q1)*y5+q0).
do if (pv <= .5).
compute xp = -xp.
end if.
compute cilow=rnd(nrow(temp)*(cdfnorm(2*xp-xp2))).
compute cihigh=trunc(nrow(temp)*(cdfnorm(2*xp+(xp2))))+1.
do if (cilow < 1).
compute cilow = 1.
compute booterr=1.
compute badlo = 1.
end if.
do if (cihigh > nrow(temp)).
compute cihigh = boot.
compute booterr=1.
compute badhi = 1.
end if.
compute llcit=temp(cilow,1).
compute ulcit=temp(cihigh,1).
do if (badlo = 1 and llcit <> priorlo).
compute badend={badend, llcit}.
compute priorlo = llcit.
end if.
do if (badhi = 1 and ulcit <> priorhi).
compute badend={badend, ulcit}.
compute priorhi = ulcit.
end if.
end if.
do if ( (indtotal(1,1)*bc)+(9999*(1-bc)) = 9999).
compute llcit=temp(cilow,1).
compute ulcit=temp(cihigh,1).
end if.
compute bootse=t(sqrt((cssq(temp)-((csum(temp)&**2)/nrow(temp)))/(nrow(temp)-1)))
.
compute indtemp={indtemp, bootse,llcit,ulcit}.
end if.
do if (mc > 0).
compute obtmc=indtab(:,1).
compute indtemp=csum(obtmc).

.
compute temp = indtotal(:,1).
compute temp(GRADE( indtotal(:,1) )) = indtotal(:,1).
compute badlo = 0.
compute badhi = 0.
do if ( 9999 <> 9999).
compute pv=csum(temp < 9999 )/nrow(temp).
compute ppv = pv.
do if (pv > .5).
compute ppv = 1-pv.
end if.
compute y5=sqrt(-2*ln(ppv)).
compute xp=y5+((((y5*p4+p3)*y5+p2)*y5+p1)*y5+p0)/((((y5*q4+q3)*y5+q2)*y5+q1)*y5+q0).
do if (pv <= .5).
compute xp = -xp.
end if.
compute cilow=rnd(nrow(temp)*(cdfnorm(2*xp-xp2))).
compute cihigh=trunc(nrow(temp)*(cdfnorm(2*xp+(xp2))))+1.
do if (cilow < 1).
compute cilow = 1.
compute booterr=1.
compute badlo = 1.
end if.
do if (cihigh > nrow(temp)).
compute cihigh = boot.
compute booterr=1.
compute badhi = 1.
end if.
compute llcit=temp(cilow,1).
compute ulcit=temp(cihigh,1).
do if (badlo = 1 and llcit <> priorlo).
compute badend={badend, llcit}.
compute priorlo = llcit.
end if.
do if (badhi = 1 and ulcit <> priorhi).
compute badend={badend, ulcit}.
compute priorhi = ulcit.
end if.
end if.
do if ( 9999 = 9999).
compute llcit=temp(cilow,1).
compute ulcit=temp(cihigh,1).
end if.
compute bootse=t(sqrt((cssq(temp)-((csum(temp)&**2)/nrow(temp)))/(nrow(temp)-1)))
.
compute indtemp={indtemp, bootse,llcit,ulcit}.
end if.
compute indtab={indtemp;indtab}.
end if.
compute bootlbs={'Effect', 'BootSE','BootLLCI','BootULCI'}.
do if (mc > 0).
compute bootlbs={'Effect', 'MC SE','MC LLCI','MC ULCI'}.
end if.
do if (nxvls = 1).
do if (contrast <> 0).
compute inddiff=inddiff(:,2:ncol(inddiff)).
do if (mc > 0).
compute inddiff={t(obtmc);inddiff}.
end if.
do if (contrast = 4).
compute inddifft=inddiff*t(contvec).
compute indtemp=inddifft(1,1).
do if (boot > 0 or mc > 0).
do if (mc > 0).

.
compute temp = inddifft(2:nrow(inddifft),1).
compute temp(GRADE( inddifft(2:nrow(inddifft),1) )) = inddifft(2:nrow(inddifft),1).
compute badlo = 0.
compute badhi = 0.
do if ( 9999 <> 9999).
compute pv=csum(temp < 9999 )/nrow(temp).
compute ppv = pv.
do if (pv > .5).
compute ppv = 1-pv.
end if.
compute y5=sqrt(-2*ln(ppv)).
compute xp=y5+((((y5*p4+p3)*y5+p2)*y5+p1)*y5+p0)/((((y5*q4+q3)*y5+q2)*y5+q1)*y5+q0).
do if (pv <= .5).
compute xp = -xp.
end if.
compute cilow=rnd(nrow(temp)*(cdfnorm(2*xp-xp2))).
compute cihigh=trunc(nrow(temp)*(cdfnorm(2*xp+(xp2))))+1.
do if (cilow < 1).
compute cilow = 1.
compute booterr=1.
compute badlo = 1.
end if.
do if (cihigh > nrow(temp)).
compute cihigh = boot.
compute booterr=1.
compute badhi = 1.
end if.
compute llcit=temp(cilow,1).
compute ulcit=temp(cihigh,1).
do if (badlo = 1 and llcit <> priorlo).
compute badend={badend, llcit}.
compute priorlo = llcit.
end if.
do if (badhi = 1 and ulcit <> priorhi).
compute badend={badend, ulcit}.
compute priorhi = ulcit.
end if.
end if.
do if ( 9999 = 9999).
compute llcit=temp(cilow,1).
compute ulcit=temp(cihigh,1).
end if.
compute bootse=t(sqrt((cssq(temp)-((csum(temp)&**2)/nrow(temp)))/(nrow(temp)-1)))
.
end if.
do if (boot > 0).

.
compute temp = inddifft(2:nrow(inddifft),1).
compute temp(GRADE( inddifft(2:nrow(inddifft),1) )) = inddifft(2:nrow(inddifft),1).
compute badlo = 0.
compute badhi = 0.
do if ( (inddifft(1,1)*bc)+(9999* (1-bc)) <> 9999).
compute pv=csum(temp < (inddifft(1,1)*bc)+(9999* (1-bc)) )/nrow(temp).
compute ppv = pv.
do if (pv > .5).
compute ppv = 1-pv.
end if.
compute y5=sqrt(-2*ln(ppv)).
compute xp=y5+((((y5*p4+p3)*y5+p2)*y5+p1)*y5+p0)/((((y5*q4+q3)*y5+q2)*y5+q1)*y5+q0).
do if (pv <= .5).
compute xp = -xp.
end if.
compute cilow=rnd(nrow(temp)*(cdfnorm(2*xp-xp2))).
compute cihigh=trunc(nrow(temp)*(cdfnorm(2*xp+(xp2))))+1.
do if (cilow < 1).
compute cilow = 1.
compute booterr=1.
compute badlo = 1.
end if.
do if (cihigh > nrow(temp)).
compute cihigh = boot.
compute booterr=1.
compute badhi = 1.
end if.
compute llcit=temp(cilow,1).
compute ulcit=temp(cihigh,1).
do if (badlo = 1 and llcit <> priorlo).
compute badend={badend, llcit}.
compute priorlo = llcit.
end if.
do if (badhi = 1 and ulcit <> priorhi).
compute badend={badend, ulcit}.
compute priorhi = ulcit.
end if.
end if.
do if ( (inddifft(1,1)*bc)+(9999* (1-bc)) = 9999).
compute llcit=temp(cilow,1).
compute ulcit=temp(cihigh,1).
end if.
compute bootse=t(sqrt((cssq(temp)-((csum(temp)&**2)/nrow(temp)))/(nrow(temp)-1)))
.
end if.
compute indtemp={indtemp, bootse,llcit,ulcit}.
end if.
compute indtab={indtab;indtemp}.
end if.
do if (contrast = 1 or contrast = 2 or contrast=3).
compute conkey=make(1,4,' ').
loop i = 1 to ncol(inddiff)-1.
loop j = (i+1) to ncol(inddiff).
compute inddifft=inddiff(:,i)-inddiff(:,j).
do if (contrast=2).
compute inddifft=abs(inddiff(:,i))-abs(inddiff(:,j)).
end if.
do if (contrast=3).
compute inddifft=inddiff(:,i)+inddiff(:,j).
end if.
compute indtemp=inddifft(1,1).
do if (contrast=1 or contrast=2).
compute conkeyt={' ', rowlbs((i+1),1),' minus  ',rowlbs((j+1),1)}.
end if.
do if (contrast=3).
compute conkeyt={' ', rowlbs((i+1),1),'  plus  ',rowlbs((j+1),1)}.
end if.
compute conkey={conkey;conkeyt}.
do if (boot > 0 or mc > 0).
do if (mc > 0).

.
compute temp = inddifft(2:nrow(inddifft),1).
compute temp(GRADE( inddifft(2:nrow(inddifft),1) )) = inddifft(2:nrow(inddifft),1).
compute badlo = 0.
compute badhi = 0.
do if ( 9999 <> 9999).
compute pv=csum(temp < 9999 )/nrow(temp).
compute ppv = pv.
do if (pv > .5).
compute ppv = 1-pv.
end if.
compute y5=sqrt(-2*ln(ppv)).
compute xp=y5+((((y5*p4+p3)*y5+p2)*y5+p1)*y5+p0)/((((y5*q4+q3)*y5+q2)*y5+q1)*y5+q0).
do if (pv <= .5).
compute xp = -xp.
end if.
compute cilow=rnd(nrow(temp)*(cdfnorm(2*xp-xp2))).
compute cihigh=trunc(nrow(temp)*(cdfnorm(2*xp+(xp2))))+1.
do if (cilow < 1).
compute cilow = 1.
compute booterr=1.
compute badlo = 1.
end if.
do if (cihigh > nrow(temp)).
compute cihigh = boot.
compute booterr=1.
compute badhi = 1.
end if.
compute llcit=temp(cilow,1).
compute ulcit=temp(cihigh,1).
do if (badlo = 1 and llcit <> priorlo).
compute badend={badend, llcit}.
compute priorlo = llcit.
end if.
do if (badhi = 1 and ulcit <> priorhi).
compute badend={badend, ulcit}.
compute priorhi = ulcit.
end if.
end if.
do if ( 9999 = 9999).
compute llcit=temp(cilow,1).
compute ulcit=temp(cihigh,1).
end if.
compute bootse=t(sqrt((cssq(temp)-((csum(temp)&**2)/nrow(temp)))/(nrow(temp)-1)))
.
end if.
do if (boot > 0).

.
compute temp = inddifft(2:nrow(inddifft),1).
compute temp(GRADE( inddifft(2:nrow(inddifft),1) )) = inddifft(2:nrow(inddifft),1).
compute badlo = 0.
compute badhi = 0.
do if ( (inddifft(1,1)*bc)+(9999* (1-bc)) <> 9999).
compute pv=csum(temp < (inddifft(1,1)*bc)+(9999* (1-bc)) )/nrow(temp).
compute ppv = pv.
do if (pv > .5).
compute ppv = 1-pv.
end if.
compute y5=sqrt(-2*ln(ppv)).
compute xp=y5+((((y5*p4+p3)*y5+p2)*y5+p1)*y5+p0)/((((y5*q4+q3)*y5+q2)*y5+q1)*y5+q0).
do if (pv <= .5).
compute xp = -xp.
end if.
compute cilow=rnd(nrow(temp)*(cdfnorm(2*xp-xp2))).
compute cihigh=trunc(nrow(temp)*(cdfnorm(2*xp+(xp2))))+1.
do if (cilow < 1).
compute cilow = 1.
compute booterr=1.
compute badlo = 1.
end if.
do if (cihigh > nrow(temp)).
compute cihigh = boot.
compute booterr=1.
compute badhi = 1.
end if.
compute llcit=temp(cilow,1).
compute ulcit=temp(cihigh,1).
do if (badlo = 1 and llcit <> priorlo).
compute badend={badend, llcit}.
compute priorlo = llcit.
end if.
do if (badhi = 1 and ulcit <> priorhi).
compute badend={badend, ulcit}.
compute priorhi = ulcit.
end if.
end if.
do if ( (inddifft(1,1)*bc)+(9999* (1-bc)) = 9999).
compute llcit=temp(cilow,1).
compute ulcit=temp(cihigh,1).
end if.
compute bootse=t(sqrt((cssq(temp)-((csum(temp)&**2)/nrow(temp)))/(nrow(temp)-1)))
.
end if.
compute indtemp={indtemp, bootse,llcit,ulcit}.
end if.
compute indtab={indtab;indtemp}.
end loop.
end loop.
end if.
release inddiff.
compute contlbs=cntname(1:(((nindfx)*(nindfx-1))/2),1).
compute rowlbs={rowlbs;contlbs}.
end if.
compute resultm2=make(nrow(indtab),maxresm,99999).
compute resultm2(1:nrow(indtab),1:ncol(indtab))=indtab.
compute resultm={resultm;resultm2}.
do if (outscree=1).
do if (kk=1).
print indtab/title = 'Indirect effect(s) of X on Y:'/rnames=rowlbs/cnames=bootlbs/format= F10.4.
end if.
do if (kk = 2 and (xdich=1 or mcx > 0)).
print indtab/title = 'Partially standardized indirect effect(s) of X on Y:'/rnames=rowlbs/cnames=bootlbs/format= F10.4.
end if.
do if (kk = 3).
print indtab/title = 'Completely standardized indirect effect(s) of X on Y:'/rnames=rowlbs/cnames=bootlbs/format= F10.4.
end if.
end if.
do if (normal=1 and sobelok=1 and kk=1).
compute resultm2=make(nrow(sobelmat),maxresm,99999).
compute resultm2(1:nrow(sobelmat),1:ncol(sobelmat))=sobelmat.
compute resultm={resultm;resultm2}.
compute sobellab={'Effect',hclab,'Z','p'}.
compute sobelrlb=rowlbs.
do if (nms > 1).
compute sobelrlb=rowlbs(2:(1+nms),1).
end if.
do if (outscree=1).
print sobelmat/title='   Normal theory test for indirect effect(s):'/cnames=sobellab/rnames=sobelrlb/format= F10.4.
end if.
end if.
do if (contrast <> 0).
do if ((contrast=1 or contrast = 2 or contrast=3) and kk=efloop ).
compute conkey=conkey(2:nrow(conkey),:).
do if (outscree=1).
print conkey/title = 'Specific indirect effect contrast definition(s):'/rnames=contlbs/format=A8.
end if.
end if.
do if (outscree=1).
do if (contrast = 4 and kk=efloop).
compute crowlbs=rowlbs(2:(nindfx+1),1).
print contvec/title = 'Specific indirect effect contrast weights:'/cnames=crowlbs/rlabels='(C1)'/format= F10.4.
end if.
do if (contrast = 2 and kk=efloop).
print/title = 'Contrasts are differences between absolute values of indirect effects'.
end if.
end if.
end if.
do if (serial = 1 and kk=efloop).
compute rowlbst=rowlbs(2:nrow(rowlbs),1).
do if (outscree=1).
print indkey/title = 'Indirect effect key:'/rnames=rowlbst/format = A8.
end if.
end if.
else.
do if (outscree=1).
do if (kk = 1).
print/title = 'Relative indirect effects of X on Y'.
end if.
do if (kk = 2).
print/title = 'Partially standardized relative indirect effect(s) of X on Y:'.
end if.
do if (kk = 3).
print/title = 'Completely standardized relative indirect effect(s) of X on Y:'.
end if.
end if.
loop i = 1 to nrow(indmake).
compute indtabsm=indtab((((i-1)*nxvls)+1):(nxvls*i),:).
compute indkeyt=indkey(i,:).
do if (outscree=1).
print indkeyt/title=' '/space=0/format=A8.
end if.
do if (bcmat(nrow(bcmat),1)=0)).
compute direffl2=xcatlab(1:nxvls,1).
end if.
compute resultm2=make(nrow(indtabsm),maxresm,99999).
compute resultm2(1:nrow(indtabsm),1:ncol(indtabsm))=indtabsm.
compute resultm={resultm;resultm2}.
do if (outscree=1).
print indtabsm/title = ' '/cnames=bootlbs/rnames=direffl2/format= F10.4 /space=0.
end if.
do if (normal=1 and sobelok=1 and kk=1).
compute sobelsm=sobelmat((((i-1)*nxvls)+1):(nxvls*i),:).
compute sobellab={'Effect',hclab,'Z','p'}.
compute resultm2=make(nrow(sobelsm),maxresm,99999).
compute resultm2(1:nrow(sobelsm),1:ncol(sobelsm))=sobelsm.
compute resultm={resultm;resultm2}.
do if (outscree=1).
print sobelsm/title='      Normal theory test for relative indirect effects:'/cnames=sobellab/rnames=direffl2/format= F10.4.
end if.
end if.
end loop.
end if.
do if (effsize = 1 and boot > 0).
compute bootres=bootres(2:nrow(bootres),:).
end if.
end loop.
do if (alttotal=1).
compute altcnms='Effect'.
compute totbtvec=totbtvec+bootdir.
compute alttotfx=t(totbtvec(1,:)).
do if (boot > 0).
compute alttotfx=make(ncol(totbtvec),4,0).
compute alttotfx(:,1)=t(totbtvec(1,:)).
loop cec=1 to ncol(totbtvec).

.
compute temp = totbtvec(2:nrow(totbtvec),cec).
compute temp(GRADE( totbtvec(2:nrow(totbtvec),cec) )) = totbtvec(2:nrow(totbtvec),cec).
compute badlo = 0.
compute badhi = 0.
do if ( (totbtvec(1,cec)*bc)+(9999* (1-bc)) <> 9999).
compute pv=csum(temp < (totbtvec(1,cec)*bc)+(9999* (1-bc)) )/nrow(temp).
compute ppv = pv.
do if (pv > .5).
compute ppv = 1-pv.
end if.
compute y5=sqrt(-2*ln(ppv)).
compute xp=y5+((((y5*p4+p3)*y5+p2)*y5+p1)*y5+p0)/((((y5*q4+q3)*y5+q2)*y5+q1)*y5+q0).
do if (pv <= .5).
compute xp = -xp.
end if.
compute cilow=rnd(nrow(temp)*(cdfnorm(2*xp-xp2))).
compute cihigh=trunc(nrow(temp)*(cdfnorm(2*xp+(xp2))))+1.
do if (cilow < 1).
compute cilow = 1.
compute booterr=1.
compute badlo = 1.
end if.
do if (cihigh > nrow(temp)).
compute cihigh = boot.
compute booterr=1.
compute badhi = 1.
end if.
compute llcit=temp(cilow,1).
compute ulcit=temp(cihigh,1).
do if (badlo = 1 and llcit <> priorlo).
compute badend={badend, llcit}.
compute priorlo = llcit.
end if.
do if (badhi = 1 and ulcit <> priorhi).
compute badend={badend, ulcit}.
compute priorhi = ulcit.
end if.
end if.
do if ( (totbtvec(1,cec)*bc)+(9999* (1-bc)) = 9999).
compute llcit=temp(cilow,1).
compute ulcit=temp(cihigh,1).
end if.
compute bootse=t(sqrt((cssq(temp)-((csum(temp)&**2)/nrow(temp)))/(nrow(temp)-1)))
.
compute alttotfx(cec,2:4)={bootse,llcit,ulcit}.
end loop.
compute altcnms={altcnms,'BootSE', 'BootLLCI','BootULCI'}.
end if.
compute resultm2=make(nrow(alttotfx),maxresm,99999).
compute resultm2(1:nrow(alttotfx),1:ncol(alttotfx))=alttotfx.
compute resultm={resultm;resultm2}.
do if (outscree=1).
do if (nxvls > 1).
print/title= '----------'/space=0.
print alttotfx/title='Relative total effects of X on Y (sum of direct and indirect effects)'/rnames=direffl2/cnames=altcnms/format= F10.4.
end if.
do if (nxvls = 1).
print alttotfx/title='Total effect of X on Y (sum of direct and indirect effects)'/cnames=altcnms/format= F10.4.
end if.
end if.
end if.
end if.
do if (anymod > 0).
do if (boot = 0).
compute bootres=obscoeff.
compute indtab=999.
end if.
do if (boot > 0).
compute bootres={obscoeff;bootres}.
compute indtab=make(1,4,999).
end if.
do if (csum((indmod > 0))=nrow(indmod)).
do if (outscree=1).
do if (nxvls > 1).
do if (xmint=0).
print/title= 'Relative conditional indirect effects of X on Y:'.
end if.
do if (xmint=1).
print/title= 'Relative (total) natural indirect effects of X on Y:'.
end if.
end if.
do if (nxvls = 1).
do if (xmint=0).
print/title= 'Conditional indirect effects of X on Y:'.
end if.
do if (xmint=1).
print/title= '(Total) Natural indirect effect(s) of X on Y:'.
end if.
end if.
end if.
end if.
do if (csum((indmod > 0)) < nrow(indmod)).
do if (outscree=1).
do if (nxvls > 1).
print/title= 'Relative conditional and unconditional indirect effects of X on Y:'.
end if.
do if (nxvls = 1).
print/title= 'Conditional and unconditional indirect effects of X on Y:'.
end if.
end if.
end if.
compute cftotfx=make(nrow(bootres),nxvls,0).
loop i = 1 to nrow(indmake).
compute indtab=indtab(1,:)*0.
do if (outscree=1).
do if (xmint=0).
print indkey(i,:)/title = 'INDIRECT EFFECT:'/format=A8.
end if.
do if (xmint=1).
print indkey(i,:)/title = ' '/format=A8/space=0.
end if.
end if.
do if (indmod(i,1)=0).
loop j = 1 to nxvls.
compute indtemp=make(nrow(bootres),1,1).
loop k = 1 to indmake(i,1).
compute jtemp=1.
do if (j > 1 and k=1).
compute jtemp=j.
end if.
compute indtemp=indtemp&*bootres(:,pathsfoc(jtemp,indmake(i,(k+1)))).
end loop.
compute indeff=indtemp(1,1).
do if (boot > 0).

.
compute temp = indtemp(2:nrow(indtemp),1).
compute temp(GRADE( indtemp(2:nrow(indtemp),1) )) = indtemp(2:nrow(indtemp),1).
compute badlo = 0.
compute badhi = 0.
do if ( (indtemp(1,1)*bc)+(9999*(1-bc)) <> 9999).
compute pv=csum(temp < (indtemp(1,1)*bc)+(9999*(1-bc)) )/nrow(temp).
compute ppv = pv.
do if (pv > .5).
compute ppv = 1-pv.
end if.
compute y5=sqrt(-2*ln(ppv)).
compute xp=y5+((((y5*p4+p3)*y5+p2)*y5+p1)*y5+p0)/((((y5*q4+q3)*y5+q2)*y5+q1)*y5+q0).
do if (pv <= .5).
compute xp = -xp.
end if.
compute cilow=rnd(nrow(temp)*(cdfnorm(2*xp-xp2))).
compute cihigh=trunc(nrow(temp)*(cdfnorm(2*xp+(xp2))))+1.
do if (cilow < 1).
compute cilow = 1.
compute booterr=1.
compute badlo = 1.
end if.
do if (cihigh > nrow(temp)).
compute cihigh = boot.
compute booterr=1.
compute badhi = 1.
end if.
compute llcit=temp(cilow,1).
compute ulcit=temp(cihigh,1).
do if (badlo = 1 and llcit <> priorlo).
compute badend={badend, llcit}.
compute priorlo = llcit.
end if.
do if (badhi = 1 and ulcit <> priorhi).
compute badend={badend, ulcit}.
compute priorhi = ulcit.
end if.
end if.
do if ( (indtemp(1,1)*bc)+(9999*(1-bc)) = 9999).
compute llcit=temp(cilow,1).
compute ulcit=temp(cihigh,1).
end if.
compute bootse=t(sqrt((cssq(temp)-((csum(temp)&**2)/nrow(temp)))/(nrow(temp)-1)))
.
compute indeff={indeff,bootse,llcit,ulcit}.
end if.
compute indtab={indtab;indeff}.
end loop.
compute resultm2=make(nrow(indtab),maxresm,99999).
compute resultm2(1:nrow(indtab),1:ncol(indtab))=indtab.
compute resultm={resultm;resultm2}.
do if (outscree=1).
do if (nxvls > 1).
compute indefflb=xcatlab(1:nxvls,1).
print indtab(2:nrow(indtab),:)/title = ' '/clabels='Effect', 'BootSE', 'BootLLCI', 'BootULCI'/rnames=indefflb/format= F10.4 /space=0.
end if.
do if (nxvls = 1).
print indtab(2:nrow(indtab),:)/title = ' '/clabels='Effect', 'BootSE', 'BootLLCI', 'BootULCI'/format= F10.4 /space=0.
end if.
end if.
end if.
do if (indmod(i,1)>0).
do if (indmod(i,1)=1).
compute indmodva=wmodvals.
compute indprova=wprobval.
compute condlbs={wnames}.
compute printw=1.
else if (indmod(i,1)=2).
compute indmodva=zmodvals.
compute indprova=zprobval.
compute condlbs={znames}.
compute printz=1.
else if (indmod(i,1)=3).
compute cntmp=1.
compute printz=1.
compute printw=1.
compute indmodva=make((nrow(wmodvals)*nrow(zmodvals)),2,999).
loop k7 = 1 to nrow(wmodvals).
loop k8 = 1 to nrow(zmodvals).
compute indmodva(cntmp,:)={wmodvals(k7,1),zmodvals(k8,1)}.
compute cntmp=cntmp+1.
end loop.
end loop.
compute condlbs={wnames,znames}.
end if.
compute condres=make(nrow(indmodva),1,999).
do if (boot > 0).
compute condres=make(nrow(indmodva),4,999).
end if.
compute condres={indmodva,condres}.
loop k4 = 1 to nxvls.
compute imm3=make(nrow(bootres),1,1).
compute imm4=make(nrow(bootres),1,1).
compute indcontr=0.
do if (indmod(i,1)=3).
compute tihsw=wprobval.
compute tihsz=zprobval.
end if.
loop k1=1 to nrow(indmodva).
compute tucker2=make(nrow(bootres),1,1).
compute imm2=make(nrow(bootres),1,1).
compute wfirst=0.
compute zfirst=0.
compute immset=0.
loop k2=1 to indmake(i,1).
compute colnumb=indmake(i,(k2+1)).
do if (k2=1).
compute wbb=make(nrow(bootres),(nwvls*nxvls),0).
compute zbb=make(nrow(bootres),(nzvls*nxvls),0).
compute wzbb=make(nrow(bootres),(nwvls*nzvls*nxvls),0).
end if.
do if (k2<>1).
compute wbb=make(nrow(bootres),nwvls,0).
compute zbb=make(nrow(bootres),nzvls,0).
compute wzbb=make(nrow(bootres),(nwvls*nzvls),0).
end if.
compute cnt=1.
compute tihs=indlocs(2:((indlocs(1,colnumb))+1),colnumb).
do if (k2 = 1).
compute focbb=tihs(1:nxvls,1).
compute focbb=bootres(:,focbb).
do if (indmmm(i,1)>0).
compute imm=focbb(:,k4).
compute condbb=imm.
end if.
compute focaddon=make(1,nxvls,0).
compute focaddon(1,k4)=1.
compute cnt=cnt+nxvls.
compute placeh=nxvls.
do if (indmod(i,1)=1).
compute tihsz=make(nrow(wprobval),(nzvls*nxvls),0).
compute tihswz=make(nrow(wprobval),(nwvls*nzvls*nxvls),0).
do if (pathsw(1,colnumb)=1).
compute temp=make(nrow(wprobval),(nxvls*nwvls),0).
loop k5 = 1 to nrow(wprobval).
loop k6=1 to nwvls.
compute temp(k5, (((k4-1)*nwvls)+k6))=wprobval(k5,k6).
end loop.
end loop.
compute indprova={temp,tihsz,tihswz}.
else.
compute indprova={wprobval,tihsz,tihswz}.
end if.
end if.
do if (indmod(i,1)=2).
compute tihsw=make(nrow(zprobval),(nwvls*nxvls),0).
compute tihswz=make(nrow(zprobval),(nwvls*nzvls*nxvls),0).
do if (pathsz(1,colnumb)=1).
compute temp=make(nrow(zprobval),(nxvls*nzvls),0).
loop k5 = 1 to nrow(zprobval).
loop k6 =1 to nzvls.
compute temp(k5,(((k4-1)*nzvls)+k6))=zprobval(k5,k6).
end loop.
end loop.
compute indprova={tihsw,temp,tihswz}.
else.
compute indprova={tihsw,zprobval,tihswz}.
end if.
end if.
do if (indmod(i,1)=3).
compute indprova=make((nrow(wprobval)*nrow(zprobval)),((ncol(wprobval)*nxvls)+ (ncol(zprobval)*nxvls)+(nwvls*nzvls*nxvls)),0).
compute cntemp=1.
loop k7=1 to nrow(wprobval).
loop k8 =1 to nrow(zprobval).
compute temp=wprobval(k7,:)*focaddon(1,k4).
compute indprova(cntemp,(((k4-1)*nwvls)+1):(k4*(nwvls)))=temp.
compute temp=zprobval(k8,:)*focaddon(1,k4).
compute indprova(cntemp, ((((k4-1)*nzvls)+1)+(nxvls*nwvls)) : ((((k4-1)*nzvls)+1)+(nxvls*nwvls)+(nzvls-1)))=temp.
compute cntemp=cntemp+1.
end loop.
end loop.
do if (pathsz(1,colnumb)=0).
compute temp=make(nrow(indprova),(ncol(zprobval)*nxvls),0).
compute indprova(:,((ncol(wprobval)*nxvls)+1):((ncol(wprobval)+ncol(zprobval))* nxvls))=temp.
end if.
do if (pathsw(1,colnumb)=0).
compute temp=make(nrow(indprova),(ncol(wprobval)*nxvls),0).
compute indprova(:,1:(ncol(wprobval)*nxvls))=temp.
end if.
do if (pathswz(1,colnumb)=1).
compute cntemp=(ncol(wprobval)*nxvls)+(ncol(zprobval)*nxvls)+((k4-1)* ncol(wprobval)*ncol(zprobval))+1.
loop k7=1 to ncol(wprobval).
loop k8=1 to ncol(zprobval).
compute indprova(:,cntemp)=indprova(:,((ncol(wprobval)*(k4-1))+k7))&* indprova(:,((((k4-1)*ncol(zprobval))+k8)+(nxvls*ncol(wprobval)))).
compute cntemp=cntemp+1.
end loop.
end loop.
end if.
end if.
end if.
do if (k2 > 1).
compute focbb=tihs(1,1).
compute focbb=bootres(:,focbb).
do if (indmmm(i,1)>0).
compute imm=focbb(:,1).
compute condbb=imm.
end if.
compute focaddon=1.
compute cnt=cnt+1.
compute placeh=1.
do if (indmod(i,1)=1).
compute tihsz=make(nrow(wprobval),nzvls,0).
compute tihswz=make(nrow(wprobval),(nwvls*nzvls),0).
compute indprova={wprobval,tihsz,tihswz}.
end if.
do if (indmod(i,1)=2).
compute tihsw=make(nrow(zprobval),nwvls,0).
compute tihswz=make(nrow(zprobval),(nwvls*nzvls),0).
compute indprova={tihsw,zprobval,tihswz}.
end if.
do if (indmod(i,1)=3).
compute indprova=make((nrow(wprobval)*nrow(zprobval)),((ncol(wprobval)+ ncol(zprobval))+(nwvls*nzvls)),0).
compute cntemp=1.
loop k7=1 to nrow(wprobval).
loop k8 =1 to nrow(zprobval).
compute indprova(cntemp,1:(ncol(wprobval)+ncol(zprobval)))={wprobval(k7,:), zprobval(k8,:)}.
compute cntemp=cntemp+1.
end loop.
end loop.
do if (pathsz(1,colnumb)=0).
compute temp=make(nrow(indprova),ncol(zprobval),0).
compute indprova(:,(ncol(wprobval)+1):(ncol(wprobval)+ncol(zprobval)))=temp.
end if.
do if (pathsw(1,colnumb)=0).
compute temp=make(nrow(indprova),ncol(wprobval),0).
compute indprova(:,1:ncol(wprobval))=temp.
end if.
do if (pathswz(1,colnumb)=1).
compute cntemp=ncol(wprobval)+ncol(zprobval)+1.
loop k7=1 to ncol(wprobval).
loop k8=1 to ncol(zprobval).
compute indprova(:,cntemp)=indprova(:,k7)&*indprova(:,(ncol(wprobval)+k8)).
compute cntemp=cntemp+1.
end loop.
end loop.
end if.
end if.
end if.
do if (pathsw(1,colnumb)) = 1.
compute wbb=tihs(cnt:(cnt+(placeh*nwvls)-1),1).
compute wbb=bootres(:,wbb).
compute immlbs2=wcatlab(1:nwvls,1).
do if (zfirst=0).
compute wfirst=1.
end if.
do if (indmmm(i,1)=1 or indmmm(i,1)=31 or indmmm(i,1)=51).
compute imm=wbb(:,1).
loop k7 = 1 to nwvls.
compute imm={imm,wbb(:,(((k4-1)*nwvls*(k2=1))+k7))}.
end loop.
compute imm=imm(:,2:ncol(imm)).
end if.
do if (indmmm(i,1)=41 or indmmm(i,1)=51).
compute condbb=make(nrow(bootres),1,0).
loop k7 = 1 to nwvls.
compute condbb={condbb,wbb(:,(((k4-1)*nwvls*(k2=1))+k7))}.
end loop.
compute condbb=condbb(:,2:ncol(condbb)).
end if.
compute cnt=cnt+(placeh*nwvls).
end if.
do if (pathsz(1,colnumb)) = 1.
compute zbb=tihs(cnt:(cnt+(placeh*nzvls)-1),1).
compute zbb=bootres(:,zbb).
do if (wfirst=0).
compute zfirst=1.
end if.
do if (indmmm(i,1) <> 31).
compute immlbs2=zcatlab(1:nzvls,1).
end if.
do if (indmmm(i,1)=2 or indmmm(i,1)=31 or indmmm(i,1)=51).
do if (indmmm(i,1)=2).
compute imm=zbb(:,1).
end if.
loop k7 = 1 to nzvls.
compute imm={imm,zbb(:,(((k4-1)*nzvls*(k2=1))+k7))}.
end loop.
do if (indmmm(i,1)=2 or indmmm(i,1)=51).
compute imm=imm(:,2:ncol(imm)).
do if (indmmm(i,1)=51).
compute condbb={condbb,imm}.
end if.
end if.
end if.
compute cnt=cnt+(placeh*nzvls).
end if.
do if (pathswz(1,colnumb)) = 1.
compute wzbb=tihs(cnt:(cnt+(placeh*nwvls*nzvls)-1),1).
compute wzbb=bootres(:,wzbb).
do if (indmmm(i,1)=41).
compute imm=wzbb(:,1).
loop k7=1 to nwvls*nzvls.
compute imm={imm,wzbb(:,(((k4-1)*nzvls*nwvls*(k2=1))+k7))}.
end loop.
end if.
do if (indmmm(i,1)=41).
compute imm=imm(:,2:ncol(imm)).
compute condbb={condbb,imm(:,(ncol(imm)-(nwvls*nzvls)+1):ncol(imm))}.
end if.
compute cnt=cnt+(placeh*nzvls*nwvls).
end if.
compute indprobe={focaddon,indprova(k1,:)}.
compute tucker={focbb,wbb,zbb,wzbb}.
loop k3=1 to ncol(indprobe).
compute tucker(:,k3)=tucker(:,k3)*indprobe(1,k3).
end loop.
compute tucker2=tucker2&*rsum(tucker).
do if (indmmm(i,1) = 1 or indmmm(i,1)=2 or indmmm(i,1)=31 or indmmm(i,1)=41 or indmmm(i,1)=51).
do if (immset=1).
do if (ncol(imm2)=1 and ncol(imm) = 1).
compute imm2=imm2&*imm.
end if.
do if (indmmm(i,1)=41 or indmmm(i,1)=51).
do if ((ncol(condbb2) > 1) and (ncol(condbb)>1)).
compute condbb2t=make(nrow(condbb2),(ncol(condbb2)*ncol(condbb)),-999999).
compute k9=1.
do if (wfirst=1).
loop k7=1 to ncol(condbb2).
loop k8 = 1 to ncol(condbb).
compute condbb2t(:,k9)=condbb2(:,k7)&*condbb(:,k8).
compute k9=k9+1.
end loop.
end loop.
end if.
do if (zfirst=1).
loop k7=1 to ncol(condbb).
loop k8 = 1 to ncol(condbb2).
compute condbb2t(:,k9)=condbb(:,k7)&*condbb2(:,k8).
compute k9=k9+1.
end loop.
end loop.
end if.
compute condbb2=condbb2t.
release condbb2t.
end if.
do if ((ncol(condbb2) > 1) and (ncol(condbb)=1)).
loop k7 = 1 to ncol(condbb2).
compute condbb2(:,k7)=condbb2(:,k7)&*condbb.
end loop.
end if.
do if ((ncol(condbb2) = 1) and (ncol(condbb)>1)).
loop k7 = 1 to ncol(condbb).
compute condbb(:,k7)=condbb2&*condbb(:,k7).
end loop.
compute condbb2=condbb.
end if.
end if.
do if (ncol(imm2) <> 1 and ncol(imm) <> 1).
compute imm2t=make(nrow(imm2),(ncol(imm2)*ncol(imm)),-999999).
compute k9=1.
do if (wfirst=1).
loop k7=1 to ncol(imm2).
loop k8 = 1 to ncol(imm).
compute imm2t(:,k9)=imm2(:,k7)&*imm(:,k8).
compute k9=k9+1.
end loop.
end loop.
end if.
do if (zfirst=1).
loop k7=1 to ncol(imm).
loop k8 = 1 to ncol(imm2).
compute imm2t(:,k9)=imm(:,k7)&*imm2(:,k8).
compute k9=k9+1.
end loop.
end loop.
end if.
compute imm2=imm2t.
release imm2t.
end if.
do if ((ncol(imm2) > 1) and (ncol(imm)=1)).
loop k7=1 to ncol(imm2).
compute imm2(:,k7)=imm2(:,k7)&*imm.
end loop.
end if.
do if ((ncol(imm2) = 1) and (ncol(imm) > 1)).
loop k7=1 to ncol(imm).
compute imm(:,k7)=imm2&*imm(:,k7).
end loop.
compute imm2=imm.
end if.
end if.
do if (immset=0).
compute imm2=imm.
do if (indmmm(i,1)=41 or indmmm(i,1)=51).
compute condbb2=condbb.
end if.
compute immset=1.
end if.
end if.
end loop.
compute indtemp=tucker2(1,1).
do if (indmmm(i,1)=12 or indmmm(i,1)=22).
compute imm3={imm3,tucker2}.
do if (k1=nrow(indmodva)).
compute imm3=imm3(:,2:ncol(imm3)).
compute immstop=ncol(imm3).
loop k8=2 to immstop.
do if (indmmmt(i,1)=1).
compute imm3={imm3,(imm3(:,k8)-imm3(:,1))}.
end if.
do if (indmmmt(i,1)=2).
compute imm3={imm3,(imm3(:,k8)-imm3(:,(k8-1)))}.
end if.
do if (indmmmt(i,1)=3).
compute imm3={imm3,((rsum(imm3(:,(k8:immstop)))/(immstop-k8+1))-imm3(:,(k8-1))) }.
end if.
do if (indmmmt(i,1)=4).
compute imm3={imm3,(imm3(:,k8)-(rsum(imm3(:,1:immstop))/immstop))}.
end if.
end loop.
do if (indmmmt(i,1)<5).
compute imm2=imm3(:,(immstop+1):ncol(imm3)).
release imm3.
end if.
end if.
end if.
do if (indmmm(i,1)>-1 and (contrast = 1 or contrast = 2 or contrast=3)).
compute imm4={imm4,tucker2}.
do if (k1=nrow(indmodva) and k1 > 1).
compute imm4=imm4(:,2:ncol(imm4)).
compute immstop=ncol(imm4).
compute condcont=make((immstop*(immstop-1)/2),6,-999).
loop k8 = 1 to (immstop-1).
loop k9 = (k8+1) to immstop.
do if (contrast=1).
compute imm4={imm4,(imm4(:,k9)-imm4(:,k8))}.
end if.
do if (contrast=2).
compute imm4={imm4,(abs(imm4(:,k9))-abs(imm4(:,k8)))}.
end if.
do if (contrast=3).
compute imm4={imm4,(imm4(:,k9)+imm4(:,k8))}.
end if.
compute condcont((ncol(imm4)-immstop),1)=imm4(1,k9).
compute condcont((ncol(imm4)-immstop),2)=imm4(1,k8).
end loop.
end loop.
compute imm4=imm4(:,(immstop+1):ncol(imm4)).
loop k8=1 to ncol(imm4).
compute condcont(k8,3)=imm4(1,k8).
do if (boot > 0).

.
compute temp = imm4(2:nrow(imm4),k8).
compute temp(GRADE( imm4(2:nrow(imm4),k8) )) = imm4(2:nrow(imm4),k8).
compute badlo = 0.
compute badhi = 0.
do if ( (imm4(1,k8)*bc)+(9999*(1-bc)) <> 9999).
compute pv=csum(temp < (imm4(1,k8)*bc)+(9999*(1-bc)) )/nrow(temp).
compute ppv = pv.
do if (pv > .5).
compute ppv = 1-pv.
end if.
compute y5=sqrt(-2*ln(ppv)).
compute xp=y5+((((y5*p4+p3)*y5+p2)*y5+p1)*y5+p0)/((((y5*q4+q3)*y5+q2)*y5+q1)*y5+q0).
do if (pv <= .5).
compute xp = -xp.
end if.
compute cilow=rnd(nrow(temp)*(cdfnorm(2*xp-xp2))).
compute cihigh=trunc(nrow(temp)*(cdfnorm(2*xp+(xp2))))+1.
do if (cilow < 1).
compute cilow = 1.
compute booterr=1.
compute badlo = 1.
end if.
do if (cihigh > nrow(temp)).
compute cihigh = boot.
compute booterr=1.
compute badhi = 1.
end if.
compute llcit=temp(cilow,1).
compute ulcit=temp(cihigh,1).
do if (badlo = 1 and llcit <> priorlo).
compute badend={badend, llcit}.
compute priorlo = llcit.
end if.
do if (badhi = 1 and ulcit <> priorhi).
compute badend={badend, ulcit}.
compute priorhi = ulcit.
end if.
end if.
do if ( (imm4(1,k8)*bc)+(9999*(1-bc)) = 9999).
compute llcit=temp(cilow,1).
compute ulcit=temp(cihigh,1).
end if.
compute bootse=t(sqrt((cssq(temp)-((csum(temp)&**2)/nrow(temp)))/(nrow(temp)-1)))
.
compute condcont(k8,4:6)={bootse,llcit,ulcit}.
end if.
end loop.
do if (boot=0).
compute condcont=condcont(:,1:3).
end if.
compute indcontr=1.
end if.
end if.
do if (xmint=1).
do if (k1=(k4+1)).
compute cftotfx(:,k4)=cftotfx(:,k4)+(tucker2*xscaling).
end if.
end if.
do if (boot > 0).
compute tucker2=tucker2*xscaling.

.
compute temp = tucker2(2:nrow(tucker2),1).
compute temp(GRADE( tucker2(2:nrow(tucker2),1) )) = tucker2(2:nrow(tucker2),1).
compute badlo = 0.
compute badhi = 0.
do if ( (tucker2(1,1)*bc)+(9999*(1-bc)) <> 9999).
compute pv=csum(temp < (tucker2(1,1)*bc)+(9999*(1-bc)) )/nrow(temp).
compute ppv = pv.
do if (pv > .5).
compute ppv = 1-pv.
end if.
compute y5=sqrt(-2*ln(ppv)).
compute xp=y5+((((y5*p4+p3)*y5+p2)*y5+p1)*y5+p0)/((((y5*q4+q3)*y5+q2)*y5+q1)*y5+q0).
do if (pv <= .5).
compute xp = -xp.
end if.
compute cilow=rnd(nrow(temp)*(cdfnorm(2*xp-xp2))).
compute cihigh=trunc(nrow(temp)*(cdfnorm(2*xp+(xp2))))+1.
do if (cilow < 1).
compute cilow = 1.
compute booterr=1.
compute badlo = 1.
end if.
do if (cihigh > nrow(temp)).
compute cihigh = boot.
compute booterr=1.
compute badhi = 1.
end if.
compute llcit=temp(cilow,1).
compute ulcit=temp(cihigh,1).
do if (badlo = 1 and llcit <> priorlo).
compute badend={badend, llcit}.
compute priorlo = llcit.
end if.
do if (badhi = 1 and ulcit <> priorhi).
compute badend={badend, ulcit}.
compute priorhi = ulcit.
end if.
end if.
do if ( (tucker2(1,1)*bc)+(9999*(1-bc)) = 9999).
compute llcit=temp(cilow,1).
compute ulcit=temp(cihigh,1).
end if.
compute bootse=t(sqrt((cssq(temp)-((csum(temp)&**2)/nrow(temp)))/(nrow(temp)-1)))
.
compute indtemp={indtemp*xscaling, bootse,llcit,ulcit}.
end if.
do if (boot=0).
compute indtemp=indtemp*xscaling.
end if.
compute condres(k1,(ncol(indmodva)+1):ncol(condres))=indtemp.
end loop.
do if (xmint=0).
compute resultm2=make(nrow(condres),maxresm,99999).
compute resultm2(1:nrow(condres),1:ncol(condres))=condres.
compute resultm={resultm;resultm2}.
end if.
compute condlbs={condlbs,'Effect'}.
do if (boot > 0).
compute condlbs={condlbs,'BootSE', 'BootLLCI', 'BootULCI'}.
end if.
do if (xmint = 1).
do if (k4 = 1).
compute natindfx=condres((2+(k4-1)),2:ncol(condres)).
end if.
do if (k4 > 1).
compute natindfx={natindfx;condres((2+(k4-1)),2:ncol(condres))}.
end if.
do if (k4=nxvls).
compute resultm2=make(nrow(natindfx),maxresm,99999).
compute resultm2(1:nrow(natindfx),1:ncol(natindfx))=natindfx.
compute resultm={resultm;resultm2}.
end if.
end if.
do if (outscree=1 and xmint=0).
do if (nxvls=1).
print condres/title=' '/cnames=condlbs/format= F10.4 /space=0.
else.
compute condrlb=make(nrow(condres),1,xcatlab(k4,1)).
print condres/title=' '/cnames=condlbs/rnames=condrlb/format= F10.4 /space=0.
end if.
end if.
do if (outscree=1 and xmint=1 and k4=nxvls).
do if (nxvls=1).
print natindfx/title=' '/clabels='Effect', 'BootSE', 'BootLLCI', 'BootULCI'/format= F10.4 /space=0.
else.
compute condrlb=make(nrow(condres),1,xcatlab(k4,1)).
print natindfx/title=' '/clabels='Effect', 'BootSE', 'BootLLCI', 'BootULCI'/rnames=direffl2/format= F10.4 /space=0.
end if.
end if.
compute dichadj=0.
compute immcat=0.
do if ((indmmm(i,1)>0) and (xmint=0)).
do if (indmmm(i,1)=1 or indmmm(i,1)=12 or indmmm(i,1)=31).
do if (wdich=1 and mcw=0).
do if (indmmm(i,1) <> 12).
compute imm2(:,1)=imm2(:,1)*(wmax-wmin).
end if.
do if (indmmm(i,1) <> 31).
compute dichadj=1.
end if.
end if.
do if ((mcw = 1 or mcw = 2) and indmmm(i,1) <> 31)).
compute immcat=1.
end if.
end if.
do if (indmmm(i,1)=2 or indmmm(i,1)=22 or indmmm(i,1)=31).
do if (zdich=1 and mcz=0).
do if (indmmm(i,1) = 31).
compute imm2(:,(nwvls+1):ncol(imm2))=imm2(:,(nwvls+1):ncol(imm2))*(zmax-zmin).
end if.
do if (indmmm(i,1) = 2).
compute imm2(:,1)=imm2(:,1)*(zmax-zmin).
end if.
do if (indmmm(i,1) <> 31).
compute dichadj=1.
end if.
end if.
do if ((mcz = 1 or mcz = 2) and indmmm(i,1) <> 31)).
compute immcat=1.
end if.
end if.
compute immtemp2=t(imm2(1,:)).
compute immtemp=immtemp2.
compute immlbs={'Index'}.
do if (boot > 0).
compute immtemp=make(1,3,0).
loop k7=1 to ncol(imm2).

.
compute temp = imm2(2:nrow(imm2),k7).
compute temp(GRADE( imm2(2:nrow(imm2),k7) )) = imm2(2:nrow(imm2),k7).
compute badlo = 0.
compute badhi = 0.
do if ( (imm2(1,k7)*bc)+(9999*(1-bc)) <> 9999).
compute pv=csum(temp < (imm2(1,k7)*bc)+(9999*(1-bc)) )/nrow(temp).
compute ppv = pv.
do if (pv > .5).
compute ppv = 1-pv.
end if.
compute y5=sqrt(-2*ln(ppv)).
compute xp=y5+((((y5*p4+p3)*y5+p2)*y5+p1)*y5+p0)/((((y5*q4+q3)*y5+q2)*y5+q1)*y5+q0).
do if (pv <= .5).
compute xp = -xp.
end if.
compute cilow=rnd(nrow(temp)*(cdfnorm(2*xp-xp2))).
compute cihigh=trunc(nrow(temp)*(cdfnorm(2*xp+(xp2))))+1.
do if (cilow < 1).
compute cilow = 1.
compute booterr=1.
compute badlo = 1.
end if.
do if (cihigh > nrow(temp)).
compute cihigh = boot.
compute booterr=1.
compute badhi = 1.
end if.
compute llcit=temp(cilow,1).
compute ulcit=temp(cihigh,1).
do if (badlo = 1 and llcit <> priorlo).
compute badend={badend, llcit}.
compute priorlo = llcit.
end if.
do if (badhi = 1 and ulcit <> priorhi).
compute badend={badend, ulcit}.
compute priorhi = ulcit.
end if.
end if.
do if ( (imm2(1,k7)*bc)+(9999*(1-bc)) = 9999).
compute llcit=temp(cilow,1).
compute ulcit=temp(cihigh,1).
end if.
compute bootse=t(sqrt((cssq(temp)-((csum(temp)&**2)/nrow(temp)))/(nrow(temp)-1)))
.
compute temp={bootse,llcit,ulcit}.
compute immtemp={immtemp;temp}.
end loop.
compute immtemp=immtemp(2:nrow(immtemp),:).
compute immtemp={immtemp2,immtemp}.
compute immlbs={immlbs,'BootSE', 'BootLLCI', 'BootULCI'}.
end if.
compute resultm2=make(nrow(immtemp),maxresm,99999).
compute resultm2(1:nrow(immtemp),1:ncol(immtemp))=immtemp.
compute resultm={resultm;resultm2}.
do if (dichadj=0 and immcat=0 and indmmmt(i,1)<>5 and indmmm(i,1) < 100 ).
do if (outscree=1).
do if (indmmm(i,1) < 30).
print immtemp/title='      Index of moderated mediation:'/cnames=immlbs/rnames=immlbs2/format= F10.4.
end if.
do if (indmmm(i,1) = 31).
compute immlbs2={immlbs2;zcatlab(1:nzvls,1)}.
print immtemp/title='      Indices of partial moderated mediation:'/cnames=immlbs/rnames=immlbs2/format= F10.4.
end if.
end if.
do if ((nzvls = 1) and (nwvls = 1)).
do if (indmmm(i,1) = 41 or indmmm(i,1)=51).
loop k7=1 to nwvls.
compute immlbs2=zcatlab(1:nzvls,1).
compute immtemp2=immtemp((((k7-1)*nzvls)+1):(((k7-1)*nzvls)+nzvls),:).
do if (outscree=1).
do if (nwvls > 1).
compute primodv={'        ', wcatlab(k7,1)}.
print primodv/title='      Primary moderator:'/format=A8.
end if.
do if (nzvls=1).
print immtemp2/title='      Index of moderated moderated mediation'/cnames=immlbs/format= F10.4.
else.
print immtemp2/title='      Indices of moderated moderated mediation'/cnames=immlbs/rnames=immlbs2/format= F10.4.
end if.
end if.
compute cmmtemp=make(nrow(zprobval),4,0).
loop k8=1 to nrow(zprobval).
compute condbb3=condbb2(:,((nwvls+1)+((k7-1)*nzvls)):((nwvls+1)+((k7-1)*nzvls)+ (nzvls-1))).
do if (ncol(zprobval) > 1).
compute condbb3=condbb3*mdiag(zprobval(k8,:)).
else.
compute condbb3=condbb3&*zprobval(k8,:).
end if.
compute condbb3={condbb2(:,k7),condbb3}.
compute icmm=rsum(condbb3).
compute cmmtemp(k8,1)=icmm(1,1).
do if (boot > 0).

.
compute temp = icmm(2:nrow(icmm),1).
compute temp(GRADE( icmm(2:nrow(icmm),1) )) = icmm(2:nrow(icmm),1).
compute badlo = 0.
compute badhi = 0.
do if ( (icmm(1,1)*bc)+(9999*(1-bc)) <> 9999).
compute pv=csum(temp < (icmm(1,1)*bc)+(9999*(1-bc)) )/nrow(temp).
compute ppv = pv.
do if (pv > .5).
compute ppv = 1-pv.
end if.
compute y5=sqrt(-2*ln(ppv)).
compute xp=y5+((((y5*p4+p3)*y5+p2)*y5+p1)*y5+p0)/((((y5*q4+q3)*y5+q2)*y5+q1)*y5+q0).
do if (pv <= .5).
compute xp = -xp.
end if.
compute cilow=rnd(nrow(temp)*(cdfnorm(2*xp-xp2))).
compute cihigh=trunc(nrow(temp)*(cdfnorm(2*xp+(xp2))))+1.
do if (cilow < 1).
compute cilow = 1.
compute booterr=1.
compute badlo = 1.
end if.
do if (cihigh > nrow(temp)).
compute cihigh = boot.
compute booterr=1.
compute badhi = 1.
end if.
compute llcit=temp(cilow,1).
compute ulcit=temp(cihigh,1).
do if (badlo = 1 and llcit <> priorlo).
compute badend={badend, llcit}.
compute priorlo = llcit.
end if.
do if (badhi = 1 and ulcit <> priorhi).
compute badend={badend, ulcit}.
compute priorhi = ulcit.
end if.
end if.
do if ( (icmm(1,1)*bc)+(9999*(1-bc)) = 9999).
compute llcit=temp(cilow,1).
compute ulcit=temp(cihigh,1).
end if.
compute bootse=t(sqrt((cssq(temp)-((csum(temp)&**2)/nrow(temp)))/(nrow(temp)-1)))
.
compute cmmtemp(k8,2:4)={bootse,llcit,ulcit}.
end if.
end loop.
compute cmmtemp={zmodvals,cmmtemp}.
do if (boot=0).
compute cmmtemp=cmmtemp(:,1:2).
end if.
compute cmmlbs={znames,immlbs}.
compute resultm2=make(nrow(cmmtemp),maxresm,99999).
compute resultm2(1:nrow(cmmtemp),1:ncol(cmmtemp))=cmmtemp.
compute resultm={resultm;resultm2}.
do if (outscree=1).
print cmmtemp/title='      Indices of conditional moderated mediation by W'/cnames=cmmlbs/format= F10.4.
end if.
end loop.
end if.
end if.
end if.
do if (dichadj=1 or immcat=1 and indmmm(i,1) < 30).
do if (outscree=1).
print immtemp/title='Index of moderated mediation (difference between conditional indirect effects):'/cnames=immlbs/rnames=immlbs2/format= F10.4.
end if.
end if.
end if.
do if (indcontr=1).
compute resultm2=make(nrow(condcont),maxresm,99999).
compute resultm2(1:nrow(condcont),1:ncol(condcont))=condcont.
compute resultm={resultm;resultm2}.
compute condctlb={'Effect1','Effect2','Contrast','BootSE', 'BootLLCI','BootULCI'}.
do if (outscree=1).
print condcont/title=' Pairwise contrasts between conditional indirect effects (Effect1 minus Effect2)'/cnames=condctlb/format= F10.4.
end if.
end if.
end loop.
end if.
end loop.
do if (alttotal=1).
compute altcnms='Effect'.
compute alttotfx=t(cftotfx(1,:))+obnatdfx.
do if (boot > 0).
compute alttotfx=make(ncol(cftotfx),4,0).
compute alttotfx(:,1)=t(cftotfx(1,:))+obnatdfx.
compute natdirbt(1,:)=t(obnatdfx).
compute cftotfx=cftotfx+natdirbt.
loop cec=1 to ncol(cftotfx).

.
compute temp = cftotfx(2:nrow(cftotfx),cec).
compute temp(GRADE( cftotfx(2:nrow(cftotfx),cec) )) = cftotfx(2:nrow(cftotfx),cec).
compute badlo = 0.
compute badhi = 0.
do if ( (cftotfx(1,cec)*bc)+(9999*(1-bc)) <> 9999).
compute pv=csum(temp < (cftotfx(1,cec)*bc)+(9999*(1-bc)) )/nrow(temp).
compute ppv = pv.
do if (pv > .5).
compute ppv = 1-pv.
end if.
compute y5=sqrt(-2*ln(ppv)).
compute xp=y5+((((y5*p4+p3)*y5+p2)*y5+p1)*y5+p0)/((((y5*q4+q3)*y5+q2)*y5+q1)*y5+q0).
do if (pv <= .5).
compute xp = -xp.
end if.
compute cilow=rnd(nrow(temp)*(cdfnorm(2*xp-xp2))).
compute cihigh=trunc(nrow(temp)*(cdfnorm(2*xp+(xp2))))+1.
do if (cilow < 1).
compute cilow = 1.
compute booterr=1.
compute badlo = 1.
end if.
do if (cihigh > nrow(temp)).
compute cihigh = boot.
compute booterr=1.
compute badhi = 1.
end if.
compute llcit=temp(cilow,1).
compute ulcit=temp(cihigh,1).
do if (badlo = 1 and llcit <> priorlo).
compute badend={badend, llcit}.
compute priorlo = llcit.
end if.
do if (badhi = 1 and ulcit <> priorhi).
compute badend={badend, ulcit}.
compute priorhi = ulcit.
end if.
end if.
do if ( (cftotfx(1,cec)*bc)+(9999*(1-bc)) = 9999).
compute llcit=temp(cilow,1).
compute ulcit=temp(cihigh,1).
end if.
compute bootse=t(sqrt((cssq(temp)-((csum(temp)&**2)/nrow(temp)))/(nrow(temp)-1)))
.
compute alttotfx(cec,2:4)={bootse,llcit,ulcit}.
end loop.
compute altcnms={altcnms,'BootSE', 'BootLLCI','BootULCI'}.
end if.
compute resultm2=make(nrow(alttotfx),maxresm,99999).
compute resultm2(1:nrow(alttotfx),1:ncol(alttotfx))=alttotfx.
compute resultm={resultm;resultm2}.
do if (outscree=1).
print/title='----------'.
do if (nxvls > 1).
print alttotfx/title='Relative total effects of X on Y (sum of direct and indirect effects)'/cnames=altcnms/rnames=direffl2/format= F10.4.
end if.
do if (nxvls = 1).
print alttotfx/title='Total effect of X on Y (sum of direct and indirect effects)'/cnames=altcnms/format= F10.4.
end if.
end if.
end if.
end if.
end if
.
do if (criterr=0 and (saveest = 1 or saveboot=1)).
compute coeffsav=coeffmat(2:nrow(coeffmat),:).
compute conseq=conseq(2:nrow(conseq),1).
compute dfmat=dfmat(2:nrow(dfmat),1).
compute dfmat=dfmat.
compute saven={'conseqnt','antecdnt','coeff','se','t','p','LLCI','ULCI','df'}.
do if (ydich=1).
compute saven={'conseqnt','antecdnt','coeff','se','t_or_Z','p','LLCI','ULCI','df'}.
end if.
do if (saveboot=1 and boot > 0 ).
compute colslab=make(300,1,' ').


compute colslab( 1 ,1)= 'COL1'.


compute colslab( 2 ,1)= 'COL2'.


compute colslab( 3 ,1)= 'COL3'.


compute colslab( 4 ,1)= 'COL4'.


compute colslab( 5 ,1)= 'COL5'.


compute colslab( 6 ,1)= 'COL6'.


compute colslab( 7 ,1)= 'COL7'.


compute colslab( 8 ,1)= 'COL8'.


compute colslab( 9 ,1)= 'COL9'.


compute colslab( 10 ,1)= 'COL10'.


compute colslab( 11 ,1)= 'COL11'.


compute colslab( 12 ,1)= 'COL12'.


compute colslab( 13 ,1)= 'COL13'.


compute colslab( 14 ,1)= 'COL14'.


compute colslab( 15 ,1)= 'COL15'.


compute colslab( 16 ,1)= 'COL16'.


compute colslab( 17 ,1)= 'COL17'.


compute colslab( 18 ,1)= 'COL18'.


compute colslab( 19 ,1)= 'COL19'.


compute colslab( 20 ,1)= 'COL20'.


compute colslab( 21 ,1)= 'COL21'.


compute colslab( 22 ,1)= 'COL22'.


compute colslab( 23 ,1)= 'COL23'.


compute colslab( 24 ,1)= 'COL24'.


compute colslab( 25 ,1)= 'COL25'.


compute colslab( 26 ,1)= 'COL26'.


compute colslab( 27 ,1)= 'COL27'.


compute colslab( 28 ,1)= 'COL28'.


compute colslab( 29 ,1)= 'COL29'.


compute colslab( 30 ,1)= 'COL30'.


compute colslab( 31 ,1)= 'COL31'.


compute colslab( 32 ,1)= 'COL32'.


compute colslab( 33 ,1)= 'COL33'.


compute colslab( 34 ,1)= 'COL34'.


compute colslab( 35 ,1)= 'COL35'.


compute colslab( 36 ,1)= 'COL36'.


compute colslab( 37 ,1)= 'COL37'.


compute colslab( 38 ,1)= 'COL38'.


compute colslab( 39 ,1)= 'COL39'.


compute colslab( 40 ,1)= 'COL40'.


compute colslab( 41 ,1)= 'COL41'.


compute colslab( 42 ,1)= 'COL42'.


compute colslab( 43 ,1)= 'COL43'.


compute colslab( 44 ,1)= 'COL44'.


compute colslab( 45 ,1)= 'COL45'.


compute colslab( 46 ,1)= 'COL46'.


compute colslab( 47 ,1)= 'COL47'.


compute colslab( 48 ,1)= 'COL48'.


compute colslab( 49 ,1)= 'COL49'.


compute colslab( 50 ,1)= 'COL50'.


compute colslab( 51 ,1)= 'COL51'.


compute colslab( 52 ,1)= 'COL52'.


compute colslab( 53 ,1)= 'COL53'.


compute colslab( 54 ,1)= 'COL54'.


compute colslab( 55 ,1)= 'COL55'.


compute colslab( 56 ,1)= 'COL56'.


compute colslab( 57 ,1)= 'COL57'.


compute colslab( 58 ,1)= 'COL58'.


compute colslab( 59 ,1)= 'COL59'.


compute colslab( 60 ,1)= 'COL60'.


compute colslab( 61 ,1)= 'COL61'.


compute colslab( 62 ,1)= 'COL62'.


compute colslab( 63 ,1)= 'COL63'.


compute colslab( 64 ,1)= 'COL64'.


compute colslab( 65 ,1)= 'COL65'.


compute colslab( 66 ,1)= 'COL66'.


compute colslab( 67 ,1)= 'COL67'.


compute colslab( 68 ,1)= 'COL68'.


compute colslab( 69 ,1)= 'COL69'.


compute colslab( 70 ,1)= 'COL70'.


compute colslab( 71 ,1)= 'COL71'.


compute colslab( 72 ,1)= 'COL72'.


compute colslab( 73 ,1)= 'COL73'.


compute colslab( 74 ,1)= 'COL74'.


compute colslab( 75 ,1)= 'COL75'.


compute colslab( 76 ,1)= 'COL76'.


compute colslab( 77 ,1)= 'COL77'.


compute colslab( 78 ,1)= 'COL78'.


compute colslab( 79 ,1)= 'COL79'.


compute colslab( 80 ,1)= 'COL80'.


compute colslab( 81 ,1)= 'COL81'.


compute colslab( 82 ,1)= 'COL82'.


compute colslab( 83 ,1)= 'COL83'.


compute colslab( 84 ,1)= 'COL84'.


compute colslab( 85 ,1)= 'COL85'.


compute colslab( 86 ,1)= 'COL86'.


compute colslab( 87 ,1)= 'COL87'.


compute colslab( 88 ,1)= 'COL88'.


compute colslab( 89 ,1)= 'COL89'.


compute colslab( 90 ,1)= 'COL90'.


compute colslab( 91 ,1)= 'COL91'.


compute colslab( 92 ,1)= 'COL92'.


compute colslab( 93 ,1)= 'COL93'.


compute colslab( 94 ,1)= 'COL94'.


compute colslab( 95 ,1)= 'COL95'.


compute colslab( 96 ,1)= 'COL96'.


compute colslab( 97 ,1)= 'COL97'.


compute colslab( 98 ,1)= 'COL98'.


compute colslab( 99 ,1)= 'COL99'.


compute colslab( 100 ,1)= 'COL100'.


compute colslab( 101 ,1)= 'COL101'.


compute colslab( 102 ,1)= 'COL102'.


compute colslab( 103 ,1)= 'COL103'.


compute colslab( 104 ,1)= 'COL104'.


compute colslab( 105 ,1)= 'COL105'.


compute colslab( 106 ,1)= 'COL106'.


compute colslab( 107 ,1)= 'COL107'.


compute colslab( 108 ,1)= 'COL108'.


compute colslab( 109 ,1)= 'COL109'.


compute colslab( 110 ,1)= 'COL110'.


compute colslab( 111 ,1)= 'COL111'.


compute colslab( 112 ,1)= 'COL112'.


compute colslab( 113 ,1)= 'COL113'.


compute colslab( 114 ,1)= 'COL114'.


compute colslab( 115 ,1)= 'COL115'.


compute colslab( 116 ,1)= 'COL116'.


compute colslab( 117 ,1)= 'COL117'.


compute colslab( 118 ,1)= 'COL118'.


compute colslab( 119 ,1)= 'COL119'.


compute colslab( 120 ,1)= 'COL120'.


compute colslab( 121 ,1)= 'COL121'.


compute colslab( 122 ,1)= 'COL122'.


compute colslab( 123 ,1)= 'COL123'.


compute colslab( 124 ,1)= 'COL124'.


compute colslab( 125 ,1)= 'COL125'.


compute colslab( 126 ,1)= 'COL126'.


compute colslab( 127 ,1)= 'COL127'.


compute colslab( 128 ,1)= 'COL128'.


compute colslab( 129 ,1)= 'COL129'.


compute colslab( 130 ,1)= 'COL130'.


compute colslab( 131 ,1)= 'COL131'.


compute colslab( 132 ,1)= 'COL132'.


compute colslab( 133 ,1)= 'COL133'.


compute colslab( 134 ,1)= 'COL134'.


compute colslab( 135 ,1)= 'COL135'.


compute colslab( 136 ,1)= 'COL136'.


compute colslab( 137 ,1)= 'COL137'.


compute colslab( 138 ,1)= 'COL138'.


compute colslab( 139 ,1)= 'COL139'.


compute colslab( 140 ,1)= 'COL140'.


compute colslab( 141 ,1)= 'COL141'.


compute colslab( 142 ,1)= 'COL142'.


compute colslab( 143 ,1)= 'COL143'.


compute colslab( 144 ,1)= 'COL144'.


compute colslab( 145 ,1)= 'COL145'.


compute colslab( 146 ,1)= 'COL146'.


compute colslab( 147 ,1)= 'COL147'.


compute colslab( 148 ,1)= 'COL148'.


compute colslab( 149 ,1)= 'COL149'.


compute colslab( 150 ,1)= 'COL150'.


compute colslab( 151 ,1)= 'COL151'.


compute colslab( 152 ,1)= 'COL152'.


compute colslab( 153 ,1)= 'COL153'.


compute colslab( 154 ,1)= 'COL154'.


compute colslab( 155 ,1)= 'COL155'.


compute colslab( 156 ,1)= 'COL156'.


compute colslab( 157 ,1)= 'COL157'.


compute colslab( 158 ,1)= 'COL158'.


compute colslab( 159 ,1)= 'COL159'.


compute colslab( 160 ,1)= 'COL160'.


compute colslab( 161 ,1)= 'COL161'.


compute colslab( 162 ,1)= 'COL162'.


compute colslab( 163 ,1)= 'COL163'.


compute colslab( 164 ,1)= 'COL164'.


compute colslab( 165 ,1)= 'COL165'.


compute colslab( 166 ,1)= 'COL166'.


compute colslab( 167 ,1)= 'COL167'.


compute colslab( 168 ,1)= 'COL168'.


compute colslab( 169 ,1)= 'COL169'.


compute colslab( 170 ,1)= 'COL170'.


compute colslab( 171 ,1)= 'COL171'.


compute colslab( 172 ,1)= 'COL172'.


compute colslab( 173 ,1)= 'COL173'.


compute colslab( 174 ,1)= 'COL174'.


compute colslab( 175 ,1)= 'COL175'.


compute colslab( 176 ,1)= 'COL176'.


compute colslab( 177 ,1)= 'COL177'.


compute colslab( 178 ,1)= 'COL178'.


compute colslab( 179 ,1)= 'COL179'.


compute colslab( 180 ,1)= 'COL180'.


compute colslab( 181 ,1)= 'COL181'.


compute colslab( 182 ,1)= 'COL182'.


compute colslab( 183 ,1)= 'COL183'.


compute colslab( 184 ,1)= 'COL184'.


compute colslab( 185 ,1)= 'COL185'.


compute colslab( 186 ,1)= 'COL186'.


compute colslab( 187 ,1)= 'COL187'.


compute colslab( 188 ,1)= 'COL188'.


compute colslab( 189 ,1)= 'COL189'.


compute colslab( 190 ,1)= 'COL190'.


compute colslab( 191 ,1)= 'COL191'.


compute colslab( 192 ,1)= 'COL192'.


compute colslab( 193 ,1)= 'COL193'.


compute colslab( 194 ,1)= 'COL194'.


compute colslab( 195 ,1)= 'COL195'.


compute colslab( 196 ,1)= 'COL196'.


compute colslab( 197 ,1)= 'COL197'.


compute colslab( 198 ,1)= 'COL198'.


compute colslab( 199 ,1)= 'COL199'.


compute colslab( 200 ,1)= 'COL200'.


compute colslab( 201 ,1)= 'COL201'.


compute colslab( 202 ,1)= 'COL202'.


compute colslab( 203 ,1)= 'COL203'.


compute colslab( 204 ,1)= 'COL204'.


compute colslab( 205 ,1)= 'COL205'.


compute colslab( 206 ,1)= 'COL206'.


compute colslab( 207 ,1)= 'COL207'.


compute colslab( 208 ,1)= 'COL208'.


compute colslab( 209 ,1)= 'COL209'.


compute colslab( 210 ,1)= 'COL210'.


compute colslab( 211 ,1)= 'COL211'.


compute colslab( 212 ,1)= 'COL212'.


compute colslab( 213 ,1)= 'COL213'.


compute colslab( 214 ,1)= 'COL214'.


compute colslab( 215 ,1)= 'COL215'.


compute colslab( 216 ,1)= 'COL216'.


compute colslab( 217 ,1)= 'COL217'.


compute colslab( 218 ,1)= 'COL218'.


compute colslab( 219 ,1)= 'COL219'.


compute colslab( 220 ,1)= 'COL220'.


compute colslab( 221 ,1)= 'COL221'.


compute colslab( 222 ,1)= 'COL222'.


compute colslab( 223 ,1)= 'COL223'.


compute colslab( 224 ,1)= 'COL224'.


compute colslab( 225 ,1)= 'COL225'.


compute colslab( 226 ,1)= 'COL226'.


compute colslab( 227 ,1)= 'COL227'.


compute colslab( 228 ,1)= 'COL228'.


compute colslab( 229 ,1)= 'COL229'.


compute colslab( 230 ,1)= 'COL230'.


compute colslab( 231 ,1)= 'COL231'.


compute colslab( 232 ,1)= 'COL232'.


compute colslab( 233 ,1)= 'COL233'.


compute colslab( 234 ,1)= 'COL234'.


compute colslab( 235 ,1)= 'COL235'.


compute colslab( 236 ,1)= 'COL236'.


compute colslab( 237 ,1)= 'COL237'.


compute colslab( 238 ,1)= 'COL238'.


compute colslab( 239 ,1)= 'COL239'.


compute colslab( 240 ,1)= 'COL240'.


compute colslab( 241 ,1)= 'COL241'.


compute colslab( 242 ,1)= 'COL242'.


compute colslab( 243 ,1)= 'COL243'.


compute colslab( 244 ,1)= 'COL244'.


compute colslab( 245 ,1)= 'COL245'.


compute colslab( 246 ,1)= 'COL246'.


compute colslab( 247 ,1)= 'COL247'.


compute colslab( 248 ,1)= 'COL248'.


compute colslab( 249 ,1)= 'COL249'.


compute colslab( 250 ,1)= 'COL250'.


compute colslab( 251 ,1)= 'COL251'.


compute colslab( 252 ,1)= 'COL252'.


compute colslab( 253 ,1)= 'COL253'.


compute colslab( 254 ,1)= 'COL254'.


compute colslab( 255 ,1)= 'COL255'.


compute colslab( 256 ,1)= 'COL256'.


compute colslab( 257 ,1)= 'COL257'.


compute colslab( 258 ,1)= 'COL258'.


compute colslab( 259 ,1)= 'COL259'.


compute colslab( 260 ,1)= 'COL260'.


compute colslab( 261 ,1)= 'COL261'.


compute colslab( 262 ,1)= 'COL262'.


compute colslab( 263 ,1)= 'COL263'.


compute colslab( 264 ,1)= 'COL264'.


compute colslab( 265 ,1)= 'COL265'.


compute colslab( 266 ,1)= 'COL266'.


compute colslab( 267 ,1)= 'COL267'.


compute colslab( 268 ,1)= 'COL268'.


compute colslab( 269 ,1)= 'COL269'.


compute colslab( 270 ,1)= 'COL270'.


compute colslab( 271 ,1)= 'COL271'.


compute colslab( 272 ,1)= 'COL272'.


compute colslab( 273 ,1)= 'COL273'.


compute colslab( 274 ,1)= 'COL274'.


compute colslab( 275 ,1)= 'COL275'.


compute colslab( 276 ,1)= 'COL276'.


compute colslab( 277 ,1)= 'COL277'.


compute colslab( 278 ,1)= 'COL278'.


compute colslab( 279 ,1)= 'COL279'.


compute colslab( 280 ,1)= 'COL280'.


compute colslab( 281 ,1)= 'COL281'.


compute colslab( 282 ,1)= 'COL282'.


compute colslab( 283 ,1)= 'COL283'.


compute colslab( 284 ,1)= 'COL284'.


compute colslab( 285 ,1)= 'COL285'.


compute colslab( 286 ,1)= 'COL286'.


compute colslab( 287 ,1)= 'COL287'.


compute colslab( 288 ,1)= 'COL288'.


compute colslab( 289 ,1)= 'COL289'.


compute colslab( 290 ,1)= 'COL290'.


compute colslab( 291 ,1)= 'COL291'.


compute colslab( 292 ,1)= 'COL292'.


compute colslab( 293 ,1)= 'COL293'.


compute colslab( 294 ,1)= 'COL294'.


compute colslab( 295 ,1)= 'COL295'.


compute colslab( 296 ,1)= 'COL296'.


compute colslab( 297 ,1)= 'COL297'.


compute colslab( 298 ,1)= 'COL298'.


compute colslab( 299 ,1)= 'COL299'.


compute colslab( 300 ,1)= 'COL300'.

compute colslab=colslab(1:ncol(bootres),1).
compute colslab={colslab,conseq,vlabs}.
do if (outscree=1).
print/title = '**************************************************************************'.
print/title='Bootstrap estimates were saved to a file'/space=0.
print colslab/title='Map of column names to model coefficients:'/clabels=' ','Conseqnt','Antecdnt'/format=a8.
end if.
end if.
end if.
do if (criterr=0 and boot > 0 and modelbt=1).
compute labstart=1.
do if (outscree=1).
print/title = '*********** BOOTSTRAP RESULTS FOR REGRESSION MODEL PARAMETERS ************'.
end if.
loop iboot = 1 to (nms+nys).
do if (outscree=1).
print outnames(1,iboot)/title = 'OUTCOME VARIABLE:'/format = A8.
end if.
compute vlabsm=vlabs(labstart:(labstart+(nump(1,iboot)-1)),1).
compute outnmtmp=bootcim(labstart:(labstart+(nump(1,iboot)-1)),:).
compute resultm2=make(nrow(outnmtmp),maxresm,99999).
compute resultm2(1:nrow(outnmtmp),1:ncol(outnmtmp))=outnmtmp.
compute resultm={resultm;resultm2}.
do if (outscree=1).
print bootcim(labstart:(labstart+(nump(1,iboot)-1)),:)/title=' '/rnames=vlabsm/ clabels='Coeff' 'BootMean' 'BootSE' 'BootLLCI' 'BootULCI'/format= F10.4 /space=0.
end if.
compute labstart=labstart+nump(1,iboot).
do if (iboot < (nms+nys)).
do if (outscree=1).
print/title= '----------'.
end if.
end if.
end loop.
end if.
do if (criterr=0 and saveest=1).
compute resultm=resultm(2:nrow(resultm),:).
compute bocaj=(resultm=99999).
compute bocaj=csum(bocaj).
compute bocaj=1-(bocaj=nrow(resultm)).
compute j=1.
loop i = 1 to ncol(resultm).
do if (bocaj(1,i)=1).
compute j=j+1.
end if.
end loop.
compute resultm=resultm(:,1:(j-1)).
save resultm/outfile = *.
end if.
do if (criterr = 0 and matrices=1 and outscree=1).
print/title = '************************ MODEL DEFINITION MATRICES ************************'.
print/title = 'FROM variables are columns, TO variables are rows.'.
compute temp2=make(nrow(bcmat),ncol(bcmat),'0').
loop i = 2 to nrow(bcmat).
loop j = 1 to (ncol(bcmat)-1).
do if (bcmat(i,j)=1).
compute temp2(i,j)='1'.
end if.
do if (j >= i).
compute temp2(i,j)=' '.
end if.
end loop.
end loop.
compute temp2=temp2(2:nrow(bcmat),(1:(ncol(bcmat)-1))).
do if (nms > 0).
compute cmatlabs={xnames,mnames}.
compute rmatlabs={mnames,ynames}.
end if.
do if (nms = 0).
compute cmatlabs={xnames}.
compute rmatlabs={ynames}.
end if.
print temp2/title='BMATRIX: Paths freely estimated (1) and fixed to zero (0):'/cnames=cmatlabs/rnames=rmatlabs/format A3.
compute z=0.
do if (rsum(csum(wcmat))<>0).
compute temp2=make(nrow(wcmat),ncol(wcmat),'0').
loop i = 2 to nrow(wcmat).
loop j = 1 to (ncol(wcmat)-1).
do if (wcmat(i,j)=1).
compute temp2(i,j)='1'.
end if.
do if (j >= i).
compute temp2(i,j)=' '.
end if.
end loop.
end loop.
compute temp2=temp2(2:nrow(wcmat),(1:(ncol(wcmat)-1))).
print temp2/title='WMATRIX: Paths moderated (1) and not moderated (0) by W:'/cnames=cmatlabs/rnames=rmatlabs/format A3.
end if.
do if (rsum(csum(zcmat))<>0).
compute temp2=make(nrow(zcmat),ncol(zcmat),'0').
loop i = 2 to nrow(zcmat).
loop j = 1 to (ncol(zcmat)-1).
do if (zcmat(i,j)=1).
compute temp2(i,j)='1'.
end if.
do if (j >= i).
compute temp2(i,j)=' '.
end if.
end loop.
end loop.
compute temp2=temp2(2:nrow(zcmat),(1:(ncol(zcmat)-1))).
print temp2/title='ZMATRIX: Paths moderated (1) and not moderated (0) by Z:'/cnames=cmatlabs/rnames=rmatlabs/format a3.
end if.
do if (rsum(csum(wzcmat))<>0).
compute temp2=make(nrow(wzcmat),ncol(wzcmat),'0').
loop i = 2 to nrow(wzcmat).
loop j = 1 to (ncol(wzcmat)-1).
do if (wzcmat(i,j)=1).
compute temp2(i,j)='1'.
end if.
do if (j >= i).
compute temp2(i,j)=' '.
end if.
end loop.
end loop.
compute temp2=temp2(2:nrow(wzcmat),(1:(ncol(wzcmat)-1))).
print temp2/title='WZMATRIX: W moderated paths moderated (1) and not moderated (0) by Z:'/cnames=cmatlabs/rnames=rmatlabs/format a3.
end if.
do if (ncs > 0).
print ccmat/title='CMATRIX: Covariates (columns) in (1) and not in (0) the models of M and Y (rows):'/rnames=rmatlabs/cnames=covnames.
end if.
end if.
do if (outscree=1).
do if (activate=0).
print/title = '*********************** ANALYSIS NOTES AND ERRORS ************************'.
end if.
end if.
do if (activate=1).
print/title = '**************************************************************************'.
end if.
do if (criterr=0).
do if (outscree=1).
print conf/title = 'Level of confidence for all confidence intervals in output:'/format = F8.4.
do if (boot > 0).
do if (goodboot = boot and bc=0).
print boot/title='Number of bootstrap samples for percentile bootstrap confidence intervals:'.
end if.
do if (goodboot = boot and bc=1).
print boot/title='Number of bootstrap samples for bias-corrected bootstrap confidence intervals:'.
end if.
do if (booterr = 1).
compute badend = badend(1,2:ncol(badend)).
print/title = 'WARNING: Bootstrap CI endpoints below not trustworthy. Decrease confidence'.
print badend/title='or increase the number of bootstrap samples.'/space=0/format = F10.4.
end if.
end if.
do if (mc > 0).
print mc/title='Number of samples for Monte Carlo confidence intervals:'.
end if.
do if (wnotev > 0 and printw=1).
do if (wnotev=1).
print/title = 'W values in conditional tables are the 16th, 50th, and 84th percentiles.'.
else if (wnotev=2).
do if (minwwarn=0 and maxwwarn=0).
print/title = 'W values in conditional tables are the mean and +/- SD from the mean.'.
end if.
do if (minwwarn=1).
print/title = 'W values in conditional tables are the minimum, the mean, and 1 SD above the mean.'.
end if.
do if (maxwwarn=1).
print/title = 'W values in conditional tables are 1 SD below the mean, the mean, and the maximum.'.
end if.
end if.
end if.
do if (znotev > 0 and printz=1).
do if (znotev=1).
print/title = 'Z values in conditional tables are the 16th, 50th, and 84th percentiles.'.
else if (znotev=2).
do if (minzwarn=0 and maxzwarn=0).
print/title = 'Z values in conditional tables are the mean and +/- SD from the mean.'.
end if.
do if (minzwarn=1).
print/title = 'Z values in conditional tables are the minimum, the mean, and 1 SD above the mean.'.
end if.
do if (maxzwarn=1).
print/title = 'Z values in conditional tables are 1 SD below the mean, the mean, and the maximum.'.
end if.
end if.
end if.
do if (minwwarn > 0).
print/title = 'NOTE: One SD below the mean is below the minimum observed in the data for W,'.
print/title = '      so the minimum measurement on W is used for conditioning instead.'/space=0.
end if.
do if (maxwwarn > 0).
print/title = 'NOTE: One SD above the mean is above the maximum observed in the data for W,'.
print/title = '      so the maximum measurement for W is used for conditioning instead.'/space=0.
end if.
do if (minzwarn > 0).
print/title = 'NOTE: One SD below the mean is below the minimum observed in the data for Z,'.
print/title = '      so the minimum measurement for Z is used for conditioning instead.'/space=0.
end if.
do if (maxzwarn > 0).
print/title = 'NOTE: One SD above the mean is above the maximum observed in the data for Z,'.
print/title = '      so the maximum measurement for Z is used for conditioning instead.'/space=0.
end if.
do if (pstog=1).
print/title= 'NOTE: Standardized coefficients for dichotomous or multicategorical X are in'.
print/title= '      partially standardized form.'/space=0.
end if.
loop i = 1 to 100.
do if (notecode(i,1) = 32).
print/title = 'Direct, indirect, and total effects are counterfactually defined'.
do if (xcontcf=0).
print/title = 'assuming X by M interaction.'/space=0.
end if.
do if (xcontcf=1).
print/title = 'assuming X by M interaction and with the following reference (x_ref)'/space=0.
compute states=t(xrefvals).
print states/title = 'and counterfactual (x_cf) states for X:'/rlabels='x_ref' 'x_cf'/format= F10.4 /space=0.
end if.
end if.
do if (notecode(i,1)=1).
print/title = 'NOTE: COVMY is ignored when using CMATRIX option.'.
end if.
do if (notecode(i,1)=2).
print/title = 'NOTE: Confidence level restricted to between 50 and 99.9999%.  95% confidence is provided in output'.
end if.
do if (notecode(i,1)=3).
print centvar/title = 'NOTE: The following variables were mean centered prior to analysis:'/format = a8.
end if.
do if (notecode(i,1) = 4).
print/title = 'NOTE: A heteroscedasticity consistent standard error and covariance matrix estimator was used.'.
end if.
do if (notecode(i,1) = 5).
print/title = 'NOTE: The HC3 option has been replaced with HC.  See the documentation.'.
end if.
do if (notecode(i,1) = 6).
print/title = 'NOTE: Due to estimation problems, some bootstrap samples had to be replaced.'.
print badboot/title='      The number of times this happened was:'/space=0/format=F8.0.
end if.
do if (notecode(i,1) = 7).
print/title = 'NOTE: The bootstrapping was not completed due to problematic bootstrap samples.'.
print/title = '      Bootstrap confidence intervals are therefore suppressed.'/space=0.
end if.
do if (notecode(i,1) = 8).
print/title = 'NOTE: The number of bootstrap samples was adjusted upward given your desired confidence.'.
end if.
do if (notecode(i,1) = 9).
print/title = 'NOTE: WMODVAL is ignored when W is specified as multicategorical.'.
end if.
do if (notecode(i,1) = 10).
print/title = 'NOTE: ZMODVAL is ignored when Z is specified as multicategorical.'.
end if.
do if (notecode(i,1) = 11).
print/title = 'NOTE: Total effect model generated only when all covariates are specified in all'.
print/title = '      models of M and Y.'/space=0.
end if.
do if (notecode(i,1) = 30).
print/title = 'NOTE: Your vector of linear hypothesis weights is of the wrong length for this model.'.
end if.
do if (notecode(i,1) = 12).
print/title = 'NOTE: Total effect model generated only when X is freely estimated to affect each M'.
print/title = '      and both X and M are freely estimated to affect Y'/space=0.
end if.
do if (notecode(i,1) = 13).
print/title = 'NOTE: There are too many pairwise contrasts to conduct with this model.'.
end if.
do if (notecode(i,1) = 14).
print/title = 'NOTE: The number of contrast weights must equal the number of indirect effects.'.
end if.
do if (notecode(i,1) = 15).
print/title = 'NOTE: Monte Carlo confidence intervals not available for this model.'.
print/title = '      Bootstrapping is used instead.'/space=0.
end if.
do if (notecode(i,1) = 16).
print/title = 'NOTE: The number of Monte Carlo samples was adjusted upward given your desired confidence.'.
end if.
do if (notecode(i,1) = 19).
print/title = 'NOTE: Your contrast matrix is invalid or not applicable to this model.'.
end if.
do if (notecode(i,1) = 20).
print/title = 'NOTE: One of the groups specified by your contrast matrix does not exist in the data.'.
end if.
do if (notecode(i,1) = 21).
print/title = 'NOTE: The VARORDER option is not available in this release.'.
end if.
do if (notecode(i,1) = 22).
print/title = 'NOTE: The VMODVAL and QMODVAL options are not available in this release.'.
end if.
do if (notecode(i,1) = 23).
print/title = 'NOTE: The QUANTILE option is not available in this release.'.
end if.
do if (notecode(i,1) = 24).
print/title = 'NOTE: Total effect model not available with dichotomous Y.'.
end if.
do if (notecode(i,1) = 25).
print/title = 'NOTE: STAND/EFFSIZE options not available with dichotomous Y.'.
end if.
do if ((notecode(i,1) = 26) and nms > 0).
print/title = 'NOTE: Direct and indirect effects of X on Y are on a log-odds metric.'.
end if.
do if (notecode(i,1) = 27).
print/title = 'NOTE: Standardized coefficients are not available for models with moderators.'.
end if.
do if (notecode(i,1) = 28).
print/title = 'NOTE: The contrast option is not available with a multicategorical X.'.
end if.
do if (notecode(i,1) = 31).
do if (nms > 1).
print medmeans/title = 'NOTE: Controlled direct effect(s) estimated at the following mediator values:'/cnames=mnames/format= F10.4.
end if.
do if (nms=1).
print medmeans/title = 'NOTE: Controlled direct effect(s) estimated at the following mediator value:'/format= F10.4.
end if.
end if.
do if (notecode(i,1) = 33).
print/title = 'NOTE: Sobel test is not available with the XMINT option.'.
end if.
do if (notecode(i,1) = 37).
print/title = 'NOTE: The CONTRAST option is not available when using the XMINT option.'.
end if.
do if (notecode(i,1) = 34).
print/title = 'NOTE: Standardized effects are not available when using the XMINT option.'.
end if.
do if (notecode(i,1) = 36).
print/title = 'NOTE: The XREFVAL option is ignored when X is declared as multicategorical.'.
end if.
do if (notecode(i,1) = 35).
print coval/title = 'NOTE: Counterfactual effects estimated at the following covariate values:'/cnames=covnames/format= F10.4.
end if.
do if (notecode(i,1) = 29 and (listmiss=1)).
compute a=missrow.
compute conum=ncol(a).
compute allgood=0.
compute smremain=12.
compute largesti=1.
compute smallrow=0.
do if (conum > 12).
loop ii = 1 to 12.
compute check=(conum/ii).
do if (check = trunc(check)).
compute check2=conum/ii.
compute aok=ii.
do if (aok > 2).
compute allgood=1.
end if.
else.
compute remain=conum-(ii*trunc(check)).
do if (remain <= smremain).
compute smremain=remain.
compute largesti=ii.
compute smallrow=trunc(conum/largesti).
end if.
end if.
end loop.
compute atemp=a(1,1:(aok*check2)).
compute atemp=reshape(atemp,check2,aok).
do if (ncol(atemp) > 2).
print/title='NOTE: Missing data resulted in the deletion of the following row(s) of'.
print atemp/title='      data from the analysis:'/format=F6.0/space=0.
end if.
do if (allgood=0).
compute atemp=a(1,1:(smallrow*largesti)).
compute atemp=reshape(atemp,smallrow,largesti).
compute btemp=a(1,((largesti*smallrow)+1):conum).
print/title='NOTE: Missing data resulted in the deletion of the following row(s) of'.
print atemp/title='      data from the analysis:'/format=F6.0/space=0.
print btemp/title=' '/space=0/format=f6.0.
end if.
else if (conum <= 12).
print/title='NOTE: Missing data resulted in the deletion of the following row(s) of'.
print a/title='      data from the analysis:'/format=F6.0/space=0.
end if.
end if.
end loop.
do if (toomany=1).
print/title='WARNING: Variables names longer than eight characters can produce incorrect output'.
print/title='when some variables in the data file have the same first eight characters. Shorter'/space=0.
print/title='variable names are recommended. By using this output, you are accepting all risk'/space=0.
print/title='and consequences of interpreting or reporting results that may be incorrect.'/space=0.
end if.
end if.
end if.
loop i = 1 to 100.
do if (errcode(i,1)=1).
print/title = 'ERROR: You must specify a Y and an X variable.'.
end if.
do if (errcode(i,1)=2).
print/title = 'ERROR: X, M, or Y variable used more than once or W and Z are the same variable.'.
do if (toomany = 1).
print/title = '       This could be caused by the use of variables names longer'/space=0.
print varnames/title = '       than eight characters. Here are the variables I see:'/space=0/format=A8.
end if.
end if.
do if (errcode(i,1)=3).
print/title = 'ERROR: You have specified more than one variable for W, Y, X, or Z'.
end if.
do if (errcode(i,1)=4).
print/title = 'ERROR: A variable specified as multicategorical has more than nine categories.'.
end if.
do if (errcode(i,1)=5).
print/title = 'ERROR: One of the categories contains only a single case.'.
end if.
do if (errcode(i,1)=6).
print/title = 'ERROR: Invalid model number in this version of PROCESS.'.
end if.
do if (errcode(i,1)=7).
print/title = 'ERROR: Invalid model number.'.
end if.
do if (errcode(i,1)=8).
print/title = 'ERROR: You must specify an M variable for this model.'.
end if.
do if (errcode(i,1)=9).
print/title = 'ERROR: You have specified an M variable in a model that does not use it.'.
end if.
do if (errcode(i,1)=10).
print/title = 'ERROR: You have specified a W variable in a model that does not use it.'.
end if.
do if (errcode(i,1)=11).
print/title = 'ERROR: You have not specified a W variable in a model that requires it.'.
end if.
do if (errcode(i,1)=12).
print/title = 'ERROR: You have specified a Z variable in a model that does not use it.'.
end if.
do if (errcode(i,1)=13).
print/title = 'ERROR: You have not specified a Z variable in a model that requires it.'.
end if.
do if (errcode(i,1)=14).
print/title = 'ERROR: V and Q are not proper specifications in this release of PROCESS.'.
print/title = '       Moderators must be specified as W and/or Z.'/space=0.
end if.
do if (errcode(i,1)=15).
print/title = 'ERROR: One of your model variables exhibits no variation (it is a constant).'.
end if.
do if (errcode(i,1)=16).
print/title = 'ERROR: BMATRIX is not the correct length or is otherwise invalid.'.
end if.
do if (errcode(i,1)=17).
print/title = 'ERROR: WMATRIX is not the correct length or is otherwise invalid.'.
end if.
do if (errcode(i,1)=18).
print/title = 'ERROR: ZMATRIX is not the correct length or is otherwise invalid.'.
end if.
do if (errcode(i,1)=19).
print/title = 'ERROR: WZMATRIX is not the correct length or is otherwise invalid.'.
end if.
do if (errcode(i,1)=20).
print/title = 'ERROR: A path fixed at zero cannot be moderated.'.
end if.
do if (errcode(i,1)=21).
print/title = 'ERROR: If only one moderator is specified, it must be specified as W.'.
end if.
do if (errcode(i,1)=22).
print/title = 'ERROR: In BMATRIX, X must be specified to affect at least one variable.'.
end if.
do if (errcode(i,1)=23).
print/title = 'ERROR: In BMATRIX, at least one variable must be specified to affect Y.'.
end if.
do if (errcode(i,1)=24).
print/title = 'ERROR: You must specify a model number or a custom BMATRIX specification.'.
end if.
do if (errcode(i,1)=25).
print/title = 'ERROR: BMATRIX cannot be used in conjunction with a model number.'.
end if.
do if (errcode(i,1)=26).
print/title = 'ERROR: Your model has a dangling mediator (all Ms must affect and be affected).'.
end if.
do if (errcode(i,1)=27).
print/title = 'ERROR: CLUSTER is not available on this release of PROCESS.'.
end if.
do if (errcode(i,1)=29).
print/title = 'ERROR: CMATRIX is not the correct length or is otherwise invalid.'.
end if.
do if (errcode(i,1)=30).
print/title = 'ERROR: In CMATRIX, all covariates must be assigned to an M or a Y.'.
end if.
do if (errcode(i,1)=31).
print/title = 'ERROR: A linear or near linear dependency (singularity) exists in the data.'.
end if.
do if (errcode(i,1)=32).
print/title = 'ERROR: Models 80 and 81 require between 3 and 6 mediators.'.
end if.
do if (errcode(i,1)=33).
print/title = 'ERROR: Model 82 requires 4 mediators.'.
end if.
do if (errcode(i,1)=34).
print/title = 'ERROR: This model number requires between 2 and 6 mediators.'.
end if.
do if (errcode(i,1)=35).
print/title = 'ERROR: In a model with only one moderator, that moderator must be W.'.
end if.
do if (errcode(i,1)=36).
print/title = 'ERROR: A serial mediation model cannot have more than 6 mediators.'.
end if.
do if (errcode(i,1)=37).
print/title = 'ERROR: No more than 10 mediators are allowed in a PROCESS command.'.
end if.
do if (errcode(i,1)=38).
print/title = 'ERROR: XCATCODE is not provided, not the correct length, or is otherwise invalid.'.
end if.
do if (errcode(i,1)=39).
print/title = 'ERROR: WCATCODE is not provided, not the correct length, or is otherwise invalid.'.
end if.
do if (errcode(i,1)=40).
print/title = 'ERROR: ZCATCODE is not provided, not the correct length, or is otherwise invalid.'.
end if.
do if (errcode(i,1)=41).
print/title = 'ERROR: Models 1, 2, and 3 cannot be customized.'.
end if.
do if (errcode(i,1)=42).
print/title = 'ERROR: WS option available only in PROCESS v2. Or use the MEMORE macro instead.'.
print/title = '       MEMORE can be downloaded from www.akmontoya.com.'/space=0.
end if.
do if (errcode(i,1)=43).
print/title = 'ERROR: PROCESS does not allow dichotomous mediators.'.
end if.
do if (errcode(i,1)=60).
print/title = 'PROCESS is now ready for use.'.
print/title='Copyright 2017-2022 by Andrew F. Hayes. ALL RIGHTS RESERVED.'/space=0.
print/title='Workshop schedule available at http://haskayne.ucalgary.ca/CCRAM'/space=0.
end if.
do if (errcode(i,1)=50).
print/title = 'ERROR: A multicategorical moderator cannot be specified as a covariate.'.
end if.
do if (errcode(i,1)=51).
print/title = 'ERROR: A variable you specified as a covariate is a moderator in all equations.'.
end if.
do if (errcode(i,1)=52 and mcerpt=0).
compute mcerpt=1.
print/title = 'ERROR: A variable specified as multicategorical must have at least three categories'.
end if.
do if (errcode(i,1) = 47).
do if (iterrmod=1).
print/title = 'ERROR: Iteration for Y model didn''t converge to a solution. Interpret results with caution.'.
print/title = '       Try increasing the number of iterations, though this will slow down computation.'/space=0.
end if.
do if (bootiter=1).
print/title = 'ERROR: Nonconvergence during bootstrapping. Interpet bootstrap results with caution.'.
end if.
end if.
do if (errcode(i,1) = 61).
print/title = 'ERROR: Variable names should be no more than eight characters in length.'.
end if.
do if (errcode(i,1) = 62).
print/title = 'ERROR: After listwise deletion of cases with missing data, too few cases remain.'.
end if.
do if (errcode(i,1) = 63).
print/title = 'ERROR: The XMINT option is available only for model 4.'.
end if.
do if (errcode(i,1) = 64).
print/title = 'ERROR: Incorrect number of values specified in CDEVAL option.'.
end if.
do if (errcode(i,1) = 65).
print/title = 'ERROR: Only indicator or sequential coding of X is allowed with the XMINT option.'.
end if.
do if (errcode(i,1) = 66).
print/title = 'ERROR: XMINT option not available with continuous X using PROCESS GUI. Use syntax.'.
end if.
do if (errcode(i,1) = 67).
print/title = 'ERROR: Too many elements provided in XREFVAL option.'.
end if.
do if (errcode(i,1)=68).
print/title = 'ERROR: Covariate assignment is not allowed with XMINT option.'.
end if.
do if (errcode(i,1) = 69).
print/title = 'ERROR: Incorrect number of values specified in COVAL option.'.
end if.
do if (errcode(i,1) = 70).
print/title = 'ERROR: Incorrect value(s) in XREFVAL for this dichotomous X variable.'.
end if.
do if (errcode(i,1) = 71).
print/title = 'ERROR: The CENTER option is not available when using the XMINT option.'.
end if.
do if (errcode(i,1) = 72).
print/title = 'ERROR: The XMINT option is not available for models with a dichotomous Y.'.
end if.
end loop.
END MATRIX.	
Ressources	Temps de processeur	00:00:06,97	
	Temps écoulé	00:00:06,96	


Run MATRIX procedure:

***************** PROCESS Procedure for SPSS Version 4.2 *****************

          Written by Andrew F. Hayes, Ph.D.       www.afhayes.com
    Documentation available in Hayes (2022). www.guilford.com/p/hayes3

**************************************************************************
Model  : 4
    Y  : HOMAIR
    X  : WHRSt
   M1  : TG
   M2  : HDL
   M3  : LDL
   M4  : TC

Sample
Size:  156

**************************************************************************
OUTCOME VARIABLE:
 TG

Model Summary
          R       R-sq        MSE          F        df1        df2          p
      ,1949      ,0380  2532,8006     6,0830     1,0000   154,0000      ,0147

Model
              coeff         se          t          p       LLCI       ULCI
constant    67,4783     7,4203     9,0937      ,0000    52,8195    82,1370
WHRSt       21,7945     8,8366     2,4664      ,0147     4,3378    39,2512

Standardized coefficients
           coeff
WHRSt      ,4261

**************************************************************************
OUTCOME VARIABLE:
 HDL

Model Summary
          R       R-sq        MSE          F        df1        df2          p
      ,0594      ,0035   178,9896      ,5451     1,0000   154,0000      ,4614

Model
              coeff         se          t          p       LLCI       ULCI
constant    39,4565     1,9726    20,0025      ,0000    35,5597    43,3533
WHRSt        1,7344     2,3491      ,7383      ,4614    -2,9062     6,3750

Standardized coefficients
           coeff
WHRSt      ,1298

**************************************************************************
OUTCOME VARIABLE:
 LDL

Model Summary
          R       R-sq        MSE          F        df1        df2          p
      ,0420      ,0018  1187,8985      ,2717     1,0000   154,0000      ,6029

Model
              coeff         se          t          p       LLCI       ULCI
constant    75,0000     5,0817    14,7588      ,0000    64,9611    85,0389
WHRSt        3,1545     6,0517      ,5213      ,6029    -8,8005    15,1096

Standardized coefficients
           coeff
WHRSt      ,0917

**************************************************************************
OUTCOME VARIABLE:
 TC

Model Summary
          R       R-sq        MSE          F        df1        df2          p
      ,0892      ,0080  1417,9444     1,2350     1,0000   154,0000      ,2682

Model
              coeff         se          t          p       LLCI       ULCI
constant   130,1522     5,5520    23,4423      ,0000   119,1842   141,1201
WHRSt        7,3478     6,6118     1,1113      ,2682    -5,7136    20,4093

Standardized coefficients
           coeff
WHRSt      ,1950

**************************************************************************
OUTCOME VARIABLE:
 HOMAIR

Model Summary
          R       R-sq        MSE          F        df1        df2          p
      ,2615      ,0684    22,2213     2,2016     5,0000   150,0000      ,0570

Model
              coeff         se          t          p       LLCI       ULCI
constant     1,0177     1,7472      ,5825      ,5611    -2,4345     4,4699
WHRSt        1,1360      ,8524     1,3327      ,1847     -,5483     2,8202
TG            ,0253      ,0115     2,2053      ,0290      ,0026      ,0480
HDL           ,0944      ,0443     2,1336      ,0345      ,0070      ,1819
LDL           ,0868      ,0356     2,4347      ,0161      ,0164      ,1572
TC           -,0773      ,0359    -2,1523      ,0330     -,1482     -,0063

Standardized coefficients
           coeff
WHRSt      ,2364
TG         ,2696
HDL        ,2625
LDL        ,6210
TC        -,6060

************************** TOTAL EFFECT MODEL ****************************
OUTCOME VARIABLE:
 HOMAIR

Model Summary
          R       R-sq        MSE          F        df1        df2          p
      ,1483      ,0220    22,7215     3,4636     1,0000   154,0000      ,0646

Model
              coeff         se          t          p       LLCI       ULCI
constant     2,9031      ,7028     4,1308      ,0001     1,5147     4,2915
WHRSt        1,5576      ,8370     1,8611      ,0646     -,0958     3,2111

Standardized coefficients
           coeff
WHRSt      ,3242


************** TOTAL, DIRECT, AND INDIRECT EFFECTS OF X ON Y **************

Total effect of X on Y
     Effect         se          t          p       LLCI       ULCI       c_ps
     1,5576      ,8370     1,8611      ,0646     -,0958     3,2111      ,3242

Direct effect of X on Y
     Effect         se          t          p       LLCI       ULCI      c'_ps
     1,1360      ,8524     1,3327      ,1847     -,5483     2,8202      ,2364

Indirect effect(s) of X on Y:
          Effect     BootSE   BootLLCI   BootULCI
TOTAL      ,4217      ,2595     -,0166     1,0244
TG         ,5519      ,6239      ,1098     2,5207
HDL        ,1638      ,5166     -,5226     1,4157
LDL        ,2737     1,1655    -1,0850     3,2646
TC        -,5677     1,6608    -5,3460      ,4261

Partially standardized indirect effect(s) of X on Y:
          Effect     BootSE   BootLLCI   BootULCI
TOTAL      ,0878      ,0507     -,0039      ,1997
TG         ,1149      ,1254      ,0252      ,5085
HDL        ,0341      ,1038     -,1032      ,2844
LDL        ,0570      ,2390     -,2291      ,6815
TC        -,1182      ,3400    -1,0678      ,0892

*********************** ANALYSIS NOTES AND ERRORS ************************

Level of confidence for all confidence intervals in output:
  95,0000

Number of bootstrap samples for percentile bootstrap confidence intervals:
  5000

NOTE: Standardized coefficients for dichotomous or multicategorical X are in
      partially standardized form.

------ END MATRIX -----


* Encoding: UTF-8.
preserve.
set printback=off.


Matrice


Remarques	
Sortie obtenue	10-NOV-2024 17:00:14	
Commentaires		
Entrée	Données	C:\Users\Brice SAHA\Documents\UBa\Master Thesis 2021\Fanuel\Database fanuel.sav	
	Jeu de données actif	Jeu_de_données1	
	Filtre	<sans>	
	Pondération	<sans>	
	Scinder un fichier	<sans>	
	N de lignes dans le fichier de travail	169	
Syntaxe	MATRIX.
compute wnames='xxxxx'.
compute znames='xxxxx'.
compute mcerpt=0.
compute wiscov=0.
compute ziscov=0.
compute tooman=0.
compute errcode=make(100,1,0).
compute notecode=make(100,1,0).
compute model = trunc( 4 ).
compute iterate = abs(trunc( 100 )).
compute converge = abs( 0.00001 ).
compute itprobtg=0.
compute v2tag=0.
compute ydich=0.
compute maxwwarn=0.
compute minwwarn=0.
compute maxzwarn=0.
compute minzwarn=0.
compute toomany=0.
compute wdich=0.
compute zdich=0.
compute wnotev=0.
compute znotev=0.
compute nxpval=1.
compute nwpval=1.
compute nzpval=1.
compute errs=1.
compute notes=1.
compute criterr=0.
compute novar=0.
compute adjust=0.
compute ncs=0.
compute serial=0.
compute sobelok=0.
compute hasw=0.
compute hasz=0.
compute printw=0.
compute printz=0.
compute xmint=( 0 =1).
compute wmodcust=0.
compute zmodcust=0.
compute booting=0.
compute bootiter=0.
compute iterrmod=0.
compute cov = 'xxxxx'.
compute varorder=( 0 <> 0).
compute nws=0.
compute w= 'xxxxx'.
compute nzs=0.
compute z = 'xxxxx'.
compute nms=0.
compute m = 'TG HDL LDL TC'.
compute nys=0.
compute y = 'HOMAIR'.
compute nxs=0.
compute x = 'WhtRSt'.
compute effsize=( 0 =1).
compute stand=( 1 =1).
compute intprobe = .1.
compute xrefvals={ 999 }.
compute center=trunc( 0 ).
compute xcontcf=0.
compute xscaling=1.
compute cdeval={ -999 }.
compute cuscoval=0.
do if (model=74).
compute errcode(errs,1)=7.
compute errs=errs+1.
compute criterr=1.
end if.
do if (xmint=1 and model <> 4).
compute errcode(errs,1)=63.
compute errs=errs+1.
compute criterr=1.
end if.
do if (xmint=1 and model=4).
compute w=x.
compute model=74.
compute intprobe=1.
compute notecode(notes,1) = 32.
compute notes = notes + 1.
do if (effsize=1 or stand=1).
compute notecode(notes,1) = 34.
compute notes = notes + 1.
compute stand=0.
compute effsize=0.
end if.
do if (center <> 0).
compute center=0.
compute errcode(errs,1)=71.
compute errs=errs+1.
compute criterr=1.
end if.
end if.
compute v = 'xxxxx'.
compute q = 'xxxxx'.
compute linsum={ -999 }.
compute nlinsum=ncol(linsum).
do if (linsum(1,1) = -999).
compute nlinsum=0.
end if.
compute oldvars= 'xxxxx'.
compute mcxok=0.
compute mcwok=0.
compute mczok=0.
compute xprod=0.
compute zprod=0.
compute wprod=0.
compute modcok=0.
compute alttotal=0.
compute hc3=trunc( 0 ).
compute jn=( 0 = 1).
compute listmiss=( 0 =1).
compute modelres=( 0 =1).
compute outscree=( 1 =1).
compute activate=( 0 =1).
compute booterr=0.
compute normal=( 0 =1).
compute xmtest=( 0 =1).
compute describe=( 0 =1).
compute longname=( 0 =1).
do if (stand=1).
compute effsize=1.
end if.
compute pstog=0.
compute sobelok=0.
compute normal=( 0 =1).
compute mdichok=( 0 =1).
compute contrast={ 999 }.
compute ncontr=ncol(contrast).
compute ncontrow=nrow(contrast).
do if (contrast(1,1) = 999).
compute ncontr=1.
compute contrast=0.
end if.
do if (ncontr = 1).
compute contrast=trunc(contrast).
do if (contrast > 3 or contrast < 0)).
compute ncontr=1.
compute contrast = 0.
end if.
end if.
do if (ncontr > 1).
compute contvec=contrast.
compute contrast=4.
do if (ncontrow > 1).
compute contrast=0.
compute modcok=1.
compute wcontval=contvec(:,1).
compute zcontval=contvec(:,2).
do if ((ncontr <> 2) or (ncontrow <> 2)).
compute notecode(notes,1) = 19.
compute notes = notes + 1.
compute modcok=0.
end if.
end if.
end if.
do if (xmint = 1 and contrast(1,1) <> 0).
compute contrast=0.
compute notecode(notes,1) = 37.
compute notes = notes + 1.
end if.
do if (varorder = 1).
compute notecode(notes,1) = 21.
compute notes = notes + 1.
end if.
do if ( 999 <> 999 or 999 <> 999).
compute notecode(notes,1) = 22.
compute notes = notes + 1.
end if.
compute modelbt=( 0 =1).
compute cluster= 'xxxxx'.
compute matrices=( 0 =1).
compute covcoeff=( 0 =1).
compute covmy=trunc( 0 ).
do if (covmy < 0 or covmy > 2).
compute covmy = 0.
end if.
compute boot = abs(trunc( 5000 )).
compute bc=( 0 =1).
compute mc=abs(trunc( 0 )).
compute hc=trunc( 5 ).
do if (intprobe < 0 or intprobe > 1).
compute intprobe = .10.
end if.
compute plot=trunc( 0 ).
do if (plot < 0 or plot > 2).
compute plot=0.
end if.
compute total=( 1 =1).
compute dototal=0.
compute saveboot = ( 0 = 1).
compute saveest=( 0 = 2).
do if (saveest=1).
compute intprobe=1.
end if.
do if (hc >= 0 and hc < 5).
compute notecode(notes,1) = 4.
compute notes = notes + 1.
end if.
do if (hc > 5 or hc < 0).
compute hc=5.
end if.
compute mcw=trunc( 0 ).
compute mcz=trunc( 0 ).
compute mcx=trunc( 0 ).
do if (mcx > 0 and mcx < 3 and model = 74).
compute mcw=mcx.
compute xscaling=1.
end if.
do if (mcx > 2 and model = 74).
compute errcode(errs,1)=65.
compute errs=errs+1.
compute criterr=1.
end if.
do if (model = 74 and normal=1).
compute notecode(notes,1) = 33.
compute normal=0.
compute notes = notes + 1.
end if.
do if (mcx > 0 and contrast > 0).
compute notecode(notes,1) = 28.
compute notes = notes + 1.
compute contrast=0.
end if.
compute nxvls=1.
compute nmvls=1.
compute nwvls=1.
compute nzvls=1.
compute paths=999.
compute pathsw=999.
compute pathsz=999.
compute pathswz=999.
compute pathsmod=999.
compute pathtype=999.
compute obscoeff=999.
compute pathsdv={' '}.
compute quantile=1.
do if ( 999 <>999).
compute notecode(notes,1) = 23.
compute notes = notes + 1.
end if.
compute moments=( 0 =1).
do if (moments=1).
compute quantile=0.
end if.
compute bmatrix={ -999 }.
compute wmatrix={ -999 }.
compute zmatrix={ -999 }.
compute wzmatrix={ -999 }.
compute cmatrix={ -999 }.
compute xcatcode={ -999 }.
compute wcatcode={ -999 }.
compute zcatcode={ -999 }.
compute needed=0.
compute conf= 95.
do if (trunc( 95 ) >= 100 or (trunc( 95 ) <= 50)).
compute conf = 95.
compute notecode(notes,1)=2.
compute notes=notes+1.
end if.
do if (model >= 0 and model < 4 and modelbt=0).
compute boot=0.
compute mc=0.
compute bc=0.
end if.
do if (mc > 0 and boot > 0).
compute boot=0.
compute bc=0.
end if.
do if ((boot < 1000) and (mc = 0) and (boot > 0)).
compute boot=5000.
end if.
do if ((mc < 1000) and (boot = 0) and (mc > 0)).
compute mc=5000.
end if.
compute p0=-.322232431088.
compute p1 = -1.
compute p2 = -.342242088547.
compute p3 = -.0204231210245.
compute p4 = -.0000453642210148.
compute q0 = .0993484626060.
compute q1 = .588581570495.
compute q2 = .531103462366.
compute q3 = .103537752850.
compute q4 = .0038560700634.
compute badend=0.
compute priorlo = -9999999.
compute priorhi = 9999999.
compute alpha2 = (1-(conf/100))/2.
compute cilm=alpha2*2.
compute y5=sqrt(-2*ln(alpha2)).
compute xp2=(y5+((((y5*p4+p3)*y5+p2)*y5+p1)*y5+p0)/((((y5*q4+q3)*y5+q2)*y5+q1)*y5+q0)).
compute medlb={'   M1  :';'   M2  :';'   M3  :';'   M4  :';'   M5  :';'   M6  :';'   M7  :';'   M8  :';'   M9  :';'   M10 :'}.
compute medlb2={'(M1)','(M2)','(M3)','(M4)','(M5)','(M6)','(M7)','(M8)','(M9)','(M10)'}.
compute xlb={'   X1  :';'   X2  :';'   X3  :';'   X4  :';'   X5  :';'   X6  :';'   X7  :';'   X8  :';'   X9  :'}.
compute highlbw={'M1*W'; 'M2*W'; 'M3*W'; 'M4*W'; 'M5*W'; 'M6*W'; 'M7*W'; 'M8*W'; 'M9*W'; 'M10*W'}.
do if (xmint=1).
compute highlbw={'M1*X'; 'M2*X'; 'M3*X'; 'M4*X'; 'M5*X'; 'M6*X'; 'M7*X'; 'M8*X'; 'M9*X'; 'M10*X'}.
end if.
compute highlbz={'M1*Z'; 'M2*Z'; 'M3*Z'; 'M4*Z'; 'M5*Z'; 'M6*Z'; 'M7*Z'; 'M8*Z'; 'M9*Z';'M10*Z'}.
compute highlbwz={'M1*W*Z'; 'M2*W*Z'; 'M3*W*Z'; 'M4*W*Z'; 'M5*W*Z'; 'M6*W*Z'; 'M7*W*Z'; 'M8*W*Z'; 'M9*W*Z';'M10*W*Z'}.
compute highlbbt={'BOTH(M1)'; 'BOTH(M2)'; 'BOTH(M3)'; 'BOTH(M4)'; 'BOTH(M5)'; 'BOTH(M6)'; 'BOTH(M7)'; 'BOTH(M8)'; 'BOTH(M9)';'BTH(M10)'}.
compute highlbx={'M1*X'; 'M2*X'; 'M3*X'; 'M4*X'; 'M5*X'; 'M6*X'; 'M7*X'; 'M8*X'; 'M9*X'; 'M10*X'}.
compute skipwz=0.
compute validm={1,1,1,1,1,1,1,1,1,1,1,1,1,1,1,1,1,1,1,1,1,1,0,0,0,0,0,1,1,0,0,0,0, 0,0,0,0,0,0,0,0,0,0,0,0,0,0,0,0,0,0,0,0,0,0,0,0,1,1,1,1,1,1,1,1,1,1,1,1,1,1,1,1,1,1,1,0,0,0,1,1, 1,1,1,1,1,1,1,1,1,1,1}.
do if (activate=1).
compute errcode(errs,1)=60.
compute errs=errs+1.
compute criterr=1.
end if.
do if (criterr=0).
do if (( 0 =1)=1).
compute errcode(errs,1)=42.
compute errs=errs+1.
compute criterr=1.
end if.
do if (model > 0 and model < 93).
do if (validm(1,model)=0).
compute errcode(errs,1)=6.
compute errs=errs+1.
compute criterr=1.
end if.
release validm.
end if.
do if ((model > 92 or model < 0) and model <> 999)).
compute errcode(errs,1)=7.
compute errs=errs+1.
compute criterr=1.
end if.
do if (model = 999 and bmatrix(1,1)=-999).
compute errcode(errs,1)=24.
compute errs=errs+1.
compute criterr=1.
end if.
do if (model <> 999 and bmatrix(1,1) <> -999).
compute errcode(errs,1)=25.
compute errs=errs+1.
compute criterr=1.
end if.
do if ((model = 74 or (model > 0 and model < 4)) and ((wmatrix(1,1) <> -999) or (zmatrix(1,1)<>-999) or (wzmatrix(1,1)<>-999)))).
compute errcode(errs,1)=41.
compute errs=errs+1.
compute criterr=1.
end if.
do if (hc3 <> 0).
compute notecode(notes,1) = 5.
compute notes = notes + 1.
do if (hc3 = 1).
compute hc=3.
end if.
end if.
do if ((v <> 'xxxxx') or (q <> 'xxxxx')).
compute errcode(errs,1)=14.
compute errs=errs+1.
compute errcode(errs,1)=48.
compute errs=errs+1.
compute criterr=1.
end if.
do if (oldvars <> 'xxxxx').
compute errcode(errs,1)=48.
compute errs=errs+1.
compute criterr=1.
end if.
do if (cluster <> 'xxxxx').
compute errcode(errs,1)=27.
compute errs=errs+1.
compute criterr=1.
end if.
do if ((y = 'xxxxx') or (x = 'xxxxx')).
compute errcode(errs,1)=1.
compute errs=errs+1.
compute criterr=1.
end if.
do if ((m = 'xxxxx') and model > 3).
compute errcode(errs,1)=8.
compute errs=errs+1.
compute criterr=1.
end if.
end if.
do if (criterr=0).
get ytmp/variables = HOMAIR /names = ynames/MISSING = 99999.
compute nys=ncol(ytmp).
compute needed=nys.
compute n=nrow(ytmp).
compute varnames={ynames}.
compute dat=ytmp.

.


do if (toomany=1 and longname=0).
compute criterr=1.
do if (tooman=0).
compute tooman=1.
compute errcode(errs,1) = 61.
compute errs = errs + 1.
end if.
end if
.
compute modelvar={ '4' ;t(ynames)}.
do if ( 4 =999).
compute modelvar(1,1)='CUSTOM'.
end if.
get xtmp/variables = WhtRSt /names = xnames/MISSING = 99999.
compute nxs=ncol(xtmp).
compute n=nrow(xtmp).
compute needed=needed+nxs.
compute varnames={varnames,xnames}.
compute xcatlab=t(xnames).
compute dat={dat,xtmp}.

.


do if (toomany=1 and longname=0).
compute criterr=1.
do if (tooman=0).
compute tooman=1.
compute errcode(errs,1) = 61.
compute errs = errs + 1.
end if.
end if
.
compute modelvar={modelvar;t(xnames)}.
do if (nxs = 1).
compute modelvlb={'Model  :';'    Y  :';'    X  :'}.
else.
compute modelvlb={'Model  :';'    Y  :';xlb(1:nxs,1)}.
end if.
do if (m <> 'xxxxx').
get mtmp/variables = TG HDL LDL TC /names = mnames/MISSING = 99999.
compute nms=ncol(mtmp).
compute mprod=make(1,nms,0).
compute n=nrow(mtmp).
compute needed=needed+nms.
compute varnames={varnames,mnames}.
compute dat={dat,mtmp}.
compute modelvar={modelvar;t(mnames)}.
compute x2m=make(99,nms,0).
compute m2y=make(99,nms,0).
compute onem=make(nms,1,1).

.


do if (toomany=1 and longname=0).
compute criterr=1.
do if (tooman=0).
compute tooman=1.
compute errcode(errs,1) = 61.
compute errs = errs + 1.
end if.
end if
.
do if (nms > 1 and nms < 11).
compute modelvlb={modelvlb;medlb(1:nms,1)}.
else.
compute modelvlb={modelvlb;'    M  :'}.
end if.
do if (nms > 0 and model < 4).
compute errcode(errs,1)=9.
compute errs=errs+1.
do if (model <> 0).
compute errcode(errs,1)=48.
compute errs=errs+1.
end if.
compute criterr=1.
end if.
end if.
compute wlocatet=0.
compute wlocate=0.
do if (w <> 'xxxxx').
do if (xmint=0).
get wtmp/variables = xxxxx /names = wnames/MISSING = 99999.

.


do if (toomany=1 and longname=0).
compute criterr=1.
do if (tooman=0).
compute tooman=1.
compute errcode(errs,1) = 61.
compute errs = errs + 1.
end if.
end if
.
end if.
do if (xmint=1).
get wtmp/variables = WhtRSt /names = wnames/MISSING = 99999.
end if.
compute nws=ncol(wtmp).
compute n=nrow(wtmp).
compute varnames={varnames,wnames}.
compute wlocate=ncol(varnames).
do if (model=74).
compute wlocatet=1.
do if (xnames <> wnames).
compute errcode(errs,1)=45.
compute errs=errs+1.
compute criterr=1.
end if.
end if.
compute wcatlab=t(wnames).
compute dat={dat,wtmp}.
do if (xmint <> 1).
compute modelvar={modelvar;t(wnames)}.
compute modelvlb={modelvlb;'    W  :'}.
end if.
end if.
do if (z <> 'xxxxx').
get ztmp/variables = xxxxx /names = znames/MISSING = 99999.
compute nzs=ncol(ztmp).
compute n=nrow(ztmp).

.


do if (toomany=1 and longname=0).
compute criterr=1.
do if (tooman=0).
compute tooman=1.
compute errcode(errs,1) = 61.
compute errs = errs + 1.
end if.
end if
.
compute varnames={varnames,znames}.
compute zcatlab=t(znames).
compute dat={dat,ztmp}.
compute modelvar={modelvar;t(znames)}.
compute modelvlb={modelvlb;'    Z  :'}.
end if.
do if (cov <> 'xxxxx').
get ctmp/variables = xxxxx /names = covnames/MISSING = 99999.
compute ncs=ncol(ctmp).
compute n=nrow(ctmp).

.


do if (toomany=1 and longname=0).
compute criterr=1.
do if (tooman=0).
compute tooman=1.
compute errcode(errs,1) = 61.
compute errs = errs + 1.
end if.
end if
.
compute varnames={varnames,covnames}.
compute dat={dat,ctmp}.
end if.
do if (nws > 1 or nzs > 1 or nys > 1 or nxs > 1).
compute errcode(errs,1)=3.
compute errs=errs+1.
compute criterr=1.
end if.
do if ((model = 80 or model = 81) and (nms < 3 or nms > 6)).
compute errcode(errs,1)=32.
compute errs=errs+1.
compute criterr=1.
end if.
do if (model = 82 and nms <> 4).
compute errcode(errs,1)=33.
compute errs=errs+1.
compute criterr=1.
end if.
do if (nms > 10).
compute errcode(errs,1)=37.
compute errs=errs+1.
compute criterr=1.
end if.
do if ((model = 6 or (model > 82 and model < 999)) and (nms < 2 or nms > 6)).
compute errcode(errs,1)=34.
compute errs=errs+1.
compute criterr=1.
end if.
compute match=0.
compute match2=0.
compute mcwzcov=0.
loop i = 1 to (ncol(varnames)-1).
loop j = (i+1) to ncol(varnames).
do if (varnames(i)=varnames(j)).
do if (i < (nxs+nms+nys+1)).
compute match2=match2+1.
end if.
do if (wlocatet=1 and i=2 and j=wlocate).
compute match2=match2-1.
end if.
do if ((wnames=znames) and (nws > 0 or nzs > 0))).
compute match2=match2+1.
end if.
do if (i < (ncol(varnames)-ncs+1)) and j > (ncol(varnames)-ncs)).
do if ((varnames(j)=wnames) and mcw=0)).
compute match=0.
compute wiscov=(j-(ncol(varnames)-ncs)).
end if.
do if ((varnames(j)=wnames) and mcw <>0)).
compute mcwzcov=1.
end if.
do if ((varnames(j)=znames) and mcz=0).
compute match=0.
compute ziscov=(j-(ncol(varnames)-ncs)).
end if.
do if ((varnames(j)=znames) and mcz<>0)).
compute mcwzcov=1.
end if.
end if.
end if.
end loop.
end loop.
do if (match2>0 or match=1).
compute errcode(errs,1)=2.
compute errs=errs+1.
compute criterr=1.
end if.
do if (mcwzcov=1).
compute errcode(errs,1)=50.
compute errs=errs+1.
compute criterr=1.
end if.
compute ninit=nrow(dat).
compute rownum=make(ninit,1,0).
loop i = 1 to ninit.
compute rownum(i,1)=i.
end loop.
compute dat={rownum,dat}.
compute j=1.
compute missrow=0.
loop i = 1 to n.
do if (rsum(dat(i,2:ncol(dat))=99999)=0).
compute dat(j,:)=dat(i,:).
compute j=j+1.
else.
compute missrow={missrow;dat(i,1)}.
end if.
end loop.
do if (j < 5).
compute errcode(errs,1)=62.
compute errs=errs+1.
compute criterr=1.
end if.
do if (criterr=0).
compute rownum=dat(1:(j-1),1).
do if (nrow(missrow) > 1).
compute missrow=t(missrow(2:nrow(missrow),1)).
compute notecode(notes,1) = 29.
compute notes = notes + 1.
end if.
compute dat=dat(1:(j-1),2:ncol(dat)).
compute n=nrow(dat).
compute nmiss=ninit-n.
compute ytmp=dat(:,1:nys).

.
compute desctmp=make((8-(4* 0 )),ncol( ytmp ),-999).
loop jd=1 to ncol( ytmp ).
compute descdat= ytmp (:,jd).
compute desctmp(1,jd) = csum(descdat)/nrow(descdat).
compute desctmp(2,jd) = (nrow(descdat)*sscp(descdat))-(t(csum(descdat))*(csum(descdat))).
compute desctmp(2,jd) = sqrt(desctmp(2,jd)/(nrow(descdat)*(nrow(descdat)-1))).
compute desctmp(3,jd)=cmin(descdat).
compute desctmp(4,jd)=cmax(descdat).
do if ( 0 =0).
compute minwarn=0.
compute maxwarn=0.
do if ((desctmp(3,jd)=desctmp(4,jd)) and novar=0).
compute errcode(errs,1)=15.
compute errs=errs+1.
compute criterr=1.
compute novar=1.
end if.
compute tmp=((descdat(:,1)=desctmp(3,jd))+(descdat(:,1)=desctmp(4,jd))).
compute desctmp(8,jd)=(csum(tmp)=nrow(tmp)).
compute tmp = descdat.
compute tmp(GRADE(descdat),:) = descdat.
compute descdat = tmp.
release tmp.
compute decval={.16;.5;.84}.
loop kd=1 to 3.
compute low=trunc(decval(kd,1)*(nrow(descdat)+1)).
compute lowdec=decval(kd,1)*(nrow(descdat)+1)-low.
compute value=descdat(low,1)+(descdat((low+1),1)-descdat(low,1))*lowdec.
compute desctmp((4+kd),jd)=value.
end loop.
compute mnotev=1.
compute modvals=desctmp(5:7,:).
do if (quantile <> 1).
compute desctmp(5,jd)=desctmp(1,jd)-desctmp(2,jd).
compute desctmp(6,jd)=desctmp(1,jd).
compute desctmp(7,jd)=desctmp(1,jd)+desctmp(2,jd).
compute modvals=desctmp(5:7,:).
compute mnotev=2.
do if (modvals(1,1) < desctmp(3,1)).
compute modvals(1,1)=desctmp(3,1).
compute minwarn=1.
end if.
do if (modvals(3,1) > desctmp(4,1)).
compute modvals(3,1)=desctmp(4,1).
compute maxwarn=1.
end if.
end if.
do if (desctmp(8,1)=1).
compute modvals={desctmp(3,1);desctmp(4,1)}.
compute mnotev=0.
compute minwarn=0.
compute maxwarn=0.
end if.
end if.
end loop
.
compute ysd=desctmp(2,:).
compute ovsd=ysd.
do if (desctmp(8,1)=1).
compute ydich=1.
do if (total=1).
compute total=0.
compute notecode(notes,1) = 24.
compute notes = notes + 1.
end if.
do if (effsize=1).
compute effsize=0.
compute notecode(notes,1) = 25.
compute notes = notes + 1.
end if.
do if (model=74).
compute errcode(errs,1) = 72.
compute errs=errs+1.
compute criterr=1.
end if.
compute omx = cmax(ytmp).
compute omn = cmin(ytmp).
compute ytmp = (ytmp = omx).
compute dat(:,1:nys)=(dat(:,1:nys)=omx).
compute rcd = {omn, 0; omx, 1}.
end if.
compute xtmp=dat(:,(nys+1):(nys+nxs)).

.
compute desctmp=make((8-(4* 0 )),ncol( xtmp ),-999).
loop jd=1 to ncol( xtmp ).
compute descdat= xtmp (:,jd).
compute desctmp(1,jd) = csum(descdat)/nrow(descdat).
compute desctmp(2,jd) = (nrow(descdat)*sscp(descdat))-(t(csum(descdat))*(csum(descdat))).
compute desctmp(2,jd) = sqrt(desctmp(2,jd)/(nrow(descdat)*(nrow(descdat)-1))).
compute desctmp(3,jd)=cmin(descdat).
compute desctmp(4,jd)=cmax(descdat).
do if ( 0 =0).
compute minwarn=0.
compute maxwarn=0.
do if ((desctmp(3,jd)=desctmp(4,jd)) and novar=0).
compute errcode(errs,1)=15.
compute errs=errs+1.
compute criterr=1.
compute novar=1.
end if.
compute tmp=((descdat(:,1)=desctmp(3,jd))+(descdat(:,1)=desctmp(4,jd))).
compute desctmp(8,jd)=(csum(tmp)=nrow(tmp)).
compute tmp = descdat.
compute tmp(GRADE(descdat),:) = descdat.
compute descdat = tmp.
release tmp.
compute decval={.16;.5;.84}.
loop kd=1 to 3.
compute low=trunc(decval(kd,1)*(nrow(descdat)+1)).
compute lowdec=decval(kd,1)*(nrow(descdat)+1)-low.
compute value=descdat(low,1)+(descdat((low+1),1)-descdat(low,1))*lowdec.
compute desctmp((4+kd),jd)=value.
end loop.
compute mnotev=1.
compute modvals=desctmp(5:7,:).
do if (quantile <> 1).
compute desctmp(5,jd)=desctmp(1,jd)-desctmp(2,jd).
compute desctmp(6,jd)=desctmp(1,jd).
compute desctmp(7,jd)=desctmp(1,jd)+desctmp(2,jd).
compute modvals=desctmp(5:7,:).
compute mnotev=2.
do if (modvals(1,1) < desctmp(3,1)).
compute modvals(1,1)=desctmp(3,1).
compute minwarn=1.
end if.
do if (modvals(3,1) > desctmp(4,1)).
compute modvals(3,1)=desctmp(4,1).
compute maxwarn=1.
end if.
end if.
do if (desctmp(8,1)=1).
compute modvals={desctmp(3,1);desctmp(4,1)}.
compute mnotev=0.
compute minwarn=0.
compute maxwarn=0.
end if.
end if.
end loop
.
compute xsd=desctmp(2,:).
compute xmodvals=modvals.
compute xdich=desctmp(8,1).
compute xmx=cmax(xtmp).
compute xmn=cmin(xtmp).
do if ((mcx > 0) and (xrefvals(1,1) <> 999) and (xmint=1) and (model= 74)).
compute notecode(notes,1) = 36.
compute notes = notes + 1.
end if.
do if (mcx=0).
do if (ncol(xrefvals)>2 and model=74 and xmint=1).
compute errcode(errs,1)=67.
compute errs=errs+1.
compute criterr=1.
end if.
do if ((model=74) and (xmint=1)).
do if ((xrefvals(1,1)=999) and nxvls=1 and xdich=0).
compute errcode(errs,1)=66.
compute errs=errs+1.
compute criterr=1.
end if.
do if ((xrefvals(1,1)=999) and (xdich=1)).
compute xrefvals={xmn,xmx}.
compute xscaling=xrefvals(1,2)-xrefvals(1,1).
end if.
do if (ncol(xrefvals)=1 and xrefvals(1,1) <> 999).
do if (xdich=0).
compute xrefvals={xrefvals,(xrefvals(1,1)+1)}.
compute xscaling=xrefvals(1,2)-xrefvals(1,1).
end if.
do if (xdich=1).
do if ((xrefvals(1,1) <> xmx) and (xrefvals(1,1) <> xmn)).
compute errcode(errs,1)=70.
compute errs=errs+1.
compute criterr=1.
end if.
do if (xrefvals(1,1) = xmx).
compute xrefvals={xrefvals,xmn}.
compute xscaling=xrefvals(1,2)-xrefvals(1,1).
end if.
do if (xrefvals(1,1) = xmn).
compute xrefvals={xrefvals,xmx}.
compute xscaling=xrefvals(1,2)-xrefvals(1,1).
end if.
end if.
end if.
do if (ncol(xrefvals)=2).
compute xscaling=xrefvals(1,2)-xrefvals(1,1).
do if (xdich=1).
compute xreferr=1.
do if (((xrefvals(1,1) = xmx) and (xrefvals(1,2)=xmn)) or ((xrefvals(1,1) = xmn) and (xrefvals(1,2)=xmx))).
compute xreferr=0.
end if.
do if (xreferr=1).
compute errcode(errs,1)=70.
compute errs=errs+1.
compute criterr=1.
end if.
end if.
end if.
end if.
end if.
do if (xmint=1 and model=74 and mcx=0).
compute xmodvals=t(xrefvals).
compute xcontcf=1.
end if.
compute nxpval=nrow(xmodvals).
compute xprobval=xmodvals.
do if (xdich =1 and mcx > 0).
compute mcx=0.
compute errcode(errs,1) = 52.
compute errs = errs + 1.
compute criterr = 1.
end if.
do if (nms > 0).
compute mtmp=dat(:,(nys+nxs+1):(nys+nxs+nms)).

.
compute desctmp=make((8-(4* 0 )),ncol( mtmp ),-999).
loop jd=1 to ncol( mtmp ).
compute descdat= mtmp (:,jd).
compute desctmp(1,jd) = csum(descdat)/nrow(descdat).
compute desctmp(2,jd) = (nrow(descdat)*sscp(descdat))-(t(csum(descdat))*(csum(descdat))).
compute desctmp(2,jd) = sqrt(desctmp(2,jd)/(nrow(descdat)*(nrow(descdat)-1))).
compute desctmp(3,jd)=cmin(descdat).
compute desctmp(4,jd)=cmax(descdat).
do if ( 0 =0).
compute minwarn=0.
compute maxwarn=0.
do if ((desctmp(3,jd)=desctmp(4,jd)) and novar=0).
compute errcode(errs,1)=15.
compute errs=errs+1.
compute criterr=1.
compute novar=1.
end if.
compute tmp=((descdat(:,1)=desctmp(3,jd))+(descdat(:,1)=desctmp(4,jd))).
compute desctmp(8,jd)=(csum(tmp)=nrow(tmp)).
compute tmp = descdat.
compute tmp(GRADE(descdat),:) = descdat.
compute descdat = tmp.
release tmp.
compute decval={.16;.5;.84}.
loop kd=1 to 3.
compute low=trunc(decval(kd,1)*(nrow(descdat)+1)).
compute lowdec=decval(kd,1)*(nrow(descdat)+1)-low.
compute value=descdat(low,1)+(descdat((low+1),1)-descdat(low,1))*lowdec.
compute desctmp((4+kd),jd)=value.
end loop.
compute mnotev=1.
compute modvals=desctmp(5:7,:).
do if (quantile <> 1).
compute desctmp(5,jd)=desctmp(1,jd)-desctmp(2,jd).
compute desctmp(6,jd)=desctmp(1,jd).
compute desctmp(7,jd)=desctmp(1,jd)+desctmp(2,jd).
compute modvals=desctmp(5:7,:).
compute mnotev=2.
do if (modvals(1,1) < desctmp(3,1)).
compute modvals(1,1)=desctmp(3,1).
compute minwarn=1.
end if.
do if (modvals(3,1) > desctmp(4,1)).
compute modvals(3,1)=desctmp(4,1).
compute maxwarn=1.
end if.
end if.
do if (desctmp(8,1)=1).
compute modvals={desctmp(3,1);desctmp(4,1)}.
compute mnotev=0.
compute minwarn=0.
compute maxwarn=0.
end if.
end if.
end loop
.
compute ovsd={desctmp(2,:),ysd}.
compute medmeans=cdeval.
do if ((cdeval(1,1) <> -999) and (ncol(medmeans) <> nms) and (model=74)).
compute errcode(errs,1)=64.
compute errs=errs+1.
compute criterr=1.
end if.
do if ((cdeval(1,1)=-999) and (model=74)).
compute medmeans=desctmp(1,:).
end if.
do if ((cdeval(1,1)<> -999) and (model=74) and (criterr=0)).
compute notecode(notes,1) = 31.
compute notes = notes + 1.
end if.
do if ((rsum(desctmp(8,:))>0) and (mdichok <> 1)).
compute errcode(errs,1)=43.
compute errs=errs+1.
compute criterr=1.
end if.
compute mmodvals=modvals.
compute mprobval=mmodvals.
end if.
do if (nws > 0).
compute wtmp=dat(:,(nys+nxs+nms+1):(nys+nxs+nms+nws)).

.
compute desctmp=make((8-(4* 0 )),ncol( wtmp ),-999).
loop jd=1 to ncol( wtmp ).
compute descdat= wtmp (:,jd).
compute desctmp(1,jd) = csum(descdat)/nrow(descdat).
compute desctmp(2,jd) = (nrow(descdat)*sscp(descdat))-(t(csum(descdat))*(csum(descdat))).
compute desctmp(2,jd) = sqrt(desctmp(2,jd)/(nrow(descdat)*(nrow(descdat)-1))).
compute desctmp(3,jd)=cmin(descdat).
compute desctmp(4,jd)=cmax(descdat).
do if ( 0 =0).
compute minwarn=0.
compute maxwarn=0.
do if ((desctmp(3,jd)=desctmp(4,jd)) and novar=0).
compute errcode(errs,1)=15.
compute errs=errs+1.
compute criterr=1.
compute novar=1.
end if.
compute tmp=((descdat(:,1)=desctmp(3,jd))+(descdat(:,1)=desctmp(4,jd))).
compute desctmp(8,jd)=(csum(tmp)=nrow(tmp)).
compute tmp = descdat.
compute tmp(GRADE(descdat),:) = descdat.
compute descdat = tmp.
release tmp.
compute decval={.16;.5;.84}.
loop kd=1 to 3.
compute low=trunc(decval(kd,1)*(nrow(descdat)+1)).
compute lowdec=decval(kd,1)*(nrow(descdat)+1)-low.
compute value=descdat(low,1)+(descdat((low+1),1)-descdat(low,1))*lowdec.
compute desctmp((4+kd),jd)=value.
end loop.
compute mnotev=1.
compute modvals=desctmp(5:7,:).
do if (quantile <> 1).
compute desctmp(5,jd)=desctmp(1,jd)-desctmp(2,jd).
compute desctmp(6,jd)=desctmp(1,jd).
compute desctmp(7,jd)=desctmp(1,jd)+desctmp(2,jd).
compute modvals=desctmp(5:7,:).
compute mnotev=2.
do if (modvals(1,1) < desctmp(3,1)).
compute modvals(1,1)=desctmp(3,1).
compute minwarn=1.
end if.
do if (modvals(3,1) > desctmp(4,1)).
compute modvals(3,1)=desctmp(4,1).
compute maxwarn=1.
end if.
end if.
do if (desctmp(8,1)=1).
compute modvals={desctmp(3,1);desctmp(4,1)}.
compute mnotev=0.
compute minwarn=0.
compute maxwarn=0.
end if.
end if.
end loop
.
compute wmodvals=modvals.
compute wdich=desctmp(8,1).
do if (wdich =1 and mcw > 0).
compute mcw=0.
compute errcode(errs,1) = 52.
compute errs = errs + 1.
compute criterr = 1.
end if.
compute wmin=desctmp(3,1).
compute wmax=desctmp(4,1).
compute minwwarn=minwarn.
compute maxwwarn=maxwarn.
compute wnotev=mnotev.
compute wmodval={ 999 }.
do if (xmint=1 and model=74 and mcx=0).
compute wmodval=xrefvals.
end if.
compute nwcontr=ncol(wmodval).
do if (wmodval(1,1) <> 999).
compute wmodvals=wmodval(1,1).
compute wmodcust=1.
do if (nwcontr > 1).
compute wmodvals=t(wmodval).
end if.
compute minwwarn=0.
compute maxwwarn=0.
compute wnotev=0.
end if.
compute wprobval=wmodvals.
compute nwpval=nrow(wmodvals).
end if.
do if (nzs > 0).
compute ztmp=dat(:,(nys+nxs+nms+nws+1):(nys+nxs+nms+nws+nzs)).

.
compute desctmp=make((8-(4* 0 )),ncol( ztmp ),-999).
loop jd=1 to ncol( ztmp ).
compute descdat= ztmp (:,jd).
compute desctmp(1,jd) = csum(descdat)/nrow(descdat).
compute desctmp(2,jd) = (nrow(descdat)*sscp(descdat))-(t(csum(descdat))*(csum(descdat))).
compute desctmp(2,jd) = sqrt(desctmp(2,jd)/(nrow(descdat)*(nrow(descdat)-1))).
compute desctmp(3,jd)=cmin(descdat).
compute desctmp(4,jd)=cmax(descdat).
do if ( 0 =0).
compute minwarn=0.
compute maxwarn=0.
do if ((desctmp(3,jd)=desctmp(4,jd)) and novar=0).
compute errcode(errs,1)=15.
compute errs=errs+1.
compute criterr=1.
compute novar=1.
end if.
compute tmp=((descdat(:,1)=desctmp(3,jd))+(descdat(:,1)=desctmp(4,jd))).
compute desctmp(8,jd)=(csum(tmp)=nrow(tmp)).
compute tmp = descdat.
compute tmp(GRADE(descdat),:) = descdat.
compute descdat = tmp.
release tmp.
compute decval={.16;.5;.84}.
loop kd=1 to 3.
compute low=trunc(decval(kd,1)*(nrow(descdat)+1)).
compute lowdec=decval(kd,1)*(nrow(descdat)+1)-low.
compute value=descdat(low,1)+(descdat((low+1),1)-descdat(low,1))*lowdec.
compute desctmp((4+kd),jd)=value.
end loop.
compute mnotev=1.
compute modvals=desctmp(5:7,:).
do if (quantile <> 1).
compute desctmp(5,jd)=desctmp(1,jd)-desctmp(2,jd).
compute desctmp(6,jd)=desctmp(1,jd).
compute desctmp(7,jd)=desctmp(1,jd)+desctmp(2,jd).
compute modvals=desctmp(5:7,:).
compute mnotev=2.
do if (modvals(1,1) < desctmp(3,1)).
compute modvals(1,1)=desctmp(3,1).
compute minwarn=1.
end if.
do if (modvals(3,1) > desctmp(4,1)).
compute modvals(3,1)=desctmp(4,1).
compute maxwarn=1.
end if.
end if.
do if (desctmp(8,1)=1).
compute modvals={desctmp(3,1);desctmp(4,1)}.
compute mnotev=0.
compute minwarn=0.
compute maxwarn=0.
end if.
end if.
end loop
.
compute zmodvals=modvals.
compute zdich=desctmp(8,1).
do if (zdich =1 and mcz > 0).
compute mcz=0.
compute errcode(errs,1) = 52.
compute errs = errs + 1.
compute criterr = 1.
end if.
compute zmin=desctmp(3,1).
compute zmax=desctmp(4,1).
compute minzwarn=minwarn.
compute maxzwarn=maxwarn.
compute znotev=mnotev.
compute zmodval={ 999 }.
compute nzcontr=ncol(zmodval).
do if (zmodval(1,1) <> 999).
compute zmodvals=zmodval(1,1).
compute zmodcust=1.
do if (nzcontr > 1).
compute zmodvals=t(zmodval).
end if.
compute minzwarn=0.
compute maxzwarn=0.
compute znotev=0.
end if.
compute zprobval=zmodvals.
compute nzpval=nrow(zmodvals).
end if.
do if (ncs > 0).
compute ctmp=dat(:,(nys+nxs+nms+nws+nzs+1):(nys+nxs+nms+nws+nzs+ncs)).

.
compute desctmp=make((8-(4* 0 )),ncol( ctmp ),-999).
loop jd=1 to ncol( ctmp ).
compute descdat= ctmp (:,jd).
compute desctmp(1,jd) = csum(descdat)/nrow(descdat).
compute desctmp(2,jd) = (nrow(descdat)*sscp(descdat))-(t(csum(descdat))*(csum(descdat))).
compute desctmp(2,jd) = sqrt(desctmp(2,jd)/(nrow(descdat)*(nrow(descdat)-1))).
compute desctmp(3,jd)=cmin(descdat).
compute desctmp(4,jd)=cmax(descdat).
do if ( 0 =0).
compute minwarn=0.
compute maxwarn=0.
do if ((desctmp(3,jd)=desctmp(4,jd)) and novar=0).
compute errcode(errs,1)=15.
compute errs=errs+1.
compute criterr=1.
compute novar=1.
end if.
compute tmp=((descdat(:,1)=desctmp(3,jd))+(descdat(:,1)=desctmp(4,jd))).
compute desctmp(8,jd)=(csum(tmp)=nrow(tmp)).
compute tmp = descdat.
compute tmp(GRADE(descdat),:) = descdat.
compute descdat = tmp.
release tmp.
compute decval={.16;.5;.84}.
loop kd=1 to 3.
compute low=trunc(decval(kd,1)*(nrow(descdat)+1)).
compute lowdec=decval(kd,1)*(nrow(descdat)+1)-low.
compute value=descdat(low,1)+(descdat((low+1),1)-descdat(low,1))*lowdec.
compute desctmp((4+kd),jd)=value.
end loop.
compute mnotev=1.
compute modvals=desctmp(5:7,:).
do if (quantile <> 1).
compute desctmp(5,jd)=desctmp(1,jd)-desctmp(2,jd).
compute desctmp(6,jd)=desctmp(1,jd).
compute desctmp(7,jd)=desctmp(1,jd)+desctmp(2,jd).
compute modvals=desctmp(5:7,:).
compute mnotev=2.
do if (modvals(1,1) < desctmp(3,1)).
compute modvals(1,1)=desctmp(3,1).
compute minwarn=1.
end if.
do if (modvals(3,1) > desctmp(4,1)).
compute modvals(3,1)=desctmp(4,1).
compute maxwarn=1.
end if.
end if.
do if (desctmp(8,1)=1).
compute modvals={desctmp(3,1);desctmp(4,1)}.
compute mnotev=0.
compute minwarn=0.
compute maxwarn=0.
end if.
end if.
end loop
.
compute covmeans=desctmp(1,:).
compute coval={ -999 }.
do if ((coval(1,1) <> -999) and (ncol(coval) <> ncs) and (model=74)).
compute errcode(errs,1)=69.
compute errs=errs+1.
compute criterr=1.
end if.
do if ((coval(1,1)<> -999) and (criterr=0) and (model=74)).
compute notecode(notes,1) = 35.
compute notes = notes + 1.
compute cuscoval=1.
end if.
end if.
compute n=nrow(ytmp).
compute ones=make(n,1,1).
do if (nws > 0 and mcw > 0).
compute tmp={rownum,wtmp(:,1)}.

.
compute dd= tmp.
compute temp = dd.
compute temp(GRADE(dd(:,2)),:) = dd.
compute dd = temp.
compute dummy = design(dd(:,2)).
compute nvls = ncol(dummy).
compute nnvls = csum(dummy).
compute mnvls = cmin(t(nnvls)).
compute conmat1=1.
do if (mnvls < 2).
compute errcode(errs,1) = 5.
compute errs = errs + 1.
compute criterr = 1.
end if.
do if (nvls > 9).
compute errcode(errs,1) = 4.
compute errs = errs+1.
compute criterr = 1.
end if.
do if (criterr = 0).
compute dumok = 1.
compute nnvls=make(nvls,1,0).
compute nnvls(1,1)=dd(1,2).
compute temp = 2.
loop i = 2 to n.
do if (dd(i,2) <> nnvls((temp-1),1)).
compute nnvls(temp,1)=dd(i,2).
compute temp = temp+1.
end if.
end loop.
do if ( mcw > 0).
compute x = dummy(:,2:ncol(dummy)).
compute nx = ncol(x).
compute minus1 = make(1,ncol(x),-1).
compute xdes=make((nx+1),3,0).
compute xdes(1,1)=dd(1,2).
compute xdes(1,2)=1.
compute temp = 2.
loop k = 2 to n.
do if (dd(k,2) <> dd((k-1),2)).
compute xdes(temp,2) = k.
compute xdes(temp,1) = dd(k,2).
compute xdes((temp-1),3) = k-1.
compute temp=temp+1.
end if.
end loop.
compute xdes((temp-1),3)=n.
compute xdes = {xdes, (xdes(:,3)-xdes(:,2)+1)}.
do if ( mcw = 4).
loop k = 1 to n.
do if (rsum(x(k,:)) = 0).
compute x(k,:) = minus1.
end if.
end loop.
end if.
do if ( mcw = 2 or mcw = 3 or mcw =5).
loop k = 1 to n.
do if (rsum(x(k,:)) > 0).
loop i = 1 to ncol(x).
do if (x(k,i) = 0).
compute x(k,i) = 1.
else.
break.
end if.
end loop.
end if.
end loop.
do if ( mcw = 3).
compute conmat1={-8,1,1,1,1,1,1,1,1; 0,-7,1,1,1,1,1,1,1; 0,0,-6,1,1,1,1,1,1; 0,0,0,-5,1,1,1,1,1; 0,0,0,0,-4,1,1,1,1; 0,0,0,0,0,-3,1,1,1; 0,0,0,0,0,0,-2,1,1; 0,0,0,0,0,0,0,-1,1}.
loop i = 1 to 8.
compute conmat1(i,:)=conmat1(i,:)/(10-i).
end loop.
compute conmat1=t(conmat1((10-nvls):8,(10-nvls):9)).
loop k=1 to n.
compute x(k,:)=conmat1((rsum(x(k,:))+1),:).
end loop.
end if.
end if.
do if ( mcw = 5).
compute custcode={ -999 }.
do if (ncol(custcode) <> (nvls*(nvls-1))).
compute errcode(errs,1) = (37+ 2 ).
compute errs = errs + 1.
compute criterr = 1.
end if.
do if (ncol(custcode) = (nvls*(nvls-1))).
compute conmat1=make(nvls,(nvls-1),0).
compute cnt=1.
loop i = 1 to nvls.
loop k = 1 to (nvls-1).
compute conmat1(i,k)=custcode(1,cnt).
compute cnt=cnt+1.
end loop.
end loop.
loop k=1 to n.
compute x(k,:)=conmat1((rsum(x(k,:))+1),:).
end loop.
end if.
end if.
compute xskip = 1.
compute dummat = make((nx+1),nx,0).
compute dummat((2:nrow(dummat)),:)=ident(nx).
do if ( mcw = 4).
compute dummat(1,:) = minus1.
end if.
do if ( mcw = 2).
loop i = 2 to nrow(dummat).
loop j = 1 to (i-1).
compute dummat(i,j) = 1.
end loop.
end loop.
end if.
do if ( mcw = 3).
compute dummat=conmat1.
end if.
do if ( mcw = 5 and criterr=0).
compute dummat=conmat1.
end if.
compute dummat={nnvls, dummat}.
compute x={dd(:,1),x}.
compute temp = x.
compute temp(GRADE(x(:,1)),:) = x.
compute x = temp.
release conmat1,temp,dd,xskip,xdes,dummy.
end if.
end if
.
compute wmodvals=nnvls.
compute nwpval=nrow(wmodvals).
do if (criterr=0).
compute minwwarn=0.
compute maxwwarn=0.
compute wnotev=0.
compute wtmp=x(:,2:ncol(x)).
compute wcatlab={'W1';'W2';'W3';'W4';'W5';'W6';'W7';'W8';'W9'}.
do if (xmint=1).
compute wcatlab={'X1';'X2';'X3';'X4';'X5';'X6';'X7';'X8';'X9'}.
end if.
compute nwvls=nvls-1.
compute mcwok=1.
compute dummatw=dummat.
compute wprobval=dummatw(:,2:ncol(dummatw)).
do if (modcok=1).
compute wcontval=make(2,ncol(wprobval),-999).
compute temp=0.
loop i = 1 to 2.
loop j = 1 to nrow(dummatw).
do if (contvec(i,1)=dummatw(j,1)).
compute wcontval(i,:)=wprobval(j,:).
compute temp=temp+1.
end if.
end loop.
end loop.
do if (temp < 2).
compute notecode(notes,1) = 20.
compute notes = notes + 1.
compute modcok=0.
end if.
end if.
do if ((wmodval(1,1) <> 999) and (xmint <> 1)).
compute notecode(notes,1) = 9.
compute notes = notes + 1.
end if.
release tmp, dummat.
end if.
end if.
do if (nzs > 0 and mcz > 0).
compute tmp={rownum,ztmp(:,1)}.

.
compute dd= tmp.
compute temp = dd.
compute temp(GRADE(dd(:,2)),:) = dd.
compute dd = temp.
compute dummy = design(dd(:,2)).
compute nvls = ncol(dummy).
compute nnvls = csum(dummy).
compute mnvls = cmin(t(nnvls)).
compute conmat1=1.
do if (mnvls < 2).
compute errcode(errs,1) = 5.
compute errs = errs + 1.
compute criterr = 1.
end if.
do if (nvls > 9).
compute errcode(errs,1) = 4.
compute errs = errs+1.
compute criterr = 1.
end if.
do if (criterr = 0).
compute dumok = 1.
compute nnvls=make(nvls,1,0).
compute nnvls(1,1)=dd(1,2).
compute temp = 2.
loop i = 2 to n.
do if (dd(i,2) <> nnvls((temp-1),1)).
compute nnvls(temp,1)=dd(i,2).
compute temp = temp+1.
end if.
end loop.
do if ( mcz > 0).
compute x = dummy(:,2:ncol(dummy)).
compute nx = ncol(x).
compute minus1 = make(1,ncol(x),-1).
compute xdes=make((nx+1),3,0).
compute xdes(1,1)=dd(1,2).
compute xdes(1,2)=1.
compute temp = 2.
loop k = 2 to n.
do if (dd(k,2) <> dd((k-1),2)).
compute xdes(temp,2) = k.
compute xdes(temp,1) = dd(k,2).
compute xdes((temp-1),3) = k-1.
compute temp=temp+1.
end if.
end loop.
compute xdes((temp-1),3)=n.
compute xdes = {xdes, (xdes(:,3)-xdes(:,2)+1)}.
do if ( mcz = 4).
loop k = 1 to n.
do if (rsum(x(k,:)) = 0).
compute x(k,:) = minus1.
end if.
end loop.
end if.
do if ( mcz = 2 or mcz = 3 or mcz =5).
loop k = 1 to n.
do if (rsum(x(k,:)) > 0).
loop i = 1 to ncol(x).
do if (x(k,i) = 0).
compute x(k,i) = 1.
else.
break.
end if.
end loop.
end if.
end loop.
do if ( mcz = 3).
compute conmat1={-8,1,1,1,1,1,1,1,1; 0,-7,1,1,1,1,1,1,1; 0,0,-6,1,1,1,1,1,1; 0,0,0,-5,1,1,1,1,1; 0,0,0,0,-4,1,1,1,1; 0,0,0,0,0,-3,1,1,1; 0,0,0,0,0,0,-2,1,1; 0,0,0,0,0,0,0,-1,1}.
loop i = 1 to 8.
compute conmat1(i,:)=conmat1(i,:)/(10-i).
end loop.
compute conmat1=t(conmat1((10-nvls):8,(10-nvls):9)).
loop k=1 to n.
compute x(k,:)=conmat1((rsum(x(k,:))+1),:).
end loop.
end if.
end if.
do if ( mcz = 5).
compute custcode={ -999 }.
do if (ncol(custcode) <> (nvls*(nvls-1))).
compute errcode(errs,1) = (37+ 3 ).
compute errs = errs + 1.
compute criterr = 1.
end if.
do if (ncol(custcode) = (nvls*(nvls-1))).
compute conmat1=make(nvls,(nvls-1),0).
compute cnt=1.
loop i = 1 to nvls.
loop k = 1 to (nvls-1).
compute conmat1(i,k)=custcode(1,cnt).
compute cnt=cnt+1.
end loop.
end loop.
loop k=1 to n.
compute x(k,:)=conmat1((rsum(x(k,:))+1),:).
end loop.
end if.
end if.
compute xskip = 1.
compute dummat = make((nx+1),nx,0).
compute dummat((2:nrow(dummat)),:)=ident(nx).
do if ( mcz = 4).
compute dummat(1,:) = minus1.
end if.
do if ( mcz = 2).
loop i = 2 to nrow(dummat).
loop j = 1 to (i-1).
compute dummat(i,j) = 1.
end loop.
end loop.
end if.
do if ( mcz = 3).
compute dummat=conmat1.
end if.
do if ( mcz = 5 and criterr=0).
compute dummat=conmat1.
end if.
compute dummat={nnvls, dummat}.
compute x={dd(:,1),x}.
compute temp = x.
compute temp(GRADE(x(:,1)),:) = x.
compute x = temp.
release conmat1,temp,dd,xskip,xdes,dummy.
end if.
end if
.
compute zmodvals=nnvls.
compute nzpval=nrow(zmodvals).
do if (criterr=0).
compute minzwarn=0.
compute maxzwarn=0.
compute znotev=0.
compute ztmp=x(:,2:ncol(x)).
compute zcatlab={'Z1';'Z2';'Z3';'Z4';'Z5';'Z6';'Z7';'Z8';'Z9'}.
compute nzvls=nvls-1.
compute mczok=1.
compute dummatz=dummat.
compute zprobval=dummatz(:,2:ncol(dummatz)).
do if (modcok=1).
compute zcontval=make(2,ncol(zprobval),-999).
compute temp=0.
loop i = 1 to 2.
loop j = 1 to nrow(dummatz).
do if (contvec(i,2)=dummatz(j,1)).
compute zcontval(i,:)=zprobval(j,:).
compute temp=temp+1.
end if.
end loop.
end loop.
do if (temp < 2).
compute notecode(notes,1) = 20.
compute notes = notes + 1.
compute modcok=0.
end if.
end if.
do if (zmodval(1,1) <> 999).
compute notecode(notes,1) = 10.
compute notes = notes + 1.
end if.
release tmp, dummat.
end if.
end if.
do if (nxs > 0 and mcx > 0).
compute tmp={rownum,xtmp(:,1)}.

.
compute dd= tmp.
compute temp = dd.
compute temp(GRADE(dd(:,2)),:) = dd.
compute dd = temp.
compute dummy = design(dd(:,2)).
compute nvls = ncol(dummy).
compute nnvls = csum(dummy).
compute mnvls = cmin(t(nnvls)).
compute conmat1=1.
do if (mnvls < 2).
compute errcode(errs,1) = 5.
compute errs = errs + 1.
compute criterr = 1.
end if.
do if (nvls > 9).
compute errcode(errs,1) = 4.
compute errs = errs+1.
compute criterr = 1.
end if.
do if (criterr = 0).
compute dumok = 1.
compute nnvls=make(nvls,1,0).
compute nnvls(1,1)=dd(1,2).
compute temp = 2.
loop i = 2 to n.
do if (dd(i,2) <> nnvls((temp-1),1)).
compute nnvls(temp,1)=dd(i,2).
compute temp = temp+1.
end if.
end loop.
do if ( mcx > 0).
compute x = dummy(:,2:ncol(dummy)).
compute nx = ncol(x).
compute minus1 = make(1,ncol(x),-1).
compute xdes=make((nx+1),3,0).
compute xdes(1,1)=dd(1,2).
compute xdes(1,2)=1.
compute temp = 2.
loop k = 2 to n.
do if (dd(k,2) <> dd((k-1),2)).
compute xdes(temp,2) = k.
compute xdes(temp,1) = dd(k,2).
compute xdes((temp-1),3) = k-1.
compute temp=temp+1.
end if.
end loop.
compute xdes((temp-1),3)=n.
compute xdes = {xdes, (xdes(:,3)-xdes(:,2)+1)}.
do if ( mcx = 4).
loop k = 1 to n.
do if (rsum(x(k,:)) = 0).
compute x(k,:) = minus1.
end if.
end loop.
end if.
do if ( mcx = 2 or mcx = 3 or mcx =5).
loop k = 1 to n.
do if (rsum(x(k,:)) > 0).
loop i = 1 to ncol(x).
do if (x(k,i) = 0).
compute x(k,i) = 1.
else.
break.
end if.
end loop.
end if.
end loop.
do if ( mcx = 3).
compute conmat1={-8,1,1,1,1,1,1,1,1; 0,-7,1,1,1,1,1,1,1; 0,0,-6,1,1,1,1,1,1; 0,0,0,-5,1,1,1,1,1; 0,0,0,0,-4,1,1,1,1; 0,0,0,0,0,-3,1,1,1; 0,0,0,0,0,0,-2,1,1; 0,0,0,0,0,0,0,-1,1}.
loop i = 1 to 8.
compute conmat1(i,:)=conmat1(i,:)/(10-i).
end loop.
compute conmat1=t(conmat1((10-nvls):8,(10-nvls):9)).
loop k=1 to n.
compute x(k,:)=conmat1((rsum(x(k,:))+1),:).
end loop.
end if.
end if.
do if ( mcx = 5).
compute custcode={ -999 }.
do if (ncol(custcode) <> (nvls*(nvls-1))).
compute errcode(errs,1) = (37+ 1 ).
compute errs = errs + 1.
compute criterr = 1.
end if.
do if (ncol(custcode) = (nvls*(nvls-1))).
compute conmat1=make(nvls,(nvls-1),0).
compute cnt=1.
loop i = 1 to nvls.
loop k = 1 to (nvls-1).
compute conmat1(i,k)=custcode(1,cnt).
compute cnt=cnt+1.
end loop.
end loop.
loop k=1 to n.
compute x(k,:)=conmat1((rsum(x(k,:))+1),:).
end loop.
end if.
end if.
compute xskip = 1.
compute dummat = make((nx+1),nx,0).
compute dummat((2:nrow(dummat)),:)=ident(nx).
do if ( mcx = 4).
compute dummat(1,:) = minus1.
end if.
do if ( mcx = 2).
loop i = 2 to nrow(dummat).
loop j = 1 to (i-1).
compute dummat(i,j) = 1.
end loop.
end loop.
end if.
do if ( mcx = 3).
compute dummat=conmat1.
end if.
do if ( mcx = 5 and criterr=0).
compute dummat=conmat1.
end if.
compute dummat={nnvls, dummat}.
compute x={dd(:,1),x}.
compute temp = x.
compute temp(GRADE(x(:,1)),:) = x.
compute x = temp.
release conmat1,temp,dd,xskip,xdes,dummy.
end if.
end if
.
do if (criterr=0).
compute xtmp=x(:,2:ncol(x)).
compute xcatlab={'X1';'X2';'X3';'X4';'X5';'X6';'X7';'X8';'X9'}.
compute nxvls=nvls-1.
compute xdich=(nvls=2).
compute mcxok=1.
compute dummatx=dummat.
compute xmodvals=dummatx(:,1).
compute nxpval=nrow(xmodvals).
release tmp, dummat.
end if.
end if.
compute intlab=make(100,1,' ').


compute intlab( 1 ,1)= 'Int_1'.


compute intlab( 2 ,1)= 'Int_2'.


compute intlab( 3 ,1)= 'Int_3'.


compute intlab( 4 ,1)= 'Int_4'.


compute intlab( 5 ,1)= 'Int_5'.


compute intlab( 6 ,1)= 'Int_6'.


compute intlab( 7 ,1)= 'Int_7'.


compute intlab( 8 ,1)= 'Int_8'.


compute intlab( 9 ,1)= 'Int_9'.


compute intlab( 10 ,1)= 'Int_10'.


compute intlab( 11 ,1)= 'Int_11'.


compute intlab( 12 ,1)= 'Int_12'.


compute intlab( 13 ,1)= 'Int_13'.


compute intlab( 14 ,1)= 'Int_14'.


compute intlab( 15 ,1)= 'Int_15'.


compute intlab( 16 ,1)= 'Int_16'.


compute intlab( 17 ,1)= 'Int_17'.


compute intlab( 18 ,1)= 'Int_18'.


compute intlab( 19 ,1)= 'Int_19'.


compute intlab( 20 ,1)= 'Int_20'.


compute intlab( 21 ,1)= 'Int_21'.


compute intlab( 22 ,1)= 'Int_22'.


compute intlab( 23 ,1)= 'Int_23'.


compute intlab( 24 ,1)= 'Int_24'.


compute intlab( 25 ,1)= 'Int_25'.


compute intlab( 26 ,1)= 'Int_26'.


compute intlab( 27 ,1)= 'Int_27'.


compute intlab( 28 ,1)= 'Int_28'.


compute intlab( 29 ,1)= 'Int_29'.


compute intlab( 30 ,1)= 'Int_30'.


compute intlab( 31 ,1)= 'Int_31'.


compute intlab( 32 ,1)= 'Int_32'.


compute intlab( 33 ,1)= 'Int_33'.


compute intlab( 34 ,1)= 'Int_34'.


compute intlab( 35 ,1)= 'Int_35'.


compute intlab( 36 ,1)= 'Int_36'.


compute intlab( 37 ,1)= 'Int_37'.


compute intlab( 38 ,1)= 'Int_38'.


compute intlab( 39 ,1)= 'Int_39'.


compute intlab( 40 ,1)= 'Int_40'.


compute intlab( 41 ,1)= 'Int_41'.


compute intlab( 42 ,1)= 'Int_42'.


compute intlab( 43 ,1)= 'Int_43'.


compute intlab( 44 ,1)= 'Int_44'.


compute intlab( 45 ,1)= 'Int_45'.


compute intlab( 46 ,1)= 'Int_46'.


compute intlab( 47 ,1)= 'Int_47'.


compute intlab( 48 ,1)= 'Int_48'.


compute intlab( 49 ,1)= 'Int_49'.


compute intlab( 50 ,1)= 'Int_50'.


compute intlab( 51 ,1)= 'Int_51'.


compute intlab( 52 ,1)= 'Int_52'.


compute intlab( 53 ,1)= 'Int_53'.


compute intlab( 54 ,1)= 'Int_54'.


compute intlab( 55 ,1)= 'Int_55'.


compute intlab( 56 ,1)= 'Int_56'.


compute intlab( 57 ,1)= 'Int_57'.


compute intlab( 58 ,1)= 'Int_58'.


compute intlab( 59 ,1)= 'Int_59'.


compute intlab( 60 ,1)= 'Int_60'.


compute intlab( 61 ,1)= 'Int_61'.


compute intlab( 62 ,1)= 'Int_62'.


compute intlab( 63 ,1)= 'Int_63'.


compute intlab( 64 ,1)= 'Int_64'.


compute intlab( 65 ,1)= 'Int_65'.


compute intlab( 66 ,1)= 'Int_66'.


compute intlab( 67 ,1)= 'Int_67'.


compute intlab( 68 ,1)= 'Int_68'.


compute intlab( 69 ,1)= 'Int_69'.


compute intlab( 70 ,1)= 'Int_70'.


compute intlab( 71 ,1)= 'Int_71'.


compute intlab( 72 ,1)= 'Int_72'.


compute intlab( 73 ,1)= 'Int_73'.


compute intlab( 74 ,1)= 'Int_74'.


compute intlab( 75 ,1)= 'Int_75'.


compute intlab( 76 ,1)= 'Int_76'.


compute intlab( 77 ,1)= 'Int_77'.


compute intlab( 78 ,1)= 'Int_78'.


compute intlab( 79 ,1)= 'Int_79'.


compute intlab( 80 ,1)= 'Int_80'.


compute intlab( 81 ,1)= 'Int_81'.


compute intlab( 82 ,1)= 'Int_82'.


compute intlab( 83 ,1)= 'Int_83'.


compute intlab( 84 ,1)= 'Int_84'.


compute intlab( 85 ,1)= 'Int_85'.


compute intlab( 86 ,1)= 'Int_86'.


compute intlab( 87 ,1)= 'Int_87'.


compute intlab( 88 ,1)= 'Int_88'.


compute intlab( 89 ,1)= 'Int_89'.


compute intlab( 90 ,1)= 'Int_90'.


compute intlab( 91 ,1)= 'Int_91'.


compute intlab( 92 ,1)= 'Int_92'.


compute intlab( 93 ,1)= 'Int_93'.


compute intlab( 94 ,1)= 'Int_94'.


compute intlab( 95 ,1)= 'Int_95'.


compute intlab( 96 ,1)= 'Int_96'.


compute intlab( 97 ,1)= 'Int_97'.


compute intlab( 98 ,1)= 'Int_98'.


compute intlab( 99 ,1)= 'Int_99'.


compute intlab( 100 ,1)= 'Int_100'.

compute bcmat=make(needed,needed,0).
compute wcmat=make(needed,needed,0).
compute zcmat=make(needed,needed,0).
compute wzcmat=make(needed,needed,0).
compute wsum=0.
compute zsum=0.
compute wzsum=0.
end if.
end if.
do if (criterr = 0 and model <> 999).
compute modelmat= {1,0,0,0,0,0,0,1,0,0;2,0,0,0,0,0,0,1,1,0;3,0,0,0,0,0,0,1,1,1;4,0,0,0,0,0,0,0,0,0; 5,0,0,0,0,0,0,1,0,0;6,0,0,0,0,0,0,0,0,0;7,1,0,0,0,0,0,0,0,0;8,1,0,0,0,0,0,1,0,0; 9,1,1,0,0,0,0,0,0,0;10,1,1,0,0,0,0,1,1,0;11,1,1,1,0,0,0,0,0,0;12,1,1,1,0,0,0,1,1,1; 13,1,1,1,0,0,0,1,0,0;14,0,0,0,1,0,0,0,0,0;15,0,0,0,1,0,0,1,0,0;16,0,0,0,1,1,0,0,0,0; 17,0,0,0,1,1,0,1,1,0;18,0,0,0,1,1,1,0,0,0;19,0,0,0,1,1,1,1,1,1;20,0,0,0,1,1,1,1,0,0; 21,1,0,0,0,1,0,0,0,0;22,1,0,0,0,1,0,1,0,0;23,0,0,0,0,0,0,0,0,0;24,0,0,0,0,0,0,0,0,0; 25,0,0,0,0,0,0,0,0,0;26,0,0,0,0,0,0,0,0,0;27,0,0,0,0,0,0,0,0,0;28,1,0,0,0,1,0,0,1,0; 29,1,0,0,0,1,0,1,1,0;30,0,0,0,0,0,0,0,0,0;31,0,0,0,0,0,0,0,0,0;32,0,0,0,0,0,0,0,0,0; 33,0,0,0,0,0,0,0,0,0;34,0,0,0,0,0,0,0,0,0;35,0,0,0,0,0,0,0,0,0;36,0,0,0,0,0,0,0,0,0; 37,0,0,0,0,0,0,0,0,0;38,0,0,0,0,0,0,0,0,0;39,0,0,0,0,0,0,0,0,0;40,0,0,0,0,0,0,0,0,0; 41,0,0,0,0,0,0,0,0,0;42,0,0,0,0,0,0,0,0,0;43,0,0,0,0,0,0,0,0,0;44,0,0,0,0,0,0,0,0,0; 45,0,0,0,0,0,0,0,0,0;46,0,0,0,0,0,0,0,0,0;47,0,0,0,0,0,0,0,0,0;48,0,0,0,0,0,0,0

 ,0,0; 49,0,0,0,0,0,0,0,0,0;50,0,0,0,0,0,0,0,0,0;51,0,0,0,0,0,0,0,0,0;52,0,0,0,0,0,0,0,0,0; 53,0,0,0,0,0,0,0,0,0;54,0,0,0,0,0,0,0,0,0;55,0,0,0,0,0,0,0,0,0;56,0,0,0,0,0,0,0,0,0; 57,0,0,0,0,0,0,0,0,0;58,1,0,0,1,0,0,0,0,0;59,1,0,0,1,0,0,1,0,0;60,1,1,0,1,0,0,0,0,0; 61,1,1,0,1,0,0,1,0,0;62,1,1,0,1,0,0,0,1,0;63,1,1,0,1,0,0,1,1,0;64,1,0,0,1,1,0,0,0,0; 65,1,0,0,1,1,0,1,0,0;66,1,0,0,1,1,0,0,1,0;67,1,0,0,1,1,0,1,1,0;68,1,1,1,1,0,0,0,0,0; 69,1,1,1,1,0,0,1,1,1;70,1,0,0,1,1,1,0,0,0;71,1,0,0,1,1,1,1,1,1;72,1,1,1,1,1,1,0,0,0; 73,1,1,1,1,1,1,1,1,1;74,0,0,0,1,0,0,0,0,0;75,1,1,0,1,1,0,0,0,0;76,1,1,0,1,1,0,1,1,0; 77,0,0,0,0,0,0,0,0,0;78,0,0,0,0,0,0,0,0,0;79,0,0,0,0,0,0,0,0,0;80,0,0,0,0,0,0,0,0,0; 81,0,0,0,0,0,0,0,0,0;82,0,0,0,0,0,0,0,0,0;83,1,0,0,0,0,0,0,0,0;84,1,0,0,0,0,0,0,0,0; 85,1,0,0,0,0,0,1,0,0;86,1,0,0,0,0,0,1,0,0;87,0,0,0,1,0,0,0,0,0;88,0,0,0,1,0,0,0,0,0; 89,0,0,0,1,0,0,1,0,0;90,0,0,0,1,0,0,1,0,0;91,0,0,0,0,0,0,0,0,0;92,1,0,0,1,0,0,1,0,0}.
do if (model > 0).
compute tmp=modelmat(model,2:ncol(modelmat)).
end if.
do if (model=0).
compute tmp={0,0,0,0,0,0,0,0,0}.
end if.
do if (model < 4).
compute bcmat((nxs+1),1)=1.
end if.
do if ((model > 3) and (model <> 6)).
compute bcmat((nxs+1):(nxs+nms),1)=onem.
compute bcmat(nrow(bcmat),(nxs+1):(nxs+nms))=t(onem).
compute bcmat(nrow(bcmat),1)=1.
end if.
do if ((model = 6) or (model > 82 and model < 93)).
loop j = 2 to nrow(bcmat).
loop i = 1 to (j-1).
compute bcmat(j,i)=1.
end loop.
end loop.
end if.
do if (model = 80).
loop i = 1 to nms.
compute bcmat((nrow(bcmat)-1),i)=1.
end loop.
end if.
do if (model = 81).
loop j = 3 to nrow(bcmat).
compute bcmat(j,2)=1.
end loop.
end if.
do if (model = 82).
compute bcmat(3,2)=1.
compute bcmat(5,4)=1.
end if.
do if (tmp(1,1)=1).
compute wcmat((nxs+1):(nxs+nms),1)=onem.
compute wprod=1.
compute xprod=1.
do if (model = 83 or model = 86).
compute onemsx=onem.
loop i = 1 to (nms-1).
compute onemsx(i+1,1)=0.
end loop.
compute wcmat((nxs+1):(nxs+nms),1)=onemsx.
end if.
end if.
do if (tmp(1,4)=1).
compute wcmat(nrow(wcmat),(nxs+1):(nxs+nms))=t(onem).
compute wprod=1.
do if (model = 87 or model = 90).
compute onemsx=onem.
loop i = 1 to (nms-1).
compute onemsx(i,1)=0.
end loop.
compute wcmat(nrow(wcmat),(nxs+1):(nxs+nms))=t(onemsx).
end if.
end if.
do if (tmp(1,7)=1).
compute wcmat(nrow(wcmat),1)=1.
compute wprod=1.
compute xprod=1.
end if.
do if (tmp(1,2)=1).
compute zcmat((nxs+1):(nxs+nms),1)=onem.
compute zprod=1.
compute xprod=1.
end if.
do if (tmp(1,5)=1).
compute zcmat(nrow(zcmat),(nxs+1):(nxs+nms))=t(onem).
compute zprod=1.
end if.
do if (tmp(1,8)=1).
compute zcmat(nrow(zcmat),1)=1.
compute zprod=1.
compute xprod=1.
end if.
do if (tmp(1,3)=1).
compute wzcmat((nxs+1):(nxs+nms),1)=onem.
compute xprod=1.
compute wprod=1.
compute zprod=1.
end if.
do if (tmp(1,6)=1).
compute wzcmat(nrow(wzcmat),(nxs+1):(nxs+nms))=t(onem).
compute zprod=1.
compute wprod=1.
end if.
do if (tmp(1,9)=1).
compute wzcmat(nrow(wzcmat),1)=1.
compute xprod=1.
compute wprod=1.
compute zprod=1.
end if.
do if (model = 91 or model = 92).
loop j = 1 to (nms-1).
loop i = 1 to j.
compute wcmat((nxs+1+j),(nxs+i))=1.
end loop.
end loop.
end if.
do if (nms < 0).
loop i = 1 to nms.
compute tmp=csum(wcmat(:,(1+i)))+csum(zcmat(:,(1+i)))+csum(wzcmat(:,(1+i))).
compute mprod(1,i)=(tmp>0).
end loop.
end if.
end if.
do if (ncs > 0).
compute ccmat=make((nms+nys),ncs,1).
compute ccmatoff=ccmat.
do if (covmy=1).
compute ccmat(nrow(ccmat),:)=make(1,ncs,0).
end if.
do if (covmy=2).
compute ccmat(1:nms,:)=make(nms,ncs,0).
end if.
do if (cmatrix(1,1) <> -999).
do if (ncol(cmatrix) <> ((nms+nys)*ncs)).
compute errcode(errs,1)=29.
compute errs=errs+1.
compute criterr=1.
end if.
do if (criterr = 0).
compute tmp=1.
loop i = 1 to (nms+nys).
loop j = 1 to ncs.
compute ccmat(i,j)=1-(cmatrix(1,tmp) = 0).
compute tmp=tmp+1.
end loop.
end loop.
do if (rsum((csum(ccmat)=0)) <> 0).
compute errcode(errs,1)=30.
compute errs=errs+1.
compute criterr=1.
end if.
end if.
do if (covmy <> 0).
compute notecode(notes,1)=1.
compute notes=notes+1.
end if.
end if.
do if (xmint=1 and (covmy > 0 or (csum(rsum(ccmat)) <> ((nms+nys)*ncs)))).
compute errcode(errs,1)=68.
compute errs=errs+1.
compute criterr=1.
end if.
end if.
do if (criterr=0).
compute needed=needed*(needed-1)/2.
compute nopath=0.
do if (bmatrix(1,1) <> -999).
compute tmp=1.
do if ((ncol(bmatrix) <> needed) or (csum(rsum(bmatrix))=0)).
compute errcode(errs,1)=16.
compute errs=errs+1.
compute criterr=1.
else.
loop i = 2 to nrow(bcmat).
loop j = 1 to (i-1).
compute bcmat(i,j)=1-(bmatrix(1,tmp) = 0).
compute tmp=tmp+1.
end loop.
end loop.
end if.
do if ((csum(bcmat(:,1))=0) and criterr=0).
compute errcode(errs,1)=22.
compute errs=errs+1.
compute criterr=1.
end if.
do if ((rsum(bcmat(nrow(bcmat),:))=0) and criterr=0).
compute errcode(errs,1)=23.
compute errs=errs+1.
compute criterr=1.
end if.
compute dm=0.
do if (nms > 0).
loop i = 1 to nms.
do if (((rsum(bcmat((nxs+i),:)) = 0) or (csum(bcmat(:,(nxs+i))) = 0)) and (dm=0) and (criterr=0)).
compute errcode(errs,1)=26.
compute errs=errs+1.
compute criterr=1.
compute dm=1.
end if.
end loop.
end if.
release dm.
end if.
end if.
do if (criterr=0).
do if (wmatrix(1,1) <> -999).
compute tmp=1.
do if (ncol(wmatrix) <> needed).
compute errcode(errs,1)=17.
compute errs=errs+1.
compute criterr=1.
else.
compute modelvar(1,1)='CUSTOM'.
loop i = 2 to nrow(wcmat).
loop j = 1 to (i-1).
compute wcmat(i,j)=1-(wmatrix(1,tmp) = 0).
do if ((wcmat(i,j)=1) and (bcmat(i,j)=0) and (nopath=0)).
compute errcode(errs,1)=20.
compute errs=errs+1.
compute criterr=1.
compute nopath=1.
end if.
compute tmp=tmp+1.
end loop.
end loop.
end if.
end if.
do if (zmatrix(1,1) <> -999).
compute tmp=1.
do if (ncol(zmatrix) <> needed).
compute errcode(errs,1)=18.
compute errs=errs+1.
compute criterr=1.
else.
compute modelvar(1,1)='CUSTOM'.
do if (csum(rsum(wcmat))=0 and model=999).
compute errcode(errs,1)=21.
compute errs=errs+1.
compute criterr=1.
end if.
loop i = 2 to nrow(zcmat).
loop j = 1 to (i-1).
compute zcmat(i,j)=1-(zmatrix(1,tmp) = 0).
do if ((zcmat(i,j)=1) and (bcmat(i,j)=0) and (nopath=0)).
compute errcode(errs,1)=20.
compute errs=errs+1.
compute criterr=1.
compute nopath=1.
end if.
compute tmp=tmp+1.
end loop.
end loop.
end if.
end if.
compute tmp=1.
do if (wzmatrix(1,1) <> -999).
do if (ncol(wzmatrix) <> needed).
compute errcode(errs,1)=19.
compute errs=errs+1.
compute criterr=1.
end if.
compute modelvar(1,1)='CUSTOM'.
end if.
do if (criterr=0).
loop i = 2 to nrow(wzcmat).
loop j = 1 to (i-1).
do if (wzmatrix(1,1) <> -999).
compute wzcmat(i,j)=1-(wzmatrix(1,tmp) = 0).
end if.
do if (wzcmat(i,j)=1).
compute wcmat(i,j)=1.
compute zcmat(i,j)=1.
end if.
do if ((wzcmat(i,j)=1) and (bcmat(i,j)=0) and (nopath=0)).
compute errcode(errs,1)=20.
compute errs=errs+1.
compute criterr=1.
compute nopath=1.
end if.
compute tmp=tmp+1.
end loop.
end loop.
end if.
end if.
do if (criterr=0).
compute xprod=csum(wcmat(:,1))+csum(zcmat(:,1))+csum(wzcmat(:,1)).
compute xprod=(xprod > 0).
compute wsum=csum(rsum(wcmat)).
compute wprod=(wsum > 0).
do if (nms > 0).
loop i = 1 to nms.
compute tmp=csum(wcmat(:,(1+i)))+csum(zcmat(:,(1+i)))+csum(wzcmat(:,(1+i))).
compute mprod(1,i)=(tmp>0).
end loop.
end if.
do if ((wsum > 0) and (w = 'xxxxx')).
compute errcode(errs,1)=11.
compute errs=errs+1.
compute criterr=1.
end if.
do if ((wsum = 0) and (w <> 'xxxxx')).
compute errcode(errs,1)=10.
compute errs=errs+1.
compute criterr=1.
end if.
compute zsum=csum(rsum(zcmat)).
compute zprod=(zsum > 0).
do if ((zsum > 0) and (z = 'xxxxx')).
compute errcode(errs,1)=13.
compute errs=errs+1.
compute criterr=1.
end if.
do if ((zsum = 0) and (z <> 'xxxxx')).
compute errcode(errs,1)=12.
compute errs=errs+1.
compute criterr=1.
end if.
do if ((zsum > 0) and (wsum = 0)).
compute errcode(errs,1)=35.
compute errs=errs+1.
compute criterr=1.
end if.
end if.
do if (criterr=0 and nms > 1).
compute serchk=bcmat(2:(nrow(bcmat)-1),2:ncol(bcmat)).
do if (csum(rsum(serchk))) > 0.
compute serial=1.
do if (nms > 6).
compute errcode(errs,1)=36.
compute errs=errs+1.
compute criterr=1.
end if.
end if.
end if.
do if (center > 0 and criterr=0).
compute centvar={' '}.
do if (criterr=0).
do if ((center = 1) or (center = 2 and wdich = 0)).
do if (wprod=1 and mcwok=0 and nwpval > 0).
loop i = 1 to nws.
compute wtmp(:,i)=wtmp(:,i)-(csum(wtmp(:,i))/n).
compute centvar={centvar,wnames(1,i)}.
end loop.

.
compute desctmp=make((8-(4* wmodcust )),ncol( wtmp ),-999).
loop jd=1 to ncol( wtmp ).
compute descdat= wtmp (:,jd).
compute desctmp(1,jd) = csum(descdat)/nrow(descdat).
compute desctmp(2,jd) = (nrow(descdat)*sscp(descdat))-(t(csum(descdat))*(csum(descdat))).
compute desctmp(2,jd) = sqrt(desctmp(2,jd)/(nrow(descdat)*(nrow(descdat)-1))).
compute desctmp(3,jd)=cmin(descdat).
compute desctmp(4,jd)=cmax(descdat).
do if ( wmodcust =0).
compute minwarn=0.
compute maxwarn=0.
do if ((desctmp(3,jd)=desctmp(4,jd)) and novar=0).
compute errcode(errs,1)=15.
compute errs=errs+1.
compute criterr=1.
compute novar=1.
end if.
compute tmp=((descdat(:,1)=desctmp(3,jd))+(descdat(:,1)=desctmp(4,jd))).
compute desctmp(8,jd)=(csum(tmp)=nrow(tmp)).
compute tmp = descdat.
compute tmp(GRADE(descdat),:) = descdat.
compute descdat = tmp.
release tmp.
compute decval={.16;.5;.84}.
loop kd=1 to 3.
compute low=trunc(decval(kd,1)*(nrow(descdat)+1)).
compute lowdec=decval(kd,1)*(nrow(descdat)+1)-low.
compute value=descdat(low,1)+(descdat((low+1),1)-descdat(low,1))*lowdec.
compute desctmp((4+kd),jd)=value.
end loop.
compute mnotev=1.
compute modvals=desctmp(5:7,:).
do if (quantile <> 1).
compute desctmp(5,jd)=desctmp(1,jd)-desctmp(2,jd).
compute desctmp(6,jd)=desctmp(1,jd).
compute desctmp(7,jd)=desctmp(1,jd)+desctmp(2,jd).
compute modvals=desctmp(5:7,:).
compute mnotev=2.
do if (modvals(1,1) < desctmp(3,1)).
compute modvals(1,1)=desctmp(3,1).
compute minwarn=1.
end if.
do if (modvals(3,1) > desctmp(4,1)).
compute modvals(3,1)=desctmp(4,1).
compute maxwarn=1.
end if.
end if.
do if (desctmp(8,1)=1).
compute modvals={desctmp(3,1);desctmp(4,1)}.
compute mnotev=0.
compute minwarn=0.
compute maxwarn=0.
end if.
end if.
end loop
.
compute wmin=desctmp(3,1).
compute wmax=desctmp(4,1).
do if (wmodcust=0).
compute wmodvals=modvals.
compute wprobval=wmodvals.
end if.
end if.
end if.
do if ((center = 1) or (center = 2 and zdich = 0)).
do if (zprod=1 and mczok=0 and nzpval > 0).
loop i = 1 to nzs.
compute ztmp(:,i)=ztmp(:,i)-(csum(ztmp(:,i))/n).
compute centvar={centvar,znames(1,i)}.
end loop.

.
compute desctmp=make((8-(4* zmodcust )),ncol( ztmp ),-999).
loop jd=1 to ncol( ztmp ).
compute descdat= ztmp (:,jd).
compute desctmp(1,jd) = csum(descdat)/nrow(descdat).
compute desctmp(2,jd) = (nrow(descdat)*sscp(descdat))-(t(csum(descdat))*(csum(descdat))).
compute desctmp(2,jd) = sqrt(desctmp(2,jd)/(nrow(descdat)*(nrow(descdat)-1))).
compute desctmp(3,jd)=cmin(descdat).
compute desctmp(4,jd)=cmax(descdat).
do if ( zmodcust =0).
compute minwarn=0.
compute maxwarn=0.
do if ((desctmp(3,jd)=desctmp(4,jd)) and novar=0).
compute errcode(errs,1)=15.
compute errs=errs+1.
compute criterr=1.
compute novar=1.
end if.
compute tmp=((descdat(:,1)=desctmp(3,jd))+(descdat(:,1)=desctmp(4,jd))).
compute desctmp(8,jd)=(csum(tmp)=nrow(tmp)).
compute tmp = descdat.
compute tmp(GRADE(descdat),:) = descdat.
compute descdat = tmp.
release tmp.
compute decval={.16;.5;.84}.
loop kd=1 to 3.
compute low=trunc(decval(kd,1)*(nrow(descdat)+1)).
compute lowdec=decval(kd,1)*(nrow(descdat)+1)-low.
compute value=descdat(low,1)+(descdat((low+1),1)-descdat(low,1))*lowdec.
compute desctmp((4+kd),jd)=value.
end loop.
compute mnotev=1.
compute modvals=desctmp(5:7,:).
do if (quantile <> 1).
compute desctmp(5,jd)=desctmp(1,jd)-desctmp(2,jd).
compute desctmp(6,jd)=desctmp(1,jd).
compute desctmp(7,jd)=desctmp(1,jd)+desctmp(2,jd).
compute modvals=desctmp(5:7,:).
compute mnotev=2.
do if (modvals(1,1) < desctmp(3,1)).
compute modvals(1,1)=desctmp(3,1).
compute minwarn=1.
end if.
do if (modvals(3,1) > desctmp(4,1)).
compute modvals(3,1)=desctmp(4,1).
compute maxwarn=1.
end if.
end if.
do if (desctmp(8,1)=1).
compute modvals={desctmp(3,1);desctmp(4,1)}.
compute mnotev=0.
compute minwarn=0.
compute maxwarn=0.
end if.
end if.
end loop
.
compute zmin=desctmp(3,1).
compute zmax=desctmp(4,1).
do if (zmodcust=0).
compute zmodvals=modvals.
compute zprobval=zmodvals.
end if.
end if.
end if.
do if ((center = 1) or (center = 2 and xdich = 0)).
do if (xprod=1 and mcxok=0).
loop i = 1 to nxs.
compute xtmp(:,i)=xtmp(:,i)-(csum(xtmp(:,i))/n).
compute centvar={centvar,xnames(1,i)}.
end loop.

.
compute desctmp=make((8-(4* 0 )),ncol( xtmp ),-999).
loop jd=1 to ncol( xtmp ).
compute descdat= xtmp (:,jd).
compute desctmp(1,jd) = csum(descdat)/nrow(descdat).
compute desctmp(2,jd) = (nrow(descdat)*sscp(descdat))-(t(csum(descdat))*(csum(descdat))).
compute desctmp(2,jd) = sqrt(desctmp(2,jd)/(nrow(descdat)*(nrow(descdat)-1))).
compute desctmp(3,jd)=cmin(descdat).
compute desctmp(4,jd)=cmax(descdat).
do if ( 0 =0).
compute minwarn=0.
compute maxwarn=0.
do if ((desctmp(3,jd)=desctmp(4,jd)) and novar=0).
compute errcode(errs,1)=15.
compute errs=errs+1.
compute criterr=1.
compute novar=1.
end if.
compute tmp=((descdat(:,1)=desctmp(3,jd))+(descdat(:,1)=desctmp(4,jd))).
compute desctmp(8,jd)=(csum(tmp)=nrow(tmp)).
compute tmp = descdat.
compute tmp(GRADE(descdat),:) = descdat.
compute descdat = tmp.
release tmp.
compute decval={.16;.5;.84}.
loop kd=1 to 3.
compute low=trunc(decval(kd,1)*(nrow(descdat)+1)).
compute lowdec=decval(kd,1)*(nrow(descdat)+1)-low.
compute value=descdat(low,1)+(descdat((low+1),1)-descdat(low,1))*lowdec.
compute desctmp((4+kd),jd)=value.
end loop.
compute mnotev=1.
compute modvals=desctmp(5:7,:).
do if (quantile <> 1).
compute desctmp(5,jd)=desctmp(1,jd)-desctmp(2,jd).
compute desctmp(6,jd)=desctmp(1,jd).
compute desctmp(7,jd)=desctmp(1,jd)+desctmp(2,jd).
compute modvals=desctmp(5:7,:).
compute mnotev=2.
do if (modvals(1,1) < desctmp(3,1)).
compute modvals(1,1)=desctmp(3,1).
compute minwarn=1.
end if.
do if (modvals(3,1) > desctmp(4,1)).
compute modvals(3,1)=desctmp(4,1).
compute maxwarn=1.
end if.
end if.
do if (desctmp(8,1)=1).
compute modvals={desctmp(3,1);desctmp(4,1)}.
compute mnotev=0.
compute minwarn=0.
compute maxwarn=0.
end if.
end if.
end loop
.
compute xmodvals=modvals.
compute xprobval=xmodvals.
end if.
end if.
do if (nms > 0).
loop i = 1 to nms.
do if (mprod(1,i)=1).
compute mtmp(:,i)=mtmp(:,i)-(csum(mtmp(:,i))/n).
compute centvar={centvar,mnames(1,i)}.
end if.
end loop.

.
compute desctmp=make((8-(4* 0 )),ncol( mtmp ),-999).
loop jd=1 to ncol( mtmp ).
compute descdat= mtmp (:,jd).
compute desctmp(1,jd) = csum(descdat)/nrow(descdat).
compute desctmp(2,jd) = (nrow(descdat)*sscp(descdat))-(t(csum(descdat))*(csum(descdat))).
compute desctmp(2,jd) = sqrt(desctmp(2,jd)/(nrow(descdat)*(nrow(descdat)-1))).
compute desctmp(3,jd)=cmin(descdat).
compute desctmp(4,jd)=cmax(descdat).
do if ( 0 =0).
compute minwarn=0.
compute maxwarn=0.
do if ((desctmp(3,jd)=desctmp(4,jd)) and novar=0).
compute errcode(errs,1)=15.
compute errs=errs+1.
compute criterr=1.
compute novar=1.
end if.
compute tmp=((descdat(:,1)=desctmp(3,jd))+(descdat(:,1)=desctmp(4,jd))).
compute desctmp(8,jd)=(csum(tmp)=nrow(tmp)).
compute tmp = descdat.
compute tmp(GRADE(descdat),:) = descdat.
compute descdat = tmp.
release tmp.
compute decval={.16;.5;.84}.
loop kd=1 to 3.
compute low=trunc(decval(kd,1)*(nrow(descdat)+1)).
compute lowdec=decval(kd,1)*(nrow(descdat)+1)-low.
compute value=descdat(low,1)+(descdat((low+1),1)-descdat(low,1))*lowdec.
compute desctmp((4+kd),jd)=value.
end loop.
compute mnotev=1.
compute modvals=desctmp(5:7,:).
do if (quantile <> 1).
compute desctmp(5,jd)=desctmp(1,jd)-desctmp(2,jd).
compute desctmp(6,jd)=desctmp(1,jd).
compute desctmp(7,jd)=desctmp(1,jd)+desctmp(2,jd).
compute modvals=desctmp(5:7,:).
compute mnotev=2.
do if (modvals(1,1) < desctmp(3,1)).
compute modvals(1,1)=desctmp(3,1).
compute minwarn=1.
end if.
do if (modvals(3,1) > desctmp(4,1)).
compute modvals(3,1)=desctmp(4,1).
compute maxwarn=1.
end if.
end if.
do if (desctmp(8,1)=1).
compute modvals={desctmp(3,1);desctmp(4,1)}.
compute mnotev=0.
compute minwarn=0.
compute maxwarn=0.
end if.
end if.
end loop
.
do if ((cdeval(1,1)=-999) and (model=74)).
compute medmeans=desctmp(1,:).
end if.
compute mmodvals=modvals.
compute mprobval=mmodvals.
end if.
end if.
do if (ncol(centvar) > 1).
compute notecode(notes,1)=3.
compute notes=notes+1.
end if.
end if.
do if (criterr=0).
compute wsum=rsum(csum(wcmat)).
compute zsum=rsum(csum(zcmat)).
compute wzsum=rsum(csum(wzcmat)).
compute nump=make(1,(nys+nms),-999).
compue numint=make(1,(nys+nms),0).
compute datcount=1.
compute xtmpuse=0.
compute wtmpuse=0.
compute ztmpuse=0.
compute xwtmpus=0.
compute xztmpus=0.
compute wztmpus=0.
compute xwztmpu=0.
compute xtmploc=-999.
compute wtmploc=-999.
compute xwtmplo=-999.
compute ztmploc=-999.
compute xztmplo=-999.
compute wztmplo=-999.
compute xwztmplo=-999.
compute vlabs={' '}.
do if (ncs > 0).
compute ctmpuse=make(1,ncs,0).
end if.
do if (nms > 0).
compute mtmpuse=make(1,nms,0).
compute mwtmpus=make(1,nms,0).
compute mztmpus=make(1,nms,0).
compute mwztmpu=make(1,nms,0).
compute mtmploc=make(1,nms,0).
compute mwtmplo=make(nwvls,nms,-999).
compute mztmplo=make(nzvls,nms,-999).
compute mwztmplo=make((nwvls*nzvls),nms,-999).
end if.
do if (ncs > 0).
compute ctmploc=make(1,ncs,0).
end if.
compute fulldat=make(n,1,1).
compute datindx=make(1000,(nms+nys),-999).
compute wherew=make(2,(nms+nys),-999).
compute wherex=make(2,(nms+nys),-999).
compute wherez=make(2,(nms+nys),-999).
compute wherexw=make(2,(nms+nys),-999).
compute wherexz=make(2,(nms+nys),-999).
compute wherewz=make(2,(nms+nys),-999).
compute wherexwz=make(2,(nms+nys),-999).
do if (nms > 0).
compute wherem=make(nms,(nms+nys),-999).
compute wheremw = make(nms*2,(nms+nys),-999).
compute wheremz = make(nms*2,(nms+nys),-999).
compute wheremwz = make(nms*2,(nms+nys),-999).
end if.
compute wzhigh=make(1000,(((nms+1)*(nms+2))/2),0).
compute whigh=make(1000,(((nms+1)*(nms+2))/2),0).
compute zhigh=make(1000,(((nms+1)*(nms+2))/2),0).
compute fochigh=make(1000,(((nms+1)*(nms+2))/2),0).
compute xcoefloc={1;2;3;4;5;6;7;8;9}.
compute intkey = {' ', ' ', ' ', ' ', ' ', ' ', ' '}.
compute wzhighct=0.
compute whighct=0.
compute zhighct=0.
compute foccnt=0.
loop i = 2 to nrow(bcmat).
compute wdid=0.
compute zdid=0.
compute wzdid=0.
compute cntmp=1.
compute start=1.
do if (i < nrow(bcmat)).
compute outv=mtmp(:,(i-1)).
compute modlabel={mnames(1,(i-1));'constant'}.
end if.
do if (i = nrow(bcmat)).
compute outv=ytmp.
compute modlabel={ynames;'constant'}.
end if.
loop j = 1 to (i-1).
compute foccnt=foccnt+1.
do if (j = 1 and bcmat(i,j)=1).
compute outv={outv,xtmp}.
compute modlabel={modlabel;xcatlab(1:nxvls,1)}.
do if (xtmpuse=0).
compute fulldat={fulldat,xtmp}.
compute xtmpuse=1.
loop k4=datcount to (datcount+(nxvls-1)).
compute xtmploc={xtmploc;k4}.
end loop.
compute xtmploc=xtmploc(2:nrow(xtmploc),1).
compute datcount=datcount+nxvls.
end if.
compute datindx(start:(start+nrow(xtmploc)-1),(i-1))=xtmploc.
compute wherex(1,(i-1))=start+1.
compute wherex(2,(i-1))=start+nrow(xtmploc)-1+1.
do if (model = 74).
end if.
compute onebl=make(nrow(xtmploc),1,1).
compute fochigh((start+1):(start+nrow(xtmploc)),foccnt)=onebl.
compute start=start+nrow(xtmploc).
end if.
do if (j > 1 and bcmat(i,j)=1).
compute outv={outv,mtmp(:,(j-1))}.
compute modlabel={modlabel;mnames(1,(j-1))}.
do if (mtmpuse(1,(j-1))=0).
compute fulldat={fulldat,mtmp(:,(j-1))}.
compute mtmpuse(1,(j-1))=1.
compute mtmploc(1,(j-1))=datcount.
compute datcount=datcount+1.
end if.
compute datindx(start:(start+nrow(mtmploc)-1),(i-1))=mtmploc(1,(j-1)).
compute wherem((j-1),(i-1))=start+1.
compute onebl=make(nrow(mtmploc(1,j-1)),1,1).
compute ttt=nrow(mtmploc(1,(j-1)))+start-1.
compute fochigh((start+1):(start+nrow(mtmploc(1,(j-1)))),foccnt)=onebl.
compute start=start+nrow(mtmploc(1,(j-1))).
end if.
end loop.
do if (wsum > 0).
loop j = 1 to (i-1).
compute whighct=whighct+1.
do if (j = 1 and wcmat(i,j)=1).
do if (wdid=0).
compute outv={outv,wtmp}.
do if (ncs > 0 and wiscov > 0).
compute ccmatoff((i-1),wiscov)=0.
end if.
compute modlabel={modlabel;wcatlab(1:nwvls,1)}.
compute wdid=1.
do if (wtmpuse=0).
compute fulldat={fulldat,wtmp}.
do if (ncs > 0 and wiscov > 0).
compute ccmatoff((i-1),wiscov)=0.
end if.
compute wtmpuse=1.
loop k4=datcount to (datcount+(nwvls-1)).
compute wtmploc={wtmploc;k4}.
end loop.
compute wtmploc=wtmploc(2:nrow(wtmploc),1).
compute datcount=datcount+nwvls.
end if.
end if.
compute datindx(start:(start+nrow(wtmploc)-1),(i-1))=wtmploc.
compute wherew(1,(i-1))=start+1.
compute wherew(2,(i-1))=start+nrow(wtmploc)-1+1.
compute start=start+nrow(wtmploc).
loop k1=1 to nxvls.
loop k2 = 1 to nwvls.
compute outv={outv,(xtmp(:,k1)&*wtmp(:,k2))}.
do if (ncs > 0 and wiscov > 0).
compute ccmatoff((i-1),wiscov)=0.
end if.
compute modlabel={modlabel;intlab(cntmp,1)}.
compute intkey={intkey;intlab(cntmp,1),':',xcatlab(k1,1),'x',wcatlab(k2,1),' ',' '}.
compute cntmp=cntmp+1.
end loop.
end loop.
do if (xwtmpus=0).
compute fulldat={fulldat,outv(:,(ncol(outv)-(nxvls*nwvls)+1):ncol(outv))}.
compute xwtmpus=1.
do if (ncs > 0 and wiscov > 0).
compute ccmatoff((i-1),wiscov)=0.
end if.
loop k4=datcount to (datcount+((nwvls*nxvls)-1)).
compute xwtmplo={xwtmplo;k4}.
end loop.
compute xwtmplo=xwtmplo(2:nrow(xwtmplo),1).
compute datcount=datcount+(nxvls*nwvls).
end if.
compute datindx(start:(start+nrow(xwtmplo)-1),(i-1))=xwtmplo.
compute wherexw(1,(i-1))=start+1.
compute wherexw(2,(i-1))=start+nrow(xwtmplo)-1+1.
compute onebl=make(nrow(xwtmplo),1,1).
compute whigh((start+1):(start+nrow(xwtmplo)),whighct)=onebl.
compute start=start+nrow(xwtmplo).
end if.
do if (j > 1 and wcmat(i,j)=1).
do if (wdid=0 and model <> 74).
compute outv={outv,wtmp}.
do if (ncs > 0 and wiscov > 0).
compute ccmatoff((i-1),wiscov)=0.
end if.
compute modlabel={modlabel;wcatlab(1:nwvls,1)}.
compute wdid=1.
do if (wtmpuse=0).
compute fulldat={fulldat,wtmp}.
do if (ncs > 0 and wiscov > 0).
compute ccmatoff((i-1),wiscov)=0.
end if.
compute wtmpuse=1.
loop k4=datcount to (datcount+(nwvls-1)).
compute wtmploc={wtmploc;k4}.
end loop.
compute wtmploc=wtmploc(2:nrow(wtmploc),1).
compute datcount=datcount+nwvls.
end if.
compute datindx(start:(start+nrow(wtmploc)-1),(i-1))=wtmploc.
compute wherew(1,(i-1))=start+1.
compute wherew(2,(i-1))=start+nrow(wtmploc)-1+1.
compute start=start+nrow(wtmploc).
end if.
loop k2 = 1 to nwvls.
compute outv={outv,(mtmp(:,(j-1))&*wtmp(:,k2))}.
do if (ncs > 0 and wiscov > 0).
compute ccmatoff((i-1),wiscov)=0.
end if.
compute modlabel={modlabel;intlab(cntmp,1)}.
compute intkey={intkey;intlab(cntmp,1),':', mnames(1,(j-1)),'x',wcatlab(k2,1),' ',' '}.
compute cntmp=cntmp+1.
end loop.
do if (mwtmpus(1,(j-1))=0).
compute fulldat={fulldat,outv(:,(ncol(outv)-nwvls+1):ncol(outv))}.
do if (ncs > 0 and wiscov > 0).
compute ccmatoff((i-1),wiscov)=0.
end if.
compute mwtmpus(1,(j-1))=1.
compute mw22=-999.
loop k4=datcount to (datcount+(nwvls-1)).
compute mw22={mw22;k4}.
end loop.
compute mwtmplo(:,(j-1))=mw22(2:nrow(mw22),1).
compute datcount=datcount+nwvls.
end if.
compute datindx(start:(start+nrow(mwtmplo)-1),(i-1))=mwtmplo(:,(j-1)).
compute wheremw(((2*j)-3),(i-1))=start+1.
compute wheremw(((2*j)-2),(i-1))=start+nrow(mwtmplo)-1+1.
compute onebl=make(nrow(mwtmplo),1,1).
compute whigh((start+1):(start+nrow(mwtmplo)),whighct)=onebl.
compute start=start+nrow(mwtmplo).
end if.
end loop.
end if.
do if (zsum > 0).
loop j = 1 to (i-1).
compute zhighct=zhighct+1.
do if (j = 1 and zcmat(i,j)=1).
do if (zdid=0).
compute outv={outv,ztmp}.
do if (ncs > 0 and ziscov > 0).
compute ccmatoff((i-1),ziscov)=0.
end if.
compute modlabel={modlabel;zcatlab(1:nzvls,1)}.
compute zdid=1.
do if (ztmpuse=0).
compute fulldat={fulldat,ztmp}.
do if (ncs > 0 and ziscov > 0).
compute ccmatoff((i-1),ziscov)=0.
end if.
compute ztmpuse=1.
loop k4=datcount to (datcount+(nzvls-1)).
compute ztmploc={ztmploc;k4}.
end loop.
compute ztmploc=ztmploc(2:nrow(ztmploc),1).
compute datcount=datcount+nzvls.
end if.
end if.
compute datindx(start:(start+nrow(ztmploc)-1),(i-1))=ztmploc.
compute wherez(1,(i-1))=start+1.
compute wherez(2,(i-1))=start+nrow(ztmploc)-1+1.
compute start=start+nrow(ztmploc).
loop k1=1 to nxvls.
loop k2 = 1 to nzvls.
compute outv={outv,(xtmp(:,k1)&*ztmp(:,k2))}.
do if (ncs > 0 and ziscov > 0).
compute ccmatoff((i-1),ziscov)=0.
end if.
compute modlabel={modlabel;intlab(cntmp,1)}.
compute intkey={intkey;intlab(cntmp,1),':',xcatlab(k1,1),'x',zcatlab(k2,1),' ',' '}.
compute cntmp=cntmp+1.
end loop.
end loop.
do if (xztmpus=0).
compute fulldat={fulldat,outv(:,(ncol(outv)-(nxvls*nzvls)+1):ncol(outv))}.
do if (ncs > 0 and ziscov > 0).
compute ccmatoff((i-1),ziscov)=0.
end if.
compute xztmpus=1.
loop k4=datcount to (datcount+((nzvls*nxvls)-1)).
compute xztmplo={xztmplo;k4}.
end loop.
compute xztmplo=xztmplo(2:nrow(xztmplo),1).
compute datcount=datcount+(nxvls*nzvls).
end if.
compute datindx(start:(start+nrow(xztmplo)-1),(i-1))=xztmplo.
compute wherexz(1,(i-1))=start+1.
compute wherexz(2,(i-1))=start+nrow(xztmplo)-1+1.
compute onebl=make(nrow(xztmplo),1,1).
compute zhigh((start+1):(start+nrow(xztmplo)),zhighct)=onebl.
compute start=start+nrow(xztmplo).
end if.
do if (j > 1 and zcmat(i,j)=1).
do if (zdid=0).
compute outv={outv,ztmp}.
do if (ncs > 0 and ziscov > 0).
compute ccmatoff((i-1),ziscov)=0.
end if.
compute modlabel={modlabel;zcatlab(1:nzvls,1)}.
compute zdid=1.
do if (ztmpuse=0).
compute fulldat={fulldat,ztmp}.
do if (ncs > 0 and ziscov > 0).
compute ccmatoff((i-1),ziscov)=0.
end if.
compute ztmpuse=1.
loop k4=datcount to (datcount+(nzvls-1)).
compute ztmploc={ztmploc;k4}.
end loop.
compute ztmploc=ztmploc(2:nrow(ztmploc),1).
compute datcount=datcount+nzvls.
end if.
compute datindx(start:(start+nrow(ztmploc)-1),(i-1))=ztmploc.
compute wherez(1,(i-1))=start+1.
compute wherez(2,(i-1))=start+nrow(ztmploc)-1+1.
compute start=start+nrow(ztmploc).
end if.
loop k2 = 1 to nzvls.
compute outv={outv,(mtmp(:,(j-1))&*ztmp(:,k2))}.
do if (ncs > 0 and ziscov > 0).
compute ccmatoff((i-1),ziscov)=0.
end if.
compute modlabel={modlabel;intlab(cntmp,1)}.
compute intkey={intkey;intlab(cntmp,1),':', mnames(1,(j-1)),'x',zcatlab(k2,1),' ',' '}.
compute cntmp=cntmp+1.
end loop.
do if (mztmpus(1,(j-1))=0).
compute fulldat={fulldat,outv(:,(ncol(outv)-nzvls+1):ncol(outv))}.
do if (ncs > 0 and ziscov > 0).
compute ccmatoff((i-1),ziscov)=0.
end if.
compute mztmpus(1,(j-1))=1.
compute mz22=-999.
loop k4=datcount to (datcount+(nzvls-1)).
compute mz22={mz22;k4}.
end loop.
compute mztmplo(:,(j-1))=mz22(2:nrow(mz22),1).
compute datcount=datcount+nzvls.
end if.
compute datindx(start:(start+nrow(mztmplo)-1),(i-1))=mztmplo(:,(j-1)).
compute wheremz(((2*j)-3),(i-1))=start+1.
compute wheremz(((2*j)-2),(i-1))=start+nrow(mztmplo)-1+1.
compute onebl=make(nrow(mztmplo),1,1).
compute zhigh((start+1):(start+nrow(mztmplo)),zhighct)=onebl.
compute start=start+nrow(mztmplo).
end if.
end loop.
end if.
do if (wzsum > 0).
loop j = 1 to (i-1).
compute wzhighct=wzhighct+1.
do if (j = 1 and wzcmat(i,j)=1).
do if (wzdid=0).
loop k1=1 to nwvls.
loop k2 = 1 to nzvls.
compute outv={outv,(wtmp(:,k1)&*ztmp(:,k2))}.
do if (ncs > 0 and (ziscov > 0)).
compute ccmatoff((i-1),ziscov)=0.
end if.
do if (ncs > 0 and (wiscov > 0)).
compute ccmatoff((i-1),wiscov)=0.
end if.
compute modlabel={modlabel;intlab(cntmp,1)}.
compute intkey={intkey;intlab(cntmp,1),':',wcatlab(k1,1),'x',zcatlab(k2,1),' ',' '}.
compute cntmp=cntmp+1.
end loop.
end loop.
do if (wztmpus=0).
compute fulldat={fulldat,outv(:,(ncol(outv)-(nwvls*nzvls)+1):ncol(outv))}.
do if (ncs > 0 and (ziscov > 0)).
compute ccmatoff((i-1),ziscov)=0.
end if.
do if (ncs > 0 and (wiscov > 0)).
compute ccmatoff((i-1),wiscov)=0.
end if.
compute wztmpus=1.
loop k4=datcount to (datcount+((nwvls*nzvls)-1)).
compute wztmplo={wztmplo;k4}.
end loop.
compute wztmplo=wztmplo(2:nrow(wztmplo),1).
compute datcount=datcount+(nzvls*nwvls).
end if.
compute wzdid=1.
end if.
compute datindx(start:(start+nrow(wztmplo)-1),(i-1))=wztmplo.
compute wherewz(1,(i-1))=start+1.
compute wherewz(2,(i-1))=start+nrow(wztmplo)-1+1.
compute start=start+nrow(wztmplo).
loop k1=1 to nxvls.
loop k2=1 to nwvls.
loop k3=1 to nzvls.
compute outv={outv,(xtmp(:,k1)&*wtmp(:,k2)&*ztmp(:,k3))}.
do if (ncs > 0 and (ziscov > 0)).
compute ccmatoff((i-1),ziscov)=0.
end if.
do if (ncs > 0 and (wiscov > 0)).
compute ccmatoff((i-1),wiscov)=0.
end if.
compute modlabel={modlabel;intlab(cntmp,1)}.
compute intkey={intkey;intlab(cntmp,1),':',xcatlab(k1,1),'x',wcatlab(k2,1),'x', zcatlab(k3,1)}.
compute cntmp=cntmp+1.
end loop.
end loop.
end loop.
do if (xwztmpu=0).
compute fulldat={fulldat,outv(:,(ncol(outv)-(nxvls*nwvls*nzvls)+1):ncol(outv))}.
do if (ncs > 0 and (ziscov > 0)).
compute ccmatoff((i-1),ziscov)=0.
end if.
do if (ncs > 0 and (wiscov > 0)).
compute ccmatoff((i-1),wiscov)=0.
end if.
compute xwztmpu=1.
loop k4=datcount to (datcount+((nzvls*nxvls*nwvls)-1)).
compute xwztmplo={xwztmplo;k4}.
end loop.
compute xwztmplo=xwztmplo(2:nrow(xwztmplo),1).
compute datcount=datcount+(nxvls*nzvls*nwvls).
end if.
compute datindx(start:(start+nrow(xwztmplo)-1),(i-1))=xwztmplo.
compute wherexwz(1,(i-1))=start+1.
compute wherexwz(2,(i-1))=start+nrow(xwztmplo)-1+1.
compute onebl=make(nrow(xwztmplo),1,1).
compute wzhigh((start+1):(start+nrow(xwztmplo)),wzhighct)=onebl.
compute start=start+nrow(xwztmplo).
end if.
do if (j > 1 and wzcmat(i,j)=1).
do if (wzdid=0).
loop k1=1 to nwvls.
loop k2 = 1 to nzvls.
compute outv={outv,(wtmp(:,k1)&*ztmp(:,k2))}.
do if (ncs > 0 and (ziscov > 0)).
compute ccmatoff((i-1),ziscov)=0.
end if.
do if (ncs > 0 and (wiscov > 0)).
compute ccmatoff((i-1),wiscov)=0.
end if.
compute modlabel={modlabel;intlab(cntmp,1)}.
compute intkey={intkey;intlab(cntmp,1),':',wcatlab(k1,1),'x',zcatlab(k2,1),' ',' '}.
compute cntmp=cntmp+1.
end loop.
end loop.
do if (wztmpus=0).
compute fulldat={fulldat,outv(:,(ncol(outv)-(nwvls*nzvls)+1):ncol(outv))}.
do if (ncs > 0 and (ziscov > 0)).
compute ccmatoff((i-1),ziscov)=0.
end if.
do if (ncs > 0 and (wiscov > 0)).
compute ccmatoff((i-1),wiscov)=0.
end if.
compute wztmpus=1.
loop k4=datcount to (datcount+((nwvls*nzvls)-1)).
compute wztmplo={wztmplo;k4}.
end loop.
compute wztmplo=wztmplo(2:nrow(wztmplo),1).
compute datcount=datcount+(nzvls*nwvls).
end if.
compute wzdid=1.
compute datindx(start:(start+nrow(wztmplo)-1),(i-1))=wztmplo.
compute wherewz(1,(i-1))=start+1.
compute wherewz(2,(i-1))=start+nrow(wztmplo)-1+1.
compute start=start+nrow(wztmplo).
end if.
loop k1 = 1 to nwvls.
loop k2 = 1 to nzvls.
compute outv={outv,(mtmp(:,(j-1))&*wtmp(:,k1)&*ztmp(:,k2))}.
do if (ncs > 0 and (ziscov > 0)).
compute ccmatoff((i-1),ziscov)=0.
end if.
do if (ncs > 0 and (wiscov > 0)).
compute ccmatoff((i-1),wiscov)=0.
end if.
compute modlabel={modlabel;intlab(cntmp,1)}.
compute intkey={intkey;intlab(cntmp,1),':',mnames(1,(j-1)),'x',wcatlab(k1,1),'x', zcatlab(k2,1)}.
compute cntmp=cntmp+1.
end loop.
end loop.
do if (mwztmpu(1,(j-1))=0).
compute fulldat={fulldat,outv(:,(ncol(outv)-(nwvls*nzvls)+1):ncol(outv))}.
do if (ncs > 0 and (ziscov > 0)).
compute ccmatoff((i-1),ziscov)=0.
end if.
do if (ncs > 0 and (wiscov > 0)).
compute ccmatoff((i-1),wiscov)=0.
end if.
compute mwztmpu(1,(j-1))=1.
compute mz22=-999.
loop k4=datcount to (datcount+(nwvls*nzvls)-1).
compute mz22={mz22;k4}.
end loop.
compute mwztmplo(:,(j-1))=mz22(2:nrow(mz22),1).
compute datcount=datcount+(nwvls*nzvls).
end if.
compute datindx(start:(start+nrow(mwztmplo)-1),(i-1))=mwztmplo(:,(j-1)).
compute wheremwz(((2*j)-3),(i-1))=start+1.
compute wheremwz(((2*j)-2),(i-1))=start+nrow(mwztmplo)-1+1.
compute onebl=make(nrow(mwztmplo),1,1).
compute wzhigh((start+1):(start+nrow(mwztmplo)),wzhighct)=onebl.
compute start=start+nrow(mwztmplo).
end if.
end loop.
end if.
do if (ncs > 0).
compute ccmat=ccmat&*ccmatoff.
loop j = 1 to ncs.
do if (ccmat((i-1),j))=1.
do if (j=wiscov).
compute ctmp(:,j)=wtmp.
end if.
do if (j=ziscov).
compute ctmp(:,j)=ztmp.
end if.
compute outv={outv,ctmp(:,j)}.
compute modlabel={modlabel;covnames(1,j)}.
do if (ctmpuse(1,j)=0).
compute fulldat={fulldat,ctmp(:,j)}.
compute ctmpuse(1,j)=1.
compute ctmploc(1,j)=datcount.
compute datcount=datcount+1.
end if.
compute datindx(start:(start+nrow(ctmploc)-1),(i-1))=ctmploc(1,j).
compute start=start+nrow(ctmploc(1,j)).
end if.
end loop.
end if.
compute wdid=0.
compute zdid=0.
compute wzdid=0.
compute vlabs={vlabs;modlabel(2:nrow(modlabel),1)}.
compute numint(1,(i-1))=cntmp-1.
compute nump(1,(i-1))=nrow(modlabel)-1.
end loop.
release datcount, xtmpuse, wtmpuse, ztmpuse, xwtmpus, xztmpus, wztmpus, xwztmpu.
release xtmploc, wtmploc, xwtmplo, ztmploc, xztmplo, wztmplo, xwztmplo, foccnt.
do if (modcok=1 and ((nms > 0) or (zcmat(2,1) <> 1) or (mcx <> 0))).
compute notecode(notes,1) = 19.
compute notes = notes + 1.
compute modcok=0.
end if.
do if ((serial = 1 or (rsum(numint)>0) or nms=0) and mc > 0).
compute notecode(notes,1) = 15.
compute notes = notes + 1.
compute boot=mc.
compute mc=0.
end if.
do if (boot <> 0 or mc <> 0).
compute bootsz=boot.
do if (mc > 0).
compute bootsz=mc.
compute saveboot=0.
end if.
loop.
compute cilow = rnd(bootsz*(1-(conf/100))/2).
compute cihigh = trunc((bootsz*(conf/100)+(bootsz*(1-(conf/100))/2)))+1.
do if (cilow < 1 or cihigh > bootsz).
compute bootsz=trunc((bootsz+1000)/1000)*1000.
compute adjust = 1.
end if.
end loop if (cilow gt 0 and cihigh le bootsz).
do if (boot > 0).
compute boot=bootsz.
end if.
do if (mc > 0).
compute mc=bootsz.
end if.
do if (adjust = 1 and boot > 0).
compute notecode(notes,1) = 8.
compute notes = notes + 1.
end if.
do if (adjust = 1 and mc > 0).
compute notecode(notes,1) = 16.
compute notes = notes + 1.
end if.
end if.
compute maxboot = trunc(2*boot).
do if ( 0 > maxboot).
compute maxboot=trunc( 0 ).
end if.
do if (nms > 0).
release mtmpuse, mwtmpus, mwztmpu, mtmploc, mwtmplo, mztmplo, mwztmplo.
end if.
release wdid, zdid, wzdid, start,modlabel.
compute vlabs=vlabs(2:nrow(vlabs),1).
do if (rsum(numint) > 0).
compute intkey=intkey(2:nrow(intkey),:).
end if.
compute fulldat=fulldat(:,2:ncol(fulldat)).
compute fochigh=fochigh(1:rmax(nump),:).
compute whigh=whigh(1:rmax(nump),:).
compute zhigh=zhigh(1:rmax(nump),:).
compute wzhigh=wzhigh(1:rmax(nump),:).
compute coeffs=fochigh+whigh+zhigh+wzhigh.
compute bootloc=make(rmax(nump),ncol(nump),0).
do if (nms > 0).
compute cntmp=1.
loop i = 1 to ncol(nump).
loop j = 1 to nump(1,i).
compute bootloc(j,i)=cntmp.
compute cntmp=cntmp+1.
end loop.
end loop.
compute fochighb=make(nrow(fochigh),ncol(fochigh),0).
compute whighb=fochighb.
compute zhighb=fochighb.
compute wzhighb=fochighb.
compute thetaxmb=make(nrow(fochighb),nms,0).
compute thetaxyb=make(nrow(fochighb),1,0).
compute pathsfoc=make(nxvls,1,0).
compute cntmp=1.
loop i = 1 to (nms+nys).
loop j = 1 to i.
compute fochighb(:,cntmp)=fochigh(:,cntmp)&*bootloc(:,i).
compute whighb(:,cntmp)=whigh(:,cntmp)&*bootloc(:,i).
compute zhighb(:,cntmp)=zhigh(:,cntmp)&*bootloc(:,i).
compute wzhighb(:,cntmp)=wzhigh(:,cntmp)&*bootloc(:,i).
compute coeffsb=fochighb+whighb+zhighb+wzhighb.
do if ((i < (nms+nys)) and (j = 1)).
compute thetaxmb(:,i)=coeffsb(:,cntmp).
end if.
do if ((i = (nms+nys)) and (j = 1)).
compute thetaxyb(:,1)=coeffsb(:,cntmp).
end if.
compute cntmp=cntmp+1.
end loop.
end loop.
compute thetamyb=coeffsb(:,(ncol(coeffsb)-nms+1):ncol(coeffsb)).
do if (serial = 1).
compute thetammb=make(nrow(coeffsb),((nms*(nms-1))/2),0).
end if.
compute cntmp=1.
do if (nms > 1 and serial = 1).
loop i = 1 to (nms-1).
compute start=((i+2)*(i+1))/2.
loop j = 2 to (nms-i+1).
compute thetammb(:,cntmp)=coeffsb(:,start).
compute start=start+j+i-1.
compute cntmp=cntmp+1.
end loop.
end loop.
end if.
end if.
do if ((total = 1) and (rsum(numint)=0 or (xmint=1))).
compute dototal=1.
do if ((csum(bcmat(:,1)) <> (nms+nys)) or (rsum(bcmat(nrow(bcmat),:)) <> (nms+nys))).
compute dototal=0.
compute alttotal=1.
compute notecode(notes,1) = 12.
compute notes = notes + 1.
end if.
do if (ncs > 0).
do if ((csum(rsum(ccmat))) < (nrow(ccmat)*ncol(ccmat))).
compute dototal=0.
compute alttotal=1.
compute notecode(notes,1) = 11.
compute notes = notes + 1.
end if.
end if.
do if (model=74).
do if (xdich=0 and nxvls=1).
compute dototal=0.
compute alttotal=1.
end if.
do if ((xdich=1 or nxvls > 1) and (ncs > 0) and model=74).
compute dototal=0.
compute alttotal=1.
end if.
end if.
end if.
end if.
do if (criterr=0 and ncs > 0).
do if (rsum((csum(ccmat)=0)) <> 0).
compute errcode(errs,1)=51.
compute errs=errs+1.
compute criterr=1.
end if.
end if.
compute debug= 0.
do if (outscree=1).
print/title = '***************** PROCESS Procedure for SPSS Version 4.2 *****************'.
print/title = '          Written by Andrew F. Hayes, Ph.D.       www.afhayes.com'.
print/title = '    Documentation available in Hayes (2022). www.guilford.com/p/hayes3'/space=0.
end if.
do if (criterr=0).
compute modresid=make(n,1,99999).
do if (stand=1 and ydich=1).
compute stand=0.
end if.
compute anymod2=csum(rsum(wcmat+zcmat+wzcmat)).
do if (anymod2 > 0 and stand = 1).
compute notecode(notes,1) = 27.
compute notes = notes + 1.
compute stand=0.
end if.
compute funny=1.
do if (outscree=1).
print modelvar/title = '**************************************************************************'/format = A8/rnames=modelvlb.
do if (ncs > 0).
print covnames/title='Covariates:'/format=A8.
end if.
print n/title='Sample'/rlabel='Size:'.
do if (( 'random' <> 'random')).
compute seedt= 'random'.
print seedt/title='Custom'/format=A12/rlabel = 'Seed:'.
end if.
end if.
compute maxresm=9.
compute resultm=make(1,maxresm,99999).
do if (describe=1).
compute means=csum(dat)/n.
compute sigmatal = (t(dat)*(ident(n)-(1/n)*ones*t(ones))*dat)*(1/(n-1)).
compute sdvec=sqrt(diag(sigmatal)).
compute sdall = mdiag(1/sdvec).
compute corall=sdall*sigmatal*t(sdall).
compute means={means;t(sdvec)}.
do if (ncol(means) > 9).
compute resultm=make(1,ncol(means),99999).
compute maxresm=ncol(means).
end if.
compute resultm2=make(2,maxresm,99999).
compute resultm2(1:2,1:ncol(means))=means.
compute resultm={resultm;resultm2}.
compute resultm2=make(ncol(corall),maxresm,99999).
compute resultm2(1:ncol(corall),1:ncol(corall))=corall.
compute resultm={resultm;resultm2}.
do if (outscree=1).
print means/title='Variable means and standard deviations'/cnames=varnames/rlabels='Mean','SD'/format= F10.4.
print corall/title='Variable intercorrelations (Pearson r)'/cnames=varnames/rnames=varnames/format= F10.4.
end if.
end if.
do if (outscree=1).
do if (mcxok=1).
compute labtmp={xnames,t(xcatlab(1:nxvls,1))}.
print dummatx/title = 'Coding of categorical X variable for analysis:'/cnames = labtmp/format = F6.3.
end if.
do if (mcwok=1 and xmint=0).
compute labtmp={wnames,t(wcatlab(1:nwvls,1))}.
print dummatw/title = 'Coding of categorical W variable for analysis:'/cnames = labtmp/format = F6.3.
end if.
do if (mczok=1).
compute labtmp={znames,t(zcatlab(1:nzvls,1))}.
print dummatz/title = 'Coding of categorical Z variable for analysis:'/cnames = labtmp/format = F6.3.
end if.
end if.
end if.
do if (criterr = 0).
compute outnames=ynames.
compute outvars=ytmp.
do if (nms > 0).
compute outnames={mnames,ynames}.
compute outvars={mtmp,ytmp}.
compute indcov=make(((nms*2)+(nms*(nxvls-1))),((nms*2)+(nms*(nxvls-1))),0).
compute mcsopath=make(((nms*2)+(nms*(nxvls-1))),1,0).
end if.
compute labstart=1.
compute intstart=1.
compute start=1.
compute coeffmat=make(1,6,0).
compute conseq={'        '}.
compute dfmat=0.
compute coeffcol=0.
compute pathscnt=1.
compute pathscn2=1.
loop i = 1 to (nms+nys).
do if (outscree=1).
print/title = '**************************************************************************'.
end if.
compute highf=make(1,5,0).
compute highf2=highf.
do if ((i = (nms+nys)) and (ydich=1)).
compute highf=make(1,3,0).
compute highf2=highf.
end if.
compute flabel={' '}.
compute y=outvars(:,i).
compute xindx=datindx(1:(nump(1,i)-1),i).
compute x = fulldat(:,xindx).
compute x={ones,x}.
compute xsq=t(x)*x.
compute exsq=eval(xsq).
release xsq.
compute zeroeig=csum(exsq <= 0.000000000002).
do if (outscree=1).
print outnames(1,i)/title = 'OUTCOME VARIABLE:'/format = A8/space=0.
do if (ydich=1 and (i = (nms+nys))).
compute nmsd = {outnames(1,i), 'Analysis'}.
print rcd/title = 'Coding of binary Y for logistic regression analysis:'/cnames = nmsd/format = F9.2.
end if.
end if.
do if (zeroeig > 0).
print / title = 'SINGULAR OR NEAR SINGULAR DATA MATRIX.'.
compute criterr=1.
compute errcode(errs,1)=31.
compute errs=errs+1.
end if.
compute means=csum(x)/n.
compute vlabsm=vlabs(labstart:(labstart+(nump(1,i)-1)),1).
do if (criterr=0).
do if (ydich=0 or (i < (nms+nys))).

.
do if ( 1 =1).
compute hatmat=inv(t( x )* x )*t( x ).
compute b = hatmat* y.
compute modres=b.
do if ( 1 =1).
compute n1=nrow( x ).
compute dfres=n1-(ncol( x )).
compute sstotal = t( y -(csum( y )/n1))*( y -(csum( y )/n1)).
compute resid= y - x *b.
compute ssresid = csum((resid)&**2).
compute r2 = (sstotal-ssresid)/sstotal.
do if (r2 < 0).
compute r2=0.
end if.
compute adjr2 = 1-((1-r2)*(n1-1)/(dfres)).
compute mse=ssresid/(n1-ncol( x )).

.
compute n1=nrow( x ).
compute invXtX = inv(t( x )* x ).
compute varb = mse *invXtX.
compute k3 = ncol( x ).
compute xhc=0.
do if ( hc <> 5).
compute xhc= x.
compute hat = xhc(:,1).
loop i3=1 to nrow(xhc).
compute hat(i3,1)= xhc(i3,:)*invXtX*t(xhc(i3,:)).
end loop.
do if ( hc = 0 or hc =1).
loop i3 = 1 to k3.
compute xhc(:,i3)=xhc(:,i3)&* resid.
end loop.
end if.
do if ( hc =3 or hc =2).
loop i3=1 to k3.
compute xhc(:,i3) = ( resid &/(1-hat)&**(1/(4- hc )))&*xhc(:,i3).
end loop.
end if.
do if ( hc = 4).
compute hcmn=make(n,2,4).
compute hcmn(:,2)=(n1*hat)/k3.
loop i3= 1 to k3.
compute xhc(:,i3) = ( resid &/(1-hat)&**(rmin(hcmn)/2))&*xhc(:,i3).
end loop.
end if.
compute varb=(invXtX*t(xhc)*xhc*invXtX).
do if ( hc =1).
compute varb=(n1/(n1-ncol( x )))&*varb.
end if.
end if.
compute hclab={'se(HC0)','se(HC1)','se(HC2)','se(HC3)','se(HC4)','se'}.
compute hclab=hclab(1,( hc +1)).
compute hcflab={'F(HC0)','F(HC1)','F(HC2)','F(HC3)','F(HC4)','F'}.
compute hcflab=hcflab(1,( hc +1)).
release xhc
.
compute seb=sqrt(diag(varb)).
compute trat = b&/seb.
compute p = 2*(1-tcdf(abs(trat), (dfres))).
compute tval = sqrt(dfres* (exp((dfres-(5/6))*((xp2/(dfres-(2/3)+(.11/dfres)))* (xp2/(dfres-(2/3)+(.11/dfres)))))-1)).
compute modres={modres,seb,trat,p}.
compute modres={modres,(b-tval&*seb),(b+tval&*seb)}.
compute modresl={'coeff',hclab,'t','p','LLCI','ULCI'}.
compute lmat = ident(ncol( x )).
compute lmat = lmat(:,2:ncol(lmat)).
compute fratio = (t(t(lmat)*b)*inv(t(lmat)*varb*lmat)*((t(lmat)*b)))/(ncol( x )-1).
compute pfr = 1-fcdf(fratio,(ncol( x )-1),dfres).
compute modsum={sqrt(r2),r2,mse,fratio,(ncol( x )-1),dfres,pfr}.
compute modsuml={'R','R-sq','MSE',hcflab,'df1','df2', 'p'}.
end if.
end if.
do if ( 1 = 2 or 1 =3).
compute xlp= x.
compute ylp= y.
compute pt2 = make(nrow(ylp),1,(csum(ylp)/nrow(ylp))).
do if ( 1 =2).
compute LL3 = ylp&*ln(pt2)+(1-ylp)&*ln(1-pt2).
end if.
compute LL3 = -2*csum(LL3).
compute bt1 = make(ncol(xlp),1,0).
compute LL1 = 0.
compute pt1 = make(nrow(ylp),1,0.5).
compute pt1lp=pt1.
loop jjj = 1 to iterate.
compute xlptmp=t(xlp).
compute vecprb=pt1lp&*(1-pt1lp).
loop kkk=1 to ncol(xlp).
compute xlptmp(kkk,:)=xlptmp(kkk,:)&*t(vecprb).
end loop.
compute b = bt1+inv(xlptmp*xlp)*t(xlp)*(ylp-pt1lp).
do if ( 1 =2).
compute xlpb=xlp*b.
compute xlpbt=(xlpb > -709.7).
compute xlpb709=(1-xlpbt)*(-709.7).
compute xlpb=(xlpb&*xlpbt)+xlpb709.
compute pt1lp = 1/(1+exp(-(xlpb))).
end if.
compute itprob = csum((pt1lp < .00000001) or (pt1lp > .9999999)).
do if (itprob > 0).
loop kkk = 1 to nrow(pt1lp).
do if (pt1lp(kkk,1) > .9999999).
compute pt1lp(kkk,1) = .9999999.
end if.
do if (pt1lp(kkk,1) < .00000001).
compute pt1lp(kkk,1) = .00000001.
end if.
end loop.
compute itprob = 0.
end if.
do if (itprob = 0).
do if ( 1 =2).
compute LL = ylp&*ln(pt1lp)+(1-ylp)&*ln(1-pt1lp).
end if.
compute LL2 = -2*csum(ll).
end if.
do if (abs(LL1-LL2) < converge).
do if ( 1 =1).
compute xlptmp=t(xlp).
compute vecprb=pt1lp&*(1-pt1lp).
loop kkk=1 to ncol(xlp).
compute xlptmp(kkk,:)=xlptmp(kkk,:)&*t(vecprb).
end loop.
compute varb = inv(xlptmp*xlp).
compute seb = sqrt(diag(varb)).
release xlptmp.
end if.
break.
end if.
compute bt1 = b.
compute LL1 = LL2.
end loop.
compute modres=b.
do if (jjj > iterate).
compute itprob = 2.
do if (booting=0).
compute iterrmod=1.
end if.
do if (booting=1).
compute bootiter=1.
end if.
do if (itprobtg=0).
compute itprobtg=1.
compute errcode(errs,1) = 47.
compute errs = errs + 1.
do if (booting = 0 and 1 =1).
compute vt1 = mdiag(pt1lp&*(1-pt1lp)).
compute varb = inv(t(xlp)*vt1*xlp).
compute seb = sqrt(diag(varb)).
end if.
end if.
end if.
do if ( 1 =1).
compute trat = b&/seb.
compute dfres=nrow(xlp).
compute p = 2*(1-cdfnorm(abs(trat))).
compute modres={modres,seb,trat,p}.
compute modres={modres,(b-xp2&*seb),(b+xp2&*seb)}.
compute pvchi=1-chicdf((LL3-LL2),(nrow(modres)-1)).
compute mcF = (LL3-LL2)/LL3.
compute cox = 1-exp(-(LL3-LL2)/nrow(xlp)).
compute nagel = cox/(1-exp(-(LL3)/nrow(xlp))).
compute modsum={LL2,(LL3-LL2),(nrow(modres)-1),pvchi, mcF,cox,nagel}.
compute modsuml={'-2LL','ModelLL', 'df', 'p', 'McFadden', 'CoxSnell', 'Nagelkrk'}.
compute modresl={'coeff','se','Z','p','LLCI','ULCI'}.
end if.
end if
.
compute dfmatt=make(nrow(modres),1,modsum(1,6)).
compute modresid={modresid,resid}.
end if.
do if (ydich=1 and (i = (nms+nys))).

.
do if ( 2 =1).
compute hatmat=inv(t( x )* x )*t( x ).
compute b = hatmat* y.
compute modres=b.
do if ( 1 =1).
compute n1=nrow( x ).
compute dfres=n1-(ncol( x )).
compute sstotal = t( y -(csum( y )/n1))*( y -(csum( y )/n1)).
compute resid= y - x *b.
compute ssresid = csum((resid)&**2).
compute r2 = (sstotal-ssresid)/sstotal.
do if (r2 < 0).
compute r2=0.
end if.
compute adjr2 = 1-((1-r2)*(n1-1)/(dfres)).
compute mse=ssresid/(n1-ncol( x )).

.
compute n1=nrow( x ).
compute invXtX = inv(t( x )* x ).
compute varb = mse *invXtX.
compute k3 = ncol( x ).
compute xhc=0.
do if ( hc <> 5).
compute xhc= x.
compute hat = xhc(:,1).
loop i3=1 to nrow(xhc).
compute hat(i3,1)= xhc(i3,:)*invXtX*t(xhc(i3,:)).
end loop.
do if ( hc = 0 or hc =1).
loop i3 = 1 to k3.
compute xhc(:,i3)=xhc(:,i3)&* resid.
end loop.
end if.
do if ( hc =3 or hc =2).
loop i3=1 to k3.
compute xhc(:,i3) = ( resid &/(1-hat)&**(1/(4- hc )))&*xhc(:,i3).
end loop.
end if.
do if ( hc = 4).
compute hcmn=make(n,2,4).
compute hcmn(:,2)=(n1*hat)/k3.
loop i3= 1 to k3.
compute xhc(:,i3) = ( resid &/(1-hat)&**(rmin(hcmn)/2))&*xhc(:,i3).
end loop.
end if.
compute varb=(invXtX*t(xhc)*xhc*invXtX).
do if ( hc =1).
compute varb=(n1/(n1-ncol( x )))&*varb.
end if.
end if.
compute hclab={'se(HC0)','se(HC1)','se(HC2)','se(HC3)','se(HC4)','se'}.
compute hclab=hclab(1,( hc +1)).
compute hcflab={'F(HC0)','F(HC1)','F(HC2)','F(HC3)','F(HC4)','F'}.
compute hcflab=hcflab(1,( hc +1)).
release xhc
.
compute seb=sqrt(diag(varb)).
compute trat = b&/seb.
compute p = 2*(1-tcdf(abs(trat), (dfres))).
compute tval = sqrt(dfres* (exp((dfres-(5/6))*((xp2/(dfres-(2/3)+(.11/dfres)))* (xp2/(dfres-(2/3)+(.11/dfres)))))-1)).
compute modres={modres,seb,trat,p}.
compute modres={modres,(b-tval&*seb),(b+tval&*seb)}.
compute modresl={'coeff',hclab,'t','p','LLCI','ULCI'}.
compute lmat = ident(ncol( x )).
compute lmat = lmat(:,2:ncol(lmat)).
compute fratio = (t(t(lmat)*b)*inv(t(lmat)*varb*lmat)*((t(lmat)*b)))/(ncol( x )-1).
compute pfr = 1-fcdf(fratio,(ncol( x )-1),dfres).
compute modsum={sqrt(r2),r2,mse,fratio,(ncol( x )-1),dfres,pfr}.
compute modsuml={'R','R-sq','MSE',hcflab,'df1','df2', 'p'}.
end if.
end if.
do if ( 2 = 2 or 2 =3).
compute xlp= x.
compute ylp= y.
compute pt2 = make(nrow(ylp),1,(csum(ylp)/nrow(ylp))).
do if ( 2 =2).
compute LL3 = ylp&*ln(pt2)+(1-ylp)&*ln(1-pt2).
end if.
compute LL3 = -2*csum(LL3).
compute bt1 = make(ncol(xlp),1,0).
compute LL1 = 0.
compute pt1 = make(nrow(ylp),1,0.5).
compute pt1lp=pt1.
loop jjj = 1 to iterate.
compute xlptmp=t(xlp).
compute vecprb=pt1lp&*(1-pt1lp).
loop kkk=1 to ncol(xlp).
compute xlptmp(kkk,:)=xlptmp(kkk,:)&*t(vecprb).
end loop.
compute b = bt1+inv(xlptmp*xlp)*t(xlp)*(ylp-pt1lp).
do if ( 2 =2).
compute xlpb=xlp*b.
compute xlpbt=(xlpb > -709.7).
compute xlpb709=(1-xlpbt)*(-709.7).
compute xlpb=(xlpb&*xlpbt)+xlpb709.
compute pt1lp = 1/(1+exp(-(xlpb))).
end if.
compute itprob = csum((pt1lp < .00000001) or (pt1lp > .9999999)).
do if (itprob > 0).
loop kkk = 1 to nrow(pt1lp).
do if (pt1lp(kkk,1) > .9999999).
compute pt1lp(kkk,1) = .9999999.
end if.
do if (pt1lp(kkk,1) < .00000001).
compute pt1lp(kkk,1) = .00000001.
end if.
end loop.
compute itprob = 0.
end if.
do if (itprob = 0).
do if ( 2 =2).
compute LL = ylp&*ln(pt1lp)+(1-ylp)&*ln(1-pt1lp).
end if.
compute LL2 = -2*csum(ll).
end if.
do if (abs(LL1-LL2) < converge).
do if ( 1 =1).
compute xlptmp=t(xlp).
compute vecprb=pt1lp&*(1-pt1lp).
loop kkk=1 to ncol(xlp).
compute xlptmp(kkk,:)=xlptmp(kkk,:)&*t(vecprb).
end loop.
compute varb = inv(xlptmp*xlp).
compute seb = sqrt(diag(varb)).
release xlptmp.
end if.
break.
end if.
compute bt1 = b.
compute LL1 = LL2.
end loop.
compute modres=b.
do if (jjj > iterate).
compute itprob = 2.
do if (booting=0).
compute iterrmod=1.
end if.
do if (booting=1).
compute bootiter=1.
end if.
do if (itprobtg=0).
compute itprobtg=1.
compute errcode(errs,1) = 47.
compute errs = errs + 1.
do if (booting = 0 and 1 =1).
compute vt1 = mdiag(pt1lp&*(1-pt1lp)).
compute varb = inv(t(xlp)*vt1*xlp).
compute seb = sqrt(diag(varb)).
end if.
end if.
end if.
do if ( 1 =1).
compute trat = b&/seb.
compute dfres=nrow(xlp).
compute p = 2*(1-cdfnorm(abs(trat))).
compute modres={modres,seb,trat,p}.
compute modres={modres,(b-xp2&*seb),(b+xp2&*seb)}.
compute pvchi=1-chicdf((LL3-LL2),(nrow(modres)-1)).
compute mcF = (LL3-LL2)/LL3.
compute cox = 1-exp(-(LL3-LL2)/nrow(xlp)).
compute nagel = cox/(1-exp(-(LL3)/nrow(xlp))).
compute modsum={LL2,(LL3-LL2),(nrow(modres)-1),pvchi, mcF,cox,nagel}.
compute modsuml={'-2LL','ModelLL', 'df', 'p', 'McFadden', 'CoxSnell', 'Nagelkrk'}.
compute modresl={'coeff','se','Z','p','LLCI','ULCI'}.
end if.
end if
.
compute dfmatt=make(nrow(modres),1,-999).
end if.
compute resultm2=make(1,maxresm,99999).
compute resultm2(1,1:ncol(modsum))=modsum.
compute resultm={resultm;resultm2}.
compute resultm2=make(nrow(modres),maxresm,99999).
compute resultm2(1:nrow(modres),1:ncol(modres))=modres.
compute resultm={resultm;resultm2}.
compute obscoeff={obscoeff,t(b)}.
do if (outscree=1).
print modsum/title = 'Model Summary'/cnames = modsuml/format= F10.4.
print modres/title='Model'/rnames=vlabsm/cnames=modresl/format= F10.4.
end if.
compute basemod=modsum(1,1).
compute basemodx=basemod.
do if (ydich=1 and (i = (nms+nys))).
do if (outscree=1).
print/title='These results are expressed in a log-odds metric.'.
end if.
compute notecode(notes,1) = 26.
compute notes = notes + 1.
end if.
compute coeffmat={coeffmat;modres}.
compute conseqt=make(nrow(modres),1,outnames(1,i)).
compute conseq={conseq;conseqt}.
compute dfmat={dfmat;dfmatt}.
compute labstart=labstart+nump(1,i).
do if (stand=1).
compute predsd=make(nrow(modres),1,0).
compute stdmod=modres(:,1)&/ovsd(1,i).
loop jd=1 to ncol(x).
compute descdat=x(:,jd).
compute predsd(jd,1) = (nrow(descdat)*sscp(descdat))-(t(csum(descdat))*(csum(descdat))).
compute predsd(jd,1) = sqrt(predsd(jd,1)/(nrow(descdat)*(nrow(descdat)-1))).
end loop.
do if (wherex(1,i) <> -999 and ((nxvls > 1) or (xdich=1))).
compute sdmsone=make(nxvls,1,1).
compute predsd(wherex(1,i):wherex(2,i),1)=sdmsone.
compute pstog=1.
end if.
compute predsd(1,1)=1.
compute stdmod=stdmod&*predsd.
compute stdmod=stdmod(2:nrow(stdmod),1).
compute sdvlabs=vlabsm(2:nrow(vlabsm),1).
compute resultm2=make(nrow(stdmod),maxresm,99999).
compute resultm2(1:nrow(stdmod),1:ncol(stdmod))=stdmod.
compute resultm={resultm;resultm2}.
do if (outscree=1).
print stdmod/title='Standardized coefficients'/clabels='coeff'/rnames=sdvlabs/format= F10.4.
end if.
end if.
do if (nms > 0 and serial = 0 and (rsum(numint) = 0) and (normal=1 or mc > 0)).
do if (i < (nms+nys)).
compute indcov((((i-1)*nxvls)+1):(i*nxvls),(((i-1)*nxvls)+1):(i*nxvls))=varb(2:(1+nxvls), 2:(1+nxvls)).
compute mcsopath((((i-1)*nxvls)+1):(i*nxvls) ,1)=modres(2:(1+nxvls),1).
end if.
do if (i = (nms+nys)).
compute atm=ncol(wherem).
compute indcov(((nms*nxvls)+1):nrow(mcsopath),((nms*nxvls)+1):nrow(mcsopath))= varb(wherem(1,atm):(wherem(1,atm)+nms-1),wherem(1,atm):(wherem(1,atm)+nms-1)).
compute mcsopath(((nms*nxvls)+1):nrow(mcsopath),1)=modres(wherem(1,atm):(wherem(1,atm)+ nms-1),1).
compute sobelok=1.
end if.
end if.
compute obsdirfx=make(1,nxvls,0).
compute dirzes=make(1,nxvls,0).
do if ((i = (nms+nys)) and (bcmat(nrow(bcmat),1)=1)).
compute direff=modres(2:(1+nxvls),:).
compute obsdirfx=t(direff(:,1)).
compute direfflb=modresl.
compute direffl2=vlabsm(2:(1+nxvls),:).
compute lmat=make(nrow(b),1,0).
compute lmat2=make(nxvls,1,1).
compute lmat(2:(1+nxvls),1)=lmat2.
do if (ydich <> 1).

.
compute lmat2= lmat.
do if ( 0 =0).
compute lmat2 = mdiag( lmat ).
compute lmat3=make(nrow(lmat2),1,0).
loop flp=1 to ncol(lmat2).
do if (csum(lmat2(:,flp))=1).
compute lmat3={lmat3,lmat2(:,flp)}.
end if.
end loop.
compute lmat2=lmat3(:,2:ncol(lmat3)).
end if.
compute fratio = (t(t(lmat2)* b )*inv(t(lmat2)* varb *lmat2)*((t(lmat2)* b )))/ncol(lmat2).
compute pfr = 1-fcdf(fratio,ncol(lmat2),(n-nrow( b ))).
compute fresult={fratio,ncol(lmat2),(n-nrow( b )),pfr}.
do if (i = (nms+nys) and (ydich=1)).
compute fratio=fratio*ncol(lmat2).
compute pfr=1-chicdf(fratio,ncol(lmat2)).
compute fresult={fratio,ncol(lmat2),pfr}.
end if.
do if ( 1 =1).
compute lmat3=1-rsum(lmat2).
compute xfm=make(n,csum(lmat3),0).
compute flpc=1.
loop flp=1 to nrow(lmat3).
do if (lmat3(flp,1)=1).
compute xfm(:,flpc)=x(:,flp).
compute flpc=flpc+1.
end if.
end loop.
compute bfm=inv(t(xfm)*xfm)*t(xfm)*y.
compute resid=y-(xfm*bfm).
compute sstotal=(y-(csum(y)/n)).
compute sstotal=csum(sstotal&*sstotal).
compute ssresid=csum(resid&*resid).
compute rsqch= r2 -((sstotal-ssresid)/sstotal).
compute fresult={rsqch,fresult}.
release xfm,flpc, resid, ssresid, bfm.
end if
.
compute diromni=fresult.
end if.
do if (ydich = 1 ).

.
compute btemphld=b.
compute llrdat=make(nrow(x),nrow( lmat )-csum( lmat ),-999).
compute llrdf=ncol(x)-ncol(llrdat).
compute llrcnt=0.
loop llri=1 to nrow( lmat ).
do if ( lmat (llri,1)=0).
compute llrcnt=llrcnt+1.
compute llrdat(:,llrcnt)=x(:,llri).
end if.
end loop.

.
do if ( 2 =1).
compute hatmat=inv(t( llrdat )* llrdat )*t( llrdat ).
compute b = hatmat* y.
compute modres=b.
do if ( 0 =1).
compute n1=nrow( llrdat ).
compute dfres=n1-(ncol( llrdat )).
compute sstotal = t( y -(csum( y )/n1))*( y -(csum( y )/n1)).
compute resid= y - llrdat *b.
compute ssresid = csum((resid)&**2).
compute r2 = (sstotal-ssresid)/sstotal.
do if (r2 < 0).
compute r2=0.
end if.
compute adjr2 = 1-((1-r2)*(n1-1)/(dfres)).
compute mse=ssresid/(n1-ncol( llrdat )).

.
compute n1=nrow( x ).
compute invXtX = inv(t( x )* x ).
compute varb = mse *invXtX.
compute k3 = ncol( x ).
compute xhc=0.
do if ( hc <> 5).
compute xhc= x.
compute hat = xhc(:,1).
loop i3=1 to nrow(xhc).
compute hat(i3,1)= xhc(i3,:)*invXtX*t(xhc(i3,:)).
end loop.
do if ( hc = 0 or hc =1).
loop i3 = 1 to k3.
compute xhc(:,i3)=xhc(:,i3)&* resid.
end loop.
end if.
do if ( hc =3 or hc =2).
loop i3=1 to k3.
compute xhc(:,i3) = ( resid &/(1-hat)&**(1/(4- hc )))&*xhc(:,i3).
end loop.
end if.
do if ( hc = 4).
compute hcmn=make(n,2,4).
compute hcmn(:,2)=(n1*hat)/k3.
loop i3= 1 to k3.
compute xhc(:,i3) = ( resid &/(1-hat)&**(rmin(hcmn)/2))&*xhc(:,i3).
end loop.
end if.
compute varb=(invXtX*t(xhc)*xhc*invXtX).
do if ( hc =1).
compute varb=(n1/(n1-ncol( x )))&*varb.
end if.
end if.
compute hclab={'se(HC0)','se(HC1)','se(HC2)','se(HC3)','se(HC4)','se'}.
compute hclab=hclab(1,( hc +1)).
compute hcflab={'F(HC0)','F(HC1)','F(HC2)','F(HC3)','F(HC4)','F'}.
compute hcflab=hcflab(1,( hc +1)).
release xhc
.
compute seb=sqrt(diag(varb)).
compute trat = b&/seb.
compute p = 2*(1-tcdf(abs(trat), (dfres))).
compute tval = sqrt(dfres* (exp((dfres-(5/6))*((xp2/(dfres-(2/3)+(.11/dfres)))* (xp2/(dfres-(2/3)+(.11/dfres)))))-1)).
compute modres={modres,seb,trat,p}.
compute modres={modres,(b-tval&*seb),(b+tval&*seb)}.
compute modresl={'coeff',hclab,'t','p','LLCI','ULCI'}.
compute lmat = ident(ncol( llrdat )).
compute lmat = lmat(:,2:ncol(lmat)).
compute fratio = (t(t(lmat)*b)*inv(t(lmat)*varb*lmat)*((t(lmat)*b)))/(ncol( llrdat )-1).
compute pfr = 1-fcdf(fratio,(ncol( llrdat )-1),dfres).
compute modsum={sqrt(r2),r2,mse,fratio,(ncol( llrdat )-1),dfres,pfr}.
compute modsuml={'R','R-sq','MSE',hcflab,'df1','df2', 'p'}.
end if.
end if.
do if ( 2 = 2 or 2 =3).
compute xlp= llrdat.
compute ylp= y.
compute pt2 = make(nrow(ylp),1,(csum(ylp)/nrow(ylp))).
do if ( 2 =2).
compute LL3 = ylp&*ln(pt2)+(1-ylp)&*ln(1-pt2).
end if.
compute LL3 = -2*csum(LL3).
compute bt1 = make(ncol(xlp),1,0).
compute LL1 = 0.
compute pt1 = make(nrow(ylp),1,0.5).
compute pt1lp=pt1.
loop jjj = 1 to iterate.
compute xlptmp=t(xlp).
compute vecprb=pt1lp&*(1-pt1lp).
loop kkk=1 to ncol(xlp).
compute xlptmp(kkk,:)=xlptmp(kkk,:)&*t(vecprb).
end loop.
compute b = bt1+inv(xlptmp*xlp)*t(xlp)*(ylp-pt1lp).
do if ( 2 =2).
compute xlpb=xlp*b.
compute xlpbt=(xlpb > -709.7).
compute xlpb709=(1-xlpbt)*(-709.7).
compute xlpb=(xlpb&*xlpbt)+xlpb709.
compute pt1lp = 1/(1+exp(-(xlpb))).
end if.
compute itprob = csum((pt1lp < .00000001) or (pt1lp > .9999999)).
do if (itprob > 0).
loop kkk = 1 to nrow(pt1lp).
do if (pt1lp(kkk,1) > .9999999).
compute pt1lp(kkk,1) = .9999999.
end if.
do if (pt1lp(kkk,1) < .00000001).
compute pt1lp(kkk,1) = .00000001.
end if.
end loop.
compute itprob = 0.
end if.
do if (itprob = 0).
do if ( 2 =2).
compute LL = ylp&*ln(pt1lp)+(1-ylp)&*ln(1-pt1lp).
end if.
compute LL2 = -2*csum(ll).
end if.
do if (abs(LL1-LL2) < converge).
do if ( 0 =1).
compute xlptmp=t(xlp).
compute vecprb=pt1lp&*(1-pt1lp).
loop kkk=1 to ncol(xlp).
compute xlptmp(kkk,:)=xlptmp(kkk,:)&*t(vecprb).
end loop.
compute varb = inv(xlptmp*xlp).
compute seb = sqrt(diag(varb)).
release xlptmp.
end if.
break.
end if.
compute bt1 = b.
compute LL1 = LL2.
end loop.
compute modres=b.
do if (jjj > iterate).
compute itprob = 2.
do if (booting=0).
compute iterrmod=1.
end if.
do if (booting=1).
compute bootiter=1.
end if.
do if (itprobtg=0).
compute itprobtg=1.
compute errcode(errs,1) = 47.
compute errs = errs + 1.
do if (booting = 0 and 0 =1).
compute vt1 = mdiag(pt1lp&*(1-pt1lp)).
compute varb = inv(t(xlp)*vt1*xlp).
compute seb = sqrt(diag(varb)).
end if.
end if.
end if.
do if ( 0 =1).
compute trat = b&/seb.
compute dfres=nrow(xlp).
compute p = 2*(1-cdfnorm(abs(trat))).
compute modres={modres,seb,trat,p}.
compute modres={modres,(b-xp2&*seb),(b+xp2&*seb)}.
compute pvchi=1-chicdf((LL3-LL2),(nrow(modres)-1)).
compute mcF = (LL3-LL2)/LL3.
compute cox = 1-exp(-(LL3-LL2)/nrow(xlp)).
compute nagel = cox/(1-exp(-(LL3)/nrow(xlp))).
compute modsum={LL2,(LL3-LL2),(nrow(modres)-1),pvchi, mcF,cox,nagel}.
compute modsuml={'-2LL','ModelLL', 'df', 'p', 'McFadden', 'CoxSnell', 'Nagelkrk'}.
compute modresl={'coeff','se','Z','p','LLCI','ULCI'}.
end if.
end if
.
compute b=btemphld.
compute fresult={(LL2-basemod),llrdf,1-chicdf((LL2-basemod),llrdf)}
.
compute diromni=fresult.
end if.
end if.
do if (numint(1,i) > 0).
compute intkeym=intkey(intstart:(intstart+numint(1,i)-1),:).
do if (outscree=1).
print intkeym/title='Product terms key:'/format = A8.
end if.
end if.
do if (covcoeff=1).
do if (outscree=1).
print varb/title='Covariance matrix of regression parameter estimates:'/rnames=vlabsm /cnames=vlabsm/format= F10.4.
end if.
compute resultm2=make(nrow(varb),maxresm,99999).
do if (ncol(varb) <= maxresm).
compute resultm2(1:nrow(varb),1:ncol(varb))=varb.
compute resultm={resultm;resultm2}.
end if.
do if (ncol(varb) > maxresm).
compute resultmt=make(nrow(resultm),ncol(varb),99999).
compute resultmt(1:nrow(resultm),1:ncol(resultm))=resultm.
compute resultm=resultmt.
compute resultm2=make(nrow(varb),ncol(resultm),99999).
compute resultm2(1:nrow(varb),1:ncol(varb))=varb.
compute resultm={resultm;resultm2}.
compute maxresm=ncol(resultm).
end if.
end if.
do if (model <> 74 and xmtest=1 and nms > 0).
compute r2tmp=r2.
compute btmp=b.
compute varbtmp=varb.
compute dfrestmp=dfres.
compute tvaltmp=tval.
compute xmtst=make(nms,4,0).
compute xmtstlbc={hcflab,'df1','df2','p'}.
do if ((i=(nms+nys)) and (ydich=1)).
compute xmtst=make(nms,3,0).
compute xmtstlbc={'Chi-sq','df','p'}.
end if.
compute xmtstlb={' '}.
compute xmtmat=x.
compute numxint=0.
loop xmints=2 to i.
compute x=xmtmat.
do if ((bcmat((i+1),xmints)=1) and (wzcmat((i+1),xmints) <> 1)).
do if (bcmat((i+1),1)=0).
compute x={xmtmat,xtmp}.
do if ((ydich=1) and (i=(nms+nys))).

.
do if ( 2 =1).
compute hatmat=inv(t( x )* x )*t( x ).
compute b = hatmat* y.
compute modres=b.
do if ( 1 =1).
compute n1=nrow( x ).
compute dfres=n1-(ncol( x )).
compute sstotal = t( y -(csum( y )/n1))*( y -(csum( y )/n1)).
compute resid= y - x *b.
compute ssresid = csum((resid)&**2).
compute r2 = (sstotal-ssresid)/sstotal.
do if (r2 < 0).
compute r2=0.
end if.
compute adjr2 = 1-((1-r2)*(n1-1)/(dfres)).
compute mse=ssresid/(n1-ncol( x )).

.
compute n1=nrow( x ).
compute invXtX = inv(t( x )* x ).
compute varb = mse *invXtX.
compute k3 = ncol( x ).
compute xhc=0.
do if ( hc <> 5).
compute xhc= x.
compute hat = xhc(:,1).
loop i3=1 to nrow(xhc).
compute hat(i3,1)= xhc(i3,:)*invXtX*t(xhc(i3,:)).
end loop.
do if ( hc = 0 or hc =1).
loop i3 = 1 to k3.
compute xhc(:,i3)=xhc(:,i3)&* resid.
end loop.
end if.
do if ( hc =3 or hc =2).
loop i3=1 to k3.
compute xhc(:,i3) = ( resid &/(1-hat)&**(1/(4- hc )))&*xhc(:,i3).
end loop.
end if.
do if ( hc = 4).
compute hcmn=make(n,2,4).
compute hcmn(:,2)=(n1*hat)/k3.
loop i3= 1 to k3.
compute xhc(:,i3) = ( resid &/(1-hat)&**(rmin(hcmn)/2))&*xhc(:,i3).
end loop.
end if.
compute varb=(invXtX*t(xhc)*xhc*invXtX).
do if ( hc =1).
compute varb=(n1/(n1-ncol( x )))&*varb.
end if.
end if.
compute hclab={'se(HC0)','se(HC1)','se(HC2)','se(HC3)','se(HC4)','se'}.
compute hclab=hclab(1,( hc +1)).
compute hcflab={'F(HC0)','F(HC1)','F(HC2)','F(HC3)','F(HC4)','F'}.
compute hcflab=hcflab(1,( hc +1)).
release xhc
.
compute seb=sqrt(diag(varb)).
compute trat = b&/seb.
compute p = 2*(1-tcdf(abs(trat), (dfres))).
compute tval = sqrt(dfres* (exp((dfres-(5/6))*((xp2/(dfres-(2/3)+(.11/dfres)))* (xp2/(dfres-(2/3)+(.11/dfres)))))-1)).
compute modres={modres,seb,trat,p}.
compute modres={modres,(b-tval&*seb),(b+tval&*seb)}.
compute modresl={'coeff',hclab,'t','p','LLCI','ULCI'}.
compute lmat = ident(ncol( x )).
compute lmat = lmat(:,2:ncol(lmat)).
compute fratio = (t(t(lmat)*b)*inv(t(lmat)*varb*lmat)*((t(lmat)*b)))/(ncol( x )-1).
compute pfr = 1-fcdf(fratio,(ncol( x )-1),dfres).
compute modsum={sqrt(r2),r2,mse,fratio,(ncol( x )-1),dfres,pfr}.
compute modsuml={'R','R-sq','MSE',hcflab,'df1','df2', 'p'}.
end if.
end if.
do if ( 2 = 2 or 2 =3).
compute xlp= x.
compute ylp= y.
compute pt2 = make(nrow(ylp),1,(csum(ylp)/nrow(ylp))).
do if ( 2 =2).
compute LL3 = ylp&*ln(pt2)+(1-ylp)&*ln(1-pt2).
end if.
compute LL3 = -2*csum(LL3).
compute bt1 = make(ncol(xlp),1,0).
compute LL1 = 0.
compute pt1 = make(nrow(ylp),1,0.5).
compute pt1lp=pt1.
loop jjj = 1 to iterate.
compute xlptmp=t(xlp).
compute vecprb=pt1lp&*(1-pt1lp).
loop kkk=1 to ncol(xlp).
compute xlptmp(kkk,:)=xlptmp(kkk,:)&*t(vecprb).
end loop.
compute b = bt1+inv(xlptmp*xlp)*t(xlp)*(ylp-pt1lp).
do if ( 2 =2).
compute xlpb=xlp*b.
compute xlpbt=(xlpb > -709.7).
compute xlpb709=(1-xlpbt)*(-709.7).
compute xlpb=(xlpb&*xlpbt)+xlpb709.
compute pt1lp = 1/(1+exp(-(xlpb))).
end if.
compute itprob = csum((pt1lp < .00000001) or (pt1lp > .9999999)).
do if (itprob > 0).
loop kkk = 1 to nrow(pt1lp).
do if (pt1lp(kkk,1) > .9999999).
compute pt1lp(kkk,1) = .9999999.
end if.
do if (pt1lp(kkk,1) < .00000001).
compute pt1lp(kkk,1) = .00000001.
end if.
end loop.
compute itprob = 0.
end if.
do if (itprob = 0).
do if ( 2 =2).
compute LL = ylp&*ln(pt1lp)+(1-ylp)&*ln(1-pt1lp).
end if.
compute LL2 = -2*csum(ll).
end if.
do if (abs(LL1-LL2) < converge).
do if ( 1 =1).
compute xlptmp=t(xlp).
compute vecprb=pt1lp&*(1-pt1lp).
loop kkk=1 to ncol(xlp).
compute xlptmp(kkk,:)=xlptmp(kkk,:)&*t(vecprb).
end loop.
compute varb = inv(xlptmp*xlp).
compute seb = sqrt(diag(varb)).
release xlptmp.
end if.
break.
end if.
compute bt1 = b.
compute LL1 = LL2.
end loop.
compute modres=b.
do if (jjj > iterate).
compute itprob = 2.
do if (booting=0).
compute iterrmod=1.
end if.
do if (booting=1).
compute bootiter=1.
end if.
do if (itprobtg=0).
compute itprobtg=1.
compute errcode(errs,1) = 47.
compute errs = errs + 1.
do if (booting = 0 and 1 =1).
compute vt1 = mdiag(pt1lp&*(1-pt1lp)).
compute varb = inv(t(xlp)*vt1*xlp).
compute seb = sqrt(diag(varb)).
end if.
end if.
end if.
do if ( 1 =1).
compute trat = b&/seb.
compute dfres=nrow(xlp).
compute p = 2*(1-cdfnorm(abs(trat))).
compute modres={modres,seb,trat,p}.
compute modres={modres,(b-xp2&*seb),(b+xp2&*seb)}.
compute pvchi=1-chicdf((LL3-LL2),(nrow(modres)-1)).
compute mcF = (LL3-LL2)/LL3.
compute cox = 1-exp(-(LL3-LL2)/nrow(xlp)).
compute nagel = cox/(1-exp(-(LL3)/nrow(xlp))).
compute modsum={LL2,(LL3-LL2),(nrow(modres)-1),pvchi, mcF,cox,nagel}.
compute modsuml={'-2LL','ModelLL', 'df', 'p', 'McFadden', 'CoxSnell', 'Nagelkrk'}.
compute modresl={'coeff','se','Z','p','LLCI','ULCI'}.
end if.
end if
.
compute basemodx=LL2.
end if.
end if.
loop xmtlp1=1 to nxvls.
compute x={x,xtmp(:,xmtlp1)&*(mtmp(:,(xmints-1))-(csum(mtmp(:,(xmints-1)))/nrow(mtmp)) )}.
end loop.
do if ((i < (nms+nys)) or (ydich=0)).

.
do if ( 1 =1).
compute hatmat=inv(t( x )* x )*t( x ).
compute b = hatmat* y.
compute modres=b.
do if ( 1 =1).
compute n1=nrow( x ).
compute dfres=n1-(ncol( x )).
compute sstotal = t( y -(csum( y )/n1))*( y -(csum( y )/n1)).
compute resid= y - x *b.
compute ssresid = csum((resid)&**2).
compute r2 = (sstotal-ssresid)/sstotal.
do if (r2 < 0).
compute r2=0.
end if.
compute adjr2 = 1-((1-r2)*(n1-1)/(dfres)).
compute mse=ssresid/(n1-ncol( x )).

.
compute n1=nrow( x ).
compute invXtX = inv(t( x )* x ).
compute varb = mse *invXtX.
compute k3 = ncol( x ).
compute xhc=0.
do if ( hc <> 5).
compute xhc= x.
compute hat = xhc(:,1).
loop i3=1 to nrow(xhc).
compute hat(i3,1)= xhc(i3,:)*invXtX*t(xhc(i3,:)).
end loop.
do if ( hc = 0 or hc =1).
loop i3 = 1 to k3.
compute xhc(:,i3)=xhc(:,i3)&* resid.
end loop.
end if.
do if ( hc =3 or hc =2).
loop i3=1 to k3.
compute xhc(:,i3) = ( resid &/(1-hat)&**(1/(4- hc )))&*xhc(:,i3).
end loop.
end if.
do if ( hc = 4).
compute hcmn=make(n,2,4).
compute hcmn(:,2)=(n1*hat)/k3.
loop i3= 1 to k3.
compute xhc(:,i3) = ( resid &/(1-hat)&**(rmin(hcmn)/2))&*xhc(:,i3).
end loop.
end if.
compute varb=(invXtX*t(xhc)*xhc*invXtX).
do if ( hc =1).
compute varb=(n1/(n1-ncol( x )))&*varb.
end if.
end if.
compute hclab={'se(HC0)','se(HC1)','se(HC2)','se(HC3)','se(HC4)','se'}.
compute hclab=hclab(1,( hc +1)).
compute hcflab={'F(HC0)','F(HC1)','F(HC2)','F(HC3)','F(HC4)','F'}.
compute hcflab=hcflab(1,( hc +1)).
release xhc
.
compute seb=sqrt(diag(varb)).
compute trat = b&/seb.
compute p = 2*(1-tcdf(abs(trat), (dfres))).
compute tval = sqrt(dfres* (exp((dfres-(5/6))*((xp2/(dfres-(2/3)+(.11/dfres)))* (xp2/(dfres-(2/3)+(.11/dfres)))))-1)).
compute modres={modres,seb,trat,p}.
compute modres={modres,(b-tval&*seb),(b+tval&*seb)}.
compute modresl={'coeff',hclab,'t','p','LLCI','ULCI'}.
compute lmat = ident(ncol( x )).
compute lmat = lmat(:,2:ncol(lmat)).
compute fratio = (t(t(lmat)*b)*inv(t(lmat)*varb*lmat)*((t(lmat)*b)))/(ncol( x )-1).
compute pfr = 1-fcdf(fratio,(ncol( x )-1),dfres).
compute modsum={sqrt(r2),r2,mse,fratio,(ncol( x )-1),dfres,pfr}.
compute modsuml={'R','R-sq','MSE',hcflab,'df1','df2', 'p'}.
end if.
end if.
do if ( 1 = 2 or 1 =3).
compute xlp= x.
compute ylp= y.
compute pt2 = make(nrow(ylp),1,(csum(ylp)/nrow(ylp))).
do if ( 1 =2).
compute LL3 = ylp&*ln(pt2)+(1-ylp)&*ln(1-pt2).
end if.
compute LL3 = -2*csum(LL3).
compute bt1 = make(ncol(xlp),1,0).
compute LL1 = 0.
compute pt1 = make(nrow(ylp),1,0.5).
compute pt1lp=pt1.
loop jjj = 1 to iterate.
compute xlptmp=t(xlp).
compute vecprb=pt1lp&*(1-pt1lp).
loop kkk=1 to ncol(xlp).
compute xlptmp(kkk,:)=xlptmp(kkk,:)&*t(vecprb).
end loop.
compute b = bt1+inv(xlptmp*xlp)*t(xlp)*(ylp-pt1lp).
do if ( 1 =2).
compute xlpb=xlp*b.
compute xlpbt=(xlpb > -709.7).
compute xlpb709=(1-xlpbt)*(-709.7).
compute xlpb=(xlpb&*xlpbt)+xlpb709.
compute pt1lp = 1/(1+exp(-(xlpb))).
end if.
compute itprob = csum((pt1lp < .00000001) or (pt1lp > .9999999)).
do if (itprob > 0).
loop kkk = 1 to nrow(pt1lp).
do if (pt1lp(kkk,1) > .9999999).
compute pt1lp(kkk,1) = .9999999.
end if.
do if (pt1lp(kkk,1) < .00000001).
compute pt1lp(kkk,1) = .00000001.
end if.
end loop.
compute itprob = 0.
end if.
do if (itprob = 0).
do if ( 1 =2).
compute LL = ylp&*ln(pt1lp)+(1-ylp)&*ln(1-pt1lp).
end if.
compute LL2 = -2*csum(ll).
end if.
do if (abs(LL1-LL2) < converge).
do if ( 1 =1).
compute xlptmp=t(xlp).
compute vecprb=pt1lp&*(1-pt1lp).
loop kkk=1 to ncol(xlp).
compute xlptmp(kkk,:)=xlptmp(kkk,:)&*t(vecprb).
end loop.
compute varb = inv(xlptmp*xlp).
compute seb = sqrt(diag(varb)).
release xlptmp.
end if.
break.
end if.
compute bt1 = b.
compute LL1 = LL2.
end loop.
compute modres=b.
do if (jjj > iterate).
compute itprob = 2.
do if (booting=0).
compute iterrmod=1.
end if.
do if (booting=1).
compute bootiter=1.
end if.
do if (itprobtg=0).
compute itprobtg=1.
compute errcode(errs,1) = 47.
compute errs = errs + 1.
do if (booting = 0 and 1 =1).
compute vt1 = mdiag(pt1lp&*(1-pt1lp)).
compute varb = inv(t(xlp)*vt1*xlp).
compute seb = sqrt(diag(varb)).
end if.
end if.
end if.
do if ( 1 =1).
compute trat = b&/seb.
compute dfres=nrow(xlp).
compute p = 2*(1-cdfnorm(abs(trat))).
compute modres={modres,seb,trat,p}.
compute modres={modres,(b-xp2&*seb),(b+xp2&*seb)}.
compute pvchi=1-chicdf((LL3-LL2),(nrow(modres)-1)).
compute mcF = (LL3-LL2)/LL3.
compute cox = 1-exp(-(LL3-LL2)/nrow(xlp)).
compute nagel = cox/(1-exp(-(LL3)/nrow(xlp))).
compute modsum={LL2,(LL3-LL2),(nrow(modres)-1),pvchi, mcF,cox,nagel}.
compute modsuml={'-2LL','ModelLL', 'df', 'p', 'McFadden', 'CoxSnell', 'Nagelkrk'}.
compute modresl={'coeff','se','Z','p','LLCI','ULCI'}.
end if.
end if
.
end if.
do if ((i = (nms+nys)) and (ydich=1)).

.
do if ( 2 =1).
compute hatmat=inv(t( x )* x )*t( x ).
compute b = hatmat* y.
compute modres=b.
do if ( 1 =1).
compute n1=nrow( x ).
compute dfres=n1-(ncol( x )).
compute sstotal = t( y -(csum( y )/n1))*( y -(csum( y )/n1)).
compute resid= y - x *b.
compute ssresid = csum((resid)&**2).
compute r2 = (sstotal-ssresid)/sstotal.
do if (r2 < 0).
compute r2=0.
end if.
compute adjr2 = 1-((1-r2)*(n1-1)/(dfres)).
compute mse=ssresid/(n1-ncol( x )).

.
compute n1=nrow( x ).
compute invXtX = inv(t( x )* x ).
compute varb = mse *invXtX.
compute k3 = ncol( x ).
compute xhc=0.
do if ( hc <> 5).
compute xhc= x.
compute hat = xhc(:,1).
loop i3=1 to nrow(xhc).
compute hat(i3,1)= xhc(i3,:)*invXtX*t(xhc(i3,:)).
end loop.
do if ( hc = 0 or hc =1).
loop i3 = 1 to k3.
compute xhc(:,i3)=xhc(:,i3)&* resid.
end loop.
end if.
do if ( hc =3 or hc =2).
loop i3=1 to k3.
compute xhc(:,i3) = ( resid &/(1-hat)&**(1/(4- hc )))&*xhc(:,i3).
end loop.
end if.
do if ( hc = 4).
compute hcmn=make(n,2,4).
compute hcmn(:,2)=(n1*hat)/k3.
loop i3= 1 to k3.
compute xhc(:,i3) = ( resid &/(1-hat)&**(rmin(hcmn)/2))&*xhc(:,i3).
end loop.
end if.
compute varb=(invXtX*t(xhc)*xhc*invXtX).
do if ( hc =1).
compute varb=(n1/(n1-ncol( x )))&*varb.
end if.
end if.
compute hclab={'se(HC0)','se(HC1)','se(HC2)','se(HC3)','se(HC4)','se'}.
compute hclab=hclab(1,( hc +1)).
compute hcflab={'F(HC0)','F(HC1)','F(HC2)','F(HC3)','F(HC4)','F'}.
compute hcflab=hcflab(1,( hc +1)).
release xhc
.
compute seb=sqrt(diag(varb)).
compute trat = b&/seb.
compute p = 2*(1-tcdf(abs(trat), (dfres))).
compute tval = sqrt(dfres* (exp((dfres-(5/6))*((xp2/(dfres-(2/3)+(.11/dfres)))* (xp2/(dfres-(2/3)+(.11/dfres)))))-1)).
compute modres={modres,seb,trat,p}.
compute modres={modres,(b-tval&*seb),(b+tval&*seb)}.
compute modresl={'coeff',hclab,'t','p','LLCI','ULCI'}.
compute lmat = ident(ncol( x )).
compute lmat = lmat(:,2:ncol(lmat)).
compute fratio = (t(t(lmat)*b)*inv(t(lmat)*varb*lmat)*((t(lmat)*b)))/(ncol( x )-1).
compute pfr = 1-fcdf(fratio,(ncol( x )-1),dfres).
compute modsum={sqrt(r2),r2,mse,fratio,(ncol( x )-1),dfres,pfr}.
compute modsuml={'R','R-sq','MSE',hcflab,'df1','df2', 'p'}.
end if.
end if.
do if ( 2 = 2 or 2 =3).
compute xlp= x.
compute ylp= y.
compute pt2 = make(nrow(ylp),1,(csum(ylp)/nrow(ylp))).
do if ( 2 =2).
compute LL3 = ylp&*ln(pt2)+(1-ylp)&*ln(1-pt2).
end if.
compute LL3 = -2*csum(LL3).
compute bt1 = make(ncol(xlp),1,0).
compute LL1 = 0.
compute pt1 = make(nrow(ylp),1,0.5).
compute pt1lp=pt1.
loop jjj = 1 to iterate.
compute xlptmp=t(xlp).
compute vecprb=pt1lp&*(1-pt1lp).
loop kkk=1 to ncol(xlp).
compute xlptmp(kkk,:)=xlptmp(kkk,:)&*t(vecprb).
end loop.
compute b = bt1+inv(xlptmp*xlp)*t(xlp)*(ylp-pt1lp).
do if ( 2 =2).
compute xlpb=xlp*b.
compute xlpbt=(xlpb > -709.7).
compute xlpb709=(1-xlpbt)*(-709.7).
compute xlpb=(xlpb&*xlpbt)+xlpb709.
compute pt1lp = 1/(1+exp(-(xlpb))).
end if.
compute itprob = csum((pt1lp < .00000001) or (pt1lp > .9999999)).
do if (itprob > 0).
loop kkk = 1 to nrow(pt1lp).
do if (pt1lp(kkk,1) > .9999999).
compute pt1lp(kkk,1) = .9999999.
end if.
do if (pt1lp(kkk,1) < .00000001).
compute pt1lp(kkk,1) = .00000001.
end if.
end loop.
compute itprob = 0.
end if.
do if (itprob = 0).
do if ( 2 =2).
compute LL = ylp&*ln(pt1lp)+(1-ylp)&*ln(1-pt1lp).
end if.
compute LL2 = -2*csum(ll).
end if.
do if (abs(LL1-LL2) < converge).
do if ( 1 =1).
compute xlptmp=t(xlp).
compute vecprb=pt1lp&*(1-pt1lp).
loop kkk=1 to ncol(xlp).
compute xlptmp(kkk,:)=xlptmp(kkk,:)&*t(vecprb).
end loop.
compute varb = inv(xlptmp*xlp).
compute seb = sqrt(diag(varb)).
release xlptmp.
end if.
break.
end if.
compute bt1 = b.
compute LL1 = LL2.
end loop.
compute modres=b.
do if (jjj > iterate).
compute itprob = 2.
do if (booting=0).
compute iterrmod=1.
end if.
do if (booting=1).
compute bootiter=1.
end if.
do if (itprobtg=0).
compute itprobtg=1.
compute errcode(errs,1) = 47.
compute errs = errs + 1.
do if (booting = 0 and 1 =1).
compute vt1 = mdiag(pt1lp&*(1-pt1lp)).
compute varb = inv(t(xlp)*vt1*xlp).
compute seb = sqrt(diag(varb)).
end if.
end if.
end if.
do if ( 1 =1).
compute trat = b&/seb.
compute dfres=nrow(xlp).
compute p = 2*(1-cdfnorm(abs(trat))).
compute modres={modres,seb,trat,p}.
compute modres={modres,(b-xp2&*seb),(b+xp2&*seb)}.
compute pvchi=1-chicdf((LL3-LL2),(nrow(modres)-1)).
compute mcF = (LL3-LL2)/LL3.
compute cox = 1-exp(-(LL3-LL2)/nrow(xlp)).
compute nagel = cox/(1-exp(-(LL3)/nrow(xlp))).
compute modsum={LL2,(LL3-LL2),(nrow(modres)-1),pvchi, mcF,cox,nagel}.
compute modsuml={'-2LL','ModelLL', 'df', 'p', 'McFadden', 'CoxSnell', 'Nagelkrk'}.
compute modresl={'coeff','se','Z','p','LLCI','ULCI'}.
end if.
end if
.
compute chidfxm=basemodx-LL2.
end if.
compute lmat=make(nrow(b),nxvls,0).
compute lmattmp=ident(nxvls).
compute lmat((nrow(lmat)-nxvls+1):nrow(lmat),:)=lmattmp.

.
compute lmat2= lmat.
do if ( 1 =0).
compute lmat2 = mdiag( lmat ).
compute lmat3=make(nrow(lmat2),1,0).
loop flp=1 to ncol(lmat2).
do if (csum(lmat2(:,flp))=1).
compute lmat3={lmat3,lmat2(:,flp)}.
end if.
end loop.
compute lmat2=lmat3(:,2:ncol(lmat3)).
end if.
compute fratio = (t(t(lmat2)* b )*inv(t(lmat2)* varb *lmat2)*((t(lmat2)* b )))/ncol(lmat2).
compute pfr = 1-fcdf(fratio,ncol(lmat2),(n-nrow( b ))).
compute fresult={fratio,ncol(lmat2),(n-nrow( b )),pfr}.
do if (i = (nms+nys) and (ydich=1)).
compute fratio=fratio*ncol(lmat2).
compute pfr=1-chicdf(fratio,ncol(lmat2)).
compute fresult={fratio,ncol(lmat2),pfr}.
end if.
do if ( 0 =1).
compute lmat3=1-rsum(lmat2).
compute xfm=make(n,csum(lmat3),0).
compute flpc=1.
loop flp=1 to nrow(lmat3).
do if (lmat3(flp,1)=1).
compute xfm(:,flpc)=x(:,flp).
compute flpc=flpc+1.
end if.
end loop.
compute bfm=inv(t(xfm)*xfm)*t(xfm)*y.
compute resid=y-(xfm*bfm).
compute sstotal=(y-(csum(y)/n)).
compute sstotal=csum(sstotal&*sstotal).
compute ssresid=csum(resid&*resid).
compute rsqch= 0 -((sstotal-ssresid)/sstotal).
compute fresult={rsqch,fresult}.
release xfm,flpc, resid, ssresid, bfm.
end if
.
compute numxint=numxint+1.
compute xmtst(numxint,:)=fresult.
do if ((i = (nms+nys)) and (ydich=1)).
compute xmtst(numxint,1)=chidfxm.
compute xmtst(numxint,3)=1-chicdf(chidfxm,nxvls).
end if.
compute xmtstlb={xmtstlb;highlbx((xmints-1),1)}.
end if.
end loop.
compute x=xmtmat.
release xmtmat.
do if (numxint>0).
compute xmtstlb=xmtstlb((2:(numxint+1)),:).
compute xmtst=xmtst(1:numxint,:).
compute resultm2=make(nrow(xmtst),maxresm,99999).
compute resultm2(1:nrow(xmtst),1:ncol(xmtst))=xmtst.
compute resultm={resultm;resultm2}.
do if (nms=1).
compute xmtstlb=' '.
end if.
do if (outscree=1).
do if ((i < (nms+nys)) or (ydich=0)).
print xmtst/title='Test(s) of X by M interaction:'/rnames=xmtstlb/cnames=xmtstlbc/format= F10.4.
end if.
do if ((i = (nms+nys)) and (ydich=1)).
print xmtst/title='Likelihood ratio test(s) of X by M interaction:'/rnames=xmtstlb/cnames=xmtstlbc/format= F10.4.
end if.
end if.
end if.
compute r2=r2tmp.
compute b=btmp.
compute varb=varbtmp.
compute dfres=dfrestmp.
compute tval=tvaltmp.
end if.
do if (criterr = 0).
compute jj=0.
loop j = start to ((start+i)-1).
compute dbint=0.
compute lmat=whigh(1:nump(1,i),j).
compute lmat2=wzhigh(1:nump(1,i),j).
do if ((csum(lmat) > 0) and (csum(lmat2) = 0)).
do if ((i < (nms+nys)) or (ydich <> 1)).

.
compute lmat2= lmat.
do if ( 0 =0).
compute lmat2 = mdiag( lmat ).
compute lmat3=make(nrow(lmat2),1,0).
loop flp=1 to ncol(lmat2).
do if (csum(lmat2(:,flp))=1).
compute lmat3={lmat3,lmat2(:,flp)}.
end if.
end loop.
compute lmat2=lmat3(:,2:ncol(lmat3)).
end if.
compute fratio = (t(t(lmat2)* b )*inv(t(lmat2)* varb *lmat2)*((t(lmat2)* b )))/ncol(lmat2).
compute pfr = 1-fcdf(fratio,ncol(lmat2),(n-nrow( b ))).
compute fresult={fratio,ncol(lmat2),(n-nrow( b )),pfr}.
do if (i = (nms+nys) and (ydich=1)).
compute fratio=fratio*ncol(lmat2).
compute pfr=1-chicdf(fratio,ncol(lmat2)).
compute fresult={fratio,ncol(lmat2),pfr}.
end if.
do if ( 1 =1).
compute lmat3=1-rsum(lmat2).
compute xfm=make(n,csum(lmat3),0).
compute flpc=1.
loop flp=1 to nrow(lmat3).
do if (lmat3(flp,1)=1).
compute xfm(:,flpc)=x(:,flp).
compute flpc=flpc+1.
end if.
end loop.
compute bfm=inv(t(xfm)*xfm)*t(xfm)*y.
compute resid=y-(xfm*bfm).
compute sstotal=(y-(csum(y)/n)).
compute sstotal=csum(sstotal&*sstotal).
compute ssresid=csum(resid&*resid).
compute rsqch= r2 -((sstotal-ssresid)/sstotal).
compute fresult={rsqch,fresult}.
release xfm,flpc, resid, ssresid, bfm.
end if
.
compute lmatdb=lmat.
compute dbint=dbint+1.
end if.
do if ((ydich = 1) and (i = (nms+nys))).

.
compute btemphld=b.
compute llrdat=make(nrow(x),nrow( lmat )-csum( lmat ),-999).
compute llrdf=ncol(x)-ncol(llrdat).
compute llrcnt=0.
loop llri=1 to nrow( lmat ).
do if ( lmat (llri,1)=0).
compute llrcnt=llrcnt+1.
compute llrdat(:,llrcnt)=x(:,llri).
end if.
end loop.

.
do if ( 2 =1).
compute hatmat=inv(t( llrdat )* llrdat )*t( llrdat ).
compute b = hatmat* y.
compute modres=b.
do if ( 0 =1).
compute n1=nrow( llrdat ).
compute dfres=n1-(ncol( llrdat )).
compute sstotal = t( y -(csum( y )/n1))*( y -(csum( y )/n1)).
compute resid= y - llrdat *b.
compute ssresid = csum((resid)&**2).
compute r2 = (sstotal-ssresid)/sstotal.
do if (r2 < 0).
compute r2=0.
end if.
compute adjr2 = 1-((1-r2)*(n1-1)/(dfres)).
compute mse=ssresid/(n1-ncol( llrdat )).

.
compute n1=nrow( x ).
compute invXtX = inv(t( x )* x ).
compute varb = mse *invXtX.
compute k3 = ncol( x ).
compute xhc=0.
do if ( hc <> 5).
compute xhc= x.
compute hat = xhc(:,1).
loop i3=1 to nrow(xhc).
compute hat(i3,1)= xhc(i3,:)*invXtX*t(xhc(i3,:)).
end loop.
do if ( hc = 0 or hc =1).
loop i3 = 1 to k3.
compute xhc(:,i3)=xhc(:,i3)&* resid.
end loop.
end if.
do if ( hc =3 or hc =2).
loop i3=1 to k3.
compute xhc(:,i3) = ( resid &/(1-hat)&**(1/(4- hc )))&*xhc(:,i3).
end loop.
end if.
do if ( hc = 4).
compute hcmn=make(n,2,4).
compute hcmn(:,2)=(n1*hat)/k3.
loop i3= 1 to k3.
compute xhc(:,i3) = ( resid &/(1-hat)&**(rmin(hcmn)/2))&*xhc(:,i3).
end loop.
end if.
compute varb=(invXtX*t(xhc)*xhc*invXtX).
do if ( hc =1).
compute varb=(n1/(n1-ncol( x )))&*varb.
end if.
end if.
compute hclab={'se(HC0)','se(HC1)','se(HC2)','se(HC3)','se(HC4)','se'}.
compute hclab=hclab(1,( hc +1)).
compute hcflab={'F(HC0)','F(HC1)','F(HC2)','F(HC3)','F(HC4)','F'}.
compute hcflab=hcflab(1,( hc +1)).
release xhc
.
compute seb=sqrt(diag(varb)).
compute trat = b&/seb.
compute p = 2*(1-tcdf(abs(trat), (dfres))).
compute tval = sqrt(dfres* (exp((dfres-(5/6))*((xp2/(dfres-(2/3)+(.11/dfres)))* (xp2/(dfres-(2/3)+(.11/dfres)))))-1)).
compute modres={modres,seb,trat,p}.
compute modres={modres,(b-tval&*seb),(b+tval&*seb)}.
compute modresl={'coeff',hclab,'t','p','LLCI','ULCI'}.
compute lmat = ident(ncol( llrdat )).
compute lmat = lmat(:,2:ncol(lmat)).
compute fratio = (t(t(lmat)*b)*inv(t(lmat)*varb*lmat)*((t(lmat)*b)))/(ncol( llrdat )-1).
compute pfr = 1-fcdf(fratio,(ncol( llrdat )-1),dfres).
compute modsum={sqrt(r2),r2,mse,fratio,(ncol( llrdat )-1),dfres,pfr}.
compute modsuml={'R','R-sq','MSE',hcflab,'df1','df2', 'p'}.
end if.
end if.
do if ( 2 = 2 or 2 =3).
compute xlp= llrdat.
compute ylp= y.
compute pt2 = make(nrow(ylp),1,(csum(ylp)/nrow(ylp))).
do if ( 2 =2).
compute LL3 = ylp&*ln(pt2)+(1-ylp)&*ln(1-pt2).
end if.
compute LL3 = -2*csum(LL3).
compute bt1 = make(ncol(xlp),1,0).
compute LL1 = 0.
compute pt1 = make(nrow(ylp),1,0.5).
compute pt1lp=pt1.
loop jjj = 1 to iterate.
compute xlptmp=t(xlp).
compute vecprb=pt1lp&*(1-pt1lp).
loop kkk=1 to ncol(xlp).
compute xlptmp(kkk,:)=xlptmp(kkk,:)&*t(vecprb).
end loop.
compute b = bt1+inv(xlptmp*xlp)*t(xlp)*(ylp-pt1lp).
do if ( 2 =2).
compute xlpb=xlp*b.
compute xlpbt=(xlpb > -709.7).
compute xlpb709=(1-xlpbt)*(-709.7).
compute xlpb=(xlpb&*xlpbt)+xlpb709.
compute pt1lp = 1/(1+exp(-(xlpb))).
end if.
compute itprob = csum((pt1lp < .00000001) or (pt1lp > .9999999)).
do if (itprob > 0).
loop kkk = 1 to nrow(pt1lp).
do if (pt1lp(kkk,1) > .9999999).
compute pt1lp(kkk,1) = .9999999.
end if.
do if (pt1lp(kkk,1) < .00000001).
compute pt1lp(kkk,1) = .00000001.
end if.
end loop.
compute itprob = 0.
end if.
do if (itprob = 0).
do if ( 2 =2).
compute LL = ylp&*ln(pt1lp)+(1-ylp)&*ln(1-pt1lp).
end if.
compute LL2 = -2*csum(ll).
end if.
do if (abs(LL1-LL2) < converge).
do if ( 0 =1).
compute xlptmp=t(xlp).
compute vecprb=pt1lp&*(1-pt1lp).
loop kkk=1 to ncol(xlp).
compute xlptmp(kkk,:)=xlptmp(kkk,:)&*t(vecprb).
end loop.
compute varb = inv(xlptmp*xlp).
compute seb = sqrt(diag(varb)).
release xlptmp.
end if.
break.
end if.
compute bt1 = b.
compute LL1 = LL2.
end loop.
compute modres=b.
do if (jjj > iterate).
compute itprob = 2.
do if (booting=0).
compute iterrmod=1.
end if.
do if (booting=1).
compute bootiter=1.
end if.
do if (itprobtg=0).
compute itprobtg=1.
compute errcode(errs,1) = 47.
compute errs = errs + 1.
do if (booting = 0 and 0 =1).
compute vt1 = mdiag(pt1lp&*(1-pt1lp)).
compute varb = inv(t(xlp)*vt1*xlp).
compute seb = sqrt(diag(varb)).
end if.
end if.
end if.
do if ( 0 =1).
compute trat = b&/seb.
compute dfres=nrow(xlp).
compute p = 2*(1-cdfnorm(abs(trat))).
compute modres={modres,seb,trat,p}.
compute modres={modres,(b-xp2&*seb),(b+xp2&*seb)}.
compute pvchi=1-chicdf((LL3-LL2),(nrow(modres)-1)).
compute mcF = (LL3-LL2)/LL3.
compute cox = 1-exp(-(LL3-LL2)/nrow(xlp)).
compute nagel = cox/(1-exp(-(LL3)/nrow(xlp))).
compute modsum={LL2,(LL3-LL2),(nrow(modres)-1),pvchi, mcF,cox,nagel}.
compute modsuml={'-2LL','ModelLL', 'df', 'p', 'McFadden', 'CoxSnell', 'Nagelkrk'}.
compute modresl={'coeff','se','Z','p','LLCI','ULCI'}.
end if.
end if
.
compute b=btemphld.
compute fresult={(LL2-basemod),llrdf,1-chicdf((LL2-basemod),llrdf)}
.
compute lmatdb=lmat.
compute dbint=dbint+1.
end if.
compute highf={highf;fresult}.
compute highf2={highf2;fresult}.
do if (j = start).
compute flabel={flabel;'X*W'}.
end if.
do if (j > start).
do if (nms > 1).
compute flabel={flabel;highlbw(jj,1)}.
else if (nms = 1).
do if (xmint=0).
compute flabel={flabel;'M*W'}.
end if.
do if (xmint=1).
compute flabel={flabel;'X*M'}.
end if.
end if.
end if.
end if.
compute lmat=zhigh(1:nump(1,i),j).
compute lmat2=wzhigh(1:nump(1,i),j).
do if ((csum(lmat) > 0) and (csum(lmat2) = 0)).
do if ((i < (nms+nys)) or (ydich <> 1)).

.
compute lmat2= lmat.
do if ( 0 =0).
compute lmat2 = mdiag( lmat ).
compute lmat3=make(nrow(lmat2),1,0).
loop flp=1 to ncol(lmat2).
do if (csum(lmat2(:,flp))=1).
compute lmat3={lmat3,lmat2(:,flp)}.
end if.
end loop.
compute lmat2=lmat3(:,2:ncol(lmat3)).
end if.
compute fratio = (t(t(lmat2)* b )*inv(t(lmat2)* varb *lmat2)*((t(lmat2)* b )))/ncol(lmat2).
compute pfr = 1-fcdf(fratio,ncol(lmat2),(n-nrow( b ))).
compute fresult={fratio,ncol(lmat2),(n-nrow( b )),pfr}.
do if (i = (nms+nys) and (ydich=1)).
compute fratio=fratio*ncol(lmat2).
compute pfr=1-chicdf(fratio,ncol(lmat2)).
compute fresult={fratio,ncol(lmat2),pfr}.
end if.
do if ( 1 =1).
compute lmat3=1-rsum(lmat2).
compute xfm=make(n,csum(lmat3),0).
compute flpc=1.
loop flp=1 to nrow(lmat3).
do if (lmat3(flp,1)=1).
compute xfm(:,flpc)=x(:,flp).
compute flpc=flpc+1.
end if.
end loop.
compute bfm=inv(t(xfm)*xfm)*t(xfm)*y.
compute resid=y-(xfm*bfm).
compute sstotal=(y-(csum(y)/n)).
compute sstotal=csum(sstotal&*sstotal).
compute ssresid=csum(resid&*resid).
compute rsqch= r2 -((sstotal-ssresid)/sstotal).
compute fresult={rsqch,fresult}.
release xfm,flpc, resid, ssresid, bfm.
end if
.
compute dbint=dbint+1.
end if.
do if ((ydich = 1) and (i = (nms+nys))).

.
compute btemphld=b.
compute llrdat=make(nrow(x),nrow( lmat )-csum( lmat ),-999).
compute llrdf=ncol(x)-ncol(llrdat).
compute llrcnt=0.
loop llri=1 to nrow( lmat ).
do if ( lmat (llri,1)=0).
compute llrcnt=llrcnt+1.
compute llrdat(:,llrcnt)=x(:,llri).
end if.
end loop.

.
do if ( 2 =1).
compute hatmat=inv(t( llrdat )* llrdat )*t( llrdat ).
compute b = hatmat* y.
compute modres=b.
do if ( 0 =1).
compute n1=nrow( llrdat ).
compute dfres=n1-(ncol( llrdat )).
compute sstotal = t( y -(csum( y )/n1))*( y -(csum( y )/n1)).
compute resid= y - llrdat *b.
compute ssresid = csum((resid)&**2).
compute r2 = (sstotal-ssresid)/sstotal.
do if (r2 < 0).
compute r2=0.
end if.
compute adjr2 = 1-((1-r2)*(n1-1)/(dfres)).
compute mse=ssresid/(n1-ncol( llrdat )).

.
compute n1=nrow( x ).
compute invXtX = inv(t( x )* x ).
compute varb = mse *invXtX.
compute k3 = ncol( x ).
compute xhc=0.
do if ( hc <> 5).
compute xhc= x.
compute hat = xhc(:,1).
loop i3=1 to nrow(xhc).
compute hat(i3,1)= xhc(i3,:)*invXtX*t(xhc(i3,:)).
end loop.
do if ( hc = 0 or hc =1).
loop i3 = 1 to k3.
compute xhc(:,i3)=xhc(:,i3)&* resid.
end loop.
end if.
do if ( hc =3 or hc =2).
loop i3=1 to k3.
compute xhc(:,i3) = ( resid &/(1-hat)&**(1/(4- hc )))&*xhc(:,i3).
end loop.
end if.
do if ( hc = 4).
compute hcmn=make(n,2,4).
compute hcmn(:,2)=(n1*hat)/k3.
loop i3= 1 to k3.
compute xhc(:,i3) = ( resid &/(1-hat)&**(rmin(hcmn)/2))&*xhc(:,i3).
end loop.
end if.
compute varb=(invXtX*t(xhc)*xhc*invXtX).
do if ( hc =1).
compute varb=(n1/(n1-ncol( x )))&*varb.
end if.
end if.
compute hclab={'se(HC0)','se(HC1)','se(HC2)','se(HC3)','se(HC4)','se'}.
compute hclab=hclab(1,( hc +1)).
compute hcflab={'F(HC0)','F(HC1)','F(HC2)','F(HC3)','F(HC4)','F'}.
compute hcflab=hcflab(1,( hc +1)).
release xhc
.
compute seb=sqrt(diag(varb)).
compute trat = b&/seb.
compute p = 2*(1-tcdf(abs(trat), (dfres))).
compute tval = sqrt(dfres* (exp((dfres-(5/6))*((xp2/(dfres-(2/3)+(.11/dfres)))* (xp2/(dfres-(2/3)+(.11/dfres)))))-1)).
compute modres={modres,seb,trat,p}.
compute modres={modres,(b-tval&*seb),(b+tval&*seb)}.
compute modresl={'coeff',hclab,'t','p','LLCI','ULCI'}.
compute lmat = ident(ncol( llrdat )).
compute lmat = lmat(:,2:ncol(lmat)).
compute fratio = (t(t(lmat)*b)*inv(t(lmat)*varb*lmat)*((t(lmat)*b)))/(ncol( llrdat )-1).
compute pfr = 1-fcdf(fratio,(ncol( llrdat )-1),dfres).
compute modsum={sqrt(r2),r2,mse,fratio,(ncol( llrdat )-1),dfres,pfr}.
compute modsuml={'R','R-sq','MSE',hcflab,'df1','df2', 'p'}.
end if.
end if.
do if ( 2 = 2 or 2 =3).
compute xlp= llrdat.
compute ylp= y.
compute pt2 = make(nrow(ylp),1,(csum(ylp)/nrow(ylp))).
do if ( 2 =2).
compute LL3 = ylp&*ln(pt2)+(1-ylp)&*ln(1-pt2).
end if.
compute LL3 = -2*csum(LL3).
compute bt1 = make(ncol(xlp),1,0).
compute LL1 = 0.
compute pt1 = make(nrow(ylp),1,0.5).
compute pt1lp=pt1.
loop jjj = 1 to iterate.
compute xlptmp=t(xlp).
compute vecprb=pt1lp&*(1-pt1lp).
loop kkk=1 to ncol(xlp).
compute xlptmp(kkk,:)=xlptmp(kkk,:)&*t(vecprb).
end loop.
compute b = bt1+inv(xlptmp*xlp)*t(xlp)*(ylp-pt1lp).
do if ( 2 =2).
compute xlpb=xlp*b.
compute xlpbt=(xlpb > -709.7).
compute xlpb709=(1-xlpbt)*(-709.7).
compute xlpb=(xlpb&*xlpbt)+xlpb709.
compute pt1lp = 1/(1+exp(-(xlpb))).
end if.
compute itprob = csum((pt1lp < .00000001) or (pt1lp > .9999999)).
do if (itprob > 0).
loop kkk = 1 to nrow(pt1lp).
do if (pt1lp(kkk,1) > .9999999).
compute pt1lp(kkk,1) = .9999999.
end if.
do if (pt1lp(kkk,1) < .00000001).
compute pt1lp(kkk,1) = .00000001.
end if.
end loop.
compute itprob = 0.
end if.
do if (itprob = 0).
do if ( 2 =2).
compute LL = ylp&*ln(pt1lp)+(1-ylp)&*ln(1-pt1lp).
end if.
compute LL2 = -2*csum(ll).
end if.
do if (abs(LL1-LL2) < converge).
do if ( 0 =1).
compute xlptmp=t(xlp).
compute vecprb=pt1lp&*(1-pt1lp).
loop kkk=1 to ncol(xlp).
compute xlptmp(kkk,:)=xlptmp(kkk,:)&*t(vecprb).
end loop.
compute varb = inv(xlptmp*xlp).
compute seb = sqrt(diag(varb)).
release xlptmp.
end if.
break.
end if.
compute bt1 = b.
compute LL1 = LL2.
end loop.
compute modres=b.
do if (jjj > iterate).
compute itprob = 2.
do if (booting=0).
compute iterrmod=1.
end if.
do if (booting=1).
compute bootiter=1.
end if.
do if (itprobtg=0).
compute itprobtg=1.
compute errcode(errs,1) = 47.
compute errs = errs + 1.
do if (booting = 0 and 0 =1).
compute vt1 = mdiag(pt1lp&*(1-pt1lp)).
compute varb = inv(t(xlp)*vt1*xlp).
compute seb = sqrt(diag(varb)).
end if.
end if.
end if.
do if ( 0 =1).
compute trat = b&/seb.
compute dfres=nrow(xlp).
compute p = 2*(1-cdfnorm(abs(trat))).
compute modres={modres,seb,trat,p}.
compute modres={modres,(b-xp2&*seb),(b+xp2&*seb)}.
compute pvchi=1-chicdf((LL3-LL2),(nrow(modres)-1)).
compute mcF = (LL3-LL2)/LL3.
compute cox = 1-exp(-(LL3-LL2)/nrow(xlp)).
compute nagel = cox/(1-exp(-(LL3)/nrow(xlp))).
compute modsum={LL2,(LL3-LL2),(nrow(modres)-1),pvchi, mcF,cox,nagel}.
compute modsuml={'-2LL','ModelLL', 'df', 'p', 'McFadden', 'CoxSnell', 'Nagelkrk'}.
compute modresl={'coeff','se','Z','p','LLCI','ULCI'}.
end if.
end if
.
compute b=btemphld.
compute fresult={(LL2-basemod),llrdf,1-chicdf((LL2-basemod),llrdf)}
.
compute dbint=dbint+1.
end if.
compute highf={highf;fresult}.
compute highf2={highf2;fresult}.
do if (j = start).
compute flabel={flabel;'X*Z'}.
end if.
do if (j > start).
do if (nms > 1).
compute flabel={flabel;highlbz(jj,1)}.
else if (nms = 1).
compute flabel={flabel;'M*Z'}.
end if.
end if.
end if.
do if (dbint=2).
compute lmatdb=lmatdb+lmat.
do if ((ydich = 1) and (i = (nms+nys))).

.
compute btemphld=b.
compute llrdat=make(nrow(x),nrow( lmatdb )-csum( lmatdb ),-999).
compute llrdf=ncol(x)-ncol(llrdat).
compute llrcnt=0.
loop llri=1 to nrow( lmatdb ).
do if ( lmatdb (llri,1)=0).
compute llrcnt=llrcnt+1.
compute llrdat(:,llrcnt)=x(:,llri).
end if.
end loop.

.
do if ( 2 =1).
compute hatmat=inv(t( llrdat )* llrdat )*t( llrdat ).
compute b = hatmat* y.
compute modres=b.
do if ( 0 =1).
compute n1=nrow( llrdat ).
compute dfres=n1-(ncol( llrdat )).
compute sstotal = t( y -(csum( y )/n1))*( y -(csum( y )/n1)).
compute resid= y - llrdat *b.
compute ssresid = csum((resid)&**2).
compute r2 = (sstotal-ssresid)/sstotal.
do if (r2 < 0).
compute r2=0.
end if.
compute adjr2 = 1-((1-r2)*(n1-1)/(dfres)).
compute mse=ssresid/(n1-ncol( llrdat )).

.
compute n1=nrow( x ).
compute invXtX = inv(t( x )* x ).
compute varb = mse *invXtX.
compute k3 = ncol( x ).
compute xhc=0.
do if ( hc <> 5).
compute xhc= x.
compute hat = xhc(:,1).
loop i3=1 to nrow(xhc).
compute hat(i3,1)= xhc(i3,:)*invXtX*t(xhc(i3,:)).
end loop.
do if ( hc = 0 or hc =1).
loop i3 = 1 to k3.
compute xhc(:,i3)=xhc(:,i3)&* resid.
end loop.
end if.
do if ( hc =3 or hc =2).
loop i3=1 to k3.
compute xhc(:,i3) = ( resid &/(1-hat)&**(1/(4- hc )))&*xhc(:,i3).
end loop.
end if.
do if ( hc = 4).
compute hcmn=make(n,2,4).
compute hcmn(:,2)=(n1*hat)/k3.
loop i3= 1 to k3.
compute xhc(:,i3) = ( resid &/(1-hat)&**(rmin(hcmn)/2))&*xhc(:,i3).
end loop.
end if.
compute varb=(invXtX*t(xhc)*xhc*invXtX).
do if ( hc =1).
compute varb=(n1/(n1-ncol( x )))&*varb.
end if.
end if.
compute hclab={'se(HC0)','se(HC1)','se(HC2)','se(HC3)','se(HC4)','se'}.
compute hclab=hclab(1,( hc +1)).
compute hcflab={'F(HC0)','F(HC1)','F(HC2)','F(HC3)','F(HC4)','F'}.
compute hcflab=hcflab(1,( hc +1)).
release xhc
.
compute seb=sqrt(diag(varb)).
compute trat = b&/seb.
compute p = 2*(1-tcdf(abs(trat), (dfres))).
compute tval = sqrt(dfres* (exp((dfres-(5/6))*((xp2/(dfres-(2/3)+(.11/dfres)))* (xp2/(dfres-(2/3)+(.11/dfres)))))-1)).
compute modres={modres,seb,trat,p}.
compute modres={modres,(b-tval&*seb),(b+tval&*seb)}.
compute modresl={'coeff',hclab,'t','p','LLCI','ULCI'}.
compute lmat = ident(ncol( llrdat )).
compute lmat = lmat(:,2:ncol(lmat)).
compute fratio = (t(t(lmat)*b)*inv(t(lmat)*varb*lmat)*((t(lmat)*b)))/(ncol( llrdat )-1).
compute pfr = 1-fcdf(fratio,(ncol( llrdat )-1),dfres).
compute modsum={sqrt(r2),r2,mse,fratio,(ncol( llrdat )-1),dfres,pfr}.
compute modsuml={'R','R-sq','MSE',hcflab,'df1','df2', 'p'}.
end if.
end if.
do if ( 2 = 2 or 2 =3).
compute xlp= llrdat.
compute ylp= y.
compute pt2 = make(nrow(ylp),1,(csum(ylp)/nrow(ylp))).
do if ( 2 =2).
compute LL3 = ylp&*ln(pt2)+(1-ylp)&*ln(1-pt2).
end if.
compute LL3 = -2*csum(LL3).
compute bt1 = make(ncol(xlp),1,0).
compute LL1 = 0.
compute pt1 = make(nrow(ylp),1,0.5).
compute pt1lp=pt1.
loop jjj = 1 to iterate.
compute xlptmp=t(xlp).
compute vecprb=pt1lp&*(1-pt1lp).
loop kkk=1 to ncol(xlp).
compute xlptmp(kkk,:)=xlptmp(kkk,:)&*t(vecprb).
end loop.
compute b = bt1+inv(xlptmp*xlp)*t(xlp)*(ylp-pt1lp).
do if ( 2 =2).
compute xlpb=xlp*b.
compute xlpbt=(xlpb > -709.7).
compute xlpb709=(1-xlpbt)*(-709.7).
compute xlpb=(xlpb&*xlpbt)+xlpb709.
compute pt1lp = 1/(1+exp(-(xlpb))).
end if.
compute itprob = csum((pt1lp < .00000001) or (pt1lp > .9999999)).
do if (itprob > 0).
loop kkk = 1 to nrow(pt1lp).
do if (pt1lp(kkk,1) > .9999999).
compute pt1lp(kkk,1) = .9999999.
end if.
do if (pt1lp(kkk,1) < .00000001).
compute pt1lp(kkk,1) = .00000001.
end if.
end loop.
compute itprob = 0.
end if.
do if (itprob = 0).
do if ( 2 =2).
compute LL = ylp&*ln(pt1lp)+(1-ylp)&*ln(1-pt1lp).
end if.
compute LL2 = -2*csum(ll).
end if.
do if (abs(LL1-LL2) < converge).
do if ( 0 =1).
compute xlptmp=t(xlp).
compute vecprb=pt1lp&*(1-pt1lp).
loop kkk=1 to ncol(xlp).
compute xlptmp(kkk,:)=xlptmp(kkk,:)&*t(vecprb).
end loop.
compute varb = inv(xlptmp*xlp).
compute seb = sqrt(diag(varb)).
release xlptmp.
end if.
break.
end if.
compute bt1 = b.
compute LL1 = LL2.
end loop.
compute modres=b.
do if (jjj > iterate).
compute itprob = 2.
do if (booting=0).
compute iterrmod=1.
end if.
do if (booting=1).
compute bootiter=1.
end if.
do if (itprobtg=0).
compute itprobtg=1.
compute errcode(errs,1) = 47.
compute errs = errs + 1.
do if (booting = 0 and 0 =1).
compute vt1 = mdiag(pt1lp&*(1-pt1lp)).
compute varb = inv(t(xlp)*vt1*xlp).
compute seb = sqrt(diag(varb)).
end if.
end if.
end if.
do if ( 0 =1).
compute trat = b&/seb.
compute dfres=nrow(xlp).
compute p = 2*(1-cdfnorm(abs(trat))).
compute modres={modres,seb,trat,p}.
compute modres={modres,(b-xp2&*seb),(b+xp2&*seb)}.
compute pvchi=1-chicdf((LL3-LL2),(nrow(modres)-1)).
compute mcF = (LL3-LL2)/LL3.
compute cox = 1-exp(-(LL3-LL2)/nrow(xlp)).
compute nagel = cox/(1-exp(-(LL3)/nrow(xlp))).
compute modsum={LL2,(LL3-LL2),(nrow(modres)-1),pvchi, mcF,cox,nagel}.
compute modsuml={'-2LL','ModelLL', 'df', 'p', 'McFadden', 'CoxSnell', 'Nagelkrk'}.
compute modresl={'coeff','se','Z','p','LLCI','ULCI'}.
end if.
end if
.
compute b=btemphld.
compute fresult={(LL2-basemod),llrdf,1-chicdf((LL2-basemod),llrdf)}
.
end if.
do if (ydich <> 1 or i < (nms+nys)).

.
compute lmat2= lmatdb.
do if ( 0 =0).
compute lmat2 = mdiag( lmatdb ).
compute lmat3=make(nrow(lmat2),1,0).
loop flp=1 to ncol(lmat2).
do if (csum(lmat2(:,flp))=1).
compute lmat3={lmat3,lmat2(:,flp)}.
end if.
end loop.
compute lmat2=lmat3(:,2:ncol(lmat3)).
end if.
compute fratio = (t(t(lmat2)* b )*inv(t(lmat2)* varb *lmat2)*((t(lmat2)* b )))/ncol(lmat2).
compute pfr = 1-fcdf(fratio,ncol(lmat2),(n-nrow( b ))).
compute fresult={fratio,ncol(lmat2),(n-nrow( b )),pfr}.
do if (i = (nms+nys) and (ydich=1)).
compute fratio=fratio*ncol(lmat2).
compute pfr=1-chicdf(fratio,ncol(lmat2)).
compute fresult={fratio,ncol(lmat2),pfr}.
end if.
do if ( 1 =1).
compute lmat3=1-rsum(lmat2).
compute xfm=make(n,csum(lmat3),0).
compute flpc=1.
loop flp=1 to nrow(lmat3).
do if (lmat3(flp,1)=1).
compute xfm(:,flpc)=x(:,flp).
compute flpc=flpc+1.
end if.
end loop.
compute bfm=inv(t(xfm)*xfm)*t(xfm)*y.
compute resid=y-(xfm*bfm).
compute sstotal=(y-(csum(y)/n)).
compute sstotal=csum(sstotal&*sstotal).
compute ssresid=csum(resid&*resid).
compute rsqch= r2 -((sstotal-ssresid)/sstotal).
compute fresult={rsqch,fresult}.
release xfm,flpc, resid, ssresid, bfm.
end if
.
end if.
compute dbint=0.
compute highf={highf;fresult}.
do if (jj=0 and nms > 0).
compute flabel={flabel;'BOTH(X)'}.
end if.
do if (jj=0 and nms = 0).
compute flabel={flabel;'BOTH'}.
end if.
do if (jj>0 and nms = 1).
compute flabel={flabel;'BOTH(M)'}.
end if.
do if (nms > 1 and jj > 0).
compute flabel={flabel;highlbbt(jj,1)}.
end if.
end if.
compute lmat2=wzhigh(1:nump(1,i),j).
do if (csum(lmat2) > 0).
do if ((i < (nms+nys)) or (ydich <> 1)).

.
compute lmat2= lmat2.
do if ( 0 =0).
compute lmat2 = mdiag( lmat2 ).
compute lmat3=make(nrow(lmat2),1,0).
loop flp=1 to ncol(lmat2).
do if (csum(lmat2(:,flp))=1).
compute lmat3={lmat3,lmat2(:,flp)}.
end if.
end loop.
compute lmat2=lmat3(:,2:ncol(lmat3)).
end if.
compute fratio = (t(t(lmat2)* b )*inv(t(lmat2)* varb *lmat2)*((t(lmat2)* b )))/ncol(lmat2).
compute pfr = 1-fcdf(fratio,ncol(lmat2),(n-nrow( b ))).
compute fresult={fratio,ncol(lmat2),(n-nrow( b )),pfr}.
do if (i = (nms+nys) and (ydich=1)).
compute fratio=fratio*ncol(lmat2).
compute pfr=1-chicdf(fratio,ncol(lmat2)).
compute fresult={fratio,ncol(lmat2),pfr}.
end if.
do if ( 1 =1).
compute lmat3=1-rsum(lmat2).
compute xfm=make(n,csum(lmat3),0).
compute flpc=1.
loop flp=1 to nrow(lmat3).
do if (lmat3(flp,1)=1).
compute xfm(:,flpc)=x(:,flp).
compute flpc=flpc+1.
end if.
end loop.
compute bfm=inv(t(xfm)*xfm)*t(xfm)*y.
compute resid=y-(xfm*bfm).
compute sstotal=(y-(csum(y)/n)).
compute sstotal=csum(sstotal&*sstotal).
compute ssresid=csum(resid&*resid).
compute rsqch= r2 -((sstotal-ssresid)/sstotal).
compute fresult={rsqch,fresult}.
release xfm,flpc, resid, ssresid, bfm.
end if
.
end if.
do if ((ydich = 1) and (i = (nms+nys))).

.
compute btemphld=b.
compute llrdat=make(nrow(x),nrow( lmat2 )-csum( lmat2 ),-999).
compute llrdf=ncol(x)-ncol(llrdat).
compute llrcnt=0.
loop llri=1 to nrow( lmat2 ).
do if ( lmat2 (llri,1)=0).
compute llrcnt=llrcnt+1.
compute llrdat(:,llrcnt)=x(:,llri).
end if.
end loop.

.
do if ( 2 =1).
compute hatmat=inv(t( llrdat )* llrdat )*t( llrdat ).
compute b = hatmat* y.
compute modres=b.
do if ( 0 =1).
compute n1=nrow( llrdat ).
compute dfres=n1-(ncol( llrdat )).
compute sstotal = t( y -(csum( y )/n1))*( y -(csum( y )/n1)).
compute resid= y - llrdat *b.
compute ssresid = csum((resid)&**2).
compute r2 = (sstotal-ssresid)/sstotal.
do if (r2 < 0).
compute r2=0.
end if.
compute adjr2 = 1-((1-r2)*(n1-1)/(dfres)).
compute mse=ssresid/(n1-ncol( llrdat )).

.
compute n1=nrow( x ).
compute invXtX = inv(t( x )* x ).
compute varb = mse *invXtX.
compute k3 = ncol( x ).
compute xhc=0.
do if ( hc <> 5).
compute xhc= x.
compute hat = xhc(:,1).
loop i3=1 to nrow(xhc).
compute hat(i3,1)= xhc(i3,:)*invXtX*t(xhc(i3,:)).
end loop.
do if ( hc = 0 or hc =1).
loop i3 = 1 to k3.
compute xhc(:,i3)=xhc(:,i3)&* resid.
end loop.
end if.
do if ( hc =3 or hc =2).
loop i3=1 to k3.
compute xhc(:,i3) = ( resid &/(1-hat)&**(1/(4- hc )))&*xhc(:,i3).
end loop.
end if.
do if ( hc = 4).
compute hcmn=make(n,2,4).
compute hcmn(:,2)=(n1*hat)/k3.
loop i3= 1 to k3.
compute xhc(:,i3) = ( resid &/(1-hat)&**(rmin(hcmn)/2))&*xhc(:,i3).
end loop.
end if.
compute varb=(invXtX*t(xhc)*xhc*invXtX).
do if ( hc =1).
compute varb=(n1/(n1-ncol( x )))&*varb.
end if.
end if.
compute hclab={'se(HC0)','se(HC1)','se(HC2)','se(HC3)','se(HC4)','se'}.
compute hclab=hclab(1,( hc +1)).
compute hcflab={'F(HC0)','F(HC1)','F(HC2)','F(HC3)','F(HC4)','F'}.
compute hcflab=hcflab(1,( hc +1)).
release xhc
.
compute seb=sqrt(diag(varb)).
compute trat = b&/seb.
compute p = 2*(1-tcdf(abs(trat), (dfres))).
compute tval = sqrt(dfres* (exp((dfres-(5/6))*((xp2/(dfres-(2/3)+(.11/dfres)))* (xp2/(dfres-(2/3)+(.11/dfres)))))-1)).
compute modres={modres,seb,trat,p}.
compute modres={modres,(b-tval&*seb),(b+tval&*seb)}.
compute modresl={'coeff',hclab,'t','p','LLCI','ULCI'}.
compute lmat = ident(ncol( llrdat )).
compute lmat = lmat(:,2:ncol(lmat)).
compute fratio = (t(t(lmat)*b)*inv(t(lmat)*varb*lmat)*((t(lmat)*b)))/(ncol( llrdat )-1).
compute pfr = 1-fcdf(fratio,(ncol( llrdat )-1),dfres).
compute modsum={sqrt(r2),r2,mse,fratio,(ncol( llrdat )-1),dfres,pfr}.
compute modsuml={'R','R-sq','MSE',hcflab,'df1','df2', 'p'}.
end if.
end if.
do if ( 2 = 2 or 2 =3).
compute xlp= llrdat.
compute ylp= y.
compute pt2 = make(nrow(ylp),1,(csum(ylp)/nrow(ylp))).
do if ( 2 =2).
compute LL3 = ylp&*ln(pt2)+(1-ylp)&*ln(1-pt2).
end if.
compute LL3 = -2*csum(LL3).
compute bt1 = make(ncol(xlp),1,0).
compute LL1 = 0.
compute pt1 = make(nrow(ylp),1,0.5).
compute pt1lp=pt1.
loop jjj = 1 to iterate.
compute xlptmp=t(xlp).
compute vecprb=pt1lp&*(1-pt1lp).
loop kkk=1 to ncol(xlp).
compute xlptmp(kkk,:)=xlptmp(kkk,:)&*t(vecprb).
end loop.
compute b = bt1+inv(xlptmp*xlp)*t(xlp)*(ylp-pt1lp).
do if ( 2 =2).
compute xlpb=xlp*b.
compute xlpbt=(xlpb > -709.7).
compute xlpb709=(1-xlpbt)*(-709.7).
compute xlpb=(xlpb&*xlpbt)+xlpb709.
compute pt1lp = 1/(1+exp(-(xlpb))).
end if.
compute itprob = csum((pt1lp < .00000001) or (pt1lp > .9999999)).
do if (itprob > 0).
loop kkk = 1 to nrow(pt1lp).
do if (pt1lp(kkk,1) > .9999999).
compute pt1lp(kkk,1) = .9999999.
end if.
do if (pt1lp(kkk,1) < .00000001).
compute pt1lp(kkk,1) = .00000001.
end if.
end loop.
compute itprob = 0.
end if.
do if (itprob = 0).
do if ( 2 =2).
compute LL = ylp&*ln(pt1lp)+(1-ylp)&*ln(1-pt1lp).
end if.
compute LL2 = -2*csum(ll).
end if.
do if (abs(LL1-LL2) < converge).
do if ( 0 =1).
compute xlptmp=t(xlp).
compute vecprb=pt1lp&*(1-pt1lp).
loop kkk=1 to ncol(xlp).
compute xlptmp(kkk,:)=xlptmp(kkk,:)&*t(vecprb).
end loop.
compute varb = inv(xlptmp*xlp).
compute seb = sqrt(diag(varb)).
release xlptmp.
end if.
break.
end if.
compute bt1 = b.
compute LL1 = LL2.
end loop.
compute modres=b.
do if (jjj > iterate).
compute itprob = 2.
do if (booting=0).
compute iterrmod=1.
end if.
do if (booting=1).
compute bootiter=1.
end if.
do if (itprobtg=0).
compute itprobtg=1.
compute errcode(errs,1) = 47.
compute errs = errs + 1.
do if (booting = 0 and 0 =1).
compute vt1 = mdiag(pt1lp&*(1-pt1lp)).
compute varb = inv(t(xlp)*vt1*xlp).
compute seb = sqrt(diag(varb)).
end if.
end if.
end if.
do if ( 0 =1).
compute trat = b&/seb.
compute dfres=nrow(xlp).
compute p = 2*(1-cdfnorm(abs(trat))).
compute modres={modres,seb,trat,p}.
compute modres={modres,(b-xp2&*seb),(b+xp2&*seb)}.
compute pvchi=1-chicdf((LL3-LL2),(nrow(modres)-1)).
compute mcF = (LL3-LL2)/LL3.
compute cox = 1-exp(-(LL3-LL2)/nrow(xlp)).
compute nagel = cox/(1-exp(-(LL3)/nrow(xlp))).
compute modsum={LL2,(LL3-LL2),(nrow(modres)-1),pvchi, mcF,cox,nagel}.
compute modsuml={'-2LL','ModelLL', 'df', 'p', 'McFadden', 'CoxSnell', 'Nagelkrk'}.
compute modresl={'coeff','se','Z','p','LLCI','ULCI'}.
end if.
end if
.
compute b=btemphld.
compute fresult={(LL2-basemod),llrdf,1-chicdf((LL2-basemod),llrdf)}
.
end if.
compute highf={highf;fresult}.
compute highf2={highf2;fresult}.
do if (j = start).
compute flabel={flabel;'X*W*Z'}.
end if.
do if (j > start).
do if (nms > 1).
compute flabel={flabel;highlbwz(jj,1)}.
else if (nms = 1).
compute flabel={flabel;'M*W*Z'}.
end if.
end if.
end if.
compute jj=jj+1.
end loop.
release jj.
compute start=start+i.
end if.
do if (nrow(highf) > 1).
compute highf=highf(2:nrow(highf),:).
compute highf2=highf2(2:nrow(highf2),:).
compute flabel=flabel(2:nrow(flabel),1).
compute resultm2=make(nrow(highf),maxresm,99999).
compute resultm2(1:nrow(highf),1:ncol(highf))=highf.
compute resultm={resultm;resultm2}.
do if (outscree=1).
do if ((i < nms+nys) or (ydich=0)).
compute clabtmp={'R2-chng', hcflab,'df1','df2','p'}.
print highf/format = F10.4 /rnames=flabel/cnames=clabtmp/ title = 'Test(s) of highest order unconditional interaction(s):'.
end if.
do if (ydich=1 and i=(nms+nys)).
compute clabtmp={'Chi-sq', 'df','p'}.
print/title='Likelihood ratio test(s) of highest order'.
print highf/format = F10.4 /rnames=flabel/cnames=clabtmp/ title = 'unconditional interactions(s):'/space=0.
end if.
end if.
compute intpb=highf2(:,ncol(highf2)).
end if.
compute intstart=intstart+numint(1,i).
end if.

.
do if (criterr=0).
compute threeway=0.
compute didprint=0.
compute didsome=0.
compute sigintct=0.
loop jmed =1 to (nms+1).
compute hasw=0.
compute hasz=0.
compute jnok=0.
compute nm1vls=0.
compute nm2vls=0.
compute panelgrp=0.
compute graphixs={'WITH', outnames(1,i), 'BY'}.
compute focpred4={' '}.
compute intprint=0.
compute modcat=0.
do if (jmed <= i).
do if ((jmed = 1) and ((i+1) = nrow(bcmat))).
compute pathscnt=pathscnt+1.
else.
compute paths={paths,bcmat((i+1),jmed)}.
compute pathsw={pathsw,wcmat((i+1),jmed)}.
compute pathsz={pathsz,zcmat((i+1),jmed)}.
compute pathswz={pathswz,wzcmat((i+1),jmed)}.
compute temp=fochigh(:,pathscnt)&*bootloc(:,i).
compute pathsfoc={pathsfoc,pathsfoc(:,1)}.
do if (jmed=1).
compute pathtype={pathtype,1}.
end if.
do if ((i+1)=nrow(bcmat)).
compute pathtype={pathtype,3}.
end if.
do if (jmed > 1) and ((i+1) < nrow(bcmat)).
compute pathtype={pathtype,2}.
end if.
do if (jmed=1 and nxvls > 1 and (bcmat((i+1),jmed)=1)).
compute pathsfoc(:,(pathscn2+1))=temp(2:(nxvls+1),1).
end if.
do if ((jmed > 1) or (jmed=1 and nxvls=1)).
compute temp=cmax(temp).
compute pathsfoc(1,(pathscn2+1))=temp.
end if.
compute pathscnt=pathscnt+1.
compute pathscn2=pathscn2+1.
do if (i <= nms).
compute pathsdv={pathsdv,mnames(1,i)}.
end if.
do if (i > nms).
compute pathsdv={pathsdv,ynames}.
end if.
end if.
compute coeffcol=coeffcol+1.
compute probettt=coeffs(1:nrow(b),coeffcol).
do if (jmed=1 and (bcmat((i+1),jmed)=1)).
compute omni=make(nrow(probettt),nxvls,0).
compute omnitmp=ident(nxvls).
compute omni(2:(1+nxvls),:)=omnitmp.
end if.
do if (csum(probettt)>0).
compute probvarb=make(csum(probettt),csum(probettt),999).
compute probcoef=make(csum(probettt),1,999).
compute coefflp2=1.
loop coefflp=1 to nrow(probettt).
do if (probettt(coefflp,1)=1).
compute probcoef(coefflp2,1)=b(coefflp,1).
compute coefflp2=coefflp2+1.
end if.
end loop.
compute coefflp=0.
compute coefflp2=0.
loop iclp=1 to nrow(probettt).
do if probettt(iclp,1)=1.
compute coefflp=coefflp+1.
compute coefflp2=coefflp.
compute probvarb(coefflp,coefflp) = varb(iclp,iclp).
do if (iclp < nrow(probettt)).
loop jclp=(iclp+1) to nrow(probettt).
do if (probettt(jclp,1)=1).
compute coefflp2=coefflp2+1.
compute probvarb(coefflp,coefflp2)=varb(iclp, jclp).
compute probvarb(coefflp2,coefflp)=varb(iclp, jclp).
end if.
end loop.
end if.
end if.
end loop.
end if.
end if.
compute xprobval=xmodvals.
do if (nxvls > 1 or mcx > 0).
compute xprobval=dummatx(:,2:ncol(dummatx)).
end if.
do if ((wcmat((i+1),jmed)=1) and (zcmat((i+1),jmed)=0)).
compute numplps=1.
compute modvals=wmodvals.
compute probeval=wmodvals.
compute wheremv1=wherexw.
compute nm1vls=nwvls.
compute lpstsp={1,1}.
compute modcat=0.
compute jnmod=wtmp.
compute jnmodlab=wnames.
compute jnok=1.
compute jnmin=wmin.
compute jnmax=wmax.
compute wherejn1=2.
do if (jmed=1).
compute wherejn3=wherexw(1,i).
do if (nxvls > 1).
compute jnok=0.
end if.
end if.
do if (jmed > 1).
compute wherejn1=wherem((jmed-1),i).
compute wherejn3=wheremw(((2*jmed)-3),i).
end if.
do if (nwvls > 1).
compute probeval=wprobval.
compute lpstsp(1,2)=ncol(probeval).
compute modcat=1.
compute jnok=0.
end if.
do if (wdich = 1).
compute modcat=1.
compute jnok=0.
end if.
compute problabs=wnames.
compute focpred3={wnames,'(W)'}.
do if (xmint=1).
compute focpred3={wnames,'(X)'}.
end if.
compute hasw=1.
compute modgrph=wnames.
compute intprint=1.
compute sigintct=sigintct+1.
compute printpbe=intpb(sigintct,1).
end if.
do if ((wcmat((i+1),jmed)=0) and (zcmat((i+1),jmed)=1)).
compute numplps=1.
compute modvals=zmodvals.
compute probeval=zmodvals.
compute wheremv1=wherexz.
compute nm1vls=nzvls.
compute lpstsp={1,1}.
compute jnok=1.
compute jnmod=ztmp.
compute jnmin=zmin.
compute jnmax=zmax.
compute jnmodlab=znames.
compute wherejn1=2.
do if (jmed=1).
compute wherejn3=wherexz(1,i).
do if (nxvls > 1).
compute jnok=0.
end if.
end if.
do if (jmed > 1).
compute wherejn1=wherem((jmed-1),i).
compute wherejn3=wheremz(((2*jmed)-3),i).
end if.
do if (nzvls > 1).
compute probeval=zprobval.
compute lpstsp(1,2)=ncol(probeval).
compute modcat=1.
compute jnok=0.
end if.
do if (zdich = 1).
compute modcat=1.
compute jnok=0.
end if.
compute problabs=znames.
compute focpred3={znames,'(Z)'}.
compute modgrph=znames.
compute hasz=1.
compute intprint=1.
compute sigintct=sigintct+1.
compute printpbe=intpb(sigintct,1).
end if.
do if ((wzcmat((i+1),jmed)=1) or ((wcmat((i+1),jmed)=1) and (zcmat((i+1),jmed)=1))).
compute numplps=2.
compute probecnt=1.
compute intprint=1.
do if (wzcmat((i+1),jmed)=1).
compute sigintct=sigintct+1.
compute printpbe=intpb(sigintct,1).
else.
compute sigintct=sigintct+2.
compute printpbe=cmin(intpb((sigintct-1):sigintct,1)).
end if.
compute panelgrp=1.
compute hasw=1.
compute hasz=1.
compute panelcde={'/PANEL','ROWVAR=',znames,'.'}.
compute modgrph=wnames.
compute lpstsp={1,1;1,1}.
compute wheremv1=wherexw.
compute nm1vls=nwvls.
compute wheremv2=wherexz.
compute nm2vls=nzvls.
compute jnok=0.
do if (wzcmat((i+1),jmed)=1).
compute jnok=1.
end if.
do if (jmed > 1).
compute mprobval=mmodvals.
end if.
do if (jmed=1).
do if (nxvls > 1).
compute jnok=0.
end if.
end if.
do if (nwvls > 1).
compute lpstsp(1,2)=ncol(wprobval).
compute modcat=1.
compute jnok=0.
end if.
do if (zdich=1).
compute modcat=1.
compute jnok=0.
end if.
compute lpstsp(2,1)=lpstsp(1,2)+1.
compute lpstsp(2,2)=lpstsp(1,2)+1.
do if (nzvls > 1).
compute lpstsp(2,1)=lpstsp(1,2)+1.
compute lpstsp(2,2)=lpstsp(1,2)+ncol(zprobval).
compute jnok=0.
end if.
do if (zdich=1).
compute jnok=0.
end if.
compute omni3=make(nrow(b),(nxvls*nwvls),0).
do if (jmed > 1).
compute omni3=make(nrow(b),nwvls,0).
end if.
compute focpred3={wnames,'(W)'}.
do if (xmint=1).
compute focpred3={wnames,'(X)'}.
end if.
compute focpred4={'    ', 'Mod var:', znames, '(Z)'}.
compute modvals=make((nrow(wmodvals)*nrow(zmodvals)),2,0).
compute probeval=make((nrow(wmodvals)*nrow(zmodvals)),(ncol(wprobval)+ncol(zprobval)),0).
loop probei= 1 to nrow(wmodvals).
loop probej =1 to nrow(zmodvals).
compute modvals(probecnt,1)=wmodvals(probei,1).
compute probeval(probecnt,1:nwvls)=wprobval(probei,:).
compute modvals(probecnt,2)=zmodvals(probej,1).
compute probeval(probecnt,(nwvls+1):(nwvls+nzvls))=zprobval(probej,:).
compute probecnt=probecnt+1.
end loop.
end loop.
do if (wzcmat((i+1),jmed)=1).
compute numplps=numplps+1.
compute probprod=make(1,(ncol(wprobval)*ncol(zprobval)),0).
compute lpstsp2={1,1}.
compute lpstsp={lpstsp;lpstsp2}.
compute lpstsp(3,1)=lpstsp(2,2)+1.
compute lpstsp(3,2)=lpstsp(2,2)+ncol(probprod).
compute jnmod=ztmp.
compute jnmin=zmin.
compute jnmax=zmax.
compute jnmodlab=znames.
do if (jmed = 1).
compute wherejn1=wherexw(1,i).
compute wherejn3=wherexwz(1,i).
end if.
do if (jmed > 1).
compute wherejn1=wheremw(((2*jmed)-3),i).
compute wherejn3=wheremwz(((2*jmed)-3),i).
end if.
loop probei = 1 to nrow(wmodvals).
loop probej = 1 to nrow(zmodvals).
compute probtemp=1.
loop probek = 1 to ncol(wprobval).
compute probtemp={probtemp,(wprobval(probei,probek)&*zprobval(probej,:))}.
end loop.
compute probprod={probprod;probtemp(1,2:ncol(probtemp))}.
end loop.
end loop.
compute probprod=probprod(2:nrow(probprod),:).
compute probeval={probeval,probprod}.
end if.
compute problabs={wnames,znames}.
release probecnt, probei, probej.
end if.
do if (intprint=1).
compute focpred={'   Focal', 'predict:'}.
do if (jmed=1).
compute focpred={focpred,xnames,'(X)'}.
compute focplotv=xmodvals.
end if.
do if (jmed >1).
do if (nms > 1).
compute focpred={focpred,mnames(1,(jmed-1)), medlb2(1,(jmed-1))}.
end if.
do if (nms = 1).
compute focpred={focpred,mnames(1,(jmed-1)), '(M)'}.
end if.
compute focplotv=mmodvals(:,(jmed-1)).
end if.
compute focpred2={'    ', 'Mod var:',focpred3}.
compute focpred={focpred;focpred2}.
do if (ncol(focpred4) > 1).
compute focpred={focpred;focpred4}.
compute focpred4={' '}.
end if.
release focpred2,focpred3.
do if (outscree=1).
do if ((plot = 1 or plot = 2) or (printpbe <= intprobe)).
print focpred/title='----------'/format=A8/space=0.
end if.
end if.
compute foctmp=make(nrow(modvals),1,1).
compute probexpl=1.
compute probeva2={foctmp,probeval}.
do if (jmed=1 and nxs > 0 and mcx > 0).
compute probexpl=nxvls.
end if.
compute foctmp=make(nrow(modvals),1,1).
compute modvals3=make(1,(6+ncol(problabs)),0).
compute probrown=make(nrow(probeval),1,0).
compute jtmp=1.
loop probei = 1 to nrow(probeval).
compute probrown(probei,1)=jtmp.
compute jtmp=jtmp+nxvls.
end loop.
release jtmp.
compute probrow=999.
compute modvarl=problabs.
do if (plot = 1 or plot = 2 or nxvls > 1).
compute plotvals=make((nrow(modvals)*nrow(focplotv)),(ncol(modvals)+1),999).
loop ploti=1 to nrow(modvals).
loop plotj=1 to nrow(focplotv).
compute plotvals((((ploti-1)*nrow(focplotv))+plotj),2:ncol(plotvals))=modvals(ploti,:).
compute plotvals((((ploti-1)*nrow(focplotv))+plotj),1)=focplotv(plotj,1).
end loop.
end loop.
compute focpredn=3.
do if (jmed=1).
do if (nxvls > 1).
compute focpredn=(nxvls+1).
end if.
do if (nxvls=1 and xdich=1).
compute focpredn=2.
end if.
end if.
compute meanmat=mdiag(means).
compute onesmat=make(nrow(meanmat),(nrow(probeval)*focpredn),1).
compute probeplt=t(mdiag(means)*onesmat).
do if (jmed=1).
do if ((wcmat((i+1),1)=1) or (zcmat((i+1),1)=1)).
compute plotcnt=1.
compute iloops=nwpval*nzpval.
compute plotmx=nxpval*nzpval.
do if ((wcmat((i+1),1)=1) and (zcmat((i+1),1)=0)).
compute iloops=nwpval.
compute plotmx=nxpval.
end if.
do if ((wcmat((i+1),1)=0) and (zcmat((i+1),1)=1)).
compute iloops=nzpval.
compute plotmx=nxpval.
end if.
compute xestvals=make((nxpval*iloops),ncol(xprobval),-999).
do if (wcmat((i+1),1)=1).
compute westvals=make(nrow(xestvals),ncol(wprobval),-999).
end if.
do if (zcmat((i+1),1)=1).
compute zestvals=make(nrow(xestvals),ncol(zprobval),-999).
end if.
loop ploti=1 to iloops.
loop plotj=1 to nxpval.
compute xestvals(plotcnt,:)=xprobval(plotj,:).
compute plotcnt=plotcnt+1.
end loop.
end loop.
compute plotcnt=1.
compute plotcnt1=1.
compute plotcnt2=1.
compute plotcntz=1.
loop ploti = 1 to (iloops*nxpval).
do if (wcmat((i+1),1)=1).
compute westvals(ploti,:)=wprobval(plotcnt1,:).
end if.
do if ((wcmat((i+1),1)=0) and (zcmat((i+1),1)=1)).
compute zestvals(ploti,:)=zprobval(plotcnt1,:).
end if.
do if ((wcmat((i+1),1)=1) and (zcmat((i+1),1)=1)).
compute zestvals(ploti,:)=zprobval(plotcnt2,:).
compute plotcntz=plotcntz+1.
end if.
compute plotcnt=plotcnt+1.
do if (plotcnt > plotmx).
compute plotcnt=1.
compute plotcnt1=plotcnt1+1.
end if.
do if (plotcntz > nxpval).
compute plotcnt2=plotcnt2+1.
compute plotcntz=1.
do if (plotcnt2 > nzpval).
compute plotcnt2=1.
end if.
end if.
end loop.
compute probeplt(:,2:(1+(ncol(xestvals))))=xestvals.
do if (wcmat((i+1),1)=1).
compute probeplt(:,(wherew(1,i)):(wherew(2,i)))=westvals.
end if.
do if (zcmat((i+1),1)=1).
compute probeplt(:,(wherez(1,i)):(wherez(2,i)))=zestvals.
end if.
end if.
end if.
do if (jmed > 1).
do if ((wcmat((i+1),jmed)=1) or (zcmat((i+1),jmed)=1)).
compute plotcnt=1.
compute iloops=nwpval*nzpval.
compute plotmx=3*nzpval.
do if ((wcmat((i+1),jmed)=1) and (zcmat((i+1),jmed)=0)).
compute iloops=nwpval.
compute plotmx=3.
end if.
do if ((wcmat((i+1),jmed)=0) and (zcmat((i+1),jmed)=1)).
compute iloops=nzpval.
compute plotmx=3.
end if.
compute mestvals=make((3*iloops),1,-999).
do if (wcmat((i+1),jmed)=1).
compute westvals=make(nrow(mestvals),ncol(wprobval),-999).
end if.
do if (zcmat((i+1),jmed)=1).
compute zestvals=make(nrow(mestvals),ncol(zprobval),-999).
end if.
loop ploti=1 to iloops.
loop plotj=1 to 3.
compute mestvals(plotcnt,:)=mprobval(plotj,(jmed-1)).
compute plotcnt=plotcnt+1.
end loop.
end loop.
compute plotcnt=1.
compute plotcnt1=1.
compute plotcnt2=1.
compute plotcntz=1.
loop ploti = 1 to (iloops*3).
do if (wcmat((i+1),jmed)=1).
compute westvals(ploti,:)=wprobval(plotcnt1,:).
end if.
do if ((wcmat((i+1),jmed)=0) and (zcmat((i+1),jmed)=1)).
compute zestvals(ploti,:)=zprobval(plotcnt1,:).
end if.
do if ((wcmat((i+1),jmed)=1) and (zcmat((i+1),jmed)=1)).
compute zestvals(ploti,:)=zprobval(plotcnt2,:).
compute plotcntz=plotcntz+1.
end if.
compute plotcnt=plotcnt+1.
do if (plotcnt > plotmx).
compute plotcnt=1.
compute plotcnt1=plotcnt1+1.
end if.
do if (plotcntz > 3).
compute plotcnt2=plotcnt2+1.
compute plotcntz=1.
do if (plotcnt2 > nzpval).
compute plotcnt2=1.
end if.
end if.
end loop.
compute probeplt(:,wherem((jmed-1),i))=mestvals.
do if (wcmat((i+1),jmed)=1).
do if (model <> 74).
compute probeplt(:,(wherew(1,i)):(wherew(2,i)))=westvals.
end if.
do if (model = 74).
compute probeplt(:,(wherex(1,i)):(wherex(2,i)))=westvals.
end if.
end if.
do if (zcmat((i+1),jmed)=1).
compute probeplt(:,(wherez(1,i)):(wherez(2,i)))=zestvals.
end if.
end if.
end if.
compute prodloop = 1.
do if (jmed=1).
compute prodloop=ncol(xestvals).
end if.
do if (wcmat((i+1),jmed))=1.
compute plotcnt=0.
loop ploti = 1 to prodloop.
loop plotj = 1 to ncol(westvals).
do if (jmed=1).
compute probeplt(:,(wherexw(1,i)+plotcnt))=xestvals(:,ploti)&*westvals(:,plotj).
end if.
do if (jmed > 1).
compute probeplt(:,(wheremw(((jmed*2)-3) ,i)+plotcnt))=mestvals(:,ploti)&*westvals(:,plotj).
end if.
compute plotcnt=plotcnt+1.
end loop.
end loop.
end if.
do if (zcmat((i+1),jmed))=1.
compute plotcnt=0.
loop ploti = 1 to prodloop.
loop plotj = 1 to ncol(zestvals).
do if (jmed = 1).
compute probeplt(:,(wherexz(1,i)+plotcnt))=xestvals(:,ploti)&*zestvals(:,plotj).
end if.
do if (jmed > 1).
compute probeplt(:,(wheremz(((jmed*2)-3),i)+plotcnt))=mestvals(:,ploti)&*zestvals(:, plotj).
end if.
compute plotcnt=plotcnt+1.
end loop.
end loop.
end if.
do if (wzcmat((i+1),jmed))=1.
compute plotcnt=0.
compute threeway=1.
loop ploti = 1 to ncol(westvals).
loop plotj = 1 to ncol(zestvals).
compute probeplt(:,(wherewz(1,i)+plotcnt))=westvals(:,ploti)&*zestvals(:,plotj).
compute plotcnt=plotcnt+1.
end loop.
end loop.
compute plotcnt=0.
loop plotk = 1 to prodloop.
loop ploti = 1 to ncol(westvals).
loop plotj = 1 to ncol(zestvals).
do if (jmed = 1).
compute probeplt(:,(wherexwz(1,i)+plotcnt))=xestvals(:,plotk)&*westvals(:,ploti)&* zestvals(:,plotj).
end if.
do if (jmed > 1).
compute probeplt(:,(wheremwz(((jmed*2)-3),i)+plotcnt))=mestvals(:,plotk)&* westvals(:,ploti)&*zestvals(:,plotj).
end if.
compute plotcnt=plotcnt+1.
end loop.
end loop.
end loop.
end if.
loop newplp=1 to i.
do if (newplp <> jmed).
do if (wcmat((i+1),newplp))=1.
compute prodloop=1.
do if (newplp=1).
compute prodloop=nxvls.
end if.
compute plotcnt=0.
loop ploti = 1 to prodloop.
loop plotj = 1 to nwvls.
do if (newplp = 1).
compute probeplt(:,(wherexw(1,i)+plotcnt))=probeplt(:,(1+ploti))&*probeplt(:, (wherew(1,i)+plotj-1)).
end if.
do if (newplp > 1).
do if (model <> 74).
compute probeplt(:,(wheremw(((newplp*2)-3) ,i)+plotcnt))=probeplt(:,wherem((newplp-1),i))&*probeplt(:,(wherew(1,i)+plotj-1)).
end if.
do if (model = 74).
compute probeplt(:,(wheremw(((newplp*2)-3) ,i)+plotcnt))=probeplt(:,wherem((newplp-1),i))&*probeplt(:,(wherex(1,i)+plotj-1)).
end if.
end if.
compute plotcnt=plotcnt+1.
end loop.
end loop.
end if.
do if (zcmat((i+1),newplp))=1.
compute prodloop=1.
do if (newplp=1).
compute prodloop=nxvls.
end if.
compute plotcnt=0.
loop ploti = 1 to prodloop.
loop plotj = 1 to nzvls.
do if (newplp = 1).
compute probeplt(:,(wherexz(1,i)+plotcnt))=probeplt(:,(1+ploti))&*probeplt(:, (wherez(1,i)+plotj-1)).
end if.
do if (newplp > 1).
compute probeplt(:,(wheremz(((newplp*2)-3),i)+plotcnt))=probeplt(:, wherem((newplp-1),i))&*probeplt(:,(wherez(1,i)+plotj-1)).
end if.
compute plotcnt=plotcnt+1.
end loop.
end loop.
end if.
do if (wzcmat((i+1),newplp))=1.
compute plotcnt=0.
do if (threeway=0).
loop ploti = 1 to nwvls.
loop plotj = 1 to nzvls.
compute probeplt(:,(wherewz(1,i)+plotcnt))=probeplt(:,(wherew(1,i)+ploti-1))&* probeplt(:,(wherez(1,i)+plotj-1)).
compute plotcnt=plotcnt+1.
end loop.
end loop.
end if.
compute prodloop=1.
do if (newplp=1).
compute prodloop=nxvls.
end if.
compute plotcnt=0.
loop plotk = 1 to prodloop.
loop ploti = 1 to nwvls.
loop plotj = 1 to nzvls.
do if (newplp = 1).
compute probeplt(:,(wherexwz(1,i)+plotcnt))=probeplt(:,(1+plotk))&*probeplt(:, (wherew(1,i)+ploti-1))&*probeplt(:,(wherez(1,i)+plotj-1)).
end if.
do if (newplp > 1).
compute probeplt(:,(wheremwz(((newplp*2)-3),i)+plotcnt))=probeplt(:, wherem((newplp-1),i))&*probeplt(:,(wherew(1,i)+ploti-1))&*probeplt(:,(wherez(1,i)+plotj-1)).
end if.
compute plotcnt=plotcnt+1.
end loop.
end loop.
end loop.
end if.
end if.
end loop.
compute predvals=probeplt*b.
do if (debug <> 0).
print probeplt.
print b.
end if.
do if (i = nms+nys) and (ydich=1).
compute predvalt=(predvals < 709.7).
compute prevalt7=(1-predvalt)*(709.7).
compute predvals=(predvals&*predvalt)+prevalt7.
compute expyhat=exp(predvals)&/(1+exp(predvals)).
end if.
compute sepred=make(nrow(plotvals),3,999).
loop sei=1 to nrow(plotvals).
compute ask=probeplt(sei,:).
compute sepred(sei,1)=sqrt(ask*varb*t(ask)).
do if ((i < nms+nys) or (ydich=0)).
compute sepred(sei,2)=predvals(sei,1)-tval*sepred(sei,1).
compute sepred(sei,3)=predvals(sei,1)+tval*sepred(sei,1).
end if.
do if ((i = nms+nys) and (ydich=1)).
compute sepred(sei,2)=predvals(sei,1)-xp2*sepred(sei,1).
compute sepred(sei,3)=predvals(sei,1)+xp2*sepred(sei,1).
end if.
end loop.
compute prevloc=ncol(plotvals)+1.
compute probeplt={plotvals,predvals}.
do if (plot = 2).
compute probeplt={probeplt,sepred}.
end if.
do if ((i = nms+nys) and (ydich=1)).
compute probeplt={probeplt,expyhat}.
end if.
compute didsome=0.
end if.
do if ((wzcmat((i+1),jmed)=1) and (printpbe <= intprobe)).
do if (jmed=1).
compute omnilp2=nxvls*nwvls.
compute omnitmp=ident(omnilp2).
compute omni3(wherexw(1,i):wherexw(2,i),:)=omnitmp.
end if.
do if (jmed>1).
compute omnilp2=nwvls.
compute omnitmp=ident(omnilp2).
compute omni3(wheremw(((jmed*2)-3),i):wheremw(((jmed*2)-2),i),:)=omnitmp.
end if.
compute omnif=make(1,4,0).
do if ((i = nms+nys) and (ydich=1)).
compute omnif=make(1,3,0).
end if.
compute condeff3=0.
loop omnilp1=1 to nrow(zprobval).
loop omnilp=1 to (omnilp2).
do if (jmed=1).
compute omni3((wherexwz(1,i)+((omnilp-1)*nzvls)):(wherexwz(1,i)+((omnilp-1)*nzvls)+ (nzvls-1)),omnilp)=t(zprobval(omnilp1,:)).
end if.
do if (jmed > 1).
compute omni3((wheremwz(((jmed*2)-3),i)+((omnilp-1)*nzvls)):(wheremwz(((jmed*2)-3),i)+ ((omnilp-1)*nzvls)+(nzvls-1)),omnilp)=t(zprobval(omnilp1,:)).
end if.
end loop.
compute condeff=t(omni3)*b.
compute condeff3={condeff3;condeff}.

.
compute lmat2= omni3.
do if ( 1 =0).
compute lmat2 = mdiag( omni3 ).
compute lmat3=make(nrow(lmat2),1,0).
loop flp=1 to ncol(lmat2).
do if (csum(lmat2(:,flp))=1).
compute lmat3={lmat3,lmat2(:,flp)}.
end if.
end loop.
compute lmat2=lmat3(:,2:ncol(lmat3)).
end if.
compute fratio = (t(t(lmat2)* b )*inv(t(lmat2)* varb *lmat2)*((t(lmat2)* b )))/ncol(lmat2).
compute pfr = 1-fcdf(fratio,ncol(lmat2),(n-nrow( b ))).
compute fresult={fratio,ncol(lmat2),(n-nrow( b )),pfr}.
do if (i = (nms+nys) and (ydich=1)).
compute fratio=fratio*ncol(lmat2).
compute pfr=1-chicdf(fratio,ncol(lmat2)).
compute fresult={fratio,ncol(lmat2),pfr}.
end if.
do if ( 0 =1).
compute lmat3=1-rsum(lmat2).
compute xfm=make(n,csum(lmat3),0).
compute flpc=1.
loop flp=1 to nrow(lmat3).
do if (lmat3(flp,1)=1).
compute xfm(:,flpc)=x(:,flp).
compute flpc=flpc+1.
end if.
end loop.
compute bfm=inv(t(xfm)*xfm)*t(xfm)*y.
compute resid=y-(xfm*bfm).
compute sstotal=(y-(csum(y)/n)).
compute sstotal=csum(sstotal&*sstotal).
compute ssresid=csum(resid&*resid).
compute rsqch= 0 -((sstotal-ssresid)/sstotal).
compute fresult={rsqch,fresult}.
release xfm,flpc, resid, ssresid, bfm.
end if
.
compute omnif={omnif;fresult}.
end loop.
compute omnif=omnif(2:nrow(omnif),:).
compute clabtmp=znames.
compute condeff3=condeff3(2:nrow(condeff3),:).
do if ((nxvls*nwvls)=1).
compute omnif={condeff3,omnif}.
compute clabtmp={clabtmp,'Effect'}.
end if.
compute omnif={zmodvals,omnif}.
do if ((i < nms+nys) or (ydich=0)).
compute clabtmp={clabtmp,hcflab,'df1','df2','p'}.
end if.
do if ((i = (nms+nys)) and (ydich=1)).
compute clabtmp={clabtmp,'Chi-sq','df','p'}.
end if.
compute resultm2=make(nrow(omnif),maxresm,99999).
compute resultm2(1:nrow(omnif),1:ncol(omnif))=omnif.
compute resultm={resultm;resultm2}.
do if (outscree=1).
do if (jmed=1).
print omnif/title='Test of conditional X*W interaction at value(s) of Z:'/cnames=clabtmp/format= F10.4.
end if.
do if (jmed>1).
print omnif/title='Test of conditional M*W interaction at value(s) of Z:'/cnames=clabtmp/format= F10.4.
end if.
end if.
release omni3.
end if.
loop probei = 1 to probexpl.
do if (probexpl > 1).
compute foctmp=make(nrow(modvals),probexpl,0).
compute foctmp(:,probei)=foctmp(:,probei)+1.
compute probtemp=make(nrow(modvals),1,0).
loop probem = 1 to numplps.
loop probek = 1 to nxvls.
loop probej=lpstsp(probem,1) to lpstsp(probem,2).
compute probtemp={probtemp,foctmp(:,probek)&*probeval(:,probej)}.
end loop.
end loop.
end loop.
compute probeva2=probtemp(:,2:ncol(probtemp)).
compute probeva2={foctmp,probeva2}.
end if.
compute probres=probeva2*probcoef.
compute probrese=sqrt(diag(probeva2*probvarb*t(probeva2))).
compute tratio = probres&/probrese.
compute p = 2*(1-tcdf(abs(tratio), dfres)).
do if (ydich=1 and i = (nms+nys)).
compute p = 2*(1-cdfnorm(abs(tratio))).
end if.
compute modvals2={modvals,probres,probrese,tratio, p}.
do if ((i < nms+nys) or (ydich=0)).
compute modvals2={modvals2,(probres-tval&*probrese),(probres+tval&*probrese)}.
compute problabs={problabs,'Effect',hclab,'t', 'p', 'LLCI', 'ULCI'}.
end if.
do if (ydich=1 and i = (nms+nys)).
compute modvals2={modvals2,(probres-xp2&*probrese),(probres+xp2&*probrese)}.
compute problabs={problabs,'Effect','se','Z', 'p', 'LLCI', 'ULCI'}.
end if.
do if (probexpl > 1 and (printpbe <= intprobe)).
do if (hasz = 1).
compute printz=1.
end if.
do if (hasw=1).
compute printw=1.
end if.
compute probrlab=make(nrow(modvals),1,xcatlab(probei,1)).
compute modvals3={modvals3; modvals2}.
compute probrow={probrow;probrown}.
compute probrown=probrown+1.
do if (probei=probexpl).
compute xproblab=xcatlab(1:nxvls,1).
compute probrow=probrow(2:nrow(probrow),1).
compute modvals3=modvals3(2:nrow(modvals3),:).
compute temp=modvals3.
compute temp(GRADE(probrow(:,1)),:)=modvals3.
compute modvals3=temp.
compute start2=1.
compute problabs=problabs(1,(1+(ncol(modvarl))):ncol(problabs)).
compute pstart=1.
loop probek= 1 to nrow(probeval).
compute endstart=start2+(nxvls-1).
compute temp=modvals3(start2:endstart,(1+ncol(modvarl)):ncol(modvals3)).
compute temp2=t(modvals3(start2:start2,1:ncol(modvarl))).
compute trnames=t(modvarl).
do if (outscree=1).
do if (probek > 1).
print/title='----------'/space=0.
else.
print/title = 'Conditional effects of the focal predictor at values of the moderator(s):'.
do if ((jmed=1) and (i = (nms+nys)) and (nms > 0)).
do if (nxvls = 1).
print/title = '(These are also the conditional direct effects of X on Y)'/space=0.
else.
print/title = '(These are also the relative conditional direct effects of X on Y)'/space=0.
end if.
end if.
print.
end if.
print temp2/title = 'Moderator value(s):'/rnames=trnames/format= F10.4 /space=0.
print temp/title = ' '/cnames=problabs/rnames=xproblab/format= F10.4 /space=0.
end if.
compute resultm2=make(nrow(temp2),maxresm,99999).
compute resultm2(1:nrow(temp2),1:ncol(temp2))=temp2.
compute resultm={resultm;resultm2}.
compute resultm2=make(nrow(temp),maxresm,99999).
compute resultm2(1:nrow(temp),1:ncol(temp))=temp.
compute resultm={resultm;resultm2}.
compute start2=start2+nxvls.
compute didsome=1.
do if (jmed=1).
compute mod1val=probeval(probek,1:nm1vls).
loop omnilp=1 to nxvls.
compute omni((wheremv1(1,i)+((omnilp-1)*nm1vls)):(wheremv1(1,i)+((omnilp-1)* nm1vls)+(nm1vls-1)),omnilp)=t(mod1val).
do if (nm1vls < ncol(probeval)).
compute mod2val=probeval(probek,(nm1vls+1):(nm1vls+nm2vls)).
compute omni((wheremv2(1,i)+((omnilp-1)*nm2vls)):(wheremv2(1,i)+((omnilp-1)* nm2vls)+(nm2vls-1)),omnilp)=t(mod2val).
do if ((nm1vls+nm2vls) < ncol(probeval)).
compute intlen=nm1vls*nm2vls.
compute modintvl=probeval(probek,(nm1vls+nm2vls+1):ncol(probeval)).
compute omni((wherexwz(1,i)+((omnilp-1)*intlen)):(wherexwz(1,i)+((omnilp-1)* intlen)+(intlen-1)),omnilp)=t(modintvl).
end if.
end if.
end loop.

.
compute lmat2= omni.
do if ( 1 =0).
compute lmat2 = mdiag( omni ).
compute lmat3=make(nrow(lmat2),1,0).
loop flp=1 to ncol(lmat2).
do if (csum(lmat2(:,flp))=1).
compute lmat3={lmat3,lmat2(:,flp)}.
end if.
end loop.
compute lmat2=lmat3(:,2:ncol(lmat3)).
end if.
compute fratio = (t(t(lmat2)* b )*inv(t(lmat2)* varb *lmat2)*((t(lmat2)* b )))/ncol(lmat2).
compute pfr = 1-fcdf(fratio,ncol(lmat2),(n-nrow( b ))).
compute fresult={fratio,ncol(lmat2),(n-nrow( b )),pfr}.
do if (i = (nms+nys) and (ydich=1)).
compute fratio=fratio*ncol(lmat2).
compute pfr=1-chicdf(fratio,ncol(lmat2)).
compute fresult={fratio,ncol(lmat2),pfr}.
end if.
do if ( 0 =1).
compute lmat3=1-rsum(lmat2).
compute xfm=make(n,csum(lmat3),0).
compute flpc=1.
loop flp=1 to nrow(lmat3).
do if (lmat3(flp,1)=1).
compute xfm(:,flpc)=x(:,flp).
compute flpc=flpc+1.
end if.
end loop.
compute bfm=inv(t(xfm)*xfm)*t(xfm)*y.
compute resid=y-(xfm*bfm).
compute sstotal=(y-(csum(y)/n)).
compute sstotal=csum(sstotal&*sstotal).
compute ssresid=csum(resid&*resid).
compute rsqch= 0 -((sstotal-ssresid)/sstotal).
compute fresult={rsqch,fresult}.
release xfm,flpc, resid, ssresid, bfm.
end if
.
compute resultm2=make(nrow(fresult),maxresm,99999).
compute resultm2(1:nrow(fresult),1:ncol(fresult))=fresult.
compute resultm={resultm;resultm2}.
do if (outscree=1).
do if (i < (nms + nys) or (ydich = 0)).
compute clabtmp={hcflab,'df1','df2','p'}.
print fresult/title='Test of equality of conditional means'/cnames=clabtmp/format= F10.4.
end if.
do if (i = (nms + nys) and (ydich = 1)).
compute clabtmp={'Chi-sq','df','p'}.
print fresult/title='Test of equality of conditional logits or probabilities'/cnames=clabtmp/format= F10.4.
end if.
end if.
compute probetmp=probeplt(pstart:(pstart+nxvls),1).
compute probetmp={probetmp,probeplt(pstart:(pstart+nxvls),prevloc:ncol(probeplt))}.
compute pstart=pstart+(nxvls+1).
compute resultm2=make(nrow(probetmp),maxresm,99999).
compute resultm2(1:nrow(probetmp),1:ncol(probetmp))=probetmp.
compute resultm={resultm;resultm2}.
do if (i < ((nms + nys)) or (ydich = 0)).
compute clabtmp={xnames, outnames(1,i), hclab, 'LLCI', 'ULCI'}.
do if (outscree=1).
print probetmp/title = 'Estimated conditional means being compared:'/cnames=clabtmp/format= F10.4.
end if.
end if.
do if ((i = (nms+nys)) and (ydich = 1)).
compute clabtmp={xnames, outnames(1,i), 'prob'}.
compute probetm2=probetmp(:,1:2).
compute probetm2={probetm2,probetmp(:,ncol(probetmp))}.
do if (outscree=1).
print probetm2/title = 'Estimated conditional logits and probabilities:'/cnames=clabtmp/format= F10.4.
end if.
end if.
end if.
end loop.
release probrow, start2, endstart, temp, temp2.
end if.
end if.
do if (probexpl = 1 and (printpbe <= intprobe)).
compute resultm2=make(nrow(modvals2),maxresm,99999).
compute resultm2(1:nrow(modvals2),1:ncol(modvals2))=modvals2.
compute resultm={resultm;resultm2}.
do if (outscree=1).
print/title = 'Conditional effects of the focal predictor at values of the moderator(s):'.
print modvals2/cnames=problabs/title = ' '/space=0/format= F10.4.
end if.
compute didsome=1.
do if (hasz = 1).
compute printz=1.
end if.
do if (hasw=1).
compute printw=1.
end if.
do if (jn = 1 and jnok=1).
do if (criterr = 0).
compute dfres=n-nrow(b).
compute roots=99999.
compute jncrit =(dfres* (exp((dfres-(5/6))*((xp2/(dfres-(2/3)+(.11/dfres)))* (xp2/(dfres-(2/3)+(.11/dfres)))))-1)).
do if (i = (nms+nys) and (ydich=1)).
compute jncrit=xp2*xp2.
end if.
compute jnb1=b(wherejn1,1).
compute jnb3=b(wherejn3,1).
compute jnsb1=varb(wherejn1,wherejn1).
compute jnsb3=varb(wherejn3,wherejn3).
compute jnsb1b3=varb(wherejn1,wherejn3).
compute ajn =(jncrit*jnsb3)-(jnb3*jnb3).
compute bjn = 2*((jncrit*jnsb1b3)-(jnb1*jnb3)).
compute cjn = (jncrit*jnsb1)-(jnb1*jnb1).
compute radarg = (bjn*bjn)-(4*ajn*cjn).
compute den = 2*ajn.
compute nrts = 0.
do if (radarg >= 0 and den <> 0).
compute x21 = (-bjn+sqrt(radarg))/den.
compute x22 = (-bjn-sqrt(radarg))/den.
compute roots = 0.
do if (x21 >= jnmin and x21 <= jnmax).
compute nrts = 1.
compute roots = {roots; x21}.
end if.
do if (x22 >= jnmin and x22 <= jnmax).
compute nrts = nrts + 1.
compute roots = {roots; x22}.
end if.
compute roots={roots,make(nrow(roots),2,0)}.
end if.
do if (nrts > 0).
compute roots = roots(2:nrow(roots),1:3).
compute roots(1,2)=(csum(jnmod < roots(1,1))/n)*100.
compute roots(1,3)=(csum(jnmod > roots(1,1))/n)*100.
do if (nrow(roots)=2).
compute roots(2,2)=(csum(jnmod < roots(2,1))/n)*100.
compute roots(2,3)=(csum(jnmod > roots(2,1))/n)*100.
end if.
do if (outscree=1).
print roots/title = 'Moderator value(s) defining Johnson-Neyman significance region(s):'/clabels = 'Value', '% below', '% above'/format F10.4.
end if.
do if (nrts=1).
compute tmprts=make(1,3,99999).
compute roots={roots;tmprts}.
end if.
end if.
do if (nrts = 0).
compute roots=make(2,1,99999).
do if (outscree=1).
print/title = 'There are no statistical significance transition points within the observed'.
print/title = 'range of the moderator found using the Johnson-Neyman method.'/space=0.
end if.
end if.
compute resultm2=make(nrow(roots),maxresm,99999).
compute resultm2(1:nrow(roots),1:ncol(roots))=roots.
compute resultm={resultm;resultm2}.
compute jnvals=make(23,7,0).
loop jni= 0 to (21-(nrts)).
compute jnvals((jni+1),1)=jnmin+(jni*((jnmax-jnmin)/(21-nrts))).
end loop.
do if (nrts > 0).
loop jni = 1 to nrts.
loop jnj = 2 to (nrow(jnvals)-1).
do if ((roots(jni,1) > jnvals((jnj-1),1)) and (roots(jni,1) < jnvals(jnj,1))).
compute jnvals((jnj+1):(21+jni),1)=jnvals(jnj:(20+jni),1).
compute jnvals(jnj,1)=roots(jni,1).
end if.
end loop.
end loop.
end if.
compute jnvals=jnvals(1:22,:).
loop jni = 1 to nrow(jnvals).
compute jnvals(jni,2)=jnb1+jnb3*jnvals(jni,1).
compute jnvals(jni,3)=sqrt(jnsb1+2*jnvals(jni,1)*jnsb1b3+(jnvals(jni,1)*jnvals(jni, 1))*jnsb3).
compute jnvals(jni,4)=jnvals(jni,2)/jnvals(jni,3).
compute jnvals(jni,5)=2*(1-tcdf(abs(jnvals(jni,4)), dfres)).
compute jnvals(jni,6)=jnvals(jni,2)-sqrt(jncrit)*jnvals(jni,3).
compute jnvals(jni,7)=jnvals(jni,2)+sqrt(jncrit)*jnvals(jni,3).
do if ((i = nms + nys) and (ydich=1)).
compute jnvals(jni,5)=2*(1-cdfnorm(abs(jnvals(jni,4)))).
compute jnvals(jni,6)=jnvals(jni,2)-xp2*jnvals(jni,3).
compute jnvals(jni,7)=jnvals(jni,2)+xp2*jnvals(jni,3).
end if.
end loop.
compute resultm2=make(nrow(jnvals),maxresm,99999).
compute resultm2(1:nrow(jnvals),1:ncol(jnvals))=jnvals.
compute resultm={resultm;resultm2}.
do if ((i < nms+nys) or (ydich=0)).
compute jnclbs={jnmodlab,'Effect',hclab,'t', 'p', 'LLCI', 'ULCI'}.
end if.
do if ((i = nms + nys) and (ydich=1)).
compute jnclbs={jnmodlab,'Effect','se','Z', 'p', 'LLCI', 'ULCI'}.
end if.
do if (((wcmat((i+1),jmed)=1) or (zcmat((i+1),jmed)=1)) and (wzcmat((i+1),jmed)=0)).
do if (outscree=1).
print jnvals/title = 'Conditional effect of focal predictor at values of the moderator:'/cnames =jnclbs/format = F10.4.
end if.
end if.
do if (outscree=1).
do if ((jmed = 1) and (wzcmat((i+1),jmed)=1)).
print jnvals/title = 'Conditional X*W interaction at values of the moderator Z:'/cnames =jnclbs/format = F10.4.
end if.
do if ((jmed > 1) and (wzcmat((i+1),jmed)=1)).
print jnvals/title = 'Conditional M*W interaction at values of the moderator Z:'/cnames =jnclbs/format = F10.4.
end if.
end if.
end if.
end if.
end if.
do if ((i = (nms+nys)) and (jmed=1) and (bcmat(nrow(bcmat),1)=1)).
do if (probei=1).
compute direfflb=problabs.
compute direff=modvals2.
end if.
do if (probei>1).
compute direff={direff;modvals2}.
end if.
end if.
compute intprint=0.
do if ((jmed=1) and (i=1) and nms=0) and modcok=1).
compute contvec2=make(2,1,1).
compute contvec2={contvec2,wcontval,zcontval}.
do if (wzcmat((i+1),jmed)=1).
loop conti= 1 to ncol(wcontval).
loop contj = 1 to ncol(zcontval).
compute contvec2={contvec2,wcontval(:,conti)&*zcontval(:,contj)}.
end loop.
end loop.
end if.
compute conteff=contvec2*probcoef.
compute contdiff=contvec2(1,:)-contvec2(2,:).
compute contse=sqrt(contdiff*probvarb*t(contdiff)).
compute conteffd=conteff(1,1)-conteff(2,1).
compute contvec={contvec,conteff}.
compute contvecm=contvec.
compute resultm2=make(nrow(contvecm),maxresm,99999).
compute resultm2(1:nrow(contvecm),1:ncol(contvecm))=contvecm.
compute resultm={resultm;resultm2}.
do if (outscree=1).
print/title='Contrast between conditional effects of X:'.
print contvec/title=' '/rlabels='Effect1:','Effect2:'/cnames=problabs/format = F10.4 /space=0.
end if.
do if (ydich=0).
compute p=2*(1-tcdf(abs(conteffd/contse), dfres)).
compute contvec={conteffd,contse,conteffd/contse, p}.
compute contvec={contvec,(conteffd-(tval*contse))}.
compute contvec={contvec,(conteffd+(tval*contse))}.
compute contlabs={'Contrast', hclab, 't', 'p', 'LLCI', 'ULCI'}.
end if.
do if (ydich=1).
compute p=2*(1-cdfnorm(abs(conteffd/contse))).
compute contvec={conteffd,contse,conteffd/contse, p}.
compute contvec={contvec,(conteffd-(xp2*contse))}.
compute contvec={contvec,(conteffd+(xp2*contse))}.
compute contlabs={'Contrast', 'se', 'Z', 'p', 'LLCI', 'ULCI'}.
end if.
compute resultm2=make(nrow(contvec),maxresm,99999).
compute resultm2(1:nrow(contvec),1:ncol(contvec))=contvec.
compute resultm={resultm;resultm2}.
do if (outscree=1).
print contvec/title='Test of Effect1 minus Effect2'/format= F10.4 /cnames=contlabs.
end if.
end if.
end loop.
do if (plot = 1 or plot = 2).
compute datalabs={t(focpred(:,3)),outnames(1,i)}.
do if (plot = 1).
compute datalabs={datalabs}.
end if.
do if (plot = 2).
compute datalabs={datalabs,'se', 'LLCI', 'ULCI'}.
end if.
do if ((i = nms+nys) and (ydich=1)).
compute datalabs={datalabs,'prob'}.
end if.
compute resultm2=make(nrow(probeplt),maxresm,99999).
compute resultm2(1:nrow(probeplt),1:ncol(probeplt))=probeplt.
compute resultm={resultm;resultm2}.
compute datalabs={datalabs,'.'}.
do if (outscree=1).
print/title = 'Data for visualizing the conditional effect of the focal predictor:'.
print/title = 'Paste text below into a SPSS syntax window and execute to produce plot.'/space=0.
compute dumb = {' ', ' ', ' ', ' ', ' ', ' ', ' '}.
print datalabs/title = 'DATA LIST FREE/'/format=A10.
print probeplt/title = 'BEGIN DATA.'/format= F10.4 /space=0.
print/title = 'END DATA.'/space=0.
end if.
compute focgrph=datalabs(1,1).
compute graphix={focgrph,graphixs,modgrph}.
do if (((xdich=1) or (nxvls > 1)) and ((modcat=0) and (focgrph = xnames))).
compute graphix={modgrph,graphixs,focgrph}.
end if.
do if (panelgrp = 0).
compute graphix={graphix,'.'}.
else.
compute graphix={graphix,panelcde}.
end if.
do if (outscree=1).
print graphix/title = 'GRAPH/SCATTERPLOT='/format=A8/space=0.
end if.
do if (i = (nms + nys) and ydich = 1).
compute graphixd=graphix.
compute graphixd(1,3)='prob'.
do if (outscree=1).
print graphixd/title = 'GRAPH/SCATTERPLOT='/format=A8/space=0.
end if.
end if.
end if.
end if.
end loop.
release jmed, intprint,didprint.
end if
.
do if (model = 74 and i <= nms).
compute onetemp=make(nrow(xprobval),1,1).
compute mestmt74={onetemp,xprobval}.
do if (ncs > 0).
compute ncovmdl=rsum(ccmat(i,:)).
do if (ncovmdl > 0).
compute cvmnc=make(nrow(mestmt74),ncovmdl,1).
compute cvmnctmp=csum(x(:,(ncol(x)-ncovmdl+1):ncol(x)))/nrow(x).
do if (cuscoval > 0).
compute cvmnctmp=coval.
end if.
loop mestlp=1 to ncovmdl.
compute cvmnc(:,mestlp)=cvmnc(:,mestlp)*cvmnctmp(:,mestlp).
end loop.
compute mestmt74={mestmt74,cvmnc}.
end if.
end if.
compute mest74t=mestmt74*b.
do if (i = 1).
compute mest74=mest74t.
end if.
do if (i > 1).
compute mest74={mest74,mest74t}.
end if.
end if.
do if ((i = (nms+nys)) and (model >= 0 and model < 4) and (linsum(1,1) <> -999)).
compute lhyprob=1.
compute meansub=0.
do if (((nlinsum=nrow(b)) or (nlinsum = (nrow(b)-ncs)))).
do if (nlinsum = (nrow(b)-ncs) and ncs > 0).
compute linsum={linsum,covmeans}.
compute meansub=1.
end if.
compute lhyprob=0.
compute hypest=linsum*b.
compute sehypest=sqrt(linsum*varb*t(linsum)).
compute phypest=2*(1-tcdf(abs(hypest/sehypest), (dfres))).
compute hypest={hypest,sehypest,(hypest/sehypest),phypest,(hypest-tval*sehypest),(hypest+ tval*sehypest)}.
compute resultm2=make(ncol(linsum),maxresm,99999).
compute resultm2(:,1)=t(linsum).
compute resultm={resultm;resultm2}.
compute resultm2=make(1,maxresm,99999).
compute resultm2(1,1:ncol(hypest))=hypest.
compute resultm={resultm;resultm2}.
do if (outscree=1).
compute hyplabs={'Estimate',hclab,'t','p','LLCI','ULCI'}.
print/title='----------'.
print/title='Linear Combination Estimate and Hypothesis Test'/space=0.
print t(linsum)/title= 'Weight vector:'/clabels='weight'/rnames=vlabsm/format F10.4.
print hypest/title=' '/cnames=hyplabs/format= F10.4 /space=0.
do if (meansub=1).
print/title='Covariate weight(s) set to the sample mean.'.
end if.
end if.
end if.
do if (lhyprob=1).
compute notecode(notes,1) = 30.
compute notes = notes + 1.
end if.
end if.
end loop if criterr=1.
compute lastb=b.
compute lastcov=varb.
do if (criterr=0 and dototal = 1).
compute x=xtmp.
compute vlabsm={'constant';xcatlab(1:nxvls,1)}.
do if (ncs > 0).
compute x = {x,ctmp}.
compute vlabsm={vlabsm;t(covnames)}.
end if.
compute x = {ones,x}.

.
do if ( 1 =1).
compute hatmat=inv(t( x )* x )*t( x ).
compute b = hatmat* y.
compute modres=b.
do if ( 1 =1).
compute n1=nrow( x ).
compute dfres=n1-(ncol( x )).
compute sstotal = t( y -(csum( y )/n1))*( y -(csum( y )/n1)).
compute resid= y - x *b.
compute ssresid = csum((resid)&**2).
compute r2 = (sstotal-ssresid)/sstotal.
do if (r2 < 0).
compute r2=0.
end if.
compute adjr2 = 1-((1-r2)*(n1-1)/(dfres)).
compute mse=ssresid/(n1-ncol( x )).

.
compute n1=nrow( x ).
compute invXtX = inv(t( x )* x ).
compute varb = mse *invXtX.
compute k3 = ncol( x ).
compute xhc=0.
do if ( hc <> 5).
compute xhc= x.
compute hat = xhc(:,1).
loop i3=1 to nrow(xhc).
compute hat(i3,1)= xhc(i3,:)*invXtX*t(xhc(i3,:)).
end loop.
do if ( hc = 0 or hc =1).
loop i3 = 1 to k3.
compute xhc(:,i3)=xhc(:,i3)&* resid.
end loop.
end if.
do if ( hc =3 or hc =2).
loop i3=1 to k3.
compute xhc(:,i3) = ( resid &/(1-hat)&**(1/(4- hc )))&*xhc(:,i3).
end loop.
end if.
do if ( hc = 4).
compute hcmn=make(n,2,4).
compute hcmn(:,2)=(n1*hat)/k3.
loop i3= 1 to k3.
compute xhc(:,i3) = ( resid &/(1-hat)&**(rmin(hcmn)/2))&*xhc(:,i3).
end loop.
end if.
compute varb=(invXtX*t(xhc)*xhc*invXtX).
do if ( hc =1).
compute varb=(n1/(n1-ncol( x )))&*varb.
end if.
end if.
compute hclab={'se(HC0)','se(HC1)','se(HC2)','se(HC3)','se(HC4)','se'}.
compute hclab=hclab(1,( hc +1)).
compute hcflab={'F(HC0)','F(HC1)','F(HC2)','F(HC3)','F(HC4)','F'}.
compute hcflab=hcflab(1,( hc +1)).
release xhc
.
compute seb=sqrt(diag(varb)).
compute trat = b&/seb.
compute p = 2*(1-tcdf(abs(trat), (dfres))).
compute tval = sqrt(dfres* (exp((dfres-(5/6))*((xp2/(dfres-(2/3)+(.11/dfres)))* (xp2/(dfres-(2/3)+(.11/dfres)))))-1)).
compute modres={modres,seb,trat,p}.
compute modres={modres,(b-tval&*seb),(b+tval&*seb)}.
compute modresl={'coeff',hclab,'t','p','LLCI','ULCI'}.
compute lmat = ident(ncol( x )).
compute lmat = lmat(:,2:ncol(lmat)).
compute fratio = (t(t(lmat)*b)*inv(t(lmat)*varb*lmat)*((t(lmat)*b)))/(ncol( x )-1).
compute pfr = 1-fcdf(fratio,(ncol( x )-1),dfres).
compute modsum={sqrt(r2),r2,mse,fratio,(ncol( x )-1),dfres,pfr}.
compute modsuml={'R','R-sq','MSE',hcflab,'df1','df2', 'p'}.
end if.
end if.
do if ( 1 = 2 or 1 =3).
compute xlp= x.
compute ylp= y.
compute pt2 = make(nrow(ylp),1,(csum(ylp)/nrow(ylp))).
do if ( 1 =2).
compute LL3 = ylp&*ln(pt2)+(1-ylp)&*ln(1-pt2).
end if.
compute LL3 = -2*csum(LL3).
compute bt1 = make(ncol(xlp),1,0).
compute LL1 = 0.
compute pt1 = make(nrow(ylp),1,0.5).
compute pt1lp=pt1.
loop jjj = 1 to iterate.
compute xlptmp=t(xlp).
compute vecprb=pt1lp&*(1-pt1lp).
loop kkk=1 to ncol(xlp).
compute xlptmp(kkk,:)=xlptmp(kkk,:)&*t(vecprb).
end loop.
compute b = bt1+inv(xlptmp*xlp)*t(xlp)*(ylp-pt1lp).
do if ( 1 =2).
compute xlpb=xlp*b.
compute xlpbt=(xlpb > -709.7).
compute xlpb709=(1-xlpbt)*(-709.7).
compute xlpb=(xlpb&*xlpbt)+xlpb709.
compute pt1lp = 1/(1+exp(-(xlpb))).
end if.
compute itprob = csum((pt1lp < .00000001) or (pt1lp > .9999999)).
do if (itprob > 0).
loop kkk = 1 to nrow(pt1lp).
do if (pt1lp(kkk,1) > .9999999).
compute pt1lp(kkk,1) = .9999999.
end if.
do if (pt1lp(kkk,1) < .00000001).
compute pt1lp(kkk,1) = .00000001.
end if.
end loop.
compute itprob = 0.
end if.
do if (itprob = 0).
do if ( 1 =2).
compute LL = ylp&*ln(pt1lp)+(1-ylp)&*ln(1-pt1lp).
end if.
compute LL2 = -2*csum(ll).
end if.
do if (abs(LL1-LL2) < converge).
do if ( 1 =1).
compute xlptmp=t(xlp).
compute vecprb=pt1lp&*(1-pt1lp).
loop kkk=1 to ncol(xlp).
compute xlptmp(kkk,:)=xlptmp(kkk,:)&*t(vecprb).
end loop.
compute varb = inv(xlptmp*xlp).
compute seb = sqrt(diag(varb)).
release xlptmp.
end if.
break.
end if.
compute bt1 = b.
compute LL1 = LL2.
end loop.
compute modres=b.
do if (jjj > iterate).
compute itprob = 2.
do if (booting=0).
compute iterrmod=1.
end if.
do if (booting=1).
compute bootiter=1.
end if.
do if (itprobtg=0).
compute itprobtg=1.
compute errcode(errs,1) = 47.
compute errs = errs + 1.
do if (booting = 0 and 1 =1).
compute vt1 = mdiag(pt1lp&*(1-pt1lp)).
compute varb = inv(t(xlp)*vt1*xlp).
compute seb = sqrt(diag(varb)).
end if.
end if.
end if.
do if ( 1 =1).
compute trat = b&/seb.
compute dfres=nrow(xlp).
compute p = 2*(1-cdfnorm(abs(trat))).
compute modres={modres,seb,trat,p}.
compute modres={modres,(b-xp2&*seb),(b+xp2&*seb)}.
compute pvchi=1-chicdf((LL3-LL2),(nrow(modres)-1)).
compute mcF = (LL3-LL2)/LL3.
compute cox = 1-exp(-(LL3-LL2)/nrow(xlp)).
compute nagel = cox/(1-exp(-(LL3)/nrow(xlp))).
compute modsum={LL2,(LL3-LL2),(nrow(modres)-1),pvchi, mcF,cox,nagel}.
compute modsuml={'-2LL','ModelLL', 'df', 'p', 'McFadden', 'CoxSnell', 'Nagelkrk'}.
compute modresl={'coeff','se','Z','p','LLCI','ULCI'}.
end if.
end if
.
compute toteff=modres(2:(1+nxvls),:).
compute nodotot=0.
do if (xdich=1 and xmint=1 and model=74).
compute toteff(:,1)=toteff(:,1)*xscaling.
compute toteff(:,2)=toteff(:,2)*abs(xscaling).
compute toteff(:,3)=toteff(:,3)*xscaling.
compute toteff(:,5)=toteff(:,5)*xscaling.
compute toteff(:,6)=toteff(:,6)*xscaling.
compute citmp=toteff(:,5:6).
do if (xscaling < 0).
compute toteff(:,5)=citmp(:,2).
compute toteff(:,6)=citmp(:,1).
end if.
compute nodotot=1.
end if.
compute totefflb=modresl.
do if (outscree=1).
do if (nodotot=0).
print/title = '************************** TOTAL EFFECT MODEL ****************************'.
end if.
do if (nodotot=1).
print/title = '**************************************************************************'.
end if.
print outnames(1,ncol(outnames))/title = 'OUTCOME VARIABLE:'/format = A8/space=0.
end if.
compute toteffl2=vlabsm(2:(1+nxvls),:).
compute resultm2=make(1,maxresm,99999).
compute resultm2(1,1:ncol(modsum))=modsum.
compute resultm={resultm;resultm2}.
compute resultm2=make(nrow(modres),maxresm,99999).
compute resultm2(1:nrow(modres),1:ncol(modres))=modres.
compute resultm={resultm;resultm2}.
do if (outscree=1).
print modsum/title = 'Model Summary'/cnames = modsuml/format= F10.4.
print modres/title='Model'/rnames=vlabsm/cnames=modresl/format= F10.4.
end if.
compute lmat=make(nrow(b),1,0).
compute lmat2=make(nxvls,1,1).
compute lmat(2:(1+nxvls),1)=lmat2.
do if (ydich <> 1).

.
compute lmat2= lmat.
do if ( 0 =0).
compute lmat2 = mdiag( lmat ).
compute lmat3=make(nrow(lmat2),1,0).
loop flp=1 to ncol(lmat2).
do if (csum(lmat2(:,flp))=1).
compute lmat3={lmat3,lmat2(:,flp)}.
end if.
end loop.
compute lmat2=lmat3(:,2:ncol(lmat3)).
end if.
compute fratio = (t(t(lmat2)* b )*inv(t(lmat2)* varb *lmat2)*((t(lmat2)* b )))/ncol(lmat2).
compute pfr = 1-fcdf(fratio,ncol(lmat2),(n-nrow( b ))).
compute fresult={fratio,ncol(lmat2),(n-nrow( b )),pfr}.
do if (i = (nms+nys) and (ydich=1)).
compute fratio=fratio*ncol(lmat2).
compute pfr=1-chicdf(fratio,ncol(lmat2)).
compute fresult={fratio,ncol(lmat2),pfr}.
end if.
do if ( 1 =1).
compute lmat3=1-rsum(lmat2).
compute xfm=make(n,csum(lmat3),0).
compute flpc=1.
loop flp=1 to nrow(lmat3).
do if (lmat3(flp,1)=1).
compute xfm(:,flpc)=x(:,flp).
compute flpc=flpc+1.
end if.
end loop.
compute bfm=inv(t(xfm)*xfm)*t(xfm)*y.
compute resid=y-(xfm*bfm).
compute sstotal=(y-(csum(y)/n)).
compute sstotal=csum(sstotal&*sstotal).
compute ssresid=csum(resid&*resid).
compute rsqch= r2 -((sstotal-ssresid)/sstotal).
compute fresult={rsqch,fresult}.
release xfm,flpc, resid, ssresid, bfm.
end if
.
compute totomni=fresult.
end if.
do if (stand=1).
compute predsd=make(nrow(modres),1,0).
compute stdmod=modres(:,1)&/ovsd(1,ncol(ovsd)).
loop jd=1 to ncol(x).
compute descdat=x(:,jd).
compute predsd(jd,1) = (nrow(descdat)*sscp(descdat))-(t(csum(descdat))*(csum(descdat))).
compute predsd(jd,1) = sqrt(predsd(jd,1)/(nrow(descdat)*(nrow(descdat)-1))).
end loop.
do if (wherex(1,ncol(wherex)) <> -999 and ((nxvls > 1) or (xdich=1))).
compute sdmsone=make(nxvls,1,1).
compute predsd(wherex(1,ncol(wherex)):wherex(2,ncol(wherex)),1)=sdmsone.
compute pstog=1.
end if.
compute predsd(1,1)=1.
compute stdmod=stdmod&*predsd.
compute stdmod=stdmod(2:nrow(stdmod),1).
compute sdvlabs=vlabsm(2:nrow(vlabsm),1).
compute resultm2=make(nrow(stdmod),maxresm,99999).
compute resultm2(1:nrow(stdmod),1:ncol(stdmod))=stdmod.
compute resultm={resultm;resultm2}.
do if (outscree=1).
print stdmod/title='Standardized coefficients'/clabels='coeff'/rnames=sdvlabs/format= F10.4.
end if.
end if.
do if (covcoeff=1).
do if (outscree=1).
print varb/title='Covariance matrix of regression parameter estimates:'/rnames=vlabsm/cnames=vlabsm/format= F10.4.
end if.
compute resultm2=make(nrow(varb),maxresm,99999).
do if (ncol(varb) <= maxresm).
compute resultm2(1:nrow(varb),1:ncol(varb))=varb.
compute resultm={resultm;resultm2}.
end if.
do if (ncol(varb) > maxresm).
compute resultmt=make(nrow(resultm),ncol(varb),99999).
compute resultmt(1:nrow(resultm),1:ncol(resultm))=resultm.
compute resultm=resultmt.
compute resultm2=make(nrow(varb),ncol(resultm),99999).
compute resultm2(1:nrow(varb),1:ncol(varb))=varb.
compute resultm={resultm;resultm2}.
compute maxresm=ncol(resultm).
end if.
end if.
end if.
end if.
do if (criterr=0 and nms > 0 and ydich=0 and modelres=1).
compute modresid=modresid(:,2:ncol(modresid)).
compute sigmatal = (t(modresid)*(ident(n)-(1/n)*ones*t(ones))*modresid)*(1/(n-1)).
compute sdall = mdiag(1/sqrt(diag(sigmatal))).
compute corall=sdall*sigmatal*t(sdall).
compute resultm2=make(nrow(corall),maxresm,99999).
compute resultm2(1:nrow(corall),1:ncol(corall))=corall.
compute resultm={resultm;resultm2}.
do if (outscree=1).
print/title = '****************** CORRELATIONS BETWEEN MODEL RESIDUALS ******************'.
print corall/title=' '/format= F10.4 / cnames=outnames/rnames=outnames/space=0.
end if.
end if.
do if (criterr=0 and boot > 0).
compute bootres=make(1,rsum(nump),-999).
compute bootdir=obsdirfx.
compute natdirbt=make(1,nxvls,-999).
do if (effsize=1).
compute bootysd=make(1,1,-999).
compute bootxsd=make(1,1,-999).
end if.
compute badboot=0.
compute goodboot=0.
compute smallest=1.
compute booting=1.
loop j = 1 to maxboot.
compute nobootx=1.
compute modres2=999.
compute v=trunc(uniform(n,1)*n)+1.
compute bad=0.
loop i = 1 to (nms+nys).
compute y=outvars(v,i).
compute ynovar= (nrow(y)*sscp(y))-(t(csum(y))*(csum(y))).
do if (ynovar = 0).
compute bad=1.
end if.
compute xindx=datindx(1:(nump(1,i)-1),i).
compute hello=0.
compute x = fulldat(v,xindx).
compute x={ones,x}.
compute xsq=t(x)*x.
compute exsq=eval(xsq).
release xsq.
compute holymoly=cmin(exsq).
compute zeroeig=csum(exsq <= 0.000000000002).
compute bad=bad + (zeroeig > 0).

.
compute desctmp=make((8-(4* 1 )),ncol( y ),-999).
loop jd=1 to ncol( y ).
compute descdat= y (:,jd).
compute desctmp(1,jd) = csum(descdat)/nrow(descdat).
compute desctmp(2,jd) = (nrow(descdat)*sscp(descdat))-(t(csum(descdat))*(csum(descdat))).
compute desctmp(2,jd) = sqrt(desctmp(2,jd)/(nrow(descdat)*(nrow(descdat)-1))).
compute desctmp(3,jd)=cmin(descdat).
compute desctmp(4,jd)=cmax(descdat).
do if ( 1 =0).
compute minwarn=0.
compute maxwarn=0.
do if ((desctmp(3,jd)=desctmp(4,jd)) and novar=0).
compute errcode(errs,1)=15.
compute errs=errs+1.
compute criterr=1.
compute novar=1.
end if.
compute tmp=((descdat(:,1)=desctmp(3,jd))+(descdat(:,1)=desctmp(4,jd))).
compute desctmp(8,jd)=(csum(tmp)=nrow(tmp)).
compute tmp = descdat.
compute tmp(GRADE(descdat),:) = descdat.
compute descdat = tmp.
release tmp.
compute decval={.16;.5;.84}.
loop kd=1 to 3.
compute low=trunc(decval(kd,1)*(nrow(descdat)+1)).
compute lowdec=decval(kd,1)*(nrow(descdat)+1)-low.
compute value=descdat(low,1)+(descdat((low+1),1)-descdat(low,1))*lowdec.
compute desctmp((4+kd),jd)=value.
end loop.
compute mnotev=1.
compute modvals=desctmp(5:7,:).
do if (quantile <> 1).
compute desctmp(5,jd)=desctmp(1,jd)-desctmp(2,jd).
compute desctmp(6,jd)=desctmp(1,jd).
compute desctmp(7,jd)=desctmp(1,jd)+desctmp(2,jd).
compute modvals=desctmp(5:7,:).
compute mnotev=2.
do if (modvals(1,1) < desctmp(3,1)).
compute modvals(1,1)=desctmp(3,1).
compute minwarn=1.
end if.
do if (modvals(3,1) > desctmp(4,1)).
compute modvals(3,1)=desctmp(4,1).
compute maxwarn=1.
end if.
end if.
do if (desctmp(8,1)=1).
compute modvals={desctmp(3,1);desctmp(4,1)}.
compute mnotev=0.
compute minwarn=0.
compute maxwarn=0.
end if.
end if.
end loop
.
compute bad=bad+ (desctmp(2,1) <= 0.00000000001).
do if (bad = 0).
do if (holymoly < smallest).
compute smallest=holymoly.
end if.
do if (ydich=0 or (i < (nms+nys))).

.
do if ( 1 =1).
compute hatmat=inv(t( x )* x )*t( x ).
compute b = hatmat* y.
compute modres=b.
do if ( 0 =1).
compute n1=nrow( x ).
compute dfres=n1-(ncol( x )).
compute sstotal = t( y -(csum( y )/n1))*( y -(csum( y )/n1)).
compute resid= y - x *b.
compute ssresid = csum((resid)&**2).
compute r2 = (sstotal-ssresid)/sstotal.
do if (r2 < 0).
compute r2=0.
end if.
compute adjr2 = 1-((1-r2)*(n1-1)/(dfres)).
compute mse=ssresid/(n1-ncol( x )).

.
compute n1=nrow( x ).
compute invXtX = inv(t( x )* x ).
compute varb = mse *invXtX.
compute k3 = ncol( x ).
compute xhc=0.
do if ( hc <> 5).
compute xhc= x.
compute hat = xhc(:,1).
loop i3=1 to nrow(xhc).
compute hat(i3,1)= xhc(i3,:)*invXtX*t(xhc(i3,:)).
end loop.
do if ( hc = 0 or hc =1).
loop i3 = 1 to k3.
compute xhc(:,i3)=xhc(:,i3)&* resid.
end loop.
end if.
do if ( hc =3 or hc =2).
loop i3=1 to k3.
compute xhc(:,i3) = ( resid &/(1-hat)&**(1/(4- hc )))&*xhc(:,i3).
end loop.
end if.
do if ( hc = 4).
compute hcmn=make(n,2,4).
compute hcmn(:,2)=(n1*hat)/k3.
loop i3= 1 to k3.
compute xhc(:,i3) = ( resid &/(1-hat)&**(rmin(hcmn)/2))&*xhc(:,i3).
end loop.
end if.
compute varb=(invXtX*t(xhc)*xhc*invXtX).
do if ( hc =1).
compute varb=(n1/(n1-ncol( x )))&*varb.
end if.
end if.
compute hclab={'se(HC0)','se(HC1)','se(HC2)','se(HC3)','se(HC4)','se'}.
compute hclab=hclab(1,( hc +1)).
compute hcflab={'F(HC0)','F(HC1)','F(HC2)','F(HC3)','F(HC4)','F'}.
compute hcflab=hcflab(1,( hc +1)).
release xhc
.
compute seb=sqrt(diag(varb)).
compute trat = b&/seb.
compute p = 2*(1-tcdf(abs(trat), (dfres))).
compute tval = sqrt(dfres* (exp((dfres-(5/6))*((xp2/(dfres-(2/3)+(.11/dfres)))* (xp2/(dfres-(2/3)+(.11/dfres)))))-1)).
compute modres={modres,seb,trat,p}.
compute modres={modres,(b-tval&*seb),(b+tval&*seb)}.
compute modresl={'coeff',hclab,'t','p','LLCI','ULCI'}.
compute lmat = ident(ncol( x )).
compute lmat = lmat(:,2:ncol(lmat)).
compute fratio = (t(t(lmat)*b)*inv(t(lmat)*varb*lmat)*((t(lmat)*b)))/(ncol( x )-1).
compute pfr = 1-fcdf(fratio,(ncol( x )-1),dfres).
compute modsum={sqrt(r2),r2,mse,fratio,(ncol( x )-1),dfres,pfr}.
compute modsuml={'R','R-sq','MSE',hcflab,'df1','df2', 'p'}.
end if.
end if.
do if ( 1 = 2 or 1 =3).
compute xlp= x.
compute ylp= y.
compute pt2 = make(nrow(ylp),1,(csum(ylp)/nrow(ylp))).
do if ( 1 =2).
compute LL3 = ylp&*ln(pt2)+(1-ylp)&*ln(1-pt2).
end if.
compute LL3 = -2*csum(LL3).
compute bt1 = make(ncol(xlp),1,0).
compute LL1 = 0.
compute pt1 = make(nrow(ylp),1,0.5).
compute pt1lp=pt1.
loop jjj = 1 to iterate.
compute xlptmp=t(xlp).
compute vecprb=pt1lp&*(1-pt1lp).
loop kkk=1 to ncol(xlp).
compute xlptmp(kkk,:)=xlptmp(kkk,:)&*t(vecprb).
end loop.
compute b = bt1+inv(xlptmp*xlp)*t(xlp)*(ylp-pt1lp).
do if ( 1 =2).
compute xlpb=xlp*b.
compute xlpbt=(xlpb > -709.7).
compute xlpb709=(1-xlpbt)*(-709.7).
compute xlpb=(xlpb&*xlpbt)+xlpb709.
compute pt1lp = 1/(1+exp(-(xlpb))).
end if.
compute itprob = csum((pt1lp < .00000001) or (pt1lp > .9999999)).
do if (itprob > 0).
loop kkk = 1 to nrow(pt1lp).
do if (pt1lp(kkk,1) > .9999999).
compute pt1lp(kkk,1) = .9999999.
end if.
do if (pt1lp(kkk,1) < .00000001).
compute pt1lp(kkk,1) = .00000001.
end if.
end loop.
compute itprob = 0.
end if.
do if (itprob = 0).
do if ( 1 =2).
compute LL = ylp&*ln(pt1lp)+(1-ylp)&*ln(1-pt1lp).
end if.
compute LL2 = -2*csum(ll).
end if.
do if (abs(LL1-LL2) < converge).
do if ( 0 =1).
compute xlptmp=t(xlp).
compute vecprb=pt1lp&*(1-pt1lp).
loop kkk=1 to ncol(xlp).
compute xlptmp(kkk,:)=xlptmp(kkk,:)&*t(vecprb).
end loop.
compute varb = inv(xlptmp*xlp).
compute seb = sqrt(diag(varb)).
release xlptmp.
end if.
break.
end if.
compute bt1 = b.
compute LL1 = LL2.
end loop.
compute modres=b.
do if (jjj > iterate).
compute itprob = 2.
do if (booting=0).
compute iterrmod=1.
end if.
do if (booting=1).
compute bootiter=1.
end if.
do if (itprobtg=0).
compute itprobtg=1.
compute errcode(errs,1) = 47.
compute errs = errs + 1.
do if (booting = 0 and 0 =1).
compute vt1 = mdiag(pt1lp&*(1-pt1lp)).
compute varb = inv(t(xlp)*vt1*xlp).
compute seb = sqrt(diag(varb)).
end if.
end if.
end if.
do if ( 0 =1).
compute trat = b&/seb.
compute dfres=nrow(xlp).
compute p = 2*(1-cdfnorm(abs(trat))).
compute modres={modres,seb,trat,p}.
compute modres={modres,(b-xp2&*seb),(b+xp2&*seb)}.
compute pvchi=1-chicdf((LL3-LL2),(nrow(modres)-1)).
compute mcF = (LL3-LL2)/LL3.
compute cox = 1-exp(-(LL3-LL2)/nrow(xlp)).
compute nagel = cox/(1-exp(-(LL3)/nrow(xlp))).
compute modsum={LL2,(LL3-LL2),(nrow(modres)-1),pvchi, mcF,cox,nagel}.
compute modsuml={'-2LL','ModelLL', 'df', 'p', 'McFadden', 'CoxSnell', 'Nagelkrk'}.
compute modresl={'coeff','se','Z','p','LLCI','ULCI'}.
end if.
end if
.
end if.
do if (ydich=1 and (i = (nms+nys))).

.
do if ( 2 =1).
compute hatmat=inv(t( x )* x )*t( x ).
compute b = hatmat* y.
compute modres=b.
do if ( 0 =1).
compute n1=nrow( x ).
compute dfres=n1-(ncol( x )).
compute sstotal = t( y -(csum( y )/n1))*( y -(csum( y )/n1)).
compute resid= y - x *b.
compute ssresid = csum((resid)&**2).
compute r2 = (sstotal-ssresid)/sstotal.
do if (r2 < 0).
compute r2=0.
end if.
compute adjr2 = 1-((1-r2)*(n1-1)/(dfres)).
compute mse=ssresid/(n1-ncol( x )).

.
compute n1=nrow( x ).
compute invXtX = inv(t( x )* x ).
compute varb = mse *invXtX.
compute k3 = ncol( x ).
compute xhc=0.
do if ( hc <> 5).
compute xhc= x.
compute hat = xhc(:,1).
loop i3=1 to nrow(xhc).
compute hat(i3,1)= xhc(i3,:)*invXtX*t(xhc(i3,:)).
end loop.
do if ( hc = 0 or hc =1).
loop i3 = 1 to k3.
compute xhc(:,i3)=xhc(:,i3)&* resid.
end loop.
end if.
do if ( hc =3 or hc =2).
loop i3=1 to k3.
compute xhc(:,i3) = ( resid &/(1-hat)&**(1/(4- hc )))&*xhc(:,i3).
end loop.
end if.
do if ( hc = 4).
compute hcmn=make(n,2,4).
compute hcmn(:,2)=(n1*hat)/k3.
loop i3= 1 to k3.
compute xhc(:,i3) = ( resid &/(1-hat)&**(rmin(hcmn)/2))&*xhc(:,i3).
end loop.
end if.
compute varb=(invXtX*t(xhc)*xhc*invXtX).
do if ( hc =1).
compute varb=(n1/(n1-ncol( x )))&*varb.
end if.
end if.
compute hclab={'se(HC0)','se(HC1)','se(HC2)','se(HC3)','se(HC4)','se'}.
compute hclab=hclab(1,( hc +1)).
compute hcflab={'F(HC0)','F(HC1)','F(HC2)','F(HC3)','F(HC4)','F'}.
compute hcflab=hcflab(1,( hc +1)).
release xhc
.
compute seb=sqrt(diag(varb)).
compute trat = b&/seb.
compute p = 2*(1-tcdf(abs(trat), (dfres))).
compute tval = sqrt(dfres* (exp((dfres-(5/6))*((xp2/(dfres-(2/3)+(.11/dfres)))* (xp2/(dfres-(2/3)+(.11/dfres)))))-1)).
compute modres={modres,seb,trat,p}.
compute modres={modres,(b-tval&*seb),(b+tval&*seb)}.
compute modresl={'coeff',hclab,'t','p','LLCI','ULCI'}.
compute lmat = ident(ncol( x )).
compute lmat = lmat(:,2:ncol(lmat)).
compute fratio = (t(t(lmat)*b)*inv(t(lmat)*varb*lmat)*((t(lmat)*b)))/(ncol( x )-1).
compute pfr = 1-fcdf(fratio,(ncol( x )-1),dfres).
compute modsum={sqrt(r2),r2,mse,fratio,(ncol( x )-1),dfres,pfr}.
compute modsuml={'R','R-sq','MSE',hcflab,'df1','df2', 'p'}.
end if.
end if.
do if ( 2 = 2 or 2 =3).
compute xlp= x.
compute ylp= y.
compute pt2 = make(nrow(ylp),1,(csum(ylp)/nrow(ylp))).
do if ( 2 =2).
compute LL3 = ylp&*ln(pt2)+(1-ylp)&*ln(1-pt2).
end if.
compute LL3 = -2*csum(LL3).
compute bt1 = make(ncol(xlp),1,0).
compute LL1 = 0.
compute pt1 = make(nrow(ylp),1,0.5).
compute pt1lp=pt1.
loop jjj = 1 to iterate.
compute xlptmp=t(xlp).
compute vecprb=pt1lp&*(1-pt1lp).
loop kkk=1 to ncol(xlp).
compute xlptmp(kkk,:)=xlptmp(kkk,:)&*t(vecprb).
end loop.
compute b = bt1+inv(xlptmp*xlp)*t(xlp)*(ylp-pt1lp).
do if ( 2 =2).
compute xlpb=xlp*b.
compute xlpbt=(xlpb > -709.7).
compute xlpb709=(1-xlpbt)*(-709.7).
compute xlpb=(xlpb&*xlpbt)+xlpb709.
compute pt1lp = 1/(1+exp(-(xlpb))).
end if.
compute itprob = csum((pt1lp < .00000001) or (pt1lp > .9999999)).
do if (itprob > 0).
loop kkk = 1 to nrow(pt1lp).
do if (pt1lp(kkk,1) > .9999999).
compute pt1lp(kkk,1) = .9999999.
end if.
do if (pt1lp(kkk,1) < .00000001).
compute pt1lp(kkk,1) = .00000001.
end if.
end loop.
compute itprob = 0.
end if.
do if (itprob = 0).
do if ( 2 =2).
compute LL = ylp&*ln(pt1lp)+(1-ylp)&*ln(1-pt1lp).
end if.
compute LL2 = -2*csum(ll).
end if.
do if (abs(LL1-LL2) < converge).
do if ( 0 =1).
compute xlptmp=t(xlp).
compute vecprb=pt1lp&*(1-pt1lp).
loop kkk=1 to ncol(xlp).
compute xlptmp(kkk,:)=xlptmp(kkk,:)&*t(vecprb).
end loop.
compute varb = inv(xlptmp*xlp).
compute seb = sqrt(diag(varb)).
release xlptmp.
end if.
break.
end if.
compute bt1 = b.
compute LL1 = LL2.
end loop.
compute modres=b.
do if (jjj > iterate).
compute itprob = 2.
do if (booting=0).
compute iterrmod=1.
end if.
do if (booting=1).
compute bootiter=1.
end if.
do if (itprobtg=0).
compute itprobtg=1.
compute errcode(errs,1) = 47.
compute errs = errs + 1.
do if (booting = 0 and 0 =1).
compute vt1 = mdiag(pt1lp&*(1-pt1lp)).
compute varb = inv(t(xlp)*vt1*xlp).
compute seb = sqrt(diag(varb)).
end if.
end if.
end if.
do if ( 0 =1).
compute trat = b&/seb.
compute dfres=nrow(xlp).
compute p = 2*(1-cdfnorm(abs(trat))).
compute modres={modres,seb,trat,p}.
compute modres={modres,(b-xp2&*seb),(b+xp2&*seb)}.
compute pvchi=1-chicdf((LL3-LL2),(nrow(modres)-1)).
compute mcF = (LL3-LL2)/LL3.
compute cox = 1-exp(-(LL3-LL2)/nrow(xlp)).
compute nagel = cox/(1-exp(-(LL3)/nrow(xlp))).
compute modsum={LL2,(LL3-LL2),(nrow(modres)-1),pvchi, mcF,cox,nagel}.
compute modsuml={'-2LL','ModelLL', 'df', 'p', 'McFadden', 'CoxSnell', 'Nagelkrk'}.
compute modresl={'coeff','se','Z','p','LLCI','ULCI'}.
end if.
end if
.
end if.
compute modres2={modres2,t(modres)}.
do if (i = (nms+nys)).
do if (bcmat((i+1),1) = 1).
compute bootdir={bootdir;t(modres(wherex(1,i):wherex(2,i),1))}.
end if.
do if (bcmat((i+1),1) = 0).
compute bootdir={bootdir;dirzes}.
end if.
end if.
do if (model = 74 and i <= nms).
compute onetemp=make(nrow(xprobval),1,1).
compute mestmtb={onetemp,xprobval}.
do if (ncs > 0).
compute ncovmdlb=rsum(ccmat(i,:)).
do if (ncovmdl > 0).
compute cvmncb=make(nrow(mestmtb),ncovmdlb,1).
compute cvmnctmp=csum(x(:,(ncol(x)-ncovmdlb+1):ncol(x)))/nrow(x).
do if (cuscoval > 0).
compute cvmnctmp=coval.
end if.
loop mestlp=1 to ncovmdlb.
compute cvmncb(:,mestlp)=cvmncb(:,mestlp)*cvmnctmp(:,mestlp).
end loop.
compute mestmtb={mestmtb,cvmncb}.
end if.
end if.
compute mestbt=mestmtb*modres.
do if (i = 1).
compute mestb=mestbt.
end if.
do if (i > 1).
compute mestb={mestb,mestbt}.
end if.
end if.
do if (model = 74 and i = (nms+nys)).
compute xvalptmp=make(1,nxvls,0).
compute mest74sp=make(1,nms,0).
compute mest74sp={mest74sp;mestb}.
compute xvalptmp={xvalptmp;ident(nxvls)}.
compute dirfxcf=make(nrow(mestb),1,0).
loop cfloop1=1 to nrow(mestb).
compute ndirfx=make(nrow(modres),1,0).
compute ndirfx(2:(nxvls+1),1)=t(xvalptmp(cfloop1,:)).
loop cfloop3=1 to nms.
do if (mcx=1 or mcx=0).
compute ndirfx(wheremw((1+((cfloop3-1)*2)),ncol(wheremw)):wheremw((2+((cfloop3-1)*2) ),ncol(wheremw)))=t(xvalptmp(cfloop1,:)*mestb(1,cfloop3)).
end if.
do if (mcx=2).
compute ndirfx(wheremw((1+((cfloop3-1)*2)),ncol(wheremw)):wheremw((2+((cfloop3-1)*2) ),ncol(wheremw)))=t(xvalptmp(cfloop1,:)*mest74sp(cfloop1,cfloop3)).
end if.
end loop.
do if (cfloop1 > 1).
compute dirfxcf(cfloop1,1)=t(ndirfx)*modres*xscaling.
end if.
end loop.
compute natdirbt={natdirbt;t(dirfxcf(2:nrow(dirfxcf),:))}.
end if.
do if ( (bcmat((i+1),1) = 1) and (nobootx=1) and (effsize=1)).
compute nobootx=0.
compute xsdtemp= (nrow(x)*sscp(x(:,2)))-(t(csum(x(:,2)))*(csum(x(:,2)))).
compute xsdtemp= sqrt(xsdtemp/(nrow(x)*(nrow(x)-1))).
end if.
end if.
end loop.
do if (bad = 0).
compute bootres={bootres;modres2(:,2:ncol(modres2))}.
do if (effsize=1).
compute ysdtemp= (nrow(y)*sscp(y))-(t(csum(y))*(csum(y))).
compute ysdtemp= sqrt(ysdtemp/(nrow(y)*(nrow(y)-1))).
compute bootysd={bootysd;ysdtemp}.
compute bootxsd={bootxsd;xsdtemp}.
end if.
compute goodboot=goodboot+1.
end if.
do if (bad <> 0).
compute badboot=badboot+1.
end if.
end loop if (goodboot = boot).
compute bootres=bootres(2:nrow(bootres),:).
do if (effsize=1).
compute bootysd=bootysd(2:nrow(bootysd),:).
do if (nrow(bootxsd) > 1).
compute bootxsd=bootxsd(2:nrow(bootxsd),:).
end if.
end if.
do if (goodboot < (boot)).
compute boot=0.
compute modelbt=0.
compute notecode(notes,1) = 7.
compute notes = notes + 1.
end if.
do if (boot > 0).
do if (effsize=1).
compute bootysd={ysd;bootysd}.
compute bootxsd={xsd;bootxsd}.
end if.
do if (saveboot = 1).
save bootres/outfile = *.
end if.
do if (modelbt=1).
compute bootcim=make(ncol(bootres),5,-99999).
compute bootcim(:,2) = t(csum(bootres)/nrow(bootres)).
compute bootcim(:,1) = coeffmat(2:nrow(coeffmat),1).
loop i = 1 to ncol(bootres).

.
compute temp = bootres(:,i).
compute temp(GRADE( bootres(:,i) )) = bootres(:,i).
compute badlo = 0.
compute badhi = 0.
do if ( (bootcim(i,1)*bc)+(9999*(1-bc)) <> 9999).
compute pv=csum(temp < (bootcim(i,1)*bc)+(9999*(1-bc)) )/nrow(temp).
compute ppv = pv.
do if (pv > .5).
compute ppv = 1-pv.
end if.
compute y5=sqrt(-2*ln(ppv)).
compute xp=y5+((((y5*p4+p3)*y5+p2)*y5+p1)*y5+p0)/((((y5*q4+q3)*y5+q2)*y5+q1)*y5+q0).
do if (pv <= .5).
compute xp = -xp.
end if.
compute cilow=rnd(nrow(temp)*(cdfnorm(2*xp-xp2))).
compute cihigh=trunc(nrow(temp)*(cdfnorm(2*xp+(xp2))))+1.
do if (cilow < 1).
compute cilow = 1.
compute booterr=1.
compute badlo = 1.
end if.
do if (cihigh > nrow(temp)).
compute cihigh = boot.
compute booterr=1.
compute badhi = 1.
end if.
compute llcit=temp(cilow,1).
compute ulcit=temp(cihigh,1).
do if (badlo = 1 and llcit <> priorlo).
compute badend={badend, llcit}.
compute priorlo = llcit.
end if.
do if (badhi = 1 and ulcit <> priorhi).
compute badend={badend, ulcit}.
compute priorhi = ulcit.
end if.
end if.
do if ( (bootcim(i,1)*bc)+(9999*(1-bc)) = 9999).
compute llcit=temp(cilow,1).
compute ulcit=temp(cihigh,1).
end if.
compute bootse=t(sqrt((cssq(temp)-((csum(temp)&**2)/nrow(temp)))/(nrow(temp)-1)))
.
compute bootcim(i,4:5)={llcit,ulcit}.
compute bootcim(i,3)=bootse.
end loop.
end if.
end if.
do if (badboot > 0).
compute notecode(notes,1) = 6.
compute notes = notes + 1.
end if.
end if.
do if (criterr=0).
release fulldat,x,y.
end if.
do if (xmint=1 and criterr=0).
compute xvalptmp=make(1,nxvls,0).
compute mest74sp=make(1,nms,0).
compute mest74sp={mest74sp;mest74}.
compute xvalptmp={xvalptmp;ident(nxvls)}.
loop kcfuhd=1 to 2.
compute dirfxcf=make(nrow(mest74),6,0).
loop cfloop1=1 to nrow(mest74).
compute ndirfx=make(nrow(lastb),1,0).
compute ndirfx(2:(nxvls+1),1)=t(xvalptmp(cfloop1,:)).
loop cfloop3=1 to nms.
do if (kcfuhd=1).
do if (mcx=1 or mcx=0).
compute ndirfx(wheremw((1+((cfloop3-1)*2)),ncol(wheremw)):wheremw((2+((cfloop3-1)*2)), ncol(wheremw)))=t(xvalptmp(cfloop1,:)*mest74(1,cfloop3)).
end if.
do if (mcx=2).
compute ndirfx(wheremw((1+((cfloop3-1)*2)),ncol(wheremw)):wheremw((2+((cfloop3-1)*2)), ncol(wheremw)))=t(xvalptmp(cfloop1,:)*mest74sp(cfloop1,cfloop3)).
end if.
end if.
do if (kcfuhd=2).
compute ndirfx(wheremw((1+((cfloop3-1)*2)),ncol(wheremw)):wheremw((2+((cfloop3-1)*2)), ncol(wheremw)))=t(xvalptmp(cfloop1,:)*medmeans(1,cfloop3)).
end if.
end loop.
do if (cfloop1 > 1).
compute dirfxcf(cfloop1,1)=t(ndirfx)*lastb*xscaling.
compute dirfxcf(cfloop1,2)=sqrt(diag(t(ndirfx)*lastcov*ndirfx))*abs(xscaling).
compute dirfxcf(cfloop1,3)=dirfxcf(cfloop1,1)/dirfxcf(cfloop1,2).
compute dirfxcf(cfloop1,4)=2*(1-tcdf(abs(dirfxcf(cfloop1,3)), dfres)).
compute dirfxcf(cfloop1,5)=dirfxcf(cfloop1,1)-tval*dirfxcf(cfloop1,2).
compute dirfxcf(cfloop1,6)=dirfxcf(cfloop1,1)+tval*dirfxcf(cfloop1,2).
end if.
end loop.
compute codireff=dirfxcf(2:nrow(dirfxcf),:).
do if (kcfuhd=1).
compute direff=codireff.
end if.
end loop.
end if.

.
do if (criterr = 0 and nms > 0).
compute paths=paths(:,2:ncol(paths)).
compute pathsw=pathsw(:,2:ncol(pathsw)).
compute pathsz=pathsz(:,2:ncol(pathsz)).
compute pathswz=pathswz(:,2:ncol(pathswz)).
compute pathsmod=pathsw+pathsz+pathswz.
compute pathsdv=pathsdv(:,2:ncol(pathsdv)).
compute pathsfoc=pathsfoc(:,2:ncol(pathsfoc)).
compute pathtype=pathtype(:,2:ncol(pathtype)).
compute anymod=(rsum(pathsmod) > 0).
compute obscoeff=obscoeff(1,2:ncol(obscoeff)).
do if (outscree=1).
do if (xmint=1).
print/title = '************************* COUNTERFACTUALLY DEFINED ************************'.
else.
print/title = ' '.
end if.
do if (dototal = 0 and alttotal=0).
print/title = '****************** DIRECT AND INDIRECT EFFECTS OF X ON Y *****************'/space=0.
end if.
do if (alttotal=1).
print/title = '************** TOTAL, DIRECT, AND INDIRECT EFFECTS OF X ON Y **************'/space=0.
end if.
end if.
do if (dototal = 1).
do if (outscree=1).
print/title = '************** TOTAL, DIRECT, AND INDIRECT EFFECTS OF X ON Y **************'/space=0.
end if.
compute totefflb(1,1)='Effect'.
do if (effsize=1).
compute toteffsz=toteff(:,1)/ysd.
do if (xdich=1 or mcx > 0).
compute totefflb={totefflb,'c_ps'}.
end if.
do if (xdich = 0 and mcx = 0).
compute toteffsz=toteffsz*xsd.
compute totefflb={totefflb,'c_cs'}.
end if.
compute toteff={toteff,toteffsz}.
end if.
compute resultm2=make(nrow(toteff),maxresm,99999).
compute resultm2(1:nrow(toteff),1:ncol(toteff))=toteff.
compute resultm={resultm;resultm2}.
do if (nxvls > 1).
compute resultm2=make(nrow(totomni),maxresm,99999).
compute resultm2(1:nrow(totomni),1:ncol(totomni))=totomni.
compute resultm={resultm;resultm2}.
compute clabtmp={'R2-chng', hcflab, 'df1','df2','p'}.
do if (outscree=1).
print toteff/title='Relative total effects of X on Y'/rnames=toteffl2/cnames=totefflb/format= F10.4.
print totomni/title='Omnibus test of total effect of X on Y'/cnames=clabtmp/format= F10.4.
print/title= '----------'/space=0.
end if.
else.
do if (outscree=1).
print toteff/title='Total effect of X on Y'/cnames=totefflb/format= F10.4.
end if.
end if.
end if.
compute moddir=wcmat(nrow(bcmat),1)+zcmat(nrow(bcmat),1).
do if (xmint=1).
compute moddir=1.
end if.
do if (bcmat(nrow(bcmat),1)=1).
do if (ydich=1).
compute direfflb(:,(ncol(direfflb)-5):ncol(direfflb))={'Effect','se','Z','p','LLCI','ULCI'}.
end if.
do if (moddir=0 or xmint=1).
compute direfflb(1,1)='Effect'.
end if.
do if (effsize=1 and moddir=0 and anymod = 0).
compute direffsz=direff(:,1)/ysd.
do if (xdich=1 or mcx > 0).
compute direfflb={direfflb,'c''_ps'}.
end if.
do if (xdich = 0 and mcx = 0).
compute direffsz=direffsz*xsd.
compute direfflb={direfflb,'c''_cs'}.
end if.
compute direff={direff,direffsz}.
end if.
compute resultm2=make(nrow(direff),maxresm,99999).
compute resultm2(1:nrow(direff),1:ncol(direff))=direff.
compute resultm={resultm;resultm2}.
do if (xmint=1).
compute resultm2=make(nrow(codireff),maxresm,99999).
compute resultm2(1:nrow(codireff),1:ncol(codireff))=codireff.
compute resultm={resultm;resultm2}.
end if.
do if (moddir = 0 and nxvls=1).
do if (outscree=1).
print direff/title='Direct effect of X on Y'/cnames=direfflb/format= F10.4.
end if.
end if.
do if (moddir = 0 and nxvls>1).
compute resultm2=make(nrow(diromni),maxresm,99999).
compute resultm2(1:nrow(diromni),1:ncol(diromni))=diromni.
compute resultm={resultm;resultm2}.
do if (outscree=1).
print direff/title='Relative direct effects of X on Y'/rnames=direffl2/cnames=direfflb/format= F10.4.
do if (ydich = 0).
compute clabtmp={'R2-chng', hcflab, 'df1','df2','p'}.
print diromni/title='Omnibus test of direct effect of X on Y:'/cnames=clabtmp/format= F10.4.
end if.
do if (ydich = 1).
compute clabtmp={'Chi-sq', 'df', 'p'}.
print diromni/title='Omnibus likelihood ratio test of direct effect of X on Y'/cnames=clabtmp/format= F10.4.
end if.
print/title= '----------'/space=0.
end if.
end if.
do if (moddir > 0 and nxvls=1).
do if (xmint=0 and outscree=1).
print direff/title='Conditional direct effects of X on Y'/cnames=direfflb/format= F10.4.
end if.
do if (xmint=1).
do if (outscree=1).
print direff/title='(Pure) Natural direct effect of X on Y'/cnames=direfflb/format= F10.4.
print codireff/title='Controlled direct effect of X on Y'/cnames=direfflb/format= F10.4.
print/title= '----------'/space=0.
end if.
compute obnatdfx=direff(:,1).
end if.
end if.
compute direffl4=direffl2.
do if (moddir > 0 and nxvls>1).
compute direffl2={' '}.
loop i = 1 to nxvls.
loop j = 1 to (nrow(direff)/nxvls).
compute direffl2={direffl2;xcatlab(i,1)}.
end loop.
end loop.
compute direffl2=direffl2(2:nrow(direffl2),1).
do if (xmint=0 and outscree=1).
print direff/title='Relative conditional direct effects of X on Y'/cnames=direfflb/rnames=direffl2/format= F10.4.
end if.
do if (xmint=1).
do if (outscree=1).
print direff/title='Relative (pure) natural direct effects of X on Y'/cnames=direfflb/rnames=direffl2/format= F10.4.
print codireff/title='Relative controlled direct effects of X on Y'/cnames=direfflb/rnames=direffl2/format= F10.4.
print/title= '----------'/space=0.
end if.
compute obnatdfx=direff(:,1).
end if.
end if.
compute direffl2=direffl4.
end if.
do if (bcmat(nrow(bcmat),1)=0 and xmint <> 1 and outscree=1).
print/title='The direct effect of X on Y is fixed to zero.'.
end if.
do if (nms = 1).
compute indmark={2}.
compute indsets={1,2}.
end if.
do if (nms = 2).
compute indmark={2,2,3}.
compute indsets={1,4,2,5,1,3,5}.
compute thetam=1.
end if.
do if (nms = 3).
compute indmark={2,2,2,3,3,3,4}.
compute indsets={1,7,2,8,4,9,1,3,8,1,5,9,2,6,9,1,3,6,9}.
compute thetam={1,2,3}.
end if.
do if (nms = 4).
compute indmark={2,2,2,2,3,3,3,3,3,3,4,4,4,4,5}.
compute indsets={1,11,2,12,4,13,7,14,1,3,12,1,5,13,1,8,14,2,6,13,2,9,14,4,10,14,1,3,6,13,1,3,9, 14,1,5,10,14,2,6,10,14,1,3,6,10,14}.
compute thetam={1,2,4,3,5,6}.
end if.
do if (nms = 5).
compute indmark={2,2,2,2,2,3,3,3,3,3,3,3,3,3,3,4,4,4,4,4,4,4,4,4,4,5,5,5,5,5,6}.
compute indsets={1,16,2,17,4,18,7,19,11,20,1,3,17,1,5,18,1,8,19,1,12,20,2,6,18,2,9,19,2,13,20,4, 10,19,4,14,20,7,15,20,1,3,6,18, 1,3,9,19,1,3,13,20,1,5,10,19,1,5,14,20,1,8,15,20,2,6,10,19,2,6, 14,20,2,9,15,20,4,10,15,20,1,3,6,10,19, 1,3,6,14,20,1,3,9,15,20,1,5,10,15,20,2,6,10,15,20,1,3,6,10,15, 20}.
compute thetam={1,2,5,3,6,8,4,7,9,10}.
end if.
do if (nms = 6).
compute indmark={2,2,2,2,2,2,3,3,3,3,3,3,3,3,3,3,3,3,3,3,3,4,4,4,4,4,4,4,4,4,4,4,4,4,4,4,4,4,4, 4,4,5,5,5,5,5,5,5,5,5,5,5,5,5,5,5,6,6,6,6,6,6,7}.
compute indsets={1,22,2,23,4,24,7,25,11,26,16,27,1,3,23,1,5,24,1,8,25,1,12,26,1,17,27,2,6,24,2, 9,25,2,13,26,2,18,27,4,10,25,4,14,26, 4,19,27,7,15,26,7,20,27,11,21,27,1,3,6,24,1,3,9,25,1,3,13,26,1, 3,18,27,1,5,10,25,1,5,14,26,1,5,19,27,1,8,15,26,1,8,20,27, 1,12,21,27,2,6,10,25,2,6,14,26,2,6,19,27,2,9,15,26,2,9,20,27,2, 13,21,27,4,10,15,26,4,10,20,27,4,14,21,27,7,15,21,27, 1,3,6,10,25,1,3,6,14,26,1,3,6,19,27,1,3,9,15,26,1,3,9,20,27,1,3, 13,21,27,1,5,10,15,26,1,5,10,20,27,1,5,14,21,27, 1,8,15,21,27,2,6,10,15,26,2,6,10,20,27,2,6,14,21,27,2,9,15,21, 27,4,10,15,21,27,1,3,6,10,15,26,1,3,6,10,20,27, 1,3,6,14,21,27,1,3,9,15,21,27,1,5,10,15,21,27,2,6,10,15,21,27, 1,3,6,10,15,21,27}.
compute thetam={1,2,6,3,7,10,4,8,11,13,5,9,12,14,15}.
end if.
do if (nms = 7).
compute indmark={2,2,2,2,2,2,2}.
compute indsets={1,29,2,30,4,31,7,32,11,33,16,34,22,35}.
end if.
do if (nms = 8).
compute indmark={2,2,2,2,2,2,2,2}.
compute indsets={1,37,2,38,4,39,7,40,11,41,16,42,22,43,29,44}.
end if.
do if (nms = 9).
compute indmark={2,2,2,2,2,2,2,2,2}.
compute indsets={1,46,2,47,4,48,7,49,11,50,16,51,22,52,29,53,37,54}.
end if.
do if (nms=10).
compute indmark={2,2,2,2,2,2,2,2,2,2}.
compute indsets={1,56,2,57,4,58,7,59,11,60,16,61,22,62,29,63,37,64,46,65}.
end if.
compute indlbl = {'Ind1'; 'Ind2'; 'Ind3'; 'Ind4'; 'Ind5'; 'Ind6'; 'Ind7'; 'Ind8'; 'Ind9'; 'Ind10'; 'Ind11'; 'Ind12'; 'Ind13'; 'Ind14'; 'Ind15'}.
compute indlbl = {indlbl; 'Ind16';'Ind17';'Ind18';'Ind19';'Ind20';'Ind21';'Ind22';'Ind23';'Ind24';'Ind25';'Ind26';'Ind27';'Ind28';'Ind29';'Ind30'}.
compute indlbl = {indlbl; 'Ind31';'Ind32';'Ind33';'Ind34';'Ind35';'Ind36';'Ind37';'Ind38';'Ind39';'Ind40';'Ind41';'Ind42';'Ind43';'Ind44';'Ind45'}.
compute indlbl = {indlbl; 'Ind46';'Ind47';'Ind48';'Ind49';'Ind50';'Ind51';'Ind52';'Ind53';'Ind54';'Ind55';'Ind56';'Ind57';'Ind58';'Ind59';'Ind60'}.
compute indlbl = {indlbl; 'Ind61';'Ind62';'Ind63';'Ind64';'Ind65';'Ind66';'Ind67';'Ind68';'Ind69';'Ind70';'Ind71';'Ind72';'Ind73';'Ind74';'Ind75'}.
compute indlbl = {indlbl; 'Ind76';'Ind77';'Ind78';'Ind79';'Ind80';'Ind81';'Ind82';'Ind83';'Ind84';'Ind85';'Ind86';'Ind87';'Ind88';'Ind89';'Ind90'}.
compute cntname={'(C1)';'(C2)';'(C3)';'(C4)';'(C5)';'(C6)';'(C7)';'(C8)';'(C9)';'(C10)';'(C11)';'(C12)';'(C13)';'(C14)';'(C15)';'(C16)';'(C17)'}.
compute cntname={cntname;'(C18)';'(C19)';'(C20)';'(C21)';'(C22)';'(C23)';'(C24)';'(C25)';'(C26)';'(C27)';'(C28)';'(C29)';'(C30)';'(C31)'}.
compute cntname={cntname;'(C32)';'(C33)';'(C34)';'(C35)';'(C36)';'(C37)';'(C38)';'(C39)';'(C40)';'(C41)';'(C42)';'(C43)';'(C44)';'(C45)'}.
compute cntname={cntname;'(C46)';'(C47)';'(C48)';'(C49)';'(C50)';'(C51)';'(C52)';'(C53)';'(C54)';'(C55)';'(C56)';'(C57)';'(C58)';'(C59)'}.
compute cntname={cntname;'(C60)';'(C61)';'(C62)';'(C63)';'(C64)';'(C65)';'(C66)';'(C67)';'(C68)';'(C69)';'(C70)';'(C71)';'(C72)';'(C73)'}.
compute cntname={cntname;'(C74)';'(C75)';'(C76)';'(C77)';'(C78)';'(C79)';'(C80)';'(C81)';'(C82)';'(C83)';'(C84)';'(C85)';'(C86)';'(C87)'}.
compute cntname={cntname;'(C88)';'(C89)';'(C90)';'(C91)';'(C92)';'(C93)';'(C94)';'(C95)';'(C96)';'(C97)';'(C98)';'(C99)';'(C100)';'(C101)'}.
compute cntname={cntname;'(C102)';'(C103)';'(C104)';'(C105)'}.
compute indmake=make(ncol(indmark),(nms+2),0).
compute indmod=make(ncol(indmark),1,999).
compute indmmm=make(ncol(indmark),1,0).
compute indmmmt=make(ncol(indmark),1,0).
compute start=1.
compute end=0.
compute nindfx=0.
compute indlocs=make(nrow(thetaxmb),ncol(paths),999).
compute indkey=make(ncol(indmark),1+((rmax(indmark)*2)+1),'     ').
compute c1=1.
compute c2=1.
compute c3=1.
loop i = 1 to ncol(paths).
do if (pathtype(1,i)=1).
compute indlocs(:,i)=thetaxmb(:,c1).
compute c1=c1+1.
end if.
do if (pathtype(1,i)=3).
compute indlocs(:,i)=thetamyb(:,c2).
compute c2=c2+1.
end if.
do if (pathtype(1,i)=2 and nms < 7 and serial=1).
compute indlocs(:,i)=thetammb(:,thetam(1,c3)).
compute c3=c3+1.
end if.
end loop.
loop i = 1 to ncol(indlocs).
compute c1=2.
loop j = 2 to nrow(indlocs).
do if (indlocs(j,i) <> 0).
compute indlocs(c1,i)=indlocs(j,i).
compute c1=c1+1.
end if.
end loop.
compute indlocs(1,i)=c1-2.
end loop.
compute indlocs=indlocs(1:rmax((indlocs(1,:))+1),:).
loop i = 1 to ncol(indmark).
compute numget=indmark(1,i).
compute end=end+numget.
compute gotcha=indsets(1,start:end).
compute start=end+1.
compute ok=1.
compute temp=0.
compute repoman=make(4,1,0).
loop j = 1 to ncol(gotcha).
do if paths(1,gotcha(1,j))=0.
compute ok=0.
end if.
do if (pathsmod(1,gotcha(1,j)) > 0).
compute temp=1.
compute temp2={pathsw(1,gotcha(1,j));pathsz(1,gotcha(1,j));pathswz(1,gotcha(1,j));0}.
do if (temp2(1,1)=1 and temp2(2,1)=1 and temp2(3,1)=0).
compute temp2(4,1)=1.
end if.
compute repoman=repoman+temp2.
end if.
end loop.
compute temp=0.
compute tempmmm=0.
compute typemmm=0.
do if ((repoman(1,1) > 0) and (repoman(2,1) = 0)).
compute temp=1.
do if (repoman(1,1)=1).
compute tempmmm=1.
end if.
do if (repoman(1,1) > 1 and (wdich=1 or mcw > 0)).
compute tempmmm=12.
compute typemmm=mcw.
do if (wdich=1).
compute typemmm=1.
end if.
end if.
do if (repoman(1,1) > 1 and (wdich=0 and mcw = 0)).
compute tempmmm=101.
end if.
end if.
do if ((repoman(1,1) = 0) and (repoman(2,1) > 0)).
compute temp=2.
do if (repoman(2,1)=1).
compute tempmmm=2.
end if.
do if (repoman(2,1) > 1 and (zdich = 1 or mcz > 0)).
compute tempmmm=22.
compute typemmm=mcz.
do if (zdich=1).
compute typemmm=1.
end if.
end if.
do if (repoman(2,1) > 1 and (zdich = 0 and mcw = 0)).
compute tempmmm=102.
end if.
end if.
do if (repoman(1,1)>0 and repoman(2,1)>0).
compute temp=3.
do if (repoman(1,1)=1 and repoman(2,1)=1).
do if (repoman(4,1)=1).
compute tempmmm=31.
end if.
do if (repoman(3,1)=1).
compute tempmmm=41.
end if.
end if.
end if.
do if (repoman(1,1)=1 and repoman(2,1)=1 and repoman(3,1)=0 and repoman(4,1)=0).
compute tempmmm=51.
end if.
do if (ok = 1).
compute nindfx=nindfx+1.
compute indmake(nindfx,1)=numget.
compute indmod(nindfx,1)=temp.
compute indmmm(nindfx,1)=tempmmm.
compute indmmmt(nindfx,1)=typemmm.
compute indmake(nindfx,2:(1+numget))=gotcha.
compute indkey(nindfx,1)=xnames.
loop j = 1 to numget.
compute indkey(nindfx,(j*2+1))=pathsdv(1,gotcha(1,j)).
compute indkey(nindfx,(j*2))='   ->   '.
end loop.
end if.
end loop.
compute indkey=indkey(1:nindfx,1:((cmax(indmake(:,1))*2)+1)).
compute indmake=indmake(1:nindfx,1:(cmax(indmake(:,1))+1)).
compute indmod=indmod(1:nrow(indmake),1).
compute indmmm=indmmm(1:nrow(indmake),1).
compute indmmmt=indmmmt(1:nrow(indmake),1).
compute ncpairs = (((nindfx)*(nindfx-1))/2).
do if ((contrast = 1 or contrast = 2 or contrast=3) and (ncpairs > 105)).
compute contrast=0.
compute notecode(notes,1) = 13.
compute notes = notes + 1.
end if.
do if (contrast = 4).
do if (ncol(contvec) <> nindfx).
compute contrast=0.
compute notecode(notes,1) = 14.
compute notes = notes + 1.
end if.
end if.
do if (anymod=0).
do if (nms = 1 and contrast > 0).
compute contrast=0.
end if.
compute efloop=(((1-(effsize=0))*2)+1)-(((mcx>0 or xdich=1))*(1-(effsize=0))).
loop kk=1 to efloop.
do if (boot = 0).
compute bootres=obscoeff.
do if (kk=1).
compute totbtvec=make(1,nxvls,0).
end if.
compute bootdir=obsdirfx.
compute indtab=999.
compute inddiff=999.
compute bootysd=ysd.
compute bootxsd=xsd.
end if.
do if (boot > 0).
compute bootres={obscoeff;bootres}.
do if (kk=1).
compute totbtvec=make(nrow(bootres),nxvls,0).
end if.
compute indtab=make(1,4,999).
compute inddiff=make(nrow(bootres),1,999).
end if.
compute indtotal=make(nrow(bootres),1,0).
loop i = 1 to nrow(indmake).
loop j = 1 to nxvls.
compute indtemp=make(nrow(bootres),1,1).
loop k = 1 to indmake(i,1).
compute jtemp=1.
do if (j > 1 and k=1).
compute jtemp=j.
end if.
compute indtemp=indtemp&*bootres(:,pathsfoc(jtemp,indmake(i,(k+1)))).
end loop.
do if (kk = 2).
compute indtemp=indtemp/bootysd.
end if.
do if (kk = 3).
compute indtemp=(bootxsd&*indtemp)/bootysd.
end if.
do if (contrast <> 0).
compute inddiff={inddiff,indtemp}.
end if.
do if (nxvls=1).
compute indtotal=indtotal+indtemp.
end if.
compute indeff=indtemp(1,1).
do if (kk=1).
compute totbtvec(:,j)=totbtvec(:,j)+indtemp.
end if.
do if (boot > 0).

.
compute temp = indtemp(2:nrow(indtemp),1).
compute temp(GRADE( indtemp(2:nrow(indtemp),1) )) = indtemp(2:nrow(indtemp),1).
compute badlo = 0.
compute badhi = 0.
do if ( (indtemp(1,1)*bc)+(9999*(1-bc)) <> 9999).
compute pv=csum(temp < (indtemp(1,1)*bc)+(9999*(1-bc)) )/nrow(temp).
compute ppv = pv.
do if (pv > .5).
compute ppv = 1-pv.
end if.
compute y5=sqrt(-2*ln(ppv)).
compute xp=y5+((((y5*p4+p3)*y5+p2)*y5+p1)*y5+p0)/((((y5*q4+q3)*y5+q2)*y5+q1)*y5+q0).
do if (pv <= .5).
compute xp = -xp.
end if.
compute cilow=rnd(nrow(temp)*(cdfnorm(2*xp-xp2))).
compute cihigh=trunc(nrow(temp)*(cdfnorm(2*xp+(xp2))))+1.
do if (cilow < 1).
compute cilow = 1.
compute booterr=1.
compute badlo = 1.
end if.
do if (cihigh > nrow(temp)).
compute cihigh = boot.
compute booterr=1.
compute badhi = 1.
end if.
compute llcit=temp(cilow,1).
compute ulcit=temp(cihigh,1).
do if (badlo = 1 and llcit <> priorlo).
compute badend={badend, llcit}.
compute priorlo = llcit.
end if.
do if (badhi = 1 and ulcit <> priorhi).
compute badend={badend, ulcit}.
compute priorhi = ulcit.
end if.
end if.
do if ( (indtemp(1,1)*bc)+(9999*(1-bc)) = 9999).
compute llcit=temp(cilow,1).
compute ulcit=temp(cihigh,1).
end if.
compute bootse=t(sqrt((cssq(temp)-((csum(temp)&**2)/nrow(temp)))/(nrow(temp)-1)))
.
compute indeff={indeff,bootse,llcit,ulcit}.
end if.
do if (kk=1).
compute indtabn={indtab;indeff}.
end if.
compute indtab={indtab;indeff}.
end loop.
end loop.
compute indtab=indtab(2:nrow(indtab),:).
do if (kk=1).
compute indtabn=indtabn(2:nrow(indtabn),:).
end if.
compute rowlbs=indlbl(1:nrow(indtab),1).
do if (mc > 0).
compute inddiff=make(mc,1,-999).
compute indtab2=make(nrow(indtab),4,-999).
compute indtab2(:,1)=indtab.
compute indtab=indtab2.
release indtab2.
compute mcct=0.
compute indtotal=make(mc,1,0).
do if (kk = 1).
compute x1 = sqrt(-2*ln(uniform(mc,nrow(mcsopath))))&*cos((2*3.14159265358979)*uniform(mc, nrow(mcsopath))).
compute x1=x1*chol(indcov).
loop ii=1 to nrow(x1).
compute x1(ii,:)=x1(ii,:)+t(mcsopath).
end loop.
end if.
loop ii=1 to nms.
compute tmpb=x1(:,((nms*nxvls)+ii)).
compute tmpb2=tmpb.
do if (nxvls > 1).
loop jj=1 to (nxvls-1).
compute tmpb2={tmpb2,tmpb}.
end loop.
end if.
compute indtemp=x1(:,(((ii-1)*nxvls)+1):(ii*nxvls))&*tmpb2.
loop jj=1 to ncol(indtemp).
do if (kk = 2).
compute indtemp(:,jj)=indtemp(:,jj)/ysd.
end if.
do if (kk = 3).
compute indtemp(:,jj)=(xsd*indtemp(:,jj))/ysd.
end if.

.
compute temp = indtemp(:,jj).
compute temp(GRADE( indtemp(:,jj) )) = indtemp(:,jj).
compute badlo = 0.
compute badhi = 0.
do if ( 9999 <> 9999).
compute pv=csum(temp < 9999 )/nrow(temp).
compute ppv = pv.
do if (pv > .5).
compute ppv = 1-pv.
end if.
compute y5=sqrt(-2*ln(ppv)).
compute xp=y5+((((y5*p4+p3)*y5+p2)*y5+p1)*y5+p0)/((((y5*q4+q3)*y5+q2)*y5+q1)*y5+q0).
do if (pv <= .5).
compute xp = -xp.
end if.
compute cilow=rnd(nrow(temp)*(cdfnorm(2*xp-xp2))).
compute cihigh=trunc(nrow(temp)*(cdfnorm(2*xp+(xp2))))+1.
do if (cilow < 1).
compute cilow = 1.
compute booterr=1.
compute badlo = 1.
end if.
do if (cihigh > nrow(temp)).
compute cihigh = boot.
compute booterr=1.
compute badhi = 1.
end if.
compute llcit=temp(cilow,1).
compute ulcit=temp(cihigh,1).
do if (badlo = 1 and llcit <> priorlo).
compute badend={badend, llcit}.
compute priorlo = llcit.
end if.
do if (badhi = 1 and ulcit <> priorhi).
compute badend={badend, ulcit}.
compute priorhi = ulcit.
end if.
end if.
do if ( 9999 = 9999).
compute llcit=temp(cilow,1).
compute ulcit=temp(cihigh,1).
end if.
compute bootse=t(sqrt((cssq(temp)-((csum(temp)&**2)/nrow(temp)))/(nrow(temp)-1)))
.
compute mcct=mcct+1.
compute indtab(mcct,2:4)={bootse,llcit,ulcit}.
end loop.
do if (nxvls=1).
compute indtotal=indtotal+indtemp.
do if (contrast <> 0).
compute inddiff={inddiff,indtemp}.
end if.
end if.
end loop.
release indtemp,tmpb.
end if.
do if (normal = 1 and sobelok=1).
compute sobelmat=indtab(:,1).
compute sobelmat={sobelmat,(sobelmat/2),sobelmat,sobelmat}.
loop ii=1 to nms.
compute se2b=indcov(((nms*nxvls)+ii),((nms*nxvls)+ii)).
compute bpath2=mcsopath(((nms*nxvls)+ii),1)&**2.
compute se2a=diag(indcov((((ii-1)*nxvls)+1):(ii*nxvls),(((ii-1)*nxvls)+1):(ii*nxvls))).
compute apath2=mcsopath((((ii-1)*nxvls)+1):(ii*nxvls) ,1)&**2.
compute sesobel=sqrt(apath2*se2b+bpath2*se2a+se2a*se2b).
compute sobelmat((((ii-1)*nxvls)+1):(ii*nxvls),2)=sesobel.
end loop.
release se2b,bpath2,se2a,apath2,sesobel,ii.
compute sobelmat(:,3)=sobelmat(:,1)&/sobelmat(:,2).
compute sobelmat(:,4) = 2*(1-cdfnorm(abs(sobelmat(:,3)))).
end if.
do if (serial = 0).
compute rowlbs=t(mnames).
end if.
do if (nxvls=1 and nms > 1).
compute rowlbs={'TOTAL';rowlbs}.
compute indtemp=indtotal(1,1).
do if (boot > 0 and nxvls=1).

.
compute temp = indtotal(2:nrow(indtotal),1).
compute temp(GRADE( indtotal(2:nrow(indtotal),1) )) = indtotal(2:nrow(indtotal),1).
compute badlo = 0.
compute badhi = 0.
do if ( (indtotal(1,1)*bc)+(9999*(1-bc)) <> 9999).
compute pv=csum(temp < (indtotal(1,1)*bc)+(9999*(1-bc)) )/nrow(temp).
compute ppv = pv.
do if (pv > .5).
compute ppv = 1-pv.
end if.
compute y5=sqrt(-2*ln(ppv)).
compute xp=y5+((((y5*p4+p3)*y5+p2)*y5+p1)*y5+p0)/((((y5*q4+q3)*y5+q2)*y5+q1)*y5+q0).
do if (pv <= .5).
compute xp = -xp.
end if.
compute cilow=rnd(nrow(temp)*(cdfnorm(2*xp-xp2))).
compute cihigh=trunc(nrow(temp)*(cdfnorm(2*xp+(xp2))))+1.
do if (cilow < 1).
compute cilow = 1.
compute booterr=1.
compute badlo = 1.
end if.
do if (cihigh > nrow(temp)).
compute cihigh = boot.
compute booterr=1.
compute badhi = 1.
end if.
compute llcit=temp(cilow,1).
compute ulcit=temp(cihigh,1).
do if (badlo = 1 and llcit <> priorlo).
compute badend={badend, llcit}.
compute priorlo = llcit.
end if.
do if (badhi = 1 and ulcit <> priorhi).
compute badend={badend, ulcit}.
compute priorhi = ulcit.
end if.
end if.
do if ( (indtotal(1,1)*bc)+(9999*(1-bc)) = 9999).
compute llcit=temp(cilow,1).
compute ulcit=temp(cihigh,1).
end if.
compute bootse=t(sqrt((cssq(temp)-((csum(temp)&**2)/nrow(temp)))/(nrow(temp)-1)))
.
compute indtemp={indtemp, bootse,llcit,ulcit}.
end if.
do if (mc > 0).
compute obtmc=indtab(:,1).
compute indtemp=csum(obtmc).

.
compute temp = indtotal(:,1).
compute temp(GRADE( indtotal(:,1) )) = indtotal(:,1).
compute badlo = 0.
compute badhi = 0.
do if ( 9999 <> 9999).
compute pv=csum(temp < 9999 )/nrow(temp).
compute ppv = pv.
do if (pv > .5).
compute ppv = 1-pv.
end if.
compute y5=sqrt(-2*ln(ppv)).
compute xp=y5+((((y5*p4+p3)*y5+p2)*y5+p1)*y5+p0)/((((y5*q4+q3)*y5+q2)*y5+q1)*y5+q0).
do if (pv <= .5).
compute xp = -xp.
end if.
compute cilow=rnd(nrow(temp)*(cdfnorm(2*xp-xp2))).
compute cihigh=trunc(nrow(temp)*(cdfnorm(2*xp+(xp2))))+1.
do if (cilow < 1).
compute cilow = 1.
compute booterr=1.
compute badlo = 1.
end if.
do if (cihigh > nrow(temp)).
compute cihigh = boot.
compute booterr=1.
compute badhi = 1.
end if.
compute llcit=temp(cilow,1).
compute ulcit=temp(cihigh,1).
do if (badlo = 1 and llcit <> priorlo).
compute badend={badend, llcit}.
compute priorlo = llcit.
end if.
do if (badhi = 1 and ulcit <> priorhi).
compute badend={badend, ulcit}.
compute priorhi = ulcit.
end if.
end if.
do if ( 9999 = 9999).
compute llcit=temp(cilow,1).
compute ulcit=temp(cihigh,1).
end if.
compute bootse=t(sqrt((cssq(temp)-((csum(temp)&**2)/nrow(temp)))/(nrow(temp)-1)))
.
compute indtemp={indtemp, bootse,llcit,ulcit}.
end if.
compute indtab={indtemp;indtab}.
end if.
compute bootlbs={'Effect', 'BootSE','BootLLCI','BootULCI'}.
do if (mc > 0).
compute bootlbs={'Effect', 'MC SE','MC LLCI','MC ULCI'}.
end if.
do if (nxvls = 1).
do if (contrast <> 0).
compute inddiff=inddiff(:,2:ncol(inddiff)).
do if (mc > 0).
compute inddiff={t(obtmc);inddiff}.
end if.
do if (contrast = 4).
compute inddifft=inddiff*t(contvec).
compute indtemp=inddifft(1,1).
do if (boot > 0 or mc > 0).
do if (mc > 0).

.
compute temp = inddifft(2:nrow(inddifft),1).
compute temp(GRADE( inddifft(2:nrow(inddifft),1) )) = inddifft(2:nrow(inddifft),1).
compute badlo = 0.
compute badhi = 0.
do if ( 9999 <> 9999).
compute pv=csum(temp < 9999 )/nrow(temp).
compute ppv = pv.
do if (pv > .5).
compute ppv = 1-pv.
end if.
compute y5=sqrt(-2*ln(ppv)).
compute xp=y5+((((y5*p4+p3)*y5+p2)*y5+p1)*y5+p0)/((((y5*q4+q3)*y5+q2)*y5+q1)*y5+q0).
do if (pv <= .5).
compute xp = -xp.
end if.
compute cilow=rnd(nrow(temp)*(cdfnorm(2*xp-xp2))).
compute cihigh=trunc(nrow(temp)*(cdfnorm(2*xp+(xp2))))+1.
do if (cilow < 1).
compute cilow = 1.
compute booterr=1.
compute badlo = 1.
end if.
do if (cihigh > nrow(temp)).
compute cihigh = boot.
compute booterr=1.
compute badhi = 1.
end if.
compute llcit=temp(cilow,1).
compute ulcit=temp(cihigh,1).
do if (badlo = 1 and llcit <> priorlo).
compute badend={badend, llcit}.
compute priorlo = llcit.
end if.
do if (badhi = 1 and ulcit <> priorhi).
compute badend={badend, ulcit}.
compute priorhi = ulcit.
end if.
end if.
do if ( 9999 = 9999).
compute llcit=temp(cilow,1).
compute ulcit=temp(cihigh,1).
end if.
compute bootse=t(sqrt((cssq(temp)-((csum(temp)&**2)/nrow(temp)))/(nrow(temp)-1)))
.
end if.
do if (boot > 0).

.
compute temp = inddifft(2:nrow(inddifft),1).
compute temp(GRADE( inddifft(2:nrow(inddifft),1) )) = inddifft(2:nrow(inddifft),1).
compute badlo = 0.
compute badhi = 0.
do if ( (inddifft(1,1)*bc)+(9999* (1-bc)) <> 9999).
compute pv=csum(temp < (inddifft(1,1)*bc)+(9999* (1-bc)) )/nrow(temp).
compute ppv = pv.
do if (pv > .5).
compute ppv = 1-pv.
end if.
compute y5=sqrt(-2*ln(ppv)).
compute xp=y5+((((y5*p4+p3)*y5+p2)*y5+p1)*y5+p0)/((((y5*q4+q3)*y5+q2)*y5+q1)*y5+q0).
do if (pv <= .5).
compute xp = -xp.
end if.
compute cilow=rnd(nrow(temp)*(cdfnorm(2*xp-xp2))).
compute cihigh=trunc(nrow(temp)*(cdfnorm(2*xp+(xp2))))+1.
do if (cilow < 1).
compute cilow = 1.
compute booterr=1.
compute badlo = 1.
end if.
do if (cihigh > nrow(temp)).
compute cihigh = boot.
compute booterr=1.
compute badhi = 1.
end if.
compute llcit=temp(cilow,1).
compute ulcit=temp(cihigh,1).
do if (badlo = 1 and llcit <> priorlo).
compute badend={badend, llcit}.
compute priorlo = llcit.
end if.
do if (badhi = 1 and ulcit <> priorhi).
compute badend={badend, ulcit}.
compute priorhi = ulcit.
end if.
end if.
do if ( (inddifft(1,1)*bc)+(9999* (1-bc)) = 9999).
compute llcit=temp(cilow,1).
compute ulcit=temp(cihigh,1).
end if.
compute bootse=t(sqrt((cssq(temp)-((csum(temp)&**2)/nrow(temp)))/(nrow(temp)-1)))
.
end if.
compute indtemp={indtemp, bootse,llcit,ulcit}.
end if.
compute indtab={indtab;indtemp}.
end if.
do if (contrast = 1 or contrast = 2 or contrast=3).
compute conkey=make(1,4,' ').
loop i = 1 to ncol(inddiff)-1.
loop j = (i+1) to ncol(inddiff).
compute inddifft=inddiff(:,i)-inddiff(:,j).
do if (contrast=2).
compute inddifft=abs(inddiff(:,i))-abs(inddiff(:,j)).
end if.
do if (contrast=3).
compute inddifft=inddiff(:,i)+inddiff(:,j).
end if.
compute indtemp=inddifft(1,1).
do if (contrast=1 or contrast=2).
compute conkeyt={' ', rowlbs((i+1),1),' minus  ',rowlbs((j+1),1)}.
end if.
do if (contrast=3).
compute conkeyt={' ', rowlbs((i+1),1),'  plus  ',rowlbs((j+1),1)}.
end if.
compute conkey={conkey;conkeyt}.
do if (boot > 0 or mc > 0).
do if (mc > 0).

.
compute temp = inddifft(2:nrow(inddifft),1).
compute temp(GRADE( inddifft(2:nrow(inddifft),1) )) = inddifft(2:nrow(inddifft),1).
compute badlo = 0.
compute badhi = 0.
do if ( 9999 <> 9999).
compute pv=csum(temp < 9999 )/nrow(temp).
compute ppv = pv.
do if (pv > .5).
compute ppv = 1-pv.
end if.
compute y5=sqrt(-2*ln(ppv)).
compute xp=y5+((((y5*p4+p3)*y5+p2)*y5+p1)*y5+p0)/((((y5*q4+q3)*y5+q2)*y5+q1)*y5+q0).
do if (pv <= .5).
compute xp = -xp.
end if.
compute cilow=rnd(nrow(temp)*(cdfnorm(2*xp-xp2))).
compute cihigh=trunc(nrow(temp)*(cdfnorm(2*xp+(xp2))))+1.
do if (cilow < 1).
compute cilow = 1.
compute booterr=1.
compute badlo = 1.
end if.
do if (cihigh > nrow(temp)).
compute cihigh = boot.
compute booterr=1.
compute badhi = 1.
end if.
compute llcit=temp(cilow,1).
compute ulcit=temp(cihigh,1).
do if (badlo = 1 and llcit <> priorlo).
compute badend={badend, llcit}.
compute priorlo = llcit.
end if.
do if (badhi = 1 and ulcit <> priorhi).
compute badend={badend, ulcit}.
compute priorhi = ulcit.
end if.
end if.
do if ( 9999 = 9999).
compute llcit=temp(cilow,1).
compute ulcit=temp(cihigh,1).
end if.
compute bootse=t(sqrt((cssq(temp)-((csum(temp)&**2)/nrow(temp)))/(nrow(temp)-1)))
.
end if.
do if (boot > 0).

.
compute temp = inddifft(2:nrow(inddifft),1).
compute temp(GRADE( inddifft(2:nrow(inddifft),1) )) = inddifft(2:nrow(inddifft),1).
compute badlo = 0.
compute badhi = 0.
do if ( (inddifft(1,1)*bc)+(9999* (1-bc)) <> 9999).
compute pv=csum(temp < (inddifft(1,1)*bc)+(9999* (1-bc)) )/nrow(temp).
compute ppv = pv.
do if (pv > .5).
compute ppv = 1-pv.
end if.
compute y5=sqrt(-2*ln(ppv)).
compute xp=y5+((((y5*p4+p3)*y5+p2)*y5+p1)*y5+p0)/((((y5*q4+q3)*y5+q2)*y5+q1)*y5+q0).
do if (pv <= .5).
compute xp = -xp.
end if.
compute cilow=rnd(nrow(temp)*(cdfnorm(2*xp-xp2))).
compute cihigh=trunc(nrow(temp)*(cdfnorm(2*xp+(xp2))))+1.
do if (cilow < 1).
compute cilow = 1.
compute booterr=1.
compute badlo = 1.
end if.
do if (cihigh > nrow(temp)).
compute cihigh = boot.
compute booterr=1.
compute badhi = 1.
end if.
compute llcit=temp(cilow,1).
compute ulcit=temp(cihigh,1).
do if (badlo = 1 and llcit <> priorlo).
compute badend={badend, llcit}.
compute priorlo = llcit.
end if.
do if (badhi = 1 and ulcit <> priorhi).
compute badend={badend, ulcit}.
compute priorhi = ulcit.
end if.
end if.
do if ( (inddifft(1,1)*bc)+(9999* (1-bc)) = 9999).
compute llcit=temp(cilow,1).
compute ulcit=temp(cihigh,1).
end if.
compute bootse=t(sqrt((cssq(temp)-((csum(temp)&**2)/nrow(temp)))/(nrow(temp)-1)))
.
end if.
compute indtemp={indtemp, bootse,llcit,ulcit}.
end if.
compute indtab={indtab;indtemp}.
end loop.
end loop.
end if.
release inddiff.
compute contlbs=cntname(1:(((nindfx)*(nindfx-1))/2),1).
compute rowlbs={rowlbs;contlbs}.
end if.
compute resultm2=make(nrow(indtab),maxresm,99999).
compute resultm2(1:nrow(indtab),1:ncol(indtab))=indtab.
compute resultm={resultm;resultm2}.
do if (outscree=1).
do if (kk=1).
print indtab/title = 'Indirect effect(s) of X on Y:'/rnames=rowlbs/cnames=bootlbs/format= F10.4.
end if.
do if (kk = 2 and (xdich=1 or mcx > 0)).
print indtab/title = 'Partially standardized indirect effect(s) of X on Y:'/rnames=rowlbs/cnames=bootlbs/format= F10.4.
end if.
do if (kk = 3).
print indtab/title = 'Completely standardized indirect effect(s) of X on Y:'/rnames=rowlbs/cnames=bootlbs/format= F10.4.
end if.
end if.
do if (normal=1 and sobelok=1 and kk=1).
compute resultm2=make(nrow(sobelmat),maxresm,99999).
compute resultm2(1:nrow(sobelmat),1:ncol(sobelmat))=sobelmat.
compute resultm={resultm;resultm2}.
compute sobellab={'Effect',hclab,'Z','p'}.
compute sobelrlb=rowlbs.
do if (nms > 1).
compute sobelrlb=rowlbs(2:(1+nms),1).
end if.
do if (outscree=1).
print sobelmat/title='   Normal theory test for indirect effect(s):'/cnames=sobellab/rnames=sobelrlb/format= F10.4.
end if.
end if.
do if (contrast <> 0).
do if ((contrast=1 or contrast = 2 or contrast=3) and kk=efloop ).
compute conkey=conkey(2:nrow(conkey),:).
do if (outscree=1).
print conkey/title = 'Specific indirect effect contrast definition(s):'/rnames=contlbs/format=A8.
end if.
end if.
do if (outscree=1).
do if (contrast = 4 and kk=efloop).
compute crowlbs=rowlbs(2:(nindfx+1),1).
print contvec/title = 'Specific indirect effect contrast weights:'/cnames=crowlbs/rlabels='(C1)'/format= F10.4.
end if.
do if (contrast = 2 and kk=efloop).
print/title = 'Contrasts are differences between absolute values of indirect effects'.
end if.
end if.
end if.
do if (serial = 1 and kk=efloop).
compute rowlbst=rowlbs(2:nrow(rowlbs),1).
do if (outscree=1).
print indkey/title = 'Indirect effect key:'/rnames=rowlbst/format = A8.
end if.
end if.
else.
do if (outscree=1).
do if (kk = 1).
print/title = 'Relative indirect effects of X on Y'.
end if.
do if (kk = 2).
print/title = 'Partially standardized relative indirect effect(s) of X on Y:'.
end if.
do if (kk = 3).
print/title = 'Completely standardized relative indirect effect(s) of X on Y:'.
end if.
end if.
loop i = 1 to nrow(indmake).
compute indtabsm=indtab((((i-1)*nxvls)+1):(nxvls*i),:).
compute indkeyt=indkey(i,:).
do if (outscree=1).
print indkeyt/title=' '/space=0/format=A8.
end if.
do if (bcmat(nrow(bcmat),1)=0)).
compute direffl2=xcatlab(1:nxvls,1).
end if.
compute resultm2=make(nrow(indtabsm),maxresm,99999).
compute resultm2(1:nrow(indtabsm),1:ncol(indtabsm))=indtabsm.
compute resultm={resultm;resultm2}.
do if (outscree=1).
print indtabsm/title = ' '/cnames=bootlbs/rnames=direffl2/format= F10.4 /space=0.
end if.
do if (normal=1 and sobelok=1 and kk=1).
compute sobelsm=sobelmat((((i-1)*nxvls)+1):(nxvls*i),:).
compute sobellab={'Effect',hclab,'Z','p'}.
compute resultm2=make(nrow(sobelsm),maxresm,99999).
compute resultm2(1:nrow(sobelsm),1:ncol(sobelsm))=sobelsm.
compute resultm={resultm;resultm2}.
do if (outscree=1).
print sobelsm/title='      Normal theory test for relative indirect effects:'/cnames=sobellab/rnames=direffl2/format= F10.4.
end if.
end if.
end loop.
end if.
do if (effsize = 1 and boot > 0).
compute bootres=bootres(2:nrow(bootres),:).
end if.
end loop.
do if (alttotal=1).
compute altcnms='Effect'.
compute totbtvec=totbtvec+bootdir.
compute alttotfx=t(totbtvec(1,:)).
do if (boot > 0).
compute alttotfx=make(ncol(totbtvec),4,0).
compute alttotfx(:,1)=t(totbtvec(1,:)).
loop cec=1 to ncol(totbtvec).

.
compute temp = totbtvec(2:nrow(totbtvec),cec).
compute temp(GRADE( totbtvec(2:nrow(totbtvec),cec) )) = totbtvec(2:nrow(totbtvec),cec).
compute badlo = 0.
compute badhi = 0.
do if ( (totbtvec(1,cec)*bc)+(9999* (1-bc)) <> 9999).
compute pv=csum(temp < (totbtvec(1,cec)*bc)+(9999* (1-bc)) )/nrow(temp).
compute ppv = pv.
do if (pv > .5).
compute ppv = 1-pv.
end if.
compute y5=sqrt(-2*ln(ppv)).
compute xp=y5+((((y5*p4+p3)*y5+p2)*y5+p1)*y5+p0)/((((y5*q4+q3)*y5+q2)*y5+q1)*y5+q0).
do if (pv <= .5).
compute xp = -xp.
end if.
compute cilow=rnd(nrow(temp)*(cdfnorm(2*xp-xp2))).
compute cihigh=trunc(nrow(temp)*(cdfnorm(2*xp+(xp2))))+1.
do if (cilow < 1).
compute cilow = 1.
compute booterr=1.
compute badlo = 1.
end if.
do if (cihigh > nrow(temp)).
compute cihigh = boot.
compute booterr=1.
compute badhi = 1.
end if.
compute llcit=temp(cilow,1).
compute ulcit=temp(cihigh,1).
do if (badlo = 1 and llcit <> priorlo).
compute badend={badend, llcit}.
compute priorlo = llcit.
end if.
do if (badhi = 1 and ulcit <> priorhi).
compute badend={badend, ulcit}.
compute priorhi = ulcit.
end if.
end if.
do if ( (totbtvec(1,cec)*bc)+(9999* (1-bc)) = 9999).
compute llcit=temp(cilow,1).
compute ulcit=temp(cihigh,1).
end if.
compute bootse=t(sqrt((cssq(temp)-((csum(temp)&**2)/nrow(temp)))/(nrow(temp)-1)))
.
compute alttotfx(cec,2:4)={bootse,llcit,ulcit}.
end loop.
compute altcnms={altcnms,'BootSE', 'BootLLCI','BootULCI'}.
end if.
compute resultm2=make(nrow(alttotfx),maxresm,99999).
compute resultm2(1:nrow(alttotfx),1:ncol(alttotfx))=alttotfx.
compute resultm={resultm;resultm2}.
do if (outscree=1).
do if (nxvls > 1).
print/title= '----------'/space=0.
print alttotfx/title='Relative total effects of X on Y (sum of direct and indirect effects)'/rnames=direffl2/cnames=altcnms/format= F10.4.
end if.
do if (nxvls = 1).
print alttotfx/title='Total effect of X on Y (sum of direct and indirect effects)'/cnames=altcnms/format= F10.4.
end if.
end if.
end if.
end if.
do if (anymod > 0).
do if (boot = 0).
compute bootres=obscoeff.
compute indtab=999.
end if.
do if (boot > 0).
compute bootres={obscoeff;bootres}.
compute indtab=make(1,4,999).
end if.
do if (csum((indmod > 0))=nrow(indmod)).
do if (outscree=1).
do if (nxvls > 1).
do if (xmint=0).
print/title= 'Relative conditional indirect effects of X on Y:'.
end if.
do if (xmint=1).
print/title= 'Relative (total) natural indirect effects of X on Y:'.
end if.
end if.
do if (nxvls = 1).
do if (xmint=0).
print/title= 'Conditional indirect effects of X on Y:'.
end if.
do if (xmint=1).
print/title= '(Total) Natural indirect effect(s) of X on Y:'.
end if.
end if.
end if.
end if.
do if (csum((indmod > 0)) < nrow(indmod)).
do if (outscree=1).
do if (nxvls > 1).
print/title= 'Relative conditional and unconditional indirect effects of X on Y:'.
end if.
do if (nxvls = 1).
print/title= 'Conditional and unconditional indirect effects of X on Y:'.
end if.
end if.
end if.
compute cftotfx=make(nrow(bootres),nxvls,0).
loop i = 1 to nrow(indmake).
compute indtab=indtab(1,:)*0.
do if (outscree=1).
do if (xmint=0).
print indkey(i,:)/title = 'INDIRECT EFFECT:'/format=A8.
end if.
do if (xmint=1).
print indkey(i,:)/title = ' '/format=A8/space=0.
end if.
end if.
do if (indmod(i,1)=0).
loop j = 1 to nxvls.
compute indtemp=make(nrow(bootres),1,1).
loop k = 1 to indmake(i,1).
compute jtemp=1.
do if (j > 1 and k=1).
compute jtemp=j.
end if.
compute indtemp=indtemp&*bootres(:,pathsfoc(jtemp,indmake(i,(k+1)))).
end loop.
compute indeff=indtemp(1,1).
do if (boot > 0).

.
compute temp = indtemp(2:nrow(indtemp),1).
compute temp(GRADE( indtemp(2:nrow(indtemp),1) )) = indtemp(2:nrow(indtemp),1).
compute badlo = 0.
compute badhi = 0.
do if ( (indtemp(1,1)*bc)+(9999*(1-bc)) <> 9999).
compute pv=csum(temp < (indtemp(1,1)*bc)+(9999*(1-bc)) )/nrow(temp).
compute ppv = pv.
do if (pv > .5).
compute ppv = 1-pv.
end if.
compute y5=sqrt(-2*ln(ppv)).
compute xp=y5+((((y5*p4+p3)*y5+p2)*y5+p1)*y5+p0)/((((y5*q4+q3)*y5+q2)*y5+q1)*y5+q0).
do if (pv <= .5).
compute xp = -xp.
end if.
compute cilow=rnd(nrow(temp)*(cdfnorm(2*xp-xp2))).
compute cihigh=trunc(nrow(temp)*(cdfnorm(2*xp+(xp2))))+1.
do if (cilow < 1).
compute cilow = 1.
compute booterr=1.
compute badlo = 1.
end if.
do if (cihigh > nrow(temp)).
compute cihigh = boot.
compute booterr=1.
compute badhi = 1.
end if.
compute llcit=temp(cilow,1).
compute ulcit=temp(cihigh,1).
do if (badlo = 1 and llcit <> priorlo).
compute badend={badend, llcit}.
compute priorlo = llcit.
end if.
do if (badhi = 1 and ulcit <> priorhi).
compute badend={badend, ulcit}.
compute priorhi = ulcit.
end if.
end if.
do if ( (indtemp(1,1)*bc)+(9999*(1-bc)) = 9999).
compute llcit=temp(cilow,1).
compute ulcit=temp(cihigh,1).
end if.
compute bootse=t(sqrt((cssq(temp)-((csum(temp)&**2)/nrow(temp)))/(nrow(temp)-1)))
.
compute indeff={indeff,bootse,llcit,ulcit}.
end if.
compute indtab={indtab;indeff}.
end loop.
compute resultm2=make(nrow(indtab),maxresm,99999).
compute resultm2(1:nrow(indtab),1:ncol(indtab))=indtab.
compute resultm={resultm;resultm2}.
do if (outscree=1).
do if (nxvls > 1).
compute indefflb=xcatlab(1:nxvls,1).
print indtab(2:nrow(indtab),:)/title = ' '/clabels='Effect', 'BootSE', 'BootLLCI', 'BootULCI'/rnames=indefflb/format= F10.4 /space=0.
end if.
do if (nxvls = 1).
print indtab(2:nrow(indtab),:)/title = ' '/clabels='Effect', 'BootSE', 'BootLLCI', 'BootULCI'/format= F10.4 /space=0.
end if.
end if.
end if.
do if (indmod(i,1)>0).
do if (indmod(i,1)=1).
compute indmodva=wmodvals.
compute indprova=wprobval.
compute condlbs={wnames}.
compute printw=1.
else if (indmod(i,1)=2).
compute indmodva=zmodvals.
compute indprova=zprobval.
compute condlbs={znames}.
compute printz=1.
else if (indmod(i,1)=3).
compute cntmp=1.
compute printz=1.
compute printw=1.
compute indmodva=make((nrow(wmodvals)*nrow(zmodvals)),2,999).
loop k7 = 1 to nrow(wmodvals).
loop k8 = 1 to nrow(zmodvals).
compute indmodva(cntmp,:)={wmodvals(k7,1),zmodvals(k8,1)}.
compute cntmp=cntmp+1.
end loop.
end loop.
compute condlbs={wnames,znames}.
end if.
compute condres=make(nrow(indmodva),1,999).
do if (boot > 0).
compute condres=make(nrow(indmodva),4,999).
end if.
compute condres={indmodva,condres}.
loop k4 = 1 to nxvls.
compute imm3=make(nrow(bootres),1,1).
compute imm4=make(nrow(bootres),1,1).
compute indcontr=0.
do if (indmod(i,1)=3).
compute tihsw=wprobval.
compute tihsz=zprobval.
end if.
loop k1=1 to nrow(indmodva).
compute tucker2=make(nrow(bootres),1,1).
compute imm2=make(nrow(bootres),1,1).
compute wfirst=0.
compute zfirst=0.
compute immset=0.
loop k2=1 to indmake(i,1).
compute colnumb=indmake(i,(k2+1)).
do if (k2=1).
compute wbb=make(nrow(bootres),(nwvls*nxvls),0).
compute zbb=make(nrow(bootres),(nzvls*nxvls),0).
compute wzbb=make(nrow(bootres),(nwvls*nzvls*nxvls),0).
end if.
do if (k2<>1).
compute wbb=make(nrow(bootres),nwvls,0).
compute zbb=make(nrow(bootres),nzvls,0).
compute wzbb=make(nrow(bootres),(nwvls*nzvls),0).
end if.
compute cnt=1.
compute tihs=indlocs(2:((indlocs(1,colnumb))+1),colnumb).
do if (k2 = 1).
compute focbb=tihs(1:nxvls,1).
compute focbb=bootres(:,focbb).
do if (indmmm(i,1)>0).
compute imm=focbb(:,k4).
compute condbb=imm.
end if.
compute focaddon=make(1,nxvls,0).
compute focaddon(1,k4)=1.
compute cnt=cnt+nxvls.
compute placeh=nxvls.
do if (indmod(i,1)=1).
compute tihsz=make(nrow(wprobval),(nzvls*nxvls),0).
compute tihswz=make(nrow(wprobval),(nwvls*nzvls*nxvls),0).
do if (pathsw(1,colnumb)=1).
compute temp=make(nrow(wprobval),(nxvls*nwvls),0).
loop k5 = 1 to nrow(wprobval).
loop k6=1 to nwvls.
compute temp(k5, (((k4-1)*nwvls)+k6))=wprobval(k5,k6).
end loop.
end loop.
compute indprova={temp,tihsz,tihswz}.
else.
compute indprova={wprobval,tihsz,tihswz}.
end if.
end if.
do if (indmod(i,1)=2).
compute tihsw=make(nrow(zprobval),(nwvls*nxvls),0).
compute tihswz=make(nrow(zprobval),(nwvls*nzvls*nxvls),0).
do if (pathsz(1,colnumb)=1).
compute temp=make(nrow(zprobval),(nxvls*nzvls),0).
loop k5 = 1 to nrow(zprobval).
loop k6 =1 to nzvls.
compute temp(k5,(((k4-1)*nzvls)+k6))=zprobval(k5,k6).
end loop.
end loop.
compute indprova={tihsw,temp,tihswz}.
else.
compute indprova={tihsw,zprobval,tihswz}.
end if.
end if.
do if (indmod(i,1)=3).
compute indprova=make((nrow(wprobval)*nrow(zprobval)),((ncol(wprobval)*nxvls)+ (ncol(zprobval)*nxvls)+(nwvls*nzvls*nxvls)),0).
compute cntemp=1.
loop k7=1 to nrow(wprobval).
loop k8 =1 to nrow(zprobval).
compute temp=wprobval(k7,:)*focaddon(1,k4).
compute indprova(cntemp,(((k4-1)*nwvls)+1):(k4*(nwvls)))=temp.
compute temp=zprobval(k8,:)*focaddon(1,k4).
compute indprova(cntemp, ((((k4-1)*nzvls)+1)+(nxvls*nwvls)) : ((((k4-1)*nzvls)+1)+(nxvls*nwvls)+(nzvls-1)))=temp.
compute cntemp=cntemp+1.
end loop.
end loop.
do if (pathsz(1,colnumb)=0).
compute temp=make(nrow(indprova),(ncol(zprobval)*nxvls),0).
compute indprova(:,((ncol(wprobval)*nxvls)+1):((ncol(wprobval)+ncol(zprobval))* nxvls))=temp.
end if.
do if (pathsw(1,colnumb)=0).
compute temp=make(nrow(indprova),(ncol(wprobval)*nxvls),0).
compute indprova(:,1:(ncol(wprobval)*nxvls))=temp.
end if.
do if (pathswz(1,colnumb)=1).
compute cntemp=(ncol(wprobval)*nxvls)+(ncol(zprobval)*nxvls)+((k4-1)* ncol(wprobval)*ncol(zprobval))+1.
loop k7=1 to ncol(wprobval).
loop k8=1 to ncol(zprobval).
compute indprova(:,cntemp)=indprova(:,((ncol(wprobval)*(k4-1))+k7))&* indprova(:,((((k4-1)*ncol(zprobval))+k8)+(nxvls*ncol(wprobval)))).
compute cntemp=cntemp+1.
end loop.
end loop.
end if.
end if.
end if.
do if (k2 > 1).
compute focbb=tihs(1,1).
compute focbb=bootres(:,focbb).
do if (indmmm(i,1)>0).
compute imm=focbb(:,1).
compute condbb=imm.
end if.
compute focaddon=1.
compute cnt=cnt+1.
compute placeh=1.
do if (indmod(i,1)=1).
compute tihsz=make(nrow(wprobval),nzvls,0).
compute tihswz=make(nrow(wprobval),(nwvls*nzvls),0).
compute indprova={wprobval,tihsz,tihswz}.
end if.
do if (indmod(i,1)=2).
compute tihsw=make(nrow(zprobval),nwvls,0).
compute tihswz=make(nrow(zprobval),(nwvls*nzvls),0).
compute indprova={tihsw,zprobval,tihswz}.
end if.
do if (indmod(i,1)=3).
compute indprova=make((nrow(wprobval)*nrow(zprobval)),((ncol(wprobval)+ ncol(zprobval))+(nwvls*nzvls)),0).
compute cntemp=1.
loop k7=1 to nrow(wprobval).
loop k8 =1 to nrow(zprobval).
compute indprova(cntemp,1:(ncol(wprobval)+ncol(zprobval)))={wprobval(k7,:), zprobval(k8,:)}.
compute cntemp=cntemp+1.
end loop.
end loop.
do if (pathsz(1,colnumb)=0).
compute temp=make(nrow(indprova),ncol(zprobval),0).
compute indprova(:,(ncol(wprobval)+1):(ncol(wprobval)+ncol(zprobval)))=temp.
end if.
do if (pathsw(1,colnumb)=0).
compute temp=make(nrow(indprova),ncol(wprobval),0).
compute indprova(:,1:ncol(wprobval))=temp.
end if.
do if (pathswz(1,colnumb)=1).
compute cntemp=ncol(wprobval)+ncol(zprobval)+1.
loop k7=1 to ncol(wprobval).
loop k8=1 to ncol(zprobval).
compute indprova(:,cntemp)=indprova(:,k7)&*indprova(:,(ncol(wprobval)+k8)).
compute cntemp=cntemp+1.
end loop.
end loop.
end if.
end if.
end if.
do if (pathsw(1,colnumb)) = 1.
compute wbb=tihs(cnt:(cnt+(placeh*nwvls)-1),1).
compute wbb=bootres(:,wbb).
compute immlbs2=wcatlab(1:nwvls,1).
do if (zfirst=0).
compute wfirst=1.
end if.
do if (indmmm(i,1)=1 or indmmm(i,1)=31 or indmmm(i,1)=51).
compute imm=wbb(:,1).
loop k7 = 1 to nwvls.
compute imm={imm,wbb(:,(((k4-1)*nwvls*(k2=1))+k7))}.
end loop.
compute imm=imm(:,2:ncol(imm)).
end if.
do if (indmmm(i,1)=41 or indmmm(i,1)=51).
compute condbb=make(nrow(bootres),1,0).
loop k7 = 1 to nwvls.
compute condbb={condbb,wbb(:,(((k4-1)*nwvls*(k2=1))+k7))}.
end loop.
compute condbb=condbb(:,2:ncol(condbb)).
end if.
compute cnt=cnt+(placeh*nwvls).
end if.
do if (pathsz(1,colnumb)) = 1.
compute zbb=tihs(cnt:(cnt+(placeh*nzvls)-1),1).
compute zbb=bootres(:,zbb).
do if (wfirst=0).
compute zfirst=1.
end if.
do if (indmmm(i,1) <> 31).
compute immlbs2=zcatlab(1:nzvls,1).
end if.
do if (indmmm(i,1)=2 or indmmm(i,1)=31 or indmmm(i,1)=51).
do if (indmmm(i,1)=2).
compute imm=zbb(:,1).
end if.
loop k7 = 1 to nzvls.
compute imm={imm,zbb(:,(((k4-1)*nzvls*(k2=1))+k7))}.
end loop.
do if (indmmm(i,1)=2 or indmmm(i,1)=51).
compute imm=imm(:,2:ncol(imm)).
do if (indmmm(i,1)=51).
compute condbb={condbb,imm}.
end if.
end if.
end if.
compute cnt=cnt+(placeh*nzvls).
end if.
do if (pathswz(1,colnumb)) = 1.
compute wzbb=tihs(cnt:(cnt+(placeh*nwvls*nzvls)-1),1).
compute wzbb=bootres(:,wzbb).
do if (indmmm(i,1)=41).
compute imm=wzbb(:,1).
loop k7=1 to nwvls*nzvls.
compute imm={imm,wzbb(:,(((k4-1)*nzvls*nwvls*(k2=1))+k7))}.
end loop.
end if.
do if (indmmm(i,1)=41).
compute imm=imm(:,2:ncol(imm)).
compute condbb={condbb,imm(:,(ncol(imm)-(nwvls*nzvls)+1):ncol(imm))}.
end if.
compute cnt=cnt+(placeh*nzvls*nwvls).
end if.
compute indprobe={focaddon,indprova(k1,:)}.
compute tucker={focbb,wbb,zbb,wzbb}.
loop k3=1 to ncol(indprobe).
compute tucker(:,k3)=tucker(:,k3)*indprobe(1,k3).
end loop.
compute tucker2=tucker2&*rsum(tucker).
do if (indmmm(i,1) = 1 or indmmm(i,1)=2 or indmmm(i,1)=31 or indmmm(i,1)=41 or indmmm(i,1)=51).
do if (immset=1).
do if (ncol(imm2)=1 and ncol(imm) = 1).
compute imm2=imm2&*imm.
end if.
do if (indmmm(i,1)=41 or indmmm(i,1)=51).
do if ((ncol(condbb2) > 1) and (ncol(condbb)>1)).
compute condbb2t=make(nrow(condbb2),(ncol(condbb2)*ncol(condbb)),-999999).
compute k9=1.
do if (wfirst=1).
loop k7=1 to ncol(condbb2).
loop k8 = 1 to ncol(condbb).
compute condbb2t(:,k9)=condbb2(:,k7)&*condbb(:,k8).
compute k9=k9+1.
end loop.
end loop.
end if.
do if (zfirst=1).
loop k7=1 to ncol(condbb).
loop k8 = 1 to ncol(condbb2).
compute condbb2t(:,k9)=condbb(:,k7)&*condbb2(:,k8).
compute k9=k9+1.
end loop.
end loop.
end if.
compute condbb2=condbb2t.
release condbb2t.
end if.
do if ((ncol(condbb2) > 1) and (ncol(condbb)=1)).
loop k7 = 1 to ncol(condbb2).
compute condbb2(:,k7)=condbb2(:,k7)&*condbb.
end loop.
end if.
do if ((ncol(condbb2) = 1) and (ncol(condbb)>1)).
loop k7 = 1 to ncol(condbb).
compute condbb(:,k7)=condbb2&*condbb(:,k7).
end loop.
compute condbb2=condbb.
end if.
end if.
do if (ncol(imm2) <> 1 and ncol(imm) <> 1).
compute imm2t=make(nrow(imm2),(ncol(imm2)*ncol(imm)),-999999).
compute k9=1.
do if (wfirst=1).
loop k7=1 to ncol(imm2).
loop k8 = 1 to ncol(imm).
compute imm2t(:,k9)=imm2(:,k7)&*imm(:,k8).
compute k9=k9+1.
end loop.
end loop.
end if.
do if (zfirst=1).
loop k7=1 to ncol(imm).
loop k8 = 1 to ncol(imm2).
compute imm2t(:,k9)=imm(:,k7)&*imm2(:,k8).
compute k9=k9+1.
end loop.
end loop.
end if.
compute imm2=imm2t.
release imm2t.
end if.
do if ((ncol(imm2) > 1) and (ncol(imm)=1)).
loop k7=1 to ncol(imm2).
compute imm2(:,k7)=imm2(:,k7)&*imm.
end loop.
end if.
do if ((ncol(imm2) = 1) and (ncol(imm) > 1)).
loop k7=1 to ncol(imm).
compute imm(:,k7)=imm2&*imm(:,k7).
end loop.
compute imm2=imm.
end if.
end if.
do if (immset=0).
compute imm2=imm.
do if (indmmm(i,1)=41 or indmmm(i,1)=51).
compute condbb2=condbb.
end if.
compute immset=1.
end if.
end if.
end loop.
compute indtemp=tucker2(1,1).
do if (indmmm(i,1)=12 or indmmm(i,1)=22).
compute imm3={imm3,tucker2}.
do if (k1=nrow(indmodva)).
compute imm3=imm3(:,2:ncol(imm3)).
compute immstop=ncol(imm3).
loop k8=2 to immstop.
do if (indmmmt(i,1)=1).
compute imm3={imm3,(imm3(:,k8)-imm3(:,1))}.
end if.
do if (indmmmt(i,1)=2).
compute imm3={imm3,(imm3(:,k8)-imm3(:,(k8-1)))}.
end if.
do if (indmmmt(i,1)=3).
compute imm3={imm3,((rsum(imm3(:,(k8:immstop)))/(immstop-k8+1))-imm3(:,(k8-1))) }.
end if.
do if (indmmmt(i,1)=4).
compute imm3={imm3,(imm3(:,k8)-(rsum(imm3(:,1:immstop))/immstop))}.
end if.
end loop.
do if (indmmmt(i,1)<5).
compute imm2=imm3(:,(immstop+1):ncol(imm3)).
release imm3.
end if.
end if.
end if.
do if (indmmm(i,1)>-1 and (contrast = 1 or contrast = 2 or contrast=3)).
compute imm4={imm4,tucker2}.
do if (k1=nrow(indmodva) and k1 > 1).
compute imm4=imm4(:,2:ncol(imm4)).
compute immstop=ncol(imm4).
compute condcont=make((immstop*(immstop-1)/2),6,-999).
loop k8 = 1 to (immstop-1).
loop k9 = (k8+1) to immstop.
do if (contrast=1).
compute imm4={imm4,(imm4(:,k9)-imm4(:,k8))}.
end if.
do if (contrast=2).
compute imm4={imm4,(abs(imm4(:,k9))-abs(imm4(:,k8)))}.
end if.
do if (contrast=3).
compute imm4={imm4,(imm4(:,k9)+imm4(:,k8))}.
end if.
compute condcont((ncol(imm4)-immstop),1)=imm4(1,k9).
compute condcont((ncol(imm4)-immstop),2)=imm4(1,k8).
end loop.
end loop.
compute imm4=imm4(:,(immstop+1):ncol(imm4)).
loop k8=1 to ncol(imm4).
compute condcont(k8,3)=imm4(1,k8).
do if (boot > 0).

.
compute temp = imm4(2:nrow(imm4),k8).
compute temp(GRADE( imm4(2:nrow(imm4),k8) )) = imm4(2:nrow(imm4),k8).
compute badlo = 0.
compute badhi = 0.
do if ( (imm4(1,k8)*bc)+(9999*(1-bc)) <> 9999).
compute pv=csum(temp < (imm4(1,k8)*bc)+(9999*(1-bc)) )/nrow(temp).
compute ppv = pv.
do if (pv > .5).
compute ppv = 1-pv.
end if.
compute y5=sqrt(-2*ln(ppv)).
compute xp=y5+((((y5*p4+p3)*y5+p2)*y5+p1)*y5+p0)/((((y5*q4+q3)*y5+q2)*y5+q1)*y5+q0).
do if (pv <= .5).
compute xp = -xp.
end if.
compute cilow=rnd(nrow(temp)*(cdfnorm(2*xp-xp2))).
compute cihigh=trunc(nrow(temp)*(cdfnorm(2*xp+(xp2))))+1.
do if (cilow < 1).
compute cilow = 1.
compute booterr=1.
compute badlo = 1.
end if.
do if (cihigh > nrow(temp)).
compute cihigh = boot.
compute booterr=1.
compute badhi = 1.
end if.
compute llcit=temp(cilow,1).
compute ulcit=temp(cihigh,1).
do if (badlo = 1 and llcit <> priorlo).
compute badend={badend, llcit}.
compute priorlo = llcit.
end if.
do if (badhi = 1 and ulcit <> priorhi).
compute badend={badend, ulcit}.
compute priorhi = ulcit.
end if.
end if.
do if ( (imm4(1,k8)*bc)+(9999*(1-bc)) = 9999).
compute llcit=temp(cilow,1).
compute ulcit=temp(cihigh,1).
end if.
compute bootse=t(sqrt((cssq(temp)-((csum(temp)&**2)/nrow(temp)))/(nrow(temp)-1)))
.
compute condcont(k8,4:6)={bootse,llcit,ulcit}.
end if.
end loop.
do if (boot=0).
compute condcont=condcont(:,1:3).
end if.
compute indcontr=1.
end if.
end if.
do if (xmint=1).
do if (k1=(k4+1)).
compute cftotfx(:,k4)=cftotfx(:,k4)+(tucker2*xscaling).
end if.
end if.
do if (boot > 0).
compute tucker2=tucker2*xscaling.

.
compute temp = tucker2(2:nrow(tucker2),1).
compute temp(GRADE( tucker2(2:nrow(tucker2),1) )) = tucker2(2:nrow(tucker2),1).
compute badlo = 0.
compute badhi = 0.
do if ( (tucker2(1,1)*bc)+(9999*(1-bc)) <> 9999).
compute pv=csum(temp < (tucker2(1,1)*bc)+(9999*(1-bc)) )/nrow(temp).
compute ppv = pv.
do if (pv > .5).
compute ppv = 1-pv.
end if.
compute y5=sqrt(-2*ln(ppv)).
compute xp=y5+((((y5*p4+p3)*y5+p2)*y5+p1)*y5+p0)/((((y5*q4+q3)*y5+q2)*y5+q1)*y5+q0).
do if (pv <= .5).
compute xp = -xp.
end if.
compute cilow=rnd(nrow(temp)*(cdfnorm(2*xp-xp2))).
compute cihigh=trunc(nrow(temp)*(cdfnorm(2*xp+(xp2))))+1.
do if (cilow < 1).
compute cilow = 1.
compute booterr=1.
compute badlo = 1.
end if.
do if (cihigh > nrow(temp)).
compute cihigh = boot.
compute booterr=1.
compute badhi = 1.
end if.
compute llcit=temp(cilow,1).
compute ulcit=temp(cihigh,1).
do if (badlo = 1 and llcit <> priorlo).
compute badend={badend, llcit}.
compute priorlo = llcit.
end if.
do if (badhi = 1 and ulcit <> priorhi).
compute badend={badend, ulcit}.
compute priorhi = ulcit.
end if.
end if.
do if ( (tucker2(1,1)*bc)+(9999*(1-bc)) = 9999).
compute llcit=temp(cilow,1).
compute ulcit=temp(cihigh,1).
end if.
compute bootse=t(sqrt((cssq(temp)-((csum(temp)&**2)/nrow(temp)))/(nrow(temp)-1)))
.
compute indtemp={indtemp*xscaling, bootse,llcit,ulcit}.
end if.
do if (boot=0).
compute indtemp=indtemp*xscaling.
end if.
compute condres(k1,(ncol(indmodva)+1):ncol(condres))=indtemp.
end loop.
do if (xmint=0).
compute resultm2=make(nrow(condres),maxresm,99999).
compute resultm2(1:nrow(condres),1:ncol(condres))=condres.
compute resultm={resultm;resultm2}.
end if.
compute condlbs={condlbs,'Effect'}.
do if (boot > 0).
compute condlbs={condlbs,'BootSE', 'BootLLCI', 'BootULCI'}.
end if.
do if (xmint = 1).
do if (k4 = 1).
compute natindfx=condres((2+(k4-1)),2:ncol(condres)).
end if.
do if (k4 > 1).
compute natindfx={natindfx;condres((2+(k4-1)),2:ncol(condres))}.
end if.
do if (k4=nxvls).
compute resultm2=make(nrow(natindfx),maxresm,99999).
compute resultm2(1:nrow(natindfx),1:ncol(natindfx))=natindfx.
compute resultm={resultm;resultm2}.
end if.
end if.
do if (outscree=1 and xmint=0).
do if (nxvls=1).
print condres/title=' '/cnames=condlbs/format= F10.4 /space=0.
else.
compute condrlb=make(nrow(condres),1,xcatlab(k4,1)).
print condres/title=' '/cnames=condlbs/rnames=condrlb/format= F10.4 /space=0.
end if.
end if.
do if (outscree=1 and xmint=1 and k4=nxvls).
do if (nxvls=1).
print natindfx/title=' '/clabels='Effect', 'BootSE', 'BootLLCI', 'BootULCI'/format= F10.4 /space=0.
else.
compute condrlb=make(nrow(condres),1,xcatlab(k4,1)).
print natindfx/title=' '/clabels='Effect', 'BootSE', 'BootLLCI', 'BootULCI'/rnames=direffl2/format= F10.4 /space=0.
end if.
end if.
compute dichadj=0.
compute immcat=0.
do if ((indmmm(i,1)>0) and (xmint=0)).
do if (indmmm(i,1)=1 or indmmm(i,1)=12 or indmmm(i,1)=31).
do if (wdich=1 and mcw=0).
do if (indmmm(i,1) <> 12).
compute imm2(:,1)=imm2(:,1)*(wmax-wmin).
end if.
do if (indmmm(i,1) <> 31).
compute dichadj=1.
end if.
end if.
do if ((mcw = 1 or mcw = 2) and indmmm(i,1) <> 31)).
compute immcat=1.
end if.
end if.
do if (indmmm(i,1)=2 or indmmm(i,1)=22 or indmmm(i,1)=31).
do if (zdich=1 and mcz=0).
do if (indmmm(i,1) = 31).
compute imm2(:,(nwvls+1):ncol(imm2))=imm2(:,(nwvls+1):ncol(imm2))*(zmax-zmin).
end if.
do if (indmmm(i,1) = 2).
compute imm2(:,1)=imm2(:,1)*(zmax-zmin).
end if.
do if (indmmm(i,1) <> 31).
compute dichadj=1.
end if.
end if.
do if ((mcz = 1 or mcz = 2) and indmmm(i,1) <> 31)).
compute immcat=1.
end if.
end if.
compute immtemp2=t(imm2(1,:)).
compute immtemp=immtemp2.
compute immlbs={'Index'}.
do if (boot > 0).
compute immtemp=make(1,3,0).
loop k7=1 to ncol(imm2).

.
compute temp = imm2(2:nrow(imm2),k7).
compute temp(GRADE( imm2(2:nrow(imm2),k7) )) = imm2(2:nrow(imm2),k7).
compute badlo = 0.
compute badhi = 0.
do if ( (imm2(1,k7)*bc)+(9999*(1-bc)) <> 9999).
compute pv=csum(temp < (imm2(1,k7)*bc)+(9999*(1-bc)) )/nrow(temp).
compute ppv = pv.
do if (pv > .5).
compute ppv = 1-pv.
end if.
compute y5=sqrt(-2*ln(ppv)).
compute xp=y5+((((y5*p4+p3)*y5+p2)*y5+p1)*y5+p0)/((((y5*q4+q3)*y5+q2)*y5+q1)*y5+q0).
do if (pv <= .5).
compute xp = -xp.
end if.
compute cilow=rnd(nrow(temp)*(cdfnorm(2*xp-xp2))).
compute cihigh=trunc(nrow(temp)*(cdfnorm(2*xp+(xp2))))+1.
do if (cilow < 1).
compute cilow = 1.
compute booterr=1.
compute badlo = 1.
end if.
do if (cihigh > nrow(temp)).
compute cihigh = boot.
compute booterr=1.
compute badhi = 1.
end if.
compute llcit=temp(cilow,1).
compute ulcit=temp(cihigh,1).
do if (badlo = 1 and llcit <> priorlo).
compute badend={badend, llcit}.
compute priorlo = llcit.
end if.
do if (badhi = 1 and ulcit <> priorhi).
compute badend={badend, ulcit}.
compute priorhi = ulcit.
end if.
end if.
do if ( (imm2(1,k7)*bc)+(9999*(1-bc)) = 9999).
compute llcit=temp(cilow,1).
compute ulcit=temp(cihigh,1).
end if.
compute bootse=t(sqrt((cssq(temp)-((csum(temp)&**2)/nrow(temp)))/(nrow(temp)-1)))
.
compute temp={bootse,llcit,ulcit}.
compute immtemp={immtemp;temp}.
end loop.
compute immtemp=immtemp(2:nrow(immtemp),:).
compute immtemp={immtemp2,immtemp}.
compute immlbs={immlbs,'BootSE', 'BootLLCI', 'BootULCI'}.
end if.
compute resultm2=make(nrow(immtemp),maxresm,99999).
compute resultm2(1:nrow(immtemp),1:ncol(immtemp))=immtemp.
compute resultm={resultm;resultm2}.
do if (dichadj=0 and immcat=0 and indmmmt(i,1)<>5 and indmmm(i,1) < 100 ).
do if (outscree=1).
do if (indmmm(i,1) < 30).
print immtemp/title='      Index of moderated mediation:'/cnames=immlbs/rnames=immlbs2/format= F10.4.
end if.
do if (indmmm(i,1) = 31).
compute immlbs2={immlbs2;zcatlab(1:nzvls,1)}.
print immtemp/title='      Indices of partial moderated mediation:'/cnames=immlbs/rnames=immlbs2/format= F10.4.
end if.
end if.
do if ((nzvls = 1) and (nwvls = 1)).
do if (indmmm(i,1) = 41 or indmmm(i,1)=51).
loop k7=1 to nwvls.
compute immlbs2=zcatlab(1:nzvls,1).
compute immtemp2=immtemp((((k7-1)*nzvls)+1):(((k7-1)*nzvls)+nzvls),:).
do if (outscree=1).
do if (nwvls > 1).
compute primodv={'        ', wcatlab(k7,1)}.
print primodv/title='      Primary moderator:'/format=A8.
end if.
do if (nzvls=1).
print immtemp2/title='      Index of moderated moderated mediation'/cnames=immlbs/format= F10.4.
else.
print immtemp2/title='      Indices of moderated moderated mediation'/cnames=immlbs/rnames=immlbs2/format= F10.4.
end if.
end if.
compute cmmtemp=make(nrow(zprobval),4,0).
loop k8=1 to nrow(zprobval).
compute condbb3=condbb2(:,((nwvls+1)+((k7-1)*nzvls)):((nwvls+1)+((k7-1)*nzvls)+ (nzvls-1))).
do if (ncol(zprobval) > 1).
compute condbb3=condbb3*mdiag(zprobval(k8,:)).
else.
compute condbb3=condbb3&*zprobval(k8,:).
end if.
compute condbb3={condbb2(:,k7),condbb3}.
compute icmm=rsum(condbb3).
compute cmmtemp(k8,1)=icmm(1,1).
do if (boot > 0).

.
compute temp = icmm(2:nrow(icmm),1).
compute temp(GRADE( icmm(2:nrow(icmm),1) )) = icmm(2:nrow(icmm),1).
compute badlo = 0.
compute badhi = 0.
do if ( (icmm(1,1)*bc)+(9999*(1-bc)) <> 9999).
compute pv=csum(temp < (icmm(1,1)*bc)+(9999*(1-bc)) )/nrow(temp).
compute ppv = pv.
do if (pv > .5).
compute ppv = 1-pv.
end if.
compute y5=sqrt(-2*ln(ppv)).
compute xp=y5+((((y5*p4+p3)*y5+p2)*y5+p1)*y5+p0)/((((y5*q4+q3)*y5+q2)*y5+q1)*y5+q0).
do if (pv <= .5).
compute xp = -xp.
end if.
compute cilow=rnd(nrow(temp)*(cdfnorm(2*xp-xp2))).
compute cihigh=trunc(nrow(temp)*(cdfnorm(2*xp+(xp2))))+1.
do if (cilow < 1).
compute cilow = 1.
compute booterr=1.
compute badlo = 1.
end if.
do if (cihigh > nrow(temp)).
compute cihigh = boot.
compute booterr=1.
compute badhi = 1.
end if.
compute llcit=temp(cilow,1).
compute ulcit=temp(cihigh,1).
do if (badlo = 1 and llcit <> priorlo).
compute badend={badend, llcit}.
compute priorlo = llcit.
end if.
do if (badhi = 1 and ulcit <> priorhi).
compute badend={badend, ulcit}.
compute priorhi = ulcit.
end if.
end if.
do if ( (icmm(1,1)*bc)+(9999*(1-bc)) = 9999).
compute llcit=temp(cilow,1).
compute ulcit=temp(cihigh,1).
end if.
compute bootse=t(sqrt((cssq(temp)-((csum(temp)&**2)/nrow(temp)))/(nrow(temp)-1)))
.
compute cmmtemp(k8,2:4)={bootse,llcit,ulcit}.
end if.
end loop.
compute cmmtemp={zmodvals,cmmtemp}.
do if (boot=0).
compute cmmtemp=cmmtemp(:,1:2).
end if.
compute cmmlbs={znames,immlbs}.
compute resultm2=make(nrow(cmmtemp),maxresm,99999).
compute resultm2(1:nrow(cmmtemp),1:ncol(cmmtemp))=cmmtemp.
compute resultm={resultm;resultm2}.
do if (outscree=1).
print cmmtemp/title='      Indices of conditional moderated mediation by W'/cnames=cmmlbs/format= F10.4.
end if.
end loop.
end if.
end if.
end if.
do if (dichadj=1 or immcat=1 and indmmm(i,1) < 30).
do if (outscree=1).
print immtemp/title='Index of moderated mediation (difference between conditional indirect effects):'/cnames=immlbs/rnames=immlbs2/format= F10.4.
end if.
end if.
end if.
do if (indcontr=1).
compute resultm2=make(nrow(condcont),maxresm,99999).
compute resultm2(1:nrow(condcont),1:ncol(condcont))=condcont.
compute resultm={resultm;resultm2}.
compute condctlb={'Effect1','Effect2','Contrast','BootSE', 'BootLLCI','BootULCI'}.
do if (outscree=1).
print condcont/title=' Pairwise contrasts between conditional indirect effects (Effect1 minus Effect2)'/cnames=condctlb/format= F10.4.
end if.
end if.
end loop.
end if.
end loop.
do if (alttotal=1).
compute altcnms='Effect'.
compute alttotfx=t(cftotfx(1,:))+obnatdfx.
do if (boot > 0).
compute alttotfx=make(ncol(cftotfx),4,0).
compute alttotfx(:,1)=t(cftotfx(1,:))+obnatdfx.
compute natdirbt(1,:)=t(obnatdfx).
compute cftotfx=cftotfx+natdirbt.
loop cec=1 to ncol(cftotfx).

.
compute temp = cftotfx(2:nrow(cftotfx),cec).
compute temp(GRADE( cftotfx(2:nrow(cftotfx),cec) )) = cftotfx(2:nrow(cftotfx),cec).
compute badlo = 0.
compute badhi = 0.
do if ( (cftotfx(1,cec)*bc)+(9999*(1-bc)) <> 9999).
compute pv=csum(temp < (cftotfx(1,cec)*bc)+(9999*(1-bc)) )/nrow(temp).
compute ppv = pv.
do if (pv > .5).
compute ppv = 1-pv.
end if.
compute y5=sqrt(-2*ln(ppv)).
compute xp=y5+((((y5*p4+p3)*y5+p2)*y5+p1)*y5+p0)/((((y5*q4+q3)*y5+q2)*y5+q1)*y5+q0).
do if (pv <= .5).
compute xp = -xp.
end if.
compute cilow=rnd(nrow(temp)*(cdfnorm(2*xp-xp2))).
compute cihigh=trunc(nrow(temp)*(cdfnorm(2*xp+(xp2))))+1.
do if (cilow < 1).
compute cilow = 1.
compute booterr=1.
compute badlo = 1.
end if.
do if (cihigh > nrow(temp)).
compute cihigh = boot.
compute booterr=1.
compute badhi = 1.
end if.
compute llcit=temp(cilow,1).
compute ulcit=temp(cihigh,1).
do if (badlo = 1 and llcit <> priorlo).
compute badend={badend, llcit}.
compute priorlo = llcit.
end if.
do if (badhi = 1 and ulcit <> priorhi).
compute badend={badend, ulcit}.
compute priorhi = ulcit.
end if.
end if.
do if ( (cftotfx(1,cec)*bc)+(9999*(1-bc)) = 9999).
compute llcit=temp(cilow,1).
compute ulcit=temp(cihigh,1).
end if.
compute bootse=t(sqrt((cssq(temp)-((csum(temp)&**2)/nrow(temp)))/(nrow(temp)-1)))
.
compute alttotfx(cec,2:4)={bootse,llcit,ulcit}.
end loop.
compute altcnms={altcnms,'BootSE', 'BootLLCI','BootULCI'}.
end if.
compute resultm2=make(nrow(alttotfx),maxresm,99999).
compute resultm2(1:nrow(alttotfx),1:ncol(alttotfx))=alttotfx.
compute resultm={resultm;resultm2}.
do if (outscree=1).
print/title='----------'.
do if (nxvls > 1).
print alttotfx/title='Relative total effects of X on Y (sum of direct and indirect effects)'/cnames=altcnms/rnames=direffl2/format= F10.4.
end if.
do if (nxvls = 1).
print alttotfx/title='Total effect of X on Y (sum of direct and indirect effects)'/cnames=altcnms/format= F10.4.
end if.
end if.
end if.
end if.
end if
.
do if (criterr=0 and (saveest = 1 or saveboot=1)).
compute coeffsav=coeffmat(2:nrow(coeffmat),:).
compute conseq=conseq(2:nrow(conseq),1).
compute dfmat=dfmat(2:nrow(dfmat),1).
compute dfmat=dfmat.
compute saven={'conseqnt','antecdnt','coeff','se','t','p','LLCI','ULCI','df'}.
do if (ydich=1).
compute saven={'conseqnt','antecdnt','coeff','se','t_or_Z','p','LLCI','ULCI','df'}.
end if.
do if (saveboot=1 and boot > 0 ).
compute colslab=make(300,1,' ').


compute colslab( 1 ,1)= 'COL1'.


compute colslab( 2 ,1)= 'COL2'.


compute colslab( 3 ,1)= 'COL3'.


compute colslab( 4 ,1)= 'COL4'.


compute colslab( 5 ,1)= 'COL5'.


compute colslab( 6 ,1)= 'COL6'.


compute colslab( 7 ,1)= 'COL7'.


compute colslab( 8 ,1)= 'COL8'.


compute colslab( 9 ,1)= 'COL9'.


compute colslab( 10 ,1)= 'COL10'.


compute colslab( 11 ,1)= 'COL11'.


compute colslab( 12 ,1)= 'COL12'.


compute colslab( 13 ,1)= 'COL13'.


compute colslab( 14 ,1)= 'COL14'.


compute colslab( 15 ,1)= 'COL15'.


compute colslab( 16 ,1)= 'COL16'.


compute colslab( 17 ,1)= 'COL17'.


compute colslab( 18 ,1)= 'COL18'.


compute colslab( 19 ,1)= 'COL19'.


compute colslab( 20 ,1)= 'COL20'.


compute colslab( 21 ,1)= 'COL21'.


compute colslab( 22 ,1)= 'COL22'.


compute colslab( 23 ,1)= 'COL23'.


compute colslab( 24 ,1)= 'COL24'.


compute colslab( 25 ,1)= 'COL25'.


compute colslab( 26 ,1)= 'COL26'.


compute colslab( 27 ,1)= 'COL27'.


compute colslab( 28 ,1)= 'COL28'.


compute colslab( 29 ,1)= 'COL29'.


compute colslab( 30 ,1)= 'COL30'.


compute colslab( 31 ,1)= 'COL31'.


compute colslab( 32 ,1)= 'COL32'.


compute colslab( 33 ,1)= 'COL33'.


compute colslab( 34 ,1)= 'COL34'.


compute colslab( 35 ,1)= 'COL35'.


compute colslab( 36 ,1)= 'COL36'.


compute colslab( 37 ,1)= 'COL37'.


compute colslab( 38 ,1)= 'COL38'.


compute colslab( 39 ,1)= 'COL39'.


compute colslab( 40 ,1)= 'COL40'.


compute colslab( 41 ,1)= 'COL41'.


compute colslab( 42 ,1)= 'COL42'.


compute colslab( 43 ,1)= 'COL43'.


compute colslab( 44 ,1)= 'COL44'.


compute colslab( 45 ,1)= 'COL45'.


compute colslab( 46 ,1)= 'COL46'.


compute colslab( 47 ,1)= 'COL47'.


compute colslab( 48 ,1)= 'COL48'.


compute colslab( 49 ,1)= 'COL49'.


compute colslab( 50 ,1)= 'COL50'.


compute colslab( 51 ,1)= 'COL51'.


compute colslab( 52 ,1)= 'COL52'.


compute colslab( 53 ,1)= 'COL53'.


compute colslab( 54 ,1)= 'COL54'.


compute colslab( 55 ,1)= 'COL55'.


compute colslab( 56 ,1)= 'COL56'.


compute colslab( 57 ,1)= 'COL57'.


compute colslab( 58 ,1)= 'COL58'.


compute colslab( 59 ,1)= 'COL59'.


compute colslab( 60 ,1)= 'COL60'.


compute colslab( 61 ,1)= 'COL61'.


compute colslab( 62 ,1)= 'COL62'.


compute colslab( 63 ,1)= 'COL63'.


compute colslab( 64 ,1)= 'COL64'.


compute colslab( 65 ,1)= 'COL65'.


compute colslab( 66 ,1)= 'COL66'.


compute colslab( 67 ,1)= 'COL67'.


compute colslab( 68 ,1)= 'COL68'.


compute colslab( 69 ,1)= 'COL69'.


compute colslab( 70 ,1)= 'COL70'.


compute colslab( 71 ,1)= 'COL71'.


compute colslab( 72 ,1)= 'COL72'.


compute colslab( 73 ,1)= 'COL73'.


compute colslab( 74 ,1)= 'COL74'.


compute colslab( 75 ,1)= 'COL75'.


compute colslab( 76 ,1)= 'COL76'.


compute colslab( 77 ,1)= 'COL77'.


compute colslab( 78 ,1)= 'COL78'.


compute colslab( 79 ,1)= 'COL79'.


compute colslab( 80 ,1)= 'COL80'.


compute colslab( 81 ,1)= 'COL81'.


compute colslab( 82 ,1)= 'COL82'.


compute colslab( 83 ,1)= 'COL83'.


compute colslab( 84 ,1)= 'COL84'.


compute colslab( 85 ,1)= 'COL85'.


compute colslab( 86 ,1)= 'COL86'.


compute colslab( 87 ,1)= 'COL87'.


compute colslab( 88 ,1)= 'COL88'.


compute colslab( 89 ,1)= 'COL89'.


compute colslab( 90 ,1)= 'COL90'.


compute colslab( 91 ,1)= 'COL91'.


compute colslab( 92 ,1)= 'COL92'.


compute colslab( 93 ,1)= 'COL93'.


compute colslab( 94 ,1)= 'COL94'.


compute colslab( 95 ,1)= 'COL95'.


compute colslab( 96 ,1)= 'COL96'.


compute colslab( 97 ,1)= 'COL97'.


compute colslab( 98 ,1)= 'COL98'.


compute colslab( 99 ,1)= 'COL99'.


compute colslab( 100 ,1)= 'COL100'.


compute colslab( 101 ,1)= 'COL101'.


compute colslab( 102 ,1)= 'COL102'.


compute colslab( 103 ,1)= 'COL103'.


compute colslab( 104 ,1)= 'COL104'.


compute colslab( 105 ,1)= 'COL105'.


compute colslab( 106 ,1)= 'COL106'.


compute colslab( 107 ,1)= 'COL107'.


compute colslab( 108 ,1)= 'COL108'.


compute colslab( 109 ,1)= 'COL109'.


compute colslab( 110 ,1)= 'COL110'.


compute colslab( 111 ,1)= 'COL111'.


compute colslab( 112 ,1)= 'COL112'.


compute colslab( 113 ,1)= 'COL113'.


compute colslab( 114 ,1)= 'COL114'.


compute colslab( 115 ,1)= 'COL115'.


compute colslab( 116 ,1)= 'COL116'.


compute colslab( 117 ,1)= 'COL117'.


compute colslab( 118 ,1)= 'COL118'.


compute colslab( 119 ,1)= 'COL119'.


compute colslab( 120 ,1)= 'COL120'.


compute colslab( 121 ,1)= 'COL121'.


compute colslab( 122 ,1)= 'COL122'.


compute colslab( 123 ,1)= 'COL123'.


compute colslab( 124 ,1)= 'COL124'.


compute colslab( 125 ,1)= 'COL125'.


compute colslab( 126 ,1)= 'COL126'.


compute colslab( 127 ,1)= 'COL127'.


compute colslab( 128 ,1)= 'COL128'.


compute colslab( 129 ,1)= 'COL129'.


compute colslab( 130 ,1)= 'COL130'.


compute colslab( 131 ,1)= 'COL131'.


compute colslab( 132 ,1)= 'COL132'.


compute colslab( 133 ,1)= 'COL133'.


compute colslab( 134 ,1)= 'COL134'.


compute colslab( 135 ,1)= 'COL135'.


compute colslab( 136 ,1)= 'COL136'.


compute colslab( 137 ,1)= 'COL137'.


compute colslab( 138 ,1)= 'COL138'.


compute colslab( 139 ,1)= 'COL139'.


compute colslab( 140 ,1)= 'COL140'.


compute colslab( 141 ,1)= 'COL141'.


compute colslab( 142 ,1)= 'COL142'.


compute colslab( 143 ,1)= 'COL143'.


compute colslab( 144 ,1)= 'COL144'.


compute colslab( 145 ,1)= 'COL145'.


compute colslab( 146 ,1)= 'COL146'.


compute colslab( 147 ,1)= 'COL147'.


compute colslab( 148 ,1)= 'COL148'.


compute colslab( 149 ,1)= 'COL149'.


compute colslab( 150 ,1)= 'COL150'.


compute colslab( 151 ,1)= 'COL151'.


compute colslab( 152 ,1)= 'COL152'.


compute colslab( 153 ,1)= 'COL153'.


compute colslab( 154 ,1)= 'COL154'.


compute colslab( 155 ,1)= 'COL155'.


compute colslab( 156 ,1)= 'COL156'.


compute colslab( 157 ,1)= 'COL157'.


compute colslab( 158 ,1)= 'COL158'.


compute colslab( 159 ,1)= 'COL159'.


compute colslab( 160 ,1)= 'COL160'.


compute colslab( 161 ,1)= 'COL161'.


compute colslab( 162 ,1)= 'COL162'.


compute colslab( 163 ,1)= 'COL163'.


compute colslab( 164 ,1)= 'COL164'.


compute colslab( 165 ,1)= 'COL165'.


compute colslab( 166 ,1)= 'COL166'.


compute colslab( 167 ,1)= 'COL167'.


compute colslab( 168 ,1)= 'COL168'.


compute colslab( 169 ,1)= 'COL169'.


compute colslab( 170 ,1)= 'COL170'.


compute colslab( 171 ,1)= 'COL171'.


compute colslab( 172 ,1)= 'COL172'.


compute colslab( 173 ,1)= 'COL173'.


compute colslab( 174 ,1)= 'COL174'.


compute colslab( 175 ,1)= 'COL175'.


compute colslab( 176 ,1)= 'COL176'.


compute colslab( 177 ,1)= 'COL177'.


compute colslab( 178 ,1)= 'COL178'.


compute colslab( 179 ,1)= 'COL179'.


compute colslab( 180 ,1)= 'COL180'.


compute colslab( 181 ,1)= 'COL181'.


compute colslab( 182 ,1)= 'COL182'.


compute colslab( 183 ,1)= 'COL183'.


compute colslab( 184 ,1)= 'COL184'.


compute colslab( 185 ,1)= 'COL185'.


compute colslab( 186 ,1)= 'COL186'.


compute colslab( 187 ,1)= 'COL187'.


compute colslab( 188 ,1)= 'COL188'.


compute colslab( 189 ,1)= 'COL189'.


compute colslab( 190 ,1)= 'COL190'.


compute colslab( 191 ,1)= 'COL191'.


compute colslab( 192 ,1)= 'COL192'.


compute colslab( 193 ,1)= 'COL193'.


compute colslab( 194 ,1)= 'COL194'.


compute colslab( 195 ,1)= 'COL195'.


compute colslab( 196 ,1)= 'COL196'.


compute colslab( 197 ,1)= 'COL197'.


compute colslab( 198 ,1)= 'COL198'.


compute colslab( 199 ,1)= 'COL199'.


compute colslab( 200 ,1)= 'COL200'.


compute colslab( 201 ,1)= 'COL201'.


compute colslab( 202 ,1)= 'COL202'.


compute colslab( 203 ,1)= 'COL203'.


compute colslab( 204 ,1)= 'COL204'.


compute colslab( 205 ,1)= 'COL205'.


compute colslab( 206 ,1)= 'COL206'.


compute colslab( 207 ,1)= 'COL207'.


compute colslab( 208 ,1)= 'COL208'.


compute colslab( 209 ,1)= 'COL209'.


compute colslab( 210 ,1)= 'COL210'.


compute colslab( 211 ,1)= 'COL211'.


compute colslab( 212 ,1)= 'COL212'.


compute colslab( 213 ,1)= 'COL213'.


compute colslab( 214 ,1)= 'COL214'.


compute colslab( 215 ,1)= 'COL215'.


compute colslab( 216 ,1)= 'COL216'.


compute colslab( 217 ,1)= 'COL217'.


compute colslab( 218 ,1)= 'COL218'.


compute colslab( 219 ,1)= 'COL219'.


compute colslab( 220 ,1)= 'COL220'.


compute colslab( 221 ,1)= 'COL221'.


compute colslab( 222 ,1)= 'COL222'.


compute colslab( 223 ,1)= 'COL223'.


compute colslab( 224 ,1)= 'COL224'.


compute colslab( 225 ,1)= 'COL225'.


compute colslab( 226 ,1)= 'COL226'.


compute colslab( 227 ,1)= 'COL227'.


compute colslab( 228 ,1)= 'COL228'.


compute colslab( 229 ,1)= 'COL229'.


compute colslab( 230 ,1)= 'COL230'.


compute colslab( 231 ,1)= 'COL231'.


compute colslab( 232 ,1)= 'COL232'.


compute colslab( 233 ,1)= 'COL233'.


compute colslab( 234 ,1)= 'COL234'.


compute colslab( 235 ,1)= 'COL235'.


compute colslab( 236 ,1)= 'COL236'.


compute colslab( 237 ,1)= 'COL237'.


compute colslab( 238 ,1)= 'COL238'.


compute colslab( 239 ,1)= 'COL239'.


compute colslab( 240 ,1)= 'COL240'.


compute colslab( 241 ,1)= 'COL241'.


compute colslab( 242 ,1)= 'COL242'.


compute colslab( 243 ,1)= 'COL243'.


compute colslab( 244 ,1)= 'COL244'.


compute colslab( 245 ,1)= 'COL245'.


compute colslab( 246 ,1)= 'COL246'.


compute colslab( 247 ,1)= 'COL247'.


compute colslab( 248 ,1)= 'COL248'.


compute colslab( 249 ,1)= 'COL249'.


compute colslab( 250 ,1)= 'COL250'.


compute colslab( 251 ,1)= 'COL251'.


compute colslab( 252 ,1)= 'COL252'.


compute colslab( 253 ,1)= 'COL253'.


compute colslab( 254 ,1)= 'COL254'.


compute colslab( 255 ,1)= 'COL255'.


compute colslab( 256 ,1)= 'COL256'.


compute colslab( 257 ,1)= 'COL257'.


compute colslab( 258 ,1)= 'COL258'.


compute colslab( 259 ,1)= 'COL259'.


compute colslab( 260 ,1)= 'COL260'.


compute colslab( 261 ,1)= 'COL261'.


compute colslab( 262 ,1)= 'COL262'.


compute colslab( 263 ,1)= 'COL263'.


compute colslab( 264 ,1)= 'COL264'.


compute colslab( 265 ,1)= 'COL265'.


compute colslab( 266 ,1)= 'COL266'.


compute colslab( 267 ,1)= 'COL267'.


compute colslab( 268 ,1)= 'COL268'.


compute colslab( 269 ,1)= 'COL269'.


compute colslab( 270 ,1)= 'COL270'.


compute colslab( 271 ,1)= 'COL271'.


compute colslab( 272 ,1)= 'COL272'.


compute colslab( 273 ,1)= 'COL273'.


compute colslab( 274 ,1)= 'COL274'.


compute colslab( 275 ,1)= 'COL275'.


compute colslab( 276 ,1)= 'COL276'.


compute colslab( 277 ,1)= 'COL277'.


compute colslab( 278 ,1)= 'COL278'.


compute colslab( 279 ,1)= 'COL279'.


compute colslab( 280 ,1)= 'COL280'.


compute colslab( 281 ,1)= 'COL281'.


compute colslab( 282 ,1)= 'COL282'.


compute colslab( 283 ,1)= 'COL283'.


compute colslab( 284 ,1)= 'COL284'.


compute colslab( 285 ,1)= 'COL285'.


compute colslab( 286 ,1)= 'COL286'.


compute colslab( 287 ,1)= 'COL287'.


compute colslab( 288 ,1)= 'COL288'.


compute colslab( 289 ,1)= 'COL289'.


compute colslab( 290 ,1)= 'COL290'.


compute colslab( 291 ,1)= 'COL291'.


compute colslab( 292 ,1)= 'COL292'.


compute colslab( 293 ,1)= 'COL293'.


compute colslab( 294 ,1)= 'COL294'.


compute colslab( 295 ,1)= 'COL295'.


compute colslab( 296 ,1)= 'COL296'.


compute colslab( 297 ,1)= 'COL297'.


compute colslab( 298 ,1)= 'COL298'.


compute colslab( 299 ,1)= 'COL299'.


compute colslab( 300 ,1)= 'COL300'.

compute colslab=colslab(1:ncol(bootres),1).
compute colslab={colslab,conseq,vlabs}.
do if (outscree=1).
print/title = '**************************************************************************'.
print/title='Bootstrap estimates were saved to a file'/space=0.
print colslab/title='Map of column names to model coefficients:'/clabels=' ','Conseqnt','Antecdnt'/format=a8.
end if.
end if.
end if.
do if (criterr=0 and boot > 0 and modelbt=1).
compute labstart=1.
do if (outscree=1).
print/title = '*********** BOOTSTRAP RESULTS FOR REGRESSION MODEL PARAMETERS ************'.
end if.
loop iboot = 1 to (nms+nys).
do if (outscree=1).
print outnames(1,iboot)/title = 'OUTCOME VARIABLE:'/format = A8.
end if.
compute vlabsm=vlabs(labstart:(labstart+(nump(1,iboot)-1)),1).
compute outnmtmp=bootcim(labstart:(labstart+(nump(1,iboot)-1)),:).
compute resultm2=make(nrow(outnmtmp),maxresm,99999).
compute resultm2(1:nrow(outnmtmp),1:ncol(outnmtmp))=outnmtmp.
compute resultm={resultm;resultm2}.
do if (outscree=1).
print bootcim(labstart:(labstart+(nump(1,iboot)-1)),:)/title=' '/rnames=vlabsm/ clabels='Coeff' 'BootMean' 'BootSE' 'BootLLCI' 'BootULCI'/format= F10.4 /space=0.
end if.
compute labstart=labstart+nump(1,iboot).
do if (iboot < (nms+nys)).
do if (outscree=1).
print/title= '----------'.
end if.
end if.
end loop.
end if.
do if (criterr=0 and saveest=1).
compute resultm=resultm(2:nrow(resultm),:).
compute bocaj=(resultm=99999).
compute bocaj=csum(bocaj).
compute bocaj=1-(bocaj=nrow(resultm)).
compute j=1.
loop i = 1 to ncol(resultm).
do if (bocaj(1,i)=1).
compute j=j+1.
end if.
end loop.
compute resultm=resultm(:,1:(j-1)).
save resultm/outfile = *.
end if.
do if (criterr = 0 and matrices=1 and outscree=1).
print/title = '************************ MODEL DEFINITION MATRICES ************************'.
print/title = 'FROM variables are columns, TO variables are rows.'.
compute temp2=make(nrow(bcmat),ncol(bcmat),'0').
loop i = 2 to nrow(bcmat).
loop j = 1 to (ncol(bcmat)-1).
do if (bcmat(i,j)=1).
compute temp2(i,j)='1'.
end if.
do if (j >= i).
compute temp2(i,j)=' '.
end if.
end loop.
end loop.
compute temp2=temp2(2:nrow(bcmat),(1:(ncol(bcmat)-1))).
do if (nms > 0).
compute cmatlabs={xnames,mnames}.
compute rmatlabs={mnames,ynames}.
end if.
do if (nms = 0).
compute cmatlabs={xnames}.
compute rmatlabs={ynames}.
end if.
print temp2/title='BMATRIX: Paths freely estimated (1) and fixed to zero (0):'/cnames=cmatlabs/rnames=rmatlabs/format A3.
compute z=0.
do if (rsum(csum(wcmat))<>0).
compute temp2=make(nrow(wcmat),ncol(wcmat),'0').
loop i = 2 to nrow(wcmat).
loop j = 1 to (ncol(wcmat)-1).
do if (wcmat(i,j)=1).
compute temp2(i,j)='1'.
end if.
do if (j >= i).
compute temp2(i,j)=' '.
end if.
end loop.
end loop.
compute temp2=temp2(2:nrow(wcmat),(1:(ncol(wcmat)-1))).
print temp2/title='WMATRIX: Paths moderated (1) and not moderated (0) by W:'/cnames=cmatlabs/rnames=rmatlabs/format A3.
end if.
do if (rsum(csum(zcmat))<>0).
compute temp2=make(nrow(zcmat),ncol(zcmat),'0').
loop i = 2 to nrow(zcmat).
loop j = 1 to (ncol(zcmat)-1).
do if (zcmat(i,j)=1).
compute temp2(i,j)='1'.
end if.
do if (j >= i).
compute temp2(i,j)=' '.
end if.
end loop.
end loop.
compute temp2=temp2(2:nrow(zcmat),(1:(ncol(zcmat)-1))).
print temp2/title='ZMATRIX: Paths moderated (1) and not moderated (0) by Z:'/cnames=cmatlabs/rnames=rmatlabs/format a3.
end if.
do if (rsum(csum(wzcmat))<>0).
compute temp2=make(nrow(wzcmat),ncol(wzcmat),'0').
loop i = 2 to nrow(wzcmat).
loop j = 1 to (ncol(wzcmat)-1).
do if (wzcmat(i,j)=1).
compute temp2(i,j)='1'.
end if.
do if (j >= i).
compute temp2(i,j)=' '.
end if.
end loop.
end loop.
compute temp2=temp2(2:nrow(wzcmat),(1:(ncol(wzcmat)-1))).
print temp2/title='WZMATRIX: W moderated paths moderated (1) and not moderated (0) by Z:'/cnames=cmatlabs/rnames=rmatlabs/format a3.
end if.
do if (ncs > 0).
print ccmat/title='CMATRIX: Covariates (columns) in (1) and not in (0) the models of M and Y (rows):'/rnames=rmatlabs/cnames=covnames.
end if.
end if.
do if (outscree=1).
do if (activate=0).
print/title = '*********************** ANALYSIS NOTES AND ERRORS ************************'.
end if.
end if.
do if (activate=1).
print/title = '**************************************************************************'.
end if.
do if (criterr=0).
do if (outscree=1).
print conf/title = 'Level of confidence for all confidence intervals in output:'/format = F8.4.
do if (boot > 0).
do if (goodboot = boot and bc=0).
print boot/title='Number of bootstrap samples for percentile bootstrap confidence intervals:'.
end if.
do if (goodboot = boot and bc=1).
print boot/title='Number of bootstrap samples for bias-corrected bootstrap confidence intervals:'.
end if.
do if (booterr = 1).
compute badend = badend(1,2:ncol(badend)).
print/title = 'WARNING: Bootstrap CI endpoints below not trustworthy. Decrease confidence'.
print badend/title='or increase the number of bootstrap samples.'/space=0/format = F10.4.
end if.
end if.
do if (mc > 0).
print mc/title='Number of samples for Monte Carlo confidence intervals:'.
end if.
do if (wnotev > 0 and printw=1).
do if (wnotev=1).
print/title = 'W values in conditional tables are the 16th, 50th, and 84th percentiles.'.
else if (wnotev=2).
do if (minwwarn=0 and maxwwarn=0).
print/title = 'W values in conditional tables are the mean and +/- SD from the mean.'.
end if.
do if (minwwarn=1).
print/title = 'W values in conditional tables are the minimum, the mean, and 1 SD above the mean.'.
end if.
do if (maxwwarn=1).
print/title = 'W values in conditional tables are 1 SD below the mean, the mean, and the maximum.'.
end if.
end if.
end if.
do if (znotev > 0 and printz=1).
do if (znotev=1).
print/title = 'Z values in conditional tables are the 16th, 50th, and 84th percentiles.'.
else if (znotev=2).
do if (minzwarn=0 and maxzwarn=0).
print/title = 'Z values in conditional tables are the mean and +/- SD from the mean.'.
end if.
do if (minzwarn=1).
print/title = 'Z values in conditional tables are the minimum, the mean, and 1 SD above the mean.'.
end if.
do if (maxzwarn=1).
print/title = 'Z values in conditional tables are 1 SD below the mean, the mean, and the maximum.'.
end if.
end if.
end if.
do if (minwwarn > 0).
print/title = 'NOTE: One SD below the mean is below the minimum observed in the data for W,'.
print/title = '      so the minimum measurement on W is used for conditioning instead.'/space=0.
end if.
do if (maxwwarn > 0).
print/title = 'NOTE: One SD above the mean is above the maximum observed in the data for W,'.
print/title = '      so the maximum measurement for W is used for conditioning instead.'/space=0.
end if.
do if (minzwarn > 0).
print/title = 'NOTE: One SD below the mean is below the minimum observed in the data for Z,'.
print/title = '      so the minimum measurement for Z is used for conditioning instead.'/space=0.
end if.
do if (maxzwarn > 0).
print/title = 'NOTE: One SD above the mean is above the maximum observed in the data for Z,'.
print/title = '      so the maximum measurement for Z is used for conditioning instead.'/space=0.
end if.
do if (pstog=1).
print/title= 'NOTE: Standardized coefficients for dichotomous or multicategorical X are in'.
print/title= '      partially standardized form.'/space=0.
end if.
loop i = 1 to 100.
do if (notecode(i,1) = 32).
print/title = 'Direct, indirect, and total effects are counterfactually defined'.
do if (xcontcf=0).
print/title = 'assuming X by M interaction.'/space=0.
end if.
do if (xcontcf=1).
print/title = 'assuming X by M interaction and with the following reference (x_ref)'/space=0.
compute states=t(xrefvals).
print states/title = 'and counterfactual (x_cf) states for X:'/rlabels='x_ref' 'x_cf'/format= F10.4 /space=0.
end if.
end if.
do if (notecode(i,1)=1).
print/title = 'NOTE: COVMY is ignored when using CMATRIX option.'.
end if.
do if (notecode(i,1)=2).
print/title = 'NOTE: Confidence level restricted to between 50 and 99.9999%.  95% confidence is provided in output'.
end if.
do if (notecode(i,1)=3).
print centvar/title = 'NOTE: The following variables were mean centered prior to analysis:'/format = a8.
end if.
do if (notecode(i,1) = 4).
print/title = 'NOTE: A heteroscedasticity consistent standard error and covariance matrix estimator was used.'.
end if.
do if (notecode(i,1) = 5).
print/title = 'NOTE: The HC3 option has been replaced with HC.  See the documentation.'.
end if.
do if (notecode(i,1) = 6).
print/title = 'NOTE: Due to estimation problems, some bootstrap samples had to be replaced.'.
print badboot/title='      The number of times this happened was:'/space=0/format=F8.0.
end if.
do if (notecode(i,1) = 7).
print/title = 'NOTE: The bootstrapping was not completed due to problematic bootstrap samples.'.
print/title = '      Bootstrap confidence intervals are therefore suppressed.'/space=0.
end if.
do if (notecode(i,1) = 8).
print/title = 'NOTE: The number of bootstrap samples was adjusted upward given your desired confidence.'.
end if.
do if (notecode(i,1) = 9).
print/title = 'NOTE: WMODVAL is ignored when W is specified as multicategorical.'.
end if.
do if (notecode(i,1) = 10).
print/title = 'NOTE: ZMODVAL is ignored when Z is specified as multicategorical.'.
end if.
do if (notecode(i,1) = 11).
print/title = 'NOTE: Total effect model generated only when all covariates are specified in all'.
print/title = '      models of M and Y.'/space=0.
end if.
do if (notecode(i,1) = 30).
print/title = 'NOTE: Your vector of linear hypothesis weights is of the wrong length for this model.'.
end if.
do if (notecode(i,1) = 12).
print/title = 'NOTE: Total effect model generated only when X is freely estimated to affect each M'.
print/title = '      and both X and M are freely estimated to affect Y'/space=0.
end if.
do if (notecode(i,1) = 13).
print/title = 'NOTE: There are too many pairwise contrasts to conduct with this model.'.
end if.
do if (notecode(i,1) = 14).
print/title = 'NOTE: The number of contrast weights must equal the number of indirect effects.'.
end if.
do if (notecode(i,1) = 15).
print/title = 'NOTE: Monte Carlo confidence intervals not available for this model.'.
print/title = '      Bootstrapping is used instead.'/space=0.
end if.
do if (notecode(i,1) = 16).
print/title = 'NOTE: The number of Monte Carlo samples was adjusted upward given your desired confidence.'.
end if.
do if (notecode(i,1) = 19).
print/title = 'NOTE: Your contrast matrix is invalid or not applicable to this model.'.
end if.
do if (notecode(i,1) = 20).
print/title = 'NOTE: One of the groups specified by your contrast matrix does not exist in the data.'.
end if.
do if (notecode(i,1) = 21).
print/title = 'NOTE: The VARORDER option is not available in this release.'.
end if.
do if (notecode(i,1) = 22).
print/title = 'NOTE: The VMODVAL and QMODVAL options are not available in this release.'.
end if.
do if (notecode(i,1) = 23).
print/title = 'NOTE: The QUANTILE option is not available in this release.'.
end if.
do if (notecode(i,1) = 24).
print/title = 'NOTE: Total effect model not available with dichotomous Y.'.
end if.
do if (notecode(i,1) = 25).
print/title = 'NOTE: STAND/EFFSIZE options not available with dichotomous Y.'.
end if.
do if ((notecode(i,1) = 26) and nms > 0).
print/title = 'NOTE: Direct and indirect effects of X on Y are on a log-odds metric.'.
end if.
do if (notecode(i,1) = 27).
print/title = 'NOTE: Standardized coefficients are not available for models with moderators.'.
end if.
do if (notecode(i,1) = 28).
print/title = 'NOTE: The contrast option is not available with a multicategorical X.'.
end if.
do if (notecode(i,1) = 31).
do if (nms > 1).
print medmeans/title = 'NOTE: Controlled direct effect(s) estimated at the following mediator values:'/cnames=mnames/format= F10.4.
end if.
do if (nms=1).
print medmeans/title = 'NOTE: Controlled direct effect(s) estimated at the following mediator value:'/format= F10.4.
end if.
end if.
do if (notecode(i,1) = 33).
print/title = 'NOTE: Sobel test is not available with the XMINT option.'.
end if.
do if (notecode(i,1) = 37).
print/title = 'NOTE: The CONTRAST option is not available when using the XMINT option.'.
end if.
do if (notecode(i,1) = 34).
print/title = 'NOTE: Standardized effects are not available when using the XMINT option.'.
end if.
do if (notecode(i,1) = 36).
print/title = 'NOTE: The XREFVAL option is ignored when X is declared as multicategorical.'.
end if.
do if (notecode(i,1) = 35).
print coval/title = 'NOTE: Counterfactual effects estimated at the following covariate values:'/cnames=covnames/format= F10.4.
end if.
do if (notecode(i,1) = 29 and (listmiss=1)).
compute a=missrow.
compute conum=ncol(a).
compute allgood=0.
compute smremain=12.
compute largesti=1.
compute smallrow=0.
do if (conum > 12).
loop ii = 1 to 12.
compute check=(conum/ii).
do if (check = trunc(check)).
compute check2=conum/ii.
compute aok=ii.
do if (aok > 2).
compute allgood=1.
end if.
else.
compute remain=conum-(ii*trunc(check)).
do if (remain <= smremain).
compute smremain=remain.
compute largesti=ii.
compute smallrow=trunc(conum/largesti).
end if.
end if.
end loop.
compute atemp=a(1,1:(aok*check2)).
compute atemp=reshape(atemp,check2,aok).
do if (ncol(atemp) > 2).
print/title='NOTE: Missing data resulted in the deletion of the following row(s) of'.
print atemp/title='      data from the analysis:'/format=F6.0/space=0.
end if.
do if (allgood=0).
compute atemp=a(1,1:(smallrow*largesti)).
compute atemp=reshape(atemp,smallrow,largesti).
compute btemp=a(1,((largesti*smallrow)+1):conum).
print/title='NOTE: Missing data resulted in the deletion of the following row(s) of'.
print atemp/title='      data from the analysis:'/format=F6.0/space=0.
print btemp/title=' '/space=0/format=f6.0.
end if.
else if (conum <= 12).
print/title='NOTE: Missing data resulted in the deletion of the following row(s) of'.
print a/title='      data from the analysis:'/format=F6.0/space=0.
end if.
end if.
end loop.
do if (toomany=1).
print/title='WARNING: Variables names longer than eight characters can produce incorrect output'.
print/title='when some variables in the data file have the same first eight characters. Shorter'/space=0.
print/title='variable names are recommended. By using this output, you are accepting all risk'/space=0.
print/title='and consequences of interpreting or reporting results that may be incorrect.'/space=0.
end if.
end if.
end if.
loop i = 1 to 100.
do if (errcode(i,1)=1).
print/title = 'ERROR: You must specify a Y and an X variable.'.
end if.
do if (errcode(i,1)=2).
print/title = 'ERROR: X, M, or Y variable used more than once or W and Z are the same variable.'.
do if (toomany = 1).
print/title = '       This could be caused by the use of variables names longer'/space=0.
print varnames/title = '       than eight characters. Here are the variables I see:'/space=0/format=A8.
end if.
end if.
do if (errcode(i,1)=3).
print/title = 'ERROR: You have specified more than one variable for W, Y, X, or Z'.
end if.
do if (errcode(i,1)=4).
print/title = 'ERROR: A variable specified as multicategorical has more than nine categories.'.
end if.
do if (errcode(i,1)=5).
print/title = 'ERROR: One of the categories contains only a single case.'.
end if.
do if (errcode(i,1)=6).
print/title = 'ERROR: Invalid model number in this version of PROCESS.'.
end if.
do if (errcode(i,1)=7).
print/title = 'ERROR: Invalid model number.'.
end if.
do if (errcode(i,1)=8).
print/title = 'ERROR: You must specify an M variable for this model.'.
end if.
do if (errcode(i,1)=9).
print/title = 'ERROR: You have specified an M variable in a model that does not use it.'.
end if.
do if (errcode(i,1)=10).
print/title = 'ERROR: You have specified a W variable in a model that does not use it.'.
end if.
do if (errcode(i,1)=11).
print/title = 'ERROR: You have not specified a W variable in a model that requires it.'.
end if.
do if (errcode(i,1)=12).
print/title = 'ERROR: You have specified a Z variable in a model that does not use it.'.
end if.
do if (errcode(i,1)=13).
print/title = 'ERROR: You have not specified a Z variable in a model that requires it.'.
end if.
do if (errcode(i,1)=14).
print/title = 'ERROR: V and Q are not proper specifications in this release of PROCESS.'.
print/title = '       Moderators must be specified as W and/or Z.'/space=0.
end if.
do if (errcode(i,1)=15).
print/title = 'ERROR: One of your model variables exhibits no variation (it is a constant).'.
end if.
do if (errcode(i,1)=16).
print/title = 'ERROR: BMATRIX is not the correct length or is otherwise invalid.'.
end if.
do if (errcode(i,1)=17).
print/title = 'ERROR: WMATRIX is not the correct length or is otherwise invalid.'.
end if.
do if (errcode(i,1)=18).
print/title = 'ERROR: ZMATRIX is not the correct length or is otherwise invalid.'.
end if.
do if (errcode(i,1)=19).
print/title = 'ERROR: WZMATRIX is not the correct length or is otherwise invalid.'.
end if.
do if (errcode(i,1)=20).
print/title = 'ERROR: A path fixed at zero cannot be moderated.'.
end if.
do if (errcode(i,1)=21).
print/title = 'ERROR: If only one moderator is specified, it must be specified as W.'.
end if.
do if (errcode(i,1)=22).
print/title = 'ERROR: In BMATRIX, X must be specified to affect at least one variable.'.
end if.
do if (errcode(i,1)=23).
print/title = 'ERROR: In BMATRIX, at least one variable must be specified to affect Y.'.
end if.
do if (errcode(i,1)=24).
print/title = 'ERROR: You must specify a model number or a custom BMATRIX specification.'.
end if.
do if (errcode(i,1)=25).
print/title = 'ERROR: BMATRIX cannot be used in conjunction with a model number.'.
end if.
do if (errcode(i,1)=26).
print/title = 'ERROR: Your model has a dangling mediator (all Ms must affect and be affected).'.
end if.
do if (errcode(i,1)=27).
print/title = 'ERROR: CLUSTER is not available on this release of PROCESS.'.
end if.
do if (errcode(i,1)=29).
print/title = 'ERROR: CMATRIX is not the correct length or is otherwise invalid.'.
end if.
do if (errcode(i,1)=30).
print/title = 'ERROR: In CMATRIX, all covariates must be assigned to an M or a Y.'.
end if.
do if (errcode(i,1)=31).
print/title = 'ERROR: A linear or near linear dependency (singularity) exists in the data.'.
end if.
do if (errcode(i,1)=32).
print/title = 'ERROR: Models 80 and 81 require between 3 and 6 mediators.'.
end if.
do if (errcode(i,1)=33).
print/title = 'ERROR: Model 82 requires 4 mediators.'.
end if.
do if (errcode(i,1)=34).
print/title = 'ERROR: This model number requires between 2 and 6 mediators.'.
end if.
do if (errcode(i,1)=35).
print/title = 'ERROR: In a model with only one moderator, that moderator must be W.'.
end if.
do if (errcode(i,1)=36).
print/title = 'ERROR: A serial mediation model cannot have more than 6 mediators.'.
end if.
do if (errcode(i,1)=37).
print/title = 'ERROR: No more than 10 mediators are allowed in a PROCESS command.'.
end if.
do if (errcode(i,1)=38).
print/title = 'ERROR: XCATCODE is not provided, not the correct length, or is otherwise invalid.'.
end if.
do if (errcode(i,1)=39).
print/title = 'ERROR: WCATCODE is not provided, not the correct length, or is otherwise invalid.'.
end if.
do if (errcode(i,1)=40).
print/title = 'ERROR: ZCATCODE is not provided, not the correct length, or is otherwise invalid.'.
end if.
do if (errcode(i,1)=41).
print/title = 'ERROR: Models 1, 2, and 3 cannot be customized.'.
end if.
do if (errcode(i,1)=42).
print/title = 'ERROR: WS option available only in PROCESS v2. Or use the MEMORE macro instead.'.
print/title = '       MEMORE can be downloaded from www.akmontoya.com.'/space=0.
end if.
do if (errcode(i,1)=43).
print/title = 'ERROR: PROCESS does not allow dichotomous mediators.'.
end if.
do if (errcode(i,1)=60).
print/title = 'PROCESS is now ready for use.'.
print/title='Copyright 2017-2022 by Andrew F. Hayes. ALL RIGHTS RESERVED.'/space=0.
print/title='Workshop schedule available at http://haskayne.ucalgary.ca/CCRAM'/space=0.
end if.
do if (errcode(i,1)=50).
print/title = 'ERROR: A multicategorical moderator cannot be specified as a covariate.'.
end if.
do if (errcode(i,1)=51).
print/title = 'ERROR: A variable you specified as a covariate is a moderator in all equations.'.
end if.
do if (errcode(i,1)=52 and mcerpt=0).
compute mcerpt=1.
print/title = 'ERROR: A variable specified as multicategorical must have at least three categories'.
end if.
do if (errcode(i,1) = 47).
do if (iterrmod=1).
print/title = 'ERROR: Iteration for Y model didn''t converge to a solution. Interpret results with caution.'.
print/title = '       Try increasing the number of iterations, though this will slow down computation.'/space=0.
end if.
do if (bootiter=1).
print/title = 'ERROR: Nonconvergence during bootstrapping. Interpet bootstrap results with caution.'.
end if.
end if.
do if (errcode(i,1) = 61).
print/title = 'ERROR: Variable names should be no more than eight characters in length.'.
end if.
do if (errcode(i,1) = 62).
print/title = 'ERROR: After listwise deletion of cases with missing data, too few cases remain.'.
end if.
do if (errcode(i,1) = 63).
print/title = 'ERROR: The XMINT option is available only for model 4.'.
end if.
do if (errcode(i,1) = 64).
print/title = 'ERROR: Incorrect number of values specified in CDEVAL option.'.
end if.
do if (errcode(i,1) = 65).
print/title = 'ERROR: Only indicator or sequential coding of X is allowed with the XMINT option.'.
end if.
do if (errcode(i,1) = 66).
print/title = 'ERROR: XMINT option not available with continuous X using PROCESS GUI. Use syntax.'.
end if.
do if (errcode(i,1) = 67).
print/title = 'ERROR: Too many elements provided in XREFVAL option.'.
end if.
do if (errcode(i,1)=68).
print/title = 'ERROR: Covariate assignment is not allowed with XMINT option.'.
end if.
do if (errcode(i,1) = 69).
print/title = 'ERROR: Incorrect number of values specified in COVAL option.'.
end if.
do if (errcode(i,1) = 70).
print/title = 'ERROR: Incorrect value(s) in XREFVAL for this dichotomous X variable.'.
end if.
do if (errcode(i,1) = 71).
print/title = 'ERROR: The CENTER option is not available when using the XMINT option.'.
end if.
do if (errcode(i,1) = 72).
print/title = 'ERROR: The XMINT option is not available for models with a dichotomous Y.'.
end if.
end loop.
END MATRIX.	
Ressources	Temps de processeur	00:00:07,00	
	Temps écoulé	00:00:06,98	


Run MATRIX procedure:

***************** PROCESS Procedure for SPSS Version 4.2 *****************

          Written by Andrew F. Hayes, Ph.D.       www.afhayes.com
    Documentation available in Hayes (2022). www.guilford.com/p/hayes3

**************************************************************************
Model  : 4
    Y  : HOMAIR
    X  : WhtRSt
   M1  : TG
   M2  : HDL
   M3  : LDL
   M4  : TC

Sample
Size:  169

**************************************************************************
OUTCOME VARIABLE:
 TG

Model Summary
          R       R-sq        MSE          F        df1        df2          p
      ,1506      ,0227  3959,7647     3,8753     1,0000   167,0000      ,0507

Model
              coeff         se          t          p       LLCI       ULCI
constant    81,0367     6,0273    13,4450      ,0000    69,1372    92,9362
WhtRSt      19,9133    10,1155     1,9686      ,0507     -,0575    39,8841

Standardized coefficients
            coeff
WhtRSt      ,3138

**************************************************************************
OUTCOME VARIABLE:
 HDL

Model Summary
          R       R-sq        MSE          F        df1        df2          p
      ,0136      ,0002   178,1029      ,0309     1,0000   167,0000      ,8607

Model
              coeff         se          t          p       LLCI       ULCI
constant    41,2936     1,2783    32,3043      ,0000    38,7699    43,8172
WhtRSt       -,3769     2,1453     -,1757      ,8607    -4,6123     3,8585

Standardized coefficients
            coeff
WhtRSt     -,0283

**************************************************************************
OUTCOME VARIABLE:
 LDL

Model Summary
          R       R-sq        MSE          F        df1        df2          p
      ,1594      ,0254  1159,7952     4,3564     1,0000   167,0000      ,0384

Model
              coeff         se          t          p       LLCI       ULCI
constant    72,2569     3,2620    22,1514      ,0000    65,8169    78,6969
WhtRSt      11,4265     5,4745     2,0872      ,0384      ,6183    22,2346

Standardized coefficients
            coeff
WhtRSt      ,3322

**************************************************************************
OUTCOME VARIABLE:
 TC

Model Summary
          R       R-sq        MSE          F        df1        df2          p
      ,1701      ,0290  1409,8971     4,9789     1,0000   167,0000      ,0270

Model
              coeff         se          t          p       LLCI       ULCI
constant   130,9817     3,5965    36,4192      ,0000   123,8812   138,0821
WhtRSt      13,4683     6,0360     2,2313      ,0270     1,5517    25,3850

Standardized coefficients
            coeff
WhtRSt      ,3545

**************************************************************************
OUTCOME VARIABLE:
 HOMAIR

Model Summary
          R       R-sq        MSE          F        df1        df2          p
      ,3747      ,1404    23,0467     5,3259     5,0000   163,0000      ,0001

Model
              coeff         se          t          p       LLCI       ULCI
constant     -,0239     1,6570     -,0144      ,9885    -3,2958     3,2480
WhtRSt        ,9357      ,7930     1,1799      ,2398     -,6302     2,5016
TG            ,0420      ,0098     4,2797      ,0000      ,0226      ,0614
HDL           ,1215      ,0431     2,8182      ,0054      ,0364      ,2066
LDL           ,0881      ,0358     2,4564      ,0151      ,0173      ,1588
TC           -,0849      ,0355    -2,3958      ,0177     -,1550     -,0149

Standardized coefficients
            coeff
WhtRSt      ,1834
TG          ,5224
HDL         ,3170
LDL         ,5938
TC         -,6327

************************** TOTAL EFFECT MODEL ****************************
OUTCOME VARIABLE:
 HOMAIR

Model Summary
          R       R-sq        MSE          F        df1        df2          p
      ,1494      ,0223    25,5853     3,8145     1,0000   167,0000      ,0525

Model
              coeff         se          t          p       LLCI       ULCI
constant     3,6320      ,4845     7,4965      ,0000     2,6755     4,5885
WhtRSt       1,5881      ,8131     1,9531      ,0525     -,0172     3,1934

Standardized coefficients
            coeff
WhtRSt      ,3114


************** TOTAL, DIRECT, AND INDIRECT EFFECTS OF X ON Y **************

Total effect of X on Y
     Effect         se          t          p       LLCI       ULCI       c_ps
     1,5881      ,8131     1,9531      ,0525     -,0172     3,1934      ,3114

Direct effect of X on Y
     Effect         se          t          p       LLCI       ULCI      c'_ps
      ,9357      ,7930     1,1799      ,2398     -,6302     2,5016      ,1834

Indirect effect(s) of X on Y:
          Effect     BootSE   BootLLCI   BootULCI
TOTAL      ,6524      ,4369      ,0247     1,6857
TG         ,8361      ,7861     -,0309     2,9180
HDL       -,0458      ,3831     -,8175      ,7255
LDL       1,0062     1,5357     -,0159     5,6832
TC       -1,1441     1,7679    -6,7441     -,0507

Partially standardized indirect effect(s) of X on Y:
          Effect     BootSE   BootLLCI   BootULCI
TOTAL      ,1279      ,0815      ,0049      ,3206
TG         ,1639      ,1440     -,0066      ,5468
HDL       -,0090      ,0729     -,1533      ,1395
LDL        ,1973      ,2905     -,0039     1,0711
TC        -,2243      ,3321    -1,2282     -,0113

*********************** ANALYSIS NOTES AND ERRORS ************************

Level of confidence for all confidence intervals in output:
  95,0000

Number of bootstrap samples for percentile bootstrap confidence intervals:
  5000

NOTE: Standardized coefficients for dichotomous or multicategorical X are in
      partially standardized form.

------ END MATRIX -----


* Encoding: UTF-8.
preserve.
set printback=off.


Matrice


Remarques	
Sortie obtenue	10-NOV-2024 17:01:34	
Commentaires		
Entrée	Données	C:\Users\Brice SAHA\Documents\UBa\Master Thesis 2021\Fanuel\Database fanuel.sav	
	Jeu de données actif	Jeu_de_données1	
	Filtre	<sans>	
	Pondération	<sans>	
	Scinder un fichier	<sans>	
	N de lignes dans le fichier de travail	169	
Syntaxe	MATRIX.
compute wnames='xxxxx'.
compute znames='xxxxx'.
compute mcerpt=0.
compute wiscov=0.
compute ziscov=0.
compute tooman=0.
compute errcode=make(100,1,0).
compute notecode=make(100,1,0).
compute model = trunc( 4 ).
compute iterate = abs(trunc( 100 )).
compute converge = abs( 0.00001 ).
compute itprobtg=0.
compute v2tag=0.
compute ydich=0.
compute maxwwarn=0.
compute minwwarn=0.
compute maxzwarn=0.
compute minzwarn=0.
compute toomany=0.
compute wdich=0.
compute zdich=0.
compute wnotev=0.
compute znotev=0.
compute nxpval=1.
compute nwpval=1.
compute nzpval=1.
compute errs=1.
compute notes=1.
compute criterr=0.
compute novar=0.
compute adjust=0.
compute ncs=0.
compute serial=0.
compute sobelok=0.
compute hasw=0.
compute hasz=0.
compute printw=0.
compute printz=0.
compute xmint=( 0 =1).
compute wmodcust=0.
compute zmodcust=0.
compute booting=0.
compute bootiter=0.
compute iterrmod=0.
compute cov = 'xxxxx'.
compute varorder=( 0 <> 0).
compute nws=0.
compute w= 'xxxxx'.
compute nzs=0.
compute z = 'xxxxx'.
compute nms=0.
compute m = 'TG HDL LDL TC'.
compute nys=0.
compute y = 'HOMAIR'.
compute nxs=0.
compute x = 'FGDS'.
compute effsize=( 0 =1).
compute stand=( 1 =1).
compute intprobe = .1.
compute xrefvals={ 999 }.
compute center=trunc( 0 ).
compute xcontcf=0.
compute xscaling=1.
compute cdeval={ -999 }.
compute cuscoval=0.
do if (model=74).
compute errcode(errs,1)=7.
compute errs=errs+1.
compute criterr=1.
end if.
do if (xmint=1 and model <> 4).
compute errcode(errs,1)=63.
compute errs=errs+1.
compute criterr=1.
end if.
do if (xmint=1 and model=4).
compute w=x.
compute model=74.
compute intprobe=1.
compute notecode(notes,1) = 32.
compute notes = notes + 1.
do if (effsize=1 or stand=1).
compute notecode(notes,1) = 34.
compute notes = notes + 1.
compute stand=0.
compute effsize=0.
end if.
do if (center <> 0).
compute center=0.
compute errcode(errs,1)=71.
compute errs=errs+1.
compute criterr=1.
end if.
end if.
compute v = 'xxxxx'.
compute q = 'xxxxx'.
compute linsum={ -999 }.
compute nlinsum=ncol(linsum).
do if (linsum(1,1) = -999).
compute nlinsum=0.
end if.
compute oldvars= 'xxxxx'.
compute mcxok=0.
compute mcwok=0.
compute mczok=0.
compute xprod=0.
compute zprod=0.
compute wprod=0.
compute modcok=0.
compute alttotal=0.
compute hc3=trunc( 0 ).
compute jn=( 0 = 1).
compute listmiss=( 0 =1).
compute modelres=( 0 =1).
compute outscree=( 1 =1).
compute activate=( 0 =1).
compute booterr=0.
compute normal=( 0 =1).
compute xmtest=( 0 =1).
compute describe=( 0 =1).
compute longname=( 0 =1).
do if (stand=1).
compute effsize=1.
end if.
compute pstog=0.
compute sobelok=0.
compute normal=( 0 =1).
compute mdichok=( 0 =1).
compute contrast={ 999 }.
compute ncontr=ncol(contrast).
compute ncontrow=nrow(contrast).
do if (contrast(1,1) = 999).
compute ncontr=1.
compute contrast=0.
end if.
do if (ncontr = 1).
compute contrast=trunc(contrast).
do if (contrast > 3 or contrast < 0)).
compute ncontr=1.
compute contrast = 0.
end if.
end if.
do if (ncontr > 1).
compute contvec=contrast.
compute contrast=4.
do if (ncontrow > 1).
compute contrast=0.
compute modcok=1.
compute wcontval=contvec(:,1).
compute zcontval=contvec(:,2).
do if ((ncontr <> 2) or (ncontrow <> 2)).
compute notecode(notes,1) = 19.
compute notes = notes + 1.
compute modcok=0.
end if.
end if.
end if.
do if (xmint = 1 and contrast(1,1) <> 0).
compute contrast=0.
compute notecode(notes,1) = 37.
compute notes = notes + 1.
end if.
do if (varorder = 1).
compute notecode(notes,1) = 21.
compute notes = notes + 1.
end if.
do if ( 999 <> 999 or 999 <> 999).
compute notecode(notes,1) = 22.
compute notes = notes + 1.
end if.
compute modelbt=( 0 =1).
compute cluster= 'xxxxx'.
compute matrices=( 0 =1).
compute covcoeff=( 0 =1).
compute covmy=trunc( 0 ).
do if (covmy < 0 or covmy > 2).
compute covmy = 0.
end if.
compute boot = abs(trunc( 5000 )).
compute bc=( 0 =1).
compute mc=abs(trunc( 0 )).
compute hc=trunc( 5 ).
do if (intprobe < 0 or intprobe > 1).
compute intprobe = .10.
end if.
compute plot=trunc( 0 ).
do if (plot < 0 or plot > 2).
compute plot=0.
end if.
compute total=( 1 =1).
compute dototal=0.
compute saveboot = ( 0 = 1).
compute saveest=( 0 = 2).
do if (saveest=1).
compute intprobe=1.
end if.
do if (hc >= 0 and hc < 5).
compute notecode(notes,1) = 4.
compute notes = notes + 1.
end if.
do if (hc > 5 or hc < 0).
compute hc=5.
end if.
compute mcw=trunc( 0 ).
compute mcz=trunc( 0 ).
compute mcx=trunc( 0 ).
do if (mcx > 0 and mcx < 3 and model = 74).
compute mcw=mcx.
compute xscaling=1.
end if.
do if (mcx > 2 and model = 74).
compute errcode(errs,1)=65.
compute errs=errs+1.
compute criterr=1.
end if.
do if (model = 74 and normal=1).
compute notecode(notes,1) = 33.
compute normal=0.
compute notes = notes + 1.
end if.
do if (mcx > 0 and contrast > 0).
compute notecode(notes,1) = 28.
compute notes = notes + 1.
compute contrast=0.
end if.
compute nxvls=1.
compute nmvls=1.
compute nwvls=1.
compute nzvls=1.
compute paths=999.
compute pathsw=999.
compute pathsz=999.
compute pathswz=999.
compute pathsmod=999.
compute pathtype=999.
compute obscoeff=999.
compute pathsdv={' '}.
compute quantile=1.
do if ( 999 <>999).
compute notecode(notes,1) = 23.
compute notes = notes + 1.
end if.
compute moments=( 0 =1).
do if (moments=1).
compute quantile=0.
end if.
compute bmatrix={ -999 }.
compute wmatrix={ -999 }.
compute zmatrix={ -999 }.
compute wzmatrix={ -999 }.
compute cmatrix={ -999 }.
compute xcatcode={ -999 }.
compute wcatcode={ -999 }.
compute zcatcode={ -999 }.
compute needed=0.
compute conf= 95.
do if (trunc( 95 ) >= 100 or (trunc( 95 ) <= 50)).
compute conf = 95.
compute notecode(notes,1)=2.
compute notes=notes+1.
end if.
do if (model >= 0 and model < 4 and modelbt=0).
compute boot=0.
compute mc=0.
compute bc=0.
end if.
do if (mc > 0 and boot > 0).
compute boot=0.
compute bc=0.
end if.
do if ((boot < 1000) and (mc = 0) and (boot > 0)).
compute boot=5000.
end if.
do if ((mc < 1000) and (boot = 0) and (mc > 0)).
compute mc=5000.
end if.
compute p0=-.322232431088.
compute p1 = -1.
compute p2 = -.342242088547.
compute p3 = -.0204231210245.
compute p4 = -.0000453642210148.
compute q0 = .0993484626060.
compute q1 = .588581570495.
compute q2 = .531103462366.
compute q3 = .103537752850.
compute q4 = .0038560700634.
compute badend=0.
compute priorlo = -9999999.
compute priorhi = 9999999.
compute alpha2 = (1-(conf/100))/2.
compute cilm=alpha2*2.
compute y5=sqrt(-2*ln(alpha2)).
compute xp2=(y5+((((y5*p4+p3)*y5+p2)*y5+p1)*y5+p0)/((((y5*q4+q3)*y5+q2)*y5+q1)*y5+q0)).
compute medlb={'   M1  :';'   M2  :';'   M3  :';'   M4  :';'   M5  :';'   M6  :';'   M7  :';'   M8  :';'   M9  :';'   M10 :'}.
compute medlb2={'(M1)','(M2)','(M3)','(M4)','(M5)','(M6)','(M7)','(M8)','(M9)','(M10)'}.
compute xlb={'   X1  :';'   X2  :';'   X3  :';'   X4  :';'   X5  :';'   X6  :';'   X7  :';'   X8  :';'   X9  :'}.
compute highlbw={'M1*W'; 'M2*W'; 'M3*W'; 'M4*W'; 'M5*W'; 'M6*W'; 'M7*W'; 'M8*W'; 'M9*W'; 'M10*W'}.
do if (xmint=1).
compute highlbw={'M1*X'; 'M2*X'; 'M3*X'; 'M4*X'; 'M5*X'; 'M6*X'; 'M7*X'; 'M8*X'; 'M9*X'; 'M10*X'}.
end if.
compute highlbz={'M1*Z'; 'M2*Z'; 'M3*Z'; 'M4*Z'; 'M5*Z'; 'M6*Z'; 'M7*Z'; 'M8*Z'; 'M9*Z';'M10*Z'}.
compute highlbwz={'M1*W*Z'; 'M2*W*Z'; 'M3*W*Z'; 'M4*W*Z'; 'M5*W*Z'; 'M6*W*Z'; 'M7*W*Z'; 'M8*W*Z'; 'M9*W*Z';'M10*W*Z'}.
compute highlbbt={'BOTH(M1)'; 'BOTH(M2)'; 'BOTH(M3)'; 'BOTH(M4)'; 'BOTH(M5)'; 'BOTH(M6)'; 'BOTH(M7)'; 'BOTH(M8)'; 'BOTH(M9)';'BTH(M10)'}.
compute highlbx={'M1*X'; 'M2*X'; 'M3*X'; 'M4*X'; 'M5*X'; 'M6*X'; 'M7*X'; 'M8*X'; 'M9*X'; 'M10*X'}.
compute skipwz=0.
compute validm={1,1,1,1,1,1,1,1,1,1,1,1,1,1,1,1,1,1,1,1,1,1,0,0,0,0,0,1,1,0,0,0,0, 0,0,0,0,0,0,0,0,0,0,0,0,0,0,0,0,0,0,0,0,0,0,0,0,1,1,1,1,1,1,1,1,1,1,1,1,1,1,1,1,1,1,1,0,0,0,1,1, 1,1,1,1,1,1,1,1,1,1,1}.
do if (activate=1).
compute errcode(errs,1)=60.
compute errs=errs+1.
compute criterr=1.
end if.
do if (criterr=0).
do if (( 0 =1)=1).
compute errcode(errs,1)=42.
compute errs=errs+1.
compute criterr=1.
end if.
do if (model > 0 and model < 93).
do if (validm(1,model)=0).
compute errcode(errs,1)=6.
compute errs=errs+1.
compute criterr=1.
end if.
release validm.
end if.
do if ((model > 92 or model < 0) and model <> 999)).
compute errcode(errs,1)=7.
compute errs=errs+1.
compute criterr=1.
end if.
do if (model = 999 and bmatrix(1,1)=-999).
compute errcode(errs,1)=24.
compute errs=errs+1.
compute criterr=1.
end if.
do if (model <> 999 and bmatrix(1,1) <> -999).
compute errcode(errs,1)=25.
compute errs=errs+1.
compute criterr=1.
end if.
do if ((model = 74 or (model > 0 and model < 4)) and ((wmatrix(1,1) <> -999) or (zmatrix(1,1)<>-999) or (wzmatrix(1,1)<>-999)))).
compute errcode(errs,1)=41.
compute errs=errs+1.
compute criterr=1.
end if.
do if (hc3 <> 0).
compute notecode(notes,1) = 5.
compute notes = notes + 1.
do if (hc3 = 1).
compute hc=3.
end if.
end if.
do if ((v <> 'xxxxx') or (q <> 'xxxxx')).
compute errcode(errs,1)=14.
compute errs=errs+1.
compute errcode(errs,1)=48.
compute errs=errs+1.
compute criterr=1.
end if.
do if (oldvars <> 'xxxxx').
compute errcode(errs,1)=48.
compute errs=errs+1.
compute criterr=1.
end if.
do if (cluster <> 'xxxxx').
compute errcode(errs,1)=27.
compute errs=errs+1.
compute criterr=1.
end if.
do if ((y = 'xxxxx') or (x = 'xxxxx')).
compute errcode(errs,1)=1.
compute errs=errs+1.
compute criterr=1.
end if.
do if ((m = 'xxxxx') and model > 3).
compute errcode(errs,1)=8.
compute errs=errs+1.
compute criterr=1.
end if.
end if.
do if (criterr=0).
get ytmp/variables = HOMAIR /names = ynames/MISSING = 99999.
compute nys=ncol(ytmp).
compute needed=nys.
compute n=nrow(ytmp).
compute varnames={ynames}.
compute dat=ytmp.

.


do if (toomany=1 and longname=0).
compute criterr=1.
do if (tooman=0).
compute tooman=1.
compute errcode(errs,1) = 61.
compute errs = errs + 1.
end if.
end if
.
compute modelvar={ '4' ;t(ynames)}.
do if ( 4 =999).
compute modelvar(1,1)='CUSTOM'.
end if.
get xtmp/variables = FGDS /names = xnames/MISSING = 99999.
compute nxs=ncol(xtmp).
compute n=nrow(xtmp).
compute needed=needed+nxs.
compute varnames={varnames,xnames}.
compute xcatlab=t(xnames).
compute dat={dat,xtmp}.

.


do if (toomany=1 and longname=0).
compute criterr=1.
do if (tooman=0).
compute tooman=1.
compute errcode(errs,1) = 61.
compute errs = errs + 1.
end if.
end if
.
compute modelvar={modelvar;t(xnames)}.
do if (nxs = 1).
compute modelvlb={'Model  :';'    Y  :';'    X  :'}.
else.
compute modelvlb={'Model  :';'    Y  :';xlb(1:nxs,1)}.
end if.
do if (m <> 'xxxxx').
get mtmp/variables = TG HDL LDL TC /names = mnames/MISSING = 99999.
compute nms=ncol(mtmp).
compute mprod=make(1,nms,0).
compute n=nrow(mtmp).
compute needed=needed+nms.
compute varnames={varnames,mnames}.
compute dat={dat,mtmp}.
compute modelvar={modelvar;t(mnames)}.
compute x2m=make(99,nms,0).
compute m2y=make(99,nms,0).
compute onem=make(nms,1,1).

.


do if (toomany=1 and longname=0).
compute criterr=1.
do if (tooman=0).
compute tooman=1.
compute errcode(errs,1) = 61.
compute errs = errs + 1.
end if.
end if
.
do if (nms > 1 and nms < 11).
compute modelvlb={modelvlb;medlb(1:nms,1)}.
else.
compute modelvlb={modelvlb;'    M  :'}.
end if.
do if (nms > 0 and model < 4).
compute errcode(errs,1)=9.
compute errs=errs+1.
do if (model <> 0).
compute errcode(errs,1)=48.
compute errs=errs+1.
end if.
compute criterr=1.
end if.
end if.
compute wlocatet=0.
compute wlocate=0.
do if (w <> 'xxxxx').
do if (xmint=0).
get wtmp/variables = xxxxx /names = wnames/MISSING = 99999.

.


do if (toomany=1 and longname=0).
compute criterr=1.
do if (tooman=0).
compute tooman=1.
compute errcode(errs,1) = 61.
compute errs = errs + 1.
end if.
end if
.
end if.
do if (xmint=1).
get wtmp/variables = FGDS /names = wnames/MISSING = 99999.
end if.
compute nws=ncol(wtmp).
compute n=nrow(wtmp).
compute varnames={varnames,wnames}.
compute wlocate=ncol(varnames).
do if (model=74).
compute wlocatet=1.
do if (xnames <> wnames).
compute errcode(errs,1)=45.
compute errs=errs+1.
compute criterr=1.
end if.
end if.
compute wcatlab=t(wnames).
compute dat={dat,wtmp}.
do if (xmint <> 1).
compute modelvar={modelvar;t(wnames)}.
compute modelvlb={modelvlb;'    W  :'}.
end if.
end if.
do if (z <> 'xxxxx').
get ztmp/variables = xxxxx /names = znames/MISSING = 99999.
compute nzs=ncol(ztmp).
compute n=nrow(ztmp).

.


do if (toomany=1 and longname=0).
compute criterr=1.
do if (tooman=0).
compute tooman=1.
compute errcode(errs,1) = 61.
compute errs = errs + 1.
end if.
end if
.
compute varnames={varnames,znames}.
compute zcatlab=t(znames).
compute dat={dat,ztmp}.
compute modelvar={modelvar;t(znames)}.
compute modelvlb={modelvlb;'    Z  :'}.
end if.
do if (cov <> 'xxxxx').
get ctmp/variables = xxxxx /names = covnames/MISSING = 99999.
compute ncs=ncol(ctmp).
compute n=nrow(ctmp).

.


do if (toomany=1 and longname=0).
compute criterr=1.
do if (tooman=0).
compute tooman=1.
compute errcode(errs,1) = 61.
compute errs = errs + 1.
end if.
end if
.
compute varnames={varnames,covnames}.
compute dat={dat,ctmp}.
end if.
do if (nws > 1 or nzs > 1 or nys > 1 or nxs > 1).
compute errcode(errs,1)=3.
compute errs=errs+1.
compute criterr=1.
end if.
do if ((model = 80 or model = 81) and (nms < 3 or nms > 6)).
compute errcode(errs,1)=32.
compute errs=errs+1.
compute criterr=1.
end if.
do if (model = 82 and nms <> 4).
compute errcode(errs,1)=33.
compute errs=errs+1.
compute criterr=1.
end if.
do if (nms > 10).
compute errcode(errs,1)=37.
compute errs=errs+1.
compute criterr=1.
end if.
do if ((model = 6 or (model > 82 and model < 999)) and (nms < 2 or nms > 6)).
compute errcode(errs,1)=34.
compute errs=errs+1.
compute criterr=1.
end if.
compute match=0.
compute match2=0.
compute mcwzcov=0.
loop i = 1 to (ncol(varnames)-1).
loop j = (i+1) to ncol(varnames).
do if (varnames(i)=varnames(j)).
do if (i < (nxs+nms+nys+1)).
compute match2=match2+1.
end if.
do if (wlocatet=1 and i=2 and j=wlocate).
compute match2=match2-1.
end if.
do if ((wnames=znames) and (nws > 0 or nzs > 0))).
compute match2=match2+1.
end if.
do if (i < (ncol(varnames)-ncs+1)) and j > (ncol(varnames)-ncs)).
do if ((varnames(j)=wnames) and mcw=0)).
compute match=0.
compute wiscov=(j-(ncol(varnames)-ncs)).
end if.
do if ((varnames(j)=wnames) and mcw <>0)).
compute mcwzcov=1.
end if.
do if ((varnames(j)=znames) and mcz=0).
compute match=0.
compute ziscov=(j-(ncol(varnames)-ncs)).
end if.
do if ((varnames(j)=znames) and mcz<>0)).
compute mcwzcov=1.
end if.
end if.
end if.
end loop.
end loop.
do if (match2>0 or match=1).
compute errcode(errs,1)=2.
compute errs=errs+1.
compute criterr=1.
end if.
do if (mcwzcov=1).
compute errcode(errs,1)=50.
compute errs=errs+1.
compute criterr=1.
end if.
compute ninit=nrow(dat).
compute rownum=make(ninit,1,0).
loop i = 1 to ninit.
compute rownum(i,1)=i.
end loop.
compute dat={rownum,dat}.
compute j=1.
compute missrow=0.
loop i = 1 to n.
do if (rsum(dat(i,2:ncol(dat))=99999)=0).
compute dat(j,:)=dat(i,:).
compute j=j+1.
else.
compute missrow={missrow;dat(i,1)}.
end if.
end loop.
do if (j < 5).
compute errcode(errs,1)=62.
compute errs=errs+1.
compute criterr=1.
end if.
do if (criterr=0).
compute rownum=dat(1:(j-1),1).
do if (nrow(missrow) > 1).
compute missrow=t(missrow(2:nrow(missrow),1)).
compute notecode(notes,1) = 29.
compute notes = notes + 1.
end if.
compute dat=dat(1:(j-1),2:ncol(dat)).
compute n=nrow(dat).
compute nmiss=ninit-n.
compute ytmp=dat(:,1:nys).

.
compute desctmp=make((8-(4* 0 )),ncol( ytmp ),-999).
loop jd=1 to ncol( ytmp ).
compute descdat= ytmp (:,jd).
compute desctmp(1,jd) = csum(descdat)/nrow(descdat).
compute desctmp(2,jd) = (nrow(descdat)*sscp(descdat))-(t(csum(descdat))*(csum(descdat))).
compute desctmp(2,jd) = sqrt(desctmp(2,jd)/(nrow(descdat)*(nrow(descdat)-1))).
compute desctmp(3,jd)=cmin(descdat).
compute desctmp(4,jd)=cmax(descdat).
do if ( 0 =0).
compute minwarn=0.
compute maxwarn=0.
do if ((desctmp(3,jd)=desctmp(4,jd)) and novar=0).
compute errcode(errs,1)=15.
compute errs=errs+1.
compute criterr=1.
compute novar=1.
end if.
compute tmp=((descdat(:,1)=desctmp(3,jd))+(descdat(:,1)=desctmp(4,jd))).
compute desctmp(8,jd)=(csum(tmp)=nrow(tmp)).
compute tmp = descdat.
compute tmp(GRADE(descdat),:) = descdat.
compute descdat = tmp.
release tmp.
compute decval={.16;.5;.84}.
loop kd=1 to 3.
compute low=trunc(decval(kd,1)*(nrow(descdat)+1)).
compute lowdec=decval(kd,1)*(nrow(descdat)+1)-low.
compute value=descdat(low,1)+(descdat((low+1),1)-descdat(low,1))*lowdec.
compute desctmp((4+kd),jd)=value.
end loop.
compute mnotev=1.
compute modvals=desctmp(5:7,:).
do if (quantile <> 1).
compute desctmp(5,jd)=desctmp(1,jd)-desctmp(2,jd).
compute desctmp(6,jd)=desctmp(1,jd).
compute desctmp(7,jd)=desctmp(1,jd)+desctmp(2,jd).
compute modvals=desctmp(5:7,:).
compute mnotev=2.
do if (modvals(1,1) < desctmp(3,1)).
compute modvals(1,1)=desctmp(3,1).
compute minwarn=1.
end if.
do if (modvals(3,1) > desctmp(4,1)).
compute modvals(3,1)=desctmp(4,1).
compute maxwarn=1.
end if.
end if.
do if (desctmp(8,1)=1).
compute modvals={desctmp(3,1);desctmp(4,1)}.
compute mnotev=0.
compute minwarn=0.
compute maxwarn=0.
end if.
end if.
end loop
.
compute ysd=desctmp(2,:).
compute ovsd=ysd.
do if (desctmp(8,1)=1).
compute ydich=1.
do if (total=1).
compute total=0.
compute notecode(notes,1) = 24.
compute notes = notes + 1.
end if.
do if (effsize=1).
compute effsize=0.
compute notecode(notes,1) = 25.
compute notes = notes + 1.
end if.
do if (model=74).
compute errcode(errs,1) = 72.
compute errs=errs+1.
compute criterr=1.
end if.
compute omx = cmax(ytmp).
compute omn = cmin(ytmp).
compute ytmp = (ytmp = omx).
compute dat(:,1:nys)=(dat(:,1:nys)=omx).
compute rcd = {omn, 0; omx, 1}.
end if.
compute xtmp=dat(:,(nys+1):(nys+nxs)).

.
compute desctmp=make((8-(4* 0 )),ncol( xtmp ),-999).
loop jd=1 to ncol( xtmp ).
compute descdat= xtmp (:,jd).
compute desctmp(1,jd) = csum(descdat)/nrow(descdat).
compute desctmp(2,jd) = (nrow(descdat)*sscp(descdat))-(t(csum(descdat))*(csum(descdat))).
compute desctmp(2,jd) = sqrt(desctmp(2,jd)/(nrow(descdat)*(nrow(descdat)-1))).
compute desctmp(3,jd)=cmin(descdat).
compute desctmp(4,jd)=cmax(descdat).
do if ( 0 =0).
compute minwarn=0.
compute maxwarn=0.
do if ((desctmp(3,jd)=desctmp(4,jd)) and novar=0).
compute errcode(errs,1)=15.
compute errs=errs+1.
compute criterr=1.
compute novar=1.
end if.
compute tmp=((descdat(:,1)=desctmp(3,jd))+(descdat(:,1)=desctmp(4,jd))).
compute desctmp(8,jd)=(csum(tmp)=nrow(tmp)).
compute tmp = descdat.
compute tmp(GRADE(descdat),:) = descdat.
compute descdat = tmp.
release tmp.
compute decval={.16;.5;.84}.
loop kd=1 to 3.
compute low=trunc(decval(kd,1)*(nrow(descdat)+1)).
compute lowdec=decval(kd,1)*(nrow(descdat)+1)-low.
compute value=descdat(low,1)+(descdat((low+1),1)-descdat(low,1))*lowdec.
compute desctmp((4+kd),jd)=value.
end loop.
compute mnotev=1.
compute modvals=desctmp(5:7,:).
do if (quantile <> 1).
compute desctmp(5,jd)=desctmp(1,jd)-desctmp(2,jd).
compute desctmp(6,jd)=desctmp(1,jd).
compute desctmp(7,jd)=desctmp(1,jd)+desctmp(2,jd).
compute modvals=desctmp(5:7,:).
compute mnotev=2.
do if (modvals(1,1) < desctmp(3,1)).
compute modvals(1,1)=desctmp(3,1).
compute minwarn=1.
end if.
do if (modvals(3,1) > desctmp(4,1)).
compute modvals(3,1)=desctmp(4,1).
compute maxwarn=1.
end if.
end if.
do if (desctmp(8,1)=1).
compute modvals={desctmp(3,1);desctmp(4,1)}.
compute mnotev=0.
compute minwarn=0.
compute maxwarn=0.
end if.
end if.
end loop
.
compute xsd=desctmp(2,:).
compute xmodvals=modvals.
compute xdich=desctmp(8,1).
compute xmx=cmax(xtmp).
compute xmn=cmin(xtmp).
do if ((mcx > 0) and (xrefvals(1,1) <> 999) and (xmint=1) and (model= 74)).
compute notecode(notes,1) = 36.
compute notes = notes + 1.
end if.
do if (mcx=0).
do if (ncol(xrefvals)>2 and model=74 and xmint=1).
compute errcode(errs,1)=67.
compute errs=errs+1.
compute criterr=1.
end if.
do if ((model=74) and (xmint=1)).
do if ((xrefvals(1,1)=999) and nxvls=1 and xdich=0).
compute errcode(errs,1)=66.
compute errs=errs+1.
compute criterr=1.
end if.
do if ((xrefvals(1,1)=999) and (xdich=1)).
compute xrefvals={xmn,xmx}.
compute xscaling=xrefvals(1,2)-xrefvals(1,1).
end if.
do if (ncol(xrefvals)=1 and xrefvals(1,1) <> 999).
do if (xdich=0).
compute xrefvals={xrefvals,(xrefvals(1,1)+1)}.
compute xscaling=xrefvals(1,2)-xrefvals(1,1).
end if.
do if (xdich=1).
do if ((xrefvals(1,1) <> xmx) and (xrefvals(1,1) <> xmn)).
compute errcode(errs,1)=70.
compute errs=errs+1.
compute criterr=1.
end if.
do if (xrefvals(1,1) = xmx).
compute xrefvals={xrefvals,xmn}.
compute xscaling=xrefvals(1,2)-xrefvals(1,1).
end if.
do if (xrefvals(1,1) = xmn).
compute xrefvals={xrefvals,xmx}.
compute xscaling=xrefvals(1,2)-xrefvals(1,1).
end if.
end if.
end if.
do if (ncol(xrefvals)=2).
compute xscaling=xrefvals(1,2)-xrefvals(1,1).
do if (xdich=1).
compute xreferr=1.
do if (((xrefvals(1,1) = xmx) and (xrefvals(1,2)=xmn)) or ((xrefvals(1,1) = xmn) and (xrefvals(1,2)=xmx))).
compute xreferr=0.
end if.
do if (xreferr=1).
compute errcode(errs,1)=70.
compute errs=errs+1.
compute criterr=1.
end if.
end if.
end if.
end if.
end if.
do if (xmint=1 and model=74 and mcx=0).
compute xmodvals=t(xrefvals).
compute xcontcf=1.
end if.
compute nxpval=nrow(xmodvals).
compute xprobval=xmodvals.
do if (xdich =1 and mcx > 0).
compute mcx=0.
compute errcode(errs,1) = 52.
compute errs = errs + 1.
compute criterr = 1.
end if.
do if (nms > 0).
compute mtmp=dat(:,(nys+nxs+1):(nys+nxs+nms)).

.
compute desctmp=make((8-(4* 0 )),ncol( mtmp ),-999).
loop jd=1 to ncol( mtmp ).
compute descdat= mtmp (:,jd).
compute desctmp(1,jd) = csum(descdat)/nrow(descdat).
compute desctmp(2,jd) = (nrow(descdat)*sscp(descdat))-(t(csum(descdat))*(csum(descdat))).
compute desctmp(2,jd) = sqrt(desctmp(2,jd)/(nrow(descdat)*(nrow(descdat)-1))).
compute desctmp(3,jd)=cmin(descdat).
compute desctmp(4,jd)=cmax(descdat).
do if ( 0 =0).
compute minwarn=0.
compute maxwarn=0.
do if ((desctmp(3,jd)=desctmp(4,jd)) and novar=0).
compute errcode(errs,1)=15.
compute errs=errs+1.
compute criterr=1.
compute novar=1.
end if.
compute tmp=((descdat(:,1)=desctmp(3,jd))+(descdat(:,1)=desctmp(4,jd))).
compute desctmp(8,jd)=(csum(tmp)=nrow(tmp)).
compute tmp = descdat.
compute tmp(GRADE(descdat),:) = descdat.
compute descdat = tmp.
release tmp.
compute decval={.16;.5;.84}.
loop kd=1 to 3.
compute low=trunc(decval(kd,1)*(nrow(descdat)+1)).
compute lowdec=decval(kd,1)*(nrow(descdat)+1)-low.
compute value=descdat(low,1)+(descdat((low+1),1)-descdat(low,1))*lowdec.
compute desctmp((4+kd),jd)=value.
end loop.
compute mnotev=1.
compute modvals=desctmp(5:7,:).
do if (quantile <> 1).
compute desctmp(5,jd)=desctmp(1,jd)-desctmp(2,jd).
compute desctmp(6,jd)=desctmp(1,jd).
compute desctmp(7,jd)=desctmp(1,jd)+desctmp(2,jd).
compute modvals=desctmp(5:7,:).
compute mnotev=2.
do if (modvals(1,1) < desctmp(3,1)).
compute modvals(1,1)=desctmp(3,1).
compute minwarn=1.
end if.
do if (modvals(3,1) > desctmp(4,1)).
compute modvals(3,1)=desctmp(4,1).
compute maxwarn=1.
end if.
end if.
do if (desctmp(8,1)=1).
compute modvals={desctmp(3,1);desctmp(4,1)}.
compute mnotev=0.
compute minwarn=0.
compute maxwarn=0.
end if.
end if.
end loop
.
compute ovsd={desctmp(2,:),ysd}.
compute medmeans=cdeval.
do if ((cdeval(1,1) <> -999) and (ncol(medmeans) <> nms) and (model=74)).
compute errcode(errs,1)=64.
compute errs=errs+1.
compute criterr=1.
end if.
do if ((cdeval(1,1)=-999) and (model=74)).
compute medmeans=desctmp(1,:).
end if.
do if ((cdeval(1,1)<> -999) and (model=74) and (criterr=0)).
compute notecode(notes,1) = 31.
compute notes = notes + 1.
end if.
do if ((rsum(desctmp(8,:))>0) and (mdichok <> 1)).
compute errcode(errs,1)=43.
compute errs=errs+1.
compute criterr=1.
end if.
compute mmodvals=modvals.
compute mprobval=mmodvals.
end if.
do if (nws > 0).
compute wtmp=dat(:,(nys+nxs+nms+1):(nys+nxs+nms+nws)).

.
compute desctmp=make((8-(4* 0 )),ncol( wtmp ),-999).
loop jd=1 to ncol( wtmp ).
compute descdat= wtmp (:,jd).
compute desctmp(1,jd) = csum(descdat)/nrow(descdat).
compute desctmp(2,jd) = (nrow(descdat)*sscp(descdat))-(t(csum(descdat))*(csum(descdat))).
compute desctmp(2,jd) = sqrt(desctmp(2,jd)/(nrow(descdat)*(nrow(descdat)-1))).
compute desctmp(3,jd)=cmin(descdat).
compute desctmp(4,jd)=cmax(descdat).
do if ( 0 =0).
compute minwarn=0.
compute maxwarn=0.
do if ((desctmp(3,jd)=desctmp(4,jd)) and novar=0).
compute errcode(errs,1)=15.
compute errs=errs+1.
compute criterr=1.
compute novar=1.
end if.
compute tmp=((descdat(:,1)=desctmp(3,jd))+(descdat(:,1)=desctmp(4,jd))).
compute desctmp(8,jd)=(csum(tmp)=nrow(tmp)).
compute tmp = descdat.
compute tmp(GRADE(descdat),:) = descdat.
compute descdat = tmp.
release tmp.
compute decval={.16;.5;.84}.
loop kd=1 to 3.
compute low=trunc(decval(kd,1)*(nrow(descdat)+1)).
compute lowdec=decval(kd,1)*(nrow(descdat)+1)-low.
compute value=descdat(low,1)+(descdat((low+1),1)-descdat(low,1))*lowdec.
compute desctmp((4+kd),jd)=value.
end loop.
compute mnotev=1.
compute modvals=desctmp(5:7,:).
do if (quantile <> 1).
compute desctmp(5,jd)=desctmp(1,jd)-desctmp(2,jd).
compute desctmp(6,jd)=desctmp(1,jd).
compute desctmp(7,jd)=desctmp(1,jd)+desctmp(2,jd).
compute modvals=desctmp(5:7,:).
compute mnotev=2.
do if (modvals(1,1) < desctmp(3,1)).
compute modvals(1,1)=desctmp(3,1).
compute minwarn=1.
end if.
do if (modvals(3,1) > desctmp(4,1)).
compute modvals(3,1)=desctmp(4,1).
compute maxwarn=1.
end if.
end if.
do if (desctmp(8,1)=1).
compute modvals={desctmp(3,1);desctmp(4,1)}.
compute mnotev=0.
compute minwarn=0.
compute maxwarn=0.
end if.
end if.
end loop
.
compute wmodvals=modvals.
compute wdich=desctmp(8,1).
do if (wdich =1 and mcw > 0).
compute mcw=0.
compute errcode(errs,1) = 52.
compute errs = errs + 1.
compute criterr = 1.
end if.
compute wmin=desctmp(3,1).
compute wmax=desctmp(4,1).
compute minwwarn=minwarn.
compute maxwwarn=maxwarn.
compute wnotev=mnotev.
compute wmodval={ 999 }.
do if (xmint=1 and model=74 and mcx=0).
compute wmodval=xrefvals.
end if.
compute nwcontr=ncol(wmodval).
do if (wmodval(1,1) <> 999).
compute wmodvals=wmodval(1,1).
compute wmodcust=1.
do if (nwcontr > 1).
compute wmodvals=t(wmodval).
end if.
compute minwwarn=0.
compute maxwwarn=0.
compute wnotev=0.
end if.
compute wprobval=wmodvals.
compute nwpval=nrow(wmodvals).
end if.
do if (nzs > 0).
compute ztmp=dat(:,(nys+nxs+nms+nws+1):(nys+nxs+nms+nws+nzs)).

.
compute desctmp=make((8-(4* 0 )),ncol( ztmp ),-999).
loop jd=1 to ncol( ztmp ).
compute descdat= ztmp (:,jd).
compute desctmp(1,jd) = csum(descdat)/nrow(descdat).
compute desctmp(2,jd) = (nrow(descdat)*sscp(descdat))-(t(csum(descdat))*(csum(descdat))).
compute desctmp(2,jd) = sqrt(desctmp(2,jd)/(nrow(descdat)*(nrow(descdat)-1))).
compute desctmp(3,jd)=cmin(descdat).
compute desctmp(4,jd)=cmax(descdat).
do if ( 0 =0).
compute minwarn=0.
compute maxwarn=0.
do if ((desctmp(3,jd)=desctmp(4,jd)) and novar=0).
compute errcode(errs,1)=15.
compute errs=errs+1.
compute criterr=1.
compute novar=1.
end if.
compute tmp=((descdat(:,1)=desctmp(3,jd))+(descdat(:,1)=desctmp(4,jd))).
compute desctmp(8,jd)=(csum(tmp)=nrow(tmp)).
compute tmp = descdat.
compute tmp(GRADE(descdat),:) = descdat.
compute descdat = tmp.
release tmp.
compute decval={.16;.5;.84}.
loop kd=1 to 3.
compute low=trunc(decval(kd,1)*(nrow(descdat)+1)).
compute lowdec=decval(kd,1)*(nrow(descdat)+1)-low.
compute value=descdat(low,1)+(descdat((low+1),1)-descdat(low,1))*lowdec.
compute desctmp((4+kd),jd)=value.
end loop.
compute mnotev=1.
compute modvals=desctmp(5:7,:).
do if (quantile <> 1).
compute desctmp(5,jd)=desctmp(1,jd)-desctmp(2,jd).
compute desctmp(6,jd)=desctmp(1,jd).
compute desctmp(7,jd)=desctmp(1,jd)+desctmp(2,jd).
compute modvals=desctmp(5:7,:).
compute mnotev=2.
do if (modvals(1,1) < desctmp(3,1)).
compute modvals(1,1)=desctmp(3,1).
compute minwarn=1.
end if.
do if (modvals(3,1) > desctmp(4,1)).
compute modvals(3,1)=desctmp(4,1).
compute maxwarn=1.
end if.
end if.
do if (desctmp(8,1)=1).
compute modvals={desctmp(3,1);desctmp(4,1)}.
compute mnotev=0.
compute minwarn=0.
compute maxwarn=0.
end if.
end if.
end loop
.
compute zmodvals=modvals.
compute zdich=desctmp(8,1).
do if (zdich =1 and mcz > 0).
compute mcz=0.
compute errcode(errs,1) = 52.
compute errs = errs + 1.
compute criterr = 1.
end if.
compute zmin=desctmp(3,1).
compute zmax=desctmp(4,1).
compute minzwarn=minwarn.
compute maxzwarn=maxwarn.
compute znotev=mnotev.
compute zmodval={ 999 }.
compute nzcontr=ncol(zmodval).
do if (zmodval(1,1) <> 999).
compute zmodvals=zmodval(1,1).
compute zmodcust=1.
do if (nzcontr > 1).
compute zmodvals=t(zmodval).
end if.
compute minzwarn=0.
compute maxzwarn=0.
compute znotev=0.
end if.
compute zprobval=zmodvals.
compute nzpval=nrow(zmodvals).
end if.
do if (ncs > 0).
compute ctmp=dat(:,(nys+nxs+nms+nws+nzs+1):(nys+nxs+nms+nws+nzs+ncs)).

.
compute desctmp=make((8-(4* 0 )),ncol( ctmp ),-999).
loop jd=1 to ncol( ctmp ).
compute descdat= ctmp (:,jd).
compute desctmp(1,jd) = csum(descdat)/nrow(descdat).
compute desctmp(2,jd) = (nrow(descdat)*sscp(descdat))-(t(csum(descdat))*(csum(descdat))).
compute desctmp(2,jd) = sqrt(desctmp(2,jd)/(nrow(descdat)*(nrow(descdat)-1))).
compute desctmp(3,jd)=cmin(descdat).
compute desctmp(4,jd)=cmax(descdat).
do if ( 0 =0).
compute minwarn=0.
compute maxwarn=0.
do if ((desctmp(3,jd)=desctmp(4,jd)) and novar=0).
compute errcode(errs,1)=15.
compute errs=errs+1.
compute criterr=1.
compute novar=1.
end if.
compute tmp=((descdat(:,1)=desctmp(3,jd))+(descdat(:,1)=desctmp(4,jd))).
compute desctmp(8,jd)=(csum(tmp)=nrow(tmp)).
compute tmp = descdat.
compute tmp(GRADE(descdat),:) = descdat.
compute descdat = tmp.
release tmp.
compute decval={.16;.5;.84}.
loop kd=1 to 3.
compute low=trunc(decval(kd,1)*(nrow(descdat)+1)).
compute lowdec=decval(kd,1)*(nrow(descdat)+1)-low.
compute value=descdat(low,1)+(descdat((low+1),1)-descdat(low,1))*lowdec.
compute desctmp((4+kd),jd)=value.
end loop.
compute mnotev=1.
compute modvals=desctmp(5:7,:).
do if (quantile <> 1).
compute desctmp(5,jd)=desctmp(1,jd)-desctmp(2,jd).
compute desctmp(6,jd)=desctmp(1,jd).
compute desctmp(7,jd)=desctmp(1,jd)+desctmp(2,jd).
compute modvals=desctmp(5:7,:).
compute mnotev=2.
do if (modvals(1,1) < desctmp(3,1)).
compute modvals(1,1)=desctmp(3,1).
compute minwarn=1.
end if.
do if (modvals(3,1) > desctmp(4,1)).
compute modvals(3,1)=desctmp(4,1).
compute maxwarn=1.
end if.
end if.
do if (desctmp(8,1)=1).
compute modvals={desctmp(3,1);desctmp(4,1)}.
compute mnotev=0.
compute minwarn=0.
compute maxwarn=0.
end if.
end if.
end loop
.
compute covmeans=desctmp(1,:).
compute coval={ -999 }.
do if ((coval(1,1) <> -999) and (ncol(coval) <> ncs) and (model=74)).
compute errcode(errs,1)=69.
compute errs=errs+1.
compute criterr=1.
end if.
do if ((coval(1,1)<> -999) and (criterr=0) and (model=74)).
compute notecode(notes,1) = 35.
compute notes = notes + 1.
compute cuscoval=1.
end if.
end if.
compute n=nrow(ytmp).
compute ones=make(n,1,1).
do if (nws > 0 and mcw > 0).
compute tmp={rownum,wtmp(:,1)}.

.
compute dd= tmp.
compute temp = dd.
compute temp(GRADE(dd(:,2)),:) = dd.
compute dd = temp.
compute dummy = design(dd(:,2)).
compute nvls = ncol(dummy).
compute nnvls = csum(dummy).
compute mnvls = cmin(t(nnvls)).
compute conmat1=1.
do if (mnvls < 2).
compute errcode(errs,1) = 5.
compute errs = errs + 1.
compute criterr = 1.
end if.
do if (nvls > 9).
compute errcode(errs,1) = 4.
compute errs = errs+1.
compute criterr = 1.
end if.
do if (criterr = 0).
compute dumok = 1.
compute nnvls=make(nvls,1,0).
compute nnvls(1,1)=dd(1,2).
compute temp = 2.
loop i = 2 to n.
do if (dd(i,2) <> nnvls((temp-1),1)).
compute nnvls(temp,1)=dd(i,2).
compute temp = temp+1.
end if.
end loop.
do if ( mcw > 0).
compute x = dummy(:,2:ncol(dummy)).
compute nx = ncol(x).
compute minus1 = make(1,ncol(x),-1).
compute xdes=make((nx+1),3,0).
compute xdes(1,1)=dd(1,2).
compute xdes(1,2)=1.
compute temp = 2.
loop k = 2 to n.
do if (dd(k,2) <> dd((k-1),2)).
compute xdes(temp,2) = k.
compute xdes(temp,1) = dd(k,2).
compute xdes((temp-1),3) = k-1.
compute temp=temp+1.
end if.
end loop.
compute xdes((temp-1),3)=n.
compute xdes = {xdes, (xdes(:,3)-xdes(:,2)+1)}.
do if ( mcw = 4).
loop k = 1 to n.
do if (rsum(x(k,:)) = 0).
compute x(k,:) = minus1.
end if.
end loop.
end if.
do if ( mcw = 2 or mcw = 3 or mcw =5).
loop k = 1 to n.
do if (rsum(x(k,:)) > 0).
loop i = 1 to ncol(x).
do if (x(k,i) = 0).
compute x(k,i) = 1.
else.
break.
end if.
end loop.
end if.
end loop.
do if ( mcw = 3).
compute conmat1={-8,1,1,1,1,1,1,1,1; 0,-7,1,1,1,1,1,1,1; 0,0,-6,1,1,1,1,1,1; 0,0,0,-5,1,1,1,1,1; 0,0,0,0,-4,1,1,1,1; 0,0,0,0,0,-3,1,1,1; 0,0,0,0,0,0,-2,1,1; 0,0,0,0,0,0,0,-1,1}.
loop i = 1 to 8.
compute conmat1(i,:)=conmat1(i,:)/(10-i).
end loop.
compute conmat1=t(conmat1((10-nvls):8,(10-nvls):9)).
loop k=1 to n.
compute x(k,:)=conmat1((rsum(x(k,:))+1),:).
end loop.
end if.
end if.
do if ( mcw = 5).
compute custcode={ -999 }.
do if (ncol(custcode) <> (nvls*(nvls-1))).
compute errcode(errs,1) = (37+ 2 ).
compute errs = errs + 1.
compute criterr = 1.
end if.
do if (ncol(custcode) = (nvls*(nvls-1))).
compute conmat1=make(nvls,(nvls-1),0).
compute cnt=1.
loop i = 1 to nvls.
loop k = 1 to (nvls-1).
compute conmat1(i,k)=custcode(1,cnt).
compute cnt=cnt+1.
end loop.
end loop.
loop k=1 to n.
compute x(k,:)=conmat1((rsum(x(k,:))+1),:).
end loop.
end if.
end if.
compute xskip = 1.
compute dummat = make((nx+1),nx,0).
compute dummat((2:nrow(dummat)),:)=ident(nx).
do if ( mcw = 4).
compute dummat(1,:) = minus1.
end if.
do if ( mcw = 2).
loop i = 2 to nrow(dummat).
loop j = 1 to (i-1).
compute dummat(i,j) = 1.
end loop.
end loop.
end if.
do if ( mcw = 3).
compute dummat=conmat1.
end if.
do if ( mcw = 5 and criterr=0).
compute dummat=conmat1.
end if.
compute dummat={nnvls, dummat}.
compute x={dd(:,1),x}.
compute temp = x.
compute temp(GRADE(x(:,1)),:) = x.
compute x = temp.
release conmat1,temp,dd,xskip,xdes,dummy.
end if.
end if
.
compute wmodvals=nnvls.
compute nwpval=nrow(wmodvals).
do if (criterr=0).
compute minwwarn=0.
compute maxwwarn=0.
compute wnotev=0.
compute wtmp=x(:,2:ncol(x)).
compute wcatlab={'W1';'W2';'W3';'W4';'W5';'W6';'W7';'W8';'W9'}.
do if (xmint=1).
compute wcatlab={'X1';'X2';'X3';'X4';'X5';'X6';'X7';'X8';'X9'}.
end if.
compute nwvls=nvls-1.
compute mcwok=1.
compute dummatw=dummat.
compute wprobval=dummatw(:,2:ncol(dummatw)).
do if (modcok=1).
compute wcontval=make(2,ncol(wprobval),-999).
compute temp=0.
loop i = 1 to 2.
loop j = 1 to nrow(dummatw).
do if (contvec(i,1)=dummatw(j,1)).
compute wcontval(i,:)=wprobval(j,:).
compute temp=temp+1.
end if.
end loop.
end loop.
do if (temp < 2).
compute notecode(notes,1) = 20.
compute notes = notes + 1.
compute modcok=0.
end if.
end if.
do if ((wmodval(1,1) <> 999) and (xmint <> 1)).
compute notecode(notes,1) = 9.
compute notes = notes + 1.
end if.
release tmp, dummat.
end if.
end if.
do if (nzs > 0 and mcz > 0).
compute tmp={rownum,ztmp(:,1)}.

.
compute dd= tmp.
compute temp = dd.
compute temp(GRADE(dd(:,2)),:) = dd.
compute dd = temp.
compute dummy = design(dd(:,2)).
compute nvls = ncol(dummy).
compute nnvls = csum(dummy).
compute mnvls = cmin(t(nnvls)).
compute conmat1=1.
do if (mnvls < 2).
compute errcode(errs,1) = 5.
compute errs = errs + 1.
compute criterr = 1.
end if.
do if (nvls > 9).
compute errcode(errs,1) = 4.
compute errs = errs+1.
compute criterr = 1.
end if.
do if (criterr = 0).
compute dumok = 1.
compute nnvls=make(nvls,1,0).
compute nnvls(1,1)=dd(1,2).
compute temp = 2.
loop i = 2 to n.
do if (dd(i,2) <> nnvls((temp-1),1)).
compute nnvls(temp,1)=dd(i,2).
compute temp = temp+1.
end if.
end loop.
do if ( mcz > 0).
compute x = dummy(:,2:ncol(dummy)).
compute nx = ncol(x).
compute minus1 = make(1,ncol(x),-1).
compute xdes=make((nx+1),3,0).
compute xdes(1,1)=dd(1,2).
compute xdes(1,2)=1.
compute temp = 2.
loop k = 2 to n.
do if (dd(k,2) <> dd((k-1),2)).
compute xdes(temp,2) = k.
compute xdes(temp,1) = dd(k,2).
compute xdes((temp-1),3) = k-1.
compute temp=temp+1.
end if.
end loop.
compute xdes((temp-1),3)=n.
compute xdes = {xdes, (xdes(:,3)-xdes(:,2)+1)}.
do if ( mcz = 4).
loop k = 1 to n.
do if (rsum(x(k,:)) = 0).
compute x(k,:) = minus1.
end if.
end loop.
end if.
do if ( mcz = 2 or mcz = 3 or mcz =5).
loop k = 1 to n.
do if (rsum(x(k,:)) > 0).
loop i = 1 to ncol(x).
do if (x(k,i) = 0).
compute x(k,i) = 1.
else.
break.
end if.
end loop.
end if.
end loop.
do if ( mcz = 3).
compute conmat1={-8,1,1,1,1,1,1,1,1; 0,-7,1,1,1,1,1,1,1; 0,0,-6,1,1,1,1,1,1; 0,0,0,-5,1,1,1,1,1; 0,0,0,0,-4,1,1,1,1; 0,0,0,0,0,-3,1,1,1; 0,0,0,0,0,0,-2,1,1; 0,0,0,0,0,0,0,-1,1}.
loop i = 1 to 8.
compute conmat1(i,:)=conmat1(i,:)/(10-i).
end loop.
compute conmat1=t(conmat1((10-nvls):8,(10-nvls):9)).
loop k=1 to n.
compute x(k,:)=conmat1((rsum(x(k,:))+1),:).
end loop.
end if.
end if.
do if ( mcz = 5).
compute custcode={ -999 }.
do if (ncol(custcode) <> (nvls*(nvls-1))).
compute errcode(errs,1) = (37+ 3 ).
compute errs = errs + 1.
compute criterr = 1.
end if.
do if (ncol(custcode) = (nvls*(nvls-1))).
compute conmat1=make(nvls,(nvls-1),0).
compute cnt=1.
loop i = 1 to nvls.
loop k = 1 to (nvls-1).
compute conmat1(i,k)=custcode(1,cnt).
compute cnt=cnt+1.
end loop.
end loop.
loop k=1 to n.
compute x(k,:)=conmat1((rsum(x(k,:))+1),:).
end loop.
end if.
end if.
compute xskip = 1.
compute dummat = make((nx+1),nx,0).
compute dummat((2:nrow(dummat)),:)=ident(nx).
do if ( mcz = 4).
compute dummat(1,:) = minus1.
end if.
do if ( mcz = 2).
loop i = 2 to nrow(dummat).
loop j = 1 to (i-1).
compute dummat(i,j) = 1.
end loop.
end loop.
end if.
do if ( mcz = 3).
compute dummat=conmat1.
end if.
do if ( mcz = 5 and criterr=0).
compute dummat=conmat1.
end if.
compute dummat={nnvls, dummat}.
compute x={dd(:,1),x}.
compute temp = x.
compute temp(GRADE(x(:,1)),:) = x.
compute x = temp.
release conmat1,temp,dd,xskip,xdes,dummy.
end if.
end if
.
compute zmodvals=nnvls.
compute nzpval=nrow(zmodvals).
do if (criterr=0).
compute minzwarn=0.
compute maxzwarn=0.
compute znotev=0.
compute ztmp=x(:,2:ncol(x)).
compute zcatlab={'Z1';'Z2';'Z3';'Z4';'Z5';'Z6';'Z7';'Z8';'Z9'}.
compute nzvls=nvls-1.
compute mczok=1.
compute dummatz=dummat.
compute zprobval=dummatz(:,2:ncol(dummatz)).
do if (modcok=1).
compute zcontval=make(2,ncol(zprobval),-999).
compute temp=0.
loop i = 1 to 2.
loop j = 1 to nrow(dummatz).
do if (contvec(i,2)=dummatz(j,1)).
compute zcontval(i,:)=zprobval(j,:).
compute temp=temp+1.
end if.
end loop.
end loop.
do if (temp < 2).
compute notecode(notes,1) = 20.
compute notes = notes + 1.
compute modcok=0.
end if.
end if.
do if (zmodval(1,1) <> 999).
compute notecode(notes,1) = 10.
compute notes = notes + 1.
end if.
release tmp, dummat.
end if.
end if.
do if (nxs > 0 and mcx > 0).
compute tmp={rownum,xtmp(:,1)}.

.
compute dd= tmp.
compute temp = dd.
compute temp(GRADE(dd(:,2)),:) = dd.
compute dd = temp.
compute dummy = design(dd(:,2)).
compute nvls = ncol(dummy).
compute nnvls = csum(dummy).
compute mnvls = cmin(t(nnvls)).
compute conmat1=1.
do if (mnvls < 2).
compute errcode(errs,1) = 5.
compute errs = errs + 1.
compute criterr = 1.
end if.
do if (nvls > 9).
compute errcode(errs,1) = 4.
compute errs = errs+1.
compute criterr = 1.
end if.
do if (criterr = 0).
compute dumok = 1.
compute nnvls=make(nvls,1,0).
compute nnvls(1,1)=dd(1,2).
compute temp = 2.
loop i = 2 to n.
do if (dd(i,2) <> nnvls((temp-1),1)).
compute nnvls(temp,1)=dd(i,2).
compute temp = temp+1.
end if.
end loop.
do if ( mcx > 0).
compute x = dummy(:,2:ncol(dummy)).
compute nx = ncol(x).
compute minus1 = make(1,ncol(x),-1).
compute xdes=make((nx+1),3,0).
compute xdes(1,1)=dd(1,2).
compute xdes(1,2)=1.
compute temp = 2.
loop k = 2 to n.
do if (dd(k,2) <> dd((k-1),2)).
compute xdes(temp,2) = k.
compute xdes(temp,1) = dd(k,2).
compute xdes((temp-1),3) = k-1.
compute temp=temp+1.
end if.
end loop.
compute xdes((temp-1),3)=n.
compute xdes = {xdes, (xdes(:,3)-xdes(:,2)+1)}.
do if ( mcx = 4).
loop k = 1 to n.
do if (rsum(x(k,:)) = 0).
compute x(k,:) = minus1.
end if.
end loop.
end if.
do if ( mcx = 2 or mcx = 3 or mcx =5).
loop k = 1 to n.
do if (rsum(x(k,:)) > 0).
loop i = 1 to ncol(x).
do if (x(k,i) = 0).
compute x(k,i) = 1.
else.
break.
end if.
end loop.
end if.
end loop.
do if ( mcx = 3).
compute conmat1={-8,1,1,1,1,1,1,1,1; 0,-7,1,1,1,1,1,1,1; 0,0,-6,1,1,1,1,1,1; 0,0,0,-5,1,1,1,1,1; 0,0,0,0,-4,1,1,1,1; 0,0,0,0,0,-3,1,1,1; 0,0,0,0,0,0,-2,1,1; 0,0,0,0,0,0,0,-1,1}.
loop i = 1 to 8.
compute conmat1(i,:)=conmat1(i,:)/(10-i).
end loop.
compute conmat1=t(conmat1((10-nvls):8,(10-nvls):9)).
loop k=1 to n.
compute x(k,:)=conmat1((rsum(x(k,:))+1),:).
end loop.
end if.
end if.
do if ( mcx = 5).
compute custcode={ -999 }.
do if (ncol(custcode) <> (nvls*(nvls-1))).
compute errcode(errs,1) = (37+ 1 ).
compute errs = errs + 1.
compute criterr = 1.
end if.
do if (ncol(custcode) = (nvls*(nvls-1))).
compute conmat1=make(nvls,(nvls-1),0).
compute cnt=1.
loop i = 1 to nvls.
loop k = 1 to (nvls-1).
compute conmat1(i,k)=custcode(1,cnt).
compute cnt=cnt+1.
end loop.
end loop.
loop k=1 to n.
compute x(k,:)=conmat1((rsum(x(k,:))+1),:).
end loop.
end if.
end if.
compute xskip = 1.
compute dummat = make((nx+1),nx,0).
compute dummat((2:nrow(dummat)),:)=ident(nx).
do if ( mcx = 4).
compute dummat(1,:) = minus1.
end if.
do if ( mcx = 2).
loop i = 2 to nrow(dummat).
loop j = 1 to (i-1).
compute dummat(i,j) = 1.
end loop.
end loop.
end if.
do if ( mcx = 3).
compute dummat=conmat1.
end if.
do if ( mcx = 5 and criterr=0).
compute dummat=conmat1.
end if.
compute dummat={nnvls, dummat}.
compute x={dd(:,1),x}.
compute temp = x.
compute temp(GRADE(x(:,1)),:) = x.
compute x = temp.
release conmat1,temp,dd,xskip,xdes,dummy.
end if.
end if
.
do if (criterr=0).
compute xtmp=x(:,2:ncol(x)).
compute xcatlab={'X1';'X2';'X3';'X4';'X5';'X6';'X7';'X8';'X9'}.
compute nxvls=nvls-1.
compute xdich=(nvls=2).
compute mcxok=1.
compute dummatx=dummat.
compute xmodvals=dummatx(:,1).
compute nxpval=nrow(xmodvals).
release tmp, dummat.
end if.
end if.
compute intlab=make(100,1,' ').


compute intlab( 1 ,1)= 'Int_1'.


compute intlab( 2 ,1)= 'Int_2'.


compute intlab( 3 ,1)= 'Int_3'.


compute intlab( 4 ,1)= 'Int_4'.


compute intlab( 5 ,1)= 'Int_5'.


compute intlab( 6 ,1)= 'Int_6'.


compute intlab( 7 ,1)= 'Int_7'.


compute intlab( 8 ,1)= 'Int_8'.


compute intlab( 9 ,1)= 'Int_9'.


compute intlab( 10 ,1)= 'Int_10'.


compute intlab( 11 ,1)= 'Int_11'.


compute intlab( 12 ,1)= 'Int_12'.


compute intlab( 13 ,1)= 'Int_13'.


compute intlab( 14 ,1)= 'Int_14'.


compute intlab( 15 ,1)= 'Int_15'.


compute intlab( 16 ,1)= 'Int_16'.


compute intlab( 17 ,1)= 'Int_17'.


compute intlab( 18 ,1)= 'Int_18'.


compute intlab( 19 ,1)= 'Int_19'.


compute intlab( 20 ,1)= 'Int_20'.


compute intlab( 21 ,1)= 'Int_21'.


compute intlab( 22 ,1)= 'Int_22'.


compute intlab( 23 ,1)= 'Int_23'.


compute intlab( 24 ,1)= 'Int_24'.


compute intlab( 25 ,1)= 'Int_25'.


compute intlab( 26 ,1)= 'Int_26'.


compute intlab( 27 ,1)= 'Int_27'.


compute intlab( 28 ,1)= 'Int_28'.


compute intlab( 29 ,1)= 'Int_29'.


compute intlab( 30 ,1)= 'Int_30'.


compute intlab( 31 ,1)= 'Int_31'.


compute intlab( 32 ,1)= 'Int_32'.


compute intlab( 33 ,1)= 'Int_33'.


compute intlab( 34 ,1)= 'Int_34'.


compute intlab( 35 ,1)= 'Int_35'.


compute intlab( 36 ,1)= 'Int_36'.


compute intlab( 37 ,1)= 'Int_37'.


compute intlab( 38 ,1)= 'Int_38'.


compute intlab( 39 ,1)= 'Int_39'.


compute intlab( 40 ,1)= 'Int_40'.


compute intlab( 41 ,1)= 'Int_41'.


compute intlab( 42 ,1)= 'Int_42'.


compute intlab( 43 ,1)= 'Int_43'.


compute intlab( 44 ,1)= 'Int_44'.


compute intlab( 45 ,1)= 'Int_45'.


compute intlab( 46 ,1)= 'Int_46'.


compute intlab( 47 ,1)= 'Int_47'.


compute intlab( 48 ,1)= 'Int_48'.


compute intlab( 49 ,1)= 'Int_49'.


compute intlab( 50 ,1)= 'Int_50'.


compute intlab( 51 ,1)= 'Int_51'.


compute intlab( 52 ,1)= 'Int_52'.


compute intlab( 53 ,1)= 'Int_53'.


compute intlab( 54 ,1)= 'Int_54'.


compute intlab( 55 ,1)= 'Int_55'.


compute intlab( 56 ,1)= 'Int_56'.


compute intlab( 57 ,1)= 'Int_57'.


compute intlab( 58 ,1)= 'Int_58'.


compute intlab( 59 ,1)= 'Int_59'.


compute intlab( 60 ,1)= 'Int_60'.


compute intlab( 61 ,1)= 'Int_61'.


compute intlab( 62 ,1)= 'Int_62'.


compute intlab( 63 ,1)= 'Int_63'.


compute intlab( 64 ,1)= 'Int_64'.


compute intlab( 65 ,1)= 'Int_65'.


compute intlab( 66 ,1)= 'Int_66'.


compute intlab( 67 ,1)= 'Int_67'.


compute intlab( 68 ,1)= 'Int_68'.


compute intlab( 69 ,1)= 'Int_69'.


compute intlab( 70 ,1)= 'Int_70'.


compute intlab( 71 ,1)= 'Int_71'.


compute intlab( 72 ,1)= 'Int_72'.


compute intlab( 73 ,1)= 'Int_73'.


compute intlab( 74 ,1)= 'Int_74'.


compute intlab( 75 ,1)= 'Int_75'.


compute intlab( 76 ,1)= 'Int_76'.


compute intlab( 77 ,1)= 'Int_77'.


compute intlab( 78 ,1)= 'Int_78'.


compute intlab( 79 ,1)= 'Int_79'.


compute intlab( 80 ,1)= 'Int_80'.


compute intlab( 81 ,1)= 'Int_81'.


compute intlab( 82 ,1)= 'Int_82'.


compute intlab( 83 ,1)= 'Int_83'.


compute intlab( 84 ,1)= 'Int_84'.


compute intlab( 85 ,1)= 'Int_85'.


compute intlab( 86 ,1)= 'Int_86'.


compute intlab( 87 ,1)= 'Int_87'.


compute intlab( 88 ,1)= 'Int_88'.


compute intlab( 89 ,1)= 'Int_89'.


compute intlab( 90 ,1)= 'Int_90'.


compute intlab( 91 ,1)= 'Int_91'.


compute intlab( 92 ,1)= 'Int_92'.


compute intlab( 93 ,1)= 'Int_93'.


compute intlab( 94 ,1)= 'Int_94'.


compute intlab( 95 ,1)= 'Int_95'.


compute intlab( 96 ,1)= 'Int_96'.


compute intlab( 97 ,1)= 'Int_97'.


compute intlab( 98 ,1)= 'Int_98'.


compute intlab( 99 ,1)= 'Int_99'.


compute intlab( 100 ,1)= 'Int_100'.

compute bcmat=make(needed,needed,0).
compute wcmat=make(needed,needed,0).
compute zcmat=make(needed,needed,0).
compute wzcmat=make(needed,needed,0).
compute wsum=0.
compute zsum=0.
compute wzsum=0.
end if.
end if.
do if (criterr = 0 and model <> 999).
compute modelmat= {1,0,0,0,0,0,0,1,0,0;2,0,0,0,0,0,0,1,1,0;3,0,0,0,0,0,0,1,1,1;4,0,0,0,0,0,0,0,0,0; 5,0,0,0,0,0,0,1,0,0;6,0,0,0,0,0,0,0,0,0;7,1,0,0,0,0,0,0,0,0;8,1,0,0,0,0,0,1,0,0; 9,1,1,0,0,0,0,0,0,0;10,1,1,0,0,0,0,1,1,0;11,1,1,1,0,0,0,0,0,0;12,1,1,1,0,0,0,1,1,1; 13,1,1,1,0,0,0,1,0,0;14,0,0,0,1,0,0,0,0,0;15,0,0,0,1,0,0,1,0,0;16,0,0,0,1,1,0,0,0,0; 17,0,0,0,1,1,0,1,1,0;18,0,0,0,1,1,1,0,0,0;19,0,0,0,1,1,1,1,1,1;20,0,0,0,1,1,1,1,0,0; 21,1,0,0,0,1,0,0,0,0;22,1,0,0,0,1,0,1,0,0;23,0,0,0,0,0,0,0,0,0;24,0,0,0,0,0,0,0,0,0; 25,0,0,0,0,0,0,0,0,0;26,0,0,0,0,0,0,0,0,0;27,0,0,0,0,0,0,0,0,0;28,1,0,0,0,1,0,0,1,0; 29,1,0,0,0,1,0,1,1,0;30,0,0,0,0,0,0,0,0,0;31,0,0,0,0,0,0,0,0,0;32,0,0,0,0,0,0,0,0,0; 33,0,0,0,0,0,0,0,0,0;34,0,0,0,0,0,0,0,0,0;35,0,0,0,0,0,0,0,0,0;36,0,0,0,0,0,0,0,0,0; 37,0,0,0,0,0,0,0,0,0;38,0,0,0,0,0,0,0,0,0;39,0,0,0,0,0,0,0,0,0;40,0,0,0,0,0,0,0,0,0; 41,0,0,0,0,0,0,0,0,0;42,0,0,0,0,0,0,0,0,0;43,0,0,0,0,0,0,0,0,0;44,0,0,0,0,0,0,0,0,0; 45,0,0,0,0,0,0,0,0,0;46,0,0,0,0,0,0,0,0,0;47,0,0,0,0,0,0,0,0,0;48,0,0,0,0,0,0,0

 ,0,0; 49,0,0,0,0,0,0,0,0,0;50,0,0,0,0,0,0,0,0,0;51,0,0,0,0,0,0,0,0,0;52,0,0,0,0,0,0,0,0,0; 53,0,0,0,0,0,0,0,0,0;54,0,0,0,0,0,0,0,0,0;55,0,0,0,0,0,0,0,0,0;56,0,0,0,0,0,0,0,0,0; 57,0,0,0,0,0,0,0,0,0;58,1,0,0,1,0,0,0,0,0;59,1,0,0,1,0,0,1,0,0;60,1,1,0,1,0,0,0,0,0; 61,1,1,0,1,0,0,1,0,0;62,1,1,0,1,0,0,0,1,0;63,1,1,0,1,0,0,1,1,0;64,1,0,0,1,1,0,0,0,0; 65,1,0,0,1,1,0,1,0,0;66,1,0,0,1,1,0,0,1,0;67,1,0,0,1,1,0,1,1,0;68,1,1,1,1,0,0,0,0,0; 69,1,1,1,1,0,0,1,1,1;70,1,0,0,1,1,1,0,0,0;71,1,0,0,1,1,1,1,1,1;72,1,1,1,1,1,1,0,0,0; 73,1,1,1,1,1,1,1,1,1;74,0,0,0,1,0,0,0,0,0;75,1,1,0,1,1,0,0,0,0;76,1,1,0,1,1,0,1,1,0; 77,0,0,0,0,0,0,0,0,0;78,0,0,0,0,0,0,0,0,0;79,0,0,0,0,0,0,0,0,0;80,0,0,0,0,0,0,0,0,0; 81,0,0,0,0,0,0,0,0,0;82,0,0,0,0,0,0,0,0,0;83,1,0,0,0,0,0,0,0,0;84,1,0,0,0,0,0,0,0,0; 85,1,0,0,0,0,0,1,0,0;86,1,0,0,0,0,0,1,0,0;87,0,0,0,1,0,0,0,0,0;88,0,0,0,1,0,0,0,0,0; 89,0,0,0,1,0,0,1,0,0;90,0,0,0,1,0,0,1,0,0;91,0,0,0,0,0,0,0,0,0;92,1,0,0,1,0,0,1,0,0}.
do if (model > 0).
compute tmp=modelmat(model,2:ncol(modelmat)).
end if.
do if (model=0).
compute tmp={0,0,0,0,0,0,0,0,0}.
end if.
do if (model < 4).
compute bcmat((nxs+1),1)=1.
end if.
do if ((model > 3) and (model <> 6)).
compute bcmat((nxs+1):(nxs+nms),1)=onem.
compute bcmat(nrow(bcmat),(nxs+1):(nxs+nms))=t(onem).
compute bcmat(nrow(bcmat),1)=1.
end if.
do if ((model = 6) or (model > 82 and model < 93)).
loop j = 2 to nrow(bcmat).
loop i = 1 to (j-1).
compute bcmat(j,i)=1.
end loop.
end loop.
end if.
do if (model = 80).
loop i = 1 to nms.
compute bcmat((nrow(bcmat)-1),i)=1.
end loop.
end if.
do if (model = 81).
loop j = 3 to nrow(bcmat).
compute bcmat(j,2)=1.
end loop.
end if.
do if (model = 82).
compute bcmat(3,2)=1.
compute bcmat(5,4)=1.
end if.
do if (tmp(1,1)=1).
compute wcmat((nxs+1):(nxs+nms),1)=onem.
compute wprod=1.
compute xprod=1.
do if (model = 83 or model = 86).
compute onemsx=onem.
loop i = 1 to (nms-1).
compute onemsx(i+1,1)=0.
end loop.
compute wcmat((nxs+1):(nxs+nms),1)=onemsx.
end if.
end if.
do if (tmp(1,4)=1).
compute wcmat(nrow(wcmat),(nxs+1):(nxs+nms))=t(onem).
compute wprod=1.
do if (model = 87 or model = 90).
compute onemsx=onem.
loop i = 1 to (nms-1).
compute onemsx(i,1)=0.
end loop.
compute wcmat(nrow(wcmat),(nxs+1):(nxs+nms))=t(onemsx).
end if.
end if.
do if (tmp(1,7)=1).
compute wcmat(nrow(wcmat),1)=1.
compute wprod=1.
compute xprod=1.
end if.
do if (tmp(1,2)=1).
compute zcmat((nxs+1):(nxs+nms),1)=onem.
compute zprod=1.
compute xprod=1.
end if.
do if (tmp(1,5)=1).
compute zcmat(nrow(zcmat),(nxs+1):(nxs+nms))=t(onem).
compute zprod=1.
end if.
do if (tmp(1,8)=1).
compute zcmat(nrow(zcmat),1)=1.
compute zprod=1.
compute xprod=1.
end if.
do if (tmp(1,3)=1).
compute wzcmat((nxs+1):(nxs+nms),1)=onem.
compute xprod=1.
compute wprod=1.
compute zprod=1.
end if.
do if (tmp(1,6)=1).
compute wzcmat(nrow(wzcmat),(nxs+1):(nxs+nms))=t(onem).
compute zprod=1.
compute wprod=1.
end if.
do if (tmp(1,9)=1).
compute wzcmat(nrow(wzcmat),1)=1.
compute xprod=1.
compute wprod=1.
compute zprod=1.
end if.
do if (model = 91 or model = 92).
loop j = 1 to (nms-1).
loop i = 1 to j.
compute wcmat((nxs+1+j),(nxs+i))=1.
end loop.
end loop.
end if.
do if (nms < 0).
loop i = 1 to nms.
compute tmp=csum(wcmat(:,(1+i)))+csum(zcmat(:,(1+i)))+csum(wzcmat(:,(1+i))).
compute mprod(1,i)=(tmp>0).
end loop.
end if.
end if.
do if (ncs > 0).
compute ccmat=make((nms+nys),ncs,1).
compute ccmatoff=ccmat.
do if (covmy=1).
compute ccmat(nrow(ccmat),:)=make(1,ncs,0).
end if.
do if (covmy=2).
compute ccmat(1:nms,:)=make(nms,ncs,0).
end if.
do if (cmatrix(1,1) <> -999).
do if (ncol(cmatrix) <> ((nms+nys)*ncs)).
compute errcode(errs,1)=29.
compute errs=errs+1.
compute criterr=1.
end if.
do if (criterr = 0).
compute tmp=1.
loop i = 1 to (nms+nys).
loop j = 1 to ncs.
compute ccmat(i,j)=1-(cmatrix(1,tmp) = 0).
compute tmp=tmp+1.
end loop.
end loop.
do if (rsum((csum(ccmat)=0)) <> 0).
compute errcode(errs,1)=30.
compute errs=errs+1.
compute criterr=1.
end if.
end if.
do if (covmy <> 0).
compute notecode(notes,1)=1.
compute notes=notes+1.
end if.
end if.
do if (xmint=1 and (covmy > 0 or (csum(rsum(ccmat)) <> ((nms+nys)*ncs)))).
compute errcode(errs,1)=68.
compute errs=errs+1.
compute criterr=1.
end if.
end if.
do if (criterr=0).
compute needed=needed*(needed-1)/2.
compute nopath=0.
do if (bmatrix(1,1) <> -999).
compute tmp=1.
do if ((ncol(bmatrix) <> needed) or (csum(rsum(bmatrix))=0)).
compute errcode(errs,1)=16.
compute errs=errs+1.
compute criterr=1.
else.
loop i = 2 to nrow(bcmat).
loop j = 1 to (i-1).
compute bcmat(i,j)=1-(bmatrix(1,tmp) = 0).
compute tmp=tmp+1.
end loop.
end loop.
end if.
do if ((csum(bcmat(:,1))=0) and criterr=0).
compute errcode(errs,1)=22.
compute errs=errs+1.
compute criterr=1.
end if.
do if ((rsum(bcmat(nrow(bcmat),:))=0) and criterr=0).
compute errcode(errs,1)=23.
compute errs=errs+1.
compute criterr=1.
end if.
compute dm=0.
do if (nms > 0).
loop i = 1 to nms.
do if (((rsum(bcmat((nxs+i),:)) = 0) or (csum(bcmat(:,(nxs+i))) = 0)) and (dm=0) and (criterr=0)).
compute errcode(errs,1)=26.
compute errs=errs+1.
compute criterr=1.
compute dm=1.
end if.
end loop.
end if.
release dm.
end if.
end if.
do if (criterr=0).
do if (wmatrix(1,1) <> -999).
compute tmp=1.
do if (ncol(wmatrix) <> needed).
compute errcode(errs,1)=17.
compute errs=errs+1.
compute criterr=1.
else.
compute modelvar(1,1)='CUSTOM'.
loop i = 2 to nrow(wcmat).
loop j = 1 to (i-1).
compute wcmat(i,j)=1-(wmatrix(1,tmp) = 0).
do if ((wcmat(i,j)=1) and (bcmat(i,j)=0) and (nopath=0)).
compute errcode(errs,1)=20.
compute errs=errs+1.
compute criterr=1.
compute nopath=1.
end if.
compute tmp=tmp+1.
end loop.
end loop.
end if.
end if.
do if (zmatrix(1,1) <> -999).
compute tmp=1.
do if (ncol(zmatrix) <> needed).
compute errcode(errs,1)=18.
compute errs=errs+1.
compute criterr=1.
else.
compute modelvar(1,1)='CUSTOM'.
do if (csum(rsum(wcmat))=0 and model=999).
compute errcode(errs,1)=21.
compute errs=errs+1.
compute criterr=1.
end if.
loop i = 2 to nrow(zcmat).
loop j = 1 to (i-1).
compute zcmat(i,j)=1-(zmatrix(1,tmp) = 0).
do if ((zcmat(i,j)=1) and (bcmat(i,j)=0) and (nopath=0)).
compute errcode(errs,1)=20.
compute errs=errs+1.
compute criterr=1.
compute nopath=1.
end if.
compute tmp=tmp+1.
end loop.
end loop.
end if.
end if.
compute tmp=1.
do if (wzmatrix(1,1) <> -999).
do if (ncol(wzmatrix) <> needed).
compute errcode(errs,1)=19.
compute errs=errs+1.
compute criterr=1.
end if.
compute modelvar(1,1)='CUSTOM'.
end if.
do if (criterr=0).
loop i = 2 to nrow(wzcmat).
loop j = 1 to (i-1).
do if (wzmatrix(1,1) <> -999).
compute wzcmat(i,j)=1-(wzmatrix(1,tmp) = 0).
end if.
do if (wzcmat(i,j)=1).
compute wcmat(i,j)=1.
compute zcmat(i,j)=1.
end if.
do if ((wzcmat(i,j)=1) and (bcmat(i,j)=0) and (nopath=0)).
compute errcode(errs,1)=20.
compute errs=errs+1.
compute criterr=1.
compute nopath=1.
end if.
compute tmp=tmp+1.
end loop.
end loop.
end if.
end if.
do if (criterr=0).
compute xprod=csum(wcmat(:,1))+csum(zcmat(:,1))+csum(wzcmat(:,1)).
compute xprod=(xprod > 0).
compute wsum=csum(rsum(wcmat)).
compute wprod=(wsum > 0).
do if (nms > 0).
loop i = 1 to nms.
compute tmp=csum(wcmat(:,(1+i)))+csum(zcmat(:,(1+i)))+csum(wzcmat(:,(1+i))).
compute mprod(1,i)=(tmp>0).
end loop.
end if.
do if ((wsum > 0) and (w = 'xxxxx')).
compute errcode(errs,1)=11.
compute errs=errs+1.
compute criterr=1.
end if.
do if ((wsum = 0) and (w <> 'xxxxx')).
compute errcode(errs,1)=10.
compute errs=errs+1.
compute criterr=1.
end if.
compute zsum=csum(rsum(zcmat)).
compute zprod=(zsum > 0).
do if ((zsum > 0) and (z = 'xxxxx')).
compute errcode(errs,1)=13.
compute errs=errs+1.
compute criterr=1.
end if.
do if ((zsum = 0) and (z <> 'xxxxx')).
compute errcode(errs,1)=12.
compute errs=errs+1.
compute criterr=1.
end if.
do if ((zsum > 0) and (wsum = 0)).
compute errcode(errs,1)=35.
compute errs=errs+1.
compute criterr=1.
end if.
end if.
do if (criterr=0 and nms > 1).
compute serchk=bcmat(2:(nrow(bcmat)-1),2:ncol(bcmat)).
do if (csum(rsum(serchk))) > 0.
compute serial=1.
do if (nms > 6).
compute errcode(errs,1)=36.
compute errs=errs+1.
compute criterr=1.
end if.
end if.
end if.
do if (center > 0 and criterr=0).
compute centvar={' '}.
do if (criterr=0).
do if ((center = 1) or (center = 2 and wdich = 0)).
do if (wprod=1 and mcwok=0 and nwpval > 0).
loop i = 1 to nws.
compute wtmp(:,i)=wtmp(:,i)-(csum(wtmp(:,i))/n).
compute centvar={centvar,wnames(1,i)}.
end loop.

.
compute desctmp=make((8-(4* wmodcust )),ncol( wtmp ),-999).
loop jd=1 to ncol( wtmp ).
compute descdat= wtmp (:,jd).
compute desctmp(1,jd) = csum(descdat)/nrow(descdat).
compute desctmp(2,jd) = (nrow(descdat)*sscp(descdat))-(t(csum(descdat))*(csum(descdat))).
compute desctmp(2,jd) = sqrt(desctmp(2,jd)/(nrow(descdat)*(nrow(descdat)-1))).
compute desctmp(3,jd)=cmin(descdat).
compute desctmp(4,jd)=cmax(descdat).
do if ( wmodcust =0).
compute minwarn=0.
compute maxwarn=0.
do if ((desctmp(3,jd)=desctmp(4,jd)) and novar=0).
compute errcode(errs,1)=15.
compute errs=errs+1.
compute criterr=1.
compute novar=1.
end if.
compute tmp=((descdat(:,1)=desctmp(3,jd))+(descdat(:,1)=desctmp(4,jd))).
compute desctmp(8,jd)=(csum(tmp)=nrow(tmp)).
compute tmp = descdat.
compute tmp(GRADE(descdat),:) = descdat.
compute descdat = tmp.
release tmp.
compute decval={.16;.5;.84}.
loop kd=1 to 3.
compute low=trunc(decval(kd,1)*(nrow(descdat)+1)).
compute lowdec=decval(kd,1)*(nrow(descdat)+1)-low.
compute value=descdat(low,1)+(descdat((low+1),1)-descdat(low,1))*lowdec.
compute desctmp((4+kd),jd)=value.
end loop.
compute mnotev=1.
compute modvals=desctmp(5:7,:).
do if (quantile <> 1).
compute desctmp(5,jd)=desctmp(1,jd)-desctmp(2,jd).
compute desctmp(6,jd)=desctmp(1,jd).
compute desctmp(7,jd)=desctmp(1,jd)+desctmp(2,jd).
compute modvals=desctmp(5:7,:).
compute mnotev=2.
do if (modvals(1,1) < desctmp(3,1)).
compute modvals(1,1)=desctmp(3,1).
compute minwarn=1.
end if.
do if (modvals(3,1) > desctmp(4,1)).
compute modvals(3,1)=desctmp(4,1).
compute maxwarn=1.
end if.
end if.
do if (desctmp(8,1)=1).
compute modvals={desctmp(3,1);desctmp(4,1)}.
compute mnotev=0.
compute minwarn=0.
compute maxwarn=0.
end if.
end if.
end loop
.
compute wmin=desctmp(3,1).
compute wmax=desctmp(4,1).
do if (wmodcust=0).
compute wmodvals=modvals.
compute wprobval=wmodvals.
end if.
end if.
end if.
do if ((center = 1) or (center = 2 and zdich = 0)).
do if (zprod=1 and mczok=0 and nzpval > 0).
loop i = 1 to nzs.
compute ztmp(:,i)=ztmp(:,i)-(csum(ztmp(:,i))/n).
compute centvar={centvar,znames(1,i)}.
end loop.

.
compute desctmp=make((8-(4* zmodcust )),ncol( ztmp ),-999).
loop jd=1 to ncol( ztmp ).
compute descdat= ztmp (:,jd).
compute desctmp(1,jd) = csum(descdat)/nrow(descdat).
compute desctmp(2,jd) = (nrow(descdat)*sscp(descdat))-(t(csum(descdat))*(csum(descdat))).
compute desctmp(2,jd) = sqrt(desctmp(2,jd)/(nrow(descdat)*(nrow(descdat)-1))).
compute desctmp(3,jd)=cmin(descdat).
compute desctmp(4,jd)=cmax(descdat).
do if ( zmodcust =0).
compute minwarn=0.
compute maxwarn=0.
do if ((desctmp(3,jd)=desctmp(4,jd)) and novar=0).
compute errcode(errs,1)=15.
compute errs=errs+1.
compute criterr=1.
compute novar=1.
end if.
compute tmp=((descdat(:,1)=desctmp(3,jd))+(descdat(:,1)=desctmp(4,jd))).
compute desctmp(8,jd)=(csum(tmp)=nrow(tmp)).
compute tmp = descdat.
compute tmp(GRADE(descdat),:) = descdat.
compute descdat = tmp.
release tmp.
compute decval={.16;.5;.84}.
loop kd=1 to 3.
compute low=trunc(decval(kd,1)*(nrow(descdat)+1)).
compute lowdec=decval(kd,1)*(nrow(descdat)+1)-low.
compute value=descdat(low,1)+(descdat((low+1),1)-descdat(low,1))*lowdec.
compute desctmp((4+kd),jd)=value.
end loop.
compute mnotev=1.
compute modvals=desctmp(5:7,:).
do if (quantile <> 1).
compute desctmp(5,jd)=desctmp(1,jd)-desctmp(2,jd).
compute desctmp(6,jd)=desctmp(1,jd).
compute desctmp(7,jd)=desctmp(1,jd)+desctmp(2,jd).
compute modvals=desctmp(5:7,:).
compute mnotev=2.
do if (modvals(1,1) < desctmp(3,1)).
compute modvals(1,1)=desctmp(3,1).
compute minwarn=1.
end if.
do if (modvals(3,1) > desctmp(4,1)).
compute modvals(3,1)=desctmp(4,1).
compute maxwarn=1.
end if.
end if.
do if (desctmp(8,1)=1).
compute modvals={desctmp(3,1);desctmp(4,1)}.
compute mnotev=0.
compute minwarn=0.
compute maxwarn=0.
end if.
end if.
end loop
.
compute zmin=desctmp(3,1).
compute zmax=desctmp(4,1).
do if (zmodcust=0).
compute zmodvals=modvals.
compute zprobval=zmodvals.
end if.
end if.
end if.
do if ((center = 1) or (center = 2 and xdich = 0)).
do if (xprod=1 and mcxok=0).
loop i = 1 to nxs.
compute xtmp(:,i)=xtmp(:,i)-(csum(xtmp(:,i))/n).
compute centvar={centvar,xnames(1,i)}.
end loop.

.
compute desctmp=make((8-(4* 0 )),ncol( xtmp ),-999).
loop jd=1 to ncol( xtmp ).
compute descdat= xtmp (:,jd).
compute desctmp(1,jd) = csum(descdat)/nrow(descdat).
compute desctmp(2,jd) = (nrow(descdat)*sscp(descdat))-(t(csum(descdat))*(csum(descdat))).
compute desctmp(2,jd) = sqrt(desctmp(2,jd)/(nrow(descdat)*(nrow(descdat)-1))).
compute desctmp(3,jd)=cmin(descdat).
compute desctmp(4,jd)=cmax(descdat).
do if ( 0 =0).
compute minwarn=0.
compute maxwarn=0.
do if ((desctmp(3,jd)=desctmp(4,jd)) and novar=0).
compute errcode(errs,1)=15.
compute errs=errs+1.
compute criterr=1.
compute novar=1.
end if.
compute tmp=((descdat(:,1)=desctmp(3,jd))+(descdat(:,1)=desctmp(4,jd))).
compute desctmp(8,jd)=(csum(tmp)=nrow(tmp)).
compute tmp = descdat.
compute tmp(GRADE(descdat),:) = descdat.
compute descdat = tmp.
release tmp.
compute decval={.16;.5;.84}.
loop kd=1 to 3.
compute low=trunc(decval(kd,1)*(nrow(descdat)+1)).
compute lowdec=decval(kd,1)*(nrow(descdat)+1)-low.
compute value=descdat(low,1)+(descdat((low+1),1)-descdat(low,1))*lowdec.
compute desctmp((4+kd),jd)=value.
end loop.
compute mnotev=1.
compute modvals=desctmp(5:7,:).
do if (quantile <> 1).
compute desctmp(5,jd)=desctmp(1,jd)-desctmp(2,jd).
compute desctmp(6,jd)=desctmp(1,jd).
compute desctmp(7,jd)=desctmp(1,jd)+desctmp(2,jd).
compute modvals=desctmp(5:7,:).
compute mnotev=2.
do if (modvals(1,1) < desctmp(3,1)).
compute modvals(1,1)=desctmp(3,1).
compute minwarn=1.
end if.
do if (modvals(3,1) > desctmp(4,1)).
compute modvals(3,1)=desctmp(4,1).
compute maxwarn=1.
end if.
end if.
do if (desctmp(8,1)=1).
compute modvals={desctmp(3,1);desctmp(4,1)}.
compute mnotev=0.
compute minwarn=0.
compute maxwarn=0.
end if.
end if.
end loop
.
compute xmodvals=modvals.
compute xprobval=xmodvals.
end if.
end if.
do if (nms > 0).
loop i = 1 to nms.
do if (mprod(1,i)=1).
compute mtmp(:,i)=mtmp(:,i)-(csum(mtmp(:,i))/n).
compute centvar={centvar,mnames(1,i)}.
end if.
end loop.

.
compute desctmp=make((8-(4* 0 )),ncol( mtmp ),-999).
loop jd=1 to ncol( mtmp ).
compute descdat= mtmp (:,jd).
compute desctmp(1,jd) = csum(descdat)/nrow(descdat).
compute desctmp(2,jd) = (nrow(descdat)*sscp(descdat))-(t(csum(descdat))*(csum(descdat))).
compute desctmp(2,jd) = sqrt(desctmp(2,jd)/(nrow(descdat)*(nrow(descdat)-1))).
compute desctmp(3,jd)=cmin(descdat).
compute desctmp(4,jd)=cmax(descdat).
do if ( 0 =0).
compute minwarn=0.
compute maxwarn=0.
do if ((desctmp(3,jd)=desctmp(4,jd)) and novar=0).
compute errcode(errs,1)=15.
compute errs=errs+1.
compute criterr=1.
compute novar=1.
end if.
compute tmp=((descdat(:,1)=desctmp(3,jd))+(descdat(:,1)=desctmp(4,jd))).
compute desctmp(8,jd)=(csum(tmp)=nrow(tmp)).
compute tmp = descdat.
compute tmp(GRADE(descdat),:) = descdat.
compute descdat = tmp.
release tmp.
compute decval={.16;.5;.84}.
loop kd=1 to 3.
compute low=trunc(decval(kd,1)*(nrow(descdat)+1)).
compute lowdec=decval(kd,1)*(nrow(descdat)+1)-low.
compute value=descdat(low,1)+(descdat((low+1),1)-descdat(low,1))*lowdec.
compute desctmp((4+kd),jd)=value.
end loop.
compute mnotev=1.
compute modvals=desctmp(5:7,:).
do if (quantile <> 1).
compute desctmp(5,jd)=desctmp(1,jd)-desctmp(2,jd).
compute desctmp(6,jd)=desctmp(1,jd).
compute desctmp(7,jd)=desctmp(1,jd)+desctmp(2,jd).
compute modvals=desctmp(5:7,:).
compute mnotev=2.
do if (modvals(1,1) < desctmp(3,1)).
compute modvals(1,1)=desctmp(3,1).
compute minwarn=1.
end if.
do if (modvals(3,1) > desctmp(4,1)).
compute modvals(3,1)=desctmp(4,1).
compute maxwarn=1.
end if.
end if.
do if (desctmp(8,1)=1).
compute modvals={desctmp(3,1);desctmp(4,1)}.
compute mnotev=0.
compute minwarn=0.
compute maxwarn=0.
end if.
end if.
end loop
.
do if ((cdeval(1,1)=-999) and (model=74)).
compute medmeans=desctmp(1,:).
end if.
compute mmodvals=modvals.
compute mprobval=mmodvals.
end if.
end if.
do if (ncol(centvar) > 1).
compute notecode(notes,1)=3.
compute notes=notes+1.
end if.
end if.
do if (criterr=0).
compute wsum=rsum(csum(wcmat)).
compute zsum=rsum(csum(zcmat)).
compute wzsum=rsum(csum(wzcmat)).
compute nump=make(1,(nys+nms),-999).
compue numint=make(1,(nys+nms),0).
compute datcount=1.
compute xtmpuse=0.
compute wtmpuse=0.
compute ztmpuse=0.
compute xwtmpus=0.
compute xztmpus=0.
compute wztmpus=0.
compute xwztmpu=0.
compute xtmploc=-999.
compute wtmploc=-999.
compute xwtmplo=-999.
compute ztmploc=-999.
compute xztmplo=-999.
compute wztmplo=-999.
compute xwztmplo=-999.
compute vlabs={' '}.
do if (ncs > 0).
compute ctmpuse=make(1,ncs,0).
end if.
do if (nms > 0).
compute mtmpuse=make(1,nms,0).
compute mwtmpus=make(1,nms,0).
compute mztmpus=make(1,nms,0).
compute mwztmpu=make(1,nms,0).
compute mtmploc=make(1,nms,0).
compute mwtmplo=make(nwvls,nms,-999).
compute mztmplo=make(nzvls,nms,-999).
compute mwztmplo=make((nwvls*nzvls),nms,-999).
end if.
do if (ncs > 0).
compute ctmploc=make(1,ncs,0).
end if.
compute fulldat=make(n,1,1).
compute datindx=make(1000,(nms+nys),-999).
compute wherew=make(2,(nms+nys),-999).
compute wherex=make(2,(nms+nys),-999).
compute wherez=make(2,(nms+nys),-999).
compute wherexw=make(2,(nms+nys),-999).
compute wherexz=make(2,(nms+nys),-999).
compute wherewz=make(2,(nms+nys),-999).
compute wherexwz=make(2,(nms+nys),-999).
do if (nms > 0).
compute wherem=make(nms,(nms+nys),-999).
compute wheremw = make(nms*2,(nms+nys),-999).
compute wheremz = make(nms*2,(nms+nys),-999).
compute wheremwz = make(nms*2,(nms+nys),-999).
end if.
compute wzhigh=make(1000,(((nms+1)*(nms+2))/2),0).
compute whigh=make(1000,(((nms+1)*(nms+2))/2),0).
compute zhigh=make(1000,(((nms+1)*(nms+2))/2),0).
compute fochigh=make(1000,(((nms+1)*(nms+2))/2),0).
compute xcoefloc={1;2;3;4;5;6;7;8;9}.
compute intkey = {' ', ' ', ' ', ' ', ' ', ' ', ' '}.
compute wzhighct=0.
compute whighct=0.
compute zhighct=0.
compute foccnt=0.
loop i = 2 to nrow(bcmat).
compute wdid=0.
compute zdid=0.
compute wzdid=0.
compute cntmp=1.
compute start=1.
do if (i < nrow(bcmat)).
compute outv=mtmp(:,(i-1)).
compute modlabel={mnames(1,(i-1));'constant'}.
end if.
do if (i = nrow(bcmat)).
compute outv=ytmp.
compute modlabel={ynames;'constant'}.
end if.
loop j = 1 to (i-1).
compute foccnt=foccnt+1.
do if (j = 1 and bcmat(i,j)=1).
compute outv={outv,xtmp}.
compute modlabel={modlabel;xcatlab(1:nxvls,1)}.
do if (xtmpuse=0).
compute fulldat={fulldat,xtmp}.
compute xtmpuse=1.
loop k4=datcount to (datcount+(nxvls-1)).
compute xtmploc={xtmploc;k4}.
end loop.
compute xtmploc=xtmploc(2:nrow(xtmploc),1).
compute datcount=datcount+nxvls.
end if.
compute datindx(start:(start+nrow(xtmploc)-1),(i-1))=xtmploc.
compute wherex(1,(i-1))=start+1.
compute wherex(2,(i-1))=start+nrow(xtmploc)-1+1.
do if (model = 74).
end if.
compute onebl=make(nrow(xtmploc),1,1).
compute fochigh((start+1):(start+nrow(xtmploc)),foccnt)=onebl.
compute start=start+nrow(xtmploc).
end if.
do if (j > 1 and bcmat(i,j)=1).
compute outv={outv,mtmp(:,(j-1))}.
compute modlabel={modlabel;mnames(1,(j-1))}.
do if (mtmpuse(1,(j-1))=0).
compute fulldat={fulldat,mtmp(:,(j-1))}.
compute mtmpuse(1,(j-1))=1.
compute mtmploc(1,(j-1))=datcount.
compute datcount=datcount+1.
end if.
compute datindx(start:(start+nrow(mtmploc)-1),(i-1))=mtmploc(1,(j-1)).
compute wherem((j-1),(i-1))=start+1.
compute onebl=make(nrow(mtmploc(1,j-1)),1,1).
compute ttt=nrow(mtmploc(1,(j-1)))+start-1.
compute fochigh((start+1):(start+nrow(mtmploc(1,(j-1)))),foccnt)=onebl.
compute start=start+nrow(mtmploc(1,(j-1))).
end if.
end loop.
do if (wsum > 0).
loop j = 1 to (i-1).
compute whighct=whighct+1.
do if (j = 1 and wcmat(i,j)=1).
do if (wdid=0).
compute outv={outv,wtmp}.
do if (ncs > 0 and wiscov > 0).
compute ccmatoff((i-1),wiscov)=0.
end if.
compute modlabel={modlabel;wcatlab(1:nwvls,1)}.
compute wdid=1.
do if (wtmpuse=0).
compute fulldat={fulldat,wtmp}.
do if (ncs > 0 and wiscov > 0).
compute ccmatoff((i-1),wiscov)=0.
end if.
compute wtmpuse=1.
loop k4=datcount to (datcount+(nwvls-1)).
compute wtmploc={wtmploc;k4}.
end loop.
compute wtmploc=wtmploc(2:nrow(wtmploc),1).
compute datcount=datcount+nwvls.
end if.
end if.
compute datindx(start:(start+nrow(wtmploc)-1),(i-1))=wtmploc.
compute wherew(1,(i-1))=start+1.
compute wherew(2,(i-1))=start+nrow(wtmploc)-1+1.
compute start=start+nrow(wtmploc).
loop k1=1 to nxvls.
loop k2 = 1 to nwvls.
compute outv={outv,(xtmp(:,k1)&*wtmp(:,k2))}.
do if (ncs > 0 and wiscov > 0).
compute ccmatoff((i-1),wiscov)=0.
end if.
compute modlabel={modlabel;intlab(cntmp,1)}.
compute intkey={intkey;intlab(cntmp,1),':',xcatlab(k1,1),'x',wcatlab(k2,1),' ',' '}.
compute cntmp=cntmp+1.
end loop.
end loop.
do if (xwtmpus=0).
compute fulldat={fulldat,outv(:,(ncol(outv)-(nxvls*nwvls)+1):ncol(outv))}.
compute xwtmpus=1.
do if (ncs > 0 and wiscov > 0).
compute ccmatoff((i-1),wiscov)=0.
end if.
loop k4=datcount to (datcount+((nwvls*nxvls)-1)).
compute xwtmplo={xwtmplo;k4}.
end loop.
compute xwtmplo=xwtmplo(2:nrow(xwtmplo),1).
compute datcount=datcount+(nxvls*nwvls).
end if.
compute datindx(start:(start+nrow(xwtmplo)-1),(i-1))=xwtmplo.
compute wherexw(1,(i-1))=start+1.
compute wherexw(2,(i-1))=start+nrow(xwtmplo)-1+1.
compute onebl=make(nrow(xwtmplo),1,1).
compute whigh((start+1):(start+nrow(xwtmplo)),whighct)=onebl.
compute start=start+nrow(xwtmplo).
end if.
do if (j > 1 and wcmat(i,j)=1).
do if (wdid=0 and model <> 74).
compute outv={outv,wtmp}.
do if (ncs > 0 and wiscov > 0).
compute ccmatoff((i-1),wiscov)=0.
end if.
compute modlabel={modlabel;wcatlab(1:nwvls,1)}.
compute wdid=1.
do if (wtmpuse=0).
compute fulldat={fulldat,wtmp}.
do if (ncs > 0 and wiscov > 0).
compute ccmatoff((i-1),wiscov)=0.
end if.
compute wtmpuse=1.
loop k4=datcount to (datcount+(nwvls-1)).
compute wtmploc={wtmploc;k4}.
end loop.
compute wtmploc=wtmploc(2:nrow(wtmploc),1).
compute datcount=datcount+nwvls.
end if.
compute datindx(start:(start+nrow(wtmploc)-1),(i-1))=wtmploc.
compute wherew(1,(i-1))=start+1.
compute wherew(2,(i-1))=start+nrow(wtmploc)-1+1.
compute start=start+nrow(wtmploc).
end if.
loop k2 = 1 to nwvls.
compute outv={outv,(mtmp(:,(j-1))&*wtmp(:,k2))}.
do if (ncs > 0 and wiscov > 0).
compute ccmatoff((i-1),wiscov)=0.
end if.
compute modlabel={modlabel;intlab(cntmp,1)}.
compute intkey={intkey;intlab(cntmp,1),':', mnames(1,(j-1)),'x',wcatlab(k2,1),' ',' '}.
compute cntmp=cntmp+1.
end loop.
do if (mwtmpus(1,(j-1))=0).
compute fulldat={fulldat,outv(:,(ncol(outv)-nwvls+1):ncol(outv))}.
do if (ncs > 0 and wiscov > 0).
compute ccmatoff((i-1),wiscov)=0.
end if.
compute mwtmpus(1,(j-1))=1.
compute mw22=-999.
loop k4=datcount to (datcount+(nwvls-1)).
compute mw22={mw22;k4}.
end loop.
compute mwtmplo(:,(j-1))=mw22(2:nrow(mw22),1).
compute datcount=datcount+nwvls.
end if.
compute datindx(start:(start+nrow(mwtmplo)-1),(i-1))=mwtmplo(:,(j-1)).
compute wheremw(((2*j)-3),(i-1))=start+1.
compute wheremw(((2*j)-2),(i-1))=start+nrow(mwtmplo)-1+1.
compute onebl=make(nrow(mwtmplo),1,1).
compute whigh((start+1):(start+nrow(mwtmplo)),whighct)=onebl.
compute start=start+nrow(mwtmplo).
end if.
end loop.
end if.
do if (zsum > 0).
loop j = 1 to (i-1).
compute zhighct=zhighct+1.
do if (j = 1 and zcmat(i,j)=1).
do if (zdid=0).
compute outv={outv,ztmp}.
do if (ncs > 0 and ziscov > 0).
compute ccmatoff((i-1),ziscov)=0.
end if.
compute modlabel={modlabel;zcatlab(1:nzvls,1)}.
compute zdid=1.
do if (ztmpuse=0).
compute fulldat={fulldat,ztmp}.
do if (ncs > 0 and ziscov > 0).
compute ccmatoff((i-1),ziscov)=0.
end if.
compute ztmpuse=1.
loop k4=datcount to (datcount+(nzvls-1)).
compute ztmploc={ztmploc;k4}.
end loop.
compute ztmploc=ztmploc(2:nrow(ztmploc),1).
compute datcount=datcount+nzvls.
end if.
end if.
compute datindx(start:(start+nrow(ztmploc)-1),(i-1))=ztmploc.
compute wherez(1,(i-1))=start+1.
compute wherez(2,(i-1))=start+nrow(ztmploc)-1+1.
compute start=start+nrow(ztmploc).
loop k1=1 to nxvls.
loop k2 = 1 to nzvls.
compute outv={outv,(xtmp(:,k1)&*ztmp(:,k2))}.
do if (ncs > 0 and ziscov > 0).
compute ccmatoff((i-1),ziscov)=0.
end if.
compute modlabel={modlabel;intlab(cntmp,1)}.
compute intkey={intkey;intlab(cntmp,1),':',xcatlab(k1,1),'x',zcatlab(k2,1),' ',' '}.
compute cntmp=cntmp+1.
end loop.
end loop.
do if (xztmpus=0).
compute fulldat={fulldat,outv(:,(ncol(outv)-(nxvls*nzvls)+1):ncol(outv))}.
do if (ncs > 0 and ziscov > 0).
compute ccmatoff((i-1),ziscov)=0.
end if.
compute xztmpus=1.
loop k4=datcount to (datcount+((nzvls*nxvls)-1)).
compute xztmplo={xztmplo;k4}.
end loop.
compute xztmplo=xztmplo(2:nrow(xztmplo),1).
compute datcount=datcount+(nxvls*nzvls).
end if.
compute datindx(start:(start+nrow(xztmplo)-1),(i-1))=xztmplo.
compute wherexz(1,(i-1))=start+1.
compute wherexz(2,(i-1))=start+nrow(xztmplo)-1+1.
compute onebl=make(nrow(xztmplo),1,1).
compute zhigh((start+1):(start+nrow(xztmplo)),zhighct)=onebl.
compute start=start+nrow(xztmplo).
end if.
do if (j > 1 and zcmat(i,j)=1).
do if (zdid=0).
compute outv={outv,ztmp}.
do if (ncs > 0 and ziscov > 0).
compute ccmatoff((i-1),ziscov)=0.
end if.
compute modlabel={modlabel;zcatlab(1:nzvls,1)}.
compute zdid=1.
do if (ztmpuse=0).
compute fulldat={fulldat,ztmp}.
do if (ncs > 0 and ziscov > 0).
compute ccmatoff((i-1),ziscov)=0.
end if.
compute ztmpuse=1.
loop k4=datcount to (datcount+(nzvls-1)).
compute ztmploc={ztmploc;k4}.
end loop.
compute ztmploc=ztmploc(2:nrow(ztmploc),1).
compute datcount=datcount+nzvls.
end if.
compute datindx(start:(start+nrow(ztmploc)-1),(i-1))=ztmploc.
compute wherez(1,(i-1))=start+1.
compute wherez(2,(i-1))=start+nrow(ztmploc)-1+1.
compute start=start+nrow(ztmploc).
end if.
loop k2 = 1 to nzvls.
compute outv={outv,(mtmp(:,(j-1))&*ztmp(:,k2))}.
do if (ncs > 0 and ziscov > 0).
compute ccmatoff((i-1),ziscov)=0.
end if.
compute modlabel={modlabel;intlab(cntmp,1)}.
compute intkey={intkey;intlab(cntmp,1),':', mnames(1,(j-1)),'x',zcatlab(k2,1),' ',' '}.
compute cntmp=cntmp+1.
end loop.
do if (mztmpus(1,(j-1))=0).
compute fulldat={fulldat,outv(:,(ncol(outv)-nzvls+1):ncol(outv))}.
do if (ncs > 0 and ziscov > 0).
compute ccmatoff((i-1),ziscov)=0.
end if.
compute mztmpus(1,(j-1))=1.
compute mz22=-999.
loop k4=datcount to (datcount+(nzvls-1)).
compute mz22={mz22;k4}.
end loop.
compute mztmplo(:,(j-1))=mz22(2:nrow(mz22),1).
compute datcount=datcount+nzvls.
end if.
compute datindx(start:(start+nrow(mztmplo)-1),(i-1))=mztmplo(:,(j-1)).
compute wheremz(((2*j)-3),(i-1))=start+1.
compute wheremz(((2*j)-2),(i-1))=start+nrow(mztmplo)-1+1.
compute onebl=make(nrow(mztmplo),1,1).
compute zhigh((start+1):(start+nrow(mztmplo)),zhighct)=onebl.
compute start=start+nrow(mztmplo).
end if.
end loop.
end if.
do if (wzsum > 0).
loop j = 1 to (i-1).
compute wzhighct=wzhighct+1.
do if (j = 1 and wzcmat(i,j)=1).
do if (wzdid=0).
loop k1=1 to nwvls.
loop k2 = 1 to nzvls.
compute outv={outv,(wtmp(:,k1)&*ztmp(:,k2))}.
do if (ncs > 0 and (ziscov > 0)).
compute ccmatoff((i-1),ziscov)=0.
end if.
do if (ncs > 0 and (wiscov > 0)).
compute ccmatoff((i-1),wiscov)=0.
end if.
compute modlabel={modlabel;intlab(cntmp,1)}.
compute intkey={intkey;intlab(cntmp,1),':',wcatlab(k1,1),'x',zcatlab(k2,1),' ',' '}.
compute cntmp=cntmp+1.
end loop.
end loop.
do if (wztmpus=0).
compute fulldat={fulldat,outv(:,(ncol(outv)-(nwvls*nzvls)+1):ncol(outv))}.
do if (ncs > 0 and (ziscov > 0)).
compute ccmatoff((i-1),ziscov)=0.
end if.
do if (ncs > 0 and (wiscov > 0)).
compute ccmatoff((i-1),wiscov)=0.
end if.
compute wztmpus=1.
loop k4=datcount to (datcount+((nwvls*nzvls)-1)).
compute wztmplo={wztmplo;k4}.
end loop.
compute wztmplo=wztmplo(2:nrow(wztmplo),1).
compute datcount=datcount+(nzvls*nwvls).
end if.
compute wzdid=1.
end if.
compute datindx(start:(start+nrow(wztmplo)-1),(i-1))=wztmplo.
compute wherewz(1,(i-1))=start+1.
compute wherewz(2,(i-1))=start+nrow(wztmplo)-1+1.
compute start=start+nrow(wztmplo).
loop k1=1 to nxvls.
loop k2=1 to nwvls.
loop k3=1 to nzvls.
compute outv={outv,(xtmp(:,k1)&*wtmp(:,k2)&*ztmp(:,k3))}.
do if (ncs > 0 and (ziscov > 0)).
compute ccmatoff((i-1),ziscov)=0.
end if.
do if (ncs > 0 and (wiscov > 0)).
compute ccmatoff((i-1),wiscov)=0.
end if.
compute modlabel={modlabel;intlab(cntmp,1)}.
compute intkey={intkey;intlab(cntmp,1),':',xcatlab(k1,1),'x',wcatlab(k2,1),'x', zcatlab(k3,1)}.
compute cntmp=cntmp+1.
end loop.
end loop.
end loop.
do if (xwztmpu=0).
compute fulldat={fulldat,outv(:,(ncol(outv)-(nxvls*nwvls*nzvls)+1):ncol(outv))}.
do if (ncs > 0 and (ziscov > 0)).
compute ccmatoff((i-1),ziscov)=0.
end if.
do if (ncs > 0 and (wiscov > 0)).
compute ccmatoff((i-1),wiscov)=0.
end if.
compute xwztmpu=1.
loop k4=datcount to (datcount+((nzvls*nxvls*nwvls)-1)).
compute xwztmplo={xwztmplo;k4}.
end loop.
compute xwztmplo=xwztmplo(2:nrow(xwztmplo),1).
compute datcount=datcount+(nxvls*nzvls*nwvls).
end if.
compute datindx(start:(start+nrow(xwztmplo)-1),(i-1))=xwztmplo.
compute wherexwz(1,(i-1))=start+1.
compute wherexwz(2,(i-1))=start+nrow(xwztmplo)-1+1.
compute onebl=make(nrow(xwztmplo),1,1).
compute wzhigh((start+1):(start+nrow(xwztmplo)),wzhighct)=onebl.
compute start=start+nrow(xwztmplo).
end if.
do if (j > 1 and wzcmat(i,j)=1).
do if (wzdid=0).
loop k1=1 to nwvls.
loop k2 = 1 to nzvls.
compute outv={outv,(wtmp(:,k1)&*ztmp(:,k2))}.
do if (ncs > 0 and (ziscov > 0)).
compute ccmatoff((i-1),ziscov)=0.
end if.
do if (ncs > 0 and (wiscov > 0)).
compute ccmatoff((i-1),wiscov)=0.
end if.
compute modlabel={modlabel;intlab(cntmp,1)}.
compute intkey={intkey;intlab(cntmp,1),':',wcatlab(k1,1),'x',zcatlab(k2,1),' ',' '}.
compute cntmp=cntmp+1.
end loop.
end loop.
do if (wztmpus=0).
compute fulldat={fulldat,outv(:,(ncol(outv)-(nwvls*nzvls)+1):ncol(outv))}.
do if (ncs > 0 and (ziscov > 0)).
compute ccmatoff((i-1),ziscov)=0.
end if.
do if (ncs > 0 and (wiscov > 0)).
compute ccmatoff((i-1),wiscov)=0.
end if.
compute wztmpus=1.
loop k4=datcount to (datcount+((nwvls*nzvls)-1)).
compute wztmplo={wztmplo;k4}.
end loop.
compute wztmplo=wztmplo(2:nrow(wztmplo),1).
compute datcount=datcount+(nzvls*nwvls).
end if.
compute wzdid=1.
compute datindx(start:(start+nrow(wztmplo)-1),(i-1))=wztmplo.
compute wherewz(1,(i-1))=start+1.
compute wherewz(2,(i-1))=start+nrow(wztmplo)-1+1.
compute start=start+nrow(wztmplo).
end if.
loop k1 = 1 to nwvls.
loop k2 = 1 to nzvls.
compute outv={outv,(mtmp(:,(j-1))&*wtmp(:,k1)&*ztmp(:,k2))}.
do if (ncs > 0 and (ziscov > 0)).
compute ccmatoff((i-1),ziscov)=0.
end if.
do if (ncs > 0 and (wiscov > 0)).
compute ccmatoff((i-1),wiscov)=0.
end if.
compute modlabel={modlabel;intlab(cntmp,1)}.
compute intkey={intkey;intlab(cntmp,1),':',mnames(1,(j-1)),'x',wcatlab(k1,1),'x', zcatlab(k2,1)}.
compute cntmp=cntmp+1.
end loop.
end loop.
do if (mwztmpu(1,(j-1))=0).
compute fulldat={fulldat,outv(:,(ncol(outv)-(nwvls*nzvls)+1):ncol(outv))}.
do if (ncs > 0 and (ziscov > 0)).
compute ccmatoff((i-1),ziscov)=0.
end if.
do if (ncs > 0 and (wiscov > 0)).
compute ccmatoff((i-1),wiscov)=0.
end if.
compute mwztmpu(1,(j-1))=1.
compute mz22=-999.
loop k4=datcount to (datcount+(nwvls*nzvls)-1).
compute mz22={mz22;k4}.
end loop.
compute mwztmplo(:,(j-1))=mz22(2:nrow(mz22),1).
compute datcount=datcount+(nwvls*nzvls).
end if.
compute datindx(start:(start+nrow(mwztmplo)-1),(i-1))=mwztmplo(:,(j-1)).
compute wheremwz(((2*j)-3),(i-1))=start+1.
compute wheremwz(((2*j)-2),(i-1))=start+nrow(mwztmplo)-1+1.
compute onebl=make(nrow(mwztmplo),1,1).
compute wzhigh((start+1):(start+nrow(mwztmplo)),wzhighct)=onebl.
compute start=start+nrow(mwztmplo).
end if.
end loop.
end if.
do if (ncs > 0).
compute ccmat=ccmat&*ccmatoff.
loop j = 1 to ncs.
do if (ccmat((i-1),j))=1.
do if (j=wiscov).
compute ctmp(:,j)=wtmp.
end if.
do if (j=ziscov).
compute ctmp(:,j)=ztmp.
end if.
compute outv={outv,ctmp(:,j)}.
compute modlabel={modlabel;covnames(1,j)}.
do if (ctmpuse(1,j)=0).
compute fulldat={fulldat,ctmp(:,j)}.
compute ctmpuse(1,j)=1.
compute ctmploc(1,j)=datcount.
compute datcount=datcount+1.
end if.
compute datindx(start:(start+nrow(ctmploc)-1),(i-1))=ctmploc(1,j).
compute start=start+nrow(ctmploc(1,j)).
end if.
end loop.
end if.
compute wdid=0.
compute zdid=0.
compute wzdid=0.
compute vlabs={vlabs;modlabel(2:nrow(modlabel),1)}.
compute numint(1,(i-1))=cntmp-1.
compute nump(1,(i-1))=nrow(modlabel)-1.
end loop.
release datcount, xtmpuse, wtmpuse, ztmpuse, xwtmpus, xztmpus, wztmpus, xwztmpu.
release xtmploc, wtmploc, xwtmplo, ztmploc, xztmplo, wztmplo, xwztmplo, foccnt.
do if (modcok=1 and ((nms > 0) or (zcmat(2,1) <> 1) or (mcx <> 0))).
compute notecode(notes,1) = 19.
compute notes = notes + 1.
compute modcok=0.
end if.
do if ((serial = 1 or (rsum(numint)>0) or nms=0) and mc > 0).
compute notecode(notes,1) = 15.
compute notes = notes + 1.
compute boot=mc.
compute mc=0.
end if.
do if (boot <> 0 or mc <> 0).
compute bootsz=boot.
do if (mc > 0).
compute bootsz=mc.
compute saveboot=0.
end if.
loop.
compute cilow = rnd(bootsz*(1-(conf/100))/2).
compute cihigh = trunc((bootsz*(conf/100)+(bootsz*(1-(conf/100))/2)))+1.
do if (cilow < 1 or cihigh > bootsz).
compute bootsz=trunc((bootsz+1000)/1000)*1000.
compute adjust = 1.
end if.
end loop if (cilow gt 0 and cihigh le bootsz).
do if (boot > 0).
compute boot=bootsz.
end if.
do if (mc > 0).
compute mc=bootsz.
end if.
do if (adjust = 1 and boot > 0).
compute notecode(notes,1) = 8.
compute notes = notes + 1.
end if.
do if (adjust = 1 and mc > 0).
compute notecode(notes,1) = 16.
compute notes = notes + 1.
end if.
end if.
compute maxboot = trunc(2*boot).
do if ( 0 > maxboot).
compute maxboot=trunc( 0 ).
end if.
do if (nms > 0).
release mtmpuse, mwtmpus, mwztmpu, mtmploc, mwtmplo, mztmplo, mwztmplo.
end if.
release wdid, zdid, wzdid, start,modlabel.
compute vlabs=vlabs(2:nrow(vlabs),1).
do if (rsum(numint) > 0).
compute intkey=intkey(2:nrow(intkey),:).
end if.
compute fulldat=fulldat(:,2:ncol(fulldat)).
compute fochigh=fochigh(1:rmax(nump),:).
compute whigh=whigh(1:rmax(nump),:).
compute zhigh=zhigh(1:rmax(nump),:).
compute wzhigh=wzhigh(1:rmax(nump),:).
compute coeffs=fochigh+whigh+zhigh+wzhigh.
compute bootloc=make(rmax(nump),ncol(nump),0).
do if (nms > 0).
compute cntmp=1.
loop i = 1 to ncol(nump).
loop j = 1 to nump(1,i).
compute bootloc(j,i)=cntmp.
compute cntmp=cntmp+1.
end loop.
end loop.
compute fochighb=make(nrow(fochigh),ncol(fochigh),0).
compute whighb=fochighb.
compute zhighb=fochighb.
compute wzhighb=fochighb.
compute thetaxmb=make(nrow(fochighb),nms,0).
compute thetaxyb=make(nrow(fochighb),1,0).
compute pathsfoc=make(nxvls,1,0).
compute cntmp=1.
loop i = 1 to (nms+nys).
loop j = 1 to i.
compute fochighb(:,cntmp)=fochigh(:,cntmp)&*bootloc(:,i).
compute whighb(:,cntmp)=whigh(:,cntmp)&*bootloc(:,i).
compute zhighb(:,cntmp)=zhigh(:,cntmp)&*bootloc(:,i).
compute wzhighb(:,cntmp)=wzhigh(:,cntmp)&*bootloc(:,i).
compute coeffsb=fochighb+whighb+zhighb+wzhighb.
do if ((i < (nms+nys)) and (j = 1)).
compute thetaxmb(:,i)=coeffsb(:,cntmp).
end if.
do if ((i = (nms+nys)) and (j = 1)).
compute thetaxyb(:,1)=coeffsb(:,cntmp).
end if.
compute cntmp=cntmp+1.
end loop.
end loop.
compute thetamyb=coeffsb(:,(ncol(coeffsb)-nms+1):ncol(coeffsb)).
do if (serial = 1).
compute thetammb=make(nrow(coeffsb),((nms*(nms-1))/2),0).
end if.
compute cntmp=1.
do if (nms > 1 and serial = 1).
loop i = 1 to (nms-1).
compute start=((i+2)*(i+1))/2.
loop j = 2 to (nms-i+1).
compute thetammb(:,cntmp)=coeffsb(:,start).
compute start=start+j+i-1.
compute cntmp=cntmp+1.
end loop.
end loop.
end if.
end if.
do if ((total = 1) and (rsum(numint)=0 or (xmint=1))).
compute dototal=1.
do if ((csum(bcmat(:,1)) <> (nms+nys)) or (rsum(bcmat(nrow(bcmat),:)) <> (nms+nys))).
compute dototal=0.
compute alttotal=1.
compute notecode(notes,1) = 12.
compute notes = notes + 1.
end if.
do if (ncs > 0).
do if ((csum(rsum(ccmat))) < (nrow(ccmat)*ncol(ccmat))).
compute dototal=0.
compute alttotal=1.
compute notecode(notes,1) = 11.
compute notes = notes + 1.
end if.
end if.
do if (model=74).
do if (xdich=0 and nxvls=1).
compute dototal=0.
compute alttotal=1.
end if.
do if ((xdich=1 or nxvls > 1) and (ncs > 0) and model=74).
compute dototal=0.
compute alttotal=1.
end if.
end if.
end if.
end if.
do if (criterr=0 and ncs > 0).
do if (rsum((csum(ccmat)=0)) <> 0).
compute errcode(errs,1)=51.
compute errs=errs+1.
compute criterr=1.
end if.
end if.
compute debug= 0.
do if (outscree=1).
print/title = '***************** PROCESS Procedure for SPSS Version 4.2 *****************'.
print/title = '          Written by Andrew F. Hayes, Ph.D.       www.afhayes.com'.
print/title = '    Documentation available in Hayes (2022). www.guilford.com/p/hayes3'/space=0.
end if.
do if (criterr=0).
compute modresid=make(n,1,99999).
do if (stand=1 and ydich=1).
compute stand=0.
end if.
compute anymod2=csum(rsum(wcmat+zcmat+wzcmat)).
do if (anymod2 > 0 and stand = 1).
compute notecode(notes,1) = 27.
compute notes = notes + 1.
compute stand=0.
end if.
compute funny=1.
do if (outscree=1).
print modelvar/title = '**************************************************************************'/format = A8/rnames=modelvlb.
do if (ncs > 0).
print covnames/title='Covariates:'/format=A8.
end if.
print n/title='Sample'/rlabel='Size:'.
do if (( 'random' <> 'random')).
compute seedt= 'random'.
print seedt/title='Custom'/format=A12/rlabel = 'Seed:'.
end if.
end if.
compute maxresm=9.
compute resultm=make(1,maxresm,99999).
do if (describe=1).
compute means=csum(dat)/n.
compute sigmatal = (t(dat)*(ident(n)-(1/n)*ones*t(ones))*dat)*(1/(n-1)).
compute sdvec=sqrt(diag(sigmatal)).
compute sdall = mdiag(1/sdvec).
compute corall=sdall*sigmatal*t(sdall).
compute means={means;t(sdvec)}.
do if (ncol(means) > 9).
compute resultm=make(1,ncol(means),99999).
compute maxresm=ncol(means).
end if.
compute resultm2=make(2,maxresm,99999).
compute resultm2(1:2,1:ncol(means))=means.
compute resultm={resultm;resultm2}.
compute resultm2=make(ncol(corall),maxresm,99999).
compute resultm2(1:ncol(corall),1:ncol(corall))=corall.
compute resultm={resultm;resultm2}.
do if (outscree=1).
print means/title='Variable means and standard deviations'/cnames=varnames/rlabels='Mean','SD'/format= F10.4.
print corall/title='Variable intercorrelations (Pearson r)'/cnames=varnames/rnames=varnames/format= F10.4.
end if.
end if.
do if (outscree=1).
do if (mcxok=1).
compute labtmp={xnames,t(xcatlab(1:nxvls,1))}.
print dummatx/title = 'Coding of categorical X variable for analysis:'/cnames = labtmp/format = F6.3.
end if.
do if (mcwok=1 and xmint=0).
compute labtmp={wnames,t(wcatlab(1:nwvls,1))}.
print dummatw/title = 'Coding of categorical W variable for analysis:'/cnames = labtmp/format = F6.3.
end if.
do if (mczok=1).
compute labtmp={znames,t(zcatlab(1:nzvls,1))}.
print dummatz/title = 'Coding of categorical Z variable for analysis:'/cnames = labtmp/format = F6.3.
end if.
end if.
end if.
do if (criterr = 0).
compute outnames=ynames.
compute outvars=ytmp.
do if (nms > 0).
compute outnames={mnames,ynames}.
compute outvars={mtmp,ytmp}.
compute indcov=make(((nms*2)+(nms*(nxvls-1))),((nms*2)+(nms*(nxvls-1))),0).
compute mcsopath=make(((nms*2)+(nms*(nxvls-1))),1,0).
end if.
compute labstart=1.
compute intstart=1.
compute start=1.
compute coeffmat=make(1,6,0).
compute conseq={'        '}.
compute dfmat=0.
compute coeffcol=0.
compute pathscnt=1.
compute pathscn2=1.
loop i = 1 to (nms+nys).
do if (outscree=1).
print/title = '**************************************************************************'.
end if.
compute highf=make(1,5,0).
compute highf2=highf.
do if ((i = (nms+nys)) and (ydich=1)).
compute highf=make(1,3,0).
compute highf2=highf.
end if.
compute flabel={' '}.
compute y=outvars(:,i).
compute xindx=datindx(1:(nump(1,i)-1),i).
compute x = fulldat(:,xindx).
compute x={ones,x}.
compute xsq=t(x)*x.
compute exsq=eval(xsq).
release xsq.
compute zeroeig=csum(exsq <= 0.000000000002).
do if (outscree=1).
print outnames(1,i)/title = 'OUTCOME VARIABLE:'/format = A8/space=0.
do if (ydich=1 and (i = (nms+nys))).
compute nmsd = {outnames(1,i), 'Analysis'}.
print rcd/title = 'Coding of binary Y for logistic regression analysis:'/cnames = nmsd/format = F9.2.
end if.
end if.
do if (zeroeig > 0).
print / title = 'SINGULAR OR NEAR SINGULAR DATA MATRIX.'.
compute criterr=1.
compute errcode(errs,1)=31.
compute errs=errs+1.
end if.
compute means=csum(x)/n.
compute vlabsm=vlabs(labstart:(labstart+(nump(1,i)-1)),1).
do if (criterr=0).
do if (ydich=0 or (i < (nms+nys))).

.
do if ( 1 =1).
compute hatmat=inv(t( x )* x )*t( x ).
compute b = hatmat* y.
compute modres=b.
do if ( 1 =1).
compute n1=nrow( x ).
compute dfres=n1-(ncol( x )).
compute sstotal = t( y -(csum( y )/n1))*( y -(csum( y )/n1)).
compute resid= y - x *b.
compute ssresid = csum((resid)&**2).
compute r2 = (sstotal-ssresid)/sstotal.
do if (r2 < 0).
compute r2=0.
end if.
compute adjr2 = 1-((1-r2)*(n1-1)/(dfres)).
compute mse=ssresid/(n1-ncol( x )).

.
compute n1=nrow( x ).
compute invXtX = inv(t( x )* x ).
compute varb = mse *invXtX.
compute k3 = ncol( x ).
compute xhc=0.
do if ( hc <> 5).
compute xhc= x.
compute hat = xhc(:,1).
loop i3=1 to nrow(xhc).
compute hat(i3,1)= xhc(i3,:)*invXtX*t(xhc(i3,:)).
end loop.
do if ( hc = 0 or hc =1).
loop i3 = 1 to k3.
compute xhc(:,i3)=xhc(:,i3)&* resid.
end loop.
end if.
do if ( hc =3 or hc =2).
loop i3=1 to k3.
compute xhc(:,i3) = ( resid &/(1-hat)&**(1/(4- hc )))&*xhc(:,i3).
end loop.
end if.
do if ( hc = 4).
compute hcmn=make(n,2,4).
compute hcmn(:,2)=(n1*hat)/k3.
loop i3= 1 to k3.
compute xhc(:,i3) = ( resid &/(1-hat)&**(rmin(hcmn)/2))&*xhc(:,i3).
end loop.
end if.
compute varb=(invXtX*t(xhc)*xhc*invXtX).
do if ( hc =1).
compute varb=(n1/(n1-ncol( x )))&*varb.
end if.
end if.
compute hclab={'se(HC0)','se(HC1)','se(HC2)','se(HC3)','se(HC4)','se'}.
compute hclab=hclab(1,( hc +1)).
compute hcflab={'F(HC0)','F(HC1)','F(HC2)','F(HC3)','F(HC4)','F'}.
compute hcflab=hcflab(1,( hc +1)).
release xhc
.
compute seb=sqrt(diag(varb)).
compute trat = b&/seb.
compute p = 2*(1-tcdf(abs(trat), (dfres))).
compute tval = sqrt(dfres* (exp((dfres-(5/6))*((xp2/(dfres-(2/3)+(.11/dfres)))* (xp2/(dfres-(2/3)+(.11/dfres)))))-1)).
compute modres={modres,seb,trat,p}.
compute modres={modres,(b-tval&*seb),(b+tval&*seb)}.
compute modresl={'coeff',hclab,'t','p','LLCI','ULCI'}.
compute lmat = ident(ncol( x )).
compute lmat = lmat(:,2:ncol(lmat)).
compute fratio = (t(t(lmat)*b)*inv(t(lmat)*varb*lmat)*((t(lmat)*b)))/(ncol( x )-1).
compute pfr = 1-fcdf(fratio,(ncol( x )-1),dfres).
compute modsum={sqrt(r2),r2,mse,fratio,(ncol( x )-1),dfres,pfr}.
compute modsuml={'R','R-sq','MSE',hcflab,'df1','df2', 'p'}.
end if.
end if.
do if ( 1 = 2 or 1 =3).
compute xlp= x.
compute ylp= y.
compute pt2 = make(nrow(ylp),1,(csum(ylp)/nrow(ylp))).
do if ( 1 =2).
compute LL3 = ylp&*ln(pt2)+(1-ylp)&*ln(1-pt2).
end if.
compute LL3 = -2*csum(LL3).
compute bt1 = make(ncol(xlp),1,0).
compute LL1 = 0.
compute pt1 = make(nrow(ylp),1,0.5).
compute pt1lp=pt1.
loop jjj = 1 to iterate.
compute xlptmp=t(xlp).
compute vecprb=pt1lp&*(1-pt1lp).
loop kkk=1 to ncol(xlp).
compute xlptmp(kkk,:)=xlptmp(kkk,:)&*t(vecprb).
end loop.
compute b = bt1+inv(xlptmp*xlp)*t(xlp)*(ylp-pt1lp).
do if ( 1 =2).
compute xlpb=xlp*b.
compute xlpbt=(xlpb > -709.7).
compute xlpb709=(1-xlpbt)*(-709.7).
compute xlpb=(xlpb&*xlpbt)+xlpb709.
compute pt1lp = 1/(1+exp(-(xlpb))).
end if.
compute itprob = csum((pt1lp < .00000001) or (pt1lp > .9999999)).
do if (itprob > 0).
loop kkk = 1 to nrow(pt1lp).
do if (pt1lp(kkk,1) > .9999999).
compute pt1lp(kkk,1) = .9999999.
end if.
do if (pt1lp(kkk,1) < .00000001).
compute pt1lp(kkk,1) = .00000001.
end if.
end loop.
compute itprob = 0.
end if.
do if (itprob = 0).
do if ( 1 =2).
compute LL = ylp&*ln(pt1lp)+(1-ylp)&*ln(1-pt1lp).
end if.
compute LL2 = -2*csum(ll).
end if.
do if (abs(LL1-LL2) < converge).
do if ( 1 =1).
compute xlptmp=t(xlp).
compute vecprb=pt1lp&*(1-pt1lp).
loop kkk=1 to ncol(xlp).
compute xlptmp(kkk,:)=xlptmp(kkk,:)&*t(vecprb).
end loop.
compute varb = inv(xlptmp*xlp).
compute seb = sqrt(diag(varb)).
release xlptmp.
end if.
break.
end if.
compute bt1 = b.
compute LL1 = LL2.
end loop.
compute modres=b.
do if (jjj > iterate).
compute itprob = 2.
do if (booting=0).
compute iterrmod=1.
end if.
do if (booting=1).
compute bootiter=1.
end if.
do if (itprobtg=0).
compute itprobtg=1.
compute errcode(errs,1) = 47.
compute errs = errs + 1.
do if (booting = 0 and 1 =1).
compute vt1 = mdiag(pt1lp&*(1-pt1lp)).
compute varb = inv(t(xlp)*vt1*xlp).
compute seb = sqrt(diag(varb)).
end if.
end if.
end if.
do if ( 1 =1).
compute trat = b&/seb.
compute dfres=nrow(xlp).
compute p = 2*(1-cdfnorm(abs(trat))).
compute modres={modres,seb,trat,p}.
compute modres={modres,(b-xp2&*seb),(b+xp2&*seb)}.
compute pvchi=1-chicdf((LL3-LL2),(nrow(modres)-1)).
compute mcF = (LL3-LL2)/LL3.
compute cox = 1-exp(-(LL3-LL2)/nrow(xlp)).
compute nagel = cox/(1-exp(-(LL3)/nrow(xlp))).
compute modsum={LL2,(LL3-LL2),(nrow(modres)-1),pvchi, mcF,cox,nagel}.
compute modsuml={'-2LL','ModelLL', 'df', 'p', 'McFadden', 'CoxSnell', 'Nagelkrk'}.
compute modresl={'coeff','se','Z','p','LLCI','ULCI'}.
end if.
end if
.
compute dfmatt=make(nrow(modres),1,modsum(1,6)).
compute modresid={modresid,resid}.
end if.
do if (ydich=1 and (i = (nms+nys))).

.
do if ( 2 =1).
compute hatmat=inv(t( x )* x )*t( x ).
compute b = hatmat* y.
compute modres=b.
do if ( 1 =1).
compute n1=nrow( x ).
compute dfres=n1-(ncol( x )).
compute sstotal = t( y -(csum( y )/n1))*( y -(csum( y )/n1)).
compute resid= y - x *b.
compute ssresid = csum((resid)&**2).
compute r2 = (sstotal-ssresid)/sstotal.
do if (r2 < 0).
compute r2=0.
end if.
compute adjr2 = 1-((1-r2)*(n1-1)/(dfres)).
compute mse=ssresid/(n1-ncol( x )).

.
compute n1=nrow( x ).
compute invXtX = inv(t( x )* x ).
compute varb = mse *invXtX.
compute k3 = ncol( x ).
compute xhc=0.
do if ( hc <> 5).
compute xhc= x.
compute hat = xhc(:,1).
loop i3=1 to nrow(xhc).
compute hat(i3,1)= xhc(i3,:)*invXtX*t(xhc(i3,:)).
end loop.
do if ( hc = 0 or hc =1).
loop i3 = 1 to k3.
compute xhc(:,i3)=xhc(:,i3)&* resid.
end loop.
end if.
do if ( hc =3 or hc =2).
loop i3=1 to k3.
compute xhc(:,i3) = ( resid &/(1-hat)&**(1/(4- hc )))&*xhc(:,i3).
end loop.
end if.
do if ( hc = 4).
compute hcmn=make(n,2,4).
compute hcmn(:,2)=(n1*hat)/k3.
loop i3= 1 to k3.
compute xhc(:,i3) = ( resid &/(1-hat)&**(rmin(hcmn)/2))&*xhc(:,i3).
end loop.
end if.
compute varb=(invXtX*t(xhc)*xhc*invXtX).
do if ( hc =1).
compute varb=(n1/(n1-ncol( x )))&*varb.
end if.
end if.
compute hclab={'se(HC0)','se(HC1)','se(HC2)','se(HC3)','se(HC4)','se'}.
compute hclab=hclab(1,( hc +1)).
compute hcflab={'F(HC0)','F(HC1)','F(HC2)','F(HC3)','F(HC4)','F'}.
compute hcflab=hcflab(1,( hc +1)).
release xhc
.
compute seb=sqrt(diag(varb)).
compute trat = b&/seb.
compute p = 2*(1-tcdf(abs(trat), (dfres))).
compute tval = sqrt(dfres* (exp((dfres-(5/6))*((xp2/(dfres-(2/3)+(.11/dfres)))* (xp2/(dfres-(2/3)+(.11/dfres)))))-1)).
compute modres={modres,seb,trat,p}.
compute modres={modres,(b-tval&*seb),(b+tval&*seb)}.
compute modresl={'coeff',hclab,'t','p','LLCI','ULCI'}.
compute lmat = ident(ncol( x )).
compute lmat = lmat(:,2:ncol(lmat)).
compute fratio = (t(t(lmat)*b)*inv(t(lmat)*varb*lmat)*((t(lmat)*b)))/(ncol( x )-1).
compute pfr = 1-fcdf(fratio,(ncol( x )-1),dfres).
compute modsum={sqrt(r2),r2,mse,fratio,(ncol( x )-1),dfres,pfr}.
compute modsuml={'R','R-sq','MSE',hcflab,'df1','df2', 'p'}.
end if.
end if.
do if ( 2 = 2 or 2 =3).
compute xlp= x.
compute ylp= y.
compute pt2 = make(nrow(ylp),1,(csum(ylp)/nrow(ylp))).
do if ( 2 =2).
compute LL3 = ylp&*ln(pt2)+(1-ylp)&*ln(1-pt2).
end if.
compute LL3 = -2*csum(LL3).
compute bt1 = make(ncol(xlp),1,0).
compute LL1 = 0.
compute pt1 = make(nrow(ylp),1,0.5).
compute pt1lp=pt1.
loop jjj = 1 to iterate.
compute xlptmp=t(xlp).
compute vecprb=pt1lp&*(1-pt1lp).
loop kkk=1 to ncol(xlp).
compute xlptmp(kkk,:)=xlptmp(kkk,:)&*t(vecprb).
end loop.
compute b = bt1+inv(xlptmp*xlp)*t(xlp)*(ylp-pt1lp).
do if ( 2 =2).
compute xlpb=xlp*b.
compute xlpbt=(xlpb > -709.7).
compute xlpb709=(1-xlpbt)*(-709.7).
compute xlpb=(xlpb&*xlpbt)+xlpb709.
compute pt1lp = 1/(1+exp(-(xlpb))).
end if.
compute itprob = csum((pt1lp < .00000001) or (pt1lp > .9999999)).
do if (itprob > 0).
loop kkk = 1 to nrow(pt1lp).
do if (pt1lp(kkk,1) > .9999999).
compute pt1lp(kkk,1) = .9999999.
end if.
do if (pt1lp(kkk,1) < .00000001).
compute pt1lp(kkk,1) = .00000001.
end if.
end loop.
compute itprob = 0.
end if.
do if (itprob = 0).
do if ( 2 =2).
compute LL = ylp&*ln(pt1lp)+(1-ylp)&*ln(1-pt1lp).
end if.
compute LL2 = -2*csum(ll).
end if.
do if (abs(LL1-LL2) < converge).
do if ( 1 =1).
compute xlptmp=t(xlp).
compute vecprb=pt1lp&*(1-pt1lp).
loop kkk=1 to ncol(xlp).
compute xlptmp(kkk,:)=xlptmp(kkk,:)&*t(vecprb).
end loop.
compute varb = inv(xlptmp*xlp).
compute seb = sqrt(diag(varb)).
release xlptmp.
end if.
break.
end if.
compute bt1 = b.
compute LL1 = LL2.
end loop.
compute modres=b.
do if (jjj > iterate).
compute itprob = 2.
do if (booting=0).
compute iterrmod=1.
end if.
do if (booting=1).
compute bootiter=1.
end if.
do if (itprobtg=0).
compute itprobtg=1.
compute errcode(errs,1) = 47.
compute errs = errs + 1.
do if (booting = 0 and 1 =1).
compute vt1 = mdiag(pt1lp&*(1-pt1lp)).
compute varb = inv(t(xlp)*vt1*xlp).
compute seb = sqrt(diag(varb)).
end if.
end if.
end if.
do if ( 1 =1).
compute trat = b&/seb.
compute dfres=nrow(xlp).
compute p = 2*(1-cdfnorm(abs(trat))).
compute modres={modres,seb,trat,p}.
compute modres={modres,(b-xp2&*seb),(b+xp2&*seb)}.
compute pvchi=1-chicdf((LL3-LL2),(nrow(modres)-1)).
compute mcF = (LL3-LL2)/LL3.
compute cox = 1-exp(-(LL3-LL2)/nrow(xlp)).
compute nagel = cox/(1-exp(-(LL3)/nrow(xlp))).
compute modsum={LL2,(LL3-LL2),(nrow(modres)-1),pvchi, mcF,cox,nagel}.
compute modsuml={'-2LL','ModelLL', 'df', 'p', 'McFadden', 'CoxSnell', 'Nagelkrk'}.
compute modresl={'coeff','se','Z','p','LLCI','ULCI'}.
end if.
end if
.
compute dfmatt=make(nrow(modres),1,-999).
end if.
compute resultm2=make(1,maxresm,99999).
compute resultm2(1,1:ncol(modsum))=modsum.
compute resultm={resultm;resultm2}.
compute resultm2=make(nrow(modres),maxresm,99999).
compute resultm2(1:nrow(modres),1:ncol(modres))=modres.
compute resultm={resultm;resultm2}.
compute obscoeff={obscoeff,t(b)}.
do if (outscree=1).
print modsum/title = 'Model Summary'/cnames = modsuml/format= F10.4.
print modres/title='Model'/rnames=vlabsm/cnames=modresl/format= F10.4.
end if.
compute basemod=modsum(1,1).
compute basemodx=basemod.
do if (ydich=1 and (i = (nms+nys))).
do if (outscree=1).
print/title='These results are expressed in a log-odds metric.'.
end if.
compute notecode(notes,1) = 26.
compute notes = notes + 1.
end if.
compute coeffmat={coeffmat;modres}.
compute conseqt=make(nrow(modres),1,outnames(1,i)).
compute conseq={conseq;conseqt}.
compute dfmat={dfmat;dfmatt}.
compute labstart=labstart+nump(1,i).
do if (stand=1).
compute predsd=make(nrow(modres),1,0).
compute stdmod=modres(:,1)&/ovsd(1,i).
loop jd=1 to ncol(x).
compute descdat=x(:,jd).
compute predsd(jd,1) = (nrow(descdat)*sscp(descdat))-(t(csum(descdat))*(csum(descdat))).
compute predsd(jd,1) = sqrt(predsd(jd,1)/(nrow(descdat)*(nrow(descdat)-1))).
end loop.
do if (wherex(1,i) <> -999 and ((nxvls > 1) or (xdich=1))).
compute sdmsone=make(nxvls,1,1).
compute predsd(wherex(1,i):wherex(2,i),1)=sdmsone.
compute pstog=1.
end if.
compute predsd(1,1)=1.
compute stdmod=stdmod&*predsd.
compute stdmod=stdmod(2:nrow(stdmod),1).
compute sdvlabs=vlabsm(2:nrow(vlabsm),1).
compute resultm2=make(nrow(stdmod),maxresm,99999).
compute resultm2(1:nrow(stdmod),1:ncol(stdmod))=stdmod.
compute resultm={resultm;resultm2}.
do if (outscree=1).
print stdmod/title='Standardized coefficients'/clabels='coeff'/rnames=sdvlabs/format= F10.4.
end if.
end if.
do if (nms > 0 and serial = 0 and (rsum(numint) = 0) and (normal=1 or mc > 0)).
do if (i < (nms+nys)).
compute indcov((((i-1)*nxvls)+1):(i*nxvls),(((i-1)*nxvls)+1):(i*nxvls))=varb(2:(1+nxvls), 2:(1+nxvls)).
compute mcsopath((((i-1)*nxvls)+1):(i*nxvls) ,1)=modres(2:(1+nxvls),1).
end if.
do if (i = (nms+nys)).
compute atm=ncol(wherem).
compute indcov(((nms*nxvls)+1):nrow(mcsopath),((nms*nxvls)+1):nrow(mcsopath))= varb(wherem(1,atm):(wherem(1,atm)+nms-1),wherem(1,atm):(wherem(1,atm)+nms-1)).
compute mcsopath(((nms*nxvls)+1):nrow(mcsopath),1)=modres(wherem(1,atm):(wherem(1,atm)+ nms-1),1).
compute sobelok=1.
end if.
end if.
compute obsdirfx=make(1,nxvls,0).
compute dirzes=make(1,nxvls,0).
do if ((i = (nms+nys)) and (bcmat(nrow(bcmat),1)=1)).
compute direff=modres(2:(1+nxvls),:).
compute obsdirfx=t(direff(:,1)).
compute direfflb=modresl.
compute direffl2=vlabsm(2:(1+nxvls),:).
compute lmat=make(nrow(b),1,0).
compute lmat2=make(nxvls,1,1).
compute lmat(2:(1+nxvls),1)=lmat2.
do if (ydich <> 1).

.
compute lmat2= lmat.
do if ( 0 =0).
compute lmat2 = mdiag( lmat ).
compute lmat3=make(nrow(lmat2),1,0).
loop flp=1 to ncol(lmat2).
do if (csum(lmat2(:,flp))=1).
compute lmat3={lmat3,lmat2(:,flp)}.
end if.
end loop.
compute lmat2=lmat3(:,2:ncol(lmat3)).
end if.
compute fratio = (t(t(lmat2)* b )*inv(t(lmat2)* varb *lmat2)*((t(lmat2)* b )))/ncol(lmat2).
compute pfr = 1-fcdf(fratio,ncol(lmat2),(n-nrow( b ))).
compute fresult={fratio,ncol(lmat2),(n-nrow( b )),pfr}.
do if (i = (nms+nys) and (ydich=1)).
compute fratio=fratio*ncol(lmat2).
compute pfr=1-chicdf(fratio,ncol(lmat2)).
compute fresult={fratio,ncol(lmat2),pfr}.
end if.
do if ( 1 =1).
compute lmat3=1-rsum(lmat2).
compute xfm=make(n,csum(lmat3),0).
compute flpc=1.
loop flp=1 to nrow(lmat3).
do if (lmat3(flp,1)=1).
compute xfm(:,flpc)=x(:,flp).
compute flpc=flpc+1.
end if.
end loop.
compute bfm=inv(t(xfm)*xfm)*t(xfm)*y.
compute resid=y-(xfm*bfm).
compute sstotal=(y-(csum(y)/n)).
compute sstotal=csum(sstotal&*sstotal).
compute ssresid=csum(resid&*resid).
compute rsqch= r2 -((sstotal-ssresid)/sstotal).
compute fresult={rsqch,fresult}.
release xfm,flpc, resid, ssresid, bfm.
end if
.
compute diromni=fresult.
end if.
do if (ydich = 1 ).

.
compute btemphld=b.
compute llrdat=make(nrow(x),nrow( lmat )-csum( lmat ),-999).
compute llrdf=ncol(x)-ncol(llrdat).
compute llrcnt=0.
loop llri=1 to nrow( lmat ).
do if ( lmat (llri,1)=0).
compute llrcnt=llrcnt+1.
compute llrdat(:,llrcnt)=x(:,llri).
end if.
end loop.

.
do if ( 2 =1).
compute hatmat=inv(t( llrdat )* llrdat )*t( llrdat ).
compute b = hatmat* y.
compute modres=b.
do if ( 0 =1).
compute n1=nrow( llrdat ).
compute dfres=n1-(ncol( llrdat )).
compute sstotal = t( y -(csum( y )/n1))*( y -(csum( y )/n1)).
compute resid= y - llrdat *b.
compute ssresid = csum((resid)&**2).
compute r2 = (sstotal-ssresid)/sstotal.
do if (r2 < 0).
compute r2=0.
end if.
compute adjr2 = 1-((1-r2)*(n1-1)/(dfres)).
compute mse=ssresid/(n1-ncol( llrdat )).

.
compute n1=nrow( x ).
compute invXtX = inv(t( x )* x ).
compute varb = mse *invXtX.
compute k3 = ncol( x ).
compute xhc=0.
do if ( hc <> 5).
compute xhc= x.
compute hat = xhc(:,1).
loop i3=1 to nrow(xhc).
compute hat(i3,1)= xhc(i3,:)*invXtX*t(xhc(i3,:)).
end loop.
do if ( hc = 0 or hc =1).
loop i3 = 1 to k3.
compute xhc(:,i3)=xhc(:,i3)&* resid.
end loop.
end if.
do if ( hc =3 or hc =2).
loop i3=1 to k3.
compute xhc(:,i3) = ( resid &/(1-hat)&**(1/(4- hc )))&*xhc(:,i3).
end loop.
end if.
do if ( hc = 4).
compute hcmn=make(n,2,4).
compute hcmn(:,2)=(n1*hat)/k3.
loop i3= 1 to k3.
compute xhc(:,i3) = ( resid &/(1-hat)&**(rmin(hcmn)/2))&*xhc(:,i3).
end loop.
end if.
compute varb=(invXtX*t(xhc)*xhc*invXtX).
do if ( hc =1).
compute varb=(n1/(n1-ncol( x )))&*varb.
end if.
end if.
compute hclab={'se(HC0)','se(HC1)','se(HC2)','se(HC3)','se(HC4)','se'}.
compute hclab=hclab(1,( hc +1)).
compute hcflab={'F(HC0)','F(HC1)','F(HC2)','F(HC3)','F(HC4)','F'}.
compute hcflab=hcflab(1,( hc +1)).
release xhc
.
compute seb=sqrt(diag(varb)).
compute trat = b&/seb.
compute p = 2*(1-tcdf(abs(trat), (dfres))).
compute tval = sqrt(dfres* (exp((dfres-(5/6))*((xp2/(dfres-(2/3)+(.11/dfres)))* (xp2/(dfres-(2/3)+(.11/dfres)))))-1)).
compute modres={modres,seb,trat,p}.
compute modres={modres,(b-tval&*seb),(b+tval&*seb)}.
compute modresl={'coeff',hclab,'t','p','LLCI','ULCI'}.
compute lmat = ident(ncol( llrdat )).
compute lmat = lmat(:,2:ncol(lmat)).
compute fratio = (t(t(lmat)*b)*inv(t(lmat)*varb*lmat)*((t(lmat)*b)))/(ncol( llrdat )-1).
compute pfr = 1-fcdf(fratio,(ncol( llrdat )-1),dfres).
compute modsum={sqrt(r2),r2,mse,fratio,(ncol( llrdat )-1),dfres,pfr}.
compute modsuml={'R','R-sq','MSE',hcflab,'df1','df2', 'p'}.
end if.
end if.
do if ( 2 = 2 or 2 =3).
compute xlp= llrdat.
compute ylp= y.
compute pt2 = make(nrow(ylp),1,(csum(ylp)/nrow(ylp))).
do if ( 2 =2).
compute LL3 = ylp&*ln(pt2)+(1-ylp)&*ln(1-pt2).
end if.
compute LL3 = -2*csum(LL3).
compute bt1 = make(ncol(xlp),1,0).
compute LL1 = 0.
compute pt1 = make(nrow(ylp),1,0.5).
compute pt1lp=pt1.
loop jjj = 1 to iterate.
compute xlptmp=t(xlp).
compute vecprb=pt1lp&*(1-pt1lp).
loop kkk=1 to ncol(xlp).
compute xlptmp(kkk,:)=xlptmp(kkk,:)&*t(vecprb).
end loop.
compute b = bt1+inv(xlptmp*xlp)*t(xlp)*(ylp-pt1lp).
do if ( 2 =2).
compute xlpb=xlp*b.
compute xlpbt=(xlpb > -709.7).
compute xlpb709=(1-xlpbt)*(-709.7).
compute xlpb=(xlpb&*xlpbt)+xlpb709.
compute pt1lp = 1/(1+exp(-(xlpb))).
end if.
compute itprob = csum((pt1lp < .00000001) or (pt1lp > .9999999)).
do if (itprob > 0).
loop kkk = 1 to nrow(pt1lp).
do if (pt1lp(kkk,1) > .9999999).
compute pt1lp(kkk,1) = .9999999.
end if.
do if (pt1lp(kkk,1) < .00000001).
compute pt1lp(kkk,1) = .00000001.
end if.
end loop.
compute itprob = 0.
end if.
do if (itprob = 0).
do if ( 2 =2).
compute LL = ylp&*ln(pt1lp)+(1-ylp)&*ln(1-pt1lp).
end if.
compute LL2 = -2*csum(ll).
end if.
do if (abs(LL1-LL2) < converge).
do if ( 0 =1).
compute xlptmp=t(xlp).
compute vecprb=pt1lp&*(1-pt1lp).
loop kkk=1 to ncol(xlp).
compute xlptmp(kkk,:)=xlptmp(kkk,:)&*t(vecprb).
end loop.
compute varb = inv(xlptmp*xlp).
compute seb = sqrt(diag(varb)).
release xlptmp.
end if.
break.
end if.
compute bt1 = b.
compute LL1 = LL2.
end loop.
compute modres=b.
do if (jjj > iterate).
compute itprob = 2.
do if (booting=0).
compute iterrmod=1.
end if.
do if (booting=1).
compute bootiter=1.
end if.
do if (itprobtg=0).
compute itprobtg=1.
compute errcode(errs,1) = 47.
compute errs = errs + 1.
do if (booting = 0 and 0 =1).
compute vt1 = mdiag(pt1lp&*(1-pt1lp)).
compute varb = inv(t(xlp)*vt1*xlp).
compute seb = sqrt(diag(varb)).
end if.
end if.
end if.
do if ( 0 =1).
compute trat = b&/seb.
compute dfres=nrow(xlp).
compute p = 2*(1-cdfnorm(abs(trat))).
compute modres={modres,seb,trat,p}.
compute modres={modres,(b-xp2&*seb),(b+xp2&*seb)}.
compute pvchi=1-chicdf((LL3-LL2),(nrow(modres)-1)).
compute mcF = (LL3-LL2)/LL3.
compute cox = 1-exp(-(LL3-LL2)/nrow(xlp)).
compute nagel = cox/(1-exp(-(LL3)/nrow(xlp))).
compute modsum={LL2,(LL3-LL2),(nrow(modres)-1),pvchi, mcF,cox,nagel}.
compute modsuml={'-2LL','ModelLL', 'df', 'p', 'McFadden', 'CoxSnell', 'Nagelkrk'}.
compute modresl={'coeff','se','Z','p','LLCI','ULCI'}.
end if.
end if
.
compute b=btemphld.
compute fresult={(LL2-basemod),llrdf,1-chicdf((LL2-basemod),llrdf)}
.
compute diromni=fresult.
end if.
end if.
do if (numint(1,i) > 0).
compute intkeym=intkey(intstart:(intstart+numint(1,i)-1),:).
do if (outscree=1).
print intkeym/title='Product terms key:'/format = A8.
end if.
end if.
do if (covcoeff=1).
do if (outscree=1).
print varb/title='Covariance matrix of regression parameter estimates:'/rnames=vlabsm /cnames=vlabsm/format= F10.4.
end if.
compute resultm2=make(nrow(varb),maxresm,99999).
do if (ncol(varb) <= maxresm).
compute resultm2(1:nrow(varb),1:ncol(varb))=varb.
compute resultm={resultm;resultm2}.
end if.
do if (ncol(varb) > maxresm).
compute resultmt=make(nrow(resultm),ncol(varb),99999).
compute resultmt(1:nrow(resultm),1:ncol(resultm))=resultm.
compute resultm=resultmt.
compute resultm2=make(nrow(varb),ncol(resultm),99999).
compute resultm2(1:nrow(varb),1:ncol(varb))=varb.
compute resultm={resultm;resultm2}.
compute maxresm=ncol(resultm).
end if.
end if.
do if (model <> 74 and xmtest=1 and nms > 0).
compute r2tmp=r2.
compute btmp=b.
compute varbtmp=varb.
compute dfrestmp=dfres.
compute tvaltmp=tval.
compute xmtst=make(nms,4,0).
compute xmtstlbc={hcflab,'df1','df2','p'}.
do if ((i=(nms+nys)) and (ydich=1)).
compute xmtst=make(nms,3,0).
compute xmtstlbc={'Chi-sq','df','p'}.
end if.
compute xmtstlb={' '}.
compute xmtmat=x.
compute numxint=0.
loop xmints=2 to i.
compute x=xmtmat.
do if ((bcmat((i+1),xmints)=1) and (wzcmat((i+1),xmints) <> 1)).
do if (bcmat((i+1),1)=0).
compute x={xmtmat,xtmp}.
do if ((ydich=1) and (i=(nms+nys))).

.
do if ( 2 =1).
compute hatmat=inv(t( x )* x )*t( x ).
compute b = hatmat* y.
compute modres=b.
do if ( 1 =1).
compute n1=nrow( x ).
compute dfres=n1-(ncol( x )).
compute sstotal = t( y -(csum( y )/n1))*( y -(csum( y )/n1)).
compute resid= y - x *b.
compute ssresid = csum((resid)&**2).
compute r2 = (sstotal-ssresid)/sstotal.
do if (r2 < 0).
compute r2=0.
end if.
compute adjr2 = 1-((1-r2)*(n1-1)/(dfres)).
compute mse=ssresid/(n1-ncol( x )).

.
compute n1=nrow( x ).
compute invXtX = inv(t( x )* x ).
compute varb = mse *invXtX.
compute k3 = ncol( x ).
compute xhc=0.
do if ( hc <> 5).
compute xhc= x.
compute hat = xhc(:,1).
loop i3=1 to nrow(xhc).
compute hat(i3,1)= xhc(i3,:)*invXtX*t(xhc(i3,:)).
end loop.
do if ( hc = 0 or hc =1).
loop i3 = 1 to k3.
compute xhc(:,i3)=xhc(:,i3)&* resid.
end loop.
end if.
do if ( hc =3 or hc =2).
loop i3=1 to k3.
compute xhc(:,i3) = ( resid &/(1-hat)&**(1/(4- hc )))&*xhc(:,i3).
end loop.
end if.
do if ( hc = 4).
compute hcmn=make(n,2,4).
compute hcmn(:,2)=(n1*hat)/k3.
loop i3= 1 to k3.
compute xhc(:,i3) = ( resid &/(1-hat)&**(rmin(hcmn)/2))&*xhc(:,i3).
end loop.
end if.
compute varb=(invXtX*t(xhc)*xhc*invXtX).
do if ( hc =1).
compute varb=(n1/(n1-ncol( x )))&*varb.
end if.
end if.
compute hclab={'se(HC0)','se(HC1)','se(HC2)','se(HC3)','se(HC4)','se'}.
compute hclab=hclab(1,( hc +1)).
compute hcflab={'F(HC0)','F(HC1)','F(HC2)','F(HC3)','F(HC4)','F'}.
compute hcflab=hcflab(1,( hc +1)).
release xhc
.
compute seb=sqrt(diag(varb)).
compute trat = b&/seb.
compute p = 2*(1-tcdf(abs(trat), (dfres))).
compute tval = sqrt(dfres* (exp((dfres-(5/6))*((xp2/(dfres-(2/3)+(.11/dfres)))* (xp2/(dfres-(2/3)+(.11/dfres)))))-1)).
compute modres={modres,seb,trat,p}.
compute modres={modres,(b-tval&*seb),(b+tval&*seb)}.
compute modresl={'coeff',hclab,'t','p','LLCI','ULCI'}.
compute lmat = ident(ncol( x )).
compute lmat = lmat(:,2:ncol(lmat)).
compute fratio = (t(t(lmat)*b)*inv(t(lmat)*varb*lmat)*((t(lmat)*b)))/(ncol( x )-1).
compute pfr = 1-fcdf(fratio,(ncol( x )-1),dfres).
compute modsum={sqrt(r2),r2,mse,fratio,(ncol( x )-1),dfres,pfr}.
compute modsuml={'R','R-sq','MSE',hcflab,'df1','df2', 'p'}.
end if.
end if.
do if ( 2 = 2 or 2 =3).
compute xlp= x.
compute ylp= y.
compute pt2 = make(nrow(ylp),1,(csum(ylp)/nrow(ylp))).
do if ( 2 =2).
compute LL3 = ylp&*ln(pt2)+(1-ylp)&*ln(1-pt2).
end if.
compute LL3 = -2*csum(LL3).
compute bt1 = make(ncol(xlp),1,0).
compute LL1 = 0.
compute pt1 = make(nrow(ylp),1,0.5).
compute pt1lp=pt1.
loop jjj = 1 to iterate.
compute xlptmp=t(xlp).
compute vecprb=pt1lp&*(1-pt1lp).
loop kkk=1 to ncol(xlp).
compute xlptmp(kkk,:)=xlptmp(kkk,:)&*t(vecprb).
end loop.
compute b = bt1+inv(xlptmp*xlp)*t(xlp)*(ylp-pt1lp).
do if ( 2 =2).
compute xlpb=xlp*b.
compute xlpbt=(xlpb > -709.7).
compute xlpb709=(1-xlpbt)*(-709.7).
compute xlpb=(xlpb&*xlpbt)+xlpb709.
compute pt1lp = 1/(1+exp(-(xlpb))).
end if.
compute itprob = csum((pt1lp < .00000001) or (pt1lp > .9999999)).
do if (itprob > 0).
loop kkk = 1 to nrow(pt1lp).
do if (pt1lp(kkk,1) > .9999999).
compute pt1lp(kkk,1) = .9999999.
end if.
do if (pt1lp(kkk,1) < .00000001).
compute pt1lp(kkk,1) = .00000001.
end if.
end loop.
compute itprob = 0.
end if.
do if (itprob = 0).
do if ( 2 =2).
compute LL = ylp&*ln(pt1lp)+(1-ylp)&*ln(1-pt1lp).
end if.
compute LL2 = -2*csum(ll).
end if.
do if (abs(LL1-LL2) < converge).
do if ( 1 =1).
compute xlptmp=t(xlp).
compute vecprb=pt1lp&*(1-pt1lp).
loop kkk=1 to ncol(xlp).
compute xlptmp(kkk,:)=xlptmp(kkk,:)&*t(vecprb).
end loop.
compute varb = inv(xlptmp*xlp).
compute seb = sqrt(diag(varb)).
release xlptmp.
end if.
break.
end if.
compute bt1 = b.
compute LL1 = LL2.
end loop.
compute modres=b.
do if (jjj > iterate).
compute itprob = 2.
do if (booting=0).
compute iterrmod=1.
end if.
do if (booting=1).
compute bootiter=1.
end if.
do if (itprobtg=0).
compute itprobtg=1.
compute errcode(errs,1) = 47.
compute errs = errs + 1.
do if (booting = 0 and 1 =1).
compute vt1 = mdiag(pt1lp&*(1-pt1lp)).
compute varb = inv(t(xlp)*vt1*xlp).
compute seb = sqrt(diag(varb)).
end if.
end if.
end if.
do if ( 1 =1).
compute trat = b&/seb.
compute dfres=nrow(xlp).
compute p = 2*(1-cdfnorm(abs(trat))).
compute modres={modres,seb,trat,p}.
compute modres={modres,(b-xp2&*seb),(b+xp2&*seb)}.
compute pvchi=1-chicdf((LL3-LL2),(nrow(modres)-1)).
compute mcF = (LL3-LL2)/LL3.
compute cox = 1-exp(-(LL3-LL2)/nrow(xlp)).
compute nagel = cox/(1-exp(-(LL3)/nrow(xlp))).
compute modsum={LL2,(LL3-LL2),(nrow(modres)-1),pvchi, mcF,cox,nagel}.
compute modsuml={'-2LL','ModelLL', 'df', 'p', 'McFadden', 'CoxSnell', 'Nagelkrk'}.
compute modresl={'coeff','se','Z','p','LLCI','ULCI'}.
end if.
end if
.
compute basemodx=LL2.
end if.
end if.
loop xmtlp1=1 to nxvls.
compute x={x,xtmp(:,xmtlp1)&*(mtmp(:,(xmints-1))-(csum(mtmp(:,(xmints-1)))/nrow(mtmp)) )}.
end loop.
do if ((i < (nms+nys)) or (ydich=0)).

.
do if ( 1 =1).
compute hatmat=inv(t( x )* x )*t( x ).
compute b = hatmat* y.
compute modres=b.
do if ( 1 =1).
compute n1=nrow( x ).
compute dfres=n1-(ncol( x )).
compute sstotal = t( y -(csum( y )/n1))*( y -(csum( y )/n1)).
compute resid= y - x *b.
compute ssresid = csum((resid)&**2).
compute r2 = (sstotal-ssresid)/sstotal.
do if (r2 < 0).
compute r2=0.
end if.
compute adjr2 = 1-((1-r2)*(n1-1)/(dfres)).
compute mse=ssresid/(n1-ncol( x )).

.
compute n1=nrow( x ).
compute invXtX = inv(t( x )* x ).
compute varb = mse *invXtX.
compute k3 = ncol( x ).
compute xhc=0.
do if ( hc <> 5).
compute xhc= x.
compute hat = xhc(:,1).
loop i3=1 to nrow(xhc).
compute hat(i3,1)= xhc(i3,:)*invXtX*t(xhc(i3,:)).
end loop.
do if ( hc = 0 or hc =1).
loop i3 = 1 to k3.
compute xhc(:,i3)=xhc(:,i3)&* resid.
end loop.
end if.
do if ( hc =3 or hc =2).
loop i3=1 to k3.
compute xhc(:,i3) = ( resid &/(1-hat)&**(1/(4- hc )))&*xhc(:,i3).
end loop.
end if.
do if ( hc = 4).
compute hcmn=make(n,2,4).
compute hcmn(:,2)=(n1*hat)/k3.
loop i3= 1 to k3.
compute xhc(:,i3) = ( resid &/(1-hat)&**(rmin(hcmn)/2))&*xhc(:,i3).
end loop.
end if.
compute varb=(invXtX*t(xhc)*xhc*invXtX).
do if ( hc =1).
compute varb=(n1/(n1-ncol( x )))&*varb.
end if.
end if.
compute hclab={'se(HC0)','se(HC1)','se(HC2)','se(HC3)','se(HC4)','se'}.
compute hclab=hclab(1,( hc +1)).
compute hcflab={'F(HC0)','F(HC1)','F(HC2)','F(HC3)','F(HC4)','F'}.
compute hcflab=hcflab(1,( hc +1)).
release xhc
.
compute seb=sqrt(diag(varb)).
compute trat = b&/seb.
compute p = 2*(1-tcdf(abs(trat), (dfres))).
compute tval = sqrt(dfres* (exp((dfres-(5/6))*((xp2/(dfres-(2/3)+(.11/dfres)))* (xp2/(dfres-(2/3)+(.11/dfres)))))-1)).
compute modres={modres,seb,trat,p}.
compute modres={modres,(b-tval&*seb),(b+tval&*seb)}.
compute modresl={'coeff',hclab,'t','p','LLCI','ULCI'}.
compute lmat = ident(ncol( x )).
compute lmat = lmat(:,2:ncol(lmat)).
compute fratio = (t(t(lmat)*b)*inv(t(lmat)*varb*lmat)*((t(lmat)*b)))/(ncol( x )-1).
compute pfr = 1-fcdf(fratio,(ncol( x )-1),dfres).
compute modsum={sqrt(r2),r2,mse,fratio,(ncol( x )-1),dfres,pfr}.
compute modsuml={'R','R-sq','MSE',hcflab,'df1','df2', 'p'}.
end if.
end if.
do if ( 1 = 2 or 1 =3).
compute xlp= x.
compute ylp= y.
compute pt2 = make(nrow(ylp),1,(csum(ylp)/nrow(ylp))).
do if ( 1 =2).
compute LL3 = ylp&*ln(pt2)+(1-ylp)&*ln(1-pt2).
end if.
compute LL3 = -2*csum(LL3).
compute bt1 = make(ncol(xlp),1,0).
compute LL1 = 0.
compute pt1 = make(nrow(ylp),1,0.5).
compute pt1lp=pt1.
loop jjj = 1 to iterate.
compute xlptmp=t(xlp).
compute vecprb=pt1lp&*(1-pt1lp).
loop kkk=1 to ncol(xlp).
compute xlptmp(kkk,:)=xlptmp(kkk,:)&*t(vecprb).
end loop.
compute b = bt1+inv(xlptmp*xlp)*t(xlp)*(ylp-pt1lp).
do if ( 1 =2).
compute xlpb=xlp*b.
compute xlpbt=(xlpb > -709.7).
compute xlpb709=(1-xlpbt)*(-709.7).
compute xlpb=(xlpb&*xlpbt)+xlpb709.
compute pt1lp = 1/(1+exp(-(xlpb))).
end if.
compute itprob = csum((pt1lp < .00000001) or (pt1lp > .9999999)).
do if (itprob > 0).
loop kkk = 1 to nrow(pt1lp).
do if (pt1lp(kkk,1) > .9999999).
compute pt1lp(kkk,1) = .9999999.
end if.
do if (pt1lp(kkk,1) < .00000001).
compute pt1lp(kkk,1) = .00000001.
end if.
end loop.
compute itprob = 0.
end if.
do if (itprob = 0).
do if ( 1 =2).
compute LL = ylp&*ln(pt1lp)+(1-ylp)&*ln(1-pt1lp).
end if.
compute LL2 = -2*csum(ll).
end if.
do if (abs(LL1-LL2) < converge).
do if ( 1 =1).
compute xlptmp=t(xlp).
compute vecprb=pt1lp&*(1-pt1lp).
loop kkk=1 to ncol(xlp).
compute xlptmp(kkk,:)=xlptmp(kkk,:)&*t(vecprb).
end loop.
compute varb = inv(xlptmp*xlp).
compute seb = sqrt(diag(varb)).
release xlptmp.
end if.
break.
end if.
compute bt1 = b.
compute LL1 = LL2.
end loop.
compute modres=b.
do if (jjj > iterate).
compute itprob = 2.
do if (booting=0).
compute iterrmod=1.
end if.
do if (booting=1).
compute bootiter=1.
end if.
do if (itprobtg=0).
compute itprobtg=1.
compute errcode(errs,1) = 47.
compute errs = errs + 1.
do if (booting = 0 and 1 =1).
compute vt1 = mdiag(pt1lp&*(1-pt1lp)).
compute varb = inv(t(xlp)*vt1*xlp).
compute seb = sqrt(diag(varb)).
end if.
end if.
end if.
do if ( 1 =1).
compute trat = b&/seb.
compute dfres=nrow(xlp).
compute p = 2*(1-cdfnorm(abs(trat))).
compute modres={modres,seb,trat,p}.
compute modres={modres,(b-xp2&*seb),(b+xp2&*seb)}.
compute pvchi=1-chicdf((LL3-LL2),(nrow(modres)-1)).
compute mcF = (LL3-LL2)/LL3.
compute cox = 1-exp(-(LL3-LL2)/nrow(xlp)).
compute nagel = cox/(1-exp(-(LL3)/nrow(xlp))).
compute modsum={LL2,(LL3-LL2),(nrow(modres)-1),pvchi, mcF,cox,nagel}.
compute modsuml={'-2LL','ModelLL', 'df', 'p', 'McFadden', 'CoxSnell', 'Nagelkrk'}.
compute modresl={'coeff','se','Z','p','LLCI','ULCI'}.
end if.
end if
.
end if.
do if ((i = (nms+nys)) and (ydich=1)).

.
do if ( 2 =1).
compute hatmat=inv(t( x )* x )*t( x ).
compute b = hatmat* y.
compute modres=b.
do if ( 1 =1).
compute n1=nrow( x ).
compute dfres=n1-(ncol( x )).
compute sstotal = t( y -(csum( y )/n1))*( y -(csum( y )/n1)).
compute resid= y - x *b.
compute ssresid = csum((resid)&**2).
compute r2 = (sstotal-ssresid)/sstotal.
do if (r2 < 0).
compute r2=0.
end if.
compute adjr2 = 1-((1-r2)*(n1-1)/(dfres)).
compute mse=ssresid/(n1-ncol( x )).

.
compute n1=nrow( x ).
compute invXtX = inv(t( x )* x ).
compute varb = mse *invXtX.
compute k3 = ncol( x ).
compute xhc=0.
do if ( hc <> 5).
compute xhc= x.
compute hat = xhc(:,1).
loop i3=1 to nrow(xhc).
compute hat(i3,1)= xhc(i3,:)*invXtX*t(xhc(i3,:)).
end loop.
do if ( hc = 0 or hc =1).
loop i3 = 1 to k3.
compute xhc(:,i3)=xhc(:,i3)&* resid.
end loop.
end if.
do if ( hc =3 or hc =2).
loop i3=1 to k3.
compute xhc(:,i3) = ( resid &/(1-hat)&**(1/(4- hc )))&*xhc(:,i3).
end loop.
end if.
do if ( hc = 4).
compute hcmn=make(n,2,4).
compute hcmn(:,2)=(n1*hat)/k3.
loop i3= 1 to k3.
compute xhc(:,i3) = ( resid &/(1-hat)&**(rmin(hcmn)/2))&*xhc(:,i3).
end loop.
end if.
compute varb=(invXtX*t(xhc)*xhc*invXtX).
do if ( hc =1).
compute varb=(n1/(n1-ncol( x )))&*varb.
end if.
end if.
compute hclab={'se(HC0)','se(HC1)','se(HC2)','se(HC3)','se(HC4)','se'}.
compute hclab=hclab(1,( hc +1)).
compute hcflab={'F(HC0)','F(HC1)','F(HC2)','F(HC3)','F(HC4)','F'}.
compute hcflab=hcflab(1,( hc +1)).
release xhc
.
compute seb=sqrt(diag(varb)).
compute trat = b&/seb.
compute p = 2*(1-tcdf(abs(trat), (dfres))).
compute tval = sqrt(dfres* (exp((dfres-(5/6))*((xp2/(dfres-(2/3)+(.11/dfres)))* (xp2/(dfres-(2/3)+(.11/dfres)))))-1)).
compute modres={modres,seb,trat,p}.
compute modres={modres,(b-tval&*seb),(b+tval&*seb)}.
compute modresl={'coeff',hclab,'t','p','LLCI','ULCI'}.
compute lmat = ident(ncol( x )).
compute lmat = lmat(:,2:ncol(lmat)).
compute fratio = (t(t(lmat)*b)*inv(t(lmat)*varb*lmat)*((t(lmat)*b)))/(ncol( x )-1).
compute pfr = 1-fcdf(fratio,(ncol( x )-1),dfres).
compute modsum={sqrt(r2),r2,mse,fratio,(ncol( x )-1),dfres,pfr}.
compute modsuml={'R','R-sq','MSE',hcflab,'df1','df2', 'p'}.
end if.
end if.
do if ( 2 = 2 or 2 =3).
compute xlp= x.
compute ylp= y.
compute pt2 = make(nrow(ylp),1,(csum(ylp)/nrow(ylp))).
do if ( 2 =2).
compute LL3 = ylp&*ln(pt2)+(1-ylp)&*ln(1-pt2).
end if.
compute LL3 = -2*csum(LL3).
compute bt1 = make(ncol(xlp),1,0).
compute LL1 = 0.
compute pt1 = make(nrow(ylp),1,0.5).
compute pt1lp=pt1.
loop jjj = 1 to iterate.
compute xlptmp=t(xlp).
compute vecprb=pt1lp&*(1-pt1lp).
loop kkk=1 to ncol(xlp).
compute xlptmp(kkk,:)=xlptmp(kkk,:)&*t(vecprb).
end loop.
compute b = bt1+inv(xlptmp*xlp)*t(xlp)*(ylp-pt1lp).
do if ( 2 =2).
compute xlpb=xlp*b.
compute xlpbt=(xlpb > -709.7).
compute xlpb709=(1-xlpbt)*(-709.7).
compute xlpb=(xlpb&*xlpbt)+xlpb709.
compute pt1lp = 1/(1+exp(-(xlpb))).
end if.
compute itprob = csum((pt1lp < .00000001) or (pt1lp > .9999999)).
do if (itprob > 0).
loop kkk = 1 to nrow(pt1lp).
do if (pt1lp(kkk,1) > .9999999).
compute pt1lp(kkk,1) = .9999999.
end if.
do if (pt1lp(kkk,1) < .00000001).
compute pt1lp(kkk,1) = .00000001.
end if.
end loop.
compute itprob = 0.
end if.
do if (itprob = 0).
do if ( 2 =2).
compute LL = ylp&*ln(pt1lp)+(1-ylp)&*ln(1-pt1lp).
end if.
compute LL2 = -2*csum(ll).
end if.
do if (abs(LL1-LL2) < converge).
do if ( 1 =1).
compute xlptmp=t(xlp).
compute vecprb=pt1lp&*(1-pt1lp).
loop kkk=1 to ncol(xlp).
compute xlptmp(kkk,:)=xlptmp(kkk,:)&*t(vecprb).
end loop.
compute varb = inv(xlptmp*xlp).
compute seb = sqrt(diag(varb)).
release xlptmp.
end if.
break.
end if.
compute bt1 = b.
compute LL1 = LL2.
end loop.
compute modres=b.
do if (jjj > iterate).
compute itprob = 2.
do if (booting=0).
compute iterrmod=1.
end if.
do if (booting=1).
compute bootiter=1.
end if.
do if (itprobtg=0).
compute itprobtg=1.
compute errcode(errs,1) = 47.
compute errs = errs + 1.
do if (booting = 0 and 1 =1).
compute vt1 = mdiag(pt1lp&*(1-pt1lp)).
compute varb = inv(t(xlp)*vt1*xlp).
compute seb = sqrt(diag(varb)).
end if.
end if.
end if.
do if ( 1 =1).
compute trat = b&/seb.
compute dfres=nrow(xlp).
compute p = 2*(1-cdfnorm(abs(trat))).
compute modres={modres,seb,trat,p}.
compute modres={modres,(b-xp2&*seb),(b+xp2&*seb)}.
compute pvchi=1-chicdf((LL3-LL2),(nrow(modres)-1)).
compute mcF = (LL3-LL2)/LL3.
compute cox = 1-exp(-(LL3-LL2)/nrow(xlp)).
compute nagel = cox/(1-exp(-(LL3)/nrow(xlp))).
compute modsum={LL2,(LL3-LL2),(nrow(modres)-1),pvchi, mcF,cox,nagel}.
compute modsuml={'-2LL','ModelLL', 'df', 'p', 'McFadden', 'CoxSnell', 'Nagelkrk'}.
compute modresl={'coeff','se','Z','p','LLCI','ULCI'}.
end if.
end if
.
compute chidfxm=basemodx-LL2.
end if.
compute lmat=make(nrow(b),nxvls,0).
compute lmattmp=ident(nxvls).
compute lmat((nrow(lmat)-nxvls+1):nrow(lmat),:)=lmattmp.

.
compute lmat2= lmat.
do if ( 1 =0).
compute lmat2 = mdiag( lmat ).
compute lmat3=make(nrow(lmat2),1,0).
loop flp=1 to ncol(lmat2).
do if (csum(lmat2(:,flp))=1).
compute lmat3={lmat3,lmat2(:,flp)}.
end if.
end loop.
compute lmat2=lmat3(:,2:ncol(lmat3)).
end if.
compute fratio = (t(t(lmat2)* b )*inv(t(lmat2)* varb *lmat2)*((t(lmat2)* b )))/ncol(lmat2).
compute pfr = 1-fcdf(fratio,ncol(lmat2),(n-nrow( b ))).
compute fresult={fratio,ncol(lmat2),(n-nrow( b )),pfr}.
do if (i = (nms+nys) and (ydich=1)).
compute fratio=fratio*ncol(lmat2).
compute pfr=1-chicdf(fratio,ncol(lmat2)).
compute fresult={fratio,ncol(lmat2),pfr}.
end if.
do if ( 0 =1).
compute lmat3=1-rsum(lmat2).
compute xfm=make(n,csum(lmat3),0).
compute flpc=1.
loop flp=1 to nrow(lmat3).
do if (lmat3(flp,1)=1).
compute xfm(:,flpc)=x(:,flp).
compute flpc=flpc+1.
end if.
end loop.
compute bfm=inv(t(xfm)*xfm)*t(xfm)*y.
compute resid=y-(xfm*bfm).
compute sstotal=(y-(csum(y)/n)).
compute sstotal=csum(sstotal&*sstotal).
compute ssresid=csum(resid&*resid).
compute rsqch= 0 -((sstotal-ssresid)/sstotal).
compute fresult={rsqch,fresult}.
release xfm,flpc, resid, ssresid, bfm.
end if
.
compute numxint=numxint+1.
compute xmtst(numxint,:)=fresult.
do if ((i = (nms+nys)) and (ydich=1)).
compute xmtst(numxint,1)=chidfxm.
compute xmtst(numxint,3)=1-chicdf(chidfxm,nxvls).
end if.
compute xmtstlb={xmtstlb;highlbx((xmints-1),1)}.
end if.
end loop.
compute x=xmtmat.
release xmtmat.
do if (numxint>0).
compute xmtstlb=xmtstlb((2:(numxint+1)),:).
compute xmtst=xmtst(1:numxint,:).
compute resultm2=make(nrow(xmtst),maxresm,99999).
compute resultm2(1:nrow(xmtst),1:ncol(xmtst))=xmtst.
compute resultm={resultm;resultm2}.
do if (nms=1).
compute xmtstlb=' '.
end if.
do if (outscree=1).
do if ((i < (nms+nys)) or (ydich=0)).
print xmtst/title='Test(s) of X by M interaction:'/rnames=xmtstlb/cnames=xmtstlbc/format= F10.4.
end if.
do if ((i = (nms+nys)) and (ydich=1)).
print xmtst/title='Likelihood ratio test(s) of X by M interaction:'/rnames=xmtstlb/cnames=xmtstlbc/format= F10.4.
end if.
end if.
end if.
compute r2=r2tmp.
compute b=btmp.
compute varb=varbtmp.
compute dfres=dfrestmp.
compute tval=tvaltmp.
end if.
do if (criterr = 0).
compute jj=0.
loop j = start to ((start+i)-1).
compute dbint=0.
compute lmat=whigh(1:nump(1,i),j).
compute lmat2=wzhigh(1:nump(1,i),j).
do if ((csum(lmat) > 0) and (csum(lmat2) = 0)).
do if ((i < (nms+nys)) or (ydich <> 1)).

.
compute lmat2= lmat.
do if ( 0 =0).
compute lmat2 = mdiag( lmat ).
compute lmat3=make(nrow(lmat2),1,0).
loop flp=1 to ncol(lmat2).
do if (csum(lmat2(:,flp))=1).
compute lmat3={lmat3,lmat2(:,flp)}.
end if.
end loop.
compute lmat2=lmat3(:,2:ncol(lmat3)).
end if.
compute fratio = (t(t(lmat2)* b )*inv(t(lmat2)* varb *lmat2)*((t(lmat2)* b )))/ncol(lmat2).
compute pfr = 1-fcdf(fratio,ncol(lmat2),(n-nrow( b ))).
compute fresult={fratio,ncol(lmat2),(n-nrow( b )),pfr}.
do if (i = (nms+nys) and (ydich=1)).
compute fratio=fratio*ncol(lmat2).
compute pfr=1-chicdf(fratio,ncol(lmat2)).
compute fresult={fratio,ncol(lmat2),pfr}.
end if.
do if ( 1 =1).
compute lmat3=1-rsum(lmat2).
compute xfm=make(n,csum(lmat3),0).
compute flpc=1.
loop flp=1 to nrow(lmat3).
do if (lmat3(flp,1)=1).
compute xfm(:,flpc)=x(:,flp).
compute flpc=flpc+1.
end if.
end loop.
compute bfm=inv(t(xfm)*xfm)*t(xfm)*y.
compute resid=y-(xfm*bfm).
compute sstotal=(y-(csum(y)/n)).
compute sstotal=csum(sstotal&*sstotal).
compute ssresid=csum(resid&*resid).
compute rsqch= r2 -((sstotal-ssresid)/sstotal).
compute fresult={rsqch,fresult}.
release xfm,flpc, resid, ssresid, bfm.
end if
.
compute lmatdb=lmat.
compute dbint=dbint+1.
end if.
do if ((ydich = 1) and (i = (nms+nys))).

.
compute btemphld=b.
compute llrdat=make(nrow(x),nrow( lmat )-csum( lmat ),-999).
compute llrdf=ncol(x)-ncol(llrdat).
compute llrcnt=0.
loop llri=1 to nrow( lmat ).
do if ( lmat (llri,1)=0).
compute llrcnt=llrcnt+1.
compute llrdat(:,llrcnt)=x(:,llri).
end if.
end loop.

.
do if ( 2 =1).
compute hatmat=inv(t( llrdat )* llrdat )*t( llrdat ).
compute b = hatmat* y.
compute modres=b.
do if ( 0 =1).
compute n1=nrow( llrdat ).
compute dfres=n1-(ncol( llrdat )).
compute sstotal = t( y -(csum( y )/n1))*( y -(csum( y )/n1)).
compute resid= y - llrdat *b.
compute ssresid = csum((resid)&**2).
compute r2 = (sstotal-ssresid)/sstotal.
do if (r2 < 0).
compute r2=0.
end if.
compute adjr2 = 1-((1-r2)*(n1-1)/(dfres)).
compute mse=ssresid/(n1-ncol( llrdat )).

.
compute n1=nrow( x ).
compute invXtX = inv(t( x )* x ).
compute varb = mse *invXtX.
compute k3 = ncol( x ).
compute xhc=0.
do if ( hc <> 5).
compute xhc= x.
compute hat = xhc(:,1).
loop i3=1 to nrow(xhc).
compute hat(i3,1)= xhc(i3,:)*invXtX*t(xhc(i3,:)).
end loop.
do if ( hc = 0 or hc =1).
loop i3 = 1 to k3.
compute xhc(:,i3)=xhc(:,i3)&* resid.
end loop.
end if.
do if ( hc =3 or hc =2).
loop i3=1 to k3.
compute xhc(:,i3) = ( resid &/(1-hat)&**(1/(4- hc )))&*xhc(:,i3).
end loop.
end if.
do if ( hc = 4).
compute hcmn=make(n,2,4).
compute hcmn(:,2)=(n1*hat)/k3.
loop i3= 1 to k3.
compute xhc(:,i3) = ( resid &/(1-hat)&**(rmin(hcmn)/2))&*xhc(:,i3).
end loop.
end if.
compute varb=(invXtX*t(xhc)*xhc*invXtX).
do if ( hc =1).
compute varb=(n1/(n1-ncol( x )))&*varb.
end if.
end if.
compute hclab={'se(HC0)','se(HC1)','se(HC2)','se(HC3)','se(HC4)','se'}.
compute hclab=hclab(1,( hc +1)).
compute hcflab={'F(HC0)','F(HC1)','F(HC2)','F(HC3)','F(HC4)','F'}.
compute hcflab=hcflab(1,( hc +1)).
release xhc
.
compute seb=sqrt(diag(varb)).
compute trat = b&/seb.
compute p = 2*(1-tcdf(abs(trat), (dfres))).
compute tval = sqrt(dfres* (exp((dfres-(5/6))*((xp2/(dfres-(2/3)+(.11/dfres)))* (xp2/(dfres-(2/3)+(.11/dfres)))))-1)).
compute modres={modres,seb,trat,p}.
compute modres={modres,(b-tval&*seb),(b+tval&*seb)}.
compute modresl={'coeff',hclab,'t','p','LLCI','ULCI'}.
compute lmat = ident(ncol( llrdat )).
compute lmat = lmat(:,2:ncol(lmat)).
compute fratio = (t(t(lmat)*b)*inv(t(lmat)*varb*lmat)*((t(lmat)*b)))/(ncol( llrdat )-1).
compute pfr = 1-fcdf(fratio,(ncol( llrdat )-1),dfres).
compute modsum={sqrt(r2),r2,mse,fratio,(ncol( llrdat )-1),dfres,pfr}.
compute modsuml={'R','R-sq','MSE',hcflab,'df1','df2', 'p'}.
end if.
end if.
do if ( 2 = 2 or 2 =3).
compute xlp= llrdat.
compute ylp= y.
compute pt2 = make(nrow(ylp),1,(csum(ylp)/nrow(ylp))).
do if ( 2 =2).
compute LL3 = ylp&*ln(pt2)+(1-ylp)&*ln(1-pt2).
end if.
compute LL3 = -2*csum(LL3).
compute bt1 = make(ncol(xlp),1,0).
compute LL1 = 0.
compute pt1 = make(nrow(ylp),1,0.5).
compute pt1lp=pt1.
loop jjj = 1 to iterate.
compute xlptmp=t(xlp).
compute vecprb=pt1lp&*(1-pt1lp).
loop kkk=1 to ncol(xlp).
compute xlptmp(kkk,:)=xlptmp(kkk,:)&*t(vecprb).
end loop.
compute b = bt1+inv(xlptmp*xlp)*t(xlp)*(ylp-pt1lp).
do if ( 2 =2).
compute xlpb=xlp*b.
compute xlpbt=(xlpb > -709.7).
compute xlpb709=(1-xlpbt)*(-709.7).
compute xlpb=(xlpb&*xlpbt)+xlpb709.
compute pt1lp = 1/(1+exp(-(xlpb))).
end if.
compute itprob = csum((pt1lp < .00000001) or (pt1lp > .9999999)).
do if (itprob > 0).
loop kkk = 1 to nrow(pt1lp).
do if (pt1lp(kkk,1) > .9999999).
compute pt1lp(kkk,1) = .9999999.
end if.
do if (pt1lp(kkk,1) < .00000001).
compute pt1lp(kkk,1) = .00000001.
end if.
end loop.
compute itprob = 0.
end if.
do if (itprob = 0).
do if ( 2 =2).
compute LL = ylp&*ln(pt1lp)+(1-ylp)&*ln(1-pt1lp).
end if.
compute LL2 = -2*csum(ll).
end if.
do if (abs(LL1-LL2) < converge).
do if ( 0 =1).
compute xlptmp=t(xlp).
compute vecprb=pt1lp&*(1-pt1lp).
loop kkk=1 to ncol(xlp).
compute xlptmp(kkk,:)=xlptmp(kkk,:)&*t(vecprb).
end loop.
compute varb = inv(xlptmp*xlp).
compute seb = sqrt(diag(varb)).
release xlptmp.
end if.
break.
end if.
compute bt1 = b.
compute LL1 = LL2.
end loop.
compute modres=b.
do if (jjj > iterate).
compute itprob = 2.
do if (booting=0).
compute iterrmod=1.
end if.
do if (booting=1).
compute bootiter=1.
end if.
do if (itprobtg=0).
compute itprobtg=1.
compute errcode(errs,1) = 47.
compute errs = errs + 1.
do if (booting = 0 and 0 =1).
compute vt1 = mdiag(pt1lp&*(1-pt1lp)).
compute varb = inv(t(xlp)*vt1*xlp).
compute seb = sqrt(diag(varb)).
end if.
end if.
end if.
do if ( 0 =1).
compute trat = b&/seb.
compute dfres=nrow(xlp).
compute p = 2*(1-cdfnorm(abs(trat))).
compute modres={modres,seb,trat,p}.
compute modres={modres,(b-xp2&*seb),(b+xp2&*seb)}.
compute pvchi=1-chicdf((LL3-LL2),(nrow(modres)-1)).
compute mcF = (LL3-LL2)/LL3.
compute cox = 1-exp(-(LL3-LL2)/nrow(xlp)).
compute nagel = cox/(1-exp(-(LL3)/nrow(xlp))).
compute modsum={LL2,(LL3-LL2),(nrow(modres)-1),pvchi, mcF,cox,nagel}.
compute modsuml={'-2LL','ModelLL', 'df', 'p', 'McFadden', 'CoxSnell', 'Nagelkrk'}.
compute modresl={'coeff','se','Z','p','LLCI','ULCI'}.
end if.
end if
.
compute b=btemphld.
compute fresult={(LL2-basemod),llrdf,1-chicdf((LL2-basemod),llrdf)}
.
compute lmatdb=lmat.
compute dbint=dbint+1.
end if.
compute highf={highf;fresult}.
compute highf2={highf2;fresult}.
do if (j = start).
compute flabel={flabel;'X*W'}.
end if.
do if (j > start).
do if (nms > 1).
compute flabel={flabel;highlbw(jj,1)}.
else if (nms = 1).
do if (xmint=0).
compute flabel={flabel;'M*W'}.
end if.
do if (xmint=1).
compute flabel={flabel;'X*M'}.
end if.
end if.
end if.
end if.
compute lmat=zhigh(1:nump(1,i),j).
compute lmat2=wzhigh(1:nump(1,i),j).
do if ((csum(lmat) > 0) and (csum(lmat2) = 0)).
do if ((i < (nms+nys)) or (ydich <> 1)).

.
compute lmat2= lmat.
do if ( 0 =0).
compute lmat2 = mdiag( lmat ).
compute lmat3=make(nrow(lmat2),1,0).
loop flp=1 to ncol(lmat2).
do if (csum(lmat2(:,flp))=1).
compute lmat3={lmat3,lmat2(:,flp)}.
end if.
end loop.
compute lmat2=lmat3(:,2:ncol(lmat3)).
end if.
compute fratio = (t(t(lmat2)* b )*inv(t(lmat2)* varb *lmat2)*((t(lmat2)* b )))/ncol(lmat2).
compute pfr = 1-fcdf(fratio,ncol(lmat2),(n-nrow( b ))).
compute fresult={fratio,ncol(lmat2),(n-nrow( b )),pfr}.
do if (i = (nms+nys) and (ydich=1)).
compute fratio=fratio*ncol(lmat2).
compute pfr=1-chicdf(fratio,ncol(lmat2)).
compute fresult={fratio,ncol(lmat2),pfr}.
end if.
do if ( 1 =1).
compute lmat3=1-rsum(lmat2).
compute xfm=make(n,csum(lmat3),0).
compute flpc=1.
loop flp=1 to nrow(lmat3).
do if (lmat3(flp,1)=1).
compute xfm(:,flpc)=x(:,flp).
compute flpc=flpc+1.
end if.
end loop.
compute bfm=inv(t(xfm)*xfm)*t(xfm)*y.
compute resid=y-(xfm*bfm).
compute sstotal=(y-(csum(y)/n)).
compute sstotal=csum(sstotal&*sstotal).
compute ssresid=csum(resid&*resid).
compute rsqch= r2 -((sstotal-ssresid)/sstotal).
compute fresult={rsqch,fresult}.
release xfm,flpc, resid, ssresid, bfm.
end if
.
compute dbint=dbint+1.
end if.
do if ((ydich = 1) and (i = (nms+nys))).

.
compute btemphld=b.
compute llrdat=make(nrow(x),nrow( lmat )-csum( lmat ),-999).
compute llrdf=ncol(x)-ncol(llrdat).
compute llrcnt=0.
loop llri=1 to nrow( lmat ).
do if ( lmat (llri,1)=0).
compute llrcnt=llrcnt+1.
compute llrdat(:,llrcnt)=x(:,llri).
end if.
end loop.

.
do if ( 2 =1).
compute hatmat=inv(t( llrdat )* llrdat )*t( llrdat ).
compute b = hatmat* y.
compute modres=b.
do if ( 0 =1).
compute n1=nrow( llrdat ).
compute dfres=n1-(ncol( llrdat )).
compute sstotal = t( y -(csum( y )/n1))*( y -(csum( y )/n1)).
compute resid= y - llrdat *b.
compute ssresid = csum((resid)&**2).
compute r2 = (sstotal-ssresid)/sstotal.
do if (r2 < 0).
compute r2=0.
end if.
compute adjr2 = 1-((1-r2)*(n1-1)/(dfres)).
compute mse=ssresid/(n1-ncol( llrdat )).

.
compute n1=nrow( x ).
compute invXtX = inv(t( x )* x ).
compute varb = mse *invXtX.
compute k3 = ncol( x ).
compute xhc=0.
do if ( hc <> 5).
compute xhc= x.
compute hat = xhc(:,1).
loop i3=1 to nrow(xhc).
compute hat(i3,1)= xhc(i3,:)*invXtX*t(xhc(i3,:)).
end loop.
do if ( hc = 0 or hc =1).
loop i3 = 1 to k3.
compute xhc(:,i3)=xhc(:,i3)&* resid.
end loop.
end if.
do if ( hc =3 or hc =2).
loop i3=1 to k3.
compute xhc(:,i3) = ( resid &/(1-hat)&**(1/(4- hc )))&*xhc(:,i3).
end loop.
end if.
do if ( hc = 4).
compute hcmn=make(n,2,4).
compute hcmn(:,2)=(n1*hat)/k3.
loop i3= 1 to k3.
compute xhc(:,i3) = ( resid &/(1-hat)&**(rmin(hcmn)/2))&*xhc(:,i3).
end loop.
end if.
compute varb=(invXtX*t(xhc)*xhc*invXtX).
do if ( hc =1).
compute varb=(n1/(n1-ncol( x )))&*varb.
end if.
end if.
compute hclab={'se(HC0)','se(HC1)','se(HC2)','se(HC3)','se(HC4)','se'}.
compute hclab=hclab(1,( hc +1)).
compute hcflab={'F(HC0)','F(HC1)','F(HC2)','F(HC3)','F(HC4)','F'}.
compute hcflab=hcflab(1,( hc +1)).
release xhc
.
compute seb=sqrt(diag(varb)).
compute trat = b&/seb.
compute p = 2*(1-tcdf(abs(trat), (dfres))).
compute tval = sqrt(dfres* (exp((dfres-(5/6))*((xp2/(dfres-(2/3)+(.11/dfres)))* (xp2/(dfres-(2/3)+(.11/dfres)))))-1)).
compute modres={modres,seb,trat,p}.
compute modres={modres,(b-tval&*seb),(b+tval&*seb)}.
compute modresl={'coeff',hclab,'t','p','LLCI','ULCI'}.
compute lmat = ident(ncol( llrdat )).
compute lmat = lmat(:,2:ncol(lmat)).
compute fratio = (t(t(lmat)*b)*inv(t(lmat)*varb*lmat)*((t(lmat)*b)))/(ncol( llrdat )-1).
compute pfr = 1-fcdf(fratio,(ncol( llrdat )-1),dfres).
compute modsum={sqrt(r2),r2,mse,fratio,(ncol( llrdat )-1),dfres,pfr}.
compute modsuml={'R','R-sq','MSE',hcflab,'df1','df2', 'p'}.
end if.
end if.
do if ( 2 = 2 or 2 =3).
compute xlp= llrdat.
compute ylp= y.
compute pt2 = make(nrow(ylp),1,(csum(ylp)/nrow(ylp))).
do if ( 2 =2).
compute LL3 = ylp&*ln(pt2)+(1-ylp)&*ln(1-pt2).
end if.
compute LL3 = -2*csum(LL3).
compute bt1 = make(ncol(xlp),1,0).
compute LL1 = 0.
compute pt1 = make(nrow(ylp),1,0.5).
compute pt1lp=pt1.
loop jjj = 1 to iterate.
compute xlptmp=t(xlp).
compute vecprb=pt1lp&*(1-pt1lp).
loop kkk=1 to ncol(xlp).
compute xlptmp(kkk,:)=xlptmp(kkk,:)&*t(vecprb).
end loop.
compute b = bt1+inv(xlptmp*xlp)*t(xlp)*(ylp-pt1lp).
do if ( 2 =2).
compute xlpb=xlp*b.
compute xlpbt=(xlpb > -709.7).
compute xlpb709=(1-xlpbt)*(-709.7).
compute xlpb=(xlpb&*xlpbt)+xlpb709.
compute pt1lp = 1/(1+exp(-(xlpb))).
end if.
compute itprob = csum((pt1lp < .00000001) or (pt1lp > .9999999)).
do if (itprob > 0).
loop kkk = 1 to nrow(pt1lp).
do if (pt1lp(kkk,1) > .9999999).
compute pt1lp(kkk,1) = .9999999.
end if.
do if (pt1lp(kkk,1) < .00000001).
compute pt1lp(kkk,1) = .00000001.
end if.
end loop.
compute itprob = 0.
end if.
do if (itprob = 0).
do if ( 2 =2).
compute LL = ylp&*ln(pt1lp)+(1-ylp)&*ln(1-pt1lp).
end if.
compute LL2 = -2*csum(ll).
end if.
do if (abs(LL1-LL2) < converge).
do if ( 0 =1).
compute xlptmp=t(xlp).
compute vecprb=pt1lp&*(1-pt1lp).
loop kkk=1 to ncol(xlp).
compute xlptmp(kkk,:)=xlptmp(kkk,:)&*t(vecprb).
end loop.
compute varb = inv(xlptmp*xlp).
compute seb = sqrt(diag(varb)).
release xlptmp.
end if.
break.
end if.
compute bt1 = b.
compute LL1 = LL2.
end loop.
compute modres=b.
do if (jjj > iterate).
compute itprob = 2.
do if (booting=0).
compute iterrmod=1.
end if.
do if (booting=1).
compute bootiter=1.
end if.
do if (itprobtg=0).
compute itprobtg=1.
compute errcode(errs,1) = 47.
compute errs = errs + 1.
do if (booting = 0 and 0 =1).
compute vt1 = mdiag(pt1lp&*(1-pt1lp)).
compute varb = inv(t(xlp)*vt1*xlp).
compute seb = sqrt(diag(varb)).
end if.
end if.
end if.
do if ( 0 =1).
compute trat = b&/seb.
compute dfres=nrow(xlp).
compute p = 2*(1-cdfnorm(abs(trat))).
compute modres={modres,seb,trat,p}.
compute modres={modres,(b-xp2&*seb),(b+xp2&*seb)}.
compute pvchi=1-chicdf((LL3-LL2),(nrow(modres)-1)).
compute mcF = (LL3-LL2)/LL3.
compute cox = 1-exp(-(LL3-LL2)/nrow(xlp)).
compute nagel = cox/(1-exp(-(LL3)/nrow(xlp))).
compute modsum={LL2,(LL3-LL2),(nrow(modres)-1),pvchi, mcF,cox,nagel}.
compute modsuml={'-2LL','ModelLL', 'df', 'p', 'McFadden', 'CoxSnell', 'Nagelkrk'}.
compute modresl={'coeff','se','Z','p','LLCI','ULCI'}.
end if.
end if
.
compute b=btemphld.
compute fresult={(LL2-basemod),llrdf,1-chicdf((LL2-basemod),llrdf)}
.
compute dbint=dbint+1.
end if.
compute highf={highf;fresult}.
compute highf2={highf2;fresult}.
do if (j = start).
compute flabel={flabel;'X*Z'}.
end if.
do if (j > start).
do if (nms > 1).
compute flabel={flabel;highlbz(jj,1)}.
else if (nms = 1).
compute flabel={flabel;'M*Z'}.
end if.
end if.
end if.
do if (dbint=2).
compute lmatdb=lmatdb+lmat.
do if ((ydich = 1) and (i = (nms+nys))).

.
compute btemphld=b.
compute llrdat=make(nrow(x),nrow( lmatdb )-csum( lmatdb ),-999).
compute llrdf=ncol(x)-ncol(llrdat).
compute llrcnt=0.
loop llri=1 to nrow( lmatdb ).
do if ( lmatdb (llri,1)=0).
compute llrcnt=llrcnt+1.
compute llrdat(:,llrcnt)=x(:,llri).
end if.
end loop.

.
do if ( 2 =1).
compute hatmat=inv(t( llrdat )* llrdat )*t( llrdat ).
compute b = hatmat* y.
compute modres=b.
do if ( 0 =1).
compute n1=nrow( llrdat ).
compute dfres=n1-(ncol( llrdat )).
compute sstotal = t( y -(csum( y )/n1))*( y -(csum( y )/n1)).
compute resid= y - llrdat *b.
compute ssresid = csum((resid)&**2).
compute r2 = (sstotal-ssresid)/sstotal.
do if (r2 < 0).
compute r2=0.
end if.
compute adjr2 = 1-((1-r2)*(n1-1)/(dfres)).
compute mse=ssresid/(n1-ncol( llrdat )).

.
compute n1=nrow( x ).
compute invXtX = inv(t( x )* x ).
compute varb = mse *invXtX.
compute k3 = ncol( x ).
compute xhc=0.
do if ( hc <> 5).
compute xhc= x.
compute hat = xhc(:,1).
loop i3=1 to nrow(xhc).
compute hat(i3,1)= xhc(i3,:)*invXtX*t(xhc(i3,:)).
end loop.
do if ( hc = 0 or hc =1).
loop i3 = 1 to k3.
compute xhc(:,i3)=xhc(:,i3)&* resid.
end loop.
end if.
do if ( hc =3 or hc =2).
loop i3=1 to k3.
compute xhc(:,i3) = ( resid &/(1-hat)&**(1/(4- hc )))&*xhc(:,i3).
end loop.
end if.
do if ( hc = 4).
compute hcmn=make(n,2,4).
compute hcmn(:,2)=(n1*hat)/k3.
loop i3= 1 to k3.
compute xhc(:,i3) = ( resid &/(1-hat)&**(rmin(hcmn)/2))&*xhc(:,i3).
end loop.
end if.
compute varb=(invXtX*t(xhc)*xhc*invXtX).
do if ( hc =1).
compute varb=(n1/(n1-ncol( x )))&*varb.
end if.
end if.
compute hclab={'se(HC0)','se(HC1)','se(HC2)','se(HC3)','se(HC4)','se'}.
compute hclab=hclab(1,( hc +1)).
compute hcflab={'F(HC0)','F(HC1)','F(HC2)','F(HC3)','F(HC4)','F'}.
compute hcflab=hcflab(1,( hc +1)).
release xhc
.
compute seb=sqrt(diag(varb)).
compute trat = b&/seb.
compute p = 2*(1-tcdf(abs(trat), (dfres))).
compute tval = sqrt(dfres* (exp((dfres-(5/6))*((xp2/(dfres-(2/3)+(.11/dfres)))* (xp2/(dfres-(2/3)+(.11/dfres)))))-1)).
compute modres={modres,seb,trat,p}.
compute modres={modres,(b-tval&*seb),(b+tval&*seb)}.
compute modresl={'coeff',hclab,'t','p','LLCI','ULCI'}.
compute lmat = ident(ncol( llrdat )).
compute lmat = lmat(:,2:ncol(lmat)).
compute fratio = (t(t(lmat)*b)*inv(t(lmat)*varb*lmat)*((t(lmat)*b)))/(ncol( llrdat )-1).
compute pfr = 1-fcdf(fratio,(ncol( llrdat )-1),dfres).
compute modsum={sqrt(r2),r2,mse,fratio,(ncol( llrdat )-1),dfres,pfr}.
compute modsuml={'R','R-sq','MSE',hcflab,'df1','df2', 'p'}.
end if.
end if.
do if ( 2 = 2 or 2 =3).
compute xlp= llrdat.
compute ylp= y.
compute pt2 = make(nrow(ylp),1,(csum(ylp)/nrow(ylp))).
do if ( 2 =2).
compute LL3 = ylp&*ln(pt2)+(1-ylp)&*ln(1-pt2).
end if.
compute LL3 = -2*csum(LL3).
compute bt1 = make(ncol(xlp),1,0).
compute LL1 = 0.
compute pt1 = make(nrow(ylp),1,0.5).
compute pt1lp=pt1.
loop jjj = 1 to iterate.
compute xlptmp=t(xlp).
compute vecprb=pt1lp&*(1-pt1lp).
loop kkk=1 to ncol(xlp).
compute xlptmp(kkk,:)=xlptmp(kkk,:)&*t(vecprb).
end loop.
compute b = bt1+inv(xlptmp*xlp)*t(xlp)*(ylp-pt1lp).
do if ( 2 =2).
compute xlpb=xlp*b.
compute xlpbt=(xlpb > -709.7).
compute xlpb709=(1-xlpbt)*(-709.7).
compute xlpb=(xlpb&*xlpbt)+xlpb709.
compute pt1lp = 1/(1+exp(-(xlpb))).
end if.
compute itprob = csum((pt1lp < .00000001) or (pt1lp > .9999999)).
do if (itprob > 0).
loop kkk = 1 to nrow(pt1lp).
do if (pt1lp(kkk,1) > .9999999).
compute pt1lp(kkk,1) = .9999999.
end if.
do if (pt1lp(kkk,1) < .00000001).
compute pt1lp(kkk,1) = .00000001.
end if.
end loop.
compute itprob = 0.
end if.
do if (itprob = 0).
do if ( 2 =2).
compute LL = ylp&*ln(pt1lp)+(1-ylp)&*ln(1-pt1lp).
end if.
compute LL2 = -2*csum(ll).
end if.
do if (abs(LL1-LL2) < converge).
do if ( 0 =1).
compute xlptmp=t(xlp).
compute vecprb=pt1lp&*(1-pt1lp).
loop kkk=1 to ncol(xlp).
compute xlptmp(kkk,:)=xlptmp(kkk,:)&*t(vecprb).
end loop.
compute varb = inv(xlptmp*xlp).
compute seb = sqrt(diag(varb)).
release xlptmp.
end if.
break.
end if.
compute bt1 = b.
compute LL1 = LL2.
end loop.
compute modres=b.
do if (jjj > iterate).
compute itprob = 2.
do if (booting=0).
compute iterrmod=1.
end if.
do if (booting=1).
compute bootiter=1.
end if.
do if (itprobtg=0).
compute itprobtg=1.
compute errcode(errs,1) = 47.
compute errs = errs + 1.
do if (booting = 0 and 0 =1).
compute vt1 = mdiag(pt1lp&*(1-pt1lp)).
compute varb = inv(t(xlp)*vt1*xlp).
compute seb = sqrt(diag(varb)).
end if.
end if.
end if.
do if ( 0 =1).
compute trat = b&/seb.
compute dfres=nrow(xlp).
compute p = 2*(1-cdfnorm(abs(trat))).
compute modres={modres,seb,trat,p}.
compute modres={modres,(b-xp2&*seb),(b+xp2&*seb)}.
compute pvchi=1-chicdf((LL3-LL2),(nrow(modres)-1)).
compute mcF = (LL3-LL2)/LL3.
compute cox = 1-exp(-(LL3-LL2)/nrow(xlp)).
compute nagel = cox/(1-exp(-(LL3)/nrow(xlp))).
compute modsum={LL2,(LL3-LL2),(nrow(modres)-1),pvchi, mcF,cox,nagel}.
compute modsuml={'-2LL','ModelLL', 'df', 'p', 'McFadden', 'CoxSnell', 'Nagelkrk'}.
compute modresl={'coeff','se','Z','p','LLCI','ULCI'}.
end if.
end if
.
compute b=btemphld.
compute fresult={(LL2-basemod),llrdf,1-chicdf((LL2-basemod),llrdf)}
.
end if.
do if (ydich <> 1 or i < (nms+nys)).

.
compute lmat2= lmatdb.
do if ( 0 =0).
compute lmat2 = mdiag( lmatdb ).
compute lmat3=make(nrow(lmat2),1,0).
loop flp=1 to ncol(lmat2).
do if (csum(lmat2(:,flp))=1).
compute lmat3={lmat3,lmat2(:,flp)}.
end if.
end loop.
compute lmat2=lmat3(:,2:ncol(lmat3)).
end if.
compute fratio = (t(t(lmat2)* b )*inv(t(lmat2)* varb *lmat2)*((t(lmat2)* b )))/ncol(lmat2).
compute pfr = 1-fcdf(fratio,ncol(lmat2),(n-nrow( b ))).
compute fresult={fratio,ncol(lmat2),(n-nrow( b )),pfr}.
do if (i = (nms+nys) and (ydich=1)).
compute fratio=fratio*ncol(lmat2).
compute pfr=1-chicdf(fratio,ncol(lmat2)).
compute fresult={fratio,ncol(lmat2),pfr}.
end if.
do if ( 1 =1).
compute lmat3=1-rsum(lmat2).
compute xfm=make(n,csum(lmat3),0).
compute flpc=1.
loop flp=1 to nrow(lmat3).
do if (lmat3(flp,1)=1).
compute xfm(:,flpc)=x(:,flp).
compute flpc=flpc+1.
end if.
end loop.
compute bfm=inv(t(xfm)*xfm)*t(xfm)*y.
compute resid=y-(xfm*bfm).
compute sstotal=(y-(csum(y)/n)).
compute sstotal=csum(sstotal&*sstotal).
compute ssresid=csum(resid&*resid).
compute rsqch= r2 -((sstotal-ssresid)/sstotal).
compute fresult={rsqch,fresult}.
release xfm,flpc, resid, ssresid, bfm.
end if
.
end if.
compute dbint=0.
compute highf={highf;fresult}.
do if (jj=0 and nms > 0).
compute flabel={flabel;'BOTH(X)'}.
end if.
do if (jj=0 and nms = 0).
compute flabel={flabel;'BOTH'}.
end if.
do if (jj>0 and nms = 1).
compute flabel={flabel;'BOTH(M)'}.
end if.
do if (nms > 1 and jj > 0).
compute flabel={flabel;highlbbt(jj,1)}.
end if.
end if.
compute lmat2=wzhigh(1:nump(1,i),j).
do if (csum(lmat2) > 0).
do if ((i < (nms+nys)) or (ydich <> 1)).

.
compute lmat2= lmat2.
do if ( 0 =0).
compute lmat2 = mdiag( lmat2 ).
compute lmat3=make(nrow(lmat2),1,0).
loop flp=1 to ncol(lmat2).
do if (csum(lmat2(:,flp))=1).
compute lmat3={lmat3,lmat2(:,flp)}.
end if.
end loop.
compute lmat2=lmat3(:,2:ncol(lmat3)).
end if.
compute fratio = (t(t(lmat2)* b )*inv(t(lmat2)* varb *lmat2)*((t(lmat2)* b )))/ncol(lmat2).
compute pfr = 1-fcdf(fratio,ncol(lmat2),(n-nrow( b ))).
compute fresult={fratio,ncol(lmat2),(n-nrow( b )),pfr}.
do if (i = (nms+nys) and (ydich=1)).
compute fratio=fratio*ncol(lmat2).
compute pfr=1-chicdf(fratio,ncol(lmat2)).
compute fresult={fratio,ncol(lmat2),pfr}.
end if.
do if ( 1 =1).
compute lmat3=1-rsum(lmat2).
compute xfm=make(n,csum(lmat3),0).
compute flpc=1.
loop flp=1 to nrow(lmat3).
do if (lmat3(flp,1)=1).
compute xfm(:,flpc)=x(:,flp).
compute flpc=flpc+1.
end if.
end loop.
compute bfm=inv(t(xfm)*xfm)*t(xfm)*y.
compute resid=y-(xfm*bfm).
compute sstotal=(y-(csum(y)/n)).
compute sstotal=csum(sstotal&*sstotal).
compute ssresid=csum(resid&*resid).
compute rsqch= r2 -((sstotal-ssresid)/sstotal).
compute fresult={rsqch,fresult}.
release xfm,flpc, resid, ssresid, bfm.
end if
.
end if.
do if ((ydich = 1) and (i = (nms+nys))).

.
compute btemphld=b.
compute llrdat=make(nrow(x),nrow( lmat2 )-csum( lmat2 ),-999).
compute llrdf=ncol(x)-ncol(llrdat).
compute llrcnt=0.
loop llri=1 to nrow( lmat2 ).
do if ( lmat2 (llri,1)=0).
compute llrcnt=llrcnt+1.
compute llrdat(:,llrcnt)=x(:,llri).
end if.
end loop.

.
do if ( 2 =1).
compute hatmat=inv(t( llrdat )* llrdat )*t( llrdat ).
compute b = hatmat* y.
compute modres=b.
do if ( 0 =1).
compute n1=nrow( llrdat ).
compute dfres=n1-(ncol( llrdat )).
compute sstotal = t( y -(csum( y )/n1))*( y -(csum( y )/n1)).
compute resid= y - llrdat *b.
compute ssresid = csum((resid)&**2).
compute r2 = (sstotal-ssresid)/sstotal.
do if (r2 < 0).
compute r2=0.
end if.
compute adjr2 = 1-((1-r2)*(n1-1)/(dfres)).
compute mse=ssresid/(n1-ncol( llrdat )).

.
compute n1=nrow( x ).
compute invXtX = inv(t( x )* x ).
compute varb = mse *invXtX.
compute k3 = ncol( x ).
compute xhc=0.
do if ( hc <> 5).
compute xhc= x.
compute hat = xhc(:,1).
loop i3=1 to nrow(xhc).
compute hat(i3,1)= xhc(i3,:)*invXtX*t(xhc(i3,:)).
end loop.
do if ( hc = 0 or hc =1).
loop i3 = 1 to k3.
compute xhc(:,i3)=xhc(:,i3)&* resid.
end loop.
end if.
do if ( hc =3 or hc =2).
loop i3=1 to k3.
compute xhc(:,i3) = ( resid &/(1-hat)&**(1/(4- hc )))&*xhc(:,i3).
end loop.
end if.
do if ( hc = 4).
compute hcmn=make(n,2,4).
compute hcmn(:,2)=(n1*hat)/k3.
loop i3= 1 to k3.
compute xhc(:,i3) = ( resid &/(1-hat)&**(rmin(hcmn)/2))&*xhc(:,i3).
end loop.
end if.
compute varb=(invXtX*t(xhc)*xhc*invXtX).
do if ( hc =1).
compute varb=(n1/(n1-ncol( x )))&*varb.
end if.
end if.
compute hclab={'se(HC0)','se(HC1)','se(HC2)','se(HC3)','se(HC4)','se'}.
compute hclab=hclab(1,( hc +1)).
compute hcflab={'F(HC0)','F(HC1)','F(HC2)','F(HC3)','F(HC4)','F'}.
compute hcflab=hcflab(1,( hc +1)).
release xhc
.
compute seb=sqrt(diag(varb)).
compute trat = b&/seb.
compute p = 2*(1-tcdf(abs(trat), (dfres))).
compute tval = sqrt(dfres* (exp((dfres-(5/6))*((xp2/(dfres-(2/3)+(.11/dfres)))* (xp2/(dfres-(2/3)+(.11/dfres)))))-1)).
compute modres={modres,seb,trat,p}.
compute modres={modres,(b-tval&*seb),(b+tval&*seb)}.
compute modresl={'coeff',hclab,'t','p','LLCI','ULCI'}.
compute lmat = ident(ncol( llrdat )).
compute lmat = lmat(:,2:ncol(lmat)).
compute fratio = (t(t(lmat)*b)*inv(t(lmat)*varb*lmat)*((t(lmat)*b)))/(ncol( llrdat )-1).
compute pfr = 1-fcdf(fratio,(ncol( llrdat )-1),dfres).
compute modsum={sqrt(r2),r2,mse,fratio,(ncol( llrdat )-1),dfres,pfr}.
compute modsuml={'R','R-sq','MSE',hcflab,'df1','df2', 'p'}.
end if.
end if.
do if ( 2 = 2 or 2 =3).
compute xlp= llrdat.
compute ylp= y.
compute pt2 = make(nrow(ylp),1,(csum(ylp)/nrow(ylp))).
do if ( 2 =2).
compute LL3 = ylp&*ln(pt2)+(1-ylp)&*ln(1-pt2).
end if.
compute LL3 = -2*csum(LL3).
compute bt1 = make(ncol(xlp),1,0).
compute LL1 = 0.
compute pt1 = make(nrow(ylp),1,0.5).
compute pt1lp=pt1.
loop jjj = 1 to iterate.
compute xlptmp=t(xlp).
compute vecprb=pt1lp&*(1-pt1lp).
loop kkk=1 to ncol(xlp).
compute xlptmp(kkk,:)=xlptmp(kkk,:)&*t(vecprb).
end loop.
compute b = bt1+inv(xlptmp*xlp)*t(xlp)*(ylp-pt1lp).
do if ( 2 =2).
compute xlpb=xlp*b.
compute xlpbt=(xlpb > -709.7).
compute xlpb709=(1-xlpbt)*(-709.7).
compute xlpb=(xlpb&*xlpbt)+xlpb709.
compute pt1lp = 1/(1+exp(-(xlpb))).
end if.
compute itprob = csum((pt1lp < .00000001) or (pt1lp > .9999999)).
do if (itprob > 0).
loop kkk = 1 to nrow(pt1lp).
do if (pt1lp(kkk,1) > .9999999).
compute pt1lp(kkk,1) = .9999999.
end if.
do if (pt1lp(kkk,1) < .00000001).
compute pt1lp(kkk,1) = .00000001.
end if.
end loop.
compute itprob = 0.
end if.
do if (itprob = 0).
do if ( 2 =2).
compute LL = ylp&*ln(pt1lp)+(1-ylp)&*ln(1-pt1lp).
end if.
compute LL2 = -2*csum(ll).
end if.
do if (abs(LL1-LL2) < converge).
do if ( 0 =1).
compute xlptmp=t(xlp).
compute vecprb=pt1lp&*(1-pt1lp).
loop kkk=1 to ncol(xlp).
compute xlptmp(kkk,:)=xlptmp(kkk,:)&*t(vecprb).
end loop.
compute varb = inv(xlptmp*xlp).
compute seb = sqrt(diag(varb)).
release xlptmp.
end if.
break.
end if.
compute bt1 = b.
compute LL1 = LL2.
end loop.
compute modres=b.
do if (jjj > iterate).
compute itprob = 2.
do if (booting=0).
compute iterrmod=1.
end if.
do if (booting=1).
compute bootiter=1.
end if.
do if (itprobtg=0).
compute itprobtg=1.
compute errcode(errs,1) = 47.
compute errs = errs + 1.
do if (booting = 0 and 0 =1).
compute vt1 = mdiag(pt1lp&*(1-pt1lp)).
compute varb = inv(t(xlp)*vt1*xlp).
compute seb = sqrt(diag(varb)).
end if.
end if.
end if.
do if ( 0 =1).
compute trat = b&/seb.
compute dfres=nrow(xlp).
compute p = 2*(1-cdfnorm(abs(trat))).
compute modres={modres,seb,trat,p}.
compute modres={modres,(b-xp2&*seb),(b+xp2&*seb)}.
compute pvchi=1-chicdf((LL3-LL2),(nrow(modres)-1)).
compute mcF = (LL3-LL2)/LL3.
compute cox = 1-exp(-(LL3-LL2)/nrow(xlp)).
compute nagel = cox/(1-exp(-(LL3)/nrow(xlp))).
compute modsum={LL2,(LL3-LL2),(nrow(modres)-1),pvchi, mcF,cox,nagel}.
compute modsuml={'-2LL','ModelLL', 'df', 'p', 'McFadden', 'CoxSnell', 'Nagelkrk'}.
compute modresl={'coeff','se','Z','p','LLCI','ULCI'}.
end if.
end if
.
compute b=btemphld.
compute fresult={(LL2-basemod),llrdf,1-chicdf((LL2-basemod),llrdf)}
.
end if.
compute highf={highf;fresult}.
compute highf2={highf2;fresult}.
do if (j = start).
compute flabel={flabel;'X*W*Z'}.
end if.
do if (j > start).
do if (nms > 1).
compute flabel={flabel;highlbwz(jj,1)}.
else if (nms = 1).
compute flabel={flabel;'M*W*Z'}.
end if.
end if.
end if.
compute jj=jj+1.
end loop.
release jj.
compute start=start+i.
end if.
do if (nrow(highf) > 1).
compute highf=highf(2:nrow(highf),:).
compute highf2=highf2(2:nrow(highf2),:).
compute flabel=flabel(2:nrow(flabel),1).
compute resultm2=make(nrow(highf),maxresm,99999).
compute resultm2(1:nrow(highf),1:ncol(highf))=highf.
compute resultm={resultm;resultm2}.
do if (outscree=1).
do if ((i < nms+nys) or (ydich=0)).
compute clabtmp={'R2-chng', hcflab,'df1','df2','p'}.
print highf/format = F10.4 /rnames=flabel/cnames=clabtmp/ title = 'Test(s) of highest order unconditional interaction(s):'.
end if.
do if (ydich=1 and i=(nms+nys)).
compute clabtmp={'Chi-sq', 'df','p'}.
print/title='Likelihood ratio test(s) of highest order'.
print highf/format = F10.4 /rnames=flabel/cnames=clabtmp/ title = 'unconditional interactions(s):'/space=0.
end if.
end if.
compute intpb=highf2(:,ncol(highf2)).
end if.
compute intstart=intstart+numint(1,i).
end if.

.
do if (criterr=0).
compute threeway=0.
compute didprint=0.
compute didsome=0.
compute sigintct=0.
loop jmed =1 to (nms+1).
compute hasw=0.
compute hasz=0.
compute jnok=0.
compute nm1vls=0.
compute nm2vls=0.
compute panelgrp=0.
compute graphixs={'WITH', outnames(1,i), 'BY'}.
compute focpred4={' '}.
compute intprint=0.
compute modcat=0.
do if (jmed <= i).
do if ((jmed = 1) and ((i+1) = nrow(bcmat))).
compute pathscnt=pathscnt+1.
else.
compute paths={paths,bcmat((i+1),jmed)}.
compute pathsw={pathsw,wcmat((i+1),jmed)}.
compute pathsz={pathsz,zcmat((i+1),jmed)}.
compute pathswz={pathswz,wzcmat((i+1),jmed)}.
compute temp=fochigh(:,pathscnt)&*bootloc(:,i).
compute pathsfoc={pathsfoc,pathsfoc(:,1)}.
do if (jmed=1).
compute pathtype={pathtype,1}.
end if.
do if ((i+1)=nrow(bcmat)).
compute pathtype={pathtype,3}.
end if.
do if (jmed > 1) and ((i+1) < nrow(bcmat)).
compute pathtype={pathtype,2}.
end if.
do if (jmed=1 and nxvls > 1 and (bcmat((i+1),jmed)=1)).
compute pathsfoc(:,(pathscn2+1))=temp(2:(nxvls+1),1).
end if.
do if ((jmed > 1) or (jmed=1 and nxvls=1)).
compute temp=cmax(temp).
compute pathsfoc(1,(pathscn2+1))=temp.
end if.
compute pathscnt=pathscnt+1.
compute pathscn2=pathscn2+1.
do if (i <= nms).
compute pathsdv={pathsdv,mnames(1,i)}.
end if.
do if (i > nms).
compute pathsdv={pathsdv,ynames}.
end if.
end if.
compute coeffcol=coeffcol+1.
compute probettt=coeffs(1:nrow(b),coeffcol).
do if (jmed=1 and (bcmat((i+1),jmed)=1)).
compute omni=make(nrow(probettt),nxvls,0).
compute omnitmp=ident(nxvls).
compute omni(2:(1+nxvls),:)=omnitmp.
end if.
do if (csum(probettt)>0).
compute probvarb=make(csum(probettt),csum(probettt),999).
compute probcoef=make(csum(probettt),1,999).
compute coefflp2=1.
loop coefflp=1 to nrow(probettt).
do if (probettt(coefflp,1)=1).
compute probcoef(coefflp2,1)=b(coefflp,1).
compute coefflp2=coefflp2+1.
end if.
end loop.
compute coefflp=0.
compute coefflp2=0.
loop iclp=1 to nrow(probettt).
do if probettt(iclp,1)=1.
compute coefflp=coefflp+1.
compute coefflp2=coefflp.
compute probvarb(coefflp,coefflp) = varb(iclp,iclp).
do if (iclp < nrow(probettt)).
loop jclp=(iclp+1) to nrow(probettt).
do if (probettt(jclp,1)=1).
compute coefflp2=coefflp2+1.
compute probvarb(coefflp,coefflp2)=varb(iclp, jclp).
compute probvarb(coefflp2,coefflp)=varb(iclp, jclp).
end if.
end loop.
end if.
end if.
end loop.
end if.
end if.
compute xprobval=xmodvals.
do if (nxvls > 1 or mcx > 0).
compute xprobval=dummatx(:,2:ncol(dummatx)).
end if.
do if ((wcmat((i+1),jmed)=1) and (zcmat((i+1),jmed)=0)).
compute numplps=1.
compute modvals=wmodvals.
compute probeval=wmodvals.
compute wheremv1=wherexw.
compute nm1vls=nwvls.
compute lpstsp={1,1}.
compute modcat=0.
compute jnmod=wtmp.
compute jnmodlab=wnames.
compute jnok=1.
compute jnmin=wmin.
compute jnmax=wmax.
compute wherejn1=2.
do if (jmed=1).
compute wherejn3=wherexw(1,i).
do if (nxvls > 1).
compute jnok=0.
end if.
end if.
do if (jmed > 1).
compute wherejn1=wherem((jmed-1),i).
compute wherejn3=wheremw(((2*jmed)-3),i).
end if.
do if (nwvls > 1).
compute probeval=wprobval.
compute lpstsp(1,2)=ncol(probeval).
compute modcat=1.
compute jnok=0.
end if.
do if (wdich = 1).
compute modcat=1.
compute jnok=0.
end if.
compute problabs=wnames.
compute focpred3={wnames,'(W)'}.
do if (xmint=1).
compute focpred3={wnames,'(X)'}.
end if.
compute hasw=1.
compute modgrph=wnames.
compute intprint=1.
compute sigintct=sigintct+1.
compute printpbe=intpb(sigintct,1).
end if.
do if ((wcmat((i+1),jmed)=0) and (zcmat((i+1),jmed)=1)).
compute numplps=1.
compute modvals=zmodvals.
compute probeval=zmodvals.
compute wheremv1=wherexz.
compute nm1vls=nzvls.
compute lpstsp={1,1}.
compute jnok=1.
compute jnmod=ztmp.
compute jnmin=zmin.
compute jnmax=zmax.
compute jnmodlab=znames.
compute wherejn1=2.
do if (jmed=1).
compute wherejn3=wherexz(1,i).
do if (nxvls > 1).
compute jnok=0.
end if.
end if.
do if (jmed > 1).
compute wherejn1=wherem((jmed-1),i).
compute wherejn3=wheremz(((2*jmed)-3),i).
end if.
do if (nzvls > 1).
compute probeval=zprobval.
compute lpstsp(1,2)=ncol(probeval).
compute modcat=1.
compute jnok=0.
end if.
do if (zdich = 1).
compute modcat=1.
compute jnok=0.
end if.
compute problabs=znames.
compute focpred3={znames,'(Z)'}.
compute modgrph=znames.
compute hasz=1.
compute intprint=1.
compute sigintct=sigintct+1.
compute printpbe=intpb(sigintct,1).
end if.
do if ((wzcmat((i+1),jmed)=1) or ((wcmat((i+1),jmed)=1) and (zcmat((i+1),jmed)=1))).
compute numplps=2.
compute probecnt=1.
compute intprint=1.
do if (wzcmat((i+1),jmed)=1).
compute sigintct=sigintct+1.
compute printpbe=intpb(sigintct,1).
else.
compute sigintct=sigintct+2.
compute printpbe=cmin(intpb((sigintct-1):sigintct,1)).
end if.
compute panelgrp=1.
compute hasw=1.
compute hasz=1.
compute panelcde={'/PANEL','ROWVAR=',znames,'.'}.
compute modgrph=wnames.
compute lpstsp={1,1;1,1}.
compute wheremv1=wherexw.
compute nm1vls=nwvls.
compute wheremv2=wherexz.
compute nm2vls=nzvls.
compute jnok=0.
do if (wzcmat((i+1),jmed)=1).
compute jnok=1.
end if.
do if (jmed > 1).
compute mprobval=mmodvals.
end if.
do if (jmed=1).
do if (nxvls > 1).
compute jnok=0.
end if.
end if.
do if (nwvls > 1).
compute lpstsp(1,2)=ncol(wprobval).
compute modcat=1.
compute jnok=0.
end if.
do if (zdich=1).
compute modcat=1.
compute jnok=0.
end if.
compute lpstsp(2,1)=lpstsp(1,2)+1.
compute lpstsp(2,2)=lpstsp(1,2)+1.
do if (nzvls > 1).
compute lpstsp(2,1)=lpstsp(1,2)+1.
compute lpstsp(2,2)=lpstsp(1,2)+ncol(zprobval).
compute jnok=0.
end if.
do if (zdich=1).
compute jnok=0.
end if.
compute omni3=make(nrow(b),(nxvls*nwvls),0).
do if (jmed > 1).
compute omni3=make(nrow(b),nwvls,0).
end if.
compute focpred3={wnames,'(W)'}.
do if (xmint=1).
compute focpred3={wnames,'(X)'}.
end if.
compute focpred4={'    ', 'Mod var:', znames, '(Z)'}.
compute modvals=make((nrow(wmodvals)*nrow(zmodvals)),2,0).
compute probeval=make((nrow(wmodvals)*nrow(zmodvals)),(ncol(wprobval)+ncol(zprobval)),0).
loop probei= 1 to nrow(wmodvals).
loop probej =1 to nrow(zmodvals).
compute modvals(probecnt,1)=wmodvals(probei,1).
compute probeval(probecnt,1:nwvls)=wprobval(probei,:).
compute modvals(probecnt,2)=zmodvals(probej,1).
compute probeval(probecnt,(nwvls+1):(nwvls+nzvls))=zprobval(probej,:).
compute probecnt=probecnt+1.
end loop.
end loop.
do if (wzcmat((i+1),jmed)=1).
compute numplps=numplps+1.
compute probprod=make(1,(ncol(wprobval)*ncol(zprobval)),0).
compute lpstsp2={1,1}.
compute lpstsp={lpstsp;lpstsp2}.
compute lpstsp(3,1)=lpstsp(2,2)+1.
compute lpstsp(3,2)=lpstsp(2,2)+ncol(probprod).
compute jnmod=ztmp.
compute jnmin=zmin.
compute jnmax=zmax.
compute jnmodlab=znames.
do if (jmed = 1).
compute wherejn1=wherexw(1,i).
compute wherejn3=wherexwz(1,i).
end if.
do if (jmed > 1).
compute wherejn1=wheremw(((2*jmed)-3),i).
compute wherejn3=wheremwz(((2*jmed)-3),i).
end if.
loop probei = 1 to nrow(wmodvals).
loop probej = 1 to nrow(zmodvals).
compute probtemp=1.
loop probek = 1 to ncol(wprobval).
compute probtemp={probtemp,(wprobval(probei,probek)&*zprobval(probej,:))}.
end loop.
compute probprod={probprod;probtemp(1,2:ncol(probtemp))}.
end loop.
end loop.
compute probprod=probprod(2:nrow(probprod),:).
compute probeval={probeval,probprod}.
end if.
compute problabs={wnames,znames}.
release probecnt, probei, probej.
end if.
do if (intprint=1).
compute focpred={'   Focal', 'predict:'}.
do if (jmed=1).
compute focpred={focpred,xnames,'(X)'}.
compute focplotv=xmodvals.
end if.
do if (jmed >1).
do if (nms > 1).
compute focpred={focpred,mnames(1,(jmed-1)), medlb2(1,(jmed-1))}.
end if.
do if (nms = 1).
compute focpred={focpred,mnames(1,(jmed-1)), '(M)'}.
end if.
compute focplotv=mmodvals(:,(jmed-1)).
end if.
compute focpred2={'    ', 'Mod var:',focpred3}.
compute focpred={focpred;focpred2}.
do if (ncol(focpred4) > 1).
compute focpred={focpred;focpred4}.
compute focpred4={' '}.
end if.
release focpred2,focpred3.
do if (outscree=1).
do if ((plot = 1 or plot = 2) or (printpbe <= intprobe)).
print focpred/title='----------'/format=A8/space=0.
end if.
end if.
compute foctmp=make(nrow(modvals),1,1).
compute probexpl=1.
compute probeva2={foctmp,probeval}.
do if (jmed=1 and nxs > 0 and mcx > 0).
compute probexpl=nxvls.
end if.
compute foctmp=make(nrow(modvals),1,1).
compute modvals3=make(1,(6+ncol(problabs)),0).
compute probrown=make(nrow(probeval),1,0).
compute jtmp=1.
loop probei = 1 to nrow(probeval).
compute probrown(probei,1)=jtmp.
compute jtmp=jtmp+nxvls.
end loop.
release jtmp.
compute probrow=999.
compute modvarl=problabs.
do if (plot = 1 or plot = 2 or nxvls > 1).
compute plotvals=make((nrow(modvals)*nrow(focplotv)),(ncol(modvals)+1),999).
loop ploti=1 to nrow(modvals).
loop plotj=1 to nrow(focplotv).
compute plotvals((((ploti-1)*nrow(focplotv))+plotj),2:ncol(plotvals))=modvals(ploti,:).
compute plotvals((((ploti-1)*nrow(focplotv))+plotj),1)=focplotv(plotj,1).
end loop.
end loop.
compute focpredn=3.
do if (jmed=1).
do if (nxvls > 1).
compute focpredn=(nxvls+1).
end if.
do if (nxvls=1 and xdich=1).
compute focpredn=2.
end if.
end if.
compute meanmat=mdiag(means).
compute onesmat=make(nrow(meanmat),(nrow(probeval)*focpredn),1).
compute probeplt=t(mdiag(means)*onesmat).
do if (jmed=1).
do if ((wcmat((i+1),1)=1) or (zcmat((i+1),1)=1)).
compute plotcnt=1.
compute iloops=nwpval*nzpval.
compute plotmx=nxpval*nzpval.
do if ((wcmat((i+1),1)=1) and (zcmat((i+1),1)=0)).
compute iloops=nwpval.
compute plotmx=nxpval.
end if.
do if ((wcmat((i+1),1)=0) and (zcmat((i+1),1)=1)).
compute iloops=nzpval.
compute plotmx=nxpval.
end if.
compute xestvals=make((nxpval*iloops),ncol(xprobval),-999).
do if (wcmat((i+1),1)=1).
compute westvals=make(nrow(xestvals),ncol(wprobval),-999).
end if.
do if (zcmat((i+1),1)=1).
compute zestvals=make(nrow(xestvals),ncol(zprobval),-999).
end if.
loop ploti=1 to iloops.
loop plotj=1 to nxpval.
compute xestvals(plotcnt,:)=xprobval(plotj,:).
compute plotcnt=plotcnt+1.
end loop.
end loop.
compute plotcnt=1.
compute plotcnt1=1.
compute plotcnt2=1.
compute plotcntz=1.
loop ploti = 1 to (iloops*nxpval).
do if (wcmat((i+1),1)=1).
compute westvals(ploti,:)=wprobval(plotcnt1,:).
end if.
do if ((wcmat((i+1),1)=0) and (zcmat((i+1),1)=1)).
compute zestvals(ploti,:)=zprobval(plotcnt1,:).
end if.
do if ((wcmat((i+1),1)=1) and (zcmat((i+1),1)=1)).
compute zestvals(ploti,:)=zprobval(plotcnt2,:).
compute plotcntz=plotcntz+1.
end if.
compute plotcnt=plotcnt+1.
do if (plotcnt > plotmx).
compute plotcnt=1.
compute plotcnt1=plotcnt1+1.
end if.
do if (plotcntz > nxpval).
compute plotcnt2=plotcnt2+1.
compute plotcntz=1.
do if (plotcnt2 > nzpval).
compute plotcnt2=1.
end if.
end if.
end loop.
compute probeplt(:,2:(1+(ncol(xestvals))))=xestvals.
do if (wcmat((i+1),1)=1).
compute probeplt(:,(wherew(1,i)):(wherew(2,i)))=westvals.
end if.
do if (zcmat((i+1),1)=1).
compute probeplt(:,(wherez(1,i)):(wherez(2,i)))=zestvals.
end if.
end if.
end if.
do if (jmed > 1).
do if ((wcmat((i+1),jmed)=1) or (zcmat((i+1),jmed)=1)).
compute plotcnt=1.
compute iloops=nwpval*nzpval.
compute plotmx=3*nzpval.
do if ((wcmat((i+1),jmed)=1) and (zcmat((i+1),jmed)=0)).
compute iloops=nwpval.
compute plotmx=3.
end if.
do if ((wcmat((i+1),jmed)=0) and (zcmat((i+1),jmed)=1)).
compute iloops=nzpval.
compute plotmx=3.
end if.
compute mestvals=make((3*iloops),1,-999).
do if (wcmat((i+1),jmed)=1).
compute westvals=make(nrow(mestvals),ncol(wprobval),-999).
end if.
do if (zcmat((i+1),jmed)=1).
compute zestvals=make(nrow(mestvals),ncol(zprobval),-999).
end if.
loop ploti=1 to iloops.
loop plotj=1 to 3.
compute mestvals(plotcnt,:)=mprobval(plotj,(jmed-1)).
compute plotcnt=plotcnt+1.
end loop.
end loop.
compute plotcnt=1.
compute plotcnt1=1.
compute plotcnt2=1.
compute plotcntz=1.
loop ploti = 1 to (iloops*3).
do if (wcmat((i+1),jmed)=1).
compute westvals(ploti,:)=wprobval(plotcnt1,:).
end if.
do if ((wcmat((i+1),jmed)=0) and (zcmat((i+1),jmed)=1)).
compute zestvals(ploti,:)=zprobval(plotcnt1,:).
end if.
do if ((wcmat((i+1),jmed)=1) and (zcmat((i+1),jmed)=1)).
compute zestvals(ploti,:)=zprobval(plotcnt2,:).
compute plotcntz=plotcntz+1.
end if.
compute plotcnt=plotcnt+1.
do if (plotcnt > plotmx).
compute plotcnt=1.
compute plotcnt1=plotcnt1+1.
end if.
do if (plotcntz > 3).
compute plotcnt2=plotcnt2+1.
compute plotcntz=1.
do if (plotcnt2 > nzpval).
compute plotcnt2=1.
end if.
end if.
end loop.
compute probeplt(:,wherem((jmed-1),i))=mestvals.
do if (wcmat((i+1),jmed)=1).
do if (model <> 74).
compute probeplt(:,(wherew(1,i)):(wherew(2,i)))=westvals.
end if.
do if (model = 74).
compute probeplt(:,(wherex(1,i)):(wherex(2,i)))=westvals.
end if.
end if.
do if (zcmat((i+1),jmed)=1).
compute probeplt(:,(wherez(1,i)):(wherez(2,i)))=zestvals.
end if.
end if.
end if.
compute prodloop = 1.
do if (jmed=1).
compute prodloop=ncol(xestvals).
end if.
do if (wcmat((i+1),jmed))=1.
compute plotcnt=0.
loop ploti = 1 to prodloop.
loop plotj = 1 to ncol(westvals).
do if (jmed=1).
compute probeplt(:,(wherexw(1,i)+plotcnt))=xestvals(:,ploti)&*westvals(:,plotj).
end if.
do if (jmed > 1).
compute probeplt(:,(wheremw(((jmed*2)-3) ,i)+plotcnt))=mestvals(:,ploti)&*westvals(:,plotj).
end if.
compute plotcnt=plotcnt+1.
end loop.
end loop.
end if.
do if (zcmat((i+1),jmed))=1.
compute plotcnt=0.
loop ploti = 1 to prodloop.
loop plotj = 1 to ncol(zestvals).
do if (jmed = 1).
compute probeplt(:,(wherexz(1,i)+plotcnt))=xestvals(:,ploti)&*zestvals(:,plotj).
end if.
do if (jmed > 1).
compute probeplt(:,(wheremz(((jmed*2)-3),i)+plotcnt))=mestvals(:,ploti)&*zestvals(:, plotj).
end if.
compute plotcnt=plotcnt+1.
end loop.
end loop.
end if.
do if (wzcmat((i+1),jmed))=1.
compute plotcnt=0.
compute threeway=1.
loop ploti = 1 to ncol(westvals).
loop plotj = 1 to ncol(zestvals).
compute probeplt(:,(wherewz(1,i)+plotcnt))=westvals(:,ploti)&*zestvals(:,plotj).
compute plotcnt=plotcnt+1.
end loop.
end loop.
compute plotcnt=0.
loop plotk = 1 to prodloop.
loop ploti = 1 to ncol(westvals).
loop plotj = 1 to ncol(zestvals).
do if (jmed = 1).
compute probeplt(:,(wherexwz(1,i)+plotcnt))=xestvals(:,plotk)&*westvals(:,ploti)&* zestvals(:,plotj).
end if.
do if (jmed > 1).
compute probeplt(:,(wheremwz(((jmed*2)-3),i)+plotcnt))=mestvals(:,plotk)&* westvals(:,ploti)&*zestvals(:,plotj).
end if.
compute plotcnt=plotcnt+1.
end loop.
end loop.
end loop.
end if.
loop newplp=1 to i.
do if (newplp <> jmed).
do if (wcmat((i+1),newplp))=1.
compute prodloop=1.
do if (newplp=1).
compute prodloop=nxvls.
end if.
compute plotcnt=0.
loop ploti = 1 to prodloop.
loop plotj = 1 to nwvls.
do if (newplp = 1).
compute probeplt(:,(wherexw(1,i)+plotcnt))=probeplt(:,(1+ploti))&*probeplt(:, (wherew(1,i)+plotj-1)).
end if.
do if (newplp > 1).
do if (model <> 74).
compute probeplt(:,(wheremw(((newplp*2)-3) ,i)+plotcnt))=probeplt(:,wherem((newplp-1),i))&*probeplt(:,(wherew(1,i)+plotj-1)).
end if.
do if (model = 74).
compute probeplt(:,(wheremw(((newplp*2)-3) ,i)+plotcnt))=probeplt(:,wherem((newplp-1),i))&*probeplt(:,(wherex(1,i)+plotj-1)).
end if.
end if.
compute plotcnt=plotcnt+1.
end loop.
end loop.
end if.
do if (zcmat((i+1),newplp))=1.
compute prodloop=1.
do if (newplp=1).
compute prodloop=nxvls.
end if.
compute plotcnt=0.
loop ploti = 1 to prodloop.
loop plotj = 1 to nzvls.
do if (newplp = 1).
compute probeplt(:,(wherexz(1,i)+plotcnt))=probeplt(:,(1+ploti))&*probeplt(:, (wherez(1,i)+plotj-1)).
end if.
do if (newplp > 1).
compute probeplt(:,(wheremz(((newplp*2)-3),i)+plotcnt))=probeplt(:, wherem((newplp-1),i))&*probeplt(:,(wherez(1,i)+plotj-1)).
end if.
compute plotcnt=plotcnt+1.
end loop.
end loop.
end if.
do if (wzcmat((i+1),newplp))=1.
compute plotcnt=0.
do if (threeway=0).
loop ploti = 1 to nwvls.
loop plotj = 1 to nzvls.
compute probeplt(:,(wherewz(1,i)+plotcnt))=probeplt(:,(wherew(1,i)+ploti-1))&* probeplt(:,(wherez(1,i)+plotj-1)).
compute plotcnt=plotcnt+1.
end loop.
end loop.
end if.
compute prodloop=1.
do if (newplp=1).
compute prodloop=nxvls.
end if.
compute plotcnt=0.
loop plotk = 1 to prodloop.
loop ploti = 1 to nwvls.
loop plotj = 1 to nzvls.
do if (newplp = 1).
compute probeplt(:,(wherexwz(1,i)+plotcnt))=probeplt(:,(1+plotk))&*probeplt(:, (wherew(1,i)+ploti-1))&*probeplt(:,(wherez(1,i)+plotj-1)).
end if.
do if (newplp > 1).
compute probeplt(:,(wheremwz(((newplp*2)-3),i)+plotcnt))=probeplt(:, wherem((newplp-1),i))&*probeplt(:,(wherew(1,i)+ploti-1))&*probeplt(:,(wherez(1,i)+plotj-1)).
end if.
compute plotcnt=plotcnt+1.
end loop.
end loop.
end loop.
end if.
end if.
end loop.
compute predvals=probeplt*b.
do if (debug <> 0).
print probeplt.
print b.
end if.
do if (i = nms+nys) and (ydich=1).
compute predvalt=(predvals < 709.7).
compute prevalt7=(1-predvalt)*(709.7).
compute predvals=(predvals&*predvalt)+prevalt7.
compute expyhat=exp(predvals)&/(1+exp(predvals)).
end if.
compute sepred=make(nrow(plotvals),3,999).
loop sei=1 to nrow(plotvals).
compute ask=probeplt(sei,:).
compute sepred(sei,1)=sqrt(ask*varb*t(ask)).
do if ((i < nms+nys) or (ydich=0)).
compute sepred(sei,2)=predvals(sei,1)-tval*sepred(sei,1).
compute sepred(sei,3)=predvals(sei,1)+tval*sepred(sei,1).
end if.
do if ((i = nms+nys) and (ydich=1)).
compute sepred(sei,2)=predvals(sei,1)-xp2*sepred(sei,1).
compute sepred(sei,3)=predvals(sei,1)+xp2*sepred(sei,1).
end if.
end loop.
compute prevloc=ncol(plotvals)+1.
compute probeplt={plotvals,predvals}.
do if (plot = 2).
compute probeplt={probeplt,sepred}.
end if.
do if ((i = nms+nys) and (ydich=1)).
compute probeplt={probeplt,expyhat}.
end if.
compute didsome=0.
end if.
do if ((wzcmat((i+1),jmed)=1) and (printpbe <= intprobe)).
do if (jmed=1).
compute omnilp2=nxvls*nwvls.
compute omnitmp=ident(omnilp2).
compute omni3(wherexw(1,i):wherexw(2,i),:)=omnitmp.
end if.
do if (jmed>1).
compute omnilp2=nwvls.
compute omnitmp=ident(omnilp2).
compute omni3(wheremw(((jmed*2)-3),i):wheremw(((jmed*2)-2),i),:)=omnitmp.
end if.
compute omnif=make(1,4,0).
do if ((i = nms+nys) and (ydich=1)).
compute omnif=make(1,3,0).
end if.
compute condeff3=0.
loop omnilp1=1 to nrow(zprobval).
loop omnilp=1 to (omnilp2).
do if (jmed=1).
compute omni3((wherexwz(1,i)+((omnilp-1)*nzvls)):(wherexwz(1,i)+((omnilp-1)*nzvls)+ (nzvls-1)),omnilp)=t(zprobval(omnilp1,:)).
end if.
do if (jmed > 1).
compute omni3((wheremwz(((jmed*2)-3),i)+((omnilp-1)*nzvls)):(wheremwz(((jmed*2)-3),i)+ ((omnilp-1)*nzvls)+(nzvls-1)),omnilp)=t(zprobval(omnilp1,:)).
end if.
end loop.
compute condeff=t(omni3)*b.
compute condeff3={condeff3;condeff}.

.
compute lmat2= omni3.
do if ( 1 =0).
compute lmat2 = mdiag( omni3 ).
compute lmat3=make(nrow(lmat2),1,0).
loop flp=1 to ncol(lmat2).
do if (csum(lmat2(:,flp))=1).
compute lmat3={lmat3,lmat2(:,flp)}.
end if.
end loop.
compute lmat2=lmat3(:,2:ncol(lmat3)).
end if.
compute fratio = (t(t(lmat2)* b )*inv(t(lmat2)* varb *lmat2)*((t(lmat2)* b )))/ncol(lmat2).
compute pfr = 1-fcdf(fratio,ncol(lmat2),(n-nrow( b ))).
compute fresult={fratio,ncol(lmat2),(n-nrow( b )),pfr}.
do if (i = (nms+nys) and (ydich=1)).
compute fratio=fratio*ncol(lmat2).
compute pfr=1-chicdf(fratio,ncol(lmat2)).
compute fresult={fratio,ncol(lmat2),pfr}.
end if.
do if ( 0 =1).
compute lmat3=1-rsum(lmat2).
compute xfm=make(n,csum(lmat3),0).
compute flpc=1.
loop flp=1 to nrow(lmat3).
do if (lmat3(flp,1)=1).
compute xfm(:,flpc)=x(:,flp).
compute flpc=flpc+1.
end if.
end loop.
compute bfm=inv(t(xfm)*xfm)*t(xfm)*y.
compute resid=y-(xfm*bfm).
compute sstotal=(y-(csum(y)/n)).
compute sstotal=csum(sstotal&*sstotal).
compute ssresid=csum(resid&*resid).
compute rsqch= 0 -((sstotal-ssresid)/sstotal).
compute fresult={rsqch,fresult}.
release xfm,flpc, resid, ssresid, bfm.
end if
.
compute omnif={omnif;fresult}.
end loop.
compute omnif=omnif(2:nrow(omnif),:).
compute clabtmp=znames.
compute condeff3=condeff3(2:nrow(condeff3),:).
do if ((nxvls*nwvls)=1).
compute omnif={condeff3,omnif}.
compute clabtmp={clabtmp,'Effect'}.
end if.
compute omnif={zmodvals,omnif}.
do if ((i < nms+nys) or (ydich=0)).
compute clabtmp={clabtmp,hcflab,'df1','df2','p'}.
end if.
do if ((i = (nms+nys)) and (ydich=1)).
compute clabtmp={clabtmp,'Chi-sq','df','p'}.
end if.
compute resultm2=make(nrow(omnif),maxresm,99999).
compute resultm2(1:nrow(omnif),1:ncol(omnif))=omnif.
compute resultm={resultm;resultm2}.
do if (outscree=1).
do if (jmed=1).
print omnif/title='Test of conditional X*W interaction at value(s) of Z:'/cnames=clabtmp/format= F10.4.
end if.
do if (jmed>1).
print omnif/title='Test of conditional M*W interaction at value(s) of Z:'/cnames=clabtmp/format= F10.4.
end if.
end if.
release omni3.
end if.
loop probei = 1 to probexpl.
do if (probexpl > 1).
compute foctmp=make(nrow(modvals),probexpl,0).
compute foctmp(:,probei)=foctmp(:,probei)+1.
compute probtemp=make(nrow(modvals),1,0).
loop probem = 1 to numplps.
loop probek = 1 to nxvls.
loop probej=lpstsp(probem,1) to lpstsp(probem,2).
compute probtemp={probtemp,foctmp(:,probek)&*probeval(:,probej)}.
end loop.
end loop.
end loop.
compute probeva2=probtemp(:,2:ncol(probtemp)).
compute probeva2={foctmp,probeva2}.
end if.
compute probres=probeva2*probcoef.
compute probrese=sqrt(diag(probeva2*probvarb*t(probeva2))).
compute tratio = probres&/probrese.
compute p = 2*(1-tcdf(abs(tratio), dfres)).
do if (ydich=1 and i = (nms+nys)).
compute p = 2*(1-cdfnorm(abs(tratio))).
end if.
compute modvals2={modvals,probres,probrese,tratio, p}.
do if ((i < nms+nys) or (ydich=0)).
compute modvals2={modvals2,(probres-tval&*probrese),(probres+tval&*probrese)}.
compute problabs={problabs,'Effect',hclab,'t', 'p', 'LLCI', 'ULCI'}.
end if.
do if (ydich=1 and i = (nms+nys)).
compute modvals2={modvals2,(probres-xp2&*probrese),(probres+xp2&*probrese)}.
compute problabs={problabs,'Effect','se','Z', 'p', 'LLCI', 'ULCI'}.
end if.
do if (probexpl > 1 and (printpbe <= intprobe)).
do if (hasz = 1).
compute printz=1.
end if.
do if (hasw=1).
compute printw=1.
end if.
compute probrlab=make(nrow(modvals),1,xcatlab(probei,1)).
compute modvals3={modvals3; modvals2}.
compute probrow={probrow;probrown}.
compute probrown=probrown+1.
do if (probei=probexpl).
compute xproblab=xcatlab(1:nxvls,1).
compute probrow=probrow(2:nrow(probrow),1).
compute modvals3=modvals3(2:nrow(modvals3),:).
compute temp=modvals3.
compute temp(GRADE(probrow(:,1)),:)=modvals3.
compute modvals3=temp.
compute start2=1.
compute problabs=problabs(1,(1+(ncol(modvarl))):ncol(problabs)).
compute pstart=1.
loop probek= 1 to nrow(probeval).
compute endstart=start2+(nxvls-1).
compute temp=modvals3(start2:endstart,(1+ncol(modvarl)):ncol(modvals3)).
compute temp2=t(modvals3(start2:start2,1:ncol(modvarl))).
compute trnames=t(modvarl).
do if (outscree=1).
do if (probek > 1).
print/title='----------'/space=0.
else.
print/title = 'Conditional effects of the focal predictor at values of the moderator(s):'.
do if ((jmed=1) and (i = (nms+nys)) and (nms > 0)).
do if (nxvls = 1).
print/title = '(These are also the conditional direct effects of X on Y)'/space=0.
else.
print/title = '(These are also the relative conditional direct effects of X on Y)'/space=0.
end if.
end if.
print.
end if.
print temp2/title = 'Moderator value(s):'/rnames=trnames/format= F10.4 /space=0.
print temp/title = ' '/cnames=problabs/rnames=xproblab/format= F10.4 /space=0.
end if.
compute resultm2=make(nrow(temp2),maxresm,99999).
compute resultm2(1:nrow(temp2),1:ncol(temp2))=temp2.
compute resultm={resultm;resultm2}.
compute resultm2=make(nrow(temp),maxresm,99999).
compute resultm2(1:nrow(temp),1:ncol(temp))=temp.
compute resultm={resultm;resultm2}.
compute start2=start2+nxvls.
compute didsome=1.
do if (jmed=1).
compute mod1val=probeval(probek,1:nm1vls).
loop omnilp=1 to nxvls.
compute omni((wheremv1(1,i)+((omnilp-1)*nm1vls)):(wheremv1(1,i)+((omnilp-1)* nm1vls)+(nm1vls-1)),omnilp)=t(mod1val).
do if (nm1vls < ncol(probeval)).
compute mod2val=probeval(probek,(nm1vls+1):(nm1vls+nm2vls)).
compute omni((wheremv2(1,i)+((omnilp-1)*nm2vls)):(wheremv2(1,i)+((omnilp-1)* nm2vls)+(nm2vls-1)),omnilp)=t(mod2val).
do if ((nm1vls+nm2vls) < ncol(probeval)).
compute intlen=nm1vls*nm2vls.
compute modintvl=probeval(probek,(nm1vls+nm2vls+1):ncol(probeval)).
compute omni((wherexwz(1,i)+((omnilp-1)*intlen)):(wherexwz(1,i)+((omnilp-1)* intlen)+(intlen-1)),omnilp)=t(modintvl).
end if.
end if.
end loop.

.
compute lmat2= omni.
do if ( 1 =0).
compute lmat2 = mdiag( omni ).
compute lmat3=make(nrow(lmat2),1,0).
loop flp=1 to ncol(lmat2).
do if (csum(lmat2(:,flp))=1).
compute lmat3={lmat3,lmat2(:,flp)}.
end if.
end loop.
compute lmat2=lmat3(:,2:ncol(lmat3)).
end if.
compute fratio = (t(t(lmat2)* b )*inv(t(lmat2)* varb *lmat2)*((t(lmat2)* b )))/ncol(lmat2).
compute pfr = 1-fcdf(fratio,ncol(lmat2),(n-nrow( b ))).
compute fresult={fratio,ncol(lmat2),(n-nrow( b )),pfr}.
do if (i = (nms+nys) and (ydich=1)).
compute fratio=fratio*ncol(lmat2).
compute pfr=1-chicdf(fratio,ncol(lmat2)).
compute fresult={fratio,ncol(lmat2),pfr}.
end if.
do if ( 0 =1).
compute lmat3=1-rsum(lmat2).
compute xfm=make(n,csum(lmat3),0).
compute flpc=1.
loop flp=1 to nrow(lmat3).
do if (lmat3(flp,1)=1).
compute xfm(:,flpc)=x(:,flp).
compute flpc=flpc+1.
end if.
end loop.
compute bfm=inv(t(xfm)*xfm)*t(xfm)*y.
compute resid=y-(xfm*bfm).
compute sstotal=(y-(csum(y)/n)).
compute sstotal=csum(sstotal&*sstotal).
compute ssresid=csum(resid&*resid).
compute rsqch= 0 -((sstotal-ssresid)/sstotal).
compute fresult={rsqch,fresult}.
release xfm,flpc, resid, ssresid, bfm.
end if
.
compute resultm2=make(nrow(fresult),maxresm,99999).
compute resultm2(1:nrow(fresult),1:ncol(fresult))=fresult.
compute resultm={resultm;resultm2}.
do if (outscree=1).
do if (i < (nms + nys) or (ydich = 0)).
compute clabtmp={hcflab,'df1','df2','p'}.
print fresult/title='Test of equality of conditional means'/cnames=clabtmp/format= F10.4.
end if.
do if (i = (nms + nys) and (ydich = 1)).
compute clabtmp={'Chi-sq','df','p'}.
print fresult/title='Test of equality of conditional logits or probabilities'/cnames=clabtmp/format= F10.4.
end if.
end if.
compute probetmp=probeplt(pstart:(pstart+nxvls),1).
compute probetmp={probetmp,probeplt(pstart:(pstart+nxvls),prevloc:ncol(probeplt))}.
compute pstart=pstart+(nxvls+1).
compute resultm2=make(nrow(probetmp),maxresm,99999).
compute resultm2(1:nrow(probetmp),1:ncol(probetmp))=probetmp.
compute resultm={resultm;resultm2}.
do if (i < ((nms + nys)) or (ydich = 0)).
compute clabtmp={xnames, outnames(1,i), hclab, 'LLCI', 'ULCI'}.
do if (outscree=1).
print probetmp/title = 'Estimated conditional means being compared:'/cnames=clabtmp/format= F10.4.
end if.
end if.
do if ((i = (nms+nys)) and (ydich = 1)).
compute clabtmp={xnames, outnames(1,i), 'prob'}.
compute probetm2=probetmp(:,1:2).
compute probetm2={probetm2,probetmp(:,ncol(probetmp))}.
do if (outscree=1).
print probetm2/title = 'Estimated conditional logits and probabilities:'/cnames=clabtmp/format= F10.4.
end if.
end if.
end if.
end loop.
release probrow, start2, endstart, temp, temp2.
end if.
end if.
do if (probexpl = 1 and (printpbe <= intprobe)).
compute resultm2=make(nrow(modvals2),maxresm,99999).
compute resultm2(1:nrow(modvals2),1:ncol(modvals2))=modvals2.
compute resultm={resultm;resultm2}.
do if (outscree=1).
print/title = 'Conditional effects of the focal predictor at values of the moderator(s):'.
print modvals2/cnames=problabs/title = ' '/space=0/format= F10.4.
end if.
compute didsome=1.
do if (hasz = 1).
compute printz=1.
end if.
do if (hasw=1).
compute printw=1.
end if.
do if (jn = 1 and jnok=1).
do if (criterr = 0).
compute dfres=n-nrow(b).
compute roots=99999.
compute jncrit =(dfres* (exp((dfres-(5/6))*((xp2/(dfres-(2/3)+(.11/dfres)))* (xp2/(dfres-(2/3)+(.11/dfres)))))-1)).
do if (i = (nms+nys) and (ydich=1)).
compute jncrit=xp2*xp2.
end if.
compute jnb1=b(wherejn1,1).
compute jnb3=b(wherejn3,1).
compute jnsb1=varb(wherejn1,wherejn1).
compute jnsb3=varb(wherejn3,wherejn3).
compute jnsb1b3=varb(wherejn1,wherejn3).
compute ajn =(jncrit*jnsb3)-(jnb3*jnb3).
compute bjn = 2*((jncrit*jnsb1b3)-(jnb1*jnb3)).
compute cjn = (jncrit*jnsb1)-(jnb1*jnb1).
compute radarg = (bjn*bjn)-(4*ajn*cjn).
compute den = 2*ajn.
compute nrts = 0.
do if (radarg >= 0 and den <> 0).
compute x21 = (-bjn+sqrt(radarg))/den.
compute x22 = (-bjn-sqrt(radarg))/den.
compute roots = 0.
do if (x21 >= jnmin and x21 <= jnmax).
compute nrts = 1.
compute roots = {roots; x21}.
end if.
do if (x22 >= jnmin and x22 <= jnmax).
compute nrts = nrts + 1.
compute roots = {roots; x22}.
end if.
compute roots={roots,make(nrow(roots),2,0)}.
end if.
do if (nrts > 0).
compute roots = roots(2:nrow(roots),1:3).
compute roots(1,2)=(csum(jnmod < roots(1,1))/n)*100.
compute roots(1,3)=(csum(jnmod > roots(1,1))/n)*100.
do if (nrow(roots)=2).
compute roots(2,2)=(csum(jnmod < roots(2,1))/n)*100.
compute roots(2,3)=(csum(jnmod > roots(2,1))/n)*100.
end if.
do if (outscree=1).
print roots/title = 'Moderator value(s) defining Johnson-Neyman significance region(s):'/clabels = 'Value', '% below', '% above'/format F10.4.
end if.
do if (nrts=1).
compute tmprts=make(1,3,99999).
compute roots={roots;tmprts}.
end if.
end if.
do if (nrts = 0).
compute roots=make(2,1,99999).
do if (outscree=1).
print/title = 'There are no statistical significance transition points within the observed'.
print/title = 'range of the moderator found using the Johnson-Neyman method.'/space=0.
end if.
end if.
compute resultm2=make(nrow(roots),maxresm,99999).
compute resultm2(1:nrow(roots),1:ncol(roots))=roots.
compute resultm={resultm;resultm2}.
compute jnvals=make(23,7,0).
loop jni= 0 to (21-(nrts)).
compute jnvals((jni+1),1)=jnmin+(jni*((jnmax-jnmin)/(21-nrts))).
end loop.
do if (nrts > 0).
loop jni = 1 to nrts.
loop jnj = 2 to (nrow(jnvals)-1).
do if ((roots(jni,1) > jnvals((jnj-1),1)) and (roots(jni,1) < jnvals(jnj,1))).
compute jnvals((jnj+1):(21+jni),1)=jnvals(jnj:(20+jni),1).
compute jnvals(jnj,1)=roots(jni,1).
end if.
end loop.
end loop.
end if.
compute jnvals=jnvals(1:22,:).
loop jni = 1 to nrow(jnvals).
compute jnvals(jni,2)=jnb1+jnb3*jnvals(jni,1).
compute jnvals(jni,3)=sqrt(jnsb1+2*jnvals(jni,1)*jnsb1b3+(jnvals(jni,1)*jnvals(jni, 1))*jnsb3).
compute jnvals(jni,4)=jnvals(jni,2)/jnvals(jni,3).
compute jnvals(jni,5)=2*(1-tcdf(abs(jnvals(jni,4)), dfres)).
compute jnvals(jni,6)=jnvals(jni,2)-sqrt(jncrit)*jnvals(jni,3).
compute jnvals(jni,7)=jnvals(jni,2)+sqrt(jncrit)*jnvals(jni,3).
do if ((i = nms + nys) and (ydich=1)).
compute jnvals(jni,5)=2*(1-cdfnorm(abs(jnvals(jni,4)))).
compute jnvals(jni,6)=jnvals(jni,2)-xp2*jnvals(jni,3).
compute jnvals(jni,7)=jnvals(jni,2)+xp2*jnvals(jni,3).
end if.
end loop.
compute resultm2=make(nrow(jnvals),maxresm,99999).
compute resultm2(1:nrow(jnvals),1:ncol(jnvals))=jnvals.
compute resultm={resultm;resultm2}.
do if ((i < nms+nys) or (ydich=0)).
compute jnclbs={jnmodlab,'Effect',hclab,'t', 'p', 'LLCI', 'ULCI'}.
end if.
do if ((i = nms + nys) and (ydich=1)).
compute jnclbs={jnmodlab,'Effect','se','Z', 'p', 'LLCI', 'ULCI'}.
end if.
do if (((wcmat((i+1),jmed)=1) or (zcmat((i+1),jmed)=1)) and (wzcmat((i+1),jmed)=0)).
do if (outscree=1).
print jnvals/title = 'Conditional effect of focal predictor at values of the moderator:'/cnames =jnclbs/format = F10.4.
end if.
end if.
do if (outscree=1).
do if ((jmed = 1) and (wzcmat((i+1),jmed)=1)).
print jnvals/title = 'Conditional X*W interaction at values of the moderator Z:'/cnames =jnclbs/format = F10.4.
end if.
do if ((jmed > 1) and (wzcmat((i+1),jmed)=1)).
print jnvals/title = 'Conditional M*W interaction at values of the moderator Z:'/cnames =jnclbs/format = F10.4.
end if.
end if.
end if.
end if.
end if.
do if ((i = (nms+nys)) and (jmed=1) and (bcmat(nrow(bcmat),1)=1)).
do if (probei=1).
compute direfflb=problabs.
compute direff=modvals2.
end if.
do if (probei>1).
compute direff={direff;modvals2}.
end if.
end if.
compute intprint=0.
do if ((jmed=1) and (i=1) and nms=0) and modcok=1).
compute contvec2=make(2,1,1).
compute contvec2={contvec2,wcontval,zcontval}.
do if (wzcmat((i+1),jmed)=1).
loop conti= 1 to ncol(wcontval).
loop contj = 1 to ncol(zcontval).
compute contvec2={contvec2,wcontval(:,conti)&*zcontval(:,contj)}.
end loop.
end loop.
end if.
compute conteff=contvec2*probcoef.
compute contdiff=contvec2(1,:)-contvec2(2,:).
compute contse=sqrt(contdiff*probvarb*t(contdiff)).
compute conteffd=conteff(1,1)-conteff(2,1).
compute contvec={contvec,conteff}.
compute contvecm=contvec.
compute resultm2=make(nrow(contvecm),maxresm,99999).
compute resultm2(1:nrow(contvecm),1:ncol(contvecm))=contvecm.
compute resultm={resultm;resultm2}.
do if (outscree=1).
print/title='Contrast between conditional effects of X:'.
print contvec/title=' '/rlabels='Effect1:','Effect2:'/cnames=problabs/format = F10.4 /space=0.
end if.
do if (ydich=0).
compute p=2*(1-tcdf(abs(conteffd/contse), dfres)).
compute contvec={conteffd,contse,conteffd/contse, p}.
compute contvec={contvec,(conteffd-(tval*contse))}.
compute contvec={contvec,(conteffd+(tval*contse))}.
compute contlabs={'Contrast', hclab, 't', 'p', 'LLCI', 'ULCI'}.
end if.
do if (ydich=1).
compute p=2*(1-cdfnorm(abs(conteffd/contse))).
compute contvec={conteffd,contse,conteffd/contse, p}.
compute contvec={contvec,(conteffd-(xp2*contse))}.
compute contvec={contvec,(conteffd+(xp2*contse))}.
compute contlabs={'Contrast', 'se', 'Z', 'p', 'LLCI', 'ULCI'}.
end if.
compute resultm2=make(nrow(contvec),maxresm,99999).
compute resultm2(1:nrow(contvec),1:ncol(contvec))=contvec.
compute resultm={resultm;resultm2}.
do if (outscree=1).
print contvec/title='Test of Effect1 minus Effect2'/format= F10.4 /cnames=contlabs.
end if.
end if.
end loop.
do if (plot = 1 or plot = 2).
compute datalabs={t(focpred(:,3)),outnames(1,i)}.
do if (plot = 1).
compute datalabs={datalabs}.
end if.
do if (plot = 2).
compute datalabs={datalabs,'se', 'LLCI', 'ULCI'}.
end if.
do if ((i = nms+nys) and (ydich=1)).
compute datalabs={datalabs,'prob'}.
end if.
compute resultm2=make(nrow(probeplt),maxresm,99999).
compute resultm2(1:nrow(probeplt),1:ncol(probeplt))=probeplt.
compute resultm={resultm;resultm2}.
compute datalabs={datalabs,'.'}.
do if (outscree=1).
print/title = 'Data for visualizing the conditional effect of the focal predictor:'.
print/title = 'Paste text below into a SPSS syntax window and execute to produce plot.'/space=0.
compute dumb = {' ', ' ', ' ', ' ', ' ', ' ', ' '}.
print datalabs/title = 'DATA LIST FREE/'/format=A10.
print probeplt/title = 'BEGIN DATA.'/format= F10.4 /space=0.
print/title = 'END DATA.'/space=0.
end if.
compute focgrph=datalabs(1,1).
compute graphix={focgrph,graphixs,modgrph}.
do if (((xdich=1) or (nxvls > 1)) and ((modcat=0) and (focgrph = xnames))).
compute graphix={modgrph,graphixs,focgrph}.
end if.
do if (panelgrp = 0).
compute graphix={graphix,'.'}.
else.
compute graphix={graphix,panelcde}.
end if.
do if (outscree=1).
print graphix/title = 'GRAPH/SCATTERPLOT='/format=A8/space=0.
end if.
do if (i = (nms + nys) and ydich = 1).
compute graphixd=graphix.
compute graphixd(1,3)='prob'.
do if (outscree=1).
print graphixd/title = 'GRAPH/SCATTERPLOT='/format=A8/space=0.
end if.
end if.
end if.
end if.
end loop.
release jmed, intprint,didprint.
end if
.
do if (model = 74 and i <= nms).
compute onetemp=make(nrow(xprobval),1,1).
compute mestmt74={onetemp,xprobval}.
do if (ncs > 0).
compute ncovmdl=rsum(ccmat(i,:)).
do if (ncovmdl > 0).
compute cvmnc=make(nrow(mestmt74),ncovmdl,1).
compute cvmnctmp=csum(x(:,(ncol(x)-ncovmdl+1):ncol(x)))/nrow(x).
do if (cuscoval > 0).
compute cvmnctmp=coval.
end if.
loop mestlp=1 to ncovmdl.
compute cvmnc(:,mestlp)=cvmnc(:,mestlp)*cvmnctmp(:,mestlp).
end loop.
compute mestmt74={mestmt74,cvmnc}.
end if.
end if.
compute mest74t=mestmt74*b.
do if (i = 1).
compute mest74=mest74t.
end if.
do if (i > 1).
compute mest74={mest74,mest74t}.
end if.
end if.
do if ((i = (nms+nys)) and (model >= 0 and model < 4) and (linsum(1,1) <> -999)).
compute lhyprob=1.
compute meansub=0.
do if (((nlinsum=nrow(b)) or (nlinsum = (nrow(b)-ncs)))).
do if (nlinsum = (nrow(b)-ncs) and ncs > 0).
compute linsum={linsum,covmeans}.
compute meansub=1.
end if.
compute lhyprob=0.
compute hypest=linsum*b.
compute sehypest=sqrt(linsum*varb*t(linsum)).
compute phypest=2*(1-tcdf(abs(hypest/sehypest), (dfres))).
compute hypest={hypest,sehypest,(hypest/sehypest),phypest,(hypest-tval*sehypest),(hypest+ tval*sehypest)}.
compute resultm2=make(ncol(linsum),maxresm,99999).
compute resultm2(:,1)=t(linsum).
compute resultm={resultm;resultm2}.
compute resultm2=make(1,maxresm,99999).
compute resultm2(1,1:ncol(hypest))=hypest.
compute resultm={resultm;resultm2}.
do if (outscree=1).
compute hyplabs={'Estimate',hclab,'t','p','LLCI','ULCI'}.
print/title='----------'.
print/title='Linear Combination Estimate and Hypothesis Test'/space=0.
print t(linsum)/title= 'Weight vector:'/clabels='weight'/rnames=vlabsm/format F10.4.
print hypest/title=' '/cnames=hyplabs/format= F10.4 /space=0.
do if (meansub=1).
print/title='Covariate weight(s) set to the sample mean.'.
end if.
end if.
end if.
do if (lhyprob=1).
compute notecode(notes,1) = 30.
compute notes = notes + 1.
end if.
end if.
end loop if criterr=1.
compute lastb=b.
compute lastcov=varb.
do if (criterr=0 and dototal = 1).
compute x=xtmp.
compute vlabsm={'constant';xcatlab(1:nxvls,1)}.
do if (ncs > 0).
compute x = {x,ctmp}.
compute vlabsm={vlabsm;t(covnames)}.
end if.
compute x = {ones,x}.

.
do if ( 1 =1).
compute hatmat=inv(t( x )* x )*t( x ).
compute b = hatmat* y.
compute modres=b.
do if ( 1 =1).
compute n1=nrow( x ).
compute dfres=n1-(ncol( x )).
compute sstotal = t( y -(csum( y )/n1))*( y -(csum( y )/n1)).
compute resid= y - x *b.
compute ssresid = csum((resid)&**2).
compute r2 = (sstotal-ssresid)/sstotal.
do if (r2 < 0).
compute r2=0.
end if.
compute adjr2 = 1-((1-r2)*(n1-1)/(dfres)).
compute mse=ssresid/(n1-ncol( x )).

.
compute n1=nrow( x ).
compute invXtX = inv(t( x )* x ).
compute varb = mse *invXtX.
compute k3 = ncol( x ).
compute xhc=0.
do if ( hc <> 5).
compute xhc= x.
compute hat = xhc(:,1).
loop i3=1 to nrow(xhc).
compute hat(i3,1)= xhc(i3,:)*invXtX*t(xhc(i3,:)).
end loop.
do if ( hc = 0 or hc =1).
loop i3 = 1 to k3.
compute xhc(:,i3)=xhc(:,i3)&* resid.
end loop.
end if.
do if ( hc =3 or hc =2).
loop i3=1 to k3.
compute xhc(:,i3) = ( resid &/(1-hat)&**(1/(4- hc )))&*xhc(:,i3).
end loop.
end if.
do if ( hc = 4).
compute hcmn=make(n,2,4).
compute hcmn(:,2)=(n1*hat)/k3.
loop i3= 1 to k3.
compute xhc(:,i3) = ( resid &/(1-hat)&**(rmin(hcmn)/2))&*xhc(:,i3).
end loop.
end if.
compute varb=(invXtX*t(xhc)*xhc*invXtX).
do if ( hc =1).
compute varb=(n1/(n1-ncol( x )))&*varb.
end if.
end if.
compute hclab={'se(HC0)','se(HC1)','se(HC2)','se(HC3)','se(HC4)','se'}.
compute hclab=hclab(1,( hc +1)).
compute hcflab={'F(HC0)','F(HC1)','F(HC2)','F(HC3)','F(HC4)','F'}.
compute hcflab=hcflab(1,( hc +1)).
release xhc
.
compute seb=sqrt(diag(varb)).
compute trat = b&/seb.
compute p = 2*(1-tcdf(abs(trat), (dfres))).
compute tval = sqrt(dfres* (exp((dfres-(5/6))*((xp2/(dfres-(2/3)+(.11/dfres)))* (xp2/(dfres-(2/3)+(.11/dfres)))))-1)).
compute modres={modres,seb,trat,p}.
compute modres={modres,(b-tval&*seb),(b+tval&*seb)}.
compute modresl={'coeff',hclab,'t','p','LLCI','ULCI'}.
compute lmat = ident(ncol( x )).
compute lmat = lmat(:,2:ncol(lmat)).
compute fratio = (t(t(lmat)*b)*inv(t(lmat)*varb*lmat)*((t(lmat)*b)))/(ncol( x )-1).
compute pfr = 1-fcdf(fratio,(ncol( x )-1),dfres).
compute modsum={sqrt(r2),r2,mse,fratio,(ncol( x )-1),dfres,pfr}.
compute modsuml={'R','R-sq','MSE',hcflab,'df1','df2', 'p'}.
end if.
end if.
do if ( 1 = 2 or 1 =3).
compute xlp= x.
compute ylp= y.
compute pt2 = make(nrow(ylp),1,(csum(ylp)/nrow(ylp))).
do if ( 1 =2).
compute LL3 = ylp&*ln(pt2)+(1-ylp)&*ln(1-pt2).
end if.
compute LL3 = -2*csum(LL3).
compute bt1 = make(ncol(xlp),1,0).
compute LL1 = 0.
compute pt1 = make(nrow(ylp),1,0.5).
compute pt1lp=pt1.
loop jjj = 1 to iterate.
compute xlptmp=t(xlp).
compute vecprb=pt1lp&*(1-pt1lp).
loop kkk=1 to ncol(xlp).
compute xlptmp(kkk,:)=xlptmp(kkk,:)&*t(vecprb).
end loop.
compute b = bt1+inv(xlptmp*xlp)*t(xlp)*(ylp-pt1lp).
do if ( 1 =2).
compute xlpb=xlp*b.
compute xlpbt=(xlpb > -709.7).
compute xlpb709=(1-xlpbt)*(-709.7).
compute xlpb=(xlpb&*xlpbt)+xlpb709.
compute pt1lp = 1/(1+exp(-(xlpb))).
end if.
compute itprob = csum((pt1lp < .00000001) or (pt1lp > .9999999)).
do if (itprob > 0).
loop kkk = 1 to nrow(pt1lp).
do if (pt1lp(kkk,1) > .9999999).
compute pt1lp(kkk,1) = .9999999.
end if.
do if (pt1lp(kkk,1) < .00000001).
compute pt1lp(kkk,1) = .00000001.
end if.
end loop.
compute itprob = 0.
end if.
do if (itprob = 0).
do if ( 1 =2).
compute LL = ylp&*ln(pt1lp)+(1-ylp)&*ln(1-pt1lp).
end if.
compute LL2 = -2*csum(ll).
end if.
do if (abs(LL1-LL2) < converge).
do if ( 1 =1).
compute xlptmp=t(xlp).
compute vecprb=pt1lp&*(1-pt1lp).
loop kkk=1 to ncol(xlp).
compute xlptmp(kkk,:)=xlptmp(kkk,:)&*t(vecprb).
end loop.
compute varb = inv(xlptmp*xlp).
compute seb = sqrt(diag(varb)).
release xlptmp.
end if.
break.
end if.
compute bt1 = b.
compute LL1 = LL2.
end loop.
compute modres=b.
do if (jjj > iterate).
compute itprob = 2.
do if (booting=0).
compute iterrmod=1.
end if.
do if (booting=1).
compute bootiter=1.
end if.
do if (itprobtg=0).
compute itprobtg=1.
compute errcode(errs,1) = 47.
compute errs = errs + 1.
do if (booting = 0 and 1 =1).
compute vt1 = mdiag(pt1lp&*(1-pt1lp)).
compute varb = inv(t(xlp)*vt1*xlp).
compute seb = sqrt(diag(varb)).
end if.
end if.
end if.
do if ( 1 =1).
compute trat = b&/seb.
compute dfres=nrow(xlp).
compute p = 2*(1-cdfnorm(abs(trat))).
compute modres={modres,seb,trat,p}.
compute modres={modres,(b-xp2&*seb),(b+xp2&*seb)}.
compute pvchi=1-chicdf((LL3-LL2),(nrow(modres)-1)).
compute mcF = (LL3-LL2)/LL3.
compute cox = 1-exp(-(LL3-LL2)/nrow(xlp)).
compute nagel = cox/(1-exp(-(LL3)/nrow(xlp))).
compute modsum={LL2,(LL3-LL2),(nrow(modres)-1),pvchi, mcF,cox,nagel}.
compute modsuml={'-2LL','ModelLL', 'df', 'p', 'McFadden', 'CoxSnell', 'Nagelkrk'}.
compute modresl={'coeff','se','Z','p','LLCI','ULCI'}.
end if.
end if
.
compute toteff=modres(2:(1+nxvls),:).
compute nodotot=0.
do if (xdich=1 and xmint=1 and model=74).
compute toteff(:,1)=toteff(:,1)*xscaling.
compute toteff(:,2)=toteff(:,2)*abs(xscaling).
compute toteff(:,3)=toteff(:,3)*xscaling.
compute toteff(:,5)=toteff(:,5)*xscaling.
compute toteff(:,6)=toteff(:,6)*xscaling.
compute citmp=toteff(:,5:6).
do if (xscaling < 0).
compute toteff(:,5)=citmp(:,2).
compute toteff(:,6)=citmp(:,1).
end if.
compute nodotot=1.
end if.
compute totefflb=modresl.
do if (outscree=1).
do if (nodotot=0).
print/title = '************************** TOTAL EFFECT MODEL ****************************'.
end if.
do if (nodotot=1).
print/title = '**************************************************************************'.
end if.
print outnames(1,ncol(outnames))/title = 'OUTCOME VARIABLE:'/format = A8/space=0.
end if.
compute toteffl2=vlabsm(2:(1+nxvls),:).
compute resultm2=make(1,maxresm,99999).
compute resultm2(1,1:ncol(modsum))=modsum.
compute resultm={resultm;resultm2}.
compute resultm2=make(nrow(modres),maxresm,99999).
compute resultm2(1:nrow(modres),1:ncol(modres))=modres.
compute resultm={resultm;resultm2}.
do if (outscree=1).
print modsum/title = 'Model Summary'/cnames = modsuml/format= F10.4.
print modres/title='Model'/rnames=vlabsm/cnames=modresl/format= F10.4.
end if.
compute lmat=make(nrow(b),1,0).
compute lmat2=make(nxvls,1,1).
compute lmat(2:(1+nxvls),1)=lmat2.
do if (ydich <> 1).

.
compute lmat2= lmat.
do if ( 0 =0).
compute lmat2 = mdiag( lmat ).
compute lmat3=make(nrow(lmat2),1,0).
loop flp=1 to ncol(lmat2).
do if (csum(lmat2(:,flp))=1).
compute lmat3={lmat3,lmat2(:,flp)}.
end if.
end loop.
compute lmat2=lmat3(:,2:ncol(lmat3)).
end if.
compute fratio = (t(t(lmat2)* b )*inv(t(lmat2)* varb *lmat2)*((t(lmat2)* b )))/ncol(lmat2).
compute pfr = 1-fcdf(fratio,ncol(lmat2),(n-nrow( b ))).
compute fresult={fratio,ncol(lmat2),(n-nrow( b )),pfr}.
do if (i = (nms+nys) and (ydich=1)).
compute fratio=fratio*ncol(lmat2).
compute pfr=1-chicdf(fratio,ncol(lmat2)).
compute fresult={fratio,ncol(lmat2),pfr}.
end if.
do if ( 1 =1).
compute lmat3=1-rsum(lmat2).
compute xfm=make(n,csum(lmat3),0).
compute flpc=1.
loop flp=1 to nrow(lmat3).
do if (lmat3(flp,1)=1).
compute xfm(:,flpc)=x(:,flp).
compute flpc=flpc+1.
end if.
end loop.
compute bfm=inv(t(xfm)*xfm)*t(xfm)*y.
compute resid=y-(xfm*bfm).
compute sstotal=(y-(csum(y)/n)).
compute sstotal=csum(sstotal&*sstotal).
compute ssresid=csum(resid&*resid).
compute rsqch= r2 -((sstotal-ssresid)/sstotal).
compute fresult={rsqch,fresult}.
release xfm,flpc, resid, ssresid, bfm.
end if
.
compute totomni=fresult.
end if.
do if (stand=1).
compute predsd=make(nrow(modres),1,0).
compute stdmod=modres(:,1)&/ovsd(1,ncol(ovsd)).
loop jd=1 to ncol(x).
compute descdat=x(:,jd).
compute predsd(jd,1) = (nrow(descdat)*sscp(descdat))-(t(csum(descdat))*(csum(descdat))).
compute predsd(jd,1) = sqrt(predsd(jd,1)/(nrow(descdat)*(nrow(descdat)-1))).
end loop.
do if (wherex(1,ncol(wherex)) <> -999 and ((nxvls > 1) or (xdich=1))).
compute sdmsone=make(nxvls,1,1).
compute predsd(wherex(1,ncol(wherex)):wherex(2,ncol(wherex)),1)=sdmsone.
compute pstog=1.
end if.
compute predsd(1,1)=1.
compute stdmod=stdmod&*predsd.
compute stdmod=stdmod(2:nrow(stdmod),1).
compute sdvlabs=vlabsm(2:nrow(vlabsm),1).
compute resultm2=make(nrow(stdmod),maxresm,99999).
compute resultm2(1:nrow(stdmod),1:ncol(stdmod))=stdmod.
compute resultm={resultm;resultm2}.
do if (outscree=1).
print stdmod/title='Standardized coefficients'/clabels='coeff'/rnames=sdvlabs/format= F10.4.
end if.
end if.
do if (covcoeff=1).
do if (outscree=1).
print varb/title='Covariance matrix of regression parameter estimates:'/rnames=vlabsm/cnames=vlabsm/format= F10.4.
end if.
compute resultm2=make(nrow(varb),maxresm,99999).
do if (ncol(varb) <= maxresm).
compute resultm2(1:nrow(varb),1:ncol(varb))=varb.
compute resultm={resultm;resultm2}.
end if.
do if (ncol(varb) > maxresm).
compute resultmt=make(nrow(resultm),ncol(varb),99999).
compute resultmt(1:nrow(resultm),1:ncol(resultm))=resultm.
compute resultm=resultmt.
compute resultm2=make(nrow(varb),ncol(resultm),99999).
compute resultm2(1:nrow(varb),1:ncol(varb))=varb.
compute resultm={resultm;resultm2}.
compute maxresm=ncol(resultm).
end if.
end if.
end if.
end if.
do if (criterr=0 and nms > 0 and ydich=0 and modelres=1).
compute modresid=modresid(:,2:ncol(modresid)).
compute sigmatal = (t(modresid)*(ident(n)-(1/n)*ones*t(ones))*modresid)*(1/(n-1)).
compute sdall = mdiag(1/sqrt(diag(sigmatal))).
compute corall=sdall*sigmatal*t(sdall).
compute resultm2=make(nrow(corall),maxresm,99999).
compute resultm2(1:nrow(corall),1:ncol(corall))=corall.
compute resultm={resultm;resultm2}.
do if (outscree=1).
print/title = '****************** CORRELATIONS BETWEEN MODEL RESIDUALS ******************'.
print corall/title=' '/format= F10.4 / cnames=outnames/rnames=outnames/space=0.
end if.
end if.
do if (criterr=0 and boot > 0).
compute bootres=make(1,rsum(nump),-999).
compute bootdir=obsdirfx.
compute natdirbt=make(1,nxvls,-999).
do if (effsize=1).
compute bootysd=make(1,1,-999).
compute bootxsd=make(1,1,-999).
end if.
compute badboot=0.
compute goodboot=0.
compute smallest=1.
compute booting=1.
loop j = 1 to maxboot.
compute nobootx=1.
compute modres2=999.
compute v=trunc(uniform(n,1)*n)+1.
compute bad=0.
loop i = 1 to (nms+nys).
compute y=outvars(v,i).
compute ynovar= (nrow(y)*sscp(y))-(t(csum(y))*(csum(y))).
do if (ynovar = 0).
compute bad=1.
end if.
compute xindx=datindx(1:(nump(1,i)-1),i).
compute hello=0.
compute x = fulldat(v,xindx).
compute x={ones,x}.
compute xsq=t(x)*x.
compute exsq=eval(xsq).
release xsq.
compute holymoly=cmin(exsq).
compute zeroeig=csum(exsq <= 0.000000000002).
compute bad=bad + (zeroeig > 0).

.
compute desctmp=make((8-(4* 1 )),ncol( y ),-999).
loop jd=1 to ncol( y ).
compute descdat= y (:,jd).
compute desctmp(1,jd) = csum(descdat)/nrow(descdat).
compute desctmp(2,jd) = (nrow(descdat)*sscp(descdat))-(t(csum(descdat))*(csum(descdat))).
compute desctmp(2,jd) = sqrt(desctmp(2,jd)/(nrow(descdat)*(nrow(descdat)-1))).
compute desctmp(3,jd)=cmin(descdat).
compute desctmp(4,jd)=cmax(descdat).
do if ( 1 =0).
compute minwarn=0.
compute maxwarn=0.
do if ((desctmp(3,jd)=desctmp(4,jd)) and novar=0).
compute errcode(errs,1)=15.
compute errs=errs+1.
compute criterr=1.
compute novar=1.
end if.
compute tmp=((descdat(:,1)=desctmp(3,jd))+(descdat(:,1)=desctmp(4,jd))).
compute desctmp(8,jd)=(csum(tmp)=nrow(tmp)).
compute tmp = descdat.
compute tmp(GRADE(descdat),:) = descdat.
compute descdat = tmp.
release tmp.
compute decval={.16;.5;.84}.
loop kd=1 to 3.
compute low=trunc(decval(kd,1)*(nrow(descdat)+1)).
compute lowdec=decval(kd,1)*(nrow(descdat)+1)-low.
compute value=descdat(low,1)+(descdat((low+1),1)-descdat(low,1))*lowdec.
compute desctmp((4+kd),jd)=value.
end loop.
compute mnotev=1.
compute modvals=desctmp(5:7,:).
do if (quantile <> 1).
compute desctmp(5,jd)=desctmp(1,jd)-desctmp(2,jd).
compute desctmp(6,jd)=desctmp(1,jd).
compute desctmp(7,jd)=desctmp(1,jd)+desctmp(2,jd).
compute modvals=desctmp(5:7,:).
compute mnotev=2.
do if (modvals(1,1) < desctmp(3,1)).
compute modvals(1,1)=desctmp(3,1).
compute minwarn=1.
end if.
do if (modvals(3,1) > desctmp(4,1)).
compute modvals(3,1)=desctmp(4,1).
compute maxwarn=1.
end if.
end if.
do if (desctmp(8,1)=1).
compute modvals={desctmp(3,1);desctmp(4,1)}.
compute mnotev=0.
compute minwarn=0.
compute maxwarn=0.
end if.
end if.
end loop
.
compute bad=bad+ (desctmp(2,1) <= 0.00000000001).
do if (bad = 0).
do if (holymoly < smallest).
compute smallest=holymoly.
end if.
do if (ydich=0 or (i < (nms+nys))).

.
do if ( 1 =1).
compute hatmat=inv(t( x )* x )*t( x ).
compute b = hatmat* y.
compute modres=b.
do if ( 0 =1).
compute n1=nrow( x ).
compute dfres=n1-(ncol( x )).
compute sstotal = t( y -(csum( y )/n1))*( y -(csum( y )/n1)).
compute resid= y - x *b.
compute ssresid = csum((resid)&**2).
compute r2 = (sstotal-ssresid)/sstotal.
do if (r2 < 0).
compute r2=0.
end if.
compute adjr2 = 1-((1-r2)*(n1-1)/(dfres)).
compute mse=ssresid/(n1-ncol( x )).

.
compute n1=nrow( x ).
compute invXtX = inv(t( x )* x ).
compute varb = mse *invXtX.
compute k3 = ncol( x ).
compute xhc=0.
do if ( hc <> 5).
compute xhc= x.
compute hat = xhc(:,1).
loop i3=1 to nrow(xhc).
compute hat(i3,1)= xhc(i3,:)*invXtX*t(xhc(i3,:)).
end loop.
do if ( hc = 0 or hc =1).
loop i3 = 1 to k3.
compute xhc(:,i3)=xhc(:,i3)&* resid.
end loop.
end if.
do if ( hc =3 or hc =2).
loop i3=1 to k3.
compute xhc(:,i3) = ( resid &/(1-hat)&**(1/(4- hc )))&*xhc(:,i3).
end loop.
end if.
do if ( hc = 4).
compute hcmn=make(n,2,4).
compute hcmn(:,2)=(n1*hat)/k3.
loop i3= 1 to k3.
compute xhc(:,i3) = ( resid &/(1-hat)&**(rmin(hcmn)/2))&*xhc(:,i3).
end loop.
end if.
compute varb=(invXtX*t(xhc)*xhc*invXtX).
do if ( hc =1).
compute varb=(n1/(n1-ncol( x )))&*varb.
end if.
end if.
compute hclab={'se(HC0)','se(HC1)','se(HC2)','se(HC3)','se(HC4)','se'}.
compute hclab=hclab(1,( hc +1)).
compute hcflab={'F(HC0)','F(HC1)','F(HC2)','F(HC3)','F(HC4)','F'}.
compute hcflab=hcflab(1,( hc +1)).
release xhc
.
compute seb=sqrt(diag(varb)).
compute trat = b&/seb.
compute p = 2*(1-tcdf(abs(trat), (dfres))).
compute tval = sqrt(dfres* (exp((dfres-(5/6))*((xp2/(dfres-(2/3)+(.11/dfres)))* (xp2/(dfres-(2/3)+(.11/dfres)))))-1)).
compute modres={modres,seb,trat,p}.
compute modres={modres,(b-tval&*seb),(b+tval&*seb)}.
compute modresl={'coeff',hclab,'t','p','LLCI','ULCI'}.
compute lmat = ident(ncol( x )).
compute lmat = lmat(:,2:ncol(lmat)).
compute fratio = (t(t(lmat)*b)*inv(t(lmat)*varb*lmat)*((t(lmat)*b)))/(ncol( x )-1).
compute pfr = 1-fcdf(fratio,(ncol( x )-1),dfres).
compute modsum={sqrt(r2),r2,mse,fratio,(ncol( x )-1),dfres,pfr}.
compute modsuml={'R','R-sq','MSE',hcflab,'df1','df2', 'p'}.
end if.
end if.
do if ( 1 = 2 or 1 =3).
compute xlp= x.
compute ylp= y.
compute pt2 = make(nrow(ylp),1,(csum(ylp)/nrow(ylp))).
do if ( 1 =2).
compute LL3 = ylp&*ln(pt2)+(1-ylp)&*ln(1-pt2).
end if.
compute LL3 = -2*csum(LL3).
compute bt1 = make(ncol(xlp),1,0).
compute LL1 = 0.
compute pt1 = make(nrow(ylp),1,0.5).
compute pt1lp=pt1.
loop jjj = 1 to iterate.
compute xlptmp=t(xlp).
compute vecprb=pt1lp&*(1-pt1lp).
loop kkk=1 to ncol(xlp).
compute xlptmp(kkk,:)=xlptmp(kkk,:)&*t(vecprb).
end loop.
compute b = bt1+inv(xlptmp*xlp)*t(xlp)*(ylp-pt1lp).
do if ( 1 =2).
compute xlpb=xlp*b.
compute xlpbt=(xlpb > -709.7).
compute xlpb709=(1-xlpbt)*(-709.7).
compute xlpb=(xlpb&*xlpbt)+xlpb709.
compute pt1lp = 1/(1+exp(-(xlpb))).
end if.
compute itprob = csum((pt1lp < .00000001) or (pt1lp > .9999999)).
do if (itprob > 0).
loop kkk = 1 to nrow(pt1lp).
do if (pt1lp(kkk,1) > .9999999).
compute pt1lp(kkk,1) = .9999999.
end if.
do if (pt1lp(kkk,1) < .00000001).
compute pt1lp(kkk,1) = .00000001.
end if.
end loop.
compute itprob = 0.
end if.
do if (itprob = 0).
do if ( 1 =2).
compute LL = ylp&*ln(pt1lp)+(1-ylp)&*ln(1-pt1lp).
end if.
compute LL2 = -2*csum(ll).
end if.
do if (abs(LL1-LL2) < converge).
do if ( 0 =1).
compute xlptmp=t(xlp).
compute vecprb=pt1lp&*(1-pt1lp).
loop kkk=1 to ncol(xlp).
compute xlptmp(kkk,:)=xlptmp(kkk,:)&*t(vecprb).
end loop.
compute varb = inv(xlptmp*xlp).
compute seb = sqrt(diag(varb)).
release xlptmp.
end if.
break.
end if.
compute bt1 = b.
compute LL1 = LL2.
end loop.
compute modres=b.
do if (jjj > iterate).
compute itprob = 2.
do if (booting=0).
compute iterrmod=1.
end if.
do if (booting=1).
compute bootiter=1.
end if.
do if (itprobtg=0).
compute itprobtg=1.
compute errcode(errs,1) = 47.
compute errs = errs + 1.
do if (booting = 0 and 0 =1).
compute vt1 = mdiag(pt1lp&*(1-pt1lp)).
compute varb = inv(t(xlp)*vt1*xlp).
compute seb = sqrt(diag(varb)).
end if.
end if.
end if.
do if ( 0 =1).
compute trat = b&/seb.
compute dfres=nrow(xlp).
compute p = 2*(1-cdfnorm(abs(trat))).
compute modres={modres,seb,trat,p}.
compute modres={modres,(b-xp2&*seb),(b+xp2&*seb)}.
compute pvchi=1-chicdf((LL3-LL2),(nrow(modres)-1)).
compute mcF = (LL3-LL2)/LL3.
compute cox = 1-exp(-(LL3-LL2)/nrow(xlp)).
compute nagel = cox/(1-exp(-(LL3)/nrow(xlp))).
compute modsum={LL2,(LL3-LL2),(nrow(modres)-1),pvchi, mcF,cox,nagel}.
compute modsuml={'-2LL','ModelLL', 'df', 'p', 'McFadden', 'CoxSnell', 'Nagelkrk'}.
compute modresl={'coeff','se','Z','p','LLCI','ULCI'}.
end if.
end if
.
end if.
do if (ydich=1 and (i = (nms+nys))).

.
do if ( 2 =1).
compute hatmat=inv(t( x )* x )*t( x ).
compute b = hatmat* y.
compute modres=b.
do if ( 0 =1).
compute n1=nrow( x ).
compute dfres=n1-(ncol( x )).
compute sstotal = t( y -(csum( y )/n1))*( y -(csum( y )/n1)).
compute resid= y - x *b.
compute ssresid = csum((resid)&**2).
compute r2 = (sstotal-ssresid)/sstotal.
do if (r2 < 0).
compute r2=0.
end if.
compute adjr2 = 1-((1-r2)*(n1-1)/(dfres)).
compute mse=ssresid/(n1-ncol( x )).

.
compute n1=nrow( x ).
compute invXtX = inv(t( x )* x ).
compute varb = mse *invXtX.
compute k3 = ncol( x ).
compute xhc=0.
do if ( hc <> 5).
compute xhc= x.
compute hat = xhc(:,1).
loop i3=1 to nrow(xhc).
compute hat(i3,1)= xhc(i3,:)*invXtX*t(xhc(i3,:)).
end loop.
do if ( hc = 0 or hc =1).
loop i3 = 1 to k3.
compute xhc(:,i3)=xhc(:,i3)&* resid.
end loop.
end if.
do if ( hc =3 or hc =2).
loop i3=1 to k3.
compute xhc(:,i3) = ( resid &/(1-hat)&**(1/(4- hc )))&*xhc(:,i3).
end loop.
end if.
do if ( hc = 4).
compute hcmn=make(n,2,4).
compute hcmn(:,2)=(n1*hat)/k3.
loop i3= 1 to k3.
compute xhc(:,i3) = ( resid &/(1-hat)&**(rmin(hcmn)/2))&*xhc(:,i3).
end loop.
end if.
compute varb=(invXtX*t(xhc)*xhc*invXtX).
do if ( hc =1).
compute varb=(n1/(n1-ncol( x )))&*varb.
end if.
end if.
compute hclab={'se(HC0)','se(HC1)','se(HC2)','se(HC3)','se(HC4)','se'}.
compute hclab=hclab(1,( hc +1)).
compute hcflab={'F(HC0)','F(HC1)','F(HC2)','F(HC3)','F(HC4)','F'}.
compute hcflab=hcflab(1,( hc +1)).
release xhc
.
compute seb=sqrt(diag(varb)).
compute trat = b&/seb.
compute p = 2*(1-tcdf(abs(trat), (dfres))).
compute tval = sqrt(dfres* (exp((dfres-(5/6))*((xp2/(dfres-(2/3)+(.11/dfres)))* (xp2/(dfres-(2/3)+(.11/dfres)))))-1)).
compute modres={modres,seb,trat,p}.
compute modres={modres,(b-tval&*seb),(b+tval&*seb)}.
compute modresl={'coeff',hclab,'t','p','LLCI','ULCI'}.
compute lmat = ident(ncol( x )).
compute lmat = lmat(:,2:ncol(lmat)).
compute fratio = (t(t(lmat)*b)*inv(t(lmat)*varb*lmat)*((t(lmat)*b)))/(ncol( x )-1).
compute pfr = 1-fcdf(fratio,(ncol( x )-1),dfres).
compute modsum={sqrt(r2),r2,mse,fratio,(ncol( x )-1),dfres,pfr}.
compute modsuml={'R','R-sq','MSE',hcflab,'df1','df2', 'p'}.
end if.
end if.
do if ( 2 = 2 or 2 =3).
compute xlp= x.
compute ylp= y.
compute pt2 = make(nrow(ylp),1,(csum(ylp)/nrow(ylp))).
do if ( 2 =2).
compute LL3 = ylp&*ln(pt2)+(1-ylp)&*ln(1-pt2).
end if.
compute LL3 = -2*csum(LL3).
compute bt1 = make(ncol(xlp),1,0).
compute LL1 = 0.
compute pt1 = make(nrow(ylp),1,0.5).
compute pt1lp=pt1.
loop jjj = 1 to iterate.
compute xlptmp=t(xlp).
compute vecprb=pt1lp&*(1-pt1lp).
loop kkk=1 to ncol(xlp).
compute xlptmp(kkk,:)=xlptmp(kkk,:)&*t(vecprb).
end loop.
compute b = bt1+inv(xlptmp*xlp)*t(xlp)*(ylp-pt1lp).
do if ( 2 =2).
compute xlpb=xlp*b.
compute xlpbt=(xlpb > -709.7).
compute xlpb709=(1-xlpbt)*(-709.7).
compute xlpb=(xlpb&*xlpbt)+xlpb709.
compute pt1lp = 1/(1+exp(-(xlpb))).
end if.
compute itprob = csum((pt1lp < .00000001) or (pt1lp > .9999999)).
do if (itprob > 0).
loop kkk = 1 to nrow(pt1lp).
do if (pt1lp(kkk,1) > .9999999).
compute pt1lp(kkk,1) = .9999999.
end if.
do if (pt1lp(kkk,1) < .00000001).
compute pt1lp(kkk,1) = .00000001.
end if.
end loop.
compute itprob = 0.
end if.
do if (itprob = 0).
do if ( 2 =2).
compute LL = ylp&*ln(pt1lp)+(1-ylp)&*ln(1-pt1lp).
end if.
compute LL2 = -2*csum(ll).
end if.
do if (abs(LL1-LL2) < converge).
do if ( 0 =1).
compute xlptmp=t(xlp).
compute vecprb=pt1lp&*(1-pt1lp).
loop kkk=1 to ncol(xlp).
compute xlptmp(kkk,:)=xlptmp(kkk,:)&*t(vecprb).
end loop.
compute varb = inv(xlptmp*xlp).
compute seb = sqrt(diag(varb)).
release xlptmp.
end if.
break.
end if.
compute bt1 = b.
compute LL1 = LL2.
end loop.
compute modres=b.
do if (jjj > iterate).
compute itprob = 2.
do if (booting=0).
compute iterrmod=1.
end if.
do if (booting=1).
compute bootiter=1.
end if.
do if (itprobtg=0).
compute itprobtg=1.
compute errcode(errs,1) = 47.
compute errs = errs + 1.
do if (booting = 0 and 0 =1).
compute vt1 = mdiag(pt1lp&*(1-pt1lp)).
compute varb = inv(t(xlp)*vt1*xlp).
compute seb = sqrt(diag(varb)).
end if.
end if.
end if.
do if ( 0 =1).
compute trat = b&/seb.
compute dfres=nrow(xlp).
compute p = 2*(1-cdfnorm(abs(trat))).
compute modres={modres,seb,trat,p}.
compute modres={modres,(b-xp2&*seb),(b+xp2&*seb)}.
compute pvchi=1-chicdf((LL3-LL2),(nrow(modres)-1)).
compute mcF = (LL3-LL2)/LL3.
compute cox = 1-exp(-(LL3-LL2)/nrow(xlp)).
compute nagel = cox/(1-exp(-(LL3)/nrow(xlp))).
compute modsum={LL2,(LL3-LL2),(nrow(modres)-1),pvchi, mcF,cox,nagel}.
compute modsuml={'-2LL','ModelLL', 'df', 'p', 'McFadden', 'CoxSnell', 'Nagelkrk'}.
compute modresl={'coeff','se','Z','p','LLCI','ULCI'}.
end if.
end if
.
end if.
compute modres2={modres2,t(modres)}.
do if (i = (nms+nys)).
do if (bcmat((i+1),1) = 1).
compute bootdir={bootdir;t(modres(wherex(1,i):wherex(2,i),1))}.
end if.
do if (bcmat((i+1),1) = 0).
compute bootdir={bootdir;dirzes}.
end if.
end if.
do if (model = 74 and i <= nms).
compute onetemp=make(nrow(xprobval),1,1).
compute mestmtb={onetemp,xprobval}.
do if (ncs > 0).
compute ncovmdlb=rsum(ccmat(i,:)).
do if (ncovmdl > 0).
compute cvmncb=make(nrow(mestmtb),ncovmdlb,1).
compute cvmnctmp=csum(x(:,(ncol(x)-ncovmdlb+1):ncol(x)))/nrow(x).
do if (cuscoval > 0).
compute cvmnctmp=coval.
end if.
loop mestlp=1 to ncovmdlb.
compute cvmncb(:,mestlp)=cvmncb(:,mestlp)*cvmnctmp(:,mestlp).
end loop.
compute mestmtb={mestmtb,cvmncb}.
end if.
end if.
compute mestbt=mestmtb*modres.
do if (i = 1).
compute mestb=mestbt.
end if.
do if (i > 1).
compute mestb={mestb,mestbt}.
end if.
end if.
do if (model = 74 and i = (nms+nys)).
compute xvalptmp=make(1,nxvls,0).
compute mest74sp=make(1,nms,0).
compute mest74sp={mest74sp;mestb}.
compute xvalptmp={xvalptmp;ident(nxvls)}.
compute dirfxcf=make(nrow(mestb),1,0).
loop cfloop1=1 to nrow(mestb).
compute ndirfx=make(nrow(modres),1,0).
compute ndirfx(2:(nxvls+1),1)=t(xvalptmp(cfloop1,:)).
loop cfloop3=1 to nms.
do if (mcx=1 or mcx=0).
compute ndirfx(wheremw((1+((cfloop3-1)*2)),ncol(wheremw)):wheremw((2+((cfloop3-1)*2) ),ncol(wheremw)))=t(xvalptmp(cfloop1,:)*mestb(1,cfloop3)).
end if.
do if (mcx=2).
compute ndirfx(wheremw((1+((cfloop3-1)*2)),ncol(wheremw)):wheremw((2+((cfloop3-1)*2) ),ncol(wheremw)))=t(xvalptmp(cfloop1,:)*mest74sp(cfloop1,cfloop3)).
end if.
end loop.
do if (cfloop1 > 1).
compute dirfxcf(cfloop1,1)=t(ndirfx)*modres*xscaling.
end if.
end loop.
compute natdirbt={natdirbt;t(dirfxcf(2:nrow(dirfxcf),:))}.
end if.
do if ( (bcmat((i+1),1) = 1) and (nobootx=1) and (effsize=1)).
compute nobootx=0.
compute xsdtemp= (nrow(x)*sscp(x(:,2)))-(t(csum(x(:,2)))*(csum(x(:,2)))).
compute xsdtemp= sqrt(xsdtemp/(nrow(x)*(nrow(x)-1))).
end if.
end if.
end loop.
do if (bad = 0).
compute bootres={bootres;modres2(:,2:ncol(modres2))}.
do if (effsize=1).
compute ysdtemp= (nrow(y)*sscp(y))-(t(csum(y))*(csum(y))).
compute ysdtemp= sqrt(ysdtemp/(nrow(y)*(nrow(y)-1))).
compute bootysd={bootysd;ysdtemp}.
compute bootxsd={bootxsd;xsdtemp}.
end if.
compute goodboot=goodboot+1.
end if.
do if (bad <> 0).
compute badboot=badboot+1.
end if.
end loop if (goodboot = boot).
compute bootres=bootres(2:nrow(bootres),:).
do if (effsize=1).
compute bootysd=bootysd(2:nrow(bootysd),:).
do if (nrow(bootxsd) > 1).
compute bootxsd=bootxsd(2:nrow(bootxsd),:).
end if.
end if.
do if (goodboot < (boot)).
compute boot=0.
compute modelbt=0.
compute notecode(notes,1) = 7.
compute notes = notes + 1.
end if.
do if (boot > 0).
do if (effsize=1).
compute bootysd={ysd;bootysd}.
compute bootxsd={xsd;bootxsd}.
end if.
do if (saveboot = 1).
save bootres/outfile = *.
end if.
do if (modelbt=1).
compute bootcim=make(ncol(bootres),5,-99999).
compute bootcim(:,2) = t(csum(bootres)/nrow(bootres)).
compute bootcim(:,1) = coeffmat(2:nrow(coeffmat),1).
loop i = 1 to ncol(bootres).

.
compute temp = bootres(:,i).
compute temp(GRADE( bootres(:,i) )) = bootres(:,i).
compute badlo = 0.
compute badhi = 0.
do if ( (bootcim(i,1)*bc)+(9999*(1-bc)) <> 9999).
compute pv=csum(temp < (bootcim(i,1)*bc)+(9999*(1-bc)) )/nrow(temp).
compute ppv = pv.
do if (pv > .5).
compute ppv = 1-pv.
end if.
compute y5=sqrt(-2*ln(ppv)).
compute xp=y5+((((y5*p4+p3)*y5+p2)*y5+p1)*y5+p0)/((((y5*q4+q3)*y5+q2)*y5+q1)*y5+q0).
do if (pv <= .5).
compute xp = -xp.
end if.
compute cilow=rnd(nrow(temp)*(cdfnorm(2*xp-xp2))).
compute cihigh=trunc(nrow(temp)*(cdfnorm(2*xp+(xp2))))+1.
do if (cilow < 1).
compute cilow = 1.
compute booterr=1.
compute badlo = 1.
end if.
do if (cihigh > nrow(temp)).
compute cihigh = boot.
compute booterr=1.
compute badhi = 1.
end if.
compute llcit=temp(cilow,1).
compute ulcit=temp(cihigh,1).
do if (badlo = 1 and llcit <> priorlo).
compute badend={badend, llcit}.
compute priorlo = llcit.
end if.
do if (badhi = 1 and ulcit <> priorhi).
compute badend={badend, ulcit}.
compute priorhi = ulcit.
end if.
end if.
do if ( (bootcim(i,1)*bc)+(9999*(1-bc)) = 9999).
compute llcit=temp(cilow,1).
compute ulcit=temp(cihigh,1).
end if.
compute bootse=t(sqrt((cssq(temp)-((csum(temp)&**2)/nrow(temp)))/(nrow(temp)-1)))
.
compute bootcim(i,4:5)={llcit,ulcit}.
compute bootcim(i,3)=bootse.
end loop.
end if.
end if.
do if (badboot > 0).
compute notecode(notes,1) = 6.
compute notes = notes + 1.
end if.
end if.
do if (criterr=0).
release fulldat,x,y.
end if.
do if (xmint=1 and criterr=0).
compute xvalptmp=make(1,nxvls,0).
compute mest74sp=make(1,nms,0).
compute mest74sp={mest74sp;mest74}.
compute xvalptmp={xvalptmp;ident(nxvls)}.
loop kcfuhd=1 to 2.
compute dirfxcf=make(nrow(mest74),6,0).
loop cfloop1=1 to nrow(mest74).
compute ndirfx=make(nrow(lastb),1,0).
compute ndirfx(2:(nxvls+1),1)=t(xvalptmp(cfloop1,:)).
loop cfloop3=1 to nms.
do if (kcfuhd=1).
do if (mcx=1 or mcx=0).
compute ndirfx(wheremw((1+((cfloop3-1)*2)),ncol(wheremw)):wheremw((2+((cfloop3-1)*2)), ncol(wheremw)))=t(xvalptmp(cfloop1,:)*mest74(1,cfloop3)).
end if.
do if (mcx=2).
compute ndirfx(wheremw((1+((cfloop3-1)*2)),ncol(wheremw)):wheremw((2+((cfloop3-1)*2)), ncol(wheremw)))=t(xvalptmp(cfloop1,:)*mest74sp(cfloop1,cfloop3)).
end if.
end if.
do if (kcfuhd=2).
compute ndirfx(wheremw((1+((cfloop3-1)*2)),ncol(wheremw)):wheremw((2+((cfloop3-1)*2)), ncol(wheremw)))=t(xvalptmp(cfloop1,:)*medmeans(1,cfloop3)).
end if.
end loop.
do if (cfloop1 > 1).
compute dirfxcf(cfloop1,1)=t(ndirfx)*lastb*xscaling.
compute dirfxcf(cfloop1,2)=sqrt(diag(t(ndirfx)*lastcov*ndirfx))*abs(xscaling).
compute dirfxcf(cfloop1,3)=dirfxcf(cfloop1,1)/dirfxcf(cfloop1,2).
compute dirfxcf(cfloop1,4)=2*(1-tcdf(abs(dirfxcf(cfloop1,3)), dfres)).
compute dirfxcf(cfloop1,5)=dirfxcf(cfloop1,1)-tval*dirfxcf(cfloop1,2).
compute dirfxcf(cfloop1,6)=dirfxcf(cfloop1,1)+tval*dirfxcf(cfloop1,2).
end if.
end loop.
compute codireff=dirfxcf(2:nrow(dirfxcf),:).
do if (kcfuhd=1).
compute direff=codireff.
end if.
end loop.
end if.

.
do if (criterr = 0 and nms > 0).
compute paths=paths(:,2:ncol(paths)).
compute pathsw=pathsw(:,2:ncol(pathsw)).
compute pathsz=pathsz(:,2:ncol(pathsz)).
compute pathswz=pathswz(:,2:ncol(pathswz)).
compute pathsmod=pathsw+pathsz+pathswz.
compute pathsdv=pathsdv(:,2:ncol(pathsdv)).
compute pathsfoc=pathsfoc(:,2:ncol(pathsfoc)).
compute pathtype=pathtype(:,2:ncol(pathtype)).
compute anymod=(rsum(pathsmod) > 0).
compute obscoeff=obscoeff(1,2:ncol(obscoeff)).
do if (outscree=1).
do if (xmint=1).
print/title = '************************* COUNTERFACTUALLY DEFINED ************************'.
else.
print/title = ' '.
end if.
do if (dototal = 0 and alttotal=0).
print/title = '****************** DIRECT AND INDIRECT EFFECTS OF X ON Y *****************'/space=0.
end if.
do if (alttotal=1).
print/title = '************** TOTAL, DIRECT, AND INDIRECT EFFECTS OF X ON Y **************'/space=0.
end if.
end if.
do if (dototal = 1).
do if (outscree=1).
print/title = '************** TOTAL, DIRECT, AND INDIRECT EFFECTS OF X ON Y **************'/space=0.
end if.
compute totefflb(1,1)='Effect'.
do if (effsize=1).
compute toteffsz=toteff(:,1)/ysd.
do if (xdich=1 or mcx > 0).
compute totefflb={totefflb,'c_ps'}.
end if.
do if (xdich = 0 and mcx = 0).
compute toteffsz=toteffsz*xsd.
compute totefflb={totefflb,'c_cs'}.
end if.
compute toteff={toteff,toteffsz}.
end if.
compute resultm2=make(nrow(toteff),maxresm,99999).
compute resultm2(1:nrow(toteff),1:ncol(toteff))=toteff.
compute resultm={resultm;resultm2}.
do if (nxvls > 1).
compute resultm2=make(nrow(totomni),maxresm,99999).
compute resultm2(1:nrow(totomni),1:ncol(totomni))=totomni.
compute resultm={resultm;resultm2}.
compute clabtmp={'R2-chng', hcflab, 'df1','df2','p'}.
do if (outscree=1).
print toteff/title='Relative total effects of X on Y'/rnames=toteffl2/cnames=totefflb/format= F10.4.
print totomni/title='Omnibus test of total effect of X on Y'/cnames=clabtmp/format= F10.4.
print/title= '----------'/space=0.
end if.
else.
do if (outscree=1).
print toteff/title='Total effect of X on Y'/cnames=totefflb/format= F10.4.
end if.
end if.
end if.
compute moddir=wcmat(nrow(bcmat),1)+zcmat(nrow(bcmat),1).
do if (xmint=1).
compute moddir=1.
end if.
do if (bcmat(nrow(bcmat),1)=1).
do if (ydich=1).
compute direfflb(:,(ncol(direfflb)-5):ncol(direfflb))={'Effect','se','Z','p','LLCI','ULCI'}.
end if.
do if (moddir=0 or xmint=1).
compute direfflb(1,1)='Effect'.
end if.
do if (effsize=1 and moddir=0 and anymod = 0).
compute direffsz=direff(:,1)/ysd.
do if (xdich=1 or mcx > 0).
compute direfflb={direfflb,'c''_ps'}.
end if.
do if (xdich = 0 and mcx = 0).
compute direffsz=direffsz*xsd.
compute direfflb={direfflb,'c''_cs'}.
end if.
compute direff={direff,direffsz}.
end if.
compute resultm2=make(nrow(direff),maxresm,99999).
compute resultm2(1:nrow(direff),1:ncol(direff))=direff.
compute resultm={resultm;resultm2}.
do if (xmint=1).
compute resultm2=make(nrow(codireff),maxresm,99999).
compute resultm2(1:nrow(codireff),1:ncol(codireff))=codireff.
compute resultm={resultm;resultm2}.
end if.
do if (moddir = 0 and nxvls=1).
do if (outscree=1).
print direff/title='Direct effect of X on Y'/cnames=direfflb/format= F10.4.
end if.
end if.
do if (moddir = 0 and nxvls>1).
compute resultm2=make(nrow(diromni),maxresm,99999).
compute resultm2(1:nrow(diromni),1:ncol(diromni))=diromni.
compute resultm={resultm;resultm2}.
do if (outscree=1).
print direff/title='Relative direct effects of X on Y'/rnames=direffl2/cnames=direfflb/format= F10.4.
do if (ydich = 0).
compute clabtmp={'R2-chng', hcflab, 'df1','df2','p'}.
print diromni/title='Omnibus test of direct effect of X on Y:'/cnames=clabtmp/format= F10.4.
end if.
do if (ydich = 1).
compute clabtmp={'Chi-sq', 'df', 'p'}.
print diromni/title='Omnibus likelihood ratio test of direct effect of X on Y'/cnames=clabtmp/format= F10.4.
end if.
print/title= '----------'/space=0.
end if.
end if.
do if (moddir > 0 and nxvls=1).
do if (xmint=0 and outscree=1).
print direff/title='Conditional direct effects of X on Y'/cnames=direfflb/format= F10.4.
end if.
do if (xmint=1).
do if (outscree=1).
print direff/title='(Pure) Natural direct effect of X on Y'/cnames=direfflb/format= F10.4.
print codireff/title='Controlled direct effect of X on Y'/cnames=direfflb/format= F10.4.
print/title= '----------'/space=0.
end if.
compute obnatdfx=direff(:,1).
end if.
end if.
compute direffl4=direffl2.
do if (moddir > 0 and nxvls>1).
compute direffl2={' '}.
loop i = 1 to nxvls.
loop j = 1 to (nrow(direff)/nxvls).
compute direffl2={direffl2;xcatlab(i,1)}.
end loop.
end loop.
compute direffl2=direffl2(2:nrow(direffl2),1).
do if (xmint=0 and outscree=1).
print direff/title='Relative conditional direct effects of X on Y'/cnames=direfflb/rnames=direffl2/format= F10.4.
end if.
do if (xmint=1).
do if (outscree=1).
print direff/title='Relative (pure) natural direct effects of X on Y'/cnames=direfflb/rnames=direffl2/format= F10.4.
print codireff/title='Relative controlled direct effects of X on Y'/cnames=direfflb/rnames=direffl2/format= F10.4.
print/title= '----------'/space=0.
end if.
compute obnatdfx=direff(:,1).
end if.
end if.
compute direffl2=direffl4.
end if.
do if (bcmat(nrow(bcmat),1)=0 and xmint <> 1 and outscree=1).
print/title='The direct effect of X on Y is fixed to zero.'.
end if.
do if (nms = 1).
compute indmark={2}.
compute indsets={1,2}.
end if.
do if (nms = 2).
compute indmark={2,2,3}.
compute indsets={1,4,2,5,1,3,5}.
compute thetam=1.
end if.
do if (nms = 3).
compute indmark={2,2,2,3,3,3,4}.
compute indsets={1,7,2,8,4,9,1,3,8,1,5,9,2,6,9,1,3,6,9}.
compute thetam={1,2,3}.
end if.
do if (nms = 4).
compute indmark={2,2,2,2,3,3,3,3,3,3,4,4,4,4,5}.
compute indsets={1,11,2,12,4,13,7,14,1,3,12,1,5,13,1,8,14,2,6,13,2,9,14,4,10,14,1,3,6,13,1,3,9, 14,1,5,10,14,2,6,10,14,1,3,6,10,14}.
compute thetam={1,2,4,3,5,6}.
end if.
do if (nms = 5).
compute indmark={2,2,2,2,2,3,3,3,3,3,3,3,3,3,3,4,4,4,4,4,4,4,4,4,4,5,5,5,5,5,6}.
compute indsets={1,16,2,17,4,18,7,19,11,20,1,3,17,1,5,18,1,8,19,1,12,20,2,6,18,2,9,19,2,13,20,4, 10,19,4,14,20,7,15,20,1,3,6,18, 1,3,9,19,1,3,13,20,1,5,10,19,1,5,14,20,1,8,15,20,2,6,10,19,2,6, 14,20,2,9,15,20,4,10,15,20,1,3,6,10,19, 1,3,6,14,20,1,3,9,15,20,1,5,10,15,20,2,6,10,15,20,1,3,6,10,15, 20}.
compute thetam={1,2,5,3,6,8,4,7,9,10}.
end if.
do if (nms = 6).
compute indmark={2,2,2,2,2,2,3,3,3,3,3,3,3,3,3,3,3,3,3,3,3,4,4,4,4,4,4,4,4,4,4,4,4,4,4,4,4,4,4, 4,4,5,5,5,5,5,5,5,5,5,5,5,5,5,5,5,6,6,6,6,6,6,7}.
compute indsets={1,22,2,23,4,24,7,25,11,26,16,27,1,3,23,1,5,24,1,8,25,1,12,26,1,17,27,2,6,24,2, 9,25,2,13,26,2,18,27,4,10,25,4,14,26, 4,19,27,7,15,26,7,20,27,11,21,27,1,3,6,24,1,3,9,25,1,3,13,26,1, 3,18,27,1,5,10,25,1,5,14,26,1,5,19,27,1,8,15,26,1,8,20,27, 1,12,21,27,2,6,10,25,2,6,14,26,2,6,19,27,2,9,15,26,2,9,20,27,2, 13,21,27,4,10,15,26,4,10,20,27,4,14,21,27,7,15,21,27, 1,3,6,10,25,1,3,6,14,26,1,3,6,19,27,1,3,9,15,26,1,3,9,20,27,1,3, 13,21,27,1,5,10,15,26,1,5,10,20,27,1,5,14,21,27, 1,8,15,21,27,2,6,10,15,26,2,6,10,20,27,2,6,14,21,27,2,9,15,21, 27,4,10,15,21,27,1,3,6,10,15,26,1,3,6,10,20,27, 1,3,6,14,21,27,1,3,9,15,21,27,1,5,10,15,21,27,2,6,10,15,21,27, 1,3,6,10,15,21,27}.
compute thetam={1,2,6,3,7,10,4,8,11,13,5,9,12,14,15}.
end if.
do if (nms = 7).
compute indmark={2,2,2,2,2,2,2}.
compute indsets={1,29,2,30,4,31,7,32,11,33,16,34,22,35}.
end if.
do if (nms = 8).
compute indmark={2,2,2,2,2,2,2,2}.
compute indsets={1,37,2,38,4,39,7,40,11,41,16,42,22,43,29,44}.
end if.
do if (nms = 9).
compute indmark={2,2,2,2,2,2,2,2,2}.
compute indsets={1,46,2,47,4,48,7,49,11,50,16,51,22,52,29,53,37,54}.
end if.
do if (nms=10).
compute indmark={2,2,2,2,2,2,2,2,2,2}.
compute indsets={1,56,2,57,4,58,7,59,11,60,16,61,22,62,29,63,37,64,46,65}.
end if.
compute indlbl = {'Ind1'; 'Ind2'; 'Ind3'; 'Ind4'; 'Ind5'; 'Ind6'; 'Ind7'; 'Ind8'; 'Ind9'; 'Ind10'; 'Ind11'; 'Ind12'; 'Ind13'; 'Ind14'; 'Ind15'}.
compute indlbl = {indlbl; 'Ind16';'Ind17';'Ind18';'Ind19';'Ind20';'Ind21';'Ind22';'Ind23';'Ind24';'Ind25';'Ind26';'Ind27';'Ind28';'Ind29';'Ind30'}.
compute indlbl = {indlbl; 'Ind31';'Ind32';'Ind33';'Ind34';'Ind35';'Ind36';'Ind37';'Ind38';'Ind39';'Ind40';'Ind41';'Ind42';'Ind43';'Ind44';'Ind45'}.
compute indlbl = {indlbl; 'Ind46';'Ind47';'Ind48';'Ind49';'Ind50';'Ind51';'Ind52';'Ind53';'Ind54';'Ind55';'Ind56';'Ind57';'Ind58';'Ind59';'Ind60'}.
compute indlbl = {indlbl; 'Ind61';'Ind62';'Ind63';'Ind64';'Ind65';'Ind66';'Ind67';'Ind68';'Ind69';'Ind70';'Ind71';'Ind72';'Ind73';'Ind74';'Ind75'}.
compute indlbl = {indlbl; 'Ind76';'Ind77';'Ind78';'Ind79';'Ind80';'Ind81';'Ind82';'Ind83';'Ind84';'Ind85';'Ind86';'Ind87';'Ind88';'Ind89';'Ind90'}.
compute cntname={'(C1)';'(C2)';'(C3)';'(C4)';'(C5)';'(C6)';'(C7)';'(C8)';'(C9)';'(C10)';'(C11)';'(C12)';'(C13)';'(C14)';'(C15)';'(C16)';'(C17)'}.
compute cntname={cntname;'(C18)';'(C19)';'(C20)';'(C21)';'(C22)';'(C23)';'(C24)';'(C25)';'(C26)';'(C27)';'(C28)';'(C29)';'(C30)';'(C31)'}.
compute cntname={cntname;'(C32)';'(C33)';'(C34)';'(C35)';'(C36)';'(C37)';'(C38)';'(C39)';'(C40)';'(C41)';'(C42)';'(C43)';'(C44)';'(C45)'}.
compute cntname={cntname;'(C46)';'(C47)';'(C48)';'(C49)';'(C50)';'(C51)';'(C52)';'(C53)';'(C54)';'(C55)';'(C56)';'(C57)';'(C58)';'(C59)'}.
compute cntname={cntname;'(C60)';'(C61)';'(C62)';'(C63)';'(C64)';'(C65)';'(C66)';'(C67)';'(C68)';'(C69)';'(C70)';'(C71)';'(C72)';'(C73)'}.
compute cntname={cntname;'(C74)';'(C75)';'(C76)';'(C77)';'(C78)';'(C79)';'(C80)';'(C81)';'(C82)';'(C83)';'(C84)';'(C85)';'(C86)';'(C87)'}.
compute cntname={cntname;'(C88)';'(C89)';'(C90)';'(C91)';'(C92)';'(C93)';'(C94)';'(C95)';'(C96)';'(C97)';'(C98)';'(C99)';'(C100)';'(C101)'}.
compute cntname={cntname;'(C102)';'(C103)';'(C104)';'(C105)'}.
compute indmake=make(ncol(indmark),(nms+2),0).
compute indmod=make(ncol(indmark),1,999).
compute indmmm=make(ncol(indmark),1,0).
compute indmmmt=make(ncol(indmark),1,0).
compute start=1.
compute end=0.
compute nindfx=0.
compute indlocs=make(nrow(thetaxmb),ncol(paths),999).
compute indkey=make(ncol(indmark),1+((rmax(indmark)*2)+1),'     ').
compute c1=1.
compute c2=1.
compute c3=1.
loop i = 1 to ncol(paths).
do if (pathtype(1,i)=1).
compute indlocs(:,i)=thetaxmb(:,c1).
compute c1=c1+1.
end if.
do if (pathtype(1,i)=3).
compute indlocs(:,i)=thetamyb(:,c2).
compute c2=c2+1.
end if.
do if (pathtype(1,i)=2 and nms < 7 and serial=1).
compute indlocs(:,i)=thetammb(:,thetam(1,c3)).
compute c3=c3+1.
end if.
end loop.
loop i = 1 to ncol(indlocs).
compute c1=2.
loop j = 2 to nrow(indlocs).
do if (indlocs(j,i) <> 0).
compute indlocs(c1,i)=indlocs(j,i).
compute c1=c1+1.
end if.
end loop.
compute indlocs(1,i)=c1-2.
end loop.
compute indlocs=indlocs(1:rmax((indlocs(1,:))+1),:).
loop i = 1 to ncol(indmark).
compute numget=indmark(1,i).
compute end=end+numget.
compute gotcha=indsets(1,start:end).
compute start=end+1.
compute ok=1.
compute temp=0.
compute repoman=make(4,1,0).
loop j = 1 to ncol(gotcha).
do if paths(1,gotcha(1,j))=0.
compute ok=0.
end if.
do if (pathsmod(1,gotcha(1,j)) > 0).
compute temp=1.
compute temp2={pathsw(1,gotcha(1,j));pathsz(1,gotcha(1,j));pathswz(1,gotcha(1,j));0}.
do if (temp2(1,1)=1 and temp2(2,1)=1 and temp2(3,1)=0).
compute temp2(4,1)=1.
end if.
compute repoman=repoman+temp2.
end if.
end loop.
compute temp=0.
compute tempmmm=0.
compute typemmm=0.
do if ((repoman(1,1) > 0) and (repoman(2,1) = 0)).
compute temp=1.
do if (repoman(1,1)=1).
compute tempmmm=1.
end if.
do if (repoman(1,1) > 1 and (wdich=1 or mcw > 0)).
compute tempmmm=12.
compute typemmm=mcw.
do if (wdich=1).
compute typemmm=1.
end if.
end if.
do if (repoman(1,1) > 1 and (wdich=0 and mcw = 0)).
compute tempmmm=101.
end if.
end if.
do if ((repoman(1,1) = 0) and (repoman(2,1) > 0)).
compute temp=2.
do if (repoman(2,1)=1).
compute tempmmm=2.
end if.
do if (repoman(2,1) > 1 and (zdich = 1 or mcz > 0)).
compute tempmmm=22.
compute typemmm=mcz.
do if (zdich=1).
compute typemmm=1.
end if.
end if.
do if (repoman(2,1) > 1 and (zdich = 0 and mcw = 0)).
compute tempmmm=102.
end if.
end if.
do if (repoman(1,1)>0 and repoman(2,1)>0).
compute temp=3.
do if (repoman(1,1)=1 and repoman(2,1)=1).
do if (repoman(4,1)=1).
compute tempmmm=31.
end if.
do if (repoman(3,1)=1).
compute tempmmm=41.
end if.
end if.
end if.
do if (repoman(1,1)=1 and repoman(2,1)=1 and repoman(3,1)=0 and repoman(4,1)=0).
compute tempmmm=51.
end if.
do if (ok = 1).
compute nindfx=nindfx+1.
compute indmake(nindfx,1)=numget.
compute indmod(nindfx,1)=temp.
compute indmmm(nindfx,1)=tempmmm.
compute indmmmt(nindfx,1)=typemmm.
compute indmake(nindfx,2:(1+numget))=gotcha.
compute indkey(nindfx,1)=xnames.
loop j = 1 to numget.
compute indkey(nindfx,(j*2+1))=pathsdv(1,gotcha(1,j)).
compute indkey(nindfx,(j*2))='   ->   '.
end loop.
end if.
end loop.
compute indkey=indkey(1:nindfx,1:((cmax(indmake(:,1))*2)+1)).
compute indmake=indmake(1:nindfx,1:(cmax(indmake(:,1))+1)).
compute indmod=indmod(1:nrow(indmake),1).
compute indmmm=indmmm(1:nrow(indmake),1).
compute indmmmt=indmmmt(1:nrow(indmake),1).
compute ncpairs = (((nindfx)*(nindfx-1))/2).
do if ((contrast = 1 or contrast = 2 or contrast=3) and (ncpairs > 105)).
compute contrast=0.
compute notecode(notes,1) = 13.
compute notes = notes + 1.
end if.
do if (contrast = 4).
do if (ncol(contvec) <> nindfx).
compute contrast=0.
compute notecode(notes,1) = 14.
compute notes = notes + 1.
end if.
end if.
do if (anymod=0).
do if (nms = 1 and contrast > 0).
compute contrast=0.
end if.
compute efloop=(((1-(effsize=0))*2)+1)-(((mcx>0 or xdich=1))*(1-(effsize=0))).
loop kk=1 to efloop.
do if (boot = 0).
compute bootres=obscoeff.
do if (kk=1).
compute totbtvec=make(1,nxvls,0).
end if.
compute bootdir=obsdirfx.
compute indtab=999.
compute inddiff=999.
compute bootysd=ysd.
compute bootxsd=xsd.
end if.
do if (boot > 0).
compute bootres={obscoeff;bootres}.
do if (kk=1).
compute totbtvec=make(nrow(bootres),nxvls,0).
end if.
compute indtab=make(1,4,999).
compute inddiff=make(nrow(bootres),1,999).
end if.
compute indtotal=make(nrow(bootres),1,0).
loop i = 1 to nrow(indmake).
loop j = 1 to nxvls.
compute indtemp=make(nrow(bootres),1,1).
loop k = 1 to indmake(i,1).
compute jtemp=1.
do if (j > 1 and k=1).
compute jtemp=j.
end if.
compute indtemp=indtemp&*bootres(:,pathsfoc(jtemp,indmake(i,(k+1)))).
end loop.
do if (kk = 2).
compute indtemp=indtemp/bootysd.
end if.
do if (kk = 3).
compute indtemp=(bootxsd&*indtemp)/bootysd.
end if.
do if (contrast <> 0).
compute inddiff={inddiff,indtemp}.
end if.
do if (nxvls=1).
compute indtotal=indtotal+indtemp.
end if.
compute indeff=indtemp(1,1).
do if (kk=1).
compute totbtvec(:,j)=totbtvec(:,j)+indtemp.
end if.
do if (boot > 0).

.
compute temp = indtemp(2:nrow(indtemp),1).
compute temp(GRADE( indtemp(2:nrow(indtemp),1) )) = indtemp(2:nrow(indtemp),1).
compute badlo = 0.
compute badhi = 0.
do if ( (indtemp(1,1)*bc)+(9999*(1-bc)) <> 9999).
compute pv=csum(temp < (indtemp(1,1)*bc)+(9999*(1-bc)) )/nrow(temp).
compute ppv = pv.
do if (pv > .5).
compute ppv = 1-pv.
end if.
compute y5=sqrt(-2*ln(ppv)).
compute xp=y5+((((y5*p4+p3)*y5+p2)*y5+p1)*y5+p0)/((((y5*q4+q3)*y5+q2)*y5+q1)*y5+q0).
do if (pv <= .5).
compute xp = -xp.
end if.
compute cilow=rnd(nrow(temp)*(cdfnorm(2*xp-xp2))).
compute cihigh=trunc(nrow(temp)*(cdfnorm(2*xp+(xp2))))+1.
do if (cilow < 1).
compute cilow = 1.
compute booterr=1.
compute badlo = 1.
end if.
do if (cihigh > nrow(temp)).
compute cihigh = boot.
compute booterr=1.
compute badhi = 1.
end if.
compute llcit=temp(cilow,1).
compute ulcit=temp(cihigh,1).
do if (badlo = 1 and llcit <> priorlo).
compute badend={badend, llcit}.
compute priorlo = llcit.
end if.
do if (badhi = 1 and ulcit <> priorhi).
compute badend={badend, ulcit}.
compute priorhi = ulcit.
end if.
end if.
do if ( (indtemp(1,1)*bc)+(9999*(1-bc)) = 9999).
compute llcit=temp(cilow,1).
compute ulcit=temp(cihigh,1).
end if.
compute bootse=t(sqrt((cssq(temp)-((csum(temp)&**2)/nrow(temp)))/(nrow(temp)-1)))
.
compute indeff={indeff,bootse,llcit,ulcit}.
end if.
do if (kk=1).
compute indtabn={indtab;indeff}.
end if.
compute indtab={indtab;indeff}.
end loop.
end loop.
compute indtab=indtab(2:nrow(indtab),:).
do if (kk=1).
compute indtabn=indtabn(2:nrow(indtabn),:).
end if.
compute rowlbs=indlbl(1:nrow(indtab),1).
do if (mc > 0).
compute inddiff=make(mc,1,-999).
compute indtab2=make(nrow(indtab),4,-999).
compute indtab2(:,1)=indtab.
compute indtab=indtab2.
release indtab2.
compute mcct=0.
compute indtotal=make(mc,1,0).
do if (kk = 1).
compute x1 = sqrt(-2*ln(uniform(mc,nrow(mcsopath))))&*cos((2*3.14159265358979)*uniform(mc, nrow(mcsopath))).
compute x1=x1*chol(indcov).
loop ii=1 to nrow(x1).
compute x1(ii,:)=x1(ii,:)+t(mcsopath).
end loop.
end if.
loop ii=1 to nms.
compute tmpb=x1(:,((nms*nxvls)+ii)).
compute tmpb2=tmpb.
do if (nxvls > 1).
loop jj=1 to (nxvls-1).
compute tmpb2={tmpb2,tmpb}.
end loop.
end if.
compute indtemp=x1(:,(((ii-1)*nxvls)+1):(ii*nxvls))&*tmpb2.
loop jj=1 to ncol(indtemp).
do if (kk = 2).
compute indtemp(:,jj)=indtemp(:,jj)/ysd.
end if.
do if (kk = 3).
compute indtemp(:,jj)=(xsd*indtemp(:,jj))/ysd.
end if.

.
compute temp = indtemp(:,jj).
compute temp(GRADE( indtemp(:,jj) )) = indtemp(:,jj).
compute badlo = 0.
compute badhi = 0.
do if ( 9999 <> 9999).
compute pv=csum(temp < 9999 )/nrow(temp).
compute ppv = pv.
do if (pv > .5).
compute ppv = 1-pv.
end if.
compute y5=sqrt(-2*ln(ppv)).
compute xp=y5+((((y5*p4+p3)*y5+p2)*y5+p1)*y5+p0)/((((y5*q4+q3)*y5+q2)*y5+q1)*y5+q0).
do if (pv <= .5).
compute xp = -xp.
end if.
compute cilow=rnd(nrow(temp)*(cdfnorm(2*xp-xp2))).
compute cihigh=trunc(nrow(temp)*(cdfnorm(2*xp+(xp2))))+1.
do if (cilow < 1).
compute cilow = 1.
compute booterr=1.
compute badlo = 1.
end if.
do if (cihigh > nrow(temp)).
compute cihigh = boot.
compute booterr=1.
compute badhi = 1.
end if.
compute llcit=temp(cilow,1).
compute ulcit=temp(cihigh,1).
do if (badlo = 1 and llcit <> priorlo).
compute badend={badend, llcit}.
compute priorlo = llcit.
end if.
do if (badhi = 1 and ulcit <> priorhi).
compute badend={badend, ulcit}.
compute priorhi = ulcit.
end if.
end if.
do if ( 9999 = 9999).
compute llcit=temp(cilow,1).
compute ulcit=temp(cihigh,1).
end if.
compute bootse=t(sqrt((cssq(temp)-((csum(temp)&**2)/nrow(temp)))/(nrow(temp)-1)))
.
compute mcct=mcct+1.
compute indtab(mcct,2:4)={bootse,llcit,ulcit}.
end loop.
do if (nxvls=1).
compute indtotal=indtotal+indtemp.
do if (contrast <> 0).
compute inddiff={inddiff,indtemp}.
end if.
end if.
end loop.
release indtemp,tmpb.
end if.
do if (normal = 1 and sobelok=1).
compute sobelmat=indtab(:,1).
compute sobelmat={sobelmat,(sobelmat/2),sobelmat,sobelmat}.
loop ii=1 to nms.
compute se2b=indcov(((nms*nxvls)+ii),((nms*nxvls)+ii)).
compute bpath2=mcsopath(((nms*nxvls)+ii),1)&**2.
compute se2a=diag(indcov((((ii-1)*nxvls)+1):(ii*nxvls),(((ii-1)*nxvls)+1):(ii*nxvls))).
compute apath2=mcsopath((((ii-1)*nxvls)+1):(ii*nxvls) ,1)&**2.
compute sesobel=sqrt(apath2*se2b+bpath2*se2a+se2a*se2b).
compute sobelmat((((ii-1)*nxvls)+1):(ii*nxvls),2)=sesobel.
end loop.
release se2b,bpath2,se2a,apath2,sesobel,ii.
compute sobelmat(:,3)=sobelmat(:,1)&/sobelmat(:,2).
compute sobelmat(:,4) = 2*(1-cdfnorm(abs(sobelmat(:,3)))).
end if.
do if (serial = 0).
compute rowlbs=t(mnames).
end if.
do if (nxvls=1 and nms > 1).
compute rowlbs={'TOTAL';rowlbs}.
compute indtemp=indtotal(1,1).
do if (boot > 0 and nxvls=1).

.
compute temp = indtotal(2:nrow(indtotal),1).
compute temp(GRADE( indtotal(2:nrow(indtotal),1) )) = indtotal(2:nrow(indtotal),1).
compute badlo = 0.
compute badhi = 0.
do if ( (indtotal(1,1)*bc)+(9999*(1-bc)) <> 9999).
compute pv=csum(temp < (indtotal(1,1)*bc)+(9999*(1-bc)) )/nrow(temp).
compute ppv = pv.
do if (pv > .5).
compute ppv = 1-pv.
end if.
compute y5=sqrt(-2*ln(ppv)).
compute xp=y5+((((y5*p4+p3)*y5+p2)*y5+p1)*y5+p0)/((((y5*q4+q3)*y5+q2)*y5+q1)*y5+q0).
do if (pv <= .5).
compute xp = -xp.
end if.
compute cilow=rnd(nrow(temp)*(cdfnorm(2*xp-xp2))).
compute cihigh=trunc(nrow(temp)*(cdfnorm(2*xp+(xp2))))+1.
do if (cilow < 1).
compute cilow = 1.
compute booterr=1.
compute badlo = 1.
end if.
do if (cihigh > nrow(temp)).
compute cihigh = boot.
compute booterr=1.
compute badhi = 1.
end if.
compute llcit=temp(cilow,1).
compute ulcit=temp(cihigh,1).
do if (badlo = 1 and llcit <> priorlo).
compute badend={badend, llcit}.
compute priorlo = llcit.
end if.
do if (badhi = 1 and ulcit <> priorhi).
compute badend={badend, ulcit}.
compute priorhi = ulcit.
end if.
end if.
do if ( (indtotal(1,1)*bc)+(9999*(1-bc)) = 9999).
compute llcit=temp(cilow,1).
compute ulcit=temp(cihigh,1).
end if.
compute bootse=t(sqrt((cssq(temp)-((csum(temp)&**2)/nrow(temp)))/(nrow(temp)-1)))
.
compute indtemp={indtemp, bootse,llcit,ulcit}.
end if.
do if (mc > 0).
compute obtmc=indtab(:,1).
compute indtemp=csum(obtmc).

.
compute temp = indtotal(:,1).
compute temp(GRADE( indtotal(:,1) )) = indtotal(:,1).
compute badlo = 0.
compute badhi = 0.
do if ( 9999 <> 9999).
compute pv=csum(temp < 9999 )/nrow(temp).
compute ppv = pv.
do if (pv > .5).
compute ppv = 1-pv.
end if.
compute y5=sqrt(-2*ln(ppv)).
compute xp=y5+((((y5*p4+p3)*y5+p2)*y5+p1)*y5+p0)/((((y5*q4+q3)*y5+q2)*y5+q1)*y5+q0).
do if (pv <= .5).
compute xp = -xp.
end if.
compute cilow=rnd(nrow(temp)*(cdfnorm(2*xp-xp2))).
compute cihigh=trunc(nrow(temp)*(cdfnorm(2*xp+(xp2))))+1.
do if (cilow < 1).
compute cilow = 1.
compute booterr=1.
compute badlo = 1.
end if.
do if (cihigh > nrow(temp)).
compute cihigh = boot.
compute booterr=1.
compute badhi = 1.
end if.
compute llcit=temp(cilow,1).
compute ulcit=temp(cihigh,1).
do if (badlo = 1 and llcit <> priorlo).
compute badend={badend, llcit}.
compute priorlo = llcit.
end if.
do if (badhi = 1 and ulcit <> priorhi).
compute badend={badend, ulcit}.
compute priorhi = ulcit.
end if.
end if.
do if ( 9999 = 9999).
compute llcit=temp(cilow,1).
compute ulcit=temp(cihigh,1).
end if.
compute bootse=t(sqrt((cssq(temp)-((csum(temp)&**2)/nrow(temp)))/(nrow(temp)-1)))
.
compute indtemp={indtemp, bootse,llcit,ulcit}.
end if.
compute indtab={indtemp;indtab}.
end if.
compute bootlbs={'Effect', 'BootSE','BootLLCI','BootULCI'}.
do if (mc > 0).
compute bootlbs={'Effect', 'MC SE','MC LLCI','MC ULCI'}.
end if.
do if (nxvls = 1).
do if (contrast <> 0).
compute inddiff=inddiff(:,2:ncol(inddiff)).
do if (mc > 0).
compute inddiff={t(obtmc);inddiff}.
end if.
do if (contrast = 4).
compute inddifft=inddiff*t(contvec).
compute indtemp=inddifft(1,1).
do if (boot > 0 or mc > 0).
do if (mc > 0).

.
compute temp = inddifft(2:nrow(inddifft),1).
compute temp(GRADE( inddifft(2:nrow(inddifft),1) )) = inddifft(2:nrow(inddifft),1).
compute badlo = 0.
compute badhi = 0.
do if ( 9999 <> 9999).
compute pv=csum(temp < 9999 )/nrow(temp).
compute ppv = pv.
do if (pv > .5).
compute ppv = 1-pv.
end if.
compute y5=sqrt(-2*ln(ppv)).
compute xp=y5+((((y5*p4+p3)*y5+p2)*y5+p1)*y5+p0)/((((y5*q4+q3)*y5+q2)*y5+q1)*y5+q0).
do if (pv <= .5).
compute xp = -xp.
end if.
compute cilow=rnd(nrow(temp)*(cdfnorm(2*xp-xp2))).
compute cihigh=trunc(nrow(temp)*(cdfnorm(2*xp+(xp2))))+1.
do if (cilow < 1).
compute cilow = 1.
compute booterr=1.
compute badlo = 1.
end if.
do if (cihigh > nrow(temp)).
compute cihigh = boot.
compute booterr=1.
compute badhi = 1.
end if.
compute llcit=temp(cilow,1).
compute ulcit=temp(cihigh,1).
do if (badlo = 1 and llcit <> priorlo).
compute badend={badend, llcit}.
compute priorlo = llcit.
end if.
do if (badhi = 1 and ulcit <> priorhi).
compute badend={badend, ulcit}.
compute priorhi = ulcit.
end if.
end if.
do if ( 9999 = 9999).
compute llcit=temp(cilow,1).
compute ulcit=temp(cihigh,1).
end if.
compute bootse=t(sqrt((cssq(temp)-((csum(temp)&**2)/nrow(temp)))/(nrow(temp)-1)))
.
end if.
do if (boot > 0).

.
compute temp = inddifft(2:nrow(inddifft),1).
compute temp(GRADE( inddifft(2:nrow(inddifft),1) )) = inddifft(2:nrow(inddifft),1).
compute badlo = 0.
compute badhi = 0.
do if ( (inddifft(1,1)*bc)+(9999* (1-bc)) <> 9999).
compute pv=csum(temp < (inddifft(1,1)*bc)+(9999* (1-bc)) )/nrow(temp).
compute ppv = pv.
do if (pv > .5).
compute ppv = 1-pv.
end if.
compute y5=sqrt(-2*ln(ppv)).
compute xp=y5+((((y5*p4+p3)*y5+p2)*y5+p1)*y5+p0)/((((y5*q4+q3)*y5+q2)*y5+q1)*y5+q0).
do if (pv <= .5).
compute xp = -xp.
end if.
compute cilow=rnd(nrow(temp)*(cdfnorm(2*xp-xp2))).
compute cihigh=trunc(nrow(temp)*(cdfnorm(2*xp+(xp2))))+1.
do if (cilow < 1).
compute cilow = 1.
compute booterr=1.
compute badlo = 1.
end if.
do if (cihigh > nrow(temp)).
compute cihigh = boot.
compute booterr=1.
compute badhi = 1.
end if.
compute llcit=temp(cilow,1).
compute ulcit=temp(cihigh,1).
do if (badlo = 1 and llcit <> priorlo).
compute badend={badend, llcit}.
compute priorlo = llcit.
end if.
do if (badhi = 1 and ulcit <> priorhi).
compute badend={badend, ulcit}.
compute priorhi = ulcit.
end if.
end if.
do if ( (inddifft(1,1)*bc)+(9999* (1-bc)) = 9999).
compute llcit=temp(cilow,1).
compute ulcit=temp(cihigh,1).
end if.
compute bootse=t(sqrt((cssq(temp)-((csum(temp)&**2)/nrow(temp)))/(nrow(temp)-1)))
.
end if.
compute indtemp={indtemp, bootse,llcit,ulcit}.
end if.
compute indtab={indtab;indtemp}.
end if.
do if (contrast = 1 or contrast = 2 or contrast=3).
compute conkey=make(1,4,' ').
loop i = 1 to ncol(inddiff)-1.
loop j = (i+1) to ncol(inddiff).
compute inddifft=inddiff(:,i)-inddiff(:,j).
do if (contrast=2).
compute inddifft=abs(inddiff(:,i))-abs(inddiff(:,j)).
end if.
do if (contrast=3).
compute inddifft=inddiff(:,i)+inddiff(:,j).
end if.
compute indtemp=inddifft(1,1).
do if (contrast=1 or contrast=2).
compute conkeyt={' ', rowlbs((i+1),1),' minus  ',rowlbs((j+1),1)}.
end if.
do if (contrast=3).
compute conkeyt={' ', rowlbs((i+1),1),'  plus  ',rowlbs((j+1),1)}.
end if.
compute conkey={conkey;conkeyt}.
do if (boot > 0 or mc > 0).
do if (mc > 0).

.
compute temp = inddifft(2:nrow(inddifft),1).
compute temp(GRADE( inddifft(2:nrow(inddifft),1) )) = inddifft(2:nrow(inddifft),1).
compute badlo = 0.
compute badhi = 0.
do if ( 9999 <> 9999).
compute pv=csum(temp < 9999 )/nrow(temp).
compute ppv = pv.
do if (pv > .5).
compute ppv = 1-pv.
end if.
compute y5=sqrt(-2*ln(ppv)).
compute xp=y5+((((y5*p4+p3)*y5+p2)*y5+p1)*y5+p0)/((((y5*q4+q3)*y5+q2)*y5+q1)*y5+q0).
do if (pv <= .5).
compute xp = -xp.
end if.
compute cilow=rnd(nrow(temp)*(cdfnorm(2*xp-xp2))).
compute cihigh=trunc(nrow(temp)*(cdfnorm(2*xp+(xp2))))+1.
do if (cilow < 1).
compute cilow = 1.
compute booterr=1.
compute badlo = 1.
end if.
do if (cihigh > nrow(temp)).
compute cihigh = boot.
compute booterr=1.
compute badhi = 1.
end if.
compute llcit=temp(cilow,1).
compute ulcit=temp(cihigh,1).
do if (badlo = 1 and llcit <> priorlo).
compute badend={badend, llcit}.
compute priorlo = llcit.
end if.
do if (badhi = 1 and ulcit <> priorhi).
compute badend={badend, ulcit}.
compute priorhi = ulcit.
end if.
end if.
do if ( 9999 = 9999).
compute llcit=temp(cilow,1).
compute ulcit=temp(cihigh,1).
end if.
compute bootse=t(sqrt((cssq(temp)-((csum(temp)&**2)/nrow(temp)))/(nrow(temp)-1)))
.
end if.
do if (boot > 0).

.
compute temp = inddifft(2:nrow(inddifft),1).
compute temp(GRADE( inddifft(2:nrow(inddifft),1) )) = inddifft(2:nrow(inddifft),1).
compute badlo = 0.
compute badhi = 0.
do if ( (inddifft(1,1)*bc)+(9999* (1-bc)) <> 9999).
compute pv=csum(temp < (inddifft(1,1)*bc)+(9999* (1-bc)) )/nrow(temp).
compute ppv = pv.
do if (pv > .5).
compute ppv = 1-pv.
end if.
compute y5=sqrt(-2*ln(ppv)).
compute xp=y5+((((y5*p4+p3)*y5+p2)*y5+p1)*y5+p0)/((((y5*q4+q3)*y5+q2)*y5+q1)*y5+q0).
do if (pv <= .5).
compute xp = -xp.
end if.
compute cilow=rnd(nrow(temp)*(cdfnorm(2*xp-xp2))).
compute cihigh=trunc(nrow(temp)*(cdfnorm(2*xp+(xp2))))+1.
do if (cilow < 1).
compute cilow = 1.
compute booterr=1.
compute badlo = 1.
end if.
do if (cihigh > nrow(temp)).
compute cihigh = boot.
compute booterr=1.
compute badhi = 1.
end if.
compute llcit=temp(cilow,1).
compute ulcit=temp(cihigh,1).
do if (badlo = 1 and llcit <> priorlo).
compute badend={badend, llcit}.
compute priorlo = llcit.
end if.
do if (badhi = 1 and ulcit <> priorhi).
compute badend={badend, ulcit}.
compute priorhi = ulcit.
end if.
end if.
do if ( (inddifft(1,1)*bc)+(9999* (1-bc)) = 9999).
compute llcit=temp(cilow,1).
compute ulcit=temp(cihigh,1).
end if.
compute bootse=t(sqrt((cssq(temp)-((csum(temp)&**2)/nrow(temp)))/(nrow(temp)-1)))
.
end if.
compute indtemp={indtemp, bootse,llcit,ulcit}.
end if.
compute indtab={indtab;indtemp}.
end loop.
end loop.
end if.
release inddiff.
compute contlbs=cntname(1:(((nindfx)*(nindfx-1))/2),1).
compute rowlbs={rowlbs;contlbs}.
end if.
compute resultm2=make(nrow(indtab),maxresm,99999).
compute resultm2(1:nrow(indtab),1:ncol(indtab))=indtab.
compute resultm={resultm;resultm2}.
do if (outscree=1).
do if (kk=1).
print indtab/title = 'Indirect effect(s) of X on Y:'/rnames=rowlbs/cnames=bootlbs/format= F10.4.
end if.
do if (kk = 2 and (xdich=1 or mcx > 0)).
print indtab/title = 'Partially standardized indirect effect(s) of X on Y:'/rnames=rowlbs/cnames=bootlbs/format= F10.4.
end if.
do if (kk = 3).
print indtab/title = 'Completely standardized indirect effect(s) of X on Y:'/rnames=rowlbs/cnames=bootlbs/format= F10.4.
end if.
end if.
do if (normal=1 and sobelok=1 and kk=1).
compute resultm2=make(nrow(sobelmat),maxresm,99999).
compute resultm2(1:nrow(sobelmat),1:ncol(sobelmat))=sobelmat.
compute resultm={resultm;resultm2}.
compute sobellab={'Effect',hclab,'Z','p'}.
compute sobelrlb=rowlbs.
do if (nms > 1).
compute sobelrlb=rowlbs(2:(1+nms),1).
end if.
do if (outscree=1).
print sobelmat/title='   Normal theory test for indirect effect(s):'/cnames=sobellab/rnames=sobelrlb/format= F10.4.
end if.
end if.
do if (contrast <> 0).
do if ((contrast=1 or contrast = 2 or contrast=3) and kk=efloop ).
compute conkey=conkey(2:nrow(conkey),:).
do if (outscree=1).
print conkey/title = 'Specific indirect effect contrast definition(s):'/rnames=contlbs/format=A8.
end if.
end if.
do if (outscree=1).
do if (contrast = 4 and kk=efloop).
compute crowlbs=rowlbs(2:(nindfx+1),1).
print contvec/title = 'Specific indirect effect contrast weights:'/cnames=crowlbs/rlabels='(C1)'/format= F10.4.
end if.
do if (contrast = 2 and kk=efloop).
print/title = 'Contrasts are differences between absolute values of indirect effects'.
end if.
end if.
end if.
do if (serial = 1 and kk=efloop).
compute rowlbst=rowlbs(2:nrow(rowlbs),1).
do if (outscree=1).
print indkey/title = 'Indirect effect key:'/rnames=rowlbst/format = A8.
end if.
end if.
else.
do if (outscree=1).
do if (kk = 1).
print/title = 'Relative indirect effects of X on Y'.
end if.
do if (kk = 2).
print/title = 'Partially standardized relative indirect effect(s) of X on Y:'.
end if.
do if (kk = 3).
print/title = 'Completely standardized relative indirect effect(s) of X on Y:'.
end if.
end if.
loop i = 1 to nrow(indmake).
compute indtabsm=indtab((((i-1)*nxvls)+1):(nxvls*i),:).
compute indkeyt=indkey(i,:).
do if (outscree=1).
print indkeyt/title=' '/space=0/format=A8.
end if.
do if (bcmat(nrow(bcmat),1)=0)).
compute direffl2=xcatlab(1:nxvls,1).
end if.
compute resultm2=make(nrow(indtabsm),maxresm,99999).
compute resultm2(1:nrow(indtabsm),1:ncol(indtabsm))=indtabsm.
compute resultm={resultm;resultm2}.
do if (outscree=1).
print indtabsm/title = ' '/cnames=bootlbs/rnames=direffl2/format= F10.4 /space=0.
end if.
do if (normal=1 and sobelok=1 and kk=1).
compute sobelsm=sobelmat((((i-1)*nxvls)+1):(nxvls*i),:).
compute sobellab={'Effect',hclab,'Z','p'}.
compute resultm2=make(nrow(sobelsm),maxresm,99999).
compute resultm2(1:nrow(sobelsm),1:ncol(sobelsm))=sobelsm.
compute resultm={resultm;resultm2}.
do if (outscree=1).
print sobelsm/title='      Normal theory test for relative indirect effects:'/cnames=sobellab/rnames=direffl2/format= F10.4.
end if.
end if.
end loop.
end if.
do if (effsize = 1 and boot > 0).
compute bootres=bootres(2:nrow(bootres),:).
end if.
end loop.
do if (alttotal=1).
compute altcnms='Effect'.
compute totbtvec=totbtvec+bootdir.
compute alttotfx=t(totbtvec(1,:)).
do if (boot > 0).
compute alttotfx=make(ncol(totbtvec),4,0).
compute alttotfx(:,1)=t(totbtvec(1,:)).
loop cec=1 to ncol(totbtvec).

.
compute temp = totbtvec(2:nrow(totbtvec),cec).
compute temp(GRADE( totbtvec(2:nrow(totbtvec),cec) )) = totbtvec(2:nrow(totbtvec),cec).
compute badlo = 0.
compute badhi = 0.
do if ( (totbtvec(1,cec)*bc)+(9999* (1-bc)) <> 9999).
compute pv=csum(temp < (totbtvec(1,cec)*bc)+(9999* (1-bc)) )/nrow(temp).
compute ppv = pv.
do if (pv > .5).
compute ppv = 1-pv.
end if.
compute y5=sqrt(-2*ln(ppv)).
compute xp=y5+((((y5*p4+p3)*y5+p2)*y5+p1)*y5+p0)/((((y5*q4+q3)*y5+q2)*y5+q1)*y5+q0).
do if (pv <= .5).
compute xp = -xp.
end if.
compute cilow=rnd(nrow(temp)*(cdfnorm(2*xp-xp2))).
compute cihigh=trunc(nrow(temp)*(cdfnorm(2*xp+(xp2))))+1.
do if (cilow < 1).
compute cilow = 1.
compute booterr=1.
compute badlo = 1.
end if.
do if (cihigh > nrow(temp)).
compute cihigh = boot.
compute booterr=1.
compute badhi = 1.
end if.
compute llcit=temp(cilow,1).
compute ulcit=temp(cihigh,1).
do if (badlo = 1 and llcit <> priorlo).
compute badend={badend, llcit}.
compute priorlo = llcit.
end if.
do if (badhi = 1 and ulcit <> priorhi).
compute badend={badend, ulcit}.
compute priorhi = ulcit.
end if.
end if.
do if ( (totbtvec(1,cec)*bc)+(9999* (1-bc)) = 9999).
compute llcit=temp(cilow,1).
compute ulcit=temp(cihigh,1).
end if.
compute bootse=t(sqrt((cssq(temp)-((csum(temp)&**2)/nrow(temp)))/(nrow(temp)-1)))
.
compute alttotfx(cec,2:4)={bootse,llcit,ulcit}.
end loop.
compute altcnms={altcnms,'BootSE', 'BootLLCI','BootULCI'}.
end if.
compute resultm2=make(nrow(alttotfx),maxresm,99999).
compute resultm2(1:nrow(alttotfx),1:ncol(alttotfx))=alttotfx.
compute resultm={resultm;resultm2}.
do if (outscree=1).
do if (nxvls > 1).
print/title= '----------'/space=0.
print alttotfx/title='Relative total effects of X on Y (sum of direct and indirect effects)'/rnames=direffl2/cnames=altcnms/format= F10.4.
end if.
do if (nxvls = 1).
print alttotfx/title='Total effect of X on Y (sum of direct and indirect effects)'/cnames=altcnms/format= F10.4.
end if.
end if.
end if.
end if.
do if (anymod > 0).
do if (boot = 0).
compute bootres=obscoeff.
compute indtab=999.
end if.
do if (boot > 0).
compute bootres={obscoeff;bootres}.
compute indtab=make(1,4,999).
end if.
do if (csum((indmod > 0))=nrow(indmod)).
do if (outscree=1).
do if (nxvls > 1).
do if (xmint=0).
print/title= 'Relative conditional indirect effects of X on Y:'.
end if.
do if (xmint=1).
print/title= 'Relative (total) natural indirect effects of X on Y:'.
end if.
end if.
do if (nxvls = 1).
do if (xmint=0).
print/title= 'Conditional indirect effects of X on Y:'.
end if.
do if (xmint=1).
print/title= '(Total) Natural indirect effect(s) of X on Y:'.
end if.
end if.
end if.
end if.
do if (csum((indmod > 0)) < nrow(indmod)).
do if (outscree=1).
do if (nxvls > 1).
print/title= 'Relative conditional and unconditional indirect effects of X on Y:'.
end if.
do if (nxvls = 1).
print/title= 'Conditional and unconditional indirect effects of X on Y:'.
end if.
end if.
end if.
compute cftotfx=make(nrow(bootres),nxvls,0).
loop i = 1 to nrow(indmake).
compute indtab=indtab(1,:)*0.
do if (outscree=1).
do if (xmint=0).
print indkey(i,:)/title = 'INDIRECT EFFECT:'/format=A8.
end if.
do if (xmint=1).
print indkey(i,:)/title = ' '/format=A8/space=0.
end if.
end if.
do if (indmod(i,1)=0).
loop j = 1 to nxvls.
compute indtemp=make(nrow(bootres),1,1).
loop k = 1 to indmake(i,1).
compute jtemp=1.
do if (j > 1 and k=1).
compute jtemp=j.
end if.
compute indtemp=indtemp&*bootres(:,pathsfoc(jtemp,indmake(i,(k+1)))).
end loop.
compute indeff=indtemp(1,1).
do if (boot > 0).

.
compute temp = indtemp(2:nrow(indtemp),1).
compute temp(GRADE( indtemp(2:nrow(indtemp),1) )) = indtemp(2:nrow(indtemp),1).
compute badlo = 0.
compute badhi = 0.
do if ( (indtemp(1,1)*bc)+(9999*(1-bc)) <> 9999).
compute pv=csum(temp < (indtemp(1,1)*bc)+(9999*(1-bc)) )/nrow(temp).
compute ppv = pv.
do if (pv > .5).
compute ppv = 1-pv.
end if.
compute y5=sqrt(-2*ln(ppv)).
compute xp=y5+((((y5*p4+p3)*y5+p2)*y5+p1)*y5+p0)/((((y5*q4+q3)*y5+q2)*y5+q1)*y5+q0).
do if (pv <= .5).
compute xp = -xp.
end if.
compute cilow=rnd(nrow(temp)*(cdfnorm(2*xp-xp2))).
compute cihigh=trunc(nrow(temp)*(cdfnorm(2*xp+(xp2))))+1.
do if (cilow < 1).
compute cilow = 1.
compute booterr=1.
compute badlo = 1.
end if.
do if (cihigh > nrow(temp)).
compute cihigh = boot.
compute booterr=1.
compute badhi = 1.
end if.
compute llcit=temp(cilow,1).
compute ulcit=temp(cihigh,1).
do if (badlo = 1 and llcit <> priorlo).
compute badend={badend, llcit}.
compute priorlo = llcit.
end if.
do if (badhi = 1 and ulcit <> priorhi).
compute badend={badend, ulcit}.
compute priorhi = ulcit.
end if.
end if.
do if ( (indtemp(1,1)*bc)+(9999*(1-bc)) = 9999).
compute llcit=temp(cilow,1).
compute ulcit=temp(cihigh,1).
end if.
compute bootse=t(sqrt((cssq(temp)-((csum(temp)&**2)/nrow(temp)))/(nrow(temp)-1)))
.
compute indeff={indeff,bootse,llcit,ulcit}.
end if.
compute indtab={indtab;indeff}.
end loop.
compute resultm2=make(nrow(indtab),maxresm,99999).
compute resultm2(1:nrow(indtab),1:ncol(indtab))=indtab.
compute resultm={resultm;resultm2}.
do if (outscree=1).
do if (nxvls > 1).
compute indefflb=xcatlab(1:nxvls,1).
print indtab(2:nrow(indtab),:)/title = ' '/clabels='Effect', 'BootSE', 'BootLLCI', 'BootULCI'/rnames=indefflb/format= F10.4 /space=0.
end if.
do if (nxvls = 1).
print indtab(2:nrow(indtab),:)/title = ' '/clabels='Effect', 'BootSE', 'BootLLCI', 'BootULCI'/format= F10.4 /space=0.
end if.
end if.
end if.
do if (indmod(i,1)>0).
do if (indmod(i,1)=1).
compute indmodva=wmodvals.
compute indprova=wprobval.
compute condlbs={wnames}.
compute printw=1.
else if (indmod(i,1)=2).
compute indmodva=zmodvals.
compute indprova=zprobval.
compute condlbs={znames}.
compute printz=1.
else if (indmod(i,1)=3).
compute cntmp=1.
compute printz=1.
compute printw=1.
compute indmodva=make((nrow(wmodvals)*nrow(zmodvals)),2,999).
loop k7 = 1 to nrow(wmodvals).
loop k8 = 1 to nrow(zmodvals).
compute indmodva(cntmp,:)={wmodvals(k7,1),zmodvals(k8,1)}.
compute cntmp=cntmp+1.
end loop.
end loop.
compute condlbs={wnames,znames}.
end if.
compute condres=make(nrow(indmodva),1,999).
do if (boot > 0).
compute condres=make(nrow(indmodva),4,999).
end if.
compute condres={indmodva,condres}.
loop k4 = 1 to nxvls.
compute imm3=make(nrow(bootres),1,1).
compute imm4=make(nrow(bootres),1,1).
compute indcontr=0.
do if (indmod(i,1)=3).
compute tihsw=wprobval.
compute tihsz=zprobval.
end if.
loop k1=1 to nrow(indmodva).
compute tucker2=make(nrow(bootres),1,1).
compute imm2=make(nrow(bootres),1,1).
compute wfirst=0.
compute zfirst=0.
compute immset=0.
loop k2=1 to indmake(i,1).
compute colnumb=indmake(i,(k2+1)).
do if (k2=1).
compute wbb=make(nrow(bootres),(nwvls*nxvls),0).
compute zbb=make(nrow(bootres),(nzvls*nxvls),0).
compute wzbb=make(nrow(bootres),(nwvls*nzvls*nxvls),0).
end if.
do if (k2<>1).
compute wbb=make(nrow(bootres),nwvls,0).
compute zbb=make(nrow(bootres),nzvls,0).
compute wzbb=make(nrow(bootres),(nwvls*nzvls),0).
end if.
compute cnt=1.
compute tihs=indlocs(2:((indlocs(1,colnumb))+1),colnumb).
do if (k2 = 1).
compute focbb=tihs(1:nxvls,1).
compute focbb=bootres(:,focbb).
do if (indmmm(i,1)>0).
compute imm=focbb(:,k4).
compute condbb=imm.
end if.
compute focaddon=make(1,nxvls,0).
compute focaddon(1,k4)=1.
compute cnt=cnt+nxvls.
compute placeh=nxvls.
do if (indmod(i,1)=1).
compute tihsz=make(nrow(wprobval),(nzvls*nxvls),0).
compute tihswz=make(nrow(wprobval),(nwvls*nzvls*nxvls),0).
do if (pathsw(1,colnumb)=1).
compute temp=make(nrow(wprobval),(nxvls*nwvls),0).
loop k5 = 1 to nrow(wprobval).
loop k6=1 to nwvls.
compute temp(k5, (((k4-1)*nwvls)+k6))=wprobval(k5,k6).
end loop.
end loop.
compute indprova={temp,tihsz,tihswz}.
else.
compute indprova={wprobval,tihsz,tihswz}.
end if.
end if.
do if (indmod(i,1)=2).
compute tihsw=make(nrow(zprobval),(nwvls*nxvls),0).
compute tihswz=make(nrow(zprobval),(nwvls*nzvls*nxvls),0).
do if (pathsz(1,colnumb)=1).
compute temp=make(nrow(zprobval),(nxvls*nzvls),0).
loop k5 = 1 to nrow(zprobval).
loop k6 =1 to nzvls.
compute temp(k5,(((k4-1)*nzvls)+k6))=zprobval(k5,k6).
end loop.
end loop.
compute indprova={tihsw,temp,tihswz}.
else.
compute indprova={tihsw,zprobval,tihswz}.
end if.
end if.
do if (indmod(i,1)=3).
compute indprova=make((nrow(wprobval)*nrow(zprobval)),((ncol(wprobval)*nxvls)+ (ncol(zprobval)*nxvls)+(nwvls*nzvls*nxvls)),0).
compute cntemp=1.
loop k7=1 to nrow(wprobval).
loop k8 =1 to nrow(zprobval).
compute temp=wprobval(k7,:)*focaddon(1,k4).
compute indprova(cntemp,(((k4-1)*nwvls)+1):(k4*(nwvls)))=temp.
compute temp=zprobval(k8,:)*focaddon(1,k4).
compute indprova(cntemp, ((((k4-1)*nzvls)+1)+(nxvls*nwvls)) : ((((k4-1)*nzvls)+1)+(nxvls*nwvls)+(nzvls-1)))=temp.
compute cntemp=cntemp+1.
end loop.
end loop.
do if (pathsz(1,colnumb)=0).
compute temp=make(nrow(indprova),(ncol(zprobval)*nxvls),0).
compute indprova(:,((ncol(wprobval)*nxvls)+1):((ncol(wprobval)+ncol(zprobval))* nxvls))=temp.
end if.
do if (pathsw(1,colnumb)=0).
compute temp=make(nrow(indprova),(ncol(wprobval)*nxvls),0).
compute indprova(:,1:(ncol(wprobval)*nxvls))=temp.
end if.
do if (pathswz(1,colnumb)=1).
compute cntemp=(ncol(wprobval)*nxvls)+(ncol(zprobval)*nxvls)+((k4-1)* ncol(wprobval)*ncol(zprobval))+1.
loop k7=1 to ncol(wprobval).
loop k8=1 to ncol(zprobval).
compute indprova(:,cntemp)=indprova(:,((ncol(wprobval)*(k4-1))+k7))&* indprova(:,((((k4-1)*ncol(zprobval))+k8)+(nxvls*ncol(wprobval)))).
compute cntemp=cntemp+1.
end loop.
end loop.
end if.
end if.
end if.
do if (k2 > 1).
compute focbb=tihs(1,1).
compute focbb=bootres(:,focbb).
do if (indmmm(i,1)>0).
compute imm=focbb(:,1).
compute condbb=imm.
end if.
compute focaddon=1.
compute cnt=cnt+1.
compute placeh=1.
do if (indmod(i,1)=1).
compute tihsz=make(nrow(wprobval),nzvls,0).
compute tihswz=make(nrow(wprobval),(nwvls*nzvls),0).
compute indprova={wprobval,tihsz,tihswz}.
end if.
do if (indmod(i,1)=2).
compute tihsw=make(nrow(zprobval),nwvls,0).
compute tihswz=make(nrow(zprobval),(nwvls*nzvls),0).
compute indprova={tihsw,zprobval,tihswz}.
end if.
do if (indmod(i,1)=3).
compute indprova=make((nrow(wprobval)*nrow(zprobval)),((ncol(wprobval)+ ncol(zprobval))+(nwvls*nzvls)),0).
compute cntemp=1.
loop k7=1 to nrow(wprobval).
loop k8 =1 to nrow(zprobval).
compute indprova(cntemp,1:(ncol(wprobval)+ncol(zprobval)))={wprobval(k7,:), zprobval(k8,:)}.
compute cntemp=cntemp+1.
end loop.
end loop.
do if (pathsz(1,colnumb)=0).
compute temp=make(nrow(indprova),ncol(zprobval),0).
compute indprova(:,(ncol(wprobval)+1):(ncol(wprobval)+ncol(zprobval)))=temp.
end if.
do if (pathsw(1,colnumb)=0).
compute temp=make(nrow(indprova),ncol(wprobval),0).
compute indprova(:,1:ncol(wprobval))=temp.
end if.
do if (pathswz(1,colnumb)=1).
compute cntemp=ncol(wprobval)+ncol(zprobval)+1.
loop k7=1 to ncol(wprobval).
loop k8=1 to ncol(zprobval).
compute indprova(:,cntemp)=indprova(:,k7)&*indprova(:,(ncol(wprobval)+k8)).
compute cntemp=cntemp+1.
end loop.
end loop.
end if.
end if.
end if.
do if (pathsw(1,colnumb)) = 1.
compute wbb=tihs(cnt:(cnt+(placeh*nwvls)-1),1).
compute wbb=bootres(:,wbb).
compute immlbs2=wcatlab(1:nwvls,1).
do if (zfirst=0).
compute wfirst=1.
end if.
do if (indmmm(i,1)=1 or indmmm(i,1)=31 or indmmm(i,1)=51).
compute imm=wbb(:,1).
loop k7 = 1 to nwvls.
compute imm={imm,wbb(:,(((k4-1)*nwvls*(k2=1))+k7))}.
end loop.
compute imm=imm(:,2:ncol(imm)).
end if.
do if (indmmm(i,1)=41 or indmmm(i,1)=51).
compute condbb=make(nrow(bootres),1,0).
loop k7 = 1 to nwvls.
compute condbb={condbb,wbb(:,(((k4-1)*nwvls*(k2=1))+k7))}.
end loop.
compute condbb=condbb(:,2:ncol(condbb)).
end if.
compute cnt=cnt+(placeh*nwvls).
end if.
do if (pathsz(1,colnumb)) = 1.
compute zbb=tihs(cnt:(cnt+(placeh*nzvls)-1),1).
compute zbb=bootres(:,zbb).
do if (wfirst=0).
compute zfirst=1.
end if.
do if (indmmm(i,1) <> 31).
compute immlbs2=zcatlab(1:nzvls,1).
end if.
do if (indmmm(i,1)=2 or indmmm(i,1)=31 or indmmm(i,1)=51).
do if (indmmm(i,1)=2).
compute imm=zbb(:,1).
end if.
loop k7 = 1 to nzvls.
compute imm={imm,zbb(:,(((k4-1)*nzvls*(k2=1))+k7))}.
end loop.
do if (indmmm(i,1)=2 or indmmm(i,1)=51).
compute imm=imm(:,2:ncol(imm)).
do if (indmmm(i,1)=51).
compute condbb={condbb,imm}.
end if.
end if.
end if.
compute cnt=cnt+(placeh*nzvls).
end if.
do if (pathswz(1,colnumb)) = 1.
compute wzbb=tihs(cnt:(cnt+(placeh*nwvls*nzvls)-1),1).
compute wzbb=bootres(:,wzbb).
do if (indmmm(i,1)=41).
compute imm=wzbb(:,1).
loop k7=1 to nwvls*nzvls.
compute imm={imm,wzbb(:,(((k4-1)*nzvls*nwvls*(k2=1))+k7))}.
end loop.
end if.
do if (indmmm(i,1)=41).
compute imm=imm(:,2:ncol(imm)).
compute condbb={condbb,imm(:,(ncol(imm)-(nwvls*nzvls)+1):ncol(imm))}.
end if.
compute cnt=cnt+(placeh*nzvls*nwvls).
end if.
compute indprobe={focaddon,indprova(k1,:)}.
compute tucker={focbb,wbb,zbb,wzbb}.
loop k3=1 to ncol(indprobe).
compute tucker(:,k3)=tucker(:,k3)*indprobe(1,k3).
end loop.
compute tucker2=tucker2&*rsum(tucker).
do if (indmmm(i,1) = 1 or indmmm(i,1)=2 or indmmm(i,1)=31 or indmmm(i,1)=41 or indmmm(i,1)=51).
do if (immset=1).
do if (ncol(imm2)=1 and ncol(imm) = 1).
compute imm2=imm2&*imm.
end if.
do if (indmmm(i,1)=41 or indmmm(i,1)=51).
do if ((ncol(condbb2) > 1) and (ncol(condbb)>1)).
compute condbb2t=make(nrow(condbb2),(ncol(condbb2)*ncol(condbb)),-999999).
compute k9=1.
do if (wfirst=1).
loop k7=1 to ncol(condbb2).
loop k8 = 1 to ncol(condbb).
compute condbb2t(:,k9)=condbb2(:,k7)&*condbb(:,k8).
compute k9=k9+1.
end loop.
end loop.
end if.
do if (zfirst=1).
loop k7=1 to ncol(condbb).
loop k8 = 1 to ncol(condbb2).
compute condbb2t(:,k9)=condbb(:,k7)&*condbb2(:,k8).
compute k9=k9+1.
end loop.
end loop.
end if.
compute condbb2=condbb2t.
release condbb2t.
end if.
do if ((ncol(condbb2) > 1) and (ncol(condbb)=1)).
loop k7 = 1 to ncol(condbb2).
compute condbb2(:,k7)=condbb2(:,k7)&*condbb.
end loop.
end if.
do if ((ncol(condbb2) = 1) and (ncol(condbb)>1)).
loop k7 = 1 to ncol(condbb).
compute condbb(:,k7)=condbb2&*condbb(:,k7).
end loop.
compute condbb2=condbb.
end if.
end if.
do if (ncol(imm2) <> 1 and ncol(imm) <> 1).
compute imm2t=make(nrow(imm2),(ncol(imm2)*ncol(imm)),-999999).
compute k9=1.
do if (wfirst=1).
loop k7=1 to ncol(imm2).
loop k8 = 1 to ncol(imm).
compute imm2t(:,k9)=imm2(:,k7)&*imm(:,k8).
compute k9=k9+1.
end loop.
end loop.
end if.
do if (zfirst=1).
loop k7=1 to ncol(imm).
loop k8 = 1 to ncol(imm2).
compute imm2t(:,k9)=imm(:,k7)&*imm2(:,k8).
compute k9=k9+1.
end loop.
end loop.
end if.
compute imm2=imm2t.
release imm2t.
end if.
do if ((ncol(imm2) > 1) and (ncol(imm)=1)).
loop k7=1 to ncol(imm2).
compute imm2(:,k7)=imm2(:,k7)&*imm.
end loop.
end if.
do if ((ncol(imm2) = 1) and (ncol(imm) > 1)).
loop k7=1 to ncol(imm).
compute imm(:,k7)=imm2&*imm(:,k7).
end loop.
compute imm2=imm.
end if.
end if.
do if (immset=0).
compute imm2=imm.
do if (indmmm(i,1)=41 or indmmm(i,1)=51).
compute condbb2=condbb.
end if.
compute immset=1.
end if.
end if.
end loop.
compute indtemp=tucker2(1,1).
do if (indmmm(i,1)=12 or indmmm(i,1)=22).
compute imm3={imm3,tucker2}.
do if (k1=nrow(indmodva)).
compute imm3=imm3(:,2:ncol(imm3)).
compute immstop=ncol(imm3).
loop k8=2 to immstop.
do if (indmmmt(i,1)=1).
compute imm3={imm3,(imm3(:,k8)-imm3(:,1))}.
end if.
do if (indmmmt(i,1)=2).
compute imm3={imm3,(imm3(:,k8)-imm3(:,(k8-1)))}.
end if.
do if (indmmmt(i,1)=3).
compute imm3={imm3,((rsum(imm3(:,(k8:immstop)))/(immstop-k8+1))-imm3(:,(k8-1))) }.
end if.
do if (indmmmt(i,1)=4).
compute imm3={imm3,(imm3(:,k8)-(rsum(imm3(:,1:immstop))/immstop))}.
end if.
end loop.
do if (indmmmt(i,1)<5).
compute imm2=imm3(:,(immstop+1):ncol(imm3)).
release imm3.
end if.
end if.
end if.
do if (indmmm(i,1)>-1 and (contrast = 1 or contrast = 2 or contrast=3)).
compute imm4={imm4,tucker2}.
do if (k1=nrow(indmodva) and k1 > 1).
compute imm4=imm4(:,2:ncol(imm4)).
compute immstop=ncol(imm4).
compute condcont=make((immstop*(immstop-1)/2),6,-999).
loop k8 = 1 to (immstop-1).
loop k9 = (k8+1) to immstop.
do if (contrast=1).
compute imm4={imm4,(imm4(:,k9)-imm4(:,k8))}.
end if.
do if (contrast=2).
compute imm4={imm4,(abs(imm4(:,k9))-abs(imm4(:,k8)))}.
end if.
do if (contrast=3).
compute imm4={imm4,(imm4(:,k9)+imm4(:,k8))}.
end if.
compute condcont((ncol(imm4)-immstop),1)=imm4(1,k9).
compute condcont((ncol(imm4)-immstop),2)=imm4(1,k8).
end loop.
end loop.
compute imm4=imm4(:,(immstop+1):ncol(imm4)).
loop k8=1 to ncol(imm4).
compute condcont(k8,3)=imm4(1,k8).
do if (boot > 0).

.
compute temp = imm4(2:nrow(imm4),k8).
compute temp(GRADE( imm4(2:nrow(imm4),k8) )) = imm4(2:nrow(imm4),k8).
compute badlo = 0.
compute badhi = 0.
do if ( (imm4(1,k8)*bc)+(9999*(1-bc)) <> 9999).
compute pv=csum(temp < (imm4(1,k8)*bc)+(9999*(1-bc)) )/nrow(temp).
compute ppv = pv.
do if (pv > .5).
compute ppv = 1-pv.
end if.
compute y5=sqrt(-2*ln(ppv)).
compute xp=y5+((((y5*p4+p3)*y5+p2)*y5+p1)*y5+p0)/((((y5*q4+q3)*y5+q2)*y5+q1)*y5+q0).
do if (pv <= .5).
compute xp = -xp.
end if.
compute cilow=rnd(nrow(temp)*(cdfnorm(2*xp-xp2))).
compute cihigh=trunc(nrow(temp)*(cdfnorm(2*xp+(xp2))))+1.
do if (cilow < 1).
compute cilow = 1.
compute booterr=1.
compute badlo = 1.
end if.
do if (cihigh > nrow(temp)).
compute cihigh = boot.
compute booterr=1.
compute badhi = 1.
end if.
compute llcit=temp(cilow,1).
compute ulcit=temp(cihigh,1).
do if (badlo = 1 and llcit <> priorlo).
compute badend={badend, llcit}.
compute priorlo = llcit.
end if.
do if (badhi = 1 and ulcit <> priorhi).
compute badend={badend, ulcit}.
compute priorhi = ulcit.
end if.
end if.
do if ( (imm4(1,k8)*bc)+(9999*(1-bc)) = 9999).
compute llcit=temp(cilow,1).
compute ulcit=temp(cihigh,1).
end if.
compute bootse=t(sqrt((cssq(temp)-((csum(temp)&**2)/nrow(temp)))/(nrow(temp)-1)))
.
compute condcont(k8,4:6)={bootse,llcit,ulcit}.
end if.
end loop.
do if (boot=0).
compute condcont=condcont(:,1:3).
end if.
compute indcontr=1.
end if.
end if.
do if (xmint=1).
do if (k1=(k4+1)).
compute cftotfx(:,k4)=cftotfx(:,k4)+(tucker2*xscaling).
end if.
end if.
do if (boot > 0).
compute tucker2=tucker2*xscaling.

.
compute temp = tucker2(2:nrow(tucker2),1).
compute temp(GRADE( tucker2(2:nrow(tucker2),1) )) = tucker2(2:nrow(tucker2),1).
compute badlo = 0.
compute badhi = 0.
do if ( (tucker2(1,1)*bc)+(9999*(1-bc)) <> 9999).
compute pv=csum(temp < (tucker2(1,1)*bc)+(9999*(1-bc)) )/nrow(temp).
compute ppv = pv.
do if (pv > .5).
compute ppv = 1-pv.
end if.
compute y5=sqrt(-2*ln(ppv)).
compute xp=y5+((((y5*p4+p3)*y5+p2)*y5+p1)*y5+p0)/((((y5*q4+q3)*y5+q2)*y5+q1)*y5+q0).
do if (pv <= .5).
compute xp = -xp.
end if.
compute cilow=rnd(nrow(temp)*(cdfnorm(2*xp-xp2))).
compute cihigh=trunc(nrow(temp)*(cdfnorm(2*xp+(xp2))))+1.
do if (cilow < 1).
compute cilow = 1.
compute booterr=1.
compute badlo = 1.
end if.
do if (cihigh > nrow(temp)).
compute cihigh = boot.
compute booterr=1.
compute badhi = 1.
end if.
compute llcit=temp(cilow,1).
compute ulcit=temp(cihigh,1).
do if (badlo = 1 and llcit <> priorlo).
compute badend={badend, llcit}.
compute priorlo = llcit.
end if.
do if (badhi = 1 and ulcit <> priorhi).
compute badend={badend, ulcit}.
compute priorhi = ulcit.
end if.
end if.
do if ( (tucker2(1,1)*bc)+(9999*(1-bc)) = 9999).
compute llcit=temp(cilow,1).
compute ulcit=temp(cihigh,1).
end if.
compute bootse=t(sqrt((cssq(temp)-((csum(temp)&**2)/nrow(temp)))/(nrow(temp)-1)))
.
compute indtemp={indtemp*xscaling, bootse,llcit,ulcit}.
end if.
do if (boot=0).
compute indtemp=indtemp*xscaling.
end if.
compute condres(k1,(ncol(indmodva)+1):ncol(condres))=indtemp.
end loop.
do if (xmint=0).
compute resultm2=make(nrow(condres),maxresm,99999).
compute resultm2(1:nrow(condres),1:ncol(condres))=condres.
compute resultm={resultm;resultm2}.
end if.
compute condlbs={condlbs,'Effect'}.
do if (boot > 0).
compute condlbs={condlbs,'BootSE', 'BootLLCI', 'BootULCI'}.
end if.
do if (xmint = 1).
do if (k4 = 1).
compute natindfx=condres((2+(k4-1)),2:ncol(condres)).
end if.
do if (k4 > 1).
compute natindfx={natindfx;condres((2+(k4-1)),2:ncol(condres))}.
end if.
do if (k4=nxvls).
compute resultm2=make(nrow(natindfx),maxresm,99999).
compute resultm2(1:nrow(natindfx),1:ncol(natindfx))=natindfx.
compute resultm={resultm;resultm2}.
end if.
end if.
do if (outscree=1 and xmint=0).
do if (nxvls=1).
print condres/title=' '/cnames=condlbs/format= F10.4 /space=0.
else.
compute condrlb=make(nrow(condres),1,xcatlab(k4,1)).
print condres/title=' '/cnames=condlbs/rnames=condrlb/format= F10.4 /space=0.
end if.
end if.
do if (outscree=1 and xmint=1 and k4=nxvls).
do if (nxvls=1).
print natindfx/title=' '/clabels='Effect', 'BootSE', 'BootLLCI', 'BootULCI'/format= F10.4 /space=0.
else.
compute condrlb=make(nrow(condres),1,xcatlab(k4,1)).
print natindfx/title=' '/clabels='Effect', 'BootSE', 'BootLLCI', 'BootULCI'/rnames=direffl2/format= F10.4 /space=0.
end if.
end if.
compute dichadj=0.
compute immcat=0.
do if ((indmmm(i,1)>0) and (xmint=0)).
do if (indmmm(i,1)=1 or indmmm(i,1)=12 or indmmm(i,1)=31).
do if (wdich=1 and mcw=0).
do if (indmmm(i,1) <> 12).
compute imm2(:,1)=imm2(:,1)*(wmax-wmin).
end if.
do if (indmmm(i,1) <> 31).
compute dichadj=1.
end if.
end if.
do if ((mcw = 1 or mcw = 2) and indmmm(i,1) <> 31)).
compute immcat=1.
end if.
end if.
do if (indmmm(i,1)=2 or indmmm(i,1)=22 or indmmm(i,1)=31).
do if (zdich=1 and mcz=0).
do if (indmmm(i,1) = 31).
compute imm2(:,(nwvls+1):ncol(imm2))=imm2(:,(nwvls+1):ncol(imm2))*(zmax-zmin).
end if.
do if (indmmm(i,1) = 2).
compute imm2(:,1)=imm2(:,1)*(zmax-zmin).
end if.
do if (indmmm(i,1) <> 31).
compute dichadj=1.
end if.
end if.
do if ((mcz = 1 or mcz = 2) and indmmm(i,1) <> 31)).
compute immcat=1.
end if.
end if.
compute immtemp2=t(imm2(1,:)).
compute immtemp=immtemp2.
compute immlbs={'Index'}.
do if (boot > 0).
compute immtemp=make(1,3,0).
loop k7=1 to ncol(imm2).

.
compute temp = imm2(2:nrow(imm2),k7).
compute temp(GRADE( imm2(2:nrow(imm2),k7) )) = imm2(2:nrow(imm2),k7).
compute badlo = 0.
compute badhi = 0.
do if ( (imm2(1,k7)*bc)+(9999*(1-bc)) <> 9999).
compute pv=csum(temp < (imm2(1,k7)*bc)+(9999*(1-bc)) )/nrow(temp).
compute ppv = pv.
do if (pv > .5).
compute ppv = 1-pv.
end if.
compute y5=sqrt(-2*ln(ppv)).
compute xp=y5+((((y5*p4+p3)*y5+p2)*y5+p1)*y5+p0)/((((y5*q4+q3)*y5+q2)*y5+q1)*y5+q0).
do if (pv <= .5).
compute xp = -xp.
end if.
compute cilow=rnd(nrow(temp)*(cdfnorm(2*xp-xp2))).
compute cihigh=trunc(nrow(temp)*(cdfnorm(2*xp+(xp2))))+1.
do if (cilow < 1).
compute cilow = 1.
compute booterr=1.
compute badlo = 1.
end if.
do if (cihigh > nrow(temp)).
compute cihigh = boot.
compute booterr=1.
compute badhi = 1.
end if.
compute llcit=temp(cilow,1).
compute ulcit=temp(cihigh,1).
do if (badlo = 1 and llcit <> priorlo).
compute badend={badend, llcit}.
compute priorlo = llcit.
end if.
do if (badhi = 1 and ulcit <> priorhi).
compute badend={badend, ulcit}.
compute priorhi = ulcit.
end if.
end if.
do if ( (imm2(1,k7)*bc)+(9999*(1-bc)) = 9999).
compute llcit=temp(cilow,1).
compute ulcit=temp(cihigh,1).
end if.
compute bootse=t(sqrt((cssq(temp)-((csum(temp)&**2)/nrow(temp)))/(nrow(temp)-1)))
.
compute temp={bootse,llcit,ulcit}.
compute immtemp={immtemp;temp}.
end loop.
compute immtemp=immtemp(2:nrow(immtemp),:).
compute immtemp={immtemp2,immtemp}.
compute immlbs={immlbs,'BootSE', 'BootLLCI', 'BootULCI'}.
end if.
compute resultm2=make(nrow(immtemp),maxresm,99999).
compute resultm2(1:nrow(immtemp),1:ncol(immtemp))=immtemp.
compute resultm={resultm;resultm2}.
do if (dichadj=0 and immcat=0 and indmmmt(i,1)<>5 and indmmm(i,1) < 100 ).
do if (outscree=1).
do if (indmmm(i,1) < 30).
print immtemp/title='      Index of moderated mediation:'/cnames=immlbs/rnames=immlbs2/format= F10.4.
end if.
do if (indmmm(i,1) = 31).
compute immlbs2={immlbs2;zcatlab(1:nzvls,1)}.
print immtemp/title='      Indices of partial moderated mediation:'/cnames=immlbs/rnames=immlbs2/format= F10.4.
end if.
end if.
do if ((nzvls = 1) and (nwvls = 1)).
do if (indmmm(i,1) = 41 or indmmm(i,1)=51).
loop k7=1 to nwvls.
compute immlbs2=zcatlab(1:nzvls,1).
compute immtemp2=immtemp((((k7-1)*nzvls)+1):(((k7-1)*nzvls)+nzvls),:).
do if (outscree=1).
do if (nwvls > 1).
compute primodv={'        ', wcatlab(k7,1)}.
print primodv/title='      Primary moderator:'/format=A8.
end if.
do if (nzvls=1).
print immtemp2/title='      Index of moderated moderated mediation'/cnames=immlbs/format= F10.4.
else.
print immtemp2/title='      Indices of moderated moderated mediation'/cnames=immlbs/rnames=immlbs2/format= F10.4.
end if.
end if.
compute cmmtemp=make(nrow(zprobval),4,0).
loop k8=1 to nrow(zprobval).
compute condbb3=condbb2(:,((nwvls+1)+((k7-1)*nzvls)):((nwvls+1)+((k7-1)*nzvls)+ (nzvls-1))).
do if (ncol(zprobval) > 1).
compute condbb3=condbb3*mdiag(zprobval(k8,:)).
else.
compute condbb3=condbb3&*zprobval(k8,:).
end if.
compute condbb3={condbb2(:,k7),condbb3}.
compute icmm=rsum(condbb3).
compute cmmtemp(k8,1)=icmm(1,1).
do if (boot > 0).

.
compute temp = icmm(2:nrow(icmm),1).
compute temp(GRADE( icmm(2:nrow(icmm),1) )) = icmm(2:nrow(icmm),1).
compute badlo = 0.
compute badhi = 0.
do if ( (icmm(1,1)*bc)+(9999*(1-bc)) <> 9999).
compute pv=csum(temp < (icmm(1,1)*bc)+(9999*(1-bc)) )/nrow(temp).
compute ppv = pv.
do if (pv > .5).
compute ppv = 1-pv.
end if.
compute y5=sqrt(-2*ln(ppv)).
compute xp=y5+((((y5*p4+p3)*y5+p2)*y5+p1)*y5+p0)/((((y5*q4+q3)*y5+q2)*y5+q1)*y5+q0).
do if (pv <= .5).
compute xp = -xp.
end if.
compute cilow=rnd(nrow(temp)*(cdfnorm(2*xp-xp2))).
compute cihigh=trunc(nrow(temp)*(cdfnorm(2*xp+(xp2))))+1.
do if (cilow < 1).
compute cilow = 1.
compute booterr=1.
compute badlo = 1.
end if.
do if (cihigh > nrow(temp)).
compute cihigh = boot.
compute booterr=1.
compute badhi = 1.
end if.
compute llcit=temp(cilow,1).
compute ulcit=temp(cihigh,1).
do if (badlo = 1 and llcit <> priorlo).
compute badend={badend, llcit}.
compute priorlo = llcit.
end if.
do if (badhi = 1 and ulcit <> priorhi).
compute badend={badend, ulcit}.
compute priorhi = ulcit.
end if.
end if.
do if ( (icmm(1,1)*bc)+(9999*(1-bc)) = 9999).
compute llcit=temp(cilow,1).
compute ulcit=temp(cihigh,1).
end if.
compute bootse=t(sqrt((cssq(temp)-((csum(temp)&**2)/nrow(temp)))/(nrow(temp)-1)))
.
compute cmmtemp(k8,2:4)={bootse,llcit,ulcit}.
end if.
end loop.
compute cmmtemp={zmodvals,cmmtemp}.
do if (boot=0).
compute cmmtemp=cmmtemp(:,1:2).
end if.
compute cmmlbs={znames,immlbs}.
compute resultm2=make(nrow(cmmtemp),maxresm,99999).
compute resultm2(1:nrow(cmmtemp),1:ncol(cmmtemp))=cmmtemp.
compute resultm={resultm;resultm2}.
do if (outscree=1).
print cmmtemp/title='      Indices of conditional moderated mediation by W'/cnames=cmmlbs/format= F10.4.
end if.
end loop.
end if.
end if.
end if.
do if (dichadj=1 or immcat=1 and indmmm(i,1) < 30).
do if (outscree=1).
print immtemp/title='Index of moderated mediation (difference between conditional indirect effects):'/cnames=immlbs/rnames=immlbs2/format= F10.4.
end if.
end if.
end if.
do if (indcontr=1).
compute resultm2=make(nrow(condcont),maxresm,99999).
compute resultm2(1:nrow(condcont),1:ncol(condcont))=condcont.
compute resultm={resultm;resultm2}.
compute condctlb={'Effect1','Effect2','Contrast','BootSE', 'BootLLCI','BootULCI'}.
do if (outscree=1).
print condcont/title=' Pairwise contrasts between conditional indirect effects (Effect1 minus Effect2)'/cnames=condctlb/format= F10.4.
end if.
end if.
end loop.
end if.
end loop.
do if (alttotal=1).
compute altcnms='Effect'.
compute alttotfx=t(cftotfx(1,:))+obnatdfx.
do if (boot > 0).
compute alttotfx=make(ncol(cftotfx),4,0).
compute alttotfx(:,1)=t(cftotfx(1,:))+obnatdfx.
compute natdirbt(1,:)=t(obnatdfx).
compute cftotfx=cftotfx+natdirbt.
loop cec=1 to ncol(cftotfx).

.
compute temp = cftotfx(2:nrow(cftotfx),cec).
compute temp(GRADE( cftotfx(2:nrow(cftotfx),cec) )) = cftotfx(2:nrow(cftotfx),cec).
compute badlo = 0.
compute badhi = 0.
do if ( (cftotfx(1,cec)*bc)+(9999*(1-bc)) <> 9999).
compute pv=csum(temp < (cftotfx(1,cec)*bc)+(9999*(1-bc)) )/nrow(temp).
compute ppv = pv.
do if (pv > .5).
compute ppv = 1-pv.
end if.
compute y5=sqrt(-2*ln(ppv)).
compute xp=y5+((((y5*p4+p3)*y5+p2)*y5+p1)*y5+p0)/((((y5*q4+q3)*y5+q2)*y5+q1)*y5+q0).
do if (pv <= .5).
compute xp = -xp.
end if.
compute cilow=rnd(nrow(temp)*(cdfnorm(2*xp-xp2))).
compute cihigh=trunc(nrow(temp)*(cdfnorm(2*xp+(xp2))))+1.
do if (cilow < 1).
compute cilow = 1.
compute booterr=1.
compute badlo = 1.
end if.
do if (cihigh > nrow(temp)).
compute cihigh = boot.
compute booterr=1.
compute badhi = 1.
end if.
compute llcit=temp(cilow,1).
compute ulcit=temp(cihigh,1).
do if (badlo = 1 and llcit <> priorlo).
compute badend={badend, llcit}.
compute priorlo = llcit.
end if.
do if (badhi = 1 and ulcit <> priorhi).
compute badend={badend, ulcit}.
compute priorhi = ulcit.
end if.
end if.
do if ( (cftotfx(1,cec)*bc)+(9999*(1-bc)) = 9999).
compute llcit=temp(cilow,1).
compute ulcit=temp(cihigh,1).
end if.
compute bootse=t(sqrt((cssq(temp)-((csum(temp)&**2)/nrow(temp)))/(nrow(temp)-1)))
.
compute alttotfx(cec,2:4)={bootse,llcit,ulcit}.
end loop.
compute altcnms={altcnms,'BootSE', 'BootLLCI','BootULCI'}.
end if.
compute resultm2=make(nrow(alttotfx),maxresm,99999).
compute resultm2(1:nrow(alttotfx),1:ncol(alttotfx))=alttotfx.
compute resultm={resultm;resultm2}.
do if (outscree=1).
print/title='----------'.
do if (nxvls > 1).
print alttotfx/title='Relative total effects of X on Y (sum of direct and indirect effects)'/cnames=altcnms/rnames=direffl2/format= F10.4.
end if.
do if (nxvls = 1).
print alttotfx/title='Total effect of X on Y (sum of direct and indirect effects)'/cnames=altcnms/format= F10.4.
end if.
end if.
end if.
end if.
end if
.
do if (criterr=0 and (saveest = 1 or saveboot=1)).
compute coeffsav=coeffmat(2:nrow(coeffmat),:).
compute conseq=conseq(2:nrow(conseq),1).
compute dfmat=dfmat(2:nrow(dfmat),1).
compute dfmat=dfmat.
compute saven={'conseqnt','antecdnt','coeff','se','t','p','LLCI','ULCI','df'}.
do if (ydich=1).
compute saven={'conseqnt','antecdnt','coeff','se','t_or_Z','p','LLCI','ULCI','df'}.
end if.
do if (saveboot=1 and boot > 0 ).
compute colslab=make(300,1,' ').


compute colslab( 1 ,1)= 'COL1'.


compute colslab( 2 ,1)= 'COL2'.


compute colslab( 3 ,1)= 'COL3'.


compute colslab( 4 ,1)= 'COL4'.


compute colslab( 5 ,1)= 'COL5'.


compute colslab( 6 ,1)= 'COL6'.


compute colslab( 7 ,1)= 'COL7'.


compute colslab( 8 ,1)= 'COL8'.


compute colslab( 9 ,1)= 'COL9'.


compute colslab( 10 ,1)= 'COL10'.


compute colslab( 11 ,1)= 'COL11'.


compute colslab( 12 ,1)= 'COL12'.


compute colslab( 13 ,1)= 'COL13'.


compute colslab( 14 ,1)= 'COL14'.


compute colslab( 15 ,1)= 'COL15'.


compute colslab( 16 ,1)= 'COL16'.


compute colslab( 17 ,1)= 'COL17'.


compute colslab( 18 ,1)= 'COL18'.


compute colslab( 19 ,1)= 'COL19'.


compute colslab( 20 ,1)= 'COL20'.


compute colslab( 21 ,1)= 'COL21'.


compute colslab( 22 ,1)= 'COL22'.


compute colslab( 23 ,1)= 'COL23'.


compute colslab( 24 ,1)= 'COL24'.


compute colslab( 25 ,1)= 'COL25'.


compute colslab( 26 ,1)= 'COL26'.


compute colslab( 27 ,1)= 'COL27'.


compute colslab( 28 ,1)= 'COL28'.


compute colslab( 29 ,1)= 'COL29'.


compute colslab( 30 ,1)= 'COL30'.


compute colslab( 31 ,1)= 'COL31'.


compute colslab( 32 ,1)= 'COL32'.


compute colslab( 33 ,1)= 'COL33'.


compute colslab( 34 ,1)= 'COL34'.


compute colslab( 35 ,1)= 'COL35'.


compute colslab( 36 ,1)= 'COL36'.


compute colslab( 37 ,1)= 'COL37'.


compute colslab( 38 ,1)= 'COL38'.


compute colslab( 39 ,1)= 'COL39'.


compute colslab( 40 ,1)= 'COL40'.


compute colslab( 41 ,1)= 'COL41'.


compute colslab( 42 ,1)= 'COL42'.


compute colslab( 43 ,1)= 'COL43'.


compute colslab( 44 ,1)= 'COL44'.


compute colslab( 45 ,1)= 'COL45'.


compute colslab( 46 ,1)= 'COL46'.


compute colslab( 47 ,1)= 'COL47'.


compute colslab( 48 ,1)= 'COL48'.


compute colslab( 49 ,1)= 'COL49'.


compute colslab( 50 ,1)= 'COL50'.


compute colslab( 51 ,1)= 'COL51'.


compute colslab( 52 ,1)= 'COL52'.


compute colslab( 53 ,1)= 'COL53'.


compute colslab( 54 ,1)= 'COL54'.


compute colslab( 55 ,1)= 'COL55'.


compute colslab( 56 ,1)= 'COL56'.


compute colslab( 57 ,1)= 'COL57'.


compute colslab( 58 ,1)= 'COL58'.


compute colslab( 59 ,1)= 'COL59'.


compute colslab( 60 ,1)= 'COL60'.


compute colslab( 61 ,1)= 'COL61'.


compute colslab( 62 ,1)= 'COL62'.


compute colslab( 63 ,1)= 'COL63'.


compute colslab( 64 ,1)= 'COL64'.


compute colslab( 65 ,1)= 'COL65'.


compute colslab( 66 ,1)= 'COL66'.


compute colslab( 67 ,1)= 'COL67'.


compute colslab( 68 ,1)= 'COL68'.


compute colslab( 69 ,1)= 'COL69'.


compute colslab( 70 ,1)= 'COL70'.


compute colslab( 71 ,1)= 'COL71'.


compute colslab( 72 ,1)= 'COL72'.


compute colslab( 73 ,1)= 'COL73'.


compute colslab( 74 ,1)= 'COL74'.


compute colslab( 75 ,1)= 'COL75'.


compute colslab( 76 ,1)= 'COL76'.


compute colslab( 77 ,1)= 'COL77'.


compute colslab( 78 ,1)= 'COL78'.


compute colslab( 79 ,1)= 'COL79'.


compute colslab( 80 ,1)= 'COL80'.


compute colslab( 81 ,1)= 'COL81'.


compute colslab( 82 ,1)= 'COL82'.


compute colslab( 83 ,1)= 'COL83'.


compute colslab( 84 ,1)= 'COL84'.


compute colslab( 85 ,1)= 'COL85'.


compute colslab( 86 ,1)= 'COL86'.


compute colslab( 87 ,1)= 'COL87'.


compute colslab( 88 ,1)= 'COL88'.


compute colslab( 89 ,1)= 'COL89'.


compute colslab( 90 ,1)= 'COL90'.


compute colslab( 91 ,1)= 'COL91'.


compute colslab( 92 ,1)= 'COL92'.


compute colslab( 93 ,1)= 'COL93'.


compute colslab( 94 ,1)= 'COL94'.


compute colslab( 95 ,1)= 'COL95'.


compute colslab( 96 ,1)= 'COL96'.


compute colslab( 97 ,1)= 'COL97'.


compute colslab( 98 ,1)= 'COL98'.


compute colslab( 99 ,1)= 'COL99'.


compute colslab( 100 ,1)= 'COL100'.


compute colslab( 101 ,1)= 'COL101'.


compute colslab( 102 ,1)= 'COL102'.


compute colslab( 103 ,1)= 'COL103'.


compute colslab( 104 ,1)= 'COL104'.


compute colslab( 105 ,1)= 'COL105'.


compute colslab( 106 ,1)= 'COL106'.


compute colslab( 107 ,1)= 'COL107'.


compute colslab( 108 ,1)= 'COL108'.


compute colslab( 109 ,1)= 'COL109'.


compute colslab( 110 ,1)= 'COL110'.


compute colslab( 111 ,1)= 'COL111'.


compute colslab( 112 ,1)= 'COL112'.


compute colslab( 113 ,1)= 'COL113'.


compute colslab( 114 ,1)= 'COL114'.


compute colslab( 115 ,1)= 'COL115'.


compute colslab( 116 ,1)= 'COL116'.


compute colslab( 117 ,1)= 'COL117'.


compute colslab( 118 ,1)= 'COL118'.


compute colslab( 119 ,1)= 'COL119'.


compute colslab( 120 ,1)= 'COL120'.


compute colslab( 121 ,1)= 'COL121'.


compute colslab( 122 ,1)= 'COL122'.


compute colslab( 123 ,1)= 'COL123'.


compute colslab( 124 ,1)= 'COL124'.


compute colslab( 125 ,1)= 'COL125'.


compute colslab( 126 ,1)= 'COL126'.


compute colslab( 127 ,1)= 'COL127'.


compute colslab( 128 ,1)= 'COL128'.


compute colslab( 129 ,1)= 'COL129'.


compute colslab( 130 ,1)= 'COL130'.


compute colslab( 131 ,1)= 'COL131'.


compute colslab( 132 ,1)= 'COL132'.


compute colslab( 133 ,1)= 'COL133'.


compute colslab( 134 ,1)= 'COL134'.


compute colslab( 135 ,1)= 'COL135'.


compute colslab( 136 ,1)= 'COL136'.


compute colslab( 137 ,1)= 'COL137'.


compute colslab( 138 ,1)= 'COL138'.


compute colslab( 139 ,1)= 'COL139'.


compute colslab( 140 ,1)= 'COL140'.


compute colslab( 141 ,1)= 'COL141'.


compute colslab( 142 ,1)= 'COL142'.


compute colslab( 143 ,1)= 'COL143'.


compute colslab( 144 ,1)= 'COL144'.


compute colslab( 145 ,1)= 'COL145'.


compute colslab( 146 ,1)= 'COL146'.


compute colslab( 147 ,1)= 'COL147'.


compute colslab( 148 ,1)= 'COL148'.


compute colslab( 149 ,1)= 'COL149'.


compute colslab( 150 ,1)= 'COL150'.


compute colslab( 151 ,1)= 'COL151'.


compute colslab( 152 ,1)= 'COL152'.


compute colslab( 153 ,1)= 'COL153'.


compute colslab( 154 ,1)= 'COL154'.


compute colslab( 155 ,1)= 'COL155'.


compute colslab( 156 ,1)= 'COL156'.


compute colslab( 157 ,1)= 'COL157'.


compute colslab( 158 ,1)= 'COL158'.


compute colslab( 159 ,1)= 'COL159'.


compute colslab( 160 ,1)= 'COL160'.


compute colslab( 161 ,1)= 'COL161'.


compute colslab( 162 ,1)= 'COL162'.


compute colslab( 163 ,1)= 'COL163'.


compute colslab( 164 ,1)= 'COL164'.


compute colslab( 165 ,1)= 'COL165'.


compute colslab( 166 ,1)= 'COL166'.


compute colslab( 167 ,1)= 'COL167'.


compute colslab( 168 ,1)= 'COL168'.


compute colslab( 169 ,1)= 'COL169'.


compute colslab( 170 ,1)= 'COL170'.


compute colslab( 171 ,1)= 'COL171'.


compute colslab( 172 ,1)= 'COL172'.


compute colslab( 173 ,1)= 'COL173'.


compute colslab( 174 ,1)= 'COL174'.


compute colslab( 175 ,1)= 'COL175'.


compute colslab( 176 ,1)= 'COL176'.


compute colslab( 177 ,1)= 'COL177'.


compute colslab( 178 ,1)= 'COL178'.


compute colslab( 179 ,1)= 'COL179'.


compute colslab( 180 ,1)= 'COL180'.


compute colslab( 181 ,1)= 'COL181'.


compute colslab( 182 ,1)= 'COL182'.


compute colslab( 183 ,1)= 'COL183'.


compute colslab( 184 ,1)= 'COL184'.


compute colslab( 185 ,1)= 'COL185'.


compute colslab( 186 ,1)= 'COL186'.


compute colslab( 187 ,1)= 'COL187'.


compute colslab( 188 ,1)= 'COL188'.


compute colslab( 189 ,1)= 'COL189'.


compute colslab( 190 ,1)= 'COL190'.


compute colslab( 191 ,1)= 'COL191'.


compute colslab( 192 ,1)= 'COL192'.


compute colslab( 193 ,1)= 'COL193'.


compute colslab( 194 ,1)= 'COL194'.


compute colslab( 195 ,1)= 'COL195'.


compute colslab( 196 ,1)= 'COL196'.


compute colslab( 197 ,1)= 'COL197'.


compute colslab( 198 ,1)= 'COL198'.


compute colslab( 199 ,1)= 'COL199'.


compute colslab( 200 ,1)= 'COL200'.


compute colslab( 201 ,1)= 'COL201'.


compute colslab( 202 ,1)= 'COL202'.


compute colslab( 203 ,1)= 'COL203'.


compute colslab( 204 ,1)= 'COL204'.


compute colslab( 205 ,1)= 'COL205'.


compute colslab( 206 ,1)= 'COL206'.


compute colslab( 207 ,1)= 'COL207'.


compute colslab( 208 ,1)= 'COL208'.


compute colslab( 209 ,1)= 'COL209'.


compute colslab( 210 ,1)= 'COL210'.


compute colslab( 211 ,1)= 'COL211'.


compute colslab( 212 ,1)= 'COL212'.


compute colslab( 213 ,1)= 'COL213'.


compute colslab( 214 ,1)= 'COL214'.


compute colslab( 215 ,1)= 'COL215'.


compute colslab( 216 ,1)= 'COL216'.


compute colslab( 217 ,1)= 'COL217'.


compute colslab( 218 ,1)= 'COL218'.


compute colslab( 219 ,1)= 'COL219'.


compute colslab( 220 ,1)= 'COL220'.


compute colslab( 221 ,1)= 'COL221'.


compute colslab( 222 ,1)= 'COL222'.


compute colslab( 223 ,1)= 'COL223'.


compute colslab( 224 ,1)= 'COL224'.


compute colslab( 225 ,1)= 'COL225'.


compute colslab( 226 ,1)= 'COL226'.


compute colslab( 227 ,1)= 'COL227'.


compute colslab( 228 ,1)= 'COL228'.


compute colslab( 229 ,1)= 'COL229'.


compute colslab( 230 ,1)= 'COL230'.


compute colslab( 231 ,1)= 'COL231'.


compute colslab( 232 ,1)= 'COL232'.


compute colslab( 233 ,1)= 'COL233'.


compute colslab( 234 ,1)= 'COL234'.


compute colslab( 235 ,1)= 'COL235'.


compute colslab( 236 ,1)= 'COL236'.


compute colslab( 237 ,1)= 'COL237'.


compute colslab( 238 ,1)= 'COL238'.


compute colslab( 239 ,1)= 'COL239'.


compute colslab( 240 ,1)= 'COL240'.


compute colslab( 241 ,1)= 'COL241'.


compute colslab( 242 ,1)= 'COL242'.


compute colslab( 243 ,1)= 'COL243'.


compute colslab( 244 ,1)= 'COL244'.


compute colslab( 245 ,1)= 'COL245'.


compute colslab( 246 ,1)= 'COL246'.


compute colslab( 247 ,1)= 'COL247'.


compute colslab( 248 ,1)= 'COL248'.


compute colslab( 249 ,1)= 'COL249'.


compute colslab( 250 ,1)= 'COL250'.


compute colslab( 251 ,1)= 'COL251'.


compute colslab( 252 ,1)= 'COL252'.


compute colslab( 253 ,1)= 'COL253'.


compute colslab( 254 ,1)= 'COL254'.


compute colslab( 255 ,1)= 'COL255'.


compute colslab( 256 ,1)= 'COL256'.


compute colslab( 257 ,1)= 'COL257'.


compute colslab( 258 ,1)= 'COL258'.


compute colslab( 259 ,1)= 'COL259'.


compute colslab( 260 ,1)= 'COL260'.


compute colslab( 261 ,1)= 'COL261'.


compute colslab( 262 ,1)= 'COL262'.


compute colslab( 263 ,1)= 'COL263'.


compute colslab( 264 ,1)= 'COL264'.


compute colslab( 265 ,1)= 'COL265'.


compute colslab( 266 ,1)= 'COL266'.


compute colslab( 267 ,1)= 'COL267'.


compute colslab( 268 ,1)= 'COL268'.


compute colslab( 269 ,1)= 'COL269'.


compute colslab( 270 ,1)= 'COL270'.


compute colslab( 271 ,1)= 'COL271'.


compute colslab( 272 ,1)= 'COL272'.


compute colslab( 273 ,1)= 'COL273'.


compute colslab( 274 ,1)= 'COL274'.


compute colslab( 275 ,1)= 'COL275'.


compute colslab( 276 ,1)= 'COL276'.


compute colslab( 277 ,1)= 'COL277'.


compute colslab( 278 ,1)= 'COL278'.


compute colslab( 279 ,1)= 'COL279'.


compute colslab( 280 ,1)= 'COL280'.


compute colslab( 281 ,1)= 'COL281'.


compute colslab( 282 ,1)= 'COL282'.


compute colslab( 283 ,1)= 'COL283'.


compute colslab( 284 ,1)= 'COL284'.


compute colslab( 285 ,1)= 'COL285'.


compute colslab( 286 ,1)= 'COL286'.


compute colslab( 287 ,1)= 'COL287'.


compute colslab( 288 ,1)= 'COL288'.


compute colslab( 289 ,1)= 'COL289'.


compute colslab( 290 ,1)= 'COL290'.


compute colslab( 291 ,1)= 'COL291'.


compute colslab( 292 ,1)= 'COL292'.


compute colslab( 293 ,1)= 'COL293'.


compute colslab( 294 ,1)= 'COL294'.


compute colslab( 295 ,1)= 'COL295'.


compute colslab( 296 ,1)= 'COL296'.


compute colslab( 297 ,1)= 'COL297'.


compute colslab( 298 ,1)= 'COL298'.


compute colslab( 299 ,1)= 'COL299'.


compute colslab( 300 ,1)= 'COL300'.

compute colslab=colslab(1:ncol(bootres),1).
compute colslab={colslab,conseq,vlabs}.
do if (outscree=1).
print/title = '**************************************************************************'.
print/title='Bootstrap estimates were saved to a file'/space=0.
print colslab/title='Map of column names to model coefficients:'/clabels=' ','Conseqnt','Antecdnt'/format=a8.
end if.
end if.
end if.
do if (criterr=0 and boot > 0 and modelbt=1).
compute labstart=1.
do if (outscree=1).
print/title = '*********** BOOTSTRAP RESULTS FOR REGRESSION MODEL PARAMETERS ************'.
end if.
loop iboot = 1 to (nms+nys).
do if (outscree=1).
print outnames(1,iboot)/title = 'OUTCOME VARIABLE:'/format = A8.
end if.
compute vlabsm=vlabs(labstart:(labstart+(nump(1,iboot)-1)),1).
compute outnmtmp=bootcim(labstart:(labstart+(nump(1,iboot)-1)),:).
compute resultm2=make(nrow(outnmtmp),maxresm,99999).
compute resultm2(1:nrow(outnmtmp),1:ncol(outnmtmp))=outnmtmp.
compute resultm={resultm;resultm2}.
do if (outscree=1).
print bootcim(labstart:(labstart+(nump(1,iboot)-1)),:)/title=' '/rnames=vlabsm/ clabels='Coeff' 'BootMean' 'BootSE' 'BootLLCI' 'BootULCI'/format= F10.4 /space=0.
end if.
compute labstart=labstart+nump(1,iboot).
do if (iboot < (nms+nys)).
do if (outscree=1).
print/title= '----------'.
end if.
end if.
end loop.
end if.
do if (criterr=0 and saveest=1).
compute resultm=resultm(2:nrow(resultm),:).
compute bocaj=(resultm=99999).
compute bocaj=csum(bocaj).
compute bocaj=1-(bocaj=nrow(resultm)).
compute j=1.
loop i = 1 to ncol(resultm).
do if (bocaj(1,i)=1).
compute j=j+1.
end if.
end loop.
compute resultm=resultm(:,1:(j-1)).
save resultm/outfile = *.
end if.
do if (criterr = 0 and matrices=1 and outscree=1).
print/title = '************************ MODEL DEFINITION MATRICES ************************'.
print/title = 'FROM variables are columns, TO variables are rows.'.
compute temp2=make(nrow(bcmat),ncol(bcmat),'0').
loop i = 2 to nrow(bcmat).
loop j = 1 to (ncol(bcmat)-1).
do if (bcmat(i,j)=1).
compute temp2(i,j)='1'.
end if.
do if (j >= i).
compute temp2(i,j)=' '.
end if.
end loop.
end loop.
compute temp2=temp2(2:nrow(bcmat),(1:(ncol(bcmat)-1))).
do if (nms > 0).
compute cmatlabs={xnames,mnames}.
compute rmatlabs={mnames,ynames}.
end if.
do if (nms = 0).
compute cmatlabs={xnames}.
compute rmatlabs={ynames}.
end if.
print temp2/title='BMATRIX: Paths freely estimated (1) and fixed to zero (0):'/cnames=cmatlabs/rnames=rmatlabs/format A3.
compute z=0.
do if (rsum(csum(wcmat))<>0).
compute temp2=make(nrow(wcmat),ncol(wcmat),'0').
loop i = 2 to nrow(wcmat).
loop j = 1 to (ncol(wcmat)-1).
do if (wcmat(i,j)=1).
compute temp2(i,j)='1'.
end if.
do if (j >= i).
compute temp2(i,j)=' '.
end if.
end loop.
end loop.
compute temp2=temp2(2:nrow(wcmat),(1:(ncol(wcmat)-1))).
print temp2/title='WMATRIX: Paths moderated (1) and not moderated (0) by W:'/cnames=cmatlabs/rnames=rmatlabs/format A3.
end if.
do if (rsum(csum(zcmat))<>0).
compute temp2=make(nrow(zcmat),ncol(zcmat),'0').
loop i = 2 to nrow(zcmat).
loop j = 1 to (ncol(zcmat)-1).
do if (zcmat(i,j)=1).
compute temp2(i,j)='1'.
end if.
do if (j >= i).
compute temp2(i,j)=' '.
end if.
end loop.
end loop.
compute temp2=temp2(2:nrow(zcmat),(1:(ncol(zcmat)-1))).
print temp2/title='ZMATRIX: Paths moderated (1) and not moderated (0) by Z:'/cnames=cmatlabs/rnames=rmatlabs/format a3.
end if.
do if (rsum(csum(wzcmat))<>0).
compute temp2=make(nrow(wzcmat),ncol(wzcmat),'0').
loop i = 2 to nrow(wzcmat).
loop j = 1 to (ncol(wzcmat)-1).
do if (wzcmat(i,j)=1).
compute temp2(i,j)='1'.
end if.
do if (j >= i).
compute temp2(i,j)=' '.
end if.
end loop.
end loop.
compute temp2=temp2(2:nrow(wzcmat),(1:(ncol(wzcmat)-1))).
print temp2/title='WZMATRIX: W moderated paths moderated (1) and not moderated (0) by Z:'/cnames=cmatlabs/rnames=rmatlabs/format a3.
end if.
do if (ncs > 0).
print ccmat/title='CMATRIX: Covariates (columns) in (1) and not in (0) the models of M and Y (rows):'/rnames=rmatlabs/cnames=covnames.
end if.
end if.
do if (outscree=1).
do if (activate=0).
print/title = '*********************** ANALYSIS NOTES AND ERRORS ************************'.
end if.
end if.
do if (activate=1).
print/title = '**************************************************************************'.
end if.
do if (criterr=0).
do if (outscree=1).
print conf/title = 'Level of confidence for all confidence intervals in output:'/format = F8.4.
do if (boot > 0).
do if (goodboot = boot and bc=0).
print boot/title='Number of bootstrap samples for percentile bootstrap confidence intervals:'.
end if.
do if (goodboot = boot and bc=1).
print boot/title='Number of bootstrap samples for bias-corrected bootstrap confidence intervals:'.
end if.
do if (booterr = 1).
compute badend = badend(1,2:ncol(badend)).
print/title = 'WARNING: Bootstrap CI endpoints below not trustworthy. Decrease confidence'.
print badend/title='or increase the number of bootstrap samples.'/space=0/format = F10.4.
end if.
end if.
do if (mc > 0).
print mc/title='Number of samples for Monte Carlo confidence intervals:'.
end if.
do if (wnotev > 0 and printw=1).
do if (wnotev=1).
print/title = 'W values in conditional tables are the 16th, 50th, and 84th percentiles.'.
else if (wnotev=2).
do if (minwwarn=0 and maxwwarn=0).
print/title = 'W values in conditional tables are the mean and +/- SD from the mean.'.
end if.
do if (minwwarn=1).
print/title = 'W values in conditional tables are the minimum, the mean, and 1 SD above the mean.'.
end if.
do if (maxwwarn=1).
print/title = 'W values in conditional tables are 1 SD below the mean, the mean, and the maximum.'.
end if.
end if.
end if.
do if (znotev > 0 and printz=1).
do if (znotev=1).
print/title = 'Z values in conditional tables are the 16th, 50th, and 84th percentiles.'.
else if (znotev=2).
do if (minzwarn=0 and maxzwarn=0).
print/title = 'Z values in conditional tables are the mean and +/- SD from the mean.'.
end if.
do if (minzwarn=1).
print/title = 'Z values in conditional tables are the minimum, the mean, and 1 SD above the mean.'.
end if.
do if (maxzwarn=1).
print/title = 'Z values in conditional tables are 1 SD below the mean, the mean, and the maximum.'.
end if.
end if.
end if.
do if (minwwarn > 0).
print/title = 'NOTE: One SD below the mean is below the minimum observed in the data for W,'.
print/title = '      so the minimum measurement on W is used for conditioning instead.'/space=0.
end if.
do if (maxwwarn > 0).
print/title = 'NOTE: One SD above the mean is above the maximum observed in the data for W,'.
print/title = '      so the maximum measurement for W is used for conditioning instead.'/space=0.
end if.
do if (minzwarn > 0).
print/title = 'NOTE: One SD below the mean is below the minimum observed in the data for Z,'.
print/title = '      so the minimum measurement for Z is used for conditioning instead.'/space=0.
end if.
do if (maxzwarn > 0).
print/title = 'NOTE: One SD above the mean is above the maximum observed in the data for Z,'.
print/title = '      so the maximum measurement for Z is used for conditioning instead.'/space=0.
end if.
do if (pstog=1).
print/title= 'NOTE: Standardized coefficients for dichotomous or multicategorical X are in'.
print/title= '      partially standardized form.'/space=0.
end if.
loop i = 1 to 100.
do if (notecode(i,1) = 32).
print/title = 'Direct, indirect, and total effects are counterfactually defined'.
do if (xcontcf=0).
print/title = 'assuming X by M interaction.'/space=0.
end if.
do if (xcontcf=1).
print/title = 'assuming X by M interaction and with the following reference (x_ref)'/space=0.
compute states=t(xrefvals).
print states/title = 'and counterfactual (x_cf) states for X:'/rlabels='x_ref' 'x_cf'/format= F10.4 /space=0.
end if.
end if.
do if (notecode(i,1)=1).
print/title = 'NOTE: COVMY is ignored when using CMATRIX option.'.
end if.
do if (notecode(i,1)=2).
print/title = 'NOTE: Confidence level restricted to between 50 and 99.9999%.  95% confidence is provided in output'.
end if.
do if (notecode(i,1)=3).
print centvar/title = 'NOTE: The following variables were mean centered prior to analysis:'/format = a8.
end if.
do if (notecode(i,1) = 4).
print/title = 'NOTE: A heteroscedasticity consistent standard error and covariance matrix estimator was used.'.
end if.
do if (notecode(i,1) = 5).
print/title = 'NOTE: The HC3 option has been replaced with HC.  See the documentation.'.
end if.
do if (notecode(i,1) = 6).
print/title = 'NOTE: Due to estimation problems, some bootstrap samples had to be replaced.'.
print badboot/title='      The number of times this happened was:'/space=0/format=F8.0.
end if.
do if (notecode(i,1) = 7).
print/title = 'NOTE: The bootstrapping was not completed due to problematic bootstrap samples.'.
print/title = '      Bootstrap confidence intervals are therefore suppressed.'/space=0.
end if.
do if (notecode(i,1) = 8).
print/title = 'NOTE: The number of bootstrap samples was adjusted upward given your desired confidence.'.
end if.
do if (notecode(i,1) = 9).
print/title = 'NOTE: WMODVAL is ignored when W is specified as multicategorical.'.
end if.
do if (notecode(i,1) = 10).
print/title = 'NOTE: ZMODVAL is ignored when Z is specified as multicategorical.'.
end if.
do if (notecode(i,1) = 11).
print/title = 'NOTE: Total effect model generated only when all covariates are specified in all'.
print/title = '      models of M and Y.'/space=0.
end if.
do if (notecode(i,1) = 30).
print/title = 'NOTE: Your vector of linear hypothesis weights is of the wrong length for this model.'.
end if.
do if (notecode(i,1) = 12).
print/title = 'NOTE: Total effect model generated only when X is freely estimated to affect each M'.
print/title = '      and both X and M are freely estimated to affect Y'/space=0.
end if.
do if (notecode(i,1) = 13).
print/title = 'NOTE: There are too many pairwise contrasts to conduct with this model.'.
end if.
do if (notecode(i,1) = 14).
print/title = 'NOTE: The number of contrast weights must equal the number of indirect effects.'.
end if.
do if (notecode(i,1) = 15).
print/title = 'NOTE: Monte Carlo confidence intervals not available for this model.'.
print/title = '      Bootstrapping is used instead.'/space=0.
end if.
do if (notecode(i,1) = 16).
print/title = 'NOTE: The number of Monte Carlo samples was adjusted upward given your desired confidence.'.
end if.
do if (notecode(i,1) = 19).
print/title = 'NOTE: Your contrast matrix is invalid or not applicable to this model.'.
end if.
do if (notecode(i,1) = 20).
print/title = 'NOTE: One of the groups specified by your contrast matrix does not exist in the data.'.
end if.
do if (notecode(i,1) = 21).
print/title = 'NOTE: The VARORDER option is not available in this release.'.
end if.
do if (notecode(i,1) = 22).
print/title = 'NOTE: The VMODVAL and QMODVAL options are not available in this release.'.
end if.
do if (notecode(i,1) = 23).
print/title = 'NOTE: The QUANTILE option is not available in this release.'.
end if.
do if (notecode(i,1) = 24).
print/title = 'NOTE: Total effect model not available with dichotomous Y.'.
end if.
do if (notecode(i,1) = 25).
print/title = 'NOTE: STAND/EFFSIZE options not available with dichotomous Y.'.
end if.
do if ((notecode(i,1) = 26) and nms > 0).
print/title = 'NOTE: Direct and indirect effects of X on Y are on a log-odds metric.'.
end if.
do if (notecode(i,1) = 27).
print/title = 'NOTE: Standardized coefficients are not available for models with moderators.'.
end if.
do if (notecode(i,1) = 28).
print/title = 'NOTE: The contrast option is not available with a multicategorical X.'.
end if.
do if (notecode(i,1) = 31).
do if (nms > 1).
print medmeans/title = 'NOTE: Controlled direct effect(s) estimated at the following mediator values:'/cnames=mnames/format= F10.4.
end if.
do if (nms=1).
print medmeans/title = 'NOTE: Controlled direct effect(s) estimated at the following mediator value:'/format= F10.4.
end if.
end if.
do if (notecode(i,1) = 33).
print/title = 'NOTE: Sobel test is not available with the XMINT option.'.
end if.
do if (notecode(i,1) = 37).
print/title = 'NOTE: The CONTRAST option is not available when using the XMINT option.'.
end if.
do if (notecode(i,1) = 34).
print/title = 'NOTE: Standardized effects are not available when using the XMINT option.'.
end if.
do if (notecode(i,1) = 36).
print/title = 'NOTE: The XREFVAL option is ignored when X is declared as multicategorical.'.
end if.
do if (notecode(i,1) = 35).
print coval/title = 'NOTE: Counterfactual effects estimated at the following covariate values:'/cnames=covnames/format= F10.4.
end if.
do if (notecode(i,1) = 29 and (listmiss=1)).
compute a=missrow.
compute conum=ncol(a).
compute allgood=0.
compute smremain=12.
compute largesti=1.
compute smallrow=0.
do if (conum > 12).
loop ii = 1 to 12.
compute check=(conum/ii).
do if (check = trunc(check)).
compute check2=conum/ii.
compute aok=ii.
do if (aok > 2).
compute allgood=1.
end if.
else.
compute remain=conum-(ii*trunc(check)).
do if (remain <= smremain).
compute smremain=remain.
compute largesti=ii.
compute smallrow=trunc(conum/largesti).
end if.
end if.
end loop.
compute atemp=a(1,1:(aok*check2)).
compute atemp=reshape(atemp,check2,aok).
do if (ncol(atemp) > 2).
print/title='NOTE: Missing data resulted in the deletion of the following row(s) of'.
print atemp/title='      data from the analysis:'/format=F6.0/space=0.
end if.
do if (allgood=0).
compute atemp=a(1,1:(smallrow*largesti)).
compute atemp=reshape(atemp,smallrow,largesti).
compute btemp=a(1,((largesti*smallrow)+1):conum).
print/title='NOTE: Missing data resulted in the deletion of the following row(s) of'.
print atemp/title='      data from the analysis:'/format=F6.0/space=0.
print btemp/title=' '/space=0/format=f6.0.
end if.
else if (conum <= 12).
print/title='NOTE: Missing data resulted in the deletion of the following row(s) of'.
print a/title='      data from the analysis:'/format=F6.0/space=0.
end if.
end if.
end loop.
do if (toomany=1).
print/title='WARNING: Variables names longer than eight characters can produce incorrect output'.
print/title='when some variables in the data file have the same first eight characters. Shorter'/space=0.
print/title='variable names are recommended. By using this output, you are accepting all risk'/space=0.
print/title='and consequences of interpreting or reporting results that may be incorrect.'/space=0.
end if.
end if.
end if.
loop i = 1 to 100.
do if (errcode(i,1)=1).
print/title = 'ERROR: You must specify a Y and an X variable.'.
end if.
do if (errcode(i,1)=2).
print/title = 'ERROR: X, M, or Y variable used more than once or W and Z are the same variable.'.
do if (toomany = 1).
print/title = '       This could be caused by the use of variables names longer'/space=0.
print varnames/title = '       than eight characters. Here are the variables I see:'/space=0/format=A8.
end if.
end if.
do if (errcode(i,1)=3).
print/title = 'ERROR: You have specified more than one variable for W, Y, X, or Z'.
end if.
do if (errcode(i,1)=4).
print/title = 'ERROR: A variable specified as multicategorical has more than nine categories.'.
end if.
do if (errcode(i,1)=5).
print/title = 'ERROR: One of the categories contains only a single case.'.
end if.
do if (errcode(i,1)=6).
print/title = 'ERROR: Invalid model number in this version of PROCESS.'.
end if.
do if (errcode(i,1)=7).
print/title = 'ERROR: Invalid model number.'.
end if.
do if (errcode(i,1)=8).
print/title = 'ERROR: You must specify an M variable for this model.'.
end if.
do if (errcode(i,1)=9).
print/title = 'ERROR: You have specified an M variable in a model that does not use it.'.
end if.
do if (errcode(i,1)=10).
print/title = 'ERROR: You have specified a W variable in a model that does not use it.'.
end if.
do if (errcode(i,1)=11).
print/title = 'ERROR: You have not specified a W variable in a model that requires it.'.
end if.
do if (errcode(i,1)=12).
print/title = 'ERROR: You have specified a Z variable in a model that does not use it.'.
end if.
do if (errcode(i,1)=13).
print/title = 'ERROR: You have not specified a Z variable in a model that requires it.'.
end if.
do if (errcode(i,1)=14).
print/title = 'ERROR: V and Q are not proper specifications in this release of PROCESS.'.
print/title = '       Moderators must be specified as W and/or Z.'/space=0.
end if.
do if (errcode(i,1)=15).
print/title = 'ERROR: One of your model variables exhibits no variation (it is a constant).'.
end if.
do if (errcode(i,1)=16).
print/title = 'ERROR: BMATRIX is not the correct length or is otherwise invalid.'.
end if.
do if (errcode(i,1)=17).
print/title = 'ERROR: WMATRIX is not the correct length or is otherwise invalid.'.
end if.
do if (errcode(i,1)=18).
print/title = 'ERROR: ZMATRIX is not the correct length or is otherwise invalid.'.
end if.
do if (errcode(i,1)=19).
print/title = 'ERROR: WZMATRIX is not the correct length or is otherwise invalid.'.
end if.
do if (errcode(i,1)=20).
print/title = 'ERROR: A path fixed at zero cannot be moderated.'.
end if.
do if (errcode(i,1)=21).
print/title = 'ERROR: If only one moderator is specified, it must be specified as W.'.
end if.
do if (errcode(i,1)=22).
print/title = 'ERROR: In BMATRIX, X must be specified to affect at least one variable.'.
end if.
do if (errcode(i,1)=23).
print/title = 'ERROR: In BMATRIX, at least one variable must be specified to affect Y.'.
end if.
do if (errcode(i,1)=24).
print/title = 'ERROR: You must specify a model number or a custom BMATRIX specification.'.
end if.
do if (errcode(i,1)=25).
print/title = 'ERROR: BMATRIX cannot be used in conjunction with a model number.'.
end if.
do if (errcode(i,1)=26).
print/title = 'ERROR: Your model has a dangling mediator (all Ms must affect and be affected).'.
end if.
do if (errcode(i,1)=27).
print/title = 'ERROR: CLUSTER is not available on this release of PROCESS.'.
end if.
do if (errcode(i,1)=29).
print/title = 'ERROR: CMATRIX is not the correct length or is otherwise invalid.'.
end if.
do if (errcode(i,1)=30).
print/title = 'ERROR: In CMATRIX, all covariates must be assigned to an M or a Y.'.
end if.
do if (errcode(i,1)=31).
print/title = 'ERROR: A linear or near linear dependency (singularity) exists in the data.'.
end if.
do if (errcode(i,1)=32).
print/title = 'ERROR: Models 80 and 81 require between 3 and 6 mediators.'.
end if.
do if (errcode(i,1)=33).
print/title = 'ERROR: Model 82 requires 4 mediators.'.
end if.
do if (errcode(i,1)=34).
print/title = 'ERROR: This model number requires between 2 and 6 mediators.'.
end if.
do if (errcode(i,1)=35).
print/title = 'ERROR: In a model with only one moderator, that moderator must be W.'.
end if.
do if (errcode(i,1)=36).
print/title = 'ERROR: A serial mediation model cannot have more than 6 mediators.'.
end if.
do if (errcode(i,1)=37).
print/title = 'ERROR: No more than 10 mediators are allowed in a PROCESS command.'.
end if.
do if (errcode(i,1)=38).
print/title = 'ERROR: XCATCODE is not provided, not the correct length, or is otherwise invalid.'.
end if.
do if (errcode(i,1)=39).
print/title = 'ERROR: WCATCODE is not provided, not the correct length, or is otherwise invalid.'.
end if.
do if (errcode(i,1)=40).
print/title = 'ERROR: ZCATCODE is not provided, not the correct length, or is otherwise invalid.'.
end if.
do if (errcode(i,1)=41).
print/title = 'ERROR: Models 1, 2, and 3 cannot be customized.'.
end if.
do if (errcode(i,1)=42).
print/title = 'ERROR: WS option available only in PROCESS v2. Or use the MEMORE macro instead.'.
print/title = '       MEMORE can be downloaded from www.akmontoya.com.'/space=0.
end if.
do if (errcode(i,1)=43).
print/title = 'ERROR: PROCESS does not allow dichotomous mediators.'.
end if.
do if (errcode(i,1)=60).
print/title = 'PROCESS is now ready for use.'.
print/title='Copyright 2017-2022 by Andrew F. Hayes. ALL RIGHTS RESERVED.'/space=0.
print/title='Workshop schedule available at http://haskayne.ucalgary.ca/CCRAM'/space=0.
end if.
do if (errcode(i,1)=50).
print/title = 'ERROR: A multicategorical moderator cannot be specified as a covariate.'.
end if.
do if (errcode(i,1)=51).
print/title = 'ERROR: A variable you specified as a covariate is a moderator in all equations.'.
end if.
do if (errcode(i,1)=52 and mcerpt=0).
compute mcerpt=1.
print/title = 'ERROR: A variable specified as multicategorical must have at least three categories'.
end if.
do if (errcode(i,1) = 47).
do if (iterrmod=1).
print/title = 'ERROR: Iteration for Y model didn''t converge to a solution. Interpret results with caution.'.
print/title = '       Try increasing the number of iterations, though this will slow down computation.'/space=0.
end if.
do if (bootiter=1).
print/title = 'ERROR: Nonconvergence during bootstrapping. Interpet bootstrap results with caution.'.
end if.
end if.
do if (errcode(i,1) = 61).
print/title = 'ERROR: Variable names should be no more than eight characters in length.'.
end if.
do if (errcode(i,1) = 62).
print/title = 'ERROR: After listwise deletion of cases with missing data, too few cases remain.'.
end if.
do if (errcode(i,1) = 63).
print/title = 'ERROR: The XMINT option is available only for model 4.'.
end if.
do if (errcode(i,1) = 64).
print/title = 'ERROR: Incorrect number of values specified in CDEVAL option.'.
end if.
do if (errcode(i,1) = 65).
print/title = 'ERROR: Only indicator or sequential coding of X is allowed with the XMINT option.'.
end if.
do if (errcode(i,1) = 66).
print/title = 'ERROR: XMINT option not available with continuous X using PROCESS GUI. Use syntax.'.
end if.
do if (errcode(i,1) = 67).
print/title = 'ERROR: Too many elements provided in XREFVAL option.'.
end if.
do if (errcode(i,1)=68).
print/title = 'ERROR: Covariate assignment is not allowed with XMINT option.'.
end if.
do if (errcode(i,1) = 69).
print/title = 'ERROR: Incorrect number of values specified in COVAL option.'.
end if.
do if (errcode(i,1) = 70).
print/title = 'ERROR: Incorrect value(s) in XREFVAL for this dichotomous X variable.'.
end if.
do if (errcode(i,1) = 71).
print/title = 'ERROR: The CENTER option is not available when using the XMINT option.'.
end if.
do if (errcode(i,1) = 72).
print/title = 'ERROR: The XMINT option is not available for models with a dichotomous Y.'.
end if.
end loop.
END MATRIX.	
Ressources	Temps de processeur	00:00:06,59	
	Temps écoulé	00:00:06,59	


Run MATRIX procedure:

***************** PROCESS Procedure for SPSS Version 4.2 *****************

          Written by Andrew F. Hayes, Ph.D.       www.afhayes.com
    Documentation available in Hayes (2022). www.guilford.com/p/hayes3

**************************************************************************
Model  : 4
    Y  : HOMAIR
    X  : FGDS
   M1  : TG
   M2  : HDL
   M3  : LDL
   M4  : TC

Sample
Size:  169

**************************************************************************
OUTCOME VARIABLE:
 TG

Model Summary
          R       R-sq        MSE          F        df1        df2          p
      ,0763      ,0058  4028,0808      ,9773     1,0000   167,0000      ,3243

Model
              coeff         se          t          p       LLCI       ULCI
constant   104,2896    17,0825     6,1050      ,0000    70,5640   138,0151
FGDS        -3,8575     3,9020     -,9886      ,3243   -11,5611     3,8462

Standardized coefficients
          coeff
FGDS     -,0763

**************************************************************************
OUTCOME VARIABLE:
 HDL

Model Summary
          R       R-sq        MSE          F        df1        df2          p
      ,1236      ,0153   175,4148     2,5906     1,0000   167,0000      ,1094

Model
              coeff         se          t          p       LLCI       ULCI
constant    35,6614     3,5648    10,0037      ,0000    28,6235    42,6993
FGDS         1,3106      ,8143     1,6095      ,1094     -,2970     2,9182

Standardized coefficients
          coeff
FGDS      ,1236

**************************************************************************
OUTCOME VARIABLE:
 LDL

Model Summary
          R       R-sq        MSE          F        df1        df2          p
      ,0148      ,0002  1189,7893      ,0366     1,0000   167,0000      ,8485

Model
              coeff         se          t          p       LLCI       ULCI
constant    78,0159     9,2841     8,4032      ,0000    59,6867    96,3452
FGDS         -,4058     2,1207     -,1913      ,8485    -4,5926     3,7810

Standardized coefficients
          coeff
FGDS     -,0148

**************************************************************************
OUTCOME VARIABLE:
 TC

Model Summary
          R       R-sq        MSE          F        df1        df2          p
      ,0046      ,0000  1451,9007      ,0035     1,0000   167,0000      ,9527

Model
              coeff         se          t          p       LLCI       ULCI
constant   135,1796    10,2558    13,1807      ,0000   114,9317   155,4274
FGDS          ,1391     2,3427      ,0594      ,9527    -4,4859     4,7642

Standardized coefficients
          coeff
FGDS      ,0046

**************************************************************************
OUTCOME VARIABLE:
 HOMAIR

Model Summary
          R       R-sq        MSE          F        df1        df2          p
      ,3750      ,1406    23,0424     5,3329     5,0000   163,0000      ,0001

Model
              coeff         se          t          p       LLCI       ULCI
constant    -1,3248     2,0162     -,6571      ,5121    -5,3061     2,6565
FGDS          ,3555      ,2981     1,1928      ,2347     -,2330      ,9441
TG            ,0443      ,0097     4,5627      ,0000      ,0251      ,0635
HDL           ,1216      ,0431     2,8216      ,0054      ,0365      ,2067
LDL           ,0938      ,0356     2,6354      ,0092      ,0235      ,1642
TC           -,0887      ,0354    -2,5079      ,0131     -,1586     -,0189

Standardized coefficients
          coeff
FGDS      ,0875
TG        ,5516
HDL       ,3173
LDL       ,6329
TC       -,6608

************************** TOTAL EFFECT MODEL ****************************
OUTCOME VARIABLE:
 HOMAIR

Model Summary
          R       R-sq        MSE          F        df1        df2          p
      ,0722      ,0052    26,0333      ,8753     1,0000   167,0000      ,3509

Model
              coeff         se          t          p       LLCI       ULCI
constant     2,9646     1,3733     2,1587      ,0323      ,2533     5,6758
FGDS          ,2935      ,3137      ,9356      ,3509     -,3258      ,9128

Standardized coefficients
          coeff
FGDS      ,0722


************** TOTAL, DIRECT, AND INDIRECT EFFECTS OF X ON Y **************

Total effect of X on Y
     Effect         se          t          p       LLCI       ULCI       c_cs
      ,2935      ,3137      ,9356      ,3509     -,3258      ,9128      ,0722

Direct effect of X on Y
     Effect         se          t          p       LLCI       ULCI      c'_cs
      ,3555      ,2981     1,1928      ,2347     -,2330      ,9441      ,0875

Indirect effect(s) of X on Y:
          Effect     BootSE   BootLLCI   BootULCI
TOTAL     -,0620      ,1442     -,4252      ,1821
TG        -,1710      ,1983     -,6389      ,1438
HDL        ,1594      ,2420     -,0688      ,8262
LDL       -,0381      ,4657    -1,2438      ,6137
TC        -,0123      ,4162     -,9079      ,7995

Completely standardized indirect effect(s) of X on Y:
          Effect     BootSE   BootLLCI   BootULCI
TOTAL     -,0153      ,0355     -,1057      ,0407
TG        -,0421      ,0494     -,1601      ,0331
HDL        ,0392      ,0564     -,0173      ,1970
LDL       -,0094      ,1107     -,2857      ,1569
TC        -,0030      ,0991     -,2165      ,1852

*********************** ANALYSIS NOTES AND ERRORS ************************

Level of confidence for all confidence intervals in output:
  95,0000

Number of bootstrap samples for percentile bootstrap confidence intervals:
  5000

------ END MATRIX -----


* Encoding: UTF-8.
preserve.
set printback=off.


Matrice


Remarques	
Sortie obtenue	10-NOV-2024 17:02:44	
Commentaires		
Entrée	Données	C:\Users\Brice SAHA\Documents\UBa\Master Thesis 2021\Fanuel\Database fanuel.sav	
	Jeu de données actif	Jeu_de_données1	
	Filtre	<sans>	
	Pondération	<sans>	
	Scinder un fichier	<sans>	
	N de lignes dans le fichier de travail	169	
Syntaxe	MATRIX.
compute wnames='xxxxx'.
compute znames='xxxxx'.
compute mcerpt=0.
compute wiscov=0.
compute ziscov=0.
compute tooman=0.
compute errcode=make(100,1,0).
compute notecode=make(100,1,0).
compute model = trunc( 4 ).
compute iterate = abs(trunc( 100 )).
compute converge = abs( 0.00001 ).
compute itprobtg=0.
compute v2tag=0.
compute ydich=0.
compute maxwwarn=0.
compute minwwarn=0.
compute maxzwarn=0.
compute minzwarn=0.
compute toomany=0.
compute wdich=0.
compute zdich=0.
compute wnotev=0.
compute znotev=0.
compute nxpval=1.
compute nwpval=1.
compute nzpval=1.
compute errs=1.
compute notes=1.
compute criterr=0.
compute novar=0.
compute adjust=0.
compute ncs=0.
compute serial=0.
compute sobelok=0.
compute hasw=0.
compute hasz=0.
compute printw=0.
compute printz=0.
compute xmint=( 0 =1).
compute wmodcust=0.
compute zmodcust=0.
compute booting=0.
compute bootiter=0.
compute iterrmod=0.
compute cov = 'xxxxx'.
compute varorder=( 0 <> 0).
compute nws=0.
compute w= 'xxxxx'.
compute nzs=0.
compute z = 'xxxxx'.
compute nms=0.
compute m = 'TG HDL LDL TC'.
compute nys=0.
compute y = 'HOMAIR'.
compute nxs=0.
compute x = 'ALL5'.
compute effsize=( 0 =1).
compute stand=( 1 =1).
compute intprobe = .1.
compute xrefvals={ 999 }.
compute center=trunc( 0 ).
compute xcontcf=0.
compute xscaling=1.
compute cdeval={ -999 }.
compute cuscoval=0.
do if (model=74).
compute errcode(errs,1)=7.
compute errs=errs+1.
compute criterr=1.
end if.
do if (xmint=1 and model <> 4).
compute errcode(errs,1)=63.
compute errs=errs+1.
compute criterr=1.
end if.
do if (xmint=1 and model=4).
compute w=x.
compute model=74.
compute intprobe=1.
compute notecode(notes,1) = 32.
compute notes = notes + 1.
do if (effsize=1 or stand=1).
compute notecode(notes,1) = 34.
compute notes = notes + 1.
compute stand=0.
compute effsize=0.
end if.
do if (center <> 0).
compute center=0.
compute errcode(errs,1)=71.
compute errs=errs+1.
compute criterr=1.
end if.
end if.
compute v = 'xxxxx'.
compute q = 'xxxxx'.
compute linsum={ -999 }.
compute nlinsum=ncol(linsum).
do if (linsum(1,1) = -999).
compute nlinsum=0.
end if.
compute oldvars= 'xxxxx'.
compute mcxok=0.
compute mcwok=0.
compute mczok=0.
compute xprod=0.
compute zprod=0.
compute wprod=0.
compute modcok=0.
compute alttotal=0.
compute hc3=trunc( 0 ).
compute jn=( 0 = 1).
compute listmiss=( 0 =1).
compute modelres=( 0 =1).
compute outscree=( 1 =1).
compute activate=( 0 =1).
compute booterr=0.
compute normal=( 0 =1).
compute xmtest=( 0 =1).
compute describe=( 0 =1).
compute longname=( 0 =1).
do if (stand=1).
compute effsize=1.
end if.
compute pstog=0.
compute sobelok=0.
compute normal=( 0 =1).
compute mdichok=( 0 =1).
compute contrast={ 999 }.
compute ncontr=ncol(contrast).
compute ncontrow=nrow(contrast).
do if (contrast(1,1) = 999).
compute ncontr=1.
compute contrast=0.
end if.
do if (ncontr = 1).
compute contrast=trunc(contrast).
do if (contrast > 3 or contrast < 0)).
compute ncontr=1.
compute contrast = 0.
end if.
end if.
do if (ncontr > 1).
compute contvec=contrast.
compute contrast=4.
do if (ncontrow > 1).
compute contrast=0.
compute modcok=1.
compute wcontval=contvec(:,1).
compute zcontval=contvec(:,2).
do if ((ncontr <> 2) or (ncontrow <> 2)).
compute notecode(notes,1) = 19.
compute notes = notes + 1.
compute modcok=0.
end if.
end if.
end if.
do if (xmint = 1 and contrast(1,1) <> 0).
compute contrast=0.
compute notecode(notes,1) = 37.
compute notes = notes + 1.
end if.
do if (varorder = 1).
compute notecode(notes,1) = 21.
compute notes = notes + 1.
end if.
do if ( 999 <> 999 or 999 <> 999).
compute notecode(notes,1) = 22.
compute notes = notes + 1.
end if.
compute modelbt=( 0 =1).
compute cluster= 'xxxxx'.
compute matrices=( 0 =1).
compute covcoeff=( 0 =1).
compute covmy=trunc( 0 ).
do if (covmy < 0 or covmy > 2).
compute covmy = 0.
end if.
compute boot = abs(trunc( 5000 )).
compute bc=( 0 =1).
compute mc=abs(trunc( 0 )).
compute hc=trunc( 5 ).
do if (intprobe < 0 or intprobe > 1).
compute intprobe = .10.
end if.
compute plot=trunc( 0 ).
do if (plot < 0 or plot > 2).
compute plot=0.
end if.
compute total=( 1 =1).
compute dototal=0.
compute saveboot = ( 0 = 1).
compute saveest=( 0 = 2).
do if (saveest=1).
compute intprobe=1.
end if.
do if (hc >= 0 and hc < 5).
compute notecode(notes,1) = 4.
compute notes = notes + 1.
end if.
do if (hc > 5 or hc < 0).
compute hc=5.
end if.
compute mcw=trunc( 0 ).
compute mcz=trunc( 0 ).
compute mcx=trunc( 0 ).
do if (mcx > 0 and mcx < 3 and model = 74).
compute mcw=mcx.
compute xscaling=1.
end if.
do if (mcx > 2 and model = 74).
compute errcode(errs,1)=65.
compute errs=errs+1.
compute criterr=1.
end if.
do if (model = 74 and normal=1).
compute notecode(notes,1) = 33.
compute normal=0.
compute notes = notes + 1.
end if.
do if (mcx > 0 and contrast > 0).
compute notecode(notes,1) = 28.
compute notes = notes + 1.
compute contrast=0.
end if.
compute nxvls=1.
compute nmvls=1.
compute nwvls=1.
compute nzvls=1.
compute paths=999.
compute pathsw=999.
compute pathsz=999.
compute pathswz=999.
compute pathsmod=999.
compute pathtype=999.
compute obscoeff=999.
compute pathsdv={' '}.
compute quantile=1.
do if ( 999 <>999).
compute notecode(notes,1) = 23.
compute notes = notes + 1.
end if.
compute moments=( 0 =1).
do if (moments=1).
compute quantile=0.
end if.
compute bmatrix={ -999 }.
compute wmatrix={ -999 }.
compute zmatrix={ -999 }.
compute wzmatrix={ -999 }.
compute cmatrix={ -999 }.
compute xcatcode={ -999 }.
compute wcatcode={ -999 }.
compute zcatcode={ -999 }.
compute needed=0.
compute conf= 95.
do if (trunc( 95 ) >= 100 or (trunc( 95 ) <= 50)).
compute conf = 95.
compute notecode(notes,1)=2.
compute notes=notes+1.
end if.
do if (model >= 0 and model < 4 and modelbt=0).
compute boot=0.
compute mc=0.
compute bc=0.
end if.
do if (mc > 0 and boot > 0).
compute boot=0.
compute bc=0.
end if.
do if ((boot < 1000) and (mc = 0) and (boot > 0)).
compute boot=5000.
end if.
do if ((mc < 1000) and (boot = 0) and (mc > 0)).
compute mc=5000.
end if.
compute p0=-.322232431088.
compute p1 = -1.
compute p2 = -.342242088547.
compute p3 = -.0204231210245.
compute p4 = -.0000453642210148.
compute q0 = .0993484626060.
compute q1 = .588581570495.
compute q2 = .531103462366.
compute q3 = .103537752850.
compute q4 = .0038560700634.
compute badend=0.
compute priorlo = -9999999.
compute priorhi = 9999999.
compute alpha2 = (1-(conf/100))/2.
compute cilm=alpha2*2.
compute y5=sqrt(-2*ln(alpha2)).
compute xp2=(y5+((((y5*p4+p3)*y5+p2)*y5+p1)*y5+p0)/((((y5*q4+q3)*y5+q2)*y5+q1)*y5+q0)).
compute medlb={'   M1  :';'   M2  :';'   M3  :';'   M4  :';'   M5  :';'   M6  :';'   M7  :';'   M8  :';'   M9  :';'   M10 :'}.
compute medlb2={'(M1)','(M2)','(M3)','(M4)','(M5)','(M6)','(M7)','(M8)','(M9)','(M10)'}.
compute xlb={'   X1  :';'   X2  :';'   X3  :';'   X4  :';'   X5  :';'   X6  :';'   X7  :';'   X8  :';'   X9  :'}.
compute highlbw={'M1*W'; 'M2*W'; 'M3*W'; 'M4*W'; 'M5*W'; 'M6*W'; 'M7*W'; 'M8*W'; 'M9*W'; 'M10*W'}.
do if (xmint=1).
compute highlbw={'M1*X'; 'M2*X'; 'M3*X'; 'M4*X'; 'M5*X'; 'M6*X'; 'M7*X'; 'M8*X'; 'M9*X'; 'M10*X'}.
end if.
compute highlbz={'M1*Z'; 'M2*Z'; 'M3*Z'; 'M4*Z'; 'M5*Z'; 'M6*Z'; 'M7*Z'; 'M8*Z'; 'M9*Z';'M10*Z'}.
compute highlbwz={'M1*W*Z'; 'M2*W*Z'; 'M3*W*Z'; 'M4*W*Z'; 'M5*W*Z'; 'M6*W*Z'; 'M7*W*Z'; 'M8*W*Z'; 'M9*W*Z';'M10*W*Z'}.
compute highlbbt={'BOTH(M1)'; 'BOTH(M2)'; 'BOTH(M3)'; 'BOTH(M4)'; 'BOTH(M5)'; 'BOTH(M6)'; 'BOTH(M7)'; 'BOTH(M8)'; 'BOTH(M9)';'BTH(M10)'}.
compute highlbx={'M1*X'; 'M2*X'; 'M3*X'; 'M4*X'; 'M5*X'; 'M6*X'; 'M7*X'; 'M8*X'; 'M9*X'; 'M10*X'}.
compute skipwz=0.
compute validm={1,1,1,1,1,1,1,1,1,1,1,1,1,1,1,1,1,1,1,1,1,1,0,0,0,0,0,1,1,0,0,0,0, 0,0,0,0,0,0,0,0,0,0,0,0,0,0,0,0,0,0,0,0,0,0,0,0,1,1,1,1,1,1,1,1,1,1,1,1,1,1,1,1,1,1,1,0,0,0,1,1, 1,1,1,1,1,1,1,1,1,1,1}.
do if (activate=1).
compute errcode(errs,1)=60.
compute errs=errs+1.
compute criterr=1.
end if.
do if (criterr=0).
do if (( 0 =1)=1).
compute errcode(errs,1)=42.
compute errs=errs+1.
compute criterr=1.
end if.
do if (model > 0 and model < 93).
do if (validm(1,model)=0).
compute errcode(errs,1)=6.
compute errs=errs+1.
compute criterr=1.
end if.
release validm.
end if.
do if ((model > 92 or model < 0) and model <> 999)).
compute errcode(errs,1)=7.
compute errs=errs+1.
compute criterr=1.
end if.
do if (model = 999 and bmatrix(1,1)=-999).
compute errcode(errs,1)=24.
compute errs=errs+1.
compute criterr=1.
end if.
do if (model <> 999 and bmatrix(1,1) <> -999).
compute errcode(errs,1)=25.
compute errs=errs+1.
compute criterr=1.
end if.
do if ((model = 74 or (model > 0 and model < 4)) and ((wmatrix(1,1) <> -999) or (zmatrix(1,1)<>-999) or (wzmatrix(1,1)<>-999)))).
compute errcode(errs,1)=41.
compute errs=errs+1.
compute criterr=1.
end if.
do if (hc3 <> 0).
compute notecode(notes,1) = 5.
compute notes = notes + 1.
do if (hc3 = 1).
compute hc=3.
end if.
end if.
do if ((v <> 'xxxxx') or (q <> 'xxxxx')).
compute errcode(errs,1)=14.
compute errs=errs+1.
compute errcode(errs,1)=48.
compute errs=errs+1.
compute criterr=1.
end if.
do if (oldvars <> 'xxxxx').
compute errcode(errs,1)=48.
compute errs=errs+1.
compute criterr=1.
end if.
do if (cluster <> 'xxxxx').
compute errcode(errs,1)=27.
compute errs=errs+1.
compute criterr=1.
end if.
do if ((y = 'xxxxx') or (x = 'xxxxx')).
compute errcode(errs,1)=1.
compute errs=errs+1.
compute criterr=1.
end if.
do if ((m = 'xxxxx') and model > 3).
compute errcode(errs,1)=8.
compute errs=errs+1.
compute criterr=1.
end if.
end if.
do if (criterr=0).
get ytmp/variables = HOMAIR /names = ynames/MISSING = 99999.
compute nys=ncol(ytmp).
compute needed=nys.
compute n=nrow(ytmp).
compute varnames={ynames}.
compute dat=ytmp.

.


do if (toomany=1 and longname=0).
compute criterr=1.
do if (tooman=0).
compute tooman=1.
compute errcode(errs,1) = 61.
compute errs = errs + 1.
end if.
end if
.
compute modelvar={ '4' ;t(ynames)}.
do if ( 4 =999).
compute modelvar(1,1)='CUSTOM'.
end if.
get xtmp/variables = ALL5 /names = xnames/MISSING = 99999.
compute nxs=ncol(xtmp).
compute n=nrow(xtmp).
compute needed=needed+nxs.
compute varnames={varnames,xnames}.
compute xcatlab=t(xnames).
compute dat={dat,xtmp}.

.


do if (toomany=1 and longname=0).
compute criterr=1.
do if (tooman=0).
compute tooman=1.
compute errcode(errs,1) = 61.
compute errs = errs + 1.
end if.
end if
.
compute modelvar={modelvar;t(xnames)}.
do if (nxs = 1).
compute modelvlb={'Model  :';'    Y  :';'    X  :'}.
else.
compute modelvlb={'Model  :';'    Y  :';xlb(1:nxs,1)}.
end if.
do if (m <> 'xxxxx').
get mtmp/variables = TG HDL LDL TC /names = mnames/MISSING = 99999.
compute nms=ncol(mtmp).
compute mprod=make(1,nms,0).
compute n=nrow(mtmp).
compute needed=needed+nms.
compute varnames={varnames,mnames}.
compute dat={dat,mtmp}.
compute modelvar={modelvar;t(mnames)}.
compute x2m=make(99,nms,0).
compute m2y=make(99,nms,0).
compute onem=make(nms,1,1).

.


do if (toomany=1 and longname=0).
compute criterr=1.
do if (tooman=0).
compute tooman=1.
compute errcode(errs,1) = 61.
compute errs = errs + 1.
end if.
end if
.
do if (nms > 1 and nms < 11).
compute modelvlb={modelvlb;medlb(1:nms,1)}.
else.
compute modelvlb={modelvlb;'    M  :'}.
end if.
do if (nms > 0 and model < 4).
compute errcode(errs,1)=9.
compute errs=errs+1.
do if (model <> 0).
compute errcode(errs,1)=48.
compute errs=errs+1.
end if.
compute criterr=1.
end if.
end if.
compute wlocatet=0.
compute wlocate=0.
do if (w <> 'xxxxx').
do if (xmint=0).
get wtmp/variables = xxxxx /names = wnames/MISSING = 99999.

.


do if (toomany=1 and longname=0).
compute criterr=1.
do if (tooman=0).
compute tooman=1.
compute errcode(errs,1) = 61.
compute errs = errs + 1.
end if.
end if
.
end if.
do if (xmint=1).
get wtmp/variables = ALL5 /names = wnames/MISSING = 99999.
end if.
compute nws=ncol(wtmp).
compute n=nrow(wtmp).
compute varnames={varnames,wnames}.
compute wlocate=ncol(varnames).
do if (model=74).
compute wlocatet=1.
do if (xnames <> wnames).
compute errcode(errs,1)=45.
compute errs=errs+1.
compute criterr=1.
end if.
end if.
compute wcatlab=t(wnames).
compute dat={dat,wtmp}.
do if (xmint <> 1).
compute modelvar={modelvar;t(wnames)}.
compute modelvlb={modelvlb;'    W  :'}.
end if.
end if.
do if (z <> 'xxxxx').
get ztmp/variables = xxxxx /names = znames/MISSING = 99999.
compute nzs=ncol(ztmp).
compute n=nrow(ztmp).

.


do if (toomany=1 and longname=0).
compute criterr=1.
do if (tooman=0).
compute tooman=1.
compute errcode(errs,1) = 61.
compute errs = errs + 1.
end if.
end if
.
compute varnames={varnames,znames}.
compute zcatlab=t(znames).
compute dat={dat,ztmp}.
compute modelvar={modelvar;t(znames)}.
compute modelvlb={modelvlb;'    Z  :'}.
end if.
do if (cov <> 'xxxxx').
get ctmp/variables = xxxxx /names = covnames/MISSING = 99999.
compute ncs=ncol(ctmp).
compute n=nrow(ctmp).

.


do if (toomany=1 and longname=0).
compute criterr=1.
do if (tooman=0).
compute tooman=1.
compute errcode(errs,1) = 61.
compute errs = errs + 1.
end if.
end if
.
compute varnames={varnames,covnames}.
compute dat={dat,ctmp}.
end if.
do if (nws > 1 or nzs > 1 or nys > 1 or nxs > 1).
compute errcode(errs,1)=3.
compute errs=errs+1.
compute criterr=1.
end if.
do if ((model = 80 or model = 81) and (nms < 3 or nms > 6)).
compute errcode(errs,1)=32.
compute errs=errs+1.
compute criterr=1.
end if.
do if (model = 82 and nms <> 4).
compute errcode(errs,1)=33.
compute errs=errs+1.
compute criterr=1.
end if.
do if (nms > 10).
compute errcode(errs,1)=37.
compute errs=errs+1.
compute criterr=1.
end if.
do if ((model = 6 or (model > 82 and model < 999)) and (nms < 2 or nms > 6)).
compute errcode(errs,1)=34.
compute errs=errs+1.
compute criterr=1.
end if.
compute match=0.
compute match2=0.
compute mcwzcov=0.
loop i = 1 to (ncol(varnames)-1).
loop j = (i+1) to ncol(varnames).
do if (varnames(i)=varnames(j)).
do if (i < (nxs+nms+nys+1)).
compute match2=match2+1.
end if.
do if (wlocatet=1 and i=2 and j=wlocate).
compute match2=match2-1.
end if.
do if ((wnames=znames) and (nws > 0 or nzs > 0))).
compute match2=match2+1.
end if.
do if (i < (ncol(varnames)-ncs+1)) and j > (ncol(varnames)-ncs)).
do if ((varnames(j)=wnames) and mcw=0)).
compute match=0.
compute wiscov=(j-(ncol(varnames)-ncs)).
end if.
do if ((varnames(j)=wnames) and mcw <>0)).
compute mcwzcov=1.
end if.
do if ((varnames(j)=znames) and mcz=0).
compute match=0.
compute ziscov=(j-(ncol(varnames)-ncs)).
end if.
do if ((varnames(j)=znames) and mcz<>0)).
compute mcwzcov=1.
end if.
end if.
end if.
end loop.
end loop.
do if (match2>0 or match=1).
compute errcode(errs,1)=2.
compute errs=errs+1.
compute criterr=1.
end if.
do if (mcwzcov=1).
compute errcode(errs,1)=50.
compute errs=errs+1.
compute criterr=1.
end if.
compute ninit=nrow(dat).
compute rownum=make(ninit,1,0).
loop i = 1 to ninit.
compute rownum(i,1)=i.
end loop.
compute dat={rownum,dat}.
compute j=1.
compute missrow=0.
loop i = 1 to n.
do if (rsum(dat(i,2:ncol(dat))=99999)=0).
compute dat(j,:)=dat(i,:).
compute j=j+1.
else.
compute missrow={missrow;dat(i,1)}.
end if.
end loop.
do if (j < 5).
compute errcode(errs,1)=62.
compute errs=errs+1.
compute criterr=1.
end if.
do if (criterr=0).
compute rownum=dat(1:(j-1),1).
do if (nrow(missrow) > 1).
compute missrow=t(missrow(2:nrow(missrow),1)).
compute notecode(notes,1) = 29.
compute notes = notes + 1.
end if.
compute dat=dat(1:(j-1),2:ncol(dat)).
compute n=nrow(dat).
compute nmiss=ninit-n.
compute ytmp=dat(:,1:nys).

.
compute desctmp=make((8-(4* 0 )),ncol( ytmp ),-999).
loop jd=1 to ncol( ytmp ).
compute descdat= ytmp (:,jd).
compute desctmp(1,jd) = csum(descdat)/nrow(descdat).
compute desctmp(2,jd) = (nrow(descdat)*sscp(descdat))-(t(csum(descdat))*(csum(descdat))).
compute desctmp(2,jd) = sqrt(desctmp(2,jd)/(nrow(descdat)*(nrow(descdat)-1))).
compute desctmp(3,jd)=cmin(descdat).
compute desctmp(4,jd)=cmax(descdat).
do if ( 0 =0).
compute minwarn=0.
compute maxwarn=0.
do if ((desctmp(3,jd)=desctmp(4,jd)) and novar=0).
compute errcode(errs,1)=15.
compute errs=errs+1.
compute criterr=1.
compute novar=1.
end if.
compute tmp=((descdat(:,1)=desctmp(3,jd))+(descdat(:,1)=desctmp(4,jd))).
compute desctmp(8,jd)=(csum(tmp)=nrow(tmp)).
compute tmp = descdat.
compute tmp(GRADE(descdat),:) = descdat.
compute descdat = tmp.
release tmp.
compute decval={.16;.5;.84}.
loop kd=1 to 3.
compute low=trunc(decval(kd,1)*(nrow(descdat)+1)).
compute lowdec=decval(kd,1)*(nrow(descdat)+1)-low.
compute value=descdat(low,1)+(descdat((low+1),1)-descdat(low,1))*lowdec.
compute desctmp((4+kd),jd)=value.
end loop.
compute mnotev=1.
compute modvals=desctmp(5:7,:).
do if (quantile <> 1).
compute desctmp(5,jd)=desctmp(1,jd)-desctmp(2,jd).
compute desctmp(6,jd)=desctmp(1,jd).
compute desctmp(7,jd)=desctmp(1,jd)+desctmp(2,jd).
compute modvals=desctmp(5:7,:).
compute mnotev=2.
do if (modvals(1,1) < desctmp(3,1)).
compute modvals(1,1)=desctmp(3,1).
compute minwarn=1.
end if.
do if (modvals(3,1) > desctmp(4,1)).
compute modvals(3,1)=desctmp(4,1).
compute maxwarn=1.
end if.
end if.
do if (desctmp(8,1)=1).
compute modvals={desctmp(3,1);desctmp(4,1)}.
compute mnotev=0.
compute minwarn=0.
compute maxwarn=0.
end if.
end if.
end loop
.
compute ysd=desctmp(2,:).
compute ovsd=ysd.
do if (desctmp(8,1)=1).
compute ydich=1.
do if (total=1).
compute total=0.
compute notecode(notes,1) = 24.
compute notes = notes + 1.
end if.
do if (effsize=1).
compute effsize=0.
compute notecode(notes,1) = 25.
compute notes = notes + 1.
end if.
do if (model=74).
compute errcode(errs,1) = 72.
compute errs=errs+1.
compute criterr=1.
end if.
compute omx = cmax(ytmp).
compute omn = cmin(ytmp).
compute ytmp = (ytmp = omx).
compute dat(:,1:nys)=(dat(:,1:nys)=omx).
compute rcd = {omn, 0; omx, 1}.
end if.
compute xtmp=dat(:,(nys+1):(nys+nxs)).

.
compute desctmp=make((8-(4* 0 )),ncol( xtmp ),-999).
loop jd=1 to ncol( xtmp ).
compute descdat= xtmp (:,jd).
compute desctmp(1,jd) = csum(descdat)/nrow(descdat).
compute desctmp(2,jd) = (nrow(descdat)*sscp(descdat))-(t(csum(descdat))*(csum(descdat))).
compute desctmp(2,jd) = sqrt(desctmp(2,jd)/(nrow(descdat)*(nrow(descdat)-1))).
compute desctmp(3,jd)=cmin(descdat).
compute desctmp(4,jd)=cmax(descdat).
do if ( 0 =0).
compute minwarn=0.
compute maxwarn=0.
do if ((desctmp(3,jd)=desctmp(4,jd)) and novar=0).
compute errcode(errs,1)=15.
compute errs=errs+1.
compute criterr=1.
compute novar=1.
end if.
compute tmp=((descdat(:,1)=desctmp(3,jd))+(descdat(:,1)=desctmp(4,jd))).
compute desctmp(8,jd)=(csum(tmp)=nrow(tmp)).
compute tmp = descdat.
compute tmp(GRADE(descdat),:) = descdat.
compute descdat = tmp.
release tmp.
compute decval={.16;.5;.84}.
loop kd=1 to 3.
compute low=trunc(decval(kd,1)*(nrow(descdat)+1)).
compute lowdec=decval(kd,1)*(nrow(descdat)+1)-low.
compute value=descdat(low,1)+(descdat((low+1),1)-descdat(low,1))*lowdec.
compute desctmp((4+kd),jd)=value.
end loop.
compute mnotev=1.
compute modvals=desctmp(5:7,:).
do if (quantile <> 1).
compute desctmp(5,jd)=desctmp(1,jd)-desctmp(2,jd).
compute desctmp(6,jd)=desctmp(1,jd).
compute desctmp(7,jd)=desctmp(1,jd)+desctmp(2,jd).
compute modvals=desctmp(5:7,:).
compute mnotev=2.
do if (modvals(1,1) < desctmp(3,1)).
compute modvals(1,1)=desctmp(3,1).
compute minwarn=1.
end if.
do if (modvals(3,1) > desctmp(4,1)).
compute modvals(3,1)=desctmp(4,1).
compute maxwarn=1.
end if.
end if.
do if (desctmp(8,1)=1).
compute modvals={desctmp(3,1);desctmp(4,1)}.
compute mnotev=0.
compute minwarn=0.
compute maxwarn=0.
end if.
end if.
end loop
.
compute xsd=desctmp(2,:).
compute xmodvals=modvals.
compute xdich=desctmp(8,1).
compute xmx=cmax(xtmp).
compute xmn=cmin(xtmp).
do if ((mcx > 0) and (xrefvals(1,1) <> 999) and (xmint=1) and (model= 74)).
compute notecode(notes,1) = 36.
compute notes = notes + 1.
end if.
do if (mcx=0).
do if (ncol(xrefvals)>2 and model=74 and xmint=1).
compute errcode(errs,1)=67.
compute errs=errs+1.
compute criterr=1.
end if.
do if ((model=74) and (xmint=1)).
do if ((xrefvals(1,1)=999) and nxvls=1 and xdich=0).
compute errcode(errs,1)=66.
compute errs=errs+1.
compute criterr=1.
end if.
do if ((xrefvals(1,1)=999) and (xdich=1)).
compute xrefvals={xmn,xmx}.
compute xscaling=xrefvals(1,2)-xrefvals(1,1).
end if.
do if (ncol(xrefvals)=1 and xrefvals(1,1) <> 999).
do if (xdich=0).
compute xrefvals={xrefvals,(xrefvals(1,1)+1)}.
compute xscaling=xrefvals(1,2)-xrefvals(1,1).
end if.
do if (xdich=1).
do if ((xrefvals(1,1) <> xmx) and (xrefvals(1,1) <> xmn)).
compute errcode(errs,1)=70.
compute errs=errs+1.
compute criterr=1.
end if.
do if (xrefvals(1,1) = xmx).
compute xrefvals={xrefvals,xmn}.
compute xscaling=xrefvals(1,2)-xrefvals(1,1).
end if.
do if (xrefvals(1,1) = xmn).
compute xrefvals={xrefvals,xmx}.
compute xscaling=xrefvals(1,2)-xrefvals(1,1).
end if.
end if.
end if.
do if (ncol(xrefvals)=2).
compute xscaling=xrefvals(1,2)-xrefvals(1,1).
do if (xdich=1).
compute xreferr=1.
do if (((xrefvals(1,1) = xmx) and (xrefvals(1,2)=xmn)) or ((xrefvals(1,1) = xmn) and (xrefvals(1,2)=xmx))).
compute xreferr=0.
end if.
do if (xreferr=1).
compute errcode(errs,1)=70.
compute errs=errs+1.
compute criterr=1.
end if.
end if.
end if.
end if.
end if.
do if (xmint=1 and model=74 and mcx=0).
compute xmodvals=t(xrefvals).
compute xcontcf=1.
end if.
compute nxpval=nrow(xmodvals).
compute xprobval=xmodvals.
do if (xdich =1 and mcx > 0).
compute mcx=0.
compute errcode(errs,1) = 52.
compute errs = errs + 1.
compute criterr = 1.
end if.
do if (nms > 0).
compute mtmp=dat(:,(nys+nxs+1):(nys+nxs+nms)).

.
compute desctmp=make((8-(4* 0 )),ncol( mtmp ),-999).
loop jd=1 to ncol( mtmp ).
compute descdat= mtmp (:,jd).
compute desctmp(1,jd) = csum(descdat)/nrow(descdat).
compute desctmp(2,jd) = (nrow(descdat)*sscp(descdat))-(t(csum(descdat))*(csum(descdat))).
compute desctmp(2,jd) = sqrt(desctmp(2,jd)/(nrow(descdat)*(nrow(descdat)-1))).
compute desctmp(3,jd)=cmin(descdat).
compute desctmp(4,jd)=cmax(descdat).
do if ( 0 =0).
compute minwarn=0.
compute maxwarn=0.
do if ((desctmp(3,jd)=desctmp(4,jd)) and novar=0).
compute errcode(errs,1)=15.
compute errs=errs+1.
compute criterr=1.
compute novar=1.
end if.
compute tmp=((descdat(:,1)=desctmp(3,jd))+(descdat(:,1)=desctmp(4,jd))).
compute desctmp(8,jd)=(csum(tmp)=nrow(tmp)).
compute tmp = descdat.
compute tmp(GRADE(descdat),:) = descdat.
compute descdat = tmp.
release tmp.
compute decval={.16;.5;.84}.
loop kd=1 to 3.
compute low=trunc(decval(kd,1)*(nrow(descdat)+1)).
compute lowdec=decval(kd,1)*(nrow(descdat)+1)-low.
compute value=descdat(low,1)+(descdat((low+1),1)-descdat(low,1))*lowdec.
compute desctmp((4+kd),jd)=value.
end loop.
compute mnotev=1.
compute modvals=desctmp(5:7,:).
do if (quantile <> 1).
compute desctmp(5,jd)=desctmp(1,jd)-desctmp(2,jd).
compute desctmp(6,jd)=desctmp(1,jd).
compute desctmp(7,jd)=desctmp(1,jd)+desctmp(2,jd).
compute modvals=desctmp(5:7,:).
compute mnotev=2.
do if (modvals(1,1) < desctmp(3,1)).
compute modvals(1,1)=desctmp(3,1).
compute minwarn=1.
end if.
do if (modvals(3,1) > desctmp(4,1)).
compute modvals(3,1)=desctmp(4,1).
compute maxwarn=1.
end if.
end if.
do if (desctmp(8,1)=1).
compute modvals={desctmp(3,1);desctmp(4,1)}.
compute mnotev=0.
compute minwarn=0.
compute maxwarn=0.
end if.
end if.
end loop
.
compute ovsd={desctmp(2,:),ysd}.
compute medmeans=cdeval.
do if ((cdeval(1,1) <> -999) and (ncol(medmeans) <> nms) and (model=74)).
compute errcode(errs,1)=64.
compute errs=errs+1.
compute criterr=1.
end if.
do if ((cdeval(1,1)=-999) and (model=74)).
compute medmeans=desctmp(1,:).
end if.
do if ((cdeval(1,1)<> -999) and (model=74) and (criterr=0)).
compute notecode(notes,1) = 31.
compute notes = notes + 1.
end if.
do if ((rsum(desctmp(8,:))>0) and (mdichok <> 1)).
compute errcode(errs,1)=43.
compute errs=errs+1.
compute criterr=1.
end if.
compute mmodvals=modvals.
compute mprobval=mmodvals.
end if.
do if (nws > 0).
compute wtmp=dat(:,(nys+nxs+nms+1):(nys+nxs+nms+nws)).

.
compute desctmp=make((8-(4* 0 )),ncol( wtmp ),-999).
loop jd=1 to ncol( wtmp ).
compute descdat= wtmp (:,jd).
compute desctmp(1,jd) = csum(descdat)/nrow(descdat).
compute desctmp(2,jd) = (nrow(descdat)*sscp(descdat))-(t(csum(descdat))*(csum(descdat))).
compute desctmp(2,jd) = sqrt(desctmp(2,jd)/(nrow(descdat)*(nrow(descdat)-1))).
compute desctmp(3,jd)=cmin(descdat).
compute desctmp(4,jd)=cmax(descdat).
do if ( 0 =0).
compute minwarn=0.
compute maxwarn=0.
do if ((desctmp(3,jd)=desctmp(4,jd)) and novar=0).
compute errcode(errs,1)=15.
compute errs=errs+1.
compute criterr=1.
compute novar=1.
end if.
compute tmp=((descdat(:,1)=desctmp(3,jd))+(descdat(:,1)=desctmp(4,jd))).
compute desctmp(8,jd)=(csum(tmp)=nrow(tmp)).
compute tmp = descdat.
compute tmp(GRADE(descdat),:) = descdat.
compute descdat = tmp.
release tmp.
compute decval={.16;.5;.84}.
loop kd=1 to 3.
compute low=trunc(decval(kd,1)*(nrow(descdat)+1)).
compute lowdec=decval(kd,1)*(nrow(descdat)+1)-low.
compute value=descdat(low,1)+(descdat((low+1),1)-descdat(low,1))*lowdec.
compute desctmp((4+kd),jd)=value.
end loop.
compute mnotev=1.
compute modvals=desctmp(5:7,:).
do if (quantile <> 1).
compute desctmp(5,jd)=desctmp(1,jd)-desctmp(2,jd).
compute desctmp(6,jd)=desctmp(1,jd).
compute desctmp(7,jd)=desctmp(1,jd)+desctmp(2,jd).
compute modvals=desctmp(5:7,:).
compute mnotev=2.
do if (modvals(1,1) < desctmp(3,1)).
compute modvals(1,1)=desctmp(3,1).
compute minwarn=1.
end if.
do if (modvals(3,1) > desctmp(4,1)).
compute modvals(3,1)=desctmp(4,1).
compute maxwarn=1.
end if.
end if.
do if (desctmp(8,1)=1).
compute modvals={desctmp(3,1);desctmp(4,1)}.
compute mnotev=0.
compute minwarn=0.
compute maxwarn=0.
end if.
end if.
end loop
.
compute wmodvals=modvals.
compute wdich=desctmp(8,1).
do if (wdich =1 and mcw > 0).
compute mcw=0.
compute errcode(errs,1) = 52.
compute errs = errs + 1.
compute criterr = 1.
end if.
compute wmin=desctmp(3,1).
compute wmax=desctmp(4,1).
compute minwwarn=minwarn.
compute maxwwarn=maxwarn.
compute wnotev=mnotev.
compute wmodval={ 999 }.
do if (xmint=1 and model=74 and mcx=0).
compute wmodval=xrefvals.
end if.
compute nwcontr=ncol(wmodval).
do if (wmodval(1,1) <> 999).
compute wmodvals=wmodval(1,1).
compute wmodcust=1.
do if (nwcontr > 1).
compute wmodvals=t(wmodval).
end if.
compute minwwarn=0.
compute maxwwarn=0.
compute wnotev=0.
end if.
compute wprobval=wmodvals.
compute nwpval=nrow(wmodvals).
end if.
do if (nzs > 0).
compute ztmp=dat(:,(nys+nxs+nms+nws+1):(nys+nxs+nms+nws+nzs)).

.
compute desctmp=make((8-(4* 0 )),ncol( ztmp ),-999).
loop jd=1 to ncol( ztmp ).
compute descdat= ztmp (:,jd).
compute desctmp(1,jd) = csum(descdat)/nrow(descdat).
compute desctmp(2,jd) = (nrow(descdat)*sscp(descdat))-(t(csum(descdat))*(csum(descdat))).
compute desctmp(2,jd) = sqrt(desctmp(2,jd)/(nrow(descdat)*(nrow(descdat)-1))).
compute desctmp(3,jd)=cmin(descdat).
compute desctmp(4,jd)=cmax(descdat).
do if ( 0 =0).
compute minwarn=0.
compute maxwarn=0.
do if ((desctmp(3,jd)=desctmp(4,jd)) and novar=0).
compute errcode(errs,1)=15.
compute errs=errs+1.
compute criterr=1.
compute novar=1.
end if.
compute tmp=((descdat(:,1)=desctmp(3,jd))+(descdat(:,1)=desctmp(4,jd))).
compute desctmp(8,jd)=(csum(tmp)=nrow(tmp)).
compute tmp = descdat.
compute tmp(GRADE(descdat),:) = descdat.
compute descdat = tmp.
release tmp.
compute decval={.16;.5;.84}.
loop kd=1 to 3.
compute low=trunc(decval(kd,1)*(nrow(descdat)+1)).
compute lowdec=decval(kd,1)*(nrow(descdat)+1)-low.
compute value=descdat(low,1)+(descdat((low+1),1)-descdat(low,1))*lowdec.
compute desctmp((4+kd),jd)=value.
end loop.
compute mnotev=1.
compute modvals=desctmp(5:7,:).
do if (quantile <> 1).
compute desctmp(5,jd)=desctmp(1,jd)-desctmp(2,jd).
compute desctmp(6,jd)=desctmp(1,jd).
compute desctmp(7,jd)=desctmp(1,jd)+desctmp(2,jd).
compute modvals=desctmp(5:7,:).
compute mnotev=2.
do if (modvals(1,1) < desctmp(3,1)).
compute modvals(1,1)=desctmp(3,1).
compute minwarn=1.
end if.
do if (modvals(3,1) > desctmp(4,1)).
compute modvals(3,1)=desctmp(4,1).
compute maxwarn=1.
end if.
end if.
do if (desctmp(8,1)=1).
compute modvals={desctmp(3,1);desctmp(4,1)}.
compute mnotev=0.
compute minwarn=0.
compute maxwarn=0.
end if.
end if.
end loop
.
compute zmodvals=modvals.
compute zdich=desctmp(8,1).
do if (zdich =1 and mcz > 0).
compute mcz=0.
compute errcode(errs,1) = 52.
compute errs = errs + 1.
compute criterr = 1.
end if.
compute zmin=desctmp(3,1).
compute zmax=desctmp(4,1).
compute minzwarn=minwarn.
compute maxzwarn=maxwarn.
compute znotev=mnotev.
compute zmodval={ 999 }.
compute nzcontr=ncol(zmodval).
do if (zmodval(1,1) <> 999).
compute zmodvals=zmodval(1,1).
compute zmodcust=1.
do if (nzcontr > 1).
compute zmodvals=t(zmodval).
end if.
compute minzwarn=0.
compute maxzwarn=0.
compute znotev=0.
end if.
compute zprobval=zmodvals.
compute nzpval=nrow(zmodvals).
end if.
do if (ncs > 0).
compute ctmp=dat(:,(nys+nxs+nms+nws+nzs+1):(nys+nxs+nms+nws+nzs+ncs)).

.
compute desctmp=make((8-(4* 0 )),ncol( ctmp ),-999).
loop jd=1 to ncol( ctmp ).
compute descdat= ctmp (:,jd).
compute desctmp(1,jd) = csum(descdat)/nrow(descdat).
compute desctmp(2,jd) = (nrow(descdat)*sscp(descdat))-(t(csum(descdat))*(csum(descdat))).
compute desctmp(2,jd) = sqrt(desctmp(2,jd)/(nrow(descdat)*(nrow(descdat)-1))).
compute desctmp(3,jd)=cmin(descdat).
compute desctmp(4,jd)=cmax(descdat).
do if ( 0 =0).
compute minwarn=0.
compute maxwarn=0.
do if ((desctmp(3,jd)=desctmp(4,jd)) and novar=0).
compute errcode(errs,1)=15.
compute errs=errs+1.
compute criterr=1.
compute novar=1.
end if.
compute tmp=((descdat(:,1)=desctmp(3,jd))+(descdat(:,1)=desctmp(4,jd))).
compute desctmp(8,jd)=(csum(tmp)=nrow(tmp)).
compute tmp = descdat.
compute tmp(GRADE(descdat),:) = descdat.
compute descdat = tmp.
release tmp.
compute decval={.16;.5;.84}.
loop kd=1 to 3.
compute low=trunc(decval(kd,1)*(nrow(descdat)+1)).
compute lowdec=decval(kd,1)*(nrow(descdat)+1)-low.
compute value=descdat(low,1)+(descdat((low+1),1)-descdat(low,1))*lowdec.
compute desctmp((4+kd),jd)=value.
end loop.
compute mnotev=1.
compute modvals=desctmp(5:7,:).
do if (quantile <> 1).
compute desctmp(5,jd)=desctmp(1,jd)-desctmp(2,jd).
compute desctmp(6,jd)=desctmp(1,jd).
compute desctmp(7,jd)=desctmp(1,jd)+desctmp(2,jd).
compute modvals=desctmp(5:7,:).
compute mnotev=2.
do if (modvals(1,1) < desctmp(3,1)).
compute modvals(1,1)=desctmp(3,1).
compute minwarn=1.
end if.
do if (modvals(3,1) > desctmp(4,1)).
compute modvals(3,1)=desctmp(4,1).
compute maxwarn=1.
end if.
end if.
do if (desctmp(8,1)=1).
compute modvals={desctmp(3,1);desctmp(4,1)}.
compute mnotev=0.
compute minwarn=0.
compute maxwarn=0.
end if.
end if.
end loop
.
compute covmeans=desctmp(1,:).
compute coval={ -999 }.
do if ((coval(1,1) <> -999) and (ncol(coval) <> ncs) and (model=74)).
compute errcode(errs,1)=69.
compute errs=errs+1.
compute criterr=1.
end if.
do if ((coval(1,1)<> -999) and (criterr=0) and (model=74)).
compute notecode(notes,1) = 35.
compute notes = notes + 1.
compute cuscoval=1.
end if.
end if.
compute n=nrow(ytmp).
compute ones=make(n,1,1).
do if (nws > 0 and mcw > 0).
compute tmp={rownum,wtmp(:,1)}.

.
compute dd= tmp.
compute temp = dd.
compute temp(GRADE(dd(:,2)),:) = dd.
compute dd = temp.
compute dummy = design(dd(:,2)).
compute nvls = ncol(dummy).
compute nnvls = csum(dummy).
compute mnvls = cmin(t(nnvls)).
compute conmat1=1.
do if (mnvls < 2).
compute errcode(errs,1) = 5.
compute errs = errs + 1.
compute criterr = 1.
end if.
do if (nvls > 9).
compute errcode(errs,1) = 4.
compute errs = errs+1.
compute criterr = 1.
end if.
do if (criterr = 0).
compute dumok = 1.
compute nnvls=make(nvls,1,0).
compute nnvls(1,1)=dd(1,2).
compute temp = 2.
loop i = 2 to n.
do if (dd(i,2) <> nnvls((temp-1),1)).
compute nnvls(temp,1)=dd(i,2).
compute temp = temp+1.
end if.
end loop.
do if ( mcw > 0).
compute x = dummy(:,2:ncol(dummy)).
compute nx = ncol(x).
compute minus1 = make(1,ncol(x),-1).
compute xdes=make((nx+1),3,0).
compute xdes(1,1)=dd(1,2).
compute xdes(1,2)=1.
compute temp = 2.
loop k = 2 to n.
do if (dd(k,2) <> dd((k-1),2)).
compute xdes(temp,2) = k.
compute xdes(temp,1) = dd(k,2).
compute xdes((temp-1),3) = k-1.
compute temp=temp+1.
end if.
end loop.
compute xdes((temp-1),3)=n.
compute xdes = {xdes, (xdes(:,3)-xdes(:,2)+1)}.
do if ( mcw = 4).
loop k = 1 to n.
do if (rsum(x(k,:)) = 0).
compute x(k,:) = minus1.
end if.
end loop.
end if.
do if ( mcw = 2 or mcw = 3 or mcw =5).
loop k = 1 to n.
do if (rsum(x(k,:)) > 0).
loop i = 1 to ncol(x).
do if (x(k,i) = 0).
compute x(k,i) = 1.
else.
break.
end if.
end loop.
end if.
end loop.
do if ( mcw = 3).
compute conmat1={-8,1,1,1,1,1,1,1,1; 0,-7,1,1,1,1,1,1,1; 0,0,-6,1,1,1,1,1,1; 0,0,0,-5,1,1,1,1,1; 0,0,0,0,-4,1,1,1,1; 0,0,0,0,0,-3,1,1,1; 0,0,0,0,0,0,-2,1,1; 0,0,0,0,0,0,0,-1,1}.
loop i = 1 to 8.
compute conmat1(i,:)=conmat1(i,:)/(10-i).
end loop.
compute conmat1=t(conmat1((10-nvls):8,(10-nvls):9)).
loop k=1 to n.
compute x(k,:)=conmat1((rsum(x(k,:))+1),:).
end loop.
end if.
end if.
do if ( mcw = 5).
compute custcode={ -999 }.
do if (ncol(custcode) <> (nvls*(nvls-1))).
compute errcode(errs,1) = (37+ 2 ).
compute errs = errs + 1.
compute criterr = 1.
end if.
do if (ncol(custcode) = (nvls*(nvls-1))).
compute conmat1=make(nvls,(nvls-1),0).
compute cnt=1.
loop i = 1 to nvls.
loop k = 1 to (nvls-1).
compute conmat1(i,k)=custcode(1,cnt).
compute cnt=cnt+1.
end loop.
end loop.
loop k=1 to n.
compute x(k,:)=conmat1((rsum(x(k,:))+1),:).
end loop.
end if.
end if.
compute xskip = 1.
compute dummat = make((nx+1),nx,0).
compute dummat((2:nrow(dummat)),:)=ident(nx).
do if ( mcw = 4).
compute dummat(1,:) = minus1.
end if.
do if ( mcw = 2).
loop i = 2 to nrow(dummat).
loop j = 1 to (i-1).
compute dummat(i,j) = 1.
end loop.
end loop.
end if.
do if ( mcw = 3).
compute dummat=conmat1.
end if.
do if ( mcw = 5 and criterr=0).
compute dummat=conmat1.
end if.
compute dummat={nnvls, dummat}.
compute x={dd(:,1),x}.
compute temp = x.
compute temp(GRADE(x(:,1)),:) = x.
compute x = temp.
release conmat1,temp,dd,xskip,xdes,dummy.
end if.
end if
.
compute wmodvals=nnvls.
compute nwpval=nrow(wmodvals).
do if (criterr=0).
compute minwwarn=0.
compute maxwwarn=0.
compute wnotev=0.
compute wtmp=x(:,2:ncol(x)).
compute wcatlab={'W1';'W2';'W3';'W4';'W5';'W6';'W7';'W8';'W9'}.
do if (xmint=1).
compute wcatlab={'X1';'X2';'X3';'X4';'X5';'X6';'X7';'X8';'X9'}.
end if.
compute nwvls=nvls-1.
compute mcwok=1.
compute dummatw=dummat.
compute wprobval=dummatw(:,2:ncol(dummatw)).
do if (modcok=1).
compute wcontval=make(2,ncol(wprobval),-999).
compute temp=0.
loop i = 1 to 2.
loop j = 1 to nrow(dummatw).
do if (contvec(i,1)=dummatw(j,1)).
compute wcontval(i,:)=wprobval(j,:).
compute temp=temp+1.
end if.
end loop.
end loop.
do if (temp < 2).
compute notecode(notes,1) = 20.
compute notes = notes + 1.
compute modcok=0.
end if.
end if.
do if ((wmodval(1,1) <> 999) and (xmint <> 1)).
compute notecode(notes,1) = 9.
compute notes = notes + 1.
end if.
release tmp, dummat.
end if.
end if.
do if (nzs > 0 and mcz > 0).
compute tmp={rownum,ztmp(:,1)}.

.
compute dd= tmp.
compute temp = dd.
compute temp(GRADE(dd(:,2)),:) = dd.
compute dd = temp.
compute dummy = design(dd(:,2)).
compute nvls = ncol(dummy).
compute nnvls = csum(dummy).
compute mnvls = cmin(t(nnvls)).
compute conmat1=1.
do if (mnvls < 2).
compute errcode(errs,1) = 5.
compute errs = errs + 1.
compute criterr = 1.
end if.
do if (nvls > 9).
compute errcode(errs,1) = 4.
compute errs = errs+1.
compute criterr = 1.
end if.
do if (criterr = 0).
compute dumok = 1.
compute nnvls=make(nvls,1,0).
compute nnvls(1,1)=dd(1,2).
compute temp = 2.
loop i = 2 to n.
do if (dd(i,2) <> nnvls((temp-1),1)).
compute nnvls(temp,1)=dd(i,2).
compute temp = temp+1.
end if.
end loop.
do if ( mcz > 0).
compute x = dummy(:,2:ncol(dummy)).
compute nx = ncol(x).
compute minus1 = make(1,ncol(x),-1).
compute xdes=make((nx+1),3,0).
compute xdes(1,1)=dd(1,2).
compute xdes(1,2)=1.
compute temp = 2.
loop k = 2 to n.
do if (dd(k,2) <> dd((k-1),2)).
compute xdes(temp,2) = k.
compute xdes(temp,1) = dd(k,2).
compute xdes((temp-1),3) = k-1.
compute temp=temp+1.
end if.
end loop.
compute xdes((temp-1),3)=n.
compute xdes = {xdes, (xdes(:,3)-xdes(:,2)+1)}.
do if ( mcz = 4).
loop k = 1 to n.
do if (rsum(x(k,:)) = 0).
compute x(k,:) = minus1.
end if.
end loop.
end if.
do if ( mcz = 2 or mcz = 3 or mcz =5).
loop k = 1 to n.
do if (rsum(x(k,:)) > 0).
loop i = 1 to ncol(x).
do if (x(k,i) = 0).
compute x(k,i) = 1.
else.
break.
end if.
end loop.
end if.
end loop.
do if ( mcz = 3).
compute conmat1={-8,1,1,1,1,1,1,1,1; 0,-7,1,1,1,1,1,1,1; 0,0,-6,1,1,1,1,1,1; 0,0,0,-5,1,1,1,1,1; 0,0,0,0,-4,1,1,1,1; 0,0,0,0,0,-3,1,1,1; 0,0,0,0,0,0,-2,1,1; 0,0,0,0,0,0,0,-1,1}.
loop i = 1 to 8.
compute conmat1(i,:)=conmat1(i,:)/(10-i).
end loop.
compute conmat1=t(conmat1((10-nvls):8,(10-nvls):9)).
loop k=1 to n.
compute x(k,:)=conmat1((rsum(x(k,:))+1),:).
end loop.
end if.
end if.
do if ( mcz = 5).
compute custcode={ -999 }.
do if (ncol(custcode) <> (nvls*(nvls-1))).
compute errcode(errs,1) = (37+ 3 ).
compute errs = errs + 1.
compute criterr = 1.
end if.
do if (ncol(custcode) = (nvls*(nvls-1))).
compute conmat1=make(nvls,(nvls-1),0).
compute cnt=1.
loop i = 1 to nvls.
loop k = 1 to (nvls-1).
compute conmat1(i,k)=custcode(1,cnt).
compute cnt=cnt+1.
end loop.
end loop.
loop k=1 to n.
compute x(k,:)=conmat1((rsum(x(k,:))+1),:).
end loop.
end if.
end if.
compute xskip = 1.
compute dummat = make((nx+1),nx,0).
compute dummat((2:nrow(dummat)),:)=ident(nx).
do if ( mcz = 4).
compute dummat(1,:) = minus1.
end if.
do if ( mcz = 2).
loop i = 2 to nrow(dummat).
loop j = 1 to (i-1).
compute dummat(i,j) = 1.
end loop.
end loop.
end if.
do if ( mcz = 3).
compute dummat=conmat1.
end if.
do if ( mcz = 5 and criterr=0).
compute dummat=conmat1.
end if.
compute dummat={nnvls, dummat}.
compute x={dd(:,1),x}.
compute temp = x.
compute temp(GRADE(x(:,1)),:) = x.
compute x = temp.
release conmat1,temp,dd,xskip,xdes,dummy.
end if.
end if
.
compute zmodvals=nnvls.
compute nzpval=nrow(zmodvals).
do if (criterr=0).
compute minzwarn=0.
compute maxzwarn=0.
compute znotev=0.
compute ztmp=x(:,2:ncol(x)).
compute zcatlab={'Z1';'Z2';'Z3';'Z4';'Z5';'Z6';'Z7';'Z8';'Z9'}.
compute nzvls=nvls-1.
compute mczok=1.
compute dummatz=dummat.
compute zprobval=dummatz(:,2:ncol(dummatz)).
do if (modcok=1).
compute zcontval=make(2,ncol(zprobval),-999).
compute temp=0.
loop i = 1 to 2.
loop j = 1 to nrow(dummatz).
do if (contvec(i,2)=dummatz(j,1)).
compute zcontval(i,:)=zprobval(j,:).
compute temp=temp+1.
end if.
end loop.
end loop.
do if (temp < 2).
compute notecode(notes,1) = 20.
compute notes = notes + 1.
compute modcok=0.
end if.
end if.
do if (zmodval(1,1) <> 999).
compute notecode(notes,1) = 10.
compute notes = notes + 1.
end if.
release tmp, dummat.
end if.
end if.
do if (nxs > 0 and mcx > 0).
compute tmp={rownum,xtmp(:,1)}.

.
compute dd= tmp.
compute temp = dd.
compute temp(GRADE(dd(:,2)),:) = dd.
compute dd = temp.
compute dummy = design(dd(:,2)).
compute nvls = ncol(dummy).
compute nnvls = csum(dummy).
compute mnvls = cmin(t(nnvls)).
compute conmat1=1.
do if (mnvls < 2).
compute errcode(errs,1) = 5.
compute errs = errs + 1.
compute criterr = 1.
end if.
do if (nvls > 9).
compute errcode(errs,1) = 4.
compute errs = errs+1.
compute criterr = 1.
end if.
do if (criterr = 0).
compute dumok = 1.
compute nnvls=make(nvls,1,0).
compute nnvls(1,1)=dd(1,2).
compute temp = 2.
loop i = 2 to n.
do if (dd(i,2) <> nnvls((temp-1),1)).
compute nnvls(temp,1)=dd(i,2).
compute temp = temp+1.
end if.
end loop.
do if ( mcx > 0).
compute x = dummy(:,2:ncol(dummy)).
compute nx = ncol(x).
compute minus1 = make(1,ncol(x),-1).
compute xdes=make((nx+1),3,0).
compute xdes(1,1)=dd(1,2).
compute xdes(1,2)=1.
compute temp = 2.
loop k = 2 to n.
do if (dd(k,2) <> dd((k-1),2)).
compute xdes(temp,2) = k.
compute xdes(temp,1) = dd(k,2).
compute xdes((temp-1),3) = k-1.
compute temp=temp+1.
end if.
end loop.
compute xdes((temp-1),3)=n.
compute xdes = {xdes, (xdes(:,3)-xdes(:,2)+1)}.
do if ( mcx = 4).
loop k = 1 to n.
do if (rsum(x(k,:)) = 0).
compute x(k,:) = minus1.
end if.
end loop.
end if.
do if ( mcx = 2 or mcx = 3 or mcx =5).
loop k = 1 to n.
do if (rsum(x(k,:)) > 0).
loop i = 1 to ncol(x).
do if (x(k,i) = 0).
compute x(k,i) = 1.
else.
break.
end if.
end loop.
end if.
end loop.
do if ( mcx = 3).
compute conmat1={-8,1,1,1,1,1,1,1,1; 0,-7,1,1,1,1,1,1,1; 0,0,-6,1,1,1,1,1,1; 0,0,0,-5,1,1,1,1,1; 0,0,0,0,-4,1,1,1,1; 0,0,0,0,0,-3,1,1,1; 0,0,0,0,0,0,-2,1,1; 0,0,0,0,0,0,0,-1,1}.
loop i = 1 to 8.
compute conmat1(i,:)=conmat1(i,:)/(10-i).
end loop.
compute conmat1=t(conmat1((10-nvls):8,(10-nvls):9)).
loop k=1 to n.
compute x(k,:)=conmat1((rsum(x(k,:))+1),:).
end loop.
end if.
end if.
do if ( mcx = 5).
compute custcode={ -999 }.
do if (ncol(custcode) <> (nvls*(nvls-1))).
compute errcode(errs,1) = (37+ 1 ).
compute errs = errs + 1.
compute criterr = 1.
end if.
do if (ncol(custcode) = (nvls*(nvls-1))).
compute conmat1=make(nvls,(nvls-1),0).
compute cnt=1.
loop i = 1 to nvls.
loop k = 1 to (nvls-1).
compute conmat1(i,k)=custcode(1,cnt).
compute cnt=cnt+1.
end loop.
end loop.
loop k=1 to n.
compute x(k,:)=conmat1((rsum(x(k,:))+1),:).
end loop.
end if.
end if.
compute xskip = 1.
compute dummat = make((nx+1),nx,0).
compute dummat((2:nrow(dummat)),:)=ident(nx).
do if ( mcx = 4).
compute dummat(1,:) = minus1.
end if.
do if ( mcx = 2).
loop i = 2 to nrow(dummat).
loop j = 1 to (i-1).
compute dummat(i,j) = 1.
end loop.
end loop.
end if.
do if ( mcx = 3).
compute dummat=conmat1.
end if.
do if ( mcx = 5 and criterr=0).
compute dummat=conmat1.
end if.
compute dummat={nnvls, dummat}.
compute x={dd(:,1),x}.
compute temp = x.
compute temp(GRADE(x(:,1)),:) = x.
compute x = temp.
release conmat1,temp,dd,xskip,xdes,dummy.
end if.
end if
.
do if (criterr=0).
compute xtmp=x(:,2:ncol(x)).
compute xcatlab={'X1';'X2';'X3';'X4';'X5';'X6';'X7';'X8';'X9'}.
compute nxvls=nvls-1.
compute xdich=(nvls=2).
compute mcxok=1.
compute dummatx=dummat.
compute xmodvals=dummatx(:,1).
compute nxpval=nrow(xmodvals).
release tmp, dummat.
end if.
end if.
compute intlab=make(100,1,' ').


compute intlab( 1 ,1)= 'Int_1'.


compute intlab( 2 ,1)= 'Int_2'.


compute intlab( 3 ,1)= 'Int_3'.


compute intlab( 4 ,1)= 'Int_4'.


compute intlab( 5 ,1)= 'Int_5'.


compute intlab( 6 ,1)= 'Int_6'.


compute intlab( 7 ,1)= 'Int_7'.


compute intlab( 8 ,1)= 'Int_8'.


compute intlab( 9 ,1)= 'Int_9'.


compute intlab( 10 ,1)= 'Int_10'.


compute intlab( 11 ,1)= 'Int_11'.


compute intlab( 12 ,1)= 'Int_12'.


compute intlab( 13 ,1)= 'Int_13'.


compute intlab( 14 ,1)= 'Int_14'.


compute intlab( 15 ,1)= 'Int_15'.


compute intlab( 16 ,1)= 'Int_16'.


compute intlab( 17 ,1)= 'Int_17'.


compute intlab( 18 ,1)= 'Int_18'.


compute intlab( 19 ,1)= 'Int_19'.


compute intlab( 20 ,1)= 'Int_20'.


compute intlab( 21 ,1)= 'Int_21'.


compute intlab( 22 ,1)= 'Int_22'.


compute intlab( 23 ,1)= 'Int_23'.


compute intlab( 24 ,1)= 'Int_24'.


compute intlab( 25 ,1)= 'Int_25'.


compute intlab( 26 ,1)= 'Int_26'.


compute intlab( 27 ,1)= 'Int_27'.


compute intlab( 28 ,1)= 'Int_28'.


compute intlab( 29 ,1)= 'Int_29'.


compute intlab( 30 ,1)= 'Int_30'.


compute intlab( 31 ,1)= 'Int_31'.


compute intlab( 32 ,1)= 'Int_32'.


compute intlab( 33 ,1)= 'Int_33'.


compute intlab( 34 ,1)= 'Int_34'.


compute intlab( 35 ,1)= 'Int_35'.


compute intlab( 36 ,1)= 'Int_36'.


compute intlab( 37 ,1)= 'Int_37'.


compute intlab( 38 ,1)= 'Int_38'.


compute intlab( 39 ,1)= 'Int_39'.


compute intlab( 40 ,1)= 'Int_40'.


compute intlab( 41 ,1)= 'Int_41'.


compute intlab( 42 ,1)= 'Int_42'.


compute intlab( 43 ,1)= 'Int_43'.


compute intlab( 44 ,1)= 'Int_44'.


compute intlab( 45 ,1)= 'Int_45'.


compute intlab( 46 ,1)= 'Int_46'.


compute intlab( 47 ,1)= 'Int_47'.


compute intlab( 48 ,1)= 'Int_48'.


compute intlab( 49 ,1)= 'Int_49'.


compute intlab( 50 ,1)= 'Int_50'.


compute intlab( 51 ,1)= 'Int_51'.


compute intlab( 52 ,1)= 'Int_52'.


compute intlab( 53 ,1)= 'Int_53'.


compute intlab( 54 ,1)= 'Int_54'.


compute intlab( 55 ,1)= 'Int_55'.


compute intlab( 56 ,1)= 'Int_56'.


compute intlab( 57 ,1)= 'Int_57'.


compute intlab( 58 ,1)= 'Int_58'.


compute intlab( 59 ,1)= 'Int_59'.


compute intlab( 60 ,1)= 'Int_60'.


compute intlab( 61 ,1)= 'Int_61'.


compute intlab( 62 ,1)= 'Int_62'.


compute intlab( 63 ,1)= 'Int_63'.


compute intlab( 64 ,1)= 'Int_64'.


compute intlab( 65 ,1)= 'Int_65'.


compute intlab( 66 ,1)= 'Int_66'.


compute intlab( 67 ,1)= 'Int_67'.


compute intlab( 68 ,1)= 'Int_68'.


compute intlab( 69 ,1)= 'Int_69'.


compute intlab( 70 ,1)= 'Int_70'.


compute intlab( 71 ,1)= 'Int_71'.


compute intlab( 72 ,1)= 'Int_72'.


compute intlab( 73 ,1)= 'Int_73'.


compute intlab( 74 ,1)= 'Int_74'.


compute intlab( 75 ,1)= 'Int_75'.


compute intlab( 76 ,1)= 'Int_76'.


compute intlab( 77 ,1)= 'Int_77'.


compute intlab( 78 ,1)= 'Int_78'.


compute intlab( 79 ,1)= 'Int_79'.


compute intlab( 80 ,1)= 'Int_80'.


compute intlab( 81 ,1)= 'Int_81'.


compute intlab( 82 ,1)= 'Int_82'.


compute intlab( 83 ,1)= 'Int_83'.


compute intlab( 84 ,1)= 'Int_84'.


compute intlab( 85 ,1)= 'Int_85'.


compute intlab( 86 ,1)= 'Int_86'.


compute intlab( 87 ,1)= 'Int_87'.


compute intlab( 88 ,1)= 'Int_88'.


compute intlab( 89 ,1)= 'Int_89'.


compute intlab( 90 ,1)= 'Int_90'.


compute intlab( 91 ,1)= 'Int_91'.


compute intlab( 92 ,1)= 'Int_92'.


compute intlab( 93 ,1)= 'Int_93'.


compute intlab( 94 ,1)= 'Int_94'.


compute intlab( 95 ,1)= 'Int_95'.


compute intlab( 96 ,1)= 'Int_96'.


compute intlab( 97 ,1)= 'Int_97'.


compute intlab( 98 ,1)= 'Int_98'.


compute intlab( 99 ,1)= 'Int_99'.


compute intlab( 100 ,1)= 'Int_100'.

compute bcmat=make(needed,needed,0).
compute wcmat=make(needed,needed,0).
compute zcmat=make(needed,needed,0).
compute wzcmat=make(needed,needed,0).
compute wsum=0.
compute zsum=0.
compute wzsum=0.
end if.
end if.
do if (criterr = 0 and model <> 999).
compute modelmat= {1,0,0,0,0,0,0,1,0,0;2,0,0,0,0,0,0,1,1,0;3,0,0,0,0,0,0,1,1,1;4,0,0,0,0,0,0,0,0,0; 5,0,0,0,0,0,0,1,0,0;6,0,0,0,0,0,0,0,0,0;7,1,0,0,0,0,0,0,0,0;8,1,0,0,0,0,0,1,0,0; 9,1,1,0,0,0,0,0,0,0;10,1,1,0,0,0,0,1,1,0;11,1,1,1,0,0,0,0,0,0;12,1,1,1,0,0,0,1,1,1; 13,1,1,1,0,0,0,1,0,0;14,0,0,0,1,0,0,0,0,0;15,0,0,0,1,0,0,1,0,0;16,0,0,0,1,1,0,0,0,0; 17,0,0,0,1,1,0,1,1,0;18,0,0,0,1,1,1,0,0,0;19,0,0,0,1,1,1,1,1,1;20,0,0,0,1,1,1,1,0,0; 21,1,0,0,0,1,0,0,0,0;22,1,0,0,0,1,0,1,0,0;23,0,0,0,0,0,0,0,0,0;24,0,0,0,0,0,0,0,0,0; 25,0,0,0,0,0,0,0,0,0;26,0,0,0,0,0,0,0,0,0;27,0,0,0,0,0,0,0,0,0;28,1,0,0,0,1,0,0,1,0; 29,1,0,0,0,1,0,1,1,0;30,0,0,0,0,0,0,0,0,0;31,0,0,0,0,0,0,0,0,0;32,0,0,0,0,0,0,0,0,0; 33,0,0,0,0,0,0,0,0,0;34,0,0,0,0,0,0,0,0,0;35,0,0,0,0,0,0,0,0,0;36,0,0,0,0,0,0,0,0,0; 37,0,0,0,0,0,0,0,0,0;38,0,0,0,0,0,0,0,0,0;39,0,0,0,0,0,0,0,0,0;40,0,0,0,0,0,0,0,0,0; 41,0,0,0,0,0,0,0,0,0;42,0,0,0,0,0,0,0,0,0;43,0,0,0,0,0,0,0,0,0;44,0,0,0,0,0,0,0,0,0; 45,0,0,0,0,0,0,0,0,0;46,0,0,0,0,0,0,0,0,0;47,0,0,0,0,0,0,0,0,0;48,0,0,0,0,0,0,0

 ,0,0; 49,0,0,0,0,0,0,0,0,0;50,0,0,0,0,0,0,0,0,0;51,0,0,0,0,0,0,0,0,0;52,0,0,0,0,0,0,0,0,0; 53,0,0,0,0,0,0,0,0,0;54,0,0,0,0,0,0,0,0,0;55,0,0,0,0,0,0,0,0,0;56,0,0,0,0,0,0,0,0,0; 57,0,0,0,0,0,0,0,0,0;58,1,0,0,1,0,0,0,0,0;59,1,0,0,1,0,0,1,0,0;60,1,1,0,1,0,0,0,0,0; 61,1,1,0,1,0,0,1,0,0;62,1,1,0,1,0,0,0,1,0;63,1,1,0,1,0,0,1,1,0;64,1,0,0,1,1,0,0,0,0; 65,1,0,0,1,1,0,1,0,0;66,1,0,0,1,1,0,0,1,0;67,1,0,0,1,1,0,1,1,0;68,1,1,1,1,0,0,0,0,0; 69,1,1,1,1,0,0,1,1,1;70,1,0,0,1,1,1,0,0,0;71,1,0,0,1,1,1,1,1,1;72,1,1,1,1,1,1,0,0,0; 73,1,1,1,1,1,1,1,1,1;74,0,0,0,1,0,0,0,0,0;75,1,1,0,1,1,0,0,0,0;76,1,1,0,1,1,0,1,1,0; 77,0,0,0,0,0,0,0,0,0;78,0,0,0,0,0,0,0,0,0;79,0,0,0,0,0,0,0,0,0;80,0,0,0,0,0,0,0,0,0; 81,0,0,0,0,0,0,0,0,0;82,0,0,0,0,0,0,0,0,0;83,1,0,0,0,0,0,0,0,0;84,1,0,0,0,0,0,0,0,0; 85,1,0,0,0,0,0,1,0,0;86,1,0,0,0,0,0,1,0,0;87,0,0,0,1,0,0,0,0,0;88,0,0,0,1,0,0,0,0,0; 89,0,0,0,1,0,0,1,0,0;90,0,0,0,1,0,0,1,0,0;91,0,0,0,0,0,0,0,0,0;92,1,0,0,1,0,0,1,0,0}.
do if (model > 0).
compute tmp=modelmat(model,2:ncol(modelmat)).
end if.
do if (model=0).
compute tmp={0,0,0,0,0,0,0,0,0}.
end if.
do if (model < 4).
compute bcmat((nxs+1),1)=1.
end if.
do if ((model > 3) and (model <> 6)).
compute bcmat((nxs+1):(nxs+nms),1)=onem.
compute bcmat(nrow(bcmat),(nxs+1):(nxs+nms))=t(onem).
compute bcmat(nrow(bcmat),1)=1.
end if.
do if ((model = 6) or (model > 82 and model < 93)).
loop j = 2 to nrow(bcmat).
loop i = 1 to (j-1).
compute bcmat(j,i)=1.
end loop.
end loop.
end if.
do if (model = 80).
loop i = 1 to nms.
compute bcmat((nrow(bcmat)-1),i)=1.
end loop.
end if.
do if (model = 81).
loop j = 3 to nrow(bcmat).
compute bcmat(j,2)=1.
end loop.
end if.
do if (model = 82).
compute bcmat(3,2)=1.
compute bcmat(5,4)=1.
end if.
do if (tmp(1,1)=1).
compute wcmat((nxs+1):(nxs+nms),1)=onem.
compute wprod=1.
compute xprod=1.
do if (model = 83 or model = 86).
compute onemsx=onem.
loop i = 1 to (nms-1).
compute onemsx(i+1,1)=0.
end loop.
compute wcmat((nxs+1):(nxs+nms),1)=onemsx.
end if.
end if.
do if (tmp(1,4)=1).
compute wcmat(nrow(wcmat),(nxs+1):(nxs+nms))=t(onem).
compute wprod=1.
do if (model = 87 or model = 90).
compute onemsx=onem.
loop i = 1 to (nms-1).
compute onemsx(i,1)=0.
end loop.
compute wcmat(nrow(wcmat),(nxs+1):(nxs+nms))=t(onemsx).
end if.
end if.
do if (tmp(1,7)=1).
compute wcmat(nrow(wcmat),1)=1.
compute wprod=1.
compute xprod=1.
end if.
do if (tmp(1,2)=1).
compute zcmat((nxs+1):(nxs+nms),1)=onem.
compute zprod=1.
compute xprod=1.
end if.
do if (tmp(1,5)=1).
compute zcmat(nrow(zcmat),(nxs+1):(nxs+nms))=t(onem).
compute zprod=1.
end if.
do if (tmp(1,8)=1).
compute zcmat(nrow(zcmat),1)=1.
compute zprod=1.
compute xprod=1.
end if.
do if (tmp(1,3)=1).
compute wzcmat((nxs+1):(nxs+nms),1)=onem.
compute xprod=1.
compute wprod=1.
compute zprod=1.
end if.
do if (tmp(1,6)=1).
compute wzcmat(nrow(wzcmat),(nxs+1):(nxs+nms))=t(onem).
compute zprod=1.
compute wprod=1.
end if.
do if (tmp(1,9)=1).
compute wzcmat(nrow(wzcmat),1)=1.
compute xprod=1.
compute wprod=1.
compute zprod=1.
end if.
do if (model = 91 or model = 92).
loop j = 1 to (nms-1).
loop i = 1 to j.
compute wcmat((nxs+1+j),(nxs+i))=1.
end loop.
end loop.
end if.
do if (nms < 0).
loop i = 1 to nms.
compute tmp=csum(wcmat(:,(1+i)))+csum(zcmat(:,(1+i)))+csum(wzcmat(:,(1+i))).
compute mprod(1,i)=(tmp>0).
end loop.
end if.
end if.
do if (ncs > 0).
compute ccmat=make((nms+nys),ncs,1).
compute ccmatoff=ccmat.
do if (covmy=1).
compute ccmat(nrow(ccmat),:)=make(1,ncs,0).
end if.
do if (covmy=2).
compute ccmat(1:nms,:)=make(nms,ncs,0).
end if.
do if (cmatrix(1,1) <> -999).
do if (ncol(cmatrix) <> ((nms+nys)*ncs)).
compute errcode(errs,1)=29.
compute errs=errs+1.
compute criterr=1.
end if.
do if (criterr = 0).
compute tmp=1.
loop i = 1 to (nms+nys).
loop j = 1 to ncs.
compute ccmat(i,j)=1-(cmatrix(1,tmp) = 0).
compute tmp=tmp+1.
end loop.
end loop.
do if (rsum((csum(ccmat)=0)) <> 0).
compute errcode(errs,1)=30.
compute errs=errs+1.
compute criterr=1.
end if.
end if.
do if (covmy <> 0).
compute notecode(notes,1)=1.
compute notes=notes+1.
end if.
end if.
do if (xmint=1 and (covmy > 0 or (csum(rsum(ccmat)) <> ((nms+nys)*ncs)))).
compute errcode(errs,1)=68.
compute errs=errs+1.
compute criterr=1.
end if.
end if.
do if (criterr=0).
compute needed=needed*(needed-1)/2.
compute nopath=0.
do if (bmatrix(1,1) <> -999).
compute tmp=1.
do if ((ncol(bmatrix) <> needed) or (csum(rsum(bmatrix))=0)).
compute errcode(errs,1)=16.
compute errs=errs+1.
compute criterr=1.
else.
loop i = 2 to nrow(bcmat).
loop j = 1 to (i-1).
compute bcmat(i,j)=1-(bmatrix(1,tmp) = 0).
compute tmp=tmp+1.
end loop.
end loop.
end if.
do if ((csum(bcmat(:,1))=0) and criterr=0).
compute errcode(errs,1)=22.
compute errs=errs+1.
compute criterr=1.
end if.
do if ((rsum(bcmat(nrow(bcmat),:))=0) and criterr=0).
compute errcode(errs,1)=23.
compute errs=errs+1.
compute criterr=1.
end if.
compute dm=0.
do if (nms > 0).
loop i = 1 to nms.
do if (((rsum(bcmat((nxs+i),:)) = 0) or (csum(bcmat(:,(nxs+i))) = 0)) and (dm=0) and (criterr=0)).
compute errcode(errs,1)=26.
compute errs=errs+1.
compute criterr=1.
compute dm=1.
end if.
end loop.
end if.
release dm.
end if.
end if.
do if (criterr=0).
do if (wmatrix(1,1) <> -999).
compute tmp=1.
do if (ncol(wmatrix) <> needed).
compute errcode(errs,1)=17.
compute errs=errs+1.
compute criterr=1.
else.
compute modelvar(1,1)='CUSTOM'.
loop i = 2 to nrow(wcmat).
loop j = 1 to (i-1).
compute wcmat(i,j)=1-(wmatrix(1,tmp) = 0).
do if ((wcmat(i,j)=1) and (bcmat(i,j)=0) and (nopath=0)).
compute errcode(errs,1)=20.
compute errs=errs+1.
compute criterr=1.
compute nopath=1.
end if.
compute tmp=tmp+1.
end loop.
end loop.
end if.
end if.
do if (zmatrix(1,1) <> -999).
compute tmp=1.
do if (ncol(zmatrix) <> needed).
compute errcode(errs,1)=18.
compute errs=errs+1.
compute criterr=1.
else.
compute modelvar(1,1)='CUSTOM'.
do if (csum(rsum(wcmat))=0 and model=999).
compute errcode(errs,1)=21.
compute errs=errs+1.
compute criterr=1.
end if.
loop i = 2 to nrow(zcmat).
loop j = 1 to (i-1).
compute zcmat(i,j)=1-(zmatrix(1,tmp) = 0).
do if ((zcmat(i,j)=1) and (bcmat(i,j)=0) and (nopath=0)).
compute errcode(errs,1)=20.
compute errs=errs+1.
compute criterr=1.
compute nopath=1.
end if.
compute tmp=tmp+1.
end loop.
end loop.
end if.
end if.
compute tmp=1.
do if (wzmatrix(1,1) <> -999).
do if (ncol(wzmatrix) <> needed).
compute errcode(errs,1)=19.
compute errs=errs+1.
compute criterr=1.
end if.
compute modelvar(1,1)='CUSTOM'.
end if.
do if (criterr=0).
loop i = 2 to nrow(wzcmat).
loop j = 1 to (i-1).
do if (wzmatrix(1,1) <> -999).
compute wzcmat(i,j)=1-(wzmatrix(1,tmp) = 0).
end if.
do if (wzcmat(i,j)=1).
compute wcmat(i,j)=1.
compute zcmat(i,j)=1.
end if.
do if ((wzcmat(i,j)=1) and (bcmat(i,j)=0) and (nopath=0)).
compute errcode(errs,1)=20.
compute errs=errs+1.
compute criterr=1.
compute nopath=1.
end if.
compute tmp=tmp+1.
end loop.
end loop.
end if.
end if.
do if (criterr=0).
compute xprod=csum(wcmat(:,1))+csum(zcmat(:,1))+csum(wzcmat(:,1)).
compute xprod=(xprod > 0).
compute wsum=csum(rsum(wcmat)).
compute wprod=(wsum > 0).
do if (nms > 0).
loop i = 1 to nms.
compute tmp=csum(wcmat(:,(1+i)))+csum(zcmat(:,(1+i)))+csum(wzcmat(:,(1+i))).
compute mprod(1,i)=(tmp>0).
end loop.
end if.
do if ((wsum > 0) and (w = 'xxxxx')).
compute errcode(errs,1)=11.
compute errs=errs+1.
compute criterr=1.
end if.
do if ((wsum = 0) and (w <> 'xxxxx')).
compute errcode(errs,1)=10.
compute errs=errs+1.
compute criterr=1.
end if.
compute zsum=csum(rsum(zcmat)).
compute zprod=(zsum > 0).
do if ((zsum > 0) and (z = 'xxxxx')).
compute errcode(errs,1)=13.
compute errs=errs+1.
compute criterr=1.
end if.
do if ((zsum = 0) and (z <> 'xxxxx')).
compute errcode(errs,1)=12.
compute errs=errs+1.
compute criterr=1.
end if.
do if ((zsum > 0) and (wsum = 0)).
compute errcode(errs,1)=35.
compute errs=errs+1.
compute criterr=1.
end if.
end if.
do if (criterr=0 and nms > 1).
compute serchk=bcmat(2:(nrow(bcmat)-1),2:ncol(bcmat)).
do if (csum(rsum(serchk))) > 0.
compute serial=1.
do if (nms > 6).
compute errcode(errs,1)=36.
compute errs=errs+1.
compute criterr=1.
end if.
end if.
end if.
do if (center > 0 and criterr=0).
compute centvar={' '}.
do if (criterr=0).
do if ((center = 1) or (center = 2 and wdich = 0)).
do if (wprod=1 and mcwok=0 and nwpval > 0).
loop i = 1 to nws.
compute wtmp(:,i)=wtmp(:,i)-(csum(wtmp(:,i))/n).
compute centvar={centvar,wnames(1,i)}.
end loop.

.
compute desctmp=make((8-(4* wmodcust )),ncol( wtmp ),-999).
loop jd=1 to ncol( wtmp ).
compute descdat= wtmp (:,jd).
compute desctmp(1,jd) = csum(descdat)/nrow(descdat).
compute desctmp(2,jd) = (nrow(descdat)*sscp(descdat))-(t(csum(descdat))*(csum(descdat))).
compute desctmp(2,jd) = sqrt(desctmp(2,jd)/(nrow(descdat)*(nrow(descdat)-1))).
compute desctmp(3,jd)=cmin(descdat).
compute desctmp(4,jd)=cmax(descdat).
do if ( wmodcust =0).
compute minwarn=0.
compute maxwarn=0.
do if ((desctmp(3,jd)=desctmp(4,jd)) and novar=0).
compute errcode(errs,1)=15.
compute errs=errs+1.
compute criterr=1.
compute novar=1.
end if.
compute tmp=((descdat(:,1)=desctmp(3,jd))+(descdat(:,1)=desctmp(4,jd))).
compute desctmp(8,jd)=(csum(tmp)=nrow(tmp)).
compute tmp = descdat.
compute tmp(GRADE(descdat),:) = descdat.
compute descdat = tmp.
release tmp.
compute decval={.16;.5;.84}.
loop kd=1 to 3.
compute low=trunc(decval(kd,1)*(nrow(descdat)+1)).
compute lowdec=decval(kd,1)*(nrow(descdat)+1)-low.
compute value=descdat(low,1)+(descdat((low+1),1)-descdat(low,1))*lowdec.
compute desctmp((4+kd),jd)=value.
end loop.
compute mnotev=1.
compute modvals=desctmp(5:7,:).
do if (quantile <> 1).
compute desctmp(5,jd)=desctmp(1,jd)-desctmp(2,jd).
compute desctmp(6,jd)=desctmp(1,jd).
compute desctmp(7,jd)=desctmp(1,jd)+desctmp(2,jd).
compute modvals=desctmp(5:7,:).
compute mnotev=2.
do if (modvals(1,1) < desctmp(3,1)).
compute modvals(1,1)=desctmp(3,1).
compute minwarn=1.
end if.
do if (modvals(3,1) > desctmp(4,1)).
compute modvals(3,1)=desctmp(4,1).
compute maxwarn=1.
end if.
end if.
do if (desctmp(8,1)=1).
compute modvals={desctmp(3,1);desctmp(4,1)}.
compute mnotev=0.
compute minwarn=0.
compute maxwarn=0.
end if.
end if.
end loop
.
compute wmin=desctmp(3,1).
compute wmax=desctmp(4,1).
do if (wmodcust=0).
compute wmodvals=modvals.
compute wprobval=wmodvals.
end if.
end if.
end if.
do if ((center = 1) or (center = 2 and zdich = 0)).
do if (zprod=1 and mczok=0 and nzpval > 0).
loop i = 1 to nzs.
compute ztmp(:,i)=ztmp(:,i)-(csum(ztmp(:,i))/n).
compute centvar={centvar,znames(1,i)}.
end loop.

.
compute desctmp=make((8-(4* zmodcust )),ncol( ztmp ),-999).
loop jd=1 to ncol( ztmp ).
compute descdat= ztmp (:,jd).
compute desctmp(1,jd) = csum(descdat)/nrow(descdat).
compute desctmp(2,jd) = (nrow(descdat)*sscp(descdat))-(t(csum(descdat))*(csum(descdat))).
compute desctmp(2,jd) = sqrt(desctmp(2,jd)/(nrow(descdat)*(nrow(descdat)-1))).
compute desctmp(3,jd)=cmin(descdat).
compute desctmp(4,jd)=cmax(descdat).
do if ( zmodcust =0).
compute minwarn=0.
compute maxwarn=0.
do if ((desctmp(3,jd)=desctmp(4,jd)) and novar=0).
compute errcode(errs,1)=15.
compute errs=errs+1.
compute criterr=1.
compute novar=1.
end if.
compute tmp=((descdat(:,1)=desctmp(3,jd))+(descdat(:,1)=desctmp(4,jd))).
compute desctmp(8,jd)=(csum(tmp)=nrow(tmp)).
compute tmp = descdat.
compute tmp(GRADE(descdat),:) = descdat.
compute descdat = tmp.
release tmp.
compute decval={.16;.5;.84}.
loop kd=1 to 3.
compute low=trunc(decval(kd,1)*(nrow(descdat)+1)).
compute lowdec=decval(kd,1)*(nrow(descdat)+1)-low.
compute value=descdat(low,1)+(descdat((low+1),1)-descdat(low,1))*lowdec.
compute desctmp((4+kd),jd)=value.
end loop.
compute mnotev=1.
compute modvals=desctmp(5:7,:).
do if (quantile <> 1).
compute desctmp(5,jd)=desctmp(1,jd)-desctmp(2,jd).
compute desctmp(6,jd)=desctmp(1,jd).
compute desctmp(7,jd)=desctmp(1,jd)+desctmp(2,jd).
compute modvals=desctmp(5:7,:).
compute mnotev=2.
do if (modvals(1,1) < desctmp(3,1)).
compute modvals(1,1)=desctmp(3,1).
compute minwarn=1.
end if.
do if (modvals(3,1) > desctmp(4,1)).
compute modvals(3,1)=desctmp(4,1).
compute maxwarn=1.
end if.
end if.
do if (desctmp(8,1)=1).
compute modvals={desctmp(3,1);desctmp(4,1)}.
compute mnotev=0.
compute minwarn=0.
compute maxwarn=0.
end if.
end if.
end loop
.
compute zmin=desctmp(3,1).
compute zmax=desctmp(4,1).
do if (zmodcust=0).
compute zmodvals=modvals.
compute zprobval=zmodvals.
end if.
end if.
end if.
do if ((center = 1) or (center = 2 and xdich = 0)).
do if (xprod=1 and mcxok=0).
loop i = 1 to nxs.
compute xtmp(:,i)=xtmp(:,i)-(csum(xtmp(:,i))/n).
compute centvar={centvar,xnames(1,i)}.
end loop.

.
compute desctmp=make((8-(4* 0 )),ncol( xtmp ),-999).
loop jd=1 to ncol( xtmp ).
compute descdat= xtmp (:,jd).
compute desctmp(1,jd) = csum(descdat)/nrow(descdat).
compute desctmp(2,jd) = (nrow(descdat)*sscp(descdat))-(t(csum(descdat))*(csum(descdat))).
compute desctmp(2,jd) = sqrt(desctmp(2,jd)/(nrow(descdat)*(nrow(descdat)-1))).
compute desctmp(3,jd)=cmin(descdat).
compute desctmp(4,jd)=cmax(descdat).
do if ( 0 =0).
compute minwarn=0.
compute maxwarn=0.
do if ((desctmp(3,jd)=desctmp(4,jd)) and novar=0).
compute errcode(errs,1)=15.
compute errs=errs+1.
compute criterr=1.
compute novar=1.
end if.
compute tmp=((descdat(:,1)=desctmp(3,jd))+(descdat(:,1)=desctmp(4,jd))).
compute desctmp(8,jd)=(csum(tmp)=nrow(tmp)).
compute tmp = descdat.
compute tmp(GRADE(descdat),:) = descdat.
compute descdat = tmp.
release tmp.
compute decval={.16;.5;.84}.
loop kd=1 to 3.
compute low=trunc(decval(kd,1)*(nrow(descdat)+1)).
compute lowdec=decval(kd,1)*(nrow(descdat)+1)-low.
compute value=descdat(low,1)+(descdat((low+1),1)-descdat(low,1))*lowdec.
compute desctmp((4+kd),jd)=value.
end loop.
compute mnotev=1.
compute modvals=desctmp(5:7,:).
do if (quantile <> 1).
compute desctmp(5,jd)=desctmp(1,jd)-desctmp(2,jd).
compute desctmp(6,jd)=desctmp(1,jd).
compute desctmp(7,jd)=desctmp(1,jd)+desctmp(2,jd).
compute modvals=desctmp(5:7,:).
compute mnotev=2.
do if (modvals(1,1) < desctmp(3,1)).
compute modvals(1,1)=desctmp(3,1).
compute minwarn=1.
end if.
do if (modvals(3,1) > desctmp(4,1)).
compute modvals(3,1)=desctmp(4,1).
compute maxwarn=1.
end if.
end if.
do if (desctmp(8,1)=1).
compute modvals={desctmp(3,1);desctmp(4,1)}.
compute mnotev=0.
compute minwarn=0.
compute maxwarn=0.
end if.
end if.
end loop
.
compute xmodvals=modvals.
compute xprobval=xmodvals.
end if.
end if.
do if (nms > 0).
loop i = 1 to nms.
do if (mprod(1,i)=1).
compute mtmp(:,i)=mtmp(:,i)-(csum(mtmp(:,i))/n).
compute centvar={centvar,mnames(1,i)}.
end if.
end loop.

.
compute desctmp=make((8-(4* 0 )),ncol( mtmp ),-999).
loop jd=1 to ncol( mtmp ).
compute descdat= mtmp (:,jd).
compute desctmp(1,jd) = csum(descdat)/nrow(descdat).
compute desctmp(2,jd) = (nrow(descdat)*sscp(descdat))-(t(csum(descdat))*(csum(descdat))).
compute desctmp(2,jd) = sqrt(desctmp(2,jd)/(nrow(descdat)*(nrow(descdat)-1))).
compute desctmp(3,jd)=cmin(descdat).
compute desctmp(4,jd)=cmax(descdat).
do if ( 0 =0).
compute minwarn=0.
compute maxwarn=0.
do if ((desctmp(3,jd)=desctmp(4,jd)) and novar=0).
compute errcode(errs,1)=15.
compute errs=errs+1.
compute criterr=1.
compute novar=1.
end if.
compute tmp=((descdat(:,1)=desctmp(3,jd))+(descdat(:,1)=desctmp(4,jd))).
compute desctmp(8,jd)=(csum(tmp)=nrow(tmp)).
compute tmp = descdat.
compute tmp(GRADE(descdat),:) = descdat.
compute descdat = tmp.
release tmp.
compute decval={.16;.5;.84}.
loop kd=1 to 3.
compute low=trunc(decval(kd,1)*(nrow(descdat)+1)).
compute lowdec=decval(kd,1)*(nrow(descdat)+1)-low.
compute value=descdat(low,1)+(descdat((low+1),1)-descdat(low,1))*lowdec.
compute desctmp((4+kd),jd)=value.
end loop.
compute mnotev=1.
compute modvals=desctmp(5:7,:).
do if (quantile <> 1).
compute desctmp(5,jd)=desctmp(1,jd)-desctmp(2,jd).
compute desctmp(6,jd)=desctmp(1,jd).
compute desctmp(7,jd)=desctmp(1,jd)+desctmp(2,jd).
compute modvals=desctmp(5:7,:).
compute mnotev=2.
do if (modvals(1,1) < desctmp(3,1)).
compute modvals(1,1)=desctmp(3,1).
compute minwarn=1.
end if.
do if (modvals(3,1) > desctmp(4,1)).
compute modvals(3,1)=desctmp(4,1).
compute maxwarn=1.
end if.
end if.
do if (desctmp(8,1)=1).
compute modvals={desctmp(3,1);desctmp(4,1)}.
compute mnotev=0.
compute minwarn=0.
compute maxwarn=0.
end if.
end if.
end loop
.
do if ((cdeval(1,1)=-999) and (model=74)).
compute medmeans=desctmp(1,:).
end if.
compute mmodvals=modvals.
compute mprobval=mmodvals.
end if.
end if.
do if (ncol(centvar) > 1).
compute notecode(notes,1)=3.
compute notes=notes+1.
end if.
end if.
do if (criterr=0).
compute wsum=rsum(csum(wcmat)).
compute zsum=rsum(csum(zcmat)).
compute wzsum=rsum(csum(wzcmat)).
compute nump=make(1,(nys+nms),-999).
compue numint=make(1,(nys+nms),0).
compute datcount=1.
compute xtmpuse=0.
compute wtmpuse=0.
compute ztmpuse=0.
compute xwtmpus=0.
compute xztmpus=0.
compute wztmpus=0.
compute xwztmpu=0.
compute xtmploc=-999.
compute wtmploc=-999.
compute xwtmplo=-999.
compute ztmploc=-999.
compute xztmplo=-999.
compute wztmplo=-999.
compute xwztmplo=-999.
compute vlabs={' '}.
do if (ncs > 0).
compute ctmpuse=make(1,ncs,0).
end if.
do if (nms > 0).
compute mtmpuse=make(1,nms,0).
compute mwtmpus=make(1,nms,0).
compute mztmpus=make(1,nms,0).
compute mwztmpu=make(1,nms,0).
compute mtmploc=make(1,nms,0).
compute mwtmplo=make(nwvls,nms,-999).
compute mztmplo=make(nzvls,nms,-999).
compute mwztmplo=make((nwvls*nzvls),nms,-999).
end if.
do if (ncs > 0).
compute ctmploc=make(1,ncs,0).
end if.
compute fulldat=make(n,1,1).
compute datindx=make(1000,(nms+nys),-999).
compute wherew=make(2,(nms+nys),-999).
compute wherex=make(2,(nms+nys),-999).
compute wherez=make(2,(nms+nys),-999).
compute wherexw=make(2,(nms+nys),-999).
compute wherexz=make(2,(nms+nys),-999).
compute wherewz=make(2,(nms+nys),-999).
compute wherexwz=make(2,(nms+nys),-999).
do if (nms > 0).
compute wherem=make(nms,(nms+nys),-999).
compute wheremw = make(nms*2,(nms+nys),-999).
compute wheremz = make(nms*2,(nms+nys),-999).
compute wheremwz = make(nms*2,(nms+nys),-999).
end if.
compute wzhigh=make(1000,(((nms+1)*(nms+2))/2),0).
compute whigh=make(1000,(((nms+1)*(nms+2))/2),0).
compute zhigh=make(1000,(((nms+1)*(nms+2))/2),0).
compute fochigh=make(1000,(((nms+1)*(nms+2))/2),0).
compute xcoefloc={1;2;3;4;5;6;7;8;9}.
compute intkey = {' ', ' ', ' ', ' ', ' ', ' ', ' '}.
compute wzhighct=0.
compute whighct=0.
compute zhighct=0.
compute foccnt=0.
loop i = 2 to nrow(bcmat).
compute wdid=0.
compute zdid=0.
compute wzdid=0.
compute cntmp=1.
compute start=1.
do if (i < nrow(bcmat)).
compute outv=mtmp(:,(i-1)).
compute modlabel={mnames(1,(i-1));'constant'}.
end if.
do if (i = nrow(bcmat)).
compute outv=ytmp.
compute modlabel={ynames;'constant'}.
end if.
loop j = 1 to (i-1).
compute foccnt=foccnt+1.
do if (j = 1 and bcmat(i,j)=1).
compute outv={outv,xtmp}.
compute modlabel={modlabel;xcatlab(1:nxvls,1)}.
do if (xtmpuse=0).
compute fulldat={fulldat,xtmp}.
compute xtmpuse=1.
loop k4=datcount to (datcount+(nxvls-1)).
compute xtmploc={xtmploc;k4}.
end loop.
compute xtmploc=xtmploc(2:nrow(xtmploc),1).
compute datcount=datcount+nxvls.
end if.
compute datindx(start:(start+nrow(xtmploc)-1),(i-1))=xtmploc.
compute wherex(1,(i-1))=start+1.
compute wherex(2,(i-1))=start+nrow(xtmploc)-1+1.
do if (model = 74).
end if.
compute onebl=make(nrow(xtmploc),1,1).
compute fochigh((start+1):(start+nrow(xtmploc)),foccnt)=onebl.
compute start=start+nrow(xtmploc).
end if.
do if (j > 1 and bcmat(i,j)=1).
compute outv={outv,mtmp(:,(j-1))}.
compute modlabel={modlabel;mnames(1,(j-1))}.
do if (mtmpuse(1,(j-1))=0).
compute fulldat={fulldat,mtmp(:,(j-1))}.
compute mtmpuse(1,(j-1))=1.
compute mtmploc(1,(j-1))=datcount.
compute datcount=datcount+1.
end if.
compute datindx(start:(start+nrow(mtmploc)-1),(i-1))=mtmploc(1,(j-1)).
compute wherem((j-1),(i-1))=start+1.
compute onebl=make(nrow(mtmploc(1,j-1)),1,1).
compute ttt=nrow(mtmploc(1,(j-1)))+start-1.
compute fochigh((start+1):(start+nrow(mtmploc(1,(j-1)))),foccnt)=onebl.
compute start=start+nrow(mtmploc(1,(j-1))).
end if.
end loop.
do if (wsum > 0).
loop j = 1 to (i-1).
compute whighct=whighct+1.
do if (j = 1 and wcmat(i,j)=1).
do if (wdid=0).
compute outv={outv,wtmp}.
do if (ncs > 0 and wiscov > 0).
compute ccmatoff((i-1),wiscov)=0.
end if.
compute modlabel={modlabel;wcatlab(1:nwvls,1)}.
compute wdid=1.
do if (wtmpuse=0).
compute fulldat={fulldat,wtmp}.
do if (ncs > 0 and wiscov > 0).
compute ccmatoff((i-1),wiscov)=0.
end if.
compute wtmpuse=1.
loop k4=datcount to (datcount+(nwvls-1)).
compute wtmploc={wtmploc;k4}.
end loop.
compute wtmploc=wtmploc(2:nrow(wtmploc),1).
compute datcount=datcount+nwvls.
end if.
end if.
compute datindx(start:(start+nrow(wtmploc)-1),(i-1))=wtmploc.
compute wherew(1,(i-1))=start+1.
compute wherew(2,(i-1))=start+nrow(wtmploc)-1+1.
compute start=start+nrow(wtmploc).
loop k1=1 to nxvls.
loop k2 = 1 to nwvls.
compute outv={outv,(xtmp(:,k1)&*wtmp(:,k2))}.
do if (ncs > 0 and wiscov > 0).
compute ccmatoff((i-1),wiscov)=0.
end if.
compute modlabel={modlabel;intlab(cntmp,1)}.
compute intkey={intkey;intlab(cntmp,1),':',xcatlab(k1,1),'x',wcatlab(k2,1),' ',' '}.
compute cntmp=cntmp+1.
end loop.
end loop.
do if (xwtmpus=0).
compute fulldat={fulldat,outv(:,(ncol(outv)-(nxvls*nwvls)+1):ncol(outv))}.
compute xwtmpus=1.
do if (ncs > 0 and wiscov > 0).
compute ccmatoff((i-1),wiscov)=0.
end if.
loop k4=datcount to (datcount+((nwvls*nxvls)-1)).
compute xwtmplo={xwtmplo;k4}.
end loop.
compute xwtmplo=xwtmplo(2:nrow(xwtmplo),1).
compute datcount=datcount+(nxvls*nwvls).
end if.
compute datindx(start:(start+nrow(xwtmplo)-1),(i-1))=xwtmplo.
compute wherexw(1,(i-1))=start+1.
compute wherexw(2,(i-1))=start+nrow(xwtmplo)-1+1.
compute onebl=make(nrow(xwtmplo),1,1).
compute whigh((start+1):(start+nrow(xwtmplo)),whighct)=onebl.
compute start=start+nrow(xwtmplo).
end if.
do if (j > 1 and wcmat(i,j)=1).
do if (wdid=0 and model <> 74).
compute outv={outv,wtmp}.
do if (ncs > 0 and wiscov > 0).
compute ccmatoff((i-1),wiscov)=0.
end if.
compute modlabel={modlabel;wcatlab(1:nwvls,1)}.
compute wdid=1.
do if (wtmpuse=0).
compute fulldat={fulldat,wtmp}.
do if (ncs > 0 and wiscov > 0).
compute ccmatoff((i-1),wiscov)=0.
end if.
compute wtmpuse=1.
loop k4=datcount to (datcount+(nwvls-1)).
compute wtmploc={wtmploc;k4}.
end loop.
compute wtmploc=wtmploc(2:nrow(wtmploc),1).
compute datcount=datcount+nwvls.
end if.
compute datindx(start:(start+nrow(wtmploc)-1),(i-1))=wtmploc.
compute wherew(1,(i-1))=start+1.
compute wherew(2,(i-1))=start+nrow(wtmploc)-1+1.
compute start=start+nrow(wtmploc).
end if.
loop k2 = 1 to nwvls.
compute outv={outv,(mtmp(:,(j-1))&*wtmp(:,k2))}.
do if (ncs > 0 and wiscov > 0).
compute ccmatoff((i-1),wiscov)=0.
end if.
compute modlabel={modlabel;intlab(cntmp,1)}.
compute intkey={intkey;intlab(cntmp,1),':', mnames(1,(j-1)),'x',wcatlab(k2,1),' ',' '}.
compute cntmp=cntmp+1.
end loop.
do if (mwtmpus(1,(j-1))=0).
compute fulldat={fulldat,outv(:,(ncol(outv)-nwvls+1):ncol(outv))}.
do if (ncs > 0 and wiscov > 0).
compute ccmatoff((i-1),wiscov)=0.
end if.
compute mwtmpus(1,(j-1))=1.
compute mw22=-999.
loop k4=datcount to (datcount+(nwvls-1)).
compute mw22={mw22;k4}.
end loop.
compute mwtmplo(:,(j-1))=mw22(2:nrow(mw22),1).
compute datcount=datcount+nwvls.
end if.
compute datindx(start:(start+nrow(mwtmplo)-1),(i-1))=mwtmplo(:,(j-1)).
compute wheremw(((2*j)-3),(i-1))=start+1.
compute wheremw(((2*j)-2),(i-1))=start+nrow(mwtmplo)-1+1.
compute onebl=make(nrow(mwtmplo),1,1).
compute whigh((start+1):(start+nrow(mwtmplo)),whighct)=onebl.
compute start=start+nrow(mwtmplo).
end if.
end loop.
end if.
do if (zsum > 0).
loop j = 1 to (i-1).
compute zhighct=zhighct+1.
do if (j = 1 and zcmat(i,j)=1).
do if (zdid=0).
compute outv={outv,ztmp}.
do if (ncs > 0 and ziscov > 0).
compute ccmatoff((i-1),ziscov)=0.
end if.
compute modlabel={modlabel;zcatlab(1:nzvls,1)}.
compute zdid=1.
do if (ztmpuse=0).
compute fulldat={fulldat,ztmp}.
do if (ncs > 0 and ziscov > 0).
compute ccmatoff((i-1),ziscov)=0.
end if.
compute ztmpuse=1.
loop k4=datcount to (datcount+(nzvls-1)).
compute ztmploc={ztmploc;k4}.
end loop.
compute ztmploc=ztmploc(2:nrow(ztmploc),1).
compute datcount=datcount+nzvls.
end if.
end if.
compute datindx(start:(start+nrow(ztmploc)-1),(i-1))=ztmploc.
compute wherez(1,(i-1))=start+1.
compute wherez(2,(i-1))=start+nrow(ztmploc)-1+1.
compute start=start+nrow(ztmploc).
loop k1=1 to nxvls.
loop k2 = 1 to nzvls.
compute outv={outv,(xtmp(:,k1)&*ztmp(:,k2))}.
do if (ncs > 0 and ziscov > 0).
compute ccmatoff((i-1),ziscov)=0.
end if.
compute modlabel={modlabel;intlab(cntmp,1)}.
compute intkey={intkey;intlab(cntmp,1),':',xcatlab(k1,1),'x',zcatlab(k2,1),' ',' '}.
compute cntmp=cntmp+1.
end loop.
end loop.
do if (xztmpus=0).
compute fulldat={fulldat,outv(:,(ncol(outv)-(nxvls*nzvls)+1):ncol(outv))}.
do if (ncs > 0 and ziscov > 0).
compute ccmatoff((i-1),ziscov)=0.
end if.
compute xztmpus=1.
loop k4=datcount to (datcount+((nzvls*nxvls)-1)).
compute xztmplo={xztmplo;k4}.
end loop.
compute xztmplo=xztmplo(2:nrow(xztmplo),1).
compute datcount=datcount+(nxvls*nzvls).
end if.
compute datindx(start:(start+nrow(xztmplo)-1),(i-1))=xztmplo.
compute wherexz(1,(i-1))=start+1.
compute wherexz(2,(i-1))=start+nrow(xztmplo)-1+1.
compute onebl=make(nrow(xztmplo),1,1).
compute zhigh((start+1):(start+nrow(xztmplo)),zhighct)=onebl.
compute start=start+nrow(xztmplo).
end if.
do if (j > 1 and zcmat(i,j)=1).
do if (zdid=0).
compute outv={outv,ztmp}.
do if (ncs > 0 and ziscov > 0).
compute ccmatoff((i-1),ziscov)=0.
end if.
compute modlabel={modlabel;zcatlab(1:nzvls,1)}.
compute zdid=1.
do if (ztmpuse=0).
compute fulldat={fulldat,ztmp}.
do if (ncs > 0 and ziscov > 0).
compute ccmatoff((i-1),ziscov)=0.
end if.
compute ztmpuse=1.
loop k4=datcount to (datcount+(nzvls-1)).
compute ztmploc={ztmploc;k4}.
end loop.
compute ztmploc=ztmploc(2:nrow(ztmploc),1).
compute datcount=datcount+nzvls.
end if.
compute datindx(start:(start+nrow(ztmploc)-1),(i-1))=ztmploc.
compute wherez(1,(i-1))=start+1.
compute wherez(2,(i-1))=start+nrow(ztmploc)-1+1.
compute start=start+nrow(ztmploc).
end if.
loop k2 = 1 to nzvls.
compute outv={outv,(mtmp(:,(j-1))&*ztmp(:,k2))}.
do if (ncs > 0 and ziscov > 0).
compute ccmatoff((i-1),ziscov)=0.
end if.
compute modlabel={modlabel;intlab(cntmp,1)}.
compute intkey={intkey;intlab(cntmp,1),':', mnames(1,(j-1)),'x',zcatlab(k2,1),' ',' '}.
compute cntmp=cntmp+1.
end loop.
do if (mztmpus(1,(j-1))=0).
compute fulldat={fulldat,outv(:,(ncol(outv)-nzvls+1):ncol(outv))}.
do if (ncs > 0 and ziscov > 0).
compute ccmatoff((i-1),ziscov)=0.
end if.
compute mztmpus(1,(j-1))=1.
compute mz22=-999.
loop k4=datcount to (datcount+(nzvls-1)).
compute mz22={mz22;k4}.
end loop.
compute mztmplo(:,(j-1))=mz22(2:nrow(mz22),1).
compute datcount=datcount+nzvls.
end if.
compute datindx(start:(start+nrow(mztmplo)-1),(i-1))=mztmplo(:,(j-1)).
compute wheremz(((2*j)-3),(i-1))=start+1.
compute wheremz(((2*j)-2),(i-1))=start+nrow(mztmplo)-1+1.
compute onebl=make(nrow(mztmplo),1,1).
compute zhigh((start+1):(start+nrow(mztmplo)),zhighct)=onebl.
compute start=start+nrow(mztmplo).
end if.
end loop.
end if.
do if (wzsum > 0).
loop j = 1 to (i-1).
compute wzhighct=wzhighct+1.
do if (j = 1 and wzcmat(i,j)=1).
do if (wzdid=0).
loop k1=1 to nwvls.
loop k2 = 1 to nzvls.
compute outv={outv,(wtmp(:,k1)&*ztmp(:,k2))}.
do if (ncs > 0 and (ziscov > 0)).
compute ccmatoff((i-1),ziscov)=0.
end if.
do if (ncs > 0 and (wiscov > 0)).
compute ccmatoff((i-1),wiscov)=0.
end if.
compute modlabel={modlabel;intlab(cntmp,1)}.
compute intkey={intkey;intlab(cntmp,1),':',wcatlab(k1,1),'x',zcatlab(k2,1),' ',' '}.
compute cntmp=cntmp+1.
end loop.
end loop.
do if (wztmpus=0).
compute fulldat={fulldat,outv(:,(ncol(outv)-(nwvls*nzvls)+1):ncol(outv))}.
do if (ncs > 0 and (ziscov > 0)).
compute ccmatoff((i-1),ziscov)=0.
end if.
do if (ncs > 0 and (wiscov > 0)).
compute ccmatoff((i-1),wiscov)=0.
end if.
compute wztmpus=1.
loop k4=datcount to (datcount+((nwvls*nzvls)-1)).
compute wztmplo={wztmplo;k4}.
end loop.
compute wztmplo=wztmplo(2:nrow(wztmplo),1).
compute datcount=datcount+(nzvls*nwvls).
end if.
compute wzdid=1.
end if.
compute datindx(start:(start+nrow(wztmplo)-1),(i-1))=wztmplo.
compute wherewz(1,(i-1))=start+1.
compute wherewz(2,(i-1))=start+nrow(wztmplo)-1+1.
compute start=start+nrow(wztmplo).
loop k1=1 to nxvls.
loop k2=1 to nwvls.
loop k3=1 to nzvls.
compute outv={outv,(xtmp(:,k1)&*wtmp(:,k2)&*ztmp(:,k3))}.
do if (ncs > 0 and (ziscov > 0)).
compute ccmatoff((i-1),ziscov)=0.
end if.
do if (ncs > 0 and (wiscov > 0)).
compute ccmatoff((i-1),wiscov)=0.
end if.
compute modlabel={modlabel;intlab(cntmp,1)}.
compute intkey={intkey;intlab(cntmp,1),':',xcatlab(k1,1),'x',wcatlab(k2,1),'x', zcatlab(k3,1)}.
compute cntmp=cntmp+1.
end loop.
end loop.
end loop.
do if (xwztmpu=0).
compute fulldat={fulldat,outv(:,(ncol(outv)-(nxvls*nwvls*nzvls)+1):ncol(outv))}.
do if (ncs > 0 and (ziscov > 0)).
compute ccmatoff((i-1),ziscov)=0.
end if.
do if (ncs > 0 and (wiscov > 0)).
compute ccmatoff((i-1),wiscov)=0.
end if.
compute xwztmpu=1.
loop k4=datcount to (datcount+((nzvls*nxvls*nwvls)-1)).
compute xwztmplo={xwztmplo;k4}.
end loop.
compute xwztmplo=xwztmplo(2:nrow(xwztmplo),1).
compute datcount=datcount+(nxvls*nzvls*nwvls).
end if.
compute datindx(start:(start+nrow(xwztmplo)-1),(i-1))=xwztmplo.
compute wherexwz(1,(i-1))=start+1.
compute wherexwz(2,(i-1))=start+nrow(xwztmplo)-1+1.
compute onebl=make(nrow(xwztmplo),1,1).
compute wzhigh((start+1):(start+nrow(xwztmplo)),wzhighct)=onebl.
compute start=start+nrow(xwztmplo).
end if.
do if (j > 1 and wzcmat(i,j)=1).
do if (wzdid=0).
loop k1=1 to nwvls.
loop k2 = 1 to nzvls.
compute outv={outv,(wtmp(:,k1)&*ztmp(:,k2))}.
do if (ncs > 0 and (ziscov > 0)).
compute ccmatoff((i-1),ziscov)=0.
end if.
do if (ncs > 0 and (wiscov > 0)).
compute ccmatoff((i-1),wiscov)=0.
end if.
compute modlabel={modlabel;intlab(cntmp,1)}.
compute intkey={intkey;intlab(cntmp,1),':',wcatlab(k1,1),'x',zcatlab(k2,1),' ',' '}.
compute cntmp=cntmp+1.
end loop.
end loop.
do if (wztmpus=0).
compute fulldat={fulldat,outv(:,(ncol(outv)-(nwvls*nzvls)+1):ncol(outv))}.
do if (ncs > 0 and (ziscov > 0)).
compute ccmatoff((i-1),ziscov)=0.
end if.
do if (ncs > 0 and (wiscov > 0)).
compute ccmatoff((i-1),wiscov)=0.
end if.
compute wztmpus=1.
loop k4=datcount to (datcount+((nwvls*nzvls)-1)).
compute wztmplo={wztmplo;k4}.
end loop.
compute wztmplo=wztmplo(2:nrow(wztmplo),1).
compute datcount=datcount+(nzvls*nwvls).
end if.
compute wzdid=1.
compute datindx(start:(start+nrow(wztmplo)-1),(i-1))=wztmplo.
compute wherewz(1,(i-1))=start+1.
compute wherewz(2,(i-1))=start+nrow(wztmplo)-1+1.
compute start=start+nrow(wztmplo).
end if.
loop k1 = 1 to nwvls.
loop k2 = 1 to nzvls.
compute outv={outv,(mtmp(:,(j-1))&*wtmp(:,k1)&*ztmp(:,k2))}.
do if (ncs > 0 and (ziscov > 0)).
compute ccmatoff((i-1),ziscov)=0.
end if.
do if (ncs > 0 and (wiscov > 0)).
compute ccmatoff((i-1),wiscov)=0.
end if.
compute modlabel={modlabel;intlab(cntmp,1)}.
compute intkey={intkey;intlab(cntmp,1),':',mnames(1,(j-1)),'x',wcatlab(k1,1),'x', zcatlab(k2,1)}.
compute cntmp=cntmp+1.
end loop.
end loop.
do if (mwztmpu(1,(j-1))=0).
compute fulldat={fulldat,outv(:,(ncol(outv)-(nwvls*nzvls)+1):ncol(outv))}.
do if (ncs > 0 and (ziscov > 0)).
compute ccmatoff((i-1),ziscov)=0.
end if.
do if (ncs > 0 and (wiscov > 0)).
compute ccmatoff((i-1),wiscov)=0.
end if.
compute mwztmpu(1,(j-1))=1.
compute mz22=-999.
loop k4=datcount to (datcount+(nwvls*nzvls)-1).
compute mz22={mz22;k4}.
end loop.
compute mwztmplo(:,(j-1))=mz22(2:nrow(mz22),1).
compute datcount=datcount+(nwvls*nzvls).
end if.
compute datindx(start:(start+nrow(mwztmplo)-1),(i-1))=mwztmplo(:,(j-1)).
compute wheremwz(((2*j)-3),(i-1))=start+1.
compute wheremwz(((2*j)-2),(i-1))=start+nrow(mwztmplo)-1+1.
compute onebl=make(nrow(mwztmplo),1,1).
compute wzhigh((start+1):(start+nrow(mwztmplo)),wzhighct)=onebl.
compute start=start+nrow(mwztmplo).
end if.
end loop.
end if.
do if (ncs > 0).
compute ccmat=ccmat&*ccmatoff.
loop j = 1 to ncs.
do if (ccmat((i-1),j))=1.
do if (j=wiscov).
compute ctmp(:,j)=wtmp.
end if.
do if (j=ziscov).
compute ctmp(:,j)=ztmp.
end if.
compute outv={outv,ctmp(:,j)}.
compute modlabel={modlabel;covnames(1,j)}.
do if (ctmpuse(1,j)=0).
compute fulldat={fulldat,ctmp(:,j)}.
compute ctmpuse(1,j)=1.
compute ctmploc(1,j)=datcount.
compute datcount=datcount+1.
end if.
compute datindx(start:(start+nrow(ctmploc)-1),(i-1))=ctmploc(1,j).
compute start=start+nrow(ctmploc(1,j)).
end if.
end loop.
end if.
compute wdid=0.
compute zdid=0.
compute wzdid=0.
compute vlabs={vlabs;modlabel(2:nrow(modlabel),1)}.
compute numint(1,(i-1))=cntmp-1.
compute nump(1,(i-1))=nrow(modlabel)-1.
end loop.
release datcount, xtmpuse, wtmpuse, ztmpuse, xwtmpus, xztmpus, wztmpus, xwztmpu.
release xtmploc, wtmploc, xwtmplo, ztmploc, xztmplo, wztmplo, xwztmplo, foccnt.
do if (modcok=1 and ((nms > 0) or (zcmat(2,1) <> 1) or (mcx <> 0))).
compute notecode(notes,1) = 19.
compute notes = notes + 1.
compute modcok=0.
end if.
do if ((serial = 1 or (rsum(numint)>0) or nms=0) and mc > 0).
compute notecode(notes,1) = 15.
compute notes = notes + 1.
compute boot=mc.
compute mc=0.
end if.
do if (boot <> 0 or mc <> 0).
compute bootsz=boot.
do if (mc > 0).
compute bootsz=mc.
compute saveboot=0.
end if.
loop.
compute cilow = rnd(bootsz*(1-(conf/100))/2).
compute cihigh = trunc((bootsz*(conf/100)+(bootsz*(1-(conf/100))/2)))+1.
do if (cilow < 1 or cihigh > bootsz).
compute bootsz=trunc((bootsz+1000)/1000)*1000.
compute adjust = 1.
end if.
end loop if (cilow gt 0 and cihigh le bootsz).
do if (boot > 0).
compute boot=bootsz.
end if.
do if (mc > 0).
compute mc=bootsz.
end if.
do if (adjust = 1 and boot > 0).
compute notecode(notes,1) = 8.
compute notes = notes + 1.
end if.
do if (adjust = 1 and mc > 0).
compute notecode(notes,1) = 16.
compute notes = notes + 1.
end if.
end if.
compute maxboot = trunc(2*boot).
do if ( 0 > maxboot).
compute maxboot=trunc( 0 ).
end if.
do if (nms > 0).
release mtmpuse, mwtmpus, mwztmpu, mtmploc, mwtmplo, mztmplo, mwztmplo.
end if.
release wdid, zdid, wzdid, start,modlabel.
compute vlabs=vlabs(2:nrow(vlabs),1).
do if (rsum(numint) > 0).
compute intkey=intkey(2:nrow(intkey),:).
end if.
compute fulldat=fulldat(:,2:ncol(fulldat)).
compute fochigh=fochigh(1:rmax(nump),:).
compute whigh=whigh(1:rmax(nump),:).
compute zhigh=zhigh(1:rmax(nump),:).
compute wzhigh=wzhigh(1:rmax(nump),:).
compute coeffs=fochigh+whigh+zhigh+wzhigh.
compute bootloc=make(rmax(nump),ncol(nump),0).
do if (nms > 0).
compute cntmp=1.
loop i = 1 to ncol(nump).
loop j = 1 to nump(1,i).
compute bootloc(j,i)=cntmp.
compute cntmp=cntmp+1.
end loop.
end loop.
compute fochighb=make(nrow(fochigh),ncol(fochigh),0).
compute whighb=fochighb.
compute zhighb=fochighb.
compute wzhighb=fochighb.
compute thetaxmb=make(nrow(fochighb),nms,0).
compute thetaxyb=make(nrow(fochighb),1,0).
compute pathsfoc=make(nxvls,1,0).
compute cntmp=1.
loop i = 1 to (nms+nys).
loop j = 1 to i.
compute fochighb(:,cntmp)=fochigh(:,cntmp)&*bootloc(:,i).
compute whighb(:,cntmp)=whigh(:,cntmp)&*bootloc(:,i).
compute zhighb(:,cntmp)=zhigh(:,cntmp)&*bootloc(:,i).
compute wzhighb(:,cntmp)=wzhigh(:,cntmp)&*bootloc(:,i).
compute coeffsb=fochighb+whighb+zhighb+wzhighb.
do if ((i < (nms+nys)) and (j = 1)).
compute thetaxmb(:,i)=coeffsb(:,cntmp).
end if.
do if ((i = (nms+nys)) and (j = 1)).
compute thetaxyb(:,1)=coeffsb(:,cntmp).
end if.
compute cntmp=cntmp+1.
end loop.
end loop.
compute thetamyb=coeffsb(:,(ncol(coeffsb)-nms+1):ncol(coeffsb)).
do if (serial = 1).
compute thetammb=make(nrow(coeffsb),((nms*(nms-1))/2),0).
end if.
compute cntmp=1.
do if (nms > 1 and serial = 1).
loop i = 1 to (nms-1).
compute start=((i+2)*(i+1))/2.
loop j = 2 to (nms-i+1).
compute thetammb(:,cntmp)=coeffsb(:,start).
compute start=start+j+i-1.
compute cntmp=cntmp+1.
end loop.
end loop.
end if.
end if.
do if ((total = 1) and (rsum(numint)=0 or (xmint=1))).
compute dototal=1.
do if ((csum(bcmat(:,1)) <> (nms+nys)) or (rsum(bcmat(nrow(bcmat),:)) <> (nms+nys))).
compute dototal=0.
compute alttotal=1.
compute notecode(notes,1) = 12.
compute notes = notes + 1.
end if.
do if (ncs > 0).
do if ((csum(rsum(ccmat))) < (nrow(ccmat)*ncol(ccmat))).
compute dototal=0.
compute alttotal=1.
compute notecode(notes,1) = 11.
compute notes = notes + 1.
end if.
end if.
do if (model=74).
do if (xdich=0 and nxvls=1).
compute dototal=0.
compute alttotal=1.
end if.
do if ((xdich=1 or nxvls > 1) and (ncs > 0) and model=74).
compute dototal=0.
compute alttotal=1.
end if.
end if.
end if.
end if.
do if (criterr=0 and ncs > 0).
do if (rsum((csum(ccmat)=0)) <> 0).
compute errcode(errs,1)=51.
compute errs=errs+1.
compute criterr=1.
end if.
end if.
compute debug= 0.
do if (outscree=1).
print/title = '***************** PROCESS Procedure for SPSS Version 4.2 *****************'.
print/title = '          Written by Andrew F. Hayes, Ph.D.       www.afhayes.com'.
print/title = '    Documentation available in Hayes (2022). www.guilford.com/p/hayes3'/space=0.
end if.
do if (criterr=0).
compute modresid=make(n,1,99999).
do if (stand=1 and ydich=1).
compute stand=0.
end if.
compute anymod2=csum(rsum(wcmat+zcmat+wzcmat)).
do if (anymod2 > 0 and stand = 1).
compute notecode(notes,1) = 27.
compute notes = notes + 1.
compute stand=0.
end if.
compute funny=1.
do if (outscree=1).
print modelvar/title = '**************************************************************************'/format = A8/rnames=modelvlb.
do if (ncs > 0).
print covnames/title='Covariates:'/format=A8.
end if.
print n/title='Sample'/rlabel='Size:'.
do if (( 'random' <> 'random')).
compute seedt= 'random'.
print seedt/title='Custom'/format=A12/rlabel = 'Seed:'.
end if.
end if.
compute maxresm=9.
compute resultm=make(1,maxresm,99999).
do if (describe=1).
compute means=csum(dat)/n.
compute sigmatal = (t(dat)*(ident(n)-(1/n)*ones*t(ones))*dat)*(1/(n-1)).
compute sdvec=sqrt(diag(sigmatal)).
compute sdall = mdiag(1/sdvec).
compute corall=sdall*sigmatal*t(sdall).
compute means={means;t(sdvec)}.
do if (ncol(means) > 9).
compute resultm=make(1,ncol(means),99999).
compute maxresm=ncol(means).
end if.
compute resultm2=make(2,maxresm,99999).
compute resultm2(1:2,1:ncol(means))=means.
compute resultm={resultm;resultm2}.
compute resultm2=make(ncol(corall),maxresm,99999).
compute resultm2(1:ncol(corall),1:ncol(corall))=corall.
compute resultm={resultm;resultm2}.
do if (outscree=1).
print means/title='Variable means and standard deviations'/cnames=varnames/rlabels='Mean','SD'/format= F10.4.
print corall/title='Variable intercorrelations (Pearson r)'/cnames=varnames/rnames=varnames/format= F10.4.
end if.
end if.
do if (outscree=1).
do if (mcxok=1).
compute labtmp={xnames,t(xcatlab(1:nxvls,1))}.
print dummatx/title = 'Coding of categorical X variable for analysis:'/cnames = labtmp/format = F6.3.
end if.
do if (mcwok=1 and xmint=0).
compute labtmp={wnames,t(wcatlab(1:nwvls,1))}.
print dummatw/title = 'Coding of categorical W variable for analysis:'/cnames = labtmp/format = F6.3.
end if.
do if (mczok=1).
compute labtmp={znames,t(zcatlab(1:nzvls,1))}.
print dummatz/title = 'Coding of categorical Z variable for analysis:'/cnames = labtmp/format = F6.3.
end if.
end if.
end if.
do if (criterr = 0).
compute outnames=ynames.
compute outvars=ytmp.
do if (nms > 0).
compute outnames={mnames,ynames}.
compute outvars={mtmp,ytmp}.
compute indcov=make(((nms*2)+(nms*(nxvls-1))),((nms*2)+(nms*(nxvls-1))),0).
compute mcsopath=make(((nms*2)+(nms*(nxvls-1))),1,0).
end if.
compute labstart=1.
compute intstart=1.
compute start=1.
compute coeffmat=make(1,6,0).
compute conseq={'        '}.
compute dfmat=0.
compute coeffcol=0.
compute pathscnt=1.
compute pathscn2=1.
loop i = 1 to (nms+nys).
do if (outscree=1).
print/title = '**************************************************************************'.
end if.
compute highf=make(1,5,0).
compute highf2=highf.
do if ((i = (nms+nys)) and (ydich=1)).
compute highf=make(1,3,0).
compute highf2=highf.
end if.
compute flabel={' '}.
compute y=outvars(:,i).
compute xindx=datindx(1:(nump(1,i)-1),i).
compute x = fulldat(:,xindx).
compute x={ones,x}.
compute xsq=t(x)*x.
compute exsq=eval(xsq).
release xsq.
compute zeroeig=csum(exsq <= 0.000000000002).
do if (outscree=1).
print outnames(1,i)/title = 'OUTCOME VARIABLE:'/format = A8/space=0.
do if (ydich=1 and (i = (nms+nys))).
compute nmsd = {outnames(1,i), 'Analysis'}.
print rcd/title = 'Coding of binary Y for logistic regression analysis:'/cnames = nmsd/format = F9.2.
end if.
end if.
do if (zeroeig > 0).
print / title = 'SINGULAR OR NEAR SINGULAR DATA MATRIX.'.
compute criterr=1.
compute errcode(errs,1)=31.
compute errs=errs+1.
end if.
compute means=csum(x)/n.
compute vlabsm=vlabs(labstart:(labstart+(nump(1,i)-1)),1).
do if (criterr=0).
do if (ydich=0 or (i < (nms+nys))).

.
do if ( 1 =1).
compute hatmat=inv(t( x )* x )*t( x ).
compute b = hatmat* y.
compute modres=b.
do if ( 1 =1).
compute n1=nrow( x ).
compute dfres=n1-(ncol( x )).
compute sstotal = t( y -(csum( y )/n1))*( y -(csum( y )/n1)).
compute resid= y - x *b.
compute ssresid = csum((resid)&**2).
compute r2 = (sstotal-ssresid)/sstotal.
do if (r2 < 0).
compute r2=0.
end if.
compute adjr2 = 1-((1-r2)*(n1-1)/(dfres)).
compute mse=ssresid/(n1-ncol( x )).

.
compute n1=nrow( x ).
compute invXtX = inv(t( x )* x ).
compute varb = mse *invXtX.
compute k3 = ncol( x ).
compute xhc=0.
do if ( hc <> 5).
compute xhc= x.
compute hat = xhc(:,1).
loop i3=1 to nrow(xhc).
compute hat(i3,1)= xhc(i3,:)*invXtX*t(xhc(i3,:)).
end loop.
do if ( hc = 0 or hc =1).
loop i3 = 1 to k3.
compute xhc(:,i3)=xhc(:,i3)&* resid.
end loop.
end if.
do if ( hc =3 or hc =2).
loop i3=1 to k3.
compute xhc(:,i3) = ( resid &/(1-hat)&**(1/(4- hc )))&*xhc(:,i3).
end loop.
end if.
do if ( hc = 4).
compute hcmn=make(n,2,4).
compute hcmn(:,2)=(n1*hat)/k3.
loop i3= 1 to k3.
compute xhc(:,i3) = ( resid &/(1-hat)&**(rmin(hcmn)/2))&*xhc(:,i3).
end loop.
end if.
compute varb=(invXtX*t(xhc)*xhc*invXtX).
do if ( hc =1).
compute varb=(n1/(n1-ncol( x )))&*varb.
end if.
end if.
compute hclab={'se(HC0)','se(HC1)','se(HC2)','se(HC3)','se(HC4)','se'}.
compute hclab=hclab(1,( hc +1)).
compute hcflab={'F(HC0)','F(HC1)','F(HC2)','F(HC3)','F(HC4)','F'}.
compute hcflab=hcflab(1,( hc +1)).
release xhc
.
compute seb=sqrt(diag(varb)).
compute trat = b&/seb.
compute p = 2*(1-tcdf(abs(trat), (dfres))).
compute tval = sqrt(dfres* (exp((dfres-(5/6))*((xp2/(dfres-(2/3)+(.11/dfres)))* (xp2/(dfres-(2/3)+(.11/dfres)))))-1)).
compute modres={modres,seb,trat,p}.
compute modres={modres,(b-tval&*seb),(b+tval&*seb)}.
compute modresl={'coeff',hclab,'t','p','LLCI','ULCI'}.
compute lmat = ident(ncol( x )).
compute lmat = lmat(:,2:ncol(lmat)).
compute fratio = (t(t(lmat)*b)*inv(t(lmat)*varb*lmat)*((t(lmat)*b)))/(ncol( x )-1).
compute pfr = 1-fcdf(fratio,(ncol( x )-1),dfres).
compute modsum={sqrt(r2),r2,mse,fratio,(ncol( x )-1),dfres,pfr}.
compute modsuml={'R','R-sq','MSE',hcflab,'df1','df2', 'p'}.
end if.
end if.
do if ( 1 = 2 or 1 =3).
compute xlp= x.
compute ylp= y.
compute pt2 = make(nrow(ylp),1,(csum(ylp)/nrow(ylp))).
do if ( 1 =2).
compute LL3 = ylp&*ln(pt2)+(1-ylp)&*ln(1-pt2).
end if.
compute LL3 = -2*csum(LL3).
compute bt1 = make(ncol(xlp),1,0).
compute LL1 = 0.
compute pt1 = make(nrow(ylp),1,0.5).
compute pt1lp=pt1.
loop jjj = 1 to iterate.
compute xlptmp=t(xlp).
compute vecprb=pt1lp&*(1-pt1lp).
loop kkk=1 to ncol(xlp).
compute xlptmp(kkk,:)=xlptmp(kkk,:)&*t(vecprb).
end loop.
compute b = bt1+inv(xlptmp*xlp)*t(xlp)*(ylp-pt1lp).
do if ( 1 =2).
compute xlpb=xlp*b.
compute xlpbt=(xlpb > -709.7).
compute xlpb709=(1-xlpbt)*(-709.7).
compute xlpb=(xlpb&*xlpbt)+xlpb709.
compute pt1lp = 1/(1+exp(-(xlpb))).
end if.
compute itprob = csum((pt1lp < .00000001) or (pt1lp > .9999999)).
do if (itprob > 0).
loop kkk = 1 to nrow(pt1lp).
do if (pt1lp(kkk,1) > .9999999).
compute pt1lp(kkk,1) = .9999999.
end if.
do if (pt1lp(kkk,1) < .00000001).
compute pt1lp(kkk,1) = .00000001.
end if.
end loop.
compute itprob = 0.
end if.
do if (itprob = 0).
do if ( 1 =2).
compute LL = ylp&*ln(pt1lp)+(1-ylp)&*ln(1-pt1lp).
end if.
compute LL2 = -2*csum(ll).
end if.
do if (abs(LL1-LL2) < converge).
do if ( 1 =1).
compute xlptmp=t(xlp).
compute vecprb=pt1lp&*(1-pt1lp).
loop kkk=1 to ncol(xlp).
compute xlptmp(kkk,:)=xlptmp(kkk,:)&*t(vecprb).
end loop.
compute varb = inv(xlptmp*xlp).
compute seb = sqrt(diag(varb)).
release xlptmp.
end if.
break.
end if.
compute bt1 = b.
compute LL1 = LL2.
end loop.
compute modres=b.
do if (jjj > iterate).
compute itprob = 2.
do if (booting=0).
compute iterrmod=1.
end if.
do if (booting=1).
compute bootiter=1.
end if.
do if (itprobtg=0).
compute itprobtg=1.
compute errcode(errs,1) = 47.
compute errs = errs + 1.
do if (booting = 0 and 1 =1).
compute vt1 = mdiag(pt1lp&*(1-pt1lp)).
compute varb = inv(t(xlp)*vt1*xlp).
compute seb = sqrt(diag(varb)).
end if.
end if.
end if.
do if ( 1 =1).
compute trat = b&/seb.
compute dfres=nrow(xlp).
compute p = 2*(1-cdfnorm(abs(trat))).
compute modres={modres,seb,trat,p}.
compute modres={modres,(b-xp2&*seb),(b+xp2&*seb)}.
compute pvchi=1-chicdf((LL3-LL2),(nrow(modres)-1)).
compute mcF = (LL3-LL2)/LL3.
compute cox = 1-exp(-(LL3-LL2)/nrow(xlp)).
compute nagel = cox/(1-exp(-(LL3)/nrow(xlp))).
compute modsum={LL2,(LL3-LL2),(nrow(modres)-1),pvchi, mcF,cox,nagel}.
compute modsuml={'-2LL','ModelLL', 'df', 'p', 'McFadden', 'CoxSnell', 'Nagelkrk'}.
compute modresl={'coeff','se','Z','p','LLCI','ULCI'}.
end if.
end if
.
compute dfmatt=make(nrow(modres),1,modsum(1,6)).
compute modresid={modresid,resid}.
end if.
do if (ydich=1 and (i = (nms+nys))).

.
do if ( 2 =1).
compute hatmat=inv(t( x )* x )*t( x ).
compute b = hatmat* y.
compute modres=b.
do if ( 1 =1).
compute n1=nrow( x ).
compute dfres=n1-(ncol( x )).
compute sstotal = t( y -(csum( y )/n1))*( y -(csum( y )/n1)).
compute resid= y - x *b.
compute ssresid = csum((resid)&**2).
compute r2 = (sstotal-ssresid)/sstotal.
do if (r2 < 0).
compute r2=0.
end if.
compute adjr2 = 1-((1-r2)*(n1-1)/(dfres)).
compute mse=ssresid/(n1-ncol( x )).

.
compute n1=nrow( x ).
compute invXtX = inv(t( x )* x ).
compute varb = mse *invXtX.
compute k3 = ncol( x ).
compute xhc=0.
do if ( hc <> 5).
compute xhc= x.
compute hat = xhc(:,1).
loop i3=1 to nrow(xhc).
compute hat(i3,1)= xhc(i3,:)*invXtX*t(xhc(i3,:)).
end loop.
do if ( hc = 0 or hc =1).
loop i3 = 1 to k3.
compute xhc(:,i3)=xhc(:,i3)&* resid.
end loop.
end if.
do if ( hc =3 or hc =2).
loop i3=1 to k3.
compute xhc(:,i3) = ( resid &/(1-hat)&**(1/(4- hc )))&*xhc(:,i3).
end loop.
end if.
do if ( hc = 4).
compute hcmn=make(n,2,4).
compute hcmn(:,2)=(n1*hat)/k3.
loop i3= 1 to k3.
compute xhc(:,i3) = ( resid &/(1-hat)&**(rmin(hcmn)/2))&*xhc(:,i3).
end loop.
end if.
compute varb=(invXtX*t(xhc)*xhc*invXtX).
do if ( hc =1).
compute varb=(n1/(n1-ncol( x )))&*varb.
end if.
end if.
compute hclab={'se(HC0)','se(HC1)','se(HC2)','se(HC3)','se(HC4)','se'}.
compute hclab=hclab(1,( hc +1)).
compute hcflab={'F(HC0)','F(HC1)','F(HC2)','F(HC3)','F(HC4)','F'}.
compute hcflab=hcflab(1,( hc +1)).
release xhc
.
compute seb=sqrt(diag(varb)).
compute trat = b&/seb.
compute p = 2*(1-tcdf(abs(trat), (dfres))).
compute tval = sqrt(dfres* (exp((dfres-(5/6))*((xp2/(dfres-(2/3)+(.11/dfres)))* (xp2/(dfres-(2/3)+(.11/dfres)))))-1)).
compute modres={modres,seb,trat,p}.
compute modres={modres,(b-tval&*seb),(b+tval&*seb)}.
compute modresl={'coeff',hclab,'t','p','LLCI','ULCI'}.
compute lmat = ident(ncol( x )).
compute lmat = lmat(:,2:ncol(lmat)).
compute fratio = (t(t(lmat)*b)*inv(t(lmat)*varb*lmat)*((t(lmat)*b)))/(ncol( x )-1).
compute pfr = 1-fcdf(fratio,(ncol( x )-1),dfres).
compute modsum={sqrt(r2),r2,mse,fratio,(ncol( x )-1),dfres,pfr}.
compute modsuml={'R','R-sq','MSE',hcflab,'df1','df2', 'p'}.
end if.
end if.
do if ( 2 = 2 or 2 =3).
compute xlp= x.
compute ylp= y.
compute pt2 = make(nrow(ylp),1,(csum(ylp)/nrow(ylp))).
do if ( 2 =2).
compute LL3 = ylp&*ln(pt2)+(1-ylp)&*ln(1-pt2).
end if.
compute LL3 = -2*csum(LL3).
compute bt1 = make(ncol(xlp),1,0).
compute LL1 = 0.
compute pt1 = make(nrow(ylp),1,0.5).
compute pt1lp=pt1.
loop jjj = 1 to iterate.
compute xlptmp=t(xlp).
compute vecprb=pt1lp&*(1-pt1lp).
loop kkk=1 to ncol(xlp).
compute xlptmp(kkk,:)=xlptmp(kkk,:)&*t(vecprb).
end loop.
compute b = bt1+inv(xlptmp*xlp)*t(xlp)*(ylp-pt1lp).
do if ( 2 =2).
compute xlpb=xlp*b.
compute xlpbt=(xlpb > -709.7).
compute xlpb709=(1-xlpbt)*(-709.7).
compute xlpb=(xlpb&*xlpbt)+xlpb709.
compute pt1lp = 1/(1+exp(-(xlpb))).
end if.
compute itprob = csum((pt1lp < .00000001) or (pt1lp > .9999999)).
do if (itprob > 0).
loop kkk = 1 to nrow(pt1lp).
do if (pt1lp(kkk,1) > .9999999).
compute pt1lp(kkk,1) = .9999999.
end if.
do if (pt1lp(kkk,1) < .00000001).
compute pt1lp(kkk,1) = .00000001.
end if.
end loop.
compute itprob = 0.
end if.
do if (itprob = 0).
do if ( 2 =2).
compute LL = ylp&*ln(pt1lp)+(1-ylp)&*ln(1-pt1lp).
end if.
compute LL2 = -2*csum(ll).
end if.
do if (abs(LL1-LL2) < converge).
do if ( 1 =1).
compute xlptmp=t(xlp).
compute vecprb=pt1lp&*(1-pt1lp).
loop kkk=1 to ncol(xlp).
compute xlptmp(kkk,:)=xlptmp(kkk,:)&*t(vecprb).
end loop.
compute varb = inv(xlptmp*xlp).
compute seb = sqrt(diag(varb)).
release xlptmp.
end if.
break.
end if.
compute bt1 = b.
compute LL1 = LL2.
end loop.
compute modres=b.
do if (jjj > iterate).
compute itprob = 2.
do if (booting=0).
compute iterrmod=1.
end if.
do if (booting=1).
compute bootiter=1.
end if.
do if (itprobtg=0).
compute itprobtg=1.
compute errcode(errs,1) = 47.
compute errs = errs + 1.
do if (booting = 0 and 1 =1).
compute vt1 = mdiag(pt1lp&*(1-pt1lp)).
compute varb = inv(t(xlp)*vt1*xlp).
compute seb = sqrt(diag(varb)).
end if.
end if.
end if.
do if ( 1 =1).
compute trat = b&/seb.
compute dfres=nrow(xlp).
compute p = 2*(1-cdfnorm(abs(trat))).
compute modres={modres,seb,trat,p}.
compute modres={modres,(b-xp2&*seb),(b+xp2&*seb)}.
compute pvchi=1-chicdf((LL3-LL2),(nrow(modres)-1)).
compute mcF = (LL3-LL2)/LL3.
compute cox = 1-exp(-(LL3-LL2)/nrow(xlp)).
compute nagel = cox/(1-exp(-(LL3)/nrow(xlp))).
compute modsum={LL2,(LL3-LL2),(nrow(modres)-1),pvchi, mcF,cox,nagel}.
compute modsuml={'-2LL','ModelLL', 'df', 'p', 'McFadden', 'CoxSnell', 'Nagelkrk'}.
compute modresl={'coeff','se','Z','p','LLCI','ULCI'}.
end if.
end if
.
compute dfmatt=make(nrow(modres),1,-999).
end if.
compute resultm2=make(1,maxresm,99999).
compute resultm2(1,1:ncol(modsum))=modsum.
compute resultm={resultm;resultm2}.
compute resultm2=make(nrow(modres),maxresm,99999).
compute resultm2(1:nrow(modres),1:ncol(modres))=modres.
compute resultm={resultm;resultm2}.
compute obscoeff={obscoeff,t(b)}.
do if (outscree=1).
print modsum/title = 'Model Summary'/cnames = modsuml/format= F10.4.
print modres/title='Model'/rnames=vlabsm/cnames=modresl/format= F10.4.
end if.
compute basemod=modsum(1,1).
compute basemodx=basemod.
do if (ydich=1 and (i = (nms+nys))).
do if (outscree=1).
print/title='These results are expressed in a log-odds metric.'.
end if.
compute notecode(notes,1) = 26.
compute notes = notes + 1.
end if.
compute coeffmat={coeffmat;modres}.
compute conseqt=make(nrow(modres),1,outnames(1,i)).
compute conseq={conseq;conseqt}.
compute dfmat={dfmat;dfmatt}.
compute labstart=labstart+nump(1,i).
do if (stand=1).
compute predsd=make(nrow(modres),1,0).
compute stdmod=modres(:,1)&/ovsd(1,i).
loop jd=1 to ncol(x).
compute descdat=x(:,jd).
compute predsd(jd,1) = (nrow(descdat)*sscp(descdat))-(t(csum(descdat))*(csum(descdat))).
compute predsd(jd,1) = sqrt(predsd(jd,1)/(nrow(descdat)*(nrow(descdat)-1))).
end loop.
do if (wherex(1,i) <> -999 and ((nxvls > 1) or (xdich=1))).
compute sdmsone=make(nxvls,1,1).
compute predsd(wherex(1,i):wherex(2,i),1)=sdmsone.
compute pstog=1.
end if.
compute predsd(1,1)=1.
compute stdmod=stdmod&*predsd.
compute stdmod=stdmod(2:nrow(stdmod),1).
compute sdvlabs=vlabsm(2:nrow(vlabsm),1).
compute resultm2=make(nrow(stdmod),maxresm,99999).
compute resultm2(1:nrow(stdmod),1:ncol(stdmod))=stdmod.
compute resultm={resultm;resultm2}.
do if (outscree=1).
print stdmod/title='Standardized coefficients'/clabels='coeff'/rnames=sdvlabs/format= F10.4.
end if.
end if.
do if (nms > 0 and serial = 0 and (rsum(numint) = 0) and (normal=1 or mc > 0)).
do if (i < (nms+nys)).
compute indcov((((i-1)*nxvls)+1):(i*nxvls),(((i-1)*nxvls)+1):(i*nxvls))=varb(2:(1+nxvls), 2:(1+nxvls)).
compute mcsopath((((i-1)*nxvls)+1):(i*nxvls) ,1)=modres(2:(1+nxvls),1).
end if.
do if (i = (nms+nys)).
compute atm=ncol(wherem).
compute indcov(((nms*nxvls)+1):nrow(mcsopath),((nms*nxvls)+1):nrow(mcsopath))= varb(wherem(1,atm):(wherem(1,atm)+nms-1),wherem(1,atm):(wherem(1,atm)+nms-1)).
compute mcsopath(((nms*nxvls)+1):nrow(mcsopath),1)=modres(wherem(1,atm):(wherem(1,atm)+ nms-1),1).
compute sobelok=1.
end if.
end if.
compute obsdirfx=make(1,nxvls,0).
compute dirzes=make(1,nxvls,0).
do if ((i = (nms+nys)) and (bcmat(nrow(bcmat),1)=1)).
compute direff=modres(2:(1+nxvls),:).
compute obsdirfx=t(direff(:,1)).
compute direfflb=modresl.
compute direffl2=vlabsm(2:(1+nxvls),:).
compute lmat=make(nrow(b),1,0).
compute lmat2=make(nxvls,1,1).
compute lmat(2:(1+nxvls),1)=lmat2.
do if (ydich <> 1).

.
compute lmat2= lmat.
do if ( 0 =0).
compute lmat2 = mdiag( lmat ).
compute lmat3=make(nrow(lmat2),1,0).
loop flp=1 to ncol(lmat2).
do if (csum(lmat2(:,flp))=1).
compute lmat3={lmat3,lmat2(:,flp)}.
end if.
end loop.
compute lmat2=lmat3(:,2:ncol(lmat3)).
end if.
compute fratio = (t(t(lmat2)* b )*inv(t(lmat2)* varb *lmat2)*((t(lmat2)* b )))/ncol(lmat2).
compute pfr = 1-fcdf(fratio,ncol(lmat2),(n-nrow( b ))).
compute fresult={fratio,ncol(lmat2),(n-nrow( b )),pfr}.
do if (i = (nms+nys) and (ydich=1)).
compute fratio=fratio*ncol(lmat2).
compute pfr=1-chicdf(fratio,ncol(lmat2)).
compute fresult={fratio,ncol(lmat2),pfr}.
end if.
do if ( 1 =1).
compute lmat3=1-rsum(lmat2).
compute xfm=make(n,csum(lmat3),0).
compute flpc=1.
loop flp=1 to nrow(lmat3).
do if (lmat3(flp,1)=1).
compute xfm(:,flpc)=x(:,flp).
compute flpc=flpc+1.
end if.
end loop.
compute bfm=inv(t(xfm)*xfm)*t(xfm)*y.
compute resid=y-(xfm*bfm).
compute sstotal=(y-(csum(y)/n)).
compute sstotal=csum(sstotal&*sstotal).
compute ssresid=csum(resid&*resid).
compute rsqch= r2 -((sstotal-ssresid)/sstotal).
compute fresult={rsqch,fresult}.
release xfm,flpc, resid, ssresid, bfm.
end if
.
compute diromni=fresult.
end if.
do if (ydich = 1 ).

.
compute btemphld=b.
compute llrdat=make(nrow(x),nrow( lmat )-csum( lmat ),-999).
compute llrdf=ncol(x)-ncol(llrdat).
compute llrcnt=0.
loop llri=1 to nrow( lmat ).
do if ( lmat (llri,1)=0).
compute llrcnt=llrcnt+1.
compute llrdat(:,llrcnt)=x(:,llri).
end if.
end loop.

.
do if ( 2 =1).
compute hatmat=inv(t( llrdat )* llrdat )*t( llrdat ).
compute b = hatmat* y.
compute modres=b.
do if ( 0 =1).
compute n1=nrow( llrdat ).
compute dfres=n1-(ncol( llrdat )).
compute sstotal = t( y -(csum( y )/n1))*( y -(csum( y )/n1)).
compute resid= y - llrdat *b.
compute ssresid = csum((resid)&**2).
compute r2 = (sstotal-ssresid)/sstotal.
do if (r2 < 0).
compute r2=0.
end if.
compute adjr2 = 1-((1-r2)*(n1-1)/(dfres)).
compute mse=ssresid/(n1-ncol( llrdat )).

.
compute n1=nrow( x ).
compute invXtX = inv(t( x )* x ).
compute varb = mse *invXtX.
compute k3 = ncol( x ).
compute xhc=0.
do if ( hc <> 5).
compute xhc= x.
compute hat = xhc(:,1).
loop i3=1 to nrow(xhc).
compute hat(i3,1)= xhc(i3,:)*invXtX*t(xhc(i3,:)).
end loop.
do if ( hc = 0 or hc =1).
loop i3 = 1 to k3.
compute xhc(:,i3)=xhc(:,i3)&* resid.
end loop.
end if.
do if ( hc =3 or hc =2).
loop i3=1 to k3.
compute xhc(:,i3) = ( resid &/(1-hat)&**(1/(4- hc )))&*xhc(:,i3).
end loop.
end if.
do if ( hc = 4).
compute hcmn=make(n,2,4).
compute hcmn(:,2)=(n1*hat)/k3.
loop i3= 1 to k3.
compute xhc(:,i3) = ( resid &/(1-hat)&**(rmin(hcmn)/2))&*xhc(:,i3).
end loop.
end if.
compute varb=(invXtX*t(xhc)*xhc*invXtX).
do if ( hc =1).
compute varb=(n1/(n1-ncol( x )))&*varb.
end if.
end if.
compute hclab={'se(HC0)','se(HC1)','se(HC2)','se(HC3)','se(HC4)','se'}.
compute hclab=hclab(1,( hc +1)).
compute hcflab={'F(HC0)','F(HC1)','F(HC2)','F(HC3)','F(HC4)','F'}.
compute hcflab=hcflab(1,( hc +1)).
release xhc
.
compute seb=sqrt(diag(varb)).
compute trat = b&/seb.
compute p = 2*(1-tcdf(abs(trat), (dfres))).
compute tval = sqrt(dfres* (exp((dfres-(5/6))*((xp2/(dfres-(2/3)+(.11/dfres)))* (xp2/(dfres-(2/3)+(.11/dfres)))))-1)).
compute modres={modres,seb,trat,p}.
compute modres={modres,(b-tval&*seb),(b+tval&*seb)}.
compute modresl={'coeff',hclab,'t','p','LLCI','ULCI'}.
compute lmat = ident(ncol( llrdat )).
compute lmat = lmat(:,2:ncol(lmat)).
compute fratio = (t(t(lmat)*b)*inv(t(lmat)*varb*lmat)*((t(lmat)*b)))/(ncol( llrdat )-1).
compute pfr = 1-fcdf(fratio,(ncol( llrdat )-1),dfres).
compute modsum={sqrt(r2),r2,mse,fratio,(ncol( llrdat )-1),dfres,pfr}.
compute modsuml={'R','R-sq','MSE',hcflab,'df1','df2', 'p'}.
end if.
end if.
do if ( 2 = 2 or 2 =3).
compute xlp= llrdat.
compute ylp= y.
compute pt2 = make(nrow(ylp),1,(csum(ylp)/nrow(ylp))).
do if ( 2 =2).
compute LL3 = ylp&*ln(pt2)+(1-ylp)&*ln(1-pt2).
end if.
compute LL3 = -2*csum(LL3).
compute bt1 = make(ncol(xlp),1,0).
compute LL1 = 0.
compute pt1 = make(nrow(ylp),1,0.5).
compute pt1lp=pt1.
loop jjj = 1 to iterate.
compute xlptmp=t(xlp).
compute vecprb=pt1lp&*(1-pt1lp).
loop kkk=1 to ncol(xlp).
compute xlptmp(kkk,:)=xlptmp(kkk,:)&*t(vecprb).
end loop.
compute b = bt1+inv(xlptmp*xlp)*t(xlp)*(ylp-pt1lp).
do if ( 2 =2).
compute xlpb=xlp*b.
compute xlpbt=(xlpb > -709.7).
compute xlpb709=(1-xlpbt)*(-709.7).
compute xlpb=(xlpb&*xlpbt)+xlpb709.
compute pt1lp = 1/(1+exp(-(xlpb))).
end if.
compute itprob = csum((pt1lp < .00000001) or (pt1lp > .9999999)).
do if (itprob > 0).
loop kkk = 1 to nrow(pt1lp).
do if (pt1lp(kkk,1) > .9999999).
compute pt1lp(kkk,1) = .9999999.
end if.
do if (pt1lp(kkk,1) < .00000001).
compute pt1lp(kkk,1) = .00000001.
end if.
end loop.
compute itprob = 0.
end if.
do if (itprob = 0).
do if ( 2 =2).
compute LL = ylp&*ln(pt1lp)+(1-ylp)&*ln(1-pt1lp).
end if.
compute LL2 = -2*csum(ll).
end if.
do if (abs(LL1-LL2) < converge).
do if ( 0 =1).
compute xlptmp=t(xlp).
compute vecprb=pt1lp&*(1-pt1lp).
loop kkk=1 to ncol(xlp).
compute xlptmp(kkk,:)=xlptmp(kkk,:)&*t(vecprb).
end loop.
compute varb = inv(xlptmp*xlp).
compute seb = sqrt(diag(varb)).
release xlptmp.
end if.
break.
end if.
compute bt1 = b.
compute LL1 = LL2.
end loop.
compute modres=b.
do if (jjj > iterate).
compute itprob = 2.
do if (booting=0).
compute iterrmod=1.
end if.
do if (booting=1).
compute bootiter=1.
end if.
do if (itprobtg=0).
compute itprobtg=1.
compute errcode(errs,1) = 47.
compute errs = errs + 1.
do if (booting = 0 and 0 =1).
compute vt1 = mdiag(pt1lp&*(1-pt1lp)).
compute varb = inv(t(xlp)*vt1*xlp).
compute seb = sqrt(diag(varb)).
end if.
end if.
end if.
do if ( 0 =1).
compute trat = b&/seb.
compute dfres=nrow(xlp).
compute p = 2*(1-cdfnorm(abs(trat))).
compute modres={modres,seb,trat,p}.
compute modres={modres,(b-xp2&*seb),(b+xp2&*seb)}.
compute pvchi=1-chicdf((LL3-LL2),(nrow(modres)-1)).
compute mcF = (LL3-LL2)/LL3.
compute cox = 1-exp(-(LL3-LL2)/nrow(xlp)).
compute nagel = cox/(1-exp(-(LL3)/nrow(xlp))).
compute modsum={LL2,(LL3-LL2),(nrow(modres)-1),pvchi, mcF,cox,nagel}.
compute modsuml={'-2LL','ModelLL', 'df', 'p', 'McFadden', 'CoxSnell', 'Nagelkrk'}.
compute modresl={'coeff','se','Z','p','LLCI','ULCI'}.
end if.
end if
.
compute b=btemphld.
compute fresult={(LL2-basemod),llrdf,1-chicdf((LL2-basemod),llrdf)}
.
compute diromni=fresult.
end if.
end if.
do if (numint(1,i) > 0).
compute intkeym=intkey(intstart:(intstart+numint(1,i)-1),:).
do if (outscree=1).
print intkeym/title='Product terms key:'/format = A8.
end if.
end if.
do if (covcoeff=1).
do if (outscree=1).
print varb/title='Covariance matrix of regression parameter estimates:'/rnames=vlabsm /cnames=vlabsm/format= F10.4.
end if.
compute resultm2=make(nrow(varb),maxresm,99999).
do if (ncol(varb) <= maxresm).
compute resultm2(1:nrow(varb),1:ncol(varb))=varb.
compute resultm={resultm;resultm2}.
end if.
do if (ncol(varb) > maxresm).
compute resultmt=make(nrow(resultm),ncol(varb),99999).
compute resultmt(1:nrow(resultm),1:ncol(resultm))=resultm.
compute resultm=resultmt.
compute resultm2=make(nrow(varb),ncol(resultm),99999).
compute resultm2(1:nrow(varb),1:ncol(varb))=varb.
compute resultm={resultm;resultm2}.
compute maxresm=ncol(resultm).
end if.
end if.
do if (model <> 74 and xmtest=1 and nms > 0).
compute r2tmp=r2.
compute btmp=b.
compute varbtmp=varb.
compute dfrestmp=dfres.
compute tvaltmp=tval.
compute xmtst=make(nms,4,0).
compute xmtstlbc={hcflab,'df1','df2','p'}.
do if ((i=(nms+nys)) and (ydich=1)).
compute xmtst=make(nms,3,0).
compute xmtstlbc={'Chi-sq','df','p'}.
end if.
compute xmtstlb={' '}.
compute xmtmat=x.
compute numxint=0.
loop xmints=2 to i.
compute x=xmtmat.
do if ((bcmat((i+1),xmints)=1) and (wzcmat((i+1),xmints) <> 1)).
do if (bcmat((i+1),1)=0).
compute x={xmtmat,xtmp}.
do if ((ydich=1) and (i=(nms+nys))).

.
do if ( 2 =1).
compute hatmat=inv(t( x )* x )*t( x ).
compute b = hatmat* y.
compute modres=b.
do if ( 1 =1).
compute n1=nrow( x ).
compute dfres=n1-(ncol( x )).
compute sstotal = t( y -(csum( y )/n1))*( y -(csum( y )/n1)).
compute resid= y - x *b.
compute ssresid = csum((resid)&**2).
compute r2 = (sstotal-ssresid)/sstotal.
do if (r2 < 0).
compute r2=0.
end if.
compute adjr2 = 1-((1-r2)*(n1-1)/(dfres)).
compute mse=ssresid/(n1-ncol( x )).

.
compute n1=nrow( x ).
compute invXtX = inv(t( x )* x ).
compute varb = mse *invXtX.
compute k3 = ncol( x ).
compute xhc=0.
do if ( hc <> 5).
compute xhc= x.
compute hat = xhc(:,1).
loop i3=1 to nrow(xhc).
compute hat(i3,1)= xhc(i3,:)*invXtX*t(xhc(i3,:)).
end loop.
do if ( hc = 0 or hc =1).
loop i3 = 1 to k3.
compute xhc(:,i3)=xhc(:,i3)&* resid.
end loop.
end if.
do if ( hc =3 or hc =2).
loop i3=1 to k3.
compute xhc(:,i3) = ( resid &/(1-hat)&**(1/(4- hc )))&*xhc(:,i3).
end loop.
end if.
do if ( hc = 4).
compute hcmn=make(n,2,4).
compute hcmn(:,2)=(n1*hat)/k3.
loop i3= 1 to k3.
compute xhc(:,i3) = ( resid &/(1-hat)&**(rmin(hcmn)/2))&*xhc(:,i3).
end loop.
end if.
compute varb=(invXtX*t(xhc)*xhc*invXtX).
do if ( hc =1).
compute varb=(n1/(n1-ncol( x )))&*varb.
end if.
end if.
compute hclab={'se(HC0)','se(HC1)','se(HC2)','se(HC3)','se(HC4)','se'}.
compute hclab=hclab(1,( hc +1)).
compute hcflab={'F(HC0)','F(HC1)','F(HC2)','F(HC3)','F(HC4)','F'}.
compute hcflab=hcflab(1,( hc +1)).
release xhc
.
compute seb=sqrt(diag(varb)).
compute trat = b&/seb.
compute p = 2*(1-tcdf(abs(trat), (dfres))).
compute tval = sqrt(dfres* (exp((dfres-(5/6))*((xp2/(dfres-(2/3)+(.11/dfres)))* (xp2/(dfres-(2/3)+(.11/dfres)))))-1)).
compute modres={modres,seb,trat,p}.
compute modres={modres,(b-tval&*seb),(b+tval&*seb)}.
compute modresl={'coeff',hclab,'t','p','LLCI','ULCI'}.
compute lmat = ident(ncol( x )).
compute lmat = lmat(:,2:ncol(lmat)).
compute fratio = (t(t(lmat)*b)*inv(t(lmat)*varb*lmat)*((t(lmat)*b)))/(ncol( x )-1).
compute pfr = 1-fcdf(fratio,(ncol( x )-1),dfres).
compute modsum={sqrt(r2),r2,mse,fratio,(ncol( x )-1),dfres,pfr}.
compute modsuml={'R','R-sq','MSE',hcflab,'df1','df2', 'p'}.
end if.
end if.
do if ( 2 = 2 or 2 =3).
compute xlp= x.
compute ylp= y.
compute pt2 = make(nrow(ylp),1,(csum(ylp)/nrow(ylp))).
do if ( 2 =2).
compute LL3 = ylp&*ln(pt2)+(1-ylp)&*ln(1-pt2).
end if.
compute LL3 = -2*csum(LL3).
compute bt1 = make(ncol(xlp),1,0).
compute LL1 = 0.
compute pt1 = make(nrow(ylp),1,0.5).
compute pt1lp=pt1.
loop jjj = 1 to iterate.
compute xlptmp=t(xlp).
compute vecprb=pt1lp&*(1-pt1lp).
loop kkk=1 to ncol(xlp).
compute xlptmp(kkk,:)=xlptmp(kkk,:)&*t(vecprb).
end loop.
compute b = bt1+inv(xlptmp*xlp)*t(xlp)*(ylp-pt1lp).
do if ( 2 =2).
compute xlpb=xlp*b.
compute xlpbt=(xlpb > -709.7).
compute xlpb709=(1-xlpbt)*(-709.7).
compute xlpb=(xlpb&*xlpbt)+xlpb709.
compute pt1lp = 1/(1+exp(-(xlpb))).
end if.
compute itprob = csum((pt1lp < .00000001) or (pt1lp > .9999999)).
do if (itprob > 0).
loop kkk = 1 to nrow(pt1lp).
do if (pt1lp(kkk,1) > .9999999).
compute pt1lp(kkk,1) = .9999999.
end if.
do if (pt1lp(kkk,1) < .00000001).
compute pt1lp(kkk,1) = .00000001.
end if.
end loop.
compute itprob = 0.
end if.
do if (itprob = 0).
do if ( 2 =2).
compute LL = ylp&*ln(pt1lp)+(1-ylp)&*ln(1-pt1lp).
end if.
compute LL2 = -2*csum(ll).
end if.
do if (abs(LL1-LL2) < converge).
do if ( 1 =1).
compute xlptmp=t(xlp).
compute vecprb=pt1lp&*(1-pt1lp).
loop kkk=1 to ncol(xlp).
compute xlptmp(kkk,:)=xlptmp(kkk,:)&*t(vecprb).
end loop.
compute varb = inv(xlptmp*xlp).
compute seb = sqrt(diag(varb)).
release xlptmp.
end if.
break.
end if.
compute bt1 = b.
compute LL1 = LL2.
end loop.
compute modres=b.
do if (jjj > iterate).
compute itprob = 2.
do if (booting=0).
compute iterrmod=1.
end if.
do if (booting=1).
compute bootiter=1.
end if.
do if (itprobtg=0).
compute itprobtg=1.
compute errcode(errs,1) = 47.
compute errs = errs + 1.
do if (booting = 0 and 1 =1).
compute vt1 = mdiag(pt1lp&*(1-pt1lp)).
compute varb = inv(t(xlp)*vt1*xlp).
compute seb = sqrt(diag(varb)).
end if.
end if.
end if.
do if ( 1 =1).
compute trat = b&/seb.
compute dfres=nrow(xlp).
compute p = 2*(1-cdfnorm(abs(trat))).
compute modres={modres,seb,trat,p}.
compute modres={modres,(b-xp2&*seb),(b+xp2&*seb)}.
compute pvchi=1-chicdf((LL3-LL2),(nrow(modres)-1)).
compute mcF = (LL3-LL2)/LL3.
compute cox = 1-exp(-(LL3-LL2)/nrow(xlp)).
compute nagel = cox/(1-exp(-(LL3)/nrow(xlp))).
compute modsum={LL2,(LL3-LL2),(nrow(modres)-1),pvchi, mcF,cox,nagel}.
compute modsuml={'-2LL','ModelLL', 'df', 'p', 'McFadden', 'CoxSnell', 'Nagelkrk'}.
compute modresl={'coeff','se','Z','p','LLCI','ULCI'}.
end if.
end if
.
compute basemodx=LL2.
end if.
end if.
loop xmtlp1=1 to nxvls.
compute x={x,xtmp(:,xmtlp1)&*(mtmp(:,(xmints-1))-(csum(mtmp(:,(xmints-1)))/nrow(mtmp)) )}.
end loop.
do if ((i < (nms+nys)) or (ydich=0)).

.
do if ( 1 =1).
compute hatmat=inv(t( x )* x )*t( x ).
compute b = hatmat* y.
compute modres=b.
do if ( 1 =1).
compute n1=nrow( x ).
compute dfres=n1-(ncol( x )).
compute sstotal = t( y -(csum( y )/n1))*( y -(csum( y )/n1)).
compute resid= y - x *b.
compute ssresid = csum((resid)&**2).
compute r2 = (sstotal-ssresid)/sstotal.
do if (r2 < 0).
compute r2=0.
end if.
compute adjr2 = 1-((1-r2)*(n1-1)/(dfres)).
compute mse=ssresid/(n1-ncol( x )).

.
compute n1=nrow( x ).
compute invXtX = inv(t( x )* x ).
compute varb = mse *invXtX.
compute k3 = ncol( x ).
compute xhc=0.
do if ( hc <> 5).
compute xhc= x.
compute hat = xhc(:,1).
loop i3=1 to nrow(xhc).
compute hat(i3,1)= xhc(i3,:)*invXtX*t(xhc(i3,:)).
end loop.
do if ( hc = 0 or hc =1).
loop i3 = 1 to k3.
compute xhc(:,i3)=xhc(:,i3)&* resid.
end loop.
end if.
do if ( hc =3 or hc =2).
loop i3=1 to k3.
compute xhc(:,i3) = ( resid &/(1-hat)&**(1/(4- hc )))&*xhc(:,i3).
end loop.
end if.
do if ( hc = 4).
compute hcmn=make(n,2,4).
compute hcmn(:,2)=(n1*hat)/k3.
loop i3= 1 to k3.
compute xhc(:,i3) = ( resid &/(1-hat)&**(rmin(hcmn)/2))&*xhc(:,i3).
end loop.
end if.
compute varb=(invXtX*t(xhc)*xhc*invXtX).
do if ( hc =1).
compute varb=(n1/(n1-ncol( x )))&*varb.
end if.
end if.
compute hclab={'se(HC0)','se(HC1)','se(HC2)','se(HC3)','se(HC4)','se'}.
compute hclab=hclab(1,( hc +1)).
compute hcflab={'F(HC0)','F(HC1)','F(HC2)','F(HC3)','F(HC4)','F'}.
compute hcflab=hcflab(1,( hc +1)).
release xhc
.
compute seb=sqrt(diag(varb)).
compute trat = b&/seb.
compute p = 2*(1-tcdf(abs(trat), (dfres))).
compute tval = sqrt(dfres* (exp((dfres-(5/6))*((xp2/(dfres-(2/3)+(.11/dfres)))* (xp2/(dfres-(2/3)+(.11/dfres)))))-1)).
compute modres={modres,seb,trat,p}.
compute modres={modres,(b-tval&*seb),(b+tval&*seb)}.
compute modresl={'coeff',hclab,'t','p','LLCI','ULCI'}.
compute lmat = ident(ncol( x )).
compute lmat = lmat(:,2:ncol(lmat)).
compute fratio = (t(t(lmat)*b)*inv(t(lmat)*varb*lmat)*((t(lmat)*b)))/(ncol( x )-1).
compute pfr = 1-fcdf(fratio,(ncol( x )-1),dfres).
compute modsum={sqrt(r2),r2,mse,fratio,(ncol( x )-1),dfres,pfr}.
compute modsuml={'R','R-sq','MSE',hcflab,'df1','df2', 'p'}.
end if.
end if.
do if ( 1 = 2 or 1 =3).
compute xlp= x.
compute ylp= y.
compute pt2 = make(nrow(ylp),1,(csum(ylp)/nrow(ylp))).
do if ( 1 =2).
compute LL3 = ylp&*ln(pt2)+(1-ylp)&*ln(1-pt2).
end if.
compute LL3 = -2*csum(LL3).
compute bt1 = make(ncol(xlp),1,0).
compute LL1 = 0.
compute pt1 = make(nrow(ylp),1,0.5).
compute pt1lp=pt1.
loop jjj = 1 to iterate.
compute xlptmp=t(xlp).
compute vecprb=pt1lp&*(1-pt1lp).
loop kkk=1 to ncol(xlp).
compute xlptmp(kkk,:)=xlptmp(kkk,:)&*t(vecprb).
end loop.
compute b = bt1+inv(xlptmp*xlp)*t(xlp)*(ylp-pt1lp).
do if ( 1 =2).
compute xlpb=xlp*b.
compute xlpbt=(xlpb > -709.7).
compute xlpb709=(1-xlpbt)*(-709.7).
compute xlpb=(xlpb&*xlpbt)+xlpb709.
compute pt1lp = 1/(1+exp(-(xlpb))).
end if.
compute itprob = csum((pt1lp < .00000001) or (pt1lp > .9999999)).
do if (itprob > 0).
loop kkk = 1 to nrow(pt1lp).
do if (pt1lp(kkk,1) > .9999999).
compute pt1lp(kkk,1) = .9999999.
end if.
do if (pt1lp(kkk,1) < .00000001).
compute pt1lp(kkk,1) = .00000001.
end if.
end loop.
compute itprob = 0.
end if.
do if (itprob = 0).
do if ( 1 =2).
compute LL = ylp&*ln(pt1lp)+(1-ylp)&*ln(1-pt1lp).
end if.
compute LL2 = -2*csum(ll).
end if.
do if (abs(LL1-LL2) < converge).
do if ( 1 =1).
compute xlptmp=t(xlp).
compute vecprb=pt1lp&*(1-pt1lp).
loop kkk=1 to ncol(xlp).
compute xlptmp(kkk,:)=xlptmp(kkk,:)&*t(vecprb).
end loop.
compute varb = inv(xlptmp*xlp).
compute seb = sqrt(diag(varb)).
release xlptmp.
end if.
break.
end if.
compute bt1 = b.
compute LL1 = LL2.
end loop.
compute modres=b.
do if (jjj > iterate).
compute itprob = 2.
do if (booting=0).
compute iterrmod=1.
end if.
do if (booting=1).
compute bootiter=1.
end if.
do if (itprobtg=0).
compute itprobtg=1.
compute errcode(errs,1) = 47.
compute errs = errs + 1.
do if (booting = 0 and 1 =1).
compute vt1 = mdiag(pt1lp&*(1-pt1lp)).
compute varb = inv(t(xlp)*vt1*xlp).
compute seb = sqrt(diag(varb)).
end if.
end if.
end if.
do if ( 1 =1).
compute trat = b&/seb.
compute dfres=nrow(xlp).
compute p = 2*(1-cdfnorm(abs(trat))).
compute modres={modres,seb,trat,p}.
compute modres={modres,(b-xp2&*seb),(b+xp2&*seb)}.
compute pvchi=1-chicdf((LL3-LL2),(nrow(modres)-1)).
compute mcF = (LL3-LL2)/LL3.
compute cox = 1-exp(-(LL3-LL2)/nrow(xlp)).
compute nagel = cox/(1-exp(-(LL3)/nrow(xlp))).
compute modsum={LL2,(LL3-LL2),(nrow(modres)-1),pvchi, mcF,cox,nagel}.
compute modsuml={'-2LL','ModelLL', 'df', 'p', 'McFadden', 'CoxSnell', 'Nagelkrk'}.
compute modresl={'coeff','se','Z','p','LLCI','ULCI'}.
end if.
end if
.
end if.
do if ((i = (nms+nys)) and (ydich=1)).

.
do if ( 2 =1).
compute hatmat=inv(t( x )* x )*t( x ).
compute b = hatmat* y.
compute modres=b.
do if ( 1 =1).
compute n1=nrow( x ).
compute dfres=n1-(ncol( x )).
compute sstotal = t( y -(csum( y )/n1))*( y -(csum( y )/n1)).
compute resid= y - x *b.
compute ssresid = csum((resid)&**2).
compute r2 = (sstotal-ssresid)/sstotal.
do if (r2 < 0).
compute r2=0.
end if.
compute adjr2 = 1-((1-r2)*(n1-1)/(dfres)).
compute mse=ssresid/(n1-ncol( x )).

.
compute n1=nrow( x ).
compute invXtX = inv(t( x )* x ).
compute varb = mse *invXtX.
compute k3 = ncol( x ).
compute xhc=0.
do if ( hc <> 5).
compute xhc= x.
compute hat = xhc(:,1).
loop i3=1 to nrow(xhc).
compute hat(i3,1)= xhc(i3,:)*invXtX*t(xhc(i3,:)).
end loop.
do if ( hc = 0 or hc =1).
loop i3 = 1 to k3.
compute xhc(:,i3)=xhc(:,i3)&* resid.
end loop.
end if.
do if ( hc =3 or hc =2).
loop i3=1 to k3.
compute xhc(:,i3) = ( resid &/(1-hat)&**(1/(4- hc )))&*xhc(:,i3).
end loop.
end if.
do if ( hc = 4).
compute hcmn=make(n,2,4).
compute hcmn(:,2)=(n1*hat)/k3.
loop i3= 1 to k3.
compute xhc(:,i3) = ( resid &/(1-hat)&**(rmin(hcmn)/2))&*xhc(:,i3).
end loop.
end if.
compute varb=(invXtX*t(xhc)*xhc*invXtX).
do if ( hc =1).
compute varb=(n1/(n1-ncol( x )))&*varb.
end if.
end if.
compute hclab={'se(HC0)','se(HC1)','se(HC2)','se(HC3)','se(HC4)','se'}.
compute hclab=hclab(1,( hc +1)).
compute hcflab={'F(HC0)','F(HC1)','F(HC2)','F(HC3)','F(HC4)','F'}.
compute hcflab=hcflab(1,( hc +1)).
release xhc
.
compute seb=sqrt(diag(varb)).
compute trat = b&/seb.
compute p = 2*(1-tcdf(abs(trat), (dfres))).
compute tval = sqrt(dfres* (exp((dfres-(5/6))*((xp2/(dfres-(2/3)+(.11/dfres)))* (xp2/(dfres-(2/3)+(.11/dfres)))))-1)).
compute modres={modres,seb,trat,p}.
compute modres={modres,(b-tval&*seb),(b+tval&*seb)}.
compute modresl={'coeff',hclab,'t','p','LLCI','ULCI'}.
compute lmat = ident(ncol( x )).
compute lmat = lmat(:,2:ncol(lmat)).
compute fratio = (t(t(lmat)*b)*inv(t(lmat)*varb*lmat)*((t(lmat)*b)))/(ncol( x )-1).
compute pfr = 1-fcdf(fratio,(ncol( x )-1),dfres).
compute modsum={sqrt(r2),r2,mse,fratio,(ncol( x )-1),dfres,pfr}.
compute modsuml={'R','R-sq','MSE',hcflab,'df1','df2', 'p'}.
end if.
end if.
do if ( 2 = 2 or 2 =3).
compute xlp= x.
compute ylp= y.
compute pt2 = make(nrow(ylp),1,(csum(ylp)/nrow(ylp))).
do if ( 2 =2).
compute LL3 = ylp&*ln(pt2)+(1-ylp)&*ln(1-pt2).
end if.
compute LL3 = -2*csum(LL3).
compute bt1 = make(ncol(xlp),1,0).
compute LL1 = 0.
compute pt1 = make(nrow(ylp),1,0.5).
compute pt1lp=pt1.
loop jjj = 1 to iterate.
compute xlptmp=t(xlp).
compute vecprb=pt1lp&*(1-pt1lp).
loop kkk=1 to ncol(xlp).
compute xlptmp(kkk,:)=xlptmp(kkk,:)&*t(vecprb).
end loop.
compute b = bt1+inv(xlptmp*xlp)*t(xlp)*(ylp-pt1lp).
do if ( 2 =2).
compute xlpb=xlp*b.
compute xlpbt=(xlpb > -709.7).
compute xlpb709=(1-xlpbt)*(-709.7).
compute xlpb=(xlpb&*xlpbt)+xlpb709.
compute pt1lp = 1/(1+exp(-(xlpb))).
end if.
compute itprob = csum((pt1lp < .00000001) or (pt1lp > .9999999)).
do if (itprob > 0).
loop kkk = 1 to nrow(pt1lp).
do if (pt1lp(kkk,1) > .9999999).
compute pt1lp(kkk,1) = .9999999.
end if.
do if (pt1lp(kkk,1) < .00000001).
compute pt1lp(kkk,1) = .00000001.
end if.
end loop.
compute itprob = 0.
end if.
do if (itprob = 0).
do if ( 2 =2).
compute LL = ylp&*ln(pt1lp)+(1-ylp)&*ln(1-pt1lp).
end if.
compute LL2 = -2*csum(ll).
end if.
do if (abs(LL1-LL2) < converge).
do if ( 1 =1).
compute xlptmp=t(xlp).
compute vecprb=pt1lp&*(1-pt1lp).
loop kkk=1 to ncol(xlp).
compute xlptmp(kkk,:)=xlptmp(kkk,:)&*t(vecprb).
end loop.
compute varb = inv(xlptmp*xlp).
compute seb = sqrt(diag(varb)).
release xlptmp.
end if.
break.
end if.
compute bt1 = b.
compute LL1 = LL2.
end loop.
compute modres=b.
do if (jjj > iterate).
compute itprob = 2.
do if (booting=0).
compute iterrmod=1.
end if.
do if (booting=1).
compute bootiter=1.
end if.
do if (itprobtg=0).
compute itprobtg=1.
compute errcode(errs,1) = 47.
compute errs = errs + 1.
do if (booting = 0 and 1 =1).
compute vt1 = mdiag(pt1lp&*(1-pt1lp)).
compute varb = inv(t(xlp)*vt1*xlp).
compute seb = sqrt(diag(varb)).
end if.
end if.
end if.
do if ( 1 =1).
compute trat = b&/seb.
compute dfres=nrow(xlp).
compute p = 2*(1-cdfnorm(abs(trat))).
compute modres={modres,seb,trat,p}.
compute modres={modres,(b-xp2&*seb),(b+xp2&*seb)}.
compute pvchi=1-chicdf((LL3-LL2),(nrow(modres)-1)).
compute mcF = (LL3-LL2)/LL3.
compute cox = 1-exp(-(LL3-LL2)/nrow(xlp)).
compute nagel = cox/(1-exp(-(LL3)/nrow(xlp))).
compute modsum={LL2,(LL3-LL2),(nrow(modres)-1),pvchi, mcF,cox,nagel}.
compute modsuml={'-2LL','ModelLL', 'df', 'p', 'McFadden', 'CoxSnell', 'Nagelkrk'}.
compute modresl={'coeff','se','Z','p','LLCI','ULCI'}.
end if.
end if
.
compute chidfxm=basemodx-LL2.
end if.
compute lmat=make(nrow(b),nxvls,0).
compute lmattmp=ident(nxvls).
compute lmat((nrow(lmat)-nxvls+1):nrow(lmat),:)=lmattmp.

.
compute lmat2= lmat.
do if ( 1 =0).
compute lmat2 = mdiag( lmat ).
compute lmat3=make(nrow(lmat2),1,0).
loop flp=1 to ncol(lmat2).
do if (csum(lmat2(:,flp))=1).
compute lmat3={lmat3,lmat2(:,flp)}.
end if.
end loop.
compute lmat2=lmat3(:,2:ncol(lmat3)).
end if.
compute fratio = (t(t(lmat2)* b )*inv(t(lmat2)* varb *lmat2)*((t(lmat2)* b )))/ncol(lmat2).
compute pfr = 1-fcdf(fratio,ncol(lmat2),(n-nrow( b ))).
compute fresult={fratio,ncol(lmat2),(n-nrow( b )),pfr}.
do if (i = (nms+nys) and (ydich=1)).
compute fratio=fratio*ncol(lmat2).
compute pfr=1-chicdf(fratio,ncol(lmat2)).
compute fresult={fratio,ncol(lmat2),pfr}.
end if.
do if ( 0 =1).
compute lmat3=1-rsum(lmat2).
compute xfm=make(n,csum(lmat3),0).
compute flpc=1.
loop flp=1 to nrow(lmat3).
do if (lmat3(flp,1)=1).
compute xfm(:,flpc)=x(:,flp).
compute flpc=flpc+1.
end if.
end loop.
compute bfm=inv(t(xfm)*xfm)*t(xfm)*y.
compute resid=y-(xfm*bfm).
compute sstotal=(y-(csum(y)/n)).
compute sstotal=csum(sstotal&*sstotal).
compute ssresid=csum(resid&*resid).
compute rsqch= 0 -((sstotal-ssresid)/sstotal).
compute fresult={rsqch,fresult}.
release xfm,flpc, resid, ssresid, bfm.
end if
.
compute numxint=numxint+1.
compute xmtst(numxint,:)=fresult.
do if ((i = (nms+nys)) and (ydich=1)).
compute xmtst(numxint,1)=chidfxm.
compute xmtst(numxint,3)=1-chicdf(chidfxm,nxvls).
end if.
compute xmtstlb={xmtstlb;highlbx((xmints-1),1)}.
end if.
end loop.
compute x=xmtmat.
release xmtmat.
do if (numxint>0).
compute xmtstlb=xmtstlb((2:(numxint+1)),:).
compute xmtst=xmtst(1:numxint,:).
compute resultm2=make(nrow(xmtst),maxresm,99999).
compute resultm2(1:nrow(xmtst),1:ncol(xmtst))=xmtst.
compute resultm={resultm;resultm2}.
do if (nms=1).
compute xmtstlb=' '.
end if.
do if (outscree=1).
do if ((i < (nms+nys)) or (ydich=0)).
print xmtst/title='Test(s) of X by M interaction:'/rnames=xmtstlb/cnames=xmtstlbc/format= F10.4.
end if.
do if ((i = (nms+nys)) and (ydich=1)).
print xmtst/title='Likelihood ratio test(s) of X by M interaction:'/rnames=xmtstlb/cnames=xmtstlbc/format= F10.4.
end if.
end if.
end if.
compute r2=r2tmp.
compute b=btmp.
compute varb=varbtmp.
compute dfres=dfrestmp.
compute tval=tvaltmp.
end if.
do if (criterr = 0).
compute jj=0.
loop j = start to ((start+i)-1).
compute dbint=0.
compute lmat=whigh(1:nump(1,i),j).
compute lmat2=wzhigh(1:nump(1,i),j).
do if ((csum(lmat) > 0) and (csum(lmat2) = 0)).
do if ((i < (nms+nys)) or (ydich <> 1)).

.
compute lmat2= lmat.
do if ( 0 =0).
compute lmat2 = mdiag( lmat ).
compute lmat3=make(nrow(lmat2),1,0).
loop flp=1 to ncol(lmat2).
do if (csum(lmat2(:,flp))=1).
compute lmat3={lmat3,lmat2(:,flp)}.
end if.
end loop.
compute lmat2=lmat3(:,2:ncol(lmat3)).
end if.
compute fratio = (t(t(lmat2)* b )*inv(t(lmat2)* varb *lmat2)*((t(lmat2)* b )))/ncol(lmat2).
compute pfr = 1-fcdf(fratio,ncol(lmat2),(n-nrow( b ))).
compute fresult={fratio,ncol(lmat2),(n-nrow( b )),pfr}.
do if (i = (nms+nys) and (ydich=1)).
compute fratio=fratio*ncol(lmat2).
compute pfr=1-chicdf(fratio,ncol(lmat2)).
compute fresult={fratio,ncol(lmat2),pfr}.
end if.
do if ( 1 =1).
compute lmat3=1-rsum(lmat2).
compute xfm=make(n,csum(lmat3),0).
compute flpc=1.
loop flp=1 to nrow(lmat3).
do if (lmat3(flp,1)=1).
compute xfm(:,flpc)=x(:,flp).
compute flpc=flpc+1.
end if.
end loop.
compute bfm=inv(t(xfm)*xfm)*t(xfm)*y.
compute resid=y-(xfm*bfm).
compute sstotal=(y-(csum(y)/n)).
compute sstotal=csum(sstotal&*sstotal).
compute ssresid=csum(resid&*resid).
compute rsqch= r2 -((sstotal-ssresid)/sstotal).
compute fresult={rsqch,fresult}.
release xfm,flpc, resid, ssresid, bfm.
end if
.
compute lmatdb=lmat.
compute dbint=dbint+1.
end if.
do if ((ydich = 1) and (i = (nms+nys))).

.
compute btemphld=b.
compute llrdat=make(nrow(x),nrow( lmat )-csum( lmat ),-999).
compute llrdf=ncol(x)-ncol(llrdat).
compute llrcnt=0.
loop llri=1 to nrow( lmat ).
do if ( lmat (llri,1)=0).
compute llrcnt=llrcnt+1.
compute llrdat(:,llrcnt)=x(:,llri).
end if.
end loop.

.
do if ( 2 =1).
compute hatmat=inv(t( llrdat )* llrdat )*t( llrdat ).
compute b = hatmat* y.
compute modres=b.
do if ( 0 =1).
compute n1=nrow( llrdat ).
compute dfres=n1-(ncol( llrdat )).
compute sstotal = t( y -(csum( y )/n1))*( y -(csum( y )/n1)).
compute resid= y - llrdat *b.
compute ssresid = csum((resid)&**2).
compute r2 = (sstotal-ssresid)/sstotal.
do if (r2 < 0).
compute r2=0.
end if.
compute adjr2 = 1-((1-r2)*(n1-1)/(dfres)).
compute mse=ssresid/(n1-ncol( llrdat )).

.
compute n1=nrow( x ).
compute invXtX = inv(t( x )* x ).
compute varb = mse *invXtX.
compute k3 = ncol( x ).
compute xhc=0.
do if ( hc <> 5).
compute xhc= x.
compute hat = xhc(:,1).
loop i3=1 to nrow(xhc).
compute hat(i3,1)= xhc(i3,:)*invXtX*t(xhc(i3,:)).
end loop.
do if ( hc = 0 or hc =1).
loop i3 = 1 to k3.
compute xhc(:,i3)=xhc(:,i3)&* resid.
end loop.
end if.
do if ( hc =3 or hc =2).
loop i3=1 to k3.
compute xhc(:,i3) = ( resid &/(1-hat)&**(1/(4- hc )))&*xhc(:,i3).
end loop.
end if.
do if ( hc = 4).
compute hcmn=make(n,2,4).
compute hcmn(:,2)=(n1*hat)/k3.
loop i3= 1 to k3.
compute xhc(:,i3) = ( resid &/(1-hat)&**(rmin(hcmn)/2))&*xhc(:,i3).
end loop.
end if.
compute varb=(invXtX*t(xhc)*xhc*invXtX).
do if ( hc =1).
compute varb=(n1/(n1-ncol( x )))&*varb.
end if.
end if.
compute hclab={'se(HC0)','se(HC1)','se(HC2)','se(HC3)','se(HC4)','se'}.
compute hclab=hclab(1,( hc +1)).
compute hcflab={'F(HC0)','F(HC1)','F(HC2)','F(HC3)','F(HC4)','F'}.
compute hcflab=hcflab(1,( hc +1)).
release xhc
.
compute seb=sqrt(diag(varb)).
compute trat = b&/seb.
compute p = 2*(1-tcdf(abs(trat), (dfres))).
compute tval = sqrt(dfres* (exp((dfres-(5/6))*((xp2/(dfres-(2/3)+(.11/dfres)))* (xp2/(dfres-(2/3)+(.11/dfres)))))-1)).
compute modres={modres,seb,trat,p}.
compute modres={modres,(b-tval&*seb),(b+tval&*seb)}.
compute modresl={'coeff',hclab,'t','p','LLCI','ULCI'}.
compute lmat = ident(ncol( llrdat )).
compute lmat = lmat(:,2:ncol(lmat)).
compute fratio = (t(t(lmat)*b)*inv(t(lmat)*varb*lmat)*((t(lmat)*b)))/(ncol( llrdat )-1).
compute pfr = 1-fcdf(fratio,(ncol( llrdat )-1),dfres).
compute modsum={sqrt(r2),r2,mse,fratio,(ncol( llrdat )-1),dfres,pfr}.
compute modsuml={'R','R-sq','MSE',hcflab,'df1','df2', 'p'}.
end if.
end if.
do if ( 2 = 2 or 2 =3).
compute xlp= llrdat.
compute ylp= y.
compute pt2 = make(nrow(ylp),1,(csum(ylp)/nrow(ylp))).
do if ( 2 =2).
compute LL3 = ylp&*ln(pt2)+(1-ylp)&*ln(1-pt2).
end if.
compute LL3 = -2*csum(LL3).
compute bt1 = make(ncol(xlp),1,0).
compute LL1 = 0.
compute pt1 = make(nrow(ylp),1,0.5).
compute pt1lp=pt1.
loop jjj = 1 to iterate.
compute xlptmp=t(xlp).
compute vecprb=pt1lp&*(1-pt1lp).
loop kkk=1 to ncol(xlp).
compute xlptmp(kkk,:)=xlptmp(kkk,:)&*t(vecprb).
end loop.
compute b = bt1+inv(xlptmp*xlp)*t(xlp)*(ylp-pt1lp).
do if ( 2 =2).
compute xlpb=xlp*b.
compute xlpbt=(xlpb > -709.7).
compute xlpb709=(1-xlpbt)*(-709.7).
compute xlpb=(xlpb&*xlpbt)+xlpb709.
compute pt1lp = 1/(1+exp(-(xlpb))).
end if.
compute itprob = csum((pt1lp < .00000001) or (pt1lp > .9999999)).
do if (itprob > 0).
loop kkk = 1 to nrow(pt1lp).
do if (pt1lp(kkk,1) > .9999999).
compute pt1lp(kkk,1) = .9999999.
end if.
do if (pt1lp(kkk,1) < .00000001).
compute pt1lp(kkk,1) = .00000001.
end if.
end loop.
compute itprob = 0.
end if.
do if (itprob = 0).
do if ( 2 =2).
compute LL = ylp&*ln(pt1lp)+(1-ylp)&*ln(1-pt1lp).
end if.
compute LL2 = -2*csum(ll).
end if.
do if (abs(LL1-LL2) < converge).
do if ( 0 =1).
compute xlptmp=t(xlp).
compute vecprb=pt1lp&*(1-pt1lp).
loop kkk=1 to ncol(xlp).
compute xlptmp(kkk,:)=xlptmp(kkk,:)&*t(vecprb).
end loop.
compute varb = inv(xlptmp*xlp).
compute seb = sqrt(diag(varb)).
release xlptmp.
end if.
break.
end if.
compute bt1 = b.
compute LL1 = LL2.
end loop.
compute modres=b.
do if (jjj > iterate).
compute itprob = 2.
do if (booting=0).
compute iterrmod=1.
end if.
do if (booting=1).
compute bootiter=1.
end if.
do if (itprobtg=0).
compute itprobtg=1.
compute errcode(errs,1) = 47.
compute errs = errs + 1.
do if (booting = 0 and 0 =1).
compute vt1 = mdiag(pt1lp&*(1-pt1lp)).
compute varb = inv(t(xlp)*vt1*xlp).
compute seb = sqrt(diag(varb)).
end if.
end if.
end if.
do if ( 0 =1).
compute trat = b&/seb.
compute dfres=nrow(xlp).
compute p = 2*(1-cdfnorm(abs(trat))).
compute modres={modres,seb,trat,p}.
compute modres={modres,(b-xp2&*seb),(b+xp2&*seb)}.
compute pvchi=1-chicdf((LL3-LL2),(nrow(modres)-1)).
compute mcF = (LL3-LL2)/LL3.
compute cox = 1-exp(-(LL3-LL2)/nrow(xlp)).
compute nagel = cox/(1-exp(-(LL3)/nrow(xlp))).
compute modsum={LL2,(LL3-LL2),(nrow(modres)-1),pvchi, mcF,cox,nagel}.
compute modsuml={'-2LL','ModelLL', 'df', 'p', 'McFadden', 'CoxSnell', 'Nagelkrk'}.
compute modresl={'coeff','se','Z','p','LLCI','ULCI'}.
end if.
end if
.
compute b=btemphld.
compute fresult={(LL2-basemod),llrdf,1-chicdf((LL2-basemod),llrdf)}
.
compute lmatdb=lmat.
compute dbint=dbint+1.
end if.
compute highf={highf;fresult}.
compute highf2={highf2;fresult}.
do if (j = start).
compute flabel={flabel;'X*W'}.
end if.
do if (j > start).
do if (nms > 1).
compute flabel={flabel;highlbw(jj,1)}.
else if (nms = 1).
do if (xmint=0).
compute flabel={flabel;'M*W'}.
end if.
do if (xmint=1).
compute flabel={flabel;'X*M'}.
end if.
end if.
end if.
end if.
compute lmat=zhigh(1:nump(1,i),j).
compute lmat2=wzhigh(1:nump(1,i),j).
do if ((csum(lmat) > 0) and (csum(lmat2) = 0)).
do if ((i < (nms+nys)) or (ydich <> 1)).

.
compute lmat2= lmat.
do if ( 0 =0).
compute lmat2 = mdiag( lmat ).
compute lmat3=make(nrow(lmat2),1,0).
loop flp=1 to ncol(lmat2).
do if (csum(lmat2(:,flp))=1).
compute lmat3={lmat3,lmat2(:,flp)}.
end if.
end loop.
compute lmat2=lmat3(:,2:ncol(lmat3)).
end if.
compute fratio = (t(t(lmat2)* b )*inv(t(lmat2)* varb *lmat2)*((t(lmat2)* b )))/ncol(lmat2).
compute pfr = 1-fcdf(fratio,ncol(lmat2),(n-nrow( b ))).
compute fresult={fratio,ncol(lmat2),(n-nrow( b )),pfr}.
do if (i = (nms+nys) and (ydich=1)).
compute fratio=fratio*ncol(lmat2).
compute pfr=1-chicdf(fratio,ncol(lmat2)).
compute fresult={fratio,ncol(lmat2),pfr}.
end if.
do if ( 1 =1).
compute lmat3=1-rsum(lmat2).
compute xfm=make(n,csum(lmat3),0).
compute flpc=1.
loop flp=1 to nrow(lmat3).
do if (lmat3(flp,1)=1).
compute xfm(:,flpc)=x(:,flp).
compute flpc=flpc+1.
end if.
end loop.
compute bfm=inv(t(xfm)*xfm)*t(xfm)*y.
compute resid=y-(xfm*bfm).
compute sstotal=(y-(csum(y)/n)).
compute sstotal=csum(sstotal&*sstotal).
compute ssresid=csum(resid&*resid).
compute rsqch= r2 -((sstotal-ssresid)/sstotal).
compute fresult={rsqch,fresult}.
release xfm,flpc, resid, ssresid, bfm.
end if
.
compute dbint=dbint+1.
end if.
do if ((ydich = 1) and (i = (nms+nys))).

.
compute btemphld=b.
compute llrdat=make(nrow(x),nrow( lmat )-csum( lmat ),-999).
compute llrdf=ncol(x)-ncol(llrdat).
compute llrcnt=0.
loop llri=1 to nrow( lmat ).
do if ( lmat (llri,1)=0).
compute llrcnt=llrcnt+1.
compute llrdat(:,llrcnt)=x(:,llri).
end if.
end loop.

.
do if ( 2 =1).
compute hatmat=inv(t( llrdat )* llrdat )*t( llrdat ).
compute b = hatmat* y.
compute modres=b.
do if ( 0 =1).
compute n1=nrow( llrdat ).
compute dfres=n1-(ncol( llrdat )).
compute sstotal = t( y -(csum( y )/n1))*( y -(csum( y )/n1)).
compute resid= y - llrdat *b.
compute ssresid = csum((resid)&**2).
compute r2 = (sstotal-ssresid)/sstotal.
do if (r2 < 0).
compute r2=0.
end if.
compute adjr2 = 1-((1-r2)*(n1-1)/(dfres)).
compute mse=ssresid/(n1-ncol( llrdat )).

.
compute n1=nrow( x ).
compute invXtX = inv(t( x )* x ).
compute varb = mse *invXtX.
compute k3 = ncol( x ).
compute xhc=0.
do if ( hc <> 5).
compute xhc= x.
compute hat = xhc(:,1).
loop i3=1 to nrow(xhc).
compute hat(i3,1)= xhc(i3,:)*invXtX*t(xhc(i3,:)).
end loop.
do if ( hc = 0 or hc =1).
loop i3 = 1 to k3.
compute xhc(:,i3)=xhc(:,i3)&* resid.
end loop.
end if.
do if ( hc =3 or hc =2).
loop i3=1 to k3.
compute xhc(:,i3) = ( resid &/(1-hat)&**(1/(4- hc )))&*xhc(:,i3).
end loop.
end if.
do if ( hc = 4).
compute hcmn=make(n,2,4).
compute hcmn(:,2)=(n1*hat)/k3.
loop i3= 1 to k3.
compute xhc(:,i3) = ( resid &/(1-hat)&**(rmin(hcmn)/2))&*xhc(:,i3).
end loop.
end if.
compute varb=(invXtX*t(xhc)*xhc*invXtX).
do if ( hc =1).
compute varb=(n1/(n1-ncol( x )))&*varb.
end if.
end if.
compute hclab={'se(HC0)','se(HC1)','se(HC2)','se(HC3)','se(HC4)','se'}.
compute hclab=hclab(1,( hc +1)).
compute hcflab={'F(HC0)','F(HC1)','F(HC2)','F(HC3)','F(HC4)','F'}.
compute hcflab=hcflab(1,( hc +1)).
release xhc
.
compute seb=sqrt(diag(varb)).
compute trat = b&/seb.
compute p = 2*(1-tcdf(abs(trat), (dfres))).
compute tval = sqrt(dfres* (exp((dfres-(5/6))*((xp2/(dfres-(2/3)+(.11/dfres)))* (xp2/(dfres-(2/3)+(.11/dfres)))))-1)).
compute modres={modres,seb,trat,p}.
compute modres={modres,(b-tval&*seb),(b+tval&*seb)}.
compute modresl={'coeff',hclab,'t','p','LLCI','ULCI'}.
compute lmat = ident(ncol( llrdat )).
compute lmat = lmat(:,2:ncol(lmat)).
compute fratio = (t(t(lmat)*b)*inv(t(lmat)*varb*lmat)*((t(lmat)*b)))/(ncol( llrdat )-1).
compute pfr = 1-fcdf(fratio,(ncol( llrdat )-1),dfres).
compute modsum={sqrt(r2),r2,mse,fratio,(ncol( llrdat )-1),dfres,pfr}.
compute modsuml={'R','R-sq','MSE',hcflab,'df1','df2', 'p'}.
end if.
end if.
do if ( 2 = 2 or 2 =3).
compute xlp= llrdat.
compute ylp= y.
compute pt2 = make(nrow(ylp),1,(csum(ylp)/nrow(ylp))).
do if ( 2 =2).
compute LL3 = ylp&*ln(pt2)+(1-ylp)&*ln(1-pt2).
end if.
compute LL3 = -2*csum(LL3).
compute bt1 = make(ncol(xlp),1,0).
compute LL1 = 0.
compute pt1 = make(nrow(ylp),1,0.5).
compute pt1lp=pt1.
loop jjj = 1 to iterate.
compute xlptmp=t(xlp).
compute vecprb=pt1lp&*(1-pt1lp).
loop kkk=1 to ncol(xlp).
compute xlptmp(kkk,:)=xlptmp(kkk,:)&*t(vecprb).
end loop.
compute b = bt1+inv(xlptmp*xlp)*t(xlp)*(ylp-pt1lp).
do if ( 2 =2).
compute xlpb=xlp*b.
compute xlpbt=(xlpb > -709.7).
compute xlpb709=(1-xlpbt)*(-709.7).
compute xlpb=(xlpb&*xlpbt)+xlpb709.
compute pt1lp = 1/(1+exp(-(xlpb))).
end if.
compute itprob = csum((pt1lp < .00000001) or (pt1lp > .9999999)).
do if (itprob > 0).
loop kkk = 1 to nrow(pt1lp).
do if (pt1lp(kkk,1) > .9999999).
compute pt1lp(kkk,1) = .9999999.
end if.
do if (pt1lp(kkk,1) < .00000001).
compute pt1lp(kkk,1) = .00000001.
end if.
end loop.
compute itprob = 0.
end if.
do if (itprob = 0).
do if ( 2 =2).
compute LL = ylp&*ln(pt1lp)+(1-ylp)&*ln(1-pt1lp).
end if.
compute LL2 = -2*csum(ll).
end if.
do if (abs(LL1-LL2) < converge).
do if ( 0 =1).
compute xlptmp=t(xlp).
compute vecprb=pt1lp&*(1-pt1lp).
loop kkk=1 to ncol(xlp).
compute xlptmp(kkk,:)=xlptmp(kkk,:)&*t(vecprb).
end loop.
compute varb = inv(xlptmp*xlp).
compute seb = sqrt(diag(varb)).
release xlptmp.
end if.
break.
end if.
compute bt1 = b.
compute LL1 = LL2.
end loop.
compute modres=b.
do if (jjj > iterate).
compute itprob = 2.
do if (booting=0).
compute iterrmod=1.
end if.
do if (booting=1).
compute bootiter=1.
end if.
do if (itprobtg=0).
compute itprobtg=1.
compute errcode(errs,1) = 47.
compute errs = errs + 1.
do if (booting = 0 and 0 =1).
compute vt1 = mdiag(pt1lp&*(1-pt1lp)).
compute varb = inv(t(xlp)*vt1*xlp).
compute seb = sqrt(diag(varb)).
end if.
end if.
end if.
do if ( 0 =1).
compute trat = b&/seb.
compute dfres=nrow(xlp).
compute p = 2*(1-cdfnorm(abs(trat))).
compute modres={modres,seb,trat,p}.
compute modres={modres,(b-xp2&*seb),(b+xp2&*seb)}.
compute pvchi=1-chicdf((LL3-LL2),(nrow(modres)-1)).
compute mcF = (LL3-LL2)/LL3.
compute cox = 1-exp(-(LL3-LL2)/nrow(xlp)).
compute nagel = cox/(1-exp(-(LL3)/nrow(xlp))).
compute modsum={LL2,(LL3-LL2),(nrow(modres)-1),pvchi, mcF,cox,nagel}.
compute modsuml={'-2LL','ModelLL', 'df', 'p', 'McFadden', 'CoxSnell', 'Nagelkrk'}.
compute modresl={'coeff','se','Z','p','LLCI','ULCI'}.
end if.
end if
.
compute b=btemphld.
compute fresult={(LL2-basemod),llrdf,1-chicdf((LL2-basemod),llrdf)}
.
compute dbint=dbint+1.
end if.
compute highf={highf;fresult}.
compute highf2={highf2;fresult}.
do if (j = start).
compute flabel={flabel;'X*Z'}.
end if.
do if (j > start).
do if (nms > 1).
compute flabel={flabel;highlbz(jj,1)}.
else if (nms = 1).
compute flabel={flabel;'M*Z'}.
end if.
end if.
end if.
do if (dbint=2).
compute lmatdb=lmatdb+lmat.
do if ((ydich = 1) and (i = (nms+nys))).

.
compute btemphld=b.
compute llrdat=make(nrow(x),nrow( lmatdb )-csum( lmatdb ),-999).
compute llrdf=ncol(x)-ncol(llrdat).
compute llrcnt=0.
loop llri=1 to nrow( lmatdb ).
do if ( lmatdb (llri,1)=0).
compute llrcnt=llrcnt+1.
compute llrdat(:,llrcnt)=x(:,llri).
end if.
end loop.

.
do if ( 2 =1).
compute hatmat=inv(t( llrdat )* llrdat )*t( llrdat ).
compute b = hatmat* y.
compute modres=b.
do if ( 0 =1).
compute n1=nrow( llrdat ).
compute dfres=n1-(ncol( llrdat )).
compute sstotal = t( y -(csum( y )/n1))*( y -(csum( y )/n1)).
compute resid= y - llrdat *b.
compute ssresid = csum((resid)&**2).
compute r2 = (sstotal-ssresid)/sstotal.
do if (r2 < 0).
compute r2=0.
end if.
compute adjr2 = 1-((1-r2)*(n1-1)/(dfres)).
compute mse=ssresid/(n1-ncol( llrdat )).

.
compute n1=nrow( x ).
compute invXtX = inv(t( x )* x ).
compute varb = mse *invXtX.
compute k3 = ncol( x ).
compute xhc=0.
do if ( hc <> 5).
compute xhc= x.
compute hat = xhc(:,1).
loop i3=1 to nrow(xhc).
compute hat(i3,1)= xhc(i3,:)*invXtX*t(xhc(i3,:)).
end loop.
do if ( hc = 0 or hc =1).
loop i3 = 1 to k3.
compute xhc(:,i3)=xhc(:,i3)&* resid.
end loop.
end if.
do if ( hc =3 or hc =2).
loop i3=1 to k3.
compute xhc(:,i3) = ( resid &/(1-hat)&**(1/(4- hc )))&*xhc(:,i3).
end loop.
end if.
do if ( hc = 4).
compute hcmn=make(n,2,4).
compute hcmn(:,2)=(n1*hat)/k3.
loop i3= 1 to k3.
compute xhc(:,i3) = ( resid &/(1-hat)&**(rmin(hcmn)/2))&*xhc(:,i3).
end loop.
end if.
compute varb=(invXtX*t(xhc)*xhc*invXtX).
do if ( hc =1).
compute varb=(n1/(n1-ncol( x )))&*varb.
end if.
end if.
compute hclab={'se(HC0)','se(HC1)','se(HC2)','se(HC3)','se(HC4)','se'}.
compute hclab=hclab(1,( hc +1)).
compute hcflab={'F(HC0)','F(HC1)','F(HC2)','F(HC3)','F(HC4)','F'}.
compute hcflab=hcflab(1,( hc +1)).
release xhc
.
compute seb=sqrt(diag(varb)).
compute trat = b&/seb.
compute p = 2*(1-tcdf(abs(trat), (dfres))).
compute tval = sqrt(dfres* (exp((dfres-(5/6))*((xp2/(dfres-(2/3)+(.11/dfres)))* (xp2/(dfres-(2/3)+(.11/dfres)))))-1)).
compute modres={modres,seb,trat,p}.
compute modres={modres,(b-tval&*seb),(b+tval&*seb)}.
compute modresl={'coeff',hclab,'t','p','LLCI','ULCI'}.
compute lmat = ident(ncol( llrdat )).
compute lmat = lmat(:,2:ncol(lmat)).
compute fratio = (t(t(lmat)*b)*inv(t(lmat)*varb*lmat)*((t(lmat)*b)))/(ncol( llrdat )-1).
compute pfr = 1-fcdf(fratio,(ncol( llrdat )-1),dfres).
compute modsum={sqrt(r2),r2,mse,fratio,(ncol( llrdat )-1),dfres,pfr}.
compute modsuml={'R','R-sq','MSE',hcflab,'df1','df2', 'p'}.
end if.
end if.
do if ( 2 = 2 or 2 =3).
compute xlp= llrdat.
compute ylp= y.
compute pt2 = make(nrow(ylp),1,(csum(ylp)/nrow(ylp))).
do if ( 2 =2).
compute LL3 = ylp&*ln(pt2)+(1-ylp)&*ln(1-pt2).
end if.
compute LL3 = -2*csum(LL3).
compute bt1 = make(ncol(xlp),1,0).
compute LL1 = 0.
compute pt1 = make(nrow(ylp),1,0.5).
compute pt1lp=pt1.
loop jjj = 1 to iterate.
compute xlptmp=t(xlp).
compute vecprb=pt1lp&*(1-pt1lp).
loop kkk=1 to ncol(xlp).
compute xlptmp(kkk,:)=xlptmp(kkk,:)&*t(vecprb).
end loop.
compute b = bt1+inv(xlptmp*xlp)*t(xlp)*(ylp-pt1lp).
do if ( 2 =2).
compute xlpb=xlp*b.
compute xlpbt=(xlpb > -709.7).
compute xlpb709=(1-xlpbt)*(-709.7).
compute xlpb=(xlpb&*xlpbt)+xlpb709.
compute pt1lp = 1/(1+exp(-(xlpb))).
end if.
compute itprob = csum((pt1lp < .00000001) or (pt1lp > .9999999)).
do if (itprob > 0).
loop kkk = 1 to nrow(pt1lp).
do if (pt1lp(kkk,1) > .9999999).
compute pt1lp(kkk,1) = .9999999.
end if.
do if (pt1lp(kkk,1) < .00000001).
compute pt1lp(kkk,1) = .00000001.
end if.
end loop.
compute itprob = 0.
end if.
do if (itprob = 0).
do if ( 2 =2).
compute LL = ylp&*ln(pt1lp)+(1-ylp)&*ln(1-pt1lp).
end if.
compute LL2 = -2*csum(ll).
end if.
do if (abs(LL1-LL2) < converge).
do if ( 0 =1).
compute xlptmp=t(xlp).
compute vecprb=pt1lp&*(1-pt1lp).
loop kkk=1 to ncol(xlp).
compute xlptmp(kkk,:)=xlptmp(kkk,:)&*t(vecprb).
end loop.
compute varb = inv(xlptmp*xlp).
compute seb = sqrt(diag(varb)).
release xlptmp.
end if.
break.
end if.
compute bt1 = b.
compute LL1 = LL2.
end loop.
compute modres=b.
do if (jjj > iterate).
compute itprob = 2.
do if (booting=0).
compute iterrmod=1.
end if.
do if (booting=1).
compute bootiter=1.
end if.
do if (itprobtg=0).
compute itprobtg=1.
compute errcode(errs,1) = 47.
compute errs = errs + 1.
do if (booting = 0 and 0 =1).
compute vt1 = mdiag(pt1lp&*(1-pt1lp)).
compute varb = inv(t(xlp)*vt1*xlp).
compute seb = sqrt(diag(varb)).
end if.
end if.
end if.
do if ( 0 =1).
compute trat = b&/seb.
compute dfres=nrow(xlp).
compute p = 2*(1-cdfnorm(abs(trat))).
compute modres={modres,seb,trat,p}.
compute modres={modres,(b-xp2&*seb),(b+xp2&*seb)}.
compute pvchi=1-chicdf((LL3-LL2),(nrow(modres)-1)).
compute mcF = (LL3-LL2)/LL3.
compute cox = 1-exp(-(LL3-LL2)/nrow(xlp)).
compute nagel = cox/(1-exp(-(LL3)/nrow(xlp))).
compute modsum={LL2,(LL3-LL2),(nrow(modres)-1),pvchi, mcF,cox,nagel}.
compute modsuml={'-2LL','ModelLL', 'df', 'p', 'McFadden', 'CoxSnell', 'Nagelkrk'}.
compute modresl={'coeff','se','Z','p','LLCI','ULCI'}.
end if.
end if
.
compute b=btemphld.
compute fresult={(LL2-basemod),llrdf,1-chicdf((LL2-basemod),llrdf)}
.
end if.
do if (ydich <> 1 or i < (nms+nys)).

.
compute lmat2= lmatdb.
do if ( 0 =0).
compute lmat2 = mdiag( lmatdb ).
compute lmat3=make(nrow(lmat2),1,0).
loop flp=1 to ncol(lmat2).
do if (csum(lmat2(:,flp))=1).
compute lmat3={lmat3,lmat2(:,flp)}.
end if.
end loop.
compute lmat2=lmat3(:,2:ncol(lmat3)).
end if.
compute fratio = (t(t(lmat2)* b )*inv(t(lmat2)* varb *lmat2)*((t(lmat2)* b )))/ncol(lmat2).
compute pfr = 1-fcdf(fratio,ncol(lmat2),(n-nrow( b ))).
compute fresult={fratio,ncol(lmat2),(n-nrow( b )),pfr}.
do if (i = (nms+nys) and (ydich=1)).
compute fratio=fratio*ncol(lmat2).
compute pfr=1-chicdf(fratio,ncol(lmat2)).
compute fresult={fratio,ncol(lmat2),pfr}.
end if.
do if ( 1 =1).
compute lmat3=1-rsum(lmat2).
compute xfm=make(n,csum(lmat3),0).
compute flpc=1.
loop flp=1 to nrow(lmat3).
do if (lmat3(flp,1)=1).
compute xfm(:,flpc)=x(:,flp).
compute flpc=flpc+1.
end if.
end loop.
compute bfm=inv(t(xfm)*xfm)*t(xfm)*y.
compute resid=y-(xfm*bfm).
compute sstotal=(y-(csum(y)/n)).
compute sstotal=csum(sstotal&*sstotal).
compute ssresid=csum(resid&*resid).
compute rsqch= r2 -((sstotal-ssresid)/sstotal).
compute fresult={rsqch,fresult}.
release xfm,flpc, resid, ssresid, bfm.
end if
.
end if.
compute dbint=0.
compute highf={highf;fresult}.
do if (jj=0 and nms > 0).
compute flabel={flabel;'BOTH(X)'}.
end if.
do if (jj=0 and nms = 0).
compute flabel={flabel;'BOTH'}.
end if.
do if (jj>0 and nms = 1).
compute flabel={flabel;'BOTH(M)'}.
end if.
do if (nms > 1 and jj > 0).
compute flabel={flabel;highlbbt(jj,1)}.
end if.
end if.
compute lmat2=wzhigh(1:nump(1,i),j).
do if (csum(lmat2) > 0).
do if ((i < (nms+nys)) or (ydich <> 1)).

.
compute lmat2= lmat2.
do if ( 0 =0).
compute lmat2 = mdiag( lmat2 ).
compute lmat3=make(nrow(lmat2),1,0).
loop flp=1 to ncol(lmat2).
do if (csum(lmat2(:,flp))=1).
compute lmat3={lmat3,lmat2(:,flp)}.
end if.
end loop.
compute lmat2=lmat3(:,2:ncol(lmat3)).
end if.
compute fratio = (t(t(lmat2)* b )*inv(t(lmat2)* varb *lmat2)*((t(lmat2)* b )))/ncol(lmat2).
compute pfr = 1-fcdf(fratio,ncol(lmat2),(n-nrow( b ))).
compute fresult={fratio,ncol(lmat2),(n-nrow( b )),pfr}.
do if (i = (nms+nys) and (ydich=1)).
compute fratio=fratio*ncol(lmat2).
compute pfr=1-chicdf(fratio,ncol(lmat2)).
compute fresult={fratio,ncol(lmat2),pfr}.
end if.
do if ( 1 =1).
compute lmat3=1-rsum(lmat2).
compute xfm=make(n,csum(lmat3),0).
compute flpc=1.
loop flp=1 to nrow(lmat3).
do if (lmat3(flp,1)=1).
compute xfm(:,flpc)=x(:,flp).
compute flpc=flpc+1.
end if.
end loop.
compute bfm=inv(t(xfm)*xfm)*t(xfm)*y.
compute resid=y-(xfm*bfm).
compute sstotal=(y-(csum(y)/n)).
compute sstotal=csum(sstotal&*sstotal).
compute ssresid=csum(resid&*resid).
compute rsqch= r2 -((sstotal-ssresid)/sstotal).
compute fresult={rsqch,fresult}.
release xfm,flpc, resid, ssresid, bfm.
end if
.
end if.
do if ((ydich = 1) and (i = (nms+nys))).

.
compute btemphld=b.
compute llrdat=make(nrow(x),nrow( lmat2 )-csum( lmat2 ),-999).
compute llrdf=ncol(x)-ncol(llrdat).
compute llrcnt=0.
loop llri=1 to nrow( lmat2 ).
do if ( lmat2 (llri,1)=0).
compute llrcnt=llrcnt+1.
compute llrdat(:,llrcnt)=x(:,llri).
end if.
end loop.

.
do if ( 2 =1).
compute hatmat=inv(t( llrdat )* llrdat )*t( llrdat ).
compute b = hatmat* y.
compute modres=b.
do if ( 0 =1).
compute n1=nrow( llrdat ).
compute dfres=n1-(ncol( llrdat )).
compute sstotal = t( y -(csum( y )/n1))*( y -(csum( y )/n1)).
compute resid= y - llrdat *b.
compute ssresid = csum((resid)&**2).
compute r2 = (sstotal-ssresid)/sstotal.
do if (r2 < 0).
compute r2=0.
end if.
compute adjr2 = 1-((1-r2)*(n1-1)/(dfres)).
compute mse=ssresid/(n1-ncol( llrdat )).

.
compute n1=nrow( x ).
compute invXtX = inv(t( x )* x ).
compute varb = mse *invXtX.
compute k3 = ncol( x ).
compute xhc=0.
do if ( hc <> 5).
compute xhc= x.
compute hat = xhc(:,1).
loop i3=1 to nrow(xhc).
compute hat(i3,1)= xhc(i3,:)*invXtX*t(xhc(i3,:)).
end loop.
do if ( hc = 0 or hc =1).
loop i3 = 1 to k3.
compute xhc(:,i3)=xhc(:,i3)&* resid.
end loop.
end if.
do if ( hc =3 or hc =2).
loop i3=1 to k3.
compute xhc(:,i3) = ( resid &/(1-hat)&**(1/(4- hc )))&*xhc(:,i3).
end loop.
end if.
do if ( hc = 4).
compute hcmn=make(n,2,4).
compute hcmn(:,2)=(n1*hat)/k3.
loop i3= 1 to k3.
compute xhc(:,i3) = ( resid &/(1-hat)&**(rmin(hcmn)/2))&*xhc(:,i3).
end loop.
end if.
compute varb=(invXtX*t(xhc)*xhc*invXtX).
do if ( hc =1).
compute varb=(n1/(n1-ncol( x )))&*varb.
end if.
end if.
compute hclab={'se(HC0)','se(HC1)','se(HC2)','se(HC3)','se(HC4)','se'}.
compute hclab=hclab(1,( hc +1)).
compute hcflab={'F(HC0)','F(HC1)','F(HC2)','F(HC3)','F(HC4)','F'}.
compute hcflab=hcflab(1,( hc +1)).
release xhc
.
compute seb=sqrt(diag(varb)).
compute trat = b&/seb.
compute p = 2*(1-tcdf(abs(trat), (dfres))).
compute tval = sqrt(dfres* (exp((dfres-(5/6))*((xp2/(dfres-(2/3)+(.11/dfres)))* (xp2/(dfres-(2/3)+(.11/dfres)))))-1)).
compute modres={modres,seb,trat,p}.
compute modres={modres,(b-tval&*seb),(b+tval&*seb)}.
compute modresl={'coeff',hclab,'t','p','LLCI','ULCI'}.
compute lmat = ident(ncol( llrdat )).
compute lmat = lmat(:,2:ncol(lmat)).
compute fratio = (t(t(lmat)*b)*inv(t(lmat)*varb*lmat)*((t(lmat)*b)))/(ncol( llrdat )-1).
compute pfr = 1-fcdf(fratio,(ncol( llrdat )-1),dfres).
compute modsum={sqrt(r2),r2,mse,fratio,(ncol( llrdat )-1),dfres,pfr}.
compute modsuml={'R','R-sq','MSE',hcflab,'df1','df2', 'p'}.
end if.
end if.
do if ( 2 = 2 or 2 =3).
compute xlp= llrdat.
compute ylp= y.
compute pt2 = make(nrow(ylp),1,(csum(ylp)/nrow(ylp))).
do if ( 2 =2).
compute LL3 = ylp&*ln(pt2)+(1-ylp)&*ln(1-pt2).
end if.
compute LL3 = -2*csum(LL3).
compute bt1 = make(ncol(xlp),1,0).
compute LL1 = 0.
compute pt1 = make(nrow(ylp),1,0.5).
compute pt1lp=pt1.
loop jjj = 1 to iterate.
compute xlptmp=t(xlp).
compute vecprb=pt1lp&*(1-pt1lp).
loop kkk=1 to ncol(xlp).
compute xlptmp(kkk,:)=xlptmp(kkk,:)&*t(vecprb).
end loop.
compute b = bt1+inv(xlptmp*xlp)*t(xlp)*(ylp-pt1lp).
do if ( 2 =2).
compute xlpb=xlp*b.
compute xlpbt=(xlpb > -709.7).
compute xlpb709=(1-xlpbt)*(-709.7).
compute xlpb=(xlpb&*xlpbt)+xlpb709.
compute pt1lp = 1/(1+exp(-(xlpb))).
end if.
compute itprob = csum((pt1lp < .00000001) or (pt1lp > .9999999)).
do if (itprob > 0).
loop kkk = 1 to nrow(pt1lp).
do if (pt1lp(kkk,1) > .9999999).
compute pt1lp(kkk,1) = .9999999.
end if.
do if (pt1lp(kkk,1) < .00000001).
compute pt1lp(kkk,1) = .00000001.
end if.
end loop.
compute itprob = 0.
end if.
do if (itprob = 0).
do if ( 2 =2).
compute LL = ylp&*ln(pt1lp)+(1-ylp)&*ln(1-pt1lp).
end if.
compute LL2 = -2*csum(ll).
end if.
do if (abs(LL1-LL2) < converge).
do if ( 0 =1).
compute xlptmp=t(xlp).
compute vecprb=pt1lp&*(1-pt1lp).
loop kkk=1 to ncol(xlp).
compute xlptmp(kkk,:)=xlptmp(kkk,:)&*t(vecprb).
end loop.
compute varb = inv(xlptmp*xlp).
compute seb = sqrt(diag(varb)).
release xlptmp.
end if.
break.
end if.
compute bt1 = b.
compute LL1 = LL2.
end loop.
compute modres=b.
do if (jjj > iterate).
compute itprob = 2.
do if (booting=0).
compute iterrmod=1.
end if.
do if (booting=1).
compute bootiter=1.
end if.
do if (itprobtg=0).
compute itprobtg=1.
compute errcode(errs,1) = 47.
compute errs = errs + 1.
do if (booting = 0 and 0 =1).
compute vt1 = mdiag(pt1lp&*(1-pt1lp)).
compute varb = inv(t(xlp)*vt1*xlp).
compute seb = sqrt(diag(varb)).
end if.
end if.
end if.
do if ( 0 =1).
compute trat = b&/seb.
compute dfres=nrow(xlp).
compute p = 2*(1-cdfnorm(abs(trat))).
compute modres={modres,seb,trat,p}.
compute modres={modres,(b-xp2&*seb),(b+xp2&*seb)}.
compute pvchi=1-chicdf((LL3-LL2),(nrow(modres)-1)).
compute mcF = (LL3-LL2)/LL3.
compute cox = 1-exp(-(LL3-LL2)/nrow(xlp)).
compute nagel = cox/(1-exp(-(LL3)/nrow(xlp))).
compute modsum={LL2,(LL3-LL2),(nrow(modres)-1),pvchi, mcF,cox,nagel}.
compute modsuml={'-2LL','ModelLL', 'df', 'p', 'McFadden', 'CoxSnell', 'Nagelkrk'}.
compute modresl={'coeff','se','Z','p','LLCI','ULCI'}.
end if.
end if
.
compute b=btemphld.
compute fresult={(LL2-basemod),llrdf,1-chicdf((LL2-basemod),llrdf)}
.
end if.
compute highf={highf;fresult}.
compute highf2={highf2;fresult}.
do if (j = start).
compute flabel={flabel;'X*W*Z'}.
end if.
do if (j > start).
do if (nms > 1).
compute flabel={flabel;highlbwz(jj,1)}.
else if (nms = 1).
compute flabel={flabel;'M*W*Z'}.
end if.
end if.
end if.
compute jj=jj+1.
end loop.
release jj.
compute start=start+i.
end if.
do if (nrow(highf) > 1).
compute highf=highf(2:nrow(highf),:).
compute highf2=highf2(2:nrow(highf2),:).
compute flabel=flabel(2:nrow(flabel),1).
compute resultm2=make(nrow(highf),maxresm,99999).
compute resultm2(1:nrow(highf),1:ncol(highf))=highf.
compute resultm={resultm;resultm2}.
do if (outscree=1).
do if ((i < nms+nys) or (ydich=0)).
compute clabtmp={'R2-chng', hcflab,'df1','df2','p'}.
print highf/format = F10.4 /rnames=flabel/cnames=clabtmp/ title = 'Test(s) of highest order unconditional interaction(s):'.
end if.
do if (ydich=1 and i=(nms+nys)).
compute clabtmp={'Chi-sq', 'df','p'}.
print/title='Likelihood ratio test(s) of highest order'.
print highf/format = F10.4 /rnames=flabel/cnames=clabtmp/ title = 'unconditional interactions(s):'/space=0.
end if.
end if.
compute intpb=highf2(:,ncol(highf2)).
end if.
compute intstart=intstart+numint(1,i).
end if.

.
do if (criterr=0).
compute threeway=0.
compute didprint=0.
compute didsome=0.
compute sigintct=0.
loop jmed =1 to (nms+1).
compute hasw=0.
compute hasz=0.
compute jnok=0.
compute nm1vls=0.
compute nm2vls=0.
compute panelgrp=0.
compute graphixs={'WITH', outnames(1,i), 'BY'}.
compute focpred4={' '}.
compute intprint=0.
compute modcat=0.
do if (jmed <= i).
do if ((jmed = 1) and ((i+1) = nrow(bcmat))).
compute pathscnt=pathscnt+1.
else.
compute paths={paths,bcmat((i+1),jmed)}.
compute pathsw={pathsw,wcmat((i+1),jmed)}.
compute pathsz={pathsz,zcmat((i+1),jmed)}.
compute pathswz={pathswz,wzcmat((i+1),jmed)}.
compute temp=fochigh(:,pathscnt)&*bootloc(:,i).
compute pathsfoc={pathsfoc,pathsfoc(:,1)}.
do if (jmed=1).
compute pathtype={pathtype,1}.
end if.
do if ((i+1)=nrow(bcmat)).
compute pathtype={pathtype,3}.
end if.
do if (jmed > 1) and ((i+1) < nrow(bcmat)).
compute pathtype={pathtype,2}.
end if.
do if (jmed=1 and nxvls > 1 and (bcmat((i+1),jmed)=1)).
compute pathsfoc(:,(pathscn2+1))=temp(2:(nxvls+1),1).
end if.
do if ((jmed > 1) or (jmed=1 and nxvls=1)).
compute temp=cmax(temp).
compute pathsfoc(1,(pathscn2+1))=temp.
end if.
compute pathscnt=pathscnt+1.
compute pathscn2=pathscn2+1.
do if (i <= nms).
compute pathsdv={pathsdv,mnames(1,i)}.
end if.
do if (i > nms).
compute pathsdv={pathsdv,ynames}.
end if.
end if.
compute coeffcol=coeffcol+1.
compute probettt=coeffs(1:nrow(b),coeffcol).
do if (jmed=1 and (bcmat((i+1),jmed)=1)).
compute omni=make(nrow(probettt),nxvls,0).
compute omnitmp=ident(nxvls).
compute omni(2:(1+nxvls),:)=omnitmp.
end if.
do if (csum(probettt)>0).
compute probvarb=make(csum(probettt),csum(probettt),999).
compute probcoef=make(csum(probettt),1,999).
compute coefflp2=1.
loop coefflp=1 to nrow(probettt).
do if (probettt(coefflp,1)=1).
compute probcoef(coefflp2,1)=b(coefflp,1).
compute coefflp2=coefflp2+1.
end if.
end loop.
compute coefflp=0.
compute coefflp2=0.
loop iclp=1 to nrow(probettt).
do if probettt(iclp,1)=1.
compute coefflp=coefflp+1.
compute coefflp2=coefflp.
compute probvarb(coefflp,coefflp) = varb(iclp,iclp).
do if (iclp < nrow(probettt)).
loop jclp=(iclp+1) to nrow(probettt).
do if (probettt(jclp,1)=1).
compute coefflp2=coefflp2+1.
compute probvarb(coefflp,coefflp2)=varb(iclp, jclp).
compute probvarb(coefflp2,coefflp)=varb(iclp, jclp).
end if.
end loop.
end if.
end if.
end loop.
end if.
end if.
compute xprobval=xmodvals.
do if (nxvls > 1 or mcx > 0).
compute xprobval=dummatx(:,2:ncol(dummatx)).
end if.
do if ((wcmat((i+1),jmed)=1) and (zcmat((i+1),jmed)=0)).
compute numplps=1.
compute modvals=wmodvals.
compute probeval=wmodvals.
compute wheremv1=wherexw.
compute nm1vls=nwvls.
compute lpstsp={1,1}.
compute modcat=0.
compute jnmod=wtmp.
compute jnmodlab=wnames.
compute jnok=1.
compute jnmin=wmin.
compute jnmax=wmax.
compute wherejn1=2.
do if (jmed=1).
compute wherejn3=wherexw(1,i).
do if (nxvls > 1).
compute jnok=0.
end if.
end if.
do if (jmed > 1).
compute wherejn1=wherem((jmed-1),i).
compute wherejn3=wheremw(((2*jmed)-3),i).
end if.
do if (nwvls > 1).
compute probeval=wprobval.
compute lpstsp(1,2)=ncol(probeval).
compute modcat=1.
compute jnok=0.
end if.
do if (wdich = 1).
compute modcat=1.
compute jnok=0.
end if.
compute problabs=wnames.
compute focpred3={wnames,'(W)'}.
do if (xmint=1).
compute focpred3={wnames,'(X)'}.
end if.
compute hasw=1.
compute modgrph=wnames.
compute intprint=1.
compute sigintct=sigintct+1.
compute printpbe=intpb(sigintct,1).
end if.
do if ((wcmat((i+1),jmed)=0) and (zcmat((i+1),jmed)=1)).
compute numplps=1.
compute modvals=zmodvals.
compute probeval=zmodvals.
compute wheremv1=wherexz.
compute nm1vls=nzvls.
compute lpstsp={1,1}.
compute jnok=1.
compute jnmod=ztmp.
compute jnmin=zmin.
compute jnmax=zmax.
compute jnmodlab=znames.
compute wherejn1=2.
do if (jmed=1).
compute wherejn3=wherexz(1,i).
do if (nxvls > 1).
compute jnok=0.
end if.
end if.
do if (jmed > 1).
compute wherejn1=wherem((jmed-1),i).
compute wherejn3=wheremz(((2*jmed)-3),i).
end if.
do if (nzvls > 1).
compute probeval=zprobval.
compute lpstsp(1,2)=ncol(probeval).
compute modcat=1.
compute jnok=0.
end if.
do if (zdich = 1).
compute modcat=1.
compute jnok=0.
end if.
compute problabs=znames.
compute focpred3={znames,'(Z)'}.
compute modgrph=znames.
compute hasz=1.
compute intprint=1.
compute sigintct=sigintct+1.
compute printpbe=intpb(sigintct,1).
end if.
do if ((wzcmat((i+1),jmed)=1) or ((wcmat((i+1),jmed)=1) and (zcmat((i+1),jmed)=1))).
compute numplps=2.
compute probecnt=1.
compute intprint=1.
do if (wzcmat((i+1),jmed)=1).
compute sigintct=sigintct+1.
compute printpbe=intpb(sigintct,1).
else.
compute sigintct=sigintct+2.
compute printpbe=cmin(intpb((sigintct-1):sigintct,1)).
end if.
compute panelgrp=1.
compute hasw=1.
compute hasz=1.
compute panelcde={'/PANEL','ROWVAR=',znames,'.'}.
compute modgrph=wnames.
compute lpstsp={1,1;1,1}.
compute wheremv1=wherexw.
compute nm1vls=nwvls.
compute wheremv2=wherexz.
compute nm2vls=nzvls.
compute jnok=0.
do if (wzcmat((i+1),jmed)=1).
compute jnok=1.
end if.
do if (jmed > 1).
compute mprobval=mmodvals.
end if.
do if (jmed=1).
do if (nxvls > 1).
compute jnok=0.
end if.
end if.
do if (nwvls > 1).
compute lpstsp(1,2)=ncol(wprobval).
compute modcat=1.
compute jnok=0.
end if.
do if (zdich=1).
compute modcat=1.
compute jnok=0.
end if.
compute lpstsp(2,1)=lpstsp(1,2)+1.
compute lpstsp(2,2)=lpstsp(1,2)+1.
do if (nzvls > 1).
compute lpstsp(2,1)=lpstsp(1,2)+1.
compute lpstsp(2,2)=lpstsp(1,2)+ncol(zprobval).
compute jnok=0.
end if.
do if (zdich=1).
compute jnok=0.
end if.
compute omni3=make(nrow(b),(nxvls*nwvls),0).
do if (jmed > 1).
compute omni3=make(nrow(b),nwvls,0).
end if.
compute focpred3={wnames,'(W)'}.
do if (xmint=1).
compute focpred3={wnames,'(X)'}.
end if.
compute focpred4={'    ', 'Mod var:', znames, '(Z)'}.
compute modvals=make((nrow(wmodvals)*nrow(zmodvals)),2,0).
compute probeval=make((nrow(wmodvals)*nrow(zmodvals)),(ncol(wprobval)+ncol(zprobval)),0).
loop probei= 1 to nrow(wmodvals).
loop probej =1 to nrow(zmodvals).
compute modvals(probecnt,1)=wmodvals(probei,1).
compute probeval(probecnt,1:nwvls)=wprobval(probei,:).
compute modvals(probecnt,2)=zmodvals(probej,1).
compute probeval(probecnt,(nwvls+1):(nwvls+nzvls))=zprobval(probej,:).
compute probecnt=probecnt+1.
end loop.
end loop.
do if (wzcmat((i+1),jmed)=1).
compute numplps=numplps+1.
compute probprod=make(1,(ncol(wprobval)*ncol(zprobval)),0).
compute lpstsp2={1,1}.
compute lpstsp={lpstsp;lpstsp2}.
compute lpstsp(3,1)=lpstsp(2,2)+1.
compute lpstsp(3,2)=lpstsp(2,2)+ncol(probprod).
compute jnmod=ztmp.
compute jnmin=zmin.
compute jnmax=zmax.
compute jnmodlab=znames.
do if (jmed = 1).
compute wherejn1=wherexw(1,i).
compute wherejn3=wherexwz(1,i).
end if.
do if (jmed > 1).
compute wherejn1=wheremw(((2*jmed)-3),i).
compute wherejn3=wheremwz(((2*jmed)-3),i).
end if.
loop probei = 1 to nrow(wmodvals).
loop probej = 1 to nrow(zmodvals).
compute probtemp=1.
loop probek = 1 to ncol(wprobval).
compute probtemp={probtemp,(wprobval(probei,probek)&*zprobval(probej,:))}.
end loop.
compute probprod={probprod;probtemp(1,2:ncol(probtemp))}.
end loop.
end loop.
compute probprod=probprod(2:nrow(probprod),:).
compute probeval={probeval,probprod}.
end if.
compute problabs={wnames,znames}.
release probecnt, probei, probej.
end if.
do if (intprint=1).
compute focpred={'   Focal', 'predict:'}.
do if (jmed=1).
compute focpred={focpred,xnames,'(X)'}.
compute focplotv=xmodvals.
end if.
do if (jmed >1).
do if (nms > 1).
compute focpred={focpred,mnames(1,(jmed-1)), medlb2(1,(jmed-1))}.
end if.
do if (nms = 1).
compute focpred={focpred,mnames(1,(jmed-1)), '(M)'}.
end if.
compute focplotv=mmodvals(:,(jmed-1)).
end if.
compute focpred2={'    ', 'Mod var:',focpred3}.
compute focpred={focpred;focpred2}.
do if (ncol(focpred4) > 1).
compute focpred={focpred;focpred4}.
compute focpred4={' '}.
end if.
release focpred2,focpred3.
do if (outscree=1).
do if ((plot = 1 or plot = 2) or (printpbe <= intprobe)).
print focpred/title='----------'/format=A8/space=0.
end if.
end if.
compute foctmp=make(nrow(modvals),1,1).
compute probexpl=1.
compute probeva2={foctmp,probeval}.
do if (jmed=1 and nxs > 0 and mcx > 0).
compute probexpl=nxvls.
end if.
compute foctmp=make(nrow(modvals),1,1).
compute modvals3=make(1,(6+ncol(problabs)),0).
compute probrown=make(nrow(probeval),1,0).
compute jtmp=1.
loop probei = 1 to nrow(probeval).
compute probrown(probei,1)=jtmp.
compute jtmp=jtmp+nxvls.
end loop.
release jtmp.
compute probrow=999.
compute modvarl=problabs.
do if (plot = 1 or plot = 2 or nxvls > 1).
compute plotvals=make((nrow(modvals)*nrow(focplotv)),(ncol(modvals)+1),999).
loop ploti=1 to nrow(modvals).
loop plotj=1 to nrow(focplotv).
compute plotvals((((ploti-1)*nrow(focplotv))+plotj),2:ncol(plotvals))=modvals(ploti,:).
compute plotvals((((ploti-1)*nrow(focplotv))+plotj),1)=focplotv(plotj,1).
end loop.
end loop.
compute focpredn=3.
do if (jmed=1).
do if (nxvls > 1).
compute focpredn=(nxvls+1).
end if.
do if (nxvls=1 and xdich=1).
compute focpredn=2.
end if.
end if.
compute meanmat=mdiag(means).
compute onesmat=make(nrow(meanmat),(nrow(probeval)*focpredn),1).
compute probeplt=t(mdiag(means)*onesmat).
do if (jmed=1).
do if ((wcmat((i+1),1)=1) or (zcmat((i+1),1)=1)).
compute plotcnt=1.
compute iloops=nwpval*nzpval.
compute plotmx=nxpval*nzpval.
do if ((wcmat((i+1),1)=1) and (zcmat((i+1),1)=0)).
compute iloops=nwpval.
compute plotmx=nxpval.
end if.
do if ((wcmat((i+1),1)=0) and (zcmat((i+1),1)=1)).
compute iloops=nzpval.
compute plotmx=nxpval.
end if.
compute xestvals=make((nxpval*iloops),ncol(xprobval),-999).
do if (wcmat((i+1),1)=1).
compute westvals=make(nrow(xestvals),ncol(wprobval),-999).
end if.
do if (zcmat((i+1),1)=1).
compute zestvals=make(nrow(xestvals),ncol(zprobval),-999).
end if.
loop ploti=1 to iloops.
loop plotj=1 to nxpval.
compute xestvals(plotcnt,:)=xprobval(plotj,:).
compute plotcnt=plotcnt+1.
end loop.
end loop.
compute plotcnt=1.
compute plotcnt1=1.
compute plotcnt2=1.
compute plotcntz=1.
loop ploti = 1 to (iloops*nxpval).
do if (wcmat((i+1),1)=1).
compute westvals(ploti,:)=wprobval(plotcnt1,:).
end if.
do if ((wcmat((i+1),1)=0) and (zcmat((i+1),1)=1)).
compute zestvals(ploti,:)=zprobval(plotcnt1,:).
end if.
do if ((wcmat((i+1),1)=1) and (zcmat((i+1),1)=1)).
compute zestvals(ploti,:)=zprobval(plotcnt2,:).
compute plotcntz=plotcntz+1.
end if.
compute plotcnt=plotcnt+1.
do if (plotcnt > plotmx).
compute plotcnt=1.
compute plotcnt1=plotcnt1+1.
end if.
do if (plotcntz > nxpval).
compute plotcnt2=plotcnt2+1.
compute plotcntz=1.
do if (plotcnt2 > nzpval).
compute plotcnt2=1.
end if.
end if.
end loop.
compute probeplt(:,2:(1+(ncol(xestvals))))=xestvals.
do if (wcmat((i+1),1)=1).
compute probeplt(:,(wherew(1,i)):(wherew(2,i)))=westvals.
end if.
do if (zcmat((i+1),1)=1).
compute probeplt(:,(wherez(1,i)):(wherez(2,i)))=zestvals.
end if.
end if.
end if.
do if (jmed > 1).
do if ((wcmat((i+1),jmed)=1) or (zcmat((i+1),jmed)=1)).
compute plotcnt=1.
compute iloops=nwpval*nzpval.
compute plotmx=3*nzpval.
do if ((wcmat((i+1),jmed)=1) and (zcmat((i+1),jmed)=0)).
compute iloops=nwpval.
compute plotmx=3.
end if.
do if ((wcmat((i+1),jmed)=0) and (zcmat((i+1),jmed)=1)).
compute iloops=nzpval.
compute plotmx=3.
end if.
compute mestvals=make((3*iloops),1,-999).
do if (wcmat((i+1),jmed)=1).
compute westvals=make(nrow(mestvals),ncol(wprobval),-999).
end if.
do if (zcmat((i+1),jmed)=1).
compute zestvals=make(nrow(mestvals),ncol(zprobval),-999).
end if.
loop ploti=1 to iloops.
loop plotj=1 to 3.
compute mestvals(plotcnt,:)=mprobval(plotj,(jmed-1)).
compute plotcnt=plotcnt+1.
end loop.
end loop.
compute plotcnt=1.
compute plotcnt1=1.
compute plotcnt2=1.
compute plotcntz=1.
loop ploti = 1 to (iloops*3).
do if (wcmat((i+1),jmed)=1).
compute westvals(ploti,:)=wprobval(plotcnt1,:).
end if.
do if ((wcmat((i+1),jmed)=0) and (zcmat((i+1),jmed)=1)).
compute zestvals(ploti,:)=zprobval(plotcnt1,:).
end if.
do if ((wcmat((i+1),jmed)=1) and (zcmat((i+1),jmed)=1)).
compute zestvals(ploti,:)=zprobval(plotcnt2,:).
compute plotcntz=plotcntz+1.
end if.
compute plotcnt=plotcnt+1.
do if (plotcnt > plotmx).
compute plotcnt=1.
compute plotcnt1=plotcnt1+1.
end if.
do if (plotcntz > 3).
compute plotcnt2=plotcnt2+1.
compute plotcntz=1.
do if (plotcnt2 > nzpval).
compute plotcnt2=1.
end if.
end if.
end loop.
compute probeplt(:,wherem((jmed-1),i))=mestvals.
do if (wcmat((i+1),jmed)=1).
do if (model <> 74).
compute probeplt(:,(wherew(1,i)):(wherew(2,i)))=westvals.
end if.
do if (model = 74).
compute probeplt(:,(wherex(1,i)):(wherex(2,i)))=westvals.
end if.
end if.
do if (zcmat((i+1),jmed)=1).
compute probeplt(:,(wherez(1,i)):(wherez(2,i)))=zestvals.
end if.
end if.
end if.
compute prodloop = 1.
do if (jmed=1).
compute prodloop=ncol(xestvals).
end if.
do if (wcmat((i+1),jmed))=1.
compute plotcnt=0.
loop ploti = 1 to prodloop.
loop plotj = 1 to ncol(westvals).
do if (jmed=1).
compute probeplt(:,(wherexw(1,i)+plotcnt))=xestvals(:,ploti)&*westvals(:,plotj).
end if.
do if (jmed > 1).
compute probeplt(:,(wheremw(((jmed*2)-3) ,i)+plotcnt))=mestvals(:,ploti)&*westvals(:,plotj).
end if.
compute plotcnt=plotcnt+1.
end loop.
end loop.
end if.
do if (zcmat((i+1),jmed))=1.
compute plotcnt=0.
loop ploti = 1 to prodloop.
loop plotj = 1 to ncol(zestvals).
do if (jmed = 1).
compute probeplt(:,(wherexz(1,i)+plotcnt))=xestvals(:,ploti)&*zestvals(:,plotj).
end if.
do if (jmed > 1).
compute probeplt(:,(wheremz(((jmed*2)-3),i)+plotcnt))=mestvals(:,ploti)&*zestvals(:, plotj).
end if.
compute plotcnt=plotcnt+1.
end loop.
end loop.
end if.
do if (wzcmat((i+1),jmed))=1.
compute plotcnt=0.
compute threeway=1.
loop ploti = 1 to ncol(westvals).
loop plotj = 1 to ncol(zestvals).
compute probeplt(:,(wherewz(1,i)+plotcnt))=westvals(:,ploti)&*zestvals(:,plotj).
compute plotcnt=plotcnt+1.
end loop.
end loop.
compute plotcnt=0.
loop plotk = 1 to prodloop.
loop ploti = 1 to ncol(westvals).
loop plotj = 1 to ncol(zestvals).
do if (jmed = 1).
compute probeplt(:,(wherexwz(1,i)+plotcnt))=xestvals(:,plotk)&*westvals(:,ploti)&* zestvals(:,plotj).
end if.
do if (jmed > 1).
compute probeplt(:,(wheremwz(((jmed*2)-3),i)+plotcnt))=mestvals(:,plotk)&* westvals(:,ploti)&*zestvals(:,plotj).
end if.
compute plotcnt=plotcnt+1.
end loop.
end loop.
end loop.
end if.
loop newplp=1 to i.
do if (newplp <> jmed).
do if (wcmat((i+1),newplp))=1.
compute prodloop=1.
do if (newplp=1).
compute prodloop=nxvls.
end if.
compute plotcnt=0.
loop ploti = 1 to prodloop.
loop plotj = 1 to nwvls.
do if (newplp = 1).
compute probeplt(:,(wherexw(1,i)+plotcnt))=probeplt(:,(1+ploti))&*probeplt(:, (wherew(1,i)+plotj-1)).
end if.
do if (newplp > 1).
do if (model <> 74).
compute probeplt(:,(wheremw(((newplp*2)-3) ,i)+plotcnt))=probeplt(:,wherem((newplp-1),i))&*probeplt(:,(wherew(1,i)+plotj-1)).
end if.
do if (model = 74).
compute probeplt(:,(wheremw(((newplp*2)-3) ,i)+plotcnt))=probeplt(:,wherem((newplp-1),i))&*probeplt(:,(wherex(1,i)+plotj-1)).
end if.
end if.
compute plotcnt=plotcnt+1.
end loop.
end loop.
end if.
do if (zcmat((i+1),newplp))=1.
compute prodloop=1.
do if (newplp=1).
compute prodloop=nxvls.
end if.
compute plotcnt=0.
loop ploti = 1 to prodloop.
loop plotj = 1 to nzvls.
do if (newplp = 1).
compute probeplt(:,(wherexz(1,i)+plotcnt))=probeplt(:,(1+ploti))&*probeplt(:, (wherez(1,i)+plotj-1)).
end if.
do if (newplp > 1).
compute probeplt(:,(wheremz(((newplp*2)-3),i)+plotcnt))=probeplt(:, wherem((newplp-1),i))&*probeplt(:,(wherez(1,i)+plotj-1)).
end if.
compute plotcnt=plotcnt+1.
end loop.
end loop.
end if.
do if (wzcmat((i+1),newplp))=1.
compute plotcnt=0.
do if (threeway=0).
loop ploti = 1 to nwvls.
loop plotj = 1 to nzvls.
compute probeplt(:,(wherewz(1,i)+plotcnt))=probeplt(:,(wherew(1,i)+ploti-1))&* probeplt(:,(wherez(1,i)+plotj-1)).
compute plotcnt=plotcnt+1.
end loop.
end loop.
end if.
compute prodloop=1.
do if (newplp=1).
compute prodloop=nxvls.
end if.
compute plotcnt=0.
loop plotk = 1 to prodloop.
loop ploti = 1 to nwvls.
loop plotj = 1 to nzvls.
do if (newplp = 1).
compute probeplt(:,(wherexwz(1,i)+plotcnt))=probeplt(:,(1+plotk))&*probeplt(:, (wherew(1,i)+ploti-1))&*probeplt(:,(wherez(1,i)+plotj-1)).
end if.
do if (newplp > 1).
compute probeplt(:,(wheremwz(((newplp*2)-3),i)+plotcnt))=probeplt(:, wherem((newplp-1),i))&*probeplt(:,(wherew(1,i)+ploti-1))&*probeplt(:,(wherez(1,i)+plotj-1)).
end if.
compute plotcnt=plotcnt+1.
end loop.
end loop.
end loop.
end if.
end if.
end loop.
compute predvals=probeplt*b.
do if (debug <> 0).
print probeplt.
print b.
end if.
do if (i = nms+nys) and (ydich=1).
compute predvalt=(predvals < 709.7).
compute prevalt7=(1-predvalt)*(709.7).
compute predvals=(predvals&*predvalt)+prevalt7.
compute expyhat=exp(predvals)&/(1+exp(predvals)).
end if.
compute sepred=make(nrow(plotvals),3,999).
loop sei=1 to nrow(plotvals).
compute ask=probeplt(sei,:).
compute sepred(sei,1)=sqrt(ask*varb*t(ask)).
do if ((i < nms+nys) or (ydich=0)).
compute sepred(sei,2)=predvals(sei,1)-tval*sepred(sei,1).
compute sepred(sei,3)=predvals(sei,1)+tval*sepred(sei,1).
end if.
do if ((i = nms+nys) and (ydich=1)).
compute sepred(sei,2)=predvals(sei,1)-xp2*sepred(sei,1).
compute sepred(sei,3)=predvals(sei,1)+xp2*sepred(sei,1).
end if.
end loop.
compute prevloc=ncol(plotvals)+1.
compute probeplt={plotvals,predvals}.
do if (plot = 2).
compute probeplt={probeplt,sepred}.
end if.
do if ((i = nms+nys) and (ydich=1)).
compute probeplt={probeplt,expyhat}.
end if.
compute didsome=0.
end if.
do if ((wzcmat((i+1),jmed)=1) and (printpbe <= intprobe)).
do if (jmed=1).
compute omnilp2=nxvls*nwvls.
compute omnitmp=ident(omnilp2).
compute omni3(wherexw(1,i):wherexw(2,i),:)=omnitmp.
end if.
do if (jmed>1).
compute omnilp2=nwvls.
compute omnitmp=ident(omnilp2).
compute omni3(wheremw(((jmed*2)-3),i):wheremw(((jmed*2)-2),i),:)=omnitmp.
end if.
compute omnif=make(1,4,0).
do if ((i = nms+nys) and (ydich=1)).
compute omnif=make(1,3,0).
end if.
compute condeff3=0.
loop omnilp1=1 to nrow(zprobval).
loop omnilp=1 to (omnilp2).
do if (jmed=1).
compute omni3((wherexwz(1,i)+((omnilp-1)*nzvls)):(wherexwz(1,i)+((omnilp-1)*nzvls)+ (nzvls-1)),omnilp)=t(zprobval(omnilp1,:)).
end if.
do if (jmed > 1).
compute omni3((wheremwz(((jmed*2)-3),i)+((omnilp-1)*nzvls)):(wheremwz(((jmed*2)-3),i)+ ((omnilp-1)*nzvls)+(nzvls-1)),omnilp)=t(zprobval(omnilp1,:)).
end if.
end loop.
compute condeff=t(omni3)*b.
compute condeff3={condeff3;condeff}.

.
compute lmat2= omni3.
do if ( 1 =0).
compute lmat2 = mdiag( omni3 ).
compute lmat3=make(nrow(lmat2),1,0).
loop flp=1 to ncol(lmat2).
do if (csum(lmat2(:,flp))=1).
compute lmat3={lmat3,lmat2(:,flp)}.
end if.
end loop.
compute lmat2=lmat3(:,2:ncol(lmat3)).
end if.
compute fratio = (t(t(lmat2)* b )*inv(t(lmat2)* varb *lmat2)*((t(lmat2)* b )))/ncol(lmat2).
compute pfr = 1-fcdf(fratio,ncol(lmat2),(n-nrow( b ))).
compute fresult={fratio,ncol(lmat2),(n-nrow( b )),pfr}.
do if (i = (nms+nys) and (ydich=1)).
compute fratio=fratio*ncol(lmat2).
compute pfr=1-chicdf(fratio,ncol(lmat2)).
compute fresult={fratio,ncol(lmat2),pfr}.
end if.
do if ( 0 =1).
compute lmat3=1-rsum(lmat2).
compute xfm=make(n,csum(lmat3),0).
compute flpc=1.
loop flp=1 to nrow(lmat3).
do if (lmat3(flp,1)=1).
compute xfm(:,flpc)=x(:,flp).
compute flpc=flpc+1.
end if.
end loop.
compute bfm=inv(t(xfm)*xfm)*t(xfm)*y.
compute resid=y-(xfm*bfm).
compute sstotal=(y-(csum(y)/n)).
compute sstotal=csum(sstotal&*sstotal).
compute ssresid=csum(resid&*resid).
compute rsqch= 0 -((sstotal-ssresid)/sstotal).
compute fresult={rsqch,fresult}.
release xfm,flpc, resid, ssresid, bfm.
end if
.
compute omnif={omnif;fresult}.
end loop.
compute omnif=omnif(2:nrow(omnif),:).
compute clabtmp=znames.
compute condeff3=condeff3(2:nrow(condeff3),:).
do if ((nxvls*nwvls)=1).
compute omnif={condeff3,omnif}.
compute clabtmp={clabtmp,'Effect'}.
end if.
compute omnif={zmodvals,omnif}.
do if ((i < nms+nys) or (ydich=0)).
compute clabtmp={clabtmp,hcflab,'df1','df2','p'}.
end if.
do if ((i = (nms+nys)) and (ydich=1)).
compute clabtmp={clabtmp,'Chi-sq','df','p'}.
end if.
compute resultm2=make(nrow(omnif),maxresm,99999).
compute resultm2(1:nrow(omnif),1:ncol(omnif))=omnif.
compute resultm={resultm;resultm2}.
do if (outscree=1).
do if (jmed=1).
print omnif/title='Test of conditional X*W interaction at value(s) of Z:'/cnames=clabtmp/format= F10.4.
end if.
do if (jmed>1).
print omnif/title='Test of conditional M*W interaction at value(s) of Z:'/cnames=clabtmp/format= F10.4.
end if.
end if.
release omni3.
end if.
loop probei = 1 to probexpl.
do if (probexpl > 1).
compute foctmp=make(nrow(modvals),probexpl,0).
compute foctmp(:,probei)=foctmp(:,probei)+1.
compute probtemp=make(nrow(modvals),1,0).
loop probem = 1 to numplps.
loop probek = 1 to nxvls.
loop probej=lpstsp(probem,1) to lpstsp(probem,2).
compute probtemp={probtemp,foctmp(:,probek)&*probeval(:,probej)}.
end loop.
end loop.
end loop.
compute probeva2=probtemp(:,2:ncol(probtemp)).
compute probeva2={foctmp,probeva2}.
end if.
compute probres=probeva2*probcoef.
compute probrese=sqrt(diag(probeva2*probvarb*t(probeva2))).
compute tratio = probres&/probrese.
compute p = 2*(1-tcdf(abs(tratio), dfres)).
do if (ydich=1 and i = (nms+nys)).
compute p = 2*(1-cdfnorm(abs(tratio))).
end if.
compute modvals2={modvals,probres,probrese,tratio, p}.
do if ((i < nms+nys) or (ydich=0)).
compute modvals2={modvals2,(probres-tval&*probrese),(probres+tval&*probrese)}.
compute problabs={problabs,'Effect',hclab,'t', 'p', 'LLCI', 'ULCI'}.
end if.
do if (ydich=1 and i = (nms+nys)).
compute modvals2={modvals2,(probres-xp2&*probrese),(probres+xp2&*probrese)}.
compute problabs={problabs,'Effect','se','Z', 'p', 'LLCI', 'ULCI'}.
end if.
do if (probexpl > 1 and (printpbe <= intprobe)).
do if (hasz = 1).
compute printz=1.
end if.
do if (hasw=1).
compute printw=1.
end if.
compute probrlab=make(nrow(modvals),1,xcatlab(probei,1)).
compute modvals3={modvals3; modvals2}.
compute probrow={probrow;probrown}.
compute probrown=probrown+1.
do if (probei=probexpl).
compute xproblab=xcatlab(1:nxvls,1).
compute probrow=probrow(2:nrow(probrow),1).
compute modvals3=modvals3(2:nrow(modvals3),:).
compute temp=modvals3.
compute temp(GRADE(probrow(:,1)),:)=modvals3.
compute modvals3=temp.
compute start2=1.
compute problabs=problabs(1,(1+(ncol(modvarl))):ncol(problabs)).
compute pstart=1.
loop probek= 1 to nrow(probeval).
compute endstart=start2+(nxvls-1).
compute temp=modvals3(start2:endstart,(1+ncol(modvarl)):ncol(modvals3)).
compute temp2=t(modvals3(start2:start2,1:ncol(modvarl))).
compute trnames=t(modvarl).
do if (outscree=1).
do if (probek > 1).
print/title='----------'/space=0.
else.
print/title = 'Conditional effects of the focal predictor at values of the moderator(s):'.
do if ((jmed=1) and (i = (nms+nys)) and (nms > 0)).
do if (nxvls = 1).
print/title = '(These are also the conditional direct effects of X on Y)'/space=0.
else.
print/title = '(These are also the relative conditional direct effects of X on Y)'/space=0.
end if.
end if.
print.
end if.
print temp2/title = 'Moderator value(s):'/rnames=trnames/format= F10.4 /space=0.
print temp/title = ' '/cnames=problabs/rnames=xproblab/format= F10.4 /space=0.
end if.
compute resultm2=make(nrow(temp2),maxresm,99999).
compute resultm2(1:nrow(temp2),1:ncol(temp2))=temp2.
compute resultm={resultm;resultm2}.
compute resultm2=make(nrow(temp),maxresm,99999).
compute resultm2(1:nrow(temp),1:ncol(temp))=temp.
compute resultm={resultm;resultm2}.
compute start2=start2+nxvls.
compute didsome=1.
do if (jmed=1).
compute mod1val=probeval(probek,1:nm1vls).
loop omnilp=1 to nxvls.
compute omni((wheremv1(1,i)+((omnilp-1)*nm1vls)):(wheremv1(1,i)+((omnilp-1)* nm1vls)+(nm1vls-1)),omnilp)=t(mod1val).
do if (nm1vls < ncol(probeval)).
compute mod2val=probeval(probek,(nm1vls+1):(nm1vls+nm2vls)).
compute omni((wheremv2(1,i)+((omnilp-1)*nm2vls)):(wheremv2(1,i)+((omnilp-1)* nm2vls)+(nm2vls-1)),omnilp)=t(mod2val).
do if ((nm1vls+nm2vls) < ncol(probeval)).
compute intlen=nm1vls*nm2vls.
compute modintvl=probeval(probek,(nm1vls+nm2vls+1):ncol(probeval)).
compute omni((wherexwz(1,i)+((omnilp-1)*intlen)):(wherexwz(1,i)+((omnilp-1)* intlen)+(intlen-1)),omnilp)=t(modintvl).
end if.
end if.
end loop.

.
compute lmat2= omni.
do if ( 1 =0).
compute lmat2 = mdiag( omni ).
compute lmat3=make(nrow(lmat2),1,0).
loop flp=1 to ncol(lmat2).
do if (csum(lmat2(:,flp))=1).
compute lmat3={lmat3,lmat2(:,flp)}.
end if.
end loop.
compute lmat2=lmat3(:,2:ncol(lmat3)).
end if.
compute fratio = (t(t(lmat2)* b )*inv(t(lmat2)* varb *lmat2)*((t(lmat2)* b )))/ncol(lmat2).
compute pfr = 1-fcdf(fratio,ncol(lmat2),(n-nrow( b ))).
compute fresult={fratio,ncol(lmat2),(n-nrow( b )),pfr}.
do if (i = (nms+nys) and (ydich=1)).
compute fratio=fratio*ncol(lmat2).
compute pfr=1-chicdf(fratio,ncol(lmat2)).
compute fresult={fratio,ncol(lmat2),pfr}.
end if.
do if ( 0 =1).
compute lmat3=1-rsum(lmat2).
compute xfm=make(n,csum(lmat3),0).
compute flpc=1.
loop flp=1 to nrow(lmat3).
do if (lmat3(flp,1)=1).
compute xfm(:,flpc)=x(:,flp).
compute flpc=flpc+1.
end if.
end loop.
compute bfm=inv(t(xfm)*xfm)*t(xfm)*y.
compute resid=y-(xfm*bfm).
compute sstotal=(y-(csum(y)/n)).
compute sstotal=csum(sstotal&*sstotal).
compute ssresid=csum(resid&*resid).
compute rsqch= 0 -((sstotal-ssresid)/sstotal).
compute fresult={rsqch,fresult}.
release xfm,flpc, resid, ssresid, bfm.
end if
.
compute resultm2=make(nrow(fresult),maxresm,99999).
compute resultm2(1:nrow(fresult),1:ncol(fresult))=fresult.
compute resultm={resultm;resultm2}.
do if (outscree=1).
do if (i < (nms + nys) or (ydich = 0)).
compute clabtmp={hcflab,'df1','df2','p'}.
print fresult/title='Test of equality of conditional means'/cnames=clabtmp/format= F10.4.
end if.
do if (i = (nms + nys) and (ydich = 1)).
compute clabtmp={'Chi-sq','df','p'}.
print fresult/title='Test of equality of conditional logits or probabilities'/cnames=clabtmp/format= F10.4.
end if.
end if.
compute probetmp=probeplt(pstart:(pstart+nxvls),1).
compute probetmp={probetmp,probeplt(pstart:(pstart+nxvls),prevloc:ncol(probeplt))}.
compute pstart=pstart+(nxvls+1).
compute resultm2=make(nrow(probetmp),maxresm,99999).
compute resultm2(1:nrow(probetmp),1:ncol(probetmp))=probetmp.
compute resultm={resultm;resultm2}.
do if (i < ((nms + nys)) or (ydich = 0)).
compute clabtmp={xnames, outnames(1,i), hclab, 'LLCI', 'ULCI'}.
do if (outscree=1).
print probetmp/title = 'Estimated conditional means being compared:'/cnames=clabtmp/format= F10.4.
end if.
end if.
do if ((i = (nms+nys)) and (ydich = 1)).
compute clabtmp={xnames, outnames(1,i), 'prob'}.
compute probetm2=probetmp(:,1:2).
compute probetm2={probetm2,probetmp(:,ncol(probetmp))}.
do if (outscree=1).
print probetm2/title = 'Estimated conditional logits and probabilities:'/cnames=clabtmp/format= F10.4.
end if.
end if.
end if.
end loop.
release probrow, start2, endstart, temp, temp2.
end if.
end if.
do if (probexpl = 1 and (printpbe <= intprobe)).
compute resultm2=make(nrow(modvals2),maxresm,99999).
compute resultm2(1:nrow(modvals2),1:ncol(modvals2))=modvals2.
compute resultm={resultm;resultm2}.
do if (outscree=1).
print/title = 'Conditional effects of the focal predictor at values of the moderator(s):'.
print modvals2/cnames=problabs/title = ' '/space=0/format= F10.4.
end if.
compute didsome=1.
do if (hasz = 1).
compute printz=1.
end if.
do if (hasw=1).
compute printw=1.
end if.
do if (jn = 1 and jnok=1).
do if (criterr = 0).
compute dfres=n-nrow(b).
compute roots=99999.
compute jncrit =(dfres* (exp((dfres-(5/6))*((xp2/(dfres-(2/3)+(.11/dfres)))* (xp2/(dfres-(2/3)+(.11/dfres)))))-1)).
do if (i = (nms+nys) and (ydich=1)).
compute jncrit=xp2*xp2.
end if.
compute jnb1=b(wherejn1,1).
compute jnb3=b(wherejn3,1).
compute jnsb1=varb(wherejn1,wherejn1).
compute jnsb3=varb(wherejn3,wherejn3).
compute jnsb1b3=varb(wherejn1,wherejn3).
compute ajn =(jncrit*jnsb3)-(jnb3*jnb3).
compute bjn = 2*((jncrit*jnsb1b3)-(jnb1*jnb3)).
compute cjn = (jncrit*jnsb1)-(jnb1*jnb1).
compute radarg = (bjn*bjn)-(4*ajn*cjn).
compute den = 2*ajn.
compute nrts = 0.
do if (radarg >= 0 and den <> 0).
compute x21 = (-bjn+sqrt(radarg))/den.
compute x22 = (-bjn-sqrt(radarg))/den.
compute roots = 0.
do if (x21 >= jnmin and x21 <= jnmax).
compute nrts = 1.
compute roots = {roots; x21}.
end if.
do if (x22 >= jnmin and x22 <= jnmax).
compute nrts = nrts + 1.
compute roots = {roots; x22}.
end if.
compute roots={roots,make(nrow(roots),2,0)}.
end if.
do if (nrts > 0).
compute roots = roots(2:nrow(roots),1:3).
compute roots(1,2)=(csum(jnmod < roots(1,1))/n)*100.
compute roots(1,3)=(csum(jnmod > roots(1,1))/n)*100.
do if (nrow(roots)=2).
compute roots(2,2)=(csum(jnmod < roots(2,1))/n)*100.
compute roots(2,3)=(csum(jnmod > roots(2,1))/n)*100.
end if.
do if (outscree=1).
print roots/title = 'Moderator value(s) defining Johnson-Neyman significance region(s):'/clabels = 'Value', '% below', '% above'/format F10.4.
end if.
do if (nrts=1).
compute tmprts=make(1,3,99999).
compute roots={roots;tmprts}.
end if.
end if.
do if (nrts = 0).
compute roots=make(2,1,99999).
do if (outscree=1).
print/title = 'There are no statistical significance transition points within the observed'.
print/title = 'range of the moderator found using the Johnson-Neyman method.'/space=0.
end if.
end if.
compute resultm2=make(nrow(roots),maxresm,99999).
compute resultm2(1:nrow(roots),1:ncol(roots))=roots.
compute resultm={resultm;resultm2}.
compute jnvals=make(23,7,0).
loop jni= 0 to (21-(nrts)).
compute jnvals((jni+1),1)=jnmin+(jni*((jnmax-jnmin)/(21-nrts))).
end loop.
do if (nrts > 0).
loop jni = 1 to nrts.
loop jnj = 2 to (nrow(jnvals)-1).
do if ((roots(jni,1) > jnvals((jnj-1),1)) and (roots(jni,1) < jnvals(jnj,1))).
compute jnvals((jnj+1):(21+jni),1)=jnvals(jnj:(20+jni),1).
compute jnvals(jnj,1)=roots(jni,1).
end if.
end loop.
end loop.
end if.
compute jnvals=jnvals(1:22,:).
loop jni = 1 to nrow(jnvals).
compute jnvals(jni,2)=jnb1+jnb3*jnvals(jni,1).
compute jnvals(jni,3)=sqrt(jnsb1+2*jnvals(jni,1)*jnsb1b3+(jnvals(jni,1)*jnvals(jni, 1))*jnsb3).
compute jnvals(jni,4)=jnvals(jni,2)/jnvals(jni,3).
compute jnvals(jni,5)=2*(1-tcdf(abs(jnvals(jni,4)), dfres)).
compute jnvals(jni,6)=jnvals(jni,2)-sqrt(jncrit)*jnvals(jni,3).
compute jnvals(jni,7)=jnvals(jni,2)+sqrt(jncrit)*jnvals(jni,3).
do if ((i = nms + nys) and (ydich=1)).
compute jnvals(jni,5)=2*(1-cdfnorm(abs(jnvals(jni,4)))).
compute jnvals(jni,6)=jnvals(jni,2)-xp2*jnvals(jni,3).
compute jnvals(jni,7)=jnvals(jni,2)+xp2*jnvals(jni,3).
end if.
end loop.
compute resultm2=make(nrow(jnvals),maxresm,99999).
compute resultm2(1:nrow(jnvals),1:ncol(jnvals))=jnvals.
compute resultm={resultm;resultm2}.
do if ((i < nms+nys) or (ydich=0)).
compute jnclbs={jnmodlab,'Effect',hclab,'t', 'p', 'LLCI', 'ULCI'}.
end if.
do if ((i = nms + nys) and (ydich=1)).
compute jnclbs={jnmodlab,'Effect','se','Z', 'p', 'LLCI', 'ULCI'}.
end if.
do if (((wcmat((i+1),jmed)=1) or (zcmat((i+1),jmed)=1)) and (wzcmat((i+1),jmed)=0)).
do if (outscree=1).
print jnvals/title = 'Conditional effect of focal predictor at values of the moderator:'/cnames =jnclbs/format = F10.4.
end if.
end if.
do if (outscree=1).
do if ((jmed = 1) and (wzcmat((i+1),jmed)=1)).
print jnvals/title = 'Conditional X*W interaction at values of the moderator Z:'/cnames =jnclbs/format = F10.4.
end if.
do if ((jmed > 1) and (wzcmat((i+1),jmed)=1)).
print jnvals/title = 'Conditional M*W interaction at values of the moderator Z:'/cnames =jnclbs/format = F10.4.
end if.
end if.
end if.
end if.
end if.
do if ((i = (nms+nys)) and (jmed=1) and (bcmat(nrow(bcmat),1)=1)).
do if (probei=1).
compute direfflb=problabs.
compute direff=modvals2.
end if.
do if (probei>1).
compute direff={direff;modvals2}.
end if.
end if.
compute intprint=0.
do if ((jmed=1) and (i=1) and nms=0) and modcok=1).
compute contvec2=make(2,1,1).
compute contvec2={contvec2,wcontval,zcontval}.
do if (wzcmat((i+1),jmed)=1).
loop conti= 1 to ncol(wcontval).
loop contj = 1 to ncol(zcontval).
compute contvec2={contvec2,wcontval(:,conti)&*zcontval(:,contj)}.
end loop.
end loop.
end if.
compute conteff=contvec2*probcoef.
compute contdiff=contvec2(1,:)-contvec2(2,:).
compute contse=sqrt(contdiff*probvarb*t(contdiff)).
compute conteffd=conteff(1,1)-conteff(2,1).
compute contvec={contvec,conteff}.
compute contvecm=contvec.
compute resultm2=make(nrow(contvecm),maxresm,99999).
compute resultm2(1:nrow(contvecm),1:ncol(contvecm))=contvecm.
compute resultm={resultm;resultm2}.
do if (outscree=1).
print/title='Contrast between conditional effects of X:'.
print contvec/title=' '/rlabels='Effect1:','Effect2:'/cnames=problabs/format = F10.4 /space=0.
end if.
do if (ydich=0).
compute p=2*(1-tcdf(abs(conteffd/contse), dfres)).
compute contvec={conteffd,contse,conteffd/contse, p}.
compute contvec={contvec,(conteffd-(tval*contse))}.
compute contvec={contvec,(conteffd+(tval*contse))}.
compute contlabs={'Contrast', hclab, 't', 'p', 'LLCI', 'ULCI'}.
end if.
do if (ydich=1).
compute p=2*(1-cdfnorm(abs(conteffd/contse))).
compute contvec={conteffd,contse,conteffd/contse, p}.
compute contvec={contvec,(conteffd-(xp2*contse))}.
compute contvec={contvec,(conteffd+(xp2*contse))}.
compute contlabs={'Contrast', 'se', 'Z', 'p', 'LLCI', 'ULCI'}.
end if.
compute resultm2=make(nrow(contvec),maxresm,99999).
compute resultm2(1:nrow(contvec),1:ncol(contvec))=contvec.
compute resultm={resultm;resultm2}.
do if (outscree=1).
print contvec/title='Test of Effect1 minus Effect2'/format= F10.4 /cnames=contlabs.
end if.
end if.
end loop.
do if (plot = 1 or plot = 2).
compute datalabs={t(focpred(:,3)),outnames(1,i)}.
do if (plot = 1).
compute datalabs={datalabs}.
end if.
do if (plot = 2).
compute datalabs={datalabs,'se', 'LLCI', 'ULCI'}.
end if.
do if ((i = nms+nys) and (ydich=1)).
compute datalabs={datalabs,'prob'}.
end if.
compute resultm2=make(nrow(probeplt),maxresm,99999).
compute resultm2(1:nrow(probeplt),1:ncol(probeplt))=probeplt.
compute resultm={resultm;resultm2}.
compute datalabs={datalabs,'.'}.
do if (outscree=1).
print/title = 'Data for visualizing the conditional effect of the focal predictor:'.
print/title = 'Paste text below into a SPSS syntax window and execute to produce plot.'/space=0.
compute dumb = {' ', ' ', ' ', ' ', ' ', ' ', ' '}.
print datalabs/title = 'DATA LIST FREE/'/format=A10.
print probeplt/title = 'BEGIN DATA.'/format= F10.4 /space=0.
print/title = 'END DATA.'/space=0.
end if.
compute focgrph=datalabs(1,1).
compute graphix={focgrph,graphixs,modgrph}.
do if (((xdich=1) or (nxvls > 1)) and ((modcat=0) and (focgrph = xnames))).
compute graphix={modgrph,graphixs,focgrph}.
end if.
do if (panelgrp = 0).
compute graphix={graphix,'.'}.
else.
compute graphix={graphix,panelcde}.
end if.
do if (outscree=1).
print graphix/title = 'GRAPH/SCATTERPLOT='/format=A8/space=0.
end if.
do if (i = (nms + nys) and ydich = 1).
compute graphixd=graphix.
compute graphixd(1,3)='prob'.
do if (outscree=1).
print graphixd/title = 'GRAPH/SCATTERPLOT='/format=A8/space=0.
end if.
end if.
end if.
end if.
end loop.
release jmed, intprint,didprint.
end if
.
do if (model = 74 and i <= nms).
compute onetemp=make(nrow(xprobval),1,1).
compute mestmt74={onetemp,xprobval}.
do if (ncs > 0).
compute ncovmdl=rsum(ccmat(i,:)).
do if (ncovmdl > 0).
compute cvmnc=make(nrow(mestmt74),ncovmdl,1).
compute cvmnctmp=csum(x(:,(ncol(x)-ncovmdl+1):ncol(x)))/nrow(x).
do if (cuscoval > 0).
compute cvmnctmp=coval.
end if.
loop mestlp=1 to ncovmdl.
compute cvmnc(:,mestlp)=cvmnc(:,mestlp)*cvmnctmp(:,mestlp).
end loop.
compute mestmt74={mestmt74,cvmnc}.
end if.
end if.
compute mest74t=mestmt74*b.
do if (i = 1).
compute mest74=mest74t.
end if.
do if (i > 1).
compute mest74={mest74,mest74t}.
end if.
end if.
do if ((i = (nms+nys)) and (model >= 0 and model < 4) and (linsum(1,1) <> -999)).
compute lhyprob=1.
compute meansub=0.
do if (((nlinsum=nrow(b)) or (nlinsum = (nrow(b)-ncs)))).
do if (nlinsum = (nrow(b)-ncs) and ncs > 0).
compute linsum={linsum,covmeans}.
compute meansub=1.
end if.
compute lhyprob=0.
compute hypest=linsum*b.
compute sehypest=sqrt(linsum*varb*t(linsum)).
compute phypest=2*(1-tcdf(abs(hypest/sehypest), (dfres))).
compute hypest={hypest,sehypest,(hypest/sehypest),phypest,(hypest-tval*sehypest),(hypest+ tval*sehypest)}.
compute resultm2=make(ncol(linsum),maxresm,99999).
compute resultm2(:,1)=t(linsum).
compute resultm={resultm;resultm2}.
compute resultm2=make(1,maxresm,99999).
compute resultm2(1,1:ncol(hypest))=hypest.
compute resultm={resultm;resultm2}.
do if (outscree=1).
compute hyplabs={'Estimate',hclab,'t','p','LLCI','ULCI'}.
print/title='----------'.
print/title='Linear Combination Estimate and Hypothesis Test'/space=0.
print t(linsum)/title= 'Weight vector:'/clabels='weight'/rnames=vlabsm/format F10.4.
print hypest/title=' '/cnames=hyplabs/format= F10.4 /space=0.
do if (meansub=1).
print/title='Covariate weight(s) set to the sample mean.'.
end if.
end if.
end if.
do if (lhyprob=1).
compute notecode(notes,1) = 30.
compute notes = notes + 1.
end if.
end if.
end loop if criterr=1.
compute lastb=b.
compute lastcov=varb.
do if (criterr=0 and dototal = 1).
compute x=xtmp.
compute vlabsm={'constant';xcatlab(1:nxvls,1)}.
do if (ncs > 0).
compute x = {x,ctmp}.
compute vlabsm={vlabsm;t(covnames)}.
end if.
compute x = {ones,x}.

.
do if ( 1 =1).
compute hatmat=inv(t( x )* x )*t( x ).
compute b = hatmat* y.
compute modres=b.
do if ( 1 =1).
compute n1=nrow( x ).
compute dfres=n1-(ncol( x )).
compute sstotal = t( y -(csum( y )/n1))*( y -(csum( y )/n1)).
compute resid= y - x *b.
compute ssresid = csum((resid)&**2).
compute r2 = (sstotal-ssresid)/sstotal.
do if (r2 < 0).
compute r2=0.
end if.
compute adjr2 = 1-((1-r2)*(n1-1)/(dfres)).
compute mse=ssresid/(n1-ncol( x )).

.
compute n1=nrow( x ).
compute invXtX = inv(t( x )* x ).
compute varb = mse *invXtX.
compute k3 = ncol( x ).
compute xhc=0.
do if ( hc <> 5).
compute xhc= x.
compute hat = xhc(:,1).
loop i3=1 to nrow(xhc).
compute hat(i3,1)= xhc(i3,:)*invXtX*t(xhc(i3,:)).
end loop.
do if ( hc = 0 or hc =1).
loop i3 = 1 to k3.
compute xhc(:,i3)=xhc(:,i3)&* resid.
end loop.
end if.
do if ( hc =3 or hc =2).
loop i3=1 to k3.
compute xhc(:,i3) = ( resid &/(1-hat)&**(1/(4- hc )))&*xhc(:,i3).
end loop.
end if.
do if ( hc = 4).
compute hcmn=make(n,2,4).
compute hcmn(:,2)=(n1*hat)/k3.
loop i3= 1 to k3.
compute xhc(:,i3) = ( resid &/(1-hat)&**(rmin(hcmn)/2))&*xhc(:,i3).
end loop.
end if.
compute varb=(invXtX*t(xhc)*xhc*invXtX).
do if ( hc =1).
compute varb=(n1/(n1-ncol( x )))&*varb.
end if.
end if.
compute hclab={'se(HC0)','se(HC1)','se(HC2)','se(HC3)','se(HC4)','se'}.
compute hclab=hclab(1,( hc +1)).
compute hcflab={'F(HC0)','F(HC1)','F(HC2)','F(HC3)','F(HC4)','F'}.
compute hcflab=hcflab(1,( hc +1)).
release xhc
.
compute seb=sqrt(diag(varb)).
compute trat = b&/seb.
compute p = 2*(1-tcdf(abs(trat), (dfres))).
compute tval = sqrt(dfres* (exp((dfres-(5/6))*((xp2/(dfres-(2/3)+(.11/dfres)))* (xp2/(dfres-(2/3)+(.11/dfres)))))-1)).
compute modres={modres,seb,trat,p}.
compute modres={modres,(b-tval&*seb),(b+tval&*seb)}.
compute modresl={'coeff',hclab,'t','p','LLCI','ULCI'}.
compute lmat = ident(ncol( x )).
compute lmat = lmat(:,2:ncol(lmat)).
compute fratio = (t(t(lmat)*b)*inv(t(lmat)*varb*lmat)*((t(lmat)*b)))/(ncol( x )-1).
compute pfr = 1-fcdf(fratio,(ncol( x )-1),dfres).
compute modsum={sqrt(r2),r2,mse,fratio,(ncol( x )-1),dfres,pfr}.
compute modsuml={'R','R-sq','MSE',hcflab,'df1','df2', 'p'}.
end if.
end if.
do if ( 1 = 2 or 1 =3).
compute xlp= x.
compute ylp= y.
compute pt2 = make(nrow(ylp),1,(csum(ylp)/nrow(ylp))).
do if ( 1 =2).
compute LL3 = ylp&*ln(pt2)+(1-ylp)&*ln(1-pt2).
end if.
compute LL3 = -2*csum(LL3).
compute bt1 = make(ncol(xlp),1,0).
compute LL1 = 0.
compute pt1 = make(nrow(ylp),1,0.5).
compute pt1lp=pt1.
loop jjj = 1 to iterate.
compute xlptmp=t(xlp).
compute vecprb=pt1lp&*(1-pt1lp).
loop kkk=1 to ncol(xlp).
compute xlptmp(kkk,:)=xlptmp(kkk,:)&*t(vecprb).
end loop.
compute b = bt1+inv(xlptmp*xlp)*t(xlp)*(ylp-pt1lp).
do if ( 1 =2).
compute xlpb=xlp*b.
compute xlpbt=(xlpb > -709.7).
compute xlpb709=(1-xlpbt)*(-709.7).
compute xlpb=(xlpb&*xlpbt)+xlpb709.
compute pt1lp = 1/(1+exp(-(xlpb))).
end if.
compute itprob = csum((pt1lp < .00000001) or (pt1lp > .9999999)).
do if (itprob > 0).
loop kkk = 1 to nrow(pt1lp).
do if (pt1lp(kkk,1) > .9999999).
compute pt1lp(kkk,1) = .9999999.
end if.
do if (pt1lp(kkk,1) < .00000001).
compute pt1lp(kkk,1) = .00000001.
end if.
end loop.
compute itprob = 0.
end if.
do if (itprob = 0).
do if ( 1 =2).
compute LL = ylp&*ln(pt1lp)+(1-ylp)&*ln(1-pt1lp).
end if.
compute LL2 = -2*csum(ll).
end if.
do if (abs(LL1-LL2) < converge).
do if ( 1 =1).
compute xlptmp=t(xlp).
compute vecprb=pt1lp&*(1-pt1lp).
loop kkk=1 to ncol(xlp).
compute xlptmp(kkk,:)=xlptmp(kkk,:)&*t(vecprb).
end loop.
compute varb = inv(xlptmp*xlp).
compute seb = sqrt(diag(varb)).
release xlptmp.
end if.
break.
end if.
compute bt1 = b.
compute LL1 = LL2.
end loop.
compute modres=b.
do if (jjj > iterate).
compute itprob = 2.
do if (booting=0).
compute iterrmod=1.
end if.
do if (booting=1).
compute bootiter=1.
end if.
do if (itprobtg=0).
compute itprobtg=1.
compute errcode(errs,1) = 47.
compute errs = errs + 1.
do if (booting = 0 and 1 =1).
compute vt1 = mdiag(pt1lp&*(1-pt1lp)).
compute varb = inv(t(xlp)*vt1*xlp).
compute seb = sqrt(diag(varb)).
end if.
end if.
end if.
do if ( 1 =1).
compute trat = b&/seb.
compute dfres=nrow(xlp).
compute p = 2*(1-cdfnorm(abs(trat))).
compute modres={modres,seb,trat,p}.
compute modres={modres,(b-xp2&*seb),(b+xp2&*seb)}.
compute pvchi=1-chicdf((LL3-LL2),(nrow(modres)-1)).
compute mcF = (LL3-LL2)/LL3.
compute cox = 1-exp(-(LL3-LL2)/nrow(xlp)).
compute nagel = cox/(1-exp(-(LL3)/nrow(xlp))).
compute modsum={LL2,(LL3-LL2),(nrow(modres)-1),pvchi, mcF,cox,nagel}.
compute modsuml={'-2LL','ModelLL', 'df', 'p', 'McFadden', 'CoxSnell', 'Nagelkrk'}.
compute modresl={'coeff','se','Z','p','LLCI','ULCI'}.
end if.
end if
.
compute toteff=modres(2:(1+nxvls),:).
compute nodotot=0.
do if (xdich=1 and xmint=1 and model=74).
compute toteff(:,1)=toteff(:,1)*xscaling.
compute toteff(:,2)=toteff(:,2)*abs(xscaling).
compute toteff(:,3)=toteff(:,3)*xscaling.
compute toteff(:,5)=toteff(:,5)*xscaling.
compute toteff(:,6)=toteff(:,6)*xscaling.
compute citmp=toteff(:,5:6).
do if (xscaling < 0).
compute toteff(:,5)=citmp(:,2).
compute toteff(:,6)=citmp(:,1).
end if.
compute nodotot=1.
end if.
compute totefflb=modresl.
do if (outscree=1).
do if (nodotot=0).
print/title = '************************** TOTAL EFFECT MODEL ****************************'.
end if.
do if (nodotot=1).
print/title = '**************************************************************************'.
end if.
print outnames(1,ncol(outnames))/title = 'OUTCOME VARIABLE:'/format = A8/space=0.
end if.
compute toteffl2=vlabsm(2:(1+nxvls),:).
compute resultm2=make(1,maxresm,99999).
compute resultm2(1,1:ncol(modsum))=modsum.
compute resultm={resultm;resultm2}.
compute resultm2=make(nrow(modres),maxresm,99999).
compute resultm2(1:nrow(modres),1:ncol(modres))=modres.
compute resultm={resultm;resultm2}.
do if (outscree=1).
print modsum/title = 'Model Summary'/cnames = modsuml/format= F10.4.
print modres/title='Model'/rnames=vlabsm/cnames=modresl/format= F10.4.
end if.
compute lmat=make(nrow(b),1,0).
compute lmat2=make(nxvls,1,1).
compute lmat(2:(1+nxvls),1)=lmat2.
do if (ydich <> 1).

.
compute lmat2= lmat.
do if ( 0 =0).
compute lmat2 = mdiag( lmat ).
compute lmat3=make(nrow(lmat2),1,0).
loop flp=1 to ncol(lmat2).
do if (csum(lmat2(:,flp))=1).
compute lmat3={lmat3,lmat2(:,flp)}.
end if.
end loop.
compute lmat2=lmat3(:,2:ncol(lmat3)).
end if.
compute fratio = (t(t(lmat2)* b )*inv(t(lmat2)* varb *lmat2)*((t(lmat2)* b )))/ncol(lmat2).
compute pfr = 1-fcdf(fratio,ncol(lmat2),(n-nrow( b ))).
compute fresult={fratio,ncol(lmat2),(n-nrow( b )),pfr}.
do if (i = (nms+nys) and (ydich=1)).
compute fratio=fratio*ncol(lmat2).
compute pfr=1-chicdf(fratio,ncol(lmat2)).
compute fresult={fratio,ncol(lmat2),pfr}.
end if.
do if ( 1 =1).
compute lmat3=1-rsum(lmat2).
compute xfm=make(n,csum(lmat3),0).
compute flpc=1.
loop flp=1 to nrow(lmat3).
do if (lmat3(flp,1)=1).
compute xfm(:,flpc)=x(:,flp).
compute flpc=flpc+1.
end if.
end loop.
compute bfm=inv(t(xfm)*xfm)*t(xfm)*y.
compute resid=y-(xfm*bfm).
compute sstotal=(y-(csum(y)/n)).
compute sstotal=csum(sstotal&*sstotal).
compute ssresid=csum(resid&*resid).
compute rsqch= r2 -((sstotal-ssresid)/sstotal).
compute fresult={rsqch,fresult}.
release xfm,flpc, resid, ssresid, bfm.
end if
.
compute totomni=fresult.
end if.
do if (stand=1).
compute predsd=make(nrow(modres),1,0).
compute stdmod=modres(:,1)&/ovsd(1,ncol(ovsd)).
loop jd=1 to ncol(x).
compute descdat=x(:,jd).
compute predsd(jd,1) = (nrow(descdat)*sscp(descdat))-(t(csum(descdat))*(csum(descdat))).
compute predsd(jd,1) = sqrt(predsd(jd,1)/(nrow(descdat)*(nrow(descdat)-1))).
end loop.
do if (wherex(1,ncol(wherex)) <> -999 and ((nxvls > 1) or (xdich=1))).
compute sdmsone=make(nxvls,1,1).
compute predsd(wherex(1,ncol(wherex)):wherex(2,ncol(wherex)),1)=sdmsone.
compute pstog=1.
end if.
compute predsd(1,1)=1.
compute stdmod=stdmod&*predsd.
compute stdmod=stdmod(2:nrow(stdmod),1).
compute sdvlabs=vlabsm(2:nrow(vlabsm),1).
compute resultm2=make(nrow(stdmod),maxresm,99999).
compute resultm2(1:nrow(stdmod),1:ncol(stdmod))=stdmod.
compute resultm={resultm;resultm2}.
do if (outscree=1).
print stdmod/title='Standardized coefficients'/clabels='coeff'/rnames=sdvlabs/format= F10.4.
end if.
end if.
do if (covcoeff=1).
do if (outscree=1).
print varb/title='Covariance matrix of regression parameter estimates:'/rnames=vlabsm/cnames=vlabsm/format= F10.4.
end if.
compute resultm2=make(nrow(varb),maxresm,99999).
do if (ncol(varb) <= maxresm).
compute resultm2(1:nrow(varb),1:ncol(varb))=varb.
compute resultm={resultm;resultm2}.
end if.
do if (ncol(varb) > maxresm).
compute resultmt=make(nrow(resultm),ncol(varb),99999).
compute resultmt(1:nrow(resultm),1:ncol(resultm))=resultm.
compute resultm=resultmt.
compute resultm2=make(nrow(varb),ncol(resultm),99999).
compute resultm2(1:nrow(varb),1:ncol(varb))=varb.
compute resultm={resultm;resultm2}.
compute maxresm=ncol(resultm).
end if.
end if.
end if.
end if.
do if (criterr=0 and nms > 0 and ydich=0 and modelres=1).
compute modresid=modresid(:,2:ncol(modresid)).
compute sigmatal = (t(modresid)*(ident(n)-(1/n)*ones*t(ones))*modresid)*(1/(n-1)).
compute sdall = mdiag(1/sqrt(diag(sigmatal))).
compute corall=sdall*sigmatal*t(sdall).
compute resultm2=make(nrow(corall),maxresm,99999).
compute resultm2(1:nrow(corall),1:ncol(corall))=corall.
compute resultm={resultm;resultm2}.
do if (outscree=1).
print/title = '****************** CORRELATIONS BETWEEN MODEL RESIDUALS ******************'.
print corall/title=' '/format= F10.4 / cnames=outnames/rnames=outnames/space=0.
end if.
end if.
do if (criterr=0 and boot > 0).
compute bootres=make(1,rsum(nump),-999).
compute bootdir=obsdirfx.
compute natdirbt=make(1,nxvls,-999).
do if (effsize=1).
compute bootysd=make(1,1,-999).
compute bootxsd=make(1,1,-999).
end if.
compute badboot=0.
compute goodboot=0.
compute smallest=1.
compute booting=1.
loop j = 1 to maxboot.
compute nobootx=1.
compute modres2=999.
compute v=trunc(uniform(n,1)*n)+1.
compute bad=0.
loop i = 1 to (nms+nys).
compute y=outvars(v,i).
compute ynovar= (nrow(y)*sscp(y))-(t(csum(y))*(csum(y))).
do if (ynovar = 0).
compute bad=1.
end if.
compute xindx=datindx(1:(nump(1,i)-1),i).
compute hello=0.
compute x = fulldat(v,xindx).
compute x={ones,x}.
compute xsq=t(x)*x.
compute exsq=eval(xsq).
release xsq.
compute holymoly=cmin(exsq).
compute zeroeig=csum(exsq <= 0.000000000002).
compute bad=bad + (zeroeig > 0).

.
compute desctmp=make((8-(4* 1 )),ncol( y ),-999).
loop jd=1 to ncol( y ).
compute descdat= y (:,jd).
compute desctmp(1,jd) = csum(descdat)/nrow(descdat).
compute desctmp(2,jd) = (nrow(descdat)*sscp(descdat))-(t(csum(descdat))*(csum(descdat))).
compute desctmp(2,jd) = sqrt(desctmp(2,jd)/(nrow(descdat)*(nrow(descdat)-1))).
compute desctmp(3,jd)=cmin(descdat).
compute desctmp(4,jd)=cmax(descdat).
do if ( 1 =0).
compute minwarn=0.
compute maxwarn=0.
do if ((desctmp(3,jd)=desctmp(4,jd)) and novar=0).
compute errcode(errs,1)=15.
compute errs=errs+1.
compute criterr=1.
compute novar=1.
end if.
compute tmp=((descdat(:,1)=desctmp(3,jd))+(descdat(:,1)=desctmp(4,jd))).
compute desctmp(8,jd)=(csum(tmp)=nrow(tmp)).
compute tmp = descdat.
compute tmp(GRADE(descdat),:) = descdat.
compute descdat = tmp.
release tmp.
compute decval={.16;.5;.84}.
loop kd=1 to 3.
compute low=trunc(decval(kd,1)*(nrow(descdat)+1)).
compute lowdec=decval(kd,1)*(nrow(descdat)+1)-low.
compute value=descdat(low,1)+(descdat((low+1),1)-descdat(low,1))*lowdec.
compute desctmp((4+kd),jd)=value.
end loop.
compute mnotev=1.
compute modvals=desctmp(5:7,:).
do if (quantile <> 1).
compute desctmp(5,jd)=desctmp(1,jd)-desctmp(2,jd).
compute desctmp(6,jd)=desctmp(1,jd).
compute desctmp(7,jd)=desctmp(1,jd)+desctmp(2,jd).
compute modvals=desctmp(5:7,:).
compute mnotev=2.
do if (modvals(1,1) < desctmp(3,1)).
compute modvals(1,1)=desctmp(3,1).
compute minwarn=1.
end if.
do if (modvals(3,1) > desctmp(4,1)).
compute modvals(3,1)=desctmp(4,1).
compute maxwarn=1.
end if.
end if.
do if (desctmp(8,1)=1).
compute modvals={desctmp(3,1);desctmp(4,1)}.
compute mnotev=0.
compute minwarn=0.
compute maxwarn=0.
end if.
end if.
end loop
.
compute bad=bad+ (desctmp(2,1) <= 0.00000000001).
do if (bad = 0).
do if (holymoly < smallest).
compute smallest=holymoly.
end if.
do if (ydich=0 or (i < (nms+nys))).

.
do if ( 1 =1).
compute hatmat=inv(t( x )* x )*t( x ).
compute b = hatmat* y.
compute modres=b.
do if ( 0 =1).
compute n1=nrow( x ).
compute dfres=n1-(ncol( x )).
compute sstotal = t( y -(csum( y )/n1))*( y -(csum( y )/n1)).
compute resid= y - x *b.
compute ssresid = csum((resid)&**2).
compute r2 = (sstotal-ssresid)/sstotal.
do if (r2 < 0).
compute r2=0.
end if.
compute adjr2 = 1-((1-r2)*(n1-1)/(dfres)).
compute mse=ssresid/(n1-ncol( x )).

.
compute n1=nrow( x ).
compute invXtX = inv(t( x )* x ).
compute varb = mse *invXtX.
compute k3 = ncol( x ).
compute xhc=0.
do if ( hc <> 5).
compute xhc= x.
compute hat = xhc(:,1).
loop i3=1 to nrow(xhc).
compute hat(i3,1)= xhc(i3,:)*invXtX*t(xhc(i3,:)).
end loop.
do if ( hc = 0 or hc =1).
loop i3 = 1 to k3.
compute xhc(:,i3)=xhc(:,i3)&* resid.
end loop.
end if.
do if ( hc =3 or hc =2).
loop i3=1 to k3.
compute xhc(:,i3) = ( resid &/(1-hat)&**(1/(4- hc )))&*xhc(:,i3).
end loop.
end if.
do if ( hc = 4).
compute hcmn=make(n,2,4).
compute hcmn(:,2)=(n1*hat)/k3.
loop i3= 1 to k3.
compute xhc(:,i3) = ( resid &/(1-hat)&**(rmin(hcmn)/2))&*xhc(:,i3).
end loop.
end if.
compute varb=(invXtX*t(xhc)*xhc*invXtX).
do if ( hc =1).
compute varb=(n1/(n1-ncol( x )))&*varb.
end if.
end if.
compute hclab={'se(HC0)','se(HC1)','se(HC2)','se(HC3)','se(HC4)','se'}.
compute hclab=hclab(1,( hc +1)).
compute hcflab={'F(HC0)','F(HC1)','F(HC2)','F(HC3)','F(HC4)','F'}.
compute hcflab=hcflab(1,( hc +1)).
release xhc
.
compute seb=sqrt(diag(varb)).
compute trat = b&/seb.
compute p = 2*(1-tcdf(abs(trat), (dfres))).
compute tval = sqrt(dfres* (exp((dfres-(5/6))*((xp2/(dfres-(2/3)+(.11/dfres)))* (xp2/(dfres-(2/3)+(.11/dfres)))))-1)).
compute modres={modres,seb,trat,p}.
compute modres={modres,(b-tval&*seb),(b+tval&*seb)}.
compute modresl={'coeff',hclab,'t','p','LLCI','ULCI'}.
compute lmat = ident(ncol( x )).
compute lmat = lmat(:,2:ncol(lmat)).
compute fratio = (t(t(lmat)*b)*inv(t(lmat)*varb*lmat)*((t(lmat)*b)))/(ncol( x )-1).
compute pfr = 1-fcdf(fratio,(ncol( x )-1),dfres).
compute modsum={sqrt(r2),r2,mse,fratio,(ncol( x )-1),dfres,pfr}.
compute modsuml={'R','R-sq','MSE',hcflab,'df1','df2', 'p'}.
end if.
end if.
do if ( 1 = 2 or 1 =3).
compute xlp= x.
compute ylp= y.
compute pt2 = make(nrow(ylp),1,(csum(ylp)/nrow(ylp))).
do if ( 1 =2).
compute LL3 = ylp&*ln(pt2)+(1-ylp)&*ln(1-pt2).
end if.
compute LL3 = -2*csum(LL3).
compute bt1 = make(ncol(xlp),1,0).
compute LL1 = 0.
compute pt1 = make(nrow(ylp),1,0.5).
compute pt1lp=pt1.
loop jjj = 1 to iterate.
compute xlptmp=t(xlp).
compute vecprb=pt1lp&*(1-pt1lp).
loop kkk=1 to ncol(xlp).
compute xlptmp(kkk,:)=xlptmp(kkk,:)&*t(vecprb).
end loop.
compute b = bt1+inv(xlptmp*xlp)*t(xlp)*(ylp-pt1lp).
do if ( 1 =2).
compute xlpb=xlp*b.
compute xlpbt=(xlpb > -709.7).
compute xlpb709=(1-xlpbt)*(-709.7).
compute xlpb=(xlpb&*xlpbt)+xlpb709.
compute pt1lp = 1/(1+exp(-(xlpb))).
end if.
compute itprob = csum((pt1lp < .00000001) or (pt1lp > .9999999)).
do if (itprob > 0).
loop kkk = 1 to nrow(pt1lp).
do if (pt1lp(kkk,1) > .9999999).
compute pt1lp(kkk,1) = .9999999.
end if.
do if (pt1lp(kkk,1) < .00000001).
compute pt1lp(kkk,1) = .00000001.
end if.
end loop.
compute itprob = 0.
end if.
do if (itprob = 0).
do if ( 1 =2).
compute LL = ylp&*ln(pt1lp)+(1-ylp)&*ln(1-pt1lp).
end if.
compute LL2 = -2*csum(ll).
end if.
do if (abs(LL1-LL2) < converge).
do if ( 0 =1).
compute xlptmp=t(xlp).
compute vecprb=pt1lp&*(1-pt1lp).
loop kkk=1 to ncol(xlp).
compute xlptmp(kkk,:)=xlptmp(kkk,:)&*t(vecprb).
end loop.
compute varb = inv(xlptmp*xlp).
compute seb = sqrt(diag(varb)).
release xlptmp.
end if.
break.
end if.
compute bt1 = b.
compute LL1 = LL2.
end loop.
compute modres=b.
do if (jjj > iterate).
compute itprob = 2.
do if (booting=0).
compute iterrmod=1.
end if.
do if (booting=1).
compute bootiter=1.
end if.
do if (itprobtg=0).
compute itprobtg=1.
compute errcode(errs,1) = 47.
compute errs = errs + 1.
do if (booting = 0 and 0 =1).
compute vt1 = mdiag(pt1lp&*(1-pt1lp)).
compute varb = inv(t(xlp)*vt1*xlp).
compute seb = sqrt(diag(varb)).
end if.
end if.
end if.
do if ( 0 =1).
compute trat = b&/seb.
compute dfres=nrow(xlp).
compute p = 2*(1-cdfnorm(abs(trat))).
compute modres={modres,seb,trat,p}.
compute modres={modres,(b-xp2&*seb),(b+xp2&*seb)}.
compute pvchi=1-chicdf((LL3-LL2),(nrow(modres)-1)).
compute mcF = (LL3-LL2)/LL3.
compute cox = 1-exp(-(LL3-LL2)/nrow(xlp)).
compute nagel = cox/(1-exp(-(LL3)/nrow(xlp))).
compute modsum={LL2,(LL3-LL2),(nrow(modres)-1),pvchi, mcF,cox,nagel}.
compute modsuml={'-2LL','ModelLL', 'df', 'p', 'McFadden', 'CoxSnell', 'Nagelkrk'}.
compute modresl={'coeff','se','Z','p','LLCI','ULCI'}.
end if.
end if
.
end if.
do if (ydich=1 and (i = (nms+nys))).

.
do if ( 2 =1).
compute hatmat=inv(t( x )* x )*t( x ).
compute b = hatmat* y.
compute modres=b.
do if ( 0 =1).
compute n1=nrow( x ).
compute dfres=n1-(ncol( x )).
compute sstotal = t( y -(csum( y )/n1))*( y -(csum( y )/n1)).
compute resid= y - x *b.
compute ssresid = csum((resid)&**2).
compute r2 = (sstotal-ssresid)/sstotal.
do if (r2 < 0).
compute r2=0.
end if.
compute adjr2 = 1-((1-r2)*(n1-1)/(dfres)).
compute mse=ssresid/(n1-ncol( x )).

.
compute n1=nrow( x ).
compute invXtX = inv(t( x )* x ).
compute varb = mse *invXtX.
compute k3 = ncol( x ).
compute xhc=0.
do if ( hc <> 5).
compute xhc= x.
compute hat = xhc(:,1).
loop i3=1 to nrow(xhc).
compute hat(i3,1)= xhc(i3,:)*invXtX*t(xhc(i3,:)).
end loop.
do if ( hc = 0 or hc =1).
loop i3 = 1 to k3.
compute xhc(:,i3)=xhc(:,i3)&* resid.
end loop.
end if.
do if ( hc =3 or hc =2).
loop i3=1 to k3.
compute xhc(:,i3) = ( resid &/(1-hat)&**(1/(4- hc )))&*xhc(:,i3).
end loop.
end if.
do if ( hc = 4).
compute hcmn=make(n,2,4).
compute hcmn(:,2)=(n1*hat)/k3.
loop i3= 1 to k3.
compute xhc(:,i3) = ( resid &/(1-hat)&**(rmin(hcmn)/2))&*xhc(:,i3).
end loop.
end if.
compute varb=(invXtX*t(xhc)*xhc*invXtX).
do if ( hc =1).
compute varb=(n1/(n1-ncol( x )))&*varb.
end if.
end if.
compute hclab={'se(HC0)','se(HC1)','se(HC2)','se(HC3)','se(HC4)','se'}.
compute hclab=hclab(1,( hc +1)).
compute hcflab={'F(HC0)','F(HC1)','F(HC2)','F(HC3)','F(HC4)','F'}.
compute hcflab=hcflab(1,( hc +1)).
release xhc
.
compute seb=sqrt(diag(varb)).
compute trat = b&/seb.
compute p = 2*(1-tcdf(abs(trat), (dfres))).
compute tval = sqrt(dfres* (exp((dfres-(5/6))*((xp2/(dfres-(2/3)+(.11/dfres)))* (xp2/(dfres-(2/3)+(.11/dfres)))))-1)).
compute modres={modres,seb,trat,p}.
compute modres={modres,(b-tval&*seb),(b+tval&*seb)}.
compute modresl={'coeff',hclab,'t','p','LLCI','ULCI'}.
compute lmat = ident(ncol( x )).
compute lmat = lmat(:,2:ncol(lmat)).
compute fratio = (t(t(lmat)*b)*inv(t(lmat)*varb*lmat)*((t(lmat)*b)))/(ncol( x )-1).
compute pfr = 1-fcdf(fratio,(ncol( x )-1),dfres).
compute modsum={sqrt(r2),r2,mse,fratio,(ncol( x )-1),dfres,pfr}.
compute modsuml={'R','R-sq','MSE',hcflab,'df1','df2', 'p'}.
end if.
end if.
do if ( 2 = 2 or 2 =3).
compute xlp= x.
compute ylp= y.
compute pt2 = make(nrow(ylp),1,(csum(ylp)/nrow(ylp))).
do if ( 2 =2).
compute LL3 = ylp&*ln(pt2)+(1-ylp)&*ln(1-pt2).
end if.
compute LL3 = -2*csum(LL3).
compute bt1 = make(ncol(xlp),1,0).
compute LL1 = 0.
compute pt1 = make(nrow(ylp),1,0.5).
compute pt1lp=pt1.
loop jjj = 1 to iterate.
compute xlptmp=t(xlp).
compute vecprb=pt1lp&*(1-pt1lp).
loop kkk=1 to ncol(xlp).
compute xlptmp(kkk,:)=xlptmp(kkk,:)&*t(vecprb).
end loop.
compute b = bt1+inv(xlptmp*xlp)*t(xlp)*(ylp-pt1lp).
do if ( 2 =2).
compute xlpb=xlp*b.
compute xlpbt=(xlpb > -709.7).
compute xlpb709=(1-xlpbt)*(-709.7).
compute xlpb=(xlpb&*xlpbt)+xlpb709.
compute pt1lp = 1/(1+exp(-(xlpb))).
end if.
compute itprob = csum((pt1lp < .00000001) or (pt1lp > .9999999)).
do if (itprob > 0).
loop kkk = 1 to nrow(pt1lp).
do if (pt1lp(kkk,1) > .9999999).
compute pt1lp(kkk,1) = .9999999.
end if.
do if (pt1lp(kkk,1) < .00000001).
compute pt1lp(kkk,1) = .00000001.
end if.
end loop.
compute itprob = 0.
end if.
do if (itprob = 0).
do if ( 2 =2).
compute LL = ylp&*ln(pt1lp)+(1-ylp)&*ln(1-pt1lp).
end if.
compute LL2 = -2*csum(ll).
end if.
do if (abs(LL1-LL2) < converge).
do if ( 0 =1).
compute xlptmp=t(xlp).
compute vecprb=pt1lp&*(1-pt1lp).
loop kkk=1 to ncol(xlp).
compute xlptmp(kkk,:)=xlptmp(kkk,:)&*t(vecprb).
end loop.
compute varb = inv(xlptmp*xlp).
compute seb = sqrt(diag(varb)).
release xlptmp.
end if.
break.
end if.
compute bt1 = b.
compute LL1 = LL2.
end loop.
compute modres=b.
do if (jjj > iterate).
compute itprob = 2.
do if (booting=0).
compute iterrmod=1.
end if.
do if (booting=1).
compute bootiter=1.
end if.
do if (itprobtg=0).
compute itprobtg=1.
compute errcode(errs,1) = 47.
compute errs = errs + 1.
do if (booting = 0 and 0 =1).
compute vt1 = mdiag(pt1lp&*(1-pt1lp)).
compute varb = inv(t(xlp)*vt1*xlp).
compute seb = sqrt(diag(varb)).
end if.
end if.
end if.
do if ( 0 =1).
compute trat = b&/seb.
compute dfres=nrow(xlp).
compute p = 2*(1-cdfnorm(abs(trat))).
compute modres={modres,seb,trat,p}.
compute modres={modres,(b-xp2&*seb),(b+xp2&*seb)}.
compute pvchi=1-chicdf((LL3-LL2),(nrow(modres)-1)).
compute mcF = (LL3-LL2)/LL3.
compute cox = 1-exp(-(LL3-LL2)/nrow(xlp)).
compute nagel = cox/(1-exp(-(LL3)/nrow(xlp))).
compute modsum={LL2,(LL3-LL2),(nrow(modres)-1),pvchi, mcF,cox,nagel}.
compute modsuml={'-2LL','ModelLL', 'df', 'p', 'McFadden', 'CoxSnell', 'Nagelkrk'}.
compute modresl={'coeff','se','Z','p','LLCI','ULCI'}.
end if.
end if
.
end if.
compute modres2={modres2,t(modres)}.
do if (i = (nms+nys)).
do if (bcmat((i+1),1) = 1).
compute bootdir={bootdir;t(modres(wherex(1,i):wherex(2,i),1))}.
end if.
do if (bcmat((i+1),1) = 0).
compute bootdir={bootdir;dirzes}.
end if.
end if.
do if (model = 74 and i <= nms).
compute onetemp=make(nrow(xprobval),1,1).
compute mestmtb={onetemp,xprobval}.
do if (ncs > 0).
compute ncovmdlb=rsum(ccmat(i,:)).
do if (ncovmdl > 0).
compute cvmncb=make(nrow(mestmtb),ncovmdlb,1).
compute cvmnctmp=csum(x(:,(ncol(x)-ncovmdlb+1):ncol(x)))/nrow(x).
do if (cuscoval > 0).
compute cvmnctmp=coval.
end if.
loop mestlp=1 to ncovmdlb.
compute cvmncb(:,mestlp)=cvmncb(:,mestlp)*cvmnctmp(:,mestlp).
end loop.
compute mestmtb={mestmtb,cvmncb}.
end if.
end if.
compute mestbt=mestmtb*modres.
do if (i = 1).
compute mestb=mestbt.
end if.
do if (i > 1).
compute mestb={mestb,mestbt}.
end if.
end if.
do if (model = 74 and i = (nms+nys)).
compute xvalptmp=make(1,nxvls,0).
compute mest74sp=make(1,nms,0).
compute mest74sp={mest74sp;mestb}.
compute xvalptmp={xvalptmp;ident(nxvls)}.
compute dirfxcf=make(nrow(mestb),1,0).
loop cfloop1=1 to nrow(mestb).
compute ndirfx=make(nrow(modres),1,0).
compute ndirfx(2:(nxvls+1),1)=t(xvalptmp(cfloop1,:)).
loop cfloop3=1 to nms.
do if (mcx=1 or mcx=0).
compute ndirfx(wheremw((1+((cfloop3-1)*2)),ncol(wheremw)):wheremw((2+((cfloop3-1)*2) ),ncol(wheremw)))=t(xvalptmp(cfloop1,:)*mestb(1,cfloop3)).
end if.
do if (mcx=2).
compute ndirfx(wheremw((1+((cfloop3-1)*2)),ncol(wheremw)):wheremw((2+((cfloop3-1)*2) ),ncol(wheremw)))=t(xvalptmp(cfloop1,:)*mest74sp(cfloop1,cfloop3)).
end if.
end loop.
do if (cfloop1 > 1).
compute dirfxcf(cfloop1,1)=t(ndirfx)*modres*xscaling.
end if.
end loop.
compute natdirbt={natdirbt;t(dirfxcf(2:nrow(dirfxcf),:))}.
end if.
do if ( (bcmat((i+1),1) = 1) and (nobootx=1) and (effsize=1)).
compute nobootx=0.
compute xsdtemp= (nrow(x)*sscp(x(:,2)))-(t(csum(x(:,2)))*(csum(x(:,2)))).
compute xsdtemp= sqrt(xsdtemp/(nrow(x)*(nrow(x)-1))).
end if.
end if.
end loop.
do if (bad = 0).
compute bootres={bootres;modres2(:,2:ncol(modres2))}.
do if (effsize=1).
compute ysdtemp= (nrow(y)*sscp(y))-(t(csum(y))*(csum(y))).
compute ysdtemp= sqrt(ysdtemp/(nrow(y)*(nrow(y)-1))).
compute bootysd={bootysd;ysdtemp}.
compute bootxsd={bootxsd;xsdtemp}.
end if.
compute goodboot=goodboot+1.
end if.
do if (bad <> 0).
compute badboot=badboot+1.
end if.
end loop if (goodboot = boot).
compute bootres=bootres(2:nrow(bootres),:).
do if (effsize=1).
compute bootysd=bootysd(2:nrow(bootysd),:).
do if (nrow(bootxsd) > 1).
compute bootxsd=bootxsd(2:nrow(bootxsd),:).
end if.
end if.
do if (goodboot < (boot)).
compute boot=0.
compute modelbt=0.
compute notecode(notes,1) = 7.
compute notes = notes + 1.
end if.
do if (boot > 0).
do if (effsize=1).
compute bootysd={ysd;bootysd}.
compute bootxsd={xsd;bootxsd}.
end if.
do if (saveboot = 1).
save bootres/outfile = *.
end if.
do if (modelbt=1).
compute bootcim=make(ncol(bootres),5,-99999).
compute bootcim(:,2) = t(csum(bootres)/nrow(bootres)).
compute bootcim(:,1) = coeffmat(2:nrow(coeffmat),1).
loop i = 1 to ncol(bootres).

.
compute temp = bootres(:,i).
compute temp(GRADE( bootres(:,i) )) = bootres(:,i).
compute badlo = 0.
compute badhi = 0.
do if ( (bootcim(i,1)*bc)+(9999*(1-bc)) <> 9999).
compute pv=csum(temp < (bootcim(i,1)*bc)+(9999*(1-bc)) )/nrow(temp).
compute ppv = pv.
do if (pv > .5).
compute ppv = 1-pv.
end if.
compute y5=sqrt(-2*ln(ppv)).
compute xp=y5+((((y5*p4+p3)*y5+p2)*y5+p1)*y5+p0)/((((y5*q4+q3)*y5+q2)*y5+q1)*y5+q0).
do if (pv <= .5).
compute xp = -xp.
end if.
compute cilow=rnd(nrow(temp)*(cdfnorm(2*xp-xp2))).
compute cihigh=trunc(nrow(temp)*(cdfnorm(2*xp+(xp2))))+1.
do if (cilow < 1).
compute cilow = 1.
compute booterr=1.
compute badlo = 1.
end if.
do if (cihigh > nrow(temp)).
compute cihigh = boot.
compute booterr=1.
compute badhi = 1.
end if.
compute llcit=temp(cilow,1).
compute ulcit=temp(cihigh,1).
do if (badlo = 1 and llcit <> priorlo).
compute badend={badend, llcit}.
compute priorlo = llcit.
end if.
do if (badhi = 1 and ulcit <> priorhi).
compute badend={badend, ulcit}.
compute priorhi = ulcit.
end if.
end if.
do if ( (bootcim(i,1)*bc)+(9999*(1-bc)) = 9999).
compute llcit=temp(cilow,1).
compute ulcit=temp(cihigh,1).
end if.
compute bootse=t(sqrt((cssq(temp)-((csum(temp)&**2)/nrow(temp)))/(nrow(temp)-1)))
.
compute bootcim(i,4:5)={llcit,ulcit}.
compute bootcim(i,3)=bootse.
end loop.
end if.
end if.
do if (badboot > 0).
compute notecode(notes,1) = 6.
compute notes = notes + 1.
end if.
end if.
do if (criterr=0).
release fulldat,x,y.
end if.
do if (xmint=1 and criterr=0).
compute xvalptmp=make(1,nxvls,0).
compute mest74sp=make(1,nms,0).
compute mest74sp={mest74sp;mest74}.
compute xvalptmp={xvalptmp;ident(nxvls)}.
loop kcfuhd=1 to 2.
compute dirfxcf=make(nrow(mest74),6,0).
loop cfloop1=1 to nrow(mest74).
compute ndirfx=make(nrow(lastb),1,0).
compute ndirfx(2:(nxvls+1),1)=t(xvalptmp(cfloop1,:)).
loop cfloop3=1 to nms.
do if (kcfuhd=1).
do if (mcx=1 or mcx=0).
compute ndirfx(wheremw((1+((cfloop3-1)*2)),ncol(wheremw)):wheremw((2+((cfloop3-1)*2)), ncol(wheremw)))=t(xvalptmp(cfloop1,:)*mest74(1,cfloop3)).
end if.
do if (mcx=2).
compute ndirfx(wheremw((1+((cfloop3-1)*2)),ncol(wheremw)):wheremw((2+((cfloop3-1)*2)), ncol(wheremw)))=t(xvalptmp(cfloop1,:)*mest74sp(cfloop1,cfloop3)).
end if.
end if.
do if (kcfuhd=2).
compute ndirfx(wheremw((1+((cfloop3-1)*2)),ncol(wheremw)):wheremw((2+((cfloop3-1)*2)), ncol(wheremw)))=t(xvalptmp(cfloop1,:)*medmeans(1,cfloop3)).
end if.
end loop.
do if (cfloop1 > 1).
compute dirfxcf(cfloop1,1)=t(ndirfx)*lastb*xscaling.
compute dirfxcf(cfloop1,2)=sqrt(diag(t(ndirfx)*lastcov*ndirfx))*abs(xscaling).
compute dirfxcf(cfloop1,3)=dirfxcf(cfloop1,1)/dirfxcf(cfloop1,2).
compute dirfxcf(cfloop1,4)=2*(1-tcdf(abs(dirfxcf(cfloop1,3)), dfres)).
compute dirfxcf(cfloop1,5)=dirfxcf(cfloop1,1)-tval*dirfxcf(cfloop1,2).
compute dirfxcf(cfloop1,6)=dirfxcf(cfloop1,1)+tval*dirfxcf(cfloop1,2).
end if.
end loop.
compute codireff=dirfxcf(2:nrow(dirfxcf),:).
do if (kcfuhd=1).
compute direff=codireff.
end if.
end loop.
end if.

.
do if (criterr = 0 and nms > 0).
compute paths=paths(:,2:ncol(paths)).
compute pathsw=pathsw(:,2:ncol(pathsw)).
compute pathsz=pathsz(:,2:ncol(pathsz)).
compute pathswz=pathswz(:,2:ncol(pathswz)).
compute pathsmod=pathsw+pathsz+pathswz.
compute pathsdv=pathsdv(:,2:ncol(pathsdv)).
compute pathsfoc=pathsfoc(:,2:ncol(pathsfoc)).
compute pathtype=pathtype(:,2:ncol(pathtype)).
compute anymod=(rsum(pathsmod) > 0).
compute obscoeff=obscoeff(1,2:ncol(obscoeff)).
do if (outscree=1).
do if (xmint=1).
print/title = '************************* COUNTERFACTUALLY DEFINED ************************'.
else.
print/title = ' '.
end if.
do if (dototal = 0 and alttotal=0).
print/title = '****************** DIRECT AND INDIRECT EFFECTS OF X ON Y *****************'/space=0.
end if.
do if (alttotal=1).
print/title = '************** TOTAL, DIRECT, AND INDIRECT EFFECTS OF X ON Y **************'/space=0.
end if.
end if.
do if (dototal = 1).
do if (outscree=1).
print/title = '************** TOTAL, DIRECT, AND INDIRECT EFFECTS OF X ON Y **************'/space=0.
end if.
compute totefflb(1,1)='Effect'.
do if (effsize=1).
compute toteffsz=toteff(:,1)/ysd.
do if (xdich=1 or mcx > 0).
compute totefflb={totefflb,'c_ps'}.
end if.
do if (xdich = 0 and mcx = 0).
compute toteffsz=toteffsz*xsd.
compute totefflb={totefflb,'c_cs'}.
end if.
compute toteff={toteff,toteffsz}.
end if.
compute resultm2=make(nrow(toteff),maxresm,99999).
compute resultm2(1:nrow(toteff),1:ncol(toteff))=toteff.
compute resultm={resultm;resultm2}.
do if (nxvls > 1).
compute resultm2=make(nrow(totomni),maxresm,99999).
compute resultm2(1:nrow(totomni),1:ncol(totomni))=totomni.
compute resultm={resultm;resultm2}.
compute clabtmp={'R2-chng', hcflab, 'df1','df2','p'}.
do if (outscree=1).
print toteff/title='Relative total effects of X on Y'/rnames=toteffl2/cnames=totefflb/format= F10.4.
print totomni/title='Omnibus test of total effect of X on Y'/cnames=clabtmp/format= F10.4.
print/title= '----------'/space=0.
end if.
else.
do if (outscree=1).
print toteff/title='Total effect of X on Y'/cnames=totefflb/format= F10.4.
end if.
end if.
end if.
compute moddir=wcmat(nrow(bcmat),1)+zcmat(nrow(bcmat),1).
do if (xmint=1).
compute moddir=1.
end if.
do if (bcmat(nrow(bcmat),1)=1).
do if (ydich=1).
compute direfflb(:,(ncol(direfflb)-5):ncol(direfflb))={'Effect','se','Z','p','LLCI','ULCI'}.
end if.
do if (moddir=0 or xmint=1).
compute direfflb(1,1)='Effect'.
end if.
do if (effsize=1 and moddir=0 and anymod = 0).
compute direffsz=direff(:,1)/ysd.
do if (xdich=1 or mcx > 0).
compute direfflb={direfflb,'c''_ps'}.
end if.
do if (xdich = 0 and mcx = 0).
compute direffsz=direffsz*xsd.
compute direfflb={direfflb,'c''_cs'}.
end if.
compute direff={direff,direffsz}.
end if.
compute resultm2=make(nrow(direff),maxresm,99999).
compute resultm2(1:nrow(direff),1:ncol(direff))=direff.
compute resultm={resultm;resultm2}.
do if (xmint=1).
compute resultm2=make(nrow(codireff),maxresm,99999).
compute resultm2(1:nrow(codireff),1:ncol(codireff))=codireff.
compute resultm={resultm;resultm2}.
end if.
do if (moddir = 0 and nxvls=1).
do if (outscree=1).
print direff/title='Direct effect of X on Y'/cnames=direfflb/format= F10.4.
end if.
end if.
do if (moddir = 0 and nxvls>1).
compute resultm2=make(nrow(diromni),maxresm,99999).
compute resultm2(1:nrow(diromni),1:ncol(diromni))=diromni.
compute resultm={resultm;resultm2}.
do if (outscree=1).
print direff/title='Relative direct effects of X on Y'/rnames=direffl2/cnames=direfflb/format= F10.4.
do if (ydich = 0).
compute clabtmp={'R2-chng', hcflab, 'df1','df2','p'}.
print diromni/title='Omnibus test of direct effect of X on Y:'/cnames=clabtmp/format= F10.4.
end if.
do if (ydich = 1).
compute clabtmp={'Chi-sq', 'df', 'p'}.
print diromni/title='Omnibus likelihood ratio test of direct effect of X on Y'/cnames=clabtmp/format= F10.4.
end if.
print/title= '----------'/space=0.
end if.
end if.
do if (moddir > 0 and nxvls=1).
do if (xmint=0 and outscree=1).
print direff/title='Conditional direct effects of X on Y'/cnames=direfflb/format= F10.4.
end if.
do if (xmint=1).
do if (outscree=1).
print direff/title='(Pure) Natural direct effect of X on Y'/cnames=direfflb/format= F10.4.
print codireff/title='Controlled direct effect of X on Y'/cnames=direfflb/format= F10.4.
print/title= '----------'/space=0.
end if.
compute obnatdfx=direff(:,1).
end if.
end if.
compute direffl4=direffl2.
do if (moddir > 0 and nxvls>1).
compute direffl2={' '}.
loop i = 1 to nxvls.
loop j = 1 to (nrow(direff)/nxvls).
compute direffl2={direffl2;xcatlab(i,1)}.
end loop.
end loop.
compute direffl2=direffl2(2:nrow(direffl2),1).
do if (xmint=0 and outscree=1).
print direff/title='Relative conditional direct effects of X on Y'/cnames=direfflb/rnames=direffl2/format= F10.4.
end if.
do if (xmint=1).
do if (outscree=1).
print direff/title='Relative (pure) natural direct effects of X on Y'/cnames=direfflb/rnames=direffl2/format= F10.4.
print codireff/title='Relative controlled direct effects of X on Y'/cnames=direfflb/rnames=direffl2/format= F10.4.
print/title= '----------'/space=0.
end if.
compute obnatdfx=direff(:,1).
end if.
end if.
compute direffl2=direffl4.
end if.
do if (bcmat(nrow(bcmat),1)=0 and xmint <> 1 and outscree=1).
print/title='The direct effect of X on Y is fixed to zero.'.
end if.
do if (nms = 1).
compute indmark={2}.
compute indsets={1,2}.
end if.
do if (nms = 2).
compute indmark={2,2,3}.
compute indsets={1,4,2,5,1,3,5}.
compute thetam=1.
end if.
do if (nms = 3).
compute indmark={2,2,2,3,3,3,4}.
compute indsets={1,7,2,8,4,9,1,3,8,1,5,9,2,6,9,1,3,6,9}.
compute thetam={1,2,3}.
end if.
do if (nms = 4).
compute indmark={2,2,2,2,3,3,3,3,3,3,4,4,4,4,5}.
compute indsets={1,11,2,12,4,13,7,14,1,3,12,1,5,13,1,8,14,2,6,13,2,9,14,4,10,14,1,3,6,13,1,3,9, 14,1,5,10,14,2,6,10,14,1,3,6,10,14}.
compute thetam={1,2,4,3,5,6}.
end if.
do if (nms = 5).
compute indmark={2,2,2,2,2,3,3,3,3,3,3,3,3,3,3,4,4,4,4,4,4,4,4,4,4,5,5,5,5,5,6}.
compute indsets={1,16,2,17,4,18,7,19,11,20,1,3,17,1,5,18,1,8,19,1,12,20,2,6,18,2,9,19,2,13,20,4, 10,19,4,14,20,7,15,20,1,3,6,18, 1,3,9,19,1,3,13,20,1,5,10,19,1,5,14,20,1,8,15,20,2,6,10,19,2,6, 14,20,2,9,15,20,4,10,15,20,1,3,6,10,19, 1,3,6,14,20,1,3,9,15,20,1,5,10,15,20,2,6,10,15,20,1,3,6,10,15, 20}.
compute thetam={1,2,5,3,6,8,4,7,9,10}.
end if.
do if (nms = 6).
compute indmark={2,2,2,2,2,2,3,3,3,3,3,3,3,3,3,3,3,3,3,3,3,4,4,4,4,4,4,4,4,4,4,4,4,4,4,4,4,4,4, 4,4,5,5,5,5,5,5,5,5,5,5,5,5,5,5,5,6,6,6,6,6,6,7}.
compute indsets={1,22,2,23,4,24,7,25,11,26,16,27,1,3,23,1,5,24,1,8,25,1,12,26,1,17,27,2,6,24,2, 9,25,2,13,26,2,18,27,4,10,25,4,14,26, 4,19,27,7,15,26,7,20,27,11,21,27,1,3,6,24,1,3,9,25,1,3,13,26,1, 3,18,27,1,5,10,25,1,5,14,26,1,5,19,27,1,8,15,26,1,8,20,27, 1,12,21,27,2,6,10,25,2,6,14,26,2,6,19,27,2,9,15,26,2,9,20,27,2, 13,21,27,4,10,15,26,4,10,20,27,4,14,21,27,7,15,21,27, 1,3,6,10,25,1,3,6,14,26,1,3,6,19,27,1,3,9,15,26,1,3,9,20,27,1,3, 13,21,27,1,5,10,15,26,1,5,10,20,27,1,5,14,21,27, 1,8,15,21,27,2,6,10,15,26,2,6,10,20,27,2,6,14,21,27,2,9,15,21, 27,4,10,15,21,27,1,3,6,10,15,26,1,3,6,10,20,27, 1,3,6,14,21,27,1,3,9,15,21,27,1,5,10,15,21,27,2,6,10,15,21,27, 1,3,6,10,15,21,27}.
compute thetam={1,2,6,3,7,10,4,8,11,13,5,9,12,14,15}.
end if.
do if (nms = 7).
compute indmark={2,2,2,2,2,2,2}.
compute indsets={1,29,2,30,4,31,7,32,11,33,16,34,22,35}.
end if.
do if (nms = 8).
compute indmark={2,2,2,2,2,2,2,2}.
compute indsets={1,37,2,38,4,39,7,40,11,41,16,42,22,43,29,44}.
end if.
do if (nms = 9).
compute indmark={2,2,2,2,2,2,2,2,2}.
compute indsets={1,46,2,47,4,48,7,49,11,50,16,51,22,52,29,53,37,54}.
end if.
do if (nms=10).
compute indmark={2,2,2,2,2,2,2,2,2,2}.
compute indsets={1,56,2,57,4,58,7,59,11,60,16,61,22,62,29,63,37,64,46,65}.
end if.
compute indlbl = {'Ind1'; 'Ind2'; 'Ind3'; 'Ind4'; 'Ind5'; 'Ind6'; 'Ind7'; 'Ind8'; 'Ind9'; 'Ind10'; 'Ind11'; 'Ind12'; 'Ind13'; 'Ind14'; 'Ind15'}.
compute indlbl = {indlbl; 'Ind16';'Ind17';'Ind18';'Ind19';'Ind20';'Ind21';'Ind22';'Ind23';'Ind24';'Ind25';'Ind26';'Ind27';'Ind28';'Ind29';'Ind30'}.
compute indlbl = {indlbl; 'Ind31';'Ind32';'Ind33';'Ind34';'Ind35';'Ind36';'Ind37';'Ind38';'Ind39';'Ind40';'Ind41';'Ind42';'Ind43';'Ind44';'Ind45'}.
compute indlbl = {indlbl; 'Ind46';'Ind47';'Ind48';'Ind49';'Ind50';'Ind51';'Ind52';'Ind53';'Ind54';'Ind55';'Ind56';'Ind57';'Ind58';'Ind59';'Ind60'}.
compute indlbl = {indlbl; 'Ind61';'Ind62';'Ind63';'Ind64';'Ind65';'Ind66';'Ind67';'Ind68';'Ind69';'Ind70';'Ind71';'Ind72';'Ind73';'Ind74';'Ind75'}.
compute indlbl = {indlbl; 'Ind76';'Ind77';'Ind78';'Ind79';'Ind80';'Ind81';'Ind82';'Ind83';'Ind84';'Ind85';'Ind86';'Ind87';'Ind88';'Ind89';'Ind90'}.
compute cntname={'(C1)';'(C2)';'(C3)';'(C4)';'(C5)';'(C6)';'(C7)';'(C8)';'(C9)';'(C10)';'(C11)';'(C12)';'(C13)';'(C14)';'(C15)';'(C16)';'(C17)'}.
compute cntname={cntname;'(C18)';'(C19)';'(C20)';'(C21)';'(C22)';'(C23)';'(C24)';'(C25)';'(C26)';'(C27)';'(C28)';'(C29)';'(C30)';'(C31)'}.
compute cntname={cntname;'(C32)';'(C33)';'(C34)';'(C35)';'(C36)';'(C37)';'(C38)';'(C39)';'(C40)';'(C41)';'(C42)';'(C43)';'(C44)';'(C45)'}.
compute cntname={cntname;'(C46)';'(C47)';'(C48)';'(C49)';'(C50)';'(C51)';'(C52)';'(C53)';'(C54)';'(C55)';'(C56)';'(C57)';'(C58)';'(C59)'}.
compute cntname={cntname;'(C60)';'(C61)';'(C62)';'(C63)';'(C64)';'(C65)';'(C66)';'(C67)';'(C68)';'(C69)';'(C70)';'(C71)';'(C72)';'(C73)'}.
compute cntname={cntname;'(C74)';'(C75)';'(C76)';'(C77)';'(C78)';'(C79)';'(C80)';'(C81)';'(C82)';'(C83)';'(C84)';'(C85)';'(C86)';'(C87)'}.
compute cntname={cntname;'(C88)';'(C89)';'(C90)';'(C91)';'(C92)';'(C93)';'(C94)';'(C95)';'(C96)';'(C97)';'(C98)';'(C99)';'(C100)';'(C101)'}.
compute cntname={cntname;'(C102)';'(C103)';'(C104)';'(C105)'}.
compute indmake=make(ncol(indmark),(nms+2),0).
compute indmod=make(ncol(indmark),1,999).
compute indmmm=make(ncol(indmark),1,0).
compute indmmmt=make(ncol(indmark),1,0).
compute start=1.
compute end=0.
compute nindfx=0.
compute indlocs=make(nrow(thetaxmb),ncol(paths),999).
compute indkey=make(ncol(indmark),1+((rmax(indmark)*2)+1),'     ').
compute c1=1.
compute c2=1.
compute c3=1.
loop i = 1 to ncol(paths).
do if (pathtype(1,i)=1).
compute indlocs(:,i)=thetaxmb(:,c1).
compute c1=c1+1.
end if.
do if (pathtype(1,i)=3).
compute indlocs(:,i)=thetamyb(:,c2).
compute c2=c2+1.
end if.
do if (pathtype(1,i)=2 and nms < 7 and serial=1).
compute indlocs(:,i)=thetammb(:,thetam(1,c3)).
compute c3=c3+1.
end if.
end loop.
loop i = 1 to ncol(indlocs).
compute c1=2.
loop j = 2 to nrow(indlocs).
do if (indlocs(j,i) <> 0).
compute indlocs(c1,i)=indlocs(j,i).
compute c1=c1+1.
end if.
end loop.
compute indlocs(1,i)=c1-2.
end loop.
compute indlocs=indlocs(1:rmax((indlocs(1,:))+1),:).
loop i = 1 to ncol(indmark).
compute numget=indmark(1,i).
compute end=end+numget.
compute gotcha=indsets(1,start:end).
compute start=end+1.
compute ok=1.
compute temp=0.
compute repoman=make(4,1,0).
loop j = 1 to ncol(gotcha).
do if paths(1,gotcha(1,j))=0.
compute ok=0.
end if.
do if (pathsmod(1,gotcha(1,j)) > 0).
compute temp=1.
compute temp2={pathsw(1,gotcha(1,j));pathsz(1,gotcha(1,j));pathswz(1,gotcha(1,j));0}.
do if (temp2(1,1)=1 and temp2(2,1)=1 and temp2(3,1)=0).
compute temp2(4,1)=1.
end if.
compute repoman=repoman+temp2.
end if.
end loop.
compute temp=0.
compute tempmmm=0.
compute typemmm=0.
do if ((repoman(1,1) > 0) and (repoman(2,1) = 0)).
compute temp=1.
do if (repoman(1,1)=1).
compute tempmmm=1.
end if.
do if (repoman(1,1) > 1 and (wdich=1 or mcw > 0)).
compute tempmmm=12.
compute typemmm=mcw.
do if (wdich=1).
compute typemmm=1.
end if.
end if.
do if (repoman(1,1) > 1 and (wdich=0 and mcw = 0)).
compute tempmmm=101.
end if.
end if.
do if ((repoman(1,1) = 0) and (repoman(2,1) > 0)).
compute temp=2.
do if (repoman(2,1)=1).
compute tempmmm=2.
end if.
do if (repoman(2,1) > 1 and (zdich = 1 or mcz > 0)).
compute tempmmm=22.
compute typemmm=mcz.
do if (zdich=1).
compute typemmm=1.
end if.
end if.
do if (repoman(2,1) > 1 and (zdich = 0 and mcw = 0)).
compute tempmmm=102.
end if.
end if.
do if (repoman(1,1)>0 and repoman(2,1)>0).
compute temp=3.
do if (repoman(1,1)=1 and repoman(2,1)=1).
do if (repoman(4,1)=1).
compute tempmmm=31.
end if.
do if (repoman(3,1)=1).
compute tempmmm=41.
end if.
end if.
end if.
do if (repoman(1,1)=1 and repoman(2,1)=1 and repoman(3,1)=0 and repoman(4,1)=0).
compute tempmmm=51.
end if.
do if (ok = 1).
compute nindfx=nindfx+1.
compute indmake(nindfx,1)=numget.
compute indmod(nindfx,1)=temp.
compute indmmm(nindfx,1)=tempmmm.
compute indmmmt(nindfx,1)=typemmm.
compute indmake(nindfx,2:(1+numget))=gotcha.
compute indkey(nindfx,1)=xnames.
loop j = 1 to numget.
compute indkey(nindfx,(j*2+1))=pathsdv(1,gotcha(1,j)).
compute indkey(nindfx,(j*2))='   ->   '.
end loop.
end if.
end loop.
compute indkey=indkey(1:nindfx,1:((cmax(indmake(:,1))*2)+1)).
compute indmake=indmake(1:nindfx,1:(cmax(indmake(:,1))+1)).
compute indmod=indmod(1:nrow(indmake),1).
compute indmmm=indmmm(1:nrow(indmake),1).
compute indmmmt=indmmmt(1:nrow(indmake),1).
compute ncpairs = (((nindfx)*(nindfx-1))/2).
do if ((contrast = 1 or contrast = 2 or contrast=3) and (ncpairs > 105)).
compute contrast=0.
compute notecode(notes,1) = 13.
compute notes = notes + 1.
end if.
do if (contrast = 4).
do if (ncol(contvec) <> nindfx).
compute contrast=0.
compute notecode(notes,1) = 14.
compute notes = notes + 1.
end if.
end if.
do if (anymod=0).
do if (nms = 1 and contrast > 0).
compute contrast=0.
end if.
compute efloop=(((1-(effsize=0))*2)+1)-(((mcx>0 or xdich=1))*(1-(effsize=0))).
loop kk=1 to efloop.
do if (boot = 0).
compute bootres=obscoeff.
do if (kk=1).
compute totbtvec=make(1,nxvls,0).
end if.
compute bootdir=obsdirfx.
compute indtab=999.
compute inddiff=999.
compute bootysd=ysd.
compute bootxsd=xsd.
end if.
do if (boot > 0).
compute bootres={obscoeff;bootres}.
do if (kk=1).
compute totbtvec=make(nrow(bootres),nxvls,0).
end if.
compute indtab=make(1,4,999).
compute inddiff=make(nrow(bootres),1,999).
end if.
compute indtotal=make(nrow(bootres),1,0).
loop i = 1 to nrow(indmake).
loop j = 1 to nxvls.
compute indtemp=make(nrow(bootres),1,1).
loop k = 1 to indmake(i,1).
compute jtemp=1.
do if (j > 1 and k=1).
compute jtemp=j.
end if.
compute indtemp=indtemp&*bootres(:,pathsfoc(jtemp,indmake(i,(k+1)))).
end loop.
do if (kk = 2).
compute indtemp=indtemp/bootysd.
end if.
do if (kk = 3).
compute indtemp=(bootxsd&*indtemp)/bootysd.
end if.
do if (contrast <> 0).
compute inddiff={inddiff,indtemp}.
end if.
do if (nxvls=1).
compute indtotal=indtotal+indtemp.
end if.
compute indeff=indtemp(1,1).
do if (kk=1).
compute totbtvec(:,j)=totbtvec(:,j)+indtemp.
end if.
do if (boot > 0).

.
compute temp = indtemp(2:nrow(indtemp),1).
compute temp(GRADE( indtemp(2:nrow(indtemp),1) )) = indtemp(2:nrow(indtemp),1).
compute badlo = 0.
compute badhi = 0.
do if ( (indtemp(1,1)*bc)+(9999*(1-bc)) <> 9999).
compute pv=csum(temp < (indtemp(1,1)*bc)+(9999*(1-bc)) )/nrow(temp).
compute ppv = pv.
do if (pv > .5).
compute ppv = 1-pv.
end if.
compute y5=sqrt(-2*ln(ppv)).
compute xp=y5+((((y5*p4+p3)*y5+p2)*y5+p1)*y5+p0)/((((y5*q4+q3)*y5+q2)*y5+q1)*y5+q0).
do if (pv <= .5).
compute xp = -xp.
end if.
compute cilow=rnd(nrow(temp)*(cdfnorm(2*xp-xp2))).
compute cihigh=trunc(nrow(temp)*(cdfnorm(2*xp+(xp2))))+1.
do if (cilow < 1).
compute cilow = 1.
compute booterr=1.
compute badlo = 1.
end if.
do if (cihigh > nrow(temp)).
compute cihigh = boot.
compute booterr=1.
compute badhi = 1.
end if.
compute llcit=temp(cilow,1).
compute ulcit=temp(cihigh,1).
do if (badlo = 1 and llcit <> priorlo).
compute badend={badend, llcit}.
compute priorlo = llcit.
end if.
do if (badhi = 1 and ulcit <> priorhi).
compute badend={badend, ulcit}.
compute priorhi = ulcit.
end if.
end if.
do if ( (indtemp(1,1)*bc)+(9999*(1-bc)) = 9999).
compute llcit=temp(cilow,1).
compute ulcit=temp(cihigh,1).
end if.
compute bootse=t(sqrt((cssq(temp)-((csum(temp)&**2)/nrow(temp)))/(nrow(temp)-1)))
.
compute indeff={indeff,bootse,llcit,ulcit}.
end if.
do if (kk=1).
compute indtabn={indtab;indeff}.
end if.
compute indtab={indtab;indeff}.
end loop.
end loop.
compute indtab=indtab(2:nrow(indtab),:).
do if (kk=1).
compute indtabn=indtabn(2:nrow(indtabn),:).
end if.
compute rowlbs=indlbl(1:nrow(indtab),1).
do if (mc > 0).
compute inddiff=make(mc,1,-999).
compute indtab2=make(nrow(indtab),4,-999).
compute indtab2(:,1)=indtab.
compute indtab=indtab2.
release indtab2.
compute mcct=0.
compute indtotal=make(mc,1,0).
do if (kk = 1).
compute x1 = sqrt(-2*ln(uniform(mc,nrow(mcsopath))))&*cos((2*3.14159265358979)*uniform(mc, nrow(mcsopath))).
compute x1=x1*chol(indcov).
loop ii=1 to nrow(x1).
compute x1(ii,:)=x1(ii,:)+t(mcsopath).
end loop.
end if.
loop ii=1 to nms.
compute tmpb=x1(:,((nms*nxvls)+ii)).
compute tmpb2=tmpb.
do if (nxvls > 1).
loop jj=1 to (nxvls-1).
compute tmpb2={tmpb2,tmpb}.
end loop.
end if.
compute indtemp=x1(:,(((ii-1)*nxvls)+1):(ii*nxvls))&*tmpb2.
loop jj=1 to ncol(indtemp).
do if (kk = 2).
compute indtemp(:,jj)=indtemp(:,jj)/ysd.
end if.
do if (kk = 3).
compute indtemp(:,jj)=(xsd*indtemp(:,jj))/ysd.
end if.

.
compute temp = indtemp(:,jj).
compute temp(GRADE( indtemp(:,jj) )) = indtemp(:,jj).
compute badlo = 0.
compute badhi = 0.
do if ( 9999 <> 9999).
compute pv=csum(temp < 9999 )/nrow(temp).
compute ppv = pv.
do if (pv > .5).
compute ppv = 1-pv.
end if.
compute y5=sqrt(-2*ln(ppv)).
compute xp=y5+((((y5*p4+p3)*y5+p2)*y5+p1)*y5+p0)/((((y5*q4+q3)*y5+q2)*y5+q1)*y5+q0).
do if (pv <= .5).
compute xp = -xp.
end if.
compute cilow=rnd(nrow(temp)*(cdfnorm(2*xp-xp2))).
compute cihigh=trunc(nrow(temp)*(cdfnorm(2*xp+(xp2))))+1.
do if (cilow < 1).
compute cilow = 1.
compute booterr=1.
compute badlo = 1.
end if.
do if (cihigh > nrow(temp)).
compute cihigh = boot.
compute booterr=1.
compute badhi = 1.
end if.
compute llcit=temp(cilow,1).
compute ulcit=temp(cihigh,1).
do if (badlo = 1 and llcit <> priorlo).
compute badend={badend, llcit}.
compute priorlo = llcit.
end if.
do if (badhi = 1 and ulcit <> priorhi).
compute badend={badend, ulcit}.
compute priorhi = ulcit.
end if.
end if.
do if ( 9999 = 9999).
compute llcit=temp(cilow,1).
compute ulcit=temp(cihigh,1).
end if.
compute bootse=t(sqrt((cssq(temp)-((csum(temp)&**2)/nrow(temp)))/(nrow(temp)-1)))
.
compute mcct=mcct+1.
compute indtab(mcct,2:4)={bootse,llcit,ulcit}.
end loop.
do if (nxvls=1).
compute indtotal=indtotal+indtemp.
do if (contrast <> 0).
compute inddiff={inddiff,indtemp}.
end if.
end if.
end loop.
release indtemp,tmpb.
end if.
do if (normal = 1 and sobelok=1).
compute sobelmat=indtab(:,1).
compute sobelmat={sobelmat,(sobelmat/2),sobelmat,sobelmat}.
loop ii=1 to nms.
compute se2b=indcov(((nms*nxvls)+ii),((nms*nxvls)+ii)).
compute bpath2=mcsopath(((nms*nxvls)+ii),1)&**2.
compute se2a=diag(indcov((((ii-1)*nxvls)+1):(ii*nxvls),(((ii-1)*nxvls)+1):(ii*nxvls))).
compute apath2=mcsopath((((ii-1)*nxvls)+1):(ii*nxvls) ,1)&**2.
compute sesobel=sqrt(apath2*se2b+bpath2*se2a+se2a*se2b).
compute sobelmat((((ii-1)*nxvls)+1):(ii*nxvls),2)=sesobel.
end loop.
release se2b,bpath2,se2a,apath2,sesobel,ii.
compute sobelmat(:,3)=sobelmat(:,1)&/sobelmat(:,2).
compute sobelmat(:,4) = 2*(1-cdfnorm(abs(sobelmat(:,3)))).
end if.
do if (serial = 0).
compute rowlbs=t(mnames).
end if.
do if (nxvls=1 and nms > 1).
compute rowlbs={'TOTAL';rowlbs}.
compute indtemp=indtotal(1,1).
do if (boot > 0 and nxvls=1).

.
compute temp = indtotal(2:nrow(indtotal),1).
compute temp(GRADE( indtotal(2:nrow(indtotal),1) )) = indtotal(2:nrow(indtotal),1).
compute badlo = 0.
compute badhi = 0.
do if ( (indtotal(1,1)*bc)+(9999*(1-bc)) <> 9999).
compute pv=csum(temp < (indtotal(1,1)*bc)+(9999*(1-bc)) )/nrow(temp).
compute ppv = pv.
do if (pv > .5).
compute ppv = 1-pv.
end if.
compute y5=sqrt(-2*ln(ppv)).
compute xp=y5+((((y5*p4+p3)*y5+p2)*y5+p1)*y5+p0)/((((y5*q4+q3)*y5+q2)*y5+q1)*y5+q0).
do if (pv <= .5).
compute xp = -xp.
end if.
compute cilow=rnd(nrow(temp)*(cdfnorm(2*xp-xp2))).
compute cihigh=trunc(nrow(temp)*(cdfnorm(2*xp+(xp2))))+1.
do if (cilow < 1).
compute cilow = 1.
compute booterr=1.
compute badlo = 1.
end if.
do if (cihigh > nrow(temp)).
compute cihigh = boot.
compute booterr=1.
compute badhi = 1.
end if.
compute llcit=temp(cilow,1).
compute ulcit=temp(cihigh,1).
do if (badlo = 1 and llcit <> priorlo).
compute badend={badend, llcit}.
compute priorlo = llcit.
end if.
do if (badhi = 1 and ulcit <> priorhi).
compute badend={badend, ulcit}.
compute priorhi = ulcit.
end if.
end if.
do if ( (indtotal(1,1)*bc)+(9999*(1-bc)) = 9999).
compute llcit=temp(cilow,1).
compute ulcit=temp(cihigh,1).
end if.
compute bootse=t(sqrt((cssq(temp)-((csum(temp)&**2)/nrow(temp)))/(nrow(temp)-1)))
.
compute indtemp={indtemp, bootse,llcit,ulcit}.
end if.
do if (mc > 0).
compute obtmc=indtab(:,1).
compute indtemp=csum(obtmc).

.
compute temp = indtotal(:,1).
compute temp(GRADE( indtotal(:,1) )) = indtotal(:,1).
compute badlo = 0.
compute badhi = 0.
do if ( 9999 <> 9999).
compute pv=csum(temp < 9999 )/nrow(temp).
compute ppv = pv.
do if (pv > .5).
compute ppv = 1-pv.
end if.
compute y5=sqrt(-2*ln(ppv)).
compute xp=y5+((((y5*p4+p3)*y5+p2)*y5+p1)*y5+p0)/((((y5*q4+q3)*y5+q2)*y5+q1)*y5+q0).
do if (pv <= .5).
compute xp = -xp.
end if.
compute cilow=rnd(nrow(temp)*(cdfnorm(2*xp-xp2))).
compute cihigh=trunc(nrow(temp)*(cdfnorm(2*xp+(xp2))))+1.
do if (cilow < 1).
compute cilow = 1.
compute booterr=1.
compute badlo = 1.
end if.
do if (cihigh > nrow(temp)).
compute cihigh = boot.
compute booterr=1.
compute badhi = 1.
end if.
compute llcit=temp(cilow,1).
compute ulcit=temp(cihigh,1).
do if (badlo = 1 and llcit <> priorlo).
compute badend={badend, llcit}.
compute priorlo = llcit.
end if.
do if (badhi = 1 and ulcit <> priorhi).
compute badend={badend, ulcit}.
compute priorhi = ulcit.
end if.
end if.
do if ( 9999 = 9999).
compute llcit=temp(cilow,1).
compute ulcit=temp(cihigh,1).
end if.
compute bootse=t(sqrt((cssq(temp)-((csum(temp)&**2)/nrow(temp)))/(nrow(temp)-1)))
.
compute indtemp={indtemp, bootse,llcit,ulcit}.
end if.
compute indtab={indtemp;indtab}.
end if.
compute bootlbs={'Effect', 'BootSE','BootLLCI','BootULCI'}.
do if (mc > 0).
compute bootlbs={'Effect', 'MC SE','MC LLCI','MC ULCI'}.
end if.
do if (nxvls = 1).
do if (contrast <> 0).
compute inddiff=inddiff(:,2:ncol(inddiff)).
do if (mc > 0).
compute inddiff={t(obtmc);inddiff}.
end if.
do if (contrast = 4).
compute inddifft=inddiff*t(contvec).
compute indtemp=inddifft(1,1).
do if (boot > 0 or mc > 0).
do if (mc > 0).

.
compute temp = inddifft(2:nrow(inddifft),1).
compute temp(GRADE( inddifft(2:nrow(inddifft),1) )) = inddifft(2:nrow(inddifft),1).
compute badlo = 0.
compute badhi = 0.
do if ( 9999 <> 9999).
compute pv=csum(temp < 9999 )/nrow(temp).
compute ppv = pv.
do if (pv > .5).
compute ppv = 1-pv.
end if.
compute y5=sqrt(-2*ln(ppv)).
compute xp=y5+((((y5*p4+p3)*y5+p2)*y5+p1)*y5+p0)/((((y5*q4+q3)*y5+q2)*y5+q1)*y5+q0).
do if (pv <= .5).
compute xp = -xp.
end if.
compute cilow=rnd(nrow(temp)*(cdfnorm(2*xp-xp2))).
compute cihigh=trunc(nrow(temp)*(cdfnorm(2*xp+(xp2))))+1.
do if (cilow < 1).
compute cilow = 1.
compute booterr=1.
compute badlo = 1.
end if.
do if (cihigh > nrow(temp)).
compute cihigh = boot.
compute booterr=1.
compute badhi = 1.
end if.
compute llcit=temp(cilow,1).
compute ulcit=temp(cihigh,1).
do if (badlo = 1 and llcit <> priorlo).
compute badend={badend, llcit}.
compute priorlo = llcit.
end if.
do if (badhi = 1 and ulcit <> priorhi).
compute badend={badend, ulcit}.
compute priorhi = ulcit.
end if.
end if.
do if ( 9999 = 9999).
compute llcit=temp(cilow,1).
compute ulcit=temp(cihigh,1).
end if.
compute bootse=t(sqrt((cssq(temp)-((csum(temp)&**2)/nrow(temp)))/(nrow(temp)-1)))
.
end if.
do if (boot > 0).

.
compute temp = inddifft(2:nrow(inddifft),1).
compute temp(GRADE( inddifft(2:nrow(inddifft),1) )) = inddifft(2:nrow(inddifft),1).
compute badlo = 0.
compute badhi = 0.
do if ( (inddifft(1,1)*bc)+(9999* (1-bc)) <> 9999).
compute pv=csum(temp < (inddifft(1,1)*bc)+(9999* (1-bc)) )/nrow(temp).
compute ppv = pv.
do if (pv > .5).
compute ppv = 1-pv.
end if.
compute y5=sqrt(-2*ln(ppv)).
compute xp=y5+((((y5*p4+p3)*y5+p2)*y5+p1)*y5+p0)/((((y5*q4+q3)*y5+q2)*y5+q1)*y5+q0).
do if (pv <= .5).
compute xp = -xp.
end if.
compute cilow=rnd(nrow(temp)*(cdfnorm(2*xp-xp2))).
compute cihigh=trunc(nrow(temp)*(cdfnorm(2*xp+(xp2))))+1.
do if (cilow < 1).
compute cilow = 1.
compute booterr=1.
compute badlo = 1.
end if.
do if (cihigh > nrow(temp)).
compute cihigh = boot.
compute booterr=1.
compute badhi = 1.
end if.
compute llcit=temp(cilow,1).
compute ulcit=temp(cihigh,1).
do if (badlo = 1 and llcit <> priorlo).
compute badend={badend, llcit}.
compute priorlo = llcit.
end if.
do if (badhi = 1 and ulcit <> priorhi).
compute badend={badend, ulcit}.
compute priorhi = ulcit.
end if.
end if.
do if ( (inddifft(1,1)*bc)+(9999* (1-bc)) = 9999).
compute llcit=temp(cilow,1).
compute ulcit=temp(cihigh,1).
end if.
compute bootse=t(sqrt((cssq(temp)-((csum(temp)&**2)/nrow(temp)))/(nrow(temp)-1)))
.
end if.
compute indtemp={indtemp, bootse,llcit,ulcit}.
end if.
compute indtab={indtab;indtemp}.
end if.
do if (contrast = 1 or contrast = 2 or contrast=3).
compute conkey=make(1,4,' ').
loop i = 1 to ncol(inddiff)-1.
loop j = (i+1) to ncol(inddiff).
compute inddifft=inddiff(:,i)-inddiff(:,j).
do if (contrast=2).
compute inddifft=abs(inddiff(:,i))-abs(inddiff(:,j)).
end if.
do if (contrast=3).
compute inddifft=inddiff(:,i)+inddiff(:,j).
end if.
compute indtemp=inddifft(1,1).
do if (contrast=1 or contrast=2).
compute conkeyt={' ', rowlbs((i+1),1),' minus  ',rowlbs((j+1),1)}.
end if.
do if (contrast=3).
compute conkeyt={' ', rowlbs((i+1),1),'  plus  ',rowlbs((j+1),1)}.
end if.
compute conkey={conkey;conkeyt}.
do if (boot > 0 or mc > 0).
do if (mc > 0).

.
compute temp = inddifft(2:nrow(inddifft),1).
compute temp(GRADE( inddifft(2:nrow(inddifft),1) )) = inddifft(2:nrow(inddifft),1).
compute badlo = 0.
compute badhi = 0.
do if ( 9999 <> 9999).
compute pv=csum(temp < 9999 )/nrow(temp).
compute ppv = pv.
do if (pv > .5).
compute ppv = 1-pv.
end if.
compute y5=sqrt(-2*ln(ppv)).
compute xp=y5+((((y5*p4+p3)*y5+p2)*y5+p1)*y5+p0)/((((y5*q4+q3)*y5+q2)*y5+q1)*y5+q0).
do if (pv <= .5).
compute xp = -xp.
end if.
compute cilow=rnd(nrow(temp)*(cdfnorm(2*xp-xp2))).
compute cihigh=trunc(nrow(temp)*(cdfnorm(2*xp+(xp2))))+1.
do if (cilow < 1).
compute cilow = 1.
compute booterr=1.
compute badlo = 1.
end if.
do if (cihigh > nrow(temp)).
compute cihigh = boot.
compute booterr=1.
compute badhi = 1.
end if.
compute llcit=temp(cilow,1).
compute ulcit=temp(cihigh,1).
do if (badlo = 1 and llcit <> priorlo).
compute badend={badend, llcit}.
compute priorlo = llcit.
end if.
do if (badhi = 1 and ulcit <> priorhi).
compute badend={badend, ulcit}.
compute priorhi = ulcit.
end if.
end if.
do if ( 9999 = 9999).
compute llcit=temp(cilow,1).
compute ulcit=temp(cihigh,1).
end if.
compute bootse=t(sqrt((cssq(temp)-((csum(temp)&**2)/nrow(temp)))/(nrow(temp)-1)))
.
end if.
do if (boot > 0).

.
compute temp = inddifft(2:nrow(inddifft),1).
compute temp(GRADE( inddifft(2:nrow(inddifft),1) )) = inddifft(2:nrow(inddifft),1).
compute badlo = 0.
compute badhi = 0.
do if ( (inddifft(1,1)*bc)+(9999* (1-bc)) <> 9999).
compute pv=csum(temp < (inddifft(1,1)*bc)+(9999* (1-bc)) )/nrow(temp).
compute ppv = pv.
do if (pv > .5).
compute ppv = 1-pv.
end if.
compute y5=sqrt(-2*ln(ppv)).
compute xp=y5+((((y5*p4+p3)*y5+p2)*y5+p1)*y5+p0)/((((y5*q4+q3)*y5+q2)*y5+q1)*y5+q0).
do if (pv <= .5).
compute xp = -xp.
end if.
compute cilow=rnd(nrow(temp)*(cdfnorm(2*xp-xp2))).
compute cihigh=trunc(nrow(temp)*(cdfnorm(2*xp+(xp2))))+1.
do if (cilow < 1).
compute cilow = 1.
compute booterr=1.
compute badlo = 1.
end if.
do if (cihigh > nrow(temp)).
compute cihigh = boot.
compute booterr=1.
compute badhi = 1.
end if.
compute llcit=temp(cilow,1).
compute ulcit=temp(cihigh,1).
do if (badlo = 1 and llcit <> priorlo).
compute badend={badend, llcit}.
compute priorlo = llcit.
end if.
do if (badhi = 1 and ulcit <> priorhi).
compute badend={badend, ulcit}.
compute priorhi = ulcit.
end if.
end if.
do if ( (inddifft(1,1)*bc)+(9999* (1-bc)) = 9999).
compute llcit=temp(cilow,1).
compute ulcit=temp(cihigh,1).
end if.
compute bootse=t(sqrt((cssq(temp)-((csum(temp)&**2)/nrow(temp)))/(nrow(temp)-1)))
.
end if.
compute indtemp={indtemp, bootse,llcit,ulcit}.
end if.
compute indtab={indtab;indtemp}.
end loop.
end loop.
end if.
release inddiff.
compute contlbs=cntname(1:(((nindfx)*(nindfx-1))/2),1).
compute rowlbs={rowlbs;contlbs}.
end if.
compute resultm2=make(nrow(indtab),maxresm,99999).
compute resultm2(1:nrow(indtab),1:ncol(indtab))=indtab.
compute resultm={resultm;resultm2}.
do if (outscree=1).
do if (kk=1).
print indtab/title = 'Indirect effect(s) of X on Y:'/rnames=rowlbs/cnames=bootlbs/format= F10.4.
end if.
do if (kk = 2 and (xdich=1 or mcx > 0)).
print indtab/title = 'Partially standardized indirect effect(s) of X on Y:'/rnames=rowlbs/cnames=bootlbs/format= F10.4.
end if.
do if (kk = 3).
print indtab/title = 'Completely standardized indirect effect(s) of X on Y:'/rnames=rowlbs/cnames=bootlbs/format= F10.4.
end if.
end if.
do if (normal=1 and sobelok=1 and kk=1).
compute resultm2=make(nrow(sobelmat),maxresm,99999).
compute resultm2(1:nrow(sobelmat),1:ncol(sobelmat))=sobelmat.
compute resultm={resultm;resultm2}.
compute sobellab={'Effect',hclab,'Z','p'}.
compute sobelrlb=rowlbs.
do if (nms > 1).
compute sobelrlb=rowlbs(2:(1+nms),1).
end if.
do if (outscree=1).
print sobelmat/title='   Normal theory test for indirect effect(s):'/cnames=sobellab/rnames=sobelrlb/format= F10.4.
end if.
end if.
do if (contrast <> 0).
do if ((contrast=1 or contrast = 2 or contrast=3) and kk=efloop ).
compute conkey=conkey(2:nrow(conkey),:).
do if (outscree=1).
print conkey/title = 'Specific indirect effect contrast definition(s):'/rnames=contlbs/format=A8.
end if.
end if.
do if (outscree=1).
do if (contrast = 4 and kk=efloop).
compute crowlbs=rowlbs(2:(nindfx+1),1).
print contvec/title = 'Specific indirect effect contrast weights:'/cnames=crowlbs/rlabels='(C1)'/format= F10.4.
end if.
do if (contrast = 2 and kk=efloop).
print/title = 'Contrasts are differences between absolute values of indirect effects'.
end if.
end if.
end if.
do if (serial = 1 and kk=efloop).
compute rowlbst=rowlbs(2:nrow(rowlbs),1).
do if (outscree=1).
print indkey/title = 'Indirect effect key:'/rnames=rowlbst/format = A8.
end if.
end if.
else.
do if (outscree=1).
do if (kk = 1).
print/title = 'Relative indirect effects of X on Y'.
end if.
do if (kk = 2).
print/title = 'Partially standardized relative indirect effect(s) of X on Y:'.
end if.
do if (kk = 3).
print/title = 'Completely standardized relative indirect effect(s) of X on Y:'.
end if.
end if.
loop i = 1 to nrow(indmake).
compute indtabsm=indtab((((i-1)*nxvls)+1):(nxvls*i),:).
compute indkeyt=indkey(i,:).
do if (outscree=1).
print indkeyt/title=' '/space=0/format=A8.
end if.
do if (bcmat(nrow(bcmat),1)=0)).
compute direffl2=xcatlab(1:nxvls,1).
end if.
compute resultm2=make(nrow(indtabsm),maxresm,99999).
compute resultm2(1:nrow(indtabsm),1:ncol(indtabsm))=indtabsm.
compute resultm={resultm;resultm2}.
do if (outscree=1).
print indtabsm/title = ' '/cnames=bootlbs/rnames=direffl2/format= F10.4 /space=0.
end if.
do if (normal=1 and sobelok=1 and kk=1).
compute sobelsm=sobelmat((((i-1)*nxvls)+1):(nxvls*i),:).
compute sobellab={'Effect',hclab,'Z','p'}.
compute resultm2=make(nrow(sobelsm),maxresm,99999).
compute resultm2(1:nrow(sobelsm),1:ncol(sobelsm))=sobelsm.
compute resultm={resultm;resultm2}.
do if (outscree=1).
print sobelsm/title='      Normal theory test for relative indirect effects:'/cnames=sobellab/rnames=direffl2/format= F10.4.
end if.
end if.
end loop.
end if.
do if (effsize = 1 and boot > 0).
compute bootres=bootres(2:nrow(bootres),:).
end if.
end loop.
do if (alttotal=1).
compute altcnms='Effect'.
compute totbtvec=totbtvec+bootdir.
compute alttotfx=t(totbtvec(1,:)).
do if (boot > 0).
compute alttotfx=make(ncol(totbtvec),4,0).
compute alttotfx(:,1)=t(totbtvec(1,:)).
loop cec=1 to ncol(totbtvec).

.
compute temp = totbtvec(2:nrow(totbtvec),cec).
compute temp(GRADE( totbtvec(2:nrow(totbtvec),cec) )) = totbtvec(2:nrow(totbtvec),cec).
compute badlo = 0.
compute badhi = 0.
do if ( (totbtvec(1,cec)*bc)+(9999* (1-bc)) <> 9999).
compute pv=csum(temp < (totbtvec(1,cec)*bc)+(9999* (1-bc)) )/nrow(temp).
compute ppv = pv.
do if (pv > .5).
compute ppv = 1-pv.
end if.
compute y5=sqrt(-2*ln(ppv)).
compute xp=y5+((((y5*p4+p3)*y5+p2)*y5+p1)*y5+p0)/((((y5*q4+q3)*y5+q2)*y5+q1)*y5+q0).
do if (pv <= .5).
compute xp = -xp.
end if.
compute cilow=rnd(nrow(temp)*(cdfnorm(2*xp-xp2))).
compute cihigh=trunc(nrow(temp)*(cdfnorm(2*xp+(xp2))))+1.
do if (cilow < 1).
compute cilow = 1.
compute booterr=1.
compute badlo = 1.
end if.
do if (cihigh > nrow(temp)).
compute cihigh = boot.
compute booterr=1.
compute badhi = 1.
end if.
compute llcit=temp(cilow,1).
compute ulcit=temp(cihigh,1).
do if (badlo = 1 and llcit <> priorlo).
compute badend={badend, llcit}.
compute priorlo = llcit.
end if.
do if (badhi = 1 and ulcit <> priorhi).
compute badend={badend, ulcit}.
compute priorhi = ulcit.
end if.
end if.
do if ( (totbtvec(1,cec)*bc)+(9999* (1-bc)) = 9999).
compute llcit=temp(cilow,1).
compute ulcit=temp(cihigh,1).
end if.
compute bootse=t(sqrt((cssq(temp)-((csum(temp)&**2)/nrow(temp)))/(nrow(temp)-1)))
.
compute alttotfx(cec,2:4)={bootse,llcit,ulcit}.
end loop.
compute altcnms={altcnms,'BootSE', 'BootLLCI','BootULCI'}.
end if.
compute resultm2=make(nrow(alttotfx),maxresm,99999).
compute resultm2(1:nrow(alttotfx),1:ncol(alttotfx))=alttotfx.
compute resultm={resultm;resultm2}.
do if (outscree=1).
do if (nxvls > 1).
print/title= '----------'/space=0.
print alttotfx/title='Relative total effects of X on Y (sum of direct and indirect effects)'/rnames=direffl2/cnames=altcnms/format= F10.4.
end if.
do if (nxvls = 1).
print alttotfx/title='Total effect of X on Y (sum of direct and indirect effects)'/cnames=altcnms/format= F10.4.
end if.
end if.
end if.
end if.
do if (anymod > 0).
do if (boot = 0).
compute bootres=obscoeff.
compute indtab=999.
end if.
do if (boot > 0).
compute bootres={obscoeff;bootres}.
compute indtab=make(1,4,999).
end if.
do if (csum((indmod > 0))=nrow(indmod)).
do if (outscree=1).
do if (nxvls > 1).
do if (xmint=0).
print/title= 'Relative conditional indirect effects of X on Y:'.
end if.
do if (xmint=1).
print/title= 'Relative (total) natural indirect effects of X on Y:'.
end if.
end if.
do if (nxvls = 1).
do if (xmint=0).
print/title= 'Conditional indirect effects of X on Y:'.
end if.
do if (xmint=1).
print/title= '(Total) Natural indirect effect(s) of X on Y:'.
end if.
end if.
end if.
end if.
do if (csum((indmod > 0)) < nrow(indmod)).
do if (outscree=1).
do if (nxvls > 1).
print/title= 'Relative conditional and unconditional indirect effects of X on Y:'.
end if.
do if (nxvls = 1).
print/title= 'Conditional and unconditional indirect effects of X on Y:'.
end if.
end if.
end if.
compute cftotfx=make(nrow(bootres),nxvls,0).
loop i = 1 to nrow(indmake).
compute indtab=indtab(1,:)*0.
do if (outscree=1).
do if (xmint=0).
print indkey(i,:)/title = 'INDIRECT EFFECT:'/format=A8.
end if.
do if (xmint=1).
print indkey(i,:)/title = ' '/format=A8/space=0.
end if.
end if.
do if (indmod(i,1)=0).
loop j = 1 to nxvls.
compute indtemp=make(nrow(bootres),1,1).
loop k = 1 to indmake(i,1).
compute jtemp=1.
do if (j > 1 and k=1).
compute jtemp=j.
end if.
compute indtemp=indtemp&*bootres(:,pathsfoc(jtemp,indmake(i,(k+1)))).
end loop.
compute indeff=indtemp(1,1).
do if (boot > 0).

.
compute temp = indtemp(2:nrow(indtemp),1).
compute temp(GRADE( indtemp(2:nrow(indtemp),1) )) = indtemp(2:nrow(indtemp),1).
compute badlo = 0.
compute badhi = 0.
do if ( (indtemp(1,1)*bc)+(9999*(1-bc)) <> 9999).
compute pv=csum(temp < (indtemp(1,1)*bc)+(9999*(1-bc)) )/nrow(temp).
compute ppv = pv.
do if (pv > .5).
compute ppv = 1-pv.
end if.
compute y5=sqrt(-2*ln(ppv)).
compute xp=y5+((((y5*p4+p3)*y5+p2)*y5+p1)*y5+p0)/((((y5*q4+q3)*y5+q2)*y5+q1)*y5+q0).
do if (pv <= .5).
compute xp = -xp.
end if.
compute cilow=rnd(nrow(temp)*(cdfnorm(2*xp-xp2))).
compute cihigh=trunc(nrow(temp)*(cdfnorm(2*xp+(xp2))))+1.
do if (cilow < 1).
compute cilow = 1.
compute booterr=1.
compute badlo = 1.
end if.
do if (cihigh > nrow(temp)).
compute cihigh = boot.
compute booterr=1.
compute badhi = 1.
end if.
compute llcit=temp(cilow,1).
compute ulcit=temp(cihigh,1).
do if (badlo = 1 and llcit <> priorlo).
compute badend={badend, llcit}.
compute priorlo = llcit.
end if.
do if (badhi = 1 and ulcit <> priorhi).
compute badend={badend, ulcit}.
compute priorhi = ulcit.
end if.
end if.
do if ( (indtemp(1,1)*bc)+(9999*(1-bc)) = 9999).
compute llcit=temp(cilow,1).
compute ulcit=temp(cihigh,1).
end if.
compute bootse=t(sqrt((cssq(temp)-((csum(temp)&**2)/nrow(temp)))/(nrow(temp)-1)))
.
compute indeff={indeff,bootse,llcit,ulcit}.
end if.
compute indtab={indtab;indeff}.
end loop.
compute resultm2=make(nrow(indtab),maxresm,99999).
compute resultm2(1:nrow(indtab),1:ncol(indtab))=indtab.
compute resultm={resultm;resultm2}.
do if (outscree=1).
do if (nxvls > 1).
compute indefflb=xcatlab(1:nxvls,1).
print indtab(2:nrow(indtab),:)/title = ' '/clabels='Effect', 'BootSE', 'BootLLCI', 'BootULCI'/rnames=indefflb/format= F10.4 /space=0.
end if.
do if (nxvls = 1).
print indtab(2:nrow(indtab),:)/title = ' '/clabels='Effect', 'BootSE', 'BootLLCI', 'BootULCI'/format= F10.4 /space=0.
end if.
end if.
end if.
do if (indmod(i,1)>0).
do if (indmod(i,1)=1).
compute indmodva=wmodvals.
compute indprova=wprobval.
compute condlbs={wnames}.
compute printw=1.
else if (indmod(i,1)=2).
compute indmodva=zmodvals.
compute indprova=zprobval.
compute condlbs={znames}.
compute printz=1.
else if (indmod(i,1)=3).
compute cntmp=1.
compute printz=1.
compute printw=1.
compute indmodva=make((nrow(wmodvals)*nrow(zmodvals)),2,999).
loop k7 = 1 to nrow(wmodvals).
loop k8 = 1 to nrow(zmodvals).
compute indmodva(cntmp,:)={wmodvals(k7,1),zmodvals(k8,1)}.
compute cntmp=cntmp+1.
end loop.
end loop.
compute condlbs={wnames,znames}.
end if.
compute condres=make(nrow(indmodva),1,999).
do if (boot > 0).
compute condres=make(nrow(indmodva),4,999).
end if.
compute condres={indmodva,condres}.
loop k4 = 1 to nxvls.
compute imm3=make(nrow(bootres),1,1).
compute imm4=make(nrow(bootres),1,1).
compute indcontr=0.
do if (indmod(i,1)=3).
compute tihsw=wprobval.
compute tihsz=zprobval.
end if.
loop k1=1 to nrow(indmodva).
compute tucker2=make(nrow(bootres),1,1).
compute imm2=make(nrow(bootres),1,1).
compute wfirst=0.
compute zfirst=0.
compute immset=0.
loop k2=1 to indmake(i,1).
compute colnumb=indmake(i,(k2+1)).
do if (k2=1).
compute wbb=make(nrow(bootres),(nwvls*nxvls),0).
compute zbb=make(nrow(bootres),(nzvls*nxvls),0).
compute wzbb=make(nrow(bootres),(nwvls*nzvls*nxvls),0).
end if.
do if (k2<>1).
compute wbb=make(nrow(bootres),nwvls,0).
compute zbb=make(nrow(bootres),nzvls,0).
compute wzbb=make(nrow(bootres),(nwvls*nzvls),0).
end if.
compute cnt=1.
compute tihs=indlocs(2:((indlocs(1,colnumb))+1),colnumb).
do if (k2 = 1).
compute focbb=tihs(1:nxvls,1).
compute focbb=bootres(:,focbb).
do if (indmmm(i,1)>0).
compute imm=focbb(:,k4).
compute condbb=imm.
end if.
compute focaddon=make(1,nxvls,0).
compute focaddon(1,k4)=1.
compute cnt=cnt+nxvls.
compute placeh=nxvls.
do if (indmod(i,1)=1).
compute tihsz=make(nrow(wprobval),(nzvls*nxvls),0).
compute tihswz=make(nrow(wprobval),(nwvls*nzvls*nxvls),0).
do if (pathsw(1,colnumb)=1).
compute temp=make(nrow(wprobval),(nxvls*nwvls),0).
loop k5 = 1 to nrow(wprobval).
loop k6=1 to nwvls.
compute temp(k5, (((k4-1)*nwvls)+k6))=wprobval(k5,k6).
end loop.
end loop.
compute indprova={temp,tihsz,tihswz}.
else.
compute indprova={wprobval,tihsz,tihswz}.
end if.
end if.
do if (indmod(i,1)=2).
compute tihsw=make(nrow(zprobval),(nwvls*nxvls),0).
compute tihswz=make(nrow(zprobval),(nwvls*nzvls*nxvls),0).
do if (pathsz(1,colnumb)=1).
compute temp=make(nrow(zprobval),(nxvls*nzvls),0).
loop k5 = 1 to nrow(zprobval).
loop k6 =1 to nzvls.
compute temp(k5,(((k4-1)*nzvls)+k6))=zprobval(k5,k6).
end loop.
end loop.
compute indprova={tihsw,temp,tihswz}.
else.
compute indprova={tihsw,zprobval,tihswz}.
end if.
end if.
do if (indmod(i,1)=3).
compute indprova=make((nrow(wprobval)*nrow(zprobval)),((ncol(wprobval)*nxvls)+ (ncol(zprobval)*nxvls)+(nwvls*nzvls*nxvls)),0).
compute cntemp=1.
loop k7=1 to nrow(wprobval).
loop k8 =1 to nrow(zprobval).
compute temp=wprobval(k7,:)*focaddon(1,k4).
compute indprova(cntemp,(((k4-1)*nwvls)+1):(k4*(nwvls)))=temp.
compute temp=zprobval(k8,:)*focaddon(1,k4).
compute indprova(cntemp, ((((k4-1)*nzvls)+1)+(nxvls*nwvls)) : ((((k4-1)*nzvls)+1)+(nxvls*nwvls)+(nzvls-1)))=temp.
compute cntemp=cntemp+1.
end loop.
end loop.
do if (pathsz(1,colnumb)=0).
compute temp=make(nrow(indprova),(ncol(zprobval)*nxvls),0).
compute indprova(:,((ncol(wprobval)*nxvls)+1):((ncol(wprobval)+ncol(zprobval))* nxvls))=temp.
end if.
do if (pathsw(1,colnumb)=0).
compute temp=make(nrow(indprova),(ncol(wprobval)*nxvls),0).
compute indprova(:,1:(ncol(wprobval)*nxvls))=temp.
end if.
do if (pathswz(1,colnumb)=1).
compute cntemp=(ncol(wprobval)*nxvls)+(ncol(zprobval)*nxvls)+((k4-1)* ncol(wprobval)*ncol(zprobval))+1.
loop k7=1 to ncol(wprobval).
loop k8=1 to ncol(zprobval).
compute indprova(:,cntemp)=indprova(:,((ncol(wprobval)*(k4-1))+k7))&* indprova(:,((((k4-1)*ncol(zprobval))+k8)+(nxvls*ncol(wprobval)))).
compute cntemp=cntemp+1.
end loop.
end loop.
end if.
end if.
end if.
do if (k2 > 1).
compute focbb=tihs(1,1).
compute focbb=bootres(:,focbb).
do if (indmmm(i,1)>0).
compute imm=focbb(:,1).
compute condbb=imm.
end if.
compute focaddon=1.
compute cnt=cnt+1.
compute placeh=1.
do if (indmod(i,1)=1).
compute tihsz=make(nrow(wprobval),nzvls,0).
compute tihswz=make(nrow(wprobval),(nwvls*nzvls),0).
compute indprova={wprobval,tihsz,tihswz}.
end if.
do if (indmod(i,1)=2).
compute tihsw=make(nrow(zprobval),nwvls,0).
compute tihswz=make(nrow(zprobval),(nwvls*nzvls),0).
compute indprova={tihsw,zprobval,tihswz}.
end if.
do if (indmod(i,1)=3).
compute indprova=make((nrow(wprobval)*nrow(zprobval)),((ncol(wprobval)+ ncol(zprobval))+(nwvls*nzvls)),0).
compute cntemp=1.
loop k7=1 to nrow(wprobval).
loop k8 =1 to nrow(zprobval).
compute indprova(cntemp,1:(ncol(wprobval)+ncol(zprobval)))={wprobval(k7,:), zprobval(k8,:)}.
compute cntemp=cntemp+1.
end loop.
end loop.
do if (pathsz(1,colnumb)=0).
compute temp=make(nrow(indprova),ncol(zprobval),0).
compute indprova(:,(ncol(wprobval)+1):(ncol(wprobval)+ncol(zprobval)))=temp.
end if.
do if (pathsw(1,colnumb)=0).
compute temp=make(nrow(indprova),ncol(wprobval),0).
compute indprova(:,1:ncol(wprobval))=temp.
end if.
do if (pathswz(1,colnumb)=1).
compute cntemp=ncol(wprobval)+ncol(zprobval)+1.
loop k7=1 to ncol(wprobval).
loop k8=1 to ncol(zprobval).
compute indprova(:,cntemp)=indprova(:,k7)&*indprova(:,(ncol(wprobval)+k8)).
compute cntemp=cntemp+1.
end loop.
end loop.
end if.
end if.
end if.
do if (pathsw(1,colnumb)) = 1.
compute wbb=tihs(cnt:(cnt+(placeh*nwvls)-1),1).
compute wbb=bootres(:,wbb).
compute immlbs2=wcatlab(1:nwvls,1).
do if (zfirst=0).
compute wfirst=1.
end if.
do if (indmmm(i,1)=1 or indmmm(i,1)=31 or indmmm(i,1)=51).
compute imm=wbb(:,1).
loop k7 = 1 to nwvls.
compute imm={imm,wbb(:,(((k4-1)*nwvls*(k2=1))+k7))}.
end loop.
compute imm=imm(:,2:ncol(imm)).
end if.
do if (indmmm(i,1)=41 or indmmm(i,1)=51).
compute condbb=make(nrow(bootres),1,0).
loop k7 = 1 to nwvls.
compute condbb={condbb,wbb(:,(((k4-1)*nwvls*(k2=1))+k7))}.
end loop.
compute condbb=condbb(:,2:ncol(condbb)).
end if.
compute cnt=cnt+(placeh*nwvls).
end if.
do if (pathsz(1,colnumb)) = 1.
compute zbb=tihs(cnt:(cnt+(placeh*nzvls)-1),1).
compute zbb=bootres(:,zbb).
do if (wfirst=0).
compute zfirst=1.
end if.
do if (indmmm(i,1) <> 31).
compute immlbs2=zcatlab(1:nzvls,1).
end if.
do if (indmmm(i,1)=2 or indmmm(i,1)=31 or indmmm(i,1)=51).
do if (indmmm(i,1)=2).
compute imm=zbb(:,1).
end if.
loop k7 = 1 to nzvls.
compute imm={imm,zbb(:,(((k4-1)*nzvls*(k2=1))+k7))}.
end loop.
do if (indmmm(i,1)=2 or indmmm(i,1)=51).
compute imm=imm(:,2:ncol(imm)).
do if (indmmm(i,1)=51).
compute condbb={condbb,imm}.
end if.
end if.
end if.
compute cnt=cnt+(placeh*nzvls).
end if.
do if (pathswz(1,colnumb)) = 1.
compute wzbb=tihs(cnt:(cnt+(placeh*nwvls*nzvls)-1),1).
compute wzbb=bootres(:,wzbb).
do if (indmmm(i,1)=41).
compute imm=wzbb(:,1).
loop k7=1 to nwvls*nzvls.
compute imm={imm,wzbb(:,(((k4-1)*nzvls*nwvls*(k2=1))+k7))}.
end loop.
end if.
do if (indmmm(i,1)=41).
compute imm=imm(:,2:ncol(imm)).
compute condbb={condbb,imm(:,(ncol(imm)-(nwvls*nzvls)+1):ncol(imm))}.
end if.
compute cnt=cnt+(placeh*nzvls*nwvls).
end if.
compute indprobe={focaddon,indprova(k1,:)}.
compute tucker={focbb,wbb,zbb,wzbb}.
loop k3=1 to ncol(indprobe).
compute tucker(:,k3)=tucker(:,k3)*indprobe(1,k3).
end loop.
compute tucker2=tucker2&*rsum(tucker).
do if (indmmm(i,1) = 1 or indmmm(i,1)=2 or indmmm(i,1)=31 or indmmm(i,1)=41 or indmmm(i,1)=51).
do if (immset=1).
do if (ncol(imm2)=1 and ncol(imm) = 1).
compute imm2=imm2&*imm.
end if.
do if (indmmm(i,1)=41 or indmmm(i,1)=51).
do if ((ncol(condbb2) > 1) and (ncol(condbb)>1)).
compute condbb2t=make(nrow(condbb2),(ncol(condbb2)*ncol(condbb)),-999999).
compute k9=1.
do if (wfirst=1).
loop k7=1 to ncol(condbb2).
loop k8 = 1 to ncol(condbb).
compute condbb2t(:,k9)=condbb2(:,k7)&*condbb(:,k8).
compute k9=k9+1.
end loop.
end loop.
end if.
do if (zfirst=1).
loop k7=1 to ncol(condbb).
loop k8 = 1 to ncol(condbb2).
compute condbb2t(:,k9)=condbb(:,k7)&*condbb2(:,k8).
compute k9=k9+1.
end loop.
end loop.
end if.
compute condbb2=condbb2t.
release condbb2t.
end if.
do if ((ncol(condbb2) > 1) and (ncol(condbb)=1)).
loop k7 = 1 to ncol(condbb2).
compute condbb2(:,k7)=condbb2(:,k7)&*condbb.
end loop.
end if.
do if ((ncol(condbb2) = 1) and (ncol(condbb)>1)).
loop k7 = 1 to ncol(condbb).
compute condbb(:,k7)=condbb2&*condbb(:,k7).
end loop.
compute condbb2=condbb.
end if.
end if.
do if (ncol(imm2) <> 1 and ncol(imm) <> 1).
compute imm2t=make(nrow(imm2),(ncol(imm2)*ncol(imm)),-999999).
compute k9=1.
do if (wfirst=1).
loop k7=1 to ncol(imm2).
loop k8 = 1 to ncol(imm).
compute imm2t(:,k9)=imm2(:,k7)&*imm(:,k8).
compute k9=k9+1.
end loop.
end loop.
end if.
do if (zfirst=1).
loop k7=1 to ncol(imm).
loop k8 = 1 to ncol(imm2).
compute imm2t(:,k9)=imm(:,k7)&*imm2(:,k8).
compute k9=k9+1.
end loop.
end loop.
end if.
compute imm2=imm2t.
release imm2t.
end if.
do if ((ncol(imm2) > 1) and (ncol(imm)=1)).
loop k7=1 to ncol(imm2).
compute imm2(:,k7)=imm2(:,k7)&*imm.
end loop.
end if.
do if ((ncol(imm2) = 1) and (ncol(imm) > 1)).
loop k7=1 to ncol(imm).
compute imm(:,k7)=imm2&*imm(:,k7).
end loop.
compute imm2=imm.
end if.
end if.
do if (immset=0).
compute imm2=imm.
do if (indmmm(i,1)=41 or indmmm(i,1)=51).
compute condbb2=condbb.
end if.
compute immset=1.
end if.
end if.
end loop.
compute indtemp=tucker2(1,1).
do if (indmmm(i,1)=12 or indmmm(i,1)=22).
compute imm3={imm3,tucker2}.
do if (k1=nrow(indmodva)).
compute imm3=imm3(:,2:ncol(imm3)).
compute immstop=ncol(imm3).
loop k8=2 to immstop.
do if (indmmmt(i,1)=1).
compute imm3={imm3,(imm3(:,k8)-imm3(:,1))}.
end if.
do if (indmmmt(i,1)=2).
compute imm3={imm3,(imm3(:,k8)-imm3(:,(k8-1)))}.
end if.
do if (indmmmt(i,1)=3).
compute imm3={imm3,((rsum(imm3(:,(k8:immstop)))/(immstop-k8+1))-imm3(:,(k8-1))) }.
end if.
do if (indmmmt(i,1)=4).
compute imm3={imm3,(imm3(:,k8)-(rsum(imm3(:,1:immstop))/immstop))}.
end if.
end loop.
do if (indmmmt(i,1)<5).
compute imm2=imm3(:,(immstop+1):ncol(imm3)).
release imm3.
end if.
end if.
end if.
do if (indmmm(i,1)>-1 and (contrast = 1 or contrast = 2 or contrast=3)).
compute imm4={imm4,tucker2}.
do if (k1=nrow(indmodva) and k1 > 1).
compute imm4=imm4(:,2:ncol(imm4)).
compute immstop=ncol(imm4).
compute condcont=make((immstop*(immstop-1)/2),6,-999).
loop k8 = 1 to (immstop-1).
loop k9 = (k8+1) to immstop.
do if (contrast=1).
compute imm4={imm4,(imm4(:,k9)-imm4(:,k8))}.
end if.
do if (contrast=2).
compute imm4={imm4,(abs(imm4(:,k9))-abs(imm4(:,k8)))}.
end if.
do if (contrast=3).
compute imm4={imm4,(imm4(:,k9)+imm4(:,k8))}.
end if.
compute condcont((ncol(imm4)-immstop),1)=imm4(1,k9).
compute condcont((ncol(imm4)-immstop),2)=imm4(1,k8).
end loop.
end loop.
compute imm4=imm4(:,(immstop+1):ncol(imm4)).
loop k8=1 to ncol(imm4).
compute condcont(k8,3)=imm4(1,k8).
do if (boot > 0).

.
compute temp = imm4(2:nrow(imm4),k8).
compute temp(GRADE( imm4(2:nrow(imm4),k8) )) = imm4(2:nrow(imm4),k8).
compute badlo = 0.
compute badhi = 0.
do if ( (imm4(1,k8)*bc)+(9999*(1-bc)) <> 9999).
compute pv=csum(temp < (imm4(1,k8)*bc)+(9999*(1-bc)) )/nrow(temp).
compute ppv = pv.
do if (pv > .5).
compute ppv = 1-pv.
end if.
compute y5=sqrt(-2*ln(ppv)).
compute xp=y5+((((y5*p4+p3)*y5+p2)*y5+p1)*y5+p0)/((((y5*q4+q3)*y5+q2)*y5+q1)*y5+q0).
do if (pv <= .5).
compute xp = -xp.
end if.
compute cilow=rnd(nrow(temp)*(cdfnorm(2*xp-xp2))).
compute cihigh=trunc(nrow(temp)*(cdfnorm(2*xp+(xp2))))+1.
do if (cilow < 1).
compute cilow = 1.
compute booterr=1.
compute badlo = 1.
end if.
do if (cihigh > nrow(temp)).
compute cihigh = boot.
compute booterr=1.
compute badhi = 1.
end if.
compute llcit=temp(cilow,1).
compute ulcit=temp(cihigh,1).
do if (badlo = 1 and llcit <> priorlo).
compute badend={badend, llcit}.
compute priorlo = llcit.
end if.
do if (badhi = 1 and ulcit <> priorhi).
compute badend={badend, ulcit}.
compute priorhi = ulcit.
end if.
end if.
do if ( (imm4(1,k8)*bc)+(9999*(1-bc)) = 9999).
compute llcit=temp(cilow,1).
compute ulcit=temp(cihigh,1).
end if.
compute bootse=t(sqrt((cssq(temp)-((csum(temp)&**2)/nrow(temp)))/(nrow(temp)-1)))
.
compute condcont(k8,4:6)={bootse,llcit,ulcit}.
end if.
end loop.
do if (boot=0).
compute condcont=condcont(:,1:3).
end if.
compute indcontr=1.
end if.
end if.
do if (xmint=1).
do if (k1=(k4+1)).
compute cftotfx(:,k4)=cftotfx(:,k4)+(tucker2*xscaling).
end if.
end if.
do if (boot > 0).
compute tucker2=tucker2*xscaling.

.
compute temp = tucker2(2:nrow(tucker2),1).
compute temp(GRADE( tucker2(2:nrow(tucker2),1) )) = tucker2(2:nrow(tucker2),1).
compute badlo = 0.
compute badhi = 0.
do if ( (tucker2(1,1)*bc)+(9999*(1-bc)) <> 9999).
compute pv=csum(temp < (tucker2(1,1)*bc)+(9999*(1-bc)) )/nrow(temp).
compute ppv = pv.
do if (pv > .5).
compute ppv = 1-pv.
end if.
compute y5=sqrt(-2*ln(ppv)).
compute xp=y5+((((y5*p4+p3)*y5+p2)*y5+p1)*y5+p0)/((((y5*q4+q3)*y5+q2)*y5+q1)*y5+q0).
do if (pv <= .5).
compute xp = -xp.
end if.
compute cilow=rnd(nrow(temp)*(cdfnorm(2*xp-xp2))).
compute cihigh=trunc(nrow(temp)*(cdfnorm(2*xp+(xp2))))+1.
do if (cilow < 1).
compute cilow = 1.
compute booterr=1.
compute badlo = 1.
end if.
do if (cihigh > nrow(temp)).
compute cihigh = boot.
compute booterr=1.
compute badhi = 1.
end if.
compute llcit=temp(cilow,1).
compute ulcit=temp(cihigh,1).
do if (badlo = 1 and llcit <> priorlo).
compute badend={badend, llcit}.
compute priorlo = llcit.
end if.
do if (badhi = 1 and ulcit <> priorhi).
compute badend={badend, ulcit}.
compute priorhi = ulcit.
end if.
end if.
do if ( (tucker2(1,1)*bc)+(9999*(1-bc)) = 9999).
compute llcit=temp(cilow,1).
compute ulcit=temp(cihigh,1).
end if.
compute bootse=t(sqrt((cssq(temp)-((csum(temp)&**2)/nrow(temp)))/(nrow(temp)-1)))
.
compute indtemp={indtemp*xscaling, bootse,llcit,ulcit}.
end if.
do if (boot=0).
compute indtemp=indtemp*xscaling.
end if.
compute condres(k1,(ncol(indmodva)+1):ncol(condres))=indtemp.
end loop.
do if (xmint=0).
compute resultm2=make(nrow(condres),maxresm,99999).
compute resultm2(1:nrow(condres),1:ncol(condres))=condres.
compute resultm={resultm;resultm2}.
end if.
compute condlbs={condlbs,'Effect'}.
do if (boot > 0).
compute condlbs={condlbs,'BootSE', 'BootLLCI', 'BootULCI'}.
end if.
do if (xmint = 1).
do if (k4 = 1).
compute natindfx=condres((2+(k4-1)),2:ncol(condres)).
end if.
do if (k4 > 1).
compute natindfx={natindfx;condres((2+(k4-1)),2:ncol(condres))}.
end if.
do if (k4=nxvls).
compute resultm2=make(nrow(natindfx),maxresm,99999).
compute resultm2(1:nrow(natindfx),1:ncol(natindfx))=natindfx.
compute resultm={resultm;resultm2}.
end if.
end if.
do if (outscree=1 and xmint=0).
do if (nxvls=1).
print condres/title=' '/cnames=condlbs/format= F10.4 /space=0.
else.
compute condrlb=make(nrow(condres),1,xcatlab(k4,1)).
print condres/title=' '/cnames=condlbs/rnames=condrlb/format= F10.4 /space=0.
end if.
end if.
do if (outscree=1 and xmint=1 and k4=nxvls).
do if (nxvls=1).
print natindfx/title=' '/clabels='Effect', 'BootSE', 'BootLLCI', 'BootULCI'/format= F10.4 /space=0.
else.
compute condrlb=make(nrow(condres),1,xcatlab(k4,1)).
print natindfx/title=' '/clabels='Effect', 'BootSE', 'BootLLCI', 'BootULCI'/rnames=direffl2/format= F10.4 /space=0.
end if.
end if.
compute dichadj=0.
compute immcat=0.
do if ((indmmm(i,1)>0) and (xmint=0)).
do if (indmmm(i,1)=1 or indmmm(i,1)=12 or indmmm(i,1)=31).
do if (wdich=1 and mcw=0).
do if (indmmm(i,1) <> 12).
compute imm2(:,1)=imm2(:,1)*(wmax-wmin).
end if.
do if (indmmm(i,1) <> 31).
compute dichadj=1.
end if.
end if.
do if ((mcw = 1 or mcw = 2) and indmmm(i,1) <> 31)).
compute immcat=1.
end if.
end if.
do if (indmmm(i,1)=2 or indmmm(i,1)=22 or indmmm(i,1)=31).
do if (zdich=1 and mcz=0).
do if (indmmm(i,1) = 31).
compute imm2(:,(nwvls+1):ncol(imm2))=imm2(:,(nwvls+1):ncol(imm2))*(zmax-zmin).
end if.
do if (indmmm(i,1) = 2).
compute imm2(:,1)=imm2(:,1)*(zmax-zmin).
end if.
do if (indmmm(i,1) <> 31).
compute dichadj=1.
end if.
end if.
do if ((mcz = 1 or mcz = 2) and indmmm(i,1) <> 31)).
compute immcat=1.
end if.
end if.
compute immtemp2=t(imm2(1,:)).
compute immtemp=immtemp2.
compute immlbs={'Index'}.
do if (boot > 0).
compute immtemp=make(1,3,0).
loop k7=1 to ncol(imm2).

.
compute temp = imm2(2:nrow(imm2),k7).
compute temp(GRADE( imm2(2:nrow(imm2),k7) )) = imm2(2:nrow(imm2),k7).
compute badlo = 0.
compute badhi = 0.
do if ( (imm2(1,k7)*bc)+(9999*(1-bc)) <> 9999).
compute pv=csum(temp < (imm2(1,k7)*bc)+(9999*(1-bc)) )/nrow(temp).
compute ppv = pv.
do if (pv > .5).
compute ppv = 1-pv.
end if.
compute y5=sqrt(-2*ln(ppv)).
compute xp=y5+((((y5*p4+p3)*y5+p2)*y5+p1)*y5+p0)/((((y5*q4+q3)*y5+q2)*y5+q1)*y5+q0).
do if (pv <= .5).
compute xp = -xp.
end if.
compute cilow=rnd(nrow(temp)*(cdfnorm(2*xp-xp2))).
compute cihigh=trunc(nrow(temp)*(cdfnorm(2*xp+(xp2))))+1.
do if (cilow < 1).
compute cilow = 1.
compute booterr=1.
compute badlo = 1.
end if.
do if (cihigh > nrow(temp)).
compute cihigh = boot.
compute booterr=1.
compute badhi = 1.
end if.
compute llcit=temp(cilow,1).
compute ulcit=temp(cihigh,1).
do if (badlo = 1 and llcit <> priorlo).
compute badend={badend, llcit}.
compute priorlo = llcit.
end if.
do if (badhi = 1 and ulcit <> priorhi).
compute badend={badend, ulcit}.
compute priorhi = ulcit.
end if.
end if.
do if ( (imm2(1,k7)*bc)+(9999*(1-bc)) = 9999).
compute llcit=temp(cilow,1).
compute ulcit=temp(cihigh,1).
end if.
compute bootse=t(sqrt((cssq(temp)-((csum(temp)&**2)/nrow(temp)))/(nrow(temp)-1)))
.
compute temp={bootse,llcit,ulcit}.
compute immtemp={immtemp;temp}.
end loop.
compute immtemp=immtemp(2:nrow(immtemp),:).
compute immtemp={immtemp2,immtemp}.
compute immlbs={immlbs,'BootSE', 'BootLLCI', 'BootULCI'}.
end if.
compute resultm2=make(nrow(immtemp),maxresm,99999).
compute resultm2(1:nrow(immtemp),1:ncol(immtemp))=immtemp.
compute resultm={resultm;resultm2}.
do if (dichadj=0 and immcat=0 and indmmmt(i,1)<>5 and indmmm(i,1) < 100 ).
do if (outscree=1).
do if (indmmm(i,1) < 30).
print immtemp/title='      Index of moderated mediation:'/cnames=immlbs/rnames=immlbs2/format= F10.4.
end if.
do if (indmmm(i,1) = 31).
compute immlbs2={immlbs2;zcatlab(1:nzvls,1)}.
print immtemp/title='      Indices of partial moderated mediation:'/cnames=immlbs/rnames=immlbs2/format= F10.4.
end if.
end if.
do if ((nzvls = 1) and (nwvls = 1)).
do if (indmmm(i,1) = 41 or indmmm(i,1)=51).
loop k7=1 to nwvls.
compute immlbs2=zcatlab(1:nzvls,1).
compute immtemp2=immtemp((((k7-1)*nzvls)+1):(((k7-1)*nzvls)+nzvls),:).
do if (outscree=1).
do if (nwvls > 1).
compute primodv={'        ', wcatlab(k7,1)}.
print primodv/title='      Primary moderator:'/format=A8.
end if.
do if (nzvls=1).
print immtemp2/title='      Index of moderated moderated mediation'/cnames=immlbs/format= F10.4.
else.
print immtemp2/title='      Indices of moderated moderated mediation'/cnames=immlbs/rnames=immlbs2/format= F10.4.
end if.
end if.
compute cmmtemp=make(nrow(zprobval),4,0).
loop k8=1 to nrow(zprobval).
compute condbb3=condbb2(:,((nwvls+1)+((k7-1)*nzvls)):((nwvls+1)+((k7-1)*nzvls)+ (nzvls-1))).
do if (ncol(zprobval) > 1).
compute condbb3=condbb3*mdiag(zprobval(k8,:)).
else.
compute condbb3=condbb3&*zprobval(k8,:).
end if.
compute condbb3={condbb2(:,k7),condbb3}.
compute icmm=rsum(condbb3).
compute cmmtemp(k8,1)=icmm(1,1).
do if (boot > 0).

.
compute temp = icmm(2:nrow(icmm),1).
compute temp(GRADE( icmm(2:nrow(icmm),1) )) = icmm(2:nrow(icmm),1).
compute badlo = 0.
compute badhi = 0.
do if ( (icmm(1,1)*bc)+(9999*(1-bc)) <> 9999).
compute pv=csum(temp < (icmm(1,1)*bc)+(9999*(1-bc)) )/nrow(temp).
compute ppv = pv.
do if (pv > .5).
compute ppv = 1-pv.
end if.
compute y5=sqrt(-2*ln(ppv)).
compute xp=y5+((((y5*p4+p3)*y5+p2)*y5+p1)*y5+p0)/((((y5*q4+q3)*y5+q2)*y5+q1)*y5+q0).
do if (pv <= .5).
compute xp = -xp.
end if.
compute cilow=rnd(nrow(temp)*(cdfnorm(2*xp-xp2))).
compute cihigh=trunc(nrow(temp)*(cdfnorm(2*xp+(xp2))))+1.
do if (cilow < 1).
compute cilow = 1.
compute booterr=1.
compute badlo = 1.
end if.
do if (cihigh > nrow(temp)).
compute cihigh = boot.
compute booterr=1.
compute badhi = 1.
end if.
compute llcit=temp(cilow,1).
compute ulcit=temp(cihigh,1).
do if (badlo = 1 and llcit <> priorlo).
compute badend={badend, llcit}.
compute priorlo = llcit.
end if.
do if (badhi = 1 and ulcit <> priorhi).
compute badend={badend, ulcit}.
compute priorhi = ulcit.
end if.
end if.
do if ( (icmm(1,1)*bc)+(9999*(1-bc)) = 9999).
compute llcit=temp(cilow,1).
compute ulcit=temp(cihigh,1).
end if.
compute bootse=t(sqrt((cssq(temp)-((csum(temp)&**2)/nrow(temp)))/(nrow(temp)-1)))
.
compute cmmtemp(k8,2:4)={bootse,llcit,ulcit}.
end if.
end loop.
compute cmmtemp={zmodvals,cmmtemp}.
do if (boot=0).
compute cmmtemp=cmmtemp(:,1:2).
end if.
compute cmmlbs={znames,immlbs}.
compute resultm2=make(nrow(cmmtemp),maxresm,99999).
compute resultm2(1:nrow(cmmtemp),1:ncol(cmmtemp))=cmmtemp.
compute resultm={resultm;resultm2}.
do if (outscree=1).
print cmmtemp/title='      Indices of conditional moderated mediation by W'/cnames=cmmlbs/format= F10.4.
end if.
end loop.
end if.
end if.
end if.
do if (dichadj=1 or immcat=1 and indmmm(i,1) < 30).
do if (outscree=1).
print immtemp/title='Index of moderated mediation (difference between conditional indirect effects):'/cnames=immlbs/rnames=immlbs2/format= F10.4.
end if.
end if.
end if.
do if (indcontr=1).
compute resultm2=make(nrow(condcont),maxresm,99999).
compute resultm2(1:nrow(condcont),1:ncol(condcont))=condcont.
compute resultm={resultm;resultm2}.
compute condctlb={'Effect1','Effect2','Contrast','BootSE', 'BootLLCI','BootULCI'}.
do if (outscree=1).
print condcont/title=' Pairwise contrasts between conditional indirect effects (Effect1 minus Effect2)'/cnames=condctlb/format= F10.4.
end if.
end if.
end loop.
end if.
end loop.
do if (alttotal=1).
compute altcnms='Effect'.
compute alttotfx=t(cftotfx(1,:))+obnatdfx.
do if (boot > 0).
compute alttotfx=make(ncol(cftotfx),4,0).
compute alttotfx(:,1)=t(cftotfx(1,:))+obnatdfx.
compute natdirbt(1,:)=t(obnatdfx).
compute cftotfx=cftotfx+natdirbt.
loop cec=1 to ncol(cftotfx).

.
compute temp = cftotfx(2:nrow(cftotfx),cec).
compute temp(GRADE( cftotfx(2:nrow(cftotfx),cec) )) = cftotfx(2:nrow(cftotfx),cec).
compute badlo = 0.
compute badhi = 0.
do if ( (cftotfx(1,cec)*bc)+(9999*(1-bc)) <> 9999).
compute pv=csum(temp < (cftotfx(1,cec)*bc)+(9999*(1-bc)) )/nrow(temp).
compute ppv = pv.
do if (pv > .5).
compute ppv = 1-pv.
end if.
compute y5=sqrt(-2*ln(ppv)).
compute xp=y5+((((y5*p4+p3)*y5+p2)*y5+p1)*y5+p0)/((((y5*q4+q3)*y5+q2)*y5+q1)*y5+q0).
do if (pv <= .5).
compute xp = -xp.
end if.
compute cilow=rnd(nrow(temp)*(cdfnorm(2*xp-xp2))).
compute cihigh=trunc(nrow(temp)*(cdfnorm(2*xp+(xp2))))+1.
do if (cilow < 1).
compute cilow = 1.
compute booterr=1.
compute badlo = 1.
end if.
do if (cihigh > nrow(temp)).
compute cihigh = boot.
compute booterr=1.
compute badhi = 1.
end if.
compute llcit=temp(cilow,1).
compute ulcit=temp(cihigh,1).
do if (badlo = 1 and llcit <> priorlo).
compute badend={badend, llcit}.
compute priorlo = llcit.
end if.
do if (badhi = 1 and ulcit <> priorhi).
compute badend={badend, ulcit}.
compute priorhi = ulcit.
end if.
end if.
do if ( (cftotfx(1,cec)*bc)+(9999*(1-bc)) = 9999).
compute llcit=temp(cilow,1).
compute ulcit=temp(cihigh,1).
end if.
compute bootse=t(sqrt((cssq(temp)-((csum(temp)&**2)/nrow(temp)))/(nrow(temp)-1)))
.
compute alttotfx(cec,2:4)={bootse,llcit,ulcit}.
end loop.
compute altcnms={altcnms,'BootSE', 'BootLLCI','BootULCI'}.
end if.
compute resultm2=make(nrow(alttotfx),maxresm,99999).
compute resultm2(1:nrow(alttotfx),1:ncol(alttotfx))=alttotfx.
compute resultm={resultm;resultm2}.
do if (outscree=1).
print/title='----------'.
do if (nxvls > 1).
print alttotfx/title='Relative total effects of X on Y (sum of direct and indirect effects)'/cnames=altcnms/rnames=direffl2/format= F10.4.
end if.
do if (nxvls = 1).
print alttotfx/title='Total effect of X on Y (sum of direct and indirect effects)'/cnames=altcnms/format= F10.4.
end if.
end if.
end if.
end if.
end if
.
do if (criterr=0 and (saveest = 1 or saveboot=1)).
compute coeffsav=coeffmat(2:nrow(coeffmat),:).
compute conseq=conseq(2:nrow(conseq),1).
compute dfmat=dfmat(2:nrow(dfmat),1).
compute dfmat=dfmat.
compute saven={'conseqnt','antecdnt','coeff','se','t','p','LLCI','ULCI','df'}.
do if (ydich=1).
compute saven={'conseqnt','antecdnt','coeff','se','t_or_Z','p','LLCI','ULCI','df'}.
end if.
do if (saveboot=1 and boot > 0 ).
compute colslab=make(300,1,' ').


compute colslab( 1 ,1)= 'COL1'.


compute colslab( 2 ,1)= 'COL2'.


compute colslab( 3 ,1)= 'COL3'.


compute colslab( 4 ,1)= 'COL4'.


compute colslab( 5 ,1)= 'COL5'.


compute colslab( 6 ,1)= 'COL6'.


compute colslab( 7 ,1)= 'COL7'.


compute colslab( 8 ,1)= 'COL8'.


compute colslab( 9 ,1)= 'COL9'.


compute colslab( 10 ,1)= 'COL10'.


compute colslab( 11 ,1)= 'COL11'.


compute colslab( 12 ,1)= 'COL12'.


compute colslab( 13 ,1)= 'COL13'.


compute colslab( 14 ,1)= 'COL14'.


compute colslab( 15 ,1)= 'COL15'.


compute colslab( 16 ,1)= 'COL16'.


compute colslab( 17 ,1)= 'COL17'.


compute colslab( 18 ,1)= 'COL18'.


compute colslab( 19 ,1)= 'COL19'.


compute colslab( 20 ,1)= 'COL20'.


compute colslab( 21 ,1)= 'COL21'.


compute colslab( 22 ,1)= 'COL22'.


compute colslab( 23 ,1)= 'COL23'.


compute colslab( 24 ,1)= 'COL24'.


compute colslab( 25 ,1)= 'COL25'.


compute colslab( 26 ,1)= 'COL26'.


compute colslab( 27 ,1)= 'COL27'.


compute colslab( 28 ,1)= 'COL28'.


compute colslab( 29 ,1)= 'COL29'.


compute colslab( 30 ,1)= 'COL30'.


compute colslab( 31 ,1)= 'COL31'.


compute colslab( 32 ,1)= 'COL32'.


compute colslab( 33 ,1)= 'COL33'.


compute colslab( 34 ,1)= 'COL34'.


compute colslab( 35 ,1)= 'COL35'.


compute colslab( 36 ,1)= 'COL36'.


compute colslab( 37 ,1)= 'COL37'.


compute colslab( 38 ,1)= 'COL38'.


compute colslab( 39 ,1)= 'COL39'.


compute colslab( 40 ,1)= 'COL40'.


compute colslab( 41 ,1)= 'COL41'.


compute colslab( 42 ,1)= 'COL42'.


compute colslab( 43 ,1)= 'COL43'.


compute colslab( 44 ,1)= 'COL44'.


compute colslab( 45 ,1)= 'COL45'.


compute colslab( 46 ,1)= 'COL46'.


compute colslab( 47 ,1)= 'COL47'.


compute colslab( 48 ,1)= 'COL48'.


compute colslab( 49 ,1)= 'COL49'.


compute colslab( 50 ,1)= 'COL50'.


compute colslab( 51 ,1)= 'COL51'.


compute colslab( 52 ,1)= 'COL52'.


compute colslab( 53 ,1)= 'COL53'.


compute colslab( 54 ,1)= 'COL54'.


compute colslab( 55 ,1)= 'COL55'.


compute colslab( 56 ,1)= 'COL56'.


compute colslab( 57 ,1)= 'COL57'.


compute colslab( 58 ,1)= 'COL58'.


compute colslab( 59 ,1)= 'COL59'.


compute colslab( 60 ,1)= 'COL60'.


compute colslab( 61 ,1)= 'COL61'.


compute colslab( 62 ,1)= 'COL62'.


compute colslab( 63 ,1)= 'COL63'.


compute colslab( 64 ,1)= 'COL64'.


compute colslab( 65 ,1)= 'COL65'.


compute colslab( 66 ,1)= 'COL66'.


compute colslab( 67 ,1)= 'COL67'.


compute colslab( 68 ,1)= 'COL68'.


compute colslab( 69 ,1)= 'COL69'.


compute colslab( 70 ,1)= 'COL70'.


compute colslab( 71 ,1)= 'COL71'.


compute colslab( 72 ,1)= 'COL72'.


compute colslab( 73 ,1)= 'COL73'.


compute colslab( 74 ,1)= 'COL74'.


compute colslab( 75 ,1)= 'COL75'.


compute colslab( 76 ,1)= 'COL76'.


compute colslab( 77 ,1)= 'COL77'.


compute colslab( 78 ,1)= 'COL78'.


compute colslab( 79 ,1)= 'COL79'.


compute colslab( 80 ,1)= 'COL80'.


compute colslab( 81 ,1)= 'COL81'.


compute colslab( 82 ,1)= 'COL82'.


compute colslab( 83 ,1)= 'COL83'.


compute colslab( 84 ,1)= 'COL84'.


compute colslab( 85 ,1)= 'COL85'.


compute colslab( 86 ,1)= 'COL86'.


compute colslab( 87 ,1)= 'COL87'.


compute colslab( 88 ,1)= 'COL88'.


compute colslab( 89 ,1)= 'COL89'.


compute colslab( 90 ,1)= 'COL90'.


compute colslab( 91 ,1)= 'COL91'.


compute colslab( 92 ,1)= 'COL92'.


compute colslab( 93 ,1)= 'COL93'.


compute colslab( 94 ,1)= 'COL94'.


compute colslab( 95 ,1)= 'COL95'.


compute colslab( 96 ,1)= 'COL96'.


compute colslab( 97 ,1)= 'COL97'.


compute colslab( 98 ,1)= 'COL98'.


compute colslab( 99 ,1)= 'COL99'.


compute colslab( 100 ,1)= 'COL100'.


compute colslab( 101 ,1)= 'COL101'.


compute colslab( 102 ,1)= 'COL102'.


compute colslab( 103 ,1)= 'COL103'.


compute colslab( 104 ,1)= 'COL104'.


compute colslab( 105 ,1)= 'COL105'.


compute colslab( 106 ,1)= 'COL106'.


compute colslab( 107 ,1)= 'COL107'.


compute colslab( 108 ,1)= 'COL108'.


compute colslab( 109 ,1)= 'COL109'.


compute colslab( 110 ,1)= 'COL110'.


compute colslab( 111 ,1)= 'COL111'.


compute colslab( 112 ,1)= 'COL112'.


compute colslab( 113 ,1)= 'COL113'.


compute colslab( 114 ,1)= 'COL114'.


compute colslab( 115 ,1)= 'COL115'.


compute colslab( 116 ,1)= 'COL116'.


compute colslab( 117 ,1)= 'COL117'.


compute colslab( 118 ,1)= 'COL118'.


compute colslab( 119 ,1)= 'COL119'.


compute colslab( 120 ,1)= 'COL120'.


compute colslab( 121 ,1)= 'COL121'.


compute colslab( 122 ,1)= 'COL122'.


compute colslab( 123 ,1)= 'COL123'.


compute colslab( 124 ,1)= 'COL124'.


compute colslab( 125 ,1)= 'COL125'.


compute colslab( 126 ,1)= 'COL126'.


compute colslab( 127 ,1)= 'COL127'.


compute colslab( 128 ,1)= 'COL128'.


compute colslab( 129 ,1)= 'COL129'.


compute colslab( 130 ,1)= 'COL130'.


compute colslab( 131 ,1)= 'COL131'.


compute colslab( 132 ,1)= 'COL132'.


compute colslab( 133 ,1)= 'COL133'.


compute colslab( 134 ,1)= 'COL134'.


compute colslab( 135 ,1)= 'COL135'.


compute colslab( 136 ,1)= 'COL136'.


compute colslab( 137 ,1)= 'COL137'.


compute colslab( 138 ,1)= 'COL138'.


compute colslab( 139 ,1)= 'COL139'.


compute colslab( 140 ,1)= 'COL140'.


compute colslab( 141 ,1)= 'COL141'.


compute colslab( 142 ,1)= 'COL142'.


compute colslab( 143 ,1)= 'COL143'.


compute colslab( 144 ,1)= 'COL144'.


compute colslab( 145 ,1)= 'COL145'.


compute colslab( 146 ,1)= 'COL146'.


compute colslab( 147 ,1)= 'COL147'.


compute colslab( 148 ,1)= 'COL148'.


compute colslab( 149 ,1)= 'COL149'.


compute colslab( 150 ,1)= 'COL150'.


compute colslab( 151 ,1)= 'COL151'.


compute colslab( 152 ,1)= 'COL152'.


compute colslab( 153 ,1)= 'COL153'.


compute colslab( 154 ,1)= 'COL154'.


compute colslab( 155 ,1)= 'COL155'.


compute colslab( 156 ,1)= 'COL156'.


compute colslab( 157 ,1)= 'COL157'.


compute colslab( 158 ,1)= 'COL158'.


compute colslab( 159 ,1)= 'COL159'.


compute colslab( 160 ,1)= 'COL160'.


compute colslab( 161 ,1)= 'COL161'.


compute colslab( 162 ,1)= 'COL162'.


compute colslab( 163 ,1)= 'COL163'.


compute colslab( 164 ,1)= 'COL164'.


compute colslab( 165 ,1)= 'COL165'.


compute colslab( 166 ,1)= 'COL166'.


compute colslab( 167 ,1)= 'COL167'.


compute colslab( 168 ,1)= 'COL168'.


compute colslab( 169 ,1)= 'COL169'.


compute colslab( 170 ,1)= 'COL170'.


compute colslab( 171 ,1)= 'COL171'.


compute colslab( 172 ,1)= 'COL172'.


compute colslab( 173 ,1)= 'COL173'.


compute colslab( 174 ,1)= 'COL174'.


compute colslab( 175 ,1)= 'COL175'.


compute colslab( 176 ,1)= 'COL176'.


compute colslab( 177 ,1)= 'COL177'.


compute colslab( 178 ,1)= 'COL178'.


compute colslab( 179 ,1)= 'COL179'.


compute colslab( 180 ,1)= 'COL180'.


compute colslab( 181 ,1)= 'COL181'.


compute colslab( 182 ,1)= 'COL182'.


compute colslab( 183 ,1)= 'COL183'.


compute colslab( 184 ,1)= 'COL184'.


compute colslab( 185 ,1)= 'COL185'.


compute colslab( 186 ,1)= 'COL186'.


compute colslab( 187 ,1)= 'COL187'.


compute colslab( 188 ,1)= 'COL188'.


compute colslab( 189 ,1)= 'COL189'.


compute colslab( 190 ,1)= 'COL190'.


compute colslab( 191 ,1)= 'COL191'.


compute colslab( 192 ,1)= 'COL192'.


compute colslab( 193 ,1)= 'COL193'.


compute colslab( 194 ,1)= 'COL194'.


compute colslab( 195 ,1)= 'COL195'.


compute colslab( 196 ,1)= 'COL196'.


compute colslab( 197 ,1)= 'COL197'.


compute colslab( 198 ,1)= 'COL198'.


compute colslab( 199 ,1)= 'COL199'.


compute colslab( 200 ,1)= 'COL200'.


compute colslab( 201 ,1)= 'COL201'.


compute colslab( 202 ,1)= 'COL202'.


compute colslab( 203 ,1)= 'COL203'.


compute colslab( 204 ,1)= 'COL204'.


compute colslab( 205 ,1)= 'COL205'.


compute colslab( 206 ,1)= 'COL206'.


compute colslab( 207 ,1)= 'COL207'.


compute colslab( 208 ,1)= 'COL208'.


compute colslab( 209 ,1)= 'COL209'.


compute colslab( 210 ,1)= 'COL210'.


compute colslab( 211 ,1)= 'COL211'.


compute colslab( 212 ,1)= 'COL212'.


compute colslab( 213 ,1)= 'COL213'.


compute colslab( 214 ,1)= 'COL214'.


compute colslab( 215 ,1)= 'COL215'.


compute colslab( 216 ,1)= 'COL216'.


compute colslab( 217 ,1)= 'COL217'.


compute colslab( 218 ,1)= 'COL218'.


compute colslab( 219 ,1)= 'COL219'.


compute colslab( 220 ,1)= 'COL220'.


compute colslab( 221 ,1)= 'COL221'.


compute colslab( 222 ,1)= 'COL222'.


compute colslab( 223 ,1)= 'COL223'.


compute colslab( 224 ,1)= 'COL224'.


compute colslab( 225 ,1)= 'COL225'.


compute colslab( 226 ,1)= 'COL226'.


compute colslab( 227 ,1)= 'COL227'.


compute colslab( 228 ,1)= 'COL228'.


compute colslab( 229 ,1)= 'COL229'.


compute colslab( 230 ,1)= 'COL230'.


compute colslab( 231 ,1)= 'COL231'.


compute colslab( 232 ,1)= 'COL232'.


compute colslab( 233 ,1)= 'COL233'.


compute colslab( 234 ,1)= 'COL234'.


compute colslab( 235 ,1)= 'COL235'.


compute colslab( 236 ,1)= 'COL236'.


compute colslab( 237 ,1)= 'COL237'.


compute colslab( 238 ,1)= 'COL238'.


compute colslab( 239 ,1)= 'COL239'.


compute colslab( 240 ,1)= 'COL240'.


compute colslab( 241 ,1)= 'COL241'.


compute colslab( 242 ,1)= 'COL242'.


compute colslab( 243 ,1)= 'COL243'.


compute colslab( 244 ,1)= 'COL244'.


compute colslab( 245 ,1)= 'COL245'.


compute colslab( 246 ,1)= 'COL246'.


compute colslab( 247 ,1)= 'COL247'.


compute colslab( 248 ,1)= 'COL248'.


compute colslab( 249 ,1)= 'COL249'.


compute colslab( 250 ,1)= 'COL250'.


compute colslab( 251 ,1)= 'COL251'.


compute colslab( 252 ,1)= 'COL252'.


compute colslab( 253 ,1)= 'COL253'.


compute colslab( 254 ,1)= 'COL254'.


compute colslab( 255 ,1)= 'COL255'.


compute colslab( 256 ,1)= 'COL256'.


compute colslab( 257 ,1)= 'COL257'.


compute colslab( 258 ,1)= 'COL258'.


compute colslab( 259 ,1)= 'COL259'.


compute colslab( 260 ,1)= 'COL260'.


compute colslab( 261 ,1)= 'COL261'.


compute colslab( 262 ,1)= 'COL262'.


compute colslab( 263 ,1)= 'COL263'.


compute colslab( 264 ,1)= 'COL264'.


compute colslab( 265 ,1)= 'COL265'.


compute colslab( 266 ,1)= 'COL266'.


compute colslab( 267 ,1)= 'COL267'.


compute colslab( 268 ,1)= 'COL268'.


compute colslab( 269 ,1)= 'COL269'.


compute colslab( 270 ,1)= 'COL270'.


compute colslab( 271 ,1)= 'COL271'.


compute colslab( 272 ,1)= 'COL272'.


compute colslab( 273 ,1)= 'COL273'.


compute colslab( 274 ,1)= 'COL274'.


compute colslab( 275 ,1)= 'COL275'.


compute colslab( 276 ,1)= 'COL276'.


compute colslab( 277 ,1)= 'COL277'.


compute colslab( 278 ,1)= 'COL278'.


compute colslab( 279 ,1)= 'COL279'.


compute colslab( 280 ,1)= 'COL280'.


compute colslab( 281 ,1)= 'COL281'.


compute colslab( 282 ,1)= 'COL282'.


compute colslab( 283 ,1)= 'COL283'.


compute colslab( 284 ,1)= 'COL284'.


compute colslab( 285 ,1)= 'COL285'.


compute colslab( 286 ,1)= 'COL286'.


compute colslab( 287 ,1)= 'COL287'.


compute colslab( 288 ,1)= 'COL288'.


compute colslab( 289 ,1)= 'COL289'.


compute colslab( 290 ,1)= 'COL290'.


compute colslab( 291 ,1)= 'COL291'.


compute colslab( 292 ,1)= 'COL292'.


compute colslab( 293 ,1)= 'COL293'.


compute colslab( 294 ,1)= 'COL294'.


compute colslab( 295 ,1)= 'COL295'.


compute colslab( 296 ,1)= 'COL296'.


compute colslab( 297 ,1)= 'COL297'.


compute colslab( 298 ,1)= 'COL298'.


compute colslab( 299 ,1)= 'COL299'.


compute colslab( 300 ,1)= 'COL300'.

compute colslab=colslab(1:ncol(bootres),1).
compute colslab={colslab,conseq,vlabs}.
do if (outscree=1).
print/title = '**************************************************************************'.
print/title='Bootstrap estimates were saved to a file'/space=0.
print colslab/title='Map of column names to model coefficients:'/clabels=' ','Conseqnt','Antecdnt'/format=a8.
end if.
end if.
end if.
do if (criterr=0 and boot > 0 and modelbt=1).
compute labstart=1.
do if (outscree=1).
print/title = '*********** BOOTSTRAP RESULTS FOR REGRESSION MODEL PARAMETERS ************'.
end if.
loop iboot = 1 to (nms+nys).
do if (outscree=1).
print outnames(1,iboot)/title = 'OUTCOME VARIABLE:'/format = A8.
end if.
compute vlabsm=vlabs(labstart:(labstart+(nump(1,iboot)-1)),1).
compute outnmtmp=bootcim(labstart:(labstart+(nump(1,iboot)-1)),:).
compute resultm2=make(nrow(outnmtmp),maxresm,99999).
compute resultm2(1:nrow(outnmtmp),1:ncol(outnmtmp))=outnmtmp.
compute resultm={resultm;resultm2}.
do if (outscree=1).
print bootcim(labstart:(labstart+(nump(1,iboot)-1)),:)/title=' '/rnames=vlabsm/ clabels='Coeff' 'BootMean' 'BootSE' 'BootLLCI' 'BootULCI'/format= F10.4 /space=0.
end if.
compute labstart=labstart+nump(1,iboot).
do if (iboot < (nms+nys)).
do if (outscree=1).
print/title= '----------'.
end if.
end if.
end loop.
end if.
do if (criterr=0 and saveest=1).
compute resultm=resultm(2:nrow(resultm),:).
compute bocaj=(resultm=99999).
compute bocaj=csum(bocaj).
compute bocaj=1-(bocaj=nrow(resultm)).
compute j=1.
loop i = 1 to ncol(resultm).
do if (bocaj(1,i)=1).
compute j=j+1.
end if.
end loop.
compute resultm=resultm(:,1:(j-1)).
save resultm/outfile = *.
end if.
do if (criterr = 0 and matrices=1 and outscree=1).
print/title = '************************ MODEL DEFINITION MATRICES ************************'.
print/title = 'FROM variables are columns, TO variables are rows.'.
compute temp2=make(nrow(bcmat),ncol(bcmat),'0').
loop i = 2 to nrow(bcmat).
loop j = 1 to (ncol(bcmat)-1).
do if (bcmat(i,j)=1).
compute temp2(i,j)='1'.
end if.
do if (j >= i).
compute temp2(i,j)=' '.
end if.
end loop.
end loop.
compute temp2=temp2(2:nrow(bcmat),(1:(ncol(bcmat)-1))).
do if (nms > 0).
compute cmatlabs={xnames,mnames}.
compute rmatlabs={mnames,ynames}.
end if.
do if (nms = 0).
compute cmatlabs={xnames}.
compute rmatlabs={ynames}.
end if.
print temp2/title='BMATRIX: Paths freely estimated (1) and fixed to zero (0):'/cnames=cmatlabs/rnames=rmatlabs/format A3.
compute z=0.
do if (rsum(csum(wcmat))<>0).
compute temp2=make(nrow(wcmat),ncol(wcmat),'0').
loop i = 2 to nrow(wcmat).
loop j = 1 to (ncol(wcmat)-1).
do if (wcmat(i,j)=1).
compute temp2(i,j)='1'.
end if.
do if (j >= i).
compute temp2(i,j)=' '.
end if.
end loop.
end loop.
compute temp2=temp2(2:nrow(wcmat),(1:(ncol(wcmat)-1))).
print temp2/title='WMATRIX: Paths moderated (1) and not moderated (0) by W:'/cnames=cmatlabs/rnames=rmatlabs/format A3.
end if.
do if (rsum(csum(zcmat))<>0).
compute temp2=make(nrow(zcmat),ncol(zcmat),'0').
loop i = 2 to nrow(zcmat).
loop j = 1 to (ncol(zcmat)-1).
do if (zcmat(i,j)=1).
compute temp2(i,j)='1'.
end if.
do if (j >= i).
compute temp2(i,j)=' '.
end if.
end loop.
end loop.
compute temp2=temp2(2:nrow(zcmat),(1:(ncol(zcmat)-1))).
print temp2/title='ZMATRIX: Paths moderated (1) and not moderated (0) by Z:'/cnames=cmatlabs/rnames=rmatlabs/format a3.
end if.
do if (rsum(csum(wzcmat))<>0).
compute temp2=make(nrow(wzcmat),ncol(wzcmat),'0').
loop i = 2 to nrow(wzcmat).
loop j = 1 to (ncol(wzcmat)-1).
do if (wzcmat(i,j)=1).
compute temp2(i,j)='1'.
end if.
do if (j >= i).
compute temp2(i,j)=' '.
end if.
end loop.
end loop.
compute temp2=temp2(2:nrow(wzcmat),(1:(ncol(wzcmat)-1))).
print temp2/title='WZMATRIX: W moderated paths moderated (1) and not moderated (0) by Z:'/cnames=cmatlabs/rnames=rmatlabs/format a3.
end if.
do if (ncs > 0).
print ccmat/title='CMATRIX: Covariates (columns) in (1) and not in (0) the models of M and Y (rows):'/rnames=rmatlabs/cnames=covnames.
end if.
end if.
do if (outscree=1).
do if (activate=0).
print/title = '*********************** ANALYSIS NOTES AND ERRORS ************************'.
end if.
end if.
do if (activate=1).
print/title = '**************************************************************************'.
end if.
do if (criterr=0).
do if (outscree=1).
print conf/title = 'Level of confidence for all confidence intervals in output:'/format = F8.4.
do if (boot > 0).
do if (goodboot = boot and bc=0).
print boot/title='Number of bootstrap samples for percentile bootstrap confidence intervals:'.
end if.
do if (goodboot = boot and bc=1).
print boot/title='Number of bootstrap samples for bias-corrected bootstrap confidence intervals:'.
end if.
do if (booterr = 1).
compute badend = badend(1,2:ncol(badend)).
print/title = 'WARNING: Bootstrap CI endpoints below not trustworthy. Decrease confidence'.
print badend/title='or increase the number of bootstrap samples.'/space=0/format = F10.4.
end if.
end if.
do if (mc > 0).
print mc/title='Number of samples for Monte Carlo confidence intervals:'.
end if.
do if (wnotev > 0 and printw=1).
do if (wnotev=1).
print/title = 'W values in conditional tables are the 16th, 50th, and 84th percentiles.'.
else if (wnotev=2).
do if (minwwarn=0 and maxwwarn=0).
print/title = 'W values in conditional tables are the mean and +/- SD from the mean.'.
end if.
do if (minwwarn=1).
print/title = 'W values in conditional tables are the minimum, the mean, and 1 SD above the mean.'.
end if.
do if (maxwwarn=1).
print/title = 'W values in conditional tables are 1 SD below the mean, the mean, and the maximum.'.
end if.
end if.
end if.
do if (znotev > 0 and printz=1).
do if (znotev=1).
print/title = 'Z values in conditional tables are the 16th, 50th, and 84th percentiles.'.
else if (znotev=2).
do if (minzwarn=0 and maxzwarn=0).
print/title = 'Z values in conditional tables are the mean and +/- SD from the mean.'.
end if.
do if (minzwarn=1).
print/title = 'Z values in conditional tables are the minimum, the mean, and 1 SD above the mean.'.
end if.
do if (maxzwarn=1).
print/title = 'Z values in conditional tables are 1 SD below the mean, the mean, and the maximum.'.
end if.
end if.
end if.
do if (minwwarn > 0).
print/title = 'NOTE: One SD below the mean is below the minimum observed in the data for W,'.
print/title = '      so the minimum measurement on W is used for conditioning instead.'/space=0.
end if.
do if (maxwwarn > 0).
print/title = 'NOTE: One SD above the mean is above the maximum observed in the data for W,'.
print/title = '      so the maximum measurement for W is used for conditioning instead.'/space=0.
end if.
do if (minzwarn > 0).
print/title = 'NOTE: One SD below the mean is below the minimum observed in the data for Z,'.
print/title = '      so the minimum measurement for Z is used for conditioning instead.'/space=0.
end if.
do if (maxzwarn > 0).
print/title = 'NOTE: One SD above the mean is above the maximum observed in the data for Z,'.
print/title = '      so the maximum measurement for Z is used for conditioning instead.'/space=0.
end if.
do if (pstog=1).
print/title= 'NOTE: Standardized coefficients for dichotomous or multicategorical X are in'.
print/title= '      partially standardized form.'/space=0.
end if.
loop i = 1 to 100.
do if (notecode(i,1) = 32).
print/title = 'Direct, indirect, and total effects are counterfactually defined'.
do if (xcontcf=0).
print/title = 'assuming X by M interaction.'/space=0.
end if.
do if (xcontcf=1).
print/title = 'assuming X by M interaction and with the following reference (x_ref)'/space=0.
compute states=t(xrefvals).
print states/title = 'and counterfactual (x_cf) states for X:'/rlabels='x_ref' 'x_cf'/format= F10.4 /space=0.
end if.
end if.
do if (notecode(i,1)=1).
print/title = 'NOTE: COVMY is ignored when using CMATRIX option.'.
end if.
do if (notecode(i,1)=2).
print/title = 'NOTE: Confidence level restricted to between 50 and 99.9999%.  95% confidence is provided in output'.
end if.
do if (notecode(i,1)=3).
print centvar/title = 'NOTE: The following variables were mean centered prior to analysis:'/format = a8.
end if.
do if (notecode(i,1) = 4).
print/title = 'NOTE: A heteroscedasticity consistent standard error and covariance matrix estimator was used.'.
end if.
do if (notecode(i,1) = 5).
print/title = 'NOTE: The HC3 option has been replaced with HC.  See the documentation.'.
end if.
do if (notecode(i,1) = 6).
print/title = 'NOTE: Due to estimation problems, some bootstrap samples had to be replaced.'.
print badboot/title='      The number of times this happened was:'/space=0/format=F8.0.
end if.
do if (notecode(i,1) = 7).
print/title = 'NOTE: The bootstrapping was not completed due to problematic bootstrap samples.'.
print/title = '      Bootstrap confidence intervals are therefore suppressed.'/space=0.
end if.
do if (notecode(i,1) = 8).
print/title = 'NOTE: The number of bootstrap samples was adjusted upward given your desired confidence.'.
end if.
do if (notecode(i,1) = 9).
print/title = 'NOTE: WMODVAL is ignored when W is specified as multicategorical.'.
end if.
do if (notecode(i,1) = 10).
print/title = 'NOTE: ZMODVAL is ignored when Z is specified as multicategorical.'.
end if.
do if (notecode(i,1) = 11).
print/title = 'NOTE: Total effect model generated only when all covariates are specified in all'.
print/title = '      models of M and Y.'/space=0.
end if.
do if (notecode(i,1) = 30).
print/title = 'NOTE: Your vector of linear hypothesis weights is of the wrong length for this model.'.
end if.
do if (notecode(i,1) = 12).
print/title = 'NOTE: Total effect model generated only when X is freely estimated to affect each M'.
print/title = '      and both X and M are freely estimated to affect Y'/space=0.
end if.
do if (notecode(i,1) = 13).
print/title = 'NOTE: There are too many pairwise contrasts to conduct with this model.'.
end if.
do if (notecode(i,1) = 14).
print/title = 'NOTE: The number of contrast weights must equal the number of indirect effects.'.
end if.
do if (notecode(i,1) = 15).
print/title = 'NOTE: Monte Carlo confidence intervals not available for this model.'.
print/title = '      Bootstrapping is used instead.'/space=0.
end if.
do if (notecode(i,1) = 16).
print/title = 'NOTE: The number of Monte Carlo samples was adjusted upward given your desired confidence.'.
end if.
do if (notecode(i,1) = 19).
print/title = 'NOTE: Your contrast matrix is invalid or not applicable to this model.'.
end if.
do if (notecode(i,1) = 20).
print/title = 'NOTE: One of the groups specified by your contrast matrix does not exist in the data.'.
end if.
do if (notecode(i,1) = 21).
print/title = 'NOTE: The VARORDER option is not available in this release.'.
end if.
do if (notecode(i,1) = 22).
print/title = 'NOTE: The VMODVAL and QMODVAL options are not available in this release.'.
end if.
do if (notecode(i,1) = 23).
print/title = 'NOTE: The QUANTILE option is not available in this release.'.
end if.
do if (notecode(i,1) = 24).
print/title = 'NOTE: Total effect model not available with dichotomous Y.'.
end if.
do if (notecode(i,1) = 25).
print/title = 'NOTE: STAND/EFFSIZE options not available with dichotomous Y.'.
end if.
do if ((notecode(i,1) = 26) and nms > 0).
print/title = 'NOTE: Direct and indirect effects of X on Y are on a log-odds metric.'.
end if.
do if (notecode(i,1) = 27).
print/title = 'NOTE: Standardized coefficients are not available for models with moderators.'.
end if.
do if (notecode(i,1) = 28).
print/title = 'NOTE: The contrast option is not available with a multicategorical X.'.
end if.
do if (notecode(i,1) = 31).
do if (nms > 1).
print medmeans/title = 'NOTE: Controlled direct effect(s) estimated at the following mediator values:'/cnames=mnames/format= F10.4.
end if.
do if (nms=1).
print medmeans/title = 'NOTE: Controlled direct effect(s) estimated at the following mediator value:'/format= F10.4.
end if.
end if.
do if (notecode(i,1) = 33).
print/title = 'NOTE: Sobel test is not available with the XMINT option.'.
end if.
do if (notecode(i,1) = 37).
print/title = 'NOTE: The CONTRAST option is not available when using the XMINT option.'.
end if.
do if (notecode(i,1) = 34).
print/title = 'NOTE: Standardized effects are not available when using the XMINT option.'.
end if.
do if (notecode(i,1) = 36).
print/title = 'NOTE: The XREFVAL option is ignored when X is declared as multicategorical.'.
end if.
do if (notecode(i,1) = 35).
print coval/title = 'NOTE: Counterfactual effects estimated at the following covariate values:'/cnames=covnames/format= F10.4.
end if.
do if (notecode(i,1) = 29 and (listmiss=1)).
compute a=missrow.
compute conum=ncol(a).
compute allgood=0.
compute smremain=12.
compute largesti=1.
compute smallrow=0.
do if (conum > 12).
loop ii = 1 to 12.
compute check=(conum/ii).
do if (check = trunc(check)).
compute check2=conum/ii.
compute aok=ii.
do if (aok > 2).
compute allgood=1.
end if.
else.
compute remain=conum-(ii*trunc(check)).
do if (remain <= smremain).
compute smremain=remain.
compute largesti=ii.
compute smallrow=trunc(conum/largesti).
end if.
end if.
end loop.
compute atemp=a(1,1:(aok*check2)).
compute atemp=reshape(atemp,check2,aok).
do if (ncol(atemp) > 2).
print/title='NOTE: Missing data resulted in the deletion of the following row(s) of'.
print atemp/title='      data from the analysis:'/format=F6.0/space=0.
end if.
do if (allgood=0).
compute atemp=a(1,1:(smallrow*largesti)).
compute atemp=reshape(atemp,smallrow,largesti).
compute btemp=a(1,((largesti*smallrow)+1):conum).
print/title='NOTE: Missing data resulted in the deletion of the following row(s) of'.
print atemp/title='      data from the analysis:'/format=F6.0/space=0.
print btemp/title=' '/space=0/format=f6.0.
end if.
else if (conum <= 12).
print/title='NOTE: Missing data resulted in the deletion of the following row(s) of'.
print a/title='      data from the analysis:'/format=F6.0/space=0.
end if.
end if.
end loop.
do if (toomany=1).
print/title='WARNING: Variables names longer than eight characters can produce incorrect output'.
print/title='when some variables in the data file have the same first eight characters. Shorter'/space=0.
print/title='variable names are recommended. By using this output, you are accepting all risk'/space=0.
print/title='and consequences of interpreting or reporting results that may be incorrect.'/space=0.
end if.
end if.
end if.
loop i = 1 to 100.
do if (errcode(i,1)=1).
print/title = 'ERROR: You must specify a Y and an X variable.'.
end if.
do if (errcode(i,1)=2).
print/title = 'ERROR: X, M, or Y variable used more than once or W and Z are the same variable.'.
do if (toomany = 1).
print/title = '       This could be caused by the use of variables names longer'/space=0.
print varnames/title = '       than eight characters. Here are the variables I see:'/space=0/format=A8.
end if.
end if.
do if (errcode(i,1)=3).
print/title = 'ERROR: You have specified more than one variable for W, Y, X, or Z'.
end if.
do if (errcode(i,1)=4).
print/title = 'ERROR: A variable specified as multicategorical has more than nine categories.'.
end if.
do if (errcode(i,1)=5).
print/title = 'ERROR: One of the categories contains only a single case.'.
end if.
do if (errcode(i,1)=6).
print/title = 'ERROR: Invalid model number in this version of PROCESS.'.
end if.
do if (errcode(i,1)=7).
print/title = 'ERROR: Invalid model number.'.
end if.
do if (errcode(i,1)=8).
print/title = 'ERROR: You must specify an M variable for this model.'.
end if.
do if (errcode(i,1)=9).
print/title = 'ERROR: You have specified an M variable in a model that does not use it.'.
end if.
do if (errcode(i,1)=10).
print/title = 'ERROR: You have specified a W variable in a model that does not use it.'.
end if.
do if (errcode(i,1)=11).
print/title = 'ERROR: You have not specified a W variable in a model that requires it.'.
end if.
do if (errcode(i,1)=12).
print/title = 'ERROR: You have specified a Z variable in a model that does not use it.'.
end if.
do if (errcode(i,1)=13).
print/title = 'ERROR: You have not specified a Z variable in a model that requires it.'.
end if.
do if (errcode(i,1)=14).
print/title = 'ERROR: V and Q are not proper specifications in this release of PROCESS.'.
print/title = '       Moderators must be specified as W and/or Z.'/space=0.
end if.
do if (errcode(i,1)=15).
print/title = 'ERROR: One of your model variables exhibits no variation (it is a constant).'.
end if.
do if (errcode(i,1)=16).
print/title = 'ERROR: BMATRIX is not the correct length or is otherwise invalid.'.
end if.
do if (errcode(i,1)=17).
print/title = 'ERROR: WMATRIX is not the correct length or is otherwise invalid.'.
end if.
do if (errcode(i,1)=18).
print/title = 'ERROR: ZMATRIX is not the correct length or is otherwise invalid.'.
end if.
do if (errcode(i,1)=19).
print/title = 'ERROR: WZMATRIX is not the correct length or is otherwise invalid.'.
end if.
do if (errcode(i,1)=20).
print/title = 'ERROR: A path fixed at zero cannot be moderated.'.
end if.
do if (errcode(i,1)=21).
print/title = 'ERROR: If only one moderator is specified, it must be specified as W.'.
end if.
do if (errcode(i,1)=22).
print/title = 'ERROR: In BMATRIX, X must be specified to affect at least one variable.'.
end if.
do if (errcode(i,1)=23).
print/title = 'ERROR: In BMATRIX, at least one variable must be specified to affect Y.'.
end if.
do if (errcode(i,1)=24).
print/title = 'ERROR: You must specify a model number or a custom BMATRIX specification.'.
end if.
do if (errcode(i,1)=25).
print/title = 'ERROR: BMATRIX cannot be used in conjunction with a model number.'.
end if.
do if (errcode(i,1)=26).
print/title = 'ERROR: Your model has a dangling mediator (all Ms must affect and be affected).'.
end if.
do if (errcode(i,1)=27).
print/title = 'ERROR: CLUSTER is not available on this release of PROCESS.'.
end if.
do if (errcode(i,1)=29).
print/title = 'ERROR: CMATRIX is not the correct length or is otherwise invalid.'.
end if.
do if (errcode(i,1)=30).
print/title = 'ERROR: In CMATRIX, all covariates must be assigned to an M or a Y.'.
end if.
do if (errcode(i,1)=31).
print/title = 'ERROR: A linear or near linear dependency (singularity) exists in the data.'.
end if.
do if (errcode(i,1)=32).
print/title = 'ERROR: Models 80 and 81 require between 3 and 6 mediators.'.
end if.
do if (errcode(i,1)=33).
print/title = 'ERROR: Model 82 requires 4 mediators.'.
end if.
do if (errcode(i,1)=34).
print/title = 'ERROR: This model number requires between 2 and 6 mediators.'.
end if.
do if (errcode(i,1)=35).
print/title = 'ERROR: In a model with only one moderator, that moderator must be W.'.
end if.
do if (errcode(i,1)=36).
print/title = 'ERROR: A serial mediation model cannot have more than 6 mediators.'.
end if.
do if (errcode(i,1)=37).
print/title = 'ERROR: No more than 10 mediators are allowed in a PROCESS command.'.
end if.
do if (errcode(i,1)=38).
print/title = 'ERROR: XCATCODE is not provided, not the correct length, or is otherwise invalid.'.
end if.
do if (errcode(i,1)=39).
print/title = 'ERROR: WCATCODE is not provided, not the correct length, or is otherwise invalid.'.
end if.
do if (errcode(i,1)=40).
print/title = 'ERROR: ZCATCODE is not provided, not the correct length, or is otherwise invalid.'.
end if.
do if (errcode(i,1)=41).
print/title = 'ERROR: Models 1, 2, and 3 cannot be customized.'.
end if.
do if (errcode(i,1)=42).
print/title = 'ERROR: WS option available only in PROCESS v2. Or use the MEMORE macro instead.'.
print/title = '       MEMORE can be downloaded from www.akmontoya.com.'/space=0.
end if.
do if (errcode(i,1)=43).
print/title = 'ERROR: PROCESS does not allow dichotomous mediators.'.
end if.
do if (errcode(i,1)=60).
print/title = 'PROCESS is now ready for use.'.
print/title='Copyright 2017-2022 by Andrew F. Hayes. ALL RIGHTS RESERVED.'/space=0.
print/title='Workshop schedule available at http://haskayne.ucalgary.ca/CCRAM'/space=0.
end if.
do if (errcode(i,1)=50).
print/title = 'ERROR: A multicategorical moderator cannot be specified as a covariate.'.
end if.
do if (errcode(i,1)=51).
print/title = 'ERROR: A variable you specified as a covariate is a moderator in all equations.'.
end if.
do if (errcode(i,1)=52 and mcerpt=0).
compute mcerpt=1.
print/title = 'ERROR: A variable specified as multicategorical must have at least three categories'.
end if.
do if (errcode(i,1) = 47).
do if (iterrmod=1).
print/title = 'ERROR: Iteration for Y model didn''t converge to a solution. Interpret results with caution.'.
print/title = '       Try increasing the number of iterations, though this will slow down computation.'/space=0.
end if.
do if (bootiter=1).
print/title = 'ERROR: Nonconvergence during bootstrapping. Interpet bootstrap results with caution.'.
end if.
end if.
do if (errcode(i,1) = 61).
print/title = 'ERROR: Variable names should be no more than eight characters in length.'.
end if.
do if (errcode(i,1) = 62).
print/title = 'ERROR: After listwise deletion of cases with missing data, too few cases remain.'.
end if.
do if (errcode(i,1) = 63).
print/title = 'ERROR: The XMINT option is available only for model 4.'.
end if.
do if (errcode(i,1) = 64).
print/title = 'ERROR: Incorrect number of values specified in CDEVAL option.'.
end if.
do if (errcode(i,1) = 65).
print/title = 'ERROR: Only indicator or sequential coding of X is allowed with the XMINT option.'.
end if.
do if (errcode(i,1) = 66).
print/title = 'ERROR: XMINT option not available with continuous X using PROCESS GUI. Use syntax.'.
end if.
do if (errcode(i,1) = 67).
print/title = 'ERROR: Too many elements provided in XREFVAL option.'.
end if.
do if (errcode(i,1)=68).
print/title = 'ERROR: Covariate assignment is not allowed with XMINT option.'.
end if.
do if (errcode(i,1) = 69).
print/title = 'ERROR: Incorrect number of values specified in COVAL option.'.
end if.
do if (errcode(i,1) = 70).
print/title = 'ERROR: Incorrect value(s) in XREFVAL for this dichotomous X variable.'.
end if.
do if (errcode(i,1) = 71).
print/title = 'ERROR: The CENTER option is not available when using the XMINT option.'.
end if.
do if (errcode(i,1) = 72).
print/title = 'ERROR: The XMINT option is not available for models with a dichotomous Y.'.
end if.
end loop.
END MATRIX.	
Ressources	Temps de processeur	00:00:06,37	
	Temps écoulé	00:00:06,38	


Run MATRIX procedure:

***************** PROCESS Procedure for SPSS Version 4.2 *****************

          Written by Andrew F. Hayes, Ph.D.       www.afhayes.com
    Documentation available in Hayes (2022). www.guilford.com/p/hayes3

**************************************************************************
Model  : 4
    Y  : HOMAIR
    X  : ALL5
   M1  : TG
   M2  : HDL
   M3  : LDL
   M4  : TC

Sample
Size:  169

**************************************************************************
OUTCOME VARIABLE:
 TG

Model Summary
          R       R-sq        MSE          F        df1        df2          p
      ,0723      ,0052  4030,4468      ,8787     1,0000   167,0000      ,3499

Model
              coeff         se          t          p       LLCI       ULCI
constant    90,0139     5,2905    17,0143      ,0000    79,5690   100,4587
ALL5       -12,8939    13,7553     -,9374      ,3499   -40,0505    14,2627

Standardized coefficients
          coeff
ALL5     -,2032

**************************************************************************
OUTCOME VARIABLE:
 HDL

Model Summary
          R       R-sq        MSE          F        df1        df2          p
      ,0967      ,0094   176,4689     1,5775     1,0000   167,0000      ,2109

Model
              coeff         se          t          p       LLCI       ULCI
constant    40,6250     1,1070    36,6978      ,0000    38,4395    42,8105
ALL5         3,6150     2,8782     1,2560      ,2109    -2,0674     9,2974

Standardized coefficients
          coeff
ALL5      ,2717

**************************************************************************
OUTCOME VARIABLE:
 LDL

Model Summary
          R       R-sq        MSE          F        df1        df2          p
      ,0351      ,0012  1188,5865      ,2057     1,0000   167,0000      ,6508

Model
              coeff         se          t          p       LLCI       ULCI
constant    75,8125     2,8730    26,3880      ,0000    70,1404    81,4846
ALL5         3,3875     7,4698      ,4535      ,6508   -11,3599    18,1349

Standardized coefficients
          coeff
ALL5      ,0985

**************************************************************************
OUTCOME VARIABLE:
 TC

Model Summary
          R       R-sq        MSE          F        df1        df2          p
      ,0352      ,0012  1450,1360      ,2068     1,0000   167,0000      ,6499

Model
              coeff         se          t          p       LLCI       ULCI
constant   135,2083     3,1734    42,6069      ,0000   128,9432   141,4735
ALL5         3,7517     8,2508      ,4547      ,6499   -12,5377    20,0410

Standardized coefficients
          coeff
ALL5      ,0988

**************************************************************************
OUTCOME VARIABLE:
 HOMAIR

Model Summary
          R       R-sq        MSE          F        df1        df2          p
      ,3660      ,1339    23,2212     5,0409     5,0000   163,0000      ,0003

Model
              coeff         se          t          p       LLCI       ULCI
constant      ,0294     1,6628      ,0177      ,9859    -3,2539     3,3128
ALL5          ,4168     1,0519      ,3962      ,6924    -1,6604     2,4940
TG            ,0438      ,0097     4,4993      ,0000      ,0246      ,0631
HDL           ,1237      ,0433     2,8587      ,0048      ,0383      ,2091
LDL           ,0928      ,0357     2,5957      ,0103      ,0222      ,1634
TC           -,0879      ,0355    -2,4751      ,0143     -,1580     -,0178

Standardized coefficients
          coeff
ALL5      ,0817
TG        ,5456
HDL       ,3227
LDL       ,6257
TC       -,6545

************************** TOTAL EFFECT MODEL ****************************
OUTCOME VARIABLE:
 HOMAIR

Model Summary
          R       R-sq        MSE          F        df1        df2          p
      ,0198      ,0004    26,1595      ,0653     1,0000   167,0000      ,7986

Model
              coeff         se          t          p       LLCI       ULCI
constant     4,1539      ,4262     9,7459      ,0000     3,3124     4,9954
ALL5          ,2832     1,1082      ,2556      ,7986    -1,9046     2,4711

Standardized coefficients
          coeff
ALL5      ,0555


************** TOTAL, DIRECT, AND INDIRECT EFFECTS OF X ON Y **************

Total effect of X on Y
     Effect         se          t          p       LLCI       ULCI       c_ps
      ,2832     1,1082      ,2556      ,7986    -1,9046     2,4711      ,0555

Direct effect of X on Y
     Effect         se          t          p       LLCI       ULCI      c'_ps
      ,4168     1,0519      ,3962      ,6924    -1,6604     2,4940      ,0817

Indirect effect(s) of X on Y:
          Effect     BootSE   BootLLCI   BootULCI
TOTAL     -,1336      ,3928     -,9083      ,6949
TG        -,5654      ,6407    -2,1462      ,3759
HDL        ,4471      ,6973     -,1977     2,3614
LDL        ,3143     1,2253    -1,5740     3,1308
TC        -,3297     1,2971    -3,6508     1,2904

Partially standardized indirect effect(s) of X on Y:
          Effect     BootSE   BootLLCI   BootULCI
TOTAL     -,0262      ,0767     -,1822      ,1298
TG        -,1108      ,1226     -,4131      ,0731
HDL        ,0877      ,1294     -,0412      ,4407
LDL        ,0616      ,2362     -,2883      ,5797
TC        -,0646      ,2474     -,6873      ,2466

*********************** ANALYSIS NOTES AND ERRORS ************************

Level of confidence for all confidence intervals in output:
  95,0000

Number of bootstrap samples for percentile bootstrap confidence intervals:
  5000

NOTE: Standardized coefficients for dichotomous or multicategorical X are in
      partially standardized form.

------ END MATRIX -----


* Encoding: UTF-8.
preserve.
set printback=off.


Matrice


Remarques	
Sortie obtenue	10-NOV-2024 17:04:23	
Commentaires		
Entrée	Données	C:\Users\Brice SAHA\Documents\UBa\Master Thesis 2021\Fanuel\Database fanuel.sav	
	Jeu de données actif	Jeu_de_données1	
	Filtre	<sans>	
	Pondération	<sans>	
	Scinder un fichier	<sans>	
	N de lignes dans le fichier de travail	169	
Syntaxe	MATRIX.
compute wnames='xxxxx'.
compute znames='xxxxx'.
compute mcerpt=0.
compute wiscov=0.
compute ziscov=0.
compute tooman=0.
compute errcode=make(100,1,0).
compute notecode=make(100,1,0).
compute model = trunc( 4 ).
compute iterate = abs(trunc( 100 )).
compute converge = abs( 0.00001 ).
compute itprobtg=0.
compute v2tag=0.
compute ydich=0.
compute maxwwarn=0.
compute minwwarn=0.
compute maxzwarn=0.
compute minzwarn=0.
compute toomany=0.
compute wdich=0.
compute zdich=0.
compute wnotev=0.
compute znotev=0.
compute nxpval=1.
compute nwpval=1.
compute nzpval=1.
compute errs=1.
compute notes=1.
compute criterr=0.
compute novar=0.
compute adjust=0.
compute ncs=0.
compute serial=0.
compute sobelok=0.
compute hasw=0.
compute hasz=0.
compute printw=0.
compute printz=0.
compute xmint=( 0 =1).
compute wmodcust=0.
compute zmodcust=0.
compute booting=0.
compute bootiter=0.
compute iterrmod=0.
compute cov = 'xxxxx'.
compute varorder=( 0 <> 0).
compute nws=0.
compute w= 'xxxxx'.
compute nzs=0.
compute z = 'xxxxx'.
compute nms=0.
compute m = 'TG HDL LDL TC'.
compute nys=0.
compute y = 'HOMAIR'.
compute nxs=0.
compute x = 'Starchy'.
compute effsize=( 0 =1).
compute stand=( 1 =1).
compute intprobe = .1.
compute xrefvals={ 999 }.
compute center=trunc( 0 ).
compute xcontcf=0.
compute xscaling=1.
compute cdeval={ -999 }.
compute cuscoval=0.
do if (model=74).
compute errcode(errs,1)=7.
compute errs=errs+1.
compute criterr=1.
end if.
do if (xmint=1 and model <> 4).
compute errcode(errs,1)=63.
compute errs=errs+1.
compute criterr=1.
end if.
do if (xmint=1 and model=4).
compute w=x.
compute model=74.
compute intprobe=1.
compute notecode(notes,1) = 32.
compute notes = notes + 1.
do if (effsize=1 or stand=1).
compute notecode(notes,1) = 34.
compute notes = notes + 1.
compute stand=0.
compute effsize=0.
end if.
do if (center <> 0).
compute center=0.
compute errcode(errs,1)=71.
compute errs=errs+1.
compute criterr=1.
end if.
end if.
compute v = 'xxxxx'.
compute q = 'xxxxx'.
compute linsum={ -999 }.
compute nlinsum=ncol(linsum).
do if (linsum(1,1) = -999).
compute nlinsum=0.
end if.
compute oldvars= 'xxxxx'.
compute mcxok=0.
compute mcwok=0.
compute mczok=0.
compute xprod=0.
compute zprod=0.
compute wprod=0.
compute modcok=0.
compute alttotal=0.
compute hc3=trunc( 0 ).
compute jn=( 0 = 1).
compute listmiss=( 0 =1).
compute modelres=( 0 =1).
compute outscree=( 1 =1).
compute activate=( 0 =1).
compute booterr=0.
compute normal=( 0 =1).
compute xmtest=( 0 =1).
compute describe=( 0 =1).
compute longname=( 0 =1).
do if (stand=1).
compute effsize=1.
end if.
compute pstog=0.
compute sobelok=0.
compute normal=( 0 =1).
compute mdichok=( 0 =1).
compute contrast={ 999 }.
compute ncontr=ncol(contrast).
compute ncontrow=nrow(contrast).
do if (contrast(1,1) = 999).
compute ncontr=1.
compute contrast=0.
end if.
do if (ncontr = 1).
compute contrast=trunc(contrast).
do if (contrast > 3 or contrast < 0)).
compute ncontr=1.
compute contrast = 0.
end if.
end if.
do if (ncontr > 1).
compute contvec=contrast.
compute contrast=4.
do if (ncontrow > 1).
compute contrast=0.
compute modcok=1.
compute wcontval=contvec(:,1).
compute zcontval=contvec(:,2).
do if ((ncontr <> 2) or (ncontrow <> 2)).
compute notecode(notes,1) = 19.
compute notes = notes + 1.
compute modcok=0.
end if.
end if.
end if.
do if (xmint = 1 and contrast(1,1) <> 0).
compute contrast=0.
compute notecode(notes,1) = 37.
compute notes = notes + 1.
end if.
do if (varorder = 1).
compute notecode(notes,1) = 21.
compute notes = notes + 1.
end if.
do if ( 999 <> 999 or 999 <> 999).
compute notecode(notes,1) = 22.
compute notes = notes + 1.
end if.
compute modelbt=( 0 =1).
compute cluster= 'xxxxx'.
compute matrices=( 0 =1).
compute covcoeff=( 0 =1).
compute covmy=trunc( 0 ).
do if (covmy < 0 or covmy > 2).
compute covmy = 0.
end if.
compute boot = abs(trunc( 5000 )).
compute bc=( 0 =1).
compute mc=abs(trunc( 0 )).
compute hc=trunc( 5 ).
do if (intprobe < 0 or intprobe > 1).
compute intprobe = .10.
end if.
compute plot=trunc( 0 ).
do if (plot < 0 or plot > 2).
compute plot=0.
end if.
compute total=( 1 =1).
compute dototal=0.
compute saveboot = ( 0 = 1).
compute saveest=( 0 = 2).
do if (saveest=1).
compute intprobe=1.
end if.
do if (hc >= 0 and hc < 5).
compute notecode(notes,1) = 4.
compute notes = notes + 1.
end if.
do if (hc > 5 or hc < 0).
compute hc=5.
end if.
compute mcw=trunc( 0 ).
compute mcz=trunc( 0 ).
compute mcx=trunc( 0 ).
do if (mcx > 0 and mcx < 3 and model = 74).
compute mcw=mcx.
compute xscaling=1.
end if.
do if (mcx > 2 and model = 74).
compute errcode(errs,1)=65.
compute errs=errs+1.
compute criterr=1.
end if.
do if (model = 74 and normal=1).
compute notecode(notes,1) = 33.
compute normal=0.
compute notes = notes + 1.
end if.
do if (mcx > 0 and contrast > 0).
compute notecode(notes,1) = 28.
compute notes = notes + 1.
compute contrast=0.
end if.
compute nxvls=1.
compute nmvls=1.
compute nwvls=1.
compute nzvls=1.
compute paths=999.
compute pathsw=999.
compute pathsz=999.
compute pathswz=999.
compute pathsmod=999.
compute pathtype=999.
compute obscoeff=999.
compute pathsdv={' '}.
compute quantile=1.
do if ( 999 <>999).
compute notecode(notes,1) = 23.
compute notes = notes + 1.
end if.
compute moments=( 0 =1).
do if (moments=1).
compute quantile=0.
end if.
compute bmatrix={ -999 }.
compute wmatrix={ -999 }.
compute zmatrix={ -999 }.
compute wzmatrix={ -999 }.
compute cmatrix={ -999 }.
compute xcatcode={ -999 }.
compute wcatcode={ -999 }.
compute zcatcode={ -999 }.
compute needed=0.
compute conf= 95.
do if (trunc( 95 ) >= 100 or (trunc( 95 ) <= 50)).
compute conf = 95.
compute notecode(notes,1)=2.
compute notes=notes+1.
end if.
do if (model >= 0 and model < 4 and modelbt=0).
compute boot=0.
compute mc=0.
compute bc=0.
end if.
do if (mc > 0 and boot > 0).
compute boot=0.
compute bc=0.
end if.
do if ((boot < 1000) and (mc = 0) and (boot > 0)).
compute boot=5000.
end if.
do if ((mc < 1000) and (boot = 0) and (mc > 0)).
compute mc=5000.
end if.
compute p0=-.322232431088.
compute p1 = -1.
compute p2 = -.342242088547.
compute p3 = -.0204231210245.
compute p4 = -.0000453642210148.
compute q0 = .0993484626060.
compute q1 = .588581570495.
compute q2 = .531103462366.
compute q3 = .103537752850.
compute q4 = .0038560700634.
compute badend=0.
compute priorlo = -9999999.
compute priorhi = 9999999.
compute alpha2 = (1-(conf/100))/2.
compute cilm=alpha2*2.
compute y5=sqrt(-2*ln(alpha2)).
compute xp2=(y5+((((y5*p4+p3)*y5+p2)*y5+p1)*y5+p0)/((((y5*q4+q3)*y5+q2)*y5+q1)*y5+q0)).
compute medlb={'   M1  :';'   M2  :';'   M3  :';'   M4  :';'   M5  :';'   M6  :';'   M7  :';'   M8  :';'   M9  :';'   M10 :'}.
compute medlb2={'(M1)','(M2)','(M3)','(M4)','(M5)','(M6)','(M7)','(M8)','(M9)','(M10)'}.
compute xlb={'   X1  :';'   X2  :';'   X3  :';'   X4  :';'   X5  :';'   X6  :';'   X7  :';'   X8  :';'   X9  :'}.
compute highlbw={'M1*W'; 'M2*W'; 'M3*W'; 'M4*W'; 'M5*W'; 'M6*W'; 'M7*W'; 'M8*W'; 'M9*W'; 'M10*W'}.
do if (xmint=1).
compute highlbw={'M1*X'; 'M2*X'; 'M3*X'; 'M4*X'; 'M5*X'; 'M6*X'; 'M7*X'; 'M8*X'; 'M9*X'; 'M10*X'}.
end if.
compute highlbz={'M1*Z'; 'M2*Z'; 'M3*Z'; 'M4*Z'; 'M5*Z'; 'M6*Z'; 'M7*Z'; 'M8*Z'; 'M9*Z';'M10*Z'}.
compute highlbwz={'M1*W*Z'; 'M2*W*Z'; 'M3*W*Z'; 'M4*W*Z'; 'M5*W*Z'; 'M6*W*Z'; 'M7*W*Z'; 'M8*W*Z'; 'M9*W*Z';'M10*W*Z'}.
compute highlbbt={'BOTH(M1)'; 'BOTH(M2)'; 'BOTH(M3)'; 'BOTH(M4)'; 'BOTH(M5)'; 'BOTH(M6)'; 'BOTH(M7)'; 'BOTH(M8)'; 'BOTH(M9)';'BTH(M10)'}.
compute highlbx={'M1*X'; 'M2*X'; 'M3*X'; 'M4*X'; 'M5*X'; 'M6*X'; 'M7*X'; 'M8*X'; 'M9*X'; 'M10*X'}.
compute skipwz=0.
compute validm={1,1,1,1,1,1,1,1,1,1,1,1,1,1,1,1,1,1,1,1,1,1,0,0,0,0,0,1,1,0,0,0,0, 0,0,0,0,0,0,0,0,0,0,0,0,0,0,0,0,0,0,0,0,0,0,0,0,1,1,1,1,1,1,1,1,1,1,1,1,1,1,1,1,1,1,1,0,0,0,1,1, 1,1,1,1,1,1,1,1,1,1,1}.
do if (activate=1).
compute errcode(errs,1)=60.
compute errs=errs+1.
compute criterr=1.
end if.
do if (criterr=0).
do if (( 0 =1)=1).
compute errcode(errs,1)=42.
compute errs=errs+1.
compute criterr=1.
end if.
do if (model > 0 and model < 93).
do if (validm(1,model)=0).
compute errcode(errs,1)=6.
compute errs=errs+1.
compute criterr=1.
end if.
release validm.
end if.
do if ((model > 92 or model < 0) and model <> 999)).
compute errcode(errs,1)=7.
compute errs=errs+1.
compute criterr=1.
end if.
do if (model = 999 and bmatrix(1,1)=-999).
compute errcode(errs,1)=24.
compute errs=errs+1.
compute criterr=1.
end if.
do if (model <> 999 and bmatrix(1,1) <> -999).
compute errcode(errs,1)=25.
compute errs=errs+1.
compute criterr=1.
end if.
do if ((model = 74 or (model > 0 and model < 4)) and ((wmatrix(1,1) <> -999) or (zmatrix(1,1)<>-999) or (wzmatrix(1,1)<>-999)))).
compute errcode(errs,1)=41.
compute errs=errs+1.
compute criterr=1.
end if.
do if (hc3 <> 0).
compute notecode(notes,1) = 5.
compute notes = notes + 1.
do if (hc3 = 1).
compute hc=3.
end if.
end if.
do if ((v <> 'xxxxx') or (q <> 'xxxxx')).
compute errcode(errs,1)=14.
compute errs=errs+1.
compute errcode(errs,1)=48.
compute errs=errs+1.
compute criterr=1.
end if.
do if (oldvars <> 'xxxxx').
compute errcode(errs,1)=48.
compute errs=errs+1.
compute criterr=1.
end if.
do if (cluster <> 'xxxxx').
compute errcode(errs,1)=27.
compute errs=errs+1.
compute criterr=1.
end if.
do if ((y = 'xxxxx') or (x = 'xxxxx')).
compute errcode(errs,1)=1.
compute errs=errs+1.
compute criterr=1.
end if.
do if ((m = 'xxxxx') and model > 3).
compute errcode(errs,1)=8.
compute errs=errs+1.
compute criterr=1.
end if.
end if.
do if (criterr=0).
get ytmp/variables = HOMAIR /names = ynames/MISSING = 99999.
compute nys=ncol(ytmp).
compute needed=nys.
compute n=nrow(ytmp).
compute varnames={ynames}.
compute dat=ytmp.

.


do if (toomany=1 and longname=0).
compute criterr=1.
do if (tooman=0).
compute tooman=1.
compute errcode(errs,1) = 61.
compute errs = errs + 1.
end if.
end if
.
compute modelvar={ '4' ;t(ynames)}.
do if ( 4 =999).
compute modelvar(1,1)='CUSTOM'.
end if.
get xtmp/variables = Starchy /names = xnames/MISSING = 99999.
compute nxs=ncol(xtmp).
compute n=nrow(xtmp).
compute needed=needed+nxs.
compute varnames={varnames,xnames}.
compute xcatlab=t(xnames).
compute dat={dat,xtmp}.

.


do if (toomany=1 and longname=0).
compute criterr=1.
do if (tooman=0).
compute tooman=1.
compute errcode(errs,1) = 61.
compute errs = errs + 1.
end if.
end if
.
compute modelvar={modelvar;t(xnames)}.
do if (nxs = 1).
compute modelvlb={'Model  :';'    Y  :';'    X  :'}.
else.
compute modelvlb={'Model  :';'    Y  :';xlb(1:nxs,1)}.
end if.
do if (m <> 'xxxxx').
get mtmp/variables = TG HDL LDL TC /names = mnames/MISSING = 99999.
compute nms=ncol(mtmp).
compute mprod=make(1,nms,0).
compute n=nrow(mtmp).
compute needed=needed+nms.
compute varnames={varnames,mnames}.
compute dat={dat,mtmp}.
compute modelvar={modelvar;t(mnames)}.
compute x2m=make(99,nms,0).
compute m2y=make(99,nms,0).
compute onem=make(nms,1,1).

.


do if (toomany=1 and longname=0).
compute criterr=1.
do if (tooman=0).
compute tooman=1.
compute errcode(errs,1) = 61.
compute errs = errs + 1.
end if.
end if
.
do if (nms > 1 and nms < 11).
compute modelvlb={modelvlb;medlb(1:nms,1)}.
else.
compute modelvlb={modelvlb;'    M  :'}.
end if.
do if (nms > 0 and model < 4).
compute errcode(errs,1)=9.
compute errs=errs+1.
do if (model <> 0).
compute errcode(errs,1)=48.
compute errs=errs+1.
end if.
compute criterr=1.
end if.
end if.
compute wlocatet=0.
compute wlocate=0.
do if (w <> 'xxxxx').
do if (xmint=0).
get wtmp/variables = xxxxx /names = wnames/MISSING = 99999.

.


do if (toomany=1 and longname=0).
compute criterr=1.
do if (tooman=0).
compute tooman=1.
compute errcode(errs,1) = 61.
compute errs = errs + 1.
end if.
end if
.
end if.
do if (xmint=1).
get wtmp/variables = Starchy /names = wnames/MISSING = 99999.
end if.
compute nws=ncol(wtmp).
compute n=nrow(wtmp).
compute varnames={varnames,wnames}.
compute wlocate=ncol(varnames).
do if (model=74).
compute wlocatet=1.
do if (xnames <> wnames).
compute errcode(errs,1)=45.
compute errs=errs+1.
compute criterr=1.
end if.
end if.
compute wcatlab=t(wnames).
compute dat={dat,wtmp}.
do if (xmint <> 1).
compute modelvar={modelvar;t(wnames)}.
compute modelvlb={modelvlb;'    W  :'}.
end if.
end if.
do if (z <> 'xxxxx').
get ztmp/variables = xxxxx /names = znames/MISSING = 99999.
compute nzs=ncol(ztmp).
compute n=nrow(ztmp).

.


do if (toomany=1 and longname=0).
compute criterr=1.
do if (tooman=0).
compute tooman=1.
compute errcode(errs,1) = 61.
compute errs = errs + 1.
end if.
end if
.
compute varnames={varnames,znames}.
compute zcatlab=t(znames).
compute dat={dat,ztmp}.
compute modelvar={modelvar;t(znames)}.
compute modelvlb={modelvlb;'    Z  :'}.
end if.
do if (cov <> 'xxxxx').
get ctmp/variables = xxxxx /names = covnames/MISSING = 99999.
compute ncs=ncol(ctmp).
compute n=nrow(ctmp).

.


do if (toomany=1 and longname=0).
compute criterr=1.
do if (tooman=0).
compute tooman=1.
compute errcode(errs,1) = 61.
compute errs = errs + 1.
end if.
end if
.
compute varnames={varnames,covnames}.
compute dat={dat,ctmp}.
end if.
do if (nws > 1 or nzs > 1 or nys > 1 or nxs > 1).
compute errcode(errs,1)=3.
compute errs=errs+1.
compute criterr=1.
end if.
do if ((model = 80 or model = 81) and (nms < 3 or nms > 6)).
compute errcode(errs,1)=32.
compute errs=errs+1.
compute criterr=1.
end if.
do if (model = 82 and nms <> 4).
compute errcode(errs,1)=33.
compute errs=errs+1.
compute criterr=1.
end if.
do if (nms > 10).
compute errcode(errs,1)=37.
compute errs=errs+1.
compute criterr=1.
end if.
do if ((model = 6 or (model > 82 and model < 999)) and (nms < 2 or nms > 6)).
compute errcode(errs,1)=34.
compute errs=errs+1.
compute criterr=1.
end if.
compute match=0.
compute match2=0.
compute mcwzcov=0.
loop i = 1 to (ncol(varnames)-1).
loop j = (i+1) to ncol(varnames).
do if (varnames(i)=varnames(j)).
do if (i < (nxs+nms+nys+1)).
compute match2=match2+1.
end if.
do if (wlocatet=1 and i=2 and j=wlocate).
compute match2=match2-1.
end if.
do if ((wnames=znames) and (nws > 0 or nzs > 0))).
compute match2=match2+1.
end if.
do if (i < (ncol(varnames)-ncs+1)) and j > (ncol(varnames)-ncs)).
do if ((varnames(j)=wnames) and mcw=0)).
compute match=0.
compute wiscov=(j-(ncol(varnames)-ncs)).
end if.
do if ((varnames(j)=wnames) and mcw <>0)).
compute mcwzcov=1.
end if.
do if ((varnames(j)=znames) and mcz=0).
compute match=0.
compute ziscov=(j-(ncol(varnames)-ncs)).
end if.
do if ((varnames(j)=znames) and mcz<>0)).
compute mcwzcov=1.
end if.
end if.
end if.
end loop.
end loop.
do if (match2>0 or match=1).
compute errcode(errs,1)=2.
compute errs=errs+1.
compute criterr=1.
end if.
do if (mcwzcov=1).
compute errcode(errs,1)=50.
compute errs=errs+1.
compute criterr=1.
end if.
compute ninit=nrow(dat).
compute rownum=make(ninit,1,0).
loop i = 1 to ninit.
compute rownum(i,1)=i.
end loop.
compute dat={rownum,dat}.
compute j=1.
compute missrow=0.
loop i = 1 to n.
do if (rsum(dat(i,2:ncol(dat))=99999)=0).
compute dat(j,:)=dat(i,:).
compute j=j+1.
else.
compute missrow={missrow;dat(i,1)}.
end if.
end loop.
do if (j < 5).
compute errcode(errs,1)=62.
compute errs=errs+1.
compute criterr=1.
end if.
do if (criterr=0).
compute rownum=dat(1:(j-1),1).
do if (nrow(missrow) > 1).
compute missrow=t(missrow(2:nrow(missrow),1)).
compute notecode(notes,1) = 29.
compute notes = notes + 1.
end if.
compute dat=dat(1:(j-1),2:ncol(dat)).
compute n=nrow(dat).
compute nmiss=ninit-n.
compute ytmp=dat(:,1:nys).

.
compute desctmp=make((8-(4* 0 )),ncol( ytmp ),-999).
loop jd=1 to ncol( ytmp ).
compute descdat= ytmp (:,jd).
compute desctmp(1,jd) = csum(descdat)/nrow(descdat).
compute desctmp(2,jd) = (nrow(descdat)*sscp(descdat))-(t(csum(descdat))*(csum(descdat))).
compute desctmp(2,jd) = sqrt(desctmp(2,jd)/(nrow(descdat)*(nrow(descdat)-1))).
compute desctmp(3,jd)=cmin(descdat).
compute desctmp(4,jd)=cmax(descdat).
do if ( 0 =0).
compute minwarn=0.
compute maxwarn=0.
do if ((desctmp(3,jd)=desctmp(4,jd)) and novar=0).
compute errcode(errs,1)=15.
compute errs=errs+1.
compute criterr=1.
compute novar=1.
end if.
compute tmp=((descdat(:,1)=desctmp(3,jd))+(descdat(:,1)=desctmp(4,jd))).
compute desctmp(8,jd)=(csum(tmp)=nrow(tmp)).
compute tmp = descdat.
compute tmp(GRADE(descdat),:) = descdat.
compute descdat = tmp.
release tmp.
compute decval={.16;.5;.84}.
loop kd=1 to 3.
compute low=trunc(decval(kd,1)*(nrow(descdat)+1)).
compute lowdec=decval(kd,1)*(nrow(descdat)+1)-low.
compute value=descdat(low,1)+(descdat((low+1),1)-descdat(low,1))*lowdec.
compute desctmp((4+kd),jd)=value.
end loop.
compute mnotev=1.
compute modvals=desctmp(5:7,:).
do if (quantile <> 1).
compute desctmp(5,jd)=desctmp(1,jd)-desctmp(2,jd).
compute desctmp(6,jd)=desctmp(1,jd).
compute desctmp(7,jd)=desctmp(1,jd)+desctmp(2,jd).
compute modvals=desctmp(5:7,:).
compute mnotev=2.
do if (modvals(1,1) < desctmp(3,1)).
compute modvals(1,1)=desctmp(3,1).
compute minwarn=1.
end if.
do if (modvals(3,1) > desctmp(4,1)).
compute modvals(3,1)=desctmp(4,1).
compute maxwarn=1.
end if.
end if.
do if (desctmp(8,1)=1).
compute modvals={desctmp(3,1);desctmp(4,1)}.
compute mnotev=0.
compute minwarn=0.
compute maxwarn=0.
end if.
end if.
end loop
.
compute ysd=desctmp(2,:).
compute ovsd=ysd.
do if (desctmp(8,1)=1).
compute ydich=1.
do if (total=1).
compute total=0.
compute notecode(notes,1) = 24.
compute notes = notes + 1.
end if.
do if (effsize=1).
compute effsize=0.
compute notecode(notes,1) = 25.
compute notes = notes + 1.
end if.
do if (model=74).
compute errcode(errs,1) = 72.
compute errs=errs+1.
compute criterr=1.
end if.
compute omx = cmax(ytmp).
compute omn = cmin(ytmp).
compute ytmp = (ytmp = omx).
compute dat(:,1:nys)=(dat(:,1:nys)=omx).
compute rcd = {omn, 0; omx, 1}.
end if.
compute xtmp=dat(:,(nys+1):(nys+nxs)).

.
compute desctmp=make((8-(4* 0 )),ncol( xtmp ),-999).
loop jd=1 to ncol( xtmp ).
compute descdat= xtmp (:,jd).
compute desctmp(1,jd) = csum(descdat)/nrow(descdat).
compute desctmp(2,jd) = (nrow(descdat)*sscp(descdat))-(t(csum(descdat))*(csum(descdat))).
compute desctmp(2,jd) = sqrt(desctmp(2,jd)/(nrow(descdat)*(nrow(descdat)-1))).
compute desctmp(3,jd)=cmin(descdat).
compute desctmp(4,jd)=cmax(descdat).
do if ( 0 =0).
compute minwarn=0.
compute maxwarn=0.
do if ((desctmp(3,jd)=desctmp(4,jd)) and novar=0).
compute errcode(errs,1)=15.
compute errs=errs+1.
compute criterr=1.
compute novar=1.
end if.
compute tmp=((descdat(:,1)=desctmp(3,jd))+(descdat(:,1)=desctmp(4,jd))).
compute desctmp(8,jd)=(csum(tmp)=nrow(tmp)).
compute tmp = descdat.
compute tmp(GRADE(descdat),:) = descdat.
compute descdat = tmp.
release tmp.
compute decval={.16;.5;.84}.
loop kd=1 to 3.
compute low=trunc(decval(kd,1)*(nrow(descdat)+1)).
compute lowdec=decval(kd,1)*(nrow(descdat)+1)-low.
compute value=descdat(low,1)+(descdat((low+1),1)-descdat(low,1))*lowdec.
compute desctmp((4+kd),jd)=value.
end loop.
compute mnotev=1.
compute modvals=desctmp(5:7,:).
do if (quantile <> 1).
compute desctmp(5,jd)=desctmp(1,jd)-desctmp(2,jd).
compute desctmp(6,jd)=desctmp(1,jd).
compute desctmp(7,jd)=desctmp(1,jd)+desctmp(2,jd).
compute modvals=desctmp(5:7,:).
compute mnotev=2.
do if (modvals(1,1) < desctmp(3,1)).
compute modvals(1,1)=desctmp(3,1).
compute minwarn=1.
end if.
do if (modvals(3,1) > desctmp(4,1)).
compute modvals(3,1)=desctmp(4,1).
compute maxwarn=1.
end if.
end if.
do if (desctmp(8,1)=1).
compute modvals={desctmp(3,1);desctmp(4,1)}.
compute mnotev=0.
compute minwarn=0.
compute maxwarn=0.
end if.
end if.
end loop
.
compute xsd=desctmp(2,:).
compute xmodvals=modvals.
compute xdich=desctmp(8,1).
compute xmx=cmax(xtmp).
compute xmn=cmin(xtmp).
do if ((mcx > 0) and (xrefvals(1,1) <> 999) and (xmint=1) and (model= 74)).
compute notecode(notes,1) = 36.
compute notes = notes + 1.
end if.
do if (mcx=0).
do if (ncol(xrefvals)>2 and model=74 and xmint=1).
compute errcode(errs,1)=67.
compute errs=errs+1.
compute criterr=1.
end if.
do if ((model=74) and (xmint=1)).
do if ((xrefvals(1,1)=999) and nxvls=1 and xdich=0).
compute errcode(errs,1)=66.
compute errs=errs+1.
compute criterr=1.
end if.
do if ((xrefvals(1,1)=999) and (xdich=1)).
compute xrefvals={xmn,xmx}.
compute xscaling=xrefvals(1,2)-xrefvals(1,1).
end if.
do if (ncol(xrefvals)=1 and xrefvals(1,1) <> 999).
do if (xdich=0).
compute xrefvals={xrefvals,(xrefvals(1,1)+1)}.
compute xscaling=xrefvals(1,2)-xrefvals(1,1).
end if.
do if (xdich=1).
do if ((xrefvals(1,1) <> xmx) and (xrefvals(1,1) <> xmn)).
compute errcode(errs,1)=70.
compute errs=errs+1.
compute criterr=1.
end if.
do if (xrefvals(1,1) = xmx).
compute xrefvals={xrefvals,xmn}.
compute xscaling=xrefvals(1,2)-xrefvals(1,1).
end if.
do if (xrefvals(1,1) = xmn).
compute xrefvals={xrefvals,xmx}.
compute xscaling=xrefvals(1,2)-xrefvals(1,1).
end if.
end if.
end if.
do if (ncol(xrefvals)=2).
compute xscaling=xrefvals(1,2)-xrefvals(1,1).
do if (xdich=1).
compute xreferr=1.
do if (((xrefvals(1,1) = xmx) and (xrefvals(1,2)=xmn)) or ((xrefvals(1,1) = xmn) and (xrefvals(1,2)=xmx))).
compute xreferr=0.
end if.
do if (xreferr=1).
compute errcode(errs,1)=70.
compute errs=errs+1.
compute criterr=1.
end if.
end if.
end if.
end if.
end if.
do if (xmint=1 and model=74 and mcx=0).
compute xmodvals=t(xrefvals).
compute xcontcf=1.
end if.
compute nxpval=nrow(xmodvals).
compute xprobval=xmodvals.
do if (xdich =1 and mcx > 0).
compute mcx=0.
compute errcode(errs,1) = 52.
compute errs = errs + 1.
compute criterr = 1.
end if.
do if (nms > 0).
compute mtmp=dat(:,(nys+nxs+1):(nys+nxs+nms)).

.
compute desctmp=make((8-(4* 0 )),ncol( mtmp ),-999).
loop jd=1 to ncol( mtmp ).
compute descdat= mtmp (:,jd).
compute desctmp(1,jd) = csum(descdat)/nrow(descdat).
compute desctmp(2,jd) = (nrow(descdat)*sscp(descdat))-(t(csum(descdat))*(csum(descdat))).
compute desctmp(2,jd) = sqrt(desctmp(2,jd)/(nrow(descdat)*(nrow(descdat)-1))).
compute desctmp(3,jd)=cmin(descdat).
compute desctmp(4,jd)=cmax(descdat).
do if ( 0 =0).
compute minwarn=0.
compute maxwarn=0.
do if ((desctmp(3,jd)=desctmp(4,jd)) and novar=0).
compute errcode(errs,1)=15.
compute errs=errs+1.
compute criterr=1.
compute novar=1.
end if.
compute tmp=((descdat(:,1)=desctmp(3,jd))+(descdat(:,1)=desctmp(4,jd))).
compute desctmp(8,jd)=(csum(tmp)=nrow(tmp)).
compute tmp = descdat.
compute tmp(GRADE(descdat),:) = descdat.
compute descdat = tmp.
release tmp.
compute decval={.16;.5;.84}.
loop kd=1 to 3.
compute low=trunc(decval(kd,1)*(nrow(descdat)+1)).
compute lowdec=decval(kd,1)*(nrow(descdat)+1)-low.
compute value=descdat(low,1)+(descdat((low+1),1)-descdat(low,1))*lowdec.
compute desctmp((4+kd),jd)=value.
end loop.
compute mnotev=1.
compute modvals=desctmp(5:7,:).
do if (quantile <> 1).
compute desctmp(5,jd)=desctmp(1,jd)-desctmp(2,jd).
compute desctmp(6,jd)=desctmp(1,jd).
compute desctmp(7,jd)=desctmp(1,jd)+desctmp(2,jd).
compute modvals=desctmp(5:7,:).
compute mnotev=2.
do if (modvals(1,1) < desctmp(3,1)).
compute modvals(1,1)=desctmp(3,1).
compute minwarn=1.
end if.
do if (modvals(3,1) > desctmp(4,1)).
compute modvals(3,1)=desctmp(4,1).
compute maxwarn=1.
end if.
end if.
do if (desctmp(8,1)=1).
compute modvals={desctmp(3,1);desctmp(4,1)}.
compute mnotev=0.
compute minwarn=0.
compute maxwarn=0.
end if.
end if.
end loop
.
compute ovsd={desctmp(2,:),ysd}.
compute medmeans=cdeval.
do if ((cdeval(1,1) <> -999) and (ncol(medmeans) <> nms) and (model=74)).
compute errcode(errs,1)=64.
compute errs=errs+1.
compute criterr=1.
end if.
do if ((cdeval(1,1)=-999) and (model=74)).
compute medmeans=desctmp(1,:).
end if.
do if ((cdeval(1,1)<> -999) and (model=74) and (criterr=0)).
compute notecode(notes,1) = 31.
compute notes = notes + 1.
end if.
do if ((rsum(desctmp(8,:))>0) and (mdichok <> 1)).
compute errcode(errs,1)=43.
compute errs=errs+1.
compute criterr=1.
end if.
compute mmodvals=modvals.
compute mprobval=mmodvals.
end if.
do if (nws > 0).
compute wtmp=dat(:,(nys+nxs+nms+1):(nys+nxs+nms+nws)).

.
compute desctmp=make((8-(4* 0 )),ncol( wtmp ),-999).
loop jd=1 to ncol( wtmp ).
compute descdat= wtmp (:,jd).
compute desctmp(1,jd) = csum(descdat)/nrow(descdat).
compute desctmp(2,jd) = (nrow(descdat)*sscp(descdat))-(t(csum(descdat))*(csum(descdat))).
compute desctmp(2,jd) = sqrt(desctmp(2,jd)/(nrow(descdat)*(nrow(descdat)-1))).
compute desctmp(3,jd)=cmin(descdat).
compute desctmp(4,jd)=cmax(descdat).
do if ( 0 =0).
compute minwarn=0.
compute maxwarn=0.
do if ((desctmp(3,jd)=desctmp(4,jd)) and novar=0).
compute errcode(errs,1)=15.
compute errs=errs+1.
compute criterr=1.
compute novar=1.
end if.
compute tmp=((descdat(:,1)=desctmp(3,jd))+(descdat(:,1)=desctmp(4,jd))).
compute desctmp(8,jd)=(csum(tmp)=nrow(tmp)).
compute tmp = descdat.
compute tmp(GRADE(descdat),:) = descdat.
compute descdat = tmp.
release tmp.
compute decval={.16;.5;.84}.
loop kd=1 to 3.
compute low=trunc(decval(kd,1)*(nrow(descdat)+1)).
compute lowdec=decval(kd,1)*(nrow(descdat)+1)-low.
compute value=descdat(low,1)+(descdat((low+1),1)-descdat(low,1))*lowdec.
compute desctmp((4+kd),jd)=value.
end loop.
compute mnotev=1.
compute modvals=desctmp(5:7,:).
do if (quantile <> 1).
compute desctmp(5,jd)=desctmp(1,jd)-desctmp(2,jd).
compute desctmp(6,jd)=desctmp(1,jd).
compute desctmp(7,jd)=desctmp(1,jd)+desctmp(2,jd).
compute modvals=desctmp(5:7,:).
compute mnotev=2.
do if (modvals(1,1) < desctmp(3,1)).
compute modvals(1,1)=desctmp(3,1).
compute minwarn=1.
end if.
do if (modvals(3,1) > desctmp(4,1)).
compute modvals(3,1)=desctmp(4,1).
compute maxwarn=1.
end if.
end if.
do if (desctmp(8,1)=1).
compute modvals={desctmp(3,1);desctmp(4,1)}.
compute mnotev=0.
compute minwarn=0.
compute maxwarn=0.
end if.
end if.
end loop
.
compute wmodvals=modvals.
compute wdich=desctmp(8,1).
do if (wdich =1 and mcw > 0).
compute mcw=0.
compute errcode(errs,1) = 52.
compute errs = errs + 1.
compute criterr = 1.
end if.
compute wmin=desctmp(3,1).
compute wmax=desctmp(4,1).
compute minwwarn=minwarn.
compute maxwwarn=maxwarn.
compute wnotev=mnotev.
compute wmodval={ 999 }.
do if (xmint=1 and model=74 and mcx=0).
compute wmodval=xrefvals.
end if.
compute nwcontr=ncol(wmodval).
do if (wmodval(1,1) <> 999).
compute wmodvals=wmodval(1,1).
compute wmodcust=1.
do if (nwcontr > 1).
compute wmodvals=t(wmodval).
end if.
compute minwwarn=0.
compute maxwwarn=0.
compute wnotev=0.
end if.
compute wprobval=wmodvals.
compute nwpval=nrow(wmodvals).
end if.
do if (nzs > 0).
compute ztmp=dat(:,(nys+nxs+nms+nws+1):(nys+nxs+nms+nws+nzs)).

.
compute desctmp=make((8-(4* 0 )),ncol( ztmp ),-999).
loop jd=1 to ncol( ztmp ).
compute descdat= ztmp (:,jd).
compute desctmp(1,jd) = csum(descdat)/nrow(descdat).
compute desctmp(2,jd) = (nrow(descdat)*sscp(descdat))-(t(csum(descdat))*(csum(descdat))).
compute desctmp(2,jd) = sqrt(desctmp(2,jd)/(nrow(descdat)*(nrow(descdat)-1))).
compute desctmp(3,jd)=cmin(descdat).
compute desctmp(4,jd)=cmax(descdat).
do if ( 0 =0).
compute minwarn=0.
compute maxwarn=0.
do if ((desctmp(3,jd)=desctmp(4,jd)) and novar=0).
compute errcode(errs,1)=15.
compute errs=errs+1.
compute criterr=1.
compute novar=1.
end if.
compute tmp=((descdat(:,1)=desctmp(3,jd))+(descdat(:,1)=desctmp(4,jd))).
compute desctmp(8,jd)=(csum(tmp)=nrow(tmp)).
compute tmp = descdat.
compute tmp(GRADE(descdat),:) = descdat.
compute descdat = tmp.
release tmp.
compute decval={.16;.5;.84}.
loop kd=1 to 3.
compute low=trunc(decval(kd,1)*(nrow(descdat)+1)).
compute lowdec=decval(kd,1)*(nrow(descdat)+1)-low.
compute value=descdat(low,1)+(descdat((low+1),1)-descdat(low,1))*lowdec.
compute desctmp((4+kd),jd)=value.
end loop.
compute mnotev=1.
compute modvals=desctmp(5:7,:).
do if (quantile <> 1).
compute desctmp(5,jd)=desctmp(1,jd)-desctmp(2,jd).
compute desctmp(6,jd)=desctmp(1,jd).
compute desctmp(7,jd)=desctmp(1,jd)+desctmp(2,jd).
compute modvals=desctmp(5:7,:).
compute mnotev=2.
do if (modvals(1,1) < desctmp(3,1)).
compute modvals(1,1)=desctmp(3,1).
compute minwarn=1.
end if.
do if (modvals(3,1) > desctmp(4,1)).
compute modvals(3,1)=desctmp(4,1).
compute maxwarn=1.
end if.
end if.
do if (desctmp(8,1)=1).
compute modvals={desctmp(3,1);desctmp(4,1)}.
compute mnotev=0.
compute minwarn=0.
compute maxwarn=0.
end if.
end if.
end loop
.
compute zmodvals=modvals.
compute zdich=desctmp(8,1).
do if (zdich =1 and mcz > 0).
compute mcz=0.
compute errcode(errs,1) = 52.
compute errs = errs + 1.
compute criterr = 1.
end if.
compute zmin=desctmp(3,1).
compute zmax=desctmp(4,1).
compute minzwarn=minwarn.
compute maxzwarn=maxwarn.
compute znotev=mnotev.
compute zmodval={ 999 }.
compute nzcontr=ncol(zmodval).
do if (zmodval(1,1) <> 999).
compute zmodvals=zmodval(1,1).
compute zmodcust=1.
do if (nzcontr > 1).
compute zmodvals=t(zmodval).
end if.
compute minzwarn=0.
compute maxzwarn=0.
compute znotev=0.
end if.
compute zprobval=zmodvals.
compute nzpval=nrow(zmodvals).
end if.
do if (ncs > 0).
compute ctmp=dat(:,(nys+nxs+nms+nws+nzs+1):(nys+nxs+nms+nws+nzs+ncs)).

.
compute desctmp=make((8-(4* 0 )),ncol( ctmp ),-999).
loop jd=1 to ncol( ctmp ).
compute descdat= ctmp (:,jd).
compute desctmp(1,jd) = csum(descdat)/nrow(descdat).
compute desctmp(2,jd) = (nrow(descdat)*sscp(descdat))-(t(csum(descdat))*(csum(descdat))).
compute desctmp(2,jd) = sqrt(desctmp(2,jd)/(nrow(descdat)*(nrow(descdat)-1))).
compute desctmp(3,jd)=cmin(descdat).
compute desctmp(4,jd)=cmax(descdat).
do if ( 0 =0).
compute minwarn=0.
compute maxwarn=0.
do if ((desctmp(3,jd)=desctmp(4,jd)) and novar=0).
compute errcode(errs,1)=15.
compute errs=errs+1.
compute criterr=1.
compute novar=1.
end if.
compute tmp=((descdat(:,1)=desctmp(3,jd))+(descdat(:,1)=desctmp(4,jd))).
compute desctmp(8,jd)=(csum(tmp)=nrow(tmp)).
compute tmp = descdat.
compute tmp(GRADE(descdat),:) = descdat.
compute descdat = tmp.
release tmp.
compute decval={.16;.5;.84}.
loop kd=1 to 3.
compute low=trunc(decval(kd,1)*(nrow(descdat)+1)).
compute lowdec=decval(kd,1)*(nrow(descdat)+1)-low.
compute value=descdat(low,1)+(descdat((low+1),1)-descdat(low,1))*lowdec.
compute desctmp((4+kd),jd)=value.
end loop.
compute mnotev=1.
compute modvals=desctmp(5:7,:).
do if (quantile <> 1).
compute desctmp(5,jd)=desctmp(1,jd)-desctmp(2,jd).
compute desctmp(6,jd)=desctmp(1,jd).
compute desctmp(7,jd)=desctmp(1,jd)+desctmp(2,jd).
compute modvals=desctmp(5:7,:).
compute mnotev=2.
do if (modvals(1,1) < desctmp(3,1)).
compute modvals(1,1)=desctmp(3,1).
compute minwarn=1.
end if.
do if (modvals(3,1) > desctmp(4,1)).
compute modvals(3,1)=desctmp(4,1).
compute maxwarn=1.
end if.
end if.
do if (desctmp(8,1)=1).
compute modvals={desctmp(3,1);desctmp(4,1)}.
compute mnotev=0.
compute minwarn=0.
compute maxwarn=0.
end if.
end if.
end loop
.
compute covmeans=desctmp(1,:).
compute coval={ -999 }.
do if ((coval(1,1) <> -999) and (ncol(coval) <> ncs) and (model=74)).
compute errcode(errs,1)=69.
compute errs=errs+1.
compute criterr=1.
end if.
do if ((coval(1,1)<> -999) and (criterr=0) and (model=74)).
compute notecode(notes,1) = 35.
compute notes = notes + 1.
compute cuscoval=1.
end if.
end if.
compute n=nrow(ytmp).
compute ones=make(n,1,1).
do if (nws > 0 and mcw > 0).
compute tmp={rownum,wtmp(:,1)}.

.
compute dd= tmp.
compute temp = dd.
compute temp(GRADE(dd(:,2)),:) = dd.
compute dd = temp.
compute dummy = design(dd(:,2)).
compute nvls = ncol(dummy).
compute nnvls = csum(dummy).
compute mnvls = cmin(t(nnvls)).
compute conmat1=1.
do if (mnvls < 2).
compute errcode(errs,1) = 5.
compute errs = errs + 1.
compute criterr = 1.
end if.
do if (nvls > 9).
compute errcode(errs,1) = 4.
compute errs = errs+1.
compute criterr = 1.
end if.
do if (criterr = 0).
compute dumok = 1.
compute nnvls=make(nvls,1,0).
compute nnvls(1,1)=dd(1,2).
compute temp = 2.
loop i = 2 to n.
do if (dd(i,2) <> nnvls((temp-1),1)).
compute nnvls(temp,1)=dd(i,2).
compute temp = temp+1.
end if.
end loop.
do if ( mcw > 0).
compute x = dummy(:,2:ncol(dummy)).
compute nx = ncol(x).
compute minus1 = make(1,ncol(x),-1).
compute xdes=make((nx+1),3,0).
compute xdes(1,1)=dd(1,2).
compute xdes(1,2)=1.
compute temp = 2.
loop k = 2 to n.
do if (dd(k,2) <> dd((k-1),2)).
compute xdes(temp,2) = k.
compute xdes(temp,1) = dd(k,2).
compute xdes((temp-1),3) = k-1.
compute temp=temp+1.
end if.
end loop.
compute xdes((temp-1),3)=n.
compute xdes = {xdes, (xdes(:,3)-xdes(:,2)+1)}.
do if ( mcw = 4).
loop k = 1 to n.
do if (rsum(x(k,:)) = 0).
compute x(k,:) = minus1.
end if.
end loop.
end if.
do if ( mcw = 2 or mcw = 3 or mcw =5).
loop k = 1 to n.
do if (rsum(x(k,:)) > 0).
loop i = 1 to ncol(x).
do if (x(k,i) = 0).
compute x(k,i) = 1.
else.
break.
end if.
end loop.
end if.
end loop.
do if ( mcw = 3).
compute conmat1={-8,1,1,1,1,1,1,1,1; 0,-7,1,1,1,1,1,1,1; 0,0,-6,1,1,1,1,1,1; 0,0,0,-5,1,1,1,1,1; 0,0,0,0,-4,1,1,1,1; 0,0,0,0,0,-3,1,1,1; 0,0,0,0,0,0,-2,1,1; 0,0,0,0,0,0,0,-1,1}.
loop i = 1 to 8.
compute conmat1(i,:)=conmat1(i,:)/(10-i).
end loop.
compute conmat1=t(conmat1((10-nvls):8,(10-nvls):9)).
loop k=1 to n.
compute x(k,:)=conmat1((rsum(x(k,:))+1),:).
end loop.
end if.
end if.
do if ( mcw = 5).
compute custcode={ -999 }.
do if (ncol(custcode) <> (nvls*(nvls-1))).
compute errcode(errs,1) = (37+ 2 ).
compute errs = errs + 1.
compute criterr = 1.
end if.
do if (ncol(custcode) = (nvls*(nvls-1))).
compute conmat1=make(nvls,(nvls-1),0).
compute cnt=1.
loop i = 1 to nvls.
loop k = 1 to (nvls-1).
compute conmat1(i,k)=custcode(1,cnt).
compute cnt=cnt+1.
end loop.
end loop.
loop k=1 to n.
compute x(k,:)=conmat1((rsum(x(k,:))+1),:).
end loop.
end if.
end if.
compute xskip = 1.
compute dummat = make((nx+1),nx,0).
compute dummat((2:nrow(dummat)),:)=ident(nx).
do if ( mcw = 4).
compute dummat(1,:) = minus1.
end if.
do if ( mcw = 2).
loop i = 2 to nrow(dummat).
loop j = 1 to (i-1).
compute dummat(i,j) = 1.
end loop.
end loop.
end if.
do if ( mcw = 3).
compute dummat=conmat1.
end if.
do if ( mcw = 5 and criterr=0).
compute dummat=conmat1.
end if.
compute dummat={nnvls, dummat}.
compute x={dd(:,1),x}.
compute temp = x.
compute temp(GRADE(x(:,1)),:) = x.
compute x = temp.
release conmat1,temp,dd,xskip,xdes,dummy.
end if.
end if
.
compute wmodvals=nnvls.
compute nwpval=nrow(wmodvals).
do if (criterr=0).
compute minwwarn=0.
compute maxwwarn=0.
compute wnotev=0.
compute wtmp=x(:,2:ncol(x)).
compute wcatlab={'W1';'W2';'W3';'W4';'W5';'W6';'W7';'W8';'W9'}.
do if (xmint=1).
compute wcatlab={'X1';'X2';'X3';'X4';'X5';'X6';'X7';'X8';'X9'}.
end if.
compute nwvls=nvls-1.
compute mcwok=1.
compute dummatw=dummat.
compute wprobval=dummatw(:,2:ncol(dummatw)).
do if (modcok=1).
compute wcontval=make(2,ncol(wprobval),-999).
compute temp=0.
loop i = 1 to 2.
loop j = 1 to nrow(dummatw).
do if (contvec(i,1)=dummatw(j,1)).
compute wcontval(i,:)=wprobval(j,:).
compute temp=temp+1.
end if.
end loop.
end loop.
do if (temp < 2).
compute notecode(notes,1) = 20.
compute notes = notes + 1.
compute modcok=0.
end if.
end if.
do if ((wmodval(1,1) <> 999) and (xmint <> 1)).
compute notecode(notes,1) = 9.
compute notes = notes + 1.
end if.
release tmp, dummat.
end if.
end if.
do if (nzs > 0 and mcz > 0).
compute tmp={rownum,ztmp(:,1)}.

.
compute dd= tmp.
compute temp = dd.
compute temp(GRADE(dd(:,2)),:) = dd.
compute dd = temp.
compute dummy = design(dd(:,2)).
compute nvls = ncol(dummy).
compute nnvls = csum(dummy).
compute mnvls = cmin(t(nnvls)).
compute conmat1=1.
do if (mnvls < 2).
compute errcode(errs,1) = 5.
compute errs = errs + 1.
compute criterr = 1.
end if.
do if (nvls > 9).
compute errcode(errs,1) = 4.
compute errs = errs+1.
compute criterr = 1.
end if.
do if (criterr = 0).
compute dumok = 1.
compute nnvls=make(nvls,1,0).
compute nnvls(1,1)=dd(1,2).
compute temp = 2.
loop i = 2 to n.
do if (dd(i,2) <> nnvls((temp-1),1)).
compute nnvls(temp,1)=dd(i,2).
compute temp = temp+1.
end if.
end loop.
do if ( mcz > 0).
compute x = dummy(:,2:ncol(dummy)).
compute nx = ncol(x).
compute minus1 = make(1,ncol(x),-1).
compute xdes=make((nx+1),3,0).
compute xdes(1,1)=dd(1,2).
compute xdes(1,2)=1.
compute temp = 2.
loop k = 2 to n.
do if (dd(k,2) <> dd((k-1),2)).
compute xdes(temp,2) = k.
compute xdes(temp,1) = dd(k,2).
compute xdes((temp-1),3) = k-1.
compute temp=temp+1.
end if.
end loop.
compute xdes((temp-1),3)=n.
compute xdes = {xdes, (xdes(:,3)-xdes(:,2)+1)}.
do if ( mcz = 4).
loop k = 1 to n.
do if (rsum(x(k,:)) = 0).
compute x(k,:) = minus1.
end if.
end loop.
end if.
do if ( mcz = 2 or mcz = 3 or mcz =5).
loop k = 1 to n.
do if (rsum(x(k,:)) > 0).
loop i = 1 to ncol(x).
do if (x(k,i) = 0).
compute x(k,i) = 1.
else.
break.
end if.
end loop.
end if.
end loop.
do if ( mcz = 3).
compute conmat1={-8,1,1,1,1,1,1,1,1; 0,-7,1,1,1,1,1,1,1; 0,0,-6,1,1,1,1,1,1; 0,0,0,-5,1,1,1,1,1; 0,0,0,0,-4,1,1,1,1; 0,0,0,0,0,-3,1,1,1; 0,0,0,0,0,0,-2,1,1; 0,0,0,0,0,0,0,-1,1}.
loop i = 1 to 8.
compute conmat1(i,:)=conmat1(i,:)/(10-i).
end loop.
compute conmat1=t(conmat1((10-nvls):8,(10-nvls):9)).
loop k=1 to n.
compute x(k,:)=conmat1((rsum(x(k,:))+1),:).
end loop.
end if.
end if.
do if ( mcz = 5).
compute custcode={ -999 }.
do if (ncol(custcode) <> (nvls*(nvls-1))).
compute errcode(errs,1) = (37+ 3 ).
compute errs = errs + 1.
compute criterr = 1.
end if.
do if (ncol(custcode) = (nvls*(nvls-1))).
compute conmat1=make(nvls,(nvls-1),0).
compute cnt=1.
loop i = 1 to nvls.
loop k = 1 to (nvls-1).
compute conmat1(i,k)=custcode(1,cnt).
compute cnt=cnt+1.
end loop.
end loop.
loop k=1 to n.
compute x(k,:)=conmat1((rsum(x(k,:))+1),:).
end loop.
end if.
end if.
compute xskip = 1.
compute dummat = make((nx+1),nx,0).
compute dummat((2:nrow(dummat)),:)=ident(nx).
do if ( mcz = 4).
compute dummat(1,:) = minus1.
end if.
do if ( mcz = 2).
loop i = 2 to nrow(dummat).
loop j = 1 to (i-1).
compute dummat(i,j) = 1.
end loop.
end loop.
end if.
do if ( mcz = 3).
compute dummat=conmat1.
end if.
do if ( mcz = 5 and criterr=0).
compute dummat=conmat1.
end if.
compute dummat={nnvls, dummat}.
compute x={dd(:,1),x}.
compute temp = x.
compute temp(GRADE(x(:,1)),:) = x.
compute x = temp.
release conmat1,temp,dd,xskip,xdes,dummy.
end if.
end if
.
compute zmodvals=nnvls.
compute nzpval=nrow(zmodvals).
do if (criterr=0).
compute minzwarn=0.
compute maxzwarn=0.
compute znotev=0.
compute ztmp=x(:,2:ncol(x)).
compute zcatlab={'Z1';'Z2';'Z3';'Z4';'Z5';'Z6';'Z7';'Z8';'Z9'}.
compute nzvls=nvls-1.
compute mczok=1.
compute dummatz=dummat.
compute zprobval=dummatz(:,2:ncol(dummatz)).
do if (modcok=1).
compute zcontval=make(2,ncol(zprobval),-999).
compute temp=0.
loop i = 1 to 2.
loop j = 1 to nrow(dummatz).
do if (contvec(i,2)=dummatz(j,1)).
compute zcontval(i,:)=zprobval(j,:).
compute temp=temp+1.
end if.
end loop.
end loop.
do if (temp < 2).
compute notecode(notes,1) = 20.
compute notes = notes + 1.
compute modcok=0.
end if.
end if.
do if (zmodval(1,1) <> 999).
compute notecode(notes,1) = 10.
compute notes = notes + 1.
end if.
release tmp, dummat.
end if.
end if.
do if (nxs > 0 and mcx > 0).
compute tmp={rownum,xtmp(:,1)}.

.
compute dd= tmp.
compute temp = dd.
compute temp(GRADE(dd(:,2)),:) = dd.
compute dd = temp.
compute dummy = design(dd(:,2)).
compute nvls = ncol(dummy).
compute nnvls = csum(dummy).
compute mnvls = cmin(t(nnvls)).
compute conmat1=1.
do if (mnvls < 2).
compute errcode(errs,1) = 5.
compute errs = errs + 1.
compute criterr = 1.
end if.
do if (nvls > 9).
compute errcode(errs,1) = 4.
compute errs = errs+1.
compute criterr = 1.
end if.
do if (criterr = 0).
compute dumok = 1.
compute nnvls=make(nvls,1,0).
compute nnvls(1,1)=dd(1,2).
compute temp = 2.
loop i = 2 to n.
do if (dd(i,2) <> nnvls((temp-1),1)).
compute nnvls(temp,1)=dd(i,2).
compute temp = temp+1.
end if.
end loop.
do if ( mcx > 0).
compute x = dummy(:,2:ncol(dummy)).
compute nx = ncol(x).
compute minus1 = make(1,ncol(x),-1).
compute xdes=make((nx+1),3,0).
compute xdes(1,1)=dd(1,2).
compute xdes(1,2)=1.
compute temp = 2.
loop k = 2 to n.
do if (dd(k,2) <> dd((k-1),2)).
compute xdes(temp,2) = k.
compute xdes(temp,1) = dd(k,2).
compute xdes((temp-1),3) = k-1.
compute temp=temp+1.
end if.
end loop.
compute xdes((temp-1),3)=n.
compute xdes = {xdes, (xdes(:,3)-xdes(:,2)+1)}.
do if ( mcx = 4).
loop k = 1 to n.
do if (rsum(x(k,:)) = 0).
compute x(k,:) = minus1.
end if.
end loop.
end if.
do if ( mcx = 2 or mcx = 3 or mcx =5).
loop k = 1 to n.
do if (rsum(x(k,:)) > 0).
loop i = 1 to ncol(x).
do if (x(k,i) = 0).
compute x(k,i) = 1.
else.
break.
end if.
end loop.
end if.
end loop.
do if ( mcx = 3).
compute conmat1={-8,1,1,1,1,1,1,1,1; 0,-7,1,1,1,1,1,1,1; 0,0,-6,1,1,1,1,1,1; 0,0,0,-5,1,1,1,1,1; 0,0,0,0,-4,1,1,1,1; 0,0,0,0,0,-3,1,1,1; 0,0,0,0,0,0,-2,1,1; 0,0,0,0,0,0,0,-1,1}.
loop i = 1 to 8.
compute conmat1(i,:)=conmat1(i,:)/(10-i).
end loop.
compute conmat1=t(conmat1((10-nvls):8,(10-nvls):9)).
loop k=1 to n.
compute x(k,:)=conmat1((rsum(x(k,:))+1),:).
end loop.
end if.
end if.
do if ( mcx = 5).
compute custcode={ -999 }.
do if (ncol(custcode) <> (nvls*(nvls-1))).
compute errcode(errs,1) = (37+ 1 ).
compute errs = errs + 1.
compute criterr = 1.
end if.
do if (ncol(custcode) = (nvls*(nvls-1))).
compute conmat1=make(nvls,(nvls-1),0).
compute cnt=1.
loop i = 1 to nvls.
loop k = 1 to (nvls-1).
compute conmat1(i,k)=custcode(1,cnt).
compute cnt=cnt+1.
end loop.
end loop.
loop k=1 to n.
compute x(k,:)=conmat1((rsum(x(k,:))+1),:).
end loop.
end if.
end if.
compute xskip = 1.
compute dummat = make((nx+1),nx,0).
compute dummat((2:nrow(dummat)),:)=ident(nx).
do if ( mcx = 4).
compute dummat(1,:) = minus1.
end if.
do if ( mcx = 2).
loop i = 2 to nrow(dummat).
loop j = 1 to (i-1).
compute dummat(i,j) = 1.
end loop.
end loop.
end if.
do if ( mcx = 3).
compute dummat=conmat1.
end if.
do if ( mcx = 5 and criterr=0).
compute dummat=conmat1.
end if.
compute dummat={nnvls, dummat}.
compute x={dd(:,1),x}.
compute temp = x.
compute temp(GRADE(x(:,1)),:) = x.
compute x = temp.
release conmat1,temp,dd,xskip,xdes,dummy.
end if.
end if
.
do if (criterr=0).
compute xtmp=x(:,2:ncol(x)).
compute xcatlab={'X1';'X2';'X3';'X4';'X5';'X6';'X7';'X8';'X9'}.
compute nxvls=nvls-1.
compute xdich=(nvls=2).
compute mcxok=1.
compute dummatx=dummat.
compute xmodvals=dummatx(:,1).
compute nxpval=nrow(xmodvals).
release tmp, dummat.
end if.
end if.
compute intlab=make(100,1,' ').


compute intlab( 1 ,1)= 'Int_1'.


compute intlab( 2 ,1)= 'Int_2'.


compute intlab( 3 ,1)= 'Int_3'.


compute intlab( 4 ,1)= 'Int_4'.


compute intlab( 5 ,1)= 'Int_5'.


compute intlab( 6 ,1)= 'Int_6'.


compute intlab( 7 ,1)= 'Int_7'.


compute intlab( 8 ,1)= 'Int_8'.


compute intlab( 9 ,1)= 'Int_9'.


compute intlab( 10 ,1)= 'Int_10'.


compute intlab( 11 ,1)= 'Int_11'.


compute intlab( 12 ,1)= 'Int_12'.


compute intlab( 13 ,1)= 'Int_13'.


compute intlab( 14 ,1)= 'Int_14'.


compute intlab( 15 ,1)= 'Int_15'.


compute intlab( 16 ,1)= 'Int_16'.


compute intlab( 17 ,1)= 'Int_17'.


compute intlab( 18 ,1)= 'Int_18'.


compute intlab( 19 ,1)= 'Int_19'.


compute intlab( 20 ,1)= 'Int_20'.


compute intlab( 21 ,1)= 'Int_21'.


compute intlab( 22 ,1)= 'Int_22'.


compute intlab( 23 ,1)= 'Int_23'.


compute intlab( 24 ,1)= 'Int_24'.


compute intlab( 25 ,1)= 'Int_25'.


compute intlab( 26 ,1)= 'Int_26'.


compute intlab( 27 ,1)= 'Int_27'.


compute intlab( 28 ,1)= 'Int_28'.


compute intlab( 29 ,1)= 'Int_29'.


compute intlab( 30 ,1)= 'Int_30'.


compute intlab( 31 ,1)= 'Int_31'.


compute intlab( 32 ,1)= 'Int_32'.


compute intlab( 33 ,1)= 'Int_33'.


compute intlab( 34 ,1)= 'Int_34'.


compute intlab( 35 ,1)= 'Int_35'.


compute intlab( 36 ,1)= 'Int_36'.


compute intlab( 37 ,1)= 'Int_37'.


compute intlab( 38 ,1)= 'Int_38'.


compute intlab( 39 ,1)= 'Int_39'.


compute intlab( 40 ,1)= 'Int_40'.


compute intlab( 41 ,1)= 'Int_41'.


compute intlab( 42 ,1)= 'Int_42'.


compute intlab( 43 ,1)= 'Int_43'.


compute intlab( 44 ,1)= 'Int_44'.


compute intlab( 45 ,1)= 'Int_45'.


compute intlab( 46 ,1)= 'Int_46'.


compute intlab( 47 ,1)= 'Int_47'.


compute intlab( 48 ,1)= 'Int_48'.


compute intlab( 49 ,1)= 'Int_49'.


compute intlab( 50 ,1)= 'Int_50'.


compute intlab( 51 ,1)= 'Int_51'.


compute intlab( 52 ,1)= 'Int_52'.


compute intlab( 53 ,1)= 'Int_53'.


compute intlab( 54 ,1)= 'Int_54'.


compute intlab( 55 ,1)= 'Int_55'.


compute intlab( 56 ,1)= 'Int_56'.


compute intlab( 57 ,1)= 'Int_57'.


compute intlab( 58 ,1)= 'Int_58'.


compute intlab( 59 ,1)= 'Int_59'.


compute intlab( 60 ,1)= 'Int_60'.


compute intlab( 61 ,1)= 'Int_61'.


compute intlab( 62 ,1)= 'Int_62'.


compute intlab( 63 ,1)= 'Int_63'.


compute intlab( 64 ,1)= 'Int_64'.


compute intlab( 65 ,1)= 'Int_65'.


compute intlab( 66 ,1)= 'Int_66'.


compute intlab( 67 ,1)= 'Int_67'.


compute intlab( 68 ,1)= 'Int_68'.


compute intlab( 69 ,1)= 'Int_69'.


compute intlab( 70 ,1)= 'Int_70'.


compute intlab( 71 ,1)= 'Int_71'.


compute intlab( 72 ,1)= 'Int_72'.


compute intlab( 73 ,1)= 'Int_73'.


compute intlab( 74 ,1)= 'Int_74'.


compute intlab( 75 ,1)= 'Int_75'.


compute intlab( 76 ,1)= 'Int_76'.


compute intlab( 77 ,1)= 'Int_77'.


compute intlab( 78 ,1)= 'Int_78'.


compute intlab( 79 ,1)= 'Int_79'.


compute intlab( 80 ,1)= 'Int_80'.


compute intlab( 81 ,1)= 'Int_81'.


compute intlab( 82 ,1)= 'Int_82'.


compute intlab( 83 ,1)= 'Int_83'.


compute intlab( 84 ,1)= 'Int_84'.


compute intlab( 85 ,1)= 'Int_85'.


compute intlab( 86 ,1)= 'Int_86'.


compute intlab( 87 ,1)= 'Int_87'.


compute intlab( 88 ,1)= 'Int_88'.


compute intlab( 89 ,1)= 'Int_89'.


compute intlab( 90 ,1)= 'Int_90'.


compute intlab( 91 ,1)= 'Int_91'.


compute intlab( 92 ,1)= 'Int_92'.


compute intlab( 93 ,1)= 'Int_93'.


compute intlab( 94 ,1)= 'Int_94'.


compute intlab( 95 ,1)= 'Int_95'.


compute intlab( 96 ,1)= 'Int_96'.


compute intlab( 97 ,1)= 'Int_97'.


compute intlab( 98 ,1)= 'Int_98'.


compute intlab( 99 ,1)= 'Int_99'.


compute intlab( 100 ,1)= 'Int_100'.

compute bcmat=make(needed,needed,0).
compute wcmat=make(needed,needed,0).
compute zcmat=make(needed,needed,0).
compute wzcmat=make(needed,needed,0).
compute wsum=0.
compute zsum=0.
compute wzsum=0.
end if.
end if.
do if (criterr = 0 and model <> 999).
compute modelmat= {1,0,0,0,0,0,0,1,0,0;2,0,0,0,0,0,0,1,1,0;3,0,0,0,0,0,0,1,1,1;4,0,0,0,0,0,0,0,0,0; 5,0,0,0,0,0,0,1,0,0;6,0,0,0,0,0,0,0,0,0;7,1,0,0,0,0,0,0,0,0;8,1,0,0,0,0,0,1,0,0; 9,1,1,0,0,0,0,0,0,0;10,1,1,0,0,0,0,1,1,0;11,1,1,1,0,0,0,0,0,0;12,1,1,1,0,0,0,1,1,1; 13,1,1,1,0,0,0,1,0,0;14,0,0,0,1,0,0,0,0,0;15,0,0,0,1,0,0,1,0,0;16,0,0,0,1,1,0,0,0,0; 17,0,0,0,1,1,0,1,1,0;18,0,0,0,1,1,1,0,0,0;19,0,0,0,1,1,1,1,1,1;20,0,0,0,1,1,1,1,0,0; 21,1,0,0,0,1,0,0,0,0;22,1,0,0,0,1,0,1,0,0;23,0,0,0,0,0,0,0,0,0;24,0,0,0,0,0,0,0,0,0; 25,0,0,0,0,0,0,0,0,0;26,0,0,0,0,0,0,0,0,0;27,0,0,0,0,0,0,0,0,0;28,1,0,0,0,1,0,0,1,0; 29,1,0,0,0,1,0,1,1,0;30,0,0,0,0,0,0,0,0,0;31,0,0,0,0,0,0,0,0,0;32,0,0,0,0,0,0,0,0,0; 33,0,0,0,0,0,0,0,0,0;34,0,0,0,0,0,0,0,0,0;35,0,0,0,0,0,0,0,0,0;36,0,0,0,0,0,0,0,0,0; 37,0,0,0,0,0,0,0,0,0;38,0,0,0,0,0,0,0,0,0;39,0,0,0,0,0,0,0,0,0;40,0,0,0,0,0,0,0,0,0; 41,0,0,0,0,0,0,0,0,0;42,0,0,0,0,0,0,0,0,0;43,0,0,0,0,0,0,0,0,0;44,0,0,0,0,0,0,0,0,0; 45,0,0,0,0,0,0,0,0,0;46,0,0,0,0,0,0,0,0,0;47,0,0,0,0,0,0,0,0,0;48,0,0,0,0,0,0,0

 ,0,0; 49,0,0,0,0,0,0,0,0,0;50,0,0,0,0,0,0,0,0,0;51,0,0,0,0,0,0,0,0,0;52,0,0,0,0,0,0,0,0,0; 53,0,0,0,0,0,0,0,0,0;54,0,0,0,0,0,0,0,0,0;55,0,0,0,0,0,0,0,0,0;56,0,0,0,0,0,0,0,0,0; 57,0,0,0,0,0,0,0,0,0;58,1,0,0,1,0,0,0,0,0;59,1,0,0,1,0,0,1,0,0;60,1,1,0,1,0,0,0,0,0; 61,1,1,0,1,0,0,1,0,0;62,1,1,0,1,0,0,0,1,0;63,1,1,0,1,0,0,1,1,0;64,1,0,0,1,1,0,0,0,0; 65,1,0,0,1,1,0,1,0,0;66,1,0,0,1,1,0,0,1,0;67,1,0,0,1,1,0,1,1,0;68,1,1,1,1,0,0,0,0,0; 69,1,1,1,1,0,0,1,1,1;70,1,0,0,1,1,1,0,0,0;71,1,0,0,1,1,1,1,1,1;72,1,1,1,1,1,1,0,0,0; 73,1,1,1,1,1,1,1,1,1;74,0,0,0,1,0,0,0,0,0;75,1,1,0,1,1,0,0,0,0;76,1,1,0,1,1,0,1,1,0; 77,0,0,0,0,0,0,0,0,0;78,0,0,0,0,0,0,0,0,0;79,0,0,0,0,0,0,0,0,0;80,0,0,0,0,0,0,0,0,0; 81,0,0,0,0,0,0,0,0,0;82,0,0,0,0,0,0,0,0,0;83,1,0,0,0,0,0,0,0,0;84,1,0,0,0,0,0,0,0,0; 85,1,0,0,0,0,0,1,0,0;86,1,0,0,0,0,0,1,0,0;87,0,0,0,1,0,0,0,0,0;88,0,0,0,1,0,0,0,0,0; 89,0,0,0,1,0,0,1,0,0;90,0,0,0,1,0,0,1,0,0;91,0,0,0,0,0,0,0,0,0;92,1,0,0,1,0,0,1,0,0}.
do if (model > 0).
compute tmp=modelmat(model,2:ncol(modelmat)).
end if.
do if (model=0).
compute tmp={0,0,0,0,0,0,0,0,0}.
end if.
do if (model < 4).
compute bcmat((nxs+1),1)=1.
end if.
do if ((model > 3) and (model <> 6)).
compute bcmat((nxs+1):(nxs+nms),1)=onem.
compute bcmat(nrow(bcmat),(nxs+1):(nxs+nms))=t(onem).
compute bcmat(nrow(bcmat),1)=1.
end if.
do if ((model = 6) or (model > 82 and model < 93)).
loop j = 2 to nrow(bcmat).
loop i = 1 to (j-1).
compute bcmat(j,i)=1.
end loop.
end loop.
end if.
do if (model = 80).
loop i = 1 to nms.
compute bcmat((nrow(bcmat)-1),i)=1.
end loop.
end if.
do if (model = 81).
loop j = 3 to nrow(bcmat).
compute bcmat(j,2)=1.
end loop.
end if.
do if (model = 82).
compute bcmat(3,2)=1.
compute bcmat(5,4)=1.
end if.
do if (tmp(1,1)=1).
compute wcmat((nxs+1):(nxs+nms),1)=onem.
compute wprod=1.
compute xprod=1.
do if (model = 83 or model = 86).
compute onemsx=onem.
loop i = 1 to (nms-1).
compute onemsx(i+1,1)=0.
end loop.
compute wcmat((nxs+1):(nxs+nms),1)=onemsx.
end if.
end if.
do if (tmp(1,4)=1).
compute wcmat(nrow(wcmat),(nxs+1):(nxs+nms))=t(onem).
compute wprod=1.
do if (model = 87 or model = 90).
compute onemsx=onem.
loop i = 1 to (nms-1).
compute onemsx(i,1)=0.
end loop.
compute wcmat(nrow(wcmat),(nxs+1):(nxs+nms))=t(onemsx).
end if.
end if.
do if (tmp(1,7)=1).
compute wcmat(nrow(wcmat),1)=1.
compute wprod=1.
compute xprod=1.
end if.
do if (tmp(1,2)=1).
compute zcmat((nxs+1):(nxs+nms),1)=onem.
compute zprod=1.
compute xprod=1.
end if.
do if (tmp(1,5)=1).
compute zcmat(nrow(zcmat),(nxs+1):(nxs+nms))=t(onem).
compute zprod=1.
end if.
do if (tmp(1,8)=1).
compute zcmat(nrow(zcmat),1)=1.
compute zprod=1.
compute xprod=1.
end if.
do if (tmp(1,3)=1).
compute wzcmat((nxs+1):(nxs+nms),1)=onem.
compute xprod=1.
compute wprod=1.
compute zprod=1.
end if.
do if (tmp(1,6)=1).
compute wzcmat(nrow(wzcmat),(nxs+1):(nxs+nms))=t(onem).
compute zprod=1.
compute wprod=1.
end if.
do if (tmp(1,9)=1).
compute wzcmat(nrow(wzcmat),1)=1.
compute xprod=1.
compute wprod=1.
compute zprod=1.
end if.
do if (model = 91 or model = 92).
loop j = 1 to (nms-1).
loop i = 1 to j.
compute wcmat((nxs+1+j),(nxs+i))=1.
end loop.
end loop.
end if.
do if (nms < 0).
loop i = 1 to nms.
compute tmp=csum(wcmat(:,(1+i)))+csum(zcmat(:,(1+i)))+csum(wzcmat(:,(1+i))).
compute mprod(1,i)=(tmp>0).
end loop.
end if.
end if.
do if (ncs > 0).
compute ccmat=make((nms+nys),ncs,1).
compute ccmatoff=ccmat.
do if (covmy=1).
compute ccmat(nrow(ccmat),:)=make(1,ncs,0).
end if.
do if (covmy=2).
compute ccmat(1:nms,:)=make(nms,ncs,0).
end if.
do if (cmatrix(1,1) <> -999).
do if (ncol(cmatrix) <> ((nms+nys)*ncs)).
compute errcode(errs,1)=29.
compute errs=errs+1.
compute criterr=1.
end if.
do if (criterr = 0).
compute tmp=1.
loop i = 1 to (nms+nys).
loop j = 1 to ncs.
compute ccmat(i,j)=1-(cmatrix(1,tmp) = 0).
compute tmp=tmp+1.
end loop.
end loop.
do if (rsum((csum(ccmat)=0)) <> 0).
compute errcode(errs,1)=30.
compute errs=errs+1.
compute criterr=1.
end if.
end if.
do if (covmy <> 0).
compute notecode(notes,1)=1.
compute notes=notes+1.
end if.
end if.
do if (xmint=1 and (covmy > 0 or (csum(rsum(ccmat)) <> ((nms+nys)*ncs)))).
compute errcode(errs,1)=68.
compute errs=errs+1.
compute criterr=1.
end if.
end if.
do if (criterr=0).
compute needed=needed*(needed-1)/2.
compute nopath=0.
do if (bmatrix(1,1) <> -999).
compute tmp=1.
do if ((ncol(bmatrix) <> needed) or (csum(rsum(bmatrix))=0)).
compute errcode(errs,1)=16.
compute errs=errs+1.
compute criterr=1.
else.
loop i = 2 to nrow(bcmat).
loop j = 1 to (i-1).
compute bcmat(i,j)=1-(bmatrix(1,tmp) = 0).
compute tmp=tmp+1.
end loop.
end loop.
end if.
do if ((csum(bcmat(:,1))=0) and criterr=0).
compute errcode(errs,1)=22.
compute errs=errs+1.
compute criterr=1.
end if.
do if ((rsum(bcmat(nrow(bcmat),:))=0) and criterr=0).
compute errcode(errs,1)=23.
compute errs=errs+1.
compute criterr=1.
end if.
compute dm=0.
do if (nms > 0).
loop i = 1 to nms.
do if (((rsum(bcmat((nxs+i),:)) = 0) or (csum(bcmat(:,(nxs+i))) = 0)) and (dm=0) and (criterr=0)).
compute errcode(errs,1)=26.
compute errs=errs+1.
compute criterr=1.
compute dm=1.
end if.
end loop.
end if.
release dm.
end if.
end if.
do if (criterr=0).
do if (wmatrix(1,1) <> -999).
compute tmp=1.
do if (ncol(wmatrix) <> needed).
compute errcode(errs,1)=17.
compute errs=errs+1.
compute criterr=1.
else.
compute modelvar(1,1)='CUSTOM'.
loop i = 2 to nrow(wcmat).
loop j = 1 to (i-1).
compute wcmat(i,j)=1-(wmatrix(1,tmp) = 0).
do if ((wcmat(i,j)=1) and (bcmat(i,j)=0) and (nopath=0)).
compute errcode(errs,1)=20.
compute errs=errs+1.
compute criterr=1.
compute nopath=1.
end if.
compute tmp=tmp+1.
end loop.
end loop.
end if.
end if.
do if (zmatrix(1,1) <> -999).
compute tmp=1.
do if (ncol(zmatrix) <> needed).
compute errcode(errs,1)=18.
compute errs=errs+1.
compute criterr=1.
else.
compute modelvar(1,1)='CUSTOM'.
do if (csum(rsum(wcmat))=0 and model=999).
compute errcode(errs,1)=21.
compute errs=errs+1.
compute criterr=1.
end if.
loop i = 2 to nrow(zcmat).
loop j = 1 to (i-1).
compute zcmat(i,j)=1-(zmatrix(1,tmp) = 0).
do if ((zcmat(i,j)=1) and (bcmat(i,j)=0) and (nopath=0)).
compute errcode(errs,1)=20.
compute errs=errs+1.
compute criterr=1.
compute nopath=1.
end if.
compute tmp=tmp+1.
end loop.
end loop.
end if.
end if.
compute tmp=1.
do if (wzmatrix(1,1) <> -999).
do if (ncol(wzmatrix) <> needed).
compute errcode(errs,1)=19.
compute errs=errs+1.
compute criterr=1.
end if.
compute modelvar(1,1)='CUSTOM'.
end if.
do if (criterr=0).
loop i = 2 to nrow(wzcmat).
loop j = 1 to (i-1).
do if (wzmatrix(1,1) <> -999).
compute wzcmat(i,j)=1-(wzmatrix(1,tmp) = 0).
end if.
do if (wzcmat(i,j)=1).
compute wcmat(i,j)=1.
compute zcmat(i,j)=1.
end if.
do if ((wzcmat(i,j)=1) and (bcmat(i,j)=0) and (nopath=0)).
compute errcode(errs,1)=20.
compute errs=errs+1.
compute criterr=1.
compute nopath=1.
end if.
compute tmp=tmp+1.
end loop.
end loop.
end if.
end if.
do if (criterr=0).
compute xprod=csum(wcmat(:,1))+csum(zcmat(:,1))+csum(wzcmat(:,1)).
compute xprod=(xprod > 0).
compute wsum=csum(rsum(wcmat)).
compute wprod=(wsum > 0).
do if (nms > 0).
loop i = 1 to nms.
compute tmp=csum(wcmat(:,(1+i)))+csum(zcmat(:,(1+i)))+csum(wzcmat(:,(1+i))).
compute mprod(1,i)=(tmp>0).
end loop.
end if.
do if ((wsum > 0) and (w = 'xxxxx')).
compute errcode(errs,1)=11.
compute errs=errs+1.
compute criterr=1.
end if.
do if ((wsum = 0) and (w <> 'xxxxx')).
compute errcode(errs,1)=10.
compute errs=errs+1.
compute criterr=1.
end if.
compute zsum=csum(rsum(zcmat)).
compute zprod=(zsum > 0).
do if ((zsum > 0) and (z = 'xxxxx')).
compute errcode(errs,1)=13.
compute errs=errs+1.
compute criterr=1.
end if.
do if ((zsum = 0) and (z <> 'xxxxx')).
compute errcode(errs,1)=12.
compute errs=errs+1.
compute criterr=1.
end if.
do if ((zsum > 0) and (wsum = 0)).
compute errcode(errs,1)=35.
compute errs=errs+1.
compute criterr=1.
end if.
end if.
do if (criterr=0 and nms > 1).
compute serchk=bcmat(2:(nrow(bcmat)-1),2:ncol(bcmat)).
do if (csum(rsum(serchk))) > 0.
compute serial=1.
do if (nms > 6).
compute errcode(errs,1)=36.
compute errs=errs+1.
compute criterr=1.
end if.
end if.
end if.
do if (center > 0 and criterr=0).
compute centvar={' '}.
do if (criterr=0).
do if ((center = 1) or (center = 2 and wdich = 0)).
do if (wprod=1 and mcwok=0 and nwpval > 0).
loop i = 1 to nws.
compute wtmp(:,i)=wtmp(:,i)-(csum(wtmp(:,i))/n).
compute centvar={centvar,wnames(1,i)}.
end loop.

.
compute desctmp=make((8-(4* wmodcust )),ncol( wtmp ),-999).
loop jd=1 to ncol( wtmp ).
compute descdat= wtmp (:,jd).
compute desctmp(1,jd) = csum(descdat)/nrow(descdat).
compute desctmp(2,jd) = (nrow(descdat)*sscp(descdat))-(t(csum(descdat))*(csum(descdat))).
compute desctmp(2,jd) = sqrt(desctmp(2,jd)/(nrow(descdat)*(nrow(descdat)-1))).
compute desctmp(3,jd)=cmin(descdat).
compute desctmp(4,jd)=cmax(descdat).
do if ( wmodcust =0).
compute minwarn=0.
compute maxwarn=0.
do if ((desctmp(3,jd)=desctmp(4,jd)) and novar=0).
compute errcode(errs,1)=15.
compute errs=errs+1.
compute criterr=1.
compute novar=1.
end if.
compute tmp=((descdat(:,1)=desctmp(3,jd))+(descdat(:,1)=desctmp(4,jd))).
compute desctmp(8,jd)=(csum(tmp)=nrow(tmp)).
compute tmp = descdat.
compute tmp(GRADE(descdat),:) = descdat.
compute descdat = tmp.
release tmp.
compute decval={.16;.5;.84}.
loop kd=1 to 3.
compute low=trunc(decval(kd,1)*(nrow(descdat)+1)).
compute lowdec=decval(kd,1)*(nrow(descdat)+1)-low.
compute value=descdat(low,1)+(descdat((low+1),1)-descdat(low,1))*lowdec.
compute desctmp((4+kd),jd)=value.
end loop.
compute mnotev=1.
compute modvals=desctmp(5:7,:).
do if (quantile <> 1).
compute desctmp(5,jd)=desctmp(1,jd)-desctmp(2,jd).
compute desctmp(6,jd)=desctmp(1,jd).
compute desctmp(7,jd)=desctmp(1,jd)+desctmp(2,jd).
compute modvals=desctmp(5:7,:).
compute mnotev=2.
do if (modvals(1,1) < desctmp(3,1)).
compute modvals(1,1)=desctmp(3,1).
compute minwarn=1.
end if.
do if (modvals(3,1) > desctmp(4,1)).
compute modvals(3,1)=desctmp(4,1).
compute maxwarn=1.
end if.
end if.
do if (desctmp(8,1)=1).
compute modvals={desctmp(3,1);desctmp(4,1)}.
compute mnotev=0.
compute minwarn=0.
compute maxwarn=0.
end if.
end if.
end loop
.
compute wmin=desctmp(3,1).
compute wmax=desctmp(4,1).
do if (wmodcust=0).
compute wmodvals=modvals.
compute wprobval=wmodvals.
end if.
end if.
end if.
do if ((center = 1) or (center = 2 and zdich = 0)).
do if (zprod=1 and mczok=0 and nzpval > 0).
loop i = 1 to nzs.
compute ztmp(:,i)=ztmp(:,i)-(csum(ztmp(:,i))/n).
compute centvar={centvar,znames(1,i)}.
end loop.

.
compute desctmp=make((8-(4* zmodcust )),ncol( ztmp ),-999).
loop jd=1 to ncol( ztmp ).
compute descdat= ztmp (:,jd).
compute desctmp(1,jd) = csum(descdat)/nrow(descdat).
compute desctmp(2,jd) = (nrow(descdat)*sscp(descdat))-(t(csum(descdat))*(csum(descdat))).
compute desctmp(2,jd) = sqrt(desctmp(2,jd)/(nrow(descdat)*(nrow(descdat)-1))).
compute desctmp(3,jd)=cmin(descdat).
compute desctmp(4,jd)=cmax(descdat).
do if ( zmodcust =0).
compute minwarn=0.
compute maxwarn=0.
do if ((desctmp(3,jd)=desctmp(4,jd)) and novar=0).
compute errcode(errs,1)=15.
compute errs=errs+1.
compute criterr=1.
compute novar=1.
end if.
compute tmp=((descdat(:,1)=desctmp(3,jd))+(descdat(:,1)=desctmp(4,jd))).
compute desctmp(8,jd)=(csum(tmp)=nrow(tmp)).
compute tmp = descdat.
compute tmp(GRADE(descdat),:) = descdat.
compute descdat = tmp.
release tmp.
compute decval={.16;.5;.84}.
loop kd=1 to 3.
compute low=trunc(decval(kd,1)*(nrow(descdat)+1)).
compute lowdec=decval(kd,1)*(nrow(descdat)+1)-low.
compute value=descdat(low,1)+(descdat((low+1),1)-descdat(low,1))*lowdec.
compute desctmp((4+kd),jd)=value.
end loop.
compute mnotev=1.
compute modvals=desctmp(5:7,:).
do if (quantile <> 1).
compute desctmp(5,jd)=desctmp(1,jd)-desctmp(2,jd).
compute desctmp(6,jd)=desctmp(1,jd).
compute desctmp(7,jd)=desctmp(1,jd)+desctmp(2,jd).
compute modvals=desctmp(5:7,:).
compute mnotev=2.
do if (modvals(1,1) < desctmp(3,1)).
compute modvals(1,1)=desctmp(3,1).
compute minwarn=1.
end if.
do if (modvals(3,1) > desctmp(4,1)).
compute modvals(3,1)=desctmp(4,1).
compute maxwarn=1.
end if.
end if.
do if (desctmp(8,1)=1).
compute modvals={desctmp(3,1);desctmp(4,1)}.
compute mnotev=0.
compute minwarn=0.
compute maxwarn=0.
end if.
end if.
end loop
.
compute zmin=desctmp(3,1).
compute zmax=desctmp(4,1).
do if (zmodcust=0).
compute zmodvals=modvals.
compute zprobval=zmodvals.
end if.
end if.
end if.
do if ((center = 1) or (center = 2 and xdich = 0)).
do if (xprod=1 and mcxok=0).
loop i = 1 to nxs.
compute xtmp(:,i)=xtmp(:,i)-(csum(xtmp(:,i))/n).
compute centvar={centvar,xnames(1,i)}.
end loop.

.
compute desctmp=make((8-(4* 0 )),ncol( xtmp ),-999).
loop jd=1 to ncol( xtmp ).
compute descdat= xtmp (:,jd).
compute desctmp(1,jd) = csum(descdat)/nrow(descdat).
compute desctmp(2,jd) = (nrow(descdat)*sscp(descdat))-(t(csum(descdat))*(csum(descdat))).
compute desctmp(2,jd) = sqrt(desctmp(2,jd)/(nrow(descdat)*(nrow(descdat)-1))).
compute desctmp(3,jd)=cmin(descdat).
compute desctmp(4,jd)=cmax(descdat).
do if ( 0 =0).
compute minwarn=0.
compute maxwarn=0.
do if ((desctmp(3,jd)=desctmp(4,jd)) and novar=0).
compute errcode(errs,1)=15.
compute errs=errs+1.
compute criterr=1.
compute novar=1.
end if.
compute tmp=((descdat(:,1)=desctmp(3,jd))+(descdat(:,1)=desctmp(4,jd))).
compute desctmp(8,jd)=(csum(tmp)=nrow(tmp)).
compute tmp = descdat.
compute tmp(GRADE(descdat),:) = descdat.
compute descdat = tmp.
release tmp.
compute decval={.16;.5;.84}.
loop kd=1 to 3.
compute low=trunc(decval(kd,1)*(nrow(descdat)+1)).
compute lowdec=decval(kd,1)*(nrow(descdat)+1)-low.
compute value=descdat(low,1)+(descdat((low+1),1)-descdat(low,1))*lowdec.
compute desctmp((4+kd),jd)=value.
end loop.
compute mnotev=1.
compute modvals=desctmp(5:7,:).
do if (quantile <> 1).
compute desctmp(5,jd)=desctmp(1,jd)-desctmp(2,jd).
compute desctmp(6,jd)=desctmp(1,jd).
compute desctmp(7,jd)=desctmp(1,jd)+desctmp(2,jd).
compute modvals=desctmp(5:7,:).
compute mnotev=2.
do if (modvals(1,1) < desctmp(3,1)).
compute modvals(1,1)=desctmp(3,1).
compute minwarn=1.
end if.
do if (modvals(3,1) > desctmp(4,1)).
compute modvals(3,1)=desctmp(4,1).
compute maxwarn=1.
end if.
end if.
do if (desctmp(8,1)=1).
compute modvals={desctmp(3,1);desctmp(4,1)}.
compute mnotev=0.
compute minwarn=0.
compute maxwarn=0.
end if.
end if.
end loop
.
compute xmodvals=modvals.
compute xprobval=xmodvals.
end if.
end if.
do if (nms > 0).
loop i = 1 to nms.
do if (mprod(1,i)=1).
compute mtmp(:,i)=mtmp(:,i)-(csum(mtmp(:,i))/n).
compute centvar={centvar,mnames(1,i)}.
end if.
end loop.

.
compute desctmp=make((8-(4* 0 )),ncol( mtmp ),-999).
loop jd=1 to ncol( mtmp ).
compute descdat= mtmp (:,jd).
compute desctmp(1,jd) = csum(descdat)/nrow(descdat).
compute desctmp(2,jd) = (nrow(descdat)*sscp(descdat))-(t(csum(descdat))*(csum(descdat))).
compute desctmp(2,jd) = sqrt(desctmp(2,jd)/(nrow(descdat)*(nrow(descdat)-1))).
compute desctmp(3,jd)=cmin(descdat).
compute desctmp(4,jd)=cmax(descdat).
do if ( 0 =0).
compute minwarn=0.
compute maxwarn=0.
do if ((desctmp(3,jd)=desctmp(4,jd)) and novar=0).
compute errcode(errs,1)=15.
compute errs=errs+1.
compute criterr=1.
compute novar=1.
end if.
compute tmp=((descdat(:,1)=desctmp(3,jd))+(descdat(:,1)=desctmp(4,jd))).
compute desctmp(8,jd)=(csum(tmp)=nrow(tmp)).
compute tmp = descdat.
compute tmp(GRADE(descdat),:) = descdat.
compute descdat = tmp.
release tmp.
compute decval={.16;.5;.84}.
loop kd=1 to 3.
compute low=trunc(decval(kd,1)*(nrow(descdat)+1)).
compute lowdec=decval(kd,1)*(nrow(descdat)+1)-low.
compute value=descdat(low,1)+(descdat((low+1),1)-descdat(low,1))*lowdec.
compute desctmp((4+kd),jd)=value.
end loop.
compute mnotev=1.
compute modvals=desctmp(5:7,:).
do if (quantile <> 1).
compute desctmp(5,jd)=desctmp(1,jd)-desctmp(2,jd).
compute desctmp(6,jd)=desctmp(1,jd).
compute desctmp(7,jd)=desctmp(1,jd)+desctmp(2,jd).
compute modvals=desctmp(5:7,:).
compute mnotev=2.
do if (modvals(1,1) < desctmp(3,1)).
compute modvals(1,1)=desctmp(3,1).
compute minwarn=1.
end if.
do if (modvals(3,1) > desctmp(4,1)).
compute modvals(3,1)=desctmp(4,1).
compute maxwarn=1.
end if.
end if.
do if (desctmp(8,1)=1).
compute modvals={desctmp(3,1);desctmp(4,1)}.
compute mnotev=0.
compute minwarn=0.
compute maxwarn=0.
end if.
end if.
end loop
.
do if ((cdeval(1,1)=-999) and (model=74)).
compute medmeans=desctmp(1,:).
end if.
compute mmodvals=modvals.
compute mprobval=mmodvals.
end if.
end if.
do if (ncol(centvar) > 1).
compute notecode(notes,1)=3.
compute notes=notes+1.
end if.
end if.
do if (criterr=0).
compute wsum=rsum(csum(wcmat)).
compute zsum=rsum(csum(zcmat)).
compute wzsum=rsum(csum(wzcmat)).
compute nump=make(1,(nys+nms),-999).
compue numint=make(1,(nys+nms),0).
compute datcount=1.
compute xtmpuse=0.
compute wtmpuse=0.
compute ztmpuse=0.
compute xwtmpus=0.
compute xztmpus=0.
compute wztmpus=0.
compute xwztmpu=0.
compute xtmploc=-999.
compute wtmploc=-999.
compute xwtmplo=-999.
compute ztmploc=-999.
compute xztmplo=-999.
compute wztmplo=-999.
compute xwztmplo=-999.
compute vlabs={' '}.
do if (ncs > 0).
compute ctmpuse=make(1,ncs,0).
end if.
do if (nms > 0).
compute mtmpuse=make(1,nms,0).
compute mwtmpus=make(1,nms,0).
compute mztmpus=make(1,nms,0).
compute mwztmpu=make(1,nms,0).
compute mtmploc=make(1,nms,0).
compute mwtmplo=make(nwvls,nms,-999).
compute mztmplo=make(nzvls,nms,-999).
compute mwztmplo=make((nwvls*nzvls),nms,-999).
end if.
do if (ncs > 0).
compute ctmploc=make(1,ncs,0).
end if.
compute fulldat=make(n,1,1).
compute datindx=make(1000,(nms+nys),-999).
compute wherew=make(2,(nms+nys),-999).
compute wherex=make(2,(nms+nys),-999).
compute wherez=make(2,(nms+nys),-999).
compute wherexw=make(2,(nms+nys),-999).
compute wherexz=make(2,(nms+nys),-999).
compute wherewz=make(2,(nms+nys),-999).
compute wherexwz=make(2,(nms+nys),-999).
do if (nms > 0).
compute wherem=make(nms,(nms+nys),-999).
compute wheremw = make(nms*2,(nms+nys),-999).
compute wheremz = make(nms*2,(nms+nys),-999).
compute wheremwz = make(nms*2,(nms+nys),-999).
end if.
compute wzhigh=make(1000,(((nms+1)*(nms+2))/2),0).
compute whigh=make(1000,(((nms+1)*(nms+2))/2),0).
compute zhigh=make(1000,(((nms+1)*(nms+2))/2),0).
compute fochigh=make(1000,(((nms+1)*(nms+2))/2),0).
compute xcoefloc={1;2;3;4;5;6;7;8;9}.
compute intkey = {' ', ' ', ' ', ' ', ' ', ' ', ' '}.
compute wzhighct=0.
compute whighct=0.
compute zhighct=0.
compute foccnt=0.
loop i = 2 to nrow(bcmat).
compute wdid=0.
compute zdid=0.
compute wzdid=0.
compute cntmp=1.
compute start=1.
do if (i < nrow(bcmat)).
compute outv=mtmp(:,(i-1)).
compute modlabel={mnames(1,(i-1));'constant'}.
end if.
do if (i = nrow(bcmat)).
compute outv=ytmp.
compute modlabel={ynames;'constant'}.
end if.
loop j = 1 to (i-1).
compute foccnt=foccnt+1.
do if (j = 1 and bcmat(i,j)=1).
compute outv={outv,xtmp}.
compute modlabel={modlabel;xcatlab(1:nxvls,1)}.
do if (xtmpuse=0).
compute fulldat={fulldat,xtmp}.
compute xtmpuse=1.
loop k4=datcount to (datcount+(nxvls-1)).
compute xtmploc={xtmploc;k4}.
end loop.
compute xtmploc=xtmploc(2:nrow(xtmploc),1).
compute datcount=datcount+nxvls.
end if.
compute datindx(start:(start+nrow(xtmploc)-1),(i-1))=xtmploc.
compute wherex(1,(i-1))=start+1.
compute wherex(2,(i-1))=start+nrow(xtmploc)-1+1.
do if (model = 74).
end if.
compute onebl=make(nrow(xtmploc),1,1).
compute fochigh((start+1):(start+nrow(xtmploc)),foccnt)=onebl.
compute start=start+nrow(xtmploc).
end if.
do if (j > 1 and bcmat(i,j)=1).
compute outv={outv,mtmp(:,(j-1))}.
compute modlabel={modlabel;mnames(1,(j-1))}.
do if (mtmpuse(1,(j-1))=0).
compute fulldat={fulldat,mtmp(:,(j-1))}.
compute mtmpuse(1,(j-1))=1.
compute mtmploc(1,(j-1))=datcount.
compute datcount=datcount+1.
end if.
compute datindx(start:(start+nrow(mtmploc)-1),(i-1))=mtmploc(1,(j-1)).
compute wherem((j-1),(i-1))=start+1.
compute onebl=make(nrow(mtmploc(1,j-1)),1,1).
compute ttt=nrow(mtmploc(1,(j-1)))+start-1.
compute fochigh((start+1):(start+nrow(mtmploc(1,(j-1)))),foccnt)=onebl.
compute start=start+nrow(mtmploc(1,(j-1))).
end if.
end loop.
do if (wsum > 0).
loop j = 1 to (i-1).
compute whighct=whighct+1.
do if (j = 1 and wcmat(i,j)=1).
do if (wdid=0).
compute outv={outv,wtmp}.
do if (ncs > 0 and wiscov > 0).
compute ccmatoff((i-1),wiscov)=0.
end if.
compute modlabel={modlabel;wcatlab(1:nwvls,1)}.
compute wdid=1.
do if (wtmpuse=0).
compute fulldat={fulldat,wtmp}.
do if (ncs > 0 and wiscov > 0).
compute ccmatoff((i-1),wiscov)=0.
end if.
compute wtmpuse=1.
loop k4=datcount to (datcount+(nwvls-1)).
compute wtmploc={wtmploc;k4}.
end loop.
compute wtmploc=wtmploc(2:nrow(wtmploc),1).
compute datcount=datcount+nwvls.
end if.
end if.
compute datindx(start:(start+nrow(wtmploc)-1),(i-1))=wtmploc.
compute wherew(1,(i-1))=start+1.
compute wherew(2,(i-1))=start+nrow(wtmploc)-1+1.
compute start=start+nrow(wtmploc).
loop k1=1 to nxvls.
loop k2 = 1 to nwvls.
compute outv={outv,(xtmp(:,k1)&*wtmp(:,k2))}.
do if (ncs > 0 and wiscov > 0).
compute ccmatoff((i-1),wiscov)=0.
end if.
compute modlabel={modlabel;intlab(cntmp,1)}.
compute intkey={intkey;intlab(cntmp,1),':',xcatlab(k1,1),'x',wcatlab(k2,1),' ',' '}.
compute cntmp=cntmp+1.
end loop.
end loop.
do if (xwtmpus=0).
compute fulldat={fulldat,outv(:,(ncol(outv)-(nxvls*nwvls)+1):ncol(outv))}.
compute xwtmpus=1.
do if (ncs > 0 and wiscov > 0).
compute ccmatoff((i-1),wiscov)=0.
end if.
loop k4=datcount to (datcount+((nwvls*nxvls)-1)).
compute xwtmplo={xwtmplo;k4}.
end loop.
compute xwtmplo=xwtmplo(2:nrow(xwtmplo),1).
compute datcount=datcount+(nxvls*nwvls).
end if.
compute datindx(start:(start+nrow(xwtmplo)-1),(i-1))=xwtmplo.
compute wherexw(1,(i-1))=start+1.
compute wherexw(2,(i-1))=start+nrow(xwtmplo)-1+1.
compute onebl=make(nrow(xwtmplo),1,1).
compute whigh((start+1):(start+nrow(xwtmplo)),whighct)=onebl.
compute start=start+nrow(xwtmplo).
end if.
do if (j > 1 and wcmat(i,j)=1).
do if (wdid=0 and model <> 74).
compute outv={outv,wtmp}.
do if (ncs > 0 and wiscov > 0).
compute ccmatoff((i-1),wiscov)=0.
end if.
compute modlabel={modlabel;wcatlab(1:nwvls,1)}.
compute wdid=1.
do if (wtmpuse=0).
compute fulldat={fulldat,wtmp}.
do if (ncs > 0 and wiscov > 0).
compute ccmatoff((i-1),wiscov)=0.
end if.
compute wtmpuse=1.
loop k4=datcount to (datcount+(nwvls-1)).
compute wtmploc={wtmploc;k4}.
end loop.
compute wtmploc=wtmploc(2:nrow(wtmploc),1).
compute datcount=datcount+nwvls.
end if.
compute datindx(start:(start+nrow(wtmploc)-1),(i-1))=wtmploc.
compute wherew(1,(i-1))=start+1.
compute wherew(2,(i-1))=start+nrow(wtmploc)-1+1.
compute start=start+nrow(wtmploc).
end if.
loop k2 = 1 to nwvls.
compute outv={outv,(mtmp(:,(j-1))&*wtmp(:,k2))}.
do if (ncs > 0 and wiscov > 0).
compute ccmatoff((i-1),wiscov)=0.
end if.
compute modlabel={modlabel;intlab(cntmp,1)}.
compute intkey={intkey;intlab(cntmp,1),':', mnames(1,(j-1)),'x',wcatlab(k2,1),' ',' '}.
compute cntmp=cntmp+1.
end loop.
do if (mwtmpus(1,(j-1))=0).
compute fulldat={fulldat,outv(:,(ncol(outv)-nwvls+1):ncol(outv))}.
do if (ncs > 0 and wiscov > 0).
compute ccmatoff((i-1),wiscov)=0.
end if.
compute mwtmpus(1,(j-1))=1.
compute mw22=-999.
loop k4=datcount to (datcount+(nwvls-1)).
compute mw22={mw22;k4}.
end loop.
compute mwtmplo(:,(j-1))=mw22(2:nrow(mw22),1).
compute datcount=datcount+nwvls.
end if.
compute datindx(start:(start+nrow(mwtmplo)-1),(i-1))=mwtmplo(:,(j-1)).
compute wheremw(((2*j)-3),(i-1))=start+1.
compute wheremw(((2*j)-2),(i-1))=start+nrow(mwtmplo)-1+1.
compute onebl=make(nrow(mwtmplo),1,1).
compute whigh((start+1):(start+nrow(mwtmplo)),whighct)=onebl.
compute start=start+nrow(mwtmplo).
end if.
end loop.
end if.
do if (zsum > 0).
loop j = 1 to (i-1).
compute zhighct=zhighct+1.
do if (j = 1 and zcmat(i,j)=1).
do if (zdid=0).
compute outv={outv,ztmp}.
do if (ncs > 0 and ziscov > 0).
compute ccmatoff((i-1),ziscov)=0.
end if.
compute modlabel={modlabel;zcatlab(1:nzvls,1)}.
compute zdid=1.
do if (ztmpuse=0).
compute fulldat={fulldat,ztmp}.
do if (ncs > 0 and ziscov > 0).
compute ccmatoff((i-1),ziscov)=0.
end if.
compute ztmpuse=1.
loop k4=datcount to (datcount+(nzvls-1)).
compute ztmploc={ztmploc;k4}.
end loop.
compute ztmploc=ztmploc(2:nrow(ztmploc),1).
compute datcount=datcount+nzvls.
end if.
end if.
compute datindx(start:(start+nrow(ztmploc)-1),(i-1))=ztmploc.
compute wherez(1,(i-1))=start+1.
compute wherez(2,(i-1))=start+nrow(ztmploc)-1+1.
compute start=start+nrow(ztmploc).
loop k1=1 to nxvls.
loop k2 = 1 to nzvls.
compute outv={outv,(xtmp(:,k1)&*ztmp(:,k2))}.
do if (ncs > 0 and ziscov > 0).
compute ccmatoff((i-1),ziscov)=0.
end if.
compute modlabel={modlabel;intlab(cntmp,1)}.
compute intkey={intkey;intlab(cntmp,1),':',xcatlab(k1,1),'x',zcatlab(k2,1),' ',' '}.
compute cntmp=cntmp+1.
end loop.
end loop.
do if (xztmpus=0).
compute fulldat={fulldat,outv(:,(ncol(outv)-(nxvls*nzvls)+1):ncol(outv))}.
do if (ncs > 0 and ziscov > 0).
compute ccmatoff((i-1),ziscov)=0.
end if.
compute xztmpus=1.
loop k4=datcount to (datcount+((nzvls*nxvls)-1)).
compute xztmplo={xztmplo;k4}.
end loop.
compute xztmplo=xztmplo(2:nrow(xztmplo),1).
compute datcount=datcount+(nxvls*nzvls).
end if.
compute datindx(start:(start+nrow(xztmplo)-1),(i-1))=xztmplo.
compute wherexz(1,(i-1))=start+1.
compute wherexz(2,(i-1))=start+nrow(xztmplo)-1+1.
compute onebl=make(nrow(xztmplo),1,1).
compute zhigh((start+1):(start+nrow(xztmplo)),zhighct)=onebl.
compute start=start+nrow(xztmplo).
end if.
do if (j > 1 and zcmat(i,j)=1).
do if (zdid=0).
compute outv={outv,ztmp}.
do if (ncs > 0 and ziscov > 0).
compute ccmatoff((i-1),ziscov)=0.
end if.
compute modlabel={modlabel;zcatlab(1:nzvls,1)}.
compute zdid=1.
do if (ztmpuse=0).
compute fulldat={fulldat,ztmp}.
do if (ncs > 0 and ziscov > 0).
compute ccmatoff((i-1),ziscov)=0.
end if.
compute ztmpuse=1.
loop k4=datcount to (datcount+(nzvls-1)).
compute ztmploc={ztmploc;k4}.
end loop.
compute ztmploc=ztmploc(2:nrow(ztmploc),1).
compute datcount=datcount+nzvls.
end if.
compute datindx(start:(start+nrow(ztmploc)-1),(i-1))=ztmploc.
compute wherez(1,(i-1))=start+1.
compute wherez(2,(i-1))=start+nrow(ztmploc)-1+1.
compute start=start+nrow(ztmploc).
end if.
loop k2 = 1 to nzvls.
compute outv={outv,(mtmp(:,(j-1))&*ztmp(:,k2))}.
do if (ncs > 0 and ziscov > 0).
compute ccmatoff((i-1),ziscov)=0.
end if.
compute modlabel={modlabel;intlab(cntmp,1)}.
compute intkey={intkey;intlab(cntmp,1),':', mnames(1,(j-1)),'x',zcatlab(k2,1),' ',' '}.
compute cntmp=cntmp+1.
end loop.
do if (mztmpus(1,(j-1))=0).
compute fulldat={fulldat,outv(:,(ncol(outv)-nzvls+1):ncol(outv))}.
do if (ncs > 0 and ziscov > 0).
compute ccmatoff((i-1),ziscov)=0.
end if.
compute mztmpus(1,(j-1))=1.
compute mz22=-999.
loop k4=datcount to (datcount+(nzvls-1)).
compute mz22={mz22;k4}.
end loop.
compute mztmplo(:,(j-1))=mz22(2:nrow(mz22),1).
compute datcount=datcount+nzvls.
end if.
compute datindx(start:(start+nrow(mztmplo)-1),(i-1))=mztmplo(:,(j-1)).
compute wheremz(((2*j)-3),(i-1))=start+1.
compute wheremz(((2*j)-2),(i-1))=start+nrow(mztmplo)-1+1.
compute onebl=make(nrow(mztmplo),1,1).
compute zhigh((start+1):(start+nrow(mztmplo)),zhighct)=onebl.
compute start=start+nrow(mztmplo).
end if.
end loop.
end if.
do if (wzsum > 0).
loop j = 1 to (i-1).
compute wzhighct=wzhighct+1.
do if (j = 1 and wzcmat(i,j)=1).
do if (wzdid=0).
loop k1=1 to nwvls.
loop k2 = 1 to nzvls.
compute outv={outv,(wtmp(:,k1)&*ztmp(:,k2))}.
do if (ncs > 0 and (ziscov > 0)).
compute ccmatoff((i-1),ziscov)=0.
end if.
do if (ncs > 0 and (wiscov > 0)).
compute ccmatoff((i-1),wiscov)=0.
end if.
compute modlabel={modlabel;intlab(cntmp,1)}.
compute intkey={intkey;intlab(cntmp,1),':',wcatlab(k1,1),'x',zcatlab(k2,1),' ',' '}.
compute cntmp=cntmp+1.
end loop.
end loop.
do if (wztmpus=0).
compute fulldat={fulldat,outv(:,(ncol(outv)-(nwvls*nzvls)+1):ncol(outv))}.
do if (ncs > 0 and (ziscov > 0)).
compute ccmatoff((i-1),ziscov)=0.
end if.
do if (ncs > 0 and (wiscov > 0)).
compute ccmatoff((i-1),wiscov)=0.
end if.
compute wztmpus=1.
loop k4=datcount to (datcount+((nwvls*nzvls)-1)).
compute wztmplo={wztmplo;k4}.
end loop.
compute wztmplo=wztmplo(2:nrow(wztmplo),1).
compute datcount=datcount+(nzvls*nwvls).
end if.
compute wzdid=1.
end if.
compute datindx(start:(start+nrow(wztmplo)-1),(i-1))=wztmplo.
compute wherewz(1,(i-1))=start+1.
compute wherewz(2,(i-1))=start+nrow(wztmplo)-1+1.
compute start=start+nrow(wztmplo).
loop k1=1 to nxvls.
loop k2=1 to nwvls.
loop k3=1 to nzvls.
compute outv={outv,(xtmp(:,k1)&*wtmp(:,k2)&*ztmp(:,k3))}.
do if (ncs > 0 and (ziscov > 0)).
compute ccmatoff((i-1),ziscov)=0.
end if.
do if (ncs > 0 and (wiscov > 0)).
compute ccmatoff((i-1),wiscov)=0.
end if.
compute modlabel={modlabel;intlab(cntmp,1)}.
compute intkey={intkey;intlab(cntmp,1),':',xcatlab(k1,1),'x',wcatlab(k2,1),'x', zcatlab(k3,1)}.
compute cntmp=cntmp+1.
end loop.
end loop.
end loop.
do if (xwztmpu=0).
compute fulldat={fulldat,outv(:,(ncol(outv)-(nxvls*nwvls*nzvls)+1):ncol(outv))}.
do if (ncs > 0 and (ziscov > 0)).
compute ccmatoff((i-1),ziscov)=0.
end if.
do if (ncs > 0 and (wiscov > 0)).
compute ccmatoff((i-1),wiscov)=0.
end if.
compute xwztmpu=1.
loop k4=datcount to (datcount+((nzvls*nxvls*nwvls)-1)).
compute xwztmplo={xwztmplo;k4}.
end loop.
compute xwztmplo=xwztmplo(2:nrow(xwztmplo),1).
compute datcount=datcount+(nxvls*nzvls*nwvls).
end if.
compute datindx(start:(start+nrow(xwztmplo)-1),(i-1))=xwztmplo.
compute wherexwz(1,(i-1))=start+1.
compute wherexwz(2,(i-1))=start+nrow(xwztmplo)-1+1.
compute onebl=make(nrow(xwztmplo),1,1).
compute wzhigh((start+1):(start+nrow(xwztmplo)),wzhighct)=onebl.
compute start=start+nrow(xwztmplo).
end if.
do if (j > 1 and wzcmat(i,j)=1).
do if (wzdid=0).
loop k1=1 to nwvls.
loop k2 = 1 to nzvls.
compute outv={outv,(wtmp(:,k1)&*ztmp(:,k2))}.
do if (ncs > 0 and (ziscov > 0)).
compute ccmatoff((i-1),ziscov)=0.
end if.
do if (ncs > 0 and (wiscov > 0)).
compute ccmatoff((i-1),wiscov)=0.
end if.
compute modlabel={modlabel;intlab(cntmp,1)}.
compute intkey={intkey;intlab(cntmp,1),':',wcatlab(k1,1),'x',zcatlab(k2,1),' ',' '}.
compute cntmp=cntmp+1.
end loop.
end loop.
do if (wztmpus=0).
compute fulldat={fulldat,outv(:,(ncol(outv)-(nwvls*nzvls)+1):ncol(outv))}.
do if (ncs > 0 and (ziscov > 0)).
compute ccmatoff((i-1),ziscov)=0.
end if.
do if (ncs > 0 and (wiscov > 0)).
compute ccmatoff((i-1),wiscov)=0.
end if.
compute wztmpus=1.
loop k4=datcount to (datcount+((nwvls*nzvls)-1)).
compute wztmplo={wztmplo;k4}.
end loop.
compute wztmplo=wztmplo(2:nrow(wztmplo),1).
compute datcount=datcount+(nzvls*nwvls).
end if.
compute wzdid=1.
compute datindx(start:(start+nrow(wztmplo)-1),(i-1))=wztmplo.
compute wherewz(1,(i-1))=start+1.
compute wherewz(2,(i-1))=start+nrow(wztmplo)-1+1.
compute start=start+nrow(wztmplo).
end if.
loop k1 = 1 to nwvls.
loop k2 = 1 to nzvls.
compute outv={outv,(mtmp(:,(j-1))&*wtmp(:,k1)&*ztmp(:,k2))}.
do if (ncs > 0 and (ziscov > 0)).
compute ccmatoff((i-1),ziscov)=0.
end if.
do if (ncs > 0 and (wiscov > 0)).
compute ccmatoff((i-1),wiscov)=0.
end if.
compute modlabel={modlabel;intlab(cntmp,1)}.
compute intkey={intkey;intlab(cntmp,1),':',mnames(1,(j-1)),'x',wcatlab(k1,1),'x', zcatlab(k2,1)}.
compute cntmp=cntmp+1.
end loop.
end loop.
do if (mwztmpu(1,(j-1))=0).
compute fulldat={fulldat,outv(:,(ncol(outv)-(nwvls*nzvls)+1):ncol(outv))}.
do if (ncs > 0 and (ziscov > 0)).
compute ccmatoff((i-1),ziscov)=0.
end if.
do if (ncs > 0 and (wiscov > 0)).
compute ccmatoff((i-1),wiscov)=0.
end if.
compute mwztmpu(1,(j-1))=1.
compute mz22=-999.
loop k4=datcount to (datcount+(nwvls*nzvls)-1).
compute mz22={mz22;k4}.
end loop.
compute mwztmplo(:,(j-1))=mz22(2:nrow(mz22),1).
compute datcount=datcount+(nwvls*nzvls).
end if.
compute datindx(start:(start+nrow(mwztmplo)-1),(i-1))=mwztmplo(:,(j-1)).
compute wheremwz(((2*j)-3),(i-1))=start+1.
compute wheremwz(((2*j)-2),(i-1))=start+nrow(mwztmplo)-1+1.
compute onebl=make(nrow(mwztmplo),1,1).
compute wzhigh((start+1):(start+nrow(mwztmplo)),wzhighct)=onebl.
compute start=start+nrow(mwztmplo).
end if.
end loop.
end if.
do if (ncs > 0).
compute ccmat=ccmat&*ccmatoff.
loop j = 1 to ncs.
do if (ccmat((i-1),j))=1.
do if (j=wiscov).
compute ctmp(:,j)=wtmp.
end if.
do if (j=ziscov).
compute ctmp(:,j)=ztmp.
end if.
compute outv={outv,ctmp(:,j)}.
compute modlabel={modlabel;covnames(1,j)}.
do if (ctmpuse(1,j)=0).
compute fulldat={fulldat,ctmp(:,j)}.
compute ctmpuse(1,j)=1.
compute ctmploc(1,j)=datcount.
compute datcount=datcount+1.
end if.
compute datindx(start:(start+nrow(ctmploc)-1),(i-1))=ctmploc(1,j).
compute start=start+nrow(ctmploc(1,j)).
end if.
end loop.
end if.
compute wdid=0.
compute zdid=0.
compute wzdid=0.
compute vlabs={vlabs;modlabel(2:nrow(modlabel),1)}.
compute numint(1,(i-1))=cntmp-1.
compute nump(1,(i-1))=nrow(modlabel)-1.
end loop.
release datcount, xtmpuse, wtmpuse, ztmpuse, xwtmpus, xztmpus, wztmpus, xwztmpu.
release xtmploc, wtmploc, xwtmplo, ztmploc, xztmplo, wztmplo, xwztmplo, foccnt.
do if (modcok=1 and ((nms > 0) or (zcmat(2,1) <> 1) or (mcx <> 0))).
compute notecode(notes,1) = 19.
compute notes = notes + 1.
compute modcok=0.
end if.
do if ((serial = 1 or (rsum(numint)>0) or nms=0) and mc > 0).
compute notecode(notes,1) = 15.
compute notes = notes + 1.
compute boot=mc.
compute mc=0.
end if.
do if (boot <> 0 or mc <> 0).
compute bootsz=boot.
do if (mc > 0).
compute bootsz=mc.
compute saveboot=0.
end if.
loop.
compute cilow = rnd(bootsz*(1-(conf/100))/2).
compute cihigh = trunc((bootsz*(conf/100)+(bootsz*(1-(conf/100))/2)))+1.
do if (cilow < 1 or cihigh > bootsz).
compute bootsz=trunc((bootsz+1000)/1000)*1000.
compute adjust = 1.
end if.
end loop if (cilow gt 0 and cihigh le bootsz).
do if (boot > 0).
compute boot=bootsz.
end if.
do if (mc > 0).
compute mc=bootsz.
end if.
do if (adjust = 1 and boot > 0).
compute notecode(notes,1) = 8.
compute notes = notes + 1.
end if.
do if (adjust = 1 and mc > 0).
compute notecode(notes,1) = 16.
compute notes = notes + 1.
end if.
end if.
compute maxboot = trunc(2*boot).
do if ( 0 > maxboot).
compute maxboot=trunc( 0 ).
end if.
do if (nms > 0).
release mtmpuse, mwtmpus, mwztmpu, mtmploc, mwtmplo, mztmplo, mwztmplo.
end if.
release wdid, zdid, wzdid, start,modlabel.
compute vlabs=vlabs(2:nrow(vlabs),1).
do if (rsum(numint) > 0).
compute intkey=intkey(2:nrow(intkey),:).
end if.
compute fulldat=fulldat(:,2:ncol(fulldat)).
compute fochigh=fochigh(1:rmax(nump),:).
compute whigh=whigh(1:rmax(nump),:).
compute zhigh=zhigh(1:rmax(nump),:).
compute wzhigh=wzhigh(1:rmax(nump),:).
compute coeffs=fochigh+whigh+zhigh+wzhigh.
compute bootloc=make(rmax(nump),ncol(nump),0).
do if (nms > 0).
compute cntmp=1.
loop i = 1 to ncol(nump).
loop j = 1 to nump(1,i).
compute bootloc(j,i)=cntmp.
compute cntmp=cntmp+1.
end loop.
end loop.
compute fochighb=make(nrow(fochigh),ncol(fochigh),0).
compute whighb=fochighb.
compute zhighb=fochighb.
compute wzhighb=fochighb.
compute thetaxmb=make(nrow(fochighb),nms,0).
compute thetaxyb=make(nrow(fochighb),1,0).
compute pathsfoc=make(nxvls,1,0).
compute cntmp=1.
loop i = 1 to (nms+nys).
loop j = 1 to i.
compute fochighb(:,cntmp)=fochigh(:,cntmp)&*bootloc(:,i).
compute whighb(:,cntmp)=whigh(:,cntmp)&*bootloc(:,i).
compute zhighb(:,cntmp)=zhigh(:,cntmp)&*bootloc(:,i).
compute wzhighb(:,cntmp)=wzhigh(:,cntmp)&*bootloc(:,i).
compute coeffsb=fochighb+whighb+zhighb+wzhighb.
do if ((i < (nms+nys)) and (j = 1)).
compute thetaxmb(:,i)=coeffsb(:,cntmp).
end if.
do if ((i = (nms+nys)) and (j = 1)).
compute thetaxyb(:,1)=coeffsb(:,cntmp).
end if.
compute cntmp=cntmp+1.
end loop.
end loop.
compute thetamyb=coeffsb(:,(ncol(coeffsb)-nms+1):ncol(coeffsb)).
do if (serial = 1).
compute thetammb=make(nrow(coeffsb),((nms*(nms-1))/2),0).
end if.
compute cntmp=1.
do if (nms > 1 and serial = 1).
loop i = 1 to (nms-1).
compute start=((i+2)*(i+1))/2.
loop j = 2 to (nms-i+1).
compute thetammb(:,cntmp)=coeffsb(:,start).
compute start=start+j+i-1.
compute cntmp=cntmp+1.
end loop.
end loop.
end if.
end if.
do if ((total = 1) and (rsum(numint)=0 or (xmint=1))).
compute dototal=1.
do if ((csum(bcmat(:,1)) <> (nms+nys)) or (rsum(bcmat(nrow(bcmat),:)) <> (nms+nys))).
compute dototal=0.
compute alttotal=1.
compute notecode(notes,1) = 12.
compute notes = notes + 1.
end if.
do if (ncs > 0).
do if ((csum(rsum(ccmat))) < (nrow(ccmat)*ncol(ccmat))).
compute dototal=0.
compute alttotal=1.
compute notecode(notes,1) = 11.
compute notes = notes + 1.
end if.
end if.
do if (model=74).
do if (xdich=0 and nxvls=1).
compute dototal=0.
compute alttotal=1.
end if.
do if ((xdich=1 or nxvls > 1) and (ncs > 0) and model=74).
compute dototal=0.
compute alttotal=1.
end if.
end if.
end if.
end if.
do if (criterr=0 and ncs > 0).
do if (rsum((csum(ccmat)=0)) <> 0).
compute errcode(errs,1)=51.
compute errs=errs+1.
compute criterr=1.
end if.
end if.
compute debug= 0.
do if (outscree=1).
print/title = '***************** PROCESS Procedure for SPSS Version 4.2 *****************'.
print/title = '          Written by Andrew F. Hayes, Ph.D.       www.afhayes.com'.
print/title = '    Documentation available in Hayes (2022). www.guilford.com/p/hayes3'/space=0.
end if.
do if (criterr=0).
compute modresid=make(n,1,99999).
do if (stand=1 and ydich=1).
compute stand=0.
end if.
compute anymod2=csum(rsum(wcmat+zcmat+wzcmat)).
do if (anymod2 > 0 and stand = 1).
compute notecode(notes,1) = 27.
compute notes = notes + 1.
compute stand=0.
end if.
compute funny=1.
do if (outscree=1).
print modelvar/title = '**************************************************************************'/format = A8/rnames=modelvlb.
do if (ncs > 0).
print covnames/title='Covariates:'/format=A8.
end if.
print n/title='Sample'/rlabel='Size:'.
do if (( 'random' <> 'random')).
compute seedt= 'random'.
print seedt/title='Custom'/format=A12/rlabel = 'Seed:'.
end if.
end if.
compute maxresm=9.
compute resultm=make(1,maxresm,99999).
do if (describe=1).
compute means=csum(dat)/n.
compute sigmatal = (t(dat)*(ident(n)-(1/n)*ones*t(ones))*dat)*(1/(n-1)).
compute sdvec=sqrt(diag(sigmatal)).
compute sdall = mdiag(1/sdvec).
compute corall=sdall*sigmatal*t(sdall).
compute means={means;t(sdvec)}.
do if (ncol(means) > 9).
compute resultm=make(1,ncol(means),99999).
compute maxresm=ncol(means).
end if.
compute resultm2=make(2,maxresm,99999).
compute resultm2(1:2,1:ncol(means))=means.
compute resultm={resultm;resultm2}.
compute resultm2=make(ncol(corall),maxresm,99999).
compute resultm2(1:ncol(corall),1:ncol(corall))=corall.
compute resultm={resultm;resultm2}.
do if (outscree=1).
print means/title='Variable means and standard deviations'/cnames=varnames/rlabels='Mean','SD'/format= F10.4.
print corall/title='Variable intercorrelations (Pearson r)'/cnames=varnames/rnames=varnames/format= F10.4.
end if.
end if.
do if (outscree=1).
do if (mcxok=1).
compute labtmp={xnames,t(xcatlab(1:nxvls,1))}.
print dummatx/title = 'Coding of categorical X variable for analysis:'/cnames = labtmp/format = F6.3.
end if.
do if (mcwok=1 and xmint=0).
compute labtmp={wnames,t(wcatlab(1:nwvls,1))}.
print dummatw/title = 'Coding of categorical W variable for analysis:'/cnames = labtmp/format = F6.3.
end if.
do if (mczok=1).
compute labtmp={znames,t(zcatlab(1:nzvls,1))}.
print dummatz/title = 'Coding of categorical Z variable for analysis:'/cnames = labtmp/format = F6.3.
end if.
end if.
end if.
do if (criterr = 0).
compute outnames=ynames.
compute outvars=ytmp.
do if (nms > 0).
compute outnames={mnames,ynames}.
compute outvars={mtmp,ytmp}.
compute indcov=make(((nms*2)+(nms*(nxvls-1))),((nms*2)+(nms*(nxvls-1))),0).
compute mcsopath=make(((nms*2)+(nms*(nxvls-1))),1,0).
end if.
compute labstart=1.
compute intstart=1.
compute start=1.
compute coeffmat=make(1,6,0).
compute conseq={'        '}.
compute dfmat=0.
compute coeffcol=0.
compute pathscnt=1.
compute pathscn2=1.
loop i = 1 to (nms+nys).
do if (outscree=1).
print/title = '**************************************************************************'.
end if.
compute highf=make(1,5,0).
compute highf2=highf.
do if ((i = (nms+nys)) and (ydich=1)).
compute highf=make(1,3,0).
compute highf2=highf.
end if.
compute flabel={' '}.
compute y=outvars(:,i).
compute xindx=datindx(1:(nump(1,i)-1),i).
compute x = fulldat(:,xindx).
compute x={ones,x}.
compute xsq=t(x)*x.
compute exsq=eval(xsq).
release xsq.
compute zeroeig=csum(exsq <= 0.000000000002).
do if (outscree=1).
print outnames(1,i)/title = 'OUTCOME VARIABLE:'/format = A8/space=0.
do if (ydich=1 and (i = (nms+nys))).
compute nmsd = {outnames(1,i), 'Analysis'}.
print rcd/title = 'Coding of binary Y for logistic regression analysis:'/cnames = nmsd/format = F9.2.
end if.
end if.
do if (zeroeig > 0).
print / title = 'SINGULAR OR NEAR SINGULAR DATA MATRIX.'.
compute criterr=1.
compute errcode(errs,1)=31.
compute errs=errs+1.
end if.
compute means=csum(x)/n.
compute vlabsm=vlabs(labstart:(labstart+(nump(1,i)-1)),1).
do if (criterr=0).
do if (ydich=0 or (i < (nms+nys))).

.
do if ( 1 =1).
compute hatmat=inv(t( x )* x )*t( x ).
compute b = hatmat* y.
compute modres=b.
do if ( 1 =1).
compute n1=nrow( x ).
compute dfres=n1-(ncol( x )).
compute sstotal = t( y -(csum( y )/n1))*( y -(csum( y )/n1)).
compute resid= y - x *b.
compute ssresid = csum((resid)&**2).
compute r2 = (sstotal-ssresid)/sstotal.
do if (r2 < 0).
compute r2=0.
end if.
compute adjr2 = 1-((1-r2)*(n1-1)/(dfres)).
compute mse=ssresid/(n1-ncol( x )).

.
compute n1=nrow( x ).
compute invXtX = inv(t( x )* x ).
compute varb = mse *invXtX.
compute k3 = ncol( x ).
compute xhc=0.
do if ( hc <> 5).
compute xhc= x.
compute hat = xhc(:,1).
loop i3=1 to nrow(xhc).
compute hat(i3,1)= xhc(i3,:)*invXtX*t(xhc(i3,:)).
end loop.
do if ( hc = 0 or hc =1).
loop i3 = 1 to k3.
compute xhc(:,i3)=xhc(:,i3)&* resid.
end loop.
end if.
do if ( hc =3 or hc =2).
loop i3=1 to k3.
compute xhc(:,i3) = ( resid &/(1-hat)&**(1/(4- hc )))&*xhc(:,i3).
end loop.
end if.
do if ( hc = 4).
compute hcmn=make(n,2,4).
compute hcmn(:,2)=(n1*hat)/k3.
loop i3= 1 to k3.
compute xhc(:,i3) = ( resid &/(1-hat)&**(rmin(hcmn)/2))&*xhc(:,i3).
end loop.
end if.
compute varb=(invXtX*t(xhc)*xhc*invXtX).
do if ( hc =1).
compute varb=(n1/(n1-ncol( x )))&*varb.
end if.
end if.
compute hclab={'se(HC0)','se(HC1)','se(HC2)','se(HC3)','se(HC4)','se'}.
compute hclab=hclab(1,( hc +1)).
compute hcflab={'F(HC0)','F(HC1)','F(HC2)','F(HC3)','F(HC4)','F'}.
compute hcflab=hcflab(1,( hc +1)).
release xhc
.
compute seb=sqrt(diag(varb)).
compute trat = b&/seb.
compute p = 2*(1-tcdf(abs(trat), (dfres))).
compute tval = sqrt(dfres* (exp((dfres-(5/6))*((xp2/(dfres-(2/3)+(.11/dfres)))* (xp2/(dfres-(2/3)+(.11/dfres)))))-1)).
compute modres={modres,seb,trat,p}.
compute modres={modres,(b-tval&*seb),(b+tval&*seb)}.
compute modresl={'coeff',hclab,'t','p','LLCI','ULCI'}.
compute lmat = ident(ncol( x )).
compute lmat = lmat(:,2:ncol(lmat)).
compute fratio = (t(t(lmat)*b)*inv(t(lmat)*varb*lmat)*((t(lmat)*b)))/(ncol( x )-1).
compute pfr = 1-fcdf(fratio,(ncol( x )-1),dfres).
compute modsum={sqrt(r2),r2,mse,fratio,(ncol( x )-1),dfres,pfr}.
compute modsuml={'R','R-sq','MSE',hcflab,'df1','df2', 'p'}.
end if.
end if.
do if ( 1 = 2 or 1 =3).
compute xlp= x.
compute ylp= y.
compute pt2 = make(nrow(ylp),1,(csum(ylp)/nrow(ylp))).
do if ( 1 =2).
compute LL3 = ylp&*ln(pt2)+(1-ylp)&*ln(1-pt2).
end if.
compute LL3 = -2*csum(LL3).
compute bt1 = make(ncol(xlp),1,0).
compute LL1 = 0.
compute pt1 = make(nrow(ylp),1,0.5).
compute pt1lp=pt1.
loop jjj = 1 to iterate.
compute xlptmp=t(xlp).
compute vecprb=pt1lp&*(1-pt1lp).
loop kkk=1 to ncol(xlp).
compute xlptmp(kkk,:)=xlptmp(kkk,:)&*t(vecprb).
end loop.
compute b = bt1+inv(xlptmp*xlp)*t(xlp)*(ylp-pt1lp).
do if ( 1 =2).
compute xlpb=xlp*b.
compute xlpbt=(xlpb > -709.7).
compute xlpb709=(1-xlpbt)*(-709.7).
compute xlpb=(xlpb&*xlpbt)+xlpb709.
compute pt1lp = 1/(1+exp(-(xlpb))).
end if.
compute itprob = csum((pt1lp < .00000001) or (pt1lp > .9999999)).
do if (itprob > 0).
loop kkk = 1 to nrow(pt1lp).
do if (pt1lp(kkk,1) > .9999999).
compute pt1lp(kkk,1) = .9999999.
end if.
do if (pt1lp(kkk,1) < .00000001).
compute pt1lp(kkk,1) = .00000001.
end if.
end loop.
compute itprob = 0.
end if.
do if (itprob = 0).
do if ( 1 =2).
compute LL = ylp&*ln(pt1lp)+(1-ylp)&*ln(1-pt1lp).
end if.
compute LL2 = -2*csum(ll).
end if.
do if (abs(LL1-LL2) < converge).
do if ( 1 =1).
compute xlptmp=t(xlp).
compute vecprb=pt1lp&*(1-pt1lp).
loop kkk=1 to ncol(xlp).
compute xlptmp(kkk,:)=xlptmp(kkk,:)&*t(vecprb).
end loop.
compute varb = inv(xlptmp*xlp).
compute seb = sqrt(diag(varb)).
release xlptmp.
end if.
break.
end if.
compute bt1 = b.
compute LL1 = LL2.
end loop.
compute modres=b.
do if (jjj > iterate).
compute itprob = 2.
do if (booting=0).
compute iterrmod=1.
end if.
do if (booting=1).
compute bootiter=1.
end if.
do if (itprobtg=0).
compute itprobtg=1.
compute errcode(errs,1) = 47.
compute errs = errs + 1.
do if (booting = 0 and 1 =1).
compute vt1 = mdiag(pt1lp&*(1-pt1lp)).
compute varb = inv(t(xlp)*vt1*xlp).
compute seb = sqrt(diag(varb)).
end if.
end if.
end if.
do if ( 1 =1).
compute trat = b&/seb.
compute dfres=nrow(xlp).
compute p = 2*(1-cdfnorm(abs(trat))).
compute modres={modres,seb,trat,p}.
compute modres={modres,(b-xp2&*seb),(b+xp2&*seb)}.
compute pvchi=1-chicdf((LL3-LL2),(nrow(modres)-1)).
compute mcF = (LL3-LL2)/LL3.
compute cox = 1-exp(-(LL3-LL2)/nrow(xlp)).
compute nagel = cox/(1-exp(-(LL3)/nrow(xlp))).
compute modsum={LL2,(LL3-LL2),(nrow(modres)-1),pvchi, mcF,cox,nagel}.
compute modsuml={'-2LL','ModelLL', 'df', 'p', 'McFadden', 'CoxSnell', 'Nagelkrk'}.
compute modresl={'coeff','se','Z','p','LLCI','ULCI'}.
end if.
end if
.
compute dfmatt=make(nrow(modres),1,modsum(1,6)).
compute modresid={modresid,resid}.
end if.
do if (ydich=1 and (i = (nms+nys))).

.
do if ( 2 =1).
compute hatmat=inv(t( x )* x )*t( x ).
compute b = hatmat* y.
compute modres=b.
do if ( 1 =1).
compute n1=nrow( x ).
compute dfres=n1-(ncol( x )).
compute sstotal = t( y -(csum( y )/n1))*( y -(csum( y )/n1)).
compute resid= y - x *b.
compute ssresid = csum((resid)&**2).
compute r2 = (sstotal-ssresid)/sstotal.
do if (r2 < 0).
compute r2=0.
end if.
compute adjr2 = 1-((1-r2)*(n1-1)/(dfres)).
compute mse=ssresid/(n1-ncol( x )).

.
compute n1=nrow( x ).
compute invXtX = inv(t( x )* x ).
compute varb = mse *invXtX.
compute k3 = ncol( x ).
compute xhc=0.
do if ( hc <> 5).
compute xhc= x.
compute hat = xhc(:,1).
loop i3=1 to nrow(xhc).
compute hat(i3,1)= xhc(i3,:)*invXtX*t(xhc(i3,:)).
end loop.
do if ( hc = 0 or hc =1).
loop i3 = 1 to k3.
compute xhc(:,i3)=xhc(:,i3)&* resid.
end loop.
end if.
do if ( hc =3 or hc =2).
loop i3=1 to k3.
compute xhc(:,i3) = ( resid &/(1-hat)&**(1/(4- hc )))&*xhc(:,i3).
end loop.
end if.
do if ( hc = 4).
compute hcmn=make(n,2,4).
compute hcmn(:,2)=(n1*hat)/k3.
loop i3= 1 to k3.
compute xhc(:,i3) = ( resid &/(1-hat)&**(rmin(hcmn)/2))&*xhc(:,i3).
end loop.
end if.
compute varb=(invXtX*t(xhc)*xhc*invXtX).
do if ( hc =1).
compute varb=(n1/(n1-ncol( x )))&*varb.
end if.
end if.
compute hclab={'se(HC0)','se(HC1)','se(HC2)','se(HC3)','se(HC4)','se'}.
compute hclab=hclab(1,( hc +1)).
compute hcflab={'F(HC0)','F(HC1)','F(HC2)','F(HC3)','F(HC4)','F'}.
compute hcflab=hcflab(1,( hc +1)).
release xhc
.
compute seb=sqrt(diag(varb)).
compute trat = b&/seb.
compute p = 2*(1-tcdf(abs(trat), (dfres))).
compute tval = sqrt(dfres* (exp((dfres-(5/6))*((xp2/(dfres-(2/3)+(.11/dfres)))* (xp2/(dfres-(2/3)+(.11/dfres)))))-1)).
compute modres={modres,seb,trat,p}.
compute modres={modres,(b-tval&*seb),(b+tval&*seb)}.
compute modresl={'coeff',hclab,'t','p','LLCI','ULCI'}.
compute lmat = ident(ncol( x )).
compute lmat = lmat(:,2:ncol(lmat)).
compute fratio = (t(t(lmat)*b)*inv(t(lmat)*varb*lmat)*((t(lmat)*b)))/(ncol( x )-1).
compute pfr = 1-fcdf(fratio,(ncol( x )-1),dfres).
compute modsum={sqrt(r2),r2,mse,fratio,(ncol( x )-1),dfres,pfr}.
compute modsuml={'R','R-sq','MSE',hcflab,'df1','df2', 'p'}.
end if.
end if.
do if ( 2 = 2 or 2 =3).
compute xlp= x.
compute ylp= y.
compute pt2 = make(nrow(ylp),1,(csum(ylp)/nrow(ylp))).
do if ( 2 =2).
compute LL3 = ylp&*ln(pt2)+(1-ylp)&*ln(1-pt2).
end if.
compute LL3 = -2*csum(LL3).
compute bt1 = make(ncol(xlp),1,0).
compute LL1 = 0.
compute pt1 = make(nrow(ylp),1,0.5).
compute pt1lp=pt1.
loop jjj = 1 to iterate.
compute xlptmp=t(xlp).
compute vecprb=pt1lp&*(1-pt1lp).
loop kkk=1 to ncol(xlp).
compute xlptmp(kkk,:)=xlptmp(kkk,:)&*t(vecprb).
end loop.
compute b = bt1+inv(xlptmp*xlp)*t(xlp)*(ylp-pt1lp).
do if ( 2 =2).
compute xlpb=xlp*b.
compute xlpbt=(xlpb > -709.7).
compute xlpb709=(1-xlpbt)*(-709.7).
compute xlpb=(xlpb&*xlpbt)+xlpb709.
compute pt1lp = 1/(1+exp(-(xlpb))).
end if.
compute itprob = csum((pt1lp < .00000001) or (pt1lp > .9999999)).
do if (itprob > 0).
loop kkk = 1 to nrow(pt1lp).
do if (pt1lp(kkk,1) > .9999999).
compute pt1lp(kkk,1) = .9999999.
end if.
do if (pt1lp(kkk,1) < .00000001).
compute pt1lp(kkk,1) = .00000001.
end if.
end loop.
compute itprob = 0.
end if.
do if (itprob = 0).
do if ( 2 =2).
compute LL = ylp&*ln(pt1lp)+(1-ylp)&*ln(1-pt1lp).
end if.
compute LL2 = -2*csum(ll).
end if.
do if (abs(LL1-LL2) < converge).
do if ( 1 =1).
compute xlptmp=t(xlp).
compute vecprb=pt1lp&*(1-pt1lp).
loop kkk=1 to ncol(xlp).
compute xlptmp(kkk,:)=xlptmp(kkk,:)&*t(vecprb).
end loop.
compute varb = inv(xlptmp*xlp).
compute seb = sqrt(diag(varb)).
release xlptmp.
end if.
break.
end if.
compute bt1 = b.
compute LL1 = LL2.
end loop.
compute modres=b.
do if (jjj > iterate).
compute itprob = 2.
do if (booting=0).
compute iterrmod=1.
end if.
do if (booting=1).
compute bootiter=1.
end if.
do if (itprobtg=0).
compute itprobtg=1.
compute errcode(errs,1) = 47.
compute errs = errs + 1.
do if (booting = 0 and 1 =1).
compute vt1 = mdiag(pt1lp&*(1-pt1lp)).
compute varb = inv(t(xlp)*vt1*xlp).
compute seb = sqrt(diag(varb)).
end if.
end if.
end if.
do if ( 1 =1).
compute trat = b&/seb.
compute dfres=nrow(xlp).
compute p = 2*(1-cdfnorm(abs(trat))).
compute modres={modres,seb,trat,p}.
compute modres={modres,(b-xp2&*seb),(b+xp2&*seb)}.
compute pvchi=1-chicdf((LL3-LL2),(nrow(modres)-1)).
compute mcF = (LL3-LL2)/LL3.
compute cox = 1-exp(-(LL3-LL2)/nrow(xlp)).
compute nagel = cox/(1-exp(-(LL3)/nrow(xlp))).
compute modsum={LL2,(LL3-LL2),(nrow(modres)-1),pvchi, mcF,cox,nagel}.
compute modsuml={'-2LL','ModelLL', 'df', 'p', 'McFadden', 'CoxSnell', 'Nagelkrk'}.
compute modresl={'coeff','se','Z','p','LLCI','ULCI'}.
end if.
end if
.
compute dfmatt=make(nrow(modres),1,-999).
end if.
compute resultm2=make(1,maxresm,99999).
compute resultm2(1,1:ncol(modsum))=modsum.
compute resultm={resultm;resultm2}.
compute resultm2=make(nrow(modres),maxresm,99999).
compute resultm2(1:nrow(modres),1:ncol(modres))=modres.
compute resultm={resultm;resultm2}.
compute obscoeff={obscoeff,t(b)}.
do if (outscree=1).
print modsum/title = 'Model Summary'/cnames = modsuml/format= F10.4.
print modres/title='Model'/rnames=vlabsm/cnames=modresl/format= F10.4.
end if.
compute basemod=modsum(1,1).
compute basemodx=basemod.
do if (ydich=1 and (i = (nms+nys))).
do if (outscree=1).
print/title='These results are expressed in a log-odds metric.'.
end if.
compute notecode(notes,1) = 26.
compute notes = notes + 1.
end if.
compute coeffmat={coeffmat;modres}.
compute conseqt=make(nrow(modres),1,outnames(1,i)).
compute conseq={conseq;conseqt}.
compute dfmat={dfmat;dfmatt}.
compute labstart=labstart+nump(1,i).
do if (stand=1).
compute predsd=make(nrow(modres),1,0).
compute stdmod=modres(:,1)&/ovsd(1,i).
loop jd=1 to ncol(x).
compute descdat=x(:,jd).
compute predsd(jd,1) = (nrow(descdat)*sscp(descdat))-(t(csum(descdat))*(csum(descdat))).
compute predsd(jd,1) = sqrt(predsd(jd,1)/(nrow(descdat)*(nrow(descdat)-1))).
end loop.
do if (wherex(1,i) <> -999 and ((nxvls > 1) or (xdich=1))).
compute sdmsone=make(nxvls,1,1).
compute predsd(wherex(1,i):wherex(2,i),1)=sdmsone.
compute pstog=1.
end if.
compute predsd(1,1)=1.
compute stdmod=stdmod&*predsd.
compute stdmod=stdmod(2:nrow(stdmod),1).
compute sdvlabs=vlabsm(2:nrow(vlabsm),1).
compute resultm2=make(nrow(stdmod),maxresm,99999).
compute resultm2(1:nrow(stdmod),1:ncol(stdmod))=stdmod.
compute resultm={resultm;resultm2}.
do if (outscree=1).
print stdmod/title='Standardized coefficients'/clabels='coeff'/rnames=sdvlabs/format= F10.4.
end if.
end if.
do if (nms > 0 and serial = 0 and (rsum(numint) = 0) and (normal=1 or mc > 0)).
do if (i < (nms+nys)).
compute indcov((((i-1)*nxvls)+1):(i*nxvls),(((i-1)*nxvls)+1):(i*nxvls))=varb(2:(1+nxvls), 2:(1+nxvls)).
compute mcsopath((((i-1)*nxvls)+1):(i*nxvls) ,1)=modres(2:(1+nxvls),1).
end if.
do if (i = (nms+nys)).
compute atm=ncol(wherem).
compute indcov(((nms*nxvls)+1):nrow(mcsopath),((nms*nxvls)+1):nrow(mcsopath))= varb(wherem(1,atm):(wherem(1,atm)+nms-1),wherem(1,atm):(wherem(1,atm)+nms-1)).
compute mcsopath(((nms*nxvls)+1):nrow(mcsopath),1)=modres(wherem(1,atm):(wherem(1,atm)+ nms-1),1).
compute sobelok=1.
end if.
end if.
compute obsdirfx=make(1,nxvls,0).
compute dirzes=make(1,nxvls,0).
do if ((i = (nms+nys)) and (bcmat(nrow(bcmat),1)=1)).
compute direff=modres(2:(1+nxvls),:).
compute obsdirfx=t(direff(:,1)).
compute direfflb=modresl.
compute direffl2=vlabsm(2:(1+nxvls),:).
compute lmat=make(nrow(b),1,0).
compute lmat2=make(nxvls,1,1).
compute lmat(2:(1+nxvls),1)=lmat2.
do if (ydich <> 1).

.
compute lmat2= lmat.
do if ( 0 =0).
compute lmat2 = mdiag( lmat ).
compute lmat3=make(nrow(lmat2),1,0).
loop flp=1 to ncol(lmat2).
do if (csum(lmat2(:,flp))=1).
compute lmat3={lmat3,lmat2(:,flp)}.
end if.
end loop.
compute lmat2=lmat3(:,2:ncol(lmat3)).
end if.
compute fratio = (t(t(lmat2)* b )*inv(t(lmat2)* varb *lmat2)*((t(lmat2)* b )))/ncol(lmat2).
compute pfr = 1-fcdf(fratio,ncol(lmat2),(n-nrow( b ))).
compute fresult={fratio,ncol(lmat2),(n-nrow( b )),pfr}.
do if (i = (nms+nys) and (ydich=1)).
compute fratio=fratio*ncol(lmat2).
compute pfr=1-chicdf(fratio,ncol(lmat2)).
compute fresult={fratio,ncol(lmat2),pfr}.
end if.
do if ( 1 =1).
compute lmat3=1-rsum(lmat2).
compute xfm=make(n,csum(lmat3),0).
compute flpc=1.
loop flp=1 to nrow(lmat3).
do if (lmat3(flp,1)=1).
compute xfm(:,flpc)=x(:,flp).
compute flpc=flpc+1.
end if.
end loop.
compute bfm=inv(t(xfm)*xfm)*t(xfm)*y.
compute resid=y-(xfm*bfm).
compute sstotal=(y-(csum(y)/n)).
compute sstotal=csum(sstotal&*sstotal).
compute ssresid=csum(resid&*resid).
compute rsqch= r2 -((sstotal-ssresid)/sstotal).
compute fresult={rsqch,fresult}.
release xfm,flpc, resid, ssresid, bfm.
end if
.
compute diromni=fresult.
end if.
do if (ydich = 1 ).

.
compute btemphld=b.
compute llrdat=make(nrow(x),nrow( lmat )-csum( lmat ),-999).
compute llrdf=ncol(x)-ncol(llrdat).
compute llrcnt=0.
loop llri=1 to nrow( lmat ).
do if ( lmat (llri,1)=0).
compute llrcnt=llrcnt+1.
compute llrdat(:,llrcnt)=x(:,llri).
end if.
end loop.

.
do if ( 2 =1).
compute hatmat=inv(t( llrdat )* llrdat )*t( llrdat ).
compute b = hatmat* y.
compute modres=b.
do if ( 0 =1).
compute n1=nrow( llrdat ).
compute dfres=n1-(ncol( llrdat )).
compute sstotal = t( y -(csum( y )/n1))*( y -(csum( y )/n1)).
compute resid= y - llrdat *b.
compute ssresid = csum((resid)&**2).
compute r2 = (sstotal-ssresid)/sstotal.
do if (r2 < 0).
compute r2=0.
end if.
compute adjr2 = 1-((1-r2)*(n1-1)/(dfres)).
compute mse=ssresid/(n1-ncol( llrdat )).

.
compute n1=nrow( x ).
compute invXtX = inv(t( x )* x ).
compute varb = mse *invXtX.
compute k3 = ncol( x ).
compute xhc=0.
do if ( hc <> 5).
compute xhc= x.
compute hat = xhc(:,1).
loop i3=1 to nrow(xhc).
compute hat(i3,1)= xhc(i3,:)*invXtX*t(xhc(i3,:)).
end loop.
do if ( hc = 0 or hc =1).
loop i3 = 1 to k3.
compute xhc(:,i3)=xhc(:,i3)&* resid.
end loop.
end if.
do if ( hc =3 or hc =2).
loop i3=1 to k3.
compute xhc(:,i3) = ( resid &/(1-hat)&**(1/(4- hc )))&*xhc(:,i3).
end loop.
end if.
do if ( hc = 4).
compute hcmn=make(n,2,4).
compute hcmn(:,2)=(n1*hat)/k3.
loop i3= 1 to k3.
compute xhc(:,i3) = ( resid &/(1-hat)&**(rmin(hcmn)/2))&*xhc(:,i3).
end loop.
end if.
compute varb=(invXtX*t(xhc)*xhc*invXtX).
do if ( hc =1).
compute varb=(n1/(n1-ncol( x )))&*varb.
end if.
end if.
compute hclab={'se(HC0)','se(HC1)','se(HC2)','se(HC3)','se(HC4)','se'}.
compute hclab=hclab(1,( hc +1)).
compute hcflab={'F(HC0)','F(HC1)','F(HC2)','F(HC3)','F(HC4)','F'}.
compute hcflab=hcflab(1,( hc +1)).
release xhc
.
compute seb=sqrt(diag(varb)).
compute trat = b&/seb.
compute p = 2*(1-tcdf(abs(trat), (dfres))).
compute tval = sqrt(dfres* (exp((dfres-(5/6))*((xp2/(dfres-(2/3)+(.11/dfres)))* (xp2/(dfres-(2/3)+(.11/dfres)))))-1)).
compute modres={modres,seb,trat,p}.
compute modres={modres,(b-tval&*seb),(b+tval&*seb)}.
compute modresl={'coeff',hclab,'t','p','LLCI','ULCI'}.
compute lmat = ident(ncol( llrdat )).
compute lmat = lmat(:,2:ncol(lmat)).
compute fratio = (t(t(lmat)*b)*inv(t(lmat)*varb*lmat)*((t(lmat)*b)))/(ncol( llrdat )-1).
compute pfr = 1-fcdf(fratio,(ncol( llrdat )-1),dfres).
compute modsum={sqrt(r2),r2,mse,fratio,(ncol( llrdat )-1),dfres,pfr}.
compute modsuml={'R','R-sq','MSE',hcflab,'df1','df2', 'p'}.
end if.
end if.
do if ( 2 = 2 or 2 =3).
compute xlp= llrdat.
compute ylp= y.
compute pt2 = make(nrow(ylp),1,(csum(ylp)/nrow(ylp))).
do if ( 2 =2).
compute LL3 = ylp&*ln(pt2)+(1-ylp)&*ln(1-pt2).
end if.
compute LL3 = -2*csum(LL3).
compute bt1 = make(ncol(xlp),1,0).
compute LL1 = 0.
compute pt1 = make(nrow(ylp),1,0.5).
compute pt1lp=pt1.
loop jjj = 1 to iterate.
compute xlptmp=t(xlp).
compute vecprb=pt1lp&*(1-pt1lp).
loop kkk=1 to ncol(xlp).
compute xlptmp(kkk,:)=xlptmp(kkk,:)&*t(vecprb).
end loop.
compute b = bt1+inv(xlptmp*xlp)*t(xlp)*(ylp-pt1lp).
do if ( 2 =2).
compute xlpb=xlp*b.
compute xlpbt=(xlpb > -709.7).
compute xlpb709=(1-xlpbt)*(-709.7).
compute xlpb=(xlpb&*xlpbt)+xlpb709.
compute pt1lp = 1/(1+exp(-(xlpb))).
end if.
compute itprob = csum((pt1lp < .00000001) or (pt1lp > .9999999)).
do if (itprob > 0).
loop kkk = 1 to nrow(pt1lp).
do if (pt1lp(kkk,1) > .9999999).
compute pt1lp(kkk,1) = .9999999.
end if.
do if (pt1lp(kkk,1) < .00000001).
compute pt1lp(kkk,1) = .00000001.
end if.
end loop.
compute itprob = 0.
end if.
do if (itprob = 0).
do if ( 2 =2).
compute LL = ylp&*ln(pt1lp)+(1-ylp)&*ln(1-pt1lp).
end if.
compute LL2 = -2*csum(ll).
end if.
do if (abs(LL1-LL2) < converge).
do if ( 0 =1).
compute xlptmp=t(xlp).
compute vecprb=pt1lp&*(1-pt1lp).
loop kkk=1 to ncol(xlp).
compute xlptmp(kkk,:)=xlptmp(kkk,:)&*t(vecprb).
end loop.
compute varb = inv(xlptmp*xlp).
compute seb = sqrt(diag(varb)).
release xlptmp.
end if.
break.
end if.
compute bt1 = b.
compute LL1 = LL2.
end loop.
compute modres=b.
do if (jjj > iterate).
compute itprob = 2.
do if (booting=0).
compute iterrmod=1.
end if.
do if (booting=1).
compute bootiter=1.
end if.
do if (itprobtg=0).
compute itprobtg=1.
compute errcode(errs,1) = 47.
compute errs = errs + 1.
do if (booting = 0 and 0 =1).
compute vt1 = mdiag(pt1lp&*(1-pt1lp)).
compute varb = inv(t(xlp)*vt1*xlp).
compute seb = sqrt(diag(varb)).
end if.
end if.
end if.
do if ( 0 =1).
compute trat = b&/seb.
compute dfres=nrow(xlp).
compute p = 2*(1-cdfnorm(abs(trat))).
compute modres={modres,seb,trat,p}.
compute modres={modres,(b-xp2&*seb),(b+xp2&*seb)}.
compute pvchi=1-chicdf((LL3-LL2),(nrow(modres)-1)).
compute mcF = (LL3-LL2)/LL3.
compute cox = 1-exp(-(LL3-LL2)/nrow(xlp)).
compute nagel = cox/(1-exp(-(LL3)/nrow(xlp))).
compute modsum={LL2,(LL3-LL2),(nrow(modres)-1),pvchi, mcF,cox,nagel}.
compute modsuml={'-2LL','ModelLL', 'df', 'p', 'McFadden', 'CoxSnell', 'Nagelkrk'}.
compute modresl={'coeff','se','Z','p','LLCI','ULCI'}.
end if.
end if
.
compute b=btemphld.
compute fresult={(LL2-basemod),llrdf,1-chicdf((LL2-basemod),llrdf)}
.
compute diromni=fresult.
end if.
end if.
do if (numint(1,i) > 0).
compute intkeym=intkey(intstart:(intstart+numint(1,i)-1),:).
do if (outscree=1).
print intkeym/title='Product terms key:'/format = A8.
end if.
end if.
do if (covcoeff=1).
do if (outscree=1).
print varb/title='Covariance matrix of regression parameter estimates:'/rnames=vlabsm /cnames=vlabsm/format= F10.4.
end if.
compute resultm2=make(nrow(varb),maxresm,99999).
do if (ncol(varb) <= maxresm).
compute resultm2(1:nrow(varb),1:ncol(varb))=varb.
compute resultm={resultm;resultm2}.
end if.
do if (ncol(varb) > maxresm).
compute resultmt=make(nrow(resultm),ncol(varb),99999).
compute resultmt(1:nrow(resultm),1:ncol(resultm))=resultm.
compute resultm=resultmt.
compute resultm2=make(nrow(varb),ncol(resultm),99999).
compute resultm2(1:nrow(varb),1:ncol(varb))=varb.
compute resultm={resultm;resultm2}.
compute maxresm=ncol(resultm).
end if.
end if.
do if (model <> 74 and xmtest=1 and nms > 0).
compute r2tmp=r2.
compute btmp=b.
compute varbtmp=varb.
compute dfrestmp=dfres.
compute tvaltmp=tval.
compute xmtst=make(nms,4,0).
compute xmtstlbc={hcflab,'df1','df2','p'}.
do if ((i=(nms+nys)) and (ydich=1)).
compute xmtst=make(nms,3,0).
compute xmtstlbc={'Chi-sq','df','p'}.
end if.
compute xmtstlb={' '}.
compute xmtmat=x.
compute numxint=0.
loop xmints=2 to i.
compute x=xmtmat.
do if ((bcmat((i+1),xmints)=1) and (wzcmat((i+1),xmints) <> 1)).
do if (bcmat((i+1),1)=0).
compute x={xmtmat,xtmp}.
do if ((ydich=1) and (i=(nms+nys))).

.
do if ( 2 =1).
compute hatmat=inv(t( x )* x )*t( x ).
compute b = hatmat* y.
compute modres=b.
do if ( 1 =1).
compute n1=nrow( x ).
compute dfres=n1-(ncol( x )).
compute sstotal = t( y -(csum( y )/n1))*( y -(csum( y )/n1)).
compute resid= y - x *b.
compute ssresid = csum((resid)&**2).
compute r2 = (sstotal-ssresid)/sstotal.
do if (r2 < 0).
compute r2=0.
end if.
compute adjr2 = 1-((1-r2)*(n1-1)/(dfres)).
compute mse=ssresid/(n1-ncol( x )).

.
compute n1=nrow( x ).
compute invXtX = inv(t( x )* x ).
compute varb = mse *invXtX.
compute k3 = ncol( x ).
compute xhc=0.
do if ( hc <> 5).
compute xhc= x.
compute hat = xhc(:,1).
loop i3=1 to nrow(xhc).
compute hat(i3,1)= xhc(i3,:)*invXtX*t(xhc(i3,:)).
end loop.
do if ( hc = 0 or hc =1).
loop i3 = 1 to k3.
compute xhc(:,i3)=xhc(:,i3)&* resid.
end loop.
end if.
do if ( hc =3 or hc =2).
loop i3=1 to k3.
compute xhc(:,i3) = ( resid &/(1-hat)&**(1/(4- hc )))&*xhc(:,i3).
end loop.
end if.
do if ( hc = 4).
compute hcmn=make(n,2,4).
compute hcmn(:,2)=(n1*hat)/k3.
loop i3= 1 to k3.
compute xhc(:,i3) = ( resid &/(1-hat)&**(rmin(hcmn)/2))&*xhc(:,i3).
end loop.
end if.
compute varb=(invXtX*t(xhc)*xhc*invXtX).
do if ( hc =1).
compute varb=(n1/(n1-ncol( x )))&*varb.
end if.
end if.
compute hclab={'se(HC0)','se(HC1)','se(HC2)','se(HC3)','se(HC4)','se'}.
compute hclab=hclab(1,( hc +1)).
compute hcflab={'F(HC0)','F(HC1)','F(HC2)','F(HC3)','F(HC4)','F'}.
compute hcflab=hcflab(1,( hc +1)).
release xhc
.
compute seb=sqrt(diag(varb)).
compute trat = b&/seb.
compute p = 2*(1-tcdf(abs(trat), (dfres))).
compute tval = sqrt(dfres* (exp((dfres-(5/6))*((xp2/(dfres-(2/3)+(.11/dfres)))* (xp2/(dfres-(2/3)+(.11/dfres)))))-1)).
compute modres={modres,seb,trat,p}.
compute modres={modres,(b-tval&*seb),(b+tval&*seb)}.
compute modresl={'coeff',hclab,'t','p','LLCI','ULCI'}.
compute lmat = ident(ncol( x )).
compute lmat = lmat(:,2:ncol(lmat)).
compute fratio = (t(t(lmat)*b)*inv(t(lmat)*varb*lmat)*((t(lmat)*b)))/(ncol( x )-1).
compute pfr = 1-fcdf(fratio,(ncol( x )-1),dfres).
compute modsum={sqrt(r2),r2,mse,fratio,(ncol( x )-1),dfres,pfr}.
compute modsuml={'R','R-sq','MSE',hcflab,'df1','df2', 'p'}.
end if.
end if.
do if ( 2 = 2 or 2 =3).
compute xlp= x.
compute ylp= y.
compute pt2 = make(nrow(ylp),1,(csum(ylp)/nrow(ylp))).
do if ( 2 =2).
compute LL3 = ylp&*ln(pt2)+(1-ylp)&*ln(1-pt2).
end if.
compute LL3 = -2*csum(LL3).
compute bt1 = make(ncol(xlp),1,0).
compute LL1 = 0.
compute pt1 = make(nrow(ylp),1,0.5).
compute pt1lp=pt1.
loop jjj = 1 to iterate.
compute xlptmp=t(xlp).
compute vecprb=pt1lp&*(1-pt1lp).
loop kkk=1 to ncol(xlp).
compute xlptmp(kkk,:)=xlptmp(kkk,:)&*t(vecprb).
end loop.
compute b = bt1+inv(xlptmp*xlp)*t(xlp)*(ylp-pt1lp).
do if ( 2 =2).
compute xlpb=xlp*b.
compute xlpbt=(xlpb > -709.7).
compute xlpb709=(1-xlpbt)*(-709.7).
compute xlpb=(xlpb&*xlpbt)+xlpb709.
compute pt1lp = 1/(1+exp(-(xlpb))).
end if.
compute itprob = csum((pt1lp < .00000001) or (pt1lp > .9999999)).
do if (itprob > 0).
loop kkk = 1 to nrow(pt1lp).
do if (pt1lp(kkk,1) > .9999999).
compute pt1lp(kkk,1) = .9999999.
end if.
do if (pt1lp(kkk,1) < .00000001).
compute pt1lp(kkk,1) = .00000001.
end if.
end loop.
compute itprob = 0.
end if.
do if (itprob = 0).
do if ( 2 =2).
compute LL = ylp&*ln(pt1lp)+(1-ylp)&*ln(1-pt1lp).
end if.
compute LL2 = -2*csum(ll).
end if.
do if (abs(LL1-LL2) < converge).
do if ( 1 =1).
compute xlptmp=t(xlp).
compute vecprb=pt1lp&*(1-pt1lp).
loop kkk=1 to ncol(xlp).
compute xlptmp(kkk,:)=xlptmp(kkk,:)&*t(vecprb).
end loop.
compute varb = inv(xlptmp*xlp).
compute seb = sqrt(diag(varb)).
release xlptmp.
end if.
break.
end if.
compute bt1 = b.
compute LL1 = LL2.
end loop.
compute modres=b.
do if (jjj > iterate).
compute itprob = 2.
do if (booting=0).
compute iterrmod=1.
end if.
do if (booting=1).
compute bootiter=1.
end if.
do if (itprobtg=0).
compute itprobtg=1.
compute errcode(errs,1) = 47.
compute errs = errs + 1.
do if (booting = 0 and 1 =1).
compute vt1 = mdiag(pt1lp&*(1-pt1lp)).
compute varb = inv(t(xlp)*vt1*xlp).
compute seb = sqrt(diag(varb)).
end if.
end if.
end if.
do if ( 1 =1).
compute trat = b&/seb.
compute dfres=nrow(xlp).
compute p = 2*(1-cdfnorm(abs(trat))).
compute modres={modres,seb,trat,p}.
compute modres={modres,(b-xp2&*seb),(b+xp2&*seb)}.
compute pvchi=1-chicdf((LL3-LL2),(nrow(modres)-1)).
compute mcF = (LL3-LL2)/LL3.
compute cox = 1-exp(-(LL3-LL2)/nrow(xlp)).
compute nagel = cox/(1-exp(-(LL3)/nrow(xlp))).
compute modsum={LL2,(LL3-LL2),(nrow(modres)-1),pvchi, mcF,cox,nagel}.
compute modsuml={'-2LL','ModelLL', 'df', 'p', 'McFadden', 'CoxSnell', 'Nagelkrk'}.
compute modresl={'coeff','se','Z','p','LLCI','ULCI'}.
end if.
end if
.
compute basemodx=LL2.
end if.
end if.
loop xmtlp1=1 to nxvls.
compute x={x,xtmp(:,xmtlp1)&*(mtmp(:,(xmints-1))-(csum(mtmp(:,(xmints-1)))/nrow(mtmp)) )}.
end loop.
do if ((i < (nms+nys)) or (ydich=0)).

.
do if ( 1 =1).
compute hatmat=inv(t( x )* x )*t( x ).
compute b = hatmat* y.
compute modres=b.
do if ( 1 =1).
compute n1=nrow( x ).
compute dfres=n1-(ncol( x )).
compute sstotal = t( y -(csum( y )/n1))*( y -(csum( y )/n1)).
compute resid= y - x *b.
compute ssresid = csum((resid)&**2).
compute r2 = (sstotal-ssresid)/sstotal.
do if (r2 < 0).
compute r2=0.
end if.
compute adjr2 = 1-((1-r2)*(n1-1)/(dfres)).
compute mse=ssresid/(n1-ncol( x )).

.
compute n1=nrow( x ).
compute invXtX = inv(t( x )* x ).
compute varb = mse *invXtX.
compute k3 = ncol( x ).
compute xhc=0.
do if ( hc <> 5).
compute xhc= x.
compute hat = xhc(:,1).
loop i3=1 to nrow(xhc).
compute hat(i3,1)= xhc(i3,:)*invXtX*t(xhc(i3,:)).
end loop.
do if ( hc = 0 or hc =1).
loop i3 = 1 to k3.
compute xhc(:,i3)=xhc(:,i3)&* resid.
end loop.
end if.
do if ( hc =3 or hc =2).
loop i3=1 to k3.
compute xhc(:,i3) = ( resid &/(1-hat)&**(1/(4- hc )))&*xhc(:,i3).
end loop.
end if.
do if ( hc = 4).
compute hcmn=make(n,2,4).
compute hcmn(:,2)=(n1*hat)/k3.
loop i3= 1 to k3.
compute xhc(:,i3) = ( resid &/(1-hat)&**(rmin(hcmn)/2))&*xhc(:,i3).
end loop.
end if.
compute varb=(invXtX*t(xhc)*xhc*invXtX).
do if ( hc =1).
compute varb=(n1/(n1-ncol( x )))&*varb.
end if.
end if.
compute hclab={'se(HC0)','se(HC1)','se(HC2)','se(HC3)','se(HC4)','se'}.
compute hclab=hclab(1,( hc +1)).
compute hcflab={'F(HC0)','F(HC1)','F(HC2)','F(HC3)','F(HC4)','F'}.
compute hcflab=hcflab(1,( hc +1)).
release xhc
.
compute seb=sqrt(diag(varb)).
compute trat = b&/seb.
compute p = 2*(1-tcdf(abs(trat), (dfres))).
compute tval = sqrt(dfres* (exp((dfres-(5/6))*((xp2/(dfres-(2/3)+(.11/dfres)))* (xp2/(dfres-(2/3)+(.11/dfres)))))-1)).
compute modres={modres,seb,trat,p}.
compute modres={modres,(b-tval&*seb),(b+tval&*seb)}.
compute modresl={'coeff',hclab,'t','p','LLCI','ULCI'}.
compute lmat = ident(ncol( x )).
compute lmat = lmat(:,2:ncol(lmat)).
compute fratio = (t(t(lmat)*b)*inv(t(lmat)*varb*lmat)*((t(lmat)*b)))/(ncol( x )-1).
compute pfr = 1-fcdf(fratio,(ncol( x )-1),dfres).
compute modsum={sqrt(r2),r2,mse,fratio,(ncol( x )-1),dfres,pfr}.
compute modsuml={'R','R-sq','MSE',hcflab,'df1','df2', 'p'}.
end if.
end if.
do if ( 1 = 2 or 1 =3).
compute xlp= x.
compute ylp= y.
compute pt2 = make(nrow(ylp),1,(csum(ylp)/nrow(ylp))).
do if ( 1 =2).
compute LL3 = ylp&*ln(pt2)+(1-ylp)&*ln(1-pt2).
end if.
compute LL3 = -2*csum(LL3).
compute bt1 = make(ncol(xlp),1,0).
compute LL1 = 0.
compute pt1 = make(nrow(ylp),1,0.5).
compute pt1lp=pt1.
loop jjj = 1 to iterate.
compute xlptmp=t(xlp).
compute vecprb=pt1lp&*(1-pt1lp).
loop kkk=1 to ncol(xlp).
compute xlptmp(kkk,:)=xlptmp(kkk,:)&*t(vecprb).
end loop.
compute b = bt1+inv(xlptmp*xlp)*t(xlp)*(ylp-pt1lp).
do if ( 1 =2).
compute xlpb=xlp*b.
compute xlpbt=(xlpb > -709.7).
compute xlpb709=(1-xlpbt)*(-709.7).
compute xlpb=(xlpb&*xlpbt)+xlpb709.
compute pt1lp = 1/(1+exp(-(xlpb))).
end if.
compute itprob = csum((pt1lp < .00000001) or (pt1lp > .9999999)).
do if (itprob > 0).
loop kkk = 1 to nrow(pt1lp).
do if (pt1lp(kkk,1) > .9999999).
compute pt1lp(kkk,1) = .9999999.
end if.
do if (pt1lp(kkk,1) < .00000001).
compute pt1lp(kkk,1) = .00000001.
end if.
end loop.
compute itprob = 0.
end if.
do if (itprob = 0).
do if ( 1 =2).
compute LL = ylp&*ln(pt1lp)+(1-ylp)&*ln(1-pt1lp).
end if.
compute LL2 = -2*csum(ll).
end if.
do if (abs(LL1-LL2) < converge).
do if ( 1 =1).
compute xlptmp=t(xlp).
compute vecprb=pt1lp&*(1-pt1lp).
loop kkk=1 to ncol(xlp).
compute xlptmp(kkk,:)=xlptmp(kkk,:)&*t(vecprb).
end loop.
compute varb = inv(xlptmp*xlp).
compute seb = sqrt(diag(varb)).
release xlptmp.
end if.
break.
end if.
compute bt1 = b.
compute LL1 = LL2.
end loop.
compute modres=b.
do if (jjj > iterate).
compute itprob = 2.
do if (booting=0).
compute iterrmod=1.
end if.
do if (booting=1).
compute bootiter=1.
end if.
do if (itprobtg=0).
compute itprobtg=1.
compute errcode(errs,1) = 47.
compute errs = errs + 1.
do if (booting = 0 and 1 =1).
compute vt1 = mdiag(pt1lp&*(1-pt1lp)).
compute varb = inv(t(xlp)*vt1*xlp).
compute seb = sqrt(diag(varb)).
end if.
end if.
end if.
do if ( 1 =1).
compute trat = b&/seb.
compute dfres=nrow(xlp).
compute p = 2*(1-cdfnorm(abs(trat))).
compute modres={modres,seb,trat,p}.
compute modres={modres,(b-xp2&*seb),(b+xp2&*seb)}.
compute pvchi=1-chicdf((LL3-LL2),(nrow(modres)-1)).
compute mcF = (LL3-LL2)/LL3.
compute cox = 1-exp(-(LL3-LL2)/nrow(xlp)).
compute nagel = cox/(1-exp(-(LL3)/nrow(xlp))).
compute modsum={LL2,(LL3-LL2),(nrow(modres)-1),pvchi, mcF,cox,nagel}.
compute modsuml={'-2LL','ModelLL', 'df', 'p', 'McFadden', 'CoxSnell', 'Nagelkrk'}.
compute modresl={'coeff','se','Z','p','LLCI','ULCI'}.
end if.
end if
.
end if.
do if ((i = (nms+nys)) and (ydich=1)).

.
do if ( 2 =1).
compute hatmat=inv(t( x )* x )*t( x ).
compute b = hatmat* y.
compute modres=b.
do if ( 1 =1).
compute n1=nrow( x ).
compute dfres=n1-(ncol( x )).
compute sstotal = t( y -(csum( y )/n1))*( y -(csum( y )/n1)).
compute resid= y - x *b.
compute ssresid = csum((resid)&**2).
compute r2 = (sstotal-ssresid)/sstotal.
do if (r2 < 0).
compute r2=0.
end if.
compute adjr2 = 1-((1-r2)*(n1-1)/(dfres)).
compute mse=ssresid/(n1-ncol( x )).

.
compute n1=nrow( x ).
compute invXtX = inv(t( x )* x ).
compute varb = mse *invXtX.
compute k3 = ncol( x ).
compute xhc=0.
do if ( hc <> 5).
compute xhc= x.
compute hat = xhc(:,1).
loop i3=1 to nrow(xhc).
compute hat(i3,1)= xhc(i3,:)*invXtX*t(xhc(i3,:)).
end loop.
do if ( hc = 0 or hc =1).
loop i3 = 1 to k3.
compute xhc(:,i3)=xhc(:,i3)&* resid.
end loop.
end if.
do if ( hc =3 or hc =2).
loop i3=1 to k3.
compute xhc(:,i3) = ( resid &/(1-hat)&**(1/(4- hc )))&*xhc(:,i3).
end loop.
end if.
do if ( hc = 4).
compute hcmn=make(n,2,4).
compute hcmn(:,2)=(n1*hat)/k3.
loop i3= 1 to k3.
compute xhc(:,i3) = ( resid &/(1-hat)&**(rmin(hcmn)/2))&*xhc(:,i3).
end loop.
end if.
compute varb=(invXtX*t(xhc)*xhc*invXtX).
do if ( hc =1).
compute varb=(n1/(n1-ncol( x )))&*varb.
end if.
end if.
compute hclab={'se(HC0)','se(HC1)','se(HC2)','se(HC3)','se(HC4)','se'}.
compute hclab=hclab(1,( hc +1)).
compute hcflab={'F(HC0)','F(HC1)','F(HC2)','F(HC3)','F(HC4)','F'}.
compute hcflab=hcflab(1,( hc +1)).
release xhc
.
compute seb=sqrt(diag(varb)).
compute trat = b&/seb.
compute p = 2*(1-tcdf(abs(trat), (dfres))).
compute tval = sqrt(dfres* (exp((dfres-(5/6))*((xp2/(dfres-(2/3)+(.11/dfres)))* (xp2/(dfres-(2/3)+(.11/dfres)))))-1)).
compute modres={modres,seb,trat,p}.
compute modres={modres,(b-tval&*seb),(b+tval&*seb)}.
compute modresl={'coeff',hclab,'t','p','LLCI','ULCI'}.
compute lmat = ident(ncol( x )).
compute lmat = lmat(:,2:ncol(lmat)).
compute fratio = (t(t(lmat)*b)*inv(t(lmat)*varb*lmat)*((t(lmat)*b)))/(ncol( x )-1).
compute pfr = 1-fcdf(fratio,(ncol( x )-1),dfres).
compute modsum={sqrt(r2),r2,mse,fratio,(ncol( x )-1),dfres,pfr}.
compute modsuml={'R','R-sq','MSE',hcflab,'df1','df2', 'p'}.
end if.
end if.
do if ( 2 = 2 or 2 =3).
compute xlp= x.
compute ylp= y.
compute pt2 = make(nrow(ylp),1,(csum(ylp)/nrow(ylp))).
do if ( 2 =2).
compute LL3 = ylp&*ln(pt2)+(1-ylp)&*ln(1-pt2).
end if.
compute LL3 = -2*csum(LL3).
compute bt1 = make(ncol(xlp),1,0).
compute LL1 = 0.
compute pt1 = make(nrow(ylp),1,0.5).
compute pt1lp=pt1.
loop jjj = 1 to iterate.
compute xlptmp=t(xlp).
compute vecprb=pt1lp&*(1-pt1lp).
loop kkk=1 to ncol(xlp).
compute xlptmp(kkk,:)=xlptmp(kkk,:)&*t(vecprb).
end loop.
compute b = bt1+inv(xlptmp*xlp)*t(xlp)*(ylp-pt1lp).
do if ( 2 =2).
compute xlpb=xlp*b.
compute xlpbt=(xlpb > -709.7).
compute xlpb709=(1-xlpbt)*(-709.7).
compute xlpb=(xlpb&*xlpbt)+xlpb709.
compute pt1lp = 1/(1+exp(-(xlpb))).
end if.
compute itprob = csum((pt1lp < .00000001) or (pt1lp > .9999999)).
do if (itprob > 0).
loop kkk = 1 to nrow(pt1lp).
do if (pt1lp(kkk,1) > .9999999).
compute pt1lp(kkk,1) = .9999999.
end if.
do if (pt1lp(kkk,1) < .00000001).
compute pt1lp(kkk,1) = .00000001.
end if.
end loop.
compute itprob = 0.
end if.
do if (itprob = 0).
do if ( 2 =2).
compute LL = ylp&*ln(pt1lp)+(1-ylp)&*ln(1-pt1lp).
end if.
compute LL2 = -2*csum(ll).
end if.
do if (abs(LL1-LL2) < converge).
do if ( 1 =1).
compute xlptmp=t(xlp).
compute vecprb=pt1lp&*(1-pt1lp).
loop kkk=1 to ncol(xlp).
compute xlptmp(kkk,:)=xlptmp(kkk,:)&*t(vecprb).
end loop.
compute varb = inv(xlptmp*xlp).
compute seb = sqrt(diag(varb)).
release xlptmp.
end if.
break.
end if.
compute bt1 = b.
compute LL1 = LL2.
end loop.
compute modres=b.
do if (jjj > iterate).
compute itprob = 2.
do if (booting=0).
compute iterrmod=1.
end if.
do if (booting=1).
compute bootiter=1.
end if.
do if (itprobtg=0).
compute itprobtg=1.
compute errcode(errs,1) = 47.
compute errs = errs + 1.
do if (booting = 0 and 1 =1).
compute vt1 = mdiag(pt1lp&*(1-pt1lp)).
compute varb = inv(t(xlp)*vt1*xlp).
compute seb = sqrt(diag(varb)).
end if.
end if.
end if.
do if ( 1 =1).
compute trat = b&/seb.
compute dfres=nrow(xlp).
compute p = 2*(1-cdfnorm(abs(trat))).
compute modres={modres,seb,trat,p}.
compute modres={modres,(b-xp2&*seb),(b+xp2&*seb)}.
compute pvchi=1-chicdf((LL3-LL2),(nrow(modres)-1)).
compute mcF = (LL3-LL2)/LL3.
compute cox = 1-exp(-(LL3-LL2)/nrow(xlp)).
compute nagel = cox/(1-exp(-(LL3)/nrow(xlp))).
compute modsum={LL2,(LL3-LL2),(nrow(modres)-1),pvchi, mcF,cox,nagel}.
compute modsuml={'-2LL','ModelLL', 'df', 'p', 'McFadden', 'CoxSnell', 'Nagelkrk'}.
compute modresl={'coeff','se','Z','p','LLCI','ULCI'}.
end if.
end if
.
compute chidfxm=basemodx-LL2.
end if.
compute lmat=make(nrow(b),nxvls,0).
compute lmattmp=ident(nxvls).
compute lmat((nrow(lmat)-nxvls+1):nrow(lmat),:)=lmattmp.

.
compute lmat2= lmat.
do if ( 1 =0).
compute lmat2 = mdiag( lmat ).
compute lmat3=make(nrow(lmat2),1,0).
loop flp=1 to ncol(lmat2).
do if (csum(lmat2(:,flp))=1).
compute lmat3={lmat3,lmat2(:,flp)}.
end if.
end loop.
compute lmat2=lmat3(:,2:ncol(lmat3)).
end if.
compute fratio = (t(t(lmat2)* b )*inv(t(lmat2)* varb *lmat2)*((t(lmat2)* b )))/ncol(lmat2).
compute pfr = 1-fcdf(fratio,ncol(lmat2),(n-nrow( b ))).
compute fresult={fratio,ncol(lmat2),(n-nrow( b )),pfr}.
do if (i = (nms+nys) and (ydich=1)).
compute fratio=fratio*ncol(lmat2).
compute pfr=1-chicdf(fratio,ncol(lmat2)).
compute fresult={fratio,ncol(lmat2),pfr}.
end if.
do if ( 0 =1).
compute lmat3=1-rsum(lmat2).
compute xfm=make(n,csum(lmat3),0).
compute flpc=1.
loop flp=1 to nrow(lmat3).
do if (lmat3(flp,1)=1).
compute xfm(:,flpc)=x(:,flp).
compute flpc=flpc+1.
end if.
end loop.
compute bfm=inv(t(xfm)*xfm)*t(xfm)*y.
compute resid=y-(xfm*bfm).
compute sstotal=(y-(csum(y)/n)).
compute sstotal=csum(sstotal&*sstotal).
compute ssresid=csum(resid&*resid).
compute rsqch= 0 -((sstotal-ssresid)/sstotal).
compute fresult={rsqch,fresult}.
release xfm,flpc, resid, ssresid, bfm.
end if
.
compute numxint=numxint+1.
compute xmtst(numxint,:)=fresult.
do if ((i = (nms+nys)) and (ydich=1)).
compute xmtst(numxint,1)=chidfxm.
compute xmtst(numxint,3)=1-chicdf(chidfxm,nxvls).
end if.
compute xmtstlb={xmtstlb;highlbx((xmints-1),1)}.
end if.
end loop.
compute x=xmtmat.
release xmtmat.
do if (numxint>0).
compute xmtstlb=xmtstlb((2:(numxint+1)),:).
compute xmtst=xmtst(1:numxint,:).
compute resultm2=make(nrow(xmtst),maxresm,99999).
compute resultm2(1:nrow(xmtst),1:ncol(xmtst))=xmtst.
compute resultm={resultm;resultm2}.
do if (nms=1).
compute xmtstlb=' '.
end if.
do if (outscree=1).
do if ((i < (nms+nys)) or (ydich=0)).
print xmtst/title='Test(s) of X by M interaction:'/rnames=xmtstlb/cnames=xmtstlbc/format= F10.4.
end if.
do if ((i = (nms+nys)) and (ydich=1)).
print xmtst/title='Likelihood ratio test(s) of X by M interaction:'/rnames=xmtstlb/cnames=xmtstlbc/format= F10.4.
end if.
end if.
end if.
compute r2=r2tmp.
compute b=btmp.
compute varb=varbtmp.
compute dfres=dfrestmp.
compute tval=tvaltmp.
end if.
do if (criterr = 0).
compute jj=0.
loop j = start to ((start+i)-1).
compute dbint=0.
compute lmat=whigh(1:nump(1,i),j).
compute lmat2=wzhigh(1:nump(1,i),j).
do if ((csum(lmat) > 0) and (csum(lmat2) = 0)).
do if ((i < (nms+nys)) or (ydich <> 1)).

.
compute lmat2= lmat.
do if ( 0 =0).
compute lmat2 = mdiag( lmat ).
compute lmat3=make(nrow(lmat2),1,0).
loop flp=1 to ncol(lmat2).
do if (csum(lmat2(:,flp))=1).
compute lmat3={lmat3,lmat2(:,flp)}.
end if.
end loop.
compute lmat2=lmat3(:,2:ncol(lmat3)).
end if.
compute fratio = (t(t(lmat2)* b )*inv(t(lmat2)* varb *lmat2)*((t(lmat2)* b )))/ncol(lmat2).
compute pfr = 1-fcdf(fratio,ncol(lmat2),(n-nrow( b ))).
compute fresult={fratio,ncol(lmat2),(n-nrow( b )),pfr}.
do if (i = (nms+nys) and (ydich=1)).
compute fratio=fratio*ncol(lmat2).
compute pfr=1-chicdf(fratio,ncol(lmat2)).
compute fresult={fratio,ncol(lmat2),pfr}.
end if.
do if ( 1 =1).
compute lmat3=1-rsum(lmat2).
compute xfm=make(n,csum(lmat3),0).
compute flpc=1.
loop flp=1 to nrow(lmat3).
do if (lmat3(flp,1)=1).
compute xfm(:,flpc)=x(:,flp).
compute flpc=flpc+1.
end if.
end loop.
compute bfm=inv(t(xfm)*xfm)*t(xfm)*y.
compute resid=y-(xfm*bfm).
compute sstotal=(y-(csum(y)/n)).
compute sstotal=csum(sstotal&*sstotal).
compute ssresid=csum(resid&*resid).
compute rsqch= r2 -((sstotal-ssresid)/sstotal).
compute fresult={rsqch,fresult}.
release xfm,flpc, resid, ssresid, bfm.
end if
.
compute lmatdb=lmat.
compute dbint=dbint+1.
end if.
do if ((ydich = 1) and (i = (nms+nys))).

.
compute btemphld=b.
compute llrdat=make(nrow(x),nrow( lmat )-csum( lmat ),-999).
compute llrdf=ncol(x)-ncol(llrdat).
compute llrcnt=0.
loop llri=1 to nrow( lmat ).
do if ( lmat (llri,1)=0).
compute llrcnt=llrcnt+1.
compute llrdat(:,llrcnt)=x(:,llri).
end if.
end loop.

.
do if ( 2 =1).
compute hatmat=inv(t( llrdat )* llrdat )*t( llrdat ).
compute b = hatmat* y.
compute modres=b.
do if ( 0 =1).
compute n1=nrow( llrdat ).
compute dfres=n1-(ncol( llrdat )).
compute sstotal = t( y -(csum( y )/n1))*( y -(csum( y )/n1)).
compute resid= y - llrdat *b.
compute ssresid = csum((resid)&**2).
compute r2 = (sstotal-ssresid)/sstotal.
do if (r2 < 0).
compute r2=0.
end if.
compute adjr2 = 1-((1-r2)*(n1-1)/(dfres)).
compute mse=ssresid/(n1-ncol( llrdat )).

.
compute n1=nrow( x ).
compute invXtX = inv(t( x )* x ).
compute varb = mse *invXtX.
compute k3 = ncol( x ).
compute xhc=0.
do if ( hc <> 5).
compute xhc= x.
compute hat = xhc(:,1).
loop i3=1 to nrow(xhc).
compute hat(i3,1)= xhc(i3,:)*invXtX*t(xhc(i3,:)).
end loop.
do if ( hc = 0 or hc =1).
loop i3 = 1 to k3.
compute xhc(:,i3)=xhc(:,i3)&* resid.
end loop.
end if.
do if ( hc =3 or hc =2).
loop i3=1 to k3.
compute xhc(:,i3) = ( resid &/(1-hat)&**(1/(4- hc )))&*xhc(:,i3).
end loop.
end if.
do if ( hc = 4).
compute hcmn=make(n,2,4).
compute hcmn(:,2)=(n1*hat)/k3.
loop i3= 1 to k3.
compute xhc(:,i3) = ( resid &/(1-hat)&**(rmin(hcmn)/2))&*xhc(:,i3).
end loop.
end if.
compute varb=(invXtX*t(xhc)*xhc*invXtX).
do if ( hc =1).
compute varb=(n1/(n1-ncol( x )))&*varb.
end if.
end if.
compute hclab={'se(HC0)','se(HC1)','se(HC2)','se(HC3)','se(HC4)','se'}.
compute hclab=hclab(1,( hc +1)).
compute hcflab={'F(HC0)','F(HC1)','F(HC2)','F(HC3)','F(HC4)','F'}.
compute hcflab=hcflab(1,( hc +1)).
release xhc
.
compute seb=sqrt(diag(varb)).
compute trat = b&/seb.
compute p = 2*(1-tcdf(abs(trat), (dfres))).
compute tval = sqrt(dfres* (exp((dfres-(5/6))*((xp2/(dfres-(2/3)+(.11/dfres)))* (xp2/(dfres-(2/3)+(.11/dfres)))))-1)).
compute modres={modres,seb,trat,p}.
compute modres={modres,(b-tval&*seb),(b+tval&*seb)}.
compute modresl={'coeff',hclab,'t','p','LLCI','ULCI'}.
compute lmat = ident(ncol( llrdat )).
compute lmat = lmat(:,2:ncol(lmat)).
compute fratio = (t(t(lmat)*b)*inv(t(lmat)*varb*lmat)*((t(lmat)*b)))/(ncol( llrdat )-1).
compute pfr = 1-fcdf(fratio,(ncol( llrdat )-1),dfres).
compute modsum={sqrt(r2),r2,mse,fratio,(ncol( llrdat )-1),dfres,pfr}.
compute modsuml={'R','R-sq','MSE',hcflab,'df1','df2', 'p'}.
end if.
end if.
do if ( 2 = 2 or 2 =3).
compute xlp= llrdat.
compute ylp= y.
compute pt2 = make(nrow(ylp),1,(csum(ylp)/nrow(ylp))).
do if ( 2 =2).
compute LL3 = ylp&*ln(pt2)+(1-ylp)&*ln(1-pt2).
end if.
compute LL3 = -2*csum(LL3).
compute bt1 = make(ncol(xlp),1,0).
compute LL1 = 0.
compute pt1 = make(nrow(ylp),1,0.5).
compute pt1lp=pt1.
loop jjj = 1 to iterate.
compute xlptmp=t(xlp).
compute vecprb=pt1lp&*(1-pt1lp).
loop kkk=1 to ncol(xlp).
compute xlptmp(kkk,:)=xlptmp(kkk,:)&*t(vecprb).
end loop.
compute b = bt1+inv(xlptmp*xlp)*t(xlp)*(ylp-pt1lp).
do if ( 2 =2).
compute xlpb=xlp*b.
compute xlpbt=(xlpb > -709.7).
compute xlpb709=(1-xlpbt)*(-709.7).
compute xlpb=(xlpb&*xlpbt)+xlpb709.
compute pt1lp = 1/(1+exp(-(xlpb))).
end if.
compute itprob = csum((pt1lp < .00000001) or (pt1lp > .9999999)).
do if (itprob > 0).
loop kkk = 1 to nrow(pt1lp).
do if (pt1lp(kkk,1) > .9999999).
compute pt1lp(kkk,1) = .9999999.
end if.
do if (pt1lp(kkk,1) < .00000001).
compute pt1lp(kkk,1) = .00000001.
end if.
end loop.
compute itprob = 0.
end if.
do if (itprob = 0).
do if ( 2 =2).
compute LL = ylp&*ln(pt1lp)+(1-ylp)&*ln(1-pt1lp).
end if.
compute LL2 = -2*csum(ll).
end if.
do if (abs(LL1-LL2) < converge).
do if ( 0 =1).
compute xlptmp=t(xlp).
compute vecprb=pt1lp&*(1-pt1lp).
loop kkk=1 to ncol(xlp).
compute xlptmp(kkk,:)=xlptmp(kkk,:)&*t(vecprb).
end loop.
compute varb = inv(xlptmp*xlp).
compute seb = sqrt(diag(varb)).
release xlptmp.
end if.
break.
end if.
compute bt1 = b.
compute LL1 = LL2.
end loop.
compute modres=b.
do if (jjj > iterate).
compute itprob = 2.
do if (booting=0).
compute iterrmod=1.
end if.
do if (booting=1).
compute bootiter=1.
end if.
do if (itprobtg=0).
compute itprobtg=1.
compute errcode(errs,1) = 47.
compute errs = errs + 1.
do if (booting = 0 and 0 =1).
compute vt1 = mdiag(pt1lp&*(1-pt1lp)).
compute varb = inv(t(xlp)*vt1*xlp).
compute seb = sqrt(diag(varb)).
end if.
end if.
end if.
do if ( 0 =1).
compute trat = b&/seb.
compute dfres=nrow(xlp).
compute p = 2*(1-cdfnorm(abs(trat))).
compute modres={modres,seb,trat,p}.
compute modres={modres,(b-xp2&*seb),(b+xp2&*seb)}.
compute pvchi=1-chicdf((LL3-LL2),(nrow(modres)-1)).
compute mcF = (LL3-LL2)/LL3.
compute cox = 1-exp(-(LL3-LL2)/nrow(xlp)).
compute nagel = cox/(1-exp(-(LL3)/nrow(xlp))).
compute modsum={LL2,(LL3-LL2),(nrow(modres)-1),pvchi, mcF,cox,nagel}.
compute modsuml={'-2LL','ModelLL', 'df', 'p', 'McFadden', 'CoxSnell', 'Nagelkrk'}.
compute modresl={'coeff','se','Z','p','LLCI','ULCI'}.
end if.
end if
.
compute b=btemphld.
compute fresult={(LL2-basemod),llrdf,1-chicdf((LL2-basemod),llrdf)}
.
compute lmatdb=lmat.
compute dbint=dbint+1.
end if.
compute highf={highf;fresult}.
compute highf2={highf2;fresult}.
do if (j = start).
compute flabel={flabel;'X*W'}.
end if.
do if (j > start).
do if (nms > 1).
compute flabel={flabel;highlbw(jj,1)}.
else if (nms = 1).
do if (xmint=0).
compute flabel={flabel;'M*W'}.
end if.
do if (xmint=1).
compute flabel={flabel;'X*M'}.
end if.
end if.
end if.
end if.
compute lmat=zhigh(1:nump(1,i),j).
compute lmat2=wzhigh(1:nump(1,i),j).
do if ((csum(lmat) > 0) and (csum(lmat2) = 0)).
do if ((i < (nms+nys)) or (ydich <> 1)).

.
compute lmat2= lmat.
do if ( 0 =0).
compute lmat2 = mdiag( lmat ).
compute lmat3=make(nrow(lmat2),1,0).
loop flp=1 to ncol(lmat2).
do if (csum(lmat2(:,flp))=1).
compute lmat3={lmat3,lmat2(:,flp)}.
end if.
end loop.
compute lmat2=lmat3(:,2:ncol(lmat3)).
end if.
compute fratio = (t(t(lmat2)* b )*inv(t(lmat2)* varb *lmat2)*((t(lmat2)* b )))/ncol(lmat2).
compute pfr = 1-fcdf(fratio,ncol(lmat2),(n-nrow( b ))).
compute fresult={fratio,ncol(lmat2),(n-nrow( b )),pfr}.
do if (i = (nms+nys) and (ydich=1)).
compute fratio=fratio*ncol(lmat2).
compute pfr=1-chicdf(fratio,ncol(lmat2)).
compute fresult={fratio,ncol(lmat2),pfr}.
end if.
do if ( 1 =1).
compute lmat3=1-rsum(lmat2).
compute xfm=make(n,csum(lmat3),0).
compute flpc=1.
loop flp=1 to nrow(lmat3).
do if (lmat3(flp,1)=1).
compute xfm(:,flpc)=x(:,flp).
compute flpc=flpc+1.
end if.
end loop.
compute bfm=inv(t(xfm)*xfm)*t(xfm)*y.
compute resid=y-(xfm*bfm).
compute sstotal=(y-(csum(y)/n)).
compute sstotal=csum(sstotal&*sstotal).
compute ssresid=csum(resid&*resid).
compute rsqch= r2 -((sstotal-ssresid)/sstotal).
compute fresult={rsqch,fresult}.
release xfm,flpc, resid, ssresid, bfm.
end if
.
compute dbint=dbint+1.
end if.
do if ((ydich = 1) and (i = (nms+nys))).

.
compute btemphld=b.
compute llrdat=make(nrow(x),nrow( lmat )-csum( lmat ),-999).
compute llrdf=ncol(x)-ncol(llrdat).
compute llrcnt=0.
loop llri=1 to nrow( lmat ).
do if ( lmat (llri,1)=0).
compute llrcnt=llrcnt+1.
compute llrdat(:,llrcnt)=x(:,llri).
end if.
end loop.

.
do if ( 2 =1).
compute hatmat=inv(t( llrdat )* llrdat )*t( llrdat ).
compute b = hatmat* y.
compute modres=b.
do if ( 0 =1).
compute n1=nrow( llrdat ).
compute dfres=n1-(ncol( llrdat )).
compute sstotal = t( y -(csum( y )/n1))*( y -(csum( y )/n1)).
compute resid= y - llrdat *b.
compute ssresid = csum((resid)&**2).
compute r2 = (sstotal-ssresid)/sstotal.
do if (r2 < 0).
compute r2=0.
end if.
compute adjr2 = 1-((1-r2)*(n1-1)/(dfres)).
compute mse=ssresid/(n1-ncol( llrdat )).

.
compute n1=nrow( x ).
compute invXtX = inv(t( x )* x ).
compute varb = mse *invXtX.
compute k3 = ncol( x ).
compute xhc=0.
do if ( hc <> 5).
compute xhc= x.
compute hat = xhc(:,1).
loop i3=1 to nrow(xhc).
compute hat(i3,1)= xhc(i3,:)*invXtX*t(xhc(i3,:)).
end loop.
do if ( hc = 0 or hc =1).
loop i3 = 1 to k3.
compute xhc(:,i3)=xhc(:,i3)&* resid.
end loop.
end if.
do if ( hc =3 or hc =2).
loop i3=1 to k3.
compute xhc(:,i3) = ( resid &/(1-hat)&**(1/(4- hc )))&*xhc(:,i3).
end loop.
end if.
do if ( hc = 4).
compute hcmn=make(n,2,4).
compute hcmn(:,2)=(n1*hat)/k3.
loop i3= 1 to k3.
compute xhc(:,i3) = ( resid &/(1-hat)&**(rmin(hcmn)/2))&*xhc(:,i3).
end loop.
end if.
compute varb=(invXtX*t(xhc)*xhc*invXtX).
do if ( hc =1).
compute varb=(n1/(n1-ncol( x )))&*varb.
end if.
end if.
compute hclab={'se(HC0)','se(HC1)','se(HC2)','se(HC3)','se(HC4)','se'}.
compute hclab=hclab(1,( hc +1)).
compute hcflab={'F(HC0)','F(HC1)','F(HC2)','F(HC3)','F(HC4)','F'}.
compute hcflab=hcflab(1,( hc +1)).
release xhc
.
compute seb=sqrt(diag(varb)).
compute trat = b&/seb.
compute p = 2*(1-tcdf(abs(trat), (dfres))).
compute tval = sqrt(dfres* (exp((dfres-(5/6))*((xp2/(dfres-(2/3)+(.11/dfres)))* (xp2/(dfres-(2/3)+(.11/dfres)))))-1)).
compute modres={modres,seb,trat,p}.
compute modres={modres,(b-tval&*seb),(b+tval&*seb)}.
compute modresl={'coeff',hclab,'t','p','LLCI','ULCI'}.
compute lmat = ident(ncol( llrdat )).
compute lmat = lmat(:,2:ncol(lmat)).
compute fratio = (t(t(lmat)*b)*inv(t(lmat)*varb*lmat)*((t(lmat)*b)))/(ncol( llrdat )-1).
compute pfr = 1-fcdf(fratio,(ncol( llrdat )-1),dfres).
compute modsum={sqrt(r2),r2,mse,fratio,(ncol( llrdat )-1),dfres,pfr}.
compute modsuml={'R','R-sq','MSE',hcflab,'df1','df2', 'p'}.
end if.
end if.
do if ( 2 = 2 or 2 =3).
compute xlp= llrdat.
compute ylp= y.
compute pt2 = make(nrow(ylp),1,(csum(ylp)/nrow(ylp))).
do if ( 2 =2).
compute LL3 = ylp&*ln(pt2)+(1-ylp)&*ln(1-pt2).
end if.
compute LL3 = -2*csum(LL3).
compute bt1 = make(ncol(xlp),1,0).
compute LL1 = 0.
compute pt1 = make(nrow(ylp),1,0.5).
compute pt1lp=pt1.
loop jjj = 1 to iterate.
compute xlptmp=t(xlp).
compute vecprb=pt1lp&*(1-pt1lp).
loop kkk=1 to ncol(xlp).
compute xlptmp(kkk,:)=xlptmp(kkk,:)&*t(vecprb).
end loop.
compute b = bt1+inv(xlptmp*xlp)*t(xlp)*(ylp-pt1lp).
do if ( 2 =2).
compute xlpb=xlp*b.
compute xlpbt=(xlpb > -709.7).
compute xlpb709=(1-xlpbt)*(-709.7).
compute xlpb=(xlpb&*xlpbt)+xlpb709.
compute pt1lp = 1/(1+exp(-(xlpb))).
end if.
compute itprob = csum((pt1lp < .00000001) or (pt1lp > .9999999)).
do if (itprob > 0).
loop kkk = 1 to nrow(pt1lp).
do if (pt1lp(kkk,1) > .9999999).
compute pt1lp(kkk,1) = .9999999.
end if.
do if (pt1lp(kkk,1) < .00000001).
compute pt1lp(kkk,1) = .00000001.
end if.
end loop.
compute itprob = 0.
end if.
do if (itprob = 0).
do if ( 2 =2).
compute LL = ylp&*ln(pt1lp)+(1-ylp)&*ln(1-pt1lp).
end if.
compute LL2 = -2*csum(ll).
end if.
do if (abs(LL1-LL2) < converge).
do if ( 0 =1).
compute xlptmp=t(xlp).
compute vecprb=pt1lp&*(1-pt1lp).
loop kkk=1 to ncol(xlp).
compute xlptmp(kkk,:)=xlptmp(kkk,:)&*t(vecprb).
end loop.
compute varb = inv(xlptmp*xlp).
compute seb = sqrt(diag(varb)).
release xlptmp.
end if.
break.
end if.
compute bt1 = b.
compute LL1 = LL2.
end loop.
compute modres=b.
do if (jjj > iterate).
compute itprob = 2.
do if (booting=0).
compute iterrmod=1.
end if.
do if (booting=1).
compute bootiter=1.
end if.
do if (itprobtg=0).
compute itprobtg=1.
compute errcode(errs,1) = 47.
compute errs = errs + 1.
do if (booting = 0 and 0 =1).
compute vt1 = mdiag(pt1lp&*(1-pt1lp)).
compute varb = inv(t(xlp)*vt1*xlp).
compute seb = sqrt(diag(varb)).
end if.
end if.
end if.
do if ( 0 =1).
compute trat = b&/seb.
compute dfres=nrow(xlp).
compute p = 2*(1-cdfnorm(abs(trat))).
compute modres={modres,seb,trat,p}.
compute modres={modres,(b-xp2&*seb),(b+xp2&*seb)}.
compute pvchi=1-chicdf((LL3-LL2),(nrow(modres)-1)).
compute mcF = (LL3-LL2)/LL3.
compute cox = 1-exp(-(LL3-LL2)/nrow(xlp)).
compute nagel = cox/(1-exp(-(LL3)/nrow(xlp))).
compute modsum={LL2,(LL3-LL2),(nrow(modres)-1),pvchi, mcF,cox,nagel}.
compute modsuml={'-2LL','ModelLL', 'df', 'p', 'McFadden', 'CoxSnell', 'Nagelkrk'}.
compute modresl={'coeff','se','Z','p','LLCI','ULCI'}.
end if.
end if
.
compute b=btemphld.
compute fresult={(LL2-basemod),llrdf,1-chicdf((LL2-basemod),llrdf)}
.
compute dbint=dbint+1.
end if.
compute highf={highf;fresult}.
compute highf2={highf2;fresult}.
do if (j = start).
compute flabel={flabel;'X*Z'}.
end if.
do if (j > start).
do if (nms > 1).
compute flabel={flabel;highlbz(jj,1)}.
else if (nms = 1).
compute flabel={flabel;'M*Z'}.
end if.
end if.
end if.
do if (dbint=2).
compute lmatdb=lmatdb+lmat.
do if ((ydich = 1) and (i = (nms+nys))).

.
compute btemphld=b.
compute llrdat=make(nrow(x),nrow( lmatdb )-csum( lmatdb ),-999).
compute llrdf=ncol(x)-ncol(llrdat).
compute llrcnt=0.
loop llri=1 to nrow( lmatdb ).
do if ( lmatdb (llri,1)=0).
compute llrcnt=llrcnt+1.
compute llrdat(:,llrcnt)=x(:,llri).
end if.
end loop.

.
do if ( 2 =1).
compute hatmat=inv(t( llrdat )* llrdat )*t( llrdat ).
compute b = hatmat* y.
compute modres=b.
do if ( 0 =1).
compute n1=nrow( llrdat ).
compute dfres=n1-(ncol( llrdat )).
compute sstotal = t( y -(csum( y )/n1))*( y -(csum( y )/n1)).
compute resid= y - llrdat *b.
compute ssresid = csum((resid)&**2).
compute r2 = (sstotal-ssresid)/sstotal.
do if (r2 < 0).
compute r2=0.
end if.
compute adjr2 = 1-((1-r2)*(n1-1)/(dfres)).
compute mse=ssresid/(n1-ncol( llrdat )).

.
compute n1=nrow( x ).
compute invXtX = inv(t( x )* x ).
compute varb = mse *invXtX.
compute k3 = ncol( x ).
compute xhc=0.
do if ( hc <> 5).
compute xhc= x.
compute hat = xhc(:,1).
loop i3=1 to nrow(xhc).
compute hat(i3,1)= xhc(i3,:)*invXtX*t(xhc(i3,:)).
end loop.
do if ( hc = 0 or hc =1).
loop i3 = 1 to k3.
compute xhc(:,i3)=xhc(:,i3)&* resid.
end loop.
end if.
do if ( hc =3 or hc =2).
loop i3=1 to k3.
compute xhc(:,i3) = ( resid &/(1-hat)&**(1/(4- hc )))&*xhc(:,i3).
end loop.
end if.
do if ( hc = 4).
compute hcmn=make(n,2,4).
compute hcmn(:,2)=(n1*hat)/k3.
loop i3= 1 to k3.
compute xhc(:,i3) = ( resid &/(1-hat)&**(rmin(hcmn)/2))&*xhc(:,i3).
end loop.
end if.
compute varb=(invXtX*t(xhc)*xhc*invXtX).
do if ( hc =1).
compute varb=(n1/(n1-ncol( x )))&*varb.
end if.
end if.
compute hclab={'se(HC0)','se(HC1)','se(HC2)','se(HC3)','se(HC4)','se'}.
compute hclab=hclab(1,( hc +1)).
compute hcflab={'F(HC0)','F(HC1)','F(HC2)','F(HC3)','F(HC4)','F'}.
compute hcflab=hcflab(1,( hc +1)).
release xhc
.
compute seb=sqrt(diag(varb)).
compute trat = b&/seb.
compute p = 2*(1-tcdf(abs(trat), (dfres))).
compute tval = sqrt(dfres* (exp((dfres-(5/6))*((xp2/(dfres-(2/3)+(.11/dfres)))* (xp2/(dfres-(2/3)+(.11/dfres)))))-1)).
compute modres={modres,seb,trat,p}.
compute modres={modres,(b-tval&*seb),(b+tval&*seb)}.
compute modresl={'coeff',hclab,'t','p','LLCI','ULCI'}.
compute lmat = ident(ncol( llrdat )).
compute lmat = lmat(:,2:ncol(lmat)).
compute fratio = (t(t(lmat)*b)*inv(t(lmat)*varb*lmat)*((t(lmat)*b)))/(ncol( llrdat )-1).
compute pfr = 1-fcdf(fratio,(ncol( llrdat )-1),dfres).
compute modsum={sqrt(r2),r2,mse,fratio,(ncol( llrdat )-1),dfres,pfr}.
compute modsuml={'R','R-sq','MSE',hcflab,'df1','df2', 'p'}.
end if.
end if.
do if ( 2 = 2 or 2 =3).
compute xlp= llrdat.
compute ylp= y.
compute pt2 = make(nrow(ylp),1,(csum(ylp)/nrow(ylp))).
do if ( 2 =2).
compute LL3 = ylp&*ln(pt2)+(1-ylp)&*ln(1-pt2).
end if.
compute LL3 = -2*csum(LL3).
compute bt1 = make(ncol(xlp),1,0).
compute LL1 = 0.
compute pt1 = make(nrow(ylp),1,0.5).
compute pt1lp=pt1.
loop jjj = 1 to iterate.
compute xlptmp=t(xlp).
compute vecprb=pt1lp&*(1-pt1lp).
loop kkk=1 to ncol(xlp).
compute xlptmp(kkk,:)=xlptmp(kkk,:)&*t(vecprb).
end loop.
compute b = bt1+inv(xlptmp*xlp)*t(xlp)*(ylp-pt1lp).
do if ( 2 =2).
compute xlpb=xlp*b.
compute xlpbt=(xlpb > -709.7).
compute xlpb709=(1-xlpbt)*(-709.7).
compute xlpb=(xlpb&*xlpbt)+xlpb709.
compute pt1lp = 1/(1+exp(-(xlpb))).
end if.
compute itprob = csum((pt1lp < .00000001) or (pt1lp > .9999999)).
do if (itprob > 0).
loop kkk = 1 to nrow(pt1lp).
do if (pt1lp(kkk,1) > .9999999).
compute pt1lp(kkk,1) = .9999999.
end if.
do if (pt1lp(kkk,1) < .00000001).
compute pt1lp(kkk,1) = .00000001.
end if.
end loop.
compute itprob = 0.
end if.
do if (itprob = 0).
do if ( 2 =2).
compute LL = ylp&*ln(pt1lp)+(1-ylp)&*ln(1-pt1lp).
end if.
compute LL2 = -2*csum(ll).
end if.
do if (abs(LL1-LL2) < converge).
do if ( 0 =1).
compute xlptmp=t(xlp).
compute vecprb=pt1lp&*(1-pt1lp).
loop kkk=1 to ncol(xlp).
compute xlptmp(kkk,:)=xlptmp(kkk,:)&*t(vecprb).
end loop.
compute varb = inv(xlptmp*xlp).
compute seb = sqrt(diag(varb)).
release xlptmp.
end if.
break.
end if.
compute bt1 = b.
compute LL1 = LL2.
end loop.
compute modres=b.
do if (jjj > iterate).
compute itprob = 2.
do if (booting=0).
compute iterrmod=1.
end if.
do if (booting=1).
compute bootiter=1.
end if.
do if (itprobtg=0).
compute itprobtg=1.
compute errcode(errs,1) = 47.
compute errs = errs + 1.
do if (booting = 0 and 0 =1).
compute vt1 = mdiag(pt1lp&*(1-pt1lp)).
compute varb = inv(t(xlp)*vt1*xlp).
compute seb = sqrt(diag(varb)).
end if.
end if.
end if.
do if ( 0 =1).
compute trat = b&/seb.
compute dfres=nrow(xlp).
compute p = 2*(1-cdfnorm(abs(trat))).
compute modres={modres,seb,trat,p}.
compute modres={modres,(b-xp2&*seb),(b+xp2&*seb)}.
compute pvchi=1-chicdf((LL3-LL2),(nrow(modres)-1)).
compute mcF = (LL3-LL2)/LL3.
compute cox = 1-exp(-(LL3-LL2)/nrow(xlp)).
compute nagel = cox/(1-exp(-(LL3)/nrow(xlp))).
compute modsum={LL2,(LL3-LL2),(nrow(modres)-1),pvchi, mcF,cox,nagel}.
compute modsuml={'-2LL','ModelLL', 'df', 'p', 'McFadden', 'CoxSnell', 'Nagelkrk'}.
compute modresl={'coeff','se','Z','p','LLCI','ULCI'}.
end if.
end if
.
compute b=btemphld.
compute fresult={(LL2-basemod),llrdf,1-chicdf((LL2-basemod),llrdf)}
.
end if.
do if (ydich <> 1 or i < (nms+nys)).

.
compute lmat2= lmatdb.
do if ( 0 =0).
compute lmat2 = mdiag( lmatdb ).
compute lmat3=make(nrow(lmat2),1,0).
loop flp=1 to ncol(lmat2).
do if (csum(lmat2(:,flp))=1).
compute lmat3={lmat3,lmat2(:,flp)}.
end if.
end loop.
compute lmat2=lmat3(:,2:ncol(lmat3)).
end if.
compute fratio = (t(t(lmat2)* b )*inv(t(lmat2)* varb *lmat2)*((t(lmat2)* b )))/ncol(lmat2).
compute pfr = 1-fcdf(fratio,ncol(lmat2),(n-nrow( b ))).
compute fresult={fratio,ncol(lmat2),(n-nrow( b )),pfr}.
do if (i = (nms+nys) and (ydich=1)).
compute fratio=fratio*ncol(lmat2).
compute pfr=1-chicdf(fratio,ncol(lmat2)).
compute fresult={fratio,ncol(lmat2),pfr}.
end if.
do if ( 1 =1).
compute lmat3=1-rsum(lmat2).
compute xfm=make(n,csum(lmat3),0).
compute flpc=1.
loop flp=1 to nrow(lmat3).
do if (lmat3(flp,1)=1).
compute xfm(:,flpc)=x(:,flp).
compute flpc=flpc+1.
end if.
end loop.
compute bfm=inv(t(xfm)*xfm)*t(xfm)*y.
compute resid=y-(xfm*bfm).
compute sstotal=(y-(csum(y)/n)).
compute sstotal=csum(sstotal&*sstotal).
compute ssresid=csum(resid&*resid).
compute rsqch= r2 -((sstotal-ssresid)/sstotal).
compute fresult={rsqch,fresult}.
release xfm,flpc, resid, ssresid, bfm.
end if
.
end if.
compute dbint=0.
compute highf={highf;fresult}.
do if (jj=0 and nms > 0).
compute flabel={flabel;'BOTH(X)'}.
end if.
do if (jj=0 and nms = 0).
compute flabel={flabel;'BOTH'}.
end if.
do if (jj>0 and nms = 1).
compute flabel={flabel;'BOTH(M)'}.
end if.
do if (nms > 1 and jj > 0).
compute flabel={flabel;highlbbt(jj,1)}.
end if.
end if.
compute lmat2=wzhigh(1:nump(1,i),j).
do if (csum(lmat2) > 0).
do if ((i < (nms+nys)) or (ydich <> 1)).

.
compute lmat2= lmat2.
do if ( 0 =0).
compute lmat2 = mdiag( lmat2 ).
compute lmat3=make(nrow(lmat2),1,0).
loop flp=1 to ncol(lmat2).
do if (csum(lmat2(:,flp))=1).
compute lmat3={lmat3,lmat2(:,flp)}.
end if.
end loop.
compute lmat2=lmat3(:,2:ncol(lmat3)).
end if.
compute fratio = (t(t(lmat2)* b )*inv(t(lmat2)* varb *lmat2)*((t(lmat2)* b )))/ncol(lmat2).
compute pfr = 1-fcdf(fratio,ncol(lmat2),(n-nrow( b ))).
compute fresult={fratio,ncol(lmat2),(n-nrow( b )),pfr}.
do if (i = (nms+nys) and (ydich=1)).
compute fratio=fratio*ncol(lmat2).
compute pfr=1-chicdf(fratio,ncol(lmat2)).
compute fresult={fratio,ncol(lmat2),pfr}.
end if.
do if ( 1 =1).
compute lmat3=1-rsum(lmat2).
compute xfm=make(n,csum(lmat3),0).
compute flpc=1.
loop flp=1 to nrow(lmat3).
do if (lmat3(flp,1)=1).
compute xfm(:,flpc)=x(:,flp).
compute flpc=flpc+1.
end if.
end loop.
compute bfm=inv(t(xfm)*xfm)*t(xfm)*y.
compute resid=y-(xfm*bfm).
compute sstotal=(y-(csum(y)/n)).
compute sstotal=csum(sstotal&*sstotal).
compute ssresid=csum(resid&*resid).
compute rsqch= r2 -((sstotal-ssresid)/sstotal).
compute fresult={rsqch,fresult}.
release xfm,flpc, resid, ssresid, bfm.
end if
.
end if.
do if ((ydich = 1) and (i = (nms+nys))).

.
compute btemphld=b.
compute llrdat=make(nrow(x),nrow( lmat2 )-csum( lmat2 ),-999).
compute llrdf=ncol(x)-ncol(llrdat).
compute llrcnt=0.
loop llri=1 to nrow( lmat2 ).
do if ( lmat2 (llri,1)=0).
compute llrcnt=llrcnt+1.
compute llrdat(:,llrcnt)=x(:,llri).
end if.
end loop.

.
do if ( 2 =1).
compute hatmat=inv(t( llrdat )* llrdat )*t( llrdat ).
compute b = hatmat* y.
compute modres=b.
do if ( 0 =1).
compute n1=nrow( llrdat ).
compute dfres=n1-(ncol( llrdat )).
compute sstotal = t( y -(csum( y )/n1))*( y -(csum( y )/n1)).
compute resid= y - llrdat *b.
compute ssresid = csum((resid)&**2).
compute r2 = (sstotal-ssresid)/sstotal.
do if (r2 < 0).
compute r2=0.
end if.
compute adjr2 = 1-((1-r2)*(n1-1)/(dfres)).
compute mse=ssresid/(n1-ncol( llrdat )).

.
compute n1=nrow( x ).
compute invXtX = inv(t( x )* x ).
compute varb = mse *invXtX.
compute k3 = ncol( x ).
compute xhc=0.
do if ( hc <> 5).
compute xhc= x.
compute hat = xhc(:,1).
loop i3=1 to nrow(xhc).
compute hat(i3,1)= xhc(i3,:)*invXtX*t(xhc(i3,:)).
end loop.
do if ( hc = 0 or hc =1).
loop i3 = 1 to k3.
compute xhc(:,i3)=xhc(:,i3)&* resid.
end loop.
end if.
do if ( hc =3 or hc =2).
loop i3=1 to k3.
compute xhc(:,i3) = ( resid &/(1-hat)&**(1/(4- hc )))&*xhc(:,i3).
end loop.
end if.
do if ( hc = 4).
compute hcmn=make(n,2,4).
compute hcmn(:,2)=(n1*hat)/k3.
loop i3= 1 to k3.
compute xhc(:,i3) = ( resid &/(1-hat)&**(rmin(hcmn)/2))&*xhc(:,i3).
end loop.
end if.
compute varb=(invXtX*t(xhc)*xhc*invXtX).
do if ( hc =1).
compute varb=(n1/(n1-ncol( x )))&*varb.
end if.
end if.
compute hclab={'se(HC0)','se(HC1)','se(HC2)','se(HC3)','se(HC4)','se'}.
compute hclab=hclab(1,( hc +1)).
compute hcflab={'F(HC0)','F(HC1)','F(HC2)','F(HC3)','F(HC4)','F'}.
compute hcflab=hcflab(1,( hc +1)).
release xhc
.
compute seb=sqrt(diag(varb)).
compute trat = b&/seb.
compute p = 2*(1-tcdf(abs(trat), (dfres))).
compute tval = sqrt(dfres* (exp((dfres-(5/6))*((xp2/(dfres-(2/3)+(.11/dfres)))* (xp2/(dfres-(2/3)+(.11/dfres)))))-1)).
compute modres={modres,seb,trat,p}.
compute modres={modres,(b-tval&*seb),(b+tval&*seb)}.
compute modresl={'coeff',hclab,'t','p','LLCI','ULCI'}.
compute lmat = ident(ncol( llrdat )).
compute lmat = lmat(:,2:ncol(lmat)).
compute fratio = (t(t(lmat)*b)*inv(t(lmat)*varb*lmat)*((t(lmat)*b)))/(ncol( llrdat )-1).
compute pfr = 1-fcdf(fratio,(ncol( llrdat )-1),dfres).
compute modsum={sqrt(r2),r2,mse,fratio,(ncol( llrdat )-1),dfres,pfr}.
compute modsuml={'R','R-sq','MSE',hcflab,'df1','df2', 'p'}.
end if.
end if.
do if ( 2 = 2 or 2 =3).
compute xlp= llrdat.
compute ylp= y.
compute pt2 = make(nrow(ylp),1,(csum(ylp)/nrow(ylp))).
do if ( 2 =2).
compute LL3 = ylp&*ln(pt2)+(1-ylp)&*ln(1-pt2).
end if.
compute LL3 = -2*csum(LL3).
compute bt1 = make(ncol(xlp),1,0).
compute LL1 = 0.
compute pt1 = make(nrow(ylp),1,0.5).
compute pt1lp=pt1.
loop jjj = 1 to iterate.
compute xlptmp=t(xlp).
compute vecprb=pt1lp&*(1-pt1lp).
loop kkk=1 to ncol(xlp).
compute xlptmp(kkk,:)=xlptmp(kkk,:)&*t(vecprb).
end loop.
compute b = bt1+inv(xlptmp*xlp)*t(xlp)*(ylp-pt1lp).
do if ( 2 =2).
compute xlpb=xlp*b.
compute xlpbt=(xlpb > -709.7).
compute xlpb709=(1-xlpbt)*(-709.7).
compute xlpb=(xlpb&*xlpbt)+xlpb709.
compute pt1lp = 1/(1+exp(-(xlpb))).
end if.
compute itprob = csum((pt1lp < .00000001) or (pt1lp > .9999999)).
do if (itprob > 0).
loop kkk = 1 to nrow(pt1lp).
do if (pt1lp(kkk,1) > .9999999).
compute pt1lp(kkk,1) = .9999999.
end if.
do if (pt1lp(kkk,1) < .00000001).
compute pt1lp(kkk,1) = .00000001.
end if.
end loop.
compute itprob = 0.
end if.
do if (itprob = 0).
do if ( 2 =2).
compute LL = ylp&*ln(pt1lp)+(1-ylp)&*ln(1-pt1lp).
end if.
compute LL2 = -2*csum(ll).
end if.
do if (abs(LL1-LL2) < converge).
do if ( 0 =1).
compute xlptmp=t(xlp).
compute vecprb=pt1lp&*(1-pt1lp).
loop kkk=1 to ncol(xlp).
compute xlptmp(kkk,:)=xlptmp(kkk,:)&*t(vecprb).
end loop.
compute varb = inv(xlptmp*xlp).
compute seb = sqrt(diag(varb)).
release xlptmp.
end if.
break.
end if.
compute bt1 = b.
compute LL1 = LL2.
end loop.
compute modres=b.
do if (jjj > iterate).
compute itprob = 2.
do if (booting=0).
compute iterrmod=1.
end if.
do if (booting=1).
compute bootiter=1.
end if.
do if (itprobtg=0).
compute itprobtg=1.
compute errcode(errs,1) = 47.
compute errs = errs + 1.
do if (booting = 0 and 0 =1).
compute vt1 = mdiag(pt1lp&*(1-pt1lp)).
compute varb = inv(t(xlp)*vt1*xlp).
compute seb = sqrt(diag(varb)).
end if.
end if.
end if.
do if ( 0 =1).
compute trat = b&/seb.
compute dfres=nrow(xlp).
compute p = 2*(1-cdfnorm(abs(trat))).
compute modres={modres,seb,trat,p}.
compute modres={modres,(b-xp2&*seb),(b+xp2&*seb)}.
compute pvchi=1-chicdf((LL3-LL2),(nrow(modres)-1)).
compute mcF = (LL3-LL2)/LL3.
compute cox = 1-exp(-(LL3-LL2)/nrow(xlp)).
compute nagel = cox/(1-exp(-(LL3)/nrow(xlp))).
compute modsum={LL2,(LL3-LL2),(nrow(modres)-1),pvchi, mcF,cox,nagel}.
compute modsuml={'-2LL','ModelLL', 'df', 'p', 'McFadden', 'CoxSnell', 'Nagelkrk'}.
compute modresl={'coeff','se','Z','p','LLCI','ULCI'}.
end if.
end if
.
compute b=btemphld.
compute fresult={(LL2-basemod),llrdf,1-chicdf((LL2-basemod),llrdf)}
.
end if.
compute highf={highf;fresult}.
compute highf2={highf2;fresult}.
do if (j = start).
compute flabel={flabel;'X*W*Z'}.
end if.
do if (j > start).
do if (nms > 1).
compute flabel={flabel;highlbwz(jj,1)}.
else if (nms = 1).
compute flabel={flabel;'M*W*Z'}.
end if.
end if.
end if.
compute jj=jj+1.
end loop.
release jj.
compute start=start+i.
end if.
do if (nrow(highf) > 1).
compute highf=highf(2:nrow(highf),:).
compute highf2=highf2(2:nrow(highf2),:).
compute flabel=flabel(2:nrow(flabel),1).
compute resultm2=make(nrow(highf),maxresm,99999).
compute resultm2(1:nrow(highf),1:ncol(highf))=highf.
compute resultm={resultm;resultm2}.
do if (outscree=1).
do if ((i < nms+nys) or (ydich=0)).
compute clabtmp={'R2-chng', hcflab,'df1','df2','p'}.
print highf/format = F10.4 /rnames=flabel/cnames=clabtmp/ title = 'Test(s) of highest order unconditional interaction(s):'.
end if.
do if (ydich=1 and i=(nms+nys)).
compute clabtmp={'Chi-sq', 'df','p'}.
print/title='Likelihood ratio test(s) of highest order'.
print highf/format = F10.4 /rnames=flabel/cnames=clabtmp/ title = 'unconditional interactions(s):'/space=0.
end if.
end if.
compute intpb=highf2(:,ncol(highf2)).
end if.
compute intstart=intstart+numint(1,i).
end if.

.
do if (criterr=0).
compute threeway=0.
compute didprint=0.
compute didsome=0.
compute sigintct=0.
loop jmed =1 to (nms+1).
compute hasw=0.
compute hasz=0.
compute jnok=0.
compute nm1vls=0.
compute nm2vls=0.
compute panelgrp=0.
compute graphixs={'WITH', outnames(1,i), 'BY'}.
compute focpred4={' '}.
compute intprint=0.
compute modcat=0.
do if (jmed <= i).
do if ((jmed = 1) and ((i+1) = nrow(bcmat))).
compute pathscnt=pathscnt+1.
else.
compute paths={paths,bcmat((i+1),jmed)}.
compute pathsw={pathsw,wcmat((i+1),jmed)}.
compute pathsz={pathsz,zcmat((i+1),jmed)}.
compute pathswz={pathswz,wzcmat((i+1),jmed)}.
compute temp=fochigh(:,pathscnt)&*bootloc(:,i).
compute pathsfoc={pathsfoc,pathsfoc(:,1)}.
do if (jmed=1).
compute pathtype={pathtype,1}.
end if.
do if ((i+1)=nrow(bcmat)).
compute pathtype={pathtype,3}.
end if.
do if (jmed > 1) and ((i+1) < nrow(bcmat)).
compute pathtype={pathtype,2}.
end if.
do if (jmed=1 and nxvls > 1 and (bcmat((i+1),jmed)=1)).
compute pathsfoc(:,(pathscn2+1))=temp(2:(nxvls+1),1).
end if.
do if ((jmed > 1) or (jmed=1 and nxvls=1)).
compute temp=cmax(temp).
compute pathsfoc(1,(pathscn2+1))=temp.
end if.
compute pathscnt=pathscnt+1.
compute pathscn2=pathscn2+1.
do if (i <= nms).
compute pathsdv={pathsdv,mnames(1,i)}.
end if.
do if (i > nms).
compute pathsdv={pathsdv,ynames}.
end if.
end if.
compute coeffcol=coeffcol+1.
compute probettt=coeffs(1:nrow(b),coeffcol).
do if (jmed=1 and (bcmat((i+1),jmed)=1)).
compute omni=make(nrow(probettt),nxvls,0).
compute omnitmp=ident(nxvls).
compute omni(2:(1+nxvls),:)=omnitmp.
end if.
do if (csum(probettt)>0).
compute probvarb=make(csum(probettt),csum(probettt),999).
compute probcoef=make(csum(probettt),1,999).
compute coefflp2=1.
loop coefflp=1 to nrow(probettt).
do if (probettt(coefflp,1)=1).
compute probcoef(coefflp2,1)=b(coefflp,1).
compute coefflp2=coefflp2+1.
end if.
end loop.
compute coefflp=0.
compute coefflp2=0.
loop iclp=1 to nrow(probettt).
do if probettt(iclp,1)=1.
compute coefflp=coefflp+1.
compute coefflp2=coefflp.
compute probvarb(coefflp,coefflp) = varb(iclp,iclp).
do if (iclp < nrow(probettt)).
loop jclp=(iclp+1) to nrow(probettt).
do if (probettt(jclp,1)=1).
compute coefflp2=coefflp2+1.
compute probvarb(coefflp,coefflp2)=varb(iclp, jclp).
compute probvarb(coefflp2,coefflp)=varb(iclp, jclp).
end if.
end loop.
end if.
end if.
end loop.
end if.
end if.
compute xprobval=xmodvals.
do if (nxvls > 1 or mcx > 0).
compute xprobval=dummatx(:,2:ncol(dummatx)).
end if.
do if ((wcmat((i+1),jmed)=1) and (zcmat((i+1),jmed)=0)).
compute numplps=1.
compute modvals=wmodvals.
compute probeval=wmodvals.
compute wheremv1=wherexw.
compute nm1vls=nwvls.
compute lpstsp={1,1}.
compute modcat=0.
compute jnmod=wtmp.
compute jnmodlab=wnames.
compute jnok=1.
compute jnmin=wmin.
compute jnmax=wmax.
compute wherejn1=2.
do if (jmed=1).
compute wherejn3=wherexw(1,i).
do if (nxvls > 1).
compute jnok=0.
end if.
end if.
do if (jmed > 1).
compute wherejn1=wherem((jmed-1),i).
compute wherejn3=wheremw(((2*jmed)-3),i).
end if.
do if (nwvls > 1).
compute probeval=wprobval.
compute lpstsp(1,2)=ncol(probeval).
compute modcat=1.
compute jnok=0.
end if.
do if (wdich = 1).
compute modcat=1.
compute jnok=0.
end if.
compute problabs=wnames.
compute focpred3={wnames,'(W)'}.
do if (xmint=1).
compute focpred3={wnames,'(X)'}.
end if.
compute hasw=1.
compute modgrph=wnames.
compute intprint=1.
compute sigintct=sigintct+1.
compute printpbe=intpb(sigintct,1).
end if.
do if ((wcmat((i+1),jmed)=0) and (zcmat((i+1),jmed)=1)).
compute numplps=1.
compute modvals=zmodvals.
compute probeval=zmodvals.
compute wheremv1=wherexz.
compute nm1vls=nzvls.
compute lpstsp={1,1}.
compute jnok=1.
compute jnmod=ztmp.
compute jnmin=zmin.
compute jnmax=zmax.
compute jnmodlab=znames.
compute wherejn1=2.
do if (jmed=1).
compute wherejn3=wherexz(1,i).
do if (nxvls > 1).
compute jnok=0.
end if.
end if.
do if (jmed > 1).
compute wherejn1=wherem((jmed-1),i).
compute wherejn3=wheremz(((2*jmed)-3),i).
end if.
do if (nzvls > 1).
compute probeval=zprobval.
compute lpstsp(1,2)=ncol(probeval).
compute modcat=1.
compute jnok=0.
end if.
do if (zdich = 1).
compute modcat=1.
compute jnok=0.
end if.
compute problabs=znames.
compute focpred3={znames,'(Z)'}.
compute modgrph=znames.
compute hasz=1.
compute intprint=1.
compute sigintct=sigintct+1.
compute printpbe=intpb(sigintct,1).
end if.
do if ((wzcmat((i+1),jmed)=1) or ((wcmat((i+1),jmed)=1) and (zcmat((i+1),jmed)=1))).
compute numplps=2.
compute probecnt=1.
compute intprint=1.
do if (wzcmat((i+1),jmed)=1).
compute sigintct=sigintct+1.
compute printpbe=intpb(sigintct,1).
else.
compute sigintct=sigintct+2.
compute printpbe=cmin(intpb((sigintct-1):sigintct,1)).
end if.
compute panelgrp=1.
compute hasw=1.
compute hasz=1.
compute panelcde={'/PANEL','ROWVAR=',znames,'.'}.
compute modgrph=wnames.
compute lpstsp={1,1;1,1}.
compute wheremv1=wherexw.
compute nm1vls=nwvls.
compute wheremv2=wherexz.
compute nm2vls=nzvls.
compute jnok=0.
do if (wzcmat((i+1),jmed)=1).
compute jnok=1.
end if.
do if (jmed > 1).
compute mprobval=mmodvals.
end if.
do if (jmed=1).
do if (nxvls > 1).
compute jnok=0.
end if.
end if.
do if (nwvls > 1).
compute lpstsp(1,2)=ncol(wprobval).
compute modcat=1.
compute jnok=0.
end if.
do if (zdich=1).
compute modcat=1.
compute jnok=0.
end if.
compute lpstsp(2,1)=lpstsp(1,2)+1.
compute lpstsp(2,2)=lpstsp(1,2)+1.
do if (nzvls > 1).
compute lpstsp(2,1)=lpstsp(1,2)+1.
compute lpstsp(2,2)=lpstsp(1,2)+ncol(zprobval).
compute jnok=0.
end if.
do if (zdich=1).
compute jnok=0.
end if.
compute omni3=make(nrow(b),(nxvls*nwvls),0).
do if (jmed > 1).
compute omni3=make(nrow(b),nwvls,0).
end if.
compute focpred3={wnames,'(W)'}.
do if (xmint=1).
compute focpred3={wnames,'(X)'}.
end if.
compute focpred4={'    ', 'Mod var:', znames, '(Z)'}.
compute modvals=make((nrow(wmodvals)*nrow(zmodvals)),2,0).
compute probeval=make((nrow(wmodvals)*nrow(zmodvals)),(ncol(wprobval)+ncol(zprobval)),0).
loop probei= 1 to nrow(wmodvals).
loop probej =1 to nrow(zmodvals).
compute modvals(probecnt,1)=wmodvals(probei,1).
compute probeval(probecnt,1:nwvls)=wprobval(probei,:).
compute modvals(probecnt,2)=zmodvals(probej,1).
compute probeval(probecnt,(nwvls+1):(nwvls+nzvls))=zprobval(probej,:).
compute probecnt=probecnt+1.
end loop.
end loop.
do if (wzcmat((i+1),jmed)=1).
compute numplps=numplps+1.
compute probprod=make(1,(ncol(wprobval)*ncol(zprobval)),0).
compute lpstsp2={1,1}.
compute lpstsp={lpstsp;lpstsp2}.
compute lpstsp(3,1)=lpstsp(2,2)+1.
compute lpstsp(3,2)=lpstsp(2,2)+ncol(probprod).
compute jnmod=ztmp.
compute jnmin=zmin.
compute jnmax=zmax.
compute jnmodlab=znames.
do if (jmed = 1).
compute wherejn1=wherexw(1,i).
compute wherejn3=wherexwz(1,i).
end if.
do if (jmed > 1).
compute wherejn1=wheremw(((2*jmed)-3),i).
compute wherejn3=wheremwz(((2*jmed)-3),i).
end if.
loop probei = 1 to nrow(wmodvals).
loop probej = 1 to nrow(zmodvals).
compute probtemp=1.
loop probek = 1 to ncol(wprobval).
compute probtemp={probtemp,(wprobval(probei,probek)&*zprobval(probej,:))}.
end loop.
compute probprod={probprod;probtemp(1,2:ncol(probtemp))}.
end loop.
end loop.
compute probprod=probprod(2:nrow(probprod),:).
compute probeval={probeval,probprod}.
end if.
compute problabs={wnames,znames}.
release probecnt, probei, probej.
end if.
do if (intprint=1).
compute focpred={'   Focal', 'predict:'}.
do if (jmed=1).
compute focpred={focpred,xnames,'(X)'}.
compute focplotv=xmodvals.
end if.
do if (jmed >1).
do if (nms > 1).
compute focpred={focpred,mnames(1,(jmed-1)), medlb2(1,(jmed-1))}.
end if.
do if (nms = 1).
compute focpred={focpred,mnames(1,(jmed-1)), '(M)'}.
end if.
compute focplotv=mmodvals(:,(jmed-1)).
end if.
compute focpred2={'    ', 'Mod var:',focpred3}.
compute focpred={focpred;focpred2}.
do if (ncol(focpred4) > 1).
compute focpred={focpred;focpred4}.
compute focpred4={' '}.
end if.
release focpred2,focpred3.
do if (outscree=1).
do if ((plot = 1 or plot = 2) or (printpbe <= intprobe)).
print focpred/title='----------'/format=A8/space=0.
end if.
end if.
compute foctmp=make(nrow(modvals),1,1).
compute probexpl=1.
compute probeva2={foctmp,probeval}.
do if (jmed=1 and nxs > 0 and mcx > 0).
compute probexpl=nxvls.
end if.
compute foctmp=make(nrow(modvals),1,1).
compute modvals3=make(1,(6+ncol(problabs)),0).
compute probrown=make(nrow(probeval),1,0).
compute jtmp=1.
loop probei = 1 to nrow(probeval).
compute probrown(probei,1)=jtmp.
compute jtmp=jtmp+nxvls.
end loop.
release jtmp.
compute probrow=999.
compute modvarl=problabs.
do if (plot = 1 or plot = 2 or nxvls > 1).
compute plotvals=make((nrow(modvals)*nrow(focplotv)),(ncol(modvals)+1),999).
loop ploti=1 to nrow(modvals).
loop plotj=1 to nrow(focplotv).
compute plotvals((((ploti-1)*nrow(focplotv))+plotj),2:ncol(plotvals))=modvals(ploti,:).
compute plotvals((((ploti-1)*nrow(focplotv))+plotj),1)=focplotv(plotj,1).
end loop.
end loop.
compute focpredn=3.
do if (jmed=1).
do if (nxvls > 1).
compute focpredn=(nxvls+1).
end if.
do if (nxvls=1 and xdich=1).
compute focpredn=2.
end if.
end if.
compute meanmat=mdiag(means).
compute onesmat=make(nrow(meanmat),(nrow(probeval)*focpredn),1).
compute probeplt=t(mdiag(means)*onesmat).
do if (jmed=1).
do if ((wcmat((i+1),1)=1) or (zcmat((i+1),1)=1)).
compute plotcnt=1.
compute iloops=nwpval*nzpval.
compute plotmx=nxpval*nzpval.
do if ((wcmat((i+1),1)=1) and (zcmat((i+1),1)=0)).
compute iloops=nwpval.
compute plotmx=nxpval.
end if.
do if ((wcmat((i+1),1)=0) and (zcmat((i+1),1)=1)).
compute iloops=nzpval.
compute plotmx=nxpval.
end if.
compute xestvals=make((nxpval*iloops),ncol(xprobval),-999).
do if (wcmat((i+1),1)=1).
compute westvals=make(nrow(xestvals),ncol(wprobval),-999).
end if.
do if (zcmat((i+1),1)=1).
compute zestvals=make(nrow(xestvals),ncol(zprobval),-999).
end if.
loop ploti=1 to iloops.
loop plotj=1 to nxpval.
compute xestvals(plotcnt,:)=xprobval(plotj,:).
compute plotcnt=plotcnt+1.
end loop.
end loop.
compute plotcnt=1.
compute plotcnt1=1.
compute plotcnt2=1.
compute plotcntz=1.
loop ploti = 1 to (iloops*nxpval).
do if (wcmat((i+1),1)=1).
compute westvals(ploti,:)=wprobval(plotcnt1,:).
end if.
do if ((wcmat((i+1),1)=0) and (zcmat((i+1),1)=1)).
compute zestvals(ploti,:)=zprobval(plotcnt1,:).
end if.
do if ((wcmat((i+1),1)=1) and (zcmat((i+1),1)=1)).
compute zestvals(ploti,:)=zprobval(plotcnt2,:).
compute plotcntz=plotcntz+1.
end if.
compute plotcnt=plotcnt+1.
do if (plotcnt > plotmx).
compute plotcnt=1.
compute plotcnt1=plotcnt1+1.
end if.
do if (plotcntz > nxpval).
compute plotcnt2=plotcnt2+1.
compute plotcntz=1.
do if (plotcnt2 > nzpval).
compute plotcnt2=1.
end if.
end if.
end loop.
compute probeplt(:,2:(1+(ncol(xestvals))))=xestvals.
do if (wcmat((i+1),1)=1).
compute probeplt(:,(wherew(1,i)):(wherew(2,i)))=westvals.
end if.
do if (zcmat((i+1),1)=1).
compute probeplt(:,(wherez(1,i)):(wherez(2,i)))=zestvals.
end if.
end if.
end if.
do if (jmed > 1).
do if ((wcmat((i+1),jmed)=1) or (zcmat((i+1),jmed)=1)).
compute plotcnt=1.
compute iloops=nwpval*nzpval.
compute plotmx=3*nzpval.
do if ((wcmat((i+1),jmed)=1) and (zcmat((i+1),jmed)=0)).
compute iloops=nwpval.
compute plotmx=3.
end if.
do if ((wcmat((i+1),jmed)=0) and (zcmat((i+1),jmed)=1)).
compute iloops=nzpval.
compute plotmx=3.
end if.
compute mestvals=make((3*iloops),1,-999).
do if (wcmat((i+1),jmed)=1).
compute westvals=make(nrow(mestvals),ncol(wprobval),-999).
end if.
do if (zcmat((i+1),jmed)=1).
compute zestvals=make(nrow(mestvals),ncol(zprobval),-999).
end if.
loop ploti=1 to iloops.
loop plotj=1 to 3.
compute mestvals(plotcnt,:)=mprobval(plotj,(jmed-1)).
compute plotcnt=plotcnt+1.
end loop.
end loop.
compute plotcnt=1.
compute plotcnt1=1.
compute plotcnt2=1.
compute plotcntz=1.
loop ploti = 1 to (iloops*3).
do if (wcmat((i+1),jmed)=1).
compute westvals(ploti,:)=wprobval(plotcnt1,:).
end if.
do if ((wcmat((i+1),jmed)=0) and (zcmat((i+1),jmed)=1)).
compute zestvals(ploti,:)=zprobval(plotcnt1,:).
end if.
do if ((wcmat((i+1),jmed)=1) and (zcmat((i+1),jmed)=1)).
compute zestvals(ploti,:)=zprobval(plotcnt2,:).
compute plotcntz=plotcntz+1.
end if.
compute plotcnt=plotcnt+1.
do if (plotcnt > plotmx).
compute plotcnt=1.
compute plotcnt1=plotcnt1+1.
end if.
do if (plotcntz > 3).
compute plotcnt2=plotcnt2+1.
compute plotcntz=1.
do if (plotcnt2 > nzpval).
compute plotcnt2=1.
end if.
end if.
end loop.
compute probeplt(:,wherem((jmed-1),i))=mestvals.
do if (wcmat((i+1),jmed)=1).
do if (model <> 74).
compute probeplt(:,(wherew(1,i)):(wherew(2,i)))=westvals.
end if.
do if (model = 74).
compute probeplt(:,(wherex(1,i)):(wherex(2,i)))=westvals.
end if.
end if.
do if (zcmat((i+1),jmed)=1).
compute probeplt(:,(wherez(1,i)):(wherez(2,i)))=zestvals.
end if.
end if.
end if.
compute prodloop = 1.
do if (jmed=1).
compute prodloop=ncol(xestvals).
end if.
do if (wcmat((i+1),jmed))=1.
compute plotcnt=0.
loop ploti = 1 to prodloop.
loop plotj = 1 to ncol(westvals).
do if (jmed=1).
compute probeplt(:,(wherexw(1,i)+plotcnt))=xestvals(:,ploti)&*westvals(:,plotj).
end if.
do if (jmed > 1).
compute probeplt(:,(wheremw(((jmed*2)-3) ,i)+plotcnt))=mestvals(:,ploti)&*westvals(:,plotj).
end if.
compute plotcnt=plotcnt+1.
end loop.
end loop.
end if.
do if (zcmat((i+1),jmed))=1.
compute plotcnt=0.
loop ploti = 1 to prodloop.
loop plotj = 1 to ncol(zestvals).
do if (jmed = 1).
compute probeplt(:,(wherexz(1,i)+plotcnt))=xestvals(:,ploti)&*zestvals(:,plotj).
end if.
do if (jmed > 1).
compute probeplt(:,(wheremz(((jmed*2)-3),i)+plotcnt))=mestvals(:,ploti)&*zestvals(:, plotj).
end if.
compute plotcnt=plotcnt+1.
end loop.
end loop.
end if.
do if (wzcmat((i+1),jmed))=1.
compute plotcnt=0.
compute threeway=1.
loop ploti = 1 to ncol(westvals).
loop plotj = 1 to ncol(zestvals).
compute probeplt(:,(wherewz(1,i)+plotcnt))=westvals(:,ploti)&*zestvals(:,plotj).
compute plotcnt=plotcnt+1.
end loop.
end loop.
compute plotcnt=0.
loop plotk = 1 to prodloop.
loop ploti = 1 to ncol(westvals).
loop plotj = 1 to ncol(zestvals).
do if (jmed = 1).
compute probeplt(:,(wherexwz(1,i)+plotcnt))=xestvals(:,plotk)&*westvals(:,ploti)&* zestvals(:,plotj).
end if.
do if (jmed > 1).
compute probeplt(:,(wheremwz(((jmed*2)-3),i)+plotcnt))=mestvals(:,plotk)&* westvals(:,ploti)&*zestvals(:,plotj).
end if.
compute plotcnt=plotcnt+1.
end loop.
end loop.
end loop.
end if.
loop newplp=1 to i.
do if (newplp <> jmed).
do if (wcmat((i+1),newplp))=1.
compute prodloop=1.
do if (newplp=1).
compute prodloop=nxvls.
end if.
compute plotcnt=0.
loop ploti = 1 to prodloop.
loop plotj = 1 to nwvls.
do if (newplp = 1).
compute probeplt(:,(wherexw(1,i)+plotcnt))=probeplt(:,(1+ploti))&*probeplt(:, (wherew(1,i)+plotj-1)).
end if.
do if (newplp > 1).
do if (model <> 74).
compute probeplt(:,(wheremw(((newplp*2)-3) ,i)+plotcnt))=probeplt(:,wherem((newplp-1),i))&*probeplt(:,(wherew(1,i)+plotj-1)).
end if.
do if (model = 74).
compute probeplt(:,(wheremw(((newplp*2)-3) ,i)+plotcnt))=probeplt(:,wherem((newplp-1),i))&*probeplt(:,(wherex(1,i)+plotj-1)).
end if.
end if.
compute plotcnt=plotcnt+1.
end loop.
end loop.
end if.
do if (zcmat((i+1),newplp))=1.
compute prodloop=1.
do if (newplp=1).
compute prodloop=nxvls.
end if.
compute plotcnt=0.
loop ploti = 1 to prodloop.
loop plotj = 1 to nzvls.
do if (newplp = 1).
compute probeplt(:,(wherexz(1,i)+plotcnt))=probeplt(:,(1+ploti))&*probeplt(:, (wherez(1,i)+plotj-1)).
end if.
do if (newplp > 1).
compute probeplt(:,(wheremz(((newplp*2)-3),i)+plotcnt))=probeplt(:, wherem((newplp-1),i))&*probeplt(:,(wherez(1,i)+plotj-1)).
end if.
compute plotcnt=plotcnt+1.
end loop.
end loop.
end if.
do if (wzcmat((i+1),newplp))=1.
compute plotcnt=0.
do if (threeway=0).
loop ploti = 1 to nwvls.
loop plotj = 1 to nzvls.
compute probeplt(:,(wherewz(1,i)+plotcnt))=probeplt(:,(wherew(1,i)+ploti-1))&* probeplt(:,(wherez(1,i)+plotj-1)).
compute plotcnt=plotcnt+1.
end loop.
end loop.
end if.
compute prodloop=1.
do if (newplp=1).
compute prodloop=nxvls.
end if.
compute plotcnt=0.
loop plotk = 1 to prodloop.
loop ploti = 1 to nwvls.
loop plotj = 1 to nzvls.
do if (newplp = 1).
compute probeplt(:,(wherexwz(1,i)+plotcnt))=probeplt(:,(1+plotk))&*probeplt(:, (wherew(1,i)+ploti-1))&*probeplt(:,(wherez(1,i)+plotj-1)).
end if.
do if (newplp > 1).
compute probeplt(:,(wheremwz(((newplp*2)-3),i)+plotcnt))=probeplt(:, wherem((newplp-1),i))&*probeplt(:,(wherew(1,i)+ploti-1))&*probeplt(:,(wherez(1,i)+plotj-1)).
end if.
compute plotcnt=plotcnt+1.
end loop.
end loop.
end loop.
end if.
end if.
end loop.
compute predvals=probeplt*b.
do if (debug <> 0).
print probeplt.
print b.
end if.
do if (i = nms+nys) and (ydich=1).
compute predvalt=(predvals < 709.7).
compute prevalt7=(1-predvalt)*(709.7).
compute predvals=(predvals&*predvalt)+prevalt7.
compute expyhat=exp(predvals)&/(1+exp(predvals)).
end if.
compute sepred=make(nrow(plotvals),3,999).
loop sei=1 to nrow(plotvals).
compute ask=probeplt(sei,:).
compute sepred(sei,1)=sqrt(ask*varb*t(ask)).
do if ((i < nms+nys) or (ydich=0)).
compute sepred(sei,2)=predvals(sei,1)-tval*sepred(sei,1).
compute sepred(sei,3)=predvals(sei,1)+tval*sepred(sei,1).
end if.
do if ((i = nms+nys) and (ydich=1)).
compute sepred(sei,2)=predvals(sei,1)-xp2*sepred(sei,1).
compute sepred(sei,3)=predvals(sei,1)+xp2*sepred(sei,1).
end if.
end loop.
compute prevloc=ncol(plotvals)+1.
compute probeplt={plotvals,predvals}.
do if (plot = 2).
compute probeplt={probeplt,sepred}.
end if.
do if ((i = nms+nys) and (ydich=1)).
compute probeplt={probeplt,expyhat}.
end if.
compute didsome=0.
end if.
do if ((wzcmat((i+1),jmed)=1) and (printpbe <= intprobe)).
do if (jmed=1).
compute omnilp2=nxvls*nwvls.
compute omnitmp=ident(omnilp2).
compute omni3(wherexw(1,i):wherexw(2,i),:)=omnitmp.
end if.
do if (jmed>1).
compute omnilp2=nwvls.
compute omnitmp=ident(omnilp2).
compute omni3(wheremw(((jmed*2)-3),i):wheremw(((jmed*2)-2),i),:)=omnitmp.
end if.
compute omnif=make(1,4,0).
do if ((i = nms+nys) and (ydich=1)).
compute omnif=make(1,3,0).
end if.
compute condeff3=0.
loop omnilp1=1 to nrow(zprobval).
loop omnilp=1 to (omnilp2).
do if (jmed=1).
compute omni3((wherexwz(1,i)+((omnilp-1)*nzvls)):(wherexwz(1,i)+((omnilp-1)*nzvls)+ (nzvls-1)),omnilp)=t(zprobval(omnilp1,:)).
end if.
do if (jmed > 1).
compute omni3((wheremwz(((jmed*2)-3),i)+((omnilp-1)*nzvls)):(wheremwz(((jmed*2)-3),i)+ ((omnilp-1)*nzvls)+(nzvls-1)),omnilp)=t(zprobval(omnilp1,:)).
end if.
end loop.
compute condeff=t(omni3)*b.
compute condeff3={condeff3;condeff}.

.
compute lmat2= omni3.
do if ( 1 =0).
compute lmat2 = mdiag( omni3 ).
compute lmat3=make(nrow(lmat2),1,0).
loop flp=1 to ncol(lmat2).
do if (csum(lmat2(:,flp))=1).
compute lmat3={lmat3,lmat2(:,flp)}.
end if.
end loop.
compute lmat2=lmat3(:,2:ncol(lmat3)).
end if.
compute fratio = (t(t(lmat2)* b )*inv(t(lmat2)* varb *lmat2)*((t(lmat2)* b )))/ncol(lmat2).
compute pfr = 1-fcdf(fratio,ncol(lmat2),(n-nrow( b ))).
compute fresult={fratio,ncol(lmat2),(n-nrow( b )),pfr}.
do if (i = (nms+nys) and (ydich=1)).
compute fratio=fratio*ncol(lmat2).
compute pfr=1-chicdf(fratio,ncol(lmat2)).
compute fresult={fratio,ncol(lmat2),pfr}.
end if.
do if ( 0 =1).
compute lmat3=1-rsum(lmat2).
compute xfm=make(n,csum(lmat3),0).
compute flpc=1.
loop flp=1 to nrow(lmat3).
do if (lmat3(flp,1)=1).
compute xfm(:,flpc)=x(:,flp).
compute flpc=flpc+1.
end if.
end loop.
compute bfm=inv(t(xfm)*xfm)*t(xfm)*y.
compute resid=y-(xfm*bfm).
compute sstotal=(y-(csum(y)/n)).
compute sstotal=csum(sstotal&*sstotal).
compute ssresid=csum(resid&*resid).
compute rsqch= 0 -((sstotal-ssresid)/sstotal).
compute fresult={rsqch,fresult}.
release xfm,flpc, resid, ssresid, bfm.
end if
.
compute omnif={omnif;fresult}.
end loop.
compute omnif=omnif(2:nrow(omnif),:).
compute clabtmp=znames.
compute condeff3=condeff3(2:nrow(condeff3),:).
do if ((nxvls*nwvls)=1).
compute omnif={condeff3,omnif}.
compute clabtmp={clabtmp,'Effect'}.
end if.
compute omnif={zmodvals,omnif}.
do if ((i < nms+nys) or (ydich=0)).
compute clabtmp={clabtmp,hcflab,'df1','df2','p'}.
end if.
do if ((i = (nms+nys)) and (ydich=1)).
compute clabtmp={clabtmp,'Chi-sq','df','p'}.
end if.
compute resultm2=make(nrow(omnif),maxresm,99999).
compute resultm2(1:nrow(omnif),1:ncol(omnif))=omnif.
compute resultm={resultm;resultm2}.
do if (outscree=1).
do if (jmed=1).
print omnif/title='Test of conditional X*W interaction at value(s) of Z:'/cnames=clabtmp/format= F10.4.
end if.
do if (jmed>1).
print omnif/title='Test of conditional M*W interaction at value(s) of Z:'/cnames=clabtmp/format= F10.4.
end if.
end if.
release omni3.
end if.
loop probei = 1 to probexpl.
do if (probexpl > 1).
compute foctmp=make(nrow(modvals),probexpl,0).
compute foctmp(:,probei)=foctmp(:,probei)+1.
compute probtemp=make(nrow(modvals),1,0).
loop probem = 1 to numplps.
loop probek = 1 to nxvls.
loop probej=lpstsp(probem,1) to lpstsp(probem,2).
compute probtemp={probtemp,foctmp(:,probek)&*probeval(:,probej)}.
end loop.
end loop.
end loop.
compute probeva2=probtemp(:,2:ncol(probtemp)).
compute probeva2={foctmp,probeva2}.
end if.
compute probres=probeva2*probcoef.
compute probrese=sqrt(diag(probeva2*probvarb*t(probeva2))).
compute tratio = probres&/probrese.
compute p = 2*(1-tcdf(abs(tratio), dfres)).
do if (ydich=1 and i = (nms+nys)).
compute p = 2*(1-cdfnorm(abs(tratio))).
end if.
compute modvals2={modvals,probres,probrese,tratio, p}.
do if ((i < nms+nys) or (ydich=0)).
compute modvals2={modvals2,(probres-tval&*probrese),(probres+tval&*probrese)}.
compute problabs={problabs,'Effect',hclab,'t', 'p', 'LLCI', 'ULCI'}.
end if.
do if (ydich=1 and i = (nms+nys)).
compute modvals2={modvals2,(probres-xp2&*probrese),(probres+xp2&*probrese)}.
compute problabs={problabs,'Effect','se','Z', 'p', 'LLCI', 'ULCI'}.
end if.
do if (probexpl > 1 and (printpbe <= intprobe)).
do if (hasz = 1).
compute printz=1.
end if.
do if (hasw=1).
compute printw=1.
end if.
compute probrlab=make(nrow(modvals),1,xcatlab(probei,1)).
compute modvals3={modvals3; modvals2}.
compute probrow={probrow;probrown}.
compute probrown=probrown+1.
do if (probei=probexpl).
compute xproblab=xcatlab(1:nxvls,1).
compute probrow=probrow(2:nrow(probrow),1).
compute modvals3=modvals3(2:nrow(modvals3),:).
compute temp=modvals3.
compute temp(GRADE(probrow(:,1)),:)=modvals3.
compute modvals3=temp.
compute start2=1.
compute problabs=problabs(1,(1+(ncol(modvarl))):ncol(problabs)).
compute pstart=1.
loop probek= 1 to nrow(probeval).
compute endstart=start2+(nxvls-1).
compute temp=modvals3(start2:endstart,(1+ncol(modvarl)):ncol(modvals3)).
compute temp2=t(modvals3(start2:start2,1:ncol(modvarl))).
compute trnames=t(modvarl).
do if (outscree=1).
do if (probek > 1).
print/title='----------'/space=0.
else.
print/title = 'Conditional effects of the focal predictor at values of the moderator(s):'.
do if ((jmed=1) and (i = (nms+nys)) and (nms > 0)).
do if (nxvls = 1).
print/title = '(These are also the conditional direct effects of X on Y)'/space=0.
else.
print/title = '(These are also the relative conditional direct effects of X on Y)'/space=0.
end if.
end if.
print.
end if.
print temp2/title = 'Moderator value(s):'/rnames=trnames/format= F10.4 /space=0.
print temp/title = ' '/cnames=problabs/rnames=xproblab/format= F10.4 /space=0.
end if.
compute resultm2=make(nrow(temp2),maxresm,99999).
compute resultm2(1:nrow(temp2),1:ncol(temp2))=temp2.
compute resultm={resultm;resultm2}.
compute resultm2=make(nrow(temp),maxresm,99999).
compute resultm2(1:nrow(temp),1:ncol(temp))=temp.
compute resultm={resultm;resultm2}.
compute start2=start2+nxvls.
compute didsome=1.
do if (jmed=1).
compute mod1val=probeval(probek,1:nm1vls).
loop omnilp=1 to nxvls.
compute omni((wheremv1(1,i)+((omnilp-1)*nm1vls)):(wheremv1(1,i)+((omnilp-1)* nm1vls)+(nm1vls-1)),omnilp)=t(mod1val).
do if (nm1vls < ncol(probeval)).
compute mod2val=probeval(probek,(nm1vls+1):(nm1vls+nm2vls)).
compute omni((wheremv2(1,i)+((omnilp-1)*nm2vls)):(wheremv2(1,i)+((omnilp-1)* nm2vls)+(nm2vls-1)),omnilp)=t(mod2val).
do if ((nm1vls+nm2vls) < ncol(probeval)).
compute intlen=nm1vls*nm2vls.
compute modintvl=probeval(probek,(nm1vls+nm2vls+1):ncol(probeval)).
compute omni((wherexwz(1,i)+((omnilp-1)*intlen)):(wherexwz(1,i)+((omnilp-1)* intlen)+(intlen-1)),omnilp)=t(modintvl).
end if.
end if.
end loop.

.
compute lmat2= omni.
do if ( 1 =0).
compute lmat2 = mdiag( omni ).
compute lmat3=make(nrow(lmat2),1,0).
loop flp=1 to ncol(lmat2).
do if (csum(lmat2(:,flp))=1).
compute lmat3={lmat3,lmat2(:,flp)}.
end if.
end loop.
compute lmat2=lmat3(:,2:ncol(lmat3)).
end if.
compute fratio = (t(t(lmat2)* b )*inv(t(lmat2)* varb *lmat2)*((t(lmat2)* b )))/ncol(lmat2).
compute pfr = 1-fcdf(fratio,ncol(lmat2),(n-nrow( b ))).
compute fresult={fratio,ncol(lmat2),(n-nrow( b )),pfr}.
do if (i = (nms+nys) and (ydich=1)).
compute fratio=fratio*ncol(lmat2).
compute pfr=1-chicdf(fratio,ncol(lmat2)).
compute fresult={fratio,ncol(lmat2),pfr}.
end if.
do if ( 0 =1).
compute lmat3=1-rsum(lmat2).
compute xfm=make(n,csum(lmat3),0).
compute flpc=1.
loop flp=1 to nrow(lmat3).
do if (lmat3(flp,1)=1).
compute xfm(:,flpc)=x(:,flp).
compute flpc=flpc+1.
end if.
end loop.
compute bfm=inv(t(xfm)*xfm)*t(xfm)*y.
compute resid=y-(xfm*bfm).
compute sstotal=(y-(csum(y)/n)).
compute sstotal=csum(sstotal&*sstotal).
compute ssresid=csum(resid&*resid).
compute rsqch= 0 -((sstotal-ssresid)/sstotal).
compute fresult={rsqch,fresult}.
release xfm,flpc, resid, ssresid, bfm.
end if
.
compute resultm2=make(nrow(fresult),maxresm,99999).
compute resultm2(1:nrow(fresult),1:ncol(fresult))=fresult.
compute resultm={resultm;resultm2}.
do if (outscree=1).
do if (i < (nms + nys) or (ydich = 0)).
compute clabtmp={hcflab,'df1','df2','p'}.
print fresult/title='Test of equality of conditional means'/cnames=clabtmp/format= F10.4.
end if.
do if (i = (nms + nys) and (ydich = 1)).
compute clabtmp={'Chi-sq','df','p'}.
print fresult/title='Test of equality of conditional logits or probabilities'/cnames=clabtmp/format= F10.4.
end if.
end if.
compute probetmp=probeplt(pstart:(pstart+nxvls),1).
compute probetmp={probetmp,probeplt(pstart:(pstart+nxvls),prevloc:ncol(probeplt))}.
compute pstart=pstart+(nxvls+1).
compute resultm2=make(nrow(probetmp),maxresm,99999).
compute resultm2(1:nrow(probetmp),1:ncol(probetmp))=probetmp.
compute resultm={resultm;resultm2}.
do if (i < ((nms + nys)) or (ydich = 0)).
compute clabtmp={xnames, outnames(1,i), hclab, 'LLCI', 'ULCI'}.
do if (outscree=1).
print probetmp/title = 'Estimated conditional means being compared:'/cnames=clabtmp/format= F10.4.
end if.
end if.
do if ((i = (nms+nys)) and (ydich = 1)).
compute clabtmp={xnames, outnames(1,i), 'prob'}.
compute probetm2=probetmp(:,1:2).
compute probetm2={probetm2,probetmp(:,ncol(probetmp))}.
do if (outscree=1).
print probetm2/title = 'Estimated conditional logits and probabilities:'/cnames=clabtmp/format= F10.4.
end if.
end if.
end if.
end loop.
release probrow, start2, endstart, temp, temp2.
end if.
end if.
do if (probexpl = 1 and (printpbe <= intprobe)).
compute resultm2=make(nrow(modvals2),maxresm,99999).
compute resultm2(1:nrow(modvals2),1:ncol(modvals2))=modvals2.
compute resultm={resultm;resultm2}.
do if (outscree=1).
print/title = 'Conditional effects of the focal predictor at values of the moderator(s):'.
print modvals2/cnames=problabs/title = ' '/space=0/format= F10.4.
end if.
compute didsome=1.
do if (hasz = 1).
compute printz=1.
end if.
do if (hasw=1).
compute printw=1.
end if.
do if (jn = 1 and jnok=1).
do if (criterr = 0).
compute dfres=n-nrow(b).
compute roots=99999.
compute jncrit =(dfres* (exp((dfres-(5/6))*((xp2/(dfres-(2/3)+(.11/dfres)))* (xp2/(dfres-(2/3)+(.11/dfres)))))-1)).
do if (i = (nms+nys) and (ydich=1)).
compute jncrit=xp2*xp2.
end if.
compute jnb1=b(wherejn1,1).
compute jnb3=b(wherejn3,1).
compute jnsb1=varb(wherejn1,wherejn1).
compute jnsb3=varb(wherejn3,wherejn3).
compute jnsb1b3=varb(wherejn1,wherejn3).
compute ajn =(jncrit*jnsb3)-(jnb3*jnb3).
compute bjn = 2*((jncrit*jnsb1b3)-(jnb1*jnb3)).
compute cjn = (jncrit*jnsb1)-(jnb1*jnb1).
compute radarg = (bjn*bjn)-(4*ajn*cjn).
compute den = 2*ajn.
compute nrts = 0.
do if (radarg >= 0 and den <> 0).
compute x21 = (-bjn+sqrt(radarg))/den.
compute x22 = (-bjn-sqrt(radarg))/den.
compute roots = 0.
do if (x21 >= jnmin and x21 <= jnmax).
compute nrts = 1.
compute roots = {roots; x21}.
end if.
do if (x22 >= jnmin and x22 <= jnmax).
compute nrts = nrts + 1.
compute roots = {roots; x22}.
end if.
compute roots={roots,make(nrow(roots),2,0)}.
end if.
do if (nrts > 0).
compute roots = roots(2:nrow(roots),1:3).
compute roots(1,2)=(csum(jnmod < roots(1,1))/n)*100.
compute roots(1,3)=(csum(jnmod > roots(1,1))/n)*100.
do if (nrow(roots)=2).
compute roots(2,2)=(csum(jnmod < roots(2,1))/n)*100.
compute roots(2,3)=(csum(jnmod > roots(2,1))/n)*100.
end if.
do if (outscree=1).
print roots/title = 'Moderator value(s) defining Johnson-Neyman significance region(s):'/clabels = 'Value', '% below', '% above'/format F10.4.
end if.
do if (nrts=1).
compute tmprts=make(1,3,99999).
compute roots={roots;tmprts}.
end if.
end if.
do if (nrts = 0).
compute roots=make(2,1,99999).
do if (outscree=1).
print/title = 'There are no statistical significance transition points within the observed'.
print/title = 'range of the moderator found using the Johnson-Neyman method.'/space=0.
end if.
end if.
compute resultm2=make(nrow(roots),maxresm,99999).
compute resultm2(1:nrow(roots),1:ncol(roots))=roots.
compute resultm={resultm;resultm2}.
compute jnvals=make(23,7,0).
loop jni= 0 to (21-(nrts)).
compute jnvals((jni+1),1)=jnmin+(jni*((jnmax-jnmin)/(21-nrts))).
end loop.
do if (nrts > 0).
loop jni = 1 to nrts.
loop jnj = 2 to (nrow(jnvals)-1).
do if ((roots(jni,1) > jnvals((jnj-1),1)) and (roots(jni,1) < jnvals(jnj,1))).
compute jnvals((jnj+1):(21+jni),1)=jnvals(jnj:(20+jni),1).
compute jnvals(jnj,1)=roots(jni,1).
end if.
end loop.
end loop.
end if.
compute jnvals=jnvals(1:22,:).
loop jni = 1 to nrow(jnvals).
compute jnvals(jni,2)=jnb1+jnb3*jnvals(jni,1).
compute jnvals(jni,3)=sqrt(jnsb1+2*jnvals(jni,1)*jnsb1b3+(jnvals(jni,1)*jnvals(jni, 1))*jnsb3).
compute jnvals(jni,4)=jnvals(jni,2)/jnvals(jni,3).
compute jnvals(jni,5)=2*(1-tcdf(abs(jnvals(jni,4)), dfres)).
compute jnvals(jni,6)=jnvals(jni,2)-sqrt(jncrit)*jnvals(jni,3).
compute jnvals(jni,7)=jnvals(jni,2)+sqrt(jncrit)*jnvals(jni,3).
do if ((i = nms + nys) and (ydich=1)).
compute jnvals(jni,5)=2*(1-cdfnorm(abs(jnvals(jni,4)))).
compute jnvals(jni,6)=jnvals(jni,2)-xp2*jnvals(jni,3).
compute jnvals(jni,7)=jnvals(jni,2)+xp2*jnvals(jni,3).
end if.
end loop.
compute resultm2=make(nrow(jnvals),maxresm,99999).
compute resultm2(1:nrow(jnvals),1:ncol(jnvals))=jnvals.
compute resultm={resultm;resultm2}.
do if ((i < nms+nys) or (ydich=0)).
compute jnclbs={jnmodlab,'Effect',hclab,'t', 'p', 'LLCI', 'ULCI'}.
end if.
do if ((i = nms + nys) and (ydich=1)).
compute jnclbs={jnmodlab,'Effect','se','Z', 'p', 'LLCI', 'ULCI'}.
end if.
do if (((wcmat((i+1),jmed)=1) or (zcmat((i+1),jmed)=1)) and (wzcmat((i+1),jmed)=0)).
do if (outscree=1).
print jnvals/title = 'Conditional effect of focal predictor at values of the moderator:'/cnames =jnclbs/format = F10.4.
end if.
end if.
do if (outscree=1).
do if ((jmed = 1) and (wzcmat((i+1),jmed)=1)).
print jnvals/title = 'Conditional X*W interaction at values of the moderator Z:'/cnames =jnclbs/format = F10.4.
end if.
do if ((jmed > 1) and (wzcmat((i+1),jmed)=1)).
print jnvals/title = 'Conditional M*W interaction at values of the moderator Z:'/cnames =jnclbs/format = F10.4.
end if.
end if.
end if.
end if.
end if.
do if ((i = (nms+nys)) and (jmed=1) and (bcmat(nrow(bcmat),1)=1)).
do if (probei=1).
compute direfflb=problabs.
compute direff=modvals2.
end if.
do if (probei>1).
compute direff={direff;modvals2}.
end if.
end if.
compute intprint=0.
do if ((jmed=1) and (i=1) and nms=0) and modcok=1).
compute contvec2=make(2,1,1).
compute contvec2={contvec2,wcontval,zcontval}.
do if (wzcmat((i+1),jmed)=1).
loop conti= 1 to ncol(wcontval).
loop contj = 1 to ncol(zcontval).
compute contvec2={contvec2,wcontval(:,conti)&*zcontval(:,contj)}.
end loop.
end loop.
end if.
compute conteff=contvec2*probcoef.
compute contdiff=contvec2(1,:)-contvec2(2,:).
compute contse=sqrt(contdiff*probvarb*t(contdiff)).
compute conteffd=conteff(1,1)-conteff(2,1).
compute contvec={contvec,conteff}.
compute contvecm=contvec.
compute resultm2=make(nrow(contvecm),maxresm,99999).
compute resultm2(1:nrow(contvecm),1:ncol(contvecm))=contvecm.
compute resultm={resultm;resultm2}.
do if (outscree=1).
print/title='Contrast between conditional effects of X:'.
print contvec/title=' '/rlabels='Effect1:','Effect2:'/cnames=problabs/format = F10.4 /space=0.
end if.
do if (ydich=0).
compute p=2*(1-tcdf(abs(conteffd/contse), dfres)).
compute contvec={conteffd,contse,conteffd/contse, p}.
compute contvec={contvec,(conteffd-(tval*contse))}.
compute contvec={contvec,(conteffd+(tval*contse))}.
compute contlabs={'Contrast', hclab, 't', 'p', 'LLCI', 'ULCI'}.
end if.
do if (ydich=1).
compute p=2*(1-cdfnorm(abs(conteffd/contse))).
compute contvec={conteffd,contse,conteffd/contse, p}.
compute contvec={contvec,(conteffd-(xp2*contse))}.
compute contvec={contvec,(conteffd+(xp2*contse))}.
compute contlabs={'Contrast', 'se', 'Z', 'p', 'LLCI', 'ULCI'}.
end if.
compute resultm2=make(nrow(contvec),maxresm,99999).
compute resultm2(1:nrow(contvec),1:ncol(contvec))=contvec.
compute resultm={resultm;resultm2}.
do if (outscree=1).
print contvec/title='Test of Effect1 minus Effect2'/format= F10.4 /cnames=contlabs.
end if.
end if.
end loop.
do if (plot = 1 or plot = 2).
compute datalabs={t(focpred(:,3)),outnames(1,i)}.
do if (plot = 1).
compute datalabs={datalabs}.
end if.
do if (plot = 2).
compute datalabs={datalabs,'se', 'LLCI', 'ULCI'}.
end if.
do if ((i = nms+nys) and (ydich=1)).
compute datalabs={datalabs,'prob'}.
end if.
compute resultm2=make(nrow(probeplt),maxresm,99999).
compute resultm2(1:nrow(probeplt),1:ncol(probeplt))=probeplt.
compute resultm={resultm;resultm2}.
compute datalabs={datalabs,'.'}.
do if (outscree=1).
print/title = 'Data for visualizing the conditional effect of the focal predictor:'.
print/title = 'Paste text below into a SPSS syntax window and execute to produce plot.'/space=0.
compute dumb = {' ', ' ', ' ', ' ', ' ', ' ', ' '}.
print datalabs/title = 'DATA LIST FREE/'/format=A10.
print probeplt/title = 'BEGIN DATA.'/format= F10.4 /space=0.
print/title = 'END DATA.'/space=0.
end if.
compute focgrph=datalabs(1,1).
compute graphix={focgrph,graphixs,modgrph}.
do if (((xdich=1) or (nxvls > 1)) and ((modcat=0) and (focgrph = xnames))).
compute graphix={modgrph,graphixs,focgrph}.
end if.
do if (panelgrp = 0).
compute graphix={graphix,'.'}.
else.
compute graphix={graphix,panelcde}.
end if.
do if (outscree=1).
print graphix/title = 'GRAPH/SCATTERPLOT='/format=A8/space=0.
end if.
do if (i = (nms + nys) and ydich = 1).
compute graphixd=graphix.
compute graphixd(1,3)='prob'.
do if (outscree=1).
print graphixd/title = 'GRAPH/SCATTERPLOT='/format=A8/space=0.
end if.
end if.
end if.
end if.
end loop.
release jmed, intprint,didprint.
end if
.
do if (model = 74 and i <= nms).
compute onetemp=make(nrow(xprobval),1,1).
compute mestmt74={onetemp,xprobval}.
do if (ncs > 0).
compute ncovmdl=rsum(ccmat(i,:)).
do if (ncovmdl > 0).
compute cvmnc=make(nrow(mestmt74),ncovmdl,1).
compute cvmnctmp=csum(x(:,(ncol(x)-ncovmdl+1):ncol(x)))/nrow(x).
do if (cuscoval > 0).
compute cvmnctmp=coval.
end if.
loop mestlp=1 to ncovmdl.
compute cvmnc(:,mestlp)=cvmnc(:,mestlp)*cvmnctmp(:,mestlp).
end loop.
compute mestmt74={mestmt74,cvmnc}.
end if.
end if.
compute mest74t=mestmt74*b.
do if (i = 1).
compute mest74=mest74t.
end if.
do if (i > 1).
compute mest74={mest74,mest74t}.
end if.
end if.
do if ((i = (nms+nys)) and (model >= 0 and model < 4) and (linsum(1,1) <> -999)).
compute lhyprob=1.
compute meansub=0.
do if (((nlinsum=nrow(b)) or (nlinsum = (nrow(b)-ncs)))).
do if (nlinsum = (nrow(b)-ncs) and ncs > 0).
compute linsum={linsum,covmeans}.
compute meansub=1.
end if.
compute lhyprob=0.
compute hypest=linsum*b.
compute sehypest=sqrt(linsum*varb*t(linsum)).
compute phypest=2*(1-tcdf(abs(hypest/sehypest), (dfres))).
compute hypest={hypest,sehypest,(hypest/sehypest),phypest,(hypest-tval*sehypest),(hypest+ tval*sehypest)}.
compute resultm2=make(ncol(linsum),maxresm,99999).
compute resultm2(:,1)=t(linsum).
compute resultm={resultm;resultm2}.
compute resultm2=make(1,maxresm,99999).
compute resultm2(1,1:ncol(hypest))=hypest.
compute resultm={resultm;resultm2}.
do if (outscree=1).
compute hyplabs={'Estimate',hclab,'t','p','LLCI','ULCI'}.
print/title='----------'.
print/title='Linear Combination Estimate and Hypothesis Test'/space=0.
print t(linsum)/title= 'Weight vector:'/clabels='weight'/rnames=vlabsm/format F10.4.
print hypest/title=' '/cnames=hyplabs/format= F10.4 /space=0.
do if (meansub=1).
print/title='Covariate weight(s) set to the sample mean.'.
end if.
end if.
end if.
do if (lhyprob=1).
compute notecode(notes,1) = 30.
compute notes = notes + 1.
end if.
end if.
end loop if criterr=1.
compute lastb=b.
compute lastcov=varb.
do if (criterr=0 and dototal = 1).
compute x=xtmp.
compute vlabsm={'constant';xcatlab(1:nxvls,1)}.
do if (ncs > 0).
compute x = {x,ctmp}.
compute vlabsm={vlabsm;t(covnames)}.
end if.
compute x = {ones,x}.

.
do if ( 1 =1).
compute hatmat=inv(t( x )* x )*t( x ).
compute b = hatmat* y.
compute modres=b.
do if ( 1 =1).
compute n1=nrow( x ).
compute dfres=n1-(ncol( x )).
compute sstotal = t( y -(csum( y )/n1))*( y -(csum( y )/n1)).
compute resid= y - x *b.
compute ssresid = csum((resid)&**2).
compute r2 = (sstotal-ssresid)/sstotal.
do if (r2 < 0).
compute r2=0.
end if.
compute adjr2 = 1-((1-r2)*(n1-1)/(dfres)).
compute mse=ssresid/(n1-ncol( x )).

.
compute n1=nrow( x ).
compute invXtX = inv(t( x )* x ).
compute varb = mse *invXtX.
compute k3 = ncol( x ).
compute xhc=0.
do if ( hc <> 5).
compute xhc= x.
compute hat = xhc(:,1).
loop i3=1 to nrow(xhc).
compute hat(i3,1)= xhc(i3,:)*invXtX*t(xhc(i3,:)).
end loop.
do if ( hc = 0 or hc =1).
loop i3 = 1 to k3.
compute xhc(:,i3)=xhc(:,i3)&* resid.
end loop.
end if.
do if ( hc =3 or hc =2).
loop i3=1 to k3.
compute xhc(:,i3) = ( resid &/(1-hat)&**(1/(4- hc )))&*xhc(:,i3).
end loop.
end if.
do if ( hc = 4).
compute hcmn=make(n,2,4).
compute hcmn(:,2)=(n1*hat)/k3.
loop i3= 1 to k3.
compute xhc(:,i3) = ( resid &/(1-hat)&**(rmin(hcmn)/2))&*xhc(:,i3).
end loop.
end if.
compute varb=(invXtX*t(xhc)*xhc*invXtX).
do if ( hc =1).
compute varb=(n1/(n1-ncol( x )))&*varb.
end if.
end if.
compute hclab={'se(HC0)','se(HC1)','se(HC2)','se(HC3)','se(HC4)','se'}.
compute hclab=hclab(1,( hc +1)).
compute hcflab={'F(HC0)','F(HC1)','F(HC2)','F(HC3)','F(HC4)','F'}.
compute hcflab=hcflab(1,( hc +1)).
release xhc
.
compute seb=sqrt(diag(varb)).
compute trat = b&/seb.
compute p = 2*(1-tcdf(abs(trat), (dfres))).
compute tval = sqrt(dfres* (exp((dfres-(5/6))*((xp2/(dfres-(2/3)+(.11/dfres)))* (xp2/(dfres-(2/3)+(.11/dfres)))))-1)).
compute modres={modres,seb,trat,p}.
compute modres={modres,(b-tval&*seb),(b+tval&*seb)}.
compute modresl={'coeff',hclab,'t','p','LLCI','ULCI'}.
compute lmat = ident(ncol( x )).
compute lmat = lmat(:,2:ncol(lmat)).
compute fratio = (t(t(lmat)*b)*inv(t(lmat)*varb*lmat)*((t(lmat)*b)))/(ncol( x )-1).
compute pfr = 1-fcdf(fratio,(ncol( x )-1),dfres).
compute modsum={sqrt(r2),r2,mse,fratio,(ncol( x )-1),dfres,pfr}.
compute modsuml={'R','R-sq','MSE',hcflab,'df1','df2', 'p'}.
end if.
end if.
do if ( 1 = 2 or 1 =3).
compute xlp= x.
compute ylp= y.
compute pt2 = make(nrow(ylp),1,(csum(ylp)/nrow(ylp))).
do if ( 1 =2).
compute LL3 = ylp&*ln(pt2)+(1-ylp)&*ln(1-pt2).
end if.
compute LL3 = -2*csum(LL3).
compute bt1 = make(ncol(xlp),1,0).
compute LL1 = 0.
compute pt1 = make(nrow(ylp),1,0.5).
compute pt1lp=pt1.
loop jjj = 1 to iterate.
compute xlptmp=t(xlp).
compute vecprb=pt1lp&*(1-pt1lp).
loop kkk=1 to ncol(xlp).
compute xlptmp(kkk,:)=xlptmp(kkk,:)&*t(vecprb).
end loop.
compute b = bt1+inv(xlptmp*xlp)*t(xlp)*(ylp-pt1lp).
do if ( 1 =2).
compute xlpb=xlp*b.
compute xlpbt=(xlpb > -709.7).
compute xlpb709=(1-xlpbt)*(-709.7).
compute xlpb=(xlpb&*xlpbt)+xlpb709.
compute pt1lp = 1/(1+exp(-(xlpb))).
end if.
compute itprob = csum((pt1lp < .00000001) or (pt1lp > .9999999)).
do if (itprob > 0).
loop kkk = 1 to nrow(pt1lp).
do if (pt1lp(kkk,1) > .9999999).
compute pt1lp(kkk,1) = .9999999.
end if.
do if (pt1lp(kkk,1) < .00000001).
compute pt1lp(kkk,1) = .00000001.
end if.
end loop.
compute itprob = 0.
end if.
do if (itprob = 0).
do if ( 1 =2).
compute LL = ylp&*ln(pt1lp)+(1-ylp)&*ln(1-pt1lp).
end if.
compute LL2 = -2*csum(ll).
end if.
do if (abs(LL1-LL2) < converge).
do if ( 1 =1).
compute xlptmp=t(xlp).
compute vecprb=pt1lp&*(1-pt1lp).
loop kkk=1 to ncol(xlp).
compute xlptmp(kkk,:)=xlptmp(kkk,:)&*t(vecprb).
end loop.
compute varb = inv(xlptmp*xlp).
compute seb = sqrt(diag(varb)).
release xlptmp.
end if.
break.
end if.
compute bt1 = b.
compute LL1 = LL2.
end loop.
compute modres=b.
do if (jjj > iterate).
compute itprob = 2.
do if (booting=0).
compute iterrmod=1.
end if.
do if (booting=1).
compute bootiter=1.
end if.
do if (itprobtg=0).
compute itprobtg=1.
compute errcode(errs,1) = 47.
compute errs = errs + 1.
do if (booting = 0 and 1 =1).
compute vt1 = mdiag(pt1lp&*(1-pt1lp)).
compute varb = inv(t(xlp)*vt1*xlp).
compute seb = sqrt(diag(varb)).
end if.
end if.
end if.
do if ( 1 =1).
compute trat = b&/seb.
compute dfres=nrow(xlp).
compute p = 2*(1-cdfnorm(abs(trat))).
compute modres={modres,seb,trat,p}.
compute modres={modres,(b-xp2&*seb),(b+xp2&*seb)}.
compute pvchi=1-chicdf((LL3-LL2),(nrow(modres)-1)).
compute mcF = (LL3-LL2)/LL3.
compute cox = 1-exp(-(LL3-LL2)/nrow(xlp)).
compute nagel = cox/(1-exp(-(LL3)/nrow(xlp))).
compute modsum={LL2,(LL3-LL2),(nrow(modres)-1),pvchi, mcF,cox,nagel}.
compute modsuml={'-2LL','ModelLL', 'df', 'p', 'McFadden', 'CoxSnell', 'Nagelkrk'}.
compute modresl={'coeff','se','Z','p','LLCI','ULCI'}.
end if.
end if
.
compute toteff=modres(2:(1+nxvls),:).
compute nodotot=0.
do if (xdich=1 and xmint=1 and model=74).
compute toteff(:,1)=toteff(:,1)*xscaling.
compute toteff(:,2)=toteff(:,2)*abs(xscaling).
compute toteff(:,3)=toteff(:,3)*xscaling.
compute toteff(:,5)=toteff(:,5)*xscaling.
compute toteff(:,6)=toteff(:,6)*xscaling.
compute citmp=toteff(:,5:6).
do if (xscaling < 0).
compute toteff(:,5)=citmp(:,2).
compute toteff(:,6)=citmp(:,1).
end if.
compute nodotot=1.
end if.
compute totefflb=modresl.
do if (outscree=1).
do if (nodotot=0).
print/title = '************************** TOTAL EFFECT MODEL ****************************'.
end if.
do if (nodotot=1).
print/title = '**************************************************************************'.
end if.
print outnames(1,ncol(outnames))/title = 'OUTCOME VARIABLE:'/format = A8/space=0.
end if.
compute toteffl2=vlabsm(2:(1+nxvls),:).
compute resultm2=make(1,maxresm,99999).
compute resultm2(1,1:ncol(modsum))=modsum.
compute resultm={resultm;resultm2}.
compute resultm2=make(nrow(modres),maxresm,99999).
compute resultm2(1:nrow(modres),1:ncol(modres))=modres.
compute resultm={resultm;resultm2}.
do if (outscree=1).
print modsum/title = 'Model Summary'/cnames = modsuml/format= F10.4.
print modres/title='Model'/rnames=vlabsm/cnames=modresl/format= F10.4.
end if.
compute lmat=make(nrow(b),1,0).
compute lmat2=make(nxvls,1,1).
compute lmat(2:(1+nxvls),1)=lmat2.
do if (ydich <> 1).

.
compute lmat2= lmat.
do if ( 0 =0).
compute lmat2 = mdiag( lmat ).
compute lmat3=make(nrow(lmat2),1,0).
loop flp=1 to ncol(lmat2).
do if (csum(lmat2(:,flp))=1).
compute lmat3={lmat3,lmat2(:,flp)}.
end if.
end loop.
compute lmat2=lmat3(:,2:ncol(lmat3)).
end if.
compute fratio = (t(t(lmat2)* b )*inv(t(lmat2)* varb *lmat2)*((t(lmat2)* b )))/ncol(lmat2).
compute pfr = 1-fcdf(fratio,ncol(lmat2),(n-nrow( b ))).
compute fresult={fratio,ncol(lmat2),(n-nrow( b )),pfr}.
do if (i = (nms+nys) and (ydich=1)).
compute fratio=fratio*ncol(lmat2).
compute pfr=1-chicdf(fratio,ncol(lmat2)).
compute fresult={fratio,ncol(lmat2),pfr}.
end if.
do if ( 1 =1).
compute lmat3=1-rsum(lmat2).
compute xfm=make(n,csum(lmat3),0).
compute flpc=1.
loop flp=1 to nrow(lmat3).
do if (lmat3(flp,1)=1).
compute xfm(:,flpc)=x(:,flp).
compute flpc=flpc+1.
end if.
end loop.
compute bfm=inv(t(xfm)*xfm)*t(xfm)*y.
compute resid=y-(xfm*bfm).
compute sstotal=(y-(csum(y)/n)).
compute sstotal=csum(sstotal&*sstotal).
compute ssresid=csum(resid&*resid).
compute rsqch= r2 -((sstotal-ssresid)/sstotal).
compute fresult={rsqch,fresult}.
release xfm,flpc, resid, ssresid, bfm.
end if
.
compute totomni=fresult.
end if.
do if (stand=1).
compute predsd=make(nrow(modres),1,0).
compute stdmod=modres(:,1)&/ovsd(1,ncol(ovsd)).
loop jd=1 to ncol(x).
compute descdat=x(:,jd).
compute predsd(jd,1) = (nrow(descdat)*sscp(descdat))-(t(csum(descdat))*(csum(descdat))).
compute predsd(jd,1) = sqrt(predsd(jd,1)/(nrow(descdat)*(nrow(descdat)-1))).
end loop.
do if (wherex(1,ncol(wherex)) <> -999 and ((nxvls > 1) or (xdich=1))).
compute sdmsone=make(nxvls,1,1).
compute predsd(wherex(1,ncol(wherex)):wherex(2,ncol(wherex)),1)=sdmsone.
compute pstog=1.
end if.
compute predsd(1,1)=1.
compute stdmod=stdmod&*predsd.
compute stdmod=stdmod(2:nrow(stdmod),1).
compute sdvlabs=vlabsm(2:nrow(vlabsm),1).
compute resultm2=make(nrow(stdmod),maxresm,99999).
compute resultm2(1:nrow(stdmod),1:ncol(stdmod))=stdmod.
compute resultm={resultm;resultm2}.
do if (outscree=1).
print stdmod/title='Standardized coefficients'/clabels='coeff'/rnames=sdvlabs/format= F10.4.
end if.
end if.
do if (covcoeff=1).
do if (outscree=1).
print varb/title='Covariance matrix of regression parameter estimates:'/rnames=vlabsm/cnames=vlabsm/format= F10.4.
end if.
compute resultm2=make(nrow(varb),maxresm,99999).
do if (ncol(varb) <= maxresm).
compute resultm2(1:nrow(varb),1:ncol(varb))=varb.
compute resultm={resultm;resultm2}.
end if.
do if (ncol(varb) > maxresm).
compute resultmt=make(nrow(resultm),ncol(varb),99999).
compute resultmt(1:nrow(resultm),1:ncol(resultm))=resultm.
compute resultm=resultmt.
compute resultm2=make(nrow(varb),ncol(resultm),99999).
compute resultm2(1:nrow(varb),1:ncol(varb))=varb.
compute resultm={resultm;resultm2}.
compute maxresm=ncol(resultm).
end if.
end if.
end if.
end if.
do if (criterr=0 and nms > 0 and ydich=0 and modelres=1).
compute modresid=modresid(:,2:ncol(modresid)).
compute sigmatal = (t(modresid)*(ident(n)-(1/n)*ones*t(ones))*modresid)*(1/(n-1)).
compute sdall = mdiag(1/sqrt(diag(sigmatal))).
compute corall=sdall*sigmatal*t(sdall).
compute resultm2=make(nrow(corall),maxresm,99999).
compute resultm2(1:nrow(corall),1:ncol(corall))=corall.
compute resultm={resultm;resultm2}.
do if (outscree=1).
print/title = '****************** CORRELATIONS BETWEEN MODEL RESIDUALS ******************'.
print corall/title=' '/format= F10.4 / cnames=outnames/rnames=outnames/space=0.
end if.
end if.
do if (criterr=0 and boot > 0).
compute bootres=make(1,rsum(nump),-999).
compute bootdir=obsdirfx.
compute natdirbt=make(1,nxvls,-999).
do if (effsize=1).
compute bootysd=make(1,1,-999).
compute bootxsd=make(1,1,-999).
end if.
compute badboot=0.
compute goodboot=0.
compute smallest=1.
compute booting=1.
loop j = 1 to maxboot.
compute nobootx=1.
compute modres2=999.
compute v=trunc(uniform(n,1)*n)+1.
compute bad=0.
loop i = 1 to (nms+nys).
compute y=outvars(v,i).
compute ynovar= (nrow(y)*sscp(y))-(t(csum(y))*(csum(y))).
do if (ynovar = 0).
compute bad=1.
end if.
compute xindx=datindx(1:(nump(1,i)-1),i).
compute hello=0.
compute x = fulldat(v,xindx).
compute x={ones,x}.
compute xsq=t(x)*x.
compute exsq=eval(xsq).
release xsq.
compute holymoly=cmin(exsq).
compute zeroeig=csum(exsq <= 0.000000000002).
compute bad=bad + (zeroeig > 0).

.
compute desctmp=make((8-(4* 1 )),ncol( y ),-999).
loop jd=1 to ncol( y ).
compute descdat= y (:,jd).
compute desctmp(1,jd) = csum(descdat)/nrow(descdat).
compute desctmp(2,jd) = (nrow(descdat)*sscp(descdat))-(t(csum(descdat))*(csum(descdat))).
compute desctmp(2,jd) = sqrt(desctmp(2,jd)/(nrow(descdat)*(nrow(descdat)-1))).
compute desctmp(3,jd)=cmin(descdat).
compute desctmp(4,jd)=cmax(descdat).
do if ( 1 =0).
compute minwarn=0.
compute maxwarn=0.
do if ((desctmp(3,jd)=desctmp(4,jd)) and novar=0).
compute errcode(errs,1)=15.
compute errs=errs+1.
compute criterr=1.
compute novar=1.
end if.
compute tmp=((descdat(:,1)=desctmp(3,jd))+(descdat(:,1)=desctmp(4,jd))).
compute desctmp(8,jd)=(csum(tmp)=nrow(tmp)).
compute tmp = descdat.
compute tmp(GRADE(descdat),:) = descdat.
compute descdat = tmp.
release tmp.
compute decval={.16;.5;.84}.
loop kd=1 to 3.
compute low=trunc(decval(kd,1)*(nrow(descdat)+1)).
compute lowdec=decval(kd,1)*(nrow(descdat)+1)-low.
compute value=descdat(low,1)+(descdat((low+1),1)-descdat(low,1))*lowdec.
compute desctmp((4+kd),jd)=value.
end loop.
compute mnotev=1.
compute modvals=desctmp(5:7,:).
do if (quantile <> 1).
compute desctmp(5,jd)=desctmp(1,jd)-desctmp(2,jd).
compute desctmp(6,jd)=desctmp(1,jd).
compute desctmp(7,jd)=desctmp(1,jd)+desctmp(2,jd).
compute modvals=desctmp(5:7,:).
compute mnotev=2.
do if (modvals(1,1) < desctmp(3,1)).
compute modvals(1,1)=desctmp(3,1).
compute minwarn=1.
end if.
do if (modvals(3,1) > desctmp(4,1)).
compute modvals(3,1)=desctmp(4,1).
compute maxwarn=1.
end if.
end if.
do if (desctmp(8,1)=1).
compute modvals={desctmp(3,1);desctmp(4,1)}.
compute mnotev=0.
compute minwarn=0.
compute maxwarn=0.
end if.
end if.
end loop
.
compute bad=bad+ (desctmp(2,1) <= 0.00000000001).
do if (bad = 0).
do if (holymoly < smallest).
compute smallest=holymoly.
end if.
do if (ydich=0 or (i < (nms+nys))).

.
do if ( 1 =1).
compute hatmat=inv(t( x )* x )*t( x ).
compute b = hatmat* y.
compute modres=b.
do if ( 0 =1).
compute n1=nrow( x ).
compute dfres=n1-(ncol( x )).
compute sstotal = t( y -(csum( y )/n1))*( y -(csum( y )/n1)).
compute resid= y - x *b.
compute ssresid = csum((resid)&**2).
compute r2 = (sstotal-ssresid)/sstotal.
do if (r2 < 0).
compute r2=0.
end if.
compute adjr2 = 1-((1-r2)*(n1-1)/(dfres)).
compute mse=ssresid/(n1-ncol( x )).

.
compute n1=nrow( x ).
compute invXtX = inv(t( x )* x ).
compute varb = mse *invXtX.
compute k3 = ncol( x ).
compute xhc=0.
do if ( hc <> 5).
compute xhc= x.
compute hat = xhc(:,1).
loop i3=1 to nrow(xhc).
compute hat(i3,1)= xhc(i3,:)*invXtX*t(xhc(i3,:)).
end loop.
do if ( hc = 0 or hc =1).
loop i3 = 1 to k3.
compute xhc(:,i3)=xhc(:,i3)&* resid.
end loop.
end if.
do if ( hc =3 or hc =2).
loop i3=1 to k3.
compute xhc(:,i3) = ( resid &/(1-hat)&**(1/(4- hc )))&*xhc(:,i3).
end loop.
end if.
do if ( hc = 4).
compute hcmn=make(n,2,4).
compute hcmn(:,2)=(n1*hat)/k3.
loop i3= 1 to k3.
compute xhc(:,i3) = ( resid &/(1-hat)&**(rmin(hcmn)/2))&*xhc(:,i3).
end loop.
end if.
compute varb=(invXtX*t(xhc)*xhc*invXtX).
do if ( hc =1).
compute varb=(n1/(n1-ncol( x )))&*varb.
end if.
end if.
compute hclab={'se(HC0)','se(HC1)','se(HC2)','se(HC3)','se(HC4)','se'}.
compute hclab=hclab(1,( hc +1)).
compute hcflab={'F(HC0)','F(HC1)','F(HC2)','F(HC3)','F(HC4)','F'}.
compute hcflab=hcflab(1,( hc +1)).
release xhc
.
compute seb=sqrt(diag(varb)).
compute trat = b&/seb.
compute p = 2*(1-tcdf(abs(trat), (dfres))).
compute tval = sqrt(dfres* (exp((dfres-(5/6))*((xp2/(dfres-(2/3)+(.11/dfres)))* (xp2/(dfres-(2/3)+(.11/dfres)))))-1)).
compute modres={modres,seb,trat,p}.
compute modres={modres,(b-tval&*seb),(b+tval&*seb)}.
compute modresl={'coeff',hclab,'t','p','LLCI','ULCI'}.
compute lmat = ident(ncol( x )).
compute lmat = lmat(:,2:ncol(lmat)).
compute fratio = (t(t(lmat)*b)*inv(t(lmat)*varb*lmat)*((t(lmat)*b)))/(ncol( x )-1).
compute pfr = 1-fcdf(fratio,(ncol( x )-1),dfres).
compute modsum={sqrt(r2),r2,mse,fratio,(ncol( x )-1),dfres,pfr}.
compute modsuml={'R','R-sq','MSE',hcflab,'df1','df2', 'p'}.
end if.
end if.
do if ( 1 = 2 or 1 =3).
compute xlp= x.
compute ylp= y.
compute pt2 = make(nrow(ylp),1,(csum(ylp)/nrow(ylp))).
do if ( 1 =2).
compute LL3 = ylp&*ln(pt2)+(1-ylp)&*ln(1-pt2).
end if.
compute LL3 = -2*csum(LL3).
compute bt1 = make(ncol(xlp),1,0).
compute LL1 = 0.
compute pt1 = make(nrow(ylp),1,0.5).
compute pt1lp=pt1.
loop jjj = 1 to iterate.
compute xlptmp=t(xlp).
compute vecprb=pt1lp&*(1-pt1lp).
loop kkk=1 to ncol(xlp).
compute xlptmp(kkk,:)=xlptmp(kkk,:)&*t(vecprb).
end loop.
compute b = bt1+inv(xlptmp*xlp)*t(xlp)*(ylp-pt1lp).
do if ( 1 =2).
compute xlpb=xlp*b.
compute xlpbt=(xlpb > -709.7).
compute xlpb709=(1-xlpbt)*(-709.7).
compute xlpb=(xlpb&*xlpbt)+xlpb709.
compute pt1lp = 1/(1+exp(-(xlpb))).
end if.
compute itprob = csum((pt1lp < .00000001) or (pt1lp > .9999999)).
do if (itprob > 0).
loop kkk = 1 to nrow(pt1lp).
do if (pt1lp(kkk,1) > .9999999).
compute pt1lp(kkk,1) = .9999999.
end if.
do if (pt1lp(kkk,1) < .00000001).
compute pt1lp(kkk,1) = .00000001.
end if.
end loop.
compute itprob = 0.
end if.
do if (itprob = 0).
do if ( 1 =2).
compute LL = ylp&*ln(pt1lp)+(1-ylp)&*ln(1-pt1lp).
end if.
compute LL2 = -2*csum(ll).
end if.
do if (abs(LL1-LL2) < converge).
do if ( 0 =1).
compute xlptmp=t(xlp).
compute vecprb=pt1lp&*(1-pt1lp).
loop kkk=1 to ncol(xlp).
compute xlptmp(kkk,:)=xlptmp(kkk,:)&*t(vecprb).
end loop.
compute varb = inv(xlptmp*xlp).
compute seb = sqrt(diag(varb)).
release xlptmp.
end if.
break.
end if.
compute bt1 = b.
compute LL1 = LL2.
end loop.
compute modres=b.
do if (jjj > iterate).
compute itprob = 2.
do if (booting=0).
compute iterrmod=1.
end if.
do if (booting=1).
compute bootiter=1.
end if.
do if (itprobtg=0).
compute itprobtg=1.
compute errcode(errs,1) = 47.
compute errs = errs + 1.
do if (booting = 0 and 0 =1).
compute vt1 = mdiag(pt1lp&*(1-pt1lp)).
compute varb = inv(t(xlp)*vt1*xlp).
compute seb = sqrt(diag(varb)).
end if.
end if.
end if.
do if ( 0 =1).
compute trat = b&/seb.
compute dfres=nrow(xlp).
compute p = 2*(1-cdfnorm(abs(trat))).
compute modres={modres,seb,trat,p}.
compute modres={modres,(b-xp2&*seb),(b+xp2&*seb)}.
compute pvchi=1-chicdf((LL3-LL2),(nrow(modres)-1)).
compute mcF = (LL3-LL2)/LL3.
compute cox = 1-exp(-(LL3-LL2)/nrow(xlp)).
compute nagel = cox/(1-exp(-(LL3)/nrow(xlp))).
compute modsum={LL2,(LL3-LL2),(nrow(modres)-1),pvchi, mcF,cox,nagel}.
compute modsuml={'-2LL','ModelLL', 'df', 'p', 'McFadden', 'CoxSnell', 'Nagelkrk'}.
compute modresl={'coeff','se','Z','p','LLCI','ULCI'}.
end if.
end if
.
end if.
do if (ydich=1 and (i = (nms+nys))).

.
do if ( 2 =1).
compute hatmat=inv(t( x )* x )*t( x ).
compute b = hatmat* y.
compute modres=b.
do if ( 0 =1).
compute n1=nrow( x ).
compute dfres=n1-(ncol( x )).
compute sstotal = t( y -(csum( y )/n1))*( y -(csum( y )/n1)).
compute resid= y - x *b.
compute ssresid = csum((resid)&**2).
compute r2 = (sstotal-ssresid)/sstotal.
do if (r2 < 0).
compute r2=0.
end if.
compute adjr2 = 1-((1-r2)*(n1-1)/(dfres)).
compute mse=ssresid/(n1-ncol( x )).

.
compute n1=nrow( x ).
compute invXtX = inv(t( x )* x ).
compute varb = mse *invXtX.
compute k3 = ncol( x ).
compute xhc=0.
do if ( hc <> 5).
compute xhc= x.
compute hat = xhc(:,1).
loop i3=1 to nrow(xhc).
compute hat(i3,1)= xhc(i3,:)*invXtX*t(xhc(i3,:)).
end loop.
do if ( hc = 0 or hc =1).
loop i3 = 1 to k3.
compute xhc(:,i3)=xhc(:,i3)&* resid.
end loop.
end if.
do if ( hc =3 or hc =2).
loop i3=1 to k3.
compute xhc(:,i3) = ( resid &/(1-hat)&**(1/(4- hc )))&*xhc(:,i3).
end loop.
end if.
do if ( hc = 4).
compute hcmn=make(n,2,4).
compute hcmn(:,2)=(n1*hat)/k3.
loop i3= 1 to k3.
compute xhc(:,i3) = ( resid &/(1-hat)&**(rmin(hcmn)/2))&*xhc(:,i3).
end loop.
end if.
compute varb=(invXtX*t(xhc)*xhc*invXtX).
do if ( hc =1).
compute varb=(n1/(n1-ncol( x )))&*varb.
end if.
end if.
compute hclab={'se(HC0)','se(HC1)','se(HC2)','se(HC3)','se(HC4)','se'}.
compute hclab=hclab(1,( hc +1)).
compute hcflab={'F(HC0)','F(HC1)','F(HC2)','F(HC3)','F(HC4)','F'}.
compute hcflab=hcflab(1,( hc +1)).
release xhc
.
compute seb=sqrt(diag(varb)).
compute trat = b&/seb.
compute p = 2*(1-tcdf(abs(trat), (dfres))).
compute tval = sqrt(dfres* (exp((dfres-(5/6))*((xp2/(dfres-(2/3)+(.11/dfres)))* (xp2/(dfres-(2/3)+(.11/dfres)))))-1)).
compute modres={modres,seb,trat,p}.
compute modres={modres,(b-tval&*seb),(b+tval&*seb)}.
compute modresl={'coeff',hclab,'t','p','LLCI','ULCI'}.
compute lmat = ident(ncol( x )).
compute lmat = lmat(:,2:ncol(lmat)).
compute fratio = (t(t(lmat)*b)*inv(t(lmat)*varb*lmat)*((t(lmat)*b)))/(ncol( x )-1).
compute pfr = 1-fcdf(fratio,(ncol( x )-1),dfres).
compute modsum={sqrt(r2),r2,mse,fratio,(ncol( x )-1),dfres,pfr}.
compute modsuml={'R','R-sq','MSE',hcflab,'df1','df2', 'p'}.
end if.
end if.
do if ( 2 = 2 or 2 =3).
compute xlp= x.
compute ylp= y.
compute pt2 = make(nrow(ylp),1,(csum(ylp)/nrow(ylp))).
do if ( 2 =2).
compute LL3 = ylp&*ln(pt2)+(1-ylp)&*ln(1-pt2).
end if.
compute LL3 = -2*csum(LL3).
compute bt1 = make(ncol(xlp),1,0).
compute LL1 = 0.
compute pt1 = make(nrow(ylp),1,0.5).
compute pt1lp=pt1.
loop jjj = 1 to iterate.
compute xlptmp=t(xlp).
compute vecprb=pt1lp&*(1-pt1lp).
loop kkk=1 to ncol(xlp).
compute xlptmp(kkk,:)=xlptmp(kkk,:)&*t(vecprb).
end loop.
compute b = bt1+inv(xlptmp*xlp)*t(xlp)*(ylp-pt1lp).
do if ( 2 =2).
compute xlpb=xlp*b.
compute xlpbt=(xlpb > -709.7).
compute xlpb709=(1-xlpbt)*(-709.7).
compute xlpb=(xlpb&*xlpbt)+xlpb709.
compute pt1lp = 1/(1+exp(-(xlpb))).
end if.
compute itprob = csum((pt1lp < .00000001) or (pt1lp > .9999999)).
do if (itprob > 0).
loop kkk = 1 to nrow(pt1lp).
do if (pt1lp(kkk,1) > .9999999).
compute pt1lp(kkk,1) = .9999999.
end if.
do if (pt1lp(kkk,1) < .00000001).
compute pt1lp(kkk,1) = .00000001.
end if.
end loop.
compute itprob = 0.
end if.
do if (itprob = 0).
do if ( 2 =2).
compute LL = ylp&*ln(pt1lp)+(1-ylp)&*ln(1-pt1lp).
end if.
compute LL2 = -2*csum(ll).
end if.
do if (abs(LL1-LL2) < converge).
do if ( 0 =1).
compute xlptmp=t(xlp).
compute vecprb=pt1lp&*(1-pt1lp).
loop kkk=1 to ncol(xlp).
compute xlptmp(kkk,:)=xlptmp(kkk,:)&*t(vecprb).
end loop.
compute varb = inv(xlptmp*xlp).
compute seb = sqrt(diag(varb)).
release xlptmp.
end if.
break.
end if.
compute bt1 = b.
compute LL1 = LL2.
end loop.
compute modres=b.
do if (jjj > iterate).
compute itprob = 2.
do if (booting=0).
compute iterrmod=1.
end if.
do if (booting=1).
compute bootiter=1.
end if.
do if (itprobtg=0).
compute itprobtg=1.
compute errcode(errs,1) = 47.
compute errs = errs + 1.
do if (booting = 0 and 0 =1).
compute vt1 = mdiag(pt1lp&*(1-pt1lp)).
compute varb = inv(t(xlp)*vt1*xlp).
compute seb = sqrt(diag(varb)).
end if.
end if.
end if.
do if ( 0 =1).
compute trat = b&/seb.
compute dfres=nrow(xlp).
compute p = 2*(1-cdfnorm(abs(trat))).
compute modres={modres,seb,trat,p}.
compute modres={modres,(b-xp2&*seb),(b+xp2&*seb)}.
compute pvchi=1-chicdf((LL3-LL2),(nrow(modres)-1)).
compute mcF = (LL3-LL2)/LL3.
compute cox = 1-exp(-(LL3-LL2)/nrow(xlp)).
compute nagel = cox/(1-exp(-(LL3)/nrow(xlp))).
compute modsum={LL2,(LL3-LL2),(nrow(modres)-1),pvchi, mcF,cox,nagel}.
compute modsuml={'-2LL','ModelLL', 'df', 'p', 'McFadden', 'CoxSnell', 'Nagelkrk'}.
compute modresl={'coeff','se','Z','p','LLCI','ULCI'}.
end if.
end if
.
end if.
compute modres2={modres2,t(modres)}.
do if (i = (nms+nys)).
do if (bcmat((i+1),1) = 1).
compute bootdir={bootdir;t(modres(wherex(1,i):wherex(2,i),1))}.
end if.
do if (bcmat((i+1),1) = 0).
compute bootdir={bootdir;dirzes}.
end if.
end if.
do if (model = 74 and i <= nms).
compute onetemp=make(nrow(xprobval),1,1).
compute mestmtb={onetemp,xprobval}.
do if (ncs > 0).
compute ncovmdlb=rsum(ccmat(i,:)).
do if (ncovmdl > 0).
compute cvmncb=make(nrow(mestmtb),ncovmdlb,1).
compute cvmnctmp=csum(x(:,(ncol(x)-ncovmdlb+1):ncol(x)))/nrow(x).
do if (cuscoval > 0).
compute cvmnctmp=coval.
end if.
loop mestlp=1 to ncovmdlb.
compute cvmncb(:,mestlp)=cvmncb(:,mestlp)*cvmnctmp(:,mestlp).
end loop.
compute mestmtb={mestmtb,cvmncb}.
end if.
end if.
compute mestbt=mestmtb*modres.
do if (i = 1).
compute mestb=mestbt.
end if.
do if (i > 1).
compute mestb={mestb,mestbt}.
end if.
end if.
do if (model = 74 and i = (nms+nys)).
compute xvalptmp=make(1,nxvls,0).
compute mest74sp=make(1,nms,0).
compute mest74sp={mest74sp;mestb}.
compute xvalptmp={xvalptmp;ident(nxvls)}.
compute dirfxcf=make(nrow(mestb),1,0).
loop cfloop1=1 to nrow(mestb).
compute ndirfx=make(nrow(modres),1,0).
compute ndirfx(2:(nxvls+1),1)=t(xvalptmp(cfloop1,:)).
loop cfloop3=1 to nms.
do if (mcx=1 or mcx=0).
compute ndirfx(wheremw((1+((cfloop3-1)*2)),ncol(wheremw)):wheremw((2+((cfloop3-1)*2) ),ncol(wheremw)))=t(xvalptmp(cfloop1,:)*mestb(1,cfloop3)).
end if.
do if (mcx=2).
compute ndirfx(wheremw((1+((cfloop3-1)*2)),ncol(wheremw)):wheremw((2+((cfloop3-1)*2) ),ncol(wheremw)))=t(xvalptmp(cfloop1,:)*mest74sp(cfloop1,cfloop3)).
end if.
end loop.
do if (cfloop1 > 1).
compute dirfxcf(cfloop1,1)=t(ndirfx)*modres*xscaling.
end if.
end loop.
compute natdirbt={natdirbt;t(dirfxcf(2:nrow(dirfxcf),:))}.
end if.
do if ( (bcmat((i+1),1) = 1) and (nobootx=1) and (effsize=1)).
compute nobootx=0.
compute xsdtemp= (nrow(x)*sscp(x(:,2)))-(t(csum(x(:,2)))*(csum(x(:,2)))).
compute xsdtemp= sqrt(xsdtemp/(nrow(x)*(nrow(x)-1))).
end if.
end if.
end loop.
do if (bad = 0).
compute bootres={bootres;modres2(:,2:ncol(modres2))}.
do if (effsize=1).
compute ysdtemp= (nrow(y)*sscp(y))-(t(csum(y))*(csum(y))).
compute ysdtemp= sqrt(ysdtemp/(nrow(y)*(nrow(y)-1))).
compute bootysd={bootysd;ysdtemp}.
compute bootxsd={bootxsd;xsdtemp}.
end if.
compute goodboot=goodboot+1.
end if.
do if (bad <> 0).
compute badboot=badboot+1.
end if.
end loop if (goodboot = boot).
compute bootres=bootres(2:nrow(bootres),:).
do if (effsize=1).
compute bootysd=bootysd(2:nrow(bootysd),:).
do if (nrow(bootxsd) > 1).
compute bootxsd=bootxsd(2:nrow(bootxsd),:).
end if.
end if.
do if (goodboot < (boot)).
compute boot=0.
compute modelbt=0.
compute notecode(notes,1) = 7.
compute notes = notes + 1.
end if.
do if (boot > 0).
do if (effsize=1).
compute bootysd={ysd;bootysd}.
compute bootxsd={xsd;bootxsd}.
end if.
do if (saveboot = 1).
save bootres/outfile = *.
end if.
do if (modelbt=1).
compute bootcim=make(ncol(bootres),5,-99999).
compute bootcim(:,2) = t(csum(bootres)/nrow(bootres)).
compute bootcim(:,1) = coeffmat(2:nrow(coeffmat),1).
loop i = 1 to ncol(bootres).

.
compute temp = bootres(:,i).
compute temp(GRADE( bootres(:,i) )) = bootres(:,i).
compute badlo = 0.
compute badhi = 0.
do if ( (bootcim(i,1)*bc)+(9999*(1-bc)) <> 9999).
compute pv=csum(temp < (bootcim(i,1)*bc)+(9999*(1-bc)) )/nrow(temp).
compute ppv = pv.
do if (pv > .5).
compute ppv = 1-pv.
end if.
compute y5=sqrt(-2*ln(ppv)).
compute xp=y5+((((y5*p4+p3)*y5+p2)*y5+p1)*y5+p0)/((((y5*q4+q3)*y5+q2)*y5+q1)*y5+q0).
do if (pv <= .5).
compute xp = -xp.
end if.
compute cilow=rnd(nrow(temp)*(cdfnorm(2*xp-xp2))).
compute cihigh=trunc(nrow(temp)*(cdfnorm(2*xp+(xp2))))+1.
do if (cilow < 1).
compute cilow = 1.
compute booterr=1.
compute badlo = 1.
end if.
do if (cihigh > nrow(temp)).
compute cihigh = boot.
compute booterr=1.
compute badhi = 1.
end if.
compute llcit=temp(cilow,1).
compute ulcit=temp(cihigh,1).
do if (badlo = 1 and llcit <> priorlo).
compute badend={badend, llcit}.
compute priorlo = llcit.
end if.
do if (badhi = 1 and ulcit <> priorhi).
compute badend={badend, ulcit}.
compute priorhi = ulcit.
end if.
end if.
do if ( (bootcim(i,1)*bc)+(9999*(1-bc)) = 9999).
compute llcit=temp(cilow,1).
compute ulcit=temp(cihigh,1).
end if.
compute bootse=t(sqrt((cssq(temp)-((csum(temp)&**2)/nrow(temp)))/(nrow(temp)-1)))
.
compute bootcim(i,4:5)={llcit,ulcit}.
compute bootcim(i,3)=bootse.
end loop.
end if.
end if.
do if (badboot > 0).
compute notecode(notes,1) = 6.
compute notes = notes + 1.
end if.
end if.
do if (criterr=0).
release fulldat,x,y.
end if.
do if (xmint=1 and criterr=0).
compute xvalptmp=make(1,nxvls,0).
compute mest74sp=make(1,nms,0).
compute mest74sp={mest74sp;mest74}.
compute xvalptmp={xvalptmp;ident(nxvls)}.
loop kcfuhd=1 to 2.
compute dirfxcf=make(nrow(mest74),6,0).
loop cfloop1=1 to nrow(mest74).
compute ndirfx=make(nrow(lastb),1,0).
compute ndirfx(2:(nxvls+1),1)=t(xvalptmp(cfloop1,:)).
loop cfloop3=1 to nms.
do if (kcfuhd=1).
do if (mcx=1 or mcx=0).
compute ndirfx(wheremw((1+((cfloop3-1)*2)),ncol(wheremw)):wheremw((2+((cfloop3-1)*2)), ncol(wheremw)))=t(xvalptmp(cfloop1,:)*mest74(1,cfloop3)).
end if.
do if (mcx=2).
compute ndirfx(wheremw((1+((cfloop3-1)*2)),ncol(wheremw)):wheremw((2+((cfloop3-1)*2)), ncol(wheremw)))=t(xvalptmp(cfloop1,:)*mest74sp(cfloop1,cfloop3)).
end if.
end if.
do if (kcfuhd=2).
compute ndirfx(wheremw((1+((cfloop3-1)*2)),ncol(wheremw)):wheremw((2+((cfloop3-1)*2)), ncol(wheremw)))=t(xvalptmp(cfloop1,:)*medmeans(1,cfloop3)).
end if.
end loop.
do if (cfloop1 > 1).
compute dirfxcf(cfloop1,1)=t(ndirfx)*lastb*xscaling.
compute dirfxcf(cfloop1,2)=sqrt(diag(t(ndirfx)*lastcov*ndirfx))*abs(xscaling).
compute dirfxcf(cfloop1,3)=dirfxcf(cfloop1,1)/dirfxcf(cfloop1,2).
compute dirfxcf(cfloop1,4)=2*(1-tcdf(abs(dirfxcf(cfloop1,3)), dfres)).
compute dirfxcf(cfloop1,5)=dirfxcf(cfloop1,1)-tval*dirfxcf(cfloop1,2).
compute dirfxcf(cfloop1,6)=dirfxcf(cfloop1,1)+tval*dirfxcf(cfloop1,2).
end if.
end loop.
compute codireff=dirfxcf(2:nrow(dirfxcf),:).
do if (kcfuhd=1).
compute direff=codireff.
end if.
end loop.
end if.

.
do if (criterr = 0 and nms > 0).
compute paths=paths(:,2:ncol(paths)).
compute pathsw=pathsw(:,2:ncol(pathsw)).
compute pathsz=pathsz(:,2:ncol(pathsz)).
compute pathswz=pathswz(:,2:ncol(pathswz)).
compute pathsmod=pathsw+pathsz+pathswz.
compute pathsdv=pathsdv(:,2:ncol(pathsdv)).
compute pathsfoc=pathsfoc(:,2:ncol(pathsfoc)).
compute pathtype=pathtype(:,2:ncol(pathtype)).
compute anymod=(rsum(pathsmod) > 0).
compute obscoeff=obscoeff(1,2:ncol(obscoeff)).
do if (outscree=1).
do if (xmint=1).
print/title = '************************* COUNTERFACTUALLY DEFINED ************************'.
else.
print/title = ' '.
end if.
do if (dototal = 0 and alttotal=0).
print/title = '****************** DIRECT AND INDIRECT EFFECTS OF X ON Y *****************'/space=0.
end if.
do if (alttotal=1).
print/title = '************** TOTAL, DIRECT, AND INDIRECT EFFECTS OF X ON Y **************'/space=0.
end if.
end if.
do if (dototal = 1).
do if (outscree=1).
print/title = '************** TOTAL, DIRECT, AND INDIRECT EFFECTS OF X ON Y **************'/space=0.
end if.
compute totefflb(1,1)='Effect'.
do if (effsize=1).
compute toteffsz=toteff(:,1)/ysd.
do if (xdich=1 or mcx > 0).
compute totefflb={totefflb,'c_ps'}.
end if.
do if (xdich = 0 and mcx = 0).
compute toteffsz=toteffsz*xsd.
compute totefflb={totefflb,'c_cs'}.
end if.
compute toteff={toteff,toteffsz}.
end if.
compute resultm2=make(nrow(toteff),maxresm,99999).
compute resultm2(1:nrow(toteff),1:ncol(toteff))=toteff.
compute resultm={resultm;resultm2}.
do if (nxvls > 1).
compute resultm2=make(nrow(totomni),maxresm,99999).
compute resultm2(1:nrow(totomni),1:ncol(totomni))=totomni.
compute resultm={resultm;resultm2}.
compute clabtmp={'R2-chng', hcflab, 'df1','df2','p'}.
do if (outscree=1).
print toteff/title='Relative total effects of X on Y'/rnames=toteffl2/cnames=totefflb/format= F10.4.
print totomni/title='Omnibus test of total effect of X on Y'/cnames=clabtmp/format= F10.4.
print/title= '----------'/space=0.
end if.
else.
do if (outscree=1).
print toteff/title='Total effect of X on Y'/cnames=totefflb/format= F10.4.
end if.
end if.
end if.
compute moddir=wcmat(nrow(bcmat),1)+zcmat(nrow(bcmat),1).
do if (xmint=1).
compute moddir=1.
end if.
do if (bcmat(nrow(bcmat),1)=1).
do if (ydich=1).
compute direfflb(:,(ncol(direfflb)-5):ncol(direfflb))={'Effect','se','Z','p','LLCI','ULCI'}.
end if.
do if (moddir=0 or xmint=1).
compute direfflb(1,1)='Effect'.
end if.
do if (effsize=1 and moddir=0 and anymod = 0).
compute direffsz=direff(:,1)/ysd.
do if (xdich=1 or mcx > 0).
compute direfflb={direfflb,'c''_ps'}.
end if.
do if (xdich = 0 and mcx = 0).
compute direffsz=direffsz*xsd.
compute direfflb={direfflb,'c''_cs'}.
end if.
compute direff={direff,direffsz}.
end if.
compute resultm2=make(nrow(direff),maxresm,99999).
compute resultm2(1:nrow(direff),1:ncol(direff))=direff.
compute resultm={resultm;resultm2}.
do if (xmint=1).
compute resultm2=make(nrow(codireff),maxresm,99999).
compute resultm2(1:nrow(codireff),1:ncol(codireff))=codireff.
compute resultm={resultm;resultm2}.
end if.
do if (moddir = 0 and nxvls=1).
do if (outscree=1).
print direff/title='Direct effect of X on Y'/cnames=direfflb/format= F10.4.
end if.
end if.
do if (moddir = 0 and nxvls>1).
compute resultm2=make(nrow(diromni),maxresm,99999).
compute resultm2(1:nrow(diromni),1:ncol(diromni))=diromni.
compute resultm={resultm;resultm2}.
do if (outscree=1).
print direff/title='Relative direct effects of X on Y'/rnames=direffl2/cnames=direfflb/format= F10.4.
do if (ydich = 0).
compute clabtmp={'R2-chng', hcflab, 'df1','df2','p'}.
print diromni/title='Omnibus test of direct effect of X on Y:'/cnames=clabtmp/format= F10.4.
end if.
do if (ydich = 1).
compute clabtmp={'Chi-sq', 'df', 'p'}.
print diromni/title='Omnibus likelihood ratio test of direct effect of X on Y'/cnames=clabtmp/format= F10.4.
end if.
print/title= '----------'/space=0.
end if.
end if.
do if (moddir > 0 and nxvls=1).
do if (xmint=0 and outscree=1).
print direff/title='Conditional direct effects of X on Y'/cnames=direfflb/format= F10.4.
end if.
do if (xmint=1).
do if (outscree=1).
print direff/title='(Pure) Natural direct effect of X on Y'/cnames=direfflb/format= F10.4.
print codireff/title='Controlled direct effect of X on Y'/cnames=direfflb/format= F10.4.
print/title= '----------'/space=0.
end if.
compute obnatdfx=direff(:,1).
end if.
end if.
compute direffl4=direffl2.
do if (moddir > 0 and nxvls>1).
compute direffl2={' '}.
loop i = 1 to nxvls.
loop j = 1 to (nrow(direff)/nxvls).
compute direffl2={direffl2;xcatlab(i,1)}.
end loop.
end loop.
compute direffl2=direffl2(2:nrow(direffl2),1).
do if (xmint=0 and outscree=1).
print direff/title='Relative conditional direct effects of X on Y'/cnames=direfflb/rnames=direffl2/format= F10.4.
end if.
do if (xmint=1).
do if (outscree=1).
print direff/title='Relative (pure) natural direct effects of X on Y'/cnames=direfflb/rnames=direffl2/format= F10.4.
print codireff/title='Relative controlled direct effects of X on Y'/cnames=direfflb/rnames=direffl2/format= F10.4.
print/title= '----------'/space=0.
end if.
compute obnatdfx=direff(:,1).
end if.
end if.
compute direffl2=direffl4.
end if.
do if (bcmat(nrow(bcmat),1)=0 and xmint <> 1 and outscree=1).
print/title='The direct effect of X on Y is fixed to zero.'.
end if.
do if (nms = 1).
compute indmark={2}.
compute indsets={1,2}.
end if.
do if (nms = 2).
compute indmark={2,2,3}.
compute indsets={1,4,2,5,1,3,5}.
compute thetam=1.
end if.
do if (nms = 3).
compute indmark={2,2,2,3,3,3,4}.
compute indsets={1,7,2,8,4,9,1,3,8,1,5,9,2,6,9,1,3,6,9}.
compute thetam={1,2,3}.
end if.
do if (nms = 4).
compute indmark={2,2,2,2,3,3,3,3,3,3,4,4,4,4,5}.
compute indsets={1,11,2,12,4,13,7,14,1,3,12,1,5,13,1,8,14,2,6,13,2,9,14,4,10,14,1,3,6,13,1,3,9, 14,1,5,10,14,2,6,10,14,1,3,6,10,14}.
compute thetam={1,2,4,3,5,6}.
end if.
do if (nms = 5).
compute indmark={2,2,2,2,2,3,3,3,3,3,3,3,3,3,3,4,4,4,4,4,4,4,4,4,4,5,5,5,5,5,6}.
compute indsets={1,16,2,17,4,18,7,19,11,20,1,3,17,1,5,18,1,8,19,1,12,20,2,6,18,2,9,19,2,13,20,4, 10,19,4,14,20,7,15,20,1,3,6,18, 1,3,9,19,1,3,13,20,1,5,10,19,1,5,14,20,1,8,15,20,2,6,10,19,2,6, 14,20,2,9,15,20,4,10,15,20,1,3,6,10,19, 1,3,6,14,20,1,3,9,15,20,1,5,10,15,20,2,6,10,15,20,1,3,6,10,15, 20}.
compute thetam={1,2,5,3,6,8,4,7,9,10}.
end if.
do if (nms = 6).
compute indmark={2,2,2,2,2,2,3,3,3,3,3,3,3,3,3,3,3,3,3,3,3,4,4,4,4,4,4,4,4,4,4,4,4,4,4,4,4,4,4, 4,4,5,5,5,5,5,5,5,5,5,5,5,5,5,5,5,6,6,6,6,6,6,7}.
compute indsets={1,22,2,23,4,24,7,25,11,26,16,27,1,3,23,1,5,24,1,8,25,1,12,26,1,17,27,2,6,24,2, 9,25,2,13,26,2,18,27,4,10,25,4,14,26, 4,19,27,7,15,26,7,20,27,11,21,27,1,3,6,24,1,3,9,25,1,3,13,26,1, 3,18,27,1,5,10,25,1,5,14,26,1,5,19,27,1,8,15,26,1,8,20,27, 1,12,21,27,2,6,10,25,2,6,14,26,2,6,19,27,2,9,15,26,2,9,20,27,2, 13,21,27,4,10,15,26,4,10,20,27,4,14,21,27,7,15,21,27, 1,3,6,10,25,1,3,6,14,26,1,3,6,19,27,1,3,9,15,26,1,3,9,20,27,1,3, 13,21,27,1,5,10,15,26,1,5,10,20,27,1,5,14,21,27, 1,8,15,21,27,2,6,10,15,26,2,6,10,20,27,2,6,14,21,27,2,9,15,21, 27,4,10,15,21,27,1,3,6,10,15,26,1,3,6,10,20,27, 1,3,6,14,21,27,1,3,9,15,21,27,1,5,10,15,21,27,2,6,10,15,21,27, 1,3,6,10,15,21,27}.
compute thetam={1,2,6,3,7,10,4,8,11,13,5,9,12,14,15}.
end if.
do if (nms = 7).
compute indmark={2,2,2,2,2,2,2}.
compute indsets={1,29,2,30,4,31,7,32,11,33,16,34,22,35}.
end if.
do if (nms = 8).
compute indmark={2,2,2,2,2,2,2,2}.
compute indsets={1,37,2,38,4,39,7,40,11,41,16,42,22,43,29,44}.
end if.
do if (nms = 9).
compute indmark={2,2,2,2,2,2,2,2,2}.
compute indsets={1,46,2,47,4,48,7,49,11,50,16,51,22,52,29,53,37,54}.
end if.
do if (nms=10).
compute indmark={2,2,2,2,2,2,2,2,2,2}.
compute indsets={1,56,2,57,4,58,7,59,11,60,16,61,22,62,29,63,37,64,46,65}.
end if.
compute indlbl = {'Ind1'; 'Ind2'; 'Ind3'; 'Ind4'; 'Ind5'; 'Ind6'; 'Ind7'; 'Ind8'; 'Ind9'; 'Ind10'; 'Ind11'; 'Ind12'; 'Ind13'; 'Ind14'; 'Ind15'}.
compute indlbl = {indlbl; 'Ind16';'Ind17';'Ind18';'Ind19';'Ind20';'Ind21';'Ind22';'Ind23';'Ind24';'Ind25';'Ind26';'Ind27';'Ind28';'Ind29';'Ind30'}.
compute indlbl = {indlbl; 'Ind31';'Ind32';'Ind33';'Ind34';'Ind35';'Ind36';'Ind37';'Ind38';'Ind39';'Ind40';'Ind41';'Ind42';'Ind43';'Ind44';'Ind45'}.
compute indlbl = {indlbl; 'Ind46';'Ind47';'Ind48';'Ind49';'Ind50';'Ind51';'Ind52';'Ind53';'Ind54';'Ind55';'Ind56';'Ind57';'Ind58';'Ind59';'Ind60'}.
compute indlbl = {indlbl; 'Ind61';'Ind62';'Ind63';'Ind64';'Ind65';'Ind66';'Ind67';'Ind68';'Ind69';'Ind70';'Ind71';'Ind72';'Ind73';'Ind74';'Ind75'}.
compute indlbl = {indlbl; 'Ind76';'Ind77';'Ind78';'Ind79';'Ind80';'Ind81';'Ind82';'Ind83';'Ind84';'Ind85';'Ind86';'Ind87';'Ind88';'Ind89';'Ind90'}.
compute cntname={'(C1)';'(C2)';'(C3)';'(C4)';'(C5)';'(C6)';'(C7)';'(C8)';'(C9)';'(C10)';'(C11)';'(C12)';'(C13)';'(C14)';'(C15)';'(C16)';'(C17)'}.
compute cntname={cntname;'(C18)';'(C19)';'(C20)';'(C21)';'(C22)';'(C23)';'(C24)';'(C25)';'(C26)';'(C27)';'(C28)';'(C29)';'(C30)';'(C31)'}.
compute cntname={cntname;'(C32)';'(C33)';'(C34)';'(C35)';'(C36)';'(C37)';'(C38)';'(C39)';'(C40)';'(C41)';'(C42)';'(C43)';'(C44)';'(C45)'}.
compute cntname={cntname;'(C46)';'(C47)';'(C48)';'(C49)';'(C50)';'(C51)';'(C52)';'(C53)';'(C54)';'(C55)';'(C56)';'(C57)';'(C58)';'(C59)'}.
compute cntname={cntname;'(C60)';'(C61)';'(C62)';'(C63)';'(C64)';'(C65)';'(C66)';'(C67)';'(C68)';'(C69)';'(C70)';'(C71)';'(C72)';'(C73)'}.
compute cntname={cntname;'(C74)';'(C75)';'(C76)';'(C77)';'(C78)';'(C79)';'(C80)';'(C81)';'(C82)';'(C83)';'(C84)';'(C85)';'(C86)';'(C87)'}.
compute cntname={cntname;'(C88)';'(C89)';'(C90)';'(C91)';'(C92)';'(C93)';'(C94)';'(C95)';'(C96)';'(C97)';'(C98)';'(C99)';'(C100)';'(C101)'}.
compute cntname={cntname;'(C102)';'(C103)';'(C104)';'(C105)'}.
compute indmake=make(ncol(indmark),(nms+2),0).
compute indmod=make(ncol(indmark),1,999).
compute indmmm=make(ncol(indmark),1,0).
compute indmmmt=make(ncol(indmark),1,0).
compute start=1.
compute end=0.
compute nindfx=0.
compute indlocs=make(nrow(thetaxmb),ncol(paths),999).
compute indkey=make(ncol(indmark),1+((rmax(indmark)*2)+1),'     ').
compute c1=1.
compute c2=1.
compute c3=1.
loop i = 1 to ncol(paths).
do if (pathtype(1,i)=1).
compute indlocs(:,i)=thetaxmb(:,c1).
compute c1=c1+1.
end if.
do if (pathtype(1,i)=3).
compute indlocs(:,i)=thetamyb(:,c2).
compute c2=c2+1.
end if.
do if (pathtype(1,i)=2 and nms < 7 and serial=1).
compute indlocs(:,i)=thetammb(:,thetam(1,c3)).
compute c3=c3+1.
end if.
end loop.
loop i = 1 to ncol(indlocs).
compute c1=2.
loop j = 2 to nrow(indlocs).
do if (indlocs(j,i) <> 0).
compute indlocs(c1,i)=indlocs(j,i).
compute c1=c1+1.
end if.
end loop.
compute indlocs(1,i)=c1-2.
end loop.
compute indlocs=indlocs(1:rmax((indlocs(1,:))+1),:).
loop i = 1 to ncol(indmark).
compute numget=indmark(1,i).
compute end=end+numget.
compute gotcha=indsets(1,start:end).
compute start=end+1.
compute ok=1.
compute temp=0.
compute repoman=make(4,1,0).
loop j = 1 to ncol(gotcha).
do if paths(1,gotcha(1,j))=0.
compute ok=0.
end if.
do if (pathsmod(1,gotcha(1,j)) > 0).
compute temp=1.
compute temp2={pathsw(1,gotcha(1,j));pathsz(1,gotcha(1,j));pathswz(1,gotcha(1,j));0}.
do if (temp2(1,1)=1 and temp2(2,1)=1 and temp2(3,1)=0).
compute temp2(4,1)=1.
end if.
compute repoman=repoman+temp2.
end if.
end loop.
compute temp=0.
compute tempmmm=0.
compute typemmm=0.
do if ((repoman(1,1) > 0) and (repoman(2,1) = 0)).
compute temp=1.
do if (repoman(1,1)=1).
compute tempmmm=1.
end if.
do if (repoman(1,1) > 1 and (wdich=1 or mcw > 0)).
compute tempmmm=12.
compute typemmm=mcw.
do if (wdich=1).
compute typemmm=1.
end if.
end if.
do if (repoman(1,1) > 1 and (wdich=0 and mcw = 0)).
compute tempmmm=101.
end if.
end if.
do if ((repoman(1,1) = 0) and (repoman(2,1) > 0)).
compute temp=2.
do if (repoman(2,1)=1).
compute tempmmm=2.
end if.
do if (repoman(2,1) > 1 and (zdich = 1 or mcz > 0)).
compute tempmmm=22.
compute typemmm=mcz.
do if (zdich=1).
compute typemmm=1.
end if.
end if.
do if (repoman(2,1) > 1 and (zdich = 0 and mcw = 0)).
compute tempmmm=102.
end if.
end if.
do if (repoman(1,1)>0 and repoman(2,1)>0).
compute temp=3.
do if (repoman(1,1)=1 and repoman(2,1)=1).
do if (repoman(4,1)=1).
compute tempmmm=31.
end if.
do if (repoman(3,1)=1).
compute tempmmm=41.
end if.
end if.
end if.
do if (repoman(1,1)=1 and repoman(2,1)=1 and repoman(3,1)=0 and repoman(4,1)=0).
compute tempmmm=51.
end if.
do if (ok = 1).
compute nindfx=nindfx+1.
compute indmake(nindfx,1)=numget.
compute indmod(nindfx,1)=temp.
compute indmmm(nindfx,1)=tempmmm.
compute indmmmt(nindfx,1)=typemmm.
compute indmake(nindfx,2:(1+numget))=gotcha.
compute indkey(nindfx,1)=xnames.
loop j = 1 to numget.
compute indkey(nindfx,(j*2+1))=pathsdv(1,gotcha(1,j)).
compute indkey(nindfx,(j*2))='   ->   '.
end loop.
end if.
end loop.
compute indkey=indkey(1:nindfx,1:((cmax(indmake(:,1))*2)+1)).
compute indmake=indmake(1:nindfx,1:(cmax(indmake(:,1))+1)).
compute indmod=indmod(1:nrow(indmake),1).
compute indmmm=indmmm(1:nrow(indmake),1).
compute indmmmt=indmmmt(1:nrow(indmake),1).
compute ncpairs = (((nindfx)*(nindfx-1))/2).
do if ((contrast = 1 or contrast = 2 or contrast=3) and (ncpairs > 105)).
compute contrast=0.
compute notecode(notes,1) = 13.
compute notes = notes + 1.
end if.
do if (contrast = 4).
do if (ncol(contvec) <> nindfx).
compute contrast=0.
compute notecode(notes,1) = 14.
compute notes = notes + 1.
end if.
end if.
do if (anymod=0).
do if (nms = 1 and contrast > 0).
compute contrast=0.
end if.
compute efloop=(((1-(effsize=0))*2)+1)-(((mcx>0 or xdich=1))*(1-(effsize=0))).
loop kk=1 to efloop.
do if (boot = 0).
compute bootres=obscoeff.
do if (kk=1).
compute totbtvec=make(1,nxvls,0).
end if.
compute bootdir=obsdirfx.
compute indtab=999.
compute inddiff=999.
compute bootysd=ysd.
compute bootxsd=xsd.
end if.
do if (boot > 0).
compute bootres={obscoeff;bootres}.
do if (kk=1).
compute totbtvec=make(nrow(bootres),nxvls,0).
end if.
compute indtab=make(1,4,999).
compute inddiff=make(nrow(bootres),1,999).
end if.
compute indtotal=make(nrow(bootres),1,0).
loop i = 1 to nrow(indmake).
loop j = 1 to nxvls.
compute indtemp=make(nrow(bootres),1,1).
loop k = 1 to indmake(i,1).
compute jtemp=1.
do if (j > 1 and k=1).
compute jtemp=j.
end if.
compute indtemp=indtemp&*bootres(:,pathsfoc(jtemp,indmake(i,(k+1)))).
end loop.
do if (kk = 2).
compute indtemp=indtemp/bootysd.
end if.
do if (kk = 3).
compute indtemp=(bootxsd&*indtemp)/bootysd.
end if.
do if (contrast <> 0).
compute inddiff={inddiff,indtemp}.
end if.
do if (nxvls=1).
compute indtotal=indtotal+indtemp.
end if.
compute indeff=indtemp(1,1).
do if (kk=1).
compute totbtvec(:,j)=totbtvec(:,j)+indtemp.
end if.
do if (boot > 0).

.
compute temp = indtemp(2:nrow(indtemp),1).
compute temp(GRADE( indtemp(2:nrow(indtemp),1) )) = indtemp(2:nrow(indtemp),1).
compute badlo = 0.
compute badhi = 0.
do if ( (indtemp(1,1)*bc)+(9999*(1-bc)) <> 9999).
compute pv=csum(temp < (indtemp(1,1)*bc)+(9999*(1-bc)) )/nrow(temp).
compute ppv = pv.
do if (pv > .5).
compute ppv = 1-pv.
end if.
compute y5=sqrt(-2*ln(ppv)).
compute xp=y5+((((y5*p4+p3)*y5+p2)*y5+p1)*y5+p0)/((((y5*q4+q3)*y5+q2)*y5+q1)*y5+q0).
do if (pv <= .5).
compute xp = -xp.
end if.
compute cilow=rnd(nrow(temp)*(cdfnorm(2*xp-xp2))).
compute cihigh=trunc(nrow(temp)*(cdfnorm(2*xp+(xp2))))+1.
do if (cilow < 1).
compute cilow = 1.
compute booterr=1.
compute badlo = 1.
end if.
do if (cihigh > nrow(temp)).
compute cihigh = boot.
compute booterr=1.
compute badhi = 1.
end if.
compute llcit=temp(cilow,1).
compute ulcit=temp(cihigh,1).
do if (badlo = 1 and llcit <> priorlo).
compute badend={badend, llcit}.
compute priorlo = llcit.
end if.
do if (badhi = 1 and ulcit <> priorhi).
compute badend={badend, ulcit}.
compute priorhi = ulcit.
end if.
end if.
do if ( (indtemp(1,1)*bc)+(9999*(1-bc)) = 9999).
compute llcit=temp(cilow,1).
compute ulcit=temp(cihigh,1).
end if.
compute bootse=t(sqrt((cssq(temp)-((csum(temp)&**2)/nrow(temp)))/(nrow(temp)-1)))
.
compute indeff={indeff,bootse,llcit,ulcit}.
end if.
do if (kk=1).
compute indtabn={indtab;indeff}.
end if.
compute indtab={indtab;indeff}.
end loop.
end loop.
compute indtab=indtab(2:nrow(indtab),:).
do if (kk=1).
compute indtabn=indtabn(2:nrow(indtabn),:).
end if.
compute rowlbs=indlbl(1:nrow(indtab),1).
do if (mc > 0).
compute inddiff=make(mc,1,-999).
compute indtab2=make(nrow(indtab),4,-999).
compute indtab2(:,1)=indtab.
compute indtab=indtab2.
release indtab2.
compute mcct=0.
compute indtotal=make(mc,1,0).
do if (kk = 1).
compute x1 = sqrt(-2*ln(uniform(mc,nrow(mcsopath))))&*cos((2*3.14159265358979)*uniform(mc, nrow(mcsopath))).
compute x1=x1*chol(indcov).
loop ii=1 to nrow(x1).
compute x1(ii,:)=x1(ii,:)+t(mcsopath).
end loop.
end if.
loop ii=1 to nms.
compute tmpb=x1(:,((nms*nxvls)+ii)).
compute tmpb2=tmpb.
do if (nxvls > 1).
loop jj=1 to (nxvls-1).
compute tmpb2={tmpb2,tmpb}.
end loop.
end if.
compute indtemp=x1(:,(((ii-1)*nxvls)+1):(ii*nxvls))&*tmpb2.
loop jj=1 to ncol(indtemp).
do if (kk = 2).
compute indtemp(:,jj)=indtemp(:,jj)/ysd.
end if.
do if (kk = 3).
compute indtemp(:,jj)=(xsd*indtemp(:,jj))/ysd.
end if.

.
compute temp = indtemp(:,jj).
compute temp(GRADE( indtemp(:,jj) )) = indtemp(:,jj).
compute badlo = 0.
compute badhi = 0.
do if ( 9999 <> 9999).
compute pv=csum(temp < 9999 )/nrow(temp).
compute ppv = pv.
do if (pv > .5).
compute ppv = 1-pv.
end if.
compute y5=sqrt(-2*ln(ppv)).
compute xp=y5+((((y5*p4+p3)*y5+p2)*y5+p1)*y5+p0)/((((y5*q4+q3)*y5+q2)*y5+q1)*y5+q0).
do if (pv <= .5).
compute xp = -xp.
end if.
compute cilow=rnd(nrow(temp)*(cdfnorm(2*xp-xp2))).
compute cihigh=trunc(nrow(temp)*(cdfnorm(2*xp+(xp2))))+1.
do if (cilow < 1).
compute cilow = 1.
compute booterr=1.
compute badlo = 1.
end if.
do if (cihigh > nrow(temp)).
compute cihigh = boot.
compute booterr=1.
compute badhi = 1.
end if.
compute llcit=temp(cilow,1).
compute ulcit=temp(cihigh,1).
do if (badlo = 1 and llcit <> priorlo).
compute badend={badend, llcit}.
compute priorlo = llcit.
end if.
do if (badhi = 1 and ulcit <> priorhi).
compute badend={badend, ulcit}.
compute priorhi = ulcit.
end if.
end if.
do if ( 9999 = 9999).
compute llcit=temp(cilow,1).
compute ulcit=temp(cihigh,1).
end if.
compute bootse=t(sqrt((cssq(temp)-((csum(temp)&**2)/nrow(temp)))/(nrow(temp)-1)))
.
compute mcct=mcct+1.
compute indtab(mcct,2:4)={bootse,llcit,ulcit}.
end loop.
do if (nxvls=1).
compute indtotal=indtotal+indtemp.
do if (contrast <> 0).
compute inddiff={inddiff,indtemp}.
end if.
end if.
end loop.
release indtemp,tmpb.
end if.
do if (normal = 1 and sobelok=1).
compute sobelmat=indtab(:,1).
compute sobelmat={sobelmat,(sobelmat/2),sobelmat,sobelmat}.
loop ii=1 to nms.
compute se2b=indcov(((nms*nxvls)+ii),((nms*nxvls)+ii)).
compute bpath2=mcsopath(((nms*nxvls)+ii),1)&**2.
compute se2a=diag(indcov((((ii-1)*nxvls)+1):(ii*nxvls),(((ii-1)*nxvls)+1):(ii*nxvls))).
compute apath2=mcsopath((((ii-1)*nxvls)+1):(ii*nxvls) ,1)&**2.
compute sesobel=sqrt(apath2*se2b+bpath2*se2a+se2a*se2b).
compute sobelmat((((ii-1)*nxvls)+1):(ii*nxvls),2)=sesobel.
end loop.
release se2b,bpath2,se2a,apath2,sesobel,ii.
compute sobelmat(:,3)=sobelmat(:,1)&/sobelmat(:,2).
compute sobelmat(:,4) = 2*(1-cdfnorm(abs(sobelmat(:,3)))).
end if.
do if (serial = 0).
compute rowlbs=t(mnames).
end if.
do if (nxvls=1 and nms > 1).
compute rowlbs={'TOTAL';rowlbs}.
compute indtemp=indtotal(1,1).
do if (boot > 0 and nxvls=1).

.
compute temp = indtotal(2:nrow(indtotal),1).
compute temp(GRADE( indtotal(2:nrow(indtotal),1) )) = indtotal(2:nrow(indtotal),1).
compute badlo = 0.
compute badhi = 0.
do if ( (indtotal(1,1)*bc)+(9999*(1-bc)) <> 9999).
compute pv=csum(temp < (indtotal(1,1)*bc)+(9999*(1-bc)) )/nrow(temp).
compute ppv = pv.
do if (pv > .5).
compute ppv = 1-pv.
end if.
compute y5=sqrt(-2*ln(ppv)).
compute xp=y5+((((y5*p4+p3)*y5+p2)*y5+p1)*y5+p0)/((((y5*q4+q3)*y5+q2)*y5+q1)*y5+q0).
do if (pv <= .5).
compute xp = -xp.
end if.
compute cilow=rnd(nrow(temp)*(cdfnorm(2*xp-xp2))).
compute cihigh=trunc(nrow(temp)*(cdfnorm(2*xp+(xp2))))+1.
do if (cilow < 1).
compute cilow = 1.
compute booterr=1.
compute badlo = 1.
end if.
do if (cihigh > nrow(temp)).
compute cihigh = boot.
compute booterr=1.
compute badhi = 1.
end if.
compute llcit=temp(cilow,1).
compute ulcit=temp(cihigh,1).
do if (badlo = 1 and llcit <> priorlo).
compute badend={badend, llcit}.
compute priorlo = llcit.
end if.
do if (badhi = 1 and ulcit <> priorhi).
compute badend={badend, ulcit}.
compute priorhi = ulcit.
end if.
end if.
do if ( (indtotal(1,1)*bc)+(9999*(1-bc)) = 9999).
compute llcit=temp(cilow,1).
compute ulcit=temp(cihigh,1).
end if.
compute bootse=t(sqrt((cssq(temp)-((csum(temp)&**2)/nrow(temp)))/(nrow(temp)-1)))
.
compute indtemp={indtemp, bootse,llcit,ulcit}.
end if.
do if (mc > 0).
compute obtmc=indtab(:,1).
compute indtemp=csum(obtmc).

.
compute temp = indtotal(:,1).
compute temp(GRADE( indtotal(:,1) )) = indtotal(:,1).
compute badlo = 0.
compute badhi = 0.
do if ( 9999 <> 9999).
compute pv=csum(temp < 9999 )/nrow(temp).
compute ppv = pv.
do if (pv > .5).
compute ppv = 1-pv.
end if.
compute y5=sqrt(-2*ln(ppv)).
compute xp=y5+((((y5*p4+p3)*y5+p2)*y5+p1)*y5+p0)/((((y5*q4+q3)*y5+q2)*y5+q1)*y5+q0).
do if (pv <= .5).
compute xp = -xp.
end if.
compute cilow=rnd(nrow(temp)*(cdfnorm(2*xp-xp2))).
compute cihigh=trunc(nrow(temp)*(cdfnorm(2*xp+(xp2))))+1.
do if (cilow < 1).
compute cilow = 1.
compute booterr=1.
compute badlo = 1.
end if.
do if (cihigh > nrow(temp)).
compute cihigh = boot.
compute booterr=1.
compute badhi = 1.
end if.
compute llcit=temp(cilow,1).
compute ulcit=temp(cihigh,1).
do if (badlo = 1 and llcit <> priorlo).
compute badend={badend, llcit}.
compute priorlo = llcit.
end if.
do if (badhi = 1 and ulcit <> priorhi).
compute badend={badend, ulcit}.
compute priorhi = ulcit.
end if.
end if.
do if ( 9999 = 9999).
compute llcit=temp(cilow,1).
compute ulcit=temp(cihigh,1).
end if.
compute bootse=t(sqrt((cssq(temp)-((csum(temp)&**2)/nrow(temp)))/(nrow(temp)-1)))
.
compute indtemp={indtemp, bootse,llcit,ulcit}.
end if.
compute indtab={indtemp;indtab}.
end if.
compute bootlbs={'Effect', 'BootSE','BootLLCI','BootULCI'}.
do if (mc > 0).
compute bootlbs={'Effect', 'MC SE','MC LLCI','MC ULCI'}.
end if.
do if (nxvls = 1).
do if (contrast <> 0).
compute inddiff=inddiff(:,2:ncol(inddiff)).
do if (mc > 0).
compute inddiff={t(obtmc);inddiff}.
end if.
do if (contrast = 4).
compute inddifft=inddiff*t(contvec).
compute indtemp=inddifft(1,1).
do if (boot > 0 or mc > 0).
do if (mc > 0).

.
compute temp = inddifft(2:nrow(inddifft),1).
compute temp(GRADE( inddifft(2:nrow(inddifft),1) )) = inddifft(2:nrow(inddifft),1).
compute badlo = 0.
compute badhi = 0.
do if ( 9999 <> 9999).
compute pv=csum(temp < 9999 )/nrow(temp).
compute ppv = pv.
do if (pv > .5).
compute ppv = 1-pv.
end if.
compute y5=sqrt(-2*ln(ppv)).
compute xp=y5+((((y5*p4+p3)*y5+p2)*y5+p1)*y5+p0)/((((y5*q4+q3)*y5+q2)*y5+q1)*y5+q0).
do if (pv <= .5).
compute xp = -xp.
end if.
compute cilow=rnd(nrow(temp)*(cdfnorm(2*xp-xp2))).
compute cihigh=trunc(nrow(temp)*(cdfnorm(2*xp+(xp2))))+1.
do if (cilow < 1).
compute cilow = 1.
compute booterr=1.
compute badlo = 1.
end if.
do if (cihigh > nrow(temp)).
compute cihigh = boot.
compute booterr=1.
compute badhi = 1.
end if.
compute llcit=temp(cilow,1).
compute ulcit=temp(cihigh,1).
do if (badlo = 1 and llcit <> priorlo).
compute badend={badend, llcit}.
compute priorlo = llcit.
end if.
do if (badhi = 1 and ulcit <> priorhi).
compute badend={badend, ulcit}.
compute priorhi = ulcit.
end if.
end if.
do if ( 9999 = 9999).
compute llcit=temp(cilow,1).
compute ulcit=temp(cihigh,1).
end if.
compute bootse=t(sqrt((cssq(temp)-((csum(temp)&**2)/nrow(temp)))/(nrow(temp)-1)))
.
end if.
do if (boot > 0).

.
compute temp = inddifft(2:nrow(inddifft),1).
compute temp(GRADE( inddifft(2:nrow(inddifft),1) )) = inddifft(2:nrow(inddifft),1).
compute badlo = 0.
compute badhi = 0.
do if ( (inddifft(1,1)*bc)+(9999* (1-bc)) <> 9999).
compute pv=csum(temp < (inddifft(1,1)*bc)+(9999* (1-bc)) )/nrow(temp).
compute ppv = pv.
do if (pv > .5).
compute ppv = 1-pv.
end if.
compute y5=sqrt(-2*ln(ppv)).
compute xp=y5+((((y5*p4+p3)*y5+p2)*y5+p1)*y5+p0)/((((y5*q4+q3)*y5+q2)*y5+q1)*y5+q0).
do if (pv <= .5).
compute xp = -xp.
end if.
compute cilow=rnd(nrow(temp)*(cdfnorm(2*xp-xp2))).
compute cihigh=trunc(nrow(temp)*(cdfnorm(2*xp+(xp2))))+1.
do if (cilow < 1).
compute cilow = 1.
compute booterr=1.
compute badlo = 1.
end if.
do if (cihigh > nrow(temp)).
compute cihigh = boot.
compute booterr=1.
compute badhi = 1.
end if.
compute llcit=temp(cilow,1).
compute ulcit=temp(cihigh,1).
do if (badlo = 1 and llcit <> priorlo).
compute badend={badend, llcit}.
compute priorlo = llcit.
end if.
do if (badhi = 1 and ulcit <> priorhi).
compute badend={badend, ulcit}.
compute priorhi = ulcit.
end if.
end if.
do if ( (inddifft(1,1)*bc)+(9999* (1-bc)) = 9999).
compute llcit=temp(cilow,1).
compute ulcit=temp(cihigh,1).
end if.
compute bootse=t(sqrt((cssq(temp)-((csum(temp)&**2)/nrow(temp)))/(nrow(temp)-1)))
.
end if.
compute indtemp={indtemp, bootse,llcit,ulcit}.
end if.
compute indtab={indtab;indtemp}.
end if.
do if (contrast = 1 or contrast = 2 or contrast=3).
compute conkey=make(1,4,' ').
loop i = 1 to ncol(inddiff)-1.
loop j = (i+1) to ncol(inddiff).
compute inddifft=inddiff(:,i)-inddiff(:,j).
do if (contrast=2).
compute inddifft=abs(inddiff(:,i))-abs(inddiff(:,j)).
end if.
do if (contrast=3).
compute inddifft=inddiff(:,i)+inddiff(:,j).
end if.
compute indtemp=inddifft(1,1).
do if (contrast=1 or contrast=2).
compute conkeyt={' ', rowlbs((i+1),1),' minus  ',rowlbs((j+1),1)}.
end if.
do if (contrast=3).
compute conkeyt={' ', rowlbs((i+1),1),'  plus  ',rowlbs((j+1),1)}.
end if.
compute conkey={conkey;conkeyt}.
do if (boot > 0 or mc > 0).
do if (mc > 0).

.
compute temp = inddifft(2:nrow(inddifft),1).
compute temp(GRADE( inddifft(2:nrow(inddifft),1) )) = inddifft(2:nrow(inddifft),1).
compute badlo = 0.
compute badhi = 0.
do if ( 9999 <> 9999).
compute pv=csum(temp < 9999 )/nrow(temp).
compute ppv = pv.
do if (pv > .5).
compute ppv = 1-pv.
end if.
compute y5=sqrt(-2*ln(ppv)).
compute xp=y5+((((y5*p4+p3)*y5+p2)*y5+p1)*y5+p0)/((((y5*q4+q3)*y5+q2)*y5+q1)*y5+q0).
do if (pv <= .5).
compute xp = -xp.
end if.
compute cilow=rnd(nrow(temp)*(cdfnorm(2*xp-xp2))).
compute cihigh=trunc(nrow(temp)*(cdfnorm(2*xp+(xp2))))+1.
do if (cilow < 1).
compute cilow = 1.
compute booterr=1.
compute badlo = 1.
end if.
do if (cihigh > nrow(temp)).
compute cihigh = boot.
compute booterr=1.
compute badhi = 1.
end if.
compute llcit=temp(cilow,1).
compute ulcit=temp(cihigh,1).
do if (badlo = 1 and llcit <> priorlo).
compute badend={badend, llcit}.
compute priorlo = llcit.
end if.
do if (badhi = 1 and ulcit <> priorhi).
compute badend={badend, ulcit}.
compute priorhi = ulcit.
end if.
end if.
do if ( 9999 = 9999).
compute llcit=temp(cilow,1).
compute ulcit=temp(cihigh,1).
end if.
compute bootse=t(sqrt((cssq(temp)-((csum(temp)&**2)/nrow(temp)))/(nrow(temp)-1)))
.
end if.
do if (boot > 0).

.
compute temp = inddifft(2:nrow(inddifft),1).
compute temp(GRADE( inddifft(2:nrow(inddifft),1) )) = inddifft(2:nrow(inddifft),1).
compute badlo = 0.
compute badhi = 0.
do if ( (inddifft(1,1)*bc)+(9999* (1-bc)) <> 9999).
compute pv=csum(temp < (inddifft(1,1)*bc)+(9999* (1-bc)) )/nrow(temp).
compute ppv = pv.
do if (pv > .5).
compute ppv = 1-pv.
end if.
compute y5=sqrt(-2*ln(ppv)).
compute xp=y5+((((y5*p4+p3)*y5+p2)*y5+p1)*y5+p0)/((((y5*q4+q3)*y5+q2)*y5+q1)*y5+q0).
do if (pv <= .5).
compute xp = -xp.
end if.
compute cilow=rnd(nrow(temp)*(cdfnorm(2*xp-xp2))).
compute cihigh=trunc(nrow(temp)*(cdfnorm(2*xp+(xp2))))+1.
do if (cilow < 1).
compute cilow = 1.
compute booterr=1.
compute badlo = 1.
end if.
do if (cihigh > nrow(temp)).
compute cihigh = boot.
compute booterr=1.
compute badhi = 1.
end if.
compute llcit=temp(cilow,1).
compute ulcit=temp(cihigh,1).
do if (badlo = 1 and llcit <> priorlo).
compute badend={badend, llcit}.
compute priorlo = llcit.
end if.
do if (badhi = 1 and ulcit <> priorhi).
compute badend={badend, ulcit}.
compute priorhi = ulcit.
end if.
end if.
do if ( (inddifft(1,1)*bc)+(9999* (1-bc)) = 9999).
compute llcit=temp(cilow,1).
compute ulcit=temp(cihigh,1).
end if.
compute bootse=t(sqrt((cssq(temp)-((csum(temp)&**2)/nrow(temp)))/(nrow(temp)-1)))
.
end if.
compute indtemp={indtemp, bootse,llcit,ulcit}.
end if.
compute indtab={indtab;indtemp}.
end loop.
end loop.
end if.
release inddiff.
compute contlbs=cntname(1:(((nindfx)*(nindfx-1))/2),1).
compute rowlbs={rowlbs;contlbs}.
end if.
compute resultm2=make(nrow(indtab),maxresm,99999).
compute resultm2(1:nrow(indtab),1:ncol(indtab))=indtab.
compute resultm={resultm;resultm2}.
do if (outscree=1).
do if (kk=1).
print indtab/title = 'Indirect effect(s) of X on Y:'/rnames=rowlbs/cnames=bootlbs/format= F10.4.
end if.
do if (kk = 2 and (xdich=1 or mcx > 0)).
print indtab/title = 'Partially standardized indirect effect(s) of X on Y:'/rnames=rowlbs/cnames=bootlbs/format= F10.4.
end if.
do if (kk = 3).
print indtab/title = 'Completely standardized indirect effect(s) of X on Y:'/rnames=rowlbs/cnames=bootlbs/format= F10.4.
end if.
end if.
do if (normal=1 and sobelok=1 and kk=1).
compute resultm2=make(nrow(sobelmat),maxresm,99999).
compute resultm2(1:nrow(sobelmat),1:ncol(sobelmat))=sobelmat.
compute resultm={resultm;resultm2}.
compute sobellab={'Effect',hclab,'Z','p'}.
compute sobelrlb=rowlbs.
do if (nms > 1).
compute sobelrlb=rowlbs(2:(1+nms),1).
end if.
do if (outscree=1).
print sobelmat/title='   Normal theory test for indirect effect(s):'/cnames=sobellab/rnames=sobelrlb/format= F10.4.
end if.
end if.
do if (contrast <> 0).
do if ((contrast=1 or contrast = 2 or contrast=3) and kk=efloop ).
compute conkey=conkey(2:nrow(conkey),:).
do if (outscree=1).
print conkey/title = 'Specific indirect effect contrast definition(s):'/rnames=contlbs/format=A8.
end if.
end if.
do if (outscree=1).
do if (contrast = 4 and kk=efloop).
compute crowlbs=rowlbs(2:(nindfx+1),1).
print contvec/title = 'Specific indirect effect contrast weights:'/cnames=crowlbs/rlabels='(C1)'/format= F10.4.
end if.
do if (contrast = 2 and kk=efloop).
print/title = 'Contrasts are differences between absolute values of indirect effects'.
end if.
end if.
end if.
do if (serial = 1 and kk=efloop).
compute rowlbst=rowlbs(2:nrow(rowlbs),1).
do if (outscree=1).
print indkey/title = 'Indirect effect key:'/rnames=rowlbst/format = A8.
end if.
end if.
else.
do if (outscree=1).
do if (kk = 1).
print/title = 'Relative indirect effects of X on Y'.
end if.
do if (kk = 2).
print/title = 'Partially standardized relative indirect effect(s) of X on Y:'.
end if.
do if (kk = 3).
print/title = 'Completely standardized relative indirect effect(s) of X on Y:'.
end if.
end if.
loop i = 1 to nrow(indmake).
compute indtabsm=indtab((((i-1)*nxvls)+1):(nxvls*i),:).
compute indkeyt=indkey(i,:).
do if (outscree=1).
print indkeyt/title=' '/space=0/format=A8.
end if.
do if (bcmat(nrow(bcmat),1)=0)).
compute direffl2=xcatlab(1:nxvls,1).
end if.
compute resultm2=make(nrow(indtabsm),maxresm,99999).
compute resultm2(1:nrow(indtabsm),1:ncol(indtabsm))=indtabsm.
compute resultm={resultm;resultm2}.
do if (outscree=1).
print indtabsm/title = ' '/cnames=bootlbs/rnames=direffl2/format= F10.4 /space=0.
end if.
do if (normal=1 and sobelok=1 and kk=1).
compute sobelsm=sobelmat((((i-1)*nxvls)+1):(nxvls*i),:).
compute sobellab={'Effect',hclab,'Z','p'}.
compute resultm2=make(nrow(sobelsm),maxresm,99999).
compute resultm2(1:nrow(sobelsm),1:ncol(sobelsm))=sobelsm.
compute resultm={resultm;resultm2}.
do if (outscree=1).
print sobelsm/title='      Normal theory test for relative indirect effects:'/cnames=sobellab/rnames=direffl2/format= F10.4.
end if.
end if.
end loop.
end if.
do if (effsize = 1 and boot > 0).
compute bootres=bootres(2:nrow(bootres),:).
end if.
end loop.
do if (alttotal=1).
compute altcnms='Effect'.
compute totbtvec=totbtvec+bootdir.
compute alttotfx=t(totbtvec(1,:)).
do if (boot > 0).
compute alttotfx=make(ncol(totbtvec),4,0).
compute alttotfx(:,1)=t(totbtvec(1,:)).
loop cec=1 to ncol(totbtvec).

.
compute temp = totbtvec(2:nrow(totbtvec),cec).
compute temp(GRADE( totbtvec(2:nrow(totbtvec),cec) )) = totbtvec(2:nrow(totbtvec),cec).
compute badlo = 0.
compute badhi = 0.
do if ( (totbtvec(1,cec)*bc)+(9999* (1-bc)) <> 9999).
compute pv=csum(temp < (totbtvec(1,cec)*bc)+(9999* (1-bc)) )/nrow(temp).
compute ppv = pv.
do if (pv > .5).
compute ppv = 1-pv.
end if.
compute y5=sqrt(-2*ln(ppv)).
compute xp=y5+((((y5*p4+p3)*y5+p2)*y5+p1)*y5+p0)/((((y5*q4+q3)*y5+q2)*y5+q1)*y5+q0).
do if (pv <= .5).
compute xp = -xp.
end if.
compute cilow=rnd(nrow(temp)*(cdfnorm(2*xp-xp2))).
compute cihigh=trunc(nrow(temp)*(cdfnorm(2*xp+(xp2))))+1.
do if (cilow < 1).
compute cilow = 1.
compute booterr=1.
compute badlo = 1.
end if.
do if (cihigh > nrow(temp)).
compute cihigh = boot.
compute booterr=1.
compute badhi = 1.
end if.
compute llcit=temp(cilow,1).
compute ulcit=temp(cihigh,1).
do if (badlo = 1 and llcit <> priorlo).
compute badend={badend, llcit}.
compute priorlo = llcit.
end if.
do if (badhi = 1 and ulcit <> priorhi).
compute badend={badend, ulcit}.
compute priorhi = ulcit.
end if.
end if.
do if ( (totbtvec(1,cec)*bc)+(9999* (1-bc)) = 9999).
compute llcit=temp(cilow,1).
compute ulcit=temp(cihigh,1).
end if.
compute bootse=t(sqrt((cssq(temp)-((csum(temp)&**2)/nrow(temp)))/(nrow(temp)-1)))
.
compute alttotfx(cec,2:4)={bootse,llcit,ulcit}.
end loop.
compute altcnms={altcnms,'BootSE', 'BootLLCI','BootULCI'}.
end if.
compute resultm2=make(nrow(alttotfx),maxresm,99999).
compute resultm2(1:nrow(alttotfx),1:ncol(alttotfx))=alttotfx.
compute resultm={resultm;resultm2}.
do if (outscree=1).
do if (nxvls > 1).
print/title= '----------'/space=0.
print alttotfx/title='Relative total effects of X on Y (sum of direct and indirect effects)'/rnames=direffl2/cnames=altcnms/format= F10.4.
end if.
do if (nxvls = 1).
print alttotfx/title='Total effect of X on Y (sum of direct and indirect effects)'/cnames=altcnms/format= F10.4.
end if.
end if.
end if.
end if.
do if (anymod > 0).
do if (boot = 0).
compute bootres=obscoeff.
compute indtab=999.
end if.
do if (boot > 0).
compute bootres={obscoeff;bootres}.
compute indtab=make(1,4,999).
end if.
do if (csum((indmod > 0))=nrow(indmod)).
do if (outscree=1).
do if (nxvls > 1).
do if (xmint=0).
print/title= 'Relative conditional indirect effects of X on Y:'.
end if.
do if (xmint=1).
print/title= 'Relative (total) natural indirect effects of X on Y:'.
end if.
end if.
do if (nxvls = 1).
do if (xmint=0).
print/title= 'Conditional indirect effects of X on Y:'.
end if.
do if (xmint=1).
print/title= '(Total) Natural indirect effect(s) of X on Y:'.
end if.
end if.
end if.
end if.
do if (csum((indmod > 0)) < nrow(indmod)).
do if (outscree=1).
do if (nxvls > 1).
print/title= 'Relative conditional and unconditional indirect effects of X on Y:'.
end if.
do if (nxvls = 1).
print/title= 'Conditional and unconditional indirect effects of X on Y:'.
end if.
end if.
end if.
compute cftotfx=make(nrow(bootres),nxvls,0).
loop i = 1 to nrow(indmake).
compute indtab=indtab(1,:)*0.
do if (outscree=1).
do if (xmint=0).
print indkey(i,:)/title = 'INDIRECT EFFECT:'/format=A8.
end if.
do if (xmint=1).
print indkey(i,:)/title = ' '/format=A8/space=0.
end if.
end if.
do if (indmod(i,1)=0).
loop j = 1 to nxvls.
compute indtemp=make(nrow(bootres),1,1).
loop k = 1 to indmake(i,1).
compute jtemp=1.
do if (j > 1 and k=1).
compute jtemp=j.
end if.
compute indtemp=indtemp&*bootres(:,pathsfoc(jtemp,indmake(i,(k+1)))).
end loop.
compute indeff=indtemp(1,1).
do if (boot > 0).

.
compute temp = indtemp(2:nrow(indtemp),1).
compute temp(GRADE( indtemp(2:nrow(indtemp),1) )) = indtemp(2:nrow(indtemp),1).
compute badlo = 0.
compute badhi = 0.
do if ( (indtemp(1,1)*bc)+(9999*(1-bc)) <> 9999).
compute pv=csum(temp < (indtemp(1,1)*bc)+(9999*(1-bc)) )/nrow(temp).
compute ppv = pv.
do if (pv > .5).
compute ppv = 1-pv.
end if.
compute y5=sqrt(-2*ln(ppv)).
compute xp=y5+((((y5*p4+p3)*y5+p2)*y5+p1)*y5+p0)/((((y5*q4+q3)*y5+q2)*y5+q1)*y5+q0).
do if (pv <= .5).
compute xp = -xp.
end if.
compute cilow=rnd(nrow(temp)*(cdfnorm(2*xp-xp2))).
compute cihigh=trunc(nrow(temp)*(cdfnorm(2*xp+(xp2))))+1.
do if (cilow < 1).
compute cilow = 1.
compute booterr=1.
compute badlo = 1.
end if.
do if (cihigh > nrow(temp)).
compute cihigh = boot.
compute booterr=1.
compute badhi = 1.
end if.
compute llcit=temp(cilow,1).
compute ulcit=temp(cihigh,1).
do if (badlo = 1 and llcit <> priorlo).
compute badend={badend, llcit}.
compute priorlo = llcit.
end if.
do if (badhi = 1 and ulcit <> priorhi).
compute badend={badend, ulcit}.
compute priorhi = ulcit.
end if.
end if.
do if ( (indtemp(1,1)*bc)+(9999*(1-bc)) = 9999).
compute llcit=temp(cilow,1).
compute ulcit=temp(cihigh,1).
end if.
compute bootse=t(sqrt((cssq(temp)-((csum(temp)&**2)/nrow(temp)))/(nrow(temp)-1)))
.
compute indeff={indeff,bootse,llcit,ulcit}.
end if.
compute indtab={indtab;indeff}.
end loop.
compute resultm2=make(nrow(indtab),maxresm,99999).
compute resultm2(1:nrow(indtab),1:ncol(indtab))=indtab.
compute resultm={resultm;resultm2}.
do if (outscree=1).
do if (nxvls > 1).
compute indefflb=xcatlab(1:nxvls,1).
print indtab(2:nrow(indtab),:)/title = ' '/clabels='Effect', 'BootSE', 'BootLLCI', 'BootULCI'/rnames=indefflb/format= F10.4 /space=0.
end if.
do if (nxvls = 1).
print indtab(2:nrow(indtab),:)/title = ' '/clabels='Effect', 'BootSE', 'BootLLCI', 'BootULCI'/format= F10.4 /space=0.
end if.
end if.
end if.
do if (indmod(i,1)>0).
do if (indmod(i,1)=1).
compute indmodva=wmodvals.
compute indprova=wprobval.
compute condlbs={wnames}.
compute printw=1.
else if (indmod(i,1)=2).
compute indmodva=zmodvals.
compute indprova=zprobval.
compute condlbs={znames}.
compute printz=1.
else if (indmod(i,1)=3).
compute cntmp=1.
compute printz=1.
compute printw=1.
compute indmodva=make((nrow(wmodvals)*nrow(zmodvals)),2,999).
loop k7 = 1 to nrow(wmodvals).
loop k8 = 1 to nrow(zmodvals).
compute indmodva(cntmp,:)={wmodvals(k7,1),zmodvals(k8,1)}.
compute cntmp=cntmp+1.
end loop.
end loop.
compute condlbs={wnames,znames}.
end if.
compute condres=make(nrow(indmodva),1,999).
do if (boot > 0).
compute condres=make(nrow(indmodva),4,999).
end if.
compute condres={indmodva,condres}.
loop k4 = 1 to nxvls.
compute imm3=make(nrow(bootres),1,1).
compute imm4=make(nrow(bootres),1,1).
compute indcontr=0.
do if (indmod(i,1)=3).
compute tihsw=wprobval.
compute tihsz=zprobval.
end if.
loop k1=1 to nrow(indmodva).
compute tucker2=make(nrow(bootres),1,1).
compute imm2=make(nrow(bootres),1,1).
compute wfirst=0.
compute zfirst=0.
compute immset=0.
loop k2=1 to indmake(i,1).
compute colnumb=indmake(i,(k2+1)).
do if (k2=1).
compute wbb=make(nrow(bootres),(nwvls*nxvls),0).
compute zbb=make(nrow(bootres),(nzvls*nxvls),0).
compute wzbb=make(nrow(bootres),(nwvls*nzvls*nxvls),0).
end if.
do if (k2<>1).
compute wbb=make(nrow(bootres),nwvls,0).
compute zbb=make(nrow(bootres),nzvls,0).
compute wzbb=make(nrow(bootres),(nwvls*nzvls),0).
end if.
compute cnt=1.
compute tihs=indlocs(2:((indlocs(1,colnumb))+1),colnumb).
do if (k2 = 1).
compute focbb=tihs(1:nxvls,1).
compute focbb=bootres(:,focbb).
do if (indmmm(i,1)>0).
compute imm=focbb(:,k4).
compute condbb=imm.
end if.
compute focaddon=make(1,nxvls,0).
compute focaddon(1,k4)=1.
compute cnt=cnt+nxvls.
compute placeh=nxvls.
do if (indmod(i,1)=1).
compute tihsz=make(nrow(wprobval),(nzvls*nxvls),0).
compute tihswz=make(nrow(wprobval),(nwvls*nzvls*nxvls),0).
do if (pathsw(1,colnumb)=1).
compute temp=make(nrow(wprobval),(nxvls*nwvls),0).
loop k5 = 1 to nrow(wprobval).
loop k6=1 to nwvls.
compute temp(k5, (((k4-1)*nwvls)+k6))=wprobval(k5,k6).
end loop.
end loop.
compute indprova={temp,tihsz,tihswz}.
else.
compute indprova={wprobval,tihsz,tihswz}.
end if.
end if.
do if (indmod(i,1)=2).
compute tihsw=make(nrow(zprobval),(nwvls*nxvls),0).
compute tihswz=make(nrow(zprobval),(nwvls*nzvls*nxvls),0).
do if (pathsz(1,colnumb)=1).
compute temp=make(nrow(zprobval),(nxvls*nzvls),0).
loop k5 = 1 to nrow(zprobval).
loop k6 =1 to nzvls.
compute temp(k5,(((k4-1)*nzvls)+k6))=zprobval(k5,k6).
end loop.
end loop.
compute indprova={tihsw,temp,tihswz}.
else.
compute indprova={tihsw,zprobval,tihswz}.
end if.
end if.
do if (indmod(i,1)=3).
compute indprova=make((nrow(wprobval)*nrow(zprobval)),((ncol(wprobval)*nxvls)+ (ncol(zprobval)*nxvls)+(nwvls*nzvls*nxvls)),0).
compute cntemp=1.
loop k7=1 to nrow(wprobval).
loop k8 =1 to nrow(zprobval).
compute temp=wprobval(k7,:)*focaddon(1,k4).
compute indprova(cntemp,(((k4-1)*nwvls)+1):(k4*(nwvls)))=temp.
compute temp=zprobval(k8,:)*focaddon(1,k4).
compute indprova(cntemp, ((((k4-1)*nzvls)+1)+(nxvls*nwvls)) : ((((k4-1)*nzvls)+1)+(nxvls*nwvls)+(nzvls-1)))=temp.
compute cntemp=cntemp+1.
end loop.
end loop.
do if (pathsz(1,colnumb)=0).
compute temp=make(nrow(indprova),(ncol(zprobval)*nxvls),0).
compute indprova(:,((ncol(wprobval)*nxvls)+1):((ncol(wprobval)+ncol(zprobval))* nxvls))=temp.
end if.
do if (pathsw(1,colnumb)=0).
compute temp=make(nrow(indprova),(ncol(wprobval)*nxvls),0).
compute indprova(:,1:(ncol(wprobval)*nxvls))=temp.
end if.
do if (pathswz(1,colnumb)=1).
compute cntemp=(ncol(wprobval)*nxvls)+(ncol(zprobval)*nxvls)+((k4-1)* ncol(wprobval)*ncol(zprobval))+1.
loop k7=1 to ncol(wprobval).
loop k8=1 to ncol(zprobval).
compute indprova(:,cntemp)=indprova(:,((ncol(wprobval)*(k4-1))+k7))&* indprova(:,((((k4-1)*ncol(zprobval))+k8)+(nxvls*ncol(wprobval)))).
compute cntemp=cntemp+1.
end loop.
end loop.
end if.
end if.
end if.
do if (k2 > 1).
compute focbb=tihs(1,1).
compute focbb=bootres(:,focbb).
do if (indmmm(i,1)>0).
compute imm=focbb(:,1).
compute condbb=imm.
end if.
compute focaddon=1.
compute cnt=cnt+1.
compute placeh=1.
do if (indmod(i,1)=1).
compute tihsz=make(nrow(wprobval),nzvls,0).
compute tihswz=make(nrow(wprobval),(nwvls*nzvls),0).
compute indprova={wprobval,tihsz,tihswz}.
end if.
do if (indmod(i,1)=2).
compute tihsw=make(nrow(zprobval),nwvls,0).
compute tihswz=make(nrow(zprobval),(nwvls*nzvls),0).
compute indprova={tihsw,zprobval,tihswz}.
end if.
do if (indmod(i,1)=3).
compute indprova=make((nrow(wprobval)*nrow(zprobval)),((ncol(wprobval)+ ncol(zprobval))+(nwvls*nzvls)),0).
compute cntemp=1.
loop k7=1 to nrow(wprobval).
loop k8 =1 to nrow(zprobval).
compute indprova(cntemp,1:(ncol(wprobval)+ncol(zprobval)))={wprobval(k7,:), zprobval(k8,:)}.
compute cntemp=cntemp+1.
end loop.
end loop.
do if (pathsz(1,colnumb)=0).
compute temp=make(nrow(indprova),ncol(zprobval),0).
compute indprova(:,(ncol(wprobval)+1):(ncol(wprobval)+ncol(zprobval)))=temp.
end if.
do if (pathsw(1,colnumb)=0).
compute temp=make(nrow(indprova),ncol(wprobval),0).
compute indprova(:,1:ncol(wprobval))=temp.
end if.
do if (pathswz(1,colnumb)=1).
compute cntemp=ncol(wprobval)+ncol(zprobval)+1.
loop k7=1 to ncol(wprobval).
loop k8=1 to ncol(zprobval).
compute indprova(:,cntemp)=indprova(:,k7)&*indprova(:,(ncol(wprobval)+k8)).
compute cntemp=cntemp+1.
end loop.
end loop.
end if.
end if.
end if.
do if (pathsw(1,colnumb)) = 1.
compute wbb=tihs(cnt:(cnt+(placeh*nwvls)-1),1).
compute wbb=bootres(:,wbb).
compute immlbs2=wcatlab(1:nwvls,1).
do if (zfirst=0).
compute wfirst=1.
end if.
do if (indmmm(i,1)=1 or indmmm(i,1)=31 or indmmm(i,1)=51).
compute imm=wbb(:,1).
loop k7 = 1 to nwvls.
compute imm={imm,wbb(:,(((k4-1)*nwvls*(k2=1))+k7))}.
end loop.
compute imm=imm(:,2:ncol(imm)).
end if.
do if (indmmm(i,1)=41 or indmmm(i,1)=51).
compute condbb=make(nrow(bootres),1,0).
loop k7 = 1 to nwvls.
compute condbb={condbb,wbb(:,(((k4-1)*nwvls*(k2=1))+k7))}.
end loop.
compute condbb=condbb(:,2:ncol(condbb)).
end if.
compute cnt=cnt+(placeh*nwvls).
end if.
do if (pathsz(1,colnumb)) = 1.
compute zbb=tihs(cnt:(cnt+(placeh*nzvls)-1),1).
compute zbb=bootres(:,zbb).
do if (wfirst=0).
compute zfirst=1.
end if.
do if (indmmm(i,1) <> 31).
compute immlbs2=zcatlab(1:nzvls,1).
end if.
do if (indmmm(i,1)=2 or indmmm(i,1)=31 or indmmm(i,1)=51).
do if (indmmm(i,1)=2).
compute imm=zbb(:,1).
end if.
loop k7 = 1 to nzvls.
compute imm={imm,zbb(:,(((k4-1)*nzvls*(k2=1))+k7))}.
end loop.
do if (indmmm(i,1)=2 or indmmm(i,1)=51).
compute imm=imm(:,2:ncol(imm)).
do if (indmmm(i,1)=51).
compute condbb={condbb,imm}.
end if.
end if.
end if.
compute cnt=cnt+(placeh*nzvls).
end if.
do if (pathswz(1,colnumb)) = 1.
compute wzbb=tihs(cnt:(cnt+(placeh*nwvls*nzvls)-1),1).
compute wzbb=bootres(:,wzbb).
do if (indmmm(i,1)=41).
compute imm=wzbb(:,1).
loop k7=1 to nwvls*nzvls.
compute imm={imm,wzbb(:,(((k4-1)*nzvls*nwvls*(k2=1))+k7))}.
end loop.
end if.
do if (indmmm(i,1)=41).
compute imm=imm(:,2:ncol(imm)).
compute condbb={condbb,imm(:,(ncol(imm)-(nwvls*nzvls)+1):ncol(imm))}.
end if.
compute cnt=cnt+(placeh*nzvls*nwvls).
end if.
compute indprobe={focaddon,indprova(k1,:)}.
compute tucker={focbb,wbb,zbb,wzbb}.
loop k3=1 to ncol(indprobe).
compute tucker(:,k3)=tucker(:,k3)*indprobe(1,k3).
end loop.
compute tucker2=tucker2&*rsum(tucker).
do if (indmmm(i,1) = 1 or indmmm(i,1)=2 or indmmm(i,1)=31 or indmmm(i,1)=41 or indmmm(i,1)=51).
do if (immset=1).
do if (ncol(imm2)=1 and ncol(imm) = 1).
compute imm2=imm2&*imm.
end if.
do if (indmmm(i,1)=41 or indmmm(i,1)=51).
do if ((ncol(condbb2) > 1) and (ncol(condbb)>1)).
compute condbb2t=make(nrow(condbb2),(ncol(condbb2)*ncol(condbb)),-999999).
compute k9=1.
do if (wfirst=1).
loop k7=1 to ncol(condbb2).
loop k8 = 1 to ncol(condbb).
compute condbb2t(:,k9)=condbb2(:,k7)&*condbb(:,k8).
compute k9=k9+1.
end loop.
end loop.
end if.
do if (zfirst=1).
loop k7=1 to ncol(condbb).
loop k8 = 1 to ncol(condbb2).
compute condbb2t(:,k9)=condbb(:,k7)&*condbb2(:,k8).
compute k9=k9+1.
end loop.
end loop.
end if.
compute condbb2=condbb2t.
release condbb2t.
end if.
do if ((ncol(condbb2) > 1) and (ncol(condbb)=1)).
loop k7 = 1 to ncol(condbb2).
compute condbb2(:,k7)=condbb2(:,k7)&*condbb.
end loop.
end if.
do if ((ncol(condbb2) = 1) and (ncol(condbb)>1)).
loop k7 = 1 to ncol(condbb).
compute condbb(:,k7)=condbb2&*condbb(:,k7).
end loop.
compute condbb2=condbb.
end if.
end if.
do if (ncol(imm2) <> 1 and ncol(imm) <> 1).
compute imm2t=make(nrow(imm2),(ncol(imm2)*ncol(imm)),-999999).
compute k9=1.
do if (wfirst=1).
loop k7=1 to ncol(imm2).
loop k8 = 1 to ncol(imm).
compute imm2t(:,k9)=imm2(:,k7)&*imm(:,k8).
compute k9=k9+1.
end loop.
end loop.
end if.
do if (zfirst=1).
loop k7=1 to ncol(imm).
loop k8 = 1 to ncol(imm2).
compute imm2t(:,k9)=imm(:,k7)&*imm2(:,k8).
compute k9=k9+1.
end loop.
end loop.
end if.
compute imm2=imm2t.
release imm2t.
end if.
do if ((ncol(imm2) > 1) and (ncol(imm)=1)).
loop k7=1 to ncol(imm2).
compute imm2(:,k7)=imm2(:,k7)&*imm.
end loop.
end if.
do if ((ncol(imm2) = 1) and (ncol(imm) > 1)).
loop k7=1 to ncol(imm).
compute imm(:,k7)=imm2&*imm(:,k7).
end loop.
compute imm2=imm.
end if.
end if.
do if (immset=0).
compute imm2=imm.
do if (indmmm(i,1)=41 or indmmm(i,1)=51).
compute condbb2=condbb.
end if.
compute immset=1.
end if.
end if.
end loop.
compute indtemp=tucker2(1,1).
do if (indmmm(i,1)=12 or indmmm(i,1)=22).
compute imm3={imm3,tucker2}.
do if (k1=nrow(indmodva)).
compute imm3=imm3(:,2:ncol(imm3)).
compute immstop=ncol(imm3).
loop k8=2 to immstop.
do if (indmmmt(i,1)=1).
compute imm3={imm3,(imm3(:,k8)-imm3(:,1))}.
end if.
do if (indmmmt(i,1)=2).
compute imm3={imm3,(imm3(:,k8)-imm3(:,(k8-1)))}.
end if.
do if (indmmmt(i,1)=3).
compute imm3={imm3,((rsum(imm3(:,(k8:immstop)))/(immstop-k8+1))-imm3(:,(k8-1))) }.
end if.
do if (indmmmt(i,1)=4).
compute imm3={imm3,(imm3(:,k8)-(rsum(imm3(:,1:immstop))/immstop))}.
end if.
end loop.
do if (indmmmt(i,1)<5).
compute imm2=imm3(:,(immstop+1):ncol(imm3)).
release imm3.
end if.
end if.
end if.
do if (indmmm(i,1)>-1 and (contrast = 1 or contrast = 2 or contrast=3)).
compute imm4={imm4,tucker2}.
do if (k1=nrow(indmodva) and k1 > 1).
compute imm4=imm4(:,2:ncol(imm4)).
compute immstop=ncol(imm4).
compute condcont=make((immstop*(immstop-1)/2),6,-999).
loop k8 = 1 to (immstop-1).
loop k9 = (k8+1) to immstop.
do if (contrast=1).
compute imm4={imm4,(imm4(:,k9)-imm4(:,k8))}.
end if.
do if (contrast=2).
compute imm4={imm4,(abs(imm4(:,k9))-abs(imm4(:,k8)))}.
end if.
do if (contrast=3).
compute imm4={imm4,(imm4(:,k9)+imm4(:,k8))}.
end if.
compute condcont((ncol(imm4)-immstop),1)=imm4(1,k9).
compute condcont((ncol(imm4)-immstop),2)=imm4(1,k8).
end loop.
end loop.
compute imm4=imm4(:,(immstop+1):ncol(imm4)).
loop k8=1 to ncol(imm4).
compute condcont(k8,3)=imm4(1,k8).
do if (boot > 0).

.
compute temp = imm4(2:nrow(imm4),k8).
compute temp(GRADE( imm4(2:nrow(imm4),k8) )) = imm4(2:nrow(imm4),k8).
compute badlo = 0.
compute badhi = 0.
do if ( (imm4(1,k8)*bc)+(9999*(1-bc)) <> 9999).
compute pv=csum(temp < (imm4(1,k8)*bc)+(9999*(1-bc)) )/nrow(temp).
compute ppv = pv.
do if (pv > .5).
compute ppv = 1-pv.
end if.
compute y5=sqrt(-2*ln(ppv)).
compute xp=y5+((((y5*p4+p3)*y5+p2)*y5+p1)*y5+p0)/((((y5*q4+q3)*y5+q2)*y5+q1)*y5+q0).
do if (pv <= .5).
compute xp = -xp.
end if.
compute cilow=rnd(nrow(temp)*(cdfnorm(2*xp-xp2))).
compute cihigh=trunc(nrow(temp)*(cdfnorm(2*xp+(xp2))))+1.
do if (cilow < 1).
compute cilow = 1.
compute booterr=1.
compute badlo = 1.
end if.
do if (cihigh > nrow(temp)).
compute cihigh = boot.
compute booterr=1.
compute badhi = 1.
end if.
compute llcit=temp(cilow,1).
compute ulcit=temp(cihigh,1).
do if (badlo = 1 and llcit <> priorlo).
compute badend={badend, llcit}.
compute priorlo = llcit.
end if.
do if (badhi = 1 and ulcit <> priorhi).
compute badend={badend, ulcit}.
compute priorhi = ulcit.
end if.
end if.
do if ( (imm4(1,k8)*bc)+(9999*(1-bc)) = 9999).
compute llcit=temp(cilow,1).
compute ulcit=temp(cihigh,1).
end if.
compute bootse=t(sqrt((cssq(temp)-((csum(temp)&**2)/nrow(temp)))/(nrow(temp)-1)))
.
compute condcont(k8,4:6)={bootse,llcit,ulcit}.
end if.
end loop.
do if (boot=0).
compute condcont=condcont(:,1:3).
end if.
compute indcontr=1.
end if.
end if.
do if (xmint=1).
do if (k1=(k4+1)).
compute cftotfx(:,k4)=cftotfx(:,k4)+(tucker2*xscaling).
end if.
end if.
do if (boot > 0).
compute tucker2=tucker2*xscaling.

.
compute temp = tucker2(2:nrow(tucker2),1).
compute temp(GRADE( tucker2(2:nrow(tucker2),1) )) = tucker2(2:nrow(tucker2),1).
compute badlo = 0.
compute badhi = 0.
do if ( (tucker2(1,1)*bc)+(9999*(1-bc)) <> 9999).
compute pv=csum(temp < (tucker2(1,1)*bc)+(9999*(1-bc)) )/nrow(temp).
compute ppv = pv.
do if (pv > .5).
compute ppv = 1-pv.
end if.
compute y5=sqrt(-2*ln(ppv)).
compute xp=y5+((((y5*p4+p3)*y5+p2)*y5+p1)*y5+p0)/((((y5*q4+q3)*y5+q2)*y5+q1)*y5+q0).
do if (pv <= .5).
compute xp = -xp.
end if.
compute cilow=rnd(nrow(temp)*(cdfnorm(2*xp-xp2))).
compute cihigh=trunc(nrow(temp)*(cdfnorm(2*xp+(xp2))))+1.
do if (cilow < 1).
compute cilow = 1.
compute booterr=1.
compute badlo = 1.
end if.
do if (cihigh > nrow(temp)).
compute cihigh = boot.
compute booterr=1.
compute badhi = 1.
end if.
compute llcit=temp(cilow,1).
compute ulcit=temp(cihigh,1).
do if (badlo = 1 and llcit <> priorlo).
compute badend={badend, llcit}.
compute priorlo = llcit.
end if.
do if (badhi = 1 and ulcit <> priorhi).
compute badend={badend, ulcit}.
compute priorhi = ulcit.
end if.
end if.
do if ( (tucker2(1,1)*bc)+(9999*(1-bc)) = 9999).
compute llcit=temp(cilow,1).
compute ulcit=temp(cihigh,1).
end if.
compute bootse=t(sqrt((cssq(temp)-((csum(temp)&**2)/nrow(temp)))/(nrow(temp)-1)))
.
compute indtemp={indtemp*xscaling, bootse,llcit,ulcit}.
end if.
do if (boot=0).
compute indtemp=indtemp*xscaling.
end if.
compute condres(k1,(ncol(indmodva)+1):ncol(condres))=indtemp.
end loop.
do if (xmint=0).
compute resultm2=make(nrow(condres),maxresm,99999).
compute resultm2(1:nrow(condres),1:ncol(condres))=condres.
compute resultm={resultm;resultm2}.
end if.
compute condlbs={condlbs,'Effect'}.
do if (boot > 0).
compute condlbs={condlbs,'BootSE', 'BootLLCI', 'BootULCI'}.
end if.
do if (xmint = 1).
do if (k4 = 1).
compute natindfx=condres((2+(k4-1)),2:ncol(condres)).
end if.
do if (k4 > 1).
compute natindfx={natindfx;condres((2+(k4-1)),2:ncol(condres))}.
end if.
do if (k4=nxvls).
compute resultm2=make(nrow(natindfx),maxresm,99999).
compute resultm2(1:nrow(natindfx),1:ncol(natindfx))=natindfx.
compute resultm={resultm;resultm2}.
end if.
end if.
do if (outscree=1 and xmint=0).
do if (nxvls=1).
print condres/title=' '/cnames=condlbs/format= F10.4 /space=0.
else.
compute condrlb=make(nrow(condres),1,xcatlab(k4,1)).
print condres/title=' '/cnames=condlbs/rnames=condrlb/format= F10.4 /space=0.
end if.
end if.
do if (outscree=1 and xmint=1 and k4=nxvls).
do if (nxvls=1).
print natindfx/title=' '/clabels='Effect', 'BootSE', 'BootLLCI', 'BootULCI'/format= F10.4 /space=0.
else.
compute condrlb=make(nrow(condres),1,xcatlab(k4,1)).
print natindfx/title=' '/clabels='Effect', 'BootSE', 'BootLLCI', 'BootULCI'/rnames=direffl2/format= F10.4 /space=0.
end if.
end if.
compute dichadj=0.
compute immcat=0.
do if ((indmmm(i,1)>0) and (xmint=0)).
do if (indmmm(i,1)=1 or indmmm(i,1)=12 or indmmm(i,1)=31).
do if (wdich=1 and mcw=0).
do if (indmmm(i,1) <> 12).
compute imm2(:,1)=imm2(:,1)*(wmax-wmin).
end if.
do if (indmmm(i,1) <> 31).
compute dichadj=1.
end if.
end if.
do if ((mcw = 1 or mcw = 2) and indmmm(i,1) <> 31)).
compute immcat=1.
end if.
end if.
do if (indmmm(i,1)=2 or indmmm(i,1)=22 or indmmm(i,1)=31).
do if (zdich=1 and mcz=0).
do if (indmmm(i,1) = 31).
compute imm2(:,(nwvls+1):ncol(imm2))=imm2(:,(nwvls+1):ncol(imm2))*(zmax-zmin).
end if.
do if (indmmm(i,1) = 2).
compute imm2(:,1)=imm2(:,1)*(zmax-zmin).
end if.
do if (indmmm(i,1) <> 31).
compute dichadj=1.
end if.
end if.
do if ((mcz = 1 or mcz = 2) and indmmm(i,1) <> 31)).
compute immcat=1.
end if.
end if.
compute immtemp2=t(imm2(1,:)).
compute immtemp=immtemp2.
compute immlbs={'Index'}.
do if (boot > 0).
compute immtemp=make(1,3,0).
loop k7=1 to ncol(imm2).

.
compute temp = imm2(2:nrow(imm2),k7).
compute temp(GRADE( imm2(2:nrow(imm2),k7) )) = imm2(2:nrow(imm2),k7).
compute badlo = 0.
compute badhi = 0.
do if ( (imm2(1,k7)*bc)+(9999*(1-bc)) <> 9999).
compute pv=csum(temp < (imm2(1,k7)*bc)+(9999*(1-bc)) )/nrow(temp).
compute ppv = pv.
do if (pv > .5).
compute ppv = 1-pv.
end if.
compute y5=sqrt(-2*ln(ppv)).
compute xp=y5+((((y5*p4+p3)*y5+p2)*y5+p1)*y5+p0)/((((y5*q4+q3)*y5+q2)*y5+q1)*y5+q0).
do if (pv <= .5).
compute xp = -xp.
end if.
compute cilow=rnd(nrow(temp)*(cdfnorm(2*xp-xp2))).
compute cihigh=trunc(nrow(temp)*(cdfnorm(2*xp+(xp2))))+1.
do if (cilow < 1).
compute cilow = 1.
compute booterr=1.
compute badlo = 1.
end if.
do if (cihigh > nrow(temp)).
compute cihigh = boot.
compute booterr=1.
compute badhi = 1.
end if.
compute llcit=temp(cilow,1).
compute ulcit=temp(cihigh,1).
do if (badlo = 1 and llcit <> priorlo).
compute badend={badend, llcit}.
compute priorlo = llcit.
end if.
do if (badhi = 1 and ulcit <> priorhi).
compute badend={badend, ulcit}.
compute priorhi = ulcit.
end if.
end if.
do if ( (imm2(1,k7)*bc)+(9999*(1-bc)) = 9999).
compute llcit=temp(cilow,1).
compute ulcit=temp(cihigh,1).
end if.
compute bootse=t(sqrt((cssq(temp)-((csum(temp)&**2)/nrow(temp)))/(nrow(temp)-1)))
.
compute temp={bootse,llcit,ulcit}.
compute immtemp={immtemp;temp}.
end loop.
compute immtemp=immtemp(2:nrow(immtemp),:).
compute immtemp={immtemp2,immtemp}.
compute immlbs={immlbs,'BootSE', 'BootLLCI', 'BootULCI'}.
end if.
compute resultm2=make(nrow(immtemp),maxresm,99999).
compute resultm2(1:nrow(immtemp),1:ncol(immtemp))=immtemp.
compute resultm={resultm;resultm2}.
do if (dichadj=0 and immcat=0 and indmmmt(i,1)<>5 and indmmm(i,1) < 100 ).
do if (outscree=1).
do if (indmmm(i,1) < 30).
print immtemp/title='      Index of moderated mediation:'/cnames=immlbs/rnames=immlbs2/format= F10.4.
end if.
do if (indmmm(i,1) = 31).
compute immlbs2={immlbs2;zcatlab(1:nzvls,1)}.
print immtemp/title='      Indices of partial moderated mediation:'/cnames=immlbs/rnames=immlbs2/format= F10.4.
end if.
end if.
do if ((nzvls = 1) and (nwvls = 1)).
do if (indmmm(i,1) = 41 or indmmm(i,1)=51).
loop k7=1 to nwvls.
compute immlbs2=zcatlab(1:nzvls,1).
compute immtemp2=immtemp((((k7-1)*nzvls)+1):(((k7-1)*nzvls)+nzvls),:).
do if (outscree=1).
do if (nwvls > 1).
compute primodv={'        ', wcatlab(k7,1)}.
print primodv/title='      Primary moderator:'/format=A8.
end if.
do if (nzvls=1).
print immtemp2/title='      Index of moderated moderated mediation'/cnames=immlbs/format= F10.4.
else.
print immtemp2/title='      Indices of moderated moderated mediation'/cnames=immlbs/rnames=immlbs2/format= F10.4.
end if.
end if.
compute cmmtemp=make(nrow(zprobval),4,0).
loop k8=1 to nrow(zprobval).
compute condbb3=condbb2(:,((nwvls+1)+((k7-1)*nzvls)):((nwvls+1)+((k7-1)*nzvls)+ (nzvls-1))).
do if (ncol(zprobval) > 1).
compute condbb3=condbb3*mdiag(zprobval(k8,:)).
else.
compute condbb3=condbb3&*zprobval(k8,:).
end if.
compute condbb3={condbb2(:,k7),condbb3}.
compute icmm=rsum(condbb3).
compute cmmtemp(k8,1)=icmm(1,1).
do if (boot > 0).

.
compute temp = icmm(2:nrow(icmm),1).
compute temp(GRADE( icmm(2:nrow(icmm),1) )) = icmm(2:nrow(icmm),1).
compute badlo = 0.
compute badhi = 0.
do if ( (icmm(1,1)*bc)+(9999*(1-bc)) <> 9999).
compute pv=csum(temp < (icmm(1,1)*bc)+(9999*(1-bc)) )/nrow(temp).
compute ppv = pv.
do if (pv > .5).
compute ppv = 1-pv.
end if.
compute y5=sqrt(-2*ln(ppv)).
compute xp=y5+((((y5*p4+p3)*y5+p2)*y5+p1)*y5+p0)/((((y5*q4+q3)*y5+q2)*y5+q1)*y5+q0).
do if (pv <= .5).
compute xp = -xp.
end if.
compute cilow=rnd(nrow(temp)*(cdfnorm(2*xp-xp2))).
compute cihigh=trunc(nrow(temp)*(cdfnorm(2*xp+(xp2))))+1.
do if (cilow < 1).
compute cilow = 1.
compute booterr=1.
compute badlo = 1.
end if.
do if (cihigh > nrow(temp)).
compute cihigh = boot.
compute booterr=1.
compute badhi = 1.
end if.
compute llcit=temp(cilow,1).
compute ulcit=temp(cihigh,1).
do if (badlo = 1 and llcit <> priorlo).
compute badend={badend, llcit}.
compute priorlo = llcit.
end if.
do if (badhi = 1 and ulcit <> priorhi).
compute badend={badend, ulcit}.
compute priorhi = ulcit.
end if.
end if.
do if ( (icmm(1,1)*bc)+(9999*(1-bc)) = 9999).
compute llcit=temp(cilow,1).
compute ulcit=temp(cihigh,1).
end if.
compute bootse=t(sqrt((cssq(temp)-((csum(temp)&**2)/nrow(temp)))/(nrow(temp)-1)))
.
compute cmmtemp(k8,2:4)={bootse,llcit,ulcit}.
end if.
end loop.
compute cmmtemp={zmodvals,cmmtemp}.
do if (boot=0).
compute cmmtemp=cmmtemp(:,1:2).
end if.
compute cmmlbs={znames,immlbs}.
compute resultm2=make(nrow(cmmtemp),maxresm,99999).
compute resultm2(1:nrow(cmmtemp),1:ncol(cmmtemp))=cmmtemp.
compute resultm={resultm;resultm2}.
do if (outscree=1).
print cmmtemp/title='      Indices of conditional moderated mediation by W'/cnames=cmmlbs/format= F10.4.
end if.
end loop.
end if.
end if.
end if.
do if (dichadj=1 or immcat=1 and indmmm(i,1) < 30).
do if (outscree=1).
print immtemp/title='Index of moderated mediation (difference between conditional indirect effects):'/cnames=immlbs/rnames=immlbs2/format= F10.4.
end if.
end if.
end if.
do if (indcontr=1).
compute resultm2=make(nrow(condcont),maxresm,99999).
compute resultm2(1:nrow(condcont),1:ncol(condcont))=condcont.
compute resultm={resultm;resultm2}.
compute condctlb={'Effect1','Effect2','Contrast','BootSE', 'BootLLCI','BootULCI'}.
do if (outscree=1).
print condcont/title=' Pairwise contrasts between conditional indirect effects (Effect1 minus Effect2)'/cnames=condctlb/format= F10.4.
end if.
end if.
end loop.
end if.
end loop.
do if (alttotal=1).
compute altcnms='Effect'.
compute alttotfx=t(cftotfx(1,:))+obnatdfx.
do if (boot > 0).
compute alttotfx=make(ncol(cftotfx),4,0).
compute alttotfx(:,1)=t(cftotfx(1,:))+obnatdfx.
compute natdirbt(1,:)=t(obnatdfx).
compute cftotfx=cftotfx+natdirbt.
loop cec=1 to ncol(cftotfx).

.
compute temp = cftotfx(2:nrow(cftotfx),cec).
compute temp(GRADE( cftotfx(2:nrow(cftotfx),cec) )) = cftotfx(2:nrow(cftotfx),cec).
compute badlo = 0.
compute badhi = 0.
do if ( (cftotfx(1,cec)*bc)+(9999*(1-bc)) <> 9999).
compute pv=csum(temp < (cftotfx(1,cec)*bc)+(9999*(1-bc)) )/nrow(temp).
compute ppv = pv.
do if (pv > .5).
compute ppv = 1-pv.
end if.
compute y5=sqrt(-2*ln(ppv)).
compute xp=y5+((((y5*p4+p3)*y5+p2)*y5+p1)*y5+p0)/((((y5*q4+q3)*y5+q2)*y5+q1)*y5+q0).
do if (pv <= .5).
compute xp = -xp.
end if.
compute cilow=rnd(nrow(temp)*(cdfnorm(2*xp-xp2))).
compute cihigh=trunc(nrow(temp)*(cdfnorm(2*xp+(xp2))))+1.
do if (cilow < 1).
compute cilow = 1.
compute booterr=1.
compute badlo = 1.
end if.
do if (cihigh > nrow(temp)).
compute cihigh = boot.
compute booterr=1.
compute badhi = 1.
end if.
compute llcit=temp(cilow,1).
compute ulcit=temp(cihigh,1).
do if (badlo = 1 and llcit <> priorlo).
compute badend={badend, llcit}.
compute priorlo = llcit.
end if.
do if (badhi = 1 and ulcit <> priorhi).
compute badend={badend, ulcit}.
compute priorhi = ulcit.
end if.
end if.
do if ( (cftotfx(1,cec)*bc)+(9999*(1-bc)) = 9999).
compute llcit=temp(cilow,1).
compute ulcit=temp(cihigh,1).
end if.
compute bootse=t(sqrt((cssq(temp)-((csum(temp)&**2)/nrow(temp)))/(nrow(temp)-1)))
.
compute alttotfx(cec,2:4)={bootse,llcit,ulcit}.
end loop.
compute altcnms={altcnms,'BootSE', 'BootLLCI','BootULCI'}.
end if.
compute resultm2=make(nrow(alttotfx),maxresm,99999).
compute resultm2(1:nrow(alttotfx),1:ncol(alttotfx))=alttotfx.
compute resultm={resultm;resultm2}.
do if (outscree=1).
print/title='----------'.
do if (nxvls > 1).
print alttotfx/title='Relative total effects of X on Y (sum of direct and indirect effects)'/cnames=altcnms/rnames=direffl2/format= F10.4.
end if.
do if (nxvls = 1).
print alttotfx/title='Total effect of X on Y (sum of direct and indirect effects)'/cnames=altcnms/format= F10.4.
end if.
end if.
end if.
end if.
end if
.
do if (criterr=0 and (saveest = 1 or saveboot=1)).
compute coeffsav=coeffmat(2:nrow(coeffmat),:).
compute conseq=conseq(2:nrow(conseq),1).
compute dfmat=dfmat(2:nrow(dfmat),1).
compute dfmat=dfmat.
compute saven={'conseqnt','antecdnt','coeff','se','t','p','LLCI','ULCI','df'}.
do if (ydich=1).
compute saven={'conseqnt','antecdnt','coeff','se','t_or_Z','p','LLCI','ULCI','df'}.
end if.
do if (saveboot=1 and boot > 0 ).
compute colslab=make(300,1,' ').


compute colslab( 1 ,1)= 'COL1'.


compute colslab( 2 ,1)= 'COL2'.


compute colslab( 3 ,1)= 'COL3'.


compute colslab( 4 ,1)= 'COL4'.


compute colslab( 5 ,1)= 'COL5'.


compute colslab( 6 ,1)= 'COL6'.


compute colslab( 7 ,1)= 'COL7'.


compute colslab( 8 ,1)= 'COL8'.


compute colslab( 9 ,1)= 'COL9'.


compute colslab( 10 ,1)= 'COL10'.


compute colslab( 11 ,1)= 'COL11'.


compute colslab( 12 ,1)= 'COL12'.


compute colslab( 13 ,1)= 'COL13'.


compute colslab( 14 ,1)= 'COL14'.


compute colslab( 15 ,1)= 'COL15'.


compute colslab( 16 ,1)= 'COL16'.


compute colslab( 17 ,1)= 'COL17'.


compute colslab( 18 ,1)= 'COL18'.


compute colslab( 19 ,1)= 'COL19'.


compute colslab( 20 ,1)= 'COL20'.


compute colslab( 21 ,1)= 'COL21'.


compute colslab( 22 ,1)= 'COL22'.


compute colslab( 23 ,1)= 'COL23'.


compute colslab( 24 ,1)= 'COL24'.


compute colslab( 25 ,1)= 'COL25'.


compute colslab( 26 ,1)= 'COL26'.


compute colslab( 27 ,1)= 'COL27'.


compute colslab( 28 ,1)= 'COL28'.


compute colslab( 29 ,1)= 'COL29'.


compute colslab( 30 ,1)= 'COL30'.


compute colslab( 31 ,1)= 'COL31'.


compute colslab( 32 ,1)= 'COL32'.


compute colslab( 33 ,1)= 'COL33'.


compute colslab( 34 ,1)= 'COL34'.


compute colslab( 35 ,1)= 'COL35'.


compute colslab( 36 ,1)= 'COL36'.


compute colslab( 37 ,1)= 'COL37'.


compute colslab( 38 ,1)= 'COL38'.


compute colslab( 39 ,1)= 'COL39'.


compute colslab( 40 ,1)= 'COL40'.


compute colslab( 41 ,1)= 'COL41'.


compute colslab( 42 ,1)= 'COL42'.


compute colslab( 43 ,1)= 'COL43'.


compute colslab( 44 ,1)= 'COL44'.


compute colslab( 45 ,1)= 'COL45'.


compute colslab( 46 ,1)= 'COL46'.


compute colslab( 47 ,1)= 'COL47'.


compute colslab( 48 ,1)= 'COL48'.


compute colslab( 49 ,1)= 'COL49'.


compute colslab( 50 ,1)= 'COL50'.


compute colslab( 51 ,1)= 'COL51'.


compute colslab( 52 ,1)= 'COL52'.


compute colslab( 53 ,1)= 'COL53'.


compute colslab( 54 ,1)= 'COL54'.


compute colslab( 55 ,1)= 'COL55'.


compute colslab( 56 ,1)= 'COL56'.


compute colslab( 57 ,1)= 'COL57'.


compute colslab( 58 ,1)= 'COL58'.


compute colslab( 59 ,1)= 'COL59'.


compute colslab( 60 ,1)= 'COL60'.


compute colslab( 61 ,1)= 'COL61'.


compute colslab( 62 ,1)= 'COL62'.


compute colslab( 63 ,1)= 'COL63'.


compute colslab( 64 ,1)= 'COL64'.


compute colslab( 65 ,1)= 'COL65'.


compute colslab( 66 ,1)= 'COL66'.


compute colslab( 67 ,1)= 'COL67'.


compute colslab( 68 ,1)= 'COL68'.


compute colslab( 69 ,1)= 'COL69'.


compute colslab( 70 ,1)= 'COL70'.


compute colslab( 71 ,1)= 'COL71'.


compute colslab( 72 ,1)= 'COL72'.


compute colslab( 73 ,1)= 'COL73'.


compute colslab( 74 ,1)= 'COL74'.


compute colslab( 75 ,1)= 'COL75'.


compute colslab( 76 ,1)= 'COL76'.


compute colslab( 77 ,1)= 'COL77'.


compute colslab( 78 ,1)= 'COL78'.


compute colslab( 79 ,1)= 'COL79'.


compute colslab( 80 ,1)= 'COL80'.


compute colslab( 81 ,1)= 'COL81'.


compute colslab( 82 ,1)= 'COL82'.


compute colslab( 83 ,1)= 'COL83'.


compute colslab( 84 ,1)= 'COL84'.


compute colslab( 85 ,1)= 'COL85'.


compute colslab( 86 ,1)= 'COL86'.


compute colslab( 87 ,1)= 'COL87'.


compute colslab( 88 ,1)= 'COL88'.


compute colslab( 89 ,1)= 'COL89'.


compute colslab( 90 ,1)= 'COL90'.


compute colslab( 91 ,1)= 'COL91'.


compute colslab( 92 ,1)= 'COL92'.


compute colslab( 93 ,1)= 'COL93'.


compute colslab( 94 ,1)= 'COL94'.


compute colslab( 95 ,1)= 'COL95'.


compute colslab( 96 ,1)= 'COL96'.


compute colslab( 97 ,1)= 'COL97'.


compute colslab( 98 ,1)= 'COL98'.


compute colslab( 99 ,1)= 'COL99'.


compute colslab( 100 ,1)= 'COL100'.


compute colslab( 101 ,1)= 'COL101'.


compute colslab( 102 ,1)= 'COL102'.


compute colslab( 103 ,1)= 'COL103'.


compute colslab( 104 ,1)= 'COL104'.


compute colslab( 105 ,1)= 'COL105'.


compute colslab( 106 ,1)= 'COL106'.


compute colslab( 107 ,1)= 'COL107'.


compute colslab( 108 ,1)= 'COL108'.


compute colslab( 109 ,1)= 'COL109'.


compute colslab( 110 ,1)= 'COL110'.


compute colslab( 111 ,1)= 'COL111'.


compute colslab( 112 ,1)= 'COL112'.


compute colslab( 113 ,1)= 'COL113'.


compute colslab( 114 ,1)= 'COL114'.


compute colslab( 115 ,1)= 'COL115'.


compute colslab( 116 ,1)= 'COL116'.


compute colslab( 117 ,1)= 'COL117'.


compute colslab( 118 ,1)= 'COL118'.


compute colslab( 119 ,1)= 'COL119'.


compute colslab( 120 ,1)= 'COL120'.


compute colslab( 121 ,1)= 'COL121'.


compute colslab( 122 ,1)= 'COL122'.


compute colslab( 123 ,1)= 'COL123'.


compute colslab( 124 ,1)= 'COL124'.


compute colslab( 125 ,1)= 'COL125'.


compute colslab( 126 ,1)= 'COL126'.


compute colslab( 127 ,1)= 'COL127'.


compute colslab( 128 ,1)= 'COL128'.


compute colslab( 129 ,1)= 'COL129'.


compute colslab( 130 ,1)= 'COL130'.


compute colslab( 131 ,1)= 'COL131'.


compute colslab( 132 ,1)= 'COL132'.


compute colslab( 133 ,1)= 'COL133'.


compute colslab( 134 ,1)= 'COL134'.


compute colslab( 135 ,1)= 'COL135'.


compute colslab( 136 ,1)= 'COL136'.


compute colslab( 137 ,1)= 'COL137'.


compute colslab( 138 ,1)= 'COL138'.


compute colslab( 139 ,1)= 'COL139'.


compute colslab( 140 ,1)= 'COL140'.


compute colslab( 141 ,1)= 'COL141'.


compute colslab( 142 ,1)= 'COL142'.


compute colslab( 143 ,1)= 'COL143'.


compute colslab( 144 ,1)= 'COL144'.


compute colslab( 145 ,1)= 'COL145'.


compute colslab( 146 ,1)= 'COL146'.


compute colslab( 147 ,1)= 'COL147'.


compute colslab( 148 ,1)= 'COL148'.


compute colslab( 149 ,1)= 'COL149'.


compute colslab( 150 ,1)= 'COL150'.


compute colslab( 151 ,1)= 'COL151'.


compute colslab( 152 ,1)= 'COL152'.


compute colslab( 153 ,1)= 'COL153'.


compute colslab( 154 ,1)= 'COL154'.


compute colslab( 155 ,1)= 'COL155'.


compute colslab( 156 ,1)= 'COL156'.


compute colslab( 157 ,1)= 'COL157'.


compute colslab( 158 ,1)= 'COL158'.


compute colslab( 159 ,1)= 'COL159'.


compute colslab( 160 ,1)= 'COL160'.


compute colslab( 161 ,1)= 'COL161'.


compute colslab( 162 ,1)= 'COL162'.


compute colslab( 163 ,1)= 'COL163'.


compute colslab( 164 ,1)= 'COL164'.


compute colslab( 165 ,1)= 'COL165'.


compute colslab( 166 ,1)= 'COL166'.


compute colslab( 167 ,1)= 'COL167'.


compute colslab( 168 ,1)= 'COL168'.


compute colslab( 169 ,1)= 'COL169'.


compute colslab( 170 ,1)= 'COL170'.


compute colslab( 171 ,1)= 'COL171'.


compute colslab( 172 ,1)= 'COL172'.


compute colslab( 173 ,1)= 'COL173'.


compute colslab( 174 ,1)= 'COL174'.


compute colslab( 175 ,1)= 'COL175'.


compute colslab( 176 ,1)= 'COL176'.


compute colslab( 177 ,1)= 'COL177'.


compute colslab( 178 ,1)= 'COL178'.


compute colslab( 179 ,1)= 'COL179'.


compute colslab( 180 ,1)= 'COL180'.


compute colslab( 181 ,1)= 'COL181'.


compute colslab( 182 ,1)= 'COL182'.


compute colslab( 183 ,1)= 'COL183'.


compute colslab( 184 ,1)= 'COL184'.


compute colslab( 185 ,1)= 'COL185'.


compute colslab( 186 ,1)= 'COL186'.


compute colslab( 187 ,1)= 'COL187'.


compute colslab( 188 ,1)= 'COL188'.


compute colslab( 189 ,1)= 'COL189'.


compute colslab( 190 ,1)= 'COL190'.


compute colslab( 191 ,1)= 'COL191'.


compute colslab( 192 ,1)= 'COL192'.


compute colslab( 193 ,1)= 'COL193'.


compute colslab( 194 ,1)= 'COL194'.


compute colslab( 195 ,1)= 'COL195'.


compute colslab( 196 ,1)= 'COL196'.


compute colslab( 197 ,1)= 'COL197'.


compute colslab( 198 ,1)= 'COL198'.


compute colslab( 199 ,1)= 'COL199'.


compute colslab( 200 ,1)= 'COL200'.


compute colslab( 201 ,1)= 'COL201'.


compute colslab( 202 ,1)= 'COL202'.


compute colslab( 203 ,1)= 'COL203'.


compute colslab( 204 ,1)= 'COL204'.


compute colslab( 205 ,1)= 'COL205'.


compute colslab( 206 ,1)= 'COL206'.


compute colslab( 207 ,1)= 'COL207'.


compute colslab( 208 ,1)= 'COL208'.


compute colslab( 209 ,1)= 'COL209'.


compute colslab( 210 ,1)= 'COL210'.


compute colslab( 211 ,1)= 'COL211'.


compute colslab( 212 ,1)= 'COL212'.


compute colslab( 213 ,1)= 'COL213'.


compute colslab( 214 ,1)= 'COL214'.


compute colslab( 215 ,1)= 'COL215'.


compute colslab( 216 ,1)= 'COL216'.


compute colslab( 217 ,1)= 'COL217'.


compute colslab( 218 ,1)= 'COL218'.


compute colslab( 219 ,1)= 'COL219'.


compute colslab( 220 ,1)= 'COL220'.


compute colslab( 221 ,1)= 'COL221'.


compute colslab( 222 ,1)= 'COL222'.


compute colslab( 223 ,1)= 'COL223'.


compute colslab( 224 ,1)= 'COL224'.


compute colslab( 225 ,1)= 'COL225'.


compute colslab( 226 ,1)= 'COL226'.


compute colslab( 227 ,1)= 'COL227'.


compute colslab( 228 ,1)= 'COL228'.


compute colslab( 229 ,1)= 'COL229'.


compute colslab( 230 ,1)= 'COL230'.


compute colslab( 231 ,1)= 'COL231'.


compute colslab( 232 ,1)= 'COL232'.


compute colslab( 233 ,1)= 'COL233'.


compute colslab( 234 ,1)= 'COL234'.


compute colslab( 235 ,1)= 'COL235'.


compute colslab( 236 ,1)= 'COL236'.


compute colslab( 237 ,1)= 'COL237'.


compute colslab( 238 ,1)= 'COL238'.


compute colslab( 239 ,1)= 'COL239'.


compute colslab( 240 ,1)= 'COL240'.


compute colslab( 241 ,1)= 'COL241'.


compute colslab( 242 ,1)= 'COL242'.


compute colslab( 243 ,1)= 'COL243'.


compute colslab( 244 ,1)= 'COL244'.


compute colslab( 245 ,1)= 'COL245'.


compute colslab( 246 ,1)= 'COL246'.


compute colslab( 247 ,1)= 'COL247'.


compute colslab( 248 ,1)= 'COL248'.


compute colslab( 249 ,1)= 'COL249'.


compute colslab( 250 ,1)= 'COL250'.


compute colslab( 251 ,1)= 'COL251'.


compute colslab( 252 ,1)= 'COL252'.


compute colslab( 253 ,1)= 'COL253'.


compute colslab( 254 ,1)= 'COL254'.


compute colslab( 255 ,1)= 'COL255'.


compute colslab( 256 ,1)= 'COL256'.


compute colslab( 257 ,1)= 'COL257'.


compute colslab( 258 ,1)= 'COL258'.


compute colslab( 259 ,1)= 'COL259'.


compute colslab( 260 ,1)= 'COL260'.


compute colslab( 261 ,1)= 'COL261'.


compute colslab( 262 ,1)= 'COL262'.


compute colslab( 263 ,1)= 'COL263'.


compute colslab( 264 ,1)= 'COL264'.


compute colslab( 265 ,1)= 'COL265'.


compute colslab( 266 ,1)= 'COL266'.


compute colslab( 267 ,1)= 'COL267'.


compute colslab( 268 ,1)= 'COL268'.


compute colslab( 269 ,1)= 'COL269'.


compute colslab( 270 ,1)= 'COL270'.


compute colslab( 271 ,1)= 'COL271'.


compute colslab( 272 ,1)= 'COL272'.


compute colslab( 273 ,1)= 'COL273'.


compute colslab( 274 ,1)= 'COL274'.


compute colslab( 275 ,1)= 'COL275'.


compute colslab( 276 ,1)= 'COL276'.


compute colslab( 277 ,1)= 'COL277'.


compute colslab( 278 ,1)= 'COL278'.


compute colslab( 279 ,1)= 'COL279'.


compute colslab( 280 ,1)= 'COL280'.


compute colslab( 281 ,1)= 'COL281'.


compute colslab( 282 ,1)= 'COL282'.


compute colslab( 283 ,1)= 'COL283'.


compute colslab( 284 ,1)= 'COL284'.


compute colslab( 285 ,1)= 'COL285'.


compute colslab( 286 ,1)= 'COL286'.


compute colslab( 287 ,1)= 'COL287'.


compute colslab( 288 ,1)= 'COL288'.


compute colslab( 289 ,1)= 'COL289'.


compute colslab( 290 ,1)= 'COL290'.


compute colslab( 291 ,1)= 'COL291'.


compute colslab( 292 ,1)= 'COL292'.


compute colslab( 293 ,1)= 'COL293'.


compute colslab( 294 ,1)= 'COL294'.


compute colslab( 295 ,1)= 'COL295'.


compute colslab( 296 ,1)= 'COL296'.


compute colslab( 297 ,1)= 'COL297'.


compute colslab( 298 ,1)= 'COL298'.


compute colslab( 299 ,1)= 'COL299'.


compute colslab( 300 ,1)= 'COL300'.

compute colslab=colslab(1:ncol(bootres),1).
compute colslab={colslab,conseq,vlabs}.
do if (outscree=1).
print/title = '**************************************************************************'.
print/title='Bootstrap estimates were saved to a file'/space=0.
print colslab/title='Map of column names to model coefficients:'/clabels=' ','Conseqnt','Antecdnt'/format=a8.
end if.
end if.
end if.
do if (criterr=0 and boot > 0 and modelbt=1).
compute labstart=1.
do if (outscree=1).
print/title = '*********** BOOTSTRAP RESULTS FOR REGRESSION MODEL PARAMETERS ************'.
end if.
loop iboot = 1 to (nms+nys).
do if (outscree=1).
print outnames(1,iboot)/title = 'OUTCOME VARIABLE:'/format = A8.
end if.
compute vlabsm=vlabs(labstart:(labstart+(nump(1,iboot)-1)),1).
compute outnmtmp=bootcim(labstart:(labstart+(nump(1,iboot)-1)),:).
compute resultm2=make(nrow(outnmtmp),maxresm,99999).
compute resultm2(1:nrow(outnmtmp),1:ncol(outnmtmp))=outnmtmp.
compute resultm={resultm;resultm2}.
do if (outscree=1).
print bootcim(labstart:(labstart+(nump(1,iboot)-1)),:)/title=' '/rnames=vlabsm/ clabels='Coeff' 'BootMean' 'BootSE' 'BootLLCI' 'BootULCI'/format= F10.4 /space=0.
end if.
compute labstart=labstart+nump(1,iboot).
do if (iboot < (nms+nys)).
do if (outscree=1).
print/title= '----------'.
end if.
end if.
end loop.
end if.
do if (criterr=0 and saveest=1).
compute resultm=resultm(2:nrow(resultm),:).
compute bocaj=(resultm=99999).
compute bocaj=csum(bocaj).
compute bocaj=1-(bocaj=nrow(resultm)).
compute j=1.
loop i = 1 to ncol(resultm).
do if (bocaj(1,i)=1).
compute j=j+1.
end if.
end loop.
compute resultm=resultm(:,1:(j-1)).
save resultm/outfile = *.
end if.
do if (criterr = 0 and matrices=1 and outscree=1).
print/title = '************************ MODEL DEFINITION MATRICES ************************'.
print/title = 'FROM variables are columns, TO variables are rows.'.
compute temp2=make(nrow(bcmat),ncol(bcmat),'0').
loop i = 2 to nrow(bcmat).
loop j = 1 to (ncol(bcmat)-1).
do if (bcmat(i,j)=1).
compute temp2(i,j)='1'.
end if.
do if (j >= i).
compute temp2(i,j)=' '.
end if.
end loop.
end loop.
compute temp2=temp2(2:nrow(bcmat),(1:(ncol(bcmat)-1))).
do if (nms > 0).
compute cmatlabs={xnames,mnames}.
compute rmatlabs={mnames,ynames}.
end if.
do if (nms = 0).
compute cmatlabs={xnames}.
compute rmatlabs={ynames}.
end if.
print temp2/title='BMATRIX: Paths freely estimated (1) and fixed to zero (0):'/cnames=cmatlabs/rnames=rmatlabs/format A3.
compute z=0.
do if (rsum(csum(wcmat))<>0).
compute temp2=make(nrow(wcmat),ncol(wcmat),'0').
loop i = 2 to nrow(wcmat).
loop j = 1 to (ncol(wcmat)-1).
do if (wcmat(i,j)=1).
compute temp2(i,j)='1'.
end if.
do if (j >= i).
compute temp2(i,j)=' '.
end if.
end loop.
end loop.
compute temp2=temp2(2:nrow(wcmat),(1:(ncol(wcmat)-1))).
print temp2/title='WMATRIX: Paths moderated (1) and not moderated (0) by W:'/cnames=cmatlabs/rnames=rmatlabs/format A3.
end if.
do if (rsum(csum(zcmat))<>0).
compute temp2=make(nrow(zcmat),ncol(zcmat),'0').
loop i = 2 to nrow(zcmat).
loop j = 1 to (ncol(zcmat)-1).
do if (zcmat(i,j)=1).
compute temp2(i,j)='1'.
end if.
do if (j >= i).
compute temp2(i,j)=' '.
end if.
end loop.
end loop.
compute temp2=temp2(2:nrow(zcmat),(1:(ncol(zcmat)-1))).
print temp2/title='ZMATRIX: Paths moderated (1) and not moderated (0) by Z:'/cnames=cmatlabs/rnames=rmatlabs/format a3.
end if.
do if (rsum(csum(wzcmat))<>0).
compute temp2=make(nrow(wzcmat),ncol(wzcmat),'0').
loop i = 2 to nrow(wzcmat).
loop j = 1 to (ncol(wzcmat)-1).
do if (wzcmat(i,j)=1).
compute temp2(i,j)='1'.
end if.
do if (j >= i).
compute temp2(i,j)=' '.
end if.
end loop.
end loop.
compute temp2=temp2(2:nrow(wzcmat),(1:(ncol(wzcmat)-1))).
print temp2/title='WZMATRIX: W moderated paths moderated (1) and not moderated (0) by Z:'/cnames=cmatlabs/rnames=rmatlabs/format a3.
end if.
do if (ncs > 0).
print ccmat/title='CMATRIX: Covariates (columns) in (1) and not in (0) the models of M and Y (rows):'/rnames=rmatlabs/cnames=covnames.
end if.
end if.
do if (outscree=1).
do if (activate=0).
print/title = '*********************** ANALYSIS NOTES AND ERRORS ************************'.
end if.
end if.
do if (activate=1).
print/title = '**************************************************************************'.
end if.
do if (criterr=0).
do if (outscree=1).
print conf/title = 'Level of confidence for all confidence intervals in output:'/format = F8.4.
do if (boot > 0).
do if (goodboot = boot and bc=0).
print boot/title='Number of bootstrap samples for percentile bootstrap confidence intervals:'.
end if.
do if (goodboot = boot and bc=1).
print boot/title='Number of bootstrap samples for bias-corrected bootstrap confidence intervals:'.
end if.
do if (booterr = 1).
compute badend = badend(1,2:ncol(badend)).
print/title = 'WARNING: Bootstrap CI endpoints below not trustworthy. Decrease confidence'.
print badend/title='or increase the number of bootstrap samples.'/space=0/format = F10.4.
end if.
end if.
do if (mc > 0).
print mc/title='Number of samples for Monte Carlo confidence intervals:'.
end if.
do if (wnotev > 0 and printw=1).
do if (wnotev=1).
print/title = 'W values in conditional tables are the 16th, 50th, and 84th percentiles.'.
else if (wnotev=2).
do if (minwwarn=0 and maxwwarn=0).
print/title = 'W values in conditional tables are the mean and +/- SD from the mean.'.
end if.
do if (minwwarn=1).
print/title = 'W values in conditional tables are the minimum, the mean, and 1 SD above the mean.'.
end if.
do if (maxwwarn=1).
print/title = 'W values in conditional tables are 1 SD below the mean, the mean, and the maximum.'.
end if.
end if.
end if.
do if (znotev > 0 and printz=1).
do if (znotev=1).
print/title = 'Z values in conditional tables are the 16th, 50th, and 84th percentiles.'.
else if (znotev=2).
do if (minzwarn=0 and maxzwarn=0).
print/title = 'Z values in conditional tables are the mean and +/- SD from the mean.'.
end if.
do if (minzwarn=1).
print/title = 'Z values in conditional tables are the minimum, the mean, and 1 SD above the mean.'.
end if.
do if (maxzwarn=1).
print/title = 'Z values in conditional tables are 1 SD below the mean, the mean, and the maximum.'.
end if.
end if.
end if.
do if (minwwarn > 0).
print/title = 'NOTE: One SD below the mean is below the minimum observed in the data for W,'.
print/title = '      so the minimum measurement on W is used for conditioning instead.'/space=0.
end if.
do if (maxwwarn > 0).
print/title = 'NOTE: One SD above the mean is above the maximum observed in the data for W,'.
print/title = '      so the maximum measurement for W is used for conditioning instead.'/space=0.
end if.
do if (minzwarn > 0).
print/title = 'NOTE: One SD below the mean is below the minimum observed in the data for Z,'.
print/title = '      so the minimum measurement for Z is used for conditioning instead.'/space=0.
end if.
do if (maxzwarn > 0).
print/title = 'NOTE: One SD above the mean is above the maximum observed in the data for Z,'.
print/title = '      so the maximum measurement for Z is used for conditioning instead.'/space=0.
end if.
do if (pstog=1).
print/title= 'NOTE: Standardized coefficients for dichotomous or multicategorical X are in'.
print/title= '      partially standardized form.'/space=0.
end if.
loop i = 1 to 100.
do if (notecode(i,1) = 32).
print/title = 'Direct, indirect, and total effects are counterfactually defined'.
do if (xcontcf=0).
print/title = 'assuming X by M interaction.'/space=0.
end if.
do if (xcontcf=1).
print/title = 'assuming X by M interaction and with the following reference (x_ref)'/space=0.
compute states=t(xrefvals).
print states/title = 'and counterfactual (x_cf) states for X:'/rlabels='x_ref' 'x_cf'/format= F10.4 /space=0.
end if.
end if.
do if (notecode(i,1)=1).
print/title = 'NOTE: COVMY is ignored when using CMATRIX option.'.
end if.
do if (notecode(i,1)=2).
print/title = 'NOTE: Confidence level restricted to between 50 and 99.9999%.  95% confidence is provided in output'.
end if.
do if (notecode(i,1)=3).
print centvar/title = 'NOTE: The following variables were mean centered prior to analysis:'/format = a8.
end if.
do if (notecode(i,1) = 4).
print/title = 'NOTE: A heteroscedasticity consistent standard error and covariance matrix estimator was used.'.
end if.
do if (notecode(i,1) = 5).
print/title = 'NOTE: The HC3 option has been replaced with HC.  See the documentation.'.
end if.
do if (notecode(i,1) = 6).
print/title = 'NOTE: Due to estimation problems, some bootstrap samples had to be replaced.'.
print badboot/title='      The number of times this happened was:'/space=0/format=F8.0.
end if.
do if (notecode(i,1) = 7).
print/title = 'NOTE: The bootstrapping was not completed due to problematic bootstrap samples.'.
print/title = '      Bootstrap confidence intervals are therefore suppressed.'/space=0.
end if.
do if (notecode(i,1) = 8).
print/title = 'NOTE: The number of bootstrap samples was adjusted upward given your desired confidence.'.
end if.
do if (notecode(i,1) = 9).
print/title = 'NOTE: WMODVAL is ignored when W is specified as multicategorical.'.
end if.
do if (notecode(i,1) = 10).
print/title = 'NOTE: ZMODVAL is ignored when Z is specified as multicategorical.'.
end if.
do if (notecode(i,1) = 11).
print/title = 'NOTE: Total effect model generated only when all covariates are specified in all'.
print/title = '      models of M and Y.'/space=0.
end if.
do if (notecode(i,1) = 30).
print/title = 'NOTE: Your vector of linear hypothesis weights is of the wrong length for this model.'.
end if.
do if (notecode(i,1) = 12).
print/title = 'NOTE: Total effect model generated only when X is freely estimated to affect each M'.
print/title = '      and both X and M are freely estimated to affect Y'/space=0.
end if.
do if (notecode(i,1) = 13).
print/title = 'NOTE: There are too many pairwise contrasts to conduct with this model.'.
end if.
do if (notecode(i,1) = 14).
print/title = 'NOTE: The number of contrast weights must equal the number of indirect effects.'.
end if.
do if (notecode(i,1) = 15).
print/title = 'NOTE: Monte Carlo confidence intervals not available for this model.'.
print/title = '      Bootstrapping is used instead.'/space=0.
end if.
do if (notecode(i,1) = 16).
print/title = 'NOTE: The number of Monte Carlo samples was adjusted upward given your desired confidence.'.
end if.
do if (notecode(i,1) = 19).
print/title = 'NOTE: Your contrast matrix is invalid or not applicable to this model.'.
end if.
do if (notecode(i,1) = 20).
print/title = 'NOTE: One of the groups specified by your contrast matrix does not exist in the data.'.
end if.
do if (notecode(i,1) = 21).
print/title = 'NOTE: The VARORDER option is not available in this release.'.
end if.
do if (notecode(i,1) = 22).
print/title = 'NOTE: The VMODVAL and QMODVAL options are not available in this release.'.
end if.
do if (notecode(i,1) = 23).
print/title = 'NOTE: The QUANTILE option is not available in this release.'.
end if.
do if (notecode(i,1) = 24).
print/title = 'NOTE: Total effect model not available with dichotomous Y.'.
end if.
do if (notecode(i,1) = 25).
print/title = 'NOTE: STAND/EFFSIZE options not available with dichotomous Y.'.
end if.
do if ((notecode(i,1) = 26) and nms > 0).
print/title = 'NOTE: Direct and indirect effects of X on Y are on a log-odds metric.'.
end if.
do if (notecode(i,1) = 27).
print/title = 'NOTE: Standardized coefficients are not available for models with moderators.'.
end if.
do if (notecode(i,1) = 28).
print/title = 'NOTE: The contrast option is not available with a multicategorical X.'.
end if.
do if (notecode(i,1) = 31).
do if (nms > 1).
print medmeans/title = 'NOTE: Controlled direct effect(s) estimated at the following mediator values:'/cnames=mnames/format= F10.4.
end if.
do if (nms=1).
print medmeans/title = 'NOTE: Controlled direct effect(s) estimated at the following mediator value:'/format= F10.4.
end if.
end if.
do if (notecode(i,1) = 33).
print/title = 'NOTE: Sobel test is not available with the XMINT option.'.
end if.
do if (notecode(i,1) = 37).
print/title = 'NOTE: The CONTRAST option is not available when using the XMINT option.'.
end if.
do if (notecode(i,1) = 34).
print/title = 'NOTE: Standardized effects are not available when using the XMINT option.'.
end if.
do if (notecode(i,1) = 36).
print/title = 'NOTE: The XREFVAL option is ignored when X is declared as multicategorical.'.
end if.
do if (notecode(i,1) = 35).
print coval/title = 'NOTE: Counterfactual effects estimated at the following covariate values:'/cnames=covnames/format= F10.4.
end if.
do if (notecode(i,1) = 29 and (listmiss=1)).
compute a=missrow.
compute conum=ncol(a).
compute allgood=0.
compute smremain=12.
compute largesti=1.
compute smallrow=0.
do if (conum > 12).
loop ii = 1 to 12.
compute check=(conum/ii).
do if (check = trunc(check)).
compute check2=conum/ii.
compute aok=ii.
do if (aok > 2).
compute allgood=1.
end if.
else.
compute remain=conum-(ii*trunc(check)).
do if (remain <= smremain).
compute smremain=remain.
compute largesti=ii.
compute smallrow=trunc(conum/largesti).
end if.
end if.
end loop.
compute atemp=a(1,1:(aok*check2)).
compute atemp=reshape(atemp,check2,aok).
do if (ncol(atemp) > 2).
print/title='NOTE: Missing data resulted in the deletion of the following row(s) of'.
print atemp/title='      data from the analysis:'/format=F6.0/space=0.
end if.
do if (allgood=0).
compute atemp=a(1,1:(smallrow*largesti)).
compute atemp=reshape(atemp,smallrow,largesti).
compute btemp=a(1,((largesti*smallrow)+1):conum).
print/title='NOTE: Missing data resulted in the deletion of the following row(s) of'.
print atemp/title='      data from the analysis:'/format=F6.0/space=0.
print btemp/title=' '/space=0/format=f6.0.
end if.
else if (conum <= 12).
print/title='NOTE: Missing data resulted in the deletion of the following row(s) of'.
print a/title='      data from the analysis:'/format=F6.0/space=0.
end if.
end if.
end loop.
do if (toomany=1).
print/title='WARNING: Variables names longer than eight characters can produce incorrect output'.
print/title='when some variables in the data file have the same first eight characters. Shorter'/space=0.
print/title='variable names are recommended. By using this output, you are accepting all risk'/space=0.
print/title='and consequences of interpreting or reporting results that may be incorrect.'/space=0.
end if.
end if.
end if.
loop i = 1 to 100.
do if (errcode(i,1)=1).
print/title = 'ERROR: You must specify a Y and an X variable.'.
end if.
do if (errcode(i,1)=2).
print/title = 'ERROR: X, M, or Y variable used more than once or W and Z are the same variable.'.
do if (toomany = 1).
print/title = '       This could be caused by the use of variables names longer'/space=0.
print varnames/title = '       than eight characters. Here are the variables I see:'/space=0/format=A8.
end if.
end if.
do if (errcode(i,1)=3).
print/title = 'ERROR: You have specified more than one variable for W, Y, X, or Z'.
end if.
do if (errcode(i,1)=4).
print/title = 'ERROR: A variable specified as multicategorical has more than nine categories.'.
end if.
do if (errcode(i,1)=5).
print/title = 'ERROR: One of the categories contains only a single case.'.
end if.
do if (errcode(i,1)=6).
print/title = 'ERROR: Invalid model number in this version of PROCESS.'.
end if.
do if (errcode(i,1)=7).
print/title = 'ERROR: Invalid model number.'.
end if.
do if (errcode(i,1)=8).
print/title = 'ERROR: You must specify an M variable for this model.'.
end if.
do if (errcode(i,1)=9).
print/title = 'ERROR: You have specified an M variable in a model that does not use it.'.
end if.
do if (errcode(i,1)=10).
print/title = 'ERROR: You have specified a W variable in a model that does not use it.'.
end if.
do if (errcode(i,1)=11).
print/title = 'ERROR: You have not specified a W variable in a model that requires it.'.
end if.
do if (errcode(i,1)=12).
print/title = 'ERROR: You have specified a Z variable in a model that does not use it.'.
end if.
do if (errcode(i,1)=13).
print/title = 'ERROR: You have not specified a Z variable in a model that requires it.'.
end if.
do if (errcode(i,1)=14).
print/title = 'ERROR: V and Q are not proper specifications in this release of PROCESS.'.
print/title = '       Moderators must be specified as W and/or Z.'/space=0.
end if.
do if (errcode(i,1)=15).
print/title = 'ERROR: One of your model variables exhibits no variation (it is a constant).'.
end if.
do if (errcode(i,1)=16).
print/title = 'ERROR: BMATRIX is not the correct length or is otherwise invalid.'.
end if.
do if (errcode(i,1)=17).
print/title = 'ERROR: WMATRIX is not the correct length or is otherwise invalid.'.
end if.
do if (errcode(i,1)=18).
print/title = 'ERROR: ZMATRIX is not the correct length or is otherwise invalid.'.
end if.
do if (errcode(i,1)=19).
print/title = 'ERROR: WZMATRIX is not the correct length or is otherwise invalid.'.
end if.
do if (errcode(i,1)=20).
print/title = 'ERROR: A path fixed at zero cannot be moderated.'.
end if.
do if (errcode(i,1)=21).
print/title = 'ERROR: If only one moderator is specified, it must be specified as W.'.
end if.
do if (errcode(i,1)=22).
print/title = 'ERROR: In BMATRIX, X must be specified to affect at least one variable.'.
end if.
do if (errcode(i,1)=23).
print/title = 'ERROR: In BMATRIX, at least one variable must be specified to affect Y.'.
end if.
do if (errcode(i,1)=24).
print/title = 'ERROR: You must specify a model number or a custom BMATRIX specification.'.
end if.
do if (errcode(i,1)=25).
print/title = 'ERROR: BMATRIX cannot be used in conjunction with a model number.'.
end if.
do if (errcode(i,1)=26).
print/title = 'ERROR: Your model has a dangling mediator (all Ms must affect and be affected).'.
end if.
do if (errcode(i,1)=27).
print/title = 'ERROR: CLUSTER is not available on this release of PROCESS.'.
end if.
do if (errcode(i,1)=29).
print/title = 'ERROR: CMATRIX is not the correct length or is otherwise invalid.'.
end if.
do if (errcode(i,1)=30).
print/title = 'ERROR: In CMATRIX, all covariates must be assigned to an M or a Y.'.
end if.
do if (errcode(i,1)=31).
print/title = 'ERROR: A linear or near linear dependency (singularity) exists in the data.'.
end if.
do if (errcode(i,1)=32).
print/title = 'ERROR: Models 80 and 81 require between 3 and 6 mediators.'.
end if.
do if (errcode(i,1)=33).
print/title = 'ERROR: Model 82 requires 4 mediators.'.
end if.
do if (errcode(i,1)=34).
print/title = 'ERROR: This model number requires between 2 and 6 mediators.'.
end if.
do if (errcode(i,1)=35).
print/title = 'ERROR: In a model with only one moderator, that moderator must be W.'.
end if.
do if (errcode(i,1)=36).
print/title = 'ERROR: A serial mediation model cannot have more than 6 mediators.'.
end if.
do if (errcode(i,1)=37).
print/title = 'ERROR: No more than 10 mediators are allowed in a PROCESS command.'.
end if.
do if (errcode(i,1)=38).
print/title = 'ERROR: XCATCODE is not provided, not the correct length, or is otherwise invalid.'.
end if.
do if (errcode(i,1)=39).
print/title = 'ERROR: WCATCODE is not provided, not the correct length, or is otherwise invalid.'.
end if.
do if (errcode(i,1)=40).
print/title = 'ERROR: ZCATCODE is not provided, not the correct length, or is otherwise invalid.'.
end if.
do if (errcode(i,1)=41).
print/title = 'ERROR: Models 1, 2, and 3 cannot be customized.'.
end if.
do if (errcode(i,1)=42).
print/title = 'ERROR: WS option available only in PROCESS v2. Or use the MEMORE macro instead.'.
print/title = '       MEMORE can be downloaded from www.akmontoya.com.'/space=0.
end if.
do if (errcode(i,1)=43).
print/title = 'ERROR: PROCESS does not allow dichotomous mediators.'.
end if.
do if (errcode(i,1)=60).
print/title = 'PROCESS is now ready for use.'.
print/title='Copyright 2017-2022 by Andrew F. Hayes. ALL RIGHTS RESERVED.'/space=0.
print/title='Workshop schedule available at http://haskayne.ucalgary.ca/CCRAM'/space=0.
end if.
do if (errcode(i,1)=50).
print/title = 'ERROR: A multicategorical moderator cannot be specified as a covariate.'.
end if.
do if (errcode(i,1)=51).
print/title = 'ERROR: A variable you specified as a covariate is a moderator in all equations.'.
end if.
do if (errcode(i,1)=52 and mcerpt=0).
compute mcerpt=1.
print/title = 'ERROR: A variable specified as multicategorical must have at least three categories'.
end if.
do if (errcode(i,1) = 47).
do if (iterrmod=1).
print/title = 'ERROR: Iteration for Y model didn''t converge to a solution. Interpret results with caution.'.
print/title = '       Try increasing the number of iterations, though this will slow down computation.'/space=0.
end if.
do if (bootiter=1).
print/title = 'ERROR: Nonconvergence during bootstrapping. Interpet bootstrap results with caution.'.
end if.
end if.
do if (errcode(i,1) = 61).
print/title = 'ERROR: Variable names should be no more than eight characters in length.'.
end if.
do if (errcode(i,1) = 62).
print/title = 'ERROR: After listwise deletion of cases with missing data, too few cases remain.'.
end if.
do if (errcode(i,1) = 63).
print/title = 'ERROR: The XMINT option is available only for model 4.'.
end if.
do if (errcode(i,1) = 64).
print/title = 'ERROR: Incorrect number of values specified in CDEVAL option.'.
end if.
do if (errcode(i,1) = 65).
print/title = 'ERROR: Only indicator or sequential coding of X is allowed with the XMINT option.'.
end if.
do if (errcode(i,1) = 66).
print/title = 'ERROR: XMINT option not available with continuous X using PROCESS GUI. Use syntax.'.
end if.
do if (errcode(i,1) = 67).
print/title = 'ERROR: Too many elements provided in XREFVAL option.'.
end if.
do if (errcode(i,1)=68).
print/title = 'ERROR: Covariate assignment is not allowed with XMINT option.'.
end if.
do if (errcode(i,1) = 69).
print/title = 'ERROR: Incorrect number of values specified in COVAL option.'.
end if.
do if (errcode(i,1) = 70).
print/title = 'ERROR: Incorrect value(s) in XREFVAL for this dichotomous X variable.'.
end if.
do if (errcode(i,1) = 71).
print/title = 'ERROR: The CENTER option is not available when using the XMINT option.'.
end if.
do if (errcode(i,1) = 72).
print/title = 'ERROR: The XMINT option is not available for models with a dichotomous Y.'.
end if.
end loop.
END MATRIX.	
Ressources	Temps de processeur	00:00:06,31	
	Temps écoulé	00:00:06,29	


Run MATRIX procedure:

***************** PROCESS Procedure for SPSS Version 4.2 *****************

          Written by Andrew F. Hayes, Ph.D.       www.afhayes.com
    Documentation available in Hayes (2022). www.guilford.com/p/hayes3

**************************************************************************
Model  : 4
    Y  : HOMAIR
    X  : Starchy
   M1  : TG
   M2  : HDL
   M3  : LDL
   M4  : TC

Sample
Size:  169

**************************************************************************
OUTCOME VARIABLE:
 TG

Model Summary
          R       R-sq        MSE          F        df1        df2          p
      ,0370      ,0014  4046,1183      ,2284     1,0000   167,0000      ,6333

Model
              coeff         se          t          p       LLCI       ULCI
constant    95,6000    16,4238     5,8208      ,0000    63,1749   128,0251
Starchy     -8,2234    17,2051     -,4780      ,6333   -42,1909    25,7441

Standardized coefficients
             coeff
Starchy     -,1296

**************************************************************************
OUTCOME VARIABLE:
 HDL

Model Summary
          R       R-sq        MSE          F        df1        df2          p
      ,0038      ,0000   178,1333      ,0024     1,0000   167,0000      ,9613

Model
              coeff         se          t          p       LLCI       ULCI
constant    41,0000     3,4461    11,8975      ,0000    34,1965    47,8035
Starchy       ,1753     3,6100      ,0486      ,9613    -6,9518     7,3025

Standardized coefficients
             coeff
Starchy      ,0132

**************************************************************************
OUTCOME VARIABLE:
 LDL

Model Summary
          R       R-sq        MSE          F        df1        df2          p
      ,1661      ,0276  1157,2315     4,7361     1,0000   167,0000      ,0309

Model
              coeff         se          t          p       LLCI       ULCI
constant    58,0667     8,7834     6,6109      ,0000    40,7258    75,4076
Starchy     20,0242     9,2013     2,1762      ,0309     1,8584    38,1900

Standardized coefficients
             coeff
Starchy      ,5822

**************************************************************************
OUTCOME VARIABLE:
 TC

Model Summary
          R       R-sq        MSE          F        df1        df2          p
      ,1211      ,0147  1430,6413     2,4852     1,0000   167,0000      ,1168

Model
              coeff         se          t          p       LLCI       ULCI
constant   121,0667     9,7661    12,3967      ,0000   101,7858   140,3475
Starchy     16,1281    10,2306     1,5765      ,1168    -4,0699    36,3262

Standardized coefficients
             coeff
Starchy      ,4245

**************************************************************************
OUTCOME VARIABLE:
 HOMAIR

Model Summary
          R       R-sq        MSE          F        df1        df2          p
      ,3917      ,1534    22,6985     5,9077     5,0000   163,0000      ,0000

Model
              coeff         se          t          p       LLCI       ULCI
constant     2,1586     1,9594     1,1017      ,2722    -1,7104     6,0276
Starchy     -2,5894     1,3089    -1,9784      ,0496    -5,1739     -,0049
TG            ,0439      ,0096     4,5586      ,0000      ,0249      ,0629
HDL           ,1279      ,0427     2,9941      ,0032      ,0436      ,2123
LDL           ,0992      ,0355     2,7967      ,0058      ,0292      ,1693
TC           -,0907      ,0351    -2,5807      ,0107     -,1600     -,0213

Standardized coefficients
             coeff
Starchy     -,5077
TG           ,5462
HDL          ,3338
LDL          ,6691
TC          -,6753

************************** TOTAL EFFECT MODEL ****************************
OUTCOME VARIABLE:
 HOMAIR

Model Summary
          R       R-sq        MSE          F        df1        df2          p
      ,1344      ,0181    25,6969     3,0724     1,0000   167,0000      ,0815

Model
              coeff         se          t          p       LLCI       ULCI
constant     6,3858     1,3089     4,8789      ,0000     3,8017     8,9699
Starchy     -2,4033     1,3711    -1,7528      ,0815    -5,1103      ,3036

Standardized coefficients
             coeff
Starchy     -,4712


************** TOTAL, DIRECT, AND INDIRECT EFFECTS OF X ON Y **************

Total effect of X on Y
     Effect         se          t          p       LLCI       ULCI       c_ps
    -2,4033     1,3711    -1,7528      ,0815    -5,1103      ,3036     -,4712

Direct effect of X on Y
     Effect         se          t          p       LLCI       ULCI      c'_ps
    -2,5894     1,3089    -1,9784      ,0496    -5,1739     -,0049     -,5077

Indirect effect(s) of X on Y:
          Effect     BootSE   BootLLCI   BootULCI
TOTAL      ,1861      ,6824    -1,1216     1,5392
TG        -,3610      ,8071    -2,2085     1,1258
HDL        ,0224      ,5571    -1,1118     1,1688
LDL       1,9868     2,3972      ,2359     8,5156
TC       -1,4622     2,4362    -8,4699     -,0115

Partially standardized indirect effect(s) of X on Y:
          Effect     BootSE   BootLLCI   BootULCI
TOTAL      ,0365      ,1335     -,2107      ,3130
TG        -,0708      ,1599     -,4366      ,2048
HDL        ,0044      ,1080     -,2051      ,2249
LDL        ,3895      ,4579      ,0510     1,5987
TC        -,2867      ,4630    -1,5640     -,0024

*********************** ANALYSIS NOTES AND ERRORS ************************

Level of confidence for all confidence intervals in output:
  95,0000

Number of bootstrap samples for percentile bootstrap confidence intervals:
  5000

NOTE: Standardized coefficients for dichotomous or multicategorical X are in
      partially standardized form.

------ END MATRIX -----


* Encoding: UTF-8.
preserve.
set printback=off.


Matrice


Remarques	
Sortie obtenue	10-NOV-2024 17:05:05	
Commentaires		
Entrée	Données	C:\Users\Brice SAHA\Documents\UBa\Master Thesis 2021\Fanuel\Database fanuel.sav	
	Jeu de données actif	Jeu_de_données1	
	Filtre	<sans>	
	Pondération	<sans>	
	Scinder un fichier	<sans>	
	N de lignes dans le fichier de travail	169	
Syntaxe	MATRIX.
compute wnames='xxxxx'.
compute znames='xxxxx'.
compute mcerpt=0.
compute wiscov=0.
compute ziscov=0.
compute tooman=0.
compute errcode=make(100,1,0).
compute notecode=make(100,1,0).
compute model = trunc( 4 ).
compute iterate = abs(trunc( 100 )).
compute converge = abs( 0.00001 ).
compute itprobtg=0.
compute v2tag=0.
compute ydich=0.
compute maxwwarn=0.
compute minwwarn=0.
compute maxzwarn=0.
compute minzwarn=0.
compute toomany=0.
compute wdich=0.
compute zdich=0.
compute wnotev=0.
compute znotev=0.
compute nxpval=1.
compute nwpval=1.
compute nzpval=1.
compute errs=1.
compute notes=1.
compute criterr=0.
compute novar=0.
compute adjust=0.
compute ncs=0.
compute serial=0.
compute sobelok=0.
compute hasw=0.
compute hasz=0.
compute printw=0.
compute printz=0.
compute xmint=( 0 =1).
compute wmodcust=0.
compute zmodcust=0.
compute booting=0.
compute bootiter=0.
compute iterrmod=0.
compute cov = 'xxxxx'.
compute varorder=( 0 <> 0).
compute nws=0.
compute w= 'xxxxx'.
compute nzs=0.
compute z = 'xxxxx'.
compute nms=0.
compute m = 'TG HDL LDL TC'.
compute nys=0.
compute y = 'HOMAIR'.
compute nxs=0.
compute x = 'Veg'.
compute effsize=( 0 =1).
compute stand=( 1 =1).
compute intprobe = .1.
compute xrefvals={ 999 }.
compute center=trunc( 0 ).
compute xcontcf=0.
compute xscaling=1.
compute cdeval={ -999 }.
compute cuscoval=0.
do if (model=74).
compute errcode(errs,1)=7.
compute errs=errs+1.
compute criterr=1.
end if.
do if (xmint=1 and model <> 4).
compute errcode(errs,1)=63.
compute errs=errs+1.
compute criterr=1.
end if.
do if (xmint=1 and model=4).
compute w=x.
compute model=74.
compute intprobe=1.
compute notecode(notes,1) = 32.
compute notes = notes + 1.
do if (effsize=1 or stand=1).
compute notecode(notes,1) = 34.
compute notes = notes + 1.
compute stand=0.
compute effsize=0.
end if.
do if (center <> 0).
compute center=0.
compute errcode(errs,1)=71.
compute errs=errs+1.
compute criterr=1.
end if.
end if.
compute v = 'xxxxx'.
compute q = 'xxxxx'.
compute linsum={ -999 }.
compute nlinsum=ncol(linsum).
do if (linsum(1,1) = -999).
compute nlinsum=0.
end if.
compute oldvars= 'xxxxx'.
compute mcxok=0.
compute mcwok=0.
compute mczok=0.
compute xprod=0.
compute zprod=0.
compute wprod=0.
compute modcok=0.
compute alttotal=0.
compute hc3=trunc( 0 ).
compute jn=( 0 = 1).
compute listmiss=( 0 =1).
compute modelres=( 0 =1).
compute outscree=( 1 =1).
compute activate=( 0 =1).
compute booterr=0.
compute normal=( 0 =1).
compute xmtest=( 0 =1).
compute describe=( 0 =1).
compute longname=( 0 =1).
do if (stand=1).
compute effsize=1.
end if.
compute pstog=0.
compute sobelok=0.
compute normal=( 0 =1).
compute mdichok=( 0 =1).
compute contrast={ 999 }.
compute ncontr=ncol(contrast).
compute ncontrow=nrow(contrast).
do if (contrast(1,1) = 999).
compute ncontr=1.
compute contrast=0.
end if.
do if (ncontr = 1).
compute contrast=trunc(contrast).
do if (contrast > 3 or contrast < 0)).
compute ncontr=1.
compute contrast = 0.
end if.
end if.
do if (ncontr > 1).
compute contvec=contrast.
compute contrast=4.
do if (ncontrow > 1).
compute contrast=0.
compute modcok=1.
compute wcontval=contvec(:,1).
compute zcontval=contvec(:,2).
do if ((ncontr <> 2) or (ncontrow <> 2)).
compute notecode(notes,1) = 19.
compute notes = notes + 1.
compute modcok=0.
end if.
end if.
end if.
do if (xmint = 1 and contrast(1,1) <> 0).
compute contrast=0.
compute notecode(notes,1) = 37.
compute notes = notes + 1.
end if.
do if (varorder = 1).
compute notecode(notes,1) = 21.
compute notes = notes + 1.
end if.
do if ( 999 <> 999 or 999 <> 999).
compute notecode(notes,1) = 22.
compute notes = notes + 1.
end if.
compute modelbt=( 0 =1).
compute cluster= 'xxxxx'.
compute matrices=( 0 =1).
compute covcoeff=( 0 =1).
compute covmy=trunc( 0 ).
do if (covmy < 0 or covmy > 2).
compute covmy = 0.
end if.
compute boot = abs(trunc( 5000 )).
compute bc=( 0 =1).
compute mc=abs(trunc( 0 )).
compute hc=trunc( 5 ).
do if (intprobe < 0 or intprobe > 1).
compute intprobe = .10.
end if.
compute plot=trunc( 0 ).
do if (plot < 0 or plot > 2).
compute plot=0.
end if.
compute total=( 1 =1).
compute dototal=0.
compute saveboot = ( 0 = 1).
compute saveest=( 0 = 2).
do if (saveest=1).
compute intprobe=1.
end if.
do if (hc >= 0 and hc < 5).
compute notecode(notes,1) = 4.
compute notes = notes + 1.
end if.
do if (hc > 5 or hc < 0).
compute hc=5.
end if.
compute mcw=trunc( 0 ).
compute mcz=trunc( 0 ).
compute mcx=trunc( 0 ).
do if (mcx > 0 and mcx < 3 and model = 74).
compute mcw=mcx.
compute xscaling=1.
end if.
do if (mcx > 2 and model = 74).
compute errcode(errs,1)=65.
compute errs=errs+1.
compute criterr=1.
end if.
do if (model = 74 and normal=1).
compute notecode(notes,1) = 33.
compute normal=0.
compute notes = notes + 1.
end if.
do if (mcx > 0 and contrast > 0).
compute notecode(notes,1) = 28.
compute notes = notes + 1.
compute contrast=0.
end if.
compute nxvls=1.
compute nmvls=1.
compute nwvls=1.
compute nzvls=1.
compute paths=999.
compute pathsw=999.
compute pathsz=999.
compute pathswz=999.
compute pathsmod=999.
compute pathtype=999.
compute obscoeff=999.
compute pathsdv={' '}.
compute quantile=1.
do if ( 999 <>999).
compute notecode(notes,1) = 23.
compute notes = notes + 1.
end if.
compute moments=( 0 =1).
do if (moments=1).
compute quantile=0.
end if.
compute bmatrix={ -999 }.
compute wmatrix={ -999 }.
compute zmatrix={ -999 }.
compute wzmatrix={ -999 }.
compute cmatrix={ -999 }.
compute xcatcode={ -999 }.
compute wcatcode={ -999 }.
compute zcatcode={ -999 }.
compute needed=0.
compute conf= 95.
do if (trunc( 95 ) >= 100 or (trunc( 95 ) <= 50)).
compute conf = 95.
compute notecode(notes,1)=2.
compute notes=notes+1.
end if.
do if (model >= 0 and model < 4 and modelbt=0).
compute boot=0.
compute mc=0.
compute bc=0.
end if.
do if (mc > 0 and boot > 0).
compute boot=0.
compute bc=0.
end if.
do if ((boot < 1000) and (mc = 0) and (boot > 0)).
compute boot=5000.
end if.
do if ((mc < 1000) and (boot = 0) and (mc > 0)).
compute mc=5000.
end if.
compute p0=-.322232431088.
compute p1 = -1.
compute p2 = -.342242088547.
compute p3 = -.0204231210245.
compute p4 = -.0000453642210148.
compute q0 = .0993484626060.
compute q1 = .588581570495.
compute q2 = .531103462366.
compute q3 = .103537752850.
compute q4 = .0038560700634.
compute badend=0.
compute priorlo = -9999999.
compute priorhi = 9999999.
compute alpha2 = (1-(conf/100))/2.
compute cilm=alpha2*2.
compute y5=sqrt(-2*ln(alpha2)).
compute xp2=(y5+((((y5*p4+p3)*y5+p2)*y5+p1)*y5+p0)/((((y5*q4+q3)*y5+q2)*y5+q1)*y5+q0)).
compute medlb={'   M1  :';'   M2  :';'   M3  :';'   M4  :';'   M5  :';'   M6  :';'   M7  :';'   M8  :';'   M9  :';'   M10 :'}.
compute medlb2={'(M1)','(M2)','(M3)','(M4)','(M5)','(M6)','(M7)','(M8)','(M9)','(M10)'}.
compute xlb={'   X1  :';'   X2  :';'   X3  :';'   X4  :';'   X5  :';'   X6  :';'   X7  :';'   X8  :';'   X9  :'}.
compute highlbw={'M1*W'; 'M2*W'; 'M3*W'; 'M4*W'; 'M5*W'; 'M6*W'; 'M7*W'; 'M8*W'; 'M9*W'; 'M10*W'}.
do if (xmint=1).
compute highlbw={'M1*X'; 'M2*X'; 'M3*X'; 'M4*X'; 'M5*X'; 'M6*X'; 'M7*X'; 'M8*X'; 'M9*X'; 'M10*X'}.
end if.
compute highlbz={'M1*Z'; 'M2*Z'; 'M3*Z'; 'M4*Z'; 'M5*Z'; 'M6*Z'; 'M7*Z'; 'M8*Z'; 'M9*Z';'M10*Z'}.
compute highlbwz={'M1*W*Z'; 'M2*W*Z'; 'M3*W*Z'; 'M4*W*Z'; 'M5*W*Z'; 'M6*W*Z'; 'M7*W*Z'; 'M8*W*Z'; 'M9*W*Z';'M10*W*Z'}.
compute highlbbt={'BOTH(M1)'; 'BOTH(M2)'; 'BOTH(M3)'; 'BOTH(M4)'; 'BOTH(M5)'; 'BOTH(M6)'; 'BOTH(M7)'; 'BOTH(M8)'; 'BOTH(M9)';'BTH(M10)'}.
compute highlbx={'M1*X'; 'M2*X'; 'M3*X'; 'M4*X'; 'M5*X'; 'M6*X'; 'M7*X'; 'M8*X'; 'M9*X'; 'M10*X'}.
compute skipwz=0.
compute validm={1,1,1,1,1,1,1,1,1,1,1,1,1,1,1,1,1,1,1,1,1,1,0,0,0,0,0,1,1,0,0,0,0, 0,0,0,0,0,0,0,0,0,0,0,0,0,0,0,0,0,0,0,0,0,0,0,0,1,1,1,1,1,1,1,1,1,1,1,1,1,1,1,1,1,1,1,0,0,0,1,1, 1,1,1,1,1,1,1,1,1,1,1}.
do if (activate=1).
compute errcode(errs,1)=60.
compute errs=errs+1.
compute criterr=1.
end if.
do if (criterr=0).
do if (( 0 =1)=1).
compute errcode(errs,1)=42.
compute errs=errs+1.
compute criterr=1.
end if.
do if (model > 0 and model < 93).
do if (validm(1,model)=0).
compute errcode(errs,1)=6.
compute errs=errs+1.
compute criterr=1.
end if.
release validm.
end if.
do if ((model > 92 or model < 0) and model <> 999)).
compute errcode(errs,1)=7.
compute errs=errs+1.
compute criterr=1.
end if.
do if (model = 999 and bmatrix(1,1)=-999).
compute errcode(errs,1)=24.
compute errs=errs+1.
compute criterr=1.
end if.
do if (model <> 999 and bmatrix(1,1) <> -999).
compute errcode(errs,1)=25.
compute errs=errs+1.
compute criterr=1.
end if.
do if ((model = 74 or (model > 0 and model < 4)) and ((wmatrix(1,1) <> -999) or (zmatrix(1,1)<>-999) or (wzmatrix(1,1)<>-999)))).
compute errcode(errs,1)=41.
compute errs=errs+1.
compute criterr=1.
end if.
do if (hc3 <> 0).
compute notecode(notes,1) = 5.
compute notes = notes + 1.
do if (hc3 = 1).
compute hc=3.
end if.
end if.
do if ((v <> 'xxxxx') or (q <> 'xxxxx')).
compute errcode(errs,1)=14.
compute errs=errs+1.
compute errcode(errs,1)=48.
compute errs=errs+1.
compute criterr=1.
end if.
do if (oldvars <> 'xxxxx').
compute errcode(errs,1)=48.
compute errs=errs+1.
compute criterr=1.
end if.
do if (cluster <> 'xxxxx').
compute errcode(errs,1)=27.
compute errs=errs+1.
compute criterr=1.
end if.
do if ((y = 'xxxxx') or (x = 'xxxxx')).
compute errcode(errs,1)=1.
compute errs=errs+1.
compute criterr=1.
end if.
do if ((m = 'xxxxx') and model > 3).
compute errcode(errs,1)=8.
compute errs=errs+1.
compute criterr=1.
end if.
end if.
do if (criterr=0).
get ytmp/variables = HOMAIR /names = ynames/MISSING = 99999.
compute nys=ncol(ytmp).
compute needed=nys.
compute n=nrow(ytmp).
compute varnames={ynames}.
compute dat=ytmp.

.


do if (toomany=1 and longname=0).
compute criterr=1.
do if (tooman=0).
compute tooman=1.
compute errcode(errs,1) = 61.
compute errs = errs + 1.
end if.
end if
.
compute modelvar={ '4' ;t(ynames)}.
do if ( 4 =999).
compute modelvar(1,1)='CUSTOM'.
end if.
get xtmp/variables = Veg /names = xnames/MISSING = 99999.
compute nxs=ncol(xtmp).
compute n=nrow(xtmp).
compute needed=needed+nxs.
compute varnames={varnames,xnames}.
compute xcatlab=t(xnames).
compute dat={dat,xtmp}.

.


do if (toomany=1 and longname=0).
compute criterr=1.
do if (tooman=0).
compute tooman=1.
compute errcode(errs,1) = 61.
compute errs = errs + 1.
end if.
end if
.
compute modelvar={modelvar;t(xnames)}.
do if (nxs = 1).
compute modelvlb={'Model  :';'    Y  :';'    X  :'}.
else.
compute modelvlb={'Model  :';'    Y  :';xlb(1:nxs,1)}.
end if.
do if (m <> 'xxxxx').
get mtmp/variables = TG HDL LDL TC /names = mnames/MISSING = 99999.
compute nms=ncol(mtmp).
compute mprod=make(1,nms,0).
compute n=nrow(mtmp).
compute needed=needed+nms.
compute varnames={varnames,mnames}.
compute dat={dat,mtmp}.
compute modelvar={modelvar;t(mnames)}.
compute x2m=make(99,nms,0).
compute m2y=make(99,nms,0).
compute onem=make(nms,1,1).

.


do if (toomany=1 and longname=0).
compute criterr=1.
do if (tooman=0).
compute tooman=1.
compute errcode(errs,1) = 61.
compute errs = errs + 1.
end if.
end if
.
do if (nms > 1 and nms < 11).
compute modelvlb={modelvlb;medlb(1:nms,1)}.
else.
compute modelvlb={modelvlb;'    M  :'}.
end if.
do if (nms > 0 and model < 4).
compute errcode(errs,1)=9.
compute errs=errs+1.
do if (model <> 0).
compute errcode(errs,1)=48.
compute errs=errs+1.
end if.
compute criterr=1.
end if.
end if.
compute wlocatet=0.
compute wlocate=0.
do if (w <> 'xxxxx').
do if (xmint=0).
get wtmp/variables = xxxxx /names = wnames/MISSING = 99999.

.


do if (toomany=1 and longname=0).
compute criterr=1.
do if (tooman=0).
compute tooman=1.
compute errcode(errs,1) = 61.
compute errs = errs + 1.
end if.
end if
.
end if.
do if (xmint=1).
get wtmp/variables = Veg /names = wnames/MISSING = 99999.
end if.
compute nws=ncol(wtmp).
compute n=nrow(wtmp).
compute varnames={varnames,wnames}.
compute wlocate=ncol(varnames).
do if (model=74).
compute wlocatet=1.
do if (xnames <> wnames).
compute errcode(errs,1)=45.
compute errs=errs+1.
compute criterr=1.
end if.
end if.
compute wcatlab=t(wnames).
compute dat={dat,wtmp}.
do if (xmint <> 1).
compute modelvar={modelvar;t(wnames)}.
compute modelvlb={modelvlb;'    W  :'}.
end if.
end if.
do if (z <> 'xxxxx').
get ztmp/variables = xxxxx /names = znames/MISSING = 99999.
compute nzs=ncol(ztmp).
compute n=nrow(ztmp).

.


do if (toomany=1 and longname=0).
compute criterr=1.
do if (tooman=0).
compute tooman=1.
compute errcode(errs,1) = 61.
compute errs = errs + 1.
end if.
end if
.
compute varnames={varnames,znames}.
compute zcatlab=t(znames).
compute dat={dat,ztmp}.
compute modelvar={modelvar;t(znames)}.
compute modelvlb={modelvlb;'    Z  :'}.
end if.
do if (cov <> 'xxxxx').
get ctmp/variables = xxxxx /names = covnames/MISSING = 99999.
compute ncs=ncol(ctmp).
compute n=nrow(ctmp).

.


do if (toomany=1 and longname=0).
compute criterr=1.
do if (tooman=0).
compute tooman=1.
compute errcode(errs,1) = 61.
compute errs = errs + 1.
end if.
end if
.
compute varnames={varnames,covnames}.
compute dat={dat,ctmp}.
end if.
do if (nws > 1 or nzs > 1 or nys > 1 or nxs > 1).
compute errcode(errs,1)=3.
compute errs=errs+1.
compute criterr=1.
end if.
do if ((model = 80 or model = 81) and (nms < 3 or nms > 6)).
compute errcode(errs,1)=32.
compute errs=errs+1.
compute criterr=1.
end if.
do if (model = 82 and nms <> 4).
compute errcode(errs,1)=33.
compute errs=errs+1.
compute criterr=1.
end if.
do if (nms > 10).
compute errcode(errs,1)=37.
compute errs=errs+1.
compute criterr=1.
end if.
do if ((model = 6 or (model > 82 and model < 999)) and (nms < 2 or nms > 6)).
compute errcode(errs,1)=34.
compute errs=errs+1.
compute criterr=1.
end if.
compute match=0.
compute match2=0.
compute mcwzcov=0.
loop i = 1 to (ncol(varnames)-1).
loop j = (i+1) to ncol(varnames).
do if (varnames(i)=varnames(j)).
do if (i < (nxs+nms+nys+1)).
compute match2=match2+1.
end if.
do if (wlocatet=1 and i=2 and j=wlocate).
compute match2=match2-1.
end if.
do if ((wnames=znames) and (nws > 0 or nzs > 0))).
compute match2=match2+1.
end if.
do if (i < (ncol(varnames)-ncs+1)) and j > (ncol(varnames)-ncs)).
do if ((varnames(j)=wnames) and mcw=0)).
compute match=0.
compute wiscov=(j-(ncol(varnames)-ncs)).
end if.
do if ((varnames(j)=wnames) and mcw <>0)).
compute mcwzcov=1.
end if.
do if ((varnames(j)=znames) and mcz=0).
compute match=0.
compute ziscov=(j-(ncol(varnames)-ncs)).
end if.
do if ((varnames(j)=znames) and mcz<>0)).
compute mcwzcov=1.
end if.
end if.
end if.
end loop.
end loop.
do if (match2>0 or match=1).
compute errcode(errs,1)=2.
compute errs=errs+1.
compute criterr=1.
end if.
do if (mcwzcov=1).
compute errcode(errs,1)=50.
compute errs=errs+1.
compute criterr=1.
end if.
compute ninit=nrow(dat).
compute rownum=make(ninit,1,0).
loop i = 1 to ninit.
compute rownum(i,1)=i.
end loop.
compute dat={rownum,dat}.
compute j=1.
compute missrow=0.
loop i = 1 to n.
do if (rsum(dat(i,2:ncol(dat))=99999)=0).
compute dat(j,:)=dat(i,:).
compute j=j+1.
else.
compute missrow={missrow;dat(i,1)}.
end if.
end loop.
do if (j < 5).
compute errcode(errs,1)=62.
compute errs=errs+1.
compute criterr=1.
end if.
do if (criterr=0).
compute rownum=dat(1:(j-1),1).
do if (nrow(missrow) > 1).
compute missrow=t(missrow(2:nrow(missrow),1)).
compute notecode(notes,1) = 29.
compute notes = notes + 1.
end if.
compute dat=dat(1:(j-1),2:ncol(dat)).
compute n=nrow(dat).
compute nmiss=ninit-n.
compute ytmp=dat(:,1:nys).

.
compute desctmp=make((8-(4* 0 )),ncol( ytmp ),-999).
loop jd=1 to ncol( ytmp ).
compute descdat= ytmp (:,jd).
compute desctmp(1,jd) = csum(descdat)/nrow(descdat).
compute desctmp(2,jd) = (nrow(descdat)*sscp(descdat))-(t(csum(descdat))*(csum(descdat))).
compute desctmp(2,jd) = sqrt(desctmp(2,jd)/(nrow(descdat)*(nrow(descdat)-1))).
compute desctmp(3,jd)=cmin(descdat).
compute desctmp(4,jd)=cmax(descdat).
do if ( 0 =0).
compute minwarn=0.
compute maxwarn=0.
do if ((desctmp(3,jd)=desctmp(4,jd)) and novar=0).
compute errcode(errs,1)=15.
compute errs=errs+1.
compute criterr=1.
compute novar=1.
end if.
compute tmp=((descdat(:,1)=desctmp(3,jd))+(descdat(:,1)=desctmp(4,jd))).
compute desctmp(8,jd)=(csum(tmp)=nrow(tmp)).
compute tmp = descdat.
compute tmp(GRADE(descdat),:) = descdat.
compute descdat = tmp.
release tmp.
compute decval={.16;.5;.84}.
loop kd=1 to 3.
compute low=trunc(decval(kd,1)*(nrow(descdat)+1)).
compute lowdec=decval(kd,1)*(nrow(descdat)+1)-low.
compute value=descdat(low,1)+(descdat((low+1),1)-descdat(low,1))*lowdec.
compute desctmp((4+kd),jd)=value.
end loop.
compute mnotev=1.
compute modvals=desctmp(5:7,:).
do if (quantile <> 1).
compute desctmp(5,jd)=desctmp(1,jd)-desctmp(2,jd).
compute desctmp(6,jd)=desctmp(1,jd).
compute desctmp(7,jd)=desctmp(1,jd)+desctmp(2,jd).
compute modvals=desctmp(5:7,:).
compute mnotev=2.
do if (modvals(1,1) < desctmp(3,1)).
compute modvals(1,1)=desctmp(3,1).
compute minwarn=1.
end if.
do if (modvals(3,1) > desctmp(4,1)).
compute modvals(3,1)=desctmp(4,1).
compute maxwarn=1.
end if.
end if.
do if (desctmp(8,1)=1).
compute modvals={desctmp(3,1);desctmp(4,1)}.
compute mnotev=0.
compute minwarn=0.
compute maxwarn=0.
end if.
end if.
end loop
.
compute ysd=desctmp(2,:).
compute ovsd=ysd.
do if (desctmp(8,1)=1).
compute ydich=1.
do if (total=1).
compute total=0.
compute notecode(notes,1) = 24.
compute notes = notes + 1.
end if.
do if (effsize=1).
compute effsize=0.
compute notecode(notes,1) = 25.
compute notes = notes + 1.
end if.
do if (model=74).
compute errcode(errs,1) = 72.
compute errs=errs+1.
compute criterr=1.
end if.
compute omx = cmax(ytmp).
compute omn = cmin(ytmp).
compute ytmp = (ytmp = omx).
compute dat(:,1:nys)=(dat(:,1:nys)=omx).
compute rcd = {omn, 0; omx, 1}.
end if.
compute xtmp=dat(:,(nys+1):(nys+nxs)).

.
compute desctmp=make((8-(4* 0 )),ncol( xtmp ),-999).
loop jd=1 to ncol( xtmp ).
compute descdat= xtmp (:,jd).
compute desctmp(1,jd) = csum(descdat)/nrow(descdat).
compute desctmp(2,jd) = (nrow(descdat)*sscp(descdat))-(t(csum(descdat))*(csum(descdat))).
compute desctmp(2,jd) = sqrt(desctmp(2,jd)/(nrow(descdat)*(nrow(descdat)-1))).
compute desctmp(3,jd)=cmin(descdat).
compute desctmp(4,jd)=cmax(descdat).
do if ( 0 =0).
compute minwarn=0.
compute maxwarn=0.
do if ((desctmp(3,jd)=desctmp(4,jd)) and novar=0).
compute errcode(errs,1)=15.
compute errs=errs+1.
compute criterr=1.
compute novar=1.
end if.
compute tmp=((descdat(:,1)=desctmp(3,jd))+(descdat(:,1)=desctmp(4,jd))).
compute desctmp(8,jd)=(csum(tmp)=nrow(tmp)).
compute tmp = descdat.
compute tmp(GRADE(descdat),:) = descdat.
compute descdat = tmp.
release tmp.
compute decval={.16;.5;.84}.
loop kd=1 to 3.
compute low=trunc(decval(kd,1)*(nrow(descdat)+1)).
compute lowdec=decval(kd,1)*(nrow(descdat)+1)-low.
compute value=descdat(low,1)+(descdat((low+1),1)-descdat(low,1))*lowdec.
compute desctmp((4+kd),jd)=value.
end loop.
compute mnotev=1.
compute modvals=desctmp(5:7,:).
do if (quantile <> 1).
compute desctmp(5,jd)=desctmp(1,jd)-desctmp(2,jd).
compute desctmp(6,jd)=desctmp(1,jd).
compute desctmp(7,jd)=desctmp(1,jd)+desctmp(2,jd).
compute modvals=desctmp(5:7,:).
compute mnotev=2.
do if (modvals(1,1) < desctmp(3,1)).
compute modvals(1,1)=desctmp(3,1).
compute minwarn=1.
end if.
do if (modvals(3,1) > desctmp(4,1)).
compute modvals(3,1)=desctmp(4,1).
compute maxwarn=1.
end if.
end if.
do if (desctmp(8,1)=1).
compute modvals={desctmp(3,1);desctmp(4,1)}.
compute mnotev=0.
compute minwarn=0.
compute maxwarn=0.
end if.
end if.
end loop
.
compute xsd=desctmp(2,:).
compute xmodvals=modvals.
compute xdich=desctmp(8,1).
compute xmx=cmax(xtmp).
compute xmn=cmin(xtmp).
do if ((mcx > 0) and (xrefvals(1,1) <> 999) and (xmint=1) and (model= 74)).
compute notecode(notes,1) = 36.
compute notes = notes + 1.
end if.
do if (mcx=0).
do if (ncol(xrefvals)>2 and model=74 and xmint=1).
compute errcode(errs,1)=67.
compute errs=errs+1.
compute criterr=1.
end if.
do if ((model=74) and (xmint=1)).
do if ((xrefvals(1,1)=999) and nxvls=1 and xdich=0).
compute errcode(errs,1)=66.
compute errs=errs+1.
compute criterr=1.
end if.
do if ((xrefvals(1,1)=999) and (xdich=1)).
compute xrefvals={xmn,xmx}.
compute xscaling=xrefvals(1,2)-xrefvals(1,1).
end if.
do if (ncol(xrefvals)=1 and xrefvals(1,1) <> 999).
do if (xdich=0).
compute xrefvals={xrefvals,(xrefvals(1,1)+1)}.
compute xscaling=xrefvals(1,2)-xrefvals(1,1).
end if.
do if (xdich=1).
do if ((xrefvals(1,1) <> xmx) and (xrefvals(1,1) <> xmn)).
compute errcode(errs,1)=70.
compute errs=errs+1.
compute criterr=1.
end if.
do if (xrefvals(1,1) = xmx).
compute xrefvals={xrefvals,xmn}.
compute xscaling=xrefvals(1,2)-xrefvals(1,1).
end if.
do if (xrefvals(1,1) = xmn).
compute xrefvals={xrefvals,xmx}.
compute xscaling=xrefvals(1,2)-xrefvals(1,1).
end if.
end if.
end if.
do if (ncol(xrefvals)=2).
compute xscaling=xrefvals(1,2)-xrefvals(1,1).
do if (xdich=1).
compute xreferr=1.
do if (((xrefvals(1,1) = xmx) and (xrefvals(1,2)=xmn)) or ((xrefvals(1,1) = xmn) and (xrefvals(1,2)=xmx))).
compute xreferr=0.
end if.
do if (xreferr=1).
compute errcode(errs,1)=70.
compute errs=errs+1.
compute criterr=1.
end if.
end if.
end if.
end if.
end if.
do if (xmint=1 and model=74 and mcx=0).
compute xmodvals=t(xrefvals).
compute xcontcf=1.
end if.
compute nxpval=nrow(xmodvals).
compute xprobval=xmodvals.
do if (xdich =1 and mcx > 0).
compute mcx=0.
compute errcode(errs,1) = 52.
compute errs = errs + 1.
compute criterr = 1.
end if.
do if (nms > 0).
compute mtmp=dat(:,(nys+nxs+1):(nys+nxs+nms)).

.
compute desctmp=make((8-(4* 0 )),ncol( mtmp ),-999).
loop jd=1 to ncol( mtmp ).
compute descdat= mtmp (:,jd).
compute desctmp(1,jd) = csum(descdat)/nrow(descdat).
compute desctmp(2,jd) = (nrow(descdat)*sscp(descdat))-(t(csum(descdat))*(csum(descdat))).
compute desctmp(2,jd) = sqrt(desctmp(2,jd)/(nrow(descdat)*(nrow(descdat)-1))).
compute desctmp(3,jd)=cmin(descdat).
compute desctmp(4,jd)=cmax(descdat).
do if ( 0 =0).
compute minwarn=0.
compute maxwarn=0.
do if ((desctmp(3,jd)=desctmp(4,jd)) and novar=0).
compute errcode(errs,1)=15.
compute errs=errs+1.
compute criterr=1.
compute novar=1.
end if.
compute tmp=((descdat(:,1)=desctmp(3,jd))+(descdat(:,1)=desctmp(4,jd))).
compute desctmp(8,jd)=(csum(tmp)=nrow(tmp)).
compute tmp = descdat.
compute tmp(GRADE(descdat),:) = descdat.
compute descdat = tmp.
release tmp.
compute decval={.16;.5;.84}.
loop kd=1 to 3.
compute low=trunc(decval(kd,1)*(nrow(descdat)+1)).
compute lowdec=decval(kd,1)*(nrow(descdat)+1)-low.
compute value=descdat(low,1)+(descdat((low+1),1)-descdat(low,1))*lowdec.
compute desctmp((4+kd),jd)=value.
end loop.
compute mnotev=1.
compute modvals=desctmp(5:7,:).
do if (quantile <> 1).
compute desctmp(5,jd)=desctmp(1,jd)-desctmp(2,jd).
compute desctmp(6,jd)=desctmp(1,jd).
compute desctmp(7,jd)=desctmp(1,jd)+desctmp(2,jd).
compute modvals=desctmp(5:7,:).
compute mnotev=2.
do if (modvals(1,1) < desctmp(3,1)).
compute modvals(1,1)=desctmp(3,1).
compute minwarn=1.
end if.
do if (modvals(3,1) > desctmp(4,1)).
compute modvals(3,1)=desctmp(4,1).
compute maxwarn=1.
end if.
end if.
do if (desctmp(8,1)=1).
compute modvals={desctmp(3,1);desctmp(4,1)}.
compute mnotev=0.
compute minwarn=0.
compute maxwarn=0.
end if.
end if.
end loop
.
compute ovsd={desctmp(2,:),ysd}.
compute medmeans=cdeval.
do if ((cdeval(1,1) <> -999) and (ncol(medmeans) <> nms) and (model=74)).
compute errcode(errs,1)=64.
compute errs=errs+1.
compute criterr=1.
end if.
do if ((cdeval(1,1)=-999) and (model=74)).
compute medmeans=desctmp(1,:).
end if.
do if ((cdeval(1,1)<> -999) and (model=74) and (criterr=0)).
compute notecode(notes,1) = 31.
compute notes = notes + 1.
end if.
do if ((rsum(desctmp(8,:))>0) and (mdichok <> 1)).
compute errcode(errs,1)=43.
compute errs=errs+1.
compute criterr=1.
end if.
compute mmodvals=modvals.
compute mprobval=mmodvals.
end if.
do if (nws > 0).
compute wtmp=dat(:,(nys+nxs+nms+1):(nys+nxs+nms+nws)).

.
compute desctmp=make((8-(4* 0 )),ncol( wtmp ),-999).
loop jd=1 to ncol( wtmp ).
compute descdat= wtmp (:,jd).
compute desctmp(1,jd) = csum(descdat)/nrow(descdat).
compute desctmp(2,jd) = (nrow(descdat)*sscp(descdat))-(t(csum(descdat))*(csum(descdat))).
compute desctmp(2,jd) = sqrt(desctmp(2,jd)/(nrow(descdat)*(nrow(descdat)-1))).
compute desctmp(3,jd)=cmin(descdat).
compute desctmp(4,jd)=cmax(descdat).
do if ( 0 =0).
compute minwarn=0.
compute maxwarn=0.
do if ((desctmp(3,jd)=desctmp(4,jd)) and novar=0).
compute errcode(errs,1)=15.
compute errs=errs+1.
compute criterr=1.
compute novar=1.
end if.
compute tmp=((descdat(:,1)=desctmp(3,jd))+(descdat(:,1)=desctmp(4,jd))).
compute desctmp(8,jd)=(csum(tmp)=nrow(tmp)).
compute tmp = descdat.
compute tmp(GRADE(descdat),:) = descdat.
compute descdat = tmp.
release tmp.
compute decval={.16;.5;.84}.
loop kd=1 to 3.
compute low=trunc(decval(kd,1)*(nrow(descdat)+1)).
compute lowdec=decval(kd,1)*(nrow(descdat)+1)-low.
compute value=descdat(low,1)+(descdat((low+1),1)-descdat(low,1))*lowdec.
compute desctmp((4+kd),jd)=value.
end loop.
compute mnotev=1.
compute modvals=desctmp(5:7,:).
do if (quantile <> 1).
compute desctmp(5,jd)=desctmp(1,jd)-desctmp(2,jd).
compute desctmp(6,jd)=desctmp(1,jd).
compute desctmp(7,jd)=desctmp(1,jd)+desctmp(2,jd).
compute modvals=desctmp(5:7,:).
compute mnotev=2.
do if (modvals(1,1) < desctmp(3,1)).
compute modvals(1,1)=desctmp(3,1).
compute minwarn=1.
end if.
do if (modvals(3,1) > desctmp(4,1)).
compute modvals(3,1)=desctmp(4,1).
compute maxwarn=1.
end if.
end if.
do if (desctmp(8,1)=1).
compute modvals={desctmp(3,1);desctmp(4,1)}.
compute mnotev=0.
compute minwarn=0.
compute maxwarn=0.
end if.
end if.
end loop
.
compute wmodvals=modvals.
compute wdich=desctmp(8,1).
do if (wdich =1 and mcw > 0).
compute mcw=0.
compute errcode(errs,1) = 52.
compute errs = errs + 1.
compute criterr = 1.
end if.
compute wmin=desctmp(3,1).
compute wmax=desctmp(4,1).
compute minwwarn=minwarn.
compute maxwwarn=maxwarn.
compute wnotev=mnotev.
compute wmodval={ 999 }.
do if (xmint=1 and model=74 and mcx=0).
compute wmodval=xrefvals.
end if.
compute nwcontr=ncol(wmodval).
do if (wmodval(1,1) <> 999).
compute wmodvals=wmodval(1,1).
compute wmodcust=1.
do if (nwcontr > 1).
compute wmodvals=t(wmodval).
end if.
compute minwwarn=0.
compute maxwwarn=0.
compute wnotev=0.
end if.
compute wprobval=wmodvals.
compute nwpval=nrow(wmodvals).
end if.
do if (nzs > 0).
compute ztmp=dat(:,(nys+nxs+nms+nws+1):(nys+nxs+nms+nws+nzs)).

.
compute desctmp=make((8-(4* 0 )),ncol( ztmp ),-999).
loop jd=1 to ncol( ztmp ).
compute descdat= ztmp (:,jd).
compute desctmp(1,jd) = csum(descdat)/nrow(descdat).
compute desctmp(2,jd) = (nrow(descdat)*sscp(descdat))-(t(csum(descdat))*(csum(descdat))).
compute desctmp(2,jd) = sqrt(desctmp(2,jd)/(nrow(descdat)*(nrow(descdat)-1))).
compute desctmp(3,jd)=cmin(descdat).
compute desctmp(4,jd)=cmax(descdat).
do if ( 0 =0).
compute minwarn=0.
compute maxwarn=0.
do if ((desctmp(3,jd)=desctmp(4,jd)) and novar=0).
compute errcode(errs,1)=15.
compute errs=errs+1.
compute criterr=1.
compute novar=1.
end if.
compute tmp=((descdat(:,1)=desctmp(3,jd))+(descdat(:,1)=desctmp(4,jd))).
compute desctmp(8,jd)=(csum(tmp)=nrow(tmp)).
compute tmp = descdat.
compute tmp(GRADE(descdat),:) = descdat.
compute descdat = tmp.
release tmp.
compute decval={.16;.5;.84}.
loop kd=1 to 3.
compute low=trunc(decval(kd,1)*(nrow(descdat)+1)).
compute lowdec=decval(kd,1)*(nrow(descdat)+1)-low.
compute value=descdat(low,1)+(descdat((low+1),1)-descdat(low,1))*lowdec.
compute desctmp((4+kd),jd)=value.
end loop.
compute mnotev=1.
compute modvals=desctmp(5:7,:).
do if (quantile <> 1).
compute desctmp(5,jd)=desctmp(1,jd)-desctmp(2,jd).
compute desctmp(6,jd)=desctmp(1,jd).
compute desctmp(7,jd)=desctmp(1,jd)+desctmp(2,jd).
compute modvals=desctmp(5:7,:).
compute mnotev=2.
do if (modvals(1,1) < desctmp(3,1)).
compute modvals(1,1)=desctmp(3,1).
compute minwarn=1.
end if.
do if (modvals(3,1) > desctmp(4,1)).
compute modvals(3,1)=desctmp(4,1).
compute maxwarn=1.
end if.
end if.
do if (desctmp(8,1)=1).
compute modvals={desctmp(3,1);desctmp(4,1)}.
compute mnotev=0.
compute minwarn=0.
compute maxwarn=0.
end if.
end if.
end loop
.
compute zmodvals=modvals.
compute zdich=desctmp(8,1).
do if (zdich =1 and mcz > 0).
compute mcz=0.
compute errcode(errs,1) = 52.
compute errs = errs + 1.
compute criterr = 1.
end if.
compute zmin=desctmp(3,1).
compute zmax=desctmp(4,1).
compute minzwarn=minwarn.
compute maxzwarn=maxwarn.
compute znotev=mnotev.
compute zmodval={ 999 }.
compute nzcontr=ncol(zmodval).
do if (zmodval(1,1) <> 999).
compute zmodvals=zmodval(1,1).
compute zmodcust=1.
do if (nzcontr > 1).
compute zmodvals=t(zmodval).
end if.
compute minzwarn=0.
compute maxzwarn=0.
compute znotev=0.
end if.
compute zprobval=zmodvals.
compute nzpval=nrow(zmodvals).
end if.
do if (ncs > 0).
compute ctmp=dat(:,(nys+nxs+nms+nws+nzs+1):(nys+nxs+nms+nws+nzs+ncs)).

.
compute desctmp=make((8-(4* 0 )),ncol( ctmp ),-999).
loop jd=1 to ncol( ctmp ).
compute descdat= ctmp (:,jd).
compute desctmp(1,jd) = csum(descdat)/nrow(descdat).
compute desctmp(2,jd) = (nrow(descdat)*sscp(descdat))-(t(csum(descdat))*(csum(descdat))).
compute desctmp(2,jd) = sqrt(desctmp(2,jd)/(nrow(descdat)*(nrow(descdat)-1))).
compute desctmp(3,jd)=cmin(descdat).
compute desctmp(4,jd)=cmax(descdat).
do if ( 0 =0).
compute minwarn=0.
compute maxwarn=0.
do if ((desctmp(3,jd)=desctmp(4,jd)) and novar=0).
compute errcode(errs,1)=15.
compute errs=errs+1.
compute criterr=1.
compute novar=1.
end if.
compute tmp=((descdat(:,1)=desctmp(3,jd))+(descdat(:,1)=desctmp(4,jd))).
compute desctmp(8,jd)=(csum(tmp)=nrow(tmp)).
compute tmp = descdat.
compute tmp(GRADE(descdat),:) = descdat.
compute descdat = tmp.
release tmp.
compute decval={.16;.5;.84}.
loop kd=1 to 3.
compute low=trunc(decval(kd,1)*(nrow(descdat)+1)).
compute lowdec=decval(kd,1)*(nrow(descdat)+1)-low.
compute value=descdat(low,1)+(descdat((low+1),1)-descdat(low,1))*lowdec.
compute desctmp((4+kd),jd)=value.
end loop.
compute mnotev=1.
compute modvals=desctmp(5:7,:).
do if (quantile <> 1).
compute desctmp(5,jd)=desctmp(1,jd)-desctmp(2,jd).
compute desctmp(6,jd)=desctmp(1,jd).
compute desctmp(7,jd)=desctmp(1,jd)+desctmp(2,jd).
compute modvals=desctmp(5:7,:).
compute mnotev=2.
do if (modvals(1,1) < desctmp(3,1)).
compute modvals(1,1)=desctmp(3,1).
compute minwarn=1.
end if.
do if (modvals(3,1) > desctmp(4,1)).
compute modvals(3,1)=desctmp(4,1).
compute maxwarn=1.
end if.
end if.
do if (desctmp(8,1)=1).
compute modvals={desctmp(3,1);desctmp(4,1)}.
compute mnotev=0.
compute minwarn=0.
compute maxwarn=0.
end if.
end if.
end loop
.
compute covmeans=desctmp(1,:).
compute coval={ -999 }.
do if ((coval(1,1) <> -999) and (ncol(coval) <> ncs) and (model=74)).
compute errcode(errs,1)=69.
compute errs=errs+1.
compute criterr=1.
end if.
do if ((coval(1,1)<> -999) and (criterr=0) and (model=74)).
compute notecode(notes,1) = 35.
compute notes = notes + 1.
compute cuscoval=1.
end if.
end if.
compute n=nrow(ytmp).
compute ones=make(n,1,1).
do if (nws > 0 and mcw > 0).
compute tmp={rownum,wtmp(:,1)}.

.
compute dd= tmp.
compute temp = dd.
compute temp(GRADE(dd(:,2)),:) = dd.
compute dd = temp.
compute dummy = design(dd(:,2)).
compute nvls = ncol(dummy).
compute nnvls = csum(dummy).
compute mnvls = cmin(t(nnvls)).
compute conmat1=1.
do if (mnvls < 2).
compute errcode(errs,1) = 5.
compute errs = errs + 1.
compute criterr = 1.
end if.
do if (nvls > 9).
compute errcode(errs,1) = 4.
compute errs = errs+1.
compute criterr = 1.
end if.
do if (criterr = 0).
compute dumok = 1.
compute nnvls=make(nvls,1,0).
compute nnvls(1,1)=dd(1,2).
compute temp = 2.
loop i = 2 to n.
do if (dd(i,2) <> nnvls((temp-1),1)).
compute nnvls(temp,1)=dd(i,2).
compute temp = temp+1.
end if.
end loop.
do if ( mcw > 0).
compute x = dummy(:,2:ncol(dummy)).
compute nx = ncol(x).
compute minus1 = make(1,ncol(x),-1).
compute xdes=make((nx+1),3,0).
compute xdes(1,1)=dd(1,2).
compute xdes(1,2)=1.
compute temp = 2.
loop k = 2 to n.
do if (dd(k,2) <> dd((k-1),2)).
compute xdes(temp,2) = k.
compute xdes(temp,1) = dd(k,2).
compute xdes((temp-1),3) = k-1.
compute temp=temp+1.
end if.
end loop.
compute xdes((temp-1),3)=n.
compute xdes = {xdes, (xdes(:,3)-xdes(:,2)+1)}.
do if ( mcw = 4).
loop k = 1 to n.
do if (rsum(x(k,:)) = 0).
compute x(k,:) = minus1.
end if.
end loop.
end if.
do if ( mcw = 2 or mcw = 3 or mcw =5).
loop k = 1 to n.
do if (rsum(x(k,:)) > 0).
loop i = 1 to ncol(x).
do if (x(k,i) = 0).
compute x(k,i) = 1.
else.
break.
end if.
end loop.
end if.
end loop.
do if ( mcw = 3).
compute conmat1={-8,1,1,1,1,1,1,1,1; 0,-7,1,1,1,1,1,1,1; 0,0,-6,1,1,1,1,1,1; 0,0,0,-5,1,1,1,1,1; 0,0,0,0,-4,1,1,1,1; 0,0,0,0,0,-3,1,1,1; 0,0,0,0,0,0,-2,1,1; 0,0,0,0,0,0,0,-1,1}.
loop i = 1 to 8.
compute conmat1(i,:)=conmat1(i,:)/(10-i).
end loop.
compute conmat1=t(conmat1((10-nvls):8,(10-nvls):9)).
loop k=1 to n.
compute x(k,:)=conmat1((rsum(x(k,:))+1),:).
end loop.
end if.
end if.
do if ( mcw = 5).
compute custcode={ -999 }.
do if (ncol(custcode) <> (nvls*(nvls-1))).
compute errcode(errs,1) = (37+ 2 ).
compute errs = errs + 1.
compute criterr = 1.
end if.
do if (ncol(custcode) = (nvls*(nvls-1))).
compute conmat1=make(nvls,(nvls-1),0).
compute cnt=1.
loop i = 1 to nvls.
loop k = 1 to (nvls-1).
compute conmat1(i,k)=custcode(1,cnt).
compute cnt=cnt+1.
end loop.
end loop.
loop k=1 to n.
compute x(k,:)=conmat1((rsum(x(k,:))+1),:).
end loop.
end if.
end if.
compute xskip = 1.
compute dummat = make((nx+1),nx,0).
compute dummat((2:nrow(dummat)),:)=ident(nx).
do if ( mcw = 4).
compute dummat(1,:) = minus1.
end if.
do if ( mcw = 2).
loop i = 2 to nrow(dummat).
loop j = 1 to (i-1).
compute dummat(i,j) = 1.
end loop.
end loop.
end if.
do if ( mcw = 3).
compute dummat=conmat1.
end if.
do if ( mcw = 5 and criterr=0).
compute dummat=conmat1.
end if.
compute dummat={nnvls, dummat}.
compute x={dd(:,1),x}.
compute temp = x.
compute temp(GRADE(x(:,1)),:) = x.
compute x = temp.
release conmat1,temp,dd,xskip,xdes,dummy.
end if.
end if
.
compute wmodvals=nnvls.
compute nwpval=nrow(wmodvals).
do if (criterr=0).
compute minwwarn=0.
compute maxwwarn=0.
compute wnotev=0.
compute wtmp=x(:,2:ncol(x)).
compute wcatlab={'W1';'W2';'W3';'W4';'W5';'W6';'W7';'W8';'W9'}.
do if (xmint=1).
compute wcatlab={'X1';'X2';'X3';'X4';'X5';'X6';'X7';'X8';'X9'}.
end if.
compute nwvls=nvls-1.
compute mcwok=1.
compute dummatw=dummat.
compute wprobval=dummatw(:,2:ncol(dummatw)).
do if (modcok=1).
compute wcontval=make(2,ncol(wprobval),-999).
compute temp=0.
loop i = 1 to 2.
loop j = 1 to nrow(dummatw).
do if (contvec(i,1)=dummatw(j,1)).
compute wcontval(i,:)=wprobval(j,:).
compute temp=temp+1.
end if.
end loop.
end loop.
do if (temp < 2).
compute notecode(notes,1) = 20.
compute notes = notes + 1.
compute modcok=0.
end if.
end if.
do if ((wmodval(1,1) <> 999) and (xmint <> 1)).
compute notecode(notes,1) = 9.
compute notes = notes + 1.
end if.
release tmp, dummat.
end if.
end if.
do if (nzs > 0 and mcz > 0).
compute tmp={rownum,ztmp(:,1)}.

.
compute dd= tmp.
compute temp = dd.
compute temp(GRADE(dd(:,2)),:) = dd.
compute dd = temp.
compute dummy = design(dd(:,2)).
compute nvls = ncol(dummy).
compute nnvls = csum(dummy).
compute mnvls = cmin(t(nnvls)).
compute conmat1=1.
do if (mnvls < 2).
compute errcode(errs,1) = 5.
compute errs = errs + 1.
compute criterr = 1.
end if.
do if (nvls > 9).
compute errcode(errs,1) = 4.
compute errs = errs+1.
compute criterr = 1.
end if.
do if (criterr = 0).
compute dumok = 1.
compute nnvls=make(nvls,1,0).
compute nnvls(1,1)=dd(1,2).
compute temp = 2.
loop i = 2 to n.
do if (dd(i,2) <> nnvls((temp-1),1)).
compute nnvls(temp,1)=dd(i,2).
compute temp = temp+1.
end if.
end loop.
do if ( mcz > 0).
compute x = dummy(:,2:ncol(dummy)).
compute nx = ncol(x).
compute minus1 = make(1,ncol(x),-1).
compute xdes=make((nx+1),3,0).
compute xdes(1,1)=dd(1,2).
compute xdes(1,2)=1.
compute temp = 2.
loop k = 2 to n.
do if (dd(k,2) <> dd((k-1),2)).
compute xdes(temp,2) = k.
compute xdes(temp,1) = dd(k,2).
compute xdes((temp-1),3) = k-1.
compute temp=temp+1.
end if.
end loop.
compute xdes((temp-1),3)=n.
compute xdes = {xdes, (xdes(:,3)-xdes(:,2)+1)}.
do if ( mcz = 4).
loop k = 1 to n.
do if (rsum(x(k,:)) = 0).
compute x(k,:) = minus1.
end if.
end loop.
end if.
do if ( mcz = 2 or mcz = 3 or mcz =5).
loop k = 1 to n.
do if (rsum(x(k,:)) > 0).
loop i = 1 to ncol(x).
do if (x(k,i) = 0).
compute x(k,i) = 1.
else.
break.
end if.
end loop.
end if.
end loop.
do if ( mcz = 3).
compute conmat1={-8,1,1,1,1,1,1,1,1; 0,-7,1,1,1,1,1,1,1; 0,0,-6,1,1,1,1,1,1; 0,0,0,-5,1,1,1,1,1; 0,0,0,0,-4,1,1,1,1; 0,0,0,0,0,-3,1,1,1; 0,0,0,0,0,0,-2,1,1; 0,0,0,0,0,0,0,-1,1}.
loop i = 1 to 8.
compute conmat1(i,:)=conmat1(i,:)/(10-i).
end loop.
compute conmat1=t(conmat1((10-nvls):8,(10-nvls):9)).
loop k=1 to n.
compute x(k,:)=conmat1((rsum(x(k,:))+1),:).
end loop.
end if.
end if.
do if ( mcz = 5).
compute custcode={ -999 }.
do if (ncol(custcode) <> (nvls*(nvls-1))).
compute errcode(errs,1) = (37+ 3 ).
compute errs = errs + 1.
compute criterr = 1.
end if.
do if (ncol(custcode) = (nvls*(nvls-1))).
compute conmat1=make(nvls,(nvls-1),0).
compute cnt=1.
loop i = 1 to nvls.
loop k = 1 to (nvls-1).
compute conmat1(i,k)=custcode(1,cnt).
compute cnt=cnt+1.
end loop.
end loop.
loop k=1 to n.
compute x(k,:)=conmat1((rsum(x(k,:))+1),:).
end loop.
end if.
end if.
compute xskip = 1.
compute dummat = make((nx+1),nx,0).
compute dummat((2:nrow(dummat)),:)=ident(nx).
do if ( mcz = 4).
compute dummat(1,:) = minus1.
end if.
do if ( mcz = 2).
loop i = 2 to nrow(dummat).
loop j = 1 to (i-1).
compute dummat(i,j) = 1.
end loop.
end loop.
end if.
do if ( mcz = 3).
compute dummat=conmat1.
end if.
do if ( mcz = 5 and criterr=0).
compute dummat=conmat1.
end if.
compute dummat={nnvls, dummat}.
compute x={dd(:,1),x}.
compute temp = x.
compute temp(GRADE(x(:,1)),:) = x.
compute x = temp.
release conmat1,temp,dd,xskip,xdes,dummy.
end if.
end if
.
compute zmodvals=nnvls.
compute nzpval=nrow(zmodvals).
do if (criterr=0).
compute minzwarn=0.
compute maxzwarn=0.
compute znotev=0.
compute ztmp=x(:,2:ncol(x)).
compute zcatlab={'Z1';'Z2';'Z3';'Z4';'Z5';'Z6';'Z7';'Z8';'Z9'}.
compute nzvls=nvls-1.
compute mczok=1.
compute dummatz=dummat.
compute zprobval=dummatz(:,2:ncol(dummatz)).
do if (modcok=1).
compute zcontval=make(2,ncol(zprobval),-999).
compute temp=0.
loop i = 1 to 2.
loop j = 1 to nrow(dummatz).
do if (contvec(i,2)=dummatz(j,1)).
compute zcontval(i,:)=zprobval(j,:).
compute temp=temp+1.
end if.
end loop.
end loop.
do if (temp < 2).
compute notecode(notes,1) = 20.
compute notes = notes + 1.
compute modcok=0.
end if.
end if.
do if (zmodval(1,1) <> 999).
compute notecode(notes,1) = 10.
compute notes = notes + 1.
end if.
release tmp, dummat.
end if.
end if.
do if (nxs > 0 and mcx > 0).
compute tmp={rownum,xtmp(:,1)}.

.
compute dd= tmp.
compute temp = dd.
compute temp(GRADE(dd(:,2)),:) = dd.
compute dd = temp.
compute dummy = design(dd(:,2)).
compute nvls = ncol(dummy).
compute nnvls = csum(dummy).
compute mnvls = cmin(t(nnvls)).
compute conmat1=1.
do if (mnvls < 2).
compute errcode(errs,1) = 5.
compute errs = errs + 1.
compute criterr = 1.
end if.
do if (nvls > 9).
compute errcode(errs,1) = 4.
compute errs = errs+1.
compute criterr = 1.
end if.
do if (criterr = 0).
compute dumok = 1.
compute nnvls=make(nvls,1,0).
compute nnvls(1,1)=dd(1,2).
compute temp = 2.
loop i = 2 to n.
do if (dd(i,2) <> nnvls((temp-1),1)).
compute nnvls(temp,1)=dd(i,2).
compute temp = temp+1.
end if.
end loop.
do if ( mcx > 0).
compute x = dummy(:,2:ncol(dummy)).
compute nx = ncol(x).
compute minus1 = make(1,ncol(x),-1).
compute xdes=make((nx+1),3,0).
compute xdes(1,1)=dd(1,2).
compute xdes(1,2)=1.
compute temp = 2.
loop k = 2 to n.
do if (dd(k,2) <> dd((k-1),2)).
compute xdes(temp,2) = k.
compute xdes(temp,1) = dd(k,2).
compute xdes((temp-1),3) = k-1.
compute temp=temp+1.
end if.
end loop.
compute xdes((temp-1),3)=n.
compute xdes = {xdes, (xdes(:,3)-xdes(:,2)+1)}.
do if ( mcx = 4).
loop k = 1 to n.
do if (rsum(x(k,:)) = 0).
compute x(k,:) = minus1.
end if.
end loop.
end if.
do if ( mcx = 2 or mcx = 3 or mcx =5).
loop k = 1 to n.
do if (rsum(x(k,:)) > 0).
loop i = 1 to ncol(x).
do if (x(k,i) = 0).
compute x(k,i) = 1.
else.
break.
end if.
end loop.
end if.
end loop.
do if ( mcx = 3).
compute conmat1={-8,1,1,1,1,1,1,1,1; 0,-7,1,1,1,1,1,1,1; 0,0,-6,1,1,1,1,1,1; 0,0,0,-5,1,1,1,1,1; 0,0,0,0,-4,1,1,1,1; 0,0,0,0,0,-3,1,1,1; 0,0,0,0,0,0,-2,1,1; 0,0,0,0,0,0,0,-1,1}.
loop i = 1 to 8.
compute conmat1(i,:)=conmat1(i,:)/(10-i).
end loop.
compute conmat1=t(conmat1((10-nvls):8,(10-nvls):9)).
loop k=1 to n.
compute x(k,:)=conmat1((rsum(x(k,:))+1),:).
end loop.
end if.
end if.
do if ( mcx = 5).
compute custcode={ -999 }.
do if (ncol(custcode) <> (nvls*(nvls-1))).
compute errcode(errs,1) = (37+ 1 ).
compute errs = errs + 1.
compute criterr = 1.
end if.
do if (ncol(custcode) = (nvls*(nvls-1))).
compute conmat1=make(nvls,(nvls-1),0).
compute cnt=1.
loop i = 1 to nvls.
loop k = 1 to (nvls-1).
compute conmat1(i,k)=custcode(1,cnt).
compute cnt=cnt+1.
end loop.
end loop.
loop k=1 to n.
compute x(k,:)=conmat1((rsum(x(k,:))+1),:).
end loop.
end if.
end if.
compute xskip = 1.
compute dummat = make((nx+1),nx,0).
compute dummat((2:nrow(dummat)),:)=ident(nx).
do if ( mcx = 4).
compute dummat(1,:) = minus1.
end if.
do if ( mcx = 2).
loop i = 2 to nrow(dummat).
loop j = 1 to (i-1).
compute dummat(i,j) = 1.
end loop.
end loop.
end if.
do if ( mcx = 3).
compute dummat=conmat1.
end if.
do if ( mcx = 5 and criterr=0).
compute dummat=conmat1.
end if.
compute dummat={nnvls, dummat}.
compute x={dd(:,1),x}.
compute temp = x.
compute temp(GRADE(x(:,1)),:) = x.
compute x = temp.
release conmat1,temp,dd,xskip,xdes,dummy.
end if.
end if
.
do if (criterr=0).
compute xtmp=x(:,2:ncol(x)).
compute xcatlab={'X1';'X2';'X3';'X4';'X5';'X6';'X7';'X8';'X9'}.
compute nxvls=nvls-1.
compute xdich=(nvls=2).
compute mcxok=1.
compute dummatx=dummat.
compute xmodvals=dummatx(:,1).
compute nxpval=nrow(xmodvals).
release tmp, dummat.
end if.
end if.
compute intlab=make(100,1,' ').


compute intlab( 1 ,1)= 'Int_1'.


compute intlab( 2 ,1)= 'Int_2'.


compute intlab( 3 ,1)= 'Int_3'.


compute intlab( 4 ,1)= 'Int_4'.


compute intlab( 5 ,1)= 'Int_5'.


compute intlab( 6 ,1)= 'Int_6'.


compute intlab( 7 ,1)= 'Int_7'.


compute intlab( 8 ,1)= 'Int_8'.


compute intlab( 9 ,1)= 'Int_9'.


compute intlab( 10 ,1)= 'Int_10'.


compute intlab( 11 ,1)= 'Int_11'.


compute intlab( 12 ,1)= 'Int_12'.


compute intlab( 13 ,1)= 'Int_13'.


compute intlab( 14 ,1)= 'Int_14'.


compute intlab( 15 ,1)= 'Int_15'.


compute intlab( 16 ,1)= 'Int_16'.


compute intlab( 17 ,1)= 'Int_17'.


compute intlab( 18 ,1)= 'Int_18'.


compute intlab( 19 ,1)= 'Int_19'.


compute intlab( 20 ,1)= 'Int_20'.


compute intlab( 21 ,1)= 'Int_21'.


compute intlab( 22 ,1)= 'Int_22'.


compute intlab( 23 ,1)= 'Int_23'.


compute intlab( 24 ,1)= 'Int_24'.


compute intlab( 25 ,1)= 'Int_25'.


compute intlab( 26 ,1)= 'Int_26'.


compute intlab( 27 ,1)= 'Int_27'.


compute intlab( 28 ,1)= 'Int_28'.


compute intlab( 29 ,1)= 'Int_29'.


compute intlab( 30 ,1)= 'Int_30'.


compute intlab( 31 ,1)= 'Int_31'.


compute intlab( 32 ,1)= 'Int_32'.


compute intlab( 33 ,1)= 'Int_33'.


compute intlab( 34 ,1)= 'Int_34'.


compute intlab( 35 ,1)= 'Int_35'.


compute intlab( 36 ,1)= 'Int_36'.


compute intlab( 37 ,1)= 'Int_37'.


compute intlab( 38 ,1)= 'Int_38'.


compute intlab( 39 ,1)= 'Int_39'.


compute intlab( 40 ,1)= 'Int_40'.


compute intlab( 41 ,1)= 'Int_41'.


compute intlab( 42 ,1)= 'Int_42'.


compute intlab( 43 ,1)= 'Int_43'.


compute intlab( 44 ,1)= 'Int_44'.


compute intlab( 45 ,1)= 'Int_45'.


compute intlab( 46 ,1)= 'Int_46'.


compute intlab( 47 ,1)= 'Int_47'.


compute intlab( 48 ,1)= 'Int_48'.


compute intlab( 49 ,1)= 'Int_49'.


compute intlab( 50 ,1)= 'Int_50'.


compute intlab( 51 ,1)= 'Int_51'.


compute intlab( 52 ,1)= 'Int_52'.


compute intlab( 53 ,1)= 'Int_53'.


compute intlab( 54 ,1)= 'Int_54'.


compute intlab( 55 ,1)= 'Int_55'.


compute intlab( 56 ,1)= 'Int_56'.


compute intlab( 57 ,1)= 'Int_57'.


compute intlab( 58 ,1)= 'Int_58'.


compute intlab( 59 ,1)= 'Int_59'.


compute intlab( 60 ,1)= 'Int_60'.


compute intlab( 61 ,1)= 'Int_61'.


compute intlab( 62 ,1)= 'Int_62'.


compute intlab( 63 ,1)= 'Int_63'.


compute intlab( 64 ,1)= 'Int_64'.


compute intlab( 65 ,1)= 'Int_65'.


compute intlab( 66 ,1)= 'Int_66'.


compute intlab( 67 ,1)= 'Int_67'.


compute intlab( 68 ,1)= 'Int_68'.


compute intlab( 69 ,1)= 'Int_69'.


compute intlab( 70 ,1)= 'Int_70'.


compute intlab( 71 ,1)= 'Int_71'.


compute intlab( 72 ,1)= 'Int_72'.


compute intlab( 73 ,1)= 'Int_73'.


compute intlab( 74 ,1)= 'Int_74'.


compute intlab( 75 ,1)= 'Int_75'.


compute intlab( 76 ,1)= 'Int_76'.


compute intlab( 77 ,1)= 'Int_77'.


compute intlab( 78 ,1)= 'Int_78'.


compute intlab( 79 ,1)= 'Int_79'.


compute intlab( 80 ,1)= 'Int_80'.


compute intlab( 81 ,1)= 'Int_81'.


compute intlab( 82 ,1)= 'Int_82'.


compute intlab( 83 ,1)= 'Int_83'.


compute intlab( 84 ,1)= 'Int_84'.


compute intlab( 85 ,1)= 'Int_85'.


compute intlab( 86 ,1)= 'Int_86'.


compute intlab( 87 ,1)= 'Int_87'.


compute intlab( 88 ,1)= 'Int_88'.


compute intlab( 89 ,1)= 'Int_89'.


compute intlab( 90 ,1)= 'Int_90'.


compute intlab( 91 ,1)= 'Int_91'.


compute intlab( 92 ,1)= 'Int_92'.


compute intlab( 93 ,1)= 'Int_93'.


compute intlab( 94 ,1)= 'Int_94'.


compute intlab( 95 ,1)= 'Int_95'.


compute intlab( 96 ,1)= 'Int_96'.


compute intlab( 97 ,1)= 'Int_97'.


compute intlab( 98 ,1)= 'Int_98'.


compute intlab( 99 ,1)= 'Int_99'.


compute intlab( 100 ,1)= 'Int_100'.

compute bcmat=make(needed,needed,0).
compute wcmat=make(needed,needed,0).
compute zcmat=make(needed,needed,0).
compute wzcmat=make(needed,needed,0).
compute wsum=0.
compute zsum=0.
compute wzsum=0.
end if.
end if.
do if (criterr = 0 and model <> 999).
compute modelmat= {1,0,0,0,0,0,0,1,0,0;2,0,0,0,0,0,0,1,1,0;3,0,0,0,0,0,0,1,1,1;4,0,0,0,0,0,0,0,0,0; 5,0,0,0,0,0,0,1,0,0;6,0,0,0,0,0,0,0,0,0;7,1,0,0,0,0,0,0,0,0;8,1,0,0,0,0,0,1,0,0; 9,1,1,0,0,0,0,0,0,0;10,1,1,0,0,0,0,1,1,0;11,1,1,1,0,0,0,0,0,0;12,1,1,1,0,0,0,1,1,1; 13,1,1,1,0,0,0,1,0,0;14,0,0,0,1,0,0,0,0,0;15,0,0,0,1,0,0,1,0,0;16,0,0,0,1,1,0,0,0,0; 17,0,0,0,1,1,0,1,1,0;18,0,0,0,1,1,1,0,0,0;19,0,0,0,1,1,1,1,1,1;20,0,0,0,1,1,1,1,0,0; 21,1,0,0,0,1,0,0,0,0;22,1,0,0,0,1,0,1,0,0;23,0,0,0,0,0,0,0,0,0;24,0,0,0,0,0,0,0,0,0; 25,0,0,0,0,0,0,0,0,0;26,0,0,0,0,0,0,0,0,0;27,0,0,0,0,0,0,0,0,0;28,1,0,0,0,1,0,0,1,0; 29,1,0,0,0,1,0,1,1,0;30,0,0,0,0,0,0,0,0,0;31,0,0,0,0,0,0,0,0,0;32,0,0,0,0,0,0,0,0,0; 33,0,0,0,0,0,0,0,0,0;34,0,0,0,0,0,0,0,0,0;35,0,0,0,0,0,0,0,0,0;36,0,0,0,0,0,0,0,0,0; 37,0,0,0,0,0,0,0,0,0;38,0,0,0,0,0,0,0,0,0;39,0,0,0,0,0,0,0,0,0;40,0,0,0,0,0,0,0,0,0; 41,0,0,0,0,0,0,0,0,0;42,0,0,0,0,0,0,0,0,0;43,0,0,0,0,0,0,0,0,0;44,0,0,0,0,0,0,0,0,0; 45,0,0,0,0,0,0,0,0,0;46,0,0,0,0,0,0,0,0,0;47,0,0,0,0,0,0,0,0,0;48,0,0,0,0,0,0,0

 ,0,0; 49,0,0,0,0,0,0,0,0,0;50,0,0,0,0,0,0,0,0,0;51,0,0,0,0,0,0,0,0,0;52,0,0,0,0,0,0,0,0,0; 53,0,0,0,0,0,0,0,0,0;54,0,0,0,0,0,0,0,0,0;55,0,0,0,0,0,0,0,0,0;56,0,0,0,0,0,0,0,0,0; 57,0,0,0,0,0,0,0,0,0;58,1,0,0,1,0,0,0,0,0;59,1,0,0,1,0,0,1,0,0;60,1,1,0,1,0,0,0,0,0; 61,1,1,0,1,0,0,1,0,0;62,1,1,0,1,0,0,0,1,0;63,1,1,0,1,0,0,1,1,0;64,1,0,0,1,1,0,0,0,0; 65,1,0,0,1,1,0,1,0,0;66,1,0,0,1,1,0,0,1,0;67,1,0,0,1,1,0,1,1,0;68,1,1,1,1,0,0,0,0,0; 69,1,1,1,1,0,0,1,1,1;70,1,0,0,1,1,1,0,0,0;71,1,0,0,1,1,1,1,1,1;72,1,1,1,1,1,1,0,0,0; 73,1,1,1,1,1,1,1,1,1;74,0,0,0,1,0,0,0,0,0;75,1,1,0,1,1,0,0,0,0;76,1,1,0,1,1,0,1,1,0; 77,0,0,0,0,0,0,0,0,0;78,0,0,0,0,0,0,0,0,0;79,0,0,0,0,0,0,0,0,0;80,0,0,0,0,0,0,0,0,0; 81,0,0,0,0,0,0,0,0,0;82,0,0,0,0,0,0,0,0,0;83,1,0,0,0,0,0,0,0,0;84,1,0,0,0,0,0,0,0,0; 85,1,0,0,0,0,0,1,0,0;86,1,0,0,0,0,0,1,0,0;87,0,0,0,1,0,0,0,0,0;88,0,0,0,1,0,0,0,0,0; 89,0,0,0,1,0,0,1,0,0;90,0,0,0,1,0,0,1,0,0;91,0,0,0,0,0,0,0,0,0;92,1,0,0,1,0,0,1,0,0}.
do if (model > 0).
compute tmp=modelmat(model,2:ncol(modelmat)).
end if.
do if (model=0).
compute tmp={0,0,0,0,0,0,0,0,0}.
end if.
do if (model < 4).
compute bcmat((nxs+1),1)=1.
end if.
do if ((model > 3) and (model <> 6)).
compute bcmat((nxs+1):(nxs+nms),1)=onem.
compute bcmat(nrow(bcmat),(nxs+1):(nxs+nms))=t(onem).
compute bcmat(nrow(bcmat),1)=1.
end if.
do if ((model = 6) or (model > 82 and model < 93)).
loop j = 2 to nrow(bcmat).
loop i = 1 to (j-1).
compute bcmat(j,i)=1.
end loop.
end loop.
end if.
do if (model = 80).
loop i = 1 to nms.
compute bcmat((nrow(bcmat)-1),i)=1.
end loop.
end if.
do if (model = 81).
loop j = 3 to nrow(bcmat).
compute bcmat(j,2)=1.
end loop.
end if.
do if (model = 82).
compute bcmat(3,2)=1.
compute bcmat(5,4)=1.
end if.
do if (tmp(1,1)=1).
compute wcmat((nxs+1):(nxs+nms),1)=onem.
compute wprod=1.
compute xprod=1.
do if (model = 83 or model = 86).
compute onemsx=onem.
loop i = 1 to (nms-1).
compute onemsx(i+1,1)=0.
end loop.
compute wcmat((nxs+1):(nxs+nms),1)=onemsx.
end if.
end if.
do if (tmp(1,4)=1).
compute wcmat(nrow(wcmat),(nxs+1):(nxs+nms))=t(onem).
compute wprod=1.
do if (model = 87 or model = 90).
compute onemsx=onem.
loop i = 1 to (nms-1).
compute onemsx(i,1)=0.
end loop.
compute wcmat(nrow(wcmat),(nxs+1):(nxs+nms))=t(onemsx).
end if.
end if.
do if (tmp(1,7)=1).
compute wcmat(nrow(wcmat),1)=1.
compute wprod=1.
compute xprod=1.
end if.
do if (tmp(1,2)=1).
compute zcmat((nxs+1):(nxs+nms),1)=onem.
compute zprod=1.
compute xprod=1.
end if.
do if (tmp(1,5)=1).
compute zcmat(nrow(zcmat),(nxs+1):(nxs+nms))=t(onem).
compute zprod=1.
end if.
do if (tmp(1,8)=1).
compute zcmat(nrow(zcmat),1)=1.
compute zprod=1.
compute xprod=1.
end if.
do if (tmp(1,3)=1).
compute wzcmat((nxs+1):(nxs+nms),1)=onem.
compute xprod=1.
compute wprod=1.
compute zprod=1.
end if.
do if (tmp(1,6)=1).
compute wzcmat(nrow(wzcmat),(nxs+1):(nxs+nms))=t(onem).
compute zprod=1.
compute wprod=1.
end if.
do if (tmp(1,9)=1).
compute wzcmat(nrow(wzcmat),1)=1.
compute xprod=1.
compute wprod=1.
compute zprod=1.
end if.
do if (model = 91 or model = 92).
loop j = 1 to (nms-1).
loop i = 1 to j.
compute wcmat((nxs+1+j),(nxs+i))=1.
end loop.
end loop.
end if.
do if (nms < 0).
loop i = 1 to nms.
compute tmp=csum(wcmat(:,(1+i)))+csum(zcmat(:,(1+i)))+csum(wzcmat(:,(1+i))).
compute mprod(1,i)=(tmp>0).
end loop.
end if.
end if.
do if (ncs > 0).
compute ccmat=make((nms+nys),ncs,1).
compute ccmatoff=ccmat.
do if (covmy=1).
compute ccmat(nrow(ccmat),:)=make(1,ncs,0).
end if.
do if (covmy=2).
compute ccmat(1:nms,:)=make(nms,ncs,0).
end if.
do if (cmatrix(1,1) <> -999).
do if (ncol(cmatrix) <> ((nms+nys)*ncs)).
compute errcode(errs,1)=29.
compute errs=errs+1.
compute criterr=1.
end if.
do if (criterr = 0).
compute tmp=1.
loop i = 1 to (nms+nys).
loop j = 1 to ncs.
compute ccmat(i,j)=1-(cmatrix(1,tmp) = 0).
compute tmp=tmp+1.
end loop.
end loop.
do if (rsum((csum(ccmat)=0)) <> 0).
compute errcode(errs,1)=30.
compute errs=errs+1.
compute criterr=1.
end if.
end if.
do if (covmy <> 0).
compute notecode(notes,1)=1.
compute notes=notes+1.
end if.
end if.
do if (xmint=1 and (covmy > 0 or (csum(rsum(ccmat)) <> ((nms+nys)*ncs)))).
compute errcode(errs,1)=68.
compute errs=errs+1.
compute criterr=1.
end if.
end if.
do if (criterr=0).
compute needed=needed*(needed-1)/2.
compute nopath=0.
do if (bmatrix(1,1) <> -999).
compute tmp=1.
do if ((ncol(bmatrix) <> needed) or (csum(rsum(bmatrix))=0)).
compute errcode(errs,1)=16.
compute errs=errs+1.
compute criterr=1.
else.
loop i = 2 to nrow(bcmat).
loop j = 1 to (i-1).
compute bcmat(i,j)=1-(bmatrix(1,tmp) = 0).
compute tmp=tmp+1.
end loop.
end loop.
end if.
do if ((csum(bcmat(:,1))=0) and criterr=0).
compute errcode(errs,1)=22.
compute errs=errs+1.
compute criterr=1.
end if.
do if ((rsum(bcmat(nrow(bcmat),:))=0) and criterr=0).
compute errcode(errs,1)=23.
compute errs=errs+1.
compute criterr=1.
end if.
compute dm=0.
do if (nms > 0).
loop i = 1 to nms.
do if (((rsum(bcmat((nxs+i),:)) = 0) or (csum(bcmat(:,(nxs+i))) = 0)) and (dm=0) and (criterr=0)).
compute errcode(errs,1)=26.
compute errs=errs+1.
compute criterr=1.
compute dm=1.
end if.
end loop.
end if.
release dm.
end if.
end if.
do if (criterr=0).
do if (wmatrix(1,1) <> -999).
compute tmp=1.
do if (ncol(wmatrix) <> needed).
compute errcode(errs,1)=17.
compute errs=errs+1.
compute criterr=1.
else.
compute modelvar(1,1)='CUSTOM'.
loop i = 2 to nrow(wcmat).
loop j = 1 to (i-1).
compute wcmat(i,j)=1-(wmatrix(1,tmp) = 0).
do if ((wcmat(i,j)=1) and (bcmat(i,j)=0) and (nopath=0)).
compute errcode(errs,1)=20.
compute errs=errs+1.
compute criterr=1.
compute nopath=1.
end if.
compute tmp=tmp+1.
end loop.
end loop.
end if.
end if.
do if (zmatrix(1,1) <> -999).
compute tmp=1.
do if (ncol(zmatrix) <> needed).
compute errcode(errs,1)=18.
compute errs=errs+1.
compute criterr=1.
else.
compute modelvar(1,1)='CUSTOM'.
do if (csum(rsum(wcmat))=0 and model=999).
compute errcode(errs,1)=21.
compute errs=errs+1.
compute criterr=1.
end if.
loop i = 2 to nrow(zcmat).
loop j = 1 to (i-1).
compute zcmat(i,j)=1-(zmatrix(1,tmp) = 0).
do if ((zcmat(i,j)=1) and (bcmat(i,j)=0) and (nopath=0)).
compute errcode(errs,1)=20.
compute errs=errs+1.
compute criterr=1.
compute nopath=1.
end if.
compute tmp=tmp+1.
end loop.
end loop.
end if.
end if.
compute tmp=1.
do if (wzmatrix(1,1) <> -999).
do if (ncol(wzmatrix) <> needed).
compute errcode(errs,1)=19.
compute errs=errs+1.
compute criterr=1.
end if.
compute modelvar(1,1)='CUSTOM'.
end if.
do if (criterr=0).
loop i = 2 to nrow(wzcmat).
loop j = 1 to (i-1).
do if (wzmatrix(1,1) <> -999).
compute wzcmat(i,j)=1-(wzmatrix(1,tmp) = 0).
end if.
do if (wzcmat(i,j)=1).
compute wcmat(i,j)=1.
compute zcmat(i,j)=1.
end if.
do if ((wzcmat(i,j)=1) and (bcmat(i,j)=0) and (nopath=0)).
compute errcode(errs,1)=20.
compute errs=errs+1.
compute criterr=1.
compute nopath=1.
end if.
compute tmp=tmp+1.
end loop.
end loop.
end if.
end if.
do if (criterr=0).
compute xprod=csum(wcmat(:,1))+csum(zcmat(:,1))+csum(wzcmat(:,1)).
compute xprod=(xprod > 0).
compute wsum=csum(rsum(wcmat)).
compute wprod=(wsum > 0).
do if (nms > 0).
loop i = 1 to nms.
compute tmp=csum(wcmat(:,(1+i)))+csum(zcmat(:,(1+i)))+csum(wzcmat(:,(1+i))).
compute mprod(1,i)=(tmp>0).
end loop.
end if.
do if ((wsum > 0) and (w = 'xxxxx')).
compute errcode(errs,1)=11.
compute errs=errs+1.
compute criterr=1.
end if.
do if ((wsum = 0) and (w <> 'xxxxx')).
compute errcode(errs,1)=10.
compute errs=errs+1.
compute criterr=1.
end if.
compute zsum=csum(rsum(zcmat)).
compute zprod=(zsum > 0).
do if ((zsum > 0) and (z = 'xxxxx')).
compute errcode(errs,1)=13.
compute errs=errs+1.
compute criterr=1.
end if.
do if ((zsum = 0) and (z <> 'xxxxx')).
compute errcode(errs,1)=12.
compute errs=errs+1.
compute criterr=1.
end if.
do if ((zsum > 0) and (wsum = 0)).
compute errcode(errs,1)=35.
compute errs=errs+1.
compute criterr=1.
end if.
end if.
do if (criterr=0 and nms > 1).
compute serchk=bcmat(2:(nrow(bcmat)-1),2:ncol(bcmat)).
do if (csum(rsum(serchk))) > 0.
compute serial=1.
do if (nms > 6).
compute errcode(errs,1)=36.
compute errs=errs+1.
compute criterr=1.
end if.
end if.
end if.
do if (center > 0 and criterr=0).
compute centvar={' '}.
do if (criterr=0).
do if ((center = 1) or (center = 2 and wdich = 0)).
do if (wprod=1 and mcwok=0 and nwpval > 0).
loop i = 1 to nws.
compute wtmp(:,i)=wtmp(:,i)-(csum(wtmp(:,i))/n).
compute centvar={centvar,wnames(1,i)}.
end loop.

.
compute desctmp=make((8-(4* wmodcust )),ncol( wtmp ),-999).
loop jd=1 to ncol( wtmp ).
compute descdat= wtmp (:,jd).
compute desctmp(1,jd) = csum(descdat)/nrow(descdat).
compute desctmp(2,jd) = (nrow(descdat)*sscp(descdat))-(t(csum(descdat))*(csum(descdat))).
compute desctmp(2,jd) = sqrt(desctmp(2,jd)/(nrow(descdat)*(nrow(descdat)-1))).
compute desctmp(3,jd)=cmin(descdat).
compute desctmp(4,jd)=cmax(descdat).
do if ( wmodcust =0).
compute minwarn=0.
compute maxwarn=0.
do if ((desctmp(3,jd)=desctmp(4,jd)) and novar=0).
compute errcode(errs,1)=15.
compute errs=errs+1.
compute criterr=1.
compute novar=1.
end if.
compute tmp=((descdat(:,1)=desctmp(3,jd))+(descdat(:,1)=desctmp(4,jd))).
compute desctmp(8,jd)=(csum(tmp)=nrow(tmp)).
compute tmp = descdat.
compute tmp(GRADE(descdat),:) = descdat.
compute descdat = tmp.
release tmp.
compute decval={.16;.5;.84}.
loop kd=1 to 3.
compute low=trunc(decval(kd,1)*(nrow(descdat)+1)).
compute lowdec=decval(kd,1)*(nrow(descdat)+1)-low.
compute value=descdat(low,1)+(descdat((low+1),1)-descdat(low,1))*lowdec.
compute desctmp((4+kd),jd)=value.
end loop.
compute mnotev=1.
compute modvals=desctmp(5:7,:).
do if (quantile <> 1).
compute desctmp(5,jd)=desctmp(1,jd)-desctmp(2,jd).
compute desctmp(6,jd)=desctmp(1,jd).
compute desctmp(7,jd)=desctmp(1,jd)+desctmp(2,jd).
compute modvals=desctmp(5:7,:).
compute mnotev=2.
do if (modvals(1,1) < desctmp(3,1)).
compute modvals(1,1)=desctmp(3,1).
compute minwarn=1.
end if.
do if (modvals(3,1) > desctmp(4,1)).
compute modvals(3,1)=desctmp(4,1).
compute maxwarn=1.
end if.
end if.
do if (desctmp(8,1)=1).
compute modvals={desctmp(3,1);desctmp(4,1)}.
compute mnotev=0.
compute minwarn=0.
compute maxwarn=0.
end if.
end if.
end loop
.
compute wmin=desctmp(3,1).
compute wmax=desctmp(4,1).
do if (wmodcust=0).
compute wmodvals=modvals.
compute wprobval=wmodvals.
end if.
end if.
end if.
do if ((center = 1) or (center = 2 and zdich = 0)).
do if (zprod=1 and mczok=0 and nzpval > 0).
loop i = 1 to nzs.
compute ztmp(:,i)=ztmp(:,i)-(csum(ztmp(:,i))/n).
compute centvar={centvar,znames(1,i)}.
end loop.

.
compute desctmp=make((8-(4* zmodcust )),ncol( ztmp ),-999).
loop jd=1 to ncol( ztmp ).
compute descdat= ztmp (:,jd).
compute desctmp(1,jd) = csum(descdat)/nrow(descdat).
compute desctmp(2,jd) = (nrow(descdat)*sscp(descdat))-(t(csum(descdat))*(csum(descdat))).
compute desctmp(2,jd) = sqrt(desctmp(2,jd)/(nrow(descdat)*(nrow(descdat)-1))).
compute desctmp(3,jd)=cmin(descdat).
compute desctmp(4,jd)=cmax(descdat).
do if ( zmodcust =0).
compute minwarn=0.
compute maxwarn=0.
do if ((desctmp(3,jd)=desctmp(4,jd)) and novar=0).
compute errcode(errs,1)=15.
compute errs=errs+1.
compute criterr=1.
compute novar=1.
end if.
compute tmp=((descdat(:,1)=desctmp(3,jd))+(descdat(:,1)=desctmp(4,jd))).
compute desctmp(8,jd)=(csum(tmp)=nrow(tmp)).
compute tmp = descdat.
compute tmp(GRADE(descdat),:) = descdat.
compute descdat = tmp.
release tmp.
compute decval={.16;.5;.84}.
loop kd=1 to 3.
compute low=trunc(decval(kd,1)*(nrow(descdat)+1)).
compute lowdec=decval(kd,1)*(nrow(descdat)+1)-low.
compute value=descdat(low,1)+(descdat((low+1),1)-descdat(low,1))*lowdec.
compute desctmp((4+kd),jd)=value.
end loop.
compute mnotev=1.
compute modvals=desctmp(5:7,:).
do if (quantile <> 1).
compute desctmp(5,jd)=desctmp(1,jd)-desctmp(2,jd).
compute desctmp(6,jd)=desctmp(1,jd).
compute desctmp(7,jd)=desctmp(1,jd)+desctmp(2,jd).
compute modvals=desctmp(5:7,:).
compute mnotev=2.
do if (modvals(1,1) < desctmp(3,1)).
compute modvals(1,1)=desctmp(3,1).
compute minwarn=1.
end if.
do if (modvals(3,1) > desctmp(4,1)).
compute modvals(3,1)=desctmp(4,1).
compute maxwarn=1.
end if.
end if.
do if (desctmp(8,1)=1).
compute modvals={desctmp(3,1);desctmp(4,1)}.
compute mnotev=0.
compute minwarn=0.
compute maxwarn=0.
end if.
end if.
end loop
.
compute zmin=desctmp(3,1).
compute zmax=desctmp(4,1).
do if (zmodcust=0).
compute zmodvals=modvals.
compute zprobval=zmodvals.
end if.
end if.
end if.
do if ((center = 1) or (center = 2 and xdich = 0)).
do if (xprod=1 and mcxok=0).
loop i = 1 to nxs.
compute xtmp(:,i)=xtmp(:,i)-(csum(xtmp(:,i))/n).
compute centvar={centvar,xnames(1,i)}.
end loop.

.
compute desctmp=make((8-(4* 0 )),ncol( xtmp ),-999).
loop jd=1 to ncol( xtmp ).
compute descdat= xtmp (:,jd).
compute desctmp(1,jd) = csum(descdat)/nrow(descdat).
compute desctmp(2,jd) = (nrow(descdat)*sscp(descdat))-(t(csum(descdat))*(csum(descdat))).
compute desctmp(2,jd) = sqrt(desctmp(2,jd)/(nrow(descdat)*(nrow(descdat)-1))).
compute desctmp(3,jd)=cmin(descdat).
compute desctmp(4,jd)=cmax(descdat).
do if ( 0 =0).
compute minwarn=0.
compute maxwarn=0.
do if ((desctmp(3,jd)=desctmp(4,jd)) and novar=0).
compute errcode(errs,1)=15.
compute errs=errs+1.
compute criterr=1.
compute novar=1.
end if.
compute tmp=((descdat(:,1)=desctmp(3,jd))+(descdat(:,1)=desctmp(4,jd))).
compute desctmp(8,jd)=(csum(tmp)=nrow(tmp)).
compute tmp = descdat.
compute tmp(GRADE(descdat),:) = descdat.
compute descdat = tmp.
release tmp.
compute decval={.16;.5;.84}.
loop kd=1 to 3.
compute low=trunc(decval(kd,1)*(nrow(descdat)+1)).
compute lowdec=decval(kd,1)*(nrow(descdat)+1)-low.
compute value=descdat(low,1)+(descdat((low+1),1)-descdat(low,1))*lowdec.
compute desctmp((4+kd),jd)=value.
end loop.
compute mnotev=1.
compute modvals=desctmp(5:7,:).
do if (quantile <> 1).
compute desctmp(5,jd)=desctmp(1,jd)-desctmp(2,jd).
compute desctmp(6,jd)=desctmp(1,jd).
compute desctmp(7,jd)=desctmp(1,jd)+desctmp(2,jd).
compute modvals=desctmp(5:7,:).
compute mnotev=2.
do if (modvals(1,1) < desctmp(3,1)).
compute modvals(1,1)=desctmp(3,1).
compute minwarn=1.
end if.
do if (modvals(3,1) > desctmp(4,1)).
compute modvals(3,1)=desctmp(4,1).
compute maxwarn=1.
end if.
end if.
do if (desctmp(8,1)=1).
compute modvals={desctmp(3,1);desctmp(4,1)}.
compute mnotev=0.
compute minwarn=0.
compute maxwarn=0.
end if.
end if.
end loop
.
compute xmodvals=modvals.
compute xprobval=xmodvals.
end if.
end if.
do if (nms > 0).
loop i = 1 to nms.
do if (mprod(1,i)=1).
compute mtmp(:,i)=mtmp(:,i)-(csum(mtmp(:,i))/n).
compute centvar={centvar,mnames(1,i)}.
end if.
end loop.

.
compute desctmp=make((8-(4* 0 )),ncol( mtmp ),-999).
loop jd=1 to ncol( mtmp ).
compute descdat= mtmp (:,jd).
compute desctmp(1,jd) = csum(descdat)/nrow(descdat).
compute desctmp(2,jd) = (nrow(descdat)*sscp(descdat))-(t(csum(descdat))*(csum(descdat))).
compute desctmp(2,jd) = sqrt(desctmp(2,jd)/(nrow(descdat)*(nrow(descdat)-1))).
compute desctmp(3,jd)=cmin(descdat).
compute desctmp(4,jd)=cmax(descdat).
do if ( 0 =0).
compute minwarn=0.
compute maxwarn=0.
do if ((desctmp(3,jd)=desctmp(4,jd)) and novar=0).
compute errcode(errs,1)=15.
compute errs=errs+1.
compute criterr=1.
compute novar=1.
end if.
compute tmp=((descdat(:,1)=desctmp(3,jd))+(descdat(:,1)=desctmp(4,jd))).
compute desctmp(8,jd)=(csum(tmp)=nrow(tmp)).
compute tmp = descdat.
compute tmp(GRADE(descdat),:) = descdat.
compute descdat = tmp.
release tmp.
compute decval={.16;.5;.84}.
loop kd=1 to 3.
compute low=trunc(decval(kd,1)*(nrow(descdat)+1)).
compute lowdec=decval(kd,1)*(nrow(descdat)+1)-low.
compute value=descdat(low,1)+(descdat((low+1),1)-descdat(low,1))*lowdec.
compute desctmp((4+kd),jd)=value.
end loop.
compute mnotev=1.
compute modvals=desctmp(5:7,:).
do if (quantile <> 1).
compute desctmp(5,jd)=desctmp(1,jd)-desctmp(2,jd).
compute desctmp(6,jd)=desctmp(1,jd).
compute desctmp(7,jd)=desctmp(1,jd)+desctmp(2,jd).
compute modvals=desctmp(5:7,:).
compute mnotev=2.
do if (modvals(1,1) < desctmp(3,1)).
compute modvals(1,1)=desctmp(3,1).
compute minwarn=1.
end if.
do if (modvals(3,1) > desctmp(4,1)).
compute modvals(3,1)=desctmp(4,1).
compute maxwarn=1.
end if.
end if.
do if (desctmp(8,1)=1).
compute modvals={desctmp(3,1);desctmp(4,1)}.
compute mnotev=0.
compute minwarn=0.
compute maxwarn=0.
end if.
end if.
end loop
.
do if ((cdeval(1,1)=-999) and (model=74)).
compute medmeans=desctmp(1,:).
end if.
compute mmodvals=modvals.
compute mprobval=mmodvals.
end if.
end if.
do if (ncol(centvar) > 1).
compute notecode(notes,1)=3.
compute notes=notes+1.
end if.
end if.
do if (criterr=0).
compute wsum=rsum(csum(wcmat)).
compute zsum=rsum(csum(zcmat)).
compute wzsum=rsum(csum(wzcmat)).
compute nump=make(1,(nys+nms),-999).
compue numint=make(1,(nys+nms),0).
compute datcount=1.
compute xtmpuse=0.
compute wtmpuse=0.
compute ztmpuse=0.
compute xwtmpus=0.
compute xztmpus=0.
compute wztmpus=0.
compute xwztmpu=0.
compute xtmploc=-999.
compute wtmploc=-999.
compute xwtmplo=-999.
compute ztmploc=-999.
compute xztmplo=-999.
compute wztmplo=-999.
compute xwztmplo=-999.
compute vlabs={' '}.
do if (ncs > 0).
compute ctmpuse=make(1,ncs,0).
end if.
do if (nms > 0).
compute mtmpuse=make(1,nms,0).
compute mwtmpus=make(1,nms,0).
compute mztmpus=make(1,nms,0).
compute mwztmpu=make(1,nms,0).
compute mtmploc=make(1,nms,0).
compute mwtmplo=make(nwvls,nms,-999).
compute mztmplo=make(nzvls,nms,-999).
compute mwztmplo=make((nwvls*nzvls),nms,-999).
end if.
do if (ncs > 0).
compute ctmploc=make(1,ncs,0).
end if.
compute fulldat=make(n,1,1).
compute datindx=make(1000,(nms+nys),-999).
compute wherew=make(2,(nms+nys),-999).
compute wherex=make(2,(nms+nys),-999).
compute wherez=make(2,(nms+nys),-999).
compute wherexw=make(2,(nms+nys),-999).
compute wherexz=make(2,(nms+nys),-999).
compute wherewz=make(2,(nms+nys),-999).
compute wherexwz=make(2,(nms+nys),-999).
do if (nms > 0).
compute wherem=make(nms,(nms+nys),-999).
compute wheremw = make(nms*2,(nms+nys),-999).
compute wheremz = make(nms*2,(nms+nys),-999).
compute wheremwz = make(nms*2,(nms+nys),-999).
end if.
compute wzhigh=make(1000,(((nms+1)*(nms+2))/2),0).
compute whigh=make(1000,(((nms+1)*(nms+2))/2),0).
compute zhigh=make(1000,(((nms+1)*(nms+2))/2),0).
compute fochigh=make(1000,(((nms+1)*(nms+2))/2),0).
compute xcoefloc={1;2;3;4;5;6;7;8;9}.
compute intkey = {' ', ' ', ' ', ' ', ' ', ' ', ' '}.
compute wzhighct=0.
compute whighct=0.
compute zhighct=0.
compute foccnt=0.
loop i = 2 to nrow(bcmat).
compute wdid=0.
compute zdid=0.
compute wzdid=0.
compute cntmp=1.
compute start=1.
do if (i < nrow(bcmat)).
compute outv=mtmp(:,(i-1)).
compute modlabel={mnames(1,(i-1));'constant'}.
end if.
do if (i = nrow(bcmat)).
compute outv=ytmp.
compute modlabel={ynames;'constant'}.
end if.
loop j = 1 to (i-1).
compute foccnt=foccnt+1.
do if (j = 1 and bcmat(i,j)=1).
compute outv={outv,xtmp}.
compute modlabel={modlabel;xcatlab(1:nxvls,1)}.
do if (xtmpuse=0).
compute fulldat={fulldat,xtmp}.
compute xtmpuse=1.
loop k4=datcount to (datcount+(nxvls-1)).
compute xtmploc={xtmploc;k4}.
end loop.
compute xtmploc=xtmploc(2:nrow(xtmploc),1).
compute datcount=datcount+nxvls.
end if.
compute datindx(start:(start+nrow(xtmploc)-1),(i-1))=xtmploc.
compute wherex(1,(i-1))=start+1.
compute wherex(2,(i-1))=start+nrow(xtmploc)-1+1.
do if (model = 74).
end if.
compute onebl=make(nrow(xtmploc),1,1).
compute fochigh((start+1):(start+nrow(xtmploc)),foccnt)=onebl.
compute start=start+nrow(xtmploc).
end if.
do if (j > 1 and bcmat(i,j)=1).
compute outv={outv,mtmp(:,(j-1))}.
compute modlabel={modlabel;mnames(1,(j-1))}.
do if (mtmpuse(1,(j-1))=0).
compute fulldat={fulldat,mtmp(:,(j-1))}.
compute mtmpuse(1,(j-1))=1.
compute mtmploc(1,(j-1))=datcount.
compute datcount=datcount+1.
end if.
compute datindx(start:(start+nrow(mtmploc)-1),(i-1))=mtmploc(1,(j-1)).
compute wherem((j-1),(i-1))=start+1.
compute onebl=make(nrow(mtmploc(1,j-1)),1,1).
compute ttt=nrow(mtmploc(1,(j-1)))+start-1.
compute fochigh((start+1):(start+nrow(mtmploc(1,(j-1)))),foccnt)=onebl.
compute start=start+nrow(mtmploc(1,(j-1))).
end if.
end loop.
do if (wsum > 0).
loop j = 1 to (i-1).
compute whighct=whighct+1.
do if (j = 1 and wcmat(i,j)=1).
do if (wdid=0).
compute outv={outv,wtmp}.
do if (ncs > 0 and wiscov > 0).
compute ccmatoff((i-1),wiscov)=0.
end if.
compute modlabel={modlabel;wcatlab(1:nwvls,1)}.
compute wdid=1.
do if (wtmpuse=0).
compute fulldat={fulldat,wtmp}.
do if (ncs > 0 and wiscov > 0).
compute ccmatoff((i-1),wiscov)=0.
end if.
compute wtmpuse=1.
loop k4=datcount to (datcount+(nwvls-1)).
compute wtmploc={wtmploc;k4}.
end loop.
compute wtmploc=wtmploc(2:nrow(wtmploc),1).
compute datcount=datcount+nwvls.
end if.
end if.
compute datindx(start:(start+nrow(wtmploc)-1),(i-1))=wtmploc.
compute wherew(1,(i-1))=start+1.
compute wherew(2,(i-1))=start+nrow(wtmploc)-1+1.
compute start=start+nrow(wtmploc).
loop k1=1 to nxvls.
loop k2 = 1 to nwvls.
compute outv={outv,(xtmp(:,k1)&*wtmp(:,k2))}.
do if (ncs > 0 and wiscov > 0).
compute ccmatoff((i-1),wiscov)=0.
end if.
compute modlabel={modlabel;intlab(cntmp,1)}.
compute intkey={intkey;intlab(cntmp,1),':',xcatlab(k1,1),'x',wcatlab(k2,1),' ',' '}.
compute cntmp=cntmp+1.
end loop.
end loop.
do if (xwtmpus=0).
compute fulldat={fulldat,outv(:,(ncol(outv)-(nxvls*nwvls)+1):ncol(outv))}.
compute xwtmpus=1.
do if (ncs > 0 and wiscov > 0).
compute ccmatoff((i-1),wiscov)=0.
end if.
loop k4=datcount to (datcount+((nwvls*nxvls)-1)).
compute xwtmplo={xwtmplo;k4}.
end loop.
compute xwtmplo=xwtmplo(2:nrow(xwtmplo),1).
compute datcount=datcount+(nxvls*nwvls).
end if.
compute datindx(start:(start+nrow(xwtmplo)-1),(i-1))=xwtmplo.
compute wherexw(1,(i-1))=start+1.
compute wherexw(2,(i-1))=start+nrow(xwtmplo)-1+1.
compute onebl=make(nrow(xwtmplo),1,1).
compute whigh((start+1):(start+nrow(xwtmplo)),whighct)=onebl.
compute start=start+nrow(xwtmplo).
end if.
do if (j > 1 and wcmat(i,j)=1).
do if (wdid=0 and model <> 74).
compute outv={outv,wtmp}.
do if (ncs > 0 and wiscov > 0).
compute ccmatoff((i-1),wiscov)=0.
end if.
compute modlabel={modlabel;wcatlab(1:nwvls,1)}.
compute wdid=1.
do if (wtmpuse=0).
compute fulldat={fulldat,wtmp}.
do if (ncs > 0 and wiscov > 0).
compute ccmatoff((i-1),wiscov)=0.
end if.
compute wtmpuse=1.
loop k4=datcount to (datcount+(nwvls-1)).
compute wtmploc={wtmploc;k4}.
end loop.
compute wtmploc=wtmploc(2:nrow(wtmploc),1).
compute datcount=datcount+nwvls.
end if.
compute datindx(start:(start+nrow(wtmploc)-1),(i-1))=wtmploc.
compute wherew(1,(i-1))=start+1.
compute wherew(2,(i-1))=start+nrow(wtmploc)-1+1.
compute start=start+nrow(wtmploc).
end if.
loop k2 = 1 to nwvls.
compute outv={outv,(mtmp(:,(j-1))&*wtmp(:,k2))}.
do if (ncs > 0 and wiscov > 0).
compute ccmatoff((i-1),wiscov)=0.
end if.
compute modlabel={modlabel;intlab(cntmp,1)}.
compute intkey={intkey;intlab(cntmp,1),':', mnames(1,(j-1)),'x',wcatlab(k2,1),' ',' '}.
compute cntmp=cntmp+1.
end loop.
do if (mwtmpus(1,(j-1))=0).
compute fulldat={fulldat,outv(:,(ncol(outv)-nwvls+1):ncol(outv))}.
do if (ncs > 0 and wiscov > 0).
compute ccmatoff((i-1),wiscov)=0.
end if.
compute mwtmpus(1,(j-1))=1.
compute mw22=-999.
loop k4=datcount to (datcount+(nwvls-1)).
compute mw22={mw22;k4}.
end loop.
compute mwtmplo(:,(j-1))=mw22(2:nrow(mw22),1).
compute datcount=datcount+nwvls.
end if.
compute datindx(start:(start+nrow(mwtmplo)-1),(i-1))=mwtmplo(:,(j-1)).
compute wheremw(((2*j)-3),(i-1))=start+1.
compute wheremw(((2*j)-2),(i-1))=start+nrow(mwtmplo)-1+1.
compute onebl=make(nrow(mwtmplo),1,1).
compute whigh((start+1):(start+nrow(mwtmplo)),whighct)=onebl.
compute start=start+nrow(mwtmplo).
end if.
end loop.
end if.
do if (zsum > 0).
loop j = 1 to (i-1).
compute zhighct=zhighct+1.
do if (j = 1 and zcmat(i,j)=1).
do if (zdid=0).
compute outv={outv,ztmp}.
do if (ncs > 0 and ziscov > 0).
compute ccmatoff((i-1),ziscov)=0.
end if.
compute modlabel={modlabel;zcatlab(1:nzvls,1)}.
compute zdid=1.
do if (ztmpuse=0).
compute fulldat={fulldat,ztmp}.
do if (ncs > 0 and ziscov > 0).
compute ccmatoff((i-1),ziscov)=0.
end if.
compute ztmpuse=1.
loop k4=datcount to (datcount+(nzvls-1)).
compute ztmploc={ztmploc;k4}.
end loop.
compute ztmploc=ztmploc(2:nrow(ztmploc),1).
compute datcount=datcount+nzvls.
end if.
end if.
compute datindx(start:(start+nrow(ztmploc)-1),(i-1))=ztmploc.
compute wherez(1,(i-1))=start+1.
compute wherez(2,(i-1))=start+nrow(ztmploc)-1+1.
compute start=start+nrow(ztmploc).
loop k1=1 to nxvls.
loop k2 = 1 to nzvls.
compute outv={outv,(xtmp(:,k1)&*ztmp(:,k2))}.
do if (ncs > 0 and ziscov > 0).
compute ccmatoff((i-1),ziscov)=0.
end if.
compute modlabel={modlabel;intlab(cntmp,1)}.
compute intkey={intkey;intlab(cntmp,1),':',xcatlab(k1,1),'x',zcatlab(k2,1),' ',' '}.
compute cntmp=cntmp+1.
end loop.
end loop.
do if (xztmpus=0).
compute fulldat={fulldat,outv(:,(ncol(outv)-(nxvls*nzvls)+1):ncol(outv))}.
do if (ncs > 0 and ziscov > 0).
compute ccmatoff((i-1),ziscov)=0.
end if.
compute xztmpus=1.
loop k4=datcount to (datcount+((nzvls*nxvls)-1)).
compute xztmplo={xztmplo;k4}.
end loop.
compute xztmplo=xztmplo(2:nrow(xztmplo),1).
compute datcount=datcount+(nxvls*nzvls).
end if.
compute datindx(start:(start+nrow(xztmplo)-1),(i-1))=xztmplo.
compute wherexz(1,(i-1))=start+1.
compute wherexz(2,(i-1))=start+nrow(xztmplo)-1+1.
compute onebl=make(nrow(xztmplo),1,1).
compute zhigh((start+1):(start+nrow(xztmplo)),zhighct)=onebl.
compute start=start+nrow(xztmplo).
end if.
do if (j > 1 and zcmat(i,j)=1).
do if (zdid=0).
compute outv={outv,ztmp}.
do if (ncs > 0 and ziscov > 0).
compute ccmatoff((i-1),ziscov)=0.
end if.
compute modlabel={modlabel;zcatlab(1:nzvls,1)}.
compute zdid=1.
do if (ztmpuse=0).
compute fulldat={fulldat,ztmp}.
do if (ncs > 0 and ziscov > 0).
compute ccmatoff((i-1),ziscov)=0.
end if.
compute ztmpuse=1.
loop k4=datcount to (datcount+(nzvls-1)).
compute ztmploc={ztmploc;k4}.
end loop.
compute ztmploc=ztmploc(2:nrow(ztmploc),1).
compute datcount=datcount+nzvls.
end if.
compute datindx(start:(start+nrow(ztmploc)-1),(i-1))=ztmploc.
compute wherez(1,(i-1))=start+1.
compute wherez(2,(i-1))=start+nrow(ztmploc)-1+1.
compute start=start+nrow(ztmploc).
end if.
loop k2 = 1 to nzvls.
compute outv={outv,(mtmp(:,(j-1))&*ztmp(:,k2))}.
do if (ncs > 0 and ziscov > 0).
compute ccmatoff((i-1),ziscov)=0.
end if.
compute modlabel={modlabel;intlab(cntmp,1)}.
compute intkey={intkey;intlab(cntmp,1),':', mnames(1,(j-1)),'x',zcatlab(k2,1),' ',' '}.
compute cntmp=cntmp+1.
end loop.
do if (mztmpus(1,(j-1))=0).
compute fulldat={fulldat,outv(:,(ncol(outv)-nzvls+1):ncol(outv))}.
do if (ncs > 0 and ziscov > 0).
compute ccmatoff((i-1),ziscov)=0.
end if.
compute mztmpus(1,(j-1))=1.
compute mz22=-999.
loop k4=datcount to (datcount+(nzvls-1)).
compute mz22={mz22;k4}.
end loop.
compute mztmplo(:,(j-1))=mz22(2:nrow(mz22),1).
compute datcount=datcount+nzvls.
end if.
compute datindx(start:(start+nrow(mztmplo)-1),(i-1))=mztmplo(:,(j-1)).
compute wheremz(((2*j)-3),(i-1))=start+1.
compute wheremz(((2*j)-2),(i-1))=start+nrow(mztmplo)-1+1.
compute onebl=make(nrow(mztmplo),1,1).
compute zhigh((start+1):(start+nrow(mztmplo)),zhighct)=onebl.
compute start=start+nrow(mztmplo).
end if.
end loop.
end if.
do if (wzsum > 0).
loop j = 1 to (i-1).
compute wzhighct=wzhighct+1.
do if (j = 1 and wzcmat(i,j)=1).
do if (wzdid=0).
loop k1=1 to nwvls.
loop k2 = 1 to nzvls.
compute outv={outv,(wtmp(:,k1)&*ztmp(:,k2))}.
do if (ncs > 0 and (ziscov > 0)).
compute ccmatoff((i-1),ziscov)=0.
end if.
do if (ncs > 0 and (wiscov > 0)).
compute ccmatoff((i-1),wiscov)=0.
end if.
compute modlabel={modlabel;intlab(cntmp,1)}.
compute intkey={intkey;intlab(cntmp,1),':',wcatlab(k1,1),'x',zcatlab(k2,1),' ',' '}.
compute cntmp=cntmp+1.
end loop.
end loop.
do if (wztmpus=0).
compute fulldat={fulldat,outv(:,(ncol(outv)-(nwvls*nzvls)+1):ncol(outv))}.
do if (ncs > 0 and (ziscov > 0)).
compute ccmatoff((i-1),ziscov)=0.
end if.
do if (ncs > 0 and (wiscov > 0)).
compute ccmatoff((i-1),wiscov)=0.
end if.
compute wztmpus=1.
loop k4=datcount to (datcount+((nwvls*nzvls)-1)).
compute wztmplo={wztmplo;k4}.
end loop.
compute wztmplo=wztmplo(2:nrow(wztmplo),1).
compute datcount=datcount+(nzvls*nwvls).
end if.
compute wzdid=1.
end if.
compute datindx(start:(start+nrow(wztmplo)-1),(i-1))=wztmplo.
compute wherewz(1,(i-1))=start+1.
compute wherewz(2,(i-1))=start+nrow(wztmplo)-1+1.
compute start=start+nrow(wztmplo).
loop k1=1 to nxvls.
loop k2=1 to nwvls.
loop k3=1 to nzvls.
compute outv={outv,(xtmp(:,k1)&*wtmp(:,k2)&*ztmp(:,k3))}.
do if (ncs > 0 and (ziscov > 0)).
compute ccmatoff((i-1),ziscov)=0.
end if.
do if (ncs > 0 and (wiscov > 0)).
compute ccmatoff((i-1),wiscov)=0.
end if.
compute modlabel={modlabel;intlab(cntmp,1)}.
compute intkey={intkey;intlab(cntmp,1),':',xcatlab(k1,1),'x',wcatlab(k2,1),'x', zcatlab(k3,1)}.
compute cntmp=cntmp+1.
end loop.
end loop.
end loop.
do if (xwztmpu=0).
compute fulldat={fulldat,outv(:,(ncol(outv)-(nxvls*nwvls*nzvls)+1):ncol(outv))}.
do if (ncs > 0 and (ziscov > 0)).
compute ccmatoff((i-1),ziscov)=0.
end if.
do if (ncs > 0 and (wiscov > 0)).
compute ccmatoff((i-1),wiscov)=0.
end if.
compute xwztmpu=1.
loop k4=datcount to (datcount+((nzvls*nxvls*nwvls)-1)).
compute xwztmplo={xwztmplo;k4}.
end loop.
compute xwztmplo=xwztmplo(2:nrow(xwztmplo),1).
compute datcount=datcount+(nxvls*nzvls*nwvls).
end if.
compute datindx(start:(start+nrow(xwztmplo)-1),(i-1))=xwztmplo.
compute wherexwz(1,(i-1))=start+1.
compute wherexwz(2,(i-1))=start+nrow(xwztmplo)-1+1.
compute onebl=make(nrow(xwztmplo),1,1).
compute wzhigh((start+1):(start+nrow(xwztmplo)),wzhighct)=onebl.
compute start=start+nrow(xwztmplo).
end if.
do if (j > 1 and wzcmat(i,j)=1).
do if (wzdid=0).
loop k1=1 to nwvls.
loop k2 = 1 to nzvls.
compute outv={outv,(wtmp(:,k1)&*ztmp(:,k2))}.
do if (ncs > 0 and (ziscov > 0)).
compute ccmatoff((i-1),ziscov)=0.
end if.
do if (ncs > 0 and (wiscov > 0)).
compute ccmatoff((i-1),wiscov)=0.
end if.
compute modlabel={modlabel;intlab(cntmp,1)}.
compute intkey={intkey;intlab(cntmp,1),':',wcatlab(k1,1),'x',zcatlab(k2,1),' ',' '}.
compute cntmp=cntmp+1.
end loop.
end loop.
do if (wztmpus=0).
compute fulldat={fulldat,outv(:,(ncol(outv)-(nwvls*nzvls)+1):ncol(outv))}.
do if (ncs > 0 and (ziscov > 0)).
compute ccmatoff((i-1),ziscov)=0.
end if.
do if (ncs > 0 and (wiscov > 0)).
compute ccmatoff((i-1),wiscov)=0.
end if.
compute wztmpus=1.
loop k4=datcount to (datcount+((nwvls*nzvls)-1)).
compute wztmplo={wztmplo;k4}.
end loop.
compute wztmplo=wztmplo(2:nrow(wztmplo),1).
compute datcount=datcount+(nzvls*nwvls).
end if.
compute wzdid=1.
compute datindx(start:(start+nrow(wztmplo)-1),(i-1))=wztmplo.
compute wherewz(1,(i-1))=start+1.
compute wherewz(2,(i-1))=start+nrow(wztmplo)-1+1.
compute start=start+nrow(wztmplo).
end if.
loop k1 = 1 to nwvls.
loop k2 = 1 to nzvls.
compute outv={outv,(mtmp(:,(j-1))&*wtmp(:,k1)&*ztmp(:,k2))}.
do if (ncs > 0 and (ziscov > 0)).
compute ccmatoff((i-1),ziscov)=0.
end if.
do if (ncs > 0 and (wiscov > 0)).
compute ccmatoff((i-1),wiscov)=0.
end if.
compute modlabel={modlabel;intlab(cntmp,1)}.
compute intkey={intkey;intlab(cntmp,1),':',mnames(1,(j-1)),'x',wcatlab(k1,1),'x', zcatlab(k2,1)}.
compute cntmp=cntmp+1.
end loop.
end loop.
do if (mwztmpu(1,(j-1))=0).
compute fulldat={fulldat,outv(:,(ncol(outv)-(nwvls*nzvls)+1):ncol(outv))}.
do if (ncs > 0 and (ziscov > 0)).
compute ccmatoff((i-1),ziscov)=0.
end if.
do if (ncs > 0 and (wiscov > 0)).
compute ccmatoff((i-1),wiscov)=0.
end if.
compute mwztmpu(1,(j-1))=1.
compute mz22=-999.
loop k4=datcount to (datcount+(nwvls*nzvls)-1).
compute mz22={mz22;k4}.
end loop.
compute mwztmplo(:,(j-1))=mz22(2:nrow(mz22),1).
compute datcount=datcount+(nwvls*nzvls).
end if.
compute datindx(start:(start+nrow(mwztmplo)-1),(i-1))=mwztmplo(:,(j-1)).
compute wheremwz(((2*j)-3),(i-1))=start+1.
compute wheremwz(((2*j)-2),(i-1))=start+nrow(mwztmplo)-1+1.
compute onebl=make(nrow(mwztmplo),1,1).
compute wzhigh((start+1):(start+nrow(mwztmplo)),wzhighct)=onebl.
compute start=start+nrow(mwztmplo).
end if.
end loop.
end if.
do if (ncs > 0).
compute ccmat=ccmat&*ccmatoff.
loop j = 1 to ncs.
do if (ccmat((i-1),j))=1.
do if (j=wiscov).
compute ctmp(:,j)=wtmp.
end if.
do if (j=ziscov).
compute ctmp(:,j)=ztmp.
end if.
compute outv={outv,ctmp(:,j)}.
compute modlabel={modlabel;covnames(1,j)}.
do if (ctmpuse(1,j)=0).
compute fulldat={fulldat,ctmp(:,j)}.
compute ctmpuse(1,j)=1.
compute ctmploc(1,j)=datcount.
compute datcount=datcount+1.
end if.
compute datindx(start:(start+nrow(ctmploc)-1),(i-1))=ctmploc(1,j).
compute start=start+nrow(ctmploc(1,j)).
end if.
end loop.
end if.
compute wdid=0.
compute zdid=0.
compute wzdid=0.
compute vlabs={vlabs;modlabel(2:nrow(modlabel),1)}.
compute numint(1,(i-1))=cntmp-1.
compute nump(1,(i-1))=nrow(modlabel)-1.
end loop.
release datcount, xtmpuse, wtmpuse, ztmpuse, xwtmpus, xztmpus, wztmpus, xwztmpu.
release xtmploc, wtmploc, xwtmplo, ztmploc, xztmplo, wztmplo, xwztmplo, foccnt.
do if (modcok=1 and ((nms > 0) or (zcmat(2,1) <> 1) or (mcx <> 0))).
compute notecode(notes,1) = 19.
compute notes = notes + 1.
compute modcok=0.
end if.
do if ((serial = 1 or (rsum(numint)>0) or nms=0) and mc > 0).
compute notecode(notes,1) = 15.
compute notes = notes + 1.
compute boot=mc.
compute mc=0.
end if.
do if (boot <> 0 or mc <> 0).
compute bootsz=boot.
do if (mc > 0).
compute bootsz=mc.
compute saveboot=0.
end if.
loop.
compute cilow = rnd(bootsz*(1-(conf/100))/2).
compute cihigh = trunc((bootsz*(conf/100)+(bootsz*(1-(conf/100))/2)))+1.
do if (cilow < 1 or cihigh > bootsz).
compute bootsz=trunc((bootsz+1000)/1000)*1000.
compute adjust = 1.
end if.
end loop if (cilow gt 0 and cihigh le bootsz).
do if (boot > 0).
compute boot=bootsz.
end if.
do if (mc > 0).
compute mc=bootsz.
end if.
do if (adjust = 1 and boot > 0).
compute notecode(notes,1) = 8.
compute notes = notes + 1.
end if.
do if (adjust = 1 and mc > 0).
compute notecode(notes,1) = 16.
compute notes = notes + 1.
end if.
end if.
compute maxboot = trunc(2*boot).
do if ( 0 > maxboot).
compute maxboot=trunc( 0 ).
end if.
do if (nms > 0).
release mtmpuse, mwtmpus, mwztmpu, mtmploc, mwtmplo, mztmplo, mwztmplo.
end if.
release wdid, zdid, wzdid, start,modlabel.
compute vlabs=vlabs(2:nrow(vlabs),1).
do if (rsum(numint) > 0).
compute intkey=intkey(2:nrow(intkey),:).
end if.
compute fulldat=fulldat(:,2:ncol(fulldat)).
compute fochigh=fochigh(1:rmax(nump),:).
compute whigh=whigh(1:rmax(nump),:).
compute zhigh=zhigh(1:rmax(nump),:).
compute wzhigh=wzhigh(1:rmax(nump),:).
compute coeffs=fochigh+whigh+zhigh+wzhigh.
compute bootloc=make(rmax(nump),ncol(nump),0).
do if (nms > 0).
compute cntmp=1.
loop i = 1 to ncol(nump).
loop j = 1 to nump(1,i).
compute bootloc(j,i)=cntmp.
compute cntmp=cntmp+1.
end loop.
end loop.
compute fochighb=make(nrow(fochigh),ncol(fochigh),0).
compute whighb=fochighb.
compute zhighb=fochighb.
compute wzhighb=fochighb.
compute thetaxmb=make(nrow(fochighb),nms,0).
compute thetaxyb=make(nrow(fochighb),1,0).
compute pathsfoc=make(nxvls,1,0).
compute cntmp=1.
loop i = 1 to (nms+nys).
loop j = 1 to i.
compute fochighb(:,cntmp)=fochigh(:,cntmp)&*bootloc(:,i).
compute whighb(:,cntmp)=whigh(:,cntmp)&*bootloc(:,i).
compute zhighb(:,cntmp)=zhigh(:,cntmp)&*bootloc(:,i).
compute wzhighb(:,cntmp)=wzhigh(:,cntmp)&*bootloc(:,i).
compute coeffsb=fochighb+whighb+zhighb+wzhighb.
do if ((i < (nms+nys)) and (j = 1)).
compute thetaxmb(:,i)=coeffsb(:,cntmp).
end if.
do if ((i = (nms+nys)) and (j = 1)).
compute thetaxyb(:,1)=coeffsb(:,cntmp).
end if.
compute cntmp=cntmp+1.
end loop.
end loop.
compute thetamyb=coeffsb(:,(ncol(coeffsb)-nms+1):ncol(coeffsb)).
do if (serial = 1).
compute thetammb=make(nrow(coeffsb),((nms*(nms-1))/2),0).
end if.
compute cntmp=1.
do if (nms > 1 and serial = 1).
loop i = 1 to (nms-1).
compute start=((i+2)*(i+1))/2.
loop j = 2 to (nms-i+1).
compute thetammb(:,cntmp)=coeffsb(:,start).
compute start=start+j+i-1.
compute cntmp=cntmp+1.
end loop.
end loop.
end if.
end if.
do if ((total = 1) and (rsum(numint)=0 or (xmint=1))).
compute dototal=1.
do if ((csum(bcmat(:,1)) <> (nms+nys)) or (rsum(bcmat(nrow(bcmat),:)) <> (nms+nys))).
compute dototal=0.
compute alttotal=1.
compute notecode(notes,1) = 12.
compute notes = notes + 1.
end if.
do if (ncs > 0).
do if ((csum(rsum(ccmat))) < (nrow(ccmat)*ncol(ccmat))).
compute dototal=0.
compute alttotal=1.
compute notecode(notes,1) = 11.
compute notes = notes + 1.
end if.
end if.
do if (model=74).
do if (xdich=0 and nxvls=1).
compute dototal=0.
compute alttotal=1.
end if.
do if ((xdich=1 or nxvls > 1) and (ncs > 0) and model=74).
compute dototal=0.
compute alttotal=1.
end if.
end if.
end if.
end if.
do if (criterr=0 and ncs > 0).
do if (rsum((csum(ccmat)=0)) <> 0).
compute errcode(errs,1)=51.
compute errs=errs+1.
compute criterr=1.
end if.
end if.
compute debug= 0.
do if (outscree=1).
print/title = '***************** PROCESS Procedure for SPSS Version 4.2 *****************'.
print/title = '          Written by Andrew F. Hayes, Ph.D.       www.afhayes.com'.
print/title = '    Documentation available in Hayes (2022). www.guilford.com/p/hayes3'/space=0.
end if.
do if (criterr=0).
compute modresid=make(n,1,99999).
do if (stand=1 and ydich=1).
compute stand=0.
end if.
compute anymod2=csum(rsum(wcmat+zcmat+wzcmat)).
do if (anymod2 > 0 and stand = 1).
compute notecode(notes,1) = 27.
compute notes = notes + 1.
compute stand=0.
end if.
compute funny=1.
do if (outscree=1).
print modelvar/title = '**************************************************************************'/format = A8/rnames=modelvlb.
do if (ncs > 0).
print covnames/title='Covariates:'/format=A8.
end if.
print n/title='Sample'/rlabel='Size:'.
do if (( 'random' <> 'random')).
compute seedt= 'random'.
print seedt/title='Custom'/format=A12/rlabel = 'Seed:'.
end if.
end if.
compute maxresm=9.
compute resultm=make(1,maxresm,99999).
do if (describe=1).
compute means=csum(dat)/n.
compute sigmatal = (t(dat)*(ident(n)-(1/n)*ones*t(ones))*dat)*(1/(n-1)).
compute sdvec=sqrt(diag(sigmatal)).
compute sdall = mdiag(1/sdvec).
compute corall=sdall*sigmatal*t(sdall).
compute means={means;t(sdvec)}.
do if (ncol(means) > 9).
compute resultm=make(1,ncol(means),99999).
compute maxresm=ncol(means).
end if.
compute resultm2=make(2,maxresm,99999).
compute resultm2(1:2,1:ncol(means))=means.
compute resultm={resultm;resultm2}.
compute resultm2=make(ncol(corall),maxresm,99999).
compute resultm2(1:ncol(corall),1:ncol(corall))=corall.
compute resultm={resultm;resultm2}.
do if (outscree=1).
print means/title='Variable means and standard deviations'/cnames=varnames/rlabels='Mean','SD'/format= F10.4.
print corall/title='Variable intercorrelations (Pearson r)'/cnames=varnames/rnames=varnames/format= F10.4.
end if.
end if.
do if (outscree=1).
do if (mcxok=1).
compute labtmp={xnames,t(xcatlab(1:nxvls,1))}.
print dummatx/title = 'Coding of categorical X variable for analysis:'/cnames = labtmp/format = F6.3.
end if.
do if (mcwok=1 and xmint=0).
compute labtmp={wnames,t(wcatlab(1:nwvls,1))}.
print dummatw/title = 'Coding of categorical W variable for analysis:'/cnames = labtmp/format = F6.3.
end if.
do if (mczok=1).
compute labtmp={znames,t(zcatlab(1:nzvls,1))}.
print dummatz/title = 'Coding of categorical Z variable for analysis:'/cnames = labtmp/format = F6.3.
end if.
end if.
end if.
do if (criterr = 0).
compute outnames=ynames.
compute outvars=ytmp.
do if (nms > 0).
compute outnames={mnames,ynames}.
compute outvars={mtmp,ytmp}.
compute indcov=make(((nms*2)+(nms*(nxvls-1))),((nms*2)+(nms*(nxvls-1))),0).
compute mcsopath=make(((nms*2)+(nms*(nxvls-1))),1,0).
end if.
compute labstart=1.
compute intstart=1.
compute start=1.
compute coeffmat=make(1,6,0).
compute conseq={'        '}.
compute dfmat=0.
compute coeffcol=0.
compute pathscnt=1.
compute pathscn2=1.
loop i = 1 to (nms+nys).
do if (outscree=1).
print/title = '**************************************************************************'.
end if.
compute highf=make(1,5,0).
compute highf2=highf.
do if ((i = (nms+nys)) and (ydich=1)).
compute highf=make(1,3,0).
compute highf2=highf.
end if.
compute flabel={' '}.
compute y=outvars(:,i).
compute xindx=datindx(1:(nump(1,i)-1),i).
compute x = fulldat(:,xindx).
compute x={ones,x}.
compute xsq=t(x)*x.
compute exsq=eval(xsq).
release xsq.
compute zeroeig=csum(exsq <= 0.000000000002).
do if (outscree=1).
print outnames(1,i)/title = 'OUTCOME VARIABLE:'/format = A8/space=0.
do if (ydich=1 and (i = (nms+nys))).
compute nmsd = {outnames(1,i), 'Analysis'}.
print rcd/title = 'Coding of binary Y for logistic regression analysis:'/cnames = nmsd/format = F9.2.
end if.
end if.
do if (zeroeig > 0).
print / title = 'SINGULAR OR NEAR SINGULAR DATA MATRIX.'.
compute criterr=1.
compute errcode(errs,1)=31.
compute errs=errs+1.
end if.
compute means=csum(x)/n.
compute vlabsm=vlabs(labstart:(labstart+(nump(1,i)-1)),1).
do if (criterr=0).
do if (ydich=0 or (i < (nms+nys))).

.
do if ( 1 =1).
compute hatmat=inv(t( x )* x )*t( x ).
compute b = hatmat* y.
compute modres=b.
do if ( 1 =1).
compute n1=nrow( x ).
compute dfres=n1-(ncol( x )).
compute sstotal = t( y -(csum( y )/n1))*( y -(csum( y )/n1)).
compute resid= y - x *b.
compute ssresid = csum((resid)&**2).
compute r2 = (sstotal-ssresid)/sstotal.
do if (r2 < 0).
compute r2=0.
end if.
compute adjr2 = 1-((1-r2)*(n1-1)/(dfres)).
compute mse=ssresid/(n1-ncol( x )).

.
compute n1=nrow( x ).
compute invXtX = inv(t( x )* x ).
compute varb = mse *invXtX.
compute k3 = ncol( x ).
compute xhc=0.
do if ( hc <> 5).
compute xhc= x.
compute hat = xhc(:,1).
loop i3=1 to nrow(xhc).
compute hat(i3,1)= xhc(i3,:)*invXtX*t(xhc(i3,:)).
end loop.
do if ( hc = 0 or hc =1).
loop i3 = 1 to k3.
compute xhc(:,i3)=xhc(:,i3)&* resid.
end loop.
end if.
do if ( hc =3 or hc =2).
loop i3=1 to k3.
compute xhc(:,i3) = ( resid &/(1-hat)&**(1/(4- hc )))&*xhc(:,i3).
end loop.
end if.
do if ( hc = 4).
compute hcmn=make(n,2,4).
compute hcmn(:,2)=(n1*hat)/k3.
loop i3= 1 to k3.
compute xhc(:,i3) = ( resid &/(1-hat)&**(rmin(hcmn)/2))&*xhc(:,i3).
end loop.
end if.
compute varb=(invXtX*t(xhc)*xhc*invXtX).
do if ( hc =1).
compute varb=(n1/(n1-ncol( x )))&*varb.
end if.
end if.
compute hclab={'se(HC0)','se(HC1)','se(HC2)','se(HC3)','se(HC4)','se'}.
compute hclab=hclab(1,( hc +1)).
compute hcflab={'F(HC0)','F(HC1)','F(HC2)','F(HC3)','F(HC4)','F'}.
compute hcflab=hcflab(1,( hc +1)).
release xhc
.
compute seb=sqrt(diag(varb)).
compute trat = b&/seb.
compute p = 2*(1-tcdf(abs(trat), (dfres))).
compute tval = sqrt(dfres* (exp((dfres-(5/6))*((xp2/(dfres-(2/3)+(.11/dfres)))* (xp2/(dfres-(2/3)+(.11/dfres)))))-1)).
compute modres={modres,seb,trat,p}.
compute modres={modres,(b-tval&*seb),(b+tval&*seb)}.
compute modresl={'coeff',hclab,'t','p','LLCI','ULCI'}.
compute lmat = ident(ncol( x )).
compute lmat = lmat(:,2:ncol(lmat)).
compute fratio = (t(t(lmat)*b)*inv(t(lmat)*varb*lmat)*((t(lmat)*b)))/(ncol( x )-1).
compute pfr = 1-fcdf(fratio,(ncol( x )-1),dfres).
compute modsum={sqrt(r2),r2,mse,fratio,(ncol( x )-1),dfres,pfr}.
compute modsuml={'R','R-sq','MSE',hcflab,'df1','df2', 'p'}.
end if.
end if.
do if ( 1 = 2 or 1 =3).
compute xlp= x.
compute ylp= y.
compute pt2 = make(nrow(ylp),1,(csum(ylp)/nrow(ylp))).
do if ( 1 =2).
compute LL3 = ylp&*ln(pt2)+(1-ylp)&*ln(1-pt2).
end if.
compute LL3 = -2*csum(LL3).
compute bt1 = make(ncol(xlp),1,0).
compute LL1 = 0.
compute pt1 = make(nrow(ylp),1,0.5).
compute pt1lp=pt1.
loop jjj = 1 to iterate.
compute xlptmp=t(xlp).
compute vecprb=pt1lp&*(1-pt1lp).
loop kkk=1 to ncol(xlp).
compute xlptmp(kkk,:)=xlptmp(kkk,:)&*t(vecprb).
end loop.
compute b = bt1+inv(xlptmp*xlp)*t(xlp)*(ylp-pt1lp).
do if ( 1 =2).
compute xlpb=xlp*b.
compute xlpbt=(xlpb > -709.7).
compute xlpb709=(1-xlpbt)*(-709.7).
compute xlpb=(xlpb&*xlpbt)+xlpb709.
compute pt1lp = 1/(1+exp(-(xlpb))).
end if.
compute itprob = csum((pt1lp < .00000001) or (pt1lp > .9999999)).
do if (itprob > 0).
loop kkk = 1 to nrow(pt1lp).
do if (pt1lp(kkk,1) > .9999999).
compute pt1lp(kkk,1) = .9999999.
end if.
do if (pt1lp(kkk,1) < .00000001).
compute pt1lp(kkk,1) = .00000001.
end if.
end loop.
compute itprob = 0.
end if.
do if (itprob = 0).
do if ( 1 =2).
compute LL = ylp&*ln(pt1lp)+(1-ylp)&*ln(1-pt1lp).
end if.
compute LL2 = -2*csum(ll).
end if.
do if (abs(LL1-LL2) < converge).
do if ( 1 =1).
compute xlptmp=t(xlp).
compute vecprb=pt1lp&*(1-pt1lp).
loop kkk=1 to ncol(xlp).
compute xlptmp(kkk,:)=xlptmp(kkk,:)&*t(vecprb).
end loop.
compute varb = inv(xlptmp*xlp).
compute seb = sqrt(diag(varb)).
release xlptmp.
end if.
break.
end if.
compute bt1 = b.
compute LL1 = LL2.
end loop.
compute modres=b.
do if (jjj > iterate).
compute itprob = 2.
do if (booting=0).
compute iterrmod=1.
end if.
do if (booting=1).
compute bootiter=1.
end if.
do if (itprobtg=0).
compute itprobtg=1.
compute errcode(errs,1) = 47.
compute errs = errs + 1.
do if (booting = 0 and 1 =1).
compute vt1 = mdiag(pt1lp&*(1-pt1lp)).
compute varb = inv(t(xlp)*vt1*xlp).
compute seb = sqrt(diag(varb)).
end if.
end if.
end if.
do if ( 1 =1).
compute trat = b&/seb.
compute dfres=nrow(xlp).
compute p = 2*(1-cdfnorm(abs(trat))).
compute modres={modres,seb,trat,p}.
compute modres={modres,(b-xp2&*seb),(b+xp2&*seb)}.
compute pvchi=1-chicdf((LL3-LL2),(nrow(modres)-1)).
compute mcF = (LL3-LL2)/LL3.
compute cox = 1-exp(-(LL3-LL2)/nrow(xlp)).
compute nagel = cox/(1-exp(-(LL3)/nrow(xlp))).
compute modsum={LL2,(LL3-LL2),(nrow(modres)-1),pvchi, mcF,cox,nagel}.
compute modsuml={'-2LL','ModelLL', 'df', 'p', 'McFadden', 'CoxSnell', 'Nagelkrk'}.
compute modresl={'coeff','se','Z','p','LLCI','ULCI'}.
end if.
end if
.
compute dfmatt=make(nrow(modres),1,modsum(1,6)).
compute modresid={modresid,resid}.
end if.
do if (ydich=1 and (i = (nms+nys))).

.
do if ( 2 =1).
compute hatmat=inv(t( x )* x )*t( x ).
compute b = hatmat* y.
compute modres=b.
do if ( 1 =1).
compute n1=nrow( x ).
compute dfres=n1-(ncol( x )).
compute sstotal = t( y -(csum( y )/n1))*( y -(csum( y )/n1)).
compute resid= y - x *b.
compute ssresid = csum((resid)&**2).
compute r2 = (sstotal-ssresid)/sstotal.
do if (r2 < 0).
compute r2=0.
end if.
compute adjr2 = 1-((1-r2)*(n1-1)/(dfres)).
compute mse=ssresid/(n1-ncol( x )).

.
compute n1=nrow( x ).
compute invXtX = inv(t( x )* x ).
compute varb = mse *invXtX.
compute k3 = ncol( x ).
compute xhc=0.
do if ( hc <> 5).
compute xhc= x.
compute hat = xhc(:,1).
loop i3=1 to nrow(xhc).
compute hat(i3,1)= xhc(i3,:)*invXtX*t(xhc(i3,:)).
end loop.
do if ( hc = 0 or hc =1).
loop i3 = 1 to k3.
compute xhc(:,i3)=xhc(:,i3)&* resid.
end loop.
end if.
do if ( hc =3 or hc =2).
loop i3=1 to k3.
compute xhc(:,i3) = ( resid &/(1-hat)&**(1/(4- hc )))&*xhc(:,i3).
end loop.
end if.
do if ( hc = 4).
compute hcmn=make(n,2,4).
compute hcmn(:,2)=(n1*hat)/k3.
loop i3= 1 to k3.
compute xhc(:,i3) = ( resid &/(1-hat)&**(rmin(hcmn)/2))&*xhc(:,i3).
end loop.
end if.
compute varb=(invXtX*t(xhc)*xhc*invXtX).
do if ( hc =1).
compute varb=(n1/(n1-ncol( x )))&*varb.
end if.
end if.
compute hclab={'se(HC0)','se(HC1)','se(HC2)','se(HC3)','se(HC4)','se'}.
compute hclab=hclab(1,( hc +1)).
compute hcflab={'F(HC0)','F(HC1)','F(HC2)','F(HC3)','F(HC4)','F'}.
compute hcflab=hcflab(1,( hc +1)).
release xhc
.
compute seb=sqrt(diag(varb)).
compute trat = b&/seb.
compute p = 2*(1-tcdf(abs(trat), (dfres))).
compute tval = sqrt(dfres* (exp((dfres-(5/6))*((xp2/(dfres-(2/3)+(.11/dfres)))* (xp2/(dfres-(2/3)+(.11/dfres)))))-1)).
compute modres={modres,seb,trat,p}.
compute modres={modres,(b-tval&*seb),(b+tval&*seb)}.
compute modresl={'coeff',hclab,'t','p','LLCI','ULCI'}.
compute lmat = ident(ncol( x )).
compute lmat = lmat(:,2:ncol(lmat)).
compute fratio = (t(t(lmat)*b)*inv(t(lmat)*varb*lmat)*((t(lmat)*b)))/(ncol( x )-1).
compute pfr = 1-fcdf(fratio,(ncol( x )-1),dfres).
compute modsum={sqrt(r2),r2,mse,fratio,(ncol( x )-1),dfres,pfr}.
compute modsuml={'R','R-sq','MSE',hcflab,'df1','df2', 'p'}.
end if.
end if.
do if ( 2 = 2 or 2 =3).
compute xlp= x.
compute ylp= y.
compute pt2 = make(nrow(ylp),1,(csum(ylp)/nrow(ylp))).
do if ( 2 =2).
compute LL3 = ylp&*ln(pt2)+(1-ylp)&*ln(1-pt2).
end if.
compute LL3 = -2*csum(LL3).
compute bt1 = make(ncol(xlp),1,0).
compute LL1 = 0.
compute pt1 = make(nrow(ylp),1,0.5).
compute pt1lp=pt1.
loop jjj = 1 to iterate.
compute xlptmp=t(xlp).
compute vecprb=pt1lp&*(1-pt1lp).
loop kkk=1 to ncol(xlp).
compute xlptmp(kkk,:)=xlptmp(kkk,:)&*t(vecprb).
end loop.
compute b = bt1+inv(xlptmp*xlp)*t(xlp)*(ylp-pt1lp).
do if ( 2 =2).
compute xlpb=xlp*b.
compute xlpbt=(xlpb > -709.7).
compute xlpb709=(1-xlpbt)*(-709.7).
compute xlpb=(xlpb&*xlpbt)+xlpb709.
compute pt1lp = 1/(1+exp(-(xlpb))).
end if.
compute itprob = csum((pt1lp < .00000001) or (pt1lp > .9999999)).
do if (itprob > 0).
loop kkk = 1 to nrow(pt1lp).
do if (pt1lp(kkk,1) > .9999999).
compute pt1lp(kkk,1) = .9999999.
end if.
do if (pt1lp(kkk,1) < .00000001).
compute pt1lp(kkk,1) = .00000001.
end if.
end loop.
compute itprob = 0.
end if.
do if (itprob = 0).
do if ( 2 =2).
compute LL = ylp&*ln(pt1lp)+(1-ylp)&*ln(1-pt1lp).
end if.
compute LL2 = -2*csum(ll).
end if.
do if (abs(LL1-LL2) < converge).
do if ( 1 =1).
compute xlptmp=t(xlp).
compute vecprb=pt1lp&*(1-pt1lp).
loop kkk=1 to ncol(xlp).
compute xlptmp(kkk,:)=xlptmp(kkk,:)&*t(vecprb).
end loop.
compute varb = inv(xlptmp*xlp).
compute seb = sqrt(diag(varb)).
release xlptmp.
end if.
break.
end if.
compute bt1 = b.
compute LL1 = LL2.
end loop.
compute modres=b.
do if (jjj > iterate).
compute itprob = 2.
do if (booting=0).
compute iterrmod=1.
end if.
do if (booting=1).
compute bootiter=1.
end if.
do if (itprobtg=0).
compute itprobtg=1.
compute errcode(errs,1) = 47.
compute errs = errs + 1.
do if (booting = 0 and 1 =1).
compute vt1 = mdiag(pt1lp&*(1-pt1lp)).
compute varb = inv(t(xlp)*vt1*xlp).
compute seb = sqrt(diag(varb)).
end if.
end if.
end if.
do if ( 1 =1).
compute trat = b&/seb.
compute dfres=nrow(xlp).
compute p = 2*(1-cdfnorm(abs(trat))).
compute modres={modres,seb,trat,p}.
compute modres={modres,(b-xp2&*seb),(b+xp2&*seb)}.
compute pvchi=1-chicdf((LL3-LL2),(nrow(modres)-1)).
compute mcF = (LL3-LL2)/LL3.
compute cox = 1-exp(-(LL3-LL2)/nrow(xlp)).
compute nagel = cox/(1-exp(-(LL3)/nrow(xlp))).
compute modsum={LL2,(LL3-LL2),(nrow(modres)-1),pvchi, mcF,cox,nagel}.
compute modsuml={'-2LL','ModelLL', 'df', 'p', 'McFadden', 'CoxSnell', 'Nagelkrk'}.
compute modresl={'coeff','se','Z','p','LLCI','ULCI'}.
end if.
end if
.
compute dfmatt=make(nrow(modres),1,-999).
end if.
compute resultm2=make(1,maxresm,99999).
compute resultm2(1,1:ncol(modsum))=modsum.
compute resultm={resultm;resultm2}.
compute resultm2=make(nrow(modres),maxresm,99999).
compute resultm2(1:nrow(modres),1:ncol(modres))=modres.
compute resultm={resultm;resultm2}.
compute obscoeff={obscoeff,t(b)}.
do if (outscree=1).
print modsum/title = 'Model Summary'/cnames = modsuml/format= F10.4.
print modres/title='Model'/rnames=vlabsm/cnames=modresl/format= F10.4.
end if.
compute basemod=modsum(1,1).
compute basemodx=basemod.
do if (ydich=1 and (i = (nms+nys))).
do if (outscree=1).
print/title='These results are expressed in a log-odds metric.'.
end if.
compute notecode(notes,1) = 26.
compute notes = notes + 1.
end if.
compute coeffmat={coeffmat;modres}.
compute conseqt=make(nrow(modres),1,outnames(1,i)).
compute conseq={conseq;conseqt}.
compute dfmat={dfmat;dfmatt}.
compute labstart=labstart+nump(1,i).
do if (stand=1).
compute predsd=make(nrow(modres),1,0).
compute stdmod=modres(:,1)&/ovsd(1,i).
loop jd=1 to ncol(x).
compute descdat=x(:,jd).
compute predsd(jd,1) = (nrow(descdat)*sscp(descdat))-(t(csum(descdat))*(csum(descdat))).
compute predsd(jd,1) = sqrt(predsd(jd,1)/(nrow(descdat)*(nrow(descdat)-1))).
end loop.
do if (wherex(1,i) <> -999 and ((nxvls > 1) or (xdich=1))).
compute sdmsone=make(nxvls,1,1).
compute predsd(wherex(1,i):wherex(2,i),1)=sdmsone.
compute pstog=1.
end if.
compute predsd(1,1)=1.
compute stdmod=stdmod&*predsd.
compute stdmod=stdmod(2:nrow(stdmod),1).
compute sdvlabs=vlabsm(2:nrow(vlabsm),1).
compute resultm2=make(nrow(stdmod),maxresm,99999).
compute resultm2(1:nrow(stdmod),1:ncol(stdmod))=stdmod.
compute resultm={resultm;resultm2}.
do if (outscree=1).
print stdmod/title='Standardized coefficients'/clabels='coeff'/rnames=sdvlabs/format= F10.4.
end if.
end if.
do if (nms > 0 and serial = 0 and (rsum(numint) = 0) and (normal=1 or mc > 0)).
do if (i < (nms+nys)).
compute indcov((((i-1)*nxvls)+1):(i*nxvls),(((i-1)*nxvls)+1):(i*nxvls))=varb(2:(1+nxvls), 2:(1+nxvls)).
compute mcsopath((((i-1)*nxvls)+1):(i*nxvls) ,1)=modres(2:(1+nxvls),1).
end if.
do if (i = (nms+nys)).
compute atm=ncol(wherem).
compute indcov(((nms*nxvls)+1):nrow(mcsopath),((nms*nxvls)+1):nrow(mcsopath))= varb(wherem(1,atm):(wherem(1,atm)+nms-1),wherem(1,atm):(wherem(1,atm)+nms-1)).
compute mcsopath(((nms*nxvls)+1):nrow(mcsopath),1)=modres(wherem(1,atm):(wherem(1,atm)+ nms-1),1).
compute sobelok=1.
end if.
end if.
compute obsdirfx=make(1,nxvls,0).
compute dirzes=make(1,nxvls,0).
do if ((i = (nms+nys)) and (bcmat(nrow(bcmat),1)=1)).
compute direff=modres(2:(1+nxvls),:).
compute obsdirfx=t(direff(:,1)).
compute direfflb=modresl.
compute direffl2=vlabsm(2:(1+nxvls),:).
compute lmat=make(nrow(b),1,0).
compute lmat2=make(nxvls,1,1).
compute lmat(2:(1+nxvls),1)=lmat2.
do if (ydich <> 1).

.
compute lmat2= lmat.
do if ( 0 =0).
compute lmat2 = mdiag( lmat ).
compute lmat3=make(nrow(lmat2),1,0).
loop flp=1 to ncol(lmat2).
do if (csum(lmat2(:,flp))=1).
compute lmat3={lmat3,lmat2(:,flp)}.
end if.
end loop.
compute lmat2=lmat3(:,2:ncol(lmat3)).
end if.
compute fratio = (t(t(lmat2)* b )*inv(t(lmat2)* varb *lmat2)*((t(lmat2)* b )))/ncol(lmat2).
compute pfr = 1-fcdf(fratio,ncol(lmat2),(n-nrow( b ))).
compute fresult={fratio,ncol(lmat2),(n-nrow( b )),pfr}.
do if (i = (nms+nys) and (ydich=1)).
compute fratio=fratio*ncol(lmat2).
compute pfr=1-chicdf(fratio,ncol(lmat2)).
compute fresult={fratio,ncol(lmat2),pfr}.
end if.
do if ( 1 =1).
compute lmat3=1-rsum(lmat2).
compute xfm=make(n,csum(lmat3),0).
compute flpc=1.
loop flp=1 to nrow(lmat3).
do if (lmat3(flp,1)=1).
compute xfm(:,flpc)=x(:,flp).
compute flpc=flpc+1.
end if.
end loop.
compute bfm=inv(t(xfm)*xfm)*t(xfm)*y.
compute resid=y-(xfm*bfm).
compute sstotal=(y-(csum(y)/n)).
compute sstotal=csum(sstotal&*sstotal).
compute ssresid=csum(resid&*resid).
compute rsqch= r2 -((sstotal-ssresid)/sstotal).
compute fresult={rsqch,fresult}.
release xfm,flpc, resid, ssresid, bfm.
end if
.
compute diromni=fresult.
end if.
do if (ydich = 1 ).

.
compute btemphld=b.
compute llrdat=make(nrow(x),nrow( lmat )-csum( lmat ),-999).
compute llrdf=ncol(x)-ncol(llrdat).
compute llrcnt=0.
loop llri=1 to nrow( lmat ).
do if ( lmat (llri,1)=0).
compute llrcnt=llrcnt+1.
compute llrdat(:,llrcnt)=x(:,llri).
end if.
end loop.

.
do if ( 2 =1).
compute hatmat=inv(t( llrdat )* llrdat )*t( llrdat ).
compute b = hatmat* y.
compute modres=b.
do if ( 0 =1).
compute n1=nrow( llrdat ).
compute dfres=n1-(ncol( llrdat )).
compute sstotal = t( y -(csum( y )/n1))*( y -(csum( y )/n1)).
compute resid= y - llrdat *b.
compute ssresid = csum((resid)&**2).
compute r2 = (sstotal-ssresid)/sstotal.
do if (r2 < 0).
compute r2=0.
end if.
compute adjr2 = 1-((1-r2)*(n1-1)/(dfres)).
compute mse=ssresid/(n1-ncol( llrdat )).

.
compute n1=nrow( x ).
compute invXtX = inv(t( x )* x ).
compute varb = mse *invXtX.
compute k3 = ncol( x ).
compute xhc=0.
do if ( hc <> 5).
compute xhc= x.
compute hat = xhc(:,1).
loop i3=1 to nrow(xhc).
compute hat(i3,1)= xhc(i3,:)*invXtX*t(xhc(i3,:)).
end loop.
do if ( hc = 0 or hc =1).
loop i3 = 1 to k3.
compute xhc(:,i3)=xhc(:,i3)&* resid.
end loop.
end if.
do if ( hc =3 or hc =2).
loop i3=1 to k3.
compute xhc(:,i3) = ( resid &/(1-hat)&**(1/(4- hc )))&*xhc(:,i3).
end loop.
end if.
do if ( hc = 4).
compute hcmn=make(n,2,4).
compute hcmn(:,2)=(n1*hat)/k3.
loop i3= 1 to k3.
compute xhc(:,i3) = ( resid &/(1-hat)&**(rmin(hcmn)/2))&*xhc(:,i3).
end loop.
end if.
compute varb=(invXtX*t(xhc)*xhc*invXtX).
do if ( hc =1).
compute varb=(n1/(n1-ncol( x )))&*varb.
end if.
end if.
compute hclab={'se(HC0)','se(HC1)','se(HC2)','se(HC3)','se(HC4)','se'}.
compute hclab=hclab(1,( hc +1)).
compute hcflab={'F(HC0)','F(HC1)','F(HC2)','F(HC3)','F(HC4)','F'}.
compute hcflab=hcflab(1,( hc +1)).
release xhc
.
compute seb=sqrt(diag(varb)).
compute trat = b&/seb.
compute p = 2*(1-tcdf(abs(trat), (dfres))).
compute tval = sqrt(dfres* (exp((dfres-(5/6))*((xp2/(dfres-(2/3)+(.11/dfres)))* (xp2/(dfres-(2/3)+(.11/dfres)))))-1)).
compute modres={modres,seb,trat,p}.
compute modres={modres,(b-tval&*seb),(b+tval&*seb)}.
compute modresl={'coeff',hclab,'t','p','LLCI','ULCI'}.
compute lmat = ident(ncol( llrdat )).
compute lmat = lmat(:,2:ncol(lmat)).
compute fratio = (t(t(lmat)*b)*inv(t(lmat)*varb*lmat)*((t(lmat)*b)))/(ncol( llrdat )-1).
compute pfr = 1-fcdf(fratio,(ncol( llrdat )-1),dfres).
compute modsum={sqrt(r2),r2,mse,fratio,(ncol( llrdat )-1),dfres,pfr}.
compute modsuml={'R','R-sq','MSE',hcflab,'df1','df2', 'p'}.
end if.
end if.
do if ( 2 = 2 or 2 =3).
compute xlp= llrdat.
compute ylp= y.
compute pt2 = make(nrow(ylp),1,(csum(ylp)/nrow(ylp))).
do if ( 2 =2).
compute LL3 = ylp&*ln(pt2)+(1-ylp)&*ln(1-pt2).
end if.
compute LL3 = -2*csum(LL3).
compute bt1 = make(ncol(xlp),1,0).
compute LL1 = 0.
compute pt1 = make(nrow(ylp),1,0.5).
compute pt1lp=pt1.
loop jjj = 1 to iterate.
compute xlptmp=t(xlp).
compute vecprb=pt1lp&*(1-pt1lp).
loop kkk=1 to ncol(xlp).
compute xlptmp(kkk,:)=xlptmp(kkk,:)&*t(vecprb).
end loop.
compute b = bt1+inv(xlptmp*xlp)*t(xlp)*(ylp-pt1lp).
do if ( 2 =2).
compute xlpb=xlp*b.
compute xlpbt=(xlpb > -709.7).
compute xlpb709=(1-xlpbt)*(-709.7).
compute xlpb=(xlpb&*xlpbt)+xlpb709.
compute pt1lp = 1/(1+exp(-(xlpb))).
end if.
compute itprob = csum((pt1lp < .00000001) or (pt1lp > .9999999)).
do if (itprob > 0).
loop kkk = 1 to nrow(pt1lp).
do if (pt1lp(kkk,1) > .9999999).
compute pt1lp(kkk,1) = .9999999.
end if.
do if (pt1lp(kkk,1) < .00000001).
compute pt1lp(kkk,1) = .00000001.
end if.
end loop.
compute itprob = 0.
end if.
do if (itprob = 0).
do if ( 2 =2).
compute LL = ylp&*ln(pt1lp)+(1-ylp)&*ln(1-pt1lp).
end if.
compute LL2 = -2*csum(ll).
end if.
do if (abs(LL1-LL2) < converge).
do if ( 0 =1).
compute xlptmp=t(xlp).
compute vecprb=pt1lp&*(1-pt1lp).
loop kkk=1 to ncol(xlp).
compute xlptmp(kkk,:)=xlptmp(kkk,:)&*t(vecprb).
end loop.
compute varb = inv(xlptmp*xlp).
compute seb = sqrt(diag(varb)).
release xlptmp.
end if.
break.
end if.
compute bt1 = b.
compute LL1 = LL2.
end loop.
compute modres=b.
do if (jjj > iterate).
compute itprob = 2.
do if (booting=0).
compute iterrmod=1.
end if.
do if (booting=1).
compute bootiter=1.
end if.
do if (itprobtg=0).
compute itprobtg=1.
compute errcode(errs,1) = 47.
compute errs = errs + 1.
do if (booting = 0 and 0 =1).
compute vt1 = mdiag(pt1lp&*(1-pt1lp)).
compute varb = inv(t(xlp)*vt1*xlp).
compute seb = sqrt(diag(varb)).
end if.
end if.
end if.
do if ( 0 =1).
compute trat = b&/seb.
compute dfres=nrow(xlp).
compute p = 2*(1-cdfnorm(abs(trat))).
compute modres={modres,seb,trat,p}.
compute modres={modres,(b-xp2&*seb),(b+xp2&*seb)}.
compute pvchi=1-chicdf((LL3-LL2),(nrow(modres)-1)).
compute mcF = (LL3-LL2)/LL3.
compute cox = 1-exp(-(LL3-LL2)/nrow(xlp)).
compute nagel = cox/(1-exp(-(LL3)/nrow(xlp))).
compute modsum={LL2,(LL3-LL2),(nrow(modres)-1),pvchi, mcF,cox,nagel}.
compute modsuml={'-2LL','ModelLL', 'df', 'p', 'McFadden', 'CoxSnell', 'Nagelkrk'}.
compute modresl={'coeff','se','Z','p','LLCI','ULCI'}.
end if.
end if
.
compute b=btemphld.
compute fresult={(LL2-basemod),llrdf,1-chicdf((LL2-basemod),llrdf)}
.
compute diromni=fresult.
end if.
end if.
do if (numint(1,i) > 0).
compute intkeym=intkey(intstart:(intstart+numint(1,i)-1),:).
do if (outscree=1).
print intkeym/title='Product terms key:'/format = A8.
end if.
end if.
do if (covcoeff=1).
do if (outscree=1).
print varb/title='Covariance matrix of regression parameter estimates:'/rnames=vlabsm /cnames=vlabsm/format= F10.4.
end if.
compute resultm2=make(nrow(varb),maxresm,99999).
do if (ncol(varb) <= maxresm).
compute resultm2(1:nrow(varb),1:ncol(varb))=varb.
compute resultm={resultm;resultm2}.
end if.
do if (ncol(varb) > maxresm).
compute resultmt=make(nrow(resultm),ncol(varb),99999).
compute resultmt(1:nrow(resultm),1:ncol(resultm))=resultm.
compute resultm=resultmt.
compute resultm2=make(nrow(varb),ncol(resultm),99999).
compute resultm2(1:nrow(varb),1:ncol(varb))=varb.
compute resultm={resultm;resultm2}.
compute maxresm=ncol(resultm).
end if.
end if.
do if (model <> 74 and xmtest=1 and nms > 0).
compute r2tmp=r2.
compute btmp=b.
compute varbtmp=varb.
compute dfrestmp=dfres.
compute tvaltmp=tval.
compute xmtst=make(nms,4,0).
compute xmtstlbc={hcflab,'df1','df2','p'}.
do if ((i=(nms+nys)) and (ydich=1)).
compute xmtst=make(nms,3,0).
compute xmtstlbc={'Chi-sq','df','p'}.
end if.
compute xmtstlb={' '}.
compute xmtmat=x.
compute numxint=0.
loop xmints=2 to i.
compute x=xmtmat.
do if ((bcmat((i+1),xmints)=1) and (wzcmat((i+1),xmints) <> 1)).
do if (bcmat((i+1),1)=0).
compute x={xmtmat,xtmp}.
do if ((ydich=1) and (i=(nms+nys))).

.
do if ( 2 =1).
compute hatmat=inv(t( x )* x )*t( x ).
compute b = hatmat* y.
compute modres=b.
do if ( 1 =1).
compute n1=nrow( x ).
compute dfres=n1-(ncol( x )).
compute sstotal = t( y -(csum( y )/n1))*( y -(csum( y )/n1)).
compute resid= y - x *b.
compute ssresid = csum((resid)&**2).
compute r2 = (sstotal-ssresid)/sstotal.
do if (r2 < 0).
compute r2=0.
end if.
compute adjr2 = 1-((1-r2)*(n1-1)/(dfres)).
compute mse=ssresid/(n1-ncol( x )).

.
compute n1=nrow( x ).
compute invXtX = inv(t( x )* x ).
compute varb = mse *invXtX.
compute k3 = ncol( x ).
compute xhc=0.
do if ( hc <> 5).
compute xhc= x.
compute hat = xhc(:,1).
loop i3=1 to nrow(xhc).
compute hat(i3,1)= xhc(i3,:)*invXtX*t(xhc(i3,:)).
end loop.
do if ( hc = 0 or hc =1).
loop i3 = 1 to k3.
compute xhc(:,i3)=xhc(:,i3)&* resid.
end loop.
end if.
do if ( hc =3 or hc =2).
loop i3=1 to k3.
compute xhc(:,i3) = ( resid &/(1-hat)&**(1/(4- hc )))&*xhc(:,i3).
end loop.
end if.
do if ( hc = 4).
compute hcmn=make(n,2,4).
compute hcmn(:,2)=(n1*hat)/k3.
loop i3= 1 to k3.
compute xhc(:,i3) = ( resid &/(1-hat)&**(rmin(hcmn)/2))&*xhc(:,i3).
end loop.
end if.
compute varb=(invXtX*t(xhc)*xhc*invXtX).
do if ( hc =1).
compute varb=(n1/(n1-ncol( x )))&*varb.
end if.
end if.
compute hclab={'se(HC0)','se(HC1)','se(HC2)','se(HC3)','se(HC4)','se'}.
compute hclab=hclab(1,( hc +1)).
compute hcflab={'F(HC0)','F(HC1)','F(HC2)','F(HC3)','F(HC4)','F'}.
compute hcflab=hcflab(1,( hc +1)).
release xhc
.
compute seb=sqrt(diag(varb)).
compute trat = b&/seb.
compute p = 2*(1-tcdf(abs(trat), (dfres))).
compute tval = sqrt(dfres* (exp((dfres-(5/6))*((xp2/(dfres-(2/3)+(.11/dfres)))* (xp2/(dfres-(2/3)+(.11/dfres)))))-1)).
compute modres={modres,seb,trat,p}.
compute modres={modres,(b-tval&*seb),(b+tval&*seb)}.
compute modresl={'coeff',hclab,'t','p','LLCI','ULCI'}.
compute lmat = ident(ncol( x )).
compute lmat = lmat(:,2:ncol(lmat)).
compute fratio = (t(t(lmat)*b)*inv(t(lmat)*varb*lmat)*((t(lmat)*b)))/(ncol( x )-1).
compute pfr = 1-fcdf(fratio,(ncol( x )-1),dfres).
compute modsum={sqrt(r2),r2,mse,fratio,(ncol( x )-1),dfres,pfr}.
compute modsuml={'R','R-sq','MSE',hcflab,'df1','df2', 'p'}.
end if.
end if.
do if ( 2 = 2 or 2 =3).
compute xlp= x.
compute ylp= y.
compute pt2 = make(nrow(ylp),1,(csum(ylp)/nrow(ylp))).
do if ( 2 =2).
compute LL3 = ylp&*ln(pt2)+(1-ylp)&*ln(1-pt2).
end if.
compute LL3 = -2*csum(LL3).
compute bt1 = make(ncol(xlp),1,0).
compute LL1 = 0.
compute pt1 = make(nrow(ylp),1,0.5).
compute pt1lp=pt1.
loop jjj = 1 to iterate.
compute xlptmp=t(xlp).
compute vecprb=pt1lp&*(1-pt1lp).
loop kkk=1 to ncol(xlp).
compute xlptmp(kkk,:)=xlptmp(kkk,:)&*t(vecprb).
end loop.
compute b = bt1+inv(xlptmp*xlp)*t(xlp)*(ylp-pt1lp).
do if ( 2 =2).
compute xlpb=xlp*b.
compute xlpbt=(xlpb > -709.7).
compute xlpb709=(1-xlpbt)*(-709.7).
compute xlpb=(xlpb&*xlpbt)+xlpb709.
compute pt1lp = 1/(1+exp(-(xlpb))).
end if.
compute itprob = csum((pt1lp < .00000001) or (pt1lp > .9999999)).
do if (itprob > 0).
loop kkk = 1 to nrow(pt1lp).
do if (pt1lp(kkk,1) > .9999999).
compute pt1lp(kkk,1) = .9999999.
end if.
do if (pt1lp(kkk,1) < .00000001).
compute pt1lp(kkk,1) = .00000001.
end if.
end loop.
compute itprob = 0.
end if.
do if (itprob = 0).
do if ( 2 =2).
compute LL = ylp&*ln(pt1lp)+(1-ylp)&*ln(1-pt1lp).
end if.
compute LL2 = -2*csum(ll).
end if.
do if (abs(LL1-LL2) < converge).
do if ( 1 =1).
compute xlptmp=t(xlp).
compute vecprb=pt1lp&*(1-pt1lp).
loop kkk=1 to ncol(xlp).
compute xlptmp(kkk,:)=xlptmp(kkk,:)&*t(vecprb).
end loop.
compute varb = inv(xlptmp*xlp).
compute seb = sqrt(diag(varb)).
release xlptmp.
end if.
break.
end if.
compute bt1 = b.
compute LL1 = LL2.
end loop.
compute modres=b.
do if (jjj > iterate).
compute itprob = 2.
do if (booting=0).
compute iterrmod=1.
end if.
do if (booting=1).
compute bootiter=1.
end if.
do if (itprobtg=0).
compute itprobtg=1.
compute errcode(errs,1) = 47.
compute errs = errs + 1.
do if (booting = 0 and 1 =1).
compute vt1 = mdiag(pt1lp&*(1-pt1lp)).
compute varb = inv(t(xlp)*vt1*xlp).
compute seb = sqrt(diag(varb)).
end if.
end if.
end if.
do if ( 1 =1).
compute trat = b&/seb.
compute dfres=nrow(xlp).
compute p = 2*(1-cdfnorm(abs(trat))).
compute modres={modres,seb,trat,p}.
compute modres={modres,(b-xp2&*seb),(b+xp2&*seb)}.
compute pvchi=1-chicdf((LL3-LL2),(nrow(modres)-1)).
compute mcF = (LL3-LL2)/LL3.
compute cox = 1-exp(-(LL3-LL2)/nrow(xlp)).
compute nagel = cox/(1-exp(-(LL3)/nrow(xlp))).
compute modsum={LL2,(LL3-LL2),(nrow(modres)-1),pvchi, mcF,cox,nagel}.
compute modsuml={'-2LL','ModelLL', 'df', 'p', 'McFadden', 'CoxSnell', 'Nagelkrk'}.
compute modresl={'coeff','se','Z','p','LLCI','ULCI'}.
end if.
end if
.
compute basemodx=LL2.
end if.
end if.
loop xmtlp1=1 to nxvls.
compute x={x,xtmp(:,xmtlp1)&*(mtmp(:,(xmints-1))-(csum(mtmp(:,(xmints-1)))/nrow(mtmp)) )}.
end loop.
do if ((i < (nms+nys)) or (ydich=0)).

.
do if ( 1 =1).
compute hatmat=inv(t( x )* x )*t( x ).
compute b = hatmat* y.
compute modres=b.
do if ( 1 =1).
compute n1=nrow( x ).
compute dfres=n1-(ncol( x )).
compute sstotal = t( y -(csum( y )/n1))*( y -(csum( y )/n1)).
compute resid= y - x *b.
compute ssresid = csum((resid)&**2).
compute r2 = (sstotal-ssresid)/sstotal.
do if (r2 < 0).
compute r2=0.
end if.
compute adjr2 = 1-((1-r2)*(n1-1)/(dfres)).
compute mse=ssresid/(n1-ncol( x )).

.
compute n1=nrow( x ).
compute invXtX = inv(t( x )* x ).
compute varb = mse *invXtX.
compute k3 = ncol( x ).
compute xhc=0.
do if ( hc <> 5).
compute xhc= x.
compute hat = xhc(:,1).
loop i3=1 to nrow(xhc).
compute hat(i3,1)= xhc(i3,:)*invXtX*t(xhc(i3,:)).
end loop.
do if ( hc = 0 or hc =1).
loop i3 = 1 to k3.
compute xhc(:,i3)=xhc(:,i3)&* resid.
end loop.
end if.
do if ( hc =3 or hc =2).
loop i3=1 to k3.
compute xhc(:,i3) = ( resid &/(1-hat)&**(1/(4- hc )))&*xhc(:,i3).
end loop.
end if.
do if ( hc = 4).
compute hcmn=make(n,2,4).
compute hcmn(:,2)=(n1*hat)/k3.
loop i3= 1 to k3.
compute xhc(:,i3) = ( resid &/(1-hat)&**(rmin(hcmn)/2))&*xhc(:,i3).
end loop.
end if.
compute varb=(invXtX*t(xhc)*xhc*invXtX).
do if ( hc =1).
compute varb=(n1/(n1-ncol( x )))&*varb.
end if.
end if.
compute hclab={'se(HC0)','se(HC1)','se(HC2)','se(HC3)','se(HC4)','se'}.
compute hclab=hclab(1,( hc +1)).
compute hcflab={'F(HC0)','F(HC1)','F(HC2)','F(HC3)','F(HC4)','F'}.
compute hcflab=hcflab(1,( hc +1)).
release xhc
.
compute seb=sqrt(diag(varb)).
compute trat = b&/seb.
compute p = 2*(1-tcdf(abs(trat), (dfres))).
compute tval = sqrt(dfres* (exp((dfres-(5/6))*((xp2/(dfres-(2/3)+(.11/dfres)))* (xp2/(dfres-(2/3)+(.11/dfres)))))-1)).
compute modres={modres,seb,trat,p}.
compute modres={modres,(b-tval&*seb),(b+tval&*seb)}.
compute modresl={'coeff',hclab,'t','p','LLCI','ULCI'}.
compute lmat = ident(ncol( x )).
compute lmat = lmat(:,2:ncol(lmat)).
compute fratio = (t(t(lmat)*b)*inv(t(lmat)*varb*lmat)*((t(lmat)*b)))/(ncol( x )-1).
compute pfr = 1-fcdf(fratio,(ncol( x )-1),dfres).
compute modsum={sqrt(r2),r2,mse,fratio,(ncol( x )-1),dfres,pfr}.
compute modsuml={'R','R-sq','MSE',hcflab,'df1','df2', 'p'}.
end if.
end if.
do if ( 1 = 2 or 1 =3).
compute xlp= x.
compute ylp= y.
compute pt2 = make(nrow(ylp),1,(csum(ylp)/nrow(ylp))).
do if ( 1 =2).
compute LL3 = ylp&*ln(pt2)+(1-ylp)&*ln(1-pt2).
end if.
compute LL3 = -2*csum(LL3).
compute bt1 = make(ncol(xlp),1,0).
compute LL1 = 0.
compute pt1 = make(nrow(ylp),1,0.5).
compute pt1lp=pt1.
loop jjj = 1 to iterate.
compute xlptmp=t(xlp).
compute vecprb=pt1lp&*(1-pt1lp).
loop kkk=1 to ncol(xlp).
compute xlptmp(kkk,:)=xlptmp(kkk,:)&*t(vecprb).
end loop.
compute b = bt1+inv(xlptmp*xlp)*t(xlp)*(ylp-pt1lp).
do if ( 1 =2).
compute xlpb=xlp*b.
compute xlpbt=(xlpb > -709.7).
compute xlpb709=(1-xlpbt)*(-709.7).
compute xlpb=(xlpb&*xlpbt)+xlpb709.
compute pt1lp = 1/(1+exp(-(xlpb))).
end if.
compute itprob = csum((pt1lp < .00000001) or (pt1lp > .9999999)).
do if (itprob > 0).
loop kkk = 1 to nrow(pt1lp).
do if (pt1lp(kkk,1) > .9999999).
compute pt1lp(kkk,1) = .9999999.
end if.
do if (pt1lp(kkk,1) < .00000001).
compute pt1lp(kkk,1) = .00000001.
end if.
end loop.
compute itprob = 0.
end if.
do if (itprob = 0).
do if ( 1 =2).
compute LL = ylp&*ln(pt1lp)+(1-ylp)&*ln(1-pt1lp).
end if.
compute LL2 = -2*csum(ll).
end if.
do if (abs(LL1-LL2) < converge).
do if ( 1 =1).
compute xlptmp=t(xlp).
compute vecprb=pt1lp&*(1-pt1lp).
loop kkk=1 to ncol(xlp).
compute xlptmp(kkk,:)=xlptmp(kkk,:)&*t(vecprb).
end loop.
compute varb = inv(xlptmp*xlp).
compute seb = sqrt(diag(varb)).
release xlptmp.
end if.
break.
end if.
compute bt1 = b.
compute LL1 = LL2.
end loop.
compute modres=b.
do if (jjj > iterate).
compute itprob = 2.
do if (booting=0).
compute iterrmod=1.
end if.
do if (booting=1).
compute bootiter=1.
end if.
do if (itprobtg=0).
compute itprobtg=1.
compute errcode(errs,1) = 47.
compute errs = errs + 1.
do if (booting = 0 and 1 =1).
compute vt1 = mdiag(pt1lp&*(1-pt1lp)).
compute varb = inv(t(xlp)*vt1*xlp).
compute seb = sqrt(diag(varb)).
end if.
end if.
end if.
do if ( 1 =1).
compute trat = b&/seb.
compute dfres=nrow(xlp).
compute p = 2*(1-cdfnorm(abs(trat))).
compute modres={modres,seb,trat,p}.
compute modres={modres,(b-xp2&*seb),(b+xp2&*seb)}.
compute pvchi=1-chicdf((LL3-LL2),(nrow(modres)-1)).
compute mcF = (LL3-LL2)/LL3.
compute cox = 1-exp(-(LL3-LL2)/nrow(xlp)).
compute nagel = cox/(1-exp(-(LL3)/nrow(xlp))).
compute modsum={LL2,(LL3-LL2),(nrow(modres)-1),pvchi, mcF,cox,nagel}.
compute modsuml={'-2LL','ModelLL', 'df', 'p', 'McFadden', 'CoxSnell', 'Nagelkrk'}.
compute modresl={'coeff','se','Z','p','LLCI','ULCI'}.
end if.
end if
.
end if.
do if ((i = (nms+nys)) and (ydich=1)).

.
do if ( 2 =1).
compute hatmat=inv(t( x )* x )*t( x ).
compute b = hatmat* y.
compute modres=b.
do if ( 1 =1).
compute n1=nrow( x ).
compute dfres=n1-(ncol( x )).
compute sstotal = t( y -(csum( y )/n1))*( y -(csum( y )/n1)).
compute resid= y - x *b.
compute ssresid = csum((resid)&**2).
compute r2 = (sstotal-ssresid)/sstotal.
do if (r2 < 0).
compute r2=0.
end if.
compute adjr2 = 1-((1-r2)*(n1-1)/(dfres)).
compute mse=ssresid/(n1-ncol( x )).

.
compute n1=nrow( x ).
compute invXtX = inv(t( x )* x ).
compute varb = mse *invXtX.
compute k3 = ncol( x ).
compute xhc=0.
do if ( hc <> 5).
compute xhc= x.
compute hat = xhc(:,1).
loop i3=1 to nrow(xhc).
compute hat(i3,1)= xhc(i3,:)*invXtX*t(xhc(i3,:)).
end loop.
do if ( hc = 0 or hc =1).
loop i3 = 1 to k3.
compute xhc(:,i3)=xhc(:,i3)&* resid.
end loop.
end if.
do if ( hc =3 or hc =2).
loop i3=1 to k3.
compute xhc(:,i3) = ( resid &/(1-hat)&**(1/(4- hc )))&*xhc(:,i3).
end loop.
end if.
do if ( hc = 4).
compute hcmn=make(n,2,4).
compute hcmn(:,2)=(n1*hat)/k3.
loop i3= 1 to k3.
compute xhc(:,i3) = ( resid &/(1-hat)&**(rmin(hcmn)/2))&*xhc(:,i3).
end loop.
end if.
compute varb=(invXtX*t(xhc)*xhc*invXtX).
do if ( hc =1).
compute varb=(n1/(n1-ncol( x )))&*varb.
end if.
end if.
compute hclab={'se(HC0)','se(HC1)','se(HC2)','se(HC3)','se(HC4)','se'}.
compute hclab=hclab(1,( hc +1)).
compute hcflab={'F(HC0)','F(HC1)','F(HC2)','F(HC3)','F(HC4)','F'}.
compute hcflab=hcflab(1,( hc +1)).
release xhc
.
compute seb=sqrt(diag(varb)).
compute trat = b&/seb.
compute p = 2*(1-tcdf(abs(trat), (dfres))).
compute tval = sqrt(dfres* (exp((dfres-(5/6))*((xp2/(dfres-(2/3)+(.11/dfres)))* (xp2/(dfres-(2/3)+(.11/dfres)))))-1)).
compute modres={modres,seb,trat,p}.
compute modres={modres,(b-tval&*seb),(b+tval&*seb)}.
compute modresl={'coeff',hclab,'t','p','LLCI','ULCI'}.
compute lmat = ident(ncol( x )).
compute lmat = lmat(:,2:ncol(lmat)).
compute fratio = (t(t(lmat)*b)*inv(t(lmat)*varb*lmat)*((t(lmat)*b)))/(ncol( x )-1).
compute pfr = 1-fcdf(fratio,(ncol( x )-1),dfres).
compute modsum={sqrt(r2),r2,mse,fratio,(ncol( x )-1),dfres,pfr}.
compute modsuml={'R','R-sq','MSE',hcflab,'df1','df2', 'p'}.
end if.
end if.
do if ( 2 = 2 or 2 =3).
compute xlp= x.
compute ylp= y.
compute pt2 = make(nrow(ylp),1,(csum(ylp)/nrow(ylp))).
do if ( 2 =2).
compute LL3 = ylp&*ln(pt2)+(1-ylp)&*ln(1-pt2).
end if.
compute LL3 = -2*csum(LL3).
compute bt1 = make(ncol(xlp),1,0).
compute LL1 = 0.
compute pt1 = make(nrow(ylp),1,0.5).
compute pt1lp=pt1.
loop jjj = 1 to iterate.
compute xlptmp=t(xlp).
compute vecprb=pt1lp&*(1-pt1lp).
loop kkk=1 to ncol(xlp).
compute xlptmp(kkk,:)=xlptmp(kkk,:)&*t(vecprb).
end loop.
compute b = bt1+inv(xlptmp*xlp)*t(xlp)*(ylp-pt1lp).
do if ( 2 =2).
compute xlpb=xlp*b.
compute xlpbt=(xlpb > -709.7).
compute xlpb709=(1-xlpbt)*(-709.7).
compute xlpb=(xlpb&*xlpbt)+xlpb709.
compute pt1lp = 1/(1+exp(-(xlpb))).
end if.
compute itprob = csum((pt1lp < .00000001) or (pt1lp > .9999999)).
do if (itprob > 0).
loop kkk = 1 to nrow(pt1lp).
do if (pt1lp(kkk,1) > .9999999).
compute pt1lp(kkk,1) = .9999999.
end if.
do if (pt1lp(kkk,1) < .00000001).
compute pt1lp(kkk,1) = .00000001.
end if.
end loop.
compute itprob = 0.
end if.
do if (itprob = 0).
do if ( 2 =2).
compute LL = ylp&*ln(pt1lp)+(1-ylp)&*ln(1-pt1lp).
end if.
compute LL2 = -2*csum(ll).
end if.
do if (abs(LL1-LL2) < converge).
do if ( 1 =1).
compute xlptmp=t(xlp).
compute vecprb=pt1lp&*(1-pt1lp).
loop kkk=1 to ncol(xlp).
compute xlptmp(kkk,:)=xlptmp(kkk,:)&*t(vecprb).
end loop.
compute varb = inv(xlptmp*xlp).
compute seb = sqrt(diag(varb)).
release xlptmp.
end if.
break.
end if.
compute bt1 = b.
compute LL1 = LL2.
end loop.
compute modres=b.
do if (jjj > iterate).
compute itprob = 2.
do if (booting=0).
compute iterrmod=1.
end if.
do if (booting=1).
compute bootiter=1.
end if.
do if (itprobtg=0).
compute itprobtg=1.
compute errcode(errs,1) = 47.
compute errs = errs + 1.
do if (booting = 0 and 1 =1).
compute vt1 = mdiag(pt1lp&*(1-pt1lp)).
compute varb = inv(t(xlp)*vt1*xlp).
compute seb = sqrt(diag(varb)).
end if.
end if.
end if.
do if ( 1 =1).
compute trat = b&/seb.
compute dfres=nrow(xlp).
compute p = 2*(1-cdfnorm(abs(trat))).
compute modres={modres,seb,trat,p}.
compute modres={modres,(b-xp2&*seb),(b+xp2&*seb)}.
compute pvchi=1-chicdf((LL3-LL2),(nrow(modres)-1)).
compute mcF = (LL3-LL2)/LL3.
compute cox = 1-exp(-(LL3-LL2)/nrow(xlp)).
compute nagel = cox/(1-exp(-(LL3)/nrow(xlp))).
compute modsum={LL2,(LL3-LL2),(nrow(modres)-1),pvchi, mcF,cox,nagel}.
compute modsuml={'-2LL','ModelLL', 'df', 'p', 'McFadden', 'CoxSnell', 'Nagelkrk'}.
compute modresl={'coeff','se','Z','p','LLCI','ULCI'}.
end if.
end if
.
compute chidfxm=basemodx-LL2.
end if.
compute lmat=make(nrow(b),nxvls,0).
compute lmattmp=ident(nxvls).
compute lmat((nrow(lmat)-nxvls+1):nrow(lmat),:)=lmattmp.

.
compute lmat2= lmat.
do if ( 1 =0).
compute lmat2 = mdiag( lmat ).
compute lmat3=make(nrow(lmat2),1,0).
loop flp=1 to ncol(lmat2).
do if (csum(lmat2(:,flp))=1).
compute lmat3={lmat3,lmat2(:,flp)}.
end if.
end loop.
compute lmat2=lmat3(:,2:ncol(lmat3)).
end if.
compute fratio = (t(t(lmat2)* b )*inv(t(lmat2)* varb *lmat2)*((t(lmat2)* b )))/ncol(lmat2).
compute pfr = 1-fcdf(fratio,ncol(lmat2),(n-nrow( b ))).
compute fresult={fratio,ncol(lmat2),(n-nrow( b )),pfr}.
do if (i = (nms+nys) and (ydich=1)).
compute fratio=fratio*ncol(lmat2).
compute pfr=1-chicdf(fratio,ncol(lmat2)).
compute fresult={fratio,ncol(lmat2),pfr}.
end if.
do if ( 0 =1).
compute lmat3=1-rsum(lmat2).
compute xfm=make(n,csum(lmat3),0).
compute flpc=1.
loop flp=1 to nrow(lmat3).
do if (lmat3(flp,1)=1).
compute xfm(:,flpc)=x(:,flp).
compute flpc=flpc+1.
end if.
end loop.
compute bfm=inv(t(xfm)*xfm)*t(xfm)*y.
compute resid=y-(xfm*bfm).
compute sstotal=(y-(csum(y)/n)).
compute sstotal=csum(sstotal&*sstotal).
compute ssresid=csum(resid&*resid).
compute rsqch= 0 -((sstotal-ssresid)/sstotal).
compute fresult={rsqch,fresult}.
release xfm,flpc, resid, ssresid, bfm.
end if
.
compute numxint=numxint+1.
compute xmtst(numxint,:)=fresult.
do if ((i = (nms+nys)) and (ydich=1)).
compute xmtst(numxint,1)=chidfxm.
compute xmtst(numxint,3)=1-chicdf(chidfxm,nxvls).
end if.
compute xmtstlb={xmtstlb;highlbx((xmints-1),1)}.
end if.
end loop.
compute x=xmtmat.
release xmtmat.
do if (numxint>0).
compute xmtstlb=xmtstlb((2:(numxint+1)),:).
compute xmtst=xmtst(1:numxint,:).
compute resultm2=make(nrow(xmtst),maxresm,99999).
compute resultm2(1:nrow(xmtst),1:ncol(xmtst))=xmtst.
compute resultm={resultm;resultm2}.
do if (nms=1).
compute xmtstlb=' '.
end if.
do if (outscree=1).
do if ((i < (nms+nys)) or (ydich=0)).
print xmtst/title='Test(s) of X by M interaction:'/rnames=xmtstlb/cnames=xmtstlbc/format= F10.4.
end if.
do if ((i = (nms+nys)) and (ydich=1)).
print xmtst/title='Likelihood ratio test(s) of X by M interaction:'/rnames=xmtstlb/cnames=xmtstlbc/format= F10.4.
end if.
end if.
end if.
compute r2=r2tmp.
compute b=btmp.
compute varb=varbtmp.
compute dfres=dfrestmp.
compute tval=tvaltmp.
end if.
do if (criterr = 0).
compute jj=0.
loop j = start to ((start+i)-1).
compute dbint=0.
compute lmat=whigh(1:nump(1,i),j).
compute lmat2=wzhigh(1:nump(1,i),j).
do if ((csum(lmat) > 0) and (csum(lmat2) = 0)).
do if ((i < (nms+nys)) or (ydich <> 1)).

.
compute lmat2= lmat.
do if ( 0 =0).
compute lmat2 = mdiag( lmat ).
compute lmat3=make(nrow(lmat2),1,0).
loop flp=1 to ncol(lmat2).
do if (csum(lmat2(:,flp))=1).
compute lmat3={lmat3,lmat2(:,flp)}.
end if.
end loop.
compute lmat2=lmat3(:,2:ncol(lmat3)).
end if.
compute fratio = (t(t(lmat2)* b )*inv(t(lmat2)* varb *lmat2)*((t(lmat2)* b )))/ncol(lmat2).
compute pfr = 1-fcdf(fratio,ncol(lmat2),(n-nrow( b ))).
compute fresult={fratio,ncol(lmat2),(n-nrow( b )),pfr}.
do if (i = (nms+nys) and (ydich=1)).
compute fratio=fratio*ncol(lmat2).
compute pfr=1-chicdf(fratio,ncol(lmat2)).
compute fresult={fratio,ncol(lmat2),pfr}.
end if.
do if ( 1 =1).
compute lmat3=1-rsum(lmat2).
compute xfm=make(n,csum(lmat3),0).
compute flpc=1.
loop flp=1 to nrow(lmat3).
do if (lmat3(flp,1)=1).
compute xfm(:,flpc)=x(:,flp).
compute flpc=flpc+1.
end if.
end loop.
compute bfm=inv(t(xfm)*xfm)*t(xfm)*y.
compute resid=y-(xfm*bfm).
compute sstotal=(y-(csum(y)/n)).
compute sstotal=csum(sstotal&*sstotal).
compute ssresid=csum(resid&*resid).
compute rsqch= r2 -((sstotal-ssresid)/sstotal).
compute fresult={rsqch,fresult}.
release xfm,flpc, resid, ssresid, bfm.
end if
.
compute lmatdb=lmat.
compute dbint=dbint+1.
end if.
do if ((ydich = 1) and (i = (nms+nys))).

.
compute btemphld=b.
compute llrdat=make(nrow(x),nrow( lmat )-csum( lmat ),-999).
compute llrdf=ncol(x)-ncol(llrdat).
compute llrcnt=0.
loop llri=1 to nrow( lmat ).
do if ( lmat (llri,1)=0).
compute llrcnt=llrcnt+1.
compute llrdat(:,llrcnt)=x(:,llri).
end if.
end loop.

.
do if ( 2 =1).
compute hatmat=inv(t( llrdat )* llrdat )*t( llrdat ).
compute b = hatmat* y.
compute modres=b.
do if ( 0 =1).
compute n1=nrow( llrdat ).
compute dfres=n1-(ncol( llrdat )).
compute sstotal = t( y -(csum( y )/n1))*( y -(csum( y )/n1)).
compute resid= y - llrdat *b.
compute ssresid = csum((resid)&**2).
compute r2 = (sstotal-ssresid)/sstotal.
do if (r2 < 0).
compute r2=0.
end if.
compute adjr2 = 1-((1-r2)*(n1-1)/(dfres)).
compute mse=ssresid/(n1-ncol( llrdat )).

.
compute n1=nrow( x ).
compute invXtX = inv(t( x )* x ).
compute varb = mse *invXtX.
compute k3 = ncol( x ).
compute xhc=0.
do if ( hc <> 5).
compute xhc= x.
compute hat = xhc(:,1).
loop i3=1 to nrow(xhc).
compute hat(i3,1)= xhc(i3,:)*invXtX*t(xhc(i3,:)).
end loop.
do if ( hc = 0 or hc =1).
loop i3 = 1 to k3.
compute xhc(:,i3)=xhc(:,i3)&* resid.
end loop.
end if.
do if ( hc =3 or hc =2).
loop i3=1 to k3.
compute xhc(:,i3) = ( resid &/(1-hat)&**(1/(4- hc )))&*xhc(:,i3).
end loop.
end if.
do if ( hc = 4).
compute hcmn=make(n,2,4).
compute hcmn(:,2)=(n1*hat)/k3.
loop i3= 1 to k3.
compute xhc(:,i3) = ( resid &/(1-hat)&**(rmin(hcmn)/2))&*xhc(:,i3).
end loop.
end if.
compute varb=(invXtX*t(xhc)*xhc*invXtX).
do if ( hc =1).
compute varb=(n1/(n1-ncol( x )))&*varb.
end if.
end if.
compute hclab={'se(HC0)','se(HC1)','se(HC2)','se(HC3)','se(HC4)','se'}.
compute hclab=hclab(1,( hc +1)).
compute hcflab={'F(HC0)','F(HC1)','F(HC2)','F(HC3)','F(HC4)','F'}.
compute hcflab=hcflab(1,( hc +1)).
release xhc
.
compute seb=sqrt(diag(varb)).
compute trat = b&/seb.
compute p = 2*(1-tcdf(abs(trat), (dfres))).
compute tval = sqrt(dfres* (exp((dfres-(5/6))*((xp2/(dfres-(2/3)+(.11/dfres)))* (xp2/(dfres-(2/3)+(.11/dfres)))))-1)).
compute modres={modres,seb,trat,p}.
compute modres={modres,(b-tval&*seb),(b+tval&*seb)}.
compute modresl={'coeff',hclab,'t','p','LLCI','ULCI'}.
compute lmat = ident(ncol( llrdat )).
compute lmat = lmat(:,2:ncol(lmat)).
compute fratio = (t(t(lmat)*b)*inv(t(lmat)*varb*lmat)*((t(lmat)*b)))/(ncol( llrdat )-1).
compute pfr = 1-fcdf(fratio,(ncol( llrdat )-1),dfres).
compute modsum={sqrt(r2),r2,mse,fratio,(ncol( llrdat )-1),dfres,pfr}.
compute modsuml={'R','R-sq','MSE',hcflab,'df1','df2', 'p'}.
end if.
end if.
do if ( 2 = 2 or 2 =3).
compute xlp= llrdat.
compute ylp= y.
compute pt2 = make(nrow(ylp),1,(csum(ylp)/nrow(ylp))).
do if ( 2 =2).
compute LL3 = ylp&*ln(pt2)+(1-ylp)&*ln(1-pt2).
end if.
compute LL3 = -2*csum(LL3).
compute bt1 = make(ncol(xlp),1,0).
compute LL1 = 0.
compute pt1 = make(nrow(ylp),1,0.5).
compute pt1lp=pt1.
loop jjj = 1 to iterate.
compute xlptmp=t(xlp).
compute vecprb=pt1lp&*(1-pt1lp).
loop kkk=1 to ncol(xlp).
compute xlptmp(kkk,:)=xlptmp(kkk,:)&*t(vecprb).
end loop.
compute b = bt1+inv(xlptmp*xlp)*t(xlp)*(ylp-pt1lp).
do if ( 2 =2).
compute xlpb=xlp*b.
compute xlpbt=(xlpb > -709.7).
compute xlpb709=(1-xlpbt)*(-709.7).
compute xlpb=(xlpb&*xlpbt)+xlpb709.
compute pt1lp = 1/(1+exp(-(xlpb))).
end if.
compute itprob = csum((pt1lp < .00000001) or (pt1lp > .9999999)).
do if (itprob > 0).
loop kkk = 1 to nrow(pt1lp).
do if (pt1lp(kkk,1) > .9999999).
compute pt1lp(kkk,1) = .9999999.
end if.
do if (pt1lp(kkk,1) < .00000001).
compute pt1lp(kkk,1) = .00000001.
end if.
end loop.
compute itprob = 0.
end if.
do if (itprob = 0).
do if ( 2 =2).
compute LL = ylp&*ln(pt1lp)+(1-ylp)&*ln(1-pt1lp).
end if.
compute LL2 = -2*csum(ll).
end if.
do if (abs(LL1-LL2) < converge).
do if ( 0 =1).
compute xlptmp=t(xlp).
compute vecprb=pt1lp&*(1-pt1lp).
loop kkk=1 to ncol(xlp).
compute xlptmp(kkk,:)=xlptmp(kkk,:)&*t(vecprb).
end loop.
compute varb = inv(xlptmp*xlp).
compute seb = sqrt(diag(varb)).
release xlptmp.
end if.
break.
end if.
compute bt1 = b.
compute LL1 = LL2.
end loop.
compute modres=b.
do if (jjj > iterate).
compute itprob = 2.
do if (booting=0).
compute iterrmod=1.
end if.
do if (booting=1).
compute bootiter=1.
end if.
do if (itprobtg=0).
compute itprobtg=1.
compute errcode(errs,1) = 47.
compute errs = errs + 1.
do if (booting = 0 and 0 =1).
compute vt1 = mdiag(pt1lp&*(1-pt1lp)).
compute varb = inv(t(xlp)*vt1*xlp).
compute seb = sqrt(diag(varb)).
end if.
end if.
end if.
do if ( 0 =1).
compute trat = b&/seb.
compute dfres=nrow(xlp).
compute p = 2*(1-cdfnorm(abs(trat))).
compute modres={modres,seb,trat,p}.
compute modres={modres,(b-xp2&*seb),(b+xp2&*seb)}.
compute pvchi=1-chicdf((LL3-LL2),(nrow(modres)-1)).
compute mcF = (LL3-LL2)/LL3.
compute cox = 1-exp(-(LL3-LL2)/nrow(xlp)).
compute nagel = cox/(1-exp(-(LL3)/nrow(xlp))).
compute modsum={LL2,(LL3-LL2),(nrow(modres)-1),pvchi, mcF,cox,nagel}.
compute modsuml={'-2LL','ModelLL', 'df', 'p', 'McFadden', 'CoxSnell', 'Nagelkrk'}.
compute modresl={'coeff','se','Z','p','LLCI','ULCI'}.
end if.
end if
.
compute b=btemphld.
compute fresult={(LL2-basemod),llrdf,1-chicdf((LL2-basemod),llrdf)}
.
compute lmatdb=lmat.
compute dbint=dbint+1.
end if.
compute highf={highf;fresult}.
compute highf2={highf2;fresult}.
do if (j = start).
compute flabel={flabel;'X*W'}.
end if.
do if (j > start).
do if (nms > 1).
compute flabel={flabel;highlbw(jj,1)}.
else if (nms = 1).
do if (xmint=0).
compute flabel={flabel;'M*W'}.
end if.
do if (xmint=1).
compute flabel={flabel;'X*M'}.
end if.
end if.
end if.
end if.
compute lmat=zhigh(1:nump(1,i),j).
compute lmat2=wzhigh(1:nump(1,i),j).
do if ((csum(lmat) > 0) and (csum(lmat2) = 0)).
do if ((i < (nms+nys)) or (ydich <> 1)).

.
compute lmat2= lmat.
do if ( 0 =0).
compute lmat2 = mdiag( lmat ).
compute lmat3=make(nrow(lmat2),1,0).
loop flp=1 to ncol(lmat2).
do if (csum(lmat2(:,flp))=1).
compute lmat3={lmat3,lmat2(:,flp)}.
end if.
end loop.
compute lmat2=lmat3(:,2:ncol(lmat3)).
end if.
compute fratio = (t(t(lmat2)* b )*inv(t(lmat2)* varb *lmat2)*((t(lmat2)* b )))/ncol(lmat2).
compute pfr = 1-fcdf(fratio,ncol(lmat2),(n-nrow( b ))).
compute fresult={fratio,ncol(lmat2),(n-nrow( b )),pfr}.
do if (i = (nms+nys) and (ydich=1)).
compute fratio=fratio*ncol(lmat2).
compute pfr=1-chicdf(fratio,ncol(lmat2)).
compute fresult={fratio,ncol(lmat2),pfr}.
end if.
do if ( 1 =1).
compute lmat3=1-rsum(lmat2).
compute xfm=make(n,csum(lmat3),0).
compute flpc=1.
loop flp=1 to nrow(lmat3).
do if (lmat3(flp,1)=1).
compute xfm(:,flpc)=x(:,flp).
compute flpc=flpc+1.
end if.
end loop.
compute bfm=inv(t(xfm)*xfm)*t(xfm)*y.
compute resid=y-(xfm*bfm).
compute sstotal=(y-(csum(y)/n)).
compute sstotal=csum(sstotal&*sstotal).
compute ssresid=csum(resid&*resid).
compute rsqch= r2 -((sstotal-ssresid)/sstotal).
compute fresult={rsqch,fresult}.
release xfm,flpc, resid, ssresid, bfm.
end if
.
compute dbint=dbint+1.
end if.
do if ((ydich = 1) and (i = (nms+nys))).

.
compute btemphld=b.
compute llrdat=make(nrow(x),nrow( lmat )-csum( lmat ),-999).
compute llrdf=ncol(x)-ncol(llrdat).
compute llrcnt=0.
loop llri=1 to nrow( lmat ).
do if ( lmat (llri,1)=0).
compute llrcnt=llrcnt+1.
compute llrdat(:,llrcnt)=x(:,llri).
end if.
end loop.

.
do if ( 2 =1).
compute hatmat=inv(t( llrdat )* llrdat )*t( llrdat ).
compute b = hatmat* y.
compute modres=b.
do if ( 0 =1).
compute n1=nrow( llrdat ).
compute dfres=n1-(ncol( llrdat )).
compute sstotal = t( y -(csum( y )/n1))*( y -(csum( y )/n1)).
compute resid= y - llrdat *b.
compute ssresid = csum((resid)&**2).
compute r2 = (sstotal-ssresid)/sstotal.
do if (r2 < 0).
compute r2=0.
end if.
compute adjr2 = 1-((1-r2)*(n1-1)/(dfres)).
compute mse=ssresid/(n1-ncol( llrdat )).

.
compute n1=nrow( x ).
compute invXtX = inv(t( x )* x ).
compute varb = mse *invXtX.
compute k3 = ncol( x ).
compute xhc=0.
do if ( hc <> 5).
compute xhc= x.
compute hat = xhc(:,1).
loop i3=1 to nrow(xhc).
compute hat(i3,1)= xhc(i3,:)*invXtX*t(xhc(i3,:)).
end loop.
do if ( hc = 0 or hc =1).
loop i3 = 1 to k3.
compute xhc(:,i3)=xhc(:,i3)&* resid.
end loop.
end if.
do if ( hc =3 or hc =2).
loop i3=1 to k3.
compute xhc(:,i3) = ( resid &/(1-hat)&**(1/(4- hc )))&*xhc(:,i3).
end loop.
end if.
do if ( hc = 4).
compute hcmn=make(n,2,4).
compute hcmn(:,2)=(n1*hat)/k3.
loop i3= 1 to k3.
compute xhc(:,i3) = ( resid &/(1-hat)&**(rmin(hcmn)/2))&*xhc(:,i3).
end loop.
end if.
compute varb=(invXtX*t(xhc)*xhc*invXtX).
do if ( hc =1).
compute varb=(n1/(n1-ncol( x )))&*varb.
end if.
end if.
compute hclab={'se(HC0)','se(HC1)','se(HC2)','se(HC3)','se(HC4)','se'}.
compute hclab=hclab(1,( hc +1)).
compute hcflab={'F(HC0)','F(HC1)','F(HC2)','F(HC3)','F(HC4)','F'}.
compute hcflab=hcflab(1,( hc +1)).
release xhc
.
compute seb=sqrt(diag(varb)).
compute trat = b&/seb.
compute p = 2*(1-tcdf(abs(trat), (dfres))).
compute tval = sqrt(dfres* (exp((dfres-(5/6))*((xp2/(dfres-(2/3)+(.11/dfres)))* (xp2/(dfres-(2/3)+(.11/dfres)))))-1)).
compute modres={modres,seb,trat,p}.
compute modres={modres,(b-tval&*seb),(b+tval&*seb)}.
compute modresl={'coeff',hclab,'t','p','LLCI','ULCI'}.
compute lmat = ident(ncol( llrdat )).
compute lmat = lmat(:,2:ncol(lmat)).
compute fratio = (t(t(lmat)*b)*inv(t(lmat)*varb*lmat)*((t(lmat)*b)))/(ncol( llrdat )-1).
compute pfr = 1-fcdf(fratio,(ncol( llrdat )-1),dfres).
compute modsum={sqrt(r2),r2,mse,fratio,(ncol( llrdat )-1),dfres,pfr}.
compute modsuml={'R','R-sq','MSE',hcflab,'df1','df2', 'p'}.
end if.
end if.
do if ( 2 = 2 or 2 =3).
compute xlp= llrdat.
compute ylp= y.
compute pt2 = make(nrow(ylp),1,(csum(ylp)/nrow(ylp))).
do if ( 2 =2).
compute LL3 = ylp&*ln(pt2)+(1-ylp)&*ln(1-pt2).
end if.
compute LL3 = -2*csum(LL3).
compute bt1 = make(ncol(xlp),1,0).
compute LL1 = 0.
compute pt1 = make(nrow(ylp),1,0.5).
compute pt1lp=pt1.
loop jjj = 1 to iterate.
compute xlptmp=t(xlp).
compute vecprb=pt1lp&*(1-pt1lp).
loop kkk=1 to ncol(xlp).
compute xlptmp(kkk,:)=xlptmp(kkk,:)&*t(vecprb).
end loop.
compute b = bt1+inv(xlptmp*xlp)*t(xlp)*(ylp-pt1lp).
do if ( 2 =2).
compute xlpb=xlp*b.
compute xlpbt=(xlpb > -709.7).
compute xlpb709=(1-xlpbt)*(-709.7).
compute xlpb=(xlpb&*xlpbt)+xlpb709.
compute pt1lp = 1/(1+exp(-(xlpb))).
end if.
compute itprob = csum((pt1lp < .00000001) or (pt1lp > .9999999)).
do if (itprob > 0).
loop kkk = 1 to nrow(pt1lp).
do if (pt1lp(kkk,1) > .9999999).
compute pt1lp(kkk,1) = .9999999.
end if.
do if (pt1lp(kkk,1) < .00000001).
compute pt1lp(kkk,1) = .00000001.
end if.
end loop.
compute itprob = 0.
end if.
do if (itprob = 0).
do if ( 2 =2).
compute LL = ylp&*ln(pt1lp)+(1-ylp)&*ln(1-pt1lp).
end if.
compute LL2 = -2*csum(ll).
end if.
do if (abs(LL1-LL2) < converge).
do if ( 0 =1).
compute xlptmp=t(xlp).
compute vecprb=pt1lp&*(1-pt1lp).
loop kkk=1 to ncol(xlp).
compute xlptmp(kkk,:)=xlptmp(kkk,:)&*t(vecprb).
end loop.
compute varb = inv(xlptmp*xlp).
compute seb = sqrt(diag(varb)).
release xlptmp.
end if.
break.
end if.
compute bt1 = b.
compute LL1 = LL2.
end loop.
compute modres=b.
do if (jjj > iterate).
compute itprob = 2.
do if (booting=0).
compute iterrmod=1.
end if.
do if (booting=1).
compute bootiter=1.
end if.
do if (itprobtg=0).
compute itprobtg=1.
compute errcode(errs,1) = 47.
compute errs = errs + 1.
do if (booting = 0 and 0 =1).
compute vt1 = mdiag(pt1lp&*(1-pt1lp)).
compute varb = inv(t(xlp)*vt1*xlp).
compute seb = sqrt(diag(varb)).
end if.
end if.
end if.
do if ( 0 =1).
compute trat = b&/seb.
compute dfres=nrow(xlp).
compute p = 2*(1-cdfnorm(abs(trat))).
compute modres={modres,seb,trat,p}.
compute modres={modres,(b-xp2&*seb),(b+xp2&*seb)}.
compute pvchi=1-chicdf((LL3-LL2),(nrow(modres)-1)).
compute mcF = (LL3-LL2)/LL3.
compute cox = 1-exp(-(LL3-LL2)/nrow(xlp)).
compute nagel = cox/(1-exp(-(LL3)/nrow(xlp))).
compute modsum={LL2,(LL3-LL2),(nrow(modres)-1),pvchi, mcF,cox,nagel}.
compute modsuml={'-2LL','ModelLL', 'df', 'p', 'McFadden', 'CoxSnell', 'Nagelkrk'}.
compute modresl={'coeff','se','Z','p','LLCI','ULCI'}.
end if.
end if
.
compute b=btemphld.
compute fresult={(LL2-basemod),llrdf,1-chicdf((LL2-basemod),llrdf)}
.
compute dbint=dbint+1.
end if.
compute highf={highf;fresult}.
compute highf2={highf2;fresult}.
do if (j = start).
compute flabel={flabel;'X*Z'}.
end if.
do if (j > start).
do if (nms > 1).
compute flabel={flabel;highlbz(jj,1)}.
else if (nms = 1).
compute flabel={flabel;'M*Z'}.
end if.
end if.
end if.
do if (dbint=2).
compute lmatdb=lmatdb+lmat.
do if ((ydich = 1) and (i = (nms+nys))).

.
compute btemphld=b.
compute llrdat=make(nrow(x),nrow( lmatdb )-csum( lmatdb ),-999).
compute llrdf=ncol(x)-ncol(llrdat).
compute llrcnt=0.
loop llri=1 to nrow( lmatdb ).
do if ( lmatdb (llri,1)=0).
compute llrcnt=llrcnt+1.
compute llrdat(:,llrcnt)=x(:,llri).
end if.
end loop.

.
do if ( 2 =1).
compute hatmat=inv(t( llrdat )* llrdat )*t( llrdat ).
compute b = hatmat* y.
compute modres=b.
do if ( 0 =1).
compute n1=nrow( llrdat ).
compute dfres=n1-(ncol( llrdat )).
compute sstotal = t( y -(csum( y )/n1))*( y -(csum( y )/n1)).
compute resid= y - llrdat *b.
compute ssresid = csum((resid)&**2).
compute r2 = (sstotal-ssresid)/sstotal.
do if (r2 < 0).
compute r2=0.
end if.
compute adjr2 = 1-((1-r2)*(n1-1)/(dfres)).
compute mse=ssresid/(n1-ncol( llrdat )).

.
compute n1=nrow( x ).
compute invXtX = inv(t( x )* x ).
compute varb = mse *invXtX.
compute k3 = ncol( x ).
compute xhc=0.
do if ( hc <> 5).
compute xhc= x.
compute hat = xhc(:,1).
loop i3=1 to nrow(xhc).
compute hat(i3,1)= xhc(i3,:)*invXtX*t(xhc(i3,:)).
end loop.
do if ( hc = 0 or hc =1).
loop i3 = 1 to k3.
compute xhc(:,i3)=xhc(:,i3)&* resid.
end loop.
end if.
do if ( hc =3 or hc =2).
loop i3=1 to k3.
compute xhc(:,i3) = ( resid &/(1-hat)&**(1/(4- hc )))&*xhc(:,i3).
end loop.
end if.
do if ( hc = 4).
compute hcmn=make(n,2,4).
compute hcmn(:,2)=(n1*hat)/k3.
loop i3= 1 to k3.
compute xhc(:,i3) = ( resid &/(1-hat)&**(rmin(hcmn)/2))&*xhc(:,i3).
end loop.
end if.
compute varb=(invXtX*t(xhc)*xhc*invXtX).
do if ( hc =1).
compute varb=(n1/(n1-ncol( x )))&*varb.
end if.
end if.
compute hclab={'se(HC0)','se(HC1)','se(HC2)','se(HC3)','se(HC4)','se'}.
compute hclab=hclab(1,( hc +1)).
compute hcflab={'F(HC0)','F(HC1)','F(HC2)','F(HC3)','F(HC4)','F'}.
compute hcflab=hcflab(1,( hc +1)).
release xhc
.
compute seb=sqrt(diag(varb)).
compute trat = b&/seb.
compute p = 2*(1-tcdf(abs(trat), (dfres))).
compute tval = sqrt(dfres* (exp((dfres-(5/6))*((xp2/(dfres-(2/3)+(.11/dfres)))* (xp2/(dfres-(2/3)+(.11/dfres)))))-1)).
compute modres={modres,seb,trat,p}.
compute modres={modres,(b-tval&*seb),(b+tval&*seb)}.
compute modresl={'coeff',hclab,'t','p','LLCI','ULCI'}.
compute lmat = ident(ncol( llrdat )).
compute lmat = lmat(:,2:ncol(lmat)).
compute fratio = (t(t(lmat)*b)*inv(t(lmat)*varb*lmat)*((t(lmat)*b)))/(ncol( llrdat )-1).
compute pfr = 1-fcdf(fratio,(ncol( llrdat )-1),dfres).
compute modsum={sqrt(r2),r2,mse,fratio,(ncol( llrdat )-1),dfres,pfr}.
compute modsuml={'R','R-sq','MSE',hcflab,'df1','df2', 'p'}.
end if.
end if.
do if ( 2 = 2 or 2 =3).
compute xlp= llrdat.
compute ylp= y.
compute pt2 = make(nrow(ylp),1,(csum(ylp)/nrow(ylp))).
do if ( 2 =2).
compute LL3 = ylp&*ln(pt2)+(1-ylp)&*ln(1-pt2).
end if.
compute LL3 = -2*csum(LL3).
compute bt1 = make(ncol(xlp),1,0).
compute LL1 = 0.
compute pt1 = make(nrow(ylp),1,0.5).
compute pt1lp=pt1.
loop jjj = 1 to iterate.
compute xlptmp=t(xlp).
compute vecprb=pt1lp&*(1-pt1lp).
loop kkk=1 to ncol(xlp).
compute xlptmp(kkk,:)=xlptmp(kkk,:)&*t(vecprb).
end loop.
compute b = bt1+inv(xlptmp*xlp)*t(xlp)*(ylp-pt1lp).
do if ( 2 =2).
compute xlpb=xlp*b.
compute xlpbt=(xlpb > -709.7).
compute xlpb709=(1-xlpbt)*(-709.7).
compute xlpb=(xlpb&*xlpbt)+xlpb709.
compute pt1lp = 1/(1+exp(-(xlpb))).
end if.
compute itprob = csum((pt1lp < .00000001) or (pt1lp > .9999999)).
do if (itprob > 0).
loop kkk = 1 to nrow(pt1lp).
do if (pt1lp(kkk,1) > .9999999).
compute pt1lp(kkk,1) = .9999999.
end if.
do if (pt1lp(kkk,1) < .00000001).
compute pt1lp(kkk,1) = .00000001.
end if.
end loop.
compute itprob = 0.
end if.
do if (itprob = 0).
do if ( 2 =2).
compute LL = ylp&*ln(pt1lp)+(1-ylp)&*ln(1-pt1lp).
end if.
compute LL2 = -2*csum(ll).
end if.
do if (abs(LL1-LL2) < converge).
do if ( 0 =1).
compute xlptmp=t(xlp).
compute vecprb=pt1lp&*(1-pt1lp).
loop kkk=1 to ncol(xlp).
compute xlptmp(kkk,:)=xlptmp(kkk,:)&*t(vecprb).
end loop.
compute varb = inv(xlptmp*xlp).
compute seb = sqrt(diag(varb)).
release xlptmp.
end if.
break.
end if.
compute bt1 = b.
compute LL1 = LL2.
end loop.
compute modres=b.
do if (jjj > iterate).
compute itprob = 2.
do if (booting=0).
compute iterrmod=1.
end if.
do if (booting=1).
compute bootiter=1.
end if.
do if (itprobtg=0).
compute itprobtg=1.
compute errcode(errs,1) = 47.
compute errs = errs + 1.
do if (booting = 0 and 0 =1).
compute vt1 = mdiag(pt1lp&*(1-pt1lp)).
compute varb = inv(t(xlp)*vt1*xlp).
compute seb = sqrt(diag(varb)).
end if.
end if.
end if.
do if ( 0 =1).
compute trat = b&/seb.
compute dfres=nrow(xlp).
compute p = 2*(1-cdfnorm(abs(trat))).
compute modres={modres,seb,trat,p}.
compute modres={modres,(b-xp2&*seb),(b+xp2&*seb)}.
compute pvchi=1-chicdf((LL3-LL2),(nrow(modres)-1)).
compute mcF = (LL3-LL2)/LL3.
compute cox = 1-exp(-(LL3-LL2)/nrow(xlp)).
compute nagel = cox/(1-exp(-(LL3)/nrow(xlp))).
compute modsum={LL2,(LL3-LL2),(nrow(modres)-1),pvchi, mcF,cox,nagel}.
compute modsuml={'-2LL','ModelLL', 'df', 'p', 'McFadden', 'CoxSnell', 'Nagelkrk'}.
compute modresl={'coeff','se','Z','p','LLCI','ULCI'}.
end if.
end if
.
compute b=btemphld.
compute fresult={(LL2-basemod),llrdf,1-chicdf((LL2-basemod),llrdf)}
.
end if.
do if (ydich <> 1 or i < (nms+nys)).

.
compute lmat2= lmatdb.
do if ( 0 =0).
compute lmat2 = mdiag( lmatdb ).
compute lmat3=make(nrow(lmat2),1,0).
loop flp=1 to ncol(lmat2).
do if (csum(lmat2(:,flp))=1).
compute lmat3={lmat3,lmat2(:,flp)}.
end if.
end loop.
compute lmat2=lmat3(:,2:ncol(lmat3)).
end if.
compute fratio = (t(t(lmat2)* b )*inv(t(lmat2)* varb *lmat2)*((t(lmat2)* b )))/ncol(lmat2).
compute pfr = 1-fcdf(fratio,ncol(lmat2),(n-nrow( b ))).
compute fresult={fratio,ncol(lmat2),(n-nrow( b )),pfr}.
do if (i = (nms+nys) and (ydich=1)).
compute fratio=fratio*ncol(lmat2).
compute pfr=1-chicdf(fratio,ncol(lmat2)).
compute fresult={fratio,ncol(lmat2),pfr}.
end if.
do if ( 1 =1).
compute lmat3=1-rsum(lmat2).
compute xfm=make(n,csum(lmat3),0).
compute flpc=1.
loop flp=1 to nrow(lmat3).
do if (lmat3(flp,1)=1).
compute xfm(:,flpc)=x(:,flp).
compute flpc=flpc+1.
end if.
end loop.
compute bfm=inv(t(xfm)*xfm)*t(xfm)*y.
compute resid=y-(xfm*bfm).
compute sstotal=(y-(csum(y)/n)).
compute sstotal=csum(sstotal&*sstotal).
compute ssresid=csum(resid&*resid).
compute rsqch= r2 -((sstotal-ssresid)/sstotal).
compute fresult={rsqch,fresult}.
release xfm,flpc, resid, ssresid, bfm.
end if
.
end if.
compute dbint=0.
compute highf={highf;fresult}.
do if (jj=0 and nms > 0).
compute flabel={flabel;'BOTH(X)'}.
end if.
do if (jj=0 and nms = 0).
compute flabel={flabel;'BOTH'}.
end if.
do if (jj>0 and nms = 1).
compute flabel={flabel;'BOTH(M)'}.
end if.
do if (nms > 1 and jj > 0).
compute flabel={flabel;highlbbt(jj,1)}.
end if.
end if.
compute lmat2=wzhigh(1:nump(1,i),j).
do if (csum(lmat2) > 0).
do if ((i < (nms+nys)) or (ydich <> 1)).

.
compute lmat2= lmat2.
do if ( 0 =0).
compute lmat2 = mdiag( lmat2 ).
compute lmat3=make(nrow(lmat2),1,0).
loop flp=1 to ncol(lmat2).
do if (csum(lmat2(:,flp))=1).
compute lmat3={lmat3,lmat2(:,flp)}.
end if.
end loop.
compute lmat2=lmat3(:,2:ncol(lmat3)).
end if.
compute fratio = (t(t(lmat2)* b )*inv(t(lmat2)* varb *lmat2)*((t(lmat2)* b )))/ncol(lmat2).
compute pfr = 1-fcdf(fratio,ncol(lmat2),(n-nrow( b ))).
compute fresult={fratio,ncol(lmat2),(n-nrow( b )),pfr}.
do if (i = (nms+nys) and (ydich=1)).
compute fratio=fratio*ncol(lmat2).
compute pfr=1-chicdf(fratio,ncol(lmat2)).
compute fresult={fratio,ncol(lmat2),pfr}.
end if.
do if ( 1 =1).
compute lmat3=1-rsum(lmat2).
compute xfm=make(n,csum(lmat3),0).
compute flpc=1.
loop flp=1 to nrow(lmat3).
do if (lmat3(flp,1)=1).
compute xfm(:,flpc)=x(:,flp).
compute flpc=flpc+1.
end if.
end loop.
compute bfm=inv(t(xfm)*xfm)*t(xfm)*y.
compute resid=y-(xfm*bfm).
compute sstotal=(y-(csum(y)/n)).
compute sstotal=csum(sstotal&*sstotal).
compute ssresid=csum(resid&*resid).
compute rsqch= r2 -((sstotal-ssresid)/sstotal).
compute fresult={rsqch,fresult}.
release xfm,flpc, resid, ssresid, bfm.
end if
.
end if.
do if ((ydich = 1) and (i = (nms+nys))).

.
compute btemphld=b.
compute llrdat=make(nrow(x),nrow( lmat2 )-csum( lmat2 ),-999).
compute llrdf=ncol(x)-ncol(llrdat).
compute llrcnt=0.
loop llri=1 to nrow( lmat2 ).
do if ( lmat2 (llri,1)=0).
compute llrcnt=llrcnt+1.
compute llrdat(:,llrcnt)=x(:,llri).
end if.
end loop.

.
do if ( 2 =1).
compute hatmat=inv(t( llrdat )* llrdat )*t( llrdat ).
compute b = hatmat* y.
compute modres=b.
do if ( 0 =1).
compute n1=nrow( llrdat ).
compute dfres=n1-(ncol( llrdat )).
compute sstotal = t( y -(csum( y )/n1))*( y -(csum( y )/n1)).
compute resid= y - llrdat *b.
compute ssresid = csum((resid)&**2).
compute r2 = (sstotal-ssresid)/sstotal.
do if (r2 < 0).
compute r2=0.
end if.
compute adjr2 = 1-((1-r2)*(n1-1)/(dfres)).
compute mse=ssresid/(n1-ncol( llrdat )).

.
compute n1=nrow( x ).
compute invXtX = inv(t( x )* x ).
compute varb = mse *invXtX.
compute k3 = ncol( x ).
compute xhc=0.
do if ( hc <> 5).
compute xhc= x.
compute hat = xhc(:,1).
loop i3=1 to nrow(xhc).
compute hat(i3,1)= xhc(i3,:)*invXtX*t(xhc(i3,:)).
end loop.
do if ( hc = 0 or hc =1).
loop i3 = 1 to k3.
compute xhc(:,i3)=xhc(:,i3)&* resid.
end loop.
end if.
do if ( hc =3 or hc =2).
loop i3=1 to k3.
compute xhc(:,i3) = ( resid &/(1-hat)&**(1/(4- hc )))&*xhc(:,i3).
end loop.
end if.
do if ( hc = 4).
compute hcmn=make(n,2,4).
compute hcmn(:,2)=(n1*hat)/k3.
loop i3= 1 to k3.
compute xhc(:,i3) = ( resid &/(1-hat)&**(rmin(hcmn)/2))&*xhc(:,i3).
end loop.
end if.
compute varb=(invXtX*t(xhc)*xhc*invXtX).
do if ( hc =1).
compute varb=(n1/(n1-ncol( x )))&*varb.
end if.
end if.
compute hclab={'se(HC0)','se(HC1)','se(HC2)','se(HC3)','se(HC4)','se'}.
compute hclab=hclab(1,( hc +1)).
compute hcflab={'F(HC0)','F(HC1)','F(HC2)','F(HC3)','F(HC4)','F'}.
compute hcflab=hcflab(1,( hc +1)).
release xhc
.
compute seb=sqrt(diag(varb)).
compute trat = b&/seb.
compute p = 2*(1-tcdf(abs(trat), (dfres))).
compute tval = sqrt(dfres* (exp((dfres-(5/6))*((xp2/(dfres-(2/3)+(.11/dfres)))* (xp2/(dfres-(2/3)+(.11/dfres)))))-1)).
compute modres={modres,seb,trat,p}.
compute modres={modres,(b-tval&*seb),(b+tval&*seb)}.
compute modresl={'coeff',hclab,'t','p','LLCI','ULCI'}.
compute lmat = ident(ncol( llrdat )).
compute lmat = lmat(:,2:ncol(lmat)).
compute fratio = (t(t(lmat)*b)*inv(t(lmat)*varb*lmat)*((t(lmat)*b)))/(ncol( llrdat )-1).
compute pfr = 1-fcdf(fratio,(ncol( llrdat )-1),dfres).
compute modsum={sqrt(r2),r2,mse,fratio,(ncol( llrdat )-1),dfres,pfr}.
compute modsuml={'R','R-sq','MSE',hcflab,'df1','df2', 'p'}.
end if.
end if.
do if ( 2 = 2 or 2 =3).
compute xlp= llrdat.
compute ylp= y.
compute pt2 = make(nrow(ylp),1,(csum(ylp)/nrow(ylp))).
do if ( 2 =2).
compute LL3 = ylp&*ln(pt2)+(1-ylp)&*ln(1-pt2).
end if.
compute LL3 = -2*csum(LL3).
compute bt1 = make(ncol(xlp),1,0).
compute LL1 = 0.
compute pt1 = make(nrow(ylp),1,0.5).
compute pt1lp=pt1.
loop jjj = 1 to iterate.
compute xlptmp=t(xlp).
compute vecprb=pt1lp&*(1-pt1lp).
loop kkk=1 to ncol(xlp).
compute xlptmp(kkk,:)=xlptmp(kkk,:)&*t(vecprb).
end loop.
compute b = bt1+inv(xlptmp*xlp)*t(xlp)*(ylp-pt1lp).
do if ( 2 =2).
compute xlpb=xlp*b.
compute xlpbt=(xlpb > -709.7).
compute xlpb709=(1-xlpbt)*(-709.7).
compute xlpb=(xlpb&*xlpbt)+xlpb709.
compute pt1lp = 1/(1+exp(-(xlpb))).
end if.
compute itprob = csum((pt1lp < .00000001) or (pt1lp > .9999999)).
do if (itprob > 0).
loop kkk = 1 to nrow(pt1lp).
do if (pt1lp(kkk,1) > .9999999).
compute pt1lp(kkk,1) = .9999999.
end if.
do if (pt1lp(kkk,1) < .00000001).
compute pt1lp(kkk,1) = .00000001.
end if.
end loop.
compute itprob = 0.
end if.
do if (itprob = 0).
do if ( 2 =2).
compute LL = ylp&*ln(pt1lp)+(1-ylp)&*ln(1-pt1lp).
end if.
compute LL2 = -2*csum(ll).
end if.
do if (abs(LL1-LL2) < converge).
do if ( 0 =1).
compute xlptmp=t(xlp).
compute vecprb=pt1lp&*(1-pt1lp).
loop kkk=1 to ncol(xlp).
compute xlptmp(kkk,:)=xlptmp(kkk,:)&*t(vecprb).
end loop.
compute varb = inv(xlptmp*xlp).
compute seb = sqrt(diag(varb)).
release xlptmp.
end if.
break.
end if.
compute bt1 = b.
compute LL1 = LL2.
end loop.
compute modres=b.
do if (jjj > iterate).
compute itprob = 2.
do if (booting=0).
compute iterrmod=1.
end if.
do if (booting=1).
compute bootiter=1.
end if.
do if (itprobtg=0).
compute itprobtg=1.
compute errcode(errs,1) = 47.
compute errs = errs + 1.
do if (booting = 0 and 0 =1).
compute vt1 = mdiag(pt1lp&*(1-pt1lp)).
compute varb = inv(t(xlp)*vt1*xlp).
compute seb = sqrt(diag(varb)).
end if.
end if.
end if.
do if ( 0 =1).
compute trat = b&/seb.
compute dfres=nrow(xlp).
compute p = 2*(1-cdfnorm(abs(trat))).
compute modres={modres,seb,trat,p}.
compute modres={modres,(b-xp2&*seb),(b+xp2&*seb)}.
compute pvchi=1-chicdf((LL3-LL2),(nrow(modres)-1)).
compute mcF = (LL3-LL2)/LL3.
compute cox = 1-exp(-(LL3-LL2)/nrow(xlp)).
compute nagel = cox/(1-exp(-(LL3)/nrow(xlp))).
compute modsum={LL2,(LL3-LL2),(nrow(modres)-1),pvchi, mcF,cox,nagel}.
compute modsuml={'-2LL','ModelLL', 'df', 'p', 'McFadden', 'CoxSnell', 'Nagelkrk'}.
compute modresl={'coeff','se','Z','p','LLCI','ULCI'}.
end if.
end if
.
compute b=btemphld.
compute fresult={(LL2-basemod),llrdf,1-chicdf((LL2-basemod),llrdf)}
.
end if.
compute highf={highf;fresult}.
compute highf2={highf2;fresult}.
do if (j = start).
compute flabel={flabel;'X*W*Z'}.
end if.
do if (j > start).
do if (nms > 1).
compute flabel={flabel;highlbwz(jj,1)}.
else if (nms = 1).
compute flabel={flabel;'M*W*Z'}.
end if.
end if.
end if.
compute jj=jj+1.
end loop.
release jj.
compute start=start+i.
end if.
do if (nrow(highf) > 1).
compute highf=highf(2:nrow(highf),:).
compute highf2=highf2(2:nrow(highf2),:).
compute flabel=flabel(2:nrow(flabel),1).
compute resultm2=make(nrow(highf),maxresm,99999).
compute resultm2(1:nrow(highf),1:ncol(highf))=highf.
compute resultm={resultm;resultm2}.
do if (outscree=1).
do if ((i < nms+nys) or (ydich=0)).
compute clabtmp={'R2-chng', hcflab,'df1','df2','p'}.
print highf/format = F10.4 /rnames=flabel/cnames=clabtmp/ title = 'Test(s) of highest order unconditional interaction(s):'.
end if.
do if (ydich=1 and i=(nms+nys)).
compute clabtmp={'Chi-sq', 'df','p'}.
print/title='Likelihood ratio test(s) of highest order'.
print highf/format = F10.4 /rnames=flabel/cnames=clabtmp/ title = 'unconditional interactions(s):'/space=0.
end if.
end if.
compute intpb=highf2(:,ncol(highf2)).
end if.
compute intstart=intstart+numint(1,i).
end if.

.
do if (criterr=0).
compute threeway=0.
compute didprint=0.
compute didsome=0.
compute sigintct=0.
loop jmed =1 to (nms+1).
compute hasw=0.
compute hasz=0.
compute jnok=0.
compute nm1vls=0.
compute nm2vls=0.
compute panelgrp=0.
compute graphixs={'WITH', outnames(1,i), 'BY'}.
compute focpred4={' '}.
compute intprint=0.
compute modcat=0.
do if (jmed <= i).
do if ((jmed = 1) and ((i+1) = nrow(bcmat))).
compute pathscnt=pathscnt+1.
else.
compute paths={paths,bcmat((i+1),jmed)}.
compute pathsw={pathsw,wcmat((i+1),jmed)}.
compute pathsz={pathsz,zcmat((i+1),jmed)}.
compute pathswz={pathswz,wzcmat((i+1),jmed)}.
compute temp=fochigh(:,pathscnt)&*bootloc(:,i).
compute pathsfoc={pathsfoc,pathsfoc(:,1)}.
do if (jmed=1).
compute pathtype={pathtype,1}.
end if.
do if ((i+1)=nrow(bcmat)).
compute pathtype={pathtype,3}.
end if.
do if (jmed > 1) and ((i+1) < nrow(bcmat)).
compute pathtype={pathtype,2}.
end if.
do if (jmed=1 and nxvls > 1 and (bcmat((i+1),jmed)=1)).
compute pathsfoc(:,(pathscn2+1))=temp(2:(nxvls+1),1).
end if.
do if ((jmed > 1) or (jmed=1 and nxvls=1)).
compute temp=cmax(temp).
compute pathsfoc(1,(pathscn2+1))=temp.
end if.
compute pathscnt=pathscnt+1.
compute pathscn2=pathscn2+1.
do if (i <= nms).
compute pathsdv={pathsdv,mnames(1,i)}.
end if.
do if (i > nms).
compute pathsdv={pathsdv,ynames}.
end if.
end if.
compute coeffcol=coeffcol+1.
compute probettt=coeffs(1:nrow(b),coeffcol).
do if (jmed=1 and (bcmat((i+1),jmed)=1)).
compute omni=make(nrow(probettt),nxvls,0).
compute omnitmp=ident(nxvls).
compute omni(2:(1+nxvls),:)=omnitmp.
end if.
do if (csum(probettt)>0).
compute probvarb=make(csum(probettt),csum(probettt),999).
compute probcoef=make(csum(probettt),1,999).
compute coefflp2=1.
loop coefflp=1 to nrow(probettt).
do if (probettt(coefflp,1)=1).
compute probcoef(coefflp2,1)=b(coefflp,1).
compute coefflp2=coefflp2+1.
end if.
end loop.
compute coefflp=0.
compute coefflp2=0.
loop iclp=1 to nrow(probettt).
do if probettt(iclp,1)=1.
compute coefflp=coefflp+1.
compute coefflp2=coefflp.
compute probvarb(coefflp,coefflp) = varb(iclp,iclp).
do if (iclp < nrow(probettt)).
loop jclp=(iclp+1) to nrow(probettt).
do if (probettt(jclp,1)=1).
compute coefflp2=coefflp2+1.
compute probvarb(coefflp,coefflp2)=varb(iclp, jclp).
compute probvarb(coefflp2,coefflp)=varb(iclp, jclp).
end if.
end loop.
end if.
end if.
end loop.
end if.
end if.
compute xprobval=xmodvals.
do if (nxvls > 1 or mcx > 0).
compute xprobval=dummatx(:,2:ncol(dummatx)).
end if.
do if ((wcmat((i+1),jmed)=1) and (zcmat((i+1),jmed)=0)).
compute numplps=1.
compute modvals=wmodvals.
compute probeval=wmodvals.
compute wheremv1=wherexw.
compute nm1vls=nwvls.
compute lpstsp={1,1}.
compute modcat=0.
compute jnmod=wtmp.
compute jnmodlab=wnames.
compute jnok=1.
compute jnmin=wmin.
compute jnmax=wmax.
compute wherejn1=2.
do if (jmed=1).
compute wherejn3=wherexw(1,i).
do if (nxvls > 1).
compute jnok=0.
end if.
end if.
do if (jmed > 1).
compute wherejn1=wherem((jmed-1),i).
compute wherejn3=wheremw(((2*jmed)-3),i).
end if.
do if (nwvls > 1).
compute probeval=wprobval.
compute lpstsp(1,2)=ncol(probeval).
compute modcat=1.
compute jnok=0.
end if.
do if (wdich = 1).
compute modcat=1.
compute jnok=0.
end if.
compute problabs=wnames.
compute focpred3={wnames,'(W)'}.
do if (xmint=1).
compute focpred3={wnames,'(X)'}.
end if.
compute hasw=1.
compute modgrph=wnames.
compute intprint=1.
compute sigintct=sigintct+1.
compute printpbe=intpb(sigintct,1).
end if.
do if ((wcmat((i+1),jmed)=0) and (zcmat((i+1),jmed)=1)).
compute numplps=1.
compute modvals=zmodvals.
compute probeval=zmodvals.
compute wheremv1=wherexz.
compute nm1vls=nzvls.
compute lpstsp={1,1}.
compute jnok=1.
compute jnmod=ztmp.
compute jnmin=zmin.
compute jnmax=zmax.
compute jnmodlab=znames.
compute wherejn1=2.
do if (jmed=1).
compute wherejn3=wherexz(1,i).
do if (nxvls > 1).
compute jnok=0.
end if.
end if.
do if (jmed > 1).
compute wherejn1=wherem((jmed-1),i).
compute wherejn3=wheremz(((2*jmed)-3),i).
end if.
do if (nzvls > 1).
compute probeval=zprobval.
compute lpstsp(1,2)=ncol(probeval).
compute modcat=1.
compute jnok=0.
end if.
do if (zdich = 1).
compute modcat=1.
compute jnok=0.
end if.
compute problabs=znames.
compute focpred3={znames,'(Z)'}.
compute modgrph=znames.
compute hasz=1.
compute intprint=1.
compute sigintct=sigintct+1.
compute printpbe=intpb(sigintct,1).
end if.
do if ((wzcmat((i+1),jmed)=1) or ((wcmat((i+1),jmed)=1) and (zcmat((i+1),jmed)=1))).
compute numplps=2.
compute probecnt=1.
compute intprint=1.
do if (wzcmat((i+1),jmed)=1).
compute sigintct=sigintct+1.
compute printpbe=intpb(sigintct,1).
else.
compute sigintct=sigintct+2.
compute printpbe=cmin(intpb((sigintct-1):sigintct,1)).
end if.
compute panelgrp=1.
compute hasw=1.
compute hasz=1.
compute panelcde={'/PANEL','ROWVAR=',znames,'.'}.
compute modgrph=wnames.
compute lpstsp={1,1;1,1}.
compute wheremv1=wherexw.
compute nm1vls=nwvls.
compute wheremv2=wherexz.
compute nm2vls=nzvls.
compute jnok=0.
do if (wzcmat((i+1),jmed)=1).
compute jnok=1.
end if.
do if (jmed > 1).
compute mprobval=mmodvals.
end if.
do if (jmed=1).
do if (nxvls > 1).
compute jnok=0.
end if.
end if.
do if (nwvls > 1).
compute lpstsp(1,2)=ncol(wprobval).
compute modcat=1.
compute jnok=0.
end if.
do if (zdich=1).
compute modcat=1.
compute jnok=0.
end if.
compute lpstsp(2,1)=lpstsp(1,2)+1.
compute lpstsp(2,2)=lpstsp(1,2)+1.
do if (nzvls > 1).
compute lpstsp(2,1)=lpstsp(1,2)+1.
compute lpstsp(2,2)=lpstsp(1,2)+ncol(zprobval).
compute jnok=0.
end if.
do if (zdich=1).
compute jnok=0.
end if.
compute omni3=make(nrow(b),(nxvls*nwvls),0).
do if (jmed > 1).
compute omni3=make(nrow(b),nwvls,0).
end if.
compute focpred3={wnames,'(W)'}.
do if (xmint=1).
compute focpred3={wnames,'(X)'}.
end if.
compute focpred4={'    ', 'Mod var:', znames, '(Z)'}.
compute modvals=make((nrow(wmodvals)*nrow(zmodvals)),2,0).
compute probeval=make((nrow(wmodvals)*nrow(zmodvals)),(ncol(wprobval)+ncol(zprobval)),0).
loop probei= 1 to nrow(wmodvals).
loop probej =1 to nrow(zmodvals).
compute modvals(probecnt,1)=wmodvals(probei,1).
compute probeval(probecnt,1:nwvls)=wprobval(probei,:).
compute modvals(probecnt,2)=zmodvals(probej,1).
compute probeval(probecnt,(nwvls+1):(nwvls+nzvls))=zprobval(probej,:).
compute probecnt=probecnt+1.
end loop.
end loop.
do if (wzcmat((i+1),jmed)=1).
compute numplps=numplps+1.
compute probprod=make(1,(ncol(wprobval)*ncol(zprobval)),0).
compute lpstsp2={1,1}.
compute lpstsp={lpstsp;lpstsp2}.
compute lpstsp(3,1)=lpstsp(2,2)+1.
compute lpstsp(3,2)=lpstsp(2,2)+ncol(probprod).
compute jnmod=ztmp.
compute jnmin=zmin.
compute jnmax=zmax.
compute jnmodlab=znames.
do if (jmed = 1).
compute wherejn1=wherexw(1,i).
compute wherejn3=wherexwz(1,i).
end if.
do if (jmed > 1).
compute wherejn1=wheremw(((2*jmed)-3),i).
compute wherejn3=wheremwz(((2*jmed)-3),i).
end if.
loop probei = 1 to nrow(wmodvals).
loop probej = 1 to nrow(zmodvals).
compute probtemp=1.
loop probek = 1 to ncol(wprobval).
compute probtemp={probtemp,(wprobval(probei,probek)&*zprobval(probej,:))}.
end loop.
compute probprod={probprod;probtemp(1,2:ncol(probtemp))}.
end loop.
end loop.
compute probprod=probprod(2:nrow(probprod),:).
compute probeval={probeval,probprod}.
end if.
compute problabs={wnames,znames}.
release probecnt, probei, probej.
end if.
do if (intprint=1).
compute focpred={'   Focal', 'predict:'}.
do if (jmed=1).
compute focpred={focpred,xnames,'(X)'}.
compute focplotv=xmodvals.
end if.
do if (jmed >1).
do if (nms > 1).
compute focpred={focpred,mnames(1,(jmed-1)), medlb2(1,(jmed-1))}.
end if.
do if (nms = 1).
compute focpred={focpred,mnames(1,(jmed-1)), '(M)'}.
end if.
compute focplotv=mmodvals(:,(jmed-1)).
end if.
compute focpred2={'    ', 'Mod var:',focpred3}.
compute focpred={focpred;focpred2}.
do if (ncol(focpred4) > 1).
compute focpred={focpred;focpred4}.
compute focpred4={' '}.
end if.
release focpred2,focpred3.
do if (outscree=1).
do if ((plot = 1 or plot = 2) or (printpbe <= intprobe)).
print focpred/title='----------'/format=A8/space=0.
end if.
end if.
compute foctmp=make(nrow(modvals),1,1).
compute probexpl=1.
compute probeva2={foctmp,probeval}.
do if (jmed=1 and nxs > 0 and mcx > 0).
compute probexpl=nxvls.
end if.
compute foctmp=make(nrow(modvals),1,1).
compute modvals3=make(1,(6+ncol(problabs)),0).
compute probrown=make(nrow(probeval),1,0).
compute jtmp=1.
loop probei = 1 to nrow(probeval).
compute probrown(probei,1)=jtmp.
compute jtmp=jtmp+nxvls.
end loop.
release jtmp.
compute probrow=999.
compute modvarl=problabs.
do if (plot = 1 or plot = 2 or nxvls > 1).
compute plotvals=make((nrow(modvals)*nrow(focplotv)),(ncol(modvals)+1),999).
loop ploti=1 to nrow(modvals).
loop plotj=1 to nrow(focplotv).
compute plotvals((((ploti-1)*nrow(focplotv))+plotj),2:ncol(plotvals))=modvals(ploti,:).
compute plotvals((((ploti-1)*nrow(focplotv))+plotj),1)=focplotv(plotj,1).
end loop.
end loop.
compute focpredn=3.
do if (jmed=1).
do if (nxvls > 1).
compute focpredn=(nxvls+1).
end if.
do if (nxvls=1 and xdich=1).
compute focpredn=2.
end if.
end if.
compute meanmat=mdiag(means).
compute onesmat=make(nrow(meanmat),(nrow(probeval)*focpredn),1).
compute probeplt=t(mdiag(means)*onesmat).
do if (jmed=1).
do if ((wcmat((i+1),1)=1) or (zcmat((i+1),1)=1)).
compute plotcnt=1.
compute iloops=nwpval*nzpval.
compute plotmx=nxpval*nzpval.
do if ((wcmat((i+1),1)=1) and (zcmat((i+1),1)=0)).
compute iloops=nwpval.
compute plotmx=nxpval.
end if.
do if ((wcmat((i+1),1)=0) and (zcmat((i+1),1)=1)).
compute iloops=nzpval.
compute plotmx=nxpval.
end if.
compute xestvals=make((nxpval*iloops),ncol(xprobval),-999).
do if (wcmat((i+1),1)=1).
compute westvals=make(nrow(xestvals),ncol(wprobval),-999).
end if.
do if (zcmat((i+1),1)=1).
compute zestvals=make(nrow(xestvals),ncol(zprobval),-999).
end if.
loop ploti=1 to iloops.
loop plotj=1 to nxpval.
compute xestvals(plotcnt,:)=xprobval(plotj,:).
compute plotcnt=plotcnt+1.
end loop.
end loop.
compute plotcnt=1.
compute plotcnt1=1.
compute plotcnt2=1.
compute plotcntz=1.
loop ploti = 1 to (iloops*nxpval).
do if (wcmat((i+1),1)=1).
compute westvals(ploti,:)=wprobval(plotcnt1,:).
end if.
do if ((wcmat((i+1),1)=0) and (zcmat((i+1),1)=1)).
compute zestvals(ploti,:)=zprobval(plotcnt1,:).
end if.
do if ((wcmat((i+1),1)=1) and (zcmat((i+1),1)=1)).
compute zestvals(ploti,:)=zprobval(plotcnt2,:).
compute plotcntz=plotcntz+1.
end if.
compute plotcnt=plotcnt+1.
do if (plotcnt > plotmx).
compute plotcnt=1.
compute plotcnt1=plotcnt1+1.
end if.
do if (plotcntz > nxpval).
compute plotcnt2=plotcnt2+1.
compute plotcntz=1.
do if (plotcnt2 > nzpval).
compute plotcnt2=1.
end if.
end if.
end loop.
compute probeplt(:,2:(1+(ncol(xestvals))))=xestvals.
do if (wcmat((i+1),1)=1).
compute probeplt(:,(wherew(1,i)):(wherew(2,i)))=westvals.
end if.
do if (zcmat((i+1),1)=1).
compute probeplt(:,(wherez(1,i)):(wherez(2,i)))=zestvals.
end if.
end if.
end if.
do if (jmed > 1).
do if ((wcmat((i+1),jmed)=1) or (zcmat((i+1),jmed)=1)).
compute plotcnt=1.
compute iloops=nwpval*nzpval.
compute plotmx=3*nzpval.
do if ((wcmat((i+1),jmed)=1) and (zcmat((i+1),jmed)=0)).
compute iloops=nwpval.
compute plotmx=3.
end if.
do if ((wcmat((i+1),jmed)=0) and (zcmat((i+1),jmed)=1)).
compute iloops=nzpval.
compute plotmx=3.
end if.
compute mestvals=make((3*iloops),1,-999).
do if (wcmat((i+1),jmed)=1).
compute westvals=make(nrow(mestvals),ncol(wprobval),-999).
end if.
do if (zcmat((i+1),jmed)=1).
compute zestvals=make(nrow(mestvals),ncol(zprobval),-999).
end if.
loop ploti=1 to iloops.
loop plotj=1 to 3.
compute mestvals(plotcnt,:)=mprobval(plotj,(jmed-1)).
compute plotcnt=plotcnt+1.
end loop.
end loop.
compute plotcnt=1.
compute plotcnt1=1.
compute plotcnt2=1.
compute plotcntz=1.
loop ploti = 1 to (iloops*3).
do if (wcmat((i+1),jmed)=1).
compute westvals(ploti,:)=wprobval(plotcnt1,:).
end if.
do if ((wcmat((i+1),jmed)=0) and (zcmat((i+1),jmed)=1)).
compute zestvals(ploti,:)=zprobval(plotcnt1,:).
end if.
do if ((wcmat((i+1),jmed)=1) and (zcmat((i+1),jmed)=1)).
compute zestvals(ploti,:)=zprobval(plotcnt2,:).
compute plotcntz=plotcntz+1.
end if.
compute plotcnt=plotcnt+1.
do if (plotcnt > plotmx).
compute plotcnt=1.
compute plotcnt1=plotcnt1+1.
end if.
do if (plotcntz > 3).
compute plotcnt2=plotcnt2+1.
compute plotcntz=1.
do if (plotcnt2 > nzpval).
compute plotcnt2=1.
end if.
end if.
end loop.
compute probeplt(:,wherem((jmed-1),i))=mestvals.
do if (wcmat((i+1),jmed)=1).
do if (model <> 74).
compute probeplt(:,(wherew(1,i)):(wherew(2,i)))=westvals.
end if.
do if (model = 74).
compute probeplt(:,(wherex(1,i)):(wherex(2,i)))=westvals.
end if.
end if.
do if (zcmat((i+1),jmed)=1).
compute probeplt(:,(wherez(1,i)):(wherez(2,i)))=zestvals.
end if.
end if.
end if.
compute prodloop = 1.
do if (jmed=1).
compute prodloop=ncol(xestvals).
end if.
do if (wcmat((i+1),jmed))=1.
compute plotcnt=0.
loop ploti = 1 to prodloop.
loop plotj = 1 to ncol(westvals).
do if (jmed=1).
compute probeplt(:,(wherexw(1,i)+plotcnt))=xestvals(:,ploti)&*westvals(:,plotj).
end if.
do if (jmed > 1).
compute probeplt(:,(wheremw(((jmed*2)-3) ,i)+plotcnt))=mestvals(:,ploti)&*westvals(:,plotj).
end if.
compute plotcnt=plotcnt+1.
end loop.
end loop.
end if.
do if (zcmat((i+1),jmed))=1.
compute plotcnt=0.
loop ploti = 1 to prodloop.
loop plotj = 1 to ncol(zestvals).
do if (jmed = 1).
compute probeplt(:,(wherexz(1,i)+plotcnt))=xestvals(:,ploti)&*zestvals(:,plotj).
end if.
do if (jmed > 1).
compute probeplt(:,(wheremz(((jmed*2)-3),i)+plotcnt))=mestvals(:,ploti)&*zestvals(:, plotj).
end if.
compute plotcnt=plotcnt+1.
end loop.
end loop.
end if.
do if (wzcmat((i+1),jmed))=1.
compute plotcnt=0.
compute threeway=1.
loop ploti = 1 to ncol(westvals).
loop plotj = 1 to ncol(zestvals).
compute probeplt(:,(wherewz(1,i)+plotcnt))=westvals(:,ploti)&*zestvals(:,plotj).
compute plotcnt=plotcnt+1.
end loop.
end loop.
compute plotcnt=0.
loop plotk = 1 to prodloop.
loop ploti = 1 to ncol(westvals).
loop plotj = 1 to ncol(zestvals).
do if (jmed = 1).
compute probeplt(:,(wherexwz(1,i)+plotcnt))=xestvals(:,plotk)&*westvals(:,ploti)&* zestvals(:,plotj).
end if.
do if (jmed > 1).
compute probeplt(:,(wheremwz(((jmed*2)-3),i)+plotcnt))=mestvals(:,plotk)&* westvals(:,ploti)&*zestvals(:,plotj).
end if.
compute plotcnt=plotcnt+1.
end loop.
end loop.
end loop.
end if.
loop newplp=1 to i.
do if (newplp <> jmed).
do if (wcmat((i+1),newplp))=1.
compute prodloop=1.
do if (newplp=1).
compute prodloop=nxvls.
end if.
compute plotcnt=0.
loop ploti = 1 to prodloop.
loop plotj = 1 to nwvls.
do if (newplp = 1).
compute probeplt(:,(wherexw(1,i)+plotcnt))=probeplt(:,(1+ploti))&*probeplt(:, (wherew(1,i)+plotj-1)).
end if.
do if (newplp > 1).
do if (model <> 74).
compute probeplt(:,(wheremw(((newplp*2)-3) ,i)+plotcnt))=probeplt(:,wherem((newplp-1),i))&*probeplt(:,(wherew(1,i)+plotj-1)).
end if.
do if (model = 74).
compute probeplt(:,(wheremw(((newplp*2)-3) ,i)+plotcnt))=probeplt(:,wherem((newplp-1),i))&*probeplt(:,(wherex(1,i)+plotj-1)).
end if.
end if.
compute plotcnt=plotcnt+1.
end loop.
end loop.
end if.
do if (zcmat((i+1),newplp))=1.
compute prodloop=1.
do if (newplp=1).
compute prodloop=nxvls.
end if.
compute plotcnt=0.
loop ploti = 1 to prodloop.
loop plotj = 1 to nzvls.
do if (newplp = 1).
compute probeplt(:,(wherexz(1,i)+plotcnt))=probeplt(:,(1+ploti))&*probeplt(:, (wherez(1,i)+plotj-1)).
end if.
do if (newplp > 1).
compute probeplt(:,(wheremz(((newplp*2)-3),i)+plotcnt))=probeplt(:, wherem((newplp-1),i))&*probeplt(:,(wherez(1,i)+plotj-1)).
end if.
compute plotcnt=plotcnt+1.
end loop.
end loop.
end if.
do if (wzcmat((i+1),newplp))=1.
compute plotcnt=0.
do if (threeway=0).
loop ploti = 1 to nwvls.
loop plotj = 1 to nzvls.
compute probeplt(:,(wherewz(1,i)+plotcnt))=probeplt(:,(wherew(1,i)+ploti-1))&* probeplt(:,(wherez(1,i)+plotj-1)).
compute plotcnt=plotcnt+1.
end loop.
end loop.
end if.
compute prodloop=1.
do if (newplp=1).
compute prodloop=nxvls.
end if.
compute plotcnt=0.
loop plotk = 1 to prodloop.
loop ploti = 1 to nwvls.
loop plotj = 1 to nzvls.
do if (newplp = 1).
compute probeplt(:,(wherexwz(1,i)+plotcnt))=probeplt(:,(1+plotk))&*probeplt(:, (wherew(1,i)+ploti-1))&*probeplt(:,(wherez(1,i)+plotj-1)).
end if.
do if (newplp > 1).
compute probeplt(:,(wheremwz(((newplp*2)-3),i)+plotcnt))=probeplt(:, wherem((newplp-1),i))&*probeplt(:,(wherew(1,i)+ploti-1))&*probeplt(:,(wherez(1,i)+plotj-1)).
end if.
compute plotcnt=plotcnt+1.
end loop.
end loop.
end loop.
end if.
end if.
end loop.
compute predvals=probeplt*b.
do if (debug <> 0).
print probeplt.
print b.
end if.
do if (i = nms+nys) and (ydich=1).
compute predvalt=(predvals < 709.7).
compute prevalt7=(1-predvalt)*(709.7).
compute predvals=(predvals&*predvalt)+prevalt7.
compute expyhat=exp(predvals)&/(1+exp(predvals)).
end if.
compute sepred=make(nrow(plotvals),3,999).
loop sei=1 to nrow(plotvals).
compute ask=probeplt(sei,:).
compute sepred(sei,1)=sqrt(ask*varb*t(ask)).
do if ((i < nms+nys) or (ydich=0)).
compute sepred(sei,2)=predvals(sei,1)-tval*sepred(sei,1).
compute sepred(sei,3)=predvals(sei,1)+tval*sepred(sei,1).
end if.
do if ((i = nms+nys) and (ydich=1)).
compute sepred(sei,2)=predvals(sei,1)-xp2*sepred(sei,1).
compute sepred(sei,3)=predvals(sei,1)+xp2*sepred(sei,1).
end if.
end loop.
compute prevloc=ncol(plotvals)+1.
compute probeplt={plotvals,predvals}.
do if (plot = 2).
compute probeplt={probeplt,sepred}.
end if.
do if ((i = nms+nys) and (ydich=1)).
compute probeplt={probeplt,expyhat}.
end if.
compute didsome=0.
end if.
do if ((wzcmat((i+1),jmed)=1) and (printpbe <= intprobe)).
do if (jmed=1).
compute omnilp2=nxvls*nwvls.
compute omnitmp=ident(omnilp2).
compute omni3(wherexw(1,i):wherexw(2,i),:)=omnitmp.
end if.
do if (jmed>1).
compute omnilp2=nwvls.
compute omnitmp=ident(omnilp2).
compute omni3(wheremw(((jmed*2)-3),i):wheremw(((jmed*2)-2),i),:)=omnitmp.
end if.
compute omnif=make(1,4,0).
do if ((i = nms+nys) and (ydich=1)).
compute omnif=make(1,3,0).
end if.
compute condeff3=0.
loop omnilp1=1 to nrow(zprobval).
loop omnilp=1 to (omnilp2).
do if (jmed=1).
compute omni3((wherexwz(1,i)+((omnilp-1)*nzvls)):(wherexwz(1,i)+((omnilp-1)*nzvls)+ (nzvls-1)),omnilp)=t(zprobval(omnilp1,:)).
end if.
do if (jmed > 1).
compute omni3((wheremwz(((jmed*2)-3),i)+((omnilp-1)*nzvls)):(wheremwz(((jmed*2)-3),i)+ ((omnilp-1)*nzvls)+(nzvls-1)),omnilp)=t(zprobval(omnilp1,:)).
end if.
end loop.
compute condeff=t(omni3)*b.
compute condeff3={condeff3;condeff}.

.
compute lmat2= omni3.
do if ( 1 =0).
compute lmat2 = mdiag( omni3 ).
compute lmat3=make(nrow(lmat2),1,0).
loop flp=1 to ncol(lmat2).
do if (csum(lmat2(:,flp))=1).
compute lmat3={lmat3,lmat2(:,flp)}.
end if.
end loop.
compute lmat2=lmat3(:,2:ncol(lmat3)).
end if.
compute fratio = (t(t(lmat2)* b )*inv(t(lmat2)* varb *lmat2)*((t(lmat2)* b )))/ncol(lmat2).
compute pfr = 1-fcdf(fratio,ncol(lmat2),(n-nrow( b ))).
compute fresult={fratio,ncol(lmat2),(n-nrow( b )),pfr}.
do if (i = (nms+nys) and (ydich=1)).
compute fratio=fratio*ncol(lmat2).
compute pfr=1-chicdf(fratio,ncol(lmat2)).
compute fresult={fratio,ncol(lmat2),pfr}.
end if.
do if ( 0 =1).
compute lmat3=1-rsum(lmat2).
compute xfm=make(n,csum(lmat3),0).
compute flpc=1.
loop flp=1 to nrow(lmat3).
do if (lmat3(flp,1)=1).
compute xfm(:,flpc)=x(:,flp).
compute flpc=flpc+1.
end if.
end loop.
compute bfm=inv(t(xfm)*xfm)*t(xfm)*y.
compute resid=y-(xfm*bfm).
compute sstotal=(y-(csum(y)/n)).
compute sstotal=csum(sstotal&*sstotal).
compute ssresid=csum(resid&*resid).
compute rsqch= 0 -((sstotal-ssresid)/sstotal).
compute fresult={rsqch,fresult}.
release xfm,flpc, resid, ssresid, bfm.
end if
.
compute omnif={omnif;fresult}.
end loop.
compute omnif=omnif(2:nrow(omnif),:).
compute clabtmp=znames.
compute condeff3=condeff3(2:nrow(condeff3),:).
do if ((nxvls*nwvls)=1).
compute omnif={condeff3,omnif}.
compute clabtmp={clabtmp,'Effect'}.
end if.
compute omnif={zmodvals,omnif}.
do if ((i < nms+nys) or (ydich=0)).
compute clabtmp={clabtmp,hcflab,'df1','df2','p'}.
end if.
do if ((i = (nms+nys)) and (ydich=1)).
compute clabtmp={clabtmp,'Chi-sq','df','p'}.
end if.
compute resultm2=make(nrow(omnif),maxresm,99999).
compute resultm2(1:nrow(omnif),1:ncol(omnif))=omnif.
compute resultm={resultm;resultm2}.
do if (outscree=1).
do if (jmed=1).
print omnif/title='Test of conditional X*W interaction at value(s) of Z:'/cnames=clabtmp/format= F10.4.
end if.
do if (jmed>1).
print omnif/title='Test of conditional M*W interaction at value(s) of Z:'/cnames=clabtmp/format= F10.4.
end if.
end if.
release omni3.
end if.
loop probei = 1 to probexpl.
do if (probexpl > 1).
compute foctmp=make(nrow(modvals),probexpl,0).
compute foctmp(:,probei)=foctmp(:,probei)+1.
compute probtemp=make(nrow(modvals),1,0).
loop probem = 1 to numplps.
loop probek = 1 to nxvls.
loop probej=lpstsp(probem,1) to lpstsp(probem,2).
compute probtemp={probtemp,foctmp(:,probek)&*probeval(:,probej)}.
end loop.
end loop.
end loop.
compute probeva2=probtemp(:,2:ncol(probtemp)).
compute probeva2={foctmp,probeva2}.
end if.
compute probres=probeva2*probcoef.
compute probrese=sqrt(diag(probeva2*probvarb*t(probeva2))).
compute tratio = probres&/probrese.
compute p = 2*(1-tcdf(abs(tratio), dfres)).
do if (ydich=1 and i = (nms+nys)).
compute p = 2*(1-cdfnorm(abs(tratio))).
end if.
compute modvals2={modvals,probres,probrese,tratio, p}.
do if ((i < nms+nys) or (ydich=0)).
compute modvals2={modvals2,(probres-tval&*probrese),(probres+tval&*probrese)}.
compute problabs={problabs,'Effect',hclab,'t', 'p', 'LLCI', 'ULCI'}.
end if.
do if (ydich=1 and i = (nms+nys)).
compute modvals2={modvals2,(probres-xp2&*probrese),(probres+xp2&*probrese)}.
compute problabs={problabs,'Effect','se','Z', 'p', 'LLCI', 'ULCI'}.
end if.
do if (probexpl > 1 and (printpbe <= intprobe)).
do if (hasz = 1).
compute printz=1.
end if.
do if (hasw=1).
compute printw=1.
end if.
compute probrlab=make(nrow(modvals),1,xcatlab(probei,1)).
compute modvals3={modvals3; modvals2}.
compute probrow={probrow;probrown}.
compute probrown=probrown+1.
do if (probei=probexpl).
compute xproblab=xcatlab(1:nxvls,1).
compute probrow=probrow(2:nrow(probrow),1).
compute modvals3=modvals3(2:nrow(modvals3),:).
compute temp=modvals3.
compute temp(GRADE(probrow(:,1)),:)=modvals3.
compute modvals3=temp.
compute start2=1.
compute problabs=problabs(1,(1+(ncol(modvarl))):ncol(problabs)).
compute pstart=1.
loop probek= 1 to nrow(probeval).
compute endstart=start2+(nxvls-1).
compute temp=modvals3(start2:endstart,(1+ncol(modvarl)):ncol(modvals3)).
compute temp2=t(modvals3(start2:start2,1:ncol(modvarl))).
compute trnames=t(modvarl).
do if (outscree=1).
do if (probek > 1).
print/title='----------'/space=0.
else.
print/title = 'Conditional effects of the focal predictor at values of the moderator(s):'.
do if ((jmed=1) and (i = (nms+nys)) and (nms > 0)).
do if (nxvls = 1).
print/title = '(These are also the conditional direct effects of X on Y)'/space=0.
else.
print/title = '(These are also the relative conditional direct effects of X on Y)'/space=0.
end if.
end if.
print.
end if.
print temp2/title = 'Moderator value(s):'/rnames=trnames/format= F10.4 /space=0.
print temp/title = ' '/cnames=problabs/rnames=xproblab/format= F10.4 /space=0.
end if.
compute resultm2=make(nrow(temp2),maxresm,99999).
compute resultm2(1:nrow(temp2),1:ncol(temp2))=temp2.
compute resultm={resultm;resultm2}.
compute resultm2=make(nrow(temp),maxresm,99999).
compute resultm2(1:nrow(temp),1:ncol(temp))=temp.
compute resultm={resultm;resultm2}.
compute start2=start2+nxvls.
compute didsome=1.
do if (jmed=1).
compute mod1val=probeval(probek,1:nm1vls).
loop omnilp=1 to nxvls.
compute omni((wheremv1(1,i)+((omnilp-1)*nm1vls)):(wheremv1(1,i)+((omnilp-1)* nm1vls)+(nm1vls-1)),omnilp)=t(mod1val).
do if (nm1vls < ncol(probeval)).
compute mod2val=probeval(probek,(nm1vls+1):(nm1vls+nm2vls)).
compute omni((wheremv2(1,i)+((omnilp-1)*nm2vls)):(wheremv2(1,i)+((omnilp-1)* nm2vls)+(nm2vls-1)),omnilp)=t(mod2val).
do if ((nm1vls+nm2vls) < ncol(probeval)).
compute intlen=nm1vls*nm2vls.
compute modintvl=probeval(probek,(nm1vls+nm2vls+1):ncol(probeval)).
compute omni((wherexwz(1,i)+((omnilp-1)*intlen)):(wherexwz(1,i)+((omnilp-1)* intlen)+(intlen-1)),omnilp)=t(modintvl).
end if.
end if.
end loop.

.
compute lmat2= omni.
do if ( 1 =0).
compute lmat2 = mdiag( omni ).
compute lmat3=make(nrow(lmat2),1,0).
loop flp=1 to ncol(lmat2).
do if (csum(lmat2(:,flp))=1).
compute lmat3={lmat3,lmat2(:,flp)}.
end if.
end loop.
compute lmat2=lmat3(:,2:ncol(lmat3)).
end if.
compute fratio = (t(t(lmat2)* b )*inv(t(lmat2)* varb *lmat2)*((t(lmat2)* b )))/ncol(lmat2).
compute pfr = 1-fcdf(fratio,ncol(lmat2),(n-nrow( b ))).
compute fresult={fratio,ncol(lmat2),(n-nrow( b )),pfr}.
do if (i = (nms+nys) and (ydich=1)).
compute fratio=fratio*ncol(lmat2).
compute pfr=1-chicdf(fratio,ncol(lmat2)).
compute fresult={fratio,ncol(lmat2),pfr}.
end if.
do if ( 0 =1).
compute lmat3=1-rsum(lmat2).
compute xfm=make(n,csum(lmat3),0).
compute flpc=1.
loop flp=1 to nrow(lmat3).
do if (lmat3(flp,1)=1).
compute xfm(:,flpc)=x(:,flp).
compute flpc=flpc+1.
end if.
end loop.
compute bfm=inv(t(xfm)*xfm)*t(xfm)*y.
compute resid=y-(xfm*bfm).
compute sstotal=(y-(csum(y)/n)).
compute sstotal=csum(sstotal&*sstotal).
compute ssresid=csum(resid&*resid).
compute rsqch= 0 -((sstotal-ssresid)/sstotal).
compute fresult={rsqch,fresult}.
release xfm,flpc, resid, ssresid, bfm.
end if
.
compute resultm2=make(nrow(fresult),maxresm,99999).
compute resultm2(1:nrow(fresult),1:ncol(fresult))=fresult.
compute resultm={resultm;resultm2}.
do if (outscree=1).
do if (i < (nms + nys) or (ydich = 0)).
compute clabtmp={hcflab,'df1','df2','p'}.
print fresult/title='Test of equality of conditional means'/cnames=clabtmp/format= F10.4.
end if.
do if (i = (nms + nys) and (ydich = 1)).
compute clabtmp={'Chi-sq','df','p'}.
print fresult/title='Test of equality of conditional logits or probabilities'/cnames=clabtmp/format= F10.4.
end if.
end if.
compute probetmp=probeplt(pstart:(pstart+nxvls),1).
compute probetmp={probetmp,probeplt(pstart:(pstart+nxvls),prevloc:ncol(probeplt))}.
compute pstart=pstart+(nxvls+1).
compute resultm2=make(nrow(probetmp),maxresm,99999).
compute resultm2(1:nrow(probetmp),1:ncol(probetmp))=probetmp.
compute resultm={resultm;resultm2}.
do if (i < ((nms + nys)) or (ydich = 0)).
compute clabtmp={xnames, outnames(1,i), hclab, 'LLCI', 'ULCI'}.
do if (outscree=1).
print probetmp/title = 'Estimated conditional means being compared:'/cnames=clabtmp/format= F10.4.
end if.
end if.
do if ((i = (nms+nys)) and (ydich = 1)).
compute clabtmp={xnames, outnames(1,i), 'prob'}.
compute probetm2=probetmp(:,1:2).
compute probetm2={probetm2,probetmp(:,ncol(probetmp))}.
do if (outscree=1).
print probetm2/title = 'Estimated conditional logits and probabilities:'/cnames=clabtmp/format= F10.4.
end if.
end if.
end if.
end loop.
release probrow, start2, endstart, temp, temp2.
end if.
end if.
do if (probexpl = 1 and (printpbe <= intprobe)).
compute resultm2=make(nrow(modvals2),maxresm,99999).
compute resultm2(1:nrow(modvals2),1:ncol(modvals2))=modvals2.
compute resultm={resultm;resultm2}.
do if (outscree=1).
print/title = 'Conditional effects of the focal predictor at values of the moderator(s):'.
print modvals2/cnames=problabs/title = ' '/space=0/format= F10.4.
end if.
compute didsome=1.
do if (hasz = 1).
compute printz=1.
end if.
do if (hasw=1).
compute printw=1.
end if.
do if (jn = 1 and jnok=1).
do if (criterr = 0).
compute dfres=n-nrow(b).
compute roots=99999.
compute jncrit =(dfres* (exp((dfres-(5/6))*((xp2/(dfres-(2/3)+(.11/dfres)))* (xp2/(dfres-(2/3)+(.11/dfres)))))-1)).
do if (i = (nms+nys) and (ydich=1)).
compute jncrit=xp2*xp2.
end if.
compute jnb1=b(wherejn1,1).
compute jnb3=b(wherejn3,1).
compute jnsb1=varb(wherejn1,wherejn1).
compute jnsb3=varb(wherejn3,wherejn3).
compute jnsb1b3=varb(wherejn1,wherejn3).
compute ajn =(jncrit*jnsb3)-(jnb3*jnb3).
compute bjn = 2*((jncrit*jnsb1b3)-(jnb1*jnb3)).
compute cjn = (jncrit*jnsb1)-(jnb1*jnb1).
compute radarg = (bjn*bjn)-(4*ajn*cjn).
compute den = 2*ajn.
compute nrts = 0.
do if (radarg >= 0 and den <> 0).
compute x21 = (-bjn+sqrt(radarg))/den.
compute x22 = (-bjn-sqrt(radarg))/den.
compute roots = 0.
do if (x21 >= jnmin and x21 <= jnmax).
compute nrts = 1.
compute roots = {roots; x21}.
end if.
do if (x22 >= jnmin and x22 <= jnmax).
compute nrts = nrts + 1.
compute roots = {roots; x22}.
end if.
compute roots={roots,make(nrow(roots),2,0)}.
end if.
do if (nrts > 0).
compute roots = roots(2:nrow(roots),1:3).
compute roots(1,2)=(csum(jnmod < roots(1,1))/n)*100.
compute roots(1,3)=(csum(jnmod > roots(1,1))/n)*100.
do if (nrow(roots)=2).
compute roots(2,2)=(csum(jnmod < roots(2,1))/n)*100.
compute roots(2,3)=(csum(jnmod > roots(2,1))/n)*100.
end if.
do if (outscree=1).
print roots/title = 'Moderator value(s) defining Johnson-Neyman significance region(s):'/clabels = 'Value', '% below', '% above'/format F10.4.
end if.
do if (nrts=1).
compute tmprts=make(1,3,99999).
compute roots={roots;tmprts}.
end if.
end if.
do if (nrts = 0).
compute roots=make(2,1,99999).
do if (outscree=1).
print/title = 'There are no statistical significance transition points within the observed'.
print/title = 'range of the moderator found using the Johnson-Neyman method.'/space=0.
end if.
end if.
compute resultm2=make(nrow(roots),maxresm,99999).
compute resultm2(1:nrow(roots),1:ncol(roots))=roots.
compute resultm={resultm;resultm2}.
compute jnvals=make(23,7,0).
loop jni= 0 to (21-(nrts)).
compute jnvals((jni+1),1)=jnmin+(jni*((jnmax-jnmin)/(21-nrts))).
end loop.
do if (nrts > 0).
loop jni = 1 to nrts.
loop jnj = 2 to (nrow(jnvals)-1).
do if ((roots(jni,1) > jnvals((jnj-1),1)) and (roots(jni,1) < jnvals(jnj,1))).
compute jnvals((jnj+1):(21+jni),1)=jnvals(jnj:(20+jni),1).
compute jnvals(jnj,1)=roots(jni,1).
end if.
end loop.
end loop.
end if.
compute jnvals=jnvals(1:22,:).
loop jni = 1 to nrow(jnvals).
compute jnvals(jni,2)=jnb1+jnb3*jnvals(jni,1).
compute jnvals(jni,3)=sqrt(jnsb1+2*jnvals(jni,1)*jnsb1b3+(jnvals(jni,1)*jnvals(jni, 1))*jnsb3).
compute jnvals(jni,4)=jnvals(jni,2)/jnvals(jni,3).
compute jnvals(jni,5)=2*(1-tcdf(abs(jnvals(jni,4)), dfres)).
compute jnvals(jni,6)=jnvals(jni,2)-sqrt(jncrit)*jnvals(jni,3).
compute jnvals(jni,7)=jnvals(jni,2)+sqrt(jncrit)*jnvals(jni,3).
do if ((i = nms + nys) and (ydich=1)).
compute jnvals(jni,5)=2*(1-cdfnorm(abs(jnvals(jni,4)))).
compute jnvals(jni,6)=jnvals(jni,2)-xp2*jnvals(jni,3).
compute jnvals(jni,7)=jnvals(jni,2)+xp2*jnvals(jni,3).
end if.
end loop.
compute resultm2=make(nrow(jnvals),maxresm,99999).
compute resultm2(1:nrow(jnvals),1:ncol(jnvals))=jnvals.
compute resultm={resultm;resultm2}.
do if ((i < nms+nys) or (ydich=0)).
compute jnclbs={jnmodlab,'Effect',hclab,'t', 'p', 'LLCI', 'ULCI'}.
end if.
do if ((i = nms + nys) and (ydich=1)).
compute jnclbs={jnmodlab,'Effect','se','Z', 'p', 'LLCI', 'ULCI'}.
end if.
do if (((wcmat((i+1),jmed)=1) or (zcmat((i+1),jmed)=1)) and (wzcmat((i+1),jmed)=0)).
do if (outscree=1).
print jnvals/title = 'Conditional effect of focal predictor at values of the moderator:'/cnames =jnclbs/format = F10.4.
end if.
end if.
do if (outscree=1).
do if ((jmed = 1) and (wzcmat((i+1),jmed)=1)).
print jnvals/title = 'Conditional X*W interaction at values of the moderator Z:'/cnames =jnclbs/format = F10.4.
end if.
do if ((jmed > 1) and (wzcmat((i+1),jmed)=1)).
print jnvals/title = 'Conditional M*W interaction at values of the moderator Z:'/cnames =jnclbs/format = F10.4.
end if.
end if.
end if.
end if.
end if.
do if ((i = (nms+nys)) and (jmed=1) and (bcmat(nrow(bcmat),1)=1)).
do if (probei=1).
compute direfflb=problabs.
compute direff=modvals2.
end if.
do if (probei>1).
compute direff={direff;modvals2}.
end if.
end if.
compute intprint=0.
do if ((jmed=1) and (i=1) and nms=0) and modcok=1).
compute contvec2=make(2,1,1).
compute contvec2={contvec2,wcontval,zcontval}.
do if (wzcmat((i+1),jmed)=1).
loop conti= 1 to ncol(wcontval).
loop contj = 1 to ncol(zcontval).
compute contvec2={contvec2,wcontval(:,conti)&*zcontval(:,contj)}.
end loop.
end loop.
end if.
compute conteff=contvec2*probcoef.
compute contdiff=contvec2(1,:)-contvec2(2,:).
compute contse=sqrt(contdiff*probvarb*t(contdiff)).
compute conteffd=conteff(1,1)-conteff(2,1).
compute contvec={contvec,conteff}.
compute contvecm=contvec.
compute resultm2=make(nrow(contvecm),maxresm,99999).
compute resultm2(1:nrow(contvecm),1:ncol(contvecm))=contvecm.
compute resultm={resultm;resultm2}.
do if (outscree=1).
print/title='Contrast between conditional effects of X:'.
print contvec/title=' '/rlabels='Effect1:','Effect2:'/cnames=problabs/format = F10.4 /space=0.
end if.
do if (ydich=0).
compute p=2*(1-tcdf(abs(conteffd/contse), dfres)).
compute contvec={conteffd,contse,conteffd/contse, p}.
compute contvec={contvec,(conteffd-(tval*contse))}.
compute contvec={contvec,(conteffd+(tval*contse))}.
compute contlabs={'Contrast', hclab, 't', 'p', 'LLCI', 'ULCI'}.
end if.
do if (ydich=1).
compute p=2*(1-cdfnorm(abs(conteffd/contse))).
compute contvec={conteffd,contse,conteffd/contse, p}.
compute contvec={contvec,(conteffd-(xp2*contse))}.
compute contvec={contvec,(conteffd+(xp2*contse))}.
compute contlabs={'Contrast', 'se', 'Z', 'p', 'LLCI', 'ULCI'}.
end if.
compute resultm2=make(nrow(contvec),maxresm,99999).
compute resultm2(1:nrow(contvec),1:ncol(contvec))=contvec.
compute resultm={resultm;resultm2}.
do if (outscree=1).
print contvec/title='Test of Effect1 minus Effect2'/format= F10.4 /cnames=contlabs.
end if.
end if.
end loop.
do if (plot = 1 or plot = 2).
compute datalabs={t(focpred(:,3)),outnames(1,i)}.
do if (plot = 1).
compute datalabs={datalabs}.
end if.
do if (plot = 2).
compute datalabs={datalabs,'se', 'LLCI', 'ULCI'}.
end if.
do if ((i = nms+nys) and (ydich=1)).
compute datalabs={datalabs,'prob'}.
end if.
compute resultm2=make(nrow(probeplt),maxresm,99999).
compute resultm2(1:nrow(probeplt),1:ncol(probeplt))=probeplt.
compute resultm={resultm;resultm2}.
compute datalabs={datalabs,'.'}.
do if (outscree=1).
print/title = 'Data for visualizing the conditional effect of the focal predictor:'.
print/title = 'Paste text below into a SPSS syntax window and execute to produce plot.'/space=0.
compute dumb = {' ', ' ', ' ', ' ', ' ', ' ', ' '}.
print datalabs/title = 'DATA LIST FREE/'/format=A10.
print probeplt/title = 'BEGIN DATA.'/format= F10.4 /space=0.
print/title = 'END DATA.'/space=0.
end if.
compute focgrph=datalabs(1,1).
compute graphix={focgrph,graphixs,modgrph}.
do if (((xdich=1) or (nxvls > 1)) and ((modcat=0) and (focgrph = xnames))).
compute graphix={modgrph,graphixs,focgrph}.
end if.
do if (panelgrp = 0).
compute graphix={graphix,'.'}.
else.
compute graphix={graphix,panelcde}.
end if.
do if (outscree=1).
print graphix/title = 'GRAPH/SCATTERPLOT='/format=A8/space=0.
end if.
do if (i = (nms + nys) and ydich = 1).
compute graphixd=graphix.
compute graphixd(1,3)='prob'.
do if (outscree=1).
print graphixd/title = 'GRAPH/SCATTERPLOT='/format=A8/space=0.
end if.
end if.
end if.
end if.
end loop.
release jmed, intprint,didprint.
end if
.
do if (model = 74 and i <= nms).
compute onetemp=make(nrow(xprobval),1,1).
compute mestmt74={onetemp,xprobval}.
do if (ncs > 0).
compute ncovmdl=rsum(ccmat(i,:)).
do if (ncovmdl > 0).
compute cvmnc=make(nrow(mestmt74),ncovmdl,1).
compute cvmnctmp=csum(x(:,(ncol(x)-ncovmdl+1):ncol(x)))/nrow(x).
do if (cuscoval > 0).
compute cvmnctmp=coval.
end if.
loop mestlp=1 to ncovmdl.
compute cvmnc(:,mestlp)=cvmnc(:,mestlp)*cvmnctmp(:,mestlp).
end loop.
compute mestmt74={mestmt74,cvmnc}.
end if.
end if.
compute mest74t=mestmt74*b.
do if (i = 1).
compute mest74=mest74t.
end if.
do if (i > 1).
compute mest74={mest74,mest74t}.
end if.
end if.
do if ((i = (nms+nys)) and (model >= 0 and model < 4) and (linsum(1,1) <> -999)).
compute lhyprob=1.
compute meansub=0.
do if (((nlinsum=nrow(b)) or (nlinsum = (nrow(b)-ncs)))).
do if (nlinsum = (nrow(b)-ncs) and ncs > 0).
compute linsum={linsum,covmeans}.
compute meansub=1.
end if.
compute lhyprob=0.
compute hypest=linsum*b.
compute sehypest=sqrt(linsum*varb*t(linsum)).
compute phypest=2*(1-tcdf(abs(hypest/sehypest), (dfres))).
compute hypest={hypest,sehypest,(hypest/sehypest),phypest,(hypest-tval*sehypest),(hypest+ tval*sehypest)}.
compute resultm2=make(ncol(linsum),maxresm,99999).
compute resultm2(:,1)=t(linsum).
compute resultm={resultm;resultm2}.
compute resultm2=make(1,maxresm,99999).
compute resultm2(1,1:ncol(hypest))=hypest.
compute resultm={resultm;resultm2}.
do if (outscree=1).
compute hyplabs={'Estimate',hclab,'t','p','LLCI','ULCI'}.
print/title='----------'.
print/title='Linear Combination Estimate and Hypothesis Test'/space=0.
print t(linsum)/title= 'Weight vector:'/clabels='weight'/rnames=vlabsm/format F10.4.
print hypest/title=' '/cnames=hyplabs/format= F10.4 /space=0.
do if (meansub=1).
print/title='Covariate weight(s) set to the sample mean.'.
end if.
end if.
end if.
do if (lhyprob=1).
compute notecode(notes,1) = 30.
compute notes = notes + 1.
end if.
end if.
end loop if criterr=1.
compute lastb=b.
compute lastcov=varb.
do if (criterr=0 and dototal = 1).
compute x=xtmp.
compute vlabsm={'constant';xcatlab(1:nxvls,1)}.
do if (ncs > 0).
compute x = {x,ctmp}.
compute vlabsm={vlabsm;t(covnames)}.
end if.
compute x = {ones,x}.

.
do if ( 1 =1).
compute hatmat=inv(t( x )* x )*t( x ).
compute b = hatmat* y.
compute modres=b.
do if ( 1 =1).
compute n1=nrow( x ).
compute dfres=n1-(ncol( x )).
compute sstotal = t( y -(csum( y )/n1))*( y -(csum( y )/n1)).
compute resid= y - x *b.
compute ssresid = csum((resid)&**2).
compute r2 = (sstotal-ssresid)/sstotal.
do if (r2 < 0).
compute r2=0.
end if.
compute adjr2 = 1-((1-r2)*(n1-1)/(dfres)).
compute mse=ssresid/(n1-ncol( x )).

.
compute n1=nrow( x ).
compute invXtX = inv(t( x )* x ).
compute varb = mse *invXtX.
compute k3 = ncol( x ).
compute xhc=0.
do if ( hc <> 5).
compute xhc= x.
compute hat = xhc(:,1).
loop i3=1 to nrow(xhc).
compute hat(i3,1)= xhc(i3,:)*invXtX*t(xhc(i3,:)).
end loop.
do if ( hc = 0 or hc =1).
loop i3 = 1 to k3.
compute xhc(:,i3)=xhc(:,i3)&* resid.
end loop.
end if.
do if ( hc =3 or hc =2).
loop i3=1 to k3.
compute xhc(:,i3) = ( resid &/(1-hat)&**(1/(4- hc )))&*xhc(:,i3).
end loop.
end if.
do if ( hc = 4).
compute hcmn=make(n,2,4).
compute hcmn(:,2)=(n1*hat)/k3.
loop i3= 1 to k3.
compute xhc(:,i3) = ( resid &/(1-hat)&**(rmin(hcmn)/2))&*xhc(:,i3).
end loop.
end if.
compute varb=(invXtX*t(xhc)*xhc*invXtX).
do if ( hc =1).
compute varb=(n1/(n1-ncol( x )))&*varb.
end if.
end if.
compute hclab={'se(HC0)','se(HC1)','se(HC2)','se(HC3)','se(HC4)','se'}.
compute hclab=hclab(1,( hc +1)).
compute hcflab={'F(HC0)','F(HC1)','F(HC2)','F(HC3)','F(HC4)','F'}.
compute hcflab=hcflab(1,( hc +1)).
release xhc
.
compute seb=sqrt(diag(varb)).
compute trat = b&/seb.
compute p = 2*(1-tcdf(abs(trat), (dfres))).
compute tval = sqrt(dfres* (exp((dfres-(5/6))*((xp2/(dfres-(2/3)+(.11/dfres)))* (xp2/(dfres-(2/3)+(.11/dfres)))))-1)).
compute modres={modres,seb,trat,p}.
compute modres={modres,(b-tval&*seb),(b+tval&*seb)}.
compute modresl={'coeff',hclab,'t','p','LLCI','ULCI'}.
compute lmat = ident(ncol( x )).
compute lmat = lmat(:,2:ncol(lmat)).
compute fratio = (t(t(lmat)*b)*inv(t(lmat)*varb*lmat)*((t(lmat)*b)))/(ncol( x )-1).
compute pfr = 1-fcdf(fratio,(ncol( x )-1),dfres).
compute modsum={sqrt(r2),r2,mse,fratio,(ncol( x )-1),dfres,pfr}.
compute modsuml={'R','R-sq','MSE',hcflab,'df1','df2', 'p'}.
end if.
end if.
do if ( 1 = 2 or 1 =3).
compute xlp= x.
compute ylp= y.
compute pt2 = make(nrow(ylp),1,(csum(ylp)/nrow(ylp))).
do if ( 1 =2).
compute LL3 = ylp&*ln(pt2)+(1-ylp)&*ln(1-pt2).
end if.
compute LL3 = -2*csum(LL3).
compute bt1 = make(ncol(xlp),1,0).
compute LL1 = 0.
compute pt1 = make(nrow(ylp),1,0.5).
compute pt1lp=pt1.
loop jjj = 1 to iterate.
compute xlptmp=t(xlp).
compute vecprb=pt1lp&*(1-pt1lp).
loop kkk=1 to ncol(xlp).
compute xlptmp(kkk,:)=xlptmp(kkk,:)&*t(vecprb).
end loop.
compute b = bt1+inv(xlptmp*xlp)*t(xlp)*(ylp-pt1lp).
do if ( 1 =2).
compute xlpb=xlp*b.
compute xlpbt=(xlpb > -709.7).
compute xlpb709=(1-xlpbt)*(-709.7).
compute xlpb=(xlpb&*xlpbt)+xlpb709.
compute pt1lp = 1/(1+exp(-(xlpb))).
end if.
compute itprob = csum((pt1lp < .00000001) or (pt1lp > .9999999)).
do if (itprob > 0).
loop kkk = 1 to nrow(pt1lp).
do if (pt1lp(kkk,1) > .9999999).
compute pt1lp(kkk,1) = .9999999.
end if.
do if (pt1lp(kkk,1) < .00000001).
compute pt1lp(kkk,1) = .00000001.
end if.
end loop.
compute itprob = 0.
end if.
do if (itprob = 0).
do if ( 1 =2).
compute LL = ylp&*ln(pt1lp)+(1-ylp)&*ln(1-pt1lp).
end if.
compute LL2 = -2*csum(ll).
end if.
do if (abs(LL1-LL2) < converge).
do if ( 1 =1).
compute xlptmp=t(xlp).
compute vecprb=pt1lp&*(1-pt1lp).
loop kkk=1 to ncol(xlp).
compute xlptmp(kkk,:)=xlptmp(kkk,:)&*t(vecprb).
end loop.
compute varb = inv(xlptmp*xlp).
compute seb = sqrt(diag(varb)).
release xlptmp.
end if.
break.
end if.
compute bt1 = b.
compute LL1 = LL2.
end loop.
compute modres=b.
do if (jjj > iterate).
compute itprob = 2.
do if (booting=0).
compute iterrmod=1.
end if.
do if (booting=1).
compute bootiter=1.
end if.
do if (itprobtg=0).
compute itprobtg=1.
compute errcode(errs,1) = 47.
compute errs = errs + 1.
do if (booting = 0 and 1 =1).
compute vt1 = mdiag(pt1lp&*(1-pt1lp)).
compute varb = inv(t(xlp)*vt1*xlp).
compute seb = sqrt(diag(varb)).
end if.
end if.
end if.
do if ( 1 =1).
compute trat = b&/seb.
compute dfres=nrow(xlp).
compute p = 2*(1-cdfnorm(abs(trat))).
compute modres={modres,seb,trat,p}.
compute modres={modres,(b-xp2&*seb),(b+xp2&*seb)}.
compute pvchi=1-chicdf((LL3-LL2),(nrow(modres)-1)).
compute mcF = (LL3-LL2)/LL3.
compute cox = 1-exp(-(LL3-LL2)/nrow(xlp)).
compute nagel = cox/(1-exp(-(LL3)/nrow(xlp))).
compute modsum={LL2,(LL3-LL2),(nrow(modres)-1),pvchi, mcF,cox,nagel}.
compute modsuml={'-2LL','ModelLL', 'df', 'p', 'McFadden', 'CoxSnell', 'Nagelkrk'}.
compute modresl={'coeff','se','Z','p','LLCI','ULCI'}.
end if.
end if
.
compute toteff=modres(2:(1+nxvls),:).
compute nodotot=0.
do if (xdich=1 and xmint=1 and model=74).
compute toteff(:,1)=toteff(:,1)*xscaling.
compute toteff(:,2)=toteff(:,2)*abs(xscaling).
compute toteff(:,3)=toteff(:,3)*xscaling.
compute toteff(:,5)=toteff(:,5)*xscaling.
compute toteff(:,6)=toteff(:,6)*xscaling.
compute citmp=toteff(:,5:6).
do if (xscaling < 0).
compute toteff(:,5)=citmp(:,2).
compute toteff(:,6)=citmp(:,1).
end if.
compute nodotot=1.
end if.
compute totefflb=modresl.
do if (outscree=1).
do if (nodotot=0).
print/title = '************************** TOTAL EFFECT MODEL ****************************'.
end if.
do if (nodotot=1).
print/title = '**************************************************************************'.
end if.
print outnames(1,ncol(outnames))/title = 'OUTCOME VARIABLE:'/format = A8/space=0.
end if.
compute toteffl2=vlabsm(2:(1+nxvls),:).
compute resultm2=make(1,maxresm,99999).
compute resultm2(1,1:ncol(modsum))=modsum.
compute resultm={resultm;resultm2}.
compute resultm2=make(nrow(modres),maxresm,99999).
compute resultm2(1:nrow(modres),1:ncol(modres))=modres.
compute resultm={resultm;resultm2}.
do if (outscree=1).
print modsum/title = 'Model Summary'/cnames = modsuml/format= F10.4.
print modres/title='Model'/rnames=vlabsm/cnames=modresl/format= F10.4.
end if.
compute lmat=make(nrow(b),1,0).
compute lmat2=make(nxvls,1,1).
compute lmat(2:(1+nxvls),1)=lmat2.
do if (ydich <> 1).

.
compute lmat2= lmat.
do if ( 0 =0).
compute lmat2 = mdiag( lmat ).
compute lmat3=make(nrow(lmat2),1,0).
loop flp=1 to ncol(lmat2).
do if (csum(lmat2(:,flp))=1).
compute lmat3={lmat3,lmat2(:,flp)}.
end if.
end loop.
compute lmat2=lmat3(:,2:ncol(lmat3)).
end if.
compute fratio = (t(t(lmat2)* b )*inv(t(lmat2)* varb *lmat2)*((t(lmat2)* b )))/ncol(lmat2).
compute pfr = 1-fcdf(fratio,ncol(lmat2),(n-nrow( b ))).
compute fresult={fratio,ncol(lmat2),(n-nrow( b )),pfr}.
do if (i = (nms+nys) and (ydich=1)).
compute fratio=fratio*ncol(lmat2).
compute pfr=1-chicdf(fratio,ncol(lmat2)).
compute fresult={fratio,ncol(lmat2),pfr}.
end if.
do if ( 1 =1).
compute lmat3=1-rsum(lmat2).
compute xfm=make(n,csum(lmat3),0).
compute flpc=1.
loop flp=1 to nrow(lmat3).
do if (lmat3(flp,1)=1).
compute xfm(:,flpc)=x(:,flp).
compute flpc=flpc+1.
end if.
end loop.
compute bfm=inv(t(xfm)*xfm)*t(xfm)*y.
compute resid=y-(xfm*bfm).
compute sstotal=(y-(csum(y)/n)).
compute sstotal=csum(sstotal&*sstotal).
compute ssresid=csum(resid&*resid).
compute rsqch= r2 -((sstotal-ssresid)/sstotal).
compute fresult={rsqch,fresult}.
release xfm,flpc, resid, ssresid, bfm.
end if
.
compute totomni=fresult.
end if.
do if (stand=1).
compute predsd=make(nrow(modres),1,0).
compute stdmod=modres(:,1)&/ovsd(1,ncol(ovsd)).
loop jd=1 to ncol(x).
compute descdat=x(:,jd).
compute predsd(jd,1) = (nrow(descdat)*sscp(descdat))-(t(csum(descdat))*(csum(descdat))).
compute predsd(jd,1) = sqrt(predsd(jd,1)/(nrow(descdat)*(nrow(descdat)-1))).
end loop.
do if (wherex(1,ncol(wherex)) <> -999 and ((nxvls > 1) or (xdich=1))).
compute sdmsone=make(nxvls,1,1).
compute predsd(wherex(1,ncol(wherex)):wherex(2,ncol(wherex)),1)=sdmsone.
compute pstog=1.
end if.
compute predsd(1,1)=1.
compute stdmod=stdmod&*predsd.
compute stdmod=stdmod(2:nrow(stdmod),1).
compute sdvlabs=vlabsm(2:nrow(vlabsm),1).
compute resultm2=make(nrow(stdmod),maxresm,99999).
compute resultm2(1:nrow(stdmod),1:ncol(stdmod))=stdmod.
compute resultm={resultm;resultm2}.
do if (outscree=1).
print stdmod/title='Standardized coefficients'/clabels='coeff'/rnames=sdvlabs/format= F10.4.
end if.
end if.
do if (covcoeff=1).
do if (outscree=1).
print varb/title='Covariance matrix of regression parameter estimates:'/rnames=vlabsm/cnames=vlabsm/format= F10.4.
end if.
compute resultm2=make(nrow(varb),maxresm,99999).
do if (ncol(varb) <= maxresm).
compute resultm2(1:nrow(varb),1:ncol(varb))=varb.
compute resultm={resultm;resultm2}.
end if.
do if (ncol(varb) > maxresm).
compute resultmt=make(nrow(resultm),ncol(varb),99999).
compute resultmt(1:nrow(resultm),1:ncol(resultm))=resultm.
compute resultm=resultmt.
compute resultm2=make(nrow(varb),ncol(resultm),99999).
compute resultm2(1:nrow(varb),1:ncol(varb))=varb.
compute resultm={resultm;resultm2}.
compute maxresm=ncol(resultm).
end if.
end if.
end if.
end if.
do if (criterr=0 and nms > 0 and ydich=0 and modelres=1).
compute modresid=modresid(:,2:ncol(modresid)).
compute sigmatal = (t(modresid)*(ident(n)-(1/n)*ones*t(ones))*modresid)*(1/(n-1)).
compute sdall = mdiag(1/sqrt(diag(sigmatal))).
compute corall=sdall*sigmatal*t(sdall).
compute resultm2=make(nrow(corall),maxresm,99999).
compute resultm2(1:nrow(corall),1:ncol(corall))=corall.
compute resultm={resultm;resultm2}.
do if (outscree=1).
print/title = '****************** CORRELATIONS BETWEEN MODEL RESIDUALS ******************'.
print corall/title=' '/format= F10.4 / cnames=outnames/rnames=outnames/space=0.
end if.
end if.
do if (criterr=0 and boot > 0).
compute bootres=make(1,rsum(nump),-999).
compute bootdir=obsdirfx.
compute natdirbt=make(1,nxvls,-999).
do if (effsize=1).
compute bootysd=make(1,1,-999).
compute bootxsd=make(1,1,-999).
end if.
compute badboot=0.
compute goodboot=0.
compute smallest=1.
compute booting=1.
loop j = 1 to maxboot.
compute nobootx=1.
compute modres2=999.
compute v=trunc(uniform(n,1)*n)+1.
compute bad=0.
loop i = 1 to (nms+nys).
compute y=outvars(v,i).
compute ynovar= (nrow(y)*sscp(y))-(t(csum(y))*(csum(y))).
do if (ynovar = 0).
compute bad=1.
end if.
compute xindx=datindx(1:(nump(1,i)-1),i).
compute hello=0.
compute x = fulldat(v,xindx).
compute x={ones,x}.
compute xsq=t(x)*x.
compute exsq=eval(xsq).
release xsq.
compute holymoly=cmin(exsq).
compute zeroeig=csum(exsq <= 0.000000000002).
compute bad=bad + (zeroeig > 0).

.
compute desctmp=make((8-(4* 1 )),ncol( y ),-999).
loop jd=1 to ncol( y ).
compute descdat= y (:,jd).
compute desctmp(1,jd) = csum(descdat)/nrow(descdat).
compute desctmp(2,jd) = (nrow(descdat)*sscp(descdat))-(t(csum(descdat))*(csum(descdat))).
compute desctmp(2,jd) = sqrt(desctmp(2,jd)/(nrow(descdat)*(nrow(descdat)-1))).
compute desctmp(3,jd)=cmin(descdat).
compute desctmp(4,jd)=cmax(descdat).
do if ( 1 =0).
compute minwarn=0.
compute maxwarn=0.
do if ((desctmp(3,jd)=desctmp(4,jd)) and novar=0).
compute errcode(errs,1)=15.
compute errs=errs+1.
compute criterr=1.
compute novar=1.
end if.
compute tmp=((descdat(:,1)=desctmp(3,jd))+(descdat(:,1)=desctmp(4,jd))).
compute desctmp(8,jd)=(csum(tmp)=nrow(tmp)).
compute tmp = descdat.
compute tmp(GRADE(descdat),:) = descdat.
compute descdat = tmp.
release tmp.
compute decval={.16;.5;.84}.
loop kd=1 to 3.
compute low=trunc(decval(kd,1)*(nrow(descdat)+1)).
compute lowdec=decval(kd,1)*(nrow(descdat)+1)-low.
compute value=descdat(low,1)+(descdat((low+1),1)-descdat(low,1))*lowdec.
compute desctmp((4+kd),jd)=value.
end loop.
compute mnotev=1.
compute modvals=desctmp(5:7,:).
do if (quantile <> 1).
compute desctmp(5,jd)=desctmp(1,jd)-desctmp(2,jd).
compute desctmp(6,jd)=desctmp(1,jd).
compute desctmp(7,jd)=desctmp(1,jd)+desctmp(2,jd).
compute modvals=desctmp(5:7,:).
compute mnotev=2.
do if (modvals(1,1) < desctmp(3,1)).
compute modvals(1,1)=desctmp(3,1).
compute minwarn=1.
end if.
do if (modvals(3,1) > desctmp(4,1)).
compute modvals(3,1)=desctmp(4,1).
compute maxwarn=1.
end if.
end if.
do if (desctmp(8,1)=1).
compute modvals={desctmp(3,1);desctmp(4,1)}.
compute mnotev=0.
compute minwarn=0.
compute maxwarn=0.
end if.
end if.
end loop
.
compute bad=bad+ (desctmp(2,1) <= 0.00000000001).
do if (bad = 0).
do if (holymoly < smallest).
compute smallest=holymoly.
end if.
do if (ydich=0 or (i < (nms+nys))).

.
do if ( 1 =1).
compute hatmat=inv(t( x )* x )*t( x ).
compute b = hatmat* y.
compute modres=b.
do if ( 0 =1).
compute n1=nrow( x ).
compute dfres=n1-(ncol( x )).
compute sstotal = t( y -(csum( y )/n1))*( y -(csum( y )/n1)).
compute resid= y - x *b.
compute ssresid = csum((resid)&**2).
compute r2 = (sstotal-ssresid)/sstotal.
do if (r2 < 0).
compute r2=0.
end if.
compute adjr2 = 1-((1-r2)*(n1-1)/(dfres)).
compute mse=ssresid/(n1-ncol( x )).

.
compute n1=nrow( x ).
compute invXtX = inv(t( x )* x ).
compute varb = mse *invXtX.
compute k3 = ncol( x ).
compute xhc=0.
do if ( hc <> 5).
compute xhc= x.
compute hat = xhc(:,1).
loop i3=1 to nrow(xhc).
compute hat(i3,1)= xhc(i3,:)*invXtX*t(xhc(i3,:)).
end loop.
do if ( hc = 0 or hc =1).
loop i3 = 1 to k3.
compute xhc(:,i3)=xhc(:,i3)&* resid.
end loop.
end if.
do if ( hc =3 or hc =2).
loop i3=1 to k3.
compute xhc(:,i3) = ( resid &/(1-hat)&**(1/(4- hc )))&*xhc(:,i3).
end loop.
end if.
do if ( hc = 4).
compute hcmn=make(n,2,4).
compute hcmn(:,2)=(n1*hat)/k3.
loop i3= 1 to k3.
compute xhc(:,i3) = ( resid &/(1-hat)&**(rmin(hcmn)/2))&*xhc(:,i3).
end loop.
end if.
compute varb=(invXtX*t(xhc)*xhc*invXtX).
do if ( hc =1).
compute varb=(n1/(n1-ncol( x )))&*varb.
end if.
end if.
compute hclab={'se(HC0)','se(HC1)','se(HC2)','se(HC3)','se(HC4)','se'}.
compute hclab=hclab(1,( hc +1)).
compute hcflab={'F(HC0)','F(HC1)','F(HC2)','F(HC3)','F(HC4)','F'}.
compute hcflab=hcflab(1,( hc +1)).
release xhc
.
compute seb=sqrt(diag(varb)).
compute trat = b&/seb.
compute p = 2*(1-tcdf(abs(trat), (dfres))).
compute tval = sqrt(dfres* (exp((dfres-(5/6))*((xp2/(dfres-(2/3)+(.11/dfres)))* (xp2/(dfres-(2/3)+(.11/dfres)))))-1)).
compute modres={modres,seb,trat,p}.
compute modres={modres,(b-tval&*seb),(b+tval&*seb)}.
compute modresl={'coeff',hclab,'t','p','LLCI','ULCI'}.
compute lmat = ident(ncol( x )).
compute lmat = lmat(:,2:ncol(lmat)).
compute fratio = (t(t(lmat)*b)*inv(t(lmat)*varb*lmat)*((t(lmat)*b)))/(ncol( x )-1).
compute pfr = 1-fcdf(fratio,(ncol( x )-1),dfres).
compute modsum={sqrt(r2),r2,mse,fratio,(ncol( x )-1),dfres,pfr}.
compute modsuml={'R','R-sq','MSE',hcflab,'df1','df2', 'p'}.
end if.
end if.
do if ( 1 = 2 or 1 =3).
compute xlp= x.
compute ylp= y.
compute pt2 = make(nrow(ylp),1,(csum(ylp)/nrow(ylp))).
do if ( 1 =2).
compute LL3 = ylp&*ln(pt2)+(1-ylp)&*ln(1-pt2).
end if.
compute LL3 = -2*csum(LL3).
compute bt1 = make(ncol(xlp),1,0).
compute LL1 = 0.
compute pt1 = make(nrow(ylp),1,0.5).
compute pt1lp=pt1.
loop jjj = 1 to iterate.
compute xlptmp=t(xlp).
compute vecprb=pt1lp&*(1-pt1lp).
loop kkk=1 to ncol(xlp).
compute xlptmp(kkk,:)=xlptmp(kkk,:)&*t(vecprb).
end loop.
compute b = bt1+inv(xlptmp*xlp)*t(xlp)*(ylp-pt1lp).
do if ( 1 =2).
compute xlpb=xlp*b.
compute xlpbt=(xlpb > -709.7).
compute xlpb709=(1-xlpbt)*(-709.7).
compute xlpb=(xlpb&*xlpbt)+xlpb709.
compute pt1lp = 1/(1+exp(-(xlpb))).
end if.
compute itprob = csum((pt1lp < .00000001) or (pt1lp > .9999999)).
do if (itprob > 0).
loop kkk = 1 to nrow(pt1lp).
do if (pt1lp(kkk,1) > .9999999).
compute pt1lp(kkk,1) = .9999999.
end if.
do if (pt1lp(kkk,1) < .00000001).
compute pt1lp(kkk,1) = .00000001.
end if.
end loop.
compute itprob = 0.
end if.
do if (itprob = 0).
do if ( 1 =2).
compute LL = ylp&*ln(pt1lp)+(1-ylp)&*ln(1-pt1lp).
end if.
compute LL2 = -2*csum(ll).
end if.
do if (abs(LL1-LL2) < converge).
do if ( 0 =1).
compute xlptmp=t(xlp).
compute vecprb=pt1lp&*(1-pt1lp).
loop kkk=1 to ncol(xlp).
compute xlptmp(kkk,:)=xlptmp(kkk,:)&*t(vecprb).
end loop.
compute varb = inv(xlptmp*xlp).
compute seb = sqrt(diag(varb)).
release xlptmp.
end if.
break.
end if.
compute bt1 = b.
compute LL1 = LL2.
end loop.
compute modres=b.
do if (jjj > iterate).
compute itprob = 2.
do if (booting=0).
compute iterrmod=1.
end if.
do if (booting=1).
compute bootiter=1.
end if.
do if (itprobtg=0).
compute itprobtg=1.
compute errcode(errs,1) = 47.
compute errs = errs + 1.
do if (booting = 0 and 0 =1).
compute vt1 = mdiag(pt1lp&*(1-pt1lp)).
compute varb = inv(t(xlp)*vt1*xlp).
compute seb = sqrt(diag(varb)).
end if.
end if.
end if.
do if ( 0 =1).
compute trat = b&/seb.
compute dfres=nrow(xlp).
compute p = 2*(1-cdfnorm(abs(trat))).
compute modres={modres,seb,trat,p}.
compute modres={modres,(b-xp2&*seb),(b+xp2&*seb)}.
compute pvchi=1-chicdf((LL3-LL2),(nrow(modres)-1)).
compute mcF = (LL3-LL2)/LL3.
compute cox = 1-exp(-(LL3-LL2)/nrow(xlp)).
compute nagel = cox/(1-exp(-(LL3)/nrow(xlp))).
compute modsum={LL2,(LL3-LL2),(nrow(modres)-1),pvchi, mcF,cox,nagel}.
compute modsuml={'-2LL','ModelLL', 'df', 'p', 'McFadden', 'CoxSnell', 'Nagelkrk'}.
compute modresl={'coeff','se','Z','p','LLCI','ULCI'}.
end if.
end if
.
end if.
do if (ydich=1 and (i = (nms+nys))).

.
do if ( 2 =1).
compute hatmat=inv(t( x )* x )*t( x ).
compute b = hatmat* y.
compute modres=b.
do if ( 0 =1).
compute n1=nrow( x ).
compute dfres=n1-(ncol( x )).
compute sstotal = t( y -(csum( y )/n1))*( y -(csum( y )/n1)).
compute resid= y - x *b.
compute ssresid = csum((resid)&**2).
compute r2 = (sstotal-ssresid)/sstotal.
do if (r2 < 0).
compute r2=0.
end if.
compute adjr2 = 1-((1-r2)*(n1-1)/(dfres)).
compute mse=ssresid/(n1-ncol( x )).

.
compute n1=nrow( x ).
compute invXtX = inv(t( x )* x ).
compute varb = mse *invXtX.
compute k3 = ncol( x ).
compute xhc=0.
do if ( hc <> 5).
compute xhc= x.
compute hat = xhc(:,1).
loop i3=1 to nrow(xhc).
compute hat(i3,1)= xhc(i3,:)*invXtX*t(xhc(i3,:)).
end loop.
do if ( hc = 0 or hc =1).
loop i3 = 1 to k3.
compute xhc(:,i3)=xhc(:,i3)&* resid.
end loop.
end if.
do if ( hc =3 or hc =2).
loop i3=1 to k3.
compute xhc(:,i3) = ( resid &/(1-hat)&**(1/(4- hc )))&*xhc(:,i3).
end loop.
end if.
do if ( hc = 4).
compute hcmn=make(n,2,4).
compute hcmn(:,2)=(n1*hat)/k3.
loop i3= 1 to k3.
compute xhc(:,i3) = ( resid &/(1-hat)&**(rmin(hcmn)/2))&*xhc(:,i3).
end loop.
end if.
compute varb=(invXtX*t(xhc)*xhc*invXtX).
do if ( hc =1).
compute varb=(n1/(n1-ncol( x )))&*varb.
end if.
end if.
compute hclab={'se(HC0)','se(HC1)','se(HC2)','se(HC3)','se(HC4)','se'}.
compute hclab=hclab(1,( hc +1)).
compute hcflab={'F(HC0)','F(HC1)','F(HC2)','F(HC3)','F(HC4)','F'}.
compute hcflab=hcflab(1,( hc +1)).
release xhc
.
compute seb=sqrt(diag(varb)).
compute trat = b&/seb.
compute p = 2*(1-tcdf(abs(trat), (dfres))).
compute tval = sqrt(dfres* (exp((dfres-(5/6))*((xp2/(dfres-(2/3)+(.11/dfres)))* (xp2/(dfres-(2/3)+(.11/dfres)))))-1)).
compute modres={modres,seb,trat,p}.
compute modres={modres,(b-tval&*seb),(b+tval&*seb)}.
compute modresl={'coeff',hclab,'t','p','LLCI','ULCI'}.
compute lmat = ident(ncol( x )).
compute lmat = lmat(:,2:ncol(lmat)).
compute fratio = (t(t(lmat)*b)*inv(t(lmat)*varb*lmat)*((t(lmat)*b)))/(ncol( x )-1).
compute pfr = 1-fcdf(fratio,(ncol( x )-1),dfres).
compute modsum={sqrt(r2),r2,mse,fratio,(ncol( x )-1),dfres,pfr}.
compute modsuml={'R','R-sq','MSE',hcflab,'df1','df2', 'p'}.
end if.
end if.
do if ( 2 = 2 or 2 =3).
compute xlp= x.
compute ylp= y.
compute pt2 = make(nrow(ylp),1,(csum(ylp)/nrow(ylp))).
do if ( 2 =2).
compute LL3 = ylp&*ln(pt2)+(1-ylp)&*ln(1-pt2).
end if.
compute LL3 = -2*csum(LL3).
compute bt1 = make(ncol(xlp),1,0).
compute LL1 = 0.
compute pt1 = make(nrow(ylp),1,0.5).
compute pt1lp=pt1.
loop jjj = 1 to iterate.
compute xlptmp=t(xlp).
compute vecprb=pt1lp&*(1-pt1lp).
loop kkk=1 to ncol(xlp).
compute xlptmp(kkk,:)=xlptmp(kkk,:)&*t(vecprb).
end loop.
compute b = bt1+inv(xlptmp*xlp)*t(xlp)*(ylp-pt1lp).
do if ( 2 =2).
compute xlpb=xlp*b.
compute xlpbt=(xlpb > -709.7).
compute xlpb709=(1-xlpbt)*(-709.7).
compute xlpb=(xlpb&*xlpbt)+xlpb709.
compute pt1lp = 1/(1+exp(-(xlpb))).
end if.
compute itprob = csum((pt1lp < .00000001) or (pt1lp > .9999999)).
do if (itprob > 0).
loop kkk = 1 to nrow(pt1lp).
do if (pt1lp(kkk,1) > .9999999).
compute pt1lp(kkk,1) = .9999999.
end if.
do if (pt1lp(kkk,1) < .00000001).
compute pt1lp(kkk,1) = .00000001.
end if.
end loop.
compute itprob = 0.
end if.
do if (itprob = 0).
do if ( 2 =2).
compute LL = ylp&*ln(pt1lp)+(1-ylp)&*ln(1-pt1lp).
end if.
compute LL2 = -2*csum(ll).
end if.
do if (abs(LL1-LL2) < converge).
do if ( 0 =1).
compute xlptmp=t(xlp).
compute vecprb=pt1lp&*(1-pt1lp).
loop kkk=1 to ncol(xlp).
compute xlptmp(kkk,:)=xlptmp(kkk,:)&*t(vecprb).
end loop.
compute varb = inv(xlptmp*xlp).
compute seb = sqrt(diag(varb)).
release xlptmp.
end if.
break.
end if.
compute bt1 = b.
compute LL1 = LL2.
end loop.
compute modres=b.
do if (jjj > iterate).
compute itprob = 2.
do if (booting=0).
compute iterrmod=1.
end if.
do if (booting=1).
compute bootiter=1.
end if.
do if (itprobtg=0).
compute itprobtg=1.
compute errcode(errs,1) = 47.
compute errs = errs + 1.
do if (booting = 0 and 0 =1).
compute vt1 = mdiag(pt1lp&*(1-pt1lp)).
compute varb = inv(t(xlp)*vt1*xlp).
compute seb = sqrt(diag(varb)).
end if.
end if.
end if.
do if ( 0 =1).
compute trat = b&/seb.
compute dfres=nrow(xlp).
compute p = 2*(1-cdfnorm(abs(trat))).
compute modres={modres,seb,trat,p}.
compute modres={modres,(b-xp2&*seb),(b+xp2&*seb)}.
compute pvchi=1-chicdf((LL3-LL2),(nrow(modres)-1)).
compute mcF = (LL3-LL2)/LL3.
compute cox = 1-exp(-(LL3-LL2)/nrow(xlp)).
compute nagel = cox/(1-exp(-(LL3)/nrow(xlp))).
compute modsum={LL2,(LL3-LL2),(nrow(modres)-1),pvchi, mcF,cox,nagel}.
compute modsuml={'-2LL','ModelLL', 'df', 'p', 'McFadden', 'CoxSnell', 'Nagelkrk'}.
compute modresl={'coeff','se','Z','p','LLCI','ULCI'}.
end if.
end if
.
end if.
compute modres2={modres2,t(modres)}.
do if (i = (nms+nys)).
do if (bcmat((i+1),1) = 1).
compute bootdir={bootdir;t(modres(wherex(1,i):wherex(2,i),1))}.
end if.
do if (bcmat((i+1),1) = 0).
compute bootdir={bootdir;dirzes}.
end if.
end if.
do if (model = 74 and i <= nms).
compute onetemp=make(nrow(xprobval),1,1).
compute mestmtb={onetemp,xprobval}.
do if (ncs > 0).
compute ncovmdlb=rsum(ccmat(i,:)).
do if (ncovmdl > 0).
compute cvmncb=make(nrow(mestmtb),ncovmdlb,1).
compute cvmnctmp=csum(x(:,(ncol(x)-ncovmdlb+1):ncol(x)))/nrow(x).
do if (cuscoval > 0).
compute cvmnctmp=coval.
end if.
loop mestlp=1 to ncovmdlb.
compute cvmncb(:,mestlp)=cvmncb(:,mestlp)*cvmnctmp(:,mestlp).
end loop.
compute mestmtb={mestmtb,cvmncb}.
end if.
end if.
compute mestbt=mestmtb*modres.
do if (i = 1).
compute mestb=mestbt.
end if.
do if (i > 1).
compute mestb={mestb,mestbt}.
end if.
end if.
do if (model = 74 and i = (nms+nys)).
compute xvalptmp=make(1,nxvls,0).
compute mest74sp=make(1,nms,0).
compute mest74sp={mest74sp;mestb}.
compute xvalptmp={xvalptmp;ident(nxvls)}.
compute dirfxcf=make(nrow(mestb),1,0).
loop cfloop1=1 to nrow(mestb).
compute ndirfx=make(nrow(modres),1,0).
compute ndirfx(2:(nxvls+1),1)=t(xvalptmp(cfloop1,:)).
loop cfloop3=1 to nms.
do if (mcx=1 or mcx=0).
compute ndirfx(wheremw((1+((cfloop3-1)*2)),ncol(wheremw)):wheremw((2+((cfloop3-1)*2) ),ncol(wheremw)))=t(xvalptmp(cfloop1,:)*mestb(1,cfloop3)).
end if.
do if (mcx=2).
compute ndirfx(wheremw((1+((cfloop3-1)*2)),ncol(wheremw)):wheremw((2+((cfloop3-1)*2) ),ncol(wheremw)))=t(xvalptmp(cfloop1,:)*mest74sp(cfloop1,cfloop3)).
end if.
end loop.
do if (cfloop1 > 1).
compute dirfxcf(cfloop1,1)=t(ndirfx)*modres*xscaling.
end if.
end loop.
compute natdirbt={natdirbt;t(dirfxcf(2:nrow(dirfxcf),:))}.
end if.
do if ( (bcmat((i+1),1) = 1) and (nobootx=1) and (effsize=1)).
compute nobootx=0.
compute xsdtemp= (nrow(x)*sscp(x(:,2)))-(t(csum(x(:,2)))*(csum(x(:,2)))).
compute xsdtemp= sqrt(xsdtemp/(nrow(x)*(nrow(x)-1))).
end if.
end if.
end loop.
do if (bad = 0).
compute bootres={bootres;modres2(:,2:ncol(modres2))}.
do if (effsize=1).
compute ysdtemp= (nrow(y)*sscp(y))-(t(csum(y))*(csum(y))).
compute ysdtemp= sqrt(ysdtemp/(nrow(y)*(nrow(y)-1))).
compute bootysd={bootysd;ysdtemp}.
compute bootxsd={bootxsd;xsdtemp}.
end if.
compute goodboot=goodboot+1.
end if.
do if (bad <> 0).
compute badboot=badboot+1.
end if.
end loop if (goodboot = boot).
compute bootres=bootres(2:nrow(bootres),:).
do if (effsize=1).
compute bootysd=bootysd(2:nrow(bootysd),:).
do if (nrow(bootxsd) > 1).
compute bootxsd=bootxsd(2:nrow(bootxsd),:).
end if.
end if.
do if (goodboot < (boot)).
compute boot=0.
compute modelbt=0.
compute notecode(notes,1) = 7.
compute notes = notes + 1.
end if.
do if (boot > 0).
do if (effsize=1).
compute bootysd={ysd;bootysd}.
compute bootxsd={xsd;bootxsd}.
end if.
do if (saveboot = 1).
save bootres/outfile = *.
end if.
do if (modelbt=1).
compute bootcim=make(ncol(bootres),5,-99999).
compute bootcim(:,2) = t(csum(bootres)/nrow(bootres)).
compute bootcim(:,1) = coeffmat(2:nrow(coeffmat),1).
loop i = 1 to ncol(bootres).

.
compute temp = bootres(:,i).
compute temp(GRADE( bootres(:,i) )) = bootres(:,i).
compute badlo = 0.
compute badhi = 0.
do if ( (bootcim(i,1)*bc)+(9999*(1-bc)) <> 9999).
compute pv=csum(temp < (bootcim(i,1)*bc)+(9999*(1-bc)) )/nrow(temp).
compute ppv = pv.
do if (pv > .5).
compute ppv = 1-pv.
end if.
compute y5=sqrt(-2*ln(ppv)).
compute xp=y5+((((y5*p4+p3)*y5+p2)*y5+p1)*y5+p0)/((((y5*q4+q3)*y5+q2)*y5+q1)*y5+q0).
do if (pv <= .5).
compute xp = -xp.
end if.
compute cilow=rnd(nrow(temp)*(cdfnorm(2*xp-xp2))).
compute cihigh=trunc(nrow(temp)*(cdfnorm(2*xp+(xp2))))+1.
do if (cilow < 1).
compute cilow = 1.
compute booterr=1.
compute badlo = 1.
end if.
do if (cihigh > nrow(temp)).
compute cihigh = boot.
compute booterr=1.
compute badhi = 1.
end if.
compute llcit=temp(cilow,1).
compute ulcit=temp(cihigh,1).
do if (badlo = 1 and llcit <> priorlo).
compute badend={badend, llcit}.
compute priorlo = llcit.
end if.
do if (badhi = 1 and ulcit <> priorhi).
compute badend={badend, ulcit}.
compute priorhi = ulcit.
end if.
end if.
do if ( (bootcim(i,1)*bc)+(9999*(1-bc)) = 9999).
compute llcit=temp(cilow,1).
compute ulcit=temp(cihigh,1).
end if.
compute bootse=t(sqrt((cssq(temp)-((csum(temp)&**2)/nrow(temp)))/(nrow(temp)-1)))
.
compute bootcim(i,4:5)={llcit,ulcit}.
compute bootcim(i,3)=bootse.
end loop.
end if.
end if.
do if (badboot > 0).
compute notecode(notes,1) = 6.
compute notes = notes + 1.
end if.
end if.
do if (criterr=0).
release fulldat,x,y.
end if.
do if (xmint=1 and criterr=0).
compute xvalptmp=make(1,nxvls,0).
compute mest74sp=make(1,nms,0).
compute mest74sp={mest74sp;mest74}.
compute xvalptmp={xvalptmp;ident(nxvls)}.
loop kcfuhd=1 to 2.
compute dirfxcf=make(nrow(mest74),6,0).
loop cfloop1=1 to nrow(mest74).
compute ndirfx=make(nrow(lastb),1,0).
compute ndirfx(2:(nxvls+1),1)=t(xvalptmp(cfloop1,:)).
loop cfloop3=1 to nms.
do if (kcfuhd=1).
do if (mcx=1 or mcx=0).
compute ndirfx(wheremw((1+((cfloop3-1)*2)),ncol(wheremw)):wheremw((2+((cfloop3-1)*2)), ncol(wheremw)))=t(xvalptmp(cfloop1,:)*mest74(1,cfloop3)).
end if.
do if (mcx=2).
compute ndirfx(wheremw((1+((cfloop3-1)*2)),ncol(wheremw)):wheremw((2+((cfloop3-1)*2)), ncol(wheremw)))=t(xvalptmp(cfloop1,:)*mest74sp(cfloop1,cfloop3)).
end if.
end if.
do if (kcfuhd=2).
compute ndirfx(wheremw((1+((cfloop3-1)*2)),ncol(wheremw)):wheremw((2+((cfloop3-1)*2)), ncol(wheremw)))=t(xvalptmp(cfloop1,:)*medmeans(1,cfloop3)).
end if.
end loop.
do if (cfloop1 > 1).
compute dirfxcf(cfloop1,1)=t(ndirfx)*lastb*xscaling.
compute dirfxcf(cfloop1,2)=sqrt(diag(t(ndirfx)*lastcov*ndirfx))*abs(xscaling).
compute dirfxcf(cfloop1,3)=dirfxcf(cfloop1,1)/dirfxcf(cfloop1,2).
compute dirfxcf(cfloop1,4)=2*(1-tcdf(abs(dirfxcf(cfloop1,3)), dfres)).
compute dirfxcf(cfloop1,5)=dirfxcf(cfloop1,1)-tval*dirfxcf(cfloop1,2).
compute dirfxcf(cfloop1,6)=dirfxcf(cfloop1,1)+tval*dirfxcf(cfloop1,2).
end if.
end loop.
compute codireff=dirfxcf(2:nrow(dirfxcf),:).
do if (kcfuhd=1).
compute direff=codireff.
end if.
end loop.
end if.

.
do if (criterr = 0 and nms > 0).
compute paths=paths(:,2:ncol(paths)).
compute pathsw=pathsw(:,2:ncol(pathsw)).
compute pathsz=pathsz(:,2:ncol(pathsz)).
compute pathswz=pathswz(:,2:ncol(pathswz)).
compute pathsmod=pathsw+pathsz+pathswz.
compute pathsdv=pathsdv(:,2:ncol(pathsdv)).
compute pathsfoc=pathsfoc(:,2:ncol(pathsfoc)).
compute pathtype=pathtype(:,2:ncol(pathtype)).
compute anymod=(rsum(pathsmod) > 0).
compute obscoeff=obscoeff(1,2:ncol(obscoeff)).
do if (outscree=1).
do if (xmint=1).
print/title = '************************* COUNTERFACTUALLY DEFINED ************************'.
else.
print/title = ' '.
end if.
do if (dototal = 0 and alttotal=0).
print/title = '****************** DIRECT AND INDIRECT EFFECTS OF X ON Y *****************'/space=0.
end if.
do if (alttotal=1).
print/title = '************** TOTAL, DIRECT, AND INDIRECT EFFECTS OF X ON Y **************'/space=0.
end if.
end if.
do if (dototal = 1).
do if (outscree=1).
print/title = '************** TOTAL, DIRECT, AND INDIRECT EFFECTS OF X ON Y **************'/space=0.
end if.
compute totefflb(1,1)='Effect'.
do if (effsize=1).
compute toteffsz=toteff(:,1)/ysd.
do if (xdich=1 or mcx > 0).
compute totefflb={totefflb,'c_ps'}.
end if.
do if (xdich = 0 and mcx = 0).
compute toteffsz=toteffsz*xsd.
compute totefflb={totefflb,'c_cs'}.
end if.
compute toteff={toteff,toteffsz}.
end if.
compute resultm2=make(nrow(toteff),maxresm,99999).
compute resultm2(1:nrow(toteff),1:ncol(toteff))=toteff.
compute resultm={resultm;resultm2}.
do if (nxvls > 1).
compute resultm2=make(nrow(totomni),maxresm,99999).
compute resultm2(1:nrow(totomni),1:ncol(totomni))=totomni.
compute resultm={resultm;resultm2}.
compute clabtmp={'R2-chng', hcflab, 'df1','df2','p'}.
do if (outscree=1).
print toteff/title='Relative total effects of X on Y'/rnames=toteffl2/cnames=totefflb/format= F10.4.
print totomni/title='Omnibus test of total effect of X on Y'/cnames=clabtmp/format= F10.4.
print/title= '----------'/space=0.
end if.
else.
do if (outscree=1).
print toteff/title='Total effect of X on Y'/cnames=totefflb/format= F10.4.
end if.
end if.
end if.
compute moddir=wcmat(nrow(bcmat),1)+zcmat(nrow(bcmat),1).
do if (xmint=1).
compute moddir=1.
end if.
do if (bcmat(nrow(bcmat),1)=1).
do if (ydich=1).
compute direfflb(:,(ncol(direfflb)-5):ncol(direfflb))={'Effect','se','Z','p','LLCI','ULCI'}.
end if.
do if (moddir=0 or xmint=1).
compute direfflb(1,1)='Effect'.
end if.
do if (effsize=1 and moddir=0 and anymod = 0).
compute direffsz=direff(:,1)/ysd.
do if (xdich=1 or mcx > 0).
compute direfflb={direfflb,'c''_ps'}.
end if.
do if (xdich = 0 and mcx = 0).
compute direffsz=direffsz*xsd.
compute direfflb={direfflb,'c''_cs'}.
end if.
compute direff={direff,direffsz}.
end if.
compute resultm2=make(nrow(direff),maxresm,99999).
compute resultm2(1:nrow(direff),1:ncol(direff))=direff.
compute resultm={resultm;resultm2}.
do if (xmint=1).
compute resultm2=make(nrow(codireff),maxresm,99999).
compute resultm2(1:nrow(codireff),1:ncol(codireff))=codireff.
compute resultm={resultm;resultm2}.
end if.
do if (moddir = 0 and nxvls=1).
do if (outscree=1).
print direff/title='Direct effect of X on Y'/cnames=direfflb/format= F10.4.
end if.
end if.
do if (moddir = 0 and nxvls>1).
compute resultm2=make(nrow(diromni),maxresm,99999).
compute resultm2(1:nrow(diromni),1:ncol(diromni))=diromni.
compute resultm={resultm;resultm2}.
do if (outscree=1).
print direff/title='Relative direct effects of X on Y'/rnames=direffl2/cnames=direfflb/format= F10.4.
do if (ydich = 0).
compute clabtmp={'R2-chng', hcflab, 'df1','df2','p'}.
print diromni/title='Omnibus test of direct effect of X on Y:'/cnames=clabtmp/format= F10.4.
end if.
do if (ydich = 1).
compute clabtmp={'Chi-sq', 'df', 'p'}.
print diromni/title='Omnibus likelihood ratio test of direct effect of X on Y'/cnames=clabtmp/format= F10.4.
end if.
print/title= '----------'/space=0.
end if.
end if.
do if (moddir > 0 and nxvls=1).
do if (xmint=0 and outscree=1).
print direff/title='Conditional direct effects of X on Y'/cnames=direfflb/format= F10.4.
end if.
do if (xmint=1).
do if (outscree=1).
print direff/title='(Pure) Natural direct effect of X on Y'/cnames=direfflb/format= F10.4.
print codireff/title='Controlled direct effect of X on Y'/cnames=direfflb/format= F10.4.
print/title= '----------'/space=0.
end if.
compute obnatdfx=direff(:,1).
end if.
end if.
compute direffl4=direffl2.
do if (moddir > 0 and nxvls>1).
compute direffl2={' '}.
loop i = 1 to nxvls.
loop j = 1 to (nrow(direff)/nxvls).
compute direffl2={direffl2;xcatlab(i,1)}.
end loop.
end loop.
compute direffl2=direffl2(2:nrow(direffl2),1).
do if (xmint=0 and outscree=1).
print direff/title='Relative conditional direct effects of X on Y'/cnames=direfflb/rnames=direffl2/format= F10.4.
end if.
do if (xmint=1).
do if (outscree=1).
print direff/title='Relative (pure) natural direct effects of X on Y'/cnames=direfflb/rnames=direffl2/format= F10.4.
print codireff/title='Relative controlled direct effects of X on Y'/cnames=direfflb/rnames=direffl2/format= F10.4.
print/title= '----------'/space=0.
end if.
compute obnatdfx=direff(:,1).
end if.
end if.
compute direffl2=direffl4.
end if.
do if (bcmat(nrow(bcmat),1)=0 and xmint <> 1 and outscree=1).
print/title='The direct effect of X on Y is fixed to zero.'.
end if.
do if (nms = 1).
compute indmark={2}.
compute indsets={1,2}.
end if.
do if (nms = 2).
compute indmark={2,2,3}.
compute indsets={1,4,2,5,1,3,5}.
compute thetam=1.
end if.
do if (nms = 3).
compute indmark={2,2,2,3,3,3,4}.
compute indsets={1,7,2,8,4,9,1,3,8,1,5,9,2,6,9,1,3,6,9}.
compute thetam={1,2,3}.
end if.
do if (nms = 4).
compute indmark={2,2,2,2,3,3,3,3,3,3,4,4,4,4,5}.
compute indsets={1,11,2,12,4,13,7,14,1,3,12,1,5,13,1,8,14,2,6,13,2,9,14,4,10,14,1,3,6,13,1,3,9, 14,1,5,10,14,2,6,10,14,1,3,6,10,14}.
compute thetam={1,2,4,3,5,6}.
end if.
do if (nms = 5).
compute indmark={2,2,2,2,2,3,3,3,3,3,3,3,3,3,3,4,4,4,4,4,4,4,4,4,4,5,5,5,5,5,6}.
compute indsets={1,16,2,17,4,18,7,19,11,20,1,3,17,1,5,18,1,8,19,1,12,20,2,6,18,2,9,19,2,13,20,4, 10,19,4,14,20,7,15,20,1,3,6,18, 1,3,9,19,1,3,13,20,1,5,10,19,1,5,14,20,1,8,15,20,2,6,10,19,2,6, 14,20,2,9,15,20,4,10,15,20,1,3,6,10,19, 1,3,6,14,20,1,3,9,15,20,1,5,10,15,20,2,6,10,15,20,1,3,6,10,15, 20}.
compute thetam={1,2,5,3,6,8,4,7,9,10}.
end if.
do if (nms = 6).
compute indmark={2,2,2,2,2,2,3,3,3,3,3,3,3,3,3,3,3,3,3,3,3,4,4,4,4,4,4,4,4,4,4,4,4,4,4,4,4,4,4, 4,4,5,5,5,5,5,5,5,5,5,5,5,5,5,5,5,6,6,6,6,6,6,7}.
compute indsets={1,22,2,23,4,24,7,25,11,26,16,27,1,3,23,1,5,24,1,8,25,1,12,26,1,17,27,2,6,24,2, 9,25,2,13,26,2,18,27,4,10,25,4,14,26, 4,19,27,7,15,26,7,20,27,11,21,27,1,3,6,24,1,3,9,25,1,3,13,26,1, 3,18,27,1,5,10,25,1,5,14,26,1,5,19,27,1,8,15,26,1,8,20,27, 1,12,21,27,2,6,10,25,2,6,14,26,2,6,19,27,2,9,15,26,2,9,20,27,2, 13,21,27,4,10,15,26,4,10,20,27,4,14,21,27,7,15,21,27, 1,3,6,10,25,1,3,6,14,26,1,3,6,19,27,1,3,9,15,26,1,3,9,20,27,1,3, 13,21,27,1,5,10,15,26,1,5,10,20,27,1,5,14,21,27, 1,8,15,21,27,2,6,10,15,26,2,6,10,20,27,2,6,14,21,27,2,9,15,21, 27,4,10,15,21,27,1,3,6,10,15,26,1,3,6,10,20,27, 1,3,6,14,21,27,1,3,9,15,21,27,1,5,10,15,21,27,2,6,10,15,21,27, 1,3,6,10,15,21,27}.
compute thetam={1,2,6,3,7,10,4,8,11,13,5,9,12,14,15}.
end if.
do if (nms = 7).
compute indmark={2,2,2,2,2,2,2}.
compute indsets={1,29,2,30,4,31,7,32,11,33,16,34,22,35}.
end if.
do if (nms = 8).
compute indmark={2,2,2,2,2,2,2,2}.
compute indsets={1,37,2,38,4,39,7,40,11,41,16,42,22,43,29,44}.
end if.
do if (nms = 9).
compute indmark={2,2,2,2,2,2,2,2,2}.
compute indsets={1,46,2,47,4,48,7,49,11,50,16,51,22,52,29,53,37,54}.
end if.
do if (nms=10).
compute indmark={2,2,2,2,2,2,2,2,2,2}.
compute indsets={1,56,2,57,4,58,7,59,11,60,16,61,22,62,29,63,37,64,46,65}.
end if.
compute indlbl = {'Ind1'; 'Ind2'; 'Ind3'; 'Ind4'; 'Ind5'; 'Ind6'; 'Ind7'; 'Ind8'; 'Ind9'; 'Ind10'; 'Ind11'; 'Ind12'; 'Ind13'; 'Ind14'; 'Ind15'}.
compute indlbl = {indlbl; 'Ind16';'Ind17';'Ind18';'Ind19';'Ind20';'Ind21';'Ind22';'Ind23';'Ind24';'Ind25';'Ind26';'Ind27';'Ind28';'Ind29';'Ind30'}.
compute indlbl = {indlbl; 'Ind31';'Ind32';'Ind33';'Ind34';'Ind35';'Ind36';'Ind37';'Ind38';'Ind39';'Ind40';'Ind41';'Ind42';'Ind43';'Ind44';'Ind45'}.
compute indlbl = {indlbl; 'Ind46';'Ind47';'Ind48';'Ind49';'Ind50';'Ind51';'Ind52';'Ind53';'Ind54';'Ind55';'Ind56';'Ind57';'Ind58';'Ind59';'Ind60'}.
compute indlbl = {indlbl; 'Ind61';'Ind62';'Ind63';'Ind64';'Ind65';'Ind66';'Ind67';'Ind68';'Ind69';'Ind70';'Ind71';'Ind72';'Ind73';'Ind74';'Ind75'}.
compute indlbl = {indlbl; 'Ind76';'Ind77';'Ind78';'Ind79';'Ind80';'Ind81';'Ind82';'Ind83';'Ind84';'Ind85';'Ind86';'Ind87';'Ind88';'Ind89';'Ind90'}.
compute cntname={'(C1)';'(C2)';'(C3)';'(C4)';'(C5)';'(C6)';'(C7)';'(C8)';'(C9)';'(C10)';'(C11)';'(C12)';'(C13)';'(C14)';'(C15)';'(C16)';'(C17)'}.
compute cntname={cntname;'(C18)';'(C19)';'(C20)';'(C21)';'(C22)';'(C23)';'(C24)';'(C25)';'(C26)';'(C27)';'(C28)';'(C29)';'(C30)';'(C31)'}.
compute cntname={cntname;'(C32)';'(C33)';'(C34)';'(C35)';'(C36)';'(C37)';'(C38)';'(C39)';'(C40)';'(C41)';'(C42)';'(C43)';'(C44)';'(C45)'}.
compute cntname={cntname;'(C46)';'(C47)';'(C48)';'(C49)';'(C50)';'(C51)';'(C52)';'(C53)';'(C54)';'(C55)';'(C56)';'(C57)';'(C58)';'(C59)'}.
compute cntname={cntname;'(C60)';'(C61)';'(C62)';'(C63)';'(C64)';'(C65)';'(C66)';'(C67)';'(C68)';'(C69)';'(C70)';'(C71)';'(C72)';'(C73)'}.
compute cntname={cntname;'(C74)';'(C75)';'(C76)';'(C77)';'(C78)';'(C79)';'(C80)';'(C81)';'(C82)';'(C83)';'(C84)';'(C85)';'(C86)';'(C87)'}.
compute cntname={cntname;'(C88)';'(C89)';'(C90)';'(C91)';'(C92)';'(C93)';'(C94)';'(C95)';'(C96)';'(C97)';'(C98)';'(C99)';'(C100)';'(C101)'}.
compute cntname={cntname;'(C102)';'(C103)';'(C104)';'(C105)'}.
compute indmake=make(ncol(indmark),(nms+2),0).
compute indmod=make(ncol(indmark),1,999).
compute indmmm=make(ncol(indmark),1,0).
compute indmmmt=make(ncol(indmark),1,0).
compute start=1.
compute end=0.
compute nindfx=0.
compute indlocs=make(nrow(thetaxmb),ncol(paths),999).
compute indkey=make(ncol(indmark),1+((rmax(indmark)*2)+1),'     ').
compute c1=1.
compute c2=1.
compute c3=1.
loop i = 1 to ncol(paths).
do if (pathtype(1,i)=1).
compute indlocs(:,i)=thetaxmb(:,c1).
compute c1=c1+1.
end if.
do if (pathtype(1,i)=3).
compute indlocs(:,i)=thetamyb(:,c2).
compute c2=c2+1.
end if.
do if (pathtype(1,i)=2 and nms < 7 and serial=1).
compute indlocs(:,i)=thetammb(:,thetam(1,c3)).
compute c3=c3+1.
end if.
end loop.
loop i = 1 to ncol(indlocs).
compute c1=2.
loop j = 2 to nrow(indlocs).
do if (indlocs(j,i) <> 0).
compute indlocs(c1,i)=indlocs(j,i).
compute c1=c1+1.
end if.
end loop.
compute indlocs(1,i)=c1-2.
end loop.
compute indlocs=indlocs(1:rmax((indlocs(1,:))+1),:).
loop i = 1 to ncol(indmark).
compute numget=indmark(1,i).
compute end=end+numget.
compute gotcha=indsets(1,start:end).
compute start=end+1.
compute ok=1.
compute temp=0.
compute repoman=make(4,1,0).
loop j = 1 to ncol(gotcha).
do if paths(1,gotcha(1,j))=0.
compute ok=0.
end if.
do if (pathsmod(1,gotcha(1,j)) > 0).
compute temp=1.
compute temp2={pathsw(1,gotcha(1,j));pathsz(1,gotcha(1,j));pathswz(1,gotcha(1,j));0}.
do if (temp2(1,1)=1 and temp2(2,1)=1 and temp2(3,1)=0).
compute temp2(4,1)=1.
end if.
compute repoman=repoman+temp2.
end if.
end loop.
compute temp=0.
compute tempmmm=0.
compute typemmm=0.
do if ((repoman(1,1) > 0) and (repoman(2,1) = 0)).
compute temp=1.
do if (repoman(1,1)=1).
compute tempmmm=1.
end if.
do if (repoman(1,1) > 1 and (wdich=1 or mcw > 0)).
compute tempmmm=12.
compute typemmm=mcw.
do if (wdich=1).
compute typemmm=1.
end if.
end if.
do if (repoman(1,1) > 1 and (wdich=0 and mcw = 0)).
compute tempmmm=101.
end if.
end if.
do if ((repoman(1,1) = 0) and (repoman(2,1) > 0)).
compute temp=2.
do if (repoman(2,1)=1).
compute tempmmm=2.
end if.
do if (repoman(2,1) > 1 and (zdich = 1 or mcz > 0)).
compute tempmmm=22.
compute typemmm=mcz.
do if (zdich=1).
compute typemmm=1.
end if.
end if.
do if (repoman(2,1) > 1 and (zdich = 0 and mcw = 0)).
compute tempmmm=102.
end if.
end if.
do if (repoman(1,1)>0 and repoman(2,1)>0).
compute temp=3.
do if (repoman(1,1)=1 and repoman(2,1)=1).
do if (repoman(4,1)=1).
compute tempmmm=31.
end if.
do if (repoman(3,1)=1).
compute tempmmm=41.
end if.
end if.
end if.
do if (repoman(1,1)=1 and repoman(2,1)=1 and repoman(3,1)=0 and repoman(4,1)=0).
compute tempmmm=51.
end if.
do if (ok = 1).
compute nindfx=nindfx+1.
compute indmake(nindfx,1)=numget.
compute indmod(nindfx,1)=temp.
compute indmmm(nindfx,1)=tempmmm.
compute indmmmt(nindfx,1)=typemmm.
compute indmake(nindfx,2:(1+numget))=gotcha.
compute indkey(nindfx,1)=xnames.
loop j = 1 to numget.
compute indkey(nindfx,(j*2+1))=pathsdv(1,gotcha(1,j)).
compute indkey(nindfx,(j*2))='   ->   '.
end loop.
end if.
end loop.
compute indkey=indkey(1:nindfx,1:((cmax(indmake(:,1))*2)+1)).
compute indmake=indmake(1:nindfx,1:(cmax(indmake(:,1))+1)).
compute indmod=indmod(1:nrow(indmake),1).
compute indmmm=indmmm(1:nrow(indmake),1).
compute indmmmt=indmmmt(1:nrow(indmake),1).
compute ncpairs = (((nindfx)*(nindfx-1))/2).
do if ((contrast = 1 or contrast = 2 or contrast=3) and (ncpairs > 105)).
compute contrast=0.
compute notecode(notes,1) = 13.
compute notes = notes + 1.
end if.
do if (contrast = 4).
do if (ncol(contvec) <> nindfx).
compute contrast=0.
compute notecode(notes,1) = 14.
compute notes = notes + 1.
end if.
end if.
do if (anymod=0).
do if (nms = 1 and contrast > 0).
compute contrast=0.
end if.
compute efloop=(((1-(effsize=0))*2)+1)-(((mcx>0 or xdich=1))*(1-(effsize=0))).
loop kk=1 to efloop.
do if (boot = 0).
compute bootres=obscoeff.
do if (kk=1).
compute totbtvec=make(1,nxvls,0).
end if.
compute bootdir=obsdirfx.
compute indtab=999.
compute inddiff=999.
compute bootysd=ysd.
compute bootxsd=xsd.
end if.
do if (boot > 0).
compute bootres={obscoeff;bootres}.
do if (kk=1).
compute totbtvec=make(nrow(bootres),nxvls,0).
end if.
compute indtab=make(1,4,999).
compute inddiff=make(nrow(bootres),1,999).
end if.
compute indtotal=make(nrow(bootres),1,0).
loop i = 1 to nrow(indmake).
loop j = 1 to nxvls.
compute indtemp=make(nrow(bootres),1,1).
loop k = 1 to indmake(i,1).
compute jtemp=1.
do if (j > 1 and k=1).
compute jtemp=j.
end if.
compute indtemp=indtemp&*bootres(:,pathsfoc(jtemp,indmake(i,(k+1)))).
end loop.
do if (kk = 2).
compute indtemp=indtemp/bootysd.
end if.
do if (kk = 3).
compute indtemp=(bootxsd&*indtemp)/bootysd.
end if.
do if (contrast <> 0).
compute inddiff={inddiff,indtemp}.
end if.
do if (nxvls=1).
compute indtotal=indtotal+indtemp.
end if.
compute indeff=indtemp(1,1).
do if (kk=1).
compute totbtvec(:,j)=totbtvec(:,j)+indtemp.
end if.
do if (boot > 0).

.
compute temp = indtemp(2:nrow(indtemp),1).
compute temp(GRADE( indtemp(2:nrow(indtemp),1) )) = indtemp(2:nrow(indtemp),1).
compute badlo = 0.
compute badhi = 0.
do if ( (indtemp(1,1)*bc)+(9999*(1-bc)) <> 9999).
compute pv=csum(temp < (indtemp(1,1)*bc)+(9999*(1-bc)) )/nrow(temp).
compute ppv = pv.
do if (pv > .5).
compute ppv = 1-pv.
end if.
compute y5=sqrt(-2*ln(ppv)).
compute xp=y5+((((y5*p4+p3)*y5+p2)*y5+p1)*y5+p0)/((((y5*q4+q3)*y5+q2)*y5+q1)*y5+q0).
do if (pv <= .5).
compute xp = -xp.
end if.
compute cilow=rnd(nrow(temp)*(cdfnorm(2*xp-xp2))).
compute cihigh=trunc(nrow(temp)*(cdfnorm(2*xp+(xp2))))+1.
do if (cilow < 1).
compute cilow = 1.
compute booterr=1.
compute badlo = 1.
end if.
do if (cihigh > nrow(temp)).
compute cihigh = boot.
compute booterr=1.
compute badhi = 1.
end if.
compute llcit=temp(cilow,1).
compute ulcit=temp(cihigh,1).
do if (badlo = 1 and llcit <> priorlo).
compute badend={badend, llcit}.
compute priorlo = llcit.
end if.
do if (badhi = 1 and ulcit <> priorhi).
compute badend={badend, ulcit}.
compute priorhi = ulcit.
end if.
end if.
do if ( (indtemp(1,1)*bc)+(9999*(1-bc)) = 9999).
compute llcit=temp(cilow,1).
compute ulcit=temp(cihigh,1).
end if.
compute bootse=t(sqrt((cssq(temp)-((csum(temp)&**2)/nrow(temp)))/(nrow(temp)-1)))
.
compute indeff={indeff,bootse,llcit,ulcit}.
end if.
do if (kk=1).
compute indtabn={indtab;indeff}.
end if.
compute indtab={indtab;indeff}.
end loop.
end loop.
compute indtab=indtab(2:nrow(indtab),:).
do if (kk=1).
compute indtabn=indtabn(2:nrow(indtabn),:).
end if.
compute rowlbs=indlbl(1:nrow(indtab),1).
do if (mc > 0).
compute inddiff=make(mc,1,-999).
compute indtab2=make(nrow(indtab),4,-999).
compute indtab2(:,1)=indtab.
compute indtab=indtab2.
release indtab2.
compute mcct=0.
compute indtotal=make(mc,1,0).
do if (kk = 1).
compute x1 = sqrt(-2*ln(uniform(mc,nrow(mcsopath))))&*cos((2*3.14159265358979)*uniform(mc, nrow(mcsopath))).
compute x1=x1*chol(indcov).
loop ii=1 to nrow(x1).
compute x1(ii,:)=x1(ii,:)+t(mcsopath).
end loop.
end if.
loop ii=1 to nms.
compute tmpb=x1(:,((nms*nxvls)+ii)).
compute tmpb2=tmpb.
do if (nxvls > 1).
loop jj=1 to (nxvls-1).
compute tmpb2={tmpb2,tmpb}.
end loop.
end if.
compute indtemp=x1(:,(((ii-1)*nxvls)+1):(ii*nxvls))&*tmpb2.
loop jj=1 to ncol(indtemp).
do if (kk = 2).
compute indtemp(:,jj)=indtemp(:,jj)/ysd.
end if.
do if (kk = 3).
compute indtemp(:,jj)=(xsd*indtemp(:,jj))/ysd.
end if.

.
compute temp = indtemp(:,jj).
compute temp(GRADE( indtemp(:,jj) )) = indtemp(:,jj).
compute badlo = 0.
compute badhi = 0.
do if ( 9999 <> 9999).
compute pv=csum(temp < 9999 )/nrow(temp).
compute ppv = pv.
do if (pv > .5).
compute ppv = 1-pv.
end if.
compute y5=sqrt(-2*ln(ppv)).
compute xp=y5+((((y5*p4+p3)*y5+p2)*y5+p1)*y5+p0)/((((y5*q4+q3)*y5+q2)*y5+q1)*y5+q0).
do if (pv <= .5).
compute xp = -xp.
end if.
compute cilow=rnd(nrow(temp)*(cdfnorm(2*xp-xp2))).
compute cihigh=trunc(nrow(temp)*(cdfnorm(2*xp+(xp2))))+1.
do if (cilow < 1).
compute cilow = 1.
compute booterr=1.
compute badlo = 1.
end if.
do if (cihigh > nrow(temp)).
compute cihigh = boot.
compute booterr=1.
compute badhi = 1.
end if.
compute llcit=temp(cilow,1).
compute ulcit=temp(cihigh,1).
do if (badlo = 1 and llcit <> priorlo).
compute badend={badend, llcit}.
compute priorlo = llcit.
end if.
do if (badhi = 1 and ulcit <> priorhi).
compute badend={badend, ulcit}.
compute priorhi = ulcit.
end if.
end if.
do if ( 9999 = 9999).
compute llcit=temp(cilow,1).
compute ulcit=temp(cihigh,1).
end if.
compute bootse=t(sqrt((cssq(temp)-((csum(temp)&**2)/nrow(temp)))/(nrow(temp)-1)))
.
compute mcct=mcct+1.
compute indtab(mcct,2:4)={bootse,llcit,ulcit}.
end loop.
do if (nxvls=1).
compute indtotal=indtotal+indtemp.
do if (contrast <> 0).
compute inddiff={inddiff,indtemp}.
end if.
end if.
end loop.
release indtemp,tmpb.
end if.
do if (normal = 1 and sobelok=1).
compute sobelmat=indtab(:,1).
compute sobelmat={sobelmat,(sobelmat/2),sobelmat,sobelmat}.
loop ii=1 to nms.
compute se2b=indcov(((nms*nxvls)+ii),((nms*nxvls)+ii)).
compute bpath2=mcsopath(((nms*nxvls)+ii),1)&**2.
compute se2a=diag(indcov((((ii-1)*nxvls)+1):(ii*nxvls),(((ii-1)*nxvls)+1):(ii*nxvls))).
compute apath2=mcsopath((((ii-1)*nxvls)+1):(ii*nxvls) ,1)&**2.
compute sesobel=sqrt(apath2*se2b+bpath2*se2a+se2a*se2b).
compute sobelmat((((ii-1)*nxvls)+1):(ii*nxvls),2)=sesobel.
end loop.
release se2b,bpath2,se2a,apath2,sesobel,ii.
compute sobelmat(:,3)=sobelmat(:,1)&/sobelmat(:,2).
compute sobelmat(:,4) = 2*(1-cdfnorm(abs(sobelmat(:,3)))).
end if.
do if (serial = 0).
compute rowlbs=t(mnames).
end if.
do if (nxvls=1 and nms > 1).
compute rowlbs={'TOTAL';rowlbs}.
compute indtemp=indtotal(1,1).
do if (boot > 0 and nxvls=1).

.
compute temp = indtotal(2:nrow(indtotal),1).
compute temp(GRADE( indtotal(2:nrow(indtotal),1) )) = indtotal(2:nrow(indtotal),1).
compute badlo = 0.
compute badhi = 0.
do if ( (indtotal(1,1)*bc)+(9999*(1-bc)) <> 9999).
compute pv=csum(temp < (indtotal(1,1)*bc)+(9999*(1-bc)) )/nrow(temp).
compute ppv = pv.
do if (pv > .5).
compute ppv = 1-pv.
end if.
compute y5=sqrt(-2*ln(ppv)).
compute xp=y5+((((y5*p4+p3)*y5+p2)*y5+p1)*y5+p0)/((((y5*q4+q3)*y5+q2)*y5+q1)*y5+q0).
do if (pv <= .5).
compute xp = -xp.
end if.
compute cilow=rnd(nrow(temp)*(cdfnorm(2*xp-xp2))).
compute cihigh=trunc(nrow(temp)*(cdfnorm(2*xp+(xp2))))+1.
do if (cilow < 1).
compute cilow = 1.
compute booterr=1.
compute badlo = 1.
end if.
do if (cihigh > nrow(temp)).
compute cihigh = boot.
compute booterr=1.
compute badhi = 1.
end if.
compute llcit=temp(cilow,1).
compute ulcit=temp(cihigh,1).
do if (badlo = 1 and llcit <> priorlo).
compute badend={badend, llcit}.
compute priorlo = llcit.
end if.
do if (badhi = 1 and ulcit <> priorhi).
compute badend={badend, ulcit}.
compute priorhi = ulcit.
end if.
end if.
do if ( (indtotal(1,1)*bc)+(9999*(1-bc)) = 9999).
compute llcit=temp(cilow,1).
compute ulcit=temp(cihigh,1).
end if.
compute bootse=t(sqrt((cssq(temp)-((csum(temp)&**2)/nrow(temp)))/(nrow(temp)-1)))
.
compute indtemp={indtemp, bootse,llcit,ulcit}.
end if.
do if (mc > 0).
compute obtmc=indtab(:,1).
compute indtemp=csum(obtmc).

.
compute temp = indtotal(:,1).
compute temp(GRADE( indtotal(:,1) )) = indtotal(:,1).
compute badlo = 0.
compute badhi = 0.
do if ( 9999 <> 9999).
compute pv=csum(temp < 9999 )/nrow(temp).
compute ppv = pv.
do if (pv > .5).
compute ppv = 1-pv.
end if.
compute y5=sqrt(-2*ln(ppv)).
compute xp=y5+((((y5*p4+p3)*y5+p2)*y5+p1)*y5+p0)/((((y5*q4+q3)*y5+q2)*y5+q1)*y5+q0).
do if (pv <= .5).
compute xp = -xp.
end if.
compute cilow=rnd(nrow(temp)*(cdfnorm(2*xp-xp2))).
compute cihigh=trunc(nrow(temp)*(cdfnorm(2*xp+(xp2))))+1.
do if (cilow < 1).
compute cilow = 1.
compute booterr=1.
compute badlo = 1.
end if.
do if (cihigh > nrow(temp)).
compute cihigh = boot.
compute booterr=1.
compute badhi = 1.
end if.
compute llcit=temp(cilow,1).
compute ulcit=temp(cihigh,1).
do if (badlo = 1 and llcit <> priorlo).
compute badend={badend, llcit}.
compute priorlo = llcit.
end if.
do if (badhi = 1 and ulcit <> priorhi).
compute badend={badend, ulcit}.
compute priorhi = ulcit.
end if.
end if.
do if ( 9999 = 9999).
compute llcit=temp(cilow,1).
compute ulcit=temp(cihigh,1).
end if.
compute bootse=t(sqrt((cssq(temp)-((csum(temp)&**2)/nrow(temp)))/(nrow(temp)-1)))
.
compute indtemp={indtemp, bootse,llcit,ulcit}.
end if.
compute indtab={indtemp;indtab}.
end if.
compute bootlbs={'Effect', 'BootSE','BootLLCI','BootULCI'}.
do if (mc > 0).
compute bootlbs={'Effect', 'MC SE','MC LLCI','MC ULCI'}.
end if.
do if (nxvls = 1).
do if (contrast <> 0).
compute inddiff=inddiff(:,2:ncol(inddiff)).
do if (mc > 0).
compute inddiff={t(obtmc);inddiff}.
end if.
do if (contrast = 4).
compute inddifft=inddiff*t(contvec).
compute indtemp=inddifft(1,1).
do if (boot > 0 or mc > 0).
do if (mc > 0).

.
compute temp = inddifft(2:nrow(inddifft),1).
compute temp(GRADE( inddifft(2:nrow(inddifft),1) )) = inddifft(2:nrow(inddifft),1).
compute badlo = 0.
compute badhi = 0.
do if ( 9999 <> 9999).
compute pv=csum(temp < 9999 )/nrow(temp).
compute ppv = pv.
do if (pv > .5).
compute ppv = 1-pv.
end if.
compute y5=sqrt(-2*ln(ppv)).
compute xp=y5+((((y5*p4+p3)*y5+p2)*y5+p1)*y5+p0)/((((y5*q4+q3)*y5+q2)*y5+q1)*y5+q0).
do if (pv <= .5).
compute xp = -xp.
end if.
compute cilow=rnd(nrow(temp)*(cdfnorm(2*xp-xp2))).
compute cihigh=trunc(nrow(temp)*(cdfnorm(2*xp+(xp2))))+1.
do if (cilow < 1).
compute cilow = 1.
compute booterr=1.
compute badlo = 1.
end if.
do if (cihigh > nrow(temp)).
compute cihigh = boot.
compute booterr=1.
compute badhi = 1.
end if.
compute llcit=temp(cilow,1).
compute ulcit=temp(cihigh,1).
do if (badlo = 1 and llcit <> priorlo).
compute badend={badend, llcit}.
compute priorlo = llcit.
end if.
do if (badhi = 1 and ulcit <> priorhi).
compute badend={badend, ulcit}.
compute priorhi = ulcit.
end if.
end if.
do if ( 9999 = 9999).
compute llcit=temp(cilow,1).
compute ulcit=temp(cihigh,1).
end if.
compute bootse=t(sqrt((cssq(temp)-((csum(temp)&**2)/nrow(temp)))/(nrow(temp)-1)))
.
end if.
do if (boot > 0).

.
compute temp = inddifft(2:nrow(inddifft),1).
compute temp(GRADE( inddifft(2:nrow(inddifft),1) )) = inddifft(2:nrow(inddifft),1).
compute badlo = 0.
compute badhi = 0.
do if ( (inddifft(1,1)*bc)+(9999* (1-bc)) <> 9999).
compute pv=csum(temp < (inddifft(1,1)*bc)+(9999* (1-bc)) )/nrow(temp).
compute ppv = pv.
do if (pv > .5).
compute ppv = 1-pv.
end if.
compute y5=sqrt(-2*ln(ppv)).
compute xp=y5+((((y5*p4+p3)*y5+p2)*y5+p1)*y5+p0)/((((y5*q4+q3)*y5+q2)*y5+q1)*y5+q0).
do if (pv <= .5).
compute xp = -xp.
end if.
compute cilow=rnd(nrow(temp)*(cdfnorm(2*xp-xp2))).
compute cihigh=trunc(nrow(temp)*(cdfnorm(2*xp+(xp2))))+1.
do if (cilow < 1).
compute cilow = 1.
compute booterr=1.
compute badlo = 1.
end if.
do if (cihigh > nrow(temp)).
compute cihigh = boot.
compute booterr=1.
compute badhi = 1.
end if.
compute llcit=temp(cilow,1).
compute ulcit=temp(cihigh,1).
do if (badlo = 1 and llcit <> priorlo).
compute badend={badend, llcit}.
compute priorlo = llcit.
end if.
do if (badhi = 1 and ulcit <> priorhi).
compute badend={badend, ulcit}.
compute priorhi = ulcit.
end if.
end if.
do if ( (inddifft(1,1)*bc)+(9999* (1-bc)) = 9999).
compute llcit=temp(cilow,1).
compute ulcit=temp(cihigh,1).
end if.
compute bootse=t(sqrt((cssq(temp)-((csum(temp)&**2)/nrow(temp)))/(nrow(temp)-1)))
.
end if.
compute indtemp={indtemp, bootse,llcit,ulcit}.
end if.
compute indtab={indtab;indtemp}.
end if.
do if (contrast = 1 or contrast = 2 or contrast=3).
compute conkey=make(1,4,' ').
loop i = 1 to ncol(inddiff)-1.
loop j = (i+1) to ncol(inddiff).
compute inddifft=inddiff(:,i)-inddiff(:,j).
do if (contrast=2).
compute inddifft=abs(inddiff(:,i))-abs(inddiff(:,j)).
end if.
do if (contrast=3).
compute inddifft=inddiff(:,i)+inddiff(:,j).
end if.
compute indtemp=inddifft(1,1).
do if (contrast=1 or contrast=2).
compute conkeyt={' ', rowlbs((i+1),1),' minus  ',rowlbs((j+1),1)}.
end if.
do if (contrast=3).
compute conkeyt={' ', rowlbs((i+1),1),'  plus  ',rowlbs((j+1),1)}.
end if.
compute conkey={conkey;conkeyt}.
do if (boot > 0 or mc > 0).
do if (mc > 0).

.
compute temp = inddifft(2:nrow(inddifft),1).
compute temp(GRADE( inddifft(2:nrow(inddifft),1) )) = inddifft(2:nrow(inddifft),1).
compute badlo = 0.
compute badhi = 0.
do if ( 9999 <> 9999).
compute pv=csum(temp < 9999 )/nrow(temp).
compute ppv = pv.
do if (pv > .5).
compute ppv = 1-pv.
end if.
compute y5=sqrt(-2*ln(ppv)).
compute xp=y5+((((y5*p4+p3)*y5+p2)*y5+p1)*y5+p0)/((((y5*q4+q3)*y5+q2)*y5+q1)*y5+q0).
do if (pv <= .5).
compute xp = -xp.
end if.
compute cilow=rnd(nrow(temp)*(cdfnorm(2*xp-xp2))).
compute cihigh=trunc(nrow(temp)*(cdfnorm(2*xp+(xp2))))+1.
do if (cilow < 1).
compute cilow = 1.
compute booterr=1.
compute badlo = 1.
end if.
do if (cihigh > nrow(temp)).
compute cihigh = boot.
compute booterr=1.
compute badhi = 1.
end if.
compute llcit=temp(cilow,1).
compute ulcit=temp(cihigh,1).
do if (badlo = 1 and llcit <> priorlo).
compute badend={badend, llcit}.
compute priorlo = llcit.
end if.
do if (badhi = 1 and ulcit <> priorhi).
compute badend={badend, ulcit}.
compute priorhi = ulcit.
end if.
end if.
do if ( 9999 = 9999).
compute llcit=temp(cilow,1).
compute ulcit=temp(cihigh,1).
end if.
compute bootse=t(sqrt((cssq(temp)-((csum(temp)&**2)/nrow(temp)))/(nrow(temp)-1)))
.
end if.
do if (boot > 0).

.
compute temp = inddifft(2:nrow(inddifft),1).
compute temp(GRADE( inddifft(2:nrow(inddifft),1) )) = inddifft(2:nrow(inddifft),1).
compute badlo = 0.
compute badhi = 0.
do if ( (inddifft(1,1)*bc)+(9999* (1-bc)) <> 9999).
compute pv=csum(temp < (inddifft(1,1)*bc)+(9999* (1-bc)) )/nrow(temp).
compute ppv = pv.
do if (pv > .5).
compute ppv = 1-pv.
end if.
compute y5=sqrt(-2*ln(ppv)).
compute xp=y5+((((y5*p4+p3)*y5+p2)*y5+p1)*y5+p0)/((((y5*q4+q3)*y5+q2)*y5+q1)*y5+q0).
do if (pv <= .5).
compute xp = -xp.
end if.
compute cilow=rnd(nrow(temp)*(cdfnorm(2*xp-xp2))).
compute cihigh=trunc(nrow(temp)*(cdfnorm(2*xp+(xp2))))+1.
do if (cilow < 1).
compute cilow = 1.
compute booterr=1.
compute badlo = 1.
end if.
do if (cihigh > nrow(temp)).
compute cihigh = boot.
compute booterr=1.
compute badhi = 1.
end if.
compute llcit=temp(cilow,1).
compute ulcit=temp(cihigh,1).
do if (badlo = 1 and llcit <> priorlo).
compute badend={badend, llcit}.
compute priorlo = llcit.
end if.
do if (badhi = 1 and ulcit <> priorhi).
compute badend={badend, ulcit}.
compute priorhi = ulcit.
end if.
end if.
do if ( (inddifft(1,1)*bc)+(9999* (1-bc)) = 9999).
compute llcit=temp(cilow,1).
compute ulcit=temp(cihigh,1).
end if.
compute bootse=t(sqrt((cssq(temp)-((csum(temp)&**2)/nrow(temp)))/(nrow(temp)-1)))
.
end if.
compute indtemp={indtemp, bootse,llcit,ulcit}.
end if.
compute indtab={indtab;indtemp}.
end loop.
end loop.
end if.
release inddiff.
compute contlbs=cntname(1:(((nindfx)*(nindfx-1))/2),1).
compute rowlbs={rowlbs;contlbs}.
end if.
compute resultm2=make(nrow(indtab),maxresm,99999).
compute resultm2(1:nrow(indtab),1:ncol(indtab))=indtab.
compute resultm={resultm;resultm2}.
do if (outscree=1).
do if (kk=1).
print indtab/title = 'Indirect effect(s) of X on Y:'/rnames=rowlbs/cnames=bootlbs/format= F10.4.
end if.
do if (kk = 2 and (xdich=1 or mcx > 0)).
print indtab/title = 'Partially standardized indirect effect(s) of X on Y:'/rnames=rowlbs/cnames=bootlbs/format= F10.4.
end if.
do if (kk = 3).
print indtab/title = 'Completely standardized indirect effect(s) of X on Y:'/rnames=rowlbs/cnames=bootlbs/format= F10.4.
end if.
end if.
do if (normal=1 and sobelok=1 and kk=1).
compute resultm2=make(nrow(sobelmat),maxresm,99999).
compute resultm2(1:nrow(sobelmat),1:ncol(sobelmat))=sobelmat.
compute resultm={resultm;resultm2}.
compute sobellab={'Effect',hclab,'Z','p'}.
compute sobelrlb=rowlbs.
do if (nms > 1).
compute sobelrlb=rowlbs(2:(1+nms),1).
end if.
do if (outscree=1).
print sobelmat/title='   Normal theory test for indirect effect(s):'/cnames=sobellab/rnames=sobelrlb/format= F10.4.
end if.
end if.
do if (contrast <> 0).
do if ((contrast=1 or contrast = 2 or contrast=3) and kk=efloop ).
compute conkey=conkey(2:nrow(conkey),:).
do if (outscree=1).
print conkey/title = 'Specific indirect effect contrast definition(s):'/rnames=contlbs/format=A8.
end if.
end if.
do if (outscree=1).
do if (contrast = 4 and kk=efloop).
compute crowlbs=rowlbs(2:(nindfx+1),1).
print contvec/title = 'Specific indirect effect contrast weights:'/cnames=crowlbs/rlabels='(C1)'/format= F10.4.
end if.
do if (contrast = 2 and kk=efloop).
print/title = 'Contrasts are differences between absolute values of indirect effects'.
end if.
end if.
end if.
do if (serial = 1 and kk=efloop).
compute rowlbst=rowlbs(2:nrow(rowlbs),1).
do if (outscree=1).
print indkey/title = 'Indirect effect key:'/rnames=rowlbst/format = A8.
end if.
end if.
else.
do if (outscree=1).
do if (kk = 1).
print/title = 'Relative indirect effects of X on Y'.
end if.
do if (kk = 2).
print/title = 'Partially standardized relative indirect effect(s) of X on Y:'.
end if.
do if (kk = 3).
print/title = 'Completely standardized relative indirect effect(s) of X on Y:'.
end if.
end if.
loop i = 1 to nrow(indmake).
compute indtabsm=indtab((((i-1)*nxvls)+1):(nxvls*i),:).
compute indkeyt=indkey(i,:).
do if (outscree=1).
print indkeyt/title=' '/space=0/format=A8.
end if.
do if (bcmat(nrow(bcmat),1)=0)).
compute direffl2=xcatlab(1:nxvls,1).
end if.
compute resultm2=make(nrow(indtabsm),maxresm,99999).
compute resultm2(1:nrow(indtabsm),1:ncol(indtabsm))=indtabsm.
compute resultm={resultm;resultm2}.
do if (outscree=1).
print indtabsm/title = ' '/cnames=bootlbs/rnames=direffl2/format= F10.4 /space=0.
end if.
do if (normal=1 and sobelok=1 and kk=1).
compute sobelsm=sobelmat((((i-1)*nxvls)+1):(nxvls*i),:).
compute sobellab={'Effect',hclab,'Z','p'}.
compute resultm2=make(nrow(sobelsm),maxresm,99999).
compute resultm2(1:nrow(sobelsm),1:ncol(sobelsm))=sobelsm.
compute resultm={resultm;resultm2}.
do if (outscree=1).
print sobelsm/title='      Normal theory test for relative indirect effects:'/cnames=sobellab/rnames=direffl2/format= F10.4.
end if.
end if.
end loop.
end if.
do if (effsize = 1 and boot > 0).
compute bootres=bootres(2:nrow(bootres),:).
end if.
end loop.
do if (alttotal=1).
compute altcnms='Effect'.
compute totbtvec=totbtvec+bootdir.
compute alttotfx=t(totbtvec(1,:)).
do if (boot > 0).
compute alttotfx=make(ncol(totbtvec),4,0).
compute alttotfx(:,1)=t(totbtvec(1,:)).
loop cec=1 to ncol(totbtvec).

.
compute temp = totbtvec(2:nrow(totbtvec),cec).
compute temp(GRADE( totbtvec(2:nrow(totbtvec),cec) )) = totbtvec(2:nrow(totbtvec),cec).
compute badlo = 0.
compute badhi = 0.
do if ( (totbtvec(1,cec)*bc)+(9999* (1-bc)) <> 9999).
compute pv=csum(temp < (totbtvec(1,cec)*bc)+(9999* (1-bc)) )/nrow(temp).
compute ppv = pv.
do if (pv > .5).
compute ppv = 1-pv.
end if.
compute y5=sqrt(-2*ln(ppv)).
compute xp=y5+((((y5*p4+p3)*y5+p2)*y5+p1)*y5+p0)/((((y5*q4+q3)*y5+q2)*y5+q1)*y5+q0).
do if (pv <= .5).
compute xp = -xp.
end if.
compute cilow=rnd(nrow(temp)*(cdfnorm(2*xp-xp2))).
compute cihigh=trunc(nrow(temp)*(cdfnorm(2*xp+(xp2))))+1.
do if (cilow < 1).
compute cilow = 1.
compute booterr=1.
compute badlo = 1.
end if.
do if (cihigh > nrow(temp)).
compute cihigh = boot.
compute booterr=1.
compute badhi = 1.
end if.
compute llcit=temp(cilow,1).
compute ulcit=temp(cihigh,1).
do if (badlo = 1 and llcit <> priorlo).
compute badend={badend, llcit}.
compute priorlo = llcit.
end if.
do if (badhi = 1 and ulcit <> priorhi).
compute badend={badend, ulcit}.
compute priorhi = ulcit.
end if.
end if.
do if ( (totbtvec(1,cec)*bc)+(9999* (1-bc)) = 9999).
compute llcit=temp(cilow,1).
compute ulcit=temp(cihigh,1).
end if.
compute bootse=t(sqrt((cssq(temp)-((csum(temp)&**2)/nrow(temp)))/(nrow(temp)-1)))
.
compute alttotfx(cec,2:4)={bootse,llcit,ulcit}.
end loop.
compute altcnms={altcnms,'BootSE', 'BootLLCI','BootULCI'}.
end if.
compute resultm2=make(nrow(alttotfx),maxresm,99999).
compute resultm2(1:nrow(alttotfx),1:ncol(alttotfx))=alttotfx.
compute resultm={resultm;resultm2}.
do if (outscree=1).
do if (nxvls > 1).
print/title= '----------'/space=0.
print alttotfx/title='Relative total effects of X on Y (sum of direct and indirect effects)'/rnames=direffl2/cnames=altcnms/format= F10.4.
end if.
do if (nxvls = 1).
print alttotfx/title='Total effect of X on Y (sum of direct and indirect effects)'/cnames=altcnms/format= F10.4.
end if.
end if.
end if.
end if.
do if (anymod > 0).
do if (boot = 0).
compute bootres=obscoeff.
compute indtab=999.
end if.
do if (boot > 0).
compute bootres={obscoeff;bootres}.
compute indtab=make(1,4,999).
end if.
do if (csum((indmod > 0))=nrow(indmod)).
do if (outscree=1).
do if (nxvls > 1).
do if (xmint=0).
print/title= 'Relative conditional indirect effects of X on Y:'.
end if.
do if (xmint=1).
print/title= 'Relative (total) natural indirect effects of X on Y:'.
end if.
end if.
do if (nxvls = 1).
do if (xmint=0).
print/title= 'Conditional indirect effects of X on Y:'.
end if.
do if (xmint=1).
print/title= '(Total) Natural indirect effect(s) of X on Y:'.
end if.
end if.
end if.
end if.
do if (csum((indmod > 0)) < nrow(indmod)).
do if (outscree=1).
do if (nxvls > 1).
print/title= 'Relative conditional and unconditional indirect effects of X on Y:'.
end if.
do if (nxvls = 1).
print/title= 'Conditional and unconditional indirect effects of X on Y:'.
end if.
end if.
end if.
compute cftotfx=make(nrow(bootres),nxvls,0).
loop i = 1 to nrow(indmake).
compute indtab=indtab(1,:)*0.
do if (outscree=1).
do if (xmint=0).
print indkey(i,:)/title = 'INDIRECT EFFECT:'/format=A8.
end if.
do if (xmint=1).
print indkey(i,:)/title = ' '/format=A8/space=0.
end if.
end if.
do if (indmod(i,1)=0).
loop j = 1 to nxvls.
compute indtemp=make(nrow(bootres),1,1).
loop k = 1 to indmake(i,1).
compute jtemp=1.
do if (j > 1 and k=1).
compute jtemp=j.
end if.
compute indtemp=indtemp&*bootres(:,pathsfoc(jtemp,indmake(i,(k+1)))).
end loop.
compute indeff=indtemp(1,1).
do if (boot > 0).

.
compute temp = indtemp(2:nrow(indtemp),1).
compute temp(GRADE( indtemp(2:nrow(indtemp),1) )) = indtemp(2:nrow(indtemp),1).
compute badlo = 0.
compute badhi = 0.
do if ( (indtemp(1,1)*bc)+(9999*(1-bc)) <> 9999).
compute pv=csum(temp < (indtemp(1,1)*bc)+(9999*(1-bc)) )/nrow(temp).
compute ppv = pv.
do if (pv > .5).
compute ppv = 1-pv.
end if.
compute y5=sqrt(-2*ln(ppv)).
compute xp=y5+((((y5*p4+p3)*y5+p2)*y5+p1)*y5+p0)/((((y5*q4+q3)*y5+q2)*y5+q1)*y5+q0).
do if (pv <= .5).
compute xp = -xp.
end if.
compute cilow=rnd(nrow(temp)*(cdfnorm(2*xp-xp2))).
compute cihigh=trunc(nrow(temp)*(cdfnorm(2*xp+(xp2))))+1.
do if (cilow < 1).
compute cilow = 1.
compute booterr=1.
compute badlo = 1.
end if.
do if (cihigh > nrow(temp)).
compute cihigh = boot.
compute booterr=1.
compute badhi = 1.
end if.
compute llcit=temp(cilow,1).
compute ulcit=temp(cihigh,1).
do if (badlo = 1 and llcit <> priorlo).
compute badend={badend, llcit}.
compute priorlo = llcit.
end if.
do if (badhi = 1 and ulcit <> priorhi).
compute badend={badend, ulcit}.
compute priorhi = ulcit.
end if.
end if.
do if ( (indtemp(1,1)*bc)+(9999*(1-bc)) = 9999).
compute llcit=temp(cilow,1).
compute ulcit=temp(cihigh,1).
end if.
compute bootse=t(sqrt((cssq(temp)-((csum(temp)&**2)/nrow(temp)))/(nrow(temp)-1)))
.
compute indeff={indeff,bootse,llcit,ulcit}.
end if.
compute indtab={indtab;indeff}.
end loop.
compute resultm2=make(nrow(indtab),maxresm,99999).
compute resultm2(1:nrow(indtab),1:ncol(indtab))=indtab.
compute resultm={resultm;resultm2}.
do if (outscree=1).
do if (nxvls > 1).
compute indefflb=xcatlab(1:nxvls,1).
print indtab(2:nrow(indtab),:)/title = ' '/clabels='Effect', 'BootSE', 'BootLLCI', 'BootULCI'/rnames=indefflb/format= F10.4 /space=0.
end if.
do if (nxvls = 1).
print indtab(2:nrow(indtab),:)/title = ' '/clabels='Effect', 'BootSE', 'BootLLCI', 'BootULCI'/format= F10.4 /space=0.
end if.
end if.
end if.
do if (indmod(i,1)>0).
do if (indmod(i,1)=1).
compute indmodva=wmodvals.
compute indprova=wprobval.
compute condlbs={wnames}.
compute printw=1.
else if (indmod(i,1)=2).
compute indmodva=zmodvals.
compute indprova=zprobval.
compute condlbs={znames}.
compute printz=1.
else if (indmod(i,1)=3).
compute cntmp=1.
compute printz=1.
compute printw=1.
compute indmodva=make((nrow(wmodvals)*nrow(zmodvals)),2,999).
loop k7 = 1 to nrow(wmodvals).
loop k8 = 1 to nrow(zmodvals).
compute indmodva(cntmp,:)={wmodvals(k7,1),zmodvals(k8,1)}.
compute cntmp=cntmp+1.
end loop.
end loop.
compute condlbs={wnames,znames}.
end if.
compute condres=make(nrow(indmodva),1,999).
do if (boot > 0).
compute condres=make(nrow(indmodva),4,999).
end if.
compute condres={indmodva,condres}.
loop k4 = 1 to nxvls.
compute imm3=make(nrow(bootres),1,1).
compute imm4=make(nrow(bootres),1,1).
compute indcontr=0.
do if (indmod(i,1)=3).
compute tihsw=wprobval.
compute tihsz=zprobval.
end if.
loop k1=1 to nrow(indmodva).
compute tucker2=make(nrow(bootres),1,1).
compute imm2=make(nrow(bootres),1,1).
compute wfirst=0.
compute zfirst=0.
compute immset=0.
loop k2=1 to indmake(i,1).
compute colnumb=indmake(i,(k2+1)).
do if (k2=1).
compute wbb=make(nrow(bootres),(nwvls*nxvls),0).
compute zbb=make(nrow(bootres),(nzvls*nxvls),0).
compute wzbb=make(nrow(bootres),(nwvls*nzvls*nxvls),0).
end if.
do if (k2<>1).
compute wbb=make(nrow(bootres),nwvls,0).
compute zbb=make(nrow(bootres),nzvls,0).
compute wzbb=make(nrow(bootres),(nwvls*nzvls),0).
end if.
compute cnt=1.
compute tihs=indlocs(2:((indlocs(1,colnumb))+1),colnumb).
do if (k2 = 1).
compute focbb=tihs(1:nxvls,1).
compute focbb=bootres(:,focbb).
do if (indmmm(i,1)>0).
compute imm=focbb(:,k4).
compute condbb=imm.
end if.
compute focaddon=make(1,nxvls,0).
compute focaddon(1,k4)=1.
compute cnt=cnt+nxvls.
compute placeh=nxvls.
do if (indmod(i,1)=1).
compute tihsz=make(nrow(wprobval),(nzvls*nxvls),0).
compute tihswz=make(nrow(wprobval),(nwvls*nzvls*nxvls),0).
do if (pathsw(1,colnumb)=1).
compute temp=make(nrow(wprobval),(nxvls*nwvls),0).
loop k5 = 1 to nrow(wprobval).
loop k6=1 to nwvls.
compute temp(k5, (((k4-1)*nwvls)+k6))=wprobval(k5,k6).
end loop.
end loop.
compute indprova={temp,tihsz,tihswz}.
else.
compute indprova={wprobval,tihsz,tihswz}.
end if.
end if.
do if (indmod(i,1)=2).
compute tihsw=make(nrow(zprobval),(nwvls*nxvls),0).
compute tihswz=make(nrow(zprobval),(nwvls*nzvls*nxvls),0).
do if (pathsz(1,colnumb)=1).
compute temp=make(nrow(zprobval),(nxvls*nzvls),0).
loop k5 = 1 to nrow(zprobval).
loop k6 =1 to nzvls.
compute temp(k5,(((k4-1)*nzvls)+k6))=zprobval(k5,k6).
end loop.
end loop.
compute indprova={tihsw,temp,tihswz}.
else.
compute indprova={tihsw,zprobval,tihswz}.
end if.
end if.
do if (indmod(i,1)=3).
compute indprova=make((nrow(wprobval)*nrow(zprobval)),((ncol(wprobval)*nxvls)+ (ncol(zprobval)*nxvls)+(nwvls*nzvls*nxvls)),0).
compute cntemp=1.
loop k7=1 to nrow(wprobval).
loop k8 =1 to nrow(zprobval).
compute temp=wprobval(k7,:)*focaddon(1,k4).
compute indprova(cntemp,(((k4-1)*nwvls)+1):(k4*(nwvls)))=temp.
compute temp=zprobval(k8,:)*focaddon(1,k4).
compute indprova(cntemp, ((((k4-1)*nzvls)+1)+(nxvls*nwvls)) : ((((k4-1)*nzvls)+1)+(nxvls*nwvls)+(nzvls-1)))=temp.
compute cntemp=cntemp+1.
end loop.
end loop.
do if (pathsz(1,colnumb)=0).
compute temp=make(nrow(indprova),(ncol(zprobval)*nxvls),0).
compute indprova(:,((ncol(wprobval)*nxvls)+1):((ncol(wprobval)+ncol(zprobval))* nxvls))=temp.
end if.
do if (pathsw(1,colnumb)=0).
compute temp=make(nrow(indprova),(ncol(wprobval)*nxvls),0).
compute indprova(:,1:(ncol(wprobval)*nxvls))=temp.
end if.
do if (pathswz(1,colnumb)=1).
compute cntemp=(ncol(wprobval)*nxvls)+(ncol(zprobval)*nxvls)+((k4-1)* ncol(wprobval)*ncol(zprobval))+1.
loop k7=1 to ncol(wprobval).
loop k8=1 to ncol(zprobval).
compute indprova(:,cntemp)=indprova(:,((ncol(wprobval)*(k4-1))+k7))&* indprova(:,((((k4-1)*ncol(zprobval))+k8)+(nxvls*ncol(wprobval)))).
compute cntemp=cntemp+1.
end loop.
end loop.
end if.
end if.
end if.
do if (k2 > 1).
compute focbb=tihs(1,1).
compute focbb=bootres(:,focbb).
do if (indmmm(i,1)>0).
compute imm=focbb(:,1).
compute condbb=imm.
end if.
compute focaddon=1.
compute cnt=cnt+1.
compute placeh=1.
do if (indmod(i,1)=1).
compute tihsz=make(nrow(wprobval),nzvls,0).
compute tihswz=make(nrow(wprobval),(nwvls*nzvls),0).
compute indprova={wprobval,tihsz,tihswz}.
end if.
do if (indmod(i,1)=2).
compute tihsw=make(nrow(zprobval),nwvls,0).
compute tihswz=make(nrow(zprobval),(nwvls*nzvls),0).
compute indprova={tihsw,zprobval,tihswz}.
end if.
do if (indmod(i,1)=3).
compute indprova=make((nrow(wprobval)*nrow(zprobval)),((ncol(wprobval)+ ncol(zprobval))+(nwvls*nzvls)),0).
compute cntemp=1.
loop k7=1 to nrow(wprobval).
loop k8 =1 to nrow(zprobval).
compute indprova(cntemp,1:(ncol(wprobval)+ncol(zprobval)))={wprobval(k7,:), zprobval(k8,:)}.
compute cntemp=cntemp+1.
end loop.
end loop.
do if (pathsz(1,colnumb)=0).
compute temp=make(nrow(indprova),ncol(zprobval),0).
compute indprova(:,(ncol(wprobval)+1):(ncol(wprobval)+ncol(zprobval)))=temp.
end if.
do if (pathsw(1,colnumb)=0).
compute temp=make(nrow(indprova),ncol(wprobval),0).
compute indprova(:,1:ncol(wprobval))=temp.
end if.
do if (pathswz(1,colnumb)=1).
compute cntemp=ncol(wprobval)+ncol(zprobval)+1.
loop k7=1 to ncol(wprobval).
loop k8=1 to ncol(zprobval).
compute indprova(:,cntemp)=indprova(:,k7)&*indprova(:,(ncol(wprobval)+k8)).
compute cntemp=cntemp+1.
end loop.
end loop.
end if.
end if.
end if.
do if (pathsw(1,colnumb)) = 1.
compute wbb=tihs(cnt:(cnt+(placeh*nwvls)-1),1).
compute wbb=bootres(:,wbb).
compute immlbs2=wcatlab(1:nwvls,1).
do if (zfirst=0).
compute wfirst=1.
end if.
do if (indmmm(i,1)=1 or indmmm(i,1)=31 or indmmm(i,1)=51).
compute imm=wbb(:,1).
loop k7 = 1 to nwvls.
compute imm={imm,wbb(:,(((k4-1)*nwvls*(k2=1))+k7))}.
end loop.
compute imm=imm(:,2:ncol(imm)).
end if.
do if (indmmm(i,1)=41 or indmmm(i,1)=51).
compute condbb=make(nrow(bootres),1,0).
loop k7 = 1 to nwvls.
compute condbb={condbb,wbb(:,(((k4-1)*nwvls*(k2=1))+k7))}.
end loop.
compute condbb=condbb(:,2:ncol(condbb)).
end if.
compute cnt=cnt+(placeh*nwvls).
end if.
do if (pathsz(1,colnumb)) = 1.
compute zbb=tihs(cnt:(cnt+(placeh*nzvls)-1),1).
compute zbb=bootres(:,zbb).
do if (wfirst=0).
compute zfirst=1.
end if.
do if (indmmm(i,1) <> 31).
compute immlbs2=zcatlab(1:nzvls,1).
end if.
do if (indmmm(i,1)=2 or indmmm(i,1)=31 or indmmm(i,1)=51).
do if (indmmm(i,1)=2).
compute imm=zbb(:,1).
end if.
loop k7 = 1 to nzvls.
compute imm={imm,zbb(:,(((k4-1)*nzvls*(k2=1))+k7))}.
end loop.
do if (indmmm(i,1)=2 or indmmm(i,1)=51).
compute imm=imm(:,2:ncol(imm)).
do if (indmmm(i,1)=51).
compute condbb={condbb,imm}.
end if.
end if.
end if.
compute cnt=cnt+(placeh*nzvls).
end if.
do if (pathswz(1,colnumb)) = 1.
compute wzbb=tihs(cnt:(cnt+(placeh*nwvls*nzvls)-1),1).
compute wzbb=bootres(:,wzbb).
do if (indmmm(i,1)=41).
compute imm=wzbb(:,1).
loop k7=1 to nwvls*nzvls.
compute imm={imm,wzbb(:,(((k4-1)*nzvls*nwvls*(k2=1))+k7))}.
end loop.
end if.
do if (indmmm(i,1)=41).
compute imm=imm(:,2:ncol(imm)).
compute condbb={condbb,imm(:,(ncol(imm)-(nwvls*nzvls)+1):ncol(imm))}.
end if.
compute cnt=cnt+(placeh*nzvls*nwvls).
end if.
compute indprobe={focaddon,indprova(k1,:)}.
compute tucker={focbb,wbb,zbb,wzbb}.
loop k3=1 to ncol(indprobe).
compute tucker(:,k3)=tucker(:,k3)*indprobe(1,k3).
end loop.
compute tucker2=tucker2&*rsum(tucker).
do if (indmmm(i,1) = 1 or indmmm(i,1)=2 or indmmm(i,1)=31 or indmmm(i,1)=41 or indmmm(i,1)=51).
do if (immset=1).
do if (ncol(imm2)=1 and ncol(imm) = 1).
compute imm2=imm2&*imm.
end if.
do if (indmmm(i,1)=41 or indmmm(i,1)=51).
do if ((ncol(condbb2) > 1) and (ncol(condbb)>1)).
compute condbb2t=make(nrow(condbb2),(ncol(condbb2)*ncol(condbb)),-999999).
compute k9=1.
do if (wfirst=1).
loop k7=1 to ncol(condbb2).
loop k8 = 1 to ncol(condbb).
compute condbb2t(:,k9)=condbb2(:,k7)&*condbb(:,k8).
compute k9=k9+1.
end loop.
end loop.
end if.
do if (zfirst=1).
loop k7=1 to ncol(condbb).
loop k8 = 1 to ncol(condbb2).
compute condbb2t(:,k9)=condbb(:,k7)&*condbb2(:,k8).
compute k9=k9+1.
end loop.
end loop.
end if.
compute condbb2=condbb2t.
release condbb2t.
end if.
do if ((ncol(condbb2) > 1) and (ncol(condbb)=1)).
loop k7 = 1 to ncol(condbb2).
compute condbb2(:,k7)=condbb2(:,k7)&*condbb.
end loop.
end if.
do if ((ncol(condbb2) = 1) and (ncol(condbb)>1)).
loop k7 = 1 to ncol(condbb).
compute condbb(:,k7)=condbb2&*condbb(:,k7).
end loop.
compute condbb2=condbb.
end if.
end if.
do if (ncol(imm2) <> 1 and ncol(imm) <> 1).
compute imm2t=make(nrow(imm2),(ncol(imm2)*ncol(imm)),-999999).
compute k9=1.
do if (wfirst=1).
loop k7=1 to ncol(imm2).
loop k8 = 1 to ncol(imm).
compute imm2t(:,k9)=imm2(:,k7)&*imm(:,k8).
compute k9=k9+1.
end loop.
end loop.
end if.
do if (zfirst=1).
loop k7=1 to ncol(imm).
loop k8 = 1 to ncol(imm2).
compute imm2t(:,k9)=imm(:,k7)&*imm2(:,k8).
compute k9=k9+1.
end loop.
end loop.
end if.
compute imm2=imm2t.
release imm2t.
end if.
do if ((ncol(imm2) > 1) and (ncol(imm)=1)).
loop k7=1 to ncol(imm2).
compute imm2(:,k7)=imm2(:,k7)&*imm.
end loop.
end if.
do if ((ncol(imm2) = 1) and (ncol(imm) > 1)).
loop k7=1 to ncol(imm).
compute imm(:,k7)=imm2&*imm(:,k7).
end loop.
compute imm2=imm.
end if.
end if.
do if (immset=0).
compute imm2=imm.
do if (indmmm(i,1)=41 or indmmm(i,1)=51).
compute condbb2=condbb.
end if.
compute immset=1.
end if.
end if.
end loop.
compute indtemp=tucker2(1,1).
do if (indmmm(i,1)=12 or indmmm(i,1)=22).
compute imm3={imm3,tucker2}.
do if (k1=nrow(indmodva)).
compute imm3=imm3(:,2:ncol(imm3)).
compute immstop=ncol(imm3).
loop k8=2 to immstop.
do if (indmmmt(i,1)=1).
compute imm3={imm3,(imm3(:,k8)-imm3(:,1))}.
end if.
do if (indmmmt(i,1)=2).
compute imm3={imm3,(imm3(:,k8)-imm3(:,(k8-1)))}.
end if.
do if (indmmmt(i,1)=3).
compute imm3={imm3,((rsum(imm3(:,(k8:immstop)))/(immstop-k8+1))-imm3(:,(k8-1))) }.
end if.
do if (indmmmt(i,1)=4).
compute imm3={imm3,(imm3(:,k8)-(rsum(imm3(:,1:immstop))/immstop))}.
end if.
end loop.
do if (indmmmt(i,1)<5).
compute imm2=imm3(:,(immstop+1):ncol(imm3)).
release imm3.
end if.
end if.
end if.
do if (indmmm(i,1)>-1 and (contrast = 1 or contrast = 2 or contrast=3)).
compute imm4={imm4,tucker2}.
do if (k1=nrow(indmodva) and k1 > 1).
compute imm4=imm4(:,2:ncol(imm4)).
compute immstop=ncol(imm4).
compute condcont=make((immstop*(immstop-1)/2),6,-999).
loop k8 = 1 to (immstop-1).
loop k9 = (k8+1) to immstop.
do if (contrast=1).
compute imm4={imm4,(imm4(:,k9)-imm4(:,k8))}.
end if.
do if (contrast=2).
compute imm4={imm4,(abs(imm4(:,k9))-abs(imm4(:,k8)))}.
end if.
do if (contrast=3).
compute imm4={imm4,(imm4(:,k9)+imm4(:,k8))}.
end if.
compute condcont((ncol(imm4)-immstop),1)=imm4(1,k9).
compute condcont((ncol(imm4)-immstop),2)=imm4(1,k8).
end loop.
end loop.
compute imm4=imm4(:,(immstop+1):ncol(imm4)).
loop k8=1 to ncol(imm4).
compute condcont(k8,3)=imm4(1,k8).
do if (boot > 0).

.
compute temp = imm4(2:nrow(imm4),k8).
compute temp(GRADE( imm4(2:nrow(imm4),k8) )) = imm4(2:nrow(imm4),k8).
compute badlo = 0.
compute badhi = 0.
do if ( (imm4(1,k8)*bc)+(9999*(1-bc)) <> 9999).
compute pv=csum(temp < (imm4(1,k8)*bc)+(9999*(1-bc)) )/nrow(temp).
compute ppv = pv.
do if (pv > .5).
compute ppv = 1-pv.
end if.
compute y5=sqrt(-2*ln(ppv)).
compute xp=y5+((((y5*p4+p3)*y5+p2)*y5+p1)*y5+p0)/((((y5*q4+q3)*y5+q2)*y5+q1)*y5+q0).
do if (pv <= .5).
compute xp = -xp.
end if.
compute cilow=rnd(nrow(temp)*(cdfnorm(2*xp-xp2))).
compute cihigh=trunc(nrow(temp)*(cdfnorm(2*xp+(xp2))))+1.
do if (cilow < 1).
compute cilow = 1.
compute booterr=1.
compute badlo = 1.
end if.
do if (cihigh > nrow(temp)).
compute cihigh = boot.
compute booterr=1.
compute badhi = 1.
end if.
compute llcit=temp(cilow,1).
compute ulcit=temp(cihigh,1).
do if (badlo = 1 and llcit <> priorlo).
compute badend={badend, llcit}.
compute priorlo = llcit.
end if.
do if (badhi = 1 and ulcit <> priorhi).
compute badend={badend, ulcit}.
compute priorhi = ulcit.
end if.
end if.
do if ( (imm4(1,k8)*bc)+(9999*(1-bc)) = 9999).
compute llcit=temp(cilow,1).
compute ulcit=temp(cihigh,1).
end if.
compute bootse=t(sqrt((cssq(temp)-((csum(temp)&**2)/nrow(temp)))/(nrow(temp)-1)))
.
compute condcont(k8,4:6)={bootse,llcit,ulcit}.
end if.
end loop.
do if (boot=0).
compute condcont=condcont(:,1:3).
end if.
compute indcontr=1.
end if.
end if.
do if (xmint=1).
do if (k1=(k4+1)).
compute cftotfx(:,k4)=cftotfx(:,k4)+(tucker2*xscaling).
end if.
end if.
do if (boot > 0).
compute tucker2=tucker2*xscaling.

.
compute temp = tucker2(2:nrow(tucker2),1).
compute temp(GRADE( tucker2(2:nrow(tucker2),1) )) = tucker2(2:nrow(tucker2),1).
compute badlo = 0.
compute badhi = 0.
do if ( (tucker2(1,1)*bc)+(9999*(1-bc)) <> 9999).
compute pv=csum(temp < (tucker2(1,1)*bc)+(9999*(1-bc)) )/nrow(temp).
compute ppv = pv.
do if (pv > .5).
compute ppv = 1-pv.
end if.
compute y5=sqrt(-2*ln(ppv)).
compute xp=y5+((((y5*p4+p3)*y5+p2)*y5+p1)*y5+p0)/((((y5*q4+q3)*y5+q2)*y5+q1)*y5+q0).
do if (pv <= .5).
compute xp = -xp.
end if.
compute cilow=rnd(nrow(temp)*(cdfnorm(2*xp-xp2))).
compute cihigh=trunc(nrow(temp)*(cdfnorm(2*xp+(xp2))))+1.
do if (cilow < 1).
compute cilow = 1.
compute booterr=1.
compute badlo = 1.
end if.
do if (cihigh > nrow(temp)).
compute cihigh = boot.
compute booterr=1.
compute badhi = 1.
end if.
compute llcit=temp(cilow,1).
compute ulcit=temp(cihigh,1).
do if (badlo = 1 and llcit <> priorlo).
compute badend={badend, llcit}.
compute priorlo = llcit.
end if.
do if (badhi = 1 and ulcit <> priorhi).
compute badend={badend, ulcit}.
compute priorhi = ulcit.
end if.
end if.
do if ( (tucker2(1,1)*bc)+(9999*(1-bc)) = 9999).
compute llcit=temp(cilow,1).
compute ulcit=temp(cihigh,1).
end if.
compute bootse=t(sqrt((cssq(temp)-((csum(temp)&**2)/nrow(temp)))/(nrow(temp)-1)))
.
compute indtemp={indtemp*xscaling, bootse,llcit,ulcit}.
end if.
do if (boot=0).
compute indtemp=indtemp*xscaling.
end if.
compute condres(k1,(ncol(indmodva)+1):ncol(condres))=indtemp.
end loop.
do if (xmint=0).
compute resultm2=make(nrow(condres),maxresm,99999).
compute resultm2(1:nrow(condres),1:ncol(condres))=condres.
compute resultm={resultm;resultm2}.
end if.
compute condlbs={condlbs,'Effect'}.
do if (boot > 0).
compute condlbs={condlbs,'BootSE', 'BootLLCI', 'BootULCI'}.
end if.
do if (xmint = 1).
do if (k4 = 1).
compute natindfx=condres((2+(k4-1)),2:ncol(condres)).
end if.
do if (k4 > 1).
compute natindfx={natindfx;condres((2+(k4-1)),2:ncol(condres))}.
end if.
do if (k4=nxvls).
compute resultm2=make(nrow(natindfx),maxresm,99999).
compute resultm2(1:nrow(natindfx),1:ncol(natindfx))=natindfx.
compute resultm={resultm;resultm2}.
end if.
end if.
do if (outscree=1 and xmint=0).
do if (nxvls=1).
print condres/title=' '/cnames=condlbs/format= F10.4 /space=0.
else.
compute condrlb=make(nrow(condres),1,xcatlab(k4,1)).
print condres/title=' '/cnames=condlbs/rnames=condrlb/format= F10.4 /space=0.
end if.
end if.
do if (outscree=1 and xmint=1 and k4=nxvls).
do if (nxvls=1).
print natindfx/title=' '/clabels='Effect', 'BootSE', 'BootLLCI', 'BootULCI'/format= F10.4 /space=0.
else.
compute condrlb=make(nrow(condres),1,xcatlab(k4,1)).
print natindfx/title=' '/clabels='Effect', 'BootSE', 'BootLLCI', 'BootULCI'/rnames=direffl2/format= F10.4 /space=0.
end if.
end if.
compute dichadj=0.
compute immcat=0.
do if ((indmmm(i,1)>0) and (xmint=0)).
do if (indmmm(i,1)=1 or indmmm(i,1)=12 or indmmm(i,1)=31).
do if (wdich=1 and mcw=0).
do if (indmmm(i,1) <> 12).
compute imm2(:,1)=imm2(:,1)*(wmax-wmin).
end if.
do if (indmmm(i,1) <> 31).
compute dichadj=1.
end if.
end if.
do if ((mcw = 1 or mcw = 2) and indmmm(i,1) <> 31)).
compute immcat=1.
end if.
end if.
do if (indmmm(i,1)=2 or indmmm(i,1)=22 or indmmm(i,1)=31).
do if (zdich=1 and mcz=0).
do if (indmmm(i,1) = 31).
compute imm2(:,(nwvls+1):ncol(imm2))=imm2(:,(nwvls+1):ncol(imm2))*(zmax-zmin).
end if.
do if (indmmm(i,1) = 2).
compute imm2(:,1)=imm2(:,1)*(zmax-zmin).
end if.
do if (indmmm(i,1) <> 31).
compute dichadj=1.
end if.
end if.
do if ((mcz = 1 or mcz = 2) and indmmm(i,1) <> 31)).
compute immcat=1.
end if.
end if.
compute immtemp2=t(imm2(1,:)).
compute immtemp=immtemp2.
compute immlbs={'Index'}.
do if (boot > 0).
compute immtemp=make(1,3,0).
loop k7=1 to ncol(imm2).

.
compute temp = imm2(2:nrow(imm2),k7).
compute temp(GRADE( imm2(2:nrow(imm2),k7) )) = imm2(2:nrow(imm2),k7).
compute badlo = 0.
compute badhi = 0.
do if ( (imm2(1,k7)*bc)+(9999*(1-bc)) <> 9999).
compute pv=csum(temp < (imm2(1,k7)*bc)+(9999*(1-bc)) )/nrow(temp).
compute ppv = pv.
do if (pv > .5).
compute ppv = 1-pv.
end if.
compute y5=sqrt(-2*ln(ppv)).
compute xp=y5+((((y5*p4+p3)*y5+p2)*y5+p1)*y5+p0)/((((y5*q4+q3)*y5+q2)*y5+q1)*y5+q0).
do if (pv <= .5).
compute xp = -xp.
end if.
compute cilow=rnd(nrow(temp)*(cdfnorm(2*xp-xp2))).
compute cihigh=trunc(nrow(temp)*(cdfnorm(2*xp+(xp2))))+1.
do if (cilow < 1).
compute cilow = 1.
compute booterr=1.
compute badlo = 1.
end if.
do if (cihigh > nrow(temp)).
compute cihigh = boot.
compute booterr=1.
compute badhi = 1.
end if.
compute llcit=temp(cilow,1).
compute ulcit=temp(cihigh,1).
do if (badlo = 1 and llcit <> priorlo).
compute badend={badend, llcit}.
compute priorlo = llcit.
end if.
do if (badhi = 1 and ulcit <> priorhi).
compute badend={badend, ulcit}.
compute priorhi = ulcit.
end if.
end if.
do if ( (imm2(1,k7)*bc)+(9999*(1-bc)) = 9999).
compute llcit=temp(cilow,1).
compute ulcit=temp(cihigh,1).
end if.
compute bootse=t(sqrt((cssq(temp)-((csum(temp)&**2)/nrow(temp)))/(nrow(temp)-1)))
.
compute temp={bootse,llcit,ulcit}.
compute immtemp={immtemp;temp}.
end loop.
compute immtemp=immtemp(2:nrow(immtemp),:).
compute immtemp={immtemp2,immtemp}.
compute immlbs={immlbs,'BootSE', 'BootLLCI', 'BootULCI'}.
end if.
compute resultm2=make(nrow(immtemp),maxresm,99999).
compute resultm2(1:nrow(immtemp),1:ncol(immtemp))=immtemp.
compute resultm={resultm;resultm2}.
do if (dichadj=0 and immcat=0 and indmmmt(i,1)<>5 and indmmm(i,1) < 100 ).
do if (outscree=1).
do if (indmmm(i,1) < 30).
print immtemp/title='      Index of moderated mediation:'/cnames=immlbs/rnames=immlbs2/format= F10.4.
end if.
do if (indmmm(i,1) = 31).
compute immlbs2={immlbs2;zcatlab(1:nzvls,1)}.
print immtemp/title='      Indices of partial moderated mediation:'/cnames=immlbs/rnames=immlbs2/format= F10.4.
end if.
end if.
do if ((nzvls = 1) and (nwvls = 1)).
do if (indmmm(i,1) = 41 or indmmm(i,1)=51).
loop k7=1 to nwvls.
compute immlbs2=zcatlab(1:nzvls,1).
compute immtemp2=immtemp((((k7-1)*nzvls)+1):(((k7-1)*nzvls)+nzvls),:).
do if (outscree=1).
do if (nwvls > 1).
compute primodv={'        ', wcatlab(k7,1)}.
print primodv/title='      Primary moderator:'/format=A8.
end if.
do if (nzvls=1).
print immtemp2/title='      Index of moderated moderated mediation'/cnames=immlbs/format= F10.4.
else.
print immtemp2/title='      Indices of moderated moderated mediation'/cnames=immlbs/rnames=immlbs2/format= F10.4.
end if.
end if.
compute cmmtemp=make(nrow(zprobval),4,0).
loop k8=1 to nrow(zprobval).
compute condbb3=condbb2(:,((nwvls+1)+((k7-1)*nzvls)):((nwvls+1)+((k7-1)*nzvls)+ (nzvls-1))).
do if (ncol(zprobval) > 1).
compute condbb3=condbb3*mdiag(zprobval(k8,:)).
else.
compute condbb3=condbb3&*zprobval(k8,:).
end if.
compute condbb3={condbb2(:,k7),condbb3}.
compute icmm=rsum(condbb3).
compute cmmtemp(k8,1)=icmm(1,1).
do if (boot > 0).

.
compute temp = icmm(2:nrow(icmm),1).
compute temp(GRADE( icmm(2:nrow(icmm),1) )) = icmm(2:nrow(icmm),1).
compute badlo = 0.
compute badhi = 0.
do if ( (icmm(1,1)*bc)+(9999*(1-bc)) <> 9999).
compute pv=csum(temp < (icmm(1,1)*bc)+(9999*(1-bc)) )/nrow(temp).
compute ppv = pv.
do if (pv > .5).
compute ppv = 1-pv.
end if.
compute y5=sqrt(-2*ln(ppv)).
compute xp=y5+((((y5*p4+p3)*y5+p2)*y5+p1)*y5+p0)/((((y5*q4+q3)*y5+q2)*y5+q1)*y5+q0).
do if (pv <= .5).
compute xp = -xp.
end if.
compute cilow=rnd(nrow(temp)*(cdfnorm(2*xp-xp2))).
compute cihigh=trunc(nrow(temp)*(cdfnorm(2*xp+(xp2))))+1.
do if (cilow < 1).
compute cilow = 1.
compute booterr=1.
compute badlo = 1.
end if.
do if (cihigh > nrow(temp)).
compute cihigh = boot.
compute booterr=1.
compute badhi = 1.
end if.
compute llcit=temp(cilow,1).
compute ulcit=temp(cihigh,1).
do if (badlo = 1 and llcit <> priorlo).
compute badend={badend, llcit}.
compute priorlo = llcit.
end if.
do if (badhi = 1 and ulcit <> priorhi).
compute badend={badend, ulcit}.
compute priorhi = ulcit.
end if.
end if.
do if ( (icmm(1,1)*bc)+(9999*(1-bc)) = 9999).
compute llcit=temp(cilow,1).
compute ulcit=temp(cihigh,1).
end if.
compute bootse=t(sqrt((cssq(temp)-((csum(temp)&**2)/nrow(temp)))/(nrow(temp)-1)))
.
compute cmmtemp(k8,2:4)={bootse,llcit,ulcit}.
end if.
end loop.
compute cmmtemp={zmodvals,cmmtemp}.
do if (boot=0).
compute cmmtemp=cmmtemp(:,1:2).
end if.
compute cmmlbs={znames,immlbs}.
compute resultm2=make(nrow(cmmtemp),maxresm,99999).
compute resultm2(1:nrow(cmmtemp),1:ncol(cmmtemp))=cmmtemp.
compute resultm={resultm;resultm2}.
do if (outscree=1).
print cmmtemp/title='      Indices of conditional moderated mediation by W'/cnames=cmmlbs/format= F10.4.
end if.
end loop.
end if.
end if.
end if.
do if (dichadj=1 or immcat=1 and indmmm(i,1) < 30).
do if (outscree=1).
print immtemp/title='Index of moderated mediation (difference between conditional indirect effects):'/cnames=immlbs/rnames=immlbs2/format= F10.4.
end if.
end if.
end if.
do if (indcontr=1).
compute resultm2=make(nrow(condcont),maxresm,99999).
compute resultm2(1:nrow(condcont),1:ncol(condcont))=condcont.
compute resultm={resultm;resultm2}.
compute condctlb={'Effect1','Effect2','Contrast','BootSE', 'BootLLCI','BootULCI'}.
do if (outscree=1).
print condcont/title=' Pairwise contrasts between conditional indirect effects (Effect1 minus Effect2)'/cnames=condctlb/format= F10.4.
end if.
end if.
end loop.
end if.
end loop.
do if (alttotal=1).
compute altcnms='Effect'.
compute alttotfx=t(cftotfx(1,:))+obnatdfx.
do if (boot > 0).
compute alttotfx=make(ncol(cftotfx),4,0).
compute alttotfx(:,1)=t(cftotfx(1,:))+obnatdfx.
compute natdirbt(1,:)=t(obnatdfx).
compute cftotfx=cftotfx+natdirbt.
loop cec=1 to ncol(cftotfx).

.
compute temp = cftotfx(2:nrow(cftotfx),cec).
compute temp(GRADE( cftotfx(2:nrow(cftotfx),cec) )) = cftotfx(2:nrow(cftotfx),cec).
compute badlo = 0.
compute badhi = 0.
do if ( (cftotfx(1,cec)*bc)+(9999*(1-bc)) <> 9999).
compute pv=csum(temp < (cftotfx(1,cec)*bc)+(9999*(1-bc)) )/nrow(temp).
compute ppv = pv.
do if (pv > .5).
compute ppv = 1-pv.
end if.
compute y5=sqrt(-2*ln(ppv)).
compute xp=y5+((((y5*p4+p3)*y5+p2)*y5+p1)*y5+p0)/((((y5*q4+q3)*y5+q2)*y5+q1)*y5+q0).
do if (pv <= .5).
compute xp = -xp.
end if.
compute cilow=rnd(nrow(temp)*(cdfnorm(2*xp-xp2))).
compute cihigh=trunc(nrow(temp)*(cdfnorm(2*xp+(xp2))))+1.
do if (cilow < 1).
compute cilow = 1.
compute booterr=1.
compute badlo = 1.
end if.
do if (cihigh > nrow(temp)).
compute cihigh = boot.
compute booterr=1.
compute badhi = 1.
end if.
compute llcit=temp(cilow,1).
compute ulcit=temp(cihigh,1).
do if (badlo = 1 and llcit <> priorlo).
compute badend={badend, llcit}.
compute priorlo = llcit.
end if.
do if (badhi = 1 and ulcit <> priorhi).
compute badend={badend, ulcit}.
compute priorhi = ulcit.
end if.
end if.
do if ( (cftotfx(1,cec)*bc)+(9999*(1-bc)) = 9999).
compute llcit=temp(cilow,1).
compute ulcit=temp(cihigh,1).
end if.
compute bootse=t(sqrt((cssq(temp)-((csum(temp)&**2)/nrow(temp)))/(nrow(temp)-1)))
.
compute alttotfx(cec,2:4)={bootse,llcit,ulcit}.
end loop.
compute altcnms={altcnms,'BootSE', 'BootLLCI','BootULCI'}.
end if.
compute resultm2=make(nrow(alttotfx),maxresm,99999).
compute resultm2(1:nrow(alttotfx),1:ncol(alttotfx))=alttotfx.
compute resultm={resultm;resultm2}.
do if (outscree=1).
print/title='----------'.
do if (nxvls > 1).
print alttotfx/title='Relative total effects of X on Y (sum of direct and indirect effects)'/cnames=altcnms/rnames=direffl2/format= F10.4.
end if.
do if (nxvls = 1).
print alttotfx/title='Total effect of X on Y (sum of direct and indirect effects)'/cnames=altcnms/format= F10.4.
end if.
end if.
end if.
end if.
end if
.
do if (criterr=0 and (saveest = 1 or saveboot=1)).
compute coeffsav=coeffmat(2:nrow(coeffmat),:).
compute conseq=conseq(2:nrow(conseq),1).
compute dfmat=dfmat(2:nrow(dfmat),1).
compute dfmat=dfmat.
compute saven={'conseqnt','antecdnt','coeff','se','t','p','LLCI','ULCI','df'}.
do if (ydich=1).
compute saven={'conseqnt','antecdnt','coeff','se','t_or_Z','p','LLCI','ULCI','df'}.
end if.
do if (saveboot=1 and boot > 0 ).
compute colslab=make(300,1,' ').


compute colslab( 1 ,1)= 'COL1'.


compute colslab( 2 ,1)= 'COL2'.


compute colslab( 3 ,1)= 'COL3'.


compute colslab( 4 ,1)= 'COL4'.


compute colslab( 5 ,1)= 'COL5'.


compute colslab( 6 ,1)= 'COL6'.


compute colslab( 7 ,1)= 'COL7'.


compute colslab( 8 ,1)= 'COL8'.


compute colslab( 9 ,1)= 'COL9'.


compute colslab( 10 ,1)= 'COL10'.


compute colslab( 11 ,1)= 'COL11'.


compute colslab( 12 ,1)= 'COL12'.


compute colslab( 13 ,1)= 'COL13'.


compute colslab( 14 ,1)= 'COL14'.


compute colslab( 15 ,1)= 'COL15'.


compute colslab( 16 ,1)= 'COL16'.


compute colslab( 17 ,1)= 'COL17'.


compute colslab( 18 ,1)= 'COL18'.


compute colslab( 19 ,1)= 'COL19'.


compute colslab( 20 ,1)= 'COL20'.


compute colslab( 21 ,1)= 'COL21'.


compute colslab( 22 ,1)= 'COL22'.


compute colslab( 23 ,1)= 'COL23'.


compute colslab( 24 ,1)= 'COL24'.


compute colslab( 25 ,1)= 'COL25'.


compute colslab( 26 ,1)= 'COL26'.


compute colslab( 27 ,1)= 'COL27'.


compute colslab( 28 ,1)= 'COL28'.


compute colslab( 29 ,1)= 'COL29'.


compute colslab( 30 ,1)= 'COL30'.


compute colslab( 31 ,1)= 'COL31'.


compute colslab( 32 ,1)= 'COL32'.


compute colslab( 33 ,1)= 'COL33'.


compute colslab( 34 ,1)= 'COL34'.


compute colslab( 35 ,1)= 'COL35'.


compute colslab( 36 ,1)= 'COL36'.


compute colslab( 37 ,1)= 'COL37'.


compute colslab( 38 ,1)= 'COL38'.


compute colslab( 39 ,1)= 'COL39'.


compute colslab( 40 ,1)= 'COL40'.


compute colslab( 41 ,1)= 'COL41'.


compute colslab( 42 ,1)= 'COL42'.


compute colslab( 43 ,1)= 'COL43'.


compute colslab( 44 ,1)= 'COL44'.


compute colslab( 45 ,1)= 'COL45'.


compute colslab( 46 ,1)= 'COL46'.


compute colslab( 47 ,1)= 'COL47'.


compute colslab( 48 ,1)= 'COL48'.


compute colslab( 49 ,1)= 'COL49'.


compute colslab( 50 ,1)= 'COL50'.


compute colslab( 51 ,1)= 'COL51'.


compute colslab( 52 ,1)= 'COL52'.


compute colslab( 53 ,1)= 'COL53'.


compute colslab( 54 ,1)= 'COL54'.


compute colslab( 55 ,1)= 'COL55'.


compute colslab( 56 ,1)= 'COL56'.


compute colslab( 57 ,1)= 'COL57'.


compute colslab( 58 ,1)= 'COL58'.


compute colslab( 59 ,1)= 'COL59'.


compute colslab( 60 ,1)= 'COL60'.


compute colslab( 61 ,1)= 'COL61'.


compute colslab( 62 ,1)= 'COL62'.


compute colslab( 63 ,1)= 'COL63'.


compute colslab( 64 ,1)= 'COL64'.


compute colslab( 65 ,1)= 'COL65'.


compute colslab( 66 ,1)= 'COL66'.


compute colslab( 67 ,1)= 'COL67'.


compute colslab( 68 ,1)= 'COL68'.


compute colslab( 69 ,1)= 'COL69'.


compute colslab( 70 ,1)= 'COL70'.


compute colslab( 71 ,1)= 'COL71'.


compute colslab( 72 ,1)= 'COL72'.


compute colslab( 73 ,1)= 'COL73'.


compute colslab( 74 ,1)= 'COL74'.


compute colslab( 75 ,1)= 'COL75'.


compute colslab( 76 ,1)= 'COL76'.


compute colslab( 77 ,1)= 'COL77'.


compute colslab( 78 ,1)= 'COL78'.


compute colslab( 79 ,1)= 'COL79'.


compute colslab( 80 ,1)= 'COL80'.


compute colslab( 81 ,1)= 'COL81'.


compute colslab( 82 ,1)= 'COL82'.


compute colslab( 83 ,1)= 'COL83'.


compute colslab( 84 ,1)= 'COL84'.


compute colslab( 85 ,1)= 'COL85'.


compute colslab( 86 ,1)= 'COL86'.


compute colslab( 87 ,1)= 'COL87'.


compute colslab( 88 ,1)= 'COL88'.


compute colslab( 89 ,1)= 'COL89'.


compute colslab( 90 ,1)= 'COL90'.


compute colslab( 91 ,1)= 'COL91'.


compute colslab( 92 ,1)= 'COL92'.


compute colslab( 93 ,1)= 'COL93'.


compute colslab( 94 ,1)= 'COL94'.


compute colslab( 95 ,1)= 'COL95'.


compute colslab( 96 ,1)= 'COL96'.


compute colslab( 97 ,1)= 'COL97'.


compute colslab( 98 ,1)= 'COL98'.


compute colslab( 99 ,1)= 'COL99'.


compute colslab( 100 ,1)= 'COL100'.


compute colslab( 101 ,1)= 'COL101'.


compute colslab( 102 ,1)= 'COL102'.


compute colslab( 103 ,1)= 'COL103'.


compute colslab( 104 ,1)= 'COL104'.


compute colslab( 105 ,1)= 'COL105'.


compute colslab( 106 ,1)= 'COL106'.


compute colslab( 107 ,1)= 'COL107'.


compute colslab( 108 ,1)= 'COL108'.


compute colslab( 109 ,1)= 'COL109'.


compute colslab( 110 ,1)= 'COL110'.


compute colslab( 111 ,1)= 'COL111'.


compute colslab( 112 ,1)= 'COL112'.


compute colslab( 113 ,1)= 'COL113'.


compute colslab( 114 ,1)= 'COL114'.


compute colslab( 115 ,1)= 'COL115'.


compute colslab( 116 ,1)= 'COL116'.


compute colslab( 117 ,1)= 'COL117'.


compute colslab( 118 ,1)= 'COL118'.


compute colslab( 119 ,1)= 'COL119'.


compute colslab( 120 ,1)= 'COL120'.


compute colslab( 121 ,1)= 'COL121'.


compute colslab( 122 ,1)= 'COL122'.


compute colslab( 123 ,1)= 'COL123'.


compute colslab( 124 ,1)= 'COL124'.


compute colslab( 125 ,1)= 'COL125'.


compute colslab( 126 ,1)= 'COL126'.


compute colslab( 127 ,1)= 'COL127'.


compute colslab( 128 ,1)= 'COL128'.


compute colslab( 129 ,1)= 'COL129'.


compute colslab( 130 ,1)= 'COL130'.


compute colslab( 131 ,1)= 'COL131'.


compute colslab( 132 ,1)= 'COL132'.


compute colslab( 133 ,1)= 'COL133'.


compute colslab( 134 ,1)= 'COL134'.


compute colslab( 135 ,1)= 'COL135'.


compute colslab( 136 ,1)= 'COL136'.


compute colslab( 137 ,1)= 'COL137'.


compute colslab( 138 ,1)= 'COL138'.


compute colslab( 139 ,1)= 'COL139'.


compute colslab( 140 ,1)= 'COL140'.


compute colslab( 141 ,1)= 'COL141'.


compute colslab( 142 ,1)= 'COL142'.


compute colslab( 143 ,1)= 'COL143'.


compute colslab( 144 ,1)= 'COL144'.


compute colslab( 145 ,1)= 'COL145'.


compute colslab( 146 ,1)= 'COL146'.


compute colslab( 147 ,1)= 'COL147'.


compute colslab( 148 ,1)= 'COL148'.


compute colslab( 149 ,1)= 'COL149'.


compute colslab( 150 ,1)= 'COL150'.


compute colslab( 151 ,1)= 'COL151'.


compute colslab( 152 ,1)= 'COL152'.


compute colslab( 153 ,1)= 'COL153'.


compute colslab( 154 ,1)= 'COL154'.


compute colslab( 155 ,1)= 'COL155'.


compute colslab( 156 ,1)= 'COL156'.


compute colslab( 157 ,1)= 'COL157'.


compute colslab( 158 ,1)= 'COL158'.


compute colslab( 159 ,1)= 'COL159'.


compute colslab( 160 ,1)= 'COL160'.


compute colslab( 161 ,1)= 'COL161'.


compute colslab( 162 ,1)= 'COL162'.


compute colslab( 163 ,1)= 'COL163'.


compute colslab( 164 ,1)= 'COL164'.


compute colslab( 165 ,1)= 'COL165'.


compute colslab( 166 ,1)= 'COL166'.


compute colslab( 167 ,1)= 'COL167'.


compute colslab( 168 ,1)= 'COL168'.


compute colslab( 169 ,1)= 'COL169'.


compute colslab( 170 ,1)= 'COL170'.


compute colslab( 171 ,1)= 'COL171'.


compute colslab( 172 ,1)= 'COL172'.


compute colslab( 173 ,1)= 'COL173'.


compute colslab( 174 ,1)= 'COL174'.


compute colslab( 175 ,1)= 'COL175'.


compute colslab( 176 ,1)= 'COL176'.


compute colslab( 177 ,1)= 'COL177'.


compute colslab( 178 ,1)= 'COL178'.


compute colslab( 179 ,1)= 'COL179'.


compute colslab( 180 ,1)= 'COL180'.


compute colslab( 181 ,1)= 'COL181'.


compute colslab( 182 ,1)= 'COL182'.


compute colslab( 183 ,1)= 'COL183'.


compute colslab( 184 ,1)= 'COL184'.


compute colslab( 185 ,1)= 'COL185'.


compute colslab( 186 ,1)= 'COL186'.


compute colslab( 187 ,1)= 'COL187'.


compute colslab( 188 ,1)= 'COL188'.


compute colslab( 189 ,1)= 'COL189'.


compute colslab( 190 ,1)= 'COL190'.


compute colslab( 191 ,1)= 'COL191'.


compute colslab( 192 ,1)= 'COL192'.


compute colslab( 193 ,1)= 'COL193'.


compute colslab( 194 ,1)= 'COL194'.


compute colslab( 195 ,1)= 'COL195'.


compute colslab( 196 ,1)= 'COL196'.


compute colslab( 197 ,1)= 'COL197'.


compute colslab( 198 ,1)= 'COL198'.


compute colslab( 199 ,1)= 'COL199'.


compute colslab( 200 ,1)= 'COL200'.


compute colslab( 201 ,1)= 'COL201'.


compute colslab( 202 ,1)= 'COL202'.


compute colslab( 203 ,1)= 'COL203'.


compute colslab( 204 ,1)= 'COL204'.


compute colslab( 205 ,1)= 'COL205'.


compute colslab( 206 ,1)= 'COL206'.


compute colslab( 207 ,1)= 'COL207'.


compute colslab( 208 ,1)= 'COL208'.


compute colslab( 209 ,1)= 'COL209'.


compute colslab( 210 ,1)= 'COL210'.


compute colslab( 211 ,1)= 'COL211'.


compute colslab( 212 ,1)= 'COL212'.


compute colslab( 213 ,1)= 'COL213'.


compute colslab( 214 ,1)= 'COL214'.


compute colslab( 215 ,1)= 'COL215'.


compute colslab( 216 ,1)= 'COL216'.


compute colslab( 217 ,1)= 'COL217'.


compute colslab( 218 ,1)= 'COL218'.


compute colslab( 219 ,1)= 'COL219'.


compute colslab( 220 ,1)= 'COL220'.


compute colslab( 221 ,1)= 'COL221'.


compute colslab( 222 ,1)= 'COL222'.


compute colslab( 223 ,1)= 'COL223'.


compute colslab( 224 ,1)= 'COL224'.


compute colslab( 225 ,1)= 'COL225'.


compute colslab( 226 ,1)= 'COL226'.


compute colslab( 227 ,1)= 'COL227'.


compute colslab( 228 ,1)= 'COL228'.


compute colslab( 229 ,1)= 'COL229'.


compute colslab( 230 ,1)= 'COL230'.


compute colslab( 231 ,1)= 'COL231'.


compute colslab( 232 ,1)= 'COL232'.


compute colslab( 233 ,1)= 'COL233'.


compute colslab( 234 ,1)= 'COL234'.


compute colslab( 235 ,1)= 'COL235'.


compute colslab( 236 ,1)= 'COL236'.


compute colslab( 237 ,1)= 'COL237'.


compute colslab( 238 ,1)= 'COL238'.


compute colslab( 239 ,1)= 'COL239'.


compute colslab( 240 ,1)= 'COL240'.


compute colslab( 241 ,1)= 'COL241'.


compute colslab( 242 ,1)= 'COL242'.


compute colslab( 243 ,1)= 'COL243'.


compute colslab( 244 ,1)= 'COL244'.


compute colslab( 245 ,1)= 'COL245'.


compute colslab( 246 ,1)= 'COL246'.


compute colslab( 247 ,1)= 'COL247'.


compute colslab( 248 ,1)= 'COL248'.


compute colslab( 249 ,1)= 'COL249'.


compute colslab( 250 ,1)= 'COL250'.


compute colslab( 251 ,1)= 'COL251'.


compute colslab( 252 ,1)= 'COL252'.


compute colslab( 253 ,1)= 'COL253'.


compute colslab( 254 ,1)= 'COL254'.


compute colslab( 255 ,1)= 'COL255'.


compute colslab( 256 ,1)= 'COL256'.


compute colslab( 257 ,1)= 'COL257'.


compute colslab( 258 ,1)= 'COL258'.


compute colslab( 259 ,1)= 'COL259'.


compute colslab( 260 ,1)= 'COL260'.


compute colslab( 261 ,1)= 'COL261'.


compute colslab( 262 ,1)= 'COL262'.


compute colslab( 263 ,1)= 'COL263'.


compute colslab( 264 ,1)= 'COL264'.


compute colslab( 265 ,1)= 'COL265'.


compute colslab( 266 ,1)= 'COL266'.


compute colslab( 267 ,1)= 'COL267'.


compute colslab( 268 ,1)= 'COL268'.


compute colslab( 269 ,1)= 'COL269'.


compute colslab( 270 ,1)= 'COL270'.


compute colslab( 271 ,1)= 'COL271'.


compute colslab( 272 ,1)= 'COL272'.


compute colslab( 273 ,1)= 'COL273'.


compute colslab( 274 ,1)= 'COL274'.


compute colslab( 275 ,1)= 'COL275'.


compute colslab( 276 ,1)= 'COL276'.


compute colslab( 277 ,1)= 'COL277'.


compute colslab( 278 ,1)= 'COL278'.


compute colslab( 279 ,1)= 'COL279'.


compute colslab( 280 ,1)= 'COL280'.


compute colslab( 281 ,1)= 'COL281'.


compute colslab( 282 ,1)= 'COL282'.


compute colslab( 283 ,1)= 'COL283'.


compute colslab( 284 ,1)= 'COL284'.


compute colslab( 285 ,1)= 'COL285'.


compute colslab( 286 ,1)= 'COL286'.


compute colslab( 287 ,1)= 'COL287'.


compute colslab( 288 ,1)= 'COL288'.


compute colslab( 289 ,1)= 'COL289'.


compute colslab( 290 ,1)= 'COL290'.


compute colslab( 291 ,1)= 'COL291'.


compute colslab( 292 ,1)= 'COL292'.


compute colslab( 293 ,1)= 'COL293'.


compute colslab( 294 ,1)= 'COL294'.


compute colslab( 295 ,1)= 'COL295'.


compute colslab( 296 ,1)= 'COL296'.


compute colslab( 297 ,1)= 'COL297'.


compute colslab( 298 ,1)= 'COL298'.


compute colslab( 299 ,1)= 'COL299'.


compute colslab( 300 ,1)= 'COL300'.

compute colslab=colslab(1:ncol(bootres),1).
compute colslab={colslab,conseq,vlabs}.
do if (outscree=1).
print/title = '**************************************************************************'.
print/title='Bootstrap estimates were saved to a file'/space=0.
print colslab/title='Map of column names to model coefficients:'/clabels=' ','Conseqnt','Antecdnt'/format=a8.
end if.
end if.
end if.
do if (criterr=0 and boot > 0 and modelbt=1).
compute labstart=1.
do if (outscree=1).
print/title = '*********** BOOTSTRAP RESULTS FOR REGRESSION MODEL PARAMETERS ************'.
end if.
loop iboot = 1 to (nms+nys).
do if (outscree=1).
print outnames(1,iboot)/title = 'OUTCOME VARIABLE:'/format = A8.
end if.
compute vlabsm=vlabs(labstart:(labstart+(nump(1,iboot)-1)),1).
compute outnmtmp=bootcim(labstart:(labstart+(nump(1,iboot)-1)),:).
compute resultm2=make(nrow(outnmtmp),maxresm,99999).
compute resultm2(1:nrow(outnmtmp),1:ncol(outnmtmp))=outnmtmp.
compute resultm={resultm;resultm2}.
do if (outscree=1).
print bootcim(labstart:(labstart+(nump(1,iboot)-1)),:)/title=' '/rnames=vlabsm/ clabels='Coeff' 'BootMean' 'BootSE' 'BootLLCI' 'BootULCI'/format= F10.4 /space=0.
end if.
compute labstart=labstart+nump(1,iboot).
do if (iboot < (nms+nys)).
do if (outscree=1).
print/title= '----------'.
end if.
end if.
end loop.
end if.
do if (criterr=0 and saveest=1).
compute resultm=resultm(2:nrow(resultm),:).
compute bocaj=(resultm=99999).
compute bocaj=csum(bocaj).
compute bocaj=1-(bocaj=nrow(resultm)).
compute j=1.
loop i = 1 to ncol(resultm).
do if (bocaj(1,i)=1).
compute j=j+1.
end if.
end loop.
compute resultm=resultm(:,1:(j-1)).
save resultm/outfile = *.
end if.
do if (criterr = 0 and matrices=1 and outscree=1).
print/title = '************************ MODEL DEFINITION MATRICES ************************'.
print/title = 'FROM variables are columns, TO variables are rows.'.
compute temp2=make(nrow(bcmat),ncol(bcmat),'0').
loop i = 2 to nrow(bcmat).
loop j = 1 to (ncol(bcmat)-1).
do if (bcmat(i,j)=1).
compute temp2(i,j)='1'.
end if.
do if (j >= i).
compute temp2(i,j)=' '.
end if.
end loop.
end loop.
compute temp2=temp2(2:nrow(bcmat),(1:(ncol(bcmat)-1))).
do if (nms > 0).
compute cmatlabs={xnames,mnames}.
compute rmatlabs={mnames,ynames}.
end if.
do if (nms = 0).
compute cmatlabs={xnames}.
compute rmatlabs={ynames}.
end if.
print temp2/title='BMATRIX: Paths freely estimated (1) and fixed to zero (0):'/cnames=cmatlabs/rnames=rmatlabs/format A3.
compute z=0.
do if (rsum(csum(wcmat))<>0).
compute temp2=make(nrow(wcmat),ncol(wcmat),'0').
loop i = 2 to nrow(wcmat).
loop j = 1 to (ncol(wcmat)-1).
do if (wcmat(i,j)=1).
compute temp2(i,j)='1'.
end if.
do if (j >= i).
compute temp2(i,j)=' '.
end if.
end loop.
end loop.
compute temp2=temp2(2:nrow(wcmat),(1:(ncol(wcmat)-1))).
print temp2/title='WMATRIX: Paths moderated (1) and not moderated (0) by W:'/cnames=cmatlabs/rnames=rmatlabs/format A3.
end if.
do if (rsum(csum(zcmat))<>0).
compute temp2=make(nrow(zcmat),ncol(zcmat),'0').
loop i = 2 to nrow(zcmat).
loop j = 1 to (ncol(zcmat)-1).
do if (zcmat(i,j)=1).
compute temp2(i,j)='1'.
end if.
do if (j >= i).
compute temp2(i,j)=' '.
end if.
end loop.
end loop.
compute temp2=temp2(2:nrow(zcmat),(1:(ncol(zcmat)-1))).
print temp2/title='ZMATRIX: Paths moderated (1) and not moderated (0) by Z:'/cnames=cmatlabs/rnames=rmatlabs/format a3.
end if.
do if (rsum(csum(wzcmat))<>0).
compute temp2=make(nrow(wzcmat),ncol(wzcmat),'0').
loop i = 2 to nrow(wzcmat).
loop j = 1 to (ncol(wzcmat)-1).
do if (wzcmat(i,j)=1).
compute temp2(i,j)='1'.
end if.
do if (j >= i).
compute temp2(i,j)=' '.
end if.
end loop.
end loop.
compute temp2=temp2(2:nrow(wzcmat),(1:(ncol(wzcmat)-1))).
print temp2/title='WZMATRIX: W moderated paths moderated (1) and not moderated (0) by Z:'/cnames=cmatlabs/rnames=rmatlabs/format a3.
end if.
do if (ncs > 0).
print ccmat/title='CMATRIX: Covariates (columns) in (1) and not in (0) the models of M and Y (rows):'/rnames=rmatlabs/cnames=covnames.
end if.
end if.
do if (outscree=1).
do if (activate=0).
print/title = '*********************** ANALYSIS NOTES AND ERRORS ************************'.
end if.
end if.
do if (activate=1).
print/title = '**************************************************************************'.
end if.
do if (criterr=0).
do if (outscree=1).
print conf/title = 'Level of confidence for all confidence intervals in output:'/format = F8.4.
do if (boot > 0).
do if (goodboot = boot and bc=0).
print boot/title='Number of bootstrap samples for percentile bootstrap confidence intervals:'.
end if.
do if (goodboot = boot and bc=1).
print boot/title='Number of bootstrap samples for bias-corrected bootstrap confidence intervals:'.
end if.
do if (booterr = 1).
compute badend = badend(1,2:ncol(badend)).
print/title = 'WARNING: Bootstrap CI endpoints below not trustworthy. Decrease confidence'.
print badend/title='or increase the number of bootstrap samples.'/space=0/format = F10.4.
end if.
end if.
do if (mc > 0).
print mc/title='Number of samples for Monte Carlo confidence intervals:'.
end if.
do if (wnotev > 0 and printw=1).
do if (wnotev=1).
print/title = 'W values in conditional tables are the 16th, 50th, and 84th percentiles.'.
else if (wnotev=2).
do if (minwwarn=0 and maxwwarn=0).
print/title = 'W values in conditional tables are the mean and +/- SD from the mean.'.
end if.
do if (minwwarn=1).
print/title = 'W values in conditional tables are the minimum, the mean, and 1 SD above the mean.'.
end if.
do if (maxwwarn=1).
print/title = 'W values in conditional tables are 1 SD below the mean, the mean, and the maximum.'.
end if.
end if.
end if.
do if (znotev > 0 and printz=1).
do if (znotev=1).
print/title = 'Z values in conditional tables are the 16th, 50th, and 84th percentiles.'.
else if (znotev=2).
do if (minzwarn=0 and maxzwarn=0).
print/title = 'Z values in conditional tables are the mean and +/- SD from the mean.'.
end if.
do if (minzwarn=1).
print/title = 'Z values in conditional tables are the minimum, the mean, and 1 SD above the mean.'.
end if.
do if (maxzwarn=1).
print/title = 'Z values in conditional tables are 1 SD below the mean, the mean, and the maximum.'.
end if.
end if.
end if.
do if (minwwarn > 0).
print/title = 'NOTE: One SD below the mean is below the minimum observed in the data for W,'.
print/title = '      so the minimum measurement on W is used for conditioning instead.'/space=0.
end if.
do if (maxwwarn > 0).
print/title = 'NOTE: One SD above the mean is above the maximum observed in the data for W,'.
print/title = '      so the maximum measurement for W is used for conditioning instead.'/space=0.
end if.
do if (minzwarn > 0).
print/title = 'NOTE: One SD below the mean is below the minimum observed in the data for Z,'.
print/title = '      so the minimum measurement for Z is used for conditioning instead.'/space=0.
end if.
do if (maxzwarn > 0).
print/title = 'NOTE: One SD above the mean is above the maximum observed in the data for Z,'.
print/title = '      so the maximum measurement for Z is used for conditioning instead.'/space=0.
end if.
do if (pstog=1).
print/title= 'NOTE: Standardized coefficients for dichotomous or multicategorical X are in'.
print/title= '      partially standardized form.'/space=0.
end if.
loop i = 1 to 100.
do if (notecode(i,1) = 32).
print/title = 'Direct, indirect, and total effects are counterfactually defined'.
do if (xcontcf=0).
print/title = 'assuming X by M interaction.'/space=0.
end if.
do if (xcontcf=1).
print/title = 'assuming X by M interaction and with the following reference (x_ref)'/space=0.
compute states=t(xrefvals).
print states/title = 'and counterfactual (x_cf) states for X:'/rlabels='x_ref' 'x_cf'/format= F10.4 /space=0.
end if.
end if.
do if (notecode(i,1)=1).
print/title = 'NOTE: COVMY is ignored when using CMATRIX option.'.
end if.
do if (notecode(i,1)=2).
print/title = 'NOTE: Confidence level restricted to between 50 and 99.9999%.  95% confidence is provided in output'.
end if.
do if (notecode(i,1)=3).
print centvar/title = 'NOTE: The following variables were mean centered prior to analysis:'/format = a8.
end if.
do if (notecode(i,1) = 4).
print/title = 'NOTE: A heteroscedasticity consistent standard error and covariance matrix estimator was used.'.
end if.
do if (notecode(i,1) = 5).
print/title = 'NOTE: The HC3 option has been replaced with HC.  See the documentation.'.
end if.
do if (notecode(i,1) = 6).
print/title = 'NOTE: Due to estimation problems, some bootstrap samples had to be replaced.'.
print badboot/title='      The number of times this happened was:'/space=0/format=F8.0.
end if.
do if (notecode(i,1) = 7).
print/title = 'NOTE: The bootstrapping was not completed due to problematic bootstrap samples.'.
print/title = '      Bootstrap confidence intervals are therefore suppressed.'/space=0.
end if.
do if (notecode(i,1) = 8).
print/title = 'NOTE: The number of bootstrap samples was adjusted upward given your desired confidence.'.
end if.
do if (notecode(i,1) = 9).
print/title = 'NOTE: WMODVAL is ignored when W is specified as multicategorical.'.
end if.
do if (notecode(i,1) = 10).
print/title = 'NOTE: ZMODVAL is ignored when Z is specified as multicategorical.'.
end if.
do if (notecode(i,1) = 11).
print/title = 'NOTE: Total effect model generated only when all covariates are specified in all'.
print/title = '      models of M and Y.'/space=0.
end if.
do if (notecode(i,1) = 30).
print/title = 'NOTE: Your vector of linear hypothesis weights is of the wrong length for this model.'.
end if.
do if (notecode(i,1) = 12).
print/title = 'NOTE: Total effect model generated only when X is freely estimated to affect each M'.
print/title = '      and both X and M are freely estimated to affect Y'/space=0.
end if.
do if (notecode(i,1) = 13).
print/title = 'NOTE: There are too many pairwise contrasts to conduct with this model.'.
end if.
do if (notecode(i,1) = 14).
print/title = 'NOTE: The number of contrast weights must equal the number of indirect effects.'.
end if.
do if (notecode(i,1) = 15).
print/title = 'NOTE: Monte Carlo confidence intervals not available for this model.'.
print/title = '      Bootstrapping is used instead.'/space=0.
end if.
do if (notecode(i,1) = 16).
print/title = 'NOTE: The number of Monte Carlo samples was adjusted upward given your desired confidence.'.
end if.
do if (notecode(i,1) = 19).
print/title = 'NOTE: Your contrast matrix is invalid or not applicable to this model.'.
end if.
do if (notecode(i,1) = 20).
print/title = 'NOTE: One of the groups specified by your contrast matrix does not exist in the data.'.
end if.
do if (notecode(i,1) = 21).
print/title = 'NOTE: The VARORDER option is not available in this release.'.
end if.
do if (notecode(i,1) = 22).
print/title = 'NOTE: The VMODVAL and QMODVAL options are not available in this release.'.
end if.
do if (notecode(i,1) = 23).
print/title = 'NOTE: The QUANTILE option is not available in this release.'.
end if.
do if (notecode(i,1) = 24).
print/title = 'NOTE: Total effect model not available with dichotomous Y.'.
end if.
do if (notecode(i,1) = 25).
print/title = 'NOTE: STAND/EFFSIZE options not available with dichotomous Y.'.
end if.
do if ((notecode(i,1) = 26) and nms > 0).
print/title = 'NOTE: Direct and indirect effects of X on Y are on a log-odds metric.'.
end if.
do if (notecode(i,1) = 27).
print/title = 'NOTE: Standardized coefficients are not available for models with moderators.'.
end if.
do if (notecode(i,1) = 28).
print/title = 'NOTE: The contrast option is not available with a multicategorical X.'.
end if.
do if (notecode(i,1) = 31).
do if (nms > 1).
print medmeans/title = 'NOTE: Controlled direct effect(s) estimated at the following mediator values:'/cnames=mnames/format= F10.4.
end if.
do if (nms=1).
print medmeans/title = 'NOTE: Controlled direct effect(s) estimated at the following mediator value:'/format= F10.4.
end if.
end if.
do if (notecode(i,1) = 33).
print/title = 'NOTE: Sobel test is not available with the XMINT option.'.
end if.
do if (notecode(i,1) = 37).
print/title = 'NOTE: The CONTRAST option is not available when using the XMINT option.'.
end if.
do if (notecode(i,1) = 34).
print/title = 'NOTE: Standardized effects are not available when using the XMINT option.'.
end if.
do if (notecode(i,1) = 36).
print/title = 'NOTE: The XREFVAL option is ignored when X is declared as multicategorical.'.
end if.
do if (notecode(i,1) = 35).
print coval/title = 'NOTE: Counterfactual effects estimated at the following covariate values:'/cnames=covnames/format= F10.4.
end if.
do if (notecode(i,1) = 29 and (listmiss=1)).
compute a=missrow.
compute conum=ncol(a).
compute allgood=0.
compute smremain=12.
compute largesti=1.
compute smallrow=0.
do if (conum > 12).
loop ii = 1 to 12.
compute check=(conum/ii).
do if (check = trunc(check)).
compute check2=conum/ii.
compute aok=ii.
do if (aok > 2).
compute allgood=1.
end if.
else.
compute remain=conum-(ii*trunc(check)).
do if (remain <= smremain).
compute smremain=remain.
compute largesti=ii.
compute smallrow=trunc(conum/largesti).
end if.
end if.
end loop.
compute atemp=a(1,1:(aok*check2)).
compute atemp=reshape(atemp,check2,aok).
do if (ncol(atemp) > 2).
print/title='NOTE: Missing data resulted in the deletion of the following row(s) of'.
print atemp/title='      data from the analysis:'/format=F6.0/space=0.
end if.
do if (allgood=0).
compute atemp=a(1,1:(smallrow*largesti)).
compute atemp=reshape(atemp,smallrow,largesti).
compute btemp=a(1,((largesti*smallrow)+1):conum).
print/title='NOTE: Missing data resulted in the deletion of the following row(s) of'.
print atemp/title='      data from the analysis:'/format=F6.0/space=0.
print btemp/title=' '/space=0/format=f6.0.
end if.
else if (conum <= 12).
print/title='NOTE: Missing data resulted in the deletion of the following row(s) of'.
print a/title='      data from the analysis:'/format=F6.0/space=0.
end if.
end if.
end loop.
do if (toomany=1).
print/title='WARNING: Variables names longer than eight characters can produce incorrect output'.
print/title='when some variables in the data file have the same first eight characters. Shorter'/space=0.
print/title='variable names are recommended. By using this output, you are accepting all risk'/space=0.
print/title='and consequences of interpreting or reporting results that may be incorrect.'/space=0.
end if.
end if.
end if.
loop i = 1 to 100.
do if (errcode(i,1)=1).
print/title = 'ERROR: You must specify a Y and an X variable.'.
end if.
do if (errcode(i,1)=2).
print/title = 'ERROR: X, M, or Y variable used more than once or W and Z are the same variable.'.
do if (toomany = 1).
print/title = '       This could be caused by the use of variables names longer'/space=0.
print varnames/title = '       than eight characters. Here are the variables I see:'/space=0/format=A8.
end if.
end if.
do if (errcode(i,1)=3).
print/title = 'ERROR: You have specified more than one variable for W, Y, X, or Z'.
end if.
do if (errcode(i,1)=4).
print/title = 'ERROR: A variable specified as multicategorical has more than nine categories.'.
end if.
do if (errcode(i,1)=5).
print/title = 'ERROR: One of the categories contains only a single case.'.
end if.
do if (errcode(i,1)=6).
print/title = 'ERROR: Invalid model number in this version of PROCESS.'.
end if.
do if (errcode(i,1)=7).
print/title = 'ERROR: Invalid model number.'.
end if.
do if (errcode(i,1)=8).
print/title = 'ERROR: You must specify an M variable for this model.'.
end if.
do if (errcode(i,1)=9).
print/title = 'ERROR: You have specified an M variable in a model that does not use it.'.
end if.
do if (errcode(i,1)=10).
print/title = 'ERROR: You have specified a W variable in a model that does not use it.'.
end if.
do if (errcode(i,1)=11).
print/title = 'ERROR: You have not specified a W variable in a model that requires it.'.
end if.
do if (errcode(i,1)=12).
print/title = 'ERROR: You have specified a Z variable in a model that does not use it.'.
end if.
do if (errcode(i,1)=13).
print/title = 'ERROR: You have not specified a Z variable in a model that requires it.'.
end if.
do if (errcode(i,1)=14).
print/title = 'ERROR: V and Q are not proper specifications in this release of PROCESS.'.
print/title = '       Moderators must be specified as W and/or Z.'/space=0.
end if.
do if (errcode(i,1)=15).
print/title = 'ERROR: One of your model variables exhibits no variation (it is a constant).'.
end if.
do if (errcode(i,1)=16).
print/title = 'ERROR: BMATRIX is not the correct length or is otherwise invalid.'.
end if.
do if (errcode(i,1)=17).
print/title = 'ERROR: WMATRIX is not the correct length or is otherwise invalid.'.
end if.
do if (errcode(i,1)=18).
print/title = 'ERROR: ZMATRIX is not the correct length or is otherwise invalid.'.
end if.
do if (errcode(i,1)=19).
print/title = 'ERROR: WZMATRIX is not the correct length or is otherwise invalid.'.
end if.
do if (errcode(i,1)=20).
print/title = 'ERROR: A path fixed at zero cannot be moderated.'.
end if.
do if (errcode(i,1)=21).
print/title = 'ERROR: If only one moderator is specified, it must be specified as W.'.
end if.
do if (errcode(i,1)=22).
print/title = 'ERROR: In BMATRIX, X must be specified to affect at least one variable.'.
end if.
do if (errcode(i,1)=23).
print/title = 'ERROR: In BMATRIX, at least one variable must be specified to affect Y.'.
end if.
do if (errcode(i,1)=24).
print/title = 'ERROR: You must specify a model number or a custom BMATRIX specification.'.
end if.
do if (errcode(i,1)=25).
print/title = 'ERROR: BMATRIX cannot be used in conjunction with a model number.'.
end if.
do if (errcode(i,1)=26).
print/title = 'ERROR: Your model has a dangling mediator (all Ms must affect and be affected).'.
end if.
do if (errcode(i,1)=27).
print/title = 'ERROR: CLUSTER is not available on this release of PROCESS.'.
end if.
do if (errcode(i,1)=29).
print/title = 'ERROR: CMATRIX is not the correct length or is otherwise invalid.'.
end if.
do if (errcode(i,1)=30).
print/title = 'ERROR: In CMATRIX, all covariates must be assigned to an M or a Y.'.
end if.
do if (errcode(i,1)=31).
print/title = 'ERROR: A linear or near linear dependency (singularity) exists in the data.'.
end if.
do if (errcode(i,1)=32).
print/title = 'ERROR: Models 80 and 81 require between 3 and 6 mediators.'.
end if.
do if (errcode(i,1)=33).
print/title = 'ERROR: Model 82 requires 4 mediators.'.
end if.
do if (errcode(i,1)=34).
print/title = 'ERROR: This model number requires between 2 and 6 mediators.'.
end if.
do if (errcode(i,1)=35).
print/title = 'ERROR: In a model with only one moderator, that moderator must be W.'.
end if.
do if (errcode(i,1)=36).
print/title = 'ERROR: A serial mediation model cannot have more than 6 mediators.'.
end if.
do if (errcode(i,1)=37).
print/title = 'ERROR: No more than 10 mediators are allowed in a PROCESS command.'.
end if.
do if (errcode(i,1)=38).
print/title = 'ERROR: XCATCODE is not provided, not the correct length, or is otherwise invalid.'.
end if.
do if (errcode(i,1)=39).
print/title = 'ERROR: WCATCODE is not provided, not the correct length, or is otherwise invalid.'.
end if.
do if (errcode(i,1)=40).
print/title = 'ERROR: ZCATCODE is not provided, not the correct length, or is otherwise invalid.'.
end if.
do if (errcode(i,1)=41).
print/title = 'ERROR: Models 1, 2, and 3 cannot be customized.'.
end if.
do if (errcode(i,1)=42).
print/title = 'ERROR: WS option available only in PROCESS v2. Or use the MEMORE macro instead.'.
print/title = '       MEMORE can be downloaded from www.akmontoya.com.'/space=0.
end if.
do if (errcode(i,1)=43).
print/title = 'ERROR: PROCESS does not allow dichotomous mediators.'.
end if.
do if (errcode(i,1)=60).
print/title = 'PROCESS is now ready for use.'.
print/title='Copyright 2017-2022 by Andrew F. Hayes. ALL RIGHTS RESERVED.'/space=0.
print/title='Workshop schedule available at http://haskayne.ucalgary.ca/CCRAM'/space=0.
end if.
do if (errcode(i,1)=50).
print/title = 'ERROR: A multicategorical moderator cannot be specified as a covariate.'.
end if.
do if (errcode(i,1)=51).
print/title = 'ERROR: A variable you specified as a covariate is a moderator in all equations.'.
end if.
do if (errcode(i,1)=52 and mcerpt=0).
compute mcerpt=1.
print/title = 'ERROR: A variable specified as multicategorical must have at least three categories'.
end if.
do if (errcode(i,1) = 47).
do if (iterrmod=1).
print/title = 'ERROR: Iteration for Y model didn''t converge to a solution. Interpret results with caution.'.
print/title = '       Try increasing the number of iterations, though this will slow down computation.'/space=0.
end if.
do if (bootiter=1).
print/title = 'ERROR: Nonconvergence during bootstrapping. Interpet bootstrap results with caution.'.
end if.
end if.
do if (errcode(i,1) = 61).
print/title = 'ERROR: Variable names should be no more than eight characters in length.'.
end if.
do if (errcode(i,1) = 62).
print/title = 'ERROR: After listwise deletion of cases with missing data, too few cases remain.'.
end if.
do if (errcode(i,1) = 63).
print/title = 'ERROR: The XMINT option is available only for model 4.'.
end if.
do if (errcode(i,1) = 64).
print/title = 'ERROR: Incorrect number of values specified in CDEVAL option.'.
end if.
do if (errcode(i,1) = 65).
print/title = 'ERROR: Only indicator or sequential coding of X is allowed with the XMINT option.'.
end if.
do if (errcode(i,1) = 66).
print/title = 'ERROR: XMINT option not available with continuous X using PROCESS GUI. Use syntax.'.
end if.
do if (errcode(i,1) = 67).
print/title = 'ERROR: Too many elements provided in XREFVAL option.'.
end if.
do if (errcode(i,1)=68).
print/title = 'ERROR: Covariate assignment is not allowed with XMINT option.'.
end if.
do if (errcode(i,1) = 69).
print/title = 'ERROR: Incorrect number of values specified in COVAL option.'.
end if.
do if (errcode(i,1) = 70).
print/title = 'ERROR: Incorrect value(s) in XREFVAL for this dichotomous X variable.'.
end if.
do if (errcode(i,1) = 71).
print/title = 'ERROR: The CENTER option is not available when using the XMINT option.'.
end if.
do if (errcode(i,1) = 72).
print/title = 'ERROR: The XMINT option is not available for models with a dichotomous Y.'.
end if.
end loop.
END MATRIX.	
Ressources	Temps de processeur	00:00:06,27	
	Temps écoulé	00:00:06,22	


Run MATRIX procedure:

***************** PROCESS Procedure for SPSS Version 4.2 *****************

          Written by Andrew F. Hayes, Ph.D.       www.afhayes.com
    Documentation available in Hayes (2022). www.guilford.com/p/hayes3

**************************************************************************
Model  : 4
    Y  : HOMAIR
    X  : Veg
   M1  : TG
   M2  : HDL
   M3  : LDL
   M4  : TC

Sample
Size:  169

**************************************************************************
OUTCOME VARIABLE:
 TG

Model Summary
          R       R-sq        MSE          F        df1        df2          p
      ,0090      ,0001  4051,3243      ,0136     1,0000   167,0000      ,9075

Model
              coeff         se          t          p       LLCI       ULCI
constant    86,9706    10,9159     7,9673      ,0000    65,4197   108,5215
Veg          1,4220    12,2134      ,1164      ,9075   -22,6905    25,5345

Standardized coefficients
         coeff
Veg      ,0224

**************************************************************************
OUTCOME VARIABLE:
 HDL

Model Summary
          R       R-sq        MSE          F        df1        df2          p
      ,0006      ,0000   178,1358      ,0001     1,0000   167,0000      ,9935

Model
              coeff         se          t          p       LLCI       ULCI
constant    41,1765     2,2889    17,9892      ,0000    36,6575    45,6955
Veg          -,0209     2,5610     -,0082      ,9935    -5,0771     5,0352

Standardized coefficients
         coeff
Veg     -,0016

**************************************************************************
OUTCOME VARIABLE:
 LDL

Model Summary
          R       R-sq        MSE          F        df1        df2          p
      ,0029      ,0000  1190,0404      ,0014     1,0000   167,0000      ,9705

Model
              coeff         se          t          p       LLCI       ULCI
constant    76,1176     5,9162    12,8660      ,0000    64,4375    87,7978
Veg           ,2453     6,6194      ,0371      ,9705   -12,8232    13,3138

Standardized coefficients
         coeff
Veg      ,0071

**************************************************************************
OUTCOME VARIABLE:
 TC

Model Summary
          R       R-sq        MSE          F        df1        df2          p
      ,0323      ,0010  1450,4142      ,1747     1,0000   167,0000      ,6765

Model
              coeff         se          t          p       LLCI       ULCI
constant   133,3235     6,5314    20,4127      ,0000   120,4288   146,2183
Veg          3,0542     7,3077      ,4179      ,6765   -11,3732    17,4817

Standardized coefficients
         coeff
Veg      ,0804

**************************************************************************
OUTCOME VARIABLE:
 HOMAIR

Model Summary
          R       R-sq        MSE          F        df1        df2          p
      ,3684      ,1357    23,1735     5,1184     5,0000   163,0000      ,0002

Model
              coeff         se          t          p       LLCI       ULCI
constant     -,4543     1,8078     -,2513      ,8019    -4,0240     3,1153
Veg           ,6516      ,9281      ,7021      ,4836    -1,1811     2,4843
TG            ,0442      ,0098     4,5312      ,0000      ,0249      ,0635
HDL           ,1270      ,0433     2,9347      ,0038      ,0415      ,2124
LDL           ,0953      ,0359     2,6572      ,0087      ,0245      ,1661
TC           -,0903      ,0356    -2,5342      ,0122     -,1607     -,0199

Standardized coefficients
         coeff
Veg      ,1278
TG       ,5499
HDL      ,3312
LDL      ,6425
TC      -,6727

************************** TOTAL EFFECT MODEL ****************************
OUTCOME VARIABLE:
 HOMAIR

Model Summary
          R       R-sq        MSE          F        df1        df2          p
      ,0362      ,0013    26,1354      ,2193     1,0000   167,0000      ,6402

Model
              coeff         se          t          p       LLCI       ULCI
constant     3,8288      ,8767     4,3671      ,0000     2,0979     5,5598
Veg           ,4594      ,9810      ,4683      ,6402    -1,4773     2,3961

Standardized coefficients
         coeff
Veg      ,0901


************** TOTAL, DIRECT, AND INDIRECT EFFECTS OF X ON Y **************

Total effect of X on Y
     Effect         se          t          p       LLCI       ULCI       c_ps
      ,4594      ,9810      ,4683      ,6402    -1,4773     2,3961      ,0901

Direct effect of X on Y
     Effect         se          t          p       LLCI       ULCI      c'_ps
      ,6516      ,9281      ,7021      ,4836    -1,1811     2,4843      ,1278

Indirect effect(s) of X on Y:
          Effect     BootSE   BootLLCI   BootULCI
TOTAL     -,1923      ,3952    -1,1120      ,4880
TG         ,0628      ,5752    -1,0188     1,3331
HDL       -,0027      ,5995    -1,1618     1,2626
LDL        ,0234     1,5230    -3,0101     2,7723
TC        -,2758     1,5034    -3,3949     2,4330

Partially standardized indirect effect(s) of X on Y:
          Effect     BootSE   BootLLCI   BootULCI
TOTAL     -,0377      ,0769     -,2130      ,0973
TG         ,0123      ,1102     -,2001      ,2444
HDL       -,0005      ,1138     -,2148      ,2378
LDL        ,0046      ,2887     -,5823      ,5380
TC        -,0541      ,2849     -,6385      ,4628

*********************** ANALYSIS NOTES AND ERRORS ************************

Level of confidence for all confidence intervals in output:
  95,0000

Number of bootstrap samples for percentile bootstrap confidence intervals:
  5000

NOTE: Standardized coefficients for dichotomous or multicategorical X are in
      partially standardized form.

------ END MATRIX -----


* Encoding: UTF-8.
preserve.
set printback=off.


Matrice


Remarques	
Sortie obtenue	10-NOV-2024 17:05:41	
Commentaires		
Entrée	Données	C:\Users\Brice SAHA\Documents\UBa\Master Thesis 2021\Fanuel\Database fanuel.sav	
	Jeu de données actif	Jeu_de_données1	
	Filtre	<sans>	
	Pondération	<sans>	
	Scinder un fichier	<sans>	
	N de lignes dans le fichier de travail	169	
Syntaxe	MATRIX.
compute wnames='xxxxx'.
compute znames='xxxxx'.
compute mcerpt=0.
compute wiscov=0.
compute ziscov=0.
compute tooman=0.
compute errcode=make(100,1,0).
compute notecode=make(100,1,0).
compute model = trunc( 4 ).
compute iterate = abs(trunc( 100 )).
compute converge = abs( 0.00001 ).
compute itprobtg=0.
compute v2tag=0.
compute ydich=0.
compute maxwwarn=0.
compute minwwarn=0.
compute maxzwarn=0.
compute minzwarn=0.
compute toomany=0.
compute wdich=0.
compute zdich=0.
compute wnotev=0.
compute znotev=0.
compute nxpval=1.
compute nwpval=1.
compute nzpval=1.
compute errs=1.
compute notes=1.
compute criterr=0.
compute novar=0.
compute adjust=0.
compute ncs=0.
compute serial=0.
compute sobelok=0.
compute hasw=0.
compute hasz=0.
compute printw=0.
compute printz=0.
compute xmint=( 0 =1).
compute wmodcust=0.
compute zmodcust=0.
compute booting=0.
compute bootiter=0.
compute iterrmod=0.
compute cov = 'xxxxx'.
compute varorder=( 0 <> 0).
compute nws=0.
compute w= 'xxxxx'.
compute nzs=0.
compute z = 'xxxxx'.
compute nms=0.
compute m = 'TG HDL LDL TC'.
compute nys=0.
compute y = 'HOMAIR'.
compute nxs=0.
compute x = 'Fruits'.
compute effsize=( 0 =1).
compute stand=( 1 =1).
compute intprobe = .1.
compute xrefvals={ 999 }.
compute center=trunc( 0 ).
compute xcontcf=0.
compute xscaling=1.
compute cdeval={ -999 }.
compute cuscoval=0.
do if (model=74).
compute errcode(errs,1)=7.
compute errs=errs+1.
compute criterr=1.
end if.
do if (xmint=1 and model <> 4).
compute errcode(errs,1)=63.
compute errs=errs+1.
compute criterr=1.
end if.
do if (xmint=1 and model=4).
compute w=x.
compute model=74.
compute intprobe=1.
compute notecode(notes,1) = 32.
compute notes = notes + 1.
do if (effsize=1 or stand=1).
compute notecode(notes,1) = 34.
compute notes = notes + 1.
compute stand=0.
compute effsize=0.
end if.
do if (center <> 0).
compute center=0.
compute errcode(errs,1)=71.
compute errs=errs+1.
compute criterr=1.
end if.
end if.
compute v = 'xxxxx'.
compute q = 'xxxxx'.
compute linsum={ -999 }.
compute nlinsum=ncol(linsum).
do if (linsum(1,1) = -999).
compute nlinsum=0.
end if.
compute oldvars= 'xxxxx'.
compute mcxok=0.
compute mcwok=0.
compute mczok=0.
compute xprod=0.
compute zprod=0.
compute wprod=0.
compute modcok=0.
compute alttotal=0.
compute hc3=trunc( 0 ).
compute jn=( 0 = 1).
compute listmiss=( 0 =1).
compute modelres=( 0 =1).
compute outscree=( 1 =1).
compute activate=( 0 =1).
compute booterr=0.
compute normal=( 0 =1).
compute xmtest=( 0 =1).
compute describe=( 0 =1).
compute longname=( 0 =1).
do if (stand=1).
compute effsize=1.
end if.
compute pstog=0.
compute sobelok=0.
compute normal=( 0 =1).
compute mdichok=( 0 =1).
compute contrast={ 999 }.
compute ncontr=ncol(contrast).
compute ncontrow=nrow(contrast).
do if (contrast(1,1) = 999).
compute ncontr=1.
compute contrast=0.
end if.
do if (ncontr = 1).
compute contrast=trunc(contrast).
do if (contrast > 3 or contrast < 0)).
compute ncontr=1.
compute contrast = 0.
end if.
end if.
do if (ncontr > 1).
compute contvec=contrast.
compute contrast=4.
do if (ncontrow > 1).
compute contrast=0.
compute modcok=1.
compute wcontval=contvec(:,1).
compute zcontval=contvec(:,2).
do if ((ncontr <> 2) or (ncontrow <> 2)).
compute notecode(notes,1) = 19.
compute notes = notes + 1.
compute modcok=0.
end if.
end if.
end if.
do if (xmint = 1 and contrast(1,1) <> 0).
compute contrast=0.
compute notecode(notes,1) = 37.
compute notes = notes + 1.
end if.
do if (varorder = 1).
compute notecode(notes,1) = 21.
compute notes = notes + 1.
end if.
do if ( 999 <> 999 or 999 <> 999).
compute notecode(notes,1) = 22.
compute notes = notes + 1.
end if.
compute modelbt=( 0 =1).
compute cluster= 'xxxxx'.
compute matrices=( 0 =1).
compute covcoeff=( 0 =1).
compute covmy=trunc( 0 ).
do if (covmy < 0 or covmy > 2).
compute covmy = 0.
end if.
compute boot = abs(trunc( 5000 )).
compute bc=( 0 =1).
compute mc=abs(trunc( 0 )).
compute hc=trunc( 5 ).
do if (intprobe < 0 or intprobe > 1).
compute intprobe = .10.
end if.
compute plot=trunc( 0 ).
do if (plot < 0 or plot > 2).
compute plot=0.
end if.
compute total=( 1 =1).
compute dototal=0.
compute saveboot = ( 0 = 1).
compute saveest=( 0 = 2).
do if (saveest=1).
compute intprobe=1.
end if.
do if (hc >= 0 and hc < 5).
compute notecode(notes,1) = 4.
compute notes = notes + 1.
end if.
do if (hc > 5 or hc < 0).
compute hc=5.
end if.
compute mcw=trunc( 0 ).
compute mcz=trunc( 0 ).
compute mcx=trunc( 0 ).
do if (mcx > 0 and mcx < 3 and model = 74).
compute mcw=mcx.
compute xscaling=1.
end if.
do if (mcx > 2 and model = 74).
compute errcode(errs,1)=65.
compute errs=errs+1.
compute criterr=1.
end if.
do if (model = 74 and normal=1).
compute notecode(notes,1) = 33.
compute normal=0.
compute notes = notes + 1.
end if.
do if (mcx > 0 and contrast > 0).
compute notecode(notes,1) = 28.
compute notes = notes + 1.
compute contrast=0.
end if.
compute nxvls=1.
compute nmvls=1.
compute nwvls=1.
compute nzvls=1.
compute paths=999.
compute pathsw=999.
compute pathsz=999.
compute pathswz=999.
compute pathsmod=999.
compute pathtype=999.
compute obscoeff=999.
compute pathsdv={' '}.
compute quantile=1.
do if ( 999 <>999).
compute notecode(notes,1) = 23.
compute notes = notes + 1.
end if.
compute moments=( 0 =1).
do if (moments=1).
compute quantile=0.
end if.
compute bmatrix={ -999 }.
compute wmatrix={ -999 }.
compute zmatrix={ -999 }.
compute wzmatrix={ -999 }.
compute cmatrix={ -999 }.
compute xcatcode={ -999 }.
compute wcatcode={ -999 }.
compute zcatcode={ -999 }.
compute needed=0.
compute conf= 95.
do if (trunc( 95 ) >= 100 or (trunc( 95 ) <= 50)).
compute conf = 95.
compute notecode(notes,1)=2.
compute notes=notes+1.
end if.
do if (model >= 0 and model < 4 and modelbt=0).
compute boot=0.
compute mc=0.
compute bc=0.
end if.
do if (mc > 0 and boot > 0).
compute boot=0.
compute bc=0.
end if.
do if ((boot < 1000) and (mc = 0) and (boot > 0)).
compute boot=5000.
end if.
do if ((mc < 1000) and (boot = 0) and (mc > 0)).
compute mc=5000.
end if.
compute p0=-.322232431088.
compute p1 = -1.
compute p2 = -.342242088547.
compute p3 = -.0204231210245.
compute p4 = -.0000453642210148.
compute q0 = .0993484626060.
compute q1 = .588581570495.
compute q2 = .531103462366.
compute q3 = .103537752850.
compute q4 = .0038560700634.
compute badend=0.
compute priorlo = -9999999.
compute priorhi = 9999999.
compute alpha2 = (1-(conf/100))/2.
compute cilm=alpha2*2.
compute y5=sqrt(-2*ln(alpha2)).
compute xp2=(y5+((((y5*p4+p3)*y5+p2)*y5+p1)*y5+p0)/((((y5*q4+q3)*y5+q2)*y5+q1)*y5+q0)).
compute medlb={'   M1  :';'   M2  :';'   M3  :';'   M4  :';'   M5  :';'   M6  :';'   M7  :';'   M8  :';'   M9  :';'   M10 :'}.
compute medlb2={'(M1)','(M2)','(M3)','(M4)','(M5)','(M6)','(M7)','(M8)','(M9)','(M10)'}.
compute xlb={'   X1  :';'   X2  :';'   X3  :';'   X4  :';'   X5  :';'   X6  :';'   X7  :';'   X8  :';'   X9  :'}.
compute highlbw={'M1*W'; 'M2*W'; 'M3*W'; 'M4*W'; 'M5*W'; 'M6*W'; 'M7*W'; 'M8*W'; 'M9*W'; 'M10*W'}.
do if (xmint=1).
compute highlbw={'M1*X'; 'M2*X'; 'M3*X'; 'M4*X'; 'M5*X'; 'M6*X'; 'M7*X'; 'M8*X'; 'M9*X'; 'M10*X'}.
end if.
compute highlbz={'M1*Z'; 'M2*Z'; 'M3*Z'; 'M4*Z'; 'M5*Z'; 'M6*Z'; 'M7*Z'; 'M8*Z'; 'M9*Z';'M10*Z'}.
compute highlbwz={'M1*W*Z'; 'M2*W*Z'; 'M3*W*Z'; 'M4*W*Z'; 'M5*W*Z'; 'M6*W*Z'; 'M7*W*Z'; 'M8*W*Z'; 'M9*W*Z';'M10*W*Z'}.
compute highlbbt={'BOTH(M1)'; 'BOTH(M2)'; 'BOTH(M3)'; 'BOTH(M4)'; 'BOTH(M5)'; 'BOTH(M6)'; 'BOTH(M7)'; 'BOTH(M8)'; 'BOTH(M9)';'BTH(M10)'}.
compute highlbx={'M1*X'; 'M2*X'; 'M3*X'; 'M4*X'; 'M5*X'; 'M6*X'; 'M7*X'; 'M8*X'; 'M9*X'; 'M10*X'}.
compute skipwz=0.
compute validm={1,1,1,1,1,1,1,1,1,1,1,1,1,1,1,1,1,1,1,1,1,1,0,0,0,0,0,1,1,0,0,0,0, 0,0,0,0,0,0,0,0,0,0,0,0,0,0,0,0,0,0,0,0,0,0,0,0,1,1,1,1,1,1,1,1,1,1,1,1,1,1,1,1,1,1,1,0,0,0,1,1, 1,1,1,1,1,1,1,1,1,1,1}.
do if (activate=1).
compute errcode(errs,1)=60.
compute errs=errs+1.
compute criterr=1.
end if.
do if (criterr=0).
do if (( 0 =1)=1).
compute errcode(errs,1)=42.
compute errs=errs+1.
compute criterr=1.
end if.
do if (model > 0 and model < 93).
do if (validm(1,model)=0).
compute errcode(errs,1)=6.
compute errs=errs+1.
compute criterr=1.
end if.
release validm.
end if.
do if ((model > 92 or model < 0) and model <> 999)).
compute errcode(errs,1)=7.
compute errs=errs+1.
compute criterr=1.
end if.
do if (model = 999 and bmatrix(1,1)=-999).
compute errcode(errs,1)=24.
compute errs=errs+1.
compute criterr=1.
end if.
do if (model <> 999 and bmatrix(1,1) <> -999).
compute errcode(errs,1)=25.
compute errs=errs+1.
compute criterr=1.
end if.
do if ((model = 74 or (model > 0 and model < 4)) and ((wmatrix(1,1) <> -999) or (zmatrix(1,1)<>-999) or (wzmatrix(1,1)<>-999)))).
compute errcode(errs,1)=41.
compute errs=errs+1.
compute criterr=1.
end if.
do if (hc3 <> 0).
compute notecode(notes,1) = 5.
compute notes = notes + 1.
do if (hc3 = 1).
compute hc=3.
end if.
end if.
do if ((v <> 'xxxxx') or (q <> 'xxxxx')).
compute errcode(errs,1)=14.
compute errs=errs+1.
compute errcode(errs,1)=48.
compute errs=errs+1.
compute criterr=1.
end if.
do if (oldvars <> 'xxxxx').
compute errcode(errs,1)=48.
compute errs=errs+1.
compute criterr=1.
end if.
do if (cluster <> 'xxxxx').
compute errcode(errs,1)=27.
compute errs=errs+1.
compute criterr=1.
end if.
do if ((y = 'xxxxx') or (x = 'xxxxx')).
compute errcode(errs,1)=1.
compute errs=errs+1.
compute criterr=1.
end if.
do if ((m = 'xxxxx') and model > 3).
compute errcode(errs,1)=8.
compute errs=errs+1.
compute criterr=1.
end if.
end if.
do if (criterr=0).
get ytmp/variables = HOMAIR /names = ynames/MISSING = 99999.
compute nys=ncol(ytmp).
compute needed=nys.
compute n=nrow(ytmp).
compute varnames={ynames}.
compute dat=ytmp.

.


do if (toomany=1 and longname=0).
compute criterr=1.
do if (tooman=0).
compute tooman=1.
compute errcode(errs,1) = 61.
compute errs = errs + 1.
end if.
end if
.
compute modelvar={ '4' ;t(ynames)}.
do if ( 4 =999).
compute modelvar(1,1)='CUSTOM'.
end if.
get xtmp/variables = Fruits /names = xnames/MISSING = 99999.
compute nxs=ncol(xtmp).
compute n=nrow(xtmp).
compute needed=needed+nxs.
compute varnames={varnames,xnames}.
compute xcatlab=t(xnames).
compute dat={dat,xtmp}.

.


do if (toomany=1 and longname=0).
compute criterr=1.
do if (tooman=0).
compute tooman=1.
compute errcode(errs,1) = 61.
compute errs = errs + 1.
end if.
end if
.
compute modelvar={modelvar;t(xnames)}.
do if (nxs = 1).
compute modelvlb={'Model  :';'    Y  :';'    X  :'}.
else.
compute modelvlb={'Model  :';'    Y  :';xlb(1:nxs,1)}.
end if.
do if (m <> 'xxxxx').
get mtmp/variables = TG HDL LDL TC /names = mnames/MISSING = 99999.
compute nms=ncol(mtmp).
compute mprod=make(1,nms,0).
compute n=nrow(mtmp).
compute needed=needed+nms.
compute varnames={varnames,mnames}.
compute dat={dat,mtmp}.
compute modelvar={modelvar;t(mnames)}.
compute x2m=make(99,nms,0).
compute m2y=make(99,nms,0).
compute onem=make(nms,1,1).

.


do if (toomany=1 and longname=0).
compute criterr=1.
do if (tooman=0).
compute tooman=1.
compute errcode(errs,1) = 61.
compute errs = errs + 1.
end if.
end if
.
do if (nms > 1 and nms < 11).
compute modelvlb={modelvlb;medlb(1:nms,1)}.
else.
compute modelvlb={modelvlb;'    M  :'}.
end if.
do if (nms > 0 and model < 4).
compute errcode(errs,1)=9.
compute errs=errs+1.
do if (model <> 0).
compute errcode(errs,1)=48.
compute errs=errs+1.
end if.
compute criterr=1.
end if.
end if.
compute wlocatet=0.
compute wlocate=0.
do if (w <> 'xxxxx').
do if (xmint=0).
get wtmp/variables = xxxxx /names = wnames/MISSING = 99999.

.


do if (toomany=1 and longname=0).
compute criterr=1.
do if (tooman=0).
compute tooman=1.
compute errcode(errs,1) = 61.
compute errs = errs + 1.
end if.
end if
.
end if.
do if (xmint=1).
get wtmp/variables = Fruits /names = wnames/MISSING = 99999.
end if.
compute nws=ncol(wtmp).
compute n=nrow(wtmp).
compute varnames={varnames,wnames}.
compute wlocate=ncol(varnames).
do if (model=74).
compute wlocatet=1.
do if (xnames <> wnames).
compute errcode(errs,1)=45.
compute errs=errs+1.
compute criterr=1.
end if.
end if.
compute wcatlab=t(wnames).
compute dat={dat,wtmp}.
do if (xmint <> 1).
compute modelvar={modelvar;t(wnames)}.
compute modelvlb={modelvlb;'    W  :'}.
end if.
end if.
do if (z <> 'xxxxx').
get ztmp/variables = xxxxx /names = znames/MISSING = 99999.
compute nzs=ncol(ztmp).
compute n=nrow(ztmp).

.


do if (toomany=1 and longname=0).
compute criterr=1.
do if (tooman=0).
compute tooman=1.
compute errcode(errs,1) = 61.
compute errs = errs + 1.
end if.
end if
.
compute varnames={varnames,znames}.
compute zcatlab=t(znames).
compute dat={dat,ztmp}.
compute modelvar={modelvar;t(znames)}.
compute modelvlb={modelvlb;'    Z  :'}.
end if.
do if (cov <> 'xxxxx').
get ctmp/variables = xxxxx /names = covnames/MISSING = 99999.
compute ncs=ncol(ctmp).
compute n=nrow(ctmp).

.


do if (toomany=1 and longname=0).
compute criterr=1.
do if (tooman=0).
compute tooman=1.
compute errcode(errs,1) = 61.
compute errs = errs + 1.
end if.
end if
.
compute varnames={varnames,covnames}.
compute dat={dat,ctmp}.
end if.
do if (nws > 1 or nzs > 1 or nys > 1 or nxs > 1).
compute errcode(errs,1)=3.
compute errs=errs+1.
compute criterr=1.
end if.
do if ((model = 80 or model = 81) and (nms < 3 or nms > 6)).
compute errcode(errs,1)=32.
compute errs=errs+1.
compute criterr=1.
end if.
do if (model = 82 and nms <> 4).
compute errcode(errs,1)=33.
compute errs=errs+1.
compute criterr=1.
end if.
do if (nms > 10).
compute errcode(errs,1)=37.
compute errs=errs+1.
compute criterr=1.
end if.
do if ((model = 6 or (model > 82 and model < 999)) and (nms < 2 or nms > 6)).
compute errcode(errs,1)=34.
compute errs=errs+1.
compute criterr=1.
end if.
compute match=0.
compute match2=0.
compute mcwzcov=0.
loop i = 1 to (ncol(varnames)-1).
loop j = (i+1) to ncol(varnames).
do if (varnames(i)=varnames(j)).
do if (i < (nxs+nms+nys+1)).
compute match2=match2+1.
end if.
do if (wlocatet=1 and i=2 and j=wlocate).
compute match2=match2-1.
end if.
do if ((wnames=znames) and (nws > 0 or nzs > 0))).
compute match2=match2+1.
end if.
do if (i < (ncol(varnames)-ncs+1)) and j > (ncol(varnames)-ncs)).
do if ((varnames(j)=wnames) and mcw=0)).
compute match=0.
compute wiscov=(j-(ncol(varnames)-ncs)).
end if.
do if ((varnames(j)=wnames) and mcw <>0)).
compute mcwzcov=1.
end if.
do if ((varnames(j)=znames) and mcz=0).
compute match=0.
compute ziscov=(j-(ncol(varnames)-ncs)).
end if.
do if ((varnames(j)=znames) and mcz<>0)).
compute mcwzcov=1.
end if.
end if.
end if.
end loop.
end loop.
do if (match2>0 or match=1).
compute errcode(errs,1)=2.
compute errs=errs+1.
compute criterr=1.
end if.
do if (mcwzcov=1).
compute errcode(errs,1)=50.
compute errs=errs+1.
compute criterr=1.
end if.
compute ninit=nrow(dat).
compute rownum=make(ninit,1,0).
loop i = 1 to ninit.
compute rownum(i,1)=i.
end loop.
compute dat={rownum,dat}.
compute j=1.
compute missrow=0.
loop i = 1 to n.
do if (rsum(dat(i,2:ncol(dat))=99999)=0).
compute dat(j,:)=dat(i,:).
compute j=j+1.
else.
compute missrow={missrow;dat(i,1)}.
end if.
end loop.
do if (j < 5).
compute errcode(errs,1)=62.
compute errs=errs+1.
compute criterr=1.
end if.
do if (criterr=0).
compute rownum=dat(1:(j-1),1).
do if (nrow(missrow) > 1).
compute missrow=t(missrow(2:nrow(missrow),1)).
compute notecode(notes,1) = 29.
compute notes = notes + 1.
end if.
compute dat=dat(1:(j-1),2:ncol(dat)).
compute n=nrow(dat).
compute nmiss=ninit-n.
compute ytmp=dat(:,1:nys).

.
compute desctmp=make((8-(4* 0 )),ncol( ytmp ),-999).
loop jd=1 to ncol( ytmp ).
compute descdat= ytmp (:,jd).
compute desctmp(1,jd) = csum(descdat)/nrow(descdat).
compute desctmp(2,jd) = (nrow(descdat)*sscp(descdat))-(t(csum(descdat))*(csum(descdat))).
compute desctmp(2,jd) = sqrt(desctmp(2,jd)/(nrow(descdat)*(nrow(descdat)-1))).
compute desctmp(3,jd)=cmin(descdat).
compute desctmp(4,jd)=cmax(descdat).
do if ( 0 =0).
compute minwarn=0.
compute maxwarn=0.
do if ((desctmp(3,jd)=desctmp(4,jd)) and novar=0).
compute errcode(errs,1)=15.
compute errs=errs+1.
compute criterr=1.
compute novar=1.
end if.
compute tmp=((descdat(:,1)=desctmp(3,jd))+(descdat(:,1)=desctmp(4,jd))).
compute desctmp(8,jd)=(csum(tmp)=nrow(tmp)).
compute tmp = descdat.
compute tmp(GRADE(descdat),:) = descdat.
compute descdat = tmp.
release tmp.
compute decval={.16;.5;.84}.
loop kd=1 to 3.
compute low=trunc(decval(kd,1)*(nrow(descdat)+1)).
compute lowdec=decval(kd,1)*(nrow(descdat)+1)-low.
compute value=descdat(low,1)+(descdat((low+1),1)-descdat(low,1))*lowdec.
compute desctmp((4+kd),jd)=value.
end loop.
compute mnotev=1.
compute modvals=desctmp(5:7,:).
do if (quantile <> 1).
compute desctmp(5,jd)=desctmp(1,jd)-desctmp(2,jd).
compute desctmp(6,jd)=desctmp(1,jd).
compute desctmp(7,jd)=desctmp(1,jd)+desctmp(2,jd).
compute modvals=desctmp(5:7,:).
compute mnotev=2.
do if (modvals(1,1) < desctmp(3,1)).
compute modvals(1,1)=desctmp(3,1).
compute minwarn=1.
end if.
do if (modvals(3,1) > desctmp(4,1)).
compute modvals(3,1)=desctmp(4,1).
compute maxwarn=1.
end if.
end if.
do if (desctmp(8,1)=1).
compute modvals={desctmp(3,1);desctmp(4,1)}.
compute mnotev=0.
compute minwarn=0.
compute maxwarn=0.
end if.
end if.
end loop
.
compute ysd=desctmp(2,:).
compute ovsd=ysd.
do if (desctmp(8,1)=1).
compute ydich=1.
do if (total=1).
compute total=0.
compute notecode(notes,1) = 24.
compute notes = notes + 1.
end if.
do if (effsize=1).
compute effsize=0.
compute notecode(notes,1) = 25.
compute notes = notes + 1.
end if.
do if (model=74).
compute errcode(errs,1) = 72.
compute errs=errs+1.
compute criterr=1.
end if.
compute omx = cmax(ytmp).
compute omn = cmin(ytmp).
compute ytmp = (ytmp = omx).
compute dat(:,1:nys)=(dat(:,1:nys)=omx).
compute rcd = {omn, 0; omx, 1}.
end if.
compute xtmp=dat(:,(nys+1):(nys+nxs)).

.
compute desctmp=make((8-(4* 0 )),ncol( xtmp ),-999).
loop jd=1 to ncol( xtmp ).
compute descdat= xtmp (:,jd).
compute desctmp(1,jd) = csum(descdat)/nrow(descdat).
compute desctmp(2,jd) = (nrow(descdat)*sscp(descdat))-(t(csum(descdat))*(csum(descdat))).
compute desctmp(2,jd) = sqrt(desctmp(2,jd)/(nrow(descdat)*(nrow(descdat)-1))).
compute desctmp(3,jd)=cmin(descdat).
compute desctmp(4,jd)=cmax(descdat).
do if ( 0 =0).
compute minwarn=0.
compute maxwarn=0.
do if ((desctmp(3,jd)=desctmp(4,jd)) and novar=0).
compute errcode(errs,1)=15.
compute errs=errs+1.
compute criterr=1.
compute novar=1.
end if.
compute tmp=((descdat(:,1)=desctmp(3,jd))+(descdat(:,1)=desctmp(4,jd))).
compute desctmp(8,jd)=(csum(tmp)=nrow(tmp)).
compute tmp = descdat.
compute tmp(GRADE(descdat),:) = descdat.
compute descdat = tmp.
release tmp.
compute decval={.16;.5;.84}.
loop kd=1 to 3.
compute low=trunc(decval(kd,1)*(nrow(descdat)+1)).
compute lowdec=decval(kd,1)*(nrow(descdat)+1)-low.
compute value=descdat(low,1)+(descdat((low+1),1)-descdat(low,1))*lowdec.
compute desctmp((4+kd),jd)=value.
end loop.
compute mnotev=1.
compute modvals=desctmp(5:7,:).
do if (quantile <> 1).
compute desctmp(5,jd)=desctmp(1,jd)-desctmp(2,jd).
compute desctmp(6,jd)=desctmp(1,jd).
compute desctmp(7,jd)=desctmp(1,jd)+desctmp(2,jd).
compute modvals=desctmp(5:7,:).
compute mnotev=2.
do if (modvals(1,1) < desctmp(3,1)).
compute modvals(1,1)=desctmp(3,1).
compute minwarn=1.
end if.
do if (modvals(3,1) > desctmp(4,1)).
compute modvals(3,1)=desctmp(4,1).
compute maxwarn=1.
end if.
end if.
do if (desctmp(8,1)=1).
compute modvals={desctmp(3,1);desctmp(4,1)}.
compute mnotev=0.
compute minwarn=0.
compute maxwarn=0.
end if.
end if.
end loop
.
compute xsd=desctmp(2,:).
compute xmodvals=modvals.
compute xdich=desctmp(8,1).
compute xmx=cmax(xtmp).
compute xmn=cmin(xtmp).
do if ((mcx > 0) and (xrefvals(1,1) <> 999) and (xmint=1) and (model= 74)).
compute notecode(notes,1) = 36.
compute notes = notes + 1.
end if.
do if (mcx=0).
do if (ncol(xrefvals)>2 and model=74 and xmint=1).
compute errcode(errs,1)=67.
compute errs=errs+1.
compute criterr=1.
end if.
do if ((model=74) and (xmint=1)).
do if ((xrefvals(1,1)=999) and nxvls=1 and xdich=0).
compute errcode(errs,1)=66.
compute errs=errs+1.
compute criterr=1.
end if.
do if ((xrefvals(1,1)=999) and (xdich=1)).
compute xrefvals={xmn,xmx}.
compute xscaling=xrefvals(1,2)-xrefvals(1,1).
end if.
do if (ncol(xrefvals)=1 and xrefvals(1,1) <> 999).
do if (xdich=0).
compute xrefvals={xrefvals,(xrefvals(1,1)+1)}.
compute xscaling=xrefvals(1,2)-xrefvals(1,1).
end if.
do if (xdich=1).
do if ((xrefvals(1,1) <> xmx) and (xrefvals(1,1) <> xmn)).
compute errcode(errs,1)=70.
compute errs=errs+1.
compute criterr=1.
end if.
do if (xrefvals(1,1) = xmx).
compute xrefvals={xrefvals,xmn}.
compute xscaling=xrefvals(1,2)-xrefvals(1,1).
end if.
do if (xrefvals(1,1) = xmn).
compute xrefvals={xrefvals,xmx}.
compute xscaling=xrefvals(1,2)-xrefvals(1,1).
end if.
end if.
end if.
do if (ncol(xrefvals)=2).
compute xscaling=xrefvals(1,2)-xrefvals(1,1).
do if (xdich=1).
compute xreferr=1.
do if (((xrefvals(1,1) = xmx) and (xrefvals(1,2)=xmn)) or ((xrefvals(1,1) = xmn) and (xrefvals(1,2)=xmx))).
compute xreferr=0.
end if.
do if (xreferr=1).
compute errcode(errs,1)=70.
compute errs=errs+1.
compute criterr=1.
end if.
end if.
end if.
end if.
end if.
do if (xmint=1 and model=74 and mcx=0).
compute xmodvals=t(xrefvals).
compute xcontcf=1.
end if.
compute nxpval=nrow(xmodvals).
compute xprobval=xmodvals.
do if (xdich =1 and mcx > 0).
compute mcx=0.
compute errcode(errs,1) = 52.
compute errs = errs + 1.
compute criterr = 1.
end if.
do if (nms > 0).
compute mtmp=dat(:,(nys+nxs+1):(nys+nxs+nms)).

.
compute desctmp=make((8-(4* 0 )),ncol( mtmp ),-999).
loop jd=1 to ncol( mtmp ).
compute descdat= mtmp (:,jd).
compute desctmp(1,jd) = csum(descdat)/nrow(descdat).
compute desctmp(2,jd) = (nrow(descdat)*sscp(descdat))-(t(csum(descdat))*(csum(descdat))).
compute desctmp(2,jd) = sqrt(desctmp(2,jd)/(nrow(descdat)*(nrow(descdat)-1))).
compute desctmp(3,jd)=cmin(descdat).
compute desctmp(4,jd)=cmax(descdat).
do if ( 0 =0).
compute minwarn=0.
compute maxwarn=0.
do if ((desctmp(3,jd)=desctmp(4,jd)) and novar=0).
compute errcode(errs,1)=15.
compute errs=errs+1.
compute criterr=1.
compute novar=1.
end if.
compute tmp=((descdat(:,1)=desctmp(3,jd))+(descdat(:,1)=desctmp(4,jd))).
compute desctmp(8,jd)=(csum(tmp)=nrow(tmp)).
compute tmp = descdat.
compute tmp(GRADE(descdat),:) = descdat.
compute descdat = tmp.
release tmp.
compute decval={.16;.5;.84}.
loop kd=1 to 3.
compute low=trunc(decval(kd,1)*(nrow(descdat)+1)).
compute lowdec=decval(kd,1)*(nrow(descdat)+1)-low.
compute value=descdat(low,1)+(descdat((low+1),1)-descdat(low,1))*lowdec.
compute desctmp((4+kd),jd)=value.
end loop.
compute mnotev=1.
compute modvals=desctmp(5:7,:).
do if (quantile <> 1).
compute desctmp(5,jd)=desctmp(1,jd)-desctmp(2,jd).
compute desctmp(6,jd)=desctmp(1,jd).
compute desctmp(7,jd)=desctmp(1,jd)+desctmp(2,jd).
compute modvals=desctmp(5:7,:).
compute mnotev=2.
do if (modvals(1,1) < desctmp(3,1)).
compute modvals(1,1)=desctmp(3,1).
compute minwarn=1.
end if.
do if (modvals(3,1) > desctmp(4,1)).
compute modvals(3,1)=desctmp(4,1).
compute maxwarn=1.
end if.
end if.
do if (desctmp(8,1)=1).
compute modvals={desctmp(3,1);desctmp(4,1)}.
compute mnotev=0.
compute minwarn=0.
compute maxwarn=0.
end if.
end if.
end loop
.
compute ovsd={desctmp(2,:),ysd}.
compute medmeans=cdeval.
do if ((cdeval(1,1) <> -999) and (ncol(medmeans) <> nms) and (model=74)).
compute errcode(errs,1)=64.
compute errs=errs+1.
compute criterr=1.
end if.
do if ((cdeval(1,1)=-999) and (model=74)).
compute medmeans=desctmp(1,:).
end if.
do if ((cdeval(1,1)<> -999) and (model=74) and (criterr=0)).
compute notecode(notes,1) = 31.
compute notes = notes + 1.
end if.
do if ((rsum(desctmp(8,:))>0) and (mdichok <> 1)).
compute errcode(errs,1)=43.
compute errs=errs+1.
compute criterr=1.
end if.
compute mmodvals=modvals.
compute mprobval=mmodvals.
end if.
do if (nws > 0).
compute wtmp=dat(:,(nys+nxs+nms+1):(nys+nxs+nms+nws)).

.
compute desctmp=make((8-(4* 0 )),ncol( wtmp ),-999).
loop jd=1 to ncol( wtmp ).
compute descdat= wtmp (:,jd).
compute desctmp(1,jd) = csum(descdat)/nrow(descdat).
compute desctmp(2,jd) = (nrow(descdat)*sscp(descdat))-(t(csum(descdat))*(csum(descdat))).
compute desctmp(2,jd) = sqrt(desctmp(2,jd)/(nrow(descdat)*(nrow(descdat)-1))).
compute desctmp(3,jd)=cmin(descdat).
compute desctmp(4,jd)=cmax(descdat).
do if ( 0 =0).
compute minwarn=0.
compute maxwarn=0.
do if ((desctmp(3,jd)=desctmp(4,jd)) and novar=0).
compute errcode(errs,1)=15.
compute errs=errs+1.
compute criterr=1.
compute novar=1.
end if.
compute tmp=((descdat(:,1)=desctmp(3,jd))+(descdat(:,1)=desctmp(4,jd))).
compute desctmp(8,jd)=(csum(tmp)=nrow(tmp)).
compute tmp = descdat.
compute tmp(GRADE(descdat),:) = descdat.
compute descdat = tmp.
release tmp.
compute decval={.16;.5;.84}.
loop kd=1 to 3.
compute low=trunc(decval(kd,1)*(nrow(descdat)+1)).
compute lowdec=decval(kd,1)*(nrow(descdat)+1)-low.
compute value=descdat(low,1)+(descdat((low+1),1)-descdat(low,1))*lowdec.
compute desctmp((4+kd),jd)=value.
end loop.
compute mnotev=1.
compute modvals=desctmp(5:7,:).
do if (quantile <> 1).
compute desctmp(5,jd)=desctmp(1,jd)-desctmp(2,jd).
compute desctmp(6,jd)=desctmp(1,jd).
compute desctmp(7,jd)=desctmp(1,jd)+desctmp(2,jd).
compute modvals=desctmp(5:7,:).
compute mnotev=2.
do if (modvals(1,1) < desctmp(3,1)).
compute modvals(1,1)=desctmp(3,1).
compute minwarn=1.
end if.
do if (modvals(3,1) > desctmp(4,1)).
compute modvals(3,1)=desctmp(4,1).
compute maxwarn=1.
end if.
end if.
do if (desctmp(8,1)=1).
compute modvals={desctmp(3,1);desctmp(4,1)}.
compute mnotev=0.
compute minwarn=0.
compute maxwarn=0.
end if.
end if.
end loop
.
compute wmodvals=modvals.
compute wdich=desctmp(8,1).
do if (wdich =1 and mcw > 0).
compute mcw=0.
compute errcode(errs,1) = 52.
compute errs = errs + 1.
compute criterr = 1.
end if.
compute wmin=desctmp(3,1).
compute wmax=desctmp(4,1).
compute minwwarn=minwarn.
compute maxwwarn=maxwarn.
compute wnotev=mnotev.
compute wmodval={ 999 }.
do if (xmint=1 and model=74 and mcx=0).
compute wmodval=xrefvals.
end if.
compute nwcontr=ncol(wmodval).
do if (wmodval(1,1) <> 999).
compute wmodvals=wmodval(1,1).
compute wmodcust=1.
do if (nwcontr > 1).
compute wmodvals=t(wmodval).
end if.
compute minwwarn=0.
compute maxwwarn=0.
compute wnotev=0.
end if.
compute wprobval=wmodvals.
compute nwpval=nrow(wmodvals).
end if.
do if (nzs > 0).
compute ztmp=dat(:,(nys+nxs+nms+nws+1):(nys+nxs+nms+nws+nzs)).

.
compute desctmp=make((8-(4* 0 )),ncol( ztmp ),-999).
loop jd=1 to ncol( ztmp ).
compute descdat= ztmp (:,jd).
compute desctmp(1,jd) = csum(descdat)/nrow(descdat).
compute desctmp(2,jd) = (nrow(descdat)*sscp(descdat))-(t(csum(descdat))*(csum(descdat))).
compute desctmp(2,jd) = sqrt(desctmp(2,jd)/(nrow(descdat)*(nrow(descdat)-1))).
compute desctmp(3,jd)=cmin(descdat).
compute desctmp(4,jd)=cmax(descdat).
do if ( 0 =0).
compute minwarn=0.
compute maxwarn=0.
do if ((desctmp(3,jd)=desctmp(4,jd)) and novar=0).
compute errcode(errs,1)=15.
compute errs=errs+1.
compute criterr=1.
compute novar=1.
end if.
compute tmp=((descdat(:,1)=desctmp(3,jd))+(descdat(:,1)=desctmp(4,jd))).
compute desctmp(8,jd)=(csum(tmp)=nrow(tmp)).
compute tmp = descdat.
compute tmp(GRADE(descdat),:) = descdat.
compute descdat = tmp.
release tmp.
compute decval={.16;.5;.84}.
loop kd=1 to 3.
compute low=trunc(decval(kd,1)*(nrow(descdat)+1)).
compute lowdec=decval(kd,1)*(nrow(descdat)+1)-low.
compute value=descdat(low,1)+(descdat((low+1),1)-descdat(low,1))*lowdec.
compute desctmp((4+kd),jd)=value.
end loop.
compute mnotev=1.
compute modvals=desctmp(5:7,:).
do if (quantile <> 1).
compute desctmp(5,jd)=desctmp(1,jd)-desctmp(2,jd).
compute desctmp(6,jd)=desctmp(1,jd).
compute desctmp(7,jd)=desctmp(1,jd)+desctmp(2,jd).
compute modvals=desctmp(5:7,:).
compute mnotev=2.
do if (modvals(1,1) < desctmp(3,1)).
compute modvals(1,1)=desctmp(3,1).
compute minwarn=1.
end if.
do if (modvals(3,1) > desctmp(4,1)).
compute modvals(3,1)=desctmp(4,1).
compute maxwarn=1.
end if.
end if.
do if (desctmp(8,1)=1).
compute modvals={desctmp(3,1);desctmp(4,1)}.
compute mnotev=0.
compute minwarn=0.
compute maxwarn=0.
end if.
end if.
end loop
.
compute zmodvals=modvals.
compute zdich=desctmp(8,1).
do if (zdich =1 and mcz > 0).
compute mcz=0.
compute errcode(errs,1) = 52.
compute errs = errs + 1.
compute criterr = 1.
end if.
compute zmin=desctmp(3,1).
compute zmax=desctmp(4,1).
compute minzwarn=minwarn.
compute maxzwarn=maxwarn.
compute znotev=mnotev.
compute zmodval={ 999 }.
compute nzcontr=ncol(zmodval).
do if (zmodval(1,1) <> 999).
compute zmodvals=zmodval(1,1).
compute zmodcust=1.
do if (nzcontr > 1).
compute zmodvals=t(zmodval).
end if.
compute minzwarn=0.
compute maxzwarn=0.
compute znotev=0.
end if.
compute zprobval=zmodvals.
compute nzpval=nrow(zmodvals).
end if.
do if (ncs > 0).
compute ctmp=dat(:,(nys+nxs+nms+nws+nzs+1):(nys+nxs+nms+nws+nzs+ncs)).

.
compute desctmp=make((8-(4* 0 )),ncol( ctmp ),-999).
loop jd=1 to ncol( ctmp ).
compute descdat= ctmp (:,jd).
compute desctmp(1,jd) = csum(descdat)/nrow(descdat).
compute desctmp(2,jd) = (nrow(descdat)*sscp(descdat))-(t(csum(descdat))*(csum(descdat))).
compute desctmp(2,jd) = sqrt(desctmp(2,jd)/(nrow(descdat)*(nrow(descdat)-1))).
compute desctmp(3,jd)=cmin(descdat).
compute desctmp(4,jd)=cmax(descdat).
do if ( 0 =0).
compute minwarn=0.
compute maxwarn=0.
do if ((desctmp(3,jd)=desctmp(4,jd)) and novar=0).
compute errcode(errs,1)=15.
compute errs=errs+1.
compute criterr=1.
compute novar=1.
end if.
compute tmp=((descdat(:,1)=desctmp(3,jd))+(descdat(:,1)=desctmp(4,jd))).
compute desctmp(8,jd)=(csum(tmp)=nrow(tmp)).
compute tmp = descdat.
compute tmp(GRADE(descdat),:) = descdat.
compute descdat = tmp.
release tmp.
compute decval={.16;.5;.84}.
loop kd=1 to 3.
compute low=trunc(decval(kd,1)*(nrow(descdat)+1)).
compute lowdec=decval(kd,1)*(nrow(descdat)+1)-low.
compute value=descdat(low,1)+(descdat((low+1),1)-descdat(low,1))*lowdec.
compute desctmp((4+kd),jd)=value.
end loop.
compute mnotev=1.
compute modvals=desctmp(5:7,:).
do if (quantile <> 1).
compute desctmp(5,jd)=desctmp(1,jd)-desctmp(2,jd).
compute desctmp(6,jd)=desctmp(1,jd).
compute desctmp(7,jd)=desctmp(1,jd)+desctmp(2,jd).
compute modvals=desctmp(5:7,:).
compute mnotev=2.
do if (modvals(1,1) < desctmp(3,1)).
compute modvals(1,1)=desctmp(3,1).
compute minwarn=1.
end if.
do if (modvals(3,1) > desctmp(4,1)).
compute modvals(3,1)=desctmp(4,1).
compute maxwarn=1.
end if.
end if.
do if (desctmp(8,1)=1).
compute modvals={desctmp(3,1);desctmp(4,1)}.
compute mnotev=0.
compute minwarn=0.
compute maxwarn=0.
end if.
end if.
end loop
.
compute covmeans=desctmp(1,:).
compute coval={ -999 }.
do if ((coval(1,1) <> -999) and (ncol(coval) <> ncs) and (model=74)).
compute errcode(errs,1)=69.
compute errs=errs+1.
compute criterr=1.
end if.
do if ((coval(1,1)<> -999) and (criterr=0) and (model=74)).
compute notecode(notes,1) = 35.
compute notes = notes + 1.
compute cuscoval=1.
end if.
end if.
compute n=nrow(ytmp).
compute ones=make(n,1,1).
do if (nws > 0 and mcw > 0).
compute tmp={rownum,wtmp(:,1)}.

.
compute dd= tmp.
compute temp = dd.
compute temp(GRADE(dd(:,2)),:) = dd.
compute dd = temp.
compute dummy = design(dd(:,2)).
compute nvls = ncol(dummy).
compute nnvls = csum(dummy).
compute mnvls = cmin(t(nnvls)).
compute conmat1=1.
do if (mnvls < 2).
compute errcode(errs,1) = 5.
compute errs = errs + 1.
compute criterr = 1.
end if.
do if (nvls > 9).
compute errcode(errs,1) = 4.
compute errs = errs+1.
compute criterr = 1.
end if.
do if (criterr = 0).
compute dumok = 1.
compute nnvls=make(nvls,1,0).
compute nnvls(1,1)=dd(1,2).
compute temp = 2.
loop i = 2 to n.
do if (dd(i,2) <> nnvls((temp-1),1)).
compute nnvls(temp,1)=dd(i,2).
compute temp = temp+1.
end if.
end loop.
do if ( mcw > 0).
compute x = dummy(:,2:ncol(dummy)).
compute nx = ncol(x).
compute minus1 = make(1,ncol(x),-1).
compute xdes=make((nx+1),3,0).
compute xdes(1,1)=dd(1,2).
compute xdes(1,2)=1.
compute temp = 2.
loop k = 2 to n.
do if (dd(k,2) <> dd((k-1),2)).
compute xdes(temp,2) = k.
compute xdes(temp,1) = dd(k,2).
compute xdes((temp-1),3) = k-1.
compute temp=temp+1.
end if.
end loop.
compute xdes((temp-1),3)=n.
compute xdes = {xdes, (xdes(:,3)-xdes(:,2)+1)}.
do if ( mcw = 4).
loop k = 1 to n.
do if (rsum(x(k,:)) = 0).
compute x(k,:) = minus1.
end if.
end loop.
end if.
do if ( mcw = 2 or mcw = 3 or mcw =5).
loop k = 1 to n.
do if (rsum(x(k,:)) > 0).
loop i = 1 to ncol(x).
do if (x(k,i) = 0).
compute x(k,i) = 1.
else.
break.
end if.
end loop.
end if.
end loop.
do if ( mcw = 3).
compute conmat1={-8,1,1,1,1,1,1,1,1; 0,-7,1,1,1,1,1,1,1; 0,0,-6,1,1,1,1,1,1; 0,0,0,-5,1,1,1,1,1; 0,0,0,0,-4,1,1,1,1; 0,0,0,0,0,-3,1,1,1; 0,0,0,0,0,0,-2,1,1; 0,0,0,0,0,0,0,-1,1}.
loop i = 1 to 8.
compute conmat1(i,:)=conmat1(i,:)/(10-i).
end loop.
compute conmat1=t(conmat1((10-nvls):8,(10-nvls):9)).
loop k=1 to n.
compute x(k,:)=conmat1((rsum(x(k,:))+1),:).
end loop.
end if.
end if.
do if ( mcw = 5).
compute custcode={ -999 }.
do if (ncol(custcode) <> (nvls*(nvls-1))).
compute errcode(errs,1) = (37+ 2 ).
compute errs = errs + 1.
compute criterr = 1.
end if.
do if (ncol(custcode) = (nvls*(nvls-1))).
compute conmat1=make(nvls,(nvls-1),0).
compute cnt=1.
loop i = 1 to nvls.
loop k = 1 to (nvls-1).
compute conmat1(i,k)=custcode(1,cnt).
compute cnt=cnt+1.
end loop.
end loop.
loop k=1 to n.
compute x(k,:)=conmat1((rsum(x(k,:))+1),:).
end loop.
end if.
end if.
compute xskip = 1.
compute dummat = make((nx+1),nx,0).
compute dummat((2:nrow(dummat)),:)=ident(nx).
do if ( mcw = 4).
compute dummat(1,:) = minus1.
end if.
do if ( mcw = 2).
loop i = 2 to nrow(dummat).
loop j = 1 to (i-1).
compute dummat(i,j) = 1.
end loop.
end loop.
end if.
do if ( mcw = 3).
compute dummat=conmat1.
end if.
do if ( mcw = 5 and criterr=0).
compute dummat=conmat1.
end if.
compute dummat={nnvls, dummat}.
compute x={dd(:,1),x}.
compute temp = x.
compute temp(GRADE(x(:,1)),:) = x.
compute x = temp.
release conmat1,temp,dd,xskip,xdes,dummy.
end if.
end if
.
compute wmodvals=nnvls.
compute nwpval=nrow(wmodvals).
do if (criterr=0).
compute minwwarn=0.
compute maxwwarn=0.
compute wnotev=0.
compute wtmp=x(:,2:ncol(x)).
compute wcatlab={'W1';'W2';'W3';'W4';'W5';'W6';'W7';'W8';'W9'}.
do if (xmint=1).
compute wcatlab={'X1';'X2';'X3';'X4';'X5';'X6';'X7';'X8';'X9'}.
end if.
compute nwvls=nvls-1.
compute mcwok=1.
compute dummatw=dummat.
compute wprobval=dummatw(:,2:ncol(dummatw)).
do if (modcok=1).
compute wcontval=make(2,ncol(wprobval),-999).
compute temp=0.
loop i = 1 to 2.
loop j = 1 to nrow(dummatw).
do if (contvec(i,1)=dummatw(j,1)).
compute wcontval(i,:)=wprobval(j,:).
compute temp=temp+1.
end if.
end loop.
end loop.
do if (temp < 2).
compute notecode(notes,1) = 20.
compute notes = notes + 1.
compute modcok=0.
end if.
end if.
do if ((wmodval(1,1) <> 999) and (xmint <> 1)).
compute notecode(notes,1) = 9.
compute notes = notes + 1.
end if.
release tmp, dummat.
end if.
end if.
do if (nzs > 0 and mcz > 0).
compute tmp={rownum,ztmp(:,1)}.

.
compute dd= tmp.
compute temp = dd.
compute temp(GRADE(dd(:,2)),:) = dd.
compute dd = temp.
compute dummy = design(dd(:,2)).
compute nvls = ncol(dummy).
compute nnvls = csum(dummy).
compute mnvls = cmin(t(nnvls)).
compute conmat1=1.
do if (mnvls < 2).
compute errcode(errs,1) = 5.
compute errs = errs + 1.
compute criterr = 1.
end if.
do if (nvls > 9).
compute errcode(errs,1) = 4.
compute errs = errs+1.
compute criterr = 1.
end if.
do if (criterr = 0).
compute dumok = 1.
compute nnvls=make(nvls,1,0).
compute nnvls(1,1)=dd(1,2).
compute temp = 2.
loop i = 2 to n.
do if (dd(i,2) <> nnvls((temp-1),1)).
compute nnvls(temp,1)=dd(i,2).
compute temp = temp+1.
end if.
end loop.
do if ( mcz > 0).
compute x = dummy(:,2:ncol(dummy)).
compute nx = ncol(x).
compute minus1 = make(1,ncol(x),-1).
compute xdes=make((nx+1),3,0).
compute xdes(1,1)=dd(1,2).
compute xdes(1,2)=1.
compute temp = 2.
loop k = 2 to n.
do if (dd(k,2) <> dd((k-1),2)).
compute xdes(temp,2) = k.
compute xdes(temp,1) = dd(k,2).
compute xdes((temp-1),3) = k-1.
compute temp=temp+1.
end if.
end loop.
compute xdes((temp-1),3)=n.
compute xdes = {xdes, (xdes(:,3)-xdes(:,2)+1)}.
do if ( mcz = 4).
loop k = 1 to n.
do if (rsum(x(k,:)) = 0).
compute x(k,:) = minus1.
end if.
end loop.
end if.
do if ( mcz = 2 or mcz = 3 or mcz =5).
loop k = 1 to n.
do if (rsum(x(k,:)) > 0).
loop i = 1 to ncol(x).
do if (x(k,i) = 0).
compute x(k,i) = 1.
else.
break.
end if.
end loop.
end if.
end loop.
do if ( mcz = 3).
compute conmat1={-8,1,1,1,1,1,1,1,1; 0,-7,1,1,1,1,1,1,1; 0,0,-6,1,1,1,1,1,1; 0,0,0,-5,1,1,1,1,1; 0,0,0,0,-4,1,1,1,1; 0,0,0,0,0,-3,1,1,1; 0,0,0,0,0,0,-2,1,1; 0,0,0,0,0,0,0,-1,1}.
loop i = 1 to 8.
compute conmat1(i,:)=conmat1(i,:)/(10-i).
end loop.
compute conmat1=t(conmat1((10-nvls):8,(10-nvls):9)).
loop k=1 to n.
compute x(k,:)=conmat1((rsum(x(k,:))+1),:).
end loop.
end if.
end if.
do if ( mcz = 5).
compute custcode={ -999 }.
do if (ncol(custcode) <> (nvls*(nvls-1))).
compute errcode(errs,1) = (37+ 3 ).
compute errs = errs + 1.
compute criterr = 1.
end if.
do if (ncol(custcode) = (nvls*(nvls-1))).
compute conmat1=make(nvls,(nvls-1),0).
compute cnt=1.
loop i = 1 to nvls.
loop k = 1 to (nvls-1).
compute conmat1(i,k)=custcode(1,cnt).
compute cnt=cnt+1.
end loop.
end loop.
loop k=1 to n.
compute x(k,:)=conmat1((rsum(x(k,:))+1),:).
end loop.
end if.
end if.
compute xskip = 1.
compute dummat = make((nx+1),nx,0).
compute dummat((2:nrow(dummat)),:)=ident(nx).
do if ( mcz = 4).
compute dummat(1,:) = minus1.
end if.
do if ( mcz = 2).
loop i = 2 to nrow(dummat).
loop j = 1 to (i-1).
compute dummat(i,j) = 1.
end loop.
end loop.
end if.
do if ( mcz = 3).
compute dummat=conmat1.
end if.
do if ( mcz = 5 and criterr=0).
compute dummat=conmat1.
end if.
compute dummat={nnvls, dummat}.
compute x={dd(:,1),x}.
compute temp = x.
compute temp(GRADE(x(:,1)),:) = x.
compute x = temp.
release conmat1,temp,dd,xskip,xdes,dummy.
end if.
end if
.
compute zmodvals=nnvls.
compute nzpval=nrow(zmodvals).
do if (criterr=0).
compute minzwarn=0.
compute maxzwarn=0.
compute znotev=0.
compute ztmp=x(:,2:ncol(x)).
compute zcatlab={'Z1';'Z2';'Z3';'Z4';'Z5';'Z6';'Z7';'Z8';'Z9'}.
compute nzvls=nvls-1.
compute mczok=1.
compute dummatz=dummat.
compute zprobval=dummatz(:,2:ncol(dummatz)).
do if (modcok=1).
compute zcontval=make(2,ncol(zprobval),-999).
compute temp=0.
loop i = 1 to 2.
loop j = 1 to nrow(dummatz).
do if (contvec(i,2)=dummatz(j,1)).
compute zcontval(i,:)=zprobval(j,:).
compute temp=temp+1.
end if.
end loop.
end loop.
do if (temp < 2).
compute notecode(notes,1) = 20.
compute notes = notes + 1.
compute modcok=0.
end if.
end if.
do if (zmodval(1,1) <> 999).
compute notecode(notes,1) = 10.
compute notes = notes + 1.
end if.
release tmp, dummat.
end if.
end if.
do if (nxs > 0 and mcx > 0).
compute tmp={rownum,xtmp(:,1)}.

.
compute dd= tmp.
compute temp = dd.
compute temp(GRADE(dd(:,2)),:) = dd.
compute dd = temp.
compute dummy = design(dd(:,2)).
compute nvls = ncol(dummy).
compute nnvls = csum(dummy).
compute mnvls = cmin(t(nnvls)).
compute conmat1=1.
do if (mnvls < 2).
compute errcode(errs,1) = 5.
compute errs = errs + 1.
compute criterr = 1.
end if.
do if (nvls > 9).
compute errcode(errs,1) = 4.
compute errs = errs+1.
compute criterr = 1.
end if.
do if (criterr = 0).
compute dumok = 1.
compute nnvls=make(nvls,1,0).
compute nnvls(1,1)=dd(1,2).
compute temp = 2.
loop i = 2 to n.
do if (dd(i,2) <> nnvls((temp-1),1)).
compute nnvls(temp,1)=dd(i,2).
compute temp = temp+1.
end if.
end loop.
do if ( mcx > 0).
compute x = dummy(:,2:ncol(dummy)).
compute nx = ncol(x).
compute minus1 = make(1,ncol(x),-1).
compute xdes=make((nx+1),3,0).
compute xdes(1,1)=dd(1,2).
compute xdes(1,2)=1.
compute temp = 2.
loop k = 2 to n.
do if (dd(k,2) <> dd((k-1),2)).
compute xdes(temp,2) = k.
compute xdes(temp,1) = dd(k,2).
compute xdes((temp-1),3) = k-1.
compute temp=temp+1.
end if.
end loop.
compute xdes((temp-1),3)=n.
compute xdes = {xdes, (xdes(:,3)-xdes(:,2)+1)}.
do if ( mcx = 4).
loop k = 1 to n.
do if (rsum(x(k,:)) = 0).
compute x(k,:) = minus1.
end if.
end loop.
end if.
do if ( mcx = 2 or mcx = 3 or mcx =5).
loop k = 1 to n.
do if (rsum(x(k,:)) > 0).
loop i = 1 to ncol(x).
do if (x(k,i) = 0).
compute x(k,i) = 1.
else.
break.
end if.
end loop.
end if.
end loop.
do if ( mcx = 3).
compute conmat1={-8,1,1,1,1,1,1,1,1; 0,-7,1,1,1,1,1,1,1; 0,0,-6,1,1,1,1,1,1; 0,0,0,-5,1,1,1,1,1; 0,0,0,0,-4,1,1,1,1; 0,0,0,0,0,-3,1,1,1; 0,0,0,0,0,0,-2,1,1; 0,0,0,0,0,0,0,-1,1}.
loop i = 1 to 8.
compute conmat1(i,:)=conmat1(i,:)/(10-i).
end loop.
compute conmat1=t(conmat1((10-nvls):8,(10-nvls):9)).
loop k=1 to n.
compute x(k,:)=conmat1((rsum(x(k,:))+1),:).
end loop.
end if.
end if.
do if ( mcx = 5).
compute custcode={ -999 }.
do if (ncol(custcode) <> (nvls*(nvls-1))).
compute errcode(errs,1) = (37+ 1 ).
compute errs = errs + 1.
compute criterr = 1.
end if.
do if (ncol(custcode) = (nvls*(nvls-1))).
compute conmat1=make(nvls,(nvls-1),0).
compute cnt=1.
loop i = 1 to nvls.
loop k = 1 to (nvls-1).
compute conmat1(i,k)=custcode(1,cnt).
compute cnt=cnt+1.
end loop.
end loop.
loop k=1 to n.
compute x(k,:)=conmat1((rsum(x(k,:))+1),:).
end loop.
end if.
end if.
compute xskip = 1.
compute dummat = make((nx+1),nx,0).
compute dummat((2:nrow(dummat)),:)=ident(nx).
do if ( mcx = 4).
compute dummat(1,:) = minus1.
end if.
do if ( mcx = 2).
loop i = 2 to nrow(dummat).
loop j = 1 to (i-1).
compute dummat(i,j) = 1.
end loop.
end loop.
end if.
do if ( mcx = 3).
compute dummat=conmat1.
end if.
do if ( mcx = 5 and criterr=0).
compute dummat=conmat1.
end if.
compute dummat={nnvls, dummat}.
compute x={dd(:,1),x}.
compute temp = x.
compute temp(GRADE(x(:,1)),:) = x.
compute x = temp.
release conmat1,temp,dd,xskip,xdes,dummy.
end if.
end if
.
do if (criterr=0).
compute xtmp=x(:,2:ncol(x)).
compute xcatlab={'X1';'X2';'X3';'X4';'X5';'X6';'X7';'X8';'X9'}.
compute nxvls=nvls-1.
compute xdich=(nvls=2).
compute mcxok=1.
compute dummatx=dummat.
compute xmodvals=dummatx(:,1).
compute nxpval=nrow(xmodvals).
release tmp, dummat.
end if.
end if.
compute intlab=make(100,1,' ').


compute intlab( 1 ,1)= 'Int_1'.


compute intlab( 2 ,1)= 'Int_2'.


compute intlab( 3 ,1)= 'Int_3'.


compute intlab( 4 ,1)= 'Int_4'.


compute intlab( 5 ,1)= 'Int_5'.


compute intlab( 6 ,1)= 'Int_6'.


compute intlab( 7 ,1)= 'Int_7'.


compute intlab( 8 ,1)= 'Int_8'.


compute intlab( 9 ,1)= 'Int_9'.


compute intlab( 10 ,1)= 'Int_10'.


compute intlab( 11 ,1)= 'Int_11'.


compute intlab( 12 ,1)= 'Int_12'.


compute intlab( 13 ,1)= 'Int_13'.


compute intlab( 14 ,1)= 'Int_14'.


compute intlab( 15 ,1)= 'Int_15'.


compute intlab( 16 ,1)= 'Int_16'.


compute intlab( 17 ,1)= 'Int_17'.


compute intlab( 18 ,1)= 'Int_18'.


compute intlab( 19 ,1)= 'Int_19'.


compute intlab( 20 ,1)= 'Int_20'.


compute intlab( 21 ,1)= 'Int_21'.


compute intlab( 22 ,1)= 'Int_22'.


compute intlab( 23 ,1)= 'Int_23'.


compute intlab( 24 ,1)= 'Int_24'.


compute intlab( 25 ,1)= 'Int_25'.


compute intlab( 26 ,1)= 'Int_26'.


compute intlab( 27 ,1)= 'Int_27'.


compute intlab( 28 ,1)= 'Int_28'.


compute intlab( 29 ,1)= 'Int_29'.


compute intlab( 30 ,1)= 'Int_30'.


compute intlab( 31 ,1)= 'Int_31'.


compute intlab( 32 ,1)= 'Int_32'.


compute intlab( 33 ,1)= 'Int_33'.


compute intlab( 34 ,1)= 'Int_34'.


compute intlab( 35 ,1)= 'Int_35'.


compute intlab( 36 ,1)= 'Int_36'.


compute intlab( 37 ,1)= 'Int_37'.


compute intlab( 38 ,1)= 'Int_38'.


compute intlab( 39 ,1)= 'Int_39'.


compute intlab( 40 ,1)= 'Int_40'.


compute intlab( 41 ,1)= 'Int_41'.


compute intlab( 42 ,1)= 'Int_42'.


compute intlab( 43 ,1)= 'Int_43'.


compute intlab( 44 ,1)= 'Int_44'.


compute intlab( 45 ,1)= 'Int_45'.


compute intlab( 46 ,1)= 'Int_46'.


compute intlab( 47 ,1)= 'Int_47'.


compute intlab( 48 ,1)= 'Int_48'.


compute intlab( 49 ,1)= 'Int_49'.


compute intlab( 50 ,1)= 'Int_50'.


compute intlab( 51 ,1)= 'Int_51'.


compute intlab( 52 ,1)= 'Int_52'.


compute intlab( 53 ,1)= 'Int_53'.


compute intlab( 54 ,1)= 'Int_54'.


compute intlab( 55 ,1)= 'Int_55'.


compute intlab( 56 ,1)= 'Int_56'.


compute intlab( 57 ,1)= 'Int_57'.


compute intlab( 58 ,1)= 'Int_58'.


compute intlab( 59 ,1)= 'Int_59'.


compute intlab( 60 ,1)= 'Int_60'.


compute intlab( 61 ,1)= 'Int_61'.


compute intlab( 62 ,1)= 'Int_62'.


compute intlab( 63 ,1)= 'Int_63'.


compute intlab( 64 ,1)= 'Int_64'.


compute intlab( 65 ,1)= 'Int_65'.


compute intlab( 66 ,1)= 'Int_66'.


compute intlab( 67 ,1)= 'Int_67'.


compute intlab( 68 ,1)= 'Int_68'.


compute intlab( 69 ,1)= 'Int_69'.


compute intlab( 70 ,1)= 'Int_70'.


compute intlab( 71 ,1)= 'Int_71'.


compute intlab( 72 ,1)= 'Int_72'.


compute intlab( 73 ,1)= 'Int_73'.


compute intlab( 74 ,1)= 'Int_74'.


compute intlab( 75 ,1)= 'Int_75'.


compute intlab( 76 ,1)= 'Int_76'.


compute intlab( 77 ,1)= 'Int_77'.


compute intlab( 78 ,1)= 'Int_78'.


compute intlab( 79 ,1)= 'Int_79'.


compute intlab( 80 ,1)= 'Int_80'.


compute intlab( 81 ,1)= 'Int_81'.


compute intlab( 82 ,1)= 'Int_82'.


compute intlab( 83 ,1)= 'Int_83'.


compute intlab( 84 ,1)= 'Int_84'.


compute intlab( 85 ,1)= 'Int_85'.


compute intlab( 86 ,1)= 'Int_86'.


compute intlab( 87 ,1)= 'Int_87'.


compute intlab( 88 ,1)= 'Int_88'.


compute intlab( 89 ,1)= 'Int_89'.


compute intlab( 90 ,1)= 'Int_90'.


compute intlab( 91 ,1)= 'Int_91'.


compute intlab( 92 ,1)= 'Int_92'.


compute intlab( 93 ,1)= 'Int_93'.


compute intlab( 94 ,1)= 'Int_94'.


compute intlab( 95 ,1)= 'Int_95'.


compute intlab( 96 ,1)= 'Int_96'.


compute intlab( 97 ,1)= 'Int_97'.


compute intlab( 98 ,1)= 'Int_98'.


compute intlab( 99 ,1)= 'Int_99'.


compute intlab( 100 ,1)= 'Int_100'.

compute bcmat=make(needed,needed,0).
compute wcmat=make(needed,needed,0).
compute zcmat=make(needed,needed,0).
compute wzcmat=make(needed,needed,0).
compute wsum=0.
compute zsum=0.
compute wzsum=0.
end if.
end if.
do if (criterr = 0 and model <> 999).
compute modelmat= {1,0,0,0,0,0,0,1,0,0;2,0,0,0,0,0,0,1,1,0;3,0,0,0,0,0,0,1,1,1;4,0,0,0,0,0,0,0,0,0; 5,0,0,0,0,0,0,1,0,0;6,0,0,0,0,0,0,0,0,0;7,1,0,0,0,0,0,0,0,0;8,1,0,0,0,0,0,1,0,0; 9,1,1,0,0,0,0,0,0,0;10,1,1,0,0,0,0,1,1,0;11,1,1,1,0,0,0,0,0,0;12,1,1,1,0,0,0,1,1,1; 13,1,1,1,0,0,0,1,0,0;14,0,0,0,1,0,0,0,0,0;15,0,0,0,1,0,0,1,0,0;16,0,0,0,1,1,0,0,0,0; 17,0,0,0,1,1,0,1,1,0;18,0,0,0,1,1,1,0,0,0;19,0,0,0,1,1,1,1,1,1;20,0,0,0,1,1,1,1,0,0; 21,1,0,0,0,1,0,0,0,0;22,1,0,0,0,1,0,1,0,0;23,0,0,0,0,0,0,0,0,0;24,0,0,0,0,0,0,0,0,0; 25,0,0,0,0,0,0,0,0,0;26,0,0,0,0,0,0,0,0,0;27,0,0,0,0,0,0,0,0,0;28,1,0,0,0,1,0,0,1,0; 29,1,0,0,0,1,0,1,1,0;30,0,0,0,0,0,0,0,0,0;31,0,0,0,0,0,0,0,0,0;32,0,0,0,0,0,0,0,0,0; 33,0,0,0,0,0,0,0,0,0;34,0,0,0,0,0,0,0,0,0;35,0,0,0,0,0,0,0,0,0;36,0,0,0,0,0,0,0,0,0; 37,0,0,0,0,0,0,0,0,0;38,0,0,0,0,0,0,0,0,0;39,0,0,0,0,0,0,0,0,0;40,0,0,0,0,0,0,0,0,0; 41,0,0,0,0,0,0,0,0,0;42,0,0,0,0,0,0,0,0,0;43,0,0,0,0,0,0,0,0,0;44,0,0,0,0,0,0,0,0,0; 45,0,0,0,0,0,0,0,0,0;46,0,0,0,0,0,0,0,0,0;47,0,0,0,0,0,0,0,0,0;48,0,0,0,0,0,0,0

 ,0,0; 49,0,0,0,0,0,0,0,0,0;50,0,0,0,0,0,0,0,0,0;51,0,0,0,0,0,0,0,0,0;52,0,0,0,0,0,0,0,0,0; 53,0,0,0,0,0,0,0,0,0;54,0,0,0,0,0,0,0,0,0;55,0,0,0,0,0,0,0,0,0;56,0,0,0,0,0,0,0,0,0; 57,0,0,0,0,0,0,0,0,0;58,1,0,0,1,0,0,0,0,0;59,1,0,0,1,0,0,1,0,0;60,1,1,0,1,0,0,0,0,0; 61,1,1,0,1,0,0,1,0,0;62,1,1,0,1,0,0,0,1,0;63,1,1,0,1,0,0,1,1,0;64,1,0,0,1,1,0,0,0,0; 65,1,0,0,1,1,0,1,0,0;66,1,0,0,1,1,0,0,1,0;67,1,0,0,1,1,0,1,1,0;68,1,1,1,1,0,0,0,0,0; 69,1,1,1,1,0,0,1,1,1;70,1,0,0,1,1,1,0,0,0;71,1,0,0,1,1,1,1,1,1;72,1,1,1,1,1,1,0,0,0; 73,1,1,1,1,1,1,1,1,1;74,0,0,0,1,0,0,0,0,0;75,1,1,0,1,1,0,0,0,0;76,1,1,0,1,1,0,1,1,0; 77,0,0,0,0,0,0,0,0,0;78,0,0,0,0,0,0,0,0,0;79,0,0,0,0,0,0,0,0,0;80,0,0,0,0,0,0,0,0,0; 81,0,0,0,0,0,0,0,0,0;82,0,0,0,0,0,0,0,0,0;83,1,0,0,0,0,0,0,0,0;84,1,0,0,0,0,0,0,0,0; 85,1,0,0,0,0,0,1,0,0;86,1,0,0,0,0,0,1,0,0;87,0,0,0,1,0,0,0,0,0;88,0,0,0,1,0,0,0,0,0; 89,0,0,0,1,0,0,1,0,0;90,0,0,0,1,0,0,1,0,0;91,0,0,0,0,0,0,0,0,0;92,1,0,0,1,0,0,1,0,0}.
do if (model > 0).
compute tmp=modelmat(model,2:ncol(modelmat)).
end if.
do if (model=0).
compute tmp={0,0,0,0,0,0,0,0,0}.
end if.
do if (model < 4).
compute bcmat((nxs+1),1)=1.
end if.
do if ((model > 3) and (model <> 6)).
compute bcmat((nxs+1):(nxs+nms),1)=onem.
compute bcmat(nrow(bcmat),(nxs+1):(nxs+nms))=t(onem).
compute bcmat(nrow(bcmat),1)=1.
end if.
do if ((model = 6) or (model > 82 and model < 93)).
loop j = 2 to nrow(bcmat).
loop i = 1 to (j-1).
compute bcmat(j,i)=1.
end loop.
end loop.
end if.
do if (model = 80).
loop i = 1 to nms.
compute bcmat((nrow(bcmat)-1),i)=1.
end loop.
end if.
do if (model = 81).
loop j = 3 to nrow(bcmat).
compute bcmat(j,2)=1.
end loop.
end if.
do if (model = 82).
compute bcmat(3,2)=1.
compute bcmat(5,4)=1.
end if.
do if (tmp(1,1)=1).
compute wcmat((nxs+1):(nxs+nms),1)=onem.
compute wprod=1.
compute xprod=1.
do if (model = 83 or model = 86).
compute onemsx=onem.
loop i = 1 to (nms-1).
compute onemsx(i+1,1)=0.
end loop.
compute wcmat((nxs+1):(nxs+nms),1)=onemsx.
end if.
end if.
do if (tmp(1,4)=1).
compute wcmat(nrow(wcmat),(nxs+1):(nxs+nms))=t(onem).
compute wprod=1.
do if (model = 87 or model = 90).
compute onemsx=onem.
loop i = 1 to (nms-1).
compute onemsx(i,1)=0.
end loop.
compute wcmat(nrow(wcmat),(nxs+1):(nxs+nms))=t(onemsx).
end if.
end if.
do if (tmp(1,7)=1).
compute wcmat(nrow(wcmat),1)=1.
compute wprod=1.
compute xprod=1.
end if.
do if (tmp(1,2)=1).
compute zcmat((nxs+1):(nxs+nms),1)=onem.
compute zprod=1.
compute xprod=1.
end if.
do if (tmp(1,5)=1).
compute zcmat(nrow(zcmat),(nxs+1):(nxs+nms))=t(onem).
compute zprod=1.
end if.
do if (tmp(1,8)=1).
compute zcmat(nrow(zcmat),1)=1.
compute zprod=1.
compute xprod=1.
end if.
do if (tmp(1,3)=1).
compute wzcmat((nxs+1):(nxs+nms),1)=onem.
compute xprod=1.
compute wprod=1.
compute zprod=1.
end if.
do if (tmp(1,6)=1).
compute wzcmat(nrow(wzcmat),(nxs+1):(nxs+nms))=t(onem).
compute zprod=1.
compute wprod=1.
end if.
do if (tmp(1,9)=1).
compute wzcmat(nrow(wzcmat),1)=1.
compute xprod=1.
compute wprod=1.
compute zprod=1.
end if.
do if (model = 91 or model = 92).
loop j = 1 to (nms-1).
loop i = 1 to j.
compute wcmat((nxs+1+j),(nxs+i))=1.
end loop.
end loop.
end if.
do if (nms < 0).
loop i = 1 to nms.
compute tmp=csum(wcmat(:,(1+i)))+csum(zcmat(:,(1+i)))+csum(wzcmat(:,(1+i))).
compute mprod(1,i)=(tmp>0).
end loop.
end if.
end if.
do if (ncs > 0).
compute ccmat=make((nms+nys),ncs,1).
compute ccmatoff=ccmat.
do if (covmy=1).
compute ccmat(nrow(ccmat),:)=make(1,ncs,0).
end if.
do if (covmy=2).
compute ccmat(1:nms,:)=make(nms,ncs,0).
end if.
do if (cmatrix(1,1) <> -999).
do if (ncol(cmatrix) <> ((nms+nys)*ncs)).
compute errcode(errs,1)=29.
compute errs=errs+1.
compute criterr=1.
end if.
do if (criterr = 0).
compute tmp=1.
loop i = 1 to (nms+nys).
loop j = 1 to ncs.
compute ccmat(i,j)=1-(cmatrix(1,tmp) = 0).
compute tmp=tmp+1.
end loop.
end loop.
do if (rsum((csum(ccmat)=0)) <> 0).
compute errcode(errs,1)=30.
compute errs=errs+1.
compute criterr=1.
end if.
end if.
do if (covmy <> 0).
compute notecode(notes,1)=1.
compute notes=notes+1.
end if.
end if.
do if (xmint=1 and (covmy > 0 or (csum(rsum(ccmat)) <> ((nms+nys)*ncs)))).
compute errcode(errs,1)=68.
compute errs=errs+1.
compute criterr=1.
end if.
end if.
do if (criterr=0).
compute needed=needed*(needed-1)/2.
compute nopath=0.
do if (bmatrix(1,1) <> -999).
compute tmp=1.
do if ((ncol(bmatrix) <> needed) or (csum(rsum(bmatrix))=0)).
compute errcode(errs,1)=16.
compute errs=errs+1.
compute criterr=1.
else.
loop i = 2 to nrow(bcmat).
loop j = 1 to (i-1).
compute bcmat(i,j)=1-(bmatrix(1,tmp) = 0).
compute tmp=tmp+1.
end loop.
end loop.
end if.
do if ((csum(bcmat(:,1))=0) and criterr=0).
compute errcode(errs,1)=22.
compute errs=errs+1.
compute criterr=1.
end if.
do if ((rsum(bcmat(nrow(bcmat),:))=0) and criterr=0).
compute errcode(errs,1)=23.
compute errs=errs+1.
compute criterr=1.
end if.
compute dm=0.
do if (nms > 0).
loop i = 1 to nms.
do if (((rsum(bcmat((nxs+i),:)) = 0) or (csum(bcmat(:,(nxs+i))) = 0)) and (dm=0) and (criterr=0)).
compute errcode(errs,1)=26.
compute errs=errs+1.
compute criterr=1.
compute dm=1.
end if.
end loop.
end if.
release dm.
end if.
end if.
do if (criterr=0).
do if (wmatrix(1,1) <> -999).
compute tmp=1.
do if (ncol(wmatrix) <> needed).
compute errcode(errs,1)=17.
compute errs=errs+1.
compute criterr=1.
else.
compute modelvar(1,1)='CUSTOM'.
loop i = 2 to nrow(wcmat).
loop j = 1 to (i-1).
compute wcmat(i,j)=1-(wmatrix(1,tmp) = 0).
do if ((wcmat(i,j)=1) and (bcmat(i,j)=0) and (nopath=0)).
compute errcode(errs,1)=20.
compute errs=errs+1.
compute criterr=1.
compute nopath=1.
end if.
compute tmp=tmp+1.
end loop.
end loop.
end if.
end if.
do if (zmatrix(1,1) <> -999).
compute tmp=1.
do if (ncol(zmatrix) <> needed).
compute errcode(errs,1)=18.
compute errs=errs+1.
compute criterr=1.
else.
compute modelvar(1,1)='CUSTOM'.
do if (csum(rsum(wcmat))=0 and model=999).
compute errcode(errs,1)=21.
compute errs=errs+1.
compute criterr=1.
end if.
loop i = 2 to nrow(zcmat).
loop j = 1 to (i-1).
compute zcmat(i,j)=1-(zmatrix(1,tmp) = 0).
do if ((zcmat(i,j)=1) and (bcmat(i,j)=0) and (nopath=0)).
compute errcode(errs,1)=20.
compute errs=errs+1.
compute criterr=1.
compute nopath=1.
end if.
compute tmp=tmp+1.
end loop.
end loop.
end if.
end if.
compute tmp=1.
do if (wzmatrix(1,1) <> -999).
do if (ncol(wzmatrix) <> needed).
compute errcode(errs,1)=19.
compute errs=errs+1.
compute criterr=1.
end if.
compute modelvar(1,1)='CUSTOM'.
end if.
do if (criterr=0).
loop i = 2 to nrow(wzcmat).
loop j = 1 to (i-1).
do if (wzmatrix(1,1) <> -999).
compute wzcmat(i,j)=1-(wzmatrix(1,tmp) = 0).
end if.
do if (wzcmat(i,j)=1).
compute wcmat(i,j)=1.
compute zcmat(i,j)=1.
end if.
do if ((wzcmat(i,j)=1) and (bcmat(i,j)=0) and (nopath=0)).
compute errcode(errs,1)=20.
compute errs=errs+1.
compute criterr=1.
compute nopath=1.
end if.
compute tmp=tmp+1.
end loop.
end loop.
end if.
end if.
do if (criterr=0).
compute xprod=csum(wcmat(:,1))+csum(zcmat(:,1))+csum(wzcmat(:,1)).
compute xprod=(xprod > 0).
compute wsum=csum(rsum(wcmat)).
compute wprod=(wsum > 0).
do if (nms > 0).
loop i = 1 to nms.
compute tmp=csum(wcmat(:,(1+i)))+csum(zcmat(:,(1+i)))+csum(wzcmat(:,(1+i))).
compute mprod(1,i)=(tmp>0).
end loop.
end if.
do if ((wsum > 0) and (w = 'xxxxx')).
compute errcode(errs,1)=11.
compute errs=errs+1.
compute criterr=1.
end if.
do if ((wsum = 0) and (w <> 'xxxxx')).
compute errcode(errs,1)=10.
compute errs=errs+1.
compute criterr=1.
end if.
compute zsum=csum(rsum(zcmat)).
compute zprod=(zsum > 0).
do if ((zsum > 0) and (z = 'xxxxx')).
compute errcode(errs,1)=13.
compute errs=errs+1.
compute criterr=1.
end if.
do if ((zsum = 0) and (z <> 'xxxxx')).
compute errcode(errs,1)=12.
compute errs=errs+1.
compute criterr=1.
end if.
do if ((zsum > 0) and (wsum = 0)).
compute errcode(errs,1)=35.
compute errs=errs+1.
compute criterr=1.
end if.
end if.
do if (criterr=0 and nms > 1).
compute serchk=bcmat(2:(nrow(bcmat)-1),2:ncol(bcmat)).
do if (csum(rsum(serchk))) > 0.
compute serial=1.
do if (nms > 6).
compute errcode(errs,1)=36.
compute errs=errs+1.
compute criterr=1.
end if.
end if.
end if.
do if (center > 0 and criterr=0).
compute centvar={' '}.
do if (criterr=0).
do if ((center = 1) or (center = 2 and wdich = 0)).
do if (wprod=1 and mcwok=0 and nwpval > 0).
loop i = 1 to nws.
compute wtmp(:,i)=wtmp(:,i)-(csum(wtmp(:,i))/n).
compute centvar={centvar,wnames(1,i)}.
end loop.

.
compute desctmp=make((8-(4* wmodcust )),ncol( wtmp ),-999).
loop jd=1 to ncol( wtmp ).
compute descdat= wtmp (:,jd).
compute desctmp(1,jd) = csum(descdat)/nrow(descdat).
compute desctmp(2,jd) = (nrow(descdat)*sscp(descdat))-(t(csum(descdat))*(csum(descdat))).
compute desctmp(2,jd) = sqrt(desctmp(2,jd)/(nrow(descdat)*(nrow(descdat)-1))).
compute desctmp(3,jd)=cmin(descdat).
compute desctmp(4,jd)=cmax(descdat).
do if ( wmodcust =0).
compute minwarn=0.
compute maxwarn=0.
do if ((desctmp(3,jd)=desctmp(4,jd)) and novar=0).
compute errcode(errs,1)=15.
compute errs=errs+1.
compute criterr=1.
compute novar=1.
end if.
compute tmp=((descdat(:,1)=desctmp(3,jd))+(descdat(:,1)=desctmp(4,jd))).
compute desctmp(8,jd)=(csum(tmp)=nrow(tmp)).
compute tmp = descdat.
compute tmp(GRADE(descdat),:) = descdat.
compute descdat = tmp.
release tmp.
compute decval={.16;.5;.84}.
loop kd=1 to 3.
compute low=trunc(decval(kd,1)*(nrow(descdat)+1)).
compute lowdec=decval(kd,1)*(nrow(descdat)+1)-low.
compute value=descdat(low,1)+(descdat((low+1),1)-descdat(low,1))*lowdec.
compute desctmp((4+kd),jd)=value.
end loop.
compute mnotev=1.
compute modvals=desctmp(5:7,:).
do if (quantile <> 1).
compute desctmp(5,jd)=desctmp(1,jd)-desctmp(2,jd).
compute desctmp(6,jd)=desctmp(1,jd).
compute desctmp(7,jd)=desctmp(1,jd)+desctmp(2,jd).
compute modvals=desctmp(5:7,:).
compute mnotev=2.
do if (modvals(1,1) < desctmp(3,1)).
compute modvals(1,1)=desctmp(3,1).
compute minwarn=1.
end if.
do if (modvals(3,1) > desctmp(4,1)).
compute modvals(3,1)=desctmp(4,1).
compute maxwarn=1.
end if.
end if.
do if (desctmp(8,1)=1).
compute modvals={desctmp(3,1);desctmp(4,1)}.
compute mnotev=0.
compute minwarn=0.
compute maxwarn=0.
end if.
end if.
end loop
.
compute wmin=desctmp(3,1).
compute wmax=desctmp(4,1).
do if (wmodcust=0).
compute wmodvals=modvals.
compute wprobval=wmodvals.
end if.
end if.
end if.
do if ((center = 1) or (center = 2 and zdich = 0)).
do if (zprod=1 and mczok=0 and nzpval > 0).
loop i = 1 to nzs.
compute ztmp(:,i)=ztmp(:,i)-(csum(ztmp(:,i))/n).
compute centvar={centvar,znames(1,i)}.
end loop.

.
compute desctmp=make((8-(4* zmodcust )),ncol( ztmp ),-999).
loop jd=1 to ncol( ztmp ).
compute descdat= ztmp (:,jd).
compute desctmp(1,jd) = csum(descdat)/nrow(descdat).
compute desctmp(2,jd) = (nrow(descdat)*sscp(descdat))-(t(csum(descdat))*(csum(descdat))).
compute desctmp(2,jd) = sqrt(desctmp(2,jd)/(nrow(descdat)*(nrow(descdat)-1))).
compute desctmp(3,jd)=cmin(descdat).
compute desctmp(4,jd)=cmax(descdat).
do if ( zmodcust =0).
compute minwarn=0.
compute maxwarn=0.
do if ((desctmp(3,jd)=desctmp(4,jd)) and novar=0).
compute errcode(errs,1)=15.
compute errs=errs+1.
compute criterr=1.
compute novar=1.
end if.
compute tmp=((descdat(:,1)=desctmp(3,jd))+(descdat(:,1)=desctmp(4,jd))).
compute desctmp(8,jd)=(csum(tmp)=nrow(tmp)).
compute tmp = descdat.
compute tmp(GRADE(descdat),:) = descdat.
compute descdat = tmp.
release tmp.
compute decval={.16;.5;.84}.
loop kd=1 to 3.
compute low=trunc(decval(kd,1)*(nrow(descdat)+1)).
compute lowdec=decval(kd,1)*(nrow(descdat)+1)-low.
compute value=descdat(low,1)+(descdat((low+1),1)-descdat(low,1))*lowdec.
compute desctmp((4+kd),jd)=value.
end loop.
compute mnotev=1.
compute modvals=desctmp(5:7,:).
do if (quantile <> 1).
compute desctmp(5,jd)=desctmp(1,jd)-desctmp(2,jd).
compute desctmp(6,jd)=desctmp(1,jd).
compute desctmp(7,jd)=desctmp(1,jd)+desctmp(2,jd).
compute modvals=desctmp(5:7,:).
compute mnotev=2.
do if (modvals(1,1) < desctmp(3,1)).
compute modvals(1,1)=desctmp(3,1).
compute minwarn=1.
end if.
do if (modvals(3,1) > desctmp(4,1)).
compute modvals(3,1)=desctmp(4,1).
compute maxwarn=1.
end if.
end if.
do if (desctmp(8,1)=1).
compute modvals={desctmp(3,1);desctmp(4,1)}.
compute mnotev=0.
compute minwarn=0.
compute maxwarn=0.
end if.
end if.
end loop
.
compute zmin=desctmp(3,1).
compute zmax=desctmp(4,1).
do if (zmodcust=0).
compute zmodvals=modvals.
compute zprobval=zmodvals.
end if.
end if.
end if.
do if ((center = 1) or (center = 2 and xdich = 0)).
do if (xprod=1 and mcxok=0).
loop i = 1 to nxs.
compute xtmp(:,i)=xtmp(:,i)-(csum(xtmp(:,i))/n).
compute centvar={centvar,xnames(1,i)}.
end loop.

.
compute desctmp=make((8-(4* 0 )),ncol( xtmp ),-999).
loop jd=1 to ncol( xtmp ).
compute descdat= xtmp (:,jd).
compute desctmp(1,jd) = csum(descdat)/nrow(descdat).
compute desctmp(2,jd) = (nrow(descdat)*sscp(descdat))-(t(csum(descdat))*(csum(descdat))).
compute desctmp(2,jd) = sqrt(desctmp(2,jd)/(nrow(descdat)*(nrow(descdat)-1))).
compute desctmp(3,jd)=cmin(descdat).
compute desctmp(4,jd)=cmax(descdat).
do if ( 0 =0).
compute minwarn=0.
compute maxwarn=0.
do if ((desctmp(3,jd)=desctmp(4,jd)) and novar=0).
compute errcode(errs,1)=15.
compute errs=errs+1.
compute criterr=1.
compute novar=1.
end if.
compute tmp=((descdat(:,1)=desctmp(3,jd))+(descdat(:,1)=desctmp(4,jd))).
compute desctmp(8,jd)=(csum(tmp)=nrow(tmp)).
compute tmp = descdat.
compute tmp(GRADE(descdat),:) = descdat.
compute descdat = tmp.
release tmp.
compute decval={.16;.5;.84}.
loop kd=1 to 3.
compute low=trunc(decval(kd,1)*(nrow(descdat)+1)).
compute lowdec=decval(kd,1)*(nrow(descdat)+1)-low.
compute value=descdat(low,1)+(descdat((low+1),1)-descdat(low,1))*lowdec.
compute desctmp((4+kd),jd)=value.
end loop.
compute mnotev=1.
compute modvals=desctmp(5:7,:).
do if (quantile <> 1).
compute desctmp(5,jd)=desctmp(1,jd)-desctmp(2,jd).
compute desctmp(6,jd)=desctmp(1,jd).
compute desctmp(7,jd)=desctmp(1,jd)+desctmp(2,jd).
compute modvals=desctmp(5:7,:).
compute mnotev=2.
do if (modvals(1,1) < desctmp(3,1)).
compute modvals(1,1)=desctmp(3,1).
compute minwarn=1.
end if.
do if (modvals(3,1) > desctmp(4,1)).
compute modvals(3,1)=desctmp(4,1).
compute maxwarn=1.
end if.
end if.
do if (desctmp(8,1)=1).
compute modvals={desctmp(3,1);desctmp(4,1)}.
compute mnotev=0.
compute minwarn=0.
compute maxwarn=0.
end if.
end if.
end loop
.
compute xmodvals=modvals.
compute xprobval=xmodvals.
end if.
end if.
do if (nms > 0).
loop i = 1 to nms.
do if (mprod(1,i)=1).
compute mtmp(:,i)=mtmp(:,i)-(csum(mtmp(:,i))/n).
compute centvar={centvar,mnames(1,i)}.
end if.
end loop.

.
compute desctmp=make((8-(4* 0 )),ncol( mtmp ),-999).
loop jd=1 to ncol( mtmp ).
compute descdat= mtmp (:,jd).
compute desctmp(1,jd) = csum(descdat)/nrow(descdat).
compute desctmp(2,jd) = (nrow(descdat)*sscp(descdat))-(t(csum(descdat))*(csum(descdat))).
compute desctmp(2,jd) = sqrt(desctmp(2,jd)/(nrow(descdat)*(nrow(descdat)-1))).
compute desctmp(3,jd)=cmin(descdat).
compute desctmp(4,jd)=cmax(descdat).
do if ( 0 =0).
compute minwarn=0.
compute maxwarn=0.
do if ((desctmp(3,jd)=desctmp(4,jd)) and novar=0).
compute errcode(errs,1)=15.
compute errs=errs+1.
compute criterr=1.
compute novar=1.
end if.
compute tmp=((descdat(:,1)=desctmp(3,jd))+(descdat(:,1)=desctmp(4,jd))).
compute desctmp(8,jd)=(csum(tmp)=nrow(tmp)).
compute tmp = descdat.
compute tmp(GRADE(descdat),:) = descdat.
compute descdat = tmp.
release tmp.
compute decval={.16;.5;.84}.
loop kd=1 to 3.
compute low=trunc(decval(kd,1)*(nrow(descdat)+1)).
compute lowdec=decval(kd,1)*(nrow(descdat)+1)-low.
compute value=descdat(low,1)+(descdat((low+1),1)-descdat(low,1))*lowdec.
compute desctmp((4+kd),jd)=value.
end loop.
compute mnotev=1.
compute modvals=desctmp(5:7,:).
do if (quantile <> 1).
compute desctmp(5,jd)=desctmp(1,jd)-desctmp(2,jd).
compute desctmp(6,jd)=desctmp(1,jd).
compute desctmp(7,jd)=desctmp(1,jd)+desctmp(2,jd).
compute modvals=desctmp(5:7,:).
compute mnotev=2.
do if (modvals(1,1) < desctmp(3,1)).
compute modvals(1,1)=desctmp(3,1).
compute minwarn=1.
end if.
do if (modvals(3,1) > desctmp(4,1)).
compute modvals(3,1)=desctmp(4,1).
compute maxwarn=1.
end if.
end if.
do if (desctmp(8,1)=1).
compute modvals={desctmp(3,1);desctmp(4,1)}.
compute mnotev=0.
compute minwarn=0.
compute maxwarn=0.
end if.
end if.
end loop
.
do if ((cdeval(1,1)=-999) and (model=74)).
compute medmeans=desctmp(1,:).
end if.
compute mmodvals=modvals.
compute mprobval=mmodvals.
end if.
end if.
do if (ncol(centvar) > 1).
compute notecode(notes,1)=3.
compute notes=notes+1.
end if.
end if.
do if (criterr=0).
compute wsum=rsum(csum(wcmat)).
compute zsum=rsum(csum(zcmat)).
compute wzsum=rsum(csum(wzcmat)).
compute nump=make(1,(nys+nms),-999).
compue numint=make(1,(nys+nms),0).
compute datcount=1.
compute xtmpuse=0.
compute wtmpuse=0.
compute ztmpuse=0.
compute xwtmpus=0.
compute xztmpus=0.
compute wztmpus=0.
compute xwztmpu=0.
compute xtmploc=-999.
compute wtmploc=-999.
compute xwtmplo=-999.
compute ztmploc=-999.
compute xztmplo=-999.
compute wztmplo=-999.
compute xwztmplo=-999.
compute vlabs={' '}.
do if (ncs > 0).
compute ctmpuse=make(1,ncs,0).
end if.
do if (nms > 0).
compute mtmpuse=make(1,nms,0).
compute mwtmpus=make(1,nms,0).
compute mztmpus=make(1,nms,0).
compute mwztmpu=make(1,nms,0).
compute mtmploc=make(1,nms,0).
compute mwtmplo=make(nwvls,nms,-999).
compute mztmplo=make(nzvls,nms,-999).
compute mwztmplo=make((nwvls*nzvls),nms,-999).
end if.
do if (ncs > 0).
compute ctmploc=make(1,ncs,0).
end if.
compute fulldat=make(n,1,1).
compute datindx=make(1000,(nms+nys),-999).
compute wherew=make(2,(nms+nys),-999).
compute wherex=make(2,(nms+nys),-999).
compute wherez=make(2,(nms+nys),-999).
compute wherexw=make(2,(nms+nys),-999).
compute wherexz=make(2,(nms+nys),-999).
compute wherewz=make(2,(nms+nys),-999).
compute wherexwz=make(2,(nms+nys),-999).
do if (nms > 0).
compute wherem=make(nms,(nms+nys),-999).
compute wheremw = make(nms*2,(nms+nys),-999).
compute wheremz = make(nms*2,(nms+nys),-999).
compute wheremwz = make(nms*2,(nms+nys),-999).
end if.
compute wzhigh=make(1000,(((nms+1)*(nms+2))/2),0).
compute whigh=make(1000,(((nms+1)*(nms+2))/2),0).
compute zhigh=make(1000,(((nms+1)*(nms+2))/2),0).
compute fochigh=make(1000,(((nms+1)*(nms+2))/2),0).
compute xcoefloc={1;2;3;4;5;6;7;8;9}.
compute intkey = {' ', ' ', ' ', ' ', ' ', ' ', ' '}.
compute wzhighct=0.
compute whighct=0.
compute zhighct=0.
compute foccnt=0.
loop i = 2 to nrow(bcmat).
compute wdid=0.
compute zdid=0.
compute wzdid=0.
compute cntmp=1.
compute start=1.
do if (i < nrow(bcmat)).
compute outv=mtmp(:,(i-1)).
compute modlabel={mnames(1,(i-1));'constant'}.
end if.
do if (i = nrow(bcmat)).
compute outv=ytmp.
compute modlabel={ynames;'constant'}.
end if.
loop j = 1 to (i-1).
compute foccnt=foccnt+1.
do if (j = 1 and bcmat(i,j)=1).
compute outv={outv,xtmp}.
compute modlabel={modlabel;xcatlab(1:nxvls,1)}.
do if (xtmpuse=0).
compute fulldat={fulldat,xtmp}.
compute xtmpuse=1.
loop k4=datcount to (datcount+(nxvls-1)).
compute xtmploc={xtmploc;k4}.
end loop.
compute xtmploc=xtmploc(2:nrow(xtmploc),1).
compute datcount=datcount+nxvls.
end if.
compute datindx(start:(start+nrow(xtmploc)-1),(i-1))=xtmploc.
compute wherex(1,(i-1))=start+1.
compute wherex(2,(i-1))=start+nrow(xtmploc)-1+1.
do if (model = 74).
end if.
compute onebl=make(nrow(xtmploc),1,1).
compute fochigh((start+1):(start+nrow(xtmploc)),foccnt)=onebl.
compute start=start+nrow(xtmploc).
end if.
do if (j > 1 and bcmat(i,j)=1).
compute outv={outv,mtmp(:,(j-1))}.
compute modlabel={modlabel;mnames(1,(j-1))}.
do if (mtmpuse(1,(j-1))=0).
compute fulldat={fulldat,mtmp(:,(j-1))}.
compute mtmpuse(1,(j-1))=1.
compute mtmploc(1,(j-1))=datcount.
compute datcount=datcount+1.
end if.
compute datindx(start:(start+nrow(mtmploc)-1),(i-1))=mtmploc(1,(j-1)).
compute wherem((j-1),(i-1))=start+1.
compute onebl=make(nrow(mtmploc(1,j-1)),1,1).
compute ttt=nrow(mtmploc(1,(j-1)))+start-1.
compute fochigh((start+1):(start+nrow(mtmploc(1,(j-1)))),foccnt)=onebl.
compute start=start+nrow(mtmploc(1,(j-1))).
end if.
end loop.
do if (wsum > 0).
loop j = 1 to (i-1).
compute whighct=whighct+1.
do if (j = 1 and wcmat(i,j)=1).
do if (wdid=0).
compute outv={outv,wtmp}.
do if (ncs > 0 and wiscov > 0).
compute ccmatoff((i-1),wiscov)=0.
end if.
compute modlabel={modlabel;wcatlab(1:nwvls,1)}.
compute wdid=1.
do if (wtmpuse=0).
compute fulldat={fulldat,wtmp}.
do if (ncs > 0 and wiscov > 0).
compute ccmatoff((i-1),wiscov)=0.
end if.
compute wtmpuse=1.
loop k4=datcount to (datcount+(nwvls-1)).
compute wtmploc={wtmploc;k4}.
end loop.
compute wtmploc=wtmploc(2:nrow(wtmploc),1).
compute datcount=datcount+nwvls.
end if.
end if.
compute datindx(start:(start+nrow(wtmploc)-1),(i-1))=wtmploc.
compute wherew(1,(i-1))=start+1.
compute wherew(2,(i-1))=start+nrow(wtmploc)-1+1.
compute start=start+nrow(wtmploc).
loop k1=1 to nxvls.
loop k2 = 1 to nwvls.
compute outv={outv,(xtmp(:,k1)&*wtmp(:,k2))}.
do if (ncs > 0 and wiscov > 0).
compute ccmatoff((i-1),wiscov)=0.
end if.
compute modlabel={modlabel;intlab(cntmp,1)}.
compute intkey={intkey;intlab(cntmp,1),':',xcatlab(k1,1),'x',wcatlab(k2,1),' ',' '}.
compute cntmp=cntmp+1.
end loop.
end loop.
do if (xwtmpus=0).
compute fulldat={fulldat,outv(:,(ncol(outv)-(nxvls*nwvls)+1):ncol(outv))}.
compute xwtmpus=1.
do if (ncs > 0 and wiscov > 0).
compute ccmatoff((i-1),wiscov)=0.
end if.
loop k4=datcount to (datcount+((nwvls*nxvls)-1)).
compute xwtmplo={xwtmplo;k4}.
end loop.
compute xwtmplo=xwtmplo(2:nrow(xwtmplo),1).
compute datcount=datcount+(nxvls*nwvls).
end if.
compute datindx(start:(start+nrow(xwtmplo)-1),(i-1))=xwtmplo.
compute wherexw(1,(i-1))=start+1.
compute wherexw(2,(i-1))=start+nrow(xwtmplo)-1+1.
compute onebl=make(nrow(xwtmplo),1,1).
compute whigh((start+1):(start+nrow(xwtmplo)),whighct)=onebl.
compute start=start+nrow(xwtmplo).
end if.
do if (j > 1 and wcmat(i,j)=1).
do if (wdid=0 and model <> 74).
compute outv={outv,wtmp}.
do if (ncs > 0 and wiscov > 0).
compute ccmatoff((i-1),wiscov)=0.
end if.
compute modlabel={modlabel;wcatlab(1:nwvls,1)}.
compute wdid=1.
do if (wtmpuse=0).
compute fulldat={fulldat,wtmp}.
do if (ncs > 0 and wiscov > 0).
compute ccmatoff((i-1),wiscov)=0.
end if.
compute wtmpuse=1.
loop k4=datcount to (datcount+(nwvls-1)).
compute wtmploc={wtmploc;k4}.
end loop.
compute wtmploc=wtmploc(2:nrow(wtmploc),1).
compute datcount=datcount+nwvls.
end if.
compute datindx(start:(start+nrow(wtmploc)-1),(i-1))=wtmploc.
compute wherew(1,(i-1))=start+1.
compute wherew(2,(i-1))=start+nrow(wtmploc)-1+1.
compute start=start+nrow(wtmploc).
end if.
loop k2 = 1 to nwvls.
compute outv={outv,(mtmp(:,(j-1))&*wtmp(:,k2))}.
do if (ncs > 0 and wiscov > 0).
compute ccmatoff((i-1),wiscov)=0.
end if.
compute modlabel={modlabel;intlab(cntmp,1)}.
compute intkey={intkey;intlab(cntmp,1),':', mnames(1,(j-1)),'x',wcatlab(k2,1),' ',' '}.
compute cntmp=cntmp+1.
end loop.
do if (mwtmpus(1,(j-1))=0).
compute fulldat={fulldat,outv(:,(ncol(outv)-nwvls+1):ncol(outv))}.
do if (ncs > 0 and wiscov > 0).
compute ccmatoff((i-1),wiscov)=0.
end if.
compute mwtmpus(1,(j-1))=1.
compute mw22=-999.
loop k4=datcount to (datcount+(nwvls-1)).
compute mw22={mw22;k4}.
end loop.
compute mwtmplo(:,(j-1))=mw22(2:nrow(mw22),1).
compute datcount=datcount+nwvls.
end if.
compute datindx(start:(start+nrow(mwtmplo)-1),(i-1))=mwtmplo(:,(j-1)).
compute wheremw(((2*j)-3),(i-1))=start+1.
compute wheremw(((2*j)-2),(i-1))=start+nrow(mwtmplo)-1+1.
compute onebl=make(nrow(mwtmplo),1,1).
compute whigh((start+1):(start+nrow(mwtmplo)),whighct)=onebl.
compute start=start+nrow(mwtmplo).
end if.
end loop.
end if.
do if (zsum > 0).
loop j = 1 to (i-1).
compute zhighct=zhighct+1.
do if (j = 1 and zcmat(i,j)=1).
do if (zdid=0).
compute outv={outv,ztmp}.
do if (ncs > 0 and ziscov > 0).
compute ccmatoff((i-1),ziscov)=0.
end if.
compute modlabel={modlabel;zcatlab(1:nzvls,1)}.
compute zdid=1.
do if (ztmpuse=0).
compute fulldat={fulldat,ztmp}.
do if (ncs > 0 and ziscov > 0).
compute ccmatoff((i-1),ziscov)=0.
end if.
compute ztmpuse=1.
loop k4=datcount to (datcount+(nzvls-1)).
compute ztmploc={ztmploc;k4}.
end loop.
compute ztmploc=ztmploc(2:nrow(ztmploc),1).
compute datcount=datcount+nzvls.
end if.
end if.
compute datindx(start:(start+nrow(ztmploc)-1),(i-1))=ztmploc.
compute wherez(1,(i-1))=start+1.
compute wherez(2,(i-1))=start+nrow(ztmploc)-1+1.
compute start=start+nrow(ztmploc).
loop k1=1 to nxvls.
loop k2 = 1 to nzvls.
compute outv={outv,(xtmp(:,k1)&*ztmp(:,k2))}.
do if (ncs > 0 and ziscov > 0).
compute ccmatoff((i-1),ziscov)=0.
end if.
compute modlabel={modlabel;intlab(cntmp,1)}.
compute intkey={intkey;intlab(cntmp,1),':',xcatlab(k1,1),'x',zcatlab(k2,1),' ',' '}.
compute cntmp=cntmp+1.
end loop.
end loop.
do if (xztmpus=0).
compute fulldat={fulldat,outv(:,(ncol(outv)-(nxvls*nzvls)+1):ncol(outv))}.
do if (ncs > 0 and ziscov > 0).
compute ccmatoff((i-1),ziscov)=0.
end if.
compute xztmpus=1.
loop k4=datcount to (datcount+((nzvls*nxvls)-1)).
compute xztmplo={xztmplo;k4}.
end loop.
compute xztmplo=xztmplo(2:nrow(xztmplo),1).
compute datcount=datcount+(nxvls*nzvls).
end if.
compute datindx(start:(start+nrow(xztmplo)-1),(i-1))=xztmplo.
compute wherexz(1,(i-1))=start+1.
compute wherexz(2,(i-1))=start+nrow(xztmplo)-1+1.
compute onebl=make(nrow(xztmplo),1,1).
compute zhigh((start+1):(start+nrow(xztmplo)),zhighct)=onebl.
compute start=start+nrow(xztmplo).
end if.
do if (j > 1 and zcmat(i,j)=1).
do if (zdid=0).
compute outv={outv,ztmp}.
do if (ncs > 0 and ziscov > 0).
compute ccmatoff((i-1),ziscov)=0.
end if.
compute modlabel={modlabel;zcatlab(1:nzvls,1)}.
compute zdid=1.
do if (ztmpuse=0).
compute fulldat={fulldat,ztmp}.
do if (ncs > 0 and ziscov > 0).
compute ccmatoff((i-1),ziscov)=0.
end if.
compute ztmpuse=1.
loop k4=datcount to (datcount+(nzvls-1)).
compute ztmploc={ztmploc;k4}.
end loop.
compute ztmploc=ztmploc(2:nrow(ztmploc),1).
compute datcount=datcount+nzvls.
end if.
compute datindx(start:(start+nrow(ztmploc)-1),(i-1))=ztmploc.
compute wherez(1,(i-1))=start+1.
compute wherez(2,(i-1))=start+nrow(ztmploc)-1+1.
compute start=start+nrow(ztmploc).
end if.
loop k2 = 1 to nzvls.
compute outv={outv,(mtmp(:,(j-1))&*ztmp(:,k2))}.
do if (ncs > 0 and ziscov > 0).
compute ccmatoff((i-1),ziscov)=0.
end if.
compute modlabel={modlabel;intlab(cntmp,1)}.
compute intkey={intkey;intlab(cntmp,1),':', mnames(1,(j-1)),'x',zcatlab(k2,1),' ',' '}.
compute cntmp=cntmp+1.
end loop.
do if (mztmpus(1,(j-1))=0).
compute fulldat={fulldat,outv(:,(ncol(outv)-nzvls+1):ncol(outv))}.
do if (ncs > 0 and ziscov > 0).
compute ccmatoff((i-1),ziscov)=0.
end if.
compute mztmpus(1,(j-1))=1.
compute mz22=-999.
loop k4=datcount to (datcount+(nzvls-1)).
compute mz22={mz22;k4}.
end loop.
compute mztmplo(:,(j-1))=mz22(2:nrow(mz22),1).
compute datcount=datcount+nzvls.
end if.
compute datindx(start:(start+nrow(mztmplo)-1),(i-1))=mztmplo(:,(j-1)).
compute wheremz(((2*j)-3),(i-1))=start+1.
compute wheremz(((2*j)-2),(i-1))=start+nrow(mztmplo)-1+1.
compute onebl=make(nrow(mztmplo),1,1).
compute zhigh((start+1):(start+nrow(mztmplo)),zhighct)=onebl.
compute start=start+nrow(mztmplo).
end if.
end loop.
end if.
do if (wzsum > 0).
loop j = 1 to (i-1).
compute wzhighct=wzhighct+1.
do if (j = 1 and wzcmat(i,j)=1).
do if (wzdid=0).
loop k1=1 to nwvls.
loop k2 = 1 to nzvls.
compute outv={outv,(wtmp(:,k1)&*ztmp(:,k2))}.
do if (ncs > 0 and (ziscov > 0)).
compute ccmatoff((i-1),ziscov)=0.
end if.
do if (ncs > 0 and (wiscov > 0)).
compute ccmatoff((i-1),wiscov)=0.
end if.
compute modlabel={modlabel;intlab(cntmp,1)}.
compute intkey={intkey;intlab(cntmp,1),':',wcatlab(k1,1),'x',zcatlab(k2,1),' ',' '}.
compute cntmp=cntmp+1.
end loop.
end loop.
do if (wztmpus=0).
compute fulldat={fulldat,outv(:,(ncol(outv)-(nwvls*nzvls)+1):ncol(outv))}.
do if (ncs > 0 and (ziscov > 0)).
compute ccmatoff((i-1),ziscov)=0.
end if.
do if (ncs > 0 and (wiscov > 0)).
compute ccmatoff((i-1),wiscov)=0.
end if.
compute wztmpus=1.
loop k4=datcount to (datcount+((nwvls*nzvls)-1)).
compute wztmplo={wztmplo;k4}.
end loop.
compute wztmplo=wztmplo(2:nrow(wztmplo),1).
compute datcount=datcount+(nzvls*nwvls).
end if.
compute wzdid=1.
end if.
compute datindx(start:(start+nrow(wztmplo)-1),(i-1))=wztmplo.
compute wherewz(1,(i-1))=start+1.
compute wherewz(2,(i-1))=start+nrow(wztmplo)-1+1.
compute start=start+nrow(wztmplo).
loop k1=1 to nxvls.
loop k2=1 to nwvls.
loop k3=1 to nzvls.
compute outv={outv,(xtmp(:,k1)&*wtmp(:,k2)&*ztmp(:,k3))}.
do if (ncs > 0 and (ziscov > 0)).
compute ccmatoff((i-1),ziscov)=0.
end if.
do if (ncs > 0 and (wiscov > 0)).
compute ccmatoff((i-1),wiscov)=0.
end if.
compute modlabel={modlabel;intlab(cntmp,1)}.
compute intkey={intkey;intlab(cntmp,1),':',xcatlab(k1,1),'x',wcatlab(k2,1),'x', zcatlab(k3,1)}.
compute cntmp=cntmp+1.
end loop.
end loop.
end loop.
do if (xwztmpu=0).
compute fulldat={fulldat,outv(:,(ncol(outv)-(nxvls*nwvls*nzvls)+1):ncol(outv))}.
do if (ncs > 0 and (ziscov > 0)).
compute ccmatoff((i-1),ziscov)=0.
end if.
do if (ncs > 0 and (wiscov > 0)).
compute ccmatoff((i-1),wiscov)=0.
end if.
compute xwztmpu=1.
loop k4=datcount to (datcount+((nzvls*nxvls*nwvls)-1)).
compute xwztmplo={xwztmplo;k4}.
end loop.
compute xwztmplo=xwztmplo(2:nrow(xwztmplo),1).
compute datcount=datcount+(nxvls*nzvls*nwvls).
end if.
compute datindx(start:(start+nrow(xwztmplo)-1),(i-1))=xwztmplo.
compute wherexwz(1,(i-1))=start+1.
compute wherexwz(2,(i-1))=start+nrow(xwztmplo)-1+1.
compute onebl=make(nrow(xwztmplo),1,1).
compute wzhigh((start+1):(start+nrow(xwztmplo)),wzhighct)=onebl.
compute start=start+nrow(xwztmplo).
end if.
do if (j > 1 and wzcmat(i,j)=1).
do if (wzdid=0).
loop k1=1 to nwvls.
loop k2 = 1 to nzvls.
compute outv={outv,(wtmp(:,k1)&*ztmp(:,k2))}.
do if (ncs > 0 and (ziscov > 0)).
compute ccmatoff((i-1),ziscov)=0.
end if.
do if (ncs > 0 and (wiscov > 0)).
compute ccmatoff((i-1),wiscov)=0.
end if.
compute modlabel={modlabel;intlab(cntmp,1)}.
compute intkey={intkey;intlab(cntmp,1),':',wcatlab(k1,1),'x',zcatlab(k2,1),' ',' '}.
compute cntmp=cntmp+1.
end loop.
end loop.
do if (wztmpus=0).
compute fulldat={fulldat,outv(:,(ncol(outv)-(nwvls*nzvls)+1):ncol(outv))}.
do if (ncs > 0 and (ziscov > 0)).
compute ccmatoff((i-1),ziscov)=0.
end if.
do if (ncs > 0 and (wiscov > 0)).
compute ccmatoff((i-1),wiscov)=0.
end if.
compute wztmpus=1.
loop k4=datcount to (datcount+((nwvls*nzvls)-1)).
compute wztmplo={wztmplo;k4}.
end loop.
compute wztmplo=wztmplo(2:nrow(wztmplo),1).
compute datcount=datcount+(nzvls*nwvls).
end if.
compute wzdid=1.
compute datindx(start:(start+nrow(wztmplo)-1),(i-1))=wztmplo.
compute wherewz(1,(i-1))=start+1.
compute wherewz(2,(i-1))=start+nrow(wztmplo)-1+1.
compute start=start+nrow(wztmplo).
end if.
loop k1 = 1 to nwvls.
loop k2 = 1 to nzvls.
compute outv={outv,(mtmp(:,(j-1))&*wtmp(:,k1)&*ztmp(:,k2))}.
do if (ncs > 0 and (ziscov > 0)).
compute ccmatoff((i-1),ziscov)=0.
end if.
do if (ncs > 0 and (wiscov > 0)).
compute ccmatoff((i-1),wiscov)=0.
end if.
compute modlabel={modlabel;intlab(cntmp,1)}.
compute intkey={intkey;intlab(cntmp,1),':',mnames(1,(j-1)),'x',wcatlab(k1,1),'x', zcatlab(k2,1)}.
compute cntmp=cntmp+1.
end loop.
end loop.
do if (mwztmpu(1,(j-1))=0).
compute fulldat={fulldat,outv(:,(ncol(outv)-(nwvls*nzvls)+1):ncol(outv))}.
do if (ncs > 0 and (ziscov > 0)).
compute ccmatoff((i-1),ziscov)=0.
end if.
do if (ncs > 0 and (wiscov > 0)).
compute ccmatoff((i-1),wiscov)=0.
end if.
compute mwztmpu(1,(j-1))=1.
compute mz22=-999.
loop k4=datcount to (datcount+(nwvls*nzvls)-1).
compute mz22={mz22;k4}.
end loop.
compute mwztmplo(:,(j-1))=mz22(2:nrow(mz22),1).
compute datcount=datcount+(nwvls*nzvls).
end if.
compute datindx(start:(start+nrow(mwztmplo)-1),(i-1))=mwztmplo(:,(j-1)).
compute wheremwz(((2*j)-3),(i-1))=start+1.
compute wheremwz(((2*j)-2),(i-1))=start+nrow(mwztmplo)-1+1.
compute onebl=make(nrow(mwztmplo),1,1).
compute wzhigh((start+1):(start+nrow(mwztmplo)),wzhighct)=onebl.
compute start=start+nrow(mwztmplo).
end if.
end loop.
end if.
do if (ncs > 0).
compute ccmat=ccmat&*ccmatoff.
loop j = 1 to ncs.
do if (ccmat((i-1),j))=1.
do if (j=wiscov).
compute ctmp(:,j)=wtmp.
end if.
do if (j=ziscov).
compute ctmp(:,j)=ztmp.
end if.
compute outv={outv,ctmp(:,j)}.
compute modlabel={modlabel;covnames(1,j)}.
do if (ctmpuse(1,j)=0).
compute fulldat={fulldat,ctmp(:,j)}.
compute ctmpuse(1,j)=1.
compute ctmploc(1,j)=datcount.
compute datcount=datcount+1.
end if.
compute datindx(start:(start+nrow(ctmploc)-1),(i-1))=ctmploc(1,j).
compute start=start+nrow(ctmploc(1,j)).
end if.
end loop.
end if.
compute wdid=0.
compute zdid=0.
compute wzdid=0.
compute vlabs={vlabs;modlabel(2:nrow(modlabel),1)}.
compute numint(1,(i-1))=cntmp-1.
compute nump(1,(i-1))=nrow(modlabel)-1.
end loop.
release datcount, xtmpuse, wtmpuse, ztmpuse, xwtmpus, xztmpus, wztmpus, xwztmpu.
release xtmploc, wtmploc, xwtmplo, ztmploc, xztmplo, wztmplo, xwztmplo, foccnt.
do if (modcok=1 and ((nms > 0) or (zcmat(2,1) <> 1) or (mcx <> 0))).
compute notecode(notes,1) = 19.
compute notes = notes + 1.
compute modcok=0.
end if.
do if ((serial = 1 or (rsum(numint)>0) or nms=0) and mc > 0).
compute notecode(notes,1) = 15.
compute notes = notes + 1.
compute boot=mc.
compute mc=0.
end if.
do if (boot <> 0 or mc <> 0).
compute bootsz=boot.
do if (mc > 0).
compute bootsz=mc.
compute saveboot=0.
end if.
loop.
compute cilow = rnd(bootsz*(1-(conf/100))/2).
compute cihigh = trunc((bootsz*(conf/100)+(bootsz*(1-(conf/100))/2)))+1.
do if (cilow < 1 or cihigh > bootsz).
compute bootsz=trunc((bootsz+1000)/1000)*1000.
compute adjust = 1.
end if.
end loop if (cilow gt 0 and cihigh le bootsz).
do if (boot > 0).
compute boot=bootsz.
end if.
do if (mc > 0).
compute mc=bootsz.
end if.
do if (adjust = 1 and boot > 0).
compute notecode(notes,1) = 8.
compute notes = notes + 1.
end if.
do if (adjust = 1 and mc > 0).
compute notecode(notes,1) = 16.
compute notes = notes + 1.
end if.
end if.
compute maxboot = trunc(2*boot).
do if ( 0 > maxboot).
compute maxboot=trunc( 0 ).
end if.
do if (nms > 0).
release mtmpuse, mwtmpus, mwztmpu, mtmploc, mwtmplo, mztmplo, mwztmplo.
end if.
release wdid, zdid, wzdid, start,modlabel.
compute vlabs=vlabs(2:nrow(vlabs),1).
do if (rsum(numint) > 0).
compute intkey=intkey(2:nrow(intkey),:).
end if.
compute fulldat=fulldat(:,2:ncol(fulldat)).
compute fochigh=fochigh(1:rmax(nump),:).
compute whigh=whigh(1:rmax(nump),:).
compute zhigh=zhigh(1:rmax(nump),:).
compute wzhigh=wzhigh(1:rmax(nump),:).
compute coeffs=fochigh+whigh+zhigh+wzhigh.
compute bootloc=make(rmax(nump),ncol(nump),0).
do if (nms > 0).
compute cntmp=1.
loop i = 1 to ncol(nump).
loop j = 1 to nump(1,i).
compute bootloc(j,i)=cntmp.
compute cntmp=cntmp+1.
end loop.
end loop.
compute fochighb=make(nrow(fochigh),ncol(fochigh),0).
compute whighb=fochighb.
compute zhighb=fochighb.
compute wzhighb=fochighb.
compute thetaxmb=make(nrow(fochighb),nms,0).
compute thetaxyb=make(nrow(fochighb),1,0).
compute pathsfoc=make(nxvls,1,0).
compute cntmp=1.
loop i = 1 to (nms+nys).
loop j = 1 to i.
compute fochighb(:,cntmp)=fochigh(:,cntmp)&*bootloc(:,i).
compute whighb(:,cntmp)=whigh(:,cntmp)&*bootloc(:,i).
compute zhighb(:,cntmp)=zhigh(:,cntmp)&*bootloc(:,i).
compute wzhighb(:,cntmp)=wzhigh(:,cntmp)&*bootloc(:,i).
compute coeffsb=fochighb+whighb+zhighb+wzhighb.
do if ((i < (nms+nys)) and (j = 1)).
compute thetaxmb(:,i)=coeffsb(:,cntmp).
end if.
do if ((i = (nms+nys)) and (j = 1)).
compute thetaxyb(:,1)=coeffsb(:,cntmp).
end if.
compute cntmp=cntmp+1.
end loop.
end loop.
compute thetamyb=coeffsb(:,(ncol(coeffsb)-nms+1):ncol(coeffsb)).
do if (serial = 1).
compute thetammb=make(nrow(coeffsb),((nms*(nms-1))/2),0).
end if.
compute cntmp=1.
do if (nms > 1 and serial = 1).
loop i = 1 to (nms-1).
compute start=((i+2)*(i+1))/2.
loop j = 2 to (nms-i+1).
compute thetammb(:,cntmp)=coeffsb(:,start).
compute start=start+j+i-1.
compute cntmp=cntmp+1.
end loop.
end loop.
end if.
end if.
do if ((total = 1) and (rsum(numint)=0 or (xmint=1))).
compute dototal=1.
do if ((csum(bcmat(:,1)) <> (nms+nys)) or (rsum(bcmat(nrow(bcmat),:)) <> (nms+nys))).
compute dototal=0.
compute alttotal=1.
compute notecode(notes,1) = 12.
compute notes = notes + 1.
end if.
do if (ncs > 0).
do if ((csum(rsum(ccmat))) < (nrow(ccmat)*ncol(ccmat))).
compute dototal=0.
compute alttotal=1.
compute notecode(notes,1) = 11.
compute notes = notes + 1.
end if.
end if.
do if (model=74).
do if (xdich=0 and nxvls=1).
compute dototal=0.
compute alttotal=1.
end if.
do if ((xdich=1 or nxvls > 1) and (ncs > 0) and model=74).
compute dototal=0.
compute alttotal=1.
end if.
end if.
end if.
end if.
do if (criterr=0 and ncs > 0).
do if (rsum((csum(ccmat)=0)) <> 0).
compute errcode(errs,1)=51.
compute errs=errs+1.
compute criterr=1.
end if.
end if.
compute debug= 0.
do if (outscree=1).
print/title = '***************** PROCESS Procedure for SPSS Version 4.2 *****************'.
print/title = '          Written by Andrew F. Hayes, Ph.D.       www.afhayes.com'.
print/title = '    Documentation available in Hayes (2022). www.guilford.com/p/hayes3'/space=0.
end if.
do if (criterr=0).
compute modresid=make(n,1,99999).
do if (stand=1 and ydich=1).
compute stand=0.
end if.
compute anymod2=csum(rsum(wcmat+zcmat+wzcmat)).
do if (anymod2 > 0 and stand = 1).
compute notecode(notes,1) = 27.
compute notes = notes + 1.
compute stand=0.
end if.
compute funny=1.
do if (outscree=1).
print modelvar/title = '**************************************************************************'/format = A8/rnames=modelvlb.
do if (ncs > 0).
print covnames/title='Covariates:'/format=A8.
end if.
print n/title='Sample'/rlabel='Size:'.
do if (( 'random' <> 'random')).
compute seedt= 'random'.
print seedt/title='Custom'/format=A12/rlabel = 'Seed:'.
end if.
end if.
compute maxresm=9.
compute resultm=make(1,maxresm,99999).
do if (describe=1).
compute means=csum(dat)/n.
compute sigmatal = (t(dat)*(ident(n)-(1/n)*ones*t(ones))*dat)*(1/(n-1)).
compute sdvec=sqrt(diag(sigmatal)).
compute sdall = mdiag(1/sdvec).
compute corall=sdall*sigmatal*t(sdall).
compute means={means;t(sdvec)}.
do if (ncol(means) > 9).
compute resultm=make(1,ncol(means),99999).
compute maxresm=ncol(means).
end if.
compute resultm2=make(2,maxresm,99999).
compute resultm2(1:2,1:ncol(means))=means.
compute resultm={resultm;resultm2}.
compute resultm2=make(ncol(corall),maxresm,99999).
compute resultm2(1:ncol(corall),1:ncol(corall))=corall.
compute resultm={resultm;resultm2}.
do if (outscree=1).
print means/title='Variable means and standard deviations'/cnames=varnames/rlabels='Mean','SD'/format= F10.4.
print corall/title='Variable intercorrelations (Pearson r)'/cnames=varnames/rnames=varnames/format= F10.4.
end if.
end if.
do if (outscree=1).
do if (mcxok=1).
compute labtmp={xnames,t(xcatlab(1:nxvls,1))}.
print dummatx/title = 'Coding of categorical X variable for analysis:'/cnames = labtmp/format = F6.3.
end if.
do if (mcwok=1 and xmint=0).
compute labtmp={wnames,t(wcatlab(1:nwvls,1))}.
print dummatw/title = 'Coding of categorical W variable for analysis:'/cnames = labtmp/format = F6.3.
end if.
do if (mczok=1).
compute labtmp={znames,t(zcatlab(1:nzvls,1))}.
print dummatz/title = 'Coding of categorical Z variable for analysis:'/cnames = labtmp/format = F6.3.
end if.
end if.
end if.
do if (criterr = 0).
compute outnames=ynames.
compute outvars=ytmp.
do if (nms > 0).
compute outnames={mnames,ynames}.
compute outvars={mtmp,ytmp}.
compute indcov=make(((nms*2)+(nms*(nxvls-1))),((nms*2)+(nms*(nxvls-1))),0).
compute mcsopath=make(((nms*2)+(nms*(nxvls-1))),1,0).
end if.
compute labstart=1.
compute intstart=1.
compute start=1.
compute coeffmat=make(1,6,0).
compute conseq={'        '}.
compute dfmat=0.
compute coeffcol=0.
compute pathscnt=1.
compute pathscn2=1.
loop i = 1 to (nms+nys).
do if (outscree=1).
print/title = '**************************************************************************'.
end if.
compute highf=make(1,5,0).
compute highf2=highf.
do if ((i = (nms+nys)) and (ydich=1)).
compute highf=make(1,3,0).
compute highf2=highf.
end if.
compute flabel={' '}.
compute y=outvars(:,i).
compute xindx=datindx(1:(nump(1,i)-1),i).
compute x = fulldat(:,xindx).
compute x={ones,x}.
compute xsq=t(x)*x.
compute exsq=eval(xsq).
release xsq.
compute zeroeig=csum(exsq <= 0.000000000002).
do if (outscree=1).
print outnames(1,i)/title = 'OUTCOME VARIABLE:'/format = A8/space=0.
do if (ydich=1 and (i = (nms+nys))).
compute nmsd = {outnames(1,i), 'Analysis'}.
print rcd/title = 'Coding of binary Y for logistic regression analysis:'/cnames = nmsd/format = F9.2.
end if.
end if.
do if (zeroeig > 0).
print / title = 'SINGULAR OR NEAR SINGULAR DATA MATRIX.'.
compute criterr=1.
compute errcode(errs,1)=31.
compute errs=errs+1.
end if.
compute means=csum(x)/n.
compute vlabsm=vlabs(labstart:(labstart+(nump(1,i)-1)),1).
do if (criterr=0).
do if (ydich=0 or (i < (nms+nys))).

.
do if ( 1 =1).
compute hatmat=inv(t( x )* x )*t( x ).
compute b = hatmat* y.
compute modres=b.
do if ( 1 =1).
compute n1=nrow( x ).
compute dfres=n1-(ncol( x )).
compute sstotal = t( y -(csum( y )/n1))*( y -(csum( y )/n1)).
compute resid= y - x *b.
compute ssresid = csum((resid)&**2).
compute r2 = (sstotal-ssresid)/sstotal.
do if (r2 < 0).
compute r2=0.
end if.
compute adjr2 = 1-((1-r2)*(n1-1)/(dfres)).
compute mse=ssresid/(n1-ncol( x )).

.
compute n1=nrow( x ).
compute invXtX = inv(t( x )* x ).
compute varb = mse *invXtX.
compute k3 = ncol( x ).
compute xhc=0.
do if ( hc <> 5).
compute xhc= x.
compute hat = xhc(:,1).
loop i3=1 to nrow(xhc).
compute hat(i3,1)= xhc(i3,:)*invXtX*t(xhc(i3,:)).
end loop.
do if ( hc = 0 or hc =1).
loop i3 = 1 to k3.
compute xhc(:,i3)=xhc(:,i3)&* resid.
end loop.
end if.
do if ( hc =3 or hc =2).
loop i3=1 to k3.
compute xhc(:,i3) = ( resid &/(1-hat)&**(1/(4- hc )))&*xhc(:,i3).
end loop.
end if.
do if ( hc = 4).
compute hcmn=make(n,2,4).
compute hcmn(:,2)=(n1*hat)/k3.
loop i3= 1 to k3.
compute xhc(:,i3) = ( resid &/(1-hat)&**(rmin(hcmn)/2))&*xhc(:,i3).
end loop.
end if.
compute varb=(invXtX*t(xhc)*xhc*invXtX).
do if ( hc =1).
compute varb=(n1/(n1-ncol( x )))&*varb.
end if.
end if.
compute hclab={'se(HC0)','se(HC1)','se(HC2)','se(HC3)','se(HC4)','se'}.
compute hclab=hclab(1,( hc +1)).
compute hcflab={'F(HC0)','F(HC1)','F(HC2)','F(HC3)','F(HC4)','F'}.
compute hcflab=hcflab(1,( hc +1)).
release xhc
.
compute seb=sqrt(diag(varb)).
compute trat = b&/seb.
compute p = 2*(1-tcdf(abs(trat), (dfres))).
compute tval = sqrt(dfres* (exp((dfres-(5/6))*((xp2/(dfres-(2/3)+(.11/dfres)))* (xp2/(dfres-(2/3)+(.11/dfres)))))-1)).
compute modres={modres,seb,trat,p}.
compute modres={modres,(b-tval&*seb),(b+tval&*seb)}.
compute modresl={'coeff',hclab,'t','p','LLCI','ULCI'}.
compute lmat = ident(ncol( x )).
compute lmat = lmat(:,2:ncol(lmat)).
compute fratio = (t(t(lmat)*b)*inv(t(lmat)*varb*lmat)*((t(lmat)*b)))/(ncol( x )-1).
compute pfr = 1-fcdf(fratio,(ncol( x )-1),dfres).
compute modsum={sqrt(r2),r2,mse,fratio,(ncol( x )-1),dfres,pfr}.
compute modsuml={'R','R-sq','MSE',hcflab,'df1','df2', 'p'}.
end if.
end if.
do if ( 1 = 2 or 1 =3).
compute xlp= x.
compute ylp= y.
compute pt2 = make(nrow(ylp),1,(csum(ylp)/nrow(ylp))).
do if ( 1 =2).
compute LL3 = ylp&*ln(pt2)+(1-ylp)&*ln(1-pt2).
end if.
compute LL3 = -2*csum(LL3).
compute bt1 = make(ncol(xlp),1,0).
compute LL1 = 0.
compute pt1 = make(nrow(ylp),1,0.5).
compute pt1lp=pt1.
loop jjj = 1 to iterate.
compute xlptmp=t(xlp).
compute vecprb=pt1lp&*(1-pt1lp).
loop kkk=1 to ncol(xlp).
compute xlptmp(kkk,:)=xlptmp(kkk,:)&*t(vecprb).
end loop.
compute b = bt1+inv(xlptmp*xlp)*t(xlp)*(ylp-pt1lp).
do if ( 1 =2).
compute xlpb=xlp*b.
compute xlpbt=(xlpb > -709.7).
compute xlpb709=(1-xlpbt)*(-709.7).
compute xlpb=(xlpb&*xlpbt)+xlpb709.
compute pt1lp = 1/(1+exp(-(xlpb))).
end if.
compute itprob = csum((pt1lp < .00000001) or (pt1lp > .9999999)).
do if (itprob > 0).
loop kkk = 1 to nrow(pt1lp).
do if (pt1lp(kkk,1) > .9999999).
compute pt1lp(kkk,1) = .9999999.
end if.
do if (pt1lp(kkk,1) < .00000001).
compute pt1lp(kkk,1) = .00000001.
end if.
end loop.
compute itprob = 0.
end if.
do if (itprob = 0).
do if ( 1 =2).
compute LL = ylp&*ln(pt1lp)+(1-ylp)&*ln(1-pt1lp).
end if.
compute LL2 = -2*csum(ll).
end if.
do if (abs(LL1-LL2) < converge).
do if ( 1 =1).
compute xlptmp=t(xlp).
compute vecprb=pt1lp&*(1-pt1lp).
loop kkk=1 to ncol(xlp).
compute xlptmp(kkk,:)=xlptmp(kkk,:)&*t(vecprb).
end loop.
compute varb = inv(xlptmp*xlp).
compute seb = sqrt(diag(varb)).
release xlptmp.
end if.
break.
end if.
compute bt1 = b.
compute LL1 = LL2.
end loop.
compute modres=b.
do if (jjj > iterate).
compute itprob = 2.
do if (booting=0).
compute iterrmod=1.
end if.
do if (booting=1).
compute bootiter=1.
end if.
do if (itprobtg=0).
compute itprobtg=1.
compute errcode(errs,1) = 47.
compute errs = errs + 1.
do if (booting = 0 and 1 =1).
compute vt1 = mdiag(pt1lp&*(1-pt1lp)).
compute varb = inv(t(xlp)*vt1*xlp).
compute seb = sqrt(diag(varb)).
end if.
end if.
end if.
do if ( 1 =1).
compute trat = b&/seb.
compute dfres=nrow(xlp).
compute p = 2*(1-cdfnorm(abs(trat))).
compute modres={modres,seb,trat,p}.
compute modres={modres,(b-xp2&*seb),(b+xp2&*seb)}.
compute pvchi=1-chicdf((LL3-LL2),(nrow(modres)-1)).
compute mcF = (LL3-LL2)/LL3.
compute cox = 1-exp(-(LL3-LL2)/nrow(xlp)).
compute nagel = cox/(1-exp(-(LL3)/nrow(xlp))).
compute modsum={LL2,(LL3-LL2),(nrow(modres)-1),pvchi, mcF,cox,nagel}.
compute modsuml={'-2LL','ModelLL', 'df', 'p', 'McFadden', 'CoxSnell', 'Nagelkrk'}.
compute modresl={'coeff','se','Z','p','LLCI','ULCI'}.
end if.
end if
.
compute dfmatt=make(nrow(modres),1,modsum(1,6)).
compute modresid={modresid,resid}.
end if.
do if (ydich=1 and (i = (nms+nys))).

.
do if ( 2 =1).
compute hatmat=inv(t( x )* x )*t( x ).
compute b = hatmat* y.
compute modres=b.
do if ( 1 =1).
compute n1=nrow( x ).
compute dfres=n1-(ncol( x )).
compute sstotal = t( y -(csum( y )/n1))*( y -(csum( y )/n1)).
compute resid= y - x *b.
compute ssresid = csum((resid)&**2).
compute r2 = (sstotal-ssresid)/sstotal.
do if (r2 < 0).
compute r2=0.
end if.
compute adjr2 = 1-((1-r2)*(n1-1)/(dfres)).
compute mse=ssresid/(n1-ncol( x )).

.
compute n1=nrow( x ).
compute invXtX = inv(t( x )* x ).
compute varb = mse *invXtX.
compute k3 = ncol( x ).
compute xhc=0.
do if ( hc <> 5).
compute xhc= x.
compute hat = xhc(:,1).
loop i3=1 to nrow(xhc).
compute hat(i3,1)= xhc(i3,:)*invXtX*t(xhc(i3,:)).
end loop.
do if ( hc = 0 or hc =1).
loop i3 = 1 to k3.
compute xhc(:,i3)=xhc(:,i3)&* resid.
end loop.
end if.
do if ( hc =3 or hc =2).
loop i3=1 to k3.
compute xhc(:,i3) = ( resid &/(1-hat)&**(1/(4- hc )))&*xhc(:,i3).
end loop.
end if.
do if ( hc = 4).
compute hcmn=make(n,2,4).
compute hcmn(:,2)=(n1*hat)/k3.
loop i3= 1 to k3.
compute xhc(:,i3) = ( resid &/(1-hat)&**(rmin(hcmn)/2))&*xhc(:,i3).
end loop.
end if.
compute varb=(invXtX*t(xhc)*xhc*invXtX).
do if ( hc =1).
compute varb=(n1/(n1-ncol( x )))&*varb.
end if.
end if.
compute hclab={'se(HC0)','se(HC1)','se(HC2)','se(HC3)','se(HC4)','se'}.
compute hclab=hclab(1,( hc +1)).
compute hcflab={'F(HC0)','F(HC1)','F(HC2)','F(HC3)','F(HC4)','F'}.
compute hcflab=hcflab(1,( hc +1)).
release xhc
.
compute seb=sqrt(diag(varb)).
compute trat = b&/seb.
compute p = 2*(1-tcdf(abs(trat), (dfres))).
compute tval = sqrt(dfres* (exp((dfres-(5/6))*((xp2/(dfres-(2/3)+(.11/dfres)))* (xp2/(dfres-(2/3)+(.11/dfres)))))-1)).
compute modres={modres,seb,trat,p}.
compute modres={modres,(b-tval&*seb),(b+tval&*seb)}.
compute modresl={'coeff',hclab,'t','p','LLCI','ULCI'}.
compute lmat = ident(ncol( x )).
compute lmat = lmat(:,2:ncol(lmat)).
compute fratio = (t(t(lmat)*b)*inv(t(lmat)*varb*lmat)*((t(lmat)*b)))/(ncol( x )-1).
compute pfr = 1-fcdf(fratio,(ncol( x )-1),dfres).
compute modsum={sqrt(r2),r2,mse,fratio,(ncol( x )-1),dfres,pfr}.
compute modsuml={'R','R-sq','MSE',hcflab,'df1','df2', 'p'}.
end if.
end if.
do if ( 2 = 2 or 2 =3).
compute xlp= x.
compute ylp= y.
compute pt2 = make(nrow(ylp),1,(csum(ylp)/nrow(ylp))).
do if ( 2 =2).
compute LL3 = ylp&*ln(pt2)+(1-ylp)&*ln(1-pt2).
end if.
compute LL3 = -2*csum(LL3).
compute bt1 = make(ncol(xlp),1,0).
compute LL1 = 0.
compute pt1 = make(nrow(ylp),1,0.5).
compute pt1lp=pt1.
loop jjj = 1 to iterate.
compute xlptmp=t(xlp).
compute vecprb=pt1lp&*(1-pt1lp).
loop kkk=1 to ncol(xlp).
compute xlptmp(kkk,:)=xlptmp(kkk,:)&*t(vecprb).
end loop.
compute b = bt1+inv(xlptmp*xlp)*t(xlp)*(ylp-pt1lp).
do if ( 2 =2).
compute xlpb=xlp*b.
compute xlpbt=(xlpb > -709.7).
compute xlpb709=(1-xlpbt)*(-709.7).
compute xlpb=(xlpb&*xlpbt)+xlpb709.
compute pt1lp = 1/(1+exp(-(xlpb))).
end if.
compute itprob = csum((pt1lp < .00000001) or (pt1lp > .9999999)).
do if (itprob > 0).
loop kkk = 1 to nrow(pt1lp).
do if (pt1lp(kkk,1) > .9999999).
compute pt1lp(kkk,1) = .9999999.
end if.
do if (pt1lp(kkk,1) < .00000001).
compute pt1lp(kkk,1) = .00000001.
end if.
end loop.
compute itprob = 0.
end if.
do if (itprob = 0).
do if ( 2 =2).
compute LL = ylp&*ln(pt1lp)+(1-ylp)&*ln(1-pt1lp).
end if.
compute LL2 = -2*csum(ll).
end if.
do if (abs(LL1-LL2) < converge).
do if ( 1 =1).
compute xlptmp=t(xlp).
compute vecprb=pt1lp&*(1-pt1lp).
loop kkk=1 to ncol(xlp).
compute xlptmp(kkk,:)=xlptmp(kkk,:)&*t(vecprb).
end loop.
compute varb = inv(xlptmp*xlp).
compute seb = sqrt(diag(varb)).
release xlptmp.
end if.
break.
end if.
compute bt1 = b.
compute LL1 = LL2.
end loop.
compute modres=b.
do if (jjj > iterate).
compute itprob = 2.
do if (booting=0).
compute iterrmod=1.
end if.
do if (booting=1).
compute bootiter=1.
end if.
do if (itprobtg=0).
compute itprobtg=1.
compute errcode(errs,1) = 47.
compute errs = errs + 1.
do if (booting = 0 and 1 =1).
compute vt1 = mdiag(pt1lp&*(1-pt1lp)).
compute varb = inv(t(xlp)*vt1*xlp).
compute seb = sqrt(diag(varb)).
end if.
end if.
end if.
do if ( 1 =1).
compute trat = b&/seb.
compute dfres=nrow(xlp).
compute p = 2*(1-cdfnorm(abs(trat))).
compute modres={modres,seb,trat,p}.
compute modres={modres,(b-xp2&*seb),(b+xp2&*seb)}.
compute pvchi=1-chicdf((LL3-LL2),(nrow(modres)-1)).
compute mcF = (LL3-LL2)/LL3.
compute cox = 1-exp(-(LL3-LL2)/nrow(xlp)).
compute nagel = cox/(1-exp(-(LL3)/nrow(xlp))).
compute modsum={LL2,(LL3-LL2),(nrow(modres)-1),pvchi, mcF,cox,nagel}.
compute modsuml={'-2LL','ModelLL', 'df', 'p', 'McFadden', 'CoxSnell', 'Nagelkrk'}.
compute modresl={'coeff','se','Z','p','LLCI','ULCI'}.
end if.
end if
.
compute dfmatt=make(nrow(modres),1,-999).
end if.
compute resultm2=make(1,maxresm,99999).
compute resultm2(1,1:ncol(modsum))=modsum.
compute resultm={resultm;resultm2}.
compute resultm2=make(nrow(modres),maxresm,99999).
compute resultm2(1:nrow(modres),1:ncol(modres))=modres.
compute resultm={resultm;resultm2}.
compute obscoeff={obscoeff,t(b)}.
do if (outscree=1).
print modsum/title = 'Model Summary'/cnames = modsuml/format= F10.4.
print modres/title='Model'/rnames=vlabsm/cnames=modresl/format= F10.4.
end if.
compute basemod=modsum(1,1).
compute basemodx=basemod.
do if (ydich=1 and (i = (nms+nys))).
do if (outscree=1).
print/title='These results are expressed in a log-odds metric.'.
end if.
compute notecode(notes,1) = 26.
compute notes = notes + 1.
end if.
compute coeffmat={coeffmat;modres}.
compute conseqt=make(nrow(modres),1,outnames(1,i)).
compute conseq={conseq;conseqt}.
compute dfmat={dfmat;dfmatt}.
compute labstart=labstart+nump(1,i).
do if (stand=1).
compute predsd=make(nrow(modres),1,0).
compute stdmod=modres(:,1)&/ovsd(1,i).
loop jd=1 to ncol(x).
compute descdat=x(:,jd).
compute predsd(jd,1) = (nrow(descdat)*sscp(descdat))-(t(csum(descdat))*(csum(descdat))).
compute predsd(jd,1) = sqrt(predsd(jd,1)/(nrow(descdat)*(nrow(descdat)-1))).
end loop.
do if (wherex(1,i) <> -999 and ((nxvls > 1) or (xdich=1))).
compute sdmsone=make(nxvls,1,1).
compute predsd(wherex(1,i):wherex(2,i),1)=sdmsone.
compute pstog=1.
end if.
compute predsd(1,1)=1.
compute stdmod=stdmod&*predsd.
compute stdmod=stdmod(2:nrow(stdmod),1).
compute sdvlabs=vlabsm(2:nrow(vlabsm),1).
compute resultm2=make(nrow(stdmod),maxresm,99999).
compute resultm2(1:nrow(stdmod),1:ncol(stdmod))=stdmod.
compute resultm={resultm;resultm2}.
do if (outscree=1).
print stdmod/title='Standardized coefficients'/clabels='coeff'/rnames=sdvlabs/format= F10.4.
end if.
end if.
do if (nms > 0 and serial = 0 and (rsum(numint) = 0) and (normal=1 or mc > 0)).
do if (i < (nms+nys)).
compute indcov((((i-1)*nxvls)+1):(i*nxvls),(((i-1)*nxvls)+1):(i*nxvls))=varb(2:(1+nxvls), 2:(1+nxvls)).
compute mcsopath((((i-1)*nxvls)+1):(i*nxvls) ,1)=modres(2:(1+nxvls),1).
end if.
do if (i = (nms+nys)).
compute atm=ncol(wherem).
compute indcov(((nms*nxvls)+1):nrow(mcsopath),((nms*nxvls)+1):nrow(mcsopath))= varb(wherem(1,atm):(wherem(1,atm)+nms-1),wherem(1,atm):(wherem(1,atm)+nms-1)).
compute mcsopath(((nms*nxvls)+1):nrow(mcsopath),1)=modres(wherem(1,atm):(wherem(1,atm)+ nms-1),1).
compute sobelok=1.
end if.
end if.
compute obsdirfx=make(1,nxvls,0).
compute dirzes=make(1,nxvls,0).
do if ((i = (nms+nys)) and (bcmat(nrow(bcmat),1)=1)).
compute direff=modres(2:(1+nxvls),:).
compute obsdirfx=t(direff(:,1)).
compute direfflb=modresl.
compute direffl2=vlabsm(2:(1+nxvls),:).
compute lmat=make(nrow(b),1,0).
compute lmat2=make(nxvls,1,1).
compute lmat(2:(1+nxvls),1)=lmat2.
do if (ydich <> 1).

.
compute lmat2= lmat.
do if ( 0 =0).
compute lmat2 = mdiag( lmat ).
compute lmat3=make(nrow(lmat2),1,0).
loop flp=1 to ncol(lmat2).
do if (csum(lmat2(:,flp))=1).
compute lmat3={lmat3,lmat2(:,flp)}.
end if.
end loop.
compute lmat2=lmat3(:,2:ncol(lmat3)).
end if.
compute fratio = (t(t(lmat2)* b )*inv(t(lmat2)* varb *lmat2)*((t(lmat2)* b )))/ncol(lmat2).
compute pfr = 1-fcdf(fratio,ncol(lmat2),(n-nrow( b ))).
compute fresult={fratio,ncol(lmat2),(n-nrow( b )),pfr}.
do if (i = (nms+nys) and (ydich=1)).
compute fratio=fratio*ncol(lmat2).
compute pfr=1-chicdf(fratio,ncol(lmat2)).
compute fresult={fratio,ncol(lmat2),pfr}.
end if.
do if ( 1 =1).
compute lmat3=1-rsum(lmat2).
compute xfm=make(n,csum(lmat3),0).
compute flpc=1.
loop flp=1 to nrow(lmat3).
do if (lmat3(flp,1)=1).
compute xfm(:,flpc)=x(:,flp).
compute flpc=flpc+1.
end if.
end loop.
compute bfm=inv(t(xfm)*xfm)*t(xfm)*y.
compute resid=y-(xfm*bfm).
compute sstotal=(y-(csum(y)/n)).
compute sstotal=csum(sstotal&*sstotal).
compute ssresid=csum(resid&*resid).
compute rsqch= r2 -((sstotal-ssresid)/sstotal).
compute fresult={rsqch,fresult}.
release xfm,flpc, resid, ssresid, bfm.
end if
.
compute diromni=fresult.
end if.
do if (ydich = 1 ).

.
compute btemphld=b.
compute llrdat=make(nrow(x),nrow( lmat )-csum( lmat ),-999).
compute llrdf=ncol(x)-ncol(llrdat).
compute llrcnt=0.
loop llri=1 to nrow( lmat ).
do if ( lmat (llri,1)=0).
compute llrcnt=llrcnt+1.
compute llrdat(:,llrcnt)=x(:,llri).
end if.
end loop.

.
do if ( 2 =1).
compute hatmat=inv(t( llrdat )* llrdat )*t( llrdat ).
compute b = hatmat* y.
compute modres=b.
do if ( 0 =1).
compute n1=nrow( llrdat ).
compute dfres=n1-(ncol( llrdat )).
compute sstotal = t( y -(csum( y )/n1))*( y -(csum( y )/n1)).
compute resid= y - llrdat *b.
compute ssresid = csum((resid)&**2).
compute r2 = (sstotal-ssresid)/sstotal.
do if (r2 < 0).
compute r2=0.
end if.
compute adjr2 = 1-((1-r2)*(n1-1)/(dfres)).
compute mse=ssresid/(n1-ncol( llrdat )).

.
compute n1=nrow( x ).
compute invXtX = inv(t( x )* x ).
compute varb = mse *invXtX.
compute k3 = ncol( x ).
compute xhc=0.
do if ( hc <> 5).
compute xhc= x.
compute hat = xhc(:,1).
loop i3=1 to nrow(xhc).
compute hat(i3,1)= xhc(i3,:)*invXtX*t(xhc(i3,:)).
end loop.
do if ( hc = 0 or hc =1).
loop i3 = 1 to k3.
compute xhc(:,i3)=xhc(:,i3)&* resid.
end loop.
end if.
do if ( hc =3 or hc =2).
loop i3=1 to k3.
compute xhc(:,i3) = ( resid &/(1-hat)&**(1/(4- hc )))&*xhc(:,i3).
end loop.
end if.
do if ( hc = 4).
compute hcmn=make(n,2,4).
compute hcmn(:,2)=(n1*hat)/k3.
loop i3= 1 to k3.
compute xhc(:,i3) = ( resid &/(1-hat)&**(rmin(hcmn)/2))&*xhc(:,i3).
end loop.
end if.
compute varb=(invXtX*t(xhc)*xhc*invXtX).
do if ( hc =1).
compute varb=(n1/(n1-ncol( x )))&*varb.
end if.
end if.
compute hclab={'se(HC0)','se(HC1)','se(HC2)','se(HC3)','se(HC4)','se'}.
compute hclab=hclab(1,( hc +1)).
compute hcflab={'F(HC0)','F(HC1)','F(HC2)','F(HC3)','F(HC4)','F'}.
compute hcflab=hcflab(1,( hc +1)).
release xhc
.
compute seb=sqrt(diag(varb)).
compute trat = b&/seb.
compute p = 2*(1-tcdf(abs(trat), (dfres))).
compute tval = sqrt(dfres* (exp((dfres-(5/6))*((xp2/(dfres-(2/3)+(.11/dfres)))* (xp2/(dfres-(2/3)+(.11/dfres)))))-1)).
compute modres={modres,seb,trat,p}.
compute modres={modres,(b-tval&*seb),(b+tval&*seb)}.
compute modresl={'coeff',hclab,'t','p','LLCI','ULCI'}.
compute lmat = ident(ncol( llrdat )).
compute lmat = lmat(:,2:ncol(lmat)).
compute fratio = (t(t(lmat)*b)*inv(t(lmat)*varb*lmat)*((t(lmat)*b)))/(ncol( llrdat )-1).
compute pfr = 1-fcdf(fratio,(ncol( llrdat )-1),dfres).
compute modsum={sqrt(r2),r2,mse,fratio,(ncol( llrdat )-1),dfres,pfr}.
compute modsuml={'R','R-sq','MSE',hcflab,'df1','df2', 'p'}.
end if.
end if.
do if ( 2 = 2 or 2 =3).
compute xlp= llrdat.
compute ylp= y.
compute pt2 = make(nrow(ylp),1,(csum(ylp)/nrow(ylp))).
do if ( 2 =2).
compute LL3 = ylp&*ln(pt2)+(1-ylp)&*ln(1-pt2).
end if.
compute LL3 = -2*csum(LL3).
compute bt1 = make(ncol(xlp),1,0).
compute LL1 = 0.
compute pt1 = make(nrow(ylp),1,0.5).
compute pt1lp=pt1.
loop jjj = 1 to iterate.
compute xlptmp=t(xlp).
compute vecprb=pt1lp&*(1-pt1lp).
loop kkk=1 to ncol(xlp).
compute xlptmp(kkk,:)=xlptmp(kkk,:)&*t(vecprb).
end loop.
compute b = bt1+inv(xlptmp*xlp)*t(xlp)*(ylp-pt1lp).
do if ( 2 =2).
compute xlpb=xlp*b.
compute xlpbt=(xlpb > -709.7).
compute xlpb709=(1-xlpbt)*(-709.7).
compute xlpb=(xlpb&*xlpbt)+xlpb709.
compute pt1lp = 1/(1+exp(-(xlpb))).
end if.
compute itprob = csum((pt1lp < .00000001) or (pt1lp > .9999999)).
do if (itprob > 0).
loop kkk = 1 to nrow(pt1lp).
do if (pt1lp(kkk,1) > .9999999).
compute pt1lp(kkk,1) = .9999999.
end if.
do if (pt1lp(kkk,1) < .00000001).
compute pt1lp(kkk,1) = .00000001.
end if.
end loop.
compute itprob = 0.
end if.
do if (itprob = 0).
do if ( 2 =2).
compute LL = ylp&*ln(pt1lp)+(1-ylp)&*ln(1-pt1lp).
end if.
compute LL2 = -2*csum(ll).
end if.
do if (abs(LL1-LL2) < converge).
do if ( 0 =1).
compute xlptmp=t(xlp).
compute vecprb=pt1lp&*(1-pt1lp).
loop kkk=1 to ncol(xlp).
compute xlptmp(kkk,:)=xlptmp(kkk,:)&*t(vecprb).
end loop.
compute varb = inv(xlptmp*xlp).
compute seb = sqrt(diag(varb)).
release xlptmp.
end if.
break.
end if.
compute bt1 = b.
compute LL1 = LL2.
end loop.
compute modres=b.
do if (jjj > iterate).
compute itprob = 2.
do if (booting=0).
compute iterrmod=1.
end if.
do if (booting=1).
compute bootiter=1.
end if.
do if (itprobtg=0).
compute itprobtg=1.
compute errcode(errs,1) = 47.
compute errs = errs + 1.
do if (booting = 0 and 0 =1).
compute vt1 = mdiag(pt1lp&*(1-pt1lp)).
compute varb = inv(t(xlp)*vt1*xlp).
compute seb = sqrt(diag(varb)).
end if.
end if.
end if.
do if ( 0 =1).
compute trat = b&/seb.
compute dfres=nrow(xlp).
compute p = 2*(1-cdfnorm(abs(trat))).
compute modres={modres,seb,trat,p}.
compute modres={modres,(b-xp2&*seb),(b+xp2&*seb)}.
compute pvchi=1-chicdf((LL3-LL2),(nrow(modres)-1)).
compute mcF = (LL3-LL2)/LL3.
compute cox = 1-exp(-(LL3-LL2)/nrow(xlp)).
compute nagel = cox/(1-exp(-(LL3)/nrow(xlp))).
compute modsum={LL2,(LL3-LL2),(nrow(modres)-1),pvchi, mcF,cox,nagel}.
compute modsuml={'-2LL','ModelLL', 'df', 'p', 'McFadden', 'CoxSnell', 'Nagelkrk'}.
compute modresl={'coeff','se','Z','p','LLCI','ULCI'}.
end if.
end if
.
compute b=btemphld.
compute fresult={(LL2-basemod),llrdf,1-chicdf((LL2-basemod),llrdf)}
.
compute diromni=fresult.
end if.
end if.
do if (numint(1,i) > 0).
compute intkeym=intkey(intstart:(intstart+numint(1,i)-1),:).
do if (outscree=1).
print intkeym/title='Product terms key:'/format = A8.
end if.
end if.
do if (covcoeff=1).
do if (outscree=1).
print varb/title='Covariance matrix of regression parameter estimates:'/rnames=vlabsm /cnames=vlabsm/format= F10.4.
end if.
compute resultm2=make(nrow(varb),maxresm,99999).
do if (ncol(varb) <= maxresm).
compute resultm2(1:nrow(varb),1:ncol(varb))=varb.
compute resultm={resultm;resultm2}.
end if.
do if (ncol(varb) > maxresm).
compute resultmt=make(nrow(resultm),ncol(varb),99999).
compute resultmt(1:nrow(resultm),1:ncol(resultm))=resultm.
compute resultm=resultmt.
compute resultm2=make(nrow(varb),ncol(resultm),99999).
compute resultm2(1:nrow(varb),1:ncol(varb))=varb.
compute resultm={resultm;resultm2}.
compute maxresm=ncol(resultm).
end if.
end if.
do if (model <> 74 and xmtest=1 and nms > 0).
compute r2tmp=r2.
compute btmp=b.
compute varbtmp=varb.
compute dfrestmp=dfres.
compute tvaltmp=tval.
compute xmtst=make(nms,4,0).
compute xmtstlbc={hcflab,'df1','df2','p'}.
do if ((i=(nms+nys)) and (ydich=1)).
compute xmtst=make(nms,3,0).
compute xmtstlbc={'Chi-sq','df','p'}.
end if.
compute xmtstlb={' '}.
compute xmtmat=x.
compute numxint=0.
loop xmints=2 to i.
compute x=xmtmat.
do if ((bcmat((i+1),xmints)=1) and (wzcmat((i+1),xmints) <> 1)).
do if (bcmat((i+1),1)=0).
compute x={xmtmat,xtmp}.
do if ((ydich=1) and (i=(nms+nys))).

.
do if ( 2 =1).
compute hatmat=inv(t( x )* x )*t( x ).
compute b = hatmat* y.
compute modres=b.
do if ( 1 =1).
compute n1=nrow( x ).
compute dfres=n1-(ncol( x )).
compute sstotal = t( y -(csum( y )/n1))*( y -(csum( y )/n1)).
compute resid= y - x *b.
compute ssresid = csum((resid)&**2).
compute r2 = (sstotal-ssresid)/sstotal.
do if (r2 < 0).
compute r2=0.
end if.
compute adjr2 = 1-((1-r2)*(n1-1)/(dfres)).
compute mse=ssresid/(n1-ncol( x )).

.
compute n1=nrow( x ).
compute invXtX = inv(t( x )* x ).
compute varb = mse *invXtX.
compute k3 = ncol( x ).
compute xhc=0.
do if ( hc <> 5).
compute xhc= x.
compute hat = xhc(:,1).
loop i3=1 to nrow(xhc).
compute hat(i3,1)= xhc(i3,:)*invXtX*t(xhc(i3,:)).
end loop.
do if ( hc = 0 or hc =1).
loop i3 = 1 to k3.
compute xhc(:,i3)=xhc(:,i3)&* resid.
end loop.
end if.
do if ( hc =3 or hc =2).
loop i3=1 to k3.
compute xhc(:,i3) = ( resid &/(1-hat)&**(1/(4- hc )))&*xhc(:,i3).
end loop.
end if.
do if ( hc = 4).
compute hcmn=make(n,2,4).
compute hcmn(:,2)=(n1*hat)/k3.
loop i3= 1 to k3.
compute xhc(:,i3) = ( resid &/(1-hat)&**(rmin(hcmn)/2))&*xhc(:,i3).
end loop.
end if.
compute varb=(invXtX*t(xhc)*xhc*invXtX).
do if ( hc =1).
compute varb=(n1/(n1-ncol( x )))&*varb.
end if.
end if.
compute hclab={'se(HC0)','se(HC1)','se(HC2)','se(HC3)','se(HC4)','se'}.
compute hclab=hclab(1,( hc +1)).
compute hcflab={'F(HC0)','F(HC1)','F(HC2)','F(HC3)','F(HC4)','F'}.
compute hcflab=hcflab(1,( hc +1)).
release xhc
.
compute seb=sqrt(diag(varb)).
compute trat = b&/seb.
compute p = 2*(1-tcdf(abs(trat), (dfres))).
compute tval = sqrt(dfres* (exp((dfres-(5/6))*((xp2/(dfres-(2/3)+(.11/dfres)))* (xp2/(dfres-(2/3)+(.11/dfres)))))-1)).
compute modres={modres,seb,trat,p}.
compute modres={modres,(b-tval&*seb),(b+tval&*seb)}.
compute modresl={'coeff',hclab,'t','p','LLCI','ULCI'}.
compute lmat = ident(ncol( x )).
compute lmat = lmat(:,2:ncol(lmat)).
compute fratio = (t(t(lmat)*b)*inv(t(lmat)*varb*lmat)*((t(lmat)*b)))/(ncol( x )-1).
compute pfr = 1-fcdf(fratio,(ncol( x )-1),dfres).
compute modsum={sqrt(r2),r2,mse,fratio,(ncol( x )-1),dfres,pfr}.
compute modsuml={'R','R-sq','MSE',hcflab,'df1','df2', 'p'}.
end if.
end if.
do if ( 2 = 2 or 2 =3).
compute xlp= x.
compute ylp= y.
compute pt2 = make(nrow(ylp),1,(csum(ylp)/nrow(ylp))).
do if ( 2 =2).
compute LL3 = ylp&*ln(pt2)+(1-ylp)&*ln(1-pt2).
end if.
compute LL3 = -2*csum(LL3).
compute bt1 = make(ncol(xlp),1,0).
compute LL1 = 0.
compute pt1 = make(nrow(ylp),1,0.5).
compute pt1lp=pt1.
loop jjj = 1 to iterate.
compute xlptmp=t(xlp).
compute vecprb=pt1lp&*(1-pt1lp).
loop kkk=1 to ncol(xlp).
compute xlptmp(kkk,:)=xlptmp(kkk,:)&*t(vecprb).
end loop.
compute b = bt1+inv(xlptmp*xlp)*t(xlp)*(ylp-pt1lp).
do if ( 2 =2).
compute xlpb=xlp*b.
compute xlpbt=(xlpb > -709.7).
compute xlpb709=(1-xlpbt)*(-709.7).
compute xlpb=(xlpb&*xlpbt)+xlpb709.
compute pt1lp = 1/(1+exp(-(xlpb))).
end if.
compute itprob = csum((pt1lp < .00000001) or (pt1lp > .9999999)).
do if (itprob > 0).
loop kkk = 1 to nrow(pt1lp).
do if (pt1lp(kkk,1) > .9999999).
compute pt1lp(kkk,1) = .9999999.
end if.
do if (pt1lp(kkk,1) < .00000001).
compute pt1lp(kkk,1) = .00000001.
end if.
end loop.
compute itprob = 0.
end if.
do if (itprob = 0).
do if ( 2 =2).
compute LL = ylp&*ln(pt1lp)+(1-ylp)&*ln(1-pt1lp).
end if.
compute LL2 = -2*csum(ll).
end if.
do if (abs(LL1-LL2) < converge).
do if ( 1 =1).
compute xlptmp=t(xlp).
compute vecprb=pt1lp&*(1-pt1lp).
loop kkk=1 to ncol(xlp).
compute xlptmp(kkk,:)=xlptmp(kkk,:)&*t(vecprb).
end loop.
compute varb = inv(xlptmp*xlp).
compute seb = sqrt(diag(varb)).
release xlptmp.
end if.
break.
end if.
compute bt1 = b.
compute LL1 = LL2.
end loop.
compute modres=b.
do if (jjj > iterate).
compute itprob = 2.
do if (booting=0).
compute iterrmod=1.
end if.
do if (booting=1).
compute bootiter=1.
end if.
do if (itprobtg=0).
compute itprobtg=1.
compute errcode(errs,1) = 47.
compute errs = errs + 1.
do if (booting = 0 and 1 =1).
compute vt1 = mdiag(pt1lp&*(1-pt1lp)).
compute varb = inv(t(xlp)*vt1*xlp).
compute seb = sqrt(diag(varb)).
end if.
end if.
end if.
do if ( 1 =1).
compute trat = b&/seb.
compute dfres=nrow(xlp).
compute p = 2*(1-cdfnorm(abs(trat))).
compute modres={modres,seb,trat,p}.
compute modres={modres,(b-xp2&*seb),(b+xp2&*seb)}.
compute pvchi=1-chicdf((LL3-LL2),(nrow(modres)-1)).
compute mcF = (LL3-LL2)/LL3.
compute cox = 1-exp(-(LL3-LL2)/nrow(xlp)).
compute nagel = cox/(1-exp(-(LL3)/nrow(xlp))).
compute modsum={LL2,(LL3-LL2),(nrow(modres)-1),pvchi, mcF,cox,nagel}.
compute modsuml={'-2LL','ModelLL', 'df', 'p', 'McFadden', 'CoxSnell', 'Nagelkrk'}.
compute modresl={'coeff','se','Z','p','LLCI','ULCI'}.
end if.
end if
.
compute basemodx=LL2.
end if.
end if.
loop xmtlp1=1 to nxvls.
compute x={x,xtmp(:,xmtlp1)&*(mtmp(:,(xmints-1))-(csum(mtmp(:,(xmints-1)))/nrow(mtmp)) )}.
end loop.
do if ((i < (nms+nys)) or (ydich=0)).

.
do if ( 1 =1).
compute hatmat=inv(t( x )* x )*t( x ).
compute b = hatmat* y.
compute modres=b.
do if ( 1 =1).
compute n1=nrow( x ).
compute dfres=n1-(ncol( x )).
compute sstotal = t( y -(csum( y )/n1))*( y -(csum( y )/n1)).
compute resid= y - x *b.
compute ssresid = csum((resid)&**2).
compute r2 = (sstotal-ssresid)/sstotal.
do if (r2 < 0).
compute r2=0.
end if.
compute adjr2 = 1-((1-r2)*(n1-1)/(dfres)).
compute mse=ssresid/(n1-ncol( x )).

.
compute n1=nrow( x ).
compute invXtX = inv(t( x )* x ).
compute varb = mse *invXtX.
compute k3 = ncol( x ).
compute xhc=0.
do if ( hc <> 5).
compute xhc= x.
compute hat = xhc(:,1).
loop i3=1 to nrow(xhc).
compute hat(i3,1)= xhc(i3,:)*invXtX*t(xhc(i3,:)).
end loop.
do if ( hc = 0 or hc =1).
loop i3 = 1 to k3.
compute xhc(:,i3)=xhc(:,i3)&* resid.
end loop.
end if.
do if ( hc =3 or hc =2).
loop i3=1 to k3.
compute xhc(:,i3) = ( resid &/(1-hat)&**(1/(4- hc )))&*xhc(:,i3).
end loop.
end if.
do if ( hc = 4).
compute hcmn=make(n,2,4).
compute hcmn(:,2)=(n1*hat)/k3.
loop i3= 1 to k3.
compute xhc(:,i3) = ( resid &/(1-hat)&**(rmin(hcmn)/2))&*xhc(:,i3).
end loop.
end if.
compute varb=(invXtX*t(xhc)*xhc*invXtX).
do if ( hc =1).
compute varb=(n1/(n1-ncol( x )))&*varb.
end if.
end if.
compute hclab={'se(HC0)','se(HC1)','se(HC2)','se(HC3)','se(HC4)','se'}.
compute hclab=hclab(1,( hc +1)).
compute hcflab={'F(HC0)','F(HC1)','F(HC2)','F(HC3)','F(HC4)','F'}.
compute hcflab=hcflab(1,( hc +1)).
release xhc
.
compute seb=sqrt(diag(varb)).
compute trat = b&/seb.
compute p = 2*(1-tcdf(abs(trat), (dfres))).
compute tval = sqrt(dfres* (exp((dfres-(5/6))*((xp2/(dfres-(2/3)+(.11/dfres)))* (xp2/(dfres-(2/3)+(.11/dfres)))))-1)).
compute modres={modres,seb,trat,p}.
compute modres={modres,(b-tval&*seb),(b+tval&*seb)}.
compute modresl={'coeff',hclab,'t','p','LLCI','ULCI'}.
compute lmat = ident(ncol( x )).
compute lmat = lmat(:,2:ncol(lmat)).
compute fratio = (t(t(lmat)*b)*inv(t(lmat)*varb*lmat)*((t(lmat)*b)))/(ncol( x )-1).
compute pfr = 1-fcdf(fratio,(ncol( x )-1),dfres).
compute modsum={sqrt(r2),r2,mse,fratio,(ncol( x )-1),dfres,pfr}.
compute modsuml={'R','R-sq','MSE',hcflab,'df1','df2', 'p'}.
end if.
end if.
do if ( 1 = 2 or 1 =3).
compute xlp= x.
compute ylp= y.
compute pt2 = make(nrow(ylp),1,(csum(ylp)/nrow(ylp))).
do if ( 1 =2).
compute LL3 = ylp&*ln(pt2)+(1-ylp)&*ln(1-pt2).
end if.
compute LL3 = -2*csum(LL3).
compute bt1 = make(ncol(xlp),1,0).
compute LL1 = 0.
compute pt1 = make(nrow(ylp),1,0.5).
compute pt1lp=pt1.
loop jjj = 1 to iterate.
compute xlptmp=t(xlp).
compute vecprb=pt1lp&*(1-pt1lp).
loop kkk=1 to ncol(xlp).
compute xlptmp(kkk,:)=xlptmp(kkk,:)&*t(vecprb).
end loop.
compute b = bt1+inv(xlptmp*xlp)*t(xlp)*(ylp-pt1lp).
do if ( 1 =2).
compute xlpb=xlp*b.
compute xlpbt=(xlpb > -709.7).
compute xlpb709=(1-xlpbt)*(-709.7).
compute xlpb=(xlpb&*xlpbt)+xlpb709.
compute pt1lp = 1/(1+exp(-(xlpb))).
end if.
compute itprob = csum((pt1lp < .00000001) or (pt1lp > .9999999)).
do if (itprob > 0).
loop kkk = 1 to nrow(pt1lp).
do if (pt1lp(kkk,1) > .9999999).
compute pt1lp(kkk,1) = .9999999.
end if.
do if (pt1lp(kkk,1) < .00000001).
compute pt1lp(kkk,1) = .00000001.
end if.
end loop.
compute itprob = 0.
end if.
do if (itprob = 0).
do if ( 1 =2).
compute LL = ylp&*ln(pt1lp)+(1-ylp)&*ln(1-pt1lp).
end if.
compute LL2 = -2*csum(ll).
end if.
do if (abs(LL1-LL2) < converge).
do if ( 1 =1).
compute xlptmp=t(xlp).
compute vecprb=pt1lp&*(1-pt1lp).
loop kkk=1 to ncol(xlp).
compute xlptmp(kkk,:)=xlptmp(kkk,:)&*t(vecprb).
end loop.
compute varb = inv(xlptmp*xlp).
compute seb = sqrt(diag(varb)).
release xlptmp.
end if.
break.
end if.
compute bt1 = b.
compute LL1 = LL2.
end loop.
compute modres=b.
do if (jjj > iterate).
compute itprob = 2.
do if (booting=0).
compute iterrmod=1.
end if.
do if (booting=1).
compute bootiter=1.
end if.
do if (itprobtg=0).
compute itprobtg=1.
compute errcode(errs,1) = 47.
compute errs = errs + 1.
do if (booting = 0 and 1 =1).
compute vt1 = mdiag(pt1lp&*(1-pt1lp)).
compute varb = inv(t(xlp)*vt1*xlp).
compute seb = sqrt(diag(varb)).
end if.
end if.
end if.
do if ( 1 =1).
compute trat = b&/seb.
compute dfres=nrow(xlp).
compute p = 2*(1-cdfnorm(abs(trat))).
compute modres={modres,seb,trat,p}.
compute modres={modres,(b-xp2&*seb),(b+xp2&*seb)}.
compute pvchi=1-chicdf((LL3-LL2),(nrow(modres)-1)).
compute mcF = (LL3-LL2)/LL3.
compute cox = 1-exp(-(LL3-LL2)/nrow(xlp)).
compute nagel = cox/(1-exp(-(LL3)/nrow(xlp))).
compute modsum={LL2,(LL3-LL2),(nrow(modres)-1),pvchi, mcF,cox,nagel}.
compute modsuml={'-2LL','ModelLL', 'df', 'p', 'McFadden', 'CoxSnell', 'Nagelkrk'}.
compute modresl={'coeff','se','Z','p','LLCI','ULCI'}.
end if.
end if
.
end if.
do if ((i = (nms+nys)) and (ydich=1)).

.
do if ( 2 =1).
compute hatmat=inv(t( x )* x )*t( x ).
compute b = hatmat* y.
compute modres=b.
do if ( 1 =1).
compute n1=nrow( x ).
compute dfres=n1-(ncol( x )).
compute sstotal = t( y -(csum( y )/n1))*( y -(csum( y )/n1)).
compute resid= y - x *b.
compute ssresid = csum((resid)&**2).
compute r2 = (sstotal-ssresid)/sstotal.
do if (r2 < 0).
compute r2=0.
end if.
compute adjr2 = 1-((1-r2)*(n1-1)/(dfres)).
compute mse=ssresid/(n1-ncol( x )).

.
compute n1=nrow( x ).
compute invXtX = inv(t( x )* x ).
compute varb = mse *invXtX.
compute k3 = ncol( x ).
compute xhc=0.
do if ( hc <> 5).
compute xhc= x.
compute hat = xhc(:,1).
loop i3=1 to nrow(xhc).
compute hat(i3,1)= xhc(i3,:)*invXtX*t(xhc(i3,:)).
end loop.
do if ( hc = 0 or hc =1).
loop i3 = 1 to k3.
compute xhc(:,i3)=xhc(:,i3)&* resid.
end loop.
end if.
do if ( hc =3 or hc =2).
loop i3=1 to k3.
compute xhc(:,i3) = ( resid &/(1-hat)&**(1/(4- hc )))&*xhc(:,i3).
end loop.
end if.
do if ( hc = 4).
compute hcmn=make(n,2,4).
compute hcmn(:,2)=(n1*hat)/k3.
loop i3= 1 to k3.
compute xhc(:,i3) = ( resid &/(1-hat)&**(rmin(hcmn)/2))&*xhc(:,i3).
end loop.
end if.
compute varb=(invXtX*t(xhc)*xhc*invXtX).
do if ( hc =1).
compute varb=(n1/(n1-ncol( x )))&*varb.
end if.
end if.
compute hclab={'se(HC0)','se(HC1)','se(HC2)','se(HC3)','se(HC4)','se'}.
compute hclab=hclab(1,( hc +1)).
compute hcflab={'F(HC0)','F(HC1)','F(HC2)','F(HC3)','F(HC4)','F'}.
compute hcflab=hcflab(1,( hc +1)).
release xhc
.
compute seb=sqrt(diag(varb)).
compute trat = b&/seb.
compute p = 2*(1-tcdf(abs(trat), (dfres))).
compute tval = sqrt(dfres* (exp((dfres-(5/6))*((xp2/(dfres-(2/3)+(.11/dfres)))* (xp2/(dfres-(2/3)+(.11/dfres)))))-1)).
compute modres={modres,seb,trat,p}.
compute modres={modres,(b-tval&*seb),(b+tval&*seb)}.
compute modresl={'coeff',hclab,'t','p','LLCI','ULCI'}.
compute lmat = ident(ncol( x )).
compute lmat = lmat(:,2:ncol(lmat)).
compute fratio = (t(t(lmat)*b)*inv(t(lmat)*varb*lmat)*((t(lmat)*b)))/(ncol( x )-1).
compute pfr = 1-fcdf(fratio,(ncol( x )-1),dfres).
compute modsum={sqrt(r2),r2,mse,fratio,(ncol( x )-1),dfres,pfr}.
compute modsuml={'R','R-sq','MSE',hcflab,'df1','df2', 'p'}.
end if.
end if.
do if ( 2 = 2 or 2 =3).
compute xlp= x.
compute ylp= y.
compute pt2 = make(nrow(ylp),1,(csum(ylp)/nrow(ylp))).
do if ( 2 =2).
compute LL3 = ylp&*ln(pt2)+(1-ylp)&*ln(1-pt2).
end if.
compute LL3 = -2*csum(LL3).
compute bt1 = make(ncol(xlp),1,0).
compute LL1 = 0.
compute pt1 = make(nrow(ylp),1,0.5).
compute pt1lp=pt1.
loop jjj = 1 to iterate.
compute xlptmp=t(xlp).
compute vecprb=pt1lp&*(1-pt1lp).
loop kkk=1 to ncol(xlp).
compute xlptmp(kkk,:)=xlptmp(kkk,:)&*t(vecprb).
end loop.
compute b = bt1+inv(xlptmp*xlp)*t(xlp)*(ylp-pt1lp).
do if ( 2 =2).
compute xlpb=xlp*b.
compute xlpbt=(xlpb > -709.7).
compute xlpb709=(1-xlpbt)*(-709.7).
compute xlpb=(xlpb&*xlpbt)+xlpb709.
compute pt1lp = 1/(1+exp(-(xlpb))).
end if.
compute itprob = csum((pt1lp < .00000001) or (pt1lp > .9999999)).
do if (itprob > 0).
loop kkk = 1 to nrow(pt1lp).
do if (pt1lp(kkk,1) > .9999999).
compute pt1lp(kkk,1) = .9999999.
end if.
do if (pt1lp(kkk,1) < .00000001).
compute pt1lp(kkk,1) = .00000001.
end if.
end loop.
compute itprob = 0.
end if.
do if (itprob = 0).
do if ( 2 =2).
compute LL = ylp&*ln(pt1lp)+(1-ylp)&*ln(1-pt1lp).
end if.
compute LL2 = -2*csum(ll).
end if.
do if (abs(LL1-LL2) < converge).
do if ( 1 =1).
compute xlptmp=t(xlp).
compute vecprb=pt1lp&*(1-pt1lp).
loop kkk=1 to ncol(xlp).
compute xlptmp(kkk,:)=xlptmp(kkk,:)&*t(vecprb).
end loop.
compute varb = inv(xlptmp*xlp).
compute seb = sqrt(diag(varb)).
release xlptmp.
end if.
break.
end if.
compute bt1 = b.
compute LL1 = LL2.
end loop.
compute modres=b.
do if (jjj > iterate).
compute itprob = 2.
do if (booting=0).
compute iterrmod=1.
end if.
do if (booting=1).
compute bootiter=1.
end if.
do if (itprobtg=0).
compute itprobtg=1.
compute errcode(errs,1) = 47.
compute errs = errs + 1.
do if (booting = 0 and 1 =1).
compute vt1 = mdiag(pt1lp&*(1-pt1lp)).
compute varb = inv(t(xlp)*vt1*xlp).
compute seb = sqrt(diag(varb)).
end if.
end if.
end if.
do if ( 1 =1).
compute trat = b&/seb.
compute dfres=nrow(xlp).
compute p = 2*(1-cdfnorm(abs(trat))).
compute modres={modres,seb,trat,p}.
compute modres={modres,(b-xp2&*seb),(b+xp2&*seb)}.
compute pvchi=1-chicdf((LL3-LL2),(nrow(modres)-1)).
compute mcF = (LL3-LL2)/LL3.
compute cox = 1-exp(-(LL3-LL2)/nrow(xlp)).
compute nagel = cox/(1-exp(-(LL3)/nrow(xlp))).
compute modsum={LL2,(LL3-LL2),(nrow(modres)-1),pvchi, mcF,cox,nagel}.
compute modsuml={'-2LL','ModelLL', 'df', 'p', 'McFadden', 'CoxSnell', 'Nagelkrk'}.
compute modresl={'coeff','se','Z','p','LLCI','ULCI'}.
end if.
end if
.
compute chidfxm=basemodx-LL2.
end if.
compute lmat=make(nrow(b),nxvls,0).
compute lmattmp=ident(nxvls).
compute lmat((nrow(lmat)-nxvls+1):nrow(lmat),:)=lmattmp.

.
compute lmat2= lmat.
do if ( 1 =0).
compute lmat2 = mdiag( lmat ).
compute lmat3=make(nrow(lmat2),1,0).
loop flp=1 to ncol(lmat2).
do if (csum(lmat2(:,flp))=1).
compute lmat3={lmat3,lmat2(:,flp)}.
end if.
end loop.
compute lmat2=lmat3(:,2:ncol(lmat3)).
end if.
compute fratio = (t(t(lmat2)* b )*inv(t(lmat2)* varb *lmat2)*((t(lmat2)* b )))/ncol(lmat2).
compute pfr = 1-fcdf(fratio,ncol(lmat2),(n-nrow( b ))).
compute fresult={fratio,ncol(lmat2),(n-nrow( b )),pfr}.
do if (i = (nms+nys) and (ydich=1)).
compute fratio=fratio*ncol(lmat2).
compute pfr=1-chicdf(fratio,ncol(lmat2)).
compute fresult={fratio,ncol(lmat2),pfr}.
end if.
do if ( 0 =1).
compute lmat3=1-rsum(lmat2).
compute xfm=make(n,csum(lmat3),0).
compute flpc=1.
loop flp=1 to nrow(lmat3).
do if (lmat3(flp,1)=1).
compute xfm(:,flpc)=x(:,flp).
compute flpc=flpc+1.
end if.
end loop.
compute bfm=inv(t(xfm)*xfm)*t(xfm)*y.
compute resid=y-(xfm*bfm).
compute sstotal=(y-(csum(y)/n)).
compute sstotal=csum(sstotal&*sstotal).
compute ssresid=csum(resid&*resid).
compute rsqch= 0 -((sstotal-ssresid)/sstotal).
compute fresult={rsqch,fresult}.
release xfm,flpc, resid, ssresid, bfm.
end if
.
compute numxint=numxint+1.
compute xmtst(numxint,:)=fresult.
do if ((i = (nms+nys)) and (ydich=1)).
compute xmtst(numxint,1)=chidfxm.
compute xmtst(numxint,3)=1-chicdf(chidfxm,nxvls).
end if.
compute xmtstlb={xmtstlb;highlbx((xmints-1),1)}.
end if.
end loop.
compute x=xmtmat.
release xmtmat.
do if (numxint>0).
compute xmtstlb=xmtstlb((2:(numxint+1)),:).
compute xmtst=xmtst(1:numxint,:).
compute resultm2=make(nrow(xmtst),maxresm,99999).
compute resultm2(1:nrow(xmtst),1:ncol(xmtst))=xmtst.
compute resultm={resultm;resultm2}.
do if (nms=1).
compute xmtstlb=' '.
end if.
do if (outscree=1).
do if ((i < (nms+nys)) or (ydich=0)).
print xmtst/title='Test(s) of X by M interaction:'/rnames=xmtstlb/cnames=xmtstlbc/format= F10.4.
end if.
do if ((i = (nms+nys)) and (ydich=1)).
print xmtst/title='Likelihood ratio test(s) of X by M interaction:'/rnames=xmtstlb/cnames=xmtstlbc/format= F10.4.
end if.
end if.
end if.
compute r2=r2tmp.
compute b=btmp.
compute varb=varbtmp.
compute dfres=dfrestmp.
compute tval=tvaltmp.
end if.
do if (criterr = 0).
compute jj=0.
loop j = start to ((start+i)-1).
compute dbint=0.
compute lmat=whigh(1:nump(1,i),j).
compute lmat2=wzhigh(1:nump(1,i),j).
do if ((csum(lmat) > 0) and (csum(lmat2) = 0)).
do if ((i < (nms+nys)) or (ydich <> 1)).

.
compute lmat2= lmat.
do if ( 0 =0).
compute lmat2 = mdiag( lmat ).
compute lmat3=make(nrow(lmat2),1,0).
loop flp=1 to ncol(lmat2).
do if (csum(lmat2(:,flp))=1).
compute lmat3={lmat3,lmat2(:,flp)}.
end if.
end loop.
compute lmat2=lmat3(:,2:ncol(lmat3)).
end if.
compute fratio = (t(t(lmat2)* b )*inv(t(lmat2)* varb *lmat2)*((t(lmat2)* b )))/ncol(lmat2).
compute pfr = 1-fcdf(fratio,ncol(lmat2),(n-nrow( b ))).
compute fresult={fratio,ncol(lmat2),(n-nrow( b )),pfr}.
do if (i = (nms+nys) and (ydich=1)).
compute fratio=fratio*ncol(lmat2).
compute pfr=1-chicdf(fratio,ncol(lmat2)).
compute fresult={fratio,ncol(lmat2),pfr}.
end if.
do if ( 1 =1).
compute lmat3=1-rsum(lmat2).
compute xfm=make(n,csum(lmat3),0).
compute flpc=1.
loop flp=1 to nrow(lmat3).
do if (lmat3(flp,1)=1).
compute xfm(:,flpc)=x(:,flp).
compute flpc=flpc+1.
end if.
end loop.
compute bfm=inv(t(xfm)*xfm)*t(xfm)*y.
compute resid=y-(xfm*bfm).
compute sstotal=(y-(csum(y)/n)).
compute sstotal=csum(sstotal&*sstotal).
compute ssresid=csum(resid&*resid).
compute rsqch= r2 -((sstotal-ssresid)/sstotal).
compute fresult={rsqch,fresult}.
release xfm,flpc, resid, ssresid, bfm.
end if
.
compute lmatdb=lmat.
compute dbint=dbint+1.
end if.
do if ((ydich = 1) and (i = (nms+nys))).

.
compute btemphld=b.
compute llrdat=make(nrow(x),nrow( lmat )-csum( lmat ),-999).
compute llrdf=ncol(x)-ncol(llrdat).
compute llrcnt=0.
loop llri=1 to nrow( lmat ).
do if ( lmat (llri,1)=0).
compute llrcnt=llrcnt+1.
compute llrdat(:,llrcnt)=x(:,llri).
end if.
end loop.

.
do if ( 2 =1).
compute hatmat=inv(t( llrdat )* llrdat )*t( llrdat ).
compute b = hatmat* y.
compute modres=b.
do if ( 0 =1).
compute n1=nrow( llrdat ).
compute dfres=n1-(ncol( llrdat )).
compute sstotal = t( y -(csum( y )/n1))*( y -(csum( y )/n1)).
compute resid= y - llrdat *b.
compute ssresid = csum((resid)&**2).
compute r2 = (sstotal-ssresid)/sstotal.
do if (r2 < 0).
compute r2=0.
end if.
compute adjr2 = 1-((1-r2)*(n1-1)/(dfres)).
compute mse=ssresid/(n1-ncol( llrdat )).

.
compute n1=nrow( x ).
compute invXtX = inv(t( x )* x ).
compute varb = mse *invXtX.
compute k3 = ncol( x ).
compute xhc=0.
do if ( hc <> 5).
compute xhc= x.
compute hat = xhc(:,1).
loop i3=1 to nrow(xhc).
compute hat(i3,1)= xhc(i3,:)*invXtX*t(xhc(i3,:)).
end loop.
do if ( hc = 0 or hc =1).
loop i3 = 1 to k3.
compute xhc(:,i3)=xhc(:,i3)&* resid.
end loop.
end if.
do if ( hc =3 or hc =2).
loop i3=1 to k3.
compute xhc(:,i3) = ( resid &/(1-hat)&**(1/(4- hc )))&*xhc(:,i3).
end loop.
end if.
do if ( hc = 4).
compute hcmn=make(n,2,4).
compute hcmn(:,2)=(n1*hat)/k3.
loop i3= 1 to k3.
compute xhc(:,i3) = ( resid &/(1-hat)&**(rmin(hcmn)/2))&*xhc(:,i3).
end loop.
end if.
compute varb=(invXtX*t(xhc)*xhc*invXtX).
do if ( hc =1).
compute varb=(n1/(n1-ncol( x )))&*varb.
end if.
end if.
compute hclab={'se(HC0)','se(HC1)','se(HC2)','se(HC3)','se(HC4)','se'}.
compute hclab=hclab(1,( hc +1)).
compute hcflab={'F(HC0)','F(HC1)','F(HC2)','F(HC3)','F(HC4)','F'}.
compute hcflab=hcflab(1,( hc +1)).
release xhc
.
compute seb=sqrt(diag(varb)).
compute trat = b&/seb.
compute p = 2*(1-tcdf(abs(trat), (dfres))).
compute tval = sqrt(dfres* (exp((dfres-(5/6))*((xp2/(dfres-(2/3)+(.11/dfres)))* (xp2/(dfres-(2/3)+(.11/dfres)))))-1)).
compute modres={modres,seb,trat,p}.
compute modres={modres,(b-tval&*seb),(b+tval&*seb)}.
compute modresl={'coeff',hclab,'t','p','LLCI','ULCI'}.
compute lmat = ident(ncol( llrdat )).
compute lmat = lmat(:,2:ncol(lmat)).
compute fratio = (t(t(lmat)*b)*inv(t(lmat)*varb*lmat)*((t(lmat)*b)))/(ncol( llrdat )-1).
compute pfr = 1-fcdf(fratio,(ncol( llrdat )-1),dfres).
compute modsum={sqrt(r2),r2,mse,fratio,(ncol( llrdat )-1),dfres,pfr}.
compute modsuml={'R','R-sq','MSE',hcflab,'df1','df2', 'p'}.
end if.
end if.
do if ( 2 = 2 or 2 =3).
compute xlp= llrdat.
compute ylp= y.
compute pt2 = make(nrow(ylp),1,(csum(ylp)/nrow(ylp))).
do if ( 2 =2).
compute LL3 = ylp&*ln(pt2)+(1-ylp)&*ln(1-pt2).
end if.
compute LL3 = -2*csum(LL3).
compute bt1 = make(ncol(xlp),1,0).
compute LL1 = 0.
compute pt1 = make(nrow(ylp),1,0.5).
compute pt1lp=pt1.
loop jjj = 1 to iterate.
compute xlptmp=t(xlp).
compute vecprb=pt1lp&*(1-pt1lp).
loop kkk=1 to ncol(xlp).
compute xlptmp(kkk,:)=xlptmp(kkk,:)&*t(vecprb).
end loop.
compute b = bt1+inv(xlptmp*xlp)*t(xlp)*(ylp-pt1lp).
do if ( 2 =2).
compute xlpb=xlp*b.
compute xlpbt=(xlpb > -709.7).
compute xlpb709=(1-xlpbt)*(-709.7).
compute xlpb=(xlpb&*xlpbt)+xlpb709.
compute pt1lp = 1/(1+exp(-(xlpb))).
end if.
compute itprob = csum((pt1lp < .00000001) or (pt1lp > .9999999)).
do if (itprob > 0).
loop kkk = 1 to nrow(pt1lp).
do if (pt1lp(kkk,1) > .9999999).
compute pt1lp(kkk,1) = .9999999.
end if.
do if (pt1lp(kkk,1) < .00000001).
compute pt1lp(kkk,1) = .00000001.
end if.
end loop.
compute itprob = 0.
end if.
do if (itprob = 0).
do if ( 2 =2).
compute LL = ylp&*ln(pt1lp)+(1-ylp)&*ln(1-pt1lp).
end if.
compute LL2 = -2*csum(ll).
end if.
do if (abs(LL1-LL2) < converge).
do if ( 0 =1).
compute xlptmp=t(xlp).
compute vecprb=pt1lp&*(1-pt1lp).
loop kkk=1 to ncol(xlp).
compute xlptmp(kkk,:)=xlptmp(kkk,:)&*t(vecprb).
end loop.
compute varb = inv(xlptmp*xlp).
compute seb = sqrt(diag(varb)).
release xlptmp.
end if.
break.
end if.
compute bt1 = b.
compute LL1 = LL2.
end loop.
compute modres=b.
do if (jjj > iterate).
compute itprob = 2.
do if (booting=0).
compute iterrmod=1.
end if.
do if (booting=1).
compute bootiter=1.
end if.
do if (itprobtg=0).
compute itprobtg=1.
compute errcode(errs,1) = 47.
compute errs = errs + 1.
do if (booting = 0 and 0 =1).
compute vt1 = mdiag(pt1lp&*(1-pt1lp)).
compute varb = inv(t(xlp)*vt1*xlp).
compute seb = sqrt(diag(varb)).
end if.
end if.
end if.
do if ( 0 =1).
compute trat = b&/seb.
compute dfres=nrow(xlp).
compute p = 2*(1-cdfnorm(abs(trat))).
compute modres={modres,seb,trat,p}.
compute modres={modres,(b-xp2&*seb),(b+xp2&*seb)}.
compute pvchi=1-chicdf((LL3-LL2),(nrow(modres)-1)).
compute mcF = (LL3-LL2)/LL3.
compute cox = 1-exp(-(LL3-LL2)/nrow(xlp)).
compute nagel = cox/(1-exp(-(LL3)/nrow(xlp))).
compute modsum={LL2,(LL3-LL2),(nrow(modres)-1),pvchi, mcF,cox,nagel}.
compute modsuml={'-2LL','ModelLL', 'df', 'p', 'McFadden', 'CoxSnell', 'Nagelkrk'}.
compute modresl={'coeff','se','Z','p','LLCI','ULCI'}.
end if.
end if
.
compute b=btemphld.
compute fresult={(LL2-basemod),llrdf,1-chicdf((LL2-basemod),llrdf)}
.
compute lmatdb=lmat.
compute dbint=dbint+1.
end if.
compute highf={highf;fresult}.
compute highf2={highf2;fresult}.
do if (j = start).
compute flabel={flabel;'X*W'}.
end if.
do if (j > start).
do if (nms > 1).
compute flabel={flabel;highlbw(jj,1)}.
else if (nms = 1).
do if (xmint=0).
compute flabel={flabel;'M*W'}.
end if.
do if (xmint=1).
compute flabel={flabel;'X*M'}.
end if.
end if.
end if.
end if.
compute lmat=zhigh(1:nump(1,i),j).
compute lmat2=wzhigh(1:nump(1,i),j).
do if ((csum(lmat) > 0) and (csum(lmat2) = 0)).
do if ((i < (nms+nys)) or (ydich <> 1)).

.
compute lmat2= lmat.
do if ( 0 =0).
compute lmat2 = mdiag( lmat ).
compute lmat3=make(nrow(lmat2),1,0).
loop flp=1 to ncol(lmat2).
do if (csum(lmat2(:,flp))=1).
compute lmat3={lmat3,lmat2(:,flp)}.
end if.
end loop.
compute lmat2=lmat3(:,2:ncol(lmat3)).
end if.
compute fratio = (t(t(lmat2)* b )*inv(t(lmat2)* varb *lmat2)*((t(lmat2)* b )))/ncol(lmat2).
compute pfr = 1-fcdf(fratio,ncol(lmat2),(n-nrow( b ))).
compute fresult={fratio,ncol(lmat2),(n-nrow( b )),pfr}.
do if (i = (nms+nys) and (ydich=1)).
compute fratio=fratio*ncol(lmat2).
compute pfr=1-chicdf(fratio,ncol(lmat2)).
compute fresult={fratio,ncol(lmat2),pfr}.
end if.
do if ( 1 =1).
compute lmat3=1-rsum(lmat2).
compute xfm=make(n,csum(lmat3),0).
compute flpc=1.
loop flp=1 to nrow(lmat3).
do if (lmat3(flp,1)=1).
compute xfm(:,flpc)=x(:,flp).
compute flpc=flpc+1.
end if.
end loop.
compute bfm=inv(t(xfm)*xfm)*t(xfm)*y.
compute resid=y-(xfm*bfm).
compute sstotal=(y-(csum(y)/n)).
compute sstotal=csum(sstotal&*sstotal).
compute ssresid=csum(resid&*resid).
compute rsqch= r2 -((sstotal-ssresid)/sstotal).
compute fresult={rsqch,fresult}.
release xfm,flpc, resid, ssresid, bfm.
end if
.
compute dbint=dbint+1.
end if.
do if ((ydich = 1) and (i = (nms+nys))).

.
compute btemphld=b.
compute llrdat=make(nrow(x),nrow( lmat )-csum( lmat ),-999).
compute llrdf=ncol(x)-ncol(llrdat).
compute llrcnt=0.
loop llri=1 to nrow( lmat ).
do if ( lmat (llri,1)=0).
compute llrcnt=llrcnt+1.
compute llrdat(:,llrcnt)=x(:,llri).
end if.
end loop.

.
do if ( 2 =1).
compute hatmat=inv(t( llrdat )* llrdat )*t( llrdat ).
compute b = hatmat* y.
compute modres=b.
do if ( 0 =1).
compute n1=nrow( llrdat ).
compute dfres=n1-(ncol( llrdat )).
compute sstotal = t( y -(csum( y )/n1))*( y -(csum( y )/n1)).
compute resid= y - llrdat *b.
compute ssresid = csum((resid)&**2).
compute r2 = (sstotal-ssresid)/sstotal.
do if (r2 < 0).
compute r2=0.
end if.
compute adjr2 = 1-((1-r2)*(n1-1)/(dfres)).
compute mse=ssresid/(n1-ncol( llrdat )).

.
compute n1=nrow( x ).
compute invXtX = inv(t( x )* x ).
compute varb = mse *invXtX.
compute k3 = ncol( x ).
compute xhc=0.
do if ( hc <> 5).
compute xhc= x.
compute hat = xhc(:,1).
loop i3=1 to nrow(xhc).
compute hat(i3,1)= xhc(i3,:)*invXtX*t(xhc(i3,:)).
end loop.
do if ( hc = 0 or hc =1).
loop i3 = 1 to k3.
compute xhc(:,i3)=xhc(:,i3)&* resid.
end loop.
end if.
do if ( hc =3 or hc =2).
loop i3=1 to k3.
compute xhc(:,i3) = ( resid &/(1-hat)&**(1/(4- hc )))&*xhc(:,i3).
end loop.
end if.
do if ( hc = 4).
compute hcmn=make(n,2,4).
compute hcmn(:,2)=(n1*hat)/k3.
loop i3= 1 to k3.
compute xhc(:,i3) = ( resid &/(1-hat)&**(rmin(hcmn)/2))&*xhc(:,i3).
end loop.
end if.
compute varb=(invXtX*t(xhc)*xhc*invXtX).
do if ( hc =1).
compute varb=(n1/(n1-ncol( x )))&*varb.
end if.
end if.
compute hclab={'se(HC0)','se(HC1)','se(HC2)','se(HC3)','se(HC4)','se'}.
compute hclab=hclab(1,( hc +1)).
compute hcflab={'F(HC0)','F(HC1)','F(HC2)','F(HC3)','F(HC4)','F'}.
compute hcflab=hcflab(1,( hc +1)).
release xhc
.
compute seb=sqrt(diag(varb)).
compute trat = b&/seb.
compute p = 2*(1-tcdf(abs(trat), (dfres))).
compute tval = sqrt(dfres* (exp((dfres-(5/6))*((xp2/(dfres-(2/3)+(.11/dfres)))* (xp2/(dfres-(2/3)+(.11/dfres)))))-1)).
compute modres={modres,seb,trat,p}.
compute modres={modres,(b-tval&*seb),(b+tval&*seb)}.
compute modresl={'coeff',hclab,'t','p','LLCI','ULCI'}.
compute lmat = ident(ncol( llrdat )).
compute lmat = lmat(:,2:ncol(lmat)).
compute fratio = (t(t(lmat)*b)*inv(t(lmat)*varb*lmat)*((t(lmat)*b)))/(ncol( llrdat )-1).
compute pfr = 1-fcdf(fratio,(ncol( llrdat )-1),dfres).
compute modsum={sqrt(r2),r2,mse,fratio,(ncol( llrdat )-1),dfres,pfr}.
compute modsuml={'R','R-sq','MSE',hcflab,'df1','df2', 'p'}.
end if.
end if.
do if ( 2 = 2 or 2 =3).
compute xlp= llrdat.
compute ylp= y.
compute pt2 = make(nrow(ylp),1,(csum(ylp)/nrow(ylp))).
do if ( 2 =2).
compute LL3 = ylp&*ln(pt2)+(1-ylp)&*ln(1-pt2).
end if.
compute LL3 = -2*csum(LL3).
compute bt1 = make(ncol(xlp),1,0).
compute LL1 = 0.
compute pt1 = make(nrow(ylp),1,0.5).
compute pt1lp=pt1.
loop jjj = 1 to iterate.
compute xlptmp=t(xlp).
compute vecprb=pt1lp&*(1-pt1lp).
loop kkk=1 to ncol(xlp).
compute xlptmp(kkk,:)=xlptmp(kkk,:)&*t(vecprb).
end loop.
compute b = bt1+inv(xlptmp*xlp)*t(xlp)*(ylp-pt1lp).
do if ( 2 =2).
compute xlpb=xlp*b.
compute xlpbt=(xlpb > -709.7).
compute xlpb709=(1-xlpbt)*(-709.7).
compute xlpb=(xlpb&*xlpbt)+xlpb709.
compute pt1lp = 1/(1+exp(-(xlpb))).
end if.
compute itprob = csum((pt1lp < .00000001) or (pt1lp > .9999999)).
do if (itprob > 0).
loop kkk = 1 to nrow(pt1lp).
do if (pt1lp(kkk,1) > .9999999).
compute pt1lp(kkk,1) = .9999999.
end if.
do if (pt1lp(kkk,1) < .00000001).
compute pt1lp(kkk,1) = .00000001.
end if.
end loop.
compute itprob = 0.
end if.
do if (itprob = 0).
do if ( 2 =2).
compute LL = ylp&*ln(pt1lp)+(1-ylp)&*ln(1-pt1lp).
end if.
compute LL2 = -2*csum(ll).
end if.
do if (abs(LL1-LL2) < converge).
do if ( 0 =1).
compute xlptmp=t(xlp).
compute vecprb=pt1lp&*(1-pt1lp).
loop kkk=1 to ncol(xlp).
compute xlptmp(kkk,:)=xlptmp(kkk,:)&*t(vecprb).
end loop.
compute varb = inv(xlptmp*xlp).
compute seb = sqrt(diag(varb)).
release xlptmp.
end if.
break.
end if.
compute bt1 = b.
compute LL1 = LL2.
end loop.
compute modres=b.
do if (jjj > iterate).
compute itprob = 2.
do if (booting=0).
compute iterrmod=1.
end if.
do if (booting=1).
compute bootiter=1.
end if.
do if (itprobtg=0).
compute itprobtg=1.
compute errcode(errs,1) = 47.
compute errs = errs + 1.
do if (booting = 0 and 0 =1).
compute vt1 = mdiag(pt1lp&*(1-pt1lp)).
compute varb = inv(t(xlp)*vt1*xlp).
compute seb = sqrt(diag(varb)).
end if.
end if.
end if.
do if ( 0 =1).
compute trat = b&/seb.
compute dfres=nrow(xlp).
compute p = 2*(1-cdfnorm(abs(trat))).
compute modres={modres,seb,trat,p}.
compute modres={modres,(b-xp2&*seb),(b+xp2&*seb)}.
compute pvchi=1-chicdf((LL3-LL2),(nrow(modres)-1)).
compute mcF = (LL3-LL2)/LL3.
compute cox = 1-exp(-(LL3-LL2)/nrow(xlp)).
compute nagel = cox/(1-exp(-(LL3)/nrow(xlp))).
compute modsum={LL2,(LL3-LL2),(nrow(modres)-1),pvchi, mcF,cox,nagel}.
compute modsuml={'-2LL','ModelLL', 'df', 'p', 'McFadden', 'CoxSnell', 'Nagelkrk'}.
compute modresl={'coeff','se','Z','p','LLCI','ULCI'}.
end if.
end if
.
compute b=btemphld.
compute fresult={(LL2-basemod),llrdf,1-chicdf((LL2-basemod),llrdf)}
.
compute dbint=dbint+1.
end if.
compute highf={highf;fresult}.
compute highf2={highf2;fresult}.
do if (j = start).
compute flabel={flabel;'X*Z'}.
end if.
do if (j > start).
do if (nms > 1).
compute flabel={flabel;highlbz(jj,1)}.
else if (nms = 1).
compute flabel={flabel;'M*Z'}.
end if.
end if.
end if.
do if (dbint=2).
compute lmatdb=lmatdb+lmat.
do if ((ydich = 1) and (i = (nms+nys))).

.
compute btemphld=b.
compute llrdat=make(nrow(x),nrow( lmatdb )-csum( lmatdb ),-999).
compute llrdf=ncol(x)-ncol(llrdat).
compute llrcnt=0.
loop llri=1 to nrow( lmatdb ).
do if ( lmatdb (llri,1)=0).
compute llrcnt=llrcnt+1.
compute llrdat(:,llrcnt)=x(:,llri).
end if.
end loop.

.
do if ( 2 =1).
compute hatmat=inv(t( llrdat )* llrdat )*t( llrdat ).
compute b = hatmat* y.
compute modres=b.
do if ( 0 =1).
compute n1=nrow( llrdat ).
compute dfres=n1-(ncol( llrdat )).
compute sstotal = t( y -(csum( y )/n1))*( y -(csum( y )/n1)).
compute resid= y - llrdat *b.
compute ssresid = csum((resid)&**2).
compute r2 = (sstotal-ssresid)/sstotal.
do if (r2 < 0).
compute r2=0.
end if.
compute adjr2 = 1-((1-r2)*(n1-1)/(dfres)).
compute mse=ssresid/(n1-ncol( llrdat )).

.
compute n1=nrow( x ).
compute invXtX = inv(t( x )* x ).
compute varb = mse *invXtX.
compute k3 = ncol( x ).
compute xhc=0.
do if ( hc <> 5).
compute xhc= x.
compute hat = xhc(:,1).
loop i3=1 to nrow(xhc).
compute hat(i3,1)= xhc(i3,:)*invXtX*t(xhc(i3,:)).
end loop.
do if ( hc = 0 or hc =1).
loop i3 = 1 to k3.
compute xhc(:,i3)=xhc(:,i3)&* resid.
end loop.
end if.
do if ( hc =3 or hc =2).
loop i3=1 to k3.
compute xhc(:,i3) = ( resid &/(1-hat)&**(1/(4- hc )))&*xhc(:,i3).
end loop.
end if.
do if ( hc = 4).
compute hcmn=make(n,2,4).
compute hcmn(:,2)=(n1*hat)/k3.
loop i3= 1 to k3.
compute xhc(:,i3) = ( resid &/(1-hat)&**(rmin(hcmn)/2))&*xhc(:,i3).
end loop.
end if.
compute varb=(invXtX*t(xhc)*xhc*invXtX).
do if ( hc =1).
compute varb=(n1/(n1-ncol( x )))&*varb.
end if.
end if.
compute hclab={'se(HC0)','se(HC1)','se(HC2)','se(HC3)','se(HC4)','se'}.
compute hclab=hclab(1,( hc +1)).
compute hcflab={'F(HC0)','F(HC1)','F(HC2)','F(HC3)','F(HC4)','F'}.
compute hcflab=hcflab(1,( hc +1)).
release xhc
.
compute seb=sqrt(diag(varb)).
compute trat = b&/seb.
compute p = 2*(1-tcdf(abs(trat), (dfres))).
compute tval = sqrt(dfres* (exp((dfres-(5/6))*((xp2/(dfres-(2/3)+(.11/dfres)))* (xp2/(dfres-(2/3)+(.11/dfres)))))-1)).
compute modres={modres,seb,trat,p}.
compute modres={modres,(b-tval&*seb),(b+tval&*seb)}.
compute modresl={'coeff',hclab,'t','p','LLCI','ULCI'}.
compute lmat = ident(ncol( llrdat )).
compute lmat = lmat(:,2:ncol(lmat)).
compute fratio = (t(t(lmat)*b)*inv(t(lmat)*varb*lmat)*((t(lmat)*b)))/(ncol( llrdat )-1).
compute pfr = 1-fcdf(fratio,(ncol( llrdat )-1),dfres).
compute modsum={sqrt(r2),r2,mse,fratio,(ncol( llrdat )-1),dfres,pfr}.
compute modsuml={'R','R-sq','MSE',hcflab,'df1','df2', 'p'}.
end if.
end if.
do if ( 2 = 2 or 2 =3).
compute xlp= llrdat.
compute ylp= y.
compute pt2 = make(nrow(ylp),1,(csum(ylp)/nrow(ylp))).
do if ( 2 =2).
compute LL3 = ylp&*ln(pt2)+(1-ylp)&*ln(1-pt2).
end if.
compute LL3 = -2*csum(LL3).
compute bt1 = make(ncol(xlp),1,0).
compute LL1 = 0.
compute pt1 = make(nrow(ylp),1,0.5).
compute pt1lp=pt1.
loop jjj = 1 to iterate.
compute xlptmp=t(xlp).
compute vecprb=pt1lp&*(1-pt1lp).
loop kkk=1 to ncol(xlp).
compute xlptmp(kkk,:)=xlptmp(kkk,:)&*t(vecprb).
end loop.
compute b = bt1+inv(xlptmp*xlp)*t(xlp)*(ylp-pt1lp).
do if ( 2 =2).
compute xlpb=xlp*b.
compute xlpbt=(xlpb > -709.7).
compute xlpb709=(1-xlpbt)*(-709.7).
compute xlpb=(xlpb&*xlpbt)+xlpb709.
compute pt1lp = 1/(1+exp(-(xlpb))).
end if.
compute itprob = csum((pt1lp < .00000001) or (pt1lp > .9999999)).
do if (itprob > 0).
loop kkk = 1 to nrow(pt1lp).
do if (pt1lp(kkk,1) > .9999999).
compute pt1lp(kkk,1) = .9999999.
end if.
do if (pt1lp(kkk,1) < .00000001).
compute pt1lp(kkk,1) = .00000001.
end if.
end loop.
compute itprob = 0.
end if.
do if (itprob = 0).
do if ( 2 =2).
compute LL = ylp&*ln(pt1lp)+(1-ylp)&*ln(1-pt1lp).
end if.
compute LL2 = -2*csum(ll).
end if.
do if (abs(LL1-LL2) < converge).
do if ( 0 =1).
compute xlptmp=t(xlp).
compute vecprb=pt1lp&*(1-pt1lp).
loop kkk=1 to ncol(xlp).
compute xlptmp(kkk,:)=xlptmp(kkk,:)&*t(vecprb).
end loop.
compute varb = inv(xlptmp*xlp).
compute seb = sqrt(diag(varb)).
release xlptmp.
end if.
break.
end if.
compute bt1 = b.
compute LL1 = LL2.
end loop.
compute modres=b.
do if (jjj > iterate).
compute itprob = 2.
do if (booting=0).
compute iterrmod=1.
end if.
do if (booting=1).
compute bootiter=1.
end if.
do if (itprobtg=0).
compute itprobtg=1.
compute errcode(errs,1) = 47.
compute errs = errs + 1.
do if (booting = 0 and 0 =1).
compute vt1 = mdiag(pt1lp&*(1-pt1lp)).
compute varb = inv(t(xlp)*vt1*xlp).
compute seb = sqrt(diag(varb)).
end if.
end if.
end if.
do if ( 0 =1).
compute trat = b&/seb.
compute dfres=nrow(xlp).
compute p = 2*(1-cdfnorm(abs(trat))).
compute modres={modres,seb,trat,p}.
compute modres={modres,(b-xp2&*seb),(b+xp2&*seb)}.
compute pvchi=1-chicdf((LL3-LL2),(nrow(modres)-1)).
compute mcF = (LL3-LL2)/LL3.
compute cox = 1-exp(-(LL3-LL2)/nrow(xlp)).
compute nagel = cox/(1-exp(-(LL3)/nrow(xlp))).
compute modsum={LL2,(LL3-LL2),(nrow(modres)-1),pvchi, mcF,cox,nagel}.
compute modsuml={'-2LL','ModelLL', 'df', 'p', 'McFadden', 'CoxSnell', 'Nagelkrk'}.
compute modresl={'coeff','se','Z','p','LLCI','ULCI'}.
end if.
end if
.
compute b=btemphld.
compute fresult={(LL2-basemod),llrdf,1-chicdf((LL2-basemod),llrdf)}
.
end if.
do if (ydich <> 1 or i < (nms+nys)).

.
compute lmat2= lmatdb.
do if ( 0 =0).
compute lmat2 = mdiag( lmatdb ).
compute lmat3=make(nrow(lmat2),1,0).
loop flp=1 to ncol(lmat2).
do if (csum(lmat2(:,flp))=1).
compute lmat3={lmat3,lmat2(:,flp)}.
end if.
end loop.
compute lmat2=lmat3(:,2:ncol(lmat3)).
end if.
compute fratio = (t(t(lmat2)* b )*inv(t(lmat2)* varb *lmat2)*((t(lmat2)* b )))/ncol(lmat2).
compute pfr = 1-fcdf(fratio,ncol(lmat2),(n-nrow( b ))).
compute fresult={fratio,ncol(lmat2),(n-nrow( b )),pfr}.
do if (i = (nms+nys) and (ydich=1)).
compute fratio=fratio*ncol(lmat2).
compute pfr=1-chicdf(fratio,ncol(lmat2)).
compute fresult={fratio,ncol(lmat2),pfr}.
end if.
do if ( 1 =1).
compute lmat3=1-rsum(lmat2).
compute xfm=make(n,csum(lmat3),0).
compute flpc=1.
loop flp=1 to nrow(lmat3).
do if (lmat3(flp,1)=1).
compute xfm(:,flpc)=x(:,flp).
compute flpc=flpc+1.
end if.
end loop.
compute bfm=inv(t(xfm)*xfm)*t(xfm)*y.
compute resid=y-(xfm*bfm).
compute sstotal=(y-(csum(y)/n)).
compute sstotal=csum(sstotal&*sstotal).
compute ssresid=csum(resid&*resid).
compute rsqch= r2 -((sstotal-ssresid)/sstotal).
compute fresult={rsqch,fresult}.
release xfm,flpc, resid, ssresid, bfm.
end if
.
end if.
compute dbint=0.
compute highf={highf;fresult}.
do if (jj=0 and nms > 0).
compute flabel={flabel;'BOTH(X)'}.
end if.
do if (jj=0 and nms = 0).
compute flabel={flabel;'BOTH'}.
end if.
do if (jj>0 and nms = 1).
compute flabel={flabel;'BOTH(M)'}.
end if.
do if (nms > 1 and jj > 0).
compute flabel={flabel;highlbbt(jj,1)}.
end if.
end if.
compute lmat2=wzhigh(1:nump(1,i),j).
do if (csum(lmat2) > 0).
do if ((i < (nms+nys)) or (ydich <> 1)).

.
compute lmat2= lmat2.
do if ( 0 =0).
compute lmat2 = mdiag( lmat2 ).
compute lmat3=make(nrow(lmat2),1,0).
loop flp=1 to ncol(lmat2).
do if (csum(lmat2(:,flp))=1).
compute lmat3={lmat3,lmat2(:,flp)}.
end if.
end loop.
compute lmat2=lmat3(:,2:ncol(lmat3)).
end if.
compute fratio = (t(t(lmat2)* b )*inv(t(lmat2)* varb *lmat2)*((t(lmat2)* b )))/ncol(lmat2).
compute pfr = 1-fcdf(fratio,ncol(lmat2),(n-nrow( b ))).
compute fresult={fratio,ncol(lmat2),(n-nrow( b )),pfr}.
do if (i = (nms+nys) and (ydich=1)).
compute fratio=fratio*ncol(lmat2).
compute pfr=1-chicdf(fratio,ncol(lmat2)).
compute fresult={fratio,ncol(lmat2),pfr}.
end if.
do if ( 1 =1).
compute lmat3=1-rsum(lmat2).
compute xfm=make(n,csum(lmat3),0).
compute flpc=1.
loop flp=1 to nrow(lmat3).
do if (lmat3(flp,1)=1).
compute xfm(:,flpc)=x(:,flp).
compute flpc=flpc+1.
end if.
end loop.
compute bfm=inv(t(xfm)*xfm)*t(xfm)*y.
compute resid=y-(xfm*bfm).
compute sstotal=(y-(csum(y)/n)).
compute sstotal=csum(sstotal&*sstotal).
compute ssresid=csum(resid&*resid).
compute rsqch= r2 -((sstotal-ssresid)/sstotal).
compute fresult={rsqch,fresult}.
release xfm,flpc, resid, ssresid, bfm.
end if
.
end if.
do if ((ydich = 1) and (i = (nms+nys))).

.
compute btemphld=b.
compute llrdat=make(nrow(x),nrow( lmat2 )-csum( lmat2 ),-999).
compute llrdf=ncol(x)-ncol(llrdat).
compute llrcnt=0.
loop llri=1 to nrow( lmat2 ).
do if ( lmat2 (llri,1)=0).
compute llrcnt=llrcnt+1.
compute llrdat(:,llrcnt)=x(:,llri).
end if.
end loop.

.
do if ( 2 =1).
compute hatmat=inv(t( llrdat )* llrdat )*t( llrdat ).
compute b = hatmat* y.
compute modres=b.
do if ( 0 =1).
compute n1=nrow( llrdat ).
compute dfres=n1-(ncol( llrdat )).
compute sstotal = t( y -(csum( y )/n1))*( y -(csum( y )/n1)).
compute resid= y - llrdat *b.
compute ssresid = csum((resid)&**2).
compute r2 = (sstotal-ssresid)/sstotal.
do if (r2 < 0).
compute r2=0.
end if.
compute adjr2 = 1-((1-r2)*(n1-1)/(dfres)).
compute mse=ssresid/(n1-ncol( llrdat )).

.
compute n1=nrow( x ).
compute invXtX = inv(t( x )* x ).
compute varb = mse *invXtX.
compute k3 = ncol( x ).
compute xhc=0.
do if ( hc <> 5).
compute xhc= x.
compute hat = xhc(:,1).
loop i3=1 to nrow(xhc).
compute hat(i3,1)= xhc(i3,:)*invXtX*t(xhc(i3,:)).
end loop.
do if ( hc = 0 or hc =1).
loop i3 = 1 to k3.
compute xhc(:,i3)=xhc(:,i3)&* resid.
end loop.
end if.
do if ( hc =3 or hc =2).
loop i3=1 to k3.
compute xhc(:,i3) = ( resid &/(1-hat)&**(1/(4- hc )))&*xhc(:,i3).
end loop.
end if.
do if ( hc = 4).
compute hcmn=make(n,2,4).
compute hcmn(:,2)=(n1*hat)/k3.
loop i3= 1 to k3.
compute xhc(:,i3) = ( resid &/(1-hat)&**(rmin(hcmn)/2))&*xhc(:,i3).
end loop.
end if.
compute varb=(invXtX*t(xhc)*xhc*invXtX).
do if ( hc =1).
compute varb=(n1/(n1-ncol( x )))&*varb.
end if.
end if.
compute hclab={'se(HC0)','se(HC1)','se(HC2)','se(HC3)','se(HC4)','se'}.
compute hclab=hclab(1,( hc +1)).
compute hcflab={'F(HC0)','F(HC1)','F(HC2)','F(HC3)','F(HC4)','F'}.
compute hcflab=hcflab(1,( hc +1)).
release xhc
.
compute seb=sqrt(diag(varb)).
compute trat = b&/seb.
compute p = 2*(1-tcdf(abs(trat), (dfres))).
compute tval = sqrt(dfres* (exp((dfres-(5/6))*((xp2/(dfres-(2/3)+(.11/dfres)))* (xp2/(dfres-(2/3)+(.11/dfres)))))-1)).
compute modres={modres,seb,trat,p}.
compute modres={modres,(b-tval&*seb),(b+tval&*seb)}.
compute modresl={'coeff',hclab,'t','p','LLCI','ULCI'}.
compute lmat = ident(ncol( llrdat )).
compute lmat = lmat(:,2:ncol(lmat)).
compute fratio = (t(t(lmat)*b)*inv(t(lmat)*varb*lmat)*((t(lmat)*b)))/(ncol( llrdat )-1).
compute pfr = 1-fcdf(fratio,(ncol( llrdat )-1),dfres).
compute modsum={sqrt(r2),r2,mse,fratio,(ncol( llrdat )-1),dfres,pfr}.
compute modsuml={'R','R-sq','MSE',hcflab,'df1','df2', 'p'}.
end if.
end if.
do if ( 2 = 2 or 2 =3).
compute xlp= llrdat.
compute ylp= y.
compute pt2 = make(nrow(ylp),1,(csum(ylp)/nrow(ylp))).
do if ( 2 =2).
compute LL3 = ylp&*ln(pt2)+(1-ylp)&*ln(1-pt2).
end if.
compute LL3 = -2*csum(LL3).
compute bt1 = make(ncol(xlp),1,0).
compute LL1 = 0.
compute pt1 = make(nrow(ylp),1,0.5).
compute pt1lp=pt1.
loop jjj = 1 to iterate.
compute xlptmp=t(xlp).
compute vecprb=pt1lp&*(1-pt1lp).
loop kkk=1 to ncol(xlp).
compute xlptmp(kkk,:)=xlptmp(kkk,:)&*t(vecprb).
end loop.
compute b = bt1+inv(xlptmp*xlp)*t(xlp)*(ylp-pt1lp).
do if ( 2 =2).
compute xlpb=xlp*b.
compute xlpbt=(xlpb > -709.7).
compute xlpb709=(1-xlpbt)*(-709.7).
compute xlpb=(xlpb&*xlpbt)+xlpb709.
compute pt1lp = 1/(1+exp(-(xlpb))).
end if.
compute itprob = csum((pt1lp < .00000001) or (pt1lp > .9999999)).
do if (itprob > 0).
loop kkk = 1 to nrow(pt1lp).
do if (pt1lp(kkk,1) > .9999999).
compute pt1lp(kkk,1) = .9999999.
end if.
do if (pt1lp(kkk,1) < .00000001).
compute pt1lp(kkk,1) = .00000001.
end if.
end loop.
compute itprob = 0.
end if.
do if (itprob = 0).
do if ( 2 =2).
compute LL = ylp&*ln(pt1lp)+(1-ylp)&*ln(1-pt1lp).
end if.
compute LL2 = -2*csum(ll).
end if.
do if (abs(LL1-LL2) < converge).
do if ( 0 =1).
compute xlptmp=t(xlp).
compute vecprb=pt1lp&*(1-pt1lp).
loop kkk=1 to ncol(xlp).
compute xlptmp(kkk,:)=xlptmp(kkk,:)&*t(vecprb).
end loop.
compute varb = inv(xlptmp*xlp).
compute seb = sqrt(diag(varb)).
release xlptmp.
end if.
break.
end if.
compute bt1 = b.
compute LL1 = LL2.
end loop.
compute modres=b.
do if (jjj > iterate).
compute itprob = 2.
do if (booting=0).
compute iterrmod=1.
end if.
do if (booting=1).
compute bootiter=1.
end if.
do if (itprobtg=0).
compute itprobtg=1.
compute errcode(errs,1) = 47.
compute errs = errs + 1.
do if (booting = 0 and 0 =1).
compute vt1 = mdiag(pt1lp&*(1-pt1lp)).
compute varb = inv(t(xlp)*vt1*xlp).
compute seb = sqrt(diag(varb)).
end if.
end if.
end if.
do if ( 0 =1).
compute trat = b&/seb.
compute dfres=nrow(xlp).
compute p = 2*(1-cdfnorm(abs(trat))).
compute modres={modres,seb,trat,p}.
compute modres={modres,(b-xp2&*seb),(b+xp2&*seb)}.
compute pvchi=1-chicdf((LL3-LL2),(nrow(modres)-1)).
compute mcF = (LL3-LL2)/LL3.
compute cox = 1-exp(-(LL3-LL2)/nrow(xlp)).
compute nagel = cox/(1-exp(-(LL3)/nrow(xlp))).
compute modsum={LL2,(LL3-LL2),(nrow(modres)-1),pvchi, mcF,cox,nagel}.
compute modsuml={'-2LL','ModelLL', 'df', 'p', 'McFadden', 'CoxSnell', 'Nagelkrk'}.
compute modresl={'coeff','se','Z','p','LLCI','ULCI'}.
end if.
end if
.
compute b=btemphld.
compute fresult={(LL2-basemod),llrdf,1-chicdf((LL2-basemod),llrdf)}
.
end if.
compute highf={highf;fresult}.
compute highf2={highf2;fresult}.
do if (j = start).
compute flabel={flabel;'X*W*Z'}.
end if.
do if (j > start).
do if (nms > 1).
compute flabel={flabel;highlbwz(jj,1)}.
else if (nms = 1).
compute flabel={flabel;'M*W*Z'}.
end if.
end if.
end if.
compute jj=jj+1.
end loop.
release jj.
compute start=start+i.
end if.
do if (nrow(highf) > 1).
compute highf=highf(2:nrow(highf),:).
compute highf2=highf2(2:nrow(highf2),:).
compute flabel=flabel(2:nrow(flabel),1).
compute resultm2=make(nrow(highf),maxresm,99999).
compute resultm2(1:nrow(highf),1:ncol(highf))=highf.
compute resultm={resultm;resultm2}.
do if (outscree=1).
do if ((i < nms+nys) or (ydich=0)).
compute clabtmp={'R2-chng', hcflab,'df1','df2','p'}.
print highf/format = F10.4 /rnames=flabel/cnames=clabtmp/ title = 'Test(s) of highest order unconditional interaction(s):'.
end if.
do if (ydich=1 and i=(nms+nys)).
compute clabtmp={'Chi-sq', 'df','p'}.
print/title='Likelihood ratio test(s) of highest order'.
print highf/format = F10.4 /rnames=flabel/cnames=clabtmp/ title = 'unconditional interactions(s):'/space=0.
end if.
end if.
compute intpb=highf2(:,ncol(highf2)).
end if.
compute intstart=intstart+numint(1,i).
end if.

.
do if (criterr=0).
compute threeway=0.
compute didprint=0.
compute didsome=0.
compute sigintct=0.
loop jmed =1 to (nms+1).
compute hasw=0.
compute hasz=0.
compute jnok=0.
compute nm1vls=0.
compute nm2vls=0.
compute panelgrp=0.
compute graphixs={'WITH', outnames(1,i), 'BY'}.
compute focpred4={' '}.
compute intprint=0.
compute modcat=0.
do if (jmed <= i).
do if ((jmed = 1) and ((i+1) = nrow(bcmat))).
compute pathscnt=pathscnt+1.
else.
compute paths={paths,bcmat((i+1),jmed)}.
compute pathsw={pathsw,wcmat((i+1),jmed)}.
compute pathsz={pathsz,zcmat((i+1),jmed)}.
compute pathswz={pathswz,wzcmat((i+1),jmed)}.
compute temp=fochigh(:,pathscnt)&*bootloc(:,i).
compute pathsfoc={pathsfoc,pathsfoc(:,1)}.
do if (jmed=1).
compute pathtype={pathtype,1}.
end if.
do if ((i+1)=nrow(bcmat)).
compute pathtype={pathtype,3}.
end if.
do if (jmed > 1) and ((i+1) < nrow(bcmat)).
compute pathtype={pathtype,2}.
end if.
do if (jmed=1 and nxvls > 1 and (bcmat((i+1),jmed)=1)).
compute pathsfoc(:,(pathscn2+1))=temp(2:(nxvls+1),1).
end if.
do if ((jmed > 1) or (jmed=1 and nxvls=1)).
compute temp=cmax(temp).
compute pathsfoc(1,(pathscn2+1))=temp.
end if.
compute pathscnt=pathscnt+1.
compute pathscn2=pathscn2+1.
do if (i <= nms).
compute pathsdv={pathsdv,mnames(1,i)}.
end if.
do if (i > nms).
compute pathsdv={pathsdv,ynames}.
end if.
end if.
compute coeffcol=coeffcol+1.
compute probettt=coeffs(1:nrow(b),coeffcol).
do if (jmed=1 and (bcmat((i+1),jmed)=1)).
compute omni=make(nrow(probettt),nxvls,0).
compute omnitmp=ident(nxvls).
compute omni(2:(1+nxvls),:)=omnitmp.
end if.
do if (csum(probettt)>0).
compute probvarb=make(csum(probettt),csum(probettt),999).
compute probcoef=make(csum(probettt),1,999).
compute coefflp2=1.
loop coefflp=1 to nrow(probettt).
do if (probettt(coefflp,1)=1).
compute probcoef(coefflp2,1)=b(coefflp,1).
compute coefflp2=coefflp2+1.
end if.
end loop.
compute coefflp=0.
compute coefflp2=0.
loop iclp=1 to nrow(probettt).
do if probettt(iclp,1)=1.
compute coefflp=coefflp+1.
compute coefflp2=coefflp.
compute probvarb(coefflp,coefflp) = varb(iclp,iclp).
do if (iclp < nrow(probettt)).
loop jclp=(iclp+1) to nrow(probettt).
do if (probettt(jclp,1)=1).
compute coefflp2=coefflp2+1.
compute probvarb(coefflp,coefflp2)=varb(iclp, jclp).
compute probvarb(coefflp2,coefflp)=varb(iclp, jclp).
end if.
end loop.
end if.
end if.
end loop.
end if.
end if.
compute xprobval=xmodvals.
do if (nxvls > 1 or mcx > 0).
compute xprobval=dummatx(:,2:ncol(dummatx)).
end if.
do if ((wcmat((i+1),jmed)=1) and (zcmat((i+1),jmed)=0)).
compute numplps=1.
compute modvals=wmodvals.
compute probeval=wmodvals.
compute wheremv1=wherexw.
compute nm1vls=nwvls.
compute lpstsp={1,1}.
compute modcat=0.
compute jnmod=wtmp.
compute jnmodlab=wnames.
compute jnok=1.
compute jnmin=wmin.
compute jnmax=wmax.
compute wherejn1=2.
do if (jmed=1).
compute wherejn3=wherexw(1,i).
do if (nxvls > 1).
compute jnok=0.
end if.
end if.
do if (jmed > 1).
compute wherejn1=wherem((jmed-1),i).
compute wherejn3=wheremw(((2*jmed)-3),i).
end if.
do if (nwvls > 1).
compute probeval=wprobval.
compute lpstsp(1,2)=ncol(probeval).
compute modcat=1.
compute jnok=0.
end if.
do if (wdich = 1).
compute modcat=1.
compute jnok=0.
end if.
compute problabs=wnames.
compute focpred3={wnames,'(W)'}.
do if (xmint=1).
compute focpred3={wnames,'(X)'}.
end if.
compute hasw=1.
compute modgrph=wnames.
compute intprint=1.
compute sigintct=sigintct+1.
compute printpbe=intpb(sigintct,1).
end if.
do if ((wcmat((i+1),jmed)=0) and (zcmat((i+1),jmed)=1)).
compute numplps=1.
compute modvals=zmodvals.
compute probeval=zmodvals.
compute wheremv1=wherexz.
compute nm1vls=nzvls.
compute lpstsp={1,1}.
compute jnok=1.
compute jnmod=ztmp.
compute jnmin=zmin.
compute jnmax=zmax.
compute jnmodlab=znames.
compute wherejn1=2.
do if (jmed=1).
compute wherejn3=wherexz(1,i).
do if (nxvls > 1).
compute jnok=0.
end if.
end if.
do if (jmed > 1).
compute wherejn1=wherem((jmed-1),i).
compute wherejn3=wheremz(((2*jmed)-3),i).
end if.
do if (nzvls > 1).
compute probeval=zprobval.
compute lpstsp(1,2)=ncol(probeval).
compute modcat=1.
compute jnok=0.
end if.
do if (zdich = 1).
compute modcat=1.
compute jnok=0.
end if.
compute problabs=znames.
compute focpred3={znames,'(Z)'}.
compute modgrph=znames.
compute hasz=1.
compute intprint=1.
compute sigintct=sigintct+1.
compute printpbe=intpb(sigintct,1).
end if.
do if ((wzcmat((i+1),jmed)=1) or ((wcmat((i+1),jmed)=1) and (zcmat((i+1),jmed)=1))).
compute numplps=2.
compute probecnt=1.
compute intprint=1.
do if (wzcmat((i+1),jmed)=1).
compute sigintct=sigintct+1.
compute printpbe=intpb(sigintct,1).
else.
compute sigintct=sigintct+2.
compute printpbe=cmin(intpb((sigintct-1):sigintct,1)).
end if.
compute panelgrp=1.
compute hasw=1.
compute hasz=1.
compute panelcde={'/PANEL','ROWVAR=',znames,'.'}.
compute modgrph=wnames.
compute lpstsp={1,1;1,1}.
compute wheremv1=wherexw.
compute nm1vls=nwvls.
compute wheremv2=wherexz.
compute nm2vls=nzvls.
compute jnok=0.
do if (wzcmat((i+1),jmed)=1).
compute jnok=1.
end if.
do if (jmed > 1).
compute mprobval=mmodvals.
end if.
do if (jmed=1).
do if (nxvls > 1).
compute jnok=0.
end if.
end if.
do if (nwvls > 1).
compute lpstsp(1,2)=ncol(wprobval).
compute modcat=1.
compute jnok=0.
end if.
do if (zdich=1).
compute modcat=1.
compute jnok=0.
end if.
compute lpstsp(2,1)=lpstsp(1,2)+1.
compute lpstsp(2,2)=lpstsp(1,2)+1.
do if (nzvls > 1).
compute lpstsp(2,1)=lpstsp(1,2)+1.
compute lpstsp(2,2)=lpstsp(1,2)+ncol(zprobval).
compute jnok=0.
end if.
do if (zdich=1).
compute jnok=0.
end if.
compute omni3=make(nrow(b),(nxvls*nwvls),0).
do if (jmed > 1).
compute omni3=make(nrow(b),nwvls,0).
end if.
compute focpred3={wnames,'(W)'}.
do if (xmint=1).
compute focpred3={wnames,'(X)'}.
end if.
compute focpred4={'    ', 'Mod var:', znames, '(Z)'}.
compute modvals=make((nrow(wmodvals)*nrow(zmodvals)),2,0).
compute probeval=make((nrow(wmodvals)*nrow(zmodvals)),(ncol(wprobval)+ncol(zprobval)),0).
loop probei= 1 to nrow(wmodvals).
loop probej =1 to nrow(zmodvals).
compute modvals(probecnt,1)=wmodvals(probei,1).
compute probeval(probecnt,1:nwvls)=wprobval(probei,:).
compute modvals(probecnt,2)=zmodvals(probej,1).
compute probeval(probecnt,(nwvls+1):(nwvls+nzvls))=zprobval(probej,:).
compute probecnt=probecnt+1.
end loop.
end loop.
do if (wzcmat((i+1),jmed)=1).
compute numplps=numplps+1.
compute probprod=make(1,(ncol(wprobval)*ncol(zprobval)),0).
compute lpstsp2={1,1}.
compute lpstsp={lpstsp;lpstsp2}.
compute lpstsp(3,1)=lpstsp(2,2)+1.
compute lpstsp(3,2)=lpstsp(2,2)+ncol(probprod).
compute jnmod=ztmp.
compute jnmin=zmin.
compute jnmax=zmax.
compute jnmodlab=znames.
do if (jmed = 1).
compute wherejn1=wherexw(1,i).
compute wherejn3=wherexwz(1,i).
end if.
do if (jmed > 1).
compute wherejn1=wheremw(((2*jmed)-3),i).
compute wherejn3=wheremwz(((2*jmed)-3),i).
end if.
loop probei = 1 to nrow(wmodvals).
loop probej = 1 to nrow(zmodvals).
compute probtemp=1.
loop probek = 1 to ncol(wprobval).
compute probtemp={probtemp,(wprobval(probei,probek)&*zprobval(probej,:))}.
end loop.
compute probprod={probprod;probtemp(1,2:ncol(probtemp))}.
end loop.
end loop.
compute probprod=probprod(2:nrow(probprod),:).
compute probeval={probeval,probprod}.
end if.
compute problabs={wnames,znames}.
release probecnt, probei, probej.
end if.
do if (intprint=1).
compute focpred={'   Focal', 'predict:'}.
do if (jmed=1).
compute focpred={focpred,xnames,'(X)'}.
compute focplotv=xmodvals.
end if.
do if (jmed >1).
do if (nms > 1).
compute focpred={focpred,mnames(1,(jmed-1)), medlb2(1,(jmed-1))}.
end if.
do if (nms = 1).
compute focpred={focpred,mnames(1,(jmed-1)), '(M)'}.
end if.
compute focplotv=mmodvals(:,(jmed-1)).
end if.
compute focpred2={'    ', 'Mod var:',focpred3}.
compute focpred={focpred;focpred2}.
do if (ncol(focpred4) > 1).
compute focpred={focpred;focpred4}.
compute focpred4={' '}.
end if.
release focpred2,focpred3.
do if (outscree=1).
do if ((plot = 1 or plot = 2) or (printpbe <= intprobe)).
print focpred/title='----------'/format=A8/space=0.
end if.
end if.
compute foctmp=make(nrow(modvals),1,1).
compute probexpl=1.
compute probeva2={foctmp,probeval}.
do if (jmed=1 and nxs > 0 and mcx > 0).
compute probexpl=nxvls.
end if.
compute foctmp=make(nrow(modvals),1,1).
compute modvals3=make(1,(6+ncol(problabs)),0).
compute probrown=make(nrow(probeval),1,0).
compute jtmp=1.
loop probei = 1 to nrow(probeval).
compute probrown(probei,1)=jtmp.
compute jtmp=jtmp+nxvls.
end loop.
release jtmp.
compute probrow=999.
compute modvarl=problabs.
do if (plot = 1 or plot = 2 or nxvls > 1).
compute plotvals=make((nrow(modvals)*nrow(focplotv)),(ncol(modvals)+1),999).
loop ploti=1 to nrow(modvals).
loop plotj=1 to nrow(focplotv).
compute plotvals((((ploti-1)*nrow(focplotv))+plotj),2:ncol(plotvals))=modvals(ploti,:).
compute plotvals((((ploti-1)*nrow(focplotv))+plotj),1)=focplotv(plotj,1).
end loop.
end loop.
compute focpredn=3.
do if (jmed=1).
do if (nxvls > 1).
compute focpredn=(nxvls+1).
end if.
do if (nxvls=1 and xdich=1).
compute focpredn=2.
end if.
end if.
compute meanmat=mdiag(means).
compute onesmat=make(nrow(meanmat),(nrow(probeval)*focpredn),1).
compute probeplt=t(mdiag(means)*onesmat).
do if (jmed=1).
do if ((wcmat((i+1),1)=1) or (zcmat((i+1),1)=1)).
compute plotcnt=1.
compute iloops=nwpval*nzpval.
compute plotmx=nxpval*nzpval.
do if ((wcmat((i+1),1)=1) and (zcmat((i+1),1)=0)).
compute iloops=nwpval.
compute plotmx=nxpval.
end if.
do if ((wcmat((i+1),1)=0) and (zcmat((i+1),1)=1)).
compute iloops=nzpval.
compute plotmx=nxpval.
end if.
compute xestvals=make((nxpval*iloops),ncol(xprobval),-999).
do if (wcmat((i+1),1)=1).
compute westvals=make(nrow(xestvals),ncol(wprobval),-999).
end if.
do if (zcmat((i+1),1)=1).
compute zestvals=make(nrow(xestvals),ncol(zprobval),-999).
end if.
loop ploti=1 to iloops.
loop plotj=1 to nxpval.
compute xestvals(plotcnt,:)=xprobval(plotj,:).
compute plotcnt=plotcnt+1.
end loop.
end loop.
compute plotcnt=1.
compute plotcnt1=1.
compute plotcnt2=1.
compute plotcntz=1.
loop ploti = 1 to (iloops*nxpval).
do if (wcmat((i+1),1)=1).
compute westvals(ploti,:)=wprobval(plotcnt1,:).
end if.
do if ((wcmat((i+1),1)=0) and (zcmat((i+1),1)=1)).
compute zestvals(ploti,:)=zprobval(plotcnt1,:).
end if.
do if ((wcmat((i+1),1)=1) and (zcmat((i+1),1)=1)).
compute zestvals(ploti,:)=zprobval(plotcnt2,:).
compute plotcntz=plotcntz+1.
end if.
compute plotcnt=plotcnt+1.
do if (plotcnt > plotmx).
compute plotcnt=1.
compute plotcnt1=plotcnt1+1.
end if.
do if (plotcntz > nxpval).
compute plotcnt2=plotcnt2+1.
compute plotcntz=1.
do if (plotcnt2 > nzpval).
compute plotcnt2=1.
end if.
end if.
end loop.
compute probeplt(:,2:(1+(ncol(xestvals))))=xestvals.
do if (wcmat((i+1),1)=1).
compute probeplt(:,(wherew(1,i)):(wherew(2,i)))=westvals.
end if.
do if (zcmat((i+1),1)=1).
compute probeplt(:,(wherez(1,i)):(wherez(2,i)))=zestvals.
end if.
end if.
end if.
do if (jmed > 1).
do if ((wcmat((i+1),jmed)=1) or (zcmat((i+1),jmed)=1)).
compute plotcnt=1.
compute iloops=nwpval*nzpval.
compute plotmx=3*nzpval.
do if ((wcmat((i+1),jmed)=1) and (zcmat((i+1),jmed)=0)).
compute iloops=nwpval.
compute plotmx=3.
end if.
do if ((wcmat((i+1),jmed)=0) and (zcmat((i+1),jmed)=1)).
compute iloops=nzpval.
compute plotmx=3.
end if.
compute mestvals=make((3*iloops),1,-999).
do if (wcmat((i+1),jmed)=1).
compute westvals=make(nrow(mestvals),ncol(wprobval),-999).
end if.
do if (zcmat((i+1),jmed)=1).
compute zestvals=make(nrow(mestvals),ncol(zprobval),-999).
end if.
loop ploti=1 to iloops.
loop plotj=1 to 3.
compute mestvals(plotcnt,:)=mprobval(plotj,(jmed-1)).
compute plotcnt=plotcnt+1.
end loop.
end loop.
compute plotcnt=1.
compute plotcnt1=1.
compute plotcnt2=1.
compute plotcntz=1.
loop ploti = 1 to (iloops*3).
do if (wcmat((i+1),jmed)=1).
compute westvals(ploti,:)=wprobval(plotcnt1,:).
end if.
do if ((wcmat((i+1),jmed)=0) and (zcmat((i+1),jmed)=1)).
compute zestvals(ploti,:)=zprobval(plotcnt1,:).
end if.
do if ((wcmat((i+1),jmed)=1) and (zcmat((i+1),jmed)=1)).
compute zestvals(ploti,:)=zprobval(plotcnt2,:).
compute plotcntz=plotcntz+1.
end if.
compute plotcnt=plotcnt+1.
do if (plotcnt > plotmx).
compute plotcnt=1.
compute plotcnt1=plotcnt1+1.
end if.
do if (plotcntz > 3).
compute plotcnt2=plotcnt2+1.
compute plotcntz=1.
do if (plotcnt2 > nzpval).
compute plotcnt2=1.
end if.
end if.
end loop.
compute probeplt(:,wherem((jmed-1),i))=mestvals.
do if (wcmat((i+1),jmed)=1).
do if (model <> 74).
compute probeplt(:,(wherew(1,i)):(wherew(2,i)))=westvals.
end if.
do if (model = 74).
compute probeplt(:,(wherex(1,i)):(wherex(2,i)))=westvals.
end if.
end if.
do if (zcmat((i+1),jmed)=1).
compute probeplt(:,(wherez(1,i)):(wherez(2,i)))=zestvals.
end if.
end if.
end if.
compute prodloop = 1.
do if (jmed=1).
compute prodloop=ncol(xestvals).
end if.
do if (wcmat((i+1),jmed))=1.
compute plotcnt=0.
loop ploti = 1 to prodloop.
loop plotj = 1 to ncol(westvals).
do if (jmed=1).
compute probeplt(:,(wherexw(1,i)+plotcnt))=xestvals(:,ploti)&*westvals(:,plotj).
end if.
do if (jmed > 1).
compute probeplt(:,(wheremw(((jmed*2)-3) ,i)+plotcnt))=mestvals(:,ploti)&*westvals(:,plotj).
end if.
compute plotcnt=plotcnt+1.
end loop.
end loop.
end if.
do if (zcmat((i+1),jmed))=1.
compute plotcnt=0.
loop ploti = 1 to prodloop.
loop plotj = 1 to ncol(zestvals).
do if (jmed = 1).
compute probeplt(:,(wherexz(1,i)+plotcnt))=xestvals(:,ploti)&*zestvals(:,plotj).
end if.
do if (jmed > 1).
compute probeplt(:,(wheremz(((jmed*2)-3),i)+plotcnt))=mestvals(:,ploti)&*zestvals(:, plotj).
end if.
compute plotcnt=plotcnt+1.
end loop.
end loop.
end if.
do if (wzcmat((i+1),jmed))=1.
compute plotcnt=0.
compute threeway=1.
loop ploti = 1 to ncol(westvals).
loop plotj = 1 to ncol(zestvals).
compute probeplt(:,(wherewz(1,i)+plotcnt))=westvals(:,ploti)&*zestvals(:,plotj).
compute plotcnt=plotcnt+1.
end loop.
end loop.
compute plotcnt=0.
loop plotk = 1 to prodloop.
loop ploti = 1 to ncol(westvals).
loop plotj = 1 to ncol(zestvals).
do if (jmed = 1).
compute probeplt(:,(wherexwz(1,i)+plotcnt))=xestvals(:,plotk)&*westvals(:,ploti)&* zestvals(:,plotj).
end if.
do if (jmed > 1).
compute probeplt(:,(wheremwz(((jmed*2)-3),i)+plotcnt))=mestvals(:,plotk)&* westvals(:,ploti)&*zestvals(:,plotj).
end if.
compute plotcnt=plotcnt+1.
end loop.
end loop.
end loop.
end if.
loop newplp=1 to i.
do if (newplp <> jmed).
do if (wcmat((i+1),newplp))=1.
compute prodloop=1.
do if (newplp=1).
compute prodloop=nxvls.
end if.
compute plotcnt=0.
loop ploti = 1 to prodloop.
loop plotj = 1 to nwvls.
do if (newplp = 1).
compute probeplt(:,(wherexw(1,i)+plotcnt))=probeplt(:,(1+ploti))&*probeplt(:, (wherew(1,i)+plotj-1)).
end if.
do if (newplp > 1).
do if (model <> 74).
compute probeplt(:,(wheremw(((newplp*2)-3) ,i)+plotcnt))=probeplt(:,wherem((newplp-1),i))&*probeplt(:,(wherew(1,i)+plotj-1)).
end if.
do if (model = 74).
compute probeplt(:,(wheremw(((newplp*2)-3) ,i)+plotcnt))=probeplt(:,wherem((newplp-1),i))&*probeplt(:,(wherex(1,i)+plotj-1)).
end if.
end if.
compute plotcnt=plotcnt+1.
end loop.
end loop.
end if.
do if (zcmat((i+1),newplp))=1.
compute prodloop=1.
do if (newplp=1).
compute prodloop=nxvls.
end if.
compute plotcnt=0.
loop ploti = 1 to prodloop.
loop plotj = 1 to nzvls.
do if (newplp = 1).
compute probeplt(:,(wherexz(1,i)+plotcnt))=probeplt(:,(1+ploti))&*probeplt(:, (wherez(1,i)+plotj-1)).
end if.
do if (newplp > 1).
compute probeplt(:,(wheremz(((newplp*2)-3),i)+plotcnt))=probeplt(:, wherem((newplp-1),i))&*probeplt(:,(wherez(1,i)+plotj-1)).
end if.
compute plotcnt=plotcnt+1.
end loop.
end loop.
end if.
do if (wzcmat((i+1),newplp))=1.
compute plotcnt=0.
do if (threeway=0).
loop ploti = 1 to nwvls.
loop plotj = 1 to nzvls.
compute probeplt(:,(wherewz(1,i)+plotcnt))=probeplt(:,(wherew(1,i)+ploti-1))&* probeplt(:,(wherez(1,i)+plotj-1)).
compute plotcnt=plotcnt+1.
end loop.
end loop.
end if.
compute prodloop=1.
do if (newplp=1).
compute prodloop=nxvls.
end if.
compute plotcnt=0.
loop plotk = 1 to prodloop.
loop ploti = 1 to nwvls.
loop plotj = 1 to nzvls.
do if (newplp = 1).
compute probeplt(:,(wherexwz(1,i)+plotcnt))=probeplt(:,(1+plotk))&*probeplt(:, (wherew(1,i)+ploti-1))&*probeplt(:,(wherez(1,i)+plotj-1)).
end if.
do if (newplp > 1).
compute probeplt(:,(wheremwz(((newplp*2)-3),i)+plotcnt))=probeplt(:, wherem((newplp-1),i))&*probeplt(:,(wherew(1,i)+ploti-1))&*probeplt(:,(wherez(1,i)+plotj-1)).
end if.
compute plotcnt=plotcnt+1.
end loop.
end loop.
end loop.
end if.
end if.
end loop.
compute predvals=probeplt*b.
do if (debug <> 0).
print probeplt.
print b.
end if.
do if (i = nms+nys) and (ydich=1).
compute predvalt=(predvals < 709.7).
compute prevalt7=(1-predvalt)*(709.7).
compute predvals=(predvals&*predvalt)+prevalt7.
compute expyhat=exp(predvals)&/(1+exp(predvals)).
end if.
compute sepred=make(nrow(plotvals),3,999).
loop sei=1 to nrow(plotvals).
compute ask=probeplt(sei,:).
compute sepred(sei,1)=sqrt(ask*varb*t(ask)).
do if ((i < nms+nys) or (ydich=0)).
compute sepred(sei,2)=predvals(sei,1)-tval*sepred(sei,1).
compute sepred(sei,3)=predvals(sei,1)+tval*sepred(sei,1).
end if.
do if ((i = nms+nys) and (ydich=1)).
compute sepred(sei,2)=predvals(sei,1)-xp2*sepred(sei,1).
compute sepred(sei,3)=predvals(sei,1)+xp2*sepred(sei,1).
end if.
end loop.
compute prevloc=ncol(plotvals)+1.
compute probeplt={plotvals,predvals}.
do if (plot = 2).
compute probeplt={probeplt,sepred}.
end if.
do if ((i = nms+nys) and (ydich=1)).
compute probeplt={probeplt,expyhat}.
end if.
compute didsome=0.
end if.
do if ((wzcmat((i+1),jmed)=1) and (printpbe <= intprobe)).
do if (jmed=1).
compute omnilp2=nxvls*nwvls.
compute omnitmp=ident(omnilp2).
compute omni3(wherexw(1,i):wherexw(2,i),:)=omnitmp.
end if.
do if (jmed>1).
compute omnilp2=nwvls.
compute omnitmp=ident(omnilp2).
compute omni3(wheremw(((jmed*2)-3),i):wheremw(((jmed*2)-2),i),:)=omnitmp.
end if.
compute omnif=make(1,4,0).
do if ((i = nms+nys) and (ydich=1)).
compute omnif=make(1,3,0).
end if.
compute condeff3=0.
loop omnilp1=1 to nrow(zprobval).
loop omnilp=1 to (omnilp2).
do if (jmed=1).
compute omni3((wherexwz(1,i)+((omnilp-1)*nzvls)):(wherexwz(1,i)+((omnilp-1)*nzvls)+ (nzvls-1)),omnilp)=t(zprobval(omnilp1,:)).
end if.
do if (jmed > 1).
compute omni3((wheremwz(((jmed*2)-3),i)+((omnilp-1)*nzvls)):(wheremwz(((jmed*2)-3),i)+ ((omnilp-1)*nzvls)+(nzvls-1)),omnilp)=t(zprobval(omnilp1,:)).
end if.
end loop.
compute condeff=t(omni3)*b.
compute condeff3={condeff3;condeff}.

.
compute lmat2= omni3.
do if ( 1 =0).
compute lmat2 = mdiag( omni3 ).
compute lmat3=make(nrow(lmat2),1,0).
loop flp=1 to ncol(lmat2).
do if (csum(lmat2(:,flp))=1).
compute lmat3={lmat3,lmat2(:,flp)}.
end if.
end loop.
compute lmat2=lmat3(:,2:ncol(lmat3)).
end if.
compute fratio = (t(t(lmat2)* b )*inv(t(lmat2)* varb *lmat2)*((t(lmat2)* b )))/ncol(lmat2).
compute pfr = 1-fcdf(fratio,ncol(lmat2),(n-nrow( b ))).
compute fresult={fratio,ncol(lmat2),(n-nrow( b )),pfr}.
do if (i = (nms+nys) and (ydich=1)).
compute fratio=fratio*ncol(lmat2).
compute pfr=1-chicdf(fratio,ncol(lmat2)).
compute fresult={fratio,ncol(lmat2),pfr}.
end if.
do if ( 0 =1).
compute lmat3=1-rsum(lmat2).
compute xfm=make(n,csum(lmat3),0).
compute flpc=1.
loop flp=1 to nrow(lmat3).
do if (lmat3(flp,1)=1).
compute xfm(:,flpc)=x(:,flp).
compute flpc=flpc+1.
end if.
end loop.
compute bfm=inv(t(xfm)*xfm)*t(xfm)*y.
compute resid=y-(xfm*bfm).
compute sstotal=(y-(csum(y)/n)).
compute sstotal=csum(sstotal&*sstotal).
compute ssresid=csum(resid&*resid).
compute rsqch= 0 -((sstotal-ssresid)/sstotal).
compute fresult={rsqch,fresult}.
release xfm,flpc, resid, ssresid, bfm.
end if
.
compute omnif={omnif;fresult}.
end loop.
compute omnif=omnif(2:nrow(omnif),:).
compute clabtmp=znames.
compute condeff3=condeff3(2:nrow(condeff3),:).
do if ((nxvls*nwvls)=1).
compute omnif={condeff3,omnif}.
compute clabtmp={clabtmp,'Effect'}.
end if.
compute omnif={zmodvals,omnif}.
do if ((i < nms+nys) or (ydich=0)).
compute clabtmp={clabtmp,hcflab,'df1','df2','p'}.
end if.
do if ((i = (nms+nys)) and (ydich=1)).
compute clabtmp={clabtmp,'Chi-sq','df','p'}.
end if.
compute resultm2=make(nrow(omnif),maxresm,99999).
compute resultm2(1:nrow(omnif),1:ncol(omnif))=omnif.
compute resultm={resultm;resultm2}.
do if (outscree=1).
do if (jmed=1).
print omnif/title='Test of conditional X*W interaction at value(s) of Z:'/cnames=clabtmp/format= F10.4.
end if.
do if (jmed>1).
print omnif/title='Test of conditional M*W interaction at value(s) of Z:'/cnames=clabtmp/format= F10.4.
end if.
end if.
release omni3.
end if.
loop probei = 1 to probexpl.
do if (probexpl > 1).
compute foctmp=make(nrow(modvals),probexpl,0).
compute foctmp(:,probei)=foctmp(:,probei)+1.
compute probtemp=make(nrow(modvals),1,0).
loop probem = 1 to numplps.
loop probek = 1 to nxvls.
loop probej=lpstsp(probem,1) to lpstsp(probem,2).
compute probtemp={probtemp,foctmp(:,probek)&*probeval(:,probej)}.
end loop.
end loop.
end loop.
compute probeva2=probtemp(:,2:ncol(probtemp)).
compute probeva2={foctmp,probeva2}.
end if.
compute probres=probeva2*probcoef.
compute probrese=sqrt(diag(probeva2*probvarb*t(probeva2))).
compute tratio = probres&/probrese.
compute p = 2*(1-tcdf(abs(tratio), dfres)).
do if (ydich=1 and i = (nms+nys)).
compute p = 2*(1-cdfnorm(abs(tratio))).
end if.
compute modvals2={modvals,probres,probrese,tratio, p}.
do if ((i < nms+nys) or (ydich=0)).
compute modvals2={modvals2,(probres-tval&*probrese),(probres+tval&*probrese)}.
compute problabs={problabs,'Effect',hclab,'t', 'p', 'LLCI', 'ULCI'}.
end if.
do if (ydich=1 and i = (nms+nys)).
compute modvals2={modvals2,(probres-xp2&*probrese),(probres+xp2&*probrese)}.
compute problabs={problabs,'Effect','se','Z', 'p', 'LLCI', 'ULCI'}.
end if.
do if (probexpl > 1 and (printpbe <= intprobe)).
do if (hasz = 1).
compute printz=1.
end if.
do if (hasw=1).
compute printw=1.
end if.
compute probrlab=make(nrow(modvals),1,xcatlab(probei,1)).
compute modvals3={modvals3; modvals2}.
compute probrow={probrow;probrown}.
compute probrown=probrown+1.
do if (probei=probexpl).
compute xproblab=xcatlab(1:nxvls,1).
compute probrow=probrow(2:nrow(probrow),1).
compute modvals3=modvals3(2:nrow(modvals3),:).
compute temp=modvals3.
compute temp(GRADE(probrow(:,1)),:)=modvals3.
compute modvals3=temp.
compute start2=1.
compute problabs=problabs(1,(1+(ncol(modvarl))):ncol(problabs)).
compute pstart=1.
loop probek= 1 to nrow(probeval).
compute endstart=start2+(nxvls-1).
compute temp=modvals3(start2:endstart,(1+ncol(modvarl)):ncol(modvals3)).
compute temp2=t(modvals3(start2:start2,1:ncol(modvarl))).
compute trnames=t(modvarl).
do if (outscree=1).
do if (probek > 1).
print/title='----------'/space=0.
else.
print/title = 'Conditional effects of the focal predictor at values of the moderator(s):'.
do if ((jmed=1) and (i = (nms+nys)) and (nms > 0)).
do if (nxvls = 1).
print/title = '(These are also the conditional direct effects of X on Y)'/space=0.
else.
print/title = '(These are also the relative conditional direct effects of X on Y)'/space=0.
end if.
end if.
print.
end if.
print temp2/title = 'Moderator value(s):'/rnames=trnames/format= F10.4 /space=0.
print temp/title = ' '/cnames=problabs/rnames=xproblab/format= F10.4 /space=0.
end if.
compute resultm2=make(nrow(temp2),maxresm,99999).
compute resultm2(1:nrow(temp2),1:ncol(temp2))=temp2.
compute resultm={resultm;resultm2}.
compute resultm2=make(nrow(temp),maxresm,99999).
compute resultm2(1:nrow(temp),1:ncol(temp))=temp.
compute resultm={resultm;resultm2}.
compute start2=start2+nxvls.
compute didsome=1.
do if (jmed=1).
compute mod1val=probeval(probek,1:nm1vls).
loop omnilp=1 to nxvls.
compute omni((wheremv1(1,i)+((omnilp-1)*nm1vls)):(wheremv1(1,i)+((omnilp-1)* nm1vls)+(nm1vls-1)),omnilp)=t(mod1val).
do if (nm1vls < ncol(probeval)).
compute mod2val=probeval(probek,(nm1vls+1):(nm1vls+nm2vls)).
compute omni((wheremv2(1,i)+((omnilp-1)*nm2vls)):(wheremv2(1,i)+((omnilp-1)* nm2vls)+(nm2vls-1)),omnilp)=t(mod2val).
do if ((nm1vls+nm2vls) < ncol(probeval)).
compute intlen=nm1vls*nm2vls.
compute modintvl=probeval(probek,(nm1vls+nm2vls+1):ncol(probeval)).
compute omni((wherexwz(1,i)+((omnilp-1)*intlen)):(wherexwz(1,i)+((omnilp-1)* intlen)+(intlen-1)),omnilp)=t(modintvl).
end if.
end if.
end loop.

.
compute lmat2= omni.
do if ( 1 =0).
compute lmat2 = mdiag( omni ).
compute lmat3=make(nrow(lmat2),1,0).
loop flp=1 to ncol(lmat2).
do if (csum(lmat2(:,flp))=1).
compute lmat3={lmat3,lmat2(:,flp)}.
end if.
end loop.
compute lmat2=lmat3(:,2:ncol(lmat3)).
end if.
compute fratio = (t(t(lmat2)* b )*inv(t(lmat2)* varb *lmat2)*((t(lmat2)* b )))/ncol(lmat2).
compute pfr = 1-fcdf(fratio,ncol(lmat2),(n-nrow( b ))).
compute fresult={fratio,ncol(lmat2),(n-nrow( b )),pfr}.
do if (i = (nms+nys) and (ydich=1)).
compute fratio=fratio*ncol(lmat2).
compute pfr=1-chicdf(fratio,ncol(lmat2)).
compute fresult={fratio,ncol(lmat2),pfr}.
end if.
do if ( 0 =1).
compute lmat3=1-rsum(lmat2).
compute xfm=make(n,csum(lmat3),0).
compute flpc=1.
loop flp=1 to nrow(lmat3).
do if (lmat3(flp,1)=1).
compute xfm(:,flpc)=x(:,flp).
compute flpc=flpc+1.
end if.
end loop.
compute bfm=inv(t(xfm)*xfm)*t(xfm)*y.
compute resid=y-(xfm*bfm).
compute sstotal=(y-(csum(y)/n)).
compute sstotal=csum(sstotal&*sstotal).
compute ssresid=csum(resid&*resid).
compute rsqch= 0 -((sstotal-ssresid)/sstotal).
compute fresult={rsqch,fresult}.
release xfm,flpc, resid, ssresid, bfm.
end if
.
compute resultm2=make(nrow(fresult),maxresm,99999).
compute resultm2(1:nrow(fresult),1:ncol(fresult))=fresult.
compute resultm={resultm;resultm2}.
do if (outscree=1).
do if (i < (nms + nys) or (ydich = 0)).
compute clabtmp={hcflab,'df1','df2','p'}.
print fresult/title='Test of equality of conditional means'/cnames=clabtmp/format= F10.4.
end if.
do if (i = (nms + nys) and (ydich = 1)).
compute clabtmp={'Chi-sq','df','p'}.
print fresult/title='Test of equality of conditional logits or probabilities'/cnames=clabtmp/format= F10.4.
end if.
end if.
compute probetmp=probeplt(pstart:(pstart+nxvls),1).
compute probetmp={probetmp,probeplt(pstart:(pstart+nxvls),prevloc:ncol(probeplt))}.
compute pstart=pstart+(nxvls+1).
compute resultm2=make(nrow(probetmp),maxresm,99999).
compute resultm2(1:nrow(probetmp),1:ncol(probetmp))=probetmp.
compute resultm={resultm;resultm2}.
do if (i < ((nms + nys)) or (ydich = 0)).
compute clabtmp={xnames, outnames(1,i), hclab, 'LLCI', 'ULCI'}.
do if (outscree=1).
print probetmp/title = 'Estimated conditional means being compared:'/cnames=clabtmp/format= F10.4.
end if.
end if.
do if ((i = (nms+nys)) and (ydich = 1)).
compute clabtmp={xnames, outnames(1,i), 'prob'}.
compute probetm2=probetmp(:,1:2).
compute probetm2={probetm2,probetmp(:,ncol(probetmp))}.
do if (outscree=1).
print probetm2/title = 'Estimated conditional logits and probabilities:'/cnames=clabtmp/format= F10.4.
end if.
end if.
end if.
end loop.
release probrow, start2, endstart, temp, temp2.
end if.
end if.
do if (probexpl = 1 and (printpbe <= intprobe)).
compute resultm2=make(nrow(modvals2),maxresm,99999).
compute resultm2(1:nrow(modvals2),1:ncol(modvals2))=modvals2.
compute resultm={resultm;resultm2}.
do if (outscree=1).
print/title = 'Conditional effects of the focal predictor at values of the moderator(s):'.
print modvals2/cnames=problabs/title = ' '/space=0/format= F10.4.
end if.
compute didsome=1.
do if (hasz = 1).
compute printz=1.
end if.
do if (hasw=1).
compute printw=1.
end if.
do if (jn = 1 and jnok=1).
do if (criterr = 0).
compute dfres=n-nrow(b).
compute roots=99999.
compute jncrit =(dfres* (exp((dfres-(5/6))*((xp2/(dfres-(2/3)+(.11/dfres)))* (xp2/(dfres-(2/3)+(.11/dfres)))))-1)).
do if (i = (nms+nys) and (ydich=1)).
compute jncrit=xp2*xp2.
end if.
compute jnb1=b(wherejn1,1).
compute jnb3=b(wherejn3,1).
compute jnsb1=varb(wherejn1,wherejn1).
compute jnsb3=varb(wherejn3,wherejn3).
compute jnsb1b3=varb(wherejn1,wherejn3).
compute ajn =(jncrit*jnsb3)-(jnb3*jnb3).
compute bjn = 2*((jncrit*jnsb1b3)-(jnb1*jnb3)).
compute cjn = (jncrit*jnsb1)-(jnb1*jnb1).
compute radarg = (bjn*bjn)-(4*ajn*cjn).
compute den = 2*ajn.
compute nrts = 0.
do if (radarg >= 0 and den <> 0).
compute x21 = (-bjn+sqrt(radarg))/den.
compute x22 = (-bjn-sqrt(radarg))/den.
compute roots = 0.
do if (x21 >= jnmin and x21 <= jnmax).
compute nrts = 1.
compute roots = {roots; x21}.
end if.
do if (x22 >= jnmin and x22 <= jnmax).
compute nrts = nrts + 1.
compute roots = {roots; x22}.
end if.
compute roots={roots,make(nrow(roots),2,0)}.
end if.
do if (nrts > 0).
compute roots = roots(2:nrow(roots),1:3).
compute roots(1,2)=(csum(jnmod < roots(1,1))/n)*100.
compute roots(1,3)=(csum(jnmod > roots(1,1))/n)*100.
do if (nrow(roots)=2).
compute roots(2,2)=(csum(jnmod < roots(2,1))/n)*100.
compute roots(2,3)=(csum(jnmod > roots(2,1))/n)*100.
end if.
do if (outscree=1).
print roots/title = 'Moderator value(s) defining Johnson-Neyman significance region(s):'/clabels = 'Value', '% below', '% above'/format F10.4.
end if.
do if (nrts=1).
compute tmprts=make(1,3,99999).
compute roots={roots;tmprts}.
end if.
end if.
do if (nrts = 0).
compute roots=make(2,1,99999).
do if (outscree=1).
print/title = 'There are no statistical significance transition points within the observed'.
print/title = 'range of the moderator found using the Johnson-Neyman method.'/space=0.
end if.
end if.
compute resultm2=make(nrow(roots),maxresm,99999).
compute resultm2(1:nrow(roots),1:ncol(roots))=roots.
compute resultm={resultm;resultm2}.
compute jnvals=make(23,7,0).
loop jni= 0 to (21-(nrts)).
compute jnvals((jni+1),1)=jnmin+(jni*((jnmax-jnmin)/(21-nrts))).
end loop.
do if (nrts > 0).
loop jni = 1 to nrts.
loop jnj = 2 to (nrow(jnvals)-1).
do if ((roots(jni,1) > jnvals((jnj-1),1)) and (roots(jni,1) < jnvals(jnj,1))).
compute jnvals((jnj+1):(21+jni),1)=jnvals(jnj:(20+jni),1).
compute jnvals(jnj,1)=roots(jni,1).
end if.
end loop.
end loop.
end if.
compute jnvals=jnvals(1:22,:).
loop jni = 1 to nrow(jnvals).
compute jnvals(jni,2)=jnb1+jnb3*jnvals(jni,1).
compute jnvals(jni,3)=sqrt(jnsb1+2*jnvals(jni,1)*jnsb1b3+(jnvals(jni,1)*jnvals(jni, 1))*jnsb3).
compute jnvals(jni,4)=jnvals(jni,2)/jnvals(jni,3).
compute jnvals(jni,5)=2*(1-tcdf(abs(jnvals(jni,4)), dfres)).
compute jnvals(jni,6)=jnvals(jni,2)-sqrt(jncrit)*jnvals(jni,3).
compute jnvals(jni,7)=jnvals(jni,2)+sqrt(jncrit)*jnvals(jni,3).
do if ((i = nms + nys) and (ydich=1)).
compute jnvals(jni,5)=2*(1-cdfnorm(abs(jnvals(jni,4)))).
compute jnvals(jni,6)=jnvals(jni,2)-xp2*jnvals(jni,3).
compute jnvals(jni,7)=jnvals(jni,2)+xp2*jnvals(jni,3).
end if.
end loop.
compute resultm2=make(nrow(jnvals),maxresm,99999).
compute resultm2(1:nrow(jnvals),1:ncol(jnvals))=jnvals.
compute resultm={resultm;resultm2}.
do if ((i < nms+nys) or (ydich=0)).
compute jnclbs={jnmodlab,'Effect',hclab,'t', 'p', 'LLCI', 'ULCI'}.
end if.
do if ((i = nms + nys) and (ydich=1)).
compute jnclbs={jnmodlab,'Effect','se','Z', 'p', 'LLCI', 'ULCI'}.
end if.
do if (((wcmat((i+1),jmed)=1) or (zcmat((i+1),jmed)=1)) and (wzcmat((i+1),jmed)=0)).
do if (outscree=1).
print jnvals/title = 'Conditional effect of focal predictor at values of the moderator:'/cnames =jnclbs/format = F10.4.
end if.
end if.
do if (outscree=1).
do if ((jmed = 1) and (wzcmat((i+1),jmed)=1)).
print jnvals/title = 'Conditional X*W interaction at values of the moderator Z:'/cnames =jnclbs/format = F10.4.
end if.
do if ((jmed > 1) and (wzcmat((i+1),jmed)=1)).
print jnvals/title = 'Conditional M*W interaction at values of the moderator Z:'/cnames =jnclbs/format = F10.4.
end if.
end if.
end if.
end if.
end if.
do if ((i = (nms+nys)) and (jmed=1) and (bcmat(nrow(bcmat),1)=1)).
do if (probei=1).
compute direfflb=problabs.
compute direff=modvals2.
end if.
do if (probei>1).
compute direff={direff;modvals2}.
end if.
end if.
compute intprint=0.
do if ((jmed=1) and (i=1) and nms=0) and modcok=1).
compute contvec2=make(2,1,1).
compute contvec2={contvec2,wcontval,zcontval}.
do if (wzcmat((i+1),jmed)=1).
loop conti= 1 to ncol(wcontval).
loop contj = 1 to ncol(zcontval).
compute contvec2={contvec2,wcontval(:,conti)&*zcontval(:,contj)}.
end loop.
end loop.
end if.
compute conteff=contvec2*probcoef.
compute contdiff=contvec2(1,:)-contvec2(2,:).
compute contse=sqrt(contdiff*probvarb*t(contdiff)).
compute conteffd=conteff(1,1)-conteff(2,1).
compute contvec={contvec,conteff}.
compute contvecm=contvec.
compute resultm2=make(nrow(contvecm),maxresm,99999).
compute resultm2(1:nrow(contvecm),1:ncol(contvecm))=contvecm.
compute resultm={resultm;resultm2}.
do if (outscree=1).
print/title='Contrast between conditional effects of X:'.
print contvec/title=' '/rlabels='Effect1:','Effect2:'/cnames=problabs/format = F10.4 /space=0.
end if.
do if (ydich=0).
compute p=2*(1-tcdf(abs(conteffd/contse), dfres)).
compute contvec={conteffd,contse,conteffd/contse, p}.
compute contvec={contvec,(conteffd-(tval*contse))}.
compute contvec={contvec,(conteffd+(tval*contse))}.
compute contlabs={'Contrast', hclab, 't', 'p', 'LLCI', 'ULCI'}.
end if.
do if (ydich=1).
compute p=2*(1-cdfnorm(abs(conteffd/contse))).
compute contvec={conteffd,contse,conteffd/contse, p}.
compute contvec={contvec,(conteffd-(xp2*contse))}.
compute contvec={contvec,(conteffd+(xp2*contse))}.
compute contlabs={'Contrast', 'se', 'Z', 'p', 'LLCI', 'ULCI'}.
end if.
compute resultm2=make(nrow(contvec),maxresm,99999).
compute resultm2(1:nrow(contvec),1:ncol(contvec))=contvec.
compute resultm={resultm;resultm2}.
do if (outscree=1).
print contvec/title='Test of Effect1 minus Effect2'/format= F10.4 /cnames=contlabs.
end if.
end if.
end loop.
do if (plot = 1 or plot = 2).
compute datalabs={t(focpred(:,3)),outnames(1,i)}.
do if (plot = 1).
compute datalabs={datalabs}.
end if.
do if (plot = 2).
compute datalabs={datalabs,'se', 'LLCI', 'ULCI'}.
end if.
do if ((i = nms+nys) and (ydich=1)).
compute datalabs={datalabs,'prob'}.
end if.
compute resultm2=make(nrow(probeplt),maxresm,99999).
compute resultm2(1:nrow(probeplt),1:ncol(probeplt))=probeplt.
compute resultm={resultm;resultm2}.
compute datalabs={datalabs,'.'}.
do if (outscree=1).
print/title = 'Data for visualizing the conditional effect of the focal predictor:'.
print/title = 'Paste text below into a SPSS syntax window and execute to produce plot.'/space=0.
compute dumb = {' ', ' ', ' ', ' ', ' ', ' ', ' '}.
print datalabs/title = 'DATA LIST FREE/'/format=A10.
print probeplt/title = 'BEGIN DATA.'/format= F10.4 /space=0.
print/title = 'END DATA.'/space=0.
end if.
compute focgrph=datalabs(1,1).
compute graphix={focgrph,graphixs,modgrph}.
do if (((xdich=1) or (nxvls > 1)) and ((modcat=0) and (focgrph = xnames))).
compute graphix={modgrph,graphixs,focgrph}.
end if.
do if (panelgrp = 0).
compute graphix={graphix,'.'}.
else.
compute graphix={graphix,panelcde}.
end if.
do if (outscree=1).
print graphix/title = 'GRAPH/SCATTERPLOT='/format=A8/space=0.
end if.
do if (i = (nms + nys) and ydich = 1).
compute graphixd=graphix.
compute graphixd(1,3)='prob'.
do if (outscree=1).
print graphixd/title = 'GRAPH/SCATTERPLOT='/format=A8/space=0.
end if.
end if.
end if.
end if.
end loop.
release jmed, intprint,didprint.
end if
.
do if (model = 74 and i <= nms).
compute onetemp=make(nrow(xprobval),1,1).
compute mestmt74={onetemp,xprobval}.
do if (ncs > 0).
compute ncovmdl=rsum(ccmat(i,:)).
do if (ncovmdl > 0).
compute cvmnc=make(nrow(mestmt74),ncovmdl,1).
compute cvmnctmp=csum(x(:,(ncol(x)-ncovmdl+1):ncol(x)))/nrow(x).
do if (cuscoval > 0).
compute cvmnctmp=coval.
end if.
loop mestlp=1 to ncovmdl.
compute cvmnc(:,mestlp)=cvmnc(:,mestlp)*cvmnctmp(:,mestlp).
end loop.
compute mestmt74={mestmt74,cvmnc}.
end if.
end if.
compute mest74t=mestmt74*b.
do if (i = 1).
compute mest74=mest74t.
end if.
do if (i > 1).
compute mest74={mest74,mest74t}.
end if.
end if.
do if ((i = (nms+nys)) and (model >= 0 and model < 4) and (linsum(1,1) <> -999)).
compute lhyprob=1.
compute meansub=0.
do if (((nlinsum=nrow(b)) or (nlinsum = (nrow(b)-ncs)))).
do if (nlinsum = (nrow(b)-ncs) and ncs > 0).
compute linsum={linsum,covmeans}.
compute meansub=1.
end if.
compute lhyprob=0.
compute hypest=linsum*b.
compute sehypest=sqrt(linsum*varb*t(linsum)).
compute phypest=2*(1-tcdf(abs(hypest/sehypest), (dfres))).
compute hypest={hypest,sehypest,(hypest/sehypest),phypest,(hypest-tval*sehypest),(hypest+ tval*sehypest)}.
compute resultm2=make(ncol(linsum),maxresm,99999).
compute resultm2(:,1)=t(linsum).
compute resultm={resultm;resultm2}.
compute resultm2=make(1,maxresm,99999).
compute resultm2(1,1:ncol(hypest))=hypest.
compute resultm={resultm;resultm2}.
do if (outscree=1).
compute hyplabs={'Estimate',hclab,'t','p','LLCI','ULCI'}.
print/title='----------'.
print/title='Linear Combination Estimate and Hypothesis Test'/space=0.
print t(linsum)/title= 'Weight vector:'/clabels='weight'/rnames=vlabsm/format F10.4.
print hypest/title=' '/cnames=hyplabs/format= F10.4 /space=0.
do if (meansub=1).
print/title='Covariate weight(s) set to the sample mean.'.
end if.
end if.
end if.
do if (lhyprob=1).
compute notecode(notes,1) = 30.
compute notes = notes + 1.
end if.
end if.
end loop if criterr=1.
compute lastb=b.
compute lastcov=varb.
do if (criterr=0 and dototal = 1).
compute x=xtmp.
compute vlabsm={'constant';xcatlab(1:nxvls,1)}.
do if (ncs > 0).
compute x = {x,ctmp}.
compute vlabsm={vlabsm;t(covnames)}.
end if.
compute x = {ones,x}.

.
do if ( 1 =1).
compute hatmat=inv(t( x )* x )*t( x ).
compute b = hatmat* y.
compute modres=b.
do if ( 1 =1).
compute n1=nrow( x ).
compute dfres=n1-(ncol( x )).
compute sstotal = t( y -(csum( y )/n1))*( y -(csum( y )/n1)).
compute resid= y - x *b.
compute ssresid = csum((resid)&**2).
compute r2 = (sstotal-ssresid)/sstotal.
do if (r2 < 0).
compute r2=0.
end if.
compute adjr2 = 1-((1-r2)*(n1-1)/(dfres)).
compute mse=ssresid/(n1-ncol( x )).

.
compute n1=nrow( x ).
compute invXtX = inv(t( x )* x ).
compute varb = mse *invXtX.
compute k3 = ncol( x ).
compute xhc=0.
do if ( hc <> 5).
compute xhc= x.
compute hat = xhc(:,1).
loop i3=1 to nrow(xhc).
compute hat(i3,1)= xhc(i3,:)*invXtX*t(xhc(i3,:)).
end loop.
do if ( hc = 0 or hc =1).
loop i3 = 1 to k3.
compute xhc(:,i3)=xhc(:,i3)&* resid.
end loop.
end if.
do if ( hc =3 or hc =2).
loop i3=1 to k3.
compute xhc(:,i3) = ( resid &/(1-hat)&**(1/(4- hc )))&*xhc(:,i3).
end loop.
end if.
do if ( hc = 4).
compute hcmn=make(n,2,4).
compute hcmn(:,2)=(n1*hat)/k3.
loop i3= 1 to k3.
compute xhc(:,i3) = ( resid &/(1-hat)&**(rmin(hcmn)/2))&*xhc(:,i3).
end loop.
end if.
compute varb=(invXtX*t(xhc)*xhc*invXtX).
do if ( hc =1).
compute varb=(n1/(n1-ncol( x )))&*varb.
end if.
end if.
compute hclab={'se(HC0)','se(HC1)','se(HC2)','se(HC3)','se(HC4)','se'}.
compute hclab=hclab(1,( hc +1)).
compute hcflab={'F(HC0)','F(HC1)','F(HC2)','F(HC3)','F(HC4)','F'}.
compute hcflab=hcflab(1,( hc +1)).
release xhc
.
compute seb=sqrt(diag(varb)).
compute trat = b&/seb.
compute p = 2*(1-tcdf(abs(trat), (dfres))).
compute tval = sqrt(dfres* (exp((dfres-(5/6))*((xp2/(dfres-(2/3)+(.11/dfres)))* (xp2/(dfres-(2/3)+(.11/dfres)))))-1)).
compute modres={modres,seb,trat,p}.
compute modres={modres,(b-tval&*seb),(b+tval&*seb)}.
compute modresl={'coeff',hclab,'t','p','LLCI','ULCI'}.
compute lmat = ident(ncol( x )).
compute lmat = lmat(:,2:ncol(lmat)).
compute fratio = (t(t(lmat)*b)*inv(t(lmat)*varb*lmat)*((t(lmat)*b)))/(ncol( x )-1).
compute pfr = 1-fcdf(fratio,(ncol( x )-1),dfres).
compute modsum={sqrt(r2),r2,mse,fratio,(ncol( x )-1),dfres,pfr}.
compute modsuml={'R','R-sq','MSE',hcflab,'df1','df2', 'p'}.
end if.
end if.
do if ( 1 = 2 or 1 =3).
compute xlp= x.
compute ylp= y.
compute pt2 = make(nrow(ylp),1,(csum(ylp)/nrow(ylp))).
do if ( 1 =2).
compute LL3 = ylp&*ln(pt2)+(1-ylp)&*ln(1-pt2).
end if.
compute LL3 = -2*csum(LL3).
compute bt1 = make(ncol(xlp),1,0).
compute LL1 = 0.
compute pt1 = make(nrow(ylp),1,0.5).
compute pt1lp=pt1.
loop jjj = 1 to iterate.
compute xlptmp=t(xlp).
compute vecprb=pt1lp&*(1-pt1lp).
loop kkk=1 to ncol(xlp).
compute xlptmp(kkk,:)=xlptmp(kkk,:)&*t(vecprb).
end loop.
compute b = bt1+inv(xlptmp*xlp)*t(xlp)*(ylp-pt1lp).
do if ( 1 =2).
compute xlpb=xlp*b.
compute xlpbt=(xlpb > -709.7).
compute xlpb709=(1-xlpbt)*(-709.7).
compute xlpb=(xlpb&*xlpbt)+xlpb709.
compute pt1lp = 1/(1+exp(-(xlpb))).
end if.
compute itprob = csum((pt1lp < .00000001) or (pt1lp > .9999999)).
do if (itprob > 0).
loop kkk = 1 to nrow(pt1lp).
do if (pt1lp(kkk,1) > .9999999).
compute pt1lp(kkk,1) = .9999999.
end if.
do if (pt1lp(kkk,1) < .00000001).
compute pt1lp(kkk,1) = .00000001.
end if.
end loop.
compute itprob = 0.
end if.
do if (itprob = 0).
do if ( 1 =2).
compute LL = ylp&*ln(pt1lp)+(1-ylp)&*ln(1-pt1lp).
end if.
compute LL2 = -2*csum(ll).
end if.
do if (abs(LL1-LL2) < converge).
do if ( 1 =1).
compute xlptmp=t(xlp).
compute vecprb=pt1lp&*(1-pt1lp).
loop kkk=1 to ncol(xlp).
compute xlptmp(kkk,:)=xlptmp(kkk,:)&*t(vecprb).
end loop.
compute varb = inv(xlptmp*xlp).
compute seb = sqrt(diag(varb)).
release xlptmp.
end if.
break.
end if.
compute bt1 = b.
compute LL1 = LL2.
end loop.
compute modres=b.
do if (jjj > iterate).
compute itprob = 2.
do if (booting=0).
compute iterrmod=1.
end if.
do if (booting=1).
compute bootiter=1.
end if.
do if (itprobtg=0).
compute itprobtg=1.
compute errcode(errs,1) = 47.
compute errs = errs + 1.
do if (booting = 0 and 1 =1).
compute vt1 = mdiag(pt1lp&*(1-pt1lp)).
compute varb = inv(t(xlp)*vt1*xlp).
compute seb = sqrt(diag(varb)).
end if.
end if.
end if.
do if ( 1 =1).
compute trat = b&/seb.
compute dfres=nrow(xlp).
compute p = 2*(1-cdfnorm(abs(trat))).
compute modres={modres,seb,trat,p}.
compute modres={modres,(b-xp2&*seb),(b+xp2&*seb)}.
compute pvchi=1-chicdf((LL3-LL2),(nrow(modres)-1)).
compute mcF = (LL3-LL2)/LL3.
compute cox = 1-exp(-(LL3-LL2)/nrow(xlp)).
compute nagel = cox/(1-exp(-(LL3)/nrow(xlp))).
compute modsum={LL2,(LL3-LL2),(nrow(modres)-1),pvchi, mcF,cox,nagel}.
compute modsuml={'-2LL','ModelLL', 'df', 'p', 'McFadden', 'CoxSnell', 'Nagelkrk'}.
compute modresl={'coeff','se','Z','p','LLCI','ULCI'}.
end if.
end if
.
compute toteff=modres(2:(1+nxvls),:).
compute nodotot=0.
do if (xdich=1 and xmint=1 and model=74).
compute toteff(:,1)=toteff(:,1)*xscaling.
compute toteff(:,2)=toteff(:,2)*abs(xscaling).
compute toteff(:,3)=toteff(:,3)*xscaling.
compute toteff(:,5)=toteff(:,5)*xscaling.
compute toteff(:,6)=toteff(:,6)*xscaling.
compute citmp=toteff(:,5:6).
do if (xscaling < 0).
compute toteff(:,5)=citmp(:,2).
compute toteff(:,6)=citmp(:,1).
end if.
compute nodotot=1.
end if.
compute totefflb=modresl.
do if (outscree=1).
do if (nodotot=0).
print/title = '************************** TOTAL EFFECT MODEL ****************************'.
end if.
do if (nodotot=1).
print/title = '**************************************************************************'.
end if.
print outnames(1,ncol(outnames))/title = 'OUTCOME VARIABLE:'/format = A8/space=0.
end if.
compute toteffl2=vlabsm(2:(1+nxvls),:).
compute resultm2=make(1,maxresm,99999).
compute resultm2(1,1:ncol(modsum))=modsum.
compute resultm={resultm;resultm2}.
compute resultm2=make(nrow(modres),maxresm,99999).
compute resultm2(1:nrow(modres),1:ncol(modres))=modres.
compute resultm={resultm;resultm2}.
do if (outscree=1).
print modsum/title = 'Model Summary'/cnames = modsuml/format= F10.4.
print modres/title='Model'/rnames=vlabsm/cnames=modresl/format= F10.4.
end if.
compute lmat=make(nrow(b),1,0).
compute lmat2=make(nxvls,1,1).
compute lmat(2:(1+nxvls),1)=lmat2.
do if (ydich <> 1).

.
compute lmat2= lmat.
do if ( 0 =0).
compute lmat2 = mdiag( lmat ).
compute lmat3=make(nrow(lmat2),1,0).
loop flp=1 to ncol(lmat2).
do if (csum(lmat2(:,flp))=1).
compute lmat3={lmat3,lmat2(:,flp)}.
end if.
end loop.
compute lmat2=lmat3(:,2:ncol(lmat3)).
end if.
compute fratio = (t(t(lmat2)* b )*inv(t(lmat2)* varb *lmat2)*((t(lmat2)* b )))/ncol(lmat2).
compute pfr = 1-fcdf(fratio,ncol(lmat2),(n-nrow( b ))).
compute fresult={fratio,ncol(lmat2),(n-nrow( b )),pfr}.
do if (i = (nms+nys) and (ydich=1)).
compute fratio=fratio*ncol(lmat2).
compute pfr=1-chicdf(fratio,ncol(lmat2)).
compute fresult={fratio,ncol(lmat2),pfr}.
end if.
do if ( 1 =1).
compute lmat3=1-rsum(lmat2).
compute xfm=make(n,csum(lmat3),0).
compute flpc=1.
loop flp=1 to nrow(lmat3).
do if (lmat3(flp,1)=1).
compute xfm(:,flpc)=x(:,flp).
compute flpc=flpc+1.
end if.
end loop.
compute bfm=inv(t(xfm)*xfm)*t(xfm)*y.
compute resid=y-(xfm*bfm).
compute sstotal=(y-(csum(y)/n)).
compute sstotal=csum(sstotal&*sstotal).
compute ssresid=csum(resid&*resid).
compute rsqch= r2 -((sstotal-ssresid)/sstotal).
compute fresult={rsqch,fresult}.
release xfm,flpc, resid, ssresid, bfm.
end if
.
compute totomni=fresult.
end if.
do if (stand=1).
compute predsd=make(nrow(modres),1,0).
compute stdmod=modres(:,1)&/ovsd(1,ncol(ovsd)).
loop jd=1 to ncol(x).
compute descdat=x(:,jd).
compute predsd(jd,1) = (nrow(descdat)*sscp(descdat))-(t(csum(descdat))*(csum(descdat))).
compute predsd(jd,1) = sqrt(predsd(jd,1)/(nrow(descdat)*(nrow(descdat)-1))).
end loop.
do if (wherex(1,ncol(wherex)) <> -999 and ((nxvls > 1) or (xdich=1))).
compute sdmsone=make(nxvls,1,1).
compute predsd(wherex(1,ncol(wherex)):wherex(2,ncol(wherex)),1)=sdmsone.
compute pstog=1.
end if.
compute predsd(1,1)=1.
compute stdmod=stdmod&*predsd.
compute stdmod=stdmod(2:nrow(stdmod),1).
compute sdvlabs=vlabsm(2:nrow(vlabsm),1).
compute resultm2=make(nrow(stdmod),maxresm,99999).
compute resultm2(1:nrow(stdmod),1:ncol(stdmod))=stdmod.
compute resultm={resultm;resultm2}.
do if (outscree=1).
print stdmod/title='Standardized coefficients'/clabels='coeff'/rnames=sdvlabs/format= F10.4.
end if.
end if.
do if (covcoeff=1).
do if (outscree=1).
print varb/title='Covariance matrix of regression parameter estimates:'/rnames=vlabsm/cnames=vlabsm/format= F10.4.
end if.
compute resultm2=make(nrow(varb),maxresm,99999).
do if (ncol(varb) <= maxresm).
compute resultm2(1:nrow(varb),1:ncol(varb))=varb.
compute resultm={resultm;resultm2}.
end if.
do if (ncol(varb) > maxresm).
compute resultmt=make(nrow(resultm),ncol(varb),99999).
compute resultmt(1:nrow(resultm),1:ncol(resultm))=resultm.
compute resultm=resultmt.
compute resultm2=make(nrow(varb),ncol(resultm),99999).
compute resultm2(1:nrow(varb),1:ncol(varb))=varb.
compute resultm={resultm;resultm2}.
compute maxresm=ncol(resultm).
end if.
end if.
end if.
end if.
do if (criterr=0 and nms > 0 and ydich=0 and modelres=1).
compute modresid=modresid(:,2:ncol(modresid)).
compute sigmatal = (t(modresid)*(ident(n)-(1/n)*ones*t(ones))*modresid)*(1/(n-1)).
compute sdall = mdiag(1/sqrt(diag(sigmatal))).
compute corall=sdall*sigmatal*t(sdall).
compute resultm2=make(nrow(corall),maxresm,99999).
compute resultm2(1:nrow(corall),1:ncol(corall))=corall.
compute resultm={resultm;resultm2}.
do if (outscree=1).
print/title = '****************** CORRELATIONS BETWEEN MODEL RESIDUALS ******************'.
print corall/title=' '/format= F10.4 / cnames=outnames/rnames=outnames/space=0.
end if.
end if.
do if (criterr=0 and boot > 0).
compute bootres=make(1,rsum(nump),-999).
compute bootdir=obsdirfx.
compute natdirbt=make(1,nxvls,-999).
do if (effsize=1).
compute bootysd=make(1,1,-999).
compute bootxsd=make(1,1,-999).
end if.
compute badboot=0.
compute goodboot=0.
compute smallest=1.
compute booting=1.
loop j = 1 to maxboot.
compute nobootx=1.
compute modres2=999.
compute v=trunc(uniform(n,1)*n)+1.
compute bad=0.
loop i = 1 to (nms+nys).
compute y=outvars(v,i).
compute ynovar= (nrow(y)*sscp(y))-(t(csum(y))*(csum(y))).
do if (ynovar = 0).
compute bad=1.
end if.
compute xindx=datindx(1:(nump(1,i)-1),i).
compute hello=0.
compute x = fulldat(v,xindx).
compute x={ones,x}.
compute xsq=t(x)*x.
compute exsq=eval(xsq).
release xsq.
compute holymoly=cmin(exsq).
compute zeroeig=csum(exsq <= 0.000000000002).
compute bad=bad + (zeroeig > 0).

.
compute desctmp=make((8-(4* 1 )),ncol( y ),-999).
loop jd=1 to ncol( y ).
compute descdat= y (:,jd).
compute desctmp(1,jd) = csum(descdat)/nrow(descdat).
compute desctmp(2,jd) = (nrow(descdat)*sscp(descdat))-(t(csum(descdat))*(csum(descdat))).
compute desctmp(2,jd) = sqrt(desctmp(2,jd)/(nrow(descdat)*(nrow(descdat)-1))).
compute desctmp(3,jd)=cmin(descdat).
compute desctmp(4,jd)=cmax(descdat).
do if ( 1 =0).
compute minwarn=0.
compute maxwarn=0.
do if ((desctmp(3,jd)=desctmp(4,jd)) and novar=0).
compute errcode(errs,1)=15.
compute errs=errs+1.
compute criterr=1.
compute novar=1.
end if.
compute tmp=((descdat(:,1)=desctmp(3,jd))+(descdat(:,1)=desctmp(4,jd))).
compute desctmp(8,jd)=(csum(tmp)=nrow(tmp)).
compute tmp = descdat.
compute tmp(GRADE(descdat),:) = descdat.
compute descdat = tmp.
release tmp.
compute decval={.16;.5;.84}.
loop kd=1 to 3.
compute low=trunc(decval(kd,1)*(nrow(descdat)+1)).
compute lowdec=decval(kd,1)*(nrow(descdat)+1)-low.
compute value=descdat(low,1)+(descdat((low+1),1)-descdat(low,1))*lowdec.
compute desctmp((4+kd),jd)=value.
end loop.
compute mnotev=1.
compute modvals=desctmp(5:7,:).
do if (quantile <> 1).
compute desctmp(5,jd)=desctmp(1,jd)-desctmp(2,jd).
compute desctmp(6,jd)=desctmp(1,jd).
compute desctmp(7,jd)=desctmp(1,jd)+desctmp(2,jd).
compute modvals=desctmp(5:7,:).
compute mnotev=2.
do if (modvals(1,1) < desctmp(3,1)).
compute modvals(1,1)=desctmp(3,1).
compute minwarn=1.
end if.
do if (modvals(3,1) > desctmp(4,1)).
compute modvals(3,1)=desctmp(4,1).
compute maxwarn=1.
end if.
end if.
do if (desctmp(8,1)=1).
compute modvals={desctmp(3,1);desctmp(4,1)}.
compute mnotev=0.
compute minwarn=0.
compute maxwarn=0.
end if.
end if.
end loop
.
compute bad=bad+ (desctmp(2,1) <= 0.00000000001).
do if (bad = 0).
do if (holymoly < smallest).
compute smallest=holymoly.
end if.
do if (ydich=0 or (i < (nms+nys))).

.
do if ( 1 =1).
compute hatmat=inv(t( x )* x )*t( x ).
compute b = hatmat* y.
compute modres=b.
do if ( 0 =1).
compute n1=nrow( x ).
compute dfres=n1-(ncol( x )).
compute sstotal = t( y -(csum( y )/n1))*( y -(csum( y )/n1)).
compute resid= y - x *b.
compute ssresid = csum((resid)&**2).
compute r2 = (sstotal-ssresid)/sstotal.
do if (r2 < 0).
compute r2=0.
end if.
compute adjr2 = 1-((1-r2)*(n1-1)/(dfres)).
compute mse=ssresid/(n1-ncol( x )).

.
compute n1=nrow( x ).
compute invXtX = inv(t( x )* x ).
compute varb = mse *invXtX.
compute k3 = ncol( x ).
compute xhc=0.
do if ( hc <> 5).
compute xhc= x.
compute hat = xhc(:,1).
loop i3=1 to nrow(xhc).
compute hat(i3,1)= xhc(i3,:)*invXtX*t(xhc(i3,:)).
end loop.
do if ( hc = 0 or hc =1).
loop i3 = 1 to k3.
compute xhc(:,i3)=xhc(:,i3)&* resid.
end loop.
end if.
do if ( hc =3 or hc =2).
loop i3=1 to k3.
compute xhc(:,i3) = ( resid &/(1-hat)&**(1/(4- hc )))&*xhc(:,i3).
end loop.
end if.
do if ( hc = 4).
compute hcmn=make(n,2,4).
compute hcmn(:,2)=(n1*hat)/k3.
loop i3= 1 to k3.
compute xhc(:,i3) = ( resid &/(1-hat)&**(rmin(hcmn)/2))&*xhc(:,i3).
end loop.
end if.
compute varb=(invXtX*t(xhc)*xhc*invXtX).
do if ( hc =1).
compute varb=(n1/(n1-ncol( x )))&*varb.
end if.
end if.
compute hclab={'se(HC0)','se(HC1)','se(HC2)','se(HC3)','se(HC4)','se'}.
compute hclab=hclab(1,( hc +1)).
compute hcflab={'F(HC0)','F(HC1)','F(HC2)','F(HC3)','F(HC4)','F'}.
compute hcflab=hcflab(1,( hc +1)).
release xhc
.
compute seb=sqrt(diag(varb)).
compute trat = b&/seb.
compute p = 2*(1-tcdf(abs(trat), (dfres))).
compute tval = sqrt(dfres* (exp((dfres-(5/6))*((xp2/(dfres-(2/3)+(.11/dfres)))* (xp2/(dfres-(2/3)+(.11/dfres)))))-1)).
compute modres={modres,seb,trat,p}.
compute modres={modres,(b-tval&*seb),(b+tval&*seb)}.
compute modresl={'coeff',hclab,'t','p','LLCI','ULCI'}.
compute lmat = ident(ncol( x )).
compute lmat = lmat(:,2:ncol(lmat)).
compute fratio = (t(t(lmat)*b)*inv(t(lmat)*varb*lmat)*((t(lmat)*b)))/(ncol( x )-1).
compute pfr = 1-fcdf(fratio,(ncol( x )-1),dfres).
compute modsum={sqrt(r2),r2,mse,fratio,(ncol( x )-1),dfres,pfr}.
compute modsuml={'R','R-sq','MSE',hcflab,'df1','df2', 'p'}.
end if.
end if.
do if ( 1 = 2 or 1 =3).
compute xlp= x.
compute ylp= y.
compute pt2 = make(nrow(ylp),1,(csum(ylp)/nrow(ylp))).
do if ( 1 =2).
compute LL3 = ylp&*ln(pt2)+(1-ylp)&*ln(1-pt2).
end if.
compute LL3 = -2*csum(LL3).
compute bt1 = make(ncol(xlp),1,0).
compute LL1 = 0.
compute pt1 = make(nrow(ylp),1,0.5).
compute pt1lp=pt1.
loop jjj = 1 to iterate.
compute xlptmp=t(xlp).
compute vecprb=pt1lp&*(1-pt1lp).
loop kkk=1 to ncol(xlp).
compute xlptmp(kkk,:)=xlptmp(kkk,:)&*t(vecprb).
end loop.
compute b = bt1+inv(xlptmp*xlp)*t(xlp)*(ylp-pt1lp).
do if ( 1 =2).
compute xlpb=xlp*b.
compute xlpbt=(xlpb > -709.7).
compute xlpb709=(1-xlpbt)*(-709.7).
compute xlpb=(xlpb&*xlpbt)+xlpb709.
compute pt1lp = 1/(1+exp(-(xlpb))).
end if.
compute itprob = csum((pt1lp < .00000001) or (pt1lp > .9999999)).
do if (itprob > 0).
loop kkk = 1 to nrow(pt1lp).
do if (pt1lp(kkk,1) > .9999999).
compute pt1lp(kkk,1) = .9999999.
end if.
do if (pt1lp(kkk,1) < .00000001).
compute pt1lp(kkk,1) = .00000001.
end if.
end loop.
compute itprob = 0.
end if.
do if (itprob = 0).
do if ( 1 =2).
compute LL = ylp&*ln(pt1lp)+(1-ylp)&*ln(1-pt1lp).
end if.
compute LL2 = -2*csum(ll).
end if.
do if (abs(LL1-LL2) < converge).
do if ( 0 =1).
compute xlptmp=t(xlp).
compute vecprb=pt1lp&*(1-pt1lp).
loop kkk=1 to ncol(xlp).
compute xlptmp(kkk,:)=xlptmp(kkk,:)&*t(vecprb).
end loop.
compute varb = inv(xlptmp*xlp).
compute seb = sqrt(diag(varb)).
release xlptmp.
end if.
break.
end if.
compute bt1 = b.
compute LL1 = LL2.
end loop.
compute modres=b.
do if (jjj > iterate).
compute itprob = 2.
do if (booting=0).
compute iterrmod=1.
end if.
do if (booting=1).
compute bootiter=1.
end if.
do if (itprobtg=0).
compute itprobtg=1.
compute errcode(errs,1) = 47.
compute errs = errs + 1.
do if (booting = 0 and 0 =1).
compute vt1 = mdiag(pt1lp&*(1-pt1lp)).
compute varb = inv(t(xlp)*vt1*xlp).
compute seb = sqrt(diag(varb)).
end if.
end if.
end if.
do if ( 0 =1).
compute trat = b&/seb.
compute dfres=nrow(xlp).
compute p = 2*(1-cdfnorm(abs(trat))).
compute modres={modres,seb,trat,p}.
compute modres={modres,(b-xp2&*seb),(b+xp2&*seb)}.
compute pvchi=1-chicdf((LL3-LL2),(nrow(modres)-1)).
compute mcF = (LL3-LL2)/LL3.
compute cox = 1-exp(-(LL3-LL2)/nrow(xlp)).
compute nagel = cox/(1-exp(-(LL3)/nrow(xlp))).
compute modsum={LL2,(LL3-LL2),(nrow(modres)-1),pvchi, mcF,cox,nagel}.
compute modsuml={'-2LL','ModelLL', 'df', 'p', 'McFadden', 'CoxSnell', 'Nagelkrk'}.
compute modresl={'coeff','se','Z','p','LLCI','ULCI'}.
end if.
end if
.
end if.
do if (ydich=1 and (i = (nms+nys))).

.
do if ( 2 =1).
compute hatmat=inv(t( x )* x )*t( x ).
compute b = hatmat* y.
compute modres=b.
do if ( 0 =1).
compute n1=nrow( x ).
compute dfres=n1-(ncol( x )).
compute sstotal = t( y -(csum( y )/n1))*( y -(csum( y )/n1)).
compute resid= y - x *b.
compute ssresid = csum((resid)&**2).
compute r2 = (sstotal-ssresid)/sstotal.
do if (r2 < 0).
compute r2=0.
end if.
compute adjr2 = 1-((1-r2)*(n1-1)/(dfres)).
compute mse=ssresid/(n1-ncol( x )).

.
compute n1=nrow( x ).
compute invXtX = inv(t( x )* x ).
compute varb = mse *invXtX.
compute k3 = ncol( x ).
compute xhc=0.
do if ( hc <> 5).
compute xhc= x.
compute hat = xhc(:,1).
loop i3=1 to nrow(xhc).
compute hat(i3,1)= xhc(i3,:)*invXtX*t(xhc(i3,:)).
end loop.
do if ( hc = 0 or hc =1).
loop i3 = 1 to k3.
compute xhc(:,i3)=xhc(:,i3)&* resid.
end loop.
end if.
do if ( hc =3 or hc =2).
loop i3=1 to k3.
compute xhc(:,i3) = ( resid &/(1-hat)&**(1/(4- hc )))&*xhc(:,i3).
end loop.
end if.
do if ( hc = 4).
compute hcmn=make(n,2,4).
compute hcmn(:,2)=(n1*hat)/k3.
loop i3= 1 to k3.
compute xhc(:,i3) = ( resid &/(1-hat)&**(rmin(hcmn)/2))&*xhc(:,i3).
end loop.
end if.
compute varb=(invXtX*t(xhc)*xhc*invXtX).
do if ( hc =1).
compute varb=(n1/(n1-ncol( x )))&*varb.
end if.
end if.
compute hclab={'se(HC0)','se(HC1)','se(HC2)','se(HC3)','se(HC4)','se'}.
compute hclab=hclab(1,( hc +1)).
compute hcflab={'F(HC0)','F(HC1)','F(HC2)','F(HC3)','F(HC4)','F'}.
compute hcflab=hcflab(1,( hc +1)).
release xhc
.
compute seb=sqrt(diag(varb)).
compute trat = b&/seb.
compute p = 2*(1-tcdf(abs(trat), (dfres))).
compute tval = sqrt(dfres* (exp((dfres-(5/6))*((xp2/(dfres-(2/3)+(.11/dfres)))* (xp2/(dfres-(2/3)+(.11/dfres)))))-1)).
compute modres={modres,seb,trat,p}.
compute modres={modres,(b-tval&*seb),(b+tval&*seb)}.
compute modresl={'coeff',hclab,'t','p','LLCI','ULCI'}.
compute lmat = ident(ncol( x )).
compute lmat = lmat(:,2:ncol(lmat)).
compute fratio = (t(t(lmat)*b)*inv(t(lmat)*varb*lmat)*((t(lmat)*b)))/(ncol( x )-1).
compute pfr = 1-fcdf(fratio,(ncol( x )-1),dfres).
compute modsum={sqrt(r2),r2,mse,fratio,(ncol( x )-1),dfres,pfr}.
compute modsuml={'R','R-sq','MSE',hcflab,'df1','df2', 'p'}.
end if.
end if.
do if ( 2 = 2 or 2 =3).
compute xlp= x.
compute ylp= y.
compute pt2 = make(nrow(ylp),1,(csum(ylp)/nrow(ylp))).
do if ( 2 =2).
compute LL3 = ylp&*ln(pt2)+(1-ylp)&*ln(1-pt2).
end if.
compute LL3 = -2*csum(LL3).
compute bt1 = make(ncol(xlp),1,0).
compute LL1 = 0.
compute pt1 = make(nrow(ylp),1,0.5).
compute pt1lp=pt1.
loop jjj = 1 to iterate.
compute xlptmp=t(xlp).
compute vecprb=pt1lp&*(1-pt1lp).
loop kkk=1 to ncol(xlp).
compute xlptmp(kkk,:)=xlptmp(kkk,:)&*t(vecprb).
end loop.
compute b = bt1+inv(xlptmp*xlp)*t(xlp)*(ylp-pt1lp).
do if ( 2 =2).
compute xlpb=xlp*b.
compute xlpbt=(xlpb > -709.7).
compute xlpb709=(1-xlpbt)*(-709.7).
compute xlpb=(xlpb&*xlpbt)+xlpb709.
compute pt1lp = 1/(1+exp(-(xlpb))).
end if.
compute itprob = csum((pt1lp < .00000001) or (pt1lp > .9999999)).
do if (itprob > 0).
loop kkk = 1 to nrow(pt1lp).
do if (pt1lp(kkk,1) > .9999999).
compute pt1lp(kkk,1) = .9999999.
end if.
do if (pt1lp(kkk,1) < .00000001).
compute pt1lp(kkk,1) = .00000001.
end if.
end loop.
compute itprob = 0.
end if.
do if (itprob = 0).
do if ( 2 =2).
compute LL = ylp&*ln(pt1lp)+(1-ylp)&*ln(1-pt1lp).
end if.
compute LL2 = -2*csum(ll).
end if.
do if (abs(LL1-LL2) < converge).
do if ( 0 =1).
compute xlptmp=t(xlp).
compute vecprb=pt1lp&*(1-pt1lp).
loop kkk=1 to ncol(xlp).
compute xlptmp(kkk,:)=xlptmp(kkk,:)&*t(vecprb).
end loop.
compute varb = inv(xlptmp*xlp).
compute seb = sqrt(diag(varb)).
release xlptmp.
end if.
break.
end if.
compute bt1 = b.
compute LL1 = LL2.
end loop.
compute modres=b.
do if (jjj > iterate).
compute itprob = 2.
do if (booting=0).
compute iterrmod=1.
end if.
do if (booting=1).
compute bootiter=1.
end if.
do if (itprobtg=0).
compute itprobtg=1.
compute errcode(errs,1) = 47.
compute errs = errs + 1.
do if (booting = 0 and 0 =1).
compute vt1 = mdiag(pt1lp&*(1-pt1lp)).
compute varb = inv(t(xlp)*vt1*xlp).
compute seb = sqrt(diag(varb)).
end if.
end if.
end if.
do if ( 0 =1).
compute trat = b&/seb.
compute dfres=nrow(xlp).
compute p = 2*(1-cdfnorm(abs(trat))).
compute modres={modres,seb,trat,p}.
compute modres={modres,(b-xp2&*seb),(b+xp2&*seb)}.
compute pvchi=1-chicdf((LL3-LL2),(nrow(modres)-1)).
compute mcF = (LL3-LL2)/LL3.
compute cox = 1-exp(-(LL3-LL2)/nrow(xlp)).
compute nagel = cox/(1-exp(-(LL3)/nrow(xlp))).
compute modsum={LL2,(LL3-LL2),(nrow(modres)-1),pvchi, mcF,cox,nagel}.
compute modsuml={'-2LL','ModelLL', 'df', 'p', 'McFadden', 'CoxSnell', 'Nagelkrk'}.
compute modresl={'coeff','se','Z','p','LLCI','ULCI'}.
end if.
end if
.
end if.
compute modres2={modres2,t(modres)}.
do if (i = (nms+nys)).
do if (bcmat((i+1),1) = 1).
compute bootdir={bootdir;t(modres(wherex(1,i):wherex(2,i),1))}.
end if.
do if (bcmat((i+1),1) = 0).
compute bootdir={bootdir;dirzes}.
end if.
end if.
do if (model = 74 and i <= nms).
compute onetemp=make(nrow(xprobval),1,1).
compute mestmtb={onetemp,xprobval}.
do if (ncs > 0).
compute ncovmdlb=rsum(ccmat(i,:)).
do if (ncovmdl > 0).
compute cvmncb=make(nrow(mestmtb),ncovmdlb,1).
compute cvmnctmp=csum(x(:,(ncol(x)-ncovmdlb+1):ncol(x)))/nrow(x).
do if (cuscoval > 0).
compute cvmnctmp=coval.
end if.
loop mestlp=1 to ncovmdlb.
compute cvmncb(:,mestlp)=cvmncb(:,mestlp)*cvmnctmp(:,mestlp).
end loop.
compute mestmtb={mestmtb,cvmncb}.
end if.
end if.
compute mestbt=mestmtb*modres.
do if (i = 1).
compute mestb=mestbt.
end if.
do if (i > 1).
compute mestb={mestb,mestbt}.
end if.
end if.
do if (model = 74 and i = (nms+nys)).
compute xvalptmp=make(1,nxvls,0).
compute mest74sp=make(1,nms,0).
compute mest74sp={mest74sp;mestb}.
compute xvalptmp={xvalptmp;ident(nxvls)}.
compute dirfxcf=make(nrow(mestb),1,0).
loop cfloop1=1 to nrow(mestb).
compute ndirfx=make(nrow(modres),1,0).
compute ndirfx(2:(nxvls+1),1)=t(xvalptmp(cfloop1,:)).
loop cfloop3=1 to nms.
do if (mcx=1 or mcx=0).
compute ndirfx(wheremw((1+((cfloop3-1)*2)),ncol(wheremw)):wheremw((2+((cfloop3-1)*2) ),ncol(wheremw)))=t(xvalptmp(cfloop1,:)*mestb(1,cfloop3)).
end if.
do if (mcx=2).
compute ndirfx(wheremw((1+((cfloop3-1)*2)),ncol(wheremw)):wheremw((2+((cfloop3-1)*2) ),ncol(wheremw)))=t(xvalptmp(cfloop1,:)*mest74sp(cfloop1,cfloop3)).
end if.
end loop.
do if (cfloop1 > 1).
compute dirfxcf(cfloop1,1)=t(ndirfx)*modres*xscaling.
end if.
end loop.
compute natdirbt={natdirbt;t(dirfxcf(2:nrow(dirfxcf),:))}.
end if.
do if ( (bcmat((i+1),1) = 1) and (nobootx=1) and (effsize=1)).
compute nobootx=0.
compute xsdtemp= (nrow(x)*sscp(x(:,2)))-(t(csum(x(:,2)))*(csum(x(:,2)))).
compute xsdtemp= sqrt(xsdtemp/(nrow(x)*(nrow(x)-1))).
end if.
end if.
end loop.
do if (bad = 0).
compute bootres={bootres;modres2(:,2:ncol(modres2))}.
do if (effsize=1).
compute ysdtemp= (nrow(y)*sscp(y))-(t(csum(y))*(csum(y))).
compute ysdtemp= sqrt(ysdtemp/(nrow(y)*(nrow(y)-1))).
compute bootysd={bootysd;ysdtemp}.
compute bootxsd={bootxsd;xsdtemp}.
end if.
compute goodboot=goodboot+1.
end if.
do if (bad <> 0).
compute badboot=badboot+1.
end if.
end loop if (goodboot = boot).
compute bootres=bootres(2:nrow(bootres),:).
do if (effsize=1).
compute bootysd=bootysd(2:nrow(bootysd),:).
do if (nrow(bootxsd) > 1).
compute bootxsd=bootxsd(2:nrow(bootxsd),:).
end if.
end if.
do if (goodboot < (boot)).
compute boot=0.
compute modelbt=0.
compute notecode(notes,1) = 7.
compute notes = notes + 1.
end if.
do if (boot > 0).
do if (effsize=1).
compute bootysd={ysd;bootysd}.
compute bootxsd={xsd;bootxsd}.
end if.
do if (saveboot = 1).
save bootres/outfile = *.
end if.
do if (modelbt=1).
compute bootcim=make(ncol(bootres),5,-99999).
compute bootcim(:,2) = t(csum(bootres)/nrow(bootres)).
compute bootcim(:,1) = coeffmat(2:nrow(coeffmat),1).
loop i = 1 to ncol(bootres).

.
compute temp = bootres(:,i).
compute temp(GRADE( bootres(:,i) )) = bootres(:,i).
compute badlo = 0.
compute badhi = 0.
do if ( (bootcim(i,1)*bc)+(9999*(1-bc)) <> 9999).
compute pv=csum(temp < (bootcim(i,1)*bc)+(9999*(1-bc)) )/nrow(temp).
compute ppv = pv.
do if (pv > .5).
compute ppv = 1-pv.
end if.
compute y5=sqrt(-2*ln(ppv)).
compute xp=y5+((((y5*p4+p3)*y5+p2)*y5+p1)*y5+p0)/((((y5*q4+q3)*y5+q2)*y5+q1)*y5+q0).
do if (pv <= .5).
compute xp = -xp.
end if.
compute cilow=rnd(nrow(temp)*(cdfnorm(2*xp-xp2))).
compute cihigh=trunc(nrow(temp)*(cdfnorm(2*xp+(xp2))))+1.
do if (cilow < 1).
compute cilow = 1.
compute booterr=1.
compute badlo = 1.
end if.
do if (cihigh > nrow(temp)).
compute cihigh = boot.
compute booterr=1.
compute badhi = 1.
end if.
compute llcit=temp(cilow,1).
compute ulcit=temp(cihigh,1).
do if (badlo = 1 and llcit <> priorlo).
compute badend={badend, llcit}.
compute priorlo = llcit.
end if.
do if (badhi = 1 and ulcit <> priorhi).
compute badend={badend, ulcit}.
compute priorhi = ulcit.
end if.
end if.
do if ( (bootcim(i,1)*bc)+(9999*(1-bc)) = 9999).
compute llcit=temp(cilow,1).
compute ulcit=temp(cihigh,1).
end if.
compute bootse=t(sqrt((cssq(temp)-((csum(temp)&**2)/nrow(temp)))/(nrow(temp)-1)))
.
compute bootcim(i,4:5)={llcit,ulcit}.
compute bootcim(i,3)=bootse.
end loop.
end if.
end if.
do if (badboot > 0).
compute notecode(notes,1) = 6.
compute notes = notes + 1.
end if.
end if.
do if (criterr=0).
release fulldat,x,y.
end if.
do if (xmint=1 and criterr=0).
compute xvalptmp=make(1,nxvls,0).
compute mest74sp=make(1,nms,0).
compute mest74sp={mest74sp;mest74}.
compute xvalptmp={xvalptmp;ident(nxvls)}.
loop kcfuhd=1 to 2.
compute dirfxcf=make(nrow(mest74),6,0).
loop cfloop1=1 to nrow(mest74).
compute ndirfx=make(nrow(lastb),1,0).
compute ndirfx(2:(nxvls+1),1)=t(xvalptmp(cfloop1,:)).
loop cfloop3=1 to nms.
do if (kcfuhd=1).
do if (mcx=1 or mcx=0).
compute ndirfx(wheremw((1+((cfloop3-1)*2)),ncol(wheremw)):wheremw((2+((cfloop3-1)*2)), ncol(wheremw)))=t(xvalptmp(cfloop1,:)*mest74(1,cfloop3)).
end if.
do if (mcx=2).
compute ndirfx(wheremw((1+((cfloop3-1)*2)),ncol(wheremw)):wheremw((2+((cfloop3-1)*2)), ncol(wheremw)))=t(xvalptmp(cfloop1,:)*mest74sp(cfloop1,cfloop3)).
end if.
end if.
do if (kcfuhd=2).
compute ndirfx(wheremw((1+((cfloop3-1)*2)),ncol(wheremw)):wheremw((2+((cfloop3-1)*2)), ncol(wheremw)))=t(xvalptmp(cfloop1,:)*medmeans(1,cfloop3)).
end if.
end loop.
do if (cfloop1 > 1).
compute dirfxcf(cfloop1,1)=t(ndirfx)*lastb*xscaling.
compute dirfxcf(cfloop1,2)=sqrt(diag(t(ndirfx)*lastcov*ndirfx))*abs(xscaling).
compute dirfxcf(cfloop1,3)=dirfxcf(cfloop1,1)/dirfxcf(cfloop1,2).
compute dirfxcf(cfloop1,4)=2*(1-tcdf(abs(dirfxcf(cfloop1,3)), dfres)).
compute dirfxcf(cfloop1,5)=dirfxcf(cfloop1,1)-tval*dirfxcf(cfloop1,2).
compute dirfxcf(cfloop1,6)=dirfxcf(cfloop1,1)+tval*dirfxcf(cfloop1,2).
end if.
end loop.
compute codireff=dirfxcf(2:nrow(dirfxcf),:).
do if (kcfuhd=1).
compute direff=codireff.
end if.
end loop.
end if.

.
do if (criterr = 0 and nms > 0).
compute paths=paths(:,2:ncol(paths)).
compute pathsw=pathsw(:,2:ncol(pathsw)).
compute pathsz=pathsz(:,2:ncol(pathsz)).
compute pathswz=pathswz(:,2:ncol(pathswz)).
compute pathsmod=pathsw+pathsz+pathswz.
compute pathsdv=pathsdv(:,2:ncol(pathsdv)).
compute pathsfoc=pathsfoc(:,2:ncol(pathsfoc)).
compute pathtype=pathtype(:,2:ncol(pathtype)).
compute anymod=(rsum(pathsmod) > 0).
compute obscoeff=obscoeff(1,2:ncol(obscoeff)).
do if (outscree=1).
do if (xmint=1).
print/title = '************************* COUNTERFACTUALLY DEFINED ************************'.
else.
print/title = ' '.
end if.
do if (dototal = 0 and alttotal=0).
print/title = '****************** DIRECT AND INDIRECT EFFECTS OF X ON Y *****************'/space=0.
end if.
do if (alttotal=1).
print/title = '************** TOTAL, DIRECT, AND INDIRECT EFFECTS OF X ON Y **************'/space=0.
end if.
end if.
do if (dototal = 1).
do if (outscree=1).
print/title = '************** TOTAL, DIRECT, AND INDIRECT EFFECTS OF X ON Y **************'/space=0.
end if.
compute totefflb(1,1)='Effect'.
do if (effsize=1).
compute toteffsz=toteff(:,1)/ysd.
do if (xdich=1 or mcx > 0).
compute totefflb={totefflb,'c_ps'}.
end if.
do if (xdich = 0 and mcx = 0).
compute toteffsz=toteffsz*xsd.
compute totefflb={totefflb,'c_cs'}.
end if.
compute toteff={toteff,toteffsz}.
end if.
compute resultm2=make(nrow(toteff),maxresm,99999).
compute resultm2(1:nrow(toteff),1:ncol(toteff))=toteff.
compute resultm={resultm;resultm2}.
do if (nxvls > 1).
compute resultm2=make(nrow(totomni),maxresm,99999).
compute resultm2(1:nrow(totomni),1:ncol(totomni))=totomni.
compute resultm={resultm;resultm2}.
compute clabtmp={'R2-chng', hcflab, 'df1','df2','p'}.
do if (outscree=1).
print toteff/title='Relative total effects of X on Y'/rnames=toteffl2/cnames=totefflb/format= F10.4.
print totomni/title='Omnibus test of total effect of X on Y'/cnames=clabtmp/format= F10.4.
print/title= '----------'/space=0.
end if.
else.
do if (outscree=1).
print toteff/title='Total effect of X on Y'/cnames=totefflb/format= F10.4.
end if.
end if.
end if.
compute moddir=wcmat(nrow(bcmat),1)+zcmat(nrow(bcmat),1).
do if (xmint=1).
compute moddir=1.
end if.
do if (bcmat(nrow(bcmat),1)=1).
do if (ydich=1).
compute direfflb(:,(ncol(direfflb)-5):ncol(direfflb))={'Effect','se','Z','p','LLCI','ULCI'}.
end if.
do if (moddir=0 or xmint=1).
compute direfflb(1,1)='Effect'.
end if.
do if (effsize=1 and moddir=0 and anymod = 0).
compute direffsz=direff(:,1)/ysd.
do if (xdich=1 or mcx > 0).
compute direfflb={direfflb,'c''_ps'}.
end if.
do if (xdich = 0 and mcx = 0).
compute direffsz=direffsz*xsd.
compute direfflb={direfflb,'c''_cs'}.
end if.
compute direff={direff,direffsz}.
end if.
compute resultm2=make(nrow(direff),maxresm,99999).
compute resultm2(1:nrow(direff),1:ncol(direff))=direff.
compute resultm={resultm;resultm2}.
do if (xmint=1).
compute resultm2=make(nrow(codireff),maxresm,99999).
compute resultm2(1:nrow(codireff),1:ncol(codireff))=codireff.
compute resultm={resultm;resultm2}.
end if.
do if (moddir = 0 and nxvls=1).
do if (outscree=1).
print direff/title='Direct effect of X on Y'/cnames=direfflb/format= F10.4.
end if.
end if.
do if (moddir = 0 and nxvls>1).
compute resultm2=make(nrow(diromni),maxresm,99999).
compute resultm2(1:nrow(diromni),1:ncol(diromni))=diromni.
compute resultm={resultm;resultm2}.
do if (outscree=1).
print direff/title='Relative direct effects of X on Y'/rnames=direffl2/cnames=direfflb/format= F10.4.
do if (ydich = 0).
compute clabtmp={'R2-chng', hcflab, 'df1','df2','p'}.
print diromni/title='Omnibus test of direct effect of X on Y:'/cnames=clabtmp/format= F10.4.
end if.
do if (ydich = 1).
compute clabtmp={'Chi-sq', 'df', 'p'}.
print diromni/title='Omnibus likelihood ratio test of direct effect of X on Y'/cnames=clabtmp/format= F10.4.
end if.
print/title= '----------'/space=0.
end if.
end if.
do if (moddir > 0 and nxvls=1).
do if (xmint=0 and outscree=1).
print direff/title='Conditional direct effects of X on Y'/cnames=direfflb/format= F10.4.
end if.
do if (xmint=1).
do if (outscree=1).
print direff/title='(Pure) Natural direct effect of X on Y'/cnames=direfflb/format= F10.4.
print codireff/title='Controlled direct effect of X on Y'/cnames=direfflb/format= F10.4.
print/title= '----------'/space=0.
end if.
compute obnatdfx=direff(:,1).
end if.
end if.
compute direffl4=direffl2.
do if (moddir > 0 and nxvls>1).
compute direffl2={' '}.
loop i = 1 to nxvls.
loop j = 1 to (nrow(direff)/nxvls).
compute direffl2={direffl2;xcatlab(i,1)}.
end loop.
end loop.
compute direffl2=direffl2(2:nrow(direffl2),1).
do if (xmint=0 and outscree=1).
print direff/title='Relative conditional direct effects of X on Y'/cnames=direfflb/rnames=direffl2/format= F10.4.
end if.
do if (xmint=1).
do if (outscree=1).
print direff/title='Relative (pure) natural direct effects of X on Y'/cnames=direfflb/rnames=direffl2/format= F10.4.
print codireff/title='Relative controlled direct effects of X on Y'/cnames=direfflb/rnames=direffl2/format= F10.4.
print/title= '----------'/space=0.
end if.
compute obnatdfx=direff(:,1).
end if.
end if.
compute direffl2=direffl4.
end if.
do if (bcmat(nrow(bcmat),1)=0 and xmint <> 1 and outscree=1).
print/title='The direct effect of X on Y is fixed to zero.'.
end if.
do if (nms = 1).
compute indmark={2}.
compute indsets={1,2}.
end if.
do if (nms = 2).
compute indmark={2,2,3}.
compute indsets={1,4,2,5,1,3,5}.
compute thetam=1.
end if.
do if (nms = 3).
compute indmark={2,2,2,3,3,3,4}.
compute indsets={1,7,2,8,4,9,1,3,8,1,5,9,2,6,9,1,3,6,9}.
compute thetam={1,2,3}.
end if.
do if (nms = 4).
compute indmark={2,2,2,2,3,3,3,3,3,3,4,4,4,4,5}.
compute indsets={1,11,2,12,4,13,7,14,1,3,12,1,5,13,1,8,14,2,6,13,2,9,14,4,10,14,1,3,6,13,1,3,9, 14,1,5,10,14,2,6,10,14,1,3,6,10,14}.
compute thetam={1,2,4,3,5,6}.
end if.
do if (nms = 5).
compute indmark={2,2,2,2,2,3,3,3,3,3,3,3,3,3,3,4,4,4,4,4,4,4,4,4,4,5,5,5,5,5,6}.
compute indsets={1,16,2,17,4,18,7,19,11,20,1,3,17,1,5,18,1,8,19,1,12,20,2,6,18,2,9,19,2,13,20,4, 10,19,4,14,20,7,15,20,1,3,6,18, 1,3,9,19,1,3,13,20,1,5,10,19,1,5,14,20,1,8,15,20,2,6,10,19,2,6, 14,20,2,9,15,20,4,10,15,20,1,3,6,10,19, 1,3,6,14,20,1,3,9,15,20,1,5,10,15,20,2,6,10,15,20,1,3,6,10,15, 20}.
compute thetam={1,2,5,3,6,8,4,7,9,10}.
end if.
do if (nms = 6).
compute indmark={2,2,2,2,2,2,3,3,3,3,3,3,3,3,3,3,3,3,3,3,3,4,4,4,4,4,4,4,4,4,4,4,4,4,4,4,4,4,4, 4,4,5,5,5,5,5,5,5,5,5,5,5,5,5,5,5,6,6,6,6,6,6,7}.
compute indsets={1,22,2,23,4,24,7,25,11,26,16,27,1,3,23,1,5,24,1,8,25,1,12,26,1,17,27,2,6,24,2, 9,25,2,13,26,2,18,27,4,10,25,4,14,26, 4,19,27,7,15,26,7,20,27,11,21,27,1,3,6,24,1,3,9,25,1,3,13,26,1, 3,18,27,1,5,10,25,1,5,14,26,1,5,19,27,1,8,15,26,1,8,20,27, 1,12,21,27,2,6,10,25,2,6,14,26,2,6,19,27,2,9,15,26,2,9,20,27,2, 13,21,27,4,10,15,26,4,10,20,27,4,14,21,27,7,15,21,27, 1,3,6,10,25,1,3,6,14,26,1,3,6,19,27,1,3,9,15,26,1,3,9,20,27,1,3, 13,21,27,1,5,10,15,26,1,5,10,20,27,1,5,14,21,27, 1,8,15,21,27,2,6,10,15,26,2,6,10,20,27,2,6,14,21,27,2,9,15,21, 27,4,10,15,21,27,1,3,6,10,15,26,1,3,6,10,20,27, 1,3,6,14,21,27,1,3,9,15,21,27,1,5,10,15,21,27,2,6,10,15,21,27, 1,3,6,10,15,21,27}.
compute thetam={1,2,6,3,7,10,4,8,11,13,5,9,12,14,15}.
end if.
do if (nms = 7).
compute indmark={2,2,2,2,2,2,2}.
compute indsets={1,29,2,30,4,31,7,32,11,33,16,34,22,35}.
end if.
do if (nms = 8).
compute indmark={2,2,2,2,2,2,2,2}.
compute indsets={1,37,2,38,4,39,7,40,11,41,16,42,22,43,29,44}.
end if.
do if (nms = 9).
compute indmark={2,2,2,2,2,2,2,2,2}.
compute indsets={1,46,2,47,4,48,7,49,11,50,16,51,22,52,29,53,37,54}.
end if.
do if (nms=10).
compute indmark={2,2,2,2,2,2,2,2,2,2}.
compute indsets={1,56,2,57,4,58,7,59,11,60,16,61,22,62,29,63,37,64,46,65}.
end if.
compute indlbl = {'Ind1'; 'Ind2'; 'Ind3'; 'Ind4'; 'Ind5'; 'Ind6'; 'Ind7'; 'Ind8'; 'Ind9'; 'Ind10'; 'Ind11'; 'Ind12'; 'Ind13'; 'Ind14'; 'Ind15'}.
compute indlbl = {indlbl; 'Ind16';'Ind17';'Ind18';'Ind19';'Ind20';'Ind21';'Ind22';'Ind23';'Ind24';'Ind25';'Ind26';'Ind27';'Ind28';'Ind29';'Ind30'}.
compute indlbl = {indlbl; 'Ind31';'Ind32';'Ind33';'Ind34';'Ind35';'Ind36';'Ind37';'Ind38';'Ind39';'Ind40';'Ind41';'Ind42';'Ind43';'Ind44';'Ind45'}.
compute indlbl = {indlbl; 'Ind46';'Ind47';'Ind48';'Ind49';'Ind50';'Ind51';'Ind52';'Ind53';'Ind54';'Ind55';'Ind56';'Ind57';'Ind58';'Ind59';'Ind60'}.
compute indlbl = {indlbl; 'Ind61';'Ind62';'Ind63';'Ind64';'Ind65';'Ind66';'Ind67';'Ind68';'Ind69';'Ind70';'Ind71';'Ind72';'Ind73';'Ind74';'Ind75'}.
compute indlbl = {indlbl; 'Ind76';'Ind77';'Ind78';'Ind79';'Ind80';'Ind81';'Ind82';'Ind83';'Ind84';'Ind85';'Ind86';'Ind87';'Ind88';'Ind89';'Ind90'}.
compute cntname={'(C1)';'(C2)';'(C3)';'(C4)';'(C5)';'(C6)';'(C7)';'(C8)';'(C9)';'(C10)';'(C11)';'(C12)';'(C13)';'(C14)';'(C15)';'(C16)';'(C17)'}.
compute cntname={cntname;'(C18)';'(C19)';'(C20)';'(C21)';'(C22)';'(C23)';'(C24)';'(C25)';'(C26)';'(C27)';'(C28)';'(C29)';'(C30)';'(C31)'}.
compute cntname={cntname;'(C32)';'(C33)';'(C34)';'(C35)';'(C36)';'(C37)';'(C38)';'(C39)';'(C40)';'(C41)';'(C42)';'(C43)';'(C44)';'(C45)'}.
compute cntname={cntname;'(C46)';'(C47)';'(C48)';'(C49)';'(C50)';'(C51)';'(C52)';'(C53)';'(C54)';'(C55)';'(C56)';'(C57)';'(C58)';'(C59)'}.
compute cntname={cntname;'(C60)';'(C61)';'(C62)';'(C63)';'(C64)';'(C65)';'(C66)';'(C67)';'(C68)';'(C69)';'(C70)';'(C71)';'(C72)';'(C73)'}.
compute cntname={cntname;'(C74)';'(C75)';'(C76)';'(C77)';'(C78)';'(C79)';'(C80)';'(C81)';'(C82)';'(C83)';'(C84)';'(C85)';'(C86)';'(C87)'}.
compute cntname={cntname;'(C88)';'(C89)';'(C90)';'(C91)';'(C92)';'(C93)';'(C94)';'(C95)';'(C96)';'(C97)';'(C98)';'(C99)';'(C100)';'(C101)'}.
compute cntname={cntname;'(C102)';'(C103)';'(C104)';'(C105)'}.
compute indmake=make(ncol(indmark),(nms+2),0).
compute indmod=make(ncol(indmark),1,999).
compute indmmm=make(ncol(indmark),1,0).
compute indmmmt=make(ncol(indmark),1,0).
compute start=1.
compute end=0.
compute nindfx=0.
compute indlocs=make(nrow(thetaxmb),ncol(paths),999).
compute indkey=make(ncol(indmark),1+((rmax(indmark)*2)+1),'     ').
compute c1=1.
compute c2=1.
compute c3=1.
loop i = 1 to ncol(paths).
do if (pathtype(1,i)=1).
compute indlocs(:,i)=thetaxmb(:,c1).
compute c1=c1+1.
end if.
do if (pathtype(1,i)=3).
compute indlocs(:,i)=thetamyb(:,c2).
compute c2=c2+1.
end if.
do if (pathtype(1,i)=2 and nms < 7 and serial=1).
compute indlocs(:,i)=thetammb(:,thetam(1,c3)).
compute c3=c3+1.
end if.
end loop.
loop i = 1 to ncol(indlocs).
compute c1=2.
loop j = 2 to nrow(indlocs).
do if (indlocs(j,i) <> 0).
compute indlocs(c1,i)=indlocs(j,i).
compute c1=c1+1.
end if.
end loop.
compute indlocs(1,i)=c1-2.
end loop.
compute indlocs=indlocs(1:rmax((indlocs(1,:))+1),:).
loop i = 1 to ncol(indmark).
compute numget=indmark(1,i).
compute end=end+numget.
compute gotcha=indsets(1,start:end).
compute start=end+1.
compute ok=1.
compute temp=0.
compute repoman=make(4,1,0).
loop j = 1 to ncol(gotcha).
do if paths(1,gotcha(1,j))=0.
compute ok=0.
end if.
do if (pathsmod(1,gotcha(1,j)) > 0).
compute temp=1.
compute temp2={pathsw(1,gotcha(1,j));pathsz(1,gotcha(1,j));pathswz(1,gotcha(1,j));0}.
do if (temp2(1,1)=1 and temp2(2,1)=1 and temp2(3,1)=0).
compute temp2(4,1)=1.
end if.
compute repoman=repoman+temp2.
end if.
end loop.
compute temp=0.
compute tempmmm=0.
compute typemmm=0.
do if ((repoman(1,1) > 0) and (repoman(2,1) = 0)).
compute temp=1.
do if (repoman(1,1)=1).
compute tempmmm=1.
end if.
do if (repoman(1,1) > 1 and (wdich=1 or mcw > 0)).
compute tempmmm=12.
compute typemmm=mcw.
do if (wdich=1).
compute typemmm=1.
end if.
end if.
do if (repoman(1,1) > 1 and (wdich=0 and mcw = 0)).
compute tempmmm=101.
end if.
end if.
do if ((repoman(1,1) = 0) and (repoman(2,1) > 0)).
compute temp=2.
do if (repoman(2,1)=1).
compute tempmmm=2.
end if.
do if (repoman(2,1) > 1 and (zdich = 1 or mcz > 0)).
compute tempmmm=22.
compute typemmm=mcz.
do if (zdich=1).
compute typemmm=1.
end if.
end if.
do if (repoman(2,1) > 1 and (zdich = 0 and mcw = 0)).
compute tempmmm=102.
end if.
end if.
do if (repoman(1,1)>0 and repoman(2,1)>0).
compute temp=3.
do if (repoman(1,1)=1 and repoman(2,1)=1).
do if (repoman(4,1)=1).
compute tempmmm=31.
end if.
do if (repoman(3,1)=1).
compute tempmmm=41.
end if.
end if.
end if.
do if (repoman(1,1)=1 and repoman(2,1)=1 and repoman(3,1)=0 and repoman(4,1)=0).
compute tempmmm=51.
end if.
do if (ok = 1).
compute nindfx=nindfx+1.
compute indmake(nindfx,1)=numget.
compute indmod(nindfx,1)=temp.
compute indmmm(nindfx,1)=tempmmm.
compute indmmmt(nindfx,1)=typemmm.
compute indmake(nindfx,2:(1+numget))=gotcha.
compute indkey(nindfx,1)=xnames.
loop j = 1 to numget.
compute indkey(nindfx,(j*2+1))=pathsdv(1,gotcha(1,j)).
compute indkey(nindfx,(j*2))='   ->   '.
end loop.
end if.
end loop.
compute indkey=indkey(1:nindfx,1:((cmax(indmake(:,1))*2)+1)).
compute indmake=indmake(1:nindfx,1:(cmax(indmake(:,1))+1)).
compute indmod=indmod(1:nrow(indmake),1).
compute indmmm=indmmm(1:nrow(indmake),1).
compute indmmmt=indmmmt(1:nrow(indmake),1).
compute ncpairs = (((nindfx)*(nindfx-1))/2).
do if ((contrast = 1 or contrast = 2 or contrast=3) and (ncpairs > 105)).
compute contrast=0.
compute notecode(notes,1) = 13.
compute notes = notes + 1.
end if.
do if (contrast = 4).
do if (ncol(contvec) <> nindfx).
compute contrast=0.
compute notecode(notes,1) = 14.
compute notes = notes + 1.
end if.
end if.
do if (anymod=0).
do if (nms = 1 and contrast > 0).
compute contrast=0.
end if.
compute efloop=(((1-(effsize=0))*2)+1)-(((mcx>0 or xdich=1))*(1-(effsize=0))).
loop kk=1 to efloop.
do if (boot = 0).
compute bootres=obscoeff.
do if (kk=1).
compute totbtvec=make(1,nxvls,0).
end if.
compute bootdir=obsdirfx.
compute indtab=999.
compute inddiff=999.
compute bootysd=ysd.
compute bootxsd=xsd.
end if.
do if (boot > 0).
compute bootres={obscoeff;bootres}.
do if (kk=1).
compute totbtvec=make(nrow(bootres),nxvls,0).
end if.
compute indtab=make(1,4,999).
compute inddiff=make(nrow(bootres),1,999).
end if.
compute indtotal=make(nrow(bootres),1,0).
loop i = 1 to nrow(indmake).
loop j = 1 to nxvls.
compute indtemp=make(nrow(bootres),1,1).
loop k = 1 to indmake(i,1).
compute jtemp=1.
do if (j > 1 and k=1).
compute jtemp=j.
end if.
compute indtemp=indtemp&*bootres(:,pathsfoc(jtemp,indmake(i,(k+1)))).
end loop.
do if (kk = 2).
compute indtemp=indtemp/bootysd.
end if.
do if (kk = 3).
compute indtemp=(bootxsd&*indtemp)/bootysd.
end if.
do if (contrast <> 0).
compute inddiff={inddiff,indtemp}.
end if.
do if (nxvls=1).
compute indtotal=indtotal+indtemp.
end if.
compute indeff=indtemp(1,1).
do if (kk=1).
compute totbtvec(:,j)=totbtvec(:,j)+indtemp.
end if.
do if (boot > 0).

.
compute temp = indtemp(2:nrow(indtemp),1).
compute temp(GRADE( indtemp(2:nrow(indtemp),1) )) = indtemp(2:nrow(indtemp),1).
compute badlo = 0.
compute badhi = 0.
do if ( (indtemp(1,1)*bc)+(9999*(1-bc)) <> 9999).
compute pv=csum(temp < (indtemp(1,1)*bc)+(9999*(1-bc)) )/nrow(temp).
compute ppv = pv.
do if (pv > .5).
compute ppv = 1-pv.
end if.
compute y5=sqrt(-2*ln(ppv)).
compute xp=y5+((((y5*p4+p3)*y5+p2)*y5+p1)*y5+p0)/((((y5*q4+q3)*y5+q2)*y5+q1)*y5+q0).
do if (pv <= .5).
compute xp = -xp.
end if.
compute cilow=rnd(nrow(temp)*(cdfnorm(2*xp-xp2))).
compute cihigh=trunc(nrow(temp)*(cdfnorm(2*xp+(xp2))))+1.
do if (cilow < 1).
compute cilow = 1.
compute booterr=1.
compute badlo = 1.
end if.
do if (cihigh > nrow(temp)).
compute cihigh = boot.
compute booterr=1.
compute badhi = 1.
end if.
compute llcit=temp(cilow,1).
compute ulcit=temp(cihigh,1).
do if (badlo = 1 and llcit <> priorlo).
compute badend={badend, llcit}.
compute priorlo = llcit.
end if.
do if (badhi = 1 and ulcit <> priorhi).
compute badend={badend, ulcit}.
compute priorhi = ulcit.
end if.
end if.
do if ( (indtemp(1,1)*bc)+(9999*(1-bc)) = 9999).
compute llcit=temp(cilow,1).
compute ulcit=temp(cihigh,1).
end if.
compute bootse=t(sqrt((cssq(temp)-((csum(temp)&**2)/nrow(temp)))/(nrow(temp)-1)))
.
compute indeff={indeff,bootse,llcit,ulcit}.
end if.
do if (kk=1).
compute indtabn={indtab;indeff}.
end if.
compute indtab={indtab;indeff}.
end loop.
end loop.
compute indtab=indtab(2:nrow(indtab),:).
do if (kk=1).
compute indtabn=indtabn(2:nrow(indtabn),:).
end if.
compute rowlbs=indlbl(1:nrow(indtab),1).
do if (mc > 0).
compute inddiff=make(mc,1,-999).
compute indtab2=make(nrow(indtab),4,-999).
compute indtab2(:,1)=indtab.
compute indtab=indtab2.
release indtab2.
compute mcct=0.
compute indtotal=make(mc,1,0).
do if (kk = 1).
compute x1 = sqrt(-2*ln(uniform(mc,nrow(mcsopath))))&*cos((2*3.14159265358979)*uniform(mc, nrow(mcsopath))).
compute x1=x1*chol(indcov).
loop ii=1 to nrow(x1).
compute x1(ii,:)=x1(ii,:)+t(mcsopath).
end loop.
end if.
loop ii=1 to nms.
compute tmpb=x1(:,((nms*nxvls)+ii)).
compute tmpb2=tmpb.
do if (nxvls > 1).
loop jj=1 to (nxvls-1).
compute tmpb2={tmpb2,tmpb}.
end loop.
end if.
compute indtemp=x1(:,(((ii-1)*nxvls)+1):(ii*nxvls))&*tmpb2.
loop jj=1 to ncol(indtemp).
do if (kk = 2).
compute indtemp(:,jj)=indtemp(:,jj)/ysd.
end if.
do if (kk = 3).
compute indtemp(:,jj)=(xsd*indtemp(:,jj))/ysd.
end if.

.
compute temp = indtemp(:,jj).
compute temp(GRADE( indtemp(:,jj) )) = indtemp(:,jj).
compute badlo = 0.
compute badhi = 0.
do if ( 9999 <> 9999).
compute pv=csum(temp < 9999 )/nrow(temp).
compute ppv = pv.
do if (pv > .5).
compute ppv = 1-pv.
end if.
compute y5=sqrt(-2*ln(ppv)).
compute xp=y5+((((y5*p4+p3)*y5+p2)*y5+p1)*y5+p0)/((((y5*q4+q3)*y5+q2)*y5+q1)*y5+q0).
do if (pv <= .5).
compute xp = -xp.
end if.
compute cilow=rnd(nrow(temp)*(cdfnorm(2*xp-xp2))).
compute cihigh=trunc(nrow(temp)*(cdfnorm(2*xp+(xp2))))+1.
do if (cilow < 1).
compute cilow = 1.
compute booterr=1.
compute badlo = 1.
end if.
do if (cihigh > nrow(temp)).
compute cihigh = boot.
compute booterr=1.
compute badhi = 1.
end if.
compute llcit=temp(cilow,1).
compute ulcit=temp(cihigh,1).
do if (badlo = 1 and llcit <> priorlo).
compute badend={badend, llcit}.
compute priorlo = llcit.
end if.
do if (badhi = 1 and ulcit <> priorhi).
compute badend={badend, ulcit}.
compute priorhi = ulcit.
end if.
end if.
do if ( 9999 = 9999).
compute llcit=temp(cilow,1).
compute ulcit=temp(cihigh,1).
end if.
compute bootse=t(sqrt((cssq(temp)-((csum(temp)&**2)/nrow(temp)))/(nrow(temp)-1)))
.
compute mcct=mcct+1.
compute indtab(mcct,2:4)={bootse,llcit,ulcit}.
end loop.
do if (nxvls=1).
compute indtotal=indtotal+indtemp.
do if (contrast <> 0).
compute inddiff={inddiff,indtemp}.
end if.
end if.
end loop.
release indtemp,tmpb.
end if.
do if (normal = 1 and sobelok=1).
compute sobelmat=indtab(:,1).
compute sobelmat={sobelmat,(sobelmat/2),sobelmat,sobelmat}.
loop ii=1 to nms.
compute se2b=indcov(((nms*nxvls)+ii),((nms*nxvls)+ii)).
compute bpath2=mcsopath(((nms*nxvls)+ii),1)&**2.
compute se2a=diag(indcov((((ii-1)*nxvls)+1):(ii*nxvls),(((ii-1)*nxvls)+1):(ii*nxvls))).
compute apath2=mcsopath((((ii-1)*nxvls)+1):(ii*nxvls) ,1)&**2.
compute sesobel=sqrt(apath2*se2b+bpath2*se2a+se2a*se2b).
compute sobelmat((((ii-1)*nxvls)+1):(ii*nxvls),2)=sesobel.
end loop.
release se2b,bpath2,se2a,apath2,sesobel,ii.
compute sobelmat(:,3)=sobelmat(:,1)&/sobelmat(:,2).
compute sobelmat(:,4) = 2*(1-cdfnorm(abs(sobelmat(:,3)))).
end if.
do if (serial = 0).
compute rowlbs=t(mnames).
end if.
do if (nxvls=1 and nms > 1).
compute rowlbs={'TOTAL';rowlbs}.
compute indtemp=indtotal(1,1).
do if (boot > 0 and nxvls=1).

.
compute temp = indtotal(2:nrow(indtotal),1).
compute temp(GRADE( indtotal(2:nrow(indtotal),1) )) = indtotal(2:nrow(indtotal),1).
compute badlo = 0.
compute badhi = 0.
do if ( (indtotal(1,1)*bc)+(9999*(1-bc)) <> 9999).
compute pv=csum(temp < (indtotal(1,1)*bc)+(9999*(1-bc)) )/nrow(temp).
compute ppv = pv.
do if (pv > .5).
compute ppv = 1-pv.
end if.
compute y5=sqrt(-2*ln(ppv)).
compute xp=y5+((((y5*p4+p3)*y5+p2)*y5+p1)*y5+p0)/((((y5*q4+q3)*y5+q2)*y5+q1)*y5+q0).
do if (pv <= .5).
compute xp = -xp.
end if.
compute cilow=rnd(nrow(temp)*(cdfnorm(2*xp-xp2))).
compute cihigh=trunc(nrow(temp)*(cdfnorm(2*xp+(xp2))))+1.
do if (cilow < 1).
compute cilow = 1.
compute booterr=1.
compute badlo = 1.
end if.
do if (cihigh > nrow(temp)).
compute cihigh = boot.
compute booterr=1.
compute badhi = 1.
end if.
compute llcit=temp(cilow,1).
compute ulcit=temp(cihigh,1).
do if (badlo = 1 and llcit <> priorlo).
compute badend={badend, llcit}.
compute priorlo = llcit.
end if.
do if (badhi = 1 and ulcit <> priorhi).
compute badend={badend, ulcit}.
compute priorhi = ulcit.
end if.
end if.
do if ( (indtotal(1,1)*bc)+(9999*(1-bc)) = 9999).
compute llcit=temp(cilow,1).
compute ulcit=temp(cihigh,1).
end if.
compute bootse=t(sqrt((cssq(temp)-((csum(temp)&**2)/nrow(temp)))/(nrow(temp)-1)))
.
compute indtemp={indtemp, bootse,llcit,ulcit}.
end if.
do if (mc > 0).
compute obtmc=indtab(:,1).
compute indtemp=csum(obtmc).

.
compute temp = indtotal(:,1).
compute temp(GRADE( indtotal(:,1) )) = indtotal(:,1).
compute badlo = 0.
compute badhi = 0.
do if ( 9999 <> 9999).
compute pv=csum(temp < 9999 )/nrow(temp).
compute ppv = pv.
do if (pv > .5).
compute ppv = 1-pv.
end if.
compute y5=sqrt(-2*ln(ppv)).
compute xp=y5+((((y5*p4+p3)*y5+p2)*y5+p1)*y5+p0)/((((y5*q4+q3)*y5+q2)*y5+q1)*y5+q0).
do if (pv <= .5).
compute xp = -xp.
end if.
compute cilow=rnd(nrow(temp)*(cdfnorm(2*xp-xp2))).
compute cihigh=trunc(nrow(temp)*(cdfnorm(2*xp+(xp2))))+1.
do if (cilow < 1).
compute cilow = 1.
compute booterr=1.
compute badlo = 1.
end if.
do if (cihigh > nrow(temp)).
compute cihigh = boot.
compute booterr=1.
compute badhi = 1.
end if.
compute llcit=temp(cilow,1).
compute ulcit=temp(cihigh,1).
do if (badlo = 1 and llcit <> priorlo).
compute badend={badend, llcit}.
compute priorlo = llcit.
end if.
do if (badhi = 1 and ulcit <> priorhi).
compute badend={badend, ulcit}.
compute priorhi = ulcit.
end if.
end if.
do if ( 9999 = 9999).
compute llcit=temp(cilow,1).
compute ulcit=temp(cihigh,1).
end if.
compute bootse=t(sqrt((cssq(temp)-((csum(temp)&**2)/nrow(temp)))/(nrow(temp)-1)))
.
compute indtemp={indtemp, bootse,llcit,ulcit}.
end if.
compute indtab={indtemp;indtab}.
end if.
compute bootlbs={'Effect', 'BootSE','BootLLCI','BootULCI'}.
do if (mc > 0).
compute bootlbs={'Effect', 'MC SE','MC LLCI','MC ULCI'}.
end if.
do if (nxvls = 1).
do if (contrast <> 0).
compute inddiff=inddiff(:,2:ncol(inddiff)).
do if (mc > 0).
compute inddiff={t(obtmc);inddiff}.
end if.
do if (contrast = 4).
compute inddifft=inddiff*t(contvec).
compute indtemp=inddifft(1,1).
do if (boot > 0 or mc > 0).
do if (mc > 0).

.
compute temp = inddifft(2:nrow(inddifft),1).
compute temp(GRADE( inddifft(2:nrow(inddifft),1) )) = inddifft(2:nrow(inddifft),1).
compute badlo = 0.
compute badhi = 0.
do if ( 9999 <> 9999).
compute pv=csum(temp < 9999 )/nrow(temp).
compute ppv = pv.
do if (pv > .5).
compute ppv = 1-pv.
end if.
compute y5=sqrt(-2*ln(ppv)).
compute xp=y5+((((y5*p4+p3)*y5+p2)*y5+p1)*y5+p0)/((((y5*q4+q3)*y5+q2)*y5+q1)*y5+q0).
do if (pv <= .5).
compute xp = -xp.
end if.
compute cilow=rnd(nrow(temp)*(cdfnorm(2*xp-xp2))).
compute cihigh=trunc(nrow(temp)*(cdfnorm(2*xp+(xp2))))+1.
do if (cilow < 1).
compute cilow = 1.
compute booterr=1.
compute badlo = 1.
end if.
do if (cihigh > nrow(temp)).
compute cihigh = boot.
compute booterr=1.
compute badhi = 1.
end if.
compute llcit=temp(cilow,1).
compute ulcit=temp(cihigh,1).
do if (badlo = 1 and llcit <> priorlo).
compute badend={badend, llcit}.
compute priorlo = llcit.
end if.
do if (badhi = 1 and ulcit <> priorhi).
compute badend={badend, ulcit}.
compute priorhi = ulcit.
end if.
end if.
do if ( 9999 = 9999).
compute llcit=temp(cilow,1).
compute ulcit=temp(cihigh,1).
end if.
compute bootse=t(sqrt((cssq(temp)-((csum(temp)&**2)/nrow(temp)))/(nrow(temp)-1)))
.
end if.
do if (boot > 0).

.
compute temp = inddifft(2:nrow(inddifft),1).
compute temp(GRADE( inddifft(2:nrow(inddifft),1) )) = inddifft(2:nrow(inddifft),1).
compute badlo = 0.
compute badhi = 0.
do if ( (inddifft(1,1)*bc)+(9999* (1-bc)) <> 9999).
compute pv=csum(temp < (inddifft(1,1)*bc)+(9999* (1-bc)) )/nrow(temp).
compute ppv = pv.
do if (pv > .5).
compute ppv = 1-pv.
end if.
compute y5=sqrt(-2*ln(ppv)).
compute xp=y5+((((y5*p4+p3)*y5+p2)*y5+p1)*y5+p0)/((((y5*q4+q3)*y5+q2)*y5+q1)*y5+q0).
do if (pv <= .5).
compute xp = -xp.
end if.
compute cilow=rnd(nrow(temp)*(cdfnorm(2*xp-xp2))).
compute cihigh=trunc(nrow(temp)*(cdfnorm(2*xp+(xp2))))+1.
do if (cilow < 1).
compute cilow = 1.
compute booterr=1.
compute badlo = 1.
end if.
do if (cihigh > nrow(temp)).
compute cihigh = boot.
compute booterr=1.
compute badhi = 1.
end if.
compute llcit=temp(cilow,1).
compute ulcit=temp(cihigh,1).
do if (badlo = 1 and llcit <> priorlo).
compute badend={badend, llcit}.
compute priorlo = llcit.
end if.
do if (badhi = 1 and ulcit <> priorhi).
compute badend={badend, ulcit}.
compute priorhi = ulcit.
end if.
end if.
do if ( (inddifft(1,1)*bc)+(9999* (1-bc)) = 9999).
compute llcit=temp(cilow,1).
compute ulcit=temp(cihigh,1).
end if.
compute bootse=t(sqrt((cssq(temp)-((csum(temp)&**2)/nrow(temp)))/(nrow(temp)-1)))
.
end if.
compute indtemp={indtemp, bootse,llcit,ulcit}.
end if.
compute indtab={indtab;indtemp}.
end if.
do if (contrast = 1 or contrast = 2 or contrast=3).
compute conkey=make(1,4,' ').
loop i = 1 to ncol(inddiff)-1.
loop j = (i+1) to ncol(inddiff).
compute inddifft=inddiff(:,i)-inddiff(:,j).
do if (contrast=2).
compute inddifft=abs(inddiff(:,i))-abs(inddiff(:,j)).
end if.
do if (contrast=3).
compute inddifft=inddiff(:,i)+inddiff(:,j).
end if.
compute indtemp=inddifft(1,1).
do if (contrast=1 or contrast=2).
compute conkeyt={' ', rowlbs((i+1),1),' minus  ',rowlbs((j+1),1)}.
end if.
do if (contrast=3).
compute conkeyt={' ', rowlbs((i+1),1),'  plus  ',rowlbs((j+1),1)}.
end if.
compute conkey={conkey;conkeyt}.
do if (boot > 0 or mc > 0).
do if (mc > 0).

.
compute temp = inddifft(2:nrow(inddifft),1).
compute temp(GRADE( inddifft(2:nrow(inddifft),1) )) = inddifft(2:nrow(inddifft),1).
compute badlo = 0.
compute badhi = 0.
do if ( 9999 <> 9999).
compute pv=csum(temp < 9999 )/nrow(temp).
compute ppv = pv.
do if (pv > .5).
compute ppv = 1-pv.
end if.
compute y5=sqrt(-2*ln(ppv)).
compute xp=y5+((((y5*p4+p3)*y5+p2)*y5+p1)*y5+p0)/((((y5*q4+q3)*y5+q2)*y5+q1)*y5+q0).
do if (pv <= .5).
compute xp = -xp.
end if.
compute cilow=rnd(nrow(temp)*(cdfnorm(2*xp-xp2))).
compute cihigh=trunc(nrow(temp)*(cdfnorm(2*xp+(xp2))))+1.
do if (cilow < 1).
compute cilow = 1.
compute booterr=1.
compute badlo = 1.
end if.
do if (cihigh > nrow(temp)).
compute cihigh = boot.
compute booterr=1.
compute badhi = 1.
end if.
compute llcit=temp(cilow,1).
compute ulcit=temp(cihigh,1).
do if (badlo = 1 and llcit <> priorlo).
compute badend={badend, llcit}.
compute priorlo = llcit.
end if.
do if (badhi = 1 and ulcit <> priorhi).
compute badend={badend, ulcit}.
compute priorhi = ulcit.
end if.
end if.
do if ( 9999 = 9999).
compute llcit=temp(cilow,1).
compute ulcit=temp(cihigh,1).
end if.
compute bootse=t(sqrt((cssq(temp)-((csum(temp)&**2)/nrow(temp)))/(nrow(temp)-1)))
.
end if.
do if (boot > 0).

.
compute temp = inddifft(2:nrow(inddifft),1).
compute temp(GRADE( inddifft(2:nrow(inddifft),1) )) = inddifft(2:nrow(inddifft),1).
compute badlo = 0.
compute badhi = 0.
do if ( (inddifft(1,1)*bc)+(9999* (1-bc)) <> 9999).
compute pv=csum(temp < (inddifft(1,1)*bc)+(9999* (1-bc)) )/nrow(temp).
compute ppv = pv.
do if (pv > .5).
compute ppv = 1-pv.
end if.
compute y5=sqrt(-2*ln(ppv)).
compute xp=y5+((((y5*p4+p3)*y5+p2)*y5+p1)*y5+p0)/((((y5*q4+q3)*y5+q2)*y5+q1)*y5+q0).
do if (pv <= .5).
compute xp = -xp.
end if.
compute cilow=rnd(nrow(temp)*(cdfnorm(2*xp-xp2))).
compute cihigh=trunc(nrow(temp)*(cdfnorm(2*xp+(xp2))))+1.
do if (cilow < 1).
compute cilow = 1.
compute booterr=1.
compute badlo = 1.
end if.
do if (cihigh > nrow(temp)).
compute cihigh = boot.
compute booterr=1.
compute badhi = 1.
end if.
compute llcit=temp(cilow,1).
compute ulcit=temp(cihigh,1).
do if (badlo = 1 and llcit <> priorlo).
compute badend={badend, llcit}.
compute priorlo = llcit.
end if.
do if (badhi = 1 and ulcit <> priorhi).
compute badend={badend, ulcit}.
compute priorhi = ulcit.
end if.
end if.
do if ( (inddifft(1,1)*bc)+(9999* (1-bc)) = 9999).
compute llcit=temp(cilow,1).
compute ulcit=temp(cihigh,1).
end if.
compute bootse=t(sqrt((cssq(temp)-((csum(temp)&**2)/nrow(temp)))/(nrow(temp)-1)))
.
end if.
compute indtemp={indtemp, bootse,llcit,ulcit}.
end if.
compute indtab={indtab;indtemp}.
end loop.
end loop.
end if.
release inddiff.
compute contlbs=cntname(1:(((nindfx)*(nindfx-1))/2),1).
compute rowlbs={rowlbs;contlbs}.
end if.
compute resultm2=make(nrow(indtab),maxresm,99999).
compute resultm2(1:nrow(indtab),1:ncol(indtab))=indtab.
compute resultm={resultm;resultm2}.
do if (outscree=1).
do if (kk=1).
print indtab/title = 'Indirect effect(s) of X on Y:'/rnames=rowlbs/cnames=bootlbs/format= F10.4.
end if.
do if (kk = 2 and (xdich=1 or mcx > 0)).
print indtab/title = 'Partially standardized indirect effect(s) of X on Y:'/rnames=rowlbs/cnames=bootlbs/format= F10.4.
end if.
do if (kk = 3).
print indtab/title = 'Completely standardized indirect effect(s) of X on Y:'/rnames=rowlbs/cnames=bootlbs/format= F10.4.
end if.
end if.
do if (normal=1 and sobelok=1 and kk=1).
compute resultm2=make(nrow(sobelmat),maxresm,99999).
compute resultm2(1:nrow(sobelmat),1:ncol(sobelmat))=sobelmat.
compute resultm={resultm;resultm2}.
compute sobellab={'Effect',hclab,'Z','p'}.
compute sobelrlb=rowlbs.
do if (nms > 1).
compute sobelrlb=rowlbs(2:(1+nms),1).
end if.
do if (outscree=1).
print sobelmat/title='   Normal theory test for indirect effect(s):'/cnames=sobellab/rnames=sobelrlb/format= F10.4.
end if.
end if.
do if (contrast <> 0).
do if ((contrast=1 or contrast = 2 or contrast=3) and kk=efloop ).
compute conkey=conkey(2:nrow(conkey),:).
do if (outscree=1).
print conkey/title = 'Specific indirect effect contrast definition(s):'/rnames=contlbs/format=A8.
end if.
end if.
do if (outscree=1).
do if (contrast = 4 and kk=efloop).
compute crowlbs=rowlbs(2:(nindfx+1),1).
print contvec/title = 'Specific indirect effect contrast weights:'/cnames=crowlbs/rlabels='(C1)'/format= F10.4.
end if.
do if (contrast = 2 and kk=efloop).
print/title = 'Contrasts are differences between absolute values of indirect effects'.
end if.
end if.
end if.
do if (serial = 1 and kk=efloop).
compute rowlbst=rowlbs(2:nrow(rowlbs),1).
do if (outscree=1).
print indkey/title = 'Indirect effect key:'/rnames=rowlbst/format = A8.
end if.
end if.
else.
do if (outscree=1).
do if (kk = 1).
print/title = 'Relative indirect effects of X on Y'.
end if.
do if (kk = 2).
print/title = 'Partially standardized relative indirect effect(s) of X on Y:'.
end if.
do if (kk = 3).
print/title = 'Completely standardized relative indirect effect(s) of X on Y:'.
end if.
end if.
loop i = 1 to nrow(indmake).
compute indtabsm=indtab((((i-1)*nxvls)+1):(nxvls*i),:).
compute indkeyt=indkey(i,:).
do if (outscree=1).
print indkeyt/title=' '/space=0/format=A8.
end if.
do if (bcmat(nrow(bcmat),1)=0)).
compute direffl2=xcatlab(1:nxvls,1).
end if.
compute resultm2=make(nrow(indtabsm),maxresm,99999).
compute resultm2(1:nrow(indtabsm),1:ncol(indtabsm))=indtabsm.
compute resultm={resultm;resultm2}.
do if (outscree=1).
print indtabsm/title = ' '/cnames=bootlbs/rnames=direffl2/format= F10.4 /space=0.
end if.
do if (normal=1 and sobelok=1 and kk=1).
compute sobelsm=sobelmat((((i-1)*nxvls)+1):(nxvls*i),:).
compute sobellab={'Effect',hclab,'Z','p'}.
compute resultm2=make(nrow(sobelsm),maxresm,99999).
compute resultm2(1:nrow(sobelsm),1:ncol(sobelsm))=sobelsm.
compute resultm={resultm;resultm2}.
do if (outscree=1).
print sobelsm/title='      Normal theory test for relative indirect effects:'/cnames=sobellab/rnames=direffl2/format= F10.4.
end if.
end if.
end loop.
end if.
do if (effsize = 1 and boot > 0).
compute bootres=bootres(2:nrow(bootres),:).
end if.
end loop.
do if (alttotal=1).
compute altcnms='Effect'.
compute totbtvec=totbtvec+bootdir.
compute alttotfx=t(totbtvec(1,:)).
do if (boot > 0).
compute alttotfx=make(ncol(totbtvec),4,0).
compute alttotfx(:,1)=t(totbtvec(1,:)).
loop cec=1 to ncol(totbtvec).

.
compute temp = totbtvec(2:nrow(totbtvec),cec).
compute temp(GRADE( totbtvec(2:nrow(totbtvec),cec) )) = totbtvec(2:nrow(totbtvec),cec).
compute badlo = 0.
compute badhi = 0.
do if ( (totbtvec(1,cec)*bc)+(9999* (1-bc)) <> 9999).
compute pv=csum(temp < (totbtvec(1,cec)*bc)+(9999* (1-bc)) )/nrow(temp).
compute ppv = pv.
do if (pv > .5).
compute ppv = 1-pv.
end if.
compute y5=sqrt(-2*ln(ppv)).
compute xp=y5+((((y5*p4+p3)*y5+p2)*y5+p1)*y5+p0)/((((y5*q4+q3)*y5+q2)*y5+q1)*y5+q0).
do if (pv <= .5).
compute xp = -xp.
end if.
compute cilow=rnd(nrow(temp)*(cdfnorm(2*xp-xp2))).
compute cihigh=trunc(nrow(temp)*(cdfnorm(2*xp+(xp2))))+1.
do if (cilow < 1).
compute cilow = 1.
compute booterr=1.
compute badlo = 1.
end if.
do if (cihigh > nrow(temp)).
compute cihigh = boot.
compute booterr=1.
compute badhi = 1.
end if.
compute llcit=temp(cilow,1).
compute ulcit=temp(cihigh,1).
do if (badlo = 1 and llcit <> priorlo).
compute badend={badend, llcit}.
compute priorlo = llcit.
end if.
do if (badhi = 1 and ulcit <> priorhi).
compute badend={badend, ulcit}.
compute priorhi = ulcit.
end if.
end if.
do if ( (totbtvec(1,cec)*bc)+(9999* (1-bc)) = 9999).
compute llcit=temp(cilow,1).
compute ulcit=temp(cihigh,1).
end if.
compute bootse=t(sqrt((cssq(temp)-((csum(temp)&**2)/nrow(temp)))/(nrow(temp)-1)))
.
compute alttotfx(cec,2:4)={bootse,llcit,ulcit}.
end loop.
compute altcnms={altcnms,'BootSE', 'BootLLCI','BootULCI'}.
end if.
compute resultm2=make(nrow(alttotfx),maxresm,99999).
compute resultm2(1:nrow(alttotfx),1:ncol(alttotfx))=alttotfx.
compute resultm={resultm;resultm2}.
do if (outscree=1).
do if (nxvls > 1).
print/title= '----------'/space=0.
print alttotfx/title='Relative total effects of X on Y (sum of direct and indirect effects)'/rnames=direffl2/cnames=altcnms/format= F10.4.
end if.
do if (nxvls = 1).
print alttotfx/title='Total effect of X on Y (sum of direct and indirect effects)'/cnames=altcnms/format= F10.4.
end if.
end if.
end if.
end if.
do if (anymod > 0).
do if (boot = 0).
compute bootres=obscoeff.
compute indtab=999.
end if.
do if (boot > 0).
compute bootres={obscoeff;bootres}.
compute indtab=make(1,4,999).
end if.
do if (csum((indmod > 0))=nrow(indmod)).
do if (outscree=1).
do if (nxvls > 1).
do if (xmint=0).
print/title= 'Relative conditional indirect effects of X on Y:'.
end if.
do if (xmint=1).
print/title= 'Relative (total) natural indirect effects of X on Y:'.
end if.
end if.
do if (nxvls = 1).
do if (xmint=0).
print/title= 'Conditional indirect effects of X on Y:'.
end if.
do if (xmint=1).
print/title= '(Total) Natural indirect effect(s) of X on Y:'.
end if.
end if.
end if.
end if.
do if (csum((indmod > 0)) < nrow(indmod)).
do if (outscree=1).
do if (nxvls > 1).
print/title= 'Relative conditional and unconditional indirect effects of X on Y:'.
end if.
do if (nxvls = 1).
print/title= 'Conditional and unconditional indirect effects of X on Y:'.
end if.
end if.
end if.
compute cftotfx=make(nrow(bootres),nxvls,0).
loop i = 1 to nrow(indmake).
compute indtab=indtab(1,:)*0.
do if (outscree=1).
do if (xmint=0).
print indkey(i,:)/title = 'INDIRECT EFFECT:'/format=A8.
end if.
do if (xmint=1).
print indkey(i,:)/title = ' '/format=A8/space=0.
end if.
end if.
do if (indmod(i,1)=0).
loop j = 1 to nxvls.
compute indtemp=make(nrow(bootres),1,1).
loop k = 1 to indmake(i,1).
compute jtemp=1.
do if (j > 1 and k=1).
compute jtemp=j.
end if.
compute indtemp=indtemp&*bootres(:,pathsfoc(jtemp,indmake(i,(k+1)))).
end loop.
compute indeff=indtemp(1,1).
do if (boot > 0).

.
compute temp = indtemp(2:nrow(indtemp),1).
compute temp(GRADE( indtemp(2:nrow(indtemp),1) )) = indtemp(2:nrow(indtemp),1).
compute badlo = 0.
compute badhi = 0.
do if ( (indtemp(1,1)*bc)+(9999*(1-bc)) <> 9999).
compute pv=csum(temp < (indtemp(1,1)*bc)+(9999*(1-bc)) )/nrow(temp).
compute ppv = pv.
do if (pv > .5).
compute ppv = 1-pv.
end if.
compute y5=sqrt(-2*ln(ppv)).
compute xp=y5+((((y5*p4+p3)*y5+p2)*y5+p1)*y5+p0)/((((y5*q4+q3)*y5+q2)*y5+q1)*y5+q0).
do if (pv <= .5).
compute xp = -xp.
end if.
compute cilow=rnd(nrow(temp)*(cdfnorm(2*xp-xp2))).
compute cihigh=trunc(nrow(temp)*(cdfnorm(2*xp+(xp2))))+1.
do if (cilow < 1).
compute cilow = 1.
compute booterr=1.
compute badlo = 1.
end if.
do if (cihigh > nrow(temp)).
compute cihigh = boot.
compute booterr=1.
compute badhi = 1.
end if.
compute llcit=temp(cilow,1).
compute ulcit=temp(cihigh,1).
do if (badlo = 1 and llcit <> priorlo).
compute badend={badend, llcit}.
compute priorlo = llcit.
end if.
do if (badhi = 1 and ulcit <> priorhi).
compute badend={badend, ulcit}.
compute priorhi = ulcit.
end if.
end if.
do if ( (indtemp(1,1)*bc)+(9999*(1-bc)) = 9999).
compute llcit=temp(cilow,1).
compute ulcit=temp(cihigh,1).
end if.
compute bootse=t(sqrt((cssq(temp)-((csum(temp)&**2)/nrow(temp)))/(nrow(temp)-1)))
.
compute indeff={indeff,bootse,llcit,ulcit}.
end if.
compute indtab={indtab;indeff}.
end loop.
compute resultm2=make(nrow(indtab),maxresm,99999).
compute resultm2(1:nrow(indtab),1:ncol(indtab))=indtab.
compute resultm={resultm;resultm2}.
do if (outscree=1).
do if (nxvls > 1).
compute indefflb=xcatlab(1:nxvls,1).
print indtab(2:nrow(indtab),:)/title = ' '/clabels='Effect', 'BootSE', 'BootLLCI', 'BootULCI'/rnames=indefflb/format= F10.4 /space=0.
end if.
do if (nxvls = 1).
print indtab(2:nrow(indtab),:)/title = ' '/clabels='Effect', 'BootSE', 'BootLLCI', 'BootULCI'/format= F10.4 /space=0.
end if.
end if.
end if.
do if (indmod(i,1)>0).
do if (indmod(i,1)=1).
compute indmodva=wmodvals.
compute indprova=wprobval.
compute condlbs={wnames}.
compute printw=1.
else if (indmod(i,1)=2).
compute indmodva=zmodvals.
compute indprova=zprobval.
compute condlbs={znames}.
compute printz=1.
else if (indmod(i,1)=3).
compute cntmp=1.
compute printz=1.
compute printw=1.
compute indmodva=make((nrow(wmodvals)*nrow(zmodvals)),2,999).
loop k7 = 1 to nrow(wmodvals).
loop k8 = 1 to nrow(zmodvals).
compute indmodva(cntmp,:)={wmodvals(k7,1),zmodvals(k8,1)}.
compute cntmp=cntmp+1.
end loop.
end loop.
compute condlbs={wnames,znames}.
end if.
compute condres=make(nrow(indmodva),1,999).
do if (boot > 0).
compute condres=make(nrow(indmodva),4,999).
end if.
compute condres={indmodva,condres}.
loop k4 = 1 to nxvls.
compute imm3=make(nrow(bootres),1,1).
compute imm4=make(nrow(bootres),1,1).
compute indcontr=0.
do if (indmod(i,1)=3).
compute tihsw=wprobval.
compute tihsz=zprobval.
end if.
loop k1=1 to nrow(indmodva).
compute tucker2=make(nrow(bootres),1,1).
compute imm2=make(nrow(bootres),1,1).
compute wfirst=0.
compute zfirst=0.
compute immset=0.
loop k2=1 to indmake(i,1).
compute colnumb=indmake(i,(k2+1)).
do if (k2=1).
compute wbb=make(nrow(bootres),(nwvls*nxvls),0).
compute zbb=make(nrow(bootres),(nzvls*nxvls),0).
compute wzbb=make(nrow(bootres),(nwvls*nzvls*nxvls),0).
end if.
do if (k2<>1).
compute wbb=make(nrow(bootres),nwvls,0).
compute zbb=make(nrow(bootres),nzvls,0).
compute wzbb=make(nrow(bootres),(nwvls*nzvls),0).
end if.
compute cnt=1.
compute tihs=indlocs(2:((indlocs(1,colnumb))+1),colnumb).
do if (k2 = 1).
compute focbb=tihs(1:nxvls,1).
compute focbb=bootres(:,focbb).
do if (indmmm(i,1)>0).
compute imm=focbb(:,k4).
compute condbb=imm.
end if.
compute focaddon=make(1,nxvls,0).
compute focaddon(1,k4)=1.
compute cnt=cnt+nxvls.
compute placeh=nxvls.
do if (indmod(i,1)=1).
compute tihsz=make(nrow(wprobval),(nzvls*nxvls),0).
compute tihswz=make(nrow(wprobval),(nwvls*nzvls*nxvls),0).
do if (pathsw(1,colnumb)=1).
compute temp=make(nrow(wprobval),(nxvls*nwvls),0).
loop k5 = 1 to nrow(wprobval).
loop k6=1 to nwvls.
compute temp(k5, (((k4-1)*nwvls)+k6))=wprobval(k5,k6).
end loop.
end loop.
compute indprova={temp,tihsz,tihswz}.
else.
compute indprova={wprobval,tihsz,tihswz}.
end if.
end if.
do if (indmod(i,1)=2).
compute tihsw=make(nrow(zprobval),(nwvls*nxvls),0).
compute tihswz=make(nrow(zprobval),(nwvls*nzvls*nxvls),0).
do if (pathsz(1,colnumb)=1).
compute temp=make(nrow(zprobval),(nxvls*nzvls),0).
loop k5 = 1 to nrow(zprobval).
loop k6 =1 to nzvls.
compute temp(k5,(((k4-1)*nzvls)+k6))=zprobval(k5,k6).
end loop.
end loop.
compute indprova={tihsw,temp,tihswz}.
else.
compute indprova={tihsw,zprobval,tihswz}.
end if.
end if.
do if (indmod(i,1)=3).
compute indprova=make((nrow(wprobval)*nrow(zprobval)),((ncol(wprobval)*nxvls)+ (ncol(zprobval)*nxvls)+(nwvls*nzvls*nxvls)),0).
compute cntemp=1.
loop k7=1 to nrow(wprobval).
loop k8 =1 to nrow(zprobval).
compute temp=wprobval(k7,:)*focaddon(1,k4).
compute indprova(cntemp,(((k4-1)*nwvls)+1):(k4*(nwvls)))=temp.
compute temp=zprobval(k8,:)*focaddon(1,k4).
compute indprova(cntemp, ((((k4-1)*nzvls)+1)+(nxvls*nwvls)) : ((((k4-1)*nzvls)+1)+(nxvls*nwvls)+(nzvls-1)))=temp.
compute cntemp=cntemp+1.
end loop.
end loop.
do if (pathsz(1,colnumb)=0).
compute temp=make(nrow(indprova),(ncol(zprobval)*nxvls),0).
compute indprova(:,((ncol(wprobval)*nxvls)+1):((ncol(wprobval)+ncol(zprobval))* nxvls))=temp.
end if.
do if (pathsw(1,colnumb)=0).
compute temp=make(nrow(indprova),(ncol(wprobval)*nxvls),0).
compute indprova(:,1:(ncol(wprobval)*nxvls))=temp.
end if.
do if (pathswz(1,colnumb)=1).
compute cntemp=(ncol(wprobval)*nxvls)+(ncol(zprobval)*nxvls)+((k4-1)* ncol(wprobval)*ncol(zprobval))+1.
loop k7=1 to ncol(wprobval).
loop k8=1 to ncol(zprobval).
compute indprova(:,cntemp)=indprova(:,((ncol(wprobval)*(k4-1))+k7))&* indprova(:,((((k4-1)*ncol(zprobval))+k8)+(nxvls*ncol(wprobval)))).
compute cntemp=cntemp+1.
end loop.
end loop.
end if.
end if.
end if.
do if (k2 > 1).
compute focbb=tihs(1,1).
compute focbb=bootres(:,focbb).
do if (indmmm(i,1)>0).
compute imm=focbb(:,1).
compute condbb=imm.
end if.
compute focaddon=1.
compute cnt=cnt+1.
compute placeh=1.
do if (indmod(i,1)=1).
compute tihsz=make(nrow(wprobval),nzvls,0).
compute tihswz=make(nrow(wprobval),(nwvls*nzvls),0).
compute indprova={wprobval,tihsz,tihswz}.
end if.
do if (indmod(i,1)=2).
compute tihsw=make(nrow(zprobval),nwvls,0).
compute tihswz=make(nrow(zprobval),(nwvls*nzvls),0).
compute indprova={tihsw,zprobval,tihswz}.
end if.
do if (indmod(i,1)=3).
compute indprova=make((nrow(wprobval)*nrow(zprobval)),((ncol(wprobval)+ ncol(zprobval))+(nwvls*nzvls)),0).
compute cntemp=1.
loop k7=1 to nrow(wprobval).
loop k8 =1 to nrow(zprobval).
compute indprova(cntemp,1:(ncol(wprobval)+ncol(zprobval)))={wprobval(k7,:), zprobval(k8,:)}.
compute cntemp=cntemp+1.
end loop.
end loop.
do if (pathsz(1,colnumb)=0).
compute temp=make(nrow(indprova),ncol(zprobval),0).
compute indprova(:,(ncol(wprobval)+1):(ncol(wprobval)+ncol(zprobval)))=temp.
end if.
do if (pathsw(1,colnumb)=0).
compute temp=make(nrow(indprova),ncol(wprobval),0).
compute indprova(:,1:ncol(wprobval))=temp.
end if.
do if (pathswz(1,colnumb)=1).
compute cntemp=ncol(wprobval)+ncol(zprobval)+1.
loop k7=1 to ncol(wprobval).
loop k8=1 to ncol(zprobval).
compute indprova(:,cntemp)=indprova(:,k7)&*indprova(:,(ncol(wprobval)+k8)).
compute cntemp=cntemp+1.
end loop.
end loop.
end if.
end if.
end if.
do if (pathsw(1,colnumb)) = 1.
compute wbb=tihs(cnt:(cnt+(placeh*nwvls)-1),1).
compute wbb=bootres(:,wbb).
compute immlbs2=wcatlab(1:nwvls,1).
do if (zfirst=0).
compute wfirst=1.
end if.
do if (indmmm(i,1)=1 or indmmm(i,1)=31 or indmmm(i,1)=51).
compute imm=wbb(:,1).
loop k7 = 1 to nwvls.
compute imm={imm,wbb(:,(((k4-1)*nwvls*(k2=1))+k7))}.
end loop.
compute imm=imm(:,2:ncol(imm)).
end if.
do if (indmmm(i,1)=41 or indmmm(i,1)=51).
compute condbb=make(nrow(bootres),1,0).
loop k7 = 1 to nwvls.
compute condbb={condbb,wbb(:,(((k4-1)*nwvls*(k2=1))+k7))}.
end loop.
compute condbb=condbb(:,2:ncol(condbb)).
end if.
compute cnt=cnt+(placeh*nwvls).
end if.
do if (pathsz(1,colnumb)) = 1.
compute zbb=tihs(cnt:(cnt+(placeh*nzvls)-1),1).
compute zbb=bootres(:,zbb).
do if (wfirst=0).
compute zfirst=1.
end if.
do if (indmmm(i,1) <> 31).
compute immlbs2=zcatlab(1:nzvls,1).
end if.
do if (indmmm(i,1)=2 or indmmm(i,1)=31 or indmmm(i,1)=51).
do if (indmmm(i,1)=2).
compute imm=zbb(:,1).
end if.
loop k7 = 1 to nzvls.
compute imm={imm,zbb(:,(((k4-1)*nzvls*(k2=1))+k7))}.
end loop.
do if (indmmm(i,1)=2 or indmmm(i,1)=51).
compute imm=imm(:,2:ncol(imm)).
do if (indmmm(i,1)=51).
compute condbb={condbb,imm}.
end if.
end if.
end if.
compute cnt=cnt+(placeh*nzvls).
end if.
do if (pathswz(1,colnumb)) = 1.
compute wzbb=tihs(cnt:(cnt+(placeh*nwvls*nzvls)-1),1).
compute wzbb=bootres(:,wzbb).
do if (indmmm(i,1)=41).
compute imm=wzbb(:,1).
loop k7=1 to nwvls*nzvls.
compute imm={imm,wzbb(:,(((k4-1)*nzvls*nwvls*(k2=1))+k7))}.
end loop.
end if.
do if (indmmm(i,1)=41).
compute imm=imm(:,2:ncol(imm)).
compute condbb={condbb,imm(:,(ncol(imm)-(nwvls*nzvls)+1):ncol(imm))}.
end if.
compute cnt=cnt+(placeh*nzvls*nwvls).
end if.
compute indprobe={focaddon,indprova(k1,:)}.
compute tucker={focbb,wbb,zbb,wzbb}.
loop k3=1 to ncol(indprobe).
compute tucker(:,k3)=tucker(:,k3)*indprobe(1,k3).
end loop.
compute tucker2=tucker2&*rsum(tucker).
do if (indmmm(i,1) = 1 or indmmm(i,1)=2 or indmmm(i,1)=31 or indmmm(i,1)=41 or indmmm(i,1)=51).
do if (immset=1).
do if (ncol(imm2)=1 and ncol(imm) = 1).
compute imm2=imm2&*imm.
end if.
do if (indmmm(i,1)=41 or indmmm(i,1)=51).
do if ((ncol(condbb2) > 1) and (ncol(condbb)>1)).
compute condbb2t=make(nrow(condbb2),(ncol(condbb2)*ncol(condbb)),-999999).
compute k9=1.
do if (wfirst=1).
loop k7=1 to ncol(condbb2).
loop k8 = 1 to ncol(condbb).
compute condbb2t(:,k9)=condbb2(:,k7)&*condbb(:,k8).
compute k9=k9+1.
end loop.
end loop.
end if.
do if (zfirst=1).
loop k7=1 to ncol(condbb).
loop k8 = 1 to ncol(condbb2).
compute condbb2t(:,k9)=condbb(:,k7)&*condbb2(:,k8).
compute k9=k9+1.
end loop.
end loop.
end if.
compute condbb2=condbb2t.
release condbb2t.
end if.
do if ((ncol(condbb2) > 1) and (ncol(condbb)=1)).
loop k7 = 1 to ncol(condbb2).
compute condbb2(:,k7)=condbb2(:,k7)&*condbb.
end loop.
end if.
do if ((ncol(condbb2) = 1) and (ncol(condbb)>1)).
loop k7 = 1 to ncol(condbb).
compute condbb(:,k7)=condbb2&*condbb(:,k7).
end loop.
compute condbb2=condbb.
end if.
end if.
do if (ncol(imm2) <> 1 and ncol(imm) <> 1).
compute imm2t=make(nrow(imm2),(ncol(imm2)*ncol(imm)),-999999).
compute k9=1.
do if (wfirst=1).
loop k7=1 to ncol(imm2).
loop k8 = 1 to ncol(imm).
compute imm2t(:,k9)=imm2(:,k7)&*imm(:,k8).
compute k9=k9+1.
end loop.
end loop.
end if.
do if (zfirst=1).
loop k7=1 to ncol(imm).
loop k8 = 1 to ncol(imm2).
compute imm2t(:,k9)=imm(:,k7)&*imm2(:,k8).
compute k9=k9+1.
end loop.
end loop.
end if.
compute imm2=imm2t.
release imm2t.
end if.
do if ((ncol(imm2) > 1) and (ncol(imm)=1)).
loop k7=1 to ncol(imm2).
compute imm2(:,k7)=imm2(:,k7)&*imm.
end loop.
end if.
do if ((ncol(imm2) = 1) and (ncol(imm) > 1)).
loop k7=1 to ncol(imm).
compute imm(:,k7)=imm2&*imm(:,k7).
end loop.
compute imm2=imm.
end if.
end if.
do if (immset=0).
compute imm2=imm.
do if (indmmm(i,1)=41 or indmmm(i,1)=51).
compute condbb2=condbb.
end if.
compute immset=1.
end if.
end if.
end loop.
compute indtemp=tucker2(1,1).
do if (indmmm(i,1)=12 or indmmm(i,1)=22).
compute imm3={imm3,tucker2}.
do if (k1=nrow(indmodva)).
compute imm3=imm3(:,2:ncol(imm3)).
compute immstop=ncol(imm3).
loop k8=2 to immstop.
do if (indmmmt(i,1)=1).
compute imm3={imm3,(imm3(:,k8)-imm3(:,1))}.
end if.
do if (indmmmt(i,1)=2).
compute imm3={imm3,(imm3(:,k8)-imm3(:,(k8-1)))}.
end if.
do if (indmmmt(i,1)=3).
compute imm3={imm3,((rsum(imm3(:,(k8:immstop)))/(immstop-k8+1))-imm3(:,(k8-1))) }.
end if.
do if (indmmmt(i,1)=4).
compute imm3={imm3,(imm3(:,k8)-(rsum(imm3(:,1:immstop))/immstop))}.
end if.
end loop.
do if (indmmmt(i,1)<5).
compute imm2=imm3(:,(immstop+1):ncol(imm3)).
release imm3.
end if.
end if.
end if.
do if (indmmm(i,1)>-1 and (contrast = 1 or contrast = 2 or contrast=3)).
compute imm4={imm4,tucker2}.
do if (k1=nrow(indmodva) and k1 > 1).
compute imm4=imm4(:,2:ncol(imm4)).
compute immstop=ncol(imm4).
compute condcont=make((immstop*(immstop-1)/2),6,-999).
loop k8 = 1 to (immstop-1).
loop k9 = (k8+1) to immstop.
do if (contrast=1).
compute imm4={imm4,(imm4(:,k9)-imm4(:,k8))}.
end if.
do if (contrast=2).
compute imm4={imm4,(abs(imm4(:,k9))-abs(imm4(:,k8)))}.
end if.
do if (contrast=3).
compute imm4={imm4,(imm4(:,k9)+imm4(:,k8))}.
end if.
compute condcont((ncol(imm4)-immstop),1)=imm4(1,k9).
compute condcont((ncol(imm4)-immstop),2)=imm4(1,k8).
end loop.
end loop.
compute imm4=imm4(:,(immstop+1):ncol(imm4)).
loop k8=1 to ncol(imm4).
compute condcont(k8,3)=imm4(1,k8).
do if (boot > 0).

.
compute temp = imm4(2:nrow(imm4),k8).
compute temp(GRADE( imm4(2:nrow(imm4),k8) )) = imm4(2:nrow(imm4),k8).
compute badlo = 0.
compute badhi = 0.
do if ( (imm4(1,k8)*bc)+(9999*(1-bc)) <> 9999).
compute pv=csum(temp < (imm4(1,k8)*bc)+(9999*(1-bc)) )/nrow(temp).
compute ppv = pv.
do if (pv > .5).
compute ppv = 1-pv.
end if.
compute y5=sqrt(-2*ln(ppv)).
compute xp=y5+((((y5*p4+p3)*y5+p2)*y5+p1)*y5+p0)/((((y5*q4+q3)*y5+q2)*y5+q1)*y5+q0).
do if (pv <= .5).
compute xp = -xp.
end if.
compute cilow=rnd(nrow(temp)*(cdfnorm(2*xp-xp2))).
compute cihigh=trunc(nrow(temp)*(cdfnorm(2*xp+(xp2))))+1.
do if (cilow < 1).
compute cilow = 1.
compute booterr=1.
compute badlo = 1.
end if.
do if (cihigh > nrow(temp)).
compute cihigh = boot.
compute booterr=1.
compute badhi = 1.
end if.
compute llcit=temp(cilow,1).
compute ulcit=temp(cihigh,1).
do if (badlo = 1 and llcit <> priorlo).
compute badend={badend, llcit}.
compute priorlo = llcit.
end if.
do if (badhi = 1 and ulcit <> priorhi).
compute badend={badend, ulcit}.
compute priorhi = ulcit.
end if.
end if.
do if ( (imm4(1,k8)*bc)+(9999*(1-bc)) = 9999).
compute llcit=temp(cilow,1).
compute ulcit=temp(cihigh,1).
end if.
compute bootse=t(sqrt((cssq(temp)-((csum(temp)&**2)/nrow(temp)))/(nrow(temp)-1)))
.
compute condcont(k8,4:6)={bootse,llcit,ulcit}.
end if.
end loop.
do if (boot=0).
compute condcont=condcont(:,1:3).
end if.
compute indcontr=1.
end if.
end if.
do if (xmint=1).
do if (k1=(k4+1)).
compute cftotfx(:,k4)=cftotfx(:,k4)+(tucker2*xscaling).
end if.
end if.
do if (boot > 0).
compute tucker2=tucker2*xscaling.

.
compute temp = tucker2(2:nrow(tucker2),1).
compute temp(GRADE( tucker2(2:nrow(tucker2),1) )) = tucker2(2:nrow(tucker2),1).
compute badlo = 0.
compute badhi = 0.
do if ( (tucker2(1,1)*bc)+(9999*(1-bc)) <> 9999).
compute pv=csum(temp < (tucker2(1,1)*bc)+(9999*(1-bc)) )/nrow(temp).
compute ppv = pv.
do if (pv > .5).
compute ppv = 1-pv.
end if.
compute y5=sqrt(-2*ln(ppv)).
compute xp=y5+((((y5*p4+p3)*y5+p2)*y5+p1)*y5+p0)/((((y5*q4+q3)*y5+q2)*y5+q1)*y5+q0).
do if (pv <= .5).
compute xp = -xp.
end if.
compute cilow=rnd(nrow(temp)*(cdfnorm(2*xp-xp2))).
compute cihigh=trunc(nrow(temp)*(cdfnorm(2*xp+(xp2))))+1.
do if (cilow < 1).
compute cilow = 1.
compute booterr=1.
compute badlo = 1.
end if.
do if (cihigh > nrow(temp)).
compute cihigh = boot.
compute booterr=1.
compute badhi = 1.
end if.
compute llcit=temp(cilow,1).
compute ulcit=temp(cihigh,1).
do if (badlo = 1 and llcit <> priorlo).
compute badend={badend, llcit}.
compute priorlo = llcit.
end if.
do if (badhi = 1 and ulcit <> priorhi).
compute badend={badend, ulcit}.
compute priorhi = ulcit.
end if.
end if.
do if ( (tucker2(1,1)*bc)+(9999*(1-bc)) = 9999).
compute llcit=temp(cilow,1).
compute ulcit=temp(cihigh,1).
end if.
compute bootse=t(sqrt((cssq(temp)-((csum(temp)&**2)/nrow(temp)))/(nrow(temp)-1)))
.
compute indtemp={indtemp*xscaling, bootse,llcit,ulcit}.
end if.
do if (boot=0).
compute indtemp=indtemp*xscaling.
end if.
compute condres(k1,(ncol(indmodva)+1):ncol(condres))=indtemp.
end loop.
do if (xmint=0).
compute resultm2=make(nrow(condres),maxresm,99999).
compute resultm2(1:nrow(condres),1:ncol(condres))=condres.
compute resultm={resultm;resultm2}.
end if.
compute condlbs={condlbs,'Effect'}.
do if (boot > 0).
compute condlbs={condlbs,'BootSE', 'BootLLCI', 'BootULCI'}.
end if.
do if (xmint = 1).
do if (k4 = 1).
compute natindfx=condres((2+(k4-1)),2:ncol(condres)).
end if.
do if (k4 > 1).
compute natindfx={natindfx;condres((2+(k4-1)),2:ncol(condres))}.
end if.
do if (k4=nxvls).
compute resultm2=make(nrow(natindfx),maxresm,99999).
compute resultm2(1:nrow(natindfx),1:ncol(natindfx))=natindfx.
compute resultm={resultm;resultm2}.
end if.
end if.
do if (outscree=1 and xmint=0).
do if (nxvls=1).
print condres/title=' '/cnames=condlbs/format= F10.4 /space=0.
else.
compute condrlb=make(nrow(condres),1,xcatlab(k4,1)).
print condres/title=' '/cnames=condlbs/rnames=condrlb/format= F10.4 /space=0.
end if.
end if.
do if (outscree=1 and xmint=1 and k4=nxvls).
do if (nxvls=1).
print natindfx/title=' '/clabels='Effect', 'BootSE', 'BootLLCI', 'BootULCI'/format= F10.4 /space=0.
else.
compute condrlb=make(nrow(condres),1,xcatlab(k4,1)).
print natindfx/title=' '/clabels='Effect', 'BootSE', 'BootLLCI', 'BootULCI'/rnames=direffl2/format= F10.4 /space=0.
end if.
end if.
compute dichadj=0.
compute immcat=0.
do if ((indmmm(i,1)>0) and (xmint=0)).
do if (indmmm(i,1)=1 or indmmm(i,1)=12 or indmmm(i,1)=31).
do if (wdich=1 and mcw=0).
do if (indmmm(i,1) <> 12).
compute imm2(:,1)=imm2(:,1)*(wmax-wmin).
end if.
do if (indmmm(i,1) <> 31).
compute dichadj=1.
end if.
end if.
do if ((mcw = 1 or mcw = 2) and indmmm(i,1) <> 31)).
compute immcat=1.
end if.
end if.
do if (indmmm(i,1)=2 or indmmm(i,1)=22 or indmmm(i,1)=31).
do if (zdich=1 and mcz=0).
do if (indmmm(i,1) = 31).
compute imm2(:,(nwvls+1):ncol(imm2))=imm2(:,(nwvls+1):ncol(imm2))*(zmax-zmin).
end if.
do if (indmmm(i,1) = 2).
compute imm2(:,1)=imm2(:,1)*(zmax-zmin).
end if.
do if (indmmm(i,1) <> 31).
compute dichadj=1.
end if.
end if.
do if ((mcz = 1 or mcz = 2) and indmmm(i,1) <> 31)).
compute immcat=1.
end if.
end if.
compute immtemp2=t(imm2(1,:)).
compute immtemp=immtemp2.
compute immlbs={'Index'}.
do if (boot > 0).
compute immtemp=make(1,3,0).
loop k7=1 to ncol(imm2).

.
compute temp = imm2(2:nrow(imm2),k7).
compute temp(GRADE( imm2(2:nrow(imm2),k7) )) = imm2(2:nrow(imm2),k7).
compute badlo = 0.
compute badhi = 0.
do if ( (imm2(1,k7)*bc)+(9999*(1-bc)) <> 9999).
compute pv=csum(temp < (imm2(1,k7)*bc)+(9999*(1-bc)) )/nrow(temp).
compute ppv = pv.
do if (pv > .5).
compute ppv = 1-pv.
end if.
compute y5=sqrt(-2*ln(ppv)).
compute xp=y5+((((y5*p4+p3)*y5+p2)*y5+p1)*y5+p0)/((((y5*q4+q3)*y5+q2)*y5+q1)*y5+q0).
do if (pv <= .5).
compute xp = -xp.
end if.
compute cilow=rnd(nrow(temp)*(cdfnorm(2*xp-xp2))).
compute cihigh=trunc(nrow(temp)*(cdfnorm(2*xp+(xp2))))+1.
do if (cilow < 1).
compute cilow = 1.
compute booterr=1.
compute badlo = 1.
end if.
do if (cihigh > nrow(temp)).
compute cihigh = boot.
compute booterr=1.
compute badhi = 1.
end if.
compute llcit=temp(cilow,1).
compute ulcit=temp(cihigh,1).
do if (badlo = 1 and llcit <> priorlo).
compute badend={badend, llcit}.
compute priorlo = llcit.
end if.
do if (badhi = 1 and ulcit <> priorhi).
compute badend={badend, ulcit}.
compute priorhi = ulcit.
end if.
end if.
do if ( (imm2(1,k7)*bc)+(9999*(1-bc)) = 9999).
compute llcit=temp(cilow,1).
compute ulcit=temp(cihigh,1).
end if.
compute bootse=t(sqrt((cssq(temp)-((csum(temp)&**2)/nrow(temp)))/(nrow(temp)-1)))
.
compute temp={bootse,llcit,ulcit}.
compute immtemp={immtemp;temp}.
end loop.
compute immtemp=immtemp(2:nrow(immtemp),:).
compute immtemp={immtemp2,immtemp}.
compute immlbs={immlbs,'BootSE', 'BootLLCI', 'BootULCI'}.
end if.
compute resultm2=make(nrow(immtemp),maxresm,99999).
compute resultm2(1:nrow(immtemp),1:ncol(immtemp))=immtemp.
compute resultm={resultm;resultm2}.
do if (dichadj=0 and immcat=0 and indmmmt(i,1)<>5 and indmmm(i,1) < 100 ).
do if (outscree=1).
do if (indmmm(i,1) < 30).
print immtemp/title='      Index of moderated mediation:'/cnames=immlbs/rnames=immlbs2/format= F10.4.
end if.
do if (indmmm(i,1) = 31).
compute immlbs2={immlbs2;zcatlab(1:nzvls,1)}.
print immtemp/title='      Indices of partial moderated mediation:'/cnames=immlbs/rnames=immlbs2/format= F10.4.
end if.
end if.
do if ((nzvls = 1) and (nwvls = 1)).
do if (indmmm(i,1) = 41 or indmmm(i,1)=51).
loop k7=1 to nwvls.
compute immlbs2=zcatlab(1:nzvls,1).
compute immtemp2=immtemp((((k7-1)*nzvls)+1):(((k7-1)*nzvls)+nzvls),:).
do if (outscree=1).
do if (nwvls > 1).
compute primodv={'        ', wcatlab(k7,1)}.
print primodv/title='      Primary moderator:'/format=A8.
end if.
do if (nzvls=1).
print immtemp2/title='      Index of moderated moderated mediation'/cnames=immlbs/format= F10.4.
else.
print immtemp2/title='      Indices of moderated moderated mediation'/cnames=immlbs/rnames=immlbs2/format= F10.4.
end if.
end if.
compute cmmtemp=make(nrow(zprobval),4,0).
loop k8=1 to nrow(zprobval).
compute condbb3=condbb2(:,((nwvls+1)+((k7-1)*nzvls)):((nwvls+1)+((k7-1)*nzvls)+ (nzvls-1))).
do if (ncol(zprobval) > 1).
compute condbb3=condbb3*mdiag(zprobval(k8,:)).
else.
compute condbb3=condbb3&*zprobval(k8,:).
end if.
compute condbb3={condbb2(:,k7),condbb3}.
compute icmm=rsum(condbb3).
compute cmmtemp(k8,1)=icmm(1,1).
do if (boot > 0).

.
compute temp = icmm(2:nrow(icmm),1).
compute temp(GRADE( icmm(2:nrow(icmm),1) )) = icmm(2:nrow(icmm),1).
compute badlo = 0.
compute badhi = 0.
do if ( (icmm(1,1)*bc)+(9999*(1-bc)) <> 9999).
compute pv=csum(temp < (icmm(1,1)*bc)+(9999*(1-bc)) )/nrow(temp).
compute ppv = pv.
do if (pv > .5).
compute ppv = 1-pv.
end if.
compute y5=sqrt(-2*ln(ppv)).
compute xp=y5+((((y5*p4+p3)*y5+p2)*y5+p1)*y5+p0)/((((y5*q4+q3)*y5+q2)*y5+q1)*y5+q0).
do if (pv <= .5).
compute xp = -xp.
end if.
compute cilow=rnd(nrow(temp)*(cdfnorm(2*xp-xp2))).
compute cihigh=trunc(nrow(temp)*(cdfnorm(2*xp+(xp2))))+1.
do if (cilow < 1).
compute cilow = 1.
compute booterr=1.
compute badlo = 1.
end if.
do if (cihigh > nrow(temp)).
compute cihigh = boot.
compute booterr=1.
compute badhi = 1.
end if.
compute llcit=temp(cilow,1).
compute ulcit=temp(cihigh,1).
do if (badlo = 1 and llcit <> priorlo).
compute badend={badend, llcit}.
compute priorlo = llcit.
end if.
do if (badhi = 1 and ulcit <> priorhi).
compute badend={badend, ulcit}.
compute priorhi = ulcit.
end if.
end if.
do if ( (icmm(1,1)*bc)+(9999*(1-bc)) = 9999).
compute llcit=temp(cilow,1).
compute ulcit=temp(cihigh,1).
end if.
compute bootse=t(sqrt((cssq(temp)-((csum(temp)&**2)/nrow(temp)))/(nrow(temp)-1)))
.
compute cmmtemp(k8,2:4)={bootse,llcit,ulcit}.
end if.
end loop.
compute cmmtemp={zmodvals,cmmtemp}.
do if (boot=0).
compute cmmtemp=cmmtemp(:,1:2).
end if.
compute cmmlbs={znames,immlbs}.
compute resultm2=make(nrow(cmmtemp),maxresm,99999).
compute resultm2(1:nrow(cmmtemp),1:ncol(cmmtemp))=cmmtemp.
compute resultm={resultm;resultm2}.
do if (outscree=1).
print cmmtemp/title='      Indices of conditional moderated mediation by W'/cnames=cmmlbs/format= F10.4.
end if.
end loop.
end if.
end if.
end if.
do if (dichadj=1 or immcat=1 and indmmm(i,1) < 30).
do if (outscree=1).
print immtemp/title='Index of moderated mediation (difference between conditional indirect effects):'/cnames=immlbs/rnames=immlbs2/format= F10.4.
end if.
end if.
end if.
do if (indcontr=1).
compute resultm2=make(nrow(condcont),maxresm,99999).
compute resultm2(1:nrow(condcont),1:ncol(condcont))=condcont.
compute resultm={resultm;resultm2}.
compute condctlb={'Effect1','Effect2','Contrast','BootSE', 'BootLLCI','BootULCI'}.
do if (outscree=1).
print condcont/title=' Pairwise contrasts between conditional indirect effects (Effect1 minus Effect2)'/cnames=condctlb/format= F10.4.
end if.
end if.
end loop.
end if.
end loop.
do if (alttotal=1).
compute altcnms='Effect'.
compute alttotfx=t(cftotfx(1,:))+obnatdfx.
do if (boot > 0).
compute alttotfx=make(ncol(cftotfx),4,0).
compute alttotfx(:,1)=t(cftotfx(1,:))+obnatdfx.
compute natdirbt(1,:)=t(obnatdfx).
compute cftotfx=cftotfx+natdirbt.
loop cec=1 to ncol(cftotfx).

.
compute temp = cftotfx(2:nrow(cftotfx),cec).
compute temp(GRADE( cftotfx(2:nrow(cftotfx),cec) )) = cftotfx(2:nrow(cftotfx),cec).
compute badlo = 0.
compute badhi = 0.
do if ( (cftotfx(1,cec)*bc)+(9999*(1-bc)) <> 9999).
compute pv=csum(temp < (cftotfx(1,cec)*bc)+(9999*(1-bc)) )/nrow(temp).
compute ppv = pv.
do if (pv > .5).
compute ppv = 1-pv.
end if.
compute y5=sqrt(-2*ln(ppv)).
compute xp=y5+((((y5*p4+p3)*y5+p2)*y5+p1)*y5+p0)/((((y5*q4+q3)*y5+q2)*y5+q1)*y5+q0).
do if (pv <= .5).
compute xp = -xp.
end if.
compute cilow=rnd(nrow(temp)*(cdfnorm(2*xp-xp2))).
compute cihigh=trunc(nrow(temp)*(cdfnorm(2*xp+(xp2))))+1.
do if (cilow < 1).
compute cilow = 1.
compute booterr=1.
compute badlo = 1.
end if.
do if (cihigh > nrow(temp)).
compute cihigh = boot.
compute booterr=1.
compute badhi = 1.
end if.
compute llcit=temp(cilow,1).
compute ulcit=temp(cihigh,1).
do if (badlo = 1 and llcit <> priorlo).
compute badend={badend, llcit}.
compute priorlo = llcit.
end if.
do if (badhi = 1 and ulcit <> priorhi).
compute badend={badend, ulcit}.
compute priorhi = ulcit.
end if.
end if.
do if ( (cftotfx(1,cec)*bc)+(9999*(1-bc)) = 9999).
compute llcit=temp(cilow,1).
compute ulcit=temp(cihigh,1).
end if.
compute bootse=t(sqrt((cssq(temp)-((csum(temp)&**2)/nrow(temp)))/(nrow(temp)-1)))
.
compute alttotfx(cec,2:4)={bootse,llcit,ulcit}.
end loop.
compute altcnms={altcnms,'BootSE', 'BootLLCI','BootULCI'}.
end if.
compute resultm2=make(nrow(alttotfx),maxresm,99999).
compute resultm2(1:nrow(alttotfx),1:ncol(alttotfx))=alttotfx.
compute resultm={resultm;resultm2}.
do if (outscree=1).
print/title='----------'.
do if (nxvls > 1).
print alttotfx/title='Relative total effects of X on Y (sum of direct and indirect effects)'/cnames=altcnms/rnames=direffl2/format= F10.4.
end if.
do if (nxvls = 1).
print alttotfx/title='Total effect of X on Y (sum of direct and indirect effects)'/cnames=altcnms/format= F10.4.
end if.
end if.
end if.
end if.
end if
.
do if (criterr=0 and (saveest = 1 or saveboot=1)).
compute coeffsav=coeffmat(2:nrow(coeffmat),:).
compute conseq=conseq(2:nrow(conseq),1).
compute dfmat=dfmat(2:nrow(dfmat),1).
compute dfmat=dfmat.
compute saven={'conseqnt','antecdnt','coeff','se','t','p','LLCI','ULCI','df'}.
do if (ydich=1).
compute saven={'conseqnt','antecdnt','coeff','se','t_or_Z','p','LLCI','ULCI','df'}.
end if.
do if (saveboot=1 and boot > 0 ).
compute colslab=make(300,1,' ').


compute colslab( 1 ,1)= 'COL1'.


compute colslab( 2 ,1)= 'COL2'.


compute colslab( 3 ,1)= 'COL3'.


compute colslab( 4 ,1)= 'COL4'.


compute colslab( 5 ,1)= 'COL5'.


compute colslab( 6 ,1)= 'COL6'.


compute colslab( 7 ,1)= 'COL7'.


compute colslab( 8 ,1)= 'COL8'.


compute colslab( 9 ,1)= 'COL9'.


compute colslab( 10 ,1)= 'COL10'.


compute colslab( 11 ,1)= 'COL11'.


compute colslab( 12 ,1)= 'COL12'.


compute colslab( 13 ,1)= 'COL13'.


compute colslab( 14 ,1)= 'COL14'.


compute colslab( 15 ,1)= 'COL15'.


compute colslab( 16 ,1)= 'COL16'.


compute colslab( 17 ,1)= 'COL17'.


compute colslab( 18 ,1)= 'COL18'.


compute colslab( 19 ,1)= 'COL19'.


compute colslab( 20 ,1)= 'COL20'.


compute colslab( 21 ,1)= 'COL21'.


compute colslab( 22 ,1)= 'COL22'.


compute colslab( 23 ,1)= 'COL23'.


compute colslab( 24 ,1)= 'COL24'.


compute colslab( 25 ,1)= 'COL25'.


compute colslab( 26 ,1)= 'COL26'.


compute colslab( 27 ,1)= 'COL27'.


compute colslab( 28 ,1)= 'COL28'.


compute colslab( 29 ,1)= 'COL29'.


compute colslab( 30 ,1)= 'COL30'.


compute colslab( 31 ,1)= 'COL31'.


compute colslab( 32 ,1)= 'COL32'.


compute colslab( 33 ,1)= 'COL33'.


compute colslab( 34 ,1)= 'COL34'.


compute colslab( 35 ,1)= 'COL35'.


compute colslab( 36 ,1)= 'COL36'.


compute colslab( 37 ,1)= 'COL37'.


compute colslab( 38 ,1)= 'COL38'.


compute colslab( 39 ,1)= 'COL39'.


compute colslab( 40 ,1)= 'COL40'.


compute colslab( 41 ,1)= 'COL41'.


compute colslab( 42 ,1)= 'COL42'.


compute colslab( 43 ,1)= 'COL43'.


compute colslab( 44 ,1)= 'COL44'.


compute colslab( 45 ,1)= 'COL45'.


compute colslab( 46 ,1)= 'COL46'.


compute colslab( 47 ,1)= 'COL47'.


compute colslab( 48 ,1)= 'COL48'.


compute colslab( 49 ,1)= 'COL49'.


compute colslab( 50 ,1)= 'COL50'.


compute colslab( 51 ,1)= 'COL51'.


compute colslab( 52 ,1)= 'COL52'.


compute colslab( 53 ,1)= 'COL53'.


compute colslab( 54 ,1)= 'COL54'.


compute colslab( 55 ,1)= 'COL55'.


compute colslab( 56 ,1)= 'COL56'.


compute colslab( 57 ,1)= 'COL57'.


compute colslab( 58 ,1)= 'COL58'.


compute colslab( 59 ,1)= 'COL59'.


compute colslab( 60 ,1)= 'COL60'.


compute colslab( 61 ,1)= 'COL61'.


compute colslab( 62 ,1)= 'COL62'.


compute colslab( 63 ,1)= 'COL63'.


compute colslab( 64 ,1)= 'COL64'.


compute colslab( 65 ,1)= 'COL65'.


compute colslab( 66 ,1)= 'COL66'.


compute colslab( 67 ,1)= 'COL67'.


compute colslab( 68 ,1)= 'COL68'.


compute colslab( 69 ,1)= 'COL69'.


compute colslab( 70 ,1)= 'COL70'.


compute colslab( 71 ,1)= 'COL71'.


compute colslab( 72 ,1)= 'COL72'.


compute colslab( 73 ,1)= 'COL73'.


compute colslab( 74 ,1)= 'COL74'.


compute colslab( 75 ,1)= 'COL75'.


compute colslab( 76 ,1)= 'COL76'.


compute colslab( 77 ,1)= 'COL77'.


compute colslab( 78 ,1)= 'COL78'.


compute colslab( 79 ,1)= 'COL79'.


compute colslab( 80 ,1)= 'COL80'.


compute colslab( 81 ,1)= 'COL81'.


compute colslab( 82 ,1)= 'COL82'.


compute colslab( 83 ,1)= 'COL83'.


compute colslab( 84 ,1)= 'COL84'.


compute colslab( 85 ,1)= 'COL85'.


compute colslab( 86 ,1)= 'COL86'.


compute colslab( 87 ,1)= 'COL87'.


compute colslab( 88 ,1)= 'COL88'.


compute colslab( 89 ,1)= 'COL89'.


compute colslab( 90 ,1)= 'COL90'.


compute colslab( 91 ,1)= 'COL91'.


compute colslab( 92 ,1)= 'COL92'.


compute colslab( 93 ,1)= 'COL93'.


compute colslab( 94 ,1)= 'COL94'.


compute colslab( 95 ,1)= 'COL95'.


compute colslab( 96 ,1)= 'COL96'.


compute colslab( 97 ,1)= 'COL97'.


compute colslab( 98 ,1)= 'COL98'.


compute colslab( 99 ,1)= 'COL99'.


compute colslab( 100 ,1)= 'COL100'.


compute colslab( 101 ,1)= 'COL101'.


compute colslab( 102 ,1)= 'COL102'.


compute colslab( 103 ,1)= 'COL103'.


compute colslab( 104 ,1)= 'COL104'.


compute colslab( 105 ,1)= 'COL105'.


compute colslab( 106 ,1)= 'COL106'.


compute colslab( 107 ,1)= 'COL107'.


compute colslab( 108 ,1)= 'COL108'.


compute colslab( 109 ,1)= 'COL109'.


compute colslab( 110 ,1)= 'COL110'.


compute colslab( 111 ,1)= 'COL111'.


compute colslab( 112 ,1)= 'COL112'.


compute colslab( 113 ,1)= 'COL113'.


compute colslab( 114 ,1)= 'COL114'.


compute colslab( 115 ,1)= 'COL115'.


compute colslab( 116 ,1)= 'COL116'.


compute colslab( 117 ,1)= 'COL117'.


compute colslab( 118 ,1)= 'COL118'.


compute colslab( 119 ,1)= 'COL119'.


compute colslab( 120 ,1)= 'COL120'.


compute colslab( 121 ,1)= 'COL121'.


compute colslab( 122 ,1)= 'COL122'.


compute colslab( 123 ,1)= 'COL123'.


compute colslab( 124 ,1)= 'COL124'.


compute colslab( 125 ,1)= 'COL125'.


compute colslab( 126 ,1)= 'COL126'.


compute colslab( 127 ,1)= 'COL127'.


compute colslab( 128 ,1)= 'COL128'.


compute colslab( 129 ,1)= 'COL129'.


compute colslab( 130 ,1)= 'COL130'.


compute colslab( 131 ,1)= 'COL131'.


compute colslab( 132 ,1)= 'COL132'.


compute colslab( 133 ,1)= 'COL133'.


compute colslab( 134 ,1)= 'COL134'.


compute colslab( 135 ,1)= 'COL135'.


compute colslab( 136 ,1)= 'COL136'.


compute colslab( 137 ,1)= 'COL137'.


compute colslab( 138 ,1)= 'COL138'.


compute colslab( 139 ,1)= 'COL139'.


compute colslab( 140 ,1)= 'COL140'.


compute colslab( 141 ,1)= 'COL141'.


compute colslab( 142 ,1)= 'COL142'.


compute colslab( 143 ,1)= 'COL143'.


compute colslab( 144 ,1)= 'COL144'.


compute colslab( 145 ,1)= 'COL145'.


compute colslab( 146 ,1)= 'COL146'.


compute colslab( 147 ,1)= 'COL147'.


compute colslab( 148 ,1)= 'COL148'.


compute colslab( 149 ,1)= 'COL149'.


compute colslab( 150 ,1)= 'COL150'.


compute colslab( 151 ,1)= 'COL151'.


compute colslab( 152 ,1)= 'COL152'.


compute colslab( 153 ,1)= 'COL153'.


compute colslab( 154 ,1)= 'COL154'.


compute colslab( 155 ,1)= 'COL155'.


compute colslab( 156 ,1)= 'COL156'.


compute colslab( 157 ,1)= 'COL157'.


compute colslab( 158 ,1)= 'COL158'.


compute colslab( 159 ,1)= 'COL159'.


compute colslab( 160 ,1)= 'COL160'.


compute colslab( 161 ,1)= 'COL161'.


compute colslab( 162 ,1)= 'COL162'.


compute colslab( 163 ,1)= 'COL163'.


compute colslab( 164 ,1)= 'COL164'.


compute colslab( 165 ,1)= 'COL165'.


compute colslab( 166 ,1)= 'COL166'.


compute colslab( 167 ,1)= 'COL167'.


compute colslab( 168 ,1)= 'COL168'.


compute colslab( 169 ,1)= 'COL169'.


compute colslab( 170 ,1)= 'COL170'.


compute colslab( 171 ,1)= 'COL171'.


compute colslab( 172 ,1)= 'COL172'.


compute colslab( 173 ,1)= 'COL173'.


compute colslab( 174 ,1)= 'COL174'.


compute colslab( 175 ,1)= 'COL175'.


compute colslab( 176 ,1)= 'COL176'.


compute colslab( 177 ,1)= 'COL177'.


compute colslab( 178 ,1)= 'COL178'.


compute colslab( 179 ,1)= 'COL179'.


compute colslab( 180 ,1)= 'COL180'.


compute colslab( 181 ,1)= 'COL181'.


compute colslab( 182 ,1)= 'COL182'.


compute colslab( 183 ,1)= 'COL183'.


compute colslab( 184 ,1)= 'COL184'.


compute colslab( 185 ,1)= 'COL185'.


compute colslab( 186 ,1)= 'COL186'.


compute colslab( 187 ,1)= 'COL187'.


compute colslab( 188 ,1)= 'COL188'.


compute colslab( 189 ,1)= 'COL189'.


compute colslab( 190 ,1)= 'COL190'.


compute colslab( 191 ,1)= 'COL191'.


compute colslab( 192 ,1)= 'COL192'.


compute colslab( 193 ,1)= 'COL193'.


compute colslab( 194 ,1)= 'COL194'.


compute colslab( 195 ,1)= 'COL195'.


compute colslab( 196 ,1)= 'COL196'.


compute colslab( 197 ,1)= 'COL197'.


compute colslab( 198 ,1)= 'COL198'.


compute colslab( 199 ,1)= 'COL199'.


compute colslab( 200 ,1)= 'COL200'.


compute colslab( 201 ,1)= 'COL201'.


compute colslab( 202 ,1)= 'COL202'.


compute colslab( 203 ,1)= 'COL203'.


compute colslab( 204 ,1)= 'COL204'.


compute colslab( 205 ,1)= 'COL205'.


compute colslab( 206 ,1)= 'COL206'.


compute colslab( 207 ,1)= 'COL207'.


compute colslab( 208 ,1)= 'COL208'.


compute colslab( 209 ,1)= 'COL209'.


compute colslab( 210 ,1)= 'COL210'.


compute colslab( 211 ,1)= 'COL211'.


compute colslab( 212 ,1)= 'COL212'.


compute colslab( 213 ,1)= 'COL213'.


compute colslab( 214 ,1)= 'COL214'.


compute colslab( 215 ,1)= 'COL215'.


compute colslab( 216 ,1)= 'COL216'.


compute colslab( 217 ,1)= 'COL217'.


compute colslab( 218 ,1)= 'COL218'.


compute colslab( 219 ,1)= 'COL219'.


compute colslab( 220 ,1)= 'COL220'.


compute colslab( 221 ,1)= 'COL221'.


compute colslab( 222 ,1)= 'COL222'.


compute colslab( 223 ,1)= 'COL223'.


compute colslab( 224 ,1)= 'COL224'.


compute colslab( 225 ,1)= 'COL225'.


compute colslab( 226 ,1)= 'COL226'.


compute colslab( 227 ,1)= 'COL227'.


compute colslab( 228 ,1)= 'COL228'.


compute colslab( 229 ,1)= 'COL229'.


compute colslab( 230 ,1)= 'COL230'.


compute colslab( 231 ,1)= 'COL231'.


compute colslab( 232 ,1)= 'COL232'.


compute colslab( 233 ,1)= 'COL233'.


compute colslab( 234 ,1)= 'COL234'.


compute colslab( 235 ,1)= 'COL235'.


compute colslab( 236 ,1)= 'COL236'.


compute colslab( 237 ,1)= 'COL237'.


compute colslab( 238 ,1)= 'COL238'.


compute colslab( 239 ,1)= 'COL239'.


compute colslab( 240 ,1)= 'COL240'.


compute colslab( 241 ,1)= 'COL241'.


compute colslab( 242 ,1)= 'COL242'.


compute colslab( 243 ,1)= 'COL243'.


compute colslab( 244 ,1)= 'COL244'.


compute colslab( 245 ,1)= 'COL245'.


compute colslab( 246 ,1)= 'COL246'.


compute colslab( 247 ,1)= 'COL247'.


compute colslab( 248 ,1)= 'COL248'.


compute colslab( 249 ,1)= 'COL249'.


compute colslab( 250 ,1)= 'COL250'.


compute colslab( 251 ,1)= 'COL251'.


compute colslab( 252 ,1)= 'COL252'.


compute colslab( 253 ,1)= 'COL253'.


compute colslab( 254 ,1)= 'COL254'.


compute colslab( 255 ,1)= 'COL255'.


compute colslab( 256 ,1)= 'COL256'.


compute colslab( 257 ,1)= 'COL257'.


compute colslab( 258 ,1)= 'COL258'.


compute colslab( 259 ,1)= 'COL259'.


compute colslab( 260 ,1)= 'COL260'.


compute colslab( 261 ,1)= 'COL261'.


compute colslab( 262 ,1)= 'COL262'.


compute colslab( 263 ,1)= 'COL263'.


compute colslab( 264 ,1)= 'COL264'.


compute colslab( 265 ,1)= 'COL265'.


compute colslab( 266 ,1)= 'COL266'.


compute colslab( 267 ,1)= 'COL267'.


compute colslab( 268 ,1)= 'COL268'.


compute colslab( 269 ,1)= 'COL269'.


compute colslab( 270 ,1)= 'COL270'.


compute colslab( 271 ,1)= 'COL271'.


compute colslab( 272 ,1)= 'COL272'.


compute colslab( 273 ,1)= 'COL273'.


compute colslab( 274 ,1)= 'COL274'.


compute colslab( 275 ,1)= 'COL275'.


compute colslab( 276 ,1)= 'COL276'.


compute colslab( 277 ,1)= 'COL277'.


compute colslab( 278 ,1)= 'COL278'.


compute colslab( 279 ,1)= 'COL279'.


compute colslab( 280 ,1)= 'COL280'.


compute colslab( 281 ,1)= 'COL281'.


compute colslab( 282 ,1)= 'COL282'.


compute colslab( 283 ,1)= 'COL283'.


compute colslab( 284 ,1)= 'COL284'.


compute colslab( 285 ,1)= 'COL285'.


compute colslab( 286 ,1)= 'COL286'.


compute colslab( 287 ,1)= 'COL287'.


compute colslab( 288 ,1)= 'COL288'.


compute colslab( 289 ,1)= 'COL289'.


compute colslab( 290 ,1)= 'COL290'.


compute colslab( 291 ,1)= 'COL291'.


compute colslab( 292 ,1)= 'COL292'.


compute colslab( 293 ,1)= 'COL293'.


compute colslab( 294 ,1)= 'COL294'.


compute colslab( 295 ,1)= 'COL295'.


compute colslab( 296 ,1)= 'COL296'.


compute colslab( 297 ,1)= 'COL297'.


compute colslab( 298 ,1)= 'COL298'.


compute colslab( 299 ,1)= 'COL299'.


compute colslab( 300 ,1)= 'COL300'.

compute colslab=colslab(1:ncol(bootres),1).
compute colslab={colslab,conseq,vlabs}.
do if (outscree=1).
print/title = '**************************************************************************'.
print/title='Bootstrap estimates were saved to a file'/space=0.
print colslab/title='Map of column names to model coefficients:'/clabels=' ','Conseqnt','Antecdnt'/format=a8.
end if.
end if.
end if.
do if (criterr=0 and boot > 0 and modelbt=1).
compute labstart=1.
do if (outscree=1).
print/title = '*********** BOOTSTRAP RESULTS FOR REGRESSION MODEL PARAMETERS ************'.
end if.
loop iboot = 1 to (nms+nys).
do if (outscree=1).
print outnames(1,iboot)/title = 'OUTCOME VARIABLE:'/format = A8.
end if.
compute vlabsm=vlabs(labstart:(labstart+(nump(1,iboot)-1)),1).
compute outnmtmp=bootcim(labstart:(labstart+(nump(1,iboot)-1)),:).
compute resultm2=make(nrow(outnmtmp),maxresm,99999).
compute resultm2(1:nrow(outnmtmp),1:ncol(outnmtmp))=outnmtmp.
compute resultm={resultm;resultm2}.
do if (outscree=1).
print bootcim(labstart:(labstart+(nump(1,iboot)-1)),:)/title=' '/rnames=vlabsm/ clabels='Coeff' 'BootMean' 'BootSE' 'BootLLCI' 'BootULCI'/format= F10.4 /space=0.
end if.
compute labstart=labstart+nump(1,iboot).
do if (iboot < (nms+nys)).
do if (outscree=1).
print/title= '----------'.
end if.
end if.
end loop.
end if.
do if (criterr=0 and saveest=1).
compute resultm=resultm(2:nrow(resultm),:).
compute bocaj=(resultm=99999).
compute bocaj=csum(bocaj).
compute bocaj=1-(bocaj=nrow(resultm)).
compute j=1.
loop i = 1 to ncol(resultm).
do if (bocaj(1,i)=1).
compute j=j+1.
end if.
end loop.
compute resultm=resultm(:,1:(j-1)).
save resultm/outfile = *.
end if.
do if (criterr = 0 and matrices=1 and outscree=1).
print/title = '************************ MODEL DEFINITION MATRICES ************************'.
print/title = 'FROM variables are columns, TO variables are rows.'.
compute temp2=make(nrow(bcmat),ncol(bcmat),'0').
loop i = 2 to nrow(bcmat).
loop j = 1 to (ncol(bcmat)-1).
do if (bcmat(i,j)=1).
compute temp2(i,j)='1'.
end if.
do if (j >= i).
compute temp2(i,j)=' '.
end if.
end loop.
end loop.
compute temp2=temp2(2:nrow(bcmat),(1:(ncol(bcmat)-1))).
do if (nms > 0).
compute cmatlabs={xnames,mnames}.
compute rmatlabs={mnames,ynames}.
end if.
do if (nms = 0).
compute cmatlabs={xnames}.
compute rmatlabs={ynames}.
end if.
print temp2/title='BMATRIX: Paths freely estimated (1) and fixed to zero (0):'/cnames=cmatlabs/rnames=rmatlabs/format A3.
compute z=0.
do if (rsum(csum(wcmat))<>0).
compute temp2=make(nrow(wcmat),ncol(wcmat),'0').
loop i = 2 to nrow(wcmat).
loop j = 1 to (ncol(wcmat)-1).
do if (wcmat(i,j)=1).
compute temp2(i,j)='1'.
end if.
do if (j >= i).
compute temp2(i,j)=' '.
end if.
end loop.
end loop.
compute temp2=temp2(2:nrow(wcmat),(1:(ncol(wcmat)-1))).
print temp2/title='WMATRIX: Paths moderated (1) and not moderated (0) by W:'/cnames=cmatlabs/rnames=rmatlabs/format A3.
end if.
do if (rsum(csum(zcmat))<>0).
compute temp2=make(nrow(zcmat),ncol(zcmat),'0').
loop i = 2 to nrow(zcmat).
loop j = 1 to (ncol(zcmat)-1).
do if (zcmat(i,j)=1).
compute temp2(i,j)='1'.
end if.
do if (j >= i).
compute temp2(i,j)=' '.
end if.
end loop.
end loop.
compute temp2=temp2(2:nrow(zcmat),(1:(ncol(zcmat)-1))).
print temp2/title='ZMATRIX: Paths moderated (1) and not moderated (0) by Z:'/cnames=cmatlabs/rnames=rmatlabs/format a3.
end if.
do if (rsum(csum(wzcmat))<>0).
compute temp2=make(nrow(wzcmat),ncol(wzcmat),'0').
loop i = 2 to nrow(wzcmat).
loop j = 1 to (ncol(wzcmat)-1).
do if (wzcmat(i,j)=1).
compute temp2(i,j)='1'.
end if.
do if (j >= i).
compute temp2(i,j)=' '.
end if.
end loop.
end loop.
compute temp2=temp2(2:nrow(wzcmat),(1:(ncol(wzcmat)-1))).
print temp2/title='WZMATRIX: W moderated paths moderated (1) and not moderated (0) by Z:'/cnames=cmatlabs/rnames=rmatlabs/format a3.
end if.
do if (ncs > 0).
print ccmat/title='CMATRIX: Covariates (columns) in (1) and not in (0) the models of M and Y (rows):'/rnames=rmatlabs/cnames=covnames.
end if.
end if.
do if (outscree=1).
do if (activate=0).
print/title = '*********************** ANALYSIS NOTES AND ERRORS ************************'.
end if.
end if.
do if (activate=1).
print/title = '**************************************************************************'.
end if.
do if (criterr=0).
do if (outscree=1).
print conf/title = 'Level of confidence for all confidence intervals in output:'/format = F8.4.
do if (boot > 0).
do if (goodboot = boot and bc=0).
print boot/title='Number of bootstrap samples for percentile bootstrap confidence intervals:'.
end if.
do if (goodboot = boot and bc=1).
print boot/title='Number of bootstrap samples for bias-corrected bootstrap confidence intervals:'.
end if.
do if (booterr = 1).
compute badend = badend(1,2:ncol(badend)).
print/title = 'WARNING: Bootstrap CI endpoints below not trustworthy. Decrease confidence'.
print badend/title='or increase the number of bootstrap samples.'/space=0/format = F10.4.
end if.
end if.
do if (mc > 0).
print mc/title='Number of samples for Monte Carlo confidence intervals:'.
end if.
do if (wnotev > 0 and printw=1).
do if (wnotev=1).
print/title = 'W values in conditional tables are the 16th, 50th, and 84th percentiles.'.
else if (wnotev=2).
do if (minwwarn=0 and maxwwarn=0).
print/title = 'W values in conditional tables are the mean and +/- SD from the mean.'.
end if.
do if (minwwarn=1).
print/title = 'W values in conditional tables are the minimum, the mean, and 1 SD above the mean.'.
end if.
do if (maxwwarn=1).
print/title = 'W values in conditional tables are 1 SD below the mean, the mean, and the maximum.'.
end if.
end if.
end if.
do if (znotev > 0 and printz=1).
do if (znotev=1).
print/title = 'Z values in conditional tables are the 16th, 50th, and 84th percentiles.'.
else if (znotev=2).
do if (minzwarn=0 and maxzwarn=0).
print/title = 'Z values in conditional tables are the mean and +/- SD from the mean.'.
end if.
do if (minzwarn=1).
print/title = 'Z values in conditional tables are the minimum, the mean, and 1 SD above the mean.'.
end if.
do if (maxzwarn=1).
print/title = 'Z values in conditional tables are 1 SD below the mean, the mean, and the maximum.'.
end if.
end if.
end if.
do if (minwwarn > 0).
print/title = 'NOTE: One SD below the mean is below the minimum observed in the data for W,'.
print/title = '      so the minimum measurement on W is used for conditioning instead.'/space=0.
end if.
do if (maxwwarn > 0).
print/title = 'NOTE: One SD above the mean is above the maximum observed in the data for W,'.
print/title = '      so the maximum measurement for W is used for conditioning instead.'/space=0.
end if.
do if (minzwarn > 0).
print/title = 'NOTE: One SD below the mean is below the minimum observed in the data for Z,'.
print/title = '      so the minimum measurement for Z is used for conditioning instead.'/space=0.
end if.
do if (maxzwarn > 0).
print/title = 'NOTE: One SD above the mean is above the maximum observed in the data for Z,'.
print/title = '      so the maximum measurement for Z is used for conditioning instead.'/space=0.
end if.
do if (pstog=1).
print/title= 'NOTE: Standardized coefficients for dichotomous or multicategorical X are in'.
print/title= '      partially standardized form.'/space=0.
end if.
loop i = 1 to 100.
do if (notecode(i,1) = 32).
print/title = 'Direct, indirect, and total effects are counterfactually defined'.
do if (xcontcf=0).
print/title = 'assuming X by M interaction.'/space=0.
end if.
do if (xcontcf=1).
print/title = 'assuming X by M interaction and with the following reference (x_ref)'/space=0.
compute states=t(xrefvals).
print states/title = 'and counterfactual (x_cf) states for X:'/rlabels='x_ref' 'x_cf'/format= F10.4 /space=0.
end if.
end if.
do if (notecode(i,1)=1).
print/title = 'NOTE: COVMY is ignored when using CMATRIX option.'.
end if.
do if (notecode(i,1)=2).
print/title = 'NOTE: Confidence level restricted to between 50 and 99.9999%.  95% confidence is provided in output'.
end if.
do if (notecode(i,1)=3).
print centvar/title = 'NOTE: The following variables were mean centered prior to analysis:'/format = a8.
end if.
do if (notecode(i,1) = 4).
print/title = 'NOTE: A heteroscedasticity consistent standard error and covariance matrix estimator was used.'.
end if.
do if (notecode(i,1) = 5).
print/title = 'NOTE: The HC3 option has been replaced with HC.  See the documentation.'.
end if.
do if (notecode(i,1) = 6).
print/title = 'NOTE: Due to estimation problems, some bootstrap samples had to be replaced.'.
print badboot/title='      The number of times this happened was:'/space=0/format=F8.0.
end if.
do if (notecode(i,1) = 7).
print/title = 'NOTE: The bootstrapping was not completed due to problematic bootstrap samples.'.
print/title = '      Bootstrap confidence intervals are therefore suppressed.'/space=0.
end if.
do if (notecode(i,1) = 8).
print/title = 'NOTE: The number of bootstrap samples was adjusted upward given your desired confidence.'.
end if.
do if (notecode(i,1) = 9).
print/title = 'NOTE: WMODVAL is ignored when W is specified as multicategorical.'.
end if.
do if (notecode(i,1) = 10).
print/title = 'NOTE: ZMODVAL is ignored when Z is specified as multicategorical.'.
end if.
do if (notecode(i,1) = 11).
print/title = 'NOTE: Total effect model generated only when all covariates are specified in all'.
print/title = '      models of M and Y.'/space=0.
end if.
do if (notecode(i,1) = 30).
print/title = 'NOTE: Your vector of linear hypothesis weights is of the wrong length for this model.'.
end if.
do if (notecode(i,1) = 12).
print/title = 'NOTE: Total effect model generated only when X is freely estimated to affect each M'.
print/title = '      and both X and M are freely estimated to affect Y'/space=0.
end if.
do if (notecode(i,1) = 13).
print/title = 'NOTE: There are too many pairwise contrasts to conduct with this model.'.
end if.
do if (notecode(i,1) = 14).
print/title = 'NOTE: The number of contrast weights must equal the number of indirect effects.'.
end if.
do if (notecode(i,1) = 15).
print/title = 'NOTE: Monte Carlo confidence intervals not available for this model.'.
print/title = '      Bootstrapping is used instead.'/space=0.
end if.
do if (notecode(i,1) = 16).
print/title = 'NOTE: The number of Monte Carlo samples was adjusted upward given your desired confidence.'.
end if.
do if (notecode(i,1) = 19).
print/title = 'NOTE: Your contrast matrix is invalid or not applicable to this model.'.
end if.
do if (notecode(i,1) = 20).
print/title = 'NOTE: One of the groups specified by your contrast matrix does not exist in the data.'.
end if.
do if (notecode(i,1) = 21).
print/title = 'NOTE: The VARORDER option is not available in this release.'.
end if.
do if (notecode(i,1) = 22).
print/title = 'NOTE: The VMODVAL and QMODVAL options are not available in this release.'.
end if.
do if (notecode(i,1) = 23).
print/title = 'NOTE: The QUANTILE option is not available in this release.'.
end if.
do if (notecode(i,1) = 24).
print/title = 'NOTE: Total effect model not available with dichotomous Y.'.
end if.
do if (notecode(i,1) = 25).
print/title = 'NOTE: STAND/EFFSIZE options not available with dichotomous Y.'.
end if.
do if ((notecode(i,1) = 26) and nms > 0).
print/title = 'NOTE: Direct and indirect effects of X on Y are on a log-odds metric.'.
end if.
do if (notecode(i,1) = 27).
print/title = 'NOTE: Standardized coefficients are not available for models with moderators.'.
end if.
do if (notecode(i,1) = 28).
print/title = 'NOTE: The contrast option is not available with a multicategorical X.'.
end if.
do if (notecode(i,1) = 31).
do if (nms > 1).
print medmeans/title = 'NOTE: Controlled direct effect(s) estimated at the following mediator values:'/cnames=mnames/format= F10.4.
end if.
do if (nms=1).
print medmeans/title = 'NOTE: Controlled direct effect(s) estimated at the following mediator value:'/format= F10.4.
end if.
end if.
do if (notecode(i,1) = 33).
print/title = 'NOTE: Sobel test is not available with the XMINT option.'.
end if.
do if (notecode(i,1) = 37).
print/title = 'NOTE: The CONTRAST option is not available when using the XMINT option.'.
end if.
do if (notecode(i,1) = 34).
print/title = 'NOTE: Standardized effects are not available when using the XMINT option.'.
end if.
do if (notecode(i,1) = 36).
print/title = 'NOTE: The XREFVAL option is ignored when X is declared as multicategorical.'.
end if.
do if (notecode(i,1) = 35).
print coval/title = 'NOTE: Counterfactual effects estimated at the following covariate values:'/cnames=covnames/format= F10.4.
end if.
do if (notecode(i,1) = 29 and (listmiss=1)).
compute a=missrow.
compute conum=ncol(a).
compute allgood=0.
compute smremain=12.
compute largesti=1.
compute smallrow=0.
do if (conum > 12).
loop ii = 1 to 12.
compute check=(conum/ii).
do if (check = trunc(check)).
compute check2=conum/ii.
compute aok=ii.
do if (aok > 2).
compute allgood=1.
end if.
else.
compute remain=conum-(ii*trunc(check)).
do if (remain <= smremain).
compute smremain=remain.
compute largesti=ii.
compute smallrow=trunc(conum/largesti).
end if.
end if.
end loop.
compute atemp=a(1,1:(aok*check2)).
compute atemp=reshape(atemp,check2,aok).
do if (ncol(atemp) > 2).
print/title='NOTE: Missing data resulted in the deletion of the following row(s) of'.
print atemp/title='      data from the analysis:'/format=F6.0/space=0.
end if.
do if (allgood=0).
compute atemp=a(1,1:(smallrow*largesti)).
compute atemp=reshape(atemp,smallrow,largesti).
compute btemp=a(1,((largesti*smallrow)+1):conum).
print/title='NOTE: Missing data resulted in the deletion of the following row(s) of'.
print atemp/title='      data from the analysis:'/format=F6.0/space=0.
print btemp/title=' '/space=0/format=f6.0.
end if.
else if (conum <= 12).
print/title='NOTE: Missing data resulted in the deletion of the following row(s) of'.
print a/title='      data from the analysis:'/format=F6.0/space=0.
end if.
end if.
end loop.
do if (toomany=1).
print/title='WARNING: Variables names longer than eight characters can produce incorrect output'.
print/title='when some variables in the data file have the same first eight characters. Shorter'/space=0.
print/title='variable names are recommended. By using this output, you are accepting all risk'/space=0.
print/title='and consequences of interpreting or reporting results that may be incorrect.'/space=0.
end if.
end if.
end if.
loop i = 1 to 100.
do if (errcode(i,1)=1).
print/title = 'ERROR: You must specify a Y and an X variable.'.
end if.
do if (errcode(i,1)=2).
print/title = 'ERROR: X, M, or Y variable used more than once or W and Z are the same variable.'.
do if (toomany = 1).
print/title = '       This could be caused by the use of variables names longer'/space=0.
print varnames/title = '       than eight characters. Here are the variables I see:'/space=0/format=A8.
end if.
end if.
do if (errcode(i,1)=3).
print/title = 'ERROR: You have specified more than one variable for W, Y, X, or Z'.
end if.
do if (errcode(i,1)=4).
print/title = 'ERROR: A variable specified as multicategorical has more than nine categories.'.
end if.
do if (errcode(i,1)=5).
print/title = 'ERROR: One of the categories contains only a single case.'.
end if.
do if (errcode(i,1)=6).
print/title = 'ERROR: Invalid model number in this version of PROCESS.'.
end if.
do if (errcode(i,1)=7).
print/title = 'ERROR: Invalid model number.'.
end if.
do if (errcode(i,1)=8).
print/title = 'ERROR: You must specify an M variable for this model.'.
end if.
do if (errcode(i,1)=9).
print/title = 'ERROR: You have specified an M variable in a model that does not use it.'.
end if.
do if (errcode(i,1)=10).
print/title = 'ERROR: You have specified a W variable in a model that does not use it.'.
end if.
do if (errcode(i,1)=11).
print/title = 'ERROR: You have not specified a W variable in a model that requires it.'.
end if.
do if (errcode(i,1)=12).
print/title = 'ERROR: You have specified a Z variable in a model that does not use it.'.
end if.
do if (errcode(i,1)=13).
print/title = 'ERROR: You have not specified a Z variable in a model that requires it.'.
end if.
do if (errcode(i,1)=14).
print/title = 'ERROR: V and Q are not proper specifications in this release of PROCESS.'.
print/title = '       Moderators must be specified as W and/or Z.'/space=0.
end if.
do if (errcode(i,1)=15).
print/title = 'ERROR: One of your model variables exhibits no variation (it is a constant).'.
end if.
do if (errcode(i,1)=16).
print/title = 'ERROR: BMATRIX is not the correct length or is otherwise invalid.'.
end if.
do if (errcode(i,1)=17).
print/title = 'ERROR: WMATRIX is not the correct length or is otherwise invalid.'.
end if.
do if (errcode(i,1)=18).
print/title = 'ERROR: ZMATRIX is not the correct length or is otherwise invalid.'.
end if.
do if (errcode(i,1)=19).
print/title = 'ERROR: WZMATRIX is not the correct length or is otherwise invalid.'.
end if.
do if (errcode(i,1)=20).
print/title = 'ERROR: A path fixed at zero cannot be moderated.'.
end if.
do if (errcode(i,1)=21).
print/title = 'ERROR: If only one moderator is specified, it must be specified as W.'.
end if.
do if (errcode(i,1)=22).
print/title = 'ERROR: In BMATRIX, X must be specified to affect at least one variable.'.
end if.
do if (errcode(i,1)=23).
print/title = 'ERROR: In BMATRIX, at least one variable must be specified to affect Y.'.
end if.
do if (errcode(i,1)=24).
print/title = 'ERROR: You must specify a model number or a custom BMATRIX specification.'.
end if.
do if (errcode(i,1)=25).
print/title = 'ERROR: BMATRIX cannot be used in conjunction with a model number.'.
end if.
do if (errcode(i,1)=26).
print/title = 'ERROR: Your model has a dangling mediator (all Ms must affect and be affected).'.
end if.
do if (errcode(i,1)=27).
print/title = 'ERROR: CLUSTER is not available on this release of PROCESS.'.
end if.
do if (errcode(i,1)=29).
print/title = 'ERROR: CMATRIX is not the correct length or is otherwise invalid.'.
end if.
do if (errcode(i,1)=30).
print/title = 'ERROR: In CMATRIX, all covariates must be assigned to an M or a Y.'.
end if.
do if (errcode(i,1)=31).
print/title = 'ERROR: A linear or near linear dependency (singularity) exists in the data.'.
end if.
do if (errcode(i,1)=32).
print/title = 'ERROR: Models 80 and 81 require between 3 and 6 mediators.'.
end if.
do if (errcode(i,1)=33).
print/title = 'ERROR: Model 82 requires 4 mediators.'.
end if.
do if (errcode(i,1)=34).
print/title = 'ERROR: This model number requires between 2 and 6 mediators.'.
end if.
do if (errcode(i,1)=35).
print/title = 'ERROR: In a model with only one moderator, that moderator must be W.'.
end if.
do if (errcode(i,1)=36).
print/title = 'ERROR: A serial mediation model cannot have more than 6 mediators.'.
end if.
do if (errcode(i,1)=37).
print/title = 'ERROR: No more than 10 mediators are allowed in a PROCESS command.'.
end if.
do if (errcode(i,1)=38).
print/title = 'ERROR: XCATCODE is not provided, not the correct length, or is otherwise invalid.'.
end if.
do if (errcode(i,1)=39).
print/title = 'ERROR: WCATCODE is not provided, not the correct length, or is otherwise invalid.'.
end if.
do if (errcode(i,1)=40).
print/title = 'ERROR: ZCATCODE is not provided, not the correct length, or is otherwise invalid.'.
end if.
do if (errcode(i,1)=41).
print/title = 'ERROR: Models 1, 2, and 3 cannot be customized.'.
end if.
do if (errcode(i,1)=42).
print/title = 'ERROR: WS option available only in PROCESS v2. Or use the MEMORE macro instead.'.
print/title = '       MEMORE can be downloaded from www.akmontoya.com.'/space=0.
end if.
do if (errcode(i,1)=43).
print/title = 'ERROR: PROCESS does not allow dichotomous mediators.'.
end if.
do if (errcode(i,1)=60).
print/title = 'PROCESS is now ready for use.'.
print/title='Copyright 2017-2022 by Andrew F. Hayes. ALL RIGHTS RESERVED.'/space=0.
print/title='Workshop schedule available at http://haskayne.ucalgary.ca/CCRAM'/space=0.
end if.
do if (errcode(i,1)=50).
print/title = 'ERROR: A multicategorical moderator cannot be specified as a covariate.'.
end if.
do if (errcode(i,1)=51).
print/title = 'ERROR: A variable you specified as a covariate is a moderator in all equations.'.
end if.
do if (errcode(i,1)=52 and mcerpt=0).
compute mcerpt=1.
print/title = 'ERROR: A variable specified as multicategorical must have at least three categories'.
end if.
do if (errcode(i,1) = 47).
do if (iterrmod=1).
print/title = 'ERROR: Iteration for Y model didn''t converge to a solution. Interpret results with caution.'.
print/title = '       Try increasing the number of iterations, though this will slow down computation.'/space=0.
end if.
do if (bootiter=1).
print/title = 'ERROR: Nonconvergence during bootstrapping. Interpet bootstrap results with caution.'.
end if.
end if.
do if (errcode(i,1) = 61).
print/title = 'ERROR: Variable names should be no more than eight characters in length.'.
end if.
do if (errcode(i,1) = 62).
print/title = 'ERROR: After listwise deletion of cases with missing data, too few cases remain.'.
end if.
do if (errcode(i,1) = 63).
print/title = 'ERROR: The XMINT option is available only for model 4.'.
end if.
do if (errcode(i,1) = 64).
print/title = 'ERROR: Incorrect number of values specified in CDEVAL option.'.
end if.
do if (errcode(i,1) = 65).
print/title = 'ERROR: Only indicator or sequential coding of X is allowed with the XMINT option.'.
end if.
do if (errcode(i,1) = 66).
print/title = 'ERROR: XMINT option not available with continuous X using PROCESS GUI. Use syntax.'.
end if.
do if (errcode(i,1) = 67).
print/title = 'ERROR: Too many elements provided in XREFVAL option.'.
end if.
do if (errcode(i,1)=68).
print/title = 'ERROR: Covariate assignment is not allowed with XMINT option.'.
end if.
do if (errcode(i,1) = 69).
print/title = 'ERROR: Incorrect number of values specified in COVAL option.'.
end if.
do if (errcode(i,1) = 70).
print/title = 'ERROR: Incorrect value(s) in XREFVAL for this dichotomous X variable.'.
end if.
do if (errcode(i,1) = 71).
print/title = 'ERROR: The CENTER option is not available when using the XMINT option.'.
end if.
do if (errcode(i,1) = 72).
print/title = 'ERROR: The XMINT option is not available for models with a dichotomous Y.'.
end if.
end loop.
END MATRIX.	
Ressources	Temps de processeur	00:00:06,33	
	Temps écoulé	00:00:06,31	


Run MATRIX procedure:

***************** PROCESS Procedure for SPSS Version 4.2 *****************

          Written by Andrew F. Hayes, Ph.D.       www.afhayes.com
    Documentation available in Hayes (2022). www.guilford.com/p/hayes3

**************************************************************************
Model  : 4
    Y  : HOMAIR
    X  : Fruits
   M1  : TG
   M2  : HDL
   M3  : LDL
   M4  : TC

Sample
Size:  169

**************************************************************************
OUTCOME VARIABLE:
 TG

Model Summary
          R       R-sq        MSE          F        df1        df2          p
      ,1392      ,0194  3973,1844     3,2982     1,0000   167,0000      ,0712

Model
              coeff         se          t          p       LLCI       ULCI
constant    98,8382     7,6439    12,9303      ,0000    83,7471   113,9294
Fruits     -17,9570     9,8878    -1,8161      ,0712   -37,4782     1,5641

Standardized coefficients
            coeff
Fruits     -,2830

**************************************************************************
OUTCOME VARIABLE:
 HDL

Model Summary
          R       R-sq        MSE          F        df1        df2          p
      ,1763      ,0311   172,5981     5,3581     1,0000   167,0000      ,0218

Model
              coeff         se          t          p       LLCI       ULCI
constant    38,3088     1,5932    24,0456      ,0000    35,1635    41,4542
Fruits       4,7704     2,0608     2,3148      ,0218      ,7017     8,8391

Standardized coefficients
            coeff
Fruits      ,3585

**************************************************************************
OUTCOME VARIABLE:
 LDL

Model Summary
          R       R-sq        MSE          F        df1        df2          p
      ,0864      ,0075  1181,1569     1,2574     1,0000   167,0000      ,2638

Model
              coeff         se          t          p       LLCI       ULCI
constant    79,9265     4,1677    19,1775      ,0000    71,6982    88,1547
Fruits      -6,0453     5,3912    -1,1213      ,2638   -16,6889     4,5983

Standardized coefficients
            coeff
Fruits     -,1758

**************************************************************************
OUTCOME VARIABLE:
 TC

Model Summary
          R       R-sq        MSE          F        df1        df2          p
      ,0727      ,0053  1444,2653      ,8864     1,0000   167,0000      ,3478

Model
              coeff         se          t          p       LLCI       ULCI
constant   139,1176     4,6086    30,1865      ,0000   130,0190   148,2163
Fruits      -5,6127     5,9614     -,9415      ,3478   -17,3822     6,1568

Standardized coefficients
            coeff
Fruits     -,1477

**************************************************************************
OUTCOME VARIABLE:
 HOMAIR

Model Summary
          R       R-sq        MSE          F        df1        df2          p
      ,3934      ,1548    22,6622     5,9693     5,0000   163,0000      ,0000

Model
              coeff         se          t          p       LLCI       ULCI
constant     -,7783     1,6909     -,4603      ,6459    -4,1173     2,5607
Fruits       1,5676      ,7666     2,0448      ,0425      ,0538     3,0814
TG            ,0450      ,0096     4,6633      ,0000      ,0259      ,0640
HDL           ,1147      ,0429     2,6714      ,0083      ,0299      ,1995
LDL           ,0937      ,0353     2,6534      ,0088      ,0240      ,1634
TC           -,0869      ,0351    -2,4769      ,0143     -,1561     -,0176

Standardized coefficients
            coeff
Fruits      ,3073
TG          ,5594
HDL         ,2993
LDL         ,6318
TC         -,6471

************************** TOTAL EFFECT MODEL ****************************
OUTCOME VARIABLE:
 HOMAIR

Model Summary
          R       R-sq        MSE          F        df1        df2          p
      ,1185      ,0140    25,8022     2,3783     1,0000   167,0000      ,1249

Model
              coeff         se          t          p       LLCI       ULCI
constant     3,4614      ,6160     5,6192      ,0000     2,2453     4,6775
Fruits       1,2288      ,7968     1,5422      ,1249     -,3443     2,8020

Standardized coefficients
            coeff
Fruits      ,2409


************** TOTAL, DIRECT, AND INDIRECT EFFECTS OF X ON Y **************

Total effect of X on Y
     Effect         se          t          p       LLCI       ULCI       c_ps
     1,2288      ,7968     1,5422      ,1249     -,3443     2,8020      ,2409

Direct effect of X on Y
     Effect         se          t          p       LLCI       ULCI      c'_ps
     1,5676      ,7666     2,0448      ,0425      ,0538     3,0814      ,3073

Indirect effect(s) of X on Y:
          Effect     BootSE   BootLLCI   BootULCI
TOTAL     -,3387      ,4399    -1,3706      ,3706
TG        -,8073      ,8384    -2,9830      ,0591
HDL        ,5473      ,6642      ,0510     2,3786
LDL       -,5664     1,1368    -3,5532      ,5800
TC         ,4876     1,1716     -,9040     3,4137

Partially standardized indirect effect(s) of X on Y:
          Effect     BootSE   BootLLCI   BootULCI
TOTAL     -,0664      ,0861     -,2671      ,0694
TG        -,1583      ,1550     -,5580      ,0116
HDL        ,1073      ,1239      ,0120      ,4476
LDL       -,1110      ,2160     -,6710      ,1078
TC         ,0956      ,2205     -,1748      ,6499

*********************** ANALYSIS NOTES AND ERRORS ************************

Level of confidence for all confidence intervals in output:
  95,0000

Number of bootstrap samples for percentile bootstrap confidence intervals:
  5000

NOTE: Standardized coefficients for dichotomous or multicategorical X are in
      partially standardized form.

------ END MATRIX -----


* Encoding: UTF-8.
preserve.
set printback=off.


Matrice


Remarques	
Sortie obtenue	10-NOV-2024 17:06:16	
Commentaires		
Entrée	Données	C:\Users\Brice SAHA\Documents\UBa\Master Thesis 2021\Fanuel\Database fanuel.sav	
	Jeu de données actif	Jeu_de_données1	
	Filtre	<sans>	
	Pondération	<sans>	
	Scinder un fichier	<sans>	
	N de lignes dans le fichier de travail	169	
Syntaxe	MATRIX.
compute wnames='xxxxx'.
compute znames='xxxxx'.
compute mcerpt=0.
compute wiscov=0.
compute ziscov=0.
compute tooman=0.
compute errcode=make(100,1,0).
compute notecode=make(100,1,0).
compute model = trunc( 4 ).
compute iterate = abs(trunc( 100 )).
compute converge = abs( 0.00001 ).
compute itprobtg=0.
compute v2tag=0.
compute ydich=0.
compute maxwwarn=0.
compute minwwarn=0.
compute maxzwarn=0.
compute minzwarn=0.
compute toomany=0.
compute wdich=0.
compute zdich=0.
compute wnotev=0.
compute znotev=0.
compute nxpval=1.
compute nwpval=1.
compute nzpval=1.
compute errs=1.
compute notes=1.
compute criterr=0.
compute novar=0.
compute adjust=0.
compute ncs=0.
compute serial=0.
compute sobelok=0.
compute hasw=0.
compute hasz=0.
compute printw=0.
compute printz=0.
compute xmint=( 0 =1).
compute wmodcust=0.
compute zmodcust=0.
compute booting=0.
compute bootiter=0.
compute iterrmod=0.
compute cov = 'xxxxx'.
compute varorder=( 0 <> 0).
compute nws=0.
compute w= 'xxxxx'.
compute nzs=0.
compute z = 'xxxxx'.
compute nms=0.
compute m = 'TG HDL LDL TC'.
compute nys=0.
compute y = 'HOMAIR'.
compute nxs=0.
compute x = 'Pulsenut'.
compute effsize=( 0 =1).
compute stand=( 1 =1).
compute intprobe = .1.
compute xrefvals={ 999 }.
compute center=trunc( 0 ).
compute xcontcf=0.
compute xscaling=1.
compute cdeval={ -999 }.
compute cuscoval=0.
do if (model=74).
compute errcode(errs,1)=7.
compute errs=errs+1.
compute criterr=1.
end if.
do if (xmint=1 and model <> 4).
compute errcode(errs,1)=63.
compute errs=errs+1.
compute criterr=1.
end if.
do if (xmint=1 and model=4).
compute w=x.
compute model=74.
compute intprobe=1.
compute notecode(notes,1) = 32.
compute notes = notes + 1.
do if (effsize=1 or stand=1).
compute notecode(notes,1) = 34.
compute notes = notes + 1.
compute stand=0.
compute effsize=0.
end if.
do if (center <> 0).
compute center=0.
compute errcode(errs,1)=71.
compute errs=errs+1.
compute criterr=1.
end if.
end if.
compute v = 'xxxxx'.
compute q = 'xxxxx'.
compute linsum={ -999 }.
compute nlinsum=ncol(linsum).
do if (linsum(1,1) = -999).
compute nlinsum=0.
end if.
compute oldvars= 'xxxxx'.
compute mcxok=0.
compute mcwok=0.
compute mczok=0.
compute xprod=0.
compute zprod=0.
compute wprod=0.
compute modcok=0.
compute alttotal=0.
compute hc3=trunc( 0 ).
compute jn=( 0 = 1).
compute listmiss=( 0 =1).
compute modelres=( 0 =1).
compute outscree=( 1 =1).
compute activate=( 0 =1).
compute booterr=0.
compute normal=( 0 =1).
compute xmtest=( 0 =1).
compute describe=( 0 =1).
compute longname=( 0 =1).
do if (stand=1).
compute effsize=1.
end if.
compute pstog=0.
compute sobelok=0.
compute normal=( 0 =1).
compute mdichok=( 0 =1).
compute contrast={ 999 }.
compute ncontr=ncol(contrast).
compute ncontrow=nrow(contrast).
do if (contrast(1,1) = 999).
compute ncontr=1.
compute contrast=0.
end if.
do if (ncontr = 1).
compute contrast=trunc(contrast).
do if (contrast > 3 or contrast < 0)).
compute ncontr=1.
compute contrast = 0.
end if.
end if.
do if (ncontr > 1).
compute contvec=contrast.
compute contrast=4.
do if (ncontrow > 1).
compute contrast=0.
compute modcok=1.
compute wcontval=contvec(:,1).
compute zcontval=contvec(:,2).
do if ((ncontr <> 2) or (ncontrow <> 2)).
compute notecode(notes,1) = 19.
compute notes = notes + 1.
compute modcok=0.
end if.
end if.
end if.
do if (xmint = 1 and contrast(1,1) <> 0).
compute contrast=0.
compute notecode(notes,1) = 37.
compute notes = notes + 1.
end if.
do if (varorder = 1).
compute notecode(notes,1) = 21.
compute notes = notes + 1.
end if.
do if ( 999 <> 999 or 999 <> 999).
compute notecode(notes,1) = 22.
compute notes = notes + 1.
end if.
compute modelbt=( 0 =1).
compute cluster= 'xxxxx'.
compute matrices=( 0 =1).
compute covcoeff=( 0 =1).
compute covmy=trunc( 0 ).
do if (covmy < 0 or covmy > 2).
compute covmy = 0.
end if.
compute boot = abs(trunc( 5000 )).
compute bc=( 0 =1).
compute mc=abs(trunc( 0 )).
compute hc=trunc( 5 ).
do if (intprobe < 0 or intprobe > 1).
compute intprobe = .10.
end if.
compute plot=trunc( 0 ).
do if (plot < 0 or plot > 2).
compute plot=0.
end if.
compute total=( 1 =1).
compute dototal=0.
compute saveboot = ( 0 = 1).
compute saveest=( 0 = 2).
do if (saveest=1).
compute intprobe=1.
end if.
do if (hc >= 0 and hc < 5).
compute notecode(notes,1) = 4.
compute notes = notes + 1.
end if.
do if (hc > 5 or hc < 0).
compute hc=5.
end if.
compute mcw=trunc( 0 ).
compute mcz=trunc( 0 ).
compute mcx=trunc( 0 ).
do if (mcx > 0 and mcx < 3 and model = 74).
compute mcw=mcx.
compute xscaling=1.
end if.
do if (mcx > 2 and model = 74).
compute errcode(errs,1)=65.
compute errs=errs+1.
compute criterr=1.
end if.
do if (model = 74 and normal=1).
compute notecode(notes,1) = 33.
compute normal=0.
compute notes = notes + 1.
end if.
do if (mcx > 0 and contrast > 0).
compute notecode(notes,1) = 28.
compute notes = notes + 1.
compute contrast=0.
end if.
compute nxvls=1.
compute nmvls=1.
compute nwvls=1.
compute nzvls=1.
compute paths=999.
compute pathsw=999.
compute pathsz=999.
compute pathswz=999.
compute pathsmod=999.
compute pathtype=999.
compute obscoeff=999.
compute pathsdv={' '}.
compute quantile=1.
do if ( 999 <>999).
compute notecode(notes,1) = 23.
compute notes = notes + 1.
end if.
compute moments=( 0 =1).
do if (moments=1).
compute quantile=0.
end if.
compute bmatrix={ -999 }.
compute wmatrix={ -999 }.
compute zmatrix={ -999 }.
compute wzmatrix={ -999 }.
compute cmatrix={ -999 }.
compute xcatcode={ -999 }.
compute wcatcode={ -999 }.
compute zcatcode={ -999 }.
compute needed=0.
compute conf= 95.
do if (trunc( 95 ) >= 100 or (trunc( 95 ) <= 50)).
compute conf = 95.
compute notecode(notes,1)=2.
compute notes=notes+1.
end if.
do if (model >= 0 and model < 4 and modelbt=0).
compute boot=0.
compute mc=0.
compute bc=0.
end if.
do if (mc > 0 and boot > 0).
compute boot=0.
compute bc=0.
end if.
do if ((boot < 1000) and (mc = 0) and (boot > 0)).
compute boot=5000.
end if.
do if ((mc < 1000) and (boot = 0) and (mc > 0)).
compute mc=5000.
end if.
compute p0=-.322232431088.
compute p1 = -1.
compute p2 = -.342242088547.
compute p3 = -.0204231210245.
compute p4 = -.0000453642210148.
compute q0 = .0993484626060.
compute q1 = .588581570495.
compute q2 = .531103462366.
compute q3 = .103537752850.
compute q4 = .0038560700634.
compute badend=0.
compute priorlo = -9999999.
compute priorhi = 9999999.
compute alpha2 = (1-(conf/100))/2.
compute cilm=alpha2*2.
compute y5=sqrt(-2*ln(alpha2)).
compute xp2=(y5+((((y5*p4+p3)*y5+p2)*y5+p1)*y5+p0)/((((y5*q4+q3)*y5+q2)*y5+q1)*y5+q0)).
compute medlb={'   M1  :';'   M2  :';'   M3  :';'   M4  :';'   M5  :';'   M6  :';'   M7  :';'   M8  :';'   M9  :';'   M10 :'}.
compute medlb2={'(M1)','(M2)','(M3)','(M4)','(M5)','(M6)','(M7)','(M8)','(M9)','(M10)'}.
compute xlb={'   X1  :';'   X2  :';'   X3  :';'   X4  :';'   X5  :';'   X6  :';'   X7  :';'   X8  :';'   X9  :'}.
compute highlbw={'M1*W'; 'M2*W'; 'M3*W'; 'M4*W'; 'M5*W'; 'M6*W'; 'M7*W'; 'M8*W'; 'M9*W'; 'M10*W'}.
do if (xmint=1).
compute highlbw={'M1*X'; 'M2*X'; 'M3*X'; 'M4*X'; 'M5*X'; 'M6*X'; 'M7*X'; 'M8*X'; 'M9*X'; 'M10*X'}.
end if.
compute highlbz={'M1*Z'; 'M2*Z'; 'M3*Z'; 'M4*Z'; 'M5*Z'; 'M6*Z'; 'M7*Z'; 'M8*Z'; 'M9*Z';'M10*Z'}.
compute highlbwz={'M1*W*Z'; 'M2*W*Z'; 'M3*W*Z'; 'M4*W*Z'; 'M5*W*Z'; 'M6*W*Z'; 'M7*W*Z'; 'M8*W*Z'; 'M9*W*Z';'M10*W*Z'}.
compute highlbbt={'BOTH(M1)'; 'BOTH(M2)'; 'BOTH(M3)'; 'BOTH(M4)'; 'BOTH(M5)'; 'BOTH(M6)'; 'BOTH(M7)'; 'BOTH(M8)'; 'BOTH(M9)';'BTH(M10)'}.
compute highlbx={'M1*X'; 'M2*X'; 'M3*X'; 'M4*X'; 'M5*X'; 'M6*X'; 'M7*X'; 'M8*X'; 'M9*X'; 'M10*X'}.
compute skipwz=0.
compute validm={1,1,1,1,1,1,1,1,1,1,1,1,1,1,1,1,1,1,1,1,1,1,0,0,0,0,0,1,1,0,0,0,0, 0,0,0,0,0,0,0,0,0,0,0,0,0,0,0,0,0,0,0,0,0,0,0,0,1,1,1,1,1,1,1,1,1,1,1,1,1,1,1,1,1,1,1,0,0,0,1,1, 1,1,1,1,1,1,1,1,1,1,1}.
do if (activate=1).
compute errcode(errs,1)=60.
compute errs=errs+1.
compute criterr=1.
end if.
do if (criterr=0).
do if (( 0 =1)=1).
compute errcode(errs,1)=42.
compute errs=errs+1.
compute criterr=1.
end if.
do if (model > 0 and model < 93).
do if (validm(1,model)=0).
compute errcode(errs,1)=6.
compute errs=errs+1.
compute criterr=1.
end if.
release validm.
end if.
do if ((model > 92 or model < 0) and model <> 999)).
compute errcode(errs,1)=7.
compute errs=errs+1.
compute criterr=1.
end if.
do if (model = 999 and bmatrix(1,1)=-999).
compute errcode(errs,1)=24.
compute errs=errs+1.
compute criterr=1.
end if.
do if (model <> 999 and bmatrix(1,1) <> -999).
compute errcode(errs,1)=25.
compute errs=errs+1.
compute criterr=1.
end if.
do if ((model = 74 or (model > 0 and model < 4)) and ((wmatrix(1,1) <> -999) or (zmatrix(1,1)<>-999) or (wzmatrix(1,1)<>-999)))).
compute errcode(errs,1)=41.
compute errs=errs+1.
compute criterr=1.
end if.
do if (hc3 <> 0).
compute notecode(notes,1) = 5.
compute notes = notes + 1.
do if (hc3 = 1).
compute hc=3.
end if.
end if.
do if ((v <> 'xxxxx') or (q <> 'xxxxx')).
compute errcode(errs,1)=14.
compute errs=errs+1.
compute errcode(errs,1)=48.
compute errs=errs+1.
compute criterr=1.
end if.
do if (oldvars <> 'xxxxx').
compute errcode(errs,1)=48.
compute errs=errs+1.
compute criterr=1.
end if.
do if (cluster <> 'xxxxx').
compute errcode(errs,1)=27.
compute errs=errs+1.
compute criterr=1.
end if.
do if ((y = 'xxxxx') or (x = 'xxxxx')).
compute errcode(errs,1)=1.
compute errs=errs+1.
compute criterr=1.
end if.
do if ((m = 'xxxxx') and model > 3).
compute errcode(errs,1)=8.
compute errs=errs+1.
compute criterr=1.
end if.
end if.
do if (criterr=0).
get ytmp/variables = HOMAIR /names = ynames/MISSING = 99999.
compute nys=ncol(ytmp).
compute needed=nys.
compute n=nrow(ytmp).
compute varnames={ynames}.
compute dat=ytmp.

.


do if (toomany=1 and longname=0).
compute criterr=1.
do if (tooman=0).
compute tooman=1.
compute errcode(errs,1) = 61.
compute errs = errs + 1.
end if.
end if
.
compute modelvar={ '4' ;t(ynames)}.
do if ( 4 =999).
compute modelvar(1,1)='CUSTOM'.
end if.
get xtmp/variables = Pulsenut /names = xnames/MISSING = 99999.
compute nxs=ncol(xtmp).
compute n=nrow(xtmp).
compute needed=needed+nxs.
compute varnames={varnames,xnames}.
compute xcatlab=t(xnames).
compute dat={dat,xtmp}.

.


do if (toomany=1 and longname=0).
compute criterr=1.
do if (tooman=0).
compute tooman=1.
compute errcode(errs,1) = 61.
compute errs = errs + 1.
end if.
end if
.
compute modelvar={modelvar;t(xnames)}.
do if (nxs = 1).
compute modelvlb={'Model  :';'    Y  :';'    X  :'}.
else.
compute modelvlb={'Model  :';'    Y  :';xlb(1:nxs,1)}.
end if.
do if (m <> 'xxxxx').
get mtmp/variables = TG HDL LDL TC /names = mnames/MISSING = 99999.
compute nms=ncol(mtmp).
compute mprod=make(1,nms,0).
compute n=nrow(mtmp).
compute needed=needed+nms.
compute varnames={varnames,mnames}.
compute dat={dat,mtmp}.
compute modelvar={modelvar;t(mnames)}.
compute x2m=make(99,nms,0).
compute m2y=make(99,nms,0).
compute onem=make(nms,1,1).

.


do if (toomany=1 and longname=0).
compute criterr=1.
do if (tooman=0).
compute tooman=1.
compute errcode(errs,1) = 61.
compute errs = errs + 1.
end if.
end if
.
do if (nms > 1 and nms < 11).
compute modelvlb={modelvlb;medlb(1:nms,1)}.
else.
compute modelvlb={modelvlb;'    M  :'}.
end if.
do if (nms > 0 and model < 4).
compute errcode(errs,1)=9.
compute errs=errs+1.
do if (model <> 0).
compute errcode(errs,1)=48.
compute errs=errs+1.
end if.
compute criterr=1.
end if.
end if.
compute wlocatet=0.
compute wlocate=0.
do if (w <> 'xxxxx').
do if (xmint=0).
get wtmp/variables = xxxxx /names = wnames/MISSING = 99999.

.


do if (toomany=1 and longname=0).
compute criterr=1.
do if (tooman=0).
compute tooman=1.
compute errcode(errs,1) = 61.
compute errs = errs + 1.
end if.
end if
.
end if.
do if (xmint=1).
get wtmp/variables = Pulsenut /names = wnames/MISSING = 99999.
end if.
compute nws=ncol(wtmp).
compute n=nrow(wtmp).
compute varnames={varnames,wnames}.
compute wlocate=ncol(varnames).
do if (model=74).
compute wlocatet=1.
do if (xnames <> wnames).
compute errcode(errs,1)=45.
compute errs=errs+1.
compute criterr=1.
end if.
end if.
compute wcatlab=t(wnames).
compute dat={dat,wtmp}.
do if (xmint <> 1).
compute modelvar={modelvar;t(wnames)}.
compute modelvlb={modelvlb;'    W  :'}.
end if.
end if.
do if (z <> 'xxxxx').
get ztmp/variables = xxxxx /names = znames/MISSING = 99999.
compute nzs=ncol(ztmp).
compute n=nrow(ztmp).

.


do if (toomany=1 and longname=0).
compute criterr=1.
do if (tooman=0).
compute tooman=1.
compute errcode(errs,1) = 61.
compute errs = errs + 1.
end if.
end if
.
compute varnames={varnames,znames}.
compute zcatlab=t(znames).
compute dat={dat,ztmp}.
compute modelvar={modelvar;t(znames)}.
compute modelvlb={modelvlb;'    Z  :'}.
end if.
do if (cov <> 'xxxxx').
get ctmp/variables = xxxxx /names = covnames/MISSING = 99999.
compute ncs=ncol(ctmp).
compute n=nrow(ctmp).

.


do if (toomany=1 and longname=0).
compute criterr=1.
do if (tooman=0).
compute tooman=1.
compute errcode(errs,1) = 61.
compute errs = errs + 1.
end if.
end if
.
compute varnames={varnames,covnames}.
compute dat={dat,ctmp}.
end if.
do if (nws > 1 or nzs > 1 or nys > 1 or nxs > 1).
compute errcode(errs,1)=3.
compute errs=errs+1.
compute criterr=1.
end if.
do if ((model = 80 or model = 81) and (nms < 3 or nms > 6)).
compute errcode(errs,1)=32.
compute errs=errs+1.
compute criterr=1.
end if.
do if (model = 82 and nms <> 4).
compute errcode(errs,1)=33.
compute errs=errs+1.
compute criterr=1.
end if.
do if (nms > 10).
compute errcode(errs,1)=37.
compute errs=errs+1.
compute criterr=1.
end if.
do if ((model = 6 or (model > 82 and model < 999)) and (nms < 2 or nms > 6)).
compute errcode(errs,1)=34.
compute errs=errs+1.
compute criterr=1.
end if.
compute match=0.
compute match2=0.
compute mcwzcov=0.
loop i = 1 to (ncol(varnames)-1).
loop j = (i+1) to ncol(varnames).
do if (varnames(i)=varnames(j)).
do if (i < (nxs+nms+nys+1)).
compute match2=match2+1.
end if.
do if (wlocatet=1 and i=2 and j=wlocate).
compute match2=match2-1.
end if.
do if ((wnames=znames) and (nws > 0 or nzs > 0))).
compute match2=match2+1.
end if.
do if (i < (ncol(varnames)-ncs+1)) and j > (ncol(varnames)-ncs)).
do if ((varnames(j)=wnames) and mcw=0)).
compute match=0.
compute wiscov=(j-(ncol(varnames)-ncs)).
end if.
do if ((varnames(j)=wnames) and mcw <>0)).
compute mcwzcov=1.
end if.
do if ((varnames(j)=znames) and mcz=0).
compute match=0.
compute ziscov=(j-(ncol(varnames)-ncs)).
end if.
do if ((varnames(j)=znames) and mcz<>0)).
compute mcwzcov=1.
end if.
end if.
end if.
end loop.
end loop.
do if (match2>0 or match=1).
compute errcode(errs,1)=2.
compute errs=errs+1.
compute criterr=1.
end if.
do if (mcwzcov=1).
compute errcode(errs,1)=50.
compute errs=errs+1.
compute criterr=1.
end if.
compute ninit=nrow(dat).
compute rownum=make(ninit,1,0).
loop i = 1 to ninit.
compute rownum(i,1)=i.
end loop.
compute dat={rownum,dat}.
compute j=1.
compute missrow=0.
loop i = 1 to n.
do if (rsum(dat(i,2:ncol(dat))=99999)=0).
compute dat(j,:)=dat(i,:).
compute j=j+1.
else.
compute missrow={missrow;dat(i,1)}.
end if.
end loop.
do if (j < 5).
compute errcode(errs,1)=62.
compute errs=errs+1.
compute criterr=1.
end if.
do if (criterr=0).
compute rownum=dat(1:(j-1),1).
do if (nrow(missrow) > 1).
compute missrow=t(missrow(2:nrow(missrow),1)).
compute notecode(notes,1) = 29.
compute notes = notes + 1.
end if.
compute dat=dat(1:(j-1),2:ncol(dat)).
compute n=nrow(dat).
compute nmiss=ninit-n.
compute ytmp=dat(:,1:nys).

.
compute desctmp=make((8-(4* 0 )),ncol( ytmp ),-999).
loop jd=1 to ncol( ytmp ).
compute descdat= ytmp (:,jd).
compute desctmp(1,jd) = csum(descdat)/nrow(descdat).
compute desctmp(2,jd) = (nrow(descdat)*sscp(descdat))-(t(csum(descdat))*(csum(descdat))).
compute desctmp(2,jd) = sqrt(desctmp(2,jd)/(nrow(descdat)*(nrow(descdat)-1))).
compute desctmp(3,jd)=cmin(descdat).
compute desctmp(4,jd)=cmax(descdat).
do if ( 0 =0).
compute minwarn=0.
compute maxwarn=0.
do if ((desctmp(3,jd)=desctmp(4,jd)) and novar=0).
compute errcode(errs,1)=15.
compute errs=errs+1.
compute criterr=1.
compute novar=1.
end if.
compute tmp=((descdat(:,1)=desctmp(3,jd))+(descdat(:,1)=desctmp(4,jd))).
compute desctmp(8,jd)=(csum(tmp)=nrow(tmp)).
compute tmp = descdat.
compute tmp(GRADE(descdat),:) = descdat.
compute descdat = tmp.
release tmp.
compute decval={.16;.5;.84}.
loop kd=1 to 3.
compute low=trunc(decval(kd,1)*(nrow(descdat)+1)).
compute lowdec=decval(kd,1)*(nrow(descdat)+1)-low.
compute value=descdat(low,1)+(descdat((low+1),1)-descdat(low,1))*lowdec.
compute desctmp((4+kd),jd)=value.
end loop.
compute mnotev=1.
compute modvals=desctmp(5:7,:).
do if (quantile <> 1).
compute desctmp(5,jd)=desctmp(1,jd)-desctmp(2,jd).
compute desctmp(6,jd)=desctmp(1,jd).
compute desctmp(7,jd)=desctmp(1,jd)+desctmp(2,jd).
compute modvals=desctmp(5:7,:).
compute mnotev=2.
do if (modvals(1,1) < desctmp(3,1)).
compute modvals(1,1)=desctmp(3,1).
compute minwarn=1.
end if.
do if (modvals(3,1) > desctmp(4,1)).
compute modvals(3,1)=desctmp(4,1).
compute maxwarn=1.
end if.
end if.
do if (desctmp(8,1)=1).
compute modvals={desctmp(3,1);desctmp(4,1)}.
compute mnotev=0.
compute minwarn=0.
compute maxwarn=0.
end if.
end if.
end loop
.
compute ysd=desctmp(2,:).
compute ovsd=ysd.
do if (desctmp(8,1)=1).
compute ydich=1.
do if (total=1).
compute total=0.
compute notecode(notes,1) = 24.
compute notes = notes + 1.
end if.
do if (effsize=1).
compute effsize=0.
compute notecode(notes,1) = 25.
compute notes = notes + 1.
end if.
do if (model=74).
compute errcode(errs,1) = 72.
compute errs=errs+1.
compute criterr=1.
end if.
compute omx = cmax(ytmp).
compute omn = cmin(ytmp).
compute ytmp = (ytmp = omx).
compute dat(:,1:nys)=(dat(:,1:nys)=omx).
compute rcd = {omn, 0; omx, 1}.
end if.
compute xtmp=dat(:,(nys+1):(nys+nxs)).

.
compute desctmp=make((8-(4* 0 )),ncol( xtmp ),-999).
loop jd=1 to ncol( xtmp ).
compute descdat= xtmp (:,jd).
compute desctmp(1,jd) = csum(descdat)/nrow(descdat).
compute desctmp(2,jd) = (nrow(descdat)*sscp(descdat))-(t(csum(descdat))*(csum(descdat))).
compute desctmp(2,jd) = sqrt(desctmp(2,jd)/(nrow(descdat)*(nrow(descdat)-1))).
compute desctmp(3,jd)=cmin(descdat).
compute desctmp(4,jd)=cmax(descdat).
do if ( 0 =0).
compute minwarn=0.
compute maxwarn=0.
do if ((desctmp(3,jd)=desctmp(4,jd)) and novar=0).
compute errcode(errs,1)=15.
compute errs=errs+1.
compute criterr=1.
compute novar=1.
end if.
compute tmp=((descdat(:,1)=desctmp(3,jd))+(descdat(:,1)=desctmp(4,jd))).
compute desctmp(8,jd)=(csum(tmp)=nrow(tmp)).
compute tmp = descdat.
compute tmp(GRADE(descdat),:) = descdat.
compute descdat = tmp.
release tmp.
compute decval={.16;.5;.84}.
loop kd=1 to 3.
compute low=trunc(decval(kd,1)*(nrow(descdat)+1)).
compute lowdec=decval(kd,1)*(nrow(descdat)+1)-low.
compute value=descdat(low,1)+(descdat((low+1),1)-descdat(low,1))*lowdec.
compute desctmp((4+kd),jd)=value.
end loop.
compute mnotev=1.
compute modvals=desctmp(5:7,:).
do if (quantile <> 1).
compute desctmp(5,jd)=desctmp(1,jd)-desctmp(2,jd).
compute desctmp(6,jd)=desctmp(1,jd).
compute desctmp(7,jd)=desctmp(1,jd)+desctmp(2,jd).
compute modvals=desctmp(5:7,:).
compute mnotev=2.
do if (modvals(1,1) < desctmp(3,1)).
compute modvals(1,1)=desctmp(3,1).
compute minwarn=1.
end if.
do if (modvals(3,1) > desctmp(4,1)).
compute modvals(3,1)=desctmp(4,1).
compute maxwarn=1.
end if.
end if.
do if (desctmp(8,1)=1).
compute modvals={desctmp(3,1);desctmp(4,1)}.
compute mnotev=0.
compute minwarn=0.
compute maxwarn=0.
end if.
end if.
end loop
.
compute xsd=desctmp(2,:).
compute xmodvals=modvals.
compute xdich=desctmp(8,1).
compute xmx=cmax(xtmp).
compute xmn=cmin(xtmp).
do if ((mcx > 0) and (xrefvals(1,1) <> 999) and (xmint=1) and (model= 74)).
compute notecode(notes,1) = 36.
compute notes = notes + 1.
end if.
do if (mcx=0).
do if (ncol(xrefvals)>2 and model=74 and xmint=1).
compute errcode(errs,1)=67.
compute errs=errs+1.
compute criterr=1.
end if.
do if ((model=74) and (xmint=1)).
do if ((xrefvals(1,1)=999) and nxvls=1 and xdich=0).
compute errcode(errs,1)=66.
compute errs=errs+1.
compute criterr=1.
end if.
do if ((xrefvals(1,1)=999) and (xdich=1)).
compute xrefvals={xmn,xmx}.
compute xscaling=xrefvals(1,2)-xrefvals(1,1).
end if.
do if (ncol(xrefvals)=1 and xrefvals(1,1) <> 999).
do if (xdich=0).
compute xrefvals={xrefvals,(xrefvals(1,1)+1)}.
compute xscaling=xrefvals(1,2)-xrefvals(1,1).
end if.
do if (xdich=1).
do if ((xrefvals(1,1) <> xmx) and (xrefvals(1,1) <> xmn)).
compute errcode(errs,1)=70.
compute errs=errs+1.
compute criterr=1.
end if.
do if (xrefvals(1,1) = xmx).
compute xrefvals={xrefvals,xmn}.
compute xscaling=xrefvals(1,2)-xrefvals(1,1).
end if.
do if (xrefvals(1,1) = xmn).
compute xrefvals={xrefvals,xmx}.
compute xscaling=xrefvals(1,2)-xrefvals(1,1).
end if.
end if.
end if.
do if (ncol(xrefvals)=2).
compute xscaling=xrefvals(1,2)-xrefvals(1,1).
do if (xdich=1).
compute xreferr=1.
do if (((xrefvals(1,1) = xmx) and (xrefvals(1,2)=xmn)) or ((xrefvals(1,1) = xmn) and (xrefvals(1,2)=xmx))).
compute xreferr=0.
end if.
do if (xreferr=1).
compute errcode(errs,1)=70.
compute errs=errs+1.
compute criterr=1.
end if.
end if.
end if.
end if.
end if.
do if (xmint=1 and model=74 and mcx=0).
compute xmodvals=t(xrefvals).
compute xcontcf=1.
end if.
compute nxpval=nrow(xmodvals).
compute xprobval=xmodvals.
do if (xdich =1 and mcx > 0).
compute mcx=0.
compute errcode(errs,1) = 52.
compute errs = errs + 1.
compute criterr = 1.
end if.
do if (nms > 0).
compute mtmp=dat(:,(nys+nxs+1):(nys+nxs+nms)).

.
compute desctmp=make((8-(4* 0 )),ncol( mtmp ),-999).
loop jd=1 to ncol( mtmp ).
compute descdat= mtmp (:,jd).
compute desctmp(1,jd) = csum(descdat)/nrow(descdat).
compute desctmp(2,jd) = (nrow(descdat)*sscp(descdat))-(t(csum(descdat))*(csum(descdat))).
compute desctmp(2,jd) = sqrt(desctmp(2,jd)/(nrow(descdat)*(nrow(descdat)-1))).
compute desctmp(3,jd)=cmin(descdat).
compute desctmp(4,jd)=cmax(descdat).
do if ( 0 =0).
compute minwarn=0.
compute maxwarn=0.
do if ((desctmp(3,jd)=desctmp(4,jd)) and novar=0).
compute errcode(errs,1)=15.
compute errs=errs+1.
compute criterr=1.
compute novar=1.
end if.
compute tmp=((descdat(:,1)=desctmp(3,jd))+(descdat(:,1)=desctmp(4,jd))).
compute desctmp(8,jd)=(csum(tmp)=nrow(tmp)).
compute tmp = descdat.
compute tmp(GRADE(descdat),:) = descdat.
compute descdat = tmp.
release tmp.
compute decval={.16;.5;.84}.
loop kd=1 to 3.
compute low=trunc(decval(kd,1)*(nrow(descdat)+1)).
compute lowdec=decval(kd,1)*(nrow(descdat)+1)-low.
compute value=descdat(low,1)+(descdat((low+1),1)-descdat(low,1))*lowdec.
compute desctmp((4+kd),jd)=value.
end loop.
compute mnotev=1.
compute modvals=desctmp(5:7,:).
do if (quantile <> 1).
compute desctmp(5,jd)=desctmp(1,jd)-desctmp(2,jd).
compute desctmp(6,jd)=desctmp(1,jd).
compute desctmp(7,jd)=desctmp(1,jd)+desctmp(2,jd).
compute modvals=desctmp(5:7,:).
compute mnotev=2.
do if (modvals(1,1) < desctmp(3,1)).
compute modvals(1,1)=desctmp(3,1).
compute minwarn=1.
end if.
do if (modvals(3,1) > desctmp(4,1)).
compute modvals(3,1)=desctmp(4,1).
compute maxwarn=1.
end if.
end if.
do if (desctmp(8,1)=1).
compute modvals={desctmp(3,1);desctmp(4,1)}.
compute mnotev=0.
compute minwarn=0.
compute maxwarn=0.
end if.
end if.
end loop
.
compute ovsd={desctmp(2,:),ysd}.
compute medmeans=cdeval.
do if ((cdeval(1,1) <> -999) and (ncol(medmeans) <> nms) and (model=74)).
compute errcode(errs,1)=64.
compute errs=errs+1.
compute criterr=1.
end if.
do if ((cdeval(1,1)=-999) and (model=74)).
compute medmeans=desctmp(1,:).
end if.
do if ((cdeval(1,1)<> -999) and (model=74) and (criterr=0)).
compute notecode(notes,1) = 31.
compute notes = notes + 1.
end if.
do if ((rsum(desctmp(8,:))>0) and (mdichok <> 1)).
compute errcode(errs,1)=43.
compute errs=errs+1.
compute criterr=1.
end if.
compute mmodvals=modvals.
compute mprobval=mmodvals.
end if.
do if (nws > 0).
compute wtmp=dat(:,(nys+nxs+nms+1):(nys+nxs+nms+nws)).

.
compute desctmp=make((8-(4* 0 )),ncol( wtmp ),-999).
loop jd=1 to ncol( wtmp ).
compute descdat= wtmp (:,jd).
compute desctmp(1,jd) = csum(descdat)/nrow(descdat).
compute desctmp(2,jd) = (nrow(descdat)*sscp(descdat))-(t(csum(descdat))*(csum(descdat))).
compute desctmp(2,jd) = sqrt(desctmp(2,jd)/(nrow(descdat)*(nrow(descdat)-1))).
compute desctmp(3,jd)=cmin(descdat).
compute desctmp(4,jd)=cmax(descdat).
do if ( 0 =0).
compute minwarn=0.
compute maxwarn=0.
do if ((desctmp(3,jd)=desctmp(4,jd)) and novar=0).
compute errcode(errs,1)=15.
compute errs=errs+1.
compute criterr=1.
compute novar=1.
end if.
compute tmp=((descdat(:,1)=desctmp(3,jd))+(descdat(:,1)=desctmp(4,jd))).
compute desctmp(8,jd)=(csum(tmp)=nrow(tmp)).
compute tmp = descdat.
compute tmp(GRADE(descdat),:) = descdat.
compute descdat = tmp.
release tmp.
compute decval={.16;.5;.84}.
loop kd=1 to 3.
compute low=trunc(decval(kd,1)*(nrow(descdat)+1)).
compute lowdec=decval(kd,1)*(nrow(descdat)+1)-low.
compute value=descdat(low,1)+(descdat((low+1),1)-descdat(low,1))*lowdec.
compute desctmp((4+kd),jd)=value.
end loop.
compute mnotev=1.
compute modvals=desctmp(5:7,:).
do if (quantile <> 1).
compute desctmp(5,jd)=desctmp(1,jd)-desctmp(2,jd).
compute desctmp(6,jd)=desctmp(1,jd).
compute desctmp(7,jd)=desctmp(1,jd)+desctmp(2,jd).
compute modvals=desctmp(5:7,:).
compute mnotev=2.
do if (modvals(1,1) < desctmp(3,1)).
compute modvals(1,1)=desctmp(3,1).
compute minwarn=1.
end if.
do if (modvals(3,1) > desctmp(4,1)).
compute modvals(3,1)=desctmp(4,1).
compute maxwarn=1.
end if.
end if.
do if (desctmp(8,1)=1).
compute modvals={desctmp(3,1);desctmp(4,1)}.
compute mnotev=0.
compute minwarn=0.
compute maxwarn=0.
end if.
end if.
end loop
.
compute wmodvals=modvals.
compute wdich=desctmp(8,1).
do if (wdich =1 and mcw > 0).
compute mcw=0.
compute errcode(errs,1) = 52.
compute errs = errs + 1.
compute criterr = 1.
end if.
compute wmin=desctmp(3,1).
compute wmax=desctmp(4,1).
compute minwwarn=minwarn.
compute maxwwarn=maxwarn.
compute wnotev=mnotev.
compute wmodval={ 999 }.
do if (xmint=1 and model=74 and mcx=0).
compute wmodval=xrefvals.
end if.
compute nwcontr=ncol(wmodval).
do if (wmodval(1,1) <> 999).
compute wmodvals=wmodval(1,1).
compute wmodcust=1.
do if (nwcontr > 1).
compute wmodvals=t(wmodval).
end if.
compute minwwarn=0.
compute maxwwarn=0.
compute wnotev=0.
end if.
compute wprobval=wmodvals.
compute nwpval=nrow(wmodvals).
end if.
do if (nzs > 0).
compute ztmp=dat(:,(nys+nxs+nms+nws+1):(nys+nxs+nms+nws+nzs)).

.
compute desctmp=make((8-(4* 0 )),ncol( ztmp ),-999).
loop jd=1 to ncol( ztmp ).
compute descdat= ztmp (:,jd).
compute desctmp(1,jd) = csum(descdat)/nrow(descdat).
compute desctmp(2,jd) = (nrow(descdat)*sscp(descdat))-(t(csum(descdat))*(csum(descdat))).
compute desctmp(2,jd) = sqrt(desctmp(2,jd)/(nrow(descdat)*(nrow(descdat)-1))).
compute desctmp(3,jd)=cmin(descdat).
compute desctmp(4,jd)=cmax(descdat).
do if ( 0 =0).
compute minwarn=0.
compute maxwarn=0.
do if ((desctmp(3,jd)=desctmp(4,jd)) and novar=0).
compute errcode(errs,1)=15.
compute errs=errs+1.
compute criterr=1.
compute novar=1.
end if.
compute tmp=((descdat(:,1)=desctmp(3,jd))+(descdat(:,1)=desctmp(4,jd))).
compute desctmp(8,jd)=(csum(tmp)=nrow(tmp)).
compute tmp = descdat.
compute tmp(GRADE(descdat),:) = descdat.
compute descdat = tmp.
release tmp.
compute decval={.16;.5;.84}.
loop kd=1 to 3.
compute low=trunc(decval(kd,1)*(nrow(descdat)+1)).
compute lowdec=decval(kd,1)*(nrow(descdat)+1)-low.
compute value=descdat(low,1)+(descdat((low+1),1)-descdat(low,1))*lowdec.
compute desctmp((4+kd),jd)=value.
end loop.
compute mnotev=1.
compute modvals=desctmp(5:7,:).
do if (quantile <> 1).
compute desctmp(5,jd)=desctmp(1,jd)-desctmp(2,jd).
compute desctmp(6,jd)=desctmp(1,jd).
compute desctmp(7,jd)=desctmp(1,jd)+desctmp(2,jd).
compute modvals=desctmp(5:7,:).
compute mnotev=2.
do if (modvals(1,1) < desctmp(3,1)).
compute modvals(1,1)=desctmp(3,1).
compute minwarn=1.
end if.
do if (modvals(3,1) > desctmp(4,1)).
compute modvals(3,1)=desctmp(4,1).
compute maxwarn=1.
end if.
end if.
do if (desctmp(8,1)=1).
compute modvals={desctmp(3,1);desctmp(4,1)}.
compute mnotev=0.
compute minwarn=0.
compute maxwarn=0.
end if.
end if.
end loop
.
compute zmodvals=modvals.
compute zdich=desctmp(8,1).
do if (zdich =1 and mcz > 0).
compute mcz=0.
compute errcode(errs,1) = 52.
compute errs = errs + 1.
compute criterr = 1.
end if.
compute zmin=desctmp(3,1).
compute zmax=desctmp(4,1).
compute minzwarn=minwarn.
compute maxzwarn=maxwarn.
compute znotev=mnotev.
compute zmodval={ 999 }.
compute nzcontr=ncol(zmodval).
do if (zmodval(1,1) <> 999).
compute zmodvals=zmodval(1,1).
compute zmodcust=1.
do if (nzcontr > 1).
compute zmodvals=t(zmodval).
end if.
compute minzwarn=0.
compute maxzwarn=0.
compute znotev=0.
end if.
compute zprobval=zmodvals.
compute nzpval=nrow(zmodvals).
end if.
do if (ncs > 0).
compute ctmp=dat(:,(nys+nxs+nms+nws+nzs+1):(nys+nxs+nms+nws+nzs+ncs)).

.
compute desctmp=make((8-(4* 0 )),ncol( ctmp ),-999).
loop jd=1 to ncol( ctmp ).
compute descdat= ctmp (:,jd).
compute desctmp(1,jd) = csum(descdat)/nrow(descdat).
compute desctmp(2,jd) = (nrow(descdat)*sscp(descdat))-(t(csum(descdat))*(csum(descdat))).
compute desctmp(2,jd) = sqrt(desctmp(2,jd)/(nrow(descdat)*(nrow(descdat)-1))).
compute desctmp(3,jd)=cmin(descdat).
compute desctmp(4,jd)=cmax(descdat).
do if ( 0 =0).
compute minwarn=0.
compute maxwarn=0.
do if ((desctmp(3,jd)=desctmp(4,jd)) and novar=0).
compute errcode(errs,1)=15.
compute errs=errs+1.
compute criterr=1.
compute novar=1.
end if.
compute tmp=((descdat(:,1)=desctmp(3,jd))+(descdat(:,1)=desctmp(4,jd))).
compute desctmp(8,jd)=(csum(tmp)=nrow(tmp)).
compute tmp = descdat.
compute tmp(GRADE(descdat),:) = descdat.
compute descdat = tmp.
release tmp.
compute decval={.16;.5;.84}.
loop kd=1 to 3.
compute low=trunc(decval(kd,1)*(nrow(descdat)+1)).
compute lowdec=decval(kd,1)*(nrow(descdat)+1)-low.
compute value=descdat(low,1)+(descdat((low+1),1)-descdat(low,1))*lowdec.
compute desctmp((4+kd),jd)=value.
end loop.
compute mnotev=1.
compute modvals=desctmp(5:7,:).
do if (quantile <> 1).
compute desctmp(5,jd)=desctmp(1,jd)-desctmp(2,jd).
compute desctmp(6,jd)=desctmp(1,jd).
compute desctmp(7,jd)=desctmp(1,jd)+desctmp(2,jd).
compute modvals=desctmp(5:7,:).
compute mnotev=2.
do if (modvals(1,1) < desctmp(3,1)).
compute modvals(1,1)=desctmp(3,1).
compute minwarn=1.
end if.
do if (modvals(3,1) > desctmp(4,1)).
compute modvals(3,1)=desctmp(4,1).
compute maxwarn=1.
end if.
end if.
do if (desctmp(8,1)=1).
compute modvals={desctmp(3,1);desctmp(4,1)}.
compute mnotev=0.
compute minwarn=0.
compute maxwarn=0.
end if.
end if.
end loop
.
compute covmeans=desctmp(1,:).
compute coval={ -999 }.
do if ((coval(1,1) <> -999) and (ncol(coval) <> ncs) and (model=74)).
compute errcode(errs,1)=69.
compute errs=errs+1.
compute criterr=1.
end if.
do if ((coval(1,1)<> -999) and (criterr=0) and (model=74)).
compute notecode(notes,1) = 35.
compute notes = notes + 1.
compute cuscoval=1.
end if.
end if.
compute n=nrow(ytmp).
compute ones=make(n,1,1).
do if (nws > 0 and mcw > 0).
compute tmp={rownum,wtmp(:,1)}.

.
compute dd= tmp.
compute temp = dd.
compute temp(GRADE(dd(:,2)),:) = dd.
compute dd = temp.
compute dummy = design(dd(:,2)).
compute nvls = ncol(dummy).
compute nnvls = csum(dummy).
compute mnvls = cmin(t(nnvls)).
compute conmat1=1.
do if (mnvls < 2).
compute errcode(errs,1) = 5.
compute errs = errs + 1.
compute criterr = 1.
end if.
do if (nvls > 9).
compute errcode(errs,1) = 4.
compute errs = errs+1.
compute criterr = 1.
end if.
do if (criterr = 0).
compute dumok = 1.
compute nnvls=make(nvls,1,0).
compute nnvls(1,1)=dd(1,2).
compute temp = 2.
loop i = 2 to n.
do if (dd(i,2) <> nnvls((temp-1),1)).
compute nnvls(temp,1)=dd(i,2).
compute temp = temp+1.
end if.
end loop.
do if ( mcw > 0).
compute x = dummy(:,2:ncol(dummy)).
compute nx = ncol(x).
compute minus1 = make(1,ncol(x),-1).
compute xdes=make((nx+1),3,0).
compute xdes(1,1)=dd(1,2).
compute xdes(1,2)=1.
compute temp = 2.
loop k = 2 to n.
do if (dd(k,2) <> dd((k-1),2)).
compute xdes(temp,2) = k.
compute xdes(temp,1) = dd(k,2).
compute xdes((temp-1),3) = k-1.
compute temp=temp+1.
end if.
end loop.
compute xdes((temp-1),3)=n.
compute xdes = {xdes, (xdes(:,3)-xdes(:,2)+1)}.
do if ( mcw = 4).
loop k = 1 to n.
do if (rsum(x(k,:)) = 0).
compute x(k,:) = minus1.
end if.
end loop.
end if.
do if ( mcw = 2 or mcw = 3 or mcw =5).
loop k = 1 to n.
do if (rsum(x(k,:)) > 0).
loop i = 1 to ncol(x).
do if (x(k,i) = 0).
compute x(k,i) = 1.
else.
break.
end if.
end loop.
end if.
end loop.
do if ( mcw = 3).
compute conmat1={-8,1,1,1,1,1,1,1,1; 0,-7,1,1,1,1,1,1,1; 0,0,-6,1,1,1,1,1,1; 0,0,0,-5,1,1,1,1,1; 0,0,0,0,-4,1,1,1,1; 0,0,0,0,0,-3,1,1,1; 0,0,0,0,0,0,-2,1,1; 0,0,0,0,0,0,0,-1,1}.
loop i = 1 to 8.
compute conmat1(i,:)=conmat1(i,:)/(10-i).
end loop.
compute conmat1=t(conmat1((10-nvls):8,(10-nvls):9)).
loop k=1 to n.
compute x(k,:)=conmat1((rsum(x(k,:))+1),:).
end loop.
end if.
end if.
do if ( mcw = 5).
compute custcode={ -999 }.
do if (ncol(custcode) <> (nvls*(nvls-1))).
compute errcode(errs,1) = (37+ 2 ).
compute errs = errs + 1.
compute criterr = 1.
end if.
do if (ncol(custcode) = (nvls*(nvls-1))).
compute conmat1=make(nvls,(nvls-1),0).
compute cnt=1.
loop i = 1 to nvls.
loop k = 1 to (nvls-1).
compute conmat1(i,k)=custcode(1,cnt).
compute cnt=cnt+1.
end loop.
end loop.
loop k=1 to n.
compute x(k,:)=conmat1((rsum(x(k,:))+1),:).
end loop.
end if.
end if.
compute xskip = 1.
compute dummat = make((nx+1),nx,0).
compute dummat((2:nrow(dummat)),:)=ident(nx).
do if ( mcw = 4).
compute dummat(1,:) = minus1.
end if.
do if ( mcw = 2).
loop i = 2 to nrow(dummat).
loop j = 1 to (i-1).
compute dummat(i,j) = 1.
end loop.
end loop.
end if.
do if ( mcw = 3).
compute dummat=conmat1.
end if.
do if ( mcw = 5 and criterr=0).
compute dummat=conmat1.
end if.
compute dummat={nnvls, dummat}.
compute x={dd(:,1),x}.
compute temp = x.
compute temp(GRADE(x(:,1)),:) = x.
compute x = temp.
release conmat1,temp,dd,xskip,xdes,dummy.
end if.
end if
.
compute wmodvals=nnvls.
compute nwpval=nrow(wmodvals).
do if (criterr=0).
compute minwwarn=0.
compute maxwwarn=0.
compute wnotev=0.
compute wtmp=x(:,2:ncol(x)).
compute wcatlab={'W1';'W2';'W3';'W4';'W5';'W6';'W7';'W8';'W9'}.
do if (xmint=1).
compute wcatlab={'X1';'X2';'X3';'X4';'X5';'X6';'X7';'X8';'X9'}.
end if.
compute nwvls=nvls-1.
compute mcwok=1.
compute dummatw=dummat.
compute wprobval=dummatw(:,2:ncol(dummatw)).
do if (modcok=1).
compute wcontval=make(2,ncol(wprobval),-999).
compute temp=0.
loop i = 1 to 2.
loop j = 1 to nrow(dummatw).
do if (contvec(i,1)=dummatw(j,1)).
compute wcontval(i,:)=wprobval(j,:).
compute temp=temp+1.
end if.
end loop.
end loop.
do if (temp < 2).
compute notecode(notes,1) = 20.
compute notes = notes + 1.
compute modcok=0.
end if.
end if.
do if ((wmodval(1,1) <> 999) and (xmint <> 1)).
compute notecode(notes,1) = 9.
compute notes = notes + 1.
end if.
release tmp, dummat.
end if.
end if.
do if (nzs > 0 and mcz > 0).
compute tmp={rownum,ztmp(:,1)}.

.
compute dd= tmp.
compute temp = dd.
compute temp(GRADE(dd(:,2)),:) = dd.
compute dd = temp.
compute dummy = design(dd(:,2)).
compute nvls = ncol(dummy).
compute nnvls = csum(dummy).
compute mnvls = cmin(t(nnvls)).
compute conmat1=1.
do if (mnvls < 2).
compute errcode(errs,1) = 5.
compute errs = errs + 1.
compute criterr = 1.
end if.
do if (nvls > 9).
compute errcode(errs,1) = 4.
compute errs = errs+1.
compute criterr = 1.
end if.
do if (criterr = 0).
compute dumok = 1.
compute nnvls=make(nvls,1,0).
compute nnvls(1,1)=dd(1,2).
compute temp = 2.
loop i = 2 to n.
do if (dd(i,2) <> nnvls((temp-1),1)).
compute nnvls(temp,1)=dd(i,2).
compute temp = temp+1.
end if.
end loop.
do if ( mcz > 0).
compute x = dummy(:,2:ncol(dummy)).
compute nx = ncol(x).
compute minus1 = make(1,ncol(x),-1).
compute xdes=make((nx+1),3,0).
compute xdes(1,1)=dd(1,2).
compute xdes(1,2)=1.
compute temp = 2.
loop k = 2 to n.
do if (dd(k,2) <> dd((k-1),2)).
compute xdes(temp,2) = k.
compute xdes(temp,1) = dd(k,2).
compute xdes((temp-1),3) = k-1.
compute temp=temp+1.
end if.
end loop.
compute xdes((temp-1),3)=n.
compute xdes = {xdes, (xdes(:,3)-xdes(:,2)+1)}.
do if ( mcz = 4).
loop k = 1 to n.
do if (rsum(x(k,:)) = 0).
compute x(k,:) = minus1.
end if.
end loop.
end if.
do if ( mcz = 2 or mcz = 3 or mcz =5).
loop k = 1 to n.
do if (rsum(x(k,:)) > 0).
loop i = 1 to ncol(x).
do if (x(k,i) = 0).
compute x(k,i) = 1.
else.
break.
end if.
end loop.
end if.
end loop.
do if ( mcz = 3).
compute conmat1={-8,1,1,1,1,1,1,1,1; 0,-7,1,1,1,1,1,1,1; 0,0,-6,1,1,1,1,1,1; 0,0,0,-5,1,1,1,1,1; 0,0,0,0,-4,1,1,1,1; 0,0,0,0,0,-3,1,1,1; 0,0,0,0,0,0,-2,1,1; 0,0,0,0,0,0,0,-1,1}.
loop i = 1 to 8.
compute conmat1(i,:)=conmat1(i,:)/(10-i).
end loop.
compute conmat1=t(conmat1((10-nvls):8,(10-nvls):9)).
loop k=1 to n.
compute x(k,:)=conmat1((rsum(x(k,:))+1),:).
end loop.
end if.
end if.
do if ( mcz = 5).
compute custcode={ -999 }.
do if (ncol(custcode) <> (nvls*(nvls-1))).
compute errcode(errs,1) = (37+ 3 ).
compute errs = errs + 1.
compute criterr = 1.
end if.
do if (ncol(custcode) = (nvls*(nvls-1))).
compute conmat1=make(nvls,(nvls-1),0).
compute cnt=1.
loop i = 1 to nvls.
loop k = 1 to (nvls-1).
compute conmat1(i,k)=custcode(1,cnt).
compute cnt=cnt+1.
end loop.
end loop.
loop k=1 to n.
compute x(k,:)=conmat1((rsum(x(k,:))+1),:).
end loop.
end if.
end if.
compute xskip = 1.
compute dummat = make((nx+1),nx,0).
compute dummat((2:nrow(dummat)),:)=ident(nx).
do if ( mcz = 4).
compute dummat(1,:) = minus1.
end if.
do if ( mcz = 2).
loop i = 2 to nrow(dummat).
loop j = 1 to (i-1).
compute dummat(i,j) = 1.
end loop.
end loop.
end if.
do if ( mcz = 3).
compute dummat=conmat1.
end if.
do if ( mcz = 5 and criterr=0).
compute dummat=conmat1.
end if.
compute dummat={nnvls, dummat}.
compute x={dd(:,1),x}.
compute temp = x.
compute temp(GRADE(x(:,1)),:) = x.
compute x = temp.
release conmat1,temp,dd,xskip,xdes,dummy.
end if.
end if
.
compute zmodvals=nnvls.
compute nzpval=nrow(zmodvals).
do if (criterr=0).
compute minzwarn=0.
compute maxzwarn=0.
compute znotev=0.
compute ztmp=x(:,2:ncol(x)).
compute zcatlab={'Z1';'Z2';'Z3';'Z4';'Z5';'Z6';'Z7';'Z8';'Z9'}.
compute nzvls=nvls-1.
compute mczok=1.
compute dummatz=dummat.
compute zprobval=dummatz(:,2:ncol(dummatz)).
do if (modcok=1).
compute zcontval=make(2,ncol(zprobval),-999).
compute temp=0.
loop i = 1 to 2.
loop j = 1 to nrow(dummatz).
do if (contvec(i,2)=dummatz(j,1)).
compute zcontval(i,:)=zprobval(j,:).
compute temp=temp+1.
end if.
end loop.
end loop.
do if (temp < 2).
compute notecode(notes,1) = 20.
compute notes = notes + 1.
compute modcok=0.
end if.
end if.
do if (zmodval(1,1) <> 999).
compute notecode(notes,1) = 10.
compute notes = notes + 1.
end if.
release tmp, dummat.
end if.
end if.
do if (nxs > 0 and mcx > 0).
compute tmp={rownum,xtmp(:,1)}.

.
compute dd= tmp.
compute temp = dd.
compute temp(GRADE(dd(:,2)),:) = dd.
compute dd = temp.
compute dummy = design(dd(:,2)).
compute nvls = ncol(dummy).
compute nnvls = csum(dummy).
compute mnvls = cmin(t(nnvls)).
compute conmat1=1.
do if (mnvls < 2).
compute errcode(errs,1) = 5.
compute errs = errs + 1.
compute criterr = 1.
end if.
do if (nvls > 9).
compute errcode(errs,1) = 4.
compute errs = errs+1.
compute criterr = 1.
end if.
do if (criterr = 0).
compute dumok = 1.
compute nnvls=make(nvls,1,0).
compute nnvls(1,1)=dd(1,2).
compute temp = 2.
loop i = 2 to n.
do if (dd(i,2) <> nnvls((temp-1),1)).
compute nnvls(temp,1)=dd(i,2).
compute temp = temp+1.
end if.
end loop.
do if ( mcx > 0).
compute x = dummy(:,2:ncol(dummy)).
compute nx = ncol(x).
compute minus1 = make(1,ncol(x),-1).
compute xdes=make((nx+1),3,0).
compute xdes(1,1)=dd(1,2).
compute xdes(1,2)=1.
compute temp = 2.
loop k = 2 to n.
do if (dd(k,2) <> dd((k-1),2)).
compute xdes(temp,2) = k.
compute xdes(temp,1) = dd(k,2).
compute xdes((temp-1),3) = k-1.
compute temp=temp+1.
end if.
end loop.
compute xdes((temp-1),3)=n.
compute xdes = {xdes, (xdes(:,3)-xdes(:,2)+1)}.
do if ( mcx = 4).
loop k = 1 to n.
do if (rsum(x(k,:)) = 0).
compute x(k,:) = minus1.
end if.
end loop.
end if.
do if ( mcx = 2 or mcx = 3 or mcx =5).
loop k = 1 to n.
do if (rsum(x(k,:)) > 0).
loop i = 1 to ncol(x).
do if (x(k,i) = 0).
compute x(k,i) = 1.
else.
break.
end if.
end loop.
end if.
end loop.
do if ( mcx = 3).
compute conmat1={-8,1,1,1,1,1,1,1,1; 0,-7,1,1,1,1,1,1,1; 0,0,-6,1,1,1,1,1,1; 0,0,0,-5,1,1,1,1,1; 0,0,0,0,-4,1,1,1,1; 0,0,0,0,0,-3,1,1,1; 0,0,0,0,0,0,-2,1,1; 0,0,0,0,0,0,0,-1,1}.
loop i = 1 to 8.
compute conmat1(i,:)=conmat1(i,:)/(10-i).
end loop.
compute conmat1=t(conmat1((10-nvls):8,(10-nvls):9)).
loop k=1 to n.
compute x(k,:)=conmat1((rsum(x(k,:))+1),:).
end loop.
end if.
end if.
do if ( mcx = 5).
compute custcode={ -999 }.
do if (ncol(custcode) <> (nvls*(nvls-1))).
compute errcode(errs,1) = (37+ 1 ).
compute errs = errs + 1.
compute criterr = 1.
end if.
do if (ncol(custcode) = (nvls*(nvls-1))).
compute conmat1=make(nvls,(nvls-1),0).
compute cnt=1.
loop i = 1 to nvls.
loop k = 1 to (nvls-1).
compute conmat1(i,k)=custcode(1,cnt).
compute cnt=cnt+1.
end loop.
end loop.
loop k=1 to n.
compute x(k,:)=conmat1((rsum(x(k,:))+1),:).
end loop.
end if.
end if.
compute xskip = 1.
compute dummat = make((nx+1),nx,0).
compute dummat((2:nrow(dummat)),:)=ident(nx).
do if ( mcx = 4).
compute dummat(1,:) = minus1.
end if.
do if ( mcx = 2).
loop i = 2 to nrow(dummat).
loop j = 1 to (i-1).
compute dummat(i,j) = 1.
end loop.
end loop.
end if.
do if ( mcx = 3).
compute dummat=conmat1.
end if.
do if ( mcx = 5 and criterr=0).
compute dummat=conmat1.
end if.
compute dummat={nnvls, dummat}.
compute x={dd(:,1),x}.
compute temp = x.
compute temp(GRADE(x(:,1)),:) = x.
compute x = temp.
release conmat1,temp,dd,xskip,xdes,dummy.
end if.
end if
.
do if (criterr=0).
compute xtmp=x(:,2:ncol(x)).
compute xcatlab={'X1';'X2';'X3';'X4';'X5';'X6';'X7';'X8';'X9'}.
compute nxvls=nvls-1.
compute xdich=(nvls=2).
compute mcxok=1.
compute dummatx=dummat.
compute xmodvals=dummatx(:,1).
compute nxpval=nrow(xmodvals).
release tmp, dummat.
end if.
end if.
compute intlab=make(100,1,' ').


compute intlab( 1 ,1)= 'Int_1'.


compute intlab( 2 ,1)= 'Int_2'.


compute intlab( 3 ,1)= 'Int_3'.


compute intlab( 4 ,1)= 'Int_4'.


compute intlab( 5 ,1)= 'Int_5'.


compute intlab( 6 ,1)= 'Int_6'.


compute intlab( 7 ,1)= 'Int_7'.


compute intlab( 8 ,1)= 'Int_8'.


compute intlab( 9 ,1)= 'Int_9'.


compute intlab( 10 ,1)= 'Int_10'.


compute intlab( 11 ,1)= 'Int_11'.


compute intlab( 12 ,1)= 'Int_12'.


compute intlab( 13 ,1)= 'Int_13'.


compute intlab( 14 ,1)= 'Int_14'.


compute intlab( 15 ,1)= 'Int_15'.


compute intlab( 16 ,1)= 'Int_16'.


compute intlab( 17 ,1)= 'Int_17'.


compute intlab( 18 ,1)= 'Int_18'.


compute intlab( 19 ,1)= 'Int_19'.


compute intlab( 20 ,1)= 'Int_20'.


compute intlab( 21 ,1)= 'Int_21'.


compute intlab( 22 ,1)= 'Int_22'.


compute intlab( 23 ,1)= 'Int_23'.


compute intlab( 24 ,1)= 'Int_24'.


compute intlab( 25 ,1)= 'Int_25'.


compute intlab( 26 ,1)= 'Int_26'.


compute intlab( 27 ,1)= 'Int_27'.


compute intlab( 28 ,1)= 'Int_28'.


compute intlab( 29 ,1)= 'Int_29'.


compute intlab( 30 ,1)= 'Int_30'.


compute intlab( 31 ,1)= 'Int_31'.


compute intlab( 32 ,1)= 'Int_32'.


compute intlab( 33 ,1)= 'Int_33'.


compute intlab( 34 ,1)= 'Int_34'.


compute intlab( 35 ,1)= 'Int_35'.


compute intlab( 36 ,1)= 'Int_36'.


compute intlab( 37 ,1)= 'Int_37'.


compute intlab( 38 ,1)= 'Int_38'.


compute intlab( 39 ,1)= 'Int_39'.


compute intlab( 40 ,1)= 'Int_40'.


compute intlab( 41 ,1)= 'Int_41'.


compute intlab( 42 ,1)= 'Int_42'.


compute intlab( 43 ,1)= 'Int_43'.


compute intlab( 44 ,1)= 'Int_44'.


compute intlab( 45 ,1)= 'Int_45'.


compute intlab( 46 ,1)= 'Int_46'.


compute intlab( 47 ,1)= 'Int_47'.


compute intlab( 48 ,1)= 'Int_48'.


compute intlab( 49 ,1)= 'Int_49'.


compute intlab( 50 ,1)= 'Int_50'.


compute intlab( 51 ,1)= 'Int_51'.


compute intlab( 52 ,1)= 'Int_52'.


compute intlab( 53 ,1)= 'Int_53'.


compute intlab( 54 ,1)= 'Int_54'.


compute intlab( 55 ,1)= 'Int_55'.


compute intlab( 56 ,1)= 'Int_56'.


compute intlab( 57 ,1)= 'Int_57'.


compute intlab( 58 ,1)= 'Int_58'.


compute intlab( 59 ,1)= 'Int_59'.


compute intlab( 60 ,1)= 'Int_60'.


compute intlab( 61 ,1)= 'Int_61'.


compute intlab( 62 ,1)= 'Int_62'.


compute intlab( 63 ,1)= 'Int_63'.


compute intlab( 64 ,1)= 'Int_64'.


compute intlab( 65 ,1)= 'Int_65'.


compute intlab( 66 ,1)= 'Int_66'.


compute intlab( 67 ,1)= 'Int_67'.


compute intlab( 68 ,1)= 'Int_68'.


compute intlab( 69 ,1)= 'Int_69'.


compute intlab( 70 ,1)= 'Int_70'.


compute intlab( 71 ,1)= 'Int_71'.


compute intlab( 72 ,1)= 'Int_72'.


compute intlab( 73 ,1)= 'Int_73'.


compute intlab( 74 ,1)= 'Int_74'.


compute intlab( 75 ,1)= 'Int_75'.


compute intlab( 76 ,1)= 'Int_76'.


compute intlab( 77 ,1)= 'Int_77'.


compute intlab( 78 ,1)= 'Int_78'.


compute intlab( 79 ,1)= 'Int_79'.


compute intlab( 80 ,1)= 'Int_80'.


compute intlab( 81 ,1)= 'Int_81'.


compute intlab( 82 ,1)= 'Int_82'.


compute intlab( 83 ,1)= 'Int_83'.


compute intlab( 84 ,1)= 'Int_84'.


compute intlab( 85 ,1)= 'Int_85'.


compute intlab( 86 ,1)= 'Int_86'.


compute intlab( 87 ,1)= 'Int_87'.


compute intlab( 88 ,1)= 'Int_88'.


compute intlab( 89 ,1)= 'Int_89'.


compute intlab( 90 ,1)= 'Int_90'.


compute intlab( 91 ,1)= 'Int_91'.


compute intlab( 92 ,1)= 'Int_92'.


compute intlab( 93 ,1)= 'Int_93'.


compute intlab( 94 ,1)= 'Int_94'.


compute intlab( 95 ,1)= 'Int_95'.


compute intlab( 96 ,1)= 'Int_96'.


compute intlab( 97 ,1)= 'Int_97'.


compute intlab( 98 ,1)= 'Int_98'.


compute intlab( 99 ,1)= 'Int_99'.


compute intlab( 100 ,1)= 'Int_100'.

compute bcmat=make(needed,needed,0).
compute wcmat=make(needed,needed,0).
compute zcmat=make(needed,needed,0).
compute wzcmat=make(needed,needed,0).
compute wsum=0.
compute zsum=0.
compute wzsum=0.
end if.
end if.
do if (criterr = 0 and model <> 999).
compute modelmat= {1,0,0,0,0,0,0,1,0,0;2,0,0,0,0,0,0,1,1,0;3,0,0,0,0,0,0,1,1,1;4,0,0,0,0,0,0,0,0,0; 5,0,0,0,0,0,0,1,0,0;6,0,0,0,0,0,0,0,0,0;7,1,0,0,0,0,0,0,0,0;8,1,0,0,0,0,0,1,0,0; 9,1,1,0,0,0,0,0,0,0;10,1,1,0,0,0,0,1,1,0;11,1,1,1,0,0,0,0,0,0;12,1,1,1,0,0,0,1,1,1; 13,1,1,1,0,0,0,1,0,0;14,0,0,0,1,0,0,0,0,0;15,0,0,0,1,0,0,1,0,0;16,0,0,0,1,1,0,0,0,0; 17,0,0,0,1,1,0,1,1,0;18,0,0,0,1,1,1,0,0,0;19,0,0,0,1,1,1,1,1,1;20,0,0,0,1,1,1,1,0,0; 21,1,0,0,0,1,0,0,0,0;22,1,0,0,0,1,0,1,0,0;23,0,0,0,0,0,0,0,0,0;24,0,0,0,0,0,0,0,0,0; 25,0,0,0,0,0,0,0,0,0;26,0,0,0,0,0,0,0,0,0;27,0,0,0,0,0,0,0,0,0;28,1,0,0,0,1,0,0,1,0; 29,1,0,0,0,1,0,1,1,0;30,0,0,0,0,0,0,0,0,0;31,0,0,0,0,0,0,0,0,0;32,0,0,0,0,0,0,0,0,0; 33,0,0,0,0,0,0,0,0,0;34,0,0,0,0,0,0,0,0,0;35,0,0,0,0,0,0,0,0,0;36,0,0,0,0,0,0,0,0,0; 37,0,0,0,0,0,0,0,0,0;38,0,0,0,0,0,0,0,0,0;39,0,0,0,0,0,0,0,0,0;40,0,0,0,0,0,0,0,0,0; 41,0,0,0,0,0,0,0,0,0;42,0,0,0,0,0,0,0,0,0;43,0,0,0,0,0,0,0,0,0;44,0,0,0,0,0,0,0,0,0; 45,0,0,0,0,0,0,0,0,0;46,0,0,0,0,0,0,0,0,0;47,0,0,0,0,0,0,0,0,0;48,0,0,0,0,0,0,0

 ,0,0; 49,0,0,0,0,0,0,0,0,0;50,0,0,0,0,0,0,0,0,0;51,0,0,0,0,0,0,0,0,0;52,0,0,0,0,0,0,0,0,0; 53,0,0,0,0,0,0,0,0,0;54,0,0,0,0,0,0,0,0,0;55,0,0,0,0,0,0,0,0,0;56,0,0,0,0,0,0,0,0,0; 57,0,0,0,0,0,0,0,0,0;58,1,0,0,1,0,0,0,0,0;59,1,0,0,1,0,0,1,0,0;60,1,1,0,1,0,0,0,0,0; 61,1,1,0,1,0,0,1,0,0;62,1,1,0,1,0,0,0,1,0;63,1,1,0,1,0,0,1,1,0;64,1,0,0,1,1,0,0,0,0; 65,1,0,0,1,1,0,1,0,0;66,1,0,0,1,1,0,0,1,0;67,1,0,0,1,1,0,1,1,0;68,1,1,1,1,0,0,0,0,0; 69,1,1,1,1,0,0,1,1,1;70,1,0,0,1,1,1,0,0,0;71,1,0,0,1,1,1,1,1,1;72,1,1,1,1,1,1,0,0,0; 73,1,1,1,1,1,1,1,1,1;74,0,0,0,1,0,0,0,0,0;75,1,1,0,1,1,0,0,0,0;76,1,1,0,1,1,0,1,1,0; 77,0,0,0,0,0,0,0,0,0;78,0,0,0,0,0,0,0,0,0;79,0,0,0,0,0,0,0,0,0;80,0,0,0,0,0,0,0,0,0; 81,0,0,0,0,0,0,0,0,0;82,0,0,0,0,0,0,0,0,0;83,1,0,0,0,0,0,0,0,0;84,1,0,0,0,0,0,0,0,0; 85,1,0,0,0,0,0,1,0,0;86,1,0,0,0,0,0,1,0,0;87,0,0,0,1,0,0,0,0,0;88,0,0,0,1,0,0,0,0,0; 89,0,0,0,1,0,0,1,0,0;90,0,0,0,1,0,0,1,0,0;91,0,0,0,0,0,0,0,0,0;92,1,0,0,1,0,0,1,0,0}.
do if (model > 0).
compute tmp=modelmat(model,2:ncol(modelmat)).
end if.
do if (model=0).
compute tmp={0,0,0,0,0,0,0,0,0}.
end if.
do if (model < 4).
compute bcmat((nxs+1),1)=1.
end if.
do if ((model > 3) and (model <> 6)).
compute bcmat((nxs+1):(nxs+nms),1)=onem.
compute bcmat(nrow(bcmat),(nxs+1):(nxs+nms))=t(onem).
compute bcmat(nrow(bcmat),1)=1.
end if.
do if ((model = 6) or (model > 82 and model < 93)).
loop j = 2 to nrow(bcmat).
loop i = 1 to (j-1).
compute bcmat(j,i)=1.
end loop.
end loop.
end if.
do if (model = 80).
loop i = 1 to nms.
compute bcmat((nrow(bcmat)-1),i)=1.
end loop.
end if.
do if (model = 81).
loop j = 3 to nrow(bcmat).
compute bcmat(j,2)=1.
end loop.
end if.
do if (model = 82).
compute bcmat(3,2)=1.
compute bcmat(5,4)=1.
end if.
do if (tmp(1,1)=1).
compute wcmat((nxs+1):(nxs+nms),1)=onem.
compute wprod=1.
compute xprod=1.
do if (model = 83 or model = 86).
compute onemsx=onem.
loop i = 1 to (nms-1).
compute onemsx(i+1,1)=0.
end loop.
compute wcmat((nxs+1):(nxs+nms),1)=onemsx.
end if.
end if.
do if (tmp(1,4)=1).
compute wcmat(nrow(wcmat),(nxs+1):(nxs+nms))=t(onem).
compute wprod=1.
do if (model = 87 or model = 90).
compute onemsx=onem.
loop i = 1 to (nms-1).
compute onemsx(i,1)=0.
end loop.
compute wcmat(nrow(wcmat),(nxs+1):(nxs+nms))=t(onemsx).
end if.
end if.
do if (tmp(1,7)=1).
compute wcmat(nrow(wcmat),1)=1.
compute wprod=1.
compute xprod=1.
end if.
do if (tmp(1,2)=1).
compute zcmat((nxs+1):(nxs+nms),1)=onem.
compute zprod=1.
compute xprod=1.
end if.
do if (tmp(1,5)=1).
compute zcmat(nrow(zcmat),(nxs+1):(nxs+nms))=t(onem).
compute zprod=1.
end if.
do if (tmp(1,8)=1).
compute zcmat(nrow(zcmat),1)=1.
compute zprod=1.
compute xprod=1.
end if.
do if (tmp(1,3)=1).
compute wzcmat((nxs+1):(nxs+nms),1)=onem.
compute xprod=1.
compute wprod=1.
compute zprod=1.
end if.
do if (tmp(1,6)=1).
compute wzcmat(nrow(wzcmat),(nxs+1):(nxs+nms))=t(onem).
compute zprod=1.
compute wprod=1.
end if.
do if (tmp(1,9)=1).
compute wzcmat(nrow(wzcmat),1)=1.
compute xprod=1.
compute wprod=1.
compute zprod=1.
end if.
do if (model = 91 or model = 92).
loop j = 1 to (nms-1).
loop i = 1 to j.
compute wcmat((nxs+1+j),(nxs+i))=1.
end loop.
end loop.
end if.
do if (nms < 0).
loop i = 1 to nms.
compute tmp=csum(wcmat(:,(1+i)))+csum(zcmat(:,(1+i)))+csum(wzcmat(:,(1+i))).
compute mprod(1,i)=(tmp>0).
end loop.
end if.
end if.
do if (ncs > 0).
compute ccmat=make((nms+nys),ncs,1).
compute ccmatoff=ccmat.
do if (covmy=1).
compute ccmat(nrow(ccmat),:)=make(1,ncs,0).
end if.
do if (covmy=2).
compute ccmat(1:nms,:)=make(nms,ncs,0).
end if.
do if (cmatrix(1,1) <> -999).
do if (ncol(cmatrix) <> ((nms+nys)*ncs)).
compute errcode(errs,1)=29.
compute errs=errs+1.
compute criterr=1.
end if.
do if (criterr = 0).
compute tmp=1.
loop i = 1 to (nms+nys).
loop j = 1 to ncs.
compute ccmat(i,j)=1-(cmatrix(1,tmp) = 0).
compute tmp=tmp+1.
end loop.
end loop.
do if (rsum((csum(ccmat)=0)) <> 0).
compute errcode(errs,1)=30.
compute errs=errs+1.
compute criterr=1.
end if.
end if.
do if (covmy <> 0).
compute notecode(notes,1)=1.
compute notes=notes+1.
end if.
end if.
do if (xmint=1 and (covmy > 0 or (csum(rsum(ccmat)) <> ((nms+nys)*ncs)))).
compute errcode(errs,1)=68.
compute errs=errs+1.
compute criterr=1.
end if.
end if.
do if (criterr=0).
compute needed=needed*(needed-1)/2.
compute nopath=0.
do if (bmatrix(1,1) <> -999).
compute tmp=1.
do if ((ncol(bmatrix) <> needed) or (csum(rsum(bmatrix))=0)).
compute errcode(errs,1)=16.
compute errs=errs+1.
compute criterr=1.
else.
loop i = 2 to nrow(bcmat).
loop j = 1 to (i-1).
compute bcmat(i,j)=1-(bmatrix(1,tmp) = 0).
compute tmp=tmp+1.
end loop.
end loop.
end if.
do if ((csum(bcmat(:,1))=0) and criterr=0).
compute errcode(errs,1)=22.
compute errs=errs+1.
compute criterr=1.
end if.
do if ((rsum(bcmat(nrow(bcmat),:))=0) and criterr=0).
compute errcode(errs,1)=23.
compute errs=errs+1.
compute criterr=1.
end if.
compute dm=0.
do if (nms > 0).
loop i = 1 to nms.
do if (((rsum(bcmat((nxs+i),:)) = 0) or (csum(bcmat(:,(nxs+i))) = 0)) and (dm=0) and (criterr=0)).
compute errcode(errs,1)=26.
compute errs=errs+1.
compute criterr=1.
compute dm=1.
end if.
end loop.
end if.
release dm.
end if.
end if.
do if (criterr=0).
do if (wmatrix(1,1) <> -999).
compute tmp=1.
do if (ncol(wmatrix) <> needed).
compute errcode(errs,1)=17.
compute errs=errs+1.
compute criterr=1.
else.
compute modelvar(1,1)='CUSTOM'.
loop i = 2 to nrow(wcmat).
loop j = 1 to (i-1).
compute wcmat(i,j)=1-(wmatrix(1,tmp) = 0).
do if ((wcmat(i,j)=1) and (bcmat(i,j)=0) and (nopath=0)).
compute errcode(errs,1)=20.
compute errs=errs+1.
compute criterr=1.
compute nopath=1.
end if.
compute tmp=tmp+1.
end loop.
end loop.
end if.
end if.
do if (zmatrix(1,1) <> -999).
compute tmp=1.
do if (ncol(zmatrix) <> needed).
compute errcode(errs,1)=18.
compute errs=errs+1.
compute criterr=1.
else.
compute modelvar(1,1)='CUSTOM'.
do if (csum(rsum(wcmat))=0 and model=999).
compute errcode(errs,1)=21.
compute errs=errs+1.
compute criterr=1.
end if.
loop i = 2 to nrow(zcmat).
loop j = 1 to (i-1).
compute zcmat(i,j)=1-(zmatrix(1,tmp) = 0).
do if ((zcmat(i,j)=1) and (bcmat(i,j)=0) and (nopath=0)).
compute errcode(errs,1)=20.
compute errs=errs+1.
compute criterr=1.
compute nopath=1.
end if.
compute tmp=tmp+1.
end loop.
end loop.
end if.
end if.
compute tmp=1.
do if (wzmatrix(1,1) <> -999).
do if (ncol(wzmatrix) <> needed).
compute errcode(errs,1)=19.
compute errs=errs+1.
compute criterr=1.
end if.
compute modelvar(1,1)='CUSTOM'.
end if.
do if (criterr=0).
loop i = 2 to nrow(wzcmat).
loop j = 1 to (i-1).
do if (wzmatrix(1,1) <> -999).
compute wzcmat(i,j)=1-(wzmatrix(1,tmp) = 0).
end if.
do if (wzcmat(i,j)=1).
compute wcmat(i,j)=1.
compute zcmat(i,j)=1.
end if.
do if ((wzcmat(i,j)=1) and (bcmat(i,j)=0) and (nopath=0)).
compute errcode(errs,1)=20.
compute errs=errs+1.
compute criterr=1.
compute nopath=1.
end if.
compute tmp=tmp+1.
end loop.
end loop.
end if.
end if.
do if (criterr=0).
compute xprod=csum(wcmat(:,1))+csum(zcmat(:,1))+csum(wzcmat(:,1)).
compute xprod=(xprod > 0).
compute wsum=csum(rsum(wcmat)).
compute wprod=(wsum > 0).
do if (nms > 0).
loop i = 1 to nms.
compute tmp=csum(wcmat(:,(1+i)))+csum(zcmat(:,(1+i)))+csum(wzcmat(:,(1+i))).
compute mprod(1,i)=(tmp>0).
end loop.
end if.
do if ((wsum > 0) and (w = 'xxxxx')).
compute errcode(errs,1)=11.
compute errs=errs+1.
compute criterr=1.
end if.
do if ((wsum = 0) and (w <> 'xxxxx')).
compute errcode(errs,1)=10.
compute errs=errs+1.
compute criterr=1.
end if.
compute zsum=csum(rsum(zcmat)).
compute zprod=(zsum > 0).
do if ((zsum > 0) and (z = 'xxxxx')).
compute errcode(errs,1)=13.
compute errs=errs+1.
compute criterr=1.
end if.
do if ((zsum = 0) and (z <> 'xxxxx')).
compute errcode(errs,1)=12.
compute errs=errs+1.
compute criterr=1.
end if.
do if ((zsum > 0) and (wsum = 0)).
compute errcode(errs,1)=35.
compute errs=errs+1.
compute criterr=1.
end if.
end if.
do if (criterr=0 and nms > 1).
compute serchk=bcmat(2:(nrow(bcmat)-1),2:ncol(bcmat)).
do if (csum(rsum(serchk))) > 0.
compute serial=1.
do if (nms > 6).
compute errcode(errs,1)=36.
compute errs=errs+1.
compute criterr=1.
end if.
end if.
end if.
do if (center > 0 and criterr=0).
compute centvar={' '}.
do if (criterr=0).
do if ((center = 1) or (center = 2 and wdich = 0)).
do if (wprod=1 and mcwok=0 and nwpval > 0).
loop i = 1 to nws.
compute wtmp(:,i)=wtmp(:,i)-(csum(wtmp(:,i))/n).
compute centvar={centvar,wnames(1,i)}.
end loop.

.
compute desctmp=make((8-(4* wmodcust )),ncol( wtmp ),-999).
loop jd=1 to ncol( wtmp ).
compute descdat= wtmp (:,jd).
compute desctmp(1,jd) = csum(descdat)/nrow(descdat).
compute desctmp(2,jd) = (nrow(descdat)*sscp(descdat))-(t(csum(descdat))*(csum(descdat))).
compute desctmp(2,jd) = sqrt(desctmp(2,jd)/(nrow(descdat)*(nrow(descdat)-1))).
compute desctmp(3,jd)=cmin(descdat).
compute desctmp(4,jd)=cmax(descdat).
do if ( wmodcust =0).
compute minwarn=0.
compute maxwarn=0.
do if ((desctmp(3,jd)=desctmp(4,jd)) and novar=0).
compute errcode(errs,1)=15.
compute errs=errs+1.
compute criterr=1.
compute novar=1.
end if.
compute tmp=((descdat(:,1)=desctmp(3,jd))+(descdat(:,1)=desctmp(4,jd))).
compute desctmp(8,jd)=(csum(tmp)=nrow(tmp)).
compute tmp = descdat.
compute tmp(GRADE(descdat),:) = descdat.
compute descdat = tmp.
release tmp.
compute decval={.16;.5;.84}.
loop kd=1 to 3.
compute low=trunc(decval(kd,1)*(nrow(descdat)+1)).
compute lowdec=decval(kd,1)*(nrow(descdat)+1)-low.
compute value=descdat(low,1)+(descdat((low+1),1)-descdat(low,1))*lowdec.
compute desctmp((4+kd),jd)=value.
end loop.
compute mnotev=1.
compute modvals=desctmp(5:7,:).
do if (quantile <> 1).
compute desctmp(5,jd)=desctmp(1,jd)-desctmp(2,jd).
compute desctmp(6,jd)=desctmp(1,jd).
compute desctmp(7,jd)=desctmp(1,jd)+desctmp(2,jd).
compute modvals=desctmp(5:7,:).
compute mnotev=2.
do if (modvals(1,1) < desctmp(3,1)).
compute modvals(1,1)=desctmp(3,1).
compute minwarn=1.
end if.
do if (modvals(3,1) > desctmp(4,1)).
compute modvals(3,1)=desctmp(4,1).
compute maxwarn=1.
end if.
end if.
do if (desctmp(8,1)=1).
compute modvals={desctmp(3,1);desctmp(4,1)}.
compute mnotev=0.
compute minwarn=0.
compute maxwarn=0.
end if.
end if.
end loop
.
compute wmin=desctmp(3,1).
compute wmax=desctmp(4,1).
do if (wmodcust=0).
compute wmodvals=modvals.
compute wprobval=wmodvals.
end if.
end if.
end if.
do if ((center = 1) or (center = 2 and zdich = 0)).
do if (zprod=1 and mczok=0 and nzpval > 0).
loop i = 1 to nzs.
compute ztmp(:,i)=ztmp(:,i)-(csum(ztmp(:,i))/n).
compute centvar={centvar,znames(1,i)}.
end loop.

.
compute desctmp=make((8-(4* zmodcust )),ncol( ztmp ),-999).
loop jd=1 to ncol( ztmp ).
compute descdat= ztmp (:,jd).
compute desctmp(1,jd) = csum(descdat)/nrow(descdat).
compute desctmp(2,jd) = (nrow(descdat)*sscp(descdat))-(t(csum(descdat))*(csum(descdat))).
compute desctmp(2,jd) = sqrt(desctmp(2,jd)/(nrow(descdat)*(nrow(descdat)-1))).
compute desctmp(3,jd)=cmin(descdat).
compute desctmp(4,jd)=cmax(descdat).
do if ( zmodcust =0).
compute minwarn=0.
compute maxwarn=0.
do if ((desctmp(3,jd)=desctmp(4,jd)) and novar=0).
compute errcode(errs,1)=15.
compute errs=errs+1.
compute criterr=1.
compute novar=1.
end if.
compute tmp=((descdat(:,1)=desctmp(3,jd))+(descdat(:,1)=desctmp(4,jd))).
compute desctmp(8,jd)=(csum(tmp)=nrow(tmp)).
compute tmp = descdat.
compute tmp(GRADE(descdat),:) = descdat.
compute descdat = tmp.
release tmp.
compute decval={.16;.5;.84}.
loop kd=1 to 3.
compute low=trunc(decval(kd,1)*(nrow(descdat)+1)).
compute lowdec=decval(kd,1)*(nrow(descdat)+1)-low.
compute value=descdat(low,1)+(descdat((low+1),1)-descdat(low,1))*lowdec.
compute desctmp((4+kd),jd)=value.
end loop.
compute mnotev=1.
compute modvals=desctmp(5:7,:).
do if (quantile <> 1).
compute desctmp(5,jd)=desctmp(1,jd)-desctmp(2,jd).
compute desctmp(6,jd)=desctmp(1,jd).
compute desctmp(7,jd)=desctmp(1,jd)+desctmp(2,jd).
compute modvals=desctmp(5:7,:).
compute mnotev=2.
do if (modvals(1,1) < desctmp(3,1)).
compute modvals(1,1)=desctmp(3,1).
compute minwarn=1.
end if.
do if (modvals(3,1) > desctmp(4,1)).
compute modvals(3,1)=desctmp(4,1).
compute maxwarn=1.
end if.
end if.
do if (desctmp(8,1)=1).
compute modvals={desctmp(3,1);desctmp(4,1)}.
compute mnotev=0.
compute minwarn=0.
compute maxwarn=0.
end if.
end if.
end loop
.
compute zmin=desctmp(3,1).
compute zmax=desctmp(4,1).
do if (zmodcust=0).
compute zmodvals=modvals.
compute zprobval=zmodvals.
end if.
end if.
end if.
do if ((center = 1) or (center = 2 and xdich = 0)).
do if (xprod=1 and mcxok=0).
loop i = 1 to nxs.
compute xtmp(:,i)=xtmp(:,i)-(csum(xtmp(:,i))/n).
compute centvar={centvar,xnames(1,i)}.
end loop.

.
compute desctmp=make((8-(4* 0 )),ncol( xtmp ),-999).
loop jd=1 to ncol( xtmp ).
compute descdat= xtmp (:,jd).
compute desctmp(1,jd) = csum(descdat)/nrow(descdat).
compute desctmp(2,jd) = (nrow(descdat)*sscp(descdat))-(t(csum(descdat))*(csum(descdat))).
compute desctmp(2,jd) = sqrt(desctmp(2,jd)/(nrow(descdat)*(nrow(descdat)-1))).
compute desctmp(3,jd)=cmin(descdat).
compute desctmp(4,jd)=cmax(descdat).
do if ( 0 =0).
compute minwarn=0.
compute maxwarn=0.
do if ((desctmp(3,jd)=desctmp(4,jd)) and novar=0).
compute errcode(errs,1)=15.
compute errs=errs+1.
compute criterr=1.
compute novar=1.
end if.
compute tmp=((descdat(:,1)=desctmp(3,jd))+(descdat(:,1)=desctmp(4,jd))).
compute desctmp(8,jd)=(csum(tmp)=nrow(tmp)).
compute tmp = descdat.
compute tmp(GRADE(descdat),:) = descdat.
compute descdat = tmp.
release tmp.
compute decval={.16;.5;.84}.
loop kd=1 to 3.
compute low=trunc(decval(kd,1)*(nrow(descdat)+1)).
compute lowdec=decval(kd,1)*(nrow(descdat)+1)-low.
compute value=descdat(low,1)+(descdat((low+1),1)-descdat(low,1))*lowdec.
compute desctmp((4+kd),jd)=value.
end loop.
compute mnotev=1.
compute modvals=desctmp(5:7,:).
do if (quantile <> 1).
compute desctmp(5,jd)=desctmp(1,jd)-desctmp(2,jd).
compute desctmp(6,jd)=desctmp(1,jd).
compute desctmp(7,jd)=desctmp(1,jd)+desctmp(2,jd).
compute modvals=desctmp(5:7,:).
compute mnotev=2.
do if (modvals(1,1) < desctmp(3,1)).
compute modvals(1,1)=desctmp(3,1).
compute minwarn=1.
end if.
do if (modvals(3,1) > desctmp(4,1)).
compute modvals(3,1)=desctmp(4,1).
compute maxwarn=1.
end if.
end if.
do if (desctmp(8,1)=1).
compute modvals={desctmp(3,1);desctmp(4,1)}.
compute mnotev=0.
compute minwarn=0.
compute maxwarn=0.
end if.
end if.
end loop
.
compute xmodvals=modvals.
compute xprobval=xmodvals.
end if.
end if.
do if (nms > 0).
loop i = 1 to nms.
do if (mprod(1,i)=1).
compute mtmp(:,i)=mtmp(:,i)-(csum(mtmp(:,i))/n).
compute centvar={centvar,mnames(1,i)}.
end if.
end loop.

.
compute desctmp=make((8-(4* 0 )),ncol( mtmp ),-999).
loop jd=1 to ncol( mtmp ).
compute descdat= mtmp (:,jd).
compute desctmp(1,jd) = csum(descdat)/nrow(descdat).
compute desctmp(2,jd) = (nrow(descdat)*sscp(descdat))-(t(csum(descdat))*(csum(descdat))).
compute desctmp(2,jd) = sqrt(desctmp(2,jd)/(nrow(descdat)*(nrow(descdat)-1))).
compute desctmp(3,jd)=cmin(descdat).
compute desctmp(4,jd)=cmax(descdat).
do if ( 0 =0).
compute minwarn=0.
compute maxwarn=0.
do if ((desctmp(3,jd)=desctmp(4,jd)) and novar=0).
compute errcode(errs,1)=15.
compute errs=errs+1.
compute criterr=1.
compute novar=1.
end if.
compute tmp=((descdat(:,1)=desctmp(3,jd))+(descdat(:,1)=desctmp(4,jd))).
compute desctmp(8,jd)=(csum(tmp)=nrow(tmp)).
compute tmp = descdat.
compute tmp(GRADE(descdat),:) = descdat.
compute descdat = tmp.
release tmp.
compute decval={.16;.5;.84}.
loop kd=1 to 3.
compute low=trunc(decval(kd,1)*(nrow(descdat)+1)).
compute lowdec=decval(kd,1)*(nrow(descdat)+1)-low.
compute value=descdat(low,1)+(descdat((low+1),1)-descdat(low,1))*lowdec.
compute desctmp((4+kd),jd)=value.
end loop.
compute mnotev=1.
compute modvals=desctmp(5:7,:).
do if (quantile <> 1).
compute desctmp(5,jd)=desctmp(1,jd)-desctmp(2,jd).
compute desctmp(6,jd)=desctmp(1,jd).
compute desctmp(7,jd)=desctmp(1,jd)+desctmp(2,jd).
compute modvals=desctmp(5:7,:).
compute mnotev=2.
do if (modvals(1,1) < desctmp(3,1)).
compute modvals(1,1)=desctmp(3,1).
compute minwarn=1.
end if.
do if (modvals(3,1) > desctmp(4,1)).
compute modvals(3,1)=desctmp(4,1).
compute maxwarn=1.
end if.
end if.
do if (desctmp(8,1)=1).
compute modvals={desctmp(3,1);desctmp(4,1)}.
compute mnotev=0.
compute minwarn=0.
compute maxwarn=0.
end if.
end if.
end loop
.
do if ((cdeval(1,1)=-999) and (model=74)).
compute medmeans=desctmp(1,:).
end if.
compute mmodvals=modvals.
compute mprobval=mmodvals.
end if.
end if.
do if (ncol(centvar) > 1).
compute notecode(notes,1)=3.
compute notes=notes+1.
end if.
end if.
do if (criterr=0).
compute wsum=rsum(csum(wcmat)).
compute zsum=rsum(csum(zcmat)).
compute wzsum=rsum(csum(wzcmat)).
compute nump=make(1,(nys+nms),-999).
compue numint=make(1,(nys+nms),0).
compute datcount=1.
compute xtmpuse=0.
compute wtmpuse=0.
compute ztmpuse=0.
compute xwtmpus=0.
compute xztmpus=0.
compute wztmpus=0.
compute xwztmpu=0.
compute xtmploc=-999.
compute wtmploc=-999.
compute xwtmplo=-999.
compute ztmploc=-999.
compute xztmplo=-999.
compute wztmplo=-999.
compute xwztmplo=-999.
compute vlabs={' '}.
do if (ncs > 0).
compute ctmpuse=make(1,ncs,0).
end if.
do if (nms > 0).
compute mtmpuse=make(1,nms,0).
compute mwtmpus=make(1,nms,0).
compute mztmpus=make(1,nms,0).
compute mwztmpu=make(1,nms,0).
compute mtmploc=make(1,nms,0).
compute mwtmplo=make(nwvls,nms,-999).
compute mztmplo=make(nzvls,nms,-999).
compute mwztmplo=make((nwvls*nzvls),nms,-999).
end if.
do if (ncs > 0).
compute ctmploc=make(1,ncs,0).
end if.
compute fulldat=make(n,1,1).
compute datindx=make(1000,(nms+nys),-999).
compute wherew=make(2,(nms+nys),-999).
compute wherex=make(2,(nms+nys),-999).
compute wherez=make(2,(nms+nys),-999).
compute wherexw=make(2,(nms+nys),-999).
compute wherexz=make(2,(nms+nys),-999).
compute wherewz=make(2,(nms+nys),-999).
compute wherexwz=make(2,(nms+nys),-999).
do if (nms > 0).
compute wherem=make(nms,(nms+nys),-999).
compute wheremw = make(nms*2,(nms+nys),-999).
compute wheremz = make(nms*2,(nms+nys),-999).
compute wheremwz = make(nms*2,(nms+nys),-999).
end if.
compute wzhigh=make(1000,(((nms+1)*(nms+2))/2),0).
compute whigh=make(1000,(((nms+1)*(nms+2))/2),0).
compute zhigh=make(1000,(((nms+1)*(nms+2))/2),0).
compute fochigh=make(1000,(((nms+1)*(nms+2))/2),0).
compute xcoefloc={1;2;3;4;5;6;7;8;9}.
compute intkey = {' ', ' ', ' ', ' ', ' ', ' ', ' '}.
compute wzhighct=0.
compute whighct=0.
compute zhighct=0.
compute foccnt=0.
loop i = 2 to nrow(bcmat).
compute wdid=0.
compute zdid=0.
compute wzdid=0.
compute cntmp=1.
compute start=1.
do if (i < nrow(bcmat)).
compute outv=mtmp(:,(i-1)).
compute modlabel={mnames(1,(i-1));'constant'}.
end if.
do if (i = nrow(bcmat)).
compute outv=ytmp.
compute modlabel={ynames;'constant'}.
end if.
loop j = 1 to (i-1).
compute foccnt=foccnt+1.
do if (j = 1 and bcmat(i,j)=1).
compute outv={outv,xtmp}.
compute modlabel={modlabel;xcatlab(1:nxvls,1)}.
do if (xtmpuse=0).
compute fulldat={fulldat,xtmp}.
compute xtmpuse=1.
loop k4=datcount to (datcount+(nxvls-1)).
compute xtmploc={xtmploc;k4}.
end loop.
compute xtmploc=xtmploc(2:nrow(xtmploc),1).
compute datcount=datcount+nxvls.
end if.
compute datindx(start:(start+nrow(xtmploc)-1),(i-1))=xtmploc.
compute wherex(1,(i-1))=start+1.
compute wherex(2,(i-1))=start+nrow(xtmploc)-1+1.
do if (model = 74).
end if.
compute onebl=make(nrow(xtmploc),1,1).
compute fochigh((start+1):(start+nrow(xtmploc)),foccnt)=onebl.
compute start=start+nrow(xtmploc).
end if.
do if (j > 1 and bcmat(i,j)=1).
compute outv={outv,mtmp(:,(j-1))}.
compute modlabel={modlabel;mnames(1,(j-1))}.
do if (mtmpuse(1,(j-1))=0).
compute fulldat={fulldat,mtmp(:,(j-1))}.
compute mtmpuse(1,(j-1))=1.
compute mtmploc(1,(j-1))=datcount.
compute datcount=datcount+1.
end if.
compute datindx(start:(start+nrow(mtmploc)-1),(i-1))=mtmploc(1,(j-1)).
compute wherem((j-1),(i-1))=start+1.
compute onebl=make(nrow(mtmploc(1,j-1)),1,1).
compute ttt=nrow(mtmploc(1,(j-1)))+start-1.
compute fochigh((start+1):(start+nrow(mtmploc(1,(j-1)))),foccnt)=onebl.
compute start=start+nrow(mtmploc(1,(j-1))).
end if.
end loop.
do if (wsum > 0).
loop j = 1 to (i-1).
compute whighct=whighct+1.
do if (j = 1 and wcmat(i,j)=1).
do if (wdid=0).
compute outv={outv,wtmp}.
do if (ncs > 0 and wiscov > 0).
compute ccmatoff((i-1),wiscov)=0.
end if.
compute modlabel={modlabel;wcatlab(1:nwvls,1)}.
compute wdid=1.
do if (wtmpuse=0).
compute fulldat={fulldat,wtmp}.
do if (ncs > 0 and wiscov > 0).
compute ccmatoff((i-1),wiscov)=0.
end if.
compute wtmpuse=1.
loop k4=datcount to (datcount+(nwvls-1)).
compute wtmploc={wtmploc;k4}.
end loop.
compute wtmploc=wtmploc(2:nrow(wtmploc),1).
compute datcount=datcount+nwvls.
end if.
end if.
compute datindx(start:(start+nrow(wtmploc)-1),(i-1))=wtmploc.
compute wherew(1,(i-1))=start+1.
compute wherew(2,(i-1))=start+nrow(wtmploc)-1+1.
compute start=start+nrow(wtmploc).
loop k1=1 to nxvls.
loop k2 = 1 to nwvls.
compute outv={outv,(xtmp(:,k1)&*wtmp(:,k2))}.
do if (ncs > 0 and wiscov > 0).
compute ccmatoff((i-1),wiscov)=0.
end if.
compute modlabel={modlabel;intlab(cntmp,1)}.
compute intkey={intkey;intlab(cntmp,1),':',xcatlab(k1,1),'x',wcatlab(k2,1),' ',' '}.
compute cntmp=cntmp+1.
end loop.
end loop.
do if (xwtmpus=0).
compute fulldat={fulldat,outv(:,(ncol(outv)-(nxvls*nwvls)+1):ncol(outv))}.
compute xwtmpus=1.
do if (ncs > 0 and wiscov > 0).
compute ccmatoff((i-1),wiscov)=0.
end if.
loop k4=datcount to (datcount+((nwvls*nxvls)-1)).
compute xwtmplo={xwtmplo;k4}.
end loop.
compute xwtmplo=xwtmplo(2:nrow(xwtmplo),1).
compute datcount=datcount+(nxvls*nwvls).
end if.
compute datindx(start:(start+nrow(xwtmplo)-1),(i-1))=xwtmplo.
compute wherexw(1,(i-1))=start+1.
compute wherexw(2,(i-1))=start+nrow(xwtmplo)-1+1.
compute onebl=make(nrow(xwtmplo),1,1).
compute whigh((start+1):(start+nrow(xwtmplo)),whighct)=onebl.
compute start=start+nrow(xwtmplo).
end if.
do if (j > 1 and wcmat(i,j)=1).
do if (wdid=0 and model <> 74).
compute outv={outv,wtmp}.
do if (ncs > 0 and wiscov > 0).
compute ccmatoff((i-1),wiscov)=0.
end if.
compute modlabel={modlabel;wcatlab(1:nwvls,1)}.
compute wdid=1.
do if (wtmpuse=0).
compute fulldat={fulldat,wtmp}.
do if (ncs > 0 and wiscov > 0).
compute ccmatoff((i-1),wiscov)=0.
end if.
compute wtmpuse=1.
loop k4=datcount to (datcount+(nwvls-1)).
compute wtmploc={wtmploc;k4}.
end loop.
compute wtmploc=wtmploc(2:nrow(wtmploc),1).
compute datcount=datcount+nwvls.
end if.
compute datindx(start:(start+nrow(wtmploc)-1),(i-1))=wtmploc.
compute wherew(1,(i-1))=start+1.
compute wherew(2,(i-1))=start+nrow(wtmploc)-1+1.
compute start=start+nrow(wtmploc).
end if.
loop k2 = 1 to nwvls.
compute outv={outv,(mtmp(:,(j-1))&*wtmp(:,k2))}.
do if (ncs > 0 and wiscov > 0).
compute ccmatoff((i-1),wiscov)=0.
end if.
compute modlabel={modlabel;intlab(cntmp,1)}.
compute intkey={intkey;intlab(cntmp,1),':', mnames(1,(j-1)),'x',wcatlab(k2,1),' ',' '}.
compute cntmp=cntmp+1.
end loop.
do if (mwtmpus(1,(j-1))=0).
compute fulldat={fulldat,outv(:,(ncol(outv)-nwvls+1):ncol(outv))}.
do if (ncs > 0 and wiscov > 0).
compute ccmatoff((i-1),wiscov)=0.
end if.
compute mwtmpus(1,(j-1))=1.
compute mw22=-999.
loop k4=datcount to (datcount+(nwvls-1)).
compute mw22={mw22;k4}.
end loop.
compute mwtmplo(:,(j-1))=mw22(2:nrow(mw22),1).
compute datcount=datcount+nwvls.
end if.
compute datindx(start:(start+nrow(mwtmplo)-1),(i-1))=mwtmplo(:,(j-1)).
compute wheremw(((2*j)-3),(i-1))=start+1.
compute wheremw(((2*j)-2),(i-1))=start+nrow(mwtmplo)-1+1.
compute onebl=make(nrow(mwtmplo),1,1).
compute whigh((start+1):(start+nrow(mwtmplo)),whighct)=onebl.
compute start=start+nrow(mwtmplo).
end if.
end loop.
end if.
do if (zsum > 0).
loop j = 1 to (i-1).
compute zhighct=zhighct+1.
do if (j = 1 and zcmat(i,j)=1).
do if (zdid=0).
compute outv={outv,ztmp}.
do if (ncs > 0 and ziscov > 0).
compute ccmatoff((i-1),ziscov)=0.
end if.
compute modlabel={modlabel;zcatlab(1:nzvls,1)}.
compute zdid=1.
do if (ztmpuse=0).
compute fulldat={fulldat,ztmp}.
do if (ncs > 0 and ziscov > 0).
compute ccmatoff((i-1),ziscov)=0.
end if.
compute ztmpuse=1.
loop k4=datcount to (datcount+(nzvls-1)).
compute ztmploc={ztmploc;k4}.
end loop.
compute ztmploc=ztmploc(2:nrow(ztmploc),1).
compute datcount=datcount+nzvls.
end if.
end if.
compute datindx(start:(start+nrow(ztmploc)-1),(i-1))=ztmploc.
compute wherez(1,(i-1))=start+1.
compute wherez(2,(i-1))=start+nrow(ztmploc)-1+1.
compute start=start+nrow(ztmploc).
loop k1=1 to nxvls.
loop k2 = 1 to nzvls.
compute outv={outv,(xtmp(:,k1)&*ztmp(:,k2))}.
do if (ncs > 0 and ziscov > 0).
compute ccmatoff((i-1),ziscov)=0.
end if.
compute modlabel={modlabel;intlab(cntmp,1)}.
compute intkey={intkey;intlab(cntmp,1),':',xcatlab(k1,1),'x',zcatlab(k2,1),' ',' '}.
compute cntmp=cntmp+1.
end loop.
end loop.
do if (xztmpus=0).
compute fulldat={fulldat,outv(:,(ncol(outv)-(nxvls*nzvls)+1):ncol(outv))}.
do if (ncs > 0 and ziscov > 0).
compute ccmatoff((i-1),ziscov)=0.
end if.
compute xztmpus=1.
loop k4=datcount to (datcount+((nzvls*nxvls)-1)).
compute xztmplo={xztmplo;k4}.
end loop.
compute xztmplo=xztmplo(2:nrow(xztmplo),1).
compute datcount=datcount+(nxvls*nzvls).
end if.
compute datindx(start:(start+nrow(xztmplo)-1),(i-1))=xztmplo.
compute wherexz(1,(i-1))=start+1.
compute wherexz(2,(i-1))=start+nrow(xztmplo)-1+1.
compute onebl=make(nrow(xztmplo),1,1).
compute zhigh((start+1):(start+nrow(xztmplo)),zhighct)=onebl.
compute start=start+nrow(xztmplo).
end if.
do if (j > 1 and zcmat(i,j)=1).
do if (zdid=0).
compute outv={outv,ztmp}.
do if (ncs > 0 and ziscov > 0).
compute ccmatoff((i-1),ziscov)=0.
end if.
compute modlabel={modlabel;zcatlab(1:nzvls,1)}.
compute zdid=1.
do if (ztmpuse=0).
compute fulldat={fulldat,ztmp}.
do if (ncs > 0 and ziscov > 0).
compute ccmatoff((i-1),ziscov)=0.
end if.
compute ztmpuse=1.
loop k4=datcount to (datcount+(nzvls-1)).
compute ztmploc={ztmploc;k4}.
end loop.
compute ztmploc=ztmploc(2:nrow(ztmploc),1).
compute datcount=datcount+nzvls.
end if.
compute datindx(start:(start+nrow(ztmploc)-1),(i-1))=ztmploc.
compute wherez(1,(i-1))=start+1.
compute wherez(2,(i-1))=start+nrow(ztmploc)-1+1.
compute start=start+nrow(ztmploc).
end if.
loop k2 = 1 to nzvls.
compute outv={outv,(mtmp(:,(j-1))&*ztmp(:,k2))}.
do if (ncs > 0 and ziscov > 0).
compute ccmatoff((i-1),ziscov)=0.
end if.
compute modlabel={modlabel;intlab(cntmp,1)}.
compute intkey={intkey;intlab(cntmp,1),':', mnames(1,(j-1)),'x',zcatlab(k2,1),' ',' '}.
compute cntmp=cntmp+1.
end loop.
do if (mztmpus(1,(j-1))=0).
compute fulldat={fulldat,outv(:,(ncol(outv)-nzvls+1):ncol(outv))}.
do if (ncs > 0 and ziscov > 0).
compute ccmatoff((i-1),ziscov)=0.
end if.
compute mztmpus(1,(j-1))=1.
compute mz22=-999.
loop k4=datcount to (datcount+(nzvls-1)).
compute mz22={mz22;k4}.
end loop.
compute mztmplo(:,(j-1))=mz22(2:nrow(mz22),1).
compute datcount=datcount+nzvls.
end if.
compute datindx(start:(start+nrow(mztmplo)-1),(i-1))=mztmplo(:,(j-1)).
compute wheremz(((2*j)-3),(i-1))=start+1.
compute wheremz(((2*j)-2),(i-1))=start+nrow(mztmplo)-1+1.
compute onebl=make(nrow(mztmplo),1,1).
compute zhigh((start+1):(start+nrow(mztmplo)),zhighct)=onebl.
compute start=start+nrow(mztmplo).
end if.
end loop.
end if.
do if (wzsum > 0).
loop j = 1 to (i-1).
compute wzhighct=wzhighct+1.
do if (j = 1 and wzcmat(i,j)=1).
do if (wzdid=0).
loop k1=1 to nwvls.
loop k2 = 1 to nzvls.
compute outv={outv,(wtmp(:,k1)&*ztmp(:,k2))}.
do if (ncs > 0 and (ziscov > 0)).
compute ccmatoff((i-1),ziscov)=0.
end if.
do if (ncs > 0 and (wiscov > 0)).
compute ccmatoff((i-1),wiscov)=0.
end if.
compute modlabel={modlabel;intlab(cntmp,1)}.
compute intkey={intkey;intlab(cntmp,1),':',wcatlab(k1,1),'x',zcatlab(k2,1),' ',' '}.
compute cntmp=cntmp+1.
end loop.
end loop.
do if (wztmpus=0).
compute fulldat={fulldat,outv(:,(ncol(outv)-(nwvls*nzvls)+1):ncol(outv))}.
do if (ncs > 0 and (ziscov > 0)).
compute ccmatoff((i-1),ziscov)=0.
end if.
do if (ncs > 0 and (wiscov > 0)).
compute ccmatoff((i-1),wiscov)=0.
end if.
compute wztmpus=1.
loop k4=datcount to (datcount+((nwvls*nzvls)-1)).
compute wztmplo={wztmplo;k4}.
end loop.
compute wztmplo=wztmplo(2:nrow(wztmplo),1).
compute datcount=datcount+(nzvls*nwvls).
end if.
compute wzdid=1.
end if.
compute datindx(start:(start+nrow(wztmplo)-1),(i-1))=wztmplo.
compute wherewz(1,(i-1))=start+1.
compute wherewz(2,(i-1))=start+nrow(wztmplo)-1+1.
compute start=start+nrow(wztmplo).
loop k1=1 to nxvls.
loop k2=1 to nwvls.
loop k3=1 to nzvls.
compute outv={outv,(xtmp(:,k1)&*wtmp(:,k2)&*ztmp(:,k3))}.
do if (ncs > 0 and (ziscov > 0)).
compute ccmatoff((i-1),ziscov)=0.
end if.
do if (ncs > 0 and (wiscov > 0)).
compute ccmatoff((i-1),wiscov)=0.
end if.
compute modlabel={modlabel;intlab(cntmp,1)}.
compute intkey={intkey;intlab(cntmp,1),':',xcatlab(k1,1),'x',wcatlab(k2,1),'x', zcatlab(k3,1)}.
compute cntmp=cntmp+1.
end loop.
end loop.
end loop.
do if (xwztmpu=0).
compute fulldat={fulldat,outv(:,(ncol(outv)-(nxvls*nwvls*nzvls)+1):ncol(outv))}.
do if (ncs > 0 and (ziscov > 0)).
compute ccmatoff((i-1),ziscov)=0.
end if.
do if (ncs > 0 and (wiscov > 0)).
compute ccmatoff((i-1),wiscov)=0.
end if.
compute xwztmpu=1.
loop k4=datcount to (datcount+((nzvls*nxvls*nwvls)-1)).
compute xwztmplo={xwztmplo;k4}.
end loop.
compute xwztmplo=xwztmplo(2:nrow(xwztmplo),1).
compute datcount=datcount+(nxvls*nzvls*nwvls).
end if.
compute datindx(start:(start+nrow(xwztmplo)-1),(i-1))=xwztmplo.
compute wherexwz(1,(i-1))=start+1.
compute wherexwz(2,(i-1))=start+nrow(xwztmplo)-1+1.
compute onebl=make(nrow(xwztmplo),1,1).
compute wzhigh((start+1):(start+nrow(xwztmplo)),wzhighct)=onebl.
compute start=start+nrow(xwztmplo).
end if.
do if (j > 1 and wzcmat(i,j)=1).
do if (wzdid=0).
loop k1=1 to nwvls.
loop k2 = 1 to nzvls.
compute outv={outv,(wtmp(:,k1)&*ztmp(:,k2))}.
do if (ncs > 0 and (ziscov > 0)).
compute ccmatoff((i-1),ziscov)=0.
end if.
do if (ncs > 0 and (wiscov > 0)).
compute ccmatoff((i-1),wiscov)=0.
end if.
compute modlabel={modlabel;intlab(cntmp,1)}.
compute intkey={intkey;intlab(cntmp,1),':',wcatlab(k1,1),'x',zcatlab(k2,1),' ',' '}.
compute cntmp=cntmp+1.
end loop.
end loop.
do if (wztmpus=0).
compute fulldat={fulldat,outv(:,(ncol(outv)-(nwvls*nzvls)+1):ncol(outv))}.
do if (ncs > 0 and (ziscov > 0)).
compute ccmatoff((i-1),ziscov)=0.
end if.
do if (ncs > 0 and (wiscov > 0)).
compute ccmatoff((i-1),wiscov)=0.
end if.
compute wztmpus=1.
loop k4=datcount to (datcount+((nwvls*nzvls)-1)).
compute wztmplo={wztmplo;k4}.
end loop.
compute wztmplo=wztmplo(2:nrow(wztmplo),1).
compute datcount=datcount+(nzvls*nwvls).
end if.
compute wzdid=1.
compute datindx(start:(start+nrow(wztmplo)-1),(i-1))=wztmplo.
compute wherewz(1,(i-1))=start+1.
compute wherewz(2,(i-1))=start+nrow(wztmplo)-1+1.
compute start=start+nrow(wztmplo).
end if.
loop k1 = 1 to nwvls.
loop k2 = 1 to nzvls.
compute outv={outv,(mtmp(:,(j-1))&*wtmp(:,k1)&*ztmp(:,k2))}.
do if (ncs > 0 and (ziscov > 0)).
compute ccmatoff((i-1),ziscov)=0.
end if.
do if (ncs > 0 and (wiscov > 0)).
compute ccmatoff((i-1),wiscov)=0.
end if.
compute modlabel={modlabel;intlab(cntmp,1)}.
compute intkey={intkey;intlab(cntmp,1),':',mnames(1,(j-1)),'x',wcatlab(k1,1),'x', zcatlab(k2,1)}.
compute cntmp=cntmp+1.
end loop.
end loop.
do if (mwztmpu(1,(j-1))=0).
compute fulldat={fulldat,outv(:,(ncol(outv)-(nwvls*nzvls)+1):ncol(outv))}.
do if (ncs > 0 and (ziscov > 0)).
compute ccmatoff((i-1),ziscov)=0.
end if.
do if (ncs > 0 and (wiscov > 0)).
compute ccmatoff((i-1),wiscov)=0.
end if.
compute mwztmpu(1,(j-1))=1.
compute mz22=-999.
loop k4=datcount to (datcount+(nwvls*nzvls)-1).
compute mz22={mz22;k4}.
end loop.
compute mwztmplo(:,(j-1))=mz22(2:nrow(mz22),1).
compute datcount=datcount+(nwvls*nzvls).
end if.
compute datindx(start:(start+nrow(mwztmplo)-1),(i-1))=mwztmplo(:,(j-1)).
compute wheremwz(((2*j)-3),(i-1))=start+1.
compute wheremwz(((2*j)-2),(i-1))=start+nrow(mwztmplo)-1+1.
compute onebl=make(nrow(mwztmplo),1,1).
compute wzhigh((start+1):(start+nrow(mwztmplo)),wzhighct)=onebl.
compute start=start+nrow(mwztmplo).
end if.
end loop.
end if.
do if (ncs > 0).
compute ccmat=ccmat&*ccmatoff.
loop j = 1 to ncs.
do if (ccmat((i-1),j))=1.
do if (j=wiscov).
compute ctmp(:,j)=wtmp.
end if.
do if (j=ziscov).
compute ctmp(:,j)=ztmp.
end if.
compute outv={outv,ctmp(:,j)}.
compute modlabel={modlabel;covnames(1,j)}.
do if (ctmpuse(1,j)=0).
compute fulldat={fulldat,ctmp(:,j)}.
compute ctmpuse(1,j)=1.
compute ctmploc(1,j)=datcount.
compute datcount=datcount+1.
end if.
compute datindx(start:(start+nrow(ctmploc)-1),(i-1))=ctmploc(1,j).
compute start=start+nrow(ctmploc(1,j)).
end if.
end loop.
end if.
compute wdid=0.
compute zdid=0.
compute wzdid=0.
compute vlabs={vlabs;modlabel(2:nrow(modlabel),1)}.
compute numint(1,(i-1))=cntmp-1.
compute nump(1,(i-1))=nrow(modlabel)-1.
end loop.
release datcount, xtmpuse, wtmpuse, ztmpuse, xwtmpus, xztmpus, wztmpus, xwztmpu.
release xtmploc, wtmploc, xwtmplo, ztmploc, xztmplo, wztmplo, xwztmplo, foccnt.
do if (modcok=1 and ((nms > 0) or (zcmat(2,1) <> 1) or (mcx <> 0))).
compute notecode(notes,1) = 19.
compute notes = notes + 1.
compute modcok=0.
end if.
do if ((serial = 1 or (rsum(numint)>0) or nms=0) and mc > 0).
compute notecode(notes,1) = 15.
compute notes = notes + 1.
compute boot=mc.
compute mc=0.
end if.
do if (boot <> 0 or mc <> 0).
compute bootsz=boot.
do if (mc > 0).
compute bootsz=mc.
compute saveboot=0.
end if.
loop.
compute cilow = rnd(bootsz*(1-(conf/100))/2).
compute cihigh = trunc((bootsz*(conf/100)+(bootsz*(1-(conf/100))/2)))+1.
do if (cilow < 1 or cihigh > bootsz).
compute bootsz=trunc((bootsz+1000)/1000)*1000.
compute adjust = 1.
end if.
end loop if (cilow gt 0 and cihigh le bootsz).
do if (boot > 0).
compute boot=bootsz.
end if.
do if (mc > 0).
compute mc=bootsz.
end if.
do if (adjust = 1 and boot > 0).
compute notecode(notes,1) = 8.
compute notes = notes + 1.
end if.
do if (adjust = 1 and mc > 0).
compute notecode(notes,1) = 16.
compute notes = notes + 1.
end if.
end if.
compute maxboot = trunc(2*boot).
do if ( 0 > maxboot).
compute maxboot=trunc( 0 ).
end if.
do if (nms > 0).
release mtmpuse, mwtmpus, mwztmpu, mtmploc, mwtmplo, mztmplo, mwztmplo.
end if.
release wdid, zdid, wzdid, start,modlabel.
compute vlabs=vlabs(2:nrow(vlabs),1).
do if (rsum(numint) > 0).
compute intkey=intkey(2:nrow(intkey),:).
end if.
compute fulldat=fulldat(:,2:ncol(fulldat)).
compute fochigh=fochigh(1:rmax(nump),:).
compute whigh=whigh(1:rmax(nump),:).
compute zhigh=zhigh(1:rmax(nump),:).
compute wzhigh=wzhigh(1:rmax(nump),:).
compute coeffs=fochigh+whigh+zhigh+wzhigh.
compute bootloc=make(rmax(nump),ncol(nump),0).
do if (nms > 0).
compute cntmp=1.
loop i = 1 to ncol(nump).
loop j = 1 to nump(1,i).
compute bootloc(j,i)=cntmp.
compute cntmp=cntmp+1.
end loop.
end loop.
compute fochighb=make(nrow(fochigh),ncol(fochigh),0).
compute whighb=fochighb.
compute zhighb=fochighb.
compute wzhighb=fochighb.
compute thetaxmb=make(nrow(fochighb),nms,0).
compute thetaxyb=make(nrow(fochighb),1,0).
compute pathsfoc=make(nxvls,1,0).
compute cntmp=1.
loop i = 1 to (nms+nys).
loop j = 1 to i.
compute fochighb(:,cntmp)=fochigh(:,cntmp)&*bootloc(:,i).
compute whighb(:,cntmp)=whigh(:,cntmp)&*bootloc(:,i).
compute zhighb(:,cntmp)=zhigh(:,cntmp)&*bootloc(:,i).
compute wzhighb(:,cntmp)=wzhigh(:,cntmp)&*bootloc(:,i).
compute coeffsb=fochighb+whighb+zhighb+wzhighb.
do if ((i < (nms+nys)) and (j = 1)).
compute thetaxmb(:,i)=coeffsb(:,cntmp).
end if.
do if ((i = (nms+nys)) and (j = 1)).
compute thetaxyb(:,1)=coeffsb(:,cntmp).
end if.
compute cntmp=cntmp+1.
end loop.
end loop.
compute thetamyb=coeffsb(:,(ncol(coeffsb)-nms+1):ncol(coeffsb)).
do if (serial = 1).
compute thetammb=make(nrow(coeffsb),((nms*(nms-1))/2),0).
end if.
compute cntmp=1.
do if (nms > 1 and serial = 1).
loop i = 1 to (nms-1).
compute start=((i+2)*(i+1))/2.
loop j = 2 to (nms-i+1).
compute thetammb(:,cntmp)=coeffsb(:,start).
compute start=start+j+i-1.
compute cntmp=cntmp+1.
end loop.
end loop.
end if.
end if.
do if ((total = 1) and (rsum(numint)=0 or (xmint=1))).
compute dototal=1.
do if ((csum(bcmat(:,1)) <> (nms+nys)) or (rsum(bcmat(nrow(bcmat),:)) <> (nms+nys))).
compute dototal=0.
compute alttotal=1.
compute notecode(notes,1) = 12.
compute notes = notes + 1.
end if.
do if (ncs > 0).
do if ((csum(rsum(ccmat))) < (nrow(ccmat)*ncol(ccmat))).
compute dototal=0.
compute alttotal=1.
compute notecode(notes,1) = 11.
compute notes = notes + 1.
end if.
end if.
do if (model=74).
do if (xdich=0 and nxvls=1).
compute dototal=0.
compute alttotal=1.
end if.
do if ((xdich=1 or nxvls > 1) and (ncs > 0) and model=74).
compute dototal=0.
compute alttotal=1.
end if.
end if.
end if.
end if.
do if (criterr=0 and ncs > 0).
do if (rsum((csum(ccmat)=0)) <> 0).
compute errcode(errs,1)=51.
compute errs=errs+1.
compute criterr=1.
end if.
end if.
compute debug= 0.
do if (outscree=1).
print/title = '***************** PROCESS Procedure for SPSS Version 4.2 *****************'.
print/title = '          Written by Andrew F. Hayes, Ph.D.       www.afhayes.com'.
print/title = '    Documentation available in Hayes (2022). www.guilford.com/p/hayes3'/space=0.
end if.
do if (criterr=0).
compute modresid=make(n,1,99999).
do if (stand=1 and ydich=1).
compute stand=0.
end if.
compute anymod2=csum(rsum(wcmat+zcmat+wzcmat)).
do if (anymod2 > 0 and stand = 1).
compute notecode(notes,1) = 27.
compute notes = notes + 1.
compute stand=0.
end if.
compute funny=1.
do if (outscree=1).
print modelvar/title = '**************************************************************************'/format = A8/rnames=modelvlb.
do if (ncs > 0).
print covnames/title='Covariates:'/format=A8.
end if.
print n/title='Sample'/rlabel='Size:'.
do if (( 'random' <> 'random')).
compute seedt= 'random'.
print seedt/title='Custom'/format=A12/rlabel = 'Seed:'.
end if.
end if.
compute maxresm=9.
compute resultm=make(1,maxresm,99999).
do if (describe=1).
compute means=csum(dat)/n.
compute sigmatal = (t(dat)*(ident(n)-(1/n)*ones*t(ones))*dat)*(1/(n-1)).
compute sdvec=sqrt(diag(sigmatal)).
compute sdall = mdiag(1/sdvec).
compute corall=sdall*sigmatal*t(sdall).
compute means={means;t(sdvec)}.
do if (ncol(means) > 9).
compute resultm=make(1,ncol(means),99999).
compute maxresm=ncol(means).
end if.
compute resultm2=make(2,maxresm,99999).
compute resultm2(1:2,1:ncol(means))=means.
compute resultm={resultm;resultm2}.
compute resultm2=make(ncol(corall),maxresm,99999).
compute resultm2(1:ncol(corall),1:ncol(corall))=corall.
compute resultm={resultm;resultm2}.
do if (outscree=1).
print means/title='Variable means and standard deviations'/cnames=varnames/rlabels='Mean','SD'/format= F10.4.
print corall/title='Variable intercorrelations (Pearson r)'/cnames=varnames/rnames=varnames/format= F10.4.
end if.
end if.
do if (outscree=1).
do if (mcxok=1).
compute labtmp={xnames,t(xcatlab(1:nxvls,1))}.
print dummatx/title = 'Coding of categorical X variable for analysis:'/cnames = labtmp/format = F6.3.
end if.
do if (mcwok=1 and xmint=0).
compute labtmp={wnames,t(wcatlab(1:nwvls,1))}.
print dummatw/title = 'Coding of categorical W variable for analysis:'/cnames = labtmp/format = F6.3.
end if.
do if (mczok=1).
compute labtmp={znames,t(zcatlab(1:nzvls,1))}.
print dummatz/title = 'Coding of categorical Z variable for analysis:'/cnames = labtmp/format = F6.3.
end if.
end if.
end if.
do if (criterr = 0).
compute outnames=ynames.
compute outvars=ytmp.
do if (nms > 0).
compute outnames={mnames,ynames}.
compute outvars={mtmp,ytmp}.
compute indcov=make(((nms*2)+(nms*(nxvls-1))),((nms*2)+(nms*(nxvls-1))),0).
compute mcsopath=make(((nms*2)+(nms*(nxvls-1))),1,0).
end if.
compute labstart=1.
compute intstart=1.
compute start=1.
compute coeffmat=make(1,6,0).
compute conseq={'        '}.
compute dfmat=0.
compute coeffcol=0.
compute pathscnt=1.
compute pathscn2=1.
loop i = 1 to (nms+nys).
do if (outscree=1).
print/title = '**************************************************************************'.
end if.
compute highf=make(1,5,0).
compute highf2=highf.
do if ((i = (nms+nys)) and (ydich=1)).
compute highf=make(1,3,0).
compute highf2=highf.
end if.
compute flabel={' '}.
compute y=outvars(:,i).
compute xindx=datindx(1:(nump(1,i)-1),i).
compute x = fulldat(:,xindx).
compute x={ones,x}.
compute xsq=t(x)*x.
compute exsq=eval(xsq).
release xsq.
compute zeroeig=csum(exsq <= 0.000000000002).
do if (outscree=1).
print outnames(1,i)/title = 'OUTCOME VARIABLE:'/format = A8/space=0.
do if (ydich=1 and (i = (nms+nys))).
compute nmsd = {outnames(1,i), 'Analysis'}.
print rcd/title = 'Coding of binary Y for logistic regression analysis:'/cnames = nmsd/format = F9.2.
end if.
end if.
do if (zeroeig > 0).
print / title = 'SINGULAR OR NEAR SINGULAR DATA MATRIX.'.
compute criterr=1.
compute errcode(errs,1)=31.
compute errs=errs+1.
end if.
compute means=csum(x)/n.
compute vlabsm=vlabs(labstart:(labstart+(nump(1,i)-1)),1).
do if (criterr=0).
do if (ydich=0 or (i < (nms+nys))).

.
do if ( 1 =1).
compute hatmat=inv(t( x )* x )*t( x ).
compute b = hatmat* y.
compute modres=b.
do if ( 1 =1).
compute n1=nrow( x ).
compute dfres=n1-(ncol( x )).
compute sstotal = t( y -(csum( y )/n1))*( y -(csum( y )/n1)).
compute resid= y - x *b.
compute ssresid = csum((resid)&**2).
compute r2 = (sstotal-ssresid)/sstotal.
do if (r2 < 0).
compute r2=0.
end if.
compute adjr2 = 1-((1-r2)*(n1-1)/(dfres)).
compute mse=ssresid/(n1-ncol( x )).

.
compute n1=nrow( x ).
compute invXtX = inv(t( x )* x ).
compute varb = mse *invXtX.
compute k3 = ncol( x ).
compute xhc=0.
do if ( hc <> 5).
compute xhc= x.
compute hat = xhc(:,1).
loop i3=1 to nrow(xhc).
compute hat(i3,1)= xhc(i3,:)*invXtX*t(xhc(i3,:)).
end loop.
do if ( hc = 0 or hc =1).
loop i3 = 1 to k3.
compute xhc(:,i3)=xhc(:,i3)&* resid.
end loop.
end if.
do if ( hc =3 or hc =2).
loop i3=1 to k3.
compute xhc(:,i3) = ( resid &/(1-hat)&**(1/(4- hc )))&*xhc(:,i3).
end loop.
end if.
do if ( hc = 4).
compute hcmn=make(n,2,4).
compute hcmn(:,2)=(n1*hat)/k3.
loop i3= 1 to k3.
compute xhc(:,i3) = ( resid &/(1-hat)&**(rmin(hcmn)/2))&*xhc(:,i3).
end loop.
end if.
compute varb=(invXtX*t(xhc)*xhc*invXtX).
do if ( hc =1).
compute varb=(n1/(n1-ncol( x )))&*varb.
end if.
end if.
compute hclab={'se(HC0)','se(HC1)','se(HC2)','se(HC3)','se(HC4)','se'}.
compute hclab=hclab(1,( hc +1)).
compute hcflab={'F(HC0)','F(HC1)','F(HC2)','F(HC3)','F(HC4)','F'}.
compute hcflab=hcflab(1,( hc +1)).
release xhc
.
compute seb=sqrt(diag(varb)).
compute trat = b&/seb.
compute p = 2*(1-tcdf(abs(trat), (dfres))).
compute tval = sqrt(dfres* (exp((dfres-(5/6))*((xp2/(dfres-(2/3)+(.11/dfres)))* (xp2/(dfres-(2/3)+(.11/dfres)))))-1)).
compute modres={modres,seb,trat,p}.
compute modres={modres,(b-tval&*seb),(b+tval&*seb)}.
compute modresl={'coeff',hclab,'t','p','LLCI','ULCI'}.
compute lmat = ident(ncol( x )).
compute lmat = lmat(:,2:ncol(lmat)).
compute fratio = (t(t(lmat)*b)*inv(t(lmat)*varb*lmat)*((t(lmat)*b)))/(ncol( x )-1).
compute pfr = 1-fcdf(fratio,(ncol( x )-1),dfres).
compute modsum={sqrt(r2),r2,mse,fratio,(ncol( x )-1),dfres,pfr}.
compute modsuml={'R','R-sq','MSE',hcflab,'df1','df2', 'p'}.
end if.
end if.
do if ( 1 = 2 or 1 =3).
compute xlp= x.
compute ylp= y.
compute pt2 = make(nrow(ylp),1,(csum(ylp)/nrow(ylp))).
do if ( 1 =2).
compute LL3 = ylp&*ln(pt2)+(1-ylp)&*ln(1-pt2).
end if.
compute LL3 = -2*csum(LL3).
compute bt1 = make(ncol(xlp),1,0).
compute LL1 = 0.
compute pt1 = make(nrow(ylp),1,0.5).
compute pt1lp=pt1.
loop jjj = 1 to iterate.
compute xlptmp=t(xlp).
compute vecprb=pt1lp&*(1-pt1lp).
loop kkk=1 to ncol(xlp).
compute xlptmp(kkk,:)=xlptmp(kkk,:)&*t(vecprb).
end loop.
compute b = bt1+inv(xlptmp*xlp)*t(xlp)*(ylp-pt1lp).
do if ( 1 =2).
compute xlpb=xlp*b.
compute xlpbt=(xlpb > -709.7).
compute xlpb709=(1-xlpbt)*(-709.7).
compute xlpb=(xlpb&*xlpbt)+xlpb709.
compute pt1lp = 1/(1+exp(-(xlpb))).
end if.
compute itprob = csum((pt1lp < .00000001) or (pt1lp > .9999999)).
do if (itprob > 0).
loop kkk = 1 to nrow(pt1lp).
do if (pt1lp(kkk,1) > .9999999).
compute pt1lp(kkk,1) = .9999999.
end if.
do if (pt1lp(kkk,1) < .00000001).
compute pt1lp(kkk,1) = .00000001.
end if.
end loop.
compute itprob = 0.
end if.
do if (itprob = 0).
do if ( 1 =2).
compute LL = ylp&*ln(pt1lp)+(1-ylp)&*ln(1-pt1lp).
end if.
compute LL2 = -2*csum(ll).
end if.
do if (abs(LL1-LL2) < converge).
do if ( 1 =1).
compute xlptmp=t(xlp).
compute vecprb=pt1lp&*(1-pt1lp).
loop kkk=1 to ncol(xlp).
compute xlptmp(kkk,:)=xlptmp(kkk,:)&*t(vecprb).
end loop.
compute varb = inv(xlptmp*xlp).
compute seb = sqrt(diag(varb)).
release xlptmp.
end if.
break.
end if.
compute bt1 = b.
compute LL1 = LL2.
end loop.
compute modres=b.
do if (jjj > iterate).
compute itprob = 2.
do if (booting=0).
compute iterrmod=1.
end if.
do if (booting=1).
compute bootiter=1.
end if.
do if (itprobtg=0).
compute itprobtg=1.
compute errcode(errs,1) = 47.
compute errs = errs + 1.
do if (booting = 0 and 1 =1).
compute vt1 = mdiag(pt1lp&*(1-pt1lp)).
compute varb = inv(t(xlp)*vt1*xlp).
compute seb = sqrt(diag(varb)).
end if.
end if.
end if.
do if ( 1 =1).
compute trat = b&/seb.
compute dfres=nrow(xlp).
compute p = 2*(1-cdfnorm(abs(trat))).
compute modres={modres,seb,trat,p}.
compute modres={modres,(b-xp2&*seb),(b+xp2&*seb)}.
compute pvchi=1-chicdf((LL3-LL2),(nrow(modres)-1)).
compute mcF = (LL3-LL2)/LL3.
compute cox = 1-exp(-(LL3-LL2)/nrow(xlp)).
compute nagel = cox/(1-exp(-(LL3)/nrow(xlp))).
compute modsum={LL2,(LL3-LL2),(nrow(modres)-1),pvchi, mcF,cox,nagel}.
compute modsuml={'-2LL','ModelLL', 'df', 'p', 'McFadden', 'CoxSnell', 'Nagelkrk'}.
compute modresl={'coeff','se','Z','p','LLCI','ULCI'}.
end if.
end if
.
compute dfmatt=make(nrow(modres),1,modsum(1,6)).
compute modresid={modresid,resid}.
end if.
do if (ydich=1 and (i = (nms+nys))).

.
do if ( 2 =1).
compute hatmat=inv(t( x )* x )*t( x ).
compute b = hatmat* y.
compute modres=b.
do if ( 1 =1).
compute n1=nrow( x ).
compute dfres=n1-(ncol( x )).
compute sstotal = t( y -(csum( y )/n1))*( y -(csum( y )/n1)).
compute resid= y - x *b.
compute ssresid = csum((resid)&**2).
compute r2 = (sstotal-ssresid)/sstotal.
do if (r2 < 0).
compute r2=0.
end if.
compute adjr2 = 1-((1-r2)*(n1-1)/(dfres)).
compute mse=ssresid/(n1-ncol( x )).

.
compute n1=nrow( x ).
compute invXtX = inv(t( x )* x ).
compute varb = mse *invXtX.
compute k3 = ncol( x ).
compute xhc=0.
do if ( hc <> 5).
compute xhc= x.
compute hat = xhc(:,1).
loop i3=1 to nrow(xhc).
compute hat(i3,1)= xhc(i3,:)*invXtX*t(xhc(i3,:)).
end loop.
do if ( hc = 0 or hc =1).
loop i3 = 1 to k3.
compute xhc(:,i3)=xhc(:,i3)&* resid.
end loop.
end if.
do if ( hc =3 or hc =2).
loop i3=1 to k3.
compute xhc(:,i3) = ( resid &/(1-hat)&**(1/(4- hc )))&*xhc(:,i3).
end loop.
end if.
do if ( hc = 4).
compute hcmn=make(n,2,4).
compute hcmn(:,2)=(n1*hat)/k3.
loop i3= 1 to k3.
compute xhc(:,i3) = ( resid &/(1-hat)&**(rmin(hcmn)/2))&*xhc(:,i3).
end loop.
end if.
compute varb=(invXtX*t(xhc)*xhc*invXtX).
do if ( hc =1).
compute varb=(n1/(n1-ncol( x )))&*varb.
end if.
end if.
compute hclab={'se(HC0)','se(HC1)','se(HC2)','se(HC3)','se(HC4)','se'}.
compute hclab=hclab(1,( hc +1)).
compute hcflab={'F(HC0)','F(HC1)','F(HC2)','F(HC3)','F(HC4)','F'}.
compute hcflab=hcflab(1,( hc +1)).
release xhc
.
compute seb=sqrt(diag(varb)).
compute trat = b&/seb.
compute p = 2*(1-tcdf(abs(trat), (dfres))).
compute tval = sqrt(dfres* (exp((dfres-(5/6))*((xp2/(dfres-(2/3)+(.11/dfres)))* (xp2/(dfres-(2/3)+(.11/dfres)))))-1)).
compute modres={modres,seb,trat,p}.
compute modres={modres,(b-tval&*seb),(b+tval&*seb)}.
compute modresl={'coeff',hclab,'t','p','LLCI','ULCI'}.
compute lmat = ident(ncol( x )).
compute lmat = lmat(:,2:ncol(lmat)).
compute fratio = (t(t(lmat)*b)*inv(t(lmat)*varb*lmat)*((t(lmat)*b)))/(ncol( x )-1).
compute pfr = 1-fcdf(fratio,(ncol( x )-1),dfres).
compute modsum={sqrt(r2),r2,mse,fratio,(ncol( x )-1),dfres,pfr}.
compute modsuml={'R','R-sq','MSE',hcflab,'df1','df2', 'p'}.
end if.
end if.
do if ( 2 = 2 or 2 =3).
compute xlp= x.
compute ylp= y.
compute pt2 = make(nrow(ylp),1,(csum(ylp)/nrow(ylp))).
do if ( 2 =2).
compute LL3 = ylp&*ln(pt2)+(1-ylp)&*ln(1-pt2).
end if.
compute LL3 = -2*csum(LL3).
compute bt1 = make(ncol(xlp),1,0).
compute LL1 = 0.
compute pt1 = make(nrow(ylp),1,0.5).
compute pt1lp=pt1.
loop jjj = 1 to iterate.
compute xlptmp=t(xlp).
compute vecprb=pt1lp&*(1-pt1lp).
loop kkk=1 to ncol(xlp).
compute xlptmp(kkk,:)=xlptmp(kkk,:)&*t(vecprb).
end loop.
compute b = bt1+inv(xlptmp*xlp)*t(xlp)*(ylp-pt1lp).
do if ( 2 =2).
compute xlpb=xlp*b.
compute xlpbt=(xlpb > -709.7).
compute xlpb709=(1-xlpbt)*(-709.7).
compute xlpb=(xlpb&*xlpbt)+xlpb709.
compute pt1lp = 1/(1+exp(-(xlpb))).
end if.
compute itprob = csum((pt1lp < .00000001) or (pt1lp > .9999999)).
do if (itprob > 0).
loop kkk = 1 to nrow(pt1lp).
do if (pt1lp(kkk,1) > .9999999).
compute pt1lp(kkk,1) = .9999999.
end if.
do if (pt1lp(kkk,1) < .00000001).
compute pt1lp(kkk,1) = .00000001.
end if.
end loop.
compute itprob = 0.
end if.
do if (itprob = 0).
do if ( 2 =2).
compute LL = ylp&*ln(pt1lp)+(1-ylp)&*ln(1-pt1lp).
end if.
compute LL2 = -2*csum(ll).
end if.
do if (abs(LL1-LL2) < converge).
do if ( 1 =1).
compute xlptmp=t(xlp).
compute vecprb=pt1lp&*(1-pt1lp).
loop kkk=1 to ncol(xlp).
compute xlptmp(kkk,:)=xlptmp(kkk,:)&*t(vecprb).
end loop.
compute varb = inv(xlptmp*xlp).
compute seb = sqrt(diag(varb)).
release xlptmp.
end if.
break.
end if.
compute bt1 = b.
compute LL1 = LL2.
end loop.
compute modres=b.
do if (jjj > iterate).
compute itprob = 2.
do if (booting=0).
compute iterrmod=1.
end if.
do if (booting=1).
compute bootiter=1.
end if.
do if (itprobtg=0).
compute itprobtg=1.
compute errcode(errs,1) = 47.
compute errs = errs + 1.
do if (booting = 0 and 1 =1).
compute vt1 = mdiag(pt1lp&*(1-pt1lp)).
compute varb = inv(t(xlp)*vt1*xlp).
compute seb = sqrt(diag(varb)).
end if.
end if.
end if.
do if ( 1 =1).
compute trat = b&/seb.
compute dfres=nrow(xlp).
compute p = 2*(1-cdfnorm(abs(trat))).
compute modres={modres,seb,trat,p}.
compute modres={modres,(b-xp2&*seb),(b+xp2&*seb)}.
compute pvchi=1-chicdf((LL3-LL2),(nrow(modres)-1)).
compute mcF = (LL3-LL2)/LL3.
compute cox = 1-exp(-(LL3-LL2)/nrow(xlp)).
compute nagel = cox/(1-exp(-(LL3)/nrow(xlp))).
compute modsum={LL2,(LL3-LL2),(nrow(modres)-1),pvchi, mcF,cox,nagel}.
compute modsuml={'-2LL','ModelLL', 'df', 'p', 'McFadden', 'CoxSnell', 'Nagelkrk'}.
compute modresl={'coeff','se','Z','p','LLCI','ULCI'}.
end if.
end if
.
compute dfmatt=make(nrow(modres),1,-999).
end if.
compute resultm2=make(1,maxresm,99999).
compute resultm2(1,1:ncol(modsum))=modsum.
compute resultm={resultm;resultm2}.
compute resultm2=make(nrow(modres),maxresm,99999).
compute resultm2(1:nrow(modres),1:ncol(modres))=modres.
compute resultm={resultm;resultm2}.
compute obscoeff={obscoeff,t(b)}.
do if (outscree=1).
print modsum/title = 'Model Summary'/cnames = modsuml/format= F10.4.
print modres/title='Model'/rnames=vlabsm/cnames=modresl/format= F10.4.
end if.
compute basemod=modsum(1,1).
compute basemodx=basemod.
do if (ydich=1 and (i = (nms+nys))).
do if (outscree=1).
print/title='These results are expressed in a log-odds metric.'.
end if.
compute notecode(notes,1) = 26.
compute notes = notes + 1.
end if.
compute coeffmat={coeffmat;modres}.
compute conseqt=make(nrow(modres),1,outnames(1,i)).
compute conseq={conseq;conseqt}.
compute dfmat={dfmat;dfmatt}.
compute labstart=labstart+nump(1,i).
do if (stand=1).
compute predsd=make(nrow(modres),1,0).
compute stdmod=modres(:,1)&/ovsd(1,i).
loop jd=1 to ncol(x).
compute descdat=x(:,jd).
compute predsd(jd,1) = (nrow(descdat)*sscp(descdat))-(t(csum(descdat))*(csum(descdat))).
compute predsd(jd,1) = sqrt(predsd(jd,1)/(nrow(descdat)*(nrow(descdat)-1))).
end loop.
do if (wherex(1,i) <> -999 and ((nxvls > 1) or (xdich=1))).
compute sdmsone=make(nxvls,1,1).
compute predsd(wherex(1,i):wherex(2,i),1)=sdmsone.
compute pstog=1.
end if.
compute predsd(1,1)=1.
compute stdmod=stdmod&*predsd.
compute stdmod=stdmod(2:nrow(stdmod),1).
compute sdvlabs=vlabsm(2:nrow(vlabsm),1).
compute resultm2=make(nrow(stdmod),maxresm,99999).
compute resultm2(1:nrow(stdmod),1:ncol(stdmod))=stdmod.
compute resultm={resultm;resultm2}.
do if (outscree=1).
print stdmod/title='Standardized coefficients'/clabels='coeff'/rnames=sdvlabs/format= F10.4.
end if.
end if.
do if (nms > 0 and serial = 0 and (rsum(numint) = 0) and (normal=1 or mc > 0)).
do if (i < (nms+nys)).
compute indcov((((i-1)*nxvls)+1):(i*nxvls),(((i-1)*nxvls)+1):(i*nxvls))=varb(2:(1+nxvls), 2:(1+nxvls)).
compute mcsopath((((i-1)*nxvls)+1):(i*nxvls) ,1)=modres(2:(1+nxvls),1).
end if.
do if (i = (nms+nys)).
compute atm=ncol(wherem).
compute indcov(((nms*nxvls)+1):nrow(mcsopath),((nms*nxvls)+1):nrow(mcsopath))= varb(wherem(1,atm):(wherem(1,atm)+nms-1),wherem(1,atm):(wherem(1,atm)+nms-1)).
compute mcsopath(((nms*nxvls)+1):nrow(mcsopath),1)=modres(wherem(1,atm):(wherem(1,atm)+ nms-1),1).
compute sobelok=1.
end if.
end if.
compute obsdirfx=make(1,nxvls,0).
compute dirzes=make(1,nxvls,0).
do if ((i = (nms+nys)) and (bcmat(nrow(bcmat),1)=1)).
compute direff=modres(2:(1+nxvls),:).
compute obsdirfx=t(direff(:,1)).
compute direfflb=modresl.
compute direffl2=vlabsm(2:(1+nxvls),:).
compute lmat=make(nrow(b),1,0).
compute lmat2=make(nxvls,1,1).
compute lmat(2:(1+nxvls),1)=lmat2.
do if (ydich <> 1).

.
compute lmat2= lmat.
do if ( 0 =0).
compute lmat2 = mdiag( lmat ).
compute lmat3=make(nrow(lmat2),1,0).
loop flp=1 to ncol(lmat2).
do if (csum(lmat2(:,flp))=1).
compute lmat3={lmat3,lmat2(:,flp)}.
end if.
end loop.
compute lmat2=lmat3(:,2:ncol(lmat3)).
end if.
compute fratio = (t(t(lmat2)* b )*inv(t(lmat2)* varb *lmat2)*((t(lmat2)* b )))/ncol(lmat2).
compute pfr = 1-fcdf(fratio,ncol(lmat2),(n-nrow( b ))).
compute fresult={fratio,ncol(lmat2),(n-nrow( b )),pfr}.
do if (i = (nms+nys) and (ydich=1)).
compute fratio=fratio*ncol(lmat2).
compute pfr=1-chicdf(fratio,ncol(lmat2)).
compute fresult={fratio,ncol(lmat2),pfr}.
end if.
do if ( 1 =1).
compute lmat3=1-rsum(lmat2).
compute xfm=make(n,csum(lmat3),0).
compute flpc=1.
loop flp=1 to nrow(lmat3).
do if (lmat3(flp,1)=1).
compute xfm(:,flpc)=x(:,flp).
compute flpc=flpc+1.
end if.
end loop.
compute bfm=inv(t(xfm)*xfm)*t(xfm)*y.
compute resid=y-(xfm*bfm).
compute sstotal=(y-(csum(y)/n)).
compute sstotal=csum(sstotal&*sstotal).
compute ssresid=csum(resid&*resid).
compute rsqch= r2 -((sstotal-ssresid)/sstotal).
compute fresult={rsqch,fresult}.
release xfm,flpc, resid, ssresid, bfm.
end if
.
compute diromni=fresult.
end if.
do if (ydich = 1 ).

.
compute btemphld=b.
compute llrdat=make(nrow(x),nrow( lmat )-csum( lmat ),-999).
compute llrdf=ncol(x)-ncol(llrdat).
compute llrcnt=0.
loop llri=1 to nrow( lmat ).
do if ( lmat (llri,1)=0).
compute llrcnt=llrcnt+1.
compute llrdat(:,llrcnt)=x(:,llri).
end if.
end loop.

.
do if ( 2 =1).
compute hatmat=inv(t( llrdat )* llrdat )*t( llrdat ).
compute b = hatmat* y.
compute modres=b.
do if ( 0 =1).
compute n1=nrow( llrdat ).
compute dfres=n1-(ncol( llrdat )).
compute sstotal = t( y -(csum( y )/n1))*( y -(csum( y )/n1)).
compute resid= y - llrdat *b.
compute ssresid = csum((resid)&**2).
compute r2 = (sstotal-ssresid)/sstotal.
do if (r2 < 0).
compute r2=0.
end if.
compute adjr2 = 1-((1-r2)*(n1-1)/(dfres)).
compute mse=ssresid/(n1-ncol( llrdat )).

.
compute n1=nrow( x ).
compute invXtX = inv(t( x )* x ).
compute varb = mse *invXtX.
compute k3 = ncol( x ).
compute xhc=0.
do if ( hc <> 5).
compute xhc= x.
compute hat = xhc(:,1).
loop i3=1 to nrow(xhc).
compute hat(i3,1)= xhc(i3,:)*invXtX*t(xhc(i3,:)).
end loop.
do if ( hc = 0 or hc =1).
loop i3 = 1 to k3.
compute xhc(:,i3)=xhc(:,i3)&* resid.
end loop.
end if.
do if ( hc =3 or hc =2).
loop i3=1 to k3.
compute xhc(:,i3) = ( resid &/(1-hat)&**(1/(4- hc )))&*xhc(:,i3).
end loop.
end if.
do if ( hc = 4).
compute hcmn=make(n,2,4).
compute hcmn(:,2)=(n1*hat)/k3.
loop i3= 1 to k3.
compute xhc(:,i3) = ( resid &/(1-hat)&**(rmin(hcmn)/2))&*xhc(:,i3).
end loop.
end if.
compute varb=(invXtX*t(xhc)*xhc*invXtX).
do if ( hc =1).
compute varb=(n1/(n1-ncol( x )))&*varb.
end if.
end if.
compute hclab={'se(HC0)','se(HC1)','se(HC2)','se(HC3)','se(HC4)','se'}.
compute hclab=hclab(1,( hc +1)).
compute hcflab={'F(HC0)','F(HC1)','F(HC2)','F(HC3)','F(HC4)','F'}.
compute hcflab=hcflab(1,( hc +1)).
release xhc
.
compute seb=sqrt(diag(varb)).
compute trat = b&/seb.
compute p = 2*(1-tcdf(abs(trat), (dfres))).
compute tval = sqrt(dfres* (exp((dfres-(5/6))*((xp2/(dfres-(2/3)+(.11/dfres)))* (xp2/(dfres-(2/3)+(.11/dfres)))))-1)).
compute modres={modres,seb,trat,p}.
compute modres={modres,(b-tval&*seb),(b+tval&*seb)}.
compute modresl={'coeff',hclab,'t','p','LLCI','ULCI'}.
compute lmat = ident(ncol( llrdat )).
compute lmat = lmat(:,2:ncol(lmat)).
compute fratio = (t(t(lmat)*b)*inv(t(lmat)*varb*lmat)*((t(lmat)*b)))/(ncol( llrdat )-1).
compute pfr = 1-fcdf(fratio,(ncol( llrdat )-1),dfres).
compute modsum={sqrt(r2),r2,mse,fratio,(ncol( llrdat )-1),dfres,pfr}.
compute modsuml={'R','R-sq','MSE',hcflab,'df1','df2', 'p'}.
end if.
end if.
do if ( 2 = 2 or 2 =3).
compute xlp= llrdat.
compute ylp= y.
compute pt2 = make(nrow(ylp),1,(csum(ylp)/nrow(ylp))).
do if ( 2 =2).
compute LL3 = ylp&*ln(pt2)+(1-ylp)&*ln(1-pt2).
end if.
compute LL3 = -2*csum(LL3).
compute bt1 = make(ncol(xlp),1,0).
compute LL1 = 0.
compute pt1 = make(nrow(ylp),1,0.5).
compute pt1lp=pt1.
loop jjj = 1 to iterate.
compute xlptmp=t(xlp).
compute vecprb=pt1lp&*(1-pt1lp).
loop kkk=1 to ncol(xlp).
compute xlptmp(kkk,:)=xlptmp(kkk,:)&*t(vecprb).
end loop.
compute b = bt1+inv(xlptmp*xlp)*t(xlp)*(ylp-pt1lp).
do if ( 2 =2).
compute xlpb=xlp*b.
compute xlpbt=(xlpb > -709.7).
compute xlpb709=(1-xlpbt)*(-709.7).
compute xlpb=(xlpb&*xlpbt)+xlpb709.
compute pt1lp = 1/(1+exp(-(xlpb))).
end if.
compute itprob = csum((pt1lp < .00000001) or (pt1lp > .9999999)).
do if (itprob > 0).
loop kkk = 1 to nrow(pt1lp).
do if (pt1lp(kkk,1) > .9999999).
compute pt1lp(kkk,1) = .9999999.
end if.
do if (pt1lp(kkk,1) < .00000001).
compute pt1lp(kkk,1) = .00000001.
end if.
end loop.
compute itprob = 0.
end if.
do if (itprob = 0).
do if ( 2 =2).
compute LL = ylp&*ln(pt1lp)+(1-ylp)&*ln(1-pt1lp).
end if.
compute LL2 = -2*csum(ll).
end if.
do if (abs(LL1-LL2) < converge).
do if ( 0 =1).
compute xlptmp=t(xlp).
compute vecprb=pt1lp&*(1-pt1lp).
loop kkk=1 to ncol(xlp).
compute xlptmp(kkk,:)=xlptmp(kkk,:)&*t(vecprb).
end loop.
compute varb = inv(xlptmp*xlp).
compute seb = sqrt(diag(varb)).
release xlptmp.
end if.
break.
end if.
compute bt1 = b.
compute LL1 = LL2.
end loop.
compute modres=b.
do if (jjj > iterate).
compute itprob = 2.
do if (booting=0).
compute iterrmod=1.
end if.
do if (booting=1).
compute bootiter=1.
end if.
do if (itprobtg=0).
compute itprobtg=1.
compute errcode(errs,1) = 47.
compute errs = errs + 1.
do if (booting = 0 and 0 =1).
compute vt1 = mdiag(pt1lp&*(1-pt1lp)).
compute varb = inv(t(xlp)*vt1*xlp).
compute seb = sqrt(diag(varb)).
end if.
end if.
end if.
do if ( 0 =1).
compute trat = b&/seb.
compute dfres=nrow(xlp).
compute p = 2*(1-cdfnorm(abs(trat))).
compute modres={modres,seb,trat,p}.
compute modres={modres,(b-xp2&*seb),(b+xp2&*seb)}.
compute pvchi=1-chicdf((LL3-LL2),(nrow(modres)-1)).
compute mcF = (LL3-LL2)/LL3.
compute cox = 1-exp(-(LL3-LL2)/nrow(xlp)).
compute nagel = cox/(1-exp(-(LL3)/nrow(xlp))).
compute modsum={LL2,(LL3-LL2),(nrow(modres)-1),pvchi, mcF,cox,nagel}.
compute modsuml={'-2LL','ModelLL', 'df', 'p', 'McFadden', 'CoxSnell', 'Nagelkrk'}.
compute modresl={'coeff','se','Z','p','LLCI','ULCI'}.
end if.
end if
.
compute b=btemphld.
compute fresult={(LL2-basemod),llrdf,1-chicdf((LL2-basemod),llrdf)}
.
compute diromni=fresult.
end if.
end if.
do if (numint(1,i) > 0).
compute intkeym=intkey(intstart:(intstart+numint(1,i)-1),:).
do if (outscree=1).
print intkeym/title='Product terms key:'/format = A8.
end if.
end if.
do if (covcoeff=1).
do if (outscree=1).
print varb/title='Covariance matrix of regression parameter estimates:'/rnames=vlabsm /cnames=vlabsm/format= F10.4.
end if.
compute resultm2=make(nrow(varb),maxresm,99999).
do if (ncol(varb) <= maxresm).
compute resultm2(1:nrow(varb),1:ncol(varb))=varb.
compute resultm={resultm;resultm2}.
end if.
do if (ncol(varb) > maxresm).
compute resultmt=make(nrow(resultm),ncol(varb),99999).
compute resultmt(1:nrow(resultm),1:ncol(resultm))=resultm.
compute resultm=resultmt.
compute resultm2=make(nrow(varb),ncol(resultm),99999).
compute resultm2(1:nrow(varb),1:ncol(varb))=varb.
compute resultm={resultm;resultm2}.
compute maxresm=ncol(resultm).
end if.
end if.
do if (model <> 74 and xmtest=1 and nms > 0).
compute r2tmp=r2.
compute btmp=b.
compute varbtmp=varb.
compute dfrestmp=dfres.
compute tvaltmp=tval.
compute xmtst=make(nms,4,0).
compute xmtstlbc={hcflab,'df1','df2','p'}.
do if ((i=(nms+nys)) and (ydich=1)).
compute xmtst=make(nms,3,0).
compute xmtstlbc={'Chi-sq','df','p'}.
end if.
compute xmtstlb={' '}.
compute xmtmat=x.
compute numxint=0.
loop xmints=2 to i.
compute x=xmtmat.
do if ((bcmat((i+1),xmints)=1) and (wzcmat((i+1),xmints) <> 1)).
do if (bcmat((i+1),1)=0).
compute x={xmtmat,xtmp}.
do if ((ydich=1) and (i=(nms+nys))).

.
do if ( 2 =1).
compute hatmat=inv(t( x )* x )*t( x ).
compute b = hatmat* y.
compute modres=b.
do if ( 1 =1).
compute n1=nrow( x ).
compute dfres=n1-(ncol( x )).
compute sstotal = t( y -(csum( y )/n1))*( y -(csum( y )/n1)).
compute resid= y - x *b.
compute ssresid = csum((resid)&**2).
compute r2 = (sstotal-ssresid)/sstotal.
do if (r2 < 0).
compute r2=0.
end if.
compute adjr2 = 1-((1-r2)*(n1-1)/(dfres)).
compute mse=ssresid/(n1-ncol( x )).

.
compute n1=nrow( x ).
compute invXtX = inv(t( x )* x ).
compute varb = mse *invXtX.
compute k3 = ncol( x ).
compute xhc=0.
do if ( hc <> 5).
compute xhc= x.
compute hat = xhc(:,1).
loop i3=1 to nrow(xhc).
compute hat(i3,1)= xhc(i3,:)*invXtX*t(xhc(i3,:)).
end loop.
do if ( hc = 0 or hc =1).
loop i3 = 1 to k3.
compute xhc(:,i3)=xhc(:,i3)&* resid.
end loop.
end if.
do if ( hc =3 or hc =2).
loop i3=1 to k3.
compute xhc(:,i3) = ( resid &/(1-hat)&**(1/(4- hc )))&*xhc(:,i3).
end loop.
end if.
do if ( hc = 4).
compute hcmn=make(n,2,4).
compute hcmn(:,2)=(n1*hat)/k3.
loop i3= 1 to k3.
compute xhc(:,i3) = ( resid &/(1-hat)&**(rmin(hcmn)/2))&*xhc(:,i3).
end loop.
end if.
compute varb=(invXtX*t(xhc)*xhc*invXtX).
do if ( hc =1).
compute varb=(n1/(n1-ncol( x )))&*varb.
end if.
end if.
compute hclab={'se(HC0)','se(HC1)','se(HC2)','se(HC3)','se(HC4)','se'}.
compute hclab=hclab(1,( hc +1)).
compute hcflab={'F(HC0)','F(HC1)','F(HC2)','F(HC3)','F(HC4)','F'}.
compute hcflab=hcflab(1,( hc +1)).
release xhc
.
compute seb=sqrt(diag(varb)).
compute trat = b&/seb.
compute p = 2*(1-tcdf(abs(trat), (dfres))).
compute tval = sqrt(dfres* (exp((dfres-(5/6))*((xp2/(dfres-(2/3)+(.11/dfres)))* (xp2/(dfres-(2/3)+(.11/dfres)))))-1)).
compute modres={modres,seb,trat,p}.
compute modres={modres,(b-tval&*seb),(b+tval&*seb)}.
compute modresl={'coeff',hclab,'t','p','LLCI','ULCI'}.
compute lmat = ident(ncol( x )).
compute lmat = lmat(:,2:ncol(lmat)).
compute fratio = (t(t(lmat)*b)*inv(t(lmat)*varb*lmat)*((t(lmat)*b)))/(ncol( x )-1).
compute pfr = 1-fcdf(fratio,(ncol( x )-1),dfres).
compute modsum={sqrt(r2),r2,mse,fratio,(ncol( x )-1),dfres,pfr}.
compute modsuml={'R','R-sq','MSE',hcflab,'df1','df2', 'p'}.
end if.
end if.
do if ( 2 = 2 or 2 =3).
compute xlp= x.
compute ylp= y.
compute pt2 = make(nrow(ylp),1,(csum(ylp)/nrow(ylp))).
do if ( 2 =2).
compute LL3 = ylp&*ln(pt2)+(1-ylp)&*ln(1-pt2).
end if.
compute LL3 = -2*csum(LL3).
compute bt1 = make(ncol(xlp),1,0).
compute LL1 = 0.
compute pt1 = make(nrow(ylp),1,0.5).
compute pt1lp=pt1.
loop jjj = 1 to iterate.
compute xlptmp=t(xlp).
compute vecprb=pt1lp&*(1-pt1lp).
loop kkk=1 to ncol(xlp).
compute xlptmp(kkk,:)=xlptmp(kkk,:)&*t(vecprb).
end loop.
compute b = bt1+inv(xlptmp*xlp)*t(xlp)*(ylp-pt1lp).
do if ( 2 =2).
compute xlpb=xlp*b.
compute xlpbt=(xlpb > -709.7).
compute xlpb709=(1-xlpbt)*(-709.7).
compute xlpb=(xlpb&*xlpbt)+xlpb709.
compute pt1lp = 1/(1+exp(-(xlpb))).
end if.
compute itprob = csum((pt1lp < .00000001) or (pt1lp > .9999999)).
do if (itprob > 0).
loop kkk = 1 to nrow(pt1lp).
do if (pt1lp(kkk,1) > .9999999).
compute pt1lp(kkk,1) = .9999999.
end if.
do if (pt1lp(kkk,1) < .00000001).
compute pt1lp(kkk,1) = .00000001.
end if.
end loop.
compute itprob = 0.
end if.
do if (itprob = 0).
do if ( 2 =2).
compute LL = ylp&*ln(pt1lp)+(1-ylp)&*ln(1-pt1lp).
end if.
compute LL2 = -2*csum(ll).
end if.
do if (abs(LL1-LL2) < converge).
do if ( 1 =1).
compute xlptmp=t(xlp).
compute vecprb=pt1lp&*(1-pt1lp).
loop kkk=1 to ncol(xlp).
compute xlptmp(kkk,:)=xlptmp(kkk,:)&*t(vecprb).
end loop.
compute varb = inv(xlptmp*xlp).
compute seb = sqrt(diag(varb)).
release xlptmp.
end if.
break.
end if.
compute bt1 = b.
compute LL1 = LL2.
end loop.
compute modres=b.
do if (jjj > iterate).
compute itprob = 2.
do if (booting=0).
compute iterrmod=1.
end if.
do if (booting=1).
compute bootiter=1.
end if.
do if (itprobtg=0).
compute itprobtg=1.
compute errcode(errs,1) = 47.
compute errs = errs + 1.
do if (booting = 0 and 1 =1).
compute vt1 = mdiag(pt1lp&*(1-pt1lp)).
compute varb = inv(t(xlp)*vt1*xlp).
compute seb = sqrt(diag(varb)).
end if.
end if.
end if.
do if ( 1 =1).
compute trat = b&/seb.
compute dfres=nrow(xlp).
compute p = 2*(1-cdfnorm(abs(trat))).
compute modres={modres,seb,trat,p}.
compute modres={modres,(b-xp2&*seb),(b+xp2&*seb)}.
compute pvchi=1-chicdf((LL3-LL2),(nrow(modres)-1)).
compute mcF = (LL3-LL2)/LL3.
compute cox = 1-exp(-(LL3-LL2)/nrow(xlp)).
compute nagel = cox/(1-exp(-(LL3)/nrow(xlp))).
compute modsum={LL2,(LL3-LL2),(nrow(modres)-1),pvchi, mcF,cox,nagel}.
compute modsuml={'-2LL','ModelLL', 'df', 'p', 'McFadden', 'CoxSnell', 'Nagelkrk'}.
compute modresl={'coeff','se','Z','p','LLCI','ULCI'}.
end if.
end if
.
compute basemodx=LL2.
end if.
end if.
loop xmtlp1=1 to nxvls.
compute x={x,xtmp(:,xmtlp1)&*(mtmp(:,(xmints-1))-(csum(mtmp(:,(xmints-1)))/nrow(mtmp)) )}.
end loop.
do if ((i < (nms+nys)) or (ydich=0)).

.
do if ( 1 =1).
compute hatmat=inv(t( x )* x )*t( x ).
compute b = hatmat* y.
compute modres=b.
do if ( 1 =1).
compute n1=nrow( x ).
compute dfres=n1-(ncol( x )).
compute sstotal = t( y -(csum( y )/n1))*( y -(csum( y )/n1)).
compute resid= y - x *b.
compute ssresid = csum((resid)&**2).
compute r2 = (sstotal-ssresid)/sstotal.
do if (r2 < 0).
compute r2=0.
end if.
compute adjr2 = 1-((1-r2)*(n1-1)/(dfres)).
compute mse=ssresid/(n1-ncol( x )).

.
compute n1=nrow( x ).
compute invXtX = inv(t( x )* x ).
compute varb = mse *invXtX.
compute k3 = ncol( x ).
compute xhc=0.
do if ( hc <> 5).
compute xhc= x.
compute hat = xhc(:,1).
loop i3=1 to nrow(xhc).
compute hat(i3,1)= xhc(i3,:)*invXtX*t(xhc(i3,:)).
end loop.
do if ( hc = 0 or hc =1).
loop i3 = 1 to k3.
compute xhc(:,i3)=xhc(:,i3)&* resid.
end loop.
end if.
do if ( hc =3 or hc =2).
loop i3=1 to k3.
compute xhc(:,i3) = ( resid &/(1-hat)&**(1/(4- hc )))&*xhc(:,i3).
end loop.
end if.
do if ( hc = 4).
compute hcmn=make(n,2,4).
compute hcmn(:,2)=(n1*hat)/k3.
loop i3= 1 to k3.
compute xhc(:,i3) = ( resid &/(1-hat)&**(rmin(hcmn)/2))&*xhc(:,i3).
end loop.
end if.
compute varb=(invXtX*t(xhc)*xhc*invXtX).
do if ( hc =1).
compute varb=(n1/(n1-ncol( x )))&*varb.
end if.
end if.
compute hclab={'se(HC0)','se(HC1)','se(HC2)','se(HC3)','se(HC4)','se'}.
compute hclab=hclab(1,( hc +1)).
compute hcflab={'F(HC0)','F(HC1)','F(HC2)','F(HC3)','F(HC4)','F'}.
compute hcflab=hcflab(1,( hc +1)).
release xhc
.
compute seb=sqrt(diag(varb)).
compute trat = b&/seb.
compute p = 2*(1-tcdf(abs(trat), (dfres))).
compute tval = sqrt(dfres* (exp((dfres-(5/6))*((xp2/(dfres-(2/3)+(.11/dfres)))* (xp2/(dfres-(2/3)+(.11/dfres)))))-1)).
compute modres={modres,seb,trat,p}.
compute modres={modres,(b-tval&*seb),(b+tval&*seb)}.
compute modresl={'coeff',hclab,'t','p','LLCI','ULCI'}.
compute lmat = ident(ncol( x )).
compute lmat = lmat(:,2:ncol(lmat)).
compute fratio = (t(t(lmat)*b)*inv(t(lmat)*varb*lmat)*((t(lmat)*b)))/(ncol( x )-1).
compute pfr = 1-fcdf(fratio,(ncol( x )-1),dfres).
compute modsum={sqrt(r2),r2,mse,fratio,(ncol( x )-1),dfres,pfr}.
compute modsuml={'R','R-sq','MSE',hcflab,'df1','df2', 'p'}.
end if.
end if.
do if ( 1 = 2 or 1 =3).
compute xlp= x.
compute ylp= y.
compute pt2 = make(nrow(ylp),1,(csum(ylp)/nrow(ylp))).
do if ( 1 =2).
compute LL3 = ylp&*ln(pt2)+(1-ylp)&*ln(1-pt2).
end if.
compute LL3 = -2*csum(LL3).
compute bt1 = make(ncol(xlp),1,0).
compute LL1 = 0.
compute pt1 = make(nrow(ylp),1,0.5).
compute pt1lp=pt1.
loop jjj = 1 to iterate.
compute xlptmp=t(xlp).
compute vecprb=pt1lp&*(1-pt1lp).
loop kkk=1 to ncol(xlp).
compute xlptmp(kkk,:)=xlptmp(kkk,:)&*t(vecprb).
end loop.
compute b = bt1+inv(xlptmp*xlp)*t(xlp)*(ylp-pt1lp).
do if ( 1 =2).
compute xlpb=xlp*b.
compute xlpbt=(xlpb > -709.7).
compute xlpb709=(1-xlpbt)*(-709.7).
compute xlpb=(xlpb&*xlpbt)+xlpb709.
compute pt1lp = 1/(1+exp(-(xlpb))).
end if.
compute itprob = csum((pt1lp < .00000001) or (pt1lp > .9999999)).
do if (itprob > 0).
loop kkk = 1 to nrow(pt1lp).
do if (pt1lp(kkk,1) > .9999999).
compute pt1lp(kkk,1) = .9999999.
end if.
do if (pt1lp(kkk,1) < .00000001).
compute pt1lp(kkk,1) = .00000001.
end if.
end loop.
compute itprob = 0.
end if.
do if (itprob = 0).
do if ( 1 =2).
compute LL = ylp&*ln(pt1lp)+(1-ylp)&*ln(1-pt1lp).
end if.
compute LL2 = -2*csum(ll).
end if.
do if (abs(LL1-LL2) < converge).
do if ( 1 =1).
compute xlptmp=t(xlp).
compute vecprb=pt1lp&*(1-pt1lp).
loop kkk=1 to ncol(xlp).
compute xlptmp(kkk,:)=xlptmp(kkk,:)&*t(vecprb).
end loop.
compute varb = inv(xlptmp*xlp).
compute seb = sqrt(diag(varb)).
release xlptmp.
end if.
break.
end if.
compute bt1 = b.
compute LL1 = LL2.
end loop.
compute modres=b.
do if (jjj > iterate).
compute itprob = 2.
do if (booting=0).
compute iterrmod=1.
end if.
do if (booting=1).
compute bootiter=1.
end if.
do if (itprobtg=0).
compute itprobtg=1.
compute errcode(errs,1) = 47.
compute errs = errs + 1.
do if (booting = 0 and 1 =1).
compute vt1 = mdiag(pt1lp&*(1-pt1lp)).
compute varb = inv(t(xlp)*vt1*xlp).
compute seb = sqrt(diag(varb)).
end if.
end if.
end if.
do if ( 1 =1).
compute trat = b&/seb.
compute dfres=nrow(xlp).
compute p = 2*(1-cdfnorm(abs(trat))).
compute modres={modres,seb,trat,p}.
compute modres={modres,(b-xp2&*seb),(b+xp2&*seb)}.
compute pvchi=1-chicdf((LL3-LL2),(nrow(modres)-1)).
compute mcF = (LL3-LL2)/LL3.
compute cox = 1-exp(-(LL3-LL2)/nrow(xlp)).
compute nagel = cox/(1-exp(-(LL3)/nrow(xlp))).
compute modsum={LL2,(LL3-LL2),(nrow(modres)-1),pvchi, mcF,cox,nagel}.
compute modsuml={'-2LL','ModelLL', 'df', 'p', 'McFadden', 'CoxSnell', 'Nagelkrk'}.
compute modresl={'coeff','se','Z','p','LLCI','ULCI'}.
end if.
end if
.
end if.
do if ((i = (nms+nys)) and (ydich=1)).

.
do if ( 2 =1).
compute hatmat=inv(t( x )* x )*t( x ).
compute b = hatmat* y.
compute modres=b.
do if ( 1 =1).
compute n1=nrow( x ).
compute dfres=n1-(ncol( x )).
compute sstotal = t( y -(csum( y )/n1))*( y -(csum( y )/n1)).
compute resid= y - x *b.
compute ssresid = csum((resid)&**2).
compute r2 = (sstotal-ssresid)/sstotal.
do if (r2 < 0).
compute r2=0.
end if.
compute adjr2 = 1-((1-r2)*(n1-1)/(dfres)).
compute mse=ssresid/(n1-ncol( x )).

.
compute n1=nrow( x ).
compute invXtX = inv(t( x )* x ).
compute varb = mse *invXtX.
compute k3 = ncol( x ).
compute xhc=0.
do if ( hc <> 5).
compute xhc= x.
compute hat = xhc(:,1).
loop i3=1 to nrow(xhc).
compute hat(i3,1)= xhc(i3,:)*invXtX*t(xhc(i3,:)).
end loop.
do if ( hc = 0 or hc =1).
loop i3 = 1 to k3.
compute xhc(:,i3)=xhc(:,i3)&* resid.
end loop.
end if.
do if ( hc =3 or hc =2).
loop i3=1 to k3.
compute xhc(:,i3) = ( resid &/(1-hat)&**(1/(4- hc )))&*xhc(:,i3).
end loop.
end if.
do if ( hc = 4).
compute hcmn=make(n,2,4).
compute hcmn(:,2)=(n1*hat)/k3.
loop i3= 1 to k3.
compute xhc(:,i3) = ( resid &/(1-hat)&**(rmin(hcmn)/2))&*xhc(:,i3).
end loop.
end if.
compute varb=(invXtX*t(xhc)*xhc*invXtX).
do if ( hc =1).
compute varb=(n1/(n1-ncol( x )))&*varb.
end if.
end if.
compute hclab={'se(HC0)','se(HC1)','se(HC2)','se(HC3)','se(HC4)','se'}.
compute hclab=hclab(1,( hc +1)).
compute hcflab={'F(HC0)','F(HC1)','F(HC2)','F(HC3)','F(HC4)','F'}.
compute hcflab=hcflab(1,( hc +1)).
release xhc
.
compute seb=sqrt(diag(varb)).
compute trat = b&/seb.
compute p = 2*(1-tcdf(abs(trat), (dfres))).
compute tval = sqrt(dfres* (exp((dfres-(5/6))*((xp2/(dfres-(2/3)+(.11/dfres)))* (xp2/(dfres-(2/3)+(.11/dfres)))))-1)).
compute modres={modres,seb,trat,p}.
compute modres={modres,(b-tval&*seb),(b+tval&*seb)}.
compute modresl={'coeff',hclab,'t','p','LLCI','ULCI'}.
compute lmat = ident(ncol( x )).
compute lmat = lmat(:,2:ncol(lmat)).
compute fratio = (t(t(lmat)*b)*inv(t(lmat)*varb*lmat)*((t(lmat)*b)))/(ncol( x )-1).
compute pfr = 1-fcdf(fratio,(ncol( x )-1),dfres).
compute modsum={sqrt(r2),r2,mse,fratio,(ncol( x )-1),dfres,pfr}.
compute modsuml={'R','R-sq','MSE',hcflab,'df1','df2', 'p'}.
end if.
end if.
do if ( 2 = 2 or 2 =3).
compute xlp= x.
compute ylp= y.
compute pt2 = make(nrow(ylp),1,(csum(ylp)/nrow(ylp))).
do if ( 2 =2).
compute LL3 = ylp&*ln(pt2)+(1-ylp)&*ln(1-pt2).
end if.
compute LL3 = -2*csum(LL3).
compute bt1 = make(ncol(xlp),1,0).
compute LL1 = 0.
compute pt1 = make(nrow(ylp),1,0.5).
compute pt1lp=pt1.
loop jjj = 1 to iterate.
compute xlptmp=t(xlp).
compute vecprb=pt1lp&*(1-pt1lp).
loop kkk=1 to ncol(xlp).
compute xlptmp(kkk,:)=xlptmp(kkk,:)&*t(vecprb).
end loop.
compute b = bt1+inv(xlptmp*xlp)*t(xlp)*(ylp-pt1lp).
do if ( 2 =2).
compute xlpb=xlp*b.
compute xlpbt=(xlpb > -709.7).
compute xlpb709=(1-xlpbt)*(-709.7).
compute xlpb=(xlpb&*xlpbt)+xlpb709.
compute pt1lp = 1/(1+exp(-(xlpb))).
end if.
compute itprob = csum((pt1lp < .00000001) or (pt1lp > .9999999)).
do if (itprob > 0).
loop kkk = 1 to nrow(pt1lp).
do if (pt1lp(kkk,1) > .9999999).
compute pt1lp(kkk,1) = .9999999.
end if.
do if (pt1lp(kkk,1) < .00000001).
compute pt1lp(kkk,1) = .00000001.
end if.
end loop.
compute itprob = 0.
end if.
do if (itprob = 0).
do if ( 2 =2).
compute LL = ylp&*ln(pt1lp)+(1-ylp)&*ln(1-pt1lp).
end if.
compute LL2 = -2*csum(ll).
end if.
do if (abs(LL1-LL2) < converge).
do if ( 1 =1).
compute xlptmp=t(xlp).
compute vecprb=pt1lp&*(1-pt1lp).
loop kkk=1 to ncol(xlp).
compute xlptmp(kkk,:)=xlptmp(kkk,:)&*t(vecprb).
end loop.
compute varb = inv(xlptmp*xlp).
compute seb = sqrt(diag(varb)).
release xlptmp.
end if.
break.
end if.
compute bt1 = b.
compute LL1 = LL2.
end loop.
compute modres=b.
do if (jjj > iterate).
compute itprob = 2.
do if (booting=0).
compute iterrmod=1.
end if.
do if (booting=1).
compute bootiter=1.
end if.
do if (itprobtg=0).
compute itprobtg=1.
compute errcode(errs,1) = 47.
compute errs = errs + 1.
do if (booting = 0 and 1 =1).
compute vt1 = mdiag(pt1lp&*(1-pt1lp)).
compute varb = inv(t(xlp)*vt1*xlp).
compute seb = sqrt(diag(varb)).
end if.
end if.
end if.
do if ( 1 =1).
compute trat = b&/seb.
compute dfres=nrow(xlp).
compute p = 2*(1-cdfnorm(abs(trat))).
compute modres={modres,seb,trat,p}.
compute modres={modres,(b-xp2&*seb),(b+xp2&*seb)}.
compute pvchi=1-chicdf((LL3-LL2),(nrow(modres)-1)).
compute mcF = (LL3-LL2)/LL3.
compute cox = 1-exp(-(LL3-LL2)/nrow(xlp)).
compute nagel = cox/(1-exp(-(LL3)/nrow(xlp))).
compute modsum={LL2,(LL3-LL2),(nrow(modres)-1),pvchi, mcF,cox,nagel}.
compute modsuml={'-2LL','ModelLL', 'df', 'p', 'McFadden', 'CoxSnell', 'Nagelkrk'}.
compute modresl={'coeff','se','Z','p','LLCI','ULCI'}.
end if.
end if
.
compute chidfxm=basemodx-LL2.
end if.
compute lmat=make(nrow(b),nxvls,0).
compute lmattmp=ident(nxvls).
compute lmat((nrow(lmat)-nxvls+1):nrow(lmat),:)=lmattmp.

.
compute lmat2= lmat.
do if ( 1 =0).
compute lmat2 = mdiag( lmat ).
compute lmat3=make(nrow(lmat2),1,0).
loop flp=1 to ncol(lmat2).
do if (csum(lmat2(:,flp))=1).
compute lmat3={lmat3,lmat2(:,flp)}.
end if.
end loop.
compute lmat2=lmat3(:,2:ncol(lmat3)).
end if.
compute fratio = (t(t(lmat2)* b )*inv(t(lmat2)* varb *lmat2)*((t(lmat2)* b )))/ncol(lmat2).
compute pfr = 1-fcdf(fratio,ncol(lmat2),(n-nrow( b ))).
compute fresult={fratio,ncol(lmat2),(n-nrow( b )),pfr}.
do if (i = (nms+nys) and (ydich=1)).
compute fratio=fratio*ncol(lmat2).
compute pfr=1-chicdf(fratio,ncol(lmat2)).
compute fresult={fratio,ncol(lmat2),pfr}.
end if.
do if ( 0 =1).
compute lmat3=1-rsum(lmat2).
compute xfm=make(n,csum(lmat3),0).
compute flpc=1.
loop flp=1 to nrow(lmat3).
do if (lmat3(flp,1)=1).
compute xfm(:,flpc)=x(:,flp).
compute flpc=flpc+1.
end if.
end loop.
compute bfm=inv(t(xfm)*xfm)*t(xfm)*y.
compute resid=y-(xfm*bfm).
compute sstotal=(y-(csum(y)/n)).
compute sstotal=csum(sstotal&*sstotal).
compute ssresid=csum(resid&*resid).
compute rsqch= 0 -((sstotal-ssresid)/sstotal).
compute fresult={rsqch,fresult}.
release xfm,flpc, resid, ssresid, bfm.
end if
.
compute numxint=numxint+1.
compute xmtst(numxint,:)=fresult.
do if ((i = (nms+nys)) and (ydich=1)).
compute xmtst(numxint,1)=chidfxm.
compute xmtst(numxint,3)=1-chicdf(chidfxm,nxvls).
end if.
compute xmtstlb={xmtstlb;highlbx((xmints-1),1)}.
end if.
end loop.
compute x=xmtmat.
release xmtmat.
do if (numxint>0).
compute xmtstlb=xmtstlb((2:(numxint+1)),:).
compute xmtst=xmtst(1:numxint,:).
compute resultm2=make(nrow(xmtst),maxresm,99999).
compute resultm2(1:nrow(xmtst),1:ncol(xmtst))=xmtst.
compute resultm={resultm;resultm2}.
do if (nms=1).
compute xmtstlb=' '.
end if.
do if (outscree=1).
do if ((i < (nms+nys)) or (ydich=0)).
print xmtst/title='Test(s) of X by M interaction:'/rnames=xmtstlb/cnames=xmtstlbc/format= F10.4.
end if.
do if ((i = (nms+nys)) and (ydich=1)).
print xmtst/title='Likelihood ratio test(s) of X by M interaction:'/rnames=xmtstlb/cnames=xmtstlbc/format= F10.4.
end if.
end if.
end if.
compute r2=r2tmp.
compute b=btmp.
compute varb=varbtmp.
compute dfres=dfrestmp.
compute tval=tvaltmp.
end if.
do if (criterr = 0).
compute jj=0.
loop j = start to ((start+i)-1).
compute dbint=0.
compute lmat=whigh(1:nump(1,i),j).
compute lmat2=wzhigh(1:nump(1,i),j).
do if ((csum(lmat) > 0) and (csum(lmat2) = 0)).
do if ((i < (nms+nys)) or (ydich <> 1)).

.
compute lmat2= lmat.
do if ( 0 =0).
compute lmat2 = mdiag( lmat ).
compute lmat3=make(nrow(lmat2),1,0).
loop flp=1 to ncol(lmat2).
do if (csum(lmat2(:,flp))=1).
compute lmat3={lmat3,lmat2(:,flp)}.
end if.
end loop.
compute lmat2=lmat3(:,2:ncol(lmat3)).
end if.
compute fratio = (t(t(lmat2)* b )*inv(t(lmat2)* varb *lmat2)*((t(lmat2)* b )))/ncol(lmat2).
compute pfr = 1-fcdf(fratio,ncol(lmat2),(n-nrow( b ))).
compute fresult={fratio,ncol(lmat2),(n-nrow( b )),pfr}.
do if (i = (nms+nys) and (ydich=1)).
compute fratio=fratio*ncol(lmat2).
compute pfr=1-chicdf(fratio,ncol(lmat2)).
compute fresult={fratio,ncol(lmat2),pfr}.
end if.
do if ( 1 =1).
compute lmat3=1-rsum(lmat2).
compute xfm=make(n,csum(lmat3),0).
compute flpc=1.
loop flp=1 to nrow(lmat3).
do if (lmat3(flp,1)=1).
compute xfm(:,flpc)=x(:,flp).
compute flpc=flpc+1.
end if.
end loop.
compute bfm=inv(t(xfm)*xfm)*t(xfm)*y.
compute resid=y-(xfm*bfm).
compute sstotal=(y-(csum(y)/n)).
compute sstotal=csum(sstotal&*sstotal).
compute ssresid=csum(resid&*resid).
compute rsqch= r2 -((sstotal-ssresid)/sstotal).
compute fresult={rsqch,fresult}.
release xfm,flpc, resid, ssresid, bfm.
end if
.
compute lmatdb=lmat.
compute dbint=dbint+1.
end if.
do if ((ydich = 1) and (i = (nms+nys))).

.
compute btemphld=b.
compute llrdat=make(nrow(x),nrow( lmat )-csum( lmat ),-999).
compute llrdf=ncol(x)-ncol(llrdat).
compute llrcnt=0.
loop llri=1 to nrow( lmat ).
do if ( lmat (llri,1)=0).
compute llrcnt=llrcnt+1.
compute llrdat(:,llrcnt)=x(:,llri).
end if.
end loop.

.
do if ( 2 =1).
compute hatmat=inv(t( llrdat )* llrdat )*t( llrdat ).
compute b = hatmat* y.
compute modres=b.
do if ( 0 =1).
compute n1=nrow( llrdat ).
compute dfres=n1-(ncol( llrdat )).
compute sstotal = t( y -(csum( y )/n1))*( y -(csum( y )/n1)).
compute resid= y - llrdat *b.
compute ssresid = csum((resid)&**2).
compute r2 = (sstotal-ssresid)/sstotal.
do if (r2 < 0).
compute r2=0.
end if.
compute adjr2 = 1-((1-r2)*(n1-1)/(dfres)).
compute mse=ssresid/(n1-ncol( llrdat )).

.
compute n1=nrow( x ).
compute invXtX = inv(t( x )* x ).
compute varb = mse *invXtX.
compute k3 = ncol( x ).
compute xhc=0.
do if ( hc <> 5).
compute xhc= x.
compute hat = xhc(:,1).
loop i3=1 to nrow(xhc).
compute hat(i3,1)= xhc(i3,:)*invXtX*t(xhc(i3,:)).
end loop.
do if ( hc = 0 or hc =1).
loop i3 = 1 to k3.
compute xhc(:,i3)=xhc(:,i3)&* resid.
end loop.
end if.
do if ( hc =3 or hc =2).
loop i3=1 to k3.
compute xhc(:,i3) = ( resid &/(1-hat)&**(1/(4- hc )))&*xhc(:,i3).
end loop.
end if.
do if ( hc = 4).
compute hcmn=make(n,2,4).
compute hcmn(:,2)=(n1*hat)/k3.
loop i3= 1 to k3.
compute xhc(:,i3) = ( resid &/(1-hat)&**(rmin(hcmn)/2))&*xhc(:,i3).
end loop.
end if.
compute varb=(invXtX*t(xhc)*xhc*invXtX).
do if ( hc =1).
compute varb=(n1/(n1-ncol( x )))&*varb.
end if.
end if.
compute hclab={'se(HC0)','se(HC1)','se(HC2)','se(HC3)','se(HC4)','se'}.
compute hclab=hclab(1,( hc +1)).
compute hcflab={'F(HC0)','F(HC1)','F(HC2)','F(HC3)','F(HC4)','F'}.
compute hcflab=hcflab(1,( hc +1)).
release xhc
.
compute seb=sqrt(diag(varb)).
compute trat = b&/seb.
compute p = 2*(1-tcdf(abs(trat), (dfres))).
compute tval = sqrt(dfres* (exp((dfres-(5/6))*((xp2/(dfres-(2/3)+(.11/dfres)))* (xp2/(dfres-(2/3)+(.11/dfres)))))-1)).
compute modres={modres,seb,trat,p}.
compute modres={modres,(b-tval&*seb),(b+tval&*seb)}.
compute modresl={'coeff',hclab,'t','p','LLCI','ULCI'}.
compute lmat = ident(ncol( llrdat )).
compute lmat = lmat(:,2:ncol(lmat)).
compute fratio = (t(t(lmat)*b)*inv(t(lmat)*varb*lmat)*((t(lmat)*b)))/(ncol( llrdat )-1).
compute pfr = 1-fcdf(fratio,(ncol( llrdat )-1),dfres).
compute modsum={sqrt(r2),r2,mse,fratio,(ncol( llrdat )-1),dfres,pfr}.
compute modsuml={'R','R-sq','MSE',hcflab,'df1','df2', 'p'}.
end if.
end if.
do if ( 2 = 2 or 2 =3).
compute xlp= llrdat.
compute ylp= y.
compute pt2 = make(nrow(ylp),1,(csum(ylp)/nrow(ylp))).
do if ( 2 =2).
compute LL3 = ylp&*ln(pt2)+(1-ylp)&*ln(1-pt2).
end if.
compute LL3 = -2*csum(LL3).
compute bt1 = make(ncol(xlp),1,0).
compute LL1 = 0.
compute pt1 = make(nrow(ylp),1,0.5).
compute pt1lp=pt1.
loop jjj = 1 to iterate.
compute xlptmp=t(xlp).
compute vecprb=pt1lp&*(1-pt1lp).
loop kkk=1 to ncol(xlp).
compute xlptmp(kkk,:)=xlptmp(kkk,:)&*t(vecprb).
end loop.
compute b = bt1+inv(xlptmp*xlp)*t(xlp)*(ylp-pt1lp).
do if ( 2 =2).
compute xlpb=xlp*b.
compute xlpbt=(xlpb > -709.7).
compute xlpb709=(1-xlpbt)*(-709.7).
compute xlpb=(xlpb&*xlpbt)+xlpb709.
compute pt1lp = 1/(1+exp(-(xlpb))).
end if.
compute itprob = csum((pt1lp < .00000001) or (pt1lp > .9999999)).
do if (itprob > 0).
loop kkk = 1 to nrow(pt1lp).
do if (pt1lp(kkk,1) > .9999999).
compute pt1lp(kkk,1) = .9999999.
end if.
do if (pt1lp(kkk,1) < .00000001).
compute pt1lp(kkk,1) = .00000001.
end if.
end loop.
compute itprob = 0.
end if.
do if (itprob = 0).
do if ( 2 =2).
compute LL = ylp&*ln(pt1lp)+(1-ylp)&*ln(1-pt1lp).
end if.
compute LL2 = -2*csum(ll).
end if.
do if (abs(LL1-LL2) < converge).
do if ( 0 =1).
compute xlptmp=t(xlp).
compute vecprb=pt1lp&*(1-pt1lp).
loop kkk=1 to ncol(xlp).
compute xlptmp(kkk,:)=xlptmp(kkk,:)&*t(vecprb).
end loop.
compute varb = inv(xlptmp*xlp).
compute seb = sqrt(diag(varb)).
release xlptmp.
end if.
break.
end if.
compute bt1 = b.
compute LL1 = LL2.
end loop.
compute modres=b.
do if (jjj > iterate).
compute itprob = 2.
do if (booting=0).
compute iterrmod=1.
end if.
do if (booting=1).
compute bootiter=1.
end if.
do if (itprobtg=0).
compute itprobtg=1.
compute errcode(errs,1) = 47.
compute errs = errs + 1.
do if (booting = 0 and 0 =1).
compute vt1 = mdiag(pt1lp&*(1-pt1lp)).
compute varb = inv(t(xlp)*vt1*xlp).
compute seb = sqrt(diag(varb)).
end if.
end if.
end if.
do if ( 0 =1).
compute trat = b&/seb.
compute dfres=nrow(xlp).
compute p = 2*(1-cdfnorm(abs(trat))).
compute modres={modres,seb,trat,p}.
compute modres={modres,(b-xp2&*seb),(b+xp2&*seb)}.
compute pvchi=1-chicdf((LL3-LL2),(nrow(modres)-1)).
compute mcF = (LL3-LL2)/LL3.
compute cox = 1-exp(-(LL3-LL2)/nrow(xlp)).
compute nagel = cox/(1-exp(-(LL3)/nrow(xlp))).
compute modsum={LL2,(LL3-LL2),(nrow(modres)-1),pvchi, mcF,cox,nagel}.
compute modsuml={'-2LL','ModelLL', 'df', 'p', 'McFadden', 'CoxSnell', 'Nagelkrk'}.
compute modresl={'coeff','se','Z','p','LLCI','ULCI'}.
end if.
end if
.
compute b=btemphld.
compute fresult={(LL2-basemod),llrdf,1-chicdf((LL2-basemod),llrdf)}
.
compute lmatdb=lmat.
compute dbint=dbint+1.
end if.
compute highf={highf;fresult}.
compute highf2={highf2;fresult}.
do if (j = start).
compute flabel={flabel;'X*W'}.
end if.
do if (j > start).
do if (nms > 1).
compute flabel={flabel;highlbw(jj,1)}.
else if (nms = 1).
do if (xmint=0).
compute flabel={flabel;'M*W'}.
end if.
do if (xmint=1).
compute flabel={flabel;'X*M'}.
end if.
end if.
end if.
end if.
compute lmat=zhigh(1:nump(1,i),j).
compute lmat2=wzhigh(1:nump(1,i),j).
do if ((csum(lmat) > 0) and (csum(lmat2) = 0)).
do if ((i < (nms+nys)) or (ydich <> 1)).

.
compute lmat2= lmat.
do if ( 0 =0).
compute lmat2 = mdiag( lmat ).
compute lmat3=make(nrow(lmat2),1,0).
loop flp=1 to ncol(lmat2).
do if (csum(lmat2(:,flp))=1).
compute lmat3={lmat3,lmat2(:,flp)}.
end if.
end loop.
compute lmat2=lmat3(:,2:ncol(lmat3)).
end if.
compute fratio = (t(t(lmat2)* b )*inv(t(lmat2)* varb *lmat2)*((t(lmat2)* b )))/ncol(lmat2).
compute pfr = 1-fcdf(fratio,ncol(lmat2),(n-nrow( b ))).
compute fresult={fratio,ncol(lmat2),(n-nrow( b )),pfr}.
do if (i = (nms+nys) and (ydich=1)).
compute fratio=fratio*ncol(lmat2).
compute pfr=1-chicdf(fratio,ncol(lmat2)).
compute fresult={fratio,ncol(lmat2),pfr}.
end if.
do if ( 1 =1).
compute lmat3=1-rsum(lmat2).
compute xfm=make(n,csum(lmat3),0).
compute flpc=1.
loop flp=1 to nrow(lmat3).
do if (lmat3(flp,1)=1).
compute xfm(:,flpc)=x(:,flp).
compute flpc=flpc+1.
end if.
end loop.
compute bfm=inv(t(xfm)*xfm)*t(xfm)*y.
compute resid=y-(xfm*bfm).
compute sstotal=(y-(csum(y)/n)).
compute sstotal=csum(sstotal&*sstotal).
compute ssresid=csum(resid&*resid).
compute rsqch= r2 -((sstotal-ssresid)/sstotal).
compute fresult={rsqch,fresult}.
release xfm,flpc, resid, ssresid, bfm.
end if
.
compute dbint=dbint+1.
end if.
do if ((ydich = 1) and (i = (nms+nys))).

.
compute btemphld=b.
compute llrdat=make(nrow(x),nrow( lmat )-csum( lmat ),-999).
compute llrdf=ncol(x)-ncol(llrdat).
compute llrcnt=0.
loop llri=1 to nrow( lmat ).
do if ( lmat (llri,1)=0).
compute llrcnt=llrcnt+1.
compute llrdat(:,llrcnt)=x(:,llri).
end if.
end loop.

.
do if ( 2 =1).
compute hatmat=inv(t( llrdat )* llrdat )*t( llrdat ).
compute b = hatmat* y.
compute modres=b.
do if ( 0 =1).
compute n1=nrow( llrdat ).
compute dfres=n1-(ncol( llrdat )).
compute sstotal = t( y -(csum( y )/n1))*( y -(csum( y )/n1)).
compute resid= y - llrdat *b.
compute ssresid = csum((resid)&**2).
compute r2 = (sstotal-ssresid)/sstotal.
do if (r2 < 0).
compute r2=0.
end if.
compute adjr2 = 1-((1-r2)*(n1-1)/(dfres)).
compute mse=ssresid/(n1-ncol( llrdat )).

.
compute n1=nrow( x ).
compute invXtX = inv(t( x )* x ).
compute varb = mse *invXtX.
compute k3 = ncol( x ).
compute xhc=0.
do if ( hc <> 5).
compute xhc= x.
compute hat = xhc(:,1).
loop i3=1 to nrow(xhc).
compute hat(i3,1)= xhc(i3,:)*invXtX*t(xhc(i3,:)).
end loop.
do if ( hc = 0 or hc =1).
loop i3 = 1 to k3.
compute xhc(:,i3)=xhc(:,i3)&* resid.
end loop.
end if.
do if ( hc =3 or hc =2).
loop i3=1 to k3.
compute xhc(:,i3) = ( resid &/(1-hat)&**(1/(4- hc )))&*xhc(:,i3).
end loop.
end if.
do if ( hc = 4).
compute hcmn=make(n,2,4).
compute hcmn(:,2)=(n1*hat)/k3.
loop i3= 1 to k3.
compute xhc(:,i3) = ( resid &/(1-hat)&**(rmin(hcmn)/2))&*xhc(:,i3).
end loop.
end if.
compute varb=(invXtX*t(xhc)*xhc*invXtX).
do if ( hc =1).
compute varb=(n1/(n1-ncol( x )))&*varb.
end if.
end if.
compute hclab={'se(HC0)','se(HC1)','se(HC2)','se(HC3)','se(HC4)','se'}.
compute hclab=hclab(1,( hc +1)).
compute hcflab={'F(HC0)','F(HC1)','F(HC2)','F(HC3)','F(HC4)','F'}.
compute hcflab=hcflab(1,( hc +1)).
release xhc
.
compute seb=sqrt(diag(varb)).
compute trat = b&/seb.
compute p = 2*(1-tcdf(abs(trat), (dfres))).
compute tval = sqrt(dfres* (exp((dfres-(5/6))*((xp2/(dfres-(2/3)+(.11/dfres)))* (xp2/(dfres-(2/3)+(.11/dfres)))))-1)).
compute modres={modres,seb,trat,p}.
compute modres={modres,(b-tval&*seb),(b+tval&*seb)}.
compute modresl={'coeff',hclab,'t','p','LLCI','ULCI'}.
compute lmat = ident(ncol( llrdat )).
compute lmat = lmat(:,2:ncol(lmat)).
compute fratio = (t(t(lmat)*b)*inv(t(lmat)*varb*lmat)*((t(lmat)*b)))/(ncol( llrdat )-1).
compute pfr = 1-fcdf(fratio,(ncol( llrdat )-1),dfres).
compute modsum={sqrt(r2),r2,mse,fratio,(ncol( llrdat )-1),dfres,pfr}.
compute modsuml={'R','R-sq','MSE',hcflab,'df1','df2', 'p'}.
end if.
end if.
do if ( 2 = 2 or 2 =3).
compute xlp= llrdat.
compute ylp= y.
compute pt2 = make(nrow(ylp),1,(csum(ylp)/nrow(ylp))).
do if ( 2 =2).
compute LL3 = ylp&*ln(pt2)+(1-ylp)&*ln(1-pt2).
end if.
compute LL3 = -2*csum(LL3).
compute bt1 = make(ncol(xlp),1,0).
compute LL1 = 0.
compute pt1 = make(nrow(ylp),1,0.5).
compute pt1lp=pt1.
loop jjj = 1 to iterate.
compute xlptmp=t(xlp).
compute vecprb=pt1lp&*(1-pt1lp).
loop kkk=1 to ncol(xlp).
compute xlptmp(kkk,:)=xlptmp(kkk,:)&*t(vecprb).
end loop.
compute b = bt1+inv(xlptmp*xlp)*t(xlp)*(ylp-pt1lp).
do if ( 2 =2).
compute xlpb=xlp*b.
compute xlpbt=(xlpb > -709.7).
compute xlpb709=(1-xlpbt)*(-709.7).
compute xlpb=(xlpb&*xlpbt)+xlpb709.
compute pt1lp = 1/(1+exp(-(xlpb))).
end if.
compute itprob = csum((pt1lp < .00000001) or (pt1lp > .9999999)).
do if (itprob > 0).
loop kkk = 1 to nrow(pt1lp).
do if (pt1lp(kkk,1) > .9999999).
compute pt1lp(kkk,1) = .9999999.
end if.
do if (pt1lp(kkk,1) < .00000001).
compute pt1lp(kkk,1) = .00000001.
end if.
end loop.
compute itprob = 0.
end if.
do if (itprob = 0).
do if ( 2 =2).
compute LL = ylp&*ln(pt1lp)+(1-ylp)&*ln(1-pt1lp).
end if.
compute LL2 = -2*csum(ll).
end if.
do if (abs(LL1-LL2) < converge).
do if ( 0 =1).
compute xlptmp=t(xlp).
compute vecprb=pt1lp&*(1-pt1lp).
loop kkk=1 to ncol(xlp).
compute xlptmp(kkk,:)=xlptmp(kkk,:)&*t(vecprb).
end loop.
compute varb = inv(xlptmp*xlp).
compute seb = sqrt(diag(varb)).
release xlptmp.
end if.
break.
end if.
compute bt1 = b.
compute LL1 = LL2.
end loop.
compute modres=b.
do if (jjj > iterate).
compute itprob = 2.
do if (booting=0).
compute iterrmod=1.
end if.
do if (booting=1).
compute bootiter=1.
end if.
do if (itprobtg=0).
compute itprobtg=1.
compute errcode(errs,1) = 47.
compute errs = errs + 1.
do if (booting = 0 and 0 =1).
compute vt1 = mdiag(pt1lp&*(1-pt1lp)).
compute varb = inv(t(xlp)*vt1*xlp).
compute seb = sqrt(diag(varb)).
end if.
end if.
end if.
do if ( 0 =1).
compute trat = b&/seb.
compute dfres=nrow(xlp).
compute p = 2*(1-cdfnorm(abs(trat))).
compute modres={modres,seb,trat,p}.
compute modres={modres,(b-xp2&*seb),(b+xp2&*seb)}.
compute pvchi=1-chicdf((LL3-LL2),(nrow(modres)-1)).
compute mcF = (LL3-LL2)/LL3.
compute cox = 1-exp(-(LL3-LL2)/nrow(xlp)).
compute nagel = cox/(1-exp(-(LL3)/nrow(xlp))).
compute modsum={LL2,(LL3-LL2),(nrow(modres)-1),pvchi, mcF,cox,nagel}.
compute modsuml={'-2LL','ModelLL', 'df', 'p', 'McFadden', 'CoxSnell', 'Nagelkrk'}.
compute modresl={'coeff','se','Z','p','LLCI','ULCI'}.
end if.
end if
.
compute b=btemphld.
compute fresult={(LL2-basemod),llrdf,1-chicdf((LL2-basemod),llrdf)}
.
compute dbint=dbint+1.
end if.
compute highf={highf;fresult}.
compute highf2={highf2;fresult}.
do if (j = start).
compute flabel={flabel;'X*Z'}.
end if.
do if (j > start).
do if (nms > 1).
compute flabel={flabel;highlbz(jj,1)}.
else if (nms = 1).
compute flabel={flabel;'M*Z'}.
end if.
end if.
end if.
do if (dbint=2).
compute lmatdb=lmatdb+lmat.
do if ((ydich = 1) and (i = (nms+nys))).

.
compute btemphld=b.
compute llrdat=make(nrow(x),nrow( lmatdb )-csum( lmatdb ),-999).
compute llrdf=ncol(x)-ncol(llrdat).
compute llrcnt=0.
loop llri=1 to nrow( lmatdb ).
do if ( lmatdb (llri,1)=0).
compute llrcnt=llrcnt+1.
compute llrdat(:,llrcnt)=x(:,llri).
end if.
end loop.

.
do if ( 2 =1).
compute hatmat=inv(t( llrdat )* llrdat )*t( llrdat ).
compute b = hatmat* y.
compute modres=b.
do if ( 0 =1).
compute n1=nrow( llrdat ).
compute dfres=n1-(ncol( llrdat )).
compute sstotal = t( y -(csum( y )/n1))*( y -(csum( y )/n1)).
compute resid= y - llrdat *b.
compute ssresid = csum((resid)&**2).
compute r2 = (sstotal-ssresid)/sstotal.
do if (r2 < 0).
compute r2=0.
end if.
compute adjr2 = 1-((1-r2)*(n1-1)/(dfres)).
compute mse=ssresid/(n1-ncol( llrdat )).

.
compute n1=nrow( x ).
compute invXtX = inv(t( x )* x ).
compute varb = mse *invXtX.
compute k3 = ncol( x ).
compute xhc=0.
do if ( hc <> 5).
compute xhc= x.
compute hat = xhc(:,1).
loop i3=1 to nrow(xhc).
compute hat(i3,1)= xhc(i3,:)*invXtX*t(xhc(i3,:)).
end loop.
do if ( hc = 0 or hc =1).
loop i3 = 1 to k3.
compute xhc(:,i3)=xhc(:,i3)&* resid.
end loop.
end if.
do if ( hc =3 or hc =2).
loop i3=1 to k3.
compute xhc(:,i3) = ( resid &/(1-hat)&**(1/(4- hc )))&*xhc(:,i3).
end loop.
end if.
do if ( hc = 4).
compute hcmn=make(n,2,4).
compute hcmn(:,2)=(n1*hat)/k3.
loop i3= 1 to k3.
compute xhc(:,i3) = ( resid &/(1-hat)&**(rmin(hcmn)/2))&*xhc(:,i3).
end loop.
end if.
compute varb=(invXtX*t(xhc)*xhc*invXtX).
do if ( hc =1).
compute varb=(n1/(n1-ncol( x )))&*varb.
end if.
end if.
compute hclab={'se(HC0)','se(HC1)','se(HC2)','se(HC3)','se(HC4)','se'}.
compute hclab=hclab(1,( hc +1)).
compute hcflab={'F(HC0)','F(HC1)','F(HC2)','F(HC3)','F(HC4)','F'}.
compute hcflab=hcflab(1,( hc +1)).
release xhc
.
compute seb=sqrt(diag(varb)).
compute trat = b&/seb.
compute p = 2*(1-tcdf(abs(trat), (dfres))).
compute tval = sqrt(dfres* (exp((dfres-(5/6))*((xp2/(dfres-(2/3)+(.11/dfres)))* (xp2/(dfres-(2/3)+(.11/dfres)))))-1)).
compute modres={modres,seb,trat,p}.
compute modres={modres,(b-tval&*seb),(b+tval&*seb)}.
compute modresl={'coeff',hclab,'t','p','LLCI','ULCI'}.
compute lmat = ident(ncol( llrdat )).
compute lmat = lmat(:,2:ncol(lmat)).
compute fratio = (t(t(lmat)*b)*inv(t(lmat)*varb*lmat)*((t(lmat)*b)))/(ncol( llrdat )-1).
compute pfr = 1-fcdf(fratio,(ncol( llrdat )-1),dfres).
compute modsum={sqrt(r2),r2,mse,fratio,(ncol( llrdat )-1),dfres,pfr}.
compute modsuml={'R','R-sq','MSE',hcflab,'df1','df2', 'p'}.
end if.
end if.
do if ( 2 = 2 or 2 =3).
compute xlp= llrdat.
compute ylp= y.
compute pt2 = make(nrow(ylp),1,(csum(ylp)/nrow(ylp))).
do if ( 2 =2).
compute LL3 = ylp&*ln(pt2)+(1-ylp)&*ln(1-pt2).
end if.
compute LL3 = -2*csum(LL3).
compute bt1 = make(ncol(xlp),1,0).
compute LL1 = 0.
compute pt1 = make(nrow(ylp),1,0.5).
compute pt1lp=pt1.
loop jjj = 1 to iterate.
compute xlptmp=t(xlp).
compute vecprb=pt1lp&*(1-pt1lp).
loop kkk=1 to ncol(xlp).
compute xlptmp(kkk,:)=xlptmp(kkk,:)&*t(vecprb).
end loop.
compute b = bt1+inv(xlptmp*xlp)*t(xlp)*(ylp-pt1lp).
do if ( 2 =2).
compute xlpb=xlp*b.
compute xlpbt=(xlpb > -709.7).
compute xlpb709=(1-xlpbt)*(-709.7).
compute xlpb=(xlpb&*xlpbt)+xlpb709.
compute pt1lp = 1/(1+exp(-(xlpb))).
end if.
compute itprob = csum((pt1lp < .00000001) or (pt1lp > .9999999)).
do if (itprob > 0).
loop kkk = 1 to nrow(pt1lp).
do if (pt1lp(kkk,1) > .9999999).
compute pt1lp(kkk,1) = .9999999.
end if.
do if (pt1lp(kkk,1) < .00000001).
compute pt1lp(kkk,1) = .00000001.
end if.
end loop.
compute itprob = 0.
end if.
do if (itprob = 0).
do if ( 2 =2).
compute LL = ylp&*ln(pt1lp)+(1-ylp)&*ln(1-pt1lp).
end if.
compute LL2 = -2*csum(ll).
end if.
do if (abs(LL1-LL2) < converge).
do if ( 0 =1).
compute xlptmp=t(xlp).
compute vecprb=pt1lp&*(1-pt1lp).
loop kkk=1 to ncol(xlp).
compute xlptmp(kkk,:)=xlptmp(kkk,:)&*t(vecprb).
end loop.
compute varb = inv(xlptmp*xlp).
compute seb = sqrt(diag(varb)).
release xlptmp.
end if.
break.
end if.
compute bt1 = b.
compute LL1 = LL2.
end loop.
compute modres=b.
do if (jjj > iterate).
compute itprob = 2.
do if (booting=0).
compute iterrmod=1.
end if.
do if (booting=1).
compute bootiter=1.
end if.
do if (itprobtg=0).
compute itprobtg=1.
compute errcode(errs,1) = 47.
compute errs = errs + 1.
do if (booting = 0 and 0 =1).
compute vt1 = mdiag(pt1lp&*(1-pt1lp)).
compute varb = inv(t(xlp)*vt1*xlp).
compute seb = sqrt(diag(varb)).
end if.
end if.
end if.
do if ( 0 =1).
compute trat = b&/seb.
compute dfres=nrow(xlp).
compute p = 2*(1-cdfnorm(abs(trat))).
compute modres={modres,seb,trat,p}.
compute modres={modres,(b-xp2&*seb),(b+xp2&*seb)}.
compute pvchi=1-chicdf((LL3-LL2),(nrow(modres)-1)).
compute mcF = (LL3-LL2)/LL3.
compute cox = 1-exp(-(LL3-LL2)/nrow(xlp)).
compute nagel = cox/(1-exp(-(LL3)/nrow(xlp))).
compute modsum={LL2,(LL3-LL2),(nrow(modres)-1),pvchi, mcF,cox,nagel}.
compute modsuml={'-2LL','ModelLL', 'df', 'p', 'McFadden', 'CoxSnell', 'Nagelkrk'}.
compute modresl={'coeff','se','Z','p','LLCI','ULCI'}.
end if.
end if
.
compute b=btemphld.
compute fresult={(LL2-basemod),llrdf,1-chicdf((LL2-basemod),llrdf)}
.
end if.
do if (ydich <> 1 or i < (nms+nys)).

.
compute lmat2= lmatdb.
do if ( 0 =0).
compute lmat2 = mdiag( lmatdb ).
compute lmat3=make(nrow(lmat2),1,0).
loop flp=1 to ncol(lmat2).
do if (csum(lmat2(:,flp))=1).
compute lmat3={lmat3,lmat2(:,flp)}.
end if.
end loop.
compute lmat2=lmat3(:,2:ncol(lmat3)).
end if.
compute fratio = (t(t(lmat2)* b )*inv(t(lmat2)* varb *lmat2)*((t(lmat2)* b )))/ncol(lmat2).
compute pfr = 1-fcdf(fratio,ncol(lmat2),(n-nrow( b ))).
compute fresult={fratio,ncol(lmat2),(n-nrow( b )),pfr}.
do if (i = (nms+nys) and (ydich=1)).
compute fratio=fratio*ncol(lmat2).
compute pfr=1-chicdf(fratio,ncol(lmat2)).
compute fresult={fratio,ncol(lmat2),pfr}.
end if.
do if ( 1 =1).
compute lmat3=1-rsum(lmat2).
compute xfm=make(n,csum(lmat3),0).
compute flpc=1.
loop flp=1 to nrow(lmat3).
do if (lmat3(flp,1)=1).
compute xfm(:,flpc)=x(:,flp).
compute flpc=flpc+1.
end if.
end loop.
compute bfm=inv(t(xfm)*xfm)*t(xfm)*y.
compute resid=y-(xfm*bfm).
compute sstotal=(y-(csum(y)/n)).
compute sstotal=csum(sstotal&*sstotal).
compute ssresid=csum(resid&*resid).
compute rsqch= r2 -((sstotal-ssresid)/sstotal).
compute fresult={rsqch,fresult}.
release xfm,flpc, resid, ssresid, bfm.
end if
.
end if.
compute dbint=0.
compute highf={highf;fresult}.
do if (jj=0 and nms > 0).
compute flabel={flabel;'BOTH(X)'}.
end if.
do if (jj=0 and nms = 0).
compute flabel={flabel;'BOTH'}.
end if.
do if (jj>0 and nms = 1).
compute flabel={flabel;'BOTH(M)'}.
end if.
do if (nms > 1 and jj > 0).
compute flabel={flabel;highlbbt(jj,1)}.
end if.
end if.
compute lmat2=wzhigh(1:nump(1,i),j).
do if (csum(lmat2) > 0).
do if ((i < (nms+nys)) or (ydich <> 1)).

.
compute lmat2= lmat2.
do if ( 0 =0).
compute lmat2 = mdiag( lmat2 ).
compute lmat3=make(nrow(lmat2),1,0).
loop flp=1 to ncol(lmat2).
do if (csum(lmat2(:,flp))=1).
compute lmat3={lmat3,lmat2(:,flp)}.
end if.
end loop.
compute lmat2=lmat3(:,2:ncol(lmat3)).
end if.
compute fratio = (t(t(lmat2)* b )*inv(t(lmat2)* varb *lmat2)*((t(lmat2)* b )))/ncol(lmat2).
compute pfr = 1-fcdf(fratio,ncol(lmat2),(n-nrow( b ))).
compute fresult={fratio,ncol(lmat2),(n-nrow( b )),pfr}.
do if (i = (nms+nys) and (ydich=1)).
compute fratio=fratio*ncol(lmat2).
compute pfr=1-chicdf(fratio,ncol(lmat2)).
compute fresult={fratio,ncol(lmat2),pfr}.
end if.
do if ( 1 =1).
compute lmat3=1-rsum(lmat2).
compute xfm=make(n,csum(lmat3),0).
compute flpc=1.
loop flp=1 to nrow(lmat3).
do if (lmat3(flp,1)=1).
compute xfm(:,flpc)=x(:,flp).
compute flpc=flpc+1.
end if.
end loop.
compute bfm=inv(t(xfm)*xfm)*t(xfm)*y.
compute resid=y-(xfm*bfm).
compute sstotal=(y-(csum(y)/n)).
compute sstotal=csum(sstotal&*sstotal).
compute ssresid=csum(resid&*resid).
compute rsqch= r2 -((sstotal-ssresid)/sstotal).
compute fresult={rsqch,fresult}.
release xfm,flpc, resid, ssresid, bfm.
end if
.
end if.
do if ((ydich = 1) and (i = (nms+nys))).

.
compute btemphld=b.
compute llrdat=make(nrow(x),nrow( lmat2 )-csum( lmat2 ),-999).
compute llrdf=ncol(x)-ncol(llrdat).
compute llrcnt=0.
loop llri=1 to nrow( lmat2 ).
do if ( lmat2 (llri,1)=0).
compute llrcnt=llrcnt+1.
compute llrdat(:,llrcnt)=x(:,llri).
end if.
end loop.

.
do if ( 2 =1).
compute hatmat=inv(t( llrdat )* llrdat )*t( llrdat ).
compute b = hatmat* y.
compute modres=b.
do if ( 0 =1).
compute n1=nrow( llrdat ).
compute dfres=n1-(ncol( llrdat )).
compute sstotal = t( y -(csum( y )/n1))*( y -(csum( y )/n1)).
compute resid= y - llrdat *b.
compute ssresid = csum((resid)&**2).
compute r2 = (sstotal-ssresid)/sstotal.
do if (r2 < 0).
compute r2=0.
end if.
compute adjr2 = 1-((1-r2)*(n1-1)/(dfres)).
compute mse=ssresid/(n1-ncol( llrdat )).

.
compute n1=nrow( x ).
compute invXtX = inv(t( x )* x ).
compute varb = mse *invXtX.
compute k3 = ncol( x ).
compute xhc=0.
do if ( hc <> 5).
compute xhc= x.
compute hat = xhc(:,1).
loop i3=1 to nrow(xhc).
compute hat(i3,1)= xhc(i3,:)*invXtX*t(xhc(i3,:)).
end loop.
do if ( hc = 0 or hc =1).
loop i3 = 1 to k3.
compute xhc(:,i3)=xhc(:,i3)&* resid.
end loop.
end if.
do if ( hc =3 or hc =2).
loop i3=1 to k3.
compute xhc(:,i3) = ( resid &/(1-hat)&**(1/(4- hc )))&*xhc(:,i3).
end loop.
end if.
do if ( hc = 4).
compute hcmn=make(n,2,4).
compute hcmn(:,2)=(n1*hat)/k3.
loop i3= 1 to k3.
compute xhc(:,i3) = ( resid &/(1-hat)&**(rmin(hcmn)/2))&*xhc(:,i3).
end loop.
end if.
compute varb=(invXtX*t(xhc)*xhc*invXtX).
do if ( hc =1).
compute varb=(n1/(n1-ncol( x )))&*varb.
end if.
end if.
compute hclab={'se(HC0)','se(HC1)','se(HC2)','se(HC3)','se(HC4)','se'}.
compute hclab=hclab(1,( hc +1)).
compute hcflab={'F(HC0)','F(HC1)','F(HC2)','F(HC3)','F(HC4)','F'}.
compute hcflab=hcflab(1,( hc +1)).
release xhc
.
compute seb=sqrt(diag(varb)).
compute trat = b&/seb.
compute p = 2*(1-tcdf(abs(trat), (dfres))).
compute tval = sqrt(dfres* (exp((dfres-(5/6))*((xp2/(dfres-(2/3)+(.11/dfres)))* (xp2/(dfres-(2/3)+(.11/dfres)))))-1)).
compute modres={modres,seb,trat,p}.
compute modres={modres,(b-tval&*seb),(b+tval&*seb)}.
compute modresl={'coeff',hclab,'t','p','LLCI','ULCI'}.
compute lmat = ident(ncol( llrdat )).
compute lmat = lmat(:,2:ncol(lmat)).
compute fratio = (t(t(lmat)*b)*inv(t(lmat)*varb*lmat)*((t(lmat)*b)))/(ncol( llrdat )-1).
compute pfr = 1-fcdf(fratio,(ncol( llrdat )-1),dfres).
compute modsum={sqrt(r2),r2,mse,fratio,(ncol( llrdat )-1),dfres,pfr}.
compute modsuml={'R','R-sq','MSE',hcflab,'df1','df2', 'p'}.
end if.
end if.
do if ( 2 = 2 or 2 =3).
compute xlp= llrdat.
compute ylp= y.
compute pt2 = make(nrow(ylp),1,(csum(ylp)/nrow(ylp))).
do if ( 2 =2).
compute LL3 = ylp&*ln(pt2)+(1-ylp)&*ln(1-pt2).
end if.
compute LL3 = -2*csum(LL3).
compute bt1 = make(ncol(xlp),1,0).
compute LL1 = 0.
compute pt1 = make(nrow(ylp),1,0.5).
compute pt1lp=pt1.
loop jjj = 1 to iterate.
compute xlptmp=t(xlp).
compute vecprb=pt1lp&*(1-pt1lp).
loop kkk=1 to ncol(xlp).
compute xlptmp(kkk,:)=xlptmp(kkk,:)&*t(vecprb).
end loop.
compute b = bt1+inv(xlptmp*xlp)*t(xlp)*(ylp-pt1lp).
do if ( 2 =2).
compute xlpb=xlp*b.
compute xlpbt=(xlpb > -709.7).
compute xlpb709=(1-xlpbt)*(-709.7).
compute xlpb=(xlpb&*xlpbt)+xlpb709.
compute pt1lp = 1/(1+exp(-(xlpb))).
end if.
compute itprob = csum((pt1lp < .00000001) or (pt1lp > .9999999)).
do if (itprob > 0).
loop kkk = 1 to nrow(pt1lp).
do if (pt1lp(kkk,1) > .9999999).
compute pt1lp(kkk,1) = .9999999.
end if.
do if (pt1lp(kkk,1) < .00000001).
compute pt1lp(kkk,1) = .00000001.
end if.
end loop.
compute itprob = 0.
end if.
do if (itprob = 0).
do if ( 2 =2).
compute LL = ylp&*ln(pt1lp)+(1-ylp)&*ln(1-pt1lp).
end if.
compute LL2 = -2*csum(ll).
end if.
do if (abs(LL1-LL2) < converge).
do if ( 0 =1).
compute xlptmp=t(xlp).
compute vecprb=pt1lp&*(1-pt1lp).
loop kkk=1 to ncol(xlp).
compute xlptmp(kkk,:)=xlptmp(kkk,:)&*t(vecprb).
end loop.
compute varb = inv(xlptmp*xlp).
compute seb = sqrt(diag(varb)).
release xlptmp.
end if.
break.
end if.
compute bt1 = b.
compute LL1 = LL2.
end loop.
compute modres=b.
do if (jjj > iterate).
compute itprob = 2.
do if (booting=0).
compute iterrmod=1.
end if.
do if (booting=1).
compute bootiter=1.
end if.
do if (itprobtg=0).
compute itprobtg=1.
compute errcode(errs,1) = 47.
compute errs = errs + 1.
do if (booting = 0 and 0 =1).
compute vt1 = mdiag(pt1lp&*(1-pt1lp)).
compute varb = inv(t(xlp)*vt1*xlp).
compute seb = sqrt(diag(varb)).
end if.
end if.
end if.
do if ( 0 =1).
compute trat = b&/seb.
compute dfres=nrow(xlp).
compute p = 2*(1-cdfnorm(abs(trat))).
compute modres={modres,seb,trat,p}.
compute modres={modres,(b-xp2&*seb),(b+xp2&*seb)}.
compute pvchi=1-chicdf((LL3-LL2),(nrow(modres)-1)).
compute mcF = (LL3-LL2)/LL3.
compute cox = 1-exp(-(LL3-LL2)/nrow(xlp)).
compute nagel = cox/(1-exp(-(LL3)/nrow(xlp))).
compute modsum={LL2,(LL3-LL2),(nrow(modres)-1),pvchi, mcF,cox,nagel}.
compute modsuml={'-2LL','ModelLL', 'df', 'p', 'McFadden', 'CoxSnell', 'Nagelkrk'}.
compute modresl={'coeff','se','Z','p','LLCI','ULCI'}.
end if.
end if
.
compute b=btemphld.
compute fresult={(LL2-basemod),llrdf,1-chicdf((LL2-basemod),llrdf)}
.
end if.
compute highf={highf;fresult}.
compute highf2={highf2;fresult}.
do if (j = start).
compute flabel={flabel;'X*W*Z'}.
end if.
do if (j > start).
do if (nms > 1).
compute flabel={flabel;highlbwz(jj,1)}.
else if (nms = 1).
compute flabel={flabel;'M*W*Z'}.
end if.
end if.
end if.
compute jj=jj+1.
end loop.
release jj.
compute start=start+i.
end if.
do if (nrow(highf) > 1).
compute highf=highf(2:nrow(highf),:).
compute highf2=highf2(2:nrow(highf2),:).
compute flabel=flabel(2:nrow(flabel),1).
compute resultm2=make(nrow(highf),maxresm,99999).
compute resultm2(1:nrow(highf),1:ncol(highf))=highf.
compute resultm={resultm;resultm2}.
do if (outscree=1).
do if ((i < nms+nys) or (ydich=0)).
compute clabtmp={'R2-chng', hcflab,'df1','df2','p'}.
print highf/format = F10.4 /rnames=flabel/cnames=clabtmp/ title = 'Test(s) of highest order unconditional interaction(s):'.
end if.
do if (ydich=1 and i=(nms+nys)).
compute clabtmp={'Chi-sq', 'df','p'}.
print/title='Likelihood ratio test(s) of highest order'.
print highf/format = F10.4 /rnames=flabel/cnames=clabtmp/ title = 'unconditional interactions(s):'/space=0.
end if.
end if.
compute intpb=highf2(:,ncol(highf2)).
end if.
compute intstart=intstart+numint(1,i).
end if.

.
do if (criterr=0).
compute threeway=0.
compute didprint=0.
compute didsome=0.
compute sigintct=0.
loop jmed =1 to (nms+1).
compute hasw=0.
compute hasz=0.
compute jnok=0.
compute nm1vls=0.
compute nm2vls=0.
compute panelgrp=0.
compute graphixs={'WITH', outnames(1,i), 'BY'}.
compute focpred4={' '}.
compute intprint=0.
compute modcat=0.
do if (jmed <= i).
do if ((jmed = 1) and ((i+1) = nrow(bcmat))).
compute pathscnt=pathscnt+1.
else.
compute paths={paths,bcmat((i+1),jmed)}.
compute pathsw={pathsw,wcmat((i+1),jmed)}.
compute pathsz={pathsz,zcmat((i+1),jmed)}.
compute pathswz={pathswz,wzcmat((i+1),jmed)}.
compute temp=fochigh(:,pathscnt)&*bootloc(:,i).
compute pathsfoc={pathsfoc,pathsfoc(:,1)}.
do if (jmed=1).
compute pathtype={pathtype,1}.
end if.
do if ((i+1)=nrow(bcmat)).
compute pathtype={pathtype,3}.
end if.
do if (jmed > 1) and ((i+1) < nrow(bcmat)).
compute pathtype={pathtype,2}.
end if.
do if (jmed=1 and nxvls > 1 and (bcmat((i+1),jmed)=1)).
compute pathsfoc(:,(pathscn2+1))=temp(2:(nxvls+1),1).
end if.
do if ((jmed > 1) or (jmed=1 and nxvls=1)).
compute temp=cmax(temp).
compute pathsfoc(1,(pathscn2+1))=temp.
end if.
compute pathscnt=pathscnt+1.
compute pathscn2=pathscn2+1.
do if (i <= nms).
compute pathsdv={pathsdv,mnames(1,i)}.
end if.
do if (i > nms).
compute pathsdv={pathsdv,ynames}.
end if.
end if.
compute coeffcol=coeffcol+1.
compute probettt=coeffs(1:nrow(b),coeffcol).
do if (jmed=1 and (bcmat((i+1),jmed)=1)).
compute omni=make(nrow(probettt),nxvls,0).
compute omnitmp=ident(nxvls).
compute omni(2:(1+nxvls),:)=omnitmp.
end if.
do if (csum(probettt)>0).
compute probvarb=make(csum(probettt),csum(probettt),999).
compute probcoef=make(csum(probettt),1,999).
compute coefflp2=1.
loop coefflp=1 to nrow(probettt).
do if (probettt(coefflp,1)=1).
compute probcoef(coefflp2,1)=b(coefflp,1).
compute coefflp2=coefflp2+1.
end if.
end loop.
compute coefflp=0.
compute coefflp2=0.
loop iclp=1 to nrow(probettt).
do if probettt(iclp,1)=1.
compute coefflp=coefflp+1.
compute coefflp2=coefflp.
compute probvarb(coefflp,coefflp) = varb(iclp,iclp).
do if (iclp < nrow(probettt)).
loop jclp=(iclp+1) to nrow(probettt).
do if (probettt(jclp,1)=1).
compute coefflp2=coefflp2+1.
compute probvarb(coefflp,coefflp2)=varb(iclp, jclp).
compute probvarb(coefflp2,coefflp)=varb(iclp, jclp).
end if.
end loop.
end if.
end if.
end loop.
end if.
end if.
compute xprobval=xmodvals.
do if (nxvls > 1 or mcx > 0).
compute xprobval=dummatx(:,2:ncol(dummatx)).
end if.
do if ((wcmat((i+1),jmed)=1) and (zcmat((i+1),jmed)=0)).
compute numplps=1.
compute modvals=wmodvals.
compute probeval=wmodvals.
compute wheremv1=wherexw.
compute nm1vls=nwvls.
compute lpstsp={1,1}.
compute modcat=0.
compute jnmod=wtmp.
compute jnmodlab=wnames.
compute jnok=1.
compute jnmin=wmin.
compute jnmax=wmax.
compute wherejn1=2.
do if (jmed=1).
compute wherejn3=wherexw(1,i).
do if (nxvls > 1).
compute jnok=0.
end if.
end if.
do if (jmed > 1).
compute wherejn1=wherem((jmed-1),i).
compute wherejn3=wheremw(((2*jmed)-3),i).
end if.
do if (nwvls > 1).
compute probeval=wprobval.
compute lpstsp(1,2)=ncol(probeval).
compute modcat=1.
compute jnok=0.
end if.
do if (wdich = 1).
compute modcat=1.
compute jnok=0.
end if.
compute problabs=wnames.
compute focpred3={wnames,'(W)'}.
do if (xmint=1).
compute focpred3={wnames,'(X)'}.
end if.
compute hasw=1.
compute modgrph=wnames.
compute intprint=1.
compute sigintct=sigintct+1.
compute printpbe=intpb(sigintct,1).
end if.
do if ((wcmat((i+1),jmed)=0) and (zcmat((i+1),jmed)=1)).
compute numplps=1.
compute modvals=zmodvals.
compute probeval=zmodvals.
compute wheremv1=wherexz.
compute nm1vls=nzvls.
compute lpstsp={1,1}.
compute jnok=1.
compute jnmod=ztmp.
compute jnmin=zmin.
compute jnmax=zmax.
compute jnmodlab=znames.
compute wherejn1=2.
do if (jmed=1).
compute wherejn3=wherexz(1,i).
do if (nxvls > 1).
compute jnok=0.
end if.
end if.
do if (jmed > 1).
compute wherejn1=wherem((jmed-1),i).
compute wherejn3=wheremz(((2*jmed)-3),i).
end if.
do if (nzvls > 1).
compute probeval=zprobval.
compute lpstsp(1,2)=ncol(probeval).
compute modcat=1.
compute jnok=0.
end if.
do if (zdich = 1).
compute modcat=1.
compute jnok=0.
end if.
compute problabs=znames.
compute focpred3={znames,'(Z)'}.
compute modgrph=znames.
compute hasz=1.
compute intprint=1.
compute sigintct=sigintct+1.
compute printpbe=intpb(sigintct,1).
end if.
do if ((wzcmat((i+1),jmed)=1) or ((wcmat((i+1),jmed)=1) and (zcmat((i+1),jmed)=1))).
compute numplps=2.
compute probecnt=1.
compute intprint=1.
do if (wzcmat((i+1),jmed)=1).
compute sigintct=sigintct+1.
compute printpbe=intpb(sigintct,1).
else.
compute sigintct=sigintct+2.
compute printpbe=cmin(intpb((sigintct-1):sigintct,1)).
end if.
compute panelgrp=1.
compute hasw=1.
compute hasz=1.
compute panelcde={'/PANEL','ROWVAR=',znames,'.'}.
compute modgrph=wnames.
compute lpstsp={1,1;1,1}.
compute wheremv1=wherexw.
compute nm1vls=nwvls.
compute wheremv2=wherexz.
compute nm2vls=nzvls.
compute jnok=0.
do if (wzcmat((i+1),jmed)=1).
compute jnok=1.
end if.
do if (jmed > 1).
compute mprobval=mmodvals.
end if.
do if (jmed=1).
do if (nxvls > 1).
compute jnok=0.
end if.
end if.
do if (nwvls > 1).
compute lpstsp(1,2)=ncol(wprobval).
compute modcat=1.
compute jnok=0.
end if.
do if (zdich=1).
compute modcat=1.
compute jnok=0.
end if.
compute lpstsp(2,1)=lpstsp(1,2)+1.
compute lpstsp(2,2)=lpstsp(1,2)+1.
do if (nzvls > 1).
compute lpstsp(2,1)=lpstsp(1,2)+1.
compute lpstsp(2,2)=lpstsp(1,2)+ncol(zprobval).
compute jnok=0.
end if.
do if (zdich=1).
compute jnok=0.
end if.
compute omni3=make(nrow(b),(nxvls*nwvls),0).
do if (jmed > 1).
compute omni3=make(nrow(b),nwvls,0).
end if.
compute focpred3={wnames,'(W)'}.
do if (xmint=1).
compute focpred3={wnames,'(X)'}.
end if.
compute focpred4={'    ', 'Mod var:', znames, '(Z)'}.
compute modvals=make((nrow(wmodvals)*nrow(zmodvals)),2,0).
compute probeval=make((nrow(wmodvals)*nrow(zmodvals)),(ncol(wprobval)+ncol(zprobval)),0).
loop probei= 1 to nrow(wmodvals).
loop probej =1 to nrow(zmodvals).
compute modvals(probecnt,1)=wmodvals(probei,1).
compute probeval(probecnt,1:nwvls)=wprobval(probei,:).
compute modvals(probecnt,2)=zmodvals(probej,1).
compute probeval(probecnt,(nwvls+1):(nwvls+nzvls))=zprobval(probej,:).
compute probecnt=probecnt+1.
end loop.
end loop.
do if (wzcmat((i+1),jmed)=1).
compute numplps=numplps+1.
compute probprod=make(1,(ncol(wprobval)*ncol(zprobval)),0).
compute lpstsp2={1,1}.
compute lpstsp={lpstsp;lpstsp2}.
compute lpstsp(3,1)=lpstsp(2,2)+1.
compute lpstsp(3,2)=lpstsp(2,2)+ncol(probprod).
compute jnmod=ztmp.
compute jnmin=zmin.
compute jnmax=zmax.
compute jnmodlab=znames.
do if (jmed = 1).
compute wherejn1=wherexw(1,i).
compute wherejn3=wherexwz(1,i).
end if.
do if (jmed > 1).
compute wherejn1=wheremw(((2*jmed)-3),i).
compute wherejn3=wheremwz(((2*jmed)-3),i).
end if.
loop probei = 1 to nrow(wmodvals).
loop probej = 1 to nrow(zmodvals).
compute probtemp=1.
loop probek = 1 to ncol(wprobval).
compute probtemp={probtemp,(wprobval(probei,probek)&*zprobval(probej,:))}.
end loop.
compute probprod={probprod;probtemp(1,2:ncol(probtemp))}.
end loop.
end loop.
compute probprod=probprod(2:nrow(probprod),:).
compute probeval={probeval,probprod}.
end if.
compute problabs={wnames,znames}.
release probecnt, probei, probej.
end if.
do if (intprint=1).
compute focpred={'   Focal', 'predict:'}.
do if (jmed=1).
compute focpred={focpred,xnames,'(X)'}.
compute focplotv=xmodvals.
end if.
do if (jmed >1).
do if (nms > 1).
compute focpred={focpred,mnames(1,(jmed-1)), medlb2(1,(jmed-1))}.
end if.
do if (nms = 1).
compute focpred={focpred,mnames(1,(jmed-1)), '(M)'}.
end if.
compute focplotv=mmodvals(:,(jmed-1)).
end if.
compute focpred2={'    ', 'Mod var:',focpred3}.
compute focpred={focpred;focpred2}.
do if (ncol(focpred4) > 1).
compute focpred={focpred;focpred4}.
compute focpred4={' '}.
end if.
release focpred2,focpred3.
do if (outscree=1).
do if ((plot = 1 or plot = 2) or (printpbe <= intprobe)).
print focpred/title='----------'/format=A8/space=0.
end if.
end if.
compute foctmp=make(nrow(modvals),1,1).
compute probexpl=1.
compute probeva2={foctmp,probeval}.
do if (jmed=1 and nxs > 0 and mcx > 0).
compute probexpl=nxvls.
end if.
compute foctmp=make(nrow(modvals),1,1).
compute modvals3=make(1,(6+ncol(problabs)),0).
compute probrown=make(nrow(probeval),1,0).
compute jtmp=1.
loop probei = 1 to nrow(probeval).
compute probrown(probei,1)=jtmp.
compute jtmp=jtmp+nxvls.
end loop.
release jtmp.
compute probrow=999.
compute modvarl=problabs.
do if (plot = 1 or plot = 2 or nxvls > 1).
compute plotvals=make((nrow(modvals)*nrow(focplotv)),(ncol(modvals)+1),999).
loop ploti=1 to nrow(modvals).
loop plotj=1 to nrow(focplotv).
compute plotvals((((ploti-1)*nrow(focplotv))+plotj),2:ncol(plotvals))=modvals(ploti,:).
compute plotvals((((ploti-1)*nrow(focplotv))+plotj),1)=focplotv(plotj,1).
end loop.
end loop.
compute focpredn=3.
do if (jmed=1).
do if (nxvls > 1).
compute focpredn=(nxvls+1).
end if.
do if (nxvls=1 and xdich=1).
compute focpredn=2.
end if.
end if.
compute meanmat=mdiag(means).
compute onesmat=make(nrow(meanmat),(nrow(probeval)*focpredn),1).
compute probeplt=t(mdiag(means)*onesmat).
do if (jmed=1).
do if ((wcmat((i+1),1)=1) or (zcmat((i+1),1)=1)).
compute plotcnt=1.
compute iloops=nwpval*nzpval.
compute plotmx=nxpval*nzpval.
do if ((wcmat((i+1),1)=1) and (zcmat((i+1),1)=0)).
compute iloops=nwpval.
compute plotmx=nxpval.
end if.
do if ((wcmat((i+1),1)=0) and (zcmat((i+1),1)=1)).
compute iloops=nzpval.
compute plotmx=nxpval.
end if.
compute xestvals=make((nxpval*iloops),ncol(xprobval),-999).
do if (wcmat((i+1),1)=1).
compute westvals=make(nrow(xestvals),ncol(wprobval),-999).
end if.
do if (zcmat((i+1),1)=1).
compute zestvals=make(nrow(xestvals),ncol(zprobval),-999).
end if.
loop ploti=1 to iloops.
loop plotj=1 to nxpval.
compute xestvals(plotcnt,:)=xprobval(plotj,:).
compute plotcnt=plotcnt+1.
end loop.
end loop.
compute plotcnt=1.
compute plotcnt1=1.
compute plotcnt2=1.
compute plotcntz=1.
loop ploti = 1 to (iloops*nxpval).
do if (wcmat((i+1),1)=1).
compute westvals(ploti,:)=wprobval(plotcnt1,:).
end if.
do if ((wcmat((i+1),1)=0) and (zcmat((i+1),1)=1)).
compute zestvals(ploti,:)=zprobval(plotcnt1,:).
end if.
do if ((wcmat((i+1),1)=1) and (zcmat((i+1),1)=1)).
compute zestvals(ploti,:)=zprobval(plotcnt2,:).
compute plotcntz=plotcntz+1.
end if.
compute plotcnt=plotcnt+1.
do if (plotcnt > plotmx).
compute plotcnt=1.
compute plotcnt1=plotcnt1+1.
end if.
do if (plotcntz > nxpval).
compute plotcnt2=plotcnt2+1.
compute plotcntz=1.
do if (plotcnt2 > nzpval).
compute plotcnt2=1.
end if.
end if.
end loop.
compute probeplt(:,2:(1+(ncol(xestvals))))=xestvals.
do if (wcmat((i+1),1)=1).
compute probeplt(:,(wherew(1,i)):(wherew(2,i)))=westvals.
end if.
do if (zcmat((i+1),1)=1).
compute probeplt(:,(wherez(1,i)):(wherez(2,i)))=zestvals.
end if.
end if.
end if.
do if (jmed > 1).
do if ((wcmat((i+1),jmed)=1) or (zcmat((i+1),jmed)=1)).
compute plotcnt=1.
compute iloops=nwpval*nzpval.
compute plotmx=3*nzpval.
do if ((wcmat((i+1),jmed)=1) and (zcmat((i+1),jmed)=0)).
compute iloops=nwpval.
compute plotmx=3.
end if.
do if ((wcmat((i+1),jmed)=0) and (zcmat((i+1),jmed)=1)).
compute iloops=nzpval.
compute plotmx=3.
end if.
compute mestvals=make((3*iloops),1,-999).
do if (wcmat((i+1),jmed)=1).
compute westvals=make(nrow(mestvals),ncol(wprobval),-999).
end if.
do if (zcmat((i+1),jmed)=1).
compute zestvals=make(nrow(mestvals),ncol(zprobval),-999).
end if.
loop ploti=1 to iloops.
loop plotj=1 to 3.
compute mestvals(plotcnt,:)=mprobval(plotj,(jmed-1)).
compute plotcnt=plotcnt+1.
end loop.
end loop.
compute plotcnt=1.
compute plotcnt1=1.
compute plotcnt2=1.
compute plotcntz=1.
loop ploti = 1 to (iloops*3).
do if (wcmat((i+1),jmed)=1).
compute westvals(ploti,:)=wprobval(plotcnt1,:).
end if.
do if ((wcmat((i+1),jmed)=0) and (zcmat((i+1),jmed)=1)).
compute zestvals(ploti,:)=zprobval(plotcnt1,:).
end if.
do if ((wcmat((i+1),jmed)=1) and (zcmat((i+1),jmed)=1)).
compute zestvals(ploti,:)=zprobval(plotcnt2,:).
compute plotcntz=plotcntz+1.
end if.
compute plotcnt=plotcnt+1.
do if (plotcnt > plotmx).
compute plotcnt=1.
compute plotcnt1=plotcnt1+1.
end if.
do if (plotcntz > 3).
compute plotcnt2=plotcnt2+1.
compute plotcntz=1.
do if (plotcnt2 > nzpval).
compute plotcnt2=1.
end if.
end if.
end loop.
compute probeplt(:,wherem((jmed-1),i))=mestvals.
do if (wcmat((i+1),jmed)=1).
do if (model <> 74).
compute probeplt(:,(wherew(1,i)):(wherew(2,i)))=westvals.
end if.
do if (model = 74).
compute probeplt(:,(wherex(1,i)):(wherex(2,i)))=westvals.
end if.
end if.
do if (zcmat((i+1),jmed)=1).
compute probeplt(:,(wherez(1,i)):(wherez(2,i)))=zestvals.
end if.
end if.
end if.
compute prodloop = 1.
do if (jmed=1).
compute prodloop=ncol(xestvals).
end if.
do if (wcmat((i+1),jmed))=1.
compute plotcnt=0.
loop ploti = 1 to prodloop.
loop plotj = 1 to ncol(westvals).
do if (jmed=1).
compute probeplt(:,(wherexw(1,i)+plotcnt))=xestvals(:,ploti)&*westvals(:,plotj).
end if.
do if (jmed > 1).
compute probeplt(:,(wheremw(((jmed*2)-3) ,i)+plotcnt))=mestvals(:,ploti)&*westvals(:,plotj).
end if.
compute plotcnt=plotcnt+1.
end loop.
end loop.
end if.
do if (zcmat((i+1),jmed))=1.
compute plotcnt=0.
loop ploti = 1 to prodloop.
loop plotj = 1 to ncol(zestvals).
do if (jmed = 1).
compute probeplt(:,(wherexz(1,i)+plotcnt))=xestvals(:,ploti)&*zestvals(:,plotj).
end if.
do if (jmed > 1).
compute probeplt(:,(wheremz(((jmed*2)-3),i)+plotcnt))=mestvals(:,ploti)&*zestvals(:, plotj).
end if.
compute plotcnt=plotcnt+1.
end loop.
end loop.
end if.
do if (wzcmat((i+1),jmed))=1.
compute plotcnt=0.
compute threeway=1.
loop ploti = 1 to ncol(westvals).
loop plotj = 1 to ncol(zestvals).
compute probeplt(:,(wherewz(1,i)+plotcnt))=westvals(:,ploti)&*zestvals(:,plotj).
compute plotcnt=plotcnt+1.
end loop.
end loop.
compute plotcnt=0.
loop plotk = 1 to prodloop.
loop ploti = 1 to ncol(westvals).
loop plotj = 1 to ncol(zestvals).
do if (jmed = 1).
compute probeplt(:,(wherexwz(1,i)+plotcnt))=xestvals(:,plotk)&*westvals(:,ploti)&* zestvals(:,plotj).
end if.
do if (jmed > 1).
compute probeplt(:,(wheremwz(((jmed*2)-3),i)+plotcnt))=mestvals(:,plotk)&* westvals(:,ploti)&*zestvals(:,plotj).
end if.
compute plotcnt=plotcnt+1.
end loop.
end loop.
end loop.
end if.
loop newplp=1 to i.
do if (newplp <> jmed).
do if (wcmat((i+1),newplp))=1.
compute prodloop=1.
do if (newplp=1).
compute prodloop=nxvls.
end if.
compute plotcnt=0.
loop ploti = 1 to prodloop.
loop plotj = 1 to nwvls.
do if (newplp = 1).
compute probeplt(:,(wherexw(1,i)+plotcnt))=probeplt(:,(1+ploti))&*probeplt(:, (wherew(1,i)+plotj-1)).
end if.
do if (newplp > 1).
do if (model <> 74).
compute probeplt(:,(wheremw(((newplp*2)-3) ,i)+plotcnt))=probeplt(:,wherem((newplp-1),i))&*probeplt(:,(wherew(1,i)+plotj-1)).
end if.
do if (model = 74).
compute probeplt(:,(wheremw(((newplp*2)-3) ,i)+plotcnt))=probeplt(:,wherem((newplp-1),i))&*probeplt(:,(wherex(1,i)+plotj-1)).
end if.
end if.
compute plotcnt=plotcnt+1.
end loop.
end loop.
end if.
do if (zcmat((i+1),newplp))=1.
compute prodloop=1.
do if (newplp=1).
compute prodloop=nxvls.
end if.
compute plotcnt=0.
loop ploti = 1 to prodloop.
loop plotj = 1 to nzvls.
do if (newplp = 1).
compute probeplt(:,(wherexz(1,i)+plotcnt))=probeplt(:,(1+ploti))&*probeplt(:, (wherez(1,i)+plotj-1)).
end if.
do if (newplp > 1).
compute probeplt(:,(wheremz(((newplp*2)-3),i)+plotcnt))=probeplt(:, wherem((newplp-1),i))&*probeplt(:,(wherez(1,i)+plotj-1)).
end if.
compute plotcnt=plotcnt+1.
end loop.
end loop.
end if.
do if (wzcmat((i+1),newplp))=1.
compute plotcnt=0.
do if (threeway=0).
loop ploti = 1 to nwvls.
loop plotj = 1 to nzvls.
compute probeplt(:,(wherewz(1,i)+plotcnt))=probeplt(:,(wherew(1,i)+ploti-1))&* probeplt(:,(wherez(1,i)+plotj-1)).
compute plotcnt=plotcnt+1.
end loop.
end loop.
end if.
compute prodloop=1.
do if (newplp=1).
compute prodloop=nxvls.
end if.
compute plotcnt=0.
loop plotk = 1 to prodloop.
loop ploti = 1 to nwvls.
loop plotj = 1 to nzvls.
do if (newplp = 1).
compute probeplt(:,(wherexwz(1,i)+plotcnt))=probeplt(:,(1+plotk))&*probeplt(:, (wherew(1,i)+ploti-1))&*probeplt(:,(wherez(1,i)+plotj-1)).
end if.
do if (newplp > 1).
compute probeplt(:,(wheremwz(((newplp*2)-3),i)+plotcnt))=probeplt(:, wherem((newplp-1),i))&*probeplt(:,(wherew(1,i)+ploti-1))&*probeplt(:,(wherez(1,i)+plotj-1)).
end if.
compute plotcnt=plotcnt+1.
end loop.
end loop.
end loop.
end if.
end if.
end loop.
compute predvals=probeplt*b.
do if (debug <> 0).
print probeplt.
print b.
end if.
do if (i = nms+nys) and (ydich=1).
compute predvalt=(predvals < 709.7).
compute prevalt7=(1-predvalt)*(709.7).
compute predvals=(predvals&*predvalt)+prevalt7.
compute expyhat=exp(predvals)&/(1+exp(predvals)).
end if.
compute sepred=make(nrow(plotvals),3,999).
loop sei=1 to nrow(plotvals).
compute ask=probeplt(sei,:).
compute sepred(sei,1)=sqrt(ask*varb*t(ask)).
do if ((i < nms+nys) or (ydich=0)).
compute sepred(sei,2)=predvals(sei,1)-tval*sepred(sei,1).
compute sepred(sei,3)=predvals(sei,1)+tval*sepred(sei,1).
end if.
do if ((i = nms+nys) and (ydich=1)).
compute sepred(sei,2)=predvals(sei,1)-xp2*sepred(sei,1).
compute sepred(sei,3)=predvals(sei,1)+xp2*sepred(sei,1).
end if.
end loop.
compute prevloc=ncol(plotvals)+1.
compute probeplt={plotvals,predvals}.
do if (plot = 2).
compute probeplt={probeplt,sepred}.
end if.
do if ((i = nms+nys) and (ydich=1)).
compute probeplt={probeplt,expyhat}.
end if.
compute didsome=0.
end if.
do if ((wzcmat((i+1),jmed)=1) and (printpbe <= intprobe)).
do if (jmed=1).
compute omnilp2=nxvls*nwvls.
compute omnitmp=ident(omnilp2).
compute omni3(wherexw(1,i):wherexw(2,i),:)=omnitmp.
end if.
do if (jmed>1).
compute omnilp2=nwvls.
compute omnitmp=ident(omnilp2).
compute omni3(wheremw(((jmed*2)-3),i):wheremw(((jmed*2)-2),i),:)=omnitmp.
end if.
compute omnif=make(1,4,0).
do if ((i = nms+nys) and (ydich=1)).
compute omnif=make(1,3,0).
end if.
compute condeff3=0.
loop omnilp1=1 to nrow(zprobval).
loop omnilp=1 to (omnilp2).
do if (jmed=1).
compute omni3((wherexwz(1,i)+((omnilp-1)*nzvls)):(wherexwz(1,i)+((omnilp-1)*nzvls)+ (nzvls-1)),omnilp)=t(zprobval(omnilp1,:)).
end if.
do if (jmed > 1).
compute omni3((wheremwz(((jmed*2)-3),i)+((omnilp-1)*nzvls)):(wheremwz(((jmed*2)-3),i)+ ((omnilp-1)*nzvls)+(nzvls-1)),omnilp)=t(zprobval(omnilp1,:)).
end if.
end loop.
compute condeff=t(omni3)*b.
compute condeff3={condeff3;condeff}.

.
compute lmat2= omni3.
do if ( 1 =0).
compute lmat2 = mdiag( omni3 ).
compute lmat3=make(nrow(lmat2),1,0).
loop flp=1 to ncol(lmat2).
do if (csum(lmat2(:,flp))=1).
compute lmat3={lmat3,lmat2(:,flp)}.
end if.
end loop.
compute lmat2=lmat3(:,2:ncol(lmat3)).
end if.
compute fratio = (t(t(lmat2)* b )*inv(t(lmat2)* varb *lmat2)*((t(lmat2)* b )))/ncol(lmat2).
compute pfr = 1-fcdf(fratio,ncol(lmat2),(n-nrow( b ))).
compute fresult={fratio,ncol(lmat2),(n-nrow( b )),pfr}.
do if (i = (nms+nys) and (ydich=1)).
compute fratio=fratio*ncol(lmat2).
compute pfr=1-chicdf(fratio,ncol(lmat2)).
compute fresult={fratio,ncol(lmat2),pfr}.
end if.
do if ( 0 =1).
compute lmat3=1-rsum(lmat2).
compute xfm=make(n,csum(lmat3),0).
compute flpc=1.
loop flp=1 to nrow(lmat3).
do if (lmat3(flp,1)=1).
compute xfm(:,flpc)=x(:,flp).
compute flpc=flpc+1.
end if.
end loop.
compute bfm=inv(t(xfm)*xfm)*t(xfm)*y.
compute resid=y-(xfm*bfm).
compute sstotal=(y-(csum(y)/n)).
compute sstotal=csum(sstotal&*sstotal).
compute ssresid=csum(resid&*resid).
compute rsqch= 0 -((sstotal-ssresid)/sstotal).
compute fresult={rsqch,fresult}.
release xfm,flpc, resid, ssresid, bfm.
end if
.
compute omnif={omnif;fresult}.
end loop.
compute omnif=omnif(2:nrow(omnif),:).
compute clabtmp=znames.
compute condeff3=condeff3(2:nrow(condeff3),:).
do if ((nxvls*nwvls)=1).
compute omnif={condeff3,omnif}.
compute clabtmp={clabtmp,'Effect'}.
end if.
compute omnif={zmodvals,omnif}.
do if ((i < nms+nys) or (ydich=0)).
compute clabtmp={clabtmp,hcflab,'df1','df2','p'}.
end if.
do if ((i = (nms+nys)) and (ydich=1)).
compute clabtmp={clabtmp,'Chi-sq','df','p'}.
end if.
compute resultm2=make(nrow(omnif),maxresm,99999).
compute resultm2(1:nrow(omnif),1:ncol(omnif))=omnif.
compute resultm={resultm;resultm2}.
do if (outscree=1).
do if (jmed=1).
print omnif/title='Test of conditional X*W interaction at value(s) of Z:'/cnames=clabtmp/format= F10.4.
end if.
do if (jmed>1).
print omnif/title='Test of conditional M*W interaction at value(s) of Z:'/cnames=clabtmp/format= F10.4.
end if.
end if.
release omni3.
end if.
loop probei = 1 to probexpl.
do if (probexpl > 1).
compute foctmp=make(nrow(modvals),probexpl,0).
compute foctmp(:,probei)=foctmp(:,probei)+1.
compute probtemp=make(nrow(modvals),1,0).
loop probem = 1 to numplps.
loop probek = 1 to nxvls.
loop probej=lpstsp(probem,1) to lpstsp(probem,2).
compute probtemp={probtemp,foctmp(:,probek)&*probeval(:,probej)}.
end loop.
end loop.
end loop.
compute probeva2=probtemp(:,2:ncol(probtemp)).
compute probeva2={foctmp,probeva2}.
end if.
compute probres=probeva2*probcoef.
compute probrese=sqrt(diag(probeva2*probvarb*t(probeva2))).
compute tratio = probres&/probrese.
compute p = 2*(1-tcdf(abs(tratio), dfres)).
do if (ydich=1 and i = (nms+nys)).
compute p = 2*(1-cdfnorm(abs(tratio))).
end if.
compute modvals2={modvals,probres,probrese,tratio, p}.
do if ((i < nms+nys) or (ydich=0)).
compute modvals2={modvals2,(probres-tval&*probrese),(probres+tval&*probrese)}.
compute problabs={problabs,'Effect',hclab,'t', 'p', 'LLCI', 'ULCI'}.
end if.
do if (ydich=1 and i = (nms+nys)).
compute modvals2={modvals2,(probres-xp2&*probrese),(probres+xp2&*probrese)}.
compute problabs={problabs,'Effect','se','Z', 'p', 'LLCI', 'ULCI'}.
end if.
do if (probexpl > 1 and (printpbe <= intprobe)).
do if (hasz = 1).
compute printz=1.
end if.
do if (hasw=1).
compute printw=1.
end if.
compute probrlab=make(nrow(modvals),1,xcatlab(probei,1)).
compute modvals3={modvals3; modvals2}.
compute probrow={probrow;probrown}.
compute probrown=probrown+1.
do if (probei=probexpl).
compute xproblab=xcatlab(1:nxvls,1).
compute probrow=probrow(2:nrow(probrow),1).
compute modvals3=modvals3(2:nrow(modvals3),:).
compute temp=modvals3.
compute temp(GRADE(probrow(:,1)),:)=modvals3.
compute modvals3=temp.
compute start2=1.
compute problabs=problabs(1,(1+(ncol(modvarl))):ncol(problabs)).
compute pstart=1.
loop probek= 1 to nrow(probeval).
compute endstart=start2+(nxvls-1).
compute temp=modvals3(start2:endstart,(1+ncol(modvarl)):ncol(modvals3)).
compute temp2=t(modvals3(start2:start2,1:ncol(modvarl))).
compute trnames=t(modvarl).
do if (outscree=1).
do if (probek > 1).
print/title='----------'/space=0.
else.
print/title = 'Conditional effects of the focal predictor at values of the moderator(s):'.
do if ((jmed=1) and (i = (nms+nys)) and (nms > 0)).
do if (nxvls = 1).
print/title = '(These are also the conditional direct effects of X on Y)'/space=0.
else.
print/title = '(These are also the relative conditional direct effects of X on Y)'/space=0.
end if.
end if.
print.
end if.
print temp2/title = 'Moderator value(s):'/rnames=trnames/format= F10.4 /space=0.
print temp/title = ' '/cnames=problabs/rnames=xproblab/format= F10.4 /space=0.
end if.
compute resultm2=make(nrow(temp2),maxresm,99999).
compute resultm2(1:nrow(temp2),1:ncol(temp2))=temp2.
compute resultm={resultm;resultm2}.
compute resultm2=make(nrow(temp),maxresm,99999).
compute resultm2(1:nrow(temp),1:ncol(temp))=temp.
compute resultm={resultm;resultm2}.
compute start2=start2+nxvls.
compute didsome=1.
do if (jmed=1).
compute mod1val=probeval(probek,1:nm1vls).
loop omnilp=1 to nxvls.
compute omni((wheremv1(1,i)+((omnilp-1)*nm1vls)):(wheremv1(1,i)+((omnilp-1)* nm1vls)+(nm1vls-1)),omnilp)=t(mod1val).
do if (nm1vls < ncol(probeval)).
compute mod2val=probeval(probek,(nm1vls+1):(nm1vls+nm2vls)).
compute omni((wheremv2(1,i)+((omnilp-1)*nm2vls)):(wheremv2(1,i)+((omnilp-1)* nm2vls)+(nm2vls-1)),omnilp)=t(mod2val).
do if ((nm1vls+nm2vls) < ncol(probeval)).
compute intlen=nm1vls*nm2vls.
compute modintvl=probeval(probek,(nm1vls+nm2vls+1):ncol(probeval)).
compute omni((wherexwz(1,i)+((omnilp-1)*intlen)):(wherexwz(1,i)+((omnilp-1)* intlen)+(intlen-1)),omnilp)=t(modintvl).
end if.
end if.
end loop.

.
compute lmat2= omni.
do if ( 1 =0).
compute lmat2 = mdiag( omni ).
compute lmat3=make(nrow(lmat2),1,0).
loop flp=1 to ncol(lmat2).
do if (csum(lmat2(:,flp))=1).
compute lmat3={lmat3,lmat2(:,flp)}.
end if.
end loop.
compute lmat2=lmat3(:,2:ncol(lmat3)).
end if.
compute fratio = (t(t(lmat2)* b )*inv(t(lmat2)* varb *lmat2)*((t(lmat2)* b )))/ncol(lmat2).
compute pfr = 1-fcdf(fratio,ncol(lmat2),(n-nrow( b ))).
compute fresult={fratio,ncol(lmat2),(n-nrow( b )),pfr}.
do if (i = (nms+nys) and (ydich=1)).
compute fratio=fratio*ncol(lmat2).
compute pfr=1-chicdf(fratio,ncol(lmat2)).
compute fresult={fratio,ncol(lmat2),pfr}.
end if.
do if ( 0 =1).
compute lmat3=1-rsum(lmat2).
compute xfm=make(n,csum(lmat3),0).
compute flpc=1.
loop flp=1 to nrow(lmat3).
do if (lmat3(flp,1)=1).
compute xfm(:,flpc)=x(:,flp).
compute flpc=flpc+1.
end if.
end loop.
compute bfm=inv(t(xfm)*xfm)*t(xfm)*y.
compute resid=y-(xfm*bfm).
compute sstotal=(y-(csum(y)/n)).
compute sstotal=csum(sstotal&*sstotal).
compute ssresid=csum(resid&*resid).
compute rsqch= 0 -((sstotal-ssresid)/sstotal).
compute fresult={rsqch,fresult}.
release xfm,flpc, resid, ssresid, bfm.
end if
.
compute resultm2=make(nrow(fresult),maxresm,99999).
compute resultm2(1:nrow(fresult),1:ncol(fresult))=fresult.
compute resultm={resultm;resultm2}.
do if (outscree=1).
do if (i < (nms + nys) or (ydich = 0)).
compute clabtmp={hcflab,'df1','df2','p'}.
print fresult/title='Test of equality of conditional means'/cnames=clabtmp/format= F10.4.
end if.
do if (i = (nms + nys) and (ydich = 1)).
compute clabtmp={'Chi-sq','df','p'}.
print fresult/title='Test of equality of conditional logits or probabilities'/cnames=clabtmp/format= F10.4.
end if.
end if.
compute probetmp=probeplt(pstart:(pstart+nxvls),1).
compute probetmp={probetmp,probeplt(pstart:(pstart+nxvls),prevloc:ncol(probeplt))}.
compute pstart=pstart+(nxvls+1).
compute resultm2=make(nrow(probetmp),maxresm,99999).
compute resultm2(1:nrow(probetmp),1:ncol(probetmp))=probetmp.
compute resultm={resultm;resultm2}.
do if (i < ((nms + nys)) or (ydich = 0)).
compute clabtmp={xnames, outnames(1,i), hclab, 'LLCI', 'ULCI'}.
do if (outscree=1).
print probetmp/title = 'Estimated conditional means being compared:'/cnames=clabtmp/format= F10.4.
end if.
end if.
do if ((i = (nms+nys)) and (ydich = 1)).
compute clabtmp={xnames, outnames(1,i), 'prob'}.
compute probetm2=probetmp(:,1:2).
compute probetm2={probetm2,probetmp(:,ncol(probetmp))}.
do if (outscree=1).
print probetm2/title = 'Estimated conditional logits and probabilities:'/cnames=clabtmp/format= F10.4.
end if.
end if.
end if.
end loop.
release probrow, start2, endstart, temp, temp2.
end if.
end if.
do if (probexpl = 1 and (printpbe <= intprobe)).
compute resultm2=make(nrow(modvals2),maxresm,99999).
compute resultm2(1:nrow(modvals2),1:ncol(modvals2))=modvals2.
compute resultm={resultm;resultm2}.
do if (outscree=1).
print/title = 'Conditional effects of the focal predictor at values of the moderator(s):'.
print modvals2/cnames=problabs/title = ' '/space=0/format= F10.4.
end if.
compute didsome=1.
do if (hasz = 1).
compute printz=1.
end if.
do if (hasw=1).
compute printw=1.
end if.
do if (jn = 1 and jnok=1).
do if (criterr = 0).
compute dfres=n-nrow(b).
compute roots=99999.
compute jncrit =(dfres* (exp((dfres-(5/6))*((xp2/(dfres-(2/3)+(.11/dfres)))* (xp2/(dfres-(2/3)+(.11/dfres)))))-1)).
do if (i = (nms+nys) and (ydich=1)).
compute jncrit=xp2*xp2.
end if.
compute jnb1=b(wherejn1,1).
compute jnb3=b(wherejn3,1).
compute jnsb1=varb(wherejn1,wherejn1).
compute jnsb3=varb(wherejn3,wherejn3).
compute jnsb1b3=varb(wherejn1,wherejn3).
compute ajn =(jncrit*jnsb3)-(jnb3*jnb3).
compute bjn = 2*((jncrit*jnsb1b3)-(jnb1*jnb3)).
compute cjn = (jncrit*jnsb1)-(jnb1*jnb1).
compute radarg = (bjn*bjn)-(4*ajn*cjn).
compute den = 2*ajn.
compute nrts = 0.
do if (radarg >= 0 and den <> 0).
compute x21 = (-bjn+sqrt(radarg))/den.
compute x22 = (-bjn-sqrt(radarg))/den.
compute roots = 0.
do if (x21 >= jnmin and x21 <= jnmax).
compute nrts = 1.
compute roots = {roots; x21}.
end if.
do if (x22 >= jnmin and x22 <= jnmax).
compute nrts = nrts + 1.
compute roots = {roots; x22}.
end if.
compute roots={roots,make(nrow(roots),2,0)}.
end if.
do if (nrts > 0).
compute roots = roots(2:nrow(roots),1:3).
compute roots(1,2)=(csum(jnmod < roots(1,1))/n)*100.
compute roots(1,3)=(csum(jnmod > roots(1,1))/n)*100.
do if (nrow(roots)=2).
compute roots(2,2)=(csum(jnmod < roots(2,1))/n)*100.
compute roots(2,3)=(csum(jnmod > roots(2,1))/n)*100.
end if.
do if (outscree=1).
print roots/title = 'Moderator value(s) defining Johnson-Neyman significance region(s):'/clabels = 'Value', '% below', '% above'/format F10.4.
end if.
do if (nrts=1).
compute tmprts=make(1,3,99999).
compute roots={roots;tmprts}.
end if.
end if.
do if (nrts = 0).
compute roots=make(2,1,99999).
do if (outscree=1).
print/title = 'There are no statistical significance transition points within the observed'.
print/title = 'range of the moderator found using the Johnson-Neyman method.'/space=0.
end if.
end if.
compute resultm2=make(nrow(roots),maxresm,99999).
compute resultm2(1:nrow(roots),1:ncol(roots))=roots.
compute resultm={resultm;resultm2}.
compute jnvals=make(23,7,0).
loop jni= 0 to (21-(nrts)).
compute jnvals((jni+1),1)=jnmin+(jni*((jnmax-jnmin)/(21-nrts))).
end loop.
do if (nrts > 0).
loop jni = 1 to nrts.
loop jnj = 2 to (nrow(jnvals)-1).
do if ((roots(jni,1) > jnvals((jnj-1),1)) and (roots(jni,1) < jnvals(jnj,1))).
compute jnvals((jnj+1):(21+jni),1)=jnvals(jnj:(20+jni),1).
compute jnvals(jnj,1)=roots(jni,1).
end if.
end loop.
end loop.
end if.
compute jnvals=jnvals(1:22,:).
loop jni = 1 to nrow(jnvals).
compute jnvals(jni,2)=jnb1+jnb3*jnvals(jni,1).
compute jnvals(jni,3)=sqrt(jnsb1+2*jnvals(jni,1)*jnsb1b3+(jnvals(jni,1)*jnvals(jni, 1))*jnsb3).
compute jnvals(jni,4)=jnvals(jni,2)/jnvals(jni,3).
compute jnvals(jni,5)=2*(1-tcdf(abs(jnvals(jni,4)), dfres)).
compute jnvals(jni,6)=jnvals(jni,2)-sqrt(jncrit)*jnvals(jni,3).
compute jnvals(jni,7)=jnvals(jni,2)+sqrt(jncrit)*jnvals(jni,3).
do if ((i = nms + nys) and (ydich=1)).
compute jnvals(jni,5)=2*(1-cdfnorm(abs(jnvals(jni,4)))).
compute jnvals(jni,6)=jnvals(jni,2)-xp2*jnvals(jni,3).
compute jnvals(jni,7)=jnvals(jni,2)+xp2*jnvals(jni,3).
end if.
end loop.
compute resultm2=make(nrow(jnvals),maxresm,99999).
compute resultm2(1:nrow(jnvals),1:ncol(jnvals))=jnvals.
compute resultm={resultm;resultm2}.
do if ((i < nms+nys) or (ydich=0)).
compute jnclbs={jnmodlab,'Effect',hclab,'t', 'p', 'LLCI', 'ULCI'}.
end if.
do if ((i = nms + nys) and (ydich=1)).
compute jnclbs={jnmodlab,'Effect','se','Z', 'p', 'LLCI', 'ULCI'}.
end if.
do if (((wcmat((i+1),jmed)=1) or (zcmat((i+1),jmed)=1)) and (wzcmat((i+1),jmed)=0)).
do if (outscree=1).
print jnvals/title = 'Conditional effect of focal predictor at values of the moderator:'/cnames =jnclbs/format = F10.4.
end if.
end if.
do if (outscree=1).
do if ((jmed = 1) and (wzcmat((i+1),jmed)=1)).
print jnvals/title = 'Conditional X*W interaction at values of the moderator Z:'/cnames =jnclbs/format = F10.4.
end if.
do if ((jmed > 1) and (wzcmat((i+1),jmed)=1)).
print jnvals/title = 'Conditional M*W interaction at values of the moderator Z:'/cnames =jnclbs/format = F10.4.
end if.
end if.
end if.
end if.
end if.
do if ((i = (nms+nys)) and (jmed=1) and (bcmat(nrow(bcmat),1)=1)).
do if (probei=1).
compute direfflb=problabs.
compute direff=modvals2.
end if.
do if (probei>1).
compute direff={direff;modvals2}.
end if.
end if.
compute intprint=0.
do if ((jmed=1) and (i=1) and nms=0) and modcok=1).
compute contvec2=make(2,1,1).
compute contvec2={contvec2,wcontval,zcontval}.
do if (wzcmat((i+1),jmed)=1).
loop conti= 1 to ncol(wcontval).
loop contj = 1 to ncol(zcontval).
compute contvec2={contvec2,wcontval(:,conti)&*zcontval(:,contj)}.
end loop.
end loop.
end if.
compute conteff=contvec2*probcoef.
compute contdiff=contvec2(1,:)-contvec2(2,:).
compute contse=sqrt(contdiff*probvarb*t(contdiff)).
compute conteffd=conteff(1,1)-conteff(2,1).
compute contvec={contvec,conteff}.
compute contvecm=contvec.
compute resultm2=make(nrow(contvecm),maxresm,99999).
compute resultm2(1:nrow(contvecm),1:ncol(contvecm))=contvecm.
compute resultm={resultm;resultm2}.
do if (outscree=1).
print/title='Contrast between conditional effects of X:'.
print contvec/title=' '/rlabels='Effect1:','Effect2:'/cnames=problabs/format = F10.4 /space=0.
end if.
do if (ydich=0).
compute p=2*(1-tcdf(abs(conteffd/contse), dfres)).
compute contvec={conteffd,contse,conteffd/contse, p}.
compute contvec={contvec,(conteffd-(tval*contse))}.
compute contvec={contvec,(conteffd+(tval*contse))}.
compute contlabs={'Contrast', hclab, 't', 'p', 'LLCI', 'ULCI'}.
end if.
do if (ydich=1).
compute p=2*(1-cdfnorm(abs(conteffd/contse))).
compute contvec={conteffd,contse,conteffd/contse, p}.
compute contvec={contvec,(conteffd-(xp2*contse))}.
compute contvec={contvec,(conteffd+(xp2*contse))}.
compute contlabs={'Contrast', 'se', 'Z', 'p', 'LLCI', 'ULCI'}.
end if.
compute resultm2=make(nrow(contvec),maxresm,99999).
compute resultm2(1:nrow(contvec),1:ncol(contvec))=contvec.
compute resultm={resultm;resultm2}.
do if (outscree=1).
print contvec/title='Test of Effect1 minus Effect2'/format= F10.4 /cnames=contlabs.
end if.
end if.
end loop.
do if (plot = 1 or plot = 2).
compute datalabs={t(focpred(:,3)),outnames(1,i)}.
do if (plot = 1).
compute datalabs={datalabs}.
end if.
do if (plot = 2).
compute datalabs={datalabs,'se', 'LLCI', 'ULCI'}.
end if.
do if ((i = nms+nys) and (ydich=1)).
compute datalabs={datalabs,'prob'}.
end if.
compute resultm2=make(nrow(probeplt),maxresm,99999).
compute resultm2(1:nrow(probeplt),1:ncol(probeplt))=probeplt.
compute resultm={resultm;resultm2}.
compute datalabs={datalabs,'.'}.
do if (outscree=1).
print/title = 'Data for visualizing the conditional effect of the focal predictor:'.
print/title = 'Paste text below into a SPSS syntax window and execute to produce plot.'/space=0.
compute dumb = {' ', ' ', ' ', ' ', ' ', ' ', ' '}.
print datalabs/title = 'DATA LIST FREE/'/format=A10.
print probeplt/title = 'BEGIN DATA.'/format= F10.4 /space=0.
print/title = 'END DATA.'/space=0.
end if.
compute focgrph=datalabs(1,1).
compute graphix={focgrph,graphixs,modgrph}.
do if (((xdich=1) or (nxvls > 1)) and ((modcat=0) and (focgrph = xnames))).
compute graphix={modgrph,graphixs,focgrph}.
end if.
do if (panelgrp = 0).
compute graphix={graphix,'.'}.
else.
compute graphix={graphix,panelcde}.
end if.
do if (outscree=1).
print graphix/title = 'GRAPH/SCATTERPLOT='/format=A8/space=0.
end if.
do if (i = (nms + nys) and ydich = 1).
compute graphixd=graphix.
compute graphixd(1,3)='prob'.
do if (outscree=1).
print graphixd/title = 'GRAPH/SCATTERPLOT='/format=A8/space=0.
end if.
end if.
end if.
end if.
end loop.
release jmed, intprint,didprint.
end if
.
do if (model = 74 and i <= nms).
compute onetemp=make(nrow(xprobval),1,1).
compute mestmt74={onetemp,xprobval}.
do if (ncs > 0).
compute ncovmdl=rsum(ccmat(i,:)).
do if (ncovmdl > 0).
compute cvmnc=make(nrow(mestmt74),ncovmdl,1).
compute cvmnctmp=csum(x(:,(ncol(x)-ncovmdl+1):ncol(x)))/nrow(x).
do if (cuscoval > 0).
compute cvmnctmp=coval.
end if.
loop mestlp=1 to ncovmdl.
compute cvmnc(:,mestlp)=cvmnc(:,mestlp)*cvmnctmp(:,mestlp).
end loop.
compute mestmt74={mestmt74,cvmnc}.
end if.
end if.
compute mest74t=mestmt74*b.
do if (i = 1).
compute mest74=mest74t.
end if.
do if (i > 1).
compute mest74={mest74,mest74t}.
end if.
end if.
do if ((i = (nms+nys)) and (model >= 0 and model < 4) and (linsum(1,1) <> -999)).
compute lhyprob=1.
compute meansub=0.
do if (((nlinsum=nrow(b)) or (nlinsum = (nrow(b)-ncs)))).
do if (nlinsum = (nrow(b)-ncs) and ncs > 0).
compute linsum={linsum,covmeans}.
compute meansub=1.
end if.
compute lhyprob=0.
compute hypest=linsum*b.
compute sehypest=sqrt(linsum*varb*t(linsum)).
compute phypest=2*(1-tcdf(abs(hypest/sehypest), (dfres))).
compute hypest={hypest,sehypest,(hypest/sehypest),phypest,(hypest-tval*sehypest),(hypest+ tval*sehypest)}.
compute resultm2=make(ncol(linsum),maxresm,99999).
compute resultm2(:,1)=t(linsum).
compute resultm={resultm;resultm2}.
compute resultm2=make(1,maxresm,99999).
compute resultm2(1,1:ncol(hypest))=hypest.
compute resultm={resultm;resultm2}.
do if (outscree=1).
compute hyplabs={'Estimate',hclab,'t','p','LLCI','ULCI'}.
print/title='----------'.
print/title='Linear Combination Estimate and Hypothesis Test'/space=0.
print t(linsum)/title= 'Weight vector:'/clabels='weight'/rnames=vlabsm/format F10.4.
print hypest/title=' '/cnames=hyplabs/format= F10.4 /space=0.
do if (meansub=1).
print/title='Covariate weight(s) set to the sample mean.'.
end if.
end if.
end if.
do if (lhyprob=1).
compute notecode(notes,1) = 30.
compute notes = notes + 1.
end if.
end if.
end loop if criterr=1.
compute lastb=b.
compute lastcov=varb.
do if (criterr=0 and dototal = 1).
compute x=xtmp.
compute vlabsm={'constant';xcatlab(1:nxvls,1)}.
do if (ncs > 0).
compute x = {x,ctmp}.
compute vlabsm={vlabsm;t(covnames)}.
end if.
compute x = {ones,x}.

.
do if ( 1 =1).
compute hatmat=inv(t( x )* x )*t( x ).
compute b = hatmat* y.
compute modres=b.
do if ( 1 =1).
compute n1=nrow( x ).
compute dfres=n1-(ncol( x )).
compute sstotal = t( y -(csum( y )/n1))*( y -(csum( y )/n1)).
compute resid= y - x *b.
compute ssresid = csum((resid)&**2).
compute r2 = (sstotal-ssresid)/sstotal.
do if (r2 < 0).
compute r2=0.
end if.
compute adjr2 = 1-((1-r2)*(n1-1)/(dfres)).
compute mse=ssresid/(n1-ncol( x )).

.
compute n1=nrow( x ).
compute invXtX = inv(t( x )* x ).
compute varb = mse *invXtX.
compute k3 = ncol( x ).
compute xhc=0.
do if ( hc <> 5).
compute xhc= x.
compute hat = xhc(:,1).
loop i3=1 to nrow(xhc).
compute hat(i3,1)= xhc(i3,:)*invXtX*t(xhc(i3,:)).
end loop.
do if ( hc = 0 or hc =1).
loop i3 = 1 to k3.
compute xhc(:,i3)=xhc(:,i3)&* resid.
end loop.
end if.
do if ( hc =3 or hc =2).
loop i3=1 to k3.
compute xhc(:,i3) = ( resid &/(1-hat)&**(1/(4- hc )))&*xhc(:,i3).
end loop.
end if.
do if ( hc = 4).
compute hcmn=make(n,2,4).
compute hcmn(:,2)=(n1*hat)/k3.
loop i3= 1 to k3.
compute xhc(:,i3) = ( resid &/(1-hat)&**(rmin(hcmn)/2))&*xhc(:,i3).
end loop.
end if.
compute varb=(invXtX*t(xhc)*xhc*invXtX).
do if ( hc =1).
compute varb=(n1/(n1-ncol( x )))&*varb.
end if.
end if.
compute hclab={'se(HC0)','se(HC1)','se(HC2)','se(HC3)','se(HC4)','se'}.
compute hclab=hclab(1,( hc +1)).
compute hcflab={'F(HC0)','F(HC1)','F(HC2)','F(HC3)','F(HC4)','F'}.
compute hcflab=hcflab(1,( hc +1)).
release xhc
.
compute seb=sqrt(diag(varb)).
compute trat = b&/seb.
compute p = 2*(1-tcdf(abs(trat), (dfres))).
compute tval = sqrt(dfres* (exp((dfres-(5/6))*((xp2/(dfres-(2/3)+(.11/dfres)))* (xp2/(dfres-(2/3)+(.11/dfres)))))-1)).
compute modres={modres,seb,trat,p}.
compute modres={modres,(b-tval&*seb),(b+tval&*seb)}.
compute modresl={'coeff',hclab,'t','p','LLCI','ULCI'}.
compute lmat = ident(ncol( x )).
compute lmat = lmat(:,2:ncol(lmat)).
compute fratio = (t(t(lmat)*b)*inv(t(lmat)*varb*lmat)*((t(lmat)*b)))/(ncol( x )-1).
compute pfr = 1-fcdf(fratio,(ncol( x )-1),dfres).
compute modsum={sqrt(r2),r2,mse,fratio,(ncol( x )-1),dfres,pfr}.
compute modsuml={'R','R-sq','MSE',hcflab,'df1','df2', 'p'}.
end if.
end if.
do if ( 1 = 2 or 1 =3).
compute xlp= x.
compute ylp= y.
compute pt2 = make(nrow(ylp),1,(csum(ylp)/nrow(ylp))).
do if ( 1 =2).
compute LL3 = ylp&*ln(pt2)+(1-ylp)&*ln(1-pt2).
end if.
compute LL3 = -2*csum(LL3).
compute bt1 = make(ncol(xlp),1,0).
compute LL1 = 0.
compute pt1 = make(nrow(ylp),1,0.5).
compute pt1lp=pt1.
loop jjj = 1 to iterate.
compute xlptmp=t(xlp).
compute vecprb=pt1lp&*(1-pt1lp).
loop kkk=1 to ncol(xlp).
compute xlptmp(kkk,:)=xlptmp(kkk,:)&*t(vecprb).
end loop.
compute b = bt1+inv(xlptmp*xlp)*t(xlp)*(ylp-pt1lp).
do if ( 1 =2).
compute xlpb=xlp*b.
compute xlpbt=(xlpb > -709.7).
compute xlpb709=(1-xlpbt)*(-709.7).
compute xlpb=(xlpb&*xlpbt)+xlpb709.
compute pt1lp = 1/(1+exp(-(xlpb))).
end if.
compute itprob = csum((pt1lp < .00000001) or (pt1lp > .9999999)).
do if (itprob > 0).
loop kkk = 1 to nrow(pt1lp).
do if (pt1lp(kkk,1) > .9999999).
compute pt1lp(kkk,1) = .9999999.
end if.
do if (pt1lp(kkk,1) < .00000001).
compute pt1lp(kkk,1) = .00000001.
end if.
end loop.
compute itprob = 0.
end if.
do if (itprob = 0).
do if ( 1 =2).
compute LL = ylp&*ln(pt1lp)+(1-ylp)&*ln(1-pt1lp).
end if.
compute LL2 = -2*csum(ll).
end if.
do if (abs(LL1-LL2) < converge).
do if ( 1 =1).
compute xlptmp=t(xlp).
compute vecprb=pt1lp&*(1-pt1lp).
loop kkk=1 to ncol(xlp).
compute xlptmp(kkk,:)=xlptmp(kkk,:)&*t(vecprb).
end loop.
compute varb = inv(xlptmp*xlp).
compute seb = sqrt(diag(varb)).
release xlptmp.
end if.
break.
end if.
compute bt1 = b.
compute LL1 = LL2.
end loop.
compute modres=b.
do if (jjj > iterate).
compute itprob = 2.
do if (booting=0).
compute iterrmod=1.
end if.
do if (booting=1).
compute bootiter=1.
end if.
do if (itprobtg=0).
compute itprobtg=1.
compute errcode(errs,1) = 47.
compute errs = errs + 1.
do if (booting = 0 and 1 =1).
compute vt1 = mdiag(pt1lp&*(1-pt1lp)).
compute varb = inv(t(xlp)*vt1*xlp).
compute seb = sqrt(diag(varb)).
end if.
end if.
end if.
do if ( 1 =1).
compute trat = b&/seb.
compute dfres=nrow(xlp).
compute p = 2*(1-cdfnorm(abs(trat))).
compute modres={modres,seb,trat,p}.
compute modres={modres,(b-xp2&*seb),(b+xp2&*seb)}.
compute pvchi=1-chicdf((LL3-LL2),(nrow(modres)-1)).
compute mcF = (LL3-LL2)/LL3.
compute cox = 1-exp(-(LL3-LL2)/nrow(xlp)).
compute nagel = cox/(1-exp(-(LL3)/nrow(xlp))).
compute modsum={LL2,(LL3-LL2),(nrow(modres)-1),pvchi, mcF,cox,nagel}.
compute modsuml={'-2LL','ModelLL', 'df', 'p', 'McFadden', 'CoxSnell', 'Nagelkrk'}.
compute modresl={'coeff','se','Z','p','LLCI','ULCI'}.
end if.
end if
.
compute toteff=modres(2:(1+nxvls),:).
compute nodotot=0.
do if (xdich=1 and xmint=1 and model=74).
compute toteff(:,1)=toteff(:,1)*xscaling.
compute toteff(:,2)=toteff(:,2)*abs(xscaling).
compute toteff(:,3)=toteff(:,3)*xscaling.
compute toteff(:,5)=toteff(:,5)*xscaling.
compute toteff(:,6)=toteff(:,6)*xscaling.
compute citmp=toteff(:,5:6).
do if (xscaling < 0).
compute toteff(:,5)=citmp(:,2).
compute toteff(:,6)=citmp(:,1).
end if.
compute nodotot=1.
end if.
compute totefflb=modresl.
do if (outscree=1).
do if (nodotot=0).
print/title = '************************** TOTAL EFFECT MODEL ****************************'.
end if.
do if (nodotot=1).
print/title = '**************************************************************************'.
end if.
print outnames(1,ncol(outnames))/title = 'OUTCOME VARIABLE:'/format = A8/space=0.
end if.
compute toteffl2=vlabsm(2:(1+nxvls),:).
compute resultm2=make(1,maxresm,99999).
compute resultm2(1,1:ncol(modsum))=modsum.
compute resultm={resultm;resultm2}.
compute resultm2=make(nrow(modres),maxresm,99999).
compute resultm2(1:nrow(modres),1:ncol(modres))=modres.
compute resultm={resultm;resultm2}.
do if (outscree=1).
print modsum/title = 'Model Summary'/cnames = modsuml/format= F10.4.
print modres/title='Model'/rnames=vlabsm/cnames=modresl/format= F10.4.
end if.
compute lmat=make(nrow(b),1,0).
compute lmat2=make(nxvls,1,1).
compute lmat(2:(1+nxvls),1)=lmat2.
do if (ydich <> 1).

.
compute lmat2= lmat.
do if ( 0 =0).
compute lmat2 = mdiag( lmat ).
compute lmat3=make(nrow(lmat2),1,0).
loop flp=1 to ncol(lmat2).
do if (csum(lmat2(:,flp))=1).
compute lmat3={lmat3,lmat2(:,flp)}.
end if.
end loop.
compute lmat2=lmat3(:,2:ncol(lmat3)).
end if.
compute fratio = (t(t(lmat2)* b )*inv(t(lmat2)* varb *lmat2)*((t(lmat2)* b )))/ncol(lmat2).
compute pfr = 1-fcdf(fratio,ncol(lmat2),(n-nrow( b ))).
compute fresult={fratio,ncol(lmat2),(n-nrow( b )),pfr}.
do if (i = (nms+nys) and (ydich=1)).
compute fratio=fratio*ncol(lmat2).
compute pfr=1-chicdf(fratio,ncol(lmat2)).
compute fresult={fratio,ncol(lmat2),pfr}.
end if.
do if ( 1 =1).
compute lmat3=1-rsum(lmat2).
compute xfm=make(n,csum(lmat3),0).
compute flpc=1.
loop flp=1 to nrow(lmat3).
do if (lmat3(flp,1)=1).
compute xfm(:,flpc)=x(:,flp).
compute flpc=flpc+1.
end if.
end loop.
compute bfm=inv(t(xfm)*xfm)*t(xfm)*y.
compute resid=y-(xfm*bfm).
compute sstotal=(y-(csum(y)/n)).
compute sstotal=csum(sstotal&*sstotal).
compute ssresid=csum(resid&*resid).
compute rsqch= r2 -((sstotal-ssresid)/sstotal).
compute fresult={rsqch,fresult}.
release xfm,flpc, resid, ssresid, bfm.
end if
.
compute totomni=fresult.
end if.
do if (stand=1).
compute predsd=make(nrow(modres),1,0).
compute stdmod=modres(:,1)&/ovsd(1,ncol(ovsd)).
loop jd=1 to ncol(x).
compute descdat=x(:,jd).
compute predsd(jd,1) = (nrow(descdat)*sscp(descdat))-(t(csum(descdat))*(csum(descdat))).
compute predsd(jd,1) = sqrt(predsd(jd,1)/(nrow(descdat)*(nrow(descdat)-1))).
end loop.
do if (wherex(1,ncol(wherex)) <> -999 and ((nxvls > 1) or (xdich=1))).
compute sdmsone=make(nxvls,1,1).
compute predsd(wherex(1,ncol(wherex)):wherex(2,ncol(wherex)),1)=sdmsone.
compute pstog=1.
end if.
compute predsd(1,1)=1.
compute stdmod=stdmod&*predsd.
compute stdmod=stdmod(2:nrow(stdmod),1).
compute sdvlabs=vlabsm(2:nrow(vlabsm),1).
compute resultm2=make(nrow(stdmod),maxresm,99999).
compute resultm2(1:nrow(stdmod),1:ncol(stdmod))=stdmod.
compute resultm={resultm;resultm2}.
do if (outscree=1).
print stdmod/title='Standardized coefficients'/clabels='coeff'/rnames=sdvlabs/format= F10.4.
end if.
end if.
do if (covcoeff=1).
do if (outscree=1).
print varb/title='Covariance matrix of regression parameter estimates:'/rnames=vlabsm/cnames=vlabsm/format= F10.4.
end if.
compute resultm2=make(nrow(varb),maxresm,99999).
do if (ncol(varb) <= maxresm).
compute resultm2(1:nrow(varb),1:ncol(varb))=varb.
compute resultm={resultm;resultm2}.
end if.
do if (ncol(varb) > maxresm).
compute resultmt=make(nrow(resultm),ncol(varb),99999).
compute resultmt(1:nrow(resultm),1:ncol(resultm))=resultm.
compute resultm=resultmt.
compute resultm2=make(nrow(varb),ncol(resultm),99999).
compute resultm2(1:nrow(varb),1:ncol(varb))=varb.
compute resultm={resultm;resultm2}.
compute maxresm=ncol(resultm).
end if.
end if.
end if.
end if.
do if (criterr=0 and nms > 0 and ydich=0 and modelres=1).
compute modresid=modresid(:,2:ncol(modresid)).
compute sigmatal = (t(modresid)*(ident(n)-(1/n)*ones*t(ones))*modresid)*(1/(n-1)).
compute sdall = mdiag(1/sqrt(diag(sigmatal))).
compute corall=sdall*sigmatal*t(sdall).
compute resultm2=make(nrow(corall),maxresm,99999).
compute resultm2(1:nrow(corall),1:ncol(corall))=corall.
compute resultm={resultm;resultm2}.
do if (outscree=1).
print/title = '****************** CORRELATIONS BETWEEN MODEL RESIDUALS ******************'.
print corall/title=' '/format= F10.4 / cnames=outnames/rnames=outnames/space=0.
end if.
end if.
do if (criterr=0 and boot > 0).
compute bootres=make(1,rsum(nump),-999).
compute bootdir=obsdirfx.
compute natdirbt=make(1,nxvls,-999).
do if (effsize=1).
compute bootysd=make(1,1,-999).
compute bootxsd=make(1,1,-999).
end if.
compute badboot=0.
compute goodboot=0.
compute smallest=1.
compute booting=1.
loop j = 1 to maxboot.
compute nobootx=1.
compute modres2=999.
compute v=trunc(uniform(n,1)*n)+1.
compute bad=0.
loop i = 1 to (nms+nys).
compute y=outvars(v,i).
compute ynovar= (nrow(y)*sscp(y))-(t(csum(y))*(csum(y))).
do if (ynovar = 0).
compute bad=1.
end if.
compute xindx=datindx(1:(nump(1,i)-1),i).
compute hello=0.
compute x = fulldat(v,xindx).
compute x={ones,x}.
compute xsq=t(x)*x.
compute exsq=eval(xsq).
release xsq.
compute holymoly=cmin(exsq).
compute zeroeig=csum(exsq <= 0.000000000002).
compute bad=bad + (zeroeig > 0).

.
compute desctmp=make((8-(4* 1 )),ncol( y ),-999).
loop jd=1 to ncol( y ).
compute descdat= y (:,jd).
compute desctmp(1,jd) = csum(descdat)/nrow(descdat).
compute desctmp(2,jd) = (nrow(descdat)*sscp(descdat))-(t(csum(descdat))*(csum(descdat))).
compute desctmp(2,jd) = sqrt(desctmp(2,jd)/(nrow(descdat)*(nrow(descdat)-1))).
compute desctmp(3,jd)=cmin(descdat).
compute desctmp(4,jd)=cmax(descdat).
do if ( 1 =0).
compute minwarn=0.
compute maxwarn=0.
do if ((desctmp(3,jd)=desctmp(4,jd)) and novar=0).
compute errcode(errs,1)=15.
compute errs=errs+1.
compute criterr=1.
compute novar=1.
end if.
compute tmp=((descdat(:,1)=desctmp(3,jd))+(descdat(:,1)=desctmp(4,jd))).
compute desctmp(8,jd)=(csum(tmp)=nrow(tmp)).
compute tmp = descdat.
compute tmp(GRADE(descdat),:) = descdat.
compute descdat = tmp.
release tmp.
compute decval={.16;.5;.84}.
loop kd=1 to 3.
compute low=trunc(decval(kd,1)*(nrow(descdat)+1)).
compute lowdec=decval(kd,1)*(nrow(descdat)+1)-low.
compute value=descdat(low,1)+(descdat((low+1),1)-descdat(low,1))*lowdec.
compute desctmp((4+kd),jd)=value.
end loop.
compute mnotev=1.
compute modvals=desctmp(5:7,:).
do if (quantile <> 1).
compute desctmp(5,jd)=desctmp(1,jd)-desctmp(2,jd).
compute desctmp(6,jd)=desctmp(1,jd).
compute desctmp(7,jd)=desctmp(1,jd)+desctmp(2,jd).
compute modvals=desctmp(5:7,:).
compute mnotev=2.
do if (modvals(1,1) < desctmp(3,1)).
compute modvals(1,1)=desctmp(3,1).
compute minwarn=1.
end if.
do if (modvals(3,1) > desctmp(4,1)).
compute modvals(3,1)=desctmp(4,1).
compute maxwarn=1.
end if.
end if.
do if (desctmp(8,1)=1).
compute modvals={desctmp(3,1);desctmp(4,1)}.
compute mnotev=0.
compute minwarn=0.
compute maxwarn=0.
end if.
end if.
end loop
.
compute bad=bad+ (desctmp(2,1) <= 0.00000000001).
do if (bad = 0).
do if (holymoly < smallest).
compute smallest=holymoly.
end if.
do if (ydich=0 or (i < (nms+nys))).

.
do if ( 1 =1).
compute hatmat=inv(t( x )* x )*t( x ).
compute b = hatmat* y.
compute modres=b.
do if ( 0 =1).
compute n1=nrow( x ).
compute dfres=n1-(ncol( x )).
compute sstotal = t( y -(csum( y )/n1))*( y -(csum( y )/n1)).
compute resid= y - x *b.
compute ssresid = csum((resid)&**2).
compute r2 = (sstotal-ssresid)/sstotal.
do if (r2 < 0).
compute r2=0.
end if.
compute adjr2 = 1-((1-r2)*(n1-1)/(dfres)).
compute mse=ssresid/(n1-ncol( x )).

.
compute n1=nrow( x ).
compute invXtX = inv(t( x )* x ).
compute varb = mse *invXtX.
compute k3 = ncol( x ).
compute xhc=0.
do if ( hc <> 5).
compute xhc= x.
compute hat = xhc(:,1).
loop i3=1 to nrow(xhc).
compute hat(i3,1)= xhc(i3,:)*invXtX*t(xhc(i3,:)).
end loop.
do if ( hc = 0 or hc =1).
loop i3 = 1 to k3.
compute xhc(:,i3)=xhc(:,i3)&* resid.
end loop.
end if.
do if ( hc =3 or hc =2).
loop i3=1 to k3.
compute xhc(:,i3) = ( resid &/(1-hat)&**(1/(4- hc )))&*xhc(:,i3).
end loop.
end if.
do if ( hc = 4).
compute hcmn=make(n,2,4).
compute hcmn(:,2)=(n1*hat)/k3.
loop i3= 1 to k3.
compute xhc(:,i3) = ( resid &/(1-hat)&**(rmin(hcmn)/2))&*xhc(:,i3).
end loop.
end if.
compute varb=(invXtX*t(xhc)*xhc*invXtX).
do if ( hc =1).
compute varb=(n1/(n1-ncol( x )))&*varb.
end if.
end if.
compute hclab={'se(HC0)','se(HC1)','se(HC2)','se(HC3)','se(HC4)','se'}.
compute hclab=hclab(1,( hc +1)).
compute hcflab={'F(HC0)','F(HC1)','F(HC2)','F(HC3)','F(HC4)','F'}.
compute hcflab=hcflab(1,( hc +1)).
release xhc
.
compute seb=sqrt(diag(varb)).
compute trat = b&/seb.
compute p = 2*(1-tcdf(abs(trat), (dfres))).
compute tval = sqrt(dfres* (exp((dfres-(5/6))*((xp2/(dfres-(2/3)+(.11/dfres)))* (xp2/(dfres-(2/3)+(.11/dfres)))))-1)).
compute modres={modres,seb,trat,p}.
compute modres={modres,(b-tval&*seb),(b+tval&*seb)}.
compute modresl={'coeff',hclab,'t','p','LLCI','ULCI'}.
compute lmat = ident(ncol( x )).
compute lmat = lmat(:,2:ncol(lmat)).
compute fratio = (t(t(lmat)*b)*inv(t(lmat)*varb*lmat)*((t(lmat)*b)))/(ncol( x )-1).
compute pfr = 1-fcdf(fratio,(ncol( x )-1),dfres).
compute modsum={sqrt(r2),r2,mse,fratio,(ncol( x )-1),dfres,pfr}.
compute modsuml={'R','R-sq','MSE',hcflab,'df1','df2', 'p'}.
end if.
end if.
do if ( 1 = 2 or 1 =3).
compute xlp= x.
compute ylp= y.
compute pt2 = make(nrow(ylp),1,(csum(ylp)/nrow(ylp))).
do if ( 1 =2).
compute LL3 = ylp&*ln(pt2)+(1-ylp)&*ln(1-pt2).
end if.
compute LL3 = -2*csum(LL3).
compute bt1 = make(ncol(xlp),1,0).
compute LL1 = 0.
compute pt1 = make(nrow(ylp),1,0.5).
compute pt1lp=pt1.
loop jjj = 1 to iterate.
compute xlptmp=t(xlp).
compute vecprb=pt1lp&*(1-pt1lp).
loop kkk=1 to ncol(xlp).
compute xlptmp(kkk,:)=xlptmp(kkk,:)&*t(vecprb).
end loop.
compute b = bt1+inv(xlptmp*xlp)*t(xlp)*(ylp-pt1lp).
do if ( 1 =2).
compute xlpb=xlp*b.
compute xlpbt=(xlpb > -709.7).
compute xlpb709=(1-xlpbt)*(-709.7).
compute xlpb=(xlpb&*xlpbt)+xlpb709.
compute pt1lp = 1/(1+exp(-(xlpb))).
end if.
compute itprob = csum((pt1lp < .00000001) or (pt1lp > .9999999)).
do if (itprob > 0).
loop kkk = 1 to nrow(pt1lp).
do if (pt1lp(kkk,1) > .9999999).
compute pt1lp(kkk,1) = .9999999.
end if.
do if (pt1lp(kkk,1) < .00000001).
compute pt1lp(kkk,1) = .00000001.
end if.
end loop.
compute itprob = 0.
end if.
do if (itprob = 0).
do if ( 1 =2).
compute LL = ylp&*ln(pt1lp)+(1-ylp)&*ln(1-pt1lp).
end if.
compute LL2 = -2*csum(ll).
end if.
do if (abs(LL1-LL2) < converge).
do if ( 0 =1).
compute xlptmp=t(xlp).
compute vecprb=pt1lp&*(1-pt1lp).
loop kkk=1 to ncol(xlp).
compute xlptmp(kkk,:)=xlptmp(kkk,:)&*t(vecprb).
end loop.
compute varb = inv(xlptmp*xlp).
compute seb = sqrt(diag(varb)).
release xlptmp.
end if.
break.
end if.
compute bt1 = b.
compute LL1 = LL2.
end loop.
compute modres=b.
do if (jjj > iterate).
compute itprob = 2.
do if (booting=0).
compute iterrmod=1.
end if.
do if (booting=1).
compute bootiter=1.
end if.
do if (itprobtg=0).
compute itprobtg=1.
compute errcode(errs,1) = 47.
compute errs = errs + 1.
do if (booting = 0 and 0 =1).
compute vt1 = mdiag(pt1lp&*(1-pt1lp)).
compute varb = inv(t(xlp)*vt1*xlp).
compute seb = sqrt(diag(varb)).
end if.
end if.
end if.
do if ( 0 =1).
compute trat = b&/seb.
compute dfres=nrow(xlp).
compute p = 2*(1-cdfnorm(abs(trat))).
compute modres={modres,seb,trat,p}.
compute modres={modres,(b-xp2&*seb),(b+xp2&*seb)}.
compute pvchi=1-chicdf((LL3-LL2),(nrow(modres)-1)).
compute mcF = (LL3-LL2)/LL3.
compute cox = 1-exp(-(LL3-LL2)/nrow(xlp)).
compute nagel = cox/(1-exp(-(LL3)/nrow(xlp))).
compute modsum={LL2,(LL3-LL2),(nrow(modres)-1),pvchi, mcF,cox,nagel}.
compute modsuml={'-2LL','ModelLL', 'df', 'p', 'McFadden', 'CoxSnell', 'Nagelkrk'}.
compute modresl={'coeff','se','Z','p','LLCI','ULCI'}.
end if.
end if
.
end if.
do if (ydich=1 and (i = (nms+nys))).

.
do if ( 2 =1).
compute hatmat=inv(t( x )* x )*t( x ).
compute b = hatmat* y.
compute modres=b.
do if ( 0 =1).
compute n1=nrow( x ).
compute dfres=n1-(ncol( x )).
compute sstotal = t( y -(csum( y )/n1))*( y -(csum( y )/n1)).
compute resid= y - x *b.
compute ssresid = csum((resid)&**2).
compute r2 = (sstotal-ssresid)/sstotal.
do if (r2 < 0).
compute r2=0.
end if.
compute adjr2 = 1-((1-r2)*(n1-1)/(dfres)).
compute mse=ssresid/(n1-ncol( x )).

.
compute n1=nrow( x ).
compute invXtX = inv(t( x )* x ).
compute varb = mse *invXtX.
compute k3 = ncol( x ).
compute xhc=0.
do if ( hc <> 5).
compute xhc= x.
compute hat = xhc(:,1).
loop i3=1 to nrow(xhc).
compute hat(i3,1)= xhc(i3,:)*invXtX*t(xhc(i3,:)).
end loop.
do if ( hc = 0 or hc =1).
loop i3 = 1 to k3.
compute xhc(:,i3)=xhc(:,i3)&* resid.
end loop.
end if.
do if ( hc =3 or hc =2).
loop i3=1 to k3.
compute xhc(:,i3) = ( resid &/(1-hat)&**(1/(4- hc )))&*xhc(:,i3).
end loop.
end if.
do if ( hc = 4).
compute hcmn=make(n,2,4).
compute hcmn(:,2)=(n1*hat)/k3.
loop i3= 1 to k3.
compute xhc(:,i3) = ( resid &/(1-hat)&**(rmin(hcmn)/2))&*xhc(:,i3).
end loop.
end if.
compute varb=(invXtX*t(xhc)*xhc*invXtX).
do if ( hc =1).
compute varb=(n1/(n1-ncol( x )))&*varb.
end if.
end if.
compute hclab={'se(HC0)','se(HC1)','se(HC2)','se(HC3)','se(HC4)','se'}.
compute hclab=hclab(1,( hc +1)).
compute hcflab={'F(HC0)','F(HC1)','F(HC2)','F(HC3)','F(HC4)','F'}.
compute hcflab=hcflab(1,( hc +1)).
release xhc
.
compute seb=sqrt(diag(varb)).
compute trat = b&/seb.
compute p = 2*(1-tcdf(abs(trat), (dfres))).
compute tval = sqrt(dfres* (exp((dfres-(5/6))*((xp2/(dfres-(2/3)+(.11/dfres)))* (xp2/(dfres-(2/3)+(.11/dfres)))))-1)).
compute modres={modres,seb,trat,p}.
compute modres={modres,(b-tval&*seb),(b+tval&*seb)}.
compute modresl={'coeff',hclab,'t','p','LLCI','ULCI'}.
compute lmat = ident(ncol( x )).
compute lmat = lmat(:,2:ncol(lmat)).
compute fratio = (t(t(lmat)*b)*inv(t(lmat)*varb*lmat)*((t(lmat)*b)))/(ncol( x )-1).
compute pfr = 1-fcdf(fratio,(ncol( x )-1),dfres).
compute modsum={sqrt(r2),r2,mse,fratio,(ncol( x )-1),dfres,pfr}.
compute modsuml={'R','R-sq','MSE',hcflab,'df1','df2', 'p'}.
end if.
end if.
do if ( 2 = 2 or 2 =3).
compute xlp= x.
compute ylp= y.
compute pt2 = make(nrow(ylp),1,(csum(ylp)/nrow(ylp))).
do if ( 2 =2).
compute LL3 = ylp&*ln(pt2)+(1-ylp)&*ln(1-pt2).
end if.
compute LL3 = -2*csum(LL3).
compute bt1 = make(ncol(xlp),1,0).
compute LL1 = 0.
compute pt1 = make(nrow(ylp),1,0.5).
compute pt1lp=pt1.
loop jjj = 1 to iterate.
compute xlptmp=t(xlp).
compute vecprb=pt1lp&*(1-pt1lp).
loop kkk=1 to ncol(xlp).
compute xlptmp(kkk,:)=xlptmp(kkk,:)&*t(vecprb).
end loop.
compute b = bt1+inv(xlptmp*xlp)*t(xlp)*(ylp-pt1lp).
do if ( 2 =2).
compute xlpb=xlp*b.
compute xlpbt=(xlpb > -709.7).
compute xlpb709=(1-xlpbt)*(-709.7).
compute xlpb=(xlpb&*xlpbt)+xlpb709.
compute pt1lp = 1/(1+exp(-(xlpb))).
end if.
compute itprob = csum((pt1lp < .00000001) or (pt1lp > .9999999)).
do if (itprob > 0).
loop kkk = 1 to nrow(pt1lp).
do if (pt1lp(kkk,1) > .9999999).
compute pt1lp(kkk,1) = .9999999.
end if.
do if (pt1lp(kkk,1) < .00000001).
compute pt1lp(kkk,1) = .00000001.
end if.
end loop.
compute itprob = 0.
end if.
do if (itprob = 0).
do if ( 2 =2).
compute LL = ylp&*ln(pt1lp)+(1-ylp)&*ln(1-pt1lp).
end if.
compute LL2 = -2*csum(ll).
end if.
do if (abs(LL1-LL2) < converge).
do if ( 0 =1).
compute xlptmp=t(xlp).
compute vecprb=pt1lp&*(1-pt1lp).
loop kkk=1 to ncol(xlp).
compute xlptmp(kkk,:)=xlptmp(kkk,:)&*t(vecprb).
end loop.
compute varb = inv(xlptmp*xlp).
compute seb = sqrt(diag(varb)).
release xlptmp.
end if.
break.
end if.
compute bt1 = b.
compute LL1 = LL2.
end loop.
compute modres=b.
do if (jjj > iterate).
compute itprob = 2.
do if (booting=0).
compute iterrmod=1.
end if.
do if (booting=1).
compute bootiter=1.
end if.
do if (itprobtg=0).
compute itprobtg=1.
compute errcode(errs,1) = 47.
compute errs = errs + 1.
do if (booting = 0 and 0 =1).
compute vt1 = mdiag(pt1lp&*(1-pt1lp)).
compute varb = inv(t(xlp)*vt1*xlp).
compute seb = sqrt(diag(varb)).
end if.
end if.
end if.
do if ( 0 =1).
compute trat = b&/seb.
compute dfres=nrow(xlp).
compute p = 2*(1-cdfnorm(abs(trat))).
compute modres={modres,seb,trat,p}.
compute modres={modres,(b-xp2&*seb),(b+xp2&*seb)}.
compute pvchi=1-chicdf((LL3-LL2),(nrow(modres)-1)).
compute mcF = (LL3-LL2)/LL3.
compute cox = 1-exp(-(LL3-LL2)/nrow(xlp)).
compute nagel = cox/(1-exp(-(LL3)/nrow(xlp))).
compute modsum={LL2,(LL3-LL2),(nrow(modres)-1),pvchi, mcF,cox,nagel}.
compute modsuml={'-2LL','ModelLL', 'df', 'p', 'McFadden', 'CoxSnell', 'Nagelkrk'}.
compute modresl={'coeff','se','Z','p','LLCI','ULCI'}.
end if.
end if
.
end if.
compute modres2={modres2,t(modres)}.
do if (i = (nms+nys)).
do if (bcmat((i+1),1) = 1).
compute bootdir={bootdir;t(modres(wherex(1,i):wherex(2,i),1))}.
end if.
do if (bcmat((i+1),1) = 0).
compute bootdir={bootdir;dirzes}.
end if.
end if.
do if (model = 74 and i <= nms).
compute onetemp=make(nrow(xprobval),1,1).
compute mestmtb={onetemp,xprobval}.
do if (ncs > 0).
compute ncovmdlb=rsum(ccmat(i,:)).
do if (ncovmdl > 0).
compute cvmncb=make(nrow(mestmtb),ncovmdlb,1).
compute cvmnctmp=csum(x(:,(ncol(x)-ncovmdlb+1):ncol(x)))/nrow(x).
do if (cuscoval > 0).
compute cvmnctmp=coval.
end if.
loop mestlp=1 to ncovmdlb.
compute cvmncb(:,mestlp)=cvmncb(:,mestlp)*cvmnctmp(:,mestlp).
end loop.
compute mestmtb={mestmtb,cvmncb}.
end if.
end if.
compute mestbt=mestmtb*modres.
do if (i = 1).
compute mestb=mestbt.
end if.
do if (i > 1).
compute mestb={mestb,mestbt}.
end if.
end if.
do if (model = 74 and i = (nms+nys)).
compute xvalptmp=make(1,nxvls,0).
compute mest74sp=make(1,nms,0).
compute mest74sp={mest74sp;mestb}.
compute xvalptmp={xvalptmp;ident(nxvls)}.
compute dirfxcf=make(nrow(mestb),1,0).
loop cfloop1=1 to nrow(mestb).
compute ndirfx=make(nrow(modres),1,0).
compute ndirfx(2:(nxvls+1),1)=t(xvalptmp(cfloop1,:)).
loop cfloop3=1 to nms.
do if (mcx=1 or mcx=0).
compute ndirfx(wheremw((1+((cfloop3-1)*2)),ncol(wheremw)):wheremw((2+((cfloop3-1)*2) ),ncol(wheremw)))=t(xvalptmp(cfloop1,:)*mestb(1,cfloop3)).
end if.
do if (mcx=2).
compute ndirfx(wheremw((1+((cfloop3-1)*2)),ncol(wheremw)):wheremw((2+((cfloop3-1)*2) ),ncol(wheremw)))=t(xvalptmp(cfloop1,:)*mest74sp(cfloop1,cfloop3)).
end if.
end loop.
do if (cfloop1 > 1).
compute dirfxcf(cfloop1,1)=t(ndirfx)*modres*xscaling.
end if.
end loop.
compute natdirbt={natdirbt;t(dirfxcf(2:nrow(dirfxcf),:))}.
end if.
do if ( (bcmat((i+1),1) = 1) and (nobootx=1) and (effsize=1)).
compute nobootx=0.
compute xsdtemp= (nrow(x)*sscp(x(:,2)))-(t(csum(x(:,2)))*(csum(x(:,2)))).
compute xsdtemp= sqrt(xsdtemp/(nrow(x)*(nrow(x)-1))).
end if.
end if.
end loop.
do if (bad = 0).
compute bootres={bootres;modres2(:,2:ncol(modres2))}.
do if (effsize=1).
compute ysdtemp= (nrow(y)*sscp(y))-(t(csum(y))*(csum(y))).
compute ysdtemp= sqrt(ysdtemp/(nrow(y)*(nrow(y)-1))).
compute bootysd={bootysd;ysdtemp}.
compute bootxsd={bootxsd;xsdtemp}.
end if.
compute goodboot=goodboot+1.
end if.
do if (bad <> 0).
compute badboot=badboot+1.
end if.
end loop if (goodboot = boot).
compute bootres=bootres(2:nrow(bootres),:).
do if (effsize=1).
compute bootysd=bootysd(2:nrow(bootysd),:).
do if (nrow(bootxsd) > 1).
compute bootxsd=bootxsd(2:nrow(bootxsd),:).
end if.
end if.
do if (goodboot < (boot)).
compute boot=0.
compute modelbt=0.
compute notecode(notes,1) = 7.
compute notes = notes + 1.
end if.
do if (boot > 0).
do if (effsize=1).
compute bootysd={ysd;bootysd}.
compute bootxsd={xsd;bootxsd}.
end if.
do if (saveboot = 1).
save bootres/outfile = *.
end if.
do if (modelbt=1).
compute bootcim=make(ncol(bootres),5,-99999).
compute bootcim(:,2) = t(csum(bootres)/nrow(bootres)).
compute bootcim(:,1) = coeffmat(2:nrow(coeffmat),1).
loop i = 1 to ncol(bootres).

.
compute temp = bootres(:,i).
compute temp(GRADE( bootres(:,i) )) = bootres(:,i).
compute badlo = 0.
compute badhi = 0.
do if ( (bootcim(i,1)*bc)+(9999*(1-bc)) <> 9999).
compute pv=csum(temp < (bootcim(i,1)*bc)+(9999*(1-bc)) )/nrow(temp).
compute ppv = pv.
do if (pv > .5).
compute ppv = 1-pv.
end if.
compute y5=sqrt(-2*ln(ppv)).
compute xp=y5+((((y5*p4+p3)*y5+p2)*y5+p1)*y5+p0)/((((y5*q4+q3)*y5+q2)*y5+q1)*y5+q0).
do if (pv <= .5).
compute xp = -xp.
end if.
compute cilow=rnd(nrow(temp)*(cdfnorm(2*xp-xp2))).
compute cihigh=trunc(nrow(temp)*(cdfnorm(2*xp+(xp2))))+1.
do if (cilow < 1).
compute cilow = 1.
compute booterr=1.
compute badlo = 1.
end if.
do if (cihigh > nrow(temp)).
compute cihigh = boot.
compute booterr=1.
compute badhi = 1.
end if.
compute llcit=temp(cilow,1).
compute ulcit=temp(cihigh,1).
do if (badlo = 1 and llcit <> priorlo).
compute badend={badend, llcit}.
compute priorlo = llcit.
end if.
do if (badhi = 1 and ulcit <> priorhi).
compute badend={badend, ulcit}.
compute priorhi = ulcit.
end if.
end if.
do if ( (bootcim(i,1)*bc)+(9999*(1-bc)) = 9999).
compute llcit=temp(cilow,1).
compute ulcit=temp(cihigh,1).
end if.
compute bootse=t(sqrt((cssq(temp)-((csum(temp)&**2)/nrow(temp)))/(nrow(temp)-1)))
.
compute bootcim(i,4:5)={llcit,ulcit}.
compute bootcim(i,3)=bootse.
end loop.
end if.
end if.
do if (badboot > 0).
compute notecode(notes,1) = 6.
compute notes = notes + 1.
end if.
end if.
do if (criterr=0).
release fulldat,x,y.
end if.
do if (xmint=1 and criterr=0).
compute xvalptmp=make(1,nxvls,0).
compute mest74sp=make(1,nms,0).
compute mest74sp={mest74sp;mest74}.
compute xvalptmp={xvalptmp;ident(nxvls)}.
loop kcfuhd=1 to 2.
compute dirfxcf=make(nrow(mest74),6,0).
loop cfloop1=1 to nrow(mest74).
compute ndirfx=make(nrow(lastb),1,0).
compute ndirfx(2:(nxvls+1),1)=t(xvalptmp(cfloop1,:)).
loop cfloop3=1 to nms.
do if (kcfuhd=1).
do if (mcx=1 or mcx=0).
compute ndirfx(wheremw((1+((cfloop3-1)*2)),ncol(wheremw)):wheremw((2+((cfloop3-1)*2)), ncol(wheremw)))=t(xvalptmp(cfloop1,:)*mest74(1,cfloop3)).
end if.
do if (mcx=2).
compute ndirfx(wheremw((1+((cfloop3-1)*2)),ncol(wheremw)):wheremw((2+((cfloop3-1)*2)), ncol(wheremw)))=t(xvalptmp(cfloop1,:)*mest74sp(cfloop1,cfloop3)).
end if.
end if.
do if (kcfuhd=2).
compute ndirfx(wheremw((1+((cfloop3-1)*2)),ncol(wheremw)):wheremw((2+((cfloop3-1)*2)), ncol(wheremw)))=t(xvalptmp(cfloop1,:)*medmeans(1,cfloop3)).
end if.
end loop.
do if (cfloop1 > 1).
compute dirfxcf(cfloop1,1)=t(ndirfx)*lastb*xscaling.
compute dirfxcf(cfloop1,2)=sqrt(diag(t(ndirfx)*lastcov*ndirfx))*abs(xscaling).
compute dirfxcf(cfloop1,3)=dirfxcf(cfloop1,1)/dirfxcf(cfloop1,2).
compute dirfxcf(cfloop1,4)=2*(1-tcdf(abs(dirfxcf(cfloop1,3)), dfres)).
compute dirfxcf(cfloop1,5)=dirfxcf(cfloop1,1)-tval*dirfxcf(cfloop1,2).
compute dirfxcf(cfloop1,6)=dirfxcf(cfloop1,1)+tval*dirfxcf(cfloop1,2).
end if.
end loop.
compute codireff=dirfxcf(2:nrow(dirfxcf),:).
do if (kcfuhd=1).
compute direff=codireff.
end if.
end loop.
end if.

.
do if (criterr = 0 and nms > 0).
compute paths=paths(:,2:ncol(paths)).
compute pathsw=pathsw(:,2:ncol(pathsw)).
compute pathsz=pathsz(:,2:ncol(pathsz)).
compute pathswz=pathswz(:,2:ncol(pathswz)).
compute pathsmod=pathsw+pathsz+pathswz.
compute pathsdv=pathsdv(:,2:ncol(pathsdv)).
compute pathsfoc=pathsfoc(:,2:ncol(pathsfoc)).
compute pathtype=pathtype(:,2:ncol(pathtype)).
compute anymod=(rsum(pathsmod) > 0).
compute obscoeff=obscoeff(1,2:ncol(obscoeff)).
do if (outscree=1).
do if (xmint=1).
print/title = '************************* COUNTERFACTUALLY DEFINED ************************'.
else.
print/title = ' '.
end if.
do if (dototal = 0 and alttotal=0).
print/title = '****************** DIRECT AND INDIRECT EFFECTS OF X ON Y *****************'/space=0.
end if.
do if (alttotal=1).
print/title = '************** TOTAL, DIRECT, AND INDIRECT EFFECTS OF X ON Y **************'/space=0.
end if.
end if.
do if (dototal = 1).
do if (outscree=1).
print/title = '************** TOTAL, DIRECT, AND INDIRECT EFFECTS OF X ON Y **************'/space=0.
end if.
compute totefflb(1,1)='Effect'.
do if (effsize=1).
compute toteffsz=toteff(:,1)/ysd.
do if (xdich=1 or mcx > 0).
compute totefflb={totefflb,'c_ps'}.
end if.
do if (xdich = 0 and mcx = 0).
compute toteffsz=toteffsz*xsd.
compute totefflb={totefflb,'c_cs'}.
end if.
compute toteff={toteff,toteffsz}.
end if.
compute resultm2=make(nrow(toteff),maxresm,99999).
compute resultm2(1:nrow(toteff),1:ncol(toteff))=toteff.
compute resultm={resultm;resultm2}.
do if (nxvls > 1).
compute resultm2=make(nrow(totomni),maxresm,99999).
compute resultm2(1:nrow(totomni),1:ncol(totomni))=totomni.
compute resultm={resultm;resultm2}.
compute clabtmp={'R2-chng', hcflab, 'df1','df2','p'}.
do if (outscree=1).
print toteff/title='Relative total effects of X on Y'/rnames=toteffl2/cnames=totefflb/format= F10.4.
print totomni/title='Omnibus test of total effect of X on Y'/cnames=clabtmp/format= F10.4.
print/title= '----------'/space=0.
end if.
else.
do if (outscree=1).
print toteff/title='Total effect of X on Y'/cnames=totefflb/format= F10.4.
end if.
end if.
end if.
compute moddir=wcmat(nrow(bcmat),1)+zcmat(nrow(bcmat),1).
do if (xmint=1).
compute moddir=1.
end if.
do if (bcmat(nrow(bcmat),1)=1).
do if (ydich=1).
compute direfflb(:,(ncol(direfflb)-5):ncol(direfflb))={'Effect','se','Z','p','LLCI','ULCI'}.
end if.
do if (moddir=0 or xmint=1).
compute direfflb(1,1)='Effect'.
end if.
do if (effsize=1 and moddir=0 and anymod = 0).
compute direffsz=direff(:,1)/ysd.
do if (xdich=1 or mcx > 0).
compute direfflb={direfflb,'c''_ps'}.
end if.
do if (xdich = 0 and mcx = 0).
compute direffsz=direffsz*xsd.
compute direfflb={direfflb,'c''_cs'}.
end if.
compute direff={direff,direffsz}.
end if.
compute resultm2=make(nrow(direff),maxresm,99999).
compute resultm2(1:nrow(direff),1:ncol(direff))=direff.
compute resultm={resultm;resultm2}.
do if (xmint=1).
compute resultm2=make(nrow(codireff),maxresm,99999).
compute resultm2(1:nrow(codireff),1:ncol(codireff))=codireff.
compute resultm={resultm;resultm2}.
end if.
do if (moddir = 0 and nxvls=1).
do if (outscree=1).
print direff/title='Direct effect of X on Y'/cnames=direfflb/format= F10.4.
end if.
end if.
do if (moddir = 0 and nxvls>1).
compute resultm2=make(nrow(diromni),maxresm,99999).
compute resultm2(1:nrow(diromni),1:ncol(diromni))=diromni.
compute resultm={resultm;resultm2}.
do if (outscree=1).
print direff/title='Relative direct effects of X on Y'/rnames=direffl2/cnames=direfflb/format= F10.4.
do if (ydich = 0).
compute clabtmp={'R2-chng', hcflab, 'df1','df2','p'}.
print diromni/title='Omnibus test of direct effect of X on Y:'/cnames=clabtmp/format= F10.4.
end if.
do if (ydich = 1).
compute clabtmp={'Chi-sq', 'df', 'p'}.
print diromni/title='Omnibus likelihood ratio test of direct effect of X on Y'/cnames=clabtmp/format= F10.4.
end if.
print/title= '----------'/space=0.
end if.
end if.
do if (moddir > 0 and nxvls=1).
do if (xmint=0 and outscree=1).
print direff/title='Conditional direct effects of X on Y'/cnames=direfflb/format= F10.4.
end if.
do if (xmint=1).
do if (outscree=1).
print direff/title='(Pure) Natural direct effect of X on Y'/cnames=direfflb/format= F10.4.
print codireff/title='Controlled direct effect of X on Y'/cnames=direfflb/format= F10.4.
print/title= '----------'/space=0.
end if.
compute obnatdfx=direff(:,1).
end if.
end if.
compute direffl4=direffl2.
do if (moddir > 0 and nxvls>1).
compute direffl2={' '}.
loop i = 1 to nxvls.
loop j = 1 to (nrow(direff)/nxvls).
compute direffl2={direffl2;xcatlab(i,1)}.
end loop.
end loop.
compute direffl2=direffl2(2:nrow(direffl2),1).
do if (xmint=0 and outscree=1).
print direff/title='Relative conditional direct effects of X on Y'/cnames=direfflb/rnames=direffl2/format= F10.4.
end if.
do if (xmint=1).
do if (outscree=1).
print direff/title='Relative (pure) natural direct effects of X on Y'/cnames=direfflb/rnames=direffl2/format= F10.4.
print codireff/title='Relative controlled direct effects of X on Y'/cnames=direfflb/rnames=direffl2/format= F10.4.
print/title= '----------'/space=0.
end if.
compute obnatdfx=direff(:,1).
end if.
end if.
compute direffl2=direffl4.
end if.
do if (bcmat(nrow(bcmat),1)=0 and xmint <> 1 and outscree=1).
print/title='The direct effect of X on Y is fixed to zero.'.
end if.
do if (nms = 1).
compute indmark={2}.
compute indsets={1,2}.
end if.
do if (nms = 2).
compute indmark={2,2,3}.
compute indsets={1,4,2,5,1,3,5}.
compute thetam=1.
end if.
do if (nms = 3).
compute indmark={2,2,2,3,3,3,4}.
compute indsets={1,7,2,8,4,9,1,3,8,1,5,9,2,6,9,1,3,6,9}.
compute thetam={1,2,3}.
end if.
do if (nms = 4).
compute indmark={2,2,2,2,3,3,3,3,3,3,4,4,4,4,5}.
compute indsets={1,11,2,12,4,13,7,14,1,3,12,1,5,13,1,8,14,2,6,13,2,9,14,4,10,14,1,3,6,13,1,3,9, 14,1,5,10,14,2,6,10,14,1,3,6,10,14}.
compute thetam={1,2,4,3,5,6}.
end if.
do if (nms = 5).
compute indmark={2,2,2,2,2,3,3,3,3,3,3,3,3,3,3,4,4,4,4,4,4,4,4,4,4,5,5,5,5,5,6}.
compute indsets={1,16,2,17,4,18,7,19,11,20,1,3,17,1,5,18,1,8,19,1,12,20,2,6,18,2,9,19,2,13,20,4, 10,19,4,14,20,7,15,20,1,3,6,18, 1,3,9,19,1,3,13,20,1,5,10,19,1,5,14,20,1,8,15,20,2,6,10,19,2,6, 14,20,2,9,15,20,4,10,15,20,1,3,6,10,19, 1,3,6,14,20,1,3,9,15,20,1,5,10,15,20,2,6,10,15,20,1,3,6,10,15, 20}.
compute thetam={1,2,5,3,6,8,4,7,9,10}.
end if.
do if (nms = 6).
compute indmark={2,2,2,2,2,2,3,3,3,3,3,3,3,3,3,3,3,3,3,3,3,4,4,4,4,4,4,4,4,4,4,4,4,4,4,4,4,4,4, 4,4,5,5,5,5,5,5,5,5,5,5,5,5,5,5,5,6,6,6,6,6,6,7}.
compute indsets={1,22,2,23,4,24,7,25,11,26,16,27,1,3,23,1,5,24,1,8,25,1,12,26,1,17,27,2,6,24,2, 9,25,2,13,26,2,18,27,4,10,25,4,14,26, 4,19,27,7,15,26,7,20,27,11,21,27,1,3,6,24,1,3,9,25,1,3,13,26,1, 3,18,27,1,5,10,25,1,5,14,26,1,5,19,27,1,8,15,26,1,8,20,27, 1,12,21,27,2,6,10,25,2,6,14,26,2,6,19,27,2,9,15,26,2,9,20,27,2, 13,21,27,4,10,15,26,4,10,20,27,4,14,21,27,7,15,21,27, 1,3,6,10,25,1,3,6,14,26,1,3,6,19,27,1,3,9,15,26,1,3,9,20,27,1,3, 13,21,27,1,5,10,15,26,1,5,10,20,27,1,5,14,21,27, 1,8,15,21,27,2,6,10,15,26,2,6,10,20,27,2,6,14,21,27,2,9,15,21, 27,4,10,15,21,27,1,3,6,10,15,26,1,3,6,10,20,27, 1,3,6,14,21,27,1,3,9,15,21,27,1,5,10,15,21,27,2,6,10,15,21,27, 1,3,6,10,15,21,27}.
compute thetam={1,2,6,3,7,10,4,8,11,13,5,9,12,14,15}.
end if.
do if (nms = 7).
compute indmark={2,2,2,2,2,2,2}.
compute indsets={1,29,2,30,4,31,7,32,11,33,16,34,22,35}.
end if.
do if (nms = 8).
compute indmark={2,2,2,2,2,2,2,2}.
compute indsets={1,37,2,38,4,39,7,40,11,41,16,42,22,43,29,44}.
end if.
do if (nms = 9).
compute indmark={2,2,2,2,2,2,2,2,2}.
compute indsets={1,46,2,47,4,48,7,49,11,50,16,51,22,52,29,53,37,54}.
end if.
do if (nms=10).
compute indmark={2,2,2,2,2,2,2,2,2,2}.
compute indsets={1,56,2,57,4,58,7,59,11,60,16,61,22,62,29,63,37,64,46,65}.
end if.
compute indlbl = {'Ind1'; 'Ind2'; 'Ind3'; 'Ind4'; 'Ind5'; 'Ind6'; 'Ind7'; 'Ind8'; 'Ind9'; 'Ind10'; 'Ind11'; 'Ind12'; 'Ind13'; 'Ind14'; 'Ind15'}.
compute indlbl = {indlbl; 'Ind16';'Ind17';'Ind18';'Ind19';'Ind20';'Ind21';'Ind22';'Ind23';'Ind24';'Ind25';'Ind26';'Ind27';'Ind28';'Ind29';'Ind30'}.
compute indlbl = {indlbl; 'Ind31';'Ind32';'Ind33';'Ind34';'Ind35';'Ind36';'Ind37';'Ind38';'Ind39';'Ind40';'Ind41';'Ind42';'Ind43';'Ind44';'Ind45'}.
compute indlbl = {indlbl; 'Ind46';'Ind47';'Ind48';'Ind49';'Ind50';'Ind51';'Ind52';'Ind53';'Ind54';'Ind55';'Ind56';'Ind57';'Ind58';'Ind59';'Ind60'}.
compute indlbl = {indlbl; 'Ind61';'Ind62';'Ind63';'Ind64';'Ind65';'Ind66';'Ind67';'Ind68';'Ind69';'Ind70';'Ind71';'Ind72';'Ind73';'Ind74';'Ind75'}.
compute indlbl = {indlbl; 'Ind76';'Ind77';'Ind78';'Ind79';'Ind80';'Ind81';'Ind82';'Ind83';'Ind84';'Ind85';'Ind86';'Ind87';'Ind88';'Ind89';'Ind90'}.
compute cntname={'(C1)';'(C2)';'(C3)';'(C4)';'(C5)';'(C6)';'(C7)';'(C8)';'(C9)';'(C10)';'(C11)';'(C12)';'(C13)';'(C14)';'(C15)';'(C16)';'(C17)'}.
compute cntname={cntname;'(C18)';'(C19)';'(C20)';'(C21)';'(C22)';'(C23)';'(C24)';'(C25)';'(C26)';'(C27)';'(C28)';'(C29)';'(C30)';'(C31)'}.
compute cntname={cntname;'(C32)';'(C33)';'(C34)';'(C35)';'(C36)';'(C37)';'(C38)';'(C39)';'(C40)';'(C41)';'(C42)';'(C43)';'(C44)';'(C45)'}.
compute cntname={cntname;'(C46)';'(C47)';'(C48)';'(C49)';'(C50)';'(C51)';'(C52)';'(C53)';'(C54)';'(C55)';'(C56)';'(C57)';'(C58)';'(C59)'}.
compute cntname={cntname;'(C60)';'(C61)';'(C62)';'(C63)';'(C64)';'(C65)';'(C66)';'(C67)';'(C68)';'(C69)';'(C70)';'(C71)';'(C72)';'(C73)'}.
compute cntname={cntname;'(C74)';'(C75)';'(C76)';'(C77)';'(C78)';'(C79)';'(C80)';'(C81)';'(C82)';'(C83)';'(C84)';'(C85)';'(C86)';'(C87)'}.
compute cntname={cntname;'(C88)';'(C89)';'(C90)';'(C91)';'(C92)';'(C93)';'(C94)';'(C95)';'(C96)';'(C97)';'(C98)';'(C99)';'(C100)';'(C101)'}.
compute cntname={cntname;'(C102)';'(C103)';'(C104)';'(C105)'}.
compute indmake=make(ncol(indmark),(nms+2),0).
compute indmod=make(ncol(indmark),1,999).
compute indmmm=make(ncol(indmark),1,0).
compute indmmmt=make(ncol(indmark),1,0).
compute start=1.
compute end=0.
compute nindfx=0.
compute indlocs=make(nrow(thetaxmb),ncol(paths),999).
compute indkey=make(ncol(indmark),1+((rmax(indmark)*2)+1),'     ').
compute c1=1.
compute c2=1.
compute c3=1.
loop i = 1 to ncol(paths).
do if (pathtype(1,i)=1).
compute indlocs(:,i)=thetaxmb(:,c1).
compute c1=c1+1.
end if.
do if (pathtype(1,i)=3).
compute indlocs(:,i)=thetamyb(:,c2).
compute c2=c2+1.
end if.
do if (pathtype(1,i)=2 and nms < 7 and serial=1).
compute indlocs(:,i)=thetammb(:,thetam(1,c3)).
compute c3=c3+1.
end if.
end loop.
loop i = 1 to ncol(indlocs).
compute c1=2.
loop j = 2 to nrow(indlocs).
do if (indlocs(j,i) <> 0).
compute indlocs(c1,i)=indlocs(j,i).
compute c1=c1+1.
end if.
end loop.
compute indlocs(1,i)=c1-2.
end loop.
compute indlocs=indlocs(1:rmax((indlocs(1,:))+1),:).
loop i = 1 to ncol(indmark).
compute numget=indmark(1,i).
compute end=end+numget.
compute gotcha=indsets(1,start:end).
compute start=end+1.
compute ok=1.
compute temp=0.
compute repoman=make(4,1,0).
loop j = 1 to ncol(gotcha).
do if paths(1,gotcha(1,j))=0.
compute ok=0.
end if.
do if (pathsmod(1,gotcha(1,j)) > 0).
compute temp=1.
compute temp2={pathsw(1,gotcha(1,j));pathsz(1,gotcha(1,j));pathswz(1,gotcha(1,j));0}.
do if (temp2(1,1)=1 and temp2(2,1)=1 and temp2(3,1)=0).
compute temp2(4,1)=1.
end if.
compute repoman=repoman+temp2.
end if.
end loop.
compute temp=0.
compute tempmmm=0.
compute typemmm=0.
do if ((repoman(1,1) > 0) and (repoman(2,1) = 0)).
compute temp=1.
do if (repoman(1,1)=1).
compute tempmmm=1.
end if.
do if (repoman(1,1) > 1 and (wdich=1 or mcw > 0)).
compute tempmmm=12.
compute typemmm=mcw.
do if (wdich=1).
compute typemmm=1.
end if.
end if.
do if (repoman(1,1) > 1 and (wdich=0 and mcw = 0)).
compute tempmmm=101.
end if.
end if.
do if ((repoman(1,1) = 0) and (repoman(2,1) > 0)).
compute temp=2.
do if (repoman(2,1)=1).
compute tempmmm=2.
end if.
do if (repoman(2,1) > 1 and (zdich = 1 or mcz > 0)).
compute tempmmm=22.
compute typemmm=mcz.
do if (zdich=1).
compute typemmm=1.
end if.
end if.
do if (repoman(2,1) > 1 and (zdich = 0 and mcw = 0)).
compute tempmmm=102.
end if.
end if.
do if (repoman(1,1)>0 and repoman(2,1)>0).
compute temp=3.
do if (repoman(1,1)=1 and repoman(2,1)=1).
do if (repoman(4,1)=1).
compute tempmmm=31.
end if.
do if (repoman(3,1)=1).
compute tempmmm=41.
end if.
end if.
end if.
do if (repoman(1,1)=1 and repoman(2,1)=1 and repoman(3,1)=0 and repoman(4,1)=0).
compute tempmmm=51.
end if.
do if (ok = 1).
compute nindfx=nindfx+1.
compute indmake(nindfx,1)=numget.
compute indmod(nindfx,1)=temp.
compute indmmm(nindfx,1)=tempmmm.
compute indmmmt(nindfx,1)=typemmm.
compute indmake(nindfx,2:(1+numget))=gotcha.
compute indkey(nindfx,1)=xnames.
loop j = 1 to numget.
compute indkey(nindfx,(j*2+1))=pathsdv(1,gotcha(1,j)).
compute indkey(nindfx,(j*2))='   ->   '.
end loop.
end if.
end loop.
compute indkey=indkey(1:nindfx,1:((cmax(indmake(:,1))*2)+1)).
compute indmake=indmake(1:nindfx,1:(cmax(indmake(:,1))+1)).
compute indmod=indmod(1:nrow(indmake),1).
compute indmmm=indmmm(1:nrow(indmake),1).
compute indmmmt=indmmmt(1:nrow(indmake),1).
compute ncpairs = (((nindfx)*(nindfx-1))/2).
do if ((contrast = 1 or contrast = 2 or contrast=3) and (ncpairs > 105)).
compute contrast=0.
compute notecode(notes,1) = 13.
compute notes = notes + 1.
end if.
do if (contrast = 4).
do if (ncol(contvec) <> nindfx).
compute contrast=0.
compute notecode(notes,1) = 14.
compute notes = notes + 1.
end if.
end if.
do if (anymod=0).
do if (nms = 1 and contrast > 0).
compute contrast=0.
end if.
compute efloop=(((1-(effsize=0))*2)+1)-(((mcx>0 or xdich=1))*(1-(effsize=0))).
loop kk=1 to efloop.
do if (boot = 0).
compute bootres=obscoeff.
do if (kk=1).
compute totbtvec=make(1,nxvls,0).
end if.
compute bootdir=obsdirfx.
compute indtab=999.
compute inddiff=999.
compute bootysd=ysd.
compute bootxsd=xsd.
end if.
do if (boot > 0).
compute bootres={obscoeff;bootres}.
do if (kk=1).
compute totbtvec=make(nrow(bootres),nxvls,0).
end if.
compute indtab=make(1,4,999).
compute inddiff=make(nrow(bootres),1,999).
end if.
compute indtotal=make(nrow(bootres),1,0).
loop i = 1 to nrow(indmake).
loop j = 1 to nxvls.
compute indtemp=make(nrow(bootres),1,1).
loop k = 1 to indmake(i,1).
compute jtemp=1.
do if (j > 1 and k=1).
compute jtemp=j.
end if.
compute indtemp=indtemp&*bootres(:,pathsfoc(jtemp,indmake(i,(k+1)))).
end loop.
do if (kk = 2).
compute indtemp=indtemp/bootysd.
end if.
do if (kk = 3).
compute indtemp=(bootxsd&*indtemp)/bootysd.
end if.
do if (contrast <> 0).
compute inddiff={inddiff,indtemp}.
end if.
do if (nxvls=1).
compute indtotal=indtotal+indtemp.
end if.
compute indeff=indtemp(1,1).
do if (kk=1).
compute totbtvec(:,j)=totbtvec(:,j)+indtemp.
end if.
do if (boot > 0).

.
compute temp = indtemp(2:nrow(indtemp),1).
compute temp(GRADE( indtemp(2:nrow(indtemp),1) )) = indtemp(2:nrow(indtemp),1).
compute badlo = 0.
compute badhi = 0.
do if ( (indtemp(1,1)*bc)+(9999*(1-bc)) <> 9999).
compute pv=csum(temp < (indtemp(1,1)*bc)+(9999*(1-bc)) )/nrow(temp).
compute ppv = pv.
do if (pv > .5).
compute ppv = 1-pv.
end if.
compute y5=sqrt(-2*ln(ppv)).
compute xp=y5+((((y5*p4+p3)*y5+p2)*y5+p1)*y5+p0)/((((y5*q4+q3)*y5+q2)*y5+q1)*y5+q0).
do if (pv <= .5).
compute xp = -xp.
end if.
compute cilow=rnd(nrow(temp)*(cdfnorm(2*xp-xp2))).
compute cihigh=trunc(nrow(temp)*(cdfnorm(2*xp+(xp2))))+1.
do if (cilow < 1).
compute cilow = 1.
compute booterr=1.
compute badlo = 1.
end if.
do if (cihigh > nrow(temp)).
compute cihigh = boot.
compute booterr=1.
compute badhi = 1.
end if.
compute llcit=temp(cilow,1).
compute ulcit=temp(cihigh,1).
do if (badlo = 1 and llcit <> priorlo).
compute badend={badend, llcit}.
compute priorlo = llcit.
end if.
do if (badhi = 1 and ulcit <> priorhi).
compute badend={badend, ulcit}.
compute priorhi = ulcit.
end if.
end if.
do if ( (indtemp(1,1)*bc)+(9999*(1-bc)) = 9999).
compute llcit=temp(cilow,1).
compute ulcit=temp(cihigh,1).
end if.
compute bootse=t(sqrt((cssq(temp)-((csum(temp)&**2)/nrow(temp)))/(nrow(temp)-1)))
.
compute indeff={indeff,bootse,llcit,ulcit}.
end if.
do if (kk=1).
compute indtabn={indtab;indeff}.
end if.
compute indtab={indtab;indeff}.
end loop.
end loop.
compute indtab=indtab(2:nrow(indtab),:).
do if (kk=1).
compute indtabn=indtabn(2:nrow(indtabn),:).
end if.
compute rowlbs=indlbl(1:nrow(indtab),1).
do if (mc > 0).
compute inddiff=make(mc,1,-999).
compute indtab2=make(nrow(indtab),4,-999).
compute indtab2(:,1)=indtab.
compute indtab=indtab2.
release indtab2.
compute mcct=0.
compute indtotal=make(mc,1,0).
do if (kk = 1).
compute x1 = sqrt(-2*ln(uniform(mc,nrow(mcsopath))))&*cos((2*3.14159265358979)*uniform(mc, nrow(mcsopath))).
compute x1=x1*chol(indcov).
loop ii=1 to nrow(x1).
compute x1(ii,:)=x1(ii,:)+t(mcsopath).
end loop.
end if.
loop ii=1 to nms.
compute tmpb=x1(:,((nms*nxvls)+ii)).
compute tmpb2=tmpb.
do if (nxvls > 1).
loop jj=1 to (nxvls-1).
compute tmpb2={tmpb2,tmpb}.
end loop.
end if.
compute indtemp=x1(:,(((ii-1)*nxvls)+1):(ii*nxvls))&*tmpb2.
loop jj=1 to ncol(indtemp).
do if (kk = 2).
compute indtemp(:,jj)=indtemp(:,jj)/ysd.
end if.
do if (kk = 3).
compute indtemp(:,jj)=(xsd*indtemp(:,jj))/ysd.
end if.

.
compute temp = indtemp(:,jj).
compute temp(GRADE( indtemp(:,jj) )) = indtemp(:,jj).
compute badlo = 0.
compute badhi = 0.
do if ( 9999 <> 9999).
compute pv=csum(temp < 9999 )/nrow(temp).
compute ppv = pv.
do if (pv > .5).
compute ppv = 1-pv.
end if.
compute y5=sqrt(-2*ln(ppv)).
compute xp=y5+((((y5*p4+p3)*y5+p2)*y5+p1)*y5+p0)/((((y5*q4+q3)*y5+q2)*y5+q1)*y5+q0).
do if (pv <= .5).
compute xp = -xp.
end if.
compute cilow=rnd(nrow(temp)*(cdfnorm(2*xp-xp2))).
compute cihigh=trunc(nrow(temp)*(cdfnorm(2*xp+(xp2))))+1.
do if (cilow < 1).
compute cilow = 1.
compute booterr=1.
compute badlo = 1.
end if.
do if (cihigh > nrow(temp)).
compute cihigh = boot.
compute booterr=1.
compute badhi = 1.
end if.
compute llcit=temp(cilow,1).
compute ulcit=temp(cihigh,1).
do if (badlo = 1 and llcit <> priorlo).
compute badend={badend, llcit}.
compute priorlo = llcit.
end if.
do if (badhi = 1 and ulcit <> priorhi).
compute badend={badend, ulcit}.
compute priorhi = ulcit.
end if.
end if.
do if ( 9999 = 9999).
compute llcit=temp(cilow,1).
compute ulcit=temp(cihigh,1).
end if.
compute bootse=t(sqrt((cssq(temp)-((csum(temp)&**2)/nrow(temp)))/(nrow(temp)-1)))
.
compute mcct=mcct+1.
compute indtab(mcct,2:4)={bootse,llcit,ulcit}.
end loop.
do if (nxvls=1).
compute indtotal=indtotal+indtemp.
do if (contrast <> 0).
compute inddiff={inddiff,indtemp}.
end if.
end if.
end loop.
release indtemp,tmpb.
end if.
do if (normal = 1 and sobelok=1).
compute sobelmat=indtab(:,1).
compute sobelmat={sobelmat,(sobelmat/2),sobelmat,sobelmat}.
loop ii=1 to nms.
compute se2b=indcov(((nms*nxvls)+ii),((nms*nxvls)+ii)).
compute bpath2=mcsopath(((nms*nxvls)+ii),1)&**2.
compute se2a=diag(indcov((((ii-1)*nxvls)+1):(ii*nxvls),(((ii-1)*nxvls)+1):(ii*nxvls))).
compute apath2=mcsopath((((ii-1)*nxvls)+1):(ii*nxvls) ,1)&**2.
compute sesobel=sqrt(apath2*se2b+bpath2*se2a+se2a*se2b).
compute sobelmat((((ii-1)*nxvls)+1):(ii*nxvls),2)=sesobel.
end loop.
release se2b,bpath2,se2a,apath2,sesobel,ii.
compute sobelmat(:,3)=sobelmat(:,1)&/sobelmat(:,2).
compute sobelmat(:,4) = 2*(1-cdfnorm(abs(sobelmat(:,3)))).
end if.
do if (serial = 0).
compute rowlbs=t(mnames).
end if.
do if (nxvls=1 and nms > 1).
compute rowlbs={'TOTAL';rowlbs}.
compute indtemp=indtotal(1,1).
do if (boot > 0 and nxvls=1).

.
compute temp = indtotal(2:nrow(indtotal),1).
compute temp(GRADE( indtotal(2:nrow(indtotal),1) )) = indtotal(2:nrow(indtotal),1).
compute badlo = 0.
compute badhi = 0.
do if ( (indtotal(1,1)*bc)+(9999*(1-bc)) <> 9999).
compute pv=csum(temp < (indtotal(1,1)*bc)+(9999*(1-bc)) )/nrow(temp).
compute ppv = pv.
do if (pv > .5).
compute ppv = 1-pv.
end if.
compute y5=sqrt(-2*ln(ppv)).
compute xp=y5+((((y5*p4+p3)*y5+p2)*y5+p1)*y5+p0)/((((y5*q4+q3)*y5+q2)*y5+q1)*y5+q0).
do if (pv <= .5).
compute xp = -xp.
end if.
compute cilow=rnd(nrow(temp)*(cdfnorm(2*xp-xp2))).
compute cihigh=trunc(nrow(temp)*(cdfnorm(2*xp+(xp2))))+1.
do if (cilow < 1).
compute cilow = 1.
compute booterr=1.
compute badlo = 1.
end if.
do if (cihigh > nrow(temp)).
compute cihigh = boot.
compute booterr=1.
compute badhi = 1.
end if.
compute llcit=temp(cilow,1).
compute ulcit=temp(cihigh,1).
do if (badlo = 1 and llcit <> priorlo).
compute badend={badend, llcit}.
compute priorlo = llcit.
end if.
do if (badhi = 1 and ulcit <> priorhi).
compute badend={badend, ulcit}.
compute priorhi = ulcit.
end if.
end if.
do if ( (indtotal(1,1)*bc)+(9999*(1-bc)) = 9999).
compute llcit=temp(cilow,1).
compute ulcit=temp(cihigh,1).
end if.
compute bootse=t(sqrt((cssq(temp)-((csum(temp)&**2)/nrow(temp)))/(nrow(temp)-1)))
.
compute indtemp={indtemp, bootse,llcit,ulcit}.
end if.
do if (mc > 0).
compute obtmc=indtab(:,1).
compute indtemp=csum(obtmc).

.
compute temp = indtotal(:,1).
compute temp(GRADE( indtotal(:,1) )) = indtotal(:,1).
compute badlo = 0.
compute badhi = 0.
do if ( 9999 <> 9999).
compute pv=csum(temp < 9999 )/nrow(temp).
compute ppv = pv.
do if (pv > .5).
compute ppv = 1-pv.
end if.
compute y5=sqrt(-2*ln(ppv)).
compute xp=y5+((((y5*p4+p3)*y5+p2)*y5+p1)*y5+p0)/((((y5*q4+q3)*y5+q2)*y5+q1)*y5+q0).
do if (pv <= .5).
compute xp = -xp.
end if.
compute cilow=rnd(nrow(temp)*(cdfnorm(2*xp-xp2))).
compute cihigh=trunc(nrow(temp)*(cdfnorm(2*xp+(xp2))))+1.
do if (cilow < 1).
compute cilow = 1.
compute booterr=1.
compute badlo = 1.
end if.
do if (cihigh > nrow(temp)).
compute cihigh = boot.
compute booterr=1.
compute badhi = 1.
end if.
compute llcit=temp(cilow,1).
compute ulcit=temp(cihigh,1).
do if (badlo = 1 and llcit <> priorlo).
compute badend={badend, llcit}.
compute priorlo = llcit.
end if.
do if (badhi = 1 and ulcit <> priorhi).
compute badend={badend, ulcit}.
compute priorhi = ulcit.
end if.
end if.
do if ( 9999 = 9999).
compute llcit=temp(cilow,1).
compute ulcit=temp(cihigh,1).
end if.
compute bootse=t(sqrt((cssq(temp)-((csum(temp)&**2)/nrow(temp)))/(nrow(temp)-1)))
.
compute indtemp={indtemp, bootse,llcit,ulcit}.
end if.
compute indtab={indtemp;indtab}.
end if.
compute bootlbs={'Effect', 'BootSE','BootLLCI','BootULCI'}.
do if (mc > 0).
compute bootlbs={'Effect', 'MC SE','MC LLCI','MC ULCI'}.
end if.
do if (nxvls = 1).
do if (contrast <> 0).
compute inddiff=inddiff(:,2:ncol(inddiff)).
do if (mc > 0).
compute inddiff={t(obtmc);inddiff}.
end if.
do if (contrast = 4).
compute inddifft=inddiff*t(contvec).
compute indtemp=inddifft(1,1).
do if (boot > 0 or mc > 0).
do if (mc > 0).

.
compute temp = inddifft(2:nrow(inddifft),1).
compute temp(GRADE( inddifft(2:nrow(inddifft),1) )) = inddifft(2:nrow(inddifft),1).
compute badlo = 0.
compute badhi = 0.
do if ( 9999 <> 9999).
compute pv=csum(temp < 9999 )/nrow(temp).
compute ppv = pv.
do if (pv > .5).
compute ppv = 1-pv.
end if.
compute y5=sqrt(-2*ln(ppv)).
compute xp=y5+((((y5*p4+p3)*y5+p2)*y5+p1)*y5+p0)/((((y5*q4+q3)*y5+q2)*y5+q1)*y5+q0).
do if (pv <= .5).
compute xp = -xp.
end if.
compute cilow=rnd(nrow(temp)*(cdfnorm(2*xp-xp2))).
compute cihigh=trunc(nrow(temp)*(cdfnorm(2*xp+(xp2))))+1.
do if (cilow < 1).
compute cilow = 1.
compute booterr=1.
compute badlo = 1.
end if.
do if (cihigh > nrow(temp)).
compute cihigh = boot.
compute booterr=1.
compute badhi = 1.
end if.
compute llcit=temp(cilow,1).
compute ulcit=temp(cihigh,1).
do if (badlo = 1 and llcit <> priorlo).
compute badend={badend, llcit}.
compute priorlo = llcit.
end if.
do if (badhi = 1 and ulcit <> priorhi).
compute badend={badend, ulcit}.
compute priorhi = ulcit.
end if.
end if.
do if ( 9999 = 9999).
compute llcit=temp(cilow,1).
compute ulcit=temp(cihigh,1).
end if.
compute bootse=t(sqrt((cssq(temp)-((csum(temp)&**2)/nrow(temp)))/(nrow(temp)-1)))
.
end if.
do if (boot > 0).

.
compute temp = inddifft(2:nrow(inddifft),1).
compute temp(GRADE( inddifft(2:nrow(inddifft),1) )) = inddifft(2:nrow(inddifft),1).
compute badlo = 0.
compute badhi = 0.
do if ( (inddifft(1,1)*bc)+(9999* (1-bc)) <> 9999).
compute pv=csum(temp < (inddifft(1,1)*bc)+(9999* (1-bc)) )/nrow(temp).
compute ppv = pv.
do if (pv > .5).
compute ppv = 1-pv.
end if.
compute y5=sqrt(-2*ln(ppv)).
compute xp=y5+((((y5*p4+p3)*y5+p2)*y5+p1)*y5+p0)/((((y5*q4+q3)*y5+q2)*y5+q1)*y5+q0).
do if (pv <= .5).
compute xp = -xp.
end if.
compute cilow=rnd(nrow(temp)*(cdfnorm(2*xp-xp2))).
compute cihigh=trunc(nrow(temp)*(cdfnorm(2*xp+(xp2))))+1.
do if (cilow < 1).
compute cilow = 1.
compute booterr=1.
compute badlo = 1.
end if.
do if (cihigh > nrow(temp)).
compute cihigh = boot.
compute booterr=1.
compute badhi = 1.
end if.
compute llcit=temp(cilow,1).
compute ulcit=temp(cihigh,1).
do if (badlo = 1 and llcit <> priorlo).
compute badend={badend, llcit}.
compute priorlo = llcit.
end if.
do if (badhi = 1 and ulcit <> priorhi).
compute badend={badend, ulcit}.
compute priorhi = ulcit.
end if.
end if.
do if ( (inddifft(1,1)*bc)+(9999* (1-bc)) = 9999).
compute llcit=temp(cilow,1).
compute ulcit=temp(cihigh,1).
end if.
compute bootse=t(sqrt((cssq(temp)-((csum(temp)&**2)/nrow(temp)))/(nrow(temp)-1)))
.
end if.
compute indtemp={indtemp, bootse,llcit,ulcit}.
end if.
compute indtab={indtab;indtemp}.
end if.
do if (contrast = 1 or contrast = 2 or contrast=3).
compute conkey=make(1,4,' ').
loop i = 1 to ncol(inddiff)-1.
loop j = (i+1) to ncol(inddiff).
compute inddifft=inddiff(:,i)-inddiff(:,j).
do if (contrast=2).
compute inddifft=abs(inddiff(:,i))-abs(inddiff(:,j)).
end if.
do if (contrast=3).
compute inddifft=inddiff(:,i)+inddiff(:,j).
end if.
compute indtemp=inddifft(1,1).
do if (contrast=1 or contrast=2).
compute conkeyt={' ', rowlbs((i+1),1),' minus  ',rowlbs((j+1),1)}.
end if.
do if (contrast=3).
compute conkeyt={' ', rowlbs((i+1),1),'  plus  ',rowlbs((j+1),1)}.
end if.
compute conkey={conkey;conkeyt}.
do if (boot > 0 or mc > 0).
do if (mc > 0).

.
compute temp = inddifft(2:nrow(inddifft),1).
compute temp(GRADE( inddifft(2:nrow(inddifft),1) )) = inddifft(2:nrow(inddifft),1).
compute badlo = 0.
compute badhi = 0.
do if ( 9999 <> 9999).
compute pv=csum(temp < 9999 )/nrow(temp).
compute ppv = pv.
do if (pv > .5).
compute ppv = 1-pv.
end if.
compute y5=sqrt(-2*ln(ppv)).
compute xp=y5+((((y5*p4+p3)*y5+p2)*y5+p1)*y5+p0)/((((y5*q4+q3)*y5+q2)*y5+q1)*y5+q0).
do if (pv <= .5).
compute xp = -xp.
end if.
compute cilow=rnd(nrow(temp)*(cdfnorm(2*xp-xp2))).
compute cihigh=trunc(nrow(temp)*(cdfnorm(2*xp+(xp2))))+1.
do if (cilow < 1).
compute cilow = 1.
compute booterr=1.
compute badlo = 1.
end if.
do if (cihigh > nrow(temp)).
compute cihigh = boot.
compute booterr=1.
compute badhi = 1.
end if.
compute llcit=temp(cilow,1).
compute ulcit=temp(cihigh,1).
do if (badlo = 1 and llcit <> priorlo).
compute badend={badend, llcit}.
compute priorlo = llcit.
end if.
do if (badhi = 1 and ulcit <> priorhi).
compute badend={badend, ulcit}.
compute priorhi = ulcit.
end if.
end if.
do if ( 9999 = 9999).
compute llcit=temp(cilow,1).
compute ulcit=temp(cihigh,1).
end if.
compute bootse=t(sqrt((cssq(temp)-((csum(temp)&**2)/nrow(temp)))/(nrow(temp)-1)))
.
end if.
do if (boot > 0).

.
compute temp = inddifft(2:nrow(inddifft),1).
compute temp(GRADE( inddifft(2:nrow(inddifft),1) )) = inddifft(2:nrow(inddifft),1).
compute badlo = 0.
compute badhi = 0.
do if ( (inddifft(1,1)*bc)+(9999* (1-bc)) <> 9999).
compute pv=csum(temp < (inddifft(1,1)*bc)+(9999* (1-bc)) )/nrow(temp).
compute ppv = pv.
do if (pv > .5).
compute ppv = 1-pv.
end if.
compute y5=sqrt(-2*ln(ppv)).
compute xp=y5+((((y5*p4+p3)*y5+p2)*y5+p1)*y5+p0)/((((y5*q4+q3)*y5+q2)*y5+q1)*y5+q0).
do if (pv <= .5).
compute xp = -xp.
end if.
compute cilow=rnd(nrow(temp)*(cdfnorm(2*xp-xp2))).
compute cihigh=trunc(nrow(temp)*(cdfnorm(2*xp+(xp2))))+1.
do if (cilow < 1).
compute cilow = 1.
compute booterr=1.
compute badlo = 1.
end if.
do if (cihigh > nrow(temp)).
compute cihigh = boot.
compute booterr=1.
compute badhi = 1.
end if.
compute llcit=temp(cilow,1).
compute ulcit=temp(cihigh,1).
do if (badlo = 1 and llcit <> priorlo).
compute badend={badend, llcit}.
compute priorlo = llcit.
end if.
do if (badhi = 1 and ulcit <> priorhi).
compute badend={badend, ulcit}.
compute priorhi = ulcit.
end if.
end if.
do if ( (inddifft(1,1)*bc)+(9999* (1-bc)) = 9999).
compute llcit=temp(cilow,1).
compute ulcit=temp(cihigh,1).
end if.
compute bootse=t(sqrt((cssq(temp)-((csum(temp)&**2)/nrow(temp)))/(nrow(temp)-1)))
.
end if.
compute indtemp={indtemp, bootse,llcit,ulcit}.
end if.
compute indtab={indtab;indtemp}.
end loop.
end loop.
end if.
release inddiff.
compute contlbs=cntname(1:(((nindfx)*(nindfx-1))/2),1).
compute rowlbs={rowlbs;contlbs}.
end if.
compute resultm2=make(nrow(indtab),maxresm,99999).
compute resultm2(1:nrow(indtab),1:ncol(indtab))=indtab.
compute resultm={resultm;resultm2}.
do if (outscree=1).
do if (kk=1).
print indtab/title = 'Indirect effect(s) of X on Y:'/rnames=rowlbs/cnames=bootlbs/format= F10.4.
end if.
do if (kk = 2 and (xdich=1 or mcx > 0)).
print indtab/title = 'Partially standardized indirect effect(s) of X on Y:'/rnames=rowlbs/cnames=bootlbs/format= F10.4.
end if.
do if (kk = 3).
print indtab/title = 'Completely standardized indirect effect(s) of X on Y:'/rnames=rowlbs/cnames=bootlbs/format= F10.4.
end if.
end if.
do if (normal=1 and sobelok=1 and kk=1).
compute resultm2=make(nrow(sobelmat),maxresm,99999).
compute resultm2(1:nrow(sobelmat),1:ncol(sobelmat))=sobelmat.
compute resultm={resultm;resultm2}.
compute sobellab={'Effect',hclab,'Z','p'}.
compute sobelrlb=rowlbs.
do if (nms > 1).
compute sobelrlb=rowlbs(2:(1+nms),1).
end if.
do if (outscree=1).
print sobelmat/title='   Normal theory test for indirect effect(s):'/cnames=sobellab/rnames=sobelrlb/format= F10.4.
end if.
end if.
do if (contrast <> 0).
do if ((contrast=1 or contrast = 2 or contrast=3) and kk=efloop ).
compute conkey=conkey(2:nrow(conkey),:).
do if (outscree=1).
print conkey/title = 'Specific indirect effect contrast definition(s):'/rnames=contlbs/format=A8.
end if.
end if.
do if (outscree=1).
do if (contrast = 4 and kk=efloop).
compute crowlbs=rowlbs(2:(nindfx+1),1).
print contvec/title = 'Specific indirect effect contrast weights:'/cnames=crowlbs/rlabels='(C1)'/format= F10.4.
end if.
do if (contrast = 2 and kk=efloop).
print/title = 'Contrasts are differences between absolute values of indirect effects'.
end if.
end if.
end if.
do if (serial = 1 and kk=efloop).
compute rowlbst=rowlbs(2:nrow(rowlbs),1).
do if (outscree=1).
print indkey/title = 'Indirect effect key:'/rnames=rowlbst/format = A8.
end if.
end if.
else.
do if (outscree=1).
do if (kk = 1).
print/title = 'Relative indirect effects of X on Y'.
end if.
do if (kk = 2).
print/title = 'Partially standardized relative indirect effect(s) of X on Y:'.
end if.
do if (kk = 3).
print/title = 'Completely standardized relative indirect effect(s) of X on Y:'.
end if.
end if.
loop i = 1 to nrow(indmake).
compute indtabsm=indtab((((i-1)*nxvls)+1):(nxvls*i),:).
compute indkeyt=indkey(i,:).
do if (outscree=1).
print indkeyt/title=' '/space=0/format=A8.
end if.
do if (bcmat(nrow(bcmat),1)=0)).
compute direffl2=xcatlab(1:nxvls,1).
end if.
compute resultm2=make(nrow(indtabsm),maxresm,99999).
compute resultm2(1:nrow(indtabsm),1:ncol(indtabsm))=indtabsm.
compute resultm={resultm;resultm2}.
do if (outscree=1).
print indtabsm/title = ' '/cnames=bootlbs/rnames=direffl2/format= F10.4 /space=0.
end if.
do if (normal=1 and sobelok=1 and kk=1).
compute sobelsm=sobelmat((((i-1)*nxvls)+1):(nxvls*i),:).
compute sobellab={'Effect',hclab,'Z','p'}.
compute resultm2=make(nrow(sobelsm),maxresm,99999).
compute resultm2(1:nrow(sobelsm),1:ncol(sobelsm))=sobelsm.
compute resultm={resultm;resultm2}.
do if (outscree=1).
print sobelsm/title='      Normal theory test for relative indirect effects:'/cnames=sobellab/rnames=direffl2/format= F10.4.
end if.
end if.
end loop.
end if.
do if (effsize = 1 and boot > 0).
compute bootres=bootres(2:nrow(bootres),:).
end if.
end loop.
do if (alttotal=1).
compute altcnms='Effect'.
compute totbtvec=totbtvec+bootdir.
compute alttotfx=t(totbtvec(1,:)).
do if (boot > 0).
compute alttotfx=make(ncol(totbtvec),4,0).
compute alttotfx(:,1)=t(totbtvec(1,:)).
loop cec=1 to ncol(totbtvec).

.
compute temp = totbtvec(2:nrow(totbtvec),cec).
compute temp(GRADE( totbtvec(2:nrow(totbtvec),cec) )) = totbtvec(2:nrow(totbtvec),cec).
compute badlo = 0.
compute badhi = 0.
do if ( (totbtvec(1,cec)*bc)+(9999* (1-bc)) <> 9999).
compute pv=csum(temp < (totbtvec(1,cec)*bc)+(9999* (1-bc)) )/nrow(temp).
compute ppv = pv.
do if (pv > .5).
compute ppv = 1-pv.
end if.
compute y5=sqrt(-2*ln(ppv)).
compute xp=y5+((((y5*p4+p3)*y5+p2)*y5+p1)*y5+p0)/((((y5*q4+q3)*y5+q2)*y5+q1)*y5+q0).
do if (pv <= .5).
compute xp = -xp.
end if.
compute cilow=rnd(nrow(temp)*(cdfnorm(2*xp-xp2))).
compute cihigh=trunc(nrow(temp)*(cdfnorm(2*xp+(xp2))))+1.
do if (cilow < 1).
compute cilow = 1.
compute booterr=1.
compute badlo = 1.
end if.
do if (cihigh > nrow(temp)).
compute cihigh = boot.
compute booterr=1.
compute badhi = 1.
end if.
compute llcit=temp(cilow,1).
compute ulcit=temp(cihigh,1).
do if (badlo = 1 and llcit <> priorlo).
compute badend={badend, llcit}.
compute priorlo = llcit.
end if.
do if (badhi = 1 and ulcit <> priorhi).
compute badend={badend, ulcit}.
compute priorhi = ulcit.
end if.
end if.
do if ( (totbtvec(1,cec)*bc)+(9999* (1-bc)) = 9999).
compute llcit=temp(cilow,1).
compute ulcit=temp(cihigh,1).
end if.
compute bootse=t(sqrt((cssq(temp)-((csum(temp)&**2)/nrow(temp)))/(nrow(temp)-1)))
.
compute alttotfx(cec,2:4)={bootse,llcit,ulcit}.
end loop.
compute altcnms={altcnms,'BootSE', 'BootLLCI','BootULCI'}.
end if.
compute resultm2=make(nrow(alttotfx),maxresm,99999).
compute resultm2(1:nrow(alttotfx),1:ncol(alttotfx))=alttotfx.
compute resultm={resultm;resultm2}.
do if (outscree=1).
do if (nxvls > 1).
print/title= '----------'/space=0.
print alttotfx/title='Relative total effects of X on Y (sum of direct and indirect effects)'/rnames=direffl2/cnames=altcnms/format= F10.4.
end if.
do if (nxvls = 1).
print alttotfx/title='Total effect of X on Y (sum of direct and indirect effects)'/cnames=altcnms/format= F10.4.
end if.
end if.
end if.
end if.
do if (anymod > 0).
do if (boot = 0).
compute bootres=obscoeff.
compute indtab=999.
end if.
do if (boot > 0).
compute bootres={obscoeff;bootres}.
compute indtab=make(1,4,999).
end if.
do if (csum((indmod > 0))=nrow(indmod)).
do if (outscree=1).
do if (nxvls > 1).
do if (xmint=0).
print/title= 'Relative conditional indirect effects of X on Y:'.
end if.
do if (xmint=1).
print/title= 'Relative (total) natural indirect effects of X on Y:'.
end if.
end if.
do if (nxvls = 1).
do if (xmint=0).
print/title= 'Conditional indirect effects of X on Y:'.
end if.
do if (xmint=1).
print/title= '(Total) Natural indirect effect(s) of X on Y:'.
end if.
end if.
end if.
end if.
do if (csum((indmod > 0)) < nrow(indmod)).
do if (outscree=1).
do if (nxvls > 1).
print/title= 'Relative conditional and unconditional indirect effects of X on Y:'.
end if.
do if (nxvls = 1).
print/title= 'Conditional and unconditional indirect effects of X on Y:'.
end if.
end if.
end if.
compute cftotfx=make(nrow(bootres),nxvls,0).
loop i = 1 to nrow(indmake).
compute indtab=indtab(1,:)*0.
do if (outscree=1).
do if (xmint=0).
print indkey(i,:)/title = 'INDIRECT EFFECT:'/format=A8.
end if.
do if (xmint=1).
print indkey(i,:)/title = ' '/format=A8/space=0.
end if.
end if.
do if (indmod(i,1)=0).
loop j = 1 to nxvls.
compute indtemp=make(nrow(bootres),1,1).
loop k = 1 to indmake(i,1).
compute jtemp=1.
do if (j > 1 and k=1).
compute jtemp=j.
end if.
compute indtemp=indtemp&*bootres(:,pathsfoc(jtemp,indmake(i,(k+1)))).
end loop.
compute indeff=indtemp(1,1).
do if (boot > 0).

.
compute temp = indtemp(2:nrow(indtemp),1).
compute temp(GRADE( indtemp(2:nrow(indtemp),1) )) = indtemp(2:nrow(indtemp),1).
compute badlo = 0.
compute badhi = 0.
do if ( (indtemp(1,1)*bc)+(9999*(1-bc)) <> 9999).
compute pv=csum(temp < (indtemp(1,1)*bc)+(9999*(1-bc)) )/nrow(temp).
compute ppv = pv.
do if (pv > .5).
compute ppv = 1-pv.
end if.
compute y5=sqrt(-2*ln(ppv)).
compute xp=y5+((((y5*p4+p3)*y5+p2)*y5+p1)*y5+p0)/((((y5*q4+q3)*y5+q2)*y5+q1)*y5+q0).
do if (pv <= .5).
compute xp = -xp.
end if.
compute cilow=rnd(nrow(temp)*(cdfnorm(2*xp-xp2))).
compute cihigh=trunc(nrow(temp)*(cdfnorm(2*xp+(xp2))))+1.
do if (cilow < 1).
compute cilow = 1.
compute booterr=1.
compute badlo = 1.
end if.
do if (cihigh > nrow(temp)).
compute cihigh = boot.
compute booterr=1.
compute badhi = 1.
end if.
compute llcit=temp(cilow,1).
compute ulcit=temp(cihigh,1).
do if (badlo = 1 and llcit <> priorlo).
compute badend={badend, llcit}.
compute priorlo = llcit.
end if.
do if (badhi = 1 and ulcit <> priorhi).
compute badend={badend, ulcit}.
compute priorhi = ulcit.
end if.
end if.
do if ( (indtemp(1,1)*bc)+(9999*(1-bc)) = 9999).
compute llcit=temp(cilow,1).
compute ulcit=temp(cihigh,1).
end if.
compute bootse=t(sqrt((cssq(temp)-((csum(temp)&**2)/nrow(temp)))/(nrow(temp)-1)))
.
compute indeff={indeff,bootse,llcit,ulcit}.
end if.
compute indtab={indtab;indeff}.
end loop.
compute resultm2=make(nrow(indtab),maxresm,99999).
compute resultm2(1:nrow(indtab),1:ncol(indtab))=indtab.
compute resultm={resultm;resultm2}.
do if (outscree=1).
do if (nxvls > 1).
compute indefflb=xcatlab(1:nxvls,1).
print indtab(2:nrow(indtab),:)/title = ' '/clabels='Effect', 'BootSE', 'BootLLCI', 'BootULCI'/rnames=indefflb/format= F10.4 /space=0.
end if.
do if (nxvls = 1).
print indtab(2:nrow(indtab),:)/title = ' '/clabels='Effect', 'BootSE', 'BootLLCI', 'BootULCI'/format= F10.4 /space=0.
end if.
end if.
end if.
do if (indmod(i,1)>0).
do if (indmod(i,1)=1).
compute indmodva=wmodvals.
compute indprova=wprobval.
compute condlbs={wnames}.
compute printw=1.
else if (indmod(i,1)=2).
compute indmodva=zmodvals.
compute indprova=zprobval.
compute condlbs={znames}.
compute printz=1.
else if (indmod(i,1)=3).
compute cntmp=1.
compute printz=1.
compute printw=1.
compute indmodva=make((nrow(wmodvals)*nrow(zmodvals)),2,999).
loop k7 = 1 to nrow(wmodvals).
loop k8 = 1 to nrow(zmodvals).
compute indmodva(cntmp,:)={wmodvals(k7,1),zmodvals(k8,1)}.
compute cntmp=cntmp+1.
end loop.
end loop.
compute condlbs={wnames,znames}.
end if.
compute condres=make(nrow(indmodva),1,999).
do if (boot > 0).
compute condres=make(nrow(indmodva),4,999).
end if.
compute condres={indmodva,condres}.
loop k4 = 1 to nxvls.
compute imm3=make(nrow(bootres),1,1).
compute imm4=make(nrow(bootres),1,1).
compute indcontr=0.
do if (indmod(i,1)=3).
compute tihsw=wprobval.
compute tihsz=zprobval.
end if.
loop k1=1 to nrow(indmodva).
compute tucker2=make(nrow(bootres),1,1).
compute imm2=make(nrow(bootres),1,1).
compute wfirst=0.
compute zfirst=0.
compute immset=0.
loop k2=1 to indmake(i,1).
compute colnumb=indmake(i,(k2+1)).
do if (k2=1).
compute wbb=make(nrow(bootres),(nwvls*nxvls),0).
compute zbb=make(nrow(bootres),(nzvls*nxvls),0).
compute wzbb=make(nrow(bootres),(nwvls*nzvls*nxvls),0).
end if.
do if (k2<>1).
compute wbb=make(nrow(bootres),nwvls,0).
compute zbb=make(nrow(bootres),nzvls,0).
compute wzbb=make(nrow(bootres),(nwvls*nzvls),0).
end if.
compute cnt=1.
compute tihs=indlocs(2:((indlocs(1,colnumb))+1),colnumb).
do if (k2 = 1).
compute focbb=tihs(1:nxvls,1).
compute focbb=bootres(:,focbb).
do if (indmmm(i,1)>0).
compute imm=focbb(:,k4).
compute condbb=imm.
end if.
compute focaddon=make(1,nxvls,0).
compute focaddon(1,k4)=1.
compute cnt=cnt+nxvls.
compute placeh=nxvls.
do if (indmod(i,1)=1).
compute tihsz=make(nrow(wprobval),(nzvls*nxvls),0).
compute tihswz=make(nrow(wprobval),(nwvls*nzvls*nxvls),0).
do if (pathsw(1,colnumb)=1).
compute temp=make(nrow(wprobval),(nxvls*nwvls),0).
loop k5 = 1 to nrow(wprobval).
loop k6=1 to nwvls.
compute temp(k5, (((k4-1)*nwvls)+k6))=wprobval(k5,k6).
end loop.
end loop.
compute indprova={temp,tihsz,tihswz}.
else.
compute indprova={wprobval,tihsz,tihswz}.
end if.
end if.
do if (indmod(i,1)=2).
compute tihsw=make(nrow(zprobval),(nwvls*nxvls),0).
compute tihswz=make(nrow(zprobval),(nwvls*nzvls*nxvls),0).
do if (pathsz(1,colnumb)=1).
compute temp=make(nrow(zprobval),(nxvls*nzvls),0).
loop k5 = 1 to nrow(zprobval).
loop k6 =1 to nzvls.
compute temp(k5,(((k4-1)*nzvls)+k6))=zprobval(k5,k6).
end loop.
end loop.
compute indprova={tihsw,temp,tihswz}.
else.
compute indprova={tihsw,zprobval,tihswz}.
end if.
end if.
do if (indmod(i,1)=3).
compute indprova=make((nrow(wprobval)*nrow(zprobval)),((ncol(wprobval)*nxvls)+ (ncol(zprobval)*nxvls)+(nwvls*nzvls*nxvls)),0).
compute cntemp=1.
loop k7=1 to nrow(wprobval).
loop k8 =1 to nrow(zprobval).
compute temp=wprobval(k7,:)*focaddon(1,k4).
compute indprova(cntemp,(((k4-1)*nwvls)+1):(k4*(nwvls)))=temp.
compute temp=zprobval(k8,:)*focaddon(1,k4).
compute indprova(cntemp, ((((k4-1)*nzvls)+1)+(nxvls*nwvls)) : ((((k4-1)*nzvls)+1)+(nxvls*nwvls)+(nzvls-1)))=temp.
compute cntemp=cntemp+1.
end loop.
end loop.
do if (pathsz(1,colnumb)=0).
compute temp=make(nrow(indprova),(ncol(zprobval)*nxvls),0).
compute indprova(:,((ncol(wprobval)*nxvls)+1):((ncol(wprobval)+ncol(zprobval))* nxvls))=temp.
end if.
do if (pathsw(1,colnumb)=0).
compute temp=make(nrow(indprova),(ncol(wprobval)*nxvls),0).
compute indprova(:,1:(ncol(wprobval)*nxvls))=temp.
end if.
do if (pathswz(1,colnumb)=1).
compute cntemp=(ncol(wprobval)*nxvls)+(ncol(zprobval)*nxvls)+((k4-1)* ncol(wprobval)*ncol(zprobval))+1.
loop k7=1 to ncol(wprobval).
loop k8=1 to ncol(zprobval).
compute indprova(:,cntemp)=indprova(:,((ncol(wprobval)*(k4-1))+k7))&* indprova(:,((((k4-1)*ncol(zprobval))+k8)+(nxvls*ncol(wprobval)))).
compute cntemp=cntemp+1.
end loop.
end loop.
end if.
end if.
end if.
do if (k2 > 1).
compute focbb=tihs(1,1).
compute focbb=bootres(:,focbb).
do if (indmmm(i,1)>0).
compute imm=focbb(:,1).
compute condbb=imm.
end if.
compute focaddon=1.
compute cnt=cnt+1.
compute placeh=1.
do if (indmod(i,1)=1).
compute tihsz=make(nrow(wprobval),nzvls,0).
compute tihswz=make(nrow(wprobval),(nwvls*nzvls),0).
compute indprova={wprobval,tihsz,tihswz}.
end if.
do if (indmod(i,1)=2).
compute tihsw=make(nrow(zprobval),nwvls,0).
compute tihswz=make(nrow(zprobval),(nwvls*nzvls),0).
compute indprova={tihsw,zprobval,tihswz}.
end if.
do if (indmod(i,1)=3).
compute indprova=make((nrow(wprobval)*nrow(zprobval)),((ncol(wprobval)+ ncol(zprobval))+(nwvls*nzvls)),0).
compute cntemp=1.
loop k7=1 to nrow(wprobval).
loop k8 =1 to nrow(zprobval).
compute indprova(cntemp,1:(ncol(wprobval)+ncol(zprobval)))={wprobval(k7,:), zprobval(k8,:)}.
compute cntemp=cntemp+1.
end loop.
end loop.
do if (pathsz(1,colnumb)=0).
compute temp=make(nrow(indprova),ncol(zprobval),0).
compute indprova(:,(ncol(wprobval)+1):(ncol(wprobval)+ncol(zprobval)))=temp.
end if.
do if (pathsw(1,colnumb)=0).
compute temp=make(nrow(indprova),ncol(wprobval),0).
compute indprova(:,1:ncol(wprobval))=temp.
end if.
do if (pathswz(1,colnumb)=1).
compute cntemp=ncol(wprobval)+ncol(zprobval)+1.
loop k7=1 to ncol(wprobval).
loop k8=1 to ncol(zprobval).
compute indprova(:,cntemp)=indprova(:,k7)&*indprova(:,(ncol(wprobval)+k8)).
compute cntemp=cntemp+1.
end loop.
end loop.
end if.
end if.
end if.
do if (pathsw(1,colnumb)) = 1.
compute wbb=tihs(cnt:(cnt+(placeh*nwvls)-1),1).
compute wbb=bootres(:,wbb).
compute immlbs2=wcatlab(1:nwvls,1).
do if (zfirst=0).
compute wfirst=1.
end if.
do if (indmmm(i,1)=1 or indmmm(i,1)=31 or indmmm(i,1)=51).
compute imm=wbb(:,1).
loop k7 = 1 to nwvls.
compute imm={imm,wbb(:,(((k4-1)*nwvls*(k2=1))+k7))}.
end loop.
compute imm=imm(:,2:ncol(imm)).
end if.
do if (indmmm(i,1)=41 or indmmm(i,1)=51).
compute condbb=make(nrow(bootres),1,0).
loop k7 = 1 to nwvls.
compute condbb={condbb,wbb(:,(((k4-1)*nwvls*(k2=1))+k7))}.
end loop.
compute condbb=condbb(:,2:ncol(condbb)).
end if.
compute cnt=cnt+(placeh*nwvls).
end if.
do if (pathsz(1,colnumb)) = 1.
compute zbb=tihs(cnt:(cnt+(placeh*nzvls)-1),1).
compute zbb=bootres(:,zbb).
do if (wfirst=0).
compute zfirst=1.
end if.
do if (indmmm(i,1) <> 31).
compute immlbs2=zcatlab(1:nzvls,1).
end if.
do if (indmmm(i,1)=2 or indmmm(i,1)=31 or indmmm(i,1)=51).
do if (indmmm(i,1)=2).
compute imm=zbb(:,1).
end if.
loop k7 = 1 to nzvls.
compute imm={imm,zbb(:,(((k4-1)*nzvls*(k2=1))+k7))}.
end loop.
do if (indmmm(i,1)=2 or indmmm(i,1)=51).
compute imm=imm(:,2:ncol(imm)).
do if (indmmm(i,1)=51).
compute condbb={condbb,imm}.
end if.
end if.
end if.
compute cnt=cnt+(placeh*nzvls).
end if.
do if (pathswz(1,colnumb)) = 1.
compute wzbb=tihs(cnt:(cnt+(placeh*nwvls*nzvls)-1),1).
compute wzbb=bootres(:,wzbb).
do if (indmmm(i,1)=41).
compute imm=wzbb(:,1).
loop k7=1 to nwvls*nzvls.
compute imm={imm,wzbb(:,(((k4-1)*nzvls*nwvls*(k2=1))+k7))}.
end loop.
end if.
do if (indmmm(i,1)=41).
compute imm=imm(:,2:ncol(imm)).
compute condbb={condbb,imm(:,(ncol(imm)-(nwvls*nzvls)+1):ncol(imm))}.
end if.
compute cnt=cnt+(placeh*nzvls*nwvls).
end if.
compute indprobe={focaddon,indprova(k1,:)}.
compute tucker={focbb,wbb,zbb,wzbb}.
loop k3=1 to ncol(indprobe).
compute tucker(:,k3)=tucker(:,k3)*indprobe(1,k3).
end loop.
compute tucker2=tucker2&*rsum(tucker).
do if (indmmm(i,1) = 1 or indmmm(i,1)=2 or indmmm(i,1)=31 or indmmm(i,1)=41 or indmmm(i,1)=51).
do if (immset=1).
do if (ncol(imm2)=1 and ncol(imm) = 1).
compute imm2=imm2&*imm.
end if.
do if (indmmm(i,1)=41 or indmmm(i,1)=51).
do if ((ncol(condbb2) > 1) and (ncol(condbb)>1)).
compute condbb2t=make(nrow(condbb2),(ncol(condbb2)*ncol(condbb)),-999999).
compute k9=1.
do if (wfirst=1).
loop k7=1 to ncol(condbb2).
loop k8 = 1 to ncol(condbb).
compute condbb2t(:,k9)=condbb2(:,k7)&*condbb(:,k8).
compute k9=k9+1.
end loop.
end loop.
end if.
do if (zfirst=1).
loop k7=1 to ncol(condbb).
loop k8 = 1 to ncol(condbb2).
compute condbb2t(:,k9)=condbb(:,k7)&*condbb2(:,k8).
compute k9=k9+1.
end loop.
end loop.
end if.
compute condbb2=condbb2t.
release condbb2t.
end if.
do if ((ncol(condbb2) > 1) and (ncol(condbb)=1)).
loop k7 = 1 to ncol(condbb2).
compute condbb2(:,k7)=condbb2(:,k7)&*condbb.
end loop.
end if.
do if ((ncol(condbb2) = 1) and (ncol(condbb)>1)).
loop k7 = 1 to ncol(condbb).
compute condbb(:,k7)=condbb2&*condbb(:,k7).
end loop.
compute condbb2=condbb.
end if.
end if.
do if (ncol(imm2) <> 1 and ncol(imm) <> 1).
compute imm2t=make(nrow(imm2),(ncol(imm2)*ncol(imm)),-999999).
compute k9=1.
do if (wfirst=1).
loop k7=1 to ncol(imm2).
loop k8 = 1 to ncol(imm).
compute imm2t(:,k9)=imm2(:,k7)&*imm(:,k8).
compute k9=k9+1.
end loop.
end loop.
end if.
do if (zfirst=1).
loop k7=1 to ncol(imm).
loop k8 = 1 to ncol(imm2).
compute imm2t(:,k9)=imm(:,k7)&*imm2(:,k8).
compute k9=k9+1.
end loop.
end loop.
end if.
compute imm2=imm2t.
release imm2t.
end if.
do if ((ncol(imm2) > 1) and (ncol(imm)=1)).
loop k7=1 to ncol(imm2).
compute imm2(:,k7)=imm2(:,k7)&*imm.
end loop.
end if.
do if ((ncol(imm2) = 1) and (ncol(imm) > 1)).
loop k7=1 to ncol(imm).
compute imm(:,k7)=imm2&*imm(:,k7).
end loop.
compute imm2=imm.
end if.
end if.
do if (immset=0).
compute imm2=imm.
do if (indmmm(i,1)=41 or indmmm(i,1)=51).
compute condbb2=condbb.
end if.
compute immset=1.
end if.
end if.
end loop.
compute indtemp=tucker2(1,1).
do if (indmmm(i,1)=12 or indmmm(i,1)=22).
compute imm3={imm3,tucker2}.
do if (k1=nrow(indmodva)).
compute imm3=imm3(:,2:ncol(imm3)).
compute immstop=ncol(imm3).
loop k8=2 to immstop.
do if (indmmmt(i,1)=1).
compute imm3={imm3,(imm3(:,k8)-imm3(:,1))}.
end if.
do if (indmmmt(i,1)=2).
compute imm3={imm3,(imm3(:,k8)-imm3(:,(k8-1)))}.
end if.
do if (indmmmt(i,1)=3).
compute imm3={imm3,((rsum(imm3(:,(k8:immstop)))/(immstop-k8+1))-imm3(:,(k8-1))) }.
end if.
do if (indmmmt(i,1)=4).
compute imm3={imm3,(imm3(:,k8)-(rsum(imm3(:,1:immstop))/immstop))}.
end if.
end loop.
do if (indmmmt(i,1)<5).
compute imm2=imm3(:,(immstop+1):ncol(imm3)).
release imm3.
end if.
end if.
end if.
do if (indmmm(i,1)>-1 and (contrast = 1 or contrast = 2 or contrast=3)).
compute imm4={imm4,tucker2}.
do if (k1=nrow(indmodva) and k1 > 1).
compute imm4=imm4(:,2:ncol(imm4)).
compute immstop=ncol(imm4).
compute condcont=make((immstop*(immstop-1)/2),6,-999).
loop k8 = 1 to (immstop-1).
loop k9 = (k8+1) to immstop.
do if (contrast=1).
compute imm4={imm4,(imm4(:,k9)-imm4(:,k8))}.
end if.
do if (contrast=2).
compute imm4={imm4,(abs(imm4(:,k9))-abs(imm4(:,k8)))}.
end if.
do if (contrast=3).
compute imm4={imm4,(imm4(:,k9)+imm4(:,k8))}.
end if.
compute condcont((ncol(imm4)-immstop),1)=imm4(1,k9).
compute condcont((ncol(imm4)-immstop),2)=imm4(1,k8).
end loop.
end loop.
compute imm4=imm4(:,(immstop+1):ncol(imm4)).
loop k8=1 to ncol(imm4).
compute condcont(k8,3)=imm4(1,k8).
do if (boot > 0).

.
compute temp = imm4(2:nrow(imm4),k8).
compute temp(GRADE( imm4(2:nrow(imm4),k8) )) = imm4(2:nrow(imm4),k8).
compute badlo = 0.
compute badhi = 0.
do if ( (imm4(1,k8)*bc)+(9999*(1-bc)) <> 9999).
compute pv=csum(temp < (imm4(1,k8)*bc)+(9999*(1-bc)) )/nrow(temp).
compute ppv = pv.
do if (pv > .5).
compute ppv = 1-pv.
end if.
compute y5=sqrt(-2*ln(ppv)).
compute xp=y5+((((y5*p4+p3)*y5+p2)*y5+p1)*y5+p0)/((((y5*q4+q3)*y5+q2)*y5+q1)*y5+q0).
do if (pv <= .5).
compute xp = -xp.
end if.
compute cilow=rnd(nrow(temp)*(cdfnorm(2*xp-xp2))).
compute cihigh=trunc(nrow(temp)*(cdfnorm(2*xp+(xp2))))+1.
do if (cilow < 1).
compute cilow = 1.
compute booterr=1.
compute badlo = 1.
end if.
do if (cihigh > nrow(temp)).
compute cihigh = boot.
compute booterr=1.
compute badhi = 1.
end if.
compute llcit=temp(cilow,1).
compute ulcit=temp(cihigh,1).
do if (badlo = 1 and llcit <> priorlo).
compute badend={badend, llcit}.
compute priorlo = llcit.
end if.
do if (badhi = 1 and ulcit <> priorhi).
compute badend={badend, ulcit}.
compute priorhi = ulcit.
end if.
end if.
do if ( (imm4(1,k8)*bc)+(9999*(1-bc)) = 9999).
compute llcit=temp(cilow,1).
compute ulcit=temp(cihigh,1).
end if.
compute bootse=t(sqrt((cssq(temp)-((csum(temp)&**2)/nrow(temp)))/(nrow(temp)-1)))
.
compute condcont(k8,4:6)={bootse,llcit,ulcit}.
end if.
end loop.
do if (boot=0).
compute condcont=condcont(:,1:3).
end if.
compute indcontr=1.
end if.
end if.
do if (xmint=1).
do if (k1=(k4+1)).
compute cftotfx(:,k4)=cftotfx(:,k4)+(tucker2*xscaling).
end if.
end if.
do if (boot > 0).
compute tucker2=tucker2*xscaling.

.
compute temp = tucker2(2:nrow(tucker2),1).
compute temp(GRADE( tucker2(2:nrow(tucker2),1) )) = tucker2(2:nrow(tucker2),1).
compute badlo = 0.
compute badhi = 0.
do if ( (tucker2(1,1)*bc)+(9999*(1-bc)) <> 9999).
compute pv=csum(temp < (tucker2(1,1)*bc)+(9999*(1-bc)) )/nrow(temp).
compute ppv = pv.
do if (pv > .5).
compute ppv = 1-pv.
end if.
compute y5=sqrt(-2*ln(ppv)).
compute xp=y5+((((y5*p4+p3)*y5+p2)*y5+p1)*y5+p0)/((((y5*q4+q3)*y5+q2)*y5+q1)*y5+q0).
do if (pv <= .5).
compute xp = -xp.
end if.
compute cilow=rnd(nrow(temp)*(cdfnorm(2*xp-xp2))).
compute cihigh=trunc(nrow(temp)*(cdfnorm(2*xp+(xp2))))+1.
do if (cilow < 1).
compute cilow = 1.
compute booterr=1.
compute badlo = 1.
end if.
do if (cihigh > nrow(temp)).
compute cihigh = boot.
compute booterr=1.
compute badhi = 1.
end if.
compute llcit=temp(cilow,1).
compute ulcit=temp(cihigh,1).
do if (badlo = 1 and llcit <> priorlo).
compute badend={badend, llcit}.
compute priorlo = llcit.
end if.
do if (badhi = 1 and ulcit <> priorhi).
compute badend={badend, ulcit}.
compute priorhi = ulcit.
end if.
end if.
do if ( (tucker2(1,1)*bc)+(9999*(1-bc)) = 9999).
compute llcit=temp(cilow,1).
compute ulcit=temp(cihigh,1).
end if.
compute bootse=t(sqrt((cssq(temp)-((csum(temp)&**2)/nrow(temp)))/(nrow(temp)-1)))
.
compute indtemp={indtemp*xscaling, bootse,llcit,ulcit}.
end if.
do if (boot=0).
compute indtemp=indtemp*xscaling.
end if.
compute condres(k1,(ncol(indmodva)+1):ncol(condres))=indtemp.
end loop.
do if (xmint=0).
compute resultm2=make(nrow(condres),maxresm,99999).
compute resultm2(1:nrow(condres),1:ncol(condres))=condres.
compute resultm={resultm;resultm2}.
end if.
compute condlbs={condlbs,'Effect'}.
do if (boot > 0).
compute condlbs={condlbs,'BootSE', 'BootLLCI', 'BootULCI'}.
end if.
do if (xmint = 1).
do if (k4 = 1).
compute natindfx=condres((2+(k4-1)),2:ncol(condres)).
end if.
do if (k4 > 1).
compute natindfx={natindfx;condres((2+(k4-1)),2:ncol(condres))}.
end if.
do if (k4=nxvls).
compute resultm2=make(nrow(natindfx),maxresm,99999).
compute resultm2(1:nrow(natindfx),1:ncol(natindfx))=natindfx.
compute resultm={resultm;resultm2}.
end if.
end if.
do if (outscree=1 and xmint=0).
do if (nxvls=1).
print condres/title=' '/cnames=condlbs/format= F10.4 /space=0.
else.
compute condrlb=make(nrow(condres),1,xcatlab(k4,1)).
print condres/title=' '/cnames=condlbs/rnames=condrlb/format= F10.4 /space=0.
end if.
end if.
do if (outscree=1 and xmint=1 and k4=nxvls).
do if (nxvls=1).
print natindfx/title=' '/clabels='Effect', 'BootSE', 'BootLLCI', 'BootULCI'/format= F10.4 /space=0.
else.
compute condrlb=make(nrow(condres),1,xcatlab(k4,1)).
print natindfx/title=' '/clabels='Effect', 'BootSE', 'BootLLCI', 'BootULCI'/rnames=direffl2/format= F10.4 /space=0.
end if.
end if.
compute dichadj=0.
compute immcat=0.
do if ((indmmm(i,1)>0) and (xmint=0)).
do if (indmmm(i,1)=1 or indmmm(i,1)=12 or indmmm(i,1)=31).
do if (wdich=1 and mcw=0).
do if (indmmm(i,1) <> 12).
compute imm2(:,1)=imm2(:,1)*(wmax-wmin).
end if.
do if (indmmm(i,1) <> 31).
compute dichadj=1.
end if.
end if.
do if ((mcw = 1 or mcw = 2) and indmmm(i,1) <> 31)).
compute immcat=1.
end if.
end if.
do if (indmmm(i,1)=2 or indmmm(i,1)=22 or indmmm(i,1)=31).
do if (zdich=1 and mcz=0).
do if (indmmm(i,1) = 31).
compute imm2(:,(nwvls+1):ncol(imm2))=imm2(:,(nwvls+1):ncol(imm2))*(zmax-zmin).
end if.
do if (indmmm(i,1) = 2).
compute imm2(:,1)=imm2(:,1)*(zmax-zmin).
end if.
do if (indmmm(i,1) <> 31).
compute dichadj=1.
end if.
end if.
do if ((mcz = 1 or mcz = 2) and indmmm(i,1) <> 31)).
compute immcat=1.
end if.
end if.
compute immtemp2=t(imm2(1,:)).
compute immtemp=immtemp2.
compute immlbs={'Index'}.
do if (boot > 0).
compute immtemp=make(1,3,0).
loop k7=1 to ncol(imm2).

.
compute temp = imm2(2:nrow(imm2),k7).
compute temp(GRADE( imm2(2:nrow(imm2),k7) )) = imm2(2:nrow(imm2),k7).
compute badlo = 0.
compute badhi = 0.
do if ( (imm2(1,k7)*bc)+(9999*(1-bc)) <> 9999).
compute pv=csum(temp < (imm2(1,k7)*bc)+(9999*(1-bc)) )/nrow(temp).
compute ppv = pv.
do if (pv > .5).
compute ppv = 1-pv.
end if.
compute y5=sqrt(-2*ln(ppv)).
compute xp=y5+((((y5*p4+p3)*y5+p2)*y5+p1)*y5+p0)/((((y5*q4+q3)*y5+q2)*y5+q1)*y5+q0).
do if (pv <= .5).
compute xp = -xp.
end if.
compute cilow=rnd(nrow(temp)*(cdfnorm(2*xp-xp2))).
compute cihigh=trunc(nrow(temp)*(cdfnorm(2*xp+(xp2))))+1.
do if (cilow < 1).
compute cilow = 1.
compute booterr=1.
compute badlo = 1.
end if.
do if (cihigh > nrow(temp)).
compute cihigh = boot.
compute booterr=1.
compute badhi = 1.
end if.
compute llcit=temp(cilow,1).
compute ulcit=temp(cihigh,1).
do if (badlo = 1 and llcit <> priorlo).
compute badend={badend, llcit}.
compute priorlo = llcit.
end if.
do if (badhi = 1 and ulcit <> priorhi).
compute badend={badend, ulcit}.
compute priorhi = ulcit.
end if.
end if.
do if ( (imm2(1,k7)*bc)+(9999*(1-bc)) = 9999).
compute llcit=temp(cilow,1).
compute ulcit=temp(cihigh,1).
end if.
compute bootse=t(sqrt((cssq(temp)-((csum(temp)&**2)/nrow(temp)))/(nrow(temp)-1)))
.
compute temp={bootse,llcit,ulcit}.
compute immtemp={immtemp;temp}.
end loop.
compute immtemp=immtemp(2:nrow(immtemp),:).
compute immtemp={immtemp2,immtemp}.
compute immlbs={immlbs,'BootSE', 'BootLLCI', 'BootULCI'}.
end if.
compute resultm2=make(nrow(immtemp),maxresm,99999).
compute resultm2(1:nrow(immtemp),1:ncol(immtemp))=immtemp.
compute resultm={resultm;resultm2}.
do if (dichadj=0 and immcat=0 and indmmmt(i,1)<>5 and indmmm(i,1) < 100 ).
do if (outscree=1).
do if (indmmm(i,1) < 30).
print immtemp/title='      Index of moderated mediation:'/cnames=immlbs/rnames=immlbs2/format= F10.4.
end if.
do if (indmmm(i,1) = 31).
compute immlbs2={immlbs2;zcatlab(1:nzvls,1)}.
print immtemp/title='      Indices of partial moderated mediation:'/cnames=immlbs/rnames=immlbs2/format= F10.4.
end if.
end if.
do if ((nzvls = 1) and (nwvls = 1)).
do if (indmmm(i,1) = 41 or indmmm(i,1)=51).
loop k7=1 to nwvls.
compute immlbs2=zcatlab(1:nzvls,1).
compute immtemp2=immtemp((((k7-1)*nzvls)+1):(((k7-1)*nzvls)+nzvls),:).
do if (outscree=1).
do if (nwvls > 1).
compute primodv={'        ', wcatlab(k7,1)}.
print primodv/title='      Primary moderator:'/format=A8.
end if.
do if (nzvls=1).
print immtemp2/title='      Index of moderated moderated mediation'/cnames=immlbs/format= F10.4.
else.
print immtemp2/title='      Indices of moderated moderated mediation'/cnames=immlbs/rnames=immlbs2/format= F10.4.
end if.
end if.
compute cmmtemp=make(nrow(zprobval),4,0).
loop k8=1 to nrow(zprobval).
compute condbb3=condbb2(:,((nwvls+1)+((k7-1)*nzvls)):((nwvls+1)+((k7-1)*nzvls)+ (nzvls-1))).
do if (ncol(zprobval) > 1).
compute condbb3=condbb3*mdiag(zprobval(k8,:)).
else.
compute condbb3=condbb3&*zprobval(k8,:).
end if.
compute condbb3={condbb2(:,k7),condbb3}.
compute icmm=rsum(condbb3).
compute cmmtemp(k8,1)=icmm(1,1).
do if (boot > 0).

.
compute temp = icmm(2:nrow(icmm),1).
compute temp(GRADE( icmm(2:nrow(icmm),1) )) = icmm(2:nrow(icmm),1).
compute badlo = 0.
compute badhi = 0.
do if ( (icmm(1,1)*bc)+(9999*(1-bc)) <> 9999).
compute pv=csum(temp < (icmm(1,1)*bc)+(9999*(1-bc)) )/nrow(temp).
compute ppv = pv.
do if (pv > .5).
compute ppv = 1-pv.
end if.
compute y5=sqrt(-2*ln(ppv)).
compute xp=y5+((((y5*p4+p3)*y5+p2)*y5+p1)*y5+p0)/((((y5*q4+q3)*y5+q2)*y5+q1)*y5+q0).
do if (pv <= .5).
compute xp = -xp.
end if.
compute cilow=rnd(nrow(temp)*(cdfnorm(2*xp-xp2))).
compute cihigh=trunc(nrow(temp)*(cdfnorm(2*xp+(xp2))))+1.
do if (cilow < 1).
compute cilow = 1.
compute booterr=1.
compute badlo = 1.
end if.
do if (cihigh > nrow(temp)).
compute cihigh = boot.
compute booterr=1.
compute badhi = 1.
end if.
compute llcit=temp(cilow,1).
compute ulcit=temp(cihigh,1).
do if (badlo = 1 and llcit <> priorlo).
compute badend={badend, llcit}.
compute priorlo = llcit.
end if.
do if (badhi = 1 and ulcit <> priorhi).
compute badend={badend, ulcit}.
compute priorhi = ulcit.
end if.
end if.
do if ( (icmm(1,1)*bc)+(9999*(1-bc)) = 9999).
compute llcit=temp(cilow,1).
compute ulcit=temp(cihigh,1).
end if.
compute bootse=t(sqrt((cssq(temp)-((csum(temp)&**2)/nrow(temp)))/(nrow(temp)-1)))
.
compute cmmtemp(k8,2:4)={bootse,llcit,ulcit}.
end if.
end loop.
compute cmmtemp={zmodvals,cmmtemp}.
do if (boot=0).
compute cmmtemp=cmmtemp(:,1:2).
end if.
compute cmmlbs={znames,immlbs}.
compute resultm2=make(nrow(cmmtemp),maxresm,99999).
compute resultm2(1:nrow(cmmtemp),1:ncol(cmmtemp))=cmmtemp.
compute resultm={resultm;resultm2}.
do if (outscree=1).
print cmmtemp/title='      Indices of conditional moderated mediation by W'/cnames=cmmlbs/format= F10.4.
end if.
end loop.
end if.
end if.
end if.
do if (dichadj=1 or immcat=1 and indmmm(i,1) < 30).
do if (outscree=1).
print immtemp/title='Index of moderated mediation (difference between conditional indirect effects):'/cnames=immlbs/rnames=immlbs2/format= F10.4.
end if.
end if.
end if.
do if (indcontr=1).
compute resultm2=make(nrow(condcont),maxresm,99999).
compute resultm2(1:nrow(condcont),1:ncol(condcont))=condcont.
compute resultm={resultm;resultm2}.
compute condctlb={'Effect1','Effect2','Contrast','BootSE', 'BootLLCI','BootULCI'}.
do if (outscree=1).
print condcont/title=' Pairwise contrasts between conditional indirect effects (Effect1 minus Effect2)'/cnames=condctlb/format= F10.4.
end if.
end if.
end loop.
end if.
end loop.
do if (alttotal=1).
compute altcnms='Effect'.
compute alttotfx=t(cftotfx(1,:))+obnatdfx.
do if (boot > 0).
compute alttotfx=make(ncol(cftotfx),4,0).
compute alttotfx(:,1)=t(cftotfx(1,:))+obnatdfx.
compute natdirbt(1,:)=t(obnatdfx).
compute cftotfx=cftotfx+natdirbt.
loop cec=1 to ncol(cftotfx).

.
compute temp = cftotfx(2:nrow(cftotfx),cec).
compute temp(GRADE( cftotfx(2:nrow(cftotfx),cec) )) = cftotfx(2:nrow(cftotfx),cec).
compute badlo = 0.
compute badhi = 0.
do if ( (cftotfx(1,cec)*bc)+(9999*(1-bc)) <> 9999).
compute pv=csum(temp < (cftotfx(1,cec)*bc)+(9999*(1-bc)) )/nrow(temp).
compute ppv = pv.
do if (pv > .5).
compute ppv = 1-pv.
end if.
compute y5=sqrt(-2*ln(ppv)).
compute xp=y5+((((y5*p4+p3)*y5+p2)*y5+p1)*y5+p0)/((((y5*q4+q3)*y5+q2)*y5+q1)*y5+q0).
do if (pv <= .5).
compute xp = -xp.
end if.
compute cilow=rnd(nrow(temp)*(cdfnorm(2*xp-xp2))).
compute cihigh=trunc(nrow(temp)*(cdfnorm(2*xp+(xp2))))+1.
do if (cilow < 1).
compute cilow = 1.
compute booterr=1.
compute badlo = 1.
end if.
do if (cihigh > nrow(temp)).
compute cihigh = boot.
compute booterr=1.
compute badhi = 1.
end if.
compute llcit=temp(cilow,1).
compute ulcit=temp(cihigh,1).
do if (badlo = 1 and llcit <> priorlo).
compute badend={badend, llcit}.
compute priorlo = llcit.
end if.
do if (badhi = 1 and ulcit <> priorhi).
compute badend={badend, ulcit}.
compute priorhi = ulcit.
end if.
end if.
do if ( (cftotfx(1,cec)*bc)+(9999*(1-bc)) = 9999).
compute llcit=temp(cilow,1).
compute ulcit=temp(cihigh,1).
end if.
compute bootse=t(sqrt((cssq(temp)-((csum(temp)&**2)/nrow(temp)))/(nrow(temp)-1)))
.
compute alttotfx(cec,2:4)={bootse,llcit,ulcit}.
end loop.
compute altcnms={altcnms,'BootSE', 'BootLLCI','BootULCI'}.
end if.
compute resultm2=make(nrow(alttotfx),maxresm,99999).
compute resultm2(1:nrow(alttotfx),1:ncol(alttotfx))=alttotfx.
compute resultm={resultm;resultm2}.
do if (outscree=1).
print/title='----------'.
do if (nxvls > 1).
print alttotfx/title='Relative total effects of X on Y (sum of direct and indirect effects)'/cnames=altcnms/rnames=direffl2/format= F10.4.
end if.
do if (nxvls = 1).
print alttotfx/title='Total effect of X on Y (sum of direct and indirect effects)'/cnames=altcnms/format= F10.4.
end if.
end if.
end if.
end if.
end if
.
do if (criterr=0 and (saveest = 1 or saveboot=1)).
compute coeffsav=coeffmat(2:nrow(coeffmat),:).
compute conseq=conseq(2:nrow(conseq),1).
compute dfmat=dfmat(2:nrow(dfmat),1).
compute dfmat=dfmat.
compute saven={'conseqnt','antecdnt','coeff','se','t','p','LLCI','ULCI','df'}.
do if (ydich=1).
compute saven={'conseqnt','antecdnt','coeff','se','t_or_Z','p','LLCI','ULCI','df'}.
end if.
do if (saveboot=1 and boot > 0 ).
compute colslab=make(300,1,' ').


compute colslab( 1 ,1)= 'COL1'.


compute colslab( 2 ,1)= 'COL2'.


compute colslab( 3 ,1)= 'COL3'.


compute colslab( 4 ,1)= 'COL4'.


compute colslab( 5 ,1)= 'COL5'.


compute colslab( 6 ,1)= 'COL6'.


compute colslab( 7 ,1)= 'COL7'.


compute colslab( 8 ,1)= 'COL8'.


compute colslab( 9 ,1)= 'COL9'.


compute colslab( 10 ,1)= 'COL10'.


compute colslab( 11 ,1)= 'COL11'.


compute colslab( 12 ,1)= 'COL12'.


compute colslab( 13 ,1)= 'COL13'.


compute colslab( 14 ,1)= 'COL14'.


compute colslab( 15 ,1)= 'COL15'.


compute colslab( 16 ,1)= 'COL16'.


compute colslab( 17 ,1)= 'COL17'.


compute colslab( 18 ,1)= 'COL18'.


compute colslab( 19 ,1)= 'COL19'.


compute colslab( 20 ,1)= 'COL20'.


compute colslab( 21 ,1)= 'COL21'.


compute colslab( 22 ,1)= 'COL22'.


compute colslab( 23 ,1)= 'COL23'.


compute colslab( 24 ,1)= 'COL24'.


compute colslab( 25 ,1)= 'COL25'.


compute colslab( 26 ,1)= 'COL26'.


compute colslab( 27 ,1)= 'COL27'.


compute colslab( 28 ,1)= 'COL28'.


compute colslab( 29 ,1)= 'COL29'.


compute colslab( 30 ,1)= 'COL30'.


compute colslab( 31 ,1)= 'COL31'.


compute colslab( 32 ,1)= 'COL32'.


compute colslab( 33 ,1)= 'COL33'.


compute colslab( 34 ,1)= 'COL34'.


compute colslab( 35 ,1)= 'COL35'.


compute colslab( 36 ,1)= 'COL36'.


compute colslab( 37 ,1)= 'COL37'.


compute colslab( 38 ,1)= 'COL38'.


compute colslab( 39 ,1)= 'COL39'.


compute colslab( 40 ,1)= 'COL40'.


compute colslab( 41 ,1)= 'COL41'.


compute colslab( 42 ,1)= 'COL42'.


compute colslab( 43 ,1)= 'COL43'.


compute colslab( 44 ,1)= 'COL44'.


compute colslab( 45 ,1)= 'COL45'.


compute colslab( 46 ,1)= 'COL46'.


compute colslab( 47 ,1)= 'COL47'.


compute colslab( 48 ,1)= 'COL48'.


compute colslab( 49 ,1)= 'COL49'.


compute colslab( 50 ,1)= 'COL50'.


compute colslab( 51 ,1)= 'COL51'.


compute colslab( 52 ,1)= 'COL52'.


compute colslab( 53 ,1)= 'COL53'.


compute colslab( 54 ,1)= 'COL54'.


compute colslab( 55 ,1)= 'COL55'.


compute colslab( 56 ,1)= 'COL56'.


compute colslab( 57 ,1)= 'COL57'.


compute colslab( 58 ,1)= 'COL58'.


compute colslab( 59 ,1)= 'COL59'.


compute colslab( 60 ,1)= 'COL60'.


compute colslab( 61 ,1)= 'COL61'.


compute colslab( 62 ,1)= 'COL62'.


compute colslab( 63 ,1)= 'COL63'.


compute colslab( 64 ,1)= 'COL64'.


compute colslab( 65 ,1)= 'COL65'.


compute colslab( 66 ,1)= 'COL66'.


compute colslab( 67 ,1)= 'COL67'.


compute colslab( 68 ,1)= 'COL68'.


compute colslab( 69 ,1)= 'COL69'.


compute colslab( 70 ,1)= 'COL70'.


compute colslab( 71 ,1)= 'COL71'.


compute colslab( 72 ,1)= 'COL72'.


compute colslab( 73 ,1)= 'COL73'.


compute colslab( 74 ,1)= 'COL74'.


compute colslab( 75 ,1)= 'COL75'.


compute colslab( 76 ,1)= 'COL76'.


compute colslab( 77 ,1)= 'COL77'.


compute colslab( 78 ,1)= 'COL78'.


compute colslab( 79 ,1)= 'COL79'.


compute colslab( 80 ,1)= 'COL80'.


compute colslab( 81 ,1)= 'COL81'.


compute colslab( 82 ,1)= 'COL82'.


compute colslab( 83 ,1)= 'COL83'.


compute colslab( 84 ,1)= 'COL84'.


compute colslab( 85 ,1)= 'COL85'.


compute colslab( 86 ,1)= 'COL86'.


compute colslab( 87 ,1)= 'COL87'.


compute colslab( 88 ,1)= 'COL88'.


compute colslab( 89 ,1)= 'COL89'.


compute colslab( 90 ,1)= 'COL90'.


compute colslab( 91 ,1)= 'COL91'.


compute colslab( 92 ,1)= 'COL92'.


compute colslab( 93 ,1)= 'COL93'.


compute colslab( 94 ,1)= 'COL94'.


compute colslab( 95 ,1)= 'COL95'.


compute colslab( 96 ,1)= 'COL96'.


compute colslab( 97 ,1)= 'COL97'.


compute colslab( 98 ,1)= 'COL98'.


compute colslab( 99 ,1)= 'COL99'.


compute colslab( 100 ,1)= 'COL100'.


compute colslab( 101 ,1)= 'COL101'.


compute colslab( 102 ,1)= 'COL102'.


compute colslab( 103 ,1)= 'COL103'.


compute colslab( 104 ,1)= 'COL104'.


compute colslab( 105 ,1)= 'COL105'.


compute colslab( 106 ,1)= 'COL106'.


compute colslab( 107 ,1)= 'COL107'.


compute colslab( 108 ,1)= 'COL108'.


compute colslab( 109 ,1)= 'COL109'.


compute colslab( 110 ,1)= 'COL110'.


compute colslab( 111 ,1)= 'COL111'.


compute colslab( 112 ,1)= 'COL112'.


compute colslab( 113 ,1)= 'COL113'.


compute colslab( 114 ,1)= 'COL114'.


compute colslab( 115 ,1)= 'COL115'.


compute colslab( 116 ,1)= 'COL116'.


compute colslab( 117 ,1)= 'COL117'.


compute colslab( 118 ,1)= 'COL118'.


compute colslab( 119 ,1)= 'COL119'.


compute colslab( 120 ,1)= 'COL120'.


compute colslab( 121 ,1)= 'COL121'.


compute colslab( 122 ,1)= 'COL122'.


compute colslab( 123 ,1)= 'COL123'.


compute colslab( 124 ,1)= 'COL124'.


compute colslab( 125 ,1)= 'COL125'.


compute colslab( 126 ,1)= 'COL126'.


compute colslab( 127 ,1)= 'COL127'.


compute colslab( 128 ,1)= 'COL128'.


compute colslab( 129 ,1)= 'COL129'.


compute colslab( 130 ,1)= 'COL130'.


compute colslab( 131 ,1)= 'COL131'.


compute colslab( 132 ,1)= 'COL132'.


compute colslab( 133 ,1)= 'COL133'.


compute colslab( 134 ,1)= 'COL134'.


compute colslab( 135 ,1)= 'COL135'.


compute colslab( 136 ,1)= 'COL136'.


compute colslab( 137 ,1)= 'COL137'.


compute colslab( 138 ,1)= 'COL138'.


compute colslab( 139 ,1)= 'COL139'.


compute colslab( 140 ,1)= 'COL140'.


compute colslab( 141 ,1)= 'COL141'.


compute colslab( 142 ,1)= 'COL142'.


compute colslab( 143 ,1)= 'COL143'.


compute colslab( 144 ,1)= 'COL144'.


compute colslab( 145 ,1)= 'COL145'.


compute colslab( 146 ,1)= 'COL146'.


compute colslab( 147 ,1)= 'COL147'.


compute colslab( 148 ,1)= 'COL148'.


compute colslab( 149 ,1)= 'COL149'.


compute colslab( 150 ,1)= 'COL150'.


compute colslab( 151 ,1)= 'COL151'.


compute colslab( 152 ,1)= 'COL152'.


compute colslab( 153 ,1)= 'COL153'.


compute colslab( 154 ,1)= 'COL154'.


compute colslab( 155 ,1)= 'COL155'.


compute colslab( 156 ,1)= 'COL156'.


compute colslab( 157 ,1)= 'COL157'.


compute colslab( 158 ,1)= 'COL158'.


compute colslab( 159 ,1)= 'COL159'.


compute colslab( 160 ,1)= 'COL160'.


compute colslab( 161 ,1)= 'COL161'.


compute colslab( 162 ,1)= 'COL162'.


compute colslab( 163 ,1)= 'COL163'.


compute colslab( 164 ,1)= 'COL164'.


compute colslab( 165 ,1)= 'COL165'.


compute colslab( 166 ,1)= 'COL166'.


compute colslab( 167 ,1)= 'COL167'.


compute colslab( 168 ,1)= 'COL168'.


compute colslab( 169 ,1)= 'COL169'.


compute colslab( 170 ,1)= 'COL170'.


compute colslab( 171 ,1)= 'COL171'.


compute colslab( 172 ,1)= 'COL172'.


compute colslab( 173 ,1)= 'COL173'.


compute colslab( 174 ,1)= 'COL174'.


compute colslab( 175 ,1)= 'COL175'.


compute colslab( 176 ,1)= 'COL176'.


compute colslab( 177 ,1)= 'COL177'.


compute colslab( 178 ,1)= 'COL178'.


compute colslab( 179 ,1)= 'COL179'.


compute colslab( 180 ,1)= 'COL180'.


compute colslab( 181 ,1)= 'COL181'.


compute colslab( 182 ,1)= 'COL182'.


compute colslab( 183 ,1)= 'COL183'.


compute colslab( 184 ,1)= 'COL184'.


compute colslab( 185 ,1)= 'COL185'.


compute colslab( 186 ,1)= 'COL186'.


compute colslab( 187 ,1)= 'COL187'.


compute colslab( 188 ,1)= 'COL188'.


compute colslab( 189 ,1)= 'COL189'.


compute colslab( 190 ,1)= 'COL190'.


compute colslab( 191 ,1)= 'COL191'.


compute colslab( 192 ,1)= 'COL192'.


compute colslab( 193 ,1)= 'COL193'.


compute colslab( 194 ,1)= 'COL194'.


compute colslab( 195 ,1)= 'COL195'.


compute colslab( 196 ,1)= 'COL196'.


compute colslab( 197 ,1)= 'COL197'.


compute colslab( 198 ,1)= 'COL198'.


compute colslab( 199 ,1)= 'COL199'.


compute colslab( 200 ,1)= 'COL200'.


compute colslab( 201 ,1)= 'COL201'.


compute colslab( 202 ,1)= 'COL202'.


compute colslab( 203 ,1)= 'COL203'.


compute colslab( 204 ,1)= 'COL204'.


compute colslab( 205 ,1)= 'COL205'.


compute colslab( 206 ,1)= 'COL206'.


compute colslab( 207 ,1)= 'COL207'.


compute colslab( 208 ,1)= 'COL208'.


compute colslab( 209 ,1)= 'COL209'.


compute colslab( 210 ,1)= 'COL210'.


compute colslab( 211 ,1)= 'COL211'.


compute colslab( 212 ,1)= 'COL212'.


compute colslab( 213 ,1)= 'COL213'.


compute colslab( 214 ,1)= 'COL214'.


compute colslab( 215 ,1)= 'COL215'.


compute colslab( 216 ,1)= 'COL216'.


compute colslab( 217 ,1)= 'COL217'.


compute colslab( 218 ,1)= 'COL218'.


compute colslab( 219 ,1)= 'COL219'.


compute colslab( 220 ,1)= 'COL220'.


compute colslab( 221 ,1)= 'COL221'.


compute colslab( 222 ,1)= 'COL222'.


compute colslab( 223 ,1)= 'COL223'.


compute colslab( 224 ,1)= 'COL224'.


compute colslab( 225 ,1)= 'COL225'.


compute colslab( 226 ,1)= 'COL226'.


compute colslab( 227 ,1)= 'COL227'.


compute colslab( 228 ,1)= 'COL228'.


compute colslab( 229 ,1)= 'COL229'.


compute colslab( 230 ,1)= 'COL230'.


compute colslab( 231 ,1)= 'COL231'.


compute colslab( 232 ,1)= 'COL232'.


compute colslab( 233 ,1)= 'COL233'.


compute colslab( 234 ,1)= 'COL234'.


compute colslab( 235 ,1)= 'COL235'.


compute colslab( 236 ,1)= 'COL236'.


compute colslab( 237 ,1)= 'COL237'.


compute colslab( 238 ,1)= 'COL238'.


compute colslab( 239 ,1)= 'COL239'.


compute colslab( 240 ,1)= 'COL240'.


compute colslab( 241 ,1)= 'COL241'.


compute colslab( 242 ,1)= 'COL242'.


compute colslab( 243 ,1)= 'COL243'.


compute colslab( 244 ,1)= 'COL244'.


compute colslab( 245 ,1)= 'COL245'.


compute colslab( 246 ,1)= 'COL246'.


compute colslab( 247 ,1)= 'COL247'.


compute colslab( 248 ,1)= 'COL248'.


compute colslab( 249 ,1)= 'COL249'.


compute colslab( 250 ,1)= 'COL250'.


compute colslab( 251 ,1)= 'COL251'.


compute colslab( 252 ,1)= 'COL252'.


compute colslab( 253 ,1)= 'COL253'.


compute colslab( 254 ,1)= 'COL254'.


compute colslab( 255 ,1)= 'COL255'.


compute colslab( 256 ,1)= 'COL256'.


compute colslab( 257 ,1)= 'COL257'.


compute colslab( 258 ,1)= 'COL258'.


compute colslab( 259 ,1)= 'COL259'.


compute colslab( 260 ,1)= 'COL260'.


compute colslab( 261 ,1)= 'COL261'.


compute colslab( 262 ,1)= 'COL262'.


compute colslab( 263 ,1)= 'COL263'.


compute colslab( 264 ,1)= 'COL264'.


compute colslab( 265 ,1)= 'COL265'.


compute colslab( 266 ,1)= 'COL266'.


compute colslab( 267 ,1)= 'COL267'.


compute colslab( 268 ,1)= 'COL268'.


compute colslab( 269 ,1)= 'COL269'.


compute colslab( 270 ,1)= 'COL270'.


compute colslab( 271 ,1)= 'COL271'.


compute colslab( 272 ,1)= 'COL272'.


compute colslab( 273 ,1)= 'COL273'.


compute colslab( 274 ,1)= 'COL274'.


compute colslab( 275 ,1)= 'COL275'.


compute colslab( 276 ,1)= 'COL276'.


compute colslab( 277 ,1)= 'COL277'.


compute colslab( 278 ,1)= 'COL278'.


compute colslab( 279 ,1)= 'COL279'.


compute colslab( 280 ,1)= 'COL280'.


compute colslab( 281 ,1)= 'COL281'.


compute colslab( 282 ,1)= 'COL282'.


compute colslab( 283 ,1)= 'COL283'.


compute colslab( 284 ,1)= 'COL284'.


compute colslab( 285 ,1)= 'COL285'.


compute colslab( 286 ,1)= 'COL286'.


compute colslab( 287 ,1)= 'COL287'.


compute colslab( 288 ,1)= 'COL288'.


compute colslab( 289 ,1)= 'COL289'.


compute colslab( 290 ,1)= 'COL290'.


compute colslab( 291 ,1)= 'COL291'.


compute colslab( 292 ,1)= 'COL292'.


compute colslab( 293 ,1)= 'COL293'.


compute colslab( 294 ,1)= 'COL294'.


compute colslab( 295 ,1)= 'COL295'.


compute colslab( 296 ,1)= 'COL296'.


compute colslab( 297 ,1)= 'COL297'.


compute colslab( 298 ,1)= 'COL298'.


compute colslab( 299 ,1)= 'COL299'.


compute colslab( 300 ,1)= 'COL300'.

compute colslab=colslab(1:ncol(bootres),1).
compute colslab={colslab,conseq,vlabs}.
do if (outscree=1).
print/title = '**************************************************************************'.
print/title='Bootstrap estimates were saved to a file'/space=0.
print colslab/title='Map of column names to model coefficients:'/clabels=' ','Conseqnt','Antecdnt'/format=a8.
end if.
end if.
end if.
do if (criterr=0 and boot > 0 and modelbt=1).
compute labstart=1.
do if (outscree=1).
print/title = '*********** BOOTSTRAP RESULTS FOR REGRESSION MODEL PARAMETERS ************'.
end if.
loop iboot = 1 to (nms+nys).
do if (outscree=1).
print outnames(1,iboot)/title = 'OUTCOME VARIABLE:'/format = A8.
end if.
compute vlabsm=vlabs(labstart:(labstart+(nump(1,iboot)-1)),1).
compute outnmtmp=bootcim(labstart:(labstart+(nump(1,iboot)-1)),:).
compute resultm2=make(nrow(outnmtmp),maxresm,99999).
compute resultm2(1:nrow(outnmtmp),1:ncol(outnmtmp))=outnmtmp.
compute resultm={resultm;resultm2}.
do if (outscree=1).
print bootcim(labstart:(labstart+(nump(1,iboot)-1)),:)/title=' '/rnames=vlabsm/ clabels='Coeff' 'BootMean' 'BootSE' 'BootLLCI' 'BootULCI'/format= F10.4 /space=0.
end if.
compute labstart=labstart+nump(1,iboot).
do if (iboot < (nms+nys)).
do if (outscree=1).
print/title= '----------'.
end if.
end if.
end loop.
end if.
do if (criterr=0 and saveest=1).
compute resultm=resultm(2:nrow(resultm),:).
compute bocaj=(resultm=99999).
compute bocaj=csum(bocaj).
compute bocaj=1-(bocaj=nrow(resultm)).
compute j=1.
loop i = 1 to ncol(resultm).
do if (bocaj(1,i)=1).
compute j=j+1.
end if.
end loop.
compute resultm=resultm(:,1:(j-1)).
save resultm/outfile = *.
end if.
do if (criterr = 0 and matrices=1 and outscree=1).
print/title = '************************ MODEL DEFINITION MATRICES ************************'.
print/title = 'FROM variables are columns, TO variables are rows.'.
compute temp2=make(nrow(bcmat),ncol(bcmat),'0').
loop i = 2 to nrow(bcmat).
loop j = 1 to (ncol(bcmat)-1).
do if (bcmat(i,j)=1).
compute temp2(i,j)='1'.
end if.
do if (j >= i).
compute temp2(i,j)=' '.
end if.
end loop.
end loop.
compute temp2=temp2(2:nrow(bcmat),(1:(ncol(bcmat)-1))).
do if (nms > 0).
compute cmatlabs={xnames,mnames}.
compute rmatlabs={mnames,ynames}.
end if.
do if (nms = 0).
compute cmatlabs={xnames}.
compute rmatlabs={ynames}.
end if.
print temp2/title='BMATRIX: Paths freely estimated (1) and fixed to zero (0):'/cnames=cmatlabs/rnames=rmatlabs/format A3.
compute z=0.
do if (rsum(csum(wcmat))<>0).
compute temp2=make(nrow(wcmat),ncol(wcmat),'0').
loop i = 2 to nrow(wcmat).
loop j = 1 to (ncol(wcmat)-1).
do if (wcmat(i,j)=1).
compute temp2(i,j)='1'.
end if.
do if (j >= i).
compute temp2(i,j)=' '.
end if.
end loop.
end loop.
compute temp2=temp2(2:nrow(wcmat),(1:(ncol(wcmat)-1))).
print temp2/title='WMATRIX: Paths moderated (1) and not moderated (0) by W:'/cnames=cmatlabs/rnames=rmatlabs/format A3.
end if.
do if (rsum(csum(zcmat))<>0).
compute temp2=make(nrow(zcmat),ncol(zcmat),'0').
loop i = 2 to nrow(zcmat).
loop j = 1 to (ncol(zcmat)-1).
do if (zcmat(i,j)=1).
compute temp2(i,j)='1'.
end if.
do if (j >= i).
compute temp2(i,j)=' '.
end if.
end loop.
end loop.
compute temp2=temp2(2:nrow(zcmat),(1:(ncol(zcmat)-1))).
print temp2/title='ZMATRIX: Paths moderated (1) and not moderated (0) by Z:'/cnames=cmatlabs/rnames=rmatlabs/format a3.
end if.
do if (rsum(csum(wzcmat))<>0).
compute temp2=make(nrow(wzcmat),ncol(wzcmat),'0').
loop i = 2 to nrow(wzcmat).
loop j = 1 to (ncol(wzcmat)-1).
do if (wzcmat(i,j)=1).
compute temp2(i,j)='1'.
end if.
do if (j >= i).
compute temp2(i,j)=' '.
end if.
end loop.
end loop.
compute temp2=temp2(2:nrow(wzcmat),(1:(ncol(wzcmat)-1))).
print temp2/title='WZMATRIX: W moderated paths moderated (1) and not moderated (0) by Z:'/cnames=cmatlabs/rnames=rmatlabs/format a3.
end if.
do if (ncs > 0).
print ccmat/title='CMATRIX: Covariates (columns) in (1) and not in (0) the models of M and Y (rows):'/rnames=rmatlabs/cnames=covnames.
end if.
end if.
do if (outscree=1).
do if (activate=0).
print/title = '*********************** ANALYSIS NOTES AND ERRORS ************************'.
end if.
end if.
do if (activate=1).
print/title = '**************************************************************************'.
end if.
do if (criterr=0).
do if (outscree=1).
print conf/title = 'Level of confidence for all confidence intervals in output:'/format = F8.4.
do if (boot > 0).
do if (goodboot = boot and bc=0).
print boot/title='Number of bootstrap samples for percentile bootstrap confidence intervals:'.
end if.
do if (goodboot = boot and bc=1).
print boot/title='Number of bootstrap samples for bias-corrected bootstrap confidence intervals:'.
end if.
do if (booterr = 1).
compute badend = badend(1,2:ncol(badend)).
print/title = 'WARNING: Bootstrap CI endpoints below not trustworthy. Decrease confidence'.
print badend/title='or increase the number of bootstrap samples.'/space=0/format = F10.4.
end if.
end if.
do if (mc > 0).
print mc/title='Number of samples for Monte Carlo confidence intervals:'.
end if.
do if (wnotev > 0 and printw=1).
do if (wnotev=1).
print/title = 'W values in conditional tables are the 16th, 50th, and 84th percentiles.'.
else if (wnotev=2).
do if (minwwarn=0 and maxwwarn=0).
print/title = 'W values in conditional tables are the mean and +/- SD from the mean.'.
end if.
do if (minwwarn=1).
print/title = 'W values in conditional tables are the minimum, the mean, and 1 SD above the mean.'.
end if.
do if (maxwwarn=1).
print/title = 'W values in conditional tables are 1 SD below the mean, the mean, and the maximum.'.
end if.
end if.
end if.
do if (znotev > 0 and printz=1).
do if (znotev=1).
print/title = 'Z values in conditional tables are the 16th, 50th, and 84th percentiles.'.
else if (znotev=2).
do if (minzwarn=0 and maxzwarn=0).
print/title = 'Z values in conditional tables are the mean and +/- SD from the mean.'.
end if.
do if (minzwarn=1).
print/title = 'Z values in conditional tables are the minimum, the mean, and 1 SD above the mean.'.
end if.
do if (maxzwarn=1).
print/title = 'Z values in conditional tables are 1 SD below the mean, the mean, and the maximum.'.
end if.
end if.
end if.
do if (minwwarn > 0).
print/title = 'NOTE: One SD below the mean is below the minimum observed in the data for W,'.
print/title = '      so the minimum measurement on W is used for conditioning instead.'/space=0.
end if.
do if (maxwwarn > 0).
print/title = 'NOTE: One SD above the mean is above the maximum observed in the data for W,'.
print/title = '      so the maximum measurement for W is used for conditioning instead.'/space=0.
end if.
do if (minzwarn > 0).
print/title = 'NOTE: One SD below the mean is below the minimum observed in the data for Z,'.
print/title = '      so the minimum measurement for Z is used for conditioning instead.'/space=0.
end if.
do if (maxzwarn > 0).
print/title = 'NOTE: One SD above the mean is above the maximum observed in the data for Z,'.
print/title = '      so the maximum measurement for Z is used for conditioning instead.'/space=0.
end if.
do if (pstog=1).
print/title= 'NOTE: Standardized coefficients for dichotomous or multicategorical X are in'.
print/title= '      partially standardized form.'/space=0.
end if.
loop i = 1 to 100.
do if (notecode(i,1) = 32).
print/title = 'Direct, indirect, and total effects are counterfactually defined'.
do if (xcontcf=0).
print/title = 'assuming X by M interaction.'/space=0.
end if.
do if (xcontcf=1).
print/title = 'assuming X by M interaction and with the following reference (x_ref)'/space=0.
compute states=t(xrefvals).
print states/title = 'and counterfactual (x_cf) states for X:'/rlabels='x_ref' 'x_cf'/format= F10.4 /space=0.
end if.
end if.
do if (notecode(i,1)=1).
print/title = 'NOTE: COVMY is ignored when using CMATRIX option.'.
end if.
do if (notecode(i,1)=2).
print/title = 'NOTE: Confidence level restricted to between 50 and 99.9999%.  95% confidence is provided in output'.
end if.
do if (notecode(i,1)=3).
print centvar/title = 'NOTE: The following variables were mean centered prior to analysis:'/format = a8.
end if.
do if (notecode(i,1) = 4).
print/title = 'NOTE: A heteroscedasticity consistent standard error and covariance matrix estimator was used.'.
end if.
do if (notecode(i,1) = 5).
print/title = 'NOTE: The HC3 option has been replaced with HC.  See the documentation.'.
end if.
do if (notecode(i,1) = 6).
print/title = 'NOTE: Due to estimation problems, some bootstrap samples had to be replaced.'.
print badboot/title='      The number of times this happened was:'/space=0/format=F8.0.
end if.
do if (notecode(i,1) = 7).
print/title = 'NOTE: The bootstrapping was not completed due to problematic bootstrap samples.'.
print/title = '      Bootstrap confidence intervals are therefore suppressed.'/space=0.
end if.
do if (notecode(i,1) = 8).
print/title = 'NOTE: The number of bootstrap samples was adjusted upward given your desired confidence.'.
end if.
do if (notecode(i,1) = 9).
print/title = 'NOTE: WMODVAL is ignored when W is specified as multicategorical.'.
end if.
do if (notecode(i,1) = 10).
print/title = 'NOTE: ZMODVAL is ignored when Z is specified as multicategorical.'.
end if.
do if (notecode(i,1) = 11).
print/title = 'NOTE: Total effect model generated only when all covariates are specified in all'.
print/title = '      models of M and Y.'/space=0.
end if.
do if (notecode(i,1) = 30).
print/title = 'NOTE: Your vector of linear hypothesis weights is of the wrong length for this model.'.
end if.
do if (notecode(i,1) = 12).
print/title = 'NOTE: Total effect model generated only when X is freely estimated to affect each M'.
print/title = '      and both X and M are freely estimated to affect Y'/space=0.
end if.
do if (notecode(i,1) = 13).
print/title = 'NOTE: There are too many pairwise contrasts to conduct with this model.'.
end if.
do if (notecode(i,1) = 14).
print/title = 'NOTE: The number of contrast weights must equal the number of indirect effects.'.
end if.
do if (notecode(i,1) = 15).
print/title = 'NOTE: Monte Carlo confidence intervals not available for this model.'.
print/title = '      Bootstrapping is used instead.'/space=0.
end if.
do if (notecode(i,1) = 16).
print/title = 'NOTE: The number of Monte Carlo samples was adjusted upward given your desired confidence.'.
end if.
do if (notecode(i,1) = 19).
print/title = 'NOTE: Your contrast matrix is invalid or not applicable to this model.'.
end if.
do if (notecode(i,1) = 20).
print/title = 'NOTE: One of the groups specified by your contrast matrix does not exist in the data.'.
end if.
do if (notecode(i,1) = 21).
print/title = 'NOTE: The VARORDER option is not available in this release.'.
end if.
do if (notecode(i,1) = 22).
print/title = 'NOTE: The VMODVAL and QMODVAL options are not available in this release.'.
end if.
do if (notecode(i,1) = 23).
print/title = 'NOTE: The QUANTILE option is not available in this release.'.
end if.
do if (notecode(i,1) = 24).
print/title = 'NOTE: Total effect model not available with dichotomous Y.'.
end if.
do if (notecode(i,1) = 25).
print/title = 'NOTE: STAND/EFFSIZE options not available with dichotomous Y.'.
end if.
do if ((notecode(i,1) = 26) and nms > 0).
print/title = 'NOTE: Direct and indirect effects of X on Y are on a log-odds metric.'.
end if.
do if (notecode(i,1) = 27).
print/title = 'NOTE: Standardized coefficients are not available for models with moderators.'.
end if.
do if (notecode(i,1) = 28).
print/title = 'NOTE: The contrast option is not available with a multicategorical X.'.
end if.
do if (notecode(i,1) = 31).
do if (nms > 1).
print medmeans/title = 'NOTE: Controlled direct effect(s) estimated at the following mediator values:'/cnames=mnames/format= F10.4.
end if.
do if (nms=1).
print medmeans/title = 'NOTE: Controlled direct effect(s) estimated at the following mediator value:'/format= F10.4.
end if.
end if.
do if (notecode(i,1) = 33).
print/title = 'NOTE: Sobel test is not available with the XMINT option.'.
end if.
do if (notecode(i,1) = 37).
print/title = 'NOTE: The CONTRAST option is not available when using the XMINT option.'.
end if.
do if (notecode(i,1) = 34).
print/title = 'NOTE: Standardized effects are not available when using the XMINT option.'.
end if.
do if (notecode(i,1) = 36).
print/title = 'NOTE: The XREFVAL option is ignored when X is declared as multicategorical.'.
end if.
do if (notecode(i,1) = 35).
print coval/title = 'NOTE: Counterfactual effects estimated at the following covariate values:'/cnames=covnames/format= F10.4.
end if.
do if (notecode(i,1) = 29 and (listmiss=1)).
compute a=missrow.
compute conum=ncol(a).
compute allgood=0.
compute smremain=12.
compute largesti=1.
compute smallrow=0.
do if (conum > 12).
loop ii = 1 to 12.
compute check=(conum/ii).
do if (check = trunc(check)).
compute check2=conum/ii.
compute aok=ii.
do if (aok > 2).
compute allgood=1.
end if.
else.
compute remain=conum-(ii*trunc(check)).
do if (remain <= smremain).
compute smremain=remain.
compute largesti=ii.
compute smallrow=trunc(conum/largesti).
end if.
end if.
end loop.
compute atemp=a(1,1:(aok*check2)).
compute atemp=reshape(atemp,check2,aok).
do if (ncol(atemp) > 2).
print/title='NOTE: Missing data resulted in the deletion of the following row(s) of'.
print atemp/title='      data from the analysis:'/format=F6.0/space=0.
end if.
do if (allgood=0).
compute atemp=a(1,1:(smallrow*largesti)).
compute atemp=reshape(atemp,smallrow,largesti).
compute btemp=a(1,((largesti*smallrow)+1):conum).
print/title='NOTE: Missing data resulted in the deletion of the following row(s) of'.
print atemp/title='      data from the analysis:'/format=F6.0/space=0.
print btemp/title=' '/space=0/format=f6.0.
end if.
else if (conum <= 12).
print/title='NOTE: Missing data resulted in the deletion of the following row(s) of'.
print a/title='      data from the analysis:'/format=F6.0/space=0.
end if.
end if.
end loop.
do if (toomany=1).
print/title='WARNING: Variables names longer than eight characters can produce incorrect output'.
print/title='when some variables in the data file have the same first eight characters. Shorter'/space=0.
print/title='variable names are recommended. By using this output, you are accepting all risk'/space=0.
print/title='and consequences of interpreting or reporting results that may be incorrect.'/space=0.
end if.
end if.
end if.
loop i = 1 to 100.
do if (errcode(i,1)=1).
print/title = 'ERROR: You must specify a Y and an X variable.'.
end if.
do if (errcode(i,1)=2).
print/title = 'ERROR: X, M, or Y variable used more than once or W and Z are the same variable.'.
do if (toomany = 1).
print/title = '       This could be caused by the use of variables names longer'/space=0.
print varnames/title = '       than eight characters. Here are the variables I see:'/space=0/format=A8.
end if.
end if.
do if (errcode(i,1)=3).
print/title = 'ERROR: You have specified more than one variable for W, Y, X, or Z'.
end if.
do if (errcode(i,1)=4).
print/title = 'ERROR: A variable specified as multicategorical has more than nine categories.'.
end if.
do if (errcode(i,1)=5).
print/title = 'ERROR: One of the categories contains only a single case.'.
end if.
do if (errcode(i,1)=6).
print/title = 'ERROR: Invalid model number in this version of PROCESS.'.
end if.
do if (errcode(i,1)=7).
print/title = 'ERROR: Invalid model number.'.
end if.
do if (errcode(i,1)=8).
print/title = 'ERROR: You must specify an M variable for this model.'.
end if.
do if (errcode(i,1)=9).
print/title = 'ERROR: You have specified an M variable in a model that does not use it.'.
end if.
do if (errcode(i,1)=10).
print/title = 'ERROR: You have specified a W variable in a model that does not use it.'.
end if.
do if (errcode(i,1)=11).
print/title = 'ERROR: You have not specified a W variable in a model that requires it.'.
end if.
do if (errcode(i,1)=12).
print/title = 'ERROR: You have specified a Z variable in a model that does not use it.'.
end if.
do if (errcode(i,1)=13).
print/title = 'ERROR: You have not specified a Z variable in a model that requires it.'.
end if.
do if (errcode(i,1)=14).
print/title = 'ERROR: V and Q are not proper specifications in this release of PROCESS.'.
print/title = '       Moderators must be specified as W and/or Z.'/space=0.
end if.
do if (errcode(i,1)=15).
print/title = 'ERROR: One of your model variables exhibits no variation (it is a constant).'.
end if.
do if (errcode(i,1)=16).
print/title = 'ERROR: BMATRIX is not the correct length or is otherwise invalid.'.
end if.
do if (errcode(i,1)=17).
print/title = 'ERROR: WMATRIX is not the correct length or is otherwise invalid.'.
end if.
do if (errcode(i,1)=18).
print/title = 'ERROR: ZMATRIX is not the correct length or is otherwise invalid.'.
end if.
do if (errcode(i,1)=19).
print/title = 'ERROR: WZMATRIX is not the correct length or is otherwise invalid.'.
end if.
do if (errcode(i,1)=20).
print/title = 'ERROR: A path fixed at zero cannot be moderated.'.
end if.
do if (errcode(i,1)=21).
print/title = 'ERROR: If only one moderator is specified, it must be specified as W.'.
end if.
do if (errcode(i,1)=22).
print/title = 'ERROR: In BMATRIX, X must be specified to affect at least one variable.'.
end if.
do if (errcode(i,1)=23).
print/title = 'ERROR: In BMATRIX, at least one variable must be specified to affect Y.'.
end if.
do if (errcode(i,1)=24).
print/title = 'ERROR: You must specify a model number or a custom BMATRIX specification.'.
end if.
do if (errcode(i,1)=25).
print/title = 'ERROR: BMATRIX cannot be used in conjunction with a model number.'.
end if.
do if (errcode(i,1)=26).
print/title = 'ERROR: Your model has a dangling mediator (all Ms must affect and be affected).'.
end if.
do if (errcode(i,1)=27).
print/title = 'ERROR: CLUSTER is not available on this release of PROCESS.'.
end if.
do if (errcode(i,1)=29).
print/title = 'ERROR: CMATRIX is not the correct length or is otherwise invalid.'.
end if.
do if (errcode(i,1)=30).
print/title = 'ERROR: In CMATRIX, all covariates must be assigned to an M or a Y.'.
end if.
do if (errcode(i,1)=31).
print/title = 'ERROR: A linear or near linear dependency (singularity) exists in the data.'.
end if.
do if (errcode(i,1)=32).
print/title = 'ERROR: Models 80 and 81 require between 3 and 6 mediators.'.
end if.
do if (errcode(i,1)=33).
print/title = 'ERROR: Model 82 requires 4 mediators.'.
end if.
do if (errcode(i,1)=34).
print/title = 'ERROR: This model number requires between 2 and 6 mediators.'.
end if.
do if (errcode(i,1)=35).
print/title = 'ERROR: In a model with only one moderator, that moderator must be W.'.
end if.
do if (errcode(i,1)=36).
print/title = 'ERROR: A serial mediation model cannot have more than 6 mediators.'.
end if.
do if (errcode(i,1)=37).
print/title = 'ERROR: No more than 10 mediators are allowed in a PROCESS command.'.
end if.
do if (errcode(i,1)=38).
print/title = 'ERROR: XCATCODE is not provided, not the correct length, or is otherwise invalid.'.
end if.
do if (errcode(i,1)=39).
print/title = 'ERROR: WCATCODE is not provided, not the correct length, or is otherwise invalid.'.
end if.
do if (errcode(i,1)=40).
print/title = 'ERROR: ZCATCODE is not provided, not the correct length, or is otherwise invalid.'.
end if.
do if (errcode(i,1)=41).
print/title = 'ERROR: Models 1, 2, and 3 cannot be customized.'.
end if.
do if (errcode(i,1)=42).
print/title = 'ERROR: WS option available only in PROCESS v2. Or use the MEMORE macro instead.'.
print/title = '       MEMORE can be downloaded from www.akmontoya.com.'/space=0.
end if.
do if (errcode(i,1)=43).
print/title = 'ERROR: PROCESS does not allow dichotomous mediators.'.
end if.
do if (errcode(i,1)=60).
print/title = 'PROCESS is now ready for use.'.
print/title='Copyright 2017-2022 by Andrew F. Hayes. ALL RIGHTS RESERVED.'/space=0.
print/title='Workshop schedule available at http://haskayne.ucalgary.ca/CCRAM'/space=0.
end if.
do if (errcode(i,1)=50).
print/title = 'ERROR: A multicategorical moderator cannot be specified as a covariate.'.
end if.
do if (errcode(i,1)=51).
print/title = 'ERROR: A variable you specified as a covariate is a moderator in all equations.'.
end if.
do if (errcode(i,1)=52 and mcerpt=0).
compute mcerpt=1.
print/title = 'ERROR: A variable specified as multicategorical must have at least three categories'.
end if.
do if (errcode(i,1) = 47).
do if (iterrmod=1).
print/title = 'ERROR: Iteration for Y model didn''t converge to a solution. Interpret results with caution.'.
print/title = '       Try increasing the number of iterations, though this will slow down computation.'/space=0.
end if.
do if (bootiter=1).
print/title = 'ERROR: Nonconvergence during bootstrapping. Interpet bootstrap results with caution.'.
end if.
end if.
do if (errcode(i,1) = 61).
print/title = 'ERROR: Variable names should be no more than eight characters in length.'.
end if.
do if (errcode(i,1) = 62).
print/title = 'ERROR: After listwise deletion of cases with missing data, too few cases remain.'.
end if.
do if (errcode(i,1) = 63).
print/title = 'ERROR: The XMINT option is available only for model 4.'.
end if.
do if (errcode(i,1) = 64).
print/title = 'ERROR: Incorrect number of values specified in CDEVAL option.'.
end if.
do if (errcode(i,1) = 65).
print/title = 'ERROR: Only indicator or sequential coding of X is allowed with the XMINT option.'.
end if.
do if (errcode(i,1) = 66).
print/title = 'ERROR: XMINT option not available with continuous X using PROCESS GUI. Use syntax.'.
end if.
do if (errcode(i,1) = 67).
print/title = 'ERROR: Too many elements provided in XREFVAL option.'.
end if.
do if (errcode(i,1)=68).
print/title = 'ERROR: Covariate assignment is not allowed with XMINT option.'.
end if.
do if (errcode(i,1) = 69).
print/title = 'ERROR: Incorrect number of values specified in COVAL option.'.
end if.
do if (errcode(i,1) = 70).
print/title = 'ERROR: Incorrect value(s) in XREFVAL for this dichotomous X variable.'.
end if.
do if (errcode(i,1) = 71).
print/title = 'ERROR: The CENTER option is not available when using the XMINT option.'.
end if.
do if (errcode(i,1) = 72).
print/title = 'ERROR: The XMINT option is not available for models with a dichotomous Y.'.
end if.
end loop.
END MATRIX.	
Ressources	Temps de processeur	00:00:06,17	
	Temps écoulé	00:00:06,16	


Run MATRIX procedure:

***************** PROCESS Procedure for SPSS Version 4.2 *****************

          Written by Andrew F. Hayes, Ph.D.       www.afhayes.com
    Documentation available in Hayes (2022). www.guilford.com/p/hayes3

**************************************************************************
Model  : 4
    Y  : HOMAIR
    X  : Pulsenut
   M1  : TG
   M2  : HDL
   M3  : LDL
   M4  : TC

Sample
Size:  169

**************************************************************************
OUTCOME VARIABLE:
 TG

Model Summary
          R       R-sq        MSE          F        df1        df2          p
      ,1345      ,0181  3978,3065     3,0789     1,0000   167,0000      ,0811

Model
              coeff         se          t          p       LLCI       ULCI
constant    94,6698     6,1263    15,4531      ,0000    82,5749   106,7647
Pulsenut   -17,6063    10,0339    -1,7547      ,0811   -37,4159     2,2033

Standardized coefficients
              coeff
Pulsenut     -,2774

**************************************************************************
OUTCOME VARIABLE:
 HDL

Model Summary
          R       R-sq        MSE          F        df1        df2          p
      ,0084      ,0001   178,1234      ,0117     1,0000   167,0000      ,9141

Model
              coeff         se          t          p       LLCI       ULCI
constant    41,2453     1,2963    31,8176      ,0000    38,6860    43,8045
Pulsenut     -,2294     2,1231     -,1081      ,9141    -4,4211     3,9623

Standardized coefficients
              coeff
Pulsenut     -,0172

**************************************************************************
OUTCOME VARIABLE:
 LDL

Model Summary
          R       R-sq        MSE          F        df1        df2          p
      ,0281      ,0008  1189,1102      ,1320     1,0000   167,0000      ,7168

Model
              coeff         se          t          p       LLCI       ULCI
constant    77,0566     3,3493    23,0066      ,0000    70,4441    83,6691
Pulsenut    -1,9931     5,4857     -,3633      ,7168   -12,8234     8,8371

Standardized coefficients
              coeff
Pulsenut     -,0579

**************************************************************************
OUTCOME VARIABLE:
 TC

Model Summary
          R       R-sq        MSE          F        df1        df2          p
      ,0517      ,0027  1448,0476      ,4479     1,0000   167,0000      ,5043

Model
              coeff         se          t          p       LLCI       ULCI
constant   137,2736     3,6961    37,1406      ,0000   129,9766   144,5706
Pulsenut    -4,0514     6,0536     -,6693      ,5043   -16,0028     7,9000

Standardized coefficients
              coeff
Pulsenut     -,1066

**************************************************************************
OUTCOME VARIABLE:
 HOMAIR

Model Summary
          R       R-sq        MSE          F        df1        df2          p
      ,3663      ,1342    23,2139     5,0528     5,0000   163,0000      ,0002

Model
              coeff         se          t          p       LLCI       ULCI
constant      ,2304     1,7094      ,1348      ,8930    -3,1451     3,6059
Pulsenut     -,3549      ,7772     -,4567      ,6485    -1,8896     1,1797
TG            ,0430      ,0099     4,3649      ,0000      ,0236      ,0625
HDL           ,1230      ,0433     2,8391      ,0051      ,0375      ,2086
LDL           ,0914      ,0359     2,5468      ,0118      ,0205      ,1623
TC           -,0864      ,0356    -2,4248      ,0164     -,1568     -,0160

Standardized coefficients
              coeff
Pulsenut     -,0696
TG            ,5352
HDL           ,3210
LDL           ,6165
TC           -,6437

************************** TOTAL EFFECT MODEL ****************************
OUTCOME VARIABLE:
 HOMAIR

Model Summary
          R       R-sq        MSE          F        df1        df2          p
      ,0925      ,0086    25,9459     1,4405     1,0000   167,0000      ,2318

Model
              coeff         se          t          p       LLCI       ULCI
constant     4,5583      ,4947     9,2135      ,0000     3,5816     5,5351
Pulsenut     -,9725      ,8103    -1,2002      ,2318    -2,5723      ,6272

Standardized coefficients
              coeff
Pulsenut     -,1907


************** TOTAL, DIRECT, AND INDIRECT EFFECTS OF X ON Y **************

Total effect of X on Y
     Effect         se          t          p       LLCI       ULCI       c_ps
     -,9725      ,8103    -1,2002      ,2318    -2,5723      ,6272     -,1907

Direct effect of X on Y
     Effect         se          t          p       LLCI       ULCI      c'_ps
     -,3549      ,7772     -,4567      ,6485    -1,8896     1,1797     -,0696

Indirect effect(s) of X on Y:
          Effect     BootSE   BootLLCI   BootULCI
TOTAL     -,6176      ,3299    -1,3266     -,0389
TG        -,7573      ,6374    -2,4177     -,0335
HDL       -,0282      ,3766     -,7352      ,8030
LDL       -,1822      ,8967    -2,4143     1,0368
TC         ,3501     1,0869     -,6547     3,3901

Partially standardized indirect effect(s) of X on Y:
          Effect     BootSE   BootLLCI   BootULCI
TOTAL     -,1211      ,0613     -,2509     -,0073
TG        -,1485      ,1188     -,4562     -,0070
HDL       -,0055      ,0718     -,1370      ,1508
LDL       -,0357      ,1731     -,4613      ,1960
TC         ,0686      ,2090     -,1288      ,6399

*********************** ANALYSIS NOTES AND ERRORS ************************

Level of confidence for all confidence intervals in output:
  95,0000

Number of bootstrap samples for percentile bootstrap confidence intervals:
  5000

NOTE: Standardized coefficients for dichotomous or multicategorical X are in
      partially standardized form.

------ END MATRIX -----


* Encoding: UTF-8.
preserve.
set printback=off.


Matrice


Remarques	
Sortie obtenue	10-NOV-2024 17:06:47	
Commentaires		
Entrée	Données	C:\Users\Brice SAHA\Documents\UBa\Master Thesis 2021\Fanuel\Database fanuel.sav	
	Jeu de données actif	Jeu_de_données1	
	Filtre	<sans>	
	Pondération	<sans>	
	Scinder un fichier	<sans>	
	N de lignes dans le fichier de travail	169	
Syntaxe	MATRIX.
compute wnames='xxxxx'.
compute znames='xxxxx'.
compute mcerpt=0.
compute wiscov=0.
compute ziscov=0.
compute tooman=0.
compute errcode=make(100,1,0).
compute notecode=make(100,1,0).
compute model = trunc( 4 ).
compute iterate = abs(trunc( 100 )).
compute converge = abs( 0.00001 ).
compute itprobtg=0.
compute v2tag=0.
compute ydich=0.
compute maxwwarn=0.
compute minwwarn=0.
compute maxzwarn=0.
compute minzwarn=0.
compute toomany=0.
compute wdich=0.
compute zdich=0.
compute wnotev=0.
compute znotev=0.
compute nxpval=1.
compute nwpval=1.
compute nzpval=1.
compute errs=1.
compute notes=1.
compute criterr=0.
compute novar=0.
compute adjust=0.
compute ncs=0.
compute serial=0.
compute sobelok=0.
compute hasw=0.
compute hasz=0.
compute printw=0.
compute printz=0.
compute xmint=( 0 =1).
compute wmodcust=0.
compute zmodcust=0.
compute booting=0.
compute bootiter=0.
compute iterrmod=0.
compute cov = 'xxxxx'.
compute varorder=( 0 <> 0).
compute nws=0.
compute w= 'xxxxx'.
compute nzs=0.
compute z = 'xxxxx'.
compute nms=0.
compute m = 'TG HDL LDL TC'.
compute nys=0.
compute y = 'HOMAIR'.
compute nxs=0.
compute x = 'AnimalF'.
compute effsize=( 0 =1).
compute stand=( 1 =1).
compute intprobe = .1.
compute xrefvals={ 999 }.
compute center=trunc( 0 ).
compute xcontcf=0.
compute xscaling=1.
compute cdeval={ -999 }.
compute cuscoval=0.
do if (model=74).
compute errcode(errs,1)=7.
compute errs=errs+1.
compute criterr=1.
end if.
do if (xmint=1 and model <> 4).
compute errcode(errs,1)=63.
compute errs=errs+1.
compute criterr=1.
end if.
do if (xmint=1 and model=4).
compute w=x.
compute model=74.
compute intprobe=1.
compute notecode(notes,1) = 32.
compute notes = notes + 1.
do if (effsize=1 or stand=1).
compute notecode(notes,1) = 34.
compute notes = notes + 1.
compute stand=0.
compute effsize=0.
end if.
do if (center <> 0).
compute center=0.
compute errcode(errs,1)=71.
compute errs=errs+1.
compute criterr=1.
end if.
end if.
compute v = 'xxxxx'.
compute q = 'xxxxx'.
compute linsum={ -999 }.
compute nlinsum=ncol(linsum).
do if (linsum(1,1) = -999).
compute nlinsum=0.
end if.
compute oldvars= 'xxxxx'.
compute mcxok=0.
compute mcwok=0.
compute mczok=0.
compute xprod=0.
compute zprod=0.
compute wprod=0.
compute modcok=0.
compute alttotal=0.
compute hc3=trunc( 0 ).
compute jn=( 0 = 1).
compute listmiss=( 0 =1).
compute modelres=( 0 =1).
compute outscree=( 1 =1).
compute activate=( 0 =1).
compute booterr=0.
compute normal=( 0 =1).
compute xmtest=( 0 =1).
compute describe=( 0 =1).
compute longname=( 0 =1).
do if (stand=1).
compute effsize=1.
end if.
compute pstog=0.
compute sobelok=0.
compute normal=( 0 =1).
compute mdichok=( 0 =1).
compute contrast={ 999 }.
compute ncontr=ncol(contrast).
compute ncontrow=nrow(contrast).
do if (contrast(1,1) = 999).
compute ncontr=1.
compute contrast=0.
end if.
do if (ncontr = 1).
compute contrast=trunc(contrast).
do if (contrast > 3 or contrast < 0)).
compute ncontr=1.
compute contrast = 0.
end if.
end if.
do if (ncontr > 1).
compute contvec=contrast.
compute contrast=4.
do if (ncontrow > 1).
compute contrast=0.
compute modcok=1.
compute wcontval=contvec(:,1).
compute zcontval=contvec(:,2).
do if ((ncontr <> 2) or (ncontrow <> 2)).
compute notecode(notes,1) = 19.
compute notes = notes + 1.
compute modcok=0.
end if.
end if.
end if.
do if (xmint = 1 and contrast(1,1) <> 0).
compute contrast=0.
compute notecode(notes,1) = 37.
compute notes = notes + 1.
end if.
do if (varorder = 1).
compute notecode(notes,1) = 21.
compute notes = notes + 1.
end if.
do if ( 999 <> 999 or 999 <> 999).
compute notecode(notes,1) = 22.
compute notes = notes + 1.
end if.
compute modelbt=( 0 =1).
compute cluster= 'xxxxx'.
compute matrices=( 0 =1).
compute covcoeff=( 0 =1).
compute covmy=trunc( 0 ).
do if (covmy < 0 or covmy > 2).
compute covmy = 0.
end if.
compute boot = abs(trunc( 5000 )).
compute bc=( 0 =1).
compute mc=abs(trunc( 0 )).
compute hc=trunc( 5 ).
do if (intprobe < 0 or intprobe > 1).
compute intprobe = .10.
end if.
compute plot=trunc( 0 ).
do if (plot < 0 or plot > 2).
compute plot=0.
end if.
compute total=( 1 =1).
compute dototal=0.
compute saveboot = ( 0 = 1).
compute saveest=( 0 = 2).
do if (saveest=1).
compute intprobe=1.
end if.
do if (hc >= 0 and hc < 5).
compute notecode(notes,1) = 4.
compute notes = notes + 1.
end if.
do if (hc > 5 or hc < 0).
compute hc=5.
end if.
compute mcw=trunc( 0 ).
compute mcz=trunc( 0 ).
compute mcx=trunc( 0 ).
do if (mcx > 0 and mcx < 3 and model = 74).
compute mcw=mcx.
compute xscaling=1.
end if.
do if (mcx > 2 and model = 74).
compute errcode(errs,1)=65.
compute errs=errs+1.
compute criterr=1.
end if.
do if (model = 74 and normal=1).
compute notecode(notes,1) = 33.
compute normal=0.
compute notes = notes + 1.
end if.
do if (mcx > 0 and contrast > 0).
compute notecode(notes,1) = 28.
compute notes = notes + 1.
compute contrast=0.
end if.
compute nxvls=1.
compute nmvls=1.
compute nwvls=1.
compute nzvls=1.
compute paths=999.
compute pathsw=999.
compute pathsz=999.
compute pathswz=999.
compute pathsmod=999.
compute pathtype=999.
compute obscoeff=999.
compute pathsdv={' '}.
compute quantile=1.
do if ( 999 <>999).
compute notecode(notes,1) = 23.
compute notes = notes + 1.
end if.
compute moments=( 0 =1).
do if (moments=1).
compute quantile=0.
end if.
compute bmatrix={ -999 }.
compute wmatrix={ -999 }.
compute zmatrix={ -999 }.
compute wzmatrix={ -999 }.
compute cmatrix={ -999 }.
compute xcatcode={ -999 }.
compute wcatcode={ -999 }.
compute zcatcode={ -999 }.
compute needed=0.
compute conf= 95.
do if (trunc( 95 ) >= 100 or (trunc( 95 ) <= 50)).
compute conf = 95.
compute notecode(notes,1)=2.
compute notes=notes+1.
end if.
do if (model >= 0 and model < 4 and modelbt=0).
compute boot=0.
compute mc=0.
compute bc=0.
end if.
do if (mc > 0 and boot > 0).
compute boot=0.
compute bc=0.
end if.
do if ((boot < 1000) and (mc = 0) and (boot > 0)).
compute boot=5000.
end if.
do if ((mc < 1000) and (boot = 0) and (mc > 0)).
compute mc=5000.
end if.
compute p0=-.322232431088.
compute p1 = -1.
compute p2 = -.342242088547.
compute p3 = -.0204231210245.
compute p4 = -.0000453642210148.
compute q0 = .0993484626060.
compute q1 = .588581570495.
compute q2 = .531103462366.
compute q3 = .103537752850.
compute q4 = .0038560700634.
compute badend=0.
compute priorlo = -9999999.
compute priorhi = 9999999.
compute alpha2 = (1-(conf/100))/2.
compute cilm=alpha2*2.
compute y5=sqrt(-2*ln(alpha2)).
compute xp2=(y5+((((y5*p4+p3)*y5+p2)*y5+p1)*y5+p0)/((((y5*q4+q3)*y5+q2)*y5+q1)*y5+q0)).
compute medlb={'   M1  :';'   M2  :';'   M3  :';'   M4  :';'   M5  :';'   M6  :';'   M7  :';'   M8  :';'   M9  :';'   M10 :'}.
compute medlb2={'(M1)','(M2)','(M3)','(M4)','(M5)','(M6)','(M7)','(M8)','(M9)','(M10)'}.
compute xlb={'   X1  :';'   X2  :';'   X3  :';'   X4  :';'   X5  :';'   X6  :';'   X7  :';'   X8  :';'   X9  :'}.
compute highlbw={'M1*W'; 'M2*W'; 'M3*W'; 'M4*W'; 'M5*W'; 'M6*W'; 'M7*W'; 'M8*W'; 'M9*W'; 'M10*W'}.
do if (xmint=1).
compute highlbw={'M1*X'; 'M2*X'; 'M3*X'; 'M4*X'; 'M5*X'; 'M6*X'; 'M7*X'; 'M8*X'; 'M9*X'; 'M10*X'}.
end if.
compute highlbz={'M1*Z'; 'M2*Z'; 'M3*Z'; 'M4*Z'; 'M5*Z'; 'M6*Z'; 'M7*Z'; 'M8*Z'; 'M9*Z';'M10*Z'}.
compute highlbwz={'M1*W*Z'; 'M2*W*Z'; 'M3*W*Z'; 'M4*W*Z'; 'M5*W*Z'; 'M6*W*Z'; 'M7*W*Z'; 'M8*W*Z'; 'M9*W*Z';'M10*W*Z'}.
compute highlbbt={'BOTH(M1)'; 'BOTH(M2)'; 'BOTH(M3)'; 'BOTH(M4)'; 'BOTH(M5)'; 'BOTH(M6)'; 'BOTH(M7)'; 'BOTH(M8)'; 'BOTH(M9)';'BTH(M10)'}.
compute highlbx={'M1*X'; 'M2*X'; 'M3*X'; 'M4*X'; 'M5*X'; 'M6*X'; 'M7*X'; 'M8*X'; 'M9*X'; 'M10*X'}.
compute skipwz=0.
compute validm={1,1,1,1,1,1,1,1,1,1,1,1,1,1,1,1,1,1,1,1,1,1,0,0,0,0,0,1,1,0,0,0,0, 0,0,0,0,0,0,0,0,0,0,0,0,0,0,0,0,0,0,0,0,0,0,0,0,1,1,1,1,1,1,1,1,1,1,1,1,1,1,1,1,1,1,1,0,0,0,1,1, 1,1,1,1,1,1,1,1,1,1,1}.
do if (activate=1).
compute errcode(errs,1)=60.
compute errs=errs+1.
compute criterr=1.
end if.
do if (criterr=0).
do if (( 0 =1)=1).
compute errcode(errs,1)=42.
compute errs=errs+1.
compute criterr=1.
end if.
do if (model > 0 and model < 93).
do if (validm(1,model)=0).
compute errcode(errs,1)=6.
compute errs=errs+1.
compute criterr=1.
end if.
release validm.
end if.
do if ((model > 92 or model < 0) and model <> 999)).
compute errcode(errs,1)=7.
compute errs=errs+1.
compute criterr=1.
end if.
do if (model = 999 and bmatrix(1,1)=-999).
compute errcode(errs,1)=24.
compute errs=errs+1.
compute criterr=1.
end if.
do if (model <> 999 and bmatrix(1,1) <> -999).
compute errcode(errs,1)=25.
compute errs=errs+1.
compute criterr=1.
end if.
do if ((model = 74 or (model > 0 and model < 4)) and ((wmatrix(1,1) <> -999) or (zmatrix(1,1)<>-999) or (wzmatrix(1,1)<>-999)))).
compute errcode(errs,1)=41.
compute errs=errs+1.
compute criterr=1.
end if.
do if (hc3 <> 0).
compute notecode(notes,1) = 5.
compute notes = notes + 1.
do if (hc3 = 1).
compute hc=3.
end if.
end if.
do if ((v <> 'xxxxx') or (q <> 'xxxxx')).
compute errcode(errs,1)=14.
compute errs=errs+1.
compute errcode(errs,1)=48.
compute errs=errs+1.
compute criterr=1.
end if.
do if (oldvars <> 'xxxxx').
compute errcode(errs,1)=48.
compute errs=errs+1.
compute criterr=1.
end if.
do if (cluster <> 'xxxxx').
compute errcode(errs,1)=27.
compute errs=errs+1.
compute criterr=1.
end if.
do if ((y = 'xxxxx') or (x = 'xxxxx')).
compute errcode(errs,1)=1.
compute errs=errs+1.
compute criterr=1.
end if.
do if ((m = 'xxxxx') and model > 3).
compute errcode(errs,1)=8.
compute errs=errs+1.
compute criterr=1.
end if.
end if.
do if (criterr=0).
get ytmp/variables = HOMAIR /names = ynames/MISSING = 99999.
compute nys=ncol(ytmp).
compute needed=nys.
compute n=nrow(ytmp).
compute varnames={ynames}.
compute dat=ytmp.

.


do if (toomany=1 and longname=0).
compute criterr=1.
do if (tooman=0).
compute tooman=1.
compute errcode(errs,1) = 61.
compute errs = errs + 1.
end if.
end if
.
compute modelvar={ '4' ;t(ynames)}.
do if ( 4 =999).
compute modelvar(1,1)='CUSTOM'.
end if.
get xtmp/variables = AnimalF /names = xnames/MISSING = 99999.
compute nxs=ncol(xtmp).
compute n=nrow(xtmp).
compute needed=needed+nxs.
compute varnames={varnames,xnames}.
compute xcatlab=t(xnames).
compute dat={dat,xtmp}.

.


do if (toomany=1 and longname=0).
compute criterr=1.
do if (tooman=0).
compute tooman=1.
compute errcode(errs,1) = 61.
compute errs = errs + 1.
end if.
end if
.
compute modelvar={modelvar;t(xnames)}.
do if (nxs = 1).
compute modelvlb={'Model  :';'    Y  :';'    X  :'}.
else.
compute modelvlb={'Model  :';'    Y  :';xlb(1:nxs,1)}.
end if.
do if (m <> 'xxxxx').
get mtmp/variables = TG HDL LDL TC /names = mnames/MISSING = 99999.
compute nms=ncol(mtmp).
compute mprod=make(1,nms,0).
compute n=nrow(mtmp).
compute needed=needed+nms.
compute varnames={varnames,mnames}.
compute dat={dat,mtmp}.
compute modelvar={modelvar;t(mnames)}.
compute x2m=make(99,nms,0).
compute m2y=make(99,nms,0).
compute onem=make(nms,1,1).

.


do if (toomany=1 and longname=0).
compute criterr=1.
do if (tooman=0).
compute tooman=1.
compute errcode(errs,1) = 61.
compute errs = errs + 1.
end if.
end if
.
do if (nms > 1 and nms < 11).
compute modelvlb={modelvlb;medlb(1:nms,1)}.
else.
compute modelvlb={modelvlb;'    M  :'}.
end if.
do if (nms > 0 and model < 4).
compute errcode(errs,1)=9.
compute errs=errs+1.
do if (model <> 0).
compute errcode(errs,1)=48.
compute errs=errs+1.
end if.
compute criterr=1.
end if.
end if.
compute wlocatet=0.
compute wlocate=0.
do if (w <> 'xxxxx').
do if (xmint=0).
get wtmp/variables = xxxxx /names = wnames/MISSING = 99999.

.


do if (toomany=1 and longname=0).
compute criterr=1.
do if (tooman=0).
compute tooman=1.
compute errcode(errs,1) = 61.
compute errs = errs + 1.
end if.
end if
.
end if.
do if (xmint=1).
get wtmp/variables = AnimalF /names = wnames/MISSING = 99999.
end if.
compute nws=ncol(wtmp).
compute n=nrow(wtmp).
compute varnames={varnames,wnames}.
compute wlocate=ncol(varnames).
do if (model=74).
compute wlocatet=1.
do if (xnames <> wnames).
compute errcode(errs,1)=45.
compute errs=errs+1.
compute criterr=1.
end if.
end if.
compute wcatlab=t(wnames).
compute dat={dat,wtmp}.
do if (xmint <> 1).
compute modelvar={modelvar;t(wnames)}.
compute modelvlb={modelvlb;'    W  :'}.
end if.
end if.
do if (z <> 'xxxxx').
get ztmp/variables = xxxxx /names = znames/MISSING = 99999.
compute nzs=ncol(ztmp).
compute n=nrow(ztmp).

.


do if (toomany=1 and longname=0).
compute criterr=1.
do if (tooman=0).
compute tooman=1.
compute errcode(errs,1) = 61.
compute errs = errs + 1.
end if.
end if
.
compute varnames={varnames,znames}.
compute zcatlab=t(znames).
compute dat={dat,ztmp}.
compute modelvar={modelvar;t(znames)}.
compute modelvlb={modelvlb;'    Z  :'}.
end if.
do if (cov <> 'xxxxx').
get ctmp/variables = xxxxx /names = covnames/MISSING = 99999.
compute ncs=ncol(ctmp).
compute n=nrow(ctmp).

.


do if (toomany=1 and longname=0).
compute criterr=1.
do if (tooman=0).
compute tooman=1.
compute errcode(errs,1) = 61.
compute errs = errs + 1.
end if.
end if
.
compute varnames={varnames,covnames}.
compute dat={dat,ctmp}.
end if.
do if (nws > 1 or nzs > 1 or nys > 1 or nxs > 1).
compute errcode(errs,1)=3.
compute errs=errs+1.
compute criterr=1.
end if.
do if ((model = 80 or model = 81) and (nms < 3 or nms > 6)).
compute errcode(errs,1)=32.
compute errs=errs+1.
compute criterr=1.
end if.
do if (model = 82 and nms <> 4).
compute errcode(errs,1)=33.
compute errs=errs+1.
compute criterr=1.
end if.
do if (nms > 10).
compute errcode(errs,1)=37.
compute errs=errs+1.
compute criterr=1.
end if.
do if ((model = 6 or (model > 82 and model < 999)) and (nms < 2 or nms > 6)).
compute errcode(errs,1)=34.
compute errs=errs+1.
compute criterr=1.
end if.
compute match=0.
compute match2=0.
compute mcwzcov=0.
loop i = 1 to (ncol(varnames)-1).
loop j = (i+1) to ncol(varnames).
do if (varnames(i)=varnames(j)).
do if (i < (nxs+nms+nys+1)).
compute match2=match2+1.
end if.
do if (wlocatet=1 and i=2 and j=wlocate).
compute match2=match2-1.
end if.
do if ((wnames=znames) and (nws > 0 or nzs > 0))).
compute match2=match2+1.
end if.
do if (i < (ncol(varnames)-ncs+1)) and j > (ncol(varnames)-ncs)).
do if ((varnames(j)=wnames) and mcw=0)).
compute match=0.
compute wiscov=(j-(ncol(varnames)-ncs)).
end if.
do if ((varnames(j)=wnames) and mcw <>0)).
compute mcwzcov=1.
end if.
do if ((varnames(j)=znames) and mcz=0).
compute match=0.
compute ziscov=(j-(ncol(varnames)-ncs)).
end if.
do if ((varnames(j)=znames) and mcz<>0)).
compute mcwzcov=1.
end if.
end if.
end if.
end loop.
end loop.
do if (match2>0 or match=1).
compute errcode(errs,1)=2.
compute errs=errs+1.
compute criterr=1.
end if.
do if (mcwzcov=1).
compute errcode(errs,1)=50.
compute errs=errs+1.
compute criterr=1.
end if.
compute ninit=nrow(dat).
compute rownum=make(ninit,1,0).
loop i = 1 to ninit.
compute rownum(i,1)=i.
end loop.
compute dat={rownum,dat}.
compute j=1.
compute missrow=0.
loop i = 1 to n.
do if (rsum(dat(i,2:ncol(dat))=99999)=0).
compute dat(j,:)=dat(i,:).
compute j=j+1.
else.
compute missrow={missrow;dat(i,1)}.
end if.
end loop.
do if (j < 5).
compute errcode(errs,1)=62.
compute errs=errs+1.
compute criterr=1.
end if.
do if (criterr=0).
compute rownum=dat(1:(j-1),1).
do if (nrow(missrow) > 1).
compute missrow=t(missrow(2:nrow(missrow),1)).
compute notecode(notes,1) = 29.
compute notes = notes + 1.
end if.
compute dat=dat(1:(j-1),2:ncol(dat)).
compute n=nrow(dat).
compute nmiss=ninit-n.
compute ytmp=dat(:,1:nys).

.
compute desctmp=make((8-(4* 0 )),ncol( ytmp ),-999).
loop jd=1 to ncol( ytmp ).
compute descdat= ytmp (:,jd).
compute desctmp(1,jd) = csum(descdat)/nrow(descdat).
compute desctmp(2,jd) = (nrow(descdat)*sscp(descdat))-(t(csum(descdat))*(csum(descdat))).
compute desctmp(2,jd) = sqrt(desctmp(2,jd)/(nrow(descdat)*(nrow(descdat)-1))).
compute desctmp(3,jd)=cmin(descdat).
compute desctmp(4,jd)=cmax(descdat).
do if ( 0 =0).
compute minwarn=0.
compute maxwarn=0.
do if ((desctmp(3,jd)=desctmp(4,jd)) and novar=0).
compute errcode(errs,1)=15.
compute errs=errs+1.
compute criterr=1.
compute novar=1.
end if.
compute tmp=((descdat(:,1)=desctmp(3,jd))+(descdat(:,1)=desctmp(4,jd))).
compute desctmp(8,jd)=(csum(tmp)=nrow(tmp)).
compute tmp = descdat.
compute tmp(GRADE(descdat),:) = descdat.
compute descdat = tmp.
release tmp.
compute decval={.16;.5;.84}.
loop kd=1 to 3.
compute low=trunc(decval(kd,1)*(nrow(descdat)+1)).
compute lowdec=decval(kd,1)*(nrow(descdat)+1)-low.
compute value=descdat(low,1)+(descdat((low+1),1)-descdat(low,1))*lowdec.
compute desctmp((4+kd),jd)=value.
end loop.
compute mnotev=1.
compute modvals=desctmp(5:7,:).
do if (quantile <> 1).
compute desctmp(5,jd)=desctmp(1,jd)-desctmp(2,jd).
compute desctmp(6,jd)=desctmp(1,jd).
compute desctmp(7,jd)=desctmp(1,jd)+desctmp(2,jd).
compute modvals=desctmp(5:7,:).
compute mnotev=2.
do if (modvals(1,1) < desctmp(3,1)).
compute modvals(1,1)=desctmp(3,1).
compute minwarn=1.
end if.
do if (modvals(3,1) > desctmp(4,1)).
compute modvals(3,1)=desctmp(4,1).
compute maxwarn=1.
end if.
end if.
do if (desctmp(8,1)=1).
compute modvals={desctmp(3,1);desctmp(4,1)}.
compute mnotev=0.
compute minwarn=0.
compute maxwarn=0.
end if.
end if.
end loop
.
compute ysd=desctmp(2,:).
compute ovsd=ysd.
do if (desctmp(8,1)=1).
compute ydich=1.
do if (total=1).
compute total=0.
compute notecode(notes,1) = 24.
compute notes = notes + 1.
end if.
do if (effsize=1).
compute effsize=0.
compute notecode(notes,1) = 25.
compute notes = notes + 1.
end if.
do if (model=74).
compute errcode(errs,1) = 72.
compute errs=errs+1.
compute criterr=1.
end if.
compute omx = cmax(ytmp).
compute omn = cmin(ytmp).
compute ytmp = (ytmp = omx).
compute dat(:,1:nys)=(dat(:,1:nys)=omx).
compute rcd = {omn, 0; omx, 1}.
end if.
compute xtmp=dat(:,(nys+1):(nys+nxs)).

.
compute desctmp=make((8-(4* 0 )),ncol( xtmp ),-999).
loop jd=1 to ncol( xtmp ).
compute descdat= xtmp (:,jd).
compute desctmp(1,jd) = csum(descdat)/nrow(descdat).
compute desctmp(2,jd) = (nrow(descdat)*sscp(descdat))-(t(csum(descdat))*(csum(descdat))).
compute desctmp(2,jd) = sqrt(desctmp(2,jd)/(nrow(descdat)*(nrow(descdat)-1))).
compute desctmp(3,jd)=cmin(descdat).
compute desctmp(4,jd)=cmax(descdat).
do if ( 0 =0).
compute minwarn=0.
compute maxwarn=0.
do if ((desctmp(3,jd)=desctmp(4,jd)) and novar=0).
compute errcode(errs,1)=15.
compute errs=errs+1.
compute criterr=1.
compute novar=1.
end if.
compute tmp=((descdat(:,1)=desctmp(3,jd))+(descdat(:,1)=desctmp(4,jd))).
compute desctmp(8,jd)=(csum(tmp)=nrow(tmp)).
compute tmp = descdat.
compute tmp(GRADE(descdat),:) = descdat.
compute descdat = tmp.
release tmp.
compute decval={.16;.5;.84}.
loop kd=1 to 3.
compute low=trunc(decval(kd,1)*(nrow(descdat)+1)).
compute lowdec=decval(kd,1)*(nrow(descdat)+1)-low.
compute value=descdat(low,1)+(descdat((low+1),1)-descdat(low,1))*lowdec.
compute desctmp((4+kd),jd)=value.
end loop.
compute mnotev=1.
compute modvals=desctmp(5:7,:).
do if (quantile <> 1).
compute desctmp(5,jd)=desctmp(1,jd)-desctmp(2,jd).
compute desctmp(6,jd)=desctmp(1,jd).
compute desctmp(7,jd)=desctmp(1,jd)+desctmp(2,jd).
compute modvals=desctmp(5:7,:).
compute mnotev=2.
do if (modvals(1,1) < desctmp(3,1)).
compute modvals(1,1)=desctmp(3,1).
compute minwarn=1.
end if.
do if (modvals(3,1) > desctmp(4,1)).
compute modvals(3,1)=desctmp(4,1).
compute maxwarn=1.
end if.
end if.
do if (desctmp(8,1)=1).
compute modvals={desctmp(3,1);desctmp(4,1)}.
compute mnotev=0.
compute minwarn=0.
compute maxwarn=0.
end if.
end if.
end loop
.
compute xsd=desctmp(2,:).
compute xmodvals=modvals.
compute xdich=desctmp(8,1).
compute xmx=cmax(xtmp).
compute xmn=cmin(xtmp).
do if ((mcx > 0) and (xrefvals(1,1) <> 999) and (xmint=1) and (model= 74)).
compute notecode(notes,1) = 36.
compute notes = notes + 1.
end if.
do if (mcx=0).
do if (ncol(xrefvals)>2 and model=74 and xmint=1).
compute errcode(errs,1)=67.
compute errs=errs+1.
compute criterr=1.
end if.
do if ((model=74) and (xmint=1)).
do if ((xrefvals(1,1)=999) and nxvls=1 and xdich=0).
compute errcode(errs,1)=66.
compute errs=errs+1.
compute criterr=1.
end if.
do if ((xrefvals(1,1)=999) and (xdich=1)).
compute xrefvals={xmn,xmx}.
compute xscaling=xrefvals(1,2)-xrefvals(1,1).
end if.
do if (ncol(xrefvals)=1 and xrefvals(1,1) <> 999).
do if (xdich=0).
compute xrefvals={xrefvals,(xrefvals(1,1)+1)}.
compute xscaling=xrefvals(1,2)-xrefvals(1,1).
end if.
do if (xdich=1).
do if ((xrefvals(1,1) <> xmx) and (xrefvals(1,1) <> xmn)).
compute errcode(errs,1)=70.
compute errs=errs+1.
compute criterr=1.
end if.
do if (xrefvals(1,1) = xmx).
compute xrefvals={xrefvals,xmn}.
compute xscaling=xrefvals(1,2)-xrefvals(1,1).
end if.
do if (xrefvals(1,1) = xmn).
compute xrefvals={xrefvals,xmx}.
compute xscaling=xrefvals(1,2)-xrefvals(1,1).
end if.
end if.
end if.
do if (ncol(xrefvals)=2).
compute xscaling=xrefvals(1,2)-xrefvals(1,1).
do if (xdich=1).
compute xreferr=1.
do if (((xrefvals(1,1) = xmx) and (xrefvals(1,2)=xmn)) or ((xrefvals(1,1) = xmn) and (xrefvals(1,2)=xmx))).
compute xreferr=0.
end if.
do if (xreferr=1).
compute errcode(errs,1)=70.
compute errs=errs+1.
compute criterr=1.
end if.
end if.
end if.
end if.
end if.
do if (xmint=1 and model=74 and mcx=0).
compute xmodvals=t(xrefvals).
compute xcontcf=1.
end if.
compute nxpval=nrow(xmodvals).
compute xprobval=xmodvals.
do if (xdich =1 and mcx > 0).
compute mcx=0.
compute errcode(errs,1) = 52.
compute errs = errs + 1.
compute criterr = 1.
end if.
do if (nms > 0).
compute mtmp=dat(:,(nys+nxs+1):(nys+nxs+nms)).

.
compute desctmp=make((8-(4* 0 )),ncol( mtmp ),-999).
loop jd=1 to ncol( mtmp ).
compute descdat= mtmp (:,jd).
compute desctmp(1,jd) = csum(descdat)/nrow(descdat).
compute desctmp(2,jd) = (nrow(descdat)*sscp(descdat))-(t(csum(descdat))*(csum(descdat))).
compute desctmp(2,jd) = sqrt(desctmp(2,jd)/(nrow(descdat)*(nrow(descdat)-1))).
compute desctmp(3,jd)=cmin(descdat).
compute desctmp(4,jd)=cmax(descdat).
do if ( 0 =0).
compute minwarn=0.
compute maxwarn=0.
do if ((desctmp(3,jd)=desctmp(4,jd)) and novar=0).
compute errcode(errs,1)=15.
compute errs=errs+1.
compute criterr=1.
compute novar=1.
end if.
compute tmp=((descdat(:,1)=desctmp(3,jd))+(descdat(:,1)=desctmp(4,jd))).
compute desctmp(8,jd)=(csum(tmp)=nrow(tmp)).
compute tmp = descdat.
compute tmp(GRADE(descdat),:) = descdat.
compute descdat = tmp.
release tmp.
compute decval={.16;.5;.84}.
loop kd=1 to 3.
compute low=trunc(decval(kd,1)*(nrow(descdat)+1)).
compute lowdec=decval(kd,1)*(nrow(descdat)+1)-low.
compute value=descdat(low,1)+(descdat((low+1),1)-descdat(low,1))*lowdec.
compute desctmp((4+kd),jd)=value.
end loop.
compute mnotev=1.
compute modvals=desctmp(5:7,:).
do if (quantile <> 1).
compute desctmp(5,jd)=desctmp(1,jd)-desctmp(2,jd).
compute desctmp(6,jd)=desctmp(1,jd).
compute desctmp(7,jd)=desctmp(1,jd)+desctmp(2,jd).
compute modvals=desctmp(5:7,:).
compute mnotev=2.
do if (modvals(1,1) < desctmp(3,1)).
compute modvals(1,1)=desctmp(3,1).
compute minwarn=1.
end if.
do if (modvals(3,1) > desctmp(4,1)).
compute modvals(3,1)=desctmp(4,1).
compute maxwarn=1.
end if.
end if.
do if (desctmp(8,1)=1).
compute modvals={desctmp(3,1);desctmp(4,1)}.
compute mnotev=0.
compute minwarn=0.
compute maxwarn=0.
end if.
end if.
end loop
.
compute ovsd={desctmp(2,:),ysd}.
compute medmeans=cdeval.
do if ((cdeval(1,1) <> -999) and (ncol(medmeans) <> nms) and (model=74)).
compute errcode(errs,1)=64.
compute errs=errs+1.
compute criterr=1.
end if.
do if ((cdeval(1,1)=-999) and (model=74)).
compute medmeans=desctmp(1,:).
end if.
do if ((cdeval(1,1)<> -999) and (model=74) and (criterr=0)).
compute notecode(notes,1) = 31.
compute notes = notes + 1.
end if.
do if ((rsum(desctmp(8,:))>0) and (mdichok <> 1)).
compute errcode(errs,1)=43.
compute errs=errs+1.
compute criterr=1.
end if.
compute mmodvals=modvals.
compute mprobval=mmodvals.
end if.
do if (nws > 0).
compute wtmp=dat(:,(nys+nxs+nms+1):(nys+nxs+nms+nws)).

.
compute desctmp=make((8-(4* 0 )),ncol( wtmp ),-999).
loop jd=1 to ncol( wtmp ).
compute descdat= wtmp (:,jd).
compute desctmp(1,jd) = csum(descdat)/nrow(descdat).
compute desctmp(2,jd) = (nrow(descdat)*sscp(descdat))-(t(csum(descdat))*(csum(descdat))).
compute desctmp(2,jd) = sqrt(desctmp(2,jd)/(nrow(descdat)*(nrow(descdat)-1))).
compute desctmp(3,jd)=cmin(descdat).
compute desctmp(4,jd)=cmax(descdat).
do if ( 0 =0).
compute minwarn=0.
compute maxwarn=0.
do if ((desctmp(3,jd)=desctmp(4,jd)) and novar=0).
compute errcode(errs,1)=15.
compute errs=errs+1.
compute criterr=1.
compute novar=1.
end if.
compute tmp=((descdat(:,1)=desctmp(3,jd))+(descdat(:,1)=desctmp(4,jd))).
compute desctmp(8,jd)=(csum(tmp)=nrow(tmp)).
compute tmp = descdat.
compute tmp(GRADE(descdat),:) = descdat.
compute descdat = tmp.
release tmp.
compute decval={.16;.5;.84}.
loop kd=1 to 3.
compute low=trunc(decval(kd,1)*(nrow(descdat)+1)).
compute lowdec=decval(kd,1)*(nrow(descdat)+1)-low.
compute value=descdat(low,1)+(descdat((low+1),1)-descdat(low,1))*lowdec.
compute desctmp((4+kd),jd)=value.
end loop.
compute mnotev=1.
compute modvals=desctmp(5:7,:).
do if (quantile <> 1).
compute desctmp(5,jd)=desctmp(1,jd)-desctmp(2,jd).
compute desctmp(6,jd)=desctmp(1,jd).
compute desctmp(7,jd)=desctmp(1,jd)+desctmp(2,jd).
compute modvals=desctmp(5:7,:).
compute mnotev=2.
do if (modvals(1,1) < desctmp(3,1)).
compute modvals(1,1)=desctmp(3,1).
compute minwarn=1.
end if.
do if (modvals(3,1) > desctmp(4,1)).
compute modvals(3,1)=desctmp(4,1).
compute maxwarn=1.
end if.
end if.
do if (desctmp(8,1)=1).
compute modvals={desctmp(3,1);desctmp(4,1)}.
compute mnotev=0.
compute minwarn=0.
compute maxwarn=0.
end if.
end if.
end loop
.
compute wmodvals=modvals.
compute wdich=desctmp(8,1).
do if (wdich =1 and mcw > 0).
compute mcw=0.
compute errcode(errs,1) = 52.
compute errs = errs + 1.
compute criterr = 1.
end if.
compute wmin=desctmp(3,1).
compute wmax=desctmp(4,1).
compute minwwarn=minwarn.
compute maxwwarn=maxwarn.
compute wnotev=mnotev.
compute wmodval={ 999 }.
do if (xmint=1 and model=74 and mcx=0).
compute wmodval=xrefvals.
end if.
compute nwcontr=ncol(wmodval).
do if (wmodval(1,1) <> 999).
compute wmodvals=wmodval(1,1).
compute wmodcust=1.
do if (nwcontr > 1).
compute wmodvals=t(wmodval).
end if.
compute minwwarn=0.
compute maxwwarn=0.
compute wnotev=0.
end if.
compute wprobval=wmodvals.
compute nwpval=nrow(wmodvals).
end if.
do if (nzs > 0).
compute ztmp=dat(:,(nys+nxs+nms+nws+1):(nys+nxs+nms+nws+nzs)).

.
compute desctmp=make((8-(4* 0 )),ncol( ztmp ),-999).
loop jd=1 to ncol( ztmp ).
compute descdat= ztmp (:,jd).
compute desctmp(1,jd) = csum(descdat)/nrow(descdat).
compute desctmp(2,jd) = (nrow(descdat)*sscp(descdat))-(t(csum(descdat))*(csum(descdat))).
compute desctmp(2,jd) = sqrt(desctmp(2,jd)/(nrow(descdat)*(nrow(descdat)-1))).
compute desctmp(3,jd)=cmin(descdat).
compute desctmp(4,jd)=cmax(descdat).
do if ( 0 =0).
compute minwarn=0.
compute maxwarn=0.
do if ((desctmp(3,jd)=desctmp(4,jd)) and novar=0).
compute errcode(errs,1)=15.
compute errs=errs+1.
compute criterr=1.
compute novar=1.
end if.
compute tmp=((descdat(:,1)=desctmp(3,jd))+(descdat(:,1)=desctmp(4,jd))).
compute desctmp(8,jd)=(csum(tmp)=nrow(tmp)).
compute tmp = descdat.
compute tmp(GRADE(descdat),:) = descdat.
compute descdat = tmp.
release tmp.
compute decval={.16;.5;.84}.
loop kd=1 to 3.
compute low=trunc(decval(kd,1)*(nrow(descdat)+1)).
compute lowdec=decval(kd,1)*(nrow(descdat)+1)-low.
compute value=descdat(low,1)+(descdat((low+1),1)-descdat(low,1))*lowdec.
compute desctmp((4+kd),jd)=value.
end loop.
compute mnotev=1.
compute modvals=desctmp(5:7,:).
do if (quantile <> 1).
compute desctmp(5,jd)=desctmp(1,jd)-desctmp(2,jd).
compute desctmp(6,jd)=desctmp(1,jd).
compute desctmp(7,jd)=desctmp(1,jd)+desctmp(2,jd).
compute modvals=desctmp(5:7,:).
compute mnotev=2.
do if (modvals(1,1) < desctmp(3,1)).
compute modvals(1,1)=desctmp(3,1).
compute minwarn=1.
end if.
do if (modvals(3,1) > desctmp(4,1)).
compute modvals(3,1)=desctmp(4,1).
compute maxwarn=1.
end if.
end if.
do if (desctmp(8,1)=1).
compute modvals={desctmp(3,1);desctmp(4,1)}.
compute mnotev=0.
compute minwarn=0.
compute maxwarn=0.
end if.
end if.
end loop
.
compute zmodvals=modvals.
compute zdich=desctmp(8,1).
do if (zdich =1 and mcz > 0).
compute mcz=0.
compute errcode(errs,1) = 52.
compute errs = errs + 1.
compute criterr = 1.
end if.
compute zmin=desctmp(3,1).
compute zmax=desctmp(4,1).
compute minzwarn=minwarn.
compute maxzwarn=maxwarn.
compute znotev=mnotev.
compute zmodval={ 999 }.
compute nzcontr=ncol(zmodval).
do if (zmodval(1,1) <> 999).
compute zmodvals=zmodval(1,1).
compute zmodcust=1.
do if (nzcontr > 1).
compute zmodvals=t(zmodval).
end if.
compute minzwarn=0.
compute maxzwarn=0.
compute znotev=0.
end if.
compute zprobval=zmodvals.
compute nzpval=nrow(zmodvals).
end if.
do if (ncs > 0).
compute ctmp=dat(:,(nys+nxs+nms+nws+nzs+1):(nys+nxs+nms+nws+nzs+ncs)).

.
compute desctmp=make((8-(4* 0 )),ncol( ctmp ),-999).
loop jd=1 to ncol( ctmp ).
compute descdat= ctmp (:,jd).
compute desctmp(1,jd) = csum(descdat)/nrow(descdat).
compute desctmp(2,jd) = (nrow(descdat)*sscp(descdat))-(t(csum(descdat))*(csum(descdat))).
compute desctmp(2,jd) = sqrt(desctmp(2,jd)/(nrow(descdat)*(nrow(descdat)-1))).
compute desctmp(3,jd)=cmin(descdat).
compute desctmp(4,jd)=cmax(descdat).
do if ( 0 =0).
compute minwarn=0.
compute maxwarn=0.
do if ((desctmp(3,jd)=desctmp(4,jd)) and novar=0).
compute errcode(errs,1)=15.
compute errs=errs+1.
compute criterr=1.
compute novar=1.
end if.
compute tmp=((descdat(:,1)=desctmp(3,jd))+(descdat(:,1)=desctmp(4,jd))).
compute desctmp(8,jd)=(csum(tmp)=nrow(tmp)).
compute tmp = descdat.
compute tmp(GRADE(descdat),:) = descdat.
compute descdat = tmp.
release tmp.
compute decval={.16;.5;.84}.
loop kd=1 to 3.
compute low=trunc(decval(kd,1)*(nrow(descdat)+1)).
compute lowdec=decval(kd,1)*(nrow(descdat)+1)-low.
compute value=descdat(low,1)+(descdat((low+1),1)-descdat(low,1))*lowdec.
compute desctmp((4+kd),jd)=value.
end loop.
compute mnotev=1.
compute modvals=desctmp(5:7,:).
do if (quantile <> 1).
compute desctmp(5,jd)=desctmp(1,jd)-desctmp(2,jd).
compute desctmp(6,jd)=desctmp(1,jd).
compute desctmp(7,jd)=desctmp(1,jd)+desctmp(2,jd).
compute modvals=desctmp(5:7,:).
compute mnotev=2.
do if (modvals(1,1) < desctmp(3,1)).
compute modvals(1,1)=desctmp(3,1).
compute minwarn=1.
end if.
do if (modvals(3,1) > desctmp(4,1)).
compute modvals(3,1)=desctmp(4,1).
compute maxwarn=1.
end if.
end if.
do if (desctmp(8,1)=1).
compute modvals={desctmp(3,1);desctmp(4,1)}.
compute mnotev=0.
compute minwarn=0.
compute maxwarn=0.
end if.
end if.
end loop
.
compute covmeans=desctmp(1,:).
compute coval={ -999 }.
do if ((coval(1,1) <> -999) and (ncol(coval) <> ncs) and (model=74)).
compute errcode(errs,1)=69.
compute errs=errs+1.
compute criterr=1.
end if.
do if ((coval(1,1)<> -999) and (criterr=0) and (model=74)).
compute notecode(notes,1) = 35.
compute notes = notes + 1.
compute cuscoval=1.
end if.
end if.
compute n=nrow(ytmp).
compute ones=make(n,1,1).
do if (nws > 0 and mcw > 0).
compute tmp={rownum,wtmp(:,1)}.

.
compute dd= tmp.
compute temp = dd.
compute temp(GRADE(dd(:,2)),:) = dd.
compute dd = temp.
compute dummy = design(dd(:,2)).
compute nvls = ncol(dummy).
compute nnvls = csum(dummy).
compute mnvls = cmin(t(nnvls)).
compute conmat1=1.
do if (mnvls < 2).
compute errcode(errs,1) = 5.
compute errs = errs + 1.
compute criterr = 1.
end if.
do if (nvls > 9).
compute errcode(errs,1) = 4.
compute errs = errs+1.
compute criterr = 1.
end if.
do if (criterr = 0).
compute dumok = 1.
compute nnvls=make(nvls,1,0).
compute nnvls(1,1)=dd(1,2).
compute temp = 2.
loop i = 2 to n.
do if (dd(i,2) <> nnvls((temp-1),1)).
compute nnvls(temp,1)=dd(i,2).
compute temp = temp+1.
end if.
end loop.
do if ( mcw > 0).
compute x = dummy(:,2:ncol(dummy)).
compute nx = ncol(x).
compute minus1 = make(1,ncol(x),-1).
compute xdes=make((nx+1),3,0).
compute xdes(1,1)=dd(1,2).
compute xdes(1,2)=1.
compute temp = 2.
loop k = 2 to n.
do if (dd(k,2) <> dd((k-1),2)).
compute xdes(temp,2) = k.
compute xdes(temp,1) = dd(k,2).
compute xdes((temp-1),3) = k-1.
compute temp=temp+1.
end if.
end loop.
compute xdes((temp-1),3)=n.
compute xdes = {xdes, (xdes(:,3)-xdes(:,2)+1)}.
do if ( mcw = 4).
loop k = 1 to n.
do if (rsum(x(k,:)) = 0).
compute x(k,:) = minus1.
end if.
end loop.
end if.
do if ( mcw = 2 or mcw = 3 or mcw =5).
loop k = 1 to n.
do if (rsum(x(k,:)) > 0).
loop i = 1 to ncol(x).
do if (x(k,i) = 0).
compute x(k,i) = 1.
else.
break.
end if.
end loop.
end if.
end loop.
do if ( mcw = 3).
compute conmat1={-8,1,1,1,1,1,1,1,1; 0,-7,1,1,1,1,1,1,1; 0,0,-6,1,1,1,1,1,1; 0,0,0,-5,1,1,1,1,1; 0,0,0,0,-4,1,1,1,1; 0,0,0,0,0,-3,1,1,1; 0,0,0,0,0,0,-2,1,1; 0,0,0,0,0,0,0,-1,1}.
loop i = 1 to 8.
compute conmat1(i,:)=conmat1(i,:)/(10-i).
end loop.
compute conmat1=t(conmat1((10-nvls):8,(10-nvls):9)).
loop k=1 to n.
compute x(k,:)=conmat1((rsum(x(k,:))+1),:).
end loop.
end if.
end if.
do if ( mcw = 5).
compute custcode={ -999 }.
do if (ncol(custcode) <> (nvls*(nvls-1))).
compute errcode(errs,1) = (37+ 2 ).
compute errs = errs + 1.
compute criterr = 1.
end if.
do if (ncol(custcode) = (nvls*(nvls-1))).
compute conmat1=make(nvls,(nvls-1),0).
compute cnt=1.
loop i = 1 to nvls.
loop k = 1 to (nvls-1).
compute conmat1(i,k)=custcode(1,cnt).
compute cnt=cnt+1.
end loop.
end loop.
loop k=1 to n.
compute x(k,:)=conmat1((rsum(x(k,:))+1),:).
end loop.
end if.
end if.
compute xskip = 1.
compute dummat = make((nx+1),nx,0).
compute dummat((2:nrow(dummat)),:)=ident(nx).
do if ( mcw = 4).
compute dummat(1,:) = minus1.
end if.
do if ( mcw = 2).
loop i = 2 to nrow(dummat).
loop j = 1 to (i-1).
compute dummat(i,j) = 1.
end loop.
end loop.
end if.
do if ( mcw = 3).
compute dummat=conmat1.
end if.
do if ( mcw = 5 and criterr=0).
compute dummat=conmat1.
end if.
compute dummat={nnvls, dummat}.
compute x={dd(:,1),x}.
compute temp = x.
compute temp(GRADE(x(:,1)),:) = x.
compute x = temp.
release conmat1,temp,dd,xskip,xdes,dummy.
end if.
end if
.
compute wmodvals=nnvls.
compute nwpval=nrow(wmodvals).
do if (criterr=0).
compute minwwarn=0.
compute maxwwarn=0.
compute wnotev=0.
compute wtmp=x(:,2:ncol(x)).
compute wcatlab={'W1';'W2';'W3';'W4';'W5';'W6';'W7';'W8';'W9'}.
do if (xmint=1).
compute wcatlab={'X1';'X2';'X3';'X4';'X5';'X6';'X7';'X8';'X9'}.
end if.
compute nwvls=nvls-1.
compute mcwok=1.
compute dummatw=dummat.
compute wprobval=dummatw(:,2:ncol(dummatw)).
do if (modcok=1).
compute wcontval=make(2,ncol(wprobval),-999).
compute temp=0.
loop i = 1 to 2.
loop j = 1 to nrow(dummatw).
do if (contvec(i,1)=dummatw(j,1)).
compute wcontval(i,:)=wprobval(j,:).
compute temp=temp+1.
end if.
end loop.
end loop.
do if (temp < 2).
compute notecode(notes,1) = 20.
compute notes = notes + 1.
compute modcok=0.
end if.
end if.
do if ((wmodval(1,1) <> 999) and (xmint <> 1)).
compute notecode(notes,1) = 9.
compute notes = notes + 1.
end if.
release tmp, dummat.
end if.
end if.
do if (nzs > 0 and mcz > 0).
compute tmp={rownum,ztmp(:,1)}.

.
compute dd= tmp.
compute temp = dd.
compute temp(GRADE(dd(:,2)),:) = dd.
compute dd = temp.
compute dummy = design(dd(:,2)).
compute nvls = ncol(dummy).
compute nnvls = csum(dummy).
compute mnvls = cmin(t(nnvls)).
compute conmat1=1.
do if (mnvls < 2).
compute errcode(errs,1) = 5.
compute errs = errs + 1.
compute criterr = 1.
end if.
do if (nvls > 9).
compute errcode(errs,1) = 4.
compute errs = errs+1.
compute criterr = 1.
end if.
do if (criterr = 0).
compute dumok = 1.
compute nnvls=make(nvls,1,0).
compute nnvls(1,1)=dd(1,2).
compute temp = 2.
loop i = 2 to n.
do if (dd(i,2) <> nnvls((temp-1),1)).
compute nnvls(temp,1)=dd(i,2).
compute temp = temp+1.
end if.
end loop.
do if ( mcz > 0).
compute x = dummy(:,2:ncol(dummy)).
compute nx = ncol(x).
compute minus1 = make(1,ncol(x),-1).
compute xdes=make((nx+1),3,0).
compute xdes(1,1)=dd(1,2).
compute xdes(1,2)=1.
compute temp = 2.
loop k = 2 to n.
do if (dd(k,2) <> dd((k-1),2)).
compute xdes(temp,2) = k.
compute xdes(temp,1) = dd(k,2).
compute xdes((temp-1),3) = k-1.
compute temp=temp+1.
end if.
end loop.
compute xdes((temp-1),3)=n.
compute xdes = {xdes, (xdes(:,3)-xdes(:,2)+1)}.
do if ( mcz = 4).
loop k = 1 to n.
do if (rsum(x(k,:)) = 0).
compute x(k,:) = minus1.
end if.
end loop.
end if.
do if ( mcz = 2 or mcz = 3 or mcz =5).
loop k = 1 to n.
do if (rsum(x(k,:)) > 0).
loop i = 1 to ncol(x).
do if (x(k,i) = 0).
compute x(k,i) = 1.
else.
break.
end if.
end loop.
end if.
end loop.
do if ( mcz = 3).
compute conmat1={-8,1,1,1,1,1,1,1,1; 0,-7,1,1,1,1,1,1,1; 0,0,-6,1,1,1,1,1,1; 0,0,0,-5,1,1,1,1,1; 0,0,0,0,-4,1,1,1,1; 0,0,0,0,0,-3,1,1,1; 0,0,0,0,0,0,-2,1,1; 0,0,0,0,0,0,0,-1,1}.
loop i = 1 to 8.
compute conmat1(i,:)=conmat1(i,:)/(10-i).
end loop.
compute conmat1=t(conmat1((10-nvls):8,(10-nvls):9)).
loop k=1 to n.
compute x(k,:)=conmat1((rsum(x(k,:))+1),:).
end loop.
end if.
end if.
do if ( mcz = 5).
compute custcode={ -999 }.
do if (ncol(custcode) <> (nvls*(nvls-1))).
compute errcode(errs,1) = (37+ 3 ).
compute errs = errs + 1.
compute criterr = 1.
end if.
do if (ncol(custcode) = (nvls*(nvls-1))).
compute conmat1=make(nvls,(nvls-1),0).
compute cnt=1.
loop i = 1 to nvls.
loop k = 1 to (nvls-1).
compute conmat1(i,k)=custcode(1,cnt).
compute cnt=cnt+1.
end loop.
end loop.
loop k=1 to n.
compute x(k,:)=conmat1((rsum(x(k,:))+1),:).
end loop.
end if.
end if.
compute xskip = 1.
compute dummat = make((nx+1),nx,0).
compute dummat((2:nrow(dummat)),:)=ident(nx).
do if ( mcz = 4).
compute dummat(1,:) = minus1.
end if.
do if ( mcz = 2).
loop i = 2 to nrow(dummat).
loop j = 1 to (i-1).
compute dummat(i,j) = 1.
end loop.
end loop.
end if.
do if ( mcz = 3).
compute dummat=conmat1.
end if.
do if ( mcz = 5 and criterr=0).
compute dummat=conmat1.
end if.
compute dummat={nnvls, dummat}.
compute x={dd(:,1),x}.
compute temp = x.
compute temp(GRADE(x(:,1)),:) = x.
compute x = temp.
release conmat1,temp,dd,xskip,xdes,dummy.
end if.
end if
.
compute zmodvals=nnvls.
compute nzpval=nrow(zmodvals).
do if (criterr=0).
compute minzwarn=0.
compute maxzwarn=0.
compute znotev=0.
compute ztmp=x(:,2:ncol(x)).
compute zcatlab={'Z1';'Z2';'Z3';'Z4';'Z5';'Z6';'Z7';'Z8';'Z9'}.
compute nzvls=nvls-1.
compute mczok=1.
compute dummatz=dummat.
compute zprobval=dummatz(:,2:ncol(dummatz)).
do if (modcok=1).
compute zcontval=make(2,ncol(zprobval),-999).
compute temp=0.
loop i = 1 to 2.
loop j = 1 to nrow(dummatz).
do if (contvec(i,2)=dummatz(j,1)).
compute zcontval(i,:)=zprobval(j,:).
compute temp=temp+1.
end if.
end loop.
end loop.
do if (temp < 2).
compute notecode(notes,1) = 20.
compute notes = notes + 1.
compute modcok=0.
end if.
end if.
do if (zmodval(1,1) <> 999).
compute notecode(notes,1) = 10.
compute notes = notes + 1.
end if.
release tmp, dummat.
end if.
end if.
do if (nxs > 0 and mcx > 0).
compute tmp={rownum,xtmp(:,1)}.

.
compute dd= tmp.
compute temp = dd.
compute temp(GRADE(dd(:,2)),:) = dd.
compute dd = temp.
compute dummy = design(dd(:,2)).
compute nvls = ncol(dummy).
compute nnvls = csum(dummy).
compute mnvls = cmin(t(nnvls)).
compute conmat1=1.
do if (mnvls < 2).
compute errcode(errs,1) = 5.
compute errs = errs + 1.
compute criterr = 1.
end if.
do if (nvls > 9).
compute errcode(errs,1) = 4.
compute errs = errs+1.
compute criterr = 1.
end if.
do if (criterr = 0).
compute dumok = 1.
compute nnvls=make(nvls,1,0).
compute nnvls(1,1)=dd(1,2).
compute temp = 2.
loop i = 2 to n.
do if (dd(i,2) <> nnvls((temp-1),1)).
compute nnvls(temp,1)=dd(i,2).
compute temp = temp+1.
end if.
end loop.
do if ( mcx > 0).
compute x = dummy(:,2:ncol(dummy)).
compute nx = ncol(x).
compute minus1 = make(1,ncol(x),-1).
compute xdes=make((nx+1),3,0).
compute xdes(1,1)=dd(1,2).
compute xdes(1,2)=1.
compute temp = 2.
loop k = 2 to n.
do if (dd(k,2) <> dd((k-1),2)).
compute xdes(temp,2) = k.
compute xdes(temp,1) = dd(k,2).
compute xdes((temp-1),3) = k-1.
compute temp=temp+1.
end if.
end loop.
compute xdes((temp-1),3)=n.
compute xdes = {xdes, (xdes(:,3)-xdes(:,2)+1)}.
do if ( mcx = 4).
loop k = 1 to n.
do if (rsum(x(k,:)) = 0).
compute x(k,:) = minus1.
end if.
end loop.
end if.
do if ( mcx = 2 or mcx = 3 or mcx =5).
loop k = 1 to n.
do if (rsum(x(k,:)) > 0).
loop i = 1 to ncol(x).
do if (x(k,i) = 0).
compute x(k,i) = 1.
else.
break.
end if.
end loop.
end if.
end loop.
do if ( mcx = 3).
compute conmat1={-8,1,1,1,1,1,1,1,1; 0,-7,1,1,1,1,1,1,1; 0,0,-6,1,1,1,1,1,1; 0,0,0,-5,1,1,1,1,1; 0,0,0,0,-4,1,1,1,1; 0,0,0,0,0,-3,1,1,1; 0,0,0,0,0,0,-2,1,1; 0,0,0,0,0,0,0,-1,1}.
loop i = 1 to 8.
compute conmat1(i,:)=conmat1(i,:)/(10-i).
end loop.
compute conmat1=t(conmat1((10-nvls):8,(10-nvls):9)).
loop k=1 to n.
compute x(k,:)=conmat1((rsum(x(k,:))+1),:).
end loop.
end if.
end if.
do if ( mcx = 5).
compute custcode={ -999 }.
do if (ncol(custcode) <> (nvls*(nvls-1))).
compute errcode(errs,1) = (37+ 1 ).
compute errs = errs + 1.
compute criterr = 1.
end if.
do if (ncol(custcode) = (nvls*(nvls-1))).
compute conmat1=make(nvls,(nvls-1),0).
compute cnt=1.
loop i = 1 to nvls.
loop k = 1 to (nvls-1).
compute conmat1(i,k)=custcode(1,cnt).
compute cnt=cnt+1.
end loop.
end loop.
loop k=1 to n.
compute x(k,:)=conmat1((rsum(x(k,:))+1),:).
end loop.
end if.
end if.
compute xskip = 1.
compute dummat = make((nx+1),nx,0).
compute dummat((2:nrow(dummat)),:)=ident(nx).
do if ( mcx = 4).
compute dummat(1,:) = minus1.
end if.
do if ( mcx = 2).
loop i = 2 to nrow(dummat).
loop j = 1 to (i-1).
compute dummat(i,j) = 1.
end loop.
end loop.
end if.
do if ( mcx = 3).
compute dummat=conmat1.
end if.
do if ( mcx = 5 and criterr=0).
compute dummat=conmat1.
end if.
compute dummat={nnvls, dummat}.
compute x={dd(:,1),x}.
compute temp = x.
compute temp(GRADE(x(:,1)),:) = x.
compute x = temp.
release conmat1,temp,dd,xskip,xdes,dummy.
end if.
end if
.
do if (criterr=0).
compute xtmp=x(:,2:ncol(x)).
compute xcatlab={'X1';'X2';'X3';'X4';'X5';'X6';'X7';'X8';'X9'}.
compute nxvls=nvls-1.
compute xdich=(nvls=2).
compute mcxok=1.
compute dummatx=dummat.
compute xmodvals=dummatx(:,1).
compute nxpval=nrow(xmodvals).
release tmp, dummat.
end if.
end if.
compute intlab=make(100,1,' ').


compute intlab( 1 ,1)= 'Int_1'.


compute intlab( 2 ,1)= 'Int_2'.


compute intlab( 3 ,1)= 'Int_3'.


compute intlab( 4 ,1)= 'Int_4'.


compute intlab( 5 ,1)= 'Int_5'.


compute intlab( 6 ,1)= 'Int_6'.


compute intlab( 7 ,1)= 'Int_7'.


compute intlab( 8 ,1)= 'Int_8'.


compute intlab( 9 ,1)= 'Int_9'.


compute intlab( 10 ,1)= 'Int_10'.


compute intlab( 11 ,1)= 'Int_11'.


compute intlab( 12 ,1)= 'Int_12'.


compute intlab( 13 ,1)= 'Int_13'.


compute intlab( 14 ,1)= 'Int_14'.


compute intlab( 15 ,1)= 'Int_15'.


compute intlab( 16 ,1)= 'Int_16'.


compute intlab( 17 ,1)= 'Int_17'.


compute intlab( 18 ,1)= 'Int_18'.


compute intlab( 19 ,1)= 'Int_19'.


compute intlab( 20 ,1)= 'Int_20'.


compute intlab( 21 ,1)= 'Int_21'.


compute intlab( 22 ,1)= 'Int_22'.


compute intlab( 23 ,1)= 'Int_23'.


compute intlab( 24 ,1)= 'Int_24'.


compute intlab( 25 ,1)= 'Int_25'.


compute intlab( 26 ,1)= 'Int_26'.


compute intlab( 27 ,1)= 'Int_27'.


compute intlab( 28 ,1)= 'Int_28'.


compute intlab( 29 ,1)= 'Int_29'.


compute intlab( 30 ,1)= 'Int_30'.


compute intlab( 31 ,1)= 'Int_31'.


compute intlab( 32 ,1)= 'Int_32'.


compute intlab( 33 ,1)= 'Int_33'.


compute intlab( 34 ,1)= 'Int_34'.


compute intlab( 35 ,1)= 'Int_35'.


compute intlab( 36 ,1)= 'Int_36'.


compute intlab( 37 ,1)= 'Int_37'.


compute intlab( 38 ,1)= 'Int_38'.


compute intlab( 39 ,1)= 'Int_39'.


compute intlab( 40 ,1)= 'Int_40'.


compute intlab( 41 ,1)= 'Int_41'.


compute intlab( 42 ,1)= 'Int_42'.


compute intlab( 43 ,1)= 'Int_43'.


compute intlab( 44 ,1)= 'Int_44'.


compute intlab( 45 ,1)= 'Int_45'.


compute intlab( 46 ,1)= 'Int_46'.


compute intlab( 47 ,1)= 'Int_47'.


compute intlab( 48 ,1)= 'Int_48'.


compute intlab( 49 ,1)= 'Int_49'.


compute intlab( 50 ,1)= 'Int_50'.


compute intlab( 51 ,1)= 'Int_51'.


compute intlab( 52 ,1)= 'Int_52'.


compute intlab( 53 ,1)= 'Int_53'.


compute intlab( 54 ,1)= 'Int_54'.


compute intlab( 55 ,1)= 'Int_55'.


compute intlab( 56 ,1)= 'Int_56'.


compute intlab( 57 ,1)= 'Int_57'.


compute intlab( 58 ,1)= 'Int_58'.


compute intlab( 59 ,1)= 'Int_59'.


compute intlab( 60 ,1)= 'Int_60'.


compute intlab( 61 ,1)= 'Int_61'.


compute intlab( 62 ,1)= 'Int_62'.


compute intlab( 63 ,1)= 'Int_63'.


compute intlab( 64 ,1)= 'Int_64'.


compute intlab( 65 ,1)= 'Int_65'.


compute intlab( 66 ,1)= 'Int_66'.


compute intlab( 67 ,1)= 'Int_67'.


compute intlab( 68 ,1)= 'Int_68'.


compute intlab( 69 ,1)= 'Int_69'.


compute intlab( 70 ,1)= 'Int_70'.


compute intlab( 71 ,1)= 'Int_71'.


compute intlab( 72 ,1)= 'Int_72'.


compute intlab( 73 ,1)= 'Int_73'.


compute intlab( 74 ,1)= 'Int_74'.


compute intlab( 75 ,1)= 'Int_75'.


compute intlab( 76 ,1)= 'Int_76'.


compute intlab( 77 ,1)= 'Int_77'.


compute intlab( 78 ,1)= 'Int_78'.


compute intlab( 79 ,1)= 'Int_79'.


compute intlab( 80 ,1)= 'Int_80'.


compute intlab( 81 ,1)= 'Int_81'.


compute intlab( 82 ,1)= 'Int_82'.


compute intlab( 83 ,1)= 'Int_83'.


compute intlab( 84 ,1)= 'Int_84'.


compute intlab( 85 ,1)= 'Int_85'.


compute intlab( 86 ,1)= 'Int_86'.


compute intlab( 87 ,1)= 'Int_87'.


compute intlab( 88 ,1)= 'Int_88'.


compute intlab( 89 ,1)= 'Int_89'.


compute intlab( 90 ,1)= 'Int_90'.


compute intlab( 91 ,1)= 'Int_91'.


compute intlab( 92 ,1)= 'Int_92'.


compute intlab( 93 ,1)= 'Int_93'.


compute intlab( 94 ,1)= 'Int_94'.


compute intlab( 95 ,1)= 'Int_95'.


compute intlab( 96 ,1)= 'Int_96'.


compute intlab( 97 ,1)= 'Int_97'.


compute intlab( 98 ,1)= 'Int_98'.


compute intlab( 99 ,1)= 'Int_99'.


compute intlab( 100 ,1)= 'Int_100'.

compute bcmat=make(needed,needed,0).
compute wcmat=make(needed,needed,0).
compute zcmat=make(needed,needed,0).
compute wzcmat=make(needed,needed,0).
compute wsum=0.
compute zsum=0.
compute wzsum=0.
end if.
end if.
do if (criterr = 0 and model <> 999).
compute modelmat= {1,0,0,0,0,0,0,1,0,0;2,0,0,0,0,0,0,1,1,0;3,0,0,0,0,0,0,1,1,1;4,0,0,0,0,0,0,0,0,0; 5,0,0,0,0,0,0,1,0,0;6,0,0,0,0,0,0,0,0,0;7,1,0,0,0,0,0,0,0,0;8,1,0,0,0,0,0,1,0,0; 9,1,1,0,0,0,0,0,0,0;10,1,1,0,0,0,0,1,1,0;11,1,1,1,0,0,0,0,0,0;12,1,1,1,0,0,0,1,1,1; 13,1,1,1,0,0,0,1,0,0;14,0,0,0,1,0,0,0,0,0;15,0,0,0,1,0,0,1,0,0;16,0,0,0,1,1,0,0,0,0; 17,0,0,0,1,1,0,1,1,0;18,0,0,0,1,1,1,0,0,0;19,0,0,0,1,1,1,1,1,1;20,0,0,0,1,1,1,1,0,0; 21,1,0,0,0,1,0,0,0,0;22,1,0,0,0,1,0,1,0,0;23,0,0,0,0,0,0,0,0,0;24,0,0,0,0,0,0,0,0,0; 25,0,0,0,0,0,0,0,0,0;26,0,0,0,0,0,0,0,0,0;27,0,0,0,0,0,0,0,0,0;28,1,0,0,0,1,0,0,1,0; 29,1,0,0,0,1,0,1,1,0;30,0,0,0,0,0,0,0,0,0;31,0,0,0,0,0,0,0,0,0;32,0,0,0,0,0,0,0,0,0; 33,0,0,0,0,0,0,0,0,0;34,0,0,0,0,0,0,0,0,0;35,0,0,0,0,0,0,0,0,0;36,0,0,0,0,0,0,0,0,0; 37,0,0,0,0,0,0,0,0,0;38,0,0,0,0,0,0,0,0,0;39,0,0,0,0,0,0,0,0,0;40,0,0,0,0,0,0,0,0,0; 41,0,0,0,0,0,0,0,0,0;42,0,0,0,0,0,0,0,0,0;43,0,0,0,0,0,0,0,0,0;44,0,0,0,0,0,0,0,0,0; 45,0,0,0,0,0,0,0,0,0;46,0,0,0,0,0,0,0,0,0;47,0,0,0,0,0,0,0,0,0;48,0,0,0,0,0,0,0

 ,0,0; 49,0,0,0,0,0,0,0,0,0;50,0,0,0,0,0,0,0,0,0;51,0,0,0,0,0,0,0,0,0;52,0,0,0,0,0,0,0,0,0; 53,0,0,0,0,0,0,0,0,0;54,0,0,0,0,0,0,0,0,0;55,0,0,0,0,0,0,0,0,0;56,0,0,0,0,0,0,0,0,0; 57,0,0,0,0,0,0,0,0,0;58,1,0,0,1,0,0,0,0,0;59,1,0,0,1,0,0,1,0,0;60,1,1,0,1,0,0,0,0,0; 61,1,1,0,1,0,0,1,0,0;62,1,1,0,1,0,0,0,1,0;63,1,1,0,1,0,0,1,1,0;64,1,0,0,1,1,0,0,0,0; 65,1,0,0,1,1,0,1,0,0;66,1,0,0,1,1,0,0,1,0;67,1,0,0,1,1,0,1,1,0;68,1,1,1,1,0,0,0,0,0; 69,1,1,1,1,0,0,1,1,1;70,1,0,0,1,1,1,0,0,0;71,1,0,0,1,1,1,1,1,1;72,1,1,1,1,1,1,0,0,0; 73,1,1,1,1,1,1,1,1,1;74,0,0,0,1,0,0,0,0,0;75,1,1,0,1,1,0,0,0,0;76,1,1,0,1,1,0,1,1,0; 77,0,0,0,0,0,0,0,0,0;78,0,0,0,0,0,0,0,0,0;79,0,0,0,0,0,0,0,0,0;80,0,0,0,0,0,0,0,0,0; 81,0,0,0,0,0,0,0,0,0;82,0,0,0,0,0,0,0,0,0;83,1,0,0,0,0,0,0,0,0;84,1,0,0,0,0,0,0,0,0; 85,1,0,0,0,0,0,1,0,0;86,1,0,0,0,0,0,1,0,0;87,0,0,0,1,0,0,0,0,0;88,0,0,0,1,0,0,0,0,0; 89,0,0,0,1,0,0,1,0,0;90,0,0,0,1,0,0,1,0,0;91,0,0,0,0,0,0,0,0,0;92,1,0,0,1,0,0,1,0,0}.
do if (model > 0).
compute tmp=modelmat(model,2:ncol(modelmat)).
end if.
do if (model=0).
compute tmp={0,0,0,0,0,0,0,0,0}.
end if.
do if (model < 4).
compute bcmat((nxs+1),1)=1.
end if.
do if ((model > 3) and (model <> 6)).
compute bcmat((nxs+1):(nxs+nms),1)=onem.
compute bcmat(nrow(bcmat),(nxs+1):(nxs+nms))=t(onem).
compute bcmat(nrow(bcmat),1)=1.
end if.
do if ((model = 6) or (model > 82 and model < 93)).
loop j = 2 to nrow(bcmat).
loop i = 1 to (j-1).
compute bcmat(j,i)=1.
end loop.
end loop.
end if.
do if (model = 80).
loop i = 1 to nms.
compute bcmat((nrow(bcmat)-1),i)=1.
end loop.
end if.
do if (model = 81).
loop j = 3 to nrow(bcmat).
compute bcmat(j,2)=1.
end loop.
end if.
do if (model = 82).
compute bcmat(3,2)=1.
compute bcmat(5,4)=1.
end if.
do if (tmp(1,1)=1).
compute wcmat((nxs+1):(nxs+nms),1)=onem.
compute wprod=1.
compute xprod=1.
do if (model = 83 or model = 86).
compute onemsx=onem.
loop i = 1 to (nms-1).
compute onemsx(i+1,1)=0.
end loop.
compute wcmat((nxs+1):(nxs+nms),1)=onemsx.
end if.
end if.
do if (tmp(1,4)=1).
compute wcmat(nrow(wcmat),(nxs+1):(nxs+nms))=t(onem).
compute wprod=1.
do if (model = 87 or model = 90).
compute onemsx=onem.
loop i = 1 to (nms-1).
compute onemsx(i,1)=0.
end loop.
compute wcmat(nrow(wcmat),(nxs+1):(nxs+nms))=t(onemsx).
end if.
end if.
do if (tmp(1,7)=1).
compute wcmat(nrow(wcmat),1)=1.
compute wprod=1.
compute xprod=1.
end if.
do if (tmp(1,2)=1).
compute zcmat((nxs+1):(nxs+nms),1)=onem.
compute zprod=1.
compute xprod=1.
end if.
do if (tmp(1,5)=1).
compute zcmat(nrow(zcmat),(nxs+1):(nxs+nms))=t(onem).
compute zprod=1.
end if.
do if (tmp(1,8)=1).
compute zcmat(nrow(zcmat),1)=1.
compute zprod=1.
compute xprod=1.
end if.
do if (tmp(1,3)=1).
compute wzcmat((nxs+1):(nxs+nms),1)=onem.
compute xprod=1.
compute wprod=1.
compute zprod=1.
end if.
do if (tmp(1,6)=1).
compute wzcmat(nrow(wzcmat),(nxs+1):(nxs+nms))=t(onem).
compute zprod=1.
compute wprod=1.
end if.
do if (tmp(1,9)=1).
compute wzcmat(nrow(wzcmat),1)=1.
compute xprod=1.
compute wprod=1.
compute zprod=1.
end if.
do if (model = 91 or model = 92).
loop j = 1 to (nms-1).
loop i = 1 to j.
compute wcmat((nxs+1+j),(nxs+i))=1.
end loop.
end loop.
end if.
do if (nms < 0).
loop i = 1 to nms.
compute tmp=csum(wcmat(:,(1+i)))+csum(zcmat(:,(1+i)))+csum(wzcmat(:,(1+i))).
compute mprod(1,i)=(tmp>0).
end loop.
end if.
end if.
do if (ncs > 0).
compute ccmat=make((nms+nys),ncs,1).
compute ccmatoff=ccmat.
do if (covmy=1).
compute ccmat(nrow(ccmat),:)=make(1,ncs,0).
end if.
do if (covmy=2).
compute ccmat(1:nms,:)=make(nms,ncs,0).
end if.
do if (cmatrix(1,1) <> -999).
do if (ncol(cmatrix) <> ((nms+nys)*ncs)).
compute errcode(errs,1)=29.
compute errs=errs+1.
compute criterr=1.
end if.
do if (criterr = 0).
compute tmp=1.
loop i = 1 to (nms+nys).
loop j = 1 to ncs.
compute ccmat(i,j)=1-(cmatrix(1,tmp) = 0).
compute tmp=tmp+1.
end loop.
end loop.
do if (rsum((csum(ccmat)=0)) <> 0).
compute errcode(errs,1)=30.
compute errs=errs+1.
compute criterr=1.
end if.
end if.
do if (covmy <> 0).
compute notecode(notes,1)=1.
compute notes=notes+1.
end if.
end if.
do if (xmint=1 and (covmy > 0 or (csum(rsum(ccmat)) <> ((nms+nys)*ncs)))).
compute errcode(errs,1)=68.
compute errs=errs+1.
compute criterr=1.
end if.
end if.
do if (criterr=0).
compute needed=needed*(needed-1)/2.
compute nopath=0.
do if (bmatrix(1,1) <> -999).
compute tmp=1.
do if ((ncol(bmatrix) <> needed) or (csum(rsum(bmatrix))=0)).
compute errcode(errs,1)=16.
compute errs=errs+1.
compute criterr=1.
else.
loop i = 2 to nrow(bcmat).
loop j = 1 to (i-1).
compute bcmat(i,j)=1-(bmatrix(1,tmp) = 0).
compute tmp=tmp+1.
end loop.
end loop.
end if.
do if ((csum(bcmat(:,1))=0) and criterr=0).
compute errcode(errs,1)=22.
compute errs=errs+1.
compute criterr=1.
end if.
do if ((rsum(bcmat(nrow(bcmat),:))=0) and criterr=0).
compute errcode(errs,1)=23.
compute errs=errs+1.
compute criterr=1.
end if.
compute dm=0.
do if (nms > 0).
loop i = 1 to nms.
do if (((rsum(bcmat((nxs+i),:)) = 0) or (csum(bcmat(:,(nxs+i))) = 0)) and (dm=0) and (criterr=0)).
compute errcode(errs,1)=26.
compute errs=errs+1.
compute criterr=1.
compute dm=1.
end if.
end loop.
end if.
release dm.
end if.
end if.
do if (criterr=0).
do if (wmatrix(1,1) <> -999).
compute tmp=1.
do if (ncol(wmatrix) <> needed).
compute errcode(errs,1)=17.
compute errs=errs+1.
compute criterr=1.
else.
compute modelvar(1,1)='CUSTOM'.
loop i = 2 to nrow(wcmat).
loop j = 1 to (i-1).
compute wcmat(i,j)=1-(wmatrix(1,tmp) = 0).
do if ((wcmat(i,j)=1) and (bcmat(i,j)=0) and (nopath=0)).
compute errcode(errs,1)=20.
compute errs=errs+1.
compute criterr=1.
compute nopath=1.
end if.
compute tmp=tmp+1.
end loop.
end loop.
end if.
end if.
do if (zmatrix(1,1) <> -999).
compute tmp=1.
do if (ncol(zmatrix) <> needed).
compute errcode(errs,1)=18.
compute errs=errs+1.
compute criterr=1.
else.
compute modelvar(1,1)='CUSTOM'.
do if (csum(rsum(wcmat))=0 and model=999).
compute errcode(errs,1)=21.
compute errs=errs+1.
compute criterr=1.
end if.
loop i = 2 to nrow(zcmat).
loop j = 1 to (i-1).
compute zcmat(i,j)=1-(zmatrix(1,tmp) = 0).
do if ((zcmat(i,j)=1) and (bcmat(i,j)=0) and (nopath=0)).
compute errcode(errs,1)=20.
compute errs=errs+1.
compute criterr=1.
compute nopath=1.
end if.
compute tmp=tmp+1.
end loop.
end loop.
end if.
end if.
compute tmp=1.
do if (wzmatrix(1,1) <> -999).
do if (ncol(wzmatrix) <> needed).
compute errcode(errs,1)=19.
compute errs=errs+1.
compute criterr=1.
end if.
compute modelvar(1,1)='CUSTOM'.
end if.
do if (criterr=0).
loop i = 2 to nrow(wzcmat).
loop j = 1 to (i-1).
do if (wzmatrix(1,1) <> -999).
compute wzcmat(i,j)=1-(wzmatrix(1,tmp) = 0).
end if.
do if (wzcmat(i,j)=1).
compute wcmat(i,j)=1.
compute zcmat(i,j)=1.
end if.
do if ((wzcmat(i,j)=1) and (bcmat(i,j)=0) and (nopath=0)).
compute errcode(errs,1)=20.
compute errs=errs+1.
compute criterr=1.
compute nopath=1.
end if.
compute tmp=tmp+1.
end loop.
end loop.
end if.
end if.
do if (criterr=0).
compute xprod=csum(wcmat(:,1))+csum(zcmat(:,1))+csum(wzcmat(:,1)).
compute xprod=(xprod > 0).
compute wsum=csum(rsum(wcmat)).
compute wprod=(wsum > 0).
do if (nms > 0).
loop i = 1 to nms.
compute tmp=csum(wcmat(:,(1+i)))+csum(zcmat(:,(1+i)))+csum(wzcmat(:,(1+i))).
compute mprod(1,i)=(tmp>0).
end loop.
end if.
do if ((wsum > 0) and (w = 'xxxxx')).
compute errcode(errs,1)=11.
compute errs=errs+1.
compute criterr=1.
end if.
do if ((wsum = 0) and (w <> 'xxxxx')).
compute errcode(errs,1)=10.
compute errs=errs+1.
compute criterr=1.
end if.
compute zsum=csum(rsum(zcmat)).
compute zprod=(zsum > 0).
do if ((zsum > 0) and (z = 'xxxxx')).
compute errcode(errs,1)=13.
compute errs=errs+1.
compute criterr=1.
end if.
do if ((zsum = 0) and (z <> 'xxxxx')).
compute errcode(errs,1)=12.
compute errs=errs+1.
compute criterr=1.
end if.
do if ((zsum > 0) and (wsum = 0)).
compute errcode(errs,1)=35.
compute errs=errs+1.
compute criterr=1.
end if.
end if.
do if (criterr=0 and nms > 1).
compute serchk=bcmat(2:(nrow(bcmat)-1),2:ncol(bcmat)).
do if (csum(rsum(serchk))) > 0.
compute serial=1.
do if (nms > 6).
compute errcode(errs,1)=36.
compute errs=errs+1.
compute criterr=1.
end if.
end if.
end if.
do if (center > 0 and criterr=0).
compute centvar={' '}.
do if (criterr=0).
do if ((center = 1) or (center = 2 and wdich = 0)).
do if (wprod=1 and mcwok=0 and nwpval > 0).
loop i = 1 to nws.
compute wtmp(:,i)=wtmp(:,i)-(csum(wtmp(:,i))/n).
compute centvar={centvar,wnames(1,i)}.
end loop.

.
compute desctmp=make((8-(4* wmodcust )),ncol( wtmp ),-999).
loop jd=1 to ncol( wtmp ).
compute descdat= wtmp (:,jd).
compute desctmp(1,jd) = csum(descdat)/nrow(descdat).
compute desctmp(2,jd) = (nrow(descdat)*sscp(descdat))-(t(csum(descdat))*(csum(descdat))).
compute desctmp(2,jd) = sqrt(desctmp(2,jd)/(nrow(descdat)*(nrow(descdat)-1))).
compute desctmp(3,jd)=cmin(descdat).
compute desctmp(4,jd)=cmax(descdat).
do if ( wmodcust =0).
compute minwarn=0.
compute maxwarn=0.
do if ((desctmp(3,jd)=desctmp(4,jd)) and novar=0).
compute errcode(errs,1)=15.
compute errs=errs+1.
compute criterr=1.
compute novar=1.
end if.
compute tmp=((descdat(:,1)=desctmp(3,jd))+(descdat(:,1)=desctmp(4,jd))).
compute desctmp(8,jd)=(csum(tmp)=nrow(tmp)).
compute tmp = descdat.
compute tmp(GRADE(descdat),:) = descdat.
compute descdat = tmp.
release tmp.
compute decval={.16;.5;.84}.
loop kd=1 to 3.
compute low=trunc(decval(kd,1)*(nrow(descdat)+1)).
compute lowdec=decval(kd,1)*(nrow(descdat)+1)-low.
compute value=descdat(low,1)+(descdat((low+1),1)-descdat(low,1))*lowdec.
compute desctmp((4+kd),jd)=value.
end loop.
compute mnotev=1.
compute modvals=desctmp(5:7,:).
do if (quantile <> 1).
compute desctmp(5,jd)=desctmp(1,jd)-desctmp(2,jd).
compute desctmp(6,jd)=desctmp(1,jd).
compute desctmp(7,jd)=desctmp(1,jd)+desctmp(2,jd).
compute modvals=desctmp(5:7,:).
compute mnotev=2.
do if (modvals(1,1) < desctmp(3,1)).
compute modvals(1,1)=desctmp(3,1).
compute minwarn=1.
end if.
do if (modvals(3,1) > desctmp(4,1)).
compute modvals(3,1)=desctmp(4,1).
compute maxwarn=1.
end if.
end if.
do if (desctmp(8,1)=1).
compute modvals={desctmp(3,1);desctmp(4,1)}.
compute mnotev=0.
compute minwarn=0.
compute maxwarn=0.
end if.
end if.
end loop
.
compute wmin=desctmp(3,1).
compute wmax=desctmp(4,1).
do if (wmodcust=0).
compute wmodvals=modvals.
compute wprobval=wmodvals.
end if.
end if.
end if.
do if ((center = 1) or (center = 2 and zdich = 0)).
do if (zprod=1 and mczok=0 and nzpval > 0).
loop i = 1 to nzs.
compute ztmp(:,i)=ztmp(:,i)-(csum(ztmp(:,i))/n).
compute centvar={centvar,znames(1,i)}.
end loop.

.
compute desctmp=make((8-(4* zmodcust )),ncol( ztmp ),-999).
loop jd=1 to ncol( ztmp ).
compute descdat= ztmp (:,jd).
compute desctmp(1,jd) = csum(descdat)/nrow(descdat).
compute desctmp(2,jd) = (nrow(descdat)*sscp(descdat))-(t(csum(descdat))*(csum(descdat))).
compute desctmp(2,jd) = sqrt(desctmp(2,jd)/(nrow(descdat)*(nrow(descdat)-1))).
compute desctmp(3,jd)=cmin(descdat).
compute desctmp(4,jd)=cmax(descdat).
do if ( zmodcust =0).
compute minwarn=0.
compute maxwarn=0.
do if ((desctmp(3,jd)=desctmp(4,jd)) and novar=0).
compute errcode(errs,1)=15.
compute errs=errs+1.
compute criterr=1.
compute novar=1.
end if.
compute tmp=((descdat(:,1)=desctmp(3,jd))+(descdat(:,1)=desctmp(4,jd))).
compute desctmp(8,jd)=(csum(tmp)=nrow(tmp)).
compute tmp = descdat.
compute tmp(GRADE(descdat),:) = descdat.
compute descdat = tmp.
release tmp.
compute decval={.16;.5;.84}.
loop kd=1 to 3.
compute low=trunc(decval(kd,1)*(nrow(descdat)+1)).
compute lowdec=decval(kd,1)*(nrow(descdat)+1)-low.
compute value=descdat(low,1)+(descdat((low+1),1)-descdat(low,1))*lowdec.
compute desctmp((4+kd),jd)=value.
end loop.
compute mnotev=1.
compute modvals=desctmp(5:7,:).
do if (quantile <> 1).
compute desctmp(5,jd)=desctmp(1,jd)-desctmp(2,jd).
compute desctmp(6,jd)=desctmp(1,jd).
compute desctmp(7,jd)=desctmp(1,jd)+desctmp(2,jd).
compute modvals=desctmp(5:7,:).
compute mnotev=2.
do if (modvals(1,1) < desctmp(3,1)).
compute modvals(1,1)=desctmp(3,1).
compute minwarn=1.
end if.
do if (modvals(3,1) > desctmp(4,1)).
compute modvals(3,1)=desctmp(4,1).
compute maxwarn=1.
end if.
end if.
do if (desctmp(8,1)=1).
compute modvals={desctmp(3,1);desctmp(4,1)}.
compute mnotev=0.
compute minwarn=0.
compute maxwarn=0.
end if.
end if.
end loop
.
compute zmin=desctmp(3,1).
compute zmax=desctmp(4,1).
do if (zmodcust=0).
compute zmodvals=modvals.
compute zprobval=zmodvals.
end if.
end if.
end if.
do if ((center = 1) or (center = 2 and xdich = 0)).
do if (xprod=1 and mcxok=0).
loop i = 1 to nxs.
compute xtmp(:,i)=xtmp(:,i)-(csum(xtmp(:,i))/n).
compute centvar={centvar,xnames(1,i)}.
end loop.

.
compute desctmp=make((8-(4* 0 )),ncol( xtmp ),-999).
loop jd=1 to ncol( xtmp ).
compute descdat= xtmp (:,jd).
compute desctmp(1,jd) = csum(descdat)/nrow(descdat).
compute desctmp(2,jd) = (nrow(descdat)*sscp(descdat))-(t(csum(descdat))*(csum(descdat))).
compute desctmp(2,jd) = sqrt(desctmp(2,jd)/(nrow(descdat)*(nrow(descdat)-1))).
compute desctmp(3,jd)=cmin(descdat).
compute desctmp(4,jd)=cmax(descdat).
do if ( 0 =0).
compute minwarn=0.
compute maxwarn=0.
do if ((desctmp(3,jd)=desctmp(4,jd)) and novar=0).
compute errcode(errs,1)=15.
compute errs=errs+1.
compute criterr=1.
compute novar=1.
end if.
compute tmp=((descdat(:,1)=desctmp(3,jd))+(descdat(:,1)=desctmp(4,jd))).
compute desctmp(8,jd)=(csum(tmp)=nrow(tmp)).
compute tmp = descdat.
compute tmp(GRADE(descdat),:) = descdat.
compute descdat = tmp.
release tmp.
compute decval={.16;.5;.84}.
loop kd=1 to 3.
compute low=trunc(decval(kd,1)*(nrow(descdat)+1)).
compute lowdec=decval(kd,1)*(nrow(descdat)+1)-low.
compute value=descdat(low,1)+(descdat((low+1),1)-descdat(low,1))*lowdec.
compute desctmp((4+kd),jd)=value.
end loop.
compute mnotev=1.
compute modvals=desctmp(5:7,:).
do if (quantile <> 1).
compute desctmp(5,jd)=desctmp(1,jd)-desctmp(2,jd).
compute desctmp(6,jd)=desctmp(1,jd).
compute desctmp(7,jd)=desctmp(1,jd)+desctmp(2,jd).
compute modvals=desctmp(5:7,:).
compute mnotev=2.
do if (modvals(1,1) < desctmp(3,1)).
compute modvals(1,1)=desctmp(3,1).
compute minwarn=1.
end if.
do if (modvals(3,1) > desctmp(4,1)).
compute modvals(3,1)=desctmp(4,1).
compute maxwarn=1.
end if.
end if.
do if (desctmp(8,1)=1).
compute modvals={desctmp(3,1);desctmp(4,1)}.
compute mnotev=0.
compute minwarn=0.
compute maxwarn=0.
end if.
end if.
end loop
.
compute xmodvals=modvals.
compute xprobval=xmodvals.
end if.
end if.
do if (nms > 0).
loop i = 1 to nms.
do if (mprod(1,i)=1).
compute mtmp(:,i)=mtmp(:,i)-(csum(mtmp(:,i))/n).
compute centvar={centvar,mnames(1,i)}.
end if.
end loop.

.
compute desctmp=make((8-(4* 0 )),ncol( mtmp ),-999).
loop jd=1 to ncol( mtmp ).
compute descdat= mtmp (:,jd).
compute desctmp(1,jd) = csum(descdat)/nrow(descdat).
compute desctmp(2,jd) = (nrow(descdat)*sscp(descdat))-(t(csum(descdat))*(csum(descdat))).
compute desctmp(2,jd) = sqrt(desctmp(2,jd)/(nrow(descdat)*(nrow(descdat)-1))).
compute desctmp(3,jd)=cmin(descdat).
compute desctmp(4,jd)=cmax(descdat).
do if ( 0 =0).
compute minwarn=0.
compute maxwarn=0.
do if ((desctmp(3,jd)=desctmp(4,jd)) and novar=0).
compute errcode(errs,1)=15.
compute errs=errs+1.
compute criterr=1.
compute novar=1.
end if.
compute tmp=((descdat(:,1)=desctmp(3,jd))+(descdat(:,1)=desctmp(4,jd))).
compute desctmp(8,jd)=(csum(tmp)=nrow(tmp)).
compute tmp = descdat.
compute tmp(GRADE(descdat),:) = descdat.
compute descdat = tmp.
release tmp.
compute decval={.16;.5;.84}.
loop kd=1 to 3.
compute low=trunc(decval(kd,1)*(nrow(descdat)+1)).
compute lowdec=decval(kd,1)*(nrow(descdat)+1)-low.
compute value=descdat(low,1)+(descdat((low+1),1)-descdat(low,1))*lowdec.
compute desctmp((4+kd),jd)=value.
end loop.
compute mnotev=1.
compute modvals=desctmp(5:7,:).
do if (quantile <> 1).
compute desctmp(5,jd)=desctmp(1,jd)-desctmp(2,jd).
compute desctmp(6,jd)=desctmp(1,jd).
compute desctmp(7,jd)=desctmp(1,jd)+desctmp(2,jd).
compute modvals=desctmp(5:7,:).
compute mnotev=2.
do if (modvals(1,1) < desctmp(3,1)).
compute modvals(1,1)=desctmp(3,1).
compute minwarn=1.
end if.
do if (modvals(3,1) > desctmp(4,1)).
compute modvals(3,1)=desctmp(4,1).
compute maxwarn=1.
end if.
end if.
do if (desctmp(8,1)=1).
compute modvals={desctmp(3,1);desctmp(4,1)}.
compute mnotev=0.
compute minwarn=0.
compute maxwarn=0.
end if.
end if.
end loop
.
do if ((cdeval(1,1)=-999) and (model=74)).
compute medmeans=desctmp(1,:).
end if.
compute mmodvals=modvals.
compute mprobval=mmodvals.
end if.
end if.
do if (ncol(centvar) > 1).
compute notecode(notes,1)=3.
compute notes=notes+1.
end if.
end if.
do if (criterr=0).
compute wsum=rsum(csum(wcmat)).
compute zsum=rsum(csum(zcmat)).
compute wzsum=rsum(csum(wzcmat)).
compute nump=make(1,(nys+nms),-999).
compue numint=make(1,(nys+nms),0).
compute datcount=1.
compute xtmpuse=0.
compute wtmpuse=0.
compute ztmpuse=0.
compute xwtmpus=0.
compute xztmpus=0.
compute wztmpus=0.
compute xwztmpu=0.
compute xtmploc=-999.
compute wtmploc=-999.
compute xwtmplo=-999.
compute ztmploc=-999.
compute xztmplo=-999.
compute wztmplo=-999.
compute xwztmplo=-999.
compute vlabs={' '}.
do if (ncs > 0).
compute ctmpuse=make(1,ncs,0).
end if.
do if (nms > 0).
compute mtmpuse=make(1,nms,0).
compute mwtmpus=make(1,nms,0).
compute mztmpus=make(1,nms,0).
compute mwztmpu=make(1,nms,0).
compute mtmploc=make(1,nms,0).
compute mwtmplo=make(nwvls,nms,-999).
compute mztmplo=make(nzvls,nms,-999).
compute mwztmplo=make((nwvls*nzvls),nms,-999).
end if.
do if (ncs > 0).
compute ctmploc=make(1,ncs,0).
end if.
compute fulldat=make(n,1,1).
compute datindx=make(1000,(nms+nys),-999).
compute wherew=make(2,(nms+nys),-999).
compute wherex=make(2,(nms+nys),-999).
compute wherez=make(2,(nms+nys),-999).
compute wherexw=make(2,(nms+nys),-999).
compute wherexz=make(2,(nms+nys),-999).
compute wherewz=make(2,(nms+nys),-999).
compute wherexwz=make(2,(nms+nys),-999).
do if (nms > 0).
compute wherem=make(nms,(nms+nys),-999).
compute wheremw = make(nms*2,(nms+nys),-999).
compute wheremz = make(nms*2,(nms+nys),-999).
compute wheremwz = make(nms*2,(nms+nys),-999).
end if.
compute wzhigh=make(1000,(((nms+1)*(nms+2))/2),0).
compute whigh=make(1000,(((nms+1)*(nms+2))/2),0).
compute zhigh=make(1000,(((nms+1)*(nms+2))/2),0).
compute fochigh=make(1000,(((nms+1)*(nms+2))/2),0).
compute xcoefloc={1;2;3;4;5;6;7;8;9}.
compute intkey = {' ', ' ', ' ', ' ', ' ', ' ', ' '}.
compute wzhighct=0.
compute whighct=0.
compute zhighct=0.
compute foccnt=0.
loop i = 2 to nrow(bcmat).
compute wdid=0.
compute zdid=0.
compute wzdid=0.
compute cntmp=1.
compute start=1.
do if (i < nrow(bcmat)).
compute outv=mtmp(:,(i-1)).
compute modlabel={mnames(1,(i-1));'constant'}.
end if.
do if (i = nrow(bcmat)).
compute outv=ytmp.
compute modlabel={ynames;'constant'}.
end if.
loop j = 1 to (i-1).
compute foccnt=foccnt+1.
do if (j = 1 and bcmat(i,j)=1).
compute outv={outv,xtmp}.
compute modlabel={modlabel;xcatlab(1:nxvls,1)}.
do if (xtmpuse=0).
compute fulldat={fulldat,xtmp}.
compute xtmpuse=1.
loop k4=datcount to (datcount+(nxvls-1)).
compute xtmploc={xtmploc;k4}.
end loop.
compute xtmploc=xtmploc(2:nrow(xtmploc),1).
compute datcount=datcount+nxvls.
end if.
compute datindx(start:(start+nrow(xtmploc)-1),(i-1))=xtmploc.
compute wherex(1,(i-1))=start+1.
compute wherex(2,(i-1))=start+nrow(xtmploc)-1+1.
do if (model = 74).
end if.
compute onebl=make(nrow(xtmploc),1,1).
compute fochigh((start+1):(start+nrow(xtmploc)),foccnt)=onebl.
compute start=start+nrow(xtmploc).
end if.
do if (j > 1 and bcmat(i,j)=1).
compute outv={outv,mtmp(:,(j-1))}.
compute modlabel={modlabel;mnames(1,(j-1))}.
do if (mtmpuse(1,(j-1))=0).
compute fulldat={fulldat,mtmp(:,(j-1))}.
compute mtmpuse(1,(j-1))=1.
compute mtmploc(1,(j-1))=datcount.
compute datcount=datcount+1.
end if.
compute datindx(start:(start+nrow(mtmploc)-1),(i-1))=mtmploc(1,(j-1)).
compute wherem((j-1),(i-1))=start+1.
compute onebl=make(nrow(mtmploc(1,j-1)),1,1).
compute ttt=nrow(mtmploc(1,(j-1)))+start-1.
compute fochigh((start+1):(start+nrow(mtmploc(1,(j-1)))),foccnt)=onebl.
compute start=start+nrow(mtmploc(1,(j-1))).
end if.
end loop.
do if (wsum > 0).
loop j = 1 to (i-1).
compute whighct=whighct+1.
do if (j = 1 and wcmat(i,j)=1).
do if (wdid=0).
compute outv={outv,wtmp}.
do if (ncs > 0 and wiscov > 0).
compute ccmatoff((i-1),wiscov)=0.
end if.
compute modlabel={modlabel;wcatlab(1:nwvls,1)}.
compute wdid=1.
do if (wtmpuse=0).
compute fulldat={fulldat,wtmp}.
do if (ncs > 0 and wiscov > 0).
compute ccmatoff((i-1),wiscov)=0.
end if.
compute wtmpuse=1.
loop k4=datcount to (datcount+(nwvls-1)).
compute wtmploc={wtmploc;k4}.
end loop.
compute wtmploc=wtmploc(2:nrow(wtmploc),1).
compute datcount=datcount+nwvls.
end if.
end if.
compute datindx(start:(start+nrow(wtmploc)-1),(i-1))=wtmploc.
compute wherew(1,(i-1))=start+1.
compute wherew(2,(i-1))=start+nrow(wtmploc)-1+1.
compute start=start+nrow(wtmploc).
loop k1=1 to nxvls.
loop k2 = 1 to nwvls.
compute outv={outv,(xtmp(:,k1)&*wtmp(:,k2))}.
do if (ncs > 0 and wiscov > 0).
compute ccmatoff((i-1),wiscov)=0.
end if.
compute modlabel={modlabel;intlab(cntmp,1)}.
compute intkey={intkey;intlab(cntmp,1),':',xcatlab(k1,1),'x',wcatlab(k2,1),' ',' '}.
compute cntmp=cntmp+1.
end loop.
end loop.
do if (xwtmpus=0).
compute fulldat={fulldat,outv(:,(ncol(outv)-(nxvls*nwvls)+1):ncol(outv))}.
compute xwtmpus=1.
do if (ncs > 0 and wiscov > 0).
compute ccmatoff((i-1),wiscov)=0.
end if.
loop k4=datcount to (datcount+((nwvls*nxvls)-1)).
compute xwtmplo={xwtmplo;k4}.
end loop.
compute xwtmplo=xwtmplo(2:nrow(xwtmplo),1).
compute datcount=datcount+(nxvls*nwvls).
end if.
compute datindx(start:(start+nrow(xwtmplo)-1),(i-1))=xwtmplo.
compute wherexw(1,(i-1))=start+1.
compute wherexw(2,(i-1))=start+nrow(xwtmplo)-1+1.
compute onebl=make(nrow(xwtmplo),1,1).
compute whigh((start+1):(start+nrow(xwtmplo)),whighct)=onebl.
compute start=start+nrow(xwtmplo).
end if.
do if (j > 1 and wcmat(i,j)=1).
do if (wdid=0 and model <> 74).
compute outv={outv,wtmp}.
do if (ncs > 0 and wiscov > 0).
compute ccmatoff((i-1),wiscov)=0.
end if.
compute modlabel={modlabel;wcatlab(1:nwvls,1)}.
compute wdid=1.
do if (wtmpuse=0).
compute fulldat={fulldat,wtmp}.
do if (ncs > 0 and wiscov > 0).
compute ccmatoff((i-1),wiscov)=0.
end if.
compute wtmpuse=1.
loop k4=datcount to (datcount+(nwvls-1)).
compute wtmploc={wtmploc;k4}.
end loop.
compute wtmploc=wtmploc(2:nrow(wtmploc),1).
compute datcount=datcount+nwvls.
end if.
compute datindx(start:(start+nrow(wtmploc)-1),(i-1))=wtmploc.
compute wherew(1,(i-1))=start+1.
compute wherew(2,(i-1))=start+nrow(wtmploc)-1+1.
compute start=start+nrow(wtmploc).
end if.
loop k2 = 1 to nwvls.
compute outv={outv,(mtmp(:,(j-1))&*wtmp(:,k2))}.
do if (ncs > 0 and wiscov > 0).
compute ccmatoff((i-1),wiscov)=0.
end if.
compute modlabel={modlabel;intlab(cntmp,1)}.
compute intkey={intkey;intlab(cntmp,1),':', mnames(1,(j-1)),'x',wcatlab(k2,1),' ',' '}.
compute cntmp=cntmp+1.
end loop.
do if (mwtmpus(1,(j-1))=0).
compute fulldat={fulldat,outv(:,(ncol(outv)-nwvls+1):ncol(outv))}.
do if (ncs > 0 and wiscov > 0).
compute ccmatoff((i-1),wiscov)=0.
end if.
compute mwtmpus(1,(j-1))=1.
compute mw22=-999.
loop k4=datcount to (datcount+(nwvls-1)).
compute mw22={mw22;k4}.
end loop.
compute mwtmplo(:,(j-1))=mw22(2:nrow(mw22),1).
compute datcount=datcount+nwvls.
end if.
compute datindx(start:(start+nrow(mwtmplo)-1),(i-1))=mwtmplo(:,(j-1)).
compute wheremw(((2*j)-3),(i-1))=start+1.
compute wheremw(((2*j)-2),(i-1))=start+nrow(mwtmplo)-1+1.
compute onebl=make(nrow(mwtmplo),1,1).
compute whigh((start+1):(start+nrow(mwtmplo)),whighct)=onebl.
compute start=start+nrow(mwtmplo).
end if.
end loop.
end if.
do if (zsum > 0).
loop j = 1 to (i-1).
compute zhighct=zhighct+1.
do if (j = 1 and zcmat(i,j)=1).
do if (zdid=0).
compute outv={outv,ztmp}.
do if (ncs > 0 and ziscov > 0).
compute ccmatoff((i-1),ziscov)=0.
end if.
compute modlabel={modlabel;zcatlab(1:nzvls,1)}.
compute zdid=1.
do if (ztmpuse=0).
compute fulldat={fulldat,ztmp}.
do if (ncs > 0 and ziscov > 0).
compute ccmatoff((i-1),ziscov)=0.
end if.
compute ztmpuse=1.
loop k4=datcount to (datcount+(nzvls-1)).
compute ztmploc={ztmploc;k4}.
end loop.
compute ztmploc=ztmploc(2:nrow(ztmploc),1).
compute datcount=datcount+nzvls.
end if.
end if.
compute datindx(start:(start+nrow(ztmploc)-1),(i-1))=ztmploc.
compute wherez(1,(i-1))=start+1.
compute wherez(2,(i-1))=start+nrow(ztmploc)-1+1.
compute start=start+nrow(ztmploc).
loop k1=1 to nxvls.
loop k2 = 1 to nzvls.
compute outv={outv,(xtmp(:,k1)&*ztmp(:,k2))}.
do if (ncs > 0 and ziscov > 0).
compute ccmatoff((i-1),ziscov)=0.
end if.
compute modlabel={modlabel;intlab(cntmp,1)}.
compute intkey={intkey;intlab(cntmp,1),':',xcatlab(k1,1),'x',zcatlab(k2,1),' ',' '}.
compute cntmp=cntmp+1.
end loop.
end loop.
do if (xztmpus=0).
compute fulldat={fulldat,outv(:,(ncol(outv)-(nxvls*nzvls)+1):ncol(outv))}.
do if (ncs > 0 and ziscov > 0).
compute ccmatoff((i-1),ziscov)=0.
end if.
compute xztmpus=1.
loop k4=datcount to (datcount+((nzvls*nxvls)-1)).
compute xztmplo={xztmplo;k4}.
end loop.
compute xztmplo=xztmplo(2:nrow(xztmplo),1).
compute datcount=datcount+(nxvls*nzvls).
end if.
compute datindx(start:(start+nrow(xztmplo)-1),(i-1))=xztmplo.
compute wherexz(1,(i-1))=start+1.
compute wherexz(2,(i-1))=start+nrow(xztmplo)-1+1.
compute onebl=make(nrow(xztmplo),1,1).
compute zhigh((start+1):(start+nrow(xztmplo)),zhighct)=onebl.
compute start=start+nrow(xztmplo).
end if.
do if (j > 1 and zcmat(i,j)=1).
do if (zdid=0).
compute outv={outv,ztmp}.
do if (ncs > 0 and ziscov > 0).
compute ccmatoff((i-1),ziscov)=0.
end if.
compute modlabel={modlabel;zcatlab(1:nzvls,1)}.
compute zdid=1.
do if (ztmpuse=0).
compute fulldat={fulldat,ztmp}.
do if (ncs > 0 and ziscov > 0).
compute ccmatoff((i-1),ziscov)=0.
end if.
compute ztmpuse=1.
loop k4=datcount to (datcount+(nzvls-1)).
compute ztmploc={ztmploc;k4}.
end loop.
compute ztmploc=ztmploc(2:nrow(ztmploc),1).
compute datcount=datcount+nzvls.
end if.
compute datindx(start:(start+nrow(ztmploc)-1),(i-1))=ztmploc.
compute wherez(1,(i-1))=start+1.
compute wherez(2,(i-1))=start+nrow(ztmploc)-1+1.
compute start=start+nrow(ztmploc).
end if.
loop k2 = 1 to nzvls.
compute outv={outv,(mtmp(:,(j-1))&*ztmp(:,k2))}.
do if (ncs > 0 and ziscov > 0).
compute ccmatoff((i-1),ziscov)=0.
end if.
compute modlabel={modlabel;intlab(cntmp,1)}.
compute intkey={intkey;intlab(cntmp,1),':', mnames(1,(j-1)),'x',zcatlab(k2,1),' ',' '}.
compute cntmp=cntmp+1.
end loop.
do if (mztmpus(1,(j-1))=0).
compute fulldat={fulldat,outv(:,(ncol(outv)-nzvls+1):ncol(outv))}.
do if (ncs > 0 and ziscov > 0).
compute ccmatoff((i-1),ziscov)=0.
end if.
compute mztmpus(1,(j-1))=1.
compute mz22=-999.
loop k4=datcount to (datcount+(nzvls-1)).
compute mz22={mz22;k4}.
end loop.
compute mztmplo(:,(j-1))=mz22(2:nrow(mz22),1).
compute datcount=datcount+nzvls.
end if.
compute datindx(start:(start+nrow(mztmplo)-1),(i-1))=mztmplo(:,(j-1)).
compute wheremz(((2*j)-3),(i-1))=start+1.
compute wheremz(((2*j)-2),(i-1))=start+nrow(mztmplo)-1+1.
compute onebl=make(nrow(mztmplo),1,1).
compute zhigh((start+1):(start+nrow(mztmplo)),zhighct)=onebl.
compute start=start+nrow(mztmplo).
end if.
end loop.
end if.
do if (wzsum > 0).
loop j = 1 to (i-1).
compute wzhighct=wzhighct+1.
do if (j = 1 and wzcmat(i,j)=1).
do if (wzdid=0).
loop k1=1 to nwvls.
loop k2 = 1 to nzvls.
compute outv={outv,(wtmp(:,k1)&*ztmp(:,k2))}.
do if (ncs > 0 and (ziscov > 0)).
compute ccmatoff((i-1),ziscov)=0.
end if.
do if (ncs > 0 and (wiscov > 0)).
compute ccmatoff((i-1),wiscov)=0.
end if.
compute modlabel={modlabel;intlab(cntmp,1)}.
compute intkey={intkey;intlab(cntmp,1),':',wcatlab(k1,1),'x',zcatlab(k2,1),' ',' '}.
compute cntmp=cntmp+1.
end loop.
end loop.
do if (wztmpus=0).
compute fulldat={fulldat,outv(:,(ncol(outv)-(nwvls*nzvls)+1):ncol(outv))}.
do if (ncs > 0 and (ziscov > 0)).
compute ccmatoff((i-1),ziscov)=0.
end if.
do if (ncs > 0 and (wiscov > 0)).
compute ccmatoff((i-1),wiscov)=0.
end if.
compute wztmpus=1.
loop k4=datcount to (datcount+((nwvls*nzvls)-1)).
compute wztmplo={wztmplo;k4}.
end loop.
compute wztmplo=wztmplo(2:nrow(wztmplo),1).
compute datcount=datcount+(nzvls*nwvls).
end if.
compute wzdid=1.
end if.
compute datindx(start:(start+nrow(wztmplo)-1),(i-1))=wztmplo.
compute wherewz(1,(i-1))=start+1.
compute wherewz(2,(i-1))=start+nrow(wztmplo)-1+1.
compute start=start+nrow(wztmplo).
loop k1=1 to nxvls.
loop k2=1 to nwvls.
loop k3=1 to nzvls.
compute outv={outv,(xtmp(:,k1)&*wtmp(:,k2)&*ztmp(:,k3))}.
do if (ncs > 0 and (ziscov > 0)).
compute ccmatoff((i-1),ziscov)=0.
end if.
do if (ncs > 0 and (wiscov > 0)).
compute ccmatoff((i-1),wiscov)=0.
end if.
compute modlabel={modlabel;intlab(cntmp,1)}.
compute intkey={intkey;intlab(cntmp,1),':',xcatlab(k1,1),'x',wcatlab(k2,1),'x', zcatlab(k3,1)}.
compute cntmp=cntmp+1.
end loop.
end loop.
end loop.
do if (xwztmpu=0).
compute fulldat={fulldat,outv(:,(ncol(outv)-(nxvls*nwvls*nzvls)+1):ncol(outv))}.
do if (ncs > 0 and (ziscov > 0)).
compute ccmatoff((i-1),ziscov)=0.
end if.
do if (ncs > 0 and (wiscov > 0)).
compute ccmatoff((i-1),wiscov)=0.
end if.
compute xwztmpu=1.
loop k4=datcount to (datcount+((nzvls*nxvls*nwvls)-1)).
compute xwztmplo={xwztmplo;k4}.
end loop.
compute xwztmplo=xwztmplo(2:nrow(xwztmplo),1).
compute datcount=datcount+(nxvls*nzvls*nwvls).
end if.
compute datindx(start:(start+nrow(xwztmplo)-1),(i-1))=xwztmplo.
compute wherexwz(1,(i-1))=start+1.
compute wherexwz(2,(i-1))=start+nrow(xwztmplo)-1+1.
compute onebl=make(nrow(xwztmplo),1,1).
compute wzhigh((start+1):(start+nrow(xwztmplo)),wzhighct)=onebl.
compute start=start+nrow(xwztmplo).
end if.
do if (j > 1 and wzcmat(i,j)=1).
do if (wzdid=0).
loop k1=1 to nwvls.
loop k2 = 1 to nzvls.
compute outv={outv,(wtmp(:,k1)&*ztmp(:,k2))}.
do if (ncs > 0 and (ziscov > 0)).
compute ccmatoff((i-1),ziscov)=0.
end if.
do if (ncs > 0 and (wiscov > 0)).
compute ccmatoff((i-1),wiscov)=0.
end if.
compute modlabel={modlabel;intlab(cntmp,1)}.
compute intkey={intkey;intlab(cntmp,1),':',wcatlab(k1,1),'x',zcatlab(k2,1),' ',' '}.
compute cntmp=cntmp+1.
end loop.
end loop.
do if (wztmpus=0).
compute fulldat={fulldat,outv(:,(ncol(outv)-(nwvls*nzvls)+1):ncol(outv))}.
do if (ncs > 0 and (ziscov > 0)).
compute ccmatoff((i-1),ziscov)=0.
end if.
do if (ncs > 0 and (wiscov > 0)).
compute ccmatoff((i-1),wiscov)=0.
end if.
compute wztmpus=1.
loop k4=datcount to (datcount+((nwvls*nzvls)-1)).
compute wztmplo={wztmplo;k4}.
end loop.
compute wztmplo=wztmplo(2:nrow(wztmplo),1).
compute datcount=datcount+(nzvls*nwvls).
end if.
compute wzdid=1.
compute datindx(start:(start+nrow(wztmplo)-1),(i-1))=wztmplo.
compute wherewz(1,(i-1))=start+1.
compute wherewz(2,(i-1))=start+nrow(wztmplo)-1+1.
compute start=start+nrow(wztmplo).
end if.
loop k1 = 1 to nwvls.
loop k2 = 1 to nzvls.
compute outv={outv,(mtmp(:,(j-1))&*wtmp(:,k1)&*ztmp(:,k2))}.
do if (ncs > 0 and (ziscov > 0)).
compute ccmatoff((i-1),ziscov)=0.
end if.
do if (ncs > 0 and (wiscov > 0)).
compute ccmatoff((i-1),wiscov)=0.
end if.
compute modlabel={modlabel;intlab(cntmp,1)}.
compute intkey={intkey;intlab(cntmp,1),':',mnames(1,(j-1)),'x',wcatlab(k1,1),'x', zcatlab(k2,1)}.
compute cntmp=cntmp+1.
end loop.
end loop.
do if (mwztmpu(1,(j-1))=0).
compute fulldat={fulldat,outv(:,(ncol(outv)-(nwvls*nzvls)+1):ncol(outv))}.
do if (ncs > 0 and (ziscov > 0)).
compute ccmatoff((i-1),ziscov)=0.
end if.
do if (ncs > 0 and (wiscov > 0)).
compute ccmatoff((i-1),wiscov)=0.
end if.
compute mwztmpu(1,(j-1))=1.
compute mz22=-999.
loop k4=datcount to (datcount+(nwvls*nzvls)-1).
compute mz22={mz22;k4}.
end loop.
compute mwztmplo(:,(j-1))=mz22(2:nrow(mz22),1).
compute datcount=datcount+(nwvls*nzvls).
end if.
compute datindx(start:(start+nrow(mwztmplo)-1),(i-1))=mwztmplo(:,(j-1)).
compute wheremwz(((2*j)-3),(i-1))=start+1.
compute wheremwz(((2*j)-2),(i-1))=start+nrow(mwztmplo)-1+1.
compute onebl=make(nrow(mwztmplo),1,1).
compute wzhigh((start+1):(start+nrow(mwztmplo)),wzhighct)=onebl.
compute start=start+nrow(mwztmplo).
end if.
end loop.
end if.
do if (ncs > 0).
compute ccmat=ccmat&*ccmatoff.
loop j = 1 to ncs.
do if (ccmat((i-1),j))=1.
do if (j=wiscov).
compute ctmp(:,j)=wtmp.
end if.
do if (j=ziscov).
compute ctmp(:,j)=ztmp.
end if.
compute outv={outv,ctmp(:,j)}.
compute modlabel={modlabel;covnames(1,j)}.
do if (ctmpuse(1,j)=0).
compute fulldat={fulldat,ctmp(:,j)}.
compute ctmpuse(1,j)=1.
compute ctmploc(1,j)=datcount.
compute datcount=datcount+1.
end if.
compute datindx(start:(start+nrow(ctmploc)-1),(i-1))=ctmploc(1,j).
compute start=start+nrow(ctmploc(1,j)).
end if.
end loop.
end if.
compute wdid=0.
compute zdid=0.
compute wzdid=0.
compute vlabs={vlabs;modlabel(2:nrow(modlabel),1)}.
compute numint(1,(i-1))=cntmp-1.
compute nump(1,(i-1))=nrow(modlabel)-1.
end loop.
release datcount, xtmpuse, wtmpuse, ztmpuse, xwtmpus, xztmpus, wztmpus, xwztmpu.
release xtmploc, wtmploc, xwtmplo, ztmploc, xztmplo, wztmplo, xwztmplo, foccnt.
do if (modcok=1 and ((nms > 0) or (zcmat(2,1) <> 1) or (mcx <> 0))).
compute notecode(notes,1) = 19.
compute notes = notes + 1.
compute modcok=0.
end if.
do if ((serial = 1 or (rsum(numint)>0) or nms=0) and mc > 0).
compute notecode(notes,1) = 15.
compute notes = notes + 1.
compute boot=mc.
compute mc=0.
end if.
do if (boot <> 0 or mc <> 0).
compute bootsz=boot.
do if (mc > 0).
compute bootsz=mc.
compute saveboot=0.
end if.
loop.
compute cilow = rnd(bootsz*(1-(conf/100))/2).
compute cihigh = trunc((bootsz*(conf/100)+(bootsz*(1-(conf/100))/2)))+1.
do if (cilow < 1 or cihigh > bootsz).
compute bootsz=trunc((bootsz+1000)/1000)*1000.
compute adjust = 1.
end if.
end loop if (cilow gt 0 and cihigh le bootsz).
do if (boot > 0).
compute boot=bootsz.
end if.
do if (mc > 0).
compute mc=bootsz.
end if.
do if (adjust = 1 and boot > 0).
compute notecode(notes,1) = 8.
compute notes = notes + 1.
end if.
do if (adjust = 1 and mc > 0).
compute notecode(notes,1) = 16.
compute notes = notes + 1.
end if.
end if.
compute maxboot = trunc(2*boot).
do if ( 0 > maxboot).
compute maxboot=trunc( 0 ).
end if.
do if (nms > 0).
release mtmpuse, mwtmpus, mwztmpu, mtmploc, mwtmplo, mztmplo, mwztmplo.
end if.
release wdid, zdid, wzdid, start,modlabel.
compute vlabs=vlabs(2:nrow(vlabs),1).
do if (rsum(numint) > 0).
compute intkey=intkey(2:nrow(intkey),:).
end if.
compute fulldat=fulldat(:,2:ncol(fulldat)).
compute fochigh=fochigh(1:rmax(nump),:).
compute whigh=whigh(1:rmax(nump),:).
compute zhigh=zhigh(1:rmax(nump),:).
compute wzhigh=wzhigh(1:rmax(nump),:).
compute coeffs=fochigh+whigh+zhigh+wzhigh.
compute bootloc=make(rmax(nump),ncol(nump),0).
do if (nms > 0).
compute cntmp=1.
loop i = 1 to ncol(nump).
loop j = 1 to nump(1,i).
compute bootloc(j,i)=cntmp.
compute cntmp=cntmp+1.
end loop.
end loop.
compute fochighb=make(nrow(fochigh),ncol(fochigh),0).
compute whighb=fochighb.
compute zhighb=fochighb.
compute wzhighb=fochighb.
compute thetaxmb=make(nrow(fochighb),nms,0).
compute thetaxyb=make(nrow(fochighb),1,0).
compute pathsfoc=make(nxvls,1,0).
compute cntmp=1.
loop i = 1 to (nms+nys).
loop j = 1 to i.
compute fochighb(:,cntmp)=fochigh(:,cntmp)&*bootloc(:,i).
compute whighb(:,cntmp)=whigh(:,cntmp)&*bootloc(:,i).
compute zhighb(:,cntmp)=zhigh(:,cntmp)&*bootloc(:,i).
compute wzhighb(:,cntmp)=wzhigh(:,cntmp)&*bootloc(:,i).
compute coeffsb=fochighb+whighb+zhighb+wzhighb.
do if ((i < (nms+nys)) and (j = 1)).
compute thetaxmb(:,i)=coeffsb(:,cntmp).
end if.
do if ((i = (nms+nys)) and (j = 1)).
compute thetaxyb(:,1)=coeffsb(:,cntmp).
end if.
compute cntmp=cntmp+1.
end loop.
end loop.
compute thetamyb=coeffsb(:,(ncol(coeffsb)-nms+1):ncol(coeffsb)).
do if (serial = 1).
compute thetammb=make(nrow(coeffsb),((nms*(nms-1))/2),0).
end if.
compute cntmp=1.
do if (nms > 1 and serial = 1).
loop i = 1 to (nms-1).
compute start=((i+2)*(i+1))/2.
loop j = 2 to (nms-i+1).
compute thetammb(:,cntmp)=coeffsb(:,start).
compute start=start+j+i-1.
compute cntmp=cntmp+1.
end loop.
end loop.
end if.
end if.
do if ((total = 1) and (rsum(numint)=0 or (xmint=1))).
compute dototal=1.
do if ((csum(bcmat(:,1)) <> (nms+nys)) or (rsum(bcmat(nrow(bcmat),:)) <> (nms+nys))).
compute dototal=0.
compute alttotal=1.
compute notecode(notes,1) = 12.
compute notes = notes + 1.
end if.
do if (ncs > 0).
do if ((csum(rsum(ccmat))) < (nrow(ccmat)*ncol(ccmat))).
compute dototal=0.
compute alttotal=1.
compute notecode(notes,1) = 11.
compute notes = notes + 1.
end if.
end if.
do if (model=74).
do if (xdich=0 and nxvls=1).
compute dototal=0.
compute alttotal=1.
end if.
do if ((xdich=1 or nxvls > 1) and (ncs > 0) and model=74).
compute dototal=0.
compute alttotal=1.
end if.
end if.
end if.
end if.
do if (criterr=0 and ncs > 0).
do if (rsum((csum(ccmat)=0)) <> 0).
compute errcode(errs,1)=51.
compute errs=errs+1.
compute criterr=1.
end if.
end if.
compute debug= 0.
do if (outscree=1).
print/title = '***************** PROCESS Procedure for SPSS Version 4.2 *****************'.
print/title = '          Written by Andrew F. Hayes, Ph.D.       www.afhayes.com'.
print/title = '    Documentation available in Hayes (2022). www.guilford.com/p/hayes3'/space=0.
end if.
do if (criterr=0).
compute modresid=make(n,1,99999).
do if (stand=1 and ydich=1).
compute stand=0.
end if.
compute anymod2=csum(rsum(wcmat+zcmat+wzcmat)).
do if (anymod2 > 0 and stand = 1).
compute notecode(notes,1) = 27.
compute notes = notes + 1.
compute stand=0.
end if.
compute funny=1.
do if (outscree=1).
print modelvar/title = '**************************************************************************'/format = A8/rnames=modelvlb.
do if (ncs > 0).
print covnames/title='Covariates:'/format=A8.
end if.
print n/title='Sample'/rlabel='Size:'.
do if (( 'random' <> 'random')).
compute seedt= 'random'.
print seedt/title='Custom'/format=A12/rlabel = 'Seed:'.
end if.
end if.
compute maxresm=9.
compute resultm=make(1,maxresm,99999).
do if (describe=1).
compute means=csum(dat)/n.
compute sigmatal = (t(dat)*(ident(n)-(1/n)*ones*t(ones))*dat)*(1/(n-1)).
compute sdvec=sqrt(diag(sigmatal)).
compute sdall = mdiag(1/sdvec).
compute corall=sdall*sigmatal*t(sdall).
compute means={means;t(sdvec)}.
do if (ncol(means) > 9).
compute resultm=make(1,ncol(means),99999).
compute maxresm=ncol(means).
end if.
compute resultm2=make(2,maxresm,99999).
compute resultm2(1:2,1:ncol(means))=means.
compute resultm={resultm;resultm2}.
compute resultm2=make(ncol(corall),maxresm,99999).
compute resultm2(1:ncol(corall),1:ncol(corall))=corall.
compute resultm={resultm;resultm2}.
do if (outscree=1).
print means/title='Variable means and standard deviations'/cnames=varnames/rlabels='Mean','SD'/format= F10.4.
print corall/title='Variable intercorrelations (Pearson r)'/cnames=varnames/rnames=varnames/format= F10.4.
end if.
end if.
do if (outscree=1).
do if (mcxok=1).
compute labtmp={xnames,t(xcatlab(1:nxvls,1))}.
print dummatx/title = 'Coding of categorical X variable for analysis:'/cnames = labtmp/format = F6.3.
end if.
do if (mcwok=1 and xmint=0).
compute labtmp={wnames,t(wcatlab(1:nwvls,1))}.
print dummatw/title = 'Coding of categorical W variable for analysis:'/cnames = labtmp/format = F6.3.
end if.
do if (mczok=1).
compute labtmp={znames,t(zcatlab(1:nzvls,1))}.
print dummatz/title = 'Coding of categorical Z variable for analysis:'/cnames = labtmp/format = F6.3.
end if.
end if.
end if.
do if (criterr = 0).
compute outnames=ynames.
compute outvars=ytmp.
do if (nms > 0).
compute outnames={mnames,ynames}.
compute outvars={mtmp,ytmp}.
compute indcov=make(((nms*2)+(nms*(nxvls-1))),((nms*2)+(nms*(nxvls-1))),0).
compute mcsopath=make(((nms*2)+(nms*(nxvls-1))),1,0).
end if.
compute labstart=1.
compute intstart=1.
compute start=1.
compute coeffmat=make(1,6,0).
compute conseq={'        '}.
compute dfmat=0.
compute coeffcol=0.
compute pathscnt=1.
compute pathscn2=1.
loop i = 1 to (nms+nys).
do if (outscree=1).
print/title = '**************************************************************************'.
end if.
compute highf=make(1,5,0).
compute highf2=highf.
do if ((i = (nms+nys)) and (ydich=1)).
compute highf=make(1,3,0).
compute highf2=highf.
end if.
compute flabel={' '}.
compute y=outvars(:,i).
compute xindx=datindx(1:(nump(1,i)-1),i).
compute x = fulldat(:,xindx).
compute x={ones,x}.
compute xsq=t(x)*x.
compute exsq=eval(xsq).
release xsq.
compute zeroeig=csum(exsq <= 0.000000000002).
do if (outscree=1).
print outnames(1,i)/title = 'OUTCOME VARIABLE:'/format = A8/space=0.
do if (ydich=1 and (i = (nms+nys))).
compute nmsd = {outnames(1,i), 'Analysis'}.
print rcd/title = 'Coding of binary Y for logistic regression analysis:'/cnames = nmsd/format = F9.2.
end if.
end if.
do if (zeroeig > 0).
print / title = 'SINGULAR OR NEAR SINGULAR DATA MATRIX.'.
compute criterr=1.
compute errcode(errs,1)=31.
compute errs=errs+1.
end if.
compute means=csum(x)/n.
compute vlabsm=vlabs(labstart:(labstart+(nump(1,i)-1)),1).
do if (criterr=0).
do if (ydich=0 or (i < (nms+nys))).

.
do if ( 1 =1).
compute hatmat=inv(t( x )* x )*t( x ).
compute b = hatmat* y.
compute modres=b.
do if ( 1 =1).
compute n1=nrow( x ).
compute dfres=n1-(ncol( x )).
compute sstotal = t( y -(csum( y )/n1))*( y -(csum( y )/n1)).
compute resid= y - x *b.
compute ssresid = csum((resid)&**2).
compute r2 = (sstotal-ssresid)/sstotal.
do if (r2 < 0).
compute r2=0.
end if.
compute adjr2 = 1-((1-r2)*(n1-1)/(dfres)).
compute mse=ssresid/(n1-ncol( x )).

.
compute n1=nrow( x ).
compute invXtX = inv(t( x )* x ).
compute varb = mse *invXtX.
compute k3 = ncol( x ).
compute xhc=0.
do if ( hc <> 5).
compute xhc= x.
compute hat = xhc(:,1).
loop i3=1 to nrow(xhc).
compute hat(i3,1)= xhc(i3,:)*invXtX*t(xhc(i3,:)).
end loop.
do if ( hc = 0 or hc =1).
loop i3 = 1 to k3.
compute xhc(:,i3)=xhc(:,i3)&* resid.
end loop.
end if.
do if ( hc =3 or hc =2).
loop i3=1 to k3.
compute xhc(:,i3) = ( resid &/(1-hat)&**(1/(4- hc )))&*xhc(:,i3).
end loop.
end if.
do if ( hc = 4).
compute hcmn=make(n,2,4).
compute hcmn(:,2)=(n1*hat)/k3.
loop i3= 1 to k3.
compute xhc(:,i3) = ( resid &/(1-hat)&**(rmin(hcmn)/2))&*xhc(:,i3).
end loop.
end if.
compute varb=(invXtX*t(xhc)*xhc*invXtX).
do if ( hc =1).
compute varb=(n1/(n1-ncol( x )))&*varb.
end if.
end if.
compute hclab={'se(HC0)','se(HC1)','se(HC2)','se(HC3)','se(HC4)','se'}.
compute hclab=hclab(1,( hc +1)).
compute hcflab={'F(HC0)','F(HC1)','F(HC2)','F(HC3)','F(HC4)','F'}.
compute hcflab=hcflab(1,( hc +1)).
release xhc
.
compute seb=sqrt(diag(varb)).
compute trat = b&/seb.
compute p = 2*(1-tcdf(abs(trat), (dfres))).
compute tval = sqrt(dfres* (exp((dfres-(5/6))*((xp2/(dfres-(2/3)+(.11/dfres)))* (xp2/(dfres-(2/3)+(.11/dfres)))))-1)).
compute modres={modres,seb,trat,p}.
compute modres={modres,(b-tval&*seb),(b+tval&*seb)}.
compute modresl={'coeff',hclab,'t','p','LLCI','ULCI'}.
compute lmat = ident(ncol( x )).
compute lmat = lmat(:,2:ncol(lmat)).
compute fratio = (t(t(lmat)*b)*inv(t(lmat)*varb*lmat)*((t(lmat)*b)))/(ncol( x )-1).
compute pfr = 1-fcdf(fratio,(ncol( x )-1),dfres).
compute modsum={sqrt(r2),r2,mse,fratio,(ncol( x )-1),dfres,pfr}.
compute modsuml={'R','R-sq','MSE',hcflab,'df1','df2', 'p'}.
end if.
end if.
do if ( 1 = 2 or 1 =3).
compute xlp= x.
compute ylp= y.
compute pt2 = make(nrow(ylp),1,(csum(ylp)/nrow(ylp))).
do if ( 1 =2).
compute LL3 = ylp&*ln(pt2)+(1-ylp)&*ln(1-pt2).
end if.
compute LL3 = -2*csum(LL3).
compute bt1 = make(ncol(xlp),1,0).
compute LL1 = 0.
compute pt1 = make(nrow(ylp),1,0.5).
compute pt1lp=pt1.
loop jjj = 1 to iterate.
compute xlptmp=t(xlp).
compute vecprb=pt1lp&*(1-pt1lp).
loop kkk=1 to ncol(xlp).
compute xlptmp(kkk,:)=xlptmp(kkk,:)&*t(vecprb).
end loop.
compute b = bt1+inv(xlptmp*xlp)*t(xlp)*(ylp-pt1lp).
do if ( 1 =2).
compute xlpb=xlp*b.
compute xlpbt=(xlpb > -709.7).
compute xlpb709=(1-xlpbt)*(-709.7).
compute xlpb=(xlpb&*xlpbt)+xlpb709.
compute pt1lp = 1/(1+exp(-(xlpb))).
end if.
compute itprob = csum((pt1lp < .00000001) or (pt1lp > .9999999)).
do if (itprob > 0).
loop kkk = 1 to nrow(pt1lp).
do if (pt1lp(kkk,1) > .9999999).
compute pt1lp(kkk,1) = .9999999.
end if.
do if (pt1lp(kkk,1) < .00000001).
compute pt1lp(kkk,1) = .00000001.
end if.
end loop.
compute itprob = 0.
end if.
do if (itprob = 0).
do if ( 1 =2).
compute LL = ylp&*ln(pt1lp)+(1-ylp)&*ln(1-pt1lp).
end if.
compute LL2 = -2*csum(ll).
end if.
do if (abs(LL1-LL2) < converge).
do if ( 1 =1).
compute xlptmp=t(xlp).
compute vecprb=pt1lp&*(1-pt1lp).
loop kkk=1 to ncol(xlp).
compute xlptmp(kkk,:)=xlptmp(kkk,:)&*t(vecprb).
end loop.
compute varb = inv(xlptmp*xlp).
compute seb = sqrt(diag(varb)).
release xlptmp.
end if.
break.
end if.
compute bt1 = b.
compute LL1 = LL2.
end loop.
compute modres=b.
do if (jjj > iterate).
compute itprob = 2.
do if (booting=0).
compute iterrmod=1.
end if.
do if (booting=1).
compute bootiter=1.
end if.
do if (itprobtg=0).
compute itprobtg=1.
compute errcode(errs,1) = 47.
compute errs = errs + 1.
do if (booting = 0 and 1 =1).
compute vt1 = mdiag(pt1lp&*(1-pt1lp)).
compute varb = inv(t(xlp)*vt1*xlp).
compute seb = sqrt(diag(varb)).
end if.
end if.
end if.
do if ( 1 =1).
compute trat = b&/seb.
compute dfres=nrow(xlp).
compute p = 2*(1-cdfnorm(abs(trat))).
compute modres={modres,seb,trat,p}.
compute modres={modres,(b-xp2&*seb),(b+xp2&*seb)}.
compute pvchi=1-chicdf((LL3-LL2),(nrow(modres)-1)).
compute mcF = (LL3-LL2)/LL3.
compute cox = 1-exp(-(LL3-LL2)/nrow(xlp)).
compute nagel = cox/(1-exp(-(LL3)/nrow(xlp))).
compute modsum={LL2,(LL3-LL2),(nrow(modres)-1),pvchi, mcF,cox,nagel}.
compute modsuml={'-2LL','ModelLL', 'df', 'p', 'McFadden', 'CoxSnell', 'Nagelkrk'}.
compute modresl={'coeff','se','Z','p','LLCI','ULCI'}.
end if.
end if
.
compute dfmatt=make(nrow(modres),1,modsum(1,6)).
compute modresid={modresid,resid}.
end if.
do if (ydich=1 and (i = (nms+nys))).

.
do if ( 2 =1).
compute hatmat=inv(t( x )* x )*t( x ).
compute b = hatmat* y.
compute modres=b.
do if ( 1 =1).
compute n1=nrow( x ).
compute dfres=n1-(ncol( x )).
compute sstotal = t( y -(csum( y )/n1))*( y -(csum( y )/n1)).
compute resid= y - x *b.
compute ssresid = csum((resid)&**2).
compute r2 = (sstotal-ssresid)/sstotal.
do if (r2 < 0).
compute r2=0.
end if.
compute adjr2 = 1-((1-r2)*(n1-1)/(dfres)).
compute mse=ssresid/(n1-ncol( x )).

.
compute n1=nrow( x ).
compute invXtX = inv(t( x )* x ).
compute varb = mse *invXtX.
compute k3 = ncol( x ).
compute xhc=0.
do if ( hc <> 5).
compute xhc= x.
compute hat = xhc(:,1).
loop i3=1 to nrow(xhc).
compute hat(i3,1)= xhc(i3,:)*invXtX*t(xhc(i3,:)).
end loop.
do if ( hc = 0 or hc =1).
loop i3 = 1 to k3.
compute xhc(:,i3)=xhc(:,i3)&* resid.
end loop.
end if.
do if ( hc =3 or hc =2).
loop i3=1 to k3.
compute xhc(:,i3) = ( resid &/(1-hat)&**(1/(4- hc )))&*xhc(:,i3).
end loop.
end if.
do if ( hc = 4).
compute hcmn=make(n,2,4).
compute hcmn(:,2)=(n1*hat)/k3.
loop i3= 1 to k3.
compute xhc(:,i3) = ( resid &/(1-hat)&**(rmin(hcmn)/2))&*xhc(:,i3).
end loop.
end if.
compute varb=(invXtX*t(xhc)*xhc*invXtX).
do if ( hc =1).
compute varb=(n1/(n1-ncol( x )))&*varb.
end if.
end if.
compute hclab={'se(HC0)','se(HC1)','se(HC2)','se(HC3)','se(HC4)','se'}.
compute hclab=hclab(1,( hc +1)).
compute hcflab={'F(HC0)','F(HC1)','F(HC2)','F(HC3)','F(HC4)','F'}.
compute hcflab=hcflab(1,( hc +1)).
release xhc
.
compute seb=sqrt(diag(varb)).
compute trat = b&/seb.
compute p = 2*(1-tcdf(abs(trat), (dfres))).
compute tval = sqrt(dfres* (exp((dfres-(5/6))*((xp2/(dfres-(2/3)+(.11/dfres)))* (xp2/(dfres-(2/3)+(.11/dfres)))))-1)).
compute modres={modres,seb,trat,p}.
compute modres={modres,(b-tval&*seb),(b+tval&*seb)}.
compute modresl={'coeff',hclab,'t','p','LLCI','ULCI'}.
compute lmat = ident(ncol( x )).
compute lmat = lmat(:,2:ncol(lmat)).
compute fratio = (t(t(lmat)*b)*inv(t(lmat)*varb*lmat)*((t(lmat)*b)))/(ncol( x )-1).
compute pfr = 1-fcdf(fratio,(ncol( x )-1),dfres).
compute modsum={sqrt(r2),r2,mse,fratio,(ncol( x )-1),dfres,pfr}.
compute modsuml={'R','R-sq','MSE',hcflab,'df1','df2', 'p'}.
end if.
end if.
do if ( 2 = 2 or 2 =3).
compute xlp= x.
compute ylp= y.
compute pt2 = make(nrow(ylp),1,(csum(ylp)/nrow(ylp))).
do if ( 2 =2).
compute LL3 = ylp&*ln(pt2)+(1-ylp)&*ln(1-pt2).
end if.
compute LL3 = -2*csum(LL3).
compute bt1 = make(ncol(xlp),1,0).
compute LL1 = 0.
compute pt1 = make(nrow(ylp),1,0.5).
compute pt1lp=pt1.
loop jjj = 1 to iterate.
compute xlptmp=t(xlp).
compute vecprb=pt1lp&*(1-pt1lp).
loop kkk=1 to ncol(xlp).
compute xlptmp(kkk,:)=xlptmp(kkk,:)&*t(vecprb).
end loop.
compute b = bt1+inv(xlptmp*xlp)*t(xlp)*(ylp-pt1lp).
do if ( 2 =2).
compute xlpb=xlp*b.
compute xlpbt=(xlpb > -709.7).
compute xlpb709=(1-xlpbt)*(-709.7).
compute xlpb=(xlpb&*xlpbt)+xlpb709.
compute pt1lp = 1/(1+exp(-(xlpb))).
end if.
compute itprob = csum((pt1lp < .00000001) or (pt1lp > .9999999)).
do if (itprob > 0).
loop kkk = 1 to nrow(pt1lp).
do if (pt1lp(kkk,1) > .9999999).
compute pt1lp(kkk,1) = .9999999.
end if.
do if (pt1lp(kkk,1) < .00000001).
compute pt1lp(kkk,1) = .00000001.
end if.
end loop.
compute itprob = 0.
end if.
do if (itprob = 0).
do if ( 2 =2).
compute LL = ylp&*ln(pt1lp)+(1-ylp)&*ln(1-pt1lp).
end if.
compute LL2 = -2*csum(ll).
end if.
do if (abs(LL1-LL2) < converge).
do if ( 1 =1).
compute xlptmp=t(xlp).
compute vecprb=pt1lp&*(1-pt1lp).
loop kkk=1 to ncol(xlp).
compute xlptmp(kkk,:)=xlptmp(kkk,:)&*t(vecprb).
end loop.
compute varb = inv(xlptmp*xlp).
compute seb = sqrt(diag(varb)).
release xlptmp.
end if.
break.
end if.
compute bt1 = b.
compute LL1 = LL2.
end loop.
compute modres=b.
do if (jjj > iterate).
compute itprob = 2.
do if (booting=0).
compute iterrmod=1.
end if.
do if (booting=1).
compute bootiter=1.
end if.
do if (itprobtg=0).
compute itprobtg=1.
compute errcode(errs,1) = 47.
compute errs = errs + 1.
do if (booting = 0 and 1 =1).
compute vt1 = mdiag(pt1lp&*(1-pt1lp)).
compute varb = inv(t(xlp)*vt1*xlp).
compute seb = sqrt(diag(varb)).
end if.
end if.
end if.
do if ( 1 =1).
compute trat = b&/seb.
compute dfres=nrow(xlp).
compute p = 2*(1-cdfnorm(abs(trat))).
compute modres={modres,seb,trat,p}.
compute modres={modres,(b-xp2&*seb),(b+xp2&*seb)}.
compute pvchi=1-chicdf((LL3-LL2),(nrow(modres)-1)).
compute mcF = (LL3-LL2)/LL3.
compute cox = 1-exp(-(LL3-LL2)/nrow(xlp)).
compute nagel = cox/(1-exp(-(LL3)/nrow(xlp))).
compute modsum={LL2,(LL3-LL2),(nrow(modres)-1),pvchi, mcF,cox,nagel}.
compute modsuml={'-2LL','ModelLL', 'df', 'p', 'McFadden', 'CoxSnell', 'Nagelkrk'}.
compute modresl={'coeff','se','Z','p','LLCI','ULCI'}.
end if.
end if
.
compute dfmatt=make(nrow(modres),1,-999).
end if.
compute resultm2=make(1,maxresm,99999).
compute resultm2(1,1:ncol(modsum))=modsum.
compute resultm={resultm;resultm2}.
compute resultm2=make(nrow(modres),maxresm,99999).
compute resultm2(1:nrow(modres),1:ncol(modres))=modres.
compute resultm={resultm;resultm2}.
compute obscoeff={obscoeff,t(b)}.
do if (outscree=1).
print modsum/title = 'Model Summary'/cnames = modsuml/format= F10.4.
print modres/title='Model'/rnames=vlabsm/cnames=modresl/format= F10.4.
end if.
compute basemod=modsum(1,1).
compute basemodx=basemod.
do if (ydich=1 and (i = (nms+nys))).
do if (outscree=1).
print/title='These results are expressed in a log-odds metric.'.
end if.
compute notecode(notes,1) = 26.
compute notes = notes + 1.
end if.
compute coeffmat={coeffmat;modres}.
compute conseqt=make(nrow(modres),1,outnames(1,i)).
compute conseq={conseq;conseqt}.
compute dfmat={dfmat;dfmatt}.
compute labstart=labstart+nump(1,i).
do if (stand=1).
compute predsd=make(nrow(modres),1,0).
compute stdmod=modres(:,1)&/ovsd(1,i).
loop jd=1 to ncol(x).
compute descdat=x(:,jd).
compute predsd(jd,1) = (nrow(descdat)*sscp(descdat))-(t(csum(descdat))*(csum(descdat))).
compute predsd(jd,1) = sqrt(predsd(jd,1)/(nrow(descdat)*(nrow(descdat)-1))).
end loop.
do if (wherex(1,i) <> -999 and ((nxvls > 1) or (xdich=1))).
compute sdmsone=make(nxvls,1,1).
compute predsd(wherex(1,i):wherex(2,i),1)=sdmsone.
compute pstog=1.
end if.
compute predsd(1,1)=1.
compute stdmod=stdmod&*predsd.
compute stdmod=stdmod(2:nrow(stdmod),1).
compute sdvlabs=vlabsm(2:nrow(vlabsm),1).
compute resultm2=make(nrow(stdmod),maxresm,99999).
compute resultm2(1:nrow(stdmod),1:ncol(stdmod))=stdmod.
compute resultm={resultm;resultm2}.
do if (outscree=1).
print stdmod/title='Standardized coefficients'/clabels='coeff'/rnames=sdvlabs/format= F10.4.
end if.
end if.
do if (nms > 0 and serial = 0 and (rsum(numint) = 0) and (normal=1 or mc > 0)).
do if (i < (nms+nys)).
compute indcov((((i-1)*nxvls)+1):(i*nxvls),(((i-1)*nxvls)+1):(i*nxvls))=varb(2:(1+nxvls), 2:(1+nxvls)).
compute mcsopath((((i-1)*nxvls)+1):(i*nxvls) ,1)=modres(2:(1+nxvls),1).
end if.
do if (i = (nms+nys)).
compute atm=ncol(wherem).
compute indcov(((nms*nxvls)+1):nrow(mcsopath),((nms*nxvls)+1):nrow(mcsopath))= varb(wherem(1,atm):(wherem(1,atm)+nms-1),wherem(1,atm):(wherem(1,atm)+nms-1)).
compute mcsopath(((nms*nxvls)+1):nrow(mcsopath),1)=modres(wherem(1,atm):(wherem(1,atm)+ nms-1),1).
compute sobelok=1.
end if.
end if.
compute obsdirfx=make(1,nxvls,0).
compute dirzes=make(1,nxvls,0).
do if ((i = (nms+nys)) and (bcmat(nrow(bcmat),1)=1)).
compute direff=modres(2:(1+nxvls),:).
compute obsdirfx=t(direff(:,1)).
compute direfflb=modresl.
compute direffl2=vlabsm(2:(1+nxvls),:).
compute lmat=make(nrow(b),1,0).
compute lmat2=make(nxvls,1,1).
compute lmat(2:(1+nxvls),1)=lmat2.
do if (ydich <> 1).

.
compute lmat2= lmat.
do if ( 0 =0).
compute lmat2 = mdiag( lmat ).
compute lmat3=make(nrow(lmat2),1,0).
loop flp=1 to ncol(lmat2).
do if (csum(lmat2(:,flp))=1).
compute lmat3={lmat3,lmat2(:,flp)}.
end if.
end loop.
compute lmat2=lmat3(:,2:ncol(lmat3)).
end if.
compute fratio = (t(t(lmat2)* b )*inv(t(lmat2)* varb *lmat2)*((t(lmat2)* b )))/ncol(lmat2).
compute pfr = 1-fcdf(fratio,ncol(lmat2),(n-nrow( b ))).
compute fresult={fratio,ncol(lmat2),(n-nrow( b )),pfr}.
do if (i = (nms+nys) and (ydich=1)).
compute fratio=fratio*ncol(lmat2).
compute pfr=1-chicdf(fratio,ncol(lmat2)).
compute fresult={fratio,ncol(lmat2),pfr}.
end if.
do if ( 1 =1).
compute lmat3=1-rsum(lmat2).
compute xfm=make(n,csum(lmat3),0).
compute flpc=1.
loop flp=1 to nrow(lmat3).
do if (lmat3(flp,1)=1).
compute xfm(:,flpc)=x(:,flp).
compute flpc=flpc+1.
end if.
end loop.
compute bfm=inv(t(xfm)*xfm)*t(xfm)*y.
compute resid=y-(xfm*bfm).
compute sstotal=(y-(csum(y)/n)).
compute sstotal=csum(sstotal&*sstotal).
compute ssresid=csum(resid&*resid).
compute rsqch= r2 -((sstotal-ssresid)/sstotal).
compute fresult={rsqch,fresult}.
release xfm,flpc, resid, ssresid, bfm.
end if
.
compute diromni=fresult.
end if.
do if (ydich = 1 ).

.
compute btemphld=b.
compute llrdat=make(nrow(x),nrow( lmat )-csum( lmat ),-999).
compute llrdf=ncol(x)-ncol(llrdat).
compute llrcnt=0.
loop llri=1 to nrow( lmat ).
do if ( lmat (llri,1)=0).
compute llrcnt=llrcnt+1.
compute llrdat(:,llrcnt)=x(:,llri).
end if.
end loop.

.
do if ( 2 =1).
compute hatmat=inv(t( llrdat )* llrdat )*t( llrdat ).
compute b = hatmat* y.
compute modres=b.
do if ( 0 =1).
compute n1=nrow( llrdat ).
compute dfres=n1-(ncol( llrdat )).
compute sstotal = t( y -(csum( y )/n1))*( y -(csum( y )/n1)).
compute resid= y - llrdat *b.
compute ssresid = csum((resid)&**2).
compute r2 = (sstotal-ssresid)/sstotal.
do if (r2 < 0).
compute r2=0.
end if.
compute adjr2 = 1-((1-r2)*(n1-1)/(dfres)).
compute mse=ssresid/(n1-ncol( llrdat )).

.
compute n1=nrow( x ).
compute invXtX = inv(t( x )* x ).
compute varb = mse *invXtX.
compute k3 = ncol( x ).
compute xhc=0.
do if ( hc <> 5).
compute xhc= x.
compute hat = xhc(:,1).
loop i3=1 to nrow(xhc).
compute hat(i3,1)= xhc(i3,:)*invXtX*t(xhc(i3,:)).
end loop.
do if ( hc = 0 or hc =1).
loop i3 = 1 to k3.
compute xhc(:,i3)=xhc(:,i3)&* resid.
end loop.
end if.
do if ( hc =3 or hc =2).
loop i3=1 to k3.
compute xhc(:,i3) = ( resid &/(1-hat)&**(1/(4- hc )))&*xhc(:,i3).
end loop.
end if.
do if ( hc = 4).
compute hcmn=make(n,2,4).
compute hcmn(:,2)=(n1*hat)/k3.
loop i3= 1 to k3.
compute xhc(:,i3) = ( resid &/(1-hat)&**(rmin(hcmn)/2))&*xhc(:,i3).
end loop.
end if.
compute varb=(invXtX*t(xhc)*xhc*invXtX).
do if ( hc =1).
compute varb=(n1/(n1-ncol( x )))&*varb.
end if.
end if.
compute hclab={'se(HC0)','se(HC1)','se(HC2)','se(HC3)','se(HC4)','se'}.
compute hclab=hclab(1,( hc +1)).
compute hcflab={'F(HC0)','F(HC1)','F(HC2)','F(HC3)','F(HC4)','F'}.
compute hcflab=hcflab(1,( hc +1)).
release xhc
.
compute seb=sqrt(diag(varb)).
compute trat = b&/seb.
compute p = 2*(1-tcdf(abs(trat), (dfres))).
compute tval = sqrt(dfres* (exp((dfres-(5/6))*((xp2/(dfres-(2/3)+(.11/dfres)))* (xp2/(dfres-(2/3)+(.11/dfres)))))-1)).
compute modres={modres,seb,trat,p}.
compute modres={modres,(b-tval&*seb),(b+tval&*seb)}.
compute modresl={'coeff',hclab,'t','p','LLCI','ULCI'}.
compute lmat = ident(ncol( llrdat )).
compute lmat = lmat(:,2:ncol(lmat)).
compute fratio = (t(t(lmat)*b)*inv(t(lmat)*varb*lmat)*((t(lmat)*b)))/(ncol( llrdat )-1).
compute pfr = 1-fcdf(fratio,(ncol( llrdat )-1),dfres).
compute modsum={sqrt(r2),r2,mse,fratio,(ncol( llrdat )-1),dfres,pfr}.
compute modsuml={'R','R-sq','MSE',hcflab,'df1','df2', 'p'}.
end if.
end if.
do if ( 2 = 2 or 2 =3).
compute xlp= llrdat.
compute ylp= y.
compute pt2 = make(nrow(ylp),1,(csum(ylp)/nrow(ylp))).
do if ( 2 =2).
compute LL3 = ylp&*ln(pt2)+(1-ylp)&*ln(1-pt2).
end if.
compute LL3 = -2*csum(LL3).
compute bt1 = make(ncol(xlp),1,0).
compute LL1 = 0.
compute pt1 = make(nrow(ylp),1,0.5).
compute pt1lp=pt1.
loop jjj = 1 to iterate.
compute xlptmp=t(xlp).
compute vecprb=pt1lp&*(1-pt1lp).
loop kkk=1 to ncol(xlp).
compute xlptmp(kkk,:)=xlptmp(kkk,:)&*t(vecprb).
end loop.
compute b = bt1+inv(xlptmp*xlp)*t(xlp)*(ylp-pt1lp).
do if ( 2 =2).
compute xlpb=xlp*b.
compute xlpbt=(xlpb > -709.7).
compute xlpb709=(1-xlpbt)*(-709.7).
compute xlpb=(xlpb&*xlpbt)+xlpb709.
compute pt1lp = 1/(1+exp(-(xlpb))).
end if.
compute itprob = csum((pt1lp < .00000001) or (pt1lp > .9999999)).
do if (itprob > 0).
loop kkk = 1 to nrow(pt1lp).
do if (pt1lp(kkk,1) > .9999999).
compute pt1lp(kkk,1) = .9999999.
end if.
do if (pt1lp(kkk,1) < .00000001).
compute pt1lp(kkk,1) = .00000001.
end if.
end loop.
compute itprob = 0.
end if.
do if (itprob = 0).
do if ( 2 =2).
compute LL = ylp&*ln(pt1lp)+(1-ylp)&*ln(1-pt1lp).
end if.
compute LL2 = -2*csum(ll).
end if.
do if (abs(LL1-LL2) < converge).
do if ( 0 =1).
compute xlptmp=t(xlp).
compute vecprb=pt1lp&*(1-pt1lp).
loop kkk=1 to ncol(xlp).
compute xlptmp(kkk,:)=xlptmp(kkk,:)&*t(vecprb).
end loop.
compute varb = inv(xlptmp*xlp).
compute seb = sqrt(diag(varb)).
release xlptmp.
end if.
break.
end if.
compute bt1 = b.
compute LL1 = LL2.
end loop.
compute modres=b.
do if (jjj > iterate).
compute itprob = 2.
do if (booting=0).
compute iterrmod=1.
end if.
do if (booting=1).
compute bootiter=1.
end if.
do if (itprobtg=0).
compute itprobtg=1.
compute errcode(errs,1) = 47.
compute errs = errs + 1.
do if (booting = 0 and 0 =1).
compute vt1 = mdiag(pt1lp&*(1-pt1lp)).
compute varb = inv(t(xlp)*vt1*xlp).
compute seb = sqrt(diag(varb)).
end if.
end if.
end if.
do if ( 0 =1).
compute trat = b&/seb.
compute dfres=nrow(xlp).
compute p = 2*(1-cdfnorm(abs(trat))).
compute modres={modres,seb,trat,p}.
compute modres={modres,(b-xp2&*seb),(b+xp2&*seb)}.
compute pvchi=1-chicdf((LL3-LL2),(nrow(modres)-1)).
compute mcF = (LL3-LL2)/LL3.
compute cox = 1-exp(-(LL3-LL2)/nrow(xlp)).
compute nagel = cox/(1-exp(-(LL3)/nrow(xlp))).
compute modsum={LL2,(LL3-LL2),(nrow(modres)-1),pvchi, mcF,cox,nagel}.
compute modsuml={'-2LL','ModelLL', 'df', 'p', 'McFadden', 'CoxSnell', 'Nagelkrk'}.
compute modresl={'coeff','se','Z','p','LLCI','ULCI'}.
end if.
end if
.
compute b=btemphld.
compute fresult={(LL2-basemod),llrdf,1-chicdf((LL2-basemod),llrdf)}
.
compute diromni=fresult.
end if.
end if.
do if (numint(1,i) > 0).
compute intkeym=intkey(intstart:(intstart+numint(1,i)-1),:).
do if (outscree=1).
print intkeym/title='Product terms key:'/format = A8.
end if.
end if.
do if (covcoeff=1).
do if (outscree=1).
print varb/title='Covariance matrix of regression parameter estimates:'/rnames=vlabsm /cnames=vlabsm/format= F10.4.
end if.
compute resultm2=make(nrow(varb),maxresm,99999).
do if (ncol(varb) <= maxresm).
compute resultm2(1:nrow(varb),1:ncol(varb))=varb.
compute resultm={resultm;resultm2}.
end if.
do if (ncol(varb) > maxresm).
compute resultmt=make(nrow(resultm),ncol(varb),99999).
compute resultmt(1:nrow(resultm),1:ncol(resultm))=resultm.
compute resultm=resultmt.
compute resultm2=make(nrow(varb),ncol(resultm),99999).
compute resultm2(1:nrow(varb),1:ncol(varb))=varb.
compute resultm={resultm;resultm2}.
compute maxresm=ncol(resultm).
end if.
end if.
do if (model <> 74 and xmtest=1 and nms > 0).
compute r2tmp=r2.
compute btmp=b.
compute varbtmp=varb.
compute dfrestmp=dfres.
compute tvaltmp=tval.
compute xmtst=make(nms,4,0).
compute xmtstlbc={hcflab,'df1','df2','p'}.
do if ((i=(nms+nys)) and (ydich=1)).
compute xmtst=make(nms,3,0).
compute xmtstlbc={'Chi-sq','df','p'}.
end if.
compute xmtstlb={' '}.
compute xmtmat=x.
compute numxint=0.
loop xmints=2 to i.
compute x=xmtmat.
do if ((bcmat((i+1),xmints)=1) and (wzcmat((i+1),xmints) <> 1)).
do if (bcmat((i+1),1)=0).
compute x={xmtmat,xtmp}.
do if ((ydich=1) and (i=(nms+nys))).

.
do if ( 2 =1).
compute hatmat=inv(t( x )* x )*t( x ).
compute b = hatmat* y.
compute modres=b.
do if ( 1 =1).
compute n1=nrow( x ).
compute dfres=n1-(ncol( x )).
compute sstotal = t( y -(csum( y )/n1))*( y -(csum( y )/n1)).
compute resid= y - x *b.
compute ssresid = csum((resid)&**2).
compute r2 = (sstotal-ssresid)/sstotal.
do if (r2 < 0).
compute r2=0.
end if.
compute adjr2 = 1-((1-r2)*(n1-1)/(dfres)).
compute mse=ssresid/(n1-ncol( x )).

.
compute n1=nrow( x ).
compute invXtX = inv(t( x )* x ).
compute varb = mse *invXtX.
compute k3 = ncol( x ).
compute xhc=0.
do if ( hc <> 5).
compute xhc= x.
compute hat = xhc(:,1).
loop i3=1 to nrow(xhc).
compute hat(i3,1)= xhc(i3,:)*invXtX*t(xhc(i3,:)).
end loop.
do if ( hc = 0 or hc =1).
loop i3 = 1 to k3.
compute xhc(:,i3)=xhc(:,i3)&* resid.
end loop.
end if.
do if ( hc =3 or hc =2).
loop i3=1 to k3.
compute xhc(:,i3) = ( resid &/(1-hat)&**(1/(4- hc )))&*xhc(:,i3).
end loop.
end if.
do if ( hc = 4).
compute hcmn=make(n,2,4).
compute hcmn(:,2)=(n1*hat)/k3.
loop i3= 1 to k3.
compute xhc(:,i3) = ( resid &/(1-hat)&**(rmin(hcmn)/2))&*xhc(:,i3).
end loop.
end if.
compute varb=(invXtX*t(xhc)*xhc*invXtX).
do if ( hc =1).
compute varb=(n1/(n1-ncol( x )))&*varb.
end if.
end if.
compute hclab={'se(HC0)','se(HC1)','se(HC2)','se(HC3)','se(HC4)','se'}.
compute hclab=hclab(1,( hc +1)).
compute hcflab={'F(HC0)','F(HC1)','F(HC2)','F(HC3)','F(HC4)','F'}.
compute hcflab=hcflab(1,( hc +1)).
release xhc
.
compute seb=sqrt(diag(varb)).
compute trat = b&/seb.
compute p = 2*(1-tcdf(abs(trat), (dfres))).
compute tval = sqrt(dfres* (exp((dfres-(5/6))*((xp2/(dfres-(2/3)+(.11/dfres)))* (xp2/(dfres-(2/3)+(.11/dfres)))))-1)).
compute modres={modres,seb,trat,p}.
compute modres={modres,(b-tval&*seb),(b+tval&*seb)}.
compute modresl={'coeff',hclab,'t','p','LLCI','ULCI'}.
compute lmat = ident(ncol( x )).
compute lmat = lmat(:,2:ncol(lmat)).
compute fratio = (t(t(lmat)*b)*inv(t(lmat)*varb*lmat)*((t(lmat)*b)))/(ncol( x )-1).
compute pfr = 1-fcdf(fratio,(ncol( x )-1),dfres).
compute modsum={sqrt(r2),r2,mse,fratio,(ncol( x )-1),dfres,pfr}.
compute modsuml={'R','R-sq','MSE',hcflab,'df1','df2', 'p'}.
end if.
end if.
do if ( 2 = 2 or 2 =3).
compute xlp= x.
compute ylp= y.
compute pt2 = make(nrow(ylp),1,(csum(ylp)/nrow(ylp))).
do if ( 2 =2).
compute LL3 = ylp&*ln(pt2)+(1-ylp)&*ln(1-pt2).
end if.
compute LL3 = -2*csum(LL3).
compute bt1 = make(ncol(xlp),1,0).
compute LL1 = 0.
compute pt1 = make(nrow(ylp),1,0.5).
compute pt1lp=pt1.
loop jjj = 1 to iterate.
compute xlptmp=t(xlp).
compute vecprb=pt1lp&*(1-pt1lp).
loop kkk=1 to ncol(xlp).
compute xlptmp(kkk,:)=xlptmp(kkk,:)&*t(vecprb).
end loop.
compute b = bt1+inv(xlptmp*xlp)*t(xlp)*(ylp-pt1lp).
do if ( 2 =2).
compute xlpb=xlp*b.
compute xlpbt=(xlpb > -709.7).
compute xlpb709=(1-xlpbt)*(-709.7).
compute xlpb=(xlpb&*xlpbt)+xlpb709.
compute pt1lp = 1/(1+exp(-(xlpb))).
end if.
compute itprob = csum((pt1lp < .00000001) or (pt1lp > .9999999)).
do if (itprob > 0).
loop kkk = 1 to nrow(pt1lp).
do if (pt1lp(kkk,1) > .9999999).
compute pt1lp(kkk,1) = .9999999.
end if.
do if (pt1lp(kkk,1) < .00000001).
compute pt1lp(kkk,1) = .00000001.
end if.
end loop.
compute itprob = 0.
end if.
do if (itprob = 0).
do if ( 2 =2).
compute LL = ylp&*ln(pt1lp)+(1-ylp)&*ln(1-pt1lp).
end if.
compute LL2 = -2*csum(ll).
end if.
do if (abs(LL1-LL2) < converge).
do if ( 1 =1).
compute xlptmp=t(xlp).
compute vecprb=pt1lp&*(1-pt1lp).
loop kkk=1 to ncol(xlp).
compute xlptmp(kkk,:)=xlptmp(kkk,:)&*t(vecprb).
end loop.
compute varb = inv(xlptmp*xlp).
compute seb = sqrt(diag(varb)).
release xlptmp.
end if.
break.
end if.
compute bt1 = b.
compute LL1 = LL2.
end loop.
compute modres=b.
do if (jjj > iterate).
compute itprob = 2.
do if (booting=0).
compute iterrmod=1.
end if.
do if (booting=1).
compute bootiter=1.
end if.
do if (itprobtg=0).
compute itprobtg=1.
compute errcode(errs,1) = 47.
compute errs = errs + 1.
do if (booting = 0 and 1 =1).
compute vt1 = mdiag(pt1lp&*(1-pt1lp)).
compute varb = inv(t(xlp)*vt1*xlp).
compute seb = sqrt(diag(varb)).
end if.
end if.
end if.
do if ( 1 =1).
compute trat = b&/seb.
compute dfres=nrow(xlp).
compute p = 2*(1-cdfnorm(abs(trat))).
compute modres={modres,seb,trat,p}.
compute modres={modres,(b-xp2&*seb),(b+xp2&*seb)}.
compute pvchi=1-chicdf((LL3-LL2),(nrow(modres)-1)).
compute mcF = (LL3-LL2)/LL3.
compute cox = 1-exp(-(LL3-LL2)/nrow(xlp)).
compute nagel = cox/(1-exp(-(LL3)/nrow(xlp))).
compute modsum={LL2,(LL3-LL2),(nrow(modres)-1),pvchi, mcF,cox,nagel}.
compute modsuml={'-2LL','ModelLL', 'df', 'p', 'McFadden', 'CoxSnell', 'Nagelkrk'}.
compute modresl={'coeff','se','Z','p','LLCI','ULCI'}.
end if.
end if
.
compute basemodx=LL2.
end if.
end if.
loop xmtlp1=1 to nxvls.
compute x={x,xtmp(:,xmtlp1)&*(mtmp(:,(xmints-1))-(csum(mtmp(:,(xmints-1)))/nrow(mtmp)) )}.
end loop.
do if ((i < (nms+nys)) or (ydich=0)).

.
do if ( 1 =1).
compute hatmat=inv(t( x )* x )*t( x ).
compute b = hatmat* y.
compute modres=b.
do if ( 1 =1).
compute n1=nrow( x ).
compute dfres=n1-(ncol( x )).
compute sstotal = t( y -(csum( y )/n1))*( y -(csum( y )/n1)).
compute resid= y - x *b.
compute ssresid = csum((resid)&**2).
compute r2 = (sstotal-ssresid)/sstotal.
do if (r2 < 0).
compute r2=0.
end if.
compute adjr2 = 1-((1-r2)*(n1-1)/(dfres)).
compute mse=ssresid/(n1-ncol( x )).

.
compute n1=nrow( x ).
compute invXtX = inv(t( x )* x ).
compute varb = mse *invXtX.
compute k3 = ncol( x ).
compute xhc=0.
do if ( hc <> 5).
compute xhc= x.
compute hat = xhc(:,1).
loop i3=1 to nrow(xhc).
compute hat(i3,1)= xhc(i3,:)*invXtX*t(xhc(i3,:)).
end loop.
do if ( hc = 0 or hc =1).
loop i3 = 1 to k3.
compute xhc(:,i3)=xhc(:,i3)&* resid.
end loop.
end if.
do if ( hc =3 or hc =2).
loop i3=1 to k3.
compute xhc(:,i3) = ( resid &/(1-hat)&**(1/(4- hc )))&*xhc(:,i3).
end loop.
end if.
do if ( hc = 4).
compute hcmn=make(n,2,4).
compute hcmn(:,2)=(n1*hat)/k3.
loop i3= 1 to k3.
compute xhc(:,i3) = ( resid &/(1-hat)&**(rmin(hcmn)/2))&*xhc(:,i3).
end loop.
end if.
compute varb=(invXtX*t(xhc)*xhc*invXtX).
do if ( hc =1).
compute varb=(n1/(n1-ncol( x )))&*varb.
end if.
end if.
compute hclab={'se(HC0)','se(HC1)','se(HC2)','se(HC3)','se(HC4)','se'}.
compute hclab=hclab(1,( hc +1)).
compute hcflab={'F(HC0)','F(HC1)','F(HC2)','F(HC3)','F(HC4)','F'}.
compute hcflab=hcflab(1,( hc +1)).
release xhc
.
compute seb=sqrt(diag(varb)).
compute trat = b&/seb.
compute p = 2*(1-tcdf(abs(trat), (dfres))).
compute tval = sqrt(dfres* (exp((dfres-(5/6))*((xp2/(dfres-(2/3)+(.11/dfres)))* (xp2/(dfres-(2/3)+(.11/dfres)))))-1)).
compute modres={modres,seb,trat,p}.
compute modres={modres,(b-tval&*seb),(b+tval&*seb)}.
compute modresl={'coeff',hclab,'t','p','LLCI','ULCI'}.
compute lmat = ident(ncol( x )).
compute lmat = lmat(:,2:ncol(lmat)).
compute fratio = (t(t(lmat)*b)*inv(t(lmat)*varb*lmat)*((t(lmat)*b)))/(ncol( x )-1).
compute pfr = 1-fcdf(fratio,(ncol( x )-1),dfres).
compute modsum={sqrt(r2),r2,mse,fratio,(ncol( x )-1),dfres,pfr}.
compute modsuml={'R','R-sq','MSE',hcflab,'df1','df2', 'p'}.
end if.
end if.
do if ( 1 = 2 or 1 =3).
compute xlp= x.
compute ylp= y.
compute pt2 = make(nrow(ylp),1,(csum(ylp)/nrow(ylp))).
do if ( 1 =2).
compute LL3 = ylp&*ln(pt2)+(1-ylp)&*ln(1-pt2).
end if.
compute LL3 = -2*csum(LL3).
compute bt1 = make(ncol(xlp),1,0).
compute LL1 = 0.
compute pt1 = make(nrow(ylp),1,0.5).
compute pt1lp=pt1.
loop jjj = 1 to iterate.
compute xlptmp=t(xlp).
compute vecprb=pt1lp&*(1-pt1lp).
loop kkk=1 to ncol(xlp).
compute xlptmp(kkk,:)=xlptmp(kkk,:)&*t(vecprb).
end loop.
compute b = bt1+inv(xlptmp*xlp)*t(xlp)*(ylp-pt1lp).
do if ( 1 =2).
compute xlpb=xlp*b.
compute xlpbt=(xlpb > -709.7).
compute xlpb709=(1-xlpbt)*(-709.7).
compute xlpb=(xlpb&*xlpbt)+xlpb709.
compute pt1lp = 1/(1+exp(-(xlpb))).
end if.
compute itprob = csum((pt1lp < .00000001) or (pt1lp > .9999999)).
do if (itprob > 0).
loop kkk = 1 to nrow(pt1lp).
do if (pt1lp(kkk,1) > .9999999).
compute pt1lp(kkk,1) = .9999999.
end if.
do if (pt1lp(kkk,1) < .00000001).
compute pt1lp(kkk,1) = .00000001.
end if.
end loop.
compute itprob = 0.
end if.
do if (itprob = 0).
do if ( 1 =2).
compute LL = ylp&*ln(pt1lp)+(1-ylp)&*ln(1-pt1lp).
end if.
compute LL2 = -2*csum(ll).
end if.
do if (abs(LL1-LL2) < converge).
do if ( 1 =1).
compute xlptmp=t(xlp).
compute vecprb=pt1lp&*(1-pt1lp).
loop kkk=1 to ncol(xlp).
compute xlptmp(kkk,:)=xlptmp(kkk,:)&*t(vecprb).
end loop.
compute varb = inv(xlptmp*xlp).
compute seb = sqrt(diag(varb)).
release xlptmp.
end if.
break.
end if.
compute bt1 = b.
compute LL1 = LL2.
end loop.
compute modres=b.
do if (jjj > iterate).
compute itprob = 2.
do if (booting=0).
compute iterrmod=1.
end if.
do if (booting=1).
compute bootiter=1.
end if.
do if (itprobtg=0).
compute itprobtg=1.
compute errcode(errs,1) = 47.
compute errs = errs + 1.
do if (booting = 0 and 1 =1).
compute vt1 = mdiag(pt1lp&*(1-pt1lp)).
compute varb = inv(t(xlp)*vt1*xlp).
compute seb = sqrt(diag(varb)).
end if.
end if.
end if.
do if ( 1 =1).
compute trat = b&/seb.
compute dfres=nrow(xlp).
compute p = 2*(1-cdfnorm(abs(trat))).
compute modres={modres,seb,trat,p}.
compute modres={modres,(b-xp2&*seb),(b+xp2&*seb)}.
compute pvchi=1-chicdf((LL3-LL2),(nrow(modres)-1)).
compute mcF = (LL3-LL2)/LL3.
compute cox = 1-exp(-(LL3-LL2)/nrow(xlp)).
compute nagel = cox/(1-exp(-(LL3)/nrow(xlp))).
compute modsum={LL2,(LL3-LL2),(nrow(modres)-1),pvchi, mcF,cox,nagel}.
compute modsuml={'-2LL','ModelLL', 'df', 'p', 'McFadden', 'CoxSnell', 'Nagelkrk'}.
compute modresl={'coeff','se','Z','p','LLCI','ULCI'}.
end if.
end if
.
end if.
do if ((i = (nms+nys)) and (ydich=1)).

.
do if ( 2 =1).
compute hatmat=inv(t( x )* x )*t( x ).
compute b = hatmat* y.
compute modres=b.
do if ( 1 =1).
compute n1=nrow( x ).
compute dfres=n1-(ncol( x )).
compute sstotal = t( y -(csum( y )/n1))*( y -(csum( y )/n1)).
compute resid= y - x *b.
compute ssresid = csum((resid)&**2).
compute r2 = (sstotal-ssresid)/sstotal.
do if (r2 < 0).
compute r2=0.
end if.
compute adjr2 = 1-((1-r2)*(n1-1)/(dfres)).
compute mse=ssresid/(n1-ncol( x )).

.
compute n1=nrow( x ).
compute invXtX = inv(t( x )* x ).
compute varb = mse *invXtX.
compute k3 = ncol( x ).
compute xhc=0.
do if ( hc <> 5).
compute xhc= x.
compute hat = xhc(:,1).
loop i3=1 to nrow(xhc).
compute hat(i3,1)= xhc(i3,:)*invXtX*t(xhc(i3,:)).
end loop.
do if ( hc = 0 or hc =1).
loop i3 = 1 to k3.
compute xhc(:,i3)=xhc(:,i3)&* resid.
end loop.
end if.
do if ( hc =3 or hc =2).
loop i3=1 to k3.
compute xhc(:,i3) = ( resid &/(1-hat)&**(1/(4- hc )))&*xhc(:,i3).
end loop.
end if.
do if ( hc = 4).
compute hcmn=make(n,2,4).
compute hcmn(:,2)=(n1*hat)/k3.
loop i3= 1 to k3.
compute xhc(:,i3) = ( resid &/(1-hat)&**(rmin(hcmn)/2))&*xhc(:,i3).
end loop.
end if.
compute varb=(invXtX*t(xhc)*xhc*invXtX).
do if ( hc =1).
compute varb=(n1/(n1-ncol( x )))&*varb.
end if.
end if.
compute hclab={'se(HC0)','se(HC1)','se(HC2)','se(HC3)','se(HC4)','se'}.
compute hclab=hclab(1,( hc +1)).
compute hcflab={'F(HC0)','F(HC1)','F(HC2)','F(HC3)','F(HC4)','F'}.
compute hcflab=hcflab(1,( hc +1)).
release xhc
.
compute seb=sqrt(diag(varb)).
compute trat = b&/seb.
compute p = 2*(1-tcdf(abs(trat), (dfres))).
compute tval = sqrt(dfres* (exp((dfres-(5/6))*((xp2/(dfres-(2/3)+(.11/dfres)))* (xp2/(dfres-(2/3)+(.11/dfres)))))-1)).
compute modres={modres,seb,trat,p}.
compute modres={modres,(b-tval&*seb),(b+tval&*seb)}.
compute modresl={'coeff',hclab,'t','p','LLCI','ULCI'}.
compute lmat = ident(ncol( x )).
compute lmat = lmat(:,2:ncol(lmat)).
compute fratio = (t(t(lmat)*b)*inv(t(lmat)*varb*lmat)*((t(lmat)*b)))/(ncol( x )-1).
compute pfr = 1-fcdf(fratio,(ncol( x )-1),dfres).
compute modsum={sqrt(r2),r2,mse,fratio,(ncol( x )-1),dfres,pfr}.
compute modsuml={'R','R-sq','MSE',hcflab,'df1','df2', 'p'}.
end if.
end if.
do if ( 2 = 2 or 2 =3).
compute xlp= x.
compute ylp= y.
compute pt2 = make(nrow(ylp),1,(csum(ylp)/nrow(ylp))).
do if ( 2 =2).
compute LL3 = ylp&*ln(pt2)+(1-ylp)&*ln(1-pt2).
end if.
compute LL3 = -2*csum(LL3).
compute bt1 = make(ncol(xlp),1,0).
compute LL1 = 0.
compute pt1 = make(nrow(ylp),1,0.5).
compute pt1lp=pt1.
loop jjj = 1 to iterate.
compute xlptmp=t(xlp).
compute vecprb=pt1lp&*(1-pt1lp).
loop kkk=1 to ncol(xlp).
compute xlptmp(kkk,:)=xlptmp(kkk,:)&*t(vecprb).
end loop.
compute b = bt1+inv(xlptmp*xlp)*t(xlp)*(ylp-pt1lp).
do if ( 2 =2).
compute xlpb=xlp*b.
compute xlpbt=(xlpb > -709.7).
compute xlpb709=(1-xlpbt)*(-709.7).
compute xlpb=(xlpb&*xlpbt)+xlpb709.
compute pt1lp = 1/(1+exp(-(xlpb))).
end if.
compute itprob = csum((pt1lp < .00000001) or (pt1lp > .9999999)).
do if (itprob > 0).
loop kkk = 1 to nrow(pt1lp).
do if (pt1lp(kkk,1) > .9999999).
compute pt1lp(kkk,1) = .9999999.
end if.
do if (pt1lp(kkk,1) < .00000001).
compute pt1lp(kkk,1) = .00000001.
end if.
end loop.
compute itprob = 0.
end if.
do if (itprob = 0).
do if ( 2 =2).
compute LL = ylp&*ln(pt1lp)+(1-ylp)&*ln(1-pt1lp).
end if.
compute LL2 = -2*csum(ll).
end if.
do if (abs(LL1-LL2) < converge).
do if ( 1 =1).
compute xlptmp=t(xlp).
compute vecprb=pt1lp&*(1-pt1lp).
loop kkk=1 to ncol(xlp).
compute xlptmp(kkk,:)=xlptmp(kkk,:)&*t(vecprb).
end loop.
compute varb = inv(xlptmp*xlp).
compute seb = sqrt(diag(varb)).
release xlptmp.
end if.
break.
end if.
compute bt1 = b.
compute LL1 = LL2.
end loop.
compute modres=b.
do if (jjj > iterate).
compute itprob = 2.
do if (booting=0).
compute iterrmod=1.
end if.
do if (booting=1).
compute bootiter=1.
end if.
do if (itprobtg=0).
compute itprobtg=1.
compute errcode(errs,1) = 47.
compute errs = errs + 1.
do if (booting = 0 and 1 =1).
compute vt1 = mdiag(pt1lp&*(1-pt1lp)).
compute varb = inv(t(xlp)*vt1*xlp).
compute seb = sqrt(diag(varb)).
end if.
end if.
end if.
do if ( 1 =1).
compute trat = b&/seb.
compute dfres=nrow(xlp).
compute p = 2*(1-cdfnorm(abs(trat))).
compute modres={modres,seb,trat,p}.
compute modres={modres,(b-xp2&*seb),(b+xp2&*seb)}.
compute pvchi=1-chicdf((LL3-LL2),(nrow(modres)-1)).
compute mcF = (LL3-LL2)/LL3.
compute cox = 1-exp(-(LL3-LL2)/nrow(xlp)).
compute nagel = cox/(1-exp(-(LL3)/nrow(xlp))).
compute modsum={LL2,(LL3-LL2),(nrow(modres)-1),pvchi, mcF,cox,nagel}.
compute modsuml={'-2LL','ModelLL', 'df', 'p', 'McFadden', 'CoxSnell', 'Nagelkrk'}.
compute modresl={'coeff','se','Z','p','LLCI','ULCI'}.
end if.
end if
.
compute chidfxm=basemodx-LL2.
end if.
compute lmat=make(nrow(b),nxvls,0).
compute lmattmp=ident(nxvls).
compute lmat((nrow(lmat)-nxvls+1):nrow(lmat),:)=lmattmp.

.
compute lmat2= lmat.
do if ( 1 =0).
compute lmat2 = mdiag( lmat ).
compute lmat3=make(nrow(lmat2),1,0).
loop flp=1 to ncol(lmat2).
do if (csum(lmat2(:,flp))=1).
compute lmat3={lmat3,lmat2(:,flp)}.
end if.
end loop.
compute lmat2=lmat3(:,2:ncol(lmat3)).
end if.
compute fratio = (t(t(lmat2)* b )*inv(t(lmat2)* varb *lmat2)*((t(lmat2)* b )))/ncol(lmat2).
compute pfr = 1-fcdf(fratio,ncol(lmat2),(n-nrow( b ))).
compute fresult={fratio,ncol(lmat2),(n-nrow( b )),pfr}.
do if (i = (nms+nys) and (ydich=1)).
compute fratio=fratio*ncol(lmat2).
compute pfr=1-chicdf(fratio,ncol(lmat2)).
compute fresult={fratio,ncol(lmat2),pfr}.
end if.
do if ( 0 =1).
compute lmat3=1-rsum(lmat2).
compute xfm=make(n,csum(lmat3),0).
compute flpc=1.
loop flp=1 to nrow(lmat3).
do if (lmat3(flp,1)=1).
compute xfm(:,flpc)=x(:,flp).
compute flpc=flpc+1.
end if.
end loop.
compute bfm=inv(t(xfm)*xfm)*t(xfm)*y.
compute resid=y-(xfm*bfm).
compute sstotal=(y-(csum(y)/n)).
compute sstotal=csum(sstotal&*sstotal).
compute ssresid=csum(resid&*resid).
compute rsqch= 0 -((sstotal-ssresid)/sstotal).
compute fresult={rsqch,fresult}.
release xfm,flpc, resid, ssresid, bfm.
end if
.
compute numxint=numxint+1.
compute xmtst(numxint,:)=fresult.
do if ((i = (nms+nys)) and (ydich=1)).
compute xmtst(numxint,1)=chidfxm.
compute xmtst(numxint,3)=1-chicdf(chidfxm,nxvls).
end if.
compute xmtstlb={xmtstlb;highlbx((xmints-1),1)}.
end if.
end loop.
compute x=xmtmat.
release xmtmat.
do if (numxint>0).
compute xmtstlb=xmtstlb((2:(numxint+1)),:).
compute xmtst=xmtst(1:numxint,:).
compute resultm2=make(nrow(xmtst),maxresm,99999).
compute resultm2(1:nrow(xmtst),1:ncol(xmtst))=xmtst.
compute resultm={resultm;resultm2}.
do if (nms=1).
compute xmtstlb=' '.
end if.
do if (outscree=1).
do if ((i < (nms+nys)) or (ydich=0)).
print xmtst/title='Test(s) of X by M interaction:'/rnames=xmtstlb/cnames=xmtstlbc/format= F10.4.
end if.
do if ((i = (nms+nys)) and (ydich=1)).
print xmtst/title='Likelihood ratio test(s) of X by M interaction:'/rnames=xmtstlb/cnames=xmtstlbc/format= F10.4.
end if.
end if.
end if.
compute r2=r2tmp.
compute b=btmp.
compute varb=varbtmp.
compute dfres=dfrestmp.
compute tval=tvaltmp.
end if.
do if (criterr = 0).
compute jj=0.
loop j = start to ((start+i)-1).
compute dbint=0.
compute lmat=whigh(1:nump(1,i),j).
compute lmat2=wzhigh(1:nump(1,i),j).
do if ((csum(lmat) > 0) and (csum(lmat2) = 0)).
do if ((i < (nms+nys)) or (ydich <> 1)).

.
compute lmat2= lmat.
do if ( 0 =0).
compute lmat2 = mdiag( lmat ).
compute lmat3=make(nrow(lmat2),1,0).
loop flp=1 to ncol(lmat2).
do if (csum(lmat2(:,flp))=1).
compute lmat3={lmat3,lmat2(:,flp)}.
end if.
end loop.
compute lmat2=lmat3(:,2:ncol(lmat3)).
end if.
compute fratio = (t(t(lmat2)* b )*inv(t(lmat2)* varb *lmat2)*((t(lmat2)* b )))/ncol(lmat2).
compute pfr = 1-fcdf(fratio,ncol(lmat2),(n-nrow( b ))).
compute fresult={fratio,ncol(lmat2),(n-nrow( b )),pfr}.
do if (i = (nms+nys) and (ydich=1)).
compute fratio=fratio*ncol(lmat2).
compute pfr=1-chicdf(fratio,ncol(lmat2)).
compute fresult={fratio,ncol(lmat2),pfr}.
end if.
do if ( 1 =1).
compute lmat3=1-rsum(lmat2).
compute xfm=make(n,csum(lmat3),0).
compute flpc=1.
loop flp=1 to nrow(lmat3).
do if (lmat3(flp,1)=1).
compute xfm(:,flpc)=x(:,flp).
compute flpc=flpc+1.
end if.
end loop.
compute bfm=inv(t(xfm)*xfm)*t(xfm)*y.
compute resid=y-(xfm*bfm).
compute sstotal=(y-(csum(y)/n)).
compute sstotal=csum(sstotal&*sstotal).
compute ssresid=csum(resid&*resid).
compute rsqch= r2 -((sstotal-ssresid)/sstotal).
compute fresult={rsqch,fresult}.
release xfm,flpc, resid, ssresid, bfm.
end if
.
compute lmatdb=lmat.
compute dbint=dbint+1.
end if.
do if ((ydich = 1) and (i = (nms+nys))).

.
compute btemphld=b.
compute llrdat=make(nrow(x),nrow( lmat )-csum( lmat ),-999).
compute llrdf=ncol(x)-ncol(llrdat).
compute llrcnt=0.
loop llri=1 to nrow( lmat ).
do if ( lmat (llri,1)=0).
compute llrcnt=llrcnt+1.
compute llrdat(:,llrcnt)=x(:,llri).
end if.
end loop.

.
do if ( 2 =1).
compute hatmat=inv(t( llrdat )* llrdat )*t( llrdat ).
compute b = hatmat* y.
compute modres=b.
do if ( 0 =1).
compute n1=nrow( llrdat ).
compute dfres=n1-(ncol( llrdat )).
compute sstotal = t( y -(csum( y )/n1))*( y -(csum( y )/n1)).
compute resid= y - llrdat *b.
compute ssresid = csum((resid)&**2).
compute r2 = (sstotal-ssresid)/sstotal.
do if (r2 < 0).
compute r2=0.
end if.
compute adjr2 = 1-((1-r2)*(n1-1)/(dfres)).
compute mse=ssresid/(n1-ncol( llrdat )).

.
compute n1=nrow( x ).
compute invXtX = inv(t( x )* x ).
compute varb = mse *invXtX.
compute k3 = ncol( x ).
compute xhc=0.
do if ( hc <> 5).
compute xhc= x.
compute hat = xhc(:,1).
loop i3=1 to nrow(xhc).
compute hat(i3,1)= xhc(i3,:)*invXtX*t(xhc(i3,:)).
end loop.
do if ( hc = 0 or hc =1).
loop i3 = 1 to k3.
compute xhc(:,i3)=xhc(:,i3)&* resid.
end loop.
end if.
do if ( hc =3 or hc =2).
loop i3=1 to k3.
compute xhc(:,i3) = ( resid &/(1-hat)&**(1/(4- hc )))&*xhc(:,i3).
end loop.
end if.
do if ( hc = 4).
compute hcmn=make(n,2,4).
compute hcmn(:,2)=(n1*hat)/k3.
loop i3= 1 to k3.
compute xhc(:,i3) = ( resid &/(1-hat)&**(rmin(hcmn)/2))&*xhc(:,i3).
end loop.
end if.
compute varb=(invXtX*t(xhc)*xhc*invXtX).
do if ( hc =1).
compute varb=(n1/(n1-ncol( x )))&*varb.
end if.
end if.
compute hclab={'se(HC0)','se(HC1)','se(HC2)','se(HC3)','se(HC4)','se'}.
compute hclab=hclab(1,( hc +1)).
compute hcflab={'F(HC0)','F(HC1)','F(HC2)','F(HC3)','F(HC4)','F'}.
compute hcflab=hcflab(1,( hc +1)).
release xhc
.
compute seb=sqrt(diag(varb)).
compute trat = b&/seb.
compute p = 2*(1-tcdf(abs(trat), (dfres))).
compute tval = sqrt(dfres* (exp((dfres-(5/6))*((xp2/(dfres-(2/3)+(.11/dfres)))* (xp2/(dfres-(2/3)+(.11/dfres)))))-1)).
compute modres={modres,seb,trat,p}.
compute modres={modres,(b-tval&*seb),(b+tval&*seb)}.
compute modresl={'coeff',hclab,'t','p','LLCI','ULCI'}.
compute lmat = ident(ncol( llrdat )).
compute lmat = lmat(:,2:ncol(lmat)).
compute fratio = (t(t(lmat)*b)*inv(t(lmat)*varb*lmat)*((t(lmat)*b)))/(ncol( llrdat )-1).
compute pfr = 1-fcdf(fratio,(ncol( llrdat )-1),dfres).
compute modsum={sqrt(r2),r2,mse,fratio,(ncol( llrdat )-1),dfres,pfr}.
compute modsuml={'R','R-sq','MSE',hcflab,'df1','df2', 'p'}.
end if.
end if.
do if ( 2 = 2 or 2 =3).
compute xlp= llrdat.
compute ylp= y.
compute pt2 = make(nrow(ylp),1,(csum(ylp)/nrow(ylp))).
do if ( 2 =2).
compute LL3 = ylp&*ln(pt2)+(1-ylp)&*ln(1-pt2).
end if.
compute LL3 = -2*csum(LL3).
compute bt1 = make(ncol(xlp),1,0).
compute LL1 = 0.
compute pt1 = make(nrow(ylp),1,0.5).
compute pt1lp=pt1.
loop jjj = 1 to iterate.
compute xlptmp=t(xlp).
compute vecprb=pt1lp&*(1-pt1lp).
loop kkk=1 to ncol(xlp).
compute xlptmp(kkk,:)=xlptmp(kkk,:)&*t(vecprb).
end loop.
compute b = bt1+inv(xlptmp*xlp)*t(xlp)*(ylp-pt1lp).
do if ( 2 =2).
compute xlpb=xlp*b.
compute xlpbt=(xlpb > -709.7).
compute xlpb709=(1-xlpbt)*(-709.7).
compute xlpb=(xlpb&*xlpbt)+xlpb709.
compute pt1lp = 1/(1+exp(-(xlpb))).
end if.
compute itprob = csum((pt1lp < .00000001) or (pt1lp > .9999999)).
do if (itprob > 0).
loop kkk = 1 to nrow(pt1lp).
do if (pt1lp(kkk,1) > .9999999).
compute pt1lp(kkk,1) = .9999999.
end if.
do if (pt1lp(kkk,1) < .00000001).
compute pt1lp(kkk,1) = .00000001.
end if.
end loop.
compute itprob = 0.
end if.
do if (itprob = 0).
do if ( 2 =2).
compute LL = ylp&*ln(pt1lp)+(1-ylp)&*ln(1-pt1lp).
end if.
compute LL2 = -2*csum(ll).
end if.
do if (abs(LL1-LL2) < converge).
do if ( 0 =1).
compute xlptmp=t(xlp).
compute vecprb=pt1lp&*(1-pt1lp).
loop kkk=1 to ncol(xlp).
compute xlptmp(kkk,:)=xlptmp(kkk,:)&*t(vecprb).
end loop.
compute varb = inv(xlptmp*xlp).
compute seb = sqrt(diag(varb)).
release xlptmp.
end if.
break.
end if.
compute bt1 = b.
compute LL1 = LL2.
end loop.
compute modres=b.
do if (jjj > iterate).
compute itprob = 2.
do if (booting=0).
compute iterrmod=1.
end if.
do if (booting=1).
compute bootiter=1.
end if.
do if (itprobtg=0).
compute itprobtg=1.
compute errcode(errs,1) = 47.
compute errs = errs + 1.
do if (booting = 0 and 0 =1).
compute vt1 = mdiag(pt1lp&*(1-pt1lp)).
compute varb = inv(t(xlp)*vt1*xlp).
compute seb = sqrt(diag(varb)).
end if.
end if.
end if.
do if ( 0 =1).
compute trat = b&/seb.
compute dfres=nrow(xlp).
compute p = 2*(1-cdfnorm(abs(trat))).
compute modres={modres,seb,trat,p}.
compute modres={modres,(b-xp2&*seb),(b+xp2&*seb)}.
compute pvchi=1-chicdf((LL3-LL2),(nrow(modres)-1)).
compute mcF = (LL3-LL2)/LL3.
compute cox = 1-exp(-(LL3-LL2)/nrow(xlp)).
compute nagel = cox/(1-exp(-(LL3)/nrow(xlp))).
compute modsum={LL2,(LL3-LL2),(nrow(modres)-1),pvchi, mcF,cox,nagel}.
compute modsuml={'-2LL','ModelLL', 'df', 'p', 'McFadden', 'CoxSnell', 'Nagelkrk'}.
compute modresl={'coeff','se','Z','p','LLCI','ULCI'}.
end if.
end if
.
compute b=btemphld.
compute fresult={(LL2-basemod),llrdf,1-chicdf((LL2-basemod),llrdf)}
.
compute lmatdb=lmat.
compute dbint=dbint+1.
end if.
compute highf={highf;fresult}.
compute highf2={highf2;fresult}.
do if (j = start).
compute flabel={flabel;'X*W'}.
end if.
do if (j > start).
do if (nms > 1).
compute flabel={flabel;highlbw(jj,1)}.
else if (nms = 1).
do if (xmint=0).
compute flabel={flabel;'M*W'}.
end if.
do if (xmint=1).
compute flabel={flabel;'X*M'}.
end if.
end if.
end if.
end if.
compute lmat=zhigh(1:nump(1,i),j).
compute lmat2=wzhigh(1:nump(1,i),j).
do if ((csum(lmat) > 0) and (csum(lmat2) = 0)).
do if ((i < (nms+nys)) or (ydich <> 1)).

.
compute lmat2= lmat.
do if ( 0 =0).
compute lmat2 = mdiag( lmat ).
compute lmat3=make(nrow(lmat2),1,0).
loop flp=1 to ncol(lmat2).
do if (csum(lmat2(:,flp))=1).
compute lmat3={lmat3,lmat2(:,flp)}.
end if.
end loop.
compute lmat2=lmat3(:,2:ncol(lmat3)).
end if.
compute fratio = (t(t(lmat2)* b )*inv(t(lmat2)* varb *lmat2)*((t(lmat2)* b )))/ncol(lmat2).
compute pfr = 1-fcdf(fratio,ncol(lmat2),(n-nrow( b ))).
compute fresult={fratio,ncol(lmat2),(n-nrow( b )),pfr}.
do if (i = (nms+nys) and (ydich=1)).
compute fratio=fratio*ncol(lmat2).
compute pfr=1-chicdf(fratio,ncol(lmat2)).
compute fresult={fratio,ncol(lmat2),pfr}.
end if.
do if ( 1 =1).
compute lmat3=1-rsum(lmat2).
compute xfm=make(n,csum(lmat3),0).
compute flpc=1.
loop flp=1 to nrow(lmat3).
do if (lmat3(flp,1)=1).
compute xfm(:,flpc)=x(:,flp).
compute flpc=flpc+1.
end if.
end loop.
compute bfm=inv(t(xfm)*xfm)*t(xfm)*y.
compute resid=y-(xfm*bfm).
compute sstotal=(y-(csum(y)/n)).
compute sstotal=csum(sstotal&*sstotal).
compute ssresid=csum(resid&*resid).
compute rsqch= r2 -((sstotal-ssresid)/sstotal).
compute fresult={rsqch,fresult}.
release xfm,flpc, resid, ssresid, bfm.
end if
.
compute dbint=dbint+1.
end if.
do if ((ydich = 1) and (i = (nms+nys))).

.
compute btemphld=b.
compute llrdat=make(nrow(x),nrow( lmat )-csum( lmat ),-999).
compute llrdf=ncol(x)-ncol(llrdat).
compute llrcnt=0.
loop llri=1 to nrow( lmat ).
do if ( lmat (llri,1)=0).
compute llrcnt=llrcnt+1.
compute llrdat(:,llrcnt)=x(:,llri).
end if.
end loop.

.
do if ( 2 =1).
compute hatmat=inv(t( llrdat )* llrdat )*t( llrdat ).
compute b = hatmat* y.
compute modres=b.
do if ( 0 =1).
compute n1=nrow( llrdat ).
compute dfres=n1-(ncol( llrdat )).
compute sstotal = t( y -(csum( y )/n1))*( y -(csum( y )/n1)).
compute resid= y - llrdat *b.
compute ssresid = csum((resid)&**2).
compute r2 = (sstotal-ssresid)/sstotal.
do if (r2 < 0).
compute r2=0.
end if.
compute adjr2 = 1-((1-r2)*(n1-1)/(dfres)).
compute mse=ssresid/(n1-ncol( llrdat )).

.
compute n1=nrow( x ).
compute invXtX = inv(t( x )* x ).
compute varb = mse *invXtX.
compute k3 = ncol( x ).
compute xhc=0.
do if ( hc <> 5).
compute xhc= x.
compute hat = xhc(:,1).
loop i3=1 to nrow(xhc).
compute hat(i3,1)= xhc(i3,:)*invXtX*t(xhc(i3,:)).
end loop.
do if ( hc = 0 or hc =1).
loop i3 = 1 to k3.
compute xhc(:,i3)=xhc(:,i3)&* resid.
end loop.
end if.
do if ( hc =3 or hc =2).
loop i3=1 to k3.
compute xhc(:,i3) = ( resid &/(1-hat)&**(1/(4- hc )))&*xhc(:,i3).
end loop.
end if.
do if ( hc = 4).
compute hcmn=make(n,2,4).
compute hcmn(:,2)=(n1*hat)/k3.
loop i3= 1 to k3.
compute xhc(:,i3) = ( resid &/(1-hat)&**(rmin(hcmn)/2))&*xhc(:,i3).
end loop.
end if.
compute varb=(invXtX*t(xhc)*xhc*invXtX).
do if ( hc =1).
compute varb=(n1/(n1-ncol( x )))&*varb.
end if.
end if.
compute hclab={'se(HC0)','se(HC1)','se(HC2)','se(HC3)','se(HC4)','se'}.
compute hclab=hclab(1,( hc +1)).
compute hcflab={'F(HC0)','F(HC1)','F(HC2)','F(HC3)','F(HC4)','F'}.
compute hcflab=hcflab(1,( hc +1)).
release xhc
.
compute seb=sqrt(diag(varb)).
compute trat = b&/seb.
compute p = 2*(1-tcdf(abs(trat), (dfres))).
compute tval = sqrt(dfres* (exp((dfres-(5/6))*((xp2/(dfres-(2/3)+(.11/dfres)))* (xp2/(dfres-(2/3)+(.11/dfres)))))-1)).
compute modres={modres,seb,trat,p}.
compute modres={modres,(b-tval&*seb),(b+tval&*seb)}.
compute modresl={'coeff',hclab,'t','p','LLCI','ULCI'}.
compute lmat = ident(ncol( llrdat )).
compute lmat = lmat(:,2:ncol(lmat)).
compute fratio = (t(t(lmat)*b)*inv(t(lmat)*varb*lmat)*((t(lmat)*b)))/(ncol( llrdat )-1).
compute pfr = 1-fcdf(fratio,(ncol( llrdat )-1),dfres).
compute modsum={sqrt(r2),r2,mse,fratio,(ncol( llrdat )-1),dfres,pfr}.
compute modsuml={'R','R-sq','MSE',hcflab,'df1','df2', 'p'}.
end if.
end if.
do if ( 2 = 2 or 2 =3).
compute xlp= llrdat.
compute ylp= y.
compute pt2 = make(nrow(ylp),1,(csum(ylp)/nrow(ylp))).
do if ( 2 =2).
compute LL3 = ylp&*ln(pt2)+(1-ylp)&*ln(1-pt2).
end if.
compute LL3 = -2*csum(LL3).
compute bt1 = make(ncol(xlp),1,0).
compute LL1 = 0.
compute pt1 = make(nrow(ylp),1,0.5).
compute pt1lp=pt1.
loop jjj = 1 to iterate.
compute xlptmp=t(xlp).
compute vecprb=pt1lp&*(1-pt1lp).
loop kkk=1 to ncol(xlp).
compute xlptmp(kkk,:)=xlptmp(kkk,:)&*t(vecprb).
end loop.
compute b = bt1+inv(xlptmp*xlp)*t(xlp)*(ylp-pt1lp).
do if ( 2 =2).
compute xlpb=xlp*b.
compute xlpbt=(xlpb > -709.7).
compute xlpb709=(1-xlpbt)*(-709.7).
compute xlpb=(xlpb&*xlpbt)+xlpb709.
compute pt1lp = 1/(1+exp(-(xlpb))).
end if.
compute itprob = csum((pt1lp < .00000001) or (pt1lp > .9999999)).
do if (itprob > 0).
loop kkk = 1 to nrow(pt1lp).
do if (pt1lp(kkk,1) > .9999999).
compute pt1lp(kkk,1) = .9999999.
end if.
do if (pt1lp(kkk,1) < .00000001).
compute pt1lp(kkk,1) = .00000001.
end if.
end loop.
compute itprob = 0.
end if.
do if (itprob = 0).
do if ( 2 =2).
compute LL = ylp&*ln(pt1lp)+(1-ylp)&*ln(1-pt1lp).
end if.
compute LL2 = -2*csum(ll).
end if.
do if (abs(LL1-LL2) < converge).
do if ( 0 =1).
compute xlptmp=t(xlp).
compute vecprb=pt1lp&*(1-pt1lp).
loop kkk=1 to ncol(xlp).
compute xlptmp(kkk,:)=xlptmp(kkk,:)&*t(vecprb).
end loop.
compute varb = inv(xlptmp*xlp).
compute seb = sqrt(diag(varb)).
release xlptmp.
end if.
break.
end if.
compute bt1 = b.
compute LL1 = LL2.
end loop.
compute modres=b.
do if (jjj > iterate).
compute itprob = 2.
do if (booting=0).
compute iterrmod=1.
end if.
do if (booting=1).
compute bootiter=1.
end if.
do if (itprobtg=0).
compute itprobtg=1.
compute errcode(errs,1) = 47.
compute errs = errs + 1.
do if (booting = 0 and 0 =1).
compute vt1 = mdiag(pt1lp&*(1-pt1lp)).
compute varb = inv(t(xlp)*vt1*xlp).
compute seb = sqrt(diag(varb)).
end if.
end if.
end if.
do if ( 0 =1).
compute trat = b&/seb.
compute dfres=nrow(xlp).
compute p = 2*(1-cdfnorm(abs(trat))).
compute modres={modres,seb,trat,p}.
compute modres={modres,(b-xp2&*seb),(b+xp2&*seb)}.
compute pvchi=1-chicdf((LL3-LL2),(nrow(modres)-1)).
compute mcF = (LL3-LL2)/LL3.
compute cox = 1-exp(-(LL3-LL2)/nrow(xlp)).
compute nagel = cox/(1-exp(-(LL3)/nrow(xlp))).
compute modsum={LL2,(LL3-LL2),(nrow(modres)-1),pvchi, mcF,cox,nagel}.
compute modsuml={'-2LL','ModelLL', 'df', 'p', 'McFadden', 'CoxSnell', 'Nagelkrk'}.
compute modresl={'coeff','se','Z','p','LLCI','ULCI'}.
end if.
end if
.
compute b=btemphld.
compute fresult={(LL2-basemod),llrdf,1-chicdf((LL2-basemod),llrdf)}
.
compute dbint=dbint+1.
end if.
compute highf={highf;fresult}.
compute highf2={highf2;fresult}.
do if (j = start).
compute flabel={flabel;'X*Z'}.
end if.
do if (j > start).
do if (nms > 1).
compute flabel={flabel;highlbz(jj,1)}.
else if (nms = 1).
compute flabel={flabel;'M*Z'}.
end if.
end if.
end if.
do if (dbint=2).
compute lmatdb=lmatdb+lmat.
do if ((ydich = 1) and (i = (nms+nys))).

.
compute btemphld=b.
compute llrdat=make(nrow(x),nrow( lmatdb )-csum( lmatdb ),-999).
compute llrdf=ncol(x)-ncol(llrdat).
compute llrcnt=0.
loop llri=1 to nrow( lmatdb ).
do if ( lmatdb (llri,1)=0).
compute llrcnt=llrcnt+1.
compute llrdat(:,llrcnt)=x(:,llri).
end if.
end loop.

.
do if ( 2 =1).
compute hatmat=inv(t( llrdat )* llrdat )*t( llrdat ).
compute b = hatmat* y.
compute modres=b.
do if ( 0 =1).
compute n1=nrow( llrdat ).
compute dfres=n1-(ncol( llrdat )).
compute sstotal = t( y -(csum( y )/n1))*( y -(csum( y )/n1)).
compute resid= y - llrdat *b.
compute ssresid = csum((resid)&**2).
compute r2 = (sstotal-ssresid)/sstotal.
do if (r2 < 0).
compute r2=0.
end if.
compute adjr2 = 1-((1-r2)*(n1-1)/(dfres)).
compute mse=ssresid/(n1-ncol( llrdat )).

.
compute n1=nrow( x ).
compute invXtX = inv(t( x )* x ).
compute varb = mse *invXtX.
compute k3 = ncol( x ).
compute xhc=0.
do if ( hc <> 5).
compute xhc= x.
compute hat = xhc(:,1).
loop i3=1 to nrow(xhc).
compute hat(i3,1)= xhc(i3,:)*invXtX*t(xhc(i3,:)).
end loop.
do if ( hc = 0 or hc =1).
loop i3 = 1 to k3.
compute xhc(:,i3)=xhc(:,i3)&* resid.
end loop.
end if.
do if ( hc =3 or hc =2).
loop i3=1 to k3.
compute xhc(:,i3) = ( resid &/(1-hat)&**(1/(4- hc )))&*xhc(:,i3).
end loop.
end if.
do if ( hc = 4).
compute hcmn=make(n,2,4).
compute hcmn(:,2)=(n1*hat)/k3.
loop i3= 1 to k3.
compute xhc(:,i3) = ( resid &/(1-hat)&**(rmin(hcmn)/2))&*xhc(:,i3).
end loop.
end if.
compute varb=(invXtX*t(xhc)*xhc*invXtX).
do if ( hc =1).
compute varb=(n1/(n1-ncol( x )))&*varb.
end if.
end if.
compute hclab={'se(HC0)','se(HC1)','se(HC2)','se(HC3)','se(HC4)','se'}.
compute hclab=hclab(1,( hc +1)).
compute hcflab={'F(HC0)','F(HC1)','F(HC2)','F(HC3)','F(HC4)','F'}.
compute hcflab=hcflab(1,( hc +1)).
release xhc
.
compute seb=sqrt(diag(varb)).
compute trat = b&/seb.
compute p = 2*(1-tcdf(abs(trat), (dfres))).
compute tval = sqrt(dfres* (exp((dfres-(5/6))*((xp2/(dfres-(2/3)+(.11/dfres)))* (xp2/(dfres-(2/3)+(.11/dfres)))))-1)).
compute modres={modres,seb,trat,p}.
compute modres={modres,(b-tval&*seb),(b+tval&*seb)}.
compute modresl={'coeff',hclab,'t','p','LLCI','ULCI'}.
compute lmat = ident(ncol( llrdat )).
compute lmat = lmat(:,2:ncol(lmat)).
compute fratio = (t(t(lmat)*b)*inv(t(lmat)*varb*lmat)*((t(lmat)*b)))/(ncol( llrdat )-1).
compute pfr = 1-fcdf(fratio,(ncol( llrdat )-1),dfres).
compute modsum={sqrt(r2),r2,mse,fratio,(ncol( llrdat )-1),dfres,pfr}.
compute modsuml={'R','R-sq','MSE',hcflab,'df1','df2', 'p'}.
end if.
end if.
do if ( 2 = 2 or 2 =3).
compute xlp= llrdat.
compute ylp= y.
compute pt2 = make(nrow(ylp),1,(csum(ylp)/nrow(ylp))).
do if ( 2 =2).
compute LL3 = ylp&*ln(pt2)+(1-ylp)&*ln(1-pt2).
end if.
compute LL3 = -2*csum(LL3).
compute bt1 = make(ncol(xlp),1,0).
compute LL1 = 0.
compute pt1 = make(nrow(ylp),1,0.5).
compute pt1lp=pt1.
loop jjj = 1 to iterate.
compute xlptmp=t(xlp).
compute vecprb=pt1lp&*(1-pt1lp).
loop kkk=1 to ncol(xlp).
compute xlptmp(kkk,:)=xlptmp(kkk,:)&*t(vecprb).
end loop.
compute b = bt1+inv(xlptmp*xlp)*t(xlp)*(ylp-pt1lp).
do if ( 2 =2).
compute xlpb=xlp*b.
compute xlpbt=(xlpb > -709.7).
compute xlpb709=(1-xlpbt)*(-709.7).
compute xlpb=(xlpb&*xlpbt)+xlpb709.
compute pt1lp = 1/(1+exp(-(xlpb))).
end if.
compute itprob = csum((pt1lp < .00000001) or (pt1lp > .9999999)).
do if (itprob > 0).
loop kkk = 1 to nrow(pt1lp).
do if (pt1lp(kkk,1) > .9999999).
compute pt1lp(kkk,1) = .9999999.
end if.
do if (pt1lp(kkk,1) < .00000001).
compute pt1lp(kkk,1) = .00000001.
end if.
end loop.
compute itprob = 0.
end if.
do if (itprob = 0).
do if ( 2 =2).
compute LL = ylp&*ln(pt1lp)+(1-ylp)&*ln(1-pt1lp).
end if.
compute LL2 = -2*csum(ll).
end if.
do if (abs(LL1-LL2) < converge).
do if ( 0 =1).
compute xlptmp=t(xlp).
compute vecprb=pt1lp&*(1-pt1lp).
loop kkk=1 to ncol(xlp).
compute xlptmp(kkk,:)=xlptmp(kkk,:)&*t(vecprb).
end loop.
compute varb = inv(xlptmp*xlp).
compute seb = sqrt(diag(varb)).
release xlptmp.
end if.
break.
end if.
compute bt1 = b.
compute LL1 = LL2.
end loop.
compute modres=b.
do if (jjj > iterate).
compute itprob = 2.
do if (booting=0).
compute iterrmod=1.
end if.
do if (booting=1).
compute bootiter=1.
end if.
do if (itprobtg=0).
compute itprobtg=1.
compute errcode(errs,1) = 47.
compute errs = errs + 1.
do if (booting = 0 and 0 =1).
compute vt1 = mdiag(pt1lp&*(1-pt1lp)).
compute varb = inv(t(xlp)*vt1*xlp).
compute seb = sqrt(diag(varb)).
end if.
end if.
end if.
do if ( 0 =1).
compute trat = b&/seb.
compute dfres=nrow(xlp).
compute p = 2*(1-cdfnorm(abs(trat))).
compute modres={modres,seb,trat,p}.
compute modres={modres,(b-xp2&*seb),(b+xp2&*seb)}.
compute pvchi=1-chicdf((LL3-LL2),(nrow(modres)-1)).
compute mcF = (LL3-LL2)/LL3.
compute cox = 1-exp(-(LL3-LL2)/nrow(xlp)).
compute nagel = cox/(1-exp(-(LL3)/nrow(xlp))).
compute modsum={LL2,(LL3-LL2),(nrow(modres)-1),pvchi, mcF,cox,nagel}.
compute modsuml={'-2LL','ModelLL', 'df', 'p', 'McFadden', 'CoxSnell', 'Nagelkrk'}.
compute modresl={'coeff','se','Z','p','LLCI','ULCI'}.
end if.
end if
.
compute b=btemphld.
compute fresult={(LL2-basemod),llrdf,1-chicdf((LL2-basemod),llrdf)}
.
end if.
do if (ydich <> 1 or i < (nms+nys)).

.
compute lmat2= lmatdb.
do if ( 0 =0).
compute lmat2 = mdiag( lmatdb ).
compute lmat3=make(nrow(lmat2),1,0).
loop flp=1 to ncol(lmat2).
do if (csum(lmat2(:,flp))=1).
compute lmat3={lmat3,lmat2(:,flp)}.
end if.
end loop.
compute lmat2=lmat3(:,2:ncol(lmat3)).
end if.
compute fratio = (t(t(lmat2)* b )*inv(t(lmat2)* varb *lmat2)*((t(lmat2)* b )))/ncol(lmat2).
compute pfr = 1-fcdf(fratio,ncol(lmat2),(n-nrow( b ))).
compute fresult={fratio,ncol(lmat2),(n-nrow( b )),pfr}.
do if (i = (nms+nys) and (ydich=1)).
compute fratio=fratio*ncol(lmat2).
compute pfr=1-chicdf(fratio,ncol(lmat2)).
compute fresult={fratio,ncol(lmat2),pfr}.
end if.
do if ( 1 =1).
compute lmat3=1-rsum(lmat2).
compute xfm=make(n,csum(lmat3),0).
compute flpc=1.
loop flp=1 to nrow(lmat3).
do if (lmat3(flp,1)=1).
compute xfm(:,flpc)=x(:,flp).
compute flpc=flpc+1.
end if.
end loop.
compute bfm=inv(t(xfm)*xfm)*t(xfm)*y.
compute resid=y-(xfm*bfm).
compute sstotal=(y-(csum(y)/n)).
compute sstotal=csum(sstotal&*sstotal).
compute ssresid=csum(resid&*resid).
compute rsqch= r2 -((sstotal-ssresid)/sstotal).
compute fresult={rsqch,fresult}.
release xfm,flpc, resid, ssresid, bfm.
end if
.
end if.
compute dbint=0.
compute highf={highf;fresult}.
do if (jj=0 and nms > 0).
compute flabel={flabel;'BOTH(X)'}.
end if.
do if (jj=0 and nms = 0).
compute flabel={flabel;'BOTH'}.
end if.
do if (jj>0 and nms = 1).
compute flabel={flabel;'BOTH(M)'}.
end if.
do if (nms > 1 and jj > 0).
compute flabel={flabel;highlbbt(jj,1)}.
end if.
end if.
compute lmat2=wzhigh(1:nump(1,i),j).
do if (csum(lmat2) > 0).
do if ((i < (nms+nys)) or (ydich <> 1)).

.
compute lmat2= lmat2.
do if ( 0 =0).
compute lmat2 = mdiag( lmat2 ).
compute lmat3=make(nrow(lmat2),1,0).
loop flp=1 to ncol(lmat2).
do if (csum(lmat2(:,flp))=1).
compute lmat3={lmat3,lmat2(:,flp)}.
end if.
end loop.
compute lmat2=lmat3(:,2:ncol(lmat3)).
end if.
compute fratio = (t(t(lmat2)* b )*inv(t(lmat2)* varb *lmat2)*((t(lmat2)* b )))/ncol(lmat2).
compute pfr = 1-fcdf(fratio,ncol(lmat2),(n-nrow( b ))).
compute fresult={fratio,ncol(lmat2),(n-nrow( b )),pfr}.
do if (i = (nms+nys) and (ydich=1)).
compute fratio=fratio*ncol(lmat2).
compute pfr=1-chicdf(fratio,ncol(lmat2)).
compute fresult={fratio,ncol(lmat2),pfr}.
end if.
do if ( 1 =1).
compute lmat3=1-rsum(lmat2).
compute xfm=make(n,csum(lmat3),0).
compute flpc=1.
loop flp=1 to nrow(lmat3).
do if (lmat3(flp,1)=1).
compute xfm(:,flpc)=x(:,flp).
compute flpc=flpc+1.
end if.
end loop.
compute bfm=inv(t(xfm)*xfm)*t(xfm)*y.
compute resid=y-(xfm*bfm).
compute sstotal=(y-(csum(y)/n)).
compute sstotal=csum(sstotal&*sstotal).
compute ssresid=csum(resid&*resid).
compute rsqch= r2 -((sstotal-ssresid)/sstotal).
compute fresult={rsqch,fresult}.
release xfm,flpc, resid, ssresid, bfm.
end if
.
end if.
do if ((ydich = 1) and (i = (nms+nys))).

.
compute btemphld=b.
compute llrdat=make(nrow(x),nrow( lmat2 )-csum( lmat2 ),-999).
compute llrdf=ncol(x)-ncol(llrdat).
compute llrcnt=0.
loop llri=1 to nrow( lmat2 ).
do if ( lmat2 (llri,1)=0).
compute llrcnt=llrcnt+1.
compute llrdat(:,llrcnt)=x(:,llri).
end if.
end loop.

.
do if ( 2 =1).
compute hatmat=inv(t( llrdat )* llrdat )*t( llrdat ).
compute b = hatmat* y.
compute modres=b.
do if ( 0 =1).
compute n1=nrow( llrdat ).
compute dfres=n1-(ncol( llrdat )).
compute sstotal = t( y -(csum( y )/n1))*( y -(csum( y )/n1)).
compute resid= y - llrdat *b.
compute ssresid = csum((resid)&**2).
compute r2 = (sstotal-ssresid)/sstotal.
do if (r2 < 0).
compute r2=0.
end if.
compute adjr2 = 1-((1-r2)*(n1-1)/(dfres)).
compute mse=ssresid/(n1-ncol( llrdat )).

.
compute n1=nrow( x ).
compute invXtX = inv(t( x )* x ).
compute varb = mse *invXtX.
compute k3 = ncol( x ).
compute xhc=0.
do if ( hc <> 5).
compute xhc= x.
compute hat = xhc(:,1).
loop i3=1 to nrow(xhc).
compute hat(i3,1)= xhc(i3,:)*invXtX*t(xhc(i3,:)).
end loop.
do if ( hc = 0 or hc =1).
loop i3 = 1 to k3.
compute xhc(:,i3)=xhc(:,i3)&* resid.
end loop.
end if.
do if ( hc =3 or hc =2).
loop i3=1 to k3.
compute xhc(:,i3) = ( resid &/(1-hat)&**(1/(4- hc )))&*xhc(:,i3).
end loop.
end if.
do if ( hc = 4).
compute hcmn=make(n,2,4).
compute hcmn(:,2)=(n1*hat)/k3.
loop i3= 1 to k3.
compute xhc(:,i3) = ( resid &/(1-hat)&**(rmin(hcmn)/2))&*xhc(:,i3).
end loop.
end if.
compute varb=(invXtX*t(xhc)*xhc*invXtX).
do if ( hc =1).
compute varb=(n1/(n1-ncol( x )))&*varb.
end if.
end if.
compute hclab={'se(HC0)','se(HC1)','se(HC2)','se(HC3)','se(HC4)','se'}.
compute hclab=hclab(1,( hc +1)).
compute hcflab={'F(HC0)','F(HC1)','F(HC2)','F(HC3)','F(HC4)','F'}.
compute hcflab=hcflab(1,( hc +1)).
release xhc
.
compute seb=sqrt(diag(varb)).
compute trat = b&/seb.
compute p = 2*(1-tcdf(abs(trat), (dfres))).
compute tval = sqrt(dfres* (exp((dfres-(5/6))*((xp2/(dfres-(2/3)+(.11/dfres)))* (xp2/(dfres-(2/3)+(.11/dfres)))))-1)).
compute modres={modres,seb,trat,p}.
compute modres={modres,(b-tval&*seb),(b+tval&*seb)}.
compute modresl={'coeff',hclab,'t','p','LLCI','ULCI'}.
compute lmat = ident(ncol( llrdat )).
compute lmat = lmat(:,2:ncol(lmat)).
compute fratio = (t(t(lmat)*b)*inv(t(lmat)*varb*lmat)*((t(lmat)*b)))/(ncol( llrdat )-1).
compute pfr = 1-fcdf(fratio,(ncol( llrdat )-1),dfres).
compute modsum={sqrt(r2),r2,mse,fratio,(ncol( llrdat )-1),dfres,pfr}.
compute modsuml={'R','R-sq','MSE',hcflab,'df1','df2', 'p'}.
end if.
end if.
do if ( 2 = 2 or 2 =3).
compute xlp= llrdat.
compute ylp= y.
compute pt2 = make(nrow(ylp),1,(csum(ylp)/nrow(ylp))).
do if ( 2 =2).
compute LL3 = ylp&*ln(pt2)+(1-ylp)&*ln(1-pt2).
end if.
compute LL3 = -2*csum(LL3).
compute bt1 = make(ncol(xlp),1,0).
compute LL1 = 0.
compute pt1 = make(nrow(ylp),1,0.5).
compute pt1lp=pt1.
loop jjj = 1 to iterate.
compute xlptmp=t(xlp).
compute vecprb=pt1lp&*(1-pt1lp).
loop kkk=1 to ncol(xlp).
compute xlptmp(kkk,:)=xlptmp(kkk,:)&*t(vecprb).
end loop.
compute b = bt1+inv(xlptmp*xlp)*t(xlp)*(ylp-pt1lp).
do if ( 2 =2).
compute xlpb=xlp*b.
compute xlpbt=(xlpb > -709.7).
compute xlpb709=(1-xlpbt)*(-709.7).
compute xlpb=(xlpb&*xlpbt)+xlpb709.
compute pt1lp = 1/(1+exp(-(xlpb))).
end if.
compute itprob = csum((pt1lp < .00000001) or (pt1lp > .9999999)).
do if (itprob > 0).
loop kkk = 1 to nrow(pt1lp).
do if (pt1lp(kkk,1) > .9999999).
compute pt1lp(kkk,1) = .9999999.
end if.
do if (pt1lp(kkk,1) < .00000001).
compute pt1lp(kkk,1) = .00000001.
end if.
end loop.
compute itprob = 0.
end if.
do if (itprob = 0).
do if ( 2 =2).
compute LL = ylp&*ln(pt1lp)+(1-ylp)&*ln(1-pt1lp).
end if.
compute LL2 = -2*csum(ll).
end if.
do if (abs(LL1-LL2) < converge).
do if ( 0 =1).
compute xlptmp=t(xlp).
compute vecprb=pt1lp&*(1-pt1lp).
loop kkk=1 to ncol(xlp).
compute xlptmp(kkk,:)=xlptmp(kkk,:)&*t(vecprb).
end loop.
compute varb = inv(xlptmp*xlp).
compute seb = sqrt(diag(varb)).
release xlptmp.
end if.
break.
end if.
compute bt1 = b.
compute LL1 = LL2.
end loop.
compute modres=b.
do if (jjj > iterate).
compute itprob = 2.
do if (booting=0).
compute iterrmod=1.
end if.
do if (booting=1).
compute bootiter=1.
end if.
do if (itprobtg=0).
compute itprobtg=1.
compute errcode(errs,1) = 47.
compute errs = errs + 1.
do if (booting = 0 and 0 =1).
compute vt1 = mdiag(pt1lp&*(1-pt1lp)).
compute varb = inv(t(xlp)*vt1*xlp).
compute seb = sqrt(diag(varb)).
end if.
end if.
end if.
do if ( 0 =1).
compute trat = b&/seb.
compute dfres=nrow(xlp).
compute p = 2*(1-cdfnorm(abs(trat))).
compute modres={modres,seb,trat,p}.
compute modres={modres,(b-xp2&*seb),(b+xp2&*seb)}.
compute pvchi=1-chicdf((LL3-LL2),(nrow(modres)-1)).
compute mcF = (LL3-LL2)/LL3.
compute cox = 1-exp(-(LL3-LL2)/nrow(xlp)).
compute nagel = cox/(1-exp(-(LL3)/nrow(xlp))).
compute modsum={LL2,(LL3-LL2),(nrow(modres)-1),pvchi, mcF,cox,nagel}.
compute modsuml={'-2LL','ModelLL', 'df', 'p', 'McFadden', 'CoxSnell', 'Nagelkrk'}.
compute modresl={'coeff','se','Z','p','LLCI','ULCI'}.
end if.
end if
.
compute b=btemphld.
compute fresult={(LL2-basemod),llrdf,1-chicdf((LL2-basemod),llrdf)}
.
end if.
compute highf={highf;fresult}.
compute highf2={highf2;fresult}.
do if (j = start).
compute flabel={flabel;'X*W*Z'}.
end if.
do if (j > start).
do if (nms > 1).
compute flabel={flabel;highlbwz(jj,1)}.
else if (nms = 1).
compute flabel={flabel;'M*W*Z'}.
end if.
end if.
end if.
compute jj=jj+1.
end loop.
release jj.
compute start=start+i.
end if.
do if (nrow(highf) > 1).
compute highf=highf(2:nrow(highf),:).
compute highf2=highf2(2:nrow(highf2),:).
compute flabel=flabel(2:nrow(flabel),1).
compute resultm2=make(nrow(highf),maxresm,99999).
compute resultm2(1:nrow(highf),1:ncol(highf))=highf.
compute resultm={resultm;resultm2}.
do if (outscree=1).
do if ((i < nms+nys) or (ydich=0)).
compute clabtmp={'R2-chng', hcflab,'df1','df2','p'}.
print highf/format = F10.4 /rnames=flabel/cnames=clabtmp/ title = 'Test(s) of highest order unconditional interaction(s):'.
end if.
do if (ydich=1 and i=(nms+nys)).
compute clabtmp={'Chi-sq', 'df','p'}.
print/title='Likelihood ratio test(s) of highest order'.
print highf/format = F10.4 /rnames=flabel/cnames=clabtmp/ title = 'unconditional interactions(s):'/space=0.
end if.
end if.
compute intpb=highf2(:,ncol(highf2)).
end if.
compute intstart=intstart+numint(1,i).
end if.

.
do if (criterr=0).
compute threeway=0.
compute didprint=0.
compute didsome=0.
compute sigintct=0.
loop jmed =1 to (nms+1).
compute hasw=0.
compute hasz=0.
compute jnok=0.
compute nm1vls=0.
compute nm2vls=0.
compute panelgrp=0.
compute graphixs={'WITH', outnames(1,i), 'BY'}.
compute focpred4={' '}.
compute intprint=0.
compute modcat=0.
do if (jmed <= i).
do if ((jmed = 1) and ((i+1) = nrow(bcmat))).
compute pathscnt=pathscnt+1.
else.
compute paths={paths,bcmat((i+1),jmed)}.
compute pathsw={pathsw,wcmat((i+1),jmed)}.
compute pathsz={pathsz,zcmat((i+1),jmed)}.
compute pathswz={pathswz,wzcmat((i+1),jmed)}.
compute temp=fochigh(:,pathscnt)&*bootloc(:,i).
compute pathsfoc={pathsfoc,pathsfoc(:,1)}.
do if (jmed=1).
compute pathtype={pathtype,1}.
end if.
do if ((i+1)=nrow(bcmat)).
compute pathtype={pathtype,3}.
end if.
do if (jmed > 1) and ((i+1) < nrow(bcmat)).
compute pathtype={pathtype,2}.
end if.
do if (jmed=1 and nxvls > 1 and (bcmat((i+1),jmed)=1)).
compute pathsfoc(:,(pathscn2+1))=temp(2:(nxvls+1),1).
end if.
do if ((jmed > 1) or (jmed=1 and nxvls=1)).
compute temp=cmax(temp).
compute pathsfoc(1,(pathscn2+1))=temp.
end if.
compute pathscnt=pathscnt+1.
compute pathscn2=pathscn2+1.
do if (i <= nms).
compute pathsdv={pathsdv,mnames(1,i)}.
end if.
do if (i > nms).
compute pathsdv={pathsdv,ynames}.
end if.
end if.
compute coeffcol=coeffcol+1.
compute probettt=coeffs(1:nrow(b),coeffcol).
do if (jmed=1 and (bcmat((i+1),jmed)=1)).
compute omni=make(nrow(probettt),nxvls,0).
compute omnitmp=ident(nxvls).
compute omni(2:(1+nxvls),:)=omnitmp.
end if.
do if (csum(probettt)>0).
compute probvarb=make(csum(probettt),csum(probettt),999).
compute probcoef=make(csum(probettt),1,999).
compute coefflp2=1.
loop coefflp=1 to nrow(probettt).
do if (probettt(coefflp,1)=1).
compute probcoef(coefflp2,1)=b(coefflp,1).
compute coefflp2=coefflp2+1.
end if.
end loop.
compute coefflp=0.
compute coefflp2=0.
loop iclp=1 to nrow(probettt).
do if probettt(iclp,1)=1.
compute coefflp=coefflp+1.
compute coefflp2=coefflp.
compute probvarb(coefflp,coefflp) = varb(iclp,iclp).
do if (iclp < nrow(probettt)).
loop jclp=(iclp+1) to nrow(probettt).
do if (probettt(jclp,1)=1).
compute coefflp2=coefflp2+1.
compute probvarb(coefflp,coefflp2)=varb(iclp, jclp).
compute probvarb(coefflp2,coefflp)=varb(iclp, jclp).
end if.
end loop.
end if.
end if.
end loop.
end if.
end if.
compute xprobval=xmodvals.
do if (nxvls > 1 or mcx > 0).
compute xprobval=dummatx(:,2:ncol(dummatx)).
end if.
do if ((wcmat((i+1),jmed)=1) and (zcmat((i+1),jmed)=0)).
compute numplps=1.
compute modvals=wmodvals.
compute probeval=wmodvals.
compute wheremv1=wherexw.
compute nm1vls=nwvls.
compute lpstsp={1,1}.
compute modcat=0.
compute jnmod=wtmp.
compute jnmodlab=wnames.
compute jnok=1.
compute jnmin=wmin.
compute jnmax=wmax.
compute wherejn1=2.
do if (jmed=1).
compute wherejn3=wherexw(1,i).
do if (nxvls > 1).
compute jnok=0.
end if.
end if.
do if (jmed > 1).
compute wherejn1=wherem((jmed-1),i).
compute wherejn3=wheremw(((2*jmed)-3),i).
end if.
do if (nwvls > 1).
compute probeval=wprobval.
compute lpstsp(1,2)=ncol(probeval).
compute modcat=1.
compute jnok=0.
end if.
do if (wdich = 1).
compute modcat=1.
compute jnok=0.
end if.
compute problabs=wnames.
compute focpred3={wnames,'(W)'}.
do if (xmint=1).
compute focpred3={wnames,'(X)'}.
end if.
compute hasw=1.
compute modgrph=wnames.
compute intprint=1.
compute sigintct=sigintct+1.
compute printpbe=intpb(sigintct,1).
end if.
do if ((wcmat((i+1),jmed)=0) and (zcmat((i+1),jmed)=1)).
compute numplps=1.
compute modvals=zmodvals.
compute probeval=zmodvals.
compute wheremv1=wherexz.
compute nm1vls=nzvls.
compute lpstsp={1,1}.
compute jnok=1.
compute jnmod=ztmp.
compute jnmin=zmin.
compute jnmax=zmax.
compute jnmodlab=znames.
compute wherejn1=2.
do if (jmed=1).
compute wherejn3=wherexz(1,i).
do if (nxvls > 1).
compute jnok=0.
end if.
end if.
do if (jmed > 1).
compute wherejn1=wherem((jmed-1),i).
compute wherejn3=wheremz(((2*jmed)-3),i).
end if.
do if (nzvls > 1).
compute probeval=zprobval.
compute lpstsp(1,2)=ncol(probeval).
compute modcat=1.
compute jnok=0.
end if.
do if (zdich = 1).
compute modcat=1.
compute jnok=0.
end if.
compute problabs=znames.
compute focpred3={znames,'(Z)'}.
compute modgrph=znames.
compute hasz=1.
compute intprint=1.
compute sigintct=sigintct+1.
compute printpbe=intpb(sigintct,1).
end if.
do if ((wzcmat((i+1),jmed)=1) or ((wcmat((i+1),jmed)=1) and (zcmat((i+1),jmed)=1))).
compute numplps=2.
compute probecnt=1.
compute intprint=1.
do if (wzcmat((i+1),jmed)=1).
compute sigintct=sigintct+1.
compute printpbe=intpb(sigintct,1).
else.
compute sigintct=sigintct+2.
compute printpbe=cmin(intpb((sigintct-1):sigintct,1)).
end if.
compute panelgrp=1.
compute hasw=1.
compute hasz=1.
compute panelcde={'/PANEL','ROWVAR=',znames,'.'}.
compute modgrph=wnames.
compute lpstsp={1,1;1,1}.
compute wheremv1=wherexw.
compute nm1vls=nwvls.
compute wheremv2=wherexz.
compute nm2vls=nzvls.
compute jnok=0.
do if (wzcmat((i+1),jmed)=1).
compute jnok=1.
end if.
do if (jmed > 1).
compute mprobval=mmodvals.
end if.
do if (jmed=1).
do if (nxvls > 1).
compute jnok=0.
end if.
end if.
do if (nwvls > 1).
compute lpstsp(1,2)=ncol(wprobval).
compute modcat=1.
compute jnok=0.
end if.
do if (zdich=1).
compute modcat=1.
compute jnok=0.
end if.
compute lpstsp(2,1)=lpstsp(1,2)+1.
compute lpstsp(2,2)=lpstsp(1,2)+1.
do if (nzvls > 1).
compute lpstsp(2,1)=lpstsp(1,2)+1.
compute lpstsp(2,2)=lpstsp(1,2)+ncol(zprobval).
compute jnok=0.
end if.
do if (zdich=1).
compute jnok=0.
end if.
compute omni3=make(nrow(b),(nxvls*nwvls),0).
do if (jmed > 1).
compute omni3=make(nrow(b),nwvls,0).
end if.
compute focpred3={wnames,'(W)'}.
do if (xmint=1).
compute focpred3={wnames,'(X)'}.
end if.
compute focpred4={'    ', 'Mod var:', znames, '(Z)'}.
compute modvals=make((nrow(wmodvals)*nrow(zmodvals)),2,0).
compute probeval=make((nrow(wmodvals)*nrow(zmodvals)),(ncol(wprobval)+ncol(zprobval)),0).
loop probei= 1 to nrow(wmodvals).
loop probej =1 to nrow(zmodvals).
compute modvals(probecnt,1)=wmodvals(probei,1).
compute probeval(probecnt,1:nwvls)=wprobval(probei,:).
compute modvals(probecnt,2)=zmodvals(probej,1).
compute probeval(probecnt,(nwvls+1):(nwvls+nzvls))=zprobval(probej,:).
compute probecnt=probecnt+1.
end loop.
end loop.
do if (wzcmat((i+1),jmed)=1).
compute numplps=numplps+1.
compute probprod=make(1,(ncol(wprobval)*ncol(zprobval)),0).
compute lpstsp2={1,1}.
compute lpstsp={lpstsp;lpstsp2}.
compute lpstsp(3,1)=lpstsp(2,2)+1.
compute lpstsp(3,2)=lpstsp(2,2)+ncol(probprod).
compute jnmod=ztmp.
compute jnmin=zmin.
compute jnmax=zmax.
compute jnmodlab=znames.
do if (jmed = 1).
compute wherejn1=wherexw(1,i).
compute wherejn3=wherexwz(1,i).
end if.
do if (jmed > 1).
compute wherejn1=wheremw(((2*jmed)-3),i).
compute wherejn3=wheremwz(((2*jmed)-3),i).
end if.
loop probei = 1 to nrow(wmodvals).
loop probej = 1 to nrow(zmodvals).
compute probtemp=1.
loop probek = 1 to ncol(wprobval).
compute probtemp={probtemp,(wprobval(probei,probek)&*zprobval(probej,:))}.
end loop.
compute probprod={probprod;probtemp(1,2:ncol(probtemp))}.
end loop.
end loop.
compute probprod=probprod(2:nrow(probprod),:).
compute probeval={probeval,probprod}.
end if.
compute problabs={wnames,znames}.
release probecnt, probei, probej.
end if.
do if (intprint=1).
compute focpred={'   Focal', 'predict:'}.
do if (jmed=1).
compute focpred={focpred,xnames,'(X)'}.
compute focplotv=xmodvals.
end if.
do if (jmed >1).
do if (nms > 1).
compute focpred={focpred,mnames(1,(jmed-1)), medlb2(1,(jmed-1))}.
end if.
do if (nms = 1).
compute focpred={focpred,mnames(1,(jmed-1)), '(M)'}.
end if.
compute focplotv=mmodvals(:,(jmed-1)).
end if.
compute focpred2={'    ', 'Mod var:',focpred3}.
compute focpred={focpred;focpred2}.
do if (ncol(focpred4) > 1).
compute focpred={focpred;focpred4}.
compute focpred4={' '}.
end if.
release focpred2,focpred3.
do if (outscree=1).
do if ((plot = 1 or plot = 2) or (printpbe <= intprobe)).
print focpred/title='----------'/format=A8/space=0.
end if.
end if.
compute foctmp=make(nrow(modvals),1,1).
compute probexpl=1.
compute probeva2={foctmp,probeval}.
do if (jmed=1 and nxs > 0 and mcx > 0).
compute probexpl=nxvls.
end if.
compute foctmp=make(nrow(modvals),1,1).
compute modvals3=make(1,(6+ncol(problabs)),0).
compute probrown=make(nrow(probeval),1,0).
compute jtmp=1.
loop probei = 1 to nrow(probeval).
compute probrown(probei,1)=jtmp.
compute jtmp=jtmp+nxvls.
end loop.
release jtmp.
compute probrow=999.
compute modvarl=problabs.
do if (plot = 1 or plot = 2 or nxvls > 1).
compute plotvals=make((nrow(modvals)*nrow(focplotv)),(ncol(modvals)+1),999).
loop ploti=1 to nrow(modvals).
loop plotj=1 to nrow(focplotv).
compute plotvals((((ploti-1)*nrow(focplotv))+plotj),2:ncol(plotvals))=modvals(ploti,:).
compute plotvals((((ploti-1)*nrow(focplotv))+plotj),1)=focplotv(plotj,1).
end loop.
end loop.
compute focpredn=3.
do if (jmed=1).
do if (nxvls > 1).
compute focpredn=(nxvls+1).
end if.
do if (nxvls=1 and xdich=1).
compute focpredn=2.
end if.
end if.
compute meanmat=mdiag(means).
compute onesmat=make(nrow(meanmat),(nrow(probeval)*focpredn),1).
compute probeplt=t(mdiag(means)*onesmat).
do if (jmed=1).
do if ((wcmat((i+1),1)=1) or (zcmat((i+1),1)=1)).
compute plotcnt=1.
compute iloops=nwpval*nzpval.
compute plotmx=nxpval*nzpval.
do if ((wcmat((i+1),1)=1) and (zcmat((i+1),1)=0)).
compute iloops=nwpval.
compute plotmx=nxpval.
end if.
do if ((wcmat((i+1),1)=0) and (zcmat((i+1),1)=1)).
compute iloops=nzpval.
compute plotmx=nxpval.
end if.
compute xestvals=make((nxpval*iloops),ncol(xprobval),-999).
do if (wcmat((i+1),1)=1).
compute westvals=make(nrow(xestvals),ncol(wprobval),-999).
end if.
do if (zcmat((i+1),1)=1).
compute zestvals=make(nrow(xestvals),ncol(zprobval),-999).
end if.
loop ploti=1 to iloops.
loop plotj=1 to nxpval.
compute xestvals(plotcnt,:)=xprobval(plotj,:).
compute plotcnt=plotcnt+1.
end loop.
end loop.
compute plotcnt=1.
compute plotcnt1=1.
compute plotcnt2=1.
compute plotcntz=1.
loop ploti = 1 to (iloops*nxpval).
do if (wcmat((i+1),1)=1).
compute westvals(ploti,:)=wprobval(plotcnt1,:).
end if.
do if ((wcmat((i+1),1)=0) and (zcmat((i+1),1)=1)).
compute zestvals(ploti,:)=zprobval(plotcnt1,:).
end if.
do if ((wcmat((i+1),1)=1) and (zcmat((i+1),1)=1)).
compute zestvals(ploti,:)=zprobval(plotcnt2,:).
compute plotcntz=plotcntz+1.
end if.
compute plotcnt=plotcnt+1.
do if (plotcnt > plotmx).
compute plotcnt=1.
compute plotcnt1=plotcnt1+1.
end if.
do if (plotcntz > nxpval).
compute plotcnt2=plotcnt2+1.
compute plotcntz=1.
do if (plotcnt2 > nzpval).
compute plotcnt2=1.
end if.
end if.
end loop.
compute probeplt(:,2:(1+(ncol(xestvals))))=xestvals.
do if (wcmat((i+1),1)=1).
compute probeplt(:,(wherew(1,i)):(wherew(2,i)))=westvals.
end if.
do if (zcmat((i+1),1)=1).
compute probeplt(:,(wherez(1,i)):(wherez(2,i)))=zestvals.
end if.
end if.
end if.
do if (jmed > 1).
do if ((wcmat((i+1),jmed)=1) or (zcmat((i+1),jmed)=1)).
compute plotcnt=1.
compute iloops=nwpval*nzpval.
compute plotmx=3*nzpval.
do if ((wcmat((i+1),jmed)=1) and (zcmat((i+1),jmed)=0)).
compute iloops=nwpval.
compute plotmx=3.
end if.
do if ((wcmat((i+1),jmed)=0) and (zcmat((i+1),jmed)=1)).
compute iloops=nzpval.
compute plotmx=3.
end if.
compute mestvals=make((3*iloops),1,-999).
do if (wcmat((i+1),jmed)=1).
compute westvals=make(nrow(mestvals),ncol(wprobval),-999).
end if.
do if (zcmat((i+1),jmed)=1).
compute zestvals=make(nrow(mestvals),ncol(zprobval),-999).
end if.
loop ploti=1 to iloops.
loop plotj=1 to 3.
compute mestvals(plotcnt,:)=mprobval(plotj,(jmed-1)).
compute plotcnt=plotcnt+1.
end loop.
end loop.
compute plotcnt=1.
compute plotcnt1=1.
compute plotcnt2=1.
compute plotcntz=1.
loop ploti = 1 to (iloops*3).
do if (wcmat((i+1),jmed)=1).
compute westvals(ploti,:)=wprobval(plotcnt1,:).
end if.
do if ((wcmat((i+1),jmed)=0) and (zcmat((i+1),jmed)=1)).
compute zestvals(ploti,:)=zprobval(plotcnt1,:).
end if.
do if ((wcmat((i+1),jmed)=1) and (zcmat((i+1),jmed)=1)).
compute zestvals(ploti,:)=zprobval(plotcnt2,:).
compute plotcntz=plotcntz+1.
end if.
compute plotcnt=plotcnt+1.
do if (plotcnt > plotmx).
compute plotcnt=1.
compute plotcnt1=plotcnt1+1.
end if.
do if (plotcntz > 3).
compute plotcnt2=plotcnt2+1.
compute plotcntz=1.
do if (plotcnt2 > nzpval).
compute plotcnt2=1.
end if.
end if.
end loop.
compute probeplt(:,wherem((jmed-1),i))=mestvals.
do if (wcmat((i+1),jmed)=1).
do if (model <> 74).
compute probeplt(:,(wherew(1,i)):(wherew(2,i)))=westvals.
end if.
do if (model = 74).
compute probeplt(:,(wherex(1,i)):(wherex(2,i)))=westvals.
end if.
end if.
do if (zcmat((i+1),jmed)=1).
compute probeplt(:,(wherez(1,i)):(wherez(2,i)))=zestvals.
end if.
end if.
end if.
compute prodloop = 1.
do if (jmed=1).
compute prodloop=ncol(xestvals).
end if.
do if (wcmat((i+1),jmed))=1.
compute plotcnt=0.
loop ploti = 1 to prodloop.
loop plotj = 1 to ncol(westvals).
do if (jmed=1).
compute probeplt(:,(wherexw(1,i)+plotcnt))=xestvals(:,ploti)&*westvals(:,plotj).
end if.
do if (jmed > 1).
compute probeplt(:,(wheremw(((jmed*2)-3) ,i)+plotcnt))=mestvals(:,ploti)&*westvals(:,plotj).
end if.
compute plotcnt=plotcnt+1.
end loop.
end loop.
end if.
do if (zcmat((i+1),jmed))=1.
compute plotcnt=0.
loop ploti = 1 to prodloop.
loop plotj = 1 to ncol(zestvals).
do if (jmed = 1).
compute probeplt(:,(wherexz(1,i)+plotcnt))=xestvals(:,ploti)&*zestvals(:,plotj).
end if.
do if (jmed > 1).
compute probeplt(:,(wheremz(((jmed*2)-3),i)+plotcnt))=mestvals(:,ploti)&*zestvals(:, plotj).
end if.
compute plotcnt=plotcnt+1.
end loop.
end loop.
end if.
do if (wzcmat((i+1),jmed))=1.
compute plotcnt=0.
compute threeway=1.
loop ploti = 1 to ncol(westvals).
loop plotj = 1 to ncol(zestvals).
compute probeplt(:,(wherewz(1,i)+plotcnt))=westvals(:,ploti)&*zestvals(:,plotj).
compute plotcnt=plotcnt+1.
end loop.
end loop.
compute plotcnt=0.
loop plotk = 1 to prodloop.
loop ploti = 1 to ncol(westvals).
loop plotj = 1 to ncol(zestvals).
do if (jmed = 1).
compute probeplt(:,(wherexwz(1,i)+plotcnt))=xestvals(:,plotk)&*westvals(:,ploti)&* zestvals(:,plotj).
end if.
do if (jmed > 1).
compute probeplt(:,(wheremwz(((jmed*2)-3),i)+plotcnt))=mestvals(:,plotk)&* westvals(:,ploti)&*zestvals(:,plotj).
end if.
compute plotcnt=plotcnt+1.
end loop.
end loop.
end loop.
end if.
loop newplp=1 to i.
do if (newplp <> jmed).
do if (wcmat((i+1),newplp))=1.
compute prodloop=1.
do if (newplp=1).
compute prodloop=nxvls.
end if.
compute plotcnt=0.
loop ploti = 1 to prodloop.
loop plotj = 1 to nwvls.
do if (newplp = 1).
compute probeplt(:,(wherexw(1,i)+plotcnt))=probeplt(:,(1+ploti))&*probeplt(:, (wherew(1,i)+plotj-1)).
end if.
do if (newplp > 1).
do if (model <> 74).
compute probeplt(:,(wheremw(((newplp*2)-3) ,i)+plotcnt))=probeplt(:,wherem((newplp-1),i))&*probeplt(:,(wherew(1,i)+plotj-1)).
end if.
do if (model = 74).
compute probeplt(:,(wheremw(((newplp*2)-3) ,i)+plotcnt))=probeplt(:,wherem((newplp-1),i))&*probeplt(:,(wherex(1,i)+plotj-1)).
end if.
end if.
compute plotcnt=plotcnt+1.
end loop.
end loop.
end if.
do if (zcmat((i+1),newplp))=1.
compute prodloop=1.
do if (newplp=1).
compute prodloop=nxvls.
end if.
compute plotcnt=0.
loop ploti = 1 to prodloop.
loop plotj = 1 to nzvls.
do if (newplp = 1).
compute probeplt(:,(wherexz(1,i)+plotcnt))=probeplt(:,(1+ploti))&*probeplt(:, (wherez(1,i)+plotj-1)).
end if.
do if (newplp > 1).
compute probeplt(:,(wheremz(((newplp*2)-3),i)+plotcnt))=probeplt(:, wherem((newplp-1),i))&*probeplt(:,(wherez(1,i)+plotj-1)).
end if.
compute plotcnt=plotcnt+1.
end loop.
end loop.
end if.
do if (wzcmat((i+1),newplp))=1.
compute plotcnt=0.
do if (threeway=0).
loop ploti = 1 to nwvls.
loop plotj = 1 to nzvls.
compute probeplt(:,(wherewz(1,i)+plotcnt))=probeplt(:,(wherew(1,i)+ploti-1))&* probeplt(:,(wherez(1,i)+plotj-1)).
compute plotcnt=plotcnt+1.
end loop.
end loop.
end if.
compute prodloop=1.
do if (newplp=1).
compute prodloop=nxvls.
end if.
compute plotcnt=0.
loop plotk = 1 to prodloop.
loop ploti = 1 to nwvls.
loop plotj = 1 to nzvls.
do if (newplp = 1).
compute probeplt(:,(wherexwz(1,i)+plotcnt))=probeplt(:,(1+plotk))&*probeplt(:, (wherew(1,i)+ploti-1))&*probeplt(:,(wherez(1,i)+plotj-1)).
end if.
do if (newplp > 1).
compute probeplt(:,(wheremwz(((newplp*2)-3),i)+plotcnt))=probeplt(:, wherem((newplp-1),i))&*probeplt(:,(wherew(1,i)+ploti-1))&*probeplt(:,(wherez(1,i)+plotj-1)).
end if.
compute plotcnt=plotcnt+1.
end loop.
end loop.
end loop.
end if.
end if.
end loop.
compute predvals=probeplt*b.
do if (debug <> 0).
print probeplt.
print b.
end if.
do if (i = nms+nys) and (ydich=1).
compute predvalt=(predvals < 709.7).
compute prevalt7=(1-predvalt)*(709.7).
compute predvals=(predvals&*predvalt)+prevalt7.
compute expyhat=exp(predvals)&/(1+exp(predvals)).
end if.
compute sepred=make(nrow(plotvals),3,999).
loop sei=1 to nrow(plotvals).
compute ask=probeplt(sei,:).
compute sepred(sei,1)=sqrt(ask*varb*t(ask)).
do if ((i < nms+nys) or (ydich=0)).
compute sepred(sei,2)=predvals(sei,1)-tval*sepred(sei,1).
compute sepred(sei,3)=predvals(sei,1)+tval*sepred(sei,1).
end if.
do if ((i = nms+nys) and (ydich=1)).
compute sepred(sei,2)=predvals(sei,1)-xp2*sepred(sei,1).
compute sepred(sei,3)=predvals(sei,1)+xp2*sepred(sei,1).
end if.
end loop.
compute prevloc=ncol(plotvals)+1.
compute probeplt={plotvals,predvals}.
do if (plot = 2).
compute probeplt={probeplt,sepred}.
end if.
do if ((i = nms+nys) and (ydich=1)).
compute probeplt={probeplt,expyhat}.
end if.
compute didsome=0.
end if.
do if ((wzcmat((i+1),jmed)=1) and (printpbe <= intprobe)).
do if (jmed=1).
compute omnilp2=nxvls*nwvls.
compute omnitmp=ident(omnilp2).
compute omni3(wherexw(1,i):wherexw(2,i),:)=omnitmp.
end if.
do if (jmed>1).
compute omnilp2=nwvls.
compute omnitmp=ident(omnilp2).
compute omni3(wheremw(((jmed*2)-3),i):wheremw(((jmed*2)-2),i),:)=omnitmp.
end if.
compute omnif=make(1,4,0).
do if ((i = nms+nys) and (ydich=1)).
compute omnif=make(1,3,0).
end if.
compute condeff3=0.
loop omnilp1=1 to nrow(zprobval).
loop omnilp=1 to (omnilp2).
do if (jmed=1).
compute omni3((wherexwz(1,i)+((omnilp-1)*nzvls)):(wherexwz(1,i)+((omnilp-1)*nzvls)+ (nzvls-1)),omnilp)=t(zprobval(omnilp1,:)).
end if.
do if (jmed > 1).
compute omni3((wheremwz(((jmed*2)-3),i)+((omnilp-1)*nzvls)):(wheremwz(((jmed*2)-3),i)+ ((omnilp-1)*nzvls)+(nzvls-1)),omnilp)=t(zprobval(omnilp1,:)).
end if.
end loop.
compute condeff=t(omni3)*b.
compute condeff3={condeff3;condeff}.

.
compute lmat2= omni3.
do if ( 1 =0).
compute lmat2 = mdiag( omni3 ).
compute lmat3=make(nrow(lmat2),1,0).
loop flp=1 to ncol(lmat2).
do if (csum(lmat2(:,flp))=1).
compute lmat3={lmat3,lmat2(:,flp)}.
end if.
end loop.
compute lmat2=lmat3(:,2:ncol(lmat3)).
end if.
compute fratio = (t(t(lmat2)* b )*inv(t(lmat2)* varb *lmat2)*((t(lmat2)* b )))/ncol(lmat2).
compute pfr = 1-fcdf(fratio,ncol(lmat2),(n-nrow( b ))).
compute fresult={fratio,ncol(lmat2),(n-nrow( b )),pfr}.
do if (i = (nms+nys) and (ydich=1)).
compute fratio=fratio*ncol(lmat2).
compute pfr=1-chicdf(fratio,ncol(lmat2)).
compute fresult={fratio,ncol(lmat2),pfr}.
end if.
do if ( 0 =1).
compute lmat3=1-rsum(lmat2).
compute xfm=make(n,csum(lmat3),0).
compute flpc=1.
loop flp=1 to nrow(lmat3).
do if (lmat3(flp,1)=1).
compute xfm(:,flpc)=x(:,flp).
compute flpc=flpc+1.
end if.
end loop.
compute bfm=inv(t(xfm)*xfm)*t(xfm)*y.
compute resid=y-(xfm*bfm).
compute sstotal=(y-(csum(y)/n)).
compute sstotal=csum(sstotal&*sstotal).
compute ssresid=csum(resid&*resid).
compute rsqch= 0 -((sstotal-ssresid)/sstotal).
compute fresult={rsqch,fresult}.
release xfm,flpc, resid, ssresid, bfm.
end if
.
compute omnif={omnif;fresult}.
end loop.
compute omnif=omnif(2:nrow(omnif),:).
compute clabtmp=znames.
compute condeff3=condeff3(2:nrow(condeff3),:).
do if ((nxvls*nwvls)=1).
compute omnif={condeff3,omnif}.
compute clabtmp={clabtmp,'Effect'}.
end if.
compute omnif={zmodvals,omnif}.
do if ((i < nms+nys) or (ydich=0)).
compute clabtmp={clabtmp,hcflab,'df1','df2','p'}.
end if.
do if ((i = (nms+nys)) and (ydich=1)).
compute clabtmp={clabtmp,'Chi-sq','df','p'}.
end if.
compute resultm2=make(nrow(omnif),maxresm,99999).
compute resultm2(1:nrow(omnif),1:ncol(omnif))=omnif.
compute resultm={resultm;resultm2}.
do if (outscree=1).
do if (jmed=1).
print omnif/title='Test of conditional X*W interaction at value(s) of Z:'/cnames=clabtmp/format= F10.4.
end if.
do if (jmed>1).
print omnif/title='Test of conditional M*W interaction at value(s) of Z:'/cnames=clabtmp/format= F10.4.
end if.
end if.
release omni3.
end if.
loop probei = 1 to probexpl.
do if (probexpl > 1).
compute foctmp=make(nrow(modvals),probexpl,0).
compute foctmp(:,probei)=foctmp(:,probei)+1.
compute probtemp=make(nrow(modvals),1,0).
loop probem = 1 to numplps.
loop probek = 1 to nxvls.
loop probej=lpstsp(probem,1) to lpstsp(probem,2).
compute probtemp={probtemp,foctmp(:,probek)&*probeval(:,probej)}.
end loop.
end loop.
end loop.
compute probeva2=probtemp(:,2:ncol(probtemp)).
compute probeva2={foctmp,probeva2}.
end if.
compute probres=probeva2*probcoef.
compute probrese=sqrt(diag(probeva2*probvarb*t(probeva2))).
compute tratio = probres&/probrese.
compute p = 2*(1-tcdf(abs(tratio), dfres)).
do if (ydich=1 and i = (nms+nys)).
compute p = 2*(1-cdfnorm(abs(tratio))).
end if.
compute modvals2={modvals,probres,probrese,tratio, p}.
do if ((i < nms+nys) or (ydich=0)).
compute modvals2={modvals2,(probres-tval&*probrese),(probres+tval&*probrese)}.
compute problabs={problabs,'Effect',hclab,'t', 'p', 'LLCI', 'ULCI'}.
end if.
do if (ydich=1 and i = (nms+nys)).
compute modvals2={modvals2,(probres-xp2&*probrese),(probres+xp2&*probrese)}.
compute problabs={problabs,'Effect','se','Z', 'p', 'LLCI', 'ULCI'}.
end if.
do if (probexpl > 1 and (printpbe <= intprobe)).
do if (hasz = 1).
compute printz=1.
end if.
do if (hasw=1).
compute printw=1.
end if.
compute probrlab=make(nrow(modvals),1,xcatlab(probei,1)).
compute modvals3={modvals3; modvals2}.
compute probrow={probrow;probrown}.
compute probrown=probrown+1.
do if (probei=probexpl).
compute xproblab=xcatlab(1:nxvls,1).
compute probrow=probrow(2:nrow(probrow),1).
compute modvals3=modvals3(2:nrow(modvals3),:).
compute temp=modvals3.
compute temp(GRADE(probrow(:,1)),:)=modvals3.
compute modvals3=temp.
compute start2=1.
compute problabs=problabs(1,(1+(ncol(modvarl))):ncol(problabs)).
compute pstart=1.
loop probek= 1 to nrow(probeval).
compute endstart=start2+(nxvls-1).
compute temp=modvals3(start2:endstart,(1+ncol(modvarl)):ncol(modvals3)).
compute temp2=t(modvals3(start2:start2,1:ncol(modvarl))).
compute trnames=t(modvarl).
do if (outscree=1).
do if (probek > 1).
print/title='----------'/space=0.
else.
print/title = 'Conditional effects of the focal predictor at values of the moderator(s):'.
do if ((jmed=1) and (i = (nms+nys)) and (nms > 0)).
do if (nxvls = 1).
print/title = '(These are also the conditional direct effects of X on Y)'/space=0.
else.
print/title = '(These are also the relative conditional direct effects of X on Y)'/space=0.
end if.
end if.
print.
end if.
print temp2/title = 'Moderator value(s):'/rnames=trnames/format= F10.4 /space=0.
print temp/title = ' '/cnames=problabs/rnames=xproblab/format= F10.4 /space=0.
end if.
compute resultm2=make(nrow(temp2),maxresm,99999).
compute resultm2(1:nrow(temp2),1:ncol(temp2))=temp2.
compute resultm={resultm;resultm2}.
compute resultm2=make(nrow(temp),maxresm,99999).
compute resultm2(1:nrow(temp),1:ncol(temp))=temp.
compute resultm={resultm;resultm2}.
compute start2=start2+nxvls.
compute didsome=1.
do if (jmed=1).
compute mod1val=probeval(probek,1:nm1vls).
loop omnilp=1 to nxvls.
compute omni((wheremv1(1,i)+((omnilp-1)*nm1vls)):(wheremv1(1,i)+((omnilp-1)* nm1vls)+(nm1vls-1)),omnilp)=t(mod1val).
do if (nm1vls < ncol(probeval)).
compute mod2val=probeval(probek,(nm1vls+1):(nm1vls+nm2vls)).
compute omni((wheremv2(1,i)+((omnilp-1)*nm2vls)):(wheremv2(1,i)+((omnilp-1)* nm2vls)+(nm2vls-1)),omnilp)=t(mod2val).
do if ((nm1vls+nm2vls) < ncol(probeval)).
compute intlen=nm1vls*nm2vls.
compute modintvl=probeval(probek,(nm1vls+nm2vls+1):ncol(probeval)).
compute omni((wherexwz(1,i)+((omnilp-1)*intlen)):(wherexwz(1,i)+((omnilp-1)* intlen)+(intlen-1)),omnilp)=t(modintvl).
end if.
end if.
end loop.

.
compute lmat2= omni.
do if ( 1 =0).
compute lmat2 = mdiag( omni ).
compute lmat3=make(nrow(lmat2),1,0).
loop flp=1 to ncol(lmat2).
do if (csum(lmat2(:,flp))=1).
compute lmat3={lmat3,lmat2(:,flp)}.
end if.
end loop.
compute lmat2=lmat3(:,2:ncol(lmat3)).
end if.
compute fratio = (t(t(lmat2)* b )*inv(t(lmat2)* varb *lmat2)*((t(lmat2)* b )))/ncol(lmat2).
compute pfr = 1-fcdf(fratio,ncol(lmat2),(n-nrow( b ))).
compute fresult={fratio,ncol(lmat2),(n-nrow( b )),pfr}.
do if (i = (nms+nys) and (ydich=1)).
compute fratio=fratio*ncol(lmat2).
compute pfr=1-chicdf(fratio,ncol(lmat2)).
compute fresult={fratio,ncol(lmat2),pfr}.
end if.
do if ( 0 =1).
compute lmat3=1-rsum(lmat2).
compute xfm=make(n,csum(lmat3),0).
compute flpc=1.
loop flp=1 to nrow(lmat3).
do if (lmat3(flp,1)=1).
compute xfm(:,flpc)=x(:,flp).
compute flpc=flpc+1.
end if.
end loop.
compute bfm=inv(t(xfm)*xfm)*t(xfm)*y.
compute resid=y-(xfm*bfm).
compute sstotal=(y-(csum(y)/n)).
compute sstotal=csum(sstotal&*sstotal).
compute ssresid=csum(resid&*resid).
compute rsqch= 0 -((sstotal-ssresid)/sstotal).
compute fresult={rsqch,fresult}.
release xfm,flpc, resid, ssresid, bfm.
end if
.
compute resultm2=make(nrow(fresult),maxresm,99999).
compute resultm2(1:nrow(fresult),1:ncol(fresult))=fresult.
compute resultm={resultm;resultm2}.
do if (outscree=1).
do if (i < (nms + nys) or (ydich = 0)).
compute clabtmp={hcflab,'df1','df2','p'}.
print fresult/title='Test of equality of conditional means'/cnames=clabtmp/format= F10.4.
end if.
do if (i = (nms + nys) and (ydich = 1)).
compute clabtmp={'Chi-sq','df','p'}.
print fresult/title='Test of equality of conditional logits or probabilities'/cnames=clabtmp/format= F10.4.
end if.
end if.
compute probetmp=probeplt(pstart:(pstart+nxvls),1).
compute probetmp={probetmp,probeplt(pstart:(pstart+nxvls),prevloc:ncol(probeplt))}.
compute pstart=pstart+(nxvls+1).
compute resultm2=make(nrow(probetmp),maxresm,99999).
compute resultm2(1:nrow(probetmp),1:ncol(probetmp))=probetmp.
compute resultm={resultm;resultm2}.
do if (i < ((nms + nys)) or (ydich = 0)).
compute clabtmp={xnames, outnames(1,i), hclab, 'LLCI', 'ULCI'}.
do if (outscree=1).
print probetmp/title = 'Estimated conditional means being compared:'/cnames=clabtmp/format= F10.4.
end if.
end if.
do if ((i = (nms+nys)) and (ydich = 1)).
compute clabtmp={xnames, outnames(1,i), 'prob'}.
compute probetm2=probetmp(:,1:2).
compute probetm2={probetm2,probetmp(:,ncol(probetmp))}.
do if (outscree=1).
print probetm2/title = 'Estimated conditional logits and probabilities:'/cnames=clabtmp/format= F10.4.
end if.
end if.
end if.
end loop.
release probrow, start2, endstart, temp, temp2.
end if.
end if.
do if (probexpl = 1 and (printpbe <= intprobe)).
compute resultm2=make(nrow(modvals2),maxresm,99999).
compute resultm2(1:nrow(modvals2),1:ncol(modvals2))=modvals2.
compute resultm={resultm;resultm2}.
do if (outscree=1).
print/title = 'Conditional effects of the focal predictor at values of the moderator(s):'.
print modvals2/cnames=problabs/title = ' '/space=0/format= F10.4.
end if.
compute didsome=1.
do if (hasz = 1).
compute printz=1.
end if.
do if (hasw=1).
compute printw=1.
end if.
do if (jn = 1 and jnok=1).
do if (criterr = 0).
compute dfres=n-nrow(b).
compute roots=99999.
compute jncrit =(dfres* (exp((dfres-(5/6))*((xp2/(dfres-(2/3)+(.11/dfres)))* (xp2/(dfres-(2/3)+(.11/dfres)))))-1)).
do if (i = (nms+nys) and (ydich=1)).
compute jncrit=xp2*xp2.
end if.
compute jnb1=b(wherejn1,1).
compute jnb3=b(wherejn3,1).
compute jnsb1=varb(wherejn1,wherejn1).
compute jnsb3=varb(wherejn3,wherejn3).
compute jnsb1b3=varb(wherejn1,wherejn3).
compute ajn =(jncrit*jnsb3)-(jnb3*jnb3).
compute bjn = 2*((jncrit*jnsb1b3)-(jnb1*jnb3)).
compute cjn = (jncrit*jnsb1)-(jnb1*jnb1).
compute radarg = (bjn*bjn)-(4*ajn*cjn).
compute den = 2*ajn.
compute nrts = 0.
do if (radarg >= 0 and den <> 0).
compute x21 = (-bjn+sqrt(radarg))/den.
compute x22 = (-bjn-sqrt(radarg))/den.
compute roots = 0.
do if (x21 >= jnmin and x21 <= jnmax).
compute nrts = 1.
compute roots = {roots; x21}.
end if.
do if (x22 >= jnmin and x22 <= jnmax).
compute nrts = nrts + 1.
compute roots = {roots; x22}.
end if.
compute roots={roots,make(nrow(roots),2,0)}.
end if.
do if (nrts > 0).
compute roots = roots(2:nrow(roots),1:3).
compute roots(1,2)=(csum(jnmod < roots(1,1))/n)*100.
compute roots(1,3)=(csum(jnmod > roots(1,1))/n)*100.
do if (nrow(roots)=2).
compute roots(2,2)=(csum(jnmod < roots(2,1))/n)*100.
compute roots(2,3)=(csum(jnmod > roots(2,1))/n)*100.
end if.
do if (outscree=1).
print roots/title = 'Moderator value(s) defining Johnson-Neyman significance region(s):'/clabels = 'Value', '% below', '% above'/format F10.4.
end if.
do if (nrts=1).
compute tmprts=make(1,3,99999).
compute roots={roots;tmprts}.
end if.
end if.
do if (nrts = 0).
compute roots=make(2,1,99999).
do if (outscree=1).
print/title = 'There are no statistical significance transition points within the observed'.
print/title = 'range of the moderator found using the Johnson-Neyman method.'/space=0.
end if.
end if.
compute resultm2=make(nrow(roots),maxresm,99999).
compute resultm2(1:nrow(roots),1:ncol(roots))=roots.
compute resultm={resultm;resultm2}.
compute jnvals=make(23,7,0).
loop jni= 0 to (21-(nrts)).
compute jnvals((jni+1),1)=jnmin+(jni*((jnmax-jnmin)/(21-nrts))).
end loop.
do if (nrts > 0).
loop jni = 1 to nrts.
loop jnj = 2 to (nrow(jnvals)-1).
do if ((roots(jni,1) > jnvals((jnj-1),1)) and (roots(jni,1) < jnvals(jnj,1))).
compute jnvals((jnj+1):(21+jni),1)=jnvals(jnj:(20+jni),1).
compute jnvals(jnj,1)=roots(jni,1).
end if.
end loop.
end loop.
end if.
compute jnvals=jnvals(1:22,:).
loop jni = 1 to nrow(jnvals).
compute jnvals(jni,2)=jnb1+jnb3*jnvals(jni,1).
compute jnvals(jni,3)=sqrt(jnsb1+2*jnvals(jni,1)*jnsb1b3+(jnvals(jni,1)*jnvals(jni, 1))*jnsb3).
compute jnvals(jni,4)=jnvals(jni,2)/jnvals(jni,3).
compute jnvals(jni,5)=2*(1-tcdf(abs(jnvals(jni,4)), dfres)).
compute jnvals(jni,6)=jnvals(jni,2)-sqrt(jncrit)*jnvals(jni,3).
compute jnvals(jni,7)=jnvals(jni,2)+sqrt(jncrit)*jnvals(jni,3).
do if ((i = nms + nys) and (ydich=1)).
compute jnvals(jni,5)=2*(1-cdfnorm(abs(jnvals(jni,4)))).
compute jnvals(jni,6)=jnvals(jni,2)-xp2*jnvals(jni,3).
compute jnvals(jni,7)=jnvals(jni,2)+xp2*jnvals(jni,3).
end if.
end loop.
compute resultm2=make(nrow(jnvals),maxresm,99999).
compute resultm2(1:nrow(jnvals),1:ncol(jnvals))=jnvals.
compute resultm={resultm;resultm2}.
do if ((i < nms+nys) or (ydich=0)).
compute jnclbs={jnmodlab,'Effect',hclab,'t', 'p', 'LLCI', 'ULCI'}.
end if.
do if ((i = nms + nys) and (ydich=1)).
compute jnclbs={jnmodlab,'Effect','se','Z', 'p', 'LLCI', 'ULCI'}.
end if.
do if (((wcmat((i+1),jmed)=1) or (zcmat((i+1),jmed)=1)) and (wzcmat((i+1),jmed)=0)).
do if (outscree=1).
print jnvals/title = 'Conditional effect of focal predictor at values of the moderator:'/cnames =jnclbs/format = F10.4.
end if.
end if.
do if (outscree=1).
do if ((jmed = 1) and (wzcmat((i+1),jmed)=1)).
print jnvals/title = 'Conditional X*W interaction at values of the moderator Z:'/cnames =jnclbs/format = F10.4.
end if.
do if ((jmed > 1) and (wzcmat((i+1),jmed)=1)).
print jnvals/title = 'Conditional M*W interaction at values of the moderator Z:'/cnames =jnclbs/format = F10.4.
end if.
end if.
end if.
end if.
end if.
do if ((i = (nms+nys)) and (jmed=1) and (bcmat(nrow(bcmat),1)=1)).
do if (probei=1).
compute direfflb=problabs.
compute direff=modvals2.
end if.
do if (probei>1).
compute direff={direff;modvals2}.
end if.
end if.
compute intprint=0.
do if ((jmed=1) and (i=1) and nms=0) and modcok=1).
compute contvec2=make(2,1,1).
compute contvec2={contvec2,wcontval,zcontval}.
do if (wzcmat((i+1),jmed)=1).
loop conti= 1 to ncol(wcontval).
loop contj = 1 to ncol(zcontval).
compute contvec2={contvec2,wcontval(:,conti)&*zcontval(:,contj)}.
end loop.
end loop.
end if.
compute conteff=contvec2*probcoef.
compute contdiff=contvec2(1,:)-contvec2(2,:).
compute contse=sqrt(contdiff*probvarb*t(contdiff)).
compute conteffd=conteff(1,1)-conteff(2,1).
compute contvec={contvec,conteff}.
compute contvecm=contvec.
compute resultm2=make(nrow(contvecm),maxresm,99999).
compute resultm2(1:nrow(contvecm),1:ncol(contvecm))=contvecm.
compute resultm={resultm;resultm2}.
do if (outscree=1).
print/title='Contrast between conditional effects of X:'.
print contvec/title=' '/rlabels='Effect1:','Effect2:'/cnames=problabs/format = F10.4 /space=0.
end if.
do if (ydich=0).
compute p=2*(1-tcdf(abs(conteffd/contse), dfres)).
compute contvec={conteffd,contse,conteffd/contse, p}.
compute contvec={contvec,(conteffd-(tval*contse))}.
compute contvec={contvec,(conteffd+(tval*contse))}.
compute contlabs={'Contrast', hclab, 't', 'p', 'LLCI', 'ULCI'}.
end if.
do if (ydich=1).
compute p=2*(1-cdfnorm(abs(conteffd/contse))).
compute contvec={conteffd,contse,conteffd/contse, p}.
compute contvec={contvec,(conteffd-(xp2*contse))}.
compute contvec={contvec,(conteffd+(xp2*contse))}.
compute contlabs={'Contrast', 'se', 'Z', 'p', 'LLCI', 'ULCI'}.
end if.
compute resultm2=make(nrow(contvec),maxresm,99999).
compute resultm2(1:nrow(contvec),1:ncol(contvec))=contvec.
compute resultm={resultm;resultm2}.
do if (outscree=1).
print contvec/title='Test of Effect1 minus Effect2'/format= F10.4 /cnames=contlabs.
end if.
end if.
end loop.
do if (plot = 1 or plot = 2).
compute datalabs={t(focpred(:,3)),outnames(1,i)}.
do if (plot = 1).
compute datalabs={datalabs}.
end if.
do if (plot = 2).
compute datalabs={datalabs,'se', 'LLCI', 'ULCI'}.
end if.
do if ((i = nms+nys) and (ydich=1)).
compute datalabs={datalabs,'prob'}.
end if.
compute resultm2=make(nrow(probeplt),maxresm,99999).
compute resultm2(1:nrow(probeplt),1:ncol(probeplt))=probeplt.
compute resultm={resultm;resultm2}.
compute datalabs={datalabs,'.'}.
do if (outscree=1).
print/title = 'Data for visualizing the conditional effect of the focal predictor:'.
print/title = 'Paste text below into a SPSS syntax window and execute to produce plot.'/space=0.
compute dumb = {' ', ' ', ' ', ' ', ' ', ' ', ' '}.
print datalabs/title = 'DATA LIST FREE/'/format=A10.
print probeplt/title = 'BEGIN DATA.'/format= F10.4 /space=0.
print/title = 'END DATA.'/space=0.
end if.
compute focgrph=datalabs(1,1).
compute graphix={focgrph,graphixs,modgrph}.
do if (((xdich=1) or (nxvls > 1)) and ((modcat=0) and (focgrph = xnames))).
compute graphix={modgrph,graphixs,focgrph}.
end if.
do if (panelgrp = 0).
compute graphix={graphix,'.'}.
else.
compute graphix={graphix,panelcde}.
end if.
do if (outscree=1).
print graphix/title = 'GRAPH/SCATTERPLOT='/format=A8/space=0.
end if.
do if (i = (nms + nys) and ydich = 1).
compute graphixd=graphix.
compute graphixd(1,3)='prob'.
do if (outscree=1).
print graphixd/title = 'GRAPH/SCATTERPLOT='/format=A8/space=0.
end if.
end if.
end if.
end if.
end loop.
release jmed, intprint,didprint.
end if
.
do if (model = 74 and i <= nms).
compute onetemp=make(nrow(xprobval),1,1).
compute mestmt74={onetemp,xprobval}.
do if (ncs > 0).
compute ncovmdl=rsum(ccmat(i,:)).
do if (ncovmdl > 0).
compute cvmnc=make(nrow(mestmt74),ncovmdl,1).
compute cvmnctmp=csum(x(:,(ncol(x)-ncovmdl+1):ncol(x)))/nrow(x).
do if (cuscoval > 0).
compute cvmnctmp=coval.
end if.
loop mestlp=1 to ncovmdl.
compute cvmnc(:,mestlp)=cvmnc(:,mestlp)*cvmnctmp(:,mestlp).
end loop.
compute mestmt74={mestmt74,cvmnc}.
end if.
end if.
compute mest74t=mestmt74*b.
do if (i = 1).
compute mest74=mest74t.
end if.
do if (i > 1).
compute mest74={mest74,mest74t}.
end if.
end if.
do if ((i = (nms+nys)) and (model >= 0 and model < 4) and (linsum(1,1) <> -999)).
compute lhyprob=1.
compute meansub=0.
do if (((nlinsum=nrow(b)) or (nlinsum = (nrow(b)-ncs)))).
do if (nlinsum = (nrow(b)-ncs) and ncs > 0).
compute linsum={linsum,covmeans}.
compute meansub=1.
end if.
compute lhyprob=0.
compute hypest=linsum*b.
compute sehypest=sqrt(linsum*varb*t(linsum)).
compute phypest=2*(1-tcdf(abs(hypest/sehypest), (dfres))).
compute hypest={hypest,sehypest,(hypest/sehypest),phypest,(hypest-tval*sehypest),(hypest+ tval*sehypest)}.
compute resultm2=make(ncol(linsum),maxresm,99999).
compute resultm2(:,1)=t(linsum).
compute resultm={resultm;resultm2}.
compute resultm2=make(1,maxresm,99999).
compute resultm2(1,1:ncol(hypest))=hypest.
compute resultm={resultm;resultm2}.
do if (outscree=1).
compute hyplabs={'Estimate',hclab,'t','p','LLCI','ULCI'}.
print/title='----------'.
print/title='Linear Combination Estimate and Hypothesis Test'/space=0.
print t(linsum)/title= 'Weight vector:'/clabels='weight'/rnames=vlabsm/format F10.4.
print hypest/title=' '/cnames=hyplabs/format= F10.4 /space=0.
do if (meansub=1).
print/title='Covariate weight(s) set to the sample mean.'.
end if.
end if.
end if.
do if (lhyprob=1).
compute notecode(notes,1) = 30.
compute notes = notes + 1.
end if.
end if.
end loop if criterr=1.
compute lastb=b.
compute lastcov=varb.
do if (criterr=0 and dototal = 1).
compute x=xtmp.
compute vlabsm={'constant';xcatlab(1:nxvls,1)}.
do if (ncs > 0).
compute x = {x,ctmp}.
compute vlabsm={vlabsm;t(covnames)}.
end if.
compute x = {ones,x}.

.
do if ( 1 =1).
compute hatmat=inv(t( x )* x )*t( x ).
compute b = hatmat* y.
compute modres=b.
do if ( 1 =1).
compute n1=nrow( x ).
compute dfres=n1-(ncol( x )).
compute sstotal = t( y -(csum( y )/n1))*( y -(csum( y )/n1)).
compute resid= y - x *b.
compute ssresid = csum((resid)&**2).
compute r2 = (sstotal-ssresid)/sstotal.
do if (r2 < 0).
compute r2=0.
end if.
compute adjr2 = 1-((1-r2)*(n1-1)/(dfres)).
compute mse=ssresid/(n1-ncol( x )).

.
compute n1=nrow( x ).
compute invXtX = inv(t( x )* x ).
compute varb = mse *invXtX.
compute k3 = ncol( x ).
compute xhc=0.
do if ( hc <> 5).
compute xhc= x.
compute hat = xhc(:,1).
loop i3=1 to nrow(xhc).
compute hat(i3,1)= xhc(i3,:)*invXtX*t(xhc(i3,:)).
end loop.
do if ( hc = 0 or hc =1).
loop i3 = 1 to k3.
compute xhc(:,i3)=xhc(:,i3)&* resid.
end loop.
end if.
do if ( hc =3 or hc =2).
loop i3=1 to k3.
compute xhc(:,i3) = ( resid &/(1-hat)&**(1/(4- hc )))&*xhc(:,i3).
end loop.
end if.
do if ( hc = 4).
compute hcmn=make(n,2,4).
compute hcmn(:,2)=(n1*hat)/k3.
loop i3= 1 to k3.
compute xhc(:,i3) = ( resid &/(1-hat)&**(rmin(hcmn)/2))&*xhc(:,i3).
end loop.
end if.
compute varb=(invXtX*t(xhc)*xhc*invXtX).
do if ( hc =1).
compute varb=(n1/(n1-ncol( x )))&*varb.
end if.
end if.
compute hclab={'se(HC0)','se(HC1)','se(HC2)','se(HC3)','se(HC4)','se'}.
compute hclab=hclab(1,( hc +1)).
compute hcflab={'F(HC0)','F(HC1)','F(HC2)','F(HC3)','F(HC4)','F'}.
compute hcflab=hcflab(1,( hc +1)).
release xhc
.
compute seb=sqrt(diag(varb)).
compute trat = b&/seb.
compute p = 2*(1-tcdf(abs(trat), (dfres))).
compute tval = sqrt(dfres* (exp((dfres-(5/6))*((xp2/(dfres-(2/3)+(.11/dfres)))* (xp2/(dfres-(2/3)+(.11/dfres)))))-1)).
compute modres={modres,seb,trat,p}.
compute modres={modres,(b-tval&*seb),(b+tval&*seb)}.
compute modresl={'coeff',hclab,'t','p','LLCI','ULCI'}.
compute lmat = ident(ncol( x )).
compute lmat = lmat(:,2:ncol(lmat)).
compute fratio = (t(t(lmat)*b)*inv(t(lmat)*varb*lmat)*((t(lmat)*b)))/(ncol( x )-1).
compute pfr = 1-fcdf(fratio,(ncol( x )-1),dfres).
compute modsum={sqrt(r2),r2,mse,fratio,(ncol( x )-1),dfres,pfr}.
compute modsuml={'R','R-sq','MSE',hcflab,'df1','df2', 'p'}.
end if.
end if.
do if ( 1 = 2 or 1 =3).
compute xlp= x.
compute ylp= y.
compute pt2 = make(nrow(ylp),1,(csum(ylp)/nrow(ylp))).
do if ( 1 =2).
compute LL3 = ylp&*ln(pt2)+(1-ylp)&*ln(1-pt2).
end if.
compute LL3 = -2*csum(LL3).
compute bt1 = make(ncol(xlp),1,0).
compute LL1 = 0.
compute pt1 = make(nrow(ylp),1,0.5).
compute pt1lp=pt1.
loop jjj = 1 to iterate.
compute xlptmp=t(xlp).
compute vecprb=pt1lp&*(1-pt1lp).
loop kkk=1 to ncol(xlp).
compute xlptmp(kkk,:)=xlptmp(kkk,:)&*t(vecprb).
end loop.
compute b = bt1+inv(xlptmp*xlp)*t(xlp)*(ylp-pt1lp).
do if ( 1 =2).
compute xlpb=xlp*b.
compute xlpbt=(xlpb > -709.7).
compute xlpb709=(1-xlpbt)*(-709.7).
compute xlpb=(xlpb&*xlpbt)+xlpb709.
compute pt1lp = 1/(1+exp(-(xlpb))).
end if.
compute itprob = csum((pt1lp < .00000001) or (pt1lp > .9999999)).
do if (itprob > 0).
loop kkk = 1 to nrow(pt1lp).
do if (pt1lp(kkk,1) > .9999999).
compute pt1lp(kkk,1) = .9999999.
end if.
do if (pt1lp(kkk,1) < .00000001).
compute pt1lp(kkk,1) = .00000001.
end if.
end loop.
compute itprob = 0.
end if.
do if (itprob = 0).
do if ( 1 =2).
compute LL = ylp&*ln(pt1lp)+(1-ylp)&*ln(1-pt1lp).
end if.
compute LL2 = -2*csum(ll).
end if.
do if (abs(LL1-LL2) < converge).
do if ( 1 =1).
compute xlptmp=t(xlp).
compute vecprb=pt1lp&*(1-pt1lp).
loop kkk=1 to ncol(xlp).
compute xlptmp(kkk,:)=xlptmp(kkk,:)&*t(vecprb).
end loop.
compute varb = inv(xlptmp*xlp).
compute seb = sqrt(diag(varb)).
release xlptmp.
end if.
break.
end if.
compute bt1 = b.
compute LL1 = LL2.
end loop.
compute modres=b.
do if (jjj > iterate).
compute itprob = 2.
do if (booting=0).
compute iterrmod=1.
end if.
do if (booting=1).
compute bootiter=1.
end if.
do if (itprobtg=0).
compute itprobtg=1.
compute errcode(errs,1) = 47.
compute errs = errs + 1.
do if (booting = 0 and 1 =1).
compute vt1 = mdiag(pt1lp&*(1-pt1lp)).
compute varb = inv(t(xlp)*vt1*xlp).
compute seb = sqrt(diag(varb)).
end if.
end if.
end if.
do if ( 1 =1).
compute trat = b&/seb.
compute dfres=nrow(xlp).
compute p = 2*(1-cdfnorm(abs(trat))).
compute modres={modres,seb,trat,p}.
compute modres={modres,(b-xp2&*seb),(b+xp2&*seb)}.
compute pvchi=1-chicdf((LL3-LL2),(nrow(modres)-1)).
compute mcF = (LL3-LL2)/LL3.
compute cox = 1-exp(-(LL3-LL2)/nrow(xlp)).
compute nagel = cox/(1-exp(-(LL3)/nrow(xlp))).
compute modsum={LL2,(LL3-LL2),(nrow(modres)-1),pvchi, mcF,cox,nagel}.
compute modsuml={'-2LL','ModelLL', 'df', 'p', 'McFadden', 'CoxSnell', 'Nagelkrk'}.
compute modresl={'coeff','se','Z','p','LLCI','ULCI'}.
end if.
end if
.
compute toteff=modres(2:(1+nxvls),:).
compute nodotot=0.
do if (xdich=1 and xmint=1 and model=74).
compute toteff(:,1)=toteff(:,1)*xscaling.
compute toteff(:,2)=toteff(:,2)*abs(xscaling).
compute toteff(:,3)=toteff(:,3)*xscaling.
compute toteff(:,5)=toteff(:,5)*xscaling.
compute toteff(:,6)=toteff(:,6)*xscaling.
compute citmp=toteff(:,5:6).
do if (xscaling < 0).
compute toteff(:,5)=citmp(:,2).
compute toteff(:,6)=citmp(:,1).
end if.
compute nodotot=1.
end if.
compute totefflb=modresl.
do if (outscree=1).
do if (nodotot=0).
print/title = '************************** TOTAL EFFECT MODEL ****************************'.
end if.
do if (nodotot=1).
print/title = '**************************************************************************'.
end if.
print outnames(1,ncol(outnames))/title = 'OUTCOME VARIABLE:'/format = A8/space=0.
end if.
compute toteffl2=vlabsm(2:(1+nxvls),:).
compute resultm2=make(1,maxresm,99999).
compute resultm2(1,1:ncol(modsum))=modsum.
compute resultm={resultm;resultm2}.
compute resultm2=make(nrow(modres),maxresm,99999).
compute resultm2(1:nrow(modres),1:ncol(modres))=modres.
compute resultm={resultm;resultm2}.
do if (outscree=1).
print modsum/title = 'Model Summary'/cnames = modsuml/format= F10.4.
print modres/title='Model'/rnames=vlabsm/cnames=modresl/format= F10.4.
end if.
compute lmat=make(nrow(b),1,0).
compute lmat2=make(nxvls,1,1).
compute lmat(2:(1+nxvls),1)=lmat2.
do if (ydich <> 1).

.
compute lmat2= lmat.
do if ( 0 =0).
compute lmat2 = mdiag( lmat ).
compute lmat3=make(nrow(lmat2),1,0).
loop flp=1 to ncol(lmat2).
do if (csum(lmat2(:,flp))=1).
compute lmat3={lmat3,lmat2(:,flp)}.
end if.
end loop.
compute lmat2=lmat3(:,2:ncol(lmat3)).
end if.
compute fratio = (t(t(lmat2)* b )*inv(t(lmat2)* varb *lmat2)*((t(lmat2)* b )))/ncol(lmat2).
compute pfr = 1-fcdf(fratio,ncol(lmat2),(n-nrow( b ))).
compute fresult={fratio,ncol(lmat2),(n-nrow( b )),pfr}.
do if (i = (nms+nys) and (ydich=1)).
compute fratio=fratio*ncol(lmat2).
compute pfr=1-chicdf(fratio,ncol(lmat2)).
compute fresult={fratio,ncol(lmat2),pfr}.
end if.
do if ( 1 =1).
compute lmat3=1-rsum(lmat2).
compute xfm=make(n,csum(lmat3),0).
compute flpc=1.
loop flp=1 to nrow(lmat3).
do if (lmat3(flp,1)=1).
compute xfm(:,flpc)=x(:,flp).
compute flpc=flpc+1.
end if.
end loop.
compute bfm=inv(t(xfm)*xfm)*t(xfm)*y.
compute resid=y-(xfm*bfm).
compute sstotal=(y-(csum(y)/n)).
compute sstotal=csum(sstotal&*sstotal).
compute ssresid=csum(resid&*resid).
compute rsqch= r2 -((sstotal-ssresid)/sstotal).
compute fresult={rsqch,fresult}.
release xfm,flpc, resid, ssresid, bfm.
end if
.
compute totomni=fresult.
end if.
do if (stand=1).
compute predsd=make(nrow(modres),1,0).
compute stdmod=modres(:,1)&/ovsd(1,ncol(ovsd)).
loop jd=1 to ncol(x).
compute descdat=x(:,jd).
compute predsd(jd,1) = (nrow(descdat)*sscp(descdat))-(t(csum(descdat))*(csum(descdat))).
compute predsd(jd,1) = sqrt(predsd(jd,1)/(nrow(descdat)*(nrow(descdat)-1))).
end loop.
do if (wherex(1,ncol(wherex)) <> -999 and ((nxvls > 1) or (xdich=1))).
compute sdmsone=make(nxvls,1,1).
compute predsd(wherex(1,ncol(wherex)):wherex(2,ncol(wherex)),1)=sdmsone.
compute pstog=1.
end if.
compute predsd(1,1)=1.
compute stdmod=stdmod&*predsd.
compute stdmod=stdmod(2:nrow(stdmod),1).
compute sdvlabs=vlabsm(2:nrow(vlabsm),1).
compute resultm2=make(nrow(stdmod),maxresm,99999).
compute resultm2(1:nrow(stdmod),1:ncol(stdmod))=stdmod.
compute resultm={resultm;resultm2}.
do if (outscree=1).
print stdmod/title='Standardized coefficients'/clabels='coeff'/rnames=sdvlabs/format= F10.4.
end if.
end if.
do if (covcoeff=1).
do if (outscree=1).
print varb/title='Covariance matrix of regression parameter estimates:'/rnames=vlabsm/cnames=vlabsm/format= F10.4.
end if.
compute resultm2=make(nrow(varb),maxresm,99999).
do if (ncol(varb) <= maxresm).
compute resultm2(1:nrow(varb),1:ncol(varb))=varb.
compute resultm={resultm;resultm2}.
end if.
do if (ncol(varb) > maxresm).
compute resultmt=make(nrow(resultm),ncol(varb),99999).
compute resultmt(1:nrow(resultm),1:ncol(resultm))=resultm.
compute resultm=resultmt.
compute resultm2=make(nrow(varb),ncol(resultm),99999).
compute resultm2(1:nrow(varb),1:ncol(varb))=varb.
compute resultm={resultm;resultm2}.
compute maxresm=ncol(resultm).
end if.
end if.
end if.
end if.
do if (criterr=0 and nms > 0 and ydich=0 and modelres=1).
compute modresid=modresid(:,2:ncol(modresid)).
compute sigmatal = (t(modresid)*(ident(n)-(1/n)*ones*t(ones))*modresid)*(1/(n-1)).
compute sdall = mdiag(1/sqrt(diag(sigmatal))).
compute corall=sdall*sigmatal*t(sdall).
compute resultm2=make(nrow(corall),maxresm,99999).
compute resultm2(1:nrow(corall),1:ncol(corall))=corall.
compute resultm={resultm;resultm2}.
do if (outscree=1).
print/title = '****************** CORRELATIONS BETWEEN MODEL RESIDUALS ******************'.
print corall/title=' '/format= F10.4 / cnames=outnames/rnames=outnames/space=0.
end if.
end if.
do if (criterr=0 and boot > 0).
compute bootres=make(1,rsum(nump),-999).
compute bootdir=obsdirfx.
compute natdirbt=make(1,nxvls,-999).
do if (effsize=1).
compute bootysd=make(1,1,-999).
compute bootxsd=make(1,1,-999).
end if.
compute badboot=0.
compute goodboot=0.
compute smallest=1.
compute booting=1.
loop j = 1 to maxboot.
compute nobootx=1.
compute modres2=999.
compute v=trunc(uniform(n,1)*n)+1.
compute bad=0.
loop i = 1 to (nms+nys).
compute y=outvars(v,i).
compute ynovar= (nrow(y)*sscp(y))-(t(csum(y))*(csum(y))).
do if (ynovar = 0).
compute bad=1.
end if.
compute xindx=datindx(1:(nump(1,i)-1),i).
compute hello=0.
compute x = fulldat(v,xindx).
compute x={ones,x}.
compute xsq=t(x)*x.
compute exsq=eval(xsq).
release xsq.
compute holymoly=cmin(exsq).
compute zeroeig=csum(exsq <= 0.000000000002).
compute bad=bad + (zeroeig > 0).

.
compute desctmp=make((8-(4* 1 )),ncol( y ),-999).
loop jd=1 to ncol( y ).
compute descdat= y (:,jd).
compute desctmp(1,jd) = csum(descdat)/nrow(descdat).
compute desctmp(2,jd) = (nrow(descdat)*sscp(descdat))-(t(csum(descdat))*(csum(descdat))).
compute desctmp(2,jd) = sqrt(desctmp(2,jd)/(nrow(descdat)*(nrow(descdat)-1))).
compute desctmp(3,jd)=cmin(descdat).
compute desctmp(4,jd)=cmax(descdat).
do if ( 1 =0).
compute minwarn=0.
compute maxwarn=0.
do if ((desctmp(3,jd)=desctmp(4,jd)) and novar=0).
compute errcode(errs,1)=15.
compute errs=errs+1.
compute criterr=1.
compute novar=1.
end if.
compute tmp=((descdat(:,1)=desctmp(3,jd))+(descdat(:,1)=desctmp(4,jd))).
compute desctmp(8,jd)=(csum(tmp)=nrow(tmp)).
compute tmp = descdat.
compute tmp(GRADE(descdat),:) = descdat.
compute descdat = tmp.
release tmp.
compute decval={.16;.5;.84}.
loop kd=1 to 3.
compute low=trunc(decval(kd,1)*(nrow(descdat)+1)).
compute lowdec=decval(kd,1)*(nrow(descdat)+1)-low.
compute value=descdat(low,1)+(descdat((low+1),1)-descdat(low,1))*lowdec.
compute desctmp((4+kd),jd)=value.
end loop.
compute mnotev=1.
compute modvals=desctmp(5:7,:).
do if (quantile <> 1).
compute desctmp(5,jd)=desctmp(1,jd)-desctmp(2,jd).
compute desctmp(6,jd)=desctmp(1,jd).
compute desctmp(7,jd)=desctmp(1,jd)+desctmp(2,jd).
compute modvals=desctmp(5:7,:).
compute mnotev=2.
do if (modvals(1,1) < desctmp(3,1)).
compute modvals(1,1)=desctmp(3,1).
compute minwarn=1.
end if.
do if (modvals(3,1) > desctmp(4,1)).
compute modvals(3,1)=desctmp(4,1).
compute maxwarn=1.
end if.
end if.
do if (desctmp(8,1)=1).
compute modvals={desctmp(3,1);desctmp(4,1)}.
compute mnotev=0.
compute minwarn=0.
compute maxwarn=0.
end if.
end if.
end loop
.
compute bad=bad+ (desctmp(2,1) <= 0.00000000001).
do if (bad = 0).
do if (holymoly < smallest).
compute smallest=holymoly.
end if.
do if (ydich=0 or (i < (nms+nys))).

.
do if ( 1 =1).
compute hatmat=inv(t( x )* x )*t( x ).
compute b = hatmat* y.
compute modres=b.
do if ( 0 =1).
compute n1=nrow( x ).
compute dfres=n1-(ncol( x )).
compute sstotal = t( y -(csum( y )/n1))*( y -(csum( y )/n1)).
compute resid= y - x *b.
compute ssresid = csum((resid)&**2).
compute r2 = (sstotal-ssresid)/sstotal.
do if (r2 < 0).
compute r2=0.
end if.
compute adjr2 = 1-((1-r2)*(n1-1)/(dfres)).
compute mse=ssresid/(n1-ncol( x )).

.
compute n1=nrow( x ).
compute invXtX = inv(t( x )* x ).
compute varb = mse *invXtX.
compute k3 = ncol( x ).
compute xhc=0.
do if ( hc <> 5).
compute xhc= x.
compute hat = xhc(:,1).
loop i3=1 to nrow(xhc).
compute hat(i3,1)= xhc(i3,:)*invXtX*t(xhc(i3,:)).
end loop.
do if ( hc = 0 or hc =1).
loop i3 = 1 to k3.
compute xhc(:,i3)=xhc(:,i3)&* resid.
end loop.
end if.
do if ( hc =3 or hc =2).
loop i3=1 to k3.
compute xhc(:,i3) = ( resid &/(1-hat)&**(1/(4- hc )))&*xhc(:,i3).
end loop.
end if.
do if ( hc = 4).
compute hcmn=make(n,2,4).
compute hcmn(:,2)=(n1*hat)/k3.
loop i3= 1 to k3.
compute xhc(:,i3) = ( resid &/(1-hat)&**(rmin(hcmn)/2))&*xhc(:,i3).
end loop.
end if.
compute varb=(invXtX*t(xhc)*xhc*invXtX).
do if ( hc =1).
compute varb=(n1/(n1-ncol( x )))&*varb.
end if.
end if.
compute hclab={'se(HC0)','se(HC1)','se(HC2)','se(HC3)','se(HC4)','se'}.
compute hclab=hclab(1,( hc +1)).
compute hcflab={'F(HC0)','F(HC1)','F(HC2)','F(HC3)','F(HC4)','F'}.
compute hcflab=hcflab(1,( hc +1)).
release xhc
.
compute seb=sqrt(diag(varb)).
compute trat = b&/seb.
compute p = 2*(1-tcdf(abs(trat), (dfres))).
compute tval = sqrt(dfres* (exp((dfres-(5/6))*((xp2/(dfres-(2/3)+(.11/dfres)))* (xp2/(dfres-(2/3)+(.11/dfres)))))-1)).
compute modres={modres,seb,trat,p}.
compute modres={modres,(b-tval&*seb),(b+tval&*seb)}.
compute modresl={'coeff',hclab,'t','p','LLCI','ULCI'}.
compute lmat = ident(ncol( x )).
compute lmat = lmat(:,2:ncol(lmat)).
compute fratio = (t(t(lmat)*b)*inv(t(lmat)*varb*lmat)*((t(lmat)*b)))/(ncol( x )-1).
compute pfr = 1-fcdf(fratio,(ncol( x )-1),dfres).
compute modsum={sqrt(r2),r2,mse,fratio,(ncol( x )-1),dfres,pfr}.
compute modsuml={'R','R-sq','MSE',hcflab,'df1','df2', 'p'}.
end if.
end if.
do if ( 1 = 2 or 1 =3).
compute xlp= x.
compute ylp= y.
compute pt2 = make(nrow(ylp),1,(csum(ylp)/nrow(ylp))).
do if ( 1 =2).
compute LL3 = ylp&*ln(pt2)+(1-ylp)&*ln(1-pt2).
end if.
compute LL3 = -2*csum(LL3).
compute bt1 = make(ncol(xlp),1,0).
compute LL1 = 0.
compute pt1 = make(nrow(ylp),1,0.5).
compute pt1lp=pt1.
loop jjj = 1 to iterate.
compute xlptmp=t(xlp).
compute vecprb=pt1lp&*(1-pt1lp).
loop kkk=1 to ncol(xlp).
compute xlptmp(kkk,:)=xlptmp(kkk,:)&*t(vecprb).
end loop.
compute b = bt1+inv(xlptmp*xlp)*t(xlp)*(ylp-pt1lp).
do if ( 1 =2).
compute xlpb=xlp*b.
compute xlpbt=(xlpb > -709.7).
compute xlpb709=(1-xlpbt)*(-709.7).
compute xlpb=(xlpb&*xlpbt)+xlpb709.
compute pt1lp = 1/(1+exp(-(xlpb))).
end if.
compute itprob = csum((pt1lp < .00000001) or (pt1lp > .9999999)).
do if (itprob > 0).
loop kkk = 1 to nrow(pt1lp).
do if (pt1lp(kkk,1) > .9999999).
compute pt1lp(kkk,1) = .9999999.
end if.
do if (pt1lp(kkk,1) < .00000001).
compute pt1lp(kkk,1) = .00000001.
end if.
end loop.
compute itprob = 0.
end if.
do if (itprob = 0).
do if ( 1 =2).
compute LL = ylp&*ln(pt1lp)+(1-ylp)&*ln(1-pt1lp).
end if.
compute LL2 = -2*csum(ll).
end if.
do if (abs(LL1-LL2) < converge).
do if ( 0 =1).
compute xlptmp=t(xlp).
compute vecprb=pt1lp&*(1-pt1lp).
loop kkk=1 to ncol(xlp).
compute xlptmp(kkk,:)=xlptmp(kkk,:)&*t(vecprb).
end loop.
compute varb = inv(xlptmp*xlp).
compute seb = sqrt(diag(varb)).
release xlptmp.
end if.
break.
end if.
compute bt1 = b.
compute LL1 = LL2.
end loop.
compute modres=b.
do if (jjj > iterate).
compute itprob = 2.
do if (booting=0).
compute iterrmod=1.
end if.
do if (booting=1).
compute bootiter=1.
end if.
do if (itprobtg=0).
compute itprobtg=1.
compute errcode(errs,1) = 47.
compute errs = errs + 1.
do if (booting = 0 and 0 =1).
compute vt1 = mdiag(pt1lp&*(1-pt1lp)).
compute varb = inv(t(xlp)*vt1*xlp).
compute seb = sqrt(diag(varb)).
end if.
end if.
end if.
do if ( 0 =1).
compute trat = b&/seb.
compute dfres=nrow(xlp).
compute p = 2*(1-cdfnorm(abs(trat))).
compute modres={modres,seb,trat,p}.
compute modres={modres,(b-xp2&*seb),(b+xp2&*seb)}.
compute pvchi=1-chicdf((LL3-LL2),(nrow(modres)-1)).
compute mcF = (LL3-LL2)/LL3.
compute cox = 1-exp(-(LL3-LL2)/nrow(xlp)).
compute nagel = cox/(1-exp(-(LL3)/nrow(xlp))).
compute modsum={LL2,(LL3-LL2),(nrow(modres)-1),pvchi, mcF,cox,nagel}.
compute modsuml={'-2LL','ModelLL', 'df', 'p', 'McFadden', 'CoxSnell', 'Nagelkrk'}.
compute modresl={'coeff','se','Z','p','LLCI','ULCI'}.
end if.
end if
.
end if.
do if (ydich=1 and (i = (nms+nys))).

.
do if ( 2 =1).
compute hatmat=inv(t( x )* x )*t( x ).
compute b = hatmat* y.
compute modres=b.
do if ( 0 =1).
compute n1=nrow( x ).
compute dfres=n1-(ncol( x )).
compute sstotal = t( y -(csum( y )/n1))*( y -(csum( y )/n1)).
compute resid= y - x *b.
compute ssresid = csum((resid)&**2).
compute r2 = (sstotal-ssresid)/sstotal.
do if (r2 < 0).
compute r2=0.
end if.
compute adjr2 = 1-((1-r2)*(n1-1)/(dfres)).
compute mse=ssresid/(n1-ncol( x )).

.
compute n1=nrow( x ).
compute invXtX = inv(t( x )* x ).
compute varb = mse *invXtX.
compute k3 = ncol( x ).
compute xhc=0.
do if ( hc <> 5).
compute xhc= x.
compute hat = xhc(:,1).
loop i3=1 to nrow(xhc).
compute hat(i3,1)= xhc(i3,:)*invXtX*t(xhc(i3,:)).
end loop.
do if ( hc = 0 or hc =1).
loop i3 = 1 to k3.
compute xhc(:,i3)=xhc(:,i3)&* resid.
end loop.
end if.
do if ( hc =3 or hc =2).
loop i3=1 to k3.
compute xhc(:,i3) = ( resid &/(1-hat)&**(1/(4- hc )))&*xhc(:,i3).
end loop.
end if.
do if ( hc = 4).
compute hcmn=make(n,2,4).
compute hcmn(:,2)=(n1*hat)/k3.
loop i3= 1 to k3.
compute xhc(:,i3) = ( resid &/(1-hat)&**(rmin(hcmn)/2))&*xhc(:,i3).
end loop.
end if.
compute varb=(invXtX*t(xhc)*xhc*invXtX).
do if ( hc =1).
compute varb=(n1/(n1-ncol( x )))&*varb.
end if.
end if.
compute hclab={'se(HC0)','se(HC1)','se(HC2)','se(HC3)','se(HC4)','se'}.
compute hclab=hclab(1,( hc +1)).
compute hcflab={'F(HC0)','F(HC1)','F(HC2)','F(HC3)','F(HC4)','F'}.
compute hcflab=hcflab(1,( hc +1)).
release xhc
.
compute seb=sqrt(diag(varb)).
compute trat = b&/seb.
compute p = 2*(1-tcdf(abs(trat), (dfres))).
compute tval = sqrt(dfres* (exp((dfres-(5/6))*((xp2/(dfres-(2/3)+(.11/dfres)))* (xp2/(dfres-(2/3)+(.11/dfres)))))-1)).
compute modres={modres,seb,trat,p}.
compute modres={modres,(b-tval&*seb),(b+tval&*seb)}.
compute modresl={'coeff',hclab,'t','p','LLCI','ULCI'}.
compute lmat = ident(ncol( x )).
compute lmat = lmat(:,2:ncol(lmat)).
compute fratio = (t(t(lmat)*b)*inv(t(lmat)*varb*lmat)*((t(lmat)*b)))/(ncol( x )-1).
compute pfr = 1-fcdf(fratio,(ncol( x )-1),dfres).
compute modsum={sqrt(r2),r2,mse,fratio,(ncol( x )-1),dfres,pfr}.
compute modsuml={'R','R-sq','MSE',hcflab,'df1','df2', 'p'}.
end if.
end if.
do if ( 2 = 2 or 2 =3).
compute xlp= x.
compute ylp= y.
compute pt2 = make(nrow(ylp),1,(csum(ylp)/nrow(ylp))).
do if ( 2 =2).
compute LL3 = ylp&*ln(pt2)+(1-ylp)&*ln(1-pt2).
end if.
compute LL3 = -2*csum(LL3).
compute bt1 = make(ncol(xlp),1,0).
compute LL1 = 0.
compute pt1 = make(nrow(ylp),1,0.5).
compute pt1lp=pt1.
loop jjj = 1 to iterate.
compute xlptmp=t(xlp).
compute vecprb=pt1lp&*(1-pt1lp).
loop kkk=1 to ncol(xlp).
compute xlptmp(kkk,:)=xlptmp(kkk,:)&*t(vecprb).
end loop.
compute b = bt1+inv(xlptmp*xlp)*t(xlp)*(ylp-pt1lp).
do if ( 2 =2).
compute xlpb=xlp*b.
compute xlpbt=(xlpb > -709.7).
compute xlpb709=(1-xlpbt)*(-709.7).
compute xlpb=(xlpb&*xlpbt)+xlpb709.
compute pt1lp = 1/(1+exp(-(xlpb))).
end if.
compute itprob = csum((pt1lp < .00000001) or (pt1lp > .9999999)).
do if (itprob > 0).
loop kkk = 1 to nrow(pt1lp).
do if (pt1lp(kkk,1) > .9999999).
compute pt1lp(kkk,1) = .9999999.
end if.
do if (pt1lp(kkk,1) < .00000001).
compute pt1lp(kkk,1) = .00000001.
end if.
end loop.
compute itprob = 0.
end if.
do if (itprob = 0).
do if ( 2 =2).
compute LL = ylp&*ln(pt1lp)+(1-ylp)&*ln(1-pt1lp).
end if.
compute LL2 = -2*csum(ll).
end if.
do if (abs(LL1-LL2) < converge).
do if ( 0 =1).
compute xlptmp=t(xlp).
compute vecprb=pt1lp&*(1-pt1lp).
loop kkk=1 to ncol(xlp).
compute xlptmp(kkk,:)=xlptmp(kkk,:)&*t(vecprb).
end loop.
compute varb = inv(xlptmp*xlp).
compute seb = sqrt(diag(varb)).
release xlptmp.
end if.
break.
end if.
compute bt1 = b.
compute LL1 = LL2.
end loop.
compute modres=b.
do if (jjj > iterate).
compute itprob = 2.
do if (booting=0).
compute iterrmod=1.
end if.
do if (booting=1).
compute bootiter=1.
end if.
do if (itprobtg=0).
compute itprobtg=1.
compute errcode(errs,1) = 47.
compute errs = errs + 1.
do if (booting = 0 and 0 =1).
compute vt1 = mdiag(pt1lp&*(1-pt1lp)).
compute varb = inv(t(xlp)*vt1*xlp).
compute seb = sqrt(diag(varb)).
end if.
end if.
end if.
do if ( 0 =1).
compute trat = b&/seb.
compute dfres=nrow(xlp).
compute p = 2*(1-cdfnorm(abs(trat))).
compute modres={modres,seb,trat,p}.
compute modres={modres,(b-xp2&*seb),(b+xp2&*seb)}.
compute pvchi=1-chicdf((LL3-LL2),(nrow(modres)-1)).
compute mcF = (LL3-LL2)/LL3.
compute cox = 1-exp(-(LL3-LL2)/nrow(xlp)).
compute nagel = cox/(1-exp(-(LL3)/nrow(xlp))).
compute modsum={LL2,(LL3-LL2),(nrow(modres)-1),pvchi, mcF,cox,nagel}.
compute modsuml={'-2LL','ModelLL', 'df', 'p', 'McFadden', 'CoxSnell', 'Nagelkrk'}.
compute modresl={'coeff','se','Z','p','LLCI','ULCI'}.
end if.
end if
.
end if.
compute modres2={modres2,t(modres)}.
do if (i = (nms+nys)).
do if (bcmat((i+1),1) = 1).
compute bootdir={bootdir;t(modres(wherex(1,i):wherex(2,i),1))}.
end if.
do if (bcmat((i+1),1) = 0).
compute bootdir={bootdir;dirzes}.
end if.
end if.
do if (model = 74 and i <= nms).
compute onetemp=make(nrow(xprobval),1,1).
compute mestmtb={onetemp,xprobval}.
do if (ncs > 0).
compute ncovmdlb=rsum(ccmat(i,:)).
do if (ncovmdl > 0).
compute cvmncb=make(nrow(mestmtb),ncovmdlb,1).
compute cvmnctmp=csum(x(:,(ncol(x)-ncovmdlb+1):ncol(x)))/nrow(x).
do if (cuscoval > 0).
compute cvmnctmp=coval.
end if.
loop mestlp=1 to ncovmdlb.
compute cvmncb(:,mestlp)=cvmncb(:,mestlp)*cvmnctmp(:,mestlp).
end loop.
compute mestmtb={mestmtb,cvmncb}.
end if.
end if.
compute mestbt=mestmtb*modres.
do if (i = 1).
compute mestb=mestbt.
end if.
do if (i > 1).
compute mestb={mestb,mestbt}.
end if.
end if.
do if (model = 74 and i = (nms+nys)).
compute xvalptmp=make(1,nxvls,0).
compute mest74sp=make(1,nms,0).
compute mest74sp={mest74sp;mestb}.
compute xvalptmp={xvalptmp;ident(nxvls)}.
compute dirfxcf=make(nrow(mestb),1,0).
loop cfloop1=1 to nrow(mestb).
compute ndirfx=make(nrow(modres),1,0).
compute ndirfx(2:(nxvls+1),1)=t(xvalptmp(cfloop1,:)).
loop cfloop3=1 to nms.
do if (mcx=1 or mcx=0).
compute ndirfx(wheremw((1+((cfloop3-1)*2)),ncol(wheremw)):wheremw((2+((cfloop3-1)*2) ),ncol(wheremw)))=t(xvalptmp(cfloop1,:)*mestb(1,cfloop3)).
end if.
do if (mcx=2).
compute ndirfx(wheremw((1+((cfloop3-1)*2)),ncol(wheremw)):wheremw((2+((cfloop3-1)*2) ),ncol(wheremw)))=t(xvalptmp(cfloop1,:)*mest74sp(cfloop1,cfloop3)).
end if.
end loop.
do if (cfloop1 > 1).
compute dirfxcf(cfloop1,1)=t(ndirfx)*modres*xscaling.
end if.
end loop.
compute natdirbt={natdirbt;t(dirfxcf(2:nrow(dirfxcf),:))}.
end if.
do if ( (bcmat((i+1),1) = 1) and (nobootx=1) and (effsize=1)).
compute nobootx=0.
compute xsdtemp= (nrow(x)*sscp(x(:,2)))-(t(csum(x(:,2)))*(csum(x(:,2)))).
compute xsdtemp= sqrt(xsdtemp/(nrow(x)*(nrow(x)-1))).
end if.
end if.
end loop.
do if (bad = 0).
compute bootres={bootres;modres2(:,2:ncol(modres2))}.
do if (effsize=1).
compute ysdtemp= (nrow(y)*sscp(y))-(t(csum(y))*(csum(y))).
compute ysdtemp= sqrt(ysdtemp/(nrow(y)*(nrow(y)-1))).
compute bootysd={bootysd;ysdtemp}.
compute bootxsd={bootxsd;xsdtemp}.
end if.
compute goodboot=goodboot+1.
end if.
do if (bad <> 0).
compute badboot=badboot+1.
end if.
end loop if (goodboot = boot).
compute bootres=bootres(2:nrow(bootres),:).
do if (effsize=1).
compute bootysd=bootysd(2:nrow(bootysd),:).
do if (nrow(bootxsd) > 1).
compute bootxsd=bootxsd(2:nrow(bootxsd),:).
end if.
end if.
do if (goodboot < (boot)).
compute boot=0.
compute modelbt=0.
compute notecode(notes,1) = 7.
compute notes = notes + 1.
end if.
do if (boot > 0).
do if (effsize=1).
compute bootysd={ysd;bootysd}.
compute bootxsd={xsd;bootxsd}.
end if.
do if (saveboot = 1).
save bootres/outfile = *.
end if.
do if (modelbt=1).
compute bootcim=make(ncol(bootres),5,-99999).
compute bootcim(:,2) = t(csum(bootres)/nrow(bootres)).
compute bootcim(:,1) = coeffmat(2:nrow(coeffmat),1).
loop i = 1 to ncol(bootres).

.
compute temp = bootres(:,i).
compute temp(GRADE( bootres(:,i) )) = bootres(:,i).
compute badlo = 0.
compute badhi = 0.
do if ( (bootcim(i,1)*bc)+(9999*(1-bc)) <> 9999).
compute pv=csum(temp < (bootcim(i,1)*bc)+(9999*(1-bc)) )/nrow(temp).
compute ppv = pv.
do if (pv > .5).
compute ppv = 1-pv.
end if.
compute y5=sqrt(-2*ln(ppv)).
compute xp=y5+((((y5*p4+p3)*y5+p2)*y5+p1)*y5+p0)/((((y5*q4+q3)*y5+q2)*y5+q1)*y5+q0).
do if (pv <= .5).
compute xp = -xp.
end if.
compute cilow=rnd(nrow(temp)*(cdfnorm(2*xp-xp2))).
compute cihigh=trunc(nrow(temp)*(cdfnorm(2*xp+(xp2))))+1.
do if (cilow < 1).
compute cilow = 1.
compute booterr=1.
compute badlo = 1.
end if.
do if (cihigh > nrow(temp)).
compute cihigh = boot.
compute booterr=1.
compute badhi = 1.
end if.
compute llcit=temp(cilow,1).
compute ulcit=temp(cihigh,1).
do if (badlo = 1 and llcit <> priorlo).
compute badend={badend, llcit}.
compute priorlo = llcit.
end if.
do if (badhi = 1 and ulcit <> priorhi).
compute badend={badend, ulcit}.
compute priorhi = ulcit.
end if.
end if.
do if ( (bootcim(i,1)*bc)+(9999*(1-bc)) = 9999).
compute llcit=temp(cilow,1).
compute ulcit=temp(cihigh,1).
end if.
compute bootse=t(sqrt((cssq(temp)-((csum(temp)&**2)/nrow(temp)))/(nrow(temp)-1)))
.
compute bootcim(i,4:5)={llcit,ulcit}.
compute bootcim(i,3)=bootse.
end loop.
end if.
end if.
do if (badboot > 0).
compute notecode(notes,1) = 6.
compute notes = notes + 1.
end if.
end if.
do if (criterr=0).
release fulldat,x,y.
end if.
do if (xmint=1 and criterr=0).
compute xvalptmp=make(1,nxvls,0).
compute mest74sp=make(1,nms,0).
compute mest74sp={mest74sp;mest74}.
compute xvalptmp={xvalptmp;ident(nxvls)}.
loop kcfuhd=1 to 2.
compute dirfxcf=make(nrow(mest74),6,0).
loop cfloop1=1 to nrow(mest74).
compute ndirfx=make(nrow(lastb),1,0).
compute ndirfx(2:(nxvls+1),1)=t(xvalptmp(cfloop1,:)).
loop cfloop3=1 to nms.
do if (kcfuhd=1).
do if (mcx=1 or mcx=0).
compute ndirfx(wheremw((1+((cfloop3-1)*2)),ncol(wheremw)):wheremw((2+((cfloop3-1)*2)), ncol(wheremw)))=t(xvalptmp(cfloop1,:)*mest74(1,cfloop3)).
end if.
do if (mcx=2).
compute ndirfx(wheremw((1+((cfloop3-1)*2)),ncol(wheremw)):wheremw((2+((cfloop3-1)*2)), ncol(wheremw)))=t(xvalptmp(cfloop1,:)*mest74sp(cfloop1,cfloop3)).
end if.
end if.
do if (kcfuhd=2).
compute ndirfx(wheremw((1+((cfloop3-1)*2)),ncol(wheremw)):wheremw((2+((cfloop3-1)*2)), ncol(wheremw)))=t(xvalptmp(cfloop1,:)*medmeans(1,cfloop3)).
end if.
end loop.
do if (cfloop1 > 1).
compute dirfxcf(cfloop1,1)=t(ndirfx)*lastb*xscaling.
compute dirfxcf(cfloop1,2)=sqrt(diag(t(ndirfx)*lastcov*ndirfx))*abs(xscaling).
compute dirfxcf(cfloop1,3)=dirfxcf(cfloop1,1)/dirfxcf(cfloop1,2).
compute dirfxcf(cfloop1,4)=2*(1-tcdf(abs(dirfxcf(cfloop1,3)), dfres)).
compute dirfxcf(cfloop1,5)=dirfxcf(cfloop1,1)-tval*dirfxcf(cfloop1,2).
compute dirfxcf(cfloop1,6)=dirfxcf(cfloop1,1)+tval*dirfxcf(cfloop1,2).
end if.
end loop.
compute codireff=dirfxcf(2:nrow(dirfxcf),:).
do if (kcfuhd=1).
compute direff=codireff.
end if.
end loop.
end if.

.
do if (criterr = 0 and nms > 0).
compute paths=paths(:,2:ncol(paths)).
compute pathsw=pathsw(:,2:ncol(pathsw)).
compute pathsz=pathsz(:,2:ncol(pathsz)).
compute pathswz=pathswz(:,2:ncol(pathswz)).
compute pathsmod=pathsw+pathsz+pathswz.
compute pathsdv=pathsdv(:,2:ncol(pathsdv)).
compute pathsfoc=pathsfoc(:,2:ncol(pathsfoc)).
compute pathtype=pathtype(:,2:ncol(pathtype)).
compute anymod=(rsum(pathsmod) > 0).
compute obscoeff=obscoeff(1,2:ncol(obscoeff)).
do if (outscree=1).
do if (xmint=1).
print/title = '************************* COUNTERFACTUALLY DEFINED ************************'.
else.
print/title = ' '.
end if.
do if (dototal = 0 and alttotal=0).
print/title = '****************** DIRECT AND INDIRECT EFFECTS OF X ON Y *****************'/space=0.
end if.
do if (alttotal=1).
print/title = '************** TOTAL, DIRECT, AND INDIRECT EFFECTS OF X ON Y **************'/space=0.
end if.
end if.
do if (dototal = 1).
do if (outscree=1).
print/title = '************** TOTAL, DIRECT, AND INDIRECT EFFECTS OF X ON Y **************'/space=0.
end if.
compute totefflb(1,1)='Effect'.
do if (effsize=1).
compute toteffsz=toteff(:,1)/ysd.
do if (xdich=1 or mcx > 0).
compute totefflb={totefflb,'c_ps'}.
end if.
do if (xdich = 0 and mcx = 0).
compute toteffsz=toteffsz*xsd.
compute totefflb={totefflb,'c_cs'}.
end if.
compute toteff={toteff,toteffsz}.
end if.
compute resultm2=make(nrow(toteff),maxresm,99999).
compute resultm2(1:nrow(toteff),1:ncol(toteff))=toteff.
compute resultm={resultm;resultm2}.
do if (nxvls > 1).
compute resultm2=make(nrow(totomni),maxresm,99999).
compute resultm2(1:nrow(totomni),1:ncol(totomni))=totomni.
compute resultm={resultm;resultm2}.
compute clabtmp={'R2-chng', hcflab, 'df1','df2','p'}.
do if (outscree=1).
print toteff/title='Relative total effects of X on Y'/rnames=toteffl2/cnames=totefflb/format= F10.4.
print totomni/title='Omnibus test of total effect of X on Y'/cnames=clabtmp/format= F10.4.
print/title= '----------'/space=0.
end if.
else.
do if (outscree=1).
print toteff/title='Total effect of X on Y'/cnames=totefflb/format= F10.4.
end if.
end if.
end if.
compute moddir=wcmat(nrow(bcmat),1)+zcmat(nrow(bcmat),1).
do if (xmint=1).
compute moddir=1.
end if.
do if (bcmat(nrow(bcmat),1)=1).
do if (ydich=1).
compute direfflb(:,(ncol(direfflb)-5):ncol(direfflb))={'Effect','se','Z','p','LLCI','ULCI'}.
end if.
do if (moddir=0 or xmint=1).
compute direfflb(1,1)='Effect'.
end if.
do if (effsize=1 and moddir=0 and anymod = 0).
compute direffsz=direff(:,1)/ysd.
do if (xdich=1 or mcx > 0).
compute direfflb={direfflb,'c''_ps'}.
end if.
do if (xdich = 0 and mcx = 0).
compute direffsz=direffsz*xsd.
compute direfflb={direfflb,'c''_cs'}.
end if.
compute direff={direff,direffsz}.
end if.
compute resultm2=make(nrow(direff),maxresm,99999).
compute resultm2(1:nrow(direff),1:ncol(direff))=direff.
compute resultm={resultm;resultm2}.
do if (xmint=1).
compute resultm2=make(nrow(codireff),maxresm,99999).
compute resultm2(1:nrow(codireff),1:ncol(codireff))=codireff.
compute resultm={resultm;resultm2}.
end if.
do if (moddir = 0 and nxvls=1).
do if (outscree=1).
print direff/title='Direct effect of X on Y'/cnames=direfflb/format= F10.4.
end if.
end if.
do if (moddir = 0 and nxvls>1).
compute resultm2=make(nrow(diromni),maxresm,99999).
compute resultm2(1:nrow(diromni),1:ncol(diromni))=diromni.
compute resultm={resultm;resultm2}.
do if (outscree=1).
print direff/title='Relative direct effects of X on Y'/rnames=direffl2/cnames=direfflb/format= F10.4.
do if (ydich = 0).
compute clabtmp={'R2-chng', hcflab, 'df1','df2','p'}.
print diromni/title='Omnibus test of direct effect of X on Y:'/cnames=clabtmp/format= F10.4.
end if.
do if (ydich = 1).
compute clabtmp={'Chi-sq', 'df', 'p'}.
print diromni/title='Omnibus likelihood ratio test of direct effect of X on Y'/cnames=clabtmp/format= F10.4.
end if.
print/title= '----------'/space=0.
end if.
end if.
do if (moddir > 0 and nxvls=1).
do if (xmint=0 and outscree=1).
print direff/title='Conditional direct effects of X on Y'/cnames=direfflb/format= F10.4.
end if.
do if (xmint=1).
do if (outscree=1).
print direff/title='(Pure) Natural direct effect of X on Y'/cnames=direfflb/format= F10.4.
print codireff/title='Controlled direct effect of X on Y'/cnames=direfflb/format= F10.4.
print/title= '----------'/space=0.
end if.
compute obnatdfx=direff(:,1).
end if.
end if.
compute direffl4=direffl2.
do if (moddir > 0 and nxvls>1).
compute direffl2={' '}.
loop i = 1 to nxvls.
loop j = 1 to (nrow(direff)/nxvls).
compute direffl2={direffl2;xcatlab(i,1)}.
end loop.
end loop.
compute direffl2=direffl2(2:nrow(direffl2),1).
do if (xmint=0 and outscree=1).
print direff/title='Relative conditional direct effects of X on Y'/cnames=direfflb/rnames=direffl2/format= F10.4.
end if.
do if (xmint=1).
do if (outscree=1).
print direff/title='Relative (pure) natural direct effects of X on Y'/cnames=direfflb/rnames=direffl2/format= F10.4.
print codireff/title='Relative controlled direct effects of X on Y'/cnames=direfflb/rnames=direffl2/format= F10.4.
print/title= '----------'/space=0.
end if.
compute obnatdfx=direff(:,1).
end if.
end if.
compute direffl2=direffl4.
end if.
do if (bcmat(nrow(bcmat),1)=0 and xmint <> 1 and outscree=1).
print/title='The direct effect of X on Y is fixed to zero.'.
end if.
do if (nms = 1).
compute indmark={2}.
compute indsets={1,2}.
end if.
do if (nms = 2).
compute indmark={2,2,3}.
compute indsets={1,4,2,5,1,3,5}.
compute thetam=1.
end if.
do if (nms = 3).
compute indmark={2,2,2,3,3,3,4}.
compute indsets={1,7,2,8,4,9,1,3,8,1,5,9,2,6,9,1,3,6,9}.
compute thetam={1,2,3}.
end if.
do if (nms = 4).
compute indmark={2,2,2,2,3,3,3,3,3,3,4,4,4,4,5}.
compute indsets={1,11,2,12,4,13,7,14,1,3,12,1,5,13,1,8,14,2,6,13,2,9,14,4,10,14,1,3,6,13,1,3,9, 14,1,5,10,14,2,6,10,14,1,3,6,10,14}.
compute thetam={1,2,4,3,5,6}.
end if.
do if (nms = 5).
compute indmark={2,2,2,2,2,3,3,3,3,3,3,3,3,3,3,4,4,4,4,4,4,4,4,4,4,5,5,5,5,5,6}.
compute indsets={1,16,2,17,4,18,7,19,11,20,1,3,17,1,5,18,1,8,19,1,12,20,2,6,18,2,9,19,2,13,20,4, 10,19,4,14,20,7,15,20,1,3,6,18, 1,3,9,19,1,3,13,20,1,5,10,19,1,5,14,20,1,8,15,20,2,6,10,19,2,6, 14,20,2,9,15,20,4,10,15,20,1,3,6,10,19, 1,3,6,14,20,1,3,9,15,20,1,5,10,15,20,2,6,10,15,20,1,3,6,10,15, 20}.
compute thetam={1,2,5,3,6,8,4,7,9,10}.
end if.
do if (nms = 6).
compute indmark={2,2,2,2,2,2,3,3,3,3,3,3,3,3,3,3,3,3,3,3,3,4,4,4,4,4,4,4,4,4,4,4,4,4,4,4,4,4,4, 4,4,5,5,5,5,5,5,5,5,5,5,5,5,5,5,5,6,6,6,6,6,6,7}.
compute indsets={1,22,2,23,4,24,7,25,11,26,16,27,1,3,23,1,5,24,1,8,25,1,12,26,1,17,27,2,6,24,2, 9,25,2,13,26,2,18,27,4,10,25,4,14,26, 4,19,27,7,15,26,7,20,27,11,21,27,1,3,6,24,1,3,9,25,1,3,13,26,1, 3,18,27,1,5,10,25,1,5,14,26,1,5,19,27,1,8,15,26,1,8,20,27, 1,12,21,27,2,6,10,25,2,6,14,26,2,6,19,27,2,9,15,26,2,9,20,27,2, 13,21,27,4,10,15,26,4,10,20,27,4,14,21,27,7,15,21,27, 1,3,6,10,25,1,3,6,14,26,1,3,6,19,27,1,3,9,15,26,1,3,9,20,27,1,3, 13,21,27,1,5,10,15,26,1,5,10,20,27,1,5,14,21,27, 1,8,15,21,27,2,6,10,15,26,2,6,10,20,27,2,6,14,21,27,2,9,15,21, 27,4,10,15,21,27,1,3,6,10,15,26,1,3,6,10,20,27, 1,3,6,14,21,27,1,3,9,15,21,27,1,5,10,15,21,27,2,6,10,15,21,27, 1,3,6,10,15,21,27}.
compute thetam={1,2,6,3,7,10,4,8,11,13,5,9,12,14,15}.
end if.
do if (nms = 7).
compute indmark={2,2,2,2,2,2,2}.
compute indsets={1,29,2,30,4,31,7,32,11,33,16,34,22,35}.
end if.
do if (nms = 8).
compute indmark={2,2,2,2,2,2,2,2}.
compute indsets={1,37,2,38,4,39,7,40,11,41,16,42,22,43,29,44}.
end if.
do if (nms = 9).
compute indmark={2,2,2,2,2,2,2,2,2}.
compute indsets={1,46,2,47,4,48,7,49,11,50,16,51,22,52,29,53,37,54}.
end if.
do if (nms=10).
compute indmark={2,2,2,2,2,2,2,2,2,2}.
compute indsets={1,56,2,57,4,58,7,59,11,60,16,61,22,62,29,63,37,64,46,65}.
end if.
compute indlbl = {'Ind1'; 'Ind2'; 'Ind3'; 'Ind4'; 'Ind5'; 'Ind6'; 'Ind7'; 'Ind8'; 'Ind9'; 'Ind10'; 'Ind11'; 'Ind12'; 'Ind13'; 'Ind14'; 'Ind15'}.
compute indlbl = {indlbl; 'Ind16';'Ind17';'Ind18';'Ind19';'Ind20';'Ind21';'Ind22';'Ind23';'Ind24';'Ind25';'Ind26';'Ind27';'Ind28';'Ind29';'Ind30'}.
compute indlbl = {indlbl; 'Ind31';'Ind32';'Ind33';'Ind34';'Ind35';'Ind36';'Ind37';'Ind38';'Ind39';'Ind40';'Ind41';'Ind42';'Ind43';'Ind44';'Ind45'}.
compute indlbl = {indlbl; 'Ind46';'Ind47';'Ind48';'Ind49';'Ind50';'Ind51';'Ind52';'Ind53';'Ind54';'Ind55';'Ind56';'Ind57';'Ind58';'Ind59';'Ind60'}.
compute indlbl = {indlbl; 'Ind61';'Ind62';'Ind63';'Ind64';'Ind65';'Ind66';'Ind67';'Ind68';'Ind69';'Ind70';'Ind71';'Ind72';'Ind73';'Ind74';'Ind75'}.
compute indlbl = {indlbl; 'Ind76';'Ind77';'Ind78';'Ind79';'Ind80';'Ind81';'Ind82';'Ind83';'Ind84';'Ind85';'Ind86';'Ind87';'Ind88';'Ind89';'Ind90'}.
compute cntname={'(C1)';'(C2)';'(C3)';'(C4)';'(C5)';'(C6)';'(C7)';'(C8)';'(C9)';'(C10)';'(C11)';'(C12)';'(C13)';'(C14)';'(C15)';'(C16)';'(C17)'}.
compute cntname={cntname;'(C18)';'(C19)';'(C20)';'(C21)';'(C22)';'(C23)';'(C24)';'(C25)';'(C26)';'(C27)';'(C28)';'(C29)';'(C30)';'(C31)'}.
compute cntname={cntname;'(C32)';'(C33)';'(C34)';'(C35)';'(C36)';'(C37)';'(C38)';'(C39)';'(C40)';'(C41)';'(C42)';'(C43)';'(C44)';'(C45)'}.
compute cntname={cntname;'(C46)';'(C47)';'(C48)';'(C49)';'(C50)';'(C51)';'(C52)';'(C53)';'(C54)';'(C55)';'(C56)';'(C57)';'(C58)';'(C59)'}.
compute cntname={cntname;'(C60)';'(C61)';'(C62)';'(C63)';'(C64)';'(C65)';'(C66)';'(C67)';'(C68)';'(C69)';'(C70)';'(C71)';'(C72)';'(C73)'}.
compute cntname={cntname;'(C74)';'(C75)';'(C76)';'(C77)';'(C78)';'(C79)';'(C80)';'(C81)';'(C82)';'(C83)';'(C84)';'(C85)';'(C86)';'(C87)'}.
compute cntname={cntname;'(C88)';'(C89)';'(C90)';'(C91)';'(C92)';'(C93)';'(C94)';'(C95)';'(C96)';'(C97)';'(C98)';'(C99)';'(C100)';'(C101)'}.
compute cntname={cntname;'(C102)';'(C103)';'(C104)';'(C105)'}.
compute indmake=make(ncol(indmark),(nms+2),0).
compute indmod=make(ncol(indmark),1,999).
compute indmmm=make(ncol(indmark),1,0).
compute indmmmt=make(ncol(indmark),1,0).
compute start=1.
compute end=0.
compute nindfx=0.
compute indlocs=make(nrow(thetaxmb),ncol(paths),999).
compute indkey=make(ncol(indmark),1+((rmax(indmark)*2)+1),'     ').
compute c1=1.
compute c2=1.
compute c3=1.
loop i = 1 to ncol(paths).
do if (pathtype(1,i)=1).
compute indlocs(:,i)=thetaxmb(:,c1).
compute c1=c1+1.
end if.
do if (pathtype(1,i)=3).
compute indlocs(:,i)=thetamyb(:,c2).
compute c2=c2+1.
end if.
do if (pathtype(1,i)=2 and nms < 7 and serial=1).
compute indlocs(:,i)=thetammb(:,thetam(1,c3)).
compute c3=c3+1.
end if.
end loop.
loop i = 1 to ncol(indlocs).
compute c1=2.
loop j = 2 to nrow(indlocs).
do if (indlocs(j,i) <> 0).
compute indlocs(c1,i)=indlocs(j,i).
compute c1=c1+1.
end if.
end loop.
compute indlocs(1,i)=c1-2.
end loop.
compute indlocs=indlocs(1:rmax((indlocs(1,:))+1),:).
loop i = 1 to ncol(indmark).
compute numget=indmark(1,i).
compute end=end+numget.
compute gotcha=indsets(1,start:end).
compute start=end+1.
compute ok=1.
compute temp=0.
compute repoman=make(4,1,0).
loop j = 1 to ncol(gotcha).
do if paths(1,gotcha(1,j))=0.
compute ok=0.
end if.
do if (pathsmod(1,gotcha(1,j)) > 0).
compute temp=1.
compute temp2={pathsw(1,gotcha(1,j));pathsz(1,gotcha(1,j));pathswz(1,gotcha(1,j));0}.
do if (temp2(1,1)=1 and temp2(2,1)=1 and temp2(3,1)=0).
compute temp2(4,1)=1.
end if.
compute repoman=repoman+temp2.
end if.
end loop.
compute temp=0.
compute tempmmm=0.
compute typemmm=0.
do if ((repoman(1,1) > 0) and (repoman(2,1) = 0)).
compute temp=1.
do if (repoman(1,1)=1).
compute tempmmm=1.
end if.
do if (repoman(1,1) > 1 and (wdich=1 or mcw > 0)).
compute tempmmm=12.
compute typemmm=mcw.
do if (wdich=1).
compute typemmm=1.
end if.
end if.
do if (repoman(1,1) > 1 and (wdich=0 and mcw = 0)).
compute tempmmm=101.
end if.
end if.
do if ((repoman(1,1) = 0) and (repoman(2,1) > 0)).
compute temp=2.
do if (repoman(2,1)=1).
compute tempmmm=2.
end if.
do if (repoman(2,1) > 1 and (zdich = 1 or mcz > 0)).
compute tempmmm=22.
compute typemmm=mcz.
do if (zdich=1).
compute typemmm=1.
end if.
end if.
do if (repoman(2,1) > 1 and (zdich = 0 and mcw = 0)).
compute tempmmm=102.
end if.
end if.
do if (repoman(1,1)>0 and repoman(2,1)>0).
compute temp=3.
do if (repoman(1,1)=1 and repoman(2,1)=1).
do if (repoman(4,1)=1).
compute tempmmm=31.
end if.
do if (repoman(3,1)=1).
compute tempmmm=41.
end if.
end if.
end if.
do if (repoman(1,1)=1 and repoman(2,1)=1 and repoman(3,1)=0 and repoman(4,1)=0).
compute tempmmm=51.
end if.
do if (ok = 1).
compute nindfx=nindfx+1.
compute indmake(nindfx,1)=numget.
compute indmod(nindfx,1)=temp.
compute indmmm(nindfx,1)=tempmmm.
compute indmmmt(nindfx,1)=typemmm.
compute indmake(nindfx,2:(1+numget))=gotcha.
compute indkey(nindfx,1)=xnames.
loop j = 1 to numget.
compute indkey(nindfx,(j*2+1))=pathsdv(1,gotcha(1,j)).
compute indkey(nindfx,(j*2))='   ->   '.
end loop.
end if.
end loop.
compute indkey=indkey(1:nindfx,1:((cmax(indmake(:,1))*2)+1)).
compute indmake=indmake(1:nindfx,1:(cmax(indmake(:,1))+1)).
compute indmod=indmod(1:nrow(indmake),1).
compute indmmm=indmmm(1:nrow(indmake),1).
compute indmmmt=indmmmt(1:nrow(indmake),1).
compute ncpairs = (((nindfx)*(nindfx-1))/2).
do if ((contrast = 1 or contrast = 2 or contrast=3) and (ncpairs > 105)).
compute contrast=0.
compute notecode(notes,1) = 13.
compute notes = notes + 1.
end if.
do if (contrast = 4).
do if (ncol(contvec) <> nindfx).
compute contrast=0.
compute notecode(notes,1) = 14.
compute notes = notes + 1.
end if.
end if.
do if (anymod=0).
do if (nms = 1 and contrast > 0).
compute contrast=0.
end if.
compute efloop=(((1-(effsize=0))*2)+1)-(((mcx>0 or xdich=1))*(1-(effsize=0))).
loop kk=1 to efloop.
do if (boot = 0).
compute bootres=obscoeff.
do if (kk=1).
compute totbtvec=make(1,nxvls,0).
end if.
compute bootdir=obsdirfx.
compute indtab=999.
compute inddiff=999.
compute bootysd=ysd.
compute bootxsd=xsd.
end if.
do if (boot > 0).
compute bootres={obscoeff;bootres}.
do if (kk=1).
compute totbtvec=make(nrow(bootres),nxvls,0).
end if.
compute indtab=make(1,4,999).
compute inddiff=make(nrow(bootres),1,999).
end if.
compute indtotal=make(nrow(bootres),1,0).
loop i = 1 to nrow(indmake).
loop j = 1 to nxvls.
compute indtemp=make(nrow(bootres),1,1).
loop k = 1 to indmake(i,1).
compute jtemp=1.
do if (j > 1 and k=1).
compute jtemp=j.
end if.
compute indtemp=indtemp&*bootres(:,pathsfoc(jtemp,indmake(i,(k+1)))).
end loop.
do if (kk = 2).
compute indtemp=indtemp/bootysd.
end if.
do if (kk = 3).
compute indtemp=(bootxsd&*indtemp)/bootysd.
end if.
do if (contrast <> 0).
compute inddiff={inddiff,indtemp}.
end if.
do if (nxvls=1).
compute indtotal=indtotal+indtemp.
end if.
compute indeff=indtemp(1,1).
do if (kk=1).
compute totbtvec(:,j)=totbtvec(:,j)+indtemp.
end if.
do if (boot > 0).

.
compute temp = indtemp(2:nrow(indtemp),1).
compute temp(GRADE( indtemp(2:nrow(indtemp),1) )) = indtemp(2:nrow(indtemp),1).
compute badlo = 0.
compute badhi = 0.
do if ( (indtemp(1,1)*bc)+(9999*(1-bc)) <> 9999).
compute pv=csum(temp < (indtemp(1,1)*bc)+(9999*(1-bc)) )/nrow(temp).
compute ppv = pv.
do if (pv > .5).
compute ppv = 1-pv.
end if.
compute y5=sqrt(-2*ln(ppv)).
compute xp=y5+((((y5*p4+p3)*y5+p2)*y5+p1)*y5+p0)/((((y5*q4+q3)*y5+q2)*y5+q1)*y5+q0).
do if (pv <= .5).
compute xp = -xp.
end if.
compute cilow=rnd(nrow(temp)*(cdfnorm(2*xp-xp2))).
compute cihigh=trunc(nrow(temp)*(cdfnorm(2*xp+(xp2))))+1.
do if (cilow < 1).
compute cilow = 1.
compute booterr=1.
compute badlo = 1.
end if.
do if (cihigh > nrow(temp)).
compute cihigh = boot.
compute booterr=1.
compute badhi = 1.
end if.
compute llcit=temp(cilow,1).
compute ulcit=temp(cihigh,1).
do if (badlo = 1 and llcit <> priorlo).
compute badend={badend, llcit}.
compute priorlo = llcit.
end if.
do if (badhi = 1 and ulcit <> priorhi).
compute badend={badend, ulcit}.
compute priorhi = ulcit.
end if.
end if.
do if ( (indtemp(1,1)*bc)+(9999*(1-bc)) = 9999).
compute llcit=temp(cilow,1).
compute ulcit=temp(cihigh,1).
end if.
compute bootse=t(sqrt((cssq(temp)-((csum(temp)&**2)/nrow(temp)))/(nrow(temp)-1)))
.
compute indeff={indeff,bootse,llcit,ulcit}.
end if.
do if (kk=1).
compute indtabn={indtab;indeff}.
end if.
compute indtab={indtab;indeff}.
end loop.
end loop.
compute indtab=indtab(2:nrow(indtab),:).
do if (kk=1).
compute indtabn=indtabn(2:nrow(indtabn),:).
end if.
compute rowlbs=indlbl(1:nrow(indtab),1).
do if (mc > 0).
compute inddiff=make(mc,1,-999).
compute indtab2=make(nrow(indtab),4,-999).
compute indtab2(:,1)=indtab.
compute indtab=indtab2.
release indtab2.
compute mcct=0.
compute indtotal=make(mc,1,0).
do if (kk = 1).
compute x1 = sqrt(-2*ln(uniform(mc,nrow(mcsopath))))&*cos((2*3.14159265358979)*uniform(mc, nrow(mcsopath))).
compute x1=x1*chol(indcov).
loop ii=1 to nrow(x1).
compute x1(ii,:)=x1(ii,:)+t(mcsopath).
end loop.
end if.
loop ii=1 to nms.
compute tmpb=x1(:,((nms*nxvls)+ii)).
compute tmpb2=tmpb.
do if (nxvls > 1).
loop jj=1 to (nxvls-1).
compute tmpb2={tmpb2,tmpb}.
end loop.
end if.
compute indtemp=x1(:,(((ii-1)*nxvls)+1):(ii*nxvls))&*tmpb2.
loop jj=1 to ncol(indtemp).
do if (kk = 2).
compute indtemp(:,jj)=indtemp(:,jj)/ysd.
end if.
do if (kk = 3).
compute indtemp(:,jj)=(xsd*indtemp(:,jj))/ysd.
end if.

.
compute temp = indtemp(:,jj).
compute temp(GRADE( indtemp(:,jj) )) = indtemp(:,jj).
compute badlo = 0.
compute badhi = 0.
do if ( 9999 <> 9999).
compute pv=csum(temp < 9999 )/nrow(temp).
compute ppv = pv.
do if (pv > .5).
compute ppv = 1-pv.
end if.
compute y5=sqrt(-2*ln(ppv)).
compute xp=y5+((((y5*p4+p3)*y5+p2)*y5+p1)*y5+p0)/((((y5*q4+q3)*y5+q2)*y5+q1)*y5+q0).
do if (pv <= .5).
compute xp = -xp.
end if.
compute cilow=rnd(nrow(temp)*(cdfnorm(2*xp-xp2))).
compute cihigh=trunc(nrow(temp)*(cdfnorm(2*xp+(xp2))))+1.
do if (cilow < 1).
compute cilow = 1.
compute booterr=1.
compute badlo = 1.
end if.
do if (cihigh > nrow(temp)).
compute cihigh = boot.
compute booterr=1.
compute badhi = 1.
end if.
compute llcit=temp(cilow,1).
compute ulcit=temp(cihigh,1).
do if (badlo = 1 and llcit <> priorlo).
compute badend={badend, llcit}.
compute priorlo = llcit.
end if.
do if (badhi = 1 and ulcit <> priorhi).
compute badend={badend, ulcit}.
compute priorhi = ulcit.
end if.
end if.
do if ( 9999 = 9999).
compute llcit=temp(cilow,1).
compute ulcit=temp(cihigh,1).
end if.
compute bootse=t(sqrt((cssq(temp)-((csum(temp)&**2)/nrow(temp)))/(nrow(temp)-1)))
.
compute mcct=mcct+1.
compute indtab(mcct,2:4)={bootse,llcit,ulcit}.
end loop.
do if (nxvls=1).
compute indtotal=indtotal+indtemp.
do if (contrast <> 0).
compute inddiff={inddiff,indtemp}.
end if.
end if.
end loop.
release indtemp,tmpb.
end if.
do if (normal = 1 and sobelok=1).
compute sobelmat=indtab(:,1).
compute sobelmat={sobelmat,(sobelmat/2),sobelmat,sobelmat}.
loop ii=1 to nms.
compute se2b=indcov(((nms*nxvls)+ii),((nms*nxvls)+ii)).
compute bpath2=mcsopath(((nms*nxvls)+ii),1)&**2.
compute se2a=diag(indcov((((ii-1)*nxvls)+1):(ii*nxvls),(((ii-1)*nxvls)+1):(ii*nxvls))).
compute apath2=mcsopath((((ii-1)*nxvls)+1):(ii*nxvls) ,1)&**2.
compute sesobel=sqrt(apath2*se2b+bpath2*se2a+se2a*se2b).
compute sobelmat((((ii-1)*nxvls)+1):(ii*nxvls),2)=sesobel.
end loop.
release se2b,bpath2,se2a,apath2,sesobel,ii.
compute sobelmat(:,3)=sobelmat(:,1)&/sobelmat(:,2).
compute sobelmat(:,4) = 2*(1-cdfnorm(abs(sobelmat(:,3)))).
end if.
do if (serial = 0).
compute rowlbs=t(mnames).
end if.
do if (nxvls=1 and nms > 1).
compute rowlbs={'TOTAL';rowlbs}.
compute indtemp=indtotal(1,1).
do if (boot > 0 and nxvls=1).

.
compute temp = indtotal(2:nrow(indtotal),1).
compute temp(GRADE( indtotal(2:nrow(indtotal),1) )) = indtotal(2:nrow(indtotal),1).
compute badlo = 0.
compute badhi = 0.
do if ( (indtotal(1,1)*bc)+(9999*(1-bc)) <> 9999).
compute pv=csum(temp < (indtotal(1,1)*bc)+(9999*(1-bc)) )/nrow(temp).
compute ppv = pv.
do if (pv > .5).
compute ppv = 1-pv.
end if.
compute y5=sqrt(-2*ln(ppv)).
compute xp=y5+((((y5*p4+p3)*y5+p2)*y5+p1)*y5+p0)/((((y5*q4+q3)*y5+q2)*y5+q1)*y5+q0).
do if (pv <= .5).
compute xp = -xp.
end if.
compute cilow=rnd(nrow(temp)*(cdfnorm(2*xp-xp2))).
compute cihigh=trunc(nrow(temp)*(cdfnorm(2*xp+(xp2))))+1.
do if (cilow < 1).
compute cilow = 1.
compute booterr=1.
compute badlo = 1.
end if.
do if (cihigh > nrow(temp)).
compute cihigh = boot.
compute booterr=1.
compute badhi = 1.
end if.
compute llcit=temp(cilow,1).
compute ulcit=temp(cihigh,1).
do if (badlo = 1 and llcit <> priorlo).
compute badend={badend, llcit}.
compute priorlo = llcit.
end if.
do if (badhi = 1 and ulcit <> priorhi).
compute badend={badend, ulcit}.
compute priorhi = ulcit.
end if.
end if.
do if ( (indtotal(1,1)*bc)+(9999*(1-bc)) = 9999).
compute llcit=temp(cilow,1).
compute ulcit=temp(cihigh,1).
end if.
compute bootse=t(sqrt((cssq(temp)-((csum(temp)&**2)/nrow(temp)))/(nrow(temp)-1)))
.
compute indtemp={indtemp, bootse,llcit,ulcit}.
end if.
do if (mc > 0).
compute obtmc=indtab(:,1).
compute indtemp=csum(obtmc).

.
compute temp = indtotal(:,1).
compute temp(GRADE( indtotal(:,1) )) = indtotal(:,1).
compute badlo = 0.
compute badhi = 0.
do if ( 9999 <> 9999).
compute pv=csum(temp < 9999 )/nrow(temp).
compute ppv = pv.
do if (pv > .5).
compute ppv = 1-pv.
end if.
compute y5=sqrt(-2*ln(ppv)).
compute xp=y5+((((y5*p4+p3)*y5+p2)*y5+p1)*y5+p0)/((((y5*q4+q3)*y5+q2)*y5+q1)*y5+q0).
do if (pv <= .5).
compute xp = -xp.
end if.
compute cilow=rnd(nrow(temp)*(cdfnorm(2*xp-xp2))).
compute cihigh=trunc(nrow(temp)*(cdfnorm(2*xp+(xp2))))+1.
do if (cilow < 1).
compute cilow = 1.
compute booterr=1.
compute badlo = 1.
end if.
do if (cihigh > nrow(temp)).
compute cihigh = boot.
compute booterr=1.
compute badhi = 1.
end if.
compute llcit=temp(cilow,1).
compute ulcit=temp(cihigh,1).
do if (badlo = 1 and llcit <> priorlo).
compute badend={badend, llcit}.
compute priorlo = llcit.
end if.
do if (badhi = 1 and ulcit <> priorhi).
compute badend={badend, ulcit}.
compute priorhi = ulcit.
end if.
end if.
do if ( 9999 = 9999).
compute llcit=temp(cilow,1).
compute ulcit=temp(cihigh,1).
end if.
compute bootse=t(sqrt((cssq(temp)-((csum(temp)&**2)/nrow(temp)))/(nrow(temp)-1)))
.
compute indtemp={indtemp, bootse,llcit,ulcit}.
end if.
compute indtab={indtemp;indtab}.
end if.
compute bootlbs={'Effect', 'BootSE','BootLLCI','BootULCI'}.
do if (mc > 0).
compute bootlbs={'Effect', 'MC SE','MC LLCI','MC ULCI'}.
end if.
do if (nxvls = 1).
do if (contrast <> 0).
compute inddiff=inddiff(:,2:ncol(inddiff)).
do if (mc > 0).
compute inddiff={t(obtmc);inddiff}.
end if.
do if (contrast = 4).
compute inddifft=inddiff*t(contvec).
compute indtemp=inddifft(1,1).
do if (boot > 0 or mc > 0).
do if (mc > 0).

.
compute temp = inddifft(2:nrow(inddifft),1).
compute temp(GRADE( inddifft(2:nrow(inddifft),1) )) = inddifft(2:nrow(inddifft),1).
compute badlo = 0.
compute badhi = 0.
do if ( 9999 <> 9999).
compute pv=csum(temp < 9999 )/nrow(temp).
compute ppv = pv.
do if (pv > .5).
compute ppv = 1-pv.
end if.
compute y5=sqrt(-2*ln(ppv)).
compute xp=y5+((((y5*p4+p3)*y5+p2)*y5+p1)*y5+p0)/((((y5*q4+q3)*y5+q2)*y5+q1)*y5+q0).
do if (pv <= .5).
compute xp = -xp.
end if.
compute cilow=rnd(nrow(temp)*(cdfnorm(2*xp-xp2))).
compute cihigh=trunc(nrow(temp)*(cdfnorm(2*xp+(xp2))))+1.
do if (cilow < 1).
compute cilow = 1.
compute booterr=1.
compute badlo = 1.
end if.
do if (cihigh > nrow(temp)).
compute cihigh = boot.
compute booterr=1.
compute badhi = 1.
end if.
compute llcit=temp(cilow,1).
compute ulcit=temp(cihigh,1).
do if (badlo = 1 and llcit <> priorlo).
compute badend={badend, llcit}.
compute priorlo = llcit.
end if.
do if (badhi = 1 and ulcit <> priorhi).
compute badend={badend, ulcit}.
compute priorhi = ulcit.
end if.
end if.
do if ( 9999 = 9999).
compute llcit=temp(cilow,1).
compute ulcit=temp(cihigh,1).
end if.
compute bootse=t(sqrt((cssq(temp)-((csum(temp)&**2)/nrow(temp)))/(nrow(temp)-1)))
.
end if.
do if (boot > 0).

.
compute temp = inddifft(2:nrow(inddifft),1).
compute temp(GRADE( inddifft(2:nrow(inddifft),1) )) = inddifft(2:nrow(inddifft),1).
compute badlo = 0.
compute badhi = 0.
do if ( (inddifft(1,1)*bc)+(9999* (1-bc)) <> 9999).
compute pv=csum(temp < (inddifft(1,1)*bc)+(9999* (1-bc)) )/nrow(temp).
compute ppv = pv.
do if (pv > .5).
compute ppv = 1-pv.
end if.
compute y5=sqrt(-2*ln(ppv)).
compute xp=y5+((((y5*p4+p3)*y5+p2)*y5+p1)*y5+p0)/((((y5*q4+q3)*y5+q2)*y5+q1)*y5+q0).
do if (pv <= .5).
compute xp = -xp.
end if.
compute cilow=rnd(nrow(temp)*(cdfnorm(2*xp-xp2))).
compute cihigh=trunc(nrow(temp)*(cdfnorm(2*xp+(xp2))))+1.
do if (cilow < 1).
compute cilow = 1.
compute booterr=1.
compute badlo = 1.
end if.
do if (cihigh > nrow(temp)).
compute cihigh = boot.
compute booterr=1.
compute badhi = 1.
end if.
compute llcit=temp(cilow,1).
compute ulcit=temp(cihigh,1).
do if (badlo = 1 and llcit <> priorlo).
compute badend={badend, llcit}.
compute priorlo = llcit.
end if.
do if (badhi = 1 and ulcit <> priorhi).
compute badend={badend, ulcit}.
compute priorhi = ulcit.
end if.
end if.
do if ( (inddifft(1,1)*bc)+(9999* (1-bc)) = 9999).
compute llcit=temp(cilow,1).
compute ulcit=temp(cihigh,1).
end if.
compute bootse=t(sqrt((cssq(temp)-((csum(temp)&**2)/nrow(temp)))/(nrow(temp)-1)))
.
end if.
compute indtemp={indtemp, bootse,llcit,ulcit}.
end if.
compute indtab={indtab;indtemp}.
end if.
do if (contrast = 1 or contrast = 2 or contrast=3).
compute conkey=make(1,4,' ').
loop i = 1 to ncol(inddiff)-1.
loop j = (i+1) to ncol(inddiff).
compute inddifft=inddiff(:,i)-inddiff(:,j).
do if (contrast=2).
compute inddifft=abs(inddiff(:,i))-abs(inddiff(:,j)).
end if.
do if (contrast=3).
compute inddifft=inddiff(:,i)+inddiff(:,j).
end if.
compute indtemp=inddifft(1,1).
do if (contrast=1 or contrast=2).
compute conkeyt={' ', rowlbs((i+1),1),' minus  ',rowlbs((j+1),1)}.
end if.
do if (contrast=3).
compute conkeyt={' ', rowlbs((i+1),1),'  plus  ',rowlbs((j+1),1)}.
end if.
compute conkey={conkey;conkeyt}.
do if (boot > 0 or mc > 0).
do if (mc > 0).

.
compute temp = inddifft(2:nrow(inddifft),1).
compute temp(GRADE( inddifft(2:nrow(inddifft),1) )) = inddifft(2:nrow(inddifft),1).
compute badlo = 0.
compute badhi = 0.
do if ( 9999 <> 9999).
compute pv=csum(temp < 9999 )/nrow(temp).
compute ppv = pv.
do if (pv > .5).
compute ppv = 1-pv.
end if.
compute y5=sqrt(-2*ln(ppv)).
compute xp=y5+((((y5*p4+p3)*y5+p2)*y5+p1)*y5+p0)/((((y5*q4+q3)*y5+q2)*y5+q1)*y5+q0).
do if (pv <= .5).
compute xp = -xp.
end if.
compute cilow=rnd(nrow(temp)*(cdfnorm(2*xp-xp2))).
compute cihigh=trunc(nrow(temp)*(cdfnorm(2*xp+(xp2))))+1.
do if (cilow < 1).
compute cilow = 1.
compute booterr=1.
compute badlo = 1.
end if.
do if (cihigh > nrow(temp)).
compute cihigh = boot.
compute booterr=1.
compute badhi = 1.
end if.
compute llcit=temp(cilow,1).
compute ulcit=temp(cihigh,1).
do if (badlo = 1 and llcit <> priorlo).
compute badend={badend, llcit}.
compute priorlo = llcit.
end if.
do if (badhi = 1 and ulcit <> priorhi).
compute badend={badend, ulcit}.
compute priorhi = ulcit.
end if.
end if.
do if ( 9999 = 9999).
compute llcit=temp(cilow,1).
compute ulcit=temp(cihigh,1).
end if.
compute bootse=t(sqrt((cssq(temp)-((csum(temp)&**2)/nrow(temp)))/(nrow(temp)-1)))
.
end if.
do if (boot > 0).

.
compute temp = inddifft(2:nrow(inddifft),1).
compute temp(GRADE( inddifft(2:nrow(inddifft),1) )) = inddifft(2:nrow(inddifft),1).
compute badlo = 0.
compute badhi = 0.
do if ( (inddifft(1,1)*bc)+(9999* (1-bc)) <> 9999).
compute pv=csum(temp < (inddifft(1,1)*bc)+(9999* (1-bc)) )/nrow(temp).
compute ppv = pv.
do if (pv > .5).
compute ppv = 1-pv.
end if.
compute y5=sqrt(-2*ln(ppv)).
compute xp=y5+((((y5*p4+p3)*y5+p2)*y5+p1)*y5+p0)/((((y5*q4+q3)*y5+q2)*y5+q1)*y5+q0).
do if (pv <= .5).
compute xp = -xp.
end if.
compute cilow=rnd(nrow(temp)*(cdfnorm(2*xp-xp2))).
compute cihigh=trunc(nrow(temp)*(cdfnorm(2*xp+(xp2))))+1.
do if (cilow < 1).
compute cilow = 1.
compute booterr=1.
compute badlo = 1.
end if.
do if (cihigh > nrow(temp)).
compute cihigh = boot.
compute booterr=1.
compute badhi = 1.
end if.
compute llcit=temp(cilow,1).
compute ulcit=temp(cihigh,1).
do if (badlo = 1 and llcit <> priorlo).
compute badend={badend, llcit}.
compute priorlo = llcit.
end if.
do if (badhi = 1 and ulcit <> priorhi).
compute badend={badend, ulcit}.
compute priorhi = ulcit.
end if.
end if.
do if ( (inddifft(1,1)*bc)+(9999* (1-bc)) = 9999).
compute llcit=temp(cilow,1).
compute ulcit=temp(cihigh,1).
end if.
compute bootse=t(sqrt((cssq(temp)-((csum(temp)&**2)/nrow(temp)))/(nrow(temp)-1)))
.
end if.
compute indtemp={indtemp, bootse,llcit,ulcit}.
end if.
compute indtab={indtab;indtemp}.
end loop.
end loop.
end if.
release inddiff.
compute contlbs=cntname(1:(((nindfx)*(nindfx-1))/2),1).
compute rowlbs={rowlbs;contlbs}.
end if.
compute resultm2=make(nrow(indtab),maxresm,99999).
compute resultm2(1:nrow(indtab),1:ncol(indtab))=indtab.
compute resultm={resultm;resultm2}.
do if (outscree=1).
do if (kk=1).
print indtab/title = 'Indirect effect(s) of X on Y:'/rnames=rowlbs/cnames=bootlbs/format= F10.4.
end if.
do if (kk = 2 and (xdich=1 or mcx > 0)).
print indtab/title = 'Partially standardized indirect effect(s) of X on Y:'/rnames=rowlbs/cnames=bootlbs/format= F10.4.
end if.
do if (kk = 3).
print indtab/title = 'Completely standardized indirect effect(s) of X on Y:'/rnames=rowlbs/cnames=bootlbs/format= F10.4.
end if.
end if.
do if (normal=1 and sobelok=1 and kk=1).
compute resultm2=make(nrow(sobelmat),maxresm,99999).
compute resultm2(1:nrow(sobelmat),1:ncol(sobelmat))=sobelmat.
compute resultm={resultm;resultm2}.
compute sobellab={'Effect',hclab,'Z','p'}.
compute sobelrlb=rowlbs.
do if (nms > 1).
compute sobelrlb=rowlbs(2:(1+nms),1).
end if.
do if (outscree=1).
print sobelmat/title='   Normal theory test for indirect effect(s):'/cnames=sobellab/rnames=sobelrlb/format= F10.4.
end if.
end if.
do if (contrast <> 0).
do if ((contrast=1 or contrast = 2 or contrast=3) and kk=efloop ).
compute conkey=conkey(2:nrow(conkey),:).
do if (outscree=1).
print conkey/title = 'Specific indirect effect contrast definition(s):'/rnames=contlbs/format=A8.
end if.
end if.
do if (outscree=1).
do if (contrast = 4 and kk=efloop).
compute crowlbs=rowlbs(2:(nindfx+1),1).
print contvec/title = 'Specific indirect effect contrast weights:'/cnames=crowlbs/rlabels='(C1)'/format= F10.4.
end if.
do if (contrast = 2 and kk=efloop).
print/title = 'Contrasts are differences between absolute values of indirect effects'.
end if.
end if.
end if.
do if (serial = 1 and kk=efloop).
compute rowlbst=rowlbs(2:nrow(rowlbs),1).
do if (outscree=1).
print indkey/title = 'Indirect effect key:'/rnames=rowlbst/format = A8.
end if.
end if.
else.
do if (outscree=1).
do if (kk = 1).
print/title = 'Relative indirect effects of X on Y'.
end if.
do if (kk = 2).
print/title = 'Partially standardized relative indirect effect(s) of X on Y:'.
end if.
do if (kk = 3).
print/title = 'Completely standardized relative indirect effect(s) of X on Y:'.
end if.
end if.
loop i = 1 to nrow(indmake).
compute indtabsm=indtab((((i-1)*nxvls)+1):(nxvls*i),:).
compute indkeyt=indkey(i,:).
do if (outscree=1).
print indkeyt/title=' '/space=0/format=A8.
end if.
do if (bcmat(nrow(bcmat),1)=0)).
compute direffl2=xcatlab(1:nxvls,1).
end if.
compute resultm2=make(nrow(indtabsm),maxresm,99999).
compute resultm2(1:nrow(indtabsm),1:ncol(indtabsm))=indtabsm.
compute resultm={resultm;resultm2}.
do if (outscree=1).
print indtabsm/title = ' '/cnames=bootlbs/rnames=direffl2/format= F10.4 /space=0.
end if.
do if (normal=1 and sobelok=1 and kk=1).
compute sobelsm=sobelmat((((i-1)*nxvls)+1):(nxvls*i),:).
compute sobellab={'Effect',hclab,'Z','p'}.
compute resultm2=make(nrow(sobelsm),maxresm,99999).
compute resultm2(1:nrow(sobelsm),1:ncol(sobelsm))=sobelsm.
compute resultm={resultm;resultm2}.
do if (outscree=1).
print sobelsm/title='      Normal theory test for relative indirect effects:'/cnames=sobellab/rnames=direffl2/format= F10.4.
end if.
end if.
end loop.
end if.
do if (effsize = 1 and boot > 0).
compute bootres=bootres(2:nrow(bootres),:).
end if.
end loop.
do if (alttotal=1).
compute altcnms='Effect'.
compute totbtvec=totbtvec+bootdir.
compute alttotfx=t(totbtvec(1,:)).
do if (boot > 0).
compute alttotfx=make(ncol(totbtvec),4,0).
compute alttotfx(:,1)=t(totbtvec(1,:)).
loop cec=1 to ncol(totbtvec).

.
compute temp = totbtvec(2:nrow(totbtvec),cec).
compute temp(GRADE( totbtvec(2:nrow(totbtvec),cec) )) = totbtvec(2:nrow(totbtvec),cec).
compute badlo = 0.
compute badhi = 0.
do if ( (totbtvec(1,cec)*bc)+(9999* (1-bc)) <> 9999).
compute pv=csum(temp < (totbtvec(1,cec)*bc)+(9999* (1-bc)) )/nrow(temp).
compute ppv = pv.
do if (pv > .5).
compute ppv = 1-pv.
end if.
compute y5=sqrt(-2*ln(ppv)).
compute xp=y5+((((y5*p4+p3)*y5+p2)*y5+p1)*y5+p0)/((((y5*q4+q3)*y5+q2)*y5+q1)*y5+q0).
do if (pv <= .5).
compute xp = -xp.
end if.
compute cilow=rnd(nrow(temp)*(cdfnorm(2*xp-xp2))).
compute cihigh=trunc(nrow(temp)*(cdfnorm(2*xp+(xp2))))+1.
do if (cilow < 1).
compute cilow = 1.
compute booterr=1.
compute badlo = 1.
end if.
do if (cihigh > nrow(temp)).
compute cihigh = boot.
compute booterr=1.
compute badhi = 1.
end if.
compute llcit=temp(cilow,1).
compute ulcit=temp(cihigh,1).
do if (badlo = 1 and llcit <> priorlo).
compute badend={badend, llcit}.
compute priorlo = llcit.
end if.
do if (badhi = 1 and ulcit <> priorhi).
compute badend={badend, ulcit}.
compute priorhi = ulcit.
end if.
end if.
do if ( (totbtvec(1,cec)*bc)+(9999* (1-bc)) = 9999).
compute llcit=temp(cilow,1).
compute ulcit=temp(cihigh,1).
end if.
compute bootse=t(sqrt((cssq(temp)-((csum(temp)&**2)/nrow(temp)))/(nrow(temp)-1)))
.
compute alttotfx(cec,2:4)={bootse,llcit,ulcit}.
end loop.
compute altcnms={altcnms,'BootSE', 'BootLLCI','BootULCI'}.
end if.
compute resultm2=make(nrow(alttotfx),maxresm,99999).
compute resultm2(1:nrow(alttotfx),1:ncol(alttotfx))=alttotfx.
compute resultm={resultm;resultm2}.
do if (outscree=1).
do if (nxvls > 1).
print/title= '----------'/space=0.
print alttotfx/title='Relative total effects of X on Y (sum of direct and indirect effects)'/rnames=direffl2/cnames=altcnms/format= F10.4.
end if.
do if (nxvls = 1).
print alttotfx/title='Total effect of X on Y (sum of direct and indirect effects)'/cnames=altcnms/format= F10.4.
end if.
end if.
end if.
end if.
do if (anymod > 0).
do if (boot = 0).
compute bootres=obscoeff.
compute indtab=999.
end if.
do if (boot > 0).
compute bootres={obscoeff;bootres}.
compute indtab=make(1,4,999).
end if.
do if (csum((indmod > 0))=nrow(indmod)).
do if (outscree=1).
do if (nxvls > 1).
do if (xmint=0).
print/title= 'Relative conditional indirect effects of X on Y:'.
end if.
do if (xmint=1).
print/title= 'Relative (total) natural indirect effects of X on Y:'.
end if.
end if.
do if (nxvls = 1).
do if (xmint=0).
print/title= 'Conditional indirect effects of X on Y:'.
end if.
do if (xmint=1).
print/title= '(Total) Natural indirect effect(s) of X on Y:'.
end if.
end if.
end if.
end if.
do if (csum((indmod > 0)) < nrow(indmod)).
do if (outscree=1).
do if (nxvls > 1).
print/title= 'Relative conditional and unconditional indirect effects of X on Y:'.
end if.
do if (nxvls = 1).
print/title= 'Conditional and unconditional indirect effects of X on Y:'.
end if.
end if.
end if.
compute cftotfx=make(nrow(bootres),nxvls,0).
loop i = 1 to nrow(indmake).
compute indtab=indtab(1,:)*0.
do if (outscree=1).
do if (xmint=0).
print indkey(i,:)/title = 'INDIRECT EFFECT:'/format=A8.
end if.
do if (xmint=1).
print indkey(i,:)/title = ' '/format=A8/space=0.
end if.
end if.
do if (indmod(i,1)=0).
loop j = 1 to nxvls.
compute indtemp=make(nrow(bootres),1,1).
loop k = 1 to indmake(i,1).
compute jtemp=1.
do if (j > 1 and k=1).
compute jtemp=j.
end if.
compute indtemp=indtemp&*bootres(:,pathsfoc(jtemp,indmake(i,(k+1)))).
end loop.
compute indeff=indtemp(1,1).
do if (boot > 0).

.
compute temp = indtemp(2:nrow(indtemp),1).
compute temp(GRADE( indtemp(2:nrow(indtemp),1) )) = indtemp(2:nrow(indtemp),1).
compute badlo = 0.
compute badhi = 0.
do if ( (indtemp(1,1)*bc)+(9999*(1-bc)) <> 9999).
compute pv=csum(temp < (indtemp(1,1)*bc)+(9999*(1-bc)) )/nrow(temp).
compute ppv = pv.
do if (pv > .5).
compute ppv = 1-pv.
end if.
compute y5=sqrt(-2*ln(ppv)).
compute xp=y5+((((y5*p4+p3)*y5+p2)*y5+p1)*y5+p0)/((((y5*q4+q3)*y5+q2)*y5+q1)*y5+q0).
do if (pv <= .5).
compute xp = -xp.
end if.
compute cilow=rnd(nrow(temp)*(cdfnorm(2*xp-xp2))).
compute cihigh=trunc(nrow(temp)*(cdfnorm(2*xp+(xp2))))+1.
do if (cilow < 1).
compute cilow = 1.
compute booterr=1.
compute badlo = 1.
end if.
do if (cihigh > nrow(temp)).
compute cihigh = boot.
compute booterr=1.
compute badhi = 1.
end if.
compute llcit=temp(cilow,1).
compute ulcit=temp(cihigh,1).
do if (badlo = 1 and llcit <> priorlo).
compute badend={badend, llcit}.
compute priorlo = llcit.
end if.
do if (badhi = 1 and ulcit <> priorhi).
compute badend={badend, ulcit}.
compute priorhi = ulcit.
end if.
end if.
do if ( (indtemp(1,1)*bc)+(9999*(1-bc)) = 9999).
compute llcit=temp(cilow,1).
compute ulcit=temp(cihigh,1).
end if.
compute bootse=t(sqrt((cssq(temp)-((csum(temp)&**2)/nrow(temp)))/(nrow(temp)-1)))
.
compute indeff={indeff,bootse,llcit,ulcit}.
end if.
compute indtab={indtab;indeff}.
end loop.
compute resultm2=make(nrow(indtab),maxresm,99999).
compute resultm2(1:nrow(indtab),1:ncol(indtab))=indtab.
compute resultm={resultm;resultm2}.
do if (outscree=1).
do if (nxvls > 1).
compute indefflb=xcatlab(1:nxvls,1).
print indtab(2:nrow(indtab),:)/title = ' '/clabels='Effect', 'BootSE', 'BootLLCI', 'BootULCI'/rnames=indefflb/format= F10.4 /space=0.
end if.
do if (nxvls = 1).
print indtab(2:nrow(indtab),:)/title = ' '/clabels='Effect', 'BootSE', 'BootLLCI', 'BootULCI'/format= F10.4 /space=0.
end if.
end if.
end if.
do if (indmod(i,1)>0).
do if (indmod(i,1)=1).
compute indmodva=wmodvals.
compute indprova=wprobval.
compute condlbs={wnames}.
compute printw=1.
else if (indmod(i,1)=2).
compute indmodva=zmodvals.
compute indprova=zprobval.
compute condlbs={znames}.
compute printz=1.
else if (indmod(i,1)=3).
compute cntmp=1.
compute printz=1.
compute printw=1.
compute indmodva=make((nrow(wmodvals)*nrow(zmodvals)),2,999).
loop k7 = 1 to nrow(wmodvals).
loop k8 = 1 to nrow(zmodvals).
compute indmodva(cntmp,:)={wmodvals(k7,1),zmodvals(k8,1)}.
compute cntmp=cntmp+1.
end loop.
end loop.
compute condlbs={wnames,znames}.
end if.
compute condres=make(nrow(indmodva),1,999).
do if (boot > 0).
compute condres=make(nrow(indmodva),4,999).
end if.
compute condres={indmodva,condres}.
loop k4 = 1 to nxvls.
compute imm3=make(nrow(bootres),1,1).
compute imm4=make(nrow(bootres),1,1).
compute indcontr=0.
do if (indmod(i,1)=3).
compute tihsw=wprobval.
compute tihsz=zprobval.
end if.
loop k1=1 to nrow(indmodva).
compute tucker2=make(nrow(bootres),1,1).
compute imm2=make(nrow(bootres),1,1).
compute wfirst=0.
compute zfirst=0.
compute immset=0.
loop k2=1 to indmake(i,1).
compute colnumb=indmake(i,(k2+1)).
do if (k2=1).
compute wbb=make(nrow(bootres),(nwvls*nxvls),0).
compute zbb=make(nrow(bootres),(nzvls*nxvls),0).
compute wzbb=make(nrow(bootres),(nwvls*nzvls*nxvls),0).
end if.
do if (k2<>1).
compute wbb=make(nrow(bootres),nwvls,0).
compute zbb=make(nrow(bootres),nzvls,0).
compute wzbb=make(nrow(bootres),(nwvls*nzvls),0).
end if.
compute cnt=1.
compute tihs=indlocs(2:((indlocs(1,colnumb))+1),colnumb).
do if (k2 = 1).
compute focbb=tihs(1:nxvls,1).
compute focbb=bootres(:,focbb).
do if (indmmm(i,1)>0).
compute imm=focbb(:,k4).
compute condbb=imm.
end if.
compute focaddon=make(1,nxvls,0).
compute focaddon(1,k4)=1.
compute cnt=cnt+nxvls.
compute placeh=nxvls.
do if (indmod(i,1)=1).
compute tihsz=make(nrow(wprobval),(nzvls*nxvls),0).
compute tihswz=make(nrow(wprobval),(nwvls*nzvls*nxvls),0).
do if (pathsw(1,colnumb)=1).
compute temp=make(nrow(wprobval),(nxvls*nwvls),0).
loop k5 = 1 to nrow(wprobval).
loop k6=1 to nwvls.
compute temp(k5, (((k4-1)*nwvls)+k6))=wprobval(k5,k6).
end loop.
end loop.
compute indprova={temp,tihsz,tihswz}.
else.
compute indprova={wprobval,tihsz,tihswz}.
end if.
end if.
do if (indmod(i,1)=2).
compute tihsw=make(nrow(zprobval),(nwvls*nxvls),0).
compute tihswz=make(nrow(zprobval),(nwvls*nzvls*nxvls),0).
do if (pathsz(1,colnumb)=1).
compute temp=make(nrow(zprobval),(nxvls*nzvls),0).
loop k5 = 1 to nrow(zprobval).
loop k6 =1 to nzvls.
compute temp(k5,(((k4-1)*nzvls)+k6))=zprobval(k5,k6).
end loop.
end loop.
compute indprova={tihsw,temp,tihswz}.
else.
compute indprova={tihsw,zprobval,tihswz}.
end if.
end if.
do if (indmod(i,1)=3).
compute indprova=make((nrow(wprobval)*nrow(zprobval)),((ncol(wprobval)*nxvls)+ (ncol(zprobval)*nxvls)+(nwvls*nzvls*nxvls)),0).
compute cntemp=1.
loop k7=1 to nrow(wprobval).
loop k8 =1 to nrow(zprobval).
compute temp=wprobval(k7,:)*focaddon(1,k4).
compute indprova(cntemp,(((k4-1)*nwvls)+1):(k4*(nwvls)))=temp.
compute temp=zprobval(k8,:)*focaddon(1,k4).
compute indprova(cntemp, ((((k4-1)*nzvls)+1)+(nxvls*nwvls)) : ((((k4-1)*nzvls)+1)+(nxvls*nwvls)+(nzvls-1)))=temp.
compute cntemp=cntemp+1.
end loop.
end loop.
do if (pathsz(1,colnumb)=0).
compute temp=make(nrow(indprova),(ncol(zprobval)*nxvls),0).
compute indprova(:,((ncol(wprobval)*nxvls)+1):((ncol(wprobval)+ncol(zprobval))* nxvls))=temp.
end if.
do if (pathsw(1,colnumb)=0).
compute temp=make(nrow(indprova),(ncol(wprobval)*nxvls),0).
compute indprova(:,1:(ncol(wprobval)*nxvls))=temp.
end if.
do if (pathswz(1,colnumb)=1).
compute cntemp=(ncol(wprobval)*nxvls)+(ncol(zprobval)*nxvls)+((k4-1)* ncol(wprobval)*ncol(zprobval))+1.
loop k7=1 to ncol(wprobval).
loop k8=1 to ncol(zprobval).
compute indprova(:,cntemp)=indprova(:,((ncol(wprobval)*(k4-1))+k7))&* indprova(:,((((k4-1)*ncol(zprobval))+k8)+(nxvls*ncol(wprobval)))).
compute cntemp=cntemp+1.
end loop.
end loop.
end if.
end if.
end if.
do if (k2 > 1).
compute focbb=tihs(1,1).
compute focbb=bootres(:,focbb).
do if (indmmm(i,1)>0).
compute imm=focbb(:,1).
compute condbb=imm.
end if.
compute focaddon=1.
compute cnt=cnt+1.
compute placeh=1.
do if (indmod(i,1)=1).
compute tihsz=make(nrow(wprobval),nzvls,0).
compute tihswz=make(nrow(wprobval),(nwvls*nzvls),0).
compute indprova={wprobval,tihsz,tihswz}.
end if.
do if (indmod(i,1)=2).
compute tihsw=make(nrow(zprobval),nwvls,0).
compute tihswz=make(nrow(zprobval),(nwvls*nzvls),0).
compute indprova={tihsw,zprobval,tihswz}.
end if.
do if (indmod(i,1)=3).
compute indprova=make((nrow(wprobval)*nrow(zprobval)),((ncol(wprobval)+ ncol(zprobval))+(nwvls*nzvls)),0).
compute cntemp=1.
loop k7=1 to nrow(wprobval).
loop k8 =1 to nrow(zprobval).
compute indprova(cntemp,1:(ncol(wprobval)+ncol(zprobval)))={wprobval(k7,:), zprobval(k8,:)}.
compute cntemp=cntemp+1.
end loop.
end loop.
do if (pathsz(1,colnumb)=0).
compute temp=make(nrow(indprova),ncol(zprobval),0).
compute indprova(:,(ncol(wprobval)+1):(ncol(wprobval)+ncol(zprobval)))=temp.
end if.
do if (pathsw(1,colnumb)=0).
compute temp=make(nrow(indprova),ncol(wprobval),0).
compute indprova(:,1:ncol(wprobval))=temp.
end if.
do if (pathswz(1,colnumb)=1).
compute cntemp=ncol(wprobval)+ncol(zprobval)+1.
loop k7=1 to ncol(wprobval).
loop k8=1 to ncol(zprobval).
compute indprova(:,cntemp)=indprova(:,k7)&*indprova(:,(ncol(wprobval)+k8)).
compute cntemp=cntemp+1.
end loop.
end loop.
end if.
end if.
end if.
do if (pathsw(1,colnumb)) = 1.
compute wbb=tihs(cnt:(cnt+(placeh*nwvls)-1),1).
compute wbb=bootres(:,wbb).
compute immlbs2=wcatlab(1:nwvls,1).
do if (zfirst=0).
compute wfirst=1.
end if.
do if (indmmm(i,1)=1 or indmmm(i,1)=31 or indmmm(i,1)=51).
compute imm=wbb(:,1).
loop k7 = 1 to nwvls.
compute imm={imm,wbb(:,(((k4-1)*nwvls*(k2=1))+k7))}.
end loop.
compute imm=imm(:,2:ncol(imm)).
end if.
do if (indmmm(i,1)=41 or indmmm(i,1)=51).
compute condbb=make(nrow(bootres),1,0).
loop k7 = 1 to nwvls.
compute condbb={condbb,wbb(:,(((k4-1)*nwvls*(k2=1))+k7))}.
end loop.
compute condbb=condbb(:,2:ncol(condbb)).
end if.
compute cnt=cnt+(placeh*nwvls).
end if.
do if (pathsz(1,colnumb)) = 1.
compute zbb=tihs(cnt:(cnt+(placeh*nzvls)-1),1).
compute zbb=bootres(:,zbb).
do if (wfirst=0).
compute zfirst=1.
end if.
do if (indmmm(i,1) <> 31).
compute immlbs2=zcatlab(1:nzvls,1).
end if.
do if (indmmm(i,1)=2 or indmmm(i,1)=31 or indmmm(i,1)=51).
do if (indmmm(i,1)=2).
compute imm=zbb(:,1).
end if.
loop k7 = 1 to nzvls.
compute imm={imm,zbb(:,(((k4-1)*nzvls*(k2=1))+k7))}.
end loop.
do if (indmmm(i,1)=2 or indmmm(i,1)=51).
compute imm=imm(:,2:ncol(imm)).
do if (indmmm(i,1)=51).
compute condbb={condbb,imm}.
end if.
end if.
end if.
compute cnt=cnt+(placeh*nzvls).
end if.
do if (pathswz(1,colnumb)) = 1.
compute wzbb=tihs(cnt:(cnt+(placeh*nwvls*nzvls)-1),1).
compute wzbb=bootres(:,wzbb).
do if (indmmm(i,1)=41).
compute imm=wzbb(:,1).
loop k7=1 to nwvls*nzvls.
compute imm={imm,wzbb(:,(((k4-1)*nzvls*nwvls*(k2=1))+k7))}.
end loop.
end if.
do if (indmmm(i,1)=41).
compute imm=imm(:,2:ncol(imm)).
compute condbb={condbb,imm(:,(ncol(imm)-(nwvls*nzvls)+1):ncol(imm))}.
end if.
compute cnt=cnt+(placeh*nzvls*nwvls).
end if.
compute indprobe={focaddon,indprova(k1,:)}.
compute tucker={focbb,wbb,zbb,wzbb}.
loop k3=1 to ncol(indprobe).
compute tucker(:,k3)=tucker(:,k3)*indprobe(1,k3).
end loop.
compute tucker2=tucker2&*rsum(tucker).
do if (indmmm(i,1) = 1 or indmmm(i,1)=2 or indmmm(i,1)=31 or indmmm(i,1)=41 or indmmm(i,1)=51).
do if (immset=1).
do if (ncol(imm2)=1 and ncol(imm) = 1).
compute imm2=imm2&*imm.
end if.
do if (indmmm(i,1)=41 or indmmm(i,1)=51).
do if ((ncol(condbb2) > 1) and (ncol(condbb)>1)).
compute condbb2t=make(nrow(condbb2),(ncol(condbb2)*ncol(condbb)),-999999).
compute k9=1.
do if (wfirst=1).
loop k7=1 to ncol(condbb2).
loop k8 = 1 to ncol(condbb).
compute condbb2t(:,k9)=condbb2(:,k7)&*condbb(:,k8).
compute k9=k9+1.
end loop.
end loop.
end if.
do if (zfirst=1).
loop k7=1 to ncol(condbb).
loop k8 = 1 to ncol(condbb2).
compute condbb2t(:,k9)=condbb(:,k7)&*condbb2(:,k8).
compute k9=k9+1.
end loop.
end loop.
end if.
compute condbb2=condbb2t.
release condbb2t.
end if.
do if ((ncol(condbb2) > 1) and (ncol(condbb)=1)).
loop k7 = 1 to ncol(condbb2).
compute condbb2(:,k7)=condbb2(:,k7)&*condbb.
end loop.
end if.
do if ((ncol(condbb2) = 1) and (ncol(condbb)>1)).
loop k7 = 1 to ncol(condbb).
compute condbb(:,k7)=condbb2&*condbb(:,k7).
end loop.
compute condbb2=condbb.
end if.
end if.
do if (ncol(imm2) <> 1 and ncol(imm) <> 1).
compute imm2t=make(nrow(imm2),(ncol(imm2)*ncol(imm)),-999999).
compute k9=1.
do if (wfirst=1).
loop k7=1 to ncol(imm2).
loop k8 = 1 to ncol(imm).
compute imm2t(:,k9)=imm2(:,k7)&*imm(:,k8).
compute k9=k9+1.
end loop.
end loop.
end if.
do if (zfirst=1).
loop k7=1 to ncol(imm).
loop k8 = 1 to ncol(imm2).
compute imm2t(:,k9)=imm(:,k7)&*imm2(:,k8).
compute k9=k9+1.
end loop.
end loop.
end if.
compute imm2=imm2t.
release imm2t.
end if.
do if ((ncol(imm2) > 1) and (ncol(imm)=1)).
loop k7=1 to ncol(imm2).
compute imm2(:,k7)=imm2(:,k7)&*imm.
end loop.
end if.
do if ((ncol(imm2) = 1) and (ncol(imm) > 1)).
loop k7=1 to ncol(imm).
compute imm(:,k7)=imm2&*imm(:,k7).
end loop.
compute imm2=imm.
end if.
end if.
do if (immset=0).
compute imm2=imm.
do if (indmmm(i,1)=41 or indmmm(i,1)=51).
compute condbb2=condbb.
end if.
compute immset=1.
end if.
end if.
end loop.
compute indtemp=tucker2(1,1).
do if (indmmm(i,1)=12 or indmmm(i,1)=22).
compute imm3={imm3,tucker2}.
do if (k1=nrow(indmodva)).
compute imm3=imm3(:,2:ncol(imm3)).
compute immstop=ncol(imm3).
loop k8=2 to immstop.
do if (indmmmt(i,1)=1).
compute imm3={imm3,(imm3(:,k8)-imm3(:,1))}.
end if.
do if (indmmmt(i,1)=2).
compute imm3={imm3,(imm3(:,k8)-imm3(:,(k8-1)))}.
end if.
do if (indmmmt(i,1)=3).
compute imm3={imm3,((rsum(imm3(:,(k8:immstop)))/(immstop-k8+1))-imm3(:,(k8-1))) }.
end if.
do if (indmmmt(i,1)=4).
compute imm3={imm3,(imm3(:,k8)-(rsum(imm3(:,1:immstop))/immstop))}.
end if.
end loop.
do if (indmmmt(i,1)<5).
compute imm2=imm3(:,(immstop+1):ncol(imm3)).
release imm3.
end if.
end if.
end if.
do if (indmmm(i,1)>-1 and (contrast = 1 or contrast = 2 or contrast=3)).
compute imm4={imm4,tucker2}.
do if (k1=nrow(indmodva) and k1 > 1).
compute imm4=imm4(:,2:ncol(imm4)).
compute immstop=ncol(imm4).
compute condcont=make((immstop*(immstop-1)/2),6,-999).
loop k8 = 1 to (immstop-1).
loop k9 = (k8+1) to immstop.
do if (contrast=1).
compute imm4={imm4,(imm4(:,k9)-imm4(:,k8))}.
end if.
do if (contrast=2).
compute imm4={imm4,(abs(imm4(:,k9))-abs(imm4(:,k8)))}.
end if.
do if (contrast=3).
compute imm4={imm4,(imm4(:,k9)+imm4(:,k8))}.
end if.
compute condcont((ncol(imm4)-immstop),1)=imm4(1,k9).
compute condcont((ncol(imm4)-immstop),2)=imm4(1,k8).
end loop.
end loop.
compute imm4=imm4(:,(immstop+1):ncol(imm4)).
loop k8=1 to ncol(imm4).
compute condcont(k8,3)=imm4(1,k8).
do if (boot > 0).

.
compute temp = imm4(2:nrow(imm4),k8).
compute temp(GRADE( imm4(2:nrow(imm4),k8) )) = imm4(2:nrow(imm4),k8).
compute badlo = 0.
compute badhi = 0.
do if ( (imm4(1,k8)*bc)+(9999*(1-bc)) <> 9999).
compute pv=csum(temp < (imm4(1,k8)*bc)+(9999*(1-bc)) )/nrow(temp).
compute ppv = pv.
do if (pv > .5).
compute ppv = 1-pv.
end if.
compute y5=sqrt(-2*ln(ppv)).
compute xp=y5+((((y5*p4+p3)*y5+p2)*y5+p1)*y5+p0)/((((y5*q4+q3)*y5+q2)*y5+q1)*y5+q0).
do if (pv <= .5).
compute xp = -xp.
end if.
compute cilow=rnd(nrow(temp)*(cdfnorm(2*xp-xp2))).
compute cihigh=trunc(nrow(temp)*(cdfnorm(2*xp+(xp2))))+1.
do if (cilow < 1).
compute cilow = 1.
compute booterr=1.
compute badlo = 1.
end if.
do if (cihigh > nrow(temp)).
compute cihigh = boot.
compute booterr=1.
compute badhi = 1.
end if.
compute llcit=temp(cilow,1).
compute ulcit=temp(cihigh,1).
do if (badlo = 1 and llcit <> priorlo).
compute badend={badend, llcit}.
compute priorlo = llcit.
end if.
do if (badhi = 1 and ulcit <> priorhi).
compute badend={badend, ulcit}.
compute priorhi = ulcit.
end if.
end if.
do if ( (imm4(1,k8)*bc)+(9999*(1-bc)) = 9999).
compute llcit=temp(cilow,1).
compute ulcit=temp(cihigh,1).
end if.
compute bootse=t(sqrt((cssq(temp)-((csum(temp)&**2)/nrow(temp)))/(nrow(temp)-1)))
.
compute condcont(k8,4:6)={bootse,llcit,ulcit}.
end if.
end loop.
do if (boot=0).
compute condcont=condcont(:,1:3).
end if.
compute indcontr=1.
end if.
end if.
do if (xmint=1).
do if (k1=(k4+1)).
compute cftotfx(:,k4)=cftotfx(:,k4)+(tucker2*xscaling).
end if.
end if.
do if (boot > 0).
compute tucker2=tucker2*xscaling.

.
compute temp = tucker2(2:nrow(tucker2),1).
compute temp(GRADE( tucker2(2:nrow(tucker2),1) )) = tucker2(2:nrow(tucker2),1).
compute badlo = 0.
compute badhi = 0.
do if ( (tucker2(1,1)*bc)+(9999*(1-bc)) <> 9999).
compute pv=csum(temp < (tucker2(1,1)*bc)+(9999*(1-bc)) )/nrow(temp).
compute ppv = pv.
do if (pv > .5).
compute ppv = 1-pv.
end if.
compute y5=sqrt(-2*ln(ppv)).
compute xp=y5+((((y5*p4+p3)*y5+p2)*y5+p1)*y5+p0)/((((y5*q4+q3)*y5+q2)*y5+q1)*y5+q0).
do if (pv <= .5).
compute xp = -xp.
end if.
compute cilow=rnd(nrow(temp)*(cdfnorm(2*xp-xp2))).
compute cihigh=trunc(nrow(temp)*(cdfnorm(2*xp+(xp2))))+1.
do if (cilow < 1).
compute cilow = 1.
compute booterr=1.
compute badlo = 1.
end if.
do if (cihigh > nrow(temp)).
compute cihigh = boot.
compute booterr=1.
compute badhi = 1.
end if.
compute llcit=temp(cilow,1).
compute ulcit=temp(cihigh,1).
do if (badlo = 1 and llcit <> priorlo).
compute badend={badend, llcit}.
compute priorlo = llcit.
end if.
do if (badhi = 1 and ulcit <> priorhi).
compute badend={badend, ulcit}.
compute priorhi = ulcit.
end if.
end if.
do if ( (tucker2(1,1)*bc)+(9999*(1-bc)) = 9999).
compute llcit=temp(cilow,1).
compute ulcit=temp(cihigh,1).
end if.
compute bootse=t(sqrt((cssq(temp)-((csum(temp)&**2)/nrow(temp)))/(nrow(temp)-1)))
.
compute indtemp={indtemp*xscaling, bootse,llcit,ulcit}.
end if.
do if (boot=0).
compute indtemp=indtemp*xscaling.
end if.
compute condres(k1,(ncol(indmodva)+1):ncol(condres))=indtemp.
end loop.
do if (xmint=0).
compute resultm2=make(nrow(condres),maxresm,99999).
compute resultm2(1:nrow(condres),1:ncol(condres))=condres.
compute resultm={resultm;resultm2}.
end if.
compute condlbs={condlbs,'Effect'}.
do if (boot > 0).
compute condlbs={condlbs,'BootSE', 'BootLLCI', 'BootULCI'}.
end if.
do if (xmint = 1).
do if (k4 = 1).
compute natindfx=condres((2+(k4-1)),2:ncol(condres)).
end if.
do if (k4 > 1).
compute natindfx={natindfx;condres((2+(k4-1)),2:ncol(condres))}.
end if.
do if (k4=nxvls).
compute resultm2=make(nrow(natindfx),maxresm,99999).
compute resultm2(1:nrow(natindfx),1:ncol(natindfx))=natindfx.
compute resultm={resultm;resultm2}.
end if.
end if.
do if (outscree=1 and xmint=0).
do if (nxvls=1).
print condres/title=' '/cnames=condlbs/format= F10.4 /space=0.
else.
compute condrlb=make(nrow(condres),1,xcatlab(k4,1)).
print condres/title=' '/cnames=condlbs/rnames=condrlb/format= F10.4 /space=0.
end if.
end if.
do if (outscree=1 and xmint=1 and k4=nxvls).
do if (nxvls=1).
print natindfx/title=' '/clabels='Effect', 'BootSE', 'BootLLCI', 'BootULCI'/format= F10.4 /space=0.
else.
compute condrlb=make(nrow(condres),1,xcatlab(k4,1)).
print natindfx/title=' '/clabels='Effect', 'BootSE', 'BootLLCI', 'BootULCI'/rnames=direffl2/format= F10.4 /space=0.
end if.
end if.
compute dichadj=0.
compute immcat=0.
do if ((indmmm(i,1)>0) and (xmint=0)).
do if (indmmm(i,1)=1 or indmmm(i,1)=12 or indmmm(i,1)=31).
do if (wdich=1 and mcw=0).
do if (indmmm(i,1) <> 12).
compute imm2(:,1)=imm2(:,1)*(wmax-wmin).
end if.
do if (indmmm(i,1) <> 31).
compute dichadj=1.
end if.
end if.
do if ((mcw = 1 or mcw = 2) and indmmm(i,1) <> 31)).
compute immcat=1.
end if.
end if.
do if (indmmm(i,1)=2 or indmmm(i,1)=22 or indmmm(i,1)=31).
do if (zdich=1 and mcz=0).
do if (indmmm(i,1) = 31).
compute imm2(:,(nwvls+1):ncol(imm2))=imm2(:,(nwvls+1):ncol(imm2))*(zmax-zmin).
end if.
do if (indmmm(i,1) = 2).
compute imm2(:,1)=imm2(:,1)*(zmax-zmin).
end if.
do if (indmmm(i,1) <> 31).
compute dichadj=1.
end if.
end if.
do if ((mcz = 1 or mcz = 2) and indmmm(i,1) <> 31)).
compute immcat=1.
end if.
end if.
compute immtemp2=t(imm2(1,:)).
compute immtemp=immtemp2.
compute immlbs={'Index'}.
do if (boot > 0).
compute immtemp=make(1,3,0).
loop k7=1 to ncol(imm2).

.
compute temp = imm2(2:nrow(imm2),k7).
compute temp(GRADE( imm2(2:nrow(imm2),k7) )) = imm2(2:nrow(imm2),k7).
compute badlo = 0.
compute badhi = 0.
do if ( (imm2(1,k7)*bc)+(9999*(1-bc)) <> 9999).
compute pv=csum(temp < (imm2(1,k7)*bc)+(9999*(1-bc)) )/nrow(temp).
compute ppv = pv.
do if (pv > .5).
compute ppv = 1-pv.
end if.
compute y5=sqrt(-2*ln(ppv)).
compute xp=y5+((((y5*p4+p3)*y5+p2)*y5+p1)*y5+p0)/((((y5*q4+q3)*y5+q2)*y5+q1)*y5+q0).
do if (pv <= .5).
compute xp = -xp.
end if.
compute cilow=rnd(nrow(temp)*(cdfnorm(2*xp-xp2))).
compute cihigh=trunc(nrow(temp)*(cdfnorm(2*xp+(xp2))))+1.
do if (cilow < 1).
compute cilow = 1.
compute booterr=1.
compute badlo = 1.
end if.
do if (cihigh > nrow(temp)).
compute cihigh = boot.
compute booterr=1.
compute badhi = 1.
end if.
compute llcit=temp(cilow,1).
compute ulcit=temp(cihigh,1).
do if (badlo = 1 and llcit <> priorlo).
compute badend={badend, llcit}.
compute priorlo = llcit.
end if.
do if (badhi = 1 and ulcit <> priorhi).
compute badend={badend, ulcit}.
compute priorhi = ulcit.
end if.
end if.
do if ( (imm2(1,k7)*bc)+(9999*(1-bc)) = 9999).
compute llcit=temp(cilow,1).
compute ulcit=temp(cihigh,1).
end if.
compute bootse=t(sqrt((cssq(temp)-((csum(temp)&**2)/nrow(temp)))/(nrow(temp)-1)))
.
compute temp={bootse,llcit,ulcit}.
compute immtemp={immtemp;temp}.
end loop.
compute immtemp=immtemp(2:nrow(immtemp),:).
compute immtemp={immtemp2,immtemp}.
compute immlbs={immlbs,'BootSE', 'BootLLCI', 'BootULCI'}.
end if.
compute resultm2=make(nrow(immtemp),maxresm,99999).
compute resultm2(1:nrow(immtemp),1:ncol(immtemp))=immtemp.
compute resultm={resultm;resultm2}.
do if (dichadj=0 and immcat=0 and indmmmt(i,1)<>5 and indmmm(i,1) < 100 ).
do if (outscree=1).
do if (indmmm(i,1) < 30).
print immtemp/title='      Index of moderated mediation:'/cnames=immlbs/rnames=immlbs2/format= F10.4.
end if.
do if (indmmm(i,1) = 31).
compute immlbs2={immlbs2;zcatlab(1:nzvls,1)}.
print immtemp/title='      Indices of partial moderated mediation:'/cnames=immlbs/rnames=immlbs2/format= F10.4.
end if.
end if.
do if ((nzvls = 1) and (nwvls = 1)).
do if (indmmm(i,1) = 41 or indmmm(i,1)=51).
loop k7=1 to nwvls.
compute immlbs2=zcatlab(1:nzvls,1).
compute immtemp2=immtemp((((k7-1)*nzvls)+1):(((k7-1)*nzvls)+nzvls),:).
do if (outscree=1).
do if (nwvls > 1).
compute primodv={'        ', wcatlab(k7,1)}.
print primodv/title='      Primary moderator:'/format=A8.
end if.
do if (nzvls=1).
print immtemp2/title='      Index of moderated moderated mediation'/cnames=immlbs/format= F10.4.
else.
print immtemp2/title='      Indices of moderated moderated mediation'/cnames=immlbs/rnames=immlbs2/format= F10.4.
end if.
end if.
compute cmmtemp=make(nrow(zprobval),4,0).
loop k8=1 to nrow(zprobval).
compute condbb3=condbb2(:,((nwvls+1)+((k7-1)*nzvls)):((nwvls+1)+((k7-1)*nzvls)+ (nzvls-1))).
do if (ncol(zprobval) > 1).
compute condbb3=condbb3*mdiag(zprobval(k8,:)).
else.
compute condbb3=condbb3&*zprobval(k8,:).
end if.
compute condbb3={condbb2(:,k7),condbb3}.
compute icmm=rsum(condbb3).
compute cmmtemp(k8,1)=icmm(1,1).
do if (boot > 0).

.
compute temp = icmm(2:nrow(icmm),1).
compute temp(GRADE( icmm(2:nrow(icmm),1) )) = icmm(2:nrow(icmm),1).
compute badlo = 0.
compute badhi = 0.
do if ( (icmm(1,1)*bc)+(9999*(1-bc)) <> 9999).
compute pv=csum(temp < (icmm(1,1)*bc)+(9999*(1-bc)) )/nrow(temp).
compute ppv = pv.
do if (pv > .5).
compute ppv = 1-pv.
end if.
compute y5=sqrt(-2*ln(ppv)).
compute xp=y5+((((y5*p4+p3)*y5+p2)*y5+p1)*y5+p0)/((((y5*q4+q3)*y5+q2)*y5+q1)*y5+q0).
do if (pv <= .5).
compute xp = -xp.
end if.
compute cilow=rnd(nrow(temp)*(cdfnorm(2*xp-xp2))).
compute cihigh=trunc(nrow(temp)*(cdfnorm(2*xp+(xp2))))+1.
do if (cilow < 1).
compute cilow = 1.
compute booterr=1.
compute badlo = 1.
end if.
do if (cihigh > nrow(temp)).
compute cihigh = boot.
compute booterr=1.
compute badhi = 1.
end if.
compute llcit=temp(cilow,1).
compute ulcit=temp(cihigh,1).
do if (badlo = 1 and llcit <> priorlo).
compute badend={badend, llcit}.
compute priorlo = llcit.
end if.
do if (badhi = 1 and ulcit <> priorhi).
compute badend={badend, ulcit}.
compute priorhi = ulcit.
end if.
end if.
do if ( (icmm(1,1)*bc)+(9999*(1-bc)) = 9999).
compute llcit=temp(cilow,1).
compute ulcit=temp(cihigh,1).
end if.
compute bootse=t(sqrt((cssq(temp)-((csum(temp)&**2)/nrow(temp)))/(nrow(temp)-1)))
.
compute cmmtemp(k8,2:4)={bootse,llcit,ulcit}.
end if.
end loop.
compute cmmtemp={zmodvals,cmmtemp}.
do if (boot=0).
compute cmmtemp=cmmtemp(:,1:2).
end if.
compute cmmlbs={znames,immlbs}.
compute resultm2=make(nrow(cmmtemp),maxresm,99999).
compute resultm2(1:nrow(cmmtemp),1:ncol(cmmtemp))=cmmtemp.
compute resultm={resultm;resultm2}.
do if (outscree=1).
print cmmtemp/title='      Indices of conditional moderated mediation by W'/cnames=cmmlbs/format= F10.4.
end if.
end loop.
end if.
end if.
end if.
do if (dichadj=1 or immcat=1 and indmmm(i,1) < 30).
do if (outscree=1).
print immtemp/title='Index of moderated mediation (difference between conditional indirect effects):'/cnames=immlbs/rnames=immlbs2/format= F10.4.
end if.
end if.
end if.
do if (indcontr=1).
compute resultm2=make(nrow(condcont),maxresm,99999).
compute resultm2(1:nrow(condcont),1:ncol(condcont))=condcont.
compute resultm={resultm;resultm2}.
compute condctlb={'Effect1','Effect2','Contrast','BootSE', 'BootLLCI','BootULCI'}.
do if (outscree=1).
print condcont/title=' Pairwise contrasts between conditional indirect effects (Effect1 minus Effect2)'/cnames=condctlb/format= F10.4.
end if.
end if.
end loop.
end if.
end loop.
do if (alttotal=1).
compute altcnms='Effect'.
compute alttotfx=t(cftotfx(1,:))+obnatdfx.
do if (boot > 0).
compute alttotfx=make(ncol(cftotfx),4,0).
compute alttotfx(:,1)=t(cftotfx(1,:))+obnatdfx.
compute natdirbt(1,:)=t(obnatdfx).
compute cftotfx=cftotfx+natdirbt.
loop cec=1 to ncol(cftotfx).

.
compute temp = cftotfx(2:nrow(cftotfx),cec).
compute temp(GRADE( cftotfx(2:nrow(cftotfx),cec) )) = cftotfx(2:nrow(cftotfx),cec).
compute badlo = 0.
compute badhi = 0.
do if ( (cftotfx(1,cec)*bc)+(9999*(1-bc)) <> 9999).
compute pv=csum(temp < (cftotfx(1,cec)*bc)+(9999*(1-bc)) )/nrow(temp).
compute ppv = pv.
do if (pv > .5).
compute ppv = 1-pv.
end if.
compute y5=sqrt(-2*ln(ppv)).
compute xp=y5+((((y5*p4+p3)*y5+p2)*y5+p1)*y5+p0)/((((y5*q4+q3)*y5+q2)*y5+q1)*y5+q0).
do if (pv <= .5).
compute xp = -xp.
end if.
compute cilow=rnd(nrow(temp)*(cdfnorm(2*xp-xp2))).
compute cihigh=trunc(nrow(temp)*(cdfnorm(2*xp+(xp2))))+1.
do if (cilow < 1).
compute cilow = 1.
compute booterr=1.
compute badlo = 1.
end if.
do if (cihigh > nrow(temp)).
compute cihigh = boot.
compute booterr=1.
compute badhi = 1.
end if.
compute llcit=temp(cilow,1).
compute ulcit=temp(cihigh,1).
do if (badlo = 1 and llcit <> priorlo).
compute badend={badend, llcit}.
compute priorlo = llcit.
end if.
do if (badhi = 1 and ulcit <> priorhi).
compute badend={badend, ulcit}.
compute priorhi = ulcit.
end if.
end if.
do if ( (cftotfx(1,cec)*bc)+(9999*(1-bc)) = 9999).
compute llcit=temp(cilow,1).
compute ulcit=temp(cihigh,1).
end if.
compute bootse=t(sqrt((cssq(temp)-((csum(temp)&**2)/nrow(temp)))/(nrow(temp)-1)))
.
compute alttotfx(cec,2:4)={bootse,llcit,ulcit}.
end loop.
compute altcnms={altcnms,'BootSE', 'BootLLCI','BootULCI'}.
end if.
compute resultm2=make(nrow(alttotfx),maxresm,99999).
compute resultm2(1:nrow(alttotfx),1:ncol(alttotfx))=alttotfx.
compute resultm={resultm;resultm2}.
do if (outscree=1).
print/title='----------'.
do if (nxvls > 1).
print alttotfx/title='Relative total effects of X on Y (sum of direct and indirect effects)'/cnames=altcnms/rnames=direffl2/format= F10.4.
end if.
do if (nxvls = 1).
print alttotfx/title='Total effect of X on Y (sum of direct and indirect effects)'/cnames=altcnms/format= F10.4.
end if.
end if.
end if.
end if.
end if
.
do if (criterr=0 and (saveest = 1 or saveboot=1)).
compute coeffsav=coeffmat(2:nrow(coeffmat),:).
compute conseq=conseq(2:nrow(conseq),1).
compute dfmat=dfmat(2:nrow(dfmat),1).
compute dfmat=dfmat.
compute saven={'conseqnt','antecdnt','coeff','se','t','p','LLCI','ULCI','df'}.
do if (ydich=1).
compute saven={'conseqnt','antecdnt','coeff','se','t_or_Z','p','LLCI','ULCI','df'}.
end if.
do if (saveboot=1 and boot > 0 ).
compute colslab=make(300,1,' ').


compute colslab( 1 ,1)= 'COL1'.


compute colslab( 2 ,1)= 'COL2'.


compute colslab( 3 ,1)= 'COL3'.


compute colslab( 4 ,1)= 'COL4'.


compute colslab( 5 ,1)= 'COL5'.


compute colslab( 6 ,1)= 'COL6'.


compute colslab( 7 ,1)= 'COL7'.


compute colslab( 8 ,1)= 'COL8'.


compute colslab( 9 ,1)= 'COL9'.


compute colslab( 10 ,1)= 'COL10'.


compute colslab( 11 ,1)= 'COL11'.


compute colslab( 12 ,1)= 'COL12'.


compute colslab( 13 ,1)= 'COL13'.


compute colslab( 14 ,1)= 'COL14'.


compute colslab( 15 ,1)= 'COL15'.


compute colslab( 16 ,1)= 'COL16'.


compute colslab( 17 ,1)= 'COL17'.


compute colslab( 18 ,1)= 'COL18'.


compute colslab( 19 ,1)= 'COL19'.


compute colslab( 20 ,1)= 'COL20'.


compute colslab( 21 ,1)= 'COL21'.


compute colslab( 22 ,1)= 'COL22'.


compute colslab( 23 ,1)= 'COL23'.


compute colslab( 24 ,1)= 'COL24'.


compute colslab( 25 ,1)= 'COL25'.


compute colslab( 26 ,1)= 'COL26'.


compute colslab( 27 ,1)= 'COL27'.


compute colslab( 28 ,1)= 'COL28'.


compute colslab( 29 ,1)= 'COL29'.


compute colslab( 30 ,1)= 'COL30'.


compute colslab( 31 ,1)= 'COL31'.


compute colslab( 32 ,1)= 'COL32'.


compute colslab( 33 ,1)= 'COL33'.


compute colslab( 34 ,1)= 'COL34'.


compute colslab( 35 ,1)= 'COL35'.


compute colslab( 36 ,1)= 'COL36'.


compute colslab( 37 ,1)= 'COL37'.


compute colslab( 38 ,1)= 'COL38'.


compute colslab( 39 ,1)= 'COL39'.


compute colslab( 40 ,1)= 'COL40'.


compute colslab( 41 ,1)= 'COL41'.


compute colslab( 42 ,1)= 'COL42'.


compute colslab( 43 ,1)= 'COL43'.


compute colslab( 44 ,1)= 'COL44'.


compute colslab( 45 ,1)= 'COL45'.


compute colslab( 46 ,1)= 'COL46'.


compute colslab( 47 ,1)= 'COL47'.


compute colslab( 48 ,1)= 'COL48'.


compute colslab( 49 ,1)= 'COL49'.


compute colslab( 50 ,1)= 'COL50'.


compute colslab( 51 ,1)= 'COL51'.


compute colslab( 52 ,1)= 'COL52'.


compute colslab( 53 ,1)= 'COL53'.


compute colslab( 54 ,1)= 'COL54'.


compute colslab( 55 ,1)= 'COL55'.


compute colslab( 56 ,1)= 'COL56'.


compute colslab( 57 ,1)= 'COL57'.


compute colslab( 58 ,1)= 'COL58'.


compute colslab( 59 ,1)= 'COL59'.


compute colslab( 60 ,1)= 'COL60'.


compute colslab( 61 ,1)= 'COL61'.


compute colslab( 62 ,1)= 'COL62'.


compute colslab( 63 ,1)= 'COL63'.


compute colslab( 64 ,1)= 'COL64'.


compute colslab( 65 ,1)= 'COL65'.


compute colslab( 66 ,1)= 'COL66'.


compute colslab( 67 ,1)= 'COL67'.


compute colslab( 68 ,1)= 'COL68'.


compute colslab( 69 ,1)= 'COL69'.


compute colslab( 70 ,1)= 'COL70'.


compute colslab( 71 ,1)= 'COL71'.


compute colslab( 72 ,1)= 'COL72'.


compute colslab( 73 ,1)= 'COL73'.


compute colslab( 74 ,1)= 'COL74'.


compute colslab( 75 ,1)= 'COL75'.


compute colslab( 76 ,1)= 'COL76'.


compute colslab( 77 ,1)= 'COL77'.


compute colslab( 78 ,1)= 'COL78'.


compute colslab( 79 ,1)= 'COL79'.


compute colslab( 80 ,1)= 'COL80'.


compute colslab( 81 ,1)= 'COL81'.


compute colslab( 82 ,1)= 'COL82'.


compute colslab( 83 ,1)= 'COL83'.


compute colslab( 84 ,1)= 'COL84'.


compute colslab( 85 ,1)= 'COL85'.


compute colslab( 86 ,1)= 'COL86'.


compute colslab( 87 ,1)= 'COL87'.


compute colslab( 88 ,1)= 'COL88'.


compute colslab( 89 ,1)= 'COL89'.


compute colslab( 90 ,1)= 'COL90'.


compute colslab( 91 ,1)= 'COL91'.


compute colslab( 92 ,1)= 'COL92'.


compute colslab( 93 ,1)= 'COL93'.


compute colslab( 94 ,1)= 'COL94'.


compute colslab( 95 ,1)= 'COL95'.


compute colslab( 96 ,1)= 'COL96'.


compute colslab( 97 ,1)= 'COL97'.


compute colslab( 98 ,1)= 'COL98'.


compute colslab( 99 ,1)= 'COL99'.


compute colslab( 100 ,1)= 'COL100'.


compute colslab( 101 ,1)= 'COL101'.


compute colslab( 102 ,1)= 'COL102'.


compute colslab( 103 ,1)= 'COL103'.


compute colslab( 104 ,1)= 'COL104'.


compute colslab( 105 ,1)= 'COL105'.


compute colslab( 106 ,1)= 'COL106'.


compute colslab( 107 ,1)= 'COL107'.


compute colslab( 108 ,1)= 'COL108'.


compute colslab( 109 ,1)= 'COL109'.


compute colslab( 110 ,1)= 'COL110'.


compute colslab( 111 ,1)= 'COL111'.


compute colslab( 112 ,1)= 'COL112'.


compute colslab( 113 ,1)= 'COL113'.


compute colslab( 114 ,1)= 'COL114'.


compute colslab( 115 ,1)= 'COL115'.


compute colslab( 116 ,1)= 'COL116'.


compute colslab( 117 ,1)= 'COL117'.


compute colslab( 118 ,1)= 'COL118'.


compute colslab( 119 ,1)= 'COL119'.


compute colslab( 120 ,1)= 'COL120'.


compute colslab( 121 ,1)= 'COL121'.


compute colslab( 122 ,1)= 'COL122'.


compute colslab( 123 ,1)= 'COL123'.


compute colslab( 124 ,1)= 'COL124'.


compute colslab( 125 ,1)= 'COL125'.


compute colslab( 126 ,1)= 'COL126'.


compute colslab( 127 ,1)= 'COL127'.


compute colslab( 128 ,1)= 'COL128'.


compute colslab( 129 ,1)= 'COL129'.


compute colslab( 130 ,1)= 'COL130'.


compute colslab( 131 ,1)= 'COL131'.


compute colslab( 132 ,1)= 'COL132'.


compute colslab( 133 ,1)= 'COL133'.


compute colslab( 134 ,1)= 'COL134'.


compute colslab( 135 ,1)= 'COL135'.


compute colslab( 136 ,1)= 'COL136'.


compute colslab( 137 ,1)= 'COL137'.


compute colslab( 138 ,1)= 'COL138'.


compute colslab( 139 ,1)= 'COL139'.


compute colslab( 140 ,1)= 'COL140'.


compute colslab( 141 ,1)= 'COL141'.


compute colslab( 142 ,1)= 'COL142'.


compute colslab( 143 ,1)= 'COL143'.


compute colslab( 144 ,1)= 'COL144'.


compute colslab( 145 ,1)= 'COL145'.


compute colslab( 146 ,1)= 'COL146'.


compute colslab( 147 ,1)= 'COL147'.


compute colslab( 148 ,1)= 'COL148'.


compute colslab( 149 ,1)= 'COL149'.


compute colslab( 150 ,1)= 'COL150'.


compute colslab( 151 ,1)= 'COL151'.


compute colslab( 152 ,1)= 'COL152'.


compute colslab( 153 ,1)= 'COL153'.


compute colslab( 154 ,1)= 'COL154'.


compute colslab( 155 ,1)= 'COL155'.


compute colslab( 156 ,1)= 'COL156'.


compute colslab( 157 ,1)= 'COL157'.


compute colslab( 158 ,1)= 'COL158'.


compute colslab( 159 ,1)= 'COL159'.


compute colslab( 160 ,1)= 'COL160'.


compute colslab( 161 ,1)= 'COL161'.


compute colslab( 162 ,1)= 'COL162'.


compute colslab( 163 ,1)= 'COL163'.


compute colslab( 164 ,1)= 'COL164'.


compute colslab( 165 ,1)= 'COL165'.


compute colslab( 166 ,1)= 'COL166'.


compute colslab( 167 ,1)= 'COL167'.


compute colslab( 168 ,1)= 'COL168'.


compute colslab( 169 ,1)= 'COL169'.


compute colslab( 170 ,1)= 'COL170'.


compute colslab( 171 ,1)= 'COL171'.


compute colslab( 172 ,1)= 'COL172'.


compute colslab( 173 ,1)= 'COL173'.


compute colslab( 174 ,1)= 'COL174'.


compute colslab( 175 ,1)= 'COL175'.


compute colslab( 176 ,1)= 'COL176'.


compute colslab( 177 ,1)= 'COL177'.


compute colslab( 178 ,1)= 'COL178'.


compute colslab( 179 ,1)= 'COL179'.


compute colslab( 180 ,1)= 'COL180'.


compute colslab( 181 ,1)= 'COL181'.


compute colslab( 182 ,1)= 'COL182'.


compute colslab( 183 ,1)= 'COL183'.


compute colslab( 184 ,1)= 'COL184'.


compute colslab( 185 ,1)= 'COL185'.


compute colslab( 186 ,1)= 'COL186'.


compute colslab( 187 ,1)= 'COL187'.


compute colslab( 188 ,1)= 'COL188'.


compute colslab( 189 ,1)= 'COL189'.


compute colslab( 190 ,1)= 'COL190'.


compute colslab( 191 ,1)= 'COL191'.


compute colslab( 192 ,1)= 'COL192'.


compute colslab( 193 ,1)= 'COL193'.


compute colslab( 194 ,1)= 'COL194'.


compute colslab( 195 ,1)= 'COL195'.


compute colslab( 196 ,1)= 'COL196'.


compute colslab( 197 ,1)= 'COL197'.


compute colslab( 198 ,1)= 'COL198'.


compute colslab( 199 ,1)= 'COL199'.


compute colslab( 200 ,1)= 'COL200'.


compute colslab( 201 ,1)= 'COL201'.


compute colslab( 202 ,1)= 'COL202'.


compute colslab( 203 ,1)= 'COL203'.


compute colslab( 204 ,1)= 'COL204'.


compute colslab( 205 ,1)= 'COL205'.


compute colslab( 206 ,1)= 'COL206'.


compute colslab( 207 ,1)= 'COL207'.


compute colslab( 208 ,1)= 'COL208'.


compute colslab( 209 ,1)= 'COL209'.


compute colslab( 210 ,1)= 'COL210'.


compute colslab( 211 ,1)= 'COL211'.


compute colslab( 212 ,1)= 'COL212'.


compute colslab( 213 ,1)= 'COL213'.


compute colslab( 214 ,1)= 'COL214'.


compute colslab( 215 ,1)= 'COL215'.


compute colslab( 216 ,1)= 'COL216'.


compute colslab( 217 ,1)= 'COL217'.


compute colslab( 218 ,1)= 'COL218'.


compute colslab( 219 ,1)= 'COL219'.


compute colslab( 220 ,1)= 'COL220'.


compute colslab( 221 ,1)= 'COL221'.


compute colslab( 222 ,1)= 'COL222'.


compute colslab( 223 ,1)= 'COL223'.


compute colslab( 224 ,1)= 'COL224'.


compute colslab( 225 ,1)= 'COL225'.


compute colslab( 226 ,1)= 'COL226'.


compute colslab( 227 ,1)= 'COL227'.


compute colslab( 228 ,1)= 'COL228'.


compute colslab( 229 ,1)= 'COL229'.


compute colslab( 230 ,1)= 'COL230'.


compute colslab( 231 ,1)= 'COL231'.


compute colslab( 232 ,1)= 'COL232'.


compute colslab( 233 ,1)= 'COL233'.


compute colslab( 234 ,1)= 'COL234'.


compute colslab( 235 ,1)= 'COL235'.


compute colslab( 236 ,1)= 'COL236'.


compute colslab( 237 ,1)= 'COL237'.


compute colslab( 238 ,1)= 'COL238'.


compute colslab( 239 ,1)= 'COL239'.


compute colslab( 240 ,1)= 'COL240'.


compute colslab( 241 ,1)= 'COL241'.


compute colslab( 242 ,1)= 'COL242'.


compute colslab( 243 ,1)= 'COL243'.


compute colslab( 244 ,1)= 'COL244'.


compute colslab( 245 ,1)= 'COL245'.


compute colslab( 246 ,1)= 'COL246'.


compute colslab( 247 ,1)= 'COL247'.


compute colslab( 248 ,1)= 'COL248'.


compute colslab( 249 ,1)= 'COL249'.


compute colslab( 250 ,1)= 'COL250'.


compute colslab( 251 ,1)= 'COL251'.


compute colslab( 252 ,1)= 'COL252'.


compute colslab( 253 ,1)= 'COL253'.


compute colslab( 254 ,1)= 'COL254'.


compute colslab( 255 ,1)= 'COL255'.


compute colslab( 256 ,1)= 'COL256'.


compute colslab( 257 ,1)= 'COL257'.


compute colslab( 258 ,1)= 'COL258'.


compute colslab( 259 ,1)= 'COL259'.


compute colslab( 260 ,1)= 'COL260'.


compute colslab( 261 ,1)= 'COL261'.


compute colslab( 262 ,1)= 'COL262'.


compute colslab( 263 ,1)= 'COL263'.


compute colslab( 264 ,1)= 'COL264'.


compute colslab( 265 ,1)= 'COL265'.


compute colslab( 266 ,1)= 'COL266'.


compute colslab( 267 ,1)= 'COL267'.


compute colslab( 268 ,1)= 'COL268'.


compute colslab( 269 ,1)= 'COL269'.


compute colslab( 270 ,1)= 'COL270'.


compute colslab( 271 ,1)= 'COL271'.


compute colslab( 272 ,1)= 'COL272'.


compute colslab( 273 ,1)= 'COL273'.


compute colslab( 274 ,1)= 'COL274'.


compute colslab( 275 ,1)= 'COL275'.


compute colslab( 276 ,1)= 'COL276'.


compute colslab( 277 ,1)= 'COL277'.


compute colslab( 278 ,1)= 'COL278'.


compute colslab( 279 ,1)= 'COL279'.


compute colslab( 280 ,1)= 'COL280'.


compute colslab( 281 ,1)= 'COL281'.


compute colslab( 282 ,1)= 'COL282'.


compute colslab( 283 ,1)= 'COL283'.


compute colslab( 284 ,1)= 'COL284'.


compute colslab( 285 ,1)= 'COL285'.


compute colslab( 286 ,1)= 'COL286'.


compute colslab( 287 ,1)= 'COL287'.


compute colslab( 288 ,1)= 'COL288'.


compute colslab( 289 ,1)= 'COL289'.


compute colslab( 290 ,1)= 'COL290'.


compute colslab( 291 ,1)= 'COL291'.


compute colslab( 292 ,1)= 'COL292'.


compute colslab( 293 ,1)= 'COL293'.


compute colslab( 294 ,1)= 'COL294'.


compute colslab( 295 ,1)= 'COL295'.


compute colslab( 296 ,1)= 'COL296'.


compute colslab( 297 ,1)= 'COL297'.


compute colslab( 298 ,1)= 'COL298'.


compute colslab( 299 ,1)= 'COL299'.


compute colslab( 300 ,1)= 'COL300'.

compute colslab=colslab(1:ncol(bootres),1).
compute colslab={colslab,conseq,vlabs}.
do if (outscree=1).
print/title = '**************************************************************************'.
print/title='Bootstrap estimates were saved to a file'/space=0.
print colslab/title='Map of column names to model coefficients:'/clabels=' ','Conseqnt','Antecdnt'/format=a8.
end if.
end if.
end if.
do if (criterr=0 and boot > 0 and modelbt=1).
compute labstart=1.
do if (outscree=1).
print/title = '*********** BOOTSTRAP RESULTS FOR REGRESSION MODEL PARAMETERS ************'.
end if.
loop iboot = 1 to (nms+nys).
do if (outscree=1).
print outnames(1,iboot)/title = 'OUTCOME VARIABLE:'/format = A8.
end if.
compute vlabsm=vlabs(labstart:(labstart+(nump(1,iboot)-1)),1).
compute outnmtmp=bootcim(labstart:(labstart+(nump(1,iboot)-1)),:).
compute resultm2=make(nrow(outnmtmp),maxresm,99999).
compute resultm2(1:nrow(outnmtmp),1:ncol(outnmtmp))=outnmtmp.
compute resultm={resultm;resultm2}.
do if (outscree=1).
print bootcim(labstart:(labstart+(nump(1,iboot)-1)),:)/title=' '/rnames=vlabsm/ clabels='Coeff' 'BootMean' 'BootSE' 'BootLLCI' 'BootULCI'/format= F10.4 /space=0.
end if.
compute labstart=labstart+nump(1,iboot).
do if (iboot < (nms+nys)).
do if (outscree=1).
print/title= '----------'.
end if.
end if.
end loop.
end if.
do if (criterr=0 and saveest=1).
compute resultm=resultm(2:nrow(resultm),:).
compute bocaj=(resultm=99999).
compute bocaj=csum(bocaj).
compute bocaj=1-(bocaj=nrow(resultm)).
compute j=1.
loop i = 1 to ncol(resultm).
do if (bocaj(1,i)=1).
compute j=j+1.
end if.
end loop.
compute resultm=resultm(:,1:(j-1)).
save resultm/outfile = *.
end if.
do if (criterr = 0 and matrices=1 and outscree=1).
print/title = '************************ MODEL DEFINITION MATRICES ************************'.
print/title = 'FROM variables are columns, TO variables are rows.'.
compute temp2=make(nrow(bcmat),ncol(bcmat),'0').
loop i = 2 to nrow(bcmat).
loop j = 1 to (ncol(bcmat)-1).
do if (bcmat(i,j)=1).
compute temp2(i,j)='1'.
end if.
do if (j >= i).
compute temp2(i,j)=' '.
end if.
end loop.
end loop.
compute temp2=temp2(2:nrow(bcmat),(1:(ncol(bcmat)-1))).
do if (nms > 0).
compute cmatlabs={xnames,mnames}.
compute rmatlabs={mnames,ynames}.
end if.
do if (nms = 0).
compute cmatlabs={xnames}.
compute rmatlabs={ynames}.
end if.
print temp2/title='BMATRIX: Paths freely estimated (1) and fixed to zero (0):'/cnames=cmatlabs/rnames=rmatlabs/format A3.
compute z=0.
do if (rsum(csum(wcmat))<>0).
compute temp2=make(nrow(wcmat),ncol(wcmat),'0').
loop i = 2 to nrow(wcmat).
loop j = 1 to (ncol(wcmat)-1).
do if (wcmat(i,j)=1).
compute temp2(i,j)='1'.
end if.
do if (j >= i).
compute temp2(i,j)=' '.
end if.
end loop.
end loop.
compute temp2=temp2(2:nrow(wcmat),(1:(ncol(wcmat)-1))).
print temp2/title='WMATRIX: Paths moderated (1) and not moderated (0) by W:'/cnames=cmatlabs/rnames=rmatlabs/format A3.
end if.
do if (rsum(csum(zcmat))<>0).
compute temp2=make(nrow(zcmat),ncol(zcmat),'0').
loop i = 2 to nrow(zcmat).
loop j = 1 to (ncol(zcmat)-1).
do if (zcmat(i,j)=1).
compute temp2(i,j)='1'.
end if.
do if (j >= i).
compute temp2(i,j)=' '.
end if.
end loop.
end loop.
compute temp2=temp2(2:nrow(zcmat),(1:(ncol(zcmat)-1))).
print temp2/title='ZMATRIX: Paths moderated (1) and not moderated (0) by Z:'/cnames=cmatlabs/rnames=rmatlabs/format a3.
end if.
do if (rsum(csum(wzcmat))<>0).
compute temp2=make(nrow(wzcmat),ncol(wzcmat),'0').
loop i = 2 to nrow(wzcmat).
loop j = 1 to (ncol(wzcmat)-1).
do if (wzcmat(i,j)=1).
compute temp2(i,j)='1'.
end if.
do if (j >= i).
compute temp2(i,j)=' '.
end if.
end loop.
end loop.
compute temp2=temp2(2:nrow(wzcmat),(1:(ncol(wzcmat)-1))).
print temp2/title='WZMATRIX: W moderated paths moderated (1) and not moderated (0) by Z:'/cnames=cmatlabs/rnames=rmatlabs/format a3.
end if.
do if (ncs > 0).
print ccmat/title='CMATRIX: Covariates (columns) in (1) and not in (0) the models of M and Y (rows):'/rnames=rmatlabs/cnames=covnames.
end if.
end if.
do if (outscree=1).
do if (activate=0).
print/title = '*********************** ANALYSIS NOTES AND ERRORS ************************'.
end if.
end if.
do if (activate=1).
print/title = '**************************************************************************'.
end if.
do if (criterr=0).
do if (outscree=1).
print conf/title = 'Level of confidence for all confidence intervals in output:'/format = F8.4.
do if (boot > 0).
do if (goodboot = boot and bc=0).
print boot/title='Number of bootstrap samples for percentile bootstrap confidence intervals:'.
end if.
do if (goodboot = boot and bc=1).
print boot/title='Number of bootstrap samples for bias-corrected bootstrap confidence intervals:'.
end if.
do if (booterr = 1).
compute badend = badend(1,2:ncol(badend)).
print/title = 'WARNING: Bootstrap CI endpoints below not trustworthy. Decrease confidence'.
print badend/title='or increase the number of bootstrap samples.'/space=0/format = F10.4.
end if.
end if.
do if (mc > 0).
print mc/title='Number of samples for Monte Carlo confidence intervals:'.
end if.
do if (wnotev > 0 and printw=1).
do if (wnotev=1).
print/title = 'W values in conditional tables are the 16th, 50th, and 84th percentiles.'.
else if (wnotev=2).
do if (minwwarn=0 and maxwwarn=0).
print/title = 'W values in conditional tables are the mean and +/- SD from the mean.'.
end if.
do if (minwwarn=1).
print/title = 'W values in conditional tables are the minimum, the mean, and 1 SD above the mean.'.
end if.
do if (maxwwarn=1).
print/title = 'W values in conditional tables are 1 SD below the mean, the mean, and the maximum.'.
end if.
end if.
end if.
do if (znotev > 0 and printz=1).
do if (znotev=1).
print/title = 'Z values in conditional tables are the 16th, 50th, and 84th percentiles.'.
else if (znotev=2).
do if (minzwarn=0 and maxzwarn=0).
print/title = 'Z values in conditional tables are the mean and +/- SD from the mean.'.
end if.
do if (minzwarn=1).
print/title = 'Z values in conditional tables are the minimum, the mean, and 1 SD above the mean.'.
end if.
do if (maxzwarn=1).
print/title = 'Z values in conditional tables are 1 SD below the mean, the mean, and the maximum.'.
end if.
end if.
end if.
do if (minwwarn > 0).
print/title = 'NOTE: One SD below the mean is below the minimum observed in the data for W,'.
print/title = '      so the minimum measurement on W is used for conditioning instead.'/space=0.
end if.
do if (maxwwarn > 0).
print/title = 'NOTE: One SD above the mean is above the maximum observed in the data for W,'.
print/title = '      so the maximum measurement for W is used for conditioning instead.'/space=0.
end if.
do if (minzwarn > 0).
print/title = 'NOTE: One SD below the mean is below the minimum observed in the data for Z,'.
print/title = '      so the minimum measurement for Z is used for conditioning instead.'/space=0.
end if.
do if (maxzwarn > 0).
print/title = 'NOTE: One SD above the mean is above the maximum observed in the data for Z,'.
print/title = '      so the maximum measurement for Z is used for conditioning instead.'/space=0.
end if.
do if (pstog=1).
print/title= 'NOTE: Standardized coefficients for dichotomous or multicategorical X are in'.
print/title= '      partially standardized form.'/space=0.
end if.
loop i = 1 to 100.
do if (notecode(i,1) = 32).
print/title = 'Direct, indirect, and total effects are counterfactually defined'.
do if (xcontcf=0).
print/title = 'assuming X by M interaction.'/space=0.
end if.
do if (xcontcf=1).
print/title = 'assuming X by M interaction and with the following reference (x_ref)'/space=0.
compute states=t(xrefvals).
print states/title = 'and counterfactual (x_cf) states for X:'/rlabels='x_ref' 'x_cf'/format= F10.4 /space=0.
end if.
end if.
do if (notecode(i,1)=1).
print/title = 'NOTE: COVMY is ignored when using CMATRIX option.'.
end if.
do if (notecode(i,1)=2).
print/title = 'NOTE: Confidence level restricted to between 50 and 99.9999%.  95% confidence is provided in output'.
end if.
do if (notecode(i,1)=3).
print centvar/title = 'NOTE: The following variables were mean centered prior to analysis:'/format = a8.
end if.
do if (notecode(i,1) = 4).
print/title = 'NOTE: A heteroscedasticity consistent standard error and covariance matrix estimator was used.'.
end if.
do if (notecode(i,1) = 5).
print/title = 'NOTE: The HC3 option has been replaced with HC.  See the documentation.'.
end if.
do if (notecode(i,1) = 6).
print/title = 'NOTE: Due to estimation problems, some bootstrap samples had to be replaced.'.
print badboot/title='      The number of times this happened was:'/space=0/format=F8.0.
end if.
do if (notecode(i,1) = 7).
print/title = 'NOTE: The bootstrapping was not completed due to problematic bootstrap samples.'.
print/title = '      Bootstrap confidence intervals are therefore suppressed.'/space=0.
end if.
do if (notecode(i,1) = 8).
print/title = 'NOTE: The number of bootstrap samples was adjusted upward given your desired confidence.'.
end if.
do if (notecode(i,1) = 9).
print/title = 'NOTE: WMODVAL is ignored when W is specified as multicategorical.'.
end if.
do if (notecode(i,1) = 10).
print/title = 'NOTE: ZMODVAL is ignored when Z is specified as multicategorical.'.
end if.
do if (notecode(i,1) = 11).
print/title = 'NOTE: Total effect model generated only when all covariates are specified in all'.
print/title = '      models of M and Y.'/space=0.
end if.
do if (notecode(i,1) = 30).
print/title = 'NOTE: Your vector of linear hypothesis weights is of the wrong length for this model.'.
end if.
do if (notecode(i,1) = 12).
print/title = 'NOTE: Total effect model generated only when X is freely estimated to affect each M'.
print/title = '      and both X and M are freely estimated to affect Y'/space=0.
end if.
do if (notecode(i,1) = 13).
print/title = 'NOTE: There are too many pairwise contrasts to conduct with this model.'.
end if.
do if (notecode(i,1) = 14).
print/title = 'NOTE: The number of contrast weights must equal the number of indirect effects.'.
end if.
do if (notecode(i,1) = 15).
print/title = 'NOTE: Monte Carlo confidence intervals not available for this model.'.
print/title = '      Bootstrapping is used instead.'/space=0.
end if.
do if (notecode(i,1) = 16).
print/title = 'NOTE: The number of Monte Carlo samples was adjusted upward given your desired confidence.'.
end if.
do if (notecode(i,1) = 19).
print/title = 'NOTE: Your contrast matrix is invalid or not applicable to this model.'.
end if.
do if (notecode(i,1) = 20).
print/title = 'NOTE: One of the groups specified by your contrast matrix does not exist in the data.'.
end if.
do if (notecode(i,1) = 21).
print/title = 'NOTE: The VARORDER option is not available in this release.'.
end if.
do if (notecode(i,1) = 22).
print/title = 'NOTE: The VMODVAL and QMODVAL options are not available in this release.'.
end if.
do if (notecode(i,1) = 23).
print/title = 'NOTE: The QUANTILE option is not available in this release.'.
end if.
do if (notecode(i,1) = 24).
print/title = 'NOTE: Total effect model not available with dichotomous Y.'.
end if.
do if (notecode(i,1) = 25).
print/title = 'NOTE: STAND/EFFSIZE options not available with dichotomous Y.'.
end if.
do if ((notecode(i,1) = 26) and nms > 0).
print/title = 'NOTE: Direct and indirect effects of X on Y are on a log-odds metric.'.
end if.
do if (notecode(i,1) = 27).
print/title = 'NOTE: Standardized coefficients are not available for models with moderators.'.
end if.
do if (notecode(i,1) = 28).
print/title = 'NOTE: The contrast option is not available with a multicategorical X.'.
end if.
do if (notecode(i,1) = 31).
do if (nms > 1).
print medmeans/title = 'NOTE: Controlled direct effect(s) estimated at the following mediator values:'/cnames=mnames/format= F10.4.
end if.
do if (nms=1).
print medmeans/title = 'NOTE: Controlled direct effect(s) estimated at the following mediator value:'/format= F10.4.
end if.
end if.
do if (notecode(i,1) = 33).
print/title = 'NOTE: Sobel test is not available with the XMINT option.'.
end if.
do if (notecode(i,1) = 37).
print/title = 'NOTE: The CONTRAST option is not available when using the XMINT option.'.
end if.
do if (notecode(i,1) = 34).
print/title = 'NOTE: Standardized effects are not available when using the XMINT option.'.
end if.
do if (notecode(i,1) = 36).
print/title = 'NOTE: The XREFVAL option is ignored when X is declared as multicategorical.'.
end if.
do if (notecode(i,1) = 35).
print coval/title = 'NOTE: Counterfactual effects estimated at the following covariate values:'/cnames=covnames/format= F10.4.
end if.
do if (notecode(i,1) = 29 and (listmiss=1)).
compute a=missrow.
compute conum=ncol(a).
compute allgood=0.
compute smremain=12.
compute largesti=1.
compute smallrow=0.
do if (conum > 12).
loop ii = 1 to 12.
compute check=(conum/ii).
do if (check = trunc(check)).
compute check2=conum/ii.
compute aok=ii.
do if (aok > 2).
compute allgood=1.
end if.
else.
compute remain=conum-(ii*trunc(check)).
do if (remain <= smremain).
compute smremain=remain.
compute largesti=ii.
compute smallrow=trunc(conum/largesti).
end if.
end if.
end loop.
compute atemp=a(1,1:(aok*check2)).
compute atemp=reshape(atemp,check2,aok).
do if (ncol(atemp) > 2).
print/title='NOTE: Missing data resulted in the deletion of the following row(s) of'.
print atemp/title='      data from the analysis:'/format=F6.0/space=0.
end if.
do if (allgood=0).
compute atemp=a(1,1:(smallrow*largesti)).
compute atemp=reshape(atemp,smallrow,largesti).
compute btemp=a(1,((largesti*smallrow)+1):conum).
print/title='NOTE: Missing data resulted in the deletion of the following row(s) of'.
print atemp/title='      data from the analysis:'/format=F6.0/space=0.
print btemp/title=' '/space=0/format=f6.0.
end if.
else if (conum <= 12).
print/title='NOTE: Missing data resulted in the deletion of the following row(s) of'.
print a/title='      data from the analysis:'/format=F6.0/space=0.
end if.
end if.
end loop.
do if (toomany=1).
print/title='WARNING: Variables names longer than eight characters can produce incorrect output'.
print/title='when some variables in the data file have the same first eight characters. Shorter'/space=0.
print/title='variable names are recommended. By using this output, you are accepting all risk'/space=0.
print/title='and consequences of interpreting or reporting results that may be incorrect.'/space=0.
end if.
end if.
end if.
loop i = 1 to 100.
do if (errcode(i,1)=1).
print/title = 'ERROR: You must specify a Y and an X variable.'.
end if.
do if (errcode(i,1)=2).
print/title = 'ERROR: X, M, or Y variable used more than once or W and Z are the same variable.'.
do if (toomany = 1).
print/title = '       This could be caused by the use of variables names longer'/space=0.
print varnames/title = '       than eight characters. Here are the variables I see:'/space=0/format=A8.
end if.
end if.
do if (errcode(i,1)=3).
print/title = 'ERROR: You have specified more than one variable for W, Y, X, or Z'.
end if.
do if (errcode(i,1)=4).
print/title = 'ERROR: A variable specified as multicategorical has more than nine categories.'.
end if.
do if (errcode(i,1)=5).
print/title = 'ERROR: One of the categories contains only a single case.'.
end if.
do if (errcode(i,1)=6).
print/title = 'ERROR: Invalid model number in this version of PROCESS.'.
end if.
do if (errcode(i,1)=7).
print/title = 'ERROR: Invalid model number.'.
end if.
do if (errcode(i,1)=8).
print/title = 'ERROR: You must specify an M variable for this model.'.
end if.
do if (errcode(i,1)=9).
print/title = 'ERROR: You have specified an M variable in a model that does not use it.'.
end if.
do if (errcode(i,1)=10).
print/title = 'ERROR: You have specified a W variable in a model that does not use it.'.
end if.
do if (errcode(i,1)=11).
print/title = 'ERROR: You have not specified a W variable in a model that requires it.'.
end if.
do if (errcode(i,1)=12).
print/title = 'ERROR: You have specified a Z variable in a model that does not use it.'.
end if.
do if (errcode(i,1)=13).
print/title = 'ERROR: You have not specified a Z variable in a model that requires it.'.
end if.
do if (errcode(i,1)=14).
print/title = 'ERROR: V and Q are not proper specifications in this release of PROCESS.'.
print/title = '       Moderators must be specified as W and/or Z.'/space=0.
end if.
do if (errcode(i,1)=15).
print/title = 'ERROR: One of your model variables exhibits no variation (it is a constant).'.
end if.
do if (errcode(i,1)=16).
print/title = 'ERROR: BMATRIX is not the correct length or is otherwise invalid.'.
end if.
do if (errcode(i,1)=17).
print/title = 'ERROR: WMATRIX is not the correct length or is otherwise invalid.'.
end if.
do if (errcode(i,1)=18).
print/title = 'ERROR: ZMATRIX is not the correct length or is otherwise invalid.'.
end if.
do if (errcode(i,1)=19).
print/title = 'ERROR: WZMATRIX is not the correct length or is otherwise invalid.'.
end if.
do if (errcode(i,1)=20).
print/title = 'ERROR: A path fixed at zero cannot be moderated.'.
end if.
do if (errcode(i,1)=21).
print/title = 'ERROR: If only one moderator is specified, it must be specified as W.'.
end if.
do if (errcode(i,1)=22).
print/title = 'ERROR: In BMATRIX, X must be specified to affect at least one variable.'.
end if.
do if (errcode(i,1)=23).
print/title = 'ERROR: In BMATRIX, at least one variable must be specified to affect Y.'.
end if.
do if (errcode(i,1)=24).
print/title = 'ERROR: You must specify a model number or a custom BMATRIX specification.'.
end if.
do if (errcode(i,1)=25).
print/title = 'ERROR: BMATRIX cannot be used in conjunction with a model number.'.
end if.
do if (errcode(i,1)=26).
print/title = 'ERROR: Your model has a dangling mediator (all Ms must affect and be affected).'.
end if.
do if (errcode(i,1)=27).
print/title = 'ERROR: CLUSTER is not available on this release of PROCESS.'.
end if.
do if (errcode(i,1)=29).
print/title = 'ERROR: CMATRIX is not the correct length or is otherwise invalid.'.
end if.
do if (errcode(i,1)=30).
print/title = 'ERROR: In CMATRIX, all covariates must be assigned to an M or a Y.'.
end if.
do if (errcode(i,1)=31).
print/title = 'ERROR: A linear or near linear dependency (singularity) exists in the data.'.
end if.
do if (errcode(i,1)=32).
print/title = 'ERROR: Models 80 and 81 require between 3 and 6 mediators.'.
end if.
do if (errcode(i,1)=33).
print/title = 'ERROR: Model 82 requires 4 mediators.'.
end if.
do if (errcode(i,1)=34).
print/title = 'ERROR: This model number requires between 2 and 6 mediators.'.
end if.
do if (errcode(i,1)=35).
print/title = 'ERROR: In a model with only one moderator, that moderator must be W.'.
end if.
do if (errcode(i,1)=36).
print/title = 'ERROR: A serial mediation model cannot have more than 6 mediators.'.
end if.
do if (errcode(i,1)=37).
print/title = 'ERROR: No more than 10 mediators are allowed in a PROCESS command.'.
end if.
do if (errcode(i,1)=38).
print/title = 'ERROR: XCATCODE is not provided, not the correct length, or is otherwise invalid.'.
end if.
do if (errcode(i,1)=39).
print/title = 'ERROR: WCATCODE is not provided, not the correct length, or is otherwise invalid.'.
end if.
do if (errcode(i,1)=40).
print/title = 'ERROR: ZCATCODE is not provided, not the correct length, or is otherwise invalid.'.
end if.
do if (errcode(i,1)=41).
print/title = 'ERROR: Models 1, 2, and 3 cannot be customized.'.
end if.
do if (errcode(i,1)=42).
print/title = 'ERROR: WS option available only in PROCESS v2. Or use the MEMORE macro instead.'.
print/title = '       MEMORE can be downloaded from www.akmontoya.com.'/space=0.
end if.
do if (errcode(i,1)=43).
print/title = 'ERROR: PROCESS does not allow dichotomous mediators.'.
end if.
do if (errcode(i,1)=60).
print/title = 'PROCESS is now ready for use.'.
print/title='Copyright 2017-2022 by Andrew F. Hayes. ALL RIGHTS RESERVED.'/space=0.
print/title='Workshop schedule available at http://haskayne.ucalgary.ca/CCRAM'/space=0.
end if.
do if (errcode(i,1)=50).
print/title = 'ERROR: A multicategorical moderator cannot be specified as a covariate.'.
end if.
do if (errcode(i,1)=51).
print/title = 'ERROR: A variable you specified as a covariate is a moderator in all equations.'.
end if.
do if (errcode(i,1)=52 and mcerpt=0).
compute mcerpt=1.
print/title = 'ERROR: A variable specified as multicategorical must have at least three categories'.
end if.
do if (errcode(i,1) = 47).
do if (iterrmod=1).
print/title = 'ERROR: Iteration for Y model didn''t converge to a solution. Interpret results with caution.'.
print/title = '       Try increasing the number of iterations, though this will slow down computation.'/space=0.
end if.
do if (bootiter=1).
print/title = 'ERROR: Nonconvergence during bootstrapping. Interpet bootstrap results with caution.'.
end if.
end if.
do if (errcode(i,1) = 61).
print/title = 'ERROR: Variable names should be no more than eight characters in length.'.
end if.
do if (errcode(i,1) = 62).
print/title = 'ERROR: After listwise deletion of cases with missing data, too few cases remain.'.
end if.
do if (errcode(i,1) = 63).
print/title = 'ERROR: The XMINT option is available only for model 4.'.
end if.
do if (errcode(i,1) = 64).
print/title = 'ERROR: Incorrect number of values specified in CDEVAL option.'.
end if.
do if (errcode(i,1) = 65).
print/title = 'ERROR: Only indicator or sequential coding of X is allowed with the XMINT option.'.
end if.
do if (errcode(i,1) = 66).
print/title = 'ERROR: XMINT option not available with continuous X using PROCESS GUI. Use syntax.'.
end if.
do if (errcode(i,1) = 67).
print/title = 'ERROR: Too many elements provided in XREFVAL option.'.
end if.
do if (errcode(i,1)=68).
print/title = 'ERROR: Covariate assignment is not allowed with XMINT option.'.
end if.
do if (errcode(i,1) = 69).
print/title = 'ERROR: Incorrect number of values specified in COVAL option.'.
end if.
do if (errcode(i,1) = 70).
print/title = 'ERROR: Incorrect value(s) in XREFVAL for this dichotomous X variable.'.
end if.
do if (errcode(i,1) = 71).
print/title = 'ERROR: The CENTER option is not available when using the XMINT option.'.
end if.
do if (errcode(i,1) = 72).
print/title = 'ERROR: The XMINT option is not available for models with a dichotomous Y.'.
end if.
end loop.
END MATRIX.	
Ressources	Temps de processeur	00:00:06,23	
	Temps écoulé	00:00:06,21	


Run MATRIX procedure:

***************** PROCESS Procedure for SPSS Version 4.2 *****************

          Written by Andrew F. Hayes, Ph.D.       www.afhayes.com
    Documentation available in Hayes (2022). www.guilford.com/p/hayes3

**************************************************************************
Model  : 4
    Y  : HOMAIR
    X  : AnimalF
   M1  : TG
   M2  : HDL
   M3  : LDL
   M4  : TC

Sample
Size:  169

**************************************************************************
OUTCOME VARIABLE:
 TG

Model Summary
          R       R-sq        MSE          F        df1        df2          p
      ,0888      ,0079  4019,6791     1,3284     1,0000   167,0000      ,2507

Model
              coeff         se          t          p       LLCI       ULCI
constant   102,6364    13,5171     7,5931      ,0000    75,9499   129,3229
AnimalF    -16,7044    14,4934    -1,1526      ,2507   -45,3182    11,9094

Standardized coefficients
             coeff
AnimalF     -,2632

**************************************************************************
OUTCOME VARIABLE:
 HDL

Model Summary
          R       R-sq        MSE          F        df1        df2          p
      ,1319      ,0174   175,0370     2,9566     1,0000   167,0000      ,0874

Model
              coeff         se          t          p       LLCI       ULCI
constant    36,6364     2,8207    12,9885      ,0000    31,0676    42,2052
AnimalF      5,2004     3,0244     1,7195      ,0874     -,7706    11,1713

Standardized coefficients
             coeff
AnimalF      ,3908

**************************************************************************
OUTCOME VARIABLE:
 LDL

Model Summary
          R       R-sq        MSE          F        df1        df2          p
      ,0929      ,0086  1179,7873     1,4527     1,0000   167,0000      ,2298

Model
              coeff         se          t          p       LLCI       ULCI
constant    84,5455     7,3230    11,5452      ,0000    70,0878    99,0031
AnimalF     -9,4638     7,8519    -1,2053      ,2298   -24,9656     6,0380

Standardized coefficients
             coeff
AnimalF     -,2752

**************************************************************************
OUTCOME VARIABLE:
 TC

Model Summary
          R       R-sq        MSE          F        df1        df2          p
      ,1069      ,0114  1435,3482     1,9294     1,0000   167,0000      ,1667

Model
              coeff         se          t          p       LLCI       ULCI
constant   146,2273     8,0773    18,1034      ,0000   130,2804   162,1741
AnimalF    -12,0300     8,6607    -1,3890      ,1667   -29,1285     5,0685

Standardized coefficients
             coeff
AnimalF     -,3167

**************************************************************************
OUTCOME VARIABLE:
 HOMAIR

Model Summary
          R       R-sq        MSE          F        df1        df2          p
      ,3656      ,1337    23,2274     5,0309     5,0000   163,0000      ,0003

Model
              coeff         se          t          p       LLCI       ULCI
constant      ,3780     1,9302      ,1959      ,8450    -3,4333     4,1894
AnimalF      -,3803     1,1288     -,3369      ,7366    -2,6092     1,8486
TG            ,0439      ,0098     4,4993      ,0000      ,0246      ,0632
HDL           ,1273      ,0439     2,9017      ,0042      ,0407      ,2140
LDL           ,0942      ,0359     2,6216      ,0096      ,0232      ,1652
TC           -,0895      ,0358    -2,4977      ,0135     -,1603     -,0187

Standardized coefficients
             coeff
AnimalF     -,0746
TG           ,5463
HDL          ,3322
LDL          ,6353
TC          -,6666

************************** TOTAL EFFECT MODEL ****************************
OUTCOME VARIABLE:
 HOMAIR

Model Summary
          R       R-sq        MSE          F        df1        df2          p
      ,0176      ,0003    26,1616      ,0519     1,0000   167,0000      ,8201

Model
              coeff         se          t          p       LLCI       ULCI
constant     4,4275     1,0905     4,0601      ,0001     2,2746     6,5804
AnimalF      -,2664     1,1692     -,2278      ,8201    -2,5748     2,0420

Standardized coefficients
             coeff
AnimalF     -,0522


************** TOTAL, DIRECT, AND INDIRECT EFFECTS OF X ON Y **************

Total effect of X on Y
     Effect         se          t          p       LLCI       ULCI       c_ps
     -,2664     1,1692     -,2278      ,8201    -2,5748     2,0420     -,0522

Direct effect of X on Y
     Effect         se          t          p       LLCI       ULCI      c'_ps
     -,3803     1,1288     -,3369      ,7366    -2,6092     1,8486     -,0746

Indirect effect(s) of X on Y:
          Effect     BootSE   BootLLCI   BootULCI
TOTAL      ,1140      ,7396    -1,6167     1,3407
TG        -,7334     1,0799    -3,2736      ,9981
HDL        ,6622      ,7831     -,1427     2,8026
LDL       -,8915     2,3186    -7,6922     1,0469
TC        1,0767     2,0843    -1,3761     6,7039

Partially standardized indirect effect(s) of X on Y:
          Effect     BootSE   BootLLCI   BootULCI
TOTAL      ,0223      ,1431     -,3277      ,2455
TG        -,1438      ,2077     -,6330      ,1934
HDL        ,1298      ,1436     -,0335      ,5103
LDL       -,1748      ,4338    -1,4068      ,1993
TC         ,2111      ,3918     -,2799     1,2357

*********************** ANALYSIS NOTES AND ERRORS ************************

Level of confidence for all confidence intervals in output:
  95,0000

Number of bootstrap samples for percentile bootstrap confidence intervals:
  5000

NOTE: Standardized coefficients for dichotomous or multicategorical X are in
      partially standardized form.

------ END MATRIX -----


* Encoding: UTF-8.
preserve.
set printback=off.


Matrice


Remarques	
Sortie obtenue	10-NOV-2024 17:07:28	
Commentaires		
Entrée	Données	C:\Users\Brice SAHA\Documents\UBa\Master Thesis 2021\Fanuel\Database fanuel.sav	
	Jeu de données actif	Jeu_de_données1	
	Filtre	<sans>	
	Pondération	<sans>	
	Scinder un fichier	<sans>	
	N de lignes dans le fichier de travail	169	
Syntaxe	MATRIX.
compute wnames='xxxxx'.
compute znames='xxxxx'.
compute mcerpt=0.
compute wiscov=0.
compute ziscov=0.
compute tooman=0.
compute errcode=make(100,1,0).
compute notecode=make(100,1,0).
compute model = trunc( 4 ).
compute iterate = abs(trunc( 100 )).
compute converge = abs( 0.00001 ).
compute itprobtg=0.
compute v2tag=0.
compute ydich=0.
compute maxwwarn=0.
compute minwwarn=0.
compute maxzwarn=0.
compute minzwarn=0.
compute toomany=0.
compute wdich=0.
compute zdich=0.
compute wnotev=0.
compute znotev=0.
compute nxpval=1.
compute nwpval=1.
compute nzpval=1.
compute errs=1.
compute notes=1.
compute criterr=0.
compute novar=0.
compute adjust=0.
compute ncs=0.
compute serial=0.
compute sobelok=0.
compute hasw=0.
compute hasz=0.
compute printw=0.
compute printz=0.
compute xmint=( 0 =1).
compute wmodcust=0.
compute zmodcust=0.
compute booting=0.
compute bootiter=0.
compute iterrmod=0.
compute cov = 'xxxxx'.
compute varorder=( 0 <> 0).
compute nws=0.
compute w= 'xxxxx'.
compute nzs=0.
compute z = 'xxxxx'.
compute nms=0.
compute m = 'TG HDL LDL TC'.
compute nys=0.
compute y = 'HOMAIR'.
compute nxs=0.
compute x = 'NCDProt'.
compute effsize=( 0 =1).
compute stand=( 1 =1).
compute intprobe = .1.
compute xrefvals={ 999 }.
compute center=trunc( 0 ).
compute xcontcf=0.
compute xscaling=1.
compute cdeval={ -999 }.
compute cuscoval=0.
do if (model=74).
compute errcode(errs,1)=7.
compute errs=errs+1.
compute criterr=1.
end if.
do if (xmint=1 and model <> 4).
compute errcode(errs,1)=63.
compute errs=errs+1.
compute criterr=1.
end if.
do if (xmint=1 and model=4).
compute w=x.
compute model=74.
compute intprobe=1.
compute notecode(notes,1) = 32.
compute notes = notes + 1.
do if (effsize=1 or stand=1).
compute notecode(notes,1) = 34.
compute notes = notes + 1.
compute stand=0.
compute effsize=0.
end if.
do if (center <> 0).
compute center=0.
compute errcode(errs,1)=71.
compute errs=errs+1.
compute criterr=1.
end if.
end if.
compute v = 'xxxxx'.
compute q = 'xxxxx'.
compute linsum={ -999 }.
compute nlinsum=ncol(linsum).
do if (linsum(1,1) = -999).
compute nlinsum=0.
end if.
compute oldvars= 'xxxxx'.
compute mcxok=0.
compute mcwok=0.
compute mczok=0.
compute xprod=0.
compute zprod=0.
compute wprod=0.
compute modcok=0.
compute alttotal=0.
compute hc3=trunc( 0 ).
compute jn=( 0 = 1).
compute listmiss=( 0 =1).
compute modelres=( 0 =1).
compute outscree=( 1 =1).
compute activate=( 0 =1).
compute booterr=0.
compute normal=( 0 =1).
compute xmtest=( 0 =1).
compute describe=( 0 =1).
compute longname=( 0 =1).
do if (stand=1).
compute effsize=1.
end if.
compute pstog=0.
compute sobelok=0.
compute normal=( 0 =1).
compute mdichok=( 0 =1).
compute contrast={ 999 }.
compute ncontr=ncol(contrast).
compute ncontrow=nrow(contrast).
do if (contrast(1,1) = 999).
compute ncontr=1.
compute contrast=0.
end if.
do if (ncontr = 1).
compute contrast=trunc(contrast).
do if (contrast > 3 or contrast < 0)).
compute ncontr=1.
compute contrast = 0.
end if.
end if.
do if (ncontr > 1).
compute contvec=contrast.
compute contrast=4.
do if (ncontrow > 1).
compute contrast=0.
compute modcok=1.
compute wcontval=contvec(:,1).
compute zcontval=contvec(:,2).
do if ((ncontr <> 2) or (ncontrow <> 2)).
compute notecode(notes,1) = 19.
compute notes = notes + 1.
compute modcok=0.
end if.
end if.
end if.
do if (xmint = 1 and contrast(1,1) <> 0).
compute contrast=0.
compute notecode(notes,1) = 37.
compute notes = notes + 1.
end if.
do if (varorder = 1).
compute notecode(notes,1) = 21.
compute notes = notes + 1.
end if.
do if ( 999 <> 999 or 999 <> 999).
compute notecode(notes,1) = 22.
compute notes = notes + 1.
end if.
compute modelbt=( 0 =1).
compute cluster= 'xxxxx'.
compute matrices=( 0 =1).
compute covcoeff=( 0 =1).
compute covmy=trunc( 0 ).
do if (covmy < 0 or covmy > 2).
compute covmy = 0.
end if.
compute boot = abs(trunc( 5000 )).
compute bc=( 0 =1).
compute mc=abs(trunc( 0 )).
compute hc=trunc( 5 ).
do if (intprobe < 0 or intprobe > 1).
compute intprobe = .10.
end if.
compute plot=trunc( 0 ).
do if (plot < 0 or plot > 2).
compute plot=0.
end if.
compute total=( 1 =1).
compute dototal=0.
compute saveboot = ( 0 = 1).
compute saveest=( 0 = 2).
do if (saveest=1).
compute intprobe=1.
end if.
do if (hc >= 0 and hc < 5).
compute notecode(notes,1) = 4.
compute notes = notes + 1.
end if.
do if (hc > 5 or hc < 0).
compute hc=5.
end if.
compute mcw=trunc( 0 ).
compute mcz=trunc( 0 ).
compute mcx=trunc( 0 ).
do if (mcx > 0 and mcx < 3 and model = 74).
compute mcw=mcx.
compute xscaling=1.
end if.
do if (mcx > 2 and model = 74).
compute errcode(errs,1)=65.
compute errs=errs+1.
compute criterr=1.
end if.
do if (model = 74 and normal=1).
compute notecode(notes,1) = 33.
compute normal=0.
compute notes = notes + 1.
end if.
do if (mcx > 0 and contrast > 0).
compute notecode(notes,1) = 28.
compute notes = notes + 1.
compute contrast=0.
end if.
compute nxvls=1.
compute nmvls=1.
compute nwvls=1.
compute nzvls=1.
compute paths=999.
compute pathsw=999.
compute pathsz=999.
compute pathswz=999.
compute pathsmod=999.
compute pathtype=999.
compute obscoeff=999.
compute pathsdv={' '}.
compute quantile=1.
do if ( 999 <>999).
compute notecode(notes,1) = 23.
compute notes = notes + 1.
end if.
compute moments=( 0 =1).
do if (moments=1).
compute quantile=0.
end if.
compute bmatrix={ -999 }.
compute wmatrix={ -999 }.
compute zmatrix={ -999 }.
compute wzmatrix={ -999 }.
compute cmatrix={ -999 }.
compute xcatcode={ -999 }.
compute wcatcode={ -999 }.
compute zcatcode={ -999 }.
compute needed=0.
compute conf= 95.
do if (trunc( 95 ) >= 100 or (trunc( 95 ) <= 50)).
compute conf = 95.
compute notecode(notes,1)=2.
compute notes=notes+1.
end if.
do if (model >= 0 and model < 4 and modelbt=0).
compute boot=0.
compute mc=0.
compute bc=0.
end if.
do if (mc > 0 and boot > 0).
compute boot=0.
compute bc=0.
end if.
do if ((boot < 1000) and (mc = 0) and (boot > 0)).
compute boot=5000.
end if.
do if ((mc < 1000) and (boot = 0) and (mc > 0)).
compute mc=5000.
end if.
compute p0=-.322232431088.
compute p1 = -1.
compute p2 = -.342242088547.
compute p3 = -.0204231210245.
compute p4 = -.0000453642210148.
compute q0 = .0993484626060.
compute q1 = .588581570495.
compute q2 = .531103462366.
compute q3 = .103537752850.
compute q4 = .0038560700634.
compute badend=0.
compute priorlo = -9999999.
compute priorhi = 9999999.
compute alpha2 = (1-(conf/100))/2.
compute cilm=alpha2*2.
compute y5=sqrt(-2*ln(alpha2)).
compute xp2=(y5+((((y5*p4+p3)*y5+p2)*y5+p1)*y5+p0)/((((y5*q4+q3)*y5+q2)*y5+q1)*y5+q0)).
compute medlb={'   M1  :';'   M2  :';'   M3  :';'   M4  :';'   M5  :';'   M6  :';'   M7  :';'   M8  :';'   M9  :';'   M10 :'}.
compute medlb2={'(M1)','(M2)','(M3)','(M4)','(M5)','(M6)','(M7)','(M8)','(M9)','(M10)'}.
compute xlb={'   X1  :';'   X2  :';'   X3  :';'   X4  :';'   X5  :';'   X6  :';'   X7  :';'   X8  :';'   X9  :'}.
compute highlbw={'M1*W'; 'M2*W'; 'M3*W'; 'M4*W'; 'M5*W'; 'M6*W'; 'M7*W'; 'M8*W'; 'M9*W'; 'M10*W'}.
do if (xmint=1).
compute highlbw={'M1*X'; 'M2*X'; 'M3*X'; 'M4*X'; 'M5*X'; 'M6*X'; 'M7*X'; 'M8*X'; 'M9*X'; 'M10*X'}.
end if.
compute highlbz={'M1*Z'; 'M2*Z'; 'M3*Z'; 'M4*Z'; 'M5*Z'; 'M6*Z'; 'M7*Z'; 'M8*Z'; 'M9*Z';'M10*Z'}.
compute highlbwz={'M1*W*Z'; 'M2*W*Z'; 'M3*W*Z'; 'M4*W*Z'; 'M5*W*Z'; 'M6*W*Z'; 'M7*W*Z'; 'M8*W*Z'; 'M9*W*Z';'M10*W*Z'}.
compute highlbbt={'BOTH(M1)'; 'BOTH(M2)'; 'BOTH(M3)'; 'BOTH(M4)'; 'BOTH(M5)'; 'BOTH(M6)'; 'BOTH(M7)'; 'BOTH(M8)'; 'BOTH(M9)';'BTH(M10)'}.
compute highlbx={'M1*X'; 'M2*X'; 'M3*X'; 'M4*X'; 'M5*X'; 'M6*X'; 'M7*X'; 'M8*X'; 'M9*X'; 'M10*X'}.
compute skipwz=0.
compute validm={1,1,1,1,1,1,1,1,1,1,1,1,1,1,1,1,1,1,1,1,1,1,0,0,0,0,0,1,1,0,0,0,0, 0,0,0,0,0,0,0,0,0,0,0,0,0,0,0,0,0,0,0,0,0,0,0,0,1,1,1,1,1,1,1,1,1,1,1,1,1,1,1,1,1,1,1,0,0,0,1,1, 1,1,1,1,1,1,1,1,1,1,1}.
do if (activate=1).
compute errcode(errs,1)=60.
compute errs=errs+1.
compute criterr=1.
end if.
do if (criterr=0).
do if (( 0 =1)=1).
compute errcode(errs,1)=42.
compute errs=errs+1.
compute criterr=1.
end if.
do if (model > 0 and model < 93).
do if (validm(1,model)=0).
compute errcode(errs,1)=6.
compute errs=errs+1.
compute criterr=1.
end if.
release validm.
end if.
do if ((model > 92 or model < 0) and model <> 999)).
compute errcode(errs,1)=7.
compute errs=errs+1.
compute criterr=1.
end if.
do if (model = 999 and bmatrix(1,1)=-999).
compute errcode(errs,1)=24.
compute errs=errs+1.
compute criterr=1.
end if.
do if (model <> 999 and bmatrix(1,1) <> -999).
compute errcode(errs,1)=25.
compute errs=errs+1.
compute criterr=1.
end if.
do if ((model = 74 or (model > 0 and model < 4)) and ((wmatrix(1,1) <> -999) or (zmatrix(1,1)<>-999) or (wzmatrix(1,1)<>-999)))).
compute errcode(errs,1)=41.
compute errs=errs+1.
compute criterr=1.
end if.
do if (hc3 <> 0).
compute notecode(notes,1) = 5.
compute notes = notes + 1.
do if (hc3 = 1).
compute hc=3.
end if.
end if.
do if ((v <> 'xxxxx') or (q <> 'xxxxx')).
compute errcode(errs,1)=14.
compute errs=errs+1.
compute errcode(errs,1)=48.
compute errs=errs+1.
compute criterr=1.
end if.
do if (oldvars <> 'xxxxx').
compute errcode(errs,1)=48.
compute errs=errs+1.
compute criterr=1.
end if.
do if (cluster <> 'xxxxx').
compute errcode(errs,1)=27.
compute errs=errs+1.
compute criterr=1.
end if.
do if ((y = 'xxxxx') or (x = 'xxxxx')).
compute errcode(errs,1)=1.
compute errs=errs+1.
compute criterr=1.
end if.
do if ((m = 'xxxxx') and model > 3).
compute errcode(errs,1)=8.
compute errs=errs+1.
compute criterr=1.
end if.
end if.
do if (criterr=0).
get ytmp/variables = HOMAIR /names = ynames/MISSING = 99999.
compute nys=ncol(ytmp).
compute needed=nys.
compute n=nrow(ytmp).
compute varnames={ynames}.
compute dat=ytmp.

.


do if (toomany=1 and longname=0).
compute criterr=1.
do if (tooman=0).
compute tooman=1.
compute errcode(errs,1) = 61.
compute errs = errs + 1.
end if.
end if
.
compute modelvar={ '4' ;t(ynames)}.
do if ( 4 =999).
compute modelvar(1,1)='CUSTOM'.
end if.
get xtmp/variables = NCDProt /names = xnames/MISSING = 99999.
compute nxs=ncol(xtmp).
compute n=nrow(xtmp).
compute needed=needed+nxs.
compute varnames={varnames,xnames}.
compute xcatlab=t(xnames).
compute dat={dat,xtmp}.

.


do if (toomany=1 and longname=0).
compute criterr=1.
do if (tooman=0).
compute tooman=1.
compute errcode(errs,1) = 61.
compute errs = errs + 1.
end if.
end if
.
compute modelvar={modelvar;t(xnames)}.
do if (nxs = 1).
compute modelvlb={'Model  :';'    Y  :';'    X  :'}.
else.
compute modelvlb={'Model  :';'    Y  :';xlb(1:nxs,1)}.
end if.
do if (m <> 'xxxxx').
get mtmp/variables = TG HDL LDL TC /names = mnames/MISSING = 99999.
compute nms=ncol(mtmp).
compute mprod=make(1,nms,0).
compute n=nrow(mtmp).
compute needed=needed+nms.
compute varnames={varnames,mnames}.
compute dat={dat,mtmp}.
compute modelvar={modelvar;t(mnames)}.
compute x2m=make(99,nms,0).
compute m2y=make(99,nms,0).
compute onem=make(nms,1,1).

.


do if (toomany=1 and longname=0).
compute criterr=1.
do if (tooman=0).
compute tooman=1.
compute errcode(errs,1) = 61.
compute errs = errs + 1.
end if.
end if
.
do if (nms > 1 and nms < 11).
compute modelvlb={modelvlb;medlb(1:nms,1)}.
else.
compute modelvlb={modelvlb;'    M  :'}.
end if.
do if (nms > 0 and model < 4).
compute errcode(errs,1)=9.
compute errs=errs+1.
do if (model <> 0).
compute errcode(errs,1)=48.
compute errs=errs+1.
end if.
compute criterr=1.
end if.
end if.
compute wlocatet=0.
compute wlocate=0.
do if (w <> 'xxxxx').
do if (xmint=0).
get wtmp/variables = xxxxx /names = wnames/MISSING = 99999.

.


do if (toomany=1 and longname=0).
compute criterr=1.
do if (tooman=0).
compute tooman=1.
compute errcode(errs,1) = 61.
compute errs = errs + 1.
end if.
end if
.
end if.
do if (xmint=1).
get wtmp/variables = NCDProt /names = wnames/MISSING = 99999.
end if.
compute nws=ncol(wtmp).
compute n=nrow(wtmp).
compute varnames={varnames,wnames}.
compute wlocate=ncol(varnames).
do if (model=74).
compute wlocatet=1.
do if (xnames <> wnames).
compute errcode(errs,1)=45.
compute errs=errs+1.
compute criterr=1.
end if.
end if.
compute wcatlab=t(wnames).
compute dat={dat,wtmp}.
do if (xmint <> 1).
compute modelvar={modelvar;t(wnames)}.
compute modelvlb={modelvlb;'    W  :'}.
end if.
end if.
do if (z <> 'xxxxx').
get ztmp/variables = xxxxx /names = znames/MISSING = 99999.
compute nzs=ncol(ztmp).
compute n=nrow(ztmp).

.


do if (toomany=1 and longname=0).
compute criterr=1.
do if (tooman=0).
compute tooman=1.
compute errcode(errs,1) = 61.
compute errs = errs + 1.
end if.
end if
.
compute varnames={varnames,znames}.
compute zcatlab=t(znames).
compute dat={dat,ztmp}.
compute modelvar={modelvar;t(znames)}.
compute modelvlb={modelvlb;'    Z  :'}.
end if.
do if (cov <> 'xxxxx').
get ctmp/variables = xxxxx /names = covnames/MISSING = 99999.
compute ncs=ncol(ctmp).
compute n=nrow(ctmp).

.


do if (toomany=1 and longname=0).
compute criterr=1.
do if (tooman=0).
compute tooman=1.
compute errcode(errs,1) = 61.
compute errs = errs + 1.
end if.
end if
.
compute varnames={varnames,covnames}.
compute dat={dat,ctmp}.
end if.
do if (nws > 1 or nzs > 1 or nys > 1 or nxs > 1).
compute errcode(errs,1)=3.
compute errs=errs+1.
compute criterr=1.
end if.
do if ((model = 80 or model = 81) and (nms < 3 or nms > 6)).
compute errcode(errs,1)=32.
compute errs=errs+1.
compute criterr=1.
end if.
do if (model = 82 and nms <> 4).
compute errcode(errs,1)=33.
compute errs=errs+1.
compute criterr=1.
end if.
do if (nms > 10).
compute errcode(errs,1)=37.
compute errs=errs+1.
compute criterr=1.
end if.
do if ((model = 6 or (model > 82 and model < 999)) and (nms < 2 or nms > 6)).
compute errcode(errs,1)=34.
compute errs=errs+1.
compute criterr=1.
end if.
compute match=0.
compute match2=0.
compute mcwzcov=0.
loop i = 1 to (ncol(varnames)-1).
loop j = (i+1) to ncol(varnames).
do if (varnames(i)=varnames(j)).
do if (i < (nxs+nms+nys+1)).
compute match2=match2+1.
end if.
do if (wlocatet=1 and i=2 and j=wlocate).
compute match2=match2-1.
end if.
do if ((wnames=znames) and (nws > 0 or nzs > 0))).
compute match2=match2+1.
end if.
do if (i < (ncol(varnames)-ncs+1)) and j > (ncol(varnames)-ncs)).
do if ((varnames(j)=wnames) and mcw=0)).
compute match=0.
compute wiscov=(j-(ncol(varnames)-ncs)).
end if.
do if ((varnames(j)=wnames) and mcw <>0)).
compute mcwzcov=1.
end if.
do if ((varnames(j)=znames) and mcz=0).
compute match=0.
compute ziscov=(j-(ncol(varnames)-ncs)).
end if.
do if ((varnames(j)=znames) and mcz<>0)).
compute mcwzcov=1.
end if.
end if.
end if.
end loop.
end loop.
do if (match2>0 or match=1).
compute errcode(errs,1)=2.
compute errs=errs+1.
compute criterr=1.
end if.
do if (mcwzcov=1).
compute errcode(errs,1)=50.
compute errs=errs+1.
compute criterr=1.
end if.
compute ninit=nrow(dat).
compute rownum=make(ninit,1,0).
loop i = 1 to ninit.
compute rownum(i,1)=i.
end loop.
compute dat={rownum,dat}.
compute j=1.
compute missrow=0.
loop i = 1 to n.
do if (rsum(dat(i,2:ncol(dat))=99999)=0).
compute dat(j,:)=dat(i,:).
compute j=j+1.
else.
compute missrow={missrow;dat(i,1)}.
end if.
end loop.
do if (j < 5).
compute errcode(errs,1)=62.
compute errs=errs+1.
compute criterr=1.
end if.
do if (criterr=0).
compute rownum=dat(1:(j-1),1).
do if (nrow(missrow) > 1).
compute missrow=t(missrow(2:nrow(missrow),1)).
compute notecode(notes,1) = 29.
compute notes = notes + 1.
end if.
compute dat=dat(1:(j-1),2:ncol(dat)).
compute n=nrow(dat).
compute nmiss=ninit-n.
compute ytmp=dat(:,1:nys).

.
compute desctmp=make((8-(4* 0 )),ncol( ytmp ),-999).
loop jd=1 to ncol( ytmp ).
compute descdat= ytmp (:,jd).
compute desctmp(1,jd) = csum(descdat)/nrow(descdat).
compute desctmp(2,jd) = (nrow(descdat)*sscp(descdat))-(t(csum(descdat))*(csum(descdat))).
compute desctmp(2,jd) = sqrt(desctmp(2,jd)/(nrow(descdat)*(nrow(descdat)-1))).
compute desctmp(3,jd)=cmin(descdat).
compute desctmp(4,jd)=cmax(descdat).
do if ( 0 =0).
compute minwarn=0.
compute maxwarn=0.
do if ((desctmp(3,jd)=desctmp(4,jd)) and novar=0).
compute errcode(errs,1)=15.
compute errs=errs+1.
compute criterr=1.
compute novar=1.
end if.
compute tmp=((descdat(:,1)=desctmp(3,jd))+(descdat(:,1)=desctmp(4,jd))).
compute desctmp(8,jd)=(csum(tmp)=nrow(tmp)).
compute tmp = descdat.
compute tmp(GRADE(descdat),:) = descdat.
compute descdat = tmp.
release tmp.
compute decval={.16;.5;.84}.
loop kd=1 to 3.
compute low=trunc(decval(kd,1)*(nrow(descdat)+1)).
compute lowdec=decval(kd,1)*(nrow(descdat)+1)-low.
compute value=descdat(low,1)+(descdat((low+1),1)-descdat(low,1))*lowdec.
compute desctmp((4+kd),jd)=value.
end loop.
compute mnotev=1.
compute modvals=desctmp(5:7,:).
do if (quantile <> 1).
compute desctmp(5,jd)=desctmp(1,jd)-desctmp(2,jd).
compute desctmp(6,jd)=desctmp(1,jd).
compute desctmp(7,jd)=desctmp(1,jd)+desctmp(2,jd).
compute modvals=desctmp(5:7,:).
compute mnotev=2.
do if (modvals(1,1) < desctmp(3,1)).
compute modvals(1,1)=desctmp(3,1).
compute minwarn=1.
end if.
do if (modvals(3,1) > desctmp(4,1)).
compute modvals(3,1)=desctmp(4,1).
compute maxwarn=1.
end if.
end if.
do if (desctmp(8,1)=1).
compute modvals={desctmp(3,1);desctmp(4,1)}.
compute mnotev=0.
compute minwarn=0.
compute maxwarn=0.
end if.
end if.
end loop
.
compute ysd=desctmp(2,:).
compute ovsd=ysd.
do if (desctmp(8,1)=1).
compute ydich=1.
do if (total=1).
compute total=0.
compute notecode(notes,1) = 24.
compute notes = notes + 1.
end if.
do if (effsize=1).
compute effsize=0.
compute notecode(notes,1) = 25.
compute notes = notes + 1.
end if.
do if (model=74).
compute errcode(errs,1) = 72.
compute errs=errs+1.
compute criterr=1.
end if.
compute omx = cmax(ytmp).
compute omn = cmin(ytmp).
compute ytmp = (ytmp = omx).
compute dat(:,1:nys)=(dat(:,1:nys)=omx).
compute rcd = {omn, 0; omx, 1}.
end if.
compute xtmp=dat(:,(nys+1):(nys+nxs)).

.
compute desctmp=make((8-(4* 0 )),ncol( xtmp ),-999).
loop jd=1 to ncol( xtmp ).
compute descdat= xtmp (:,jd).
compute desctmp(1,jd) = csum(descdat)/nrow(descdat).
compute desctmp(2,jd) = (nrow(descdat)*sscp(descdat))-(t(csum(descdat))*(csum(descdat))).
compute desctmp(2,jd) = sqrt(desctmp(2,jd)/(nrow(descdat)*(nrow(descdat)-1))).
compute desctmp(3,jd)=cmin(descdat).
compute desctmp(4,jd)=cmax(descdat).
do if ( 0 =0).
compute minwarn=0.
compute maxwarn=0.
do if ((desctmp(3,jd)=desctmp(4,jd)) and novar=0).
compute errcode(errs,1)=15.
compute errs=errs+1.
compute criterr=1.
compute novar=1.
end if.
compute tmp=((descdat(:,1)=desctmp(3,jd))+(descdat(:,1)=desctmp(4,jd))).
compute desctmp(8,jd)=(csum(tmp)=nrow(tmp)).
compute tmp = descdat.
compute tmp(GRADE(descdat),:) = descdat.
compute descdat = tmp.
release tmp.
compute decval={.16;.5;.84}.
loop kd=1 to 3.
compute low=trunc(decval(kd,1)*(nrow(descdat)+1)).
compute lowdec=decval(kd,1)*(nrow(descdat)+1)-low.
compute value=descdat(low,1)+(descdat((low+1),1)-descdat(low,1))*lowdec.
compute desctmp((4+kd),jd)=value.
end loop.
compute mnotev=1.
compute modvals=desctmp(5:7,:).
do if (quantile <> 1).
compute desctmp(5,jd)=desctmp(1,jd)-desctmp(2,jd).
compute desctmp(6,jd)=desctmp(1,jd).
compute desctmp(7,jd)=desctmp(1,jd)+desctmp(2,jd).
compute modvals=desctmp(5:7,:).
compute mnotev=2.
do if (modvals(1,1) < desctmp(3,1)).
compute modvals(1,1)=desctmp(3,1).
compute minwarn=1.
end if.
do if (modvals(3,1) > desctmp(4,1)).
compute modvals(3,1)=desctmp(4,1).
compute maxwarn=1.
end if.
end if.
do if (desctmp(8,1)=1).
compute modvals={desctmp(3,1);desctmp(4,1)}.
compute mnotev=0.
compute minwarn=0.
compute maxwarn=0.
end if.
end if.
end loop
.
compute xsd=desctmp(2,:).
compute xmodvals=modvals.
compute xdich=desctmp(8,1).
compute xmx=cmax(xtmp).
compute xmn=cmin(xtmp).
do if ((mcx > 0) and (xrefvals(1,1) <> 999) and (xmint=1) and (model= 74)).
compute notecode(notes,1) = 36.
compute notes = notes + 1.
end if.
do if (mcx=0).
do if (ncol(xrefvals)>2 and model=74 and xmint=1).
compute errcode(errs,1)=67.
compute errs=errs+1.
compute criterr=1.
end if.
do if ((model=74) and (xmint=1)).
do if ((xrefvals(1,1)=999) and nxvls=1 and xdich=0).
compute errcode(errs,1)=66.
compute errs=errs+1.
compute criterr=1.
end if.
do if ((xrefvals(1,1)=999) and (xdich=1)).
compute xrefvals={xmn,xmx}.
compute xscaling=xrefvals(1,2)-xrefvals(1,1).
end if.
do if (ncol(xrefvals)=1 and xrefvals(1,1) <> 999).
do if (xdich=0).
compute xrefvals={xrefvals,(xrefvals(1,1)+1)}.
compute xscaling=xrefvals(1,2)-xrefvals(1,1).
end if.
do if (xdich=1).
do if ((xrefvals(1,1) <> xmx) and (xrefvals(1,1) <> xmn)).
compute errcode(errs,1)=70.
compute errs=errs+1.
compute criterr=1.
end if.
do if (xrefvals(1,1) = xmx).
compute xrefvals={xrefvals,xmn}.
compute xscaling=xrefvals(1,2)-xrefvals(1,1).
end if.
do if (xrefvals(1,1) = xmn).
compute xrefvals={xrefvals,xmx}.
compute xscaling=xrefvals(1,2)-xrefvals(1,1).
end if.
end if.
end if.
do if (ncol(xrefvals)=2).
compute xscaling=xrefvals(1,2)-xrefvals(1,1).
do if (xdich=1).
compute xreferr=1.
do if (((xrefvals(1,1) = xmx) and (xrefvals(1,2)=xmn)) or ((xrefvals(1,1) = xmn) and (xrefvals(1,2)=xmx))).
compute xreferr=0.
end if.
do if (xreferr=1).
compute errcode(errs,1)=70.
compute errs=errs+1.
compute criterr=1.
end if.
end if.
end if.
end if.
end if.
do if (xmint=1 and model=74 and mcx=0).
compute xmodvals=t(xrefvals).
compute xcontcf=1.
end if.
compute nxpval=nrow(xmodvals).
compute xprobval=xmodvals.
do if (xdich =1 and mcx > 0).
compute mcx=0.
compute errcode(errs,1) = 52.
compute errs = errs + 1.
compute criterr = 1.
end if.
do if (nms > 0).
compute mtmp=dat(:,(nys+nxs+1):(nys+nxs+nms)).

.
compute desctmp=make((8-(4* 0 )),ncol( mtmp ),-999).
loop jd=1 to ncol( mtmp ).
compute descdat= mtmp (:,jd).
compute desctmp(1,jd) = csum(descdat)/nrow(descdat).
compute desctmp(2,jd) = (nrow(descdat)*sscp(descdat))-(t(csum(descdat))*(csum(descdat))).
compute desctmp(2,jd) = sqrt(desctmp(2,jd)/(nrow(descdat)*(nrow(descdat)-1))).
compute desctmp(3,jd)=cmin(descdat).
compute desctmp(4,jd)=cmax(descdat).
do if ( 0 =0).
compute minwarn=0.
compute maxwarn=0.
do if ((desctmp(3,jd)=desctmp(4,jd)) and novar=0).
compute errcode(errs,1)=15.
compute errs=errs+1.
compute criterr=1.
compute novar=1.
end if.
compute tmp=((descdat(:,1)=desctmp(3,jd))+(descdat(:,1)=desctmp(4,jd))).
compute desctmp(8,jd)=(csum(tmp)=nrow(tmp)).
compute tmp = descdat.
compute tmp(GRADE(descdat),:) = descdat.
compute descdat = tmp.
release tmp.
compute decval={.16;.5;.84}.
loop kd=1 to 3.
compute low=trunc(decval(kd,1)*(nrow(descdat)+1)).
compute lowdec=decval(kd,1)*(nrow(descdat)+1)-low.
compute value=descdat(low,1)+(descdat((low+1),1)-descdat(low,1))*lowdec.
compute desctmp((4+kd),jd)=value.
end loop.
compute mnotev=1.
compute modvals=desctmp(5:7,:).
do if (quantile <> 1).
compute desctmp(5,jd)=desctmp(1,jd)-desctmp(2,jd).
compute desctmp(6,jd)=desctmp(1,jd).
compute desctmp(7,jd)=desctmp(1,jd)+desctmp(2,jd).
compute modvals=desctmp(5:7,:).
compute mnotev=2.
do if (modvals(1,1) < desctmp(3,1)).
compute modvals(1,1)=desctmp(3,1).
compute minwarn=1.
end if.
do if (modvals(3,1) > desctmp(4,1)).
compute modvals(3,1)=desctmp(4,1).
compute maxwarn=1.
end if.
end if.
do if (desctmp(8,1)=1).
compute modvals={desctmp(3,1);desctmp(4,1)}.
compute mnotev=0.
compute minwarn=0.
compute maxwarn=0.
end if.
end if.
end loop
.
compute ovsd={desctmp(2,:),ysd}.
compute medmeans=cdeval.
do if ((cdeval(1,1) <> -999) and (ncol(medmeans) <> nms) and (model=74)).
compute errcode(errs,1)=64.
compute errs=errs+1.
compute criterr=1.
end if.
do if ((cdeval(1,1)=-999) and (model=74)).
compute medmeans=desctmp(1,:).
end if.
do if ((cdeval(1,1)<> -999) and (model=74) and (criterr=0)).
compute notecode(notes,1) = 31.
compute notes = notes + 1.
end if.
do if ((rsum(desctmp(8,:))>0) and (mdichok <> 1)).
compute errcode(errs,1)=43.
compute errs=errs+1.
compute criterr=1.
end if.
compute mmodvals=modvals.
compute mprobval=mmodvals.
end if.
do if (nws > 0).
compute wtmp=dat(:,(nys+nxs+nms+1):(nys+nxs+nms+nws)).

.
compute desctmp=make((8-(4* 0 )),ncol( wtmp ),-999).
loop jd=1 to ncol( wtmp ).
compute descdat= wtmp (:,jd).
compute desctmp(1,jd) = csum(descdat)/nrow(descdat).
compute desctmp(2,jd) = (nrow(descdat)*sscp(descdat))-(t(csum(descdat))*(csum(descdat))).
compute desctmp(2,jd) = sqrt(desctmp(2,jd)/(nrow(descdat)*(nrow(descdat)-1))).
compute desctmp(3,jd)=cmin(descdat).
compute desctmp(4,jd)=cmax(descdat).
do if ( 0 =0).
compute minwarn=0.
compute maxwarn=0.
do if ((desctmp(3,jd)=desctmp(4,jd)) and novar=0).
compute errcode(errs,1)=15.
compute errs=errs+1.
compute criterr=1.
compute novar=1.
end if.
compute tmp=((descdat(:,1)=desctmp(3,jd))+(descdat(:,1)=desctmp(4,jd))).
compute desctmp(8,jd)=(csum(tmp)=nrow(tmp)).
compute tmp = descdat.
compute tmp(GRADE(descdat),:) = descdat.
compute descdat = tmp.
release tmp.
compute decval={.16;.5;.84}.
loop kd=1 to 3.
compute low=trunc(decval(kd,1)*(nrow(descdat)+1)).
compute lowdec=decval(kd,1)*(nrow(descdat)+1)-low.
compute value=descdat(low,1)+(descdat((low+1),1)-descdat(low,1))*lowdec.
compute desctmp((4+kd),jd)=value.
end loop.
compute mnotev=1.
compute modvals=desctmp(5:7,:).
do if (quantile <> 1).
compute desctmp(5,jd)=desctmp(1,jd)-desctmp(2,jd).
compute desctmp(6,jd)=desctmp(1,jd).
compute desctmp(7,jd)=desctmp(1,jd)+desctmp(2,jd).
compute modvals=desctmp(5:7,:).
compute mnotev=2.
do if (modvals(1,1) < desctmp(3,1)).
compute modvals(1,1)=desctmp(3,1).
compute minwarn=1.
end if.
do if (modvals(3,1) > desctmp(4,1)).
compute modvals(3,1)=desctmp(4,1).
compute maxwarn=1.
end if.
end if.
do if (desctmp(8,1)=1).
compute modvals={desctmp(3,1);desctmp(4,1)}.
compute mnotev=0.
compute minwarn=0.
compute maxwarn=0.
end if.
end if.
end loop
.
compute wmodvals=modvals.
compute wdich=desctmp(8,1).
do if (wdich =1 and mcw > 0).
compute mcw=0.
compute errcode(errs,1) = 52.
compute errs = errs + 1.
compute criterr = 1.
end if.
compute wmin=desctmp(3,1).
compute wmax=desctmp(4,1).
compute minwwarn=minwarn.
compute maxwwarn=maxwarn.
compute wnotev=mnotev.
compute wmodval={ 999 }.
do if (xmint=1 and model=74 and mcx=0).
compute wmodval=xrefvals.
end if.
compute nwcontr=ncol(wmodval).
do if (wmodval(1,1) <> 999).
compute wmodvals=wmodval(1,1).
compute wmodcust=1.
do if (nwcontr > 1).
compute wmodvals=t(wmodval).
end if.
compute minwwarn=0.
compute maxwwarn=0.
compute wnotev=0.
end if.
compute wprobval=wmodvals.
compute nwpval=nrow(wmodvals).
end if.
do if (nzs > 0).
compute ztmp=dat(:,(nys+nxs+nms+nws+1):(nys+nxs+nms+nws+nzs)).

.
compute desctmp=make((8-(4* 0 )),ncol( ztmp ),-999).
loop jd=1 to ncol( ztmp ).
compute descdat= ztmp (:,jd).
compute desctmp(1,jd) = csum(descdat)/nrow(descdat).
compute desctmp(2,jd) = (nrow(descdat)*sscp(descdat))-(t(csum(descdat))*(csum(descdat))).
compute desctmp(2,jd) = sqrt(desctmp(2,jd)/(nrow(descdat)*(nrow(descdat)-1))).
compute desctmp(3,jd)=cmin(descdat).
compute desctmp(4,jd)=cmax(descdat).
do if ( 0 =0).
compute minwarn=0.
compute maxwarn=0.
do if ((desctmp(3,jd)=desctmp(4,jd)) and novar=0).
compute errcode(errs,1)=15.
compute errs=errs+1.
compute criterr=1.
compute novar=1.
end if.
compute tmp=((descdat(:,1)=desctmp(3,jd))+(descdat(:,1)=desctmp(4,jd))).
compute desctmp(8,jd)=(csum(tmp)=nrow(tmp)).
compute tmp = descdat.
compute tmp(GRADE(descdat),:) = descdat.
compute descdat = tmp.
release tmp.
compute decval={.16;.5;.84}.
loop kd=1 to 3.
compute low=trunc(decval(kd,1)*(nrow(descdat)+1)).
compute lowdec=decval(kd,1)*(nrow(descdat)+1)-low.
compute value=descdat(low,1)+(descdat((low+1),1)-descdat(low,1))*lowdec.
compute desctmp((4+kd),jd)=value.
end loop.
compute mnotev=1.
compute modvals=desctmp(5:7,:).
do if (quantile <> 1).
compute desctmp(5,jd)=desctmp(1,jd)-desctmp(2,jd).
compute desctmp(6,jd)=desctmp(1,jd).
compute desctmp(7,jd)=desctmp(1,jd)+desctmp(2,jd).
compute modvals=desctmp(5:7,:).
compute mnotev=2.
do if (modvals(1,1) < desctmp(3,1)).
compute modvals(1,1)=desctmp(3,1).
compute minwarn=1.
end if.
do if (modvals(3,1) > desctmp(4,1)).
compute modvals(3,1)=desctmp(4,1).
compute maxwarn=1.
end if.
end if.
do if (desctmp(8,1)=1).
compute modvals={desctmp(3,1);desctmp(4,1)}.
compute mnotev=0.
compute minwarn=0.
compute maxwarn=0.
end if.
end if.
end loop
.
compute zmodvals=modvals.
compute zdich=desctmp(8,1).
do if (zdich =1 and mcz > 0).
compute mcz=0.
compute errcode(errs,1) = 52.
compute errs = errs + 1.
compute criterr = 1.
end if.
compute zmin=desctmp(3,1).
compute zmax=desctmp(4,1).
compute minzwarn=minwarn.
compute maxzwarn=maxwarn.
compute znotev=mnotev.
compute zmodval={ 999 }.
compute nzcontr=ncol(zmodval).
do if (zmodval(1,1) <> 999).
compute zmodvals=zmodval(1,1).
compute zmodcust=1.
do if (nzcontr > 1).
compute zmodvals=t(zmodval).
end if.
compute minzwarn=0.
compute maxzwarn=0.
compute znotev=0.
end if.
compute zprobval=zmodvals.
compute nzpval=nrow(zmodvals).
end if.
do if (ncs > 0).
compute ctmp=dat(:,(nys+nxs+nms+nws+nzs+1):(nys+nxs+nms+nws+nzs+ncs)).

.
compute desctmp=make((8-(4* 0 )),ncol( ctmp ),-999).
loop jd=1 to ncol( ctmp ).
compute descdat= ctmp (:,jd).
compute desctmp(1,jd) = csum(descdat)/nrow(descdat).
compute desctmp(2,jd) = (nrow(descdat)*sscp(descdat))-(t(csum(descdat))*(csum(descdat))).
compute desctmp(2,jd) = sqrt(desctmp(2,jd)/(nrow(descdat)*(nrow(descdat)-1))).
compute desctmp(3,jd)=cmin(descdat).
compute desctmp(4,jd)=cmax(descdat).
do if ( 0 =0).
compute minwarn=0.
compute maxwarn=0.
do if ((desctmp(3,jd)=desctmp(4,jd)) and novar=0).
compute errcode(errs,1)=15.
compute errs=errs+1.
compute criterr=1.
compute novar=1.
end if.
compute tmp=((descdat(:,1)=desctmp(3,jd))+(descdat(:,1)=desctmp(4,jd))).
compute desctmp(8,jd)=(csum(tmp)=nrow(tmp)).
compute tmp = descdat.
compute tmp(GRADE(descdat),:) = descdat.
compute descdat = tmp.
release tmp.
compute decval={.16;.5;.84}.
loop kd=1 to 3.
compute low=trunc(decval(kd,1)*(nrow(descdat)+1)).
compute lowdec=decval(kd,1)*(nrow(descdat)+1)-low.
compute value=descdat(low,1)+(descdat((low+1),1)-descdat(low,1))*lowdec.
compute desctmp((4+kd),jd)=value.
end loop.
compute mnotev=1.
compute modvals=desctmp(5:7,:).
do if (quantile <> 1).
compute desctmp(5,jd)=desctmp(1,jd)-desctmp(2,jd).
compute desctmp(6,jd)=desctmp(1,jd).
compute desctmp(7,jd)=desctmp(1,jd)+desctmp(2,jd).
compute modvals=desctmp(5:7,:).
compute mnotev=2.
do if (modvals(1,1) < desctmp(3,1)).
compute modvals(1,1)=desctmp(3,1).
compute minwarn=1.
end if.
do if (modvals(3,1) > desctmp(4,1)).
compute modvals(3,1)=desctmp(4,1).
compute maxwarn=1.
end if.
end if.
do if (desctmp(8,1)=1).
compute modvals={desctmp(3,1);desctmp(4,1)}.
compute mnotev=0.
compute minwarn=0.
compute maxwarn=0.
end if.
end if.
end loop
.
compute covmeans=desctmp(1,:).
compute coval={ -999 }.
do if ((coval(1,1) <> -999) and (ncol(coval) <> ncs) and (model=74)).
compute errcode(errs,1)=69.
compute errs=errs+1.
compute criterr=1.
end if.
do if ((coval(1,1)<> -999) and (criterr=0) and (model=74)).
compute notecode(notes,1) = 35.
compute notes = notes + 1.
compute cuscoval=1.
end if.
end if.
compute n=nrow(ytmp).
compute ones=make(n,1,1).
do if (nws > 0 and mcw > 0).
compute tmp={rownum,wtmp(:,1)}.

.
compute dd= tmp.
compute temp = dd.
compute temp(GRADE(dd(:,2)),:) = dd.
compute dd = temp.
compute dummy = design(dd(:,2)).
compute nvls = ncol(dummy).
compute nnvls = csum(dummy).
compute mnvls = cmin(t(nnvls)).
compute conmat1=1.
do if (mnvls < 2).
compute errcode(errs,1) = 5.
compute errs = errs + 1.
compute criterr = 1.
end if.
do if (nvls > 9).
compute errcode(errs,1) = 4.
compute errs = errs+1.
compute criterr = 1.
end if.
do if (criterr = 0).
compute dumok = 1.
compute nnvls=make(nvls,1,0).
compute nnvls(1,1)=dd(1,2).
compute temp = 2.
loop i = 2 to n.
do if (dd(i,2) <> nnvls((temp-1),1)).
compute nnvls(temp,1)=dd(i,2).
compute temp = temp+1.
end if.
end loop.
do if ( mcw > 0).
compute x = dummy(:,2:ncol(dummy)).
compute nx = ncol(x).
compute minus1 = make(1,ncol(x),-1).
compute xdes=make((nx+1),3,0).
compute xdes(1,1)=dd(1,2).
compute xdes(1,2)=1.
compute temp = 2.
loop k = 2 to n.
do if (dd(k,2) <> dd((k-1),2)).
compute xdes(temp,2) = k.
compute xdes(temp,1) = dd(k,2).
compute xdes((temp-1),3) = k-1.
compute temp=temp+1.
end if.
end loop.
compute xdes((temp-1),3)=n.
compute xdes = {xdes, (xdes(:,3)-xdes(:,2)+1)}.
do if ( mcw = 4).
loop k = 1 to n.
do if (rsum(x(k,:)) = 0).
compute x(k,:) = minus1.
end if.
end loop.
end if.
do if ( mcw = 2 or mcw = 3 or mcw =5).
loop k = 1 to n.
do if (rsum(x(k,:)) > 0).
loop i = 1 to ncol(x).
do if (x(k,i) = 0).
compute x(k,i) = 1.
else.
break.
end if.
end loop.
end if.
end loop.
do if ( mcw = 3).
compute conmat1={-8,1,1,1,1,1,1,1,1; 0,-7,1,1,1,1,1,1,1; 0,0,-6,1,1,1,1,1,1; 0,0,0,-5,1,1,1,1,1; 0,0,0,0,-4,1,1,1,1; 0,0,0,0,0,-3,1,1,1; 0,0,0,0,0,0,-2,1,1; 0,0,0,0,0,0,0,-1,1}.
loop i = 1 to 8.
compute conmat1(i,:)=conmat1(i,:)/(10-i).
end loop.
compute conmat1=t(conmat1((10-nvls):8,(10-nvls):9)).
loop k=1 to n.
compute x(k,:)=conmat1((rsum(x(k,:))+1),:).
end loop.
end if.
end if.
do if ( mcw = 5).
compute custcode={ -999 }.
do if (ncol(custcode) <> (nvls*(nvls-1))).
compute errcode(errs,1) = (37+ 2 ).
compute errs = errs + 1.
compute criterr = 1.
end if.
do if (ncol(custcode) = (nvls*(nvls-1))).
compute conmat1=make(nvls,(nvls-1),0).
compute cnt=1.
loop i = 1 to nvls.
loop k = 1 to (nvls-1).
compute conmat1(i,k)=custcode(1,cnt).
compute cnt=cnt+1.
end loop.
end loop.
loop k=1 to n.
compute x(k,:)=conmat1((rsum(x(k,:))+1),:).
end loop.
end if.
end if.
compute xskip = 1.
compute dummat = make((nx+1),nx,0).
compute dummat((2:nrow(dummat)),:)=ident(nx).
do if ( mcw = 4).
compute dummat(1,:) = minus1.
end if.
do if ( mcw = 2).
loop i = 2 to nrow(dummat).
loop j = 1 to (i-1).
compute dummat(i,j) = 1.
end loop.
end loop.
end if.
do if ( mcw = 3).
compute dummat=conmat1.
end if.
do if ( mcw = 5 and criterr=0).
compute dummat=conmat1.
end if.
compute dummat={nnvls, dummat}.
compute x={dd(:,1),x}.
compute temp = x.
compute temp(GRADE(x(:,1)),:) = x.
compute x = temp.
release conmat1,temp,dd,xskip,xdes,dummy.
end if.
end if
.
compute wmodvals=nnvls.
compute nwpval=nrow(wmodvals).
do if (criterr=0).
compute minwwarn=0.
compute maxwwarn=0.
compute wnotev=0.
compute wtmp=x(:,2:ncol(x)).
compute wcatlab={'W1';'W2';'W3';'W4';'W5';'W6';'W7';'W8';'W9'}.
do if (xmint=1).
compute wcatlab={'X1';'X2';'X3';'X4';'X5';'X6';'X7';'X8';'X9'}.
end if.
compute nwvls=nvls-1.
compute mcwok=1.
compute dummatw=dummat.
compute wprobval=dummatw(:,2:ncol(dummatw)).
do if (modcok=1).
compute wcontval=make(2,ncol(wprobval),-999).
compute temp=0.
loop i = 1 to 2.
loop j = 1 to nrow(dummatw).
do if (contvec(i,1)=dummatw(j,1)).
compute wcontval(i,:)=wprobval(j,:).
compute temp=temp+1.
end if.
end loop.
end loop.
do if (temp < 2).
compute notecode(notes,1) = 20.
compute notes = notes + 1.
compute modcok=0.
end if.
end if.
do if ((wmodval(1,1) <> 999) and (xmint <> 1)).
compute notecode(notes,1) = 9.
compute notes = notes + 1.
end if.
release tmp, dummat.
end if.
end if.
do if (nzs > 0 and mcz > 0).
compute tmp={rownum,ztmp(:,1)}.

.
compute dd= tmp.
compute temp = dd.
compute temp(GRADE(dd(:,2)),:) = dd.
compute dd = temp.
compute dummy = design(dd(:,2)).
compute nvls = ncol(dummy).
compute nnvls = csum(dummy).
compute mnvls = cmin(t(nnvls)).
compute conmat1=1.
do if (mnvls < 2).
compute errcode(errs,1) = 5.
compute errs = errs + 1.
compute criterr = 1.
end if.
do if (nvls > 9).
compute errcode(errs,1) = 4.
compute errs = errs+1.
compute criterr = 1.
end if.
do if (criterr = 0).
compute dumok = 1.
compute nnvls=make(nvls,1,0).
compute nnvls(1,1)=dd(1,2).
compute temp = 2.
loop i = 2 to n.
do if (dd(i,2) <> nnvls((temp-1),1)).
compute nnvls(temp,1)=dd(i,2).
compute temp = temp+1.
end if.
end loop.
do if ( mcz > 0).
compute x = dummy(:,2:ncol(dummy)).
compute nx = ncol(x).
compute minus1 = make(1,ncol(x),-1).
compute xdes=make((nx+1),3,0).
compute xdes(1,1)=dd(1,2).
compute xdes(1,2)=1.
compute temp = 2.
loop k = 2 to n.
do if (dd(k,2) <> dd((k-1),2)).
compute xdes(temp,2) = k.
compute xdes(temp,1) = dd(k,2).
compute xdes((temp-1),3) = k-1.
compute temp=temp+1.
end if.
end loop.
compute xdes((temp-1),3)=n.
compute xdes = {xdes, (xdes(:,3)-xdes(:,2)+1)}.
do if ( mcz = 4).
loop k = 1 to n.
do if (rsum(x(k,:)) = 0).
compute x(k,:) = minus1.
end if.
end loop.
end if.
do if ( mcz = 2 or mcz = 3 or mcz =5).
loop k = 1 to n.
do if (rsum(x(k,:)) > 0).
loop i = 1 to ncol(x).
do if (x(k,i) = 0).
compute x(k,i) = 1.
else.
break.
end if.
end loop.
end if.
end loop.
do if ( mcz = 3).
compute conmat1={-8,1,1,1,1,1,1,1,1; 0,-7,1,1,1,1,1,1,1; 0,0,-6,1,1,1,1,1,1; 0,0,0,-5,1,1,1,1,1; 0,0,0,0,-4,1,1,1,1; 0,0,0,0,0,-3,1,1,1; 0,0,0,0,0,0,-2,1,1; 0,0,0,0,0,0,0,-1,1}.
loop i = 1 to 8.
compute conmat1(i,:)=conmat1(i,:)/(10-i).
end loop.
compute conmat1=t(conmat1((10-nvls):8,(10-nvls):9)).
loop k=1 to n.
compute x(k,:)=conmat1((rsum(x(k,:))+1),:).
end loop.
end if.
end if.
do if ( mcz = 5).
compute custcode={ -999 }.
do if (ncol(custcode) <> (nvls*(nvls-1))).
compute errcode(errs,1) = (37+ 3 ).
compute errs = errs + 1.
compute criterr = 1.
end if.
do if (ncol(custcode) = (nvls*(nvls-1))).
compute conmat1=make(nvls,(nvls-1),0).
compute cnt=1.
loop i = 1 to nvls.
loop k = 1 to (nvls-1).
compute conmat1(i,k)=custcode(1,cnt).
compute cnt=cnt+1.
end loop.
end loop.
loop k=1 to n.
compute x(k,:)=conmat1((rsum(x(k,:))+1),:).
end loop.
end if.
end if.
compute xskip = 1.
compute dummat = make((nx+1),nx,0).
compute dummat((2:nrow(dummat)),:)=ident(nx).
do if ( mcz = 4).
compute dummat(1,:) = minus1.
end if.
do if ( mcz = 2).
loop i = 2 to nrow(dummat).
loop j = 1 to (i-1).
compute dummat(i,j) = 1.
end loop.
end loop.
end if.
do if ( mcz = 3).
compute dummat=conmat1.
end if.
do if ( mcz = 5 and criterr=0).
compute dummat=conmat1.
end if.
compute dummat={nnvls, dummat}.
compute x={dd(:,1),x}.
compute temp = x.
compute temp(GRADE(x(:,1)),:) = x.
compute x = temp.
release conmat1,temp,dd,xskip,xdes,dummy.
end if.
end if
.
compute zmodvals=nnvls.
compute nzpval=nrow(zmodvals).
do if (criterr=0).
compute minzwarn=0.
compute maxzwarn=0.
compute znotev=0.
compute ztmp=x(:,2:ncol(x)).
compute zcatlab={'Z1';'Z2';'Z3';'Z4';'Z5';'Z6';'Z7';'Z8';'Z9'}.
compute nzvls=nvls-1.
compute mczok=1.
compute dummatz=dummat.
compute zprobval=dummatz(:,2:ncol(dummatz)).
do if (modcok=1).
compute zcontval=make(2,ncol(zprobval),-999).
compute temp=0.
loop i = 1 to 2.
loop j = 1 to nrow(dummatz).
do if (contvec(i,2)=dummatz(j,1)).
compute zcontval(i,:)=zprobval(j,:).
compute temp=temp+1.
end if.
end loop.
end loop.
do if (temp < 2).
compute notecode(notes,1) = 20.
compute notes = notes + 1.
compute modcok=0.
end if.
end if.
do if (zmodval(1,1) <> 999).
compute notecode(notes,1) = 10.
compute notes = notes + 1.
end if.
release tmp, dummat.
end if.
end if.
do if (nxs > 0 and mcx > 0).
compute tmp={rownum,xtmp(:,1)}.

.
compute dd= tmp.
compute temp = dd.
compute temp(GRADE(dd(:,2)),:) = dd.
compute dd = temp.
compute dummy = design(dd(:,2)).
compute nvls = ncol(dummy).
compute nnvls = csum(dummy).
compute mnvls = cmin(t(nnvls)).
compute conmat1=1.
do if (mnvls < 2).
compute errcode(errs,1) = 5.
compute errs = errs + 1.
compute criterr = 1.
end if.
do if (nvls > 9).
compute errcode(errs,1) = 4.
compute errs = errs+1.
compute criterr = 1.
end if.
do if (criterr = 0).
compute dumok = 1.
compute nnvls=make(nvls,1,0).
compute nnvls(1,1)=dd(1,2).
compute temp = 2.
loop i = 2 to n.
do if (dd(i,2) <> nnvls((temp-1),1)).
compute nnvls(temp,1)=dd(i,2).
compute temp = temp+1.
end if.
end loop.
do if ( mcx > 0).
compute x = dummy(:,2:ncol(dummy)).
compute nx = ncol(x).
compute minus1 = make(1,ncol(x),-1).
compute xdes=make((nx+1),3,0).
compute xdes(1,1)=dd(1,2).
compute xdes(1,2)=1.
compute temp = 2.
loop k = 2 to n.
do if (dd(k,2) <> dd((k-1),2)).
compute xdes(temp,2) = k.
compute xdes(temp,1) = dd(k,2).
compute xdes((temp-1),3) = k-1.
compute temp=temp+1.
end if.
end loop.
compute xdes((temp-1),3)=n.
compute xdes = {xdes, (xdes(:,3)-xdes(:,2)+1)}.
do if ( mcx = 4).
loop k = 1 to n.
do if (rsum(x(k,:)) = 0).
compute x(k,:) = minus1.
end if.
end loop.
end if.
do if ( mcx = 2 or mcx = 3 or mcx =5).
loop k = 1 to n.
do if (rsum(x(k,:)) > 0).
loop i = 1 to ncol(x).
do if (x(k,i) = 0).
compute x(k,i) = 1.
else.
break.
end if.
end loop.
end if.
end loop.
do if ( mcx = 3).
compute conmat1={-8,1,1,1,1,1,1,1,1; 0,-7,1,1,1,1,1,1,1; 0,0,-6,1,1,1,1,1,1; 0,0,0,-5,1,1,1,1,1; 0,0,0,0,-4,1,1,1,1; 0,0,0,0,0,-3,1,1,1; 0,0,0,0,0,0,-2,1,1; 0,0,0,0,0,0,0,-1,1}.
loop i = 1 to 8.
compute conmat1(i,:)=conmat1(i,:)/(10-i).
end loop.
compute conmat1=t(conmat1((10-nvls):8,(10-nvls):9)).
loop k=1 to n.
compute x(k,:)=conmat1((rsum(x(k,:))+1),:).
end loop.
end if.
end if.
do if ( mcx = 5).
compute custcode={ -999 }.
do if (ncol(custcode) <> (nvls*(nvls-1))).
compute errcode(errs,1) = (37+ 1 ).
compute errs = errs + 1.
compute criterr = 1.
end if.
do if (ncol(custcode) = (nvls*(nvls-1))).
compute conmat1=make(nvls,(nvls-1),0).
compute cnt=1.
loop i = 1 to nvls.
loop k = 1 to (nvls-1).
compute conmat1(i,k)=custcode(1,cnt).
compute cnt=cnt+1.
end loop.
end loop.
loop k=1 to n.
compute x(k,:)=conmat1((rsum(x(k,:))+1),:).
end loop.
end if.
end if.
compute xskip = 1.
compute dummat = make((nx+1),nx,0).
compute dummat((2:nrow(dummat)),:)=ident(nx).
do if ( mcx = 4).
compute dummat(1,:) = minus1.
end if.
do if ( mcx = 2).
loop i = 2 to nrow(dummat).
loop j = 1 to (i-1).
compute dummat(i,j) = 1.
end loop.
end loop.
end if.
do if ( mcx = 3).
compute dummat=conmat1.
end if.
do if ( mcx = 5 and criterr=0).
compute dummat=conmat1.
end if.
compute dummat={nnvls, dummat}.
compute x={dd(:,1),x}.
compute temp = x.
compute temp(GRADE(x(:,1)),:) = x.
compute x = temp.
release conmat1,temp,dd,xskip,xdes,dummy.
end if.
end if
.
do if (criterr=0).
compute xtmp=x(:,2:ncol(x)).
compute xcatlab={'X1';'X2';'X3';'X4';'X5';'X6';'X7';'X8';'X9'}.
compute nxvls=nvls-1.
compute xdich=(nvls=2).
compute mcxok=1.
compute dummatx=dummat.
compute xmodvals=dummatx(:,1).
compute nxpval=nrow(xmodvals).
release tmp, dummat.
end if.
end if.
compute intlab=make(100,1,' ').


compute intlab( 1 ,1)= 'Int_1'.


compute intlab( 2 ,1)= 'Int_2'.


compute intlab( 3 ,1)= 'Int_3'.


compute intlab( 4 ,1)= 'Int_4'.


compute intlab( 5 ,1)= 'Int_5'.


compute intlab( 6 ,1)= 'Int_6'.


compute intlab( 7 ,1)= 'Int_7'.


compute intlab( 8 ,1)= 'Int_8'.


compute intlab( 9 ,1)= 'Int_9'.


compute intlab( 10 ,1)= 'Int_10'.


compute intlab( 11 ,1)= 'Int_11'.


compute intlab( 12 ,1)= 'Int_12'.


compute intlab( 13 ,1)= 'Int_13'.


compute intlab( 14 ,1)= 'Int_14'.


compute intlab( 15 ,1)= 'Int_15'.


compute intlab( 16 ,1)= 'Int_16'.


compute intlab( 17 ,1)= 'Int_17'.


compute intlab( 18 ,1)= 'Int_18'.


compute intlab( 19 ,1)= 'Int_19'.


compute intlab( 20 ,1)= 'Int_20'.


compute intlab( 21 ,1)= 'Int_21'.


compute intlab( 22 ,1)= 'Int_22'.


compute intlab( 23 ,1)= 'Int_23'.


compute intlab( 24 ,1)= 'Int_24'.


compute intlab( 25 ,1)= 'Int_25'.


compute intlab( 26 ,1)= 'Int_26'.


compute intlab( 27 ,1)= 'Int_27'.


compute intlab( 28 ,1)= 'Int_28'.


compute intlab( 29 ,1)= 'Int_29'.


compute intlab( 30 ,1)= 'Int_30'.


compute intlab( 31 ,1)= 'Int_31'.


compute intlab( 32 ,1)= 'Int_32'.


compute intlab( 33 ,1)= 'Int_33'.


compute intlab( 34 ,1)= 'Int_34'.


compute intlab( 35 ,1)= 'Int_35'.


compute intlab( 36 ,1)= 'Int_36'.


compute intlab( 37 ,1)= 'Int_37'.


compute intlab( 38 ,1)= 'Int_38'.


compute intlab( 39 ,1)= 'Int_39'.


compute intlab( 40 ,1)= 'Int_40'.


compute intlab( 41 ,1)= 'Int_41'.


compute intlab( 42 ,1)= 'Int_42'.


compute intlab( 43 ,1)= 'Int_43'.


compute intlab( 44 ,1)= 'Int_44'.


compute intlab( 45 ,1)= 'Int_45'.


compute intlab( 46 ,1)= 'Int_46'.


compute intlab( 47 ,1)= 'Int_47'.


compute intlab( 48 ,1)= 'Int_48'.


compute intlab( 49 ,1)= 'Int_49'.


compute intlab( 50 ,1)= 'Int_50'.


compute intlab( 51 ,1)= 'Int_51'.


compute intlab( 52 ,1)= 'Int_52'.


compute intlab( 53 ,1)= 'Int_53'.


compute intlab( 54 ,1)= 'Int_54'.


compute intlab( 55 ,1)= 'Int_55'.


compute intlab( 56 ,1)= 'Int_56'.


compute intlab( 57 ,1)= 'Int_57'.


compute intlab( 58 ,1)= 'Int_58'.


compute intlab( 59 ,1)= 'Int_59'.


compute intlab( 60 ,1)= 'Int_60'.


compute intlab( 61 ,1)= 'Int_61'.


compute intlab( 62 ,1)= 'Int_62'.


compute intlab( 63 ,1)= 'Int_63'.


compute intlab( 64 ,1)= 'Int_64'.


compute intlab( 65 ,1)= 'Int_65'.


compute intlab( 66 ,1)= 'Int_66'.


compute intlab( 67 ,1)= 'Int_67'.


compute intlab( 68 ,1)= 'Int_68'.


compute intlab( 69 ,1)= 'Int_69'.


compute intlab( 70 ,1)= 'Int_70'.


compute intlab( 71 ,1)= 'Int_71'.


compute intlab( 72 ,1)= 'Int_72'.


compute intlab( 73 ,1)= 'Int_73'.


compute intlab( 74 ,1)= 'Int_74'.


compute intlab( 75 ,1)= 'Int_75'.


compute intlab( 76 ,1)= 'Int_76'.


compute intlab( 77 ,1)= 'Int_77'.


compute intlab( 78 ,1)= 'Int_78'.


compute intlab( 79 ,1)= 'Int_79'.


compute intlab( 80 ,1)= 'Int_80'.


compute intlab( 81 ,1)= 'Int_81'.


compute intlab( 82 ,1)= 'Int_82'.


compute intlab( 83 ,1)= 'Int_83'.


compute intlab( 84 ,1)= 'Int_84'.


compute intlab( 85 ,1)= 'Int_85'.


compute intlab( 86 ,1)= 'Int_86'.


compute intlab( 87 ,1)= 'Int_87'.


compute intlab( 88 ,1)= 'Int_88'.


compute intlab( 89 ,1)= 'Int_89'.


compute intlab( 90 ,1)= 'Int_90'.


compute intlab( 91 ,1)= 'Int_91'.


compute intlab( 92 ,1)= 'Int_92'.


compute intlab( 93 ,1)= 'Int_93'.


compute intlab( 94 ,1)= 'Int_94'.


compute intlab( 95 ,1)= 'Int_95'.


compute intlab( 96 ,1)= 'Int_96'.


compute intlab( 97 ,1)= 'Int_97'.


compute intlab( 98 ,1)= 'Int_98'.


compute intlab( 99 ,1)= 'Int_99'.


compute intlab( 100 ,1)= 'Int_100'.

compute bcmat=make(needed,needed,0).
compute wcmat=make(needed,needed,0).
compute zcmat=make(needed,needed,0).
compute wzcmat=make(needed,needed,0).
compute wsum=0.
compute zsum=0.
compute wzsum=0.
end if.
end if.
do if (criterr = 0 and model <> 999).
compute modelmat= {1,0,0,0,0,0,0,1,0,0;2,0,0,0,0,0,0,1,1,0;3,0,0,0,0,0,0,1,1,1;4,0,0,0,0,0,0,0,0,0; 5,0,0,0,0,0,0,1,0,0;6,0,0,0,0,0,0,0,0,0;7,1,0,0,0,0,0,0,0,0;8,1,0,0,0,0,0,1,0,0; 9,1,1,0,0,0,0,0,0,0;10,1,1,0,0,0,0,1,1,0;11,1,1,1,0,0,0,0,0,0;12,1,1,1,0,0,0,1,1,1; 13,1,1,1,0,0,0,1,0,0;14,0,0,0,1,0,0,0,0,0;15,0,0,0,1,0,0,1,0,0;16,0,0,0,1,1,0,0,0,0; 17,0,0,0,1,1,0,1,1,0;18,0,0,0,1,1,1,0,0,0;19,0,0,0,1,1,1,1,1,1;20,0,0,0,1,1,1,1,0,0; 21,1,0,0,0,1,0,0,0,0;22,1,0,0,0,1,0,1,0,0;23,0,0,0,0,0,0,0,0,0;24,0,0,0,0,0,0,0,0,0; 25,0,0,0,0,0,0,0,0,0;26,0,0,0,0,0,0,0,0,0;27,0,0,0,0,0,0,0,0,0;28,1,0,0,0,1,0,0,1,0; 29,1,0,0,0,1,0,1,1,0;30,0,0,0,0,0,0,0,0,0;31,0,0,0,0,0,0,0,0,0;32,0,0,0,0,0,0,0,0,0; 33,0,0,0,0,0,0,0,0,0;34,0,0,0,0,0,0,0,0,0;35,0,0,0,0,0,0,0,0,0;36,0,0,0,0,0,0,0,0,0; 37,0,0,0,0,0,0,0,0,0;38,0,0,0,0,0,0,0,0,0;39,0,0,0,0,0,0,0,0,0;40,0,0,0,0,0,0,0,0,0; 41,0,0,0,0,0,0,0,0,0;42,0,0,0,0,0,0,0,0,0;43,0,0,0,0,0,0,0,0,0;44,0,0,0,0,0,0,0,0,0; 45,0,0,0,0,0,0,0,0,0;46,0,0,0,0,0,0,0,0,0;47,0,0,0,0,0,0,0,0,0;48,0,0,0,0,0,0,0

 ,0,0; 49,0,0,0,0,0,0,0,0,0;50,0,0,0,0,0,0,0,0,0;51,0,0,0,0,0,0,0,0,0;52,0,0,0,0,0,0,0,0,0; 53,0,0,0,0,0,0,0,0,0;54,0,0,0,0,0,0,0,0,0;55,0,0,0,0,0,0,0,0,0;56,0,0,0,0,0,0,0,0,0; 57,0,0,0,0,0,0,0,0,0;58,1,0,0,1,0,0,0,0,0;59,1,0,0,1,0,0,1,0,0;60,1,1,0,1,0,0,0,0,0; 61,1,1,0,1,0,0,1,0,0;62,1,1,0,1,0,0,0,1,0;63,1,1,0,1,0,0,1,1,0;64,1,0,0,1,1,0,0,0,0; 65,1,0,0,1,1,0,1,0,0;66,1,0,0,1,1,0,0,1,0;67,1,0,0,1,1,0,1,1,0;68,1,1,1,1,0,0,0,0,0; 69,1,1,1,1,0,0,1,1,1;70,1,0,0,1,1,1,0,0,0;71,1,0,0,1,1,1,1,1,1;72,1,1,1,1,1,1,0,0,0; 73,1,1,1,1,1,1,1,1,1;74,0,0,0,1,0,0,0,0,0;75,1,1,0,1,1,0,0,0,0;76,1,1,0,1,1,0,1,1,0; 77,0,0,0,0,0,0,0,0,0;78,0,0,0,0,0,0,0,0,0;79,0,0,0,0,0,0,0,0,0;80,0,0,0,0,0,0,0,0,0; 81,0,0,0,0,0,0,0,0,0;82,0,0,0,0,0,0,0,0,0;83,1,0,0,0,0,0,0,0,0;84,1,0,0,0,0,0,0,0,0; 85,1,0,0,0,0,0,1,0,0;86,1,0,0,0,0,0,1,0,0;87,0,0,0,1,0,0,0,0,0;88,0,0,0,1,0,0,0,0,0; 89,0,0,0,1,0,0,1,0,0;90,0,0,0,1,0,0,1,0,0;91,0,0,0,0,0,0,0,0,0;92,1,0,0,1,0,0,1,0,0}.
do if (model > 0).
compute tmp=modelmat(model,2:ncol(modelmat)).
end if.
do if (model=0).
compute tmp={0,0,0,0,0,0,0,0,0}.
end if.
do if (model < 4).
compute bcmat((nxs+1),1)=1.
end if.
do if ((model > 3) and (model <> 6)).
compute bcmat((nxs+1):(nxs+nms),1)=onem.
compute bcmat(nrow(bcmat),(nxs+1):(nxs+nms))=t(onem).
compute bcmat(nrow(bcmat),1)=1.
end if.
do if ((model = 6) or (model > 82 and model < 93)).
loop j = 2 to nrow(bcmat).
loop i = 1 to (j-1).
compute bcmat(j,i)=1.
end loop.
end loop.
end if.
do if (model = 80).
loop i = 1 to nms.
compute bcmat((nrow(bcmat)-1),i)=1.
end loop.
end if.
do if (model = 81).
loop j = 3 to nrow(bcmat).
compute bcmat(j,2)=1.
end loop.
end if.
do if (model = 82).
compute bcmat(3,2)=1.
compute bcmat(5,4)=1.
end if.
do if (tmp(1,1)=1).
compute wcmat((nxs+1):(nxs+nms),1)=onem.
compute wprod=1.
compute xprod=1.
do if (model = 83 or model = 86).
compute onemsx=onem.
loop i = 1 to (nms-1).
compute onemsx(i+1,1)=0.
end loop.
compute wcmat((nxs+1):(nxs+nms),1)=onemsx.
end if.
end if.
do if (tmp(1,4)=1).
compute wcmat(nrow(wcmat),(nxs+1):(nxs+nms))=t(onem).
compute wprod=1.
do if (model = 87 or model = 90).
compute onemsx=onem.
loop i = 1 to (nms-1).
compute onemsx(i,1)=0.
end loop.
compute wcmat(nrow(wcmat),(nxs+1):(nxs+nms))=t(onemsx).
end if.
end if.
do if (tmp(1,7)=1).
compute wcmat(nrow(wcmat),1)=1.
compute wprod=1.
compute xprod=1.
end if.
do if (tmp(1,2)=1).
compute zcmat((nxs+1):(nxs+nms),1)=onem.
compute zprod=1.
compute xprod=1.
end if.
do if (tmp(1,5)=1).
compute zcmat(nrow(zcmat),(nxs+1):(nxs+nms))=t(onem).
compute zprod=1.
end if.
do if (tmp(1,8)=1).
compute zcmat(nrow(zcmat),1)=1.
compute zprod=1.
compute xprod=1.
end if.
do if (tmp(1,3)=1).
compute wzcmat((nxs+1):(nxs+nms),1)=onem.
compute xprod=1.
compute wprod=1.
compute zprod=1.
end if.
do if (tmp(1,6)=1).
compute wzcmat(nrow(wzcmat),(nxs+1):(nxs+nms))=t(onem).
compute zprod=1.
compute wprod=1.
end if.
do if (tmp(1,9)=1).
compute wzcmat(nrow(wzcmat),1)=1.
compute xprod=1.
compute wprod=1.
compute zprod=1.
end if.
do if (model = 91 or model = 92).
loop j = 1 to (nms-1).
loop i = 1 to j.
compute wcmat((nxs+1+j),(nxs+i))=1.
end loop.
end loop.
end if.
do if (nms < 0).
loop i = 1 to nms.
compute tmp=csum(wcmat(:,(1+i)))+csum(zcmat(:,(1+i)))+csum(wzcmat(:,(1+i))).
compute mprod(1,i)=(tmp>0).
end loop.
end if.
end if.
do if (ncs > 0).
compute ccmat=make((nms+nys),ncs,1).
compute ccmatoff=ccmat.
do if (covmy=1).
compute ccmat(nrow(ccmat),:)=make(1,ncs,0).
end if.
do if (covmy=2).
compute ccmat(1:nms,:)=make(nms,ncs,0).
end if.
do if (cmatrix(1,1) <> -999).
do if (ncol(cmatrix) <> ((nms+nys)*ncs)).
compute errcode(errs,1)=29.
compute errs=errs+1.
compute criterr=1.
end if.
do if (criterr = 0).
compute tmp=1.
loop i = 1 to (nms+nys).
loop j = 1 to ncs.
compute ccmat(i,j)=1-(cmatrix(1,tmp) = 0).
compute tmp=tmp+1.
end loop.
end loop.
do if (rsum((csum(ccmat)=0)) <> 0).
compute errcode(errs,1)=30.
compute errs=errs+1.
compute criterr=1.
end if.
end if.
do if (covmy <> 0).
compute notecode(notes,1)=1.
compute notes=notes+1.
end if.
end if.
do if (xmint=1 and (covmy > 0 or (csum(rsum(ccmat)) <> ((nms+nys)*ncs)))).
compute errcode(errs,1)=68.
compute errs=errs+1.
compute criterr=1.
end if.
end if.
do if (criterr=0).
compute needed=needed*(needed-1)/2.
compute nopath=0.
do if (bmatrix(1,1) <> -999).
compute tmp=1.
do if ((ncol(bmatrix) <> needed) or (csum(rsum(bmatrix))=0)).
compute errcode(errs,1)=16.
compute errs=errs+1.
compute criterr=1.
else.
loop i = 2 to nrow(bcmat).
loop j = 1 to (i-1).
compute bcmat(i,j)=1-(bmatrix(1,tmp) = 0).
compute tmp=tmp+1.
end loop.
end loop.
end if.
do if ((csum(bcmat(:,1))=0) and criterr=0).
compute errcode(errs,1)=22.
compute errs=errs+1.
compute criterr=1.
end if.
do if ((rsum(bcmat(nrow(bcmat),:))=0) and criterr=0).
compute errcode(errs,1)=23.
compute errs=errs+1.
compute criterr=1.
end if.
compute dm=0.
do if (nms > 0).
loop i = 1 to nms.
do if (((rsum(bcmat((nxs+i),:)) = 0) or (csum(bcmat(:,(nxs+i))) = 0)) and (dm=0) and (criterr=0)).
compute errcode(errs,1)=26.
compute errs=errs+1.
compute criterr=1.
compute dm=1.
end if.
end loop.
end if.
release dm.
end if.
end if.
do if (criterr=0).
do if (wmatrix(1,1) <> -999).
compute tmp=1.
do if (ncol(wmatrix) <> needed).
compute errcode(errs,1)=17.
compute errs=errs+1.
compute criterr=1.
else.
compute modelvar(1,1)='CUSTOM'.
loop i = 2 to nrow(wcmat).
loop j = 1 to (i-1).
compute wcmat(i,j)=1-(wmatrix(1,tmp) = 0).
do if ((wcmat(i,j)=1) and (bcmat(i,j)=0) and (nopath=0)).
compute errcode(errs,1)=20.
compute errs=errs+1.
compute criterr=1.
compute nopath=1.
end if.
compute tmp=tmp+1.
end loop.
end loop.
end if.
end if.
do if (zmatrix(1,1) <> -999).
compute tmp=1.
do if (ncol(zmatrix) <> needed).
compute errcode(errs,1)=18.
compute errs=errs+1.
compute criterr=1.
else.
compute modelvar(1,1)='CUSTOM'.
do if (csum(rsum(wcmat))=0 and model=999).
compute errcode(errs,1)=21.
compute errs=errs+1.
compute criterr=1.
end if.
loop i = 2 to nrow(zcmat).
loop j = 1 to (i-1).
compute zcmat(i,j)=1-(zmatrix(1,tmp) = 0).
do if ((zcmat(i,j)=1) and (bcmat(i,j)=0) and (nopath=0)).
compute errcode(errs,1)=20.
compute errs=errs+1.
compute criterr=1.
compute nopath=1.
end if.
compute tmp=tmp+1.
end loop.
end loop.
end if.
end if.
compute tmp=1.
do if (wzmatrix(1,1) <> -999).
do if (ncol(wzmatrix) <> needed).
compute errcode(errs,1)=19.
compute errs=errs+1.
compute criterr=1.
end if.
compute modelvar(1,1)='CUSTOM'.
end if.
do if (criterr=0).
loop i = 2 to nrow(wzcmat).
loop j = 1 to (i-1).
do if (wzmatrix(1,1) <> -999).
compute wzcmat(i,j)=1-(wzmatrix(1,tmp) = 0).
end if.
do if (wzcmat(i,j)=1).
compute wcmat(i,j)=1.
compute zcmat(i,j)=1.
end if.
do if ((wzcmat(i,j)=1) and (bcmat(i,j)=0) and (nopath=0)).
compute errcode(errs,1)=20.
compute errs=errs+1.
compute criterr=1.
compute nopath=1.
end if.
compute tmp=tmp+1.
end loop.
end loop.
end if.
end if.
do if (criterr=0).
compute xprod=csum(wcmat(:,1))+csum(zcmat(:,1))+csum(wzcmat(:,1)).
compute xprod=(xprod > 0).
compute wsum=csum(rsum(wcmat)).
compute wprod=(wsum > 0).
do if (nms > 0).
loop i = 1 to nms.
compute tmp=csum(wcmat(:,(1+i)))+csum(zcmat(:,(1+i)))+csum(wzcmat(:,(1+i))).
compute mprod(1,i)=(tmp>0).
end loop.
end if.
do if ((wsum > 0) and (w = 'xxxxx')).
compute errcode(errs,1)=11.
compute errs=errs+1.
compute criterr=1.
end if.
do if ((wsum = 0) and (w <> 'xxxxx')).
compute errcode(errs,1)=10.
compute errs=errs+1.
compute criterr=1.
end if.
compute zsum=csum(rsum(zcmat)).
compute zprod=(zsum > 0).
do if ((zsum > 0) and (z = 'xxxxx')).
compute errcode(errs,1)=13.
compute errs=errs+1.
compute criterr=1.
end if.
do if ((zsum = 0) and (z <> 'xxxxx')).
compute errcode(errs,1)=12.
compute errs=errs+1.
compute criterr=1.
end if.
do if ((zsum > 0) and (wsum = 0)).
compute errcode(errs,1)=35.
compute errs=errs+1.
compute criterr=1.
end if.
end if.
do if (criterr=0 and nms > 1).
compute serchk=bcmat(2:(nrow(bcmat)-1),2:ncol(bcmat)).
do if (csum(rsum(serchk))) > 0.
compute serial=1.
do if (nms > 6).
compute errcode(errs,1)=36.
compute errs=errs+1.
compute criterr=1.
end if.
end if.
end if.
do if (center > 0 and criterr=0).
compute centvar={' '}.
do if (criterr=0).
do if ((center = 1) or (center = 2 and wdich = 0)).
do if (wprod=1 and mcwok=0 and nwpval > 0).
loop i = 1 to nws.
compute wtmp(:,i)=wtmp(:,i)-(csum(wtmp(:,i))/n).
compute centvar={centvar,wnames(1,i)}.
end loop.

.
compute desctmp=make((8-(4* wmodcust )),ncol( wtmp ),-999).
loop jd=1 to ncol( wtmp ).
compute descdat= wtmp (:,jd).
compute desctmp(1,jd) = csum(descdat)/nrow(descdat).
compute desctmp(2,jd) = (nrow(descdat)*sscp(descdat))-(t(csum(descdat))*(csum(descdat))).
compute desctmp(2,jd) = sqrt(desctmp(2,jd)/(nrow(descdat)*(nrow(descdat)-1))).
compute desctmp(3,jd)=cmin(descdat).
compute desctmp(4,jd)=cmax(descdat).
do if ( wmodcust =0).
compute minwarn=0.
compute maxwarn=0.
do if ((desctmp(3,jd)=desctmp(4,jd)) and novar=0).
compute errcode(errs,1)=15.
compute errs=errs+1.
compute criterr=1.
compute novar=1.
end if.
compute tmp=((descdat(:,1)=desctmp(3,jd))+(descdat(:,1)=desctmp(4,jd))).
compute desctmp(8,jd)=(csum(tmp)=nrow(tmp)).
compute tmp = descdat.
compute tmp(GRADE(descdat),:) = descdat.
compute descdat = tmp.
release tmp.
compute decval={.16;.5;.84}.
loop kd=1 to 3.
compute low=trunc(decval(kd,1)*(nrow(descdat)+1)).
compute lowdec=decval(kd,1)*(nrow(descdat)+1)-low.
compute value=descdat(low,1)+(descdat((low+1),1)-descdat(low,1))*lowdec.
compute desctmp((4+kd),jd)=value.
end loop.
compute mnotev=1.
compute modvals=desctmp(5:7,:).
do if (quantile <> 1).
compute desctmp(5,jd)=desctmp(1,jd)-desctmp(2,jd).
compute desctmp(6,jd)=desctmp(1,jd).
compute desctmp(7,jd)=desctmp(1,jd)+desctmp(2,jd).
compute modvals=desctmp(5:7,:).
compute mnotev=2.
do if (modvals(1,1) < desctmp(3,1)).
compute modvals(1,1)=desctmp(3,1).
compute minwarn=1.
end if.
do if (modvals(3,1) > desctmp(4,1)).
compute modvals(3,1)=desctmp(4,1).
compute maxwarn=1.
end if.
end if.
do if (desctmp(8,1)=1).
compute modvals={desctmp(3,1);desctmp(4,1)}.
compute mnotev=0.
compute minwarn=0.
compute maxwarn=0.
end if.
end if.
end loop
.
compute wmin=desctmp(3,1).
compute wmax=desctmp(4,1).
do if (wmodcust=0).
compute wmodvals=modvals.
compute wprobval=wmodvals.
end if.
end if.
end if.
do if ((center = 1) or (center = 2 and zdich = 0)).
do if (zprod=1 and mczok=0 and nzpval > 0).
loop i = 1 to nzs.
compute ztmp(:,i)=ztmp(:,i)-(csum(ztmp(:,i))/n).
compute centvar={centvar,znames(1,i)}.
end loop.

.
compute desctmp=make((8-(4* zmodcust )),ncol( ztmp ),-999).
loop jd=1 to ncol( ztmp ).
compute descdat= ztmp (:,jd).
compute desctmp(1,jd) = csum(descdat)/nrow(descdat).
compute desctmp(2,jd) = (nrow(descdat)*sscp(descdat))-(t(csum(descdat))*(csum(descdat))).
compute desctmp(2,jd) = sqrt(desctmp(2,jd)/(nrow(descdat)*(nrow(descdat)-1))).
compute desctmp(3,jd)=cmin(descdat).
compute desctmp(4,jd)=cmax(descdat).
do if ( zmodcust =0).
compute minwarn=0.
compute maxwarn=0.
do if ((desctmp(3,jd)=desctmp(4,jd)) and novar=0).
compute errcode(errs,1)=15.
compute errs=errs+1.
compute criterr=1.
compute novar=1.
end if.
compute tmp=((descdat(:,1)=desctmp(3,jd))+(descdat(:,1)=desctmp(4,jd))).
compute desctmp(8,jd)=(csum(tmp)=nrow(tmp)).
compute tmp = descdat.
compute tmp(GRADE(descdat),:) = descdat.
compute descdat = tmp.
release tmp.
compute decval={.16;.5;.84}.
loop kd=1 to 3.
compute low=trunc(decval(kd,1)*(nrow(descdat)+1)).
compute lowdec=decval(kd,1)*(nrow(descdat)+1)-low.
compute value=descdat(low,1)+(descdat((low+1),1)-descdat(low,1))*lowdec.
compute desctmp((4+kd),jd)=value.
end loop.
compute mnotev=1.
compute modvals=desctmp(5:7,:).
do if (quantile <> 1).
compute desctmp(5,jd)=desctmp(1,jd)-desctmp(2,jd).
compute desctmp(6,jd)=desctmp(1,jd).
compute desctmp(7,jd)=desctmp(1,jd)+desctmp(2,jd).
compute modvals=desctmp(5:7,:).
compute mnotev=2.
do if (modvals(1,1) < desctmp(3,1)).
compute modvals(1,1)=desctmp(3,1).
compute minwarn=1.
end if.
do if (modvals(3,1) > desctmp(4,1)).
compute modvals(3,1)=desctmp(4,1).
compute maxwarn=1.
end if.
end if.
do if (desctmp(8,1)=1).
compute modvals={desctmp(3,1);desctmp(4,1)}.
compute mnotev=0.
compute minwarn=0.
compute maxwarn=0.
end if.
end if.
end loop
.
compute zmin=desctmp(3,1).
compute zmax=desctmp(4,1).
do if (zmodcust=0).
compute zmodvals=modvals.
compute zprobval=zmodvals.
end if.
end if.
end if.
do if ((center = 1) or (center = 2 and xdich = 0)).
do if (xprod=1 and mcxok=0).
loop i = 1 to nxs.
compute xtmp(:,i)=xtmp(:,i)-(csum(xtmp(:,i))/n).
compute centvar={centvar,xnames(1,i)}.
end loop.

.
compute desctmp=make((8-(4* 0 )),ncol( xtmp ),-999).
loop jd=1 to ncol( xtmp ).
compute descdat= xtmp (:,jd).
compute desctmp(1,jd) = csum(descdat)/nrow(descdat).
compute desctmp(2,jd) = (nrow(descdat)*sscp(descdat))-(t(csum(descdat))*(csum(descdat))).
compute desctmp(2,jd) = sqrt(desctmp(2,jd)/(nrow(descdat)*(nrow(descdat)-1))).
compute desctmp(3,jd)=cmin(descdat).
compute desctmp(4,jd)=cmax(descdat).
do if ( 0 =0).
compute minwarn=0.
compute maxwarn=0.
do if ((desctmp(3,jd)=desctmp(4,jd)) and novar=0).
compute errcode(errs,1)=15.
compute errs=errs+1.
compute criterr=1.
compute novar=1.
end if.
compute tmp=((descdat(:,1)=desctmp(3,jd))+(descdat(:,1)=desctmp(4,jd))).
compute desctmp(8,jd)=(csum(tmp)=nrow(tmp)).
compute tmp = descdat.
compute tmp(GRADE(descdat),:) = descdat.
compute descdat = tmp.
release tmp.
compute decval={.16;.5;.84}.
loop kd=1 to 3.
compute low=trunc(decval(kd,1)*(nrow(descdat)+1)).
compute lowdec=decval(kd,1)*(nrow(descdat)+1)-low.
compute value=descdat(low,1)+(descdat((low+1),1)-descdat(low,1))*lowdec.
compute desctmp((4+kd),jd)=value.
end loop.
compute mnotev=1.
compute modvals=desctmp(5:7,:).
do if (quantile <> 1).
compute desctmp(5,jd)=desctmp(1,jd)-desctmp(2,jd).
compute desctmp(6,jd)=desctmp(1,jd).
compute desctmp(7,jd)=desctmp(1,jd)+desctmp(2,jd).
compute modvals=desctmp(5:7,:).
compute mnotev=2.
do if (modvals(1,1) < desctmp(3,1)).
compute modvals(1,1)=desctmp(3,1).
compute minwarn=1.
end if.
do if (modvals(3,1) > desctmp(4,1)).
compute modvals(3,1)=desctmp(4,1).
compute maxwarn=1.
end if.
end if.
do if (desctmp(8,1)=1).
compute modvals={desctmp(3,1);desctmp(4,1)}.
compute mnotev=0.
compute minwarn=0.
compute maxwarn=0.
end if.
end if.
end loop
.
compute xmodvals=modvals.
compute xprobval=xmodvals.
end if.
end if.
do if (nms > 0).
loop i = 1 to nms.
do if (mprod(1,i)=1).
compute mtmp(:,i)=mtmp(:,i)-(csum(mtmp(:,i))/n).
compute centvar={centvar,mnames(1,i)}.
end if.
end loop.

.
compute desctmp=make((8-(4* 0 )),ncol( mtmp ),-999).
loop jd=1 to ncol( mtmp ).
compute descdat= mtmp (:,jd).
compute desctmp(1,jd) = csum(descdat)/nrow(descdat).
compute desctmp(2,jd) = (nrow(descdat)*sscp(descdat))-(t(csum(descdat))*(csum(descdat))).
compute desctmp(2,jd) = sqrt(desctmp(2,jd)/(nrow(descdat)*(nrow(descdat)-1))).
compute desctmp(3,jd)=cmin(descdat).
compute desctmp(4,jd)=cmax(descdat).
do if ( 0 =0).
compute minwarn=0.
compute maxwarn=0.
do if ((desctmp(3,jd)=desctmp(4,jd)) and novar=0).
compute errcode(errs,1)=15.
compute errs=errs+1.
compute criterr=1.
compute novar=1.
end if.
compute tmp=((descdat(:,1)=desctmp(3,jd))+(descdat(:,1)=desctmp(4,jd))).
compute desctmp(8,jd)=(csum(tmp)=nrow(tmp)).
compute tmp = descdat.
compute tmp(GRADE(descdat),:) = descdat.
compute descdat = tmp.
release tmp.
compute decval={.16;.5;.84}.
loop kd=1 to 3.
compute low=trunc(decval(kd,1)*(nrow(descdat)+1)).
compute lowdec=decval(kd,1)*(nrow(descdat)+1)-low.
compute value=descdat(low,1)+(descdat((low+1),1)-descdat(low,1))*lowdec.
compute desctmp((4+kd),jd)=value.
end loop.
compute mnotev=1.
compute modvals=desctmp(5:7,:).
do if (quantile <> 1).
compute desctmp(5,jd)=desctmp(1,jd)-desctmp(2,jd).
compute desctmp(6,jd)=desctmp(1,jd).
compute desctmp(7,jd)=desctmp(1,jd)+desctmp(2,jd).
compute modvals=desctmp(5:7,:).
compute mnotev=2.
do if (modvals(1,1) < desctmp(3,1)).
compute modvals(1,1)=desctmp(3,1).
compute minwarn=1.
end if.
do if (modvals(3,1) > desctmp(4,1)).
compute modvals(3,1)=desctmp(4,1).
compute maxwarn=1.
end if.
end if.
do if (desctmp(8,1)=1).
compute modvals={desctmp(3,1);desctmp(4,1)}.
compute mnotev=0.
compute minwarn=0.
compute maxwarn=0.
end if.
end if.
end loop
.
do if ((cdeval(1,1)=-999) and (model=74)).
compute medmeans=desctmp(1,:).
end if.
compute mmodvals=modvals.
compute mprobval=mmodvals.
end if.
end if.
do if (ncol(centvar) > 1).
compute notecode(notes,1)=3.
compute notes=notes+1.
end if.
end if.
do if (criterr=0).
compute wsum=rsum(csum(wcmat)).
compute zsum=rsum(csum(zcmat)).
compute wzsum=rsum(csum(wzcmat)).
compute nump=make(1,(nys+nms),-999).
compue numint=make(1,(nys+nms),0).
compute datcount=1.
compute xtmpuse=0.
compute wtmpuse=0.
compute ztmpuse=0.
compute xwtmpus=0.
compute xztmpus=0.
compute wztmpus=0.
compute xwztmpu=0.
compute xtmploc=-999.
compute wtmploc=-999.
compute xwtmplo=-999.
compute ztmploc=-999.
compute xztmplo=-999.
compute wztmplo=-999.
compute xwztmplo=-999.
compute vlabs={' '}.
do if (ncs > 0).
compute ctmpuse=make(1,ncs,0).
end if.
do if (nms > 0).
compute mtmpuse=make(1,nms,0).
compute mwtmpus=make(1,nms,0).
compute mztmpus=make(1,nms,0).
compute mwztmpu=make(1,nms,0).
compute mtmploc=make(1,nms,0).
compute mwtmplo=make(nwvls,nms,-999).
compute mztmplo=make(nzvls,nms,-999).
compute mwztmplo=make((nwvls*nzvls),nms,-999).
end if.
do if (ncs > 0).
compute ctmploc=make(1,ncs,0).
end if.
compute fulldat=make(n,1,1).
compute datindx=make(1000,(nms+nys),-999).
compute wherew=make(2,(nms+nys),-999).
compute wherex=make(2,(nms+nys),-999).
compute wherez=make(2,(nms+nys),-999).
compute wherexw=make(2,(nms+nys),-999).
compute wherexz=make(2,(nms+nys),-999).
compute wherewz=make(2,(nms+nys),-999).
compute wherexwz=make(2,(nms+nys),-999).
do if (nms > 0).
compute wherem=make(nms,(nms+nys),-999).
compute wheremw = make(nms*2,(nms+nys),-999).
compute wheremz = make(nms*2,(nms+nys),-999).
compute wheremwz = make(nms*2,(nms+nys),-999).
end if.
compute wzhigh=make(1000,(((nms+1)*(nms+2))/2),0).
compute whigh=make(1000,(((nms+1)*(nms+2))/2),0).
compute zhigh=make(1000,(((nms+1)*(nms+2))/2),0).
compute fochigh=make(1000,(((nms+1)*(nms+2))/2),0).
compute xcoefloc={1;2;3;4;5;6;7;8;9}.
compute intkey = {' ', ' ', ' ', ' ', ' ', ' ', ' '}.
compute wzhighct=0.
compute whighct=0.
compute zhighct=0.
compute foccnt=0.
loop i = 2 to nrow(bcmat).
compute wdid=0.
compute zdid=0.
compute wzdid=0.
compute cntmp=1.
compute start=1.
do if (i < nrow(bcmat)).
compute outv=mtmp(:,(i-1)).
compute modlabel={mnames(1,(i-1));'constant'}.
end if.
do if (i = nrow(bcmat)).
compute outv=ytmp.
compute modlabel={ynames;'constant'}.
end if.
loop j = 1 to (i-1).
compute foccnt=foccnt+1.
do if (j = 1 and bcmat(i,j)=1).
compute outv={outv,xtmp}.
compute modlabel={modlabel;xcatlab(1:nxvls,1)}.
do if (xtmpuse=0).
compute fulldat={fulldat,xtmp}.
compute xtmpuse=1.
loop k4=datcount to (datcount+(nxvls-1)).
compute xtmploc={xtmploc;k4}.
end loop.
compute xtmploc=xtmploc(2:nrow(xtmploc),1).
compute datcount=datcount+nxvls.
end if.
compute datindx(start:(start+nrow(xtmploc)-1),(i-1))=xtmploc.
compute wherex(1,(i-1))=start+1.
compute wherex(2,(i-1))=start+nrow(xtmploc)-1+1.
do if (model = 74).
end if.
compute onebl=make(nrow(xtmploc),1,1).
compute fochigh((start+1):(start+nrow(xtmploc)),foccnt)=onebl.
compute start=start+nrow(xtmploc).
end if.
do if (j > 1 and bcmat(i,j)=1).
compute outv={outv,mtmp(:,(j-1))}.
compute modlabel={modlabel;mnames(1,(j-1))}.
do if (mtmpuse(1,(j-1))=0).
compute fulldat={fulldat,mtmp(:,(j-1))}.
compute mtmpuse(1,(j-1))=1.
compute mtmploc(1,(j-1))=datcount.
compute datcount=datcount+1.
end if.
compute datindx(start:(start+nrow(mtmploc)-1),(i-1))=mtmploc(1,(j-1)).
compute wherem((j-1),(i-1))=start+1.
compute onebl=make(nrow(mtmploc(1,j-1)),1,1).
compute ttt=nrow(mtmploc(1,(j-1)))+start-1.
compute fochigh((start+1):(start+nrow(mtmploc(1,(j-1)))),foccnt)=onebl.
compute start=start+nrow(mtmploc(1,(j-1))).
end if.
end loop.
do if (wsum > 0).
loop j = 1 to (i-1).
compute whighct=whighct+1.
do if (j = 1 and wcmat(i,j)=1).
do if (wdid=0).
compute outv={outv,wtmp}.
do if (ncs > 0 and wiscov > 0).
compute ccmatoff((i-1),wiscov)=0.
end if.
compute modlabel={modlabel;wcatlab(1:nwvls,1)}.
compute wdid=1.
do if (wtmpuse=0).
compute fulldat={fulldat,wtmp}.
do if (ncs > 0 and wiscov > 0).
compute ccmatoff((i-1),wiscov)=0.
end if.
compute wtmpuse=1.
loop k4=datcount to (datcount+(nwvls-1)).
compute wtmploc={wtmploc;k4}.
end loop.
compute wtmploc=wtmploc(2:nrow(wtmploc),1).
compute datcount=datcount+nwvls.
end if.
end if.
compute datindx(start:(start+nrow(wtmploc)-1),(i-1))=wtmploc.
compute wherew(1,(i-1))=start+1.
compute wherew(2,(i-1))=start+nrow(wtmploc)-1+1.
compute start=start+nrow(wtmploc).
loop k1=1 to nxvls.
loop k2 = 1 to nwvls.
compute outv={outv,(xtmp(:,k1)&*wtmp(:,k2))}.
do if (ncs > 0 and wiscov > 0).
compute ccmatoff((i-1),wiscov)=0.
end if.
compute modlabel={modlabel;intlab(cntmp,1)}.
compute intkey={intkey;intlab(cntmp,1),':',xcatlab(k1,1),'x',wcatlab(k2,1),' ',' '}.
compute cntmp=cntmp+1.
end loop.
end loop.
do if (xwtmpus=0).
compute fulldat={fulldat,outv(:,(ncol(outv)-(nxvls*nwvls)+1):ncol(outv))}.
compute xwtmpus=1.
do if (ncs > 0 and wiscov > 0).
compute ccmatoff((i-1),wiscov)=0.
end if.
loop k4=datcount to (datcount+((nwvls*nxvls)-1)).
compute xwtmplo={xwtmplo;k4}.
end loop.
compute xwtmplo=xwtmplo(2:nrow(xwtmplo),1).
compute datcount=datcount+(nxvls*nwvls).
end if.
compute datindx(start:(start+nrow(xwtmplo)-1),(i-1))=xwtmplo.
compute wherexw(1,(i-1))=start+1.
compute wherexw(2,(i-1))=start+nrow(xwtmplo)-1+1.
compute onebl=make(nrow(xwtmplo),1,1).
compute whigh((start+1):(start+nrow(xwtmplo)),whighct)=onebl.
compute start=start+nrow(xwtmplo).
end if.
do if (j > 1 and wcmat(i,j)=1).
do if (wdid=0 and model <> 74).
compute outv={outv,wtmp}.
do if (ncs > 0 and wiscov > 0).
compute ccmatoff((i-1),wiscov)=0.
end if.
compute modlabel={modlabel;wcatlab(1:nwvls,1)}.
compute wdid=1.
do if (wtmpuse=0).
compute fulldat={fulldat,wtmp}.
do if (ncs > 0 and wiscov > 0).
compute ccmatoff((i-1),wiscov)=0.
end if.
compute wtmpuse=1.
loop k4=datcount to (datcount+(nwvls-1)).
compute wtmploc={wtmploc;k4}.
end loop.
compute wtmploc=wtmploc(2:nrow(wtmploc),1).
compute datcount=datcount+nwvls.
end if.
compute datindx(start:(start+nrow(wtmploc)-1),(i-1))=wtmploc.
compute wherew(1,(i-1))=start+1.
compute wherew(2,(i-1))=start+nrow(wtmploc)-1+1.
compute start=start+nrow(wtmploc).
end if.
loop k2 = 1 to nwvls.
compute outv={outv,(mtmp(:,(j-1))&*wtmp(:,k2))}.
do if (ncs > 0 and wiscov > 0).
compute ccmatoff((i-1),wiscov)=0.
end if.
compute modlabel={modlabel;intlab(cntmp,1)}.
compute intkey={intkey;intlab(cntmp,1),':', mnames(1,(j-1)),'x',wcatlab(k2,1),' ',' '}.
compute cntmp=cntmp+1.
end loop.
do if (mwtmpus(1,(j-1))=0).
compute fulldat={fulldat,outv(:,(ncol(outv)-nwvls+1):ncol(outv))}.
do if (ncs > 0 and wiscov > 0).
compute ccmatoff((i-1),wiscov)=0.
end if.
compute mwtmpus(1,(j-1))=1.
compute mw22=-999.
loop k4=datcount to (datcount+(nwvls-1)).
compute mw22={mw22;k4}.
end loop.
compute mwtmplo(:,(j-1))=mw22(2:nrow(mw22),1).
compute datcount=datcount+nwvls.
end if.
compute datindx(start:(start+nrow(mwtmplo)-1),(i-1))=mwtmplo(:,(j-1)).
compute wheremw(((2*j)-3),(i-1))=start+1.
compute wheremw(((2*j)-2),(i-1))=start+nrow(mwtmplo)-1+1.
compute onebl=make(nrow(mwtmplo),1,1).
compute whigh((start+1):(start+nrow(mwtmplo)),whighct)=onebl.
compute start=start+nrow(mwtmplo).
end if.
end loop.
end if.
do if (zsum > 0).
loop j = 1 to (i-1).
compute zhighct=zhighct+1.
do if (j = 1 and zcmat(i,j)=1).
do if (zdid=0).
compute outv={outv,ztmp}.
do if (ncs > 0 and ziscov > 0).
compute ccmatoff((i-1),ziscov)=0.
end if.
compute modlabel={modlabel;zcatlab(1:nzvls,1)}.
compute zdid=1.
do if (ztmpuse=0).
compute fulldat={fulldat,ztmp}.
do if (ncs > 0 and ziscov > 0).
compute ccmatoff((i-1),ziscov)=0.
end if.
compute ztmpuse=1.
loop k4=datcount to (datcount+(nzvls-1)).
compute ztmploc={ztmploc;k4}.
end loop.
compute ztmploc=ztmploc(2:nrow(ztmploc),1).
compute datcount=datcount+nzvls.
end if.
end if.
compute datindx(start:(start+nrow(ztmploc)-1),(i-1))=ztmploc.
compute wherez(1,(i-1))=start+1.
compute wherez(2,(i-1))=start+nrow(ztmploc)-1+1.
compute start=start+nrow(ztmploc).
loop k1=1 to nxvls.
loop k2 = 1 to nzvls.
compute outv={outv,(xtmp(:,k1)&*ztmp(:,k2))}.
do if (ncs > 0 and ziscov > 0).
compute ccmatoff((i-1),ziscov)=0.
end if.
compute modlabel={modlabel;intlab(cntmp,1)}.
compute intkey={intkey;intlab(cntmp,1),':',xcatlab(k1,1),'x',zcatlab(k2,1),' ',' '}.
compute cntmp=cntmp+1.
end loop.
end loop.
do if (xztmpus=0).
compute fulldat={fulldat,outv(:,(ncol(outv)-(nxvls*nzvls)+1):ncol(outv))}.
do if (ncs > 0 and ziscov > 0).
compute ccmatoff((i-1),ziscov)=0.
end if.
compute xztmpus=1.
loop k4=datcount to (datcount+((nzvls*nxvls)-1)).
compute xztmplo={xztmplo;k4}.
end loop.
compute xztmplo=xztmplo(2:nrow(xztmplo),1).
compute datcount=datcount+(nxvls*nzvls).
end if.
compute datindx(start:(start+nrow(xztmplo)-1),(i-1))=xztmplo.
compute wherexz(1,(i-1))=start+1.
compute wherexz(2,(i-1))=start+nrow(xztmplo)-1+1.
compute onebl=make(nrow(xztmplo),1,1).
compute zhigh((start+1):(start+nrow(xztmplo)),zhighct)=onebl.
compute start=start+nrow(xztmplo).
end if.
do if (j > 1 and zcmat(i,j)=1).
do if (zdid=0).
compute outv={outv,ztmp}.
do if (ncs > 0 and ziscov > 0).
compute ccmatoff((i-1),ziscov)=0.
end if.
compute modlabel={modlabel;zcatlab(1:nzvls,1)}.
compute zdid=1.
do if (ztmpuse=0).
compute fulldat={fulldat,ztmp}.
do if (ncs > 0 and ziscov > 0).
compute ccmatoff((i-1),ziscov)=0.
end if.
compute ztmpuse=1.
loop k4=datcount to (datcount+(nzvls-1)).
compute ztmploc={ztmploc;k4}.
end loop.
compute ztmploc=ztmploc(2:nrow(ztmploc),1).
compute datcount=datcount+nzvls.
end if.
compute datindx(start:(start+nrow(ztmploc)-1),(i-1))=ztmploc.
compute wherez(1,(i-1))=start+1.
compute wherez(2,(i-1))=start+nrow(ztmploc)-1+1.
compute start=start+nrow(ztmploc).
end if.
loop k2 = 1 to nzvls.
compute outv={outv,(mtmp(:,(j-1))&*ztmp(:,k2))}.
do if (ncs > 0 and ziscov > 0).
compute ccmatoff((i-1),ziscov)=0.
end if.
compute modlabel={modlabel;intlab(cntmp,1)}.
compute intkey={intkey;intlab(cntmp,1),':', mnames(1,(j-1)),'x',zcatlab(k2,1),' ',' '}.
compute cntmp=cntmp+1.
end loop.
do if (mztmpus(1,(j-1))=0).
compute fulldat={fulldat,outv(:,(ncol(outv)-nzvls+1):ncol(outv))}.
do if (ncs > 0 and ziscov > 0).
compute ccmatoff((i-1),ziscov)=0.
end if.
compute mztmpus(1,(j-1))=1.
compute mz22=-999.
loop k4=datcount to (datcount+(nzvls-1)).
compute mz22={mz22;k4}.
end loop.
compute mztmplo(:,(j-1))=mz22(2:nrow(mz22),1).
compute datcount=datcount+nzvls.
end if.
compute datindx(start:(start+nrow(mztmplo)-1),(i-1))=mztmplo(:,(j-1)).
compute wheremz(((2*j)-3),(i-1))=start+1.
compute wheremz(((2*j)-2),(i-1))=start+nrow(mztmplo)-1+1.
compute onebl=make(nrow(mztmplo),1,1).
compute zhigh((start+1):(start+nrow(mztmplo)),zhighct)=onebl.
compute start=start+nrow(mztmplo).
end if.
end loop.
end if.
do if (wzsum > 0).
loop j = 1 to (i-1).
compute wzhighct=wzhighct+1.
do if (j = 1 and wzcmat(i,j)=1).
do if (wzdid=0).
loop k1=1 to nwvls.
loop k2 = 1 to nzvls.
compute outv={outv,(wtmp(:,k1)&*ztmp(:,k2))}.
do if (ncs > 0 and (ziscov > 0)).
compute ccmatoff((i-1),ziscov)=0.
end if.
do if (ncs > 0 and (wiscov > 0)).
compute ccmatoff((i-1),wiscov)=0.
end if.
compute modlabel={modlabel;intlab(cntmp,1)}.
compute intkey={intkey;intlab(cntmp,1),':',wcatlab(k1,1),'x',zcatlab(k2,1),' ',' '}.
compute cntmp=cntmp+1.
end loop.
end loop.
do if (wztmpus=0).
compute fulldat={fulldat,outv(:,(ncol(outv)-(nwvls*nzvls)+1):ncol(outv))}.
do if (ncs > 0 and (ziscov > 0)).
compute ccmatoff((i-1),ziscov)=0.
end if.
do if (ncs > 0 and (wiscov > 0)).
compute ccmatoff((i-1),wiscov)=0.
end if.
compute wztmpus=1.
loop k4=datcount to (datcount+((nwvls*nzvls)-1)).
compute wztmplo={wztmplo;k4}.
end loop.
compute wztmplo=wztmplo(2:nrow(wztmplo),1).
compute datcount=datcount+(nzvls*nwvls).
end if.
compute wzdid=1.
end if.
compute datindx(start:(start+nrow(wztmplo)-1),(i-1))=wztmplo.
compute wherewz(1,(i-1))=start+1.
compute wherewz(2,(i-1))=start+nrow(wztmplo)-1+1.
compute start=start+nrow(wztmplo).
loop k1=1 to nxvls.
loop k2=1 to nwvls.
loop k3=1 to nzvls.
compute outv={outv,(xtmp(:,k1)&*wtmp(:,k2)&*ztmp(:,k3))}.
do if (ncs > 0 and (ziscov > 0)).
compute ccmatoff((i-1),ziscov)=0.
end if.
do if (ncs > 0 and (wiscov > 0)).
compute ccmatoff((i-1),wiscov)=0.
end if.
compute modlabel={modlabel;intlab(cntmp,1)}.
compute intkey={intkey;intlab(cntmp,1),':',xcatlab(k1,1),'x',wcatlab(k2,1),'x', zcatlab(k3,1)}.
compute cntmp=cntmp+1.
end loop.
end loop.
end loop.
do if (xwztmpu=0).
compute fulldat={fulldat,outv(:,(ncol(outv)-(nxvls*nwvls*nzvls)+1):ncol(outv))}.
do if (ncs > 0 and (ziscov > 0)).
compute ccmatoff((i-1),ziscov)=0.
end if.
do if (ncs > 0 and (wiscov > 0)).
compute ccmatoff((i-1),wiscov)=0.
end if.
compute xwztmpu=1.
loop k4=datcount to (datcount+((nzvls*nxvls*nwvls)-1)).
compute xwztmplo={xwztmplo;k4}.
end loop.
compute xwztmplo=xwztmplo(2:nrow(xwztmplo),1).
compute datcount=datcount+(nxvls*nzvls*nwvls).
end if.
compute datindx(start:(start+nrow(xwztmplo)-1),(i-1))=xwztmplo.
compute wherexwz(1,(i-1))=start+1.
compute wherexwz(2,(i-1))=start+nrow(xwztmplo)-1+1.
compute onebl=make(nrow(xwztmplo),1,1).
compute wzhigh((start+1):(start+nrow(xwztmplo)),wzhighct)=onebl.
compute start=start+nrow(xwztmplo).
end if.
do if (j > 1 and wzcmat(i,j)=1).
do if (wzdid=0).
loop k1=1 to nwvls.
loop k2 = 1 to nzvls.
compute outv={outv,(wtmp(:,k1)&*ztmp(:,k2))}.
do if (ncs > 0 and (ziscov > 0)).
compute ccmatoff((i-1),ziscov)=0.
end if.
do if (ncs > 0 and (wiscov > 0)).
compute ccmatoff((i-1),wiscov)=0.
end if.
compute modlabel={modlabel;intlab(cntmp,1)}.
compute intkey={intkey;intlab(cntmp,1),':',wcatlab(k1,1),'x',zcatlab(k2,1),' ',' '}.
compute cntmp=cntmp+1.
end loop.
end loop.
do if (wztmpus=0).
compute fulldat={fulldat,outv(:,(ncol(outv)-(nwvls*nzvls)+1):ncol(outv))}.
do if (ncs > 0 and (ziscov > 0)).
compute ccmatoff((i-1),ziscov)=0.
end if.
do if (ncs > 0 and (wiscov > 0)).
compute ccmatoff((i-1),wiscov)=0.
end if.
compute wztmpus=1.
loop k4=datcount to (datcount+((nwvls*nzvls)-1)).
compute wztmplo={wztmplo;k4}.
end loop.
compute wztmplo=wztmplo(2:nrow(wztmplo),1).
compute datcount=datcount+(nzvls*nwvls).
end if.
compute wzdid=1.
compute datindx(start:(start+nrow(wztmplo)-1),(i-1))=wztmplo.
compute wherewz(1,(i-1))=start+1.
compute wherewz(2,(i-1))=start+nrow(wztmplo)-1+1.
compute start=start+nrow(wztmplo).
end if.
loop k1 = 1 to nwvls.
loop k2 = 1 to nzvls.
compute outv={outv,(mtmp(:,(j-1))&*wtmp(:,k1)&*ztmp(:,k2))}.
do if (ncs > 0 and (ziscov > 0)).
compute ccmatoff((i-1),ziscov)=0.
end if.
do if (ncs > 0 and (wiscov > 0)).
compute ccmatoff((i-1),wiscov)=0.
end if.
compute modlabel={modlabel;intlab(cntmp,1)}.
compute intkey={intkey;intlab(cntmp,1),':',mnames(1,(j-1)),'x',wcatlab(k1,1),'x', zcatlab(k2,1)}.
compute cntmp=cntmp+1.
end loop.
end loop.
do if (mwztmpu(1,(j-1))=0).
compute fulldat={fulldat,outv(:,(ncol(outv)-(nwvls*nzvls)+1):ncol(outv))}.
do if (ncs > 0 and (ziscov > 0)).
compute ccmatoff((i-1),ziscov)=0.
end if.
do if (ncs > 0 and (wiscov > 0)).
compute ccmatoff((i-1),wiscov)=0.
end if.
compute mwztmpu(1,(j-1))=1.
compute mz22=-999.
loop k4=datcount to (datcount+(nwvls*nzvls)-1).
compute mz22={mz22;k4}.
end loop.
compute mwztmplo(:,(j-1))=mz22(2:nrow(mz22),1).
compute datcount=datcount+(nwvls*nzvls).
end if.
compute datindx(start:(start+nrow(mwztmplo)-1),(i-1))=mwztmplo(:,(j-1)).
compute wheremwz(((2*j)-3),(i-1))=start+1.
compute wheremwz(((2*j)-2),(i-1))=start+nrow(mwztmplo)-1+1.
compute onebl=make(nrow(mwztmplo),1,1).
compute wzhigh((start+1):(start+nrow(mwztmplo)),wzhighct)=onebl.
compute start=start+nrow(mwztmplo).
end if.
end loop.
end if.
do if (ncs > 0).
compute ccmat=ccmat&*ccmatoff.
loop j = 1 to ncs.
do if (ccmat((i-1),j))=1.
do if (j=wiscov).
compute ctmp(:,j)=wtmp.
end if.
do if (j=ziscov).
compute ctmp(:,j)=ztmp.
end if.
compute outv={outv,ctmp(:,j)}.
compute modlabel={modlabel;covnames(1,j)}.
do if (ctmpuse(1,j)=0).
compute fulldat={fulldat,ctmp(:,j)}.
compute ctmpuse(1,j)=1.
compute ctmploc(1,j)=datcount.
compute datcount=datcount+1.
end if.
compute datindx(start:(start+nrow(ctmploc)-1),(i-1))=ctmploc(1,j).
compute start=start+nrow(ctmploc(1,j)).
end if.
end loop.
end if.
compute wdid=0.
compute zdid=0.
compute wzdid=0.
compute vlabs={vlabs;modlabel(2:nrow(modlabel),1)}.
compute numint(1,(i-1))=cntmp-1.
compute nump(1,(i-1))=nrow(modlabel)-1.
end loop.
release datcount, xtmpuse, wtmpuse, ztmpuse, xwtmpus, xztmpus, wztmpus, xwztmpu.
release xtmploc, wtmploc, xwtmplo, ztmploc, xztmplo, wztmplo, xwztmplo, foccnt.
do if (modcok=1 and ((nms > 0) or (zcmat(2,1) <> 1) or (mcx <> 0))).
compute notecode(notes,1) = 19.
compute notes = notes + 1.
compute modcok=0.
end if.
do if ((serial = 1 or (rsum(numint)>0) or nms=0) and mc > 0).
compute notecode(notes,1) = 15.
compute notes = notes + 1.
compute boot=mc.
compute mc=0.
end if.
do if (boot <> 0 or mc <> 0).
compute bootsz=boot.
do if (mc > 0).
compute bootsz=mc.
compute saveboot=0.
end if.
loop.
compute cilow = rnd(bootsz*(1-(conf/100))/2).
compute cihigh = trunc((bootsz*(conf/100)+(bootsz*(1-(conf/100))/2)))+1.
do if (cilow < 1 or cihigh > bootsz).
compute bootsz=trunc((bootsz+1000)/1000)*1000.
compute adjust = 1.
end if.
end loop if (cilow gt 0 and cihigh le bootsz).
do if (boot > 0).
compute boot=bootsz.
end if.
do if (mc > 0).
compute mc=bootsz.
end if.
do if (adjust = 1 and boot > 0).
compute notecode(notes,1) = 8.
compute notes = notes + 1.
end if.
do if (adjust = 1 and mc > 0).
compute notecode(notes,1) = 16.
compute notes = notes + 1.
end if.
end if.
compute maxboot = trunc(2*boot).
do if ( 0 > maxboot).
compute maxboot=trunc( 0 ).
end if.
do if (nms > 0).
release mtmpuse, mwtmpus, mwztmpu, mtmploc, mwtmplo, mztmplo, mwztmplo.
end if.
release wdid, zdid, wzdid, start,modlabel.
compute vlabs=vlabs(2:nrow(vlabs),1).
do if (rsum(numint) > 0).
compute intkey=intkey(2:nrow(intkey),:).
end if.
compute fulldat=fulldat(:,2:ncol(fulldat)).
compute fochigh=fochigh(1:rmax(nump),:).
compute whigh=whigh(1:rmax(nump),:).
compute zhigh=zhigh(1:rmax(nump),:).
compute wzhigh=wzhigh(1:rmax(nump),:).
compute coeffs=fochigh+whigh+zhigh+wzhigh.
compute bootloc=make(rmax(nump),ncol(nump),0).
do if (nms > 0).
compute cntmp=1.
loop i = 1 to ncol(nump).
loop j = 1 to nump(1,i).
compute bootloc(j,i)=cntmp.
compute cntmp=cntmp+1.
end loop.
end loop.
compute fochighb=make(nrow(fochigh),ncol(fochigh),0).
compute whighb=fochighb.
compute zhighb=fochighb.
compute wzhighb=fochighb.
compute thetaxmb=make(nrow(fochighb),nms,0).
compute thetaxyb=make(nrow(fochighb),1,0).
compute pathsfoc=make(nxvls,1,0).
compute cntmp=1.
loop i = 1 to (nms+nys).
loop j = 1 to i.
compute fochighb(:,cntmp)=fochigh(:,cntmp)&*bootloc(:,i).
compute whighb(:,cntmp)=whigh(:,cntmp)&*bootloc(:,i).
compute zhighb(:,cntmp)=zhigh(:,cntmp)&*bootloc(:,i).
compute wzhighb(:,cntmp)=wzhigh(:,cntmp)&*bootloc(:,i).
compute coeffsb=fochighb+whighb+zhighb+wzhighb.
do if ((i < (nms+nys)) and (j = 1)).
compute thetaxmb(:,i)=coeffsb(:,cntmp).
end if.
do if ((i = (nms+nys)) and (j = 1)).
compute thetaxyb(:,1)=coeffsb(:,cntmp).
end if.
compute cntmp=cntmp+1.
end loop.
end loop.
compute thetamyb=coeffsb(:,(ncol(coeffsb)-nms+1):ncol(coeffsb)).
do if (serial = 1).
compute thetammb=make(nrow(coeffsb),((nms*(nms-1))/2),0).
end if.
compute cntmp=1.
do if (nms > 1 and serial = 1).
loop i = 1 to (nms-1).
compute start=((i+2)*(i+1))/2.
loop j = 2 to (nms-i+1).
compute thetammb(:,cntmp)=coeffsb(:,start).
compute start=start+j+i-1.
compute cntmp=cntmp+1.
end loop.
end loop.
end if.
end if.
do if ((total = 1) and (rsum(numint)=0 or (xmint=1))).
compute dototal=1.
do if ((csum(bcmat(:,1)) <> (nms+nys)) or (rsum(bcmat(nrow(bcmat),:)) <> (nms+nys))).
compute dototal=0.
compute alttotal=1.
compute notecode(notes,1) = 12.
compute notes = notes + 1.
end if.
do if (ncs > 0).
do if ((csum(rsum(ccmat))) < (nrow(ccmat)*ncol(ccmat))).
compute dototal=0.
compute alttotal=1.
compute notecode(notes,1) = 11.
compute notes = notes + 1.
end if.
end if.
do if (model=74).
do if (xdich=0 and nxvls=1).
compute dototal=0.
compute alttotal=1.
end if.
do if ((xdich=1 or nxvls > 1) and (ncs > 0) and model=74).
compute dototal=0.
compute alttotal=1.
end if.
end if.
end if.
end if.
do if (criterr=0 and ncs > 0).
do if (rsum((csum(ccmat)=0)) <> 0).
compute errcode(errs,1)=51.
compute errs=errs+1.
compute criterr=1.
end if.
end if.
compute debug= 0.
do if (outscree=1).
print/title = '***************** PROCESS Procedure for SPSS Version 4.2 *****************'.
print/title = '          Written by Andrew F. Hayes, Ph.D.       www.afhayes.com'.
print/title = '    Documentation available in Hayes (2022). www.guilford.com/p/hayes3'/space=0.
end if.
do if (criterr=0).
compute modresid=make(n,1,99999).
do if (stand=1 and ydich=1).
compute stand=0.
end if.
compute anymod2=csum(rsum(wcmat+zcmat+wzcmat)).
do if (anymod2 > 0 and stand = 1).
compute notecode(notes,1) = 27.
compute notes = notes + 1.
compute stand=0.
end if.
compute funny=1.
do if (outscree=1).
print modelvar/title = '**************************************************************************'/format = A8/rnames=modelvlb.
do if (ncs > 0).
print covnames/title='Covariates:'/format=A8.
end if.
print n/title='Sample'/rlabel='Size:'.
do if (( 'random' <> 'random')).
compute seedt= 'random'.
print seedt/title='Custom'/format=A12/rlabel = 'Seed:'.
end if.
end if.
compute maxresm=9.
compute resultm=make(1,maxresm,99999).
do if (describe=1).
compute means=csum(dat)/n.
compute sigmatal = (t(dat)*(ident(n)-(1/n)*ones*t(ones))*dat)*(1/(n-1)).
compute sdvec=sqrt(diag(sigmatal)).
compute sdall = mdiag(1/sdvec).
compute corall=sdall*sigmatal*t(sdall).
compute means={means;t(sdvec)}.
do if (ncol(means) > 9).
compute resultm=make(1,ncol(means),99999).
compute maxresm=ncol(means).
end if.
compute resultm2=make(2,maxresm,99999).
compute resultm2(1:2,1:ncol(means))=means.
compute resultm={resultm;resultm2}.
compute resultm2=make(ncol(corall),maxresm,99999).
compute resultm2(1:ncol(corall),1:ncol(corall))=corall.
compute resultm={resultm;resultm2}.
do if (outscree=1).
print means/title='Variable means and standard deviations'/cnames=varnames/rlabels='Mean','SD'/format= F10.4.
print corall/title='Variable intercorrelations (Pearson r)'/cnames=varnames/rnames=varnames/format= F10.4.
end if.
end if.
do if (outscree=1).
do if (mcxok=1).
compute labtmp={xnames,t(xcatlab(1:nxvls,1))}.
print dummatx/title = 'Coding of categorical X variable for analysis:'/cnames = labtmp/format = F6.3.
end if.
do if (mcwok=1 and xmint=0).
compute labtmp={wnames,t(wcatlab(1:nwvls,1))}.
print dummatw/title = 'Coding of categorical W variable for analysis:'/cnames = labtmp/format = F6.3.
end if.
do if (mczok=1).
compute labtmp={znames,t(zcatlab(1:nzvls,1))}.
print dummatz/title = 'Coding of categorical Z variable for analysis:'/cnames = labtmp/format = F6.3.
end if.
end if.
end if.
do if (criterr = 0).
compute outnames=ynames.
compute outvars=ytmp.
do if (nms > 0).
compute outnames={mnames,ynames}.
compute outvars={mtmp,ytmp}.
compute indcov=make(((nms*2)+(nms*(nxvls-1))),((nms*2)+(nms*(nxvls-1))),0).
compute mcsopath=make(((nms*2)+(nms*(nxvls-1))),1,0).
end if.
compute labstart=1.
compute intstart=1.
compute start=1.
compute coeffmat=make(1,6,0).
compute conseq={'        '}.
compute dfmat=0.
compute coeffcol=0.
compute pathscnt=1.
compute pathscn2=1.
loop i = 1 to (nms+nys).
do if (outscree=1).
print/title = '**************************************************************************'.
end if.
compute highf=make(1,5,0).
compute highf2=highf.
do if ((i = (nms+nys)) and (ydich=1)).
compute highf=make(1,3,0).
compute highf2=highf.
end if.
compute flabel={' '}.
compute y=outvars(:,i).
compute xindx=datindx(1:(nump(1,i)-1),i).
compute x = fulldat(:,xindx).
compute x={ones,x}.
compute xsq=t(x)*x.
compute exsq=eval(xsq).
release xsq.
compute zeroeig=csum(exsq <= 0.000000000002).
do if (outscree=1).
print outnames(1,i)/title = 'OUTCOME VARIABLE:'/format = A8/space=0.
do if (ydich=1 and (i = (nms+nys))).
compute nmsd = {outnames(1,i), 'Analysis'}.
print rcd/title = 'Coding of binary Y for logistic regression analysis:'/cnames = nmsd/format = F9.2.
end if.
end if.
do if (zeroeig > 0).
print / title = 'SINGULAR OR NEAR SINGULAR DATA MATRIX.'.
compute criterr=1.
compute errcode(errs,1)=31.
compute errs=errs+1.
end if.
compute means=csum(x)/n.
compute vlabsm=vlabs(labstart:(labstart+(nump(1,i)-1)),1).
do if (criterr=0).
do if (ydich=0 or (i < (nms+nys))).

.
do if ( 1 =1).
compute hatmat=inv(t( x )* x )*t( x ).
compute b = hatmat* y.
compute modres=b.
do if ( 1 =1).
compute n1=nrow( x ).
compute dfres=n1-(ncol( x )).
compute sstotal = t( y -(csum( y )/n1))*( y -(csum( y )/n1)).
compute resid= y - x *b.
compute ssresid = csum((resid)&**2).
compute r2 = (sstotal-ssresid)/sstotal.
do if (r2 < 0).
compute r2=0.
end if.
compute adjr2 = 1-((1-r2)*(n1-1)/(dfres)).
compute mse=ssresid/(n1-ncol( x )).

.
compute n1=nrow( x ).
compute invXtX = inv(t( x )* x ).
compute varb = mse *invXtX.
compute k3 = ncol( x ).
compute xhc=0.
do if ( hc <> 5).
compute xhc= x.
compute hat = xhc(:,1).
loop i3=1 to nrow(xhc).
compute hat(i3,1)= xhc(i3,:)*invXtX*t(xhc(i3,:)).
end loop.
do if ( hc = 0 or hc =1).
loop i3 = 1 to k3.
compute xhc(:,i3)=xhc(:,i3)&* resid.
end loop.
end if.
do if ( hc =3 or hc =2).
loop i3=1 to k3.
compute xhc(:,i3) = ( resid &/(1-hat)&**(1/(4- hc )))&*xhc(:,i3).
end loop.
end if.
do if ( hc = 4).
compute hcmn=make(n,2,4).
compute hcmn(:,2)=(n1*hat)/k3.
loop i3= 1 to k3.
compute xhc(:,i3) = ( resid &/(1-hat)&**(rmin(hcmn)/2))&*xhc(:,i3).
end loop.
end if.
compute varb=(invXtX*t(xhc)*xhc*invXtX).
do if ( hc =1).
compute varb=(n1/(n1-ncol( x )))&*varb.
end if.
end if.
compute hclab={'se(HC0)','se(HC1)','se(HC2)','se(HC3)','se(HC4)','se'}.
compute hclab=hclab(1,( hc +1)).
compute hcflab={'F(HC0)','F(HC1)','F(HC2)','F(HC3)','F(HC4)','F'}.
compute hcflab=hcflab(1,( hc +1)).
release xhc
.
compute seb=sqrt(diag(varb)).
compute trat = b&/seb.
compute p = 2*(1-tcdf(abs(trat), (dfres))).
compute tval = sqrt(dfres* (exp((dfres-(5/6))*((xp2/(dfres-(2/3)+(.11/dfres)))* (xp2/(dfres-(2/3)+(.11/dfres)))))-1)).
compute modres={modres,seb,trat,p}.
compute modres={modres,(b-tval&*seb),(b+tval&*seb)}.
compute modresl={'coeff',hclab,'t','p','LLCI','ULCI'}.
compute lmat = ident(ncol( x )).
compute lmat = lmat(:,2:ncol(lmat)).
compute fratio = (t(t(lmat)*b)*inv(t(lmat)*varb*lmat)*((t(lmat)*b)))/(ncol( x )-1).
compute pfr = 1-fcdf(fratio,(ncol( x )-1),dfres).
compute modsum={sqrt(r2),r2,mse,fratio,(ncol( x )-1),dfres,pfr}.
compute modsuml={'R','R-sq','MSE',hcflab,'df1','df2', 'p'}.
end if.
end if.
do if ( 1 = 2 or 1 =3).
compute xlp= x.
compute ylp= y.
compute pt2 = make(nrow(ylp),1,(csum(ylp)/nrow(ylp))).
do if ( 1 =2).
compute LL3 = ylp&*ln(pt2)+(1-ylp)&*ln(1-pt2).
end if.
compute LL3 = -2*csum(LL3).
compute bt1 = make(ncol(xlp),1,0).
compute LL1 = 0.
compute pt1 = make(nrow(ylp),1,0.5).
compute pt1lp=pt1.
loop jjj = 1 to iterate.
compute xlptmp=t(xlp).
compute vecprb=pt1lp&*(1-pt1lp).
loop kkk=1 to ncol(xlp).
compute xlptmp(kkk,:)=xlptmp(kkk,:)&*t(vecprb).
end loop.
compute b = bt1+inv(xlptmp*xlp)*t(xlp)*(ylp-pt1lp).
do if ( 1 =2).
compute xlpb=xlp*b.
compute xlpbt=(xlpb > -709.7).
compute xlpb709=(1-xlpbt)*(-709.7).
compute xlpb=(xlpb&*xlpbt)+xlpb709.
compute pt1lp = 1/(1+exp(-(xlpb))).
end if.
compute itprob = csum((pt1lp < .00000001) or (pt1lp > .9999999)).
do if (itprob > 0).
loop kkk = 1 to nrow(pt1lp).
do if (pt1lp(kkk,1) > .9999999).
compute pt1lp(kkk,1) = .9999999.
end if.
do if (pt1lp(kkk,1) < .00000001).
compute pt1lp(kkk,1) = .00000001.
end if.
end loop.
compute itprob = 0.
end if.
do if (itprob = 0).
do if ( 1 =2).
compute LL = ylp&*ln(pt1lp)+(1-ylp)&*ln(1-pt1lp).
end if.
compute LL2 = -2*csum(ll).
end if.
do if (abs(LL1-LL2) < converge).
do if ( 1 =1).
compute xlptmp=t(xlp).
compute vecprb=pt1lp&*(1-pt1lp).
loop kkk=1 to ncol(xlp).
compute xlptmp(kkk,:)=xlptmp(kkk,:)&*t(vecprb).
end loop.
compute varb = inv(xlptmp*xlp).
compute seb = sqrt(diag(varb)).
release xlptmp.
end if.
break.
end if.
compute bt1 = b.
compute LL1 = LL2.
end loop.
compute modres=b.
do if (jjj > iterate).
compute itprob = 2.
do if (booting=0).
compute iterrmod=1.
end if.
do if (booting=1).
compute bootiter=1.
end if.
do if (itprobtg=0).
compute itprobtg=1.
compute errcode(errs,1) = 47.
compute errs = errs + 1.
do if (booting = 0 and 1 =1).
compute vt1 = mdiag(pt1lp&*(1-pt1lp)).
compute varb = inv(t(xlp)*vt1*xlp).
compute seb = sqrt(diag(varb)).
end if.
end if.
end if.
do if ( 1 =1).
compute trat = b&/seb.
compute dfres=nrow(xlp).
compute p = 2*(1-cdfnorm(abs(trat))).
compute modres={modres,seb,trat,p}.
compute modres={modres,(b-xp2&*seb),(b+xp2&*seb)}.
compute pvchi=1-chicdf((LL3-LL2),(nrow(modres)-1)).
compute mcF = (LL3-LL2)/LL3.
compute cox = 1-exp(-(LL3-LL2)/nrow(xlp)).
compute nagel = cox/(1-exp(-(LL3)/nrow(xlp))).
compute modsum={LL2,(LL3-LL2),(nrow(modres)-1),pvchi, mcF,cox,nagel}.
compute modsuml={'-2LL','ModelLL', 'df', 'p', 'McFadden', 'CoxSnell', 'Nagelkrk'}.
compute modresl={'coeff','se','Z','p','LLCI','ULCI'}.
end if.
end if
.
compute dfmatt=make(nrow(modres),1,modsum(1,6)).
compute modresid={modresid,resid}.
end if.
do if (ydich=1 and (i = (nms+nys))).

.
do if ( 2 =1).
compute hatmat=inv(t( x )* x )*t( x ).
compute b = hatmat* y.
compute modres=b.
do if ( 1 =1).
compute n1=nrow( x ).
compute dfres=n1-(ncol( x )).
compute sstotal = t( y -(csum( y )/n1))*( y -(csum( y )/n1)).
compute resid= y - x *b.
compute ssresid = csum((resid)&**2).
compute r2 = (sstotal-ssresid)/sstotal.
do if (r2 < 0).
compute r2=0.
end if.
compute adjr2 = 1-((1-r2)*(n1-1)/(dfres)).
compute mse=ssresid/(n1-ncol( x )).

.
compute n1=nrow( x ).
compute invXtX = inv(t( x )* x ).
compute varb = mse *invXtX.
compute k3 = ncol( x ).
compute xhc=0.
do if ( hc <> 5).
compute xhc= x.
compute hat = xhc(:,1).
loop i3=1 to nrow(xhc).
compute hat(i3,1)= xhc(i3,:)*invXtX*t(xhc(i3,:)).
end loop.
do if ( hc = 0 or hc =1).
loop i3 = 1 to k3.
compute xhc(:,i3)=xhc(:,i3)&* resid.
end loop.
end if.
do if ( hc =3 or hc =2).
loop i3=1 to k3.
compute xhc(:,i3) = ( resid &/(1-hat)&**(1/(4- hc )))&*xhc(:,i3).
end loop.
end if.
do if ( hc = 4).
compute hcmn=make(n,2,4).
compute hcmn(:,2)=(n1*hat)/k3.
loop i3= 1 to k3.
compute xhc(:,i3) = ( resid &/(1-hat)&**(rmin(hcmn)/2))&*xhc(:,i3).
end loop.
end if.
compute varb=(invXtX*t(xhc)*xhc*invXtX).
do if ( hc =1).
compute varb=(n1/(n1-ncol( x )))&*varb.
end if.
end if.
compute hclab={'se(HC0)','se(HC1)','se(HC2)','se(HC3)','se(HC4)','se'}.
compute hclab=hclab(1,( hc +1)).
compute hcflab={'F(HC0)','F(HC1)','F(HC2)','F(HC3)','F(HC4)','F'}.
compute hcflab=hcflab(1,( hc +1)).
release xhc
.
compute seb=sqrt(diag(varb)).
compute trat = b&/seb.
compute p = 2*(1-tcdf(abs(trat), (dfres))).
compute tval = sqrt(dfres* (exp((dfres-(5/6))*((xp2/(dfres-(2/3)+(.11/dfres)))* (xp2/(dfres-(2/3)+(.11/dfres)))))-1)).
compute modres={modres,seb,trat,p}.
compute modres={modres,(b-tval&*seb),(b+tval&*seb)}.
compute modresl={'coeff',hclab,'t','p','LLCI','ULCI'}.
compute lmat = ident(ncol( x )).
compute lmat = lmat(:,2:ncol(lmat)).
compute fratio = (t(t(lmat)*b)*inv(t(lmat)*varb*lmat)*((t(lmat)*b)))/(ncol( x )-1).
compute pfr = 1-fcdf(fratio,(ncol( x )-1),dfres).
compute modsum={sqrt(r2),r2,mse,fratio,(ncol( x )-1),dfres,pfr}.
compute modsuml={'R','R-sq','MSE',hcflab,'df1','df2', 'p'}.
end if.
end if.
do if ( 2 = 2 or 2 =3).
compute xlp= x.
compute ylp= y.
compute pt2 = make(nrow(ylp),1,(csum(ylp)/nrow(ylp))).
do if ( 2 =2).
compute LL3 = ylp&*ln(pt2)+(1-ylp)&*ln(1-pt2).
end if.
compute LL3 = -2*csum(LL3).
compute bt1 = make(ncol(xlp),1,0).
compute LL1 = 0.
compute pt1 = make(nrow(ylp),1,0.5).
compute pt1lp=pt1.
loop jjj = 1 to iterate.
compute xlptmp=t(xlp).
compute vecprb=pt1lp&*(1-pt1lp).
loop kkk=1 to ncol(xlp).
compute xlptmp(kkk,:)=xlptmp(kkk,:)&*t(vecprb).
end loop.
compute b = bt1+inv(xlptmp*xlp)*t(xlp)*(ylp-pt1lp).
do if ( 2 =2).
compute xlpb=xlp*b.
compute xlpbt=(xlpb > -709.7).
compute xlpb709=(1-xlpbt)*(-709.7).
compute xlpb=(xlpb&*xlpbt)+xlpb709.
compute pt1lp = 1/(1+exp(-(xlpb))).
end if.
compute itprob = csum((pt1lp < .00000001) or (pt1lp > .9999999)).
do if (itprob > 0).
loop kkk = 1 to nrow(pt1lp).
do if (pt1lp(kkk,1) > .9999999).
compute pt1lp(kkk,1) = .9999999.
end if.
do if (pt1lp(kkk,1) < .00000001).
compute pt1lp(kkk,1) = .00000001.
end if.
end loop.
compute itprob = 0.
end if.
do if (itprob = 0).
do if ( 2 =2).
compute LL = ylp&*ln(pt1lp)+(1-ylp)&*ln(1-pt1lp).
end if.
compute LL2 = -2*csum(ll).
end if.
do if (abs(LL1-LL2) < converge).
do if ( 1 =1).
compute xlptmp=t(xlp).
compute vecprb=pt1lp&*(1-pt1lp).
loop kkk=1 to ncol(xlp).
compute xlptmp(kkk,:)=xlptmp(kkk,:)&*t(vecprb).
end loop.
compute varb = inv(xlptmp*xlp).
compute seb = sqrt(diag(varb)).
release xlptmp.
end if.
break.
end if.
compute bt1 = b.
compute LL1 = LL2.
end loop.
compute modres=b.
do if (jjj > iterate).
compute itprob = 2.
do if (booting=0).
compute iterrmod=1.
end if.
do if (booting=1).
compute bootiter=1.
end if.
do if (itprobtg=0).
compute itprobtg=1.
compute errcode(errs,1) = 47.
compute errs = errs + 1.
do if (booting = 0 and 1 =1).
compute vt1 = mdiag(pt1lp&*(1-pt1lp)).
compute varb = inv(t(xlp)*vt1*xlp).
compute seb = sqrt(diag(varb)).
end if.
end if.
end if.
do if ( 1 =1).
compute trat = b&/seb.
compute dfres=nrow(xlp).
compute p = 2*(1-cdfnorm(abs(trat))).
compute modres={modres,seb,trat,p}.
compute modres={modres,(b-xp2&*seb),(b+xp2&*seb)}.
compute pvchi=1-chicdf((LL3-LL2),(nrow(modres)-1)).
compute mcF = (LL3-LL2)/LL3.
compute cox = 1-exp(-(LL3-LL2)/nrow(xlp)).
compute nagel = cox/(1-exp(-(LL3)/nrow(xlp))).
compute modsum={LL2,(LL3-LL2),(nrow(modres)-1),pvchi, mcF,cox,nagel}.
compute modsuml={'-2LL','ModelLL', 'df', 'p', 'McFadden', 'CoxSnell', 'Nagelkrk'}.
compute modresl={'coeff','se','Z','p','LLCI','ULCI'}.
end if.
end if
.
compute dfmatt=make(nrow(modres),1,-999).
end if.
compute resultm2=make(1,maxresm,99999).
compute resultm2(1,1:ncol(modsum))=modsum.
compute resultm={resultm;resultm2}.
compute resultm2=make(nrow(modres),maxresm,99999).
compute resultm2(1:nrow(modres),1:ncol(modres))=modres.
compute resultm={resultm;resultm2}.
compute obscoeff={obscoeff,t(b)}.
do if (outscree=1).
print modsum/title = 'Model Summary'/cnames = modsuml/format= F10.4.
print modres/title='Model'/rnames=vlabsm/cnames=modresl/format= F10.4.
end if.
compute basemod=modsum(1,1).
compute basemodx=basemod.
do if (ydich=1 and (i = (nms+nys))).
do if (outscree=1).
print/title='These results are expressed in a log-odds metric.'.
end if.
compute notecode(notes,1) = 26.
compute notes = notes + 1.
end if.
compute coeffmat={coeffmat;modres}.
compute conseqt=make(nrow(modres),1,outnames(1,i)).
compute conseq={conseq;conseqt}.
compute dfmat={dfmat;dfmatt}.
compute labstart=labstart+nump(1,i).
do if (stand=1).
compute predsd=make(nrow(modres),1,0).
compute stdmod=modres(:,1)&/ovsd(1,i).
loop jd=1 to ncol(x).
compute descdat=x(:,jd).
compute predsd(jd,1) = (nrow(descdat)*sscp(descdat))-(t(csum(descdat))*(csum(descdat))).
compute predsd(jd,1) = sqrt(predsd(jd,1)/(nrow(descdat)*(nrow(descdat)-1))).
end loop.
do if (wherex(1,i) <> -999 and ((nxvls > 1) or (xdich=1))).
compute sdmsone=make(nxvls,1,1).
compute predsd(wherex(1,i):wherex(2,i),1)=sdmsone.
compute pstog=1.
end if.
compute predsd(1,1)=1.
compute stdmod=stdmod&*predsd.
compute stdmod=stdmod(2:nrow(stdmod),1).
compute sdvlabs=vlabsm(2:nrow(vlabsm),1).
compute resultm2=make(nrow(stdmod),maxresm,99999).
compute resultm2(1:nrow(stdmod),1:ncol(stdmod))=stdmod.
compute resultm={resultm;resultm2}.
do if (outscree=1).
print stdmod/title='Standardized coefficients'/clabels='coeff'/rnames=sdvlabs/format= F10.4.
end if.
end if.
do if (nms > 0 and serial = 0 and (rsum(numint) = 0) and (normal=1 or mc > 0)).
do if (i < (nms+nys)).
compute indcov((((i-1)*nxvls)+1):(i*nxvls),(((i-1)*nxvls)+1):(i*nxvls))=varb(2:(1+nxvls), 2:(1+nxvls)).
compute mcsopath((((i-1)*nxvls)+1):(i*nxvls) ,1)=modres(2:(1+nxvls),1).
end if.
do if (i = (nms+nys)).
compute atm=ncol(wherem).
compute indcov(((nms*nxvls)+1):nrow(mcsopath),((nms*nxvls)+1):nrow(mcsopath))= varb(wherem(1,atm):(wherem(1,atm)+nms-1),wherem(1,atm):(wherem(1,atm)+nms-1)).
compute mcsopath(((nms*nxvls)+1):nrow(mcsopath),1)=modres(wherem(1,atm):(wherem(1,atm)+ nms-1),1).
compute sobelok=1.
end if.
end if.
compute obsdirfx=make(1,nxvls,0).
compute dirzes=make(1,nxvls,0).
do if ((i = (nms+nys)) and (bcmat(nrow(bcmat),1)=1)).
compute direff=modres(2:(1+nxvls),:).
compute obsdirfx=t(direff(:,1)).
compute direfflb=modresl.
compute direffl2=vlabsm(2:(1+nxvls),:).
compute lmat=make(nrow(b),1,0).
compute lmat2=make(nxvls,1,1).
compute lmat(2:(1+nxvls),1)=lmat2.
do if (ydich <> 1).

.
compute lmat2= lmat.
do if ( 0 =0).
compute lmat2 = mdiag( lmat ).
compute lmat3=make(nrow(lmat2),1,0).
loop flp=1 to ncol(lmat2).
do if (csum(lmat2(:,flp))=1).
compute lmat3={lmat3,lmat2(:,flp)}.
end if.
end loop.
compute lmat2=lmat3(:,2:ncol(lmat3)).
end if.
compute fratio = (t(t(lmat2)* b )*inv(t(lmat2)* varb *lmat2)*((t(lmat2)* b )))/ncol(lmat2).
compute pfr = 1-fcdf(fratio,ncol(lmat2),(n-nrow( b ))).
compute fresult={fratio,ncol(lmat2),(n-nrow( b )),pfr}.
do if (i = (nms+nys) and (ydich=1)).
compute fratio=fratio*ncol(lmat2).
compute pfr=1-chicdf(fratio,ncol(lmat2)).
compute fresult={fratio,ncol(lmat2),pfr}.
end if.
do if ( 1 =1).
compute lmat3=1-rsum(lmat2).
compute xfm=make(n,csum(lmat3),0).
compute flpc=1.
loop flp=1 to nrow(lmat3).
do if (lmat3(flp,1)=1).
compute xfm(:,flpc)=x(:,flp).
compute flpc=flpc+1.
end if.
end loop.
compute bfm=inv(t(xfm)*xfm)*t(xfm)*y.
compute resid=y-(xfm*bfm).
compute sstotal=(y-(csum(y)/n)).
compute sstotal=csum(sstotal&*sstotal).
compute ssresid=csum(resid&*resid).
compute rsqch= r2 -((sstotal-ssresid)/sstotal).
compute fresult={rsqch,fresult}.
release xfm,flpc, resid, ssresid, bfm.
end if
.
compute diromni=fresult.
end if.
do if (ydich = 1 ).

.
compute btemphld=b.
compute llrdat=make(nrow(x),nrow( lmat )-csum( lmat ),-999).
compute llrdf=ncol(x)-ncol(llrdat).
compute llrcnt=0.
loop llri=1 to nrow( lmat ).
do if ( lmat (llri,1)=0).
compute llrcnt=llrcnt+1.
compute llrdat(:,llrcnt)=x(:,llri).
end if.
end loop.

.
do if ( 2 =1).
compute hatmat=inv(t( llrdat )* llrdat )*t( llrdat ).
compute b = hatmat* y.
compute modres=b.
do if ( 0 =1).
compute n1=nrow( llrdat ).
compute dfres=n1-(ncol( llrdat )).
compute sstotal = t( y -(csum( y )/n1))*( y -(csum( y )/n1)).
compute resid= y - llrdat *b.
compute ssresid = csum((resid)&**2).
compute r2 = (sstotal-ssresid)/sstotal.
do if (r2 < 0).
compute r2=0.
end if.
compute adjr2 = 1-((1-r2)*(n1-1)/(dfres)).
compute mse=ssresid/(n1-ncol( llrdat )).

.
compute n1=nrow( x ).
compute invXtX = inv(t( x )* x ).
compute varb = mse *invXtX.
compute k3 = ncol( x ).
compute xhc=0.
do if ( hc <> 5).
compute xhc= x.
compute hat = xhc(:,1).
loop i3=1 to nrow(xhc).
compute hat(i3,1)= xhc(i3,:)*invXtX*t(xhc(i3,:)).
end loop.
do if ( hc = 0 or hc =1).
loop i3 = 1 to k3.
compute xhc(:,i3)=xhc(:,i3)&* resid.
end loop.
end if.
do if ( hc =3 or hc =2).
loop i3=1 to k3.
compute xhc(:,i3) = ( resid &/(1-hat)&**(1/(4- hc )))&*xhc(:,i3).
end loop.
end if.
do if ( hc = 4).
compute hcmn=make(n,2,4).
compute hcmn(:,2)=(n1*hat)/k3.
loop i3= 1 to k3.
compute xhc(:,i3) = ( resid &/(1-hat)&**(rmin(hcmn)/2))&*xhc(:,i3).
end loop.
end if.
compute varb=(invXtX*t(xhc)*xhc*invXtX).
do if ( hc =1).
compute varb=(n1/(n1-ncol( x )))&*varb.
end if.
end if.
compute hclab={'se(HC0)','se(HC1)','se(HC2)','se(HC3)','se(HC4)','se'}.
compute hclab=hclab(1,( hc +1)).
compute hcflab={'F(HC0)','F(HC1)','F(HC2)','F(HC3)','F(HC4)','F'}.
compute hcflab=hcflab(1,( hc +1)).
release xhc
.
compute seb=sqrt(diag(varb)).
compute trat = b&/seb.
compute p = 2*(1-tcdf(abs(trat), (dfres))).
compute tval = sqrt(dfres* (exp((dfres-(5/6))*((xp2/(dfres-(2/3)+(.11/dfres)))* (xp2/(dfres-(2/3)+(.11/dfres)))))-1)).
compute modres={modres,seb,trat,p}.
compute modres={modres,(b-tval&*seb),(b+tval&*seb)}.
compute modresl={'coeff',hclab,'t','p','LLCI','ULCI'}.
compute lmat = ident(ncol( llrdat )).
compute lmat = lmat(:,2:ncol(lmat)).
compute fratio = (t(t(lmat)*b)*inv(t(lmat)*varb*lmat)*((t(lmat)*b)))/(ncol( llrdat )-1).
compute pfr = 1-fcdf(fratio,(ncol( llrdat )-1),dfres).
compute modsum={sqrt(r2),r2,mse,fratio,(ncol( llrdat )-1),dfres,pfr}.
compute modsuml={'R','R-sq','MSE',hcflab,'df1','df2', 'p'}.
end if.
end if.
do if ( 2 = 2 or 2 =3).
compute xlp= llrdat.
compute ylp= y.
compute pt2 = make(nrow(ylp),1,(csum(ylp)/nrow(ylp))).
do if ( 2 =2).
compute LL3 = ylp&*ln(pt2)+(1-ylp)&*ln(1-pt2).
end if.
compute LL3 = -2*csum(LL3).
compute bt1 = make(ncol(xlp),1,0).
compute LL1 = 0.
compute pt1 = make(nrow(ylp),1,0.5).
compute pt1lp=pt1.
loop jjj = 1 to iterate.
compute xlptmp=t(xlp).
compute vecprb=pt1lp&*(1-pt1lp).
loop kkk=1 to ncol(xlp).
compute xlptmp(kkk,:)=xlptmp(kkk,:)&*t(vecprb).
end loop.
compute b = bt1+inv(xlptmp*xlp)*t(xlp)*(ylp-pt1lp).
do if ( 2 =2).
compute xlpb=xlp*b.
compute xlpbt=(xlpb > -709.7).
compute xlpb709=(1-xlpbt)*(-709.7).
compute xlpb=(xlpb&*xlpbt)+xlpb709.
compute pt1lp = 1/(1+exp(-(xlpb))).
end if.
compute itprob = csum((pt1lp < .00000001) or (pt1lp > .9999999)).
do if (itprob > 0).
loop kkk = 1 to nrow(pt1lp).
do if (pt1lp(kkk,1) > .9999999).
compute pt1lp(kkk,1) = .9999999.
end if.
do if (pt1lp(kkk,1) < .00000001).
compute pt1lp(kkk,1) = .00000001.
end if.
end loop.
compute itprob = 0.
end if.
do if (itprob = 0).
do if ( 2 =2).
compute LL = ylp&*ln(pt1lp)+(1-ylp)&*ln(1-pt1lp).
end if.
compute LL2 = -2*csum(ll).
end if.
do if (abs(LL1-LL2) < converge).
do if ( 0 =1).
compute xlptmp=t(xlp).
compute vecprb=pt1lp&*(1-pt1lp).
loop kkk=1 to ncol(xlp).
compute xlptmp(kkk,:)=xlptmp(kkk,:)&*t(vecprb).
end loop.
compute varb = inv(xlptmp*xlp).
compute seb = sqrt(diag(varb)).
release xlptmp.
end if.
break.
end if.
compute bt1 = b.
compute LL1 = LL2.
end loop.
compute modres=b.
do if (jjj > iterate).
compute itprob = 2.
do if (booting=0).
compute iterrmod=1.
end if.
do if (booting=1).
compute bootiter=1.
end if.
do if (itprobtg=0).
compute itprobtg=1.
compute errcode(errs,1) = 47.
compute errs = errs + 1.
do if (booting = 0 and 0 =1).
compute vt1 = mdiag(pt1lp&*(1-pt1lp)).
compute varb = inv(t(xlp)*vt1*xlp).
compute seb = sqrt(diag(varb)).
end if.
end if.
end if.
do if ( 0 =1).
compute trat = b&/seb.
compute dfres=nrow(xlp).
compute p = 2*(1-cdfnorm(abs(trat))).
compute modres={modres,seb,trat,p}.
compute modres={modres,(b-xp2&*seb),(b+xp2&*seb)}.
compute pvchi=1-chicdf((LL3-LL2),(nrow(modres)-1)).
compute mcF = (LL3-LL2)/LL3.
compute cox = 1-exp(-(LL3-LL2)/nrow(xlp)).
compute nagel = cox/(1-exp(-(LL3)/nrow(xlp))).
compute modsum={LL2,(LL3-LL2),(nrow(modres)-1),pvchi, mcF,cox,nagel}.
compute modsuml={'-2LL','ModelLL', 'df', 'p', 'McFadden', 'CoxSnell', 'Nagelkrk'}.
compute modresl={'coeff','se','Z','p','LLCI','ULCI'}.
end if.
end if
.
compute b=btemphld.
compute fresult={(LL2-basemod),llrdf,1-chicdf((LL2-basemod),llrdf)}
.
compute diromni=fresult.
end if.
end if.
do if (numint(1,i) > 0).
compute intkeym=intkey(intstart:(intstart+numint(1,i)-1),:).
do if (outscree=1).
print intkeym/title='Product terms key:'/format = A8.
end if.
end if.
do if (covcoeff=1).
do if (outscree=1).
print varb/title='Covariance matrix of regression parameter estimates:'/rnames=vlabsm /cnames=vlabsm/format= F10.4.
end if.
compute resultm2=make(nrow(varb),maxresm,99999).
do if (ncol(varb) <= maxresm).
compute resultm2(1:nrow(varb),1:ncol(varb))=varb.
compute resultm={resultm;resultm2}.
end if.
do if (ncol(varb) > maxresm).
compute resultmt=make(nrow(resultm),ncol(varb),99999).
compute resultmt(1:nrow(resultm),1:ncol(resultm))=resultm.
compute resultm=resultmt.
compute resultm2=make(nrow(varb),ncol(resultm),99999).
compute resultm2(1:nrow(varb),1:ncol(varb))=varb.
compute resultm={resultm;resultm2}.
compute maxresm=ncol(resultm).
end if.
end if.
do if (model <> 74 and xmtest=1 and nms > 0).
compute r2tmp=r2.
compute btmp=b.
compute varbtmp=varb.
compute dfrestmp=dfres.
compute tvaltmp=tval.
compute xmtst=make(nms,4,0).
compute xmtstlbc={hcflab,'df1','df2','p'}.
do if ((i=(nms+nys)) and (ydich=1)).
compute xmtst=make(nms,3,0).
compute xmtstlbc={'Chi-sq','df','p'}.
end if.
compute xmtstlb={' '}.
compute xmtmat=x.
compute numxint=0.
loop xmints=2 to i.
compute x=xmtmat.
do if ((bcmat((i+1),xmints)=1) and (wzcmat((i+1),xmints) <> 1)).
do if (bcmat((i+1),1)=0).
compute x={xmtmat,xtmp}.
do if ((ydich=1) and (i=(nms+nys))).

.
do if ( 2 =1).
compute hatmat=inv(t( x )* x )*t( x ).
compute b = hatmat* y.
compute modres=b.
do if ( 1 =1).
compute n1=nrow( x ).
compute dfres=n1-(ncol( x )).
compute sstotal = t( y -(csum( y )/n1))*( y -(csum( y )/n1)).
compute resid= y - x *b.
compute ssresid = csum((resid)&**2).
compute r2 = (sstotal-ssresid)/sstotal.
do if (r2 < 0).
compute r2=0.
end if.
compute adjr2 = 1-((1-r2)*(n1-1)/(dfres)).
compute mse=ssresid/(n1-ncol( x )).

.
compute n1=nrow( x ).
compute invXtX = inv(t( x )* x ).
compute varb = mse *invXtX.
compute k3 = ncol( x ).
compute xhc=0.
do if ( hc <> 5).
compute xhc= x.
compute hat = xhc(:,1).
loop i3=1 to nrow(xhc).
compute hat(i3,1)= xhc(i3,:)*invXtX*t(xhc(i3,:)).
end loop.
do if ( hc = 0 or hc =1).
loop i3 = 1 to k3.
compute xhc(:,i3)=xhc(:,i3)&* resid.
end loop.
end if.
do if ( hc =3 or hc =2).
loop i3=1 to k3.
compute xhc(:,i3) = ( resid &/(1-hat)&**(1/(4- hc )))&*xhc(:,i3).
end loop.
end if.
do if ( hc = 4).
compute hcmn=make(n,2,4).
compute hcmn(:,2)=(n1*hat)/k3.
loop i3= 1 to k3.
compute xhc(:,i3) = ( resid &/(1-hat)&**(rmin(hcmn)/2))&*xhc(:,i3).
end loop.
end if.
compute varb=(invXtX*t(xhc)*xhc*invXtX).
do if ( hc =1).
compute varb=(n1/(n1-ncol( x )))&*varb.
end if.
end if.
compute hclab={'se(HC0)','se(HC1)','se(HC2)','se(HC3)','se(HC4)','se'}.
compute hclab=hclab(1,( hc +1)).
compute hcflab={'F(HC0)','F(HC1)','F(HC2)','F(HC3)','F(HC4)','F'}.
compute hcflab=hcflab(1,( hc +1)).
release xhc
.
compute seb=sqrt(diag(varb)).
compute trat = b&/seb.
compute p = 2*(1-tcdf(abs(trat), (dfres))).
compute tval = sqrt(dfres* (exp((dfres-(5/6))*((xp2/(dfres-(2/3)+(.11/dfres)))* (xp2/(dfres-(2/3)+(.11/dfres)))))-1)).
compute modres={modres,seb,trat,p}.
compute modres={modres,(b-tval&*seb),(b+tval&*seb)}.
compute modresl={'coeff',hclab,'t','p','LLCI','ULCI'}.
compute lmat = ident(ncol( x )).
compute lmat = lmat(:,2:ncol(lmat)).
compute fratio = (t(t(lmat)*b)*inv(t(lmat)*varb*lmat)*((t(lmat)*b)))/(ncol( x )-1).
compute pfr = 1-fcdf(fratio,(ncol( x )-1),dfres).
compute modsum={sqrt(r2),r2,mse,fratio,(ncol( x )-1),dfres,pfr}.
compute modsuml={'R','R-sq','MSE',hcflab,'df1','df2', 'p'}.
end if.
end if.
do if ( 2 = 2 or 2 =3).
compute xlp= x.
compute ylp= y.
compute pt2 = make(nrow(ylp),1,(csum(ylp)/nrow(ylp))).
do if ( 2 =2).
compute LL3 = ylp&*ln(pt2)+(1-ylp)&*ln(1-pt2).
end if.
compute LL3 = -2*csum(LL3).
compute bt1 = make(ncol(xlp),1,0).
compute LL1 = 0.
compute pt1 = make(nrow(ylp),1,0.5).
compute pt1lp=pt1.
loop jjj = 1 to iterate.
compute xlptmp=t(xlp).
compute vecprb=pt1lp&*(1-pt1lp).
loop kkk=1 to ncol(xlp).
compute xlptmp(kkk,:)=xlptmp(kkk,:)&*t(vecprb).
end loop.
compute b = bt1+inv(xlptmp*xlp)*t(xlp)*(ylp-pt1lp).
do if ( 2 =2).
compute xlpb=xlp*b.
compute xlpbt=(xlpb > -709.7).
compute xlpb709=(1-xlpbt)*(-709.7).
compute xlpb=(xlpb&*xlpbt)+xlpb709.
compute pt1lp = 1/(1+exp(-(xlpb))).
end if.
compute itprob = csum((pt1lp < .00000001) or (pt1lp > .9999999)).
do if (itprob > 0).
loop kkk = 1 to nrow(pt1lp).
do if (pt1lp(kkk,1) > .9999999).
compute pt1lp(kkk,1) = .9999999.
end if.
do if (pt1lp(kkk,1) < .00000001).
compute pt1lp(kkk,1) = .00000001.
end if.
end loop.
compute itprob = 0.
end if.
do if (itprob = 0).
do if ( 2 =2).
compute LL = ylp&*ln(pt1lp)+(1-ylp)&*ln(1-pt1lp).
end if.
compute LL2 = -2*csum(ll).
end if.
do if (abs(LL1-LL2) < converge).
do if ( 1 =1).
compute xlptmp=t(xlp).
compute vecprb=pt1lp&*(1-pt1lp).
loop kkk=1 to ncol(xlp).
compute xlptmp(kkk,:)=xlptmp(kkk,:)&*t(vecprb).
end loop.
compute varb = inv(xlptmp*xlp).
compute seb = sqrt(diag(varb)).
release xlptmp.
end if.
break.
end if.
compute bt1 = b.
compute LL1 = LL2.
end loop.
compute modres=b.
do if (jjj > iterate).
compute itprob = 2.
do if (booting=0).
compute iterrmod=1.
end if.
do if (booting=1).
compute bootiter=1.
end if.
do if (itprobtg=0).
compute itprobtg=1.
compute errcode(errs,1) = 47.
compute errs = errs + 1.
do if (booting = 0 and 1 =1).
compute vt1 = mdiag(pt1lp&*(1-pt1lp)).
compute varb = inv(t(xlp)*vt1*xlp).
compute seb = sqrt(diag(varb)).
end if.
end if.
end if.
do if ( 1 =1).
compute trat = b&/seb.
compute dfres=nrow(xlp).
compute p = 2*(1-cdfnorm(abs(trat))).
compute modres={modres,seb,trat,p}.
compute modres={modres,(b-xp2&*seb),(b+xp2&*seb)}.
compute pvchi=1-chicdf((LL3-LL2),(nrow(modres)-1)).
compute mcF = (LL3-LL2)/LL3.
compute cox = 1-exp(-(LL3-LL2)/nrow(xlp)).
compute nagel = cox/(1-exp(-(LL3)/nrow(xlp))).
compute modsum={LL2,(LL3-LL2),(nrow(modres)-1),pvchi, mcF,cox,nagel}.
compute modsuml={'-2LL','ModelLL', 'df', 'p', 'McFadden', 'CoxSnell', 'Nagelkrk'}.
compute modresl={'coeff','se','Z','p','LLCI','ULCI'}.
end if.
end if
.
compute basemodx=LL2.
end if.
end if.
loop xmtlp1=1 to nxvls.
compute x={x,xtmp(:,xmtlp1)&*(mtmp(:,(xmints-1))-(csum(mtmp(:,(xmints-1)))/nrow(mtmp)) )}.
end loop.
do if ((i < (nms+nys)) or (ydich=0)).

.
do if ( 1 =1).
compute hatmat=inv(t( x )* x )*t( x ).
compute b = hatmat* y.
compute modres=b.
do if ( 1 =1).
compute n1=nrow( x ).
compute dfres=n1-(ncol( x )).
compute sstotal = t( y -(csum( y )/n1))*( y -(csum( y )/n1)).
compute resid= y - x *b.
compute ssresid = csum((resid)&**2).
compute r2 = (sstotal-ssresid)/sstotal.
do if (r2 < 0).
compute r2=0.
end if.
compute adjr2 = 1-((1-r2)*(n1-1)/(dfres)).
compute mse=ssresid/(n1-ncol( x )).

.
compute n1=nrow( x ).
compute invXtX = inv(t( x )* x ).
compute varb = mse *invXtX.
compute k3 = ncol( x ).
compute xhc=0.
do if ( hc <> 5).
compute xhc= x.
compute hat = xhc(:,1).
loop i3=1 to nrow(xhc).
compute hat(i3,1)= xhc(i3,:)*invXtX*t(xhc(i3,:)).
end loop.
do if ( hc = 0 or hc =1).
loop i3 = 1 to k3.
compute xhc(:,i3)=xhc(:,i3)&* resid.
end loop.
end if.
do if ( hc =3 or hc =2).
loop i3=1 to k3.
compute xhc(:,i3) = ( resid &/(1-hat)&**(1/(4- hc )))&*xhc(:,i3).
end loop.
end if.
do if ( hc = 4).
compute hcmn=make(n,2,4).
compute hcmn(:,2)=(n1*hat)/k3.
loop i3= 1 to k3.
compute xhc(:,i3) = ( resid &/(1-hat)&**(rmin(hcmn)/2))&*xhc(:,i3).
end loop.
end if.
compute varb=(invXtX*t(xhc)*xhc*invXtX).
do if ( hc =1).
compute varb=(n1/(n1-ncol( x )))&*varb.
end if.
end if.
compute hclab={'se(HC0)','se(HC1)','se(HC2)','se(HC3)','se(HC4)','se'}.
compute hclab=hclab(1,( hc +1)).
compute hcflab={'F(HC0)','F(HC1)','F(HC2)','F(HC3)','F(HC4)','F'}.
compute hcflab=hcflab(1,( hc +1)).
release xhc
.
compute seb=sqrt(diag(varb)).
compute trat = b&/seb.
compute p = 2*(1-tcdf(abs(trat), (dfres))).
compute tval = sqrt(dfres* (exp((dfres-(5/6))*((xp2/(dfres-(2/3)+(.11/dfres)))* (xp2/(dfres-(2/3)+(.11/dfres)))))-1)).
compute modres={modres,seb,trat,p}.
compute modres={modres,(b-tval&*seb),(b+tval&*seb)}.
compute modresl={'coeff',hclab,'t','p','LLCI','ULCI'}.
compute lmat = ident(ncol( x )).
compute lmat = lmat(:,2:ncol(lmat)).
compute fratio = (t(t(lmat)*b)*inv(t(lmat)*varb*lmat)*((t(lmat)*b)))/(ncol( x )-1).
compute pfr = 1-fcdf(fratio,(ncol( x )-1),dfres).
compute modsum={sqrt(r2),r2,mse,fratio,(ncol( x )-1),dfres,pfr}.
compute modsuml={'R','R-sq','MSE',hcflab,'df1','df2', 'p'}.
end if.
end if.
do if ( 1 = 2 or 1 =3).
compute xlp= x.
compute ylp= y.
compute pt2 = make(nrow(ylp),1,(csum(ylp)/nrow(ylp))).
do if ( 1 =2).
compute LL3 = ylp&*ln(pt2)+(1-ylp)&*ln(1-pt2).
end if.
compute LL3 = -2*csum(LL3).
compute bt1 = make(ncol(xlp),1,0).
compute LL1 = 0.
compute pt1 = make(nrow(ylp),1,0.5).
compute pt1lp=pt1.
loop jjj = 1 to iterate.
compute xlptmp=t(xlp).
compute vecprb=pt1lp&*(1-pt1lp).
loop kkk=1 to ncol(xlp).
compute xlptmp(kkk,:)=xlptmp(kkk,:)&*t(vecprb).
end loop.
compute b = bt1+inv(xlptmp*xlp)*t(xlp)*(ylp-pt1lp).
do if ( 1 =2).
compute xlpb=xlp*b.
compute xlpbt=(xlpb > -709.7).
compute xlpb709=(1-xlpbt)*(-709.7).
compute xlpb=(xlpb&*xlpbt)+xlpb709.
compute pt1lp = 1/(1+exp(-(xlpb))).
end if.
compute itprob = csum((pt1lp < .00000001) or (pt1lp > .9999999)).
do if (itprob > 0).
loop kkk = 1 to nrow(pt1lp).
do if (pt1lp(kkk,1) > .9999999).
compute pt1lp(kkk,1) = .9999999.
end if.
do if (pt1lp(kkk,1) < .00000001).
compute pt1lp(kkk,1) = .00000001.
end if.
end loop.
compute itprob = 0.
end if.
do if (itprob = 0).
do if ( 1 =2).
compute LL = ylp&*ln(pt1lp)+(1-ylp)&*ln(1-pt1lp).
end if.
compute LL2 = -2*csum(ll).
end if.
do if (abs(LL1-LL2) < converge).
do if ( 1 =1).
compute xlptmp=t(xlp).
compute vecprb=pt1lp&*(1-pt1lp).
loop kkk=1 to ncol(xlp).
compute xlptmp(kkk,:)=xlptmp(kkk,:)&*t(vecprb).
end loop.
compute varb = inv(xlptmp*xlp).
compute seb = sqrt(diag(varb)).
release xlptmp.
end if.
break.
end if.
compute bt1 = b.
compute LL1 = LL2.
end loop.
compute modres=b.
do if (jjj > iterate).
compute itprob = 2.
do if (booting=0).
compute iterrmod=1.
end if.
do if (booting=1).
compute bootiter=1.
end if.
do if (itprobtg=0).
compute itprobtg=1.
compute errcode(errs,1) = 47.
compute errs = errs + 1.
do if (booting = 0 and 1 =1).
compute vt1 = mdiag(pt1lp&*(1-pt1lp)).
compute varb = inv(t(xlp)*vt1*xlp).
compute seb = sqrt(diag(varb)).
end if.
end if.
end if.
do if ( 1 =1).
compute trat = b&/seb.
compute dfres=nrow(xlp).
compute p = 2*(1-cdfnorm(abs(trat))).
compute modres={modres,seb,trat,p}.
compute modres={modres,(b-xp2&*seb),(b+xp2&*seb)}.
compute pvchi=1-chicdf((LL3-LL2),(nrow(modres)-1)).
compute mcF = (LL3-LL2)/LL3.
compute cox = 1-exp(-(LL3-LL2)/nrow(xlp)).
compute nagel = cox/(1-exp(-(LL3)/nrow(xlp))).
compute modsum={LL2,(LL3-LL2),(nrow(modres)-1),pvchi, mcF,cox,nagel}.
compute modsuml={'-2LL','ModelLL', 'df', 'p', 'McFadden', 'CoxSnell', 'Nagelkrk'}.
compute modresl={'coeff','se','Z','p','LLCI','ULCI'}.
end if.
end if
.
end if.
do if ((i = (nms+nys)) and (ydich=1)).

.
do if ( 2 =1).
compute hatmat=inv(t( x )* x )*t( x ).
compute b = hatmat* y.
compute modres=b.
do if ( 1 =1).
compute n1=nrow( x ).
compute dfres=n1-(ncol( x )).
compute sstotal = t( y -(csum( y )/n1))*( y -(csum( y )/n1)).
compute resid= y - x *b.
compute ssresid = csum((resid)&**2).
compute r2 = (sstotal-ssresid)/sstotal.
do if (r2 < 0).
compute r2=0.
end if.
compute adjr2 = 1-((1-r2)*(n1-1)/(dfres)).
compute mse=ssresid/(n1-ncol( x )).

.
compute n1=nrow( x ).
compute invXtX = inv(t( x )* x ).
compute varb = mse *invXtX.
compute k3 = ncol( x ).
compute xhc=0.
do if ( hc <> 5).
compute xhc= x.
compute hat = xhc(:,1).
loop i3=1 to nrow(xhc).
compute hat(i3,1)= xhc(i3,:)*invXtX*t(xhc(i3,:)).
end loop.
do if ( hc = 0 or hc =1).
loop i3 = 1 to k3.
compute xhc(:,i3)=xhc(:,i3)&* resid.
end loop.
end if.
do if ( hc =3 or hc =2).
loop i3=1 to k3.
compute xhc(:,i3) = ( resid &/(1-hat)&**(1/(4- hc )))&*xhc(:,i3).
end loop.
end if.
do if ( hc = 4).
compute hcmn=make(n,2,4).
compute hcmn(:,2)=(n1*hat)/k3.
loop i3= 1 to k3.
compute xhc(:,i3) = ( resid &/(1-hat)&**(rmin(hcmn)/2))&*xhc(:,i3).
end loop.
end if.
compute varb=(invXtX*t(xhc)*xhc*invXtX).
do if ( hc =1).
compute varb=(n1/(n1-ncol( x )))&*varb.
end if.
end if.
compute hclab={'se(HC0)','se(HC1)','se(HC2)','se(HC3)','se(HC4)','se'}.
compute hclab=hclab(1,( hc +1)).
compute hcflab={'F(HC0)','F(HC1)','F(HC2)','F(HC3)','F(HC4)','F'}.
compute hcflab=hcflab(1,( hc +1)).
release xhc
.
compute seb=sqrt(diag(varb)).
compute trat = b&/seb.
compute p = 2*(1-tcdf(abs(trat), (dfres))).
compute tval = sqrt(dfres* (exp((dfres-(5/6))*((xp2/(dfres-(2/3)+(.11/dfres)))* (xp2/(dfres-(2/3)+(.11/dfres)))))-1)).
compute modres={modres,seb,trat,p}.
compute modres={modres,(b-tval&*seb),(b+tval&*seb)}.
compute modresl={'coeff',hclab,'t','p','LLCI','ULCI'}.
compute lmat = ident(ncol( x )).
compute lmat = lmat(:,2:ncol(lmat)).
compute fratio = (t(t(lmat)*b)*inv(t(lmat)*varb*lmat)*((t(lmat)*b)))/(ncol( x )-1).
compute pfr = 1-fcdf(fratio,(ncol( x )-1),dfres).
compute modsum={sqrt(r2),r2,mse,fratio,(ncol( x )-1),dfres,pfr}.
compute modsuml={'R','R-sq','MSE',hcflab,'df1','df2', 'p'}.
end if.
end if.
do if ( 2 = 2 or 2 =3).
compute xlp= x.
compute ylp= y.
compute pt2 = make(nrow(ylp),1,(csum(ylp)/nrow(ylp))).
do if ( 2 =2).
compute LL3 = ylp&*ln(pt2)+(1-ylp)&*ln(1-pt2).
end if.
compute LL3 = -2*csum(LL3).
compute bt1 = make(ncol(xlp),1,0).
compute LL1 = 0.
compute pt1 = make(nrow(ylp),1,0.5).
compute pt1lp=pt1.
loop jjj = 1 to iterate.
compute xlptmp=t(xlp).
compute vecprb=pt1lp&*(1-pt1lp).
loop kkk=1 to ncol(xlp).
compute xlptmp(kkk,:)=xlptmp(kkk,:)&*t(vecprb).
end loop.
compute b = bt1+inv(xlptmp*xlp)*t(xlp)*(ylp-pt1lp).
do if ( 2 =2).
compute xlpb=xlp*b.
compute xlpbt=(xlpb > -709.7).
compute xlpb709=(1-xlpbt)*(-709.7).
compute xlpb=(xlpb&*xlpbt)+xlpb709.
compute pt1lp = 1/(1+exp(-(xlpb))).
end if.
compute itprob = csum((pt1lp < .00000001) or (pt1lp > .9999999)).
do if (itprob > 0).
loop kkk = 1 to nrow(pt1lp).
do if (pt1lp(kkk,1) > .9999999).
compute pt1lp(kkk,1) = .9999999.
end if.
do if (pt1lp(kkk,1) < .00000001).
compute pt1lp(kkk,1) = .00000001.
end if.
end loop.
compute itprob = 0.
end if.
do if (itprob = 0).
do if ( 2 =2).
compute LL = ylp&*ln(pt1lp)+(1-ylp)&*ln(1-pt1lp).
end if.
compute LL2 = -2*csum(ll).
end if.
do if (abs(LL1-LL2) < converge).
do if ( 1 =1).
compute xlptmp=t(xlp).
compute vecprb=pt1lp&*(1-pt1lp).
loop kkk=1 to ncol(xlp).
compute xlptmp(kkk,:)=xlptmp(kkk,:)&*t(vecprb).
end loop.
compute varb = inv(xlptmp*xlp).
compute seb = sqrt(diag(varb)).
release xlptmp.
end if.
break.
end if.
compute bt1 = b.
compute LL1 = LL2.
end loop.
compute modres=b.
do if (jjj > iterate).
compute itprob = 2.
do if (booting=0).
compute iterrmod=1.
end if.
do if (booting=1).
compute bootiter=1.
end if.
do if (itprobtg=0).
compute itprobtg=1.
compute errcode(errs,1) = 47.
compute errs = errs + 1.
do if (booting = 0 and 1 =1).
compute vt1 = mdiag(pt1lp&*(1-pt1lp)).
compute varb = inv(t(xlp)*vt1*xlp).
compute seb = sqrt(diag(varb)).
end if.
end if.
end if.
do if ( 1 =1).
compute trat = b&/seb.
compute dfres=nrow(xlp).
compute p = 2*(1-cdfnorm(abs(trat))).
compute modres={modres,seb,trat,p}.
compute modres={modres,(b-xp2&*seb),(b+xp2&*seb)}.
compute pvchi=1-chicdf((LL3-LL2),(nrow(modres)-1)).
compute mcF = (LL3-LL2)/LL3.
compute cox = 1-exp(-(LL3-LL2)/nrow(xlp)).
compute nagel = cox/(1-exp(-(LL3)/nrow(xlp))).
compute modsum={LL2,(LL3-LL2),(nrow(modres)-1),pvchi, mcF,cox,nagel}.
compute modsuml={'-2LL','ModelLL', 'df', 'p', 'McFadden', 'CoxSnell', 'Nagelkrk'}.
compute modresl={'coeff','se','Z','p','LLCI','ULCI'}.
end if.
end if
.
compute chidfxm=basemodx-LL2.
end if.
compute lmat=make(nrow(b),nxvls,0).
compute lmattmp=ident(nxvls).
compute lmat((nrow(lmat)-nxvls+1):nrow(lmat),:)=lmattmp.

.
compute lmat2= lmat.
do if ( 1 =0).
compute lmat2 = mdiag( lmat ).
compute lmat3=make(nrow(lmat2),1,0).
loop flp=1 to ncol(lmat2).
do if (csum(lmat2(:,flp))=1).
compute lmat3={lmat3,lmat2(:,flp)}.
end if.
end loop.
compute lmat2=lmat3(:,2:ncol(lmat3)).
end if.
compute fratio = (t(t(lmat2)* b )*inv(t(lmat2)* varb *lmat2)*((t(lmat2)* b )))/ncol(lmat2).
compute pfr = 1-fcdf(fratio,ncol(lmat2),(n-nrow( b ))).
compute fresult={fratio,ncol(lmat2),(n-nrow( b )),pfr}.
do if (i = (nms+nys) and (ydich=1)).
compute fratio=fratio*ncol(lmat2).
compute pfr=1-chicdf(fratio,ncol(lmat2)).
compute fresult={fratio,ncol(lmat2),pfr}.
end if.
do if ( 0 =1).
compute lmat3=1-rsum(lmat2).
compute xfm=make(n,csum(lmat3),0).
compute flpc=1.
loop flp=1 to nrow(lmat3).
do if (lmat3(flp,1)=1).
compute xfm(:,flpc)=x(:,flp).
compute flpc=flpc+1.
end if.
end loop.
compute bfm=inv(t(xfm)*xfm)*t(xfm)*y.
compute resid=y-(xfm*bfm).
compute sstotal=(y-(csum(y)/n)).
compute sstotal=csum(sstotal&*sstotal).
compute ssresid=csum(resid&*resid).
compute rsqch= 0 -((sstotal-ssresid)/sstotal).
compute fresult={rsqch,fresult}.
release xfm,flpc, resid, ssresid, bfm.
end if
.
compute numxint=numxint+1.
compute xmtst(numxint,:)=fresult.
do if ((i = (nms+nys)) and (ydich=1)).
compute xmtst(numxint,1)=chidfxm.
compute xmtst(numxint,3)=1-chicdf(chidfxm,nxvls).
end if.
compute xmtstlb={xmtstlb;highlbx((xmints-1),1)}.
end if.
end loop.
compute x=xmtmat.
release xmtmat.
do if (numxint>0).
compute xmtstlb=xmtstlb((2:(numxint+1)),:).
compute xmtst=xmtst(1:numxint,:).
compute resultm2=make(nrow(xmtst),maxresm,99999).
compute resultm2(1:nrow(xmtst),1:ncol(xmtst))=xmtst.
compute resultm={resultm;resultm2}.
do if (nms=1).
compute xmtstlb=' '.
end if.
do if (outscree=1).
do if ((i < (nms+nys)) or (ydich=0)).
print xmtst/title='Test(s) of X by M interaction:'/rnames=xmtstlb/cnames=xmtstlbc/format= F10.4.
end if.
do if ((i = (nms+nys)) and (ydich=1)).
print xmtst/title='Likelihood ratio test(s) of X by M interaction:'/rnames=xmtstlb/cnames=xmtstlbc/format= F10.4.
end if.
end if.
end if.
compute r2=r2tmp.
compute b=btmp.
compute varb=varbtmp.
compute dfres=dfrestmp.
compute tval=tvaltmp.
end if.
do if (criterr = 0).
compute jj=0.
loop j = start to ((start+i)-1).
compute dbint=0.
compute lmat=whigh(1:nump(1,i),j).
compute lmat2=wzhigh(1:nump(1,i),j).
do if ((csum(lmat) > 0) and (csum(lmat2) = 0)).
do if ((i < (nms+nys)) or (ydich <> 1)).

.
compute lmat2= lmat.
do if ( 0 =0).
compute lmat2 = mdiag( lmat ).
compute lmat3=make(nrow(lmat2),1,0).
loop flp=1 to ncol(lmat2).
do if (csum(lmat2(:,flp))=1).
compute lmat3={lmat3,lmat2(:,flp)}.
end if.
end loop.
compute lmat2=lmat3(:,2:ncol(lmat3)).
end if.
compute fratio = (t(t(lmat2)* b )*inv(t(lmat2)* varb *lmat2)*((t(lmat2)* b )))/ncol(lmat2).
compute pfr = 1-fcdf(fratio,ncol(lmat2),(n-nrow( b ))).
compute fresult={fratio,ncol(lmat2),(n-nrow( b )),pfr}.
do if (i = (nms+nys) and (ydich=1)).
compute fratio=fratio*ncol(lmat2).
compute pfr=1-chicdf(fratio,ncol(lmat2)).
compute fresult={fratio,ncol(lmat2),pfr}.
end if.
do if ( 1 =1).
compute lmat3=1-rsum(lmat2).
compute xfm=make(n,csum(lmat3),0).
compute flpc=1.
loop flp=1 to nrow(lmat3).
do if (lmat3(flp,1)=1).
compute xfm(:,flpc)=x(:,flp).
compute flpc=flpc+1.
end if.
end loop.
compute bfm=inv(t(xfm)*xfm)*t(xfm)*y.
compute resid=y-(xfm*bfm).
compute sstotal=(y-(csum(y)/n)).
compute sstotal=csum(sstotal&*sstotal).
compute ssresid=csum(resid&*resid).
compute rsqch= r2 -((sstotal-ssresid)/sstotal).
compute fresult={rsqch,fresult}.
release xfm,flpc, resid, ssresid, bfm.
end if
.
compute lmatdb=lmat.
compute dbint=dbint+1.
end if.
do if ((ydich = 1) and (i = (nms+nys))).

.
compute btemphld=b.
compute llrdat=make(nrow(x),nrow( lmat )-csum( lmat ),-999).
compute llrdf=ncol(x)-ncol(llrdat).
compute llrcnt=0.
loop llri=1 to nrow( lmat ).
do if ( lmat (llri,1)=0).
compute llrcnt=llrcnt+1.
compute llrdat(:,llrcnt)=x(:,llri).
end if.
end loop.

.
do if ( 2 =1).
compute hatmat=inv(t( llrdat )* llrdat )*t( llrdat ).
compute b = hatmat* y.
compute modres=b.
do if ( 0 =1).
compute n1=nrow( llrdat ).
compute dfres=n1-(ncol( llrdat )).
compute sstotal = t( y -(csum( y )/n1))*( y -(csum( y )/n1)).
compute resid= y - llrdat *b.
compute ssresid = csum((resid)&**2).
compute r2 = (sstotal-ssresid)/sstotal.
do if (r2 < 0).
compute r2=0.
end if.
compute adjr2 = 1-((1-r2)*(n1-1)/(dfres)).
compute mse=ssresid/(n1-ncol( llrdat )).

.
compute n1=nrow( x ).
compute invXtX = inv(t( x )* x ).
compute varb = mse *invXtX.
compute k3 = ncol( x ).
compute xhc=0.
do if ( hc <> 5).
compute xhc= x.
compute hat = xhc(:,1).
loop i3=1 to nrow(xhc).
compute hat(i3,1)= xhc(i3,:)*invXtX*t(xhc(i3,:)).
end loop.
do if ( hc = 0 or hc =1).
loop i3 = 1 to k3.
compute xhc(:,i3)=xhc(:,i3)&* resid.
end loop.
end if.
do if ( hc =3 or hc =2).
loop i3=1 to k3.
compute xhc(:,i3) = ( resid &/(1-hat)&**(1/(4- hc )))&*xhc(:,i3).
end loop.
end if.
do if ( hc = 4).
compute hcmn=make(n,2,4).
compute hcmn(:,2)=(n1*hat)/k3.
loop i3= 1 to k3.
compute xhc(:,i3) = ( resid &/(1-hat)&**(rmin(hcmn)/2))&*xhc(:,i3).
end loop.
end if.
compute varb=(invXtX*t(xhc)*xhc*invXtX).
do if ( hc =1).
compute varb=(n1/(n1-ncol( x )))&*varb.
end if.
end if.
compute hclab={'se(HC0)','se(HC1)','se(HC2)','se(HC3)','se(HC4)','se'}.
compute hclab=hclab(1,( hc +1)).
compute hcflab={'F(HC0)','F(HC1)','F(HC2)','F(HC3)','F(HC4)','F'}.
compute hcflab=hcflab(1,( hc +1)).
release xhc
.
compute seb=sqrt(diag(varb)).
compute trat = b&/seb.
compute p = 2*(1-tcdf(abs(trat), (dfres))).
compute tval = sqrt(dfres* (exp((dfres-(5/6))*((xp2/(dfres-(2/3)+(.11/dfres)))* (xp2/(dfres-(2/3)+(.11/dfres)))))-1)).
compute modres={modres,seb,trat,p}.
compute modres={modres,(b-tval&*seb),(b+tval&*seb)}.
compute modresl={'coeff',hclab,'t','p','LLCI','ULCI'}.
compute lmat = ident(ncol( llrdat )).
compute lmat = lmat(:,2:ncol(lmat)).
compute fratio = (t(t(lmat)*b)*inv(t(lmat)*varb*lmat)*((t(lmat)*b)))/(ncol( llrdat )-1).
compute pfr = 1-fcdf(fratio,(ncol( llrdat )-1),dfres).
compute modsum={sqrt(r2),r2,mse,fratio,(ncol( llrdat )-1),dfres,pfr}.
compute modsuml={'R','R-sq','MSE',hcflab,'df1','df2', 'p'}.
end if.
end if.
do if ( 2 = 2 or 2 =3).
compute xlp= llrdat.
compute ylp= y.
compute pt2 = make(nrow(ylp),1,(csum(ylp)/nrow(ylp))).
do if ( 2 =2).
compute LL3 = ylp&*ln(pt2)+(1-ylp)&*ln(1-pt2).
end if.
compute LL3 = -2*csum(LL3).
compute bt1 = make(ncol(xlp),1,0).
compute LL1 = 0.
compute pt1 = make(nrow(ylp),1,0.5).
compute pt1lp=pt1.
loop jjj = 1 to iterate.
compute xlptmp=t(xlp).
compute vecprb=pt1lp&*(1-pt1lp).
loop kkk=1 to ncol(xlp).
compute xlptmp(kkk,:)=xlptmp(kkk,:)&*t(vecprb).
end loop.
compute b = bt1+inv(xlptmp*xlp)*t(xlp)*(ylp-pt1lp).
do if ( 2 =2).
compute xlpb=xlp*b.
compute xlpbt=(xlpb > -709.7).
compute xlpb709=(1-xlpbt)*(-709.7).
compute xlpb=(xlpb&*xlpbt)+xlpb709.
compute pt1lp = 1/(1+exp(-(xlpb))).
end if.
compute itprob = csum((pt1lp < .00000001) or (pt1lp > .9999999)).
do if (itprob > 0).
loop kkk = 1 to nrow(pt1lp).
do if (pt1lp(kkk,1) > .9999999).
compute pt1lp(kkk,1) = .9999999.
end if.
do if (pt1lp(kkk,1) < .00000001).
compute pt1lp(kkk,1) = .00000001.
end if.
end loop.
compute itprob = 0.
end if.
do if (itprob = 0).
do if ( 2 =2).
compute LL = ylp&*ln(pt1lp)+(1-ylp)&*ln(1-pt1lp).
end if.
compute LL2 = -2*csum(ll).
end if.
do if (abs(LL1-LL2) < converge).
do if ( 0 =1).
compute xlptmp=t(xlp).
compute vecprb=pt1lp&*(1-pt1lp).
loop kkk=1 to ncol(xlp).
compute xlptmp(kkk,:)=xlptmp(kkk,:)&*t(vecprb).
end loop.
compute varb = inv(xlptmp*xlp).
compute seb = sqrt(diag(varb)).
release xlptmp.
end if.
break.
end if.
compute bt1 = b.
compute LL1 = LL2.
end loop.
compute modres=b.
do if (jjj > iterate).
compute itprob = 2.
do if (booting=0).
compute iterrmod=1.
end if.
do if (booting=1).
compute bootiter=1.
end if.
do if (itprobtg=0).
compute itprobtg=1.
compute errcode(errs,1) = 47.
compute errs = errs + 1.
do if (booting = 0 and 0 =1).
compute vt1 = mdiag(pt1lp&*(1-pt1lp)).
compute varb = inv(t(xlp)*vt1*xlp).
compute seb = sqrt(diag(varb)).
end if.
end if.
end if.
do if ( 0 =1).
compute trat = b&/seb.
compute dfres=nrow(xlp).
compute p = 2*(1-cdfnorm(abs(trat))).
compute modres={modres,seb,trat,p}.
compute modres={modres,(b-xp2&*seb),(b+xp2&*seb)}.
compute pvchi=1-chicdf((LL3-LL2),(nrow(modres)-1)).
compute mcF = (LL3-LL2)/LL3.
compute cox = 1-exp(-(LL3-LL2)/nrow(xlp)).
compute nagel = cox/(1-exp(-(LL3)/nrow(xlp))).
compute modsum={LL2,(LL3-LL2),(nrow(modres)-1),pvchi, mcF,cox,nagel}.
compute modsuml={'-2LL','ModelLL', 'df', 'p', 'McFadden', 'CoxSnell', 'Nagelkrk'}.
compute modresl={'coeff','se','Z','p','LLCI','ULCI'}.
end if.
end if
.
compute b=btemphld.
compute fresult={(LL2-basemod),llrdf,1-chicdf((LL2-basemod),llrdf)}
.
compute lmatdb=lmat.
compute dbint=dbint+1.
end if.
compute highf={highf;fresult}.
compute highf2={highf2;fresult}.
do if (j = start).
compute flabel={flabel;'X*W'}.
end if.
do if (j > start).
do if (nms > 1).
compute flabel={flabel;highlbw(jj,1)}.
else if (nms = 1).
do if (xmint=0).
compute flabel={flabel;'M*W'}.
end if.
do if (xmint=1).
compute flabel={flabel;'X*M'}.
end if.
end if.
end if.
end if.
compute lmat=zhigh(1:nump(1,i),j).
compute lmat2=wzhigh(1:nump(1,i),j).
do if ((csum(lmat) > 0) and (csum(lmat2) = 0)).
do if ((i < (nms+nys)) or (ydich <> 1)).

.
compute lmat2= lmat.
do if ( 0 =0).
compute lmat2 = mdiag( lmat ).
compute lmat3=make(nrow(lmat2),1,0).
loop flp=1 to ncol(lmat2).
do if (csum(lmat2(:,flp))=1).
compute lmat3={lmat3,lmat2(:,flp)}.
end if.
end loop.
compute lmat2=lmat3(:,2:ncol(lmat3)).
end if.
compute fratio = (t(t(lmat2)* b )*inv(t(lmat2)* varb *lmat2)*((t(lmat2)* b )))/ncol(lmat2).
compute pfr = 1-fcdf(fratio,ncol(lmat2),(n-nrow( b ))).
compute fresult={fratio,ncol(lmat2),(n-nrow( b )),pfr}.
do if (i = (nms+nys) and (ydich=1)).
compute fratio=fratio*ncol(lmat2).
compute pfr=1-chicdf(fratio,ncol(lmat2)).
compute fresult={fratio,ncol(lmat2),pfr}.
end if.
do if ( 1 =1).
compute lmat3=1-rsum(lmat2).
compute xfm=make(n,csum(lmat3),0).
compute flpc=1.
loop flp=1 to nrow(lmat3).
do if (lmat3(flp,1)=1).
compute xfm(:,flpc)=x(:,flp).
compute flpc=flpc+1.
end if.
end loop.
compute bfm=inv(t(xfm)*xfm)*t(xfm)*y.
compute resid=y-(xfm*bfm).
compute sstotal=(y-(csum(y)/n)).
compute sstotal=csum(sstotal&*sstotal).
compute ssresid=csum(resid&*resid).
compute rsqch= r2 -((sstotal-ssresid)/sstotal).
compute fresult={rsqch,fresult}.
release xfm,flpc, resid, ssresid, bfm.
end if
.
compute dbint=dbint+1.
end if.
do if ((ydich = 1) and (i = (nms+nys))).

.
compute btemphld=b.
compute llrdat=make(nrow(x),nrow( lmat )-csum( lmat ),-999).
compute llrdf=ncol(x)-ncol(llrdat).
compute llrcnt=0.
loop llri=1 to nrow( lmat ).
do if ( lmat (llri,1)=0).
compute llrcnt=llrcnt+1.
compute llrdat(:,llrcnt)=x(:,llri).
end if.
end loop.

.
do if ( 2 =1).
compute hatmat=inv(t( llrdat )* llrdat )*t( llrdat ).
compute b = hatmat* y.
compute modres=b.
do if ( 0 =1).
compute n1=nrow( llrdat ).
compute dfres=n1-(ncol( llrdat )).
compute sstotal = t( y -(csum( y )/n1))*( y -(csum( y )/n1)).
compute resid= y - llrdat *b.
compute ssresid = csum((resid)&**2).
compute r2 = (sstotal-ssresid)/sstotal.
do if (r2 < 0).
compute r2=0.
end if.
compute adjr2 = 1-((1-r2)*(n1-1)/(dfres)).
compute mse=ssresid/(n1-ncol( llrdat )).

.
compute n1=nrow( x ).
compute invXtX = inv(t( x )* x ).
compute varb = mse *invXtX.
compute k3 = ncol( x ).
compute xhc=0.
do if ( hc <> 5).
compute xhc= x.
compute hat = xhc(:,1).
loop i3=1 to nrow(xhc).
compute hat(i3,1)= xhc(i3,:)*invXtX*t(xhc(i3,:)).
end loop.
do if ( hc = 0 or hc =1).
loop i3 = 1 to k3.
compute xhc(:,i3)=xhc(:,i3)&* resid.
end loop.
end if.
do if ( hc =3 or hc =2).
loop i3=1 to k3.
compute xhc(:,i3) = ( resid &/(1-hat)&**(1/(4- hc )))&*xhc(:,i3).
end loop.
end if.
do if ( hc = 4).
compute hcmn=make(n,2,4).
compute hcmn(:,2)=(n1*hat)/k3.
loop i3= 1 to k3.
compute xhc(:,i3) = ( resid &/(1-hat)&**(rmin(hcmn)/2))&*xhc(:,i3).
end loop.
end if.
compute varb=(invXtX*t(xhc)*xhc*invXtX).
do if ( hc =1).
compute varb=(n1/(n1-ncol( x )))&*varb.
end if.
end if.
compute hclab={'se(HC0)','se(HC1)','se(HC2)','se(HC3)','se(HC4)','se'}.
compute hclab=hclab(1,( hc +1)).
compute hcflab={'F(HC0)','F(HC1)','F(HC2)','F(HC3)','F(HC4)','F'}.
compute hcflab=hcflab(1,( hc +1)).
release xhc
.
compute seb=sqrt(diag(varb)).
compute trat = b&/seb.
compute p = 2*(1-tcdf(abs(trat), (dfres))).
compute tval = sqrt(dfres* (exp((dfres-(5/6))*((xp2/(dfres-(2/3)+(.11/dfres)))* (xp2/(dfres-(2/3)+(.11/dfres)))))-1)).
compute modres={modres,seb,trat,p}.
compute modres={modres,(b-tval&*seb),(b+tval&*seb)}.
compute modresl={'coeff',hclab,'t','p','LLCI','ULCI'}.
compute lmat = ident(ncol( llrdat )).
compute lmat = lmat(:,2:ncol(lmat)).
compute fratio = (t(t(lmat)*b)*inv(t(lmat)*varb*lmat)*((t(lmat)*b)))/(ncol( llrdat )-1).
compute pfr = 1-fcdf(fratio,(ncol( llrdat )-1),dfres).
compute modsum={sqrt(r2),r2,mse,fratio,(ncol( llrdat )-1),dfres,pfr}.
compute modsuml={'R','R-sq','MSE',hcflab,'df1','df2', 'p'}.
end if.
end if.
do if ( 2 = 2 or 2 =3).
compute xlp= llrdat.
compute ylp= y.
compute pt2 = make(nrow(ylp),1,(csum(ylp)/nrow(ylp))).
do if ( 2 =2).
compute LL3 = ylp&*ln(pt2)+(1-ylp)&*ln(1-pt2).
end if.
compute LL3 = -2*csum(LL3).
compute bt1 = make(ncol(xlp),1,0).
compute LL1 = 0.
compute pt1 = make(nrow(ylp),1,0.5).
compute pt1lp=pt1.
loop jjj = 1 to iterate.
compute xlptmp=t(xlp).
compute vecprb=pt1lp&*(1-pt1lp).
loop kkk=1 to ncol(xlp).
compute xlptmp(kkk,:)=xlptmp(kkk,:)&*t(vecprb).
end loop.
compute b = bt1+inv(xlptmp*xlp)*t(xlp)*(ylp-pt1lp).
do if ( 2 =2).
compute xlpb=xlp*b.
compute xlpbt=(xlpb > -709.7).
compute xlpb709=(1-xlpbt)*(-709.7).
compute xlpb=(xlpb&*xlpbt)+xlpb709.
compute pt1lp = 1/(1+exp(-(xlpb))).
end if.
compute itprob = csum((pt1lp < .00000001) or (pt1lp > .9999999)).
do if (itprob > 0).
loop kkk = 1 to nrow(pt1lp).
do if (pt1lp(kkk,1) > .9999999).
compute pt1lp(kkk,1) = .9999999.
end if.
do if (pt1lp(kkk,1) < .00000001).
compute pt1lp(kkk,1) = .00000001.
end if.
end loop.
compute itprob = 0.
end if.
do if (itprob = 0).
do if ( 2 =2).
compute LL = ylp&*ln(pt1lp)+(1-ylp)&*ln(1-pt1lp).
end if.
compute LL2 = -2*csum(ll).
end if.
do if (abs(LL1-LL2) < converge).
do if ( 0 =1).
compute xlptmp=t(xlp).
compute vecprb=pt1lp&*(1-pt1lp).
loop kkk=1 to ncol(xlp).
compute xlptmp(kkk,:)=xlptmp(kkk,:)&*t(vecprb).
end loop.
compute varb = inv(xlptmp*xlp).
compute seb = sqrt(diag(varb)).
release xlptmp.
end if.
break.
end if.
compute bt1 = b.
compute LL1 = LL2.
end loop.
compute modres=b.
do if (jjj > iterate).
compute itprob = 2.
do if (booting=0).
compute iterrmod=1.
end if.
do if (booting=1).
compute bootiter=1.
end if.
do if (itprobtg=0).
compute itprobtg=1.
compute errcode(errs,1) = 47.
compute errs = errs + 1.
do if (booting = 0 and 0 =1).
compute vt1 = mdiag(pt1lp&*(1-pt1lp)).
compute varb = inv(t(xlp)*vt1*xlp).
compute seb = sqrt(diag(varb)).
end if.
end if.
end if.
do if ( 0 =1).
compute trat = b&/seb.
compute dfres=nrow(xlp).
compute p = 2*(1-cdfnorm(abs(trat))).
compute modres={modres,seb,trat,p}.
compute modres={modres,(b-xp2&*seb),(b+xp2&*seb)}.
compute pvchi=1-chicdf((LL3-LL2),(nrow(modres)-1)).
compute mcF = (LL3-LL2)/LL3.
compute cox = 1-exp(-(LL3-LL2)/nrow(xlp)).
compute nagel = cox/(1-exp(-(LL3)/nrow(xlp))).
compute modsum={LL2,(LL3-LL2),(nrow(modres)-1),pvchi, mcF,cox,nagel}.
compute modsuml={'-2LL','ModelLL', 'df', 'p', 'McFadden', 'CoxSnell', 'Nagelkrk'}.
compute modresl={'coeff','se','Z','p','LLCI','ULCI'}.
end if.
end if
.
compute b=btemphld.
compute fresult={(LL2-basemod),llrdf,1-chicdf((LL2-basemod),llrdf)}
.
compute dbint=dbint+1.
end if.
compute highf={highf;fresult}.
compute highf2={highf2;fresult}.
do if (j = start).
compute flabel={flabel;'X*Z'}.
end if.
do if (j > start).
do if (nms > 1).
compute flabel={flabel;highlbz(jj,1)}.
else if (nms = 1).
compute flabel={flabel;'M*Z'}.
end if.
end if.
end if.
do if (dbint=2).
compute lmatdb=lmatdb+lmat.
do if ((ydich = 1) and (i = (nms+nys))).

.
compute btemphld=b.
compute llrdat=make(nrow(x),nrow( lmatdb )-csum( lmatdb ),-999).
compute llrdf=ncol(x)-ncol(llrdat).
compute llrcnt=0.
loop llri=1 to nrow( lmatdb ).
do if ( lmatdb (llri,1)=0).
compute llrcnt=llrcnt+1.
compute llrdat(:,llrcnt)=x(:,llri).
end if.
end loop.

.
do if ( 2 =1).
compute hatmat=inv(t( llrdat )* llrdat )*t( llrdat ).
compute b = hatmat* y.
compute modres=b.
do if ( 0 =1).
compute n1=nrow( llrdat ).
compute dfres=n1-(ncol( llrdat )).
compute sstotal = t( y -(csum( y )/n1))*( y -(csum( y )/n1)).
compute resid= y - llrdat *b.
compute ssresid = csum((resid)&**2).
compute r2 = (sstotal-ssresid)/sstotal.
do if (r2 < 0).
compute r2=0.
end if.
compute adjr2 = 1-((1-r2)*(n1-1)/(dfres)).
compute mse=ssresid/(n1-ncol( llrdat )).

.
compute n1=nrow( x ).
compute invXtX = inv(t( x )* x ).
compute varb = mse *invXtX.
compute k3 = ncol( x ).
compute xhc=0.
do if ( hc <> 5).
compute xhc= x.
compute hat = xhc(:,1).
loop i3=1 to nrow(xhc).
compute hat(i3,1)= xhc(i3,:)*invXtX*t(xhc(i3,:)).
end loop.
do if ( hc = 0 or hc =1).
loop i3 = 1 to k3.
compute xhc(:,i3)=xhc(:,i3)&* resid.
end loop.
end if.
do if ( hc =3 or hc =2).
loop i3=1 to k3.
compute xhc(:,i3) = ( resid &/(1-hat)&**(1/(4- hc )))&*xhc(:,i3).
end loop.
end if.
do if ( hc = 4).
compute hcmn=make(n,2,4).
compute hcmn(:,2)=(n1*hat)/k3.
loop i3= 1 to k3.
compute xhc(:,i3) = ( resid &/(1-hat)&**(rmin(hcmn)/2))&*xhc(:,i3).
end loop.
end if.
compute varb=(invXtX*t(xhc)*xhc*invXtX).
do if ( hc =1).
compute varb=(n1/(n1-ncol( x )))&*varb.
end if.
end if.
compute hclab={'se(HC0)','se(HC1)','se(HC2)','se(HC3)','se(HC4)','se'}.
compute hclab=hclab(1,( hc +1)).
compute hcflab={'F(HC0)','F(HC1)','F(HC2)','F(HC3)','F(HC4)','F'}.
compute hcflab=hcflab(1,( hc +1)).
release xhc
.
compute seb=sqrt(diag(varb)).
compute trat = b&/seb.
compute p = 2*(1-tcdf(abs(trat), (dfres))).
compute tval = sqrt(dfres* (exp((dfres-(5/6))*((xp2/(dfres-(2/3)+(.11/dfres)))* (xp2/(dfres-(2/3)+(.11/dfres)))))-1)).
compute modres={modres,seb,trat,p}.
compute modres={modres,(b-tval&*seb),(b+tval&*seb)}.
compute modresl={'coeff',hclab,'t','p','LLCI','ULCI'}.
compute lmat = ident(ncol( llrdat )).
compute lmat = lmat(:,2:ncol(lmat)).
compute fratio = (t(t(lmat)*b)*inv(t(lmat)*varb*lmat)*((t(lmat)*b)))/(ncol( llrdat )-1).
compute pfr = 1-fcdf(fratio,(ncol( llrdat )-1),dfres).
compute modsum={sqrt(r2),r2,mse,fratio,(ncol( llrdat )-1),dfres,pfr}.
compute modsuml={'R','R-sq','MSE',hcflab,'df1','df2', 'p'}.
end if.
end if.
do if ( 2 = 2 or 2 =3).
compute xlp= llrdat.
compute ylp= y.
compute pt2 = make(nrow(ylp),1,(csum(ylp)/nrow(ylp))).
do if ( 2 =2).
compute LL3 = ylp&*ln(pt2)+(1-ylp)&*ln(1-pt2).
end if.
compute LL3 = -2*csum(LL3).
compute bt1 = make(ncol(xlp),1,0).
compute LL1 = 0.
compute pt1 = make(nrow(ylp),1,0.5).
compute pt1lp=pt1.
loop jjj = 1 to iterate.
compute xlptmp=t(xlp).
compute vecprb=pt1lp&*(1-pt1lp).
loop kkk=1 to ncol(xlp).
compute xlptmp(kkk,:)=xlptmp(kkk,:)&*t(vecprb).
end loop.
compute b = bt1+inv(xlptmp*xlp)*t(xlp)*(ylp-pt1lp).
do if ( 2 =2).
compute xlpb=xlp*b.
compute xlpbt=(xlpb > -709.7).
compute xlpb709=(1-xlpbt)*(-709.7).
compute xlpb=(xlpb&*xlpbt)+xlpb709.
compute pt1lp = 1/(1+exp(-(xlpb))).
end if.
compute itprob = csum((pt1lp < .00000001) or (pt1lp > .9999999)).
do if (itprob > 0).
loop kkk = 1 to nrow(pt1lp).
do if (pt1lp(kkk,1) > .9999999).
compute pt1lp(kkk,1) = .9999999.
end if.
do if (pt1lp(kkk,1) < .00000001).
compute pt1lp(kkk,1) = .00000001.
end if.
end loop.
compute itprob = 0.
end if.
do if (itprob = 0).
do if ( 2 =2).
compute LL = ylp&*ln(pt1lp)+(1-ylp)&*ln(1-pt1lp).
end if.
compute LL2 = -2*csum(ll).
end if.
do if (abs(LL1-LL2) < converge).
do if ( 0 =1).
compute xlptmp=t(xlp).
compute vecprb=pt1lp&*(1-pt1lp).
loop kkk=1 to ncol(xlp).
compute xlptmp(kkk,:)=xlptmp(kkk,:)&*t(vecprb).
end loop.
compute varb = inv(xlptmp*xlp).
compute seb = sqrt(diag(varb)).
release xlptmp.
end if.
break.
end if.
compute bt1 = b.
compute LL1 = LL2.
end loop.
compute modres=b.
do if (jjj > iterate).
compute itprob = 2.
do if (booting=0).
compute iterrmod=1.
end if.
do if (booting=1).
compute bootiter=1.
end if.
do if (itprobtg=0).
compute itprobtg=1.
compute errcode(errs,1) = 47.
compute errs = errs + 1.
do if (booting = 0 and 0 =1).
compute vt1 = mdiag(pt1lp&*(1-pt1lp)).
compute varb = inv(t(xlp)*vt1*xlp).
compute seb = sqrt(diag(varb)).
end if.
end if.
end if.
do if ( 0 =1).
compute trat = b&/seb.
compute dfres=nrow(xlp).
compute p = 2*(1-cdfnorm(abs(trat))).
compute modres={modres,seb,trat,p}.
compute modres={modres,(b-xp2&*seb),(b+xp2&*seb)}.
compute pvchi=1-chicdf((LL3-LL2),(nrow(modres)-1)).
compute mcF = (LL3-LL2)/LL3.
compute cox = 1-exp(-(LL3-LL2)/nrow(xlp)).
compute nagel = cox/(1-exp(-(LL3)/nrow(xlp))).
compute modsum={LL2,(LL3-LL2),(nrow(modres)-1),pvchi, mcF,cox,nagel}.
compute modsuml={'-2LL','ModelLL', 'df', 'p', 'McFadden', 'CoxSnell', 'Nagelkrk'}.
compute modresl={'coeff','se','Z','p','LLCI','ULCI'}.
end if.
end if
.
compute b=btemphld.
compute fresult={(LL2-basemod),llrdf,1-chicdf((LL2-basemod),llrdf)}
.
end if.
do if (ydich <> 1 or i < (nms+nys)).

.
compute lmat2= lmatdb.
do if ( 0 =0).
compute lmat2 = mdiag( lmatdb ).
compute lmat3=make(nrow(lmat2),1,0).
loop flp=1 to ncol(lmat2).
do if (csum(lmat2(:,flp))=1).
compute lmat3={lmat3,lmat2(:,flp)}.
end if.
end loop.
compute lmat2=lmat3(:,2:ncol(lmat3)).
end if.
compute fratio = (t(t(lmat2)* b )*inv(t(lmat2)* varb *lmat2)*((t(lmat2)* b )))/ncol(lmat2).
compute pfr = 1-fcdf(fratio,ncol(lmat2),(n-nrow( b ))).
compute fresult={fratio,ncol(lmat2),(n-nrow( b )),pfr}.
do if (i = (nms+nys) and (ydich=1)).
compute fratio=fratio*ncol(lmat2).
compute pfr=1-chicdf(fratio,ncol(lmat2)).
compute fresult={fratio,ncol(lmat2),pfr}.
end if.
do if ( 1 =1).
compute lmat3=1-rsum(lmat2).
compute xfm=make(n,csum(lmat3),0).
compute flpc=1.
loop flp=1 to nrow(lmat3).
do if (lmat3(flp,1)=1).
compute xfm(:,flpc)=x(:,flp).
compute flpc=flpc+1.
end if.
end loop.
compute bfm=inv(t(xfm)*xfm)*t(xfm)*y.
compute resid=y-(xfm*bfm).
compute sstotal=(y-(csum(y)/n)).
compute sstotal=csum(sstotal&*sstotal).
compute ssresid=csum(resid&*resid).
compute rsqch= r2 -((sstotal-ssresid)/sstotal).
compute fresult={rsqch,fresult}.
release xfm,flpc, resid, ssresid, bfm.
end if
.
end if.
compute dbint=0.
compute highf={highf;fresult}.
do if (jj=0 and nms > 0).
compute flabel={flabel;'BOTH(X)'}.
end if.
do if (jj=0 and nms = 0).
compute flabel={flabel;'BOTH'}.
end if.
do if (jj>0 and nms = 1).
compute flabel={flabel;'BOTH(M)'}.
end if.
do if (nms > 1 and jj > 0).
compute flabel={flabel;highlbbt(jj,1)}.
end if.
end if.
compute lmat2=wzhigh(1:nump(1,i),j).
do if (csum(lmat2) > 0).
do if ((i < (nms+nys)) or (ydich <> 1)).

.
compute lmat2= lmat2.
do if ( 0 =0).
compute lmat2 = mdiag( lmat2 ).
compute lmat3=make(nrow(lmat2),1,0).
loop flp=1 to ncol(lmat2).
do if (csum(lmat2(:,flp))=1).
compute lmat3={lmat3,lmat2(:,flp)}.
end if.
end loop.
compute lmat2=lmat3(:,2:ncol(lmat3)).
end if.
compute fratio = (t(t(lmat2)* b )*inv(t(lmat2)* varb *lmat2)*((t(lmat2)* b )))/ncol(lmat2).
compute pfr = 1-fcdf(fratio,ncol(lmat2),(n-nrow( b ))).
compute fresult={fratio,ncol(lmat2),(n-nrow( b )),pfr}.
do if (i = (nms+nys) and (ydich=1)).
compute fratio=fratio*ncol(lmat2).
compute pfr=1-chicdf(fratio,ncol(lmat2)).
compute fresult={fratio,ncol(lmat2),pfr}.
end if.
do if ( 1 =1).
compute lmat3=1-rsum(lmat2).
compute xfm=make(n,csum(lmat3),0).
compute flpc=1.
loop flp=1 to nrow(lmat3).
do if (lmat3(flp,1)=1).
compute xfm(:,flpc)=x(:,flp).
compute flpc=flpc+1.
end if.
end loop.
compute bfm=inv(t(xfm)*xfm)*t(xfm)*y.
compute resid=y-(xfm*bfm).
compute sstotal=(y-(csum(y)/n)).
compute sstotal=csum(sstotal&*sstotal).
compute ssresid=csum(resid&*resid).
compute rsqch= r2 -((sstotal-ssresid)/sstotal).
compute fresult={rsqch,fresult}.
release xfm,flpc, resid, ssresid, bfm.
end if
.
end if.
do if ((ydich = 1) and (i = (nms+nys))).

.
compute btemphld=b.
compute llrdat=make(nrow(x),nrow( lmat2 )-csum( lmat2 ),-999).
compute llrdf=ncol(x)-ncol(llrdat).
compute llrcnt=0.
loop llri=1 to nrow( lmat2 ).
do if ( lmat2 (llri,1)=0).
compute llrcnt=llrcnt+1.
compute llrdat(:,llrcnt)=x(:,llri).
end if.
end loop.

.
do if ( 2 =1).
compute hatmat=inv(t( llrdat )* llrdat )*t( llrdat ).
compute b = hatmat* y.
compute modres=b.
do if ( 0 =1).
compute n1=nrow( llrdat ).
compute dfres=n1-(ncol( llrdat )).
compute sstotal = t( y -(csum( y )/n1))*( y -(csum( y )/n1)).
compute resid= y - llrdat *b.
compute ssresid = csum((resid)&**2).
compute r2 = (sstotal-ssresid)/sstotal.
do if (r2 < 0).
compute r2=0.
end if.
compute adjr2 = 1-((1-r2)*(n1-1)/(dfres)).
compute mse=ssresid/(n1-ncol( llrdat )).

.
compute n1=nrow( x ).
compute invXtX = inv(t( x )* x ).
compute varb = mse *invXtX.
compute k3 = ncol( x ).
compute xhc=0.
do if ( hc <> 5).
compute xhc= x.
compute hat = xhc(:,1).
loop i3=1 to nrow(xhc).
compute hat(i3,1)= xhc(i3,:)*invXtX*t(xhc(i3,:)).
end loop.
do if ( hc = 0 or hc =1).
loop i3 = 1 to k3.
compute xhc(:,i3)=xhc(:,i3)&* resid.
end loop.
end if.
do if ( hc =3 or hc =2).
loop i3=1 to k3.
compute xhc(:,i3) = ( resid &/(1-hat)&**(1/(4- hc )))&*xhc(:,i3).
end loop.
end if.
do if ( hc = 4).
compute hcmn=make(n,2,4).
compute hcmn(:,2)=(n1*hat)/k3.
loop i3= 1 to k3.
compute xhc(:,i3) = ( resid &/(1-hat)&**(rmin(hcmn)/2))&*xhc(:,i3).
end loop.
end if.
compute varb=(invXtX*t(xhc)*xhc*invXtX).
do if ( hc =1).
compute varb=(n1/(n1-ncol( x )))&*varb.
end if.
end if.
compute hclab={'se(HC0)','se(HC1)','se(HC2)','se(HC3)','se(HC4)','se'}.
compute hclab=hclab(1,( hc +1)).
compute hcflab={'F(HC0)','F(HC1)','F(HC2)','F(HC3)','F(HC4)','F'}.
compute hcflab=hcflab(1,( hc +1)).
release xhc
.
compute seb=sqrt(diag(varb)).
compute trat = b&/seb.
compute p = 2*(1-tcdf(abs(trat), (dfres))).
compute tval = sqrt(dfres* (exp((dfres-(5/6))*((xp2/(dfres-(2/3)+(.11/dfres)))* (xp2/(dfres-(2/3)+(.11/dfres)))))-1)).
compute modres={modres,seb,trat,p}.
compute modres={modres,(b-tval&*seb),(b+tval&*seb)}.
compute modresl={'coeff',hclab,'t','p','LLCI','ULCI'}.
compute lmat = ident(ncol( llrdat )).
compute lmat = lmat(:,2:ncol(lmat)).
compute fratio = (t(t(lmat)*b)*inv(t(lmat)*varb*lmat)*((t(lmat)*b)))/(ncol( llrdat )-1).
compute pfr = 1-fcdf(fratio,(ncol( llrdat )-1),dfres).
compute modsum={sqrt(r2),r2,mse,fratio,(ncol( llrdat )-1),dfres,pfr}.
compute modsuml={'R','R-sq','MSE',hcflab,'df1','df2', 'p'}.
end if.
end if.
do if ( 2 = 2 or 2 =3).
compute xlp= llrdat.
compute ylp= y.
compute pt2 = make(nrow(ylp),1,(csum(ylp)/nrow(ylp))).
do if ( 2 =2).
compute LL3 = ylp&*ln(pt2)+(1-ylp)&*ln(1-pt2).
end if.
compute LL3 = -2*csum(LL3).
compute bt1 = make(ncol(xlp),1,0).
compute LL1 = 0.
compute pt1 = make(nrow(ylp),1,0.5).
compute pt1lp=pt1.
loop jjj = 1 to iterate.
compute xlptmp=t(xlp).
compute vecprb=pt1lp&*(1-pt1lp).
loop kkk=1 to ncol(xlp).
compute xlptmp(kkk,:)=xlptmp(kkk,:)&*t(vecprb).
end loop.
compute b = bt1+inv(xlptmp*xlp)*t(xlp)*(ylp-pt1lp).
do if ( 2 =2).
compute xlpb=xlp*b.
compute xlpbt=(xlpb > -709.7).
compute xlpb709=(1-xlpbt)*(-709.7).
compute xlpb=(xlpb&*xlpbt)+xlpb709.
compute pt1lp = 1/(1+exp(-(xlpb))).
end if.
compute itprob = csum((pt1lp < .00000001) or (pt1lp > .9999999)).
do if (itprob > 0).
loop kkk = 1 to nrow(pt1lp).
do if (pt1lp(kkk,1) > .9999999).
compute pt1lp(kkk,1) = .9999999.
end if.
do if (pt1lp(kkk,1) < .00000001).
compute pt1lp(kkk,1) = .00000001.
end if.
end loop.
compute itprob = 0.
end if.
do if (itprob = 0).
do if ( 2 =2).
compute LL = ylp&*ln(pt1lp)+(1-ylp)&*ln(1-pt1lp).
end if.
compute LL2 = -2*csum(ll).
end if.
do if (abs(LL1-LL2) < converge).
do if ( 0 =1).
compute xlptmp=t(xlp).
compute vecprb=pt1lp&*(1-pt1lp).
loop kkk=1 to ncol(xlp).
compute xlptmp(kkk,:)=xlptmp(kkk,:)&*t(vecprb).
end loop.
compute varb = inv(xlptmp*xlp).
compute seb = sqrt(diag(varb)).
release xlptmp.
end if.
break.
end if.
compute bt1 = b.
compute LL1 = LL2.
end loop.
compute modres=b.
do if (jjj > iterate).
compute itprob = 2.
do if (booting=0).
compute iterrmod=1.
end if.
do if (booting=1).
compute bootiter=1.
end if.
do if (itprobtg=0).
compute itprobtg=1.
compute errcode(errs,1) = 47.
compute errs = errs + 1.
do if (booting = 0 and 0 =1).
compute vt1 = mdiag(pt1lp&*(1-pt1lp)).
compute varb = inv(t(xlp)*vt1*xlp).
compute seb = sqrt(diag(varb)).
end if.
end if.
end if.
do if ( 0 =1).
compute trat = b&/seb.
compute dfres=nrow(xlp).
compute p = 2*(1-cdfnorm(abs(trat))).
compute modres={modres,seb,trat,p}.
compute modres={modres,(b-xp2&*seb),(b+xp2&*seb)}.
compute pvchi=1-chicdf((LL3-LL2),(nrow(modres)-1)).
compute mcF = (LL3-LL2)/LL3.
compute cox = 1-exp(-(LL3-LL2)/nrow(xlp)).
compute nagel = cox/(1-exp(-(LL3)/nrow(xlp))).
compute modsum={LL2,(LL3-LL2),(nrow(modres)-1),pvchi, mcF,cox,nagel}.
compute modsuml={'-2LL','ModelLL', 'df', 'p', 'McFadden', 'CoxSnell', 'Nagelkrk'}.
compute modresl={'coeff','se','Z','p','LLCI','ULCI'}.
end if.
end if
.
compute b=btemphld.
compute fresult={(LL2-basemod),llrdf,1-chicdf((LL2-basemod),llrdf)}
.
end if.
compute highf={highf;fresult}.
compute highf2={highf2;fresult}.
do if (j = start).
compute flabel={flabel;'X*W*Z'}.
end if.
do if (j > start).
do if (nms > 1).
compute flabel={flabel;highlbwz(jj,1)}.
else if (nms = 1).
compute flabel={flabel;'M*W*Z'}.
end if.
end if.
end if.
compute jj=jj+1.
end loop.
release jj.
compute start=start+i.
end if.
do if (nrow(highf) > 1).
compute highf=highf(2:nrow(highf),:).
compute highf2=highf2(2:nrow(highf2),:).
compute flabel=flabel(2:nrow(flabel),1).
compute resultm2=make(nrow(highf),maxresm,99999).
compute resultm2(1:nrow(highf),1:ncol(highf))=highf.
compute resultm={resultm;resultm2}.
do if (outscree=1).
do if ((i < nms+nys) or (ydich=0)).
compute clabtmp={'R2-chng', hcflab,'df1','df2','p'}.
print highf/format = F10.4 /rnames=flabel/cnames=clabtmp/ title = 'Test(s) of highest order unconditional interaction(s):'.
end if.
do if (ydich=1 and i=(nms+nys)).
compute clabtmp={'Chi-sq', 'df','p'}.
print/title='Likelihood ratio test(s) of highest order'.
print highf/format = F10.4 /rnames=flabel/cnames=clabtmp/ title = 'unconditional interactions(s):'/space=0.
end if.
end if.
compute intpb=highf2(:,ncol(highf2)).
end if.
compute intstart=intstart+numint(1,i).
end if.

.
do if (criterr=0).
compute threeway=0.
compute didprint=0.
compute didsome=0.
compute sigintct=0.
loop jmed =1 to (nms+1).
compute hasw=0.
compute hasz=0.
compute jnok=0.
compute nm1vls=0.
compute nm2vls=0.
compute panelgrp=0.
compute graphixs={'WITH', outnames(1,i), 'BY'}.
compute focpred4={' '}.
compute intprint=0.
compute modcat=0.
do if (jmed <= i).
do if ((jmed = 1) and ((i+1) = nrow(bcmat))).
compute pathscnt=pathscnt+1.
else.
compute paths={paths,bcmat((i+1),jmed)}.
compute pathsw={pathsw,wcmat((i+1),jmed)}.
compute pathsz={pathsz,zcmat((i+1),jmed)}.
compute pathswz={pathswz,wzcmat((i+1),jmed)}.
compute temp=fochigh(:,pathscnt)&*bootloc(:,i).
compute pathsfoc={pathsfoc,pathsfoc(:,1)}.
do if (jmed=1).
compute pathtype={pathtype,1}.
end if.
do if ((i+1)=nrow(bcmat)).
compute pathtype={pathtype,3}.
end if.
do if (jmed > 1) and ((i+1) < nrow(bcmat)).
compute pathtype={pathtype,2}.
end if.
do if (jmed=1 and nxvls > 1 and (bcmat((i+1),jmed)=1)).
compute pathsfoc(:,(pathscn2+1))=temp(2:(nxvls+1),1).
end if.
do if ((jmed > 1) or (jmed=1 and nxvls=1)).
compute temp=cmax(temp).
compute pathsfoc(1,(pathscn2+1))=temp.
end if.
compute pathscnt=pathscnt+1.
compute pathscn2=pathscn2+1.
do if (i <= nms).
compute pathsdv={pathsdv,mnames(1,i)}.
end if.
do if (i > nms).
compute pathsdv={pathsdv,ynames}.
end if.
end if.
compute coeffcol=coeffcol+1.
compute probettt=coeffs(1:nrow(b),coeffcol).
do if (jmed=1 and (bcmat((i+1),jmed)=1)).
compute omni=make(nrow(probettt),nxvls,0).
compute omnitmp=ident(nxvls).
compute omni(2:(1+nxvls),:)=omnitmp.
end if.
do if (csum(probettt)>0).
compute probvarb=make(csum(probettt),csum(probettt),999).
compute probcoef=make(csum(probettt),1,999).
compute coefflp2=1.
loop coefflp=1 to nrow(probettt).
do if (probettt(coefflp,1)=1).
compute probcoef(coefflp2,1)=b(coefflp,1).
compute coefflp2=coefflp2+1.
end if.
end loop.
compute coefflp=0.
compute coefflp2=0.
loop iclp=1 to nrow(probettt).
do if probettt(iclp,1)=1.
compute coefflp=coefflp+1.
compute coefflp2=coefflp.
compute probvarb(coefflp,coefflp) = varb(iclp,iclp).
do if (iclp < nrow(probettt)).
loop jclp=(iclp+1) to nrow(probettt).
do if (probettt(jclp,1)=1).
compute coefflp2=coefflp2+1.
compute probvarb(coefflp,coefflp2)=varb(iclp, jclp).
compute probvarb(coefflp2,coefflp)=varb(iclp, jclp).
end if.
end loop.
end if.
end if.
end loop.
end if.
end if.
compute xprobval=xmodvals.
do if (nxvls > 1 or mcx > 0).
compute xprobval=dummatx(:,2:ncol(dummatx)).
end if.
do if ((wcmat((i+1),jmed)=1) and (zcmat((i+1),jmed)=0)).
compute numplps=1.
compute modvals=wmodvals.
compute probeval=wmodvals.
compute wheremv1=wherexw.
compute nm1vls=nwvls.
compute lpstsp={1,1}.
compute modcat=0.
compute jnmod=wtmp.
compute jnmodlab=wnames.
compute jnok=1.
compute jnmin=wmin.
compute jnmax=wmax.
compute wherejn1=2.
do if (jmed=1).
compute wherejn3=wherexw(1,i).
do if (nxvls > 1).
compute jnok=0.
end if.
end if.
do if (jmed > 1).
compute wherejn1=wherem((jmed-1),i).
compute wherejn3=wheremw(((2*jmed)-3),i).
end if.
do if (nwvls > 1).
compute probeval=wprobval.
compute lpstsp(1,2)=ncol(probeval).
compute modcat=1.
compute jnok=0.
end if.
do if (wdich = 1).
compute modcat=1.
compute jnok=0.
end if.
compute problabs=wnames.
compute focpred3={wnames,'(W)'}.
do if (xmint=1).
compute focpred3={wnames,'(X)'}.
end if.
compute hasw=1.
compute modgrph=wnames.
compute intprint=1.
compute sigintct=sigintct+1.
compute printpbe=intpb(sigintct,1).
end if.
do if ((wcmat((i+1),jmed)=0) and (zcmat((i+1),jmed)=1)).
compute numplps=1.
compute modvals=zmodvals.
compute probeval=zmodvals.
compute wheremv1=wherexz.
compute nm1vls=nzvls.
compute lpstsp={1,1}.
compute jnok=1.
compute jnmod=ztmp.
compute jnmin=zmin.
compute jnmax=zmax.
compute jnmodlab=znames.
compute wherejn1=2.
do if (jmed=1).
compute wherejn3=wherexz(1,i).
do if (nxvls > 1).
compute jnok=0.
end if.
end if.
do if (jmed > 1).
compute wherejn1=wherem((jmed-1),i).
compute wherejn3=wheremz(((2*jmed)-3),i).
end if.
do if (nzvls > 1).
compute probeval=zprobval.
compute lpstsp(1,2)=ncol(probeval).
compute modcat=1.
compute jnok=0.
end if.
do if (zdich = 1).
compute modcat=1.
compute jnok=0.
end if.
compute problabs=znames.
compute focpred3={znames,'(Z)'}.
compute modgrph=znames.
compute hasz=1.
compute intprint=1.
compute sigintct=sigintct+1.
compute printpbe=intpb(sigintct,1).
end if.
do if ((wzcmat((i+1),jmed)=1) or ((wcmat((i+1),jmed)=1) and (zcmat((i+1),jmed)=1))).
compute numplps=2.
compute probecnt=1.
compute intprint=1.
do if (wzcmat((i+1),jmed)=1).
compute sigintct=sigintct+1.
compute printpbe=intpb(sigintct,1).
else.
compute sigintct=sigintct+2.
compute printpbe=cmin(intpb((sigintct-1):sigintct,1)).
end if.
compute panelgrp=1.
compute hasw=1.
compute hasz=1.
compute panelcde={'/PANEL','ROWVAR=',znames,'.'}.
compute modgrph=wnames.
compute lpstsp={1,1;1,1}.
compute wheremv1=wherexw.
compute nm1vls=nwvls.
compute wheremv2=wherexz.
compute nm2vls=nzvls.
compute jnok=0.
do if (wzcmat((i+1),jmed)=1).
compute jnok=1.
end if.
do if (jmed > 1).
compute mprobval=mmodvals.
end if.
do if (jmed=1).
do if (nxvls > 1).
compute jnok=0.
end if.
end if.
do if (nwvls > 1).
compute lpstsp(1,2)=ncol(wprobval).
compute modcat=1.
compute jnok=0.
end if.
do if (zdich=1).
compute modcat=1.
compute jnok=0.
end if.
compute lpstsp(2,1)=lpstsp(1,2)+1.
compute lpstsp(2,2)=lpstsp(1,2)+1.
do if (nzvls > 1).
compute lpstsp(2,1)=lpstsp(1,2)+1.
compute lpstsp(2,2)=lpstsp(1,2)+ncol(zprobval).
compute jnok=0.
end if.
do if (zdich=1).
compute jnok=0.
end if.
compute omni3=make(nrow(b),(nxvls*nwvls),0).
do if (jmed > 1).
compute omni3=make(nrow(b),nwvls,0).
end if.
compute focpred3={wnames,'(W)'}.
do if (xmint=1).
compute focpred3={wnames,'(X)'}.
end if.
compute focpred4={'    ', 'Mod var:', znames, '(Z)'}.
compute modvals=make((nrow(wmodvals)*nrow(zmodvals)),2,0).
compute probeval=make((nrow(wmodvals)*nrow(zmodvals)),(ncol(wprobval)+ncol(zprobval)),0).
loop probei= 1 to nrow(wmodvals).
loop probej =1 to nrow(zmodvals).
compute modvals(probecnt,1)=wmodvals(probei,1).
compute probeval(probecnt,1:nwvls)=wprobval(probei,:).
compute modvals(probecnt,2)=zmodvals(probej,1).
compute probeval(probecnt,(nwvls+1):(nwvls+nzvls))=zprobval(probej,:).
compute probecnt=probecnt+1.
end loop.
end loop.
do if (wzcmat((i+1),jmed)=1).
compute numplps=numplps+1.
compute probprod=make(1,(ncol(wprobval)*ncol(zprobval)),0).
compute lpstsp2={1,1}.
compute lpstsp={lpstsp;lpstsp2}.
compute lpstsp(3,1)=lpstsp(2,2)+1.
compute lpstsp(3,2)=lpstsp(2,2)+ncol(probprod).
compute jnmod=ztmp.
compute jnmin=zmin.
compute jnmax=zmax.
compute jnmodlab=znames.
do if (jmed = 1).
compute wherejn1=wherexw(1,i).
compute wherejn3=wherexwz(1,i).
end if.
do if (jmed > 1).
compute wherejn1=wheremw(((2*jmed)-3),i).
compute wherejn3=wheremwz(((2*jmed)-3),i).
end if.
loop probei = 1 to nrow(wmodvals).
loop probej = 1 to nrow(zmodvals).
compute probtemp=1.
loop probek = 1 to ncol(wprobval).
compute probtemp={probtemp,(wprobval(probei,probek)&*zprobval(probej,:))}.
end loop.
compute probprod={probprod;probtemp(1,2:ncol(probtemp))}.
end loop.
end loop.
compute probprod=probprod(2:nrow(probprod),:).
compute probeval={probeval,probprod}.
end if.
compute problabs={wnames,znames}.
release probecnt, probei, probej.
end if.
do if (intprint=1).
compute focpred={'   Focal', 'predict:'}.
do if (jmed=1).
compute focpred={focpred,xnames,'(X)'}.
compute focplotv=xmodvals.
end if.
do if (jmed >1).
do if (nms > 1).
compute focpred={focpred,mnames(1,(jmed-1)), medlb2(1,(jmed-1))}.
end if.
do if (nms = 1).
compute focpred={focpred,mnames(1,(jmed-1)), '(M)'}.
end if.
compute focplotv=mmodvals(:,(jmed-1)).
end if.
compute focpred2={'    ', 'Mod var:',focpred3}.
compute focpred={focpred;focpred2}.
do if (ncol(focpred4) > 1).
compute focpred={focpred;focpred4}.
compute focpred4={' '}.
end if.
release focpred2,focpred3.
do if (outscree=1).
do if ((plot = 1 or plot = 2) or (printpbe <= intprobe)).
print focpred/title='----------'/format=A8/space=0.
end if.
end if.
compute foctmp=make(nrow(modvals),1,1).
compute probexpl=1.
compute probeva2={foctmp,probeval}.
do if (jmed=1 and nxs > 0 and mcx > 0).
compute probexpl=nxvls.
end if.
compute foctmp=make(nrow(modvals),1,1).
compute modvals3=make(1,(6+ncol(problabs)),0).
compute probrown=make(nrow(probeval),1,0).
compute jtmp=1.
loop probei = 1 to nrow(probeval).
compute probrown(probei,1)=jtmp.
compute jtmp=jtmp+nxvls.
end loop.
release jtmp.
compute probrow=999.
compute modvarl=problabs.
do if (plot = 1 or plot = 2 or nxvls > 1).
compute plotvals=make((nrow(modvals)*nrow(focplotv)),(ncol(modvals)+1),999).
loop ploti=1 to nrow(modvals).
loop plotj=1 to nrow(focplotv).
compute plotvals((((ploti-1)*nrow(focplotv))+plotj),2:ncol(plotvals))=modvals(ploti,:).
compute plotvals((((ploti-1)*nrow(focplotv))+plotj),1)=focplotv(plotj,1).
end loop.
end loop.
compute focpredn=3.
do if (jmed=1).
do if (nxvls > 1).
compute focpredn=(nxvls+1).
end if.
do if (nxvls=1 and xdich=1).
compute focpredn=2.
end if.
end if.
compute meanmat=mdiag(means).
compute onesmat=make(nrow(meanmat),(nrow(probeval)*focpredn),1).
compute probeplt=t(mdiag(means)*onesmat).
do if (jmed=1).
do if ((wcmat((i+1),1)=1) or (zcmat((i+1),1)=1)).
compute plotcnt=1.
compute iloops=nwpval*nzpval.
compute plotmx=nxpval*nzpval.
do if ((wcmat((i+1),1)=1) and (zcmat((i+1),1)=0)).
compute iloops=nwpval.
compute plotmx=nxpval.
end if.
do if ((wcmat((i+1),1)=0) and (zcmat((i+1),1)=1)).
compute iloops=nzpval.
compute plotmx=nxpval.
end if.
compute xestvals=make((nxpval*iloops),ncol(xprobval),-999).
do if (wcmat((i+1),1)=1).
compute westvals=make(nrow(xestvals),ncol(wprobval),-999).
end if.
do if (zcmat((i+1),1)=1).
compute zestvals=make(nrow(xestvals),ncol(zprobval),-999).
end if.
loop ploti=1 to iloops.
loop plotj=1 to nxpval.
compute xestvals(plotcnt,:)=xprobval(plotj,:).
compute plotcnt=plotcnt+1.
end loop.
end loop.
compute plotcnt=1.
compute plotcnt1=1.
compute plotcnt2=1.
compute plotcntz=1.
loop ploti = 1 to (iloops*nxpval).
do if (wcmat((i+1),1)=1).
compute westvals(ploti,:)=wprobval(plotcnt1,:).
end if.
do if ((wcmat((i+1),1)=0) and (zcmat((i+1),1)=1)).
compute zestvals(ploti,:)=zprobval(plotcnt1,:).
end if.
do if ((wcmat((i+1),1)=1) and (zcmat((i+1),1)=1)).
compute zestvals(ploti,:)=zprobval(plotcnt2,:).
compute plotcntz=plotcntz+1.
end if.
compute plotcnt=plotcnt+1.
do if (plotcnt > plotmx).
compute plotcnt=1.
compute plotcnt1=plotcnt1+1.
end if.
do if (plotcntz > nxpval).
compute plotcnt2=plotcnt2+1.
compute plotcntz=1.
do if (plotcnt2 > nzpval).
compute plotcnt2=1.
end if.
end if.
end loop.
compute probeplt(:,2:(1+(ncol(xestvals))))=xestvals.
do if (wcmat((i+1),1)=1).
compute probeplt(:,(wherew(1,i)):(wherew(2,i)))=westvals.
end if.
do if (zcmat((i+1),1)=1).
compute probeplt(:,(wherez(1,i)):(wherez(2,i)))=zestvals.
end if.
end if.
end if.
do if (jmed > 1).
do if ((wcmat((i+1),jmed)=1) or (zcmat((i+1),jmed)=1)).
compute plotcnt=1.
compute iloops=nwpval*nzpval.
compute plotmx=3*nzpval.
do if ((wcmat((i+1),jmed)=1) and (zcmat((i+1),jmed)=0)).
compute iloops=nwpval.
compute plotmx=3.
end if.
do if ((wcmat((i+1),jmed)=0) and (zcmat((i+1),jmed)=1)).
compute iloops=nzpval.
compute plotmx=3.
end if.
compute mestvals=make((3*iloops),1,-999).
do if (wcmat((i+1),jmed)=1).
compute westvals=make(nrow(mestvals),ncol(wprobval),-999).
end if.
do if (zcmat((i+1),jmed)=1).
compute zestvals=make(nrow(mestvals),ncol(zprobval),-999).
end if.
loop ploti=1 to iloops.
loop plotj=1 to 3.
compute mestvals(plotcnt,:)=mprobval(plotj,(jmed-1)).
compute plotcnt=plotcnt+1.
end loop.
end loop.
compute plotcnt=1.
compute plotcnt1=1.
compute plotcnt2=1.
compute plotcntz=1.
loop ploti = 1 to (iloops*3).
do if (wcmat((i+1),jmed)=1).
compute westvals(ploti,:)=wprobval(plotcnt1,:).
end if.
do if ((wcmat((i+1),jmed)=0) and (zcmat((i+1),jmed)=1)).
compute zestvals(ploti,:)=zprobval(plotcnt1,:).
end if.
do if ((wcmat((i+1),jmed)=1) and (zcmat((i+1),jmed)=1)).
compute zestvals(ploti,:)=zprobval(plotcnt2,:).
compute plotcntz=plotcntz+1.
end if.
compute plotcnt=plotcnt+1.
do if (plotcnt > plotmx).
compute plotcnt=1.
compute plotcnt1=plotcnt1+1.
end if.
do if (plotcntz > 3).
compute plotcnt2=plotcnt2+1.
compute plotcntz=1.
do if (plotcnt2 > nzpval).
compute plotcnt2=1.
end if.
end if.
end loop.
compute probeplt(:,wherem((jmed-1),i))=mestvals.
do if (wcmat((i+1),jmed)=1).
do if (model <> 74).
compute probeplt(:,(wherew(1,i)):(wherew(2,i)))=westvals.
end if.
do if (model = 74).
compute probeplt(:,(wherex(1,i)):(wherex(2,i)))=westvals.
end if.
end if.
do if (zcmat((i+1),jmed)=1).
compute probeplt(:,(wherez(1,i)):(wherez(2,i)))=zestvals.
end if.
end if.
end if.
compute prodloop = 1.
do if (jmed=1).
compute prodloop=ncol(xestvals).
end if.
do if (wcmat((i+1),jmed))=1.
compute plotcnt=0.
loop ploti = 1 to prodloop.
loop plotj = 1 to ncol(westvals).
do if (jmed=1).
compute probeplt(:,(wherexw(1,i)+plotcnt))=xestvals(:,ploti)&*westvals(:,plotj).
end if.
do if (jmed > 1).
compute probeplt(:,(wheremw(((jmed*2)-3) ,i)+plotcnt))=mestvals(:,ploti)&*westvals(:,plotj).
end if.
compute plotcnt=plotcnt+1.
end loop.
end loop.
end if.
do if (zcmat((i+1),jmed))=1.
compute plotcnt=0.
loop ploti = 1 to prodloop.
loop plotj = 1 to ncol(zestvals).
do if (jmed = 1).
compute probeplt(:,(wherexz(1,i)+plotcnt))=xestvals(:,ploti)&*zestvals(:,plotj).
end if.
do if (jmed > 1).
compute probeplt(:,(wheremz(((jmed*2)-3),i)+plotcnt))=mestvals(:,ploti)&*zestvals(:, plotj).
end if.
compute plotcnt=plotcnt+1.
end loop.
end loop.
end if.
do if (wzcmat((i+1),jmed))=1.
compute plotcnt=0.
compute threeway=1.
loop ploti = 1 to ncol(westvals).
loop plotj = 1 to ncol(zestvals).
compute probeplt(:,(wherewz(1,i)+plotcnt))=westvals(:,ploti)&*zestvals(:,plotj).
compute plotcnt=plotcnt+1.
end loop.
end loop.
compute plotcnt=0.
loop plotk = 1 to prodloop.
loop ploti = 1 to ncol(westvals).
loop plotj = 1 to ncol(zestvals).
do if (jmed = 1).
compute probeplt(:,(wherexwz(1,i)+plotcnt))=xestvals(:,plotk)&*westvals(:,ploti)&* zestvals(:,plotj).
end if.
do if (jmed > 1).
compute probeplt(:,(wheremwz(((jmed*2)-3),i)+plotcnt))=mestvals(:,plotk)&* westvals(:,ploti)&*zestvals(:,plotj).
end if.
compute plotcnt=plotcnt+1.
end loop.
end loop.
end loop.
end if.
loop newplp=1 to i.
do if (newplp <> jmed).
do if (wcmat((i+1),newplp))=1.
compute prodloop=1.
do if (newplp=1).
compute prodloop=nxvls.
end if.
compute plotcnt=0.
loop ploti = 1 to prodloop.
loop plotj = 1 to nwvls.
do if (newplp = 1).
compute probeplt(:,(wherexw(1,i)+plotcnt))=probeplt(:,(1+ploti))&*probeplt(:, (wherew(1,i)+plotj-1)).
end if.
do if (newplp > 1).
do if (model <> 74).
compute probeplt(:,(wheremw(((newplp*2)-3) ,i)+plotcnt))=probeplt(:,wherem((newplp-1),i))&*probeplt(:,(wherew(1,i)+plotj-1)).
end if.
do if (model = 74).
compute probeplt(:,(wheremw(((newplp*2)-3) ,i)+plotcnt))=probeplt(:,wherem((newplp-1),i))&*probeplt(:,(wherex(1,i)+plotj-1)).
end if.
end if.
compute plotcnt=plotcnt+1.
end loop.
end loop.
end if.
do if (zcmat((i+1),newplp))=1.
compute prodloop=1.
do if (newplp=1).
compute prodloop=nxvls.
end if.
compute plotcnt=0.
loop ploti = 1 to prodloop.
loop plotj = 1 to nzvls.
do if (newplp = 1).
compute probeplt(:,(wherexz(1,i)+plotcnt))=probeplt(:,(1+ploti))&*probeplt(:, (wherez(1,i)+plotj-1)).
end if.
do if (newplp > 1).
compute probeplt(:,(wheremz(((newplp*2)-3),i)+plotcnt))=probeplt(:, wherem((newplp-1),i))&*probeplt(:,(wherez(1,i)+plotj-1)).
end if.
compute plotcnt=plotcnt+1.
end loop.
end loop.
end if.
do if (wzcmat((i+1),newplp))=1.
compute plotcnt=0.
do if (threeway=0).
loop ploti = 1 to nwvls.
loop plotj = 1 to nzvls.
compute probeplt(:,(wherewz(1,i)+plotcnt))=probeplt(:,(wherew(1,i)+ploti-1))&* probeplt(:,(wherez(1,i)+plotj-1)).
compute plotcnt=plotcnt+1.
end loop.
end loop.
end if.
compute prodloop=1.
do if (newplp=1).
compute prodloop=nxvls.
end if.
compute plotcnt=0.
loop plotk = 1 to prodloop.
loop ploti = 1 to nwvls.
loop plotj = 1 to nzvls.
do if (newplp = 1).
compute probeplt(:,(wherexwz(1,i)+plotcnt))=probeplt(:,(1+plotk))&*probeplt(:, (wherew(1,i)+ploti-1))&*probeplt(:,(wherez(1,i)+plotj-1)).
end if.
do if (newplp > 1).
compute probeplt(:,(wheremwz(((newplp*2)-3),i)+plotcnt))=probeplt(:, wherem((newplp-1),i))&*probeplt(:,(wherew(1,i)+ploti-1))&*probeplt(:,(wherez(1,i)+plotj-1)).
end if.
compute plotcnt=plotcnt+1.
end loop.
end loop.
end loop.
end if.
end if.
end loop.
compute predvals=probeplt*b.
do if (debug <> 0).
print probeplt.
print b.
end if.
do if (i = nms+nys) and (ydich=1).
compute predvalt=(predvals < 709.7).
compute prevalt7=(1-predvalt)*(709.7).
compute predvals=(predvals&*predvalt)+prevalt7.
compute expyhat=exp(predvals)&/(1+exp(predvals)).
end if.
compute sepred=make(nrow(plotvals),3,999).
loop sei=1 to nrow(plotvals).
compute ask=probeplt(sei,:).
compute sepred(sei,1)=sqrt(ask*varb*t(ask)).
do if ((i < nms+nys) or (ydich=0)).
compute sepred(sei,2)=predvals(sei,1)-tval*sepred(sei,1).
compute sepred(sei,3)=predvals(sei,1)+tval*sepred(sei,1).
end if.
do if ((i = nms+nys) and (ydich=1)).
compute sepred(sei,2)=predvals(sei,1)-xp2*sepred(sei,1).
compute sepred(sei,3)=predvals(sei,1)+xp2*sepred(sei,1).
end if.
end loop.
compute prevloc=ncol(plotvals)+1.
compute probeplt={plotvals,predvals}.
do if (plot = 2).
compute probeplt={probeplt,sepred}.
end if.
do if ((i = nms+nys) and (ydich=1)).
compute probeplt={probeplt,expyhat}.
end if.
compute didsome=0.
end if.
do if ((wzcmat((i+1),jmed)=1) and (printpbe <= intprobe)).
do if (jmed=1).
compute omnilp2=nxvls*nwvls.
compute omnitmp=ident(omnilp2).
compute omni3(wherexw(1,i):wherexw(2,i),:)=omnitmp.
end if.
do if (jmed>1).
compute omnilp2=nwvls.
compute omnitmp=ident(omnilp2).
compute omni3(wheremw(((jmed*2)-3),i):wheremw(((jmed*2)-2),i),:)=omnitmp.
end if.
compute omnif=make(1,4,0).
do if ((i = nms+nys) and (ydich=1)).
compute omnif=make(1,3,0).
end if.
compute condeff3=0.
loop omnilp1=1 to nrow(zprobval).
loop omnilp=1 to (omnilp2).
do if (jmed=1).
compute omni3((wherexwz(1,i)+((omnilp-1)*nzvls)):(wherexwz(1,i)+((omnilp-1)*nzvls)+ (nzvls-1)),omnilp)=t(zprobval(omnilp1,:)).
end if.
do if (jmed > 1).
compute omni3((wheremwz(((jmed*2)-3),i)+((omnilp-1)*nzvls)):(wheremwz(((jmed*2)-3),i)+ ((omnilp-1)*nzvls)+(nzvls-1)),omnilp)=t(zprobval(omnilp1,:)).
end if.
end loop.
compute condeff=t(omni3)*b.
compute condeff3={condeff3;condeff}.

.
compute lmat2= omni3.
do if ( 1 =0).
compute lmat2 = mdiag( omni3 ).
compute lmat3=make(nrow(lmat2),1,0).
loop flp=1 to ncol(lmat2).
do if (csum(lmat2(:,flp))=1).
compute lmat3={lmat3,lmat2(:,flp)}.
end if.
end loop.
compute lmat2=lmat3(:,2:ncol(lmat3)).
end if.
compute fratio = (t(t(lmat2)* b )*inv(t(lmat2)* varb *lmat2)*((t(lmat2)* b )))/ncol(lmat2).
compute pfr = 1-fcdf(fratio,ncol(lmat2),(n-nrow( b ))).
compute fresult={fratio,ncol(lmat2),(n-nrow( b )),pfr}.
do if (i = (nms+nys) and (ydich=1)).
compute fratio=fratio*ncol(lmat2).
compute pfr=1-chicdf(fratio,ncol(lmat2)).
compute fresult={fratio,ncol(lmat2),pfr}.
end if.
do if ( 0 =1).
compute lmat3=1-rsum(lmat2).
compute xfm=make(n,csum(lmat3),0).
compute flpc=1.
loop flp=1 to nrow(lmat3).
do if (lmat3(flp,1)=1).
compute xfm(:,flpc)=x(:,flp).
compute flpc=flpc+1.
end if.
end loop.
compute bfm=inv(t(xfm)*xfm)*t(xfm)*y.
compute resid=y-(xfm*bfm).
compute sstotal=(y-(csum(y)/n)).
compute sstotal=csum(sstotal&*sstotal).
compute ssresid=csum(resid&*resid).
compute rsqch= 0 -((sstotal-ssresid)/sstotal).
compute fresult={rsqch,fresult}.
release xfm,flpc, resid, ssresid, bfm.
end if
.
compute omnif={omnif;fresult}.
end loop.
compute omnif=omnif(2:nrow(omnif),:).
compute clabtmp=znames.
compute condeff3=condeff3(2:nrow(condeff3),:).
do if ((nxvls*nwvls)=1).
compute omnif={condeff3,omnif}.
compute clabtmp={clabtmp,'Effect'}.
end if.
compute omnif={zmodvals,omnif}.
do if ((i < nms+nys) or (ydich=0)).
compute clabtmp={clabtmp,hcflab,'df1','df2','p'}.
end if.
do if ((i = (nms+nys)) and (ydich=1)).
compute clabtmp={clabtmp,'Chi-sq','df','p'}.
end if.
compute resultm2=make(nrow(omnif),maxresm,99999).
compute resultm2(1:nrow(omnif),1:ncol(omnif))=omnif.
compute resultm={resultm;resultm2}.
do if (outscree=1).
do if (jmed=1).
print omnif/title='Test of conditional X*W interaction at value(s) of Z:'/cnames=clabtmp/format= F10.4.
end if.
do if (jmed>1).
print omnif/title='Test of conditional M*W interaction at value(s) of Z:'/cnames=clabtmp/format= F10.4.
end if.
end if.
release omni3.
end if.
loop probei = 1 to probexpl.
do if (probexpl > 1).
compute foctmp=make(nrow(modvals),probexpl,0).
compute foctmp(:,probei)=foctmp(:,probei)+1.
compute probtemp=make(nrow(modvals),1,0).
loop probem = 1 to numplps.
loop probek = 1 to nxvls.
loop probej=lpstsp(probem,1) to lpstsp(probem,2).
compute probtemp={probtemp,foctmp(:,probek)&*probeval(:,probej)}.
end loop.
end loop.
end loop.
compute probeva2=probtemp(:,2:ncol(probtemp)).
compute probeva2={foctmp,probeva2}.
end if.
compute probres=probeva2*probcoef.
compute probrese=sqrt(diag(probeva2*probvarb*t(probeva2))).
compute tratio = probres&/probrese.
compute p = 2*(1-tcdf(abs(tratio), dfres)).
do if (ydich=1 and i = (nms+nys)).
compute p = 2*(1-cdfnorm(abs(tratio))).
end if.
compute modvals2={modvals,probres,probrese,tratio, p}.
do if ((i < nms+nys) or (ydich=0)).
compute modvals2={modvals2,(probres-tval&*probrese),(probres+tval&*probrese)}.
compute problabs={problabs,'Effect',hclab,'t', 'p', 'LLCI', 'ULCI'}.
end if.
do if (ydich=1 and i = (nms+nys)).
compute modvals2={modvals2,(probres-xp2&*probrese),(probres+xp2&*probrese)}.
compute problabs={problabs,'Effect','se','Z', 'p', 'LLCI', 'ULCI'}.
end if.
do if (probexpl > 1 and (printpbe <= intprobe)).
do if (hasz = 1).
compute printz=1.
end if.
do if (hasw=1).
compute printw=1.
end if.
compute probrlab=make(nrow(modvals),1,xcatlab(probei,1)).
compute modvals3={modvals3; modvals2}.
compute probrow={probrow;probrown}.
compute probrown=probrown+1.
do if (probei=probexpl).
compute xproblab=xcatlab(1:nxvls,1).
compute probrow=probrow(2:nrow(probrow),1).
compute modvals3=modvals3(2:nrow(modvals3),:).
compute temp=modvals3.
compute temp(GRADE(probrow(:,1)),:)=modvals3.
compute modvals3=temp.
compute start2=1.
compute problabs=problabs(1,(1+(ncol(modvarl))):ncol(problabs)).
compute pstart=1.
loop probek= 1 to nrow(probeval).
compute endstart=start2+(nxvls-1).
compute temp=modvals3(start2:endstart,(1+ncol(modvarl)):ncol(modvals3)).
compute temp2=t(modvals3(start2:start2,1:ncol(modvarl))).
compute trnames=t(modvarl).
do if (outscree=1).
do if (probek > 1).
print/title='----------'/space=0.
else.
print/title = 'Conditional effects of the focal predictor at values of the moderator(s):'.
do if ((jmed=1) and (i = (nms+nys)) and (nms > 0)).
do if (nxvls = 1).
print/title = '(These are also the conditional direct effects of X on Y)'/space=0.
else.
print/title = '(These are also the relative conditional direct effects of X on Y)'/space=0.
end if.
end if.
print.
end if.
print temp2/title = 'Moderator value(s):'/rnames=trnames/format= F10.4 /space=0.
print temp/title = ' '/cnames=problabs/rnames=xproblab/format= F10.4 /space=0.
end if.
compute resultm2=make(nrow(temp2),maxresm,99999).
compute resultm2(1:nrow(temp2),1:ncol(temp2))=temp2.
compute resultm={resultm;resultm2}.
compute resultm2=make(nrow(temp),maxresm,99999).
compute resultm2(1:nrow(temp),1:ncol(temp))=temp.
compute resultm={resultm;resultm2}.
compute start2=start2+nxvls.
compute didsome=1.
do if (jmed=1).
compute mod1val=probeval(probek,1:nm1vls).
loop omnilp=1 to nxvls.
compute omni((wheremv1(1,i)+((omnilp-1)*nm1vls)):(wheremv1(1,i)+((omnilp-1)* nm1vls)+(nm1vls-1)),omnilp)=t(mod1val).
do if (nm1vls < ncol(probeval)).
compute mod2val=probeval(probek,(nm1vls+1):(nm1vls+nm2vls)).
compute omni((wheremv2(1,i)+((omnilp-1)*nm2vls)):(wheremv2(1,i)+((omnilp-1)* nm2vls)+(nm2vls-1)),omnilp)=t(mod2val).
do if ((nm1vls+nm2vls) < ncol(probeval)).
compute intlen=nm1vls*nm2vls.
compute modintvl=probeval(probek,(nm1vls+nm2vls+1):ncol(probeval)).
compute omni((wherexwz(1,i)+((omnilp-1)*intlen)):(wherexwz(1,i)+((omnilp-1)* intlen)+(intlen-1)),omnilp)=t(modintvl).
end if.
end if.
end loop.

.
compute lmat2= omni.
do if ( 1 =0).
compute lmat2 = mdiag( omni ).
compute lmat3=make(nrow(lmat2),1,0).
loop flp=1 to ncol(lmat2).
do if (csum(lmat2(:,flp))=1).
compute lmat3={lmat3,lmat2(:,flp)}.
end if.
end loop.
compute lmat2=lmat3(:,2:ncol(lmat3)).
end if.
compute fratio = (t(t(lmat2)* b )*inv(t(lmat2)* varb *lmat2)*((t(lmat2)* b )))/ncol(lmat2).
compute pfr = 1-fcdf(fratio,ncol(lmat2),(n-nrow( b ))).
compute fresult={fratio,ncol(lmat2),(n-nrow( b )),pfr}.
do if (i = (nms+nys) and (ydich=1)).
compute fratio=fratio*ncol(lmat2).
compute pfr=1-chicdf(fratio,ncol(lmat2)).
compute fresult={fratio,ncol(lmat2),pfr}.
end if.
do if ( 0 =1).
compute lmat3=1-rsum(lmat2).
compute xfm=make(n,csum(lmat3),0).
compute flpc=1.
loop flp=1 to nrow(lmat3).
do if (lmat3(flp,1)=1).
compute xfm(:,flpc)=x(:,flp).
compute flpc=flpc+1.
end if.
end loop.
compute bfm=inv(t(xfm)*xfm)*t(xfm)*y.
compute resid=y-(xfm*bfm).
compute sstotal=(y-(csum(y)/n)).
compute sstotal=csum(sstotal&*sstotal).
compute ssresid=csum(resid&*resid).
compute rsqch= 0 -((sstotal-ssresid)/sstotal).
compute fresult={rsqch,fresult}.
release xfm,flpc, resid, ssresid, bfm.
end if
.
compute resultm2=make(nrow(fresult),maxresm,99999).
compute resultm2(1:nrow(fresult),1:ncol(fresult))=fresult.
compute resultm={resultm;resultm2}.
do if (outscree=1).
do if (i < (nms + nys) or (ydich = 0)).
compute clabtmp={hcflab,'df1','df2','p'}.
print fresult/title='Test of equality of conditional means'/cnames=clabtmp/format= F10.4.
end if.
do if (i = (nms + nys) and (ydich = 1)).
compute clabtmp={'Chi-sq','df','p'}.
print fresult/title='Test of equality of conditional logits or probabilities'/cnames=clabtmp/format= F10.4.
end if.
end if.
compute probetmp=probeplt(pstart:(pstart+nxvls),1).
compute probetmp={probetmp,probeplt(pstart:(pstart+nxvls),prevloc:ncol(probeplt))}.
compute pstart=pstart+(nxvls+1).
compute resultm2=make(nrow(probetmp),maxresm,99999).
compute resultm2(1:nrow(probetmp),1:ncol(probetmp))=probetmp.
compute resultm={resultm;resultm2}.
do if (i < ((nms + nys)) or (ydich = 0)).
compute clabtmp={xnames, outnames(1,i), hclab, 'LLCI', 'ULCI'}.
do if (outscree=1).
print probetmp/title = 'Estimated conditional means being compared:'/cnames=clabtmp/format= F10.4.
end if.
end if.
do if ((i = (nms+nys)) and (ydich = 1)).
compute clabtmp={xnames, outnames(1,i), 'prob'}.
compute probetm2=probetmp(:,1:2).
compute probetm2={probetm2,probetmp(:,ncol(probetmp))}.
do if (outscree=1).
print probetm2/title = 'Estimated conditional logits and probabilities:'/cnames=clabtmp/format= F10.4.
end if.
end if.
end if.
end loop.
release probrow, start2, endstart, temp, temp2.
end if.
end if.
do if (probexpl = 1 and (printpbe <= intprobe)).
compute resultm2=make(nrow(modvals2),maxresm,99999).
compute resultm2(1:nrow(modvals2),1:ncol(modvals2))=modvals2.
compute resultm={resultm;resultm2}.
do if (outscree=1).
print/title = 'Conditional effects of the focal predictor at values of the moderator(s):'.
print modvals2/cnames=problabs/title = ' '/space=0/format= F10.4.
end if.
compute didsome=1.
do if (hasz = 1).
compute printz=1.
end if.
do if (hasw=1).
compute printw=1.
end if.
do if (jn = 1 and jnok=1).
do if (criterr = 0).
compute dfres=n-nrow(b).
compute roots=99999.
compute jncrit =(dfres* (exp((dfres-(5/6))*((xp2/(dfres-(2/3)+(.11/dfres)))* (xp2/(dfres-(2/3)+(.11/dfres)))))-1)).
do if (i = (nms+nys) and (ydich=1)).
compute jncrit=xp2*xp2.
end if.
compute jnb1=b(wherejn1,1).
compute jnb3=b(wherejn3,1).
compute jnsb1=varb(wherejn1,wherejn1).
compute jnsb3=varb(wherejn3,wherejn3).
compute jnsb1b3=varb(wherejn1,wherejn3).
compute ajn =(jncrit*jnsb3)-(jnb3*jnb3).
compute bjn = 2*((jncrit*jnsb1b3)-(jnb1*jnb3)).
compute cjn = (jncrit*jnsb1)-(jnb1*jnb1).
compute radarg = (bjn*bjn)-(4*ajn*cjn).
compute den = 2*ajn.
compute nrts = 0.
do if (radarg >= 0 and den <> 0).
compute x21 = (-bjn+sqrt(radarg))/den.
compute x22 = (-bjn-sqrt(radarg))/den.
compute roots = 0.
do if (x21 >= jnmin and x21 <= jnmax).
compute nrts = 1.
compute roots = {roots; x21}.
end if.
do if (x22 >= jnmin and x22 <= jnmax).
compute nrts = nrts + 1.
compute roots = {roots; x22}.
end if.
compute roots={roots,make(nrow(roots),2,0)}.
end if.
do if (nrts > 0).
compute roots = roots(2:nrow(roots),1:3).
compute roots(1,2)=(csum(jnmod < roots(1,1))/n)*100.
compute roots(1,3)=(csum(jnmod > roots(1,1))/n)*100.
do if (nrow(roots)=2).
compute roots(2,2)=(csum(jnmod < roots(2,1))/n)*100.
compute roots(2,3)=(csum(jnmod > roots(2,1))/n)*100.
end if.
do if (outscree=1).
print roots/title = 'Moderator value(s) defining Johnson-Neyman significance region(s):'/clabels = 'Value', '% below', '% above'/format F10.4.
end if.
do if (nrts=1).
compute tmprts=make(1,3,99999).
compute roots={roots;tmprts}.
end if.
end if.
do if (nrts = 0).
compute roots=make(2,1,99999).
do if (outscree=1).
print/title = 'There are no statistical significance transition points within the observed'.
print/title = 'range of the moderator found using the Johnson-Neyman method.'/space=0.
end if.
end if.
compute resultm2=make(nrow(roots),maxresm,99999).
compute resultm2(1:nrow(roots),1:ncol(roots))=roots.
compute resultm={resultm;resultm2}.
compute jnvals=make(23,7,0).
loop jni= 0 to (21-(nrts)).
compute jnvals((jni+1),1)=jnmin+(jni*((jnmax-jnmin)/(21-nrts))).
end loop.
do if (nrts > 0).
loop jni = 1 to nrts.
loop jnj = 2 to (nrow(jnvals)-1).
do if ((roots(jni,1) > jnvals((jnj-1),1)) and (roots(jni,1) < jnvals(jnj,1))).
compute jnvals((jnj+1):(21+jni),1)=jnvals(jnj:(20+jni),1).
compute jnvals(jnj,1)=roots(jni,1).
end if.
end loop.
end loop.
end if.
compute jnvals=jnvals(1:22,:).
loop jni = 1 to nrow(jnvals).
compute jnvals(jni,2)=jnb1+jnb3*jnvals(jni,1).
compute jnvals(jni,3)=sqrt(jnsb1+2*jnvals(jni,1)*jnsb1b3+(jnvals(jni,1)*jnvals(jni, 1))*jnsb3).
compute jnvals(jni,4)=jnvals(jni,2)/jnvals(jni,3).
compute jnvals(jni,5)=2*(1-tcdf(abs(jnvals(jni,4)), dfres)).
compute jnvals(jni,6)=jnvals(jni,2)-sqrt(jncrit)*jnvals(jni,3).
compute jnvals(jni,7)=jnvals(jni,2)+sqrt(jncrit)*jnvals(jni,3).
do if ((i = nms + nys) and (ydich=1)).
compute jnvals(jni,5)=2*(1-cdfnorm(abs(jnvals(jni,4)))).
compute jnvals(jni,6)=jnvals(jni,2)-xp2*jnvals(jni,3).
compute jnvals(jni,7)=jnvals(jni,2)+xp2*jnvals(jni,3).
end if.
end loop.
compute resultm2=make(nrow(jnvals),maxresm,99999).
compute resultm2(1:nrow(jnvals),1:ncol(jnvals))=jnvals.
compute resultm={resultm;resultm2}.
do if ((i < nms+nys) or (ydich=0)).
compute jnclbs={jnmodlab,'Effect',hclab,'t', 'p', 'LLCI', 'ULCI'}.
end if.
do if ((i = nms + nys) and (ydich=1)).
compute jnclbs={jnmodlab,'Effect','se','Z', 'p', 'LLCI', 'ULCI'}.
end if.
do if (((wcmat((i+1),jmed)=1) or (zcmat((i+1),jmed)=1)) and (wzcmat((i+1),jmed)=0)).
do if (outscree=1).
print jnvals/title = 'Conditional effect of focal predictor at values of the moderator:'/cnames =jnclbs/format = F10.4.
end if.
end if.
do if (outscree=1).
do if ((jmed = 1) and (wzcmat((i+1),jmed)=1)).
print jnvals/title = 'Conditional X*W interaction at values of the moderator Z:'/cnames =jnclbs/format = F10.4.
end if.
do if ((jmed > 1) and (wzcmat((i+1),jmed)=1)).
print jnvals/title = 'Conditional M*W interaction at values of the moderator Z:'/cnames =jnclbs/format = F10.4.
end if.
end if.
end if.
end if.
end if.
do if ((i = (nms+nys)) and (jmed=1) and (bcmat(nrow(bcmat),1)=1)).
do if (probei=1).
compute direfflb=problabs.
compute direff=modvals2.
end if.
do if (probei>1).
compute direff={direff;modvals2}.
end if.
end if.
compute intprint=0.
do if ((jmed=1) and (i=1) and nms=0) and modcok=1).
compute contvec2=make(2,1,1).
compute contvec2={contvec2,wcontval,zcontval}.
do if (wzcmat((i+1),jmed)=1).
loop conti= 1 to ncol(wcontval).
loop contj = 1 to ncol(zcontval).
compute contvec2={contvec2,wcontval(:,conti)&*zcontval(:,contj)}.
end loop.
end loop.
end if.
compute conteff=contvec2*probcoef.
compute contdiff=contvec2(1,:)-contvec2(2,:).
compute contse=sqrt(contdiff*probvarb*t(contdiff)).
compute conteffd=conteff(1,1)-conteff(2,1).
compute contvec={contvec,conteff}.
compute contvecm=contvec.
compute resultm2=make(nrow(contvecm),maxresm,99999).
compute resultm2(1:nrow(contvecm),1:ncol(contvecm))=contvecm.
compute resultm={resultm;resultm2}.
do if (outscree=1).
print/title='Contrast between conditional effects of X:'.
print contvec/title=' '/rlabels='Effect1:','Effect2:'/cnames=problabs/format = F10.4 /space=0.
end if.
do if (ydich=0).
compute p=2*(1-tcdf(abs(conteffd/contse), dfres)).
compute contvec={conteffd,contse,conteffd/contse, p}.
compute contvec={contvec,(conteffd-(tval*contse))}.
compute contvec={contvec,(conteffd+(tval*contse))}.
compute contlabs={'Contrast', hclab, 't', 'p', 'LLCI', 'ULCI'}.
end if.
do if (ydich=1).
compute p=2*(1-cdfnorm(abs(conteffd/contse))).
compute contvec={conteffd,contse,conteffd/contse, p}.
compute contvec={contvec,(conteffd-(xp2*contse))}.
compute contvec={contvec,(conteffd+(xp2*contse))}.
compute contlabs={'Contrast', 'se', 'Z', 'p', 'LLCI', 'ULCI'}.
end if.
compute resultm2=make(nrow(contvec),maxresm,99999).
compute resultm2(1:nrow(contvec),1:ncol(contvec))=contvec.
compute resultm={resultm;resultm2}.
do if (outscree=1).
print contvec/title='Test of Effect1 minus Effect2'/format= F10.4 /cnames=contlabs.
end if.
end if.
end loop.
do if (plot = 1 or plot = 2).
compute datalabs={t(focpred(:,3)),outnames(1,i)}.
do if (plot = 1).
compute datalabs={datalabs}.
end if.
do if (plot = 2).
compute datalabs={datalabs,'se', 'LLCI', 'ULCI'}.
end if.
do if ((i = nms+nys) and (ydich=1)).
compute datalabs={datalabs,'prob'}.
end if.
compute resultm2=make(nrow(probeplt),maxresm,99999).
compute resultm2(1:nrow(probeplt),1:ncol(probeplt))=probeplt.
compute resultm={resultm;resultm2}.
compute datalabs={datalabs,'.'}.
do if (outscree=1).
print/title = 'Data for visualizing the conditional effect of the focal predictor:'.
print/title = 'Paste text below into a SPSS syntax window and execute to produce plot.'/space=0.
compute dumb = {' ', ' ', ' ', ' ', ' ', ' ', ' '}.
print datalabs/title = 'DATA LIST FREE/'/format=A10.
print probeplt/title = 'BEGIN DATA.'/format= F10.4 /space=0.
print/title = 'END DATA.'/space=0.
end if.
compute focgrph=datalabs(1,1).
compute graphix={focgrph,graphixs,modgrph}.
do if (((xdich=1) or (nxvls > 1)) and ((modcat=0) and (focgrph = xnames))).
compute graphix={modgrph,graphixs,focgrph}.
end if.
do if (panelgrp = 0).
compute graphix={graphix,'.'}.
else.
compute graphix={graphix,panelcde}.
end if.
do if (outscree=1).
print graphix/title = 'GRAPH/SCATTERPLOT='/format=A8/space=0.
end if.
do if (i = (nms + nys) and ydich = 1).
compute graphixd=graphix.
compute graphixd(1,3)='prob'.
do if (outscree=1).
print graphixd/title = 'GRAPH/SCATTERPLOT='/format=A8/space=0.
end if.
end if.
end if.
end if.
end loop.
release jmed, intprint,didprint.
end if
.
do if (model = 74 and i <= nms).
compute onetemp=make(nrow(xprobval),1,1).
compute mestmt74={onetemp,xprobval}.
do if (ncs > 0).
compute ncovmdl=rsum(ccmat(i,:)).
do if (ncovmdl > 0).
compute cvmnc=make(nrow(mestmt74),ncovmdl,1).
compute cvmnctmp=csum(x(:,(ncol(x)-ncovmdl+1):ncol(x)))/nrow(x).
do if (cuscoval > 0).
compute cvmnctmp=coval.
end if.
loop mestlp=1 to ncovmdl.
compute cvmnc(:,mestlp)=cvmnc(:,mestlp)*cvmnctmp(:,mestlp).
end loop.
compute mestmt74={mestmt74,cvmnc}.
end if.
end if.
compute mest74t=mestmt74*b.
do if (i = 1).
compute mest74=mest74t.
end if.
do if (i > 1).
compute mest74={mest74,mest74t}.
end if.
end if.
do if ((i = (nms+nys)) and (model >= 0 and model < 4) and (linsum(1,1) <> -999)).
compute lhyprob=1.
compute meansub=0.
do if (((nlinsum=nrow(b)) or (nlinsum = (nrow(b)-ncs)))).
do if (nlinsum = (nrow(b)-ncs) and ncs > 0).
compute linsum={linsum,covmeans}.
compute meansub=1.
end if.
compute lhyprob=0.
compute hypest=linsum*b.
compute sehypest=sqrt(linsum*varb*t(linsum)).
compute phypest=2*(1-tcdf(abs(hypest/sehypest), (dfres))).
compute hypest={hypest,sehypest,(hypest/sehypest),phypest,(hypest-tval*sehypest),(hypest+ tval*sehypest)}.
compute resultm2=make(ncol(linsum),maxresm,99999).
compute resultm2(:,1)=t(linsum).
compute resultm={resultm;resultm2}.
compute resultm2=make(1,maxresm,99999).
compute resultm2(1,1:ncol(hypest))=hypest.
compute resultm={resultm;resultm2}.
do if (outscree=1).
compute hyplabs={'Estimate',hclab,'t','p','LLCI','ULCI'}.
print/title='----------'.
print/title='Linear Combination Estimate and Hypothesis Test'/space=0.
print t(linsum)/title= 'Weight vector:'/clabels='weight'/rnames=vlabsm/format F10.4.
print hypest/title=' '/cnames=hyplabs/format= F10.4 /space=0.
do if (meansub=1).
print/title='Covariate weight(s) set to the sample mean.'.
end if.
end if.
end if.
do if (lhyprob=1).
compute notecode(notes,1) = 30.
compute notes = notes + 1.
end if.
end if.
end loop if criterr=1.
compute lastb=b.
compute lastcov=varb.
do if (criterr=0 and dototal = 1).
compute x=xtmp.
compute vlabsm={'constant';xcatlab(1:nxvls,1)}.
do if (ncs > 0).
compute x = {x,ctmp}.
compute vlabsm={vlabsm;t(covnames)}.
end if.
compute x = {ones,x}.

.
do if ( 1 =1).
compute hatmat=inv(t( x )* x )*t( x ).
compute b = hatmat* y.
compute modres=b.
do if ( 1 =1).
compute n1=nrow( x ).
compute dfres=n1-(ncol( x )).
compute sstotal = t( y -(csum( y )/n1))*( y -(csum( y )/n1)).
compute resid= y - x *b.
compute ssresid = csum((resid)&**2).
compute r2 = (sstotal-ssresid)/sstotal.
do if (r2 < 0).
compute r2=0.
end if.
compute adjr2 = 1-((1-r2)*(n1-1)/(dfres)).
compute mse=ssresid/(n1-ncol( x )).

.
compute n1=nrow( x ).
compute invXtX = inv(t( x )* x ).
compute varb = mse *invXtX.
compute k3 = ncol( x ).
compute xhc=0.
do if ( hc <> 5).
compute xhc= x.
compute hat = xhc(:,1).
loop i3=1 to nrow(xhc).
compute hat(i3,1)= xhc(i3,:)*invXtX*t(xhc(i3,:)).
end loop.
do if ( hc = 0 or hc =1).
loop i3 = 1 to k3.
compute xhc(:,i3)=xhc(:,i3)&* resid.
end loop.
end if.
do if ( hc =3 or hc =2).
loop i3=1 to k3.
compute xhc(:,i3) = ( resid &/(1-hat)&**(1/(4- hc )))&*xhc(:,i3).
end loop.
end if.
do if ( hc = 4).
compute hcmn=make(n,2,4).
compute hcmn(:,2)=(n1*hat)/k3.
loop i3= 1 to k3.
compute xhc(:,i3) = ( resid &/(1-hat)&**(rmin(hcmn)/2))&*xhc(:,i3).
end loop.
end if.
compute varb=(invXtX*t(xhc)*xhc*invXtX).
do if ( hc =1).
compute varb=(n1/(n1-ncol( x )))&*varb.
end if.
end if.
compute hclab={'se(HC0)','se(HC1)','se(HC2)','se(HC3)','se(HC4)','se'}.
compute hclab=hclab(1,( hc +1)).
compute hcflab={'F(HC0)','F(HC1)','F(HC2)','F(HC3)','F(HC4)','F'}.
compute hcflab=hcflab(1,( hc +1)).
release xhc
.
compute seb=sqrt(diag(varb)).
compute trat = b&/seb.
compute p = 2*(1-tcdf(abs(trat), (dfres))).
compute tval = sqrt(dfres* (exp((dfres-(5/6))*((xp2/(dfres-(2/3)+(.11/dfres)))* (xp2/(dfres-(2/3)+(.11/dfres)))))-1)).
compute modres={modres,seb,trat,p}.
compute modres={modres,(b-tval&*seb),(b+tval&*seb)}.
compute modresl={'coeff',hclab,'t','p','LLCI','ULCI'}.
compute lmat = ident(ncol( x )).
compute lmat = lmat(:,2:ncol(lmat)).
compute fratio = (t(t(lmat)*b)*inv(t(lmat)*varb*lmat)*((t(lmat)*b)))/(ncol( x )-1).
compute pfr = 1-fcdf(fratio,(ncol( x )-1),dfres).
compute modsum={sqrt(r2),r2,mse,fratio,(ncol( x )-1),dfres,pfr}.
compute modsuml={'R','R-sq','MSE',hcflab,'df1','df2', 'p'}.
end if.
end if.
do if ( 1 = 2 or 1 =3).
compute xlp= x.
compute ylp= y.
compute pt2 = make(nrow(ylp),1,(csum(ylp)/nrow(ylp))).
do if ( 1 =2).
compute LL3 = ylp&*ln(pt2)+(1-ylp)&*ln(1-pt2).
end if.
compute LL3 = -2*csum(LL3).
compute bt1 = make(ncol(xlp),1,0).
compute LL1 = 0.
compute pt1 = make(nrow(ylp),1,0.5).
compute pt1lp=pt1.
loop jjj = 1 to iterate.
compute xlptmp=t(xlp).
compute vecprb=pt1lp&*(1-pt1lp).
loop kkk=1 to ncol(xlp).
compute xlptmp(kkk,:)=xlptmp(kkk,:)&*t(vecprb).
end loop.
compute b = bt1+inv(xlptmp*xlp)*t(xlp)*(ylp-pt1lp).
do if ( 1 =2).
compute xlpb=xlp*b.
compute xlpbt=(xlpb > -709.7).
compute xlpb709=(1-xlpbt)*(-709.7).
compute xlpb=(xlpb&*xlpbt)+xlpb709.
compute pt1lp = 1/(1+exp(-(xlpb))).
end if.
compute itprob = csum((pt1lp < .00000001) or (pt1lp > .9999999)).
do if (itprob > 0).
loop kkk = 1 to nrow(pt1lp).
do if (pt1lp(kkk,1) > .9999999).
compute pt1lp(kkk,1) = .9999999.
end if.
do if (pt1lp(kkk,1) < .00000001).
compute pt1lp(kkk,1) = .00000001.
end if.
end loop.
compute itprob = 0.
end if.
do if (itprob = 0).
do if ( 1 =2).
compute LL = ylp&*ln(pt1lp)+(1-ylp)&*ln(1-pt1lp).
end if.
compute LL2 = -2*csum(ll).
end if.
do if (abs(LL1-LL2) < converge).
do if ( 1 =1).
compute xlptmp=t(xlp).
compute vecprb=pt1lp&*(1-pt1lp).
loop kkk=1 to ncol(xlp).
compute xlptmp(kkk,:)=xlptmp(kkk,:)&*t(vecprb).
end loop.
compute varb = inv(xlptmp*xlp).
compute seb = sqrt(diag(varb)).
release xlptmp.
end if.
break.
end if.
compute bt1 = b.
compute LL1 = LL2.
end loop.
compute modres=b.
do if (jjj > iterate).
compute itprob = 2.
do if (booting=0).
compute iterrmod=1.
end if.
do if (booting=1).
compute bootiter=1.
end if.
do if (itprobtg=0).
compute itprobtg=1.
compute errcode(errs,1) = 47.
compute errs = errs + 1.
do if (booting = 0 and 1 =1).
compute vt1 = mdiag(pt1lp&*(1-pt1lp)).
compute varb = inv(t(xlp)*vt1*xlp).
compute seb = sqrt(diag(varb)).
end if.
end if.
end if.
do if ( 1 =1).
compute trat = b&/seb.
compute dfres=nrow(xlp).
compute p = 2*(1-cdfnorm(abs(trat))).
compute modres={modres,seb,trat,p}.
compute modres={modres,(b-xp2&*seb),(b+xp2&*seb)}.
compute pvchi=1-chicdf((LL3-LL2),(nrow(modres)-1)).
compute mcF = (LL3-LL2)/LL3.
compute cox = 1-exp(-(LL3-LL2)/nrow(xlp)).
compute nagel = cox/(1-exp(-(LL3)/nrow(xlp))).
compute modsum={LL2,(LL3-LL2),(nrow(modres)-1),pvchi, mcF,cox,nagel}.
compute modsuml={'-2LL','ModelLL', 'df', 'p', 'McFadden', 'CoxSnell', 'Nagelkrk'}.
compute modresl={'coeff','se','Z','p','LLCI','ULCI'}.
end if.
end if
.
compute toteff=modres(2:(1+nxvls),:).
compute nodotot=0.
do if (xdich=1 and xmint=1 and model=74).
compute toteff(:,1)=toteff(:,1)*xscaling.
compute toteff(:,2)=toteff(:,2)*abs(xscaling).
compute toteff(:,3)=toteff(:,3)*xscaling.
compute toteff(:,5)=toteff(:,5)*xscaling.
compute toteff(:,6)=toteff(:,6)*xscaling.
compute citmp=toteff(:,5:6).
do if (xscaling < 0).
compute toteff(:,5)=citmp(:,2).
compute toteff(:,6)=citmp(:,1).
end if.
compute nodotot=1.
end if.
compute totefflb=modresl.
do if (outscree=1).
do if (nodotot=0).
print/title = '************************** TOTAL EFFECT MODEL ****************************'.
end if.
do if (nodotot=1).
print/title = '**************************************************************************'.
end if.
print outnames(1,ncol(outnames))/title = 'OUTCOME VARIABLE:'/format = A8/space=0.
end if.
compute toteffl2=vlabsm(2:(1+nxvls),:).
compute resultm2=make(1,maxresm,99999).
compute resultm2(1,1:ncol(modsum))=modsum.
compute resultm={resultm;resultm2}.
compute resultm2=make(nrow(modres),maxresm,99999).
compute resultm2(1:nrow(modres),1:ncol(modres))=modres.
compute resultm={resultm;resultm2}.
do if (outscree=1).
print modsum/title = 'Model Summary'/cnames = modsuml/format= F10.4.
print modres/title='Model'/rnames=vlabsm/cnames=modresl/format= F10.4.
end if.
compute lmat=make(nrow(b),1,0).
compute lmat2=make(nxvls,1,1).
compute lmat(2:(1+nxvls),1)=lmat2.
do if (ydich <> 1).

.
compute lmat2= lmat.
do if ( 0 =0).
compute lmat2 = mdiag( lmat ).
compute lmat3=make(nrow(lmat2),1,0).
loop flp=1 to ncol(lmat2).
do if (csum(lmat2(:,flp))=1).
compute lmat3={lmat3,lmat2(:,flp)}.
end if.
end loop.
compute lmat2=lmat3(:,2:ncol(lmat3)).
end if.
compute fratio = (t(t(lmat2)* b )*inv(t(lmat2)* varb *lmat2)*((t(lmat2)* b )))/ncol(lmat2).
compute pfr = 1-fcdf(fratio,ncol(lmat2),(n-nrow( b ))).
compute fresult={fratio,ncol(lmat2),(n-nrow( b )),pfr}.
do if (i = (nms+nys) and (ydich=1)).
compute fratio=fratio*ncol(lmat2).
compute pfr=1-chicdf(fratio,ncol(lmat2)).
compute fresult={fratio,ncol(lmat2),pfr}.
end if.
do if ( 1 =1).
compute lmat3=1-rsum(lmat2).
compute xfm=make(n,csum(lmat3),0).
compute flpc=1.
loop flp=1 to nrow(lmat3).
do if (lmat3(flp,1)=1).
compute xfm(:,flpc)=x(:,flp).
compute flpc=flpc+1.
end if.
end loop.
compute bfm=inv(t(xfm)*xfm)*t(xfm)*y.
compute resid=y-(xfm*bfm).
compute sstotal=(y-(csum(y)/n)).
compute sstotal=csum(sstotal&*sstotal).
compute ssresid=csum(resid&*resid).
compute rsqch= r2 -((sstotal-ssresid)/sstotal).
compute fresult={rsqch,fresult}.
release xfm,flpc, resid, ssresid, bfm.
end if
.
compute totomni=fresult.
end if.
do if (stand=1).
compute predsd=make(nrow(modres),1,0).
compute stdmod=modres(:,1)&/ovsd(1,ncol(ovsd)).
loop jd=1 to ncol(x).
compute descdat=x(:,jd).
compute predsd(jd,1) = (nrow(descdat)*sscp(descdat))-(t(csum(descdat))*(csum(descdat))).
compute predsd(jd,1) = sqrt(predsd(jd,1)/(nrow(descdat)*(nrow(descdat)-1))).
end loop.
do if (wherex(1,ncol(wherex)) <> -999 and ((nxvls > 1) or (xdich=1))).
compute sdmsone=make(nxvls,1,1).
compute predsd(wherex(1,ncol(wherex)):wherex(2,ncol(wherex)),1)=sdmsone.
compute pstog=1.
end if.
compute predsd(1,1)=1.
compute stdmod=stdmod&*predsd.
compute stdmod=stdmod(2:nrow(stdmod),1).
compute sdvlabs=vlabsm(2:nrow(vlabsm),1).
compute resultm2=make(nrow(stdmod),maxresm,99999).
compute resultm2(1:nrow(stdmod),1:ncol(stdmod))=stdmod.
compute resultm={resultm;resultm2}.
do if (outscree=1).
print stdmod/title='Standardized coefficients'/clabels='coeff'/rnames=sdvlabs/format= F10.4.
end if.
end if.
do if (covcoeff=1).
do if (outscree=1).
print varb/title='Covariance matrix of regression parameter estimates:'/rnames=vlabsm/cnames=vlabsm/format= F10.4.
end if.
compute resultm2=make(nrow(varb),maxresm,99999).
do if (ncol(varb) <= maxresm).
compute resultm2(1:nrow(varb),1:ncol(varb))=varb.
compute resultm={resultm;resultm2}.
end if.
do if (ncol(varb) > maxresm).
compute resultmt=make(nrow(resultm),ncol(varb),99999).
compute resultmt(1:nrow(resultm),1:ncol(resultm))=resultm.
compute resultm=resultmt.
compute resultm2=make(nrow(varb),ncol(resultm),99999).
compute resultm2(1:nrow(varb),1:ncol(varb))=varb.
compute resultm={resultm;resultm2}.
compute maxresm=ncol(resultm).
end if.
end if.
end if.
end if.
do if (criterr=0 and nms > 0 and ydich=0 and modelres=1).
compute modresid=modresid(:,2:ncol(modresid)).
compute sigmatal = (t(modresid)*(ident(n)-(1/n)*ones*t(ones))*modresid)*(1/(n-1)).
compute sdall = mdiag(1/sqrt(diag(sigmatal))).
compute corall=sdall*sigmatal*t(sdall).
compute resultm2=make(nrow(corall),maxresm,99999).
compute resultm2(1:nrow(corall),1:ncol(corall))=corall.
compute resultm={resultm;resultm2}.
do if (outscree=1).
print/title = '****************** CORRELATIONS BETWEEN MODEL RESIDUALS ******************'.
print corall/title=' '/format= F10.4 / cnames=outnames/rnames=outnames/space=0.
end if.
end if.
do if (criterr=0 and boot > 0).
compute bootres=make(1,rsum(nump),-999).
compute bootdir=obsdirfx.
compute natdirbt=make(1,nxvls,-999).
do if (effsize=1).
compute bootysd=make(1,1,-999).
compute bootxsd=make(1,1,-999).
end if.
compute badboot=0.
compute goodboot=0.
compute smallest=1.
compute booting=1.
loop j = 1 to maxboot.
compute nobootx=1.
compute modres2=999.
compute v=trunc(uniform(n,1)*n)+1.
compute bad=0.
loop i = 1 to (nms+nys).
compute y=outvars(v,i).
compute ynovar= (nrow(y)*sscp(y))-(t(csum(y))*(csum(y))).
do if (ynovar = 0).
compute bad=1.
end if.
compute xindx=datindx(1:(nump(1,i)-1),i).
compute hello=0.
compute x = fulldat(v,xindx).
compute x={ones,x}.
compute xsq=t(x)*x.
compute exsq=eval(xsq).
release xsq.
compute holymoly=cmin(exsq).
compute zeroeig=csum(exsq <= 0.000000000002).
compute bad=bad + (zeroeig > 0).

.
compute desctmp=make((8-(4* 1 )),ncol( y ),-999).
loop jd=1 to ncol( y ).
compute descdat= y (:,jd).
compute desctmp(1,jd) = csum(descdat)/nrow(descdat).
compute desctmp(2,jd) = (nrow(descdat)*sscp(descdat))-(t(csum(descdat))*(csum(descdat))).
compute desctmp(2,jd) = sqrt(desctmp(2,jd)/(nrow(descdat)*(nrow(descdat)-1))).
compute desctmp(3,jd)=cmin(descdat).
compute desctmp(4,jd)=cmax(descdat).
do if ( 1 =0).
compute minwarn=0.
compute maxwarn=0.
do if ((desctmp(3,jd)=desctmp(4,jd)) and novar=0).
compute errcode(errs,1)=15.
compute errs=errs+1.
compute criterr=1.
compute novar=1.
end if.
compute tmp=((descdat(:,1)=desctmp(3,jd))+(descdat(:,1)=desctmp(4,jd))).
compute desctmp(8,jd)=(csum(tmp)=nrow(tmp)).
compute tmp = descdat.
compute tmp(GRADE(descdat),:) = descdat.
compute descdat = tmp.
release tmp.
compute decval={.16;.5;.84}.
loop kd=1 to 3.
compute low=trunc(decval(kd,1)*(nrow(descdat)+1)).
compute lowdec=decval(kd,1)*(nrow(descdat)+1)-low.
compute value=descdat(low,1)+(descdat((low+1),1)-descdat(low,1))*lowdec.
compute desctmp((4+kd),jd)=value.
end loop.
compute mnotev=1.
compute modvals=desctmp(5:7,:).
do if (quantile <> 1).
compute desctmp(5,jd)=desctmp(1,jd)-desctmp(2,jd).
compute desctmp(6,jd)=desctmp(1,jd).
compute desctmp(7,jd)=desctmp(1,jd)+desctmp(2,jd).
compute modvals=desctmp(5:7,:).
compute mnotev=2.
do if (modvals(1,1) < desctmp(3,1)).
compute modvals(1,1)=desctmp(3,1).
compute minwarn=1.
end if.
do if (modvals(3,1) > desctmp(4,1)).
compute modvals(3,1)=desctmp(4,1).
compute maxwarn=1.
end if.
end if.
do if (desctmp(8,1)=1).
compute modvals={desctmp(3,1);desctmp(4,1)}.
compute mnotev=0.
compute minwarn=0.
compute maxwarn=0.
end if.
end if.
end loop
.
compute bad=bad+ (desctmp(2,1) <= 0.00000000001).
do if (bad = 0).
do if (holymoly < smallest).
compute smallest=holymoly.
end if.
do if (ydich=0 or (i < (nms+nys))).

.
do if ( 1 =1).
compute hatmat=inv(t( x )* x )*t( x ).
compute b = hatmat* y.
compute modres=b.
do if ( 0 =1).
compute n1=nrow( x ).
compute dfres=n1-(ncol( x )).
compute sstotal = t( y -(csum( y )/n1))*( y -(csum( y )/n1)).
compute resid= y - x *b.
compute ssresid = csum((resid)&**2).
compute r2 = (sstotal-ssresid)/sstotal.
do if (r2 < 0).
compute r2=0.
end if.
compute adjr2 = 1-((1-r2)*(n1-1)/(dfres)).
compute mse=ssresid/(n1-ncol( x )).

.
compute n1=nrow( x ).
compute invXtX = inv(t( x )* x ).
compute varb = mse *invXtX.
compute k3 = ncol( x ).
compute xhc=0.
do if ( hc <> 5).
compute xhc= x.
compute hat = xhc(:,1).
loop i3=1 to nrow(xhc).
compute hat(i3,1)= xhc(i3,:)*invXtX*t(xhc(i3,:)).
end loop.
do if ( hc = 0 or hc =1).
loop i3 = 1 to k3.
compute xhc(:,i3)=xhc(:,i3)&* resid.
end loop.
end if.
do if ( hc =3 or hc =2).
loop i3=1 to k3.
compute xhc(:,i3) = ( resid &/(1-hat)&**(1/(4- hc )))&*xhc(:,i3).
end loop.
end if.
do if ( hc = 4).
compute hcmn=make(n,2,4).
compute hcmn(:,2)=(n1*hat)/k3.
loop i3= 1 to k3.
compute xhc(:,i3) = ( resid &/(1-hat)&**(rmin(hcmn)/2))&*xhc(:,i3).
end loop.
end if.
compute varb=(invXtX*t(xhc)*xhc*invXtX).
do if ( hc =1).
compute varb=(n1/(n1-ncol( x )))&*varb.
end if.
end if.
compute hclab={'se(HC0)','se(HC1)','se(HC2)','se(HC3)','se(HC4)','se'}.
compute hclab=hclab(1,( hc +1)).
compute hcflab={'F(HC0)','F(HC1)','F(HC2)','F(HC3)','F(HC4)','F'}.
compute hcflab=hcflab(1,( hc +1)).
release xhc
.
compute seb=sqrt(diag(varb)).
compute trat = b&/seb.
compute p = 2*(1-tcdf(abs(trat), (dfres))).
compute tval = sqrt(dfres* (exp((dfres-(5/6))*((xp2/(dfres-(2/3)+(.11/dfres)))* (xp2/(dfres-(2/3)+(.11/dfres)))))-1)).
compute modres={modres,seb,trat,p}.
compute modres={modres,(b-tval&*seb),(b+tval&*seb)}.
compute modresl={'coeff',hclab,'t','p','LLCI','ULCI'}.
compute lmat = ident(ncol( x )).
compute lmat = lmat(:,2:ncol(lmat)).
compute fratio = (t(t(lmat)*b)*inv(t(lmat)*varb*lmat)*((t(lmat)*b)))/(ncol( x )-1).
compute pfr = 1-fcdf(fratio,(ncol( x )-1),dfres).
compute modsum={sqrt(r2),r2,mse,fratio,(ncol( x )-1),dfres,pfr}.
compute modsuml={'R','R-sq','MSE',hcflab,'df1','df2', 'p'}.
end if.
end if.
do if ( 1 = 2 or 1 =3).
compute xlp= x.
compute ylp= y.
compute pt2 = make(nrow(ylp),1,(csum(ylp)/nrow(ylp))).
do if ( 1 =2).
compute LL3 = ylp&*ln(pt2)+(1-ylp)&*ln(1-pt2).
end if.
compute LL3 = -2*csum(LL3).
compute bt1 = make(ncol(xlp),1,0).
compute LL1 = 0.
compute pt1 = make(nrow(ylp),1,0.5).
compute pt1lp=pt1.
loop jjj = 1 to iterate.
compute xlptmp=t(xlp).
compute vecprb=pt1lp&*(1-pt1lp).
loop kkk=1 to ncol(xlp).
compute xlptmp(kkk,:)=xlptmp(kkk,:)&*t(vecprb).
end loop.
compute b = bt1+inv(xlptmp*xlp)*t(xlp)*(ylp-pt1lp).
do if ( 1 =2).
compute xlpb=xlp*b.
compute xlpbt=(xlpb > -709.7).
compute xlpb709=(1-xlpbt)*(-709.7).
compute xlpb=(xlpb&*xlpbt)+xlpb709.
compute pt1lp = 1/(1+exp(-(xlpb))).
end if.
compute itprob = csum((pt1lp < .00000001) or (pt1lp > .9999999)).
do if (itprob > 0).
loop kkk = 1 to nrow(pt1lp).
do if (pt1lp(kkk,1) > .9999999).
compute pt1lp(kkk,1) = .9999999.
end if.
do if (pt1lp(kkk,1) < .00000001).
compute pt1lp(kkk,1) = .00000001.
end if.
end loop.
compute itprob = 0.
end if.
do if (itprob = 0).
do if ( 1 =2).
compute LL = ylp&*ln(pt1lp)+(1-ylp)&*ln(1-pt1lp).
end if.
compute LL2 = -2*csum(ll).
end if.
do if (abs(LL1-LL2) < converge).
do if ( 0 =1).
compute xlptmp=t(xlp).
compute vecprb=pt1lp&*(1-pt1lp).
loop kkk=1 to ncol(xlp).
compute xlptmp(kkk,:)=xlptmp(kkk,:)&*t(vecprb).
end loop.
compute varb = inv(xlptmp*xlp).
compute seb = sqrt(diag(varb)).
release xlptmp.
end if.
break.
end if.
compute bt1 = b.
compute LL1 = LL2.
end loop.
compute modres=b.
do if (jjj > iterate).
compute itprob = 2.
do if (booting=0).
compute iterrmod=1.
end if.
do if (booting=1).
compute bootiter=1.
end if.
do if (itprobtg=0).
compute itprobtg=1.
compute errcode(errs,1) = 47.
compute errs = errs + 1.
do if (booting = 0 and 0 =1).
compute vt1 = mdiag(pt1lp&*(1-pt1lp)).
compute varb = inv(t(xlp)*vt1*xlp).
compute seb = sqrt(diag(varb)).
end if.
end if.
end if.
do if ( 0 =1).
compute trat = b&/seb.
compute dfres=nrow(xlp).
compute p = 2*(1-cdfnorm(abs(trat))).
compute modres={modres,seb,trat,p}.
compute modres={modres,(b-xp2&*seb),(b+xp2&*seb)}.
compute pvchi=1-chicdf((LL3-LL2),(nrow(modres)-1)).
compute mcF = (LL3-LL2)/LL3.
compute cox = 1-exp(-(LL3-LL2)/nrow(xlp)).
compute nagel = cox/(1-exp(-(LL3)/nrow(xlp))).
compute modsum={LL2,(LL3-LL2),(nrow(modres)-1),pvchi, mcF,cox,nagel}.
compute modsuml={'-2LL','ModelLL', 'df', 'p', 'McFadden', 'CoxSnell', 'Nagelkrk'}.
compute modresl={'coeff','se','Z','p','LLCI','ULCI'}.
end if.
end if
.
end if.
do if (ydich=1 and (i = (nms+nys))).

.
do if ( 2 =1).
compute hatmat=inv(t( x )* x )*t( x ).
compute b = hatmat* y.
compute modres=b.
do if ( 0 =1).
compute n1=nrow( x ).
compute dfres=n1-(ncol( x )).
compute sstotal = t( y -(csum( y )/n1))*( y -(csum( y )/n1)).
compute resid= y - x *b.
compute ssresid = csum((resid)&**2).
compute r2 = (sstotal-ssresid)/sstotal.
do if (r2 < 0).
compute r2=0.
end if.
compute adjr2 = 1-((1-r2)*(n1-1)/(dfres)).
compute mse=ssresid/(n1-ncol( x )).

.
compute n1=nrow( x ).
compute invXtX = inv(t( x )* x ).
compute varb = mse *invXtX.
compute k3 = ncol( x ).
compute xhc=0.
do if ( hc <> 5).
compute xhc= x.
compute hat = xhc(:,1).
loop i3=1 to nrow(xhc).
compute hat(i3,1)= xhc(i3,:)*invXtX*t(xhc(i3,:)).
end loop.
do if ( hc = 0 or hc =1).
loop i3 = 1 to k3.
compute xhc(:,i3)=xhc(:,i3)&* resid.
end loop.
end if.
do if ( hc =3 or hc =2).
loop i3=1 to k3.
compute xhc(:,i3) = ( resid &/(1-hat)&**(1/(4- hc )))&*xhc(:,i3).
end loop.
end if.
do if ( hc = 4).
compute hcmn=make(n,2,4).
compute hcmn(:,2)=(n1*hat)/k3.
loop i3= 1 to k3.
compute xhc(:,i3) = ( resid &/(1-hat)&**(rmin(hcmn)/2))&*xhc(:,i3).
end loop.
end if.
compute varb=(invXtX*t(xhc)*xhc*invXtX).
do if ( hc =1).
compute varb=(n1/(n1-ncol( x )))&*varb.
end if.
end if.
compute hclab={'se(HC0)','se(HC1)','se(HC2)','se(HC3)','se(HC4)','se'}.
compute hclab=hclab(1,( hc +1)).
compute hcflab={'F(HC0)','F(HC1)','F(HC2)','F(HC3)','F(HC4)','F'}.
compute hcflab=hcflab(1,( hc +1)).
release xhc
.
compute seb=sqrt(diag(varb)).
compute trat = b&/seb.
compute p = 2*(1-tcdf(abs(trat), (dfres))).
compute tval = sqrt(dfres* (exp((dfres-(5/6))*((xp2/(dfres-(2/3)+(.11/dfres)))* (xp2/(dfres-(2/3)+(.11/dfres)))))-1)).
compute modres={modres,seb,trat,p}.
compute modres={modres,(b-tval&*seb),(b+tval&*seb)}.
compute modresl={'coeff',hclab,'t','p','LLCI','ULCI'}.
compute lmat = ident(ncol( x )).
compute lmat = lmat(:,2:ncol(lmat)).
compute fratio = (t(t(lmat)*b)*inv(t(lmat)*varb*lmat)*((t(lmat)*b)))/(ncol( x )-1).
compute pfr = 1-fcdf(fratio,(ncol( x )-1),dfres).
compute modsum={sqrt(r2),r2,mse,fratio,(ncol( x )-1),dfres,pfr}.
compute modsuml={'R','R-sq','MSE',hcflab,'df1','df2', 'p'}.
end if.
end if.
do if ( 2 = 2 or 2 =3).
compute xlp= x.
compute ylp= y.
compute pt2 = make(nrow(ylp),1,(csum(ylp)/nrow(ylp))).
do if ( 2 =2).
compute LL3 = ylp&*ln(pt2)+(1-ylp)&*ln(1-pt2).
end if.
compute LL3 = -2*csum(LL3).
compute bt1 = make(ncol(xlp),1,0).
compute LL1 = 0.
compute pt1 = make(nrow(ylp),1,0.5).
compute pt1lp=pt1.
loop jjj = 1 to iterate.
compute xlptmp=t(xlp).
compute vecprb=pt1lp&*(1-pt1lp).
loop kkk=1 to ncol(xlp).
compute xlptmp(kkk,:)=xlptmp(kkk,:)&*t(vecprb).
end loop.
compute b = bt1+inv(xlptmp*xlp)*t(xlp)*(ylp-pt1lp).
do if ( 2 =2).
compute xlpb=xlp*b.
compute xlpbt=(xlpb > -709.7).
compute xlpb709=(1-xlpbt)*(-709.7).
compute xlpb=(xlpb&*xlpbt)+xlpb709.
compute pt1lp = 1/(1+exp(-(xlpb))).
end if.
compute itprob = csum((pt1lp < .00000001) or (pt1lp > .9999999)).
do if (itprob > 0).
loop kkk = 1 to nrow(pt1lp).
do if (pt1lp(kkk,1) > .9999999).
compute pt1lp(kkk,1) = .9999999.
end if.
do if (pt1lp(kkk,1) < .00000001).
compute pt1lp(kkk,1) = .00000001.
end if.
end loop.
compute itprob = 0.
end if.
do if (itprob = 0).
do if ( 2 =2).
compute LL = ylp&*ln(pt1lp)+(1-ylp)&*ln(1-pt1lp).
end if.
compute LL2 = -2*csum(ll).
end if.
do if (abs(LL1-LL2) < converge).
do if ( 0 =1).
compute xlptmp=t(xlp).
compute vecprb=pt1lp&*(1-pt1lp).
loop kkk=1 to ncol(xlp).
compute xlptmp(kkk,:)=xlptmp(kkk,:)&*t(vecprb).
end loop.
compute varb = inv(xlptmp*xlp).
compute seb = sqrt(diag(varb)).
release xlptmp.
end if.
break.
end if.
compute bt1 = b.
compute LL1 = LL2.
end loop.
compute modres=b.
do if (jjj > iterate).
compute itprob = 2.
do if (booting=0).
compute iterrmod=1.
end if.
do if (booting=1).
compute bootiter=1.
end if.
do if (itprobtg=0).
compute itprobtg=1.
compute errcode(errs,1) = 47.
compute errs = errs + 1.
do if (booting = 0 and 0 =1).
compute vt1 = mdiag(pt1lp&*(1-pt1lp)).
compute varb = inv(t(xlp)*vt1*xlp).
compute seb = sqrt(diag(varb)).
end if.
end if.
end if.
do if ( 0 =1).
compute trat = b&/seb.
compute dfres=nrow(xlp).
compute p = 2*(1-cdfnorm(abs(trat))).
compute modres={modres,seb,trat,p}.
compute modres={modres,(b-xp2&*seb),(b+xp2&*seb)}.
compute pvchi=1-chicdf((LL3-LL2),(nrow(modres)-1)).
compute mcF = (LL3-LL2)/LL3.
compute cox = 1-exp(-(LL3-LL2)/nrow(xlp)).
compute nagel = cox/(1-exp(-(LL3)/nrow(xlp))).
compute modsum={LL2,(LL3-LL2),(nrow(modres)-1),pvchi, mcF,cox,nagel}.
compute modsuml={'-2LL','ModelLL', 'df', 'p', 'McFadden', 'CoxSnell', 'Nagelkrk'}.
compute modresl={'coeff','se','Z','p','LLCI','ULCI'}.
end if.
end if
.
end if.
compute modres2={modres2,t(modres)}.
do if (i = (nms+nys)).
do if (bcmat((i+1),1) = 1).
compute bootdir={bootdir;t(modres(wherex(1,i):wherex(2,i),1))}.
end if.
do if (bcmat((i+1),1) = 0).
compute bootdir={bootdir;dirzes}.
end if.
end if.
do if (model = 74 and i <= nms).
compute onetemp=make(nrow(xprobval),1,1).
compute mestmtb={onetemp,xprobval}.
do if (ncs > 0).
compute ncovmdlb=rsum(ccmat(i,:)).
do if (ncovmdl > 0).
compute cvmncb=make(nrow(mestmtb),ncovmdlb,1).
compute cvmnctmp=csum(x(:,(ncol(x)-ncovmdlb+1):ncol(x)))/nrow(x).
do if (cuscoval > 0).
compute cvmnctmp=coval.
end if.
loop mestlp=1 to ncovmdlb.
compute cvmncb(:,mestlp)=cvmncb(:,mestlp)*cvmnctmp(:,mestlp).
end loop.
compute mestmtb={mestmtb,cvmncb}.
end if.
end if.
compute mestbt=mestmtb*modres.
do if (i = 1).
compute mestb=mestbt.
end if.
do if (i > 1).
compute mestb={mestb,mestbt}.
end if.
end if.
do if (model = 74 and i = (nms+nys)).
compute xvalptmp=make(1,nxvls,0).
compute mest74sp=make(1,nms,0).
compute mest74sp={mest74sp;mestb}.
compute xvalptmp={xvalptmp;ident(nxvls)}.
compute dirfxcf=make(nrow(mestb),1,0).
loop cfloop1=1 to nrow(mestb).
compute ndirfx=make(nrow(modres),1,0).
compute ndirfx(2:(nxvls+1),1)=t(xvalptmp(cfloop1,:)).
loop cfloop3=1 to nms.
do if (mcx=1 or mcx=0).
compute ndirfx(wheremw((1+((cfloop3-1)*2)),ncol(wheremw)):wheremw((2+((cfloop3-1)*2) ),ncol(wheremw)))=t(xvalptmp(cfloop1,:)*mestb(1,cfloop3)).
end if.
do if (mcx=2).
compute ndirfx(wheremw((1+((cfloop3-1)*2)),ncol(wheremw)):wheremw((2+((cfloop3-1)*2) ),ncol(wheremw)))=t(xvalptmp(cfloop1,:)*mest74sp(cfloop1,cfloop3)).
end if.
end loop.
do if (cfloop1 > 1).
compute dirfxcf(cfloop1,1)=t(ndirfx)*modres*xscaling.
end if.
end loop.
compute natdirbt={natdirbt;t(dirfxcf(2:nrow(dirfxcf),:))}.
end if.
do if ( (bcmat((i+1),1) = 1) and (nobootx=1) and (effsize=1)).
compute nobootx=0.
compute xsdtemp= (nrow(x)*sscp(x(:,2)))-(t(csum(x(:,2)))*(csum(x(:,2)))).
compute xsdtemp= sqrt(xsdtemp/(nrow(x)*(nrow(x)-1))).
end if.
end if.
end loop.
do if (bad = 0).
compute bootres={bootres;modres2(:,2:ncol(modres2))}.
do if (effsize=1).
compute ysdtemp= (nrow(y)*sscp(y))-(t(csum(y))*(csum(y))).
compute ysdtemp= sqrt(ysdtemp/(nrow(y)*(nrow(y)-1))).
compute bootysd={bootysd;ysdtemp}.
compute bootxsd={bootxsd;xsdtemp}.
end if.
compute goodboot=goodboot+1.
end if.
do if (bad <> 0).
compute badboot=badboot+1.
end if.
end loop if (goodboot = boot).
compute bootres=bootres(2:nrow(bootres),:).
do if (effsize=1).
compute bootysd=bootysd(2:nrow(bootysd),:).
do if (nrow(bootxsd) > 1).
compute bootxsd=bootxsd(2:nrow(bootxsd),:).
end if.
end if.
do if (goodboot < (boot)).
compute boot=0.
compute modelbt=0.
compute notecode(notes,1) = 7.
compute notes = notes + 1.
end if.
do if (boot > 0).
do if (effsize=1).
compute bootysd={ysd;bootysd}.
compute bootxsd={xsd;bootxsd}.
end if.
do if (saveboot = 1).
save bootres/outfile = *.
end if.
do if (modelbt=1).
compute bootcim=make(ncol(bootres),5,-99999).
compute bootcim(:,2) = t(csum(bootres)/nrow(bootres)).
compute bootcim(:,1) = coeffmat(2:nrow(coeffmat),1).
loop i = 1 to ncol(bootres).

.
compute temp = bootres(:,i).
compute temp(GRADE( bootres(:,i) )) = bootres(:,i).
compute badlo = 0.
compute badhi = 0.
do if ( (bootcim(i,1)*bc)+(9999*(1-bc)) <> 9999).
compute pv=csum(temp < (bootcim(i,1)*bc)+(9999*(1-bc)) )/nrow(temp).
compute ppv = pv.
do if (pv > .5).
compute ppv = 1-pv.
end if.
compute y5=sqrt(-2*ln(ppv)).
compute xp=y5+((((y5*p4+p3)*y5+p2)*y5+p1)*y5+p0)/((((y5*q4+q3)*y5+q2)*y5+q1)*y5+q0).
do if (pv <= .5).
compute xp = -xp.
end if.
compute cilow=rnd(nrow(temp)*(cdfnorm(2*xp-xp2))).
compute cihigh=trunc(nrow(temp)*(cdfnorm(2*xp+(xp2))))+1.
do if (cilow < 1).
compute cilow = 1.
compute booterr=1.
compute badlo = 1.
end if.
do if (cihigh > nrow(temp)).
compute cihigh = boot.
compute booterr=1.
compute badhi = 1.
end if.
compute llcit=temp(cilow,1).
compute ulcit=temp(cihigh,1).
do if (badlo = 1 and llcit <> priorlo).
compute badend={badend, llcit}.
compute priorlo = llcit.
end if.
do if (badhi = 1 and ulcit <> priorhi).
compute badend={badend, ulcit}.
compute priorhi = ulcit.
end if.
end if.
do if ( (bootcim(i,1)*bc)+(9999*(1-bc)) = 9999).
compute llcit=temp(cilow,1).
compute ulcit=temp(cihigh,1).
end if.
compute bootse=t(sqrt((cssq(temp)-((csum(temp)&**2)/nrow(temp)))/(nrow(temp)-1)))
.
compute bootcim(i,4:5)={llcit,ulcit}.
compute bootcim(i,3)=bootse.
end loop.
end if.
end if.
do if (badboot > 0).
compute notecode(notes,1) = 6.
compute notes = notes + 1.
end if.
end if.
do if (criterr=0).
release fulldat,x,y.
end if.
do if (xmint=1 and criterr=0).
compute xvalptmp=make(1,nxvls,0).
compute mest74sp=make(1,nms,0).
compute mest74sp={mest74sp;mest74}.
compute xvalptmp={xvalptmp;ident(nxvls)}.
loop kcfuhd=1 to 2.
compute dirfxcf=make(nrow(mest74),6,0).
loop cfloop1=1 to nrow(mest74).
compute ndirfx=make(nrow(lastb),1,0).
compute ndirfx(2:(nxvls+1),1)=t(xvalptmp(cfloop1,:)).
loop cfloop3=1 to nms.
do if (kcfuhd=1).
do if (mcx=1 or mcx=0).
compute ndirfx(wheremw((1+((cfloop3-1)*2)),ncol(wheremw)):wheremw((2+((cfloop3-1)*2)), ncol(wheremw)))=t(xvalptmp(cfloop1,:)*mest74(1,cfloop3)).
end if.
do if (mcx=2).
compute ndirfx(wheremw((1+((cfloop3-1)*2)),ncol(wheremw)):wheremw((2+((cfloop3-1)*2)), ncol(wheremw)))=t(xvalptmp(cfloop1,:)*mest74sp(cfloop1,cfloop3)).
end if.
end if.
do if (kcfuhd=2).
compute ndirfx(wheremw((1+((cfloop3-1)*2)),ncol(wheremw)):wheremw((2+((cfloop3-1)*2)), ncol(wheremw)))=t(xvalptmp(cfloop1,:)*medmeans(1,cfloop3)).
end if.
end loop.
do if (cfloop1 > 1).
compute dirfxcf(cfloop1,1)=t(ndirfx)*lastb*xscaling.
compute dirfxcf(cfloop1,2)=sqrt(diag(t(ndirfx)*lastcov*ndirfx))*abs(xscaling).
compute dirfxcf(cfloop1,3)=dirfxcf(cfloop1,1)/dirfxcf(cfloop1,2).
compute dirfxcf(cfloop1,4)=2*(1-tcdf(abs(dirfxcf(cfloop1,3)), dfres)).
compute dirfxcf(cfloop1,5)=dirfxcf(cfloop1,1)-tval*dirfxcf(cfloop1,2).
compute dirfxcf(cfloop1,6)=dirfxcf(cfloop1,1)+tval*dirfxcf(cfloop1,2).
end if.
end loop.
compute codireff=dirfxcf(2:nrow(dirfxcf),:).
do if (kcfuhd=1).
compute direff=codireff.
end if.
end loop.
end if.

.
do if (criterr = 0 and nms > 0).
compute paths=paths(:,2:ncol(paths)).
compute pathsw=pathsw(:,2:ncol(pathsw)).
compute pathsz=pathsz(:,2:ncol(pathsz)).
compute pathswz=pathswz(:,2:ncol(pathswz)).
compute pathsmod=pathsw+pathsz+pathswz.
compute pathsdv=pathsdv(:,2:ncol(pathsdv)).
compute pathsfoc=pathsfoc(:,2:ncol(pathsfoc)).
compute pathtype=pathtype(:,2:ncol(pathtype)).
compute anymod=(rsum(pathsmod) > 0).
compute obscoeff=obscoeff(1,2:ncol(obscoeff)).
do if (outscree=1).
do if (xmint=1).
print/title = '************************* COUNTERFACTUALLY DEFINED ************************'.
else.
print/title = ' '.
end if.
do if (dototal = 0 and alttotal=0).
print/title = '****************** DIRECT AND INDIRECT EFFECTS OF X ON Y *****************'/space=0.
end if.
do if (alttotal=1).
print/title = '************** TOTAL, DIRECT, AND INDIRECT EFFECTS OF X ON Y **************'/space=0.
end if.
end if.
do if (dototal = 1).
do if (outscree=1).
print/title = '************** TOTAL, DIRECT, AND INDIRECT EFFECTS OF X ON Y **************'/space=0.
end if.
compute totefflb(1,1)='Effect'.
do if (effsize=1).
compute toteffsz=toteff(:,1)/ysd.
do if (xdich=1 or mcx > 0).
compute totefflb={totefflb,'c_ps'}.
end if.
do if (xdich = 0 and mcx = 0).
compute toteffsz=toteffsz*xsd.
compute totefflb={totefflb,'c_cs'}.
end if.
compute toteff={toteff,toteffsz}.
end if.
compute resultm2=make(nrow(toteff),maxresm,99999).
compute resultm2(1:nrow(toteff),1:ncol(toteff))=toteff.
compute resultm={resultm;resultm2}.
do if (nxvls > 1).
compute resultm2=make(nrow(totomni),maxresm,99999).
compute resultm2(1:nrow(totomni),1:ncol(totomni))=totomni.
compute resultm={resultm;resultm2}.
compute clabtmp={'R2-chng', hcflab, 'df1','df2','p'}.
do if (outscree=1).
print toteff/title='Relative total effects of X on Y'/rnames=toteffl2/cnames=totefflb/format= F10.4.
print totomni/title='Omnibus test of total effect of X on Y'/cnames=clabtmp/format= F10.4.
print/title= '----------'/space=0.
end if.
else.
do if (outscree=1).
print toteff/title='Total effect of X on Y'/cnames=totefflb/format= F10.4.
end if.
end if.
end if.
compute moddir=wcmat(nrow(bcmat),1)+zcmat(nrow(bcmat),1).
do if (xmint=1).
compute moddir=1.
end if.
do if (bcmat(nrow(bcmat),1)=1).
do if (ydich=1).
compute direfflb(:,(ncol(direfflb)-5):ncol(direfflb))={'Effect','se','Z','p','LLCI','ULCI'}.
end if.
do if (moddir=0 or xmint=1).
compute direfflb(1,1)='Effect'.
end if.
do if (effsize=1 and moddir=0 and anymod = 0).
compute direffsz=direff(:,1)/ysd.
do if (xdich=1 or mcx > 0).
compute direfflb={direfflb,'c''_ps'}.
end if.
do if (xdich = 0 and mcx = 0).
compute direffsz=direffsz*xsd.
compute direfflb={direfflb,'c''_cs'}.
end if.
compute direff={direff,direffsz}.
end if.
compute resultm2=make(nrow(direff),maxresm,99999).
compute resultm2(1:nrow(direff),1:ncol(direff))=direff.
compute resultm={resultm;resultm2}.
do if (xmint=1).
compute resultm2=make(nrow(codireff),maxresm,99999).
compute resultm2(1:nrow(codireff),1:ncol(codireff))=codireff.
compute resultm={resultm;resultm2}.
end if.
do if (moddir = 0 and nxvls=1).
do if (outscree=1).
print direff/title='Direct effect of X on Y'/cnames=direfflb/format= F10.4.
end if.
end if.
do if (moddir = 0 and nxvls>1).
compute resultm2=make(nrow(diromni),maxresm,99999).
compute resultm2(1:nrow(diromni),1:ncol(diromni))=diromni.
compute resultm={resultm;resultm2}.
do if (outscree=1).
print direff/title='Relative direct effects of X on Y'/rnames=direffl2/cnames=direfflb/format= F10.4.
do if (ydich = 0).
compute clabtmp={'R2-chng', hcflab, 'df1','df2','p'}.
print diromni/title='Omnibus test of direct effect of X on Y:'/cnames=clabtmp/format= F10.4.
end if.
do if (ydich = 1).
compute clabtmp={'Chi-sq', 'df', 'p'}.
print diromni/title='Omnibus likelihood ratio test of direct effect of X on Y'/cnames=clabtmp/format= F10.4.
end if.
print/title= '----------'/space=0.
end if.
end if.
do if (moddir > 0 and nxvls=1).
do if (xmint=0 and outscree=1).
print direff/title='Conditional direct effects of X on Y'/cnames=direfflb/format= F10.4.
end if.
do if (xmint=1).
do if (outscree=1).
print direff/title='(Pure) Natural direct effect of X on Y'/cnames=direfflb/format= F10.4.
print codireff/title='Controlled direct effect of X on Y'/cnames=direfflb/format= F10.4.
print/title= '----------'/space=0.
end if.
compute obnatdfx=direff(:,1).
end if.
end if.
compute direffl4=direffl2.
do if (moddir > 0 and nxvls>1).
compute direffl2={' '}.
loop i = 1 to nxvls.
loop j = 1 to (nrow(direff)/nxvls).
compute direffl2={direffl2;xcatlab(i,1)}.
end loop.
end loop.
compute direffl2=direffl2(2:nrow(direffl2),1).
do if (xmint=0 and outscree=1).
print direff/title='Relative conditional direct effects of X on Y'/cnames=direfflb/rnames=direffl2/format= F10.4.
end if.
do if (xmint=1).
do if (outscree=1).
print direff/title='Relative (pure) natural direct effects of X on Y'/cnames=direfflb/rnames=direffl2/format= F10.4.
print codireff/title='Relative controlled direct effects of X on Y'/cnames=direfflb/rnames=direffl2/format= F10.4.
print/title= '----------'/space=0.
end if.
compute obnatdfx=direff(:,1).
end if.
end if.
compute direffl2=direffl4.
end if.
do if (bcmat(nrow(bcmat),1)=0 and xmint <> 1 and outscree=1).
print/title='The direct effect of X on Y is fixed to zero.'.
end if.
do if (nms = 1).
compute indmark={2}.
compute indsets={1,2}.
end if.
do if (nms = 2).
compute indmark={2,2,3}.
compute indsets={1,4,2,5,1,3,5}.
compute thetam=1.
end if.
do if (nms = 3).
compute indmark={2,2,2,3,3,3,4}.
compute indsets={1,7,2,8,4,9,1,3,8,1,5,9,2,6,9,1,3,6,9}.
compute thetam={1,2,3}.
end if.
do if (nms = 4).
compute indmark={2,2,2,2,3,3,3,3,3,3,4,4,4,4,5}.
compute indsets={1,11,2,12,4,13,7,14,1,3,12,1,5,13,1,8,14,2,6,13,2,9,14,4,10,14,1,3,6,13,1,3,9, 14,1,5,10,14,2,6,10,14,1,3,6,10,14}.
compute thetam={1,2,4,3,5,6}.
end if.
do if (nms = 5).
compute indmark={2,2,2,2,2,3,3,3,3,3,3,3,3,3,3,4,4,4,4,4,4,4,4,4,4,5,5,5,5,5,6}.
compute indsets={1,16,2,17,4,18,7,19,11,20,1,3,17,1,5,18,1,8,19,1,12,20,2,6,18,2,9,19,2,13,20,4, 10,19,4,14,20,7,15,20,1,3,6,18, 1,3,9,19,1,3,13,20,1,5,10,19,1,5,14,20,1,8,15,20,2,6,10,19,2,6, 14,20,2,9,15,20,4,10,15,20,1,3,6,10,19, 1,3,6,14,20,1,3,9,15,20,1,5,10,15,20,2,6,10,15,20,1,3,6,10,15, 20}.
compute thetam={1,2,5,3,6,8,4,7,9,10}.
end if.
do if (nms = 6).
compute indmark={2,2,2,2,2,2,3,3,3,3,3,3,3,3,3,3,3,3,3,3,3,4,4,4,4,4,4,4,4,4,4,4,4,4,4,4,4,4,4, 4,4,5,5,5,5,5,5,5,5,5,5,5,5,5,5,5,6,6,6,6,6,6,7}.
compute indsets={1,22,2,23,4,24,7,25,11,26,16,27,1,3,23,1,5,24,1,8,25,1,12,26,1,17,27,2,6,24,2, 9,25,2,13,26,2,18,27,4,10,25,4,14,26, 4,19,27,7,15,26,7,20,27,11,21,27,1,3,6,24,1,3,9,25,1,3,13,26,1, 3,18,27,1,5,10,25,1,5,14,26,1,5,19,27,1,8,15,26,1,8,20,27, 1,12,21,27,2,6,10,25,2,6,14,26,2,6,19,27,2,9,15,26,2,9,20,27,2, 13,21,27,4,10,15,26,4,10,20,27,4,14,21,27,7,15,21,27, 1,3,6,10,25,1,3,6,14,26,1,3,6,19,27,1,3,9,15,26,1,3,9,20,27,1,3, 13,21,27,1,5,10,15,26,1,5,10,20,27,1,5,14,21,27, 1,8,15,21,27,2,6,10,15,26,2,6,10,20,27,2,6,14,21,27,2,9,15,21, 27,4,10,15,21,27,1,3,6,10,15,26,1,3,6,10,20,27, 1,3,6,14,21,27,1,3,9,15,21,27,1,5,10,15,21,27,2,6,10,15,21,27, 1,3,6,10,15,21,27}.
compute thetam={1,2,6,3,7,10,4,8,11,13,5,9,12,14,15}.
end if.
do if (nms = 7).
compute indmark={2,2,2,2,2,2,2}.
compute indsets={1,29,2,30,4,31,7,32,11,33,16,34,22,35}.
end if.
do if (nms = 8).
compute indmark={2,2,2,2,2,2,2,2}.
compute indsets={1,37,2,38,4,39,7,40,11,41,16,42,22,43,29,44}.
end if.
do if (nms = 9).
compute indmark={2,2,2,2,2,2,2,2,2}.
compute indsets={1,46,2,47,4,48,7,49,11,50,16,51,22,52,29,53,37,54}.
end if.
do if (nms=10).
compute indmark={2,2,2,2,2,2,2,2,2,2}.
compute indsets={1,56,2,57,4,58,7,59,11,60,16,61,22,62,29,63,37,64,46,65}.
end if.
compute indlbl = {'Ind1'; 'Ind2'; 'Ind3'; 'Ind4'; 'Ind5'; 'Ind6'; 'Ind7'; 'Ind8'; 'Ind9'; 'Ind10'; 'Ind11'; 'Ind12'; 'Ind13'; 'Ind14'; 'Ind15'}.
compute indlbl = {indlbl; 'Ind16';'Ind17';'Ind18';'Ind19';'Ind20';'Ind21';'Ind22';'Ind23';'Ind24';'Ind25';'Ind26';'Ind27';'Ind28';'Ind29';'Ind30'}.
compute indlbl = {indlbl; 'Ind31';'Ind32';'Ind33';'Ind34';'Ind35';'Ind36';'Ind37';'Ind38';'Ind39';'Ind40';'Ind41';'Ind42';'Ind43';'Ind44';'Ind45'}.
compute indlbl = {indlbl; 'Ind46';'Ind47';'Ind48';'Ind49';'Ind50';'Ind51';'Ind52';'Ind53';'Ind54';'Ind55';'Ind56';'Ind57';'Ind58';'Ind59';'Ind60'}.
compute indlbl = {indlbl; 'Ind61';'Ind62';'Ind63';'Ind64';'Ind65';'Ind66';'Ind67';'Ind68';'Ind69';'Ind70';'Ind71';'Ind72';'Ind73';'Ind74';'Ind75'}.
compute indlbl = {indlbl; 'Ind76';'Ind77';'Ind78';'Ind79';'Ind80';'Ind81';'Ind82';'Ind83';'Ind84';'Ind85';'Ind86';'Ind87';'Ind88';'Ind89';'Ind90'}.
compute cntname={'(C1)';'(C2)';'(C3)';'(C4)';'(C5)';'(C6)';'(C7)';'(C8)';'(C9)';'(C10)';'(C11)';'(C12)';'(C13)';'(C14)';'(C15)';'(C16)';'(C17)'}.
compute cntname={cntname;'(C18)';'(C19)';'(C20)';'(C21)';'(C22)';'(C23)';'(C24)';'(C25)';'(C26)';'(C27)';'(C28)';'(C29)';'(C30)';'(C31)'}.
compute cntname={cntname;'(C32)';'(C33)';'(C34)';'(C35)';'(C36)';'(C37)';'(C38)';'(C39)';'(C40)';'(C41)';'(C42)';'(C43)';'(C44)';'(C45)'}.
compute cntname={cntname;'(C46)';'(C47)';'(C48)';'(C49)';'(C50)';'(C51)';'(C52)';'(C53)';'(C54)';'(C55)';'(C56)';'(C57)';'(C58)';'(C59)'}.
compute cntname={cntname;'(C60)';'(C61)';'(C62)';'(C63)';'(C64)';'(C65)';'(C66)';'(C67)';'(C68)';'(C69)';'(C70)';'(C71)';'(C72)';'(C73)'}.
compute cntname={cntname;'(C74)';'(C75)';'(C76)';'(C77)';'(C78)';'(C79)';'(C80)';'(C81)';'(C82)';'(C83)';'(C84)';'(C85)';'(C86)';'(C87)'}.
compute cntname={cntname;'(C88)';'(C89)';'(C90)';'(C91)';'(C92)';'(C93)';'(C94)';'(C95)';'(C96)';'(C97)';'(C98)';'(C99)';'(C100)';'(C101)'}.
compute cntname={cntname;'(C102)';'(C103)';'(C104)';'(C105)'}.
compute indmake=make(ncol(indmark),(nms+2),0).
compute indmod=make(ncol(indmark),1,999).
compute indmmm=make(ncol(indmark),1,0).
compute indmmmt=make(ncol(indmark),1,0).
compute start=1.
compute end=0.
compute nindfx=0.
compute indlocs=make(nrow(thetaxmb),ncol(paths),999).
compute indkey=make(ncol(indmark),1+((rmax(indmark)*2)+1),'     ').
compute c1=1.
compute c2=1.
compute c3=1.
loop i = 1 to ncol(paths).
do if (pathtype(1,i)=1).
compute indlocs(:,i)=thetaxmb(:,c1).
compute c1=c1+1.
end if.
do if (pathtype(1,i)=3).
compute indlocs(:,i)=thetamyb(:,c2).
compute c2=c2+1.
end if.
do if (pathtype(1,i)=2 and nms < 7 and serial=1).
compute indlocs(:,i)=thetammb(:,thetam(1,c3)).
compute c3=c3+1.
end if.
end loop.
loop i = 1 to ncol(indlocs).
compute c1=2.
loop j = 2 to nrow(indlocs).
do if (indlocs(j,i) <> 0).
compute indlocs(c1,i)=indlocs(j,i).
compute c1=c1+1.
end if.
end loop.
compute indlocs(1,i)=c1-2.
end loop.
compute indlocs=indlocs(1:rmax((indlocs(1,:))+1),:).
loop i = 1 to ncol(indmark).
compute numget=indmark(1,i).
compute end=end+numget.
compute gotcha=indsets(1,start:end).
compute start=end+1.
compute ok=1.
compute temp=0.
compute repoman=make(4,1,0).
loop j = 1 to ncol(gotcha).
do if paths(1,gotcha(1,j))=0.
compute ok=0.
end if.
do if (pathsmod(1,gotcha(1,j)) > 0).
compute temp=1.
compute temp2={pathsw(1,gotcha(1,j));pathsz(1,gotcha(1,j));pathswz(1,gotcha(1,j));0}.
do if (temp2(1,1)=1 and temp2(2,1)=1 and temp2(3,1)=0).
compute temp2(4,1)=1.
end if.
compute repoman=repoman+temp2.
end if.
end loop.
compute temp=0.
compute tempmmm=0.
compute typemmm=0.
do if ((repoman(1,1) > 0) and (repoman(2,1) = 0)).
compute temp=1.
do if (repoman(1,1)=1).
compute tempmmm=1.
end if.
do if (repoman(1,1) > 1 and (wdich=1 or mcw > 0)).
compute tempmmm=12.
compute typemmm=mcw.
do if (wdich=1).
compute typemmm=1.
end if.
end if.
do if (repoman(1,1) > 1 and (wdich=0 and mcw = 0)).
compute tempmmm=101.
end if.
end if.
do if ((repoman(1,1) = 0) and (repoman(2,1) > 0)).
compute temp=2.
do if (repoman(2,1)=1).
compute tempmmm=2.
end if.
do if (repoman(2,1) > 1 and (zdich = 1 or mcz > 0)).
compute tempmmm=22.
compute typemmm=mcz.
do if (zdich=1).
compute typemmm=1.
end if.
end if.
do if (repoman(2,1) > 1 and (zdich = 0 and mcw = 0)).
compute tempmmm=102.
end if.
end if.
do if (repoman(1,1)>0 and repoman(2,1)>0).
compute temp=3.
do if (repoman(1,1)=1 and repoman(2,1)=1).
do if (repoman(4,1)=1).
compute tempmmm=31.
end if.
do if (repoman(3,1)=1).
compute tempmmm=41.
end if.
end if.
end if.
do if (repoman(1,1)=1 and repoman(2,1)=1 and repoman(3,1)=0 and repoman(4,1)=0).
compute tempmmm=51.
end if.
do if (ok = 1).
compute nindfx=nindfx+1.
compute indmake(nindfx,1)=numget.
compute indmod(nindfx,1)=temp.
compute indmmm(nindfx,1)=tempmmm.
compute indmmmt(nindfx,1)=typemmm.
compute indmake(nindfx,2:(1+numget))=gotcha.
compute indkey(nindfx,1)=xnames.
loop j = 1 to numget.
compute indkey(nindfx,(j*2+1))=pathsdv(1,gotcha(1,j)).
compute indkey(nindfx,(j*2))='   ->   '.
end loop.
end if.
end loop.
compute indkey=indkey(1:nindfx,1:((cmax(indmake(:,1))*2)+1)).
compute indmake=indmake(1:nindfx,1:(cmax(indmake(:,1))+1)).
compute indmod=indmod(1:nrow(indmake),1).
compute indmmm=indmmm(1:nrow(indmake),1).
compute indmmmt=indmmmt(1:nrow(indmake),1).
compute ncpairs = (((nindfx)*(nindfx-1))/2).
do if ((contrast = 1 or contrast = 2 or contrast=3) and (ncpairs > 105)).
compute contrast=0.
compute notecode(notes,1) = 13.
compute notes = notes + 1.
end if.
do if (contrast = 4).
do if (ncol(contvec) <> nindfx).
compute contrast=0.
compute notecode(notes,1) = 14.
compute notes = notes + 1.
end if.
end if.
do if (anymod=0).
do if (nms = 1 and contrast > 0).
compute contrast=0.
end if.
compute efloop=(((1-(effsize=0))*2)+1)-(((mcx>0 or xdich=1))*(1-(effsize=0))).
loop kk=1 to efloop.
do if (boot = 0).
compute bootres=obscoeff.
do if (kk=1).
compute totbtvec=make(1,nxvls,0).
end if.
compute bootdir=obsdirfx.
compute indtab=999.
compute inddiff=999.
compute bootysd=ysd.
compute bootxsd=xsd.
end if.
do if (boot > 0).
compute bootres={obscoeff;bootres}.
do if (kk=1).
compute totbtvec=make(nrow(bootres),nxvls,0).
end if.
compute indtab=make(1,4,999).
compute inddiff=make(nrow(bootres),1,999).
end if.
compute indtotal=make(nrow(bootres),1,0).
loop i = 1 to nrow(indmake).
loop j = 1 to nxvls.
compute indtemp=make(nrow(bootres),1,1).
loop k = 1 to indmake(i,1).
compute jtemp=1.
do if (j > 1 and k=1).
compute jtemp=j.
end if.
compute indtemp=indtemp&*bootres(:,pathsfoc(jtemp,indmake(i,(k+1)))).
end loop.
do if (kk = 2).
compute indtemp=indtemp/bootysd.
end if.
do if (kk = 3).
compute indtemp=(bootxsd&*indtemp)/bootysd.
end if.
do if (contrast <> 0).
compute inddiff={inddiff,indtemp}.
end if.
do if (nxvls=1).
compute indtotal=indtotal+indtemp.
end if.
compute indeff=indtemp(1,1).
do if (kk=1).
compute totbtvec(:,j)=totbtvec(:,j)+indtemp.
end if.
do if (boot > 0).

.
compute temp = indtemp(2:nrow(indtemp),1).
compute temp(GRADE( indtemp(2:nrow(indtemp),1) )) = indtemp(2:nrow(indtemp),1).
compute badlo = 0.
compute badhi = 0.
do if ( (indtemp(1,1)*bc)+(9999*(1-bc)) <> 9999).
compute pv=csum(temp < (indtemp(1,1)*bc)+(9999*(1-bc)) )/nrow(temp).
compute ppv = pv.
do if (pv > .5).
compute ppv = 1-pv.
end if.
compute y5=sqrt(-2*ln(ppv)).
compute xp=y5+((((y5*p4+p3)*y5+p2)*y5+p1)*y5+p0)/((((y5*q4+q3)*y5+q2)*y5+q1)*y5+q0).
do if (pv <= .5).
compute xp = -xp.
end if.
compute cilow=rnd(nrow(temp)*(cdfnorm(2*xp-xp2))).
compute cihigh=trunc(nrow(temp)*(cdfnorm(2*xp+(xp2))))+1.
do if (cilow < 1).
compute cilow = 1.
compute booterr=1.
compute badlo = 1.
end if.
do if (cihigh > nrow(temp)).
compute cihigh = boot.
compute booterr=1.
compute badhi = 1.
end if.
compute llcit=temp(cilow,1).
compute ulcit=temp(cihigh,1).
do if (badlo = 1 and llcit <> priorlo).
compute badend={badend, llcit}.
compute priorlo = llcit.
end if.
do if (badhi = 1 and ulcit <> priorhi).
compute badend={badend, ulcit}.
compute priorhi = ulcit.
end if.
end if.
do if ( (indtemp(1,1)*bc)+(9999*(1-bc)) = 9999).
compute llcit=temp(cilow,1).
compute ulcit=temp(cihigh,1).
end if.
compute bootse=t(sqrt((cssq(temp)-((csum(temp)&**2)/nrow(temp)))/(nrow(temp)-1)))
.
compute indeff={indeff,bootse,llcit,ulcit}.
end if.
do if (kk=1).
compute indtabn={indtab;indeff}.
end if.
compute indtab={indtab;indeff}.
end loop.
end loop.
compute indtab=indtab(2:nrow(indtab),:).
do if (kk=1).
compute indtabn=indtabn(2:nrow(indtabn),:).
end if.
compute rowlbs=indlbl(1:nrow(indtab),1).
do if (mc > 0).
compute inddiff=make(mc,1,-999).
compute indtab2=make(nrow(indtab),4,-999).
compute indtab2(:,1)=indtab.
compute indtab=indtab2.
release indtab2.
compute mcct=0.
compute indtotal=make(mc,1,0).
do if (kk = 1).
compute x1 = sqrt(-2*ln(uniform(mc,nrow(mcsopath))))&*cos((2*3.14159265358979)*uniform(mc, nrow(mcsopath))).
compute x1=x1*chol(indcov).
loop ii=1 to nrow(x1).
compute x1(ii,:)=x1(ii,:)+t(mcsopath).
end loop.
end if.
loop ii=1 to nms.
compute tmpb=x1(:,((nms*nxvls)+ii)).
compute tmpb2=tmpb.
do if (nxvls > 1).
loop jj=1 to (nxvls-1).
compute tmpb2={tmpb2,tmpb}.
end loop.
end if.
compute indtemp=x1(:,(((ii-1)*nxvls)+1):(ii*nxvls))&*tmpb2.
loop jj=1 to ncol(indtemp).
do if (kk = 2).
compute indtemp(:,jj)=indtemp(:,jj)/ysd.
end if.
do if (kk = 3).
compute indtemp(:,jj)=(xsd*indtemp(:,jj))/ysd.
end if.

.
compute temp = indtemp(:,jj).
compute temp(GRADE( indtemp(:,jj) )) = indtemp(:,jj).
compute badlo = 0.
compute badhi = 0.
do if ( 9999 <> 9999).
compute pv=csum(temp < 9999 )/nrow(temp).
compute ppv = pv.
do if (pv > .5).
compute ppv = 1-pv.
end if.
compute y5=sqrt(-2*ln(ppv)).
compute xp=y5+((((y5*p4+p3)*y5+p2)*y5+p1)*y5+p0)/((((y5*q4+q3)*y5+q2)*y5+q1)*y5+q0).
do if (pv <= .5).
compute xp = -xp.
end if.
compute cilow=rnd(nrow(temp)*(cdfnorm(2*xp-xp2))).
compute cihigh=trunc(nrow(temp)*(cdfnorm(2*xp+(xp2))))+1.
do if (cilow < 1).
compute cilow = 1.
compute booterr=1.
compute badlo = 1.
end if.
do if (cihigh > nrow(temp)).
compute cihigh = boot.
compute booterr=1.
compute badhi = 1.
end if.
compute llcit=temp(cilow,1).
compute ulcit=temp(cihigh,1).
do if (badlo = 1 and llcit <> priorlo).
compute badend={badend, llcit}.
compute priorlo = llcit.
end if.
do if (badhi = 1 and ulcit <> priorhi).
compute badend={badend, ulcit}.
compute priorhi = ulcit.
end if.
end if.
do if ( 9999 = 9999).
compute llcit=temp(cilow,1).
compute ulcit=temp(cihigh,1).
end if.
compute bootse=t(sqrt((cssq(temp)-((csum(temp)&**2)/nrow(temp)))/(nrow(temp)-1)))
.
compute mcct=mcct+1.
compute indtab(mcct,2:4)={bootse,llcit,ulcit}.
end loop.
do if (nxvls=1).
compute indtotal=indtotal+indtemp.
do if (contrast <> 0).
compute inddiff={inddiff,indtemp}.
end if.
end if.
end loop.
release indtemp,tmpb.
end if.
do if (normal = 1 and sobelok=1).
compute sobelmat=indtab(:,1).
compute sobelmat={sobelmat,(sobelmat/2),sobelmat,sobelmat}.
loop ii=1 to nms.
compute se2b=indcov(((nms*nxvls)+ii),((nms*nxvls)+ii)).
compute bpath2=mcsopath(((nms*nxvls)+ii),1)&**2.
compute se2a=diag(indcov((((ii-1)*nxvls)+1):(ii*nxvls),(((ii-1)*nxvls)+1):(ii*nxvls))).
compute apath2=mcsopath((((ii-1)*nxvls)+1):(ii*nxvls) ,1)&**2.
compute sesobel=sqrt(apath2*se2b+bpath2*se2a+se2a*se2b).
compute sobelmat((((ii-1)*nxvls)+1):(ii*nxvls),2)=sesobel.
end loop.
release se2b,bpath2,se2a,apath2,sesobel,ii.
compute sobelmat(:,3)=sobelmat(:,1)&/sobelmat(:,2).
compute sobelmat(:,4) = 2*(1-cdfnorm(abs(sobelmat(:,3)))).
end if.
do if (serial = 0).
compute rowlbs=t(mnames).
end if.
do if (nxvls=1 and nms > 1).
compute rowlbs={'TOTAL';rowlbs}.
compute indtemp=indtotal(1,1).
do if (boot > 0 and nxvls=1).

.
compute temp = indtotal(2:nrow(indtotal),1).
compute temp(GRADE( indtotal(2:nrow(indtotal),1) )) = indtotal(2:nrow(indtotal),1).
compute badlo = 0.
compute badhi = 0.
do if ( (indtotal(1,1)*bc)+(9999*(1-bc)) <> 9999).
compute pv=csum(temp < (indtotal(1,1)*bc)+(9999*(1-bc)) )/nrow(temp).
compute ppv = pv.
do if (pv > .5).
compute ppv = 1-pv.
end if.
compute y5=sqrt(-2*ln(ppv)).
compute xp=y5+((((y5*p4+p3)*y5+p2)*y5+p1)*y5+p0)/((((y5*q4+q3)*y5+q2)*y5+q1)*y5+q0).
do if (pv <= .5).
compute xp = -xp.
end if.
compute cilow=rnd(nrow(temp)*(cdfnorm(2*xp-xp2))).
compute cihigh=trunc(nrow(temp)*(cdfnorm(2*xp+(xp2))))+1.
do if (cilow < 1).
compute cilow = 1.
compute booterr=1.
compute badlo = 1.
end if.
do if (cihigh > nrow(temp)).
compute cihigh = boot.
compute booterr=1.
compute badhi = 1.
end if.
compute llcit=temp(cilow,1).
compute ulcit=temp(cihigh,1).
do if (badlo = 1 and llcit <> priorlo).
compute badend={badend, llcit}.
compute priorlo = llcit.
end if.
do if (badhi = 1 and ulcit <> priorhi).
compute badend={badend, ulcit}.
compute priorhi = ulcit.
end if.
end if.
do if ( (indtotal(1,1)*bc)+(9999*(1-bc)) = 9999).
compute llcit=temp(cilow,1).
compute ulcit=temp(cihigh,1).
end if.
compute bootse=t(sqrt((cssq(temp)-((csum(temp)&**2)/nrow(temp)))/(nrow(temp)-1)))
.
compute indtemp={indtemp, bootse,llcit,ulcit}.
end if.
do if (mc > 0).
compute obtmc=indtab(:,1).
compute indtemp=csum(obtmc).

.
compute temp = indtotal(:,1).
compute temp(GRADE( indtotal(:,1) )) = indtotal(:,1).
compute badlo = 0.
compute badhi = 0.
do if ( 9999 <> 9999).
compute pv=csum(temp < 9999 )/nrow(temp).
compute ppv = pv.
do if (pv > .5).
compute ppv = 1-pv.
end if.
compute y5=sqrt(-2*ln(ppv)).
compute xp=y5+((((y5*p4+p3)*y5+p2)*y5+p1)*y5+p0)/((((y5*q4+q3)*y5+q2)*y5+q1)*y5+q0).
do if (pv <= .5).
compute xp = -xp.
end if.
compute cilow=rnd(nrow(temp)*(cdfnorm(2*xp-xp2))).
compute cihigh=trunc(nrow(temp)*(cdfnorm(2*xp+(xp2))))+1.
do if (cilow < 1).
compute cilow = 1.
compute booterr=1.
compute badlo = 1.
end if.
do if (cihigh > nrow(temp)).
compute cihigh = boot.
compute booterr=1.
compute badhi = 1.
end if.
compute llcit=temp(cilow,1).
compute ulcit=temp(cihigh,1).
do if (badlo = 1 and llcit <> priorlo).
compute badend={badend, llcit}.
compute priorlo = llcit.
end if.
do if (badhi = 1 and ulcit <> priorhi).
compute badend={badend, ulcit}.
compute priorhi = ulcit.
end if.
end if.
do if ( 9999 = 9999).
compute llcit=temp(cilow,1).
compute ulcit=temp(cihigh,1).
end if.
compute bootse=t(sqrt((cssq(temp)-((csum(temp)&**2)/nrow(temp)))/(nrow(temp)-1)))
.
compute indtemp={indtemp, bootse,llcit,ulcit}.
end if.
compute indtab={indtemp;indtab}.
end if.
compute bootlbs={'Effect', 'BootSE','BootLLCI','BootULCI'}.
do if (mc > 0).
compute bootlbs={'Effect', 'MC SE','MC LLCI','MC ULCI'}.
end if.
do if (nxvls = 1).
do if (contrast <> 0).
compute inddiff=inddiff(:,2:ncol(inddiff)).
do if (mc > 0).
compute inddiff={t(obtmc);inddiff}.
end if.
do if (contrast = 4).
compute inddifft=inddiff*t(contvec).
compute indtemp=inddifft(1,1).
do if (boot > 0 or mc > 0).
do if (mc > 0).

.
compute temp = inddifft(2:nrow(inddifft),1).
compute temp(GRADE( inddifft(2:nrow(inddifft),1) )) = inddifft(2:nrow(inddifft),1).
compute badlo = 0.
compute badhi = 0.
do if ( 9999 <> 9999).
compute pv=csum(temp < 9999 )/nrow(temp).
compute ppv = pv.
do if (pv > .5).
compute ppv = 1-pv.
end if.
compute y5=sqrt(-2*ln(ppv)).
compute xp=y5+((((y5*p4+p3)*y5+p2)*y5+p1)*y5+p0)/((((y5*q4+q3)*y5+q2)*y5+q1)*y5+q0).
do if (pv <= .5).
compute xp = -xp.
end if.
compute cilow=rnd(nrow(temp)*(cdfnorm(2*xp-xp2))).
compute cihigh=trunc(nrow(temp)*(cdfnorm(2*xp+(xp2))))+1.
do if (cilow < 1).
compute cilow = 1.
compute booterr=1.
compute badlo = 1.
end if.
do if (cihigh > nrow(temp)).
compute cihigh = boot.
compute booterr=1.
compute badhi = 1.
end if.
compute llcit=temp(cilow,1).
compute ulcit=temp(cihigh,1).
do if (badlo = 1 and llcit <> priorlo).
compute badend={badend, llcit}.
compute priorlo = llcit.
end if.
do if (badhi = 1 and ulcit <> priorhi).
compute badend={badend, ulcit}.
compute priorhi = ulcit.
end if.
end if.
do if ( 9999 = 9999).
compute llcit=temp(cilow,1).
compute ulcit=temp(cihigh,1).
end if.
compute bootse=t(sqrt((cssq(temp)-((csum(temp)&**2)/nrow(temp)))/(nrow(temp)-1)))
.
end if.
do if (boot > 0).

.
compute temp = inddifft(2:nrow(inddifft),1).
compute temp(GRADE( inddifft(2:nrow(inddifft),1) )) = inddifft(2:nrow(inddifft),1).
compute badlo = 0.
compute badhi = 0.
do if ( (inddifft(1,1)*bc)+(9999* (1-bc)) <> 9999).
compute pv=csum(temp < (inddifft(1,1)*bc)+(9999* (1-bc)) )/nrow(temp).
compute ppv = pv.
do if (pv > .5).
compute ppv = 1-pv.
end if.
compute y5=sqrt(-2*ln(ppv)).
compute xp=y5+((((y5*p4+p3)*y5+p2)*y5+p1)*y5+p0)/((((y5*q4+q3)*y5+q2)*y5+q1)*y5+q0).
do if (pv <= .5).
compute xp = -xp.
end if.
compute cilow=rnd(nrow(temp)*(cdfnorm(2*xp-xp2))).
compute cihigh=trunc(nrow(temp)*(cdfnorm(2*xp+(xp2))))+1.
do if (cilow < 1).
compute cilow = 1.
compute booterr=1.
compute badlo = 1.
end if.
do if (cihigh > nrow(temp)).
compute cihigh = boot.
compute booterr=1.
compute badhi = 1.
end if.
compute llcit=temp(cilow,1).
compute ulcit=temp(cihigh,1).
do if (badlo = 1 and llcit <> priorlo).
compute badend={badend, llcit}.
compute priorlo = llcit.
end if.
do if (badhi = 1 and ulcit <> priorhi).
compute badend={badend, ulcit}.
compute priorhi = ulcit.
end if.
end if.
do if ( (inddifft(1,1)*bc)+(9999* (1-bc)) = 9999).
compute llcit=temp(cilow,1).
compute ulcit=temp(cihigh,1).
end if.
compute bootse=t(sqrt((cssq(temp)-((csum(temp)&**2)/nrow(temp)))/(nrow(temp)-1)))
.
end if.
compute indtemp={indtemp, bootse,llcit,ulcit}.
end if.
compute indtab={indtab;indtemp}.
end if.
do if (contrast = 1 or contrast = 2 or contrast=3).
compute conkey=make(1,4,' ').
loop i = 1 to ncol(inddiff)-1.
loop j = (i+1) to ncol(inddiff).
compute inddifft=inddiff(:,i)-inddiff(:,j).
do if (contrast=2).
compute inddifft=abs(inddiff(:,i))-abs(inddiff(:,j)).
end if.
do if (contrast=3).
compute inddifft=inddiff(:,i)+inddiff(:,j).
end if.
compute indtemp=inddifft(1,1).
do if (contrast=1 or contrast=2).
compute conkeyt={' ', rowlbs((i+1),1),' minus  ',rowlbs((j+1),1)}.
end if.
do if (contrast=3).
compute conkeyt={' ', rowlbs((i+1),1),'  plus  ',rowlbs((j+1),1)}.
end if.
compute conkey={conkey;conkeyt}.
do if (boot > 0 or mc > 0).
do if (mc > 0).

.
compute temp = inddifft(2:nrow(inddifft),1).
compute temp(GRADE( inddifft(2:nrow(inddifft),1) )) = inddifft(2:nrow(inddifft),1).
compute badlo = 0.
compute badhi = 0.
do if ( 9999 <> 9999).
compute pv=csum(temp < 9999 )/nrow(temp).
compute ppv = pv.
do if (pv > .5).
compute ppv = 1-pv.
end if.
compute y5=sqrt(-2*ln(ppv)).
compute xp=y5+((((y5*p4+p3)*y5+p2)*y5+p1)*y5+p0)/((((y5*q4+q3)*y5+q2)*y5+q1)*y5+q0).
do if (pv <= .5).
compute xp = -xp.
end if.
compute cilow=rnd(nrow(temp)*(cdfnorm(2*xp-xp2))).
compute cihigh=trunc(nrow(temp)*(cdfnorm(2*xp+(xp2))))+1.
do if (cilow < 1).
compute cilow = 1.
compute booterr=1.
compute badlo = 1.
end if.
do if (cihigh > nrow(temp)).
compute cihigh = boot.
compute booterr=1.
compute badhi = 1.
end if.
compute llcit=temp(cilow,1).
compute ulcit=temp(cihigh,1).
do if (badlo = 1 and llcit <> priorlo).
compute badend={badend, llcit}.
compute priorlo = llcit.
end if.
do if (badhi = 1 and ulcit <> priorhi).
compute badend={badend, ulcit}.
compute priorhi = ulcit.
end if.
end if.
do if ( 9999 = 9999).
compute llcit=temp(cilow,1).
compute ulcit=temp(cihigh,1).
end if.
compute bootse=t(sqrt((cssq(temp)-((csum(temp)&**2)/nrow(temp)))/(nrow(temp)-1)))
.
end if.
do if (boot > 0).

.
compute temp = inddifft(2:nrow(inddifft),1).
compute temp(GRADE( inddifft(2:nrow(inddifft),1) )) = inddifft(2:nrow(inddifft),1).
compute badlo = 0.
compute badhi = 0.
do if ( (inddifft(1,1)*bc)+(9999* (1-bc)) <> 9999).
compute pv=csum(temp < (inddifft(1,1)*bc)+(9999* (1-bc)) )/nrow(temp).
compute ppv = pv.
do if (pv > .5).
compute ppv = 1-pv.
end if.
compute y5=sqrt(-2*ln(ppv)).
compute xp=y5+((((y5*p4+p3)*y5+p2)*y5+p1)*y5+p0)/((((y5*q4+q3)*y5+q2)*y5+q1)*y5+q0).
do if (pv <= .5).
compute xp = -xp.
end if.
compute cilow=rnd(nrow(temp)*(cdfnorm(2*xp-xp2))).
compute cihigh=trunc(nrow(temp)*(cdfnorm(2*xp+(xp2))))+1.
do if (cilow < 1).
compute cilow = 1.
compute booterr=1.
compute badlo = 1.
end if.
do if (cihigh > nrow(temp)).
compute cihigh = boot.
compute booterr=1.
compute badhi = 1.
end if.
compute llcit=temp(cilow,1).
compute ulcit=temp(cihigh,1).
do if (badlo = 1 and llcit <> priorlo).
compute badend={badend, llcit}.
compute priorlo = llcit.
end if.
do if (badhi = 1 and ulcit <> priorhi).
compute badend={badend, ulcit}.
compute priorhi = ulcit.
end if.
end if.
do if ( (inddifft(1,1)*bc)+(9999* (1-bc)) = 9999).
compute llcit=temp(cilow,1).
compute ulcit=temp(cihigh,1).
end if.
compute bootse=t(sqrt((cssq(temp)-((csum(temp)&**2)/nrow(temp)))/(nrow(temp)-1)))
.
end if.
compute indtemp={indtemp, bootse,llcit,ulcit}.
end if.
compute indtab={indtab;indtemp}.
end loop.
end loop.
end if.
release inddiff.
compute contlbs=cntname(1:(((nindfx)*(nindfx-1))/2),1).
compute rowlbs={rowlbs;contlbs}.
end if.
compute resultm2=make(nrow(indtab),maxresm,99999).
compute resultm2(1:nrow(indtab),1:ncol(indtab))=indtab.
compute resultm={resultm;resultm2}.
do if (outscree=1).
do if (kk=1).
print indtab/title = 'Indirect effect(s) of X on Y:'/rnames=rowlbs/cnames=bootlbs/format= F10.4.
end if.
do if (kk = 2 and (xdich=1 or mcx > 0)).
print indtab/title = 'Partially standardized indirect effect(s) of X on Y:'/rnames=rowlbs/cnames=bootlbs/format= F10.4.
end if.
do if (kk = 3).
print indtab/title = 'Completely standardized indirect effect(s) of X on Y:'/rnames=rowlbs/cnames=bootlbs/format= F10.4.
end if.
end if.
do if (normal=1 and sobelok=1 and kk=1).
compute resultm2=make(nrow(sobelmat),maxresm,99999).
compute resultm2(1:nrow(sobelmat),1:ncol(sobelmat))=sobelmat.
compute resultm={resultm;resultm2}.
compute sobellab={'Effect',hclab,'Z','p'}.
compute sobelrlb=rowlbs.
do if (nms > 1).
compute sobelrlb=rowlbs(2:(1+nms),1).
end if.
do if (outscree=1).
print sobelmat/title='   Normal theory test for indirect effect(s):'/cnames=sobellab/rnames=sobelrlb/format= F10.4.
end if.
end if.
do if (contrast <> 0).
do if ((contrast=1 or contrast = 2 or contrast=3) and kk=efloop ).
compute conkey=conkey(2:nrow(conkey),:).
do if (outscree=1).
print conkey/title = 'Specific indirect effect contrast definition(s):'/rnames=contlbs/format=A8.
end if.
end if.
do if (outscree=1).
do if (contrast = 4 and kk=efloop).
compute crowlbs=rowlbs(2:(nindfx+1),1).
print contvec/title = 'Specific indirect effect contrast weights:'/cnames=crowlbs/rlabels='(C1)'/format= F10.4.
end if.
do if (contrast = 2 and kk=efloop).
print/title = 'Contrasts are differences between absolute values of indirect effects'.
end if.
end if.
end if.
do if (serial = 1 and kk=efloop).
compute rowlbst=rowlbs(2:nrow(rowlbs),1).
do if (outscree=1).
print indkey/title = 'Indirect effect key:'/rnames=rowlbst/format = A8.
end if.
end if.
else.
do if (outscree=1).
do if (kk = 1).
print/title = 'Relative indirect effects of X on Y'.
end if.
do if (kk = 2).
print/title = 'Partially standardized relative indirect effect(s) of X on Y:'.
end if.
do if (kk = 3).
print/title = 'Completely standardized relative indirect effect(s) of X on Y:'.
end if.
end if.
loop i = 1 to nrow(indmake).
compute indtabsm=indtab((((i-1)*nxvls)+1):(nxvls*i),:).
compute indkeyt=indkey(i,:).
do if (outscree=1).
print indkeyt/title=' '/space=0/format=A8.
end if.
do if (bcmat(nrow(bcmat),1)=0)).
compute direffl2=xcatlab(1:nxvls,1).
end if.
compute resultm2=make(nrow(indtabsm),maxresm,99999).
compute resultm2(1:nrow(indtabsm),1:ncol(indtabsm))=indtabsm.
compute resultm={resultm;resultm2}.
do if (outscree=1).
print indtabsm/title = ' '/cnames=bootlbs/rnames=direffl2/format= F10.4 /space=0.
end if.
do if (normal=1 and sobelok=1 and kk=1).
compute sobelsm=sobelmat((((i-1)*nxvls)+1):(nxvls*i),:).
compute sobellab={'Effect',hclab,'Z','p'}.
compute resultm2=make(nrow(sobelsm),maxresm,99999).
compute resultm2(1:nrow(sobelsm),1:ncol(sobelsm))=sobelsm.
compute resultm={resultm;resultm2}.
do if (outscree=1).
print sobelsm/title='      Normal theory test for relative indirect effects:'/cnames=sobellab/rnames=direffl2/format= F10.4.
end if.
end if.
end loop.
end if.
do if (effsize = 1 and boot > 0).
compute bootres=bootres(2:nrow(bootres),:).
end if.
end loop.
do if (alttotal=1).
compute altcnms='Effect'.
compute totbtvec=totbtvec+bootdir.
compute alttotfx=t(totbtvec(1,:)).
do if (boot > 0).
compute alttotfx=make(ncol(totbtvec),4,0).
compute alttotfx(:,1)=t(totbtvec(1,:)).
loop cec=1 to ncol(totbtvec).

.
compute temp = totbtvec(2:nrow(totbtvec),cec).
compute temp(GRADE( totbtvec(2:nrow(totbtvec),cec) )) = totbtvec(2:nrow(totbtvec),cec).
compute badlo = 0.
compute badhi = 0.
do if ( (totbtvec(1,cec)*bc)+(9999* (1-bc)) <> 9999).
compute pv=csum(temp < (totbtvec(1,cec)*bc)+(9999* (1-bc)) )/nrow(temp).
compute ppv = pv.
do if (pv > .5).
compute ppv = 1-pv.
end if.
compute y5=sqrt(-2*ln(ppv)).
compute xp=y5+((((y5*p4+p3)*y5+p2)*y5+p1)*y5+p0)/((((y5*q4+q3)*y5+q2)*y5+q1)*y5+q0).
do if (pv <= .5).
compute xp = -xp.
end if.
compute cilow=rnd(nrow(temp)*(cdfnorm(2*xp-xp2))).
compute cihigh=trunc(nrow(temp)*(cdfnorm(2*xp+(xp2))))+1.
do if (cilow < 1).
compute cilow = 1.
compute booterr=1.
compute badlo = 1.
end if.
do if (cihigh > nrow(temp)).
compute cihigh = boot.
compute booterr=1.
compute badhi = 1.
end if.
compute llcit=temp(cilow,1).
compute ulcit=temp(cihigh,1).
do if (badlo = 1 and llcit <> priorlo).
compute badend={badend, llcit}.
compute priorlo = llcit.
end if.
do if (badhi = 1 and ulcit <> priorhi).
compute badend={badend, ulcit}.
compute priorhi = ulcit.
end if.
end if.
do if ( (totbtvec(1,cec)*bc)+(9999* (1-bc)) = 9999).
compute llcit=temp(cilow,1).
compute ulcit=temp(cihigh,1).
end if.
compute bootse=t(sqrt((cssq(temp)-((csum(temp)&**2)/nrow(temp)))/(nrow(temp)-1)))
.
compute alttotfx(cec,2:4)={bootse,llcit,ulcit}.
end loop.
compute altcnms={altcnms,'BootSE', 'BootLLCI','BootULCI'}.
end if.
compute resultm2=make(nrow(alttotfx),maxresm,99999).
compute resultm2(1:nrow(alttotfx),1:ncol(alttotfx))=alttotfx.
compute resultm={resultm;resultm2}.
do if (outscree=1).
do if (nxvls > 1).
print/title= '----------'/space=0.
print alttotfx/title='Relative total effects of X on Y (sum of direct and indirect effects)'/rnames=direffl2/cnames=altcnms/format= F10.4.
end if.
do if (nxvls = 1).
print alttotfx/title='Total effect of X on Y (sum of direct and indirect effects)'/cnames=altcnms/format= F10.4.
end if.
end if.
end if.
end if.
do if (anymod > 0).
do if (boot = 0).
compute bootres=obscoeff.
compute indtab=999.
end if.
do if (boot > 0).
compute bootres={obscoeff;bootres}.
compute indtab=make(1,4,999).
end if.
do if (csum((indmod > 0))=nrow(indmod)).
do if (outscree=1).
do if (nxvls > 1).
do if (xmint=0).
print/title= 'Relative conditional indirect effects of X on Y:'.
end if.
do if (xmint=1).
print/title= 'Relative (total) natural indirect effects of X on Y:'.
end if.
end if.
do if (nxvls = 1).
do if (xmint=0).
print/title= 'Conditional indirect effects of X on Y:'.
end if.
do if (xmint=1).
print/title= '(Total) Natural indirect effect(s) of X on Y:'.
end if.
end if.
end if.
end if.
do if (csum((indmod > 0)) < nrow(indmod)).
do if (outscree=1).
do if (nxvls > 1).
print/title= 'Relative conditional and unconditional indirect effects of X on Y:'.
end if.
do if (nxvls = 1).
print/title= 'Conditional and unconditional indirect effects of X on Y:'.
end if.
end if.
end if.
compute cftotfx=make(nrow(bootres),nxvls,0).
loop i = 1 to nrow(indmake).
compute indtab=indtab(1,:)*0.
do if (outscree=1).
do if (xmint=0).
print indkey(i,:)/title = 'INDIRECT EFFECT:'/format=A8.
end if.
do if (xmint=1).
print indkey(i,:)/title = ' '/format=A8/space=0.
end if.
end if.
do if (indmod(i,1)=0).
loop j = 1 to nxvls.
compute indtemp=make(nrow(bootres),1,1).
loop k = 1 to indmake(i,1).
compute jtemp=1.
do if (j > 1 and k=1).
compute jtemp=j.
end if.
compute indtemp=indtemp&*bootres(:,pathsfoc(jtemp,indmake(i,(k+1)))).
end loop.
compute indeff=indtemp(1,1).
do if (boot > 0).

.
compute temp = indtemp(2:nrow(indtemp),1).
compute temp(GRADE( indtemp(2:nrow(indtemp),1) )) = indtemp(2:nrow(indtemp),1).
compute badlo = 0.
compute badhi = 0.
do if ( (indtemp(1,1)*bc)+(9999*(1-bc)) <> 9999).
compute pv=csum(temp < (indtemp(1,1)*bc)+(9999*(1-bc)) )/nrow(temp).
compute ppv = pv.
do if (pv > .5).
compute ppv = 1-pv.
end if.
compute y5=sqrt(-2*ln(ppv)).
compute xp=y5+((((y5*p4+p3)*y5+p2)*y5+p1)*y5+p0)/((((y5*q4+q3)*y5+q2)*y5+q1)*y5+q0).
do if (pv <= .5).
compute xp = -xp.
end if.
compute cilow=rnd(nrow(temp)*(cdfnorm(2*xp-xp2))).
compute cihigh=trunc(nrow(temp)*(cdfnorm(2*xp+(xp2))))+1.
do if (cilow < 1).
compute cilow = 1.
compute booterr=1.
compute badlo = 1.
end if.
do if (cihigh > nrow(temp)).
compute cihigh = boot.
compute booterr=1.
compute badhi = 1.
end if.
compute llcit=temp(cilow,1).
compute ulcit=temp(cihigh,1).
do if (badlo = 1 and llcit <> priorlo).
compute badend={badend, llcit}.
compute priorlo = llcit.
end if.
do if (badhi = 1 and ulcit <> priorhi).
compute badend={badend, ulcit}.
compute priorhi = ulcit.
end if.
end if.
do if ( (indtemp(1,1)*bc)+(9999*(1-bc)) = 9999).
compute llcit=temp(cilow,1).
compute ulcit=temp(cihigh,1).
end if.
compute bootse=t(sqrt((cssq(temp)-((csum(temp)&**2)/nrow(temp)))/(nrow(temp)-1)))
.
compute indeff={indeff,bootse,llcit,ulcit}.
end if.
compute indtab={indtab;indeff}.
end loop.
compute resultm2=make(nrow(indtab),maxresm,99999).
compute resultm2(1:nrow(indtab),1:ncol(indtab))=indtab.
compute resultm={resultm;resultm2}.
do if (outscree=1).
do if (nxvls > 1).
compute indefflb=xcatlab(1:nxvls,1).
print indtab(2:nrow(indtab),:)/title = ' '/clabels='Effect', 'BootSE', 'BootLLCI', 'BootULCI'/rnames=indefflb/format= F10.4 /space=0.
end if.
do if (nxvls = 1).
print indtab(2:nrow(indtab),:)/title = ' '/clabels='Effect', 'BootSE', 'BootLLCI', 'BootULCI'/format= F10.4 /space=0.
end if.
end if.
end if.
do if (indmod(i,1)>0).
do if (indmod(i,1)=1).
compute indmodva=wmodvals.
compute indprova=wprobval.
compute condlbs={wnames}.
compute printw=1.
else if (indmod(i,1)=2).
compute indmodva=zmodvals.
compute indprova=zprobval.
compute condlbs={znames}.
compute printz=1.
else if (indmod(i,1)=3).
compute cntmp=1.
compute printz=1.
compute printw=1.
compute indmodva=make((nrow(wmodvals)*nrow(zmodvals)),2,999).
loop k7 = 1 to nrow(wmodvals).
loop k8 = 1 to nrow(zmodvals).
compute indmodva(cntmp,:)={wmodvals(k7,1),zmodvals(k8,1)}.
compute cntmp=cntmp+1.
end loop.
end loop.
compute condlbs={wnames,znames}.
end if.
compute condres=make(nrow(indmodva),1,999).
do if (boot > 0).
compute condres=make(nrow(indmodva),4,999).
end if.
compute condres={indmodva,condres}.
loop k4 = 1 to nxvls.
compute imm3=make(nrow(bootres),1,1).
compute imm4=make(nrow(bootres),1,1).
compute indcontr=0.
do if (indmod(i,1)=3).
compute tihsw=wprobval.
compute tihsz=zprobval.
end if.
loop k1=1 to nrow(indmodva).
compute tucker2=make(nrow(bootres),1,1).
compute imm2=make(nrow(bootres),1,1).
compute wfirst=0.
compute zfirst=0.
compute immset=0.
loop k2=1 to indmake(i,1).
compute colnumb=indmake(i,(k2+1)).
do if (k2=1).
compute wbb=make(nrow(bootres),(nwvls*nxvls),0).
compute zbb=make(nrow(bootres),(nzvls*nxvls),0).
compute wzbb=make(nrow(bootres),(nwvls*nzvls*nxvls),0).
end if.
do if (k2<>1).
compute wbb=make(nrow(bootres),nwvls,0).
compute zbb=make(nrow(bootres),nzvls,0).
compute wzbb=make(nrow(bootres),(nwvls*nzvls),0).
end if.
compute cnt=1.
compute tihs=indlocs(2:((indlocs(1,colnumb))+1),colnumb).
do if (k2 = 1).
compute focbb=tihs(1:nxvls,1).
compute focbb=bootres(:,focbb).
do if (indmmm(i,1)>0).
compute imm=focbb(:,k4).
compute condbb=imm.
end if.
compute focaddon=make(1,nxvls,0).
compute focaddon(1,k4)=1.
compute cnt=cnt+nxvls.
compute placeh=nxvls.
do if (indmod(i,1)=1).
compute tihsz=make(nrow(wprobval),(nzvls*nxvls),0).
compute tihswz=make(nrow(wprobval),(nwvls*nzvls*nxvls),0).
do if (pathsw(1,colnumb)=1).
compute temp=make(nrow(wprobval),(nxvls*nwvls),0).
loop k5 = 1 to nrow(wprobval).
loop k6=1 to nwvls.
compute temp(k5, (((k4-1)*nwvls)+k6))=wprobval(k5,k6).
end loop.
end loop.
compute indprova={temp,tihsz,tihswz}.
else.
compute indprova={wprobval,tihsz,tihswz}.
end if.
end if.
do if (indmod(i,1)=2).
compute tihsw=make(nrow(zprobval),(nwvls*nxvls),0).
compute tihswz=make(nrow(zprobval),(nwvls*nzvls*nxvls),0).
do if (pathsz(1,colnumb)=1).
compute temp=make(nrow(zprobval),(nxvls*nzvls),0).
loop k5 = 1 to nrow(zprobval).
loop k6 =1 to nzvls.
compute temp(k5,(((k4-1)*nzvls)+k6))=zprobval(k5,k6).
end loop.
end loop.
compute indprova={tihsw,temp,tihswz}.
else.
compute indprova={tihsw,zprobval,tihswz}.
end if.
end if.
do if (indmod(i,1)=3).
compute indprova=make((nrow(wprobval)*nrow(zprobval)),((ncol(wprobval)*nxvls)+ (ncol(zprobval)*nxvls)+(nwvls*nzvls*nxvls)),0).
compute cntemp=1.
loop k7=1 to nrow(wprobval).
loop k8 =1 to nrow(zprobval).
compute temp=wprobval(k7,:)*focaddon(1,k4).
compute indprova(cntemp,(((k4-1)*nwvls)+1):(k4*(nwvls)))=temp.
compute temp=zprobval(k8,:)*focaddon(1,k4).
compute indprova(cntemp, ((((k4-1)*nzvls)+1)+(nxvls*nwvls)) : ((((k4-1)*nzvls)+1)+(nxvls*nwvls)+(nzvls-1)))=temp.
compute cntemp=cntemp+1.
end loop.
end loop.
do if (pathsz(1,colnumb)=0).
compute temp=make(nrow(indprova),(ncol(zprobval)*nxvls),0).
compute indprova(:,((ncol(wprobval)*nxvls)+1):((ncol(wprobval)+ncol(zprobval))* nxvls))=temp.
end if.
do if (pathsw(1,colnumb)=0).
compute temp=make(nrow(indprova),(ncol(wprobval)*nxvls),0).
compute indprova(:,1:(ncol(wprobval)*nxvls))=temp.
end if.
do if (pathswz(1,colnumb)=1).
compute cntemp=(ncol(wprobval)*nxvls)+(ncol(zprobval)*nxvls)+((k4-1)* ncol(wprobval)*ncol(zprobval))+1.
loop k7=1 to ncol(wprobval).
loop k8=1 to ncol(zprobval).
compute indprova(:,cntemp)=indprova(:,((ncol(wprobval)*(k4-1))+k7))&* indprova(:,((((k4-1)*ncol(zprobval))+k8)+(nxvls*ncol(wprobval)))).
compute cntemp=cntemp+1.
end loop.
end loop.
end if.
end if.
end if.
do if (k2 > 1).
compute focbb=tihs(1,1).
compute focbb=bootres(:,focbb).
do if (indmmm(i,1)>0).
compute imm=focbb(:,1).
compute condbb=imm.
end if.
compute focaddon=1.
compute cnt=cnt+1.
compute placeh=1.
do if (indmod(i,1)=1).
compute tihsz=make(nrow(wprobval),nzvls,0).
compute tihswz=make(nrow(wprobval),(nwvls*nzvls),0).
compute indprova={wprobval,tihsz,tihswz}.
end if.
do if (indmod(i,1)=2).
compute tihsw=make(nrow(zprobval),nwvls,0).
compute tihswz=make(nrow(zprobval),(nwvls*nzvls),0).
compute indprova={tihsw,zprobval,tihswz}.
end if.
do if (indmod(i,1)=3).
compute indprova=make((nrow(wprobval)*nrow(zprobval)),((ncol(wprobval)+ ncol(zprobval))+(nwvls*nzvls)),0).
compute cntemp=1.
loop k7=1 to nrow(wprobval).
loop k8 =1 to nrow(zprobval).
compute indprova(cntemp,1:(ncol(wprobval)+ncol(zprobval)))={wprobval(k7,:), zprobval(k8,:)}.
compute cntemp=cntemp+1.
end loop.
end loop.
do if (pathsz(1,colnumb)=0).
compute temp=make(nrow(indprova),ncol(zprobval),0).
compute indprova(:,(ncol(wprobval)+1):(ncol(wprobval)+ncol(zprobval)))=temp.
end if.
do if (pathsw(1,colnumb)=0).
compute temp=make(nrow(indprova),ncol(wprobval),0).
compute indprova(:,1:ncol(wprobval))=temp.
end if.
do if (pathswz(1,colnumb)=1).
compute cntemp=ncol(wprobval)+ncol(zprobval)+1.
loop k7=1 to ncol(wprobval).
loop k8=1 to ncol(zprobval).
compute indprova(:,cntemp)=indprova(:,k7)&*indprova(:,(ncol(wprobval)+k8)).
compute cntemp=cntemp+1.
end loop.
end loop.
end if.
end if.
end if.
do if (pathsw(1,colnumb)) = 1.
compute wbb=tihs(cnt:(cnt+(placeh*nwvls)-1),1).
compute wbb=bootres(:,wbb).
compute immlbs2=wcatlab(1:nwvls,1).
do if (zfirst=0).
compute wfirst=1.
end if.
do if (indmmm(i,1)=1 or indmmm(i,1)=31 or indmmm(i,1)=51).
compute imm=wbb(:,1).
loop k7 = 1 to nwvls.
compute imm={imm,wbb(:,(((k4-1)*nwvls*(k2=1))+k7))}.
end loop.
compute imm=imm(:,2:ncol(imm)).
end if.
do if (indmmm(i,1)=41 or indmmm(i,1)=51).
compute condbb=make(nrow(bootres),1,0).
loop k7 = 1 to nwvls.
compute condbb={condbb,wbb(:,(((k4-1)*nwvls*(k2=1))+k7))}.
end loop.
compute condbb=condbb(:,2:ncol(condbb)).
end if.
compute cnt=cnt+(placeh*nwvls).
end if.
do if (pathsz(1,colnumb)) = 1.
compute zbb=tihs(cnt:(cnt+(placeh*nzvls)-1),1).
compute zbb=bootres(:,zbb).
do if (wfirst=0).
compute zfirst=1.
end if.
do if (indmmm(i,1) <> 31).
compute immlbs2=zcatlab(1:nzvls,1).
end if.
do if (indmmm(i,1)=2 or indmmm(i,1)=31 or indmmm(i,1)=51).
do if (indmmm(i,1)=2).
compute imm=zbb(:,1).
end if.
loop k7 = 1 to nzvls.
compute imm={imm,zbb(:,(((k4-1)*nzvls*(k2=1))+k7))}.
end loop.
do if (indmmm(i,1)=2 or indmmm(i,1)=51).
compute imm=imm(:,2:ncol(imm)).
do if (indmmm(i,1)=51).
compute condbb={condbb,imm}.
end if.
end if.
end if.
compute cnt=cnt+(placeh*nzvls).
end if.
do if (pathswz(1,colnumb)) = 1.
compute wzbb=tihs(cnt:(cnt+(placeh*nwvls*nzvls)-1),1).
compute wzbb=bootres(:,wzbb).
do if (indmmm(i,1)=41).
compute imm=wzbb(:,1).
loop k7=1 to nwvls*nzvls.
compute imm={imm,wzbb(:,(((k4-1)*nzvls*nwvls*(k2=1))+k7))}.
end loop.
end if.
do if (indmmm(i,1)=41).
compute imm=imm(:,2:ncol(imm)).
compute condbb={condbb,imm(:,(ncol(imm)-(nwvls*nzvls)+1):ncol(imm))}.
end if.
compute cnt=cnt+(placeh*nzvls*nwvls).
end if.
compute indprobe={focaddon,indprova(k1,:)}.
compute tucker={focbb,wbb,zbb,wzbb}.
loop k3=1 to ncol(indprobe).
compute tucker(:,k3)=tucker(:,k3)*indprobe(1,k3).
end loop.
compute tucker2=tucker2&*rsum(tucker).
do if (indmmm(i,1) = 1 or indmmm(i,1)=2 or indmmm(i,1)=31 or indmmm(i,1)=41 or indmmm(i,1)=51).
do if (immset=1).
do if (ncol(imm2)=1 and ncol(imm) = 1).
compute imm2=imm2&*imm.
end if.
do if (indmmm(i,1)=41 or indmmm(i,1)=51).
do if ((ncol(condbb2) > 1) and (ncol(condbb)>1)).
compute condbb2t=make(nrow(condbb2),(ncol(condbb2)*ncol(condbb)),-999999).
compute k9=1.
do if (wfirst=1).
loop k7=1 to ncol(condbb2).
loop k8 = 1 to ncol(condbb).
compute condbb2t(:,k9)=condbb2(:,k7)&*condbb(:,k8).
compute k9=k9+1.
end loop.
end loop.
end if.
do if (zfirst=1).
loop k7=1 to ncol(condbb).
loop k8 = 1 to ncol(condbb2).
compute condbb2t(:,k9)=condbb(:,k7)&*condbb2(:,k8).
compute k9=k9+1.
end loop.
end loop.
end if.
compute condbb2=condbb2t.
release condbb2t.
end if.
do if ((ncol(condbb2) > 1) and (ncol(condbb)=1)).
loop k7 = 1 to ncol(condbb2).
compute condbb2(:,k7)=condbb2(:,k7)&*condbb.
end loop.
end if.
do if ((ncol(condbb2) = 1) and (ncol(condbb)>1)).
loop k7 = 1 to ncol(condbb).
compute condbb(:,k7)=condbb2&*condbb(:,k7).
end loop.
compute condbb2=condbb.
end if.
end if.
do if (ncol(imm2) <> 1 and ncol(imm) <> 1).
compute imm2t=make(nrow(imm2),(ncol(imm2)*ncol(imm)),-999999).
compute k9=1.
do if (wfirst=1).
loop k7=1 to ncol(imm2).
loop k8 = 1 to ncol(imm).
compute imm2t(:,k9)=imm2(:,k7)&*imm(:,k8).
compute k9=k9+1.
end loop.
end loop.
end if.
do if (zfirst=1).
loop k7=1 to ncol(imm).
loop k8 = 1 to ncol(imm2).
compute imm2t(:,k9)=imm(:,k7)&*imm2(:,k8).
compute k9=k9+1.
end loop.
end loop.
end if.
compute imm2=imm2t.
release imm2t.
end if.
do if ((ncol(imm2) > 1) and (ncol(imm)=1)).
loop k7=1 to ncol(imm2).
compute imm2(:,k7)=imm2(:,k7)&*imm.
end loop.
end if.
do if ((ncol(imm2) = 1) and (ncol(imm) > 1)).
loop k7=1 to ncol(imm).
compute imm(:,k7)=imm2&*imm(:,k7).
end loop.
compute imm2=imm.
end if.
end if.
do if (immset=0).
compute imm2=imm.
do if (indmmm(i,1)=41 or indmmm(i,1)=51).
compute condbb2=condbb.
end if.
compute immset=1.
end if.
end if.
end loop.
compute indtemp=tucker2(1,1).
do if (indmmm(i,1)=12 or indmmm(i,1)=22).
compute imm3={imm3,tucker2}.
do if (k1=nrow(indmodva)).
compute imm3=imm3(:,2:ncol(imm3)).
compute immstop=ncol(imm3).
loop k8=2 to immstop.
do if (indmmmt(i,1)=1).
compute imm3={imm3,(imm3(:,k8)-imm3(:,1))}.
end if.
do if (indmmmt(i,1)=2).
compute imm3={imm3,(imm3(:,k8)-imm3(:,(k8-1)))}.
end if.
do if (indmmmt(i,1)=3).
compute imm3={imm3,((rsum(imm3(:,(k8:immstop)))/(immstop-k8+1))-imm3(:,(k8-1))) }.
end if.
do if (indmmmt(i,1)=4).
compute imm3={imm3,(imm3(:,k8)-(rsum(imm3(:,1:immstop))/immstop))}.
end if.
end loop.
do if (indmmmt(i,1)<5).
compute imm2=imm3(:,(immstop+1):ncol(imm3)).
release imm3.
end if.
end if.
end if.
do if (indmmm(i,1)>-1 and (contrast = 1 or contrast = 2 or contrast=3)).
compute imm4={imm4,tucker2}.
do if (k1=nrow(indmodva) and k1 > 1).
compute imm4=imm4(:,2:ncol(imm4)).
compute immstop=ncol(imm4).
compute condcont=make((immstop*(immstop-1)/2),6,-999).
loop k8 = 1 to (immstop-1).
loop k9 = (k8+1) to immstop.
do if (contrast=1).
compute imm4={imm4,(imm4(:,k9)-imm4(:,k8))}.
end if.
do if (contrast=2).
compute imm4={imm4,(abs(imm4(:,k9))-abs(imm4(:,k8)))}.
end if.
do if (contrast=3).
compute imm4={imm4,(imm4(:,k9)+imm4(:,k8))}.
end if.
compute condcont((ncol(imm4)-immstop),1)=imm4(1,k9).
compute condcont((ncol(imm4)-immstop),2)=imm4(1,k8).
end loop.
end loop.
compute imm4=imm4(:,(immstop+1):ncol(imm4)).
loop k8=1 to ncol(imm4).
compute condcont(k8,3)=imm4(1,k8).
do if (boot > 0).

.
compute temp = imm4(2:nrow(imm4),k8).
compute temp(GRADE( imm4(2:nrow(imm4),k8) )) = imm4(2:nrow(imm4),k8).
compute badlo = 0.
compute badhi = 0.
do if ( (imm4(1,k8)*bc)+(9999*(1-bc)) <> 9999).
compute pv=csum(temp < (imm4(1,k8)*bc)+(9999*(1-bc)) )/nrow(temp).
compute ppv = pv.
do if (pv > .5).
compute ppv = 1-pv.
end if.
compute y5=sqrt(-2*ln(ppv)).
compute xp=y5+((((y5*p4+p3)*y5+p2)*y5+p1)*y5+p0)/((((y5*q4+q3)*y5+q2)*y5+q1)*y5+q0).
do if (pv <= .5).
compute xp = -xp.
end if.
compute cilow=rnd(nrow(temp)*(cdfnorm(2*xp-xp2))).
compute cihigh=trunc(nrow(temp)*(cdfnorm(2*xp+(xp2))))+1.
do if (cilow < 1).
compute cilow = 1.
compute booterr=1.
compute badlo = 1.
end if.
do if (cihigh > nrow(temp)).
compute cihigh = boot.
compute booterr=1.
compute badhi = 1.
end if.
compute llcit=temp(cilow,1).
compute ulcit=temp(cihigh,1).
do if (badlo = 1 and llcit <> priorlo).
compute badend={badend, llcit}.
compute priorlo = llcit.
end if.
do if (badhi = 1 and ulcit <> priorhi).
compute badend={badend, ulcit}.
compute priorhi = ulcit.
end if.
end if.
do if ( (imm4(1,k8)*bc)+(9999*(1-bc)) = 9999).
compute llcit=temp(cilow,1).
compute ulcit=temp(cihigh,1).
end if.
compute bootse=t(sqrt((cssq(temp)-((csum(temp)&**2)/nrow(temp)))/(nrow(temp)-1)))
.
compute condcont(k8,4:6)={bootse,llcit,ulcit}.
end if.
end loop.
do if (boot=0).
compute condcont=condcont(:,1:3).
end if.
compute indcontr=1.
end if.
end if.
do if (xmint=1).
do if (k1=(k4+1)).
compute cftotfx(:,k4)=cftotfx(:,k4)+(tucker2*xscaling).
end if.
end if.
do if (boot > 0).
compute tucker2=tucker2*xscaling.

.
compute temp = tucker2(2:nrow(tucker2),1).
compute temp(GRADE( tucker2(2:nrow(tucker2),1) )) = tucker2(2:nrow(tucker2),1).
compute badlo = 0.
compute badhi = 0.
do if ( (tucker2(1,1)*bc)+(9999*(1-bc)) <> 9999).
compute pv=csum(temp < (tucker2(1,1)*bc)+(9999*(1-bc)) )/nrow(temp).
compute ppv = pv.
do if (pv > .5).
compute ppv = 1-pv.
end if.
compute y5=sqrt(-2*ln(ppv)).
compute xp=y5+((((y5*p4+p3)*y5+p2)*y5+p1)*y5+p0)/((((y5*q4+q3)*y5+q2)*y5+q1)*y5+q0).
do if (pv <= .5).
compute xp = -xp.
end if.
compute cilow=rnd(nrow(temp)*(cdfnorm(2*xp-xp2))).
compute cihigh=trunc(nrow(temp)*(cdfnorm(2*xp+(xp2))))+1.
do if (cilow < 1).
compute cilow = 1.
compute booterr=1.
compute badlo = 1.
end if.
do if (cihigh > nrow(temp)).
compute cihigh = boot.
compute booterr=1.
compute badhi = 1.
end if.
compute llcit=temp(cilow,1).
compute ulcit=temp(cihigh,1).
do if (badlo = 1 and llcit <> priorlo).
compute badend={badend, llcit}.
compute priorlo = llcit.
end if.
do if (badhi = 1 and ulcit <> priorhi).
compute badend={badend, ulcit}.
compute priorhi = ulcit.
end if.
end if.
do if ( (tucker2(1,1)*bc)+(9999*(1-bc)) = 9999).
compute llcit=temp(cilow,1).
compute ulcit=temp(cihigh,1).
end if.
compute bootse=t(sqrt((cssq(temp)-((csum(temp)&**2)/nrow(temp)))/(nrow(temp)-1)))
.
compute indtemp={indtemp*xscaling, bootse,llcit,ulcit}.
end if.
do if (boot=0).
compute indtemp=indtemp*xscaling.
end if.
compute condres(k1,(ncol(indmodva)+1):ncol(condres))=indtemp.
end loop.
do if (xmint=0).
compute resultm2=make(nrow(condres),maxresm,99999).
compute resultm2(1:nrow(condres),1:ncol(condres))=condres.
compute resultm={resultm;resultm2}.
end if.
compute condlbs={condlbs,'Effect'}.
do if (boot > 0).
compute condlbs={condlbs,'BootSE', 'BootLLCI', 'BootULCI'}.
end if.
do if (xmint = 1).
do if (k4 = 1).
compute natindfx=condres((2+(k4-1)),2:ncol(condres)).
end if.
do if (k4 > 1).
compute natindfx={natindfx;condres((2+(k4-1)),2:ncol(condres))}.
end if.
do if (k4=nxvls).
compute resultm2=make(nrow(natindfx),maxresm,99999).
compute resultm2(1:nrow(natindfx),1:ncol(natindfx))=natindfx.
compute resultm={resultm;resultm2}.
end if.
end if.
do if (outscree=1 and xmint=0).
do if (nxvls=1).
print condres/title=' '/cnames=condlbs/format= F10.4 /space=0.
else.
compute condrlb=make(nrow(condres),1,xcatlab(k4,1)).
print condres/title=' '/cnames=condlbs/rnames=condrlb/format= F10.4 /space=0.
end if.
end if.
do if (outscree=1 and xmint=1 and k4=nxvls).
do if (nxvls=1).
print natindfx/title=' '/clabels='Effect', 'BootSE', 'BootLLCI', 'BootULCI'/format= F10.4 /space=0.
else.
compute condrlb=make(nrow(condres),1,xcatlab(k4,1)).
print natindfx/title=' '/clabels='Effect', 'BootSE', 'BootLLCI', 'BootULCI'/rnames=direffl2/format= F10.4 /space=0.
end if.
end if.
compute dichadj=0.
compute immcat=0.
do if ((indmmm(i,1)>0) and (xmint=0)).
do if (indmmm(i,1)=1 or indmmm(i,1)=12 or indmmm(i,1)=31).
do if (wdich=1 and mcw=0).
do if (indmmm(i,1) <> 12).
compute imm2(:,1)=imm2(:,1)*(wmax-wmin).
end if.
do if (indmmm(i,1) <> 31).
compute dichadj=1.
end if.
end if.
do if ((mcw = 1 or mcw = 2) and indmmm(i,1) <> 31)).
compute immcat=1.
end if.
end if.
do if (indmmm(i,1)=2 or indmmm(i,1)=22 or indmmm(i,1)=31).
do if (zdich=1 and mcz=0).
do if (indmmm(i,1) = 31).
compute imm2(:,(nwvls+1):ncol(imm2))=imm2(:,(nwvls+1):ncol(imm2))*(zmax-zmin).
end if.
do if (indmmm(i,1) = 2).
compute imm2(:,1)=imm2(:,1)*(zmax-zmin).
end if.
do if (indmmm(i,1) <> 31).
compute dichadj=1.
end if.
end if.
do if ((mcz = 1 or mcz = 2) and indmmm(i,1) <> 31)).
compute immcat=1.
end if.
end if.
compute immtemp2=t(imm2(1,:)).
compute immtemp=immtemp2.
compute immlbs={'Index'}.
do if (boot > 0).
compute immtemp=make(1,3,0).
loop k7=1 to ncol(imm2).

.
compute temp = imm2(2:nrow(imm2),k7).
compute temp(GRADE( imm2(2:nrow(imm2),k7) )) = imm2(2:nrow(imm2),k7).
compute badlo = 0.
compute badhi = 0.
do if ( (imm2(1,k7)*bc)+(9999*(1-bc)) <> 9999).
compute pv=csum(temp < (imm2(1,k7)*bc)+(9999*(1-bc)) )/nrow(temp).
compute ppv = pv.
do if (pv > .5).
compute ppv = 1-pv.
end if.
compute y5=sqrt(-2*ln(ppv)).
compute xp=y5+((((y5*p4+p3)*y5+p2)*y5+p1)*y5+p0)/((((y5*q4+q3)*y5+q2)*y5+q1)*y5+q0).
do if (pv <= .5).
compute xp = -xp.
end if.
compute cilow=rnd(nrow(temp)*(cdfnorm(2*xp-xp2))).
compute cihigh=trunc(nrow(temp)*(cdfnorm(2*xp+(xp2))))+1.
do if (cilow < 1).
compute cilow = 1.
compute booterr=1.
compute badlo = 1.
end if.
do if (cihigh > nrow(temp)).
compute cihigh = boot.
compute booterr=1.
compute badhi = 1.
end if.
compute llcit=temp(cilow,1).
compute ulcit=temp(cihigh,1).
do if (badlo = 1 and llcit <> priorlo).
compute badend={badend, llcit}.
compute priorlo = llcit.
end if.
do if (badhi = 1 and ulcit <> priorhi).
compute badend={badend, ulcit}.
compute priorhi = ulcit.
end if.
end if.
do if ( (imm2(1,k7)*bc)+(9999*(1-bc)) = 9999).
compute llcit=temp(cilow,1).
compute ulcit=temp(cihigh,1).
end if.
compute bootse=t(sqrt((cssq(temp)-((csum(temp)&**2)/nrow(temp)))/(nrow(temp)-1)))
.
compute temp={bootse,llcit,ulcit}.
compute immtemp={immtemp;temp}.
end loop.
compute immtemp=immtemp(2:nrow(immtemp),:).
compute immtemp={immtemp2,immtemp}.
compute immlbs={immlbs,'BootSE', 'BootLLCI', 'BootULCI'}.
end if.
compute resultm2=make(nrow(immtemp),maxresm,99999).
compute resultm2(1:nrow(immtemp),1:ncol(immtemp))=immtemp.
compute resultm={resultm;resultm2}.
do if (dichadj=0 and immcat=0 and indmmmt(i,1)<>5 and indmmm(i,1) < 100 ).
do if (outscree=1).
do if (indmmm(i,1) < 30).
print immtemp/title='      Index of moderated mediation:'/cnames=immlbs/rnames=immlbs2/format= F10.4.
end if.
do if (indmmm(i,1) = 31).
compute immlbs2={immlbs2;zcatlab(1:nzvls,1)}.
print immtemp/title='      Indices of partial moderated mediation:'/cnames=immlbs/rnames=immlbs2/format= F10.4.
end if.
end if.
do if ((nzvls = 1) and (nwvls = 1)).
do if (indmmm(i,1) = 41 or indmmm(i,1)=51).
loop k7=1 to nwvls.
compute immlbs2=zcatlab(1:nzvls,1).
compute immtemp2=immtemp((((k7-1)*nzvls)+1):(((k7-1)*nzvls)+nzvls),:).
do if (outscree=1).
do if (nwvls > 1).
compute primodv={'        ', wcatlab(k7,1)}.
print primodv/title='      Primary moderator:'/format=A8.
end if.
do if (nzvls=1).
print immtemp2/title='      Index of moderated moderated mediation'/cnames=immlbs/format= F10.4.
else.
print immtemp2/title='      Indices of moderated moderated mediation'/cnames=immlbs/rnames=immlbs2/format= F10.4.
end if.
end if.
compute cmmtemp=make(nrow(zprobval),4,0).
loop k8=1 to nrow(zprobval).
compute condbb3=condbb2(:,((nwvls+1)+((k7-1)*nzvls)):((nwvls+1)+((k7-1)*nzvls)+ (nzvls-1))).
do if (ncol(zprobval) > 1).
compute condbb3=condbb3*mdiag(zprobval(k8,:)).
else.
compute condbb3=condbb3&*zprobval(k8,:).
end if.
compute condbb3={condbb2(:,k7),condbb3}.
compute icmm=rsum(condbb3).
compute cmmtemp(k8,1)=icmm(1,1).
do if (boot > 0).

.
compute temp = icmm(2:nrow(icmm),1).
compute temp(GRADE( icmm(2:nrow(icmm),1) )) = icmm(2:nrow(icmm),1).
compute badlo = 0.
compute badhi = 0.
do if ( (icmm(1,1)*bc)+(9999*(1-bc)) <> 9999).
compute pv=csum(temp < (icmm(1,1)*bc)+(9999*(1-bc)) )/nrow(temp).
compute ppv = pv.
do if (pv > .5).
compute ppv = 1-pv.
end if.
compute y5=sqrt(-2*ln(ppv)).
compute xp=y5+((((y5*p4+p3)*y5+p2)*y5+p1)*y5+p0)/((((y5*q4+q3)*y5+q2)*y5+q1)*y5+q0).
do if (pv <= .5).
compute xp = -xp.
end if.
compute cilow=rnd(nrow(temp)*(cdfnorm(2*xp-xp2))).
compute cihigh=trunc(nrow(temp)*(cdfnorm(2*xp+(xp2))))+1.
do if (cilow < 1).
compute cilow = 1.
compute booterr=1.
compute badlo = 1.
end if.
do if (cihigh > nrow(temp)).
compute cihigh = boot.
compute booterr=1.
compute badhi = 1.
end if.
compute llcit=temp(cilow,1).
compute ulcit=temp(cihigh,1).
do if (badlo = 1 and llcit <> priorlo).
compute badend={badend, llcit}.
compute priorlo = llcit.
end if.
do if (badhi = 1 and ulcit <> priorhi).
compute badend={badend, ulcit}.
compute priorhi = ulcit.
end if.
end if.
do if ( (icmm(1,1)*bc)+(9999*(1-bc)) = 9999).
compute llcit=temp(cilow,1).
compute ulcit=temp(cihigh,1).
end if.
compute bootse=t(sqrt((cssq(temp)-((csum(temp)&**2)/nrow(temp)))/(nrow(temp)-1)))
.
compute cmmtemp(k8,2:4)={bootse,llcit,ulcit}.
end if.
end loop.
compute cmmtemp={zmodvals,cmmtemp}.
do if (boot=0).
compute cmmtemp=cmmtemp(:,1:2).
end if.
compute cmmlbs={znames,immlbs}.
compute resultm2=make(nrow(cmmtemp),maxresm,99999).
compute resultm2(1:nrow(cmmtemp),1:ncol(cmmtemp))=cmmtemp.
compute resultm={resultm;resultm2}.
do if (outscree=1).
print cmmtemp/title='      Indices of conditional moderated mediation by W'/cnames=cmmlbs/format= F10.4.
end if.
end loop.
end if.
end if.
end if.
do if (dichadj=1 or immcat=1 and indmmm(i,1) < 30).
do if (outscree=1).
print immtemp/title='Index of moderated mediation (difference between conditional indirect effects):'/cnames=immlbs/rnames=immlbs2/format= F10.4.
end if.
end if.
end if.
do if (indcontr=1).
compute resultm2=make(nrow(condcont),maxresm,99999).
compute resultm2(1:nrow(condcont),1:ncol(condcont))=condcont.
compute resultm={resultm;resultm2}.
compute condctlb={'Effect1','Effect2','Contrast','BootSE', 'BootLLCI','BootULCI'}.
do if (outscree=1).
print condcont/title=' Pairwise contrasts between conditional indirect effects (Effect1 minus Effect2)'/cnames=condctlb/format= F10.4.
end if.
end if.
end loop.
end if.
end loop.
do if (alttotal=1).
compute altcnms='Effect'.
compute alttotfx=t(cftotfx(1,:))+obnatdfx.
do if (boot > 0).
compute alttotfx=make(ncol(cftotfx),4,0).
compute alttotfx(:,1)=t(cftotfx(1,:))+obnatdfx.
compute natdirbt(1,:)=t(obnatdfx).
compute cftotfx=cftotfx+natdirbt.
loop cec=1 to ncol(cftotfx).

.
compute temp = cftotfx(2:nrow(cftotfx),cec).
compute temp(GRADE( cftotfx(2:nrow(cftotfx),cec) )) = cftotfx(2:nrow(cftotfx),cec).
compute badlo = 0.
compute badhi = 0.
do if ( (cftotfx(1,cec)*bc)+(9999*(1-bc)) <> 9999).
compute pv=csum(temp < (cftotfx(1,cec)*bc)+(9999*(1-bc)) )/nrow(temp).
compute ppv = pv.
do if (pv > .5).
compute ppv = 1-pv.
end if.
compute y5=sqrt(-2*ln(ppv)).
compute xp=y5+((((y5*p4+p3)*y5+p2)*y5+p1)*y5+p0)/((((y5*q4+q3)*y5+q2)*y5+q1)*y5+q0).
do if (pv <= .5).
compute xp = -xp.
end if.
compute cilow=rnd(nrow(temp)*(cdfnorm(2*xp-xp2))).
compute cihigh=trunc(nrow(temp)*(cdfnorm(2*xp+(xp2))))+1.
do if (cilow < 1).
compute cilow = 1.
compute booterr=1.
compute badlo = 1.
end if.
do if (cihigh > nrow(temp)).
compute cihigh = boot.
compute booterr=1.
compute badhi = 1.
end if.
compute llcit=temp(cilow,1).
compute ulcit=temp(cihigh,1).
do if (badlo = 1 and llcit <> priorlo).
compute badend={badend, llcit}.
compute priorlo = llcit.
end if.
do if (badhi = 1 and ulcit <> priorhi).
compute badend={badend, ulcit}.
compute priorhi = ulcit.
end if.
end if.
do if ( (cftotfx(1,cec)*bc)+(9999*(1-bc)) = 9999).
compute llcit=temp(cilow,1).
compute ulcit=temp(cihigh,1).
end if.
compute bootse=t(sqrt((cssq(temp)-((csum(temp)&**2)/nrow(temp)))/(nrow(temp)-1)))
.
compute alttotfx(cec,2:4)={bootse,llcit,ulcit}.
end loop.
compute altcnms={altcnms,'BootSE', 'BootLLCI','BootULCI'}.
end if.
compute resultm2=make(nrow(alttotfx),maxresm,99999).
compute resultm2(1:nrow(alttotfx),1:ncol(alttotfx))=alttotfx.
compute resultm={resultm;resultm2}.
do if (outscree=1).
print/title='----------'.
do if (nxvls > 1).
print alttotfx/title='Relative total effects of X on Y (sum of direct and indirect effects)'/cnames=altcnms/rnames=direffl2/format= F10.4.
end if.
do if (nxvls = 1).
print alttotfx/title='Total effect of X on Y (sum of direct and indirect effects)'/cnames=altcnms/format= F10.4.
end if.
end if.
end if.
end if.
end if
.
do if (criterr=0 and (saveest = 1 or saveboot=1)).
compute coeffsav=coeffmat(2:nrow(coeffmat),:).
compute conseq=conseq(2:nrow(conseq),1).
compute dfmat=dfmat(2:nrow(dfmat),1).
compute dfmat=dfmat.
compute saven={'conseqnt','antecdnt','coeff','se','t','p','LLCI','ULCI','df'}.
do if (ydich=1).
compute saven={'conseqnt','antecdnt','coeff','se','t_or_Z','p','LLCI','ULCI','df'}.
end if.
do if (saveboot=1 and boot > 0 ).
compute colslab=make(300,1,' ').


compute colslab( 1 ,1)= 'COL1'.


compute colslab( 2 ,1)= 'COL2'.


compute colslab( 3 ,1)= 'COL3'.


compute colslab( 4 ,1)= 'COL4'.


compute colslab( 5 ,1)= 'COL5'.


compute colslab( 6 ,1)= 'COL6'.


compute colslab( 7 ,1)= 'COL7'.


compute colslab( 8 ,1)= 'COL8'.


compute colslab( 9 ,1)= 'COL9'.


compute colslab( 10 ,1)= 'COL10'.


compute colslab( 11 ,1)= 'COL11'.


compute colslab( 12 ,1)= 'COL12'.


compute colslab( 13 ,1)= 'COL13'.


compute colslab( 14 ,1)= 'COL14'.


compute colslab( 15 ,1)= 'COL15'.


compute colslab( 16 ,1)= 'COL16'.


compute colslab( 17 ,1)= 'COL17'.


compute colslab( 18 ,1)= 'COL18'.


compute colslab( 19 ,1)= 'COL19'.


compute colslab( 20 ,1)= 'COL20'.


compute colslab( 21 ,1)= 'COL21'.


compute colslab( 22 ,1)= 'COL22'.


compute colslab( 23 ,1)= 'COL23'.


compute colslab( 24 ,1)= 'COL24'.


compute colslab( 25 ,1)= 'COL25'.


compute colslab( 26 ,1)= 'COL26'.


compute colslab( 27 ,1)= 'COL27'.


compute colslab( 28 ,1)= 'COL28'.


compute colslab( 29 ,1)= 'COL29'.


compute colslab( 30 ,1)= 'COL30'.


compute colslab( 31 ,1)= 'COL31'.


compute colslab( 32 ,1)= 'COL32'.


compute colslab( 33 ,1)= 'COL33'.


compute colslab( 34 ,1)= 'COL34'.


compute colslab( 35 ,1)= 'COL35'.


compute colslab( 36 ,1)= 'COL36'.


compute colslab( 37 ,1)= 'COL37'.


compute colslab( 38 ,1)= 'COL38'.


compute colslab( 39 ,1)= 'COL39'.


compute colslab( 40 ,1)= 'COL40'.


compute colslab( 41 ,1)= 'COL41'.


compute colslab( 42 ,1)= 'COL42'.


compute colslab( 43 ,1)= 'COL43'.


compute colslab( 44 ,1)= 'COL44'.


compute colslab( 45 ,1)= 'COL45'.


compute colslab( 46 ,1)= 'COL46'.


compute colslab( 47 ,1)= 'COL47'.


compute colslab( 48 ,1)= 'COL48'.


compute colslab( 49 ,1)= 'COL49'.


compute colslab( 50 ,1)= 'COL50'.


compute colslab( 51 ,1)= 'COL51'.


compute colslab( 52 ,1)= 'COL52'.


compute colslab( 53 ,1)= 'COL53'.


compute colslab( 54 ,1)= 'COL54'.


compute colslab( 55 ,1)= 'COL55'.


compute colslab( 56 ,1)= 'COL56'.


compute colslab( 57 ,1)= 'COL57'.


compute colslab( 58 ,1)= 'COL58'.


compute colslab( 59 ,1)= 'COL59'.


compute colslab( 60 ,1)= 'COL60'.


compute colslab( 61 ,1)= 'COL61'.


compute colslab( 62 ,1)= 'COL62'.


compute colslab( 63 ,1)= 'COL63'.


compute colslab( 64 ,1)= 'COL64'.


compute colslab( 65 ,1)= 'COL65'.


compute colslab( 66 ,1)= 'COL66'.


compute colslab( 67 ,1)= 'COL67'.


compute colslab( 68 ,1)= 'COL68'.


compute colslab( 69 ,1)= 'COL69'.


compute colslab( 70 ,1)= 'COL70'.


compute colslab( 71 ,1)= 'COL71'.


compute colslab( 72 ,1)= 'COL72'.


compute colslab( 73 ,1)= 'COL73'.


compute colslab( 74 ,1)= 'COL74'.


compute colslab( 75 ,1)= 'COL75'.


compute colslab( 76 ,1)= 'COL76'.


compute colslab( 77 ,1)= 'COL77'.


compute colslab( 78 ,1)= 'COL78'.


compute colslab( 79 ,1)= 'COL79'.


compute colslab( 80 ,1)= 'COL80'.


compute colslab( 81 ,1)= 'COL81'.


compute colslab( 82 ,1)= 'COL82'.


compute colslab( 83 ,1)= 'COL83'.


compute colslab( 84 ,1)= 'COL84'.


compute colslab( 85 ,1)= 'COL85'.


compute colslab( 86 ,1)= 'COL86'.


compute colslab( 87 ,1)= 'COL87'.


compute colslab( 88 ,1)= 'COL88'.


compute colslab( 89 ,1)= 'COL89'.


compute colslab( 90 ,1)= 'COL90'.


compute colslab( 91 ,1)= 'COL91'.


compute colslab( 92 ,1)= 'COL92'.


compute colslab( 93 ,1)= 'COL93'.


compute colslab( 94 ,1)= 'COL94'.


compute colslab( 95 ,1)= 'COL95'.


compute colslab( 96 ,1)= 'COL96'.


compute colslab( 97 ,1)= 'COL97'.


compute colslab( 98 ,1)= 'COL98'.


compute colslab( 99 ,1)= 'COL99'.


compute colslab( 100 ,1)= 'COL100'.


compute colslab( 101 ,1)= 'COL101'.


compute colslab( 102 ,1)= 'COL102'.


compute colslab( 103 ,1)= 'COL103'.


compute colslab( 104 ,1)= 'COL104'.


compute colslab( 105 ,1)= 'COL105'.


compute colslab( 106 ,1)= 'COL106'.


compute colslab( 107 ,1)= 'COL107'.


compute colslab( 108 ,1)= 'COL108'.


compute colslab( 109 ,1)= 'COL109'.


compute colslab( 110 ,1)= 'COL110'.


compute colslab( 111 ,1)= 'COL111'.


compute colslab( 112 ,1)= 'COL112'.


compute colslab( 113 ,1)= 'COL113'.


compute colslab( 114 ,1)= 'COL114'.


compute colslab( 115 ,1)= 'COL115'.


compute colslab( 116 ,1)= 'COL116'.


compute colslab( 117 ,1)= 'COL117'.


compute colslab( 118 ,1)= 'COL118'.


compute colslab( 119 ,1)= 'COL119'.


compute colslab( 120 ,1)= 'COL120'.


compute colslab( 121 ,1)= 'COL121'.


compute colslab( 122 ,1)= 'COL122'.


compute colslab( 123 ,1)= 'COL123'.


compute colslab( 124 ,1)= 'COL124'.


compute colslab( 125 ,1)= 'COL125'.


compute colslab( 126 ,1)= 'COL126'.


compute colslab( 127 ,1)= 'COL127'.


compute colslab( 128 ,1)= 'COL128'.


compute colslab( 129 ,1)= 'COL129'.


compute colslab( 130 ,1)= 'COL130'.


compute colslab( 131 ,1)= 'COL131'.


compute colslab( 132 ,1)= 'COL132'.


compute colslab( 133 ,1)= 'COL133'.


compute colslab( 134 ,1)= 'COL134'.


compute colslab( 135 ,1)= 'COL135'.


compute colslab( 136 ,1)= 'COL136'.


compute colslab( 137 ,1)= 'COL137'.


compute colslab( 138 ,1)= 'COL138'.


compute colslab( 139 ,1)= 'COL139'.


compute colslab( 140 ,1)= 'COL140'.


compute colslab( 141 ,1)= 'COL141'.


compute colslab( 142 ,1)= 'COL142'.


compute colslab( 143 ,1)= 'COL143'.


compute colslab( 144 ,1)= 'COL144'.


compute colslab( 145 ,1)= 'COL145'.


compute colslab( 146 ,1)= 'COL146'.


compute colslab( 147 ,1)= 'COL147'.


compute colslab( 148 ,1)= 'COL148'.


compute colslab( 149 ,1)= 'COL149'.


compute colslab( 150 ,1)= 'COL150'.


compute colslab( 151 ,1)= 'COL151'.


compute colslab( 152 ,1)= 'COL152'.


compute colslab( 153 ,1)= 'COL153'.


compute colslab( 154 ,1)= 'COL154'.


compute colslab( 155 ,1)= 'COL155'.


compute colslab( 156 ,1)= 'COL156'.


compute colslab( 157 ,1)= 'COL157'.


compute colslab( 158 ,1)= 'COL158'.


compute colslab( 159 ,1)= 'COL159'.


compute colslab( 160 ,1)= 'COL160'.


compute colslab( 161 ,1)= 'COL161'.


compute colslab( 162 ,1)= 'COL162'.


compute colslab( 163 ,1)= 'COL163'.


compute colslab( 164 ,1)= 'COL164'.


compute colslab( 165 ,1)= 'COL165'.


compute colslab( 166 ,1)= 'COL166'.


compute colslab( 167 ,1)= 'COL167'.


compute colslab( 168 ,1)= 'COL168'.


compute colslab( 169 ,1)= 'COL169'.


compute colslab( 170 ,1)= 'COL170'.


compute colslab( 171 ,1)= 'COL171'.


compute colslab( 172 ,1)= 'COL172'.


compute colslab( 173 ,1)= 'COL173'.


compute colslab( 174 ,1)= 'COL174'.


compute colslab( 175 ,1)= 'COL175'.


compute colslab( 176 ,1)= 'COL176'.


compute colslab( 177 ,1)= 'COL177'.


compute colslab( 178 ,1)= 'COL178'.


compute colslab( 179 ,1)= 'COL179'.


compute colslab( 180 ,1)= 'COL180'.


compute colslab( 181 ,1)= 'COL181'.


compute colslab( 182 ,1)= 'COL182'.


compute colslab( 183 ,1)= 'COL183'.


compute colslab( 184 ,1)= 'COL184'.


compute colslab( 185 ,1)= 'COL185'.


compute colslab( 186 ,1)= 'COL186'.


compute colslab( 187 ,1)= 'COL187'.


compute colslab( 188 ,1)= 'COL188'.


compute colslab( 189 ,1)= 'COL189'.


compute colslab( 190 ,1)= 'COL190'.


compute colslab( 191 ,1)= 'COL191'.


compute colslab( 192 ,1)= 'COL192'.


compute colslab( 193 ,1)= 'COL193'.


compute colslab( 194 ,1)= 'COL194'.


compute colslab( 195 ,1)= 'COL195'.


compute colslab( 196 ,1)= 'COL196'.


compute colslab( 197 ,1)= 'COL197'.


compute colslab( 198 ,1)= 'COL198'.


compute colslab( 199 ,1)= 'COL199'.


compute colslab( 200 ,1)= 'COL200'.


compute colslab( 201 ,1)= 'COL201'.


compute colslab( 202 ,1)= 'COL202'.


compute colslab( 203 ,1)= 'COL203'.


compute colslab( 204 ,1)= 'COL204'.


compute colslab( 205 ,1)= 'COL205'.


compute colslab( 206 ,1)= 'COL206'.


compute colslab( 207 ,1)= 'COL207'.


compute colslab( 208 ,1)= 'COL208'.


compute colslab( 209 ,1)= 'COL209'.


compute colslab( 210 ,1)= 'COL210'.


compute colslab( 211 ,1)= 'COL211'.


compute colslab( 212 ,1)= 'COL212'.


compute colslab( 213 ,1)= 'COL213'.


compute colslab( 214 ,1)= 'COL214'.


compute colslab( 215 ,1)= 'COL215'.


compute colslab( 216 ,1)= 'COL216'.


compute colslab( 217 ,1)= 'COL217'.


compute colslab( 218 ,1)= 'COL218'.


compute colslab( 219 ,1)= 'COL219'.


compute colslab( 220 ,1)= 'COL220'.


compute colslab( 221 ,1)= 'COL221'.


compute colslab( 222 ,1)= 'COL222'.


compute colslab( 223 ,1)= 'COL223'.


compute colslab( 224 ,1)= 'COL224'.


compute colslab( 225 ,1)= 'COL225'.


compute colslab( 226 ,1)= 'COL226'.


compute colslab( 227 ,1)= 'COL227'.


compute colslab( 228 ,1)= 'COL228'.


compute colslab( 229 ,1)= 'COL229'.


compute colslab( 230 ,1)= 'COL230'.


compute colslab( 231 ,1)= 'COL231'.


compute colslab( 232 ,1)= 'COL232'.


compute colslab( 233 ,1)= 'COL233'.


compute colslab( 234 ,1)= 'COL234'.


compute colslab( 235 ,1)= 'COL235'.


compute colslab( 236 ,1)= 'COL236'.


compute colslab( 237 ,1)= 'COL237'.


compute colslab( 238 ,1)= 'COL238'.


compute colslab( 239 ,1)= 'COL239'.


compute colslab( 240 ,1)= 'COL240'.


compute colslab( 241 ,1)= 'COL241'.


compute colslab( 242 ,1)= 'COL242'.


compute colslab( 243 ,1)= 'COL243'.


compute colslab( 244 ,1)= 'COL244'.


compute colslab( 245 ,1)= 'COL245'.


compute colslab( 246 ,1)= 'COL246'.


compute colslab( 247 ,1)= 'COL247'.


compute colslab( 248 ,1)= 'COL248'.


compute colslab( 249 ,1)= 'COL249'.


compute colslab( 250 ,1)= 'COL250'.


compute colslab( 251 ,1)= 'COL251'.


compute colslab( 252 ,1)= 'COL252'.


compute colslab( 253 ,1)= 'COL253'.


compute colslab( 254 ,1)= 'COL254'.


compute colslab( 255 ,1)= 'COL255'.


compute colslab( 256 ,1)= 'COL256'.


compute colslab( 257 ,1)= 'COL257'.


compute colslab( 258 ,1)= 'COL258'.


compute colslab( 259 ,1)= 'COL259'.


compute colslab( 260 ,1)= 'COL260'.


compute colslab( 261 ,1)= 'COL261'.


compute colslab( 262 ,1)= 'COL262'.


compute colslab( 263 ,1)= 'COL263'.


compute colslab( 264 ,1)= 'COL264'.


compute colslab( 265 ,1)= 'COL265'.


compute colslab( 266 ,1)= 'COL266'.


compute colslab( 267 ,1)= 'COL267'.


compute colslab( 268 ,1)= 'COL268'.


compute colslab( 269 ,1)= 'COL269'.


compute colslab( 270 ,1)= 'COL270'.


compute colslab( 271 ,1)= 'COL271'.


compute colslab( 272 ,1)= 'COL272'.


compute colslab( 273 ,1)= 'COL273'.


compute colslab( 274 ,1)= 'COL274'.


compute colslab( 275 ,1)= 'COL275'.


compute colslab( 276 ,1)= 'COL276'.


compute colslab( 277 ,1)= 'COL277'.


compute colslab( 278 ,1)= 'COL278'.


compute colslab( 279 ,1)= 'COL279'.


compute colslab( 280 ,1)= 'COL280'.


compute colslab( 281 ,1)= 'COL281'.


compute colslab( 282 ,1)= 'COL282'.


compute colslab( 283 ,1)= 'COL283'.


compute colslab( 284 ,1)= 'COL284'.


compute colslab( 285 ,1)= 'COL285'.


compute colslab( 286 ,1)= 'COL286'.


compute colslab( 287 ,1)= 'COL287'.


compute colslab( 288 ,1)= 'COL288'.


compute colslab( 289 ,1)= 'COL289'.


compute colslab( 290 ,1)= 'COL290'.


compute colslab( 291 ,1)= 'COL291'.


compute colslab( 292 ,1)= 'COL292'.


compute colslab( 293 ,1)= 'COL293'.


compute colslab( 294 ,1)= 'COL294'.


compute colslab( 295 ,1)= 'COL295'.


compute colslab( 296 ,1)= 'COL296'.


compute colslab( 297 ,1)= 'COL297'.


compute colslab( 298 ,1)= 'COL298'.


compute colslab( 299 ,1)= 'COL299'.


compute colslab( 300 ,1)= 'COL300'.

compute colslab=colslab(1:ncol(bootres),1).
compute colslab={colslab,conseq,vlabs}.
do if (outscree=1).
print/title = '**************************************************************************'.
print/title='Bootstrap estimates were saved to a file'/space=0.
print colslab/title='Map of column names to model coefficients:'/clabels=' ','Conseqnt','Antecdnt'/format=a8.
end if.
end if.
end if.
do if (criterr=0 and boot > 0 and modelbt=1).
compute labstart=1.
do if (outscree=1).
print/title = '*********** BOOTSTRAP RESULTS FOR REGRESSION MODEL PARAMETERS ************'.
end if.
loop iboot = 1 to (nms+nys).
do if (outscree=1).
print outnames(1,iboot)/title = 'OUTCOME VARIABLE:'/format = A8.
end if.
compute vlabsm=vlabs(labstart:(labstart+(nump(1,iboot)-1)),1).
compute outnmtmp=bootcim(labstart:(labstart+(nump(1,iboot)-1)),:).
compute resultm2=make(nrow(outnmtmp),maxresm,99999).
compute resultm2(1:nrow(outnmtmp),1:ncol(outnmtmp))=outnmtmp.
compute resultm={resultm;resultm2}.
do if (outscree=1).
print bootcim(labstart:(labstart+(nump(1,iboot)-1)),:)/title=' '/rnames=vlabsm/ clabels='Coeff' 'BootMean' 'BootSE' 'BootLLCI' 'BootULCI'/format= F10.4 /space=0.
end if.
compute labstart=labstart+nump(1,iboot).
do if (iboot < (nms+nys)).
do if (outscree=1).
print/title= '----------'.
end if.
end if.
end loop.
end if.
do if (criterr=0 and saveest=1).
compute resultm=resultm(2:nrow(resultm),:).
compute bocaj=(resultm=99999).
compute bocaj=csum(bocaj).
compute bocaj=1-(bocaj=nrow(resultm)).
compute j=1.
loop i = 1 to ncol(resultm).
do if (bocaj(1,i)=1).
compute j=j+1.
end if.
end loop.
compute resultm=resultm(:,1:(j-1)).
save resultm/outfile = *.
end if.
do if (criterr = 0 and matrices=1 and outscree=1).
print/title = '************************ MODEL DEFINITION MATRICES ************************'.
print/title = 'FROM variables are columns, TO variables are rows.'.
compute temp2=make(nrow(bcmat),ncol(bcmat),'0').
loop i = 2 to nrow(bcmat).
loop j = 1 to (ncol(bcmat)-1).
do if (bcmat(i,j)=1).
compute temp2(i,j)='1'.
end if.
do if (j >= i).
compute temp2(i,j)=' '.
end if.
end loop.
end loop.
compute temp2=temp2(2:nrow(bcmat),(1:(ncol(bcmat)-1))).
do if (nms > 0).
compute cmatlabs={xnames,mnames}.
compute rmatlabs={mnames,ynames}.
end if.
do if (nms = 0).
compute cmatlabs={xnames}.
compute rmatlabs={ynames}.
end if.
print temp2/title='BMATRIX: Paths freely estimated (1) and fixed to zero (0):'/cnames=cmatlabs/rnames=rmatlabs/format A3.
compute z=0.
do if (rsum(csum(wcmat))<>0).
compute temp2=make(nrow(wcmat),ncol(wcmat),'0').
loop i = 2 to nrow(wcmat).
loop j = 1 to (ncol(wcmat)-1).
do if (wcmat(i,j)=1).
compute temp2(i,j)='1'.
end if.
do if (j >= i).
compute temp2(i,j)=' '.
end if.
end loop.
end loop.
compute temp2=temp2(2:nrow(wcmat),(1:(ncol(wcmat)-1))).
print temp2/title='WMATRIX: Paths moderated (1) and not moderated (0) by W:'/cnames=cmatlabs/rnames=rmatlabs/format A3.
end if.
do if (rsum(csum(zcmat))<>0).
compute temp2=make(nrow(zcmat),ncol(zcmat),'0').
loop i = 2 to nrow(zcmat).
loop j = 1 to (ncol(zcmat)-1).
do if (zcmat(i,j)=1).
compute temp2(i,j)='1'.
end if.
do if (j >= i).
compute temp2(i,j)=' '.
end if.
end loop.
end loop.
compute temp2=temp2(2:nrow(zcmat),(1:(ncol(zcmat)-1))).
print temp2/title='ZMATRIX: Paths moderated (1) and not moderated (0) by Z:'/cnames=cmatlabs/rnames=rmatlabs/format a3.
end if.
do if (rsum(csum(wzcmat))<>0).
compute temp2=make(nrow(wzcmat),ncol(wzcmat),'0').
loop i = 2 to nrow(wzcmat).
loop j = 1 to (ncol(wzcmat)-1).
do if (wzcmat(i,j)=1).
compute temp2(i,j)='1'.
end if.
do if (j >= i).
compute temp2(i,j)=' '.
end if.
end loop.
end loop.
compute temp2=temp2(2:nrow(wzcmat),(1:(ncol(wzcmat)-1))).
print temp2/title='WZMATRIX: W moderated paths moderated (1) and not moderated (0) by Z:'/cnames=cmatlabs/rnames=rmatlabs/format a3.
end if.
do if (ncs > 0).
print ccmat/title='CMATRIX: Covariates (columns) in (1) and not in (0) the models of M and Y (rows):'/rnames=rmatlabs/cnames=covnames.
end if.
end if.
do if (outscree=1).
do if (activate=0).
print/title = '*********************** ANALYSIS NOTES AND ERRORS ************************'.
end if.
end if.
do if (activate=1).
print/title = '**************************************************************************'.
end if.
do if (criterr=0).
do if (outscree=1).
print conf/title = 'Level of confidence for all confidence intervals in output:'/format = F8.4.
do if (boot > 0).
do if (goodboot = boot and bc=0).
print boot/title='Number of bootstrap samples for percentile bootstrap confidence intervals:'.
end if.
do if (goodboot = boot and bc=1).
print boot/title='Number of bootstrap samples for bias-corrected bootstrap confidence intervals:'.
end if.
do if (booterr = 1).
compute badend = badend(1,2:ncol(badend)).
print/title = 'WARNING: Bootstrap CI endpoints below not trustworthy. Decrease confidence'.
print badend/title='or increase the number of bootstrap samples.'/space=0/format = F10.4.
end if.
end if.
do if (mc > 0).
print mc/title='Number of samples for Monte Carlo confidence intervals:'.
end if.
do if (wnotev > 0 and printw=1).
do if (wnotev=1).
print/title = 'W values in conditional tables are the 16th, 50th, and 84th percentiles.'.
else if (wnotev=2).
do if (minwwarn=0 and maxwwarn=0).
print/title = 'W values in conditional tables are the mean and +/- SD from the mean.'.
end if.
do if (minwwarn=1).
print/title = 'W values in conditional tables are the minimum, the mean, and 1 SD above the mean.'.
end if.
do if (maxwwarn=1).
print/title = 'W values in conditional tables are 1 SD below the mean, the mean, and the maximum.'.
end if.
end if.
end if.
do if (znotev > 0 and printz=1).
do if (znotev=1).
print/title = 'Z values in conditional tables are the 16th, 50th, and 84th percentiles.'.
else if (znotev=2).
do if (minzwarn=0 and maxzwarn=0).
print/title = 'Z values in conditional tables are the mean and +/- SD from the mean.'.
end if.
do if (minzwarn=1).
print/title = 'Z values in conditional tables are the minimum, the mean, and 1 SD above the mean.'.
end if.
do if (maxzwarn=1).
print/title = 'Z values in conditional tables are 1 SD below the mean, the mean, and the maximum.'.
end if.
end if.
end if.
do if (minwwarn > 0).
print/title = 'NOTE: One SD below the mean is below the minimum observed in the data for W,'.
print/title = '      so the minimum measurement on W is used for conditioning instead.'/space=0.
end if.
do if (maxwwarn > 0).
print/title = 'NOTE: One SD above the mean is above the maximum observed in the data for W,'.
print/title = '      so the maximum measurement for W is used for conditioning instead.'/space=0.
end if.
do if (minzwarn > 0).
print/title = 'NOTE: One SD below the mean is below the minimum observed in the data for Z,'.
print/title = '      so the minimum measurement for Z is used for conditioning instead.'/space=0.
end if.
do if (maxzwarn > 0).
print/title = 'NOTE: One SD above the mean is above the maximum observed in the data for Z,'.
print/title = '      so the maximum measurement for Z is used for conditioning instead.'/space=0.
end if.
do if (pstog=1).
print/title= 'NOTE: Standardized coefficients for dichotomous or multicategorical X are in'.
print/title= '      partially standardized form.'/space=0.
end if.
loop i = 1 to 100.
do if (notecode(i,1) = 32).
print/title = 'Direct, indirect, and total effects are counterfactually defined'.
do if (xcontcf=0).
print/title = 'assuming X by M interaction.'/space=0.
end if.
do if (xcontcf=1).
print/title = 'assuming X by M interaction and with the following reference (x_ref)'/space=0.
compute states=t(xrefvals).
print states/title = 'and counterfactual (x_cf) states for X:'/rlabels='x_ref' 'x_cf'/format= F10.4 /space=0.
end if.
end if.
do if (notecode(i,1)=1).
print/title = 'NOTE: COVMY is ignored when using CMATRIX option.'.
end if.
do if (notecode(i,1)=2).
print/title = 'NOTE: Confidence level restricted to between 50 and 99.9999%.  95% confidence is provided in output'.
end if.
do if (notecode(i,1)=3).
print centvar/title = 'NOTE: The following variables were mean centered prior to analysis:'/format = a8.
end if.
do if (notecode(i,1) = 4).
print/title = 'NOTE: A heteroscedasticity consistent standard error and covariance matrix estimator was used.'.
end if.
do if (notecode(i,1) = 5).
print/title = 'NOTE: The HC3 option has been replaced with HC.  See the documentation.'.
end if.
do if (notecode(i,1) = 6).
print/title = 'NOTE: Due to estimation problems, some bootstrap samples had to be replaced.'.
print badboot/title='      The number of times this happened was:'/space=0/format=F8.0.
end if.
do if (notecode(i,1) = 7).
print/title = 'NOTE: The bootstrapping was not completed due to problematic bootstrap samples.'.
print/title = '      Bootstrap confidence intervals are therefore suppressed.'/space=0.
end if.
do if (notecode(i,1) = 8).
print/title = 'NOTE: The number of bootstrap samples was adjusted upward given your desired confidence.'.
end if.
do if (notecode(i,1) = 9).
print/title = 'NOTE: WMODVAL is ignored when W is specified as multicategorical.'.
end if.
do if (notecode(i,1) = 10).
print/title = 'NOTE: ZMODVAL is ignored when Z is specified as multicategorical.'.
end if.
do if (notecode(i,1) = 11).
print/title = 'NOTE: Total effect model generated only when all covariates are specified in all'.
print/title = '      models of M and Y.'/space=0.
end if.
do if (notecode(i,1) = 30).
print/title = 'NOTE: Your vector of linear hypothesis weights is of the wrong length for this model.'.
end if.
do if (notecode(i,1) = 12).
print/title = 'NOTE: Total effect model generated only when X is freely estimated to affect each M'.
print/title = '      and both X and M are freely estimated to affect Y'/space=0.
end if.
do if (notecode(i,1) = 13).
print/title = 'NOTE: There are too many pairwise contrasts to conduct with this model.'.
end if.
do if (notecode(i,1) = 14).
print/title = 'NOTE: The number of contrast weights must equal the number of indirect effects.'.
end if.
do if (notecode(i,1) = 15).
print/title = 'NOTE: Monte Carlo confidence intervals not available for this model.'.
print/title = '      Bootstrapping is used instead.'/space=0.
end if.
do if (notecode(i,1) = 16).
print/title = 'NOTE: The number of Monte Carlo samples was adjusted upward given your desired confidence.'.
end if.
do if (notecode(i,1) = 19).
print/title = 'NOTE: Your contrast matrix is invalid or not applicable to this model.'.
end if.
do if (notecode(i,1) = 20).
print/title = 'NOTE: One of the groups specified by your contrast matrix does not exist in the data.'.
end if.
do if (notecode(i,1) = 21).
print/title = 'NOTE: The VARORDER option is not available in this release.'.
end if.
do if (notecode(i,1) = 22).
print/title = 'NOTE: The VMODVAL and QMODVAL options are not available in this release.'.
end if.
do if (notecode(i,1) = 23).
print/title = 'NOTE: The QUANTILE option is not available in this release.'.
end if.
do if (notecode(i,1) = 24).
print/title = 'NOTE: Total effect model not available with dichotomous Y.'.
end if.
do if (notecode(i,1) = 25).
print/title = 'NOTE: STAND/EFFSIZE options not available with dichotomous Y.'.
end if.
do if ((notecode(i,1) = 26) and nms > 0).
print/title = 'NOTE: Direct and indirect effects of X on Y are on a log-odds metric.'.
end if.
do if (notecode(i,1) = 27).
print/title = 'NOTE: Standardized coefficients are not available for models with moderators.'.
end if.
do if (notecode(i,1) = 28).
print/title = 'NOTE: The contrast option is not available with a multicategorical X.'.
end if.
do if (notecode(i,1) = 31).
do if (nms > 1).
print medmeans/title = 'NOTE: Controlled direct effect(s) estimated at the following mediator values:'/cnames=mnames/format= F10.4.
end if.
do if (nms=1).
print medmeans/title = 'NOTE: Controlled direct effect(s) estimated at the following mediator value:'/format= F10.4.
end if.
end if.
do if (notecode(i,1) = 33).
print/title = 'NOTE: Sobel test is not available with the XMINT option.'.
end if.
do if (notecode(i,1) = 37).
print/title = 'NOTE: The CONTRAST option is not available when using the XMINT option.'.
end if.
do if (notecode(i,1) = 34).
print/title = 'NOTE: Standardized effects are not available when using the XMINT option.'.
end if.
do if (notecode(i,1) = 36).
print/title = 'NOTE: The XREFVAL option is ignored when X is declared as multicategorical.'.
end if.
do if (notecode(i,1) = 35).
print coval/title = 'NOTE: Counterfactual effects estimated at the following covariate values:'/cnames=covnames/format= F10.4.
end if.
do if (notecode(i,1) = 29 and (listmiss=1)).
compute a=missrow.
compute conum=ncol(a).
compute allgood=0.
compute smremain=12.
compute largesti=1.
compute smallrow=0.
do if (conum > 12).
loop ii = 1 to 12.
compute check=(conum/ii).
do if (check = trunc(check)).
compute check2=conum/ii.
compute aok=ii.
do if (aok > 2).
compute allgood=1.
end if.
else.
compute remain=conum-(ii*trunc(check)).
do if (remain <= smremain).
compute smremain=remain.
compute largesti=ii.
compute smallrow=trunc(conum/largesti).
end if.
end if.
end loop.
compute atemp=a(1,1:(aok*check2)).
compute atemp=reshape(atemp,check2,aok).
do if (ncol(atemp) > 2).
print/title='NOTE: Missing data resulted in the deletion of the following row(s) of'.
print atemp/title='      data from the analysis:'/format=F6.0/space=0.
end if.
do if (allgood=0).
compute atemp=a(1,1:(smallrow*largesti)).
compute atemp=reshape(atemp,smallrow,largesti).
compute btemp=a(1,((largesti*smallrow)+1):conum).
print/title='NOTE: Missing data resulted in the deletion of the following row(s) of'.
print atemp/title='      data from the analysis:'/format=F6.0/space=0.
print btemp/title=' '/space=0/format=f6.0.
end if.
else if (conum <= 12).
print/title='NOTE: Missing data resulted in the deletion of the following row(s) of'.
print a/title='      data from the analysis:'/format=F6.0/space=0.
end if.
end if.
end loop.
do if (toomany=1).
print/title='WARNING: Variables names longer than eight characters can produce incorrect output'.
print/title='when some variables in the data file have the same first eight characters. Shorter'/space=0.
print/title='variable names are recommended. By using this output, you are accepting all risk'/space=0.
print/title='and consequences of interpreting or reporting results that may be incorrect.'/space=0.
end if.
end if.
end if.
loop i = 1 to 100.
do if (errcode(i,1)=1).
print/title = 'ERROR: You must specify a Y and an X variable.'.
end if.
do if (errcode(i,1)=2).
print/title = 'ERROR: X, M, or Y variable used more than once or W and Z are the same variable.'.
do if (toomany = 1).
print/title = '       This could be caused by the use of variables names longer'/space=0.
print varnames/title = '       than eight characters. Here are the variables I see:'/space=0/format=A8.
end if.
end if.
do if (errcode(i,1)=3).
print/title = 'ERROR: You have specified more than one variable for W, Y, X, or Z'.
end if.
do if (errcode(i,1)=4).
print/title = 'ERROR: A variable specified as multicategorical has more than nine categories.'.
end if.
do if (errcode(i,1)=5).
print/title = 'ERROR: One of the categories contains only a single case.'.
end if.
do if (errcode(i,1)=6).
print/title = 'ERROR: Invalid model number in this version of PROCESS.'.
end if.
do if (errcode(i,1)=7).
print/title = 'ERROR: Invalid model number.'.
end if.
do if (errcode(i,1)=8).
print/title = 'ERROR: You must specify an M variable for this model.'.
end if.
do if (errcode(i,1)=9).
print/title = 'ERROR: You have specified an M variable in a model that does not use it.'.
end if.
do if (errcode(i,1)=10).
print/title = 'ERROR: You have specified a W variable in a model that does not use it.'.
end if.
do if (errcode(i,1)=11).
print/title = 'ERROR: You have not specified a W variable in a model that requires it.'.
end if.
do if (errcode(i,1)=12).
print/title = 'ERROR: You have specified a Z variable in a model that does not use it.'.
end if.
do if (errcode(i,1)=13).
print/title = 'ERROR: You have not specified a Z variable in a model that requires it.'.
end if.
do if (errcode(i,1)=14).
print/title = 'ERROR: V and Q are not proper specifications in this release of PROCESS.'.
print/title = '       Moderators must be specified as W and/or Z.'/space=0.
end if.
do if (errcode(i,1)=15).
print/title = 'ERROR: One of your model variables exhibits no variation (it is a constant).'.
end if.
do if (errcode(i,1)=16).
print/title = 'ERROR: BMATRIX is not the correct length or is otherwise invalid.'.
end if.
do if (errcode(i,1)=17).
print/title = 'ERROR: WMATRIX is not the correct length or is otherwise invalid.'.
end if.
do if (errcode(i,1)=18).
print/title = 'ERROR: ZMATRIX is not the correct length or is otherwise invalid.'.
end if.
do if (errcode(i,1)=19).
print/title = 'ERROR: WZMATRIX is not the correct length or is otherwise invalid.'.
end if.
do if (errcode(i,1)=20).
print/title = 'ERROR: A path fixed at zero cannot be moderated.'.
end if.
do if (errcode(i,1)=21).
print/title = 'ERROR: If only one moderator is specified, it must be specified as W.'.
end if.
do if (errcode(i,1)=22).
print/title = 'ERROR: In BMATRIX, X must be specified to affect at least one variable.'.
end if.
do if (errcode(i,1)=23).
print/title = 'ERROR: In BMATRIX, at least one variable must be specified to affect Y.'.
end if.
do if (errcode(i,1)=24).
print/title = 'ERROR: You must specify a model number or a custom BMATRIX specification.'.
end if.
do if (errcode(i,1)=25).
print/title = 'ERROR: BMATRIX cannot be used in conjunction with a model number.'.
end if.
do if (errcode(i,1)=26).
print/title = 'ERROR: Your model has a dangling mediator (all Ms must affect and be affected).'.
end if.
do if (errcode(i,1)=27).
print/title = 'ERROR: CLUSTER is not available on this release of PROCESS.'.
end if.
do if (errcode(i,1)=29).
print/title = 'ERROR: CMATRIX is not the correct length or is otherwise invalid.'.
end if.
do if (errcode(i,1)=30).
print/title = 'ERROR: In CMATRIX, all covariates must be assigned to an M or a Y.'.
end if.
do if (errcode(i,1)=31).
print/title = 'ERROR: A linear or near linear dependency (singularity) exists in the data.'.
end if.
do if (errcode(i,1)=32).
print/title = 'ERROR: Models 80 and 81 require between 3 and 6 mediators.'.
end if.
do if (errcode(i,1)=33).
print/title = 'ERROR: Model 82 requires 4 mediators.'.
end if.
do if (errcode(i,1)=34).
print/title = 'ERROR: This model number requires between 2 and 6 mediators.'.
end if.
do if (errcode(i,1)=35).
print/title = 'ERROR: In a model with only one moderator, that moderator must be W.'.
end if.
do if (errcode(i,1)=36).
print/title = 'ERROR: A serial mediation model cannot have more than 6 mediators.'.
end if.
do if (errcode(i,1)=37).
print/title = 'ERROR: No more than 10 mediators are allowed in a PROCESS command.'.
end if.
do if (errcode(i,1)=38).
print/title = 'ERROR: XCATCODE is not provided, not the correct length, or is otherwise invalid.'.
end if.
do if (errcode(i,1)=39).
print/title = 'ERROR: WCATCODE is not provided, not the correct length, or is otherwise invalid.'.
end if.
do if (errcode(i,1)=40).
print/title = 'ERROR: ZCATCODE is not provided, not the correct length, or is otherwise invalid.'.
end if.
do if (errcode(i,1)=41).
print/title = 'ERROR: Models 1, 2, and 3 cannot be customized.'.
end if.
do if (errcode(i,1)=42).
print/title = 'ERROR: WS option available only in PROCESS v2. Or use the MEMORE macro instead.'.
print/title = '       MEMORE can be downloaded from www.akmontoya.com.'/space=0.
end if.
do if (errcode(i,1)=43).
print/title = 'ERROR: PROCESS does not allow dichotomous mediators.'.
end if.
do if (errcode(i,1)=60).
print/title = 'PROCESS is now ready for use.'.
print/title='Copyright 2017-2022 by Andrew F. Hayes. ALL RIGHTS RESERVED.'/space=0.
print/title='Workshop schedule available at http://haskayne.ucalgary.ca/CCRAM'/space=0.
end if.
do if (errcode(i,1)=50).
print/title = 'ERROR: A multicategorical moderator cannot be specified as a covariate.'.
end if.
do if (errcode(i,1)=51).
print/title = 'ERROR: A variable you specified as a covariate is a moderator in all equations.'.
end if.
do if (errcode(i,1)=52 and mcerpt=0).
compute mcerpt=1.
print/title = 'ERROR: A variable specified as multicategorical must have at least three categories'.
end if.
do if (errcode(i,1) = 47).
do if (iterrmod=1).
print/title = 'ERROR: Iteration for Y model didn''t converge to a solution. Interpret results with caution.'.
print/title = '       Try increasing the number of iterations, though this will slow down computation.'/space=0.
end if.
do if (bootiter=1).
print/title = 'ERROR: Nonconvergence during bootstrapping. Interpet bootstrap results with caution.'.
end if.
end if.
do if (errcode(i,1) = 61).
print/title = 'ERROR: Variable names should be no more than eight characters in length.'.
end if.
do if (errcode(i,1) = 62).
print/title = 'ERROR: After listwise deletion of cases with missing data, too few cases remain.'.
end if.
do if (errcode(i,1) = 63).
print/title = 'ERROR: The XMINT option is available only for model 4.'.
end if.
do if (errcode(i,1) = 64).
print/title = 'ERROR: Incorrect number of values specified in CDEVAL option.'.
end if.
do if (errcode(i,1) = 65).
print/title = 'ERROR: Only indicator or sequential coding of X is allowed with the XMINT option.'.
end if.
do if (errcode(i,1) = 66).
print/title = 'ERROR: XMINT option not available with continuous X using PROCESS GUI. Use syntax.'.
end if.
do if (errcode(i,1) = 67).
print/title = 'ERROR: Too many elements provided in XREFVAL option.'.
end if.
do if (errcode(i,1)=68).
print/title = 'ERROR: Covariate assignment is not allowed with XMINT option.'.
end if.
do if (errcode(i,1) = 69).
print/title = 'ERROR: Incorrect number of values specified in COVAL option.'.
end if.
do if (errcode(i,1) = 70).
print/title = 'ERROR: Incorrect value(s) in XREFVAL for this dichotomous X variable.'.
end if.
do if (errcode(i,1) = 71).
print/title = 'ERROR: The CENTER option is not available when using the XMINT option.'.
end if.
do if (errcode(i,1) = 72).
print/title = 'ERROR: The XMINT option is not available for models with a dichotomous Y.'.
end if.
end loop.
END MATRIX.	
Ressources	Temps de processeur	00:00:06,52	
	Temps écoulé	00:00:06,52	


Run MATRIX procedure:

***************** PROCESS Procedure for SPSS Version 4.2 *****************

          Written by Andrew F. Hayes, Ph.D.       www.afhayes.com
    Documentation available in Hayes (2022). www.guilford.com/p/hayes3

**************************************************************************
Model  : 4
    Y  : HOMAIR
    X  : NCDProt
   M1  : TG
   M2  : HDL
   M3  : LDL
   M4  : TC

Sample
Size:  169

**************************************************************************
OUTCOME VARIABLE:
 TG

Model Summary
          R       R-sq        MSE          F        df1        df2          p
      ,0213      ,0005  4049,8185      ,0757     1,0000   167,0000      ,7836

Model
              coeff         se          t          p       LLCI       ULCI
constant    91,1223    12,0073     7,5889      ,0000    67,4167   114,8279
NCDProt     -1,0867     3,9508     -,2751      ,7836    -8,8867     6,7133

Standardized coefficients
             coeff
NCDProt     -,0213

**************************************************************************
OUTCOME VARIABLE:
 HDL

Model Summary
          R       R-sq        MSE          F        df1        df2          p
      ,1681      ,0283   173,1010     4,8574     1,0000   167,0000      ,0289

Model
              coeff         se          t          p       LLCI       ULCI
constant    36,1640     2,4824    14,5680      ,0000    31,2630    41,0649
NCDProt      1,8002      ,8168     2,2039      ,0289      ,1876     3,4128

Standardized coefficients
             coeff
NCDProt      ,1681

**************************************************************************
OUTCOME VARIABLE:
 LDL

Model Summary
          R       R-sq        MSE          F        df1        df2          p
      ,0031      ,0000  1190,0391      ,0016     1,0000   167,0000      ,9686

Model
              coeff         se          t          p       LLCI       ULCI
constant    76,0792     6,5089    11,6885      ,0000    63,2289    88,9295
NCDProt       ,0845     2,1417      ,0394      ,9686    -4,1437     4,3127

Standardized coefficients
             coeff
NCDProt      ,0031

**************************************************************************
OUTCOME VARIABLE:
 TC

Model Summary
          R       R-sq        MSE          F        df1        df2          p
      ,0744      ,0055  1443,8973      ,9292     1,0000   167,0000      ,3365

Model
              coeff         se          t          p       LLCI       ULCI
constant   129,4526     7,1696    18,0558      ,0000   115,2979   143,6073
NCDProt      2,2740     2,3590      ,9640      ,3365    -2,3834     6,9314

Standardized coefficients
             coeff
NCDProt      ,0744

**************************************************************************
OUTCOME VARIABLE:
 HOMAIR

Model Summary
          R       R-sq        MSE          F        df1        df2          p
      ,3804      ,1447    22,9327     5,5144     5,0000   163,0000      ,0001

Model
              coeff         se          t          p       LLCI       ULCI
constant     -,8653     1,7623     -,4910      ,6241    -4,3451     2,6145
NCDProt       ,4503      ,3029     1,4864      ,1391     -,1479     1,0485
TG            ,0448      ,0097     4,6147      ,0000      ,0256      ,0640
HDL           ,1221      ,0430     2,8432      ,0050      ,0373      ,2069
LDL           ,0974      ,0356     2,7330      ,0070      ,0270      ,1678
TC           -,0928      ,0354    -2,6181      ,0097     -,1627     -,0228

Standardized coefficients
             coeff
NCDProt      ,1097
TG           ,5573
HDL          ,3186
LDL          ,6569
TC          -,6910

************************** TOTAL EFFECT MODEL ****************************
OUTCOME VARIABLE:
 HOMAIR

Model Summary
          R       R-sq        MSE          F        df1        df2          p
      ,1020      ,0104    25,8973     1,7566     1,0000   167,0000      ,1869

Model
              coeff         se          t          p       LLCI       ULCI
constant     3,0337      ,9602     3,1595      ,0019     1,1381     4,9294
NCDProt       ,4187      ,3159     1,3254      ,1869     -,2050     1,0425

Standardized coefficients
             coeff
NCDProt      ,1020


************** TOTAL, DIRECT, AND INDIRECT EFFECTS OF X ON Y **************

Total effect of X on Y
     Effect         se          t          p       LLCI       ULCI       c_cs
      ,4187      ,3159     1,3254      ,1869     -,2050     1,0425      ,1020

Direct effect of X on Y
     Effect         se          t          p       LLCI       ULCI      c'_cs
      ,4503      ,3029     1,4864      ,1391     -,1479     1,0485      ,1097

Indirect effect(s) of X on Y:
          Effect     BootSE   BootLLCI   BootULCI
TOTAL     -,0316      ,1203     -,2825      ,2024
TG        -,0487      ,2010     -,4765      ,3236
HDL        ,2199      ,2992     -,0143     1,0863
LDL        ,0082      ,3991     -,7880      ,7891
TC        -,2110      ,4723    -1,4405      ,3922

Completely standardized indirect effect(s) of X on Y:
          Effect     BootSE   BootLLCI   BootULCI
TOTAL     -,0077      ,0296     -,0721      ,0468
TG        -,0119      ,0489     -,1152      ,0723
HDL        ,0536      ,0697     -,0036      ,2580
LDL        ,0020      ,0945     -,1849      ,1864
TC        -,0514      ,1114     -,3398      ,0936

*********************** ANALYSIS NOTES AND ERRORS ************************

Level of confidence for all confidence intervals in output:
  95,0000

Number of bootstrap samples for percentile bootstrap confidence intervals:
  5000

------ END MATRIX -----


* Encoding: UTF-8.
preserve.
set printback=off.


Matrice


Remarques	
Sortie obtenue	10-NOV-2024 17:08:18	
Commentaires		
Entrée	Données	C:\Users\Brice SAHA\Documents\UBa\Master Thesis 2021\Fanuel\Database fanuel.sav	
	Jeu de données actif	Jeu_de_données1	
	Filtre	<sans>	
	Pondération	<sans>	
	Scinder un fichier	<sans>	
	N de lignes dans le fichier de travail	169	
Syntaxe	MATRIX.
compute wnames='xxxxx'.
compute znames='xxxxx'.
compute mcerpt=0.
compute wiscov=0.
compute ziscov=0.
compute tooman=0.
compute errcode=make(100,1,0).
compute notecode=make(100,1,0).
compute model = trunc( 4 ).
compute iterate = abs(trunc( 100 )).
compute converge = abs( 0.00001 ).
compute itprobtg=0.
compute v2tag=0.
compute ydich=0.
compute maxwwarn=0.
compute minwwarn=0.
compute maxzwarn=0.
compute minzwarn=0.
compute toomany=0.
compute wdich=0.
compute zdich=0.
compute wnotev=0.
compute znotev=0.
compute nxpval=1.
compute nwpval=1.
compute nzpval=1.
compute errs=1.
compute notes=1.
compute criterr=0.
compute novar=0.
compute adjust=0.
compute ncs=0.
compute serial=0.
compute sobelok=0.
compute hasw=0.
compute hasz=0.
compute printw=0.
compute printz=0.
compute xmint=( 0 =1).
compute wmodcust=0.
compute zmodcust=0.
compute booting=0.
compute bootiter=0.
compute iterrmod=0.
compute cov = 'xxxxx'.
compute varorder=( 0 <> 0).
compute nws=0.
compute w= 'xxxxx'.
compute nzs=0.
compute z = 'xxxxx'.
compute nms=0.
compute m = 'TG HDL LDL TC'.
compute nys=0.
compute y = 'HOMAIR'.
compute nxs=0.
compute x = 'NCDrisk'.
compute effsize=( 0 =1).
compute stand=( 1 =1).
compute intprobe = .1.
compute xrefvals={ 999 }.
compute center=trunc( 0 ).
compute xcontcf=0.
compute xscaling=1.
compute cdeval={ -999 }.
compute cuscoval=0.
do if (model=74).
compute errcode(errs,1)=7.
compute errs=errs+1.
compute criterr=1.
end if.
do if (xmint=1 and model <> 4).
compute errcode(errs,1)=63.
compute errs=errs+1.
compute criterr=1.
end if.
do if (xmint=1 and model=4).
compute w=x.
compute model=74.
compute intprobe=1.
compute notecode(notes,1) = 32.
compute notes = notes + 1.
do if (effsize=1 or stand=1).
compute notecode(notes,1) = 34.
compute notes = notes + 1.
compute stand=0.
compute effsize=0.
end if.
do if (center <> 0).
compute center=0.
compute errcode(errs,1)=71.
compute errs=errs+1.
compute criterr=1.
end if.
end if.
compute v = 'xxxxx'.
compute q = 'xxxxx'.
compute linsum={ -999 }.
compute nlinsum=ncol(linsum).
do if (linsum(1,1) = -999).
compute nlinsum=0.
end if.
compute oldvars= 'xxxxx'.
compute mcxok=0.
compute mcwok=0.
compute mczok=0.
compute xprod=0.
compute zprod=0.
compute wprod=0.
compute modcok=0.
compute alttotal=0.
compute hc3=trunc( 0 ).
compute jn=( 0 = 1).
compute listmiss=( 0 =1).
compute modelres=( 0 =1).
compute outscree=( 1 =1).
compute activate=( 0 =1).
compute booterr=0.
compute normal=( 0 =1).
compute xmtest=( 0 =1).
compute describe=( 0 =1).
compute longname=( 0 =1).
do if (stand=1).
compute effsize=1.
end if.
compute pstog=0.
compute sobelok=0.
compute normal=( 0 =1).
compute mdichok=( 0 =1).
compute contrast={ 999 }.
compute ncontr=ncol(contrast).
compute ncontrow=nrow(contrast).
do if (contrast(1,1) = 999).
compute ncontr=1.
compute contrast=0.
end if.
do if (ncontr = 1).
compute contrast=trunc(contrast).
do if (contrast > 3 or contrast < 0)).
compute ncontr=1.
compute contrast = 0.
end if.
end if.
do if (ncontr > 1).
compute contvec=contrast.
compute contrast=4.
do if (ncontrow > 1).
compute contrast=0.
compute modcok=1.
compute wcontval=contvec(:,1).
compute zcontval=contvec(:,2).
do if ((ncontr <> 2) or (ncontrow <> 2)).
compute notecode(notes,1) = 19.
compute notes = notes + 1.
compute modcok=0.
end if.
end if.
end if.
do if (xmint = 1 and contrast(1,1) <> 0).
compute contrast=0.
compute notecode(notes,1) = 37.
compute notes = notes + 1.
end if.
do if (varorder = 1).
compute notecode(notes,1) = 21.
compute notes = notes + 1.
end if.
do if ( 999 <> 999 or 999 <> 999).
compute notecode(notes,1) = 22.
compute notes = notes + 1.
end if.
compute modelbt=( 0 =1).
compute cluster= 'xxxxx'.
compute matrices=( 0 =1).
compute covcoeff=( 0 =1).
compute covmy=trunc( 0 ).
do if (covmy < 0 or covmy > 2).
compute covmy = 0.
end if.
compute boot = abs(trunc( 5000 )).
compute bc=( 0 =1).
compute mc=abs(trunc( 0 )).
compute hc=trunc( 5 ).
do if (intprobe < 0 or intprobe > 1).
compute intprobe = .10.
end if.
compute plot=trunc( 0 ).
do if (plot < 0 or plot > 2).
compute plot=0.
end if.
compute total=( 1 =1).
compute dototal=0.
compute saveboot = ( 0 = 1).
compute saveest=( 0 = 2).
do if (saveest=1).
compute intprobe=1.
end if.
do if (hc >= 0 and hc < 5).
compute notecode(notes,1) = 4.
compute notes = notes + 1.
end if.
do if (hc > 5 or hc < 0).
compute hc=5.
end if.
compute mcw=trunc( 0 ).
compute mcz=trunc( 0 ).
compute mcx=trunc( 0 ).
do if (mcx > 0 and mcx < 3 and model = 74).
compute mcw=mcx.
compute xscaling=1.
end if.
do if (mcx > 2 and model = 74).
compute errcode(errs,1)=65.
compute errs=errs+1.
compute criterr=1.
end if.
do if (model = 74 and normal=1).
compute notecode(notes,1) = 33.
compute normal=0.
compute notes = notes + 1.
end if.
do if (mcx > 0 and contrast > 0).
compute notecode(notes,1) = 28.
compute notes = notes + 1.
compute contrast=0.
end if.
compute nxvls=1.
compute nmvls=1.
compute nwvls=1.
compute nzvls=1.
compute paths=999.
compute pathsw=999.
compute pathsz=999.
compute pathswz=999.
compute pathsmod=999.
compute pathtype=999.
compute obscoeff=999.
compute pathsdv={' '}.
compute quantile=1.
do if ( 999 <>999).
compute notecode(notes,1) = 23.
compute notes = notes + 1.
end if.
compute moments=( 0 =1).
do if (moments=1).
compute quantile=0.
end if.
compute bmatrix={ -999 }.
compute wmatrix={ -999 }.
compute zmatrix={ -999 }.
compute wzmatrix={ -999 }.
compute cmatrix={ -999 }.
compute xcatcode={ -999 }.
compute wcatcode={ -999 }.
compute zcatcode={ -999 }.
compute needed=0.
compute conf= 95.
do if (trunc( 95 ) >= 100 or (trunc( 95 ) <= 50)).
compute conf = 95.
compute notecode(notes,1)=2.
compute notes=notes+1.
end if.
do if (model >= 0 and model < 4 and modelbt=0).
compute boot=0.
compute mc=0.
compute bc=0.
end if.
do if (mc > 0 and boot > 0).
compute boot=0.
compute bc=0.
end if.
do if ((boot < 1000) and (mc = 0) and (boot > 0)).
compute boot=5000.
end if.
do if ((mc < 1000) and (boot = 0) and (mc > 0)).
compute mc=5000.
end if.
compute p0=-.322232431088.
compute p1 = -1.
compute p2 = -.342242088547.
compute p3 = -.0204231210245.
compute p4 = -.0000453642210148.
compute q0 = .0993484626060.
compute q1 = .588581570495.
compute q2 = .531103462366.
compute q3 = .103537752850.
compute q4 = .0038560700634.
compute badend=0.
compute priorlo = -9999999.
compute priorhi = 9999999.
compute alpha2 = (1-(conf/100))/2.
compute cilm=alpha2*2.
compute y5=sqrt(-2*ln(alpha2)).
compute xp2=(y5+((((y5*p4+p3)*y5+p2)*y5+p1)*y5+p0)/((((y5*q4+q3)*y5+q2)*y5+q1)*y5+q0)).
compute medlb={'   M1  :';'   M2  :';'   M3  :';'   M4  :';'   M5  :';'   M6  :';'   M7  :';'   M8  :';'   M9  :';'   M10 :'}.
compute medlb2={'(M1)','(M2)','(M3)','(M4)','(M5)','(M6)','(M7)','(M8)','(M9)','(M10)'}.
compute xlb={'   X1  :';'   X2  :';'   X3  :';'   X4  :';'   X5  :';'   X6  :';'   X7  :';'   X8  :';'   X9  :'}.
compute highlbw={'M1*W'; 'M2*W'; 'M3*W'; 'M4*W'; 'M5*W'; 'M6*W'; 'M7*W'; 'M8*W'; 'M9*W'; 'M10*W'}.
do if (xmint=1).
compute highlbw={'M1*X'; 'M2*X'; 'M3*X'; 'M4*X'; 'M5*X'; 'M6*X'; 'M7*X'; 'M8*X'; 'M9*X'; 'M10*X'}.
end if.
compute highlbz={'M1*Z'; 'M2*Z'; 'M3*Z'; 'M4*Z'; 'M5*Z'; 'M6*Z'; 'M7*Z'; 'M8*Z'; 'M9*Z';'M10*Z'}.
compute highlbwz={'M1*W*Z'; 'M2*W*Z'; 'M3*W*Z'; 'M4*W*Z'; 'M5*W*Z'; 'M6*W*Z'; 'M7*W*Z'; 'M8*W*Z'; 'M9*W*Z';'M10*W*Z'}.
compute highlbbt={'BOTH(M1)'; 'BOTH(M2)'; 'BOTH(M3)'; 'BOTH(M4)'; 'BOTH(M5)'; 'BOTH(M6)'; 'BOTH(M7)'; 'BOTH(M8)'; 'BOTH(M9)';'BTH(M10)'}.
compute highlbx={'M1*X'; 'M2*X'; 'M3*X'; 'M4*X'; 'M5*X'; 'M6*X'; 'M7*X'; 'M8*X'; 'M9*X'; 'M10*X'}.
compute skipwz=0.
compute validm={1,1,1,1,1,1,1,1,1,1,1,1,1,1,1,1,1,1,1,1,1,1,0,0,0,0,0,1,1,0,0,0,0, 0,0,0,0,0,0,0,0,0,0,0,0,0,0,0,0,0,0,0,0,0,0,0,0,1,1,1,1,1,1,1,1,1,1,1,1,1,1,1,1,1,1,1,0,0,0,1,1, 1,1,1,1,1,1,1,1,1,1,1}.
do if (activate=1).
compute errcode(errs,1)=60.
compute errs=errs+1.
compute criterr=1.
end if.
do if (criterr=0).
do if (( 0 =1)=1).
compute errcode(errs,1)=42.
compute errs=errs+1.
compute criterr=1.
end if.
do if (model > 0 and model < 93).
do if (validm(1,model)=0).
compute errcode(errs,1)=6.
compute errs=errs+1.
compute criterr=1.
end if.
release validm.
end if.
do if ((model > 92 or model < 0) and model <> 999)).
compute errcode(errs,1)=7.
compute errs=errs+1.
compute criterr=1.
end if.
do if (model = 999 and bmatrix(1,1)=-999).
compute errcode(errs,1)=24.
compute errs=errs+1.
compute criterr=1.
end if.
do if (model <> 999 and bmatrix(1,1) <> -999).
compute errcode(errs,1)=25.
compute errs=errs+1.
compute criterr=1.
end if.
do if ((model = 74 or (model > 0 and model < 4)) and ((wmatrix(1,1) <> -999) or (zmatrix(1,1)<>-999) or (wzmatrix(1,1)<>-999)))).
compute errcode(errs,1)=41.
compute errs=errs+1.
compute criterr=1.
end if.
do if (hc3 <> 0).
compute notecode(notes,1) = 5.
compute notes = notes + 1.
do if (hc3 = 1).
compute hc=3.
end if.
end if.
do if ((v <> 'xxxxx') or (q <> 'xxxxx')).
compute errcode(errs,1)=14.
compute errs=errs+1.
compute errcode(errs,1)=48.
compute errs=errs+1.
compute criterr=1.
end if.
do if (oldvars <> 'xxxxx').
compute errcode(errs,1)=48.
compute errs=errs+1.
compute criterr=1.
end if.
do if (cluster <> 'xxxxx').
compute errcode(errs,1)=27.
compute errs=errs+1.
compute criterr=1.
end if.
do if ((y = 'xxxxx') or (x = 'xxxxx')).
compute errcode(errs,1)=1.
compute errs=errs+1.
compute criterr=1.
end if.
do if ((m = 'xxxxx') and model > 3).
compute errcode(errs,1)=8.
compute errs=errs+1.
compute criterr=1.
end if.
end if.
do if (criterr=0).
get ytmp/variables = HOMAIR /names = ynames/MISSING = 99999.
compute nys=ncol(ytmp).
compute needed=nys.
compute n=nrow(ytmp).
compute varnames={ynames}.
compute dat=ytmp.

.


do if (toomany=1 and longname=0).
compute criterr=1.
do if (tooman=0).
compute tooman=1.
compute errcode(errs,1) = 61.
compute errs = errs + 1.
end if.
end if
.
compute modelvar={ '4' ;t(ynames)}.
do if ( 4 =999).
compute modelvar(1,1)='CUSTOM'.
end if.
get xtmp/variables = NCDrisk /names = xnames/MISSING = 99999.
compute nxs=ncol(xtmp).
compute n=nrow(xtmp).
compute needed=needed+nxs.
compute varnames={varnames,xnames}.
compute xcatlab=t(xnames).
compute dat={dat,xtmp}.

.


do if (toomany=1 and longname=0).
compute criterr=1.
do if (tooman=0).
compute tooman=1.
compute errcode(errs,1) = 61.
compute errs = errs + 1.
end if.
end if
.
compute modelvar={modelvar;t(xnames)}.
do if (nxs = 1).
compute modelvlb={'Model  :';'    Y  :';'    X  :'}.
else.
compute modelvlb={'Model  :';'    Y  :';xlb(1:nxs,1)}.
end if.
do if (m <> 'xxxxx').
get mtmp/variables = TG HDL LDL TC /names = mnames/MISSING = 99999.
compute nms=ncol(mtmp).
compute mprod=make(1,nms,0).
compute n=nrow(mtmp).
compute needed=needed+nms.
compute varnames={varnames,mnames}.
compute dat={dat,mtmp}.
compute modelvar={modelvar;t(mnames)}.
compute x2m=make(99,nms,0).
compute m2y=make(99,nms,0).
compute onem=make(nms,1,1).

.


do if (toomany=1 and longname=0).
compute criterr=1.
do if (tooman=0).
compute tooman=1.
compute errcode(errs,1) = 61.
compute errs = errs + 1.
end if.
end if
.
do if (nms > 1 and nms < 11).
compute modelvlb={modelvlb;medlb(1:nms,1)}.
else.
compute modelvlb={modelvlb;'    M  :'}.
end if.
do if (nms > 0 and model < 4).
compute errcode(errs,1)=9.
compute errs=errs+1.
do if (model <> 0).
compute errcode(errs,1)=48.
compute errs=errs+1.
end if.
compute criterr=1.
end if.
end if.
compute wlocatet=0.
compute wlocate=0.
do if (w <> 'xxxxx').
do if (xmint=0).
get wtmp/variables = xxxxx /names = wnames/MISSING = 99999.

.


do if (toomany=1 and longname=0).
compute criterr=1.
do if (tooman=0).
compute tooman=1.
compute errcode(errs,1) = 61.
compute errs = errs + 1.
end if.
end if
.
end if.
do if (xmint=1).
get wtmp/variables = NCDrisk /names = wnames/MISSING = 99999.
end if.
compute nws=ncol(wtmp).
compute n=nrow(wtmp).
compute varnames={varnames,wnames}.
compute wlocate=ncol(varnames).
do if (model=74).
compute wlocatet=1.
do if (xnames <> wnames).
compute errcode(errs,1)=45.
compute errs=errs+1.
compute criterr=1.
end if.
end if.
compute wcatlab=t(wnames).
compute dat={dat,wtmp}.
do if (xmint <> 1).
compute modelvar={modelvar;t(wnames)}.
compute modelvlb={modelvlb;'    W  :'}.
end if.
end if.
do if (z <> 'xxxxx').
get ztmp/variables = xxxxx /names = znames/MISSING = 99999.
compute nzs=ncol(ztmp).
compute n=nrow(ztmp).

.


do if (toomany=1 and longname=0).
compute criterr=1.
do if (tooman=0).
compute tooman=1.
compute errcode(errs,1) = 61.
compute errs = errs + 1.
end if.
end if
.
compute varnames={varnames,znames}.
compute zcatlab=t(znames).
compute dat={dat,ztmp}.
compute modelvar={modelvar;t(znames)}.
compute modelvlb={modelvlb;'    Z  :'}.
end if.
do if (cov <> 'xxxxx').
get ctmp/variables = xxxxx /names = covnames/MISSING = 99999.
compute ncs=ncol(ctmp).
compute n=nrow(ctmp).

.


do if (toomany=1 and longname=0).
compute criterr=1.
do if (tooman=0).
compute tooman=1.
compute errcode(errs,1) = 61.
compute errs = errs + 1.
end if.
end if
.
compute varnames={varnames,covnames}.
compute dat={dat,ctmp}.
end if.
do if (nws > 1 or nzs > 1 or nys > 1 or nxs > 1).
compute errcode(errs,1)=3.
compute errs=errs+1.
compute criterr=1.
end if.
do if ((model = 80 or model = 81) and (nms < 3 or nms > 6)).
compute errcode(errs,1)=32.
compute errs=errs+1.
compute criterr=1.
end if.
do if (model = 82 and nms <> 4).
compute errcode(errs,1)=33.
compute errs=errs+1.
compute criterr=1.
end if.
do if (nms > 10).
compute errcode(errs,1)=37.
compute errs=errs+1.
compute criterr=1.
end if.
do if ((model = 6 or (model > 82 and model < 999)) and (nms < 2 or nms > 6)).
compute errcode(errs,1)=34.
compute errs=errs+1.
compute criterr=1.
end if.
compute match=0.
compute match2=0.
compute mcwzcov=0.
loop i = 1 to (ncol(varnames)-1).
loop j = (i+1) to ncol(varnames).
do if (varnames(i)=varnames(j)).
do if (i < (nxs+nms+nys+1)).
compute match2=match2+1.
end if.
do if (wlocatet=1 and i=2 and j=wlocate).
compute match2=match2-1.
end if.
do if ((wnames=znames) and (nws > 0 or nzs > 0))).
compute match2=match2+1.
end if.
do if (i < (ncol(varnames)-ncs+1)) and j > (ncol(varnames)-ncs)).
do if ((varnames(j)=wnames) and mcw=0)).
compute match=0.
compute wiscov=(j-(ncol(varnames)-ncs)).
end if.
do if ((varnames(j)=wnames) and mcw <>0)).
compute mcwzcov=1.
end if.
do if ((varnames(j)=znames) and mcz=0).
compute match=0.
compute ziscov=(j-(ncol(varnames)-ncs)).
end if.
do if ((varnames(j)=znames) and mcz<>0)).
compute mcwzcov=1.
end if.
end if.
end if.
end loop.
end loop.
do if (match2>0 or match=1).
compute errcode(errs,1)=2.
compute errs=errs+1.
compute criterr=1.
end if.
do if (mcwzcov=1).
compute errcode(errs,1)=50.
compute errs=errs+1.
compute criterr=1.
end if.
compute ninit=nrow(dat).
compute rownum=make(ninit,1,0).
loop i = 1 to ninit.
compute rownum(i,1)=i.
end loop.
compute dat={rownum,dat}.
compute j=1.
compute missrow=0.
loop i = 1 to n.
do if (rsum(dat(i,2:ncol(dat))=99999)=0).
compute dat(j,:)=dat(i,:).
compute j=j+1.
else.
compute missrow={missrow;dat(i,1)}.
end if.
end loop.
do if (j < 5).
compute errcode(errs,1)=62.
compute errs=errs+1.
compute criterr=1.
end if.
do if (criterr=0).
compute rownum=dat(1:(j-1),1).
do if (nrow(missrow) > 1).
compute missrow=t(missrow(2:nrow(missrow),1)).
compute notecode(notes,1) = 29.
compute notes = notes + 1.
end if.
compute dat=dat(1:(j-1),2:ncol(dat)).
compute n=nrow(dat).
compute nmiss=ninit-n.
compute ytmp=dat(:,1:nys).

.
compute desctmp=make((8-(4* 0 )),ncol( ytmp ),-999).
loop jd=1 to ncol( ytmp ).
compute descdat= ytmp (:,jd).
compute desctmp(1,jd) = csum(descdat)/nrow(descdat).
compute desctmp(2,jd) = (nrow(descdat)*sscp(descdat))-(t(csum(descdat))*(csum(descdat))).
compute desctmp(2,jd) = sqrt(desctmp(2,jd)/(nrow(descdat)*(nrow(descdat)-1))).
compute desctmp(3,jd)=cmin(descdat).
compute desctmp(4,jd)=cmax(descdat).
do if ( 0 =0).
compute minwarn=0.
compute maxwarn=0.
do if ((desctmp(3,jd)=desctmp(4,jd)) and novar=0).
compute errcode(errs,1)=15.
compute errs=errs+1.
compute criterr=1.
compute novar=1.
end if.
compute tmp=((descdat(:,1)=desctmp(3,jd))+(descdat(:,1)=desctmp(4,jd))).
compute desctmp(8,jd)=(csum(tmp)=nrow(tmp)).
compute tmp = descdat.
compute tmp(GRADE(descdat),:) = descdat.
compute descdat = tmp.
release tmp.
compute decval={.16;.5;.84}.
loop kd=1 to 3.
compute low=trunc(decval(kd,1)*(nrow(descdat)+1)).
compute lowdec=decval(kd,1)*(nrow(descdat)+1)-low.
compute value=descdat(low,1)+(descdat((low+1),1)-descdat(low,1))*lowdec.
compute desctmp((4+kd),jd)=value.
end loop.
compute mnotev=1.
compute modvals=desctmp(5:7,:).
do if (quantile <> 1).
compute desctmp(5,jd)=desctmp(1,jd)-desctmp(2,jd).
compute desctmp(6,jd)=desctmp(1,jd).
compute desctmp(7,jd)=desctmp(1,jd)+desctmp(2,jd).
compute modvals=desctmp(5:7,:).
compute mnotev=2.
do if (modvals(1,1) < desctmp(3,1)).
compute modvals(1,1)=desctmp(3,1).
compute minwarn=1.
end if.
do if (modvals(3,1) > desctmp(4,1)).
compute modvals(3,1)=desctmp(4,1).
compute maxwarn=1.
end if.
end if.
do if (desctmp(8,1)=1).
compute modvals={desctmp(3,1);desctmp(4,1)}.
compute mnotev=0.
compute minwarn=0.
compute maxwarn=0.
end if.
end if.
end loop
.
compute ysd=desctmp(2,:).
compute ovsd=ysd.
do if (desctmp(8,1)=1).
compute ydich=1.
do if (total=1).
compute total=0.
compute notecode(notes,1) = 24.
compute notes = notes + 1.
end if.
do if (effsize=1).
compute effsize=0.
compute notecode(notes,1) = 25.
compute notes = notes + 1.
end if.
do if (model=74).
compute errcode(errs,1) = 72.
compute errs=errs+1.
compute criterr=1.
end if.
compute omx = cmax(ytmp).
compute omn = cmin(ytmp).
compute ytmp = (ytmp = omx).
compute dat(:,1:nys)=(dat(:,1:nys)=omx).
compute rcd = {omn, 0; omx, 1}.
end if.
compute xtmp=dat(:,(nys+1):(nys+nxs)).

.
compute desctmp=make((8-(4* 0 )),ncol( xtmp ),-999).
loop jd=1 to ncol( xtmp ).
compute descdat= xtmp (:,jd).
compute desctmp(1,jd) = csum(descdat)/nrow(descdat).
compute desctmp(2,jd) = (nrow(descdat)*sscp(descdat))-(t(csum(descdat))*(csum(descdat))).
compute desctmp(2,jd) = sqrt(desctmp(2,jd)/(nrow(descdat)*(nrow(descdat)-1))).
compute desctmp(3,jd)=cmin(descdat).
compute desctmp(4,jd)=cmax(descdat).
do if ( 0 =0).
compute minwarn=0.
compute maxwarn=0.
do if ((desctmp(3,jd)=desctmp(4,jd)) and novar=0).
compute errcode(errs,1)=15.
compute errs=errs+1.
compute criterr=1.
compute novar=1.
end if.
compute tmp=((descdat(:,1)=desctmp(3,jd))+(descdat(:,1)=desctmp(4,jd))).
compute desctmp(8,jd)=(csum(tmp)=nrow(tmp)).
compute tmp = descdat.
compute tmp(GRADE(descdat),:) = descdat.
compute descdat = tmp.
release tmp.
compute decval={.16;.5;.84}.
loop kd=1 to 3.
compute low=trunc(decval(kd,1)*(nrow(descdat)+1)).
compute lowdec=decval(kd,1)*(nrow(descdat)+1)-low.
compute value=descdat(low,1)+(descdat((low+1),1)-descdat(low,1))*lowdec.
compute desctmp((4+kd),jd)=value.
end loop.
compute mnotev=1.
compute modvals=desctmp(5:7,:).
do if (quantile <> 1).
compute desctmp(5,jd)=desctmp(1,jd)-desctmp(2,jd).
compute desctmp(6,jd)=desctmp(1,jd).
compute desctmp(7,jd)=desctmp(1,jd)+desctmp(2,jd).
compute modvals=desctmp(5:7,:).
compute mnotev=2.
do if (modvals(1,1) < desctmp(3,1)).
compute modvals(1,1)=desctmp(3,1).
compute minwarn=1.
end if.
do if (modvals(3,1) > desctmp(4,1)).
compute modvals(3,1)=desctmp(4,1).
compute maxwarn=1.
end if.
end if.
do if (desctmp(8,1)=1).
compute modvals={desctmp(3,1);desctmp(4,1)}.
compute mnotev=0.
compute minwarn=0.
compute maxwarn=0.
end if.
end if.
end loop
.
compute xsd=desctmp(2,:).
compute xmodvals=modvals.
compute xdich=desctmp(8,1).
compute xmx=cmax(xtmp).
compute xmn=cmin(xtmp).
do if ((mcx > 0) and (xrefvals(1,1) <> 999) and (xmint=1) and (model= 74)).
compute notecode(notes,1) = 36.
compute notes = notes + 1.
end if.
do if (mcx=0).
do if (ncol(xrefvals)>2 and model=74 and xmint=1).
compute errcode(errs,1)=67.
compute errs=errs+1.
compute criterr=1.
end if.
do if ((model=74) and (xmint=1)).
do if ((xrefvals(1,1)=999) and nxvls=1 and xdich=0).
compute errcode(errs,1)=66.
compute errs=errs+1.
compute criterr=1.
end if.
do if ((xrefvals(1,1)=999) and (xdich=1)).
compute xrefvals={xmn,xmx}.
compute xscaling=xrefvals(1,2)-xrefvals(1,1).
end if.
do if (ncol(xrefvals)=1 and xrefvals(1,1) <> 999).
do if (xdich=0).
compute xrefvals={xrefvals,(xrefvals(1,1)+1)}.
compute xscaling=xrefvals(1,2)-xrefvals(1,1).
end if.
do if (xdich=1).
do if ((xrefvals(1,1) <> xmx) and (xrefvals(1,1) <> xmn)).
compute errcode(errs,1)=70.
compute errs=errs+1.
compute criterr=1.
end if.
do if (xrefvals(1,1) = xmx).
compute xrefvals={xrefvals,xmn}.
compute xscaling=xrefvals(1,2)-xrefvals(1,1).
end if.
do if (xrefvals(1,1) = xmn).
compute xrefvals={xrefvals,xmx}.
compute xscaling=xrefvals(1,2)-xrefvals(1,1).
end if.
end if.
end if.
do if (ncol(xrefvals)=2).
compute xscaling=xrefvals(1,2)-xrefvals(1,1).
do if (xdich=1).
compute xreferr=1.
do if (((xrefvals(1,1) = xmx) and (xrefvals(1,2)=xmn)) or ((xrefvals(1,1) = xmn) and (xrefvals(1,2)=xmx))).
compute xreferr=0.
end if.
do if (xreferr=1).
compute errcode(errs,1)=70.
compute errs=errs+1.
compute criterr=1.
end if.
end if.
end if.
end if.
end if.
do if (xmint=1 and model=74 and mcx=0).
compute xmodvals=t(xrefvals).
compute xcontcf=1.
end if.
compute nxpval=nrow(xmodvals).
compute xprobval=xmodvals.
do if (xdich =1 and mcx > 0).
compute mcx=0.
compute errcode(errs,1) = 52.
compute errs = errs + 1.
compute criterr = 1.
end if.
do if (nms > 0).
compute mtmp=dat(:,(nys+nxs+1):(nys+nxs+nms)).

.
compute desctmp=make((8-(4* 0 )),ncol( mtmp ),-999).
loop jd=1 to ncol( mtmp ).
compute descdat= mtmp (:,jd).
compute desctmp(1,jd) = csum(descdat)/nrow(descdat).
compute desctmp(2,jd) = (nrow(descdat)*sscp(descdat))-(t(csum(descdat))*(csum(descdat))).
compute desctmp(2,jd) = sqrt(desctmp(2,jd)/(nrow(descdat)*(nrow(descdat)-1))).
compute desctmp(3,jd)=cmin(descdat).
compute desctmp(4,jd)=cmax(descdat).
do if ( 0 =0).
compute minwarn=0.
compute maxwarn=0.
do if ((desctmp(3,jd)=desctmp(4,jd)) and novar=0).
compute errcode(errs,1)=15.
compute errs=errs+1.
compute criterr=1.
compute novar=1.
end if.
compute tmp=((descdat(:,1)=desctmp(3,jd))+(descdat(:,1)=desctmp(4,jd))).
compute desctmp(8,jd)=(csum(tmp)=nrow(tmp)).
compute tmp = descdat.
compute tmp(GRADE(descdat),:) = descdat.
compute descdat = tmp.
release tmp.
compute decval={.16;.5;.84}.
loop kd=1 to 3.
compute low=trunc(decval(kd,1)*(nrow(descdat)+1)).
compute lowdec=decval(kd,1)*(nrow(descdat)+1)-low.
compute value=descdat(low,1)+(descdat((low+1),1)-descdat(low,1))*lowdec.
compute desctmp((4+kd),jd)=value.
end loop.
compute mnotev=1.
compute modvals=desctmp(5:7,:).
do if (quantile <> 1).
compute desctmp(5,jd)=desctmp(1,jd)-desctmp(2,jd).
compute desctmp(6,jd)=desctmp(1,jd).
compute desctmp(7,jd)=desctmp(1,jd)+desctmp(2,jd).
compute modvals=desctmp(5:7,:).
compute mnotev=2.
do if (modvals(1,1) < desctmp(3,1)).
compute modvals(1,1)=desctmp(3,1).
compute minwarn=1.
end if.
do if (modvals(3,1) > desctmp(4,1)).
compute modvals(3,1)=desctmp(4,1).
compute maxwarn=1.
end if.
end if.
do if (desctmp(8,1)=1).
compute modvals={desctmp(3,1);desctmp(4,1)}.
compute mnotev=0.
compute minwarn=0.
compute maxwarn=0.
end if.
end if.
end loop
.
compute ovsd={desctmp(2,:),ysd}.
compute medmeans=cdeval.
do if ((cdeval(1,1) <> -999) and (ncol(medmeans) <> nms) and (model=74)).
compute errcode(errs,1)=64.
compute errs=errs+1.
compute criterr=1.
end if.
do if ((cdeval(1,1)=-999) and (model=74)).
compute medmeans=desctmp(1,:).
end if.
do if ((cdeval(1,1)<> -999) and (model=74) and (criterr=0)).
compute notecode(notes,1) = 31.
compute notes = notes + 1.
end if.
do if ((rsum(desctmp(8,:))>0) and (mdichok <> 1)).
compute errcode(errs,1)=43.
compute errs=errs+1.
compute criterr=1.
end if.
compute mmodvals=modvals.
compute mprobval=mmodvals.
end if.
do if (nws > 0).
compute wtmp=dat(:,(nys+nxs+nms+1):(nys+nxs+nms+nws)).

.
compute desctmp=make((8-(4* 0 )),ncol( wtmp ),-999).
loop jd=1 to ncol( wtmp ).
compute descdat= wtmp (:,jd).
compute desctmp(1,jd) = csum(descdat)/nrow(descdat).
compute desctmp(2,jd) = (nrow(descdat)*sscp(descdat))-(t(csum(descdat))*(csum(descdat))).
compute desctmp(2,jd) = sqrt(desctmp(2,jd)/(nrow(descdat)*(nrow(descdat)-1))).
compute desctmp(3,jd)=cmin(descdat).
compute desctmp(4,jd)=cmax(descdat).
do if ( 0 =0).
compute minwarn=0.
compute maxwarn=0.
do if ((desctmp(3,jd)=desctmp(4,jd)) and novar=0).
compute errcode(errs,1)=15.
compute errs=errs+1.
compute criterr=1.
compute novar=1.
end if.
compute tmp=((descdat(:,1)=desctmp(3,jd))+(descdat(:,1)=desctmp(4,jd))).
compute desctmp(8,jd)=(csum(tmp)=nrow(tmp)).
compute tmp = descdat.
compute tmp(GRADE(descdat),:) = descdat.
compute descdat = tmp.
release tmp.
compute decval={.16;.5;.84}.
loop kd=1 to 3.
compute low=trunc(decval(kd,1)*(nrow(descdat)+1)).
compute lowdec=decval(kd,1)*(nrow(descdat)+1)-low.
compute value=descdat(low,1)+(descdat((low+1),1)-descdat(low,1))*lowdec.
compute desctmp((4+kd),jd)=value.
end loop.
compute mnotev=1.
compute modvals=desctmp(5:7,:).
do if (quantile <> 1).
compute desctmp(5,jd)=desctmp(1,jd)-desctmp(2,jd).
compute desctmp(6,jd)=desctmp(1,jd).
compute desctmp(7,jd)=desctmp(1,jd)+desctmp(2,jd).
compute modvals=desctmp(5:7,:).
compute mnotev=2.
do if (modvals(1,1) < desctmp(3,1)).
compute modvals(1,1)=desctmp(3,1).
compute minwarn=1.
end if.
do if (modvals(3,1) > desctmp(4,1)).
compute modvals(3,1)=desctmp(4,1).
compute maxwarn=1.
end if.
end if.
do if (desctmp(8,1)=1).
compute modvals={desctmp(3,1);desctmp(4,1)}.
compute mnotev=0.
compute minwarn=0.
compute maxwarn=0.
end if.
end if.
end loop
.
compute wmodvals=modvals.
compute wdich=desctmp(8,1).
do if (wdich =1 and mcw > 0).
compute mcw=0.
compute errcode(errs,1) = 52.
compute errs = errs + 1.
compute criterr = 1.
end if.
compute wmin=desctmp(3,1).
compute wmax=desctmp(4,1).
compute minwwarn=minwarn.
compute maxwwarn=maxwarn.
compute wnotev=mnotev.
compute wmodval={ 999 }.
do if (xmint=1 and model=74 and mcx=0).
compute wmodval=xrefvals.
end if.
compute nwcontr=ncol(wmodval).
do if (wmodval(1,1) <> 999).
compute wmodvals=wmodval(1,1).
compute wmodcust=1.
do if (nwcontr > 1).
compute wmodvals=t(wmodval).
end if.
compute minwwarn=0.
compute maxwwarn=0.
compute wnotev=0.
end if.
compute wprobval=wmodvals.
compute nwpval=nrow(wmodvals).
end if.
do if (nzs > 0).
compute ztmp=dat(:,(nys+nxs+nms+nws+1):(nys+nxs+nms+nws+nzs)).

.
compute desctmp=make((8-(4* 0 )),ncol( ztmp ),-999).
loop jd=1 to ncol( ztmp ).
compute descdat= ztmp (:,jd).
compute desctmp(1,jd) = csum(descdat)/nrow(descdat).
compute desctmp(2,jd) = (nrow(descdat)*sscp(descdat))-(t(csum(descdat))*(csum(descdat))).
compute desctmp(2,jd) = sqrt(desctmp(2,jd)/(nrow(descdat)*(nrow(descdat)-1))).
compute desctmp(3,jd)=cmin(descdat).
compute desctmp(4,jd)=cmax(descdat).
do if ( 0 =0).
compute minwarn=0.
compute maxwarn=0.
do if ((desctmp(3,jd)=desctmp(4,jd)) and novar=0).
compute errcode(errs,1)=15.
compute errs=errs+1.
compute criterr=1.
compute novar=1.
end if.
compute tmp=((descdat(:,1)=desctmp(3,jd))+(descdat(:,1)=desctmp(4,jd))).
compute desctmp(8,jd)=(csum(tmp)=nrow(tmp)).
compute tmp = descdat.
compute tmp(GRADE(descdat),:) = descdat.
compute descdat = tmp.
release tmp.
compute decval={.16;.5;.84}.
loop kd=1 to 3.
compute low=trunc(decval(kd,1)*(nrow(descdat)+1)).
compute lowdec=decval(kd,1)*(nrow(descdat)+1)-low.
compute value=descdat(low,1)+(descdat((low+1),1)-descdat(low,1))*lowdec.
compute desctmp((4+kd),jd)=value.
end loop.
compute mnotev=1.
compute modvals=desctmp(5:7,:).
do if (quantile <> 1).
compute desctmp(5,jd)=desctmp(1,jd)-desctmp(2,jd).
compute desctmp(6,jd)=desctmp(1,jd).
compute desctmp(7,jd)=desctmp(1,jd)+desctmp(2,jd).
compute modvals=desctmp(5:7,:).
compute mnotev=2.
do if (modvals(1,1) < desctmp(3,1)).
compute modvals(1,1)=desctmp(3,1).
compute minwarn=1.
end if.
do if (modvals(3,1) > desctmp(4,1)).
compute modvals(3,1)=desctmp(4,1).
compute maxwarn=1.
end if.
end if.
do if (desctmp(8,1)=1).
compute modvals={desctmp(3,1);desctmp(4,1)}.
compute mnotev=0.
compute minwarn=0.
compute maxwarn=0.
end if.
end if.
end loop
.
compute zmodvals=modvals.
compute zdich=desctmp(8,1).
do if (zdich =1 and mcz > 0).
compute mcz=0.
compute errcode(errs,1) = 52.
compute errs = errs + 1.
compute criterr = 1.
end if.
compute zmin=desctmp(3,1).
compute zmax=desctmp(4,1).
compute minzwarn=minwarn.
compute maxzwarn=maxwarn.
compute znotev=mnotev.
compute zmodval={ 999 }.
compute nzcontr=ncol(zmodval).
do if (zmodval(1,1) <> 999).
compute zmodvals=zmodval(1,1).
compute zmodcust=1.
do if (nzcontr > 1).
compute zmodvals=t(zmodval).
end if.
compute minzwarn=0.
compute maxzwarn=0.
compute znotev=0.
end if.
compute zprobval=zmodvals.
compute nzpval=nrow(zmodvals).
end if.
do if (ncs > 0).
compute ctmp=dat(:,(nys+nxs+nms+nws+nzs+1):(nys+nxs+nms+nws+nzs+ncs)).

.
compute desctmp=make((8-(4* 0 )),ncol( ctmp ),-999).
loop jd=1 to ncol( ctmp ).
compute descdat= ctmp (:,jd).
compute desctmp(1,jd) = csum(descdat)/nrow(descdat).
compute desctmp(2,jd) = (nrow(descdat)*sscp(descdat))-(t(csum(descdat))*(csum(descdat))).
compute desctmp(2,jd) = sqrt(desctmp(2,jd)/(nrow(descdat)*(nrow(descdat)-1))).
compute desctmp(3,jd)=cmin(descdat).
compute desctmp(4,jd)=cmax(descdat).
do if ( 0 =0).
compute minwarn=0.
compute maxwarn=0.
do if ((desctmp(3,jd)=desctmp(4,jd)) and novar=0).
compute errcode(errs,1)=15.
compute errs=errs+1.
compute criterr=1.
compute novar=1.
end if.
compute tmp=((descdat(:,1)=desctmp(3,jd))+(descdat(:,1)=desctmp(4,jd))).
compute desctmp(8,jd)=(csum(tmp)=nrow(tmp)).
compute tmp = descdat.
compute tmp(GRADE(descdat),:) = descdat.
compute descdat = tmp.
release tmp.
compute decval={.16;.5;.84}.
loop kd=1 to 3.
compute low=trunc(decval(kd,1)*(nrow(descdat)+1)).
compute lowdec=decval(kd,1)*(nrow(descdat)+1)-low.
compute value=descdat(low,1)+(descdat((low+1),1)-descdat(low,1))*lowdec.
compute desctmp((4+kd),jd)=value.
end loop.
compute mnotev=1.
compute modvals=desctmp(5:7,:).
do if (quantile <> 1).
compute desctmp(5,jd)=desctmp(1,jd)-desctmp(2,jd).
compute desctmp(6,jd)=desctmp(1,jd).
compute desctmp(7,jd)=desctmp(1,jd)+desctmp(2,jd).
compute modvals=desctmp(5:7,:).
compute mnotev=2.
do if (modvals(1,1) < desctmp(3,1)).
compute modvals(1,1)=desctmp(3,1).
compute minwarn=1.
end if.
do if (modvals(3,1) > desctmp(4,1)).
compute modvals(3,1)=desctmp(4,1).
compute maxwarn=1.
end if.
end if.
do if (desctmp(8,1)=1).
compute modvals={desctmp(3,1);desctmp(4,1)}.
compute mnotev=0.
compute minwarn=0.
compute maxwarn=0.
end if.
end if.
end loop
.
compute covmeans=desctmp(1,:).
compute coval={ -999 }.
do if ((coval(1,1) <> -999) and (ncol(coval) <> ncs) and (model=74)).
compute errcode(errs,1)=69.
compute errs=errs+1.
compute criterr=1.
end if.
do if ((coval(1,1)<> -999) and (criterr=0) and (model=74)).
compute notecode(notes,1) = 35.
compute notes = notes + 1.
compute cuscoval=1.
end if.
end if.
compute n=nrow(ytmp).
compute ones=make(n,1,1).
do if (nws > 0 and mcw > 0).
compute tmp={rownum,wtmp(:,1)}.

.
compute dd= tmp.
compute temp = dd.
compute temp(GRADE(dd(:,2)),:) = dd.
compute dd = temp.
compute dummy = design(dd(:,2)).
compute nvls = ncol(dummy).
compute nnvls = csum(dummy).
compute mnvls = cmin(t(nnvls)).
compute conmat1=1.
do if (mnvls < 2).
compute errcode(errs,1) = 5.
compute errs = errs + 1.
compute criterr = 1.
end if.
do if (nvls > 9).
compute errcode(errs,1) = 4.
compute errs = errs+1.
compute criterr = 1.
end if.
do if (criterr = 0).
compute dumok = 1.
compute nnvls=make(nvls,1,0).
compute nnvls(1,1)=dd(1,2).
compute temp = 2.
loop i = 2 to n.
do if (dd(i,2) <> nnvls((temp-1),1)).
compute nnvls(temp,1)=dd(i,2).
compute temp = temp+1.
end if.
end loop.
do if ( mcw > 0).
compute x = dummy(:,2:ncol(dummy)).
compute nx = ncol(x).
compute minus1 = make(1,ncol(x),-1).
compute xdes=make((nx+1),3,0).
compute xdes(1,1)=dd(1,2).
compute xdes(1,2)=1.
compute temp = 2.
loop k = 2 to n.
do if (dd(k,2) <> dd((k-1),2)).
compute xdes(temp,2) = k.
compute xdes(temp,1) = dd(k,2).
compute xdes((temp-1),3) = k-1.
compute temp=temp+1.
end if.
end loop.
compute xdes((temp-1),3)=n.
compute xdes = {xdes, (xdes(:,3)-xdes(:,2)+1)}.
do if ( mcw = 4).
loop k = 1 to n.
do if (rsum(x(k,:)) = 0).
compute x(k,:) = minus1.
end if.
end loop.
end if.
do if ( mcw = 2 or mcw = 3 or mcw =5).
loop k = 1 to n.
do if (rsum(x(k,:)) > 0).
loop i = 1 to ncol(x).
do if (x(k,i) = 0).
compute x(k,i) = 1.
else.
break.
end if.
end loop.
end if.
end loop.
do if ( mcw = 3).
compute conmat1={-8,1,1,1,1,1,1,1,1; 0,-7,1,1,1,1,1,1,1; 0,0,-6,1,1,1,1,1,1; 0,0,0,-5,1,1,1,1,1; 0,0,0,0,-4,1,1,1,1; 0,0,0,0,0,-3,1,1,1; 0,0,0,0,0,0,-2,1,1; 0,0,0,0,0,0,0,-1,1}.
loop i = 1 to 8.
compute conmat1(i,:)=conmat1(i,:)/(10-i).
end loop.
compute conmat1=t(conmat1((10-nvls):8,(10-nvls):9)).
loop k=1 to n.
compute x(k,:)=conmat1((rsum(x(k,:))+1),:).
end loop.
end if.
end if.
do if ( mcw = 5).
compute custcode={ -999 }.
do if (ncol(custcode) <> (nvls*(nvls-1))).
compute errcode(errs,1) = (37+ 2 ).
compute errs = errs + 1.
compute criterr = 1.
end if.
do if (ncol(custcode) = (nvls*(nvls-1))).
compute conmat1=make(nvls,(nvls-1),0).
compute cnt=1.
loop i = 1 to nvls.
loop k = 1 to (nvls-1).
compute conmat1(i,k)=custcode(1,cnt).
compute cnt=cnt+1.
end loop.
end loop.
loop k=1 to n.
compute x(k,:)=conmat1((rsum(x(k,:))+1),:).
end loop.
end if.
end if.
compute xskip = 1.
compute dummat = make((nx+1),nx,0).
compute dummat((2:nrow(dummat)),:)=ident(nx).
do if ( mcw = 4).
compute dummat(1,:) = minus1.
end if.
do if ( mcw = 2).
loop i = 2 to nrow(dummat).
loop j = 1 to (i-1).
compute dummat(i,j) = 1.
end loop.
end loop.
end if.
do if ( mcw = 3).
compute dummat=conmat1.
end if.
do if ( mcw = 5 and criterr=0).
compute dummat=conmat1.
end if.
compute dummat={nnvls, dummat}.
compute x={dd(:,1),x}.
compute temp = x.
compute temp(GRADE(x(:,1)),:) = x.
compute x = temp.
release conmat1,temp,dd,xskip,xdes,dummy.
end if.
end if
.
compute wmodvals=nnvls.
compute nwpval=nrow(wmodvals).
do if (criterr=0).
compute minwwarn=0.
compute maxwwarn=0.
compute wnotev=0.
compute wtmp=x(:,2:ncol(x)).
compute wcatlab={'W1';'W2';'W3';'W4';'W5';'W6';'W7';'W8';'W9'}.
do if (xmint=1).
compute wcatlab={'X1';'X2';'X3';'X4';'X5';'X6';'X7';'X8';'X9'}.
end if.
compute nwvls=nvls-1.
compute mcwok=1.
compute dummatw=dummat.
compute wprobval=dummatw(:,2:ncol(dummatw)).
do if (modcok=1).
compute wcontval=make(2,ncol(wprobval),-999).
compute temp=0.
loop i = 1 to 2.
loop j = 1 to nrow(dummatw).
do if (contvec(i,1)=dummatw(j,1)).
compute wcontval(i,:)=wprobval(j,:).
compute temp=temp+1.
end if.
end loop.
end loop.
do if (temp < 2).
compute notecode(notes,1) = 20.
compute notes = notes + 1.
compute modcok=0.
end if.
end if.
do if ((wmodval(1,1) <> 999) and (xmint <> 1)).
compute notecode(notes,1) = 9.
compute notes = notes + 1.
end if.
release tmp, dummat.
end if.
end if.
do if (nzs > 0 and mcz > 0).
compute tmp={rownum,ztmp(:,1)}.

.
compute dd= tmp.
compute temp = dd.
compute temp(GRADE(dd(:,2)),:) = dd.
compute dd = temp.
compute dummy = design(dd(:,2)).
compute nvls = ncol(dummy).
compute nnvls = csum(dummy).
compute mnvls = cmin(t(nnvls)).
compute conmat1=1.
do if (mnvls < 2).
compute errcode(errs,1) = 5.
compute errs = errs + 1.
compute criterr = 1.
end if.
do if (nvls > 9).
compute errcode(errs,1) = 4.
compute errs = errs+1.
compute criterr = 1.
end if.
do if (criterr = 0).
compute dumok = 1.
compute nnvls=make(nvls,1,0).
compute nnvls(1,1)=dd(1,2).
compute temp = 2.
loop i = 2 to n.
do if (dd(i,2) <> nnvls((temp-1),1)).
compute nnvls(temp,1)=dd(i,2).
compute temp = temp+1.
end if.
end loop.
do if ( mcz > 0).
compute x = dummy(:,2:ncol(dummy)).
compute nx = ncol(x).
compute minus1 = make(1,ncol(x),-1).
compute xdes=make((nx+1),3,0).
compute xdes(1,1)=dd(1,2).
compute xdes(1,2)=1.
compute temp = 2.
loop k = 2 to n.
do if (dd(k,2) <> dd((k-1),2)).
compute xdes(temp,2) = k.
compute xdes(temp,1) = dd(k,2).
compute xdes((temp-1),3) = k-1.
compute temp=temp+1.
end if.
end loop.
compute xdes((temp-1),3)=n.
compute xdes = {xdes, (xdes(:,3)-xdes(:,2)+1)}.
do if ( mcz = 4).
loop k = 1 to n.
do if (rsum(x(k,:)) = 0).
compute x(k,:) = minus1.
end if.
end loop.
end if.
do if ( mcz = 2 or mcz = 3 or mcz =5).
loop k = 1 to n.
do if (rsum(x(k,:)) > 0).
loop i = 1 to ncol(x).
do if (x(k,i) = 0).
compute x(k,i) = 1.
else.
break.
end if.
end loop.
end if.
end loop.
do if ( mcz = 3).
compute conmat1={-8,1,1,1,1,1,1,1,1; 0,-7,1,1,1,1,1,1,1; 0,0,-6,1,1,1,1,1,1; 0,0,0,-5,1,1,1,1,1; 0,0,0,0,-4,1,1,1,1; 0,0,0,0,0,-3,1,1,1; 0,0,0,0,0,0,-2,1,1; 0,0,0,0,0,0,0,-1,1}.
loop i = 1 to 8.
compute conmat1(i,:)=conmat1(i,:)/(10-i).
end loop.
compute conmat1=t(conmat1((10-nvls):8,(10-nvls):9)).
loop k=1 to n.
compute x(k,:)=conmat1((rsum(x(k,:))+1),:).
end loop.
end if.
end if.
do if ( mcz = 5).
compute custcode={ -999 }.
do if (ncol(custcode) <> (nvls*(nvls-1))).
compute errcode(errs,1) = (37+ 3 ).
compute errs = errs + 1.
compute criterr = 1.
end if.
do if (ncol(custcode) = (nvls*(nvls-1))).
compute conmat1=make(nvls,(nvls-1),0).
compute cnt=1.
loop i = 1 to nvls.
loop k = 1 to (nvls-1).
compute conmat1(i,k)=custcode(1,cnt).
compute cnt=cnt+1.
end loop.
end loop.
loop k=1 to n.
compute x(k,:)=conmat1((rsum(x(k,:))+1),:).
end loop.
end if.
end if.
compute xskip = 1.
compute dummat = make((nx+1),nx,0).
compute dummat((2:nrow(dummat)),:)=ident(nx).
do if ( mcz = 4).
compute dummat(1,:) = minus1.
end if.
do if ( mcz = 2).
loop i = 2 to nrow(dummat).
loop j = 1 to (i-1).
compute dummat(i,j) = 1.
end loop.
end loop.
end if.
do if ( mcz = 3).
compute dummat=conmat1.
end if.
do if ( mcz = 5 and criterr=0).
compute dummat=conmat1.
end if.
compute dummat={nnvls, dummat}.
compute x={dd(:,1),x}.
compute temp = x.
compute temp(GRADE(x(:,1)),:) = x.
compute x = temp.
release conmat1,temp,dd,xskip,xdes,dummy.
end if.
end if
.
compute zmodvals=nnvls.
compute nzpval=nrow(zmodvals).
do if (criterr=0).
compute minzwarn=0.
compute maxzwarn=0.
compute znotev=0.
compute ztmp=x(:,2:ncol(x)).
compute zcatlab={'Z1';'Z2';'Z3';'Z4';'Z5';'Z6';'Z7';'Z8';'Z9'}.
compute nzvls=nvls-1.
compute mczok=1.
compute dummatz=dummat.
compute zprobval=dummatz(:,2:ncol(dummatz)).
do if (modcok=1).
compute zcontval=make(2,ncol(zprobval),-999).
compute temp=0.
loop i = 1 to 2.
loop j = 1 to nrow(dummatz).
do if (contvec(i,2)=dummatz(j,1)).
compute zcontval(i,:)=zprobval(j,:).
compute temp=temp+1.
end if.
end loop.
end loop.
do if (temp < 2).
compute notecode(notes,1) = 20.
compute notes = notes + 1.
compute modcok=0.
end if.
end if.
do if (zmodval(1,1) <> 999).
compute notecode(notes,1) = 10.
compute notes = notes + 1.
end if.
release tmp, dummat.
end if.
end if.
do if (nxs > 0 and mcx > 0).
compute tmp={rownum,xtmp(:,1)}.

.
compute dd= tmp.
compute temp = dd.
compute temp(GRADE(dd(:,2)),:) = dd.
compute dd = temp.
compute dummy = design(dd(:,2)).
compute nvls = ncol(dummy).
compute nnvls = csum(dummy).
compute mnvls = cmin(t(nnvls)).
compute conmat1=1.
do if (mnvls < 2).
compute errcode(errs,1) = 5.
compute errs = errs + 1.
compute criterr = 1.
end if.
do if (nvls > 9).
compute errcode(errs,1) = 4.
compute errs = errs+1.
compute criterr = 1.
end if.
do if (criterr = 0).
compute dumok = 1.
compute nnvls=make(nvls,1,0).
compute nnvls(1,1)=dd(1,2).
compute temp = 2.
loop i = 2 to n.
do if (dd(i,2) <> nnvls((temp-1),1)).
compute nnvls(temp,1)=dd(i,2).
compute temp = temp+1.
end if.
end loop.
do if ( mcx > 0).
compute x = dummy(:,2:ncol(dummy)).
compute nx = ncol(x).
compute minus1 = make(1,ncol(x),-1).
compute xdes=make((nx+1),3,0).
compute xdes(1,1)=dd(1,2).
compute xdes(1,2)=1.
compute temp = 2.
loop k = 2 to n.
do if (dd(k,2) <> dd((k-1),2)).
compute xdes(temp,2) = k.
compute xdes(temp,1) = dd(k,2).
compute xdes((temp-1),3) = k-1.
compute temp=temp+1.
end if.
end loop.
compute xdes((temp-1),3)=n.
compute xdes = {xdes, (xdes(:,3)-xdes(:,2)+1)}.
do if ( mcx = 4).
loop k = 1 to n.
do if (rsum(x(k,:)) = 0).
compute x(k,:) = minus1.
end if.
end loop.
end if.
do if ( mcx = 2 or mcx = 3 or mcx =5).
loop k = 1 to n.
do if (rsum(x(k,:)) > 0).
loop i = 1 to ncol(x).
do if (x(k,i) = 0).
compute x(k,i) = 1.
else.
break.
end if.
end loop.
end if.
end loop.
do if ( mcx = 3).
compute conmat1={-8,1,1,1,1,1,1,1,1; 0,-7,1,1,1,1,1,1,1; 0,0,-6,1,1,1,1,1,1; 0,0,0,-5,1,1,1,1,1; 0,0,0,0,-4,1,1,1,1; 0,0,0,0,0,-3,1,1,1; 0,0,0,0,0,0,-2,1,1; 0,0,0,0,0,0,0,-1,1}.
loop i = 1 to 8.
compute conmat1(i,:)=conmat1(i,:)/(10-i).
end loop.
compute conmat1=t(conmat1((10-nvls):8,(10-nvls):9)).
loop k=1 to n.
compute x(k,:)=conmat1((rsum(x(k,:))+1),:).
end loop.
end if.
end if.
do if ( mcx = 5).
compute custcode={ -999 }.
do if (ncol(custcode) <> (nvls*(nvls-1))).
compute errcode(errs,1) = (37+ 1 ).
compute errs = errs + 1.
compute criterr = 1.
end if.
do if (ncol(custcode) = (nvls*(nvls-1))).
compute conmat1=make(nvls,(nvls-1),0).
compute cnt=1.
loop i = 1 to nvls.
loop k = 1 to (nvls-1).
compute conmat1(i,k)=custcode(1,cnt).
compute cnt=cnt+1.
end loop.
end loop.
loop k=1 to n.
compute x(k,:)=conmat1((rsum(x(k,:))+1),:).
end loop.
end if.
end if.
compute xskip = 1.
compute dummat = make((nx+1),nx,0).
compute dummat((2:nrow(dummat)),:)=ident(nx).
do if ( mcx = 4).
compute dummat(1,:) = minus1.
end if.
do if ( mcx = 2).
loop i = 2 to nrow(dummat).
loop j = 1 to (i-1).
compute dummat(i,j) = 1.
end loop.
end loop.
end if.
do if ( mcx = 3).
compute dummat=conmat1.
end if.
do if ( mcx = 5 and criterr=0).
compute dummat=conmat1.
end if.
compute dummat={nnvls, dummat}.
compute x={dd(:,1),x}.
compute temp = x.
compute temp(GRADE(x(:,1)),:) = x.
compute x = temp.
release conmat1,temp,dd,xskip,xdes,dummy.
end if.
end if
.
do if (criterr=0).
compute xtmp=x(:,2:ncol(x)).
compute xcatlab={'X1';'X2';'X3';'X4';'X5';'X6';'X7';'X8';'X9'}.
compute nxvls=nvls-1.
compute xdich=(nvls=2).
compute mcxok=1.
compute dummatx=dummat.
compute xmodvals=dummatx(:,1).
compute nxpval=nrow(xmodvals).
release tmp, dummat.
end if.
end if.
compute intlab=make(100,1,' ').


compute intlab( 1 ,1)= 'Int_1'.


compute intlab( 2 ,1)= 'Int_2'.


compute intlab( 3 ,1)= 'Int_3'.


compute intlab( 4 ,1)= 'Int_4'.


compute intlab( 5 ,1)= 'Int_5'.


compute intlab( 6 ,1)= 'Int_6'.


compute intlab( 7 ,1)= 'Int_7'.


compute intlab( 8 ,1)= 'Int_8'.


compute intlab( 9 ,1)= 'Int_9'.


compute intlab( 10 ,1)= 'Int_10'.


compute intlab( 11 ,1)= 'Int_11'.


compute intlab( 12 ,1)= 'Int_12'.


compute intlab( 13 ,1)= 'Int_13'.


compute intlab( 14 ,1)= 'Int_14'.


compute intlab( 15 ,1)= 'Int_15'.


compute intlab( 16 ,1)= 'Int_16'.


compute intlab( 17 ,1)= 'Int_17'.


compute intlab( 18 ,1)= 'Int_18'.


compute intlab( 19 ,1)= 'Int_19'.


compute intlab( 20 ,1)= 'Int_20'.


compute intlab( 21 ,1)= 'Int_21'.


compute intlab( 22 ,1)= 'Int_22'.


compute intlab( 23 ,1)= 'Int_23'.


compute intlab( 24 ,1)= 'Int_24'.


compute intlab( 25 ,1)= 'Int_25'.


compute intlab( 26 ,1)= 'Int_26'.


compute intlab( 27 ,1)= 'Int_27'.


compute intlab( 28 ,1)= 'Int_28'.


compute intlab( 29 ,1)= 'Int_29'.


compute intlab( 30 ,1)= 'Int_30'.


compute intlab( 31 ,1)= 'Int_31'.


compute intlab( 32 ,1)= 'Int_32'.


compute intlab( 33 ,1)= 'Int_33'.


compute intlab( 34 ,1)= 'Int_34'.


compute intlab( 35 ,1)= 'Int_35'.


compute intlab( 36 ,1)= 'Int_36'.


compute intlab( 37 ,1)= 'Int_37'.


compute intlab( 38 ,1)= 'Int_38'.


compute intlab( 39 ,1)= 'Int_39'.


compute intlab( 40 ,1)= 'Int_40'.


compute intlab( 41 ,1)= 'Int_41'.


compute intlab( 42 ,1)= 'Int_42'.


compute intlab( 43 ,1)= 'Int_43'.


compute intlab( 44 ,1)= 'Int_44'.


compute intlab( 45 ,1)= 'Int_45'.


compute intlab( 46 ,1)= 'Int_46'.


compute intlab( 47 ,1)= 'Int_47'.


compute intlab( 48 ,1)= 'Int_48'.


compute intlab( 49 ,1)= 'Int_49'.


compute intlab( 50 ,1)= 'Int_50'.


compute intlab( 51 ,1)= 'Int_51'.


compute intlab( 52 ,1)= 'Int_52'.


compute intlab( 53 ,1)= 'Int_53'.


compute intlab( 54 ,1)= 'Int_54'.


compute intlab( 55 ,1)= 'Int_55'.


compute intlab( 56 ,1)= 'Int_56'.


compute intlab( 57 ,1)= 'Int_57'.


compute intlab( 58 ,1)= 'Int_58'.


compute intlab( 59 ,1)= 'Int_59'.


compute intlab( 60 ,1)= 'Int_60'.


compute intlab( 61 ,1)= 'Int_61'.


compute intlab( 62 ,1)= 'Int_62'.


compute intlab( 63 ,1)= 'Int_63'.


compute intlab( 64 ,1)= 'Int_64'.


compute intlab( 65 ,1)= 'Int_65'.


compute intlab( 66 ,1)= 'Int_66'.


compute intlab( 67 ,1)= 'Int_67'.


compute intlab( 68 ,1)= 'Int_68'.


compute intlab( 69 ,1)= 'Int_69'.


compute intlab( 70 ,1)= 'Int_70'.


compute intlab( 71 ,1)= 'Int_71'.


compute intlab( 72 ,1)= 'Int_72'.


compute intlab( 73 ,1)= 'Int_73'.


compute intlab( 74 ,1)= 'Int_74'.


compute intlab( 75 ,1)= 'Int_75'.


compute intlab( 76 ,1)= 'Int_76'.


compute intlab( 77 ,1)= 'Int_77'.


compute intlab( 78 ,1)= 'Int_78'.


compute intlab( 79 ,1)= 'Int_79'.


compute intlab( 80 ,1)= 'Int_80'.


compute intlab( 81 ,1)= 'Int_81'.


compute intlab( 82 ,1)= 'Int_82'.


compute intlab( 83 ,1)= 'Int_83'.


compute intlab( 84 ,1)= 'Int_84'.


compute intlab( 85 ,1)= 'Int_85'.


compute intlab( 86 ,1)= 'Int_86'.


compute intlab( 87 ,1)= 'Int_87'.


compute intlab( 88 ,1)= 'Int_88'.


compute intlab( 89 ,1)= 'Int_89'.


compute intlab( 90 ,1)= 'Int_90'.


compute intlab( 91 ,1)= 'Int_91'.


compute intlab( 92 ,1)= 'Int_92'.


compute intlab( 93 ,1)= 'Int_93'.


compute intlab( 94 ,1)= 'Int_94'.


compute intlab( 95 ,1)= 'Int_95'.


compute intlab( 96 ,1)= 'Int_96'.


compute intlab( 97 ,1)= 'Int_97'.


compute intlab( 98 ,1)= 'Int_98'.


compute intlab( 99 ,1)= 'Int_99'.


compute intlab( 100 ,1)= 'Int_100'.

compute bcmat=make(needed,needed,0).
compute wcmat=make(needed,needed,0).
compute zcmat=make(needed,needed,0).
compute wzcmat=make(needed,needed,0).
compute wsum=0.
compute zsum=0.
compute wzsum=0.
end if.
end if.
do if (criterr = 0 and model <> 999).
compute modelmat= {1,0,0,0,0,0,0,1,0,0;2,0,0,0,0,0,0,1,1,0;3,0,0,0,0,0,0,1,1,1;4,0,0,0,0,0,0,0,0,0; 5,0,0,0,0,0,0,1,0,0;6,0,0,0,0,0,0,0,0,0;7,1,0,0,0,0,0,0,0,0;8,1,0,0,0,0,0,1,0,0; 9,1,1,0,0,0,0,0,0,0;10,1,1,0,0,0,0,1,1,0;11,1,1,1,0,0,0,0,0,0;12,1,1,1,0,0,0,1,1,1; 13,1,1,1,0,0,0,1,0,0;14,0,0,0,1,0,0,0,0,0;15,0,0,0,1,0,0,1,0,0;16,0,0,0,1,1,0,0,0,0; 17,0,0,0,1,1,0,1,1,0;18,0,0,0,1,1,1,0,0,0;19,0,0,0,1,1,1,1,1,1;20,0,0,0,1,1,1,1,0,0; 21,1,0,0,0,1,0,0,0,0;22,1,0,0,0,1,0,1,0,0;23,0,0,0,0,0,0,0,0,0;24,0,0,0,0,0,0,0,0,0; 25,0,0,0,0,0,0,0,0,0;26,0,0,0,0,0,0,0,0,0;27,0,0,0,0,0,0,0,0,0;28,1,0,0,0,1,0,0,1,0; 29,1,0,0,0,1,0,1,1,0;30,0,0,0,0,0,0,0,0,0;31,0,0,0,0,0,0,0,0,0;32,0,0,0,0,0,0,0,0,0; 33,0,0,0,0,0,0,0,0,0;34,0,0,0,0,0,0,0,0,0;35,0,0,0,0,0,0,0,0,0;36,0,0,0,0,0,0,0,0,0; 37,0,0,0,0,0,0,0,0,0;38,0,0,0,0,0,0,0,0,0;39,0,0,0,0,0,0,0,0,0;40,0,0,0,0,0,0,0,0,0; 41,0,0,0,0,0,0,0,0,0;42,0,0,0,0,0,0,0,0,0;43,0,0,0,0,0,0,0,0,0;44,0,0,0,0,0,0,0,0,0; 45,0,0,0,0,0,0,0,0,0;46,0,0,0,0,0,0,0,0,0;47,0,0,0,0,0,0,0,0,0;48,0,0,0,0,0,0,0

 ,0,0; 49,0,0,0,0,0,0,0,0,0;50,0,0,0,0,0,0,0,0,0;51,0,0,0,0,0,0,0,0,0;52,0,0,0,0,0,0,0,0,0; 53,0,0,0,0,0,0,0,0,0;54,0,0,0,0,0,0,0,0,0;55,0,0,0,0,0,0,0,0,0;56,0,0,0,0,0,0,0,0,0; 57,0,0,0,0,0,0,0,0,0;58,1,0,0,1,0,0,0,0,0;59,1,0,0,1,0,0,1,0,0;60,1,1,0,1,0,0,0,0,0; 61,1,1,0,1,0,0,1,0,0;62,1,1,0,1,0,0,0,1,0;63,1,1,0,1,0,0,1,1,0;64,1,0,0,1,1,0,0,0,0; 65,1,0,0,1,1,0,1,0,0;66,1,0,0,1,1,0,0,1,0;67,1,0,0,1,1,0,1,1,0;68,1,1,1,1,0,0,0,0,0; 69,1,1,1,1,0,0,1,1,1;70,1,0,0,1,1,1,0,0,0;71,1,0,0,1,1,1,1,1,1;72,1,1,1,1,1,1,0,0,0; 73,1,1,1,1,1,1,1,1,1;74,0,0,0,1,0,0,0,0,0;75,1,1,0,1,1,0,0,0,0;76,1,1,0,1,1,0,1,1,0; 77,0,0,0,0,0,0,0,0,0;78,0,0,0,0,0,0,0,0,0;79,0,0,0,0,0,0,0,0,0;80,0,0,0,0,0,0,0,0,0; 81,0,0,0,0,0,0,0,0,0;82,0,0,0,0,0,0,0,0,0;83,1,0,0,0,0,0,0,0,0;84,1,0,0,0,0,0,0,0,0; 85,1,0,0,0,0,0,1,0,0;86,1,0,0,0,0,0,1,0,0;87,0,0,0,1,0,0,0,0,0;88,0,0,0,1,0,0,0,0,0; 89,0,0,0,1,0,0,1,0,0;90,0,0,0,1,0,0,1,0,0;91,0,0,0,0,0,0,0,0,0;92,1,0,0,1,0,0,1,0,0}.
do if (model > 0).
compute tmp=modelmat(model,2:ncol(modelmat)).
end if.
do if (model=0).
compute tmp={0,0,0,0,0,0,0,0,0}.
end if.
do if (model < 4).
compute bcmat((nxs+1),1)=1.
end if.
do if ((model > 3) and (model <> 6)).
compute bcmat((nxs+1):(nxs+nms),1)=onem.
compute bcmat(nrow(bcmat),(nxs+1):(nxs+nms))=t(onem).
compute bcmat(nrow(bcmat),1)=1.
end if.
do if ((model = 6) or (model > 82 and model < 93)).
loop j = 2 to nrow(bcmat).
loop i = 1 to (j-1).
compute bcmat(j,i)=1.
end loop.
end loop.
end if.
do if (model = 80).
loop i = 1 to nms.
compute bcmat((nrow(bcmat)-1),i)=1.
end loop.
end if.
do if (model = 81).
loop j = 3 to nrow(bcmat).
compute bcmat(j,2)=1.
end loop.
end if.
do if (model = 82).
compute bcmat(3,2)=1.
compute bcmat(5,4)=1.
end if.
do if (tmp(1,1)=1).
compute wcmat((nxs+1):(nxs+nms),1)=onem.
compute wprod=1.
compute xprod=1.
do if (model = 83 or model = 86).
compute onemsx=onem.
loop i = 1 to (nms-1).
compute onemsx(i+1,1)=0.
end loop.
compute wcmat((nxs+1):(nxs+nms),1)=onemsx.
end if.
end if.
do if (tmp(1,4)=1).
compute wcmat(nrow(wcmat),(nxs+1):(nxs+nms))=t(onem).
compute wprod=1.
do if (model = 87 or model = 90).
compute onemsx=onem.
loop i = 1 to (nms-1).
compute onemsx(i,1)=0.
end loop.
compute wcmat(nrow(wcmat),(nxs+1):(nxs+nms))=t(onemsx).
end if.
end if.
do if (tmp(1,7)=1).
compute wcmat(nrow(wcmat),1)=1.
compute wprod=1.
compute xprod=1.
end if.
do if (tmp(1,2)=1).
compute zcmat((nxs+1):(nxs+nms),1)=onem.
compute zprod=1.
compute xprod=1.
end if.
do if (tmp(1,5)=1).
compute zcmat(nrow(zcmat),(nxs+1):(nxs+nms))=t(onem).
compute zprod=1.
end if.
do if (tmp(1,8)=1).
compute zcmat(nrow(zcmat),1)=1.
compute zprod=1.
compute xprod=1.
end if.
do if (tmp(1,3)=1).
compute wzcmat((nxs+1):(nxs+nms),1)=onem.
compute xprod=1.
compute wprod=1.
compute zprod=1.
end if.
do if (tmp(1,6)=1).
compute wzcmat(nrow(wzcmat),(nxs+1):(nxs+nms))=t(onem).
compute zprod=1.
compute wprod=1.
end if.
do if (tmp(1,9)=1).
compute wzcmat(nrow(wzcmat),1)=1.
compute xprod=1.
compute wprod=1.
compute zprod=1.
end if.
do if (model = 91 or model = 92).
loop j = 1 to (nms-1).
loop i = 1 to j.
compute wcmat((nxs+1+j),(nxs+i))=1.
end loop.
end loop.
end if.
do if (nms < 0).
loop i = 1 to nms.
compute tmp=csum(wcmat(:,(1+i)))+csum(zcmat(:,(1+i)))+csum(wzcmat(:,(1+i))).
compute mprod(1,i)=(tmp>0).
end loop.
end if.
end if.
do if (ncs > 0).
compute ccmat=make((nms+nys),ncs,1).
compute ccmatoff=ccmat.
do if (covmy=1).
compute ccmat(nrow(ccmat),:)=make(1,ncs,0).
end if.
do if (covmy=2).
compute ccmat(1:nms,:)=make(nms,ncs,0).
end if.
do if (cmatrix(1,1) <> -999).
do if (ncol(cmatrix) <> ((nms+nys)*ncs)).
compute errcode(errs,1)=29.
compute errs=errs+1.
compute criterr=1.
end if.
do if (criterr = 0).
compute tmp=1.
loop i = 1 to (nms+nys).
loop j = 1 to ncs.
compute ccmat(i,j)=1-(cmatrix(1,tmp) = 0).
compute tmp=tmp+1.
end loop.
end loop.
do if (rsum((csum(ccmat)=0)) <> 0).
compute errcode(errs,1)=30.
compute errs=errs+1.
compute criterr=1.
end if.
end if.
do if (covmy <> 0).
compute notecode(notes,1)=1.
compute notes=notes+1.
end if.
end if.
do if (xmint=1 and (covmy > 0 or (csum(rsum(ccmat)) <> ((nms+nys)*ncs)))).
compute errcode(errs,1)=68.
compute errs=errs+1.
compute criterr=1.
end if.
end if.
do if (criterr=0).
compute needed=needed*(needed-1)/2.
compute nopath=0.
do if (bmatrix(1,1) <> -999).
compute tmp=1.
do if ((ncol(bmatrix) <> needed) or (csum(rsum(bmatrix))=0)).
compute errcode(errs,1)=16.
compute errs=errs+1.
compute criterr=1.
else.
loop i = 2 to nrow(bcmat).
loop j = 1 to (i-1).
compute bcmat(i,j)=1-(bmatrix(1,tmp) = 0).
compute tmp=tmp+1.
end loop.
end loop.
end if.
do if ((csum(bcmat(:,1))=0) and criterr=0).
compute errcode(errs,1)=22.
compute errs=errs+1.
compute criterr=1.
end if.
do if ((rsum(bcmat(nrow(bcmat),:))=0) and criterr=0).
compute errcode(errs,1)=23.
compute errs=errs+1.
compute criterr=1.
end if.
compute dm=0.
do if (nms > 0).
loop i = 1 to nms.
do if (((rsum(bcmat((nxs+i),:)) = 0) or (csum(bcmat(:,(nxs+i))) = 0)) and (dm=0) and (criterr=0)).
compute errcode(errs,1)=26.
compute errs=errs+1.
compute criterr=1.
compute dm=1.
end if.
end loop.
end if.
release dm.
end if.
end if.
do if (criterr=0).
do if (wmatrix(1,1) <> -999).
compute tmp=1.
do if (ncol(wmatrix) <> needed).
compute errcode(errs,1)=17.
compute errs=errs+1.
compute criterr=1.
else.
compute modelvar(1,1)='CUSTOM'.
loop i = 2 to nrow(wcmat).
loop j = 1 to (i-1).
compute wcmat(i,j)=1-(wmatrix(1,tmp) = 0).
do if ((wcmat(i,j)=1) and (bcmat(i,j)=0) and (nopath=0)).
compute errcode(errs,1)=20.
compute errs=errs+1.
compute criterr=1.
compute nopath=1.
end if.
compute tmp=tmp+1.
end loop.
end loop.
end if.
end if.
do if (zmatrix(1,1) <> -999).
compute tmp=1.
do if (ncol(zmatrix) <> needed).
compute errcode(errs,1)=18.
compute errs=errs+1.
compute criterr=1.
else.
compute modelvar(1,1)='CUSTOM'.
do if (csum(rsum(wcmat))=0 and model=999).
compute errcode(errs,1)=21.
compute errs=errs+1.
compute criterr=1.
end if.
loop i = 2 to nrow(zcmat).
loop j = 1 to (i-1).
compute zcmat(i,j)=1-(zmatrix(1,tmp) = 0).
do if ((zcmat(i,j)=1) and (bcmat(i,j)=0) and (nopath=0)).
compute errcode(errs,1)=20.
compute errs=errs+1.
compute criterr=1.
compute nopath=1.
end if.
compute tmp=tmp+1.
end loop.
end loop.
end if.
end if.
compute tmp=1.
do if (wzmatrix(1,1) <> -999).
do if (ncol(wzmatrix) <> needed).
compute errcode(errs,1)=19.
compute errs=errs+1.
compute criterr=1.
end if.
compute modelvar(1,1)='CUSTOM'.
end if.
do if (criterr=0).
loop i = 2 to nrow(wzcmat).
loop j = 1 to (i-1).
do if (wzmatrix(1,1) <> -999).
compute wzcmat(i,j)=1-(wzmatrix(1,tmp) = 0).
end if.
do if (wzcmat(i,j)=1).
compute wcmat(i,j)=1.
compute zcmat(i,j)=1.
end if.
do if ((wzcmat(i,j)=1) and (bcmat(i,j)=0) and (nopath=0)).
compute errcode(errs,1)=20.
compute errs=errs+1.
compute criterr=1.
compute nopath=1.
end if.
compute tmp=tmp+1.
end loop.
end loop.
end if.
end if.
do if (criterr=0).
compute xprod=csum(wcmat(:,1))+csum(zcmat(:,1))+csum(wzcmat(:,1)).
compute xprod=(xprod > 0).
compute wsum=csum(rsum(wcmat)).
compute wprod=(wsum > 0).
do if (nms > 0).
loop i = 1 to nms.
compute tmp=csum(wcmat(:,(1+i)))+csum(zcmat(:,(1+i)))+csum(wzcmat(:,(1+i))).
compute mprod(1,i)=(tmp>0).
end loop.
end if.
do if ((wsum > 0) and (w = 'xxxxx')).
compute errcode(errs,1)=11.
compute errs=errs+1.
compute criterr=1.
end if.
do if ((wsum = 0) and (w <> 'xxxxx')).
compute errcode(errs,1)=10.
compute errs=errs+1.
compute criterr=1.
end if.
compute zsum=csum(rsum(zcmat)).
compute zprod=(zsum > 0).
do if ((zsum > 0) and (z = 'xxxxx')).
compute errcode(errs,1)=13.
compute errs=errs+1.
compute criterr=1.
end if.
do if ((zsum = 0) and (z <> 'xxxxx')).
compute errcode(errs,1)=12.
compute errs=errs+1.
compute criterr=1.
end if.
do if ((zsum > 0) and (wsum = 0)).
compute errcode(errs,1)=35.
compute errs=errs+1.
compute criterr=1.
end if.
end if.
do if (criterr=0 and nms > 1).
compute serchk=bcmat(2:(nrow(bcmat)-1),2:ncol(bcmat)).
do if (csum(rsum(serchk))) > 0.
compute serial=1.
do if (nms > 6).
compute errcode(errs,1)=36.
compute errs=errs+1.
compute criterr=1.
end if.
end if.
end if.
do if (center > 0 and criterr=0).
compute centvar={' '}.
do if (criterr=0).
do if ((center = 1) or (center = 2 and wdich = 0)).
do if (wprod=1 and mcwok=0 and nwpval > 0).
loop i = 1 to nws.
compute wtmp(:,i)=wtmp(:,i)-(csum(wtmp(:,i))/n).
compute centvar={centvar,wnames(1,i)}.
end loop.

.
compute desctmp=make((8-(4* wmodcust )),ncol( wtmp ),-999).
loop jd=1 to ncol( wtmp ).
compute descdat= wtmp (:,jd).
compute desctmp(1,jd) = csum(descdat)/nrow(descdat).
compute desctmp(2,jd) = (nrow(descdat)*sscp(descdat))-(t(csum(descdat))*(csum(descdat))).
compute desctmp(2,jd) = sqrt(desctmp(2,jd)/(nrow(descdat)*(nrow(descdat)-1))).
compute desctmp(3,jd)=cmin(descdat).
compute desctmp(4,jd)=cmax(descdat).
do if ( wmodcust =0).
compute minwarn=0.
compute maxwarn=0.
do if ((desctmp(3,jd)=desctmp(4,jd)) and novar=0).
compute errcode(errs,1)=15.
compute errs=errs+1.
compute criterr=1.
compute novar=1.
end if.
compute tmp=((descdat(:,1)=desctmp(3,jd))+(descdat(:,1)=desctmp(4,jd))).
compute desctmp(8,jd)=(csum(tmp)=nrow(tmp)).
compute tmp = descdat.
compute tmp(GRADE(descdat),:) = descdat.
compute descdat = tmp.
release tmp.
compute decval={.16;.5;.84}.
loop kd=1 to 3.
compute low=trunc(decval(kd,1)*(nrow(descdat)+1)).
compute lowdec=decval(kd,1)*(nrow(descdat)+1)-low.
compute value=descdat(low,1)+(descdat((low+1),1)-descdat(low,1))*lowdec.
compute desctmp((4+kd),jd)=value.
end loop.
compute mnotev=1.
compute modvals=desctmp(5:7,:).
do if (quantile <> 1).
compute desctmp(5,jd)=desctmp(1,jd)-desctmp(2,jd).
compute desctmp(6,jd)=desctmp(1,jd).
compute desctmp(7,jd)=desctmp(1,jd)+desctmp(2,jd).
compute modvals=desctmp(5:7,:).
compute mnotev=2.
do if (modvals(1,1) < desctmp(3,1)).
compute modvals(1,1)=desctmp(3,1).
compute minwarn=1.
end if.
do if (modvals(3,1) > desctmp(4,1)).
compute modvals(3,1)=desctmp(4,1).
compute maxwarn=1.
end if.
end if.
do if (desctmp(8,1)=1).
compute modvals={desctmp(3,1);desctmp(4,1)}.
compute mnotev=0.
compute minwarn=0.
compute maxwarn=0.
end if.
end if.
end loop
.
compute wmin=desctmp(3,1).
compute wmax=desctmp(4,1).
do if (wmodcust=0).
compute wmodvals=modvals.
compute wprobval=wmodvals.
end if.
end if.
end if.
do if ((center = 1) or (center = 2 and zdich = 0)).
do if (zprod=1 and mczok=0 and nzpval > 0).
loop i = 1 to nzs.
compute ztmp(:,i)=ztmp(:,i)-(csum(ztmp(:,i))/n).
compute centvar={centvar,znames(1,i)}.
end loop.

.
compute desctmp=make((8-(4* zmodcust )),ncol( ztmp ),-999).
loop jd=1 to ncol( ztmp ).
compute descdat= ztmp (:,jd).
compute desctmp(1,jd) = csum(descdat)/nrow(descdat).
compute desctmp(2,jd) = (nrow(descdat)*sscp(descdat))-(t(csum(descdat))*(csum(descdat))).
compute desctmp(2,jd) = sqrt(desctmp(2,jd)/(nrow(descdat)*(nrow(descdat)-1))).
compute desctmp(3,jd)=cmin(descdat).
compute desctmp(4,jd)=cmax(descdat).
do if ( zmodcust =0).
compute minwarn=0.
compute maxwarn=0.
do if ((desctmp(3,jd)=desctmp(4,jd)) and novar=0).
compute errcode(errs,1)=15.
compute errs=errs+1.
compute criterr=1.
compute novar=1.
end if.
compute tmp=((descdat(:,1)=desctmp(3,jd))+(descdat(:,1)=desctmp(4,jd))).
compute desctmp(8,jd)=(csum(tmp)=nrow(tmp)).
compute tmp = descdat.
compute tmp(GRADE(descdat),:) = descdat.
compute descdat = tmp.
release tmp.
compute decval={.16;.5;.84}.
loop kd=1 to 3.
compute low=trunc(decval(kd,1)*(nrow(descdat)+1)).
compute lowdec=decval(kd,1)*(nrow(descdat)+1)-low.
compute value=descdat(low,1)+(descdat((low+1),1)-descdat(low,1))*lowdec.
compute desctmp((4+kd),jd)=value.
end loop.
compute mnotev=1.
compute modvals=desctmp(5:7,:).
do if (quantile <> 1).
compute desctmp(5,jd)=desctmp(1,jd)-desctmp(2,jd).
compute desctmp(6,jd)=desctmp(1,jd).
compute desctmp(7,jd)=desctmp(1,jd)+desctmp(2,jd).
compute modvals=desctmp(5:7,:).
compute mnotev=2.
do if (modvals(1,1) < desctmp(3,1)).
compute modvals(1,1)=desctmp(3,1).
compute minwarn=1.
end if.
do if (modvals(3,1) > desctmp(4,1)).
compute modvals(3,1)=desctmp(4,1).
compute maxwarn=1.
end if.
end if.
do if (desctmp(8,1)=1).
compute modvals={desctmp(3,1);desctmp(4,1)}.
compute mnotev=0.
compute minwarn=0.
compute maxwarn=0.
end if.
end if.
end loop
.
compute zmin=desctmp(3,1).
compute zmax=desctmp(4,1).
do if (zmodcust=0).
compute zmodvals=modvals.
compute zprobval=zmodvals.
end if.
end if.
end if.
do if ((center = 1) or (center = 2 and xdich = 0)).
do if (xprod=1 and mcxok=0).
loop i = 1 to nxs.
compute xtmp(:,i)=xtmp(:,i)-(csum(xtmp(:,i))/n).
compute centvar={centvar,xnames(1,i)}.
end loop.

.
compute desctmp=make((8-(4* 0 )),ncol( xtmp ),-999).
loop jd=1 to ncol( xtmp ).
compute descdat= xtmp (:,jd).
compute desctmp(1,jd) = csum(descdat)/nrow(descdat).
compute desctmp(2,jd) = (nrow(descdat)*sscp(descdat))-(t(csum(descdat))*(csum(descdat))).
compute desctmp(2,jd) = sqrt(desctmp(2,jd)/(nrow(descdat)*(nrow(descdat)-1))).
compute desctmp(3,jd)=cmin(descdat).
compute desctmp(4,jd)=cmax(descdat).
do if ( 0 =0).
compute minwarn=0.
compute maxwarn=0.
do if ((desctmp(3,jd)=desctmp(4,jd)) and novar=0).
compute errcode(errs,1)=15.
compute errs=errs+1.
compute criterr=1.
compute novar=1.
end if.
compute tmp=((descdat(:,1)=desctmp(3,jd))+(descdat(:,1)=desctmp(4,jd))).
compute desctmp(8,jd)=(csum(tmp)=nrow(tmp)).
compute tmp = descdat.
compute tmp(GRADE(descdat),:) = descdat.
compute descdat = tmp.
release tmp.
compute decval={.16;.5;.84}.
loop kd=1 to 3.
compute low=trunc(decval(kd,1)*(nrow(descdat)+1)).
compute lowdec=decval(kd,1)*(nrow(descdat)+1)-low.
compute value=descdat(low,1)+(descdat((low+1),1)-descdat(low,1))*lowdec.
compute desctmp((4+kd),jd)=value.
end loop.
compute mnotev=1.
compute modvals=desctmp(5:7,:).
do if (quantile <> 1).
compute desctmp(5,jd)=desctmp(1,jd)-desctmp(2,jd).
compute desctmp(6,jd)=desctmp(1,jd).
compute desctmp(7,jd)=desctmp(1,jd)+desctmp(2,jd).
compute modvals=desctmp(5:7,:).
compute mnotev=2.
do if (modvals(1,1) < desctmp(3,1)).
compute modvals(1,1)=desctmp(3,1).
compute minwarn=1.
end if.
do if (modvals(3,1) > desctmp(4,1)).
compute modvals(3,1)=desctmp(4,1).
compute maxwarn=1.
end if.
end if.
do if (desctmp(8,1)=1).
compute modvals={desctmp(3,1);desctmp(4,1)}.
compute mnotev=0.
compute minwarn=0.
compute maxwarn=0.
end if.
end if.
end loop
.
compute xmodvals=modvals.
compute xprobval=xmodvals.
end if.
end if.
do if (nms > 0).
loop i = 1 to nms.
do if (mprod(1,i)=1).
compute mtmp(:,i)=mtmp(:,i)-(csum(mtmp(:,i))/n).
compute centvar={centvar,mnames(1,i)}.
end if.
end loop.

.
compute desctmp=make((8-(4* 0 )),ncol( mtmp ),-999).
loop jd=1 to ncol( mtmp ).
compute descdat= mtmp (:,jd).
compute desctmp(1,jd) = csum(descdat)/nrow(descdat).
compute desctmp(2,jd) = (nrow(descdat)*sscp(descdat))-(t(csum(descdat))*(csum(descdat))).
compute desctmp(2,jd) = sqrt(desctmp(2,jd)/(nrow(descdat)*(nrow(descdat)-1))).
compute desctmp(3,jd)=cmin(descdat).
compute desctmp(4,jd)=cmax(descdat).
do if ( 0 =0).
compute minwarn=0.
compute maxwarn=0.
do if ((desctmp(3,jd)=desctmp(4,jd)) and novar=0).
compute errcode(errs,1)=15.
compute errs=errs+1.
compute criterr=1.
compute novar=1.
end if.
compute tmp=((descdat(:,1)=desctmp(3,jd))+(descdat(:,1)=desctmp(4,jd))).
compute desctmp(8,jd)=(csum(tmp)=nrow(tmp)).
compute tmp = descdat.
compute tmp(GRADE(descdat),:) = descdat.
compute descdat = tmp.
release tmp.
compute decval={.16;.5;.84}.
loop kd=1 to 3.
compute low=trunc(decval(kd,1)*(nrow(descdat)+1)).
compute lowdec=decval(kd,1)*(nrow(descdat)+1)-low.
compute value=descdat(low,1)+(descdat((low+1),1)-descdat(low,1))*lowdec.
compute desctmp((4+kd),jd)=value.
end loop.
compute mnotev=1.
compute modvals=desctmp(5:7,:).
do if (quantile <> 1).
compute desctmp(5,jd)=desctmp(1,jd)-desctmp(2,jd).
compute desctmp(6,jd)=desctmp(1,jd).
compute desctmp(7,jd)=desctmp(1,jd)+desctmp(2,jd).
compute modvals=desctmp(5:7,:).
compute mnotev=2.
do if (modvals(1,1) < desctmp(3,1)).
compute modvals(1,1)=desctmp(3,1).
compute minwarn=1.
end if.
do if (modvals(3,1) > desctmp(4,1)).
compute modvals(3,1)=desctmp(4,1).
compute maxwarn=1.
end if.
end if.
do if (desctmp(8,1)=1).
compute modvals={desctmp(3,1);desctmp(4,1)}.
compute mnotev=0.
compute minwarn=0.
compute maxwarn=0.
end if.
end if.
end loop
.
do if ((cdeval(1,1)=-999) and (model=74)).
compute medmeans=desctmp(1,:).
end if.
compute mmodvals=modvals.
compute mprobval=mmodvals.
end if.
end if.
do if (ncol(centvar) > 1).
compute notecode(notes,1)=3.
compute notes=notes+1.
end if.
end if.
do if (criterr=0).
compute wsum=rsum(csum(wcmat)).
compute zsum=rsum(csum(zcmat)).
compute wzsum=rsum(csum(wzcmat)).
compute nump=make(1,(nys+nms),-999).
compue numint=make(1,(nys+nms),0).
compute datcount=1.
compute xtmpuse=0.
compute wtmpuse=0.
compute ztmpuse=0.
compute xwtmpus=0.
compute xztmpus=0.
compute wztmpus=0.
compute xwztmpu=0.
compute xtmploc=-999.
compute wtmploc=-999.
compute xwtmplo=-999.
compute ztmploc=-999.
compute xztmplo=-999.
compute wztmplo=-999.
compute xwztmplo=-999.
compute vlabs={' '}.
do if (ncs > 0).
compute ctmpuse=make(1,ncs,0).
end if.
do if (nms > 0).
compute mtmpuse=make(1,nms,0).
compute mwtmpus=make(1,nms,0).
compute mztmpus=make(1,nms,0).
compute mwztmpu=make(1,nms,0).
compute mtmploc=make(1,nms,0).
compute mwtmplo=make(nwvls,nms,-999).
compute mztmplo=make(nzvls,nms,-999).
compute mwztmplo=make((nwvls*nzvls),nms,-999).
end if.
do if (ncs > 0).
compute ctmploc=make(1,ncs,0).
end if.
compute fulldat=make(n,1,1).
compute datindx=make(1000,(nms+nys),-999).
compute wherew=make(2,(nms+nys),-999).
compute wherex=make(2,(nms+nys),-999).
compute wherez=make(2,(nms+nys),-999).
compute wherexw=make(2,(nms+nys),-999).
compute wherexz=make(2,(nms+nys),-999).
compute wherewz=make(2,(nms+nys),-999).
compute wherexwz=make(2,(nms+nys),-999).
do if (nms > 0).
compute wherem=make(nms,(nms+nys),-999).
compute wheremw = make(nms*2,(nms+nys),-999).
compute wheremz = make(nms*2,(nms+nys),-999).
compute wheremwz = make(nms*2,(nms+nys),-999).
end if.
compute wzhigh=make(1000,(((nms+1)*(nms+2))/2),0).
compute whigh=make(1000,(((nms+1)*(nms+2))/2),0).
compute zhigh=make(1000,(((nms+1)*(nms+2))/2),0).
compute fochigh=make(1000,(((nms+1)*(nms+2))/2),0).
compute xcoefloc={1;2;3;4;5;6;7;8;9}.
compute intkey = {' ', ' ', ' ', ' ', ' ', ' ', ' '}.
compute wzhighct=0.
compute whighct=0.
compute zhighct=0.
compute foccnt=0.
loop i = 2 to nrow(bcmat).
compute wdid=0.
compute zdid=0.
compute wzdid=0.
compute cntmp=1.
compute start=1.
do if (i < nrow(bcmat)).
compute outv=mtmp(:,(i-1)).
compute modlabel={mnames(1,(i-1));'constant'}.
end if.
do if (i = nrow(bcmat)).
compute outv=ytmp.
compute modlabel={ynames;'constant'}.
end if.
loop j = 1 to (i-1).
compute foccnt=foccnt+1.
do if (j = 1 and bcmat(i,j)=1).
compute outv={outv,xtmp}.
compute modlabel={modlabel;xcatlab(1:nxvls,1)}.
do if (xtmpuse=0).
compute fulldat={fulldat,xtmp}.
compute xtmpuse=1.
loop k4=datcount to (datcount+(nxvls-1)).
compute xtmploc={xtmploc;k4}.
end loop.
compute xtmploc=xtmploc(2:nrow(xtmploc),1).
compute datcount=datcount+nxvls.
end if.
compute datindx(start:(start+nrow(xtmploc)-1),(i-1))=xtmploc.
compute wherex(1,(i-1))=start+1.
compute wherex(2,(i-1))=start+nrow(xtmploc)-1+1.
do if (model = 74).
end if.
compute onebl=make(nrow(xtmploc),1,1).
compute fochigh((start+1):(start+nrow(xtmploc)),foccnt)=onebl.
compute start=start+nrow(xtmploc).
end if.
do if (j > 1 and bcmat(i,j)=1).
compute outv={outv,mtmp(:,(j-1))}.
compute modlabel={modlabel;mnames(1,(j-1))}.
do if (mtmpuse(1,(j-1))=0).
compute fulldat={fulldat,mtmp(:,(j-1))}.
compute mtmpuse(1,(j-1))=1.
compute mtmploc(1,(j-1))=datcount.
compute datcount=datcount+1.
end if.
compute datindx(start:(start+nrow(mtmploc)-1),(i-1))=mtmploc(1,(j-1)).
compute wherem((j-1),(i-1))=start+1.
compute onebl=make(nrow(mtmploc(1,j-1)),1,1).
compute ttt=nrow(mtmploc(1,(j-1)))+start-1.
compute fochigh((start+1):(start+nrow(mtmploc(1,(j-1)))),foccnt)=onebl.
compute start=start+nrow(mtmploc(1,(j-1))).
end if.
end loop.
do if (wsum > 0).
loop j = 1 to (i-1).
compute whighct=whighct+1.
do if (j = 1 and wcmat(i,j)=1).
do if (wdid=0).
compute outv={outv,wtmp}.
do if (ncs > 0 and wiscov > 0).
compute ccmatoff((i-1),wiscov)=0.
end if.
compute modlabel={modlabel;wcatlab(1:nwvls,1)}.
compute wdid=1.
do if (wtmpuse=0).
compute fulldat={fulldat,wtmp}.
do if (ncs > 0 and wiscov > 0).
compute ccmatoff((i-1),wiscov)=0.
end if.
compute wtmpuse=1.
loop k4=datcount to (datcount+(nwvls-1)).
compute wtmploc={wtmploc;k4}.
end loop.
compute wtmploc=wtmploc(2:nrow(wtmploc),1).
compute datcount=datcount+nwvls.
end if.
end if.
compute datindx(start:(start+nrow(wtmploc)-1),(i-1))=wtmploc.
compute wherew(1,(i-1))=start+1.
compute wherew(2,(i-1))=start+nrow(wtmploc)-1+1.
compute start=start+nrow(wtmploc).
loop k1=1 to nxvls.
loop k2 = 1 to nwvls.
compute outv={outv,(xtmp(:,k1)&*wtmp(:,k2))}.
do if (ncs > 0 and wiscov > 0).
compute ccmatoff((i-1),wiscov)=0.
end if.
compute modlabel={modlabel;intlab(cntmp,1)}.
compute intkey={intkey;intlab(cntmp,1),':',xcatlab(k1,1),'x',wcatlab(k2,1),' ',' '}.
compute cntmp=cntmp+1.
end loop.
end loop.
do if (xwtmpus=0).
compute fulldat={fulldat,outv(:,(ncol(outv)-(nxvls*nwvls)+1):ncol(outv))}.
compute xwtmpus=1.
do if (ncs > 0 and wiscov > 0).
compute ccmatoff((i-1),wiscov)=0.
end if.
loop k4=datcount to (datcount+((nwvls*nxvls)-1)).
compute xwtmplo={xwtmplo;k4}.
end loop.
compute xwtmplo=xwtmplo(2:nrow(xwtmplo),1).
compute datcount=datcount+(nxvls*nwvls).
end if.
compute datindx(start:(start+nrow(xwtmplo)-1),(i-1))=xwtmplo.
compute wherexw(1,(i-1))=start+1.
compute wherexw(2,(i-1))=start+nrow(xwtmplo)-1+1.
compute onebl=make(nrow(xwtmplo),1,1).
compute whigh((start+1):(start+nrow(xwtmplo)),whighct)=onebl.
compute start=start+nrow(xwtmplo).
end if.
do if (j > 1 and wcmat(i,j)=1).
do if (wdid=0 and model <> 74).
compute outv={outv,wtmp}.
do if (ncs > 0 and wiscov > 0).
compute ccmatoff((i-1),wiscov)=0.
end if.
compute modlabel={modlabel;wcatlab(1:nwvls,1)}.
compute wdid=1.
do if (wtmpuse=0).
compute fulldat={fulldat,wtmp}.
do if (ncs > 0 and wiscov > 0).
compute ccmatoff((i-1),wiscov)=0.
end if.
compute wtmpuse=1.
loop k4=datcount to (datcount+(nwvls-1)).
compute wtmploc={wtmploc;k4}.
end loop.
compute wtmploc=wtmploc(2:nrow(wtmploc),1).
compute datcount=datcount+nwvls.
end if.
compute datindx(start:(start+nrow(wtmploc)-1),(i-1))=wtmploc.
compute wherew(1,(i-1))=start+1.
compute wherew(2,(i-1))=start+nrow(wtmploc)-1+1.
compute start=start+nrow(wtmploc).
end if.
loop k2 = 1 to nwvls.
compute outv={outv,(mtmp(:,(j-1))&*wtmp(:,k2))}.
do if (ncs > 0 and wiscov > 0).
compute ccmatoff((i-1),wiscov)=0.
end if.
compute modlabel={modlabel;intlab(cntmp,1)}.
compute intkey={intkey;intlab(cntmp,1),':', mnames(1,(j-1)),'x',wcatlab(k2,1),' ',' '}.
compute cntmp=cntmp+1.
end loop.
do if (mwtmpus(1,(j-1))=0).
compute fulldat={fulldat,outv(:,(ncol(outv)-nwvls+1):ncol(outv))}.
do if (ncs > 0 and wiscov > 0).
compute ccmatoff((i-1),wiscov)=0.
end if.
compute mwtmpus(1,(j-1))=1.
compute mw22=-999.
loop k4=datcount to (datcount+(nwvls-1)).
compute mw22={mw22;k4}.
end loop.
compute mwtmplo(:,(j-1))=mw22(2:nrow(mw22),1).
compute datcount=datcount+nwvls.
end if.
compute datindx(start:(start+nrow(mwtmplo)-1),(i-1))=mwtmplo(:,(j-1)).
compute wheremw(((2*j)-3),(i-1))=start+1.
compute wheremw(((2*j)-2),(i-1))=start+nrow(mwtmplo)-1+1.
compute onebl=make(nrow(mwtmplo),1,1).
compute whigh((start+1):(start+nrow(mwtmplo)),whighct)=onebl.
compute start=start+nrow(mwtmplo).
end if.
end loop.
end if.
do if (zsum > 0).
loop j = 1 to (i-1).
compute zhighct=zhighct+1.
do if (j = 1 and zcmat(i,j)=1).
do if (zdid=0).
compute outv={outv,ztmp}.
do if (ncs > 0 and ziscov > 0).
compute ccmatoff((i-1),ziscov)=0.
end if.
compute modlabel={modlabel;zcatlab(1:nzvls,1)}.
compute zdid=1.
do if (ztmpuse=0).
compute fulldat={fulldat,ztmp}.
do if (ncs > 0 and ziscov > 0).
compute ccmatoff((i-1),ziscov)=0.
end if.
compute ztmpuse=1.
loop k4=datcount to (datcount+(nzvls-1)).
compute ztmploc={ztmploc;k4}.
end loop.
compute ztmploc=ztmploc(2:nrow(ztmploc),1).
compute datcount=datcount+nzvls.
end if.
end if.
compute datindx(start:(start+nrow(ztmploc)-1),(i-1))=ztmploc.
compute wherez(1,(i-1))=start+1.
compute wherez(2,(i-1))=start+nrow(ztmploc)-1+1.
compute start=start+nrow(ztmploc).
loop k1=1 to nxvls.
loop k2 = 1 to nzvls.
compute outv={outv,(xtmp(:,k1)&*ztmp(:,k2))}.
do if (ncs > 0 and ziscov > 0).
compute ccmatoff((i-1),ziscov)=0.
end if.
compute modlabel={modlabel;intlab(cntmp,1)}.
compute intkey={intkey;intlab(cntmp,1),':',xcatlab(k1,1),'x',zcatlab(k2,1),' ',' '}.
compute cntmp=cntmp+1.
end loop.
end loop.
do if (xztmpus=0).
compute fulldat={fulldat,outv(:,(ncol(outv)-(nxvls*nzvls)+1):ncol(outv))}.
do if (ncs > 0 and ziscov > 0).
compute ccmatoff((i-1),ziscov)=0.
end if.
compute xztmpus=1.
loop k4=datcount to (datcount+((nzvls*nxvls)-1)).
compute xztmplo={xztmplo;k4}.
end loop.
compute xztmplo=xztmplo(2:nrow(xztmplo),1).
compute datcount=datcount+(nxvls*nzvls).
end if.
compute datindx(start:(start+nrow(xztmplo)-1),(i-1))=xztmplo.
compute wherexz(1,(i-1))=start+1.
compute wherexz(2,(i-1))=start+nrow(xztmplo)-1+1.
compute onebl=make(nrow(xztmplo),1,1).
compute zhigh((start+1):(start+nrow(xztmplo)),zhighct)=onebl.
compute start=start+nrow(xztmplo).
end if.
do if (j > 1 and zcmat(i,j)=1).
do if (zdid=0).
compute outv={outv,ztmp}.
do if (ncs > 0 and ziscov > 0).
compute ccmatoff((i-1),ziscov)=0.
end if.
compute modlabel={modlabel;zcatlab(1:nzvls,1)}.
compute zdid=1.
do if (ztmpuse=0).
compute fulldat={fulldat,ztmp}.
do if (ncs > 0 and ziscov > 0).
compute ccmatoff((i-1),ziscov)=0.
end if.
compute ztmpuse=1.
loop k4=datcount to (datcount+(nzvls-1)).
compute ztmploc={ztmploc;k4}.
end loop.
compute ztmploc=ztmploc(2:nrow(ztmploc),1).
compute datcount=datcount+nzvls.
end if.
compute datindx(start:(start+nrow(ztmploc)-1),(i-1))=ztmploc.
compute wherez(1,(i-1))=start+1.
compute wherez(2,(i-1))=start+nrow(ztmploc)-1+1.
compute start=start+nrow(ztmploc).
end if.
loop k2 = 1 to nzvls.
compute outv={outv,(mtmp(:,(j-1))&*ztmp(:,k2))}.
do if (ncs > 0 and ziscov > 0).
compute ccmatoff((i-1),ziscov)=0.
end if.
compute modlabel={modlabel;intlab(cntmp,1)}.
compute intkey={intkey;intlab(cntmp,1),':', mnames(1,(j-1)),'x',zcatlab(k2,1),' ',' '}.
compute cntmp=cntmp+1.
end loop.
do if (mztmpus(1,(j-1))=0).
compute fulldat={fulldat,outv(:,(ncol(outv)-nzvls+1):ncol(outv))}.
do if (ncs > 0 and ziscov > 0).
compute ccmatoff((i-1),ziscov)=0.
end if.
compute mztmpus(1,(j-1))=1.
compute mz22=-999.
loop k4=datcount to (datcount+(nzvls-1)).
compute mz22={mz22;k4}.
end loop.
compute mztmplo(:,(j-1))=mz22(2:nrow(mz22),1).
compute datcount=datcount+nzvls.
end if.
compute datindx(start:(start+nrow(mztmplo)-1),(i-1))=mztmplo(:,(j-1)).
compute wheremz(((2*j)-3),(i-1))=start+1.
compute wheremz(((2*j)-2),(i-1))=start+nrow(mztmplo)-1+1.
compute onebl=make(nrow(mztmplo),1,1).
compute zhigh((start+1):(start+nrow(mztmplo)),zhighct)=onebl.
compute start=start+nrow(mztmplo).
end if.
end loop.
end if.
do if (wzsum > 0).
loop j = 1 to (i-1).
compute wzhighct=wzhighct+1.
do if (j = 1 and wzcmat(i,j)=1).
do if (wzdid=0).
loop k1=1 to nwvls.
loop k2 = 1 to nzvls.
compute outv={outv,(wtmp(:,k1)&*ztmp(:,k2))}.
do if (ncs > 0 and (ziscov > 0)).
compute ccmatoff((i-1),ziscov)=0.
end if.
do if (ncs > 0 and (wiscov > 0)).
compute ccmatoff((i-1),wiscov)=0.
end if.
compute modlabel={modlabel;intlab(cntmp,1)}.
compute intkey={intkey;intlab(cntmp,1),':',wcatlab(k1,1),'x',zcatlab(k2,1),' ',' '}.
compute cntmp=cntmp+1.
end loop.
end loop.
do if (wztmpus=0).
compute fulldat={fulldat,outv(:,(ncol(outv)-(nwvls*nzvls)+1):ncol(outv))}.
do if (ncs > 0 and (ziscov > 0)).
compute ccmatoff((i-1),ziscov)=0.
end if.
do if (ncs > 0 and (wiscov > 0)).
compute ccmatoff((i-1),wiscov)=0.
end if.
compute wztmpus=1.
loop k4=datcount to (datcount+((nwvls*nzvls)-1)).
compute wztmplo={wztmplo;k4}.
end loop.
compute wztmplo=wztmplo(2:nrow(wztmplo),1).
compute datcount=datcount+(nzvls*nwvls).
end if.
compute wzdid=1.
end if.
compute datindx(start:(start+nrow(wztmplo)-1),(i-1))=wztmplo.
compute wherewz(1,(i-1))=start+1.
compute wherewz(2,(i-1))=start+nrow(wztmplo)-1+1.
compute start=start+nrow(wztmplo).
loop k1=1 to nxvls.
loop k2=1 to nwvls.
loop k3=1 to nzvls.
compute outv={outv,(xtmp(:,k1)&*wtmp(:,k2)&*ztmp(:,k3))}.
do if (ncs > 0 and (ziscov > 0)).
compute ccmatoff((i-1),ziscov)=0.
end if.
do if (ncs > 0 and (wiscov > 0)).
compute ccmatoff((i-1),wiscov)=0.
end if.
compute modlabel={modlabel;intlab(cntmp,1)}.
compute intkey={intkey;intlab(cntmp,1),':',xcatlab(k1,1),'x',wcatlab(k2,1),'x', zcatlab(k3,1)}.
compute cntmp=cntmp+1.
end loop.
end loop.
end loop.
do if (xwztmpu=0).
compute fulldat={fulldat,outv(:,(ncol(outv)-(nxvls*nwvls*nzvls)+1):ncol(outv))}.
do if (ncs > 0 and (ziscov > 0)).
compute ccmatoff((i-1),ziscov)=0.
end if.
do if (ncs > 0 and (wiscov > 0)).
compute ccmatoff((i-1),wiscov)=0.
end if.
compute xwztmpu=1.
loop k4=datcount to (datcount+((nzvls*nxvls*nwvls)-1)).
compute xwztmplo={xwztmplo;k4}.
end loop.
compute xwztmplo=xwztmplo(2:nrow(xwztmplo),1).
compute datcount=datcount+(nxvls*nzvls*nwvls).
end if.
compute datindx(start:(start+nrow(xwztmplo)-1),(i-1))=xwztmplo.
compute wherexwz(1,(i-1))=start+1.
compute wherexwz(2,(i-1))=start+nrow(xwztmplo)-1+1.
compute onebl=make(nrow(xwztmplo),1,1).
compute wzhigh((start+1):(start+nrow(xwztmplo)),wzhighct)=onebl.
compute start=start+nrow(xwztmplo).
end if.
do if (j > 1 and wzcmat(i,j)=1).
do if (wzdid=0).
loop k1=1 to nwvls.
loop k2 = 1 to nzvls.
compute outv={outv,(wtmp(:,k1)&*ztmp(:,k2))}.
do if (ncs > 0 and (ziscov > 0)).
compute ccmatoff((i-1),ziscov)=0.
end if.
do if (ncs > 0 and (wiscov > 0)).
compute ccmatoff((i-1),wiscov)=0.
end if.
compute modlabel={modlabel;intlab(cntmp,1)}.
compute intkey={intkey;intlab(cntmp,1),':',wcatlab(k1,1),'x',zcatlab(k2,1),' ',' '}.
compute cntmp=cntmp+1.
end loop.
end loop.
do if (wztmpus=0).
compute fulldat={fulldat,outv(:,(ncol(outv)-(nwvls*nzvls)+1):ncol(outv))}.
do if (ncs > 0 and (ziscov > 0)).
compute ccmatoff((i-1),ziscov)=0.
end if.
do if (ncs > 0 and (wiscov > 0)).
compute ccmatoff((i-1),wiscov)=0.
end if.
compute wztmpus=1.
loop k4=datcount to (datcount+((nwvls*nzvls)-1)).
compute wztmplo={wztmplo;k4}.
end loop.
compute wztmplo=wztmplo(2:nrow(wztmplo),1).
compute datcount=datcount+(nzvls*nwvls).
end if.
compute wzdid=1.
compute datindx(start:(start+nrow(wztmplo)-1),(i-1))=wztmplo.
compute wherewz(1,(i-1))=start+1.
compute wherewz(2,(i-1))=start+nrow(wztmplo)-1+1.
compute start=start+nrow(wztmplo).
end if.
loop k1 = 1 to nwvls.
loop k2 = 1 to nzvls.
compute outv={outv,(mtmp(:,(j-1))&*wtmp(:,k1)&*ztmp(:,k2))}.
do if (ncs > 0 and (ziscov > 0)).
compute ccmatoff((i-1),ziscov)=0.
end if.
do if (ncs > 0 and (wiscov > 0)).
compute ccmatoff((i-1),wiscov)=0.
end if.
compute modlabel={modlabel;intlab(cntmp,1)}.
compute intkey={intkey;intlab(cntmp,1),':',mnames(1,(j-1)),'x',wcatlab(k1,1),'x', zcatlab(k2,1)}.
compute cntmp=cntmp+1.
end loop.
end loop.
do if (mwztmpu(1,(j-1))=0).
compute fulldat={fulldat,outv(:,(ncol(outv)-(nwvls*nzvls)+1):ncol(outv))}.
do if (ncs > 0 and (ziscov > 0)).
compute ccmatoff((i-1),ziscov)=0.
end if.
do if (ncs > 0 and (wiscov > 0)).
compute ccmatoff((i-1),wiscov)=0.
end if.
compute mwztmpu(1,(j-1))=1.
compute mz22=-999.
loop k4=datcount to (datcount+(nwvls*nzvls)-1).
compute mz22={mz22;k4}.
end loop.
compute mwztmplo(:,(j-1))=mz22(2:nrow(mz22),1).
compute datcount=datcount+(nwvls*nzvls).
end if.
compute datindx(start:(start+nrow(mwztmplo)-1),(i-1))=mwztmplo(:,(j-1)).
compute wheremwz(((2*j)-3),(i-1))=start+1.
compute wheremwz(((2*j)-2),(i-1))=start+nrow(mwztmplo)-1+1.
compute onebl=make(nrow(mwztmplo),1,1).
compute wzhigh((start+1):(start+nrow(mwztmplo)),wzhighct)=onebl.
compute start=start+nrow(mwztmplo).
end if.
end loop.
end if.
do if (ncs > 0).
compute ccmat=ccmat&*ccmatoff.
loop j = 1 to ncs.
do if (ccmat((i-1),j))=1.
do if (j=wiscov).
compute ctmp(:,j)=wtmp.
end if.
do if (j=ziscov).
compute ctmp(:,j)=ztmp.
end if.
compute outv={outv,ctmp(:,j)}.
compute modlabel={modlabel;covnames(1,j)}.
do if (ctmpuse(1,j)=0).
compute fulldat={fulldat,ctmp(:,j)}.
compute ctmpuse(1,j)=1.
compute ctmploc(1,j)=datcount.
compute datcount=datcount+1.
end if.
compute datindx(start:(start+nrow(ctmploc)-1),(i-1))=ctmploc(1,j).
compute start=start+nrow(ctmploc(1,j)).
end if.
end loop.
end if.
compute wdid=0.
compute zdid=0.
compute wzdid=0.
compute vlabs={vlabs;modlabel(2:nrow(modlabel),1)}.
compute numint(1,(i-1))=cntmp-1.
compute nump(1,(i-1))=nrow(modlabel)-1.
end loop.
release datcount, xtmpuse, wtmpuse, ztmpuse, xwtmpus, xztmpus, wztmpus, xwztmpu.
release xtmploc, wtmploc, xwtmplo, ztmploc, xztmplo, wztmplo, xwztmplo, foccnt.
do if (modcok=1 and ((nms > 0) or (zcmat(2,1) <> 1) or (mcx <> 0))).
compute notecode(notes,1) = 19.
compute notes = notes + 1.
compute modcok=0.
end if.
do if ((serial = 1 or (rsum(numint)>0) or nms=0) and mc > 0).
compute notecode(notes,1) = 15.
compute notes = notes + 1.
compute boot=mc.
compute mc=0.
end if.
do if (boot <> 0 or mc <> 0).
compute bootsz=boot.
do if (mc > 0).
compute bootsz=mc.
compute saveboot=0.
end if.
loop.
compute cilow = rnd(bootsz*(1-(conf/100))/2).
compute cihigh = trunc((bootsz*(conf/100)+(bootsz*(1-(conf/100))/2)))+1.
do if (cilow < 1 or cihigh > bootsz).
compute bootsz=trunc((bootsz+1000)/1000)*1000.
compute adjust = 1.
end if.
end loop if (cilow gt 0 and cihigh le bootsz).
do if (boot > 0).
compute boot=bootsz.
end if.
do if (mc > 0).
compute mc=bootsz.
end if.
do if (adjust = 1 and boot > 0).
compute notecode(notes,1) = 8.
compute notes = notes + 1.
end if.
do if (adjust = 1 and mc > 0).
compute notecode(notes,1) = 16.
compute notes = notes + 1.
end if.
end if.
compute maxboot = trunc(2*boot).
do if ( 0 > maxboot).
compute maxboot=trunc( 0 ).
end if.
do if (nms > 0).
release mtmpuse, mwtmpus, mwztmpu, mtmploc, mwtmplo, mztmplo, mwztmplo.
end if.
release wdid, zdid, wzdid, start,modlabel.
compute vlabs=vlabs(2:nrow(vlabs),1).
do if (rsum(numint) > 0).
compute intkey=intkey(2:nrow(intkey),:).
end if.
compute fulldat=fulldat(:,2:ncol(fulldat)).
compute fochigh=fochigh(1:rmax(nump),:).
compute whigh=whigh(1:rmax(nump),:).
compute zhigh=zhigh(1:rmax(nump),:).
compute wzhigh=wzhigh(1:rmax(nump),:).
compute coeffs=fochigh+whigh+zhigh+wzhigh.
compute bootloc=make(rmax(nump),ncol(nump),0).
do if (nms > 0).
compute cntmp=1.
loop i = 1 to ncol(nump).
loop j = 1 to nump(1,i).
compute bootloc(j,i)=cntmp.
compute cntmp=cntmp+1.
end loop.
end loop.
compute fochighb=make(nrow(fochigh),ncol(fochigh),0).
compute whighb=fochighb.
compute zhighb=fochighb.
compute wzhighb=fochighb.
compute thetaxmb=make(nrow(fochighb),nms,0).
compute thetaxyb=make(nrow(fochighb),1,0).
compute pathsfoc=make(nxvls,1,0).
compute cntmp=1.
loop i = 1 to (nms+nys).
loop j = 1 to i.
compute fochighb(:,cntmp)=fochigh(:,cntmp)&*bootloc(:,i).
compute whighb(:,cntmp)=whigh(:,cntmp)&*bootloc(:,i).
compute zhighb(:,cntmp)=zhigh(:,cntmp)&*bootloc(:,i).
compute wzhighb(:,cntmp)=wzhigh(:,cntmp)&*bootloc(:,i).
compute coeffsb=fochighb+whighb+zhighb+wzhighb.
do if ((i < (nms+nys)) and (j = 1)).
compute thetaxmb(:,i)=coeffsb(:,cntmp).
end if.
do if ((i = (nms+nys)) and (j = 1)).
compute thetaxyb(:,1)=coeffsb(:,cntmp).
end if.
compute cntmp=cntmp+1.
end loop.
end loop.
compute thetamyb=coeffsb(:,(ncol(coeffsb)-nms+1):ncol(coeffsb)).
do if (serial = 1).
compute thetammb=make(nrow(coeffsb),((nms*(nms-1))/2),0).
end if.
compute cntmp=1.
do if (nms > 1 and serial = 1).
loop i = 1 to (nms-1).
compute start=((i+2)*(i+1))/2.
loop j = 2 to (nms-i+1).
compute thetammb(:,cntmp)=coeffsb(:,start).
compute start=start+j+i-1.
compute cntmp=cntmp+1.
end loop.
end loop.
end if.
end if.
do if ((total = 1) and (rsum(numint)=0 or (xmint=1))).
compute dototal=1.
do if ((csum(bcmat(:,1)) <> (nms+nys)) or (rsum(bcmat(nrow(bcmat),:)) <> (nms+nys))).
compute dototal=0.
compute alttotal=1.
compute notecode(notes,1) = 12.
compute notes = notes + 1.
end if.
do if (ncs > 0).
do if ((csum(rsum(ccmat))) < (nrow(ccmat)*ncol(ccmat))).
compute dototal=0.
compute alttotal=1.
compute notecode(notes,1) = 11.
compute notes = notes + 1.
end if.
end if.
do if (model=74).
do if (xdich=0 and nxvls=1).
compute dototal=0.
compute alttotal=1.
end if.
do if ((xdich=1 or nxvls > 1) and (ncs > 0) and model=74).
compute dototal=0.
compute alttotal=1.
end if.
end if.
end if.
end if.
do if (criterr=0 and ncs > 0).
do if (rsum((csum(ccmat)=0)) <> 0).
compute errcode(errs,1)=51.
compute errs=errs+1.
compute criterr=1.
end if.
end if.
compute debug= 0.
do if (outscree=1).
print/title = '***************** PROCESS Procedure for SPSS Version 4.2 *****************'.
print/title = '          Written by Andrew F. Hayes, Ph.D.       www.afhayes.com'.
print/title = '    Documentation available in Hayes (2022). www.guilford.com/p/hayes3'/space=0.
end if.
do if (criterr=0).
compute modresid=make(n,1,99999).
do if (stand=1 and ydich=1).
compute stand=0.
end if.
compute anymod2=csum(rsum(wcmat+zcmat+wzcmat)).
do if (anymod2 > 0 and stand = 1).
compute notecode(notes,1) = 27.
compute notes = notes + 1.
compute stand=0.
end if.
compute funny=1.
do if (outscree=1).
print modelvar/title = '**************************************************************************'/format = A8/rnames=modelvlb.
do if (ncs > 0).
print covnames/title='Covariates:'/format=A8.
end if.
print n/title='Sample'/rlabel='Size:'.
do if (( 'random' <> 'random')).
compute seedt= 'random'.
print seedt/title='Custom'/format=A12/rlabel = 'Seed:'.
end if.
end if.
compute maxresm=9.
compute resultm=make(1,maxresm,99999).
do if (describe=1).
compute means=csum(dat)/n.
compute sigmatal = (t(dat)*(ident(n)-(1/n)*ones*t(ones))*dat)*(1/(n-1)).
compute sdvec=sqrt(diag(sigmatal)).
compute sdall = mdiag(1/sdvec).
compute corall=sdall*sigmatal*t(sdall).
compute means={means;t(sdvec)}.
do if (ncol(means) > 9).
compute resultm=make(1,ncol(means),99999).
compute maxresm=ncol(means).
end if.
compute resultm2=make(2,maxresm,99999).
compute resultm2(1:2,1:ncol(means))=means.
compute resultm={resultm;resultm2}.
compute resultm2=make(ncol(corall),maxresm,99999).
compute resultm2(1:ncol(corall),1:ncol(corall))=corall.
compute resultm={resultm;resultm2}.
do if (outscree=1).
print means/title='Variable means and standard deviations'/cnames=varnames/rlabels='Mean','SD'/format= F10.4.
print corall/title='Variable intercorrelations (Pearson r)'/cnames=varnames/rnames=varnames/format= F10.4.
end if.
end if.
do if (outscree=1).
do if (mcxok=1).
compute labtmp={xnames,t(xcatlab(1:nxvls,1))}.
print dummatx/title = 'Coding of categorical X variable for analysis:'/cnames = labtmp/format = F6.3.
end if.
do if (mcwok=1 and xmint=0).
compute labtmp={wnames,t(wcatlab(1:nwvls,1))}.
print dummatw/title = 'Coding of categorical W variable for analysis:'/cnames = labtmp/format = F6.3.
end if.
do if (mczok=1).
compute labtmp={znames,t(zcatlab(1:nzvls,1))}.
print dummatz/title = 'Coding of categorical Z variable for analysis:'/cnames = labtmp/format = F6.3.
end if.
end if.
end if.
do if (criterr = 0).
compute outnames=ynames.
compute outvars=ytmp.
do if (nms > 0).
compute outnames={mnames,ynames}.
compute outvars={mtmp,ytmp}.
compute indcov=make(((nms*2)+(nms*(nxvls-1))),((nms*2)+(nms*(nxvls-1))),0).
compute mcsopath=make(((nms*2)+(nms*(nxvls-1))),1,0).
end if.
compute labstart=1.
compute intstart=1.
compute start=1.
compute coeffmat=make(1,6,0).
compute conseq={'        '}.
compute dfmat=0.
compute coeffcol=0.
compute pathscnt=1.
compute pathscn2=1.
loop i = 1 to (nms+nys).
do if (outscree=1).
print/title = '**************************************************************************'.
end if.
compute highf=make(1,5,0).
compute highf2=highf.
do if ((i = (nms+nys)) and (ydich=1)).
compute highf=make(1,3,0).
compute highf2=highf.
end if.
compute flabel={' '}.
compute y=outvars(:,i).
compute xindx=datindx(1:(nump(1,i)-1),i).
compute x = fulldat(:,xindx).
compute x={ones,x}.
compute xsq=t(x)*x.
compute exsq=eval(xsq).
release xsq.
compute zeroeig=csum(exsq <= 0.000000000002).
do if (outscree=1).
print outnames(1,i)/title = 'OUTCOME VARIABLE:'/format = A8/space=0.
do if (ydich=1 and (i = (nms+nys))).
compute nmsd = {outnames(1,i), 'Analysis'}.
print rcd/title = 'Coding of binary Y for logistic regression analysis:'/cnames = nmsd/format = F9.2.
end if.
end if.
do if (zeroeig > 0).
print / title = 'SINGULAR OR NEAR SINGULAR DATA MATRIX.'.
compute criterr=1.
compute errcode(errs,1)=31.
compute errs=errs+1.
end if.
compute means=csum(x)/n.
compute vlabsm=vlabs(labstart:(labstart+(nump(1,i)-1)),1).
do if (criterr=0).
do if (ydich=0 or (i < (nms+nys))).

.
do if ( 1 =1).
compute hatmat=inv(t( x )* x )*t( x ).
compute b = hatmat* y.
compute modres=b.
do if ( 1 =1).
compute n1=nrow( x ).
compute dfres=n1-(ncol( x )).
compute sstotal = t( y -(csum( y )/n1))*( y -(csum( y )/n1)).
compute resid= y - x *b.
compute ssresid = csum((resid)&**2).
compute r2 = (sstotal-ssresid)/sstotal.
do if (r2 < 0).
compute r2=0.
end if.
compute adjr2 = 1-((1-r2)*(n1-1)/(dfres)).
compute mse=ssresid/(n1-ncol( x )).

.
compute n1=nrow( x ).
compute invXtX = inv(t( x )* x ).
compute varb = mse *invXtX.
compute k3 = ncol( x ).
compute xhc=0.
do if ( hc <> 5).
compute xhc= x.
compute hat = xhc(:,1).
loop i3=1 to nrow(xhc).
compute hat(i3,1)= xhc(i3,:)*invXtX*t(xhc(i3,:)).
end loop.
do if ( hc = 0 or hc =1).
loop i3 = 1 to k3.
compute xhc(:,i3)=xhc(:,i3)&* resid.
end loop.
end if.
do if ( hc =3 or hc =2).
loop i3=1 to k3.
compute xhc(:,i3) = ( resid &/(1-hat)&**(1/(4- hc )))&*xhc(:,i3).
end loop.
end if.
do if ( hc = 4).
compute hcmn=make(n,2,4).
compute hcmn(:,2)=(n1*hat)/k3.
loop i3= 1 to k3.
compute xhc(:,i3) = ( resid &/(1-hat)&**(rmin(hcmn)/2))&*xhc(:,i3).
end loop.
end if.
compute varb=(invXtX*t(xhc)*xhc*invXtX).
do if ( hc =1).
compute varb=(n1/(n1-ncol( x )))&*varb.
end if.
end if.
compute hclab={'se(HC0)','se(HC1)','se(HC2)','se(HC3)','se(HC4)','se'}.
compute hclab=hclab(1,( hc +1)).
compute hcflab={'F(HC0)','F(HC1)','F(HC2)','F(HC3)','F(HC4)','F'}.
compute hcflab=hcflab(1,( hc +1)).
release xhc
.
compute seb=sqrt(diag(varb)).
compute trat = b&/seb.
compute p = 2*(1-tcdf(abs(trat), (dfres))).
compute tval = sqrt(dfres* (exp((dfres-(5/6))*((xp2/(dfres-(2/3)+(.11/dfres)))* (xp2/(dfres-(2/3)+(.11/dfres)))))-1)).
compute modres={modres,seb,trat,p}.
compute modres={modres,(b-tval&*seb),(b+tval&*seb)}.
compute modresl={'coeff',hclab,'t','p','LLCI','ULCI'}.
compute lmat = ident(ncol( x )).
compute lmat = lmat(:,2:ncol(lmat)).
compute fratio = (t(t(lmat)*b)*inv(t(lmat)*varb*lmat)*((t(lmat)*b)))/(ncol( x )-1).
compute pfr = 1-fcdf(fratio,(ncol( x )-1),dfres).
compute modsum={sqrt(r2),r2,mse,fratio,(ncol( x )-1),dfres,pfr}.
compute modsuml={'R','R-sq','MSE',hcflab,'df1','df2', 'p'}.
end if.
end if.
do if ( 1 = 2 or 1 =3).
compute xlp= x.
compute ylp= y.
compute pt2 = make(nrow(ylp),1,(csum(ylp)/nrow(ylp))).
do if ( 1 =2).
compute LL3 = ylp&*ln(pt2)+(1-ylp)&*ln(1-pt2).
end if.
compute LL3 = -2*csum(LL3).
compute bt1 = make(ncol(xlp),1,0).
compute LL1 = 0.
compute pt1 = make(nrow(ylp),1,0.5).
compute pt1lp=pt1.
loop jjj = 1 to iterate.
compute xlptmp=t(xlp).
compute vecprb=pt1lp&*(1-pt1lp).
loop kkk=1 to ncol(xlp).
compute xlptmp(kkk,:)=xlptmp(kkk,:)&*t(vecprb).
end loop.
compute b = bt1+inv(xlptmp*xlp)*t(xlp)*(ylp-pt1lp).
do if ( 1 =2).
compute xlpb=xlp*b.
compute xlpbt=(xlpb > -709.7).
compute xlpb709=(1-xlpbt)*(-709.7).
compute xlpb=(xlpb&*xlpbt)+xlpb709.
compute pt1lp = 1/(1+exp(-(xlpb))).
end if.
compute itprob = csum((pt1lp < .00000001) or (pt1lp > .9999999)).
do if (itprob > 0).
loop kkk = 1 to nrow(pt1lp).
do if (pt1lp(kkk,1) > .9999999).
compute pt1lp(kkk,1) = .9999999.
end if.
do if (pt1lp(kkk,1) < .00000001).
compute pt1lp(kkk,1) = .00000001.
end if.
end loop.
compute itprob = 0.
end if.
do if (itprob = 0).
do if ( 1 =2).
compute LL = ylp&*ln(pt1lp)+(1-ylp)&*ln(1-pt1lp).
end if.
compute LL2 = -2*csum(ll).
end if.
do if (abs(LL1-LL2) < converge).
do if ( 1 =1).
compute xlptmp=t(xlp).
compute vecprb=pt1lp&*(1-pt1lp).
loop kkk=1 to ncol(xlp).
compute xlptmp(kkk,:)=xlptmp(kkk,:)&*t(vecprb).
end loop.
compute varb = inv(xlptmp*xlp).
compute seb = sqrt(diag(varb)).
release xlptmp.
end if.
break.
end if.
compute bt1 = b.
compute LL1 = LL2.
end loop.
compute modres=b.
do if (jjj > iterate).
compute itprob = 2.
do if (booting=0).
compute iterrmod=1.
end if.
do if (booting=1).
compute bootiter=1.
end if.
do if (itprobtg=0).
compute itprobtg=1.
compute errcode(errs,1) = 47.
compute errs = errs + 1.
do if (booting = 0 and 1 =1).
compute vt1 = mdiag(pt1lp&*(1-pt1lp)).
compute varb = inv(t(xlp)*vt1*xlp).
compute seb = sqrt(diag(varb)).
end if.
end if.
end if.
do if ( 1 =1).
compute trat = b&/seb.
compute dfres=nrow(xlp).
compute p = 2*(1-cdfnorm(abs(trat))).
compute modres={modres,seb,trat,p}.
compute modres={modres,(b-xp2&*seb),(b+xp2&*seb)}.
compute pvchi=1-chicdf((LL3-LL2),(nrow(modres)-1)).
compute mcF = (LL3-LL2)/LL3.
compute cox = 1-exp(-(LL3-LL2)/nrow(xlp)).
compute nagel = cox/(1-exp(-(LL3)/nrow(xlp))).
compute modsum={LL2,(LL3-LL2),(nrow(modres)-1),pvchi, mcF,cox,nagel}.
compute modsuml={'-2LL','ModelLL', 'df', 'p', 'McFadden', 'CoxSnell', 'Nagelkrk'}.
compute modresl={'coeff','se','Z','p','LLCI','ULCI'}.
end if.
end if
.
compute dfmatt=make(nrow(modres),1,modsum(1,6)).
compute modresid={modresid,resid}.
end if.
do if (ydich=1 and (i = (nms+nys))).

.
do if ( 2 =1).
compute hatmat=inv(t( x )* x )*t( x ).
compute b = hatmat* y.
compute modres=b.
do if ( 1 =1).
compute n1=nrow( x ).
compute dfres=n1-(ncol( x )).
compute sstotal = t( y -(csum( y )/n1))*( y -(csum( y )/n1)).
compute resid= y - x *b.
compute ssresid = csum((resid)&**2).
compute r2 = (sstotal-ssresid)/sstotal.
do if (r2 < 0).
compute r2=0.
end if.
compute adjr2 = 1-((1-r2)*(n1-1)/(dfres)).
compute mse=ssresid/(n1-ncol( x )).

.
compute n1=nrow( x ).
compute invXtX = inv(t( x )* x ).
compute varb = mse *invXtX.
compute k3 = ncol( x ).
compute xhc=0.
do if ( hc <> 5).
compute xhc= x.
compute hat = xhc(:,1).
loop i3=1 to nrow(xhc).
compute hat(i3,1)= xhc(i3,:)*invXtX*t(xhc(i3,:)).
end loop.
do if ( hc = 0 or hc =1).
loop i3 = 1 to k3.
compute xhc(:,i3)=xhc(:,i3)&* resid.
end loop.
end if.
do if ( hc =3 or hc =2).
loop i3=1 to k3.
compute xhc(:,i3) = ( resid &/(1-hat)&**(1/(4- hc )))&*xhc(:,i3).
end loop.
end if.
do if ( hc = 4).
compute hcmn=make(n,2,4).
compute hcmn(:,2)=(n1*hat)/k3.
loop i3= 1 to k3.
compute xhc(:,i3) = ( resid &/(1-hat)&**(rmin(hcmn)/2))&*xhc(:,i3).
end loop.
end if.
compute varb=(invXtX*t(xhc)*xhc*invXtX).
do if ( hc =1).
compute varb=(n1/(n1-ncol( x )))&*varb.
end if.
end if.
compute hclab={'se(HC0)','se(HC1)','se(HC2)','se(HC3)','se(HC4)','se'}.
compute hclab=hclab(1,( hc +1)).
compute hcflab={'F(HC0)','F(HC1)','F(HC2)','F(HC3)','F(HC4)','F'}.
compute hcflab=hcflab(1,( hc +1)).
release xhc
.
compute seb=sqrt(diag(varb)).
compute trat = b&/seb.
compute p = 2*(1-tcdf(abs(trat), (dfres))).
compute tval = sqrt(dfres* (exp((dfres-(5/6))*((xp2/(dfres-(2/3)+(.11/dfres)))* (xp2/(dfres-(2/3)+(.11/dfres)))))-1)).
compute modres={modres,seb,trat,p}.
compute modres={modres,(b-tval&*seb),(b+tval&*seb)}.
compute modresl={'coeff',hclab,'t','p','LLCI','ULCI'}.
compute lmat = ident(ncol( x )).
compute lmat = lmat(:,2:ncol(lmat)).
compute fratio = (t(t(lmat)*b)*inv(t(lmat)*varb*lmat)*((t(lmat)*b)))/(ncol( x )-1).
compute pfr = 1-fcdf(fratio,(ncol( x )-1),dfres).
compute modsum={sqrt(r2),r2,mse,fratio,(ncol( x )-1),dfres,pfr}.
compute modsuml={'R','R-sq','MSE',hcflab,'df1','df2', 'p'}.
end if.
end if.
do if ( 2 = 2 or 2 =3).
compute xlp= x.
compute ylp= y.
compute pt2 = make(nrow(ylp),1,(csum(ylp)/nrow(ylp))).
do if ( 2 =2).
compute LL3 = ylp&*ln(pt2)+(1-ylp)&*ln(1-pt2).
end if.
compute LL3 = -2*csum(LL3).
compute bt1 = make(ncol(xlp),1,0).
compute LL1 = 0.
compute pt1 = make(nrow(ylp),1,0.5).
compute pt1lp=pt1.
loop jjj = 1 to iterate.
compute xlptmp=t(xlp).
compute vecprb=pt1lp&*(1-pt1lp).
loop kkk=1 to ncol(xlp).
compute xlptmp(kkk,:)=xlptmp(kkk,:)&*t(vecprb).
end loop.
compute b = bt1+inv(xlptmp*xlp)*t(xlp)*(ylp-pt1lp).
do if ( 2 =2).
compute xlpb=xlp*b.
compute xlpbt=(xlpb > -709.7).
compute xlpb709=(1-xlpbt)*(-709.7).
compute xlpb=(xlpb&*xlpbt)+xlpb709.
compute pt1lp = 1/(1+exp(-(xlpb))).
end if.
compute itprob = csum((pt1lp < .00000001) or (pt1lp > .9999999)).
do if (itprob > 0).
loop kkk = 1 to nrow(pt1lp).
do if (pt1lp(kkk,1) > .9999999).
compute pt1lp(kkk,1) = .9999999.
end if.
do if (pt1lp(kkk,1) < .00000001).
compute pt1lp(kkk,1) = .00000001.
end if.
end loop.
compute itprob = 0.
end if.
do if (itprob = 0).
do if ( 2 =2).
compute LL = ylp&*ln(pt1lp)+(1-ylp)&*ln(1-pt1lp).
end if.
compute LL2 = -2*csum(ll).
end if.
do if (abs(LL1-LL2) < converge).
do if ( 1 =1).
compute xlptmp=t(xlp).
compute vecprb=pt1lp&*(1-pt1lp).
loop kkk=1 to ncol(xlp).
compute xlptmp(kkk,:)=xlptmp(kkk,:)&*t(vecprb).
end loop.
compute varb = inv(xlptmp*xlp).
compute seb = sqrt(diag(varb)).
release xlptmp.
end if.
break.
end if.
compute bt1 = b.
compute LL1 = LL2.
end loop.
compute modres=b.
do if (jjj > iterate).
compute itprob = 2.
do if (booting=0).
compute iterrmod=1.
end if.
do if (booting=1).
compute bootiter=1.
end if.
do if (itprobtg=0).
compute itprobtg=1.
compute errcode(errs,1) = 47.
compute errs = errs + 1.
do if (booting = 0 and 1 =1).
compute vt1 = mdiag(pt1lp&*(1-pt1lp)).
compute varb = inv(t(xlp)*vt1*xlp).
compute seb = sqrt(diag(varb)).
end if.
end if.
end if.
do if ( 1 =1).
compute trat = b&/seb.
compute dfres=nrow(xlp).
compute p = 2*(1-cdfnorm(abs(trat))).
compute modres={modres,seb,trat,p}.
compute modres={modres,(b-xp2&*seb),(b+xp2&*seb)}.
compute pvchi=1-chicdf((LL3-LL2),(nrow(modres)-1)).
compute mcF = (LL3-LL2)/LL3.
compute cox = 1-exp(-(LL3-LL2)/nrow(xlp)).
compute nagel = cox/(1-exp(-(LL3)/nrow(xlp))).
compute modsum={LL2,(LL3-LL2),(nrow(modres)-1),pvchi, mcF,cox,nagel}.
compute modsuml={'-2LL','ModelLL', 'df', 'p', 'McFadden', 'CoxSnell', 'Nagelkrk'}.
compute modresl={'coeff','se','Z','p','LLCI','ULCI'}.
end if.
end if
.
compute dfmatt=make(nrow(modres),1,-999).
end if.
compute resultm2=make(1,maxresm,99999).
compute resultm2(1,1:ncol(modsum))=modsum.
compute resultm={resultm;resultm2}.
compute resultm2=make(nrow(modres),maxresm,99999).
compute resultm2(1:nrow(modres),1:ncol(modres))=modres.
compute resultm={resultm;resultm2}.
compute obscoeff={obscoeff,t(b)}.
do if (outscree=1).
print modsum/title = 'Model Summary'/cnames = modsuml/format= F10.4.
print modres/title='Model'/rnames=vlabsm/cnames=modresl/format= F10.4.
end if.
compute basemod=modsum(1,1).
compute basemodx=basemod.
do if (ydich=1 and (i = (nms+nys))).
do if (outscree=1).
print/title='These results are expressed in a log-odds metric.'.
end if.
compute notecode(notes,1) = 26.
compute notes = notes + 1.
end if.
compute coeffmat={coeffmat;modres}.
compute conseqt=make(nrow(modres),1,outnames(1,i)).
compute conseq={conseq;conseqt}.
compute dfmat={dfmat;dfmatt}.
compute labstart=labstart+nump(1,i).
do if (stand=1).
compute predsd=make(nrow(modres),1,0).
compute stdmod=modres(:,1)&/ovsd(1,i).
loop jd=1 to ncol(x).
compute descdat=x(:,jd).
compute predsd(jd,1) = (nrow(descdat)*sscp(descdat))-(t(csum(descdat))*(csum(descdat))).
compute predsd(jd,1) = sqrt(predsd(jd,1)/(nrow(descdat)*(nrow(descdat)-1))).
end loop.
do if (wherex(1,i) <> -999 and ((nxvls > 1) or (xdich=1))).
compute sdmsone=make(nxvls,1,1).
compute predsd(wherex(1,i):wherex(2,i),1)=sdmsone.
compute pstog=1.
end if.
compute predsd(1,1)=1.
compute stdmod=stdmod&*predsd.
compute stdmod=stdmod(2:nrow(stdmod),1).
compute sdvlabs=vlabsm(2:nrow(vlabsm),1).
compute resultm2=make(nrow(stdmod),maxresm,99999).
compute resultm2(1:nrow(stdmod),1:ncol(stdmod))=stdmod.
compute resultm={resultm;resultm2}.
do if (outscree=1).
print stdmod/title='Standardized coefficients'/clabels='coeff'/rnames=sdvlabs/format= F10.4.
end if.
end if.
do if (nms > 0 and serial = 0 and (rsum(numint) = 0) and (normal=1 or mc > 0)).
do if (i < (nms+nys)).
compute indcov((((i-1)*nxvls)+1):(i*nxvls),(((i-1)*nxvls)+1):(i*nxvls))=varb(2:(1+nxvls), 2:(1+nxvls)).
compute mcsopath((((i-1)*nxvls)+1):(i*nxvls) ,1)=modres(2:(1+nxvls),1).
end if.
do if (i = (nms+nys)).
compute atm=ncol(wherem).
compute indcov(((nms*nxvls)+1):nrow(mcsopath),((nms*nxvls)+1):nrow(mcsopath))= varb(wherem(1,atm):(wherem(1,atm)+nms-1),wherem(1,atm):(wherem(1,atm)+nms-1)).
compute mcsopath(((nms*nxvls)+1):nrow(mcsopath),1)=modres(wherem(1,atm):(wherem(1,atm)+ nms-1),1).
compute sobelok=1.
end if.
end if.
compute obsdirfx=make(1,nxvls,0).
compute dirzes=make(1,nxvls,0).
do if ((i = (nms+nys)) and (bcmat(nrow(bcmat),1)=1)).
compute direff=modres(2:(1+nxvls),:).
compute obsdirfx=t(direff(:,1)).
compute direfflb=modresl.
compute direffl2=vlabsm(2:(1+nxvls),:).
compute lmat=make(nrow(b),1,0).
compute lmat2=make(nxvls,1,1).
compute lmat(2:(1+nxvls),1)=lmat2.
do if (ydich <> 1).

.
compute lmat2= lmat.
do if ( 0 =0).
compute lmat2 = mdiag( lmat ).
compute lmat3=make(nrow(lmat2),1,0).
loop flp=1 to ncol(lmat2).
do if (csum(lmat2(:,flp))=1).
compute lmat3={lmat3,lmat2(:,flp)}.
end if.
end loop.
compute lmat2=lmat3(:,2:ncol(lmat3)).
end if.
compute fratio = (t(t(lmat2)* b )*inv(t(lmat2)* varb *lmat2)*((t(lmat2)* b )))/ncol(lmat2).
compute pfr = 1-fcdf(fratio,ncol(lmat2),(n-nrow( b ))).
compute fresult={fratio,ncol(lmat2),(n-nrow( b )),pfr}.
do if (i = (nms+nys) and (ydich=1)).
compute fratio=fratio*ncol(lmat2).
compute pfr=1-chicdf(fratio,ncol(lmat2)).
compute fresult={fratio,ncol(lmat2),pfr}.
end if.
do if ( 1 =1).
compute lmat3=1-rsum(lmat2).
compute xfm=make(n,csum(lmat3),0).
compute flpc=1.
loop flp=1 to nrow(lmat3).
do if (lmat3(flp,1)=1).
compute xfm(:,flpc)=x(:,flp).
compute flpc=flpc+1.
end if.
end loop.
compute bfm=inv(t(xfm)*xfm)*t(xfm)*y.
compute resid=y-(xfm*bfm).
compute sstotal=(y-(csum(y)/n)).
compute sstotal=csum(sstotal&*sstotal).
compute ssresid=csum(resid&*resid).
compute rsqch= r2 -((sstotal-ssresid)/sstotal).
compute fresult={rsqch,fresult}.
release xfm,flpc, resid, ssresid, bfm.
end if
.
compute diromni=fresult.
end if.
do if (ydich = 1 ).

.
compute btemphld=b.
compute llrdat=make(nrow(x),nrow( lmat )-csum( lmat ),-999).
compute llrdf=ncol(x)-ncol(llrdat).
compute llrcnt=0.
loop llri=1 to nrow( lmat ).
do if ( lmat (llri,1)=0).
compute llrcnt=llrcnt+1.
compute llrdat(:,llrcnt)=x(:,llri).
end if.
end loop.

.
do if ( 2 =1).
compute hatmat=inv(t( llrdat )* llrdat )*t( llrdat ).
compute b = hatmat* y.
compute modres=b.
do if ( 0 =1).
compute n1=nrow( llrdat ).
compute dfres=n1-(ncol( llrdat )).
compute sstotal = t( y -(csum( y )/n1))*( y -(csum( y )/n1)).
compute resid= y - llrdat *b.
compute ssresid = csum((resid)&**2).
compute r2 = (sstotal-ssresid)/sstotal.
do if (r2 < 0).
compute r2=0.
end if.
compute adjr2 = 1-((1-r2)*(n1-1)/(dfres)).
compute mse=ssresid/(n1-ncol( llrdat )).

.
compute n1=nrow( x ).
compute invXtX = inv(t( x )* x ).
compute varb = mse *invXtX.
compute k3 = ncol( x ).
compute xhc=0.
do if ( hc <> 5).
compute xhc= x.
compute hat = xhc(:,1).
loop i3=1 to nrow(xhc).
compute hat(i3,1)= xhc(i3,:)*invXtX*t(xhc(i3,:)).
end loop.
do if ( hc = 0 or hc =1).
loop i3 = 1 to k3.
compute xhc(:,i3)=xhc(:,i3)&* resid.
end loop.
end if.
do if ( hc =3 or hc =2).
loop i3=1 to k3.
compute xhc(:,i3) = ( resid &/(1-hat)&**(1/(4- hc )))&*xhc(:,i3).
end loop.
end if.
do if ( hc = 4).
compute hcmn=make(n,2,4).
compute hcmn(:,2)=(n1*hat)/k3.
loop i3= 1 to k3.
compute xhc(:,i3) = ( resid &/(1-hat)&**(rmin(hcmn)/2))&*xhc(:,i3).
end loop.
end if.
compute varb=(invXtX*t(xhc)*xhc*invXtX).
do if ( hc =1).
compute varb=(n1/(n1-ncol( x )))&*varb.
end if.
end if.
compute hclab={'se(HC0)','se(HC1)','se(HC2)','se(HC3)','se(HC4)','se'}.
compute hclab=hclab(1,( hc +1)).
compute hcflab={'F(HC0)','F(HC1)','F(HC2)','F(HC3)','F(HC4)','F'}.
compute hcflab=hcflab(1,( hc +1)).
release xhc
.
compute seb=sqrt(diag(varb)).
compute trat = b&/seb.
compute p = 2*(1-tcdf(abs(trat), (dfres))).
compute tval = sqrt(dfres* (exp((dfres-(5/6))*((xp2/(dfres-(2/3)+(.11/dfres)))* (xp2/(dfres-(2/3)+(.11/dfres)))))-1)).
compute modres={modres,seb,trat,p}.
compute modres={modres,(b-tval&*seb),(b+tval&*seb)}.
compute modresl={'coeff',hclab,'t','p','LLCI','ULCI'}.
compute lmat = ident(ncol( llrdat )).
compute lmat = lmat(:,2:ncol(lmat)).
compute fratio = (t(t(lmat)*b)*inv(t(lmat)*varb*lmat)*((t(lmat)*b)))/(ncol( llrdat )-1).
compute pfr = 1-fcdf(fratio,(ncol( llrdat )-1),dfres).
compute modsum={sqrt(r2),r2,mse,fratio,(ncol( llrdat )-1),dfres,pfr}.
compute modsuml={'R','R-sq','MSE',hcflab,'df1','df2', 'p'}.
end if.
end if.
do if ( 2 = 2 or 2 =3).
compute xlp= llrdat.
compute ylp= y.
compute pt2 = make(nrow(ylp),1,(csum(ylp)/nrow(ylp))).
do if ( 2 =2).
compute LL3 = ylp&*ln(pt2)+(1-ylp)&*ln(1-pt2).
end if.
compute LL3 = -2*csum(LL3).
compute bt1 = make(ncol(xlp),1,0).
compute LL1 = 0.
compute pt1 = make(nrow(ylp),1,0.5).
compute pt1lp=pt1.
loop jjj = 1 to iterate.
compute xlptmp=t(xlp).
compute vecprb=pt1lp&*(1-pt1lp).
loop kkk=1 to ncol(xlp).
compute xlptmp(kkk,:)=xlptmp(kkk,:)&*t(vecprb).
end loop.
compute b = bt1+inv(xlptmp*xlp)*t(xlp)*(ylp-pt1lp).
do if ( 2 =2).
compute xlpb=xlp*b.
compute xlpbt=(xlpb > -709.7).
compute xlpb709=(1-xlpbt)*(-709.7).
compute xlpb=(xlpb&*xlpbt)+xlpb709.
compute pt1lp = 1/(1+exp(-(xlpb))).
end if.
compute itprob = csum((pt1lp < .00000001) or (pt1lp > .9999999)).
do if (itprob > 0).
loop kkk = 1 to nrow(pt1lp).
do if (pt1lp(kkk,1) > .9999999).
compute pt1lp(kkk,1) = .9999999.
end if.
do if (pt1lp(kkk,1) < .00000001).
compute pt1lp(kkk,1) = .00000001.
end if.
end loop.
compute itprob = 0.
end if.
do if (itprob = 0).
do if ( 2 =2).
compute LL = ylp&*ln(pt1lp)+(1-ylp)&*ln(1-pt1lp).
end if.
compute LL2 = -2*csum(ll).
end if.
do if (abs(LL1-LL2) < converge).
do if ( 0 =1).
compute xlptmp=t(xlp).
compute vecprb=pt1lp&*(1-pt1lp).
loop kkk=1 to ncol(xlp).
compute xlptmp(kkk,:)=xlptmp(kkk,:)&*t(vecprb).
end loop.
compute varb = inv(xlptmp*xlp).
compute seb = sqrt(diag(varb)).
release xlptmp.
end if.
break.
end if.
compute bt1 = b.
compute LL1 = LL2.
end loop.
compute modres=b.
do if (jjj > iterate).
compute itprob = 2.
do if (booting=0).
compute iterrmod=1.
end if.
do if (booting=1).
compute bootiter=1.
end if.
do if (itprobtg=0).
compute itprobtg=1.
compute errcode(errs,1) = 47.
compute errs = errs + 1.
do if (booting = 0 and 0 =1).
compute vt1 = mdiag(pt1lp&*(1-pt1lp)).
compute varb = inv(t(xlp)*vt1*xlp).
compute seb = sqrt(diag(varb)).
end if.
end if.
end if.
do if ( 0 =1).
compute trat = b&/seb.
compute dfres=nrow(xlp).
compute p = 2*(1-cdfnorm(abs(trat))).
compute modres={modres,seb,trat,p}.
compute modres={modres,(b-xp2&*seb),(b+xp2&*seb)}.
compute pvchi=1-chicdf((LL3-LL2),(nrow(modres)-1)).
compute mcF = (LL3-LL2)/LL3.
compute cox = 1-exp(-(LL3-LL2)/nrow(xlp)).
compute nagel = cox/(1-exp(-(LL3)/nrow(xlp))).
compute modsum={LL2,(LL3-LL2),(nrow(modres)-1),pvchi, mcF,cox,nagel}.
compute modsuml={'-2LL','ModelLL', 'df', 'p', 'McFadden', 'CoxSnell', 'Nagelkrk'}.
compute modresl={'coeff','se','Z','p','LLCI','ULCI'}.
end if.
end if
.
compute b=btemphld.
compute fresult={(LL2-basemod),llrdf,1-chicdf((LL2-basemod),llrdf)}
.
compute diromni=fresult.
end if.
end if.
do if (numint(1,i) > 0).
compute intkeym=intkey(intstart:(intstart+numint(1,i)-1),:).
do if (outscree=1).
print intkeym/title='Product terms key:'/format = A8.
end if.
end if.
do if (covcoeff=1).
do if (outscree=1).
print varb/title='Covariance matrix of regression parameter estimates:'/rnames=vlabsm /cnames=vlabsm/format= F10.4.
end if.
compute resultm2=make(nrow(varb),maxresm,99999).
do if (ncol(varb) <= maxresm).
compute resultm2(1:nrow(varb),1:ncol(varb))=varb.
compute resultm={resultm;resultm2}.
end if.
do if (ncol(varb) > maxresm).
compute resultmt=make(nrow(resultm),ncol(varb),99999).
compute resultmt(1:nrow(resultm),1:ncol(resultm))=resultm.
compute resultm=resultmt.
compute resultm2=make(nrow(varb),ncol(resultm),99999).
compute resultm2(1:nrow(varb),1:ncol(varb))=varb.
compute resultm={resultm;resultm2}.
compute maxresm=ncol(resultm).
end if.
end if.
do if (model <> 74 and xmtest=1 and nms > 0).
compute r2tmp=r2.
compute btmp=b.
compute varbtmp=varb.
compute dfrestmp=dfres.
compute tvaltmp=tval.
compute xmtst=make(nms,4,0).
compute xmtstlbc={hcflab,'df1','df2','p'}.
do if ((i=(nms+nys)) and (ydich=1)).
compute xmtst=make(nms,3,0).
compute xmtstlbc={'Chi-sq','df','p'}.
end if.
compute xmtstlb={' '}.
compute xmtmat=x.
compute numxint=0.
loop xmints=2 to i.
compute x=xmtmat.
do if ((bcmat((i+1),xmints)=1) and (wzcmat((i+1),xmints) <> 1)).
do if (bcmat((i+1),1)=0).
compute x={xmtmat,xtmp}.
do if ((ydich=1) and (i=(nms+nys))).

.
do if ( 2 =1).
compute hatmat=inv(t( x )* x )*t( x ).
compute b = hatmat* y.
compute modres=b.
do if ( 1 =1).
compute n1=nrow( x ).
compute dfres=n1-(ncol( x )).
compute sstotal = t( y -(csum( y )/n1))*( y -(csum( y )/n1)).
compute resid= y - x *b.
compute ssresid = csum((resid)&**2).
compute r2 = (sstotal-ssresid)/sstotal.
do if (r2 < 0).
compute r2=0.
end if.
compute adjr2 = 1-((1-r2)*(n1-1)/(dfres)).
compute mse=ssresid/(n1-ncol( x )).

.
compute n1=nrow( x ).
compute invXtX = inv(t( x )* x ).
compute varb = mse *invXtX.
compute k3 = ncol( x ).
compute xhc=0.
do if ( hc <> 5).
compute xhc= x.
compute hat = xhc(:,1).
loop i3=1 to nrow(xhc).
compute hat(i3,1)= xhc(i3,:)*invXtX*t(xhc(i3,:)).
end loop.
do if ( hc = 0 or hc =1).
loop i3 = 1 to k3.
compute xhc(:,i3)=xhc(:,i3)&* resid.
end loop.
end if.
do if ( hc =3 or hc =2).
loop i3=1 to k3.
compute xhc(:,i3) = ( resid &/(1-hat)&**(1/(4- hc )))&*xhc(:,i3).
end loop.
end if.
do if ( hc = 4).
compute hcmn=make(n,2,4).
compute hcmn(:,2)=(n1*hat)/k3.
loop i3= 1 to k3.
compute xhc(:,i3) = ( resid &/(1-hat)&**(rmin(hcmn)/2))&*xhc(:,i3).
end loop.
end if.
compute varb=(invXtX*t(xhc)*xhc*invXtX).
do if ( hc =1).
compute varb=(n1/(n1-ncol( x )))&*varb.
end if.
end if.
compute hclab={'se(HC0)','se(HC1)','se(HC2)','se(HC3)','se(HC4)','se'}.
compute hclab=hclab(1,( hc +1)).
compute hcflab={'F(HC0)','F(HC1)','F(HC2)','F(HC3)','F(HC4)','F'}.
compute hcflab=hcflab(1,( hc +1)).
release xhc
.
compute seb=sqrt(diag(varb)).
compute trat = b&/seb.
compute p = 2*(1-tcdf(abs(trat), (dfres))).
compute tval = sqrt(dfres* (exp((dfres-(5/6))*((xp2/(dfres-(2/3)+(.11/dfres)))* (xp2/(dfres-(2/3)+(.11/dfres)))))-1)).
compute modres={modres,seb,trat,p}.
compute modres={modres,(b-tval&*seb),(b+tval&*seb)}.
compute modresl={'coeff',hclab,'t','p','LLCI','ULCI'}.
compute lmat = ident(ncol( x )).
compute lmat = lmat(:,2:ncol(lmat)).
compute fratio = (t(t(lmat)*b)*inv(t(lmat)*varb*lmat)*((t(lmat)*b)))/(ncol( x )-1).
compute pfr = 1-fcdf(fratio,(ncol( x )-1),dfres).
compute modsum={sqrt(r2),r2,mse,fratio,(ncol( x )-1),dfres,pfr}.
compute modsuml={'R','R-sq','MSE',hcflab,'df1','df2', 'p'}.
end if.
end if.
do if ( 2 = 2 or 2 =3).
compute xlp= x.
compute ylp= y.
compute pt2 = make(nrow(ylp),1,(csum(ylp)/nrow(ylp))).
do if ( 2 =2).
compute LL3 = ylp&*ln(pt2)+(1-ylp)&*ln(1-pt2).
end if.
compute LL3 = -2*csum(LL3).
compute bt1 = make(ncol(xlp),1,0).
compute LL1 = 0.
compute pt1 = make(nrow(ylp),1,0.5).
compute pt1lp=pt1.
loop jjj = 1 to iterate.
compute xlptmp=t(xlp).
compute vecprb=pt1lp&*(1-pt1lp).
loop kkk=1 to ncol(xlp).
compute xlptmp(kkk,:)=xlptmp(kkk,:)&*t(vecprb).
end loop.
compute b = bt1+inv(xlptmp*xlp)*t(xlp)*(ylp-pt1lp).
do if ( 2 =2).
compute xlpb=xlp*b.
compute xlpbt=(xlpb > -709.7).
compute xlpb709=(1-xlpbt)*(-709.7).
compute xlpb=(xlpb&*xlpbt)+xlpb709.
compute pt1lp = 1/(1+exp(-(xlpb))).
end if.
compute itprob = csum((pt1lp < .00000001) or (pt1lp > .9999999)).
do if (itprob > 0).
loop kkk = 1 to nrow(pt1lp).
do if (pt1lp(kkk,1) > .9999999).
compute pt1lp(kkk,1) = .9999999.
end if.
do if (pt1lp(kkk,1) < .00000001).
compute pt1lp(kkk,1) = .00000001.
end if.
end loop.
compute itprob = 0.
end if.
do if (itprob = 0).
do if ( 2 =2).
compute LL = ylp&*ln(pt1lp)+(1-ylp)&*ln(1-pt1lp).
end if.
compute LL2 = -2*csum(ll).
end if.
do if (abs(LL1-LL2) < converge).
do if ( 1 =1).
compute xlptmp=t(xlp).
compute vecprb=pt1lp&*(1-pt1lp).
loop kkk=1 to ncol(xlp).
compute xlptmp(kkk,:)=xlptmp(kkk,:)&*t(vecprb).
end loop.
compute varb = inv(xlptmp*xlp).
compute seb = sqrt(diag(varb)).
release xlptmp.
end if.
break.
end if.
compute bt1 = b.
compute LL1 = LL2.
end loop.
compute modres=b.
do if (jjj > iterate).
compute itprob = 2.
do if (booting=0).
compute iterrmod=1.
end if.
do if (booting=1).
compute bootiter=1.
end if.
do if (itprobtg=0).
compute itprobtg=1.
compute errcode(errs,1) = 47.
compute errs = errs + 1.
do if (booting = 0 and 1 =1).
compute vt1 = mdiag(pt1lp&*(1-pt1lp)).
compute varb = inv(t(xlp)*vt1*xlp).
compute seb = sqrt(diag(varb)).
end if.
end if.
end if.
do if ( 1 =1).
compute trat = b&/seb.
compute dfres=nrow(xlp).
compute p = 2*(1-cdfnorm(abs(trat))).
compute modres={modres,seb,trat,p}.
compute modres={modres,(b-xp2&*seb),(b+xp2&*seb)}.
compute pvchi=1-chicdf((LL3-LL2),(nrow(modres)-1)).
compute mcF = (LL3-LL2)/LL3.
compute cox = 1-exp(-(LL3-LL2)/nrow(xlp)).
compute nagel = cox/(1-exp(-(LL3)/nrow(xlp))).
compute modsum={LL2,(LL3-LL2),(nrow(modres)-1),pvchi, mcF,cox,nagel}.
compute modsuml={'-2LL','ModelLL', 'df', 'p', 'McFadden', 'CoxSnell', 'Nagelkrk'}.
compute modresl={'coeff','se','Z','p','LLCI','ULCI'}.
end if.
end if
.
compute basemodx=LL2.
end if.
end if.
loop xmtlp1=1 to nxvls.
compute x={x,xtmp(:,xmtlp1)&*(mtmp(:,(xmints-1))-(csum(mtmp(:,(xmints-1)))/nrow(mtmp)) )}.
end loop.
do if ((i < (nms+nys)) or (ydich=0)).

.
do if ( 1 =1).
compute hatmat=inv(t( x )* x )*t( x ).
compute b = hatmat* y.
compute modres=b.
do if ( 1 =1).
compute n1=nrow( x ).
compute dfres=n1-(ncol( x )).
compute sstotal = t( y -(csum( y )/n1))*( y -(csum( y )/n1)).
compute resid= y - x *b.
compute ssresid = csum((resid)&**2).
compute r2 = (sstotal-ssresid)/sstotal.
do if (r2 < 0).
compute r2=0.
end if.
compute adjr2 = 1-((1-r2)*(n1-1)/(dfres)).
compute mse=ssresid/(n1-ncol( x )).

.
compute n1=nrow( x ).
compute invXtX = inv(t( x )* x ).
compute varb = mse *invXtX.
compute k3 = ncol( x ).
compute xhc=0.
do if ( hc <> 5).
compute xhc= x.
compute hat = xhc(:,1).
loop i3=1 to nrow(xhc).
compute hat(i3,1)= xhc(i3,:)*invXtX*t(xhc(i3,:)).
end loop.
do if ( hc = 0 or hc =1).
loop i3 = 1 to k3.
compute xhc(:,i3)=xhc(:,i3)&* resid.
end loop.
end if.
do if ( hc =3 or hc =2).
loop i3=1 to k3.
compute xhc(:,i3) = ( resid &/(1-hat)&**(1/(4- hc )))&*xhc(:,i3).
end loop.
end if.
do if ( hc = 4).
compute hcmn=make(n,2,4).
compute hcmn(:,2)=(n1*hat)/k3.
loop i3= 1 to k3.
compute xhc(:,i3) = ( resid &/(1-hat)&**(rmin(hcmn)/2))&*xhc(:,i3).
end loop.
end if.
compute varb=(invXtX*t(xhc)*xhc*invXtX).
do if ( hc =1).
compute varb=(n1/(n1-ncol( x )))&*varb.
end if.
end if.
compute hclab={'se(HC0)','se(HC1)','se(HC2)','se(HC3)','se(HC4)','se'}.
compute hclab=hclab(1,( hc +1)).
compute hcflab={'F(HC0)','F(HC1)','F(HC2)','F(HC3)','F(HC4)','F'}.
compute hcflab=hcflab(1,( hc +1)).
release xhc
.
compute seb=sqrt(diag(varb)).
compute trat = b&/seb.
compute p = 2*(1-tcdf(abs(trat), (dfres))).
compute tval = sqrt(dfres* (exp((dfres-(5/6))*((xp2/(dfres-(2/3)+(.11/dfres)))* (xp2/(dfres-(2/3)+(.11/dfres)))))-1)).
compute modres={modres,seb,trat,p}.
compute modres={modres,(b-tval&*seb),(b+tval&*seb)}.
compute modresl={'coeff',hclab,'t','p','LLCI','ULCI'}.
compute lmat = ident(ncol( x )).
compute lmat = lmat(:,2:ncol(lmat)).
compute fratio = (t(t(lmat)*b)*inv(t(lmat)*varb*lmat)*((t(lmat)*b)))/(ncol( x )-1).
compute pfr = 1-fcdf(fratio,(ncol( x )-1),dfres).
compute modsum={sqrt(r2),r2,mse,fratio,(ncol( x )-1),dfres,pfr}.
compute modsuml={'R','R-sq','MSE',hcflab,'df1','df2', 'p'}.
end if.
end if.
do if ( 1 = 2 or 1 =3).
compute xlp= x.
compute ylp= y.
compute pt2 = make(nrow(ylp),1,(csum(ylp)/nrow(ylp))).
do if ( 1 =2).
compute LL3 = ylp&*ln(pt2)+(1-ylp)&*ln(1-pt2).
end if.
compute LL3 = -2*csum(LL3).
compute bt1 = make(ncol(xlp),1,0).
compute LL1 = 0.
compute pt1 = make(nrow(ylp),1,0.5).
compute pt1lp=pt1.
loop jjj = 1 to iterate.
compute xlptmp=t(xlp).
compute vecprb=pt1lp&*(1-pt1lp).
loop kkk=1 to ncol(xlp).
compute xlptmp(kkk,:)=xlptmp(kkk,:)&*t(vecprb).
end loop.
compute b = bt1+inv(xlptmp*xlp)*t(xlp)*(ylp-pt1lp).
do if ( 1 =2).
compute xlpb=xlp*b.
compute xlpbt=(xlpb > -709.7).
compute xlpb709=(1-xlpbt)*(-709.7).
compute xlpb=(xlpb&*xlpbt)+xlpb709.
compute pt1lp = 1/(1+exp(-(xlpb))).
end if.
compute itprob = csum((pt1lp < .00000001) or (pt1lp > .9999999)).
do if (itprob > 0).
loop kkk = 1 to nrow(pt1lp).
do if (pt1lp(kkk,1) > .9999999).
compute pt1lp(kkk,1) = .9999999.
end if.
do if (pt1lp(kkk,1) < .00000001).
compute pt1lp(kkk,1) = .00000001.
end if.
end loop.
compute itprob = 0.
end if.
do if (itprob = 0).
do if ( 1 =2).
compute LL = ylp&*ln(pt1lp)+(1-ylp)&*ln(1-pt1lp).
end if.
compute LL2 = -2*csum(ll).
end if.
do if (abs(LL1-LL2) < converge).
do if ( 1 =1).
compute xlptmp=t(xlp).
compute vecprb=pt1lp&*(1-pt1lp).
loop kkk=1 to ncol(xlp).
compute xlptmp(kkk,:)=xlptmp(kkk,:)&*t(vecprb).
end loop.
compute varb = inv(xlptmp*xlp).
compute seb = sqrt(diag(varb)).
release xlptmp.
end if.
break.
end if.
compute bt1 = b.
compute LL1 = LL2.
end loop.
compute modres=b.
do if (jjj > iterate).
compute itprob = 2.
do if (booting=0).
compute iterrmod=1.
end if.
do if (booting=1).
compute bootiter=1.
end if.
do if (itprobtg=0).
compute itprobtg=1.
compute errcode(errs,1) = 47.
compute errs = errs + 1.
do if (booting = 0 and 1 =1).
compute vt1 = mdiag(pt1lp&*(1-pt1lp)).
compute varb = inv(t(xlp)*vt1*xlp).
compute seb = sqrt(diag(varb)).
end if.
end if.
end if.
do if ( 1 =1).
compute trat = b&/seb.
compute dfres=nrow(xlp).
compute p = 2*(1-cdfnorm(abs(trat))).
compute modres={modres,seb,trat,p}.
compute modres={modres,(b-xp2&*seb),(b+xp2&*seb)}.
compute pvchi=1-chicdf((LL3-LL2),(nrow(modres)-1)).
compute mcF = (LL3-LL2)/LL3.
compute cox = 1-exp(-(LL3-LL2)/nrow(xlp)).
compute nagel = cox/(1-exp(-(LL3)/nrow(xlp))).
compute modsum={LL2,(LL3-LL2),(nrow(modres)-1),pvchi, mcF,cox,nagel}.
compute modsuml={'-2LL','ModelLL', 'df', 'p', 'McFadden', 'CoxSnell', 'Nagelkrk'}.
compute modresl={'coeff','se','Z','p','LLCI','ULCI'}.
end if.
end if
.
end if.
do if ((i = (nms+nys)) and (ydich=1)).

.
do if ( 2 =1).
compute hatmat=inv(t( x )* x )*t( x ).
compute b = hatmat* y.
compute modres=b.
do if ( 1 =1).
compute n1=nrow( x ).
compute dfres=n1-(ncol( x )).
compute sstotal = t( y -(csum( y )/n1))*( y -(csum( y )/n1)).
compute resid= y - x *b.
compute ssresid = csum((resid)&**2).
compute r2 = (sstotal-ssresid)/sstotal.
do if (r2 < 0).
compute r2=0.
end if.
compute adjr2 = 1-((1-r2)*(n1-1)/(dfres)).
compute mse=ssresid/(n1-ncol( x )).

.
compute n1=nrow( x ).
compute invXtX = inv(t( x )* x ).
compute varb = mse *invXtX.
compute k3 = ncol( x ).
compute xhc=0.
do if ( hc <> 5).
compute xhc= x.
compute hat = xhc(:,1).
loop i3=1 to nrow(xhc).
compute hat(i3,1)= xhc(i3,:)*invXtX*t(xhc(i3,:)).
end loop.
do if ( hc = 0 or hc =1).
loop i3 = 1 to k3.
compute xhc(:,i3)=xhc(:,i3)&* resid.
end loop.
end if.
do if ( hc =3 or hc =2).
loop i3=1 to k3.
compute xhc(:,i3) = ( resid &/(1-hat)&**(1/(4- hc )))&*xhc(:,i3).
end loop.
end if.
do if ( hc = 4).
compute hcmn=make(n,2,4).
compute hcmn(:,2)=(n1*hat)/k3.
loop i3= 1 to k3.
compute xhc(:,i3) = ( resid &/(1-hat)&**(rmin(hcmn)/2))&*xhc(:,i3).
end loop.
end if.
compute varb=(invXtX*t(xhc)*xhc*invXtX).
do if ( hc =1).
compute varb=(n1/(n1-ncol( x )))&*varb.
end if.
end if.
compute hclab={'se(HC0)','se(HC1)','se(HC2)','se(HC3)','se(HC4)','se'}.
compute hclab=hclab(1,( hc +1)).
compute hcflab={'F(HC0)','F(HC1)','F(HC2)','F(HC3)','F(HC4)','F'}.
compute hcflab=hcflab(1,( hc +1)).
release xhc
.
compute seb=sqrt(diag(varb)).
compute trat = b&/seb.
compute p = 2*(1-tcdf(abs(trat), (dfres))).
compute tval = sqrt(dfres* (exp((dfres-(5/6))*((xp2/(dfres-(2/3)+(.11/dfres)))* (xp2/(dfres-(2/3)+(.11/dfres)))))-1)).
compute modres={modres,seb,trat,p}.
compute modres={modres,(b-tval&*seb),(b+tval&*seb)}.
compute modresl={'coeff',hclab,'t','p','LLCI','ULCI'}.
compute lmat = ident(ncol( x )).
compute lmat = lmat(:,2:ncol(lmat)).
compute fratio = (t(t(lmat)*b)*inv(t(lmat)*varb*lmat)*((t(lmat)*b)))/(ncol( x )-1).
compute pfr = 1-fcdf(fratio,(ncol( x )-1),dfres).
compute modsum={sqrt(r2),r2,mse,fratio,(ncol( x )-1),dfres,pfr}.
compute modsuml={'R','R-sq','MSE',hcflab,'df1','df2', 'p'}.
end if.
end if.
do if ( 2 = 2 or 2 =3).
compute xlp= x.
compute ylp= y.
compute pt2 = make(nrow(ylp),1,(csum(ylp)/nrow(ylp))).
do if ( 2 =2).
compute LL3 = ylp&*ln(pt2)+(1-ylp)&*ln(1-pt2).
end if.
compute LL3 = -2*csum(LL3).
compute bt1 = make(ncol(xlp),1,0).
compute LL1 = 0.
compute pt1 = make(nrow(ylp),1,0.5).
compute pt1lp=pt1.
loop jjj = 1 to iterate.
compute xlptmp=t(xlp).
compute vecprb=pt1lp&*(1-pt1lp).
loop kkk=1 to ncol(xlp).
compute xlptmp(kkk,:)=xlptmp(kkk,:)&*t(vecprb).
end loop.
compute b = bt1+inv(xlptmp*xlp)*t(xlp)*(ylp-pt1lp).
do if ( 2 =2).
compute xlpb=xlp*b.
compute xlpbt=(xlpb > -709.7).
compute xlpb709=(1-xlpbt)*(-709.7).
compute xlpb=(xlpb&*xlpbt)+xlpb709.
compute pt1lp = 1/(1+exp(-(xlpb))).
end if.
compute itprob = csum((pt1lp < .00000001) or (pt1lp > .9999999)).
do if (itprob > 0).
loop kkk = 1 to nrow(pt1lp).
do if (pt1lp(kkk,1) > .9999999).
compute pt1lp(kkk,1) = .9999999.
end if.
do if (pt1lp(kkk,1) < .00000001).
compute pt1lp(kkk,1) = .00000001.
end if.
end loop.
compute itprob = 0.
end if.
do if (itprob = 0).
do if ( 2 =2).
compute LL = ylp&*ln(pt1lp)+(1-ylp)&*ln(1-pt1lp).
end if.
compute LL2 = -2*csum(ll).
end if.
do if (abs(LL1-LL2) < converge).
do if ( 1 =1).
compute xlptmp=t(xlp).
compute vecprb=pt1lp&*(1-pt1lp).
loop kkk=1 to ncol(xlp).
compute xlptmp(kkk,:)=xlptmp(kkk,:)&*t(vecprb).
end loop.
compute varb = inv(xlptmp*xlp).
compute seb = sqrt(diag(varb)).
release xlptmp.
end if.
break.
end if.
compute bt1 = b.
compute LL1 = LL2.
end loop.
compute modres=b.
do if (jjj > iterate).
compute itprob = 2.
do if (booting=0).
compute iterrmod=1.
end if.
do if (booting=1).
compute bootiter=1.
end if.
do if (itprobtg=0).
compute itprobtg=1.
compute errcode(errs,1) = 47.
compute errs = errs + 1.
do if (booting = 0 and 1 =1).
compute vt1 = mdiag(pt1lp&*(1-pt1lp)).
compute varb = inv(t(xlp)*vt1*xlp).
compute seb = sqrt(diag(varb)).
end if.
end if.
end if.
do if ( 1 =1).
compute trat = b&/seb.
compute dfres=nrow(xlp).
compute p = 2*(1-cdfnorm(abs(trat))).
compute modres={modres,seb,trat,p}.
compute modres={modres,(b-xp2&*seb),(b+xp2&*seb)}.
compute pvchi=1-chicdf((LL3-LL2),(nrow(modres)-1)).
compute mcF = (LL3-LL2)/LL3.
compute cox = 1-exp(-(LL3-LL2)/nrow(xlp)).
compute nagel = cox/(1-exp(-(LL3)/nrow(xlp))).
compute modsum={LL2,(LL3-LL2),(nrow(modres)-1),pvchi, mcF,cox,nagel}.
compute modsuml={'-2LL','ModelLL', 'df', 'p', 'McFadden', 'CoxSnell', 'Nagelkrk'}.
compute modresl={'coeff','se','Z','p','LLCI','ULCI'}.
end if.
end if
.
compute chidfxm=basemodx-LL2.
end if.
compute lmat=make(nrow(b),nxvls,0).
compute lmattmp=ident(nxvls).
compute lmat((nrow(lmat)-nxvls+1):nrow(lmat),:)=lmattmp.

.
compute lmat2= lmat.
do if ( 1 =0).
compute lmat2 = mdiag( lmat ).
compute lmat3=make(nrow(lmat2),1,0).
loop flp=1 to ncol(lmat2).
do if (csum(lmat2(:,flp))=1).
compute lmat3={lmat3,lmat2(:,flp)}.
end if.
end loop.
compute lmat2=lmat3(:,2:ncol(lmat3)).
end if.
compute fratio = (t(t(lmat2)* b )*inv(t(lmat2)* varb *lmat2)*((t(lmat2)* b )))/ncol(lmat2).
compute pfr = 1-fcdf(fratio,ncol(lmat2),(n-nrow( b ))).
compute fresult={fratio,ncol(lmat2),(n-nrow( b )),pfr}.
do if (i = (nms+nys) and (ydich=1)).
compute fratio=fratio*ncol(lmat2).
compute pfr=1-chicdf(fratio,ncol(lmat2)).
compute fresult={fratio,ncol(lmat2),pfr}.
end if.
do if ( 0 =1).
compute lmat3=1-rsum(lmat2).
compute xfm=make(n,csum(lmat3),0).
compute flpc=1.
loop flp=1 to nrow(lmat3).
do if (lmat3(flp,1)=1).
compute xfm(:,flpc)=x(:,flp).
compute flpc=flpc+1.
end if.
end loop.
compute bfm=inv(t(xfm)*xfm)*t(xfm)*y.
compute resid=y-(xfm*bfm).
compute sstotal=(y-(csum(y)/n)).
compute sstotal=csum(sstotal&*sstotal).
compute ssresid=csum(resid&*resid).
compute rsqch= 0 -((sstotal-ssresid)/sstotal).
compute fresult={rsqch,fresult}.
release xfm,flpc, resid, ssresid, bfm.
end if
.
compute numxint=numxint+1.
compute xmtst(numxint,:)=fresult.
do if ((i = (nms+nys)) and (ydich=1)).
compute xmtst(numxint,1)=chidfxm.
compute xmtst(numxint,3)=1-chicdf(chidfxm,nxvls).
end if.
compute xmtstlb={xmtstlb;highlbx((xmints-1),1)}.
end if.
end loop.
compute x=xmtmat.
release xmtmat.
do if (numxint>0).
compute xmtstlb=xmtstlb((2:(numxint+1)),:).
compute xmtst=xmtst(1:numxint,:).
compute resultm2=make(nrow(xmtst),maxresm,99999).
compute resultm2(1:nrow(xmtst),1:ncol(xmtst))=xmtst.
compute resultm={resultm;resultm2}.
do if (nms=1).
compute xmtstlb=' '.
end if.
do if (outscree=1).
do if ((i < (nms+nys)) or (ydich=0)).
print xmtst/title='Test(s) of X by M interaction:'/rnames=xmtstlb/cnames=xmtstlbc/format= F10.4.
end if.
do if ((i = (nms+nys)) and (ydich=1)).
print xmtst/title='Likelihood ratio test(s) of X by M interaction:'/rnames=xmtstlb/cnames=xmtstlbc/format= F10.4.
end if.
end if.
end if.
compute r2=r2tmp.
compute b=btmp.
compute varb=varbtmp.
compute dfres=dfrestmp.
compute tval=tvaltmp.
end if.
do if (criterr = 0).
compute jj=0.
loop j = start to ((start+i)-1).
compute dbint=0.
compute lmat=whigh(1:nump(1,i),j).
compute lmat2=wzhigh(1:nump(1,i),j).
do if ((csum(lmat) > 0) and (csum(lmat2) = 0)).
do if ((i < (nms+nys)) or (ydich <> 1)).

.
compute lmat2= lmat.
do if ( 0 =0).
compute lmat2 = mdiag( lmat ).
compute lmat3=make(nrow(lmat2),1,0).
loop flp=1 to ncol(lmat2).
do if (csum(lmat2(:,flp))=1).
compute lmat3={lmat3,lmat2(:,flp)}.
end if.
end loop.
compute lmat2=lmat3(:,2:ncol(lmat3)).
end if.
compute fratio = (t(t(lmat2)* b )*inv(t(lmat2)* varb *lmat2)*((t(lmat2)* b )))/ncol(lmat2).
compute pfr = 1-fcdf(fratio,ncol(lmat2),(n-nrow( b ))).
compute fresult={fratio,ncol(lmat2),(n-nrow( b )),pfr}.
do if (i = (nms+nys) and (ydich=1)).
compute fratio=fratio*ncol(lmat2).
compute pfr=1-chicdf(fratio,ncol(lmat2)).
compute fresult={fratio,ncol(lmat2),pfr}.
end if.
do if ( 1 =1).
compute lmat3=1-rsum(lmat2).
compute xfm=make(n,csum(lmat3),0).
compute flpc=1.
loop flp=1 to nrow(lmat3).
do if (lmat3(flp,1)=1).
compute xfm(:,flpc)=x(:,flp).
compute flpc=flpc+1.
end if.
end loop.
compute bfm=inv(t(xfm)*xfm)*t(xfm)*y.
compute resid=y-(xfm*bfm).
compute sstotal=(y-(csum(y)/n)).
compute sstotal=csum(sstotal&*sstotal).
compute ssresid=csum(resid&*resid).
compute rsqch= r2 -((sstotal-ssresid)/sstotal).
compute fresult={rsqch,fresult}.
release xfm,flpc, resid, ssresid, bfm.
end if
.
compute lmatdb=lmat.
compute dbint=dbint+1.
end if.
do if ((ydich = 1) and (i = (nms+nys))).

.
compute btemphld=b.
compute llrdat=make(nrow(x),nrow( lmat )-csum( lmat ),-999).
compute llrdf=ncol(x)-ncol(llrdat).
compute llrcnt=0.
loop llri=1 to nrow( lmat ).
do if ( lmat (llri,1)=0).
compute llrcnt=llrcnt+1.
compute llrdat(:,llrcnt)=x(:,llri).
end if.
end loop.

.
do if ( 2 =1).
compute hatmat=inv(t( llrdat )* llrdat )*t( llrdat ).
compute b = hatmat* y.
compute modres=b.
do if ( 0 =1).
compute n1=nrow( llrdat ).
compute dfres=n1-(ncol( llrdat )).
compute sstotal = t( y -(csum( y )/n1))*( y -(csum( y )/n1)).
compute resid= y - llrdat *b.
compute ssresid = csum((resid)&**2).
compute r2 = (sstotal-ssresid)/sstotal.
do if (r2 < 0).
compute r2=0.
end if.
compute adjr2 = 1-((1-r2)*(n1-1)/(dfres)).
compute mse=ssresid/(n1-ncol( llrdat )).

.
compute n1=nrow( x ).
compute invXtX = inv(t( x )* x ).
compute varb = mse *invXtX.
compute k3 = ncol( x ).
compute xhc=0.
do if ( hc <> 5).
compute xhc= x.
compute hat = xhc(:,1).
loop i3=1 to nrow(xhc).
compute hat(i3,1)= xhc(i3,:)*invXtX*t(xhc(i3,:)).
end loop.
do if ( hc = 0 or hc =1).
loop i3 = 1 to k3.
compute xhc(:,i3)=xhc(:,i3)&* resid.
end loop.
end if.
do if ( hc =3 or hc =2).
loop i3=1 to k3.
compute xhc(:,i3) = ( resid &/(1-hat)&**(1/(4- hc )))&*xhc(:,i3).
end loop.
end if.
do if ( hc = 4).
compute hcmn=make(n,2,4).
compute hcmn(:,2)=(n1*hat)/k3.
loop i3= 1 to k3.
compute xhc(:,i3) = ( resid &/(1-hat)&**(rmin(hcmn)/2))&*xhc(:,i3).
end loop.
end if.
compute varb=(invXtX*t(xhc)*xhc*invXtX).
do if ( hc =1).
compute varb=(n1/(n1-ncol( x )))&*varb.
end if.
end if.
compute hclab={'se(HC0)','se(HC1)','se(HC2)','se(HC3)','se(HC4)','se'}.
compute hclab=hclab(1,( hc +1)).
compute hcflab={'F(HC0)','F(HC1)','F(HC2)','F(HC3)','F(HC4)','F'}.
compute hcflab=hcflab(1,( hc +1)).
release xhc
.
compute seb=sqrt(diag(varb)).
compute trat = b&/seb.
compute p = 2*(1-tcdf(abs(trat), (dfres))).
compute tval = sqrt(dfres* (exp((dfres-(5/6))*((xp2/(dfres-(2/3)+(.11/dfres)))* (xp2/(dfres-(2/3)+(.11/dfres)))))-1)).
compute modres={modres,seb,trat,p}.
compute modres={modres,(b-tval&*seb),(b+tval&*seb)}.
compute modresl={'coeff',hclab,'t','p','LLCI','ULCI'}.
compute lmat = ident(ncol( llrdat )).
compute lmat = lmat(:,2:ncol(lmat)).
compute fratio = (t(t(lmat)*b)*inv(t(lmat)*varb*lmat)*((t(lmat)*b)))/(ncol( llrdat )-1).
compute pfr = 1-fcdf(fratio,(ncol( llrdat )-1),dfres).
compute modsum={sqrt(r2),r2,mse,fratio,(ncol( llrdat )-1),dfres,pfr}.
compute modsuml={'R','R-sq','MSE',hcflab,'df1','df2', 'p'}.
end if.
end if.
do if ( 2 = 2 or 2 =3).
compute xlp= llrdat.
compute ylp= y.
compute pt2 = make(nrow(ylp),1,(csum(ylp)/nrow(ylp))).
do if ( 2 =2).
compute LL3 = ylp&*ln(pt2)+(1-ylp)&*ln(1-pt2).
end if.
compute LL3 = -2*csum(LL3).
compute bt1 = make(ncol(xlp),1,0).
compute LL1 = 0.
compute pt1 = make(nrow(ylp),1,0.5).
compute pt1lp=pt1.
loop jjj = 1 to iterate.
compute xlptmp=t(xlp).
compute vecprb=pt1lp&*(1-pt1lp).
loop kkk=1 to ncol(xlp).
compute xlptmp(kkk,:)=xlptmp(kkk,:)&*t(vecprb).
end loop.
compute b = bt1+inv(xlptmp*xlp)*t(xlp)*(ylp-pt1lp).
do if ( 2 =2).
compute xlpb=xlp*b.
compute xlpbt=(xlpb > -709.7).
compute xlpb709=(1-xlpbt)*(-709.7).
compute xlpb=(xlpb&*xlpbt)+xlpb709.
compute pt1lp = 1/(1+exp(-(xlpb))).
end if.
compute itprob = csum((pt1lp < .00000001) or (pt1lp > .9999999)).
do if (itprob > 0).
loop kkk = 1 to nrow(pt1lp).
do if (pt1lp(kkk,1) > .9999999).
compute pt1lp(kkk,1) = .9999999.
end if.
do if (pt1lp(kkk,1) < .00000001).
compute pt1lp(kkk,1) = .00000001.
end if.
end loop.
compute itprob = 0.
end if.
do if (itprob = 0).
do if ( 2 =2).
compute LL = ylp&*ln(pt1lp)+(1-ylp)&*ln(1-pt1lp).
end if.
compute LL2 = -2*csum(ll).
end if.
do if (abs(LL1-LL2) < converge).
do if ( 0 =1).
compute xlptmp=t(xlp).
compute vecprb=pt1lp&*(1-pt1lp).
loop kkk=1 to ncol(xlp).
compute xlptmp(kkk,:)=xlptmp(kkk,:)&*t(vecprb).
end loop.
compute varb = inv(xlptmp*xlp).
compute seb = sqrt(diag(varb)).
release xlptmp.
end if.
break.
end if.
compute bt1 = b.
compute LL1 = LL2.
end loop.
compute modres=b.
do if (jjj > iterate).
compute itprob = 2.
do if (booting=0).
compute iterrmod=1.
end if.
do if (booting=1).
compute bootiter=1.
end if.
do if (itprobtg=0).
compute itprobtg=1.
compute errcode(errs,1) = 47.
compute errs = errs + 1.
do if (booting = 0 and 0 =1).
compute vt1 = mdiag(pt1lp&*(1-pt1lp)).
compute varb = inv(t(xlp)*vt1*xlp).
compute seb = sqrt(diag(varb)).
end if.
end if.
end if.
do if ( 0 =1).
compute trat = b&/seb.
compute dfres=nrow(xlp).
compute p = 2*(1-cdfnorm(abs(trat))).
compute modres={modres,seb,trat,p}.
compute modres={modres,(b-xp2&*seb),(b+xp2&*seb)}.
compute pvchi=1-chicdf((LL3-LL2),(nrow(modres)-1)).
compute mcF = (LL3-LL2)/LL3.
compute cox = 1-exp(-(LL3-LL2)/nrow(xlp)).
compute nagel = cox/(1-exp(-(LL3)/nrow(xlp))).
compute modsum={LL2,(LL3-LL2),(nrow(modres)-1),pvchi, mcF,cox,nagel}.
compute modsuml={'-2LL','ModelLL', 'df', 'p', 'McFadden', 'CoxSnell', 'Nagelkrk'}.
compute modresl={'coeff','se','Z','p','LLCI','ULCI'}.
end if.
end if
.
compute b=btemphld.
compute fresult={(LL2-basemod),llrdf,1-chicdf((LL2-basemod),llrdf)}
.
compute lmatdb=lmat.
compute dbint=dbint+1.
end if.
compute highf={highf;fresult}.
compute highf2={highf2;fresult}.
do if (j = start).
compute flabel={flabel;'X*W'}.
end if.
do if (j > start).
do if (nms > 1).
compute flabel={flabel;highlbw(jj,1)}.
else if (nms = 1).
do if (xmint=0).
compute flabel={flabel;'M*W'}.
end if.
do if (xmint=1).
compute flabel={flabel;'X*M'}.
end if.
end if.
end if.
end if.
compute lmat=zhigh(1:nump(1,i),j).
compute lmat2=wzhigh(1:nump(1,i),j).
do if ((csum(lmat) > 0) and (csum(lmat2) = 0)).
do if ((i < (nms+nys)) or (ydich <> 1)).

.
compute lmat2= lmat.
do if ( 0 =0).
compute lmat2 = mdiag( lmat ).
compute lmat3=make(nrow(lmat2),1,0).
loop flp=1 to ncol(lmat2).
do if (csum(lmat2(:,flp))=1).
compute lmat3={lmat3,lmat2(:,flp)}.
end if.
end loop.
compute lmat2=lmat3(:,2:ncol(lmat3)).
end if.
compute fratio = (t(t(lmat2)* b )*inv(t(lmat2)* varb *lmat2)*((t(lmat2)* b )))/ncol(lmat2).
compute pfr = 1-fcdf(fratio,ncol(lmat2),(n-nrow( b ))).
compute fresult={fratio,ncol(lmat2),(n-nrow( b )),pfr}.
do if (i = (nms+nys) and (ydich=1)).
compute fratio=fratio*ncol(lmat2).
compute pfr=1-chicdf(fratio,ncol(lmat2)).
compute fresult={fratio,ncol(lmat2),pfr}.
end if.
do if ( 1 =1).
compute lmat3=1-rsum(lmat2).
compute xfm=make(n,csum(lmat3),0).
compute flpc=1.
loop flp=1 to nrow(lmat3).
do if (lmat3(flp,1)=1).
compute xfm(:,flpc)=x(:,flp).
compute flpc=flpc+1.
end if.
end loop.
compute bfm=inv(t(xfm)*xfm)*t(xfm)*y.
compute resid=y-(xfm*bfm).
compute sstotal=(y-(csum(y)/n)).
compute sstotal=csum(sstotal&*sstotal).
compute ssresid=csum(resid&*resid).
compute rsqch= r2 -((sstotal-ssresid)/sstotal).
compute fresult={rsqch,fresult}.
release xfm,flpc, resid, ssresid, bfm.
end if
.
compute dbint=dbint+1.
end if.
do if ((ydich = 1) and (i = (nms+nys))).

.
compute btemphld=b.
compute llrdat=make(nrow(x),nrow( lmat )-csum( lmat ),-999).
compute llrdf=ncol(x)-ncol(llrdat).
compute llrcnt=0.
loop llri=1 to nrow( lmat ).
do if ( lmat (llri,1)=0).
compute llrcnt=llrcnt+1.
compute llrdat(:,llrcnt)=x(:,llri).
end if.
end loop.

.
do if ( 2 =1).
compute hatmat=inv(t( llrdat )* llrdat )*t( llrdat ).
compute b = hatmat* y.
compute modres=b.
do if ( 0 =1).
compute n1=nrow( llrdat ).
compute dfres=n1-(ncol( llrdat )).
compute sstotal = t( y -(csum( y )/n1))*( y -(csum( y )/n1)).
compute resid= y - llrdat *b.
compute ssresid = csum((resid)&**2).
compute r2 = (sstotal-ssresid)/sstotal.
do if (r2 < 0).
compute r2=0.
end if.
compute adjr2 = 1-((1-r2)*(n1-1)/(dfres)).
compute mse=ssresid/(n1-ncol( llrdat )).

.
compute n1=nrow( x ).
compute invXtX = inv(t( x )* x ).
compute varb = mse *invXtX.
compute k3 = ncol( x ).
compute xhc=0.
do if ( hc <> 5).
compute xhc= x.
compute hat = xhc(:,1).
loop i3=1 to nrow(xhc).
compute hat(i3,1)= xhc(i3,:)*invXtX*t(xhc(i3,:)).
end loop.
do if ( hc = 0 or hc =1).
loop i3 = 1 to k3.
compute xhc(:,i3)=xhc(:,i3)&* resid.
end loop.
end if.
do if ( hc =3 or hc =2).
loop i3=1 to k3.
compute xhc(:,i3) = ( resid &/(1-hat)&**(1/(4- hc )))&*xhc(:,i3).
end loop.
end if.
do if ( hc = 4).
compute hcmn=make(n,2,4).
compute hcmn(:,2)=(n1*hat)/k3.
loop i3= 1 to k3.
compute xhc(:,i3) = ( resid &/(1-hat)&**(rmin(hcmn)/2))&*xhc(:,i3).
end loop.
end if.
compute varb=(invXtX*t(xhc)*xhc*invXtX).
do if ( hc =1).
compute varb=(n1/(n1-ncol( x )))&*varb.
end if.
end if.
compute hclab={'se(HC0)','se(HC1)','se(HC2)','se(HC3)','se(HC4)','se'}.
compute hclab=hclab(1,( hc +1)).
compute hcflab={'F(HC0)','F(HC1)','F(HC2)','F(HC3)','F(HC4)','F'}.
compute hcflab=hcflab(1,( hc +1)).
release xhc
.
compute seb=sqrt(diag(varb)).
compute trat = b&/seb.
compute p = 2*(1-tcdf(abs(trat), (dfres))).
compute tval = sqrt(dfres* (exp((dfres-(5/6))*((xp2/(dfres-(2/3)+(.11/dfres)))* (xp2/(dfres-(2/3)+(.11/dfres)))))-1)).
compute modres={modres,seb,trat,p}.
compute modres={modres,(b-tval&*seb),(b+tval&*seb)}.
compute modresl={'coeff',hclab,'t','p','LLCI','ULCI'}.
compute lmat = ident(ncol( llrdat )).
compute lmat = lmat(:,2:ncol(lmat)).
compute fratio = (t(t(lmat)*b)*inv(t(lmat)*varb*lmat)*((t(lmat)*b)))/(ncol( llrdat )-1).
compute pfr = 1-fcdf(fratio,(ncol( llrdat )-1),dfres).
compute modsum={sqrt(r2),r2,mse,fratio,(ncol( llrdat )-1),dfres,pfr}.
compute modsuml={'R','R-sq','MSE',hcflab,'df1','df2', 'p'}.
end if.
end if.
do if ( 2 = 2 or 2 =3).
compute xlp= llrdat.
compute ylp= y.
compute pt2 = make(nrow(ylp),1,(csum(ylp)/nrow(ylp))).
do if ( 2 =2).
compute LL3 = ylp&*ln(pt2)+(1-ylp)&*ln(1-pt2).
end if.
compute LL3 = -2*csum(LL3).
compute bt1 = make(ncol(xlp),1,0).
compute LL1 = 0.
compute pt1 = make(nrow(ylp),1,0.5).
compute pt1lp=pt1.
loop jjj = 1 to iterate.
compute xlptmp=t(xlp).
compute vecprb=pt1lp&*(1-pt1lp).
loop kkk=1 to ncol(xlp).
compute xlptmp(kkk,:)=xlptmp(kkk,:)&*t(vecprb).
end loop.
compute b = bt1+inv(xlptmp*xlp)*t(xlp)*(ylp-pt1lp).
do if ( 2 =2).
compute xlpb=xlp*b.
compute xlpbt=(xlpb > -709.7).
compute xlpb709=(1-xlpbt)*(-709.7).
compute xlpb=(xlpb&*xlpbt)+xlpb709.
compute pt1lp = 1/(1+exp(-(xlpb))).
end if.
compute itprob = csum((pt1lp < .00000001) or (pt1lp > .9999999)).
do if (itprob > 0).
loop kkk = 1 to nrow(pt1lp).
do if (pt1lp(kkk,1) > .9999999).
compute pt1lp(kkk,1) = .9999999.
end if.
do if (pt1lp(kkk,1) < .00000001).
compute pt1lp(kkk,1) = .00000001.
end if.
end loop.
compute itprob = 0.
end if.
do if (itprob = 0).
do if ( 2 =2).
compute LL = ylp&*ln(pt1lp)+(1-ylp)&*ln(1-pt1lp).
end if.
compute LL2 = -2*csum(ll).
end if.
do if (abs(LL1-LL2) < converge).
do if ( 0 =1).
compute xlptmp=t(xlp).
compute vecprb=pt1lp&*(1-pt1lp).
loop kkk=1 to ncol(xlp).
compute xlptmp(kkk,:)=xlptmp(kkk,:)&*t(vecprb).
end loop.
compute varb = inv(xlptmp*xlp).
compute seb = sqrt(diag(varb)).
release xlptmp.
end if.
break.
end if.
compute bt1 = b.
compute LL1 = LL2.
end loop.
compute modres=b.
do if (jjj > iterate).
compute itprob = 2.
do if (booting=0).
compute iterrmod=1.
end if.
do if (booting=1).
compute bootiter=1.
end if.
do if (itprobtg=0).
compute itprobtg=1.
compute errcode(errs,1) = 47.
compute errs = errs + 1.
do if (booting = 0 and 0 =1).
compute vt1 = mdiag(pt1lp&*(1-pt1lp)).
compute varb = inv(t(xlp)*vt1*xlp).
compute seb = sqrt(diag(varb)).
end if.
end if.
end if.
do if ( 0 =1).
compute trat = b&/seb.
compute dfres=nrow(xlp).
compute p = 2*(1-cdfnorm(abs(trat))).
compute modres={modres,seb,trat,p}.
compute modres={modres,(b-xp2&*seb),(b+xp2&*seb)}.
compute pvchi=1-chicdf((LL3-LL2),(nrow(modres)-1)).
compute mcF = (LL3-LL2)/LL3.
compute cox = 1-exp(-(LL3-LL2)/nrow(xlp)).
compute nagel = cox/(1-exp(-(LL3)/nrow(xlp))).
compute modsum={LL2,(LL3-LL2),(nrow(modres)-1),pvchi, mcF,cox,nagel}.
compute modsuml={'-2LL','ModelLL', 'df', 'p', 'McFadden', 'CoxSnell', 'Nagelkrk'}.
compute modresl={'coeff','se','Z','p','LLCI','ULCI'}.
end if.
end if
.
compute b=btemphld.
compute fresult={(LL2-basemod),llrdf,1-chicdf((LL2-basemod),llrdf)}
.
compute dbint=dbint+1.
end if.
compute highf={highf;fresult}.
compute highf2={highf2;fresult}.
do if (j = start).
compute flabel={flabel;'X*Z'}.
end if.
do if (j > start).
do if (nms > 1).
compute flabel={flabel;highlbz(jj,1)}.
else if (nms = 1).
compute flabel={flabel;'M*Z'}.
end if.
end if.
end if.
do if (dbint=2).
compute lmatdb=lmatdb+lmat.
do if ((ydich = 1) and (i = (nms+nys))).

.
compute btemphld=b.
compute llrdat=make(nrow(x),nrow( lmatdb )-csum( lmatdb ),-999).
compute llrdf=ncol(x)-ncol(llrdat).
compute llrcnt=0.
loop llri=1 to nrow( lmatdb ).
do if ( lmatdb (llri,1)=0).
compute llrcnt=llrcnt+1.
compute llrdat(:,llrcnt)=x(:,llri).
end if.
end loop.

.
do if ( 2 =1).
compute hatmat=inv(t( llrdat )* llrdat )*t( llrdat ).
compute b = hatmat* y.
compute modres=b.
do if ( 0 =1).
compute n1=nrow( llrdat ).
compute dfres=n1-(ncol( llrdat )).
compute sstotal = t( y -(csum( y )/n1))*( y -(csum( y )/n1)).
compute resid= y - llrdat *b.
compute ssresid = csum((resid)&**2).
compute r2 = (sstotal-ssresid)/sstotal.
do if (r2 < 0).
compute r2=0.
end if.
compute adjr2 = 1-((1-r2)*(n1-1)/(dfres)).
compute mse=ssresid/(n1-ncol( llrdat )).

.
compute n1=nrow( x ).
compute invXtX = inv(t( x )* x ).
compute varb = mse *invXtX.
compute k3 = ncol( x ).
compute xhc=0.
do if ( hc <> 5).
compute xhc= x.
compute hat = xhc(:,1).
loop i3=1 to nrow(xhc).
compute hat(i3,1)= xhc(i3,:)*invXtX*t(xhc(i3,:)).
end loop.
do if ( hc = 0 or hc =1).
loop i3 = 1 to k3.
compute xhc(:,i3)=xhc(:,i3)&* resid.
end loop.
end if.
do if ( hc =3 or hc =2).
loop i3=1 to k3.
compute xhc(:,i3) = ( resid &/(1-hat)&**(1/(4- hc )))&*xhc(:,i3).
end loop.
end if.
do if ( hc = 4).
compute hcmn=make(n,2,4).
compute hcmn(:,2)=(n1*hat)/k3.
loop i3= 1 to k3.
compute xhc(:,i3) = ( resid &/(1-hat)&**(rmin(hcmn)/2))&*xhc(:,i3).
end loop.
end if.
compute varb=(invXtX*t(xhc)*xhc*invXtX).
do if ( hc =1).
compute varb=(n1/(n1-ncol( x )))&*varb.
end if.
end if.
compute hclab={'se(HC0)','se(HC1)','se(HC2)','se(HC3)','se(HC4)','se'}.
compute hclab=hclab(1,( hc +1)).
compute hcflab={'F(HC0)','F(HC1)','F(HC2)','F(HC3)','F(HC4)','F'}.
compute hcflab=hcflab(1,( hc +1)).
release xhc
.
compute seb=sqrt(diag(varb)).
compute trat = b&/seb.
compute p = 2*(1-tcdf(abs(trat), (dfres))).
compute tval = sqrt(dfres* (exp((dfres-(5/6))*((xp2/(dfres-(2/3)+(.11/dfres)))* (xp2/(dfres-(2/3)+(.11/dfres)))))-1)).
compute modres={modres,seb,trat,p}.
compute modres={modres,(b-tval&*seb),(b+tval&*seb)}.
compute modresl={'coeff',hclab,'t','p','LLCI','ULCI'}.
compute lmat = ident(ncol( llrdat )).
compute lmat = lmat(:,2:ncol(lmat)).
compute fratio = (t(t(lmat)*b)*inv(t(lmat)*varb*lmat)*((t(lmat)*b)))/(ncol( llrdat )-1).
compute pfr = 1-fcdf(fratio,(ncol( llrdat )-1),dfres).
compute modsum={sqrt(r2),r2,mse,fratio,(ncol( llrdat )-1),dfres,pfr}.
compute modsuml={'R','R-sq','MSE',hcflab,'df1','df2', 'p'}.
end if.
end if.
do if ( 2 = 2 or 2 =3).
compute xlp= llrdat.
compute ylp= y.
compute pt2 = make(nrow(ylp),1,(csum(ylp)/nrow(ylp))).
do if ( 2 =2).
compute LL3 = ylp&*ln(pt2)+(1-ylp)&*ln(1-pt2).
end if.
compute LL3 = -2*csum(LL3).
compute bt1 = make(ncol(xlp),1,0).
compute LL1 = 0.
compute pt1 = make(nrow(ylp),1,0.5).
compute pt1lp=pt1.
loop jjj = 1 to iterate.
compute xlptmp=t(xlp).
compute vecprb=pt1lp&*(1-pt1lp).
loop kkk=1 to ncol(xlp).
compute xlptmp(kkk,:)=xlptmp(kkk,:)&*t(vecprb).
end loop.
compute b = bt1+inv(xlptmp*xlp)*t(xlp)*(ylp-pt1lp).
do if ( 2 =2).
compute xlpb=xlp*b.
compute xlpbt=(xlpb > -709.7).
compute xlpb709=(1-xlpbt)*(-709.7).
compute xlpb=(xlpb&*xlpbt)+xlpb709.
compute pt1lp = 1/(1+exp(-(xlpb))).
end if.
compute itprob = csum((pt1lp < .00000001) or (pt1lp > .9999999)).
do if (itprob > 0).
loop kkk = 1 to nrow(pt1lp).
do if (pt1lp(kkk,1) > .9999999).
compute pt1lp(kkk,1) = .9999999.
end if.
do if (pt1lp(kkk,1) < .00000001).
compute pt1lp(kkk,1) = .00000001.
end if.
end loop.
compute itprob = 0.
end if.
do if (itprob = 0).
do if ( 2 =2).
compute LL = ylp&*ln(pt1lp)+(1-ylp)&*ln(1-pt1lp).
end if.
compute LL2 = -2*csum(ll).
end if.
do if (abs(LL1-LL2) < converge).
do if ( 0 =1).
compute xlptmp=t(xlp).
compute vecprb=pt1lp&*(1-pt1lp).
loop kkk=1 to ncol(xlp).
compute xlptmp(kkk,:)=xlptmp(kkk,:)&*t(vecprb).
end loop.
compute varb = inv(xlptmp*xlp).
compute seb = sqrt(diag(varb)).
release xlptmp.
end if.
break.
end if.
compute bt1 = b.
compute LL1 = LL2.
end loop.
compute modres=b.
do if (jjj > iterate).
compute itprob = 2.
do if (booting=0).
compute iterrmod=1.
end if.
do if (booting=1).
compute bootiter=1.
end if.
do if (itprobtg=0).
compute itprobtg=1.
compute errcode(errs,1) = 47.
compute errs = errs + 1.
do if (booting = 0 and 0 =1).
compute vt1 = mdiag(pt1lp&*(1-pt1lp)).
compute varb = inv(t(xlp)*vt1*xlp).
compute seb = sqrt(diag(varb)).
end if.
end if.
end if.
do if ( 0 =1).
compute trat = b&/seb.
compute dfres=nrow(xlp).
compute p = 2*(1-cdfnorm(abs(trat))).
compute modres={modres,seb,trat,p}.
compute modres={modres,(b-xp2&*seb),(b+xp2&*seb)}.
compute pvchi=1-chicdf((LL3-LL2),(nrow(modres)-1)).
compute mcF = (LL3-LL2)/LL3.
compute cox = 1-exp(-(LL3-LL2)/nrow(xlp)).
compute nagel = cox/(1-exp(-(LL3)/nrow(xlp))).
compute modsum={LL2,(LL3-LL2),(nrow(modres)-1),pvchi, mcF,cox,nagel}.
compute modsuml={'-2LL','ModelLL', 'df', 'p', 'McFadden', 'CoxSnell', 'Nagelkrk'}.
compute modresl={'coeff','se','Z','p','LLCI','ULCI'}.
end if.
end if
.
compute b=btemphld.
compute fresult={(LL2-basemod),llrdf,1-chicdf((LL2-basemod),llrdf)}
.
end if.
do if (ydich <> 1 or i < (nms+nys)).

.
compute lmat2= lmatdb.
do if ( 0 =0).
compute lmat2 = mdiag( lmatdb ).
compute lmat3=make(nrow(lmat2),1,0).
loop flp=1 to ncol(lmat2).
do if (csum(lmat2(:,flp))=1).
compute lmat3={lmat3,lmat2(:,flp)}.
end if.
end loop.
compute lmat2=lmat3(:,2:ncol(lmat3)).
end if.
compute fratio = (t(t(lmat2)* b )*inv(t(lmat2)* varb *lmat2)*((t(lmat2)* b )))/ncol(lmat2).
compute pfr = 1-fcdf(fratio,ncol(lmat2),(n-nrow( b ))).
compute fresult={fratio,ncol(lmat2),(n-nrow( b )),pfr}.
do if (i = (nms+nys) and (ydich=1)).
compute fratio=fratio*ncol(lmat2).
compute pfr=1-chicdf(fratio,ncol(lmat2)).
compute fresult={fratio,ncol(lmat2),pfr}.
end if.
do if ( 1 =1).
compute lmat3=1-rsum(lmat2).
compute xfm=make(n,csum(lmat3),0).
compute flpc=1.
loop flp=1 to nrow(lmat3).
do if (lmat3(flp,1)=1).
compute xfm(:,flpc)=x(:,flp).
compute flpc=flpc+1.
end if.
end loop.
compute bfm=inv(t(xfm)*xfm)*t(xfm)*y.
compute resid=y-(xfm*bfm).
compute sstotal=(y-(csum(y)/n)).
compute sstotal=csum(sstotal&*sstotal).
compute ssresid=csum(resid&*resid).
compute rsqch= r2 -((sstotal-ssresid)/sstotal).
compute fresult={rsqch,fresult}.
release xfm,flpc, resid, ssresid, bfm.
end if
.
end if.
compute dbint=0.
compute highf={highf;fresult}.
do if (jj=0 and nms > 0).
compute flabel={flabel;'BOTH(X)'}.
end if.
do if (jj=0 and nms = 0).
compute flabel={flabel;'BOTH'}.
end if.
do if (jj>0 and nms = 1).
compute flabel={flabel;'BOTH(M)'}.
end if.
do if (nms > 1 and jj > 0).
compute flabel={flabel;highlbbt(jj,1)}.
end if.
end if.
compute lmat2=wzhigh(1:nump(1,i),j).
do if (csum(lmat2) > 0).
do if ((i < (nms+nys)) or (ydich <> 1)).

.
compute lmat2= lmat2.
do if ( 0 =0).
compute lmat2 = mdiag( lmat2 ).
compute lmat3=make(nrow(lmat2),1,0).
loop flp=1 to ncol(lmat2).
do if (csum(lmat2(:,flp))=1).
compute lmat3={lmat3,lmat2(:,flp)}.
end if.
end loop.
compute lmat2=lmat3(:,2:ncol(lmat3)).
end if.
compute fratio = (t(t(lmat2)* b )*inv(t(lmat2)* varb *lmat2)*((t(lmat2)* b )))/ncol(lmat2).
compute pfr = 1-fcdf(fratio,ncol(lmat2),(n-nrow( b ))).
compute fresult={fratio,ncol(lmat2),(n-nrow( b )),pfr}.
do if (i = (nms+nys) and (ydich=1)).
compute fratio=fratio*ncol(lmat2).
compute pfr=1-chicdf(fratio,ncol(lmat2)).
compute fresult={fratio,ncol(lmat2),pfr}.
end if.
do if ( 1 =1).
compute lmat3=1-rsum(lmat2).
compute xfm=make(n,csum(lmat3),0).
compute flpc=1.
loop flp=1 to nrow(lmat3).
do if (lmat3(flp,1)=1).
compute xfm(:,flpc)=x(:,flp).
compute flpc=flpc+1.
end if.
end loop.
compute bfm=inv(t(xfm)*xfm)*t(xfm)*y.
compute resid=y-(xfm*bfm).
compute sstotal=(y-(csum(y)/n)).
compute sstotal=csum(sstotal&*sstotal).
compute ssresid=csum(resid&*resid).
compute rsqch= r2 -((sstotal-ssresid)/sstotal).
compute fresult={rsqch,fresult}.
release xfm,flpc, resid, ssresid, bfm.
end if
.
end if.
do if ((ydich = 1) and (i = (nms+nys))).

.
compute btemphld=b.
compute llrdat=make(nrow(x),nrow( lmat2 )-csum( lmat2 ),-999).
compute llrdf=ncol(x)-ncol(llrdat).
compute llrcnt=0.
loop llri=1 to nrow( lmat2 ).
do if ( lmat2 (llri,1)=0).
compute llrcnt=llrcnt+1.
compute llrdat(:,llrcnt)=x(:,llri).
end if.
end loop.

.
do if ( 2 =1).
compute hatmat=inv(t( llrdat )* llrdat )*t( llrdat ).
compute b = hatmat* y.
compute modres=b.
do if ( 0 =1).
compute n1=nrow( llrdat ).
compute dfres=n1-(ncol( llrdat )).
compute sstotal = t( y -(csum( y )/n1))*( y -(csum( y )/n1)).
compute resid= y - llrdat *b.
compute ssresid = csum((resid)&**2).
compute r2 = (sstotal-ssresid)/sstotal.
do if (r2 < 0).
compute r2=0.
end if.
compute adjr2 = 1-((1-r2)*(n1-1)/(dfres)).
compute mse=ssresid/(n1-ncol( llrdat )).

.
compute n1=nrow( x ).
compute invXtX = inv(t( x )* x ).
compute varb = mse *invXtX.
compute k3 = ncol( x ).
compute xhc=0.
do if ( hc <> 5).
compute xhc= x.
compute hat = xhc(:,1).
loop i3=1 to nrow(xhc).
compute hat(i3,1)= xhc(i3,:)*invXtX*t(xhc(i3,:)).
end loop.
do if ( hc = 0 or hc =1).
loop i3 = 1 to k3.
compute xhc(:,i3)=xhc(:,i3)&* resid.
end loop.
end if.
do if ( hc =3 or hc =2).
loop i3=1 to k3.
compute xhc(:,i3) = ( resid &/(1-hat)&**(1/(4- hc )))&*xhc(:,i3).
end loop.
end if.
do if ( hc = 4).
compute hcmn=make(n,2,4).
compute hcmn(:,2)=(n1*hat)/k3.
loop i3= 1 to k3.
compute xhc(:,i3) = ( resid &/(1-hat)&**(rmin(hcmn)/2))&*xhc(:,i3).
end loop.
end if.
compute varb=(invXtX*t(xhc)*xhc*invXtX).
do if ( hc =1).
compute varb=(n1/(n1-ncol( x )))&*varb.
end if.
end if.
compute hclab={'se(HC0)','se(HC1)','se(HC2)','se(HC3)','se(HC4)','se'}.
compute hclab=hclab(1,( hc +1)).
compute hcflab={'F(HC0)','F(HC1)','F(HC2)','F(HC3)','F(HC4)','F'}.
compute hcflab=hcflab(1,( hc +1)).
release xhc
.
compute seb=sqrt(diag(varb)).
compute trat = b&/seb.
compute p = 2*(1-tcdf(abs(trat), (dfres))).
compute tval = sqrt(dfres* (exp((dfres-(5/6))*((xp2/(dfres-(2/3)+(.11/dfres)))* (xp2/(dfres-(2/3)+(.11/dfres)))))-1)).
compute modres={modres,seb,trat,p}.
compute modres={modres,(b-tval&*seb),(b+tval&*seb)}.
compute modresl={'coeff',hclab,'t','p','LLCI','ULCI'}.
compute lmat = ident(ncol( llrdat )).
compute lmat = lmat(:,2:ncol(lmat)).
compute fratio = (t(t(lmat)*b)*inv(t(lmat)*varb*lmat)*((t(lmat)*b)))/(ncol( llrdat )-1).
compute pfr = 1-fcdf(fratio,(ncol( llrdat )-1),dfres).
compute modsum={sqrt(r2),r2,mse,fratio,(ncol( llrdat )-1),dfres,pfr}.
compute modsuml={'R','R-sq','MSE',hcflab,'df1','df2', 'p'}.
end if.
end if.
do if ( 2 = 2 or 2 =3).
compute xlp= llrdat.
compute ylp= y.
compute pt2 = make(nrow(ylp),1,(csum(ylp)/nrow(ylp))).
do if ( 2 =2).
compute LL3 = ylp&*ln(pt2)+(1-ylp)&*ln(1-pt2).
end if.
compute LL3 = -2*csum(LL3).
compute bt1 = make(ncol(xlp),1,0).
compute LL1 = 0.
compute pt1 = make(nrow(ylp),1,0.5).
compute pt1lp=pt1.
loop jjj = 1 to iterate.
compute xlptmp=t(xlp).
compute vecprb=pt1lp&*(1-pt1lp).
loop kkk=1 to ncol(xlp).
compute xlptmp(kkk,:)=xlptmp(kkk,:)&*t(vecprb).
end loop.
compute b = bt1+inv(xlptmp*xlp)*t(xlp)*(ylp-pt1lp).
do if ( 2 =2).
compute xlpb=xlp*b.
compute xlpbt=(xlpb > -709.7).
compute xlpb709=(1-xlpbt)*(-709.7).
compute xlpb=(xlpb&*xlpbt)+xlpb709.
compute pt1lp = 1/(1+exp(-(xlpb))).
end if.
compute itprob = csum((pt1lp < .00000001) or (pt1lp > .9999999)).
do if (itprob > 0).
loop kkk = 1 to nrow(pt1lp).
do if (pt1lp(kkk,1) > .9999999).
compute pt1lp(kkk,1) = .9999999.
end if.
do if (pt1lp(kkk,1) < .00000001).
compute pt1lp(kkk,1) = .00000001.
end if.
end loop.
compute itprob = 0.
end if.
do if (itprob = 0).
do if ( 2 =2).
compute LL = ylp&*ln(pt1lp)+(1-ylp)&*ln(1-pt1lp).
end if.
compute LL2 = -2*csum(ll).
end if.
do if (abs(LL1-LL2) < converge).
do if ( 0 =1).
compute xlptmp=t(xlp).
compute vecprb=pt1lp&*(1-pt1lp).
loop kkk=1 to ncol(xlp).
compute xlptmp(kkk,:)=xlptmp(kkk,:)&*t(vecprb).
end loop.
compute varb = inv(xlptmp*xlp).
compute seb = sqrt(diag(varb)).
release xlptmp.
end if.
break.
end if.
compute bt1 = b.
compute LL1 = LL2.
end loop.
compute modres=b.
do if (jjj > iterate).
compute itprob = 2.
do if (booting=0).
compute iterrmod=1.
end if.
do if (booting=1).
compute bootiter=1.
end if.
do if (itprobtg=0).
compute itprobtg=1.
compute errcode(errs,1) = 47.
compute errs = errs + 1.
do if (booting = 0 and 0 =1).
compute vt1 = mdiag(pt1lp&*(1-pt1lp)).
compute varb = inv(t(xlp)*vt1*xlp).
compute seb = sqrt(diag(varb)).
end if.
end if.
end if.
do if ( 0 =1).
compute trat = b&/seb.
compute dfres=nrow(xlp).
compute p = 2*(1-cdfnorm(abs(trat))).
compute modres={modres,seb,trat,p}.
compute modres={modres,(b-xp2&*seb),(b+xp2&*seb)}.
compute pvchi=1-chicdf((LL3-LL2),(nrow(modres)-1)).
compute mcF = (LL3-LL2)/LL3.
compute cox = 1-exp(-(LL3-LL2)/nrow(xlp)).
compute nagel = cox/(1-exp(-(LL3)/nrow(xlp))).
compute modsum={LL2,(LL3-LL2),(nrow(modres)-1),pvchi, mcF,cox,nagel}.
compute modsuml={'-2LL','ModelLL', 'df', 'p', 'McFadden', 'CoxSnell', 'Nagelkrk'}.
compute modresl={'coeff','se','Z','p','LLCI','ULCI'}.
end if.
end if
.
compute b=btemphld.
compute fresult={(LL2-basemod),llrdf,1-chicdf((LL2-basemod),llrdf)}
.
end if.
compute highf={highf;fresult}.
compute highf2={highf2;fresult}.
do if (j = start).
compute flabel={flabel;'X*W*Z'}.
end if.
do if (j > start).
do if (nms > 1).
compute flabel={flabel;highlbwz(jj,1)}.
else if (nms = 1).
compute flabel={flabel;'M*W*Z'}.
end if.
end if.
end if.
compute jj=jj+1.
end loop.
release jj.
compute start=start+i.
end if.
do if (nrow(highf) > 1).
compute highf=highf(2:nrow(highf),:).
compute highf2=highf2(2:nrow(highf2),:).
compute flabel=flabel(2:nrow(flabel),1).
compute resultm2=make(nrow(highf),maxresm,99999).
compute resultm2(1:nrow(highf),1:ncol(highf))=highf.
compute resultm={resultm;resultm2}.
do if (outscree=1).
do if ((i < nms+nys) or (ydich=0)).
compute clabtmp={'R2-chng', hcflab,'df1','df2','p'}.
print highf/format = F10.4 /rnames=flabel/cnames=clabtmp/ title = 'Test(s) of highest order unconditional interaction(s):'.
end if.
do if (ydich=1 and i=(nms+nys)).
compute clabtmp={'Chi-sq', 'df','p'}.
print/title='Likelihood ratio test(s) of highest order'.
print highf/format = F10.4 /rnames=flabel/cnames=clabtmp/ title = 'unconditional interactions(s):'/space=0.
end if.
end if.
compute intpb=highf2(:,ncol(highf2)).
end if.
compute intstart=intstart+numint(1,i).
end if.

.
do if (criterr=0).
compute threeway=0.
compute didprint=0.
compute didsome=0.
compute sigintct=0.
loop jmed =1 to (nms+1).
compute hasw=0.
compute hasz=0.
compute jnok=0.
compute nm1vls=0.
compute nm2vls=0.
compute panelgrp=0.
compute graphixs={'WITH', outnames(1,i), 'BY'}.
compute focpred4={' '}.
compute intprint=0.
compute modcat=0.
do if (jmed <= i).
do if ((jmed = 1) and ((i+1) = nrow(bcmat))).
compute pathscnt=pathscnt+1.
else.
compute paths={paths,bcmat((i+1),jmed)}.
compute pathsw={pathsw,wcmat((i+1),jmed)}.
compute pathsz={pathsz,zcmat((i+1),jmed)}.
compute pathswz={pathswz,wzcmat((i+1),jmed)}.
compute temp=fochigh(:,pathscnt)&*bootloc(:,i).
compute pathsfoc={pathsfoc,pathsfoc(:,1)}.
do if (jmed=1).
compute pathtype={pathtype,1}.
end if.
do if ((i+1)=nrow(bcmat)).
compute pathtype={pathtype,3}.
end if.
do if (jmed > 1) and ((i+1) < nrow(bcmat)).
compute pathtype={pathtype,2}.
end if.
do if (jmed=1 and nxvls > 1 and (bcmat((i+1),jmed)=1)).
compute pathsfoc(:,(pathscn2+1))=temp(2:(nxvls+1),1).
end if.
do if ((jmed > 1) or (jmed=1 and nxvls=1)).
compute temp=cmax(temp).
compute pathsfoc(1,(pathscn2+1))=temp.
end if.
compute pathscnt=pathscnt+1.
compute pathscn2=pathscn2+1.
do if (i <= nms).
compute pathsdv={pathsdv,mnames(1,i)}.
end if.
do if (i > nms).
compute pathsdv={pathsdv,ynames}.
end if.
end if.
compute coeffcol=coeffcol+1.
compute probettt=coeffs(1:nrow(b),coeffcol).
do if (jmed=1 and (bcmat((i+1),jmed)=1)).
compute omni=make(nrow(probettt),nxvls,0).
compute omnitmp=ident(nxvls).
compute omni(2:(1+nxvls),:)=omnitmp.
end if.
do if (csum(probettt)>0).
compute probvarb=make(csum(probettt),csum(probettt),999).
compute probcoef=make(csum(probettt),1,999).
compute coefflp2=1.
loop coefflp=1 to nrow(probettt).
do if (probettt(coefflp,1)=1).
compute probcoef(coefflp2,1)=b(coefflp,1).
compute coefflp2=coefflp2+1.
end if.
end loop.
compute coefflp=0.
compute coefflp2=0.
loop iclp=1 to nrow(probettt).
do if probettt(iclp,1)=1.
compute coefflp=coefflp+1.
compute coefflp2=coefflp.
compute probvarb(coefflp,coefflp) = varb(iclp,iclp).
do if (iclp < nrow(probettt)).
loop jclp=(iclp+1) to nrow(probettt).
do if (probettt(jclp,1)=1).
compute coefflp2=coefflp2+1.
compute probvarb(coefflp,coefflp2)=varb(iclp, jclp).
compute probvarb(coefflp2,coefflp)=varb(iclp, jclp).
end if.
end loop.
end if.
end if.
end loop.
end if.
end if.
compute xprobval=xmodvals.
do if (nxvls > 1 or mcx > 0).
compute xprobval=dummatx(:,2:ncol(dummatx)).
end if.
do if ((wcmat((i+1),jmed)=1) and (zcmat((i+1),jmed)=0)).
compute numplps=1.
compute modvals=wmodvals.
compute probeval=wmodvals.
compute wheremv1=wherexw.
compute nm1vls=nwvls.
compute lpstsp={1,1}.
compute modcat=0.
compute jnmod=wtmp.
compute jnmodlab=wnames.
compute jnok=1.
compute jnmin=wmin.
compute jnmax=wmax.
compute wherejn1=2.
do if (jmed=1).
compute wherejn3=wherexw(1,i).
do if (nxvls > 1).
compute jnok=0.
end if.
end if.
do if (jmed > 1).
compute wherejn1=wherem((jmed-1),i).
compute wherejn3=wheremw(((2*jmed)-3),i).
end if.
do if (nwvls > 1).
compute probeval=wprobval.
compute lpstsp(1,2)=ncol(probeval).
compute modcat=1.
compute jnok=0.
end if.
do if (wdich = 1).
compute modcat=1.
compute jnok=0.
end if.
compute problabs=wnames.
compute focpred3={wnames,'(W)'}.
do if (xmint=1).
compute focpred3={wnames,'(X)'}.
end if.
compute hasw=1.
compute modgrph=wnames.
compute intprint=1.
compute sigintct=sigintct+1.
compute printpbe=intpb(sigintct,1).
end if.
do if ((wcmat((i+1),jmed)=0) and (zcmat((i+1),jmed)=1)).
compute numplps=1.
compute modvals=zmodvals.
compute probeval=zmodvals.
compute wheremv1=wherexz.
compute nm1vls=nzvls.
compute lpstsp={1,1}.
compute jnok=1.
compute jnmod=ztmp.
compute jnmin=zmin.
compute jnmax=zmax.
compute jnmodlab=znames.
compute wherejn1=2.
do if (jmed=1).
compute wherejn3=wherexz(1,i).
do if (nxvls > 1).
compute jnok=0.
end if.
end if.
do if (jmed > 1).
compute wherejn1=wherem((jmed-1),i).
compute wherejn3=wheremz(((2*jmed)-3),i).
end if.
do if (nzvls > 1).
compute probeval=zprobval.
compute lpstsp(1,2)=ncol(probeval).
compute modcat=1.
compute jnok=0.
end if.
do if (zdich = 1).
compute modcat=1.
compute jnok=0.
end if.
compute problabs=znames.
compute focpred3={znames,'(Z)'}.
compute modgrph=znames.
compute hasz=1.
compute intprint=1.
compute sigintct=sigintct+1.
compute printpbe=intpb(sigintct,1).
end if.
do if ((wzcmat((i+1),jmed)=1) or ((wcmat((i+1),jmed)=1) and (zcmat((i+1),jmed)=1))).
compute numplps=2.
compute probecnt=1.
compute intprint=1.
do if (wzcmat((i+1),jmed)=1).
compute sigintct=sigintct+1.
compute printpbe=intpb(sigintct,1).
else.
compute sigintct=sigintct+2.
compute printpbe=cmin(intpb((sigintct-1):sigintct,1)).
end if.
compute panelgrp=1.
compute hasw=1.
compute hasz=1.
compute panelcde={'/PANEL','ROWVAR=',znames,'.'}.
compute modgrph=wnames.
compute lpstsp={1,1;1,1}.
compute wheremv1=wherexw.
compute nm1vls=nwvls.
compute wheremv2=wherexz.
compute nm2vls=nzvls.
compute jnok=0.
do if (wzcmat((i+1),jmed)=1).
compute jnok=1.
end if.
do if (jmed > 1).
compute mprobval=mmodvals.
end if.
do if (jmed=1).
do if (nxvls > 1).
compute jnok=0.
end if.
end if.
do if (nwvls > 1).
compute lpstsp(1,2)=ncol(wprobval).
compute modcat=1.
compute jnok=0.
end if.
do if (zdich=1).
compute modcat=1.
compute jnok=0.
end if.
compute lpstsp(2,1)=lpstsp(1,2)+1.
compute lpstsp(2,2)=lpstsp(1,2)+1.
do if (nzvls > 1).
compute lpstsp(2,1)=lpstsp(1,2)+1.
compute lpstsp(2,2)=lpstsp(1,2)+ncol(zprobval).
compute jnok=0.
end if.
do if (zdich=1).
compute jnok=0.
end if.
compute omni3=make(nrow(b),(nxvls*nwvls),0).
do if (jmed > 1).
compute omni3=make(nrow(b),nwvls,0).
end if.
compute focpred3={wnames,'(W)'}.
do if (xmint=1).
compute focpred3={wnames,'(X)'}.
end if.
compute focpred4={'    ', 'Mod var:', znames, '(Z)'}.
compute modvals=make((nrow(wmodvals)*nrow(zmodvals)),2,0).
compute probeval=make((nrow(wmodvals)*nrow(zmodvals)),(ncol(wprobval)+ncol(zprobval)),0).
loop probei= 1 to nrow(wmodvals).
loop probej =1 to nrow(zmodvals).
compute modvals(probecnt,1)=wmodvals(probei,1).
compute probeval(probecnt,1:nwvls)=wprobval(probei,:).
compute modvals(probecnt,2)=zmodvals(probej,1).
compute probeval(probecnt,(nwvls+1):(nwvls+nzvls))=zprobval(probej,:).
compute probecnt=probecnt+1.
end loop.
end loop.
do if (wzcmat((i+1),jmed)=1).
compute numplps=numplps+1.
compute probprod=make(1,(ncol(wprobval)*ncol(zprobval)),0).
compute lpstsp2={1,1}.
compute lpstsp={lpstsp;lpstsp2}.
compute lpstsp(3,1)=lpstsp(2,2)+1.
compute lpstsp(3,2)=lpstsp(2,2)+ncol(probprod).
compute jnmod=ztmp.
compute jnmin=zmin.
compute jnmax=zmax.
compute jnmodlab=znames.
do if (jmed = 1).
compute wherejn1=wherexw(1,i).
compute wherejn3=wherexwz(1,i).
end if.
do if (jmed > 1).
compute wherejn1=wheremw(((2*jmed)-3),i).
compute wherejn3=wheremwz(((2*jmed)-3),i).
end if.
loop probei = 1 to nrow(wmodvals).
loop probej = 1 to nrow(zmodvals).
compute probtemp=1.
loop probek = 1 to ncol(wprobval).
compute probtemp={probtemp,(wprobval(probei,probek)&*zprobval(probej,:))}.
end loop.
compute probprod={probprod;probtemp(1,2:ncol(probtemp))}.
end loop.
end loop.
compute probprod=probprod(2:nrow(probprod),:).
compute probeval={probeval,probprod}.
end if.
compute problabs={wnames,znames}.
release probecnt, probei, probej.
end if.
do if (intprint=1).
compute focpred={'   Focal', 'predict:'}.
do if (jmed=1).
compute focpred={focpred,xnames,'(X)'}.
compute focplotv=xmodvals.
end if.
do if (jmed >1).
do if (nms > 1).
compute focpred={focpred,mnames(1,(jmed-1)), medlb2(1,(jmed-1))}.
end if.
do if (nms = 1).
compute focpred={focpred,mnames(1,(jmed-1)), '(M)'}.
end if.
compute focplotv=mmodvals(:,(jmed-1)).
end if.
compute focpred2={'    ', 'Mod var:',focpred3}.
compute focpred={focpred;focpred2}.
do if (ncol(focpred4) > 1).
compute focpred={focpred;focpred4}.
compute focpred4={' '}.
end if.
release focpred2,focpred3.
do if (outscree=1).
do if ((plot = 1 or plot = 2) or (printpbe <= intprobe)).
print focpred/title='----------'/format=A8/space=0.
end if.
end if.
compute foctmp=make(nrow(modvals),1,1).
compute probexpl=1.
compute probeva2={foctmp,probeval}.
do if (jmed=1 and nxs > 0 and mcx > 0).
compute probexpl=nxvls.
end if.
compute foctmp=make(nrow(modvals),1,1).
compute modvals3=make(1,(6+ncol(problabs)),0).
compute probrown=make(nrow(probeval),1,0).
compute jtmp=1.
loop probei = 1 to nrow(probeval).
compute probrown(probei,1)=jtmp.
compute jtmp=jtmp+nxvls.
end loop.
release jtmp.
compute probrow=999.
compute modvarl=problabs.
do if (plot = 1 or plot = 2 or nxvls > 1).
compute plotvals=make((nrow(modvals)*nrow(focplotv)),(ncol(modvals)+1),999).
loop ploti=1 to nrow(modvals).
loop plotj=1 to nrow(focplotv).
compute plotvals((((ploti-1)*nrow(focplotv))+plotj),2:ncol(plotvals))=modvals(ploti,:).
compute plotvals((((ploti-1)*nrow(focplotv))+plotj),1)=focplotv(plotj,1).
end loop.
end loop.
compute focpredn=3.
do if (jmed=1).
do if (nxvls > 1).
compute focpredn=(nxvls+1).
end if.
do if (nxvls=1 and xdich=1).
compute focpredn=2.
end if.
end if.
compute meanmat=mdiag(means).
compute onesmat=make(nrow(meanmat),(nrow(probeval)*focpredn),1).
compute probeplt=t(mdiag(means)*onesmat).
do if (jmed=1).
do if ((wcmat((i+1),1)=1) or (zcmat((i+1),1)=1)).
compute plotcnt=1.
compute iloops=nwpval*nzpval.
compute plotmx=nxpval*nzpval.
do if ((wcmat((i+1),1)=1) and (zcmat((i+1),1)=0)).
compute iloops=nwpval.
compute plotmx=nxpval.
end if.
do if ((wcmat((i+1),1)=0) and (zcmat((i+1),1)=1)).
compute iloops=nzpval.
compute plotmx=nxpval.
end if.
compute xestvals=make((nxpval*iloops),ncol(xprobval),-999).
do if (wcmat((i+1),1)=1).
compute westvals=make(nrow(xestvals),ncol(wprobval),-999).
end if.
do if (zcmat((i+1),1)=1).
compute zestvals=make(nrow(xestvals),ncol(zprobval),-999).
end if.
loop ploti=1 to iloops.
loop plotj=1 to nxpval.
compute xestvals(plotcnt,:)=xprobval(plotj,:).
compute plotcnt=plotcnt+1.
end loop.
end loop.
compute plotcnt=1.
compute plotcnt1=1.
compute plotcnt2=1.
compute plotcntz=1.
loop ploti = 1 to (iloops*nxpval).
do if (wcmat((i+1),1)=1).
compute westvals(ploti,:)=wprobval(plotcnt1,:).
end if.
do if ((wcmat((i+1),1)=0) and (zcmat((i+1),1)=1)).
compute zestvals(ploti,:)=zprobval(plotcnt1,:).
end if.
do if ((wcmat((i+1),1)=1) and (zcmat((i+1),1)=1)).
compute zestvals(ploti,:)=zprobval(plotcnt2,:).
compute plotcntz=plotcntz+1.
end if.
compute plotcnt=plotcnt+1.
do if (plotcnt > plotmx).
compute plotcnt=1.
compute plotcnt1=plotcnt1+1.
end if.
do if (plotcntz > nxpval).
compute plotcnt2=plotcnt2+1.
compute plotcntz=1.
do if (plotcnt2 > nzpval).
compute plotcnt2=1.
end if.
end if.
end loop.
compute probeplt(:,2:(1+(ncol(xestvals))))=xestvals.
do if (wcmat((i+1),1)=1).
compute probeplt(:,(wherew(1,i)):(wherew(2,i)))=westvals.
end if.
do if (zcmat((i+1),1)=1).
compute probeplt(:,(wherez(1,i)):(wherez(2,i)))=zestvals.
end if.
end if.
end if.
do if (jmed > 1).
do if ((wcmat((i+1),jmed)=1) or (zcmat((i+1),jmed)=1)).
compute plotcnt=1.
compute iloops=nwpval*nzpval.
compute plotmx=3*nzpval.
do if ((wcmat((i+1),jmed)=1) and (zcmat((i+1),jmed)=0)).
compute iloops=nwpval.
compute plotmx=3.
end if.
do if ((wcmat((i+1),jmed)=0) and (zcmat((i+1),jmed)=1)).
compute iloops=nzpval.
compute plotmx=3.
end if.
compute mestvals=make((3*iloops),1,-999).
do if (wcmat((i+1),jmed)=1).
compute westvals=make(nrow(mestvals),ncol(wprobval),-999).
end if.
do if (zcmat((i+1),jmed)=1).
compute zestvals=make(nrow(mestvals),ncol(zprobval),-999).
end if.
loop ploti=1 to iloops.
loop plotj=1 to 3.
compute mestvals(plotcnt,:)=mprobval(plotj,(jmed-1)).
compute plotcnt=plotcnt+1.
end loop.
end loop.
compute plotcnt=1.
compute plotcnt1=1.
compute plotcnt2=1.
compute plotcntz=1.
loop ploti = 1 to (iloops*3).
do if (wcmat((i+1),jmed)=1).
compute westvals(ploti,:)=wprobval(plotcnt1,:).
end if.
do if ((wcmat((i+1),jmed)=0) and (zcmat((i+1),jmed)=1)).
compute zestvals(ploti,:)=zprobval(plotcnt1,:).
end if.
do if ((wcmat((i+1),jmed)=1) and (zcmat((i+1),jmed)=1)).
compute zestvals(ploti,:)=zprobval(plotcnt2,:).
compute plotcntz=plotcntz+1.
end if.
compute plotcnt=plotcnt+1.
do if (plotcnt > plotmx).
compute plotcnt=1.
compute plotcnt1=plotcnt1+1.
end if.
do if (plotcntz > 3).
compute plotcnt2=plotcnt2+1.
compute plotcntz=1.
do if (plotcnt2 > nzpval).
compute plotcnt2=1.
end if.
end if.
end loop.
compute probeplt(:,wherem((jmed-1),i))=mestvals.
do if (wcmat((i+1),jmed)=1).
do if (model <> 74).
compute probeplt(:,(wherew(1,i)):(wherew(2,i)))=westvals.
end if.
do if (model = 74).
compute probeplt(:,(wherex(1,i)):(wherex(2,i)))=westvals.
end if.
end if.
do if (zcmat((i+1),jmed)=1).
compute probeplt(:,(wherez(1,i)):(wherez(2,i)))=zestvals.
end if.
end if.
end if.
compute prodloop = 1.
do if (jmed=1).
compute prodloop=ncol(xestvals).
end if.
do if (wcmat((i+1),jmed))=1.
compute plotcnt=0.
loop ploti = 1 to prodloop.
loop plotj = 1 to ncol(westvals).
do if (jmed=1).
compute probeplt(:,(wherexw(1,i)+plotcnt))=xestvals(:,ploti)&*westvals(:,plotj).
end if.
do if (jmed > 1).
compute probeplt(:,(wheremw(((jmed*2)-3) ,i)+plotcnt))=mestvals(:,ploti)&*westvals(:,plotj).
end if.
compute plotcnt=plotcnt+1.
end loop.
end loop.
end if.
do if (zcmat((i+1),jmed))=1.
compute plotcnt=0.
loop ploti = 1 to prodloop.
loop plotj = 1 to ncol(zestvals).
do if (jmed = 1).
compute probeplt(:,(wherexz(1,i)+plotcnt))=xestvals(:,ploti)&*zestvals(:,plotj).
end if.
do if (jmed > 1).
compute probeplt(:,(wheremz(((jmed*2)-3),i)+plotcnt))=mestvals(:,ploti)&*zestvals(:, plotj).
end if.
compute plotcnt=plotcnt+1.
end loop.
end loop.
end if.
do if (wzcmat((i+1),jmed))=1.
compute plotcnt=0.
compute threeway=1.
loop ploti = 1 to ncol(westvals).
loop plotj = 1 to ncol(zestvals).
compute probeplt(:,(wherewz(1,i)+plotcnt))=westvals(:,ploti)&*zestvals(:,plotj).
compute plotcnt=plotcnt+1.
end loop.
end loop.
compute plotcnt=0.
loop plotk = 1 to prodloop.
loop ploti = 1 to ncol(westvals).
loop plotj = 1 to ncol(zestvals).
do if (jmed = 1).
compute probeplt(:,(wherexwz(1,i)+plotcnt))=xestvals(:,plotk)&*westvals(:,ploti)&* zestvals(:,plotj).
end if.
do if (jmed > 1).
compute probeplt(:,(wheremwz(((jmed*2)-3),i)+plotcnt))=mestvals(:,plotk)&* westvals(:,ploti)&*zestvals(:,plotj).
end if.
compute plotcnt=plotcnt+1.
end loop.
end loop.
end loop.
end if.
loop newplp=1 to i.
do if (newplp <> jmed).
do if (wcmat((i+1),newplp))=1.
compute prodloop=1.
do if (newplp=1).
compute prodloop=nxvls.
end if.
compute plotcnt=0.
loop ploti = 1 to prodloop.
loop plotj = 1 to nwvls.
do if (newplp = 1).
compute probeplt(:,(wherexw(1,i)+plotcnt))=probeplt(:,(1+ploti))&*probeplt(:, (wherew(1,i)+plotj-1)).
end if.
do if (newplp > 1).
do if (model <> 74).
compute probeplt(:,(wheremw(((newplp*2)-3) ,i)+plotcnt))=probeplt(:,wherem((newplp-1),i))&*probeplt(:,(wherew(1,i)+plotj-1)).
end if.
do if (model = 74).
compute probeplt(:,(wheremw(((newplp*2)-3) ,i)+plotcnt))=probeplt(:,wherem((newplp-1),i))&*probeplt(:,(wherex(1,i)+plotj-1)).
end if.
end if.
compute plotcnt=plotcnt+1.
end loop.
end loop.
end if.
do if (zcmat((i+1),newplp))=1.
compute prodloop=1.
do if (newplp=1).
compute prodloop=nxvls.
end if.
compute plotcnt=0.
loop ploti = 1 to prodloop.
loop plotj = 1 to nzvls.
do if (newplp = 1).
compute probeplt(:,(wherexz(1,i)+plotcnt))=probeplt(:,(1+ploti))&*probeplt(:, (wherez(1,i)+plotj-1)).
end if.
do if (newplp > 1).
compute probeplt(:,(wheremz(((newplp*2)-3),i)+plotcnt))=probeplt(:, wherem((newplp-1),i))&*probeplt(:,(wherez(1,i)+plotj-1)).
end if.
compute plotcnt=plotcnt+1.
end loop.
end loop.
end if.
do if (wzcmat((i+1),newplp))=1.
compute plotcnt=0.
do if (threeway=0).
loop ploti = 1 to nwvls.
loop plotj = 1 to nzvls.
compute probeplt(:,(wherewz(1,i)+plotcnt))=probeplt(:,(wherew(1,i)+ploti-1))&* probeplt(:,(wherez(1,i)+plotj-1)).
compute plotcnt=plotcnt+1.
end loop.
end loop.
end if.
compute prodloop=1.
do if (newplp=1).
compute prodloop=nxvls.
end if.
compute plotcnt=0.
loop plotk = 1 to prodloop.
loop ploti = 1 to nwvls.
loop plotj = 1 to nzvls.
do if (newplp = 1).
compute probeplt(:,(wherexwz(1,i)+plotcnt))=probeplt(:,(1+plotk))&*probeplt(:, (wherew(1,i)+ploti-1))&*probeplt(:,(wherez(1,i)+plotj-1)).
end if.
do if (newplp > 1).
compute probeplt(:,(wheremwz(((newplp*2)-3),i)+plotcnt))=probeplt(:, wherem((newplp-1),i))&*probeplt(:,(wherew(1,i)+ploti-1))&*probeplt(:,(wherez(1,i)+plotj-1)).
end if.
compute plotcnt=plotcnt+1.
end loop.
end loop.
end loop.
end if.
end if.
end loop.
compute predvals=probeplt*b.
do if (debug <> 0).
print probeplt.
print b.
end if.
do if (i = nms+nys) and (ydich=1).
compute predvalt=(predvals < 709.7).
compute prevalt7=(1-predvalt)*(709.7).
compute predvals=(predvals&*predvalt)+prevalt7.
compute expyhat=exp(predvals)&/(1+exp(predvals)).
end if.
compute sepred=make(nrow(plotvals),3,999).
loop sei=1 to nrow(plotvals).
compute ask=probeplt(sei,:).
compute sepred(sei,1)=sqrt(ask*varb*t(ask)).
do if ((i < nms+nys) or (ydich=0)).
compute sepred(sei,2)=predvals(sei,1)-tval*sepred(sei,1).
compute sepred(sei,3)=predvals(sei,1)+tval*sepred(sei,1).
end if.
do if ((i = nms+nys) and (ydich=1)).
compute sepred(sei,2)=predvals(sei,1)-xp2*sepred(sei,1).
compute sepred(sei,3)=predvals(sei,1)+xp2*sepred(sei,1).
end if.
end loop.
compute prevloc=ncol(plotvals)+1.
compute probeplt={plotvals,predvals}.
do if (plot = 2).
compute probeplt={probeplt,sepred}.
end if.
do if ((i = nms+nys) and (ydich=1)).
compute probeplt={probeplt,expyhat}.
end if.
compute didsome=0.
end if.
do if ((wzcmat((i+1),jmed)=1) and (printpbe <= intprobe)).
do if (jmed=1).
compute omnilp2=nxvls*nwvls.
compute omnitmp=ident(omnilp2).
compute omni3(wherexw(1,i):wherexw(2,i),:)=omnitmp.
end if.
do if (jmed>1).
compute omnilp2=nwvls.
compute omnitmp=ident(omnilp2).
compute omni3(wheremw(((jmed*2)-3),i):wheremw(((jmed*2)-2),i),:)=omnitmp.
end if.
compute omnif=make(1,4,0).
do if ((i = nms+nys) and (ydich=1)).
compute omnif=make(1,3,0).
end if.
compute condeff3=0.
loop omnilp1=1 to nrow(zprobval).
loop omnilp=1 to (omnilp2).
do if (jmed=1).
compute omni3((wherexwz(1,i)+((omnilp-1)*nzvls)):(wherexwz(1,i)+((omnilp-1)*nzvls)+ (nzvls-1)),omnilp)=t(zprobval(omnilp1,:)).
end if.
do if (jmed > 1).
compute omni3((wheremwz(((jmed*2)-3),i)+((omnilp-1)*nzvls)):(wheremwz(((jmed*2)-3),i)+ ((omnilp-1)*nzvls)+(nzvls-1)),omnilp)=t(zprobval(omnilp1,:)).
end if.
end loop.
compute condeff=t(omni3)*b.
compute condeff3={condeff3;condeff}.

.
compute lmat2= omni3.
do if ( 1 =0).
compute lmat2 = mdiag( omni3 ).
compute lmat3=make(nrow(lmat2),1,0).
loop flp=1 to ncol(lmat2).
do if (csum(lmat2(:,flp))=1).
compute lmat3={lmat3,lmat2(:,flp)}.
end if.
end loop.
compute lmat2=lmat3(:,2:ncol(lmat3)).
end if.
compute fratio = (t(t(lmat2)* b )*inv(t(lmat2)* varb *lmat2)*((t(lmat2)* b )))/ncol(lmat2).
compute pfr = 1-fcdf(fratio,ncol(lmat2),(n-nrow( b ))).
compute fresult={fratio,ncol(lmat2),(n-nrow( b )),pfr}.
do if (i = (nms+nys) and (ydich=1)).
compute fratio=fratio*ncol(lmat2).
compute pfr=1-chicdf(fratio,ncol(lmat2)).
compute fresult={fratio,ncol(lmat2),pfr}.
end if.
do if ( 0 =1).
compute lmat3=1-rsum(lmat2).
compute xfm=make(n,csum(lmat3),0).
compute flpc=1.
loop flp=1 to nrow(lmat3).
do if (lmat3(flp,1)=1).
compute xfm(:,flpc)=x(:,flp).
compute flpc=flpc+1.
end if.
end loop.
compute bfm=inv(t(xfm)*xfm)*t(xfm)*y.
compute resid=y-(xfm*bfm).
compute sstotal=(y-(csum(y)/n)).
compute sstotal=csum(sstotal&*sstotal).
compute ssresid=csum(resid&*resid).
compute rsqch= 0 -((sstotal-ssresid)/sstotal).
compute fresult={rsqch,fresult}.
release xfm,flpc, resid, ssresid, bfm.
end if
.
compute omnif={omnif;fresult}.
end loop.
compute omnif=omnif(2:nrow(omnif),:).
compute clabtmp=znames.
compute condeff3=condeff3(2:nrow(condeff3),:).
do if ((nxvls*nwvls)=1).
compute omnif={condeff3,omnif}.
compute clabtmp={clabtmp,'Effect'}.
end if.
compute omnif={zmodvals,omnif}.
do if ((i < nms+nys) or (ydich=0)).
compute clabtmp={clabtmp,hcflab,'df1','df2','p'}.
end if.
do if ((i = (nms+nys)) and (ydich=1)).
compute clabtmp={clabtmp,'Chi-sq','df','p'}.
end if.
compute resultm2=make(nrow(omnif),maxresm,99999).
compute resultm2(1:nrow(omnif),1:ncol(omnif))=omnif.
compute resultm={resultm;resultm2}.
do if (outscree=1).
do if (jmed=1).
print omnif/title='Test of conditional X*W interaction at value(s) of Z:'/cnames=clabtmp/format= F10.4.
end if.
do if (jmed>1).
print omnif/title='Test of conditional M*W interaction at value(s) of Z:'/cnames=clabtmp/format= F10.4.
end if.
end if.
release omni3.
end if.
loop probei = 1 to probexpl.
do if (probexpl > 1).
compute foctmp=make(nrow(modvals),probexpl,0).
compute foctmp(:,probei)=foctmp(:,probei)+1.
compute probtemp=make(nrow(modvals),1,0).
loop probem = 1 to numplps.
loop probek = 1 to nxvls.
loop probej=lpstsp(probem,1) to lpstsp(probem,2).
compute probtemp={probtemp,foctmp(:,probek)&*probeval(:,probej)}.
end loop.
end loop.
end loop.
compute probeva2=probtemp(:,2:ncol(probtemp)).
compute probeva2={foctmp,probeva2}.
end if.
compute probres=probeva2*probcoef.
compute probrese=sqrt(diag(probeva2*probvarb*t(probeva2))).
compute tratio = probres&/probrese.
compute p = 2*(1-tcdf(abs(tratio), dfres)).
do if (ydich=1 and i = (nms+nys)).
compute p = 2*(1-cdfnorm(abs(tratio))).
end if.
compute modvals2={modvals,probres,probrese,tratio, p}.
do if ((i < nms+nys) or (ydich=0)).
compute modvals2={modvals2,(probres-tval&*probrese),(probres+tval&*probrese)}.
compute problabs={problabs,'Effect',hclab,'t', 'p', 'LLCI', 'ULCI'}.
end if.
do if (ydich=1 and i = (nms+nys)).
compute modvals2={modvals2,(probres-xp2&*probrese),(probres+xp2&*probrese)}.
compute problabs={problabs,'Effect','se','Z', 'p', 'LLCI', 'ULCI'}.
end if.
do if (probexpl > 1 and (printpbe <= intprobe)).
do if (hasz = 1).
compute printz=1.
end if.
do if (hasw=1).
compute printw=1.
end if.
compute probrlab=make(nrow(modvals),1,xcatlab(probei,1)).
compute modvals3={modvals3; modvals2}.
compute probrow={probrow;probrown}.
compute probrown=probrown+1.
do if (probei=probexpl).
compute xproblab=xcatlab(1:nxvls,1).
compute probrow=probrow(2:nrow(probrow),1).
compute modvals3=modvals3(2:nrow(modvals3),:).
compute temp=modvals3.
compute temp(GRADE(probrow(:,1)),:)=modvals3.
compute modvals3=temp.
compute start2=1.
compute problabs=problabs(1,(1+(ncol(modvarl))):ncol(problabs)).
compute pstart=1.
loop probek= 1 to nrow(probeval).
compute endstart=start2+(nxvls-1).
compute temp=modvals3(start2:endstart,(1+ncol(modvarl)):ncol(modvals3)).
compute temp2=t(modvals3(start2:start2,1:ncol(modvarl))).
compute trnames=t(modvarl).
do if (outscree=1).
do if (probek > 1).
print/title='----------'/space=0.
else.
print/title = 'Conditional effects of the focal predictor at values of the moderator(s):'.
do if ((jmed=1) and (i = (nms+nys)) and (nms > 0)).
do if (nxvls = 1).
print/title = '(These are also the conditional direct effects of X on Y)'/space=0.
else.
print/title = '(These are also the relative conditional direct effects of X on Y)'/space=0.
end if.
end if.
print.
end if.
print temp2/title = 'Moderator value(s):'/rnames=trnames/format= F10.4 /space=0.
print temp/title = ' '/cnames=problabs/rnames=xproblab/format= F10.4 /space=0.
end if.
compute resultm2=make(nrow(temp2),maxresm,99999).
compute resultm2(1:nrow(temp2),1:ncol(temp2))=temp2.
compute resultm={resultm;resultm2}.
compute resultm2=make(nrow(temp),maxresm,99999).
compute resultm2(1:nrow(temp),1:ncol(temp))=temp.
compute resultm={resultm;resultm2}.
compute start2=start2+nxvls.
compute didsome=1.
do if (jmed=1).
compute mod1val=probeval(probek,1:nm1vls).
loop omnilp=1 to nxvls.
compute omni((wheremv1(1,i)+((omnilp-1)*nm1vls)):(wheremv1(1,i)+((omnilp-1)* nm1vls)+(nm1vls-1)),omnilp)=t(mod1val).
do if (nm1vls < ncol(probeval)).
compute mod2val=probeval(probek,(nm1vls+1):(nm1vls+nm2vls)).
compute omni((wheremv2(1,i)+((omnilp-1)*nm2vls)):(wheremv2(1,i)+((omnilp-1)* nm2vls)+(nm2vls-1)),omnilp)=t(mod2val).
do if ((nm1vls+nm2vls) < ncol(probeval)).
compute intlen=nm1vls*nm2vls.
compute modintvl=probeval(probek,(nm1vls+nm2vls+1):ncol(probeval)).
compute omni((wherexwz(1,i)+((omnilp-1)*intlen)):(wherexwz(1,i)+((omnilp-1)* intlen)+(intlen-1)),omnilp)=t(modintvl).
end if.
end if.
end loop.

.
compute lmat2= omni.
do if ( 1 =0).
compute lmat2 = mdiag( omni ).
compute lmat3=make(nrow(lmat2),1,0).
loop flp=1 to ncol(lmat2).
do if (csum(lmat2(:,flp))=1).
compute lmat3={lmat3,lmat2(:,flp)}.
end if.
end loop.
compute lmat2=lmat3(:,2:ncol(lmat3)).
end if.
compute fratio = (t(t(lmat2)* b )*inv(t(lmat2)* varb *lmat2)*((t(lmat2)* b )))/ncol(lmat2).
compute pfr = 1-fcdf(fratio,ncol(lmat2),(n-nrow( b ))).
compute fresult={fratio,ncol(lmat2),(n-nrow( b )),pfr}.
do if (i = (nms+nys) and (ydich=1)).
compute fratio=fratio*ncol(lmat2).
compute pfr=1-chicdf(fratio,ncol(lmat2)).
compute fresult={fratio,ncol(lmat2),pfr}.
end if.
do if ( 0 =1).
compute lmat3=1-rsum(lmat2).
compute xfm=make(n,csum(lmat3),0).
compute flpc=1.
loop flp=1 to nrow(lmat3).
do if (lmat3(flp,1)=1).
compute xfm(:,flpc)=x(:,flp).
compute flpc=flpc+1.
end if.
end loop.
compute bfm=inv(t(xfm)*xfm)*t(xfm)*y.
compute resid=y-(xfm*bfm).
compute sstotal=(y-(csum(y)/n)).
compute sstotal=csum(sstotal&*sstotal).
compute ssresid=csum(resid&*resid).
compute rsqch= 0 -((sstotal-ssresid)/sstotal).
compute fresult={rsqch,fresult}.
release xfm,flpc, resid, ssresid, bfm.
end if
.
compute resultm2=make(nrow(fresult),maxresm,99999).
compute resultm2(1:nrow(fresult),1:ncol(fresult))=fresult.
compute resultm={resultm;resultm2}.
do if (outscree=1).
do if (i < (nms + nys) or (ydich = 0)).
compute clabtmp={hcflab,'df1','df2','p'}.
print fresult/title='Test of equality of conditional means'/cnames=clabtmp/format= F10.4.
end if.
do if (i = (nms + nys) and (ydich = 1)).
compute clabtmp={'Chi-sq','df','p'}.
print fresult/title='Test of equality of conditional logits or probabilities'/cnames=clabtmp/format= F10.4.
end if.
end if.
compute probetmp=probeplt(pstart:(pstart+nxvls),1).
compute probetmp={probetmp,probeplt(pstart:(pstart+nxvls),prevloc:ncol(probeplt))}.
compute pstart=pstart+(nxvls+1).
compute resultm2=make(nrow(probetmp),maxresm,99999).
compute resultm2(1:nrow(probetmp),1:ncol(probetmp))=probetmp.
compute resultm={resultm;resultm2}.
do if (i < ((nms + nys)) or (ydich = 0)).
compute clabtmp={xnames, outnames(1,i), hclab, 'LLCI', 'ULCI'}.
do if (outscree=1).
print probetmp/title = 'Estimated conditional means being compared:'/cnames=clabtmp/format= F10.4.
end if.
end if.
do if ((i = (nms+nys)) and (ydich = 1)).
compute clabtmp={xnames, outnames(1,i), 'prob'}.
compute probetm2=probetmp(:,1:2).
compute probetm2={probetm2,probetmp(:,ncol(probetmp))}.
do if (outscree=1).
print probetm2/title = 'Estimated conditional logits and probabilities:'/cnames=clabtmp/format= F10.4.
end if.
end if.
end if.
end loop.
release probrow, start2, endstart, temp, temp2.
end if.
end if.
do if (probexpl = 1 and (printpbe <= intprobe)).
compute resultm2=make(nrow(modvals2),maxresm,99999).
compute resultm2(1:nrow(modvals2),1:ncol(modvals2))=modvals2.
compute resultm={resultm;resultm2}.
do if (outscree=1).
print/title = 'Conditional effects of the focal predictor at values of the moderator(s):'.
print modvals2/cnames=problabs/title = ' '/space=0/format= F10.4.
end if.
compute didsome=1.
do if (hasz = 1).
compute printz=1.
end if.
do if (hasw=1).
compute printw=1.
end if.
do if (jn = 1 and jnok=1).
do if (criterr = 0).
compute dfres=n-nrow(b).
compute roots=99999.
compute jncrit =(dfres* (exp((dfres-(5/6))*((xp2/(dfres-(2/3)+(.11/dfres)))* (xp2/(dfres-(2/3)+(.11/dfres)))))-1)).
do if (i = (nms+nys) and (ydich=1)).
compute jncrit=xp2*xp2.
end if.
compute jnb1=b(wherejn1,1).
compute jnb3=b(wherejn3,1).
compute jnsb1=varb(wherejn1,wherejn1).
compute jnsb3=varb(wherejn3,wherejn3).
compute jnsb1b3=varb(wherejn1,wherejn3).
compute ajn =(jncrit*jnsb3)-(jnb3*jnb3).
compute bjn = 2*((jncrit*jnsb1b3)-(jnb1*jnb3)).
compute cjn = (jncrit*jnsb1)-(jnb1*jnb1).
compute radarg = (bjn*bjn)-(4*ajn*cjn).
compute den = 2*ajn.
compute nrts = 0.
do if (radarg >= 0 and den <> 0).
compute x21 = (-bjn+sqrt(radarg))/den.
compute x22 = (-bjn-sqrt(radarg))/den.
compute roots = 0.
do if (x21 >= jnmin and x21 <= jnmax).
compute nrts = 1.
compute roots = {roots; x21}.
end if.
do if (x22 >= jnmin and x22 <= jnmax).
compute nrts = nrts + 1.
compute roots = {roots; x22}.
end if.
compute roots={roots,make(nrow(roots),2,0)}.
end if.
do if (nrts > 0).
compute roots = roots(2:nrow(roots),1:3).
compute roots(1,2)=(csum(jnmod < roots(1,1))/n)*100.
compute roots(1,3)=(csum(jnmod > roots(1,1))/n)*100.
do if (nrow(roots)=2).
compute roots(2,2)=(csum(jnmod < roots(2,1))/n)*100.
compute roots(2,3)=(csum(jnmod > roots(2,1))/n)*100.
end if.
do if (outscree=1).
print roots/title = 'Moderator value(s) defining Johnson-Neyman significance region(s):'/clabels = 'Value', '% below', '% above'/format F10.4.
end if.
do if (nrts=1).
compute tmprts=make(1,3,99999).
compute roots={roots;tmprts}.
end if.
end if.
do if (nrts = 0).
compute roots=make(2,1,99999).
do if (outscree=1).
print/title = 'There are no statistical significance transition points within the observed'.
print/title = 'range of the moderator found using the Johnson-Neyman method.'/space=0.
end if.
end if.
compute resultm2=make(nrow(roots),maxresm,99999).
compute resultm2(1:nrow(roots),1:ncol(roots))=roots.
compute resultm={resultm;resultm2}.
compute jnvals=make(23,7,0).
loop jni= 0 to (21-(nrts)).
compute jnvals((jni+1),1)=jnmin+(jni*((jnmax-jnmin)/(21-nrts))).
end loop.
do if (nrts > 0).
loop jni = 1 to nrts.
loop jnj = 2 to (nrow(jnvals)-1).
do if ((roots(jni,1) > jnvals((jnj-1),1)) and (roots(jni,1) < jnvals(jnj,1))).
compute jnvals((jnj+1):(21+jni),1)=jnvals(jnj:(20+jni),1).
compute jnvals(jnj,1)=roots(jni,1).
end if.
end loop.
end loop.
end if.
compute jnvals=jnvals(1:22,:).
loop jni = 1 to nrow(jnvals).
compute jnvals(jni,2)=jnb1+jnb3*jnvals(jni,1).
compute jnvals(jni,3)=sqrt(jnsb1+2*jnvals(jni,1)*jnsb1b3+(jnvals(jni,1)*jnvals(jni, 1))*jnsb3).
compute jnvals(jni,4)=jnvals(jni,2)/jnvals(jni,3).
compute jnvals(jni,5)=2*(1-tcdf(abs(jnvals(jni,4)), dfres)).
compute jnvals(jni,6)=jnvals(jni,2)-sqrt(jncrit)*jnvals(jni,3).
compute jnvals(jni,7)=jnvals(jni,2)+sqrt(jncrit)*jnvals(jni,3).
do if ((i = nms + nys) and (ydich=1)).
compute jnvals(jni,5)=2*(1-cdfnorm(abs(jnvals(jni,4)))).
compute jnvals(jni,6)=jnvals(jni,2)-xp2*jnvals(jni,3).
compute jnvals(jni,7)=jnvals(jni,2)+xp2*jnvals(jni,3).
end if.
end loop.
compute resultm2=make(nrow(jnvals),maxresm,99999).
compute resultm2(1:nrow(jnvals),1:ncol(jnvals))=jnvals.
compute resultm={resultm;resultm2}.
do if ((i < nms+nys) or (ydich=0)).
compute jnclbs={jnmodlab,'Effect',hclab,'t', 'p', 'LLCI', 'ULCI'}.
end if.
do if ((i = nms + nys) and (ydich=1)).
compute jnclbs={jnmodlab,'Effect','se','Z', 'p', 'LLCI', 'ULCI'}.
end if.
do if (((wcmat((i+1),jmed)=1) or (zcmat((i+1),jmed)=1)) and (wzcmat((i+1),jmed)=0)).
do if (outscree=1).
print jnvals/title = 'Conditional effect of focal predictor at values of the moderator:'/cnames =jnclbs/format = F10.4.
end if.
end if.
do if (outscree=1).
do if ((jmed = 1) and (wzcmat((i+1),jmed)=1)).
print jnvals/title = 'Conditional X*W interaction at values of the moderator Z:'/cnames =jnclbs/format = F10.4.
end if.
do if ((jmed > 1) and (wzcmat((i+1),jmed)=1)).
print jnvals/title = 'Conditional M*W interaction at values of the moderator Z:'/cnames =jnclbs/format = F10.4.
end if.
end if.
end if.
end if.
end if.
do if ((i = (nms+nys)) and (jmed=1) and (bcmat(nrow(bcmat),1)=1)).
do if (probei=1).
compute direfflb=problabs.
compute direff=modvals2.
end if.
do if (probei>1).
compute direff={direff;modvals2}.
end if.
end if.
compute intprint=0.
do if ((jmed=1) and (i=1) and nms=0) and modcok=1).
compute contvec2=make(2,1,1).
compute contvec2={contvec2,wcontval,zcontval}.
do if (wzcmat((i+1),jmed)=1).
loop conti= 1 to ncol(wcontval).
loop contj = 1 to ncol(zcontval).
compute contvec2={contvec2,wcontval(:,conti)&*zcontval(:,contj)}.
end loop.
end loop.
end if.
compute conteff=contvec2*probcoef.
compute contdiff=contvec2(1,:)-contvec2(2,:).
compute contse=sqrt(contdiff*probvarb*t(contdiff)).
compute conteffd=conteff(1,1)-conteff(2,1).
compute contvec={contvec,conteff}.
compute contvecm=contvec.
compute resultm2=make(nrow(contvecm),maxresm,99999).
compute resultm2(1:nrow(contvecm),1:ncol(contvecm))=contvecm.
compute resultm={resultm;resultm2}.
do if (outscree=1).
print/title='Contrast between conditional effects of X:'.
print contvec/title=' '/rlabels='Effect1:','Effect2:'/cnames=problabs/format = F10.4 /space=0.
end if.
do if (ydich=0).
compute p=2*(1-tcdf(abs(conteffd/contse), dfres)).
compute contvec={conteffd,contse,conteffd/contse, p}.
compute contvec={contvec,(conteffd-(tval*contse))}.
compute contvec={contvec,(conteffd+(tval*contse))}.
compute contlabs={'Contrast', hclab, 't', 'p', 'LLCI', 'ULCI'}.
end if.
do if (ydich=1).
compute p=2*(1-cdfnorm(abs(conteffd/contse))).
compute contvec={conteffd,contse,conteffd/contse, p}.
compute contvec={contvec,(conteffd-(xp2*contse))}.
compute contvec={contvec,(conteffd+(xp2*contse))}.
compute contlabs={'Contrast', 'se', 'Z', 'p', 'LLCI', 'ULCI'}.
end if.
compute resultm2=make(nrow(contvec),maxresm,99999).
compute resultm2(1:nrow(contvec),1:ncol(contvec))=contvec.
compute resultm={resultm;resultm2}.
do if (outscree=1).
print contvec/title='Test of Effect1 minus Effect2'/format= F10.4 /cnames=contlabs.
end if.
end if.
end loop.
do if (plot = 1 or plot = 2).
compute datalabs={t(focpred(:,3)),outnames(1,i)}.
do if (plot = 1).
compute datalabs={datalabs}.
end if.
do if (plot = 2).
compute datalabs={datalabs,'se', 'LLCI', 'ULCI'}.
end if.
do if ((i = nms+nys) and (ydich=1)).
compute datalabs={datalabs,'prob'}.
end if.
compute resultm2=make(nrow(probeplt),maxresm,99999).
compute resultm2(1:nrow(probeplt),1:ncol(probeplt))=probeplt.
compute resultm={resultm;resultm2}.
compute datalabs={datalabs,'.'}.
do if (outscree=1).
print/title = 'Data for visualizing the conditional effect of the focal predictor:'.
print/title = 'Paste text below into a SPSS syntax window and execute to produce plot.'/space=0.
compute dumb = {' ', ' ', ' ', ' ', ' ', ' ', ' '}.
print datalabs/title = 'DATA LIST FREE/'/format=A10.
print probeplt/title = 'BEGIN DATA.'/format= F10.4 /space=0.
print/title = 'END DATA.'/space=0.
end if.
compute focgrph=datalabs(1,1).
compute graphix={focgrph,graphixs,modgrph}.
do if (((xdich=1) or (nxvls > 1)) and ((modcat=0) and (focgrph = xnames))).
compute graphix={modgrph,graphixs,focgrph}.
end if.
do if (panelgrp = 0).
compute graphix={graphix,'.'}.
else.
compute graphix={graphix,panelcde}.
end if.
do if (outscree=1).
print graphix/title = 'GRAPH/SCATTERPLOT='/format=A8/space=0.
end if.
do if (i = (nms + nys) and ydich = 1).
compute graphixd=graphix.
compute graphixd(1,3)='prob'.
do if (outscree=1).
print graphixd/title = 'GRAPH/SCATTERPLOT='/format=A8/space=0.
end if.
end if.
end if.
end if.
end loop.
release jmed, intprint,didprint.
end if
.
do if (model = 74 and i <= nms).
compute onetemp=make(nrow(xprobval),1,1).
compute mestmt74={onetemp,xprobval}.
do if (ncs > 0).
compute ncovmdl=rsum(ccmat(i,:)).
do if (ncovmdl > 0).
compute cvmnc=make(nrow(mestmt74),ncovmdl,1).
compute cvmnctmp=csum(x(:,(ncol(x)-ncovmdl+1):ncol(x)))/nrow(x).
do if (cuscoval > 0).
compute cvmnctmp=coval.
end if.
loop mestlp=1 to ncovmdl.
compute cvmnc(:,mestlp)=cvmnc(:,mestlp)*cvmnctmp(:,mestlp).
end loop.
compute mestmt74={mestmt74,cvmnc}.
end if.
end if.
compute mest74t=mestmt74*b.
do if (i = 1).
compute mest74=mest74t.
end if.
do if (i > 1).
compute mest74={mest74,mest74t}.
end if.
end if.
do if ((i = (nms+nys)) and (model >= 0 and model < 4) and (linsum(1,1) <> -999)).
compute lhyprob=1.
compute meansub=0.
do if (((nlinsum=nrow(b)) or (nlinsum = (nrow(b)-ncs)))).
do if (nlinsum = (nrow(b)-ncs) and ncs > 0).
compute linsum={linsum,covmeans}.
compute meansub=1.
end if.
compute lhyprob=0.
compute hypest=linsum*b.
compute sehypest=sqrt(linsum*varb*t(linsum)).
compute phypest=2*(1-tcdf(abs(hypest/sehypest), (dfres))).
compute hypest={hypest,sehypest,(hypest/sehypest),phypest,(hypest-tval*sehypest),(hypest+ tval*sehypest)}.
compute resultm2=make(ncol(linsum),maxresm,99999).
compute resultm2(:,1)=t(linsum).
compute resultm={resultm;resultm2}.
compute resultm2=make(1,maxresm,99999).
compute resultm2(1,1:ncol(hypest))=hypest.
compute resultm={resultm;resultm2}.
do if (outscree=1).
compute hyplabs={'Estimate',hclab,'t','p','LLCI','ULCI'}.
print/title='----------'.
print/title='Linear Combination Estimate and Hypothesis Test'/space=0.
print t(linsum)/title= 'Weight vector:'/clabels='weight'/rnames=vlabsm/format F10.4.
print hypest/title=' '/cnames=hyplabs/format= F10.4 /space=0.
do if (meansub=1).
print/title='Covariate weight(s) set to the sample mean.'.
end if.
end if.
end if.
do if (lhyprob=1).
compute notecode(notes,1) = 30.
compute notes = notes + 1.
end if.
end if.
end loop if criterr=1.
compute lastb=b.
compute lastcov=varb.
do if (criterr=0 and dototal = 1).
compute x=xtmp.
compute vlabsm={'constant';xcatlab(1:nxvls,1)}.
do if (ncs > 0).
compute x = {x,ctmp}.
compute vlabsm={vlabsm;t(covnames)}.
end if.
compute x = {ones,x}.

.
do if ( 1 =1).
compute hatmat=inv(t( x )* x )*t( x ).
compute b = hatmat* y.
compute modres=b.
do if ( 1 =1).
compute n1=nrow( x ).
compute dfres=n1-(ncol( x )).
compute sstotal = t( y -(csum( y )/n1))*( y -(csum( y )/n1)).
compute resid= y - x *b.
compute ssresid = csum((resid)&**2).
compute r2 = (sstotal-ssresid)/sstotal.
do if (r2 < 0).
compute r2=0.
end if.
compute adjr2 = 1-((1-r2)*(n1-1)/(dfres)).
compute mse=ssresid/(n1-ncol( x )).

.
compute n1=nrow( x ).
compute invXtX = inv(t( x )* x ).
compute varb = mse *invXtX.
compute k3 = ncol( x ).
compute xhc=0.
do if ( hc <> 5).
compute xhc= x.
compute hat = xhc(:,1).
loop i3=1 to nrow(xhc).
compute hat(i3,1)= xhc(i3,:)*invXtX*t(xhc(i3,:)).
end loop.
do if ( hc = 0 or hc =1).
loop i3 = 1 to k3.
compute xhc(:,i3)=xhc(:,i3)&* resid.
end loop.
end if.
do if ( hc =3 or hc =2).
loop i3=1 to k3.
compute xhc(:,i3) = ( resid &/(1-hat)&**(1/(4- hc )))&*xhc(:,i3).
end loop.
end if.
do if ( hc = 4).
compute hcmn=make(n,2,4).
compute hcmn(:,2)=(n1*hat)/k3.
loop i3= 1 to k3.
compute xhc(:,i3) = ( resid &/(1-hat)&**(rmin(hcmn)/2))&*xhc(:,i3).
end loop.
end if.
compute varb=(invXtX*t(xhc)*xhc*invXtX).
do if ( hc =1).
compute varb=(n1/(n1-ncol( x )))&*varb.
end if.
end if.
compute hclab={'se(HC0)','se(HC1)','se(HC2)','se(HC3)','se(HC4)','se'}.
compute hclab=hclab(1,( hc +1)).
compute hcflab={'F(HC0)','F(HC1)','F(HC2)','F(HC3)','F(HC4)','F'}.
compute hcflab=hcflab(1,( hc +1)).
release xhc
.
compute seb=sqrt(diag(varb)).
compute trat = b&/seb.
compute p = 2*(1-tcdf(abs(trat), (dfres))).
compute tval = sqrt(dfres* (exp((dfres-(5/6))*((xp2/(dfres-(2/3)+(.11/dfres)))* (xp2/(dfres-(2/3)+(.11/dfres)))))-1)).
compute modres={modres,seb,trat,p}.
compute modres={modres,(b-tval&*seb),(b+tval&*seb)}.
compute modresl={'coeff',hclab,'t','p','LLCI','ULCI'}.
compute lmat = ident(ncol( x )).
compute lmat = lmat(:,2:ncol(lmat)).
compute fratio = (t(t(lmat)*b)*inv(t(lmat)*varb*lmat)*((t(lmat)*b)))/(ncol( x )-1).
compute pfr = 1-fcdf(fratio,(ncol( x )-1),dfres).
compute modsum={sqrt(r2),r2,mse,fratio,(ncol( x )-1),dfres,pfr}.
compute modsuml={'R','R-sq','MSE',hcflab,'df1','df2', 'p'}.
end if.
end if.
do if ( 1 = 2 or 1 =3).
compute xlp= x.
compute ylp= y.
compute pt2 = make(nrow(ylp),1,(csum(ylp)/nrow(ylp))).
do if ( 1 =2).
compute LL3 = ylp&*ln(pt2)+(1-ylp)&*ln(1-pt2).
end if.
compute LL3 = -2*csum(LL3).
compute bt1 = make(ncol(xlp),1,0).
compute LL1 = 0.
compute pt1 = make(nrow(ylp),1,0.5).
compute pt1lp=pt1.
loop jjj = 1 to iterate.
compute xlptmp=t(xlp).
compute vecprb=pt1lp&*(1-pt1lp).
loop kkk=1 to ncol(xlp).
compute xlptmp(kkk,:)=xlptmp(kkk,:)&*t(vecprb).
end loop.
compute b = bt1+inv(xlptmp*xlp)*t(xlp)*(ylp-pt1lp).
do if ( 1 =2).
compute xlpb=xlp*b.
compute xlpbt=(xlpb > -709.7).
compute xlpb709=(1-xlpbt)*(-709.7).
compute xlpb=(xlpb&*xlpbt)+xlpb709.
compute pt1lp = 1/(1+exp(-(xlpb))).
end if.
compute itprob = csum((pt1lp < .00000001) or (pt1lp > .9999999)).
do if (itprob > 0).
loop kkk = 1 to nrow(pt1lp).
do if (pt1lp(kkk,1) > .9999999).
compute pt1lp(kkk,1) = .9999999.
end if.
do if (pt1lp(kkk,1) < .00000001).
compute pt1lp(kkk,1) = .00000001.
end if.
end loop.
compute itprob = 0.
end if.
do if (itprob = 0).
do if ( 1 =2).
compute LL = ylp&*ln(pt1lp)+(1-ylp)&*ln(1-pt1lp).
end if.
compute LL2 = -2*csum(ll).
end if.
do if (abs(LL1-LL2) < converge).
do if ( 1 =1).
compute xlptmp=t(xlp).
compute vecprb=pt1lp&*(1-pt1lp).
loop kkk=1 to ncol(xlp).
compute xlptmp(kkk,:)=xlptmp(kkk,:)&*t(vecprb).
end loop.
compute varb = inv(xlptmp*xlp).
compute seb = sqrt(diag(varb)).
release xlptmp.
end if.
break.
end if.
compute bt1 = b.
compute LL1 = LL2.
end loop.
compute modres=b.
do if (jjj > iterate).
compute itprob = 2.
do if (booting=0).
compute iterrmod=1.
end if.
do if (booting=1).
compute bootiter=1.
end if.
do if (itprobtg=0).
compute itprobtg=1.
compute errcode(errs,1) = 47.
compute errs = errs + 1.
do if (booting = 0 and 1 =1).
compute vt1 = mdiag(pt1lp&*(1-pt1lp)).
compute varb = inv(t(xlp)*vt1*xlp).
compute seb = sqrt(diag(varb)).
end if.
end if.
end if.
do if ( 1 =1).
compute trat = b&/seb.
compute dfres=nrow(xlp).
compute p = 2*(1-cdfnorm(abs(trat))).
compute modres={modres,seb,trat,p}.
compute modres={modres,(b-xp2&*seb),(b+xp2&*seb)}.
compute pvchi=1-chicdf((LL3-LL2),(nrow(modres)-1)).
compute mcF = (LL3-LL2)/LL3.
compute cox = 1-exp(-(LL3-LL2)/nrow(xlp)).
compute nagel = cox/(1-exp(-(LL3)/nrow(xlp))).
compute modsum={LL2,(LL3-LL2),(nrow(modres)-1),pvchi, mcF,cox,nagel}.
compute modsuml={'-2LL','ModelLL', 'df', 'p', 'McFadden', 'CoxSnell', 'Nagelkrk'}.
compute modresl={'coeff','se','Z','p','LLCI','ULCI'}.
end if.
end if
.
compute toteff=modres(2:(1+nxvls),:).
compute nodotot=0.
do if (xdich=1 and xmint=1 and model=74).
compute toteff(:,1)=toteff(:,1)*xscaling.
compute toteff(:,2)=toteff(:,2)*abs(xscaling).
compute toteff(:,3)=toteff(:,3)*xscaling.
compute toteff(:,5)=toteff(:,5)*xscaling.
compute toteff(:,6)=toteff(:,6)*xscaling.
compute citmp=toteff(:,5:6).
do if (xscaling < 0).
compute toteff(:,5)=citmp(:,2).
compute toteff(:,6)=citmp(:,1).
end if.
compute nodotot=1.
end if.
compute totefflb=modresl.
do if (outscree=1).
do if (nodotot=0).
print/title = '************************** TOTAL EFFECT MODEL ****************************'.
end if.
do if (nodotot=1).
print/title = '**************************************************************************'.
end if.
print outnames(1,ncol(outnames))/title = 'OUTCOME VARIABLE:'/format = A8/space=0.
end if.
compute toteffl2=vlabsm(2:(1+nxvls),:).
compute resultm2=make(1,maxresm,99999).
compute resultm2(1,1:ncol(modsum))=modsum.
compute resultm={resultm;resultm2}.
compute resultm2=make(nrow(modres),maxresm,99999).
compute resultm2(1:nrow(modres),1:ncol(modres))=modres.
compute resultm={resultm;resultm2}.
do if (outscree=1).
print modsum/title = 'Model Summary'/cnames = modsuml/format= F10.4.
print modres/title='Model'/rnames=vlabsm/cnames=modresl/format= F10.4.
end if.
compute lmat=make(nrow(b),1,0).
compute lmat2=make(nxvls,1,1).
compute lmat(2:(1+nxvls),1)=lmat2.
do if (ydich <> 1).

.
compute lmat2= lmat.
do if ( 0 =0).
compute lmat2 = mdiag( lmat ).
compute lmat3=make(nrow(lmat2),1,0).
loop flp=1 to ncol(lmat2).
do if (csum(lmat2(:,flp))=1).
compute lmat3={lmat3,lmat2(:,flp)}.
end if.
end loop.
compute lmat2=lmat3(:,2:ncol(lmat3)).
end if.
compute fratio = (t(t(lmat2)* b )*inv(t(lmat2)* varb *lmat2)*((t(lmat2)* b )))/ncol(lmat2).
compute pfr = 1-fcdf(fratio,ncol(lmat2),(n-nrow( b ))).
compute fresult={fratio,ncol(lmat2),(n-nrow( b )),pfr}.
do if (i = (nms+nys) and (ydich=1)).
compute fratio=fratio*ncol(lmat2).
compute pfr=1-chicdf(fratio,ncol(lmat2)).
compute fresult={fratio,ncol(lmat2),pfr}.
end if.
do if ( 1 =1).
compute lmat3=1-rsum(lmat2).
compute xfm=make(n,csum(lmat3),0).
compute flpc=1.
loop flp=1 to nrow(lmat3).
do if (lmat3(flp,1)=1).
compute xfm(:,flpc)=x(:,flp).
compute flpc=flpc+1.
end if.
end loop.
compute bfm=inv(t(xfm)*xfm)*t(xfm)*y.
compute resid=y-(xfm*bfm).
compute sstotal=(y-(csum(y)/n)).
compute sstotal=csum(sstotal&*sstotal).
compute ssresid=csum(resid&*resid).
compute rsqch= r2 -((sstotal-ssresid)/sstotal).
compute fresult={rsqch,fresult}.
release xfm,flpc, resid, ssresid, bfm.
end if
.
compute totomni=fresult.
end if.
do if (stand=1).
compute predsd=make(nrow(modres),1,0).
compute stdmod=modres(:,1)&/ovsd(1,ncol(ovsd)).
loop jd=1 to ncol(x).
compute descdat=x(:,jd).
compute predsd(jd,1) = (nrow(descdat)*sscp(descdat))-(t(csum(descdat))*(csum(descdat))).
compute predsd(jd,1) = sqrt(predsd(jd,1)/(nrow(descdat)*(nrow(descdat)-1))).
end loop.
do if (wherex(1,ncol(wherex)) <> -999 and ((nxvls > 1) or (xdich=1))).
compute sdmsone=make(nxvls,1,1).
compute predsd(wherex(1,ncol(wherex)):wherex(2,ncol(wherex)),1)=sdmsone.
compute pstog=1.
end if.
compute predsd(1,1)=1.
compute stdmod=stdmod&*predsd.
compute stdmod=stdmod(2:nrow(stdmod),1).
compute sdvlabs=vlabsm(2:nrow(vlabsm),1).
compute resultm2=make(nrow(stdmod),maxresm,99999).
compute resultm2(1:nrow(stdmod),1:ncol(stdmod))=stdmod.
compute resultm={resultm;resultm2}.
do if (outscree=1).
print stdmod/title='Standardized coefficients'/clabels='coeff'/rnames=sdvlabs/format= F10.4.
end if.
end if.
do if (covcoeff=1).
do if (outscree=1).
print varb/title='Covariance matrix of regression parameter estimates:'/rnames=vlabsm/cnames=vlabsm/format= F10.4.
end if.
compute resultm2=make(nrow(varb),maxresm,99999).
do if (ncol(varb) <= maxresm).
compute resultm2(1:nrow(varb),1:ncol(varb))=varb.
compute resultm={resultm;resultm2}.
end if.
do if (ncol(varb) > maxresm).
compute resultmt=make(nrow(resultm),ncol(varb),99999).
compute resultmt(1:nrow(resultm),1:ncol(resultm))=resultm.
compute resultm=resultmt.
compute resultm2=make(nrow(varb),ncol(resultm),99999).
compute resultm2(1:nrow(varb),1:ncol(varb))=varb.
compute resultm={resultm;resultm2}.
compute maxresm=ncol(resultm).
end if.
end if.
end if.
end if.
do if (criterr=0 and nms > 0 and ydich=0 and modelres=1).
compute modresid=modresid(:,2:ncol(modresid)).
compute sigmatal = (t(modresid)*(ident(n)-(1/n)*ones*t(ones))*modresid)*(1/(n-1)).
compute sdall = mdiag(1/sqrt(diag(sigmatal))).
compute corall=sdall*sigmatal*t(sdall).
compute resultm2=make(nrow(corall),maxresm,99999).
compute resultm2(1:nrow(corall),1:ncol(corall))=corall.
compute resultm={resultm;resultm2}.
do if (outscree=1).
print/title = '****************** CORRELATIONS BETWEEN MODEL RESIDUALS ******************'.
print corall/title=' '/format= F10.4 / cnames=outnames/rnames=outnames/space=0.
end if.
end if.
do if (criterr=0 and boot > 0).
compute bootres=make(1,rsum(nump),-999).
compute bootdir=obsdirfx.
compute natdirbt=make(1,nxvls,-999).
do if (effsize=1).
compute bootysd=make(1,1,-999).
compute bootxsd=make(1,1,-999).
end if.
compute badboot=0.
compute goodboot=0.
compute smallest=1.
compute booting=1.
loop j = 1 to maxboot.
compute nobootx=1.
compute modres2=999.
compute v=trunc(uniform(n,1)*n)+1.
compute bad=0.
loop i = 1 to (nms+nys).
compute y=outvars(v,i).
compute ynovar= (nrow(y)*sscp(y))-(t(csum(y))*(csum(y))).
do if (ynovar = 0).
compute bad=1.
end if.
compute xindx=datindx(1:(nump(1,i)-1),i).
compute hello=0.
compute x = fulldat(v,xindx).
compute x={ones,x}.
compute xsq=t(x)*x.
compute exsq=eval(xsq).
release xsq.
compute holymoly=cmin(exsq).
compute zeroeig=csum(exsq <= 0.000000000002).
compute bad=bad + (zeroeig > 0).

.
compute desctmp=make((8-(4* 1 )),ncol( y ),-999).
loop jd=1 to ncol( y ).
compute descdat= y (:,jd).
compute desctmp(1,jd) = csum(descdat)/nrow(descdat).
compute desctmp(2,jd) = (nrow(descdat)*sscp(descdat))-(t(csum(descdat))*(csum(descdat))).
compute desctmp(2,jd) = sqrt(desctmp(2,jd)/(nrow(descdat)*(nrow(descdat)-1))).
compute desctmp(3,jd)=cmin(descdat).
compute desctmp(4,jd)=cmax(descdat).
do if ( 1 =0).
compute minwarn=0.
compute maxwarn=0.
do if ((desctmp(3,jd)=desctmp(4,jd)) and novar=0).
compute errcode(errs,1)=15.
compute errs=errs+1.
compute criterr=1.
compute novar=1.
end if.
compute tmp=((descdat(:,1)=desctmp(3,jd))+(descdat(:,1)=desctmp(4,jd))).
compute desctmp(8,jd)=(csum(tmp)=nrow(tmp)).
compute tmp = descdat.
compute tmp(GRADE(descdat),:) = descdat.
compute descdat = tmp.
release tmp.
compute decval={.16;.5;.84}.
loop kd=1 to 3.
compute low=trunc(decval(kd,1)*(nrow(descdat)+1)).
compute lowdec=decval(kd,1)*(nrow(descdat)+1)-low.
compute value=descdat(low,1)+(descdat((low+1),1)-descdat(low,1))*lowdec.
compute desctmp((4+kd),jd)=value.
end loop.
compute mnotev=1.
compute modvals=desctmp(5:7,:).
do if (quantile <> 1).
compute desctmp(5,jd)=desctmp(1,jd)-desctmp(2,jd).
compute desctmp(6,jd)=desctmp(1,jd).
compute desctmp(7,jd)=desctmp(1,jd)+desctmp(2,jd).
compute modvals=desctmp(5:7,:).
compute mnotev=2.
do if (modvals(1,1) < desctmp(3,1)).
compute modvals(1,1)=desctmp(3,1).
compute minwarn=1.
end if.
do if (modvals(3,1) > desctmp(4,1)).
compute modvals(3,1)=desctmp(4,1).
compute maxwarn=1.
end if.
end if.
do if (desctmp(8,1)=1).
compute modvals={desctmp(3,1);desctmp(4,1)}.
compute mnotev=0.
compute minwarn=0.
compute maxwarn=0.
end if.
end if.
end loop
.
compute bad=bad+ (desctmp(2,1) <= 0.00000000001).
do if (bad = 0).
do if (holymoly < smallest).
compute smallest=holymoly.
end if.
do if (ydich=0 or (i < (nms+nys))).

.
do if ( 1 =1).
compute hatmat=inv(t( x )* x )*t( x ).
compute b = hatmat* y.
compute modres=b.
do if ( 0 =1).
compute n1=nrow( x ).
compute dfres=n1-(ncol( x )).
compute sstotal = t( y -(csum( y )/n1))*( y -(csum( y )/n1)).
compute resid= y - x *b.
compute ssresid = csum((resid)&**2).
compute r2 = (sstotal-ssresid)/sstotal.
do if (r2 < 0).
compute r2=0.
end if.
compute adjr2 = 1-((1-r2)*(n1-1)/(dfres)).
compute mse=ssresid/(n1-ncol( x )).

.
compute n1=nrow( x ).
compute invXtX = inv(t( x )* x ).
compute varb = mse *invXtX.
compute k3 = ncol( x ).
compute xhc=0.
do if ( hc <> 5).
compute xhc= x.
compute hat = xhc(:,1).
loop i3=1 to nrow(xhc).
compute hat(i3,1)= xhc(i3,:)*invXtX*t(xhc(i3,:)).
end loop.
do if ( hc = 0 or hc =1).
loop i3 = 1 to k3.
compute xhc(:,i3)=xhc(:,i3)&* resid.
end loop.
end if.
do if ( hc =3 or hc =2).
loop i3=1 to k3.
compute xhc(:,i3) = ( resid &/(1-hat)&**(1/(4- hc )))&*xhc(:,i3).
end loop.
end if.
do if ( hc = 4).
compute hcmn=make(n,2,4).
compute hcmn(:,2)=(n1*hat)/k3.
loop i3= 1 to k3.
compute xhc(:,i3) = ( resid &/(1-hat)&**(rmin(hcmn)/2))&*xhc(:,i3).
end loop.
end if.
compute varb=(invXtX*t(xhc)*xhc*invXtX).
do if ( hc =1).
compute varb=(n1/(n1-ncol( x )))&*varb.
end if.
end if.
compute hclab={'se(HC0)','se(HC1)','se(HC2)','se(HC3)','se(HC4)','se'}.
compute hclab=hclab(1,( hc +1)).
compute hcflab={'F(HC0)','F(HC1)','F(HC2)','F(HC3)','F(HC4)','F'}.
compute hcflab=hcflab(1,( hc +1)).
release xhc
.
compute seb=sqrt(diag(varb)).
compute trat = b&/seb.
compute p = 2*(1-tcdf(abs(trat), (dfres))).
compute tval = sqrt(dfres* (exp((dfres-(5/6))*((xp2/(dfres-(2/3)+(.11/dfres)))* (xp2/(dfres-(2/3)+(.11/dfres)))))-1)).
compute modres={modres,seb,trat,p}.
compute modres={modres,(b-tval&*seb),(b+tval&*seb)}.
compute modresl={'coeff',hclab,'t','p','LLCI','ULCI'}.
compute lmat = ident(ncol( x )).
compute lmat = lmat(:,2:ncol(lmat)).
compute fratio = (t(t(lmat)*b)*inv(t(lmat)*varb*lmat)*((t(lmat)*b)))/(ncol( x )-1).
compute pfr = 1-fcdf(fratio,(ncol( x )-1),dfres).
compute modsum={sqrt(r2),r2,mse,fratio,(ncol( x )-1),dfres,pfr}.
compute modsuml={'R','R-sq','MSE',hcflab,'df1','df2', 'p'}.
end if.
end if.
do if ( 1 = 2 or 1 =3).
compute xlp= x.
compute ylp= y.
compute pt2 = make(nrow(ylp),1,(csum(ylp)/nrow(ylp))).
do if ( 1 =2).
compute LL3 = ylp&*ln(pt2)+(1-ylp)&*ln(1-pt2).
end if.
compute LL3 = -2*csum(LL3).
compute bt1 = make(ncol(xlp),1,0).
compute LL1 = 0.
compute pt1 = make(nrow(ylp),1,0.5).
compute pt1lp=pt1.
loop jjj = 1 to iterate.
compute xlptmp=t(xlp).
compute vecprb=pt1lp&*(1-pt1lp).
loop kkk=1 to ncol(xlp).
compute xlptmp(kkk,:)=xlptmp(kkk,:)&*t(vecprb).
end loop.
compute b = bt1+inv(xlptmp*xlp)*t(xlp)*(ylp-pt1lp).
do if ( 1 =2).
compute xlpb=xlp*b.
compute xlpbt=(xlpb > -709.7).
compute xlpb709=(1-xlpbt)*(-709.7).
compute xlpb=(xlpb&*xlpbt)+xlpb709.
compute pt1lp = 1/(1+exp(-(xlpb))).
end if.
compute itprob = csum((pt1lp < .00000001) or (pt1lp > .9999999)).
do if (itprob > 0).
loop kkk = 1 to nrow(pt1lp).
do if (pt1lp(kkk,1) > .9999999).
compute pt1lp(kkk,1) = .9999999.
end if.
do if (pt1lp(kkk,1) < .00000001).
compute pt1lp(kkk,1) = .00000001.
end if.
end loop.
compute itprob = 0.
end if.
do if (itprob = 0).
do if ( 1 =2).
compute LL = ylp&*ln(pt1lp)+(1-ylp)&*ln(1-pt1lp).
end if.
compute LL2 = -2*csum(ll).
end if.
do if (abs(LL1-LL2) < converge).
do if ( 0 =1).
compute xlptmp=t(xlp).
compute vecprb=pt1lp&*(1-pt1lp).
loop kkk=1 to ncol(xlp).
compute xlptmp(kkk,:)=xlptmp(kkk,:)&*t(vecprb).
end loop.
compute varb = inv(xlptmp*xlp).
compute seb = sqrt(diag(varb)).
release xlptmp.
end if.
break.
end if.
compute bt1 = b.
compute LL1 = LL2.
end loop.
compute modres=b.
do if (jjj > iterate).
compute itprob = 2.
do if (booting=0).
compute iterrmod=1.
end if.
do if (booting=1).
compute bootiter=1.
end if.
do if (itprobtg=0).
compute itprobtg=1.
compute errcode(errs,1) = 47.
compute errs = errs + 1.
do if (booting = 0 and 0 =1).
compute vt1 = mdiag(pt1lp&*(1-pt1lp)).
compute varb = inv(t(xlp)*vt1*xlp).
compute seb = sqrt(diag(varb)).
end if.
end if.
end if.
do if ( 0 =1).
compute trat = b&/seb.
compute dfres=nrow(xlp).
compute p = 2*(1-cdfnorm(abs(trat))).
compute modres={modres,seb,trat,p}.
compute modres={modres,(b-xp2&*seb),(b+xp2&*seb)}.
compute pvchi=1-chicdf((LL3-LL2),(nrow(modres)-1)).
compute mcF = (LL3-LL2)/LL3.
compute cox = 1-exp(-(LL3-LL2)/nrow(xlp)).
compute nagel = cox/(1-exp(-(LL3)/nrow(xlp))).
compute modsum={LL2,(LL3-LL2),(nrow(modres)-1),pvchi, mcF,cox,nagel}.
compute modsuml={'-2LL','ModelLL', 'df', 'p', 'McFadden', 'CoxSnell', 'Nagelkrk'}.
compute modresl={'coeff','se','Z','p','LLCI','ULCI'}.
end if.
end if
.
end if.
do if (ydich=1 and (i = (nms+nys))).

.
do if ( 2 =1).
compute hatmat=inv(t( x )* x )*t( x ).
compute b = hatmat* y.
compute modres=b.
do if ( 0 =1).
compute n1=nrow( x ).
compute dfres=n1-(ncol( x )).
compute sstotal = t( y -(csum( y )/n1))*( y -(csum( y )/n1)).
compute resid= y - x *b.
compute ssresid = csum((resid)&**2).
compute r2 = (sstotal-ssresid)/sstotal.
do if (r2 < 0).
compute r2=0.
end if.
compute adjr2 = 1-((1-r2)*(n1-1)/(dfres)).
compute mse=ssresid/(n1-ncol( x )).

.
compute n1=nrow( x ).
compute invXtX = inv(t( x )* x ).
compute varb = mse *invXtX.
compute k3 = ncol( x ).
compute xhc=0.
do if ( hc <> 5).
compute xhc= x.
compute hat = xhc(:,1).
loop i3=1 to nrow(xhc).
compute hat(i3,1)= xhc(i3,:)*invXtX*t(xhc(i3,:)).
end loop.
do if ( hc = 0 or hc =1).
loop i3 = 1 to k3.
compute xhc(:,i3)=xhc(:,i3)&* resid.
end loop.
end if.
do if ( hc =3 or hc =2).
loop i3=1 to k3.
compute xhc(:,i3) = ( resid &/(1-hat)&**(1/(4- hc )))&*xhc(:,i3).
end loop.
end if.
do if ( hc = 4).
compute hcmn=make(n,2,4).
compute hcmn(:,2)=(n1*hat)/k3.
loop i3= 1 to k3.
compute xhc(:,i3) = ( resid &/(1-hat)&**(rmin(hcmn)/2))&*xhc(:,i3).
end loop.
end if.
compute varb=(invXtX*t(xhc)*xhc*invXtX).
do if ( hc =1).
compute varb=(n1/(n1-ncol( x )))&*varb.
end if.
end if.
compute hclab={'se(HC0)','se(HC1)','se(HC2)','se(HC3)','se(HC4)','se'}.
compute hclab=hclab(1,( hc +1)).
compute hcflab={'F(HC0)','F(HC1)','F(HC2)','F(HC3)','F(HC4)','F'}.
compute hcflab=hcflab(1,( hc +1)).
release xhc
.
compute seb=sqrt(diag(varb)).
compute trat = b&/seb.
compute p = 2*(1-tcdf(abs(trat), (dfres))).
compute tval = sqrt(dfres* (exp((dfres-(5/6))*((xp2/(dfres-(2/3)+(.11/dfres)))* (xp2/(dfres-(2/3)+(.11/dfres)))))-1)).
compute modres={modres,seb,trat,p}.
compute modres={modres,(b-tval&*seb),(b+tval&*seb)}.
compute modresl={'coeff',hclab,'t','p','LLCI','ULCI'}.
compute lmat = ident(ncol( x )).
compute lmat = lmat(:,2:ncol(lmat)).
compute fratio = (t(t(lmat)*b)*inv(t(lmat)*varb*lmat)*((t(lmat)*b)))/(ncol( x )-1).
compute pfr = 1-fcdf(fratio,(ncol( x )-1),dfres).
compute modsum={sqrt(r2),r2,mse,fratio,(ncol( x )-1),dfres,pfr}.
compute modsuml={'R','R-sq','MSE',hcflab,'df1','df2', 'p'}.
end if.
end if.
do if ( 2 = 2 or 2 =3).
compute xlp= x.
compute ylp= y.
compute pt2 = make(nrow(ylp),1,(csum(ylp)/nrow(ylp))).
do if ( 2 =2).
compute LL3 = ylp&*ln(pt2)+(1-ylp)&*ln(1-pt2).
end if.
compute LL3 = -2*csum(LL3).
compute bt1 = make(ncol(xlp),1,0).
compute LL1 = 0.
compute pt1 = make(nrow(ylp),1,0.5).
compute pt1lp=pt1.
loop jjj = 1 to iterate.
compute xlptmp=t(xlp).
compute vecprb=pt1lp&*(1-pt1lp).
loop kkk=1 to ncol(xlp).
compute xlptmp(kkk,:)=xlptmp(kkk,:)&*t(vecprb).
end loop.
compute b = bt1+inv(xlptmp*xlp)*t(xlp)*(ylp-pt1lp).
do if ( 2 =2).
compute xlpb=xlp*b.
compute xlpbt=(xlpb > -709.7).
compute xlpb709=(1-xlpbt)*(-709.7).
compute xlpb=(xlpb&*xlpbt)+xlpb709.
compute pt1lp = 1/(1+exp(-(xlpb))).
end if.
compute itprob = csum((pt1lp < .00000001) or (pt1lp > .9999999)).
do if (itprob > 0).
loop kkk = 1 to nrow(pt1lp).
do if (pt1lp(kkk,1) > .9999999).
compute pt1lp(kkk,1) = .9999999.
end if.
do if (pt1lp(kkk,1) < .00000001).
compute pt1lp(kkk,1) = .00000001.
end if.
end loop.
compute itprob = 0.
end if.
do if (itprob = 0).
do if ( 2 =2).
compute LL = ylp&*ln(pt1lp)+(1-ylp)&*ln(1-pt1lp).
end if.
compute LL2 = -2*csum(ll).
end if.
do if (abs(LL1-LL2) < converge).
do if ( 0 =1).
compute xlptmp=t(xlp).
compute vecprb=pt1lp&*(1-pt1lp).
loop kkk=1 to ncol(xlp).
compute xlptmp(kkk,:)=xlptmp(kkk,:)&*t(vecprb).
end loop.
compute varb = inv(xlptmp*xlp).
compute seb = sqrt(diag(varb)).
release xlptmp.
end if.
break.
end if.
compute bt1 = b.
compute LL1 = LL2.
end loop.
compute modres=b.
do if (jjj > iterate).
compute itprob = 2.
do if (booting=0).
compute iterrmod=1.
end if.
do if (booting=1).
compute bootiter=1.
end if.
do if (itprobtg=0).
compute itprobtg=1.
compute errcode(errs,1) = 47.
compute errs = errs + 1.
do if (booting = 0 and 0 =1).
compute vt1 = mdiag(pt1lp&*(1-pt1lp)).
compute varb = inv(t(xlp)*vt1*xlp).
compute seb = sqrt(diag(varb)).
end if.
end if.
end if.
do if ( 0 =1).
compute trat = b&/seb.
compute dfres=nrow(xlp).
compute p = 2*(1-cdfnorm(abs(trat))).
compute modres={modres,seb,trat,p}.
compute modres={modres,(b-xp2&*seb),(b+xp2&*seb)}.
compute pvchi=1-chicdf((LL3-LL2),(nrow(modres)-1)).
compute mcF = (LL3-LL2)/LL3.
compute cox = 1-exp(-(LL3-LL2)/nrow(xlp)).
compute nagel = cox/(1-exp(-(LL3)/nrow(xlp))).
compute modsum={LL2,(LL3-LL2),(nrow(modres)-1),pvchi, mcF,cox,nagel}.
compute modsuml={'-2LL','ModelLL', 'df', 'p', 'McFadden', 'CoxSnell', 'Nagelkrk'}.
compute modresl={'coeff','se','Z','p','LLCI','ULCI'}.
end if.
end if
.
end if.
compute modres2={modres2,t(modres)}.
do if (i = (nms+nys)).
do if (bcmat((i+1),1) = 1).
compute bootdir={bootdir;t(modres(wherex(1,i):wherex(2,i),1))}.
end if.
do if (bcmat((i+1),1) = 0).
compute bootdir={bootdir;dirzes}.
end if.
end if.
do if (model = 74 and i <= nms).
compute onetemp=make(nrow(xprobval),1,1).
compute mestmtb={onetemp,xprobval}.
do if (ncs > 0).
compute ncovmdlb=rsum(ccmat(i,:)).
do if (ncovmdl > 0).
compute cvmncb=make(nrow(mestmtb),ncovmdlb,1).
compute cvmnctmp=csum(x(:,(ncol(x)-ncovmdlb+1):ncol(x)))/nrow(x).
do if (cuscoval > 0).
compute cvmnctmp=coval.
end if.
loop mestlp=1 to ncovmdlb.
compute cvmncb(:,mestlp)=cvmncb(:,mestlp)*cvmnctmp(:,mestlp).
end loop.
compute mestmtb={mestmtb,cvmncb}.
end if.
end if.
compute mestbt=mestmtb*modres.
do if (i = 1).
compute mestb=mestbt.
end if.
do if (i > 1).
compute mestb={mestb,mestbt}.
end if.
end if.
do if (model = 74 and i = (nms+nys)).
compute xvalptmp=make(1,nxvls,0).
compute mest74sp=make(1,nms,0).
compute mest74sp={mest74sp;mestb}.
compute xvalptmp={xvalptmp;ident(nxvls)}.
compute dirfxcf=make(nrow(mestb),1,0).
loop cfloop1=1 to nrow(mestb).
compute ndirfx=make(nrow(modres),1,0).
compute ndirfx(2:(nxvls+1),1)=t(xvalptmp(cfloop1,:)).
loop cfloop3=1 to nms.
do if (mcx=1 or mcx=0).
compute ndirfx(wheremw((1+((cfloop3-1)*2)),ncol(wheremw)):wheremw((2+((cfloop3-1)*2) ),ncol(wheremw)))=t(xvalptmp(cfloop1,:)*mestb(1,cfloop3)).
end if.
do if (mcx=2).
compute ndirfx(wheremw((1+((cfloop3-1)*2)),ncol(wheremw)):wheremw((2+((cfloop3-1)*2) ),ncol(wheremw)))=t(xvalptmp(cfloop1,:)*mest74sp(cfloop1,cfloop3)).
end if.
end loop.
do if (cfloop1 > 1).
compute dirfxcf(cfloop1,1)=t(ndirfx)*modres*xscaling.
end if.
end loop.
compute natdirbt={natdirbt;t(dirfxcf(2:nrow(dirfxcf),:))}.
end if.
do if ( (bcmat((i+1),1) = 1) and (nobootx=1) and (effsize=1)).
compute nobootx=0.
compute xsdtemp= (nrow(x)*sscp(x(:,2)))-(t(csum(x(:,2)))*(csum(x(:,2)))).
compute xsdtemp= sqrt(xsdtemp/(nrow(x)*(nrow(x)-1))).
end if.
end if.
end loop.
do if (bad = 0).
compute bootres={bootres;modres2(:,2:ncol(modres2))}.
do if (effsize=1).
compute ysdtemp= (nrow(y)*sscp(y))-(t(csum(y))*(csum(y))).
compute ysdtemp= sqrt(ysdtemp/(nrow(y)*(nrow(y)-1))).
compute bootysd={bootysd;ysdtemp}.
compute bootxsd={bootxsd;xsdtemp}.
end if.
compute goodboot=goodboot+1.
end if.
do if (bad <> 0).
compute badboot=badboot+1.
end if.
end loop if (goodboot = boot).
compute bootres=bootres(2:nrow(bootres),:).
do if (effsize=1).
compute bootysd=bootysd(2:nrow(bootysd),:).
do if (nrow(bootxsd) > 1).
compute bootxsd=bootxsd(2:nrow(bootxsd),:).
end if.
end if.
do if (goodboot < (boot)).
compute boot=0.
compute modelbt=0.
compute notecode(notes,1) = 7.
compute notes = notes + 1.
end if.
do if (boot > 0).
do if (effsize=1).
compute bootysd={ysd;bootysd}.
compute bootxsd={xsd;bootxsd}.
end if.
do if (saveboot = 1).
save bootres/outfile = *.
end if.
do if (modelbt=1).
compute bootcim=make(ncol(bootres),5,-99999).
compute bootcim(:,2) = t(csum(bootres)/nrow(bootres)).
compute bootcim(:,1) = coeffmat(2:nrow(coeffmat),1).
loop i = 1 to ncol(bootres).

.
compute temp = bootres(:,i).
compute temp(GRADE( bootres(:,i) )) = bootres(:,i).
compute badlo = 0.
compute badhi = 0.
do if ( (bootcim(i,1)*bc)+(9999*(1-bc)) <> 9999).
compute pv=csum(temp < (bootcim(i,1)*bc)+(9999*(1-bc)) )/nrow(temp).
compute ppv = pv.
do if (pv > .5).
compute ppv = 1-pv.
end if.
compute y5=sqrt(-2*ln(ppv)).
compute xp=y5+((((y5*p4+p3)*y5+p2)*y5+p1)*y5+p0)/((((y5*q4+q3)*y5+q2)*y5+q1)*y5+q0).
do if (pv <= .5).
compute xp = -xp.
end if.
compute cilow=rnd(nrow(temp)*(cdfnorm(2*xp-xp2))).
compute cihigh=trunc(nrow(temp)*(cdfnorm(2*xp+(xp2))))+1.
do if (cilow < 1).
compute cilow = 1.
compute booterr=1.
compute badlo = 1.
end if.
do if (cihigh > nrow(temp)).
compute cihigh = boot.
compute booterr=1.
compute badhi = 1.
end if.
compute llcit=temp(cilow,1).
compute ulcit=temp(cihigh,1).
do if (badlo = 1 and llcit <> priorlo).
compute badend={badend, llcit}.
compute priorlo = llcit.
end if.
do if (badhi = 1 and ulcit <> priorhi).
compute badend={badend, ulcit}.
compute priorhi = ulcit.
end if.
end if.
do if ( (bootcim(i,1)*bc)+(9999*(1-bc)) = 9999).
compute llcit=temp(cilow,1).
compute ulcit=temp(cihigh,1).
end if.
compute bootse=t(sqrt((cssq(temp)-((csum(temp)&**2)/nrow(temp)))/(nrow(temp)-1)))
.
compute bootcim(i,4:5)={llcit,ulcit}.
compute bootcim(i,3)=bootse.
end loop.
end if.
end if.
do if (badboot > 0).
compute notecode(notes,1) = 6.
compute notes = notes + 1.
end if.
end if.
do if (criterr=0).
release fulldat,x,y.
end if.
do if (xmint=1 and criterr=0).
compute xvalptmp=make(1,nxvls,0).
compute mest74sp=make(1,nms,0).
compute mest74sp={mest74sp;mest74}.
compute xvalptmp={xvalptmp;ident(nxvls)}.
loop kcfuhd=1 to 2.
compute dirfxcf=make(nrow(mest74),6,0).
loop cfloop1=1 to nrow(mest74).
compute ndirfx=make(nrow(lastb),1,0).
compute ndirfx(2:(nxvls+1),1)=t(xvalptmp(cfloop1,:)).
loop cfloop3=1 to nms.
do if (kcfuhd=1).
do if (mcx=1 or mcx=0).
compute ndirfx(wheremw((1+((cfloop3-1)*2)),ncol(wheremw)):wheremw((2+((cfloop3-1)*2)), ncol(wheremw)))=t(xvalptmp(cfloop1,:)*mest74(1,cfloop3)).
end if.
do if (mcx=2).
compute ndirfx(wheremw((1+((cfloop3-1)*2)),ncol(wheremw)):wheremw((2+((cfloop3-1)*2)), ncol(wheremw)))=t(xvalptmp(cfloop1,:)*mest74sp(cfloop1,cfloop3)).
end if.
end if.
do if (kcfuhd=2).
compute ndirfx(wheremw((1+((cfloop3-1)*2)),ncol(wheremw)):wheremw((2+((cfloop3-1)*2)), ncol(wheremw)))=t(xvalptmp(cfloop1,:)*medmeans(1,cfloop3)).
end if.
end loop.
do if (cfloop1 > 1).
compute dirfxcf(cfloop1,1)=t(ndirfx)*lastb*xscaling.
compute dirfxcf(cfloop1,2)=sqrt(diag(t(ndirfx)*lastcov*ndirfx))*abs(xscaling).
compute dirfxcf(cfloop1,3)=dirfxcf(cfloop1,1)/dirfxcf(cfloop1,2).
compute dirfxcf(cfloop1,4)=2*(1-tcdf(abs(dirfxcf(cfloop1,3)), dfres)).
compute dirfxcf(cfloop1,5)=dirfxcf(cfloop1,1)-tval*dirfxcf(cfloop1,2).
compute dirfxcf(cfloop1,6)=dirfxcf(cfloop1,1)+tval*dirfxcf(cfloop1,2).
end if.
end loop.
compute codireff=dirfxcf(2:nrow(dirfxcf),:).
do if (kcfuhd=1).
compute direff=codireff.
end if.
end loop.
end if.

.
do if (criterr = 0 and nms > 0).
compute paths=paths(:,2:ncol(paths)).
compute pathsw=pathsw(:,2:ncol(pathsw)).
compute pathsz=pathsz(:,2:ncol(pathsz)).
compute pathswz=pathswz(:,2:ncol(pathswz)).
compute pathsmod=pathsw+pathsz+pathswz.
compute pathsdv=pathsdv(:,2:ncol(pathsdv)).
compute pathsfoc=pathsfoc(:,2:ncol(pathsfoc)).
compute pathtype=pathtype(:,2:ncol(pathtype)).
compute anymod=(rsum(pathsmod) > 0).
compute obscoeff=obscoeff(1,2:ncol(obscoeff)).
do if (outscree=1).
do if (xmint=1).
print/title = '************************* COUNTERFACTUALLY DEFINED ************************'.
else.
print/title = ' '.
end if.
do if (dototal = 0 and alttotal=0).
print/title = '****************** DIRECT AND INDIRECT EFFECTS OF X ON Y *****************'/space=0.
end if.
do if (alttotal=1).
print/title = '************** TOTAL, DIRECT, AND INDIRECT EFFECTS OF X ON Y **************'/space=0.
end if.
end if.
do if (dototal = 1).
do if (outscree=1).
print/title = '************** TOTAL, DIRECT, AND INDIRECT EFFECTS OF X ON Y **************'/space=0.
end if.
compute totefflb(1,1)='Effect'.
do if (effsize=1).
compute toteffsz=toteff(:,1)/ysd.
do if (xdich=1 or mcx > 0).
compute totefflb={totefflb,'c_ps'}.
end if.
do if (xdich = 0 and mcx = 0).
compute toteffsz=toteffsz*xsd.
compute totefflb={totefflb,'c_cs'}.
end if.
compute toteff={toteff,toteffsz}.
end if.
compute resultm2=make(nrow(toteff),maxresm,99999).
compute resultm2(1:nrow(toteff),1:ncol(toteff))=toteff.
compute resultm={resultm;resultm2}.
do if (nxvls > 1).
compute resultm2=make(nrow(totomni),maxresm,99999).
compute resultm2(1:nrow(totomni),1:ncol(totomni))=totomni.
compute resultm={resultm;resultm2}.
compute clabtmp={'R2-chng', hcflab, 'df1','df2','p'}.
do if (outscree=1).
print toteff/title='Relative total effects of X on Y'/rnames=toteffl2/cnames=totefflb/format= F10.4.
print totomni/title='Omnibus test of total effect of X on Y'/cnames=clabtmp/format= F10.4.
print/title= '----------'/space=0.
end if.
else.
do if (outscree=1).
print toteff/title='Total effect of X on Y'/cnames=totefflb/format= F10.4.
end if.
end if.
end if.
compute moddir=wcmat(nrow(bcmat),1)+zcmat(nrow(bcmat),1).
do if (xmint=1).
compute moddir=1.
end if.
do if (bcmat(nrow(bcmat),1)=1).
do if (ydich=1).
compute direfflb(:,(ncol(direfflb)-5):ncol(direfflb))={'Effect','se','Z','p','LLCI','ULCI'}.
end if.
do if (moddir=0 or xmint=1).
compute direfflb(1,1)='Effect'.
end if.
do if (effsize=1 and moddir=0 and anymod = 0).
compute direffsz=direff(:,1)/ysd.
do if (xdich=1 or mcx > 0).
compute direfflb={direfflb,'c''_ps'}.
end if.
do if (xdich = 0 and mcx = 0).
compute direffsz=direffsz*xsd.
compute direfflb={direfflb,'c''_cs'}.
end if.
compute direff={direff,direffsz}.
end if.
compute resultm2=make(nrow(direff),maxresm,99999).
compute resultm2(1:nrow(direff),1:ncol(direff))=direff.
compute resultm={resultm;resultm2}.
do if (xmint=1).
compute resultm2=make(nrow(codireff),maxresm,99999).
compute resultm2(1:nrow(codireff),1:ncol(codireff))=codireff.
compute resultm={resultm;resultm2}.
end if.
do if (moddir = 0 and nxvls=1).
do if (outscree=1).
print direff/title='Direct effect of X on Y'/cnames=direfflb/format= F10.4.
end if.
end if.
do if (moddir = 0 and nxvls>1).
compute resultm2=make(nrow(diromni),maxresm,99999).
compute resultm2(1:nrow(diromni),1:ncol(diromni))=diromni.
compute resultm={resultm;resultm2}.
do if (outscree=1).
print direff/title='Relative direct effects of X on Y'/rnames=direffl2/cnames=direfflb/format= F10.4.
do if (ydich = 0).
compute clabtmp={'R2-chng', hcflab, 'df1','df2','p'}.
print diromni/title='Omnibus test of direct effect of X on Y:'/cnames=clabtmp/format= F10.4.
end if.
do if (ydich = 1).
compute clabtmp={'Chi-sq', 'df', 'p'}.
print diromni/title='Omnibus likelihood ratio test of direct effect of X on Y'/cnames=clabtmp/format= F10.4.
end if.
print/title= '----------'/space=0.
end if.
end if.
do if (moddir > 0 and nxvls=1).
do if (xmint=0 and outscree=1).
print direff/title='Conditional direct effects of X on Y'/cnames=direfflb/format= F10.4.
end if.
do if (xmint=1).
do if (outscree=1).
print direff/title='(Pure) Natural direct effect of X on Y'/cnames=direfflb/format= F10.4.
print codireff/title='Controlled direct effect of X on Y'/cnames=direfflb/format= F10.4.
print/title= '----------'/space=0.
end if.
compute obnatdfx=direff(:,1).
end if.
end if.
compute direffl4=direffl2.
do if (moddir > 0 and nxvls>1).
compute direffl2={' '}.
loop i = 1 to nxvls.
loop j = 1 to (nrow(direff)/nxvls).
compute direffl2={direffl2;xcatlab(i,1)}.
end loop.
end loop.
compute direffl2=direffl2(2:nrow(direffl2),1).
do if (xmint=0 and outscree=1).
print direff/title='Relative conditional direct effects of X on Y'/cnames=direfflb/rnames=direffl2/format= F10.4.
end if.
do if (xmint=1).
do if (outscree=1).
print direff/title='Relative (pure) natural direct effects of X on Y'/cnames=direfflb/rnames=direffl2/format= F10.4.
print codireff/title='Relative controlled direct effects of X on Y'/cnames=direfflb/rnames=direffl2/format= F10.4.
print/title= '----------'/space=0.
end if.
compute obnatdfx=direff(:,1).
end if.
end if.
compute direffl2=direffl4.
end if.
do if (bcmat(nrow(bcmat),1)=0 and xmint <> 1 and outscree=1).
print/title='The direct effect of X on Y is fixed to zero.'.
end if.
do if (nms = 1).
compute indmark={2}.
compute indsets={1,2}.
end if.
do if (nms = 2).
compute indmark={2,2,3}.
compute indsets={1,4,2,5,1,3,5}.
compute thetam=1.
end if.
do if (nms = 3).
compute indmark={2,2,2,3,3,3,4}.
compute indsets={1,7,2,8,4,9,1,3,8,1,5,9,2,6,9,1,3,6,9}.
compute thetam={1,2,3}.
end if.
do if (nms = 4).
compute indmark={2,2,2,2,3,3,3,3,3,3,4,4,4,4,5}.
compute indsets={1,11,2,12,4,13,7,14,1,3,12,1,5,13,1,8,14,2,6,13,2,9,14,4,10,14,1,3,6,13,1,3,9, 14,1,5,10,14,2,6,10,14,1,3,6,10,14}.
compute thetam={1,2,4,3,5,6}.
end if.
do if (nms = 5).
compute indmark={2,2,2,2,2,3,3,3,3,3,3,3,3,3,3,4,4,4,4,4,4,4,4,4,4,5,5,5,5,5,6}.
compute indsets={1,16,2,17,4,18,7,19,11,20,1,3,17,1,5,18,1,8,19,1,12,20,2,6,18,2,9,19,2,13,20,4, 10,19,4,14,20,7,15,20,1,3,6,18, 1,3,9,19,1,3,13,20,1,5,10,19,1,5,14,20,1,8,15,20,2,6,10,19,2,6, 14,20,2,9,15,20,4,10,15,20,1,3,6,10,19, 1,3,6,14,20,1,3,9,15,20,1,5,10,15,20,2,6,10,15,20,1,3,6,10,15, 20}.
compute thetam={1,2,5,3,6,8,4,7,9,10}.
end if.
do if (nms = 6).
compute indmark={2,2,2,2,2,2,3,3,3,3,3,3,3,3,3,3,3,3,3,3,3,4,4,4,4,4,4,4,4,4,4,4,4,4,4,4,4,4,4, 4,4,5,5,5,5,5,5,5,5,5,5,5,5,5,5,5,6,6,6,6,6,6,7}.
compute indsets={1,22,2,23,4,24,7,25,11,26,16,27,1,3,23,1,5,24,1,8,25,1,12,26,1,17,27,2,6,24,2, 9,25,2,13,26,2,18,27,4,10,25,4,14,26, 4,19,27,7,15,26,7,20,27,11,21,27,1,3,6,24,1,3,9,25,1,3,13,26,1, 3,18,27,1,5,10,25,1,5,14,26,1,5,19,27,1,8,15,26,1,8,20,27, 1,12,21,27,2,6,10,25,2,6,14,26,2,6,19,27,2,9,15,26,2,9,20,27,2, 13,21,27,4,10,15,26,4,10,20,27,4,14,21,27,7,15,21,27, 1,3,6,10,25,1,3,6,14,26,1,3,6,19,27,1,3,9,15,26,1,3,9,20,27,1,3, 13,21,27,1,5,10,15,26,1,5,10,20,27,1,5,14,21,27, 1,8,15,21,27,2,6,10,15,26,2,6,10,20,27,2,6,14,21,27,2,9,15,21, 27,4,10,15,21,27,1,3,6,10,15,26,1,3,6,10,20,27, 1,3,6,14,21,27,1,3,9,15,21,27,1,5,10,15,21,27,2,6,10,15,21,27, 1,3,6,10,15,21,27}.
compute thetam={1,2,6,3,7,10,4,8,11,13,5,9,12,14,15}.
end if.
do if (nms = 7).
compute indmark={2,2,2,2,2,2,2}.
compute indsets={1,29,2,30,4,31,7,32,11,33,16,34,22,35}.
end if.
do if (nms = 8).
compute indmark={2,2,2,2,2,2,2,2}.
compute indsets={1,37,2,38,4,39,7,40,11,41,16,42,22,43,29,44}.
end if.
do if (nms = 9).
compute indmark={2,2,2,2,2,2,2,2,2}.
compute indsets={1,46,2,47,4,48,7,49,11,50,16,51,22,52,29,53,37,54}.
end if.
do if (nms=10).
compute indmark={2,2,2,2,2,2,2,2,2,2}.
compute indsets={1,56,2,57,4,58,7,59,11,60,16,61,22,62,29,63,37,64,46,65}.
end if.
compute indlbl = {'Ind1'; 'Ind2'; 'Ind3'; 'Ind4'; 'Ind5'; 'Ind6'; 'Ind7'; 'Ind8'; 'Ind9'; 'Ind10'; 'Ind11'; 'Ind12'; 'Ind13'; 'Ind14'; 'Ind15'}.
compute indlbl = {indlbl; 'Ind16';'Ind17';'Ind18';'Ind19';'Ind20';'Ind21';'Ind22';'Ind23';'Ind24';'Ind25';'Ind26';'Ind27';'Ind28';'Ind29';'Ind30'}.
compute indlbl = {indlbl; 'Ind31';'Ind32';'Ind33';'Ind34';'Ind35';'Ind36';'Ind37';'Ind38';'Ind39';'Ind40';'Ind41';'Ind42';'Ind43';'Ind44';'Ind45'}.
compute indlbl = {indlbl; 'Ind46';'Ind47';'Ind48';'Ind49';'Ind50';'Ind51';'Ind52';'Ind53';'Ind54';'Ind55';'Ind56';'Ind57';'Ind58';'Ind59';'Ind60'}.
compute indlbl = {indlbl; 'Ind61';'Ind62';'Ind63';'Ind64';'Ind65';'Ind66';'Ind67';'Ind68';'Ind69';'Ind70';'Ind71';'Ind72';'Ind73';'Ind74';'Ind75'}.
compute indlbl = {indlbl; 'Ind76';'Ind77';'Ind78';'Ind79';'Ind80';'Ind81';'Ind82';'Ind83';'Ind84';'Ind85';'Ind86';'Ind87';'Ind88';'Ind89';'Ind90'}.
compute cntname={'(C1)';'(C2)';'(C3)';'(C4)';'(C5)';'(C6)';'(C7)';'(C8)';'(C9)';'(C10)';'(C11)';'(C12)';'(C13)';'(C14)';'(C15)';'(C16)';'(C17)'}.
compute cntname={cntname;'(C18)';'(C19)';'(C20)';'(C21)';'(C22)';'(C23)';'(C24)';'(C25)';'(C26)';'(C27)';'(C28)';'(C29)';'(C30)';'(C31)'}.
compute cntname={cntname;'(C32)';'(C33)';'(C34)';'(C35)';'(C36)';'(C37)';'(C38)';'(C39)';'(C40)';'(C41)';'(C42)';'(C43)';'(C44)';'(C45)'}.
compute cntname={cntname;'(C46)';'(C47)';'(C48)';'(C49)';'(C50)';'(C51)';'(C52)';'(C53)';'(C54)';'(C55)';'(C56)';'(C57)';'(C58)';'(C59)'}.
compute cntname={cntname;'(C60)';'(C61)';'(C62)';'(C63)';'(C64)';'(C65)';'(C66)';'(C67)';'(C68)';'(C69)';'(C70)';'(C71)';'(C72)';'(C73)'}.
compute cntname={cntname;'(C74)';'(C75)';'(C76)';'(C77)';'(C78)';'(C79)';'(C80)';'(C81)';'(C82)';'(C83)';'(C84)';'(C85)';'(C86)';'(C87)'}.
compute cntname={cntname;'(C88)';'(C89)';'(C90)';'(C91)';'(C92)';'(C93)';'(C94)';'(C95)';'(C96)';'(C97)';'(C98)';'(C99)';'(C100)';'(C101)'}.
compute cntname={cntname;'(C102)';'(C103)';'(C104)';'(C105)'}.
compute indmake=make(ncol(indmark),(nms+2),0).
compute indmod=make(ncol(indmark),1,999).
compute indmmm=make(ncol(indmark),1,0).
compute indmmmt=make(ncol(indmark),1,0).
compute start=1.
compute end=0.
compute nindfx=0.
compute indlocs=make(nrow(thetaxmb),ncol(paths),999).
compute indkey=make(ncol(indmark),1+((rmax(indmark)*2)+1),'     ').
compute c1=1.
compute c2=1.
compute c3=1.
loop i = 1 to ncol(paths).
do if (pathtype(1,i)=1).
compute indlocs(:,i)=thetaxmb(:,c1).
compute c1=c1+1.
end if.
do if (pathtype(1,i)=3).
compute indlocs(:,i)=thetamyb(:,c2).
compute c2=c2+1.
end if.
do if (pathtype(1,i)=2 and nms < 7 and serial=1).
compute indlocs(:,i)=thetammb(:,thetam(1,c3)).
compute c3=c3+1.
end if.
end loop.
loop i = 1 to ncol(indlocs).
compute c1=2.
loop j = 2 to nrow(indlocs).
do if (indlocs(j,i) <> 0).
compute indlocs(c1,i)=indlocs(j,i).
compute c1=c1+1.
end if.
end loop.
compute indlocs(1,i)=c1-2.
end loop.
compute indlocs=indlocs(1:rmax((indlocs(1,:))+1),:).
loop i = 1 to ncol(indmark).
compute numget=indmark(1,i).
compute end=end+numget.
compute gotcha=indsets(1,start:end).
compute start=end+1.
compute ok=1.
compute temp=0.
compute repoman=make(4,1,0).
loop j = 1 to ncol(gotcha).
do if paths(1,gotcha(1,j))=0.
compute ok=0.
end if.
do if (pathsmod(1,gotcha(1,j)) > 0).
compute temp=1.
compute temp2={pathsw(1,gotcha(1,j));pathsz(1,gotcha(1,j));pathswz(1,gotcha(1,j));0}.
do if (temp2(1,1)=1 and temp2(2,1)=1 and temp2(3,1)=0).
compute temp2(4,1)=1.
end if.
compute repoman=repoman+temp2.
end if.
end loop.
compute temp=0.
compute tempmmm=0.
compute typemmm=0.
do if ((repoman(1,1) > 0) and (repoman(2,1) = 0)).
compute temp=1.
do if (repoman(1,1)=1).
compute tempmmm=1.
end if.
do if (repoman(1,1) > 1 and (wdich=1 or mcw > 0)).
compute tempmmm=12.
compute typemmm=mcw.
do if (wdich=1).
compute typemmm=1.
end if.
end if.
do if (repoman(1,1) > 1 and (wdich=0 and mcw = 0)).
compute tempmmm=101.
end if.
end if.
do if ((repoman(1,1) = 0) and (repoman(2,1) > 0)).
compute temp=2.
do if (repoman(2,1)=1).
compute tempmmm=2.
end if.
do if (repoman(2,1) > 1 and (zdich = 1 or mcz > 0)).
compute tempmmm=22.
compute typemmm=mcz.
do if (zdich=1).
compute typemmm=1.
end if.
end if.
do if (repoman(2,1) > 1 and (zdich = 0 and mcw = 0)).
compute tempmmm=102.
end if.
end if.
do if (repoman(1,1)>0 and repoman(2,1)>0).
compute temp=3.
do if (repoman(1,1)=1 and repoman(2,1)=1).
do if (repoman(4,1)=1).
compute tempmmm=31.
end if.
do if (repoman(3,1)=1).
compute tempmmm=41.
end if.
end if.
end if.
do if (repoman(1,1)=1 and repoman(2,1)=1 and repoman(3,1)=0 and repoman(4,1)=0).
compute tempmmm=51.
end if.
do if (ok = 1).
compute nindfx=nindfx+1.
compute indmake(nindfx,1)=numget.
compute indmod(nindfx,1)=temp.
compute indmmm(nindfx,1)=tempmmm.
compute indmmmt(nindfx,1)=typemmm.
compute indmake(nindfx,2:(1+numget))=gotcha.
compute indkey(nindfx,1)=xnames.
loop j = 1 to numget.
compute indkey(nindfx,(j*2+1))=pathsdv(1,gotcha(1,j)).
compute indkey(nindfx,(j*2))='   ->   '.
end loop.
end if.
end loop.
compute indkey=indkey(1:nindfx,1:((cmax(indmake(:,1))*2)+1)).
compute indmake=indmake(1:nindfx,1:(cmax(indmake(:,1))+1)).
compute indmod=indmod(1:nrow(indmake),1).
compute indmmm=indmmm(1:nrow(indmake),1).
compute indmmmt=indmmmt(1:nrow(indmake),1).
compute ncpairs = (((nindfx)*(nindfx-1))/2).
do if ((contrast = 1 or contrast = 2 or contrast=3) and (ncpairs > 105)).
compute contrast=0.
compute notecode(notes,1) = 13.
compute notes = notes + 1.
end if.
do if (contrast = 4).
do if (ncol(contvec) <> nindfx).
compute contrast=0.
compute notecode(notes,1) = 14.
compute notes = notes + 1.
end if.
end if.
do if (anymod=0).
do if (nms = 1 and contrast > 0).
compute contrast=0.
end if.
compute efloop=(((1-(effsize=0))*2)+1)-(((mcx>0 or xdich=1))*(1-(effsize=0))).
loop kk=1 to efloop.
do if (boot = 0).
compute bootres=obscoeff.
do if (kk=1).
compute totbtvec=make(1,nxvls,0).
end if.
compute bootdir=obsdirfx.
compute indtab=999.
compute inddiff=999.
compute bootysd=ysd.
compute bootxsd=xsd.
end if.
do if (boot > 0).
compute bootres={obscoeff;bootres}.
do if (kk=1).
compute totbtvec=make(nrow(bootres),nxvls,0).
end if.
compute indtab=make(1,4,999).
compute inddiff=make(nrow(bootres),1,999).
end if.
compute indtotal=make(nrow(bootres),1,0).
loop i = 1 to nrow(indmake).
loop j = 1 to nxvls.
compute indtemp=make(nrow(bootres),1,1).
loop k = 1 to indmake(i,1).
compute jtemp=1.
do if (j > 1 and k=1).
compute jtemp=j.
end if.
compute indtemp=indtemp&*bootres(:,pathsfoc(jtemp,indmake(i,(k+1)))).
end loop.
do if (kk = 2).
compute indtemp=indtemp/bootysd.
end if.
do if (kk = 3).
compute indtemp=(bootxsd&*indtemp)/bootysd.
end if.
do if (contrast <> 0).
compute inddiff={inddiff,indtemp}.
end if.
do if (nxvls=1).
compute indtotal=indtotal+indtemp.
end if.
compute indeff=indtemp(1,1).
do if (kk=1).
compute totbtvec(:,j)=totbtvec(:,j)+indtemp.
end if.
do if (boot > 0).

.
compute temp = indtemp(2:nrow(indtemp),1).
compute temp(GRADE( indtemp(2:nrow(indtemp),1) )) = indtemp(2:nrow(indtemp),1).
compute badlo = 0.
compute badhi = 0.
do if ( (indtemp(1,1)*bc)+(9999*(1-bc)) <> 9999).
compute pv=csum(temp < (indtemp(1,1)*bc)+(9999*(1-bc)) )/nrow(temp).
compute ppv = pv.
do if (pv > .5).
compute ppv = 1-pv.
end if.
compute y5=sqrt(-2*ln(ppv)).
compute xp=y5+((((y5*p4+p3)*y5+p2)*y5+p1)*y5+p0)/((((y5*q4+q3)*y5+q2)*y5+q1)*y5+q0).
do if (pv <= .5).
compute xp = -xp.
end if.
compute cilow=rnd(nrow(temp)*(cdfnorm(2*xp-xp2))).
compute cihigh=trunc(nrow(temp)*(cdfnorm(2*xp+(xp2))))+1.
do if (cilow < 1).
compute cilow = 1.
compute booterr=1.
compute badlo = 1.
end if.
do if (cihigh > nrow(temp)).
compute cihigh = boot.
compute booterr=1.
compute badhi = 1.
end if.
compute llcit=temp(cilow,1).
compute ulcit=temp(cihigh,1).
do if (badlo = 1 and llcit <> priorlo).
compute badend={badend, llcit}.
compute priorlo = llcit.
end if.
do if (badhi = 1 and ulcit <> priorhi).
compute badend={badend, ulcit}.
compute priorhi = ulcit.
end if.
end if.
do if ( (indtemp(1,1)*bc)+(9999*(1-bc)) = 9999).
compute llcit=temp(cilow,1).
compute ulcit=temp(cihigh,1).
end if.
compute bootse=t(sqrt((cssq(temp)-((csum(temp)&**2)/nrow(temp)))/(nrow(temp)-1)))
.
compute indeff={indeff,bootse,llcit,ulcit}.
end if.
do if (kk=1).
compute indtabn={indtab;indeff}.
end if.
compute indtab={indtab;indeff}.
end loop.
end loop.
compute indtab=indtab(2:nrow(indtab),:).
do if (kk=1).
compute indtabn=indtabn(2:nrow(indtabn),:).
end if.
compute rowlbs=indlbl(1:nrow(indtab),1).
do if (mc > 0).
compute inddiff=make(mc,1,-999).
compute indtab2=make(nrow(indtab),4,-999).
compute indtab2(:,1)=indtab.
compute indtab=indtab2.
release indtab2.
compute mcct=0.
compute indtotal=make(mc,1,0).
do if (kk = 1).
compute x1 = sqrt(-2*ln(uniform(mc,nrow(mcsopath))))&*cos((2*3.14159265358979)*uniform(mc, nrow(mcsopath))).
compute x1=x1*chol(indcov).
loop ii=1 to nrow(x1).
compute x1(ii,:)=x1(ii,:)+t(mcsopath).
end loop.
end if.
loop ii=1 to nms.
compute tmpb=x1(:,((nms*nxvls)+ii)).
compute tmpb2=tmpb.
do if (nxvls > 1).
loop jj=1 to (nxvls-1).
compute tmpb2={tmpb2,tmpb}.
end loop.
end if.
compute indtemp=x1(:,(((ii-1)*nxvls)+1):(ii*nxvls))&*tmpb2.
loop jj=1 to ncol(indtemp).
do if (kk = 2).
compute indtemp(:,jj)=indtemp(:,jj)/ysd.
end if.
do if (kk = 3).
compute indtemp(:,jj)=(xsd*indtemp(:,jj))/ysd.
end if.

.
compute temp = indtemp(:,jj).
compute temp(GRADE( indtemp(:,jj) )) = indtemp(:,jj).
compute badlo = 0.
compute badhi = 0.
do if ( 9999 <> 9999).
compute pv=csum(temp < 9999 )/nrow(temp).
compute ppv = pv.
do if (pv > .5).
compute ppv = 1-pv.
end if.
compute y5=sqrt(-2*ln(ppv)).
compute xp=y5+((((y5*p4+p3)*y5+p2)*y5+p1)*y5+p0)/((((y5*q4+q3)*y5+q2)*y5+q1)*y5+q0).
do if (pv <= .5).
compute xp = -xp.
end if.
compute cilow=rnd(nrow(temp)*(cdfnorm(2*xp-xp2))).
compute cihigh=trunc(nrow(temp)*(cdfnorm(2*xp+(xp2))))+1.
do if (cilow < 1).
compute cilow = 1.
compute booterr=1.
compute badlo = 1.
end if.
do if (cihigh > nrow(temp)).
compute cihigh = boot.
compute booterr=1.
compute badhi = 1.
end if.
compute llcit=temp(cilow,1).
compute ulcit=temp(cihigh,1).
do if (badlo = 1 and llcit <> priorlo).
compute badend={badend, llcit}.
compute priorlo = llcit.
end if.
do if (badhi = 1 and ulcit <> priorhi).
compute badend={badend, ulcit}.
compute priorhi = ulcit.
end if.
end if.
do if ( 9999 = 9999).
compute llcit=temp(cilow,1).
compute ulcit=temp(cihigh,1).
end if.
compute bootse=t(sqrt((cssq(temp)-((csum(temp)&**2)/nrow(temp)))/(nrow(temp)-1)))
.
compute mcct=mcct+1.
compute indtab(mcct,2:4)={bootse,llcit,ulcit}.
end loop.
do if (nxvls=1).
compute indtotal=indtotal+indtemp.
do if (contrast <> 0).
compute inddiff={inddiff,indtemp}.
end if.
end if.
end loop.
release indtemp,tmpb.
end if.
do if (normal = 1 and sobelok=1).
compute sobelmat=indtab(:,1).
compute sobelmat={sobelmat,(sobelmat/2),sobelmat,sobelmat}.
loop ii=1 to nms.
compute se2b=indcov(((nms*nxvls)+ii),((nms*nxvls)+ii)).
compute bpath2=mcsopath(((nms*nxvls)+ii),1)&**2.
compute se2a=diag(indcov((((ii-1)*nxvls)+1):(ii*nxvls),(((ii-1)*nxvls)+1):(ii*nxvls))).
compute apath2=mcsopath((((ii-1)*nxvls)+1):(ii*nxvls) ,1)&**2.
compute sesobel=sqrt(apath2*se2b+bpath2*se2a+se2a*se2b).
compute sobelmat((((ii-1)*nxvls)+1):(ii*nxvls),2)=sesobel.
end loop.
release se2b,bpath2,se2a,apath2,sesobel,ii.
compute sobelmat(:,3)=sobelmat(:,1)&/sobelmat(:,2).
compute sobelmat(:,4) = 2*(1-cdfnorm(abs(sobelmat(:,3)))).
end if.
do if (serial = 0).
compute rowlbs=t(mnames).
end if.
do if (nxvls=1 and nms > 1).
compute rowlbs={'TOTAL';rowlbs}.
compute indtemp=indtotal(1,1).
do if (boot > 0 and nxvls=1).

.
compute temp = indtotal(2:nrow(indtotal),1).
compute temp(GRADE( indtotal(2:nrow(indtotal),1) )) = indtotal(2:nrow(indtotal),1).
compute badlo = 0.
compute badhi = 0.
do if ( (indtotal(1,1)*bc)+(9999*(1-bc)) <> 9999).
compute pv=csum(temp < (indtotal(1,1)*bc)+(9999*(1-bc)) )/nrow(temp).
compute ppv = pv.
do if (pv > .5).
compute ppv = 1-pv.
end if.
compute y5=sqrt(-2*ln(ppv)).
compute xp=y5+((((y5*p4+p3)*y5+p2)*y5+p1)*y5+p0)/((((y5*q4+q3)*y5+q2)*y5+q1)*y5+q0).
do if (pv <= .5).
compute xp = -xp.
end if.
compute cilow=rnd(nrow(temp)*(cdfnorm(2*xp-xp2))).
compute cihigh=trunc(nrow(temp)*(cdfnorm(2*xp+(xp2))))+1.
do if (cilow < 1).
compute cilow = 1.
compute booterr=1.
compute badlo = 1.
end if.
do if (cihigh > nrow(temp)).
compute cihigh = boot.
compute booterr=1.
compute badhi = 1.
end if.
compute llcit=temp(cilow,1).
compute ulcit=temp(cihigh,1).
do if (badlo = 1 and llcit <> priorlo).
compute badend={badend, llcit}.
compute priorlo = llcit.
end if.
do if (badhi = 1 and ulcit <> priorhi).
compute badend={badend, ulcit}.
compute priorhi = ulcit.
end if.
end if.
do if ( (indtotal(1,1)*bc)+(9999*(1-bc)) = 9999).
compute llcit=temp(cilow,1).
compute ulcit=temp(cihigh,1).
end if.
compute bootse=t(sqrt((cssq(temp)-((csum(temp)&**2)/nrow(temp)))/(nrow(temp)-1)))
.
compute indtemp={indtemp, bootse,llcit,ulcit}.
end if.
do if (mc > 0).
compute obtmc=indtab(:,1).
compute indtemp=csum(obtmc).

.
compute temp = indtotal(:,1).
compute temp(GRADE( indtotal(:,1) )) = indtotal(:,1).
compute badlo = 0.
compute badhi = 0.
do if ( 9999 <> 9999).
compute pv=csum(temp < 9999 )/nrow(temp).
compute ppv = pv.
do if (pv > .5).
compute ppv = 1-pv.
end if.
compute y5=sqrt(-2*ln(ppv)).
compute xp=y5+((((y5*p4+p3)*y5+p2)*y5+p1)*y5+p0)/((((y5*q4+q3)*y5+q2)*y5+q1)*y5+q0).
do if (pv <= .5).
compute xp = -xp.
end if.
compute cilow=rnd(nrow(temp)*(cdfnorm(2*xp-xp2))).
compute cihigh=trunc(nrow(temp)*(cdfnorm(2*xp+(xp2))))+1.
do if (cilow < 1).
compute cilow = 1.
compute booterr=1.
compute badlo = 1.
end if.
do if (cihigh > nrow(temp)).
compute cihigh = boot.
compute booterr=1.
compute badhi = 1.
end if.
compute llcit=temp(cilow,1).
compute ulcit=temp(cihigh,1).
do if (badlo = 1 and llcit <> priorlo).
compute badend={badend, llcit}.
compute priorlo = llcit.
end if.
do if (badhi = 1 and ulcit <> priorhi).
compute badend={badend, ulcit}.
compute priorhi = ulcit.
end if.
end if.
do if ( 9999 = 9999).
compute llcit=temp(cilow,1).
compute ulcit=temp(cihigh,1).
end if.
compute bootse=t(sqrt((cssq(temp)-((csum(temp)&**2)/nrow(temp)))/(nrow(temp)-1)))
.
compute indtemp={indtemp, bootse,llcit,ulcit}.
end if.
compute indtab={indtemp;indtab}.
end if.
compute bootlbs={'Effect', 'BootSE','BootLLCI','BootULCI'}.
do if (mc > 0).
compute bootlbs={'Effect', 'MC SE','MC LLCI','MC ULCI'}.
end if.
do if (nxvls = 1).
do if (contrast <> 0).
compute inddiff=inddiff(:,2:ncol(inddiff)).
do if (mc > 0).
compute inddiff={t(obtmc);inddiff}.
end if.
do if (contrast = 4).
compute inddifft=inddiff*t(contvec).
compute indtemp=inddifft(1,1).
do if (boot > 0 or mc > 0).
do if (mc > 0).

.
compute temp = inddifft(2:nrow(inddifft),1).
compute temp(GRADE( inddifft(2:nrow(inddifft),1) )) = inddifft(2:nrow(inddifft),1).
compute badlo = 0.
compute badhi = 0.
do if ( 9999 <> 9999).
compute pv=csum(temp < 9999 )/nrow(temp).
compute ppv = pv.
do if (pv > .5).
compute ppv = 1-pv.
end if.
compute y5=sqrt(-2*ln(ppv)).
compute xp=y5+((((y5*p4+p3)*y5+p2)*y5+p1)*y5+p0)/((((y5*q4+q3)*y5+q2)*y5+q1)*y5+q0).
do if (pv <= .5).
compute xp = -xp.
end if.
compute cilow=rnd(nrow(temp)*(cdfnorm(2*xp-xp2))).
compute cihigh=trunc(nrow(temp)*(cdfnorm(2*xp+(xp2))))+1.
do if (cilow < 1).
compute cilow = 1.
compute booterr=1.
compute badlo = 1.
end if.
do if (cihigh > nrow(temp)).
compute cihigh = boot.
compute booterr=1.
compute badhi = 1.
end if.
compute llcit=temp(cilow,1).
compute ulcit=temp(cihigh,1).
do if (badlo = 1 and llcit <> priorlo).
compute badend={badend, llcit}.
compute priorlo = llcit.
end if.
do if (badhi = 1 and ulcit <> priorhi).
compute badend={badend, ulcit}.
compute priorhi = ulcit.
end if.
end if.
do if ( 9999 = 9999).
compute llcit=temp(cilow,1).
compute ulcit=temp(cihigh,1).
end if.
compute bootse=t(sqrt((cssq(temp)-((csum(temp)&**2)/nrow(temp)))/(nrow(temp)-1)))
.
end if.
do if (boot > 0).

.
compute temp = inddifft(2:nrow(inddifft),1).
compute temp(GRADE( inddifft(2:nrow(inddifft),1) )) = inddifft(2:nrow(inddifft),1).
compute badlo = 0.
compute badhi = 0.
do if ( (inddifft(1,1)*bc)+(9999* (1-bc)) <> 9999).
compute pv=csum(temp < (inddifft(1,1)*bc)+(9999* (1-bc)) )/nrow(temp).
compute ppv = pv.
do if (pv > .5).
compute ppv = 1-pv.
end if.
compute y5=sqrt(-2*ln(ppv)).
compute xp=y5+((((y5*p4+p3)*y5+p2)*y5+p1)*y5+p0)/((((y5*q4+q3)*y5+q2)*y5+q1)*y5+q0).
do if (pv <= .5).
compute xp = -xp.
end if.
compute cilow=rnd(nrow(temp)*(cdfnorm(2*xp-xp2))).
compute cihigh=trunc(nrow(temp)*(cdfnorm(2*xp+(xp2))))+1.
do if (cilow < 1).
compute cilow = 1.
compute booterr=1.
compute badlo = 1.
end if.
do if (cihigh > nrow(temp)).
compute cihigh = boot.
compute booterr=1.
compute badhi = 1.
end if.
compute llcit=temp(cilow,1).
compute ulcit=temp(cihigh,1).
do if (badlo = 1 and llcit <> priorlo).
compute badend={badend, llcit}.
compute priorlo = llcit.
end if.
do if (badhi = 1 and ulcit <> priorhi).
compute badend={badend, ulcit}.
compute priorhi = ulcit.
end if.
end if.
do if ( (inddifft(1,1)*bc)+(9999* (1-bc)) = 9999).
compute llcit=temp(cilow,1).
compute ulcit=temp(cihigh,1).
end if.
compute bootse=t(sqrt((cssq(temp)-((csum(temp)&**2)/nrow(temp)))/(nrow(temp)-1)))
.
end if.
compute indtemp={indtemp, bootse,llcit,ulcit}.
end if.
compute indtab={indtab;indtemp}.
end if.
do if (contrast = 1 or contrast = 2 or contrast=3).
compute conkey=make(1,4,' ').
loop i = 1 to ncol(inddiff)-1.
loop j = (i+1) to ncol(inddiff).
compute inddifft=inddiff(:,i)-inddiff(:,j).
do if (contrast=2).
compute inddifft=abs(inddiff(:,i))-abs(inddiff(:,j)).
end if.
do if (contrast=3).
compute inddifft=inddiff(:,i)+inddiff(:,j).
end if.
compute indtemp=inddifft(1,1).
do if (contrast=1 or contrast=2).
compute conkeyt={' ', rowlbs((i+1),1),' minus  ',rowlbs((j+1),1)}.
end if.
do if (contrast=3).
compute conkeyt={' ', rowlbs((i+1),1),'  plus  ',rowlbs((j+1),1)}.
end if.
compute conkey={conkey;conkeyt}.
do if (boot > 0 or mc > 0).
do if (mc > 0).

.
compute temp = inddifft(2:nrow(inddifft),1).
compute temp(GRADE( inddifft(2:nrow(inddifft),1) )) = inddifft(2:nrow(inddifft),1).
compute badlo = 0.
compute badhi = 0.
do if ( 9999 <> 9999).
compute pv=csum(temp < 9999 )/nrow(temp).
compute ppv = pv.
do if (pv > .5).
compute ppv = 1-pv.
end if.
compute y5=sqrt(-2*ln(ppv)).
compute xp=y5+((((y5*p4+p3)*y5+p2)*y5+p1)*y5+p0)/((((y5*q4+q3)*y5+q2)*y5+q1)*y5+q0).
do if (pv <= .5).
compute xp = -xp.
end if.
compute cilow=rnd(nrow(temp)*(cdfnorm(2*xp-xp2))).
compute cihigh=trunc(nrow(temp)*(cdfnorm(2*xp+(xp2))))+1.
do if (cilow < 1).
compute cilow = 1.
compute booterr=1.
compute badlo = 1.
end if.
do if (cihigh > nrow(temp)).
compute cihigh = boot.
compute booterr=1.
compute badhi = 1.
end if.
compute llcit=temp(cilow,1).
compute ulcit=temp(cihigh,1).
do if (badlo = 1 and llcit <> priorlo).
compute badend={badend, llcit}.
compute priorlo = llcit.
end if.
do if (badhi = 1 and ulcit <> priorhi).
compute badend={badend, ulcit}.
compute priorhi = ulcit.
end if.
end if.
do if ( 9999 = 9999).
compute llcit=temp(cilow,1).
compute ulcit=temp(cihigh,1).
end if.
compute bootse=t(sqrt((cssq(temp)-((csum(temp)&**2)/nrow(temp)))/(nrow(temp)-1)))
.
end if.
do if (boot > 0).

.
compute temp = inddifft(2:nrow(inddifft),1).
compute temp(GRADE( inddifft(2:nrow(inddifft),1) )) = inddifft(2:nrow(inddifft),1).
compute badlo = 0.
compute badhi = 0.
do if ( (inddifft(1,1)*bc)+(9999* (1-bc)) <> 9999).
compute pv=csum(temp < (inddifft(1,1)*bc)+(9999* (1-bc)) )/nrow(temp).
compute ppv = pv.
do if (pv > .5).
compute ppv = 1-pv.
end if.
compute y5=sqrt(-2*ln(ppv)).
compute xp=y5+((((y5*p4+p3)*y5+p2)*y5+p1)*y5+p0)/((((y5*q4+q3)*y5+q2)*y5+q1)*y5+q0).
do if (pv <= .5).
compute xp = -xp.
end if.
compute cilow=rnd(nrow(temp)*(cdfnorm(2*xp-xp2))).
compute cihigh=trunc(nrow(temp)*(cdfnorm(2*xp+(xp2))))+1.
do if (cilow < 1).
compute cilow = 1.
compute booterr=1.
compute badlo = 1.
end if.
do if (cihigh > nrow(temp)).
compute cihigh = boot.
compute booterr=1.
compute badhi = 1.
end if.
compute llcit=temp(cilow,1).
compute ulcit=temp(cihigh,1).
do if (badlo = 1 and llcit <> priorlo).
compute badend={badend, llcit}.
compute priorlo = llcit.
end if.
do if (badhi = 1 and ulcit <> priorhi).
compute badend={badend, ulcit}.
compute priorhi = ulcit.
end if.
end if.
do if ( (inddifft(1,1)*bc)+(9999* (1-bc)) = 9999).
compute llcit=temp(cilow,1).
compute ulcit=temp(cihigh,1).
end if.
compute bootse=t(sqrt((cssq(temp)-((csum(temp)&**2)/nrow(temp)))/(nrow(temp)-1)))
.
end if.
compute indtemp={indtemp, bootse,llcit,ulcit}.
end if.
compute indtab={indtab;indtemp}.
end loop.
end loop.
end if.
release inddiff.
compute contlbs=cntname(1:(((nindfx)*(nindfx-1))/2),1).
compute rowlbs={rowlbs;contlbs}.
end if.
compute resultm2=make(nrow(indtab),maxresm,99999).
compute resultm2(1:nrow(indtab),1:ncol(indtab))=indtab.
compute resultm={resultm;resultm2}.
do if (outscree=1).
do if (kk=1).
print indtab/title = 'Indirect effect(s) of X on Y:'/rnames=rowlbs/cnames=bootlbs/format= F10.4.
end if.
do if (kk = 2 and (xdich=1 or mcx > 0)).
print indtab/title = 'Partially standardized indirect effect(s) of X on Y:'/rnames=rowlbs/cnames=bootlbs/format= F10.4.
end if.
do if (kk = 3).
print indtab/title = 'Completely standardized indirect effect(s) of X on Y:'/rnames=rowlbs/cnames=bootlbs/format= F10.4.
end if.
end if.
do if (normal=1 and sobelok=1 and kk=1).
compute resultm2=make(nrow(sobelmat),maxresm,99999).
compute resultm2(1:nrow(sobelmat),1:ncol(sobelmat))=sobelmat.
compute resultm={resultm;resultm2}.
compute sobellab={'Effect',hclab,'Z','p'}.
compute sobelrlb=rowlbs.
do if (nms > 1).
compute sobelrlb=rowlbs(2:(1+nms),1).
end if.
do if (outscree=1).
print sobelmat/title='   Normal theory test for indirect effect(s):'/cnames=sobellab/rnames=sobelrlb/format= F10.4.
end if.
end if.
do if (contrast <> 0).
do if ((contrast=1 or contrast = 2 or contrast=3) and kk=efloop ).
compute conkey=conkey(2:nrow(conkey),:).
do if (outscree=1).
print conkey/title = 'Specific indirect effect contrast definition(s):'/rnames=contlbs/format=A8.
end if.
end if.
do if (outscree=1).
do if (contrast = 4 and kk=efloop).
compute crowlbs=rowlbs(2:(nindfx+1),1).
print contvec/title = 'Specific indirect effect contrast weights:'/cnames=crowlbs/rlabels='(C1)'/format= F10.4.
end if.
do if (contrast = 2 and kk=efloop).
print/title = 'Contrasts are differences between absolute values of indirect effects'.
end if.
end if.
end if.
do if (serial = 1 and kk=efloop).
compute rowlbst=rowlbs(2:nrow(rowlbs),1).
do if (outscree=1).
print indkey/title = 'Indirect effect key:'/rnames=rowlbst/format = A8.
end if.
end if.
else.
do if (outscree=1).
do if (kk = 1).
print/title = 'Relative indirect effects of X on Y'.
end if.
do if (kk = 2).
print/title = 'Partially standardized relative indirect effect(s) of X on Y:'.
end if.
do if (kk = 3).
print/title = 'Completely standardized relative indirect effect(s) of X on Y:'.
end if.
end if.
loop i = 1 to nrow(indmake).
compute indtabsm=indtab((((i-1)*nxvls)+1):(nxvls*i),:).
compute indkeyt=indkey(i,:).
do if (outscree=1).
print indkeyt/title=' '/space=0/format=A8.
end if.
do if (bcmat(nrow(bcmat),1)=0)).
compute direffl2=xcatlab(1:nxvls,1).
end if.
compute resultm2=make(nrow(indtabsm),maxresm,99999).
compute resultm2(1:nrow(indtabsm),1:ncol(indtabsm))=indtabsm.
compute resultm={resultm;resultm2}.
do if (outscree=1).
print indtabsm/title = ' '/cnames=bootlbs/rnames=direffl2/format= F10.4 /space=0.
end if.
do if (normal=1 and sobelok=1 and kk=1).
compute sobelsm=sobelmat((((i-1)*nxvls)+1):(nxvls*i),:).
compute sobellab={'Effect',hclab,'Z','p'}.
compute resultm2=make(nrow(sobelsm),maxresm,99999).
compute resultm2(1:nrow(sobelsm),1:ncol(sobelsm))=sobelsm.
compute resultm={resultm;resultm2}.
do if (outscree=1).
print sobelsm/title='      Normal theory test for relative indirect effects:'/cnames=sobellab/rnames=direffl2/format= F10.4.
end if.
end if.
end loop.
end if.
do if (effsize = 1 and boot > 0).
compute bootres=bootres(2:nrow(bootres),:).
end if.
end loop.
do if (alttotal=1).
compute altcnms='Effect'.
compute totbtvec=totbtvec+bootdir.
compute alttotfx=t(totbtvec(1,:)).
do if (boot > 0).
compute alttotfx=make(ncol(totbtvec),4,0).
compute alttotfx(:,1)=t(totbtvec(1,:)).
loop cec=1 to ncol(totbtvec).

.
compute temp = totbtvec(2:nrow(totbtvec),cec).
compute temp(GRADE( totbtvec(2:nrow(totbtvec),cec) )) = totbtvec(2:nrow(totbtvec),cec).
compute badlo = 0.
compute badhi = 0.
do if ( (totbtvec(1,cec)*bc)+(9999* (1-bc)) <> 9999).
compute pv=csum(temp < (totbtvec(1,cec)*bc)+(9999* (1-bc)) )/nrow(temp).
compute ppv = pv.
do if (pv > .5).
compute ppv = 1-pv.
end if.
compute y5=sqrt(-2*ln(ppv)).
compute xp=y5+((((y5*p4+p3)*y5+p2)*y5+p1)*y5+p0)/((((y5*q4+q3)*y5+q2)*y5+q1)*y5+q0).
do if (pv <= .5).
compute xp = -xp.
end if.
compute cilow=rnd(nrow(temp)*(cdfnorm(2*xp-xp2))).
compute cihigh=trunc(nrow(temp)*(cdfnorm(2*xp+(xp2))))+1.
do if (cilow < 1).
compute cilow = 1.
compute booterr=1.
compute badlo = 1.
end if.
do if (cihigh > nrow(temp)).
compute cihigh = boot.
compute booterr=1.
compute badhi = 1.
end if.
compute llcit=temp(cilow,1).
compute ulcit=temp(cihigh,1).
do if (badlo = 1 and llcit <> priorlo).
compute badend={badend, llcit}.
compute priorlo = llcit.
end if.
do if (badhi = 1 and ulcit <> priorhi).
compute badend={badend, ulcit}.
compute priorhi = ulcit.
end if.
end if.
do if ( (totbtvec(1,cec)*bc)+(9999* (1-bc)) = 9999).
compute llcit=temp(cilow,1).
compute ulcit=temp(cihigh,1).
end if.
compute bootse=t(sqrt((cssq(temp)-((csum(temp)&**2)/nrow(temp)))/(nrow(temp)-1)))
.
compute alttotfx(cec,2:4)={bootse,llcit,ulcit}.
end loop.
compute altcnms={altcnms,'BootSE', 'BootLLCI','BootULCI'}.
end if.
compute resultm2=make(nrow(alttotfx),maxresm,99999).
compute resultm2(1:nrow(alttotfx),1:ncol(alttotfx))=alttotfx.
compute resultm={resultm;resultm2}.
do if (outscree=1).
do if (nxvls > 1).
print/title= '----------'/space=0.
print alttotfx/title='Relative total effects of X on Y (sum of direct and indirect effects)'/rnames=direffl2/cnames=altcnms/format= F10.4.
end if.
do if (nxvls = 1).
print alttotfx/title='Total effect of X on Y (sum of direct and indirect effects)'/cnames=altcnms/format= F10.4.
end if.
end if.
end if.
end if.
do if (anymod > 0).
do if (boot = 0).
compute bootres=obscoeff.
compute indtab=999.
end if.
do if (boot > 0).
compute bootres={obscoeff;bootres}.
compute indtab=make(1,4,999).
end if.
do if (csum((indmod > 0))=nrow(indmod)).
do if (outscree=1).
do if (nxvls > 1).
do if (xmint=0).
print/title= 'Relative conditional indirect effects of X on Y:'.
end if.
do if (xmint=1).
print/title= 'Relative (total) natural indirect effects of X on Y:'.
end if.
end if.
do if (nxvls = 1).
do if (xmint=0).
print/title= 'Conditional indirect effects of X on Y:'.
end if.
do if (xmint=1).
print/title= '(Total) Natural indirect effect(s) of X on Y:'.
end if.
end if.
end if.
end if.
do if (csum((indmod > 0)) < nrow(indmod)).
do if (outscree=1).
do if (nxvls > 1).
print/title= 'Relative conditional and unconditional indirect effects of X on Y:'.
end if.
do if (nxvls = 1).
print/title= 'Conditional and unconditional indirect effects of X on Y:'.
end if.
end if.
end if.
compute cftotfx=make(nrow(bootres),nxvls,0).
loop i = 1 to nrow(indmake).
compute indtab=indtab(1,:)*0.
do if (outscree=1).
do if (xmint=0).
print indkey(i,:)/title = 'INDIRECT EFFECT:'/format=A8.
end if.
do if (xmint=1).
print indkey(i,:)/title = ' '/format=A8/space=0.
end if.
end if.
do if (indmod(i,1)=0).
loop j = 1 to nxvls.
compute indtemp=make(nrow(bootres),1,1).
loop k = 1 to indmake(i,1).
compute jtemp=1.
do if (j > 1 and k=1).
compute jtemp=j.
end if.
compute indtemp=indtemp&*bootres(:,pathsfoc(jtemp,indmake(i,(k+1)))).
end loop.
compute indeff=indtemp(1,1).
do if (boot > 0).

.
compute temp = indtemp(2:nrow(indtemp),1).
compute temp(GRADE( indtemp(2:nrow(indtemp),1) )) = indtemp(2:nrow(indtemp),1).
compute badlo = 0.
compute badhi = 0.
do if ( (indtemp(1,1)*bc)+(9999*(1-bc)) <> 9999).
compute pv=csum(temp < (indtemp(1,1)*bc)+(9999*(1-bc)) )/nrow(temp).
compute ppv = pv.
do if (pv > .5).
compute ppv = 1-pv.
end if.
compute y5=sqrt(-2*ln(ppv)).
compute xp=y5+((((y5*p4+p3)*y5+p2)*y5+p1)*y5+p0)/((((y5*q4+q3)*y5+q2)*y5+q1)*y5+q0).
do if (pv <= .5).
compute xp = -xp.
end if.
compute cilow=rnd(nrow(temp)*(cdfnorm(2*xp-xp2))).
compute cihigh=trunc(nrow(temp)*(cdfnorm(2*xp+(xp2))))+1.
do if (cilow < 1).
compute cilow = 1.
compute booterr=1.
compute badlo = 1.
end if.
do if (cihigh > nrow(temp)).
compute cihigh = boot.
compute booterr=1.
compute badhi = 1.
end if.
compute llcit=temp(cilow,1).
compute ulcit=temp(cihigh,1).
do if (badlo = 1 and llcit <> priorlo).
compute badend={badend, llcit}.
compute priorlo = llcit.
end if.
do if (badhi = 1 and ulcit <> priorhi).
compute badend={badend, ulcit}.
compute priorhi = ulcit.
end if.
end if.
do if ( (indtemp(1,1)*bc)+(9999*(1-bc)) = 9999).
compute llcit=temp(cilow,1).
compute ulcit=temp(cihigh,1).
end if.
compute bootse=t(sqrt((cssq(temp)-((csum(temp)&**2)/nrow(temp)))/(nrow(temp)-1)))
.
compute indeff={indeff,bootse,llcit,ulcit}.
end if.
compute indtab={indtab;indeff}.
end loop.
compute resultm2=make(nrow(indtab),maxresm,99999).
compute resultm2(1:nrow(indtab),1:ncol(indtab))=indtab.
compute resultm={resultm;resultm2}.
do if (outscree=1).
do if (nxvls > 1).
compute indefflb=xcatlab(1:nxvls,1).
print indtab(2:nrow(indtab),:)/title = ' '/clabels='Effect', 'BootSE', 'BootLLCI', 'BootULCI'/rnames=indefflb/format= F10.4 /space=0.
end if.
do if (nxvls = 1).
print indtab(2:nrow(indtab),:)/title = ' '/clabels='Effect', 'BootSE', 'BootLLCI', 'BootULCI'/format= F10.4 /space=0.
end if.
end if.
end if.
do if (indmod(i,1)>0).
do if (indmod(i,1)=1).
compute indmodva=wmodvals.
compute indprova=wprobval.
compute condlbs={wnames}.
compute printw=1.
else if (indmod(i,1)=2).
compute indmodva=zmodvals.
compute indprova=zprobval.
compute condlbs={znames}.
compute printz=1.
else if (indmod(i,1)=3).
compute cntmp=1.
compute printz=1.
compute printw=1.
compute indmodva=make((nrow(wmodvals)*nrow(zmodvals)),2,999).
loop k7 = 1 to nrow(wmodvals).
loop k8 = 1 to nrow(zmodvals).
compute indmodva(cntmp,:)={wmodvals(k7,1),zmodvals(k8,1)}.
compute cntmp=cntmp+1.
end loop.
end loop.
compute condlbs={wnames,znames}.
end if.
compute condres=make(nrow(indmodva),1,999).
do if (boot > 0).
compute condres=make(nrow(indmodva),4,999).
end if.
compute condres={indmodva,condres}.
loop k4 = 1 to nxvls.
compute imm3=make(nrow(bootres),1,1).
compute imm4=make(nrow(bootres),1,1).
compute indcontr=0.
do if (indmod(i,1)=3).
compute tihsw=wprobval.
compute tihsz=zprobval.
end if.
loop k1=1 to nrow(indmodva).
compute tucker2=make(nrow(bootres),1,1).
compute imm2=make(nrow(bootres),1,1).
compute wfirst=0.
compute zfirst=0.
compute immset=0.
loop k2=1 to indmake(i,1).
compute colnumb=indmake(i,(k2+1)).
do if (k2=1).
compute wbb=make(nrow(bootres),(nwvls*nxvls),0).
compute zbb=make(nrow(bootres),(nzvls*nxvls),0).
compute wzbb=make(nrow(bootres),(nwvls*nzvls*nxvls),0).
end if.
do if (k2<>1).
compute wbb=make(nrow(bootres),nwvls,0).
compute zbb=make(nrow(bootres),nzvls,0).
compute wzbb=make(nrow(bootres),(nwvls*nzvls),0).
end if.
compute cnt=1.
compute tihs=indlocs(2:((indlocs(1,colnumb))+1),colnumb).
do if (k2 = 1).
compute focbb=tihs(1:nxvls,1).
compute focbb=bootres(:,focbb).
do if (indmmm(i,1)>0).
compute imm=focbb(:,k4).
compute condbb=imm.
end if.
compute focaddon=make(1,nxvls,0).
compute focaddon(1,k4)=1.
compute cnt=cnt+nxvls.
compute placeh=nxvls.
do if (indmod(i,1)=1).
compute tihsz=make(nrow(wprobval),(nzvls*nxvls),0).
compute tihswz=make(nrow(wprobval),(nwvls*nzvls*nxvls),0).
do if (pathsw(1,colnumb)=1).
compute temp=make(nrow(wprobval),(nxvls*nwvls),0).
loop k5 = 1 to nrow(wprobval).
loop k6=1 to nwvls.
compute temp(k5, (((k4-1)*nwvls)+k6))=wprobval(k5,k6).
end loop.
end loop.
compute indprova={temp,tihsz,tihswz}.
else.
compute indprova={wprobval,tihsz,tihswz}.
end if.
end if.
do if (indmod(i,1)=2).
compute tihsw=make(nrow(zprobval),(nwvls*nxvls),0).
compute tihswz=make(nrow(zprobval),(nwvls*nzvls*nxvls),0).
do if (pathsz(1,colnumb)=1).
compute temp=make(nrow(zprobval),(nxvls*nzvls),0).
loop k5 = 1 to nrow(zprobval).
loop k6 =1 to nzvls.
compute temp(k5,(((k4-1)*nzvls)+k6))=zprobval(k5,k6).
end loop.
end loop.
compute indprova={tihsw,temp,tihswz}.
else.
compute indprova={tihsw,zprobval,tihswz}.
end if.
end if.
do if (indmod(i,1)=3).
compute indprova=make((nrow(wprobval)*nrow(zprobval)),((ncol(wprobval)*nxvls)+ (ncol(zprobval)*nxvls)+(nwvls*nzvls*nxvls)),0).
compute cntemp=1.
loop k7=1 to nrow(wprobval).
loop k8 =1 to nrow(zprobval).
compute temp=wprobval(k7,:)*focaddon(1,k4).
compute indprova(cntemp,(((k4-1)*nwvls)+1):(k4*(nwvls)))=temp.
compute temp=zprobval(k8,:)*focaddon(1,k4).
compute indprova(cntemp, ((((k4-1)*nzvls)+1)+(nxvls*nwvls)) : ((((k4-1)*nzvls)+1)+(nxvls*nwvls)+(nzvls-1)))=temp.
compute cntemp=cntemp+1.
end loop.
end loop.
do if (pathsz(1,colnumb)=0).
compute temp=make(nrow(indprova),(ncol(zprobval)*nxvls),0).
compute indprova(:,((ncol(wprobval)*nxvls)+1):((ncol(wprobval)+ncol(zprobval))* nxvls))=temp.
end if.
do if (pathsw(1,colnumb)=0).
compute temp=make(nrow(indprova),(ncol(wprobval)*nxvls),0).
compute indprova(:,1:(ncol(wprobval)*nxvls))=temp.
end if.
do if (pathswz(1,colnumb)=1).
compute cntemp=(ncol(wprobval)*nxvls)+(ncol(zprobval)*nxvls)+((k4-1)* ncol(wprobval)*ncol(zprobval))+1.
loop k7=1 to ncol(wprobval).
loop k8=1 to ncol(zprobval).
compute indprova(:,cntemp)=indprova(:,((ncol(wprobval)*(k4-1))+k7))&* indprova(:,((((k4-1)*ncol(zprobval))+k8)+(nxvls*ncol(wprobval)))).
compute cntemp=cntemp+1.
end loop.
end loop.
end if.
end if.
end if.
do if (k2 > 1).
compute focbb=tihs(1,1).
compute focbb=bootres(:,focbb).
do if (indmmm(i,1)>0).
compute imm=focbb(:,1).
compute condbb=imm.
end if.
compute focaddon=1.
compute cnt=cnt+1.
compute placeh=1.
do if (indmod(i,1)=1).
compute tihsz=make(nrow(wprobval),nzvls,0).
compute tihswz=make(nrow(wprobval),(nwvls*nzvls),0).
compute indprova={wprobval,tihsz,tihswz}.
end if.
do if (indmod(i,1)=2).
compute tihsw=make(nrow(zprobval),nwvls,0).
compute tihswz=make(nrow(zprobval),(nwvls*nzvls),0).
compute indprova={tihsw,zprobval,tihswz}.
end if.
do if (indmod(i,1)=3).
compute indprova=make((nrow(wprobval)*nrow(zprobval)),((ncol(wprobval)+ ncol(zprobval))+(nwvls*nzvls)),0).
compute cntemp=1.
loop k7=1 to nrow(wprobval).
loop k8 =1 to nrow(zprobval).
compute indprova(cntemp,1:(ncol(wprobval)+ncol(zprobval)))={wprobval(k7,:), zprobval(k8,:)}.
compute cntemp=cntemp+1.
end loop.
end loop.
do if (pathsz(1,colnumb)=0).
compute temp=make(nrow(indprova),ncol(zprobval),0).
compute indprova(:,(ncol(wprobval)+1):(ncol(wprobval)+ncol(zprobval)))=temp.
end if.
do if (pathsw(1,colnumb)=0).
compute temp=make(nrow(indprova),ncol(wprobval),0).
compute indprova(:,1:ncol(wprobval))=temp.
end if.
do if (pathswz(1,colnumb)=1).
compute cntemp=ncol(wprobval)+ncol(zprobval)+1.
loop k7=1 to ncol(wprobval).
loop k8=1 to ncol(zprobval).
compute indprova(:,cntemp)=indprova(:,k7)&*indprova(:,(ncol(wprobval)+k8)).
compute cntemp=cntemp+1.
end loop.
end loop.
end if.
end if.
end if.
do if (pathsw(1,colnumb)) = 1.
compute wbb=tihs(cnt:(cnt+(placeh*nwvls)-1),1).
compute wbb=bootres(:,wbb).
compute immlbs2=wcatlab(1:nwvls,1).
do if (zfirst=0).
compute wfirst=1.
end if.
do if (indmmm(i,1)=1 or indmmm(i,1)=31 or indmmm(i,1)=51).
compute imm=wbb(:,1).
loop k7 = 1 to nwvls.
compute imm={imm,wbb(:,(((k4-1)*nwvls*(k2=1))+k7))}.
end loop.
compute imm=imm(:,2:ncol(imm)).
end if.
do if (indmmm(i,1)=41 or indmmm(i,1)=51).
compute condbb=make(nrow(bootres),1,0).
loop k7 = 1 to nwvls.
compute condbb={condbb,wbb(:,(((k4-1)*nwvls*(k2=1))+k7))}.
end loop.
compute condbb=condbb(:,2:ncol(condbb)).
end if.
compute cnt=cnt+(placeh*nwvls).
end if.
do if (pathsz(1,colnumb)) = 1.
compute zbb=tihs(cnt:(cnt+(placeh*nzvls)-1),1).
compute zbb=bootres(:,zbb).
do if (wfirst=0).
compute zfirst=1.
end if.
do if (indmmm(i,1) <> 31).
compute immlbs2=zcatlab(1:nzvls,1).
end if.
do if (indmmm(i,1)=2 or indmmm(i,1)=31 or indmmm(i,1)=51).
do if (indmmm(i,1)=2).
compute imm=zbb(:,1).
end if.
loop k7 = 1 to nzvls.
compute imm={imm,zbb(:,(((k4-1)*nzvls*(k2=1))+k7))}.
end loop.
do if (indmmm(i,1)=2 or indmmm(i,1)=51).
compute imm=imm(:,2:ncol(imm)).
do if (indmmm(i,1)=51).
compute condbb={condbb,imm}.
end if.
end if.
end if.
compute cnt=cnt+(placeh*nzvls).
end if.
do if (pathswz(1,colnumb)) = 1.
compute wzbb=tihs(cnt:(cnt+(placeh*nwvls*nzvls)-1),1).
compute wzbb=bootres(:,wzbb).
do if (indmmm(i,1)=41).
compute imm=wzbb(:,1).
loop k7=1 to nwvls*nzvls.
compute imm={imm,wzbb(:,(((k4-1)*nzvls*nwvls*(k2=1))+k7))}.
end loop.
end if.
do if (indmmm(i,1)=41).
compute imm=imm(:,2:ncol(imm)).
compute condbb={condbb,imm(:,(ncol(imm)-(nwvls*nzvls)+1):ncol(imm))}.
end if.
compute cnt=cnt+(placeh*nzvls*nwvls).
end if.
compute indprobe={focaddon,indprova(k1,:)}.
compute tucker={focbb,wbb,zbb,wzbb}.
loop k3=1 to ncol(indprobe).
compute tucker(:,k3)=tucker(:,k3)*indprobe(1,k3).
end loop.
compute tucker2=tucker2&*rsum(tucker).
do if (indmmm(i,1) = 1 or indmmm(i,1)=2 or indmmm(i,1)=31 or indmmm(i,1)=41 or indmmm(i,1)=51).
do if (immset=1).
do if (ncol(imm2)=1 and ncol(imm) = 1).
compute imm2=imm2&*imm.
end if.
do if (indmmm(i,1)=41 or indmmm(i,1)=51).
do if ((ncol(condbb2) > 1) and (ncol(condbb)>1)).
compute condbb2t=make(nrow(condbb2),(ncol(condbb2)*ncol(condbb)),-999999).
compute k9=1.
do if (wfirst=1).
loop k7=1 to ncol(condbb2).
loop k8 = 1 to ncol(condbb).
compute condbb2t(:,k9)=condbb2(:,k7)&*condbb(:,k8).
compute k9=k9+1.
end loop.
end loop.
end if.
do if (zfirst=1).
loop k7=1 to ncol(condbb).
loop k8 = 1 to ncol(condbb2).
compute condbb2t(:,k9)=condbb(:,k7)&*condbb2(:,k8).
compute k9=k9+1.
end loop.
end loop.
end if.
compute condbb2=condbb2t.
release condbb2t.
end if.
do if ((ncol(condbb2) > 1) and (ncol(condbb)=1)).
loop k7 = 1 to ncol(condbb2).
compute condbb2(:,k7)=condbb2(:,k7)&*condbb.
end loop.
end if.
do if ((ncol(condbb2) = 1) and (ncol(condbb)>1)).
loop k7 = 1 to ncol(condbb).
compute condbb(:,k7)=condbb2&*condbb(:,k7).
end loop.
compute condbb2=condbb.
end if.
end if.
do if (ncol(imm2) <> 1 and ncol(imm) <> 1).
compute imm2t=make(nrow(imm2),(ncol(imm2)*ncol(imm)),-999999).
compute k9=1.
do if (wfirst=1).
loop k7=1 to ncol(imm2).
loop k8 = 1 to ncol(imm).
compute imm2t(:,k9)=imm2(:,k7)&*imm(:,k8).
compute k9=k9+1.
end loop.
end loop.
end if.
do if (zfirst=1).
loop k7=1 to ncol(imm).
loop k8 = 1 to ncol(imm2).
compute imm2t(:,k9)=imm(:,k7)&*imm2(:,k8).
compute k9=k9+1.
end loop.
end loop.
end if.
compute imm2=imm2t.
release imm2t.
end if.
do if ((ncol(imm2) > 1) and (ncol(imm)=1)).
loop k7=1 to ncol(imm2).
compute imm2(:,k7)=imm2(:,k7)&*imm.
end loop.
end if.
do if ((ncol(imm2) = 1) and (ncol(imm) > 1)).
loop k7=1 to ncol(imm).
compute imm(:,k7)=imm2&*imm(:,k7).
end loop.
compute imm2=imm.
end if.
end if.
do if (immset=0).
compute imm2=imm.
do if (indmmm(i,1)=41 or indmmm(i,1)=51).
compute condbb2=condbb.
end if.
compute immset=1.
end if.
end if.
end loop.
compute indtemp=tucker2(1,1).
do if (indmmm(i,1)=12 or indmmm(i,1)=22).
compute imm3={imm3,tucker2}.
do if (k1=nrow(indmodva)).
compute imm3=imm3(:,2:ncol(imm3)).
compute immstop=ncol(imm3).
loop k8=2 to immstop.
do if (indmmmt(i,1)=1).
compute imm3={imm3,(imm3(:,k8)-imm3(:,1))}.
end if.
do if (indmmmt(i,1)=2).
compute imm3={imm3,(imm3(:,k8)-imm3(:,(k8-1)))}.
end if.
do if (indmmmt(i,1)=3).
compute imm3={imm3,((rsum(imm3(:,(k8:immstop)))/(immstop-k8+1))-imm3(:,(k8-1))) }.
end if.
do if (indmmmt(i,1)=4).
compute imm3={imm3,(imm3(:,k8)-(rsum(imm3(:,1:immstop))/immstop))}.
end if.
end loop.
do if (indmmmt(i,1)<5).
compute imm2=imm3(:,(immstop+1):ncol(imm3)).
release imm3.
end if.
end if.
end if.
do if (indmmm(i,1)>-1 and (contrast = 1 or contrast = 2 or contrast=3)).
compute imm4={imm4,tucker2}.
do if (k1=nrow(indmodva) and k1 > 1).
compute imm4=imm4(:,2:ncol(imm4)).
compute immstop=ncol(imm4).
compute condcont=make((immstop*(immstop-1)/2),6,-999).
loop k8 = 1 to (immstop-1).
loop k9 = (k8+1) to immstop.
do if (contrast=1).
compute imm4={imm4,(imm4(:,k9)-imm4(:,k8))}.
end if.
do if (contrast=2).
compute imm4={imm4,(abs(imm4(:,k9))-abs(imm4(:,k8)))}.
end if.
do if (contrast=3).
compute imm4={imm4,(imm4(:,k9)+imm4(:,k8))}.
end if.
compute condcont((ncol(imm4)-immstop),1)=imm4(1,k9).
compute condcont((ncol(imm4)-immstop),2)=imm4(1,k8).
end loop.
end loop.
compute imm4=imm4(:,(immstop+1):ncol(imm4)).
loop k8=1 to ncol(imm4).
compute condcont(k8,3)=imm4(1,k8).
do if (boot > 0).

.
compute temp = imm4(2:nrow(imm4),k8).
compute temp(GRADE( imm4(2:nrow(imm4),k8) )) = imm4(2:nrow(imm4),k8).
compute badlo = 0.
compute badhi = 0.
do if ( (imm4(1,k8)*bc)+(9999*(1-bc)) <> 9999).
compute pv=csum(temp < (imm4(1,k8)*bc)+(9999*(1-bc)) )/nrow(temp).
compute ppv = pv.
do if (pv > .5).
compute ppv = 1-pv.
end if.
compute y5=sqrt(-2*ln(ppv)).
compute xp=y5+((((y5*p4+p3)*y5+p2)*y5+p1)*y5+p0)/((((y5*q4+q3)*y5+q2)*y5+q1)*y5+q0).
do if (pv <= .5).
compute xp = -xp.
end if.
compute cilow=rnd(nrow(temp)*(cdfnorm(2*xp-xp2))).
compute cihigh=trunc(nrow(temp)*(cdfnorm(2*xp+(xp2))))+1.
do if (cilow < 1).
compute cilow = 1.
compute booterr=1.
compute badlo = 1.
end if.
do if (cihigh > nrow(temp)).
compute cihigh = boot.
compute booterr=1.
compute badhi = 1.
end if.
compute llcit=temp(cilow,1).
compute ulcit=temp(cihigh,1).
do if (badlo = 1 and llcit <> priorlo).
compute badend={badend, llcit}.
compute priorlo = llcit.
end if.
do if (badhi = 1 and ulcit <> priorhi).
compute badend={badend, ulcit}.
compute priorhi = ulcit.
end if.
end if.
do if ( (imm4(1,k8)*bc)+(9999*(1-bc)) = 9999).
compute llcit=temp(cilow,1).
compute ulcit=temp(cihigh,1).
end if.
compute bootse=t(sqrt((cssq(temp)-((csum(temp)&**2)/nrow(temp)))/(nrow(temp)-1)))
.
compute condcont(k8,4:6)={bootse,llcit,ulcit}.
end if.
end loop.
do if (boot=0).
compute condcont=condcont(:,1:3).
end if.
compute indcontr=1.
end if.
end if.
do if (xmint=1).
do if (k1=(k4+1)).
compute cftotfx(:,k4)=cftotfx(:,k4)+(tucker2*xscaling).
end if.
end if.
do if (boot > 0).
compute tucker2=tucker2*xscaling.

.
compute temp = tucker2(2:nrow(tucker2),1).
compute temp(GRADE( tucker2(2:nrow(tucker2),1) )) = tucker2(2:nrow(tucker2),1).
compute badlo = 0.
compute badhi = 0.
do if ( (tucker2(1,1)*bc)+(9999*(1-bc)) <> 9999).
compute pv=csum(temp < (tucker2(1,1)*bc)+(9999*(1-bc)) )/nrow(temp).
compute ppv = pv.
do if (pv > .5).
compute ppv = 1-pv.
end if.
compute y5=sqrt(-2*ln(ppv)).
compute xp=y5+((((y5*p4+p3)*y5+p2)*y5+p1)*y5+p0)/((((y5*q4+q3)*y5+q2)*y5+q1)*y5+q0).
do if (pv <= .5).
compute xp = -xp.
end if.
compute cilow=rnd(nrow(temp)*(cdfnorm(2*xp-xp2))).
compute cihigh=trunc(nrow(temp)*(cdfnorm(2*xp+(xp2))))+1.
do if (cilow < 1).
compute cilow = 1.
compute booterr=1.
compute badlo = 1.
end if.
do if (cihigh > nrow(temp)).
compute cihigh = boot.
compute booterr=1.
compute badhi = 1.
end if.
compute llcit=temp(cilow,1).
compute ulcit=temp(cihigh,1).
do if (badlo = 1 and llcit <> priorlo).
compute badend={badend, llcit}.
compute priorlo = llcit.
end if.
do if (badhi = 1 and ulcit <> priorhi).
compute badend={badend, ulcit}.
compute priorhi = ulcit.
end if.
end if.
do if ( (tucker2(1,1)*bc)+(9999*(1-bc)) = 9999).
compute llcit=temp(cilow,1).
compute ulcit=temp(cihigh,1).
end if.
compute bootse=t(sqrt((cssq(temp)-((csum(temp)&**2)/nrow(temp)))/(nrow(temp)-1)))
.
compute indtemp={indtemp*xscaling, bootse,llcit,ulcit}.
end if.
do if (boot=0).
compute indtemp=indtemp*xscaling.
end if.
compute condres(k1,(ncol(indmodva)+1):ncol(condres))=indtemp.
end loop.
do if (xmint=0).
compute resultm2=make(nrow(condres),maxresm,99999).
compute resultm2(1:nrow(condres),1:ncol(condres))=condres.
compute resultm={resultm;resultm2}.
end if.
compute condlbs={condlbs,'Effect'}.
do if (boot > 0).
compute condlbs={condlbs,'BootSE', 'BootLLCI', 'BootULCI'}.
end if.
do if (xmint = 1).
do if (k4 = 1).
compute natindfx=condres((2+(k4-1)),2:ncol(condres)).
end if.
do if (k4 > 1).
compute natindfx={natindfx;condres((2+(k4-1)),2:ncol(condres))}.
end if.
do if (k4=nxvls).
compute resultm2=make(nrow(natindfx),maxresm,99999).
compute resultm2(1:nrow(natindfx),1:ncol(natindfx))=natindfx.
compute resultm={resultm;resultm2}.
end if.
end if.
do if (outscree=1 and xmint=0).
do if (nxvls=1).
print condres/title=' '/cnames=condlbs/format= F10.4 /space=0.
else.
compute condrlb=make(nrow(condres),1,xcatlab(k4,1)).
print condres/title=' '/cnames=condlbs/rnames=condrlb/format= F10.4 /space=0.
end if.
end if.
do if (outscree=1 and xmint=1 and k4=nxvls).
do if (nxvls=1).
print natindfx/title=' '/clabels='Effect', 'BootSE', 'BootLLCI', 'BootULCI'/format= F10.4 /space=0.
else.
compute condrlb=make(nrow(condres),1,xcatlab(k4,1)).
print natindfx/title=' '/clabels='Effect', 'BootSE', 'BootLLCI', 'BootULCI'/rnames=direffl2/format= F10.4 /space=0.
end if.
end if.
compute dichadj=0.
compute immcat=0.
do if ((indmmm(i,1)>0) and (xmint=0)).
do if (indmmm(i,1)=1 or indmmm(i,1)=12 or indmmm(i,1)=31).
do if (wdich=1 and mcw=0).
do if (indmmm(i,1) <> 12).
compute imm2(:,1)=imm2(:,1)*(wmax-wmin).
end if.
do if (indmmm(i,1) <> 31).
compute dichadj=1.
end if.
end if.
do if ((mcw = 1 or mcw = 2) and indmmm(i,1) <> 31)).
compute immcat=1.
end if.
end if.
do if (indmmm(i,1)=2 or indmmm(i,1)=22 or indmmm(i,1)=31).
do if (zdich=1 and mcz=0).
do if (indmmm(i,1) = 31).
compute imm2(:,(nwvls+1):ncol(imm2))=imm2(:,(nwvls+1):ncol(imm2))*(zmax-zmin).
end if.
do if (indmmm(i,1) = 2).
compute imm2(:,1)=imm2(:,1)*(zmax-zmin).
end if.
do if (indmmm(i,1) <> 31).
compute dichadj=1.
end if.
end if.
do if ((mcz = 1 or mcz = 2) and indmmm(i,1) <> 31)).
compute immcat=1.
end if.
end if.
compute immtemp2=t(imm2(1,:)).
compute immtemp=immtemp2.
compute immlbs={'Index'}.
do if (boot > 0).
compute immtemp=make(1,3,0).
loop k7=1 to ncol(imm2).

.
compute temp = imm2(2:nrow(imm2),k7).
compute temp(GRADE( imm2(2:nrow(imm2),k7) )) = imm2(2:nrow(imm2),k7).
compute badlo = 0.
compute badhi = 0.
do if ( (imm2(1,k7)*bc)+(9999*(1-bc)) <> 9999).
compute pv=csum(temp < (imm2(1,k7)*bc)+(9999*(1-bc)) )/nrow(temp).
compute ppv = pv.
do if (pv > .5).
compute ppv = 1-pv.
end if.
compute y5=sqrt(-2*ln(ppv)).
compute xp=y5+((((y5*p4+p3)*y5+p2)*y5+p1)*y5+p0)/((((y5*q4+q3)*y5+q2)*y5+q1)*y5+q0).
do if (pv <= .5).
compute xp = -xp.
end if.
compute cilow=rnd(nrow(temp)*(cdfnorm(2*xp-xp2))).
compute cihigh=trunc(nrow(temp)*(cdfnorm(2*xp+(xp2))))+1.
do if (cilow < 1).
compute cilow = 1.
compute booterr=1.
compute badlo = 1.
end if.
do if (cihigh > nrow(temp)).
compute cihigh = boot.
compute booterr=1.
compute badhi = 1.
end if.
compute llcit=temp(cilow,1).
compute ulcit=temp(cihigh,1).
do if (badlo = 1 and llcit <> priorlo).
compute badend={badend, llcit}.
compute priorlo = llcit.
end if.
do if (badhi = 1 and ulcit <> priorhi).
compute badend={badend, ulcit}.
compute priorhi = ulcit.
end if.
end if.
do if ( (imm2(1,k7)*bc)+(9999*(1-bc)) = 9999).
compute llcit=temp(cilow,1).
compute ulcit=temp(cihigh,1).
end if.
compute bootse=t(sqrt((cssq(temp)-((csum(temp)&**2)/nrow(temp)))/(nrow(temp)-1)))
.
compute temp={bootse,llcit,ulcit}.
compute immtemp={immtemp;temp}.
end loop.
compute immtemp=immtemp(2:nrow(immtemp),:).
compute immtemp={immtemp2,immtemp}.
compute immlbs={immlbs,'BootSE', 'BootLLCI', 'BootULCI'}.
end if.
compute resultm2=make(nrow(immtemp),maxresm,99999).
compute resultm2(1:nrow(immtemp),1:ncol(immtemp))=immtemp.
compute resultm={resultm;resultm2}.
do if (dichadj=0 and immcat=0 and indmmmt(i,1)<>5 and indmmm(i,1) < 100 ).
do if (outscree=1).
do if (indmmm(i,1) < 30).
print immtemp/title='      Index of moderated mediation:'/cnames=immlbs/rnames=immlbs2/format= F10.4.
end if.
do if (indmmm(i,1) = 31).
compute immlbs2={immlbs2;zcatlab(1:nzvls,1)}.
print immtemp/title='      Indices of partial moderated mediation:'/cnames=immlbs/rnames=immlbs2/format= F10.4.
end if.
end if.
do if ((nzvls = 1) and (nwvls = 1)).
do if (indmmm(i,1) = 41 or indmmm(i,1)=51).
loop k7=1 to nwvls.
compute immlbs2=zcatlab(1:nzvls,1).
compute immtemp2=immtemp((((k7-1)*nzvls)+1):(((k7-1)*nzvls)+nzvls),:).
do if (outscree=1).
do if (nwvls > 1).
compute primodv={'        ', wcatlab(k7,1)}.
print primodv/title='      Primary moderator:'/format=A8.
end if.
do if (nzvls=1).
print immtemp2/title='      Index of moderated moderated mediation'/cnames=immlbs/format= F10.4.
else.
print immtemp2/title='      Indices of moderated moderated mediation'/cnames=immlbs/rnames=immlbs2/format= F10.4.
end if.
end if.
compute cmmtemp=make(nrow(zprobval),4,0).
loop k8=1 to nrow(zprobval).
compute condbb3=condbb2(:,((nwvls+1)+((k7-1)*nzvls)):((nwvls+1)+((k7-1)*nzvls)+ (nzvls-1))).
do if (ncol(zprobval) > 1).
compute condbb3=condbb3*mdiag(zprobval(k8,:)).
else.
compute condbb3=condbb3&*zprobval(k8,:).
end if.
compute condbb3={condbb2(:,k7),condbb3}.
compute icmm=rsum(condbb3).
compute cmmtemp(k8,1)=icmm(1,1).
do if (boot > 0).

.
compute temp = icmm(2:nrow(icmm),1).
compute temp(GRADE( icmm(2:nrow(icmm),1) )) = icmm(2:nrow(icmm),1).
compute badlo = 0.
compute badhi = 0.
do if ( (icmm(1,1)*bc)+(9999*(1-bc)) <> 9999).
compute pv=csum(temp < (icmm(1,1)*bc)+(9999*(1-bc)) )/nrow(temp).
compute ppv = pv.
do if (pv > .5).
compute ppv = 1-pv.
end if.
compute y5=sqrt(-2*ln(ppv)).
compute xp=y5+((((y5*p4+p3)*y5+p2)*y5+p1)*y5+p0)/((((y5*q4+q3)*y5+q2)*y5+q1)*y5+q0).
do if (pv <= .5).
compute xp = -xp.
end if.
compute cilow=rnd(nrow(temp)*(cdfnorm(2*xp-xp2))).
compute cihigh=trunc(nrow(temp)*(cdfnorm(2*xp+(xp2))))+1.
do if (cilow < 1).
compute cilow = 1.
compute booterr=1.
compute badlo = 1.
end if.
do if (cihigh > nrow(temp)).
compute cihigh = boot.
compute booterr=1.
compute badhi = 1.
end if.
compute llcit=temp(cilow,1).
compute ulcit=temp(cihigh,1).
do if (badlo = 1 and llcit <> priorlo).
compute badend={badend, llcit}.
compute priorlo = llcit.
end if.
do if (badhi = 1 and ulcit <> priorhi).
compute badend={badend, ulcit}.
compute priorhi = ulcit.
end if.
end if.
do if ( (icmm(1,1)*bc)+(9999*(1-bc)) = 9999).
compute llcit=temp(cilow,1).
compute ulcit=temp(cihigh,1).
end if.
compute bootse=t(sqrt((cssq(temp)-((csum(temp)&**2)/nrow(temp)))/(nrow(temp)-1)))
.
compute cmmtemp(k8,2:4)={bootse,llcit,ulcit}.
end if.
end loop.
compute cmmtemp={zmodvals,cmmtemp}.
do if (boot=0).
compute cmmtemp=cmmtemp(:,1:2).
end if.
compute cmmlbs={znames,immlbs}.
compute resultm2=make(nrow(cmmtemp),maxresm,99999).
compute resultm2(1:nrow(cmmtemp),1:ncol(cmmtemp))=cmmtemp.
compute resultm={resultm;resultm2}.
do if (outscree=1).
print cmmtemp/title='      Indices of conditional moderated mediation by W'/cnames=cmmlbs/format= F10.4.
end if.
end loop.
end if.
end if.
end if.
do if (dichadj=1 or immcat=1 and indmmm(i,1) < 30).
do if (outscree=1).
print immtemp/title='Index of moderated mediation (difference between conditional indirect effects):'/cnames=immlbs/rnames=immlbs2/format= F10.4.
end if.
end if.
end if.
do if (indcontr=1).
compute resultm2=make(nrow(condcont),maxresm,99999).
compute resultm2(1:nrow(condcont),1:ncol(condcont))=condcont.
compute resultm={resultm;resultm2}.
compute condctlb={'Effect1','Effect2','Contrast','BootSE', 'BootLLCI','BootULCI'}.
do if (outscree=1).
print condcont/title=' Pairwise contrasts between conditional indirect effects (Effect1 minus Effect2)'/cnames=condctlb/format= F10.4.
end if.
end if.
end loop.
end if.
end loop.
do if (alttotal=1).
compute altcnms='Effect'.
compute alttotfx=t(cftotfx(1,:))+obnatdfx.
do if (boot > 0).
compute alttotfx=make(ncol(cftotfx),4,0).
compute alttotfx(:,1)=t(cftotfx(1,:))+obnatdfx.
compute natdirbt(1,:)=t(obnatdfx).
compute cftotfx=cftotfx+natdirbt.
loop cec=1 to ncol(cftotfx).

.
compute temp = cftotfx(2:nrow(cftotfx),cec).
compute temp(GRADE( cftotfx(2:nrow(cftotfx),cec) )) = cftotfx(2:nrow(cftotfx),cec).
compute badlo = 0.
compute badhi = 0.
do if ( (cftotfx(1,cec)*bc)+(9999*(1-bc)) <> 9999).
compute pv=csum(temp < (cftotfx(1,cec)*bc)+(9999*(1-bc)) )/nrow(temp).
compute ppv = pv.
do if (pv > .5).
compute ppv = 1-pv.
end if.
compute y5=sqrt(-2*ln(ppv)).
compute xp=y5+((((y5*p4+p3)*y5+p2)*y5+p1)*y5+p0)/((((y5*q4+q3)*y5+q2)*y5+q1)*y5+q0).
do if (pv <= .5).
compute xp = -xp.
end if.
compute cilow=rnd(nrow(temp)*(cdfnorm(2*xp-xp2))).
compute cihigh=trunc(nrow(temp)*(cdfnorm(2*xp+(xp2))))+1.
do if (cilow < 1).
compute cilow = 1.
compute booterr=1.
compute badlo = 1.
end if.
do if (cihigh > nrow(temp)).
compute cihigh = boot.
compute booterr=1.
compute badhi = 1.
end if.
compute llcit=temp(cilow,1).
compute ulcit=temp(cihigh,1).
do if (badlo = 1 and llcit <> priorlo).
compute badend={badend, llcit}.
compute priorlo = llcit.
end if.
do if (badhi = 1 and ulcit <> priorhi).
compute badend={badend, ulcit}.
compute priorhi = ulcit.
end if.
end if.
do if ( (cftotfx(1,cec)*bc)+(9999*(1-bc)) = 9999).
compute llcit=temp(cilow,1).
compute ulcit=temp(cihigh,1).
end if.
compute bootse=t(sqrt((cssq(temp)-((csum(temp)&**2)/nrow(temp)))/(nrow(temp)-1)))
.
compute alttotfx(cec,2:4)={bootse,llcit,ulcit}.
end loop.
compute altcnms={altcnms,'BootSE', 'BootLLCI','BootULCI'}.
end if.
compute resultm2=make(nrow(alttotfx),maxresm,99999).
compute resultm2(1:nrow(alttotfx),1:ncol(alttotfx))=alttotfx.
compute resultm={resultm;resultm2}.
do if (outscree=1).
print/title='----------'.
do if (nxvls > 1).
print alttotfx/title='Relative total effects of X on Y (sum of direct and indirect effects)'/cnames=altcnms/rnames=direffl2/format= F10.4.
end if.
do if (nxvls = 1).
print alttotfx/title='Total effect of X on Y (sum of direct and indirect effects)'/cnames=altcnms/format= F10.4.
end if.
end if.
end if.
end if.
end if
.
do if (criterr=0 and (saveest = 1 or saveboot=1)).
compute coeffsav=coeffmat(2:nrow(coeffmat),:).
compute conseq=conseq(2:nrow(conseq),1).
compute dfmat=dfmat(2:nrow(dfmat),1).
compute dfmat=dfmat.
compute saven={'conseqnt','antecdnt','coeff','se','t','p','LLCI','ULCI','df'}.
do if (ydich=1).
compute saven={'conseqnt','antecdnt','coeff','se','t_or_Z','p','LLCI','ULCI','df'}.
end if.
do if (saveboot=1 and boot > 0 ).
compute colslab=make(300,1,' ').


compute colslab( 1 ,1)= 'COL1'.


compute colslab( 2 ,1)= 'COL2'.


compute colslab( 3 ,1)= 'COL3'.


compute colslab( 4 ,1)= 'COL4'.


compute colslab( 5 ,1)= 'COL5'.


compute colslab( 6 ,1)= 'COL6'.


compute colslab( 7 ,1)= 'COL7'.


compute colslab( 8 ,1)= 'COL8'.


compute colslab( 9 ,1)= 'COL9'.


compute colslab( 10 ,1)= 'COL10'.


compute colslab( 11 ,1)= 'COL11'.


compute colslab( 12 ,1)= 'COL12'.


compute colslab( 13 ,1)= 'COL13'.


compute colslab( 14 ,1)= 'COL14'.


compute colslab( 15 ,1)= 'COL15'.


compute colslab( 16 ,1)= 'COL16'.


compute colslab( 17 ,1)= 'COL17'.


compute colslab( 18 ,1)= 'COL18'.


compute colslab( 19 ,1)= 'COL19'.


compute colslab( 20 ,1)= 'COL20'.


compute colslab( 21 ,1)= 'COL21'.


compute colslab( 22 ,1)= 'COL22'.


compute colslab( 23 ,1)= 'COL23'.


compute colslab( 24 ,1)= 'COL24'.


compute colslab( 25 ,1)= 'COL25'.


compute colslab( 26 ,1)= 'COL26'.


compute colslab( 27 ,1)= 'COL27'.


compute colslab( 28 ,1)= 'COL28'.


compute colslab( 29 ,1)= 'COL29'.


compute colslab( 30 ,1)= 'COL30'.


compute colslab( 31 ,1)= 'COL31'.


compute colslab( 32 ,1)= 'COL32'.


compute colslab( 33 ,1)= 'COL33'.


compute colslab( 34 ,1)= 'COL34'.


compute colslab( 35 ,1)= 'COL35'.


compute colslab( 36 ,1)= 'COL36'.


compute colslab( 37 ,1)= 'COL37'.


compute colslab( 38 ,1)= 'COL38'.


compute colslab( 39 ,1)= 'COL39'.


compute colslab( 40 ,1)= 'COL40'.


compute colslab( 41 ,1)= 'COL41'.


compute colslab( 42 ,1)= 'COL42'.


compute colslab( 43 ,1)= 'COL43'.


compute colslab( 44 ,1)= 'COL44'.


compute colslab( 45 ,1)= 'COL45'.


compute colslab( 46 ,1)= 'COL46'.


compute colslab( 47 ,1)= 'COL47'.


compute colslab( 48 ,1)= 'COL48'.


compute colslab( 49 ,1)= 'COL49'.


compute colslab( 50 ,1)= 'COL50'.


compute colslab( 51 ,1)= 'COL51'.


compute colslab( 52 ,1)= 'COL52'.


compute colslab( 53 ,1)= 'COL53'.


compute colslab( 54 ,1)= 'COL54'.


compute colslab( 55 ,1)= 'COL55'.


compute colslab( 56 ,1)= 'COL56'.


compute colslab( 57 ,1)= 'COL57'.


compute colslab( 58 ,1)= 'COL58'.


compute colslab( 59 ,1)= 'COL59'.


compute colslab( 60 ,1)= 'COL60'.


compute colslab( 61 ,1)= 'COL61'.


compute colslab( 62 ,1)= 'COL62'.


compute colslab( 63 ,1)= 'COL63'.


compute colslab( 64 ,1)= 'COL64'.


compute colslab( 65 ,1)= 'COL65'.


compute colslab( 66 ,1)= 'COL66'.


compute colslab( 67 ,1)= 'COL67'.


compute colslab( 68 ,1)= 'COL68'.


compute colslab( 69 ,1)= 'COL69'.


compute colslab( 70 ,1)= 'COL70'.


compute colslab( 71 ,1)= 'COL71'.


compute colslab( 72 ,1)= 'COL72'.


compute colslab( 73 ,1)= 'COL73'.


compute colslab( 74 ,1)= 'COL74'.


compute colslab( 75 ,1)= 'COL75'.


compute colslab( 76 ,1)= 'COL76'.


compute colslab( 77 ,1)= 'COL77'.


compute colslab( 78 ,1)= 'COL78'.


compute colslab( 79 ,1)= 'COL79'.


compute colslab( 80 ,1)= 'COL80'.


compute colslab( 81 ,1)= 'COL81'.


compute colslab( 82 ,1)= 'COL82'.


compute colslab( 83 ,1)= 'COL83'.


compute colslab( 84 ,1)= 'COL84'.


compute colslab( 85 ,1)= 'COL85'.


compute colslab( 86 ,1)= 'COL86'.


compute colslab( 87 ,1)= 'COL87'.


compute colslab( 88 ,1)= 'COL88'.


compute colslab( 89 ,1)= 'COL89'.


compute colslab( 90 ,1)= 'COL90'.


compute colslab( 91 ,1)= 'COL91'.


compute colslab( 92 ,1)= 'COL92'.


compute colslab( 93 ,1)= 'COL93'.


compute colslab( 94 ,1)= 'COL94'.


compute colslab( 95 ,1)= 'COL95'.


compute colslab( 96 ,1)= 'COL96'.


compute colslab( 97 ,1)= 'COL97'.


compute colslab( 98 ,1)= 'COL98'.


compute colslab( 99 ,1)= 'COL99'.


compute colslab( 100 ,1)= 'COL100'.


compute colslab( 101 ,1)= 'COL101'.


compute colslab( 102 ,1)= 'COL102'.


compute colslab( 103 ,1)= 'COL103'.


compute colslab( 104 ,1)= 'COL104'.


compute colslab( 105 ,1)= 'COL105'.


compute colslab( 106 ,1)= 'COL106'.


compute colslab( 107 ,1)= 'COL107'.


compute colslab( 108 ,1)= 'COL108'.


compute colslab( 109 ,1)= 'COL109'.


compute colslab( 110 ,1)= 'COL110'.


compute colslab( 111 ,1)= 'COL111'.


compute colslab( 112 ,1)= 'COL112'.


compute colslab( 113 ,1)= 'COL113'.


compute colslab( 114 ,1)= 'COL114'.


compute colslab( 115 ,1)= 'COL115'.


compute colslab( 116 ,1)= 'COL116'.


compute colslab( 117 ,1)= 'COL117'.


compute colslab( 118 ,1)= 'COL118'.


compute colslab( 119 ,1)= 'COL119'.


compute colslab( 120 ,1)= 'COL120'.


compute colslab( 121 ,1)= 'COL121'.


compute colslab( 122 ,1)= 'COL122'.


compute colslab( 123 ,1)= 'COL123'.


compute colslab( 124 ,1)= 'COL124'.


compute colslab( 125 ,1)= 'COL125'.


compute colslab( 126 ,1)= 'COL126'.


compute colslab( 127 ,1)= 'COL127'.


compute colslab( 128 ,1)= 'COL128'.


compute colslab( 129 ,1)= 'COL129'.


compute colslab( 130 ,1)= 'COL130'.


compute colslab( 131 ,1)= 'COL131'.


compute colslab( 132 ,1)= 'COL132'.


compute colslab( 133 ,1)= 'COL133'.


compute colslab( 134 ,1)= 'COL134'.


compute colslab( 135 ,1)= 'COL135'.


compute colslab( 136 ,1)= 'COL136'.


compute colslab( 137 ,1)= 'COL137'.


compute colslab( 138 ,1)= 'COL138'.


compute colslab( 139 ,1)= 'COL139'.


compute colslab( 140 ,1)= 'COL140'.


compute colslab( 141 ,1)= 'COL141'.


compute colslab( 142 ,1)= 'COL142'.


compute colslab( 143 ,1)= 'COL143'.


compute colslab( 144 ,1)= 'COL144'.


compute colslab( 145 ,1)= 'COL145'.


compute colslab( 146 ,1)= 'COL146'.


compute colslab( 147 ,1)= 'COL147'.


compute colslab( 148 ,1)= 'COL148'.


compute colslab( 149 ,1)= 'COL149'.


compute colslab( 150 ,1)= 'COL150'.


compute colslab( 151 ,1)= 'COL151'.


compute colslab( 152 ,1)= 'COL152'.


compute colslab( 153 ,1)= 'COL153'.


compute colslab( 154 ,1)= 'COL154'.


compute colslab( 155 ,1)= 'COL155'.


compute colslab( 156 ,1)= 'COL156'.


compute colslab( 157 ,1)= 'COL157'.


compute colslab( 158 ,1)= 'COL158'.


compute colslab( 159 ,1)= 'COL159'.


compute colslab( 160 ,1)= 'COL160'.


compute colslab( 161 ,1)= 'COL161'.


compute colslab( 162 ,1)= 'COL162'.


compute colslab( 163 ,1)= 'COL163'.


compute colslab( 164 ,1)= 'COL164'.


compute colslab( 165 ,1)= 'COL165'.


compute colslab( 166 ,1)= 'COL166'.


compute colslab( 167 ,1)= 'COL167'.


compute colslab( 168 ,1)= 'COL168'.


compute colslab( 169 ,1)= 'COL169'.


compute colslab( 170 ,1)= 'COL170'.


compute colslab( 171 ,1)= 'COL171'.


compute colslab( 172 ,1)= 'COL172'.


compute colslab( 173 ,1)= 'COL173'.


compute colslab( 174 ,1)= 'COL174'.


compute colslab( 175 ,1)= 'COL175'.


compute colslab( 176 ,1)= 'COL176'.


compute colslab( 177 ,1)= 'COL177'.


compute colslab( 178 ,1)= 'COL178'.


compute colslab( 179 ,1)= 'COL179'.


compute colslab( 180 ,1)= 'COL180'.


compute colslab( 181 ,1)= 'COL181'.


compute colslab( 182 ,1)= 'COL182'.


compute colslab( 183 ,1)= 'COL183'.


compute colslab( 184 ,1)= 'COL184'.


compute colslab( 185 ,1)= 'COL185'.


compute colslab( 186 ,1)= 'COL186'.


compute colslab( 187 ,1)= 'COL187'.


compute colslab( 188 ,1)= 'COL188'.


compute colslab( 189 ,1)= 'COL189'.


compute colslab( 190 ,1)= 'COL190'.


compute colslab( 191 ,1)= 'COL191'.


compute colslab( 192 ,1)= 'COL192'.


compute colslab( 193 ,1)= 'COL193'.


compute colslab( 194 ,1)= 'COL194'.


compute colslab( 195 ,1)= 'COL195'.


compute colslab( 196 ,1)= 'COL196'.


compute colslab( 197 ,1)= 'COL197'.


compute colslab( 198 ,1)= 'COL198'.


compute colslab( 199 ,1)= 'COL199'.


compute colslab( 200 ,1)= 'COL200'.


compute colslab( 201 ,1)= 'COL201'.


compute colslab( 202 ,1)= 'COL202'.


compute colslab( 203 ,1)= 'COL203'.


compute colslab( 204 ,1)= 'COL204'.


compute colslab( 205 ,1)= 'COL205'.


compute colslab( 206 ,1)= 'COL206'.


compute colslab( 207 ,1)= 'COL207'.


compute colslab( 208 ,1)= 'COL208'.


compute colslab( 209 ,1)= 'COL209'.


compute colslab( 210 ,1)= 'COL210'.


compute colslab( 211 ,1)= 'COL211'.


compute colslab( 212 ,1)= 'COL212'.


compute colslab( 213 ,1)= 'COL213'.


compute colslab( 214 ,1)= 'COL214'.


compute colslab( 215 ,1)= 'COL215'.


compute colslab( 216 ,1)= 'COL216'.


compute colslab( 217 ,1)= 'COL217'.


compute colslab( 218 ,1)= 'COL218'.


compute colslab( 219 ,1)= 'COL219'.


compute colslab( 220 ,1)= 'COL220'.


compute colslab( 221 ,1)= 'COL221'.


compute colslab( 222 ,1)= 'COL222'.


compute colslab( 223 ,1)= 'COL223'.


compute colslab( 224 ,1)= 'COL224'.


compute colslab( 225 ,1)= 'COL225'.


compute colslab( 226 ,1)= 'COL226'.


compute colslab( 227 ,1)= 'COL227'.


compute colslab( 228 ,1)= 'COL228'.


compute colslab( 229 ,1)= 'COL229'.


compute colslab( 230 ,1)= 'COL230'.


compute colslab( 231 ,1)= 'COL231'.


compute colslab( 232 ,1)= 'COL232'.


compute colslab( 233 ,1)= 'COL233'.


compute colslab( 234 ,1)= 'COL234'.


compute colslab( 235 ,1)= 'COL235'.


compute colslab( 236 ,1)= 'COL236'.


compute colslab( 237 ,1)= 'COL237'.


compute colslab( 238 ,1)= 'COL238'.


compute colslab( 239 ,1)= 'COL239'.


compute colslab( 240 ,1)= 'COL240'.


compute colslab( 241 ,1)= 'COL241'.


compute colslab( 242 ,1)= 'COL242'.


compute colslab( 243 ,1)= 'COL243'.


compute colslab( 244 ,1)= 'COL244'.


compute colslab( 245 ,1)= 'COL245'.


compute colslab( 246 ,1)= 'COL246'.


compute colslab( 247 ,1)= 'COL247'.


compute colslab( 248 ,1)= 'COL248'.


compute colslab( 249 ,1)= 'COL249'.


compute colslab( 250 ,1)= 'COL250'.


compute colslab( 251 ,1)= 'COL251'.


compute colslab( 252 ,1)= 'COL252'.


compute colslab( 253 ,1)= 'COL253'.


compute colslab( 254 ,1)= 'COL254'.


compute colslab( 255 ,1)= 'COL255'.


compute colslab( 256 ,1)= 'COL256'.


compute colslab( 257 ,1)= 'COL257'.


compute colslab( 258 ,1)= 'COL258'.


compute colslab( 259 ,1)= 'COL259'.


compute colslab( 260 ,1)= 'COL260'.


compute colslab( 261 ,1)= 'COL261'.


compute colslab( 262 ,1)= 'COL262'.


compute colslab( 263 ,1)= 'COL263'.


compute colslab( 264 ,1)= 'COL264'.


compute colslab( 265 ,1)= 'COL265'.


compute colslab( 266 ,1)= 'COL266'.


compute colslab( 267 ,1)= 'COL267'.


compute colslab( 268 ,1)= 'COL268'.


compute colslab( 269 ,1)= 'COL269'.


compute colslab( 270 ,1)= 'COL270'.


compute colslab( 271 ,1)= 'COL271'.


compute colslab( 272 ,1)= 'COL272'.


compute colslab( 273 ,1)= 'COL273'.


compute colslab( 274 ,1)= 'COL274'.


compute colslab( 275 ,1)= 'COL275'.


compute colslab( 276 ,1)= 'COL276'.


compute colslab( 277 ,1)= 'COL277'.


compute colslab( 278 ,1)= 'COL278'.


compute colslab( 279 ,1)= 'COL279'.


compute colslab( 280 ,1)= 'COL280'.


compute colslab( 281 ,1)= 'COL281'.


compute colslab( 282 ,1)= 'COL282'.


compute colslab( 283 ,1)= 'COL283'.


compute colslab( 284 ,1)= 'COL284'.


compute colslab( 285 ,1)= 'COL285'.


compute colslab( 286 ,1)= 'COL286'.


compute colslab( 287 ,1)= 'COL287'.


compute colslab( 288 ,1)= 'COL288'.


compute colslab( 289 ,1)= 'COL289'.


compute colslab( 290 ,1)= 'COL290'.


compute colslab( 291 ,1)= 'COL291'.


compute colslab( 292 ,1)= 'COL292'.


compute colslab( 293 ,1)= 'COL293'.


compute colslab( 294 ,1)= 'COL294'.


compute colslab( 295 ,1)= 'COL295'.


compute colslab( 296 ,1)= 'COL296'.


compute colslab( 297 ,1)= 'COL297'.


compute colslab( 298 ,1)= 'COL298'.


compute colslab( 299 ,1)= 'COL299'.


compute colslab( 300 ,1)= 'COL300'.

compute colslab=colslab(1:ncol(bootres),1).
compute colslab={colslab,conseq,vlabs}.
do if (outscree=1).
print/title = '**************************************************************************'.
print/title='Bootstrap estimates were saved to a file'/space=0.
print colslab/title='Map of column names to model coefficients:'/clabels=' ','Conseqnt','Antecdnt'/format=a8.
end if.
end if.
end if.
do if (criterr=0 and boot > 0 and modelbt=1).
compute labstart=1.
do if (outscree=1).
print/title = '*********** BOOTSTRAP RESULTS FOR REGRESSION MODEL PARAMETERS ************'.
end if.
loop iboot = 1 to (nms+nys).
do if (outscree=1).
print outnames(1,iboot)/title = 'OUTCOME VARIABLE:'/format = A8.
end if.
compute vlabsm=vlabs(labstart:(labstart+(nump(1,iboot)-1)),1).
compute outnmtmp=bootcim(labstart:(labstart+(nump(1,iboot)-1)),:).
compute resultm2=make(nrow(outnmtmp),maxresm,99999).
compute resultm2(1:nrow(outnmtmp),1:ncol(outnmtmp))=outnmtmp.
compute resultm={resultm;resultm2}.
do if (outscree=1).
print bootcim(labstart:(labstart+(nump(1,iboot)-1)),:)/title=' '/rnames=vlabsm/ clabels='Coeff' 'BootMean' 'BootSE' 'BootLLCI' 'BootULCI'/format= F10.4 /space=0.
end if.
compute labstart=labstart+nump(1,iboot).
do if (iboot < (nms+nys)).
do if (outscree=1).
print/title= '----------'.
end if.
end if.
end loop.
end if.
do if (criterr=0 and saveest=1).
compute resultm=resultm(2:nrow(resultm),:).
compute bocaj=(resultm=99999).
compute bocaj=csum(bocaj).
compute bocaj=1-(bocaj=nrow(resultm)).
compute j=1.
loop i = 1 to ncol(resultm).
do if (bocaj(1,i)=1).
compute j=j+1.
end if.
end loop.
compute resultm=resultm(:,1:(j-1)).
save resultm/outfile = *.
end if.
do if (criterr = 0 and matrices=1 and outscree=1).
print/title = '************************ MODEL DEFINITION MATRICES ************************'.
print/title = 'FROM variables are columns, TO variables are rows.'.
compute temp2=make(nrow(bcmat),ncol(bcmat),'0').
loop i = 2 to nrow(bcmat).
loop j = 1 to (ncol(bcmat)-1).
do if (bcmat(i,j)=1).
compute temp2(i,j)='1'.
end if.
do if (j >= i).
compute temp2(i,j)=' '.
end if.
end loop.
end loop.
compute temp2=temp2(2:nrow(bcmat),(1:(ncol(bcmat)-1))).
do if (nms > 0).
compute cmatlabs={xnames,mnames}.
compute rmatlabs={mnames,ynames}.
end if.
do if (nms = 0).
compute cmatlabs={xnames}.
compute rmatlabs={ynames}.
end if.
print temp2/title='BMATRIX: Paths freely estimated (1) and fixed to zero (0):'/cnames=cmatlabs/rnames=rmatlabs/format A3.
compute z=0.
do if (rsum(csum(wcmat))<>0).
compute temp2=make(nrow(wcmat),ncol(wcmat),'0').
loop i = 2 to nrow(wcmat).
loop j = 1 to (ncol(wcmat)-1).
do if (wcmat(i,j)=1).
compute temp2(i,j)='1'.
end if.
do if (j >= i).
compute temp2(i,j)=' '.
end if.
end loop.
end loop.
compute temp2=temp2(2:nrow(wcmat),(1:(ncol(wcmat)-1))).
print temp2/title='WMATRIX: Paths moderated (1) and not moderated (0) by W:'/cnames=cmatlabs/rnames=rmatlabs/format A3.
end if.
do if (rsum(csum(zcmat))<>0).
compute temp2=make(nrow(zcmat),ncol(zcmat),'0').
loop i = 2 to nrow(zcmat).
loop j = 1 to (ncol(zcmat)-1).
do if (zcmat(i,j)=1).
compute temp2(i,j)='1'.
end if.
do if (j >= i).
compute temp2(i,j)=' '.
end if.
end loop.
end loop.
compute temp2=temp2(2:nrow(zcmat),(1:(ncol(zcmat)-1))).
print temp2/title='ZMATRIX: Paths moderated (1) and not moderated (0) by Z:'/cnames=cmatlabs/rnames=rmatlabs/format a3.
end if.
do if (rsum(csum(wzcmat))<>0).
compute temp2=make(nrow(wzcmat),ncol(wzcmat),'0').
loop i = 2 to nrow(wzcmat).
loop j = 1 to (ncol(wzcmat)-1).
do if (wzcmat(i,j)=1).
compute temp2(i,j)='1'.
end if.
do if (j >= i).
compute temp2(i,j)=' '.
end if.
end loop.
end loop.
compute temp2=temp2(2:nrow(wzcmat),(1:(ncol(wzcmat)-1))).
print temp2/title='WZMATRIX: W moderated paths moderated (1) and not moderated (0) by Z:'/cnames=cmatlabs/rnames=rmatlabs/format a3.
end if.
do if (ncs > 0).
print ccmat/title='CMATRIX: Covariates (columns) in (1) and not in (0) the models of M and Y (rows):'/rnames=rmatlabs/cnames=covnames.
end if.
end if.
do if (outscree=1).
do if (activate=0).
print/title = '*********************** ANALYSIS NOTES AND ERRORS ************************'.
end if.
end if.
do if (activate=1).
print/title = '**************************************************************************'.
end if.
do if (criterr=0).
do if (outscree=1).
print conf/title = 'Level of confidence for all confidence intervals in output:'/format = F8.4.
do if (boot > 0).
do if (goodboot = boot and bc=0).
print boot/title='Number of bootstrap samples for percentile bootstrap confidence intervals:'.
end if.
do if (goodboot = boot and bc=1).
print boot/title='Number of bootstrap samples for bias-corrected bootstrap confidence intervals:'.
end if.
do if (booterr = 1).
compute badend = badend(1,2:ncol(badend)).
print/title = 'WARNING: Bootstrap CI endpoints below not trustworthy. Decrease confidence'.
print badend/title='or increase the number of bootstrap samples.'/space=0/format = F10.4.
end if.
end if.
do if (mc > 0).
print mc/title='Number of samples for Monte Carlo confidence intervals:'.
end if.
do if (wnotev > 0 and printw=1).
do if (wnotev=1).
print/title = 'W values in conditional tables are the 16th, 50th, and 84th percentiles.'.
else if (wnotev=2).
do if (minwwarn=0 and maxwwarn=0).
print/title = 'W values in conditional tables are the mean and +/- SD from the mean.'.
end if.
do if (minwwarn=1).
print/title = 'W values in conditional tables are the minimum, the mean, and 1 SD above the mean.'.
end if.
do if (maxwwarn=1).
print/title = 'W values in conditional tables are 1 SD below the mean, the mean, and the maximum.'.
end if.
end if.
end if.
do if (znotev > 0 and printz=1).
do if (znotev=1).
print/title = 'Z values in conditional tables are the 16th, 50th, and 84th percentiles.'.
else if (znotev=2).
do if (minzwarn=0 and maxzwarn=0).
print/title = 'Z values in conditional tables are the mean and +/- SD from the mean.'.
end if.
do if (minzwarn=1).
print/title = 'Z values in conditional tables are the minimum, the mean, and 1 SD above the mean.'.
end if.
do if (maxzwarn=1).
print/title = 'Z values in conditional tables are 1 SD below the mean, the mean, and the maximum.'.
end if.
end if.
end if.
do if (minwwarn > 0).
print/title = 'NOTE: One SD below the mean is below the minimum observed in the data for W,'.
print/title = '      so the minimum measurement on W is used for conditioning instead.'/space=0.
end if.
do if (maxwwarn > 0).
print/title = 'NOTE: One SD above the mean is above the maximum observed in the data for W,'.
print/title = '      so the maximum measurement for W is used for conditioning instead.'/space=0.
end if.
do if (minzwarn > 0).
print/title = 'NOTE: One SD below the mean is below the minimum observed in the data for Z,'.
print/title = '      so the minimum measurement for Z is used for conditioning instead.'/space=0.
end if.
do if (maxzwarn > 0).
print/title = 'NOTE: One SD above the mean is above the maximum observed in the data for Z,'.
print/title = '      so the maximum measurement for Z is used for conditioning instead.'/space=0.
end if.
do if (pstog=1).
print/title= 'NOTE: Standardized coefficients for dichotomous or multicategorical X are in'.
print/title= '      partially standardized form.'/space=0.
end if.
loop i = 1 to 100.
do if (notecode(i,1) = 32).
print/title = 'Direct, indirect, and total effects are counterfactually defined'.
do if (xcontcf=0).
print/title = 'assuming X by M interaction.'/space=0.
end if.
do if (xcontcf=1).
print/title = 'assuming X by M interaction and with the following reference (x_ref)'/space=0.
compute states=t(xrefvals).
print states/title = 'and counterfactual (x_cf) states for X:'/rlabels='x_ref' 'x_cf'/format= F10.4 /space=0.
end if.
end if.
do if (notecode(i,1)=1).
print/title = 'NOTE: COVMY is ignored when using CMATRIX option.'.
end if.
do if (notecode(i,1)=2).
print/title = 'NOTE: Confidence level restricted to between 50 and 99.9999%.  95% confidence is provided in output'.
end if.
do if (notecode(i,1)=3).
print centvar/title = 'NOTE: The following variables were mean centered prior to analysis:'/format = a8.
end if.
do if (notecode(i,1) = 4).
print/title = 'NOTE: A heteroscedasticity consistent standard error and covariance matrix estimator was used.'.
end if.
do if (notecode(i,1) = 5).
print/title = 'NOTE: The HC3 option has been replaced with HC.  See the documentation.'.
end if.
do if (notecode(i,1) = 6).
print/title = 'NOTE: Due to estimation problems, some bootstrap samples had to be replaced.'.
print badboot/title='      The number of times this happened was:'/space=0/format=F8.0.
end if.
do if (notecode(i,1) = 7).
print/title = 'NOTE: The bootstrapping was not completed due to problematic bootstrap samples.'.
print/title = '      Bootstrap confidence intervals are therefore suppressed.'/space=0.
end if.
do if (notecode(i,1) = 8).
print/title = 'NOTE: The number of bootstrap samples was adjusted upward given your desired confidence.'.
end if.
do if (notecode(i,1) = 9).
print/title = 'NOTE: WMODVAL is ignored when W is specified as multicategorical.'.
end if.
do if (notecode(i,1) = 10).
print/title = 'NOTE: ZMODVAL is ignored when Z is specified as multicategorical.'.
end if.
do if (notecode(i,1) = 11).
print/title = 'NOTE: Total effect model generated only when all covariates are specified in all'.
print/title = '      models of M and Y.'/space=0.
end if.
do if (notecode(i,1) = 30).
print/title = 'NOTE: Your vector of linear hypothesis weights is of the wrong length for this model.'.
end if.
do if (notecode(i,1) = 12).
print/title = 'NOTE: Total effect model generated only when X is freely estimated to affect each M'.
print/title = '      and both X and M are freely estimated to affect Y'/space=0.
end if.
do if (notecode(i,1) = 13).
print/title = 'NOTE: There are too many pairwise contrasts to conduct with this model.'.
end if.
do if (notecode(i,1) = 14).
print/title = 'NOTE: The number of contrast weights must equal the number of indirect effects.'.
end if.
do if (notecode(i,1) = 15).
print/title = 'NOTE: Monte Carlo confidence intervals not available for this model.'.
print/title = '      Bootstrapping is used instead.'/space=0.
end if.
do if (notecode(i,1) = 16).
print/title = 'NOTE: The number of Monte Carlo samples was adjusted upward given your desired confidence.'.
end if.
do if (notecode(i,1) = 19).
print/title = 'NOTE: Your contrast matrix is invalid or not applicable to this model.'.
end if.
do if (notecode(i,1) = 20).
print/title = 'NOTE: One of the groups specified by your contrast matrix does not exist in the data.'.
end if.
do if (notecode(i,1) = 21).
print/title = 'NOTE: The VARORDER option is not available in this release.'.
end if.
do if (notecode(i,1) = 22).
print/title = 'NOTE: The VMODVAL and QMODVAL options are not available in this release.'.
end if.
do if (notecode(i,1) = 23).
print/title = 'NOTE: The QUANTILE option is not available in this release.'.
end if.
do if (notecode(i,1) = 24).
print/title = 'NOTE: Total effect model not available with dichotomous Y.'.
end if.
do if (notecode(i,1) = 25).
print/title = 'NOTE: STAND/EFFSIZE options not available with dichotomous Y.'.
end if.
do if ((notecode(i,1) = 26) and nms > 0).
print/title = 'NOTE: Direct and indirect effects of X on Y are on a log-odds metric.'.
end if.
do if (notecode(i,1) = 27).
print/title = 'NOTE: Standardized coefficients are not available for models with moderators.'.
end if.
do if (notecode(i,1) = 28).
print/title = 'NOTE: The contrast option is not available with a multicategorical X.'.
end if.
do if (notecode(i,1) = 31).
do if (nms > 1).
print medmeans/title = 'NOTE: Controlled direct effect(s) estimated at the following mediator values:'/cnames=mnames/format= F10.4.
end if.
do if (nms=1).
print medmeans/title = 'NOTE: Controlled direct effect(s) estimated at the following mediator value:'/format= F10.4.
end if.
end if.
do if (notecode(i,1) = 33).
print/title = 'NOTE: Sobel test is not available with the XMINT option.'.
end if.
do if (notecode(i,1) = 37).
print/title = 'NOTE: The CONTRAST option is not available when using the XMINT option.'.
end if.
do if (notecode(i,1) = 34).
print/title = 'NOTE: Standardized effects are not available when using the XMINT option.'.
end if.
do if (notecode(i,1) = 36).
print/title = 'NOTE: The XREFVAL option is ignored when X is declared as multicategorical.'.
end if.
do if (notecode(i,1) = 35).
print coval/title = 'NOTE: Counterfactual effects estimated at the following covariate values:'/cnames=covnames/format= F10.4.
end if.
do if (notecode(i,1) = 29 and (listmiss=1)).
compute a=missrow.
compute conum=ncol(a).
compute allgood=0.
compute smremain=12.
compute largesti=1.
compute smallrow=0.
do if (conum > 12).
loop ii = 1 to 12.
compute check=(conum/ii).
do if (check = trunc(check)).
compute check2=conum/ii.
compute aok=ii.
do if (aok > 2).
compute allgood=1.
end if.
else.
compute remain=conum-(ii*trunc(check)).
do if (remain <= smremain).
compute smremain=remain.
compute largesti=ii.
compute smallrow=trunc(conum/largesti).
end if.
end if.
end loop.
compute atemp=a(1,1:(aok*check2)).
compute atemp=reshape(atemp,check2,aok).
do if (ncol(atemp) > 2).
print/title='NOTE: Missing data resulted in the deletion of the following row(s) of'.
print atemp/title='      data from the analysis:'/format=F6.0/space=0.
end if.
do if (allgood=0).
compute atemp=a(1,1:(smallrow*largesti)).
compute atemp=reshape(atemp,smallrow,largesti).
compute btemp=a(1,((largesti*smallrow)+1):conum).
print/title='NOTE: Missing data resulted in the deletion of the following row(s) of'.
print atemp/title='      data from the analysis:'/format=F6.0/space=0.
print btemp/title=' '/space=0/format=f6.0.
end if.
else if (conum <= 12).
print/title='NOTE: Missing data resulted in the deletion of the following row(s) of'.
print a/title='      data from the analysis:'/format=F6.0/space=0.
end if.
end if.
end loop.
do if (toomany=1).
print/title='WARNING: Variables names longer than eight characters can produce incorrect output'.
print/title='when some variables in the data file have the same first eight characters. Shorter'/space=0.
print/title='variable names are recommended. By using this output, you are accepting all risk'/space=0.
print/title='and consequences of interpreting or reporting results that may be incorrect.'/space=0.
end if.
end if.
end if.
loop i = 1 to 100.
do if (errcode(i,1)=1).
print/title = 'ERROR: You must specify a Y and an X variable.'.
end if.
do if (errcode(i,1)=2).
print/title = 'ERROR: X, M, or Y variable used more than once or W and Z are the same variable.'.
do if (toomany = 1).
print/title = '       This could be caused by the use of variables names longer'/space=0.
print varnames/title = '       than eight characters. Here are the variables I see:'/space=0/format=A8.
end if.
end if.
do if (errcode(i,1)=3).
print/title = 'ERROR: You have specified more than one variable for W, Y, X, or Z'.
end if.
do if (errcode(i,1)=4).
print/title = 'ERROR: A variable specified as multicategorical has more than nine categories.'.
end if.
do if (errcode(i,1)=5).
print/title = 'ERROR: One of the categories contains only a single case.'.
end if.
do if (errcode(i,1)=6).
print/title = 'ERROR: Invalid model number in this version of PROCESS.'.
end if.
do if (errcode(i,1)=7).
print/title = 'ERROR: Invalid model number.'.
end if.
do if (errcode(i,1)=8).
print/title = 'ERROR: You must specify an M variable for this model.'.
end if.
do if (errcode(i,1)=9).
print/title = 'ERROR: You have specified an M variable in a model that does not use it.'.
end if.
do if (errcode(i,1)=10).
print/title = 'ERROR: You have specified a W variable in a model that does not use it.'.
end if.
do if (errcode(i,1)=11).
print/title = 'ERROR: You have not specified a W variable in a model that requires it.'.
end if.
do if (errcode(i,1)=12).
print/title = 'ERROR: You have specified a Z variable in a model that does not use it.'.
end if.
do if (errcode(i,1)=13).
print/title = 'ERROR: You have not specified a Z variable in a model that requires it.'.
end if.
do if (errcode(i,1)=14).
print/title = 'ERROR: V and Q are not proper specifications in this release of PROCESS.'.
print/title = '       Moderators must be specified as W and/or Z.'/space=0.
end if.
do if (errcode(i,1)=15).
print/title = 'ERROR: One of your model variables exhibits no variation (it is a constant).'.
end if.
do if (errcode(i,1)=16).
print/title = 'ERROR: BMATRIX is not the correct length or is otherwise invalid.'.
end if.
do if (errcode(i,1)=17).
print/title = 'ERROR: WMATRIX is not the correct length or is otherwise invalid.'.
end if.
do if (errcode(i,1)=18).
print/title = 'ERROR: ZMATRIX is not the correct length or is otherwise invalid.'.
end if.
do if (errcode(i,1)=19).
print/title = 'ERROR: WZMATRIX is not the correct length or is otherwise invalid.'.
end if.
do if (errcode(i,1)=20).
print/title = 'ERROR: A path fixed at zero cannot be moderated.'.
end if.
do if (errcode(i,1)=21).
print/title = 'ERROR: If only one moderator is specified, it must be specified as W.'.
end if.
do if (errcode(i,1)=22).
print/title = 'ERROR: In BMATRIX, X must be specified to affect at least one variable.'.
end if.
do if (errcode(i,1)=23).
print/title = 'ERROR: In BMATRIX, at least one variable must be specified to affect Y.'.
end if.
do if (errcode(i,1)=24).
print/title = 'ERROR: You must specify a model number or a custom BMATRIX specification.'.
end if.
do if (errcode(i,1)=25).
print/title = 'ERROR: BMATRIX cannot be used in conjunction with a model number.'.
end if.
do if (errcode(i,1)=26).
print/title = 'ERROR: Your model has a dangling mediator (all Ms must affect and be affected).'.
end if.
do if (errcode(i,1)=27).
print/title = 'ERROR: CLUSTER is not available on this release of PROCESS.'.
end if.
do if (errcode(i,1)=29).
print/title = 'ERROR: CMATRIX is not the correct length or is otherwise invalid.'.
end if.
do if (errcode(i,1)=30).
print/title = 'ERROR: In CMATRIX, all covariates must be assigned to an M or a Y.'.
end if.
do if (errcode(i,1)=31).
print/title = 'ERROR: A linear or near linear dependency (singularity) exists in the data.'.
end if.
do if (errcode(i,1)=32).
print/title = 'ERROR: Models 80 and 81 require between 3 and 6 mediators.'.
end if.
do if (errcode(i,1)=33).
print/title = 'ERROR: Model 82 requires 4 mediators.'.
end if.
do if (errcode(i,1)=34).
print/title = 'ERROR: This model number requires between 2 and 6 mediators.'.
end if.
do if (errcode(i,1)=35).
print/title = 'ERROR: In a model with only one moderator, that moderator must be W.'.
end if.
do if (errcode(i,1)=36).
print/title = 'ERROR: A serial mediation model cannot have more than 6 mediators.'.
end if.
do if (errcode(i,1)=37).
print/title = 'ERROR: No more than 10 mediators are allowed in a PROCESS command.'.
end if.
do if (errcode(i,1)=38).
print/title = 'ERROR: XCATCODE is not provided, not the correct length, or is otherwise invalid.'.
end if.
do if (errcode(i,1)=39).
print/title = 'ERROR: WCATCODE is not provided, not the correct length, or is otherwise invalid.'.
end if.
do if (errcode(i,1)=40).
print/title = 'ERROR: ZCATCODE is not provided, not the correct length, or is otherwise invalid.'.
end if.
do if (errcode(i,1)=41).
print/title = 'ERROR: Models 1, 2, and 3 cannot be customized.'.
end if.
do if (errcode(i,1)=42).
print/title = 'ERROR: WS option available only in PROCESS v2. Or use the MEMORE macro instead.'.
print/title = '       MEMORE can be downloaded from www.akmontoya.com.'/space=0.
end if.
do if (errcode(i,1)=43).
print/title = 'ERROR: PROCESS does not allow dichotomous mediators.'.
end if.
do if (errcode(i,1)=60).
print/title = 'PROCESS is now ready for use.'.
print/title='Copyright 2017-2022 by Andrew F. Hayes. ALL RIGHTS RESERVED.'/space=0.
print/title='Workshop schedule available at http://haskayne.ucalgary.ca/CCRAM'/space=0.
end if.
do if (errcode(i,1)=50).
print/title = 'ERROR: A multicategorical moderator cannot be specified as a covariate.'.
end if.
do if (errcode(i,1)=51).
print/title = 'ERROR: A variable you specified as a covariate is a moderator in all equations.'.
end if.
do if (errcode(i,1)=52 and mcerpt=0).
compute mcerpt=1.
print/title = 'ERROR: A variable specified as multicategorical must have at least three categories'.
end if.
do if (errcode(i,1) = 47).
do if (iterrmod=1).
print/title = 'ERROR: Iteration for Y model didn''t converge to a solution. Interpret results with caution.'.
print/title = '       Try increasing the number of iterations, though this will slow down computation.'/space=0.
end if.
do if (bootiter=1).
print/title = 'ERROR: Nonconvergence during bootstrapping. Interpet bootstrap results with caution.'.
end if.
end if.
do if (errcode(i,1) = 61).
print/title = 'ERROR: Variable names should be no more than eight characters in length.'.
end if.
do if (errcode(i,1) = 62).
print/title = 'ERROR: After listwise deletion of cases with missing data, too few cases remain.'.
end if.
do if (errcode(i,1) = 63).
print/title = 'ERROR: The XMINT option is available only for model 4.'.
end if.
do if (errcode(i,1) = 64).
print/title = 'ERROR: Incorrect number of values specified in CDEVAL option.'.
end if.
do if (errcode(i,1) = 65).
print/title = 'ERROR: Only indicator or sequential coding of X is allowed with the XMINT option.'.
end if.
do if (errcode(i,1) = 66).
print/title = 'ERROR: XMINT option not available with continuous X using PROCESS GUI. Use syntax.'.
end if.
do if (errcode(i,1) = 67).
print/title = 'ERROR: Too many elements provided in XREFVAL option.'.
end if.
do if (errcode(i,1)=68).
print/title = 'ERROR: Covariate assignment is not allowed with XMINT option.'.
end if.
do if (errcode(i,1) = 69).
print/title = 'ERROR: Incorrect number of values specified in COVAL option.'.
end if.
do if (errcode(i,1) = 70).
print/title = 'ERROR: Incorrect value(s) in XREFVAL for this dichotomous X variable.'.
end if.
do if (errcode(i,1) = 71).
print/title = 'ERROR: The CENTER option is not available when using the XMINT option.'.
end if.
do if (errcode(i,1) = 72).
print/title = 'ERROR: The XMINT option is not available for models with a dichotomous Y.'.
end if.
end loop.
END MATRIX.	
Ressources	Temps de processeur	00:00:06,70	
	Temps écoulé	00:00:06,69	


Run MATRIX procedure:

***************** PROCESS Procedure for SPSS Version 4.2 *****************

          Written by Andrew F. Hayes, Ph.D.       www.afhayes.com
    Documentation available in Hayes (2022). www.guilford.com/p/hayes3

**************************************************************************
Model  : 4
    Y  : HOMAIR
    X  : NCDrisk
   M1  : TG
   M2  : HDL
   M3  : LDL
   M4  : TC

Sample
Size:  169

**************************************************************************
OUTCOME VARIABLE:
 TG

Model Summary
          R       R-sq        MSE          F        df1        df2          p
      ,1624      ,0264  3944,7337     4,5264     1,0000   167,0000      ,0348

Model
              coeff         se          t          p       LLCI       ULCI
constant    77,5209     6,9352    11,1778      ,0000    63,8289    91,2129
NCDrisk      9,7758     4,5949     2,1275      ,0348      ,7042    18,8473

Standardized coefficients
             coeff
NCDrisk      ,1624

**************************************************************************
OUTCOME VARIABLE:
 HDL

Model Summary
          R       R-sq        MSE          F        df1        df2          p
      ,0330      ,0011   177,9420      ,1819     1,0000   167,0000      ,6703

Model
              coeff         se          t          p       LLCI       ULCI
constant    40,7091     1,4730    27,6376      ,0000    37,8011    43,6171
NCDrisk       ,4162      ,9759      ,4265      ,6703    -1,5105     2,3429

Standardized coefficients
             coeff
NCDrisk      ,0330

**************************************************************************
OUTCOME VARIABLE:
 LDL

Model Summary
          R       R-sq        MSE          F        df1        df2          p
      ,0542      ,0029  1186,5518      ,4924     1,0000   167,0000      ,4838

Model
              coeff         se          t          p       LLCI       ULCI
constant    78,2284     3,8036    20,5669      ,0000    70,7191    85,7377
NCDrisk     -1,7683     2,5200     -,7017      ,4838    -6,7436     3,2069

Standardized coefficients
             coeff
NCDrisk     -,0542

**************************************************************************
OUTCOME VARIABLE:
 TC

Model Summary
          R       R-sq        MSE          F        df1        df2          p
      ,0006      ,0000  1451,9307      ,0001     1,0000   167,0000      ,9934

Model
              coeff         se          t          p       LLCI       ULCI
constant   135,7383     4,2075    32,2610      ,0000   127,4316   144,0451
NCDrisk       ,0231     2,7876      ,0083      ,9934    -5,4805     5,5267

Standardized coefficients
             coeff
NCDrisk      ,0006

**************************************************************************
OUTCOME VARIABLE:
 HOMAIR

Model Summary
          R       R-sq        MSE          F        df1        df2          p
      ,3694      ,1365    23,1534     5,1512     5,0000   163,0000      ,0002

Model
              coeff         se          t          p       LLCI       ULCI
constant      ,2878     1,6869      ,1706      ,8647    -3,0431     3,6188
NCDrisk      -,2860      ,3589     -,7968      ,4267     -,9948      ,4228
TG            ,0450      ,0099     4,5642      ,0000      ,0255      ,0645
HDL           ,1280      ,0433     2,9543      ,0036      ,0424      ,2135
LDL           ,0947      ,0358     2,6495      ,0089      ,0241      ,1654
TC           -,0902      ,0356    -2,5356      ,0122     -,1604     -,0200

Standardized coefficients
             coeff
NCDrisk     -,0591
TG           ,5600
HDL          ,3339
LDL          ,6389
TC          -,6718

************************** TOTAL EFFECT MODEL ****************************
OUTCOME VARIABLE:
 HOMAIR

Model Summary
          R       R-sq        MSE          F        df1        df2          p
      ,0078      ,0001    26,1681      ,0101     1,0000   167,0000      ,9200

Model
              coeff         se          t          p       LLCI       ULCI
constant     4,1550      ,5649     7,3559      ,0000     3,0399     5,2702
NCDrisk       ,0376      ,3742      ,1005      ,9200     -,7012      ,7765

Standardized coefficients
             coeff
NCDrisk      ,0078


************** TOTAL, DIRECT, AND INDIRECT EFFECTS OF X ON Y **************

Total effect of X on Y
     Effect         se          t          p       LLCI       ULCI       c_cs
      ,0376      ,3742      ,1005      ,9200     -,7012      ,7765      ,0078

Direct effect of X on Y
     Effect         se          t          p       LLCI       ULCI      c'_cs
     -,2860      ,3589     -,7968      ,4267     -,9948      ,4228     -,0591

Indirect effect(s) of X on Y:
          Effect     BootSE   BootLLCI   BootULCI
TOTAL      ,3236      ,2856     -,1182      ,9732
TG         ,4400      ,5657     -,0952     1,9898
HDL        ,0533      ,1938     -,2945      ,5176
LDL       -,1675      ,5076    -1,5086      ,4180
TC        -,0021      ,5206    -1,2703      ,8246

Completely standardized indirect effect(s) of X on Y:
          Effect     BootSE   BootLLCI   BootULCI
TOTAL      ,0669      ,0575     -,0236      ,2004
TG         ,0910      ,1100     -,0210      ,3869
HDL        ,0110      ,0387     -,0585      ,1029
LDL       -,0346      ,1021     -,3053      ,0816
TC        -,0004      ,1034     -,2446      ,1680

*********************** ANALYSIS NOTES AND ERRORS ************************

Level of confidence for all confidence intervals in output:
  95,0000

Number of bootstrap samples for percentile bootstrap confidence intervals:
  5000

------ END MATRIX -----


* Encoding: UTF-8.
preserve.
set printback=off.


Matrice


Remarques	
Sortie obtenue	10-NOV-2024 17:08:50	
Commentaires		
Entrée	Données	C:\Users\Brice SAHA\Documents\UBa\Master Thesis 2021\Fanuel\Database fanuel.sav	
	Jeu de données actif	Jeu_de_données1	
	Filtre	<sans>	
	Pondération	<sans>	
	Scinder un fichier	<sans>	
	N de lignes dans le fichier de travail	169	
Syntaxe	MATRIX.
compute wnames='xxxxx'.
compute znames='xxxxx'.
compute mcerpt=0.
compute wiscov=0.
compute ziscov=0.
compute tooman=0.
compute errcode=make(100,1,0).
compute notecode=make(100,1,0).
compute model = trunc( 4 ).
compute iterate = abs(trunc( 100 )).
compute converge = abs( 0.00001 ).
compute itprobtg=0.
compute v2tag=0.
compute ydich=0.
compute maxwwarn=0.
compute minwwarn=0.
compute maxzwarn=0.
compute minzwarn=0.
compute toomany=0.
compute wdich=0.
compute zdich=0.
compute wnotev=0.
compute znotev=0.
compute nxpval=1.
compute nwpval=1.
compute nzpval=1.
compute errs=1.
compute notes=1.
compute criterr=0.
compute novar=0.
compute adjust=0.
compute ncs=0.
compute serial=0.
compute sobelok=0.
compute hasw=0.
compute hasz=0.
compute printw=0.
compute printz=0.
compute xmint=( 0 =1).
compute wmodcust=0.
compute zmodcust=0.
compute booting=0.
compute bootiter=0.
compute iterrmod=0.
compute cov = 'xxxxx'.
compute varorder=( 0 <> 0).
compute nws=0.
compute w= 'xxxxx'.
compute nzs=0.
compute z = 'xxxxx'.
compute nms=0.
compute m = 'TG HDL LDL TC'.
compute nys=0.
compute y = 'HOMAIR'.
compute nxs=0.
compute x = 'GDR'.
compute effsize=( 0 =1).
compute stand=( 1 =1).
compute intprobe = .1.
compute xrefvals={ 999 }.
compute center=trunc( 0 ).
compute xcontcf=0.
compute xscaling=1.
compute cdeval={ -999 }.
compute cuscoval=0.
do if (model=74).
compute errcode(errs,1)=7.
compute errs=errs+1.
compute criterr=1.
end if.
do if (xmint=1 and model <> 4).
compute errcode(errs,1)=63.
compute errs=errs+1.
compute criterr=1.
end if.
do if (xmint=1 and model=4).
compute w=x.
compute model=74.
compute intprobe=1.
compute notecode(notes,1) = 32.
compute notes = notes + 1.
do if (effsize=1 or stand=1).
compute notecode(notes,1) = 34.
compute notes = notes + 1.
compute stand=0.
compute effsize=0.
end if.
do if (center <> 0).
compute center=0.
compute errcode(errs,1)=71.
compute errs=errs+1.
compute criterr=1.
end if.
end if.
compute v = 'xxxxx'.
compute q = 'xxxxx'.
compute linsum={ -999 }.
compute nlinsum=ncol(linsum).
do if (linsum(1,1) = -999).
compute nlinsum=0.
end if.
compute oldvars= 'xxxxx'.
compute mcxok=0.
compute mcwok=0.
compute mczok=0.
compute xprod=0.
compute zprod=0.
compute wprod=0.
compute modcok=0.
compute alttotal=0.
compute hc3=trunc( 0 ).
compute jn=( 0 = 1).
compute listmiss=( 0 =1).
compute modelres=( 0 =1).
compute outscree=( 1 =1).
compute activate=( 0 =1).
compute booterr=0.
compute normal=( 0 =1).
compute xmtest=( 0 =1).
compute describe=( 0 =1).
compute longname=( 0 =1).
do if (stand=1).
compute effsize=1.
end if.
compute pstog=0.
compute sobelok=0.
compute normal=( 0 =1).
compute mdichok=( 0 =1).
compute contrast={ 999 }.
compute ncontr=ncol(contrast).
compute ncontrow=nrow(contrast).
do if (contrast(1,1) = 999).
compute ncontr=1.
compute contrast=0.
end if.
do if (ncontr = 1).
compute contrast=trunc(contrast).
do if (contrast > 3 or contrast < 0)).
compute ncontr=1.
compute contrast = 0.
end if.
end if.
do if (ncontr > 1).
compute contvec=contrast.
compute contrast=4.
do if (ncontrow > 1).
compute contrast=0.
compute modcok=1.
compute wcontval=contvec(:,1).
compute zcontval=contvec(:,2).
do if ((ncontr <> 2) or (ncontrow <> 2)).
compute notecode(notes,1) = 19.
compute notes = notes + 1.
compute modcok=0.
end if.
end if.
end if.
do if (xmint = 1 and contrast(1,1) <> 0).
compute contrast=0.
compute notecode(notes,1) = 37.
compute notes = notes + 1.
end if.
do if (varorder = 1).
compute notecode(notes,1) = 21.
compute notes = notes + 1.
end if.
do if ( 999 <> 999 or 999 <> 999).
compute notecode(notes,1) = 22.
compute notes = notes + 1.
end if.
compute modelbt=( 0 =1).
compute cluster= 'xxxxx'.
compute matrices=( 0 =1).
compute covcoeff=( 0 =1).
compute covmy=trunc( 0 ).
do if (covmy < 0 or covmy > 2).
compute covmy = 0.
end if.
compute boot = abs(trunc( 5000 )).
compute bc=( 0 =1).
compute mc=abs(trunc( 0 )).
compute hc=trunc( 5 ).
do if (intprobe < 0 or intprobe > 1).
compute intprobe = .10.
end if.
compute plot=trunc( 0 ).
do if (plot < 0 or plot > 2).
compute plot=0.
end if.
compute total=( 1 =1).
compute dototal=0.
compute saveboot = ( 0 = 1).
compute saveest=( 0 = 2).
do if (saveest=1).
compute intprobe=1.
end if.
do if (hc >= 0 and hc < 5).
compute notecode(notes,1) = 4.
compute notes = notes + 1.
end if.
do if (hc > 5 or hc < 0).
compute hc=5.
end if.
compute mcw=trunc( 0 ).
compute mcz=trunc( 0 ).
compute mcx=trunc( 0 ).
do if (mcx > 0 and mcx < 3 and model = 74).
compute mcw=mcx.
compute xscaling=1.
end if.
do if (mcx > 2 and model = 74).
compute errcode(errs,1)=65.
compute errs=errs+1.
compute criterr=1.
end if.
do if (model = 74 and normal=1).
compute notecode(notes,1) = 33.
compute normal=0.
compute notes = notes + 1.
end if.
do if (mcx > 0 and contrast > 0).
compute notecode(notes,1) = 28.
compute notes = notes + 1.
compute contrast=0.
end if.
compute nxvls=1.
compute nmvls=1.
compute nwvls=1.
compute nzvls=1.
compute paths=999.
compute pathsw=999.
compute pathsz=999.
compute pathswz=999.
compute pathsmod=999.
compute pathtype=999.
compute obscoeff=999.
compute pathsdv={' '}.
compute quantile=1.
do if ( 999 <>999).
compute notecode(notes,1) = 23.
compute notes = notes + 1.
end if.
compute moments=( 0 =1).
do if (moments=1).
compute quantile=0.
end if.
compute bmatrix={ -999 }.
compute wmatrix={ -999 }.
compute zmatrix={ -999 }.
compute wzmatrix={ -999 }.
compute cmatrix={ -999 }.
compute xcatcode={ -999 }.
compute wcatcode={ -999 }.
compute zcatcode={ -999 }.
compute needed=0.
compute conf= 95.
do if (trunc( 95 ) >= 100 or (trunc( 95 ) <= 50)).
compute conf = 95.
compute notecode(notes,1)=2.
compute notes=notes+1.
end if.
do if (model >= 0 and model < 4 and modelbt=0).
compute boot=0.
compute mc=0.
compute bc=0.
end if.
do if (mc > 0 and boot > 0).
compute boot=0.
compute bc=0.
end if.
do if ((boot < 1000) and (mc = 0) and (boot > 0)).
compute boot=5000.
end if.
do if ((mc < 1000) and (boot = 0) and (mc > 0)).
compute mc=5000.
end if.
compute p0=-.322232431088.
compute p1 = -1.
compute p2 = -.342242088547.
compute p3 = -.0204231210245.
compute p4 = -.0000453642210148.
compute q0 = .0993484626060.
compute q1 = .588581570495.
compute q2 = .531103462366.
compute q3 = .103537752850.
compute q4 = .0038560700634.
compute badend=0.
compute priorlo = -9999999.
compute priorhi = 9999999.
compute alpha2 = (1-(conf/100))/2.
compute cilm=alpha2*2.
compute y5=sqrt(-2*ln(alpha2)).
compute xp2=(y5+((((y5*p4+p3)*y5+p2)*y5+p1)*y5+p0)/((((y5*q4+q3)*y5+q2)*y5+q1)*y5+q0)).
compute medlb={'   M1  :';'   M2  :';'   M3  :';'   M4  :';'   M5  :';'   M6  :';'   M7  :';'   M8  :';'   M9  :';'   M10 :'}.
compute medlb2={'(M1)','(M2)','(M3)','(M4)','(M5)','(M6)','(M7)','(M8)','(M9)','(M10)'}.
compute xlb={'   X1  :';'   X2  :';'   X3  :';'   X4  :';'   X5  :';'   X6  :';'   X7  :';'   X8  :';'   X9  :'}.
compute highlbw={'M1*W'; 'M2*W'; 'M3*W'; 'M4*W'; 'M5*W'; 'M6*W'; 'M7*W'; 'M8*W'; 'M9*W'; 'M10*W'}.
do if (xmint=1).
compute highlbw={'M1*X'; 'M2*X'; 'M3*X'; 'M4*X'; 'M5*X'; 'M6*X'; 'M7*X'; 'M8*X'; 'M9*X'; 'M10*X'}.
end if.
compute highlbz={'M1*Z'; 'M2*Z'; 'M3*Z'; 'M4*Z'; 'M5*Z'; 'M6*Z'; 'M7*Z'; 'M8*Z'; 'M9*Z';'M10*Z'}.
compute highlbwz={'M1*W*Z'; 'M2*W*Z'; 'M3*W*Z'; 'M4*W*Z'; 'M5*W*Z'; 'M6*W*Z'; 'M7*W*Z'; 'M8*W*Z'; 'M9*W*Z';'M10*W*Z'}.
compute highlbbt={'BOTH(M1)'; 'BOTH(M2)'; 'BOTH(M3)'; 'BOTH(M4)'; 'BOTH(M5)'; 'BOTH(M6)'; 'BOTH(M7)'; 'BOTH(M8)'; 'BOTH(M9)';'BTH(M10)'}.
compute highlbx={'M1*X'; 'M2*X'; 'M3*X'; 'M4*X'; 'M5*X'; 'M6*X'; 'M7*X'; 'M8*X'; 'M9*X'; 'M10*X'}.
compute skipwz=0.
compute validm={1,1,1,1,1,1,1,1,1,1,1,1,1,1,1,1,1,1,1,1,1,1,0,0,0,0,0,1,1,0,0,0,0, 0,0,0,0,0,0,0,0,0,0,0,0,0,0,0,0,0,0,0,0,0,0,0,0,1,1,1,1,1,1,1,1,1,1,1,1,1,1,1,1,1,1,1,0,0,0,1,1, 1,1,1,1,1,1,1,1,1,1,1}.
do if (activate=1).
compute errcode(errs,1)=60.
compute errs=errs+1.
compute criterr=1.
end if.
do if (criterr=0).
do if (( 0 =1)=1).
compute errcode(errs,1)=42.
compute errs=errs+1.
compute criterr=1.
end if.
do if (model > 0 and model < 93).
do if (validm(1,model)=0).
compute errcode(errs,1)=6.
compute errs=errs+1.
compute criterr=1.
end if.
release validm.
end if.
do if ((model > 92 or model < 0) and model <> 999)).
compute errcode(errs,1)=7.
compute errs=errs+1.
compute criterr=1.
end if.
do if (model = 999 and bmatrix(1,1)=-999).
compute errcode(errs,1)=24.
compute errs=errs+1.
compute criterr=1.
end if.
do if (model <> 999 and bmatrix(1,1) <> -999).
compute errcode(errs,1)=25.
compute errs=errs+1.
compute criterr=1.
end if.
do if ((model = 74 or (model > 0 and model < 4)) and ((wmatrix(1,1) <> -999) or (zmatrix(1,1)<>-999) or (wzmatrix(1,1)<>-999)))).
compute errcode(errs,1)=41.
compute errs=errs+1.
compute criterr=1.
end if.
do if (hc3 <> 0).
compute notecode(notes,1) = 5.
compute notes = notes + 1.
do if (hc3 = 1).
compute hc=3.
end if.
end if.
do if ((v <> 'xxxxx') or (q <> 'xxxxx')).
compute errcode(errs,1)=14.
compute errs=errs+1.
compute errcode(errs,1)=48.
compute errs=errs+1.
compute criterr=1.
end if.
do if (oldvars <> 'xxxxx').
compute errcode(errs,1)=48.
compute errs=errs+1.
compute criterr=1.
end if.
do if (cluster <> 'xxxxx').
compute errcode(errs,1)=27.
compute errs=errs+1.
compute criterr=1.
end if.
do if ((y = 'xxxxx') or (x = 'xxxxx')).
compute errcode(errs,1)=1.
compute errs=errs+1.
compute criterr=1.
end if.
do if ((m = 'xxxxx') and model > 3).
compute errcode(errs,1)=8.
compute errs=errs+1.
compute criterr=1.
end if.
end if.
do if (criterr=0).
get ytmp/variables = HOMAIR /names = ynames/MISSING = 99999.
compute nys=ncol(ytmp).
compute needed=nys.
compute n=nrow(ytmp).
compute varnames={ynames}.
compute dat=ytmp.

.


do if (toomany=1 and longname=0).
compute criterr=1.
do if (tooman=0).
compute tooman=1.
compute errcode(errs,1) = 61.
compute errs = errs + 1.
end if.
end if
.
compute modelvar={ '4' ;t(ynames)}.
do if ( 4 =999).
compute modelvar(1,1)='CUSTOM'.
end if.
get xtmp/variables = GDR /names = xnames/MISSING = 99999.
compute nxs=ncol(xtmp).
compute n=nrow(xtmp).
compute needed=needed+nxs.
compute varnames={varnames,xnames}.
compute xcatlab=t(xnames).
compute dat={dat,xtmp}.

.


do if (toomany=1 and longname=0).
compute criterr=1.
do if (tooman=0).
compute tooman=1.
compute errcode(errs,1) = 61.
compute errs = errs + 1.
end if.
end if
.
compute modelvar={modelvar;t(xnames)}.
do if (nxs = 1).
compute modelvlb={'Model  :';'    Y  :';'    X  :'}.
else.
compute modelvlb={'Model  :';'    Y  :';xlb(1:nxs,1)}.
end if.
do if (m <> 'xxxxx').
get mtmp/variables = TG HDL LDL TC /names = mnames/MISSING = 99999.
compute nms=ncol(mtmp).
compute mprod=make(1,nms,0).
compute n=nrow(mtmp).
compute needed=needed+nms.
compute varnames={varnames,mnames}.
compute dat={dat,mtmp}.
compute modelvar={modelvar;t(mnames)}.
compute x2m=make(99,nms,0).
compute m2y=make(99,nms,0).
compute onem=make(nms,1,1).

.


do if (toomany=1 and longname=0).
compute criterr=1.
do if (tooman=0).
compute tooman=1.
compute errcode(errs,1) = 61.
compute errs = errs + 1.
end if.
end if
.
do if (nms > 1 and nms < 11).
compute modelvlb={modelvlb;medlb(1:nms,1)}.
else.
compute modelvlb={modelvlb;'    M  :'}.
end if.
do if (nms > 0 and model < 4).
compute errcode(errs,1)=9.
compute errs=errs+1.
do if (model <> 0).
compute errcode(errs,1)=48.
compute errs=errs+1.
end if.
compute criterr=1.
end if.
end if.
compute wlocatet=0.
compute wlocate=0.
do if (w <> 'xxxxx').
do if (xmint=0).
get wtmp/variables = xxxxx /names = wnames/MISSING = 99999.

.


do if (toomany=1 and longname=0).
compute criterr=1.
do if (tooman=0).
compute tooman=1.
compute errcode(errs,1) = 61.
compute errs = errs + 1.
end if.
end if
.
end if.
do if (xmint=1).
get wtmp/variables = GDR /names = wnames/MISSING = 99999.
end if.
compute nws=ncol(wtmp).
compute n=nrow(wtmp).
compute varnames={varnames,wnames}.
compute wlocate=ncol(varnames).
do if (model=74).
compute wlocatet=1.
do if (xnames <> wnames).
compute errcode(errs,1)=45.
compute errs=errs+1.
compute criterr=1.
end if.
end if.
compute wcatlab=t(wnames).
compute dat={dat,wtmp}.
do if (xmint <> 1).
compute modelvar={modelvar;t(wnames)}.
compute modelvlb={modelvlb;'    W  :'}.
end if.
end if.
do if (z <> 'xxxxx').
get ztmp/variables = xxxxx /names = znames/MISSING = 99999.
compute nzs=ncol(ztmp).
compute n=nrow(ztmp).

.


do if (toomany=1 and longname=0).
compute criterr=1.
do if (tooman=0).
compute tooman=1.
compute errcode(errs,1) = 61.
compute errs = errs + 1.
end if.
end if
.
compute varnames={varnames,znames}.
compute zcatlab=t(znames).
compute dat={dat,ztmp}.
compute modelvar={modelvar;t(znames)}.
compute modelvlb={modelvlb;'    Z  :'}.
end if.
do if (cov <> 'xxxxx').
get ctmp/variables = xxxxx /names = covnames/MISSING = 99999.
compute ncs=ncol(ctmp).
compute n=nrow(ctmp).

.


do if (toomany=1 and longname=0).
compute criterr=1.
do if (tooman=0).
compute tooman=1.
compute errcode(errs,1) = 61.
compute errs = errs + 1.
end if.
end if
.
compute varnames={varnames,covnames}.
compute dat={dat,ctmp}.
end if.
do if (nws > 1 or nzs > 1 or nys > 1 or nxs > 1).
compute errcode(errs,1)=3.
compute errs=errs+1.
compute criterr=1.
end if.
do if ((model = 80 or model = 81) and (nms < 3 or nms > 6)).
compute errcode(errs,1)=32.
compute errs=errs+1.
compute criterr=1.
end if.
do if (model = 82 and nms <> 4).
compute errcode(errs,1)=33.
compute errs=errs+1.
compute criterr=1.
end if.
do if (nms > 10).
compute errcode(errs,1)=37.
compute errs=errs+1.
compute criterr=1.
end if.
do if ((model = 6 or (model > 82 and model < 999)) and (nms < 2 or nms > 6)).
compute errcode(errs,1)=34.
compute errs=errs+1.
compute criterr=1.
end if.
compute match=0.
compute match2=0.
compute mcwzcov=0.
loop i = 1 to (ncol(varnames)-1).
loop j = (i+1) to ncol(varnames).
do if (varnames(i)=varnames(j)).
do if (i < (nxs+nms+nys+1)).
compute match2=match2+1.
end if.
do if (wlocatet=1 and i=2 and j=wlocate).
compute match2=match2-1.
end if.
do if ((wnames=znames) and (nws > 0 or nzs > 0))).
compute match2=match2+1.
end if.
do if (i < (ncol(varnames)-ncs+1)) and j > (ncol(varnames)-ncs)).
do if ((varnames(j)=wnames) and mcw=0)).
compute match=0.
compute wiscov=(j-(ncol(varnames)-ncs)).
end if.
do if ((varnames(j)=wnames) and mcw <>0)).
compute mcwzcov=1.
end if.
do if ((varnames(j)=znames) and mcz=0).
compute match=0.
compute ziscov=(j-(ncol(varnames)-ncs)).
end if.
do if ((varnames(j)=znames) and mcz<>0)).
compute mcwzcov=1.
end if.
end if.
end if.
end loop.
end loop.
do if (match2>0 or match=1).
compute errcode(errs,1)=2.
compute errs=errs+1.
compute criterr=1.
end if.
do if (mcwzcov=1).
compute errcode(errs,1)=50.
compute errs=errs+1.
compute criterr=1.
end if.
compute ninit=nrow(dat).
compute rownum=make(ninit,1,0).
loop i = 1 to ninit.
compute rownum(i,1)=i.
end loop.
compute dat={rownum,dat}.
compute j=1.
compute missrow=0.
loop i = 1 to n.
do if (rsum(dat(i,2:ncol(dat))=99999)=0).
compute dat(j,:)=dat(i,:).
compute j=j+1.
else.
compute missrow={missrow;dat(i,1)}.
end if.
end loop.
do if (j < 5).
compute errcode(errs,1)=62.
compute errs=errs+1.
compute criterr=1.
end if.
do if (criterr=0).
compute rownum=dat(1:(j-1),1).
do if (nrow(missrow) > 1).
compute missrow=t(missrow(2:nrow(missrow),1)).
compute notecode(notes,1) = 29.
compute notes = notes + 1.
end if.
compute dat=dat(1:(j-1),2:ncol(dat)).
compute n=nrow(dat).
compute nmiss=ninit-n.
compute ytmp=dat(:,1:nys).

.
compute desctmp=make((8-(4* 0 )),ncol( ytmp ),-999).
loop jd=1 to ncol( ytmp ).
compute descdat= ytmp (:,jd).
compute desctmp(1,jd) = csum(descdat)/nrow(descdat).
compute desctmp(2,jd) = (nrow(descdat)*sscp(descdat))-(t(csum(descdat))*(csum(descdat))).
compute desctmp(2,jd) = sqrt(desctmp(2,jd)/(nrow(descdat)*(nrow(descdat)-1))).
compute desctmp(3,jd)=cmin(descdat).
compute desctmp(4,jd)=cmax(descdat).
do if ( 0 =0).
compute minwarn=0.
compute maxwarn=0.
do if ((desctmp(3,jd)=desctmp(4,jd)) and novar=0).
compute errcode(errs,1)=15.
compute errs=errs+1.
compute criterr=1.
compute novar=1.
end if.
compute tmp=((descdat(:,1)=desctmp(3,jd))+(descdat(:,1)=desctmp(4,jd))).
compute desctmp(8,jd)=(csum(tmp)=nrow(tmp)).
compute tmp = descdat.
compute tmp(GRADE(descdat),:) = descdat.
compute descdat = tmp.
release tmp.
compute decval={.16;.5;.84}.
loop kd=1 to 3.
compute low=trunc(decval(kd,1)*(nrow(descdat)+1)).
compute lowdec=decval(kd,1)*(nrow(descdat)+1)-low.
compute value=descdat(low,1)+(descdat((low+1),1)-descdat(low,1))*lowdec.
compute desctmp((4+kd),jd)=value.
end loop.
compute mnotev=1.
compute modvals=desctmp(5:7,:).
do if (quantile <> 1).
compute desctmp(5,jd)=desctmp(1,jd)-desctmp(2,jd).
compute desctmp(6,jd)=desctmp(1,jd).
compute desctmp(7,jd)=desctmp(1,jd)+desctmp(2,jd).
compute modvals=desctmp(5:7,:).
compute mnotev=2.
do if (modvals(1,1) < desctmp(3,1)).
compute modvals(1,1)=desctmp(3,1).
compute minwarn=1.
end if.
do if (modvals(3,1) > desctmp(4,1)).
compute modvals(3,1)=desctmp(4,1).
compute maxwarn=1.
end if.
end if.
do if (desctmp(8,1)=1).
compute modvals={desctmp(3,1);desctmp(4,1)}.
compute mnotev=0.
compute minwarn=0.
compute maxwarn=0.
end if.
end if.
end loop
.
compute ysd=desctmp(2,:).
compute ovsd=ysd.
do if (desctmp(8,1)=1).
compute ydich=1.
do if (total=1).
compute total=0.
compute notecode(notes,1) = 24.
compute notes = notes + 1.
end if.
do if (effsize=1).
compute effsize=0.
compute notecode(notes,1) = 25.
compute notes = notes + 1.
end if.
do if (model=74).
compute errcode(errs,1) = 72.
compute errs=errs+1.
compute criterr=1.
end if.
compute omx = cmax(ytmp).
compute omn = cmin(ytmp).
compute ytmp = (ytmp = omx).
compute dat(:,1:nys)=(dat(:,1:nys)=omx).
compute rcd = {omn, 0; omx, 1}.
end if.
compute xtmp=dat(:,(nys+1):(nys+nxs)).

.
compute desctmp=make((8-(4* 0 )),ncol( xtmp ),-999).
loop jd=1 to ncol( xtmp ).
compute descdat= xtmp (:,jd).
compute desctmp(1,jd) = csum(descdat)/nrow(descdat).
compute desctmp(2,jd) = (nrow(descdat)*sscp(descdat))-(t(csum(descdat))*(csum(descdat))).
compute desctmp(2,jd) = sqrt(desctmp(2,jd)/(nrow(descdat)*(nrow(descdat)-1))).
compute desctmp(3,jd)=cmin(descdat).
compute desctmp(4,jd)=cmax(descdat).
do if ( 0 =0).
compute minwarn=0.
compute maxwarn=0.
do if ((desctmp(3,jd)=desctmp(4,jd)) and novar=0).
compute errcode(errs,1)=15.
compute errs=errs+1.
compute criterr=1.
compute novar=1.
end if.
compute tmp=((descdat(:,1)=desctmp(3,jd))+(descdat(:,1)=desctmp(4,jd))).
compute desctmp(8,jd)=(csum(tmp)=nrow(tmp)).
compute tmp = descdat.
compute tmp(GRADE(descdat),:) = descdat.
compute descdat = tmp.
release tmp.
compute decval={.16;.5;.84}.
loop kd=1 to 3.
compute low=trunc(decval(kd,1)*(nrow(descdat)+1)).
compute lowdec=decval(kd,1)*(nrow(descdat)+1)-low.
compute value=descdat(low,1)+(descdat((low+1),1)-descdat(low,1))*lowdec.
compute desctmp((4+kd),jd)=value.
end loop.
compute mnotev=1.
compute modvals=desctmp(5:7,:).
do if (quantile <> 1).
compute desctmp(5,jd)=desctmp(1,jd)-desctmp(2,jd).
compute desctmp(6,jd)=desctmp(1,jd).
compute desctmp(7,jd)=desctmp(1,jd)+desctmp(2,jd).
compute modvals=desctmp(5:7,:).
compute mnotev=2.
do if (modvals(1,1) < desctmp(3,1)).
compute modvals(1,1)=desctmp(3,1).
compute minwarn=1.
end if.
do if (modvals(3,1) > desctmp(4,1)).
compute modvals(3,1)=desctmp(4,1).
compute maxwarn=1.
end if.
end if.
do if (desctmp(8,1)=1).
compute modvals={desctmp(3,1);desctmp(4,1)}.
compute mnotev=0.
compute minwarn=0.
compute maxwarn=0.
end if.
end if.
end loop
.
compute xsd=desctmp(2,:).
compute xmodvals=modvals.
compute xdich=desctmp(8,1).
compute xmx=cmax(xtmp).
compute xmn=cmin(xtmp).
do if ((mcx > 0) and (xrefvals(1,1) <> 999) and (xmint=1) and (model= 74)).
compute notecode(notes,1) = 36.
compute notes = notes + 1.
end if.
do if (mcx=0).
do if (ncol(xrefvals)>2 and model=74 and xmint=1).
compute errcode(errs,1)=67.
compute errs=errs+1.
compute criterr=1.
end if.
do if ((model=74) and (xmint=1)).
do if ((xrefvals(1,1)=999) and nxvls=1 and xdich=0).
compute errcode(errs,1)=66.
compute errs=errs+1.
compute criterr=1.
end if.
do if ((xrefvals(1,1)=999) and (xdich=1)).
compute xrefvals={xmn,xmx}.
compute xscaling=xrefvals(1,2)-xrefvals(1,1).
end if.
do if (ncol(xrefvals)=1 and xrefvals(1,1) <> 999).
do if (xdich=0).
compute xrefvals={xrefvals,(xrefvals(1,1)+1)}.
compute xscaling=xrefvals(1,2)-xrefvals(1,1).
end if.
do if (xdich=1).
do if ((xrefvals(1,1) <> xmx) and (xrefvals(1,1) <> xmn)).
compute errcode(errs,1)=70.
compute errs=errs+1.
compute criterr=1.
end if.
do if (xrefvals(1,1) = xmx).
compute xrefvals={xrefvals,xmn}.
compute xscaling=xrefvals(1,2)-xrefvals(1,1).
end if.
do if (xrefvals(1,1) = xmn).
compute xrefvals={xrefvals,xmx}.
compute xscaling=xrefvals(1,2)-xrefvals(1,1).
end if.
end if.
end if.
do if (ncol(xrefvals)=2).
compute xscaling=xrefvals(1,2)-xrefvals(1,1).
do if (xdich=1).
compute xreferr=1.
do if (((xrefvals(1,1) = xmx) and (xrefvals(1,2)=xmn)) or ((xrefvals(1,1) = xmn) and (xrefvals(1,2)=xmx))).
compute xreferr=0.
end if.
do if (xreferr=1).
compute errcode(errs,1)=70.
compute errs=errs+1.
compute criterr=1.
end if.
end if.
end if.
end if.
end if.
do if (xmint=1 and model=74 and mcx=0).
compute xmodvals=t(xrefvals).
compute xcontcf=1.
end if.
compute nxpval=nrow(xmodvals).
compute xprobval=xmodvals.
do if (xdich =1 and mcx > 0).
compute mcx=0.
compute errcode(errs,1) = 52.
compute errs = errs + 1.
compute criterr = 1.
end if.
do if (nms > 0).
compute mtmp=dat(:,(nys+nxs+1):(nys+nxs+nms)).

.
compute desctmp=make((8-(4* 0 )),ncol( mtmp ),-999).
loop jd=1 to ncol( mtmp ).
compute descdat= mtmp (:,jd).
compute desctmp(1,jd) = csum(descdat)/nrow(descdat).
compute desctmp(2,jd) = (nrow(descdat)*sscp(descdat))-(t(csum(descdat))*(csum(descdat))).
compute desctmp(2,jd) = sqrt(desctmp(2,jd)/(nrow(descdat)*(nrow(descdat)-1))).
compute desctmp(3,jd)=cmin(descdat).
compute desctmp(4,jd)=cmax(descdat).
do if ( 0 =0).
compute minwarn=0.
compute maxwarn=0.
do if ((desctmp(3,jd)=desctmp(4,jd)) and novar=0).
compute errcode(errs,1)=15.
compute errs=errs+1.
compute criterr=1.
compute novar=1.
end if.
compute tmp=((descdat(:,1)=desctmp(3,jd))+(descdat(:,1)=desctmp(4,jd))).
compute desctmp(8,jd)=(csum(tmp)=nrow(tmp)).
compute tmp = descdat.
compute tmp(GRADE(descdat),:) = descdat.
compute descdat = tmp.
release tmp.
compute decval={.16;.5;.84}.
loop kd=1 to 3.
compute low=trunc(decval(kd,1)*(nrow(descdat)+1)).
compute lowdec=decval(kd,1)*(nrow(descdat)+1)-low.
compute value=descdat(low,1)+(descdat((low+1),1)-descdat(low,1))*lowdec.
compute desctmp((4+kd),jd)=value.
end loop.
compute mnotev=1.
compute modvals=desctmp(5:7,:).
do if (quantile <> 1).
compute desctmp(5,jd)=desctmp(1,jd)-desctmp(2,jd).
compute desctmp(6,jd)=desctmp(1,jd).
compute desctmp(7,jd)=desctmp(1,jd)+desctmp(2,jd).
compute modvals=desctmp(5:7,:).
compute mnotev=2.
do if (modvals(1,1) < desctmp(3,1)).
compute modvals(1,1)=desctmp(3,1).
compute minwarn=1.
end if.
do if (modvals(3,1) > desctmp(4,1)).
compute modvals(3,1)=desctmp(4,1).
compute maxwarn=1.
end if.
end if.
do if (desctmp(8,1)=1).
compute modvals={desctmp(3,1);desctmp(4,1)}.
compute mnotev=0.
compute minwarn=0.
compute maxwarn=0.
end if.
end if.
end loop
.
compute ovsd={desctmp(2,:),ysd}.
compute medmeans=cdeval.
do if ((cdeval(1,1) <> -999) and (ncol(medmeans) <> nms) and (model=74)).
compute errcode(errs,1)=64.
compute errs=errs+1.
compute criterr=1.
end if.
do if ((cdeval(1,1)=-999) and (model=74)).
compute medmeans=desctmp(1,:).
end if.
do if ((cdeval(1,1)<> -999) and (model=74) and (criterr=0)).
compute notecode(notes,1) = 31.
compute notes = notes + 1.
end if.
do if ((rsum(desctmp(8,:))>0) and (mdichok <> 1)).
compute errcode(errs,1)=43.
compute errs=errs+1.
compute criterr=1.
end if.
compute mmodvals=modvals.
compute mprobval=mmodvals.
end if.
do if (nws > 0).
compute wtmp=dat(:,(nys+nxs+nms+1):(nys+nxs+nms+nws)).

.
compute desctmp=make((8-(4* 0 )),ncol( wtmp ),-999).
loop jd=1 to ncol( wtmp ).
compute descdat= wtmp (:,jd).
compute desctmp(1,jd) = csum(descdat)/nrow(descdat).
compute desctmp(2,jd) = (nrow(descdat)*sscp(descdat))-(t(csum(descdat))*(csum(descdat))).
compute desctmp(2,jd) = sqrt(desctmp(2,jd)/(nrow(descdat)*(nrow(descdat)-1))).
compute desctmp(3,jd)=cmin(descdat).
compute desctmp(4,jd)=cmax(descdat).
do if ( 0 =0).
compute minwarn=0.
compute maxwarn=0.
do if ((desctmp(3,jd)=desctmp(4,jd)) and novar=0).
compute errcode(errs,1)=15.
compute errs=errs+1.
compute criterr=1.
compute novar=1.
end if.
compute tmp=((descdat(:,1)=desctmp(3,jd))+(descdat(:,1)=desctmp(4,jd))).
compute desctmp(8,jd)=(csum(tmp)=nrow(tmp)).
compute tmp = descdat.
compute tmp(GRADE(descdat),:) = descdat.
compute descdat = tmp.
release tmp.
compute decval={.16;.5;.84}.
loop kd=1 to 3.
compute low=trunc(decval(kd,1)*(nrow(descdat)+1)).
compute lowdec=decval(kd,1)*(nrow(descdat)+1)-low.
compute value=descdat(low,1)+(descdat((low+1),1)-descdat(low,1))*lowdec.
compute desctmp((4+kd),jd)=value.
end loop.
compute mnotev=1.
compute modvals=desctmp(5:7,:).
do if (quantile <> 1).
compute desctmp(5,jd)=desctmp(1,jd)-desctmp(2,jd).
compute desctmp(6,jd)=desctmp(1,jd).
compute desctmp(7,jd)=desctmp(1,jd)+desctmp(2,jd).
compute modvals=desctmp(5:7,:).
compute mnotev=2.
do if (modvals(1,1) < desctmp(3,1)).
compute modvals(1,1)=desctmp(3,1).
compute minwarn=1.
end if.
do if (modvals(3,1) > desctmp(4,1)).
compute modvals(3,1)=desctmp(4,1).
compute maxwarn=1.
end if.
end if.
do if (desctmp(8,1)=1).
compute modvals={desctmp(3,1);desctmp(4,1)}.
compute mnotev=0.
compute minwarn=0.
compute maxwarn=0.
end if.
end if.
end loop
.
compute wmodvals=modvals.
compute wdich=desctmp(8,1).
do if (wdich =1 and mcw > 0).
compute mcw=0.
compute errcode(errs,1) = 52.
compute errs = errs + 1.
compute criterr = 1.
end if.
compute wmin=desctmp(3,1).
compute wmax=desctmp(4,1).
compute minwwarn=minwarn.
compute maxwwarn=maxwarn.
compute wnotev=mnotev.
compute wmodval={ 999 }.
do if (xmint=1 and model=74 and mcx=0).
compute wmodval=xrefvals.
end if.
compute nwcontr=ncol(wmodval).
do if (wmodval(1,1) <> 999).
compute wmodvals=wmodval(1,1).
compute wmodcust=1.
do if (nwcontr > 1).
compute wmodvals=t(wmodval).
end if.
compute minwwarn=0.
compute maxwwarn=0.
compute wnotev=0.
end if.
compute wprobval=wmodvals.
compute nwpval=nrow(wmodvals).
end if.
do if (nzs > 0).
compute ztmp=dat(:,(nys+nxs+nms+nws+1):(nys+nxs+nms+nws+nzs)).

.
compute desctmp=make((8-(4* 0 )),ncol( ztmp ),-999).
loop jd=1 to ncol( ztmp ).
compute descdat= ztmp (:,jd).
compute desctmp(1,jd) = csum(descdat)/nrow(descdat).
compute desctmp(2,jd) = (nrow(descdat)*sscp(descdat))-(t(csum(descdat))*(csum(descdat))).
compute desctmp(2,jd) = sqrt(desctmp(2,jd)/(nrow(descdat)*(nrow(descdat)-1))).
compute desctmp(3,jd)=cmin(descdat).
compute desctmp(4,jd)=cmax(descdat).
do if ( 0 =0).
compute minwarn=0.
compute maxwarn=0.
do if ((desctmp(3,jd)=desctmp(4,jd)) and novar=0).
compute errcode(errs,1)=15.
compute errs=errs+1.
compute criterr=1.
compute novar=1.
end if.
compute tmp=((descdat(:,1)=desctmp(3,jd))+(descdat(:,1)=desctmp(4,jd))).
compute desctmp(8,jd)=(csum(tmp)=nrow(tmp)).
compute tmp = descdat.
compute tmp(GRADE(descdat),:) = descdat.
compute descdat = tmp.
release tmp.
compute decval={.16;.5;.84}.
loop kd=1 to 3.
compute low=trunc(decval(kd,1)*(nrow(descdat)+1)).
compute lowdec=decval(kd,1)*(nrow(descdat)+1)-low.
compute value=descdat(low,1)+(descdat((low+1),1)-descdat(low,1))*lowdec.
compute desctmp((4+kd),jd)=value.
end loop.
compute mnotev=1.
compute modvals=desctmp(5:7,:).
do if (quantile <> 1).
compute desctmp(5,jd)=desctmp(1,jd)-desctmp(2,jd).
compute desctmp(6,jd)=desctmp(1,jd).
compute desctmp(7,jd)=desctmp(1,jd)+desctmp(2,jd).
compute modvals=desctmp(5:7,:).
compute mnotev=2.
do if (modvals(1,1) < desctmp(3,1)).
compute modvals(1,1)=desctmp(3,1).
compute minwarn=1.
end if.
do if (modvals(3,1) > desctmp(4,1)).
compute modvals(3,1)=desctmp(4,1).
compute maxwarn=1.
end if.
end if.
do if (desctmp(8,1)=1).
compute modvals={desctmp(3,1);desctmp(4,1)}.
compute mnotev=0.
compute minwarn=0.
compute maxwarn=0.
end if.
end if.
end loop
.
compute zmodvals=modvals.
compute zdich=desctmp(8,1).
do if (zdich =1 and mcz > 0).
compute mcz=0.
compute errcode(errs,1) = 52.
compute errs = errs + 1.
compute criterr = 1.
end if.
compute zmin=desctmp(3,1).
compute zmax=desctmp(4,1).
compute minzwarn=minwarn.
compute maxzwarn=maxwarn.
compute znotev=mnotev.
compute zmodval={ 999 }.
compute nzcontr=ncol(zmodval).
do if (zmodval(1,1) <> 999).
compute zmodvals=zmodval(1,1).
compute zmodcust=1.
do if (nzcontr > 1).
compute zmodvals=t(zmodval).
end if.
compute minzwarn=0.
compute maxzwarn=0.
compute znotev=0.
end if.
compute zprobval=zmodvals.
compute nzpval=nrow(zmodvals).
end if.
do if (ncs > 0).
compute ctmp=dat(:,(nys+nxs+nms+nws+nzs+1):(nys+nxs+nms+nws+nzs+ncs)).

.
compute desctmp=make((8-(4* 0 )),ncol( ctmp ),-999).
loop jd=1 to ncol( ctmp ).
compute descdat= ctmp (:,jd).
compute desctmp(1,jd) = csum(descdat)/nrow(descdat).
compute desctmp(2,jd) = (nrow(descdat)*sscp(descdat))-(t(csum(descdat))*(csum(descdat))).
compute desctmp(2,jd) = sqrt(desctmp(2,jd)/(nrow(descdat)*(nrow(descdat)-1))).
compute desctmp(3,jd)=cmin(descdat).
compute desctmp(4,jd)=cmax(descdat).
do if ( 0 =0).
compute minwarn=0.
compute maxwarn=0.
do if ((desctmp(3,jd)=desctmp(4,jd)) and novar=0).
compute errcode(errs,1)=15.
compute errs=errs+1.
compute criterr=1.
compute novar=1.
end if.
compute tmp=((descdat(:,1)=desctmp(3,jd))+(descdat(:,1)=desctmp(4,jd))).
compute desctmp(8,jd)=(csum(tmp)=nrow(tmp)).
compute tmp = descdat.
compute tmp(GRADE(descdat),:) = descdat.
compute descdat = tmp.
release tmp.
compute decval={.16;.5;.84}.
loop kd=1 to 3.
compute low=trunc(decval(kd,1)*(nrow(descdat)+1)).
compute lowdec=decval(kd,1)*(nrow(descdat)+1)-low.
compute value=descdat(low,1)+(descdat((low+1),1)-descdat(low,1))*lowdec.
compute desctmp((4+kd),jd)=value.
end loop.
compute mnotev=1.
compute modvals=desctmp(5:7,:).
do if (quantile <> 1).
compute desctmp(5,jd)=desctmp(1,jd)-desctmp(2,jd).
compute desctmp(6,jd)=desctmp(1,jd).
compute desctmp(7,jd)=desctmp(1,jd)+desctmp(2,jd).
compute modvals=desctmp(5:7,:).
compute mnotev=2.
do if (modvals(1,1) < desctmp(3,1)).
compute modvals(1,1)=desctmp(3,1).
compute minwarn=1.
end if.
do if (modvals(3,1) > desctmp(4,1)).
compute modvals(3,1)=desctmp(4,1).
compute maxwarn=1.
end if.
end if.
do if (desctmp(8,1)=1).
compute modvals={desctmp(3,1);desctmp(4,1)}.
compute mnotev=0.
compute minwarn=0.
compute maxwarn=0.
end if.
end if.
end loop
.
compute covmeans=desctmp(1,:).
compute coval={ -999 }.
do if ((coval(1,1) <> -999) and (ncol(coval) <> ncs) and (model=74)).
compute errcode(errs,1)=69.
compute errs=errs+1.
compute criterr=1.
end if.
do if ((coval(1,1)<> -999) and (criterr=0) and (model=74)).
compute notecode(notes,1) = 35.
compute notes = notes + 1.
compute cuscoval=1.
end if.
end if.
compute n=nrow(ytmp).
compute ones=make(n,1,1).
do if (nws > 0 and mcw > 0).
compute tmp={rownum,wtmp(:,1)}.

.
compute dd= tmp.
compute temp = dd.
compute temp(GRADE(dd(:,2)),:) = dd.
compute dd = temp.
compute dummy = design(dd(:,2)).
compute nvls = ncol(dummy).
compute nnvls = csum(dummy).
compute mnvls = cmin(t(nnvls)).
compute conmat1=1.
do if (mnvls < 2).
compute errcode(errs,1) = 5.
compute errs = errs + 1.
compute criterr = 1.
end if.
do if (nvls > 9).
compute errcode(errs,1) = 4.
compute errs = errs+1.
compute criterr = 1.
end if.
do if (criterr = 0).
compute dumok = 1.
compute nnvls=make(nvls,1,0).
compute nnvls(1,1)=dd(1,2).
compute temp = 2.
loop i = 2 to n.
do if (dd(i,2) <> nnvls((temp-1),1)).
compute nnvls(temp,1)=dd(i,2).
compute temp = temp+1.
end if.
end loop.
do if ( mcw > 0).
compute x = dummy(:,2:ncol(dummy)).
compute nx = ncol(x).
compute minus1 = make(1,ncol(x),-1).
compute xdes=make((nx+1),3,0).
compute xdes(1,1)=dd(1,2).
compute xdes(1,2)=1.
compute temp = 2.
loop k = 2 to n.
do if (dd(k,2) <> dd((k-1),2)).
compute xdes(temp,2) = k.
compute xdes(temp,1) = dd(k,2).
compute xdes((temp-1),3) = k-1.
compute temp=temp+1.
end if.
end loop.
compute xdes((temp-1),3)=n.
compute xdes = {xdes, (xdes(:,3)-xdes(:,2)+1)}.
do if ( mcw = 4).
loop k = 1 to n.
do if (rsum(x(k,:)) = 0).
compute x(k,:) = minus1.
end if.
end loop.
end if.
do if ( mcw = 2 or mcw = 3 or mcw =5).
loop k = 1 to n.
do if (rsum(x(k,:)) > 0).
loop i = 1 to ncol(x).
do if (x(k,i) = 0).
compute x(k,i) = 1.
else.
break.
end if.
end loop.
end if.
end loop.
do if ( mcw = 3).
compute conmat1={-8,1,1,1,1,1,1,1,1; 0,-7,1,1,1,1,1,1,1; 0,0,-6,1,1,1,1,1,1; 0,0,0,-5,1,1,1,1,1; 0,0,0,0,-4,1,1,1,1; 0,0,0,0,0,-3,1,1,1; 0,0,0,0,0,0,-2,1,1; 0,0,0,0,0,0,0,-1,1}.
loop i = 1 to 8.
compute conmat1(i,:)=conmat1(i,:)/(10-i).
end loop.
compute conmat1=t(conmat1((10-nvls):8,(10-nvls):9)).
loop k=1 to n.
compute x(k,:)=conmat1((rsum(x(k,:))+1),:).
end loop.
end if.
end if.
do if ( mcw = 5).
compute custcode={ -999 }.
do if (ncol(custcode) <> (nvls*(nvls-1))).
compute errcode(errs,1) = (37+ 2 ).
compute errs = errs + 1.
compute criterr = 1.
end if.
do if (ncol(custcode) = (nvls*(nvls-1))).
compute conmat1=make(nvls,(nvls-1),0).
compute cnt=1.
loop i = 1 to nvls.
loop k = 1 to (nvls-1).
compute conmat1(i,k)=custcode(1,cnt).
compute cnt=cnt+1.
end loop.
end loop.
loop k=1 to n.
compute x(k,:)=conmat1((rsum(x(k,:))+1),:).
end loop.
end if.
end if.
compute xskip = 1.
compute dummat = make((nx+1),nx,0).
compute dummat((2:nrow(dummat)),:)=ident(nx).
do if ( mcw = 4).
compute dummat(1,:) = minus1.
end if.
do if ( mcw = 2).
loop i = 2 to nrow(dummat).
loop j = 1 to (i-1).
compute dummat(i,j) = 1.
end loop.
end loop.
end if.
do if ( mcw = 3).
compute dummat=conmat1.
end if.
do if ( mcw = 5 and criterr=0).
compute dummat=conmat1.
end if.
compute dummat={nnvls, dummat}.
compute x={dd(:,1),x}.
compute temp = x.
compute temp(GRADE(x(:,1)),:) = x.
compute x = temp.
release conmat1,temp,dd,xskip,xdes,dummy.
end if.
end if
.
compute wmodvals=nnvls.
compute nwpval=nrow(wmodvals).
do if (criterr=0).
compute minwwarn=0.
compute maxwwarn=0.
compute wnotev=0.
compute wtmp=x(:,2:ncol(x)).
compute wcatlab={'W1';'W2';'W3';'W4';'W5';'W6';'W7';'W8';'W9'}.
do if (xmint=1).
compute wcatlab={'X1';'X2';'X3';'X4';'X5';'X6';'X7';'X8';'X9'}.
end if.
compute nwvls=nvls-1.
compute mcwok=1.
compute dummatw=dummat.
compute wprobval=dummatw(:,2:ncol(dummatw)).
do if (modcok=1).
compute wcontval=make(2,ncol(wprobval),-999).
compute temp=0.
loop i = 1 to 2.
loop j = 1 to nrow(dummatw).
do if (contvec(i,1)=dummatw(j,1)).
compute wcontval(i,:)=wprobval(j,:).
compute temp=temp+1.
end if.
end loop.
end loop.
do if (temp < 2).
compute notecode(notes,1) = 20.
compute notes = notes + 1.
compute modcok=0.
end if.
end if.
do if ((wmodval(1,1) <> 999) and (xmint <> 1)).
compute notecode(notes,1) = 9.
compute notes = notes + 1.
end if.
release tmp, dummat.
end if.
end if.
do if (nzs > 0 and mcz > 0).
compute tmp={rownum,ztmp(:,1)}.

.
compute dd= tmp.
compute temp = dd.
compute temp(GRADE(dd(:,2)),:) = dd.
compute dd = temp.
compute dummy = design(dd(:,2)).
compute nvls = ncol(dummy).
compute nnvls = csum(dummy).
compute mnvls = cmin(t(nnvls)).
compute conmat1=1.
do if (mnvls < 2).
compute errcode(errs,1) = 5.
compute errs = errs + 1.
compute criterr = 1.
end if.
do if (nvls > 9).
compute errcode(errs,1) = 4.
compute errs = errs+1.
compute criterr = 1.
end if.
do if (criterr = 0).
compute dumok = 1.
compute nnvls=make(nvls,1,0).
compute nnvls(1,1)=dd(1,2).
compute temp = 2.
loop i = 2 to n.
do if (dd(i,2) <> nnvls((temp-1),1)).
compute nnvls(temp,1)=dd(i,2).
compute temp = temp+1.
end if.
end loop.
do if ( mcz > 0).
compute x = dummy(:,2:ncol(dummy)).
compute nx = ncol(x).
compute minus1 = make(1,ncol(x),-1).
compute xdes=make((nx+1),3,0).
compute xdes(1,1)=dd(1,2).
compute xdes(1,2)=1.
compute temp = 2.
loop k = 2 to n.
do if (dd(k,2) <> dd((k-1),2)).
compute xdes(temp,2) = k.
compute xdes(temp,1) = dd(k,2).
compute xdes((temp-1),3) = k-1.
compute temp=temp+1.
end if.
end loop.
compute xdes((temp-1),3)=n.
compute xdes = {xdes, (xdes(:,3)-xdes(:,2)+1)}.
do if ( mcz = 4).
loop k = 1 to n.
do if (rsum(x(k,:)) = 0).
compute x(k,:) = minus1.
end if.
end loop.
end if.
do if ( mcz = 2 or mcz = 3 or mcz =5).
loop k = 1 to n.
do if (rsum(x(k,:)) > 0).
loop i = 1 to ncol(x).
do if (x(k,i) = 0).
compute x(k,i) = 1.
else.
break.
end if.
end loop.
end if.
end loop.
do if ( mcz = 3).
compute conmat1={-8,1,1,1,1,1,1,1,1; 0,-7,1,1,1,1,1,1,1; 0,0,-6,1,1,1,1,1,1; 0,0,0,-5,1,1,1,1,1; 0,0,0,0,-4,1,1,1,1; 0,0,0,0,0,-3,1,1,1; 0,0,0,0,0,0,-2,1,1; 0,0,0,0,0,0,0,-1,1}.
loop i = 1 to 8.
compute conmat1(i,:)=conmat1(i,:)/(10-i).
end loop.
compute conmat1=t(conmat1((10-nvls):8,(10-nvls):9)).
loop k=1 to n.
compute x(k,:)=conmat1((rsum(x(k,:))+1),:).
end loop.
end if.
end if.
do if ( mcz = 5).
compute custcode={ -999 }.
do if (ncol(custcode) <> (nvls*(nvls-1))).
compute errcode(errs,1) = (37+ 3 ).
compute errs = errs + 1.
compute criterr = 1.
end if.
do if (ncol(custcode) = (nvls*(nvls-1))).
compute conmat1=make(nvls,(nvls-1),0).
compute cnt=1.
loop i = 1 to nvls.
loop k = 1 to (nvls-1).
compute conmat1(i,k)=custcode(1,cnt).
compute cnt=cnt+1.
end loop.
end loop.
loop k=1 to n.
compute x(k,:)=conmat1((rsum(x(k,:))+1),:).
end loop.
end if.
end if.
compute xskip = 1.
compute dummat = make((nx+1),nx,0).
compute dummat((2:nrow(dummat)),:)=ident(nx).
do if ( mcz = 4).
compute dummat(1,:) = minus1.
end if.
do if ( mcz = 2).
loop i = 2 to nrow(dummat).
loop j = 1 to (i-1).
compute dummat(i,j) = 1.
end loop.
end loop.
end if.
do if ( mcz = 3).
compute dummat=conmat1.
end if.
do if ( mcz = 5 and criterr=0).
compute dummat=conmat1.
end if.
compute dummat={nnvls, dummat}.
compute x={dd(:,1),x}.
compute temp = x.
compute temp(GRADE(x(:,1)),:) = x.
compute x = temp.
release conmat1,temp,dd,xskip,xdes,dummy.
end if.
end if
.
compute zmodvals=nnvls.
compute nzpval=nrow(zmodvals).
do if (criterr=0).
compute minzwarn=0.
compute maxzwarn=0.
compute znotev=0.
compute ztmp=x(:,2:ncol(x)).
compute zcatlab={'Z1';'Z2';'Z3';'Z4';'Z5';'Z6';'Z7';'Z8';'Z9'}.
compute nzvls=nvls-1.
compute mczok=1.
compute dummatz=dummat.
compute zprobval=dummatz(:,2:ncol(dummatz)).
do if (modcok=1).
compute zcontval=make(2,ncol(zprobval),-999).
compute temp=0.
loop i = 1 to 2.
loop j = 1 to nrow(dummatz).
do if (contvec(i,2)=dummatz(j,1)).
compute zcontval(i,:)=zprobval(j,:).
compute temp=temp+1.
end if.
end loop.
end loop.
do if (temp < 2).
compute notecode(notes,1) = 20.
compute notes = notes + 1.
compute modcok=0.
end if.
end if.
do if (zmodval(1,1) <> 999).
compute notecode(notes,1) = 10.
compute notes = notes + 1.
end if.
release tmp, dummat.
end if.
end if.
do if (nxs > 0 and mcx > 0).
compute tmp={rownum,xtmp(:,1)}.

.
compute dd= tmp.
compute temp = dd.
compute temp(GRADE(dd(:,2)),:) = dd.
compute dd = temp.
compute dummy = design(dd(:,2)).
compute nvls = ncol(dummy).
compute nnvls = csum(dummy).
compute mnvls = cmin(t(nnvls)).
compute conmat1=1.
do if (mnvls < 2).
compute errcode(errs,1) = 5.
compute errs = errs + 1.
compute criterr = 1.
end if.
do if (nvls > 9).
compute errcode(errs,1) = 4.
compute errs = errs+1.
compute criterr = 1.
end if.
do if (criterr = 0).
compute dumok = 1.
compute nnvls=make(nvls,1,0).
compute nnvls(1,1)=dd(1,2).
compute temp = 2.
loop i = 2 to n.
do if (dd(i,2) <> nnvls((temp-1),1)).
compute nnvls(temp,1)=dd(i,2).
compute temp = temp+1.
end if.
end loop.
do if ( mcx > 0).
compute x = dummy(:,2:ncol(dummy)).
compute nx = ncol(x).
compute minus1 = make(1,ncol(x),-1).
compute xdes=make((nx+1),3,0).
compute xdes(1,1)=dd(1,2).
compute xdes(1,2)=1.
compute temp = 2.
loop k = 2 to n.
do if (dd(k,2) <> dd((k-1),2)).
compute xdes(temp,2) = k.
compute xdes(temp,1) = dd(k,2).
compute xdes((temp-1),3) = k-1.
compute temp=temp+1.
end if.
end loop.
compute xdes((temp-1),3)=n.
compute xdes = {xdes, (xdes(:,3)-xdes(:,2)+1)}.
do if ( mcx = 4).
loop k = 1 to n.
do if (rsum(x(k,:)) = 0).
compute x(k,:) = minus1.
end if.
end loop.
end if.
do if ( mcx = 2 or mcx = 3 or mcx =5).
loop k = 1 to n.
do if (rsum(x(k,:)) > 0).
loop i = 1 to ncol(x).
do if (x(k,i) = 0).
compute x(k,i) = 1.
else.
break.
end if.
end loop.
end if.
end loop.
do if ( mcx = 3).
compute conmat1={-8,1,1,1,1,1,1,1,1; 0,-7,1,1,1,1,1,1,1; 0,0,-6,1,1,1,1,1,1; 0,0,0,-5,1,1,1,1,1; 0,0,0,0,-4,1,1,1,1; 0,0,0,0,0,-3,1,1,1; 0,0,0,0,0,0,-2,1,1; 0,0,0,0,0,0,0,-1,1}.
loop i = 1 to 8.
compute conmat1(i,:)=conmat1(i,:)/(10-i).
end loop.
compute conmat1=t(conmat1((10-nvls):8,(10-nvls):9)).
loop k=1 to n.
compute x(k,:)=conmat1((rsum(x(k,:))+1),:).
end loop.
end if.
end if.
do if ( mcx = 5).
compute custcode={ -999 }.
do if (ncol(custcode) <> (nvls*(nvls-1))).
compute errcode(errs,1) = (37+ 1 ).
compute errs = errs + 1.
compute criterr = 1.
end if.
do if (ncol(custcode) = (nvls*(nvls-1))).
compute conmat1=make(nvls,(nvls-1),0).
compute cnt=1.
loop i = 1 to nvls.
loop k = 1 to (nvls-1).
compute conmat1(i,k)=custcode(1,cnt).
compute cnt=cnt+1.
end loop.
end loop.
loop k=1 to n.
compute x(k,:)=conmat1((rsum(x(k,:))+1),:).
end loop.
end if.
end if.
compute xskip = 1.
compute dummat = make((nx+1),nx,0).
compute dummat((2:nrow(dummat)),:)=ident(nx).
do if ( mcx = 4).
compute dummat(1,:) = minus1.
end if.
do if ( mcx = 2).
loop i = 2 to nrow(dummat).
loop j = 1 to (i-1).
compute dummat(i,j) = 1.
end loop.
end loop.
end if.
do if ( mcx = 3).
compute dummat=conmat1.
end if.
do if ( mcx = 5 and criterr=0).
compute dummat=conmat1.
end if.
compute dummat={nnvls, dummat}.
compute x={dd(:,1),x}.
compute temp = x.
compute temp(GRADE(x(:,1)),:) = x.
compute x = temp.
release conmat1,temp,dd,xskip,xdes,dummy.
end if.
end if
.
do if (criterr=0).
compute xtmp=x(:,2:ncol(x)).
compute xcatlab={'X1';'X2';'X3';'X4';'X5';'X6';'X7';'X8';'X9'}.
compute nxvls=nvls-1.
compute xdich=(nvls=2).
compute mcxok=1.
compute dummatx=dummat.
compute xmodvals=dummatx(:,1).
compute nxpval=nrow(xmodvals).
release tmp, dummat.
end if.
end if.
compute intlab=make(100,1,' ').


compute intlab( 1 ,1)= 'Int_1'.


compute intlab( 2 ,1)= 'Int_2'.


compute intlab( 3 ,1)= 'Int_3'.


compute intlab( 4 ,1)= 'Int_4'.


compute intlab( 5 ,1)= 'Int_5'.


compute intlab( 6 ,1)= 'Int_6'.


compute intlab( 7 ,1)= 'Int_7'.


compute intlab( 8 ,1)= 'Int_8'.


compute intlab( 9 ,1)= 'Int_9'.


compute intlab( 10 ,1)= 'Int_10'.


compute intlab( 11 ,1)= 'Int_11'.


compute intlab( 12 ,1)= 'Int_12'.


compute intlab( 13 ,1)= 'Int_13'.


compute intlab( 14 ,1)= 'Int_14'.


compute intlab( 15 ,1)= 'Int_15'.


compute intlab( 16 ,1)= 'Int_16'.


compute intlab( 17 ,1)= 'Int_17'.


compute intlab( 18 ,1)= 'Int_18'.


compute intlab( 19 ,1)= 'Int_19'.


compute intlab( 20 ,1)= 'Int_20'.


compute intlab( 21 ,1)= 'Int_21'.


compute intlab( 22 ,1)= 'Int_22'.


compute intlab( 23 ,1)= 'Int_23'.


compute intlab( 24 ,1)= 'Int_24'.


compute intlab( 25 ,1)= 'Int_25'.


compute intlab( 26 ,1)= 'Int_26'.


compute intlab( 27 ,1)= 'Int_27'.


compute intlab( 28 ,1)= 'Int_28'.


compute intlab( 29 ,1)= 'Int_29'.


compute intlab( 30 ,1)= 'Int_30'.


compute intlab( 31 ,1)= 'Int_31'.


compute intlab( 32 ,1)= 'Int_32'.


compute intlab( 33 ,1)= 'Int_33'.


compute intlab( 34 ,1)= 'Int_34'.


compute intlab( 35 ,1)= 'Int_35'.


compute intlab( 36 ,1)= 'Int_36'.


compute intlab( 37 ,1)= 'Int_37'.


compute intlab( 38 ,1)= 'Int_38'.


compute intlab( 39 ,1)= 'Int_39'.


compute intlab( 40 ,1)= 'Int_40'.


compute intlab( 41 ,1)= 'Int_41'.


compute intlab( 42 ,1)= 'Int_42'.


compute intlab( 43 ,1)= 'Int_43'.


compute intlab( 44 ,1)= 'Int_44'.


compute intlab( 45 ,1)= 'Int_45'.


compute intlab( 46 ,1)= 'Int_46'.


compute intlab( 47 ,1)= 'Int_47'.


compute intlab( 48 ,1)= 'Int_48'.


compute intlab( 49 ,1)= 'Int_49'.


compute intlab( 50 ,1)= 'Int_50'.


compute intlab( 51 ,1)= 'Int_51'.


compute intlab( 52 ,1)= 'Int_52'.


compute intlab( 53 ,1)= 'Int_53'.


compute intlab( 54 ,1)= 'Int_54'.


compute intlab( 55 ,1)= 'Int_55'.


compute intlab( 56 ,1)= 'Int_56'.


compute intlab( 57 ,1)= 'Int_57'.


compute intlab( 58 ,1)= 'Int_58'.


compute intlab( 59 ,1)= 'Int_59'.


compute intlab( 60 ,1)= 'Int_60'.


compute intlab( 61 ,1)= 'Int_61'.


compute intlab( 62 ,1)= 'Int_62'.


compute intlab( 63 ,1)= 'Int_63'.


compute intlab( 64 ,1)= 'Int_64'.


compute intlab( 65 ,1)= 'Int_65'.


compute intlab( 66 ,1)= 'Int_66'.


compute intlab( 67 ,1)= 'Int_67'.


compute intlab( 68 ,1)= 'Int_68'.


compute intlab( 69 ,1)= 'Int_69'.


compute intlab( 70 ,1)= 'Int_70'.


compute intlab( 71 ,1)= 'Int_71'.


compute intlab( 72 ,1)= 'Int_72'.


compute intlab( 73 ,1)= 'Int_73'.


compute intlab( 74 ,1)= 'Int_74'.


compute intlab( 75 ,1)= 'Int_75'.


compute intlab( 76 ,1)= 'Int_76'.


compute intlab( 77 ,1)= 'Int_77'.


compute intlab( 78 ,1)= 'Int_78'.


compute intlab( 79 ,1)= 'Int_79'.


compute intlab( 80 ,1)= 'Int_80'.


compute intlab( 81 ,1)= 'Int_81'.


compute intlab( 82 ,1)= 'Int_82'.


compute intlab( 83 ,1)= 'Int_83'.


compute intlab( 84 ,1)= 'Int_84'.


compute intlab( 85 ,1)= 'Int_85'.


compute intlab( 86 ,1)= 'Int_86'.


compute intlab( 87 ,1)= 'Int_87'.


compute intlab( 88 ,1)= 'Int_88'.


compute intlab( 89 ,1)= 'Int_89'.


compute intlab( 90 ,1)= 'Int_90'.


compute intlab( 91 ,1)= 'Int_91'.


compute intlab( 92 ,1)= 'Int_92'.


compute intlab( 93 ,1)= 'Int_93'.


compute intlab( 94 ,1)= 'Int_94'.


compute intlab( 95 ,1)= 'Int_95'.


compute intlab( 96 ,1)= 'Int_96'.


compute intlab( 97 ,1)= 'Int_97'.


compute intlab( 98 ,1)= 'Int_98'.


compute intlab( 99 ,1)= 'Int_99'.


compute intlab( 100 ,1)= 'Int_100'.

compute bcmat=make(needed,needed,0).
compute wcmat=make(needed,needed,0).
compute zcmat=make(needed,needed,0).
compute wzcmat=make(needed,needed,0).
compute wsum=0.
compute zsum=0.
compute wzsum=0.
end if.
end if.
do if (criterr = 0 and model <> 999).
compute modelmat= {1,0,0,0,0,0,0,1,0,0;2,0,0,0,0,0,0,1,1,0;3,0,0,0,0,0,0,1,1,1;4,0,0,0,0,0,0,0,0,0; 5,0,0,0,0,0,0,1,0,0;6,0,0,0,0,0,0,0,0,0;7,1,0,0,0,0,0,0,0,0;8,1,0,0,0,0,0,1,0,0; 9,1,1,0,0,0,0,0,0,0;10,1,1,0,0,0,0,1,1,0;11,1,1,1,0,0,0,0,0,0;12,1,1,1,0,0,0,1,1,1; 13,1,1,1,0,0,0,1,0,0;14,0,0,0,1,0,0,0,0,0;15,0,0,0,1,0,0,1,0,0;16,0,0,0,1,1,0,0,0,0; 17,0,0,0,1,1,0,1,1,0;18,0,0,0,1,1,1,0,0,0;19,0,0,0,1,1,1,1,1,1;20,0,0,0,1,1,1,1,0,0; 21,1,0,0,0,1,0,0,0,0;22,1,0,0,0,1,0,1,0,0;23,0,0,0,0,0,0,0,0,0;24,0,0,0,0,0,0,0,0,0; 25,0,0,0,0,0,0,0,0,0;26,0,0,0,0,0,0,0,0,0;27,0,0,0,0,0,0,0,0,0;28,1,0,0,0,1,0,0,1,0; 29,1,0,0,0,1,0,1,1,0;30,0,0,0,0,0,0,0,0,0;31,0,0,0,0,0,0,0,0,0;32,0,0,0,0,0,0,0,0,0; 33,0,0,0,0,0,0,0,0,0;34,0,0,0,0,0,0,0,0,0;35,0,0,0,0,0,0,0,0,0;36,0,0,0,0,0,0,0,0,0; 37,0,0,0,0,0,0,0,0,0;38,0,0,0,0,0,0,0,0,0;39,0,0,0,0,0,0,0,0,0;40,0,0,0,0,0,0,0,0,0; 41,0,0,0,0,0,0,0,0,0;42,0,0,0,0,0,0,0,0,0;43,0,0,0,0,0,0,0,0,0;44,0,0,0,0,0,0,0,0,0; 45,0,0,0,0,0,0,0,0,0;46,0,0,0,0,0,0,0,0,0;47,0,0,0,0,0,0,0,0,0;48,0,0,0,0,0,0,0

 ,0,0; 49,0,0,0,0,0,0,0,0,0;50,0,0,0,0,0,0,0,0,0;51,0,0,0,0,0,0,0,0,0;52,0,0,0,0,0,0,0,0,0; 53,0,0,0,0,0,0,0,0,0;54,0,0,0,0,0,0,0,0,0;55,0,0,0,0,0,0,0,0,0;56,0,0,0,0,0,0,0,0,0; 57,0,0,0,0,0,0,0,0,0;58,1,0,0,1,0,0,0,0,0;59,1,0,0,1,0,0,1,0,0;60,1,1,0,1,0,0,0,0,0; 61,1,1,0,1,0,0,1,0,0;62,1,1,0,1,0,0,0,1,0;63,1,1,0,1,0,0,1,1,0;64,1,0,0,1,1,0,0,0,0; 65,1,0,0,1,1,0,1,0,0;66,1,0,0,1,1,0,0,1,0;67,1,0,0,1,1,0,1,1,0;68,1,1,1,1,0,0,0,0,0; 69,1,1,1,1,0,0,1,1,1;70,1,0,0,1,1,1,0,0,0;71,1,0,0,1,1,1,1,1,1;72,1,1,1,1,1,1,0,0,0; 73,1,1,1,1,1,1,1,1,1;74,0,0,0,1,0,0,0,0,0;75,1,1,0,1,1,0,0,0,0;76,1,1,0,1,1,0,1,1,0; 77,0,0,0,0,0,0,0,0,0;78,0,0,0,0,0,0,0,0,0;79,0,0,0,0,0,0,0,0,0;80,0,0,0,0,0,0,0,0,0; 81,0,0,0,0,0,0,0,0,0;82,0,0,0,0,0,0,0,0,0;83,1,0,0,0,0,0,0,0,0;84,1,0,0,0,0,0,0,0,0; 85,1,0,0,0,0,0,1,0,0;86,1,0,0,0,0,0,1,0,0;87,0,0,0,1,0,0,0,0,0;88,0,0,0,1,0,0,0,0,0; 89,0,0,0,1,0,0,1,0,0;90,0,0,0,1,0,0,1,0,0;91,0,0,0,0,0,0,0,0,0;92,1,0,0,1,0,0,1,0,0}.
do if (model > 0).
compute tmp=modelmat(model,2:ncol(modelmat)).
end if.
do if (model=0).
compute tmp={0,0,0,0,0,0,0,0,0}.
end if.
do if (model < 4).
compute bcmat((nxs+1),1)=1.
end if.
do if ((model > 3) and (model <> 6)).
compute bcmat((nxs+1):(nxs+nms),1)=onem.
compute bcmat(nrow(bcmat),(nxs+1):(nxs+nms))=t(onem).
compute bcmat(nrow(bcmat),1)=1.
end if.
do if ((model = 6) or (model > 82 and model < 93)).
loop j = 2 to nrow(bcmat).
loop i = 1 to (j-1).
compute bcmat(j,i)=1.
end loop.
end loop.
end if.
do if (model = 80).
loop i = 1 to nms.
compute bcmat((nrow(bcmat)-1),i)=1.
end loop.
end if.
do if (model = 81).
loop j = 3 to nrow(bcmat).
compute bcmat(j,2)=1.
end loop.
end if.
do if (model = 82).
compute bcmat(3,2)=1.
compute bcmat(5,4)=1.
end if.
do if (tmp(1,1)=1).
compute wcmat((nxs+1):(nxs+nms),1)=onem.
compute wprod=1.
compute xprod=1.
do if (model = 83 or model = 86).
compute onemsx=onem.
loop i = 1 to (nms-1).
compute onemsx(i+1,1)=0.
end loop.
compute wcmat((nxs+1):(nxs+nms),1)=onemsx.
end if.
end if.
do if (tmp(1,4)=1).
compute wcmat(nrow(wcmat),(nxs+1):(nxs+nms))=t(onem).
compute wprod=1.
do if (model = 87 or model = 90).
compute onemsx=onem.
loop i = 1 to (nms-1).
compute onemsx(i,1)=0.
end loop.
compute wcmat(nrow(wcmat),(nxs+1):(nxs+nms))=t(onemsx).
end if.
end if.
do if (tmp(1,7)=1).
compute wcmat(nrow(wcmat),1)=1.
compute wprod=1.
compute xprod=1.
end if.
do if (tmp(1,2)=1).
compute zcmat((nxs+1):(nxs+nms),1)=onem.
compute zprod=1.
compute xprod=1.
end if.
do if (tmp(1,5)=1).
compute zcmat(nrow(zcmat),(nxs+1):(nxs+nms))=t(onem).
compute zprod=1.
end if.
do if (tmp(1,8)=1).
compute zcmat(nrow(zcmat),1)=1.
compute zprod=1.
compute xprod=1.
end if.
do if (tmp(1,3)=1).
compute wzcmat((nxs+1):(nxs+nms),1)=onem.
compute xprod=1.
compute wprod=1.
compute zprod=1.
end if.
do if (tmp(1,6)=1).
compute wzcmat(nrow(wzcmat),(nxs+1):(nxs+nms))=t(onem).
compute zprod=1.
compute wprod=1.
end if.
do if (tmp(1,9)=1).
compute wzcmat(nrow(wzcmat),1)=1.
compute xprod=1.
compute wprod=1.
compute zprod=1.
end if.
do if (model = 91 or model = 92).
loop j = 1 to (nms-1).
loop i = 1 to j.
compute wcmat((nxs+1+j),(nxs+i))=1.
end loop.
end loop.
end if.
do if (nms < 0).
loop i = 1 to nms.
compute tmp=csum(wcmat(:,(1+i)))+csum(zcmat(:,(1+i)))+csum(wzcmat(:,(1+i))).
compute mprod(1,i)=(tmp>0).
end loop.
end if.
end if.
do if (ncs > 0).
compute ccmat=make((nms+nys),ncs,1).
compute ccmatoff=ccmat.
do if (covmy=1).
compute ccmat(nrow(ccmat),:)=make(1,ncs,0).
end if.
do if (covmy=2).
compute ccmat(1:nms,:)=make(nms,ncs,0).
end if.
do if (cmatrix(1,1) <> -999).
do if (ncol(cmatrix) <> ((nms+nys)*ncs)).
compute errcode(errs,1)=29.
compute errs=errs+1.
compute criterr=1.
end if.
do if (criterr = 0).
compute tmp=1.
loop i = 1 to (nms+nys).
loop j = 1 to ncs.
compute ccmat(i,j)=1-(cmatrix(1,tmp) = 0).
compute tmp=tmp+1.
end loop.
end loop.
do if (rsum((csum(ccmat)=0)) <> 0).
compute errcode(errs,1)=30.
compute errs=errs+1.
compute criterr=1.
end if.
end if.
do if (covmy <> 0).
compute notecode(notes,1)=1.
compute notes=notes+1.
end if.
end if.
do if (xmint=1 and (covmy > 0 or (csum(rsum(ccmat)) <> ((nms+nys)*ncs)))).
compute errcode(errs,1)=68.
compute errs=errs+1.
compute criterr=1.
end if.
end if.
do if (criterr=0).
compute needed=needed*(needed-1)/2.
compute nopath=0.
do if (bmatrix(1,1) <> -999).
compute tmp=1.
do if ((ncol(bmatrix) <> needed) or (csum(rsum(bmatrix))=0)).
compute errcode(errs,1)=16.
compute errs=errs+1.
compute criterr=1.
else.
loop i = 2 to nrow(bcmat).
loop j = 1 to (i-1).
compute bcmat(i,j)=1-(bmatrix(1,tmp) = 0).
compute tmp=tmp+1.
end loop.
end loop.
end if.
do if ((csum(bcmat(:,1))=0) and criterr=0).
compute errcode(errs,1)=22.
compute errs=errs+1.
compute criterr=1.
end if.
do if ((rsum(bcmat(nrow(bcmat),:))=0) and criterr=0).
compute errcode(errs,1)=23.
compute errs=errs+1.
compute criterr=1.
end if.
compute dm=0.
do if (nms > 0).
loop i = 1 to nms.
do if (((rsum(bcmat((nxs+i),:)) = 0) or (csum(bcmat(:,(nxs+i))) = 0)) and (dm=0) and (criterr=0)).
compute errcode(errs,1)=26.
compute errs=errs+1.
compute criterr=1.
compute dm=1.
end if.
end loop.
end if.
release dm.
end if.
end if.
do if (criterr=0).
do if (wmatrix(1,1) <> -999).
compute tmp=1.
do if (ncol(wmatrix) <> needed).
compute errcode(errs,1)=17.
compute errs=errs+1.
compute criterr=1.
else.
compute modelvar(1,1)='CUSTOM'.
loop i = 2 to nrow(wcmat).
loop j = 1 to (i-1).
compute wcmat(i,j)=1-(wmatrix(1,tmp) = 0).
do if ((wcmat(i,j)=1) and (bcmat(i,j)=0) and (nopath=0)).
compute errcode(errs,1)=20.
compute errs=errs+1.
compute criterr=1.
compute nopath=1.
end if.
compute tmp=tmp+1.
end loop.
end loop.
end if.
end if.
do if (zmatrix(1,1) <> -999).
compute tmp=1.
do if (ncol(zmatrix) <> needed).
compute errcode(errs,1)=18.
compute errs=errs+1.
compute criterr=1.
else.
compute modelvar(1,1)='CUSTOM'.
do if (csum(rsum(wcmat))=0 and model=999).
compute errcode(errs,1)=21.
compute errs=errs+1.
compute criterr=1.
end if.
loop i = 2 to nrow(zcmat).
loop j = 1 to (i-1).
compute zcmat(i,j)=1-(zmatrix(1,tmp) = 0).
do if ((zcmat(i,j)=1) and (bcmat(i,j)=0) and (nopath=0)).
compute errcode(errs,1)=20.
compute errs=errs+1.
compute criterr=1.
compute nopath=1.
end if.
compute tmp=tmp+1.
end loop.
end loop.
end if.
end if.
compute tmp=1.
do if (wzmatrix(1,1) <> -999).
do if (ncol(wzmatrix) <> needed).
compute errcode(errs,1)=19.
compute errs=errs+1.
compute criterr=1.
end if.
compute modelvar(1,1)='CUSTOM'.
end if.
do if (criterr=0).
loop i = 2 to nrow(wzcmat).
loop j = 1 to (i-1).
do if (wzmatrix(1,1) <> -999).
compute wzcmat(i,j)=1-(wzmatrix(1,tmp) = 0).
end if.
do if (wzcmat(i,j)=1).
compute wcmat(i,j)=1.
compute zcmat(i,j)=1.
end if.
do if ((wzcmat(i,j)=1) and (bcmat(i,j)=0) and (nopath=0)).
compute errcode(errs,1)=20.
compute errs=errs+1.
compute criterr=1.
compute nopath=1.
end if.
compute tmp=tmp+1.
end loop.
end loop.
end if.
end if.
do if (criterr=0).
compute xprod=csum(wcmat(:,1))+csum(zcmat(:,1))+csum(wzcmat(:,1)).
compute xprod=(xprod > 0).
compute wsum=csum(rsum(wcmat)).
compute wprod=(wsum > 0).
do if (nms > 0).
loop i = 1 to nms.
compute tmp=csum(wcmat(:,(1+i)))+csum(zcmat(:,(1+i)))+csum(wzcmat(:,(1+i))).
compute mprod(1,i)=(tmp>0).
end loop.
end if.
do if ((wsum > 0) and (w = 'xxxxx')).
compute errcode(errs,1)=11.
compute errs=errs+1.
compute criterr=1.
end if.
do if ((wsum = 0) and (w <> 'xxxxx')).
compute errcode(errs,1)=10.
compute errs=errs+1.
compute criterr=1.
end if.
compute zsum=csum(rsum(zcmat)).
compute zprod=(zsum > 0).
do if ((zsum > 0) and (z = 'xxxxx')).
compute errcode(errs,1)=13.
compute errs=errs+1.
compute criterr=1.
end if.
do if ((zsum = 0) and (z <> 'xxxxx')).
compute errcode(errs,1)=12.
compute errs=errs+1.
compute criterr=1.
end if.
do if ((zsum > 0) and (wsum = 0)).
compute errcode(errs,1)=35.
compute errs=errs+1.
compute criterr=1.
end if.
end if.
do if (criterr=0 and nms > 1).
compute serchk=bcmat(2:(nrow(bcmat)-1),2:ncol(bcmat)).
do if (csum(rsum(serchk))) > 0.
compute serial=1.
do if (nms > 6).
compute errcode(errs,1)=36.
compute errs=errs+1.
compute criterr=1.
end if.
end if.
end if.
do if (center > 0 and criterr=0).
compute centvar={' '}.
do if (criterr=0).
do if ((center = 1) or (center = 2 and wdich = 0)).
do if (wprod=1 and mcwok=0 and nwpval > 0).
loop i = 1 to nws.
compute wtmp(:,i)=wtmp(:,i)-(csum(wtmp(:,i))/n).
compute centvar={centvar,wnames(1,i)}.
end loop.

.
compute desctmp=make((8-(4* wmodcust )),ncol( wtmp ),-999).
loop jd=1 to ncol( wtmp ).
compute descdat= wtmp (:,jd).
compute desctmp(1,jd) = csum(descdat)/nrow(descdat).
compute desctmp(2,jd) = (nrow(descdat)*sscp(descdat))-(t(csum(descdat))*(csum(descdat))).
compute desctmp(2,jd) = sqrt(desctmp(2,jd)/(nrow(descdat)*(nrow(descdat)-1))).
compute desctmp(3,jd)=cmin(descdat).
compute desctmp(4,jd)=cmax(descdat).
do if ( wmodcust =0).
compute minwarn=0.
compute maxwarn=0.
do if ((desctmp(3,jd)=desctmp(4,jd)) and novar=0).
compute errcode(errs,1)=15.
compute errs=errs+1.
compute criterr=1.
compute novar=1.
end if.
compute tmp=((descdat(:,1)=desctmp(3,jd))+(descdat(:,1)=desctmp(4,jd))).
compute desctmp(8,jd)=(csum(tmp)=nrow(tmp)).
compute tmp = descdat.
compute tmp(GRADE(descdat),:) = descdat.
compute descdat = tmp.
release tmp.
compute decval={.16;.5;.84}.
loop kd=1 to 3.
compute low=trunc(decval(kd,1)*(nrow(descdat)+1)).
compute lowdec=decval(kd,1)*(nrow(descdat)+1)-low.
compute value=descdat(low,1)+(descdat((low+1),1)-descdat(low,1))*lowdec.
compute desctmp((4+kd),jd)=value.
end loop.
compute mnotev=1.
compute modvals=desctmp(5:7,:).
do if (quantile <> 1).
compute desctmp(5,jd)=desctmp(1,jd)-desctmp(2,jd).
compute desctmp(6,jd)=desctmp(1,jd).
compute desctmp(7,jd)=desctmp(1,jd)+desctmp(2,jd).
compute modvals=desctmp(5:7,:).
compute mnotev=2.
do if (modvals(1,1) < desctmp(3,1)).
compute modvals(1,1)=desctmp(3,1).
compute minwarn=1.
end if.
do if (modvals(3,1) > desctmp(4,1)).
compute modvals(3,1)=desctmp(4,1).
compute maxwarn=1.
end if.
end if.
do if (desctmp(8,1)=1).
compute modvals={desctmp(3,1);desctmp(4,1)}.
compute mnotev=0.
compute minwarn=0.
compute maxwarn=0.
end if.
end if.
end loop
.
compute wmin=desctmp(3,1).
compute wmax=desctmp(4,1).
do if (wmodcust=0).
compute wmodvals=modvals.
compute wprobval=wmodvals.
end if.
end if.
end if.
do if ((center = 1) or (center = 2 and zdich = 0)).
do if (zprod=1 and mczok=0 and nzpval > 0).
loop i = 1 to nzs.
compute ztmp(:,i)=ztmp(:,i)-(csum(ztmp(:,i))/n).
compute centvar={centvar,znames(1,i)}.
end loop.

.
compute desctmp=make((8-(4* zmodcust )),ncol( ztmp ),-999).
loop jd=1 to ncol( ztmp ).
compute descdat= ztmp (:,jd).
compute desctmp(1,jd) = csum(descdat)/nrow(descdat).
compute desctmp(2,jd) = (nrow(descdat)*sscp(descdat))-(t(csum(descdat))*(csum(descdat))).
compute desctmp(2,jd) = sqrt(desctmp(2,jd)/(nrow(descdat)*(nrow(descdat)-1))).
compute desctmp(3,jd)=cmin(descdat).
compute desctmp(4,jd)=cmax(descdat).
do if ( zmodcust =0).
compute minwarn=0.
compute maxwarn=0.
do if ((desctmp(3,jd)=desctmp(4,jd)) and novar=0).
compute errcode(errs,1)=15.
compute errs=errs+1.
compute criterr=1.
compute novar=1.
end if.
compute tmp=((descdat(:,1)=desctmp(3,jd))+(descdat(:,1)=desctmp(4,jd))).
compute desctmp(8,jd)=(csum(tmp)=nrow(tmp)).
compute tmp = descdat.
compute tmp(GRADE(descdat),:) = descdat.
compute descdat = tmp.
release tmp.
compute decval={.16;.5;.84}.
loop kd=1 to 3.
compute low=trunc(decval(kd,1)*(nrow(descdat)+1)).
compute lowdec=decval(kd,1)*(nrow(descdat)+1)-low.
compute value=descdat(low,1)+(descdat((low+1),1)-descdat(low,1))*lowdec.
compute desctmp((4+kd),jd)=value.
end loop.
compute mnotev=1.
compute modvals=desctmp(5:7,:).
do if (quantile <> 1).
compute desctmp(5,jd)=desctmp(1,jd)-desctmp(2,jd).
compute desctmp(6,jd)=desctmp(1,jd).
compute desctmp(7,jd)=desctmp(1,jd)+desctmp(2,jd).
compute modvals=desctmp(5:7,:).
compute mnotev=2.
do if (modvals(1,1) < desctmp(3,1)).
compute modvals(1,1)=desctmp(3,1).
compute minwarn=1.
end if.
do if (modvals(3,1) > desctmp(4,1)).
compute modvals(3,1)=desctmp(4,1).
compute maxwarn=1.
end if.
end if.
do if (desctmp(8,1)=1).
compute modvals={desctmp(3,1);desctmp(4,1)}.
compute mnotev=0.
compute minwarn=0.
compute maxwarn=0.
end if.
end if.
end loop
.
compute zmin=desctmp(3,1).
compute zmax=desctmp(4,1).
do if (zmodcust=0).
compute zmodvals=modvals.
compute zprobval=zmodvals.
end if.
end if.
end if.
do if ((center = 1) or (center = 2 and xdich = 0)).
do if (xprod=1 and mcxok=0).
loop i = 1 to nxs.
compute xtmp(:,i)=xtmp(:,i)-(csum(xtmp(:,i))/n).
compute centvar={centvar,xnames(1,i)}.
end loop.

.
compute desctmp=make((8-(4* 0 )),ncol( xtmp ),-999).
loop jd=1 to ncol( xtmp ).
compute descdat= xtmp (:,jd).
compute desctmp(1,jd) = csum(descdat)/nrow(descdat).
compute desctmp(2,jd) = (nrow(descdat)*sscp(descdat))-(t(csum(descdat))*(csum(descdat))).
compute desctmp(2,jd) = sqrt(desctmp(2,jd)/(nrow(descdat)*(nrow(descdat)-1))).
compute desctmp(3,jd)=cmin(descdat).
compute desctmp(4,jd)=cmax(descdat).
do if ( 0 =0).
compute minwarn=0.
compute maxwarn=0.
do if ((desctmp(3,jd)=desctmp(4,jd)) and novar=0).
compute errcode(errs,1)=15.
compute errs=errs+1.
compute criterr=1.
compute novar=1.
end if.
compute tmp=((descdat(:,1)=desctmp(3,jd))+(descdat(:,1)=desctmp(4,jd))).
compute desctmp(8,jd)=(csum(tmp)=nrow(tmp)).
compute tmp = descdat.
compute tmp(GRADE(descdat),:) = descdat.
compute descdat = tmp.
release tmp.
compute decval={.16;.5;.84}.
loop kd=1 to 3.
compute low=trunc(decval(kd,1)*(nrow(descdat)+1)).
compute lowdec=decval(kd,1)*(nrow(descdat)+1)-low.
compute value=descdat(low,1)+(descdat((low+1),1)-descdat(low,1))*lowdec.
compute desctmp((4+kd),jd)=value.
end loop.
compute mnotev=1.
compute modvals=desctmp(5:7,:).
do if (quantile <> 1).
compute desctmp(5,jd)=desctmp(1,jd)-desctmp(2,jd).
compute desctmp(6,jd)=desctmp(1,jd).
compute desctmp(7,jd)=desctmp(1,jd)+desctmp(2,jd).
compute modvals=desctmp(5:7,:).
compute mnotev=2.
do if (modvals(1,1) < desctmp(3,1)).
compute modvals(1,1)=desctmp(3,1).
compute minwarn=1.
end if.
do if (modvals(3,1) > desctmp(4,1)).
compute modvals(3,1)=desctmp(4,1).
compute maxwarn=1.
end if.
end if.
do if (desctmp(8,1)=1).
compute modvals={desctmp(3,1);desctmp(4,1)}.
compute mnotev=0.
compute minwarn=0.
compute maxwarn=0.
end if.
end if.
end loop
.
compute xmodvals=modvals.
compute xprobval=xmodvals.
end if.
end if.
do if (nms > 0).
loop i = 1 to nms.
do if (mprod(1,i)=1).
compute mtmp(:,i)=mtmp(:,i)-(csum(mtmp(:,i))/n).
compute centvar={centvar,mnames(1,i)}.
end if.
end loop.

.
compute desctmp=make((8-(4* 0 )),ncol( mtmp ),-999).
loop jd=1 to ncol( mtmp ).
compute descdat= mtmp (:,jd).
compute desctmp(1,jd) = csum(descdat)/nrow(descdat).
compute desctmp(2,jd) = (nrow(descdat)*sscp(descdat))-(t(csum(descdat))*(csum(descdat))).
compute desctmp(2,jd) = sqrt(desctmp(2,jd)/(nrow(descdat)*(nrow(descdat)-1))).
compute desctmp(3,jd)=cmin(descdat).
compute desctmp(4,jd)=cmax(descdat).
do if ( 0 =0).
compute minwarn=0.
compute maxwarn=0.
do if ((desctmp(3,jd)=desctmp(4,jd)) and novar=0).
compute errcode(errs,1)=15.
compute errs=errs+1.
compute criterr=1.
compute novar=1.
end if.
compute tmp=((descdat(:,1)=desctmp(3,jd))+(descdat(:,1)=desctmp(4,jd))).
compute desctmp(8,jd)=(csum(tmp)=nrow(tmp)).
compute tmp = descdat.
compute tmp(GRADE(descdat),:) = descdat.
compute descdat = tmp.
release tmp.
compute decval={.16;.5;.84}.
loop kd=1 to 3.
compute low=trunc(decval(kd,1)*(nrow(descdat)+1)).
compute lowdec=decval(kd,1)*(nrow(descdat)+1)-low.
compute value=descdat(low,1)+(descdat((low+1),1)-descdat(low,1))*lowdec.
compute desctmp((4+kd),jd)=value.
end loop.
compute mnotev=1.
compute modvals=desctmp(5:7,:).
do if (quantile <> 1).
compute desctmp(5,jd)=desctmp(1,jd)-desctmp(2,jd).
compute desctmp(6,jd)=desctmp(1,jd).
compute desctmp(7,jd)=desctmp(1,jd)+desctmp(2,jd).
compute modvals=desctmp(5:7,:).
compute mnotev=2.
do if (modvals(1,1) < desctmp(3,1)).
compute modvals(1,1)=desctmp(3,1).
compute minwarn=1.
end if.
do if (modvals(3,1) > desctmp(4,1)).
compute modvals(3,1)=desctmp(4,1).
compute maxwarn=1.
end if.
end if.
do if (desctmp(8,1)=1).
compute modvals={desctmp(3,1);desctmp(4,1)}.
compute mnotev=0.
compute minwarn=0.
compute maxwarn=0.
end if.
end if.
end loop
.
do if ((cdeval(1,1)=-999) and (model=74)).
compute medmeans=desctmp(1,:).
end if.
compute mmodvals=modvals.
compute mprobval=mmodvals.
end if.
end if.
do if (ncol(centvar) > 1).
compute notecode(notes,1)=3.
compute notes=notes+1.
end if.
end if.
do if (criterr=0).
compute wsum=rsum(csum(wcmat)).
compute zsum=rsum(csum(zcmat)).
compute wzsum=rsum(csum(wzcmat)).
compute nump=make(1,(nys+nms),-999).
compue numint=make(1,(nys+nms),0).
compute datcount=1.
compute xtmpuse=0.
compute wtmpuse=0.
compute ztmpuse=0.
compute xwtmpus=0.
compute xztmpus=0.
compute wztmpus=0.
compute xwztmpu=0.
compute xtmploc=-999.
compute wtmploc=-999.
compute xwtmplo=-999.
compute ztmploc=-999.
compute xztmplo=-999.
compute wztmplo=-999.
compute xwztmplo=-999.
compute vlabs={' '}.
do if (ncs > 0).
compute ctmpuse=make(1,ncs,0).
end if.
do if (nms > 0).
compute mtmpuse=make(1,nms,0).
compute mwtmpus=make(1,nms,0).
compute mztmpus=make(1,nms,0).
compute mwztmpu=make(1,nms,0).
compute mtmploc=make(1,nms,0).
compute mwtmplo=make(nwvls,nms,-999).
compute mztmplo=make(nzvls,nms,-999).
compute mwztmplo=make((nwvls*nzvls),nms,-999).
end if.
do if (ncs > 0).
compute ctmploc=make(1,ncs,0).
end if.
compute fulldat=make(n,1,1).
compute datindx=make(1000,(nms+nys),-999).
compute wherew=make(2,(nms+nys),-999).
compute wherex=make(2,(nms+nys),-999).
compute wherez=make(2,(nms+nys),-999).
compute wherexw=make(2,(nms+nys),-999).
compute wherexz=make(2,(nms+nys),-999).
compute wherewz=make(2,(nms+nys),-999).
compute wherexwz=make(2,(nms+nys),-999).
do if (nms > 0).
compute wherem=make(nms,(nms+nys),-999).
compute wheremw = make(nms*2,(nms+nys),-999).
compute wheremz = make(nms*2,(nms+nys),-999).
compute wheremwz = make(nms*2,(nms+nys),-999).
end if.
compute wzhigh=make(1000,(((nms+1)*(nms+2))/2),0).
compute whigh=make(1000,(((nms+1)*(nms+2))/2),0).
compute zhigh=make(1000,(((nms+1)*(nms+2))/2),0).
compute fochigh=make(1000,(((nms+1)*(nms+2))/2),0).
compute xcoefloc={1;2;3;4;5;6;7;8;9}.
compute intkey = {' ', ' ', ' ', ' ', ' ', ' ', ' '}.
compute wzhighct=0.
compute whighct=0.
compute zhighct=0.
compute foccnt=0.
loop i = 2 to nrow(bcmat).
compute wdid=0.
compute zdid=0.
compute wzdid=0.
compute cntmp=1.
compute start=1.
do if (i < nrow(bcmat)).
compute outv=mtmp(:,(i-1)).
compute modlabel={mnames(1,(i-1));'constant'}.
end if.
do if (i = nrow(bcmat)).
compute outv=ytmp.
compute modlabel={ynames;'constant'}.
end if.
loop j = 1 to (i-1).
compute foccnt=foccnt+1.
do if (j = 1 and bcmat(i,j)=1).
compute outv={outv,xtmp}.
compute modlabel={modlabel;xcatlab(1:nxvls,1)}.
do if (xtmpuse=0).
compute fulldat={fulldat,xtmp}.
compute xtmpuse=1.
loop k4=datcount to (datcount+(nxvls-1)).
compute xtmploc={xtmploc;k4}.
end loop.
compute xtmploc=xtmploc(2:nrow(xtmploc),1).
compute datcount=datcount+nxvls.
end if.
compute datindx(start:(start+nrow(xtmploc)-1),(i-1))=xtmploc.
compute wherex(1,(i-1))=start+1.
compute wherex(2,(i-1))=start+nrow(xtmploc)-1+1.
do if (model = 74).
end if.
compute onebl=make(nrow(xtmploc),1,1).
compute fochigh((start+1):(start+nrow(xtmploc)),foccnt)=onebl.
compute start=start+nrow(xtmploc).
end if.
do if (j > 1 and bcmat(i,j)=1).
compute outv={outv,mtmp(:,(j-1))}.
compute modlabel={modlabel;mnames(1,(j-1))}.
do if (mtmpuse(1,(j-1))=0).
compute fulldat={fulldat,mtmp(:,(j-1))}.
compute mtmpuse(1,(j-1))=1.
compute mtmploc(1,(j-1))=datcount.
compute datcount=datcount+1.
end if.
compute datindx(start:(start+nrow(mtmploc)-1),(i-1))=mtmploc(1,(j-1)).
compute wherem((j-1),(i-1))=start+1.
compute onebl=make(nrow(mtmploc(1,j-1)),1,1).
compute ttt=nrow(mtmploc(1,(j-1)))+start-1.
compute fochigh((start+1):(start+nrow(mtmploc(1,(j-1)))),foccnt)=onebl.
compute start=start+nrow(mtmploc(1,(j-1))).
end if.
end loop.
do if (wsum > 0).
loop j = 1 to (i-1).
compute whighct=whighct+1.
do if (j = 1 and wcmat(i,j)=1).
do if (wdid=0).
compute outv={outv,wtmp}.
do if (ncs > 0 and wiscov > 0).
compute ccmatoff((i-1),wiscov)=0.
end if.
compute modlabel={modlabel;wcatlab(1:nwvls,1)}.
compute wdid=1.
do if (wtmpuse=0).
compute fulldat={fulldat,wtmp}.
do if (ncs > 0 and wiscov > 0).
compute ccmatoff((i-1),wiscov)=0.
end if.
compute wtmpuse=1.
loop k4=datcount to (datcount+(nwvls-1)).
compute wtmploc={wtmploc;k4}.
end loop.
compute wtmploc=wtmploc(2:nrow(wtmploc),1).
compute datcount=datcount+nwvls.
end if.
end if.
compute datindx(start:(start+nrow(wtmploc)-1),(i-1))=wtmploc.
compute wherew(1,(i-1))=start+1.
compute wherew(2,(i-1))=start+nrow(wtmploc)-1+1.
compute start=start+nrow(wtmploc).
loop k1=1 to nxvls.
loop k2 = 1 to nwvls.
compute outv={outv,(xtmp(:,k1)&*wtmp(:,k2))}.
do if (ncs > 0 and wiscov > 0).
compute ccmatoff((i-1),wiscov)=0.
end if.
compute modlabel={modlabel;intlab(cntmp,1)}.
compute intkey={intkey;intlab(cntmp,1),':',xcatlab(k1,1),'x',wcatlab(k2,1),' ',' '}.
compute cntmp=cntmp+1.
end loop.
end loop.
do if (xwtmpus=0).
compute fulldat={fulldat,outv(:,(ncol(outv)-(nxvls*nwvls)+1):ncol(outv))}.
compute xwtmpus=1.
do if (ncs > 0 and wiscov > 0).
compute ccmatoff((i-1),wiscov)=0.
end if.
loop k4=datcount to (datcount+((nwvls*nxvls)-1)).
compute xwtmplo={xwtmplo;k4}.
end loop.
compute xwtmplo=xwtmplo(2:nrow(xwtmplo),1).
compute datcount=datcount+(nxvls*nwvls).
end if.
compute datindx(start:(start+nrow(xwtmplo)-1),(i-1))=xwtmplo.
compute wherexw(1,(i-1))=start+1.
compute wherexw(2,(i-1))=start+nrow(xwtmplo)-1+1.
compute onebl=make(nrow(xwtmplo),1,1).
compute whigh((start+1):(start+nrow(xwtmplo)),whighct)=onebl.
compute start=start+nrow(xwtmplo).
end if.
do if (j > 1 and wcmat(i,j)=1).
do if (wdid=0 and model <> 74).
compute outv={outv,wtmp}.
do if (ncs > 0 and wiscov > 0).
compute ccmatoff((i-1),wiscov)=0.
end if.
compute modlabel={modlabel;wcatlab(1:nwvls,1)}.
compute wdid=1.
do if (wtmpuse=0).
compute fulldat={fulldat,wtmp}.
do if (ncs > 0 and wiscov > 0).
compute ccmatoff((i-1),wiscov)=0.
end if.
compute wtmpuse=1.
loop k4=datcount to (datcount+(nwvls-1)).
compute wtmploc={wtmploc;k4}.
end loop.
compute wtmploc=wtmploc(2:nrow(wtmploc),1).
compute datcount=datcount+nwvls.
end if.
compute datindx(start:(start+nrow(wtmploc)-1),(i-1))=wtmploc.
compute wherew(1,(i-1))=start+1.
compute wherew(2,(i-1))=start+nrow(wtmploc)-1+1.
compute start=start+nrow(wtmploc).
end if.
loop k2 = 1 to nwvls.
compute outv={outv,(mtmp(:,(j-1))&*wtmp(:,k2))}.
do if (ncs > 0 and wiscov > 0).
compute ccmatoff((i-1),wiscov)=0.
end if.
compute modlabel={modlabel;intlab(cntmp,1)}.
compute intkey={intkey;intlab(cntmp,1),':', mnames(1,(j-1)),'x',wcatlab(k2,1),' ',' '}.
compute cntmp=cntmp+1.
end loop.
do if (mwtmpus(1,(j-1))=0).
compute fulldat={fulldat,outv(:,(ncol(outv)-nwvls+1):ncol(outv))}.
do if (ncs > 0 and wiscov > 0).
compute ccmatoff((i-1),wiscov)=0.
end if.
compute mwtmpus(1,(j-1))=1.
compute mw22=-999.
loop k4=datcount to (datcount+(nwvls-1)).
compute mw22={mw22;k4}.
end loop.
compute mwtmplo(:,(j-1))=mw22(2:nrow(mw22),1).
compute datcount=datcount+nwvls.
end if.
compute datindx(start:(start+nrow(mwtmplo)-1),(i-1))=mwtmplo(:,(j-1)).
compute wheremw(((2*j)-3),(i-1))=start+1.
compute wheremw(((2*j)-2),(i-1))=start+nrow(mwtmplo)-1+1.
compute onebl=make(nrow(mwtmplo),1,1).
compute whigh((start+1):(start+nrow(mwtmplo)),whighct)=onebl.
compute start=start+nrow(mwtmplo).
end if.
end loop.
end if.
do if (zsum > 0).
loop j = 1 to (i-1).
compute zhighct=zhighct+1.
do if (j = 1 and zcmat(i,j)=1).
do if (zdid=0).
compute outv={outv,ztmp}.
do if (ncs > 0 and ziscov > 0).
compute ccmatoff((i-1),ziscov)=0.
end if.
compute modlabel={modlabel;zcatlab(1:nzvls,1)}.
compute zdid=1.
do if (ztmpuse=0).
compute fulldat={fulldat,ztmp}.
do if (ncs > 0 and ziscov > 0).
compute ccmatoff((i-1),ziscov)=0.
end if.
compute ztmpuse=1.
loop k4=datcount to (datcount+(nzvls-1)).
compute ztmploc={ztmploc;k4}.
end loop.
compute ztmploc=ztmploc(2:nrow(ztmploc),1).
compute datcount=datcount+nzvls.
end if.
end if.
compute datindx(start:(start+nrow(ztmploc)-1),(i-1))=ztmploc.
compute wherez(1,(i-1))=start+1.
compute wherez(2,(i-1))=start+nrow(ztmploc)-1+1.
compute start=start+nrow(ztmploc).
loop k1=1 to nxvls.
loop k2 = 1 to nzvls.
compute outv={outv,(xtmp(:,k1)&*ztmp(:,k2))}.
do if (ncs > 0 and ziscov > 0).
compute ccmatoff((i-1),ziscov)=0.
end if.
compute modlabel={modlabel;intlab(cntmp,1)}.
compute intkey={intkey;intlab(cntmp,1),':',xcatlab(k1,1),'x',zcatlab(k2,1),' ',' '}.
compute cntmp=cntmp+1.
end loop.
end loop.
do if (xztmpus=0).
compute fulldat={fulldat,outv(:,(ncol(outv)-(nxvls*nzvls)+1):ncol(outv))}.
do if (ncs > 0 and ziscov > 0).
compute ccmatoff((i-1),ziscov)=0.
end if.
compute xztmpus=1.
loop k4=datcount to (datcount+((nzvls*nxvls)-1)).
compute xztmplo={xztmplo;k4}.
end loop.
compute xztmplo=xztmplo(2:nrow(xztmplo),1).
compute datcount=datcount+(nxvls*nzvls).
end if.
compute datindx(start:(start+nrow(xztmplo)-1),(i-1))=xztmplo.
compute wherexz(1,(i-1))=start+1.
compute wherexz(2,(i-1))=start+nrow(xztmplo)-1+1.
compute onebl=make(nrow(xztmplo),1,1).
compute zhigh((start+1):(start+nrow(xztmplo)),zhighct)=onebl.
compute start=start+nrow(xztmplo).
end if.
do if (j > 1 and zcmat(i,j)=1).
do if (zdid=0).
compute outv={outv,ztmp}.
do if (ncs > 0 and ziscov > 0).
compute ccmatoff((i-1),ziscov)=0.
end if.
compute modlabel={modlabel;zcatlab(1:nzvls,1)}.
compute zdid=1.
do if (ztmpuse=0).
compute fulldat={fulldat,ztmp}.
do if (ncs > 0 and ziscov > 0).
compute ccmatoff((i-1),ziscov)=0.
end if.
compute ztmpuse=1.
loop k4=datcount to (datcount+(nzvls-1)).
compute ztmploc={ztmploc;k4}.
end loop.
compute ztmploc=ztmploc(2:nrow(ztmploc),1).
compute datcount=datcount+nzvls.
end if.
compute datindx(start:(start+nrow(ztmploc)-1),(i-1))=ztmploc.
compute wherez(1,(i-1))=start+1.
compute wherez(2,(i-1))=start+nrow(ztmploc)-1+1.
compute start=start+nrow(ztmploc).
end if.
loop k2 = 1 to nzvls.
compute outv={outv,(mtmp(:,(j-1))&*ztmp(:,k2))}.
do if (ncs > 0 and ziscov > 0).
compute ccmatoff((i-1),ziscov)=0.
end if.
compute modlabel={modlabel;intlab(cntmp,1)}.
compute intkey={intkey;intlab(cntmp,1),':', mnames(1,(j-1)),'x',zcatlab(k2,1),' ',' '}.
compute cntmp=cntmp+1.
end loop.
do if (mztmpus(1,(j-1))=0).
compute fulldat={fulldat,outv(:,(ncol(outv)-nzvls+1):ncol(outv))}.
do if (ncs > 0 and ziscov > 0).
compute ccmatoff((i-1),ziscov)=0.
end if.
compute mztmpus(1,(j-1))=1.
compute mz22=-999.
loop k4=datcount to (datcount+(nzvls-1)).
compute mz22={mz22;k4}.
end loop.
compute mztmplo(:,(j-1))=mz22(2:nrow(mz22),1).
compute datcount=datcount+nzvls.
end if.
compute datindx(start:(start+nrow(mztmplo)-1),(i-1))=mztmplo(:,(j-1)).
compute wheremz(((2*j)-3),(i-1))=start+1.
compute wheremz(((2*j)-2),(i-1))=start+nrow(mztmplo)-1+1.
compute onebl=make(nrow(mztmplo),1,1).
compute zhigh((start+1):(start+nrow(mztmplo)),zhighct)=onebl.
compute start=start+nrow(mztmplo).
end if.
end loop.
end if.
do if (wzsum > 0).
loop j = 1 to (i-1).
compute wzhighct=wzhighct+1.
do if (j = 1 and wzcmat(i,j)=1).
do if (wzdid=0).
loop k1=1 to nwvls.
loop k2 = 1 to nzvls.
compute outv={outv,(wtmp(:,k1)&*ztmp(:,k2))}.
do if (ncs > 0 and (ziscov > 0)).
compute ccmatoff((i-1),ziscov)=0.
end if.
do if (ncs > 0 and (wiscov > 0)).
compute ccmatoff((i-1),wiscov)=0.
end if.
compute modlabel={modlabel;intlab(cntmp,1)}.
compute intkey={intkey;intlab(cntmp,1),':',wcatlab(k1,1),'x',zcatlab(k2,1),' ',' '}.
compute cntmp=cntmp+1.
end loop.
end loop.
do if (wztmpus=0).
compute fulldat={fulldat,outv(:,(ncol(outv)-(nwvls*nzvls)+1):ncol(outv))}.
do if (ncs > 0 and (ziscov > 0)).
compute ccmatoff((i-1),ziscov)=0.
end if.
do if (ncs > 0 and (wiscov > 0)).
compute ccmatoff((i-1),wiscov)=0.
end if.
compute wztmpus=1.
loop k4=datcount to (datcount+((nwvls*nzvls)-1)).
compute wztmplo={wztmplo;k4}.
end loop.
compute wztmplo=wztmplo(2:nrow(wztmplo),1).
compute datcount=datcount+(nzvls*nwvls).
end if.
compute wzdid=1.
end if.
compute datindx(start:(start+nrow(wztmplo)-1),(i-1))=wztmplo.
compute wherewz(1,(i-1))=start+1.
compute wherewz(2,(i-1))=start+nrow(wztmplo)-1+1.
compute start=start+nrow(wztmplo).
loop k1=1 to nxvls.
loop k2=1 to nwvls.
loop k3=1 to nzvls.
compute outv={outv,(xtmp(:,k1)&*wtmp(:,k2)&*ztmp(:,k3))}.
do if (ncs > 0 and (ziscov > 0)).
compute ccmatoff((i-1),ziscov)=0.
end if.
do if (ncs > 0 and (wiscov > 0)).
compute ccmatoff((i-1),wiscov)=0.
end if.
compute modlabel={modlabel;intlab(cntmp,1)}.
compute intkey={intkey;intlab(cntmp,1),':',xcatlab(k1,1),'x',wcatlab(k2,1),'x', zcatlab(k3,1)}.
compute cntmp=cntmp+1.
end loop.
end loop.
end loop.
do if (xwztmpu=0).
compute fulldat={fulldat,outv(:,(ncol(outv)-(nxvls*nwvls*nzvls)+1):ncol(outv))}.
do if (ncs > 0 and (ziscov > 0)).
compute ccmatoff((i-1),ziscov)=0.
end if.
do if (ncs > 0 and (wiscov > 0)).
compute ccmatoff((i-1),wiscov)=0.
end if.
compute xwztmpu=1.
loop k4=datcount to (datcount+((nzvls*nxvls*nwvls)-1)).
compute xwztmplo={xwztmplo;k4}.
end loop.
compute xwztmplo=xwztmplo(2:nrow(xwztmplo),1).
compute datcount=datcount+(nxvls*nzvls*nwvls).
end if.
compute datindx(start:(start+nrow(xwztmplo)-1),(i-1))=xwztmplo.
compute wherexwz(1,(i-1))=start+1.
compute wherexwz(2,(i-1))=start+nrow(xwztmplo)-1+1.
compute onebl=make(nrow(xwztmplo),1,1).
compute wzhigh((start+1):(start+nrow(xwztmplo)),wzhighct)=onebl.
compute start=start+nrow(xwztmplo).
end if.
do if (j > 1 and wzcmat(i,j)=1).
do if (wzdid=0).
loop k1=1 to nwvls.
loop k2 = 1 to nzvls.
compute outv={outv,(wtmp(:,k1)&*ztmp(:,k2))}.
do if (ncs > 0 and (ziscov > 0)).
compute ccmatoff((i-1),ziscov)=0.
end if.
do if (ncs > 0 and (wiscov > 0)).
compute ccmatoff((i-1),wiscov)=0.
end if.
compute modlabel={modlabel;intlab(cntmp,1)}.
compute intkey={intkey;intlab(cntmp,1),':',wcatlab(k1,1),'x',zcatlab(k2,1),' ',' '}.
compute cntmp=cntmp+1.
end loop.
end loop.
do if (wztmpus=0).
compute fulldat={fulldat,outv(:,(ncol(outv)-(nwvls*nzvls)+1):ncol(outv))}.
do if (ncs > 0 and (ziscov > 0)).
compute ccmatoff((i-1),ziscov)=0.
end if.
do if (ncs > 0 and (wiscov > 0)).
compute ccmatoff((i-1),wiscov)=0.
end if.
compute wztmpus=1.
loop k4=datcount to (datcount+((nwvls*nzvls)-1)).
compute wztmplo={wztmplo;k4}.
end loop.
compute wztmplo=wztmplo(2:nrow(wztmplo),1).
compute datcount=datcount+(nzvls*nwvls).
end if.
compute wzdid=1.
compute datindx(start:(start+nrow(wztmplo)-1),(i-1))=wztmplo.
compute wherewz(1,(i-1))=start+1.
compute wherewz(2,(i-1))=start+nrow(wztmplo)-1+1.
compute start=start+nrow(wztmplo).
end if.
loop k1 = 1 to nwvls.
loop k2 = 1 to nzvls.
compute outv={outv,(mtmp(:,(j-1))&*wtmp(:,k1)&*ztmp(:,k2))}.
do if (ncs > 0 and (ziscov > 0)).
compute ccmatoff((i-1),ziscov)=0.
end if.
do if (ncs > 0 and (wiscov > 0)).
compute ccmatoff((i-1),wiscov)=0.
end if.
compute modlabel={modlabel;intlab(cntmp,1)}.
compute intkey={intkey;intlab(cntmp,1),':',mnames(1,(j-1)),'x',wcatlab(k1,1),'x', zcatlab(k2,1)}.
compute cntmp=cntmp+1.
end loop.
end loop.
do if (mwztmpu(1,(j-1))=0).
compute fulldat={fulldat,outv(:,(ncol(outv)-(nwvls*nzvls)+1):ncol(outv))}.
do if (ncs > 0 and (ziscov > 0)).
compute ccmatoff((i-1),ziscov)=0.
end if.
do if (ncs > 0 and (wiscov > 0)).
compute ccmatoff((i-1),wiscov)=0.
end if.
compute mwztmpu(1,(j-1))=1.
compute mz22=-999.
loop k4=datcount to (datcount+(nwvls*nzvls)-1).
compute mz22={mz22;k4}.
end loop.
compute mwztmplo(:,(j-1))=mz22(2:nrow(mz22),1).
compute datcount=datcount+(nwvls*nzvls).
end if.
compute datindx(start:(start+nrow(mwztmplo)-1),(i-1))=mwztmplo(:,(j-1)).
compute wheremwz(((2*j)-3),(i-1))=start+1.
compute wheremwz(((2*j)-2),(i-1))=start+nrow(mwztmplo)-1+1.
compute onebl=make(nrow(mwztmplo),1,1).
compute wzhigh((start+1):(start+nrow(mwztmplo)),wzhighct)=onebl.
compute start=start+nrow(mwztmplo).
end if.
end loop.
end if.
do if (ncs > 0).
compute ccmat=ccmat&*ccmatoff.
loop j = 1 to ncs.
do if (ccmat((i-1),j))=1.
do if (j=wiscov).
compute ctmp(:,j)=wtmp.
end if.
do if (j=ziscov).
compute ctmp(:,j)=ztmp.
end if.
compute outv={outv,ctmp(:,j)}.
compute modlabel={modlabel;covnames(1,j)}.
do if (ctmpuse(1,j)=0).
compute fulldat={fulldat,ctmp(:,j)}.
compute ctmpuse(1,j)=1.
compute ctmploc(1,j)=datcount.
compute datcount=datcount+1.
end if.
compute datindx(start:(start+nrow(ctmploc)-1),(i-1))=ctmploc(1,j).
compute start=start+nrow(ctmploc(1,j)).
end if.
end loop.
end if.
compute wdid=0.
compute zdid=0.
compute wzdid=0.
compute vlabs={vlabs;modlabel(2:nrow(modlabel),1)}.
compute numint(1,(i-1))=cntmp-1.
compute nump(1,(i-1))=nrow(modlabel)-1.
end loop.
release datcount, xtmpuse, wtmpuse, ztmpuse, xwtmpus, xztmpus, wztmpus, xwztmpu.
release xtmploc, wtmploc, xwtmplo, ztmploc, xztmplo, wztmplo, xwztmplo, foccnt.
do if (modcok=1 and ((nms > 0) or (zcmat(2,1) <> 1) or (mcx <> 0))).
compute notecode(notes,1) = 19.
compute notes = notes + 1.
compute modcok=0.
end if.
do if ((serial = 1 or (rsum(numint)>0) or nms=0) and mc > 0).
compute notecode(notes,1) = 15.
compute notes = notes + 1.
compute boot=mc.
compute mc=0.
end if.
do if (boot <> 0 or mc <> 0).
compute bootsz=boot.
do if (mc > 0).
compute bootsz=mc.
compute saveboot=0.
end if.
loop.
compute cilow = rnd(bootsz*(1-(conf/100))/2).
compute cihigh = trunc((bootsz*(conf/100)+(bootsz*(1-(conf/100))/2)))+1.
do if (cilow < 1 or cihigh > bootsz).
compute bootsz=trunc((bootsz+1000)/1000)*1000.
compute adjust = 1.
end if.
end loop if (cilow gt 0 and cihigh le bootsz).
do if (boot > 0).
compute boot=bootsz.
end if.
do if (mc > 0).
compute mc=bootsz.
end if.
do if (adjust = 1 and boot > 0).
compute notecode(notes,1) = 8.
compute notes = notes + 1.
end if.
do if (adjust = 1 and mc > 0).
compute notecode(notes,1) = 16.
compute notes = notes + 1.
end if.
end if.
compute maxboot = trunc(2*boot).
do if ( 0 > maxboot).
compute maxboot=trunc( 0 ).
end if.
do if (nms > 0).
release mtmpuse, mwtmpus, mwztmpu, mtmploc, mwtmplo, mztmplo, mwztmplo.
end if.
release wdid, zdid, wzdid, start,modlabel.
compute vlabs=vlabs(2:nrow(vlabs),1).
do if (rsum(numint) > 0).
compute intkey=intkey(2:nrow(intkey),:).
end if.
compute fulldat=fulldat(:,2:ncol(fulldat)).
compute fochigh=fochigh(1:rmax(nump),:).
compute whigh=whigh(1:rmax(nump),:).
compute zhigh=zhigh(1:rmax(nump),:).
compute wzhigh=wzhigh(1:rmax(nump),:).
compute coeffs=fochigh+whigh+zhigh+wzhigh.
compute bootloc=make(rmax(nump),ncol(nump),0).
do if (nms > 0).
compute cntmp=1.
loop i = 1 to ncol(nump).
loop j = 1 to nump(1,i).
compute bootloc(j,i)=cntmp.
compute cntmp=cntmp+1.
end loop.
end loop.
compute fochighb=make(nrow(fochigh),ncol(fochigh),0).
compute whighb=fochighb.
compute zhighb=fochighb.
compute wzhighb=fochighb.
compute thetaxmb=make(nrow(fochighb),nms,0).
compute thetaxyb=make(nrow(fochighb),1,0).
compute pathsfoc=make(nxvls,1,0).
compute cntmp=1.
loop i = 1 to (nms+nys).
loop j = 1 to i.
compute fochighb(:,cntmp)=fochigh(:,cntmp)&*bootloc(:,i).
compute whighb(:,cntmp)=whigh(:,cntmp)&*bootloc(:,i).
compute zhighb(:,cntmp)=zhigh(:,cntmp)&*bootloc(:,i).
compute wzhighb(:,cntmp)=wzhigh(:,cntmp)&*bootloc(:,i).
compute coeffsb=fochighb+whighb+zhighb+wzhighb.
do if ((i < (nms+nys)) and (j = 1)).
compute thetaxmb(:,i)=coeffsb(:,cntmp).
end if.
do if ((i = (nms+nys)) and (j = 1)).
compute thetaxyb(:,1)=coeffsb(:,cntmp).
end if.
compute cntmp=cntmp+1.
end loop.
end loop.
compute thetamyb=coeffsb(:,(ncol(coeffsb)-nms+1):ncol(coeffsb)).
do if (serial = 1).
compute thetammb=make(nrow(coeffsb),((nms*(nms-1))/2),0).
end if.
compute cntmp=1.
do if (nms > 1 and serial = 1).
loop i = 1 to (nms-1).
compute start=((i+2)*(i+1))/2.
loop j = 2 to (nms-i+1).
compute thetammb(:,cntmp)=coeffsb(:,start).
compute start=start+j+i-1.
compute cntmp=cntmp+1.
end loop.
end loop.
end if.
end if.
do if ((total = 1) and (rsum(numint)=0 or (xmint=1))).
compute dototal=1.
do if ((csum(bcmat(:,1)) <> (nms+nys)) or (rsum(bcmat(nrow(bcmat),:)) <> (nms+nys))).
compute dototal=0.
compute alttotal=1.
compute notecode(notes,1) = 12.
compute notes = notes + 1.
end if.
do if (ncs > 0).
do if ((csum(rsum(ccmat))) < (nrow(ccmat)*ncol(ccmat))).
compute dototal=0.
compute alttotal=1.
compute notecode(notes,1) = 11.
compute notes = notes + 1.
end if.
end if.
do if (model=74).
do if (xdich=0 and nxvls=1).
compute dototal=0.
compute alttotal=1.
end if.
do if ((xdich=1 or nxvls > 1) and (ncs > 0) and model=74).
compute dototal=0.
compute alttotal=1.
end if.
end if.
end if.
end if.
do if (criterr=0 and ncs > 0).
do if (rsum((csum(ccmat)=0)) <> 0).
compute errcode(errs,1)=51.
compute errs=errs+1.
compute criterr=1.
end if.
end if.
compute debug= 0.
do if (outscree=1).
print/title = '***************** PROCESS Procedure for SPSS Version 4.2 *****************'.
print/title = '          Written by Andrew F. Hayes, Ph.D.       www.afhayes.com'.
print/title = '    Documentation available in Hayes (2022). www.guilford.com/p/hayes3'/space=0.
end if.
do if (criterr=0).
compute modresid=make(n,1,99999).
do if (stand=1 and ydich=1).
compute stand=0.
end if.
compute anymod2=csum(rsum(wcmat+zcmat+wzcmat)).
do if (anymod2 > 0 and stand = 1).
compute notecode(notes,1) = 27.
compute notes = notes + 1.
compute stand=0.
end if.
compute funny=1.
do if (outscree=1).
print modelvar/title = '**************************************************************************'/format = A8/rnames=modelvlb.
do if (ncs > 0).
print covnames/title='Covariates:'/format=A8.
end if.
print n/title='Sample'/rlabel='Size:'.
do if (( 'random' <> 'random')).
compute seedt= 'random'.
print seedt/title='Custom'/format=A12/rlabel = 'Seed:'.
end if.
end if.
compute maxresm=9.
compute resultm=make(1,maxresm,99999).
do if (describe=1).
compute means=csum(dat)/n.
compute sigmatal = (t(dat)*(ident(n)-(1/n)*ones*t(ones))*dat)*(1/(n-1)).
compute sdvec=sqrt(diag(sigmatal)).
compute sdall = mdiag(1/sdvec).
compute corall=sdall*sigmatal*t(sdall).
compute means={means;t(sdvec)}.
do if (ncol(means) > 9).
compute resultm=make(1,ncol(means),99999).
compute maxresm=ncol(means).
end if.
compute resultm2=make(2,maxresm,99999).
compute resultm2(1:2,1:ncol(means))=means.
compute resultm={resultm;resultm2}.
compute resultm2=make(ncol(corall),maxresm,99999).
compute resultm2(1:ncol(corall),1:ncol(corall))=corall.
compute resultm={resultm;resultm2}.
do if (outscree=1).
print means/title='Variable means and standard deviations'/cnames=varnames/rlabels='Mean','SD'/format= F10.4.
print corall/title='Variable intercorrelations (Pearson r)'/cnames=varnames/rnames=varnames/format= F10.4.
end if.
end if.
do if (outscree=1).
do if (mcxok=1).
compute labtmp={xnames,t(xcatlab(1:nxvls,1))}.
print dummatx/title = 'Coding of categorical X variable for analysis:'/cnames = labtmp/format = F6.3.
end if.
do if (mcwok=1 and xmint=0).
compute labtmp={wnames,t(wcatlab(1:nwvls,1))}.
print dummatw/title = 'Coding of categorical W variable for analysis:'/cnames = labtmp/format = F6.3.
end if.
do if (mczok=1).
compute labtmp={znames,t(zcatlab(1:nzvls,1))}.
print dummatz/title = 'Coding of categorical Z variable for analysis:'/cnames = labtmp/format = F6.3.
end if.
end if.
end if.
do if (criterr = 0).
compute outnames=ynames.
compute outvars=ytmp.
do if (nms > 0).
compute outnames={mnames,ynames}.
compute outvars={mtmp,ytmp}.
compute indcov=make(((nms*2)+(nms*(nxvls-1))),((nms*2)+(nms*(nxvls-1))),0).
compute mcsopath=make(((nms*2)+(nms*(nxvls-1))),1,0).
end if.
compute labstart=1.
compute intstart=1.
compute start=1.
compute coeffmat=make(1,6,0).
compute conseq={'        '}.
compute dfmat=0.
compute coeffcol=0.
compute pathscnt=1.
compute pathscn2=1.
loop i = 1 to (nms+nys).
do if (outscree=1).
print/title = '**************************************************************************'.
end if.
compute highf=make(1,5,0).
compute highf2=highf.
do if ((i = (nms+nys)) and (ydich=1)).
compute highf=make(1,3,0).
compute highf2=highf.
end if.
compute flabel={' '}.
compute y=outvars(:,i).
compute xindx=datindx(1:(nump(1,i)-1),i).
compute x = fulldat(:,xindx).
compute x={ones,x}.
compute xsq=t(x)*x.
compute exsq=eval(xsq).
release xsq.
compute zeroeig=csum(exsq <= 0.000000000002).
do if (outscree=1).
print outnames(1,i)/title = 'OUTCOME VARIABLE:'/format = A8/space=0.
do if (ydich=1 and (i = (nms+nys))).
compute nmsd = {outnames(1,i), 'Analysis'}.
print rcd/title = 'Coding of binary Y for logistic regression analysis:'/cnames = nmsd/format = F9.2.
end if.
end if.
do if (zeroeig > 0).
print / title = 'SINGULAR OR NEAR SINGULAR DATA MATRIX.'.
compute criterr=1.
compute errcode(errs,1)=31.
compute errs=errs+1.
end if.
compute means=csum(x)/n.
compute vlabsm=vlabs(labstart:(labstart+(nump(1,i)-1)),1).
do if (criterr=0).
do if (ydich=0 or (i < (nms+nys))).

.
do if ( 1 =1).
compute hatmat=inv(t( x )* x )*t( x ).
compute b = hatmat* y.
compute modres=b.
do if ( 1 =1).
compute n1=nrow( x ).
compute dfres=n1-(ncol( x )).
compute sstotal = t( y -(csum( y )/n1))*( y -(csum( y )/n1)).
compute resid= y - x *b.
compute ssresid = csum((resid)&**2).
compute r2 = (sstotal-ssresid)/sstotal.
do if (r2 < 0).
compute r2=0.
end if.
compute adjr2 = 1-((1-r2)*(n1-1)/(dfres)).
compute mse=ssresid/(n1-ncol( x )).

.
compute n1=nrow( x ).
compute invXtX = inv(t( x )* x ).
compute varb = mse *invXtX.
compute k3 = ncol( x ).
compute xhc=0.
do if ( hc <> 5).
compute xhc= x.
compute hat = xhc(:,1).
loop i3=1 to nrow(xhc).
compute hat(i3,1)= xhc(i3,:)*invXtX*t(xhc(i3,:)).
end loop.
do if ( hc = 0 or hc =1).
loop i3 = 1 to k3.
compute xhc(:,i3)=xhc(:,i3)&* resid.
end loop.
end if.
do if ( hc =3 or hc =2).
loop i3=1 to k3.
compute xhc(:,i3) = ( resid &/(1-hat)&**(1/(4- hc )))&*xhc(:,i3).
end loop.
end if.
do if ( hc = 4).
compute hcmn=make(n,2,4).
compute hcmn(:,2)=(n1*hat)/k3.
loop i3= 1 to k3.
compute xhc(:,i3) = ( resid &/(1-hat)&**(rmin(hcmn)/2))&*xhc(:,i3).
end loop.
end if.
compute varb=(invXtX*t(xhc)*xhc*invXtX).
do if ( hc =1).
compute varb=(n1/(n1-ncol( x )))&*varb.
end if.
end if.
compute hclab={'se(HC0)','se(HC1)','se(HC2)','se(HC3)','se(HC4)','se'}.
compute hclab=hclab(1,( hc +1)).
compute hcflab={'F(HC0)','F(HC1)','F(HC2)','F(HC3)','F(HC4)','F'}.
compute hcflab=hcflab(1,( hc +1)).
release xhc
.
compute seb=sqrt(diag(varb)).
compute trat = b&/seb.
compute p = 2*(1-tcdf(abs(trat), (dfres))).
compute tval = sqrt(dfres* (exp((dfres-(5/6))*((xp2/(dfres-(2/3)+(.11/dfres)))* (xp2/(dfres-(2/3)+(.11/dfres)))))-1)).
compute modres={modres,seb,trat,p}.
compute modres={modres,(b-tval&*seb),(b+tval&*seb)}.
compute modresl={'coeff',hclab,'t','p','LLCI','ULCI'}.
compute lmat = ident(ncol( x )).
compute lmat = lmat(:,2:ncol(lmat)).
compute fratio = (t(t(lmat)*b)*inv(t(lmat)*varb*lmat)*((t(lmat)*b)))/(ncol( x )-1).
compute pfr = 1-fcdf(fratio,(ncol( x )-1),dfres).
compute modsum={sqrt(r2),r2,mse,fratio,(ncol( x )-1),dfres,pfr}.
compute modsuml={'R','R-sq','MSE',hcflab,'df1','df2', 'p'}.
end if.
end if.
do if ( 1 = 2 or 1 =3).
compute xlp= x.
compute ylp= y.
compute pt2 = make(nrow(ylp),1,(csum(ylp)/nrow(ylp))).
do if ( 1 =2).
compute LL3 = ylp&*ln(pt2)+(1-ylp)&*ln(1-pt2).
end if.
compute LL3 = -2*csum(LL3).
compute bt1 = make(ncol(xlp),1,0).
compute LL1 = 0.
compute pt1 = make(nrow(ylp),1,0.5).
compute pt1lp=pt1.
loop jjj = 1 to iterate.
compute xlptmp=t(xlp).
compute vecprb=pt1lp&*(1-pt1lp).
loop kkk=1 to ncol(xlp).
compute xlptmp(kkk,:)=xlptmp(kkk,:)&*t(vecprb).
end loop.
compute b = bt1+inv(xlptmp*xlp)*t(xlp)*(ylp-pt1lp).
do if ( 1 =2).
compute xlpb=xlp*b.
compute xlpbt=(xlpb > -709.7).
compute xlpb709=(1-xlpbt)*(-709.7).
compute xlpb=(xlpb&*xlpbt)+xlpb709.
compute pt1lp = 1/(1+exp(-(xlpb))).
end if.
compute itprob = csum((pt1lp < .00000001) or (pt1lp > .9999999)).
do if (itprob > 0).
loop kkk = 1 to nrow(pt1lp).
do if (pt1lp(kkk,1) > .9999999).
compute pt1lp(kkk,1) = .9999999.
end if.
do if (pt1lp(kkk,1) < .00000001).
compute pt1lp(kkk,1) = .00000001.
end if.
end loop.
compute itprob = 0.
end if.
do if (itprob = 0).
do if ( 1 =2).
compute LL = ylp&*ln(pt1lp)+(1-ylp)&*ln(1-pt1lp).
end if.
compute LL2 = -2*csum(ll).
end if.
do if (abs(LL1-LL2) < converge).
do if ( 1 =1).
compute xlptmp=t(xlp).
compute vecprb=pt1lp&*(1-pt1lp).
loop kkk=1 to ncol(xlp).
compute xlptmp(kkk,:)=xlptmp(kkk,:)&*t(vecprb).
end loop.
compute varb = inv(xlptmp*xlp).
compute seb = sqrt(diag(varb)).
release xlptmp.
end if.
break.
end if.
compute bt1 = b.
compute LL1 = LL2.
end loop.
compute modres=b.
do if (jjj > iterate).
compute itprob = 2.
do if (booting=0).
compute iterrmod=1.
end if.
do if (booting=1).
compute bootiter=1.
end if.
do if (itprobtg=0).
compute itprobtg=1.
compute errcode(errs,1) = 47.
compute errs = errs + 1.
do if (booting = 0 and 1 =1).
compute vt1 = mdiag(pt1lp&*(1-pt1lp)).
compute varb = inv(t(xlp)*vt1*xlp).
compute seb = sqrt(diag(varb)).
end if.
end if.
end if.
do if ( 1 =1).
compute trat = b&/seb.
compute dfres=nrow(xlp).
compute p = 2*(1-cdfnorm(abs(trat))).
compute modres={modres,seb,trat,p}.
compute modres={modres,(b-xp2&*seb),(b+xp2&*seb)}.
compute pvchi=1-chicdf((LL3-LL2),(nrow(modres)-1)).
compute mcF = (LL3-LL2)/LL3.
compute cox = 1-exp(-(LL3-LL2)/nrow(xlp)).
compute nagel = cox/(1-exp(-(LL3)/nrow(xlp))).
compute modsum={LL2,(LL3-LL2),(nrow(modres)-1),pvchi, mcF,cox,nagel}.
compute modsuml={'-2LL','ModelLL', 'df', 'p', 'McFadden', 'CoxSnell', 'Nagelkrk'}.
compute modresl={'coeff','se','Z','p','LLCI','ULCI'}.
end if.
end if
.
compute dfmatt=make(nrow(modres),1,modsum(1,6)).
compute modresid={modresid,resid}.
end if.
do if (ydich=1 and (i = (nms+nys))).

.
do if ( 2 =1).
compute hatmat=inv(t( x )* x )*t( x ).
compute b = hatmat* y.
compute modres=b.
do if ( 1 =1).
compute n1=nrow( x ).
compute dfres=n1-(ncol( x )).
compute sstotal = t( y -(csum( y )/n1))*( y -(csum( y )/n1)).
compute resid= y - x *b.
compute ssresid = csum((resid)&**2).
compute r2 = (sstotal-ssresid)/sstotal.
do if (r2 < 0).
compute r2=0.
end if.
compute adjr2 = 1-((1-r2)*(n1-1)/(dfres)).
compute mse=ssresid/(n1-ncol( x )).

.
compute n1=nrow( x ).
compute invXtX = inv(t( x )* x ).
compute varb = mse *invXtX.
compute k3 = ncol( x ).
compute xhc=0.
do if ( hc <> 5).
compute xhc= x.
compute hat = xhc(:,1).
loop i3=1 to nrow(xhc).
compute hat(i3,1)= xhc(i3,:)*invXtX*t(xhc(i3,:)).
end loop.
do if ( hc = 0 or hc =1).
loop i3 = 1 to k3.
compute xhc(:,i3)=xhc(:,i3)&* resid.
end loop.
end if.
do if ( hc =3 or hc =2).
loop i3=1 to k3.
compute xhc(:,i3) = ( resid &/(1-hat)&**(1/(4- hc )))&*xhc(:,i3).
end loop.
end if.
do if ( hc = 4).
compute hcmn=make(n,2,4).
compute hcmn(:,2)=(n1*hat)/k3.
loop i3= 1 to k3.
compute xhc(:,i3) = ( resid &/(1-hat)&**(rmin(hcmn)/2))&*xhc(:,i3).
end loop.
end if.
compute varb=(invXtX*t(xhc)*xhc*invXtX).
do if ( hc =1).
compute varb=(n1/(n1-ncol( x )))&*varb.
end if.
end if.
compute hclab={'se(HC0)','se(HC1)','se(HC2)','se(HC3)','se(HC4)','se'}.
compute hclab=hclab(1,( hc +1)).
compute hcflab={'F(HC0)','F(HC1)','F(HC2)','F(HC3)','F(HC4)','F'}.
compute hcflab=hcflab(1,( hc +1)).
release xhc
.
compute seb=sqrt(diag(varb)).
compute trat = b&/seb.
compute p = 2*(1-tcdf(abs(trat), (dfres))).
compute tval = sqrt(dfres* (exp((dfres-(5/6))*((xp2/(dfres-(2/3)+(.11/dfres)))* (xp2/(dfres-(2/3)+(.11/dfres)))))-1)).
compute modres={modres,seb,trat,p}.
compute modres={modres,(b-tval&*seb),(b+tval&*seb)}.
compute modresl={'coeff',hclab,'t','p','LLCI','ULCI'}.
compute lmat = ident(ncol( x )).
compute lmat = lmat(:,2:ncol(lmat)).
compute fratio = (t(t(lmat)*b)*inv(t(lmat)*varb*lmat)*((t(lmat)*b)))/(ncol( x )-1).
compute pfr = 1-fcdf(fratio,(ncol( x )-1),dfres).
compute modsum={sqrt(r2),r2,mse,fratio,(ncol( x )-1),dfres,pfr}.
compute modsuml={'R','R-sq','MSE',hcflab,'df1','df2', 'p'}.
end if.
end if.
do if ( 2 = 2 or 2 =3).
compute xlp= x.
compute ylp= y.
compute pt2 = make(nrow(ylp),1,(csum(ylp)/nrow(ylp))).
do if ( 2 =2).
compute LL3 = ylp&*ln(pt2)+(1-ylp)&*ln(1-pt2).
end if.
compute LL3 = -2*csum(LL3).
compute bt1 = make(ncol(xlp),1,0).
compute LL1 = 0.
compute pt1 = make(nrow(ylp),1,0.5).
compute pt1lp=pt1.
loop jjj = 1 to iterate.
compute xlptmp=t(xlp).
compute vecprb=pt1lp&*(1-pt1lp).
loop kkk=1 to ncol(xlp).
compute xlptmp(kkk,:)=xlptmp(kkk,:)&*t(vecprb).
end loop.
compute b = bt1+inv(xlptmp*xlp)*t(xlp)*(ylp-pt1lp).
do if ( 2 =2).
compute xlpb=xlp*b.
compute xlpbt=(xlpb > -709.7).
compute xlpb709=(1-xlpbt)*(-709.7).
compute xlpb=(xlpb&*xlpbt)+xlpb709.
compute pt1lp = 1/(1+exp(-(xlpb))).
end if.
compute itprob = csum((pt1lp < .00000001) or (pt1lp > .9999999)).
do if (itprob > 0).
loop kkk = 1 to nrow(pt1lp).
do if (pt1lp(kkk,1) > .9999999).
compute pt1lp(kkk,1) = .9999999.
end if.
do if (pt1lp(kkk,1) < .00000001).
compute pt1lp(kkk,1) = .00000001.
end if.
end loop.
compute itprob = 0.
end if.
do if (itprob = 0).
do if ( 2 =2).
compute LL = ylp&*ln(pt1lp)+(1-ylp)&*ln(1-pt1lp).
end if.
compute LL2 = -2*csum(ll).
end if.
do if (abs(LL1-LL2) < converge).
do if ( 1 =1).
compute xlptmp=t(xlp).
compute vecprb=pt1lp&*(1-pt1lp).
loop kkk=1 to ncol(xlp).
compute xlptmp(kkk,:)=xlptmp(kkk,:)&*t(vecprb).
end loop.
compute varb = inv(xlptmp*xlp).
compute seb = sqrt(diag(varb)).
release xlptmp.
end if.
break.
end if.
compute bt1 = b.
compute LL1 = LL2.
end loop.
compute modres=b.
do if (jjj > iterate).
compute itprob = 2.
do if (booting=0).
compute iterrmod=1.
end if.
do if (booting=1).
compute bootiter=1.
end if.
do if (itprobtg=0).
compute itprobtg=1.
compute errcode(errs,1) = 47.
compute errs = errs + 1.
do if (booting = 0 and 1 =1).
compute vt1 = mdiag(pt1lp&*(1-pt1lp)).
compute varb = inv(t(xlp)*vt1*xlp).
compute seb = sqrt(diag(varb)).
end if.
end if.
end if.
do if ( 1 =1).
compute trat = b&/seb.
compute dfres=nrow(xlp).
compute p = 2*(1-cdfnorm(abs(trat))).
compute modres={modres,seb,trat,p}.
compute modres={modres,(b-xp2&*seb),(b+xp2&*seb)}.
compute pvchi=1-chicdf((LL3-LL2),(nrow(modres)-1)).
compute mcF = (LL3-LL2)/LL3.
compute cox = 1-exp(-(LL3-LL2)/nrow(xlp)).
compute nagel = cox/(1-exp(-(LL3)/nrow(xlp))).
compute modsum={LL2,(LL3-LL2),(nrow(modres)-1),pvchi, mcF,cox,nagel}.
compute modsuml={'-2LL','ModelLL', 'df', 'p', 'McFadden', 'CoxSnell', 'Nagelkrk'}.
compute modresl={'coeff','se','Z','p','LLCI','ULCI'}.
end if.
end if
.
compute dfmatt=make(nrow(modres),1,-999).
end if.
compute resultm2=make(1,maxresm,99999).
compute resultm2(1,1:ncol(modsum))=modsum.
compute resultm={resultm;resultm2}.
compute resultm2=make(nrow(modres),maxresm,99999).
compute resultm2(1:nrow(modres),1:ncol(modres))=modres.
compute resultm={resultm;resultm2}.
compute obscoeff={obscoeff,t(b)}.
do if (outscree=1).
print modsum/title = 'Model Summary'/cnames = modsuml/format= F10.4.
print modres/title='Model'/rnames=vlabsm/cnames=modresl/format= F10.4.
end if.
compute basemod=modsum(1,1).
compute basemodx=basemod.
do if (ydich=1 and (i = (nms+nys))).
do if (outscree=1).
print/title='These results are expressed in a log-odds metric.'.
end if.
compute notecode(notes,1) = 26.
compute notes = notes + 1.
end if.
compute coeffmat={coeffmat;modres}.
compute conseqt=make(nrow(modres),1,outnames(1,i)).
compute conseq={conseq;conseqt}.
compute dfmat={dfmat;dfmatt}.
compute labstart=labstart+nump(1,i).
do if (stand=1).
compute predsd=make(nrow(modres),1,0).
compute stdmod=modres(:,1)&/ovsd(1,i).
loop jd=1 to ncol(x).
compute descdat=x(:,jd).
compute predsd(jd,1) = (nrow(descdat)*sscp(descdat))-(t(csum(descdat))*(csum(descdat))).
compute predsd(jd,1) = sqrt(predsd(jd,1)/(nrow(descdat)*(nrow(descdat)-1))).
end loop.
do if (wherex(1,i) <> -999 and ((nxvls > 1) or (xdich=1))).
compute sdmsone=make(nxvls,1,1).
compute predsd(wherex(1,i):wherex(2,i),1)=sdmsone.
compute pstog=1.
end if.
compute predsd(1,1)=1.
compute stdmod=stdmod&*predsd.
compute stdmod=stdmod(2:nrow(stdmod),1).
compute sdvlabs=vlabsm(2:nrow(vlabsm),1).
compute resultm2=make(nrow(stdmod),maxresm,99999).
compute resultm2(1:nrow(stdmod),1:ncol(stdmod))=stdmod.
compute resultm={resultm;resultm2}.
do if (outscree=1).
print stdmod/title='Standardized coefficients'/clabels='coeff'/rnames=sdvlabs/format= F10.4.
end if.
end if.
do if (nms > 0 and serial = 0 and (rsum(numint) = 0) and (normal=1 or mc > 0)).
do if (i < (nms+nys)).
compute indcov((((i-1)*nxvls)+1):(i*nxvls),(((i-1)*nxvls)+1):(i*nxvls))=varb(2:(1+nxvls), 2:(1+nxvls)).
compute mcsopath((((i-1)*nxvls)+1):(i*nxvls) ,1)=modres(2:(1+nxvls),1).
end if.
do if (i = (nms+nys)).
compute atm=ncol(wherem).
compute indcov(((nms*nxvls)+1):nrow(mcsopath),((nms*nxvls)+1):nrow(mcsopath))= varb(wherem(1,atm):(wherem(1,atm)+nms-1),wherem(1,atm):(wherem(1,atm)+nms-1)).
compute mcsopath(((nms*nxvls)+1):nrow(mcsopath),1)=modres(wherem(1,atm):(wherem(1,atm)+ nms-1),1).
compute sobelok=1.
end if.
end if.
compute obsdirfx=make(1,nxvls,0).
compute dirzes=make(1,nxvls,0).
do if ((i = (nms+nys)) and (bcmat(nrow(bcmat),1)=1)).
compute direff=modres(2:(1+nxvls),:).
compute obsdirfx=t(direff(:,1)).
compute direfflb=modresl.
compute direffl2=vlabsm(2:(1+nxvls),:).
compute lmat=make(nrow(b),1,0).
compute lmat2=make(nxvls,1,1).
compute lmat(2:(1+nxvls),1)=lmat2.
do if (ydich <> 1).

.
compute lmat2= lmat.
do if ( 0 =0).
compute lmat2 = mdiag( lmat ).
compute lmat3=make(nrow(lmat2),1,0).
loop flp=1 to ncol(lmat2).
do if (csum(lmat2(:,flp))=1).
compute lmat3={lmat3,lmat2(:,flp)}.
end if.
end loop.
compute lmat2=lmat3(:,2:ncol(lmat3)).
end if.
compute fratio = (t(t(lmat2)* b )*inv(t(lmat2)* varb *lmat2)*((t(lmat2)* b )))/ncol(lmat2).
compute pfr = 1-fcdf(fratio,ncol(lmat2),(n-nrow( b ))).
compute fresult={fratio,ncol(lmat2),(n-nrow( b )),pfr}.
do if (i = (nms+nys) and (ydich=1)).
compute fratio=fratio*ncol(lmat2).
compute pfr=1-chicdf(fratio,ncol(lmat2)).
compute fresult={fratio,ncol(lmat2),pfr}.
end if.
do if ( 1 =1).
compute lmat3=1-rsum(lmat2).
compute xfm=make(n,csum(lmat3),0).
compute flpc=1.
loop flp=1 to nrow(lmat3).
do if (lmat3(flp,1)=1).
compute xfm(:,flpc)=x(:,flp).
compute flpc=flpc+1.
end if.
end loop.
compute bfm=inv(t(xfm)*xfm)*t(xfm)*y.
compute resid=y-(xfm*bfm).
compute sstotal=(y-(csum(y)/n)).
compute sstotal=csum(sstotal&*sstotal).
compute ssresid=csum(resid&*resid).
compute rsqch= r2 -((sstotal-ssresid)/sstotal).
compute fresult={rsqch,fresult}.
release xfm,flpc, resid, ssresid, bfm.
end if
.
compute diromni=fresult.
end if.
do if (ydich = 1 ).

.
compute btemphld=b.
compute llrdat=make(nrow(x),nrow( lmat )-csum( lmat ),-999).
compute llrdf=ncol(x)-ncol(llrdat).
compute llrcnt=0.
loop llri=1 to nrow( lmat ).
do if ( lmat (llri,1)=0).
compute llrcnt=llrcnt+1.
compute llrdat(:,llrcnt)=x(:,llri).
end if.
end loop.

.
do if ( 2 =1).
compute hatmat=inv(t( llrdat )* llrdat )*t( llrdat ).
compute b = hatmat* y.
compute modres=b.
do if ( 0 =1).
compute n1=nrow( llrdat ).
compute dfres=n1-(ncol( llrdat )).
compute sstotal = t( y -(csum( y )/n1))*( y -(csum( y )/n1)).
compute resid= y - llrdat *b.
compute ssresid = csum((resid)&**2).
compute r2 = (sstotal-ssresid)/sstotal.
do if (r2 < 0).
compute r2=0.
end if.
compute adjr2 = 1-((1-r2)*(n1-1)/(dfres)).
compute mse=ssresid/(n1-ncol( llrdat )).

.
compute n1=nrow( x ).
compute invXtX = inv(t( x )* x ).
compute varb = mse *invXtX.
compute k3 = ncol( x ).
compute xhc=0.
do if ( hc <> 5).
compute xhc= x.
compute hat = xhc(:,1).
loop i3=1 to nrow(xhc).
compute hat(i3,1)= xhc(i3,:)*invXtX*t(xhc(i3,:)).
end loop.
do if ( hc = 0 or hc =1).
loop i3 = 1 to k3.
compute xhc(:,i3)=xhc(:,i3)&* resid.
end loop.
end if.
do if ( hc =3 or hc =2).
loop i3=1 to k3.
compute xhc(:,i3) = ( resid &/(1-hat)&**(1/(4- hc )))&*xhc(:,i3).
end loop.
end if.
do if ( hc = 4).
compute hcmn=make(n,2,4).
compute hcmn(:,2)=(n1*hat)/k3.
loop i3= 1 to k3.
compute xhc(:,i3) = ( resid &/(1-hat)&**(rmin(hcmn)/2))&*xhc(:,i3).
end loop.
end if.
compute varb=(invXtX*t(xhc)*xhc*invXtX).
do if ( hc =1).
compute varb=(n1/(n1-ncol( x )))&*varb.
end if.
end if.
compute hclab={'se(HC0)','se(HC1)','se(HC2)','se(HC3)','se(HC4)','se'}.
compute hclab=hclab(1,( hc +1)).
compute hcflab={'F(HC0)','F(HC1)','F(HC2)','F(HC3)','F(HC4)','F'}.
compute hcflab=hcflab(1,( hc +1)).
release xhc
.
compute seb=sqrt(diag(varb)).
compute trat = b&/seb.
compute p = 2*(1-tcdf(abs(trat), (dfres))).
compute tval = sqrt(dfres* (exp((dfres-(5/6))*((xp2/(dfres-(2/3)+(.11/dfres)))* (xp2/(dfres-(2/3)+(.11/dfres)))))-1)).
compute modres={modres,seb,trat,p}.
compute modres={modres,(b-tval&*seb),(b+tval&*seb)}.
compute modresl={'coeff',hclab,'t','p','LLCI','ULCI'}.
compute lmat = ident(ncol( llrdat )).
compute lmat = lmat(:,2:ncol(lmat)).
compute fratio = (t(t(lmat)*b)*inv(t(lmat)*varb*lmat)*((t(lmat)*b)))/(ncol( llrdat )-1).
compute pfr = 1-fcdf(fratio,(ncol( llrdat )-1),dfres).
compute modsum={sqrt(r2),r2,mse,fratio,(ncol( llrdat )-1),dfres,pfr}.
compute modsuml={'R','R-sq','MSE',hcflab,'df1','df2', 'p'}.
end if.
end if.
do if ( 2 = 2 or 2 =3).
compute xlp= llrdat.
compute ylp= y.
compute pt2 = make(nrow(ylp),1,(csum(ylp)/nrow(ylp))).
do if ( 2 =2).
compute LL3 = ylp&*ln(pt2)+(1-ylp)&*ln(1-pt2).
end if.
compute LL3 = -2*csum(LL3).
compute bt1 = make(ncol(xlp),1,0).
compute LL1 = 0.
compute pt1 = make(nrow(ylp),1,0.5).
compute pt1lp=pt1.
loop jjj = 1 to iterate.
compute xlptmp=t(xlp).
compute vecprb=pt1lp&*(1-pt1lp).
loop kkk=1 to ncol(xlp).
compute xlptmp(kkk,:)=xlptmp(kkk,:)&*t(vecprb).
end loop.
compute b = bt1+inv(xlptmp*xlp)*t(xlp)*(ylp-pt1lp).
do if ( 2 =2).
compute xlpb=xlp*b.
compute xlpbt=(xlpb > -709.7).
compute xlpb709=(1-xlpbt)*(-709.7).
compute xlpb=(xlpb&*xlpbt)+xlpb709.
compute pt1lp = 1/(1+exp(-(xlpb))).
end if.
compute itprob = csum((pt1lp < .00000001) or (pt1lp > .9999999)).
do if (itprob > 0).
loop kkk = 1 to nrow(pt1lp).
do if (pt1lp(kkk,1) > .9999999).
compute pt1lp(kkk,1) = .9999999.
end if.
do if (pt1lp(kkk,1) < .00000001).
compute pt1lp(kkk,1) = .00000001.
end if.
end loop.
compute itprob = 0.
end if.
do if (itprob = 0).
do if ( 2 =2).
compute LL = ylp&*ln(pt1lp)+(1-ylp)&*ln(1-pt1lp).
end if.
compute LL2 = -2*csum(ll).
end if.
do if (abs(LL1-LL2) < converge).
do if ( 0 =1).
compute xlptmp=t(xlp).
compute vecprb=pt1lp&*(1-pt1lp).
loop kkk=1 to ncol(xlp).
compute xlptmp(kkk,:)=xlptmp(kkk,:)&*t(vecprb).
end loop.
compute varb = inv(xlptmp*xlp).
compute seb = sqrt(diag(varb)).
release xlptmp.
end if.
break.
end if.
compute bt1 = b.
compute LL1 = LL2.
end loop.
compute modres=b.
do if (jjj > iterate).
compute itprob = 2.
do if (booting=0).
compute iterrmod=1.
end if.
do if (booting=1).
compute bootiter=1.
end if.
do if (itprobtg=0).
compute itprobtg=1.
compute errcode(errs,1) = 47.
compute errs = errs + 1.
do if (booting = 0 and 0 =1).
compute vt1 = mdiag(pt1lp&*(1-pt1lp)).
compute varb = inv(t(xlp)*vt1*xlp).
compute seb = sqrt(diag(varb)).
end if.
end if.
end if.
do if ( 0 =1).
compute trat = b&/seb.
compute dfres=nrow(xlp).
compute p = 2*(1-cdfnorm(abs(trat))).
compute modres={modres,seb,trat,p}.
compute modres={modres,(b-xp2&*seb),(b+xp2&*seb)}.
compute pvchi=1-chicdf((LL3-LL2),(nrow(modres)-1)).
compute mcF = (LL3-LL2)/LL3.
compute cox = 1-exp(-(LL3-LL2)/nrow(xlp)).
compute nagel = cox/(1-exp(-(LL3)/nrow(xlp))).
compute modsum={LL2,(LL3-LL2),(nrow(modres)-1),pvchi, mcF,cox,nagel}.
compute modsuml={'-2LL','ModelLL', 'df', 'p', 'McFadden', 'CoxSnell', 'Nagelkrk'}.
compute modresl={'coeff','se','Z','p','LLCI','ULCI'}.
end if.
end if
.
compute b=btemphld.
compute fresult={(LL2-basemod),llrdf,1-chicdf((LL2-basemod),llrdf)}
.
compute diromni=fresult.
end if.
end if.
do if (numint(1,i) > 0).
compute intkeym=intkey(intstart:(intstart+numint(1,i)-1),:).
do if (outscree=1).
print intkeym/title='Product terms key:'/format = A8.
end if.
end if.
do if (covcoeff=1).
do if (outscree=1).
print varb/title='Covariance matrix of regression parameter estimates:'/rnames=vlabsm /cnames=vlabsm/format= F10.4.
end if.
compute resultm2=make(nrow(varb),maxresm,99999).
do if (ncol(varb) <= maxresm).
compute resultm2(1:nrow(varb),1:ncol(varb))=varb.
compute resultm={resultm;resultm2}.
end if.
do if (ncol(varb) > maxresm).
compute resultmt=make(nrow(resultm),ncol(varb),99999).
compute resultmt(1:nrow(resultm),1:ncol(resultm))=resultm.
compute resultm=resultmt.
compute resultm2=make(nrow(varb),ncol(resultm),99999).
compute resultm2(1:nrow(varb),1:ncol(varb))=varb.
compute resultm={resultm;resultm2}.
compute maxresm=ncol(resultm).
end if.
end if.
do if (model <> 74 and xmtest=1 and nms > 0).
compute r2tmp=r2.
compute btmp=b.
compute varbtmp=varb.
compute dfrestmp=dfres.
compute tvaltmp=tval.
compute xmtst=make(nms,4,0).
compute xmtstlbc={hcflab,'df1','df2','p'}.
do if ((i=(nms+nys)) and (ydich=1)).
compute xmtst=make(nms,3,0).
compute xmtstlbc={'Chi-sq','df','p'}.
end if.
compute xmtstlb={' '}.
compute xmtmat=x.
compute numxint=0.
loop xmints=2 to i.
compute x=xmtmat.
do if ((bcmat((i+1),xmints)=1) and (wzcmat((i+1),xmints) <> 1)).
do if (bcmat((i+1),1)=0).
compute x={xmtmat,xtmp}.
do if ((ydich=1) and (i=(nms+nys))).

.
do if ( 2 =1).
compute hatmat=inv(t( x )* x )*t( x ).
compute b = hatmat* y.
compute modres=b.
do if ( 1 =1).
compute n1=nrow( x ).
compute dfres=n1-(ncol( x )).
compute sstotal = t( y -(csum( y )/n1))*( y -(csum( y )/n1)).
compute resid= y - x *b.
compute ssresid = csum((resid)&**2).
compute r2 = (sstotal-ssresid)/sstotal.
do if (r2 < 0).
compute r2=0.
end if.
compute adjr2 = 1-((1-r2)*(n1-1)/(dfres)).
compute mse=ssresid/(n1-ncol( x )).

.
compute n1=nrow( x ).
compute invXtX = inv(t( x )* x ).
compute varb = mse *invXtX.
compute k3 = ncol( x ).
compute xhc=0.
do if ( hc <> 5).
compute xhc= x.
compute hat = xhc(:,1).
loop i3=1 to nrow(xhc).
compute hat(i3,1)= xhc(i3,:)*invXtX*t(xhc(i3,:)).
end loop.
do if ( hc = 0 or hc =1).
loop i3 = 1 to k3.
compute xhc(:,i3)=xhc(:,i3)&* resid.
end loop.
end if.
do if ( hc =3 or hc =2).
loop i3=1 to k3.
compute xhc(:,i3) = ( resid &/(1-hat)&**(1/(4- hc )))&*xhc(:,i3).
end loop.
end if.
do if ( hc = 4).
compute hcmn=make(n,2,4).
compute hcmn(:,2)=(n1*hat)/k3.
loop i3= 1 to k3.
compute xhc(:,i3) = ( resid &/(1-hat)&**(rmin(hcmn)/2))&*xhc(:,i3).
end loop.
end if.
compute varb=(invXtX*t(xhc)*xhc*invXtX).
do if ( hc =1).
compute varb=(n1/(n1-ncol( x )))&*varb.
end if.
end if.
compute hclab={'se(HC0)','se(HC1)','se(HC2)','se(HC3)','se(HC4)','se'}.
compute hclab=hclab(1,( hc +1)).
compute hcflab={'F(HC0)','F(HC1)','F(HC2)','F(HC3)','F(HC4)','F'}.
compute hcflab=hcflab(1,( hc +1)).
release xhc
.
compute seb=sqrt(diag(varb)).
compute trat = b&/seb.
compute p = 2*(1-tcdf(abs(trat), (dfres))).
compute tval = sqrt(dfres* (exp((dfres-(5/6))*((xp2/(dfres-(2/3)+(.11/dfres)))* (xp2/(dfres-(2/3)+(.11/dfres)))))-1)).
compute modres={modres,seb,trat,p}.
compute modres={modres,(b-tval&*seb),(b+tval&*seb)}.
compute modresl={'coeff',hclab,'t','p','LLCI','ULCI'}.
compute lmat = ident(ncol( x )).
compute lmat = lmat(:,2:ncol(lmat)).
compute fratio = (t(t(lmat)*b)*inv(t(lmat)*varb*lmat)*((t(lmat)*b)))/(ncol( x )-1).
compute pfr = 1-fcdf(fratio,(ncol( x )-1),dfres).
compute modsum={sqrt(r2),r2,mse,fratio,(ncol( x )-1),dfres,pfr}.
compute modsuml={'R','R-sq','MSE',hcflab,'df1','df2', 'p'}.
end if.
end if.
do if ( 2 = 2 or 2 =3).
compute xlp= x.
compute ylp= y.
compute pt2 = make(nrow(ylp),1,(csum(ylp)/nrow(ylp))).
do if ( 2 =2).
compute LL3 = ylp&*ln(pt2)+(1-ylp)&*ln(1-pt2).
end if.
compute LL3 = -2*csum(LL3).
compute bt1 = make(ncol(xlp),1,0).
compute LL1 = 0.
compute pt1 = make(nrow(ylp),1,0.5).
compute pt1lp=pt1.
loop jjj = 1 to iterate.
compute xlptmp=t(xlp).
compute vecprb=pt1lp&*(1-pt1lp).
loop kkk=1 to ncol(xlp).
compute xlptmp(kkk,:)=xlptmp(kkk,:)&*t(vecprb).
end loop.
compute b = bt1+inv(xlptmp*xlp)*t(xlp)*(ylp-pt1lp).
do if ( 2 =2).
compute xlpb=xlp*b.
compute xlpbt=(xlpb > -709.7).
compute xlpb709=(1-xlpbt)*(-709.7).
compute xlpb=(xlpb&*xlpbt)+xlpb709.
compute pt1lp = 1/(1+exp(-(xlpb))).
end if.
compute itprob = csum((pt1lp < .00000001) or (pt1lp > .9999999)).
do if (itprob > 0).
loop kkk = 1 to nrow(pt1lp).
do if (pt1lp(kkk,1) > .9999999).
compute pt1lp(kkk,1) = .9999999.
end if.
do if (pt1lp(kkk,1) < .00000001).
compute pt1lp(kkk,1) = .00000001.
end if.
end loop.
compute itprob = 0.
end if.
do if (itprob = 0).
do if ( 2 =2).
compute LL = ylp&*ln(pt1lp)+(1-ylp)&*ln(1-pt1lp).
end if.
compute LL2 = -2*csum(ll).
end if.
do if (abs(LL1-LL2) < converge).
do if ( 1 =1).
compute xlptmp=t(xlp).
compute vecprb=pt1lp&*(1-pt1lp).
loop kkk=1 to ncol(xlp).
compute xlptmp(kkk,:)=xlptmp(kkk,:)&*t(vecprb).
end loop.
compute varb = inv(xlptmp*xlp).
compute seb = sqrt(diag(varb)).
release xlptmp.
end if.
break.
end if.
compute bt1 = b.
compute LL1 = LL2.
end loop.
compute modres=b.
do if (jjj > iterate).
compute itprob = 2.
do if (booting=0).
compute iterrmod=1.
end if.
do if (booting=1).
compute bootiter=1.
end if.
do if (itprobtg=0).
compute itprobtg=1.
compute errcode(errs,1) = 47.
compute errs = errs + 1.
do if (booting = 0 and 1 =1).
compute vt1 = mdiag(pt1lp&*(1-pt1lp)).
compute varb = inv(t(xlp)*vt1*xlp).
compute seb = sqrt(diag(varb)).
end if.
end if.
end if.
do if ( 1 =1).
compute trat = b&/seb.
compute dfres=nrow(xlp).
compute p = 2*(1-cdfnorm(abs(trat))).
compute modres={modres,seb,trat,p}.
compute modres={modres,(b-xp2&*seb),(b+xp2&*seb)}.
compute pvchi=1-chicdf((LL3-LL2),(nrow(modres)-1)).
compute mcF = (LL3-LL2)/LL3.
compute cox = 1-exp(-(LL3-LL2)/nrow(xlp)).
compute nagel = cox/(1-exp(-(LL3)/nrow(xlp))).
compute modsum={LL2,(LL3-LL2),(nrow(modres)-1),pvchi, mcF,cox,nagel}.
compute modsuml={'-2LL','ModelLL', 'df', 'p', 'McFadden', 'CoxSnell', 'Nagelkrk'}.
compute modresl={'coeff','se','Z','p','LLCI','ULCI'}.
end if.
end if
.
compute basemodx=LL2.
end if.
end if.
loop xmtlp1=1 to nxvls.
compute x={x,xtmp(:,xmtlp1)&*(mtmp(:,(xmints-1))-(csum(mtmp(:,(xmints-1)))/nrow(mtmp)) )}.
end loop.
do if ((i < (nms+nys)) or (ydich=0)).

.
do if ( 1 =1).
compute hatmat=inv(t( x )* x )*t( x ).
compute b = hatmat* y.
compute modres=b.
do if ( 1 =1).
compute n1=nrow( x ).
compute dfres=n1-(ncol( x )).
compute sstotal = t( y -(csum( y )/n1))*( y -(csum( y )/n1)).
compute resid= y - x *b.
compute ssresid = csum((resid)&**2).
compute r2 = (sstotal-ssresid)/sstotal.
do if (r2 < 0).
compute r2=0.
end if.
compute adjr2 = 1-((1-r2)*(n1-1)/(dfres)).
compute mse=ssresid/(n1-ncol( x )).

.
compute n1=nrow( x ).
compute invXtX = inv(t( x )* x ).
compute varb = mse *invXtX.
compute k3 = ncol( x ).
compute xhc=0.
do if ( hc <> 5).
compute xhc= x.
compute hat = xhc(:,1).
loop i3=1 to nrow(xhc).
compute hat(i3,1)= xhc(i3,:)*invXtX*t(xhc(i3,:)).
end loop.
do if ( hc = 0 or hc =1).
loop i3 = 1 to k3.
compute xhc(:,i3)=xhc(:,i3)&* resid.
end loop.
end if.
do if ( hc =3 or hc =2).
loop i3=1 to k3.
compute xhc(:,i3) = ( resid &/(1-hat)&**(1/(4- hc )))&*xhc(:,i3).
end loop.
end if.
do if ( hc = 4).
compute hcmn=make(n,2,4).
compute hcmn(:,2)=(n1*hat)/k3.
loop i3= 1 to k3.
compute xhc(:,i3) = ( resid &/(1-hat)&**(rmin(hcmn)/2))&*xhc(:,i3).
end loop.
end if.
compute varb=(invXtX*t(xhc)*xhc*invXtX).
do if ( hc =1).
compute varb=(n1/(n1-ncol( x )))&*varb.
end if.
end if.
compute hclab={'se(HC0)','se(HC1)','se(HC2)','se(HC3)','se(HC4)','se'}.
compute hclab=hclab(1,( hc +1)).
compute hcflab={'F(HC0)','F(HC1)','F(HC2)','F(HC3)','F(HC4)','F'}.
compute hcflab=hcflab(1,( hc +1)).
release xhc
.
compute seb=sqrt(diag(varb)).
compute trat = b&/seb.
compute p = 2*(1-tcdf(abs(trat), (dfres))).
compute tval = sqrt(dfres* (exp((dfres-(5/6))*((xp2/(dfres-(2/3)+(.11/dfres)))* (xp2/(dfres-(2/3)+(.11/dfres)))))-1)).
compute modres={modres,seb,trat,p}.
compute modres={modres,(b-tval&*seb),(b+tval&*seb)}.
compute modresl={'coeff',hclab,'t','p','LLCI','ULCI'}.
compute lmat = ident(ncol( x )).
compute lmat = lmat(:,2:ncol(lmat)).
compute fratio = (t(t(lmat)*b)*inv(t(lmat)*varb*lmat)*((t(lmat)*b)))/(ncol( x )-1).
compute pfr = 1-fcdf(fratio,(ncol( x )-1),dfres).
compute modsum={sqrt(r2),r2,mse,fratio,(ncol( x )-1),dfres,pfr}.
compute modsuml={'R','R-sq','MSE',hcflab,'df1','df2', 'p'}.
end if.
end if.
do if ( 1 = 2 or 1 =3).
compute xlp= x.
compute ylp= y.
compute pt2 = make(nrow(ylp),1,(csum(ylp)/nrow(ylp))).
do if ( 1 =2).
compute LL3 = ylp&*ln(pt2)+(1-ylp)&*ln(1-pt2).
end if.
compute LL3 = -2*csum(LL3).
compute bt1 = make(ncol(xlp),1,0).
compute LL1 = 0.
compute pt1 = make(nrow(ylp),1,0.5).
compute pt1lp=pt1.
loop jjj = 1 to iterate.
compute xlptmp=t(xlp).
compute vecprb=pt1lp&*(1-pt1lp).
loop kkk=1 to ncol(xlp).
compute xlptmp(kkk,:)=xlptmp(kkk,:)&*t(vecprb).
end loop.
compute b = bt1+inv(xlptmp*xlp)*t(xlp)*(ylp-pt1lp).
do if ( 1 =2).
compute xlpb=xlp*b.
compute xlpbt=(xlpb > -709.7).
compute xlpb709=(1-xlpbt)*(-709.7).
compute xlpb=(xlpb&*xlpbt)+xlpb709.
compute pt1lp = 1/(1+exp(-(xlpb))).
end if.
compute itprob = csum((pt1lp < .00000001) or (pt1lp > .9999999)).
do if (itprob > 0).
loop kkk = 1 to nrow(pt1lp).
do if (pt1lp(kkk,1) > .9999999).
compute pt1lp(kkk,1) = .9999999.
end if.
do if (pt1lp(kkk,1) < .00000001).
compute pt1lp(kkk,1) = .00000001.
end if.
end loop.
compute itprob = 0.
end if.
do if (itprob = 0).
do if ( 1 =2).
compute LL = ylp&*ln(pt1lp)+(1-ylp)&*ln(1-pt1lp).
end if.
compute LL2 = -2*csum(ll).
end if.
do if (abs(LL1-LL2) < converge).
do if ( 1 =1).
compute xlptmp=t(xlp).
compute vecprb=pt1lp&*(1-pt1lp).
loop kkk=1 to ncol(xlp).
compute xlptmp(kkk,:)=xlptmp(kkk,:)&*t(vecprb).
end loop.
compute varb = inv(xlptmp*xlp).
compute seb = sqrt(diag(varb)).
release xlptmp.
end if.
break.
end if.
compute bt1 = b.
compute LL1 = LL2.
end loop.
compute modres=b.
do if (jjj > iterate).
compute itprob = 2.
do if (booting=0).
compute iterrmod=1.
end if.
do if (booting=1).
compute bootiter=1.
end if.
do if (itprobtg=0).
compute itprobtg=1.
compute errcode(errs,1) = 47.
compute errs = errs + 1.
do if (booting = 0 and 1 =1).
compute vt1 = mdiag(pt1lp&*(1-pt1lp)).
compute varb = inv(t(xlp)*vt1*xlp).
compute seb = sqrt(diag(varb)).
end if.
end if.
end if.
do if ( 1 =1).
compute trat = b&/seb.
compute dfres=nrow(xlp).
compute p = 2*(1-cdfnorm(abs(trat))).
compute modres={modres,seb,trat,p}.
compute modres={modres,(b-xp2&*seb),(b+xp2&*seb)}.
compute pvchi=1-chicdf((LL3-LL2),(nrow(modres)-1)).
compute mcF = (LL3-LL2)/LL3.
compute cox = 1-exp(-(LL3-LL2)/nrow(xlp)).
compute nagel = cox/(1-exp(-(LL3)/nrow(xlp))).
compute modsum={LL2,(LL3-LL2),(nrow(modres)-1),pvchi, mcF,cox,nagel}.
compute modsuml={'-2LL','ModelLL', 'df', 'p', 'McFadden', 'CoxSnell', 'Nagelkrk'}.
compute modresl={'coeff','se','Z','p','LLCI','ULCI'}.
end if.
end if
.
end if.
do if ((i = (nms+nys)) and (ydich=1)).

.
do if ( 2 =1).
compute hatmat=inv(t( x )* x )*t( x ).
compute b = hatmat* y.
compute modres=b.
do if ( 1 =1).
compute n1=nrow( x ).
compute dfres=n1-(ncol( x )).
compute sstotal = t( y -(csum( y )/n1))*( y -(csum( y )/n1)).
compute resid= y - x *b.
compute ssresid = csum((resid)&**2).
compute r2 = (sstotal-ssresid)/sstotal.
do if (r2 < 0).
compute r2=0.
end if.
compute adjr2 = 1-((1-r2)*(n1-1)/(dfres)).
compute mse=ssresid/(n1-ncol( x )).

.
compute n1=nrow( x ).
compute invXtX = inv(t( x )* x ).
compute varb = mse *invXtX.
compute k3 = ncol( x ).
compute xhc=0.
do if ( hc <> 5).
compute xhc= x.
compute hat = xhc(:,1).
loop i3=1 to nrow(xhc).
compute hat(i3,1)= xhc(i3,:)*invXtX*t(xhc(i3,:)).
end loop.
do if ( hc = 0 or hc =1).
loop i3 = 1 to k3.
compute xhc(:,i3)=xhc(:,i3)&* resid.
end loop.
end if.
do if ( hc =3 or hc =2).
loop i3=1 to k3.
compute xhc(:,i3) = ( resid &/(1-hat)&**(1/(4- hc )))&*xhc(:,i3).
end loop.
end if.
do if ( hc = 4).
compute hcmn=make(n,2,4).
compute hcmn(:,2)=(n1*hat)/k3.
loop i3= 1 to k3.
compute xhc(:,i3) = ( resid &/(1-hat)&**(rmin(hcmn)/2))&*xhc(:,i3).
end loop.
end if.
compute varb=(invXtX*t(xhc)*xhc*invXtX).
do if ( hc =1).
compute varb=(n1/(n1-ncol( x )))&*varb.
end if.
end if.
compute hclab={'se(HC0)','se(HC1)','se(HC2)','se(HC3)','se(HC4)','se'}.
compute hclab=hclab(1,( hc +1)).
compute hcflab={'F(HC0)','F(HC1)','F(HC2)','F(HC3)','F(HC4)','F'}.
compute hcflab=hcflab(1,( hc +1)).
release xhc
.
compute seb=sqrt(diag(varb)).
compute trat = b&/seb.
compute p = 2*(1-tcdf(abs(trat), (dfres))).
compute tval = sqrt(dfres* (exp((dfres-(5/6))*((xp2/(dfres-(2/3)+(.11/dfres)))* (xp2/(dfres-(2/3)+(.11/dfres)))))-1)).
compute modres={modres,seb,trat,p}.
compute modres={modres,(b-tval&*seb),(b+tval&*seb)}.
compute modresl={'coeff',hclab,'t','p','LLCI','ULCI'}.
compute lmat = ident(ncol( x )).
compute lmat = lmat(:,2:ncol(lmat)).
compute fratio = (t(t(lmat)*b)*inv(t(lmat)*varb*lmat)*((t(lmat)*b)))/(ncol( x )-1).
compute pfr = 1-fcdf(fratio,(ncol( x )-1),dfres).
compute modsum={sqrt(r2),r2,mse,fratio,(ncol( x )-1),dfres,pfr}.
compute modsuml={'R','R-sq','MSE',hcflab,'df1','df2', 'p'}.
end if.
end if.
do if ( 2 = 2 or 2 =3).
compute xlp= x.
compute ylp= y.
compute pt2 = make(nrow(ylp),1,(csum(ylp)/nrow(ylp))).
do if ( 2 =2).
compute LL3 = ylp&*ln(pt2)+(1-ylp)&*ln(1-pt2).
end if.
compute LL3 = -2*csum(LL3).
compute bt1 = make(ncol(xlp),1,0).
compute LL1 = 0.
compute pt1 = make(nrow(ylp),1,0.5).
compute pt1lp=pt1.
loop jjj = 1 to iterate.
compute xlptmp=t(xlp).
compute vecprb=pt1lp&*(1-pt1lp).
loop kkk=1 to ncol(xlp).
compute xlptmp(kkk,:)=xlptmp(kkk,:)&*t(vecprb).
end loop.
compute b = bt1+inv(xlptmp*xlp)*t(xlp)*(ylp-pt1lp).
do if ( 2 =2).
compute xlpb=xlp*b.
compute xlpbt=(xlpb > -709.7).
compute xlpb709=(1-xlpbt)*(-709.7).
compute xlpb=(xlpb&*xlpbt)+xlpb709.
compute pt1lp = 1/(1+exp(-(xlpb))).
end if.
compute itprob = csum((pt1lp < .00000001) or (pt1lp > .9999999)).
do if (itprob > 0).
loop kkk = 1 to nrow(pt1lp).
do if (pt1lp(kkk,1) > .9999999).
compute pt1lp(kkk,1) = .9999999.
end if.
do if (pt1lp(kkk,1) < .00000001).
compute pt1lp(kkk,1) = .00000001.
end if.
end loop.
compute itprob = 0.
end if.
do if (itprob = 0).
do if ( 2 =2).
compute LL = ylp&*ln(pt1lp)+(1-ylp)&*ln(1-pt1lp).
end if.
compute LL2 = -2*csum(ll).
end if.
do if (abs(LL1-LL2) < converge).
do if ( 1 =1).
compute xlptmp=t(xlp).
compute vecprb=pt1lp&*(1-pt1lp).
loop kkk=1 to ncol(xlp).
compute xlptmp(kkk,:)=xlptmp(kkk,:)&*t(vecprb).
end loop.
compute varb = inv(xlptmp*xlp).
compute seb = sqrt(diag(varb)).
release xlptmp.
end if.
break.
end if.
compute bt1 = b.
compute LL1 = LL2.
end loop.
compute modres=b.
do if (jjj > iterate).
compute itprob = 2.
do if (booting=0).
compute iterrmod=1.
end if.
do if (booting=1).
compute bootiter=1.
end if.
do if (itprobtg=0).
compute itprobtg=1.
compute errcode(errs,1) = 47.
compute errs = errs + 1.
do if (booting = 0 and 1 =1).
compute vt1 = mdiag(pt1lp&*(1-pt1lp)).
compute varb = inv(t(xlp)*vt1*xlp).
compute seb = sqrt(diag(varb)).
end if.
end if.
end if.
do if ( 1 =1).
compute trat = b&/seb.
compute dfres=nrow(xlp).
compute p = 2*(1-cdfnorm(abs(trat))).
compute modres={modres,seb,trat,p}.
compute modres={modres,(b-xp2&*seb),(b+xp2&*seb)}.
compute pvchi=1-chicdf((LL3-LL2),(nrow(modres)-1)).
compute mcF = (LL3-LL2)/LL3.
compute cox = 1-exp(-(LL3-LL2)/nrow(xlp)).
compute nagel = cox/(1-exp(-(LL3)/nrow(xlp))).
compute modsum={LL2,(LL3-LL2),(nrow(modres)-1),pvchi, mcF,cox,nagel}.
compute modsuml={'-2LL','ModelLL', 'df', 'p', 'McFadden', 'CoxSnell', 'Nagelkrk'}.
compute modresl={'coeff','se','Z','p','LLCI','ULCI'}.
end if.
end if
.
compute chidfxm=basemodx-LL2.
end if.
compute lmat=make(nrow(b),nxvls,0).
compute lmattmp=ident(nxvls).
compute lmat((nrow(lmat)-nxvls+1):nrow(lmat),:)=lmattmp.

.
compute lmat2= lmat.
do if ( 1 =0).
compute lmat2 = mdiag( lmat ).
compute lmat3=make(nrow(lmat2),1,0).
loop flp=1 to ncol(lmat2).
do if (csum(lmat2(:,flp))=1).
compute lmat3={lmat3,lmat2(:,flp)}.
end if.
end loop.
compute lmat2=lmat3(:,2:ncol(lmat3)).
end if.
compute fratio = (t(t(lmat2)* b )*inv(t(lmat2)* varb *lmat2)*((t(lmat2)* b )))/ncol(lmat2).
compute pfr = 1-fcdf(fratio,ncol(lmat2),(n-nrow( b ))).
compute fresult={fratio,ncol(lmat2),(n-nrow( b )),pfr}.
do if (i = (nms+nys) and (ydich=1)).
compute fratio=fratio*ncol(lmat2).
compute pfr=1-chicdf(fratio,ncol(lmat2)).
compute fresult={fratio,ncol(lmat2),pfr}.
end if.
do if ( 0 =1).
compute lmat3=1-rsum(lmat2).
compute xfm=make(n,csum(lmat3),0).
compute flpc=1.
loop flp=1 to nrow(lmat3).
do if (lmat3(flp,1)=1).
compute xfm(:,flpc)=x(:,flp).
compute flpc=flpc+1.
end if.
end loop.
compute bfm=inv(t(xfm)*xfm)*t(xfm)*y.
compute resid=y-(xfm*bfm).
compute sstotal=(y-(csum(y)/n)).
compute sstotal=csum(sstotal&*sstotal).
compute ssresid=csum(resid&*resid).
compute rsqch= 0 -((sstotal-ssresid)/sstotal).
compute fresult={rsqch,fresult}.
release xfm,flpc, resid, ssresid, bfm.
end if
.
compute numxint=numxint+1.
compute xmtst(numxint,:)=fresult.
do if ((i = (nms+nys)) and (ydich=1)).
compute xmtst(numxint,1)=chidfxm.
compute xmtst(numxint,3)=1-chicdf(chidfxm,nxvls).
end if.
compute xmtstlb={xmtstlb;highlbx((xmints-1),1)}.
end if.
end loop.
compute x=xmtmat.
release xmtmat.
do if (numxint>0).
compute xmtstlb=xmtstlb((2:(numxint+1)),:).
compute xmtst=xmtst(1:numxint,:).
compute resultm2=make(nrow(xmtst),maxresm,99999).
compute resultm2(1:nrow(xmtst),1:ncol(xmtst))=xmtst.
compute resultm={resultm;resultm2}.
do if (nms=1).
compute xmtstlb=' '.
end if.
do if (outscree=1).
do if ((i < (nms+nys)) or (ydich=0)).
print xmtst/title='Test(s) of X by M interaction:'/rnames=xmtstlb/cnames=xmtstlbc/format= F10.4.
end if.
do if ((i = (nms+nys)) and (ydich=1)).
print xmtst/title='Likelihood ratio test(s) of X by M interaction:'/rnames=xmtstlb/cnames=xmtstlbc/format= F10.4.
end if.
end if.
end if.
compute r2=r2tmp.
compute b=btmp.
compute varb=varbtmp.
compute dfres=dfrestmp.
compute tval=tvaltmp.
end if.
do if (criterr = 0).
compute jj=0.
loop j = start to ((start+i)-1).
compute dbint=0.
compute lmat=whigh(1:nump(1,i),j).
compute lmat2=wzhigh(1:nump(1,i),j).
do if ((csum(lmat) > 0) and (csum(lmat2) = 0)).
do if ((i < (nms+nys)) or (ydich <> 1)).

.
compute lmat2= lmat.
do if ( 0 =0).
compute lmat2 = mdiag( lmat ).
compute lmat3=make(nrow(lmat2),1,0).
loop flp=1 to ncol(lmat2).
do if (csum(lmat2(:,flp))=1).
compute lmat3={lmat3,lmat2(:,flp)}.
end if.
end loop.
compute lmat2=lmat3(:,2:ncol(lmat3)).
end if.
compute fratio = (t(t(lmat2)* b )*inv(t(lmat2)* varb *lmat2)*((t(lmat2)* b )))/ncol(lmat2).
compute pfr = 1-fcdf(fratio,ncol(lmat2),(n-nrow( b ))).
compute fresult={fratio,ncol(lmat2),(n-nrow( b )),pfr}.
do if (i = (nms+nys) and (ydich=1)).
compute fratio=fratio*ncol(lmat2).
compute pfr=1-chicdf(fratio,ncol(lmat2)).
compute fresult={fratio,ncol(lmat2),pfr}.
end if.
do if ( 1 =1).
compute lmat3=1-rsum(lmat2).
compute xfm=make(n,csum(lmat3),0).
compute flpc=1.
loop flp=1 to nrow(lmat3).
do if (lmat3(flp,1)=1).
compute xfm(:,flpc)=x(:,flp).
compute flpc=flpc+1.
end if.
end loop.
compute bfm=inv(t(xfm)*xfm)*t(xfm)*y.
compute resid=y-(xfm*bfm).
compute sstotal=(y-(csum(y)/n)).
compute sstotal=csum(sstotal&*sstotal).
compute ssresid=csum(resid&*resid).
compute rsqch= r2 -((sstotal-ssresid)/sstotal).
compute fresult={rsqch,fresult}.
release xfm,flpc, resid, ssresid, bfm.
end if
.
compute lmatdb=lmat.
compute dbint=dbint+1.
end if.
do if ((ydich = 1) and (i = (nms+nys))).

.
compute btemphld=b.
compute llrdat=make(nrow(x),nrow( lmat )-csum( lmat ),-999).
compute llrdf=ncol(x)-ncol(llrdat).
compute llrcnt=0.
loop llri=1 to nrow( lmat ).
do if ( lmat (llri,1)=0).
compute llrcnt=llrcnt+1.
compute llrdat(:,llrcnt)=x(:,llri).
end if.
end loop.

.
do if ( 2 =1).
compute hatmat=inv(t( llrdat )* llrdat )*t( llrdat ).
compute b = hatmat* y.
compute modres=b.
do if ( 0 =1).
compute n1=nrow( llrdat ).
compute dfres=n1-(ncol( llrdat )).
compute sstotal = t( y -(csum( y )/n1))*( y -(csum( y )/n1)).
compute resid= y - llrdat *b.
compute ssresid = csum((resid)&**2).
compute r2 = (sstotal-ssresid)/sstotal.
do if (r2 < 0).
compute r2=0.
end if.
compute adjr2 = 1-((1-r2)*(n1-1)/(dfres)).
compute mse=ssresid/(n1-ncol( llrdat )).

.
compute n1=nrow( x ).
compute invXtX = inv(t( x )* x ).
compute varb = mse *invXtX.
compute k3 = ncol( x ).
compute xhc=0.
do if ( hc <> 5).
compute xhc= x.
compute hat = xhc(:,1).
loop i3=1 to nrow(xhc).
compute hat(i3,1)= xhc(i3,:)*invXtX*t(xhc(i3,:)).
end loop.
do if ( hc = 0 or hc =1).
loop i3 = 1 to k3.
compute xhc(:,i3)=xhc(:,i3)&* resid.
end loop.
end if.
do if ( hc =3 or hc =2).
loop i3=1 to k3.
compute xhc(:,i3) = ( resid &/(1-hat)&**(1/(4- hc )))&*xhc(:,i3).
end loop.
end if.
do if ( hc = 4).
compute hcmn=make(n,2,4).
compute hcmn(:,2)=(n1*hat)/k3.
loop i3= 1 to k3.
compute xhc(:,i3) = ( resid &/(1-hat)&**(rmin(hcmn)/2))&*xhc(:,i3).
end loop.
end if.
compute varb=(invXtX*t(xhc)*xhc*invXtX).
do if ( hc =1).
compute varb=(n1/(n1-ncol( x )))&*varb.
end if.
end if.
compute hclab={'se(HC0)','se(HC1)','se(HC2)','se(HC3)','se(HC4)','se'}.
compute hclab=hclab(1,( hc +1)).
compute hcflab={'F(HC0)','F(HC1)','F(HC2)','F(HC3)','F(HC4)','F'}.
compute hcflab=hcflab(1,( hc +1)).
release xhc
.
compute seb=sqrt(diag(varb)).
compute trat = b&/seb.
compute p = 2*(1-tcdf(abs(trat), (dfres))).
compute tval = sqrt(dfres* (exp((dfres-(5/6))*((xp2/(dfres-(2/3)+(.11/dfres)))* (xp2/(dfres-(2/3)+(.11/dfres)))))-1)).
compute modres={modres,seb,trat,p}.
compute modres={modres,(b-tval&*seb),(b+tval&*seb)}.
compute modresl={'coeff',hclab,'t','p','LLCI','ULCI'}.
compute lmat = ident(ncol( llrdat )).
compute lmat = lmat(:,2:ncol(lmat)).
compute fratio = (t(t(lmat)*b)*inv(t(lmat)*varb*lmat)*((t(lmat)*b)))/(ncol( llrdat )-1).
compute pfr = 1-fcdf(fratio,(ncol( llrdat )-1),dfres).
compute modsum={sqrt(r2),r2,mse,fratio,(ncol( llrdat )-1),dfres,pfr}.
compute modsuml={'R','R-sq','MSE',hcflab,'df1','df2', 'p'}.
end if.
end if.
do if ( 2 = 2 or 2 =3).
compute xlp= llrdat.
compute ylp= y.
compute pt2 = make(nrow(ylp),1,(csum(ylp)/nrow(ylp))).
do if ( 2 =2).
compute LL3 = ylp&*ln(pt2)+(1-ylp)&*ln(1-pt2).
end if.
compute LL3 = -2*csum(LL3).
compute bt1 = make(ncol(xlp),1,0).
compute LL1 = 0.
compute pt1 = make(nrow(ylp),1,0.5).
compute pt1lp=pt1.
loop jjj = 1 to iterate.
compute xlptmp=t(xlp).
compute vecprb=pt1lp&*(1-pt1lp).
loop kkk=1 to ncol(xlp).
compute xlptmp(kkk,:)=xlptmp(kkk,:)&*t(vecprb).
end loop.
compute b = bt1+inv(xlptmp*xlp)*t(xlp)*(ylp-pt1lp).
do if ( 2 =2).
compute xlpb=xlp*b.
compute xlpbt=(xlpb > -709.7).
compute xlpb709=(1-xlpbt)*(-709.7).
compute xlpb=(xlpb&*xlpbt)+xlpb709.
compute pt1lp = 1/(1+exp(-(xlpb))).
end if.
compute itprob = csum((pt1lp < .00000001) or (pt1lp > .9999999)).
do if (itprob > 0).
loop kkk = 1 to nrow(pt1lp).
do if (pt1lp(kkk,1) > .9999999).
compute pt1lp(kkk,1) = .9999999.
end if.
do if (pt1lp(kkk,1) < .00000001).
compute pt1lp(kkk,1) = .00000001.
end if.
end loop.
compute itprob = 0.
end if.
do if (itprob = 0).
do if ( 2 =2).
compute LL = ylp&*ln(pt1lp)+(1-ylp)&*ln(1-pt1lp).
end if.
compute LL2 = -2*csum(ll).
end if.
do if (abs(LL1-LL2) < converge).
do if ( 0 =1).
compute xlptmp=t(xlp).
compute vecprb=pt1lp&*(1-pt1lp).
loop kkk=1 to ncol(xlp).
compute xlptmp(kkk,:)=xlptmp(kkk,:)&*t(vecprb).
end loop.
compute varb = inv(xlptmp*xlp).
compute seb = sqrt(diag(varb)).
release xlptmp.
end if.
break.
end if.
compute bt1 = b.
compute LL1 = LL2.
end loop.
compute modres=b.
do if (jjj > iterate).
compute itprob = 2.
do if (booting=0).
compute iterrmod=1.
end if.
do if (booting=1).
compute bootiter=1.
end if.
do if (itprobtg=0).
compute itprobtg=1.
compute errcode(errs,1) = 47.
compute errs = errs + 1.
do if (booting = 0 and 0 =1).
compute vt1 = mdiag(pt1lp&*(1-pt1lp)).
compute varb = inv(t(xlp)*vt1*xlp).
compute seb = sqrt(diag(varb)).
end if.
end if.
end if.
do if ( 0 =1).
compute trat = b&/seb.
compute dfres=nrow(xlp).
compute p = 2*(1-cdfnorm(abs(trat))).
compute modres={modres,seb,trat,p}.
compute modres={modres,(b-xp2&*seb),(b+xp2&*seb)}.
compute pvchi=1-chicdf((LL3-LL2),(nrow(modres)-1)).
compute mcF = (LL3-LL2)/LL3.
compute cox = 1-exp(-(LL3-LL2)/nrow(xlp)).
compute nagel = cox/(1-exp(-(LL3)/nrow(xlp))).
compute modsum={LL2,(LL3-LL2),(nrow(modres)-1),pvchi, mcF,cox,nagel}.
compute modsuml={'-2LL','ModelLL', 'df', 'p', 'McFadden', 'CoxSnell', 'Nagelkrk'}.
compute modresl={'coeff','se','Z','p','LLCI','ULCI'}.
end if.
end if
.
compute b=btemphld.
compute fresult={(LL2-basemod),llrdf,1-chicdf((LL2-basemod),llrdf)}
.
compute lmatdb=lmat.
compute dbint=dbint+1.
end if.
compute highf={highf;fresult}.
compute highf2={highf2;fresult}.
do if (j = start).
compute flabel={flabel;'X*W'}.
end if.
do if (j > start).
do if (nms > 1).
compute flabel={flabel;highlbw(jj,1)}.
else if (nms = 1).
do if (xmint=0).
compute flabel={flabel;'M*W'}.
end if.
do if (xmint=1).
compute flabel={flabel;'X*M'}.
end if.
end if.
end if.
end if.
compute lmat=zhigh(1:nump(1,i),j).
compute lmat2=wzhigh(1:nump(1,i),j).
do if ((csum(lmat) > 0) and (csum(lmat2) = 0)).
do if ((i < (nms+nys)) or (ydich <> 1)).

.
compute lmat2= lmat.
do if ( 0 =0).
compute lmat2 = mdiag( lmat ).
compute lmat3=make(nrow(lmat2),1,0).
loop flp=1 to ncol(lmat2).
do if (csum(lmat2(:,flp))=1).
compute lmat3={lmat3,lmat2(:,flp)}.
end if.
end loop.
compute lmat2=lmat3(:,2:ncol(lmat3)).
end if.
compute fratio = (t(t(lmat2)* b )*inv(t(lmat2)* varb *lmat2)*((t(lmat2)* b )))/ncol(lmat2).
compute pfr = 1-fcdf(fratio,ncol(lmat2),(n-nrow( b ))).
compute fresult={fratio,ncol(lmat2),(n-nrow( b )),pfr}.
do if (i = (nms+nys) and (ydich=1)).
compute fratio=fratio*ncol(lmat2).
compute pfr=1-chicdf(fratio,ncol(lmat2)).
compute fresult={fratio,ncol(lmat2),pfr}.
end if.
do if ( 1 =1).
compute lmat3=1-rsum(lmat2).
compute xfm=make(n,csum(lmat3),0).
compute flpc=1.
loop flp=1 to nrow(lmat3).
do if (lmat3(flp,1)=1).
compute xfm(:,flpc)=x(:,flp).
compute flpc=flpc+1.
end if.
end loop.
compute bfm=inv(t(xfm)*xfm)*t(xfm)*y.
compute resid=y-(xfm*bfm).
compute sstotal=(y-(csum(y)/n)).
compute sstotal=csum(sstotal&*sstotal).
compute ssresid=csum(resid&*resid).
compute rsqch= r2 -((sstotal-ssresid)/sstotal).
compute fresult={rsqch,fresult}.
release xfm,flpc, resid, ssresid, bfm.
end if
.
compute dbint=dbint+1.
end if.
do if ((ydich = 1) and (i = (nms+nys))).

.
compute btemphld=b.
compute llrdat=make(nrow(x),nrow( lmat )-csum( lmat ),-999).
compute llrdf=ncol(x)-ncol(llrdat).
compute llrcnt=0.
loop llri=1 to nrow( lmat ).
do if ( lmat (llri,1)=0).
compute llrcnt=llrcnt+1.
compute llrdat(:,llrcnt)=x(:,llri).
end if.
end loop.

.
do if ( 2 =1).
compute hatmat=inv(t( llrdat )* llrdat )*t( llrdat ).
compute b = hatmat* y.
compute modres=b.
do if ( 0 =1).
compute n1=nrow( llrdat ).
compute dfres=n1-(ncol( llrdat )).
compute sstotal = t( y -(csum( y )/n1))*( y -(csum( y )/n1)).
compute resid= y - llrdat *b.
compute ssresid = csum((resid)&**2).
compute r2 = (sstotal-ssresid)/sstotal.
do if (r2 < 0).
compute r2=0.
end if.
compute adjr2 = 1-((1-r2)*(n1-1)/(dfres)).
compute mse=ssresid/(n1-ncol( llrdat )).

.
compute n1=nrow( x ).
compute invXtX = inv(t( x )* x ).
compute varb = mse *invXtX.
compute k3 = ncol( x ).
compute xhc=0.
do if ( hc <> 5).
compute xhc= x.
compute hat = xhc(:,1).
loop i3=1 to nrow(xhc).
compute hat(i3,1)= xhc(i3,:)*invXtX*t(xhc(i3,:)).
end loop.
do if ( hc = 0 or hc =1).
loop i3 = 1 to k3.
compute xhc(:,i3)=xhc(:,i3)&* resid.
end loop.
end if.
do if ( hc =3 or hc =2).
loop i3=1 to k3.
compute xhc(:,i3) = ( resid &/(1-hat)&**(1/(4- hc )))&*xhc(:,i3).
end loop.
end if.
do if ( hc = 4).
compute hcmn=make(n,2,4).
compute hcmn(:,2)=(n1*hat)/k3.
loop i3= 1 to k3.
compute xhc(:,i3) = ( resid &/(1-hat)&**(rmin(hcmn)/2))&*xhc(:,i3).
end loop.
end if.
compute varb=(invXtX*t(xhc)*xhc*invXtX).
do if ( hc =1).
compute varb=(n1/(n1-ncol( x )))&*varb.
end if.
end if.
compute hclab={'se(HC0)','se(HC1)','se(HC2)','se(HC3)','se(HC4)','se'}.
compute hclab=hclab(1,( hc +1)).
compute hcflab={'F(HC0)','F(HC1)','F(HC2)','F(HC3)','F(HC4)','F'}.
compute hcflab=hcflab(1,( hc +1)).
release xhc
.
compute seb=sqrt(diag(varb)).
compute trat = b&/seb.
compute p = 2*(1-tcdf(abs(trat), (dfres))).
compute tval = sqrt(dfres* (exp((dfres-(5/6))*((xp2/(dfres-(2/3)+(.11/dfres)))* (xp2/(dfres-(2/3)+(.11/dfres)))))-1)).
compute modres={modres,seb,trat,p}.
compute modres={modres,(b-tval&*seb),(b+tval&*seb)}.
compute modresl={'coeff',hclab,'t','p','LLCI','ULCI'}.
compute lmat = ident(ncol( llrdat )).
compute lmat = lmat(:,2:ncol(lmat)).
compute fratio = (t(t(lmat)*b)*inv(t(lmat)*varb*lmat)*((t(lmat)*b)))/(ncol( llrdat )-1).
compute pfr = 1-fcdf(fratio,(ncol( llrdat )-1),dfres).
compute modsum={sqrt(r2),r2,mse,fratio,(ncol( llrdat )-1),dfres,pfr}.
compute modsuml={'R','R-sq','MSE',hcflab,'df1','df2', 'p'}.
end if.
end if.
do if ( 2 = 2 or 2 =3).
compute xlp= llrdat.
compute ylp= y.
compute pt2 = make(nrow(ylp),1,(csum(ylp)/nrow(ylp))).
do if ( 2 =2).
compute LL3 = ylp&*ln(pt2)+(1-ylp)&*ln(1-pt2).
end if.
compute LL3 = -2*csum(LL3).
compute bt1 = make(ncol(xlp),1,0).
compute LL1 = 0.
compute pt1 = make(nrow(ylp),1,0.5).
compute pt1lp=pt1.
loop jjj = 1 to iterate.
compute xlptmp=t(xlp).
compute vecprb=pt1lp&*(1-pt1lp).
loop kkk=1 to ncol(xlp).
compute xlptmp(kkk,:)=xlptmp(kkk,:)&*t(vecprb).
end loop.
compute b = bt1+inv(xlptmp*xlp)*t(xlp)*(ylp-pt1lp).
do if ( 2 =2).
compute xlpb=xlp*b.
compute xlpbt=(xlpb > -709.7).
compute xlpb709=(1-xlpbt)*(-709.7).
compute xlpb=(xlpb&*xlpbt)+xlpb709.
compute pt1lp = 1/(1+exp(-(xlpb))).
end if.
compute itprob = csum((pt1lp < .00000001) or (pt1lp > .9999999)).
do if (itprob > 0).
loop kkk = 1 to nrow(pt1lp).
do if (pt1lp(kkk,1) > .9999999).
compute pt1lp(kkk,1) = .9999999.
end if.
do if (pt1lp(kkk,1) < .00000001).
compute pt1lp(kkk,1) = .00000001.
end if.
end loop.
compute itprob = 0.
end if.
do if (itprob = 0).
do if ( 2 =2).
compute LL = ylp&*ln(pt1lp)+(1-ylp)&*ln(1-pt1lp).
end if.
compute LL2 = -2*csum(ll).
end if.
do if (abs(LL1-LL2) < converge).
do if ( 0 =1).
compute xlptmp=t(xlp).
compute vecprb=pt1lp&*(1-pt1lp).
loop kkk=1 to ncol(xlp).
compute xlptmp(kkk,:)=xlptmp(kkk,:)&*t(vecprb).
end loop.
compute varb = inv(xlptmp*xlp).
compute seb = sqrt(diag(varb)).
release xlptmp.
end if.
break.
end if.
compute bt1 = b.
compute LL1 = LL2.
end loop.
compute modres=b.
do if (jjj > iterate).
compute itprob = 2.
do if (booting=0).
compute iterrmod=1.
end if.
do if (booting=1).
compute bootiter=1.
end if.
do if (itprobtg=0).
compute itprobtg=1.
compute errcode(errs,1) = 47.
compute errs = errs + 1.
do if (booting = 0 and 0 =1).
compute vt1 = mdiag(pt1lp&*(1-pt1lp)).
compute varb = inv(t(xlp)*vt1*xlp).
compute seb = sqrt(diag(varb)).
end if.
end if.
end if.
do if ( 0 =1).
compute trat = b&/seb.
compute dfres=nrow(xlp).
compute p = 2*(1-cdfnorm(abs(trat))).
compute modres={modres,seb,trat,p}.
compute modres={modres,(b-xp2&*seb),(b+xp2&*seb)}.
compute pvchi=1-chicdf((LL3-LL2),(nrow(modres)-1)).
compute mcF = (LL3-LL2)/LL3.
compute cox = 1-exp(-(LL3-LL2)/nrow(xlp)).
compute nagel = cox/(1-exp(-(LL3)/nrow(xlp))).
compute modsum={LL2,(LL3-LL2),(nrow(modres)-1),pvchi, mcF,cox,nagel}.
compute modsuml={'-2LL','ModelLL', 'df', 'p', 'McFadden', 'CoxSnell', 'Nagelkrk'}.
compute modresl={'coeff','se','Z','p','LLCI','ULCI'}.
end if.
end if
.
compute b=btemphld.
compute fresult={(LL2-basemod),llrdf,1-chicdf((LL2-basemod),llrdf)}
.
compute dbint=dbint+1.
end if.
compute highf={highf;fresult}.
compute highf2={highf2;fresult}.
do if (j = start).
compute flabel={flabel;'X*Z'}.
end if.
do if (j > start).
do if (nms > 1).
compute flabel={flabel;highlbz(jj,1)}.
else if (nms = 1).
compute flabel={flabel;'M*Z'}.
end if.
end if.
end if.
do if (dbint=2).
compute lmatdb=lmatdb+lmat.
do if ((ydich = 1) and (i = (nms+nys))).

.
compute btemphld=b.
compute llrdat=make(nrow(x),nrow( lmatdb )-csum( lmatdb ),-999).
compute llrdf=ncol(x)-ncol(llrdat).
compute llrcnt=0.
loop llri=1 to nrow( lmatdb ).
do if ( lmatdb (llri,1)=0).
compute llrcnt=llrcnt+1.
compute llrdat(:,llrcnt)=x(:,llri).
end if.
end loop.

.
do if ( 2 =1).
compute hatmat=inv(t( llrdat )* llrdat )*t( llrdat ).
compute b = hatmat* y.
compute modres=b.
do if ( 0 =1).
compute n1=nrow( llrdat ).
compute dfres=n1-(ncol( llrdat )).
compute sstotal = t( y -(csum( y )/n1))*( y -(csum( y )/n1)).
compute resid= y - llrdat *b.
compute ssresid = csum((resid)&**2).
compute r2 = (sstotal-ssresid)/sstotal.
do if (r2 < 0).
compute r2=0.
end if.
compute adjr2 = 1-((1-r2)*(n1-1)/(dfres)).
compute mse=ssresid/(n1-ncol( llrdat )).

.
compute n1=nrow( x ).
compute invXtX = inv(t( x )* x ).
compute varb = mse *invXtX.
compute k3 = ncol( x ).
compute xhc=0.
do if ( hc <> 5).
compute xhc= x.
compute hat = xhc(:,1).
loop i3=1 to nrow(xhc).
compute hat(i3,1)= xhc(i3,:)*invXtX*t(xhc(i3,:)).
end loop.
do if ( hc = 0 or hc =1).
loop i3 = 1 to k3.
compute xhc(:,i3)=xhc(:,i3)&* resid.
end loop.
end if.
do if ( hc =3 or hc =2).
loop i3=1 to k3.
compute xhc(:,i3) = ( resid &/(1-hat)&**(1/(4- hc )))&*xhc(:,i3).
end loop.
end if.
do if ( hc = 4).
compute hcmn=make(n,2,4).
compute hcmn(:,2)=(n1*hat)/k3.
loop i3= 1 to k3.
compute xhc(:,i3) = ( resid &/(1-hat)&**(rmin(hcmn)/2))&*xhc(:,i3).
end loop.
end if.
compute varb=(invXtX*t(xhc)*xhc*invXtX).
do if ( hc =1).
compute varb=(n1/(n1-ncol( x )))&*varb.
end if.
end if.
compute hclab={'se(HC0)','se(HC1)','se(HC2)','se(HC3)','se(HC4)','se'}.
compute hclab=hclab(1,( hc +1)).
compute hcflab={'F(HC0)','F(HC1)','F(HC2)','F(HC3)','F(HC4)','F'}.
compute hcflab=hcflab(1,( hc +1)).
release xhc
.
compute seb=sqrt(diag(varb)).
compute trat = b&/seb.
compute p = 2*(1-tcdf(abs(trat), (dfres))).
compute tval = sqrt(dfres* (exp((dfres-(5/6))*((xp2/(dfres-(2/3)+(.11/dfres)))* (xp2/(dfres-(2/3)+(.11/dfres)))))-1)).
compute modres={modres,seb,trat,p}.
compute modres={modres,(b-tval&*seb),(b+tval&*seb)}.
compute modresl={'coeff',hclab,'t','p','LLCI','ULCI'}.
compute lmat = ident(ncol( llrdat )).
compute lmat = lmat(:,2:ncol(lmat)).
compute fratio = (t(t(lmat)*b)*inv(t(lmat)*varb*lmat)*((t(lmat)*b)))/(ncol( llrdat )-1).
compute pfr = 1-fcdf(fratio,(ncol( llrdat )-1),dfres).
compute modsum={sqrt(r2),r2,mse,fratio,(ncol( llrdat )-1),dfres,pfr}.
compute modsuml={'R','R-sq','MSE',hcflab,'df1','df2', 'p'}.
end if.
end if.
do if ( 2 = 2 or 2 =3).
compute xlp= llrdat.
compute ylp= y.
compute pt2 = make(nrow(ylp),1,(csum(ylp)/nrow(ylp))).
do if ( 2 =2).
compute LL3 = ylp&*ln(pt2)+(1-ylp)&*ln(1-pt2).
end if.
compute LL3 = -2*csum(LL3).
compute bt1 = make(ncol(xlp),1,0).
compute LL1 = 0.
compute pt1 = make(nrow(ylp),1,0.5).
compute pt1lp=pt1.
loop jjj = 1 to iterate.
compute xlptmp=t(xlp).
compute vecprb=pt1lp&*(1-pt1lp).
loop kkk=1 to ncol(xlp).
compute xlptmp(kkk,:)=xlptmp(kkk,:)&*t(vecprb).
end loop.
compute b = bt1+inv(xlptmp*xlp)*t(xlp)*(ylp-pt1lp).
do if ( 2 =2).
compute xlpb=xlp*b.
compute xlpbt=(xlpb > -709.7).
compute xlpb709=(1-xlpbt)*(-709.7).
compute xlpb=(xlpb&*xlpbt)+xlpb709.
compute pt1lp = 1/(1+exp(-(xlpb))).
end if.
compute itprob = csum((pt1lp < .00000001) or (pt1lp > .9999999)).
do if (itprob > 0).
loop kkk = 1 to nrow(pt1lp).
do if (pt1lp(kkk,1) > .9999999).
compute pt1lp(kkk,1) = .9999999.
end if.
do if (pt1lp(kkk,1) < .00000001).
compute pt1lp(kkk,1) = .00000001.
end if.
end loop.
compute itprob = 0.
end if.
do if (itprob = 0).
do if ( 2 =2).
compute LL = ylp&*ln(pt1lp)+(1-ylp)&*ln(1-pt1lp).
end if.
compute LL2 = -2*csum(ll).
end if.
do if (abs(LL1-LL2) < converge).
do if ( 0 =1).
compute xlptmp=t(xlp).
compute vecprb=pt1lp&*(1-pt1lp).
loop kkk=1 to ncol(xlp).
compute xlptmp(kkk,:)=xlptmp(kkk,:)&*t(vecprb).
end loop.
compute varb = inv(xlptmp*xlp).
compute seb = sqrt(diag(varb)).
release xlptmp.
end if.
break.
end if.
compute bt1 = b.
compute LL1 = LL2.
end loop.
compute modres=b.
do if (jjj > iterate).
compute itprob = 2.
do if (booting=0).
compute iterrmod=1.
end if.
do if (booting=1).
compute bootiter=1.
end if.
do if (itprobtg=0).
compute itprobtg=1.
compute errcode(errs,1) = 47.
compute errs = errs + 1.
do if (booting = 0 and 0 =1).
compute vt1 = mdiag(pt1lp&*(1-pt1lp)).
compute varb = inv(t(xlp)*vt1*xlp).
compute seb = sqrt(diag(varb)).
end if.
end if.
end if.
do if ( 0 =1).
compute trat = b&/seb.
compute dfres=nrow(xlp).
compute p = 2*(1-cdfnorm(abs(trat))).
compute modres={modres,seb,trat,p}.
compute modres={modres,(b-xp2&*seb),(b+xp2&*seb)}.
compute pvchi=1-chicdf((LL3-LL2),(nrow(modres)-1)).
compute mcF = (LL3-LL2)/LL3.
compute cox = 1-exp(-(LL3-LL2)/nrow(xlp)).
compute nagel = cox/(1-exp(-(LL3)/nrow(xlp))).
compute modsum={LL2,(LL3-LL2),(nrow(modres)-1),pvchi, mcF,cox,nagel}.
compute modsuml={'-2LL','ModelLL', 'df', 'p', 'McFadden', 'CoxSnell', 'Nagelkrk'}.
compute modresl={'coeff','se','Z','p','LLCI','ULCI'}.
end if.
end if
.
compute b=btemphld.
compute fresult={(LL2-basemod),llrdf,1-chicdf((LL2-basemod),llrdf)}
.
end if.
do if (ydich <> 1 or i < (nms+nys)).

.
compute lmat2= lmatdb.
do if ( 0 =0).
compute lmat2 = mdiag( lmatdb ).
compute lmat3=make(nrow(lmat2),1,0).
loop flp=1 to ncol(lmat2).
do if (csum(lmat2(:,flp))=1).
compute lmat3={lmat3,lmat2(:,flp)}.
end if.
end loop.
compute lmat2=lmat3(:,2:ncol(lmat3)).
end if.
compute fratio = (t(t(lmat2)* b )*inv(t(lmat2)* varb *lmat2)*((t(lmat2)* b )))/ncol(lmat2).
compute pfr = 1-fcdf(fratio,ncol(lmat2),(n-nrow( b ))).
compute fresult={fratio,ncol(lmat2),(n-nrow( b )),pfr}.
do if (i = (nms+nys) and (ydich=1)).
compute fratio=fratio*ncol(lmat2).
compute pfr=1-chicdf(fratio,ncol(lmat2)).
compute fresult={fratio,ncol(lmat2),pfr}.
end if.
do if ( 1 =1).
compute lmat3=1-rsum(lmat2).
compute xfm=make(n,csum(lmat3),0).
compute flpc=1.
loop flp=1 to nrow(lmat3).
do if (lmat3(flp,1)=1).
compute xfm(:,flpc)=x(:,flp).
compute flpc=flpc+1.
end if.
end loop.
compute bfm=inv(t(xfm)*xfm)*t(xfm)*y.
compute resid=y-(xfm*bfm).
compute sstotal=(y-(csum(y)/n)).
compute sstotal=csum(sstotal&*sstotal).
compute ssresid=csum(resid&*resid).
compute rsqch= r2 -((sstotal-ssresid)/sstotal).
compute fresult={rsqch,fresult}.
release xfm,flpc, resid, ssresid, bfm.
end if
.
end if.
compute dbint=0.
compute highf={highf;fresult}.
do if (jj=0 and nms > 0).
compute flabel={flabel;'BOTH(X)'}.
end if.
do if (jj=0 and nms = 0).
compute flabel={flabel;'BOTH'}.
end if.
do if (jj>0 and nms = 1).
compute flabel={flabel;'BOTH(M)'}.
end if.
do if (nms > 1 and jj > 0).
compute flabel={flabel;highlbbt(jj,1)}.
end if.
end if.
compute lmat2=wzhigh(1:nump(1,i),j).
do if (csum(lmat2) > 0).
do if ((i < (nms+nys)) or (ydich <> 1)).

.
compute lmat2= lmat2.
do if ( 0 =0).
compute lmat2 = mdiag( lmat2 ).
compute lmat3=make(nrow(lmat2),1,0).
loop flp=1 to ncol(lmat2).
do if (csum(lmat2(:,flp))=1).
compute lmat3={lmat3,lmat2(:,flp)}.
end if.
end loop.
compute lmat2=lmat3(:,2:ncol(lmat3)).
end if.
compute fratio = (t(t(lmat2)* b )*inv(t(lmat2)* varb *lmat2)*((t(lmat2)* b )))/ncol(lmat2).
compute pfr = 1-fcdf(fratio,ncol(lmat2),(n-nrow( b ))).
compute fresult={fratio,ncol(lmat2),(n-nrow( b )),pfr}.
do if (i = (nms+nys) and (ydich=1)).
compute fratio=fratio*ncol(lmat2).
compute pfr=1-chicdf(fratio,ncol(lmat2)).
compute fresult={fratio,ncol(lmat2),pfr}.
end if.
do if ( 1 =1).
compute lmat3=1-rsum(lmat2).
compute xfm=make(n,csum(lmat3),0).
compute flpc=1.
loop flp=1 to nrow(lmat3).
do if (lmat3(flp,1)=1).
compute xfm(:,flpc)=x(:,flp).
compute flpc=flpc+1.
end if.
end loop.
compute bfm=inv(t(xfm)*xfm)*t(xfm)*y.
compute resid=y-(xfm*bfm).
compute sstotal=(y-(csum(y)/n)).
compute sstotal=csum(sstotal&*sstotal).
compute ssresid=csum(resid&*resid).
compute rsqch= r2 -((sstotal-ssresid)/sstotal).
compute fresult={rsqch,fresult}.
release xfm,flpc, resid, ssresid, bfm.
end if
.
end if.
do if ((ydich = 1) and (i = (nms+nys))).

.
compute btemphld=b.
compute llrdat=make(nrow(x),nrow( lmat2 )-csum( lmat2 ),-999).
compute llrdf=ncol(x)-ncol(llrdat).
compute llrcnt=0.
loop llri=1 to nrow( lmat2 ).
do if ( lmat2 (llri,1)=0).
compute llrcnt=llrcnt+1.
compute llrdat(:,llrcnt)=x(:,llri).
end if.
end loop.

.
do if ( 2 =1).
compute hatmat=inv(t( llrdat )* llrdat )*t( llrdat ).
compute b = hatmat* y.
compute modres=b.
do if ( 0 =1).
compute n1=nrow( llrdat ).
compute dfres=n1-(ncol( llrdat )).
compute sstotal = t( y -(csum( y )/n1))*( y -(csum( y )/n1)).
compute resid= y - llrdat *b.
compute ssresid = csum((resid)&**2).
compute r2 = (sstotal-ssresid)/sstotal.
do if (r2 < 0).
compute r2=0.
end if.
compute adjr2 = 1-((1-r2)*(n1-1)/(dfres)).
compute mse=ssresid/(n1-ncol( llrdat )).

.
compute n1=nrow( x ).
compute invXtX = inv(t( x )* x ).
compute varb = mse *invXtX.
compute k3 = ncol( x ).
compute xhc=0.
do if ( hc <> 5).
compute xhc= x.
compute hat = xhc(:,1).
loop i3=1 to nrow(xhc).
compute hat(i3,1)= xhc(i3,:)*invXtX*t(xhc(i3,:)).
end loop.
do if ( hc = 0 or hc =1).
loop i3 = 1 to k3.
compute xhc(:,i3)=xhc(:,i3)&* resid.
end loop.
end if.
do if ( hc =3 or hc =2).
loop i3=1 to k3.
compute xhc(:,i3) = ( resid &/(1-hat)&**(1/(4- hc )))&*xhc(:,i3).
end loop.
end if.
do if ( hc = 4).
compute hcmn=make(n,2,4).
compute hcmn(:,2)=(n1*hat)/k3.
loop i3= 1 to k3.
compute xhc(:,i3) = ( resid &/(1-hat)&**(rmin(hcmn)/2))&*xhc(:,i3).
end loop.
end if.
compute varb=(invXtX*t(xhc)*xhc*invXtX).
do if ( hc =1).
compute varb=(n1/(n1-ncol( x )))&*varb.
end if.
end if.
compute hclab={'se(HC0)','se(HC1)','se(HC2)','se(HC3)','se(HC4)','se'}.
compute hclab=hclab(1,( hc +1)).
compute hcflab={'F(HC0)','F(HC1)','F(HC2)','F(HC3)','F(HC4)','F'}.
compute hcflab=hcflab(1,( hc +1)).
release xhc
.
compute seb=sqrt(diag(varb)).
compute trat = b&/seb.
compute p = 2*(1-tcdf(abs(trat), (dfres))).
compute tval = sqrt(dfres* (exp((dfres-(5/6))*((xp2/(dfres-(2/3)+(.11/dfres)))* (xp2/(dfres-(2/3)+(.11/dfres)))))-1)).
compute modres={modres,seb,trat,p}.
compute modres={modres,(b-tval&*seb),(b+tval&*seb)}.
compute modresl={'coeff',hclab,'t','p','LLCI','ULCI'}.
compute lmat = ident(ncol( llrdat )).
compute lmat = lmat(:,2:ncol(lmat)).
compute fratio = (t(t(lmat)*b)*inv(t(lmat)*varb*lmat)*((t(lmat)*b)))/(ncol( llrdat )-1).
compute pfr = 1-fcdf(fratio,(ncol( llrdat )-1),dfres).
compute modsum={sqrt(r2),r2,mse,fratio,(ncol( llrdat )-1),dfres,pfr}.
compute modsuml={'R','R-sq','MSE',hcflab,'df1','df2', 'p'}.
end if.
end if.
do if ( 2 = 2 or 2 =3).
compute xlp= llrdat.
compute ylp= y.
compute pt2 = make(nrow(ylp),1,(csum(ylp)/nrow(ylp))).
do if ( 2 =2).
compute LL3 = ylp&*ln(pt2)+(1-ylp)&*ln(1-pt2).
end if.
compute LL3 = -2*csum(LL3).
compute bt1 = make(ncol(xlp),1,0).
compute LL1 = 0.
compute pt1 = make(nrow(ylp),1,0.5).
compute pt1lp=pt1.
loop jjj = 1 to iterate.
compute xlptmp=t(xlp).
compute vecprb=pt1lp&*(1-pt1lp).
loop kkk=1 to ncol(xlp).
compute xlptmp(kkk,:)=xlptmp(kkk,:)&*t(vecprb).
end loop.
compute b = bt1+inv(xlptmp*xlp)*t(xlp)*(ylp-pt1lp).
do if ( 2 =2).
compute xlpb=xlp*b.
compute xlpbt=(xlpb > -709.7).
compute xlpb709=(1-xlpbt)*(-709.7).
compute xlpb=(xlpb&*xlpbt)+xlpb709.
compute pt1lp = 1/(1+exp(-(xlpb))).
end if.
compute itprob = csum((pt1lp < .00000001) or (pt1lp > .9999999)).
do if (itprob > 0).
loop kkk = 1 to nrow(pt1lp).
do if (pt1lp(kkk,1) > .9999999).
compute pt1lp(kkk,1) = .9999999.
end if.
do if (pt1lp(kkk,1) < .00000001).
compute pt1lp(kkk,1) = .00000001.
end if.
end loop.
compute itprob = 0.
end if.
do if (itprob = 0).
do if ( 2 =2).
compute LL = ylp&*ln(pt1lp)+(1-ylp)&*ln(1-pt1lp).
end if.
compute LL2 = -2*csum(ll).
end if.
do if (abs(LL1-LL2) < converge).
do if ( 0 =1).
compute xlptmp=t(xlp).
compute vecprb=pt1lp&*(1-pt1lp).
loop kkk=1 to ncol(xlp).
compute xlptmp(kkk,:)=xlptmp(kkk,:)&*t(vecprb).
end loop.
compute varb = inv(xlptmp*xlp).
compute seb = sqrt(diag(varb)).
release xlptmp.
end if.
break.
end if.
compute bt1 = b.
compute LL1 = LL2.
end loop.
compute modres=b.
do if (jjj > iterate).
compute itprob = 2.
do if (booting=0).
compute iterrmod=1.
end if.
do if (booting=1).
compute bootiter=1.
end if.
do if (itprobtg=0).
compute itprobtg=1.
compute errcode(errs,1) = 47.
compute errs = errs + 1.
do if (booting = 0 and 0 =1).
compute vt1 = mdiag(pt1lp&*(1-pt1lp)).
compute varb = inv(t(xlp)*vt1*xlp).
compute seb = sqrt(diag(varb)).
end if.
end if.
end if.
do if ( 0 =1).
compute trat = b&/seb.
compute dfres=nrow(xlp).
compute p = 2*(1-cdfnorm(abs(trat))).
compute modres={modres,seb,trat,p}.
compute modres={modres,(b-xp2&*seb),(b+xp2&*seb)}.
compute pvchi=1-chicdf((LL3-LL2),(nrow(modres)-1)).
compute mcF = (LL3-LL2)/LL3.
compute cox = 1-exp(-(LL3-LL2)/nrow(xlp)).
compute nagel = cox/(1-exp(-(LL3)/nrow(xlp))).
compute modsum={LL2,(LL3-LL2),(nrow(modres)-1),pvchi, mcF,cox,nagel}.
compute modsuml={'-2LL','ModelLL', 'df', 'p', 'McFadden', 'CoxSnell', 'Nagelkrk'}.
compute modresl={'coeff','se','Z','p','LLCI','ULCI'}.
end if.
end if
.
compute b=btemphld.
compute fresult={(LL2-basemod),llrdf,1-chicdf((LL2-basemod),llrdf)}
.
end if.
compute highf={highf;fresult}.
compute highf2={highf2;fresult}.
do if (j = start).
compute flabel={flabel;'X*W*Z'}.
end if.
do if (j > start).
do if (nms > 1).
compute flabel={flabel;highlbwz(jj,1)}.
else if (nms = 1).
compute flabel={flabel;'M*W*Z'}.
end if.
end if.
end if.
compute jj=jj+1.
end loop.
release jj.
compute start=start+i.
end if.
do if (nrow(highf) > 1).
compute highf=highf(2:nrow(highf),:).
compute highf2=highf2(2:nrow(highf2),:).
compute flabel=flabel(2:nrow(flabel),1).
compute resultm2=make(nrow(highf),maxresm,99999).
compute resultm2(1:nrow(highf),1:ncol(highf))=highf.
compute resultm={resultm;resultm2}.
do if (outscree=1).
do if ((i < nms+nys) or (ydich=0)).
compute clabtmp={'R2-chng', hcflab,'df1','df2','p'}.
print highf/format = F10.4 /rnames=flabel/cnames=clabtmp/ title = 'Test(s) of highest order unconditional interaction(s):'.
end if.
do if (ydich=1 and i=(nms+nys)).
compute clabtmp={'Chi-sq', 'df','p'}.
print/title='Likelihood ratio test(s) of highest order'.
print highf/format = F10.4 /rnames=flabel/cnames=clabtmp/ title = 'unconditional interactions(s):'/space=0.
end if.
end if.
compute intpb=highf2(:,ncol(highf2)).
end if.
compute intstart=intstart+numint(1,i).
end if.

.
do if (criterr=0).
compute threeway=0.
compute didprint=0.
compute didsome=0.
compute sigintct=0.
loop jmed =1 to (nms+1).
compute hasw=0.
compute hasz=0.
compute jnok=0.
compute nm1vls=0.
compute nm2vls=0.
compute panelgrp=0.
compute graphixs={'WITH', outnames(1,i), 'BY'}.
compute focpred4={' '}.
compute intprint=0.
compute modcat=0.
do if (jmed <= i).
do if ((jmed = 1) and ((i+1) = nrow(bcmat))).
compute pathscnt=pathscnt+1.
else.
compute paths={paths,bcmat((i+1),jmed)}.
compute pathsw={pathsw,wcmat((i+1),jmed)}.
compute pathsz={pathsz,zcmat((i+1),jmed)}.
compute pathswz={pathswz,wzcmat((i+1),jmed)}.
compute temp=fochigh(:,pathscnt)&*bootloc(:,i).
compute pathsfoc={pathsfoc,pathsfoc(:,1)}.
do if (jmed=1).
compute pathtype={pathtype,1}.
end if.
do if ((i+1)=nrow(bcmat)).
compute pathtype={pathtype,3}.
end if.
do if (jmed > 1) and ((i+1) < nrow(bcmat)).
compute pathtype={pathtype,2}.
end if.
do if (jmed=1 and nxvls > 1 and (bcmat((i+1),jmed)=1)).
compute pathsfoc(:,(pathscn2+1))=temp(2:(nxvls+1),1).
end if.
do if ((jmed > 1) or (jmed=1 and nxvls=1)).
compute temp=cmax(temp).
compute pathsfoc(1,(pathscn2+1))=temp.
end if.
compute pathscnt=pathscnt+1.
compute pathscn2=pathscn2+1.
do if (i <= nms).
compute pathsdv={pathsdv,mnames(1,i)}.
end if.
do if (i > nms).
compute pathsdv={pathsdv,ynames}.
end if.
end if.
compute coeffcol=coeffcol+1.
compute probettt=coeffs(1:nrow(b),coeffcol).
do if (jmed=1 and (bcmat((i+1),jmed)=1)).
compute omni=make(nrow(probettt),nxvls,0).
compute omnitmp=ident(nxvls).
compute omni(2:(1+nxvls),:)=omnitmp.
end if.
do if (csum(probettt)>0).
compute probvarb=make(csum(probettt),csum(probettt),999).
compute probcoef=make(csum(probettt),1,999).
compute coefflp2=1.
loop coefflp=1 to nrow(probettt).
do if (probettt(coefflp,1)=1).
compute probcoef(coefflp2,1)=b(coefflp,1).
compute coefflp2=coefflp2+1.
end if.
end loop.
compute coefflp=0.
compute coefflp2=0.
loop iclp=1 to nrow(probettt).
do if probettt(iclp,1)=1.
compute coefflp=coefflp+1.
compute coefflp2=coefflp.
compute probvarb(coefflp,coefflp) = varb(iclp,iclp).
do if (iclp < nrow(probettt)).
loop jclp=(iclp+1) to nrow(probettt).
do if (probettt(jclp,1)=1).
compute coefflp2=coefflp2+1.
compute probvarb(coefflp,coefflp2)=varb(iclp, jclp).
compute probvarb(coefflp2,coefflp)=varb(iclp, jclp).
end if.
end loop.
end if.
end if.
end loop.
end if.
end if.
compute xprobval=xmodvals.
do if (nxvls > 1 or mcx > 0).
compute xprobval=dummatx(:,2:ncol(dummatx)).
end if.
do if ((wcmat((i+1),jmed)=1) and (zcmat((i+1),jmed)=0)).
compute numplps=1.
compute modvals=wmodvals.
compute probeval=wmodvals.
compute wheremv1=wherexw.
compute nm1vls=nwvls.
compute lpstsp={1,1}.
compute modcat=0.
compute jnmod=wtmp.
compute jnmodlab=wnames.
compute jnok=1.
compute jnmin=wmin.
compute jnmax=wmax.
compute wherejn1=2.
do if (jmed=1).
compute wherejn3=wherexw(1,i).
do if (nxvls > 1).
compute jnok=0.
end if.
end if.
do if (jmed > 1).
compute wherejn1=wherem((jmed-1),i).
compute wherejn3=wheremw(((2*jmed)-3),i).
end if.
do if (nwvls > 1).
compute probeval=wprobval.
compute lpstsp(1,2)=ncol(probeval).
compute modcat=1.
compute jnok=0.
end if.
do if (wdich = 1).
compute modcat=1.
compute jnok=0.
end if.
compute problabs=wnames.
compute focpred3={wnames,'(W)'}.
do if (xmint=1).
compute focpred3={wnames,'(X)'}.
end if.
compute hasw=1.
compute modgrph=wnames.
compute intprint=1.
compute sigintct=sigintct+1.
compute printpbe=intpb(sigintct,1).
end if.
do if ((wcmat((i+1),jmed)=0) and (zcmat((i+1),jmed)=1)).
compute numplps=1.
compute modvals=zmodvals.
compute probeval=zmodvals.
compute wheremv1=wherexz.
compute nm1vls=nzvls.
compute lpstsp={1,1}.
compute jnok=1.
compute jnmod=ztmp.
compute jnmin=zmin.
compute jnmax=zmax.
compute jnmodlab=znames.
compute wherejn1=2.
do if (jmed=1).
compute wherejn3=wherexz(1,i).
do if (nxvls > 1).
compute jnok=0.
end if.
end if.
do if (jmed > 1).
compute wherejn1=wherem((jmed-1),i).
compute wherejn3=wheremz(((2*jmed)-3),i).
end if.
do if (nzvls > 1).
compute probeval=zprobval.
compute lpstsp(1,2)=ncol(probeval).
compute modcat=1.
compute jnok=0.
end if.
do if (zdich = 1).
compute modcat=1.
compute jnok=0.
end if.
compute problabs=znames.
compute focpred3={znames,'(Z)'}.
compute modgrph=znames.
compute hasz=1.
compute intprint=1.
compute sigintct=sigintct+1.
compute printpbe=intpb(sigintct,1).
end if.
do if ((wzcmat((i+1),jmed)=1) or ((wcmat((i+1),jmed)=1) and (zcmat((i+1),jmed)=1))).
compute numplps=2.
compute probecnt=1.
compute intprint=1.
do if (wzcmat((i+1),jmed)=1).
compute sigintct=sigintct+1.
compute printpbe=intpb(sigintct,1).
else.
compute sigintct=sigintct+2.
compute printpbe=cmin(intpb((sigintct-1):sigintct,1)).
end if.
compute panelgrp=1.
compute hasw=1.
compute hasz=1.
compute panelcde={'/PANEL','ROWVAR=',znames,'.'}.
compute modgrph=wnames.
compute lpstsp={1,1;1,1}.
compute wheremv1=wherexw.
compute nm1vls=nwvls.
compute wheremv2=wherexz.
compute nm2vls=nzvls.
compute jnok=0.
do if (wzcmat((i+1),jmed)=1).
compute jnok=1.
end if.
do if (jmed > 1).
compute mprobval=mmodvals.
end if.
do if (jmed=1).
do if (nxvls > 1).
compute jnok=0.
end if.
end if.
do if (nwvls > 1).
compute lpstsp(1,2)=ncol(wprobval).
compute modcat=1.
compute jnok=0.
end if.
do if (zdich=1).
compute modcat=1.
compute jnok=0.
end if.
compute lpstsp(2,1)=lpstsp(1,2)+1.
compute lpstsp(2,2)=lpstsp(1,2)+1.
do if (nzvls > 1).
compute lpstsp(2,1)=lpstsp(1,2)+1.
compute lpstsp(2,2)=lpstsp(1,2)+ncol(zprobval).
compute jnok=0.
end if.
do if (zdich=1).
compute jnok=0.
end if.
compute omni3=make(nrow(b),(nxvls*nwvls),0).
do if (jmed > 1).
compute omni3=make(nrow(b),nwvls,0).
end if.
compute focpred3={wnames,'(W)'}.
do if (xmint=1).
compute focpred3={wnames,'(X)'}.
end if.
compute focpred4={'    ', 'Mod var:', znames, '(Z)'}.
compute modvals=make((nrow(wmodvals)*nrow(zmodvals)),2,0).
compute probeval=make((nrow(wmodvals)*nrow(zmodvals)),(ncol(wprobval)+ncol(zprobval)),0).
loop probei= 1 to nrow(wmodvals).
loop probej =1 to nrow(zmodvals).
compute modvals(probecnt,1)=wmodvals(probei,1).
compute probeval(probecnt,1:nwvls)=wprobval(probei,:).
compute modvals(probecnt,2)=zmodvals(probej,1).
compute probeval(probecnt,(nwvls+1):(nwvls+nzvls))=zprobval(probej,:).
compute probecnt=probecnt+1.
end loop.
end loop.
do if (wzcmat((i+1),jmed)=1).
compute numplps=numplps+1.
compute probprod=make(1,(ncol(wprobval)*ncol(zprobval)),0).
compute lpstsp2={1,1}.
compute lpstsp={lpstsp;lpstsp2}.
compute lpstsp(3,1)=lpstsp(2,2)+1.
compute lpstsp(3,2)=lpstsp(2,2)+ncol(probprod).
compute jnmod=ztmp.
compute jnmin=zmin.
compute jnmax=zmax.
compute jnmodlab=znames.
do if (jmed = 1).
compute wherejn1=wherexw(1,i).
compute wherejn3=wherexwz(1,i).
end if.
do if (jmed > 1).
compute wherejn1=wheremw(((2*jmed)-3),i).
compute wherejn3=wheremwz(((2*jmed)-3),i).
end if.
loop probei = 1 to nrow(wmodvals).
loop probej = 1 to nrow(zmodvals).
compute probtemp=1.
loop probek = 1 to ncol(wprobval).
compute probtemp={probtemp,(wprobval(probei,probek)&*zprobval(probej,:))}.
end loop.
compute probprod={probprod;probtemp(1,2:ncol(probtemp))}.
end loop.
end loop.
compute probprod=probprod(2:nrow(probprod),:).
compute probeval={probeval,probprod}.
end if.
compute problabs={wnames,znames}.
release probecnt, probei, probej.
end if.
do if (intprint=1).
compute focpred={'   Focal', 'predict:'}.
do if (jmed=1).
compute focpred={focpred,xnames,'(X)'}.
compute focplotv=xmodvals.
end if.
do if (jmed >1).
do if (nms > 1).
compute focpred={focpred,mnames(1,(jmed-1)), medlb2(1,(jmed-1))}.
end if.
do if (nms = 1).
compute focpred={focpred,mnames(1,(jmed-1)), '(M)'}.
end if.
compute focplotv=mmodvals(:,(jmed-1)).
end if.
compute focpred2={'    ', 'Mod var:',focpred3}.
compute focpred={focpred;focpred2}.
do if (ncol(focpred4) > 1).
compute focpred={focpred;focpred4}.
compute focpred4={' '}.
end if.
release focpred2,focpred3.
do if (outscree=1).
do if ((plot = 1 or plot = 2) or (printpbe <= intprobe)).
print focpred/title='----------'/format=A8/space=0.
end if.
end if.
compute foctmp=make(nrow(modvals),1,1).
compute probexpl=1.
compute probeva2={foctmp,probeval}.
do if (jmed=1 and nxs > 0 and mcx > 0).
compute probexpl=nxvls.
end if.
compute foctmp=make(nrow(modvals),1,1).
compute modvals3=make(1,(6+ncol(problabs)),0).
compute probrown=make(nrow(probeval),1,0).
compute jtmp=1.
loop probei = 1 to nrow(probeval).
compute probrown(probei,1)=jtmp.
compute jtmp=jtmp+nxvls.
end loop.
release jtmp.
compute probrow=999.
compute modvarl=problabs.
do if (plot = 1 or plot = 2 or nxvls > 1).
compute plotvals=make((nrow(modvals)*nrow(focplotv)),(ncol(modvals)+1),999).
loop ploti=1 to nrow(modvals).
loop plotj=1 to nrow(focplotv).
compute plotvals((((ploti-1)*nrow(focplotv))+plotj),2:ncol(plotvals))=modvals(ploti,:).
compute plotvals((((ploti-1)*nrow(focplotv))+plotj),1)=focplotv(plotj,1).
end loop.
end loop.
compute focpredn=3.
do if (jmed=1).
do if (nxvls > 1).
compute focpredn=(nxvls+1).
end if.
do if (nxvls=1 and xdich=1).
compute focpredn=2.
end if.
end if.
compute meanmat=mdiag(means).
compute onesmat=make(nrow(meanmat),(nrow(probeval)*focpredn),1).
compute probeplt=t(mdiag(means)*onesmat).
do if (jmed=1).
do if ((wcmat((i+1),1)=1) or (zcmat((i+1),1)=1)).
compute plotcnt=1.
compute iloops=nwpval*nzpval.
compute plotmx=nxpval*nzpval.
do if ((wcmat((i+1),1)=1) and (zcmat((i+1),1)=0)).
compute iloops=nwpval.
compute plotmx=nxpval.
end if.
do if ((wcmat((i+1),1)=0) and (zcmat((i+1),1)=1)).
compute iloops=nzpval.
compute plotmx=nxpval.
end if.
compute xestvals=make((nxpval*iloops),ncol(xprobval),-999).
do if (wcmat((i+1),1)=1).
compute westvals=make(nrow(xestvals),ncol(wprobval),-999).
end if.
do if (zcmat((i+1),1)=1).
compute zestvals=make(nrow(xestvals),ncol(zprobval),-999).
end if.
loop ploti=1 to iloops.
loop plotj=1 to nxpval.
compute xestvals(plotcnt,:)=xprobval(plotj,:).
compute plotcnt=plotcnt+1.
end loop.
end loop.
compute plotcnt=1.
compute plotcnt1=1.
compute plotcnt2=1.
compute plotcntz=1.
loop ploti = 1 to (iloops*nxpval).
do if (wcmat((i+1),1)=1).
compute westvals(ploti,:)=wprobval(plotcnt1,:).
end if.
do if ((wcmat((i+1),1)=0) and (zcmat((i+1),1)=1)).
compute zestvals(ploti,:)=zprobval(plotcnt1,:).
end if.
do if ((wcmat((i+1),1)=1) and (zcmat((i+1),1)=1)).
compute zestvals(ploti,:)=zprobval(plotcnt2,:).
compute plotcntz=plotcntz+1.
end if.
compute plotcnt=plotcnt+1.
do if (plotcnt > plotmx).
compute plotcnt=1.
compute plotcnt1=plotcnt1+1.
end if.
do if (plotcntz > nxpval).
compute plotcnt2=plotcnt2+1.
compute plotcntz=1.
do if (plotcnt2 > nzpval).
compute plotcnt2=1.
end if.
end if.
end loop.
compute probeplt(:,2:(1+(ncol(xestvals))))=xestvals.
do if (wcmat((i+1),1)=1).
compute probeplt(:,(wherew(1,i)):(wherew(2,i)))=westvals.
end if.
do if (zcmat((i+1),1)=1).
compute probeplt(:,(wherez(1,i)):(wherez(2,i)))=zestvals.
end if.
end if.
end if.
do if (jmed > 1).
do if ((wcmat((i+1),jmed)=1) or (zcmat((i+1),jmed)=1)).
compute plotcnt=1.
compute iloops=nwpval*nzpval.
compute plotmx=3*nzpval.
do if ((wcmat((i+1),jmed)=1) and (zcmat((i+1),jmed)=0)).
compute iloops=nwpval.
compute plotmx=3.
end if.
do if ((wcmat((i+1),jmed)=0) and (zcmat((i+1),jmed)=1)).
compute iloops=nzpval.
compute plotmx=3.
end if.
compute mestvals=make((3*iloops),1,-999).
do if (wcmat((i+1),jmed)=1).
compute westvals=make(nrow(mestvals),ncol(wprobval),-999).
end if.
do if (zcmat((i+1),jmed)=1).
compute zestvals=make(nrow(mestvals),ncol(zprobval),-999).
end if.
loop ploti=1 to iloops.
loop plotj=1 to 3.
compute mestvals(plotcnt,:)=mprobval(plotj,(jmed-1)).
compute plotcnt=plotcnt+1.
end loop.
end loop.
compute plotcnt=1.
compute plotcnt1=1.
compute plotcnt2=1.
compute plotcntz=1.
loop ploti = 1 to (iloops*3).
do if (wcmat((i+1),jmed)=1).
compute westvals(ploti,:)=wprobval(plotcnt1,:).
end if.
do if ((wcmat((i+1),jmed)=0) and (zcmat((i+1),jmed)=1)).
compute zestvals(ploti,:)=zprobval(plotcnt1,:).
end if.
do if ((wcmat((i+1),jmed)=1) and (zcmat((i+1),jmed)=1)).
compute zestvals(ploti,:)=zprobval(plotcnt2,:).
compute plotcntz=plotcntz+1.
end if.
compute plotcnt=plotcnt+1.
do if (plotcnt > plotmx).
compute plotcnt=1.
compute plotcnt1=plotcnt1+1.
end if.
do if (plotcntz > 3).
compute plotcnt2=plotcnt2+1.
compute plotcntz=1.
do if (plotcnt2 > nzpval).
compute plotcnt2=1.
end if.
end if.
end loop.
compute probeplt(:,wherem((jmed-1),i))=mestvals.
do if (wcmat((i+1),jmed)=1).
do if (model <> 74).
compute probeplt(:,(wherew(1,i)):(wherew(2,i)))=westvals.
end if.
do if (model = 74).
compute probeplt(:,(wherex(1,i)):(wherex(2,i)))=westvals.
end if.
end if.
do if (zcmat((i+1),jmed)=1).
compute probeplt(:,(wherez(1,i)):(wherez(2,i)))=zestvals.
end if.
end if.
end if.
compute prodloop = 1.
do if (jmed=1).
compute prodloop=ncol(xestvals).
end if.
do if (wcmat((i+1),jmed))=1.
compute plotcnt=0.
loop ploti = 1 to prodloop.
loop plotj = 1 to ncol(westvals).
do if (jmed=1).
compute probeplt(:,(wherexw(1,i)+plotcnt))=xestvals(:,ploti)&*westvals(:,plotj).
end if.
do if (jmed > 1).
compute probeplt(:,(wheremw(((jmed*2)-3) ,i)+plotcnt))=mestvals(:,ploti)&*westvals(:,plotj).
end if.
compute plotcnt=plotcnt+1.
end loop.
end loop.
end if.
do if (zcmat((i+1),jmed))=1.
compute plotcnt=0.
loop ploti = 1 to prodloop.
loop plotj = 1 to ncol(zestvals).
do if (jmed = 1).
compute probeplt(:,(wherexz(1,i)+plotcnt))=xestvals(:,ploti)&*zestvals(:,plotj).
end if.
do if (jmed > 1).
compute probeplt(:,(wheremz(((jmed*2)-3),i)+plotcnt))=mestvals(:,ploti)&*zestvals(:, plotj).
end if.
compute plotcnt=plotcnt+1.
end loop.
end loop.
end if.
do if (wzcmat((i+1),jmed))=1.
compute plotcnt=0.
compute threeway=1.
loop ploti = 1 to ncol(westvals).
loop plotj = 1 to ncol(zestvals).
compute probeplt(:,(wherewz(1,i)+plotcnt))=westvals(:,ploti)&*zestvals(:,plotj).
compute plotcnt=plotcnt+1.
end loop.
end loop.
compute plotcnt=0.
loop plotk = 1 to prodloop.
loop ploti = 1 to ncol(westvals).
loop plotj = 1 to ncol(zestvals).
do if (jmed = 1).
compute probeplt(:,(wherexwz(1,i)+plotcnt))=xestvals(:,plotk)&*westvals(:,ploti)&* zestvals(:,plotj).
end if.
do if (jmed > 1).
compute probeplt(:,(wheremwz(((jmed*2)-3),i)+plotcnt))=mestvals(:,plotk)&* westvals(:,ploti)&*zestvals(:,plotj).
end if.
compute plotcnt=plotcnt+1.
end loop.
end loop.
end loop.
end if.
loop newplp=1 to i.
do if (newplp <> jmed).
do if (wcmat((i+1),newplp))=1.
compute prodloop=1.
do if (newplp=1).
compute prodloop=nxvls.
end if.
compute plotcnt=0.
loop ploti = 1 to prodloop.
loop plotj = 1 to nwvls.
do if (newplp = 1).
compute probeplt(:,(wherexw(1,i)+plotcnt))=probeplt(:,(1+ploti))&*probeplt(:, (wherew(1,i)+plotj-1)).
end if.
do if (newplp > 1).
do if (model <> 74).
compute probeplt(:,(wheremw(((newplp*2)-3) ,i)+plotcnt))=probeplt(:,wherem((newplp-1),i))&*probeplt(:,(wherew(1,i)+plotj-1)).
end if.
do if (model = 74).
compute probeplt(:,(wheremw(((newplp*2)-3) ,i)+plotcnt))=probeplt(:,wherem((newplp-1),i))&*probeplt(:,(wherex(1,i)+plotj-1)).
end if.
end if.
compute plotcnt=plotcnt+1.
end loop.
end loop.
end if.
do if (zcmat((i+1),newplp))=1.
compute prodloop=1.
do if (newplp=1).
compute prodloop=nxvls.
end if.
compute plotcnt=0.
loop ploti = 1 to prodloop.
loop plotj = 1 to nzvls.
do if (newplp = 1).
compute probeplt(:,(wherexz(1,i)+plotcnt))=probeplt(:,(1+ploti))&*probeplt(:, (wherez(1,i)+plotj-1)).
end if.
do if (newplp > 1).
compute probeplt(:,(wheremz(((newplp*2)-3),i)+plotcnt))=probeplt(:, wherem((newplp-1),i))&*probeplt(:,(wherez(1,i)+plotj-1)).
end if.
compute plotcnt=plotcnt+1.
end loop.
end loop.
end if.
do if (wzcmat((i+1),newplp))=1.
compute plotcnt=0.
do if (threeway=0).
loop ploti = 1 to nwvls.
loop plotj = 1 to nzvls.
compute probeplt(:,(wherewz(1,i)+plotcnt))=probeplt(:,(wherew(1,i)+ploti-1))&* probeplt(:,(wherez(1,i)+plotj-1)).
compute plotcnt=plotcnt+1.
end loop.
end loop.
end if.
compute prodloop=1.
do if (newplp=1).
compute prodloop=nxvls.
end if.
compute plotcnt=0.
loop plotk = 1 to prodloop.
loop ploti = 1 to nwvls.
loop plotj = 1 to nzvls.
do if (newplp = 1).
compute probeplt(:,(wherexwz(1,i)+plotcnt))=probeplt(:,(1+plotk))&*probeplt(:, (wherew(1,i)+ploti-1))&*probeplt(:,(wherez(1,i)+plotj-1)).
end if.
do if (newplp > 1).
compute probeplt(:,(wheremwz(((newplp*2)-3),i)+plotcnt))=probeplt(:, wherem((newplp-1),i))&*probeplt(:,(wherew(1,i)+ploti-1))&*probeplt(:,(wherez(1,i)+plotj-1)).
end if.
compute plotcnt=plotcnt+1.
end loop.
end loop.
end loop.
end if.
end if.
end loop.
compute predvals=probeplt*b.
do if (debug <> 0).
print probeplt.
print b.
end if.
do if (i = nms+nys) and (ydich=1).
compute predvalt=(predvals < 709.7).
compute prevalt7=(1-predvalt)*(709.7).
compute predvals=(predvals&*predvalt)+prevalt7.
compute expyhat=exp(predvals)&/(1+exp(predvals)).
end if.
compute sepred=make(nrow(plotvals),3,999).
loop sei=1 to nrow(plotvals).
compute ask=probeplt(sei,:).
compute sepred(sei,1)=sqrt(ask*varb*t(ask)).
do if ((i < nms+nys) or (ydich=0)).
compute sepred(sei,2)=predvals(sei,1)-tval*sepred(sei,1).
compute sepred(sei,3)=predvals(sei,1)+tval*sepred(sei,1).
end if.
do if ((i = nms+nys) and (ydich=1)).
compute sepred(sei,2)=predvals(sei,1)-xp2*sepred(sei,1).
compute sepred(sei,3)=predvals(sei,1)+xp2*sepred(sei,1).
end if.
end loop.
compute prevloc=ncol(plotvals)+1.
compute probeplt={plotvals,predvals}.
do if (plot = 2).
compute probeplt={probeplt,sepred}.
end if.
do if ((i = nms+nys) and (ydich=1)).
compute probeplt={probeplt,expyhat}.
end if.
compute didsome=0.
end if.
do if ((wzcmat((i+1),jmed)=1) and (printpbe <= intprobe)).
do if (jmed=1).
compute omnilp2=nxvls*nwvls.
compute omnitmp=ident(omnilp2).
compute omni3(wherexw(1,i):wherexw(2,i),:)=omnitmp.
end if.
do if (jmed>1).
compute omnilp2=nwvls.
compute omnitmp=ident(omnilp2).
compute omni3(wheremw(((jmed*2)-3),i):wheremw(((jmed*2)-2),i),:)=omnitmp.
end if.
compute omnif=make(1,4,0).
do if ((i = nms+nys) and (ydich=1)).
compute omnif=make(1,3,0).
end if.
compute condeff3=0.
loop omnilp1=1 to nrow(zprobval).
loop omnilp=1 to (omnilp2).
do if (jmed=1).
compute omni3((wherexwz(1,i)+((omnilp-1)*nzvls)):(wherexwz(1,i)+((omnilp-1)*nzvls)+ (nzvls-1)),omnilp)=t(zprobval(omnilp1,:)).
end if.
do if (jmed > 1).
compute omni3((wheremwz(((jmed*2)-3),i)+((omnilp-1)*nzvls)):(wheremwz(((jmed*2)-3),i)+ ((omnilp-1)*nzvls)+(nzvls-1)),omnilp)=t(zprobval(omnilp1,:)).
end if.
end loop.
compute condeff=t(omni3)*b.
compute condeff3={condeff3;condeff}.

.
compute lmat2= omni3.
do if ( 1 =0).
compute lmat2 = mdiag( omni3 ).
compute lmat3=make(nrow(lmat2),1,0).
loop flp=1 to ncol(lmat2).
do if (csum(lmat2(:,flp))=1).
compute lmat3={lmat3,lmat2(:,flp)}.
end if.
end loop.
compute lmat2=lmat3(:,2:ncol(lmat3)).
end if.
compute fratio = (t(t(lmat2)* b )*inv(t(lmat2)* varb *lmat2)*((t(lmat2)* b )))/ncol(lmat2).
compute pfr = 1-fcdf(fratio,ncol(lmat2),(n-nrow( b ))).
compute fresult={fratio,ncol(lmat2),(n-nrow( b )),pfr}.
do if (i = (nms+nys) and (ydich=1)).
compute fratio=fratio*ncol(lmat2).
compute pfr=1-chicdf(fratio,ncol(lmat2)).
compute fresult={fratio,ncol(lmat2),pfr}.
end if.
do if ( 0 =1).
compute lmat3=1-rsum(lmat2).
compute xfm=make(n,csum(lmat3),0).
compute flpc=1.
loop flp=1 to nrow(lmat3).
do if (lmat3(flp,1)=1).
compute xfm(:,flpc)=x(:,flp).
compute flpc=flpc+1.
end if.
end loop.
compute bfm=inv(t(xfm)*xfm)*t(xfm)*y.
compute resid=y-(xfm*bfm).
compute sstotal=(y-(csum(y)/n)).
compute sstotal=csum(sstotal&*sstotal).
compute ssresid=csum(resid&*resid).
compute rsqch= 0 -((sstotal-ssresid)/sstotal).
compute fresult={rsqch,fresult}.
release xfm,flpc, resid, ssresid, bfm.
end if
.
compute omnif={omnif;fresult}.
end loop.
compute omnif=omnif(2:nrow(omnif),:).
compute clabtmp=znames.
compute condeff3=condeff3(2:nrow(condeff3),:).
do if ((nxvls*nwvls)=1).
compute omnif={condeff3,omnif}.
compute clabtmp={clabtmp,'Effect'}.
end if.
compute omnif={zmodvals,omnif}.
do if ((i < nms+nys) or (ydich=0)).
compute clabtmp={clabtmp,hcflab,'df1','df2','p'}.
end if.
do if ((i = (nms+nys)) and (ydich=1)).
compute clabtmp={clabtmp,'Chi-sq','df','p'}.
end if.
compute resultm2=make(nrow(omnif),maxresm,99999).
compute resultm2(1:nrow(omnif),1:ncol(omnif))=omnif.
compute resultm={resultm;resultm2}.
do if (outscree=1).
do if (jmed=1).
print omnif/title='Test of conditional X*W interaction at value(s) of Z:'/cnames=clabtmp/format= F10.4.
end if.
do if (jmed>1).
print omnif/title='Test of conditional M*W interaction at value(s) of Z:'/cnames=clabtmp/format= F10.4.
end if.
end if.
release omni3.
end if.
loop probei = 1 to probexpl.
do if (probexpl > 1).
compute foctmp=make(nrow(modvals),probexpl,0).
compute foctmp(:,probei)=foctmp(:,probei)+1.
compute probtemp=make(nrow(modvals),1,0).
loop probem = 1 to numplps.
loop probek = 1 to nxvls.
loop probej=lpstsp(probem,1) to lpstsp(probem,2).
compute probtemp={probtemp,foctmp(:,probek)&*probeval(:,probej)}.
end loop.
end loop.
end loop.
compute probeva2=probtemp(:,2:ncol(probtemp)).
compute probeva2={foctmp,probeva2}.
end if.
compute probres=probeva2*probcoef.
compute probrese=sqrt(diag(probeva2*probvarb*t(probeva2))).
compute tratio = probres&/probrese.
compute p = 2*(1-tcdf(abs(tratio), dfres)).
do if (ydich=1 and i = (nms+nys)).
compute p = 2*(1-cdfnorm(abs(tratio))).
end if.
compute modvals2={modvals,probres,probrese,tratio, p}.
do if ((i < nms+nys) or (ydich=0)).
compute modvals2={modvals2,(probres-tval&*probrese),(probres+tval&*probrese)}.
compute problabs={problabs,'Effect',hclab,'t', 'p', 'LLCI', 'ULCI'}.
end if.
do if (ydich=1 and i = (nms+nys)).
compute modvals2={modvals2,(probres-xp2&*probrese),(probres+xp2&*probrese)}.
compute problabs={problabs,'Effect','se','Z', 'p', 'LLCI', 'ULCI'}.
end if.
do if (probexpl > 1 and (printpbe <= intprobe)).
do if (hasz = 1).
compute printz=1.
end if.
do if (hasw=1).
compute printw=1.
end if.
compute probrlab=make(nrow(modvals),1,xcatlab(probei,1)).
compute modvals3={modvals3; modvals2}.
compute probrow={probrow;probrown}.
compute probrown=probrown+1.
do if (probei=probexpl).
compute xproblab=xcatlab(1:nxvls,1).
compute probrow=probrow(2:nrow(probrow),1).
compute modvals3=modvals3(2:nrow(modvals3),:).
compute temp=modvals3.
compute temp(GRADE(probrow(:,1)),:)=modvals3.
compute modvals3=temp.
compute start2=1.
compute problabs=problabs(1,(1+(ncol(modvarl))):ncol(problabs)).
compute pstart=1.
loop probek= 1 to nrow(probeval).
compute endstart=start2+(nxvls-1).
compute temp=modvals3(start2:endstart,(1+ncol(modvarl)):ncol(modvals3)).
compute temp2=t(modvals3(start2:start2,1:ncol(modvarl))).
compute trnames=t(modvarl).
do if (outscree=1).
do if (probek > 1).
print/title='----------'/space=0.
else.
print/title = 'Conditional effects of the focal predictor at values of the moderator(s):'.
do if ((jmed=1) and (i = (nms+nys)) and (nms > 0)).
do if (nxvls = 1).
print/title = '(These are also the conditional direct effects of X on Y)'/space=0.
else.
print/title = '(These are also the relative conditional direct effects of X on Y)'/space=0.
end if.
end if.
print.
end if.
print temp2/title = 'Moderator value(s):'/rnames=trnames/format= F10.4 /space=0.
print temp/title = ' '/cnames=problabs/rnames=xproblab/format= F10.4 /space=0.
end if.
compute resultm2=make(nrow(temp2),maxresm,99999).
compute resultm2(1:nrow(temp2),1:ncol(temp2))=temp2.
compute resultm={resultm;resultm2}.
compute resultm2=make(nrow(temp),maxresm,99999).
compute resultm2(1:nrow(temp),1:ncol(temp))=temp.
compute resultm={resultm;resultm2}.
compute start2=start2+nxvls.
compute didsome=1.
do if (jmed=1).
compute mod1val=probeval(probek,1:nm1vls).
loop omnilp=1 to nxvls.
compute omni((wheremv1(1,i)+((omnilp-1)*nm1vls)):(wheremv1(1,i)+((omnilp-1)* nm1vls)+(nm1vls-1)),omnilp)=t(mod1val).
do if (nm1vls < ncol(probeval)).
compute mod2val=probeval(probek,(nm1vls+1):(nm1vls+nm2vls)).
compute omni((wheremv2(1,i)+((omnilp-1)*nm2vls)):(wheremv2(1,i)+((omnilp-1)* nm2vls)+(nm2vls-1)),omnilp)=t(mod2val).
do if ((nm1vls+nm2vls) < ncol(probeval)).
compute intlen=nm1vls*nm2vls.
compute modintvl=probeval(probek,(nm1vls+nm2vls+1):ncol(probeval)).
compute omni((wherexwz(1,i)+((omnilp-1)*intlen)):(wherexwz(1,i)+((omnilp-1)* intlen)+(intlen-1)),omnilp)=t(modintvl).
end if.
end if.
end loop.

.
compute lmat2= omni.
do if ( 1 =0).
compute lmat2 = mdiag( omni ).
compute lmat3=make(nrow(lmat2),1,0).
loop flp=1 to ncol(lmat2).
do if (csum(lmat2(:,flp))=1).
compute lmat3={lmat3,lmat2(:,flp)}.
end if.
end loop.
compute lmat2=lmat3(:,2:ncol(lmat3)).
end if.
compute fratio = (t(t(lmat2)* b )*inv(t(lmat2)* varb *lmat2)*((t(lmat2)* b )))/ncol(lmat2).
compute pfr = 1-fcdf(fratio,ncol(lmat2),(n-nrow( b ))).
compute fresult={fratio,ncol(lmat2),(n-nrow( b )),pfr}.
do if (i = (nms+nys) and (ydich=1)).
compute fratio=fratio*ncol(lmat2).
compute pfr=1-chicdf(fratio,ncol(lmat2)).
compute fresult={fratio,ncol(lmat2),pfr}.
end if.
do if ( 0 =1).
compute lmat3=1-rsum(lmat2).
compute xfm=make(n,csum(lmat3),0).
compute flpc=1.
loop flp=1 to nrow(lmat3).
do if (lmat3(flp,1)=1).
compute xfm(:,flpc)=x(:,flp).
compute flpc=flpc+1.
end if.
end loop.
compute bfm=inv(t(xfm)*xfm)*t(xfm)*y.
compute resid=y-(xfm*bfm).
compute sstotal=(y-(csum(y)/n)).
compute sstotal=csum(sstotal&*sstotal).
compute ssresid=csum(resid&*resid).
compute rsqch= 0 -((sstotal-ssresid)/sstotal).
compute fresult={rsqch,fresult}.
release xfm,flpc, resid, ssresid, bfm.
end if
.
compute resultm2=make(nrow(fresult),maxresm,99999).
compute resultm2(1:nrow(fresult),1:ncol(fresult))=fresult.
compute resultm={resultm;resultm2}.
do if (outscree=1).
do if (i < (nms + nys) or (ydich = 0)).
compute clabtmp={hcflab,'df1','df2','p'}.
print fresult/title='Test of equality of conditional means'/cnames=clabtmp/format= F10.4.
end if.
do if (i = (nms + nys) and (ydich = 1)).
compute clabtmp={'Chi-sq','df','p'}.
print fresult/title='Test of equality of conditional logits or probabilities'/cnames=clabtmp/format= F10.4.
end if.
end if.
compute probetmp=probeplt(pstart:(pstart+nxvls),1).
compute probetmp={probetmp,probeplt(pstart:(pstart+nxvls),prevloc:ncol(probeplt))}.
compute pstart=pstart+(nxvls+1).
compute resultm2=make(nrow(probetmp),maxresm,99999).
compute resultm2(1:nrow(probetmp),1:ncol(probetmp))=probetmp.
compute resultm={resultm;resultm2}.
do if (i < ((nms + nys)) or (ydich = 0)).
compute clabtmp={xnames, outnames(1,i), hclab, 'LLCI', 'ULCI'}.
do if (outscree=1).
print probetmp/title = 'Estimated conditional means being compared:'/cnames=clabtmp/format= F10.4.
end if.
end if.
do if ((i = (nms+nys)) and (ydich = 1)).
compute clabtmp={xnames, outnames(1,i), 'prob'}.
compute probetm2=probetmp(:,1:2).
compute probetm2={probetm2,probetmp(:,ncol(probetmp))}.
do if (outscree=1).
print probetm2/title = 'Estimated conditional logits and probabilities:'/cnames=clabtmp/format= F10.4.
end if.
end if.
end if.
end loop.
release probrow, start2, endstart, temp, temp2.
end if.
end if.
do if (probexpl = 1 and (printpbe <= intprobe)).
compute resultm2=make(nrow(modvals2),maxresm,99999).
compute resultm2(1:nrow(modvals2),1:ncol(modvals2))=modvals2.
compute resultm={resultm;resultm2}.
do if (outscree=1).
print/title = 'Conditional effects of the focal predictor at values of the moderator(s):'.
print modvals2/cnames=problabs/title = ' '/space=0/format= F10.4.
end if.
compute didsome=1.
do if (hasz = 1).
compute printz=1.
end if.
do if (hasw=1).
compute printw=1.
end if.
do if (jn = 1 and jnok=1).
do if (criterr = 0).
compute dfres=n-nrow(b).
compute roots=99999.
compute jncrit =(dfres* (exp((dfres-(5/6))*((xp2/(dfres-(2/3)+(.11/dfres)))* (xp2/(dfres-(2/3)+(.11/dfres)))))-1)).
do if (i = (nms+nys) and (ydich=1)).
compute jncrit=xp2*xp2.
end if.
compute jnb1=b(wherejn1,1).
compute jnb3=b(wherejn3,1).
compute jnsb1=varb(wherejn1,wherejn1).
compute jnsb3=varb(wherejn3,wherejn3).
compute jnsb1b3=varb(wherejn1,wherejn3).
compute ajn =(jncrit*jnsb3)-(jnb3*jnb3).
compute bjn = 2*((jncrit*jnsb1b3)-(jnb1*jnb3)).
compute cjn = (jncrit*jnsb1)-(jnb1*jnb1).
compute radarg = (bjn*bjn)-(4*ajn*cjn).
compute den = 2*ajn.
compute nrts = 0.
do if (radarg >= 0 and den <> 0).
compute x21 = (-bjn+sqrt(radarg))/den.
compute x22 = (-bjn-sqrt(radarg))/den.
compute roots = 0.
do if (x21 >= jnmin and x21 <= jnmax).
compute nrts = 1.
compute roots = {roots; x21}.
end if.
do if (x22 >= jnmin and x22 <= jnmax).
compute nrts = nrts + 1.
compute roots = {roots; x22}.
end if.
compute roots={roots,make(nrow(roots),2,0)}.
end if.
do if (nrts > 0).
compute roots = roots(2:nrow(roots),1:3).
compute roots(1,2)=(csum(jnmod < roots(1,1))/n)*100.
compute roots(1,3)=(csum(jnmod > roots(1,1))/n)*100.
do if (nrow(roots)=2).
compute roots(2,2)=(csum(jnmod < roots(2,1))/n)*100.
compute roots(2,3)=(csum(jnmod > roots(2,1))/n)*100.
end if.
do if (outscree=1).
print roots/title = 'Moderator value(s) defining Johnson-Neyman significance region(s):'/clabels = 'Value', '% below', '% above'/format F10.4.
end if.
do if (nrts=1).
compute tmprts=make(1,3,99999).
compute roots={roots;tmprts}.
end if.
end if.
do if (nrts = 0).
compute roots=make(2,1,99999).
do if (outscree=1).
print/title = 'There are no statistical significance transition points within the observed'.
print/title = 'range of the moderator found using the Johnson-Neyman method.'/space=0.
end if.
end if.
compute resultm2=make(nrow(roots),maxresm,99999).
compute resultm2(1:nrow(roots),1:ncol(roots))=roots.
compute resultm={resultm;resultm2}.
compute jnvals=make(23,7,0).
loop jni= 0 to (21-(nrts)).
compute jnvals((jni+1),1)=jnmin+(jni*((jnmax-jnmin)/(21-nrts))).
end loop.
do if (nrts > 0).
loop jni = 1 to nrts.
loop jnj = 2 to (nrow(jnvals)-1).
do if ((roots(jni,1) > jnvals((jnj-1),1)) and (roots(jni,1) < jnvals(jnj,1))).
compute jnvals((jnj+1):(21+jni),1)=jnvals(jnj:(20+jni),1).
compute jnvals(jnj,1)=roots(jni,1).
end if.
end loop.
end loop.
end if.
compute jnvals=jnvals(1:22,:).
loop jni = 1 to nrow(jnvals).
compute jnvals(jni,2)=jnb1+jnb3*jnvals(jni,1).
compute jnvals(jni,3)=sqrt(jnsb1+2*jnvals(jni,1)*jnsb1b3+(jnvals(jni,1)*jnvals(jni, 1))*jnsb3).
compute jnvals(jni,4)=jnvals(jni,2)/jnvals(jni,3).
compute jnvals(jni,5)=2*(1-tcdf(abs(jnvals(jni,4)), dfres)).
compute jnvals(jni,6)=jnvals(jni,2)-sqrt(jncrit)*jnvals(jni,3).
compute jnvals(jni,7)=jnvals(jni,2)+sqrt(jncrit)*jnvals(jni,3).
do if ((i = nms + nys) and (ydich=1)).
compute jnvals(jni,5)=2*(1-cdfnorm(abs(jnvals(jni,4)))).
compute jnvals(jni,6)=jnvals(jni,2)-xp2*jnvals(jni,3).
compute jnvals(jni,7)=jnvals(jni,2)+xp2*jnvals(jni,3).
end if.
end loop.
compute resultm2=make(nrow(jnvals),maxresm,99999).
compute resultm2(1:nrow(jnvals),1:ncol(jnvals))=jnvals.
compute resultm={resultm;resultm2}.
do if ((i < nms+nys) or (ydich=0)).
compute jnclbs={jnmodlab,'Effect',hclab,'t', 'p', 'LLCI', 'ULCI'}.
end if.
do if ((i = nms + nys) and (ydich=1)).
compute jnclbs={jnmodlab,'Effect','se','Z', 'p', 'LLCI', 'ULCI'}.
end if.
do if (((wcmat((i+1),jmed)=1) or (zcmat((i+1),jmed)=1)) and (wzcmat((i+1),jmed)=0)).
do if (outscree=1).
print jnvals/title = 'Conditional effect of focal predictor at values of the moderator:'/cnames =jnclbs/format = F10.4.
end if.
end if.
do if (outscree=1).
do if ((jmed = 1) and (wzcmat((i+1),jmed)=1)).
print jnvals/title = 'Conditional X*W interaction at values of the moderator Z:'/cnames =jnclbs/format = F10.4.
end if.
do if ((jmed > 1) and (wzcmat((i+1),jmed)=1)).
print jnvals/title = 'Conditional M*W interaction at values of the moderator Z:'/cnames =jnclbs/format = F10.4.
end if.
end if.
end if.
end if.
end if.
do if ((i = (nms+nys)) and (jmed=1) and (bcmat(nrow(bcmat),1)=1)).
do if (probei=1).
compute direfflb=problabs.
compute direff=modvals2.
end if.
do if (probei>1).
compute direff={direff;modvals2}.
end if.
end if.
compute intprint=0.
do if ((jmed=1) and (i=1) and nms=0) and modcok=1).
compute contvec2=make(2,1,1).
compute contvec2={contvec2,wcontval,zcontval}.
do if (wzcmat((i+1),jmed)=1).
loop conti= 1 to ncol(wcontval).
loop contj = 1 to ncol(zcontval).
compute contvec2={contvec2,wcontval(:,conti)&*zcontval(:,contj)}.
end loop.
end loop.
end if.
compute conteff=contvec2*probcoef.
compute contdiff=contvec2(1,:)-contvec2(2,:).
compute contse=sqrt(contdiff*probvarb*t(contdiff)).
compute conteffd=conteff(1,1)-conteff(2,1).
compute contvec={contvec,conteff}.
compute contvecm=contvec.
compute resultm2=make(nrow(contvecm),maxresm,99999).
compute resultm2(1:nrow(contvecm),1:ncol(contvecm))=contvecm.
compute resultm={resultm;resultm2}.
do if (outscree=1).
print/title='Contrast between conditional effects of X:'.
print contvec/title=' '/rlabels='Effect1:','Effect2:'/cnames=problabs/format = F10.4 /space=0.
end if.
do if (ydich=0).
compute p=2*(1-tcdf(abs(conteffd/contse), dfres)).
compute contvec={conteffd,contse,conteffd/contse, p}.
compute contvec={contvec,(conteffd-(tval*contse))}.
compute contvec={contvec,(conteffd+(tval*contse))}.
compute contlabs={'Contrast', hclab, 't', 'p', 'LLCI', 'ULCI'}.
end if.
do if (ydich=1).
compute p=2*(1-cdfnorm(abs(conteffd/contse))).
compute contvec={conteffd,contse,conteffd/contse, p}.
compute contvec={contvec,(conteffd-(xp2*contse))}.
compute contvec={contvec,(conteffd+(xp2*contse))}.
compute contlabs={'Contrast', 'se', 'Z', 'p', 'LLCI', 'ULCI'}.
end if.
compute resultm2=make(nrow(contvec),maxresm,99999).
compute resultm2(1:nrow(contvec),1:ncol(contvec))=contvec.
compute resultm={resultm;resultm2}.
do if (outscree=1).
print contvec/title='Test of Effect1 minus Effect2'/format= F10.4 /cnames=contlabs.
end if.
end if.
end loop.
do if (plot = 1 or plot = 2).
compute datalabs={t(focpred(:,3)),outnames(1,i)}.
do if (plot = 1).
compute datalabs={datalabs}.
end if.
do if (plot = 2).
compute datalabs={datalabs,'se', 'LLCI', 'ULCI'}.
end if.
do if ((i = nms+nys) and (ydich=1)).
compute datalabs={datalabs,'prob'}.
end if.
compute resultm2=make(nrow(probeplt),maxresm,99999).
compute resultm2(1:nrow(probeplt),1:ncol(probeplt))=probeplt.
compute resultm={resultm;resultm2}.
compute datalabs={datalabs,'.'}.
do if (outscree=1).
print/title = 'Data for visualizing the conditional effect of the focal predictor:'.
print/title = 'Paste text below into a SPSS syntax window and execute to produce plot.'/space=0.
compute dumb = {' ', ' ', ' ', ' ', ' ', ' ', ' '}.
print datalabs/title = 'DATA LIST FREE/'/format=A10.
print probeplt/title = 'BEGIN DATA.'/format= F10.4 /space=0.
print/title = 'END DATA.'/space=0.
end if.
compute focgrph=datalabs(1,1).
compute graphix={focgrph,graphixs,modgrph}.
do if (((xdich=1) or (nxvls > 1)) and ((modcat=0) and (focgrph = xnames))).
compute graphix={modgrph,graphixs,focgrph}.
end if.
do if (panelgrp = 0).
compute graphix={graphix,'.'}.
else.
compute graphix={graphix,panelcde}.
end if.
do if (outscree=1).
print graphix/title = 'GRAPH/SCATTERPLOT='/format=A8/space=0.
end if.
do if (i = (nms + nys) and ydich = 1).
compute graphixd=graphix.
compute graphixd(1,3)='prob'.
do if (outscree=1).
print graphixd/title = 'GRAPH/SCATTERPLOT='/format=A8/space=0.
end if.
end if.
end if.
end if.
end loop.
release jmed, intprint,didprint.
end if
.
do if (model = 74 and i <= nms).
compute onetemp=make(nrow(xprobval),1,1).
compute mestmt74={onetemp,xprobval}.
do if (ncs > 0).
compute ncovmdl=rsum(ccmat(i,:)).
do if (ncovmdl > 0).
compute cvmnc=make(nrow(mestmt74),ncovmdl,1).
compute cvmnctmp=csum(x(:,(ncol(x)-ncovmdl+1):ncol(x)))/nrow(x).
do if (cuscoval > 0).
compute cvmnctmp=coval.
end if.
loop mestlp=1 to ncovmdl.
compute cvmnc(:,mestlp)=cvmnc(:,mestlp)*cvmnctmp(:,mestlp).
end loop.
compute mestmt74={mestmt74,cvmnc}.
end if.
end if.
compute mest74t=mestmt74*b.
do if (i = 1).
compute mest74=mest74t.
end if.
do if (i > 1).
compute mest74={mest74,mest74t}.
end if.
end if.
do if ((i = (nms+nys)) and (model >= 0 and model < 4) and (linsum(1,1) <> -999)).
compute lhyprob=1.
compute meansub=0.
do if (((nlinsum=nrow(b)) or (nlinsum = (nrow(b)-ncs)))).
do if (nlinsum = (nrow(b)-ncs) and ncs > 0).
compute linsum={linsum,covmeans}.
compute meansub=1.
end if.
compute lhyprob=0.
compute hypest=linsum*b.
compute sehypest=sqrt(linsum*varb*t(linsum)).
compute phypest=2*(1-tcdf(abs(hypest/sehypest), (dfres))).
compute hypest={hypest,sehypest,(hypest/sehypest),phypest,(hypest-tval*sehypest),(hypest+ tval*sehypest)}.
compute resultm2=make(ncol(linsum),maxresm,99999).
compute resultm2(:,1)=t(linsum).
compute resultm={resultm;resultm2}.
compute resultm2=make(1,maxresm,99999).
compute resultm2(1,1:ncol(hypest))=hypest.
compute resultm={resultm;resultm2}.
do if (outscree=1).
compute hyplabs={'Estimate',hclab,'t','p','LLCI','ULCI'}.
print/title='----------'.
print/title='Linear Combination Estimate and Hypothesis Test'/space=0.
print t(linsum)/title= 'Weight vector:'/clabels='weight'/rnames=vlabsm/format F10.4.
print hypest/title=' '/cnames=hyplabs/format= F10.4 /space=0.
do if (meansub=1).
print/title='Covariate weight(s) set to the sample mean.'.
end if.
end if.
end if.
do if (lhyprob=1).
compute notecode(notes,1) = 30.
compute notes = notes + 1.
end if.
end if.
end loop if criterr=1.
compute lastb=b.
compute lastcov=varb.
do if (criterr=0 and dototal = 1).
compute x=xtmp.
compute vlabsm={'constant';xcatlab(1:nxvls,1)}.
do if (ncs > 0).
compute x = {x,ctmp}.
compute vlabsm={vlabsm;t(covnames)}.
end if.
compute x = {ones,x}.

.
do if ( 1 =1).
compute hatmat=inv(t( x )* x )*t( x ).
compute b = hatmat* y.
compute modres=b.
do if ( 1 =1).
compute n1=nrow( x ).
compute dfres=n1-(ncol( x )).
compute sstotal = t( y -(csum( y )/n1))*( y -(csum( y )/n1)).
compute resid= y - x *b.
compute ssresid = csum((resid)&**2).
compute r2 = (sstotal-ssresid)/sstotal.
do if (r2 < 0).
compute r2=0.
end if.
compute adjr2 = 1-((1-r2)*(n1-1)/(dfres)).
compute mse=ssresid/(n1-ncol( x )).

.
compute n1=nrow( x ).
compute invXtX = inv(t( x )* x ).
compute varb = mse *invXtX.
compute k3 = ncol( x ).
compute xhc=0.
do if ( hc <> 5).
compute xhc= x.
compute hat = xhc(:,1).
loop i3=1 to nrow(xhc).
compute hat(i3,1)= xhc(i3,:)*invXtX*t(xhc(i3,:)).
end loop.
do if ( hc = 0 or hc =1).
loop i3 = 1 to k3.
compute xhc(:,i3)=xhc(:,i3)&* resid.
end loop.
end if.
do if ( hc =3 or hc =2).
loop i3=1 to k3.
compute xhc(:,i3) = ( resid &/(1-hat)&**(1/(4- hc )))&*xhc(:,i3).
end loop.
end if.
do if ( hc = 4).
compute hcmn=make(n,2,4).
compute hcmn(:,2)=(n1*hat)/k3.
loop i3= 1 to k3.
compute xhc(:,i3) = ( resid &/(1-hat)&**(rmin(hcmn)/2))&*xhc(:,i3).
end loop.
end if.
compute varb=(invXtX*t(xhc)*xhc*invXtX).
do if ( hc =1).
compute varb=(n1/(n1-ncol( x )))&*varb.
end if.
end if.
compute hclab={'se(HC0)','se(HC1)','se(HC2)','se(HC3)','se(HC4)','se'}.
compute hclab=hclab(1,( hc +1)).
compute hcflab={'F(HC0)','F(HC1)','F(HC2)','F(HC3)','F(HC4)','F'}.
compute hcflab=hcflab(1,( hc +1)).
release xhc
.
compute seb=sqrt(diag(varb)).
compute trat = b&/seb.
compute p = 2*(1-tcdf(abs(trat), (dfres))).
compute tval = sqrt(dfres* (exp((dfres-(5/6))*((xp2/(dfres-(2/3)+(.11/dfres)))* (xp2/(dfres-(2/3)+(.11/dfres)))))-1)).
compute modres={modres,seb,trat,p}.
compute modres={modres,(b-tval&*seb),(b+tval&*seb)}.
compute modresl={'coeff',hclab,'t','p','LLCI','ULCI'}.
compute lmat = ident(ncol( x )).
compute lmat = lmat(:,2:ncol(lmat)).
compute fratio = (t(t(lmat)*b)*inv(t(lmat)*varb*lmat)*((t(lmat)*b)))/(ncol( x )-1).
compute pfr = 1-fcdf(fratio,(ncol( x )-1),dfres).
compute modsum={sqrt(r2),r2,mse,fratio,(ncol( x )-1),dfres,pfr}.
compute modsuml={'R','R-sq','MSE',hcflab,'df1','df2', 'p'}.
end if.
end if.
do if ( 1 = 2 or 1 =3).
compute xlp= x.
compute ylp= y.
compute pt2 = make(nrow(ylp),1,(csum(ylp)/nrow(ylp))).
do if ( 1 =2).
compute LL3 = ylp&*ln(pt2)+(1-ylp)&*ln(1-pt2).
end if.
compute LL3 = -2*csum(LL3).
compute bt1 = make(ncol(xlp),1,0).
compute LL1 = 0.
compute pt1 = make(nrow(ylp),1,0.5).
compute pt1lp=pt1.
loop jjj = 1 to iterate.
compute xlptmp=t(xlp).
compute vecprb=pt1lp&*(1-pt1lp).
loop kkk=1 to ncol(xlp).
compute xlptmp(kkk,:)=xlptmp(kkk,:)&*t(vecprb).
end loop.
compute b = bt1+inv(xlptmp*xlp)*t(xlp)*(ylp-pt1lp).
do if ( 1 =2).
compute xlpb=xlp*b.
compute xlpbt=(xlpb > -709.7).
compute xlpb709=(1-xlpbt)*(-709.7).
compute xlpb=(xlpb&*xlpbt)+xlpb709.
compute pt1lp = 1/(1+exp(-(xlpb))).
end if.
compute itprob = csum((pt1lp < .00000001) or (pt1lp > .9999999)).
do if (itprob > 0).
loop kkk = 1 to nrow(pt1lp).
do if (pt1lp(kkk,1) > .9999999).
compute pt1lp(kkk,1) = .9999999.
end if.
do if (pt1lp(kkk,1) < .00000001).
compute pt1lp(kkk,1) = .00000001.
end if.
end loop.
compute itprob = 0.
end if.
do if (itprob = 0).
do if ( 1 =2).
compute LL = ylp&*ln(pt1lp)+(1-ylp)&*ln(1-pt1lp).
end if.
compute LL2 = -2*csum(ll).
end if.
do if (abs(LL1-LL2) < converge).
do if ( 1 =1).
compute xlptmp=t(xlp).
compute vecprb=pt1lp&*(1-pt1lp).
loop kkk=1 to ncol(xlp).
compute xlptmp(kkk,:)=xlptmp(kkk,:)&*t(vecprb).
end loop.
compute varb = inv(xlptmp*xlp).
compute seb = sqrt(diag(varb)).
release xlptmp.
end if.
break.
end if.
compute bt1 = b.
compute LL1 = LL2.
end loop.
compute modres=b.
do if (jjj > iterate).
compute itprob = 2.
do if (booting=0).
compute iterrmod=1.
end if.
do if (booting=1).
compute bootiter=1.
end if.
do if (itprobtg=0).
compute itprobtg=1.
compute errcode(errs,1) = 47.
compute errs = errs + 1.
do if (booting = 0 and 1 =1).
compute vt1 = mdiag(pt1lp&*(1-pt1lp)).
compute varb = inv(t(xlp)*vt1*xlp).
compute seb = sqrt(diag(varb)).
end if.
end if.
end if.
do if ( 1 =1).
compute trat = b&/seb.
compute dfres=nrow(xlp).
compute p = 2*(1-cdfnorm(abs(trat))).
compute modres={modres,seb,trat,p}.
compute modres={modres,(b-xp2&*seb),(b+xp2&*seb)}.
compute pvchi=1-chicdf((LL3-LL2),(nrow(modres)-1)).
compute mcF = (LL3-LL2)/LL3.
compute cox = 1-exp(-(LL3-LL2)/nrow(xlp)).
compute nagel = cox/(1-exp(-(LL3)/nrow(xlp))).
compute modsum={LL2,(LL3-LL2),(nrow(modres)-1),pvchi, mcF,cox,nagel}.
compute modsuml={'-2LL','ModelLL', 'df', 'p', 'McFadden', 'CoxSnell', 'Nagelkrk'}.
compute modresl={'coeff','se','Z','p','LLCI','ULCI'}.
end if.
end if
.
compute toteff=modres(2:(1+nxvls),:).
compute nodotot=0.
do if (xdich=1 and xmint=1 and model=74).
compute toteff(:,1)=toteff(:,1)*xscaling.
compute toteff(:,2)=toteff(:,2)*abs(xscaling).
compute toteff(:,3)=toteff(:,3)*xscaling.
compute toteff(:,5)=toteff(:,5)*xscaling.
compute toteff(:,6)=toteff(:,6)*xscaling.
compute citmp=toteff(:,5:6).
do if (xscaling < 0).
compute toteff(:,5)=citmp(:,2).
compute toteff(:,6)=citmp(:,1).
end if.
compute nodotot=1.
end if.
compute totefflb=modresl.
do if (outscree=1).
do if (nodotot=0).
print/title = '************************** TOTAL EFFECT MODEL ****************************'.
end if.
do if (nodotot=1).
print/title = '**************************************************************************'.
end if.
print outnames(1,ncol(outnames))/title = 'OUTCOME VARIABLE:'/format = A8/space=0.
end if.
compute toteffl2=vlabsm(2:(1+nxvls),:).
compute resultm2=make(1,maxresm,99999).
compute resultm2(1,1:ncol(modsum))=modsum.
compute resultm={resultm;resultm2}.
compute resultm2=make(nrow(modres),maxresm,99999).
compute resultm2(1:nrow(modres),1:ncol(modres))=modres.
compute resultm={resultm;resultm2}.
do if (outscree=1).
print modsum/title = 'Model Summary'/cnames = modsuml/format= F10.4.
print modres/title='Model'/rnames=vlabsm/cnames=modresl/format= F10.4.
end if.
compute lmat=make(nrow(b),1,0).
compute lmat2=make(nxvls,1,1).
compute lmat(2:(1+nxvls),1)=lmat2.
do if (ydich <> 1).

.
compute lmat2= lmat.
do if ( 0 =0).
compute lmat2 = mdiag( lmat ).
compute lmat3=make(nrow(lmat2),1,0).
loop flp=1 to ncol(lmat2).
do if (csum(lmat2(:,flp))=1).
compute lmat3={lmat3,lmat2(:,flp)}.
end if.
end loop.
compute lmat2=lmat3(:,2:ncol(lmat3)).
end if.
compute fratio = (t(t(lmat2)* b )*inv(t(lmat2)* varb *lmat2)*((t(lmat2)* b )))/ncol(lmat2).
compute pfr = 1-fcdf(fratio,ncol(lmat2),(n-nrow( b ))).
compute fresult={fratio,ncol(lmat2),(n-nrow( b )),pfr}.
do if (i = (nms+nys) and (ydich=1)).
compute fratio=fratio*ncol(lmat2).
compute pfr=1-chicdf(fratio,ncol(lmat2)).
compute fresult={fratio,ncol(lmat2),pfr}.
end if.
do if ( 1 =1).
compute lmat3=1-rsum(lmat2).
compute xfm=make(n,csum(lmat3),0).
compute flpc=1.
loop flp=1 to nrow(lmat3).
do if (lmat3(flp,1)=1).
compute xfm(:,flpc)=x(:,flp).
compute flpc=flpc+1.
end if.
end loop.
compute bfm=inv(t(xfm)*xfm)*t(xfm)*y.
compute resid=y-(xfm*bfm).
compute sstotal=(y-(csum(y)/n)).
compute sstotal=csum(sstotal&*sstotal).
compute ssresid=csum(resid&*resid).
compute rsqch= r2 -((sstotal-ssresid)/sstotal).
compute fresult={rsqch,fresult}.
release xfm,flpc, resid, ssresid, bfm.
end if
.
compute totomni=fresult.
end if.
do if (stand=1).
compute predsd=make(nrow(modres),1,0).
compute stdmod=modres(:,1)&/ovsd(1,ncol(ovsd)).
loop jd=1 to ncol(x).
compute descdat=x(:,jd).
compute predsd(jd,1) = (nrow(descdat)*sscp(descdat))-(t(csum(descdat))*(csum(descdat))).
compute predsd(jd,1) = sqrt(predsd(jd,1)/(nrow(descdat)*(nrow(descdat)-1))).
end loop.
do if (wherex(1,ncol(wherex)) <> -999 and ((nxvls > 1) or (xdich=1))).
compute sdmsone=make(nxvls,1,1).
compute predsd(wherex(1,ncol(wherex)):wherex(2,ncol(wherex)),1)=sdmsone.
compute pstog=1.
end if.
compute predsd(1,1)=1.
compute stdmod=stdmod&*predsd.
compute stdmod=stdmod(2:nrow(stdmod),1).
compute sdvlabs=vlabsm(2:nrow(vlabsm),1).
compute resultm2=make(nrow(stdmod),maxresm,99999).
compute resultm2(1:nrow(stdmod),1:ncol(stdmod))=stdmod.
compute resultm={resultm;resultm2}.
do if (outscree=1).
print stdmod/title='Standardized coefficients'/clabels='coeff'/rnames=sdvlabs/format= F10.4.
end if.
end if.
do if (covcoeff=1).
do if (outscree=1).
print varb/title='Covariance matrix of regression parameter estimates:'/rnames=vlabsm/cnames=vlabsm/format= F10.4.
end if.
compute resultm2=make(nrow(varb),maxresm,99999).
do if (ncol(varb) <= maxresm).
compute resultm2(1:nrow(varb),1:ncol(varb))=varb.
compute resultm={resultm;resultm2}.
end if.
do if (ncol(varb) > maxresm).
compute resultmt=make(nrow(resultm),ncol(varb),99999).
compute resultmt(1:nrow(resultm),1:ncol(resultm))=resultm.
compute resultm=resultmt.
compute resultm2=make(nrow(varb),ncol(resultm),99999).
compute resultm2(1:nrow(varb),1:ncol(varb))=varb.
compute resultm={resultm;resultm2}.
compute maxresm=ncol(resultm).
end if.
end if.
end if.
end if.
do if (criterr=0 and nms > 0 and ydich=0 and modelres=1).
compute modresid=modresid(:,2:ncol(modresid)).
compute sigmatal = (t(modresid)*(ident(n)-(1/n)*ones*t(ones))*modresid)*(1/(n-1)).
compute sdall = mdiag(1/sqrt(diag(sigmatal))).
compute corall=sdall*sigmatal*t(sdall).
compute resultm2=make(nrow(corall),maxresm,99999).
compute resultm2(1:nrow(corall),1:ncol(corall))=corall.
compute resultm={resultm;resultm2}.
do if (outscree=1).
print/title = '****************** CORRELATIONS BETWEEN MODEL RESIDUALS ******************'.
print corall/title=' '/format= F10.4 / cnames=outnames/rnames=outnames/space=0.
end if.
end if.
do if (criterr=0 and boot > 0).
compute bootres=make(1,rsum(nump),-999).
compute bootdir=obsdirfx.
compute natdirbt=make(1,nxvls,-999).
do if (effsize=1).
compute bootysd=make(1,1,-999).
compute bootxsd=make(1,1,-999).
end if.
compute badboot=0.
compute goodboot=0.
compute smallest=1.
compute booting=1.
loop j = 1 to maxboot.
compute nobootx=1.
compute modres2=999.
compute v=trunc(uniform(n,1)*n)+1.
compute bad=0.
loop i = 1 to (nms+nys).
compute y=outvars(v,i).
compute ynovar= (nrow(y)*sscp(y))-(t(csum(y))*(csum(y))).
do if (ynovar = 0).
compute bad=1.
end if.
compute xindx=datindx(1:(nump(1,i)-1),i).
compute hello=0.
compute x = fulldat(v,xindx).
compute x={ones,x}.
compute xsq=t(x)*x.
compute exsq=eval(xsq).
release xsq.
compute holymoly=cmin(exsq).
compute zeroeig=csum(exsq <= 0.000000000002).
compute bad=bad + (zeroeig > 0).

.
compute desctmp=make((8-(4* 1 )),ncol( y ),-999).
loop jd=1 to ncol( y ).
compute descdat= y (:,jd).
compute desctmp(1,jd) = csum(descdat)/nrow(descdat).
compute desctmp(2,jd) = (nrow(descdat)*sscp(descdat))-(t(csum(descdat))*(csum(descdat))).
compute desctmp(2,jd) = sqrt(desctmp(2,jd)/(nrow(descdat)*(nrow(descdat)-1))).
compute desctmp(3,jd)=cmin(descdat).
compute desctmp(4,jd)=cmax(descdat).
do if ( 1 =0).
compute minwarn=0.
compute maxwarn=0.
do if ((desctmp(3,jd)=desctmp(4,jd)) and novar=0).
compute errcode(errs,1)=15.
compute errs=errs+1.
compute criterr=1.
compute novar=1.
end if.
compute tmp=((descdat(:,1)=desctmp(3,jd))+(descdat(:,1)=desctmp(4,jd))).
compute desctmp(8,jd)=(csum(tmp)=nrow(tmp)).
compute tmp = descdat.
compute tmp(GRADE(descdat),:) = descdat.
compute descdat = tmp.
release tmp.
compute decval={.16;.5;.84}.
loop kd=1 to 3.
compute low=trunc(decval(kd,1)*(nrow(descdat)+1)).
compute lowdec=decval(kd,1)*(nrow(descdat)+1)-low.
compute value=descdat(low,1)+(descdat((low+1),1)-descdat(low,1))*lowdec.
compute desctmp((4+kd),jd)=value.
end loop.
compute mnotev=1.
compute modvals=desctmp(5:7,:).
do if (quantile <> 1).
compute desctmp(5,jd)=desctmp(1,jd)-desctmp(2,jd).
compute desctmp(6,jd)=desctmp(1,jd).
compute desctmp(7,jd)=desctmp(1,jd)+desctmp(2,jd).
compute modvals=desctmp(5:7,:).
compute mnotev=2.
do if (modvals(1,1) < desctmp(3,1)).
compute modvals(1,1)=desctmp(3,1).
compute minwarn=1.
end if.
do if (modvals(3,1) > desctmp(4,1)).
compute modvals(3,1)=desctmp(4,1).
compute maxwarn=1.
end if.
end if.
do if (desctmp(8,1)=1).
compute modvals={desctmp(3,1);desctmp(4,1)}.
compute mnotev=0.
compute minwarn=0.
compute maxwarn=0.
end if.
end if.
end loop
.
compute bad=bad+ (desctmp(2,1) <= 0.00000000001).
do if (bad = 0).
do if (holymoly < smallest).
compute smallest=holymoly.
end if.
do if (ydich=0 or (i < (nms+nys))).

.
do if ( 1 =1).
compute hatmat=inv(t( x )* x )*t( x ).
compute b = hatmat* y.
compute modres=b.
do if ( 0 =1).
compute n1=nrow( x ).
compute dfres=n1-(ncol( x )).
compute sstotal = t( y -(csum( y )/n1))*( y -(csum( y )/n1)).
compute resid= y - x *b.
compute ssresid = csum((resid)&**2).
compute r2 = (sstotal-ssresid)/sstotal.
do if (r2 < 0).
compute r2=0.
end if.
compute adjr2 = 1-((1-r2)*(n1-1)/(dfres)).
compute mse=ssresid/(n1-ncol( x )).

.
compute n1=nrow( x ).
compute invXtX = inv(t( x )* x ).
compute varb = mse *invXtX.
compute k3 = ncol( x ).
compute xhc=0.
do if ( hc <> 5).
compute xhc= x.
compute hat = xhc(:,1).
loop i3=1 to nrow(xhc).
compute hat(i3,1)= xhc(i3,:)*invXtX*t(xhc(i3,:)).
end loop.
do if ( hc = 0 or hc =1).
loop i3 = 1 to k3.
compute xhc(:,i3)=xhc(:,i3)&* resid.
end loop.
end if.
do if ( hc =3 or hc =2).
loop i3=1 to k3.
compute xhc(:,i3) = ( resid &/(1-hat)&**(1/(4- hc )))&*xhc(:,i3).
end loop.
end if.
do if ( hc = 4).
compute hcmn=make(n,2,4).
compute hcmn(:,2)=(n1*hat)/k3.
loop i3= 1 to k3.
compute xhc(:,i3) = ( resid &/(1-hat)&**(rmin(hcmn)/2))&*xhc(:,i3).
end loop.
end if.
compute varb=(invXtX*t(xhc)*xhc*invXtX).
do if ( hc =1).
compute varb=(n1/(n1-ncol( x )))&*varb.
end if.
end if.
compute hclab={'se(HC0)','se(HC1)','se(HC2)','se(HC3)','se(HC4)','se'}.
compute hclab=hclab(1,( hc +1)).
compute hcflab={'F(HC0)','F(HC1)','F(HC2)','F(HC3)','F(HC4)','F'}.
compute hcflab=hcflab(1,( hc +1)).
release xhc
.
compute seb=sqrt(diag(varb)).
compute trat = b&/seb.
compute p = 2*(1-tcdf(abs(trat), (dfres))).
compute tval = sqrt(dfres* (exp((dfres-(5/6))*((xp2/(dfres-(2/3)+(.11/dfres)))* (xp2/(dfres-(2/3)+(.11/dfres)))))-1)).
compute modres={modres,seb,trat,p}.
compute modres={modres,(b-tval&*seb),(b+tval&*seb)}.
compute modresl={'coeff',hclab,'t','p','LLCI','ULCI'}.
compute lmat = ident(ncol( x )).
compute lmat = lmat(:,2:ncol(lmat)).
compute fratio = (t(t(lmat)*b)*inv(t(lmat)*varb*lmat)*((t(lmat)*b)))/(ncol( x )-1).
compute pfr = 1-fcdf(fratio,(ncol( x )-1),dfres).
compute modsum={sqrt(r2),r2,mse,fratio,(ncol( x )-1),dfres,pfr}.
compute modsuml={'R','R-sq','MSE',hcflab,'df1','df2', 'p'}.
end if.
end if.
do if ( 1 = 2 or 1 =3).
compute xlp= x.
compute ylp= y.
compute pt2 = make(nrow(ylp),1,(csum(ylp)/nrow(ylp))).
do if ( 1 =2).
compute LL3 = ylp&*ln(pt2)+(1-ylp)&*ln(1-pt2).
end if.
compute LL3 = -2*csum(LL3).
compute bt1 = make(ncol(xlp),1,0).
compute LL1 = 0.
compute pt1 = make(nrow(ylp),1,0.5).
compute pt1lp=pt1.
loop jjj = 1 to iterate.
compute xlptmp=t(xlp).
compute vecprb=pt1lp&*(1-pt1lp).
loop kkk=1 to ncol(xlp).
compute xlptmp(kkk,:)=xlptmp(kkk,:)&*t(vecprb).
end loop.
compute b = bt1+inv(xlptmp*xlp)*t(xlp)*(ylp-pt1lp).
do if ( 1 =2).
compute xlpb=xlp*b.
compute xlpbt=(xlpb > -709.7).
compute xlpb709=(1-xlpbt)*(-709.7).
compute xlpb=(xlpb&*xlpbt)+xlpb709.
compute pt1lp = 1/(1+exp(-(xlpb))).
end if.
compute itprob = csum((pt1lp < .00000001) or (pt1lp > .9999999)).
do if (itprob > 0).
loop kkk = 1 to nrow(pt1lp).
do if (pt1lp(kkk,1) > .9999999).
compute pt1lp(kkk,1) = .9999999.
end if.
do if (pt1lp(kkk,1) < .00000001).
compute pt1lp(kkk,1) = .00000001.
end if.
end loop.
compute itprob = 0.
end if.
do if (itprob = 0).
do if ( 1 =2).
compute LL = ylp&*ln(pt1lp)+(1-ylp)&*ln(1-pt1lp).
end if.
compute LL2 = -2*csum(ll).
end if.
do if (abs(LL1-LL2) < converge).
do if ( 0 =1).
compute xlptmp=t(xlp).
compute vecprb=pt1lp&*(1-pt1lp).
loop kkk=1 to ncol(xlp).
compute xlptmp(kkk,:)=xlptmp(kkk,:)&*t(vecprb).
end loop.
compute varb = inv(xlptmp*xlp).
compute seb = sqrt(diag(varb)).
release xlptmp.
end if.
break.
end if.
compute bt1 = b.
compute LL1 = LL2.
end loop.
compute modres=b.
do if (jjj > iterate).
compute itprob = 2.
do if (booting=0).
compute iterrmod=1.
end if.
do if (booting=1).
compute bootiter=1.
end if.
do if (itprobtg=0).
compute itprobtg=1.
compute errcode(errs,1) = 47.
compute errs = errs + 1.
do if (booting = 0 and 0 =1).
compute vt1 = mdiag(pt1lp&*(1-pt1lp)).
compute varb = inv(t(xlp)*vt1*xlp).
compute seb = sqrt(diag(varb)).
end if.
end if.
end if.
do if ( 0 =1).
compute trat = b&/seb.
compute dfres=nrow(xlp).
compute p = 2*(1-cdfnorm(abs(trat))).
compute modres={modres,seb,trat,p}.
compute modres={modres,(b-xp2&*seb),(b+xp2&*seb)}.
compute pvchi=1-chicdf((LL3-LL2),(nrow(modres)-1)).
compute mcF = (LL3-LL2)/LL3.
compute cox = 1-exp(-(LL3-LL2)/nrow(xlp)).
compute nagel = cox/(1-exp(-(LL3)/nrow(xlp))).
compute modsum={LL2,(LL3-LL2),(nrow(modres)-1),pvchi, mcF,cox,nagel}.
compute modsuml={'-2LL','ModelLL', 'df', 'p', 'McFadden', 'CoxSnell', 'Nagelkrk'}.
compute modresl={'coeff','se','Z','p','LLCI','ULCI'}.
end if.
end if
.
end if.
do if (ydich=1 and (i = (nms+nys))).

.
do if ( 2 =1).
compute hatmat=inv(t( x )* x )*t( x ).
compute b = hatmat* y.
compute modres=b.
do if ( 0 =1).
compute n1=nrow( x ).
compute dfres=n1-(ncol( x )).
compute sstotal = t( y -(csum( y )/n1))*( y -(csum( y )/n1)).
compute resid= y - x *b.
compute ssresid = csum((resid)&**2).
compute r2 = (sstotal-ssresid)/sstotal.
do if (r2 < 0).
compute r2=0.
end if.
compute adjr2 = 1-((1-r2)*(n1-1)/(dfres)).
compute mse=ssresid/(n1-ncol( x )).

.
compute n1=nrow( x ).
compute invXtX = inv(t( x )* x ).
compute varb = mse *invXtX.
compute k3 = ncol( x ).
compute xhc=0.
do if ( hc <> 5).
compute xhc= x.
compute hat = xhc(:,1).
loop i3=1 to nrow(xhc).
compute hat(i3,1)= xhc(i3,:)*invXtX*t(xhc(i3,:)).
end loop.
do if ( hc = 0 or hc =1).
loop i3 = 1 to k3.
compute xhc(:,i3)=xhc(:,i3)&* resid.
end loop.
end if.
do if ( hc =3 or hc =2).
loop i3=1 to k3.
compute xhc(:,i3) = ( resid &/(1-hat)&**(1/(4- hc )))&*xhc(:,i3).
end loop.
end if.
do if ( hc = 4).
compute hcmn=make(n,2,4).
compute hcmn(:,2)=(n1*hat)/k3.
loop i3= 1 to k3.
compute xhc(:,i3) = ( resid &/(1-hat)&**(rmin(hcmn)/2))&*xhc(:,i3).
end loop.
end if.
compute varb=(invXtX*t(xhc)*xhc*invXtX).
do if ( hc =1).
compute varb=(n1/(n1-ncol( x )))&*varb.
end if.
end if.
compute hclab={'se(HC0)','se(HC1)','se(HC2)','se(HC3)','se(HC4)','se'}.
compute hclab=hclab(1,( hc +1)).
compute hcflab={'F(HC0)','F(HC1)','F(HC2)','F(HC3)','F(HC4)','F'}.
compute hcflab=hcflab(1,( hc +1)).
release xhc
.
compute seb=sqrt(diag(varb)).
compute trat = b&/seb.
compute p = 2*(1-tcdf(abs(trat), (dfres))).
compute tval = sqrt(dfres* (exp((dfres-(5/6))*((xp2/(dfres-(2/3)+(.11/dfres)))* (xp2/(dfres-(2/3)+(.11/dfres)))))-1)).
compute modres={modres,seb,trat,p}.
compute modres={modres,(b-tval&*seb),(b+tval&*seb)}.
compute modresl={'coeff',hclab,'t','p','LLCI','ULCI'}.
compute lmat = ident(ncol( x )).
compute lmat = lmat(:,2:ncol(lmat)).
compute fratio = (t(t(lmat)*b)*inv(t(lmat)*varb*lmat)*((t(lmat)*b)))/(ncol( x )-1).
compute pfr = 1-fcdf(fratio,(ncol( x )-1),dfres).
compute modsum={sqrt(r2),r2,mse,fratio,(ncol( x )-1),dfres,pfr}.
compute modsuml={'R','R-sq','MSE',hcflab,'df1','df2', 'p'}.
end if.
end if.
do if ( 2 = 2 or 2 =3).
compute xlp= x.
compute ylp= y.
compute pt2 = make(nrow(ylp),1,(csum(ylp)/nrow(ylp))).
do if ( 2 =2).
compute LL3 = ylp&*ln(pt2)+(1-ylp)&*ln(1-pt2).
end if.
compute LL3 = -2*csum(LL3).
compute bt1 = make(ncol(xlp),1,0).
compute LL1 = 0.
compute pt1 = make(nrow(ylp),1,0.5).
compute pt1lp=pt1.
loop jjj = 1 to iterate.
compute xlptmp=t(xlp).
compute vecprb=pt1lp&*(1-pt1lp).
loop kkk=1 to ncol(xlp).
compute xlptmp(kkk,:)=xlptmp(kkk,:)&*t(vecprb).
end loop.
compute b = bt1+inv(xlptmp*xlp)*t(xlp)*(ylp-pt1lp).
do if ( 2 =2).
compute xlpb=xlp*b.
compute xlpbt=(xlpb > -709.7).
compute xlpb709=(1-xlpbt)*(-709.7).
compute xlpb=(xlpb&*xlpbt)+xlpb709.
compute pt1lp = 1/(1+exp(-(xlpb))).
end if.
compute itprob = csum((pt1lp < .00000001) or (pt1lp > .9999999)).
do if (itprob > 0).
loop kkk = 1 to nrow(pt1lp).
do if (pt1lp(kkk,1) > .9999999).
compute pt1lp(kkk,1) = .9999999.
end if.
do if (pt1lp(kkk,1) < .00000001).
compute pt1lp(kkk,1) = .00000001.
end if.
end loop.
compute itprob = 0.
end if.
do if (itprob = 0).
do if ( 2 =2).
compute LL = ylp&*ln(pt1lp)+(1-ylp)&*ln(1-pt1lp).
end if.
compute LL2 = -2*csum(ll).
end if.
do if (abs(LL1-LL2) < converge).
do if ( 0 =1).
compute xlptmp=t(xlp).
compute vecprb=pt1lp&*(1-pt1lp).
loop kkk=1 to ncol(xlp).
compute xlptmp(kkk,:)=xlptmp(kkk,:)&*t(vecprb).
end loop.
compute varb = inv(xlptmp*xlp).
compute seb = sqrt(diag(varb)).
release xlptmp.
end if.
break.
end if.
compute bt1 = b.
compute LL1 = LL2.
end loop.
compute modres=b.
do if (jjj > iterate).
compute itprob = 2.
do if (booting=0).
compute iterrmod=1.
end if.
do if (booting=1).
compute bootiter=1.
end if.
do if (itprobtg=0).
compute itprobtg=1.
compute errcode(errs,1) = 47.
compute errs = errs + 1.
do if (booting = 0 and 0 =1).
compute vt1 = mdiag(pt1lp&*(1-pt1lp)).
compute varb = inv(t(xlp)*vt1*xlp).
compute seb = sqrt(diag(varb)).
end if.
end if.
end if.
do if ( 0 =1).
compute trat = b&/seb.
compute dfres=nrow(xlp).
compute p = 2*(1-cdfnorm(abs(trat))).
compute modres={modres,seb,trat,p}.
compute modres={modres,(b-xp2&*seb),(b+xp2&*seb)}.
compute pvchi=1-chicdf((LL3-LL2),(nrow(modres)-1)).
compute mcF = (LL3-LL2)/LL3.
compute cox = 1-exp(-(LL3-LL2)/nrow(xlp)).
compute nagel = cox/(1-exp(-(LL3)/nrow(xlp))).
compute modsum={LL2,(LL3-LL2),(nrow(modres)-1),pvchi, mcF,cox,nagel}.
compute modsuml={'-2LL','ModelLL', 'df', 'p', 'McFadden', 'CoxSnell', 'Nagelkrk'}.
compute modresl={'coeff','se','Z','p','LLCI','ULCI'}.
end if.
end if
.
end if.
compute modres2={modres2,t(modres)}.
do if (i = (nms+nys)).
do if (bcmat((i+1),1) = 1).
compute bootdir={bootdir;t(modres(wherex(1,i):wherex(2,i),1))}.
end if.
do if (bcmat((i+1),1) = 0).
compute bootdir={bootdir;dirzes}.
end if.
end if.
do if (model = 74 and i <= nms).
compute onetemp=make(nrow(xprobval),1,1).
compute mestmtb={onetemp,xprobval}.
do if (ncs > 0).
compute ncovmdlb=rsum(ccmat(i,:)).
do if (ncovmdl > 0).
compute cvmncb=make(nrow(mestmtb),ncovmdlb,1).
compute cvmnctmp=csum(x(:,(ncol(x)-ncovmdlb+1):ncol(x)))/nrow(x).
do if (cuscoval > 0).
compute cvmnctmp=coval.
end if.
loop mestlp=1 to ncovmdlb.
compute cvmncb(:,mestlp)=cvmncb(:,mestlp)*cvmnctmp(:,mestlp).
end loop.
compute mestmtb={mestmtb,cvmncb}.
end if.
end if.
compute mestbt=mestmtb*modres.
do if (i = 1).
compute mestb=mestbt.
end if.
do if (i > 1).
compute mestb={mestb,mestbt}.
end if.
end if.
do if (model = 74 and i = (nms+nys)).
compute xvalptmp=make(1,nxvls,0).
compute mest74sp=make(1,nms,0).
compute mest74sp={mest74sp;mestb}.
compute xvalptmp={xvalptmp;ident(nxvls)}.
compute dirfxcf=make(nrow(mestb),1,0).
loop cfloop1=1 to nrow(mestb).
compute ndirfx=make(nrow(modres),1,0).
compute ndirfx(2:(nxvls+1),1)=t(xvalptmp(cfloop1,:)).
loop cfloop3=1 to nms.
do if (mcx=1 or mcx=0).
compute ndirfx(wheremw((1+((cfloop3-1)*2)),ncol(wheremw)):wheremw((2+((cfloop3-1)*2) ),ncol(wheremw)))=t(xvalptmp(cfloop1,:)*mestb(1,cfloop3)).
end if.
do if (mcx=2).
compute ndirfx(wheremw((1+((cfloop3-1)*2)),ncol(wheremw)):wheremw((2+((cfloop3-1)*2) ),ncol(wheremw)))=t(xvalptmp(cfloop1,:)*mest74sp(cfloop1,cfloop3)).
end if.
end loop.
do if (cfloop1 > 1).
compute dirfxcf(cfloop1,1)=t(ndirfx)*modres*xscaling.
end if.
end loop.
compute natdirbt={natdirbt;t(dirfxcf(2:nrow(dirfxcf),:))}.
end if.
do if ( (bcmat((i+1),1) = 1) and (nobootx=1) and (effsize=1)).
compute nobootx=0.
compute xsdtemp= (nrow(x)*sscp(x(:,2)))-(t(csum(x(:,2)))*(csum(x(:,2)))).
compute xsdtemp= sqrt(xsdtemp/(nrow(x)*(nrow(x)-1))).
end if.
end if.
end loop.
do if (bad = 0).
compute bootres={bootres;modres2(:,2:ncol(modres2))}.
do if (effsize=1).
compute ysdtemp= (nrow(y)*sscp(y))-(t(csum(y))*(csum(y))).
compute ysdtemp= sqrt(ysdtemp/(nrow(y)*(nrow(y)-1))).
compute bootysd={bootysd;ysdtemp}.
compute bootxsd={bootxsd;xsdtemp}.
end if.
compute goodboot=goodboot+1.
end if.
do if (bad <> 0).
compute badboot=badboot+1.
end if.
end loop if (goodboot = boot).
compute bootres=bootres(2:nrow(bootres),:).
do if (effsize=1).
compute bootysd=bootysd(2:nrow(bootysd),:).
do if (nrow(bootxsd) > 1).
compute bootxsd=bootxsd(2:nrow(bootxsd),:).
end if.
end if.
do if (goodboot < (boot)).
compute boot=0.
compute modelbt=0.
compute notecode(notes,1) = 7.
compute notes = notes + 1.
end if.
do if (boot > 0).
do if (effsize=1).
compute bootysd={ysd;bootysd}.
compute bootxsd={xsd;bootxsd}.
end if.
do if (saveboot = 1).
save bootres/outfile = *.
end if.
do if (modelbt=1).
compute bootcim=make(ncol(bootres),5,-99999).
compute bootcim(:,2) = t(csum(bootres)/nrow(bootres)).
compute bootcim(:,1) = coeffmat(2:nrow(coeffmat),1).
loop i = 1 to ncol(bootres).

.
compute temp = bootres(:,i).
compute temp(GRADE( bootres(:,i) )) = bootres(:,i).
compute badlo = 0.
compute badhi = 0.
do if ( (bootcim(i,1)*bc)+(9999*(1-bc)) <> 9999).
compute pv=csum(temp < (bootcim(i,1)*bc)+(9999*(1-bc)) )/nrow(temp).
compute ppv = pv.
do if (pv > .5).
compute ppv = 1-pv.
end if.
compute y5=sqrt(-2*ln(ppv)).
compute xp=y5+((((y5*p4+p3)*y5+p2)*y5+p1)*y5+p0)/((((y5*q4+q3)*y5+q2)*y5+q1)*y5+q0).
do if (pv <= .5).
compute xp = -xp.
end if.
compute cilow=rnd(nrow(temp)*(cdfnorm(2*xp-xp2))).
compute cihigh=trunc(nrow(temp)*(cdfnorm(2*xp+(xp2))))+1.
do if (cilow < 1).
compute cilow = 1.
compute booterr=1.
compute badlo = 1.
end if.
do if (cihigh > nrow(temp)).
compute cihigh = boot.
compute booterr=1.
compute badhi = 1.
end if.
compute llcit=temp(cilow,1).
compute ulcit=temp(cihigh,1).
do if (badlo = 1 and llcit <> priorlo).
compute badend={badend, llcit}.
compute priorlo = llcit.
end if.
do if (badhi = 1 and ulcit <> priorhi).
compute badend={badend, ulcit}.
compute priorhi = ulcit.
end if.
end if.
do if ( (bootcim(i,1)*bc)+(9999*(1-bc)) = 9999).
compute llcit=temp(cilow,1).
compute ulcit=temp(cihigh,1).
end if.
compute bootse=t(sqrt((cssq(temp)-((csum(temp)&**2)/nrow(temp)))/(nrow(temp)-1)))
.
compute bootcim(i,4:5)={llcit,ulcit}.
compute bootcim(i,3)=bootse.
end loop.
end if.
end if.
do if (badboot > 0).
compute notecode(notes,1) = 6.
compute notes = notes + 1.
end if.
end if.
do if (criterr=0).
release fulldat,x,y.
end if.
do if (xmint=1 and criterr=0).
compute xvalptmp=make(1,nxvls,0).
compute mest74sp=make(1,nms,0).
compute mest74sp={mest74sp;mest74}.
compute xvalptmp={xvalptmp;ident(nxvls)}.
loop kcfuhd=1 to 2.
compute dirfxcf=make(nrow(mest74),6,0).
loop cfloop1=1 to nrow(mest74).
compute ndirfx=make(nrow(lastb),1,0).
compute ndirfx(2:(nxvls+1),1)=t(xvalptmp(cfloop1,:)).
loop cfloop3=1 to nms.
do if (kcfuhd=1).
do if (mcx=1 or mcx=0).
compute ndirfx(wheremw((1+((cfloop3-1)*2)),ncol(wheremw)):wheremw((2+((cfloop3-1)*2)), ncol(wheremw)))=t(xvalptmp(cfloop1,:)*mest74(1,cfloop3)).
end if.
do if (mcx=2).
compute ndirfx(wheremw((1+((cfloop3-1)*2)),ncol(wheremw)):wheremw((2+((cfloop3-1)*2)), ncol(wheremw)))=t(xvalptmp(cfloop1,:)*mest74sp(cfloop1,cfloop3)).
end if.
end if.
do if (kcfuhd=2).
compute ndirfx(wheremw((1+((cfloop3-1)*2)),ncol(wheremw)):wheremw((2+((cfloop3-1)*2)), ncol(wheremw)))=t(xvalptmp(cfloop1,:)*medmeans(1,cfloop3)).
end if.
end loop.
do if (cfloop1 > 1).
compute dirfxcf(cfloop1,1)=t(ndirfx)*lastb*xscaling.
compute dirfxcf(cfloop1,2)=sqrt(diag(t(ndirfx)*lastcov*ndirfx))*abs(xscaling).
compute dirfxcf(cfloop1,3)=dirfxcf(cfloop1,1)/dirfxcf(cfloop1,2).
compute dirfxcf(cfloop1,4)=2*(1-tcdf(abs(dirfxcf(cfloop1,3)), dfres)).
compute dirfxcf(cfloop1,5)=dirfxcf(cfloop1,1)-tval*dirfxcf(cfloop1,2).
compute dirfxcf(cfloop1,6)=dirfxcf(cfloop1,1)+tval*dirfxcf(cfloop1,2).
end if.
end loop.
compute codireff=dirfxcf(2:nrow(dirfxcf),:).
do if (kcfuhd=1).
compute direff=codireff.
end if.
end loop.
end if.

.
do if (criterr = 0 and nms > 0).
compute paths=paths(:,2:ncol(paths)).
compute pathsw=pathsw(:,2:ncol(pathsw)).
compute pathsz=pathsz(:,2:ncol(pathsz)).
compute pathswz=pathswz(:,2:ncol(pathswz)).
compute pathsmod=pathsw+pathsz+pathswz.
compute pathsdv=pathsdv(:,2:ncol(pathsdv)).
compute pathsfoc=pathsfoc(:,2:ncol(pathsfoc)).
compute pathtype=pathtype(:,2:ncol(pathtype)).
compute anymod=(rsum(pathsmod) > 0).
compute obscoeff=obscoeff(1,2:ncol(obscoeff)).
do if (outscree=1).
do if (xmint=1).
print/title = '************************* COUNTERFACTUALLY DEFINED ************************'.
else.
print/title = ' '.
end if.
do if (dototal = 0 and alttotal=0).
print/title = '****************** DIRECT AND INDIRECT EFFECTS OF X ON Y *****************'/space=0.
end if.
do if (alttotal=1).
print/title = '************** TOTAL, DIRECT, AND INDIRECT EFFECTS OF X ON Y **************'/space=0.
end if.
end if.
do if (dototal = 1).
do if (outscree=1).
print/title = '************** TOTAL, DIRECT, AND INDIRECT EFFECTS OF X ON Y **************'/space=0.
end if.
compute totefflb(1,1)='Effect'.
do if (effsize=1).
compute toteffsz=toteff(:,1)/ysd.
do if (xdich=1 or mcx > 0).
compute totefflb={totefflb,'c_ps'}.
end if.
do if (xdich = 0 and mcx = 0).
compute toteffsz=toteffsz*xsd.
compute totefflb={totefflb,'c_cs'}.
end if.
compute toteff={toteff,toteffsz}.
end if.
compute resultm2=make(nrow(toteff),maxresm,99999).
compute resultm2(1:nrow(toteff),1:ncol(toteff))=toteff.
compute resultm={resultm;resultm2}.
do if (nxvls > 1).
compute resultm2=make(nrow(totomni),maxresm,99999).
compute resultm2(1:nrow(totomni),1:ncol(totomni))=totomni.
compute resultm={resultm;resultm2}.
compute clabtmp={'R2-chng', hcflab, 'df1','df2','p'}.
do if (outscree=1).
print toteff/title='Relative total effects of X on Y'/rnames=toteffl2/cnames=totefflb/format= F10.4.
print totomni/title='Omnibus test of total effect of X on Y'/cnames=clabtmp/format= F10.4.
print/title= '----------'/space=0.
end if.
else.
do if (outscree=1).
print toteff/title='Total effect of X on Y'/cnames=totefflb/format= F10.4.
end if.
end if.
end if.
compute moddir=wcmat(nrow(bcmat),1)+zcmat(nrow(bcmat),1).
do if (xmint=1).
compute moddir=1.
end if.
do if (bcmat(nrow(bcmat),1)=1).
do if (ydich=1).
compute direfflb(:,(ncol(direfflb)-5):ncol(direfflb))={'Effect','se','Z','p','LLCI','ULCI'}.
end if.
do if (moddir=0 or xmint=1).
compute direfflb(1,1)='Effect'.
end if.
do if (effsize=1 and moddir=0 and anymod = 0).
compute direffsz=direff(:,1)/ysd.
do if (xdich=1 or mcx > 0).
compute direfflb={direfflb,'c''_ps'}.
end if.
do if (xdich = 0 and mcx = 0).
compute direffsz=direffsz*xsd.
compute direfflb={direfflb,'c''_cs'}.
end if.
compute direff={direff,direffsz}.
end if.
compute resultm2=make(nrow(direff),maxresm,99999).
compute resultm2(1:nrow(direff),1:ncol(direff))=direff.
compute resultm={resultm;resultm2}.
do if (xmint=1).
compute resultm2=make(nrow(codireff),maxresm,99999).
compute resultm2(1:nrow(codireff),1:ncol(codireff))=codireff.
compute resultm={resultm;resultm2}.
end if.
do if (moddir = 0 and nxvls=1).
do if (outscree=1).
print direff/title='Direct effect of X on Y'/cnames=direfflb/format= F10.4.
end if.
end if.
do if (moddir = 0 and nxvls>1).
compute resultm2=make(nrow(diromni),maxresm,99999).
compute resultm2(1:nrow(diromni),1:ncol(diromni))=diromni.
compute resultm={resultm;resultm2}.
do if (outscree=1).
print direff/title='Relative direct effects of X on Y'/rnames=direffl2/cnames=direfflb/format= F10.4.
do if (ydich = 0).
compute clabtmp={'R2-chng', hcflab, 'df1','df2','p'}.
print diromni/title='Omnibus test of direct effect of X on Y:'/cnames=clabtmp/format= F10.4.
end if.
do if (ydich = 1).
compute clabtmp={'Chi-sq', 'df', 'p'}.
print diromni/title='Omnibus likelihood ratio test of direct effect of X on Y'/cnames=clabtmp/format= F10.4.
end if.
print/title= '----------'/space=0.
end if.
end if.
do if (moddir > 0 and nxvls=1).
do if (xmint=0 and outscree=1).
print direff/title='Conditional direct effects of X on Y'/cnames=direfflb/format= F10.4.
end if.
do if (xmint=1).
do if (outscree=1).
print direff/title='(Pure) Natural direct effect of X on Y'/cnames=direfflb/format= F10.4.
print codireff/title='Controlled direct effect of X on Y'/cnames=direfflb/format= F10.4.
print/title= '----------'/space=0.
end if.
compute obnatdfx=direff(:,1).
end if.
end if.
compute direffl4=direffl2.
do if (moddir > 0 and nxvls>1).
compute direffl2={' '}.
loop i = 1 to nxvls.
loop j = 1 to (nrow(direff)/nxvls).
compute direffl2={direffl2;xcatlab(i,1)}.
end loop.
end loop.
compute direffl2=direffl2(2:nrow(direffl2),1).
do if (xmint=0 and outscree=1).
print direff/title='Relative conditional direct effects of X on Y'/cnames=direfflb/rnames=direffl2/format= F10.4.
end if.
do if (xmint=1).
do if (outscree=1).
print direff/title='Relative (pure) natural direct effects of X on Y'/cnames=direfflb/rnames=direffl2/format= F10.4.
print codireff/title='Relative controlled direct effects of X on Y'/cnames=direfflb/rnames=direffl2/format= F10.4.
print/title= '----------'/space=0.
end if.
compute obnatdfx=direff(:,1).
end if.
end if.
compute direffl2=direffl4.
end if.
do if (bcmat(nrow(bcmat),1)=0 and xmint <> 1 and outscree=1).
print/title='The direct effect of X on Y is fixed to zero.'.
end if.
do if (nms = 1).
compute indmark={2}.
compute indsets={1,2}.
end if.
do if (nms = 2).
compute indmark={2,2,3}.
compute indsets={1,4,2,5,1,3,5}.
compute thetam=1.
end if.
do if (nms = 3).
compute indmark={2,2,2,3,3,3,4}.
compute indsets={1,7,2,8,4,9,1,3,8,1,5,9,2,6,9,1,3,6,9}.
compute thetam={1,2,3}.
end if.
do if (nms = 4).
compute indmark={2,2,2,2,3,3,3,3,3,3,4,4,4,4,5}.
compute indsets={1,11,2,12,4,13,7,14,1,3,12,1,5,13,1,8,14,2,6,13,2,9,14,4,10,14,1,3,6,13,1,3,9, 14,1,5,10,14,2,6,10,14,1,3,6,10,14}.
compute thetam={1,2,4,3,5,6}.
end if.
do if (nms = 5).
compute indmark={2,2,2,2,2,3,3,3,3,3,3,3,3,3,3,4,4,4,4,4,4,4,4,4,4,5,5,5,5,5,6}.
compute indsets={1,16,2,17,4,18,7,19,11,20,1,3,17,1,5,18,1,8,19,1,12,20,2,6,18,2,9,19,2,13,20,4, 10,19,4,14,20,7,15,20,1,3,6,18, 1,3,9,19,1,3,13,20,1,5,10,19,1,5,14,20,1,8,15,20,2,6,10,19,2,6, 14,20,2,9,15,20,4,10,15,20,1,3,6,10,19, 1,3,6,14,20,1,3,9,15,20,1,5,10,15,20,2,6,10,15,20,1,3,6,10,15, 20}.
compute thetam={1,2,5,3,6,8,4,7,9,10}.
end if.
do if (nms = 6).
compute indmark={2,2,2,2,2,2,3,3,3,3,3,3,3,3,3,3,3,3,3,3,3,4,4,4,4,4,4,4,4,4,4,4,4,4,4,4,4,4,4, 4,4,5,5,5,5,5,5,5,5,5,5,5,5,5,5,5,6,6,6,6,6,6,7}.
compute indsets={1,22,2,23,4,24,7,25,11,26,16,27,1,3,23,1,5,24,1,8,25,1,12,26,1,17,27,2,6,24,2, 9,25,2,13,26,2,18,27,4,10,25,4,14,26, 4,19,27,7,15,26,7,20,27,11,21,27,1,3,6,24,1,3,9,25,1,3,13,26,1, 3,18,27,1,5,10,25,1,5,14,26,1,5,19,27,1,8,15,26,1,8,20,27, 1,12,21,27,2,6,10,25,2,6,14,26,2,6,19,27,2,9,15,26,2,9,20,27,2, 13,21,27,4,10,15,26,4,10,20,27,4,14,21,27,7,15,21,27, 1,3,6,10,25,1,3,6,14,26,1,3,6,19,27,1,3,9,15,26,1,3,9,20,27,1,3, 13,21,27,1,5,10,15,26,1,5,10,20,27,1,5,14,21,27, 1,8,15,21,27,2,6,10,15,26,2,6,10,20,27,2,6,14,21,27,2,9,15,21, 27,4,10,15,21,27,1,3,6,10,15,26,1,3,6,10,20,27, 1,3,6,14,21,27,1,3,9,15,21,27,1,5,10,15,21,27,2,6,10,15,21,27, 1,3,6,10,15,21,27}.
compute thetam={1,2,6,3,7,10,4,8,11,13,5,9,12,14,15}.
end if.
do if (nms = 7).
compute indmark={2,2,2,2,2,2,2}.
compute indsets={1,29,2,30,4,31,7,32,11,33,16,34,22,35}.
end if.
do if (nms = 8).
compute indmark={2,2,2,2,2,2,2,2}.
compute indsets={1,37,2,38,4,39,7,40,11,41,16,42,22,43,29,44}.
end if.
do if (nms = 9).
compute indmark={2,2,2,2,2,2,2,2,2}.
compute indsets={1,46,2,47,4,48,7,49,11,50,16,51,22,52,29,53,37,54}.
end if.
do if (nms=10).
compute indmark={2,2,2,2,2,2,2,2,2,2}.
compute indsets={1,56,2,57,4,58,7,59,11,60,16,61,22,62,29,63,37,64,46,65}.
end if.
compute indlbl = {'Ind1'; 'Ind2'; 'Ind3'; 'Ind4'; 'Ind5'; 'Ind6'; 'Ind7'; 'Ind8'; 'Ind9'; 'Ind10'; 'Ind11'; 'Ind12'; 'Ind13'; 'Ind14'; 'Ind15'}.
compute indlbl = {indlbl; 'Ind16';'Ind17';'Ind18';'Ind19';'Ind20';'Ind21';'Ind22';'Ind23';'Ind24';'Ind25';'Ind26';'Ind27';'Ind28';'Ind29';'Ind30'}.
compute indlbl = {indlbl; 'Ind31';'Ind32';'Ind33';'Ind34';'Ind35';'Ind36';'Ind37';'Ind38';'Ind39';'Ind40';'Ind41';'Ind42';'Ind43';'Ind44';'Ind45'}.
compute indlbl = {indlbl; 'Ind46';'Ind47';'Ind48';'Ind49';'Ind50';'Ind51';'Ind52';'Ind53';'Ind54';'Ind55';'Ind56';'Ind57';'Ind58';'Ind59';'Ind60'}.
compute indlbl = {indlbl; 'Ind61';'Ind62';'Ind63';'Ind64';'Ind65';'Ind66';'Ind67';'Ind68';'Ind69';'Ind70';'Ind71';'Ind72';'Ind73';'Ind74';'Ind75'}.
compute indlbl = {indlbl; 'Ind76';'Ind77';'Ind78';'Ind79';'Ind80';'Ind81';'Ind82';'Ind83';'Ind84';'Ind85';'Ind86';'Ind87';'Ind88';'Ind89';'Ind90'}.
compute cntname={'(C1)';'(C2)';'(C3)';'(C4)';'(C5)';'(C6)';'(C7)';'(C8)';'(C9)';'(C10)';'(C11)';'(C12)';'(C13)';'(C14)';'(C15)';'(C16)';'(C17)'}.
compute cntname={cntname;'(C18)';'(C19)';'(C20)';'(C21)';'(C22)';'(C23)';'(C24)';'(C25)';'(C26)';'(C27)';'(C28)';'(C29)';'(C30)';'(C31)'}.
compute cntname={cntname;'(C32)';'(C33)';'(C34)';'(C35)';'(C36)';'(C37)';'(C38)';'(C39)';'(C40)';'(C41)';'(C42)';'(C43)';'(C44)';'(C45)'}.
compute cntname={cntname;'(C46)';'(C47)';'(C48)';'(C49)';'(C50)';'(C51)';'(C52)';'(C53)';'(C54)';'(C55)';'(C56)';'(C57)';'(C58)';'(C59)'}.
compute cntname={cntname;'(C60)';'(C61)';'(C62)';'(C63)';'(C64)';'(C65)';'(C66)';'(C67)';'(C68)';'(C69)';'(C70)';'(C71)';'(C72)';'(C73)'}.
compute cntname={cntname;'(C74)';'(C75)';'(C76)';'(C77)';'(C78)';'(C79)';'(C80)';'(C81)';'(C82)';'(C83)';'(C84)';'(C85)';'(C86)';'(C87)'}.
compute cntname={cntname;'(C88)';'(C89)';'(C90)';'(C91)';'(C92)';'(C93)';'(C94)';'(C95)';'(C96)';'(C97)';'(C98)';'(C99)';'(C100)';'(C101)'}.
compute cntname={cntname;'(C102)';'(C103)';'(C104)';'(C105)'}.
compute indmake=make(ncol(indmark),(nms+2),0).
compute indmod=make(ncol(indmark),1,999).
compute indmmm=make(ncol(indmark),1,0).
compute indmmmt=make(ncol(indmark),1,0).
compute start=1.
compute end=0.
compute nindfx=0.
compute indlocs=make(nrow(thetaxmb),ncol(paths),999).
compute indkey=make(ncol(indmark),1+((rmax(indmark)*2)+1),'     ').
compute c1=1.
compute c2=1.
compute c3=1.
loop i = 1 to ncol(paths).
do if (pathtype(1,i)=1).
compute indlocs(:,i)=thetaxmb(:,c1).
compute c1=c1+1.
end if.
do if (pathtype(1,i)=3).
compute indlocs(:,i)=thetamyb(:,c2).
compute c2=c2+1.
end if.
do if (pathtype(1,i)=2 and nms < 7 and serial=1).
compute indlocs(:,i)=thetammb(:,thetam(1,c3)).
compute c3=c3+1.
end if.
end loop.
loop i = 1 to ncol(indlocs).
compute c1=2.
loop j = 2 to nrow(indlocs).
do if (indlocs(j,i) <> 0).
compute indlocs(c1,i)=indlocs(j,i).
compute c1=c1+1.
end if.
end loop.
compute indlocs(1,i)=c1-2.
end loop.
compute indlocs=indlocs(1:rmax((indlocs(1,:))+1),:).
loop i = 1 to ncol(indmark).
compute numget=indmark(1,i).
compute end=end+numget.
compute gotcha=indsets(1,start:end).
compute start=end+1.
compute ok=1.
compute temp=0.
compute repoman=make(4,1,0).
loop j = 1 to ncol(gotcha).
do if paths(1,gotcha(1,j))=0.
compute ok=0.
end if.
do if (pathsmod(1,gotcha(1,j)) > 0).
compute temp=1.
compute temp2={pathsw(1,gotcha(1,j));pathsz(1,gotcha(1,j));pathswz(1,gotcha(1,j));0}.
do if (temp2(1,1)=1 and temp2(2,1)=1 and temp2(3,1)=0).
compute temp2(4,1)=1.
end if.
compute repoman=repoman+temp2.
end if.
end loop.
compute temp=0.
compute tempmmm=0.
compute typemmm=0.
do if ((repoman(1,1) > 0) and (repoman(2,1) = 0)).
compute temp=1.
do if (repoman(1,1)=1).
compute tempmmm=1.
end if.
do if (repoman(1,1) > 1 and (wdich=1 or mcw > 0)).
compute tempmmm=12.
compute typemmm=mcw.
do if (wdich=1).
compute typemmm=1.
end if.
end if.
do if (repoman(1,1) > 1 and (wdich=0 and mcw = 0)).
compute tempmmm=101.
end if.
end if.
do if ((repoman(1,1) = 0) and (repoman(2,1) > 0)).
compute temp=2.
do if (repoman(2,1)=1).
compute tempmmm=2.
end if.
do if (repoman(2,1) > 1 and (zdich = 1 or mcz > 0)).
compute tempmmm=22.
compute typemmm=mcz.
do if (zdich=1).
compute typemmm=1.
end if.
end if.
do if (repoman(2,1) > 1 and (zdich = 0 and mcw = 0)).
compute tempmmm=102.
end if.
end if.
do if (repoman(1,1)>0 and repoman(2,1)>0).
compute temp=3.
do if (repoman(1,1)=1 and repoman(2,1)=1).
do if (repoman(4,1)=1).
compute tempmmm=31.
end if.
do if (repoman(3,1)=1).
compute tempmmm=41.
end if.
end if.
end if.
do if (repoman(1,1)=1 and repoman(2,1)=1 and repoman(3,1)=0 and repoman(4,1)=0).
compute tempmmm=51.
end if.
do if (ok = 1).
compute nindfx=nindfx+1.
compute indmake(nindfx,1)=numget.
compute indmod(nindfx,1)=temp.
compute indmmm(nindfx,1)=tempmmm.
compute indmmmt(nindfx,1)=typemmm.
compute indmake(nindfx,2:(1+numget))=gotcha.
compute indkey(nindfx,1)=xnames.
loop j = 1 to numget.
compute indkey(nindfx,(j*2+1))=pathsdv(1,gotcha(1,j)).
compute indkey(nindfx,(j*2))='   ->   '.
end loop.
end if.
end loop.
compute indkey=indkey(1:nindfx,1:((cmax(indmake(:,1))*2)+1)).
compute indmake=indmake(1:nindfx,1:(cmax(indmake(:,1))+1)).
compute indmod=indmod(1:nrow(indmake),1).
compute indmmm=indmmm(1:nrow(indmake),1).
compute indmmmt=indmmmt(1:nrow(indmake),1).
compute ncpairs = (((nindfx)*(nindfx-1))/2).
do if ((contrast = 1 or contrast = 2 or contrast=3) and (ncpairs > 105)).
compute contrast=0.
compute notecode(notes,1) = 13.
compute notes = notes + 1.
end if.
do if (contrast = 4).
do if (ncol(contvec) <> nindfx).
compute contrast=0.
compute notecode(notes,1) = 14.
compute notes = notes + 1.
end if.
end if.
do if (anymod=0).
do if (nms = 1 and contrast > 0).
compute contrast=0.
end if.
compute efloop=(((1-(effsize=0))*2)+1)-(((mcx>0 or xdich=1))*(1-(effsize=0))).
loop kk=1 to efloop.
do if (boot = 0).
compute bootres=obscoeff.
do if (kk=1).
compute totbtvec=make(1,nxvls,0).
end if.
compute bootdir=obsdirfx.
compute indtab=999.
compute inddiff=999.
compute bootysd=ysd.
compute bootxsd=xsd.
end if.
do if (boot > 0).
compute bootres={obscoeff;bootres}.
do if (kk=1).
compute totbtvec=make(nrow(bootres),nxvls,0).
end if.
compute indtab=make(1,4,999).
compute inddiff=make(nrow(bootres),1,999).
end if.
compute indtotal=make(nrow(bootres),1,0).
loop i = 1 to nrow(indmake).
loop j = 1 to nxvls.
compute indtemp=make(nrow(bootres),1,1).
loop k = 1 to indmake(i,1).
compute jtemp=1.
do if (j > 1 and k=1).
compute jtemp=j.
end if.
compute indtemp=indtemp&*bootres(:,pathsfoc(jtemp,indmake(i,(k+1)))).
end loop.
do if (kk = 2).
compute indtemp=indtemp/bootysd.
end if.
do if (kk = 3).
compute indtemp=(bootxsd&*indtemp)/bootysd.
end if.
do if (contrast <> 0).
compute inddiff={inddiff,indtemp}.
end if.
do if (nxvls=1).
compute indtotal=indtotal+indtemp.
end if.
compute indeff=indtemp(1,1).
do if (kk=1).
compute totbtvec(:,j)=totbtvec(:,j)+indtemp.
end if.
do if (boot > 0).

.
compute temp = indtemp(2:nrow(indtemp),1).
compute temp(GRADE( indtemp(2:nrow(indtemp),1) )) = indtemp(2:nrow(indtemp),1).
compute badlo = 0.
compute badhi = 0.
do if ( (indtemp(1,1)*bc)+(9999*(1-bc)) <> 9999).
compute pv=csum(temp < (indtemp(1,1)*bc)+(9999*(1-bc)) )/nrow(temp).
compute ppv = pv.
do if (pv > .5).
compute ppv = 1-pv.
end if.
compute y5=sqrt(-2*ln(ppv)).
compute xp=y5+((((y5*p4+p3)*y5+p2)*y5+p1)*y5+p0)/((((y5*q4+q3)*y5+q2)*y5+q1)*y5+q0).
do if (pv <= .5).
compute xp = -xp.
end if.
compute cilow=rnd(nrow(temp)*(cdfnorm(2*xp-xp2))).
compute cihigh=trunc(nrow(temp)*(cdfnorm(2*xp+(xp2))))+1.
do if (cilow < 1).
compute cilow = 1.
compute booterr=1.
compute badlo = 1.
end if.
do if (cihigh > nrow(temp)).
compute cihigh = boot.
compute booterr=1.
compute badhi = 1.
end if.
compute llcit=temp(cilow,1).
compute ulcit=temp(cihigh,1).
do if (badlo = 1 and llcit <> priorlo).
compute badend={badend, llcit}.
compute priorlo = llcit.
end if.
do if (badhi = 1 and ulcit <> priorhi).
compute badend={badend, ulcit}.
compute priorhi = ulcit.
end if.
end if.
do if ( (indtemp(1,1)*bc)+(9999*(1-bc)) = 9999).
compute llcit=temp(cilow,1).
compute ulcit=temp(cihigh,1).
end if.
compute bootse=t(sqrt((cssq(temp)-((csum(temp)&**2)/nrow(temp)))/(nrow(temp)-1)))
.
compute indeff={indeff,bootse,llcit,ulcit}.
end if.
do if (kk=1).
compute indtabn={indtab;indeff}.
end if.
compute indtab={indtab;indeff}.
end loop.
end loop.
compute indtab=indtab(2:nrow(indtab),:).
do if (kk=1).
compute indtabn=indtabn(2:nrow(indtabn),:).
end if.
compute rowlbs=indlbl(1:nrow(indtab),1).
do if (mc > 0).
compute inddiff=make(mc,1,-999).
compute indtab2=make(nrow(indtab),4,-999).
compute indtab2(:,1)=indtab.
compute indtab=indtab2.
release indtab2.
compute mcct=0.
compute indtotal=make(mc,1,0).
do if (kk = 1).
compute x1 = sqrt(-2*ln(uniform(mc,nrow(mcsopath))))&*cos((2*3.14159265358979)*uniform(mc, nrow(mcsopath))).
compute x1=x1*chol(indcov).
loop ii=1 to nrow(x1).
compute x1(ii,:)=x1(ii,:)+t(mcsopath).
end loop.
end if.
loop ii=1 to nms.
compute tmpb=x1(:,((nms*nxvls)+ii)).
compute tmpb2=tmpb.
do if (nxvls > 1).
loop jj=1 to (nxvls-1).
compute tmpb2={tmpb2,tmpb}.
end loop.
end if.
compute indtemp=x1(:,(((ii-1)*nxvls)+1):(ii*nxvls))&*tmpb2.
loop jj=1 to ncol(indtemp).
do if (kk = 2).
compute indtemp(:,jj)=indtemp(:,jj)/ysd.
end if.
do if (kk = 3).
compute indtemp(:,jj)=(xsd*indtemp(:,jj))/ysd.
end if.

.
compute temp = indtemp(:,jj).
compute temp(GRADE( indtemp(:,jj) )) = indtemp(:,jj).
compute badlo = 0.
compute badhi = 0.
do if ( 9999 <> 9999).
compute pv=csum(temp < 9999 )/nrow(temp).
compute ppv = pv.
do if (pv > .5).
compute ppv = 1-pv.
end if.
compute y5=sqrt(-2*ln(ppv)).
compute xp=y5+((((y5*p4+p3)*y5+p2)*y5+p1)*y5+p0)/((((y5*q4+q3)*y5+q2)*y5+q1)*y5+q0).
do if (pv <= .5).
compute xp = -xp.
end if.
compute cilow=rnd(nrow(temp)*(cdfnorm(2*xp-xp2))).
compute cihigh=trunc(nrow(temp)*(cdfnorm(2*xp+(xp2))))+1.
do if (cilow < 1).
compute cilow = 1.
compute booterr=1.
compute badlo = 1.
end if.
do if (cihigh > nrow(temp)).
compute cihigh = boot.
compute booterr=1.
compute badhi = 1.
end if.
compute llcit=temp(cilow,1).
compute ulcit=temp(cihigh,1).
do if (badlo = 1 and llcit <> priorlo).
compute badend={badend, llcit}.
compute priorlo = llcit.
end if.
do if (badhi = 1 and ulcit <> priorhi).
compute badend={badend, ulcit}.
compute priorhi = ulcit.
end if.
end if.
do if ( 9999 = 9999).
compute llcit=temp(cilow,1).
compute ulcit=temp(cihigh,1).
end if.
compute bootse=t(sqrt((cssq(temp)-((csum(temp)&**2)/nrow(temp)))/(nrow(temp)-1)))
.
compute mcct=mcct+1.
compute indtab(mcct,2:4)={bootse,llcit,ulcit}.
end loop.
do if (nxvls=1).
compute indtotal=indtotal+indtemp.
do if (contrast <> 0).
compute inddiff={inddiff,indtemp}.
end if.
end if.
end loop.
release indtemp,tmpb.
end if.
do if (normal = 1 and sobelok=1).
compute sobelmat=indtab(:,1).
compute sobelmat={sobelmat,(sobelmat/2),sobelmat,sobelmat}.
loop ii=1 to nms.
compute se2b=indcov(((nms*nxvls)+ii),((nms*nxvls)+ii)).
compute bpath2=mcsopath(((nms*nxvls)+ii),1)&**2.
compute se2a=diag(indcov((((ii-1)*nxvls)+1):(ii*nxvls),(((ii-1)*nxvls)+1):(ii*nxvls))).
compute apath2=mcsopath((((ii-1)*nxvls)+1):(ii*nxvls) ,1)&**2.
compute sesobel=sqrt(apath2*se2b+bpath2*se2a+se2a*se2b).
compute sobelmat((((ii-1)*nxvls)+1):(ii*nxvls),2)=sesobel.
end loop.
release se2b,bpath2,se2a,apath2,sesobel,ii.
compute sobelmat(:,3)=sobelmat(:,1)&/sobelmat(:,2).
compute sobelmat(:,4) = 2*(1-cdfnorm(abs(sobelmat(:,3)))).
end if.
do if (serial = 0).
compute rowlbs=t(mnames).
end if.
do if (nxvls=1 and nms > 1).
compute rowlbs={'TOTAL';rowlbs}.
compute indtemp=indtotal(1,1).
do if (boot > 0 and nxvls=1).

.
compute temp = indtotal(2:nrow(indtotal),1).
compute temp(GRADE( indtotal(2:nrow(indtotal),1) )) = indtotal(2:nrow(indtotal),1).
compute badlo = 0.
compute badhi = 0.
do if ( (indtotal(1,1)*bc)+(9999*(1-bc)) <> 9999).
compute pv=csum(temp < (indtotal(1,1)*bc)+(9999*(1-bc)) )/nrow(temp).
compute ppv = pv.
do if (pv > .5).
compute ppv = 1-pv.
end if.
compute y5=sqrt(-2*ln(ppv)).
compute xp=y5+((((y5*p4+p3)*y5+p2)*y5+p1)*y5+p0)/((((y5*q4+q3)*y5+q2)*y5+q1)*y5+q0).
do if (pv <= .5).
compute xp = -xp.
end if.
compute cilow=rnd(nrow(temp)*(cdfnorm(2*xp-xp2))).
compute cihigh=trunc(nrow(temp)*(cdfnorm(2*xp+(xp2))))+1.
do if (cilow < 1).
compute cilow = 1.
compute booterr=1.
compute badlo = 1.
end if.
do if (cihigh > nrow(temp)).
compute cihigh = boot.
compute booterr=1.
compute badhi = 1.
end if.
compute llcit=temp(cilow,1).
compute ulcit=temp(cihigh,1).
do if (badlo = 1 and llcit <> priorlo).
compute badend={badend, llcit}.
compute priorlo = llcit.
end if.
do if (badhi = 1 and ulcit <> priorhi).
compute badend={badend, ulcit}.
compute priorhi = ulcit.
end if.
end if.
do if ( (indtotal(1,1)*bc)+(9999*(1-bc)) = 9999).
compute llcit=temp(cilow,1).
compute ulcit=temp(cihigh,1).
end if.
compute bootse=t(sqrt((cssq(temp)-((csum(temp)&**2)/nrow(temp)))/(nrow(temp)-1)))
.
compute indtemp={indtemp, bootse,llcit,ulcit}.
end if.
do if (mc > 0).
compute obtmc=indtab(:,1).
compute indtemp=csum(obtmc).

.
compute temp = indtotal(:,1).
compute temp(GRADE( indtotal(:,1) )) = indtotal(:,1).
compute badlo = 0.
compute badhi = 0.
do if ( 9999 <> 9999).
compute pv=csum(temp < 9999 )/nrow(temp).
compute ppv = pv.
do if (pv > .5).
compute ppv = 1-pv.
end if.
compute y5=sqrt(-2*ln(ppv)).
compute xp=y5+((((y5*p4+p3)*y5+p2)*y5+p1)*y5+p0)/((((y5*q4+q3)*y5+q2)*y5+q1)*y5+q0).
do if (pv <= .5).
compute xp = -xp.
end if.
compute cilow=rnd(nrow(temp)*(cdfnorm(2*xp-xp2))).
compute cihigh=trunc(nrow(temp)*(cdfnorm(2*xp+(xp2))))+1.
do if (cilow < 1).
compute cilow = 1.
compute booterr=1.
compute badlo = 1.
end if.
do if (cihigh > nrow(temp)).
compute cihigh = boot.
compute booterr=1.
compute badhi = 1.
end if.
compute llcit=temp(cilow,1).
compute ulcit=temp(cihigh,1).
do if (badlo = 1 and llcit <> priorlo).
compute badend={badend, llcit}.
compute priorlo = llcit.
end if.
do if (badhi = 1 and ulcit <> priorhi).
compute badend={badend, ulcit}.
compute priorhi = ulcit.
end if.
end if.
do if ( 9999 = 9999).
compute llcit=temp(cilow,1).
compute ulcit=temp(cihigh,1).
end if.
compute bootse=t(sqrt((cssq(temp)-((csum(temp)&**2)/nrow(temp)))/(nrow(temp)-1)))
.
compute indtemp={indtemp, bootse,llcit,ulcit}.
end if.
compute indtab={indtemp;indtab}.
end if.
compute bootlbs={'Effect', 'BootSE','BootLLCI','BootULCI'}.
do if (mc > 0).
compute bootlbs={'Effect', 'MC SE','MC LLCI','MC ULCI'}.
end if.
do if (nxvls = 1).
do if (contrast <> 0).
compute inddiff=inddiff(:,2:ncol(inddiff)).
do if (mc > 0).
compute inddiff={t(obtmc);inddiff}.
end if.
do if (contrast = 4).
compute inddifft=inddiff*t(contvec).
compute indtemp=inddifft(1,1).
do if (boot > 0 or mc > 0).
do if (mc > 0).

.
compute temp = inddifft(2:nrow(inddifft),1).
compute temp(GRADE( inddifft(2:nrow(inddifft),1) )) = inddifft(2:nrow(inddifft),1).
compute badlo = 0.
compute badhi = 0.
do if ( 9999 <> 9999).
compute pv=csum(temp < 9999 )/nrow(temp).
compute ppv = pv.
do if (pv > .5).
compute ppv = 1-pv.
end if.
compute y5=sqrt(-2*ln(ppv)).
compute xp=y5+((((y5*p4+p3)*y5+p2)*y5+p1)*y5+p0)/((((y5*q4+q3)*y5+q2)*y5+q1)*y5+q0).
do if (pv <= .5).
compute xp = -xp.
end if.
compute cilow=rnd(nrow(temp)*(cdfnorm(2*xp-xp2))).
compute cihigh=trunc(nrow(temp)*(cdfnorm(2*xp+(xp2))))+1.
do if (cilow < 1).
compute cilow = 1.
compute booterr=1.
compute badlo = 1.
end if.
do if (cihigh > nrow(temp)).
compute cihigh = boot.
compute booterr=1.
compute badhi = 1.
end if.
compute llcit=temp(cilow,1).
compute ulcit=temp(cihigh,1).
do if (badlo = 1 and llcit <> priorlo).
compute badend={badend, llcit}.
compute priorlo = llcit.
end if.
do if (badhi = 1 and ulcit <> priorhi).
compute badend={badend, ulcit}.
compute priorhi = ulcit.
end if.
end if.
do if ( 9999 = 9999).
compute llcit=temp(cilow,1).
compute ulcit=temp(cihigh,1).
end if.
compute bootse=t(sqrt((cssq(temp)-((csum(temp)&**2)/nrow(temp)))/(nrow(temp)-1)))
.
end if.
do if (boot > 0).

.
compute temp = inddifft(2:nrow(inddifft),1).
compute temp(GRADE( inddifft(2:nrow(inddifft),1) )) = inddifft(2:nrow(inddifft),1).
compute badlo = 0.
compute badhi = 0.
do if ( (inddifft(1,1)*bc)+(9999* (1-bc)) <> 9999).
compute pv=csum(temp < (inddifft(1,1)*bc)+(9999* (1-bc)) )/nrow(temp).
compute ppv = pv.
do if (pv > .5).
compute ppv = 1-pv.
end if.
compute y5=sqrt(-2*ln(ppv)).
compute xp=y5+((((y5*p4+p3)*y5+p2)*y5+p1)*y5+p0)/((((y5*q4+q3)*y5+q2)*y5+q1)*y5+q0).
do if (pv <= .5).
compute xp = -xp.
end if.
compute cilow=rnd(nrow(temp)*(cdfnorm(2*xp-xp2))).
compute cihigh=trunc(nrow(temp)*(cdfnorm(2*xp+(xp2))))+1.
do if (cilow < 1).
compute cilow = 1.
compute booterr=1.
compute badlo = 1.
end if.
do if (cihigh > nrow(temp)).
compute cihigh = boot.
compute booterr=1.
compute badhi = 1.
end if.
compute llcit=temp(cilow,1).
compute ulcit=temp(cihigh,1).
do if (badlo = 1 and llcit <> priorlo).
compute badend={badend, llcit}.
compute priorlo = llcit.
end if.
do if (badhi = 1 and ulcit <> priorhi).
compute badend={badend, ulcit}.
compute priorhi = ulcit.
end if.
end if.
do if ( (inddifft(1,1)*bc)+(9999* (1-bc)) = 9999).
compute llcit=temp(cilow,1).
compute ulcit=temp(cihigh,1).
end if.
compute bootse=t(sqrt((cssq(temp)-((csum(temp)&**2)/nrow(temp)))/(nrow(temp)-1)))
.
end if.
compute indtemp={indtemp, bootse,llcit,ulcit}.
end if.
compute indtab={indtab;indtemp}.
end if.
do if (contrast = 1 or contrast = 2 or contrast=3).
compute conkey=make(1,4,' ').
loop i = 1 to ncol(inddiff)-1.
loop j = (i+1) to ncol(inddiff).
compute inddifft=inddiff(:,i)-inddiff(:,j).
do if (contrast=2).
compute inddifft=abs(inddiff(:,i))-abs(inddiff(:,j)).
end if.
do if (contrast=3).
compute inddifft=inddiff(:,i)+inddiff(:,j).
end if.
compute indtemp=inddifft(1,1).
do if (contrast=1 or contrast=2).
compute conkeyt={' ', rowlbs((i+1),1),' minus  ',rowlbs((j+1),1)}.
end if.
do if (contrast=3).
compute conkeyt={' ', rowlbs((i+1),1),'  plus  ',rowlbs((j+1),1)}.
end if.
compute conkey={conkey;conkeyt}.
do if (boot > 0 or mc > 0).
do if (mc > 0).

.
compute temp = inddifft(2:nrow(inddifft),1).
compute temp(GRADE( inddifft(2:nrow(inddifft),1) )) = inddifft(2:nrow(inddifft),1).
compute badlo = 0.
compute badhi = 0.
do if ( 9999 <> 9999).
compute pv=csum(temp < 9999 )/nrow(temp).
compute ppv = pv.
do if (pv > .5).
compute ppv = 1-pv.
end if.
compute y5=sqrt(-2*ln(ppv)).
compute xp=y5+((((y5*p4+p3)*y5+p2)*y5+p1)*y5+p0)/((((y5*q4+q3)*y5+q2)*y5+q1)*y5+q0).
do if (pv <= .5).
compute xp = -xp.
end if.
compute cilow=rnd(nrow(temp)*(cdfnorm(2*xp-xp2))).
compute cihigh=trunc(nrow(temp)*(cdfnorm(2*xp+(xp2))))+1.
do if (cilow < 1).
compute cilow = 1.
compute booterr=1.
compute badlo = 1.
end if.
do if (cihigh > nrow(temp)).
compute cihigh = boot.
compute booterr=1.
compute badhi = 1.
end if.
compute llcit=temp(cilow,1).
compute ulcit=temp(cihigh,1).
do if (badlo = 1 and llcit <> priorlo).
compute badend={badend, llcit}.
compute priorlo = llcit.
end if.
do if (badhi = 1 and ulcit <> priorhi).
compute badend={badend, ulcit}.
compute priorhi = ulcit.
end if.
end if.
do if ( 9999 = 9999).
compute llcit=temp(cilow,1).
compute ulcit=temp(cihigh,1).
end if.
compute bootse=t(sqrt((cssq(temp)-((csum(temp)&**2)/nrow(temp)))/(nrow(temp)-1)))
.
end if.
do if (boot > 0).

.
compute temp = inddifft(2:nrow(inddifft),1).
compute temp(GRADE( inddifft(2:nrow(inddifft),1) )) = inddifft(2:nrow(inddifft),1).
compute badlo = 0.
compute badhi = 0.
do if ( (inddifft(1,1)*bc)+(9999* (1-bc)) <> 9999).
compute pv=csum(temp < (inddifft(1,1)*bc)+(9999* (1-bc)) )/nrow(temp).
compute ppv = pv.
do if (pv > .5).
compute ppv = 1-pv.
end if.
compute y5=sqrt(-2*ln(ppv)).
compute xp=y5+((((y5*p4+p3)*y5+p2)*y5+p1)*y5+p0)/((((y5*q4+q3)*y5+q2)*y5+q1)*y5+q0).
do if (pv <= .5).
compute xp = -xp.
end if.
compute cilow=rnd(nrow(temp)*(cdfnorm(2*xp-xp2))).
compute cihigh=trunc(nrow(temp)*(cdfnorm(2*xp+(xp2))))+1.
do if (cilow < 1).
compute cilow = 1.
compute booterr=1.
compute badlo = 1.
end if.
do if (cihigh > nrow(temp)).
compute cihigh = boot.
compute booterr=1.
compute badhi = 1.
end if.
compute llcit=temp(cilow,1).
compute ulcit=temp(cihigh,1).
do if (badlo = 1 and llcit <> priorlo).
compute badend={badend, llcit}.
compute priorlo = llcit.
end if.
do if (badhi = 1 and ulcit <> priorhi).
compute badend={badend, ulcit}.
compute priorhi = ulcit.
end if.
end if.
do if ( (inddifft(1,1)*bc)+(9999* (1-bc)) = 9999).
compute llcit=temp(cilow,1).
compute ulcit=temp(cihigh,1).
end if.
compute bootse=t(sqrt((cssq(temp)-((csum(temp)&**2)/nrow(temp)))/(nrow(temp)-1)))
.
end if.
compute indtemp={indtemp, bootse,llcit,ulcit}.
end if.
compute indtab={indtab;indtemp}.
end loop.
end loop.
end if.
release inddiff.
compute contlbs=cntname(1:(((nindfx)*(nindfx-1))/2),1).
compute rowlbs={rowlbs;contlbs}.
end if.
compute resultm2=make(nrow(indtab),maxresm,99999).
compute resultm2(1:nrow(indtab),1:ncol(indtab))=indtab.
compute resultm={resultm;resultm2}.
do if (outscree=1).
do if (kk=1).
print indtab/title = 'Indirect effect(s) of X on Y:'/rnames=rowlbs/cnames=bootlbs/format= F10.4.
end if.
do if (kk = 2 and (xdich=1 or mcx > 0)).
print indtab/title = 'Partially standardized indirect effect(s) of X on Y:'/rnames=rowlbs/cnames=bootlbs/format= F10.4.
end if.
do if (kk = 3).
print indtab/title = 'Completely standardized indirect effect(s) of X on Y:'/rnames=rowlbs/cnames=bootlbs/format= F10.4.
end if.
end if.
do if (normal=1 and sobelok=1 and kk=1).
compute resultm2=make(nrow(sobelmat),maxresm,99999).
compute resultm2(1:nrow(sobelmat),1:ncol(sobelmat))=sobelmat.
compute resultm={resultm;resultm2}.
compute sobellab={'Effect',hclab,'Z','p'}.
compute sobelrlb=rowlbs.
do if (nms > 1).
compute sobelrlb=rowlbs(2:(1+nms),1).
end if.
do if (outscree=1).
print sobelmat/title='   Normal theory test for indirect effect(s):'/cnames=sobellab/rnames=sobelrlb/format= F10.4.
end if.
end if.
do if (contrast <> 0).
do if ((contrast=1 or contrast = 2 or contrast=3) and kk=efloop ).
compute conkey=conkey(2:nrow(conkey),:).
do if (outscree=1).
print conkey/title = 'Specific indirect effect contrast definition(s):'/rnames=contlbs/format=A8.
end if.
end if.
do if (outscree=1).
do if (contrast = 4 and kk=efloop).
compute crowlbs=rowlbs(2:(nindfx+1),1).
print contvec/title = 'Specific indirect effect contrast weights:'/cnames=crowlbs/rlabels='(C1)'/format= F10.4.
end if.
do if (contrast = 2 and kk=efloop).
print/title = 'Contrasts are differences between absolute values of indirect effects'.
end if.
end if.
end if.
do if (serial = 1 and kk=efloop).
compute rowlbst=rowlbs(2:nrow(rowlbs),1).
do if (outscree=1).
print indkey/title = 'Indirect effect key:'/rnames=rowlbst/format = A8.
end if.
end if.
else.
do if (outscree=1).
do if (kk = 1).
print/title = 'Relative indirect effects of X on Y'.
end if.
do if (kk = 2).
print/title = 'Partially standardized relative indirect effect(s) of X on Y:'.
end if.
do if (kk = 3).
print/title = 'Completely standardized relative indirect effect(s) of X on Y:'.
end if.
end if.
loop i = 1 to nrow(indmake).
compute indtabsm=indtab((((i-1)*nxvls)+1):(nxvls*i),:).
compute indkeyt=indkey(i,:).
do if (outscree=1).
print indkeyt/title=' '/space=0/format=A8.
end if.
do if (bcmat(nrow(bcmat),1)=0)).
compute direffl2=xcatlab(1:nxvls,1).
end if.
compute resultm2=make(nrow(indtabsm),maxresm,99999).
compute resultm2(1:nrow(indtabsm),1:ncol(indtabsm))=indtabsm.
compute resultm={resultm;resultm2}.
do if (outscree=1).
print indtabsm/title = ' '/cnames=bootlbs/rnames=direffl2/format= F10.4 /space=0.
end if.
do if (normal=1 and sobelok=1 and kk=1).
compute sobelsm=sobelmat((((i-1)*nxvls)+1):(nxvls*i),:).
compute sobellab={'Effect',hclab,'Z','p'}.
compute resultm2=make(nrow(sobelsm),maxresm,99999).
compute resultm2(1:nrow(sobelsm),1:ncol(sobelsm))=sobelsm.
compute resultm={resultm;resultm2}.
do if (outscree=1).
print sobelsm/title='      Normal theory test for relative indirect effects:'/cnames=sobellab/rnames=direffl2/format= F10.4.
end if.
end if.
end loop.
end if.
do if (effsize = 1 and boot > 0).
compute bootres=bootres(2:nrow(bootres),:).
end if.
end loop.
do if (alttotal=1).
compute altcnms='Effect'.
compute totbtvec=totbtvec+bootdir.
compute alttotfx=t(totbtvec(1,:)).
do if (boot > 0).
compute alttotfx=make(ncol(totbtvec),4,0).
compute alttotfx(:,1)=t(totbtvec(1,:)).
loop cec=1 to ncol(totbtvec).

.
compute temp = totbtvec(2:nrow(totbtvec),cec).
compute temp(GRADE( totbtvec(2:nrow(totbtvec),cec) )) = totbtvec(2:nrow(totbtvec),cec).
compute badlo = 0.
compute badhi = 0.
do if ( (totbtvec(1,cec)*bc)+(9999* (1-bc)) <> 9999).
compute pv=csum(temp < (totbtvec(1,cec)*bc)+(9999* (1-bc)) )/nrow(temp).
compute ppv = pv.
do if (pv > .5).
compute ppv = 1-pv.
end if.
compute y5=sqrt(-2*ln(ppv)).
compute xp=y5+((((y5*p4+p3)*y5+p2)*y5+p1)*y5+p0)/((((y5*q4+q3)*y5+q2)*y5+q1)*y5+q0).
do if (pv <= .5).
compute xp = -xp.
end if.
compute cilow=rnd(nrow(temp)*(cdfnorm(2*xp-xp2))).
compute cihigh=trunc(nrow(temp)*(cdfnorm(2*xp+(xp2))))+1.
do if (cilow < 1).
compute cilow = 1.
compute booterr=1.
compute badlo = 1.
end if.
do if (cihigh > nrow(temp)).
compute cihigh = boot.
compute booterr=1.
compute badhi = 1.
end if.
compute llcit=temp(cilow,1).
compute ulcit=temp(cihigh,1).
do if (badlo = 1 and llcit <> priorlo).
compute badend={badend, llcit}.
compute priorlo = llcit.
end if.
do if (badhi = 1 and ulcit <> priorhi).
compute badend={badend, ulcit}.
compute priorhi = ulcit.
end if.
end if.
do if ( (totbtvec(1,cec)*bc)+(9999* (1-bc)) = 9999).
compute llcit=temp(cilow,1).
compute ulcit=temp(cihigh,1).
end if.
compute bootse=t(sqrt((cssq(temp)-((csum(temp)&**2)/nrow(temp)))/(nrow(temp)-1)))
.
compute alttotfx(cec,2:4)={bootse,llcit,ulcit}.
end loop.
compute altcnms={altcnms,'BootSE', 'BootLLCI','BootULCI'}.
end if.
compute resultm2=make(nrow(alttotfx),maxresm,99999).
compute resultm2(1:nrow(alttotfx),1:ncol(alttotfx))=alttotfx.
compute resultm={resultm;resultm2}.
do if (outscree=1).
do if (nxvls > 1).
print/title= '----------'/space=0.
print alttotfx/title='Relative total effects of X on Y (sum of direct and indirect effects)'/rnames=direffl2/cnames=altcnms/format= F10.4.
end if.
do if (nxvls = 1).
print alttotfx/title='Total effect of X on Y (sum of direct and indirect effects)'/cnames=altcnms/format= F10.4.
end if.
end if.
end if.
end if.
do if (anymod > 0).
do if (boot = 0).
compute bootres=obscoeff.
compute indtab=999.
end if.
do if (boot > 0).
compute bootres={obscoeff;bootres}.
compute indtab=make(1,4,999).
end if.
do if (csum((indmod > 0))=nrow(indmod)).
do if (outscree=1).
do if (nxvls > 1).
do if (xmint=0).
print/title= 'Relative conditional indirect effects of X on Y:'.
end if.
do if (xmint=1).
print/title= 'Relative (total) natural indirect effects of X on Y:'.
end if.
end if.
do if (nxvls = 1).
do if (xmint=0).
print/title= 'Conditional indirect effects of X on Y:'.
end if.
do if (xmint=1).
print/title= '(Total) Natural indirect effect(s) of X on Y:'.
end if.
end if.
end if.
end if.
do if (csum((indmod > 0)) < nrow(indmod)).
do if (outscree=1).
do if (nxvls > 1).
print/title= 'Relative conditional and unconditional indirect effects of X on Y:'.
end if.
do if (nxvls = 1).
print/title= 'Conditional and unconditional indirect effects of X on Y:'.
end if.
end if.
end if.
compute cftotfx=make(nrow(bootres),nxvls,0).
loop i = 1 to nrow(indmake).
compute indtab=indtab(1,:)*0.
do if (outscree=1).
do if (xmint=0).
print indkey(i,:)/title = 'INDIRECT EFFECT:'/format=A8.
end if.
do if (xmint=1).
print indkey(i,:)/title = ' '/format=A8/space=0.
end if.
end if.
do if (indmod(i,1)=0).
loop j = 1 to nxvls.
compute indtemp=make(nrow(bootres),1,1).
loop k = 1 to indmake(i,1).
compute jtemp=1.
do if (j > 1 and k=1).
compute jtemp=j.
end if.
compute indtemp=indtemp&*bootres(:,pathsfoc(jtemp,indmake(i,(k+1)))).
end loop.
compute indeff=indtemp(1,1).
do if (boot > 0).

.
compute temp = indtemp(2:nrow(indtemp),1).
compute temp(GRADE( indtemp(2:nrow(indtemp),1) )) = indtemp(2:nrow(indtemp),1).
compute badlo = 0.
compute badhi = 0.
do if ( (indtemp(1,1)*bc)+(9999*(1-bc)) <> 9999).
compute pv=csum(temp < (indtemp(1,1)*bc)+(9999*(1-bc)) )/nrow(temp).
compute ppv = pv.
do if (pv > .5).
compute ppv = 1-pv.
end if.
compute y5=sqrt(-2*ln(ppv)).
compute xp=y5+((((y5*p4+p3)*y5+p2)*y5+p1)*y5+p0)/((((y5*q4+q3)*y5+q2)*y5+q1)*y5+q0).
do if (pv <= .5).
compute xp = -xp.
end if.
compute cilow=rnd(nrow(temp)*(cdfnorm(2*xp-xp2))).
compute cihigh=trunc(nrow(temp)*(cdfnorm(2*xp+(xp2))))+1.
do if (cilow < 1).
compute cilow = 1.
compute booterr=1.
compute badlo = 1.
end if.
do if (cihigh > nrow(temp)).
compute cihigh = boot.
compute booterr=1.
compute badhi = 1.
end if.
compute llcit=temp(cilow,1).
compute ulcit=temp(cihigh,1).
do if (badlo = 1 and llcit <> priorlo).
compute badend={badend, llcit}.
compute priorlo = llcit.
end if.
do if (badhi = 1 and ulcit <> priorhi).
compute badend={badend, ulcit}.
compute priorhi = ulcit.
end if.
end if.
do if ( (indtemp(1,1)*bc)+(9999*(1-bc)) = 9999).
compute llcit=temp(cilow,1).
compute ulcit=temp(cihigh,1).
end if.
compute bootse=t(sqrt((cssq(temp)-((csum(temp)&**2)/nrow(temp)))/(nrow(temp)-1)))
.
compute indeff={indeff,bootse,llcit,ulcit}.
end if.
compute indtab={indtab;indeff}.
end loop.
compute resultm2=make(nrow(indtab),maxresm,99999).
compute resultm2(1:nrow(indtab),1:ncol(indtab))=indtab.
compute resultm={resultm;resultm2}.
do if (outscree=1).
do if (nxvls > 1).
compute indefflb=xcatlab(1:nxvls,1).
print indtab(2:nrow(indtab),:)/title = ' '/clabels='Effect', 'BootSE', 'BootLLCI', 'BootULCI'/rnames=indefflb/format= F10.4 /space=0.
end if.
do if (nxvls = 1).
print indtab(2:nrow(indtab),:)/title = ' '/clabels='Effect', 'BootSE', 'BootLLCI', 'BootULCI'/format= F10.4 /space=0.
end if.
end if.
end if.
do if (indmod(i,1)>0).
do if (indmod(i,1)=1).
compute indmodva=wmodvals.
compute indprova=wprobval.
compute condlbs={wnames}.
compute printw=1.
else if (indmod(i,1)=2).
compute indmodva=zmodvals.
compute indprova=zprobval.
compute condlbs={znames}.
compute printz=1.
else if (indmod(i,1)=3).
compute cntmp=1.
compute printz=1.
compute printw=1.
compute indmodva=make((nrow(wmodvals)*nrow(zmodvals)),2,999).
loop k7 = 1 to nrow(wmodvals).
loop k8 = 1 to nrow(zmodvals).
compute indmodva(cntmp,:)={wmodvals(k7,1),zmodvals(k8,1)}.
compute cntmp=cntmp+1.
end loop.
end loop.
compute condlbs={wnames,znames}.
end if.
compute condres=make(nrow(indmodva),1,999).
do if (boot > 0).
compute condres=make(nrow(indmodva),4,999).
end if.
compute condres={indmodva,condres}.
loop k4 = 1 to nxvls.
compute imm3=make(nrow(bootres),1,1).
compute imm4=make(nrow(bootres),1,1).
compute indcontr=0.
do if (indmod(i,1)=3).
compute tihsw=wprobval.
compute tihsz=zprobval.
end if.
loop k1=1 to nrow(indmodva).
compute tucker2=make(nrow(bootres),1,1).
compute imm2=make(nrow(bootres),1,1).
compute wfirst=0.
compute zfirst=0.
compute immset=0.
loop k2=1 to indmake(i,1).
compute colnumb=indmake(i,(k2+1)).
do if (k2=1).
compute wbb=make(nrow(bootres),(nwvls*nxvls),0).
compute zbb=make(nrow(bootres),(nzvls*nxvls),0).
compute wzbb=make(nrow(bootres),(nwvls*nzvls*nxvls),0).
end if.
do if (k2<>1).
compute wbb=make(nrow(bootres),nwvls,0).
compute zbb=make(nrow(bootres),nzvls,0).
compute wzbb=make(nrow(bootres),(nwvls*nzvls),0).
end if.
compute cnt=1.
compute tihs=indlocs(2:((indlocs(1,colnumb))+1),colnumb).
do if (k2 = 1).
compute focbb=tihs(1:nxvls,1).
compute focbb=bootres(:,focbb).
do if (indmmm(i,1)>0).
compute imm=focbb(:,k4).
compute condbb=imm.
end if.
compute focaddon=make(1,nxvls,0).
compute focaddon(1,k4)=1.
compute cnt=cnt+nxvls.
compute placeh=nxvls.
do if (indmod(i,1)=1).
compute tihsz=make(nrow(wprobval),(nzvls*nxvls),0).
compute tihswz=make(nrow(wprobval),(nwvls*nzvls*nxvls),0).
do if (pathsw(1,colnumb)=1).
compute temp=make(nrow(wprobval),(nxvls*nwvls),0).
loop k5 = 1 to nrow(wprobval).
loop k6=1 to nwvls.
compute temp(k5, (((k4-1)*nwvls)+k6))=wprobval(k5,k6).
end loop.
end loop.
compute indprova={temp,tihsz,tihswz}.
else.
compute indprova={wprobval,tihsz,tihswz}.
end if.
end if.
do if (indmod(i,1)=2).
compute tihsw=make(nrow(zprobval),(nwvls*nxvls),0).
compute tihswz=make(nrow(zprobval),(nwvls*nzvls*nxvls),0).
do if (pathsz(1,colnumb)=1).
compute temp=make(nrow(zprobval),(nxvls*nzvls),0).
loop k5 = 1 to nrow(zprobval).
loop k6 =1 to nzvls.
compute temp(k5,(((k4-1)*nzvls)+k6))=zprobval(k5,k6).
end loop.
end loop.
compute indprova={tihsw,temp,tihswz}.
else.
compute indprova={tihsw,zprobval,tihswz}.
end if.
end if.
do if (indmod(i,1)=3).
compute indprova=make((nrow(wprobval)*nrow(zprobval)),((ncol(wprobval)*nxvls)+ (ncol(zprobval)*nxvls)+(nwvls*nzvls*nxvls)),0).
compute cntemp=1.
loop k7=1 to nrow(wprobval).
loop k8 =1 to nrow(zprobval).
compute temp=wprobval(k7,:)*focaddon(1,k4).
compute indprova(cntemp,(((k4-1)*nwvls)+1):(k4*(nwvls)))=temp.
compute temp=zprobval(k8,:)*focaddon(1,k4).
compute indprova(cntemp, ((((k4-1)*nzvls)+1)+(nxvls*nwvls)) : ((((k4-1)*nzvls)+1)+(nxvls*nwvls)+(nzvls-1)))=temp.
compute cntemp=cntemp+1.
end loop.
end loop.
do if (pathsz(1,colnumb)=0).
compute temp=make(nrow(indprova),(ncol(zprobval)*nxvls),0).
compute indprova(:,((ncol(wprobval)*nxvls)+1):((ncol(wprobval)+ncol(zprobval))* nxvls))=temp.
end if.
do if (pathsw(1,colnumb)=0).
compute temp=make(nrow(indprova),(ncol(wprobval)*nxvls),0).
compute indprova(:,1:(ncol(wprobval)*nxvls))=temp.
end if.
do if (pathswz(1,colnumb)=1).
compute cntemp=(ncol(wprobval)*nxvls)+(ncol(zprobval)*nxvls)+((k4-1)* ncol(wprobval)*ncol(zprobval))+1.
loop k7=1 to ncol(wprobval).
loop k8=1 to ncol(zprobval).
compute indprova(:,cntemp)=indprova(:,((ncol(wprobval)*(k4-1))+k7))&* indprova(:,((((k4-1)*ncol(zprobval))+k8)+(nxvls*ncol(wprobval)))).
compute cntemp=cntemp+1.
end loop.
end loop.
end if.
end if.
end if.
do if (k2 > 1).
compute focbb=tihs(1,1).
compute focbb=bootres(:,focbb).
do if (indmmm(i,1)>0).
compute imm=focbb(:,1).
compute condbb=imm.
end if.
compute focaddon=1.
compute cnt=cnt+1.
compute placeh=1.
do if (indmod(i,1)=1).
compute tihsz=make(nrow(wprobval),nzvls,0).
compute tihswz=make(nrow(wprobval),(nwvls*nzvls),0).
compute indprova={wprobval,tihsz,tihswz}.
end if.
do if (indmod(i,1)=2).
compute tihsw=make(nrow(zprobval),nwvls,0).
compute tihswz=make(nrow(zprobval),(nwvls*nzvls),0).
compute indprova={tihsw,zprobval,tihswz}.
end if.
do if (indmod(i,1)=3).
compute indprova=make((nrow(wprobval)*nrow(zprobval)),((ncol(wprobval)+ ncol(zprobval))+(nwvls*nzvls)),0).
compute cntemp=1.
loop k7=1 to nrow(wprobval).
loop k8 =1 to nrow(zprobval).
compute indprova(cntemp,1:(ncol(wprobval)+ncol(zprobval)))={wprobval(k7,:), zprobval(k8,:)}.
compute cntemp=cntemp+1.
end loop.
end loop.
do if (pathsz(1,colnumb)=0).
compute temp=make(nrow(indprova),ncol(zprobval),0).
compute indprova(:,(ncol(wprobval)+1):(ncol(wprobval)+ncol(zprobval)))=temp.
end if.
do if (pathsw(1,colnumb)=0).
compute temp=make(nrow(indprova),ncol(wprobval),0).
compute indprova(:,1:ncol(wprobval))=temp.
end if.
do if (pathswz(1,colnumb)=1).
compute cntemp=ncol(wprobval)+ncol(zprobval)+1.
loop k7=1 to ncol(wprobval).
loop k8=1 to ncol(zprobval).
compute indprova(:,cntemp)=indprova(:,k7)&*indprova(:,(ncol(wprobval)+k8)).
compute cntemp=cntemp+1.
end loop.
end loop.
end if.
end if.
end if.
do if (pathsw(1,colnumb)) = 1.
compute wbb=tihs(cnt:(cnt+(placeh*nwvls)-1),1).
compute wbb=bootres(:,wbb).
compute immlbs2=wcatlab(1:nwvls,1).
do if (zfirst=0).
compute wfirst=1.
end if.
do if (indmmm(i,1)=1 or indmmm(i,1)=31 or indmmm(i,1)=51).
compute imm=wbb(:,1).
loop k7 = 1 to nwvls.
compute imm={imm,wbb(:,(((k4-1)*nwvls*(k2=1))+k7))}.
end loop.
compute imm=imm(:,2:ncol(imm)).
end if.
do if (indmmm(i,1)=41 or indmmm(i,1)=51).
compute condbb=make(nrow(bootres),1,0).
loop k7 = 1 to nwvls.
compute condbb={condbb,wbb(:,(((k4-1)*nwvls*(k2=1))+k7))}.
end loop.
compute condbb=condbb(:,2:ncol(condbb)).
end if.
compute cnt=cnt+(placeh*nwvls).
end if.
do if (pathsz(1,colnumb)) = 1.
compute zbb=tihs(cnt:(cnt+(placeh*nzvls)-1),1).
compute zbb=bootres(:,zbb).
do if (wfirst=0).
compute zfirst=1.
end if.
do if (indmmm(i,1) <> 31).
compute immlbs2=zcatlab(1:nzvls,1).
end if.
do if (indmmm(i,1)=2 or indmmm(i,1)=31 or indmmm(i,1)=51).
do if (indmmm(i,1)=2).
compute imm=zbb(:,1).
end if.
loop k7 = 1 to nzvls.
compute imm={imm,zbb(:,(((k4-1)*nzvls*(k2=1))+k7))}.
end loop.
do if (indmmm(i,1)=2 or indmmm(i,1)=51).
compute imm=imm(:,2:ncol(imm)).
do if (indmmm(i,1)=51).
compute condbb={condbb,imm}.
end if.
end if.
end if.
compute cnt=cnt+(placeh*nzvls).
end if.
do if (pathswz(1,colnumb)) = 1.
compute wzbb=tihs(cnt:(cnt+(placeh*nwvls*nzvls)-1),1).
compute wzbb=bootres(:,wzbb).
do if (indmmm(i,1)=41).
compute imm=wzbb(:,1).
loop k7=1 to nwvls*nzvls.
compute imm={imm,wzbb(:,(((k4-1)*nzvls*nwvls*(k2=1))+k7))}.
end loop.
end if.
do if (indmmm(i,1)=41).
compute imm=imm(:,2:ncol(imm)).
compute condbb={condbb,imm(:,(ncol(imm)-(nwvls*nzvls)+1):ncol(imm))}.
end if.
compute cnt=cnt+(placeh*nzvls*nwvls).
end if.
compute indprobe={focaddon,indprova(k1,:)}.
compute tucker={focbb,wbb,zbb,wzbb}.
loop k3=1 to ncol(indprobe).
compute tucker(:,k3)=tucker(:,k3)*indprobe(1,k3).
end loop.
compute tucker2=tucker2&*rsum(tucker).
do if (indmmm(i,1) = 1 or indmmm(i,1)=2 or indmmm(i,1)=31 or indmmm(i,1)=41 or indmmm(i,1)=51).
do if (immset=1).
do if (ncol(imm2)=1 and ncol(imm) = 1).
compute imm2=imm2&*imm.
end if.
do if (indmmm(i,1)=41 or indmmm(i,1)=51).
do if ((ncol(condbb2) > 1) and (ncol(condbb)>1)).
compute condbb2t=make(nrow(condbb2),(ncol(condbb2)*ncol(condbb)),-999999).
compute k9=1.
do if (wfirst=1).
loop k7=1 to ncol(condbb2).
loop k8 = 1 to ncol(condbb).
compute condbb2t(:,k9)=condbb2(:,k7)&*condbb(:,k8).
compute k9=k9+1.
end loop.
end loop.
end if.
do if (zfirst=1).
loop k7=1 to ncol(condbb).
loop k8 = 1 to ncol(condbb2).
compute condbb2t(:,k9)=condbb(:,k7)&*condbb2(:,k8).
compute k9=k9+1.
end loop.
end loop.
end if.
compute condbb2=condbb2t.
release condbb2t.
end if.
do if ((ncol(condbb2) > 1) and (ncol(condbb)=1)).
loop k7 = 1 to ncol(condbb2).
compute condbb2(:,k7)=condbb2(:,k7)&*condbb.
end loop.
end if.
do if ((ncol(condbb2) = 1) and (ncol(condbb)>1)).
loop k7 = 1 to ncol(condbb).
compute condbb(:,k7)=condbb2&*condbb(:,k7).
end loop.
compute condbb2=condbb.
end if.
end if.
do if (ncol(imm2) <> 1 and ncol(imm) <> 1).
compute imm2t=make(nrow(imm2),(ncol(imm2)*ncol(imm)),-999999).
compute k9=1.
do if (wfirst=1).
loop k7=1 to ncol(imm2).
loop k8 = 1 to ncol(imm).
compute imm2t(:,k9)=imm2(:,k7)&*imm(:,k8).
compute k9=k9+1.
end loop.
end loop.
end if.
do if (zfirst=1).
loop k7=1 to ncol(imm).
loop k8 = 1 to ncol(imm2).
compute imm2t(:,k9)=imm(:,k7)&*imm2(:,k8).
compute k9=k9+1.
end loop.
end loop.
end if.
compute imm2=imm2t.
release imm2t.
end if.
do if ((ncol(imm2) > 1) and (ncol(imm)=1)).
loop k7=1 to ncol(imm2).
compute imm2(:,k7)=imm2(:,k7)&*imm.
end loop.
end if.
do if ((ncol(imm2) = 1) and (ncol(imm) > 1)).
loop k7=1 to ncol(imm).
compute imm(:,k7)=imm2&*imm(:,k7).
end loop.
compute imm2=imm.
end if.
end if.
do if (immset=0).
compute imm2=imm.
do if (indmmm(i,1)=41 or indmmm(i,1)=51).
compute condbb2=condbb.
end if.
compute immset=1.
end if.
end if.
end loop.
compute indtemp=tucker2(1,1).
do if (indmmm(i,1)=12 or indmmm(i,1)=22).
compute imm3={imm3,tucker2}.
do if (k1=nrow(indmodva)).
compute imm3=imm3(:,2:ncol(imm3)).
compute immstop=ncol(imm3).
loop k8=2 to immstop.
do if (indmmmt(i,1)=1).
compute imm3={imm3,(imm3(:,k8)-imm3(:,1))}.
end if.
do if (indmmmt(i,1)=2).
compute imm3={imm3,(imm3(:,k8)-imm3(:,(k8-1)))}.
end if.
do if (indmmmt(i,1)=3).
compute imm3={imm3,((rsum(imm3(:,(k8:immstop)))/(immstop-k8+1))-imm3(:,(k8-1))) }.
end if.
do if (indmmmt(i,1)=4).
compute imm3={imm3,(imm3(:,k8)-(rsum(imm3(:,1:immstop))/immstop))}.
end if.
end loop.
do if (indmmmt(i,1)<5).
compute imm2=imm3(:,(immstop+1):ncol(imm3)).
release imm3.
end if.
end if.
end if.
do if (indmmm(i,1)>-1 and (contrast = 1 or contrast = 2 or contrast=3)).
compute imm4={imm4,tucker2}.
do if (k1=nrow(indmodva) and k1 > 1).
compute imm4=imm4(:,2:ncol(imm4)).
compute immstop=ncol(imm4).
compute condcont=make((immstop*(immstop-1)/2),6,-999).
loop k8 = 1 to (immstop-1).
loop k9 = (k8+1) to immstop.
do if (contrast=1).
compute imm4={imm4,(imm4(:,k9)-imm4(:,k8))}.
end if.
do if (contrast=2).
compute imm4={imm4,(abs(imm4(:,k9))-abs(imm4(:,k8)))}.
end if.
do if (contrast=3).
compute imm4={imm4,(imm4(:,k9)+imm4(:,k8))}.
end if.
compute condcont((ncol(imm4)-immstop),1)=imm4(1,k9).
compute condcont((ncol(imm4)-immstop),2)=imm4(1,k8).
end loop.
end loop.
compute imm4=imm4(:,(immstop+1):ncol(imm4)).
loop k8=1 to ncol(imm4).
compute condcont(k8,3)=imm4(1,k8).
do if (boot > 0).

.
compute temp = imm4(2:nrow(imm4),k8).
compute temp(GRADE( imm4(2:nrow(imm4),k8) )) = imm4(2:nrow(imm4),k8).
compute badlo = 0.
compute badhi = 0.
do if ( (imm4(1,k8)*bc)+(9999*(1-bc)) <> 9999).
compute pv=csum(temp < (imm4(1,k8)*bc)+(9999*(1-bc)) )/nrow(temp).
compute ppv = pv.
do if (pv > .5).
compute ppv = 1-pv.
end if.
compute y5=sqrt(-2*ln(ppv)).
compute xp=y5+((((y5*p4+p3)*y5+p2)*y5+p1)*y5+p0)/((((y5*q4+q3)*y5+q2)*y5+q1)*y5+q0).
do if (pv <= .5).
compute xp = -xp.
end if.
compute cilow=rnd(nrow(temp)*(cdfnorm(2*xp-xp2))).
compute cihigh=trunc(nrow(temp)*(cdfnorm(2*xp+(xp2))))+1.
do if (cilow < 1).
compute cilow = 1.
compute booterr=1.
compute badlo = 1.
end if.
do if (cihigh > nrow(temp)).
compute cihigh = boot.
compute booterr=1.
compute badhi = 1.
end if.
compute llcit=temp(cilow,1).
compute ulcit=temp(cihigh,1).
do if (badlo = 1 and llcit <> priorlo).
compute badend={badend, llcit}.
compute priorlo = llcit.
end if.
do if (badhi = 1 and ulcit <> priorhi).
compute badend={badend, ulcit}.
compute priorhi = ulcit.
end if.
end if.
do if ( (imm4(1,k8)*bc)+(9999*(1-bc)) = 9999).
compute llcit=temp(cilow,1).
compute ulcit=temp(cihigh,1).
end if.
compute bootse=t(sqrt((cssq(temp)-((csum(temp)&**2)/nrow(temp)))/(nrow(temp)-1)))
.
compute condcont(k8,4:6)={bootse,llcit,ulcit}.
end if.
end loop.
do if (boot=0).
compute condcont=condcont(:,1:3).
end if.
compute indcontr=1.
end if.
end if.
do if (xmint=1).
do if (k1=(k4+1)).
compute cftotfx(:,k4)=cftotfx(:,k4)+(tucker2*xscaling).
end if.
end if.
do if (boot > 0).
compute tucker2=tucker2*xscaling.

.
compute temp = tucker2(2:nrow(tucker2),1).
compute temp(GRADE( tucker2(2:nrow(tucker2),1) )) = tucker2(2:nrow(tucker2),1).
compute badlo = 0.
compute badhi = 0.
do if ( (tucker2(1,1)*bc)+(9999*(1-bc)) <> 9999).
compute pv=csum(temp < (tucker2(1,1)*bc)+(9999*(1-bc)) )/nrow(temp).
compute ppv = pv.
do if (pv > .5).
compute ppv = 1-pv.
end if.
compute y5=sqrt(-2*ln(ppv)).
compute xp=y5+((((y5*p4+p3)*y5+p2)*y5+p1)*y5+p0)/((((y5*q4+q3)*y5+q2)*y5+q1)*y5+q0).
do if (pv <= .5).
compute xp = -xp.
end if.
compute cilow=rnd(nrow(temp)*(cdfnorm(2*xp-xp2))).
compute cihigh=trunc(nrow(temp)*(cdfnorm(2*xp+(xp2))))+1.
do if (cilow < 1).
compute cilow = 1.
compute booterr=1.
compute badlo = 1.
end if.
do if (cihigh > nrow(temp)).
compute cihigh = boot.
compute booterr=1.
compute badhi = 1.
end if.
compute llcit=temp(cilow,1).
compute ulcit=temp(cihigh,1).
do if (badlo = 1 and llcit <> priorlo).
compute badend={badend, llcit}.
compute priorlo = llcit.
end if.
do if (badhi = 1 and ulcit <> priorhi).
compute badend={badend, ulcit}.
compute priorhi = ulcit.
end if.
end if.
do if ( (tucker2(1,1)*bc)+(9999*(1-bc)) = 9999).
compute llcit=temp(cilow,1).
compute ulcit=temp(cihigh,1).
end if.
compute bootse=t(sqrt((cssq(temp)-((csum(temp)&**2)/nrow(temp)))/(nrow(temp)-1)))
.
compute indtemp={indtemp*xscaling, bootse,llcit,ulcit}.
end if.
do if (boot=0).
compute indtemp=indtemp*xscaling.
end if.
compute condres(k1,(ncol(indmodva)+1):ncol(condres))=indtemp.
end loop.
do if (xmint=0).
compute resultm2=make(nrow(condres),maxresm,99999).
compute resultm2(1:nrow(condres),1:ncol(condres))=condres.
compute resultm={resultm;resultm2}.
end if.
compute condlbs={condlbs,'Effect'}.
do if (boot > 0).
compute condlbs={condlbs,'BootSE', 'BootLLCI', 'BootULCI'}.
end if.
do if (xmint = 1).
do if (k4 = 1).
compute natindfx=condres((2+(k4-1)),2:ncol(condres)).
end if.
do if (k4 > 1).
compute natindfx={natindfx;condres((2+(k4-1)),2:ncol(condres))}.
end if.
do if (k4=nxvls).
compute resultm2=make(nrow(natindfx),maxresm,99999).
compute resultm2(1:nrow(natindfx),1:ncol(natindfx))=natindfx.
compute resultm={resultm;resultm2}.
end if.
end if.
do if (outscree=1 and xmint=0).
do if (nxvls=1).
print condres/title=' '/cnames=condlbs/format= F10.4 /space=0.
else.
compute condrlb=make(nrow(condres),1,xcatlab(k4,1)).
print condres/title=' '/cnames=condlbs/rnames=condrlb/format= F10.4 /space=0.
end if.
end if.
do if (outscree=1 and xmint=1 and k4=nxvls).
do if (nxvls=1).
print natindfx/title=' '/clabels='Effect', 'BootSE', 'BootLLCI', 'BootULCI'/format= F10.4 /space=0.
else.
compute condrlb=make(nrow(condres),1,xcatlab(k4,1)).
print natindfx/title=' '/clabels='Effect', 'BootSE', 'BootLLCI', 'BootULCI'/rnames=direffl2/format= F10.4 /space=0.
end if.
end if.
compute dichadj=0.
compute immcat=0.
do if ((indmmm(i,1)>0) and (xmint=0)).
do if (indmmm(i,1)=1 or indmmm(i,1)=12 or indmmm(i,1)=31).
do if (wdich=1 and mcw=0).
do if (indmmm(i,1) <> 12).
compute imm2(:,1)=imm2(:,1)*(wmax-wmin).
end if.
do if (indmmm(i,1) <> 31).
compute dichadj=1.
end if.
end if.
do if ((mcw = 1 or mcw = 2) and indmmm(i,1) <> 31)).
compute immcat=1.
end if.
end if.
do if (indmmm(i,1)=2 or indmmm(i,1)=22 or indmmm(i,1)=31).
do if (zdich=1 and mcz=0).
do if (indmmm(i,1) = 31).
compute imm2(:,(nwvls+1):ncol(imm2))=imm2(:,(nwvls+1):ncol(imm2))*(zmax-zmin).
end if.
do if (indmmm(i,1) = 2).
compute imm2(:,1)=imm2(:,1)*(zmax-zmin).
end if.
do if (indmmm(i,1) <> 31).
compute dichadj=1.
end if.
end if.
do if ((mcz = 1 or mcz = 2) and indmmm(i,1) <> 31)).
compute immcat=1.
end if.
end if.
compute immtemp2=t(imm2(1,:)).
compute immtemp=immtemp2.
compute immlbs={'Index'}.
do if (boot > 0).
compute immtemp=make(1,3,0).
loop k7=1 to ncol(imm2).

.
compute temp = imm2(2:nrow(imm2),k7).
compute temp(GRADE( imm2(2:nrow(imm2),k7) )) = imm2(2:nrow(imm2),k7).
compute badlo = 0.
compute badhi = 0.
do if ( (imm2(1,k7)*bc)+(9999*(1-bc)) <> 9999).
compute pv=csum(temp < (imm2(1,k7)*bc)+(9999*(1-bc)) )/nrow(temp).
compute ppv = pv.
do if (pv > .5).
compute ppv = 1-pv.
end if.
compute y5=sqrt(-2*ln(ppv)).
compute xp=y5+((((y5*p4+p3)*y5+p2)*y5+p1)*y5+p0)/((((y5*q4+q3)*y5+q2)*y5+q1)*y5+q0).
do if (pv <= .5).
compute xp = -xp.
end if.
compute cilow=rnd(nrow(temp)*(cdfnorm(2*xp-xp2))).
compute cihigh=trunc(nrow(temp)*(cdfnorm(2*xp+(xp2))))+1.
do if (cilow < 1).
compute cilow = 1.
compute booterr=1.
compute badlo = 1.
end if.
do if (cihigh > nrow(temp)).
compute cihigh = boot.
compute booterr=1.
compute badhi = 1.
end if.
compute llcit=temp(cilow,1).
compute ulcit=temp(cihigh,1).
do if (badlo = 1 and llcit <> priorlo).
compute badend={badend, llcit}.
compute priorlo = llcit.
end if.
do if (badhi = 1 and ulcit <> priorhi).
compute badend={badend, ulcit}.
compute priorhi = ulcit.
end if.
end if.
do if ( (imm2(1,k7)*bc)+(9999*(1-bc)) = 9999).
compute llcit=temp(cilow,1).
compute ulcit=temp(cihigh,1).
end if.
compute bootse=t(sqrt((cssq(temp)-((csum(temp)&**2)/nrow(temp)))/(nrow(temp)-1)))
.
compute temp={bootse,llcit,ulcit}.
compute immtemp={immtemp;temp}.
end loop.
compute immtemp=immtemp(2:nrow(immtemp),:).
compute immtemp={immtemp2,immtemp}.
compute immlbs={immlbs,'BootSE', 'BootLLCI', 'BootULCI'}.
end if.
compute resultm2=make(nrow(immtemp),maxresm,99999).
compute resultm2(1:nrow(immtemp),1:ncol(immtemp))=immtemp.
compute resultm={resultm;resultm2}.
do if (dichadj=0 and immcat=0 and indmmmt(i,1)<>5 and indmmm(i,1) < 100 ).
do if (outscree=1).
do if (indmmm(i,1) < 30).
print immtemp/title='      Index of moderated mediation:'/cnames=immlbs/rnames=immlbs2/format= F10.4.
end if.
do if (indmmm(i,1) = 31).
compute immlbs2={immlbs2;zcatlab(1:nzvls,1)}.
print immtemp/title='      Indices of partial moderated mediation:'/cnames=immlbs/rnames=immlbs2/format= F10.4.
end if.
end if.
do if ((nzvls = 1) and (nwvls = 1)).
do if (indmmm(i,1) = 41 or indmmm(i,1)=51).
loop k7=1 to nwvls.
compute immlbs2=zcatlab(1:nzvls,1).
compute immtemp2=immtemp((((k7-1)*nzvls)+1):(((k7-1)*nzvls)+nzvls),:).
do if (outscree=1).
do if (nwvls > 1).
compute primodv={'        ', wcatlab(k7,1)}.
print primodv/title='      Primary moderator:'/format=A8.
end if.
do if (nzvls=1).
print immtemp2/title='      Index of moderated moderated mediation'/cnames=immlbs/format= F10.4.
else.
print immtemp2/title='      Indices of moderated moderated mediation'/cnames=immlbs/rnames=immlbs2/format= F10.4.
end if.
end if.
compute cmmtemp=make(nrow(zprobval),4,0).
loop k8=1 to nrow(zprobval).
compute condbb3=condbb2(:,((nwvls+1)+((k7-1)*nzvls)):((nwvls+1)+((k7-1)*nzvls)+ (nzvls-1))).
do if (ncol(zprobval) > 1).
compute condbb3=condbb3*mdiag(zprobval(k8,:)).
else.
compute condbb3=condbb3&*zprobval(k8,:).
end if.
compute condbb3={condbb2(:,k7),condbb3}.
compute icmm=rsum(condbb3).
compute cmmtemp(k8,1)=icmm(1,1).
do if (boot > 0).

.
compute temp = icmm(2:nrow(icmm),1).
compute temp(GRADE( icmm(2:nrow(icmm),1) )) = icmm(2:nrow(icmm),1).
compute badlo = 0.
compute badhi = 0.
do if ( (icmm(1,1)*bc)+(9999*(1-bc)) <> 9999).
compute pv=csum(temp < (icmm(1,1)*bc)+(9999*(1-bc)) )/nrow(temp).
compute ppv = pv.
do if (pv > .5).
compute ppv = 1-pv.
end if.
compute y5=sqrt(-2*ln(ppv)).
compute xp=y5+((((y5*p4+p3)*y5+p2)*y5+p1)*y5+p0)/((((y5*q4+q3)*y5+q2)*y5+q1)*y5+q0).
do if (pv <= .5).
compute xp = -xp.
end if.
compute cilow=rnd(nrow(temp)*(cdfnorm(2*xp-xp2))).
compute cihigh=trunc(nrow(temp)*(cdfnorm(2*xp+(xp2))))+1.
do if (cilow < 1).
compute cilow = 1.
compute booterr=1.
compute badlo = 1.
end if.
do if (cihigh > nrow(temp)).
compute cihigh = boot.
compute booterr=1.
compute badhi = 1.
end if.
compute llcit=temp(cilow,1).
compute ulcit=temp(cihigh,1).
do if (badlo = 1 and llcit <> priorlo).
compute badend={badend, llcit}.
compute priorlo = llcit.
end if.
do if (badhi = 1 and ulcit <> priorhi).
compute badend={badend, ulcit}.
compute priorhi = ulcit.
end if.
end if.
do if ( (icmm(1,1)*bc)+(9999*(1-bc)) = 9999).
compute llcit=temp(cilow,1).
compute ulcit=temp(cihigh,1).
end if.
compute bootse=t(sqrt((cssq(temp)-((csum(temp)&**2)/nrow(temp)))/(nrow(temp)-1)))
.
compute cmmtemp(k8,2:4)={bootse,llcit,ulcit}.
end if.
end loop.
compute cmmtemp={zmodvals,cmmtemp}.
do if (boot=0).
compute cmmtemp=cmmtemp(:,1:2).
end if.
compute cmmlbs={znames,immlbs}.
compute resultm2=make(nrow(cmmtemp),maxresm,99999).
compute resultm2(1:nrow(cmmtemp),1:ncol(cmmtemp))=cmmtemp.
compute resultm={resultm;resultm2}.
do if (outscree=1).
print cmmtemp/title='      Indices of conditional moderated mediation by W'/cnames=cmmlbs/format= F10.4.
end if.
end loop.
end if.
end if.
end if.
do if (dichadj=1 or immcat=1 and indmmm(i,1) < 30).
do if (outscree=1).
print immtemp/title='Index of moderated mediation (difference between conditional indirect effects):'/cnames=immlbs/rnames=immlbs2/format= F10.4.
end if.
end if.
end if.
do if (indcontr=1).
compute resultm2=make(nrow(condcont),maxresm,99999).
compute resultm2(1:nrow(condcont),1:ncol(condcont))=condcont.
compute resultm={resultm;resultm2}.
compute condctlb={'Effect1','Effect2','Contrast','BootSE', 'BootLLCI','BootULCI'}.
do if (outscree=1).
print condcont/title=' Pairwise contrasts between conditional indirect effects (Effect1 minus Effect2)'/cnames=condctlb/format= F10.4.
end if.
end if.
end loop.
end if.
end loop.
do if (alttotal=1).
compute altcnms='Effect'.
compute alttotfx=t(cftotfx(1,:))+obnatdfx.
do if (boot > 0).
compute alttotfx=make(ncol(cftotfx),4,0).
compute alttotfx(:,1)=t(cftotfx(1,:))+obnatdfx.
compute natdirbt(1,:)=t(obnatdfx).
compute cftotfx=cftotfx+natdirbt.
loop cec=1 to ncol(cftotfx).

.
compute temp = cftotfx(2:nrow(cftotfx),cec).
compute temp(GRADE( cftotfx(2:nrow(cftotfx),cec) )) = cftotfx(2:nrow(cftotfx),cec).
compute badlo = 0.
compute badhi = 0.
do if ( (cftotfx(1,cec)*bc)+(9999*(1-bc)) <> 9999).
compute pv=csum(temp < (cftotfx(1,cec)*bc)+(9999*(1-bc)) )/nrow(temp).
compute ppv = pv.
do if (pv > .5).
compute ppv = 1-pv.
end if.
compute y5=sqrt(-2*ln(ppv)).
compute xp=y5+((((y5*p4+p3)*y5+p2)*y5+p1)*y5+p0)/((((y5*q4+q3)*y5+q2)*y5+q1)*y5+q0).
do if (pv <= .5).
compute xp = -xp.
end if.
compute cilow=rnd(nrow(temp)*(cdfnorm(2*xp-xp2))).
compute cihigh=trunc(nrow(temp)*(cdfnorm(2*xp+(xp2))))+1.
do if (cilow < 1).
compute cilow = 1.
compute booterr=1.
compute badlo = 1.
end if.
do if (cihigh > nrow(temp)).
compute cihigh = boot.
compute booterr=1.
compute badhi = 1.
end if.
compute llcit=temp(cilow,1).
compute ulcit=temp(cihigh,1).
do if (badlo = 1 and llcit <> priorlo).
compute badend={badend, llcit}.
compute priorlo = llcit.
end if.
do if (badhi = 1 and ulcit <> priorhi).
compute badend={badend, ulcit}.
compute priorhi = ulcit.
end if.
end if.
do if ( (cftotfx(1,cec)*bc)+(9999*(1-bc)) = 9999).
compute llcit=temp(cilow,1).
compute ulcit=temp(cihigh,1).
end if.
compute bootse=t(sqrt((cssq(temp)-((csum(temp)&**2)/nrow(temp)))/(nrow(temp)-1)))
.
compute alttotfx(cec,2:4)={bootse,llcit,ulcit}.
end loop.
compute altcnms={altcnms,'BootSE', 'BootLLCI','BootULCI'}.
end if.
compute resultm2=make(nrow(alttotfx),maxresm,99999).
compute resultm2(1:nrow(alttotfx),1:ncol(alttotfx))=alttotfx.
compute resultm={resultm;resultm2}.
do if (outscree=1).
print/title='----------'.
do if (nxvls > 1).
print alttotfx/title='Relative total effects of X on Y (sum of direct and indirect effects)'/cnames=altcnms/rnames=direffl2/format= F10.4.
end if.
do if (nxvls = 1).
print alttotfx/title='Total effect of X on Y (sum of direct and indirect effects)'/cnames=altcnms/format= F10.4.
end if.
end if.
end if.
end if.
end if
.
do if (criterr=0 and (saveest = 1 or saveboot=1)).
compute coeffsav=coeffmat(2:nrow(coeffmat),:).
compute conseq=conseq(2:nrow(conseq),1).
compute dfmat=dfmat(2:nrow(dfmat),1).
compute dfmat=dfmat.
compute saven={'conseqnt','antecdnt','coeff','se','t','p','LLCI','ULCI','df'}.
do if (ydich=1).
compute saven={'conseqnt','antecdnt','coeff','se','t_or_Z','p','LLCI','ULCI','df'}.
end if.
do if (saveboot=1 and boot > 0 ).
compute colslab=make(300,1,' ').


compute colslab( 1 ,1)= 'COL1'.


compute colslab( 2 ,1)= 'COL2'.


compute colslab( 3 ,1)= 'COL3'.


compute colslab( 4 ,1)= 'COL4'.


compute colslab( 5 ,1)= 'COL5'.


compute colslab( 6 ,1)= 'COL6'.


compute colslab( 7 ,1)= 'COL7'.


compute colslab( 8 ,1)= 'COL8'.


compute colslab( 9 ,1)= 'COL9'.


compute colslab( 10 ,1)= 'COL10'.


compute colslab( 11 ,1)= 'COL11'.


compute colslab( 12 ,1)= 'COL12'.


compute colslab( 13 ,1)= 'COL13'.


compute colslab( 14 ,1)= 'COL14'.


compute colslab( 15 ,1)= 'COL15'.


compute colslab( 16 ,1)= 'COL16'.


compute colslab( 17 ,1)= 'COL17'.


compute colslab( 18 ,1)= 'COL18'.


compute colslab( 19 ,1)= 'COL19'.


compute colslab( 20 ,1)= 'COL20'.


compute colslab( 21 ,1)= 'COL21'.


compute colslab( 22 ,1)= 'COL22'.


compute colslab( 23 ,1)= 'COL23'.


compute colslab( 24 ,1)= 'COL24'.


compute colslab( 25 ,1)= 'COL25'.


compute colslab( 26 ,1)= 'COL26'.


compute colslab( 27 ,1)= 'COL27'.


compute colslab( 28 ,1)= 'COL28'.


compute colslab( 29 ,1)= 'COL29'.


compute colslab( 30 ,1)= 'COL30'.


compute colslab( 31 ,1)= 'COL31'.


compute colslab( 32 ,1)= 'COL32'.


compute colslab( 33 ,1)= 'COL33'.


compute colslab( 34 ,1)= 'COL34'.


compute colslab( 35 ,1)= 'COL35'.


compute colslab( 36 ,1)= 'COL36'.


compute colslab( 37 ,1)= 'COL37'.


compute colslab( 38 ,1)= 'COL38'.


compute colslab( 39 ,1)= 'COL39'.


compute colslab( 40 ,1)= 'COL40'.


compute colslab( 41 ,1)= 'COL41'.


compute colslab( 42 ,1)= 'COL42'.


compute colslab( 43 ,1)= 'COL43'.


compute colslab( 44 ,1)= 'COL44'.


compute colslab( 45 ,1)= 'COL45'.


compute colslab( 46 ,1)= 'COL46'.


compute colslab( 47 ,1)= 'COL47'.


compute colslab( 48 ,1)= 'COL48'.


compute colslab( 49 ,1)= 'COL49'.


compute colslab( 50 ,1)= 'COL50'.


compute colslab( 51 ,1)= 'COL51'.


compute colslab( 52 ,1)= 'COL52'.


compute colslab( 53 ,1)= 'COL53'.


compute colslab( 54 ,1)= 'COL54'.


compute colslab( 55 ,1)= 'COL55'.


compute colslab( 56 ,1)= 'COL56'.


compute colslab( 57 ,1)= 'COL57'.


compute colslab( 58 ,1)= 'COL58'.


compute colslab( 59 ,1)= 'COL59'.


compute colslab( 60 ,1)= 'COL60'.


compute colslab( 61 ,1)= 'COL61'.


compute colslab( 62 ,1)= 'COL62'.


compute colslab( 63 ,1)= 'COL63'.


compute colslab( 64 ,1)= 'COL64'.


compute colslab( 65 ,1)= 'COL65'.


compute colslab( 66 ,1)= 'COL66'.


compute colslab( 67 ,1)= 'COL67'.


compute colslab( 68 ,1)= 'COL68'.


compute colslab( 69 ,1)= 'COL69'.


compute colslab( 70 ,1)= 'COL70'.


compute colslab( 71 ,1)= 'COL71'.


compute colslab( 72 ,1)= 'COL72'.


compute colslab( 73 ,1)= 'COL73'.


compute colslab( 74 ,1)= 'COL74'.


compute colslab( 75 ,1)= 'COL75'.


compute colslab( 76 ,1)= 'COL76'.


compute colslab( 77 ,1)= 'COL77'.


compute colslab( 78 ,1)= 'COL78'.


compute colslab( 79 ,1)= 'COL79'.


compute colslab( 80 ,1)= 'COL80'.


compute colslab( 81 ,1)= 'COL81'.


compute colslab( 82 ,1)= 'COL82'.


compute colslab( 83 ,1)= 'COL83'.


compute colslab( 84 ,1)= 'COL84'.


compute colslab( 85 ,1)= 'COL85'.


compute colslab( 86 ,1)= 'COL86'.


compute colslab( 87 ,1)= 'COL87'.


compute colslab( 88 ,1)= 'COL88'.


compute colslab( 89 ,1)= 'COL89'.


compute colslab( 90 ,1)= 'COL90'.


compute colslab( 91 ,1)= 'COL91'.


compute colslab( 92 ,1)= 'COL92'.


compute colslab( 93 ,1)= 'COL93'.


compute colslab( 94 ,1)= 'COL94'.


compute colslab( 95 ,1)= 'COL95'.


compute colslab( 96 ,1)= 'COL96'.


compute colslab( 97 ,1)= 'COL97'.


compute colslab( 98 ,1)= 'COL98'.


compute colslab( 99 ,1)= 'COL99'.


compute colslab( 100 ,1)= 'COL100'.


compute colslab( 101 ,1)= 'COL101'.


compute colslab( 102 ,1)= 'COL102'.


compute colslab( 103 ,1)= 'COL103'.


compute colslab( 104 ,1)= 'COL104'.


compute colslab( 105 ,1)= 'COL105'.


compute colslab( 106 ,1)= 'COL106'.


compute colslab( 107 ,1)= 'COL107'.


compute colslab( 108 ,1)= 'COL108'.


compute colslab( 109 ,1)= 'COL109'.


compute colslab( 110 ,1)= 'COL110'.


compute colslab( 111 ,1)= 'COL111'.


compute colslab( 112 ,1)= 'COL112'.


compute colslab( 113 ,1)= 'COL113'.


compute colslab( 114 ,1)= 'COL114'.


compute colslab( 115 ,1)= 'COL115'.


compute colslab( 116 ,1)= 'COL116'.


compute colslab( 117 ,1)= 'COL117'.


compute colslab( 118 ,1)= 'COL118'.


compute colslab( 119 ,1)= 'COL119'.


compute colslab( 120 ,1)= 'COL120'.


compute colslab( 121 ,1)= 'COL121'.


compute colslab( 122 ,1)= 'COL122'.


compute colslab( 123 ,1)= 'COL123'.


compute colslab( 124 ,1)= 'COL124'.


compute colslab( 125 ,1)= 'COL125'.


compute colslab( 126 ,1)= 'COL126'.


compute colslab( 127 ,1)= 'COL127'.


compute colslab( 128 ,1)= 'COL128'.


compute colslab( 129 ,1)= 'COL129'.


compute colslab( 130 ,1)= 'COL130'.


compute colslab( 131 ,1)= 'COL131'.


compute colslab( 132 ,1)= 'COL132'.


compute colslab( 133 ,1)= 'COL133'.


compute colslab( 134 ,1)= 'COL134'.


compute colslab( 135 ,1)= 'COL135'.


compute colslab( 136 ,1)= 'COL136'.


compute colslab( 137 ,1)= 'COL137'.


compute colslab( 138 ,1)= 'COL138'.


compute colslab( 139 ,1)= 'COL139'.


compute colslab( 140 ,1)= 'COL140'.


compute colslab( 141 ,1)= 'COL141'.


compute colslab( 142 ,1)= 'COL142'.


compute colslab( 143 ,1)= 'COL143'.


compute colslab( 144 ,1)= 'COL144'.


compute colslab( 145 ,1)= 'COL145'.


compute colslab( 146 ,1)= 'COL146'.


compute colslab( 147 ,1)= 'COL147'.


compute colslab( 148 ,1)= 'COL148'.


compute colslab( 149 ,1)= 'COL149'.


compute colslab( 150 ,1)= 'COL150'.


compute colslab( 151 ,1)= 'COL151'.


compute colslab( 152 ,1)= 'COL152'.


compute colslab( 153 ,1)= 'COL153'.


compute colslab( 154 ,1)= 'COL154'.


compute colslab( 155 ,1)= 'COL155'.


compute colslab( 156 ,1)= 'COL156'.


compute colslab( 157 ,1)= 'COL157'.


compute colslab( 158 ,1)= 'COL158'.


compute colslab( 159 ,1)= 'COL159'.


compute colslab( 160 ,1)= 'COL160'.


compute colslab( 161 ,1)= 'COL161'.


compute colslab( 162 ,1)= 'COL162'.


compute colslab( 163 ,1)= 'COL163'.


compute colslab( 164 ,1)= 'COL164'.


compute colslab( 165 ,1)= 'COL165'.


compute colslab( 166 ,1)= 'COL166'.


compute colslab( 167 ,1)= 'COL167'.


compute colslab( 168 ,1)= 'COL168'.


compute colslab( 169 ,1)= 'COL169'.


compute colslab( 170 ,1)= 'COL170'.


compute colslab( 171 ,1)= 'COL171'.


compute colslab( 172 ,1)= 'COL172'.


compute colslab( 173 ,1)= 'COL173'.


compute colslab( 174 ,1)= 'COL174'.


compute colslab( 175 ,1)= 'COL175'.


compute colslab( 176 ,1)= 'COL176'.


compute colslab( 177 ,1)= 'COL177'.


compute colslab( 178 ,1)= 'COL178'.


compute colslab( 179 ,1)= 'COL179'.


compute colslab( 180 ,1)= 'COL180'.


compute colslab( 181 ,1)= 'COL181'.


compute colslab( 182 ,1)= 'COL182'.


compute colslab( 183 ,1)= 'COL183'.


compute colslab( 184 ,1)= 'COL184'.


compute colslab( 185 ,1)= 'COL185'.


compute colslab( 186 ,1)= 'COL186'.


compute colslab( 187 ,1)= 'COL187'.


compute colslab( 188 ,1)= 'COL188'.


compute colslab( 189 ,1)= 'COL189'.


compute colslab( 190 ,1)= 'COL190'.


compute colslab( 191 ,1)= 'COL191'.


compute colslab( 192 ,1)= 'COL192'.


compute colslab( 193 ,1)= 'COL193'.


compute colslab( 194 ,1)= 'COL194'.


compute colslab( 195 ,1)= 'COL195'.


compute colslab( 196 ,1)= 'COL196'.


compute colslab( 197 ,1)= 'COL197'.


compute colslab( 198 ,1)= 'COL198'.


compute colslab( 199 ,1)= 'COL199'.


compute colslab( 200 ,1)= 'COL200'.


compute colslab( 201 ,1)= 'COL201'.


compute colslab( 202 ,1)= 'COL202'.


compute colslab( 203 ,1)= 'COL203'.


compute colslab( 204 ,1)= 'COL204'.


compute colslab( 205 ,1)= 'COL205'.


compute colslab( 206 ,1)= 'COL206'.


compute colslab( 207 ,1)= 'COL207'.


compute colslab( 208 ,1)= 'COL208'.


compute colslab( 209 ,1)= 'COL209'.


compute colslab( 210 ,1)= 'COL210'.


compute colslab( 211 ,1)= 'COL211'.


compute colslab( 212 ,1)= 'COL212'.


compute colslab( 213 ,1)= 'COL213'.


compute colslab( 214 ,1)= 'COL214'.


compute colslab( 215 ,1)= 'COL215'.


compute colslab( 216 ,1)= 'COL216'.


compute colslab( 217 ,1)= 'COL217'.


compute colslab( 218 ,1)= 'COL218'.


compute colslab( 219 ,1)= 'COL219'.


compute colslab( 220 ,1)= 'COL220'.


compute colslab( 221 ,1)= 'COL221'.


compute colslab( 222 ,1)= 'COL222'.


compute colslab( 223 ,1)= 'COL223'.


compute colslab( 224 ,1)= 'COL224'.


compute colslab( 225 ,1)= 'COL225'.


compute colslab( 226 ,1)= 'COL226'.


compute colslab( 227 ,1)= 'COL227'.


compute colslab( 228 ,1)= 'COL228'.


compute colslab( 229 ,1)= 'COL229'.


compute colslab( 230 ,1)= 'COL230'.


compute colslab( 231 ,1)= 'COL231'.


compute colslab( 232 ,1)= 'COL232'.


compute colslab( 233 ,1)= 'COL233'.


compute colslab( 234 ,1)= 'COL234'.


compute colslab( 235 ,1)= 'COL235'.


compute colslab( 236 ,1)= 'COL236'.


compute colslab( 237 ,1)= 'COL237'.


compute colslab( 238 ,1)= 'COL238'.


compute colslab( 239 ,1)= 'COL239'.


compute colslab( 240 ,1)= 'COL240'.


compute colslab( 241 ,1)= 'COL241'.


compute colslab( 242 ,1)= 'COL242'.


compute colslab( 243 ,1)= 'COL243'.


compute colslab( 244 ,1)= 'COL244'.


compute colslab( 245 ,1)= 'COL245'.


compute colslab( 246 ,1)= 'COL246'.


compute colslab( 247 ,1)= 'COL247'.


compute colslab( 248 ,1)= 'COL248'.


compute colslab( 249 ,1)= 'COL249'.


compute colslab( 250 ,1)= 'COL250'.


compute colslab( 251 ,1)= 'COL251'.


compute colslab( 252 ,1)= 'COL252'.


compute colslab( 253 ,1)= 'COL253'.


compute colslab( 254 ,1)= 'COL254'.


compute colslab( 255 ,1)= 'COL255'.


compute colslab( 256 ,1)= 'COL256'.


compute colslab( 257 ,1)= 'COL257'.


compute colslab( 258 ,1)= 'COL258'.


compute colslab( 259 ,1)= 'COL259'.


compute colslab( 260 ,1)= 'COL260'.


compute colslab( 261 ,1)= 'COL261'.


compute colslab( 262 ,1)= 'COL262'.


compute colslab( 263 ,1)= 'COL263'.


compute colslab( 264 ,1)= 'COL264'.


compute colslab( 265 ,1)= 'COL265'.


compute colslab( 266 ,1)= 'COL266'.


compute colslab( 267 ,1)= 'COL267'.


compute colslab( 268 ,1)= 'COL268'.


compute colslab( 269 ,1)= 'COL269'.


compute colslab( 270 ,1)= 'COL270'.


compute colslab( 271 ,1)= 'COL271'.


compute colslab( 272 ,1)= 'COL272'.


compute colslab( 273 ,1)= 'COL273'.


compute colslab( 274 ,1)= 'COL274'.


compute colslab( 275 ,1)= 'COL275'.


compute colslab( 276 ,1)= 'COL276'.


compute colslab( 277 ,1)= 'COL277'.


compute colslab( 278 ,1)= 'COL278'.


compute colslab( 279 ,1)= 'COL279'.


compute colslab( 280 ,1)= 'COL280'.


compute colslab( 281 ,1)= 'COL281'.


compute colslab( 282 ,1)= 'COL282'.


compute colslab( 283 ,1)= 'COL283'.


compute colslab( 284 ,1)= 'COL284'.


compute colslab( 285 ,1)= 'COL285'.


compute colslab( 286 ,1)= 'COL286'.


compute colslab( 287 ,1)= 'COL287'.


compute colslab( 288 ,1)= 'COL288'.


compute colslab( 289 ,1)= 'COL289'.


compute colslab( 290 ,1)= 'COL290'.


compute colslab( 291 ,1)= 'COL291'.


compute colslab( 292 ,1)= 'COL292'.


compute colslab( 293 ,1)= 'COL293'.


compute colslab( 294 ,1)= 'COL294'.


compute colslab( 295 ,1)= 'COL295'.


compute colslab( 296 ,1)= 'COL296'.


compute colslab( 297 ,1)= 'COL297'.


compute colslab( 298 ,1)= 'COL298'.


compute colslab( 299 ,1)= 'COL299'.


compute colslab( 300 ,1)= 'COL300'.

compute colslab=colslab(1:ncol(bootres),1).
compute colslab={colslab,conseq,vlabs}.
do if (outscree=1).
print/title = '**************************************************************************'.
print/title='Bootstrap estimates were saved to a file'/space=0.
print colslab/title='Map of column names to model coefficients:'/clabels=' ','Conseqnt','Antecdnt'/format=a8.
end if.
end if.
end if.
do if (criterr=0 and boot > 0 and modelbt=1).
compute labstart=1.
do if (outscree=1).
print/title = '*********** BOOTSTRAP RESULTS FOR REGRESSION MODEL PARAMETERS ************'.
end if.
loop iboot = 1 to (nms+nys).
do if (outscree=1).
print outnames(1,iboot)/title = 'OUTCOME VARIABLE:'/format = A8.
end if.
compute vlabsm=vlabs(labstart:(labstart+(nump(1,iboot)-1)),1).
compute outnmtmp=bootcim(labstart:(labstart+(nump(1,iboot)-1)),:).
compute resultm2=make(nrow(outnmtmp),maxresm,99999).
compute resultm2(1:nrow(outnmtmp),1:ncol(outnmtmp))=outnmtmp.
compute resultm={resultm;resultm2}.
do if (outscree=1).
print bootcim(labstart:(labstart+(nump(1,iboot)-1)),:)/title=' '/rnames=vlabsm/ clabels='Coeff' 'BootMean' 'BootSE' 'BootLLCI' 'BootULCI'/format= F10.4 /space=0.
end if.
compute labstart=labstart+nump(1,iboot).
do if (iboot < (nms+nys)).
do if (outscree=1).
print/title= '----------'.
end if.
end if.
end loop.
end if.
do if (criterr=0 and saveest=1).
compute resultm=resultm(2:nrow(resultm),:).
compute bocaj=(resultm=99999).
compute bocaj=csum(bocaj).
compute bocaj=1-(bocaj=nrow(resultm)).
compute j=1.
loop i = 1 to ncol(resultm).
do if (bocaj(1,i)=1).
compute j=j+1.
end if.
end loop.
compute resultm=resultm(:,1:(j-1)).
save resultm/outfile = *.
end if.
do if (criterr = 0 and matrices=1 and outscree=1).
print/title = '************************ MODEL DEFINITION MATRICES ************************'.
print/title = 'FROM variables are columns, TO variables are rows.'.
compute temp2=make(nrow(bcmat),ncol(bcmat),'0').
loop i = 2 to nrow(bcmat).
loop j = 1 to (ncol(bcmat)-1).
do if (bcmat(i,j)=1).
compute temp2(i,j)='1'.
end if.
do if (j >= i).
compute temp2(i,j)=' '.
end if.
end loop.
end loop.
compute temp2=temp2(2:nrow(bcmat),(1:(ncol(bcmat)-1))).
do if (nms > 0).
compute cmatlabs={xnames,mnames}.
compute rmatlabs={mnames,ynames}.
end if.
do if (nms = 0).
compute cmatlabs={xnames}.
compute rmatlabs={ynames}.
end if.
print temp2/title='BMATRIX: Paths freely estimated (1) and fixed to zero (0):'/cnames=cmatlabs/rnames=rmatlabs/format A3.
compute z=0.
do if (rsum(csum(wcmat))<>0).
compute temp2=make(nrow(wcmat),ncol(wcmat),'0').
loop i = 2 to nrow(wcmat).
loop j = 1 to (ncol(wcmat)-1).
do if (wcmat(i,j)=1).
compute temp2(i,j)='1'.
end if.
do if (j >= i).
compute temp2(i,j)=' '.
end if.
end loop.
end loop.
compute temp2=temp2(2:nrow(wcmat),(1:(ncol(wcmat)-1))).
print temp2/title='WMATRIX: Paths moderated (1) and not moderated (0) by W:'/cnames=cmatlabs/rnames=rmatlabs/format A3.
end if.
do if (rsum(csum(zcmat))<>0).
compute temp2=make(nrow(zcmat),ncol(zcmat),'0').
loop i = 2 to nrow(zcmat).
loop j = 1 to (ncol(zcmat)-1).
do if (zcmat(i,j)=1).
compute temp2(i,j)='1'.
end if.
do if (j >= i).
compute temp2(i,j)=' '.
end if.
end loop.
end loop.
compute temp2=temp2(2:nrow(zcmat),(1:(ncol(zcmat)-1))).
print temp2/title='ZMATRIX: Paths moderated (1) and not moderated (0) by Z:'/cnames=cmatlabs/rnames=rmatlabs/format a3.
end if.
do if (rsum(csum(wzcmat))<>0).
compute temp2=make(nrow(wzcmat),ncol(wzcmat),'0').
loop i = 2 to nrow(wzcmat).
loop j = 1 to (ncol(wzcmat)-1).
do if (wzcmat(i,j)=1).
compute temp2(i,j)='1'.
end if.
do if (j >= i).
compute temp2(i,j)=' '.
end if.
end loop.
end loop.
compute temp2=temp2(2:nrow(wzcmat),(1:(ncol(wzcmat)-1))).
print temp2/title='WZMATRIX: W moderated paths moderated (1) and not moderated (0) by Z:'/cnames=cmatlabs/rnames=rmatlabs/format a3.
end if.
do if (ncs > 0).
print ccmat/title='CMATRIX: Covariates (columns) in (1) and not in (0) the models of M and Y (rows):'/rnames=rmatlabs/cnames=covnames.
end if.
end if.
do if (outscree=1).
do if (activate=0).
print/title = '*********************** ANALYSIS NOTES AND ERRORS ************************'.
end if.
end if.
do if (activate=1).
print/title = '**************************************************************************'.
end if.
do if (criterr=0).
do if (outscree=1).
print conf/title = 'Level of confidence for all confidence intervals in output:'/format = F8.4.
do if (boot > 0).
do if (goodboot = boot and bc=0).
print boot/title='Number of bootstrap samples for percentile bootstrap confidence intervals:'.
end if.
do if (goodboot = boot and bc=1).
print boot/title='Number of bootstrap samples for bias-corrected bootstrap confidence intervals:'.
end if.
do if (booterr = 1).
compute badend = badend(1,2:ncol(badend)).
print/title = 'WARNING: Bootstrap CI endpoints below not trustworthy. Decrease confidence'.
print badend/title='or increase the number of bootstrap samples.'/space=0/format = F10.4.
end if.
end if.
do if (mc > 0).
print mc/title='Number of samples for Monte Carlo confidence intervals:'.
end if.
do if (wnotev > 0 and printw=1).
do if (wnotev=1).
print/title = 'W values in conditional tables are the 16th, 50th, and 84th percentiles.'.
else if (wnotev=2).
do if (minwwarn=0 and maxwwarn=0).
print/title = 'W values in conditional tables are the mean and +/- SD from the mean.'.
end if.
do if (minwwarn=1).
print/title = 'W values in conditional tables are the minimum, the mean, and 1 SD above the mean.'.
end if.
do if (maxwwarn=1).
print/title = 'W values in conditional tables are 1 SD below the mean, the mean, and the maximum.'.
end if.
end if.
end if.
do if (znotev > 0 and printz=1).
do if (znotev=1).
print/title = 'Z values in conditional tables are the 16th, 50th, and 84th percentiles.'.
else if (znotev=2).
do if (minzwarn=0 and maxzwarn=0).
print/title = 'Z values in conditional tables are the mean and +/- SD from the mean.'.
end if.
do if (minzwarn=1).
print/title = 'Z values in conditional tables are the minimum, the mean, and 1 SD above the mean.'.
end if.
do if (maxzwarn=1).
print/title = 'Z values in conditional tables are 1 SD below the mean, the mean, and the maximum.'.
end if.
end if.
end if.
do if (minwwarn > 0).
print/title = 'NOTE: One SD below the mean is below the minimum observed in the data for W,'.
print/title = '      so the minimum measurement on W is used for conditioning instead.'/space=0.
end if.
do if (maxwwarn > 0).
print/title = 'NOTE: One SD above the mean is above the maximum observed in the data for W,'.
print/title = '      so the maximum measurement for W is used for conditioning instead.'/space=0.
end if.
do if (minzwarn > 0).
print/title = 'NOTE: One SD below the mean is below the minimum observed in the data for Z,'.
print/title = '      so the minimum measurement for Z is used for conditioning instead.'/space=0.
end if.
do if (maxzwarn > 0).
print/title = 'NOTE: One SD above the mean is above the maximum observed in the data for Z,'.
print/title = '      so the maximum measurement for Z is used for conditioning instead.'/space=0.
end if.
do if (pstog=1).
print/title= 'NOTE: Standardized coefficients for dichotomous or multicategorical X are in'.
print/title= '      partially standardized form.'/space=0.
end if.
loop i = 1 to 100.
do if (notecode(i,1) = 32).
print/title = 'Direct, indirect, and total effects are counterfactually defined'.
do if (xcontcf=0).
print/title = 'assuming X by M interaction.'/space=0.
end if.
do if (xcontcf=1).
print/title = 'assuming X by M interaction and with the following reference (x_ref)'/space=0.
compute states=t(xrefvals).
print states/title = 'and counterfactual (x_cf) states for X:'/rlabels='x_ref' 'x_cf'/format= F10.4 /space=0.
end if.
end if.
do if (notecode(i,1)=1).
print/title = 'NOTE: COVMY is ignored when using CMATRIX option.'.
end if.
do if (notecode(i,1)=2).
print/title = 'NOTE: Confidence level restricted to between 50 and 99.9999%.  95% confidence is provided in output'.
end if.
do if (notecode(i,1)=3).
print centvar/title = 'NOTE: The following variables were mean centered prior to analysis:'/format = a8.
end if.
do if (notecode(i,1) = 4).
print/title = 'NOTE: A heteroscedasticity consistent standard error and covariance matrix estimator was used.'.
end if.
do if (notecode(i,1) = 5).
print/title = 'NOTE: The HC3 option has been replaced with HC.  See the documentation.'.
end if.
do if (notecode(i,1) = 6).
print/title = 'NOTE: Due to estimation problems, some bootstrap samples had to be replaced.'.
print badboot/title='      The number of times this happened was:'/space=0/format=F8.0.
end if.
do if (notecode(i,1) = 7).
print/title = 'NOTE: The bootstrapping was not completed due to problematic bootstrap samples.'.
print/title = '      Bootstrap confidence intervals are therefore suppressed.'/space=0.
end if.
do if (notecode(i,1) = 8).
print/title = 'NOTE: The number of bootstrap samples was adjusted upward given your desired confidence.'.
end if.
do if (notecode(i,1) = 9).
print/title = 'NOTE: WMODVAL is ignored when W is specified as multicategorical.'.
end if.
do if (notecode(i,1) = 10).
print/title = 'NOTE: ZMODVAL is ignored when Z is specified as multicategorical.'.
end if.
do if (notecode(i,1) = 11).
print/title = 'NOTE: Total effect model generated only when all covariates are specified in all'.
print/title = '      models of M and Y.'/space=0.
end if.
do if (notecode(i,1) = 30).
print/title = 'NOTE: Your vector of linear hypothesis weights is of the wrong length for this model.'.
end if.
do if (notecode(i,1) = 12).
print/title = 'NOTE: Total effect model generated only when X is freely estimated to affect each M'.
print/title = '      and both X and M are freely estimated to affect Y'/space=0.
end if.
do if (notecode(i,1) = 13).
print/title = 'NOTE: There are too many pairwise contrasts to conduct with this model.'.
end if.
do if (notecode(i,1) = 14).
print/title = 'NOTE: The number of contrast weights must equal the number of indirect effects.'.
end if.
do if (notecode(i,1) = 15).
print/title = 'NOTE: Monte Carlo confidence intervals not available for this model.'.
print/title = '      Bootstrapping is used instead.'/space=0.
end if.
do if (notecode(i,1) = 16).
print/title = 'NOTE: The number of Monte Carlo samples was adjusted upward given your desired confidence.'.
end if.
do if (notecode(i,1) = 19).
print/title = 'NOTE: Your contrast matrix is invalid or not applicable to this model.'.
end if.
do if (notecode(i,1) = 20).
print/title = 'NOTE: One of the groups specified by your contrast matrix does not exist in the data.'.
end if.
do if (notecode(i,1) = 21).
print/title = 'NOTE: The VARORDER option is not available in this release.'.
end if.
do if (notecode(i,1) = 22).
print/title = 'NOTE: The VMODVAL and QMODVAL options are not available in this release.'.
end if.
do if (notecode(i,1) = 23).
print/title = 'NOTE: The QUANTILE option is not available in this release.'.
end if.
do if (notecode(i,1) = 24).
print/title = 'NOTE: Total effect model not available with dichotomous Y.'.
end if.
do if (notecode(i,1) = 25).
print/title = 'NOTE: STAND/EFFSIZE options not available with dichotomous Y.'.
end if.
do if ((notecode(i,1) = 26) and nms > 0).
print/title = 'NOTE: Direct and indirect effects of X on Y are on a log-odds metric.'.
end if.
do if (notecode(i,1) = 27).
print/title = 'NOTE: Standardized coefficients are not available for models with moderators.'.
end if.
do if (notecode(i,1) = 28).
print/title = 'NOTE: The contrast option is not available with a multicategorical X.'.
end if.
do if (notecode(i,1) = 31).
do if (nms > 1).
print medmeans/title = 'NOTE: Controlled direct effect(s) estimated at the following mediator values:'/cnames=mnames/format= F10.4.
end if.
do if (nms=1).
print medmeans/title = 'NOTE: Controlled direct effect(s) estimated at the following mediator value:'/format= F10.4.
end if.
end if.
do if (notecode(i,1) = 33).
print/title = 'NOTE: Sobel test is not available with the XMINT option.'.
end if.
do if (notecode(i,1) = 37).
print/title = 'NOTE: The CONTRAST option is not available when using the XMINT option.'.
end if.
do if (notecode(i,1) = 34).
print/title = 'NOTE: Standardized effects are not available when using the XMINT option.'.
end if.
do if (notecode(i,1) = 36).
print/title = 'NOTE: The XREFVAL option is ignored when X is declared as multicategorical.'.
end if.
do if (notecode(i,1) = 35).
print coval/title = 'NOTE: Counterfactual effects estimated at the following covariate values:'/cnames=covnames/format= F10.4.
end if.
do if (notecode(i,1) = 29 and (listmiss=1)).
compute a=missrow.
compute conum=ncol(a).
compute allgood=0.
compute smremain=12.
compute largesti=1.
compute smallrow=0.
do if (conum > 12).
loop ii = 1 to 12.
compute check=(conum/ii).
do if (check = trunc(check)).
compute check2=conum/ii.
compute aok=ii.
do if (aok > 2).
compute allgood=1.
end if.
else.
compute remain=conum-(ii*trunc(check)).
do if (remain <= smremain).
compute smremain=remain.
compute largesti=ii.
compute smallrow=trunc(conum/largesti).
end if.
end if.
end loop.
compute atemp=a(1,1:(aok*check2)).
compute atemp=reshape(atemp,check2,aok).
do if (ncol(atemp) > 2).
print/title='NOTE: Missing data resulted in the deletion of the following row(s) of'.
print atemp/title='      data from the analysis:'/format=F6.0/space=0.
end if.
do if (allgood=0).
compute atemp=a(1,1:(smallrow*largesti)).
compute atemp=reshape(atemp,smallrow,largesti).
compute btemp=a(1,((largesti*smallrow)+1):conum).
print/title='NOTE: Missing data resulted in the deletion of the following row(s) of'.
print atemp/title='      data from the analysis:'/format=F6.0/space=0.
print btemp/title=' '/space=0/format=f6.0.
end if.
else if (conum <= 12).
print/title='NOTE: Missing data resulted in the deletion of the following row(s) of'.
print a/title='      data from the analysis:'/format=F6.0/space=0.
end if.
end if.
end loop.
do if (toomany=1).
print/title='WARNING: Variables names longer than eight characters can produce incorrect output'.
print/title='when some variables in the data file have the same first eight characters. Shorter'/space=0.
print/title='variable names are recommended. By using this output, you are accepting all risk'/space=0.
print/title='and consequences of interpreting or reporting results that may be incorrect.'/space=0.
end if.
end if.
end if.
loop i = 1 to 100.
do if (errcode(i,1)=1).
print/title = 'ERROR: You must specify a Y and an X variable.'.
end if.
do if (errcode(i,1)=2).
print/title = 'ERROR: X, M, or Y variable used more than once or W and Z are the same variable.'.
do if (toomany = 1).
print/title = '       This could be caused by the use of variables names longer'/space=0.
print varnames/title = '       than eight characters. Here are the variables I see:'/space=0/format=A8.
end if.
end if.
do if (errcode(i,1)=3).
print/title = 'ERROR: You have specified more than one variable for W, Y, X, or Z'.
end if.
do if (errcode(i,1)=4).
print/title = 'ERROR: A variable specified as multicategorical has more than nine categories.'.
end if.
do if (errcode(i,1)=5).
print/title = 'ERROR: One of the categories contains only a single case.'.
end if.
do if (errcode(i,1)=6).
print/title = 'ERROR: Invalid model number in this version of PROCESS.'.
end if.
do if (errcode(i,1)=7).
print/title = 'ERROR: Invalid model number.'.
end if.
do if (errcode(i,1)=8).
print/title = 'ERROR: You must specify an M variable for this model.'.
end if.
do if (errcode(i,1)=9).
print/title = 'ERROR: You have specified an M variable in a model that does not use it.'.
end if.
do if (errcode(i,1)=10).
print/title = 'ERROR: You have specified a W variable in a model that does not use it.'.
end if.
do if (errcode(i,1)=11).
print/title = 'ERROR: You have not specified a W variable in a model that requires it.'.
end if.
do if (errcode(i,1)=12).
print/title = 'ERROR: You have specified a Z variable in a model that does not use it.'.
end if.
do if (errcode(i,1)=13).
print/title = 'ERROR: You have not specified a Z variable in a model that requires it.'.
end if.
do if (errcode(i,1)=14).
print/title = 'ERROR: V and Q are not proper specifications in this release of PROCESS.'.
print/title = '       Moderators must be specified as W and/or Z.'/space=0.
end if.
do if (errcode(i,1)=15).
print/title = 'ERROR: One of your model variables exhibits no variation (it is a constant).'.
end if.
do if (errcode(i,1)=16).
print/title = 'ERROR: BMATRIX is not the correct length or is otherwise invalid.'.
end if.
do if (errcode(i,1)=17).
print/title = 'ERROR: WMATRIX is not the correct length or is otherwise invalid.'.
end if.
do if (errcode(i,1)=18).
print/title = 'ERROR: ZMATRIX is not the correct length or is otherwise invalid.'.
end if.
do if (errcode(i,1)=19).
print/title = 'ERROR: WZMATRIX is not the correct length or is otherwise invalid.'.
end if.
do if (errcode(i,1)=20).
print/title = 'ERROR: A path fixed at zero cannot be moderated.'.
end if.
do if (errcode(i,1)=21).
print/title = 'ERROR: If only one moderator is specified, it must be specified as W.'.
end if.
do if (errcode(i,1)=22).
print/title = 'ERROR: In BMATRIX, X must be specified to affect at least one variable.'.
end if.
do if (errcode(i,1)=23).
print/title = 'ERROR: In BMATRIX, at least one variable must be specified to affect Y.'.
end if.
do if (errcode(i,1)=24).
print/title = 'ERROR: You must specify a model number or a custom BMATRIX specification.'.
end if.
do if (errcode(i,1)=25).
print/title = 'ERROR: BMATRIX cannot be used in conjunction with a model number.'.
end if.
do if (errcode(i,1)=26).
print/title = 'ERROR: Your model has a dangling mediator (all Ms must affect and be affected).'.
end if.
do if (errcode(i,1)=27).
print/title = 'ERROR: CLUSTER is not available on this release of PROCESS.'.
end if.
do if (errcode(i,1)=29).
print/title = 'ERROR: CMATRIX is not the correct length or is otherwise invalid.'.
end if.
do if (errcode(i,1)=30).
print/title = 'ERROR: In CMATRIX, all covariates must be assigned to an M or a Y.'.
end if.
do if (errcode(i,1)=31).
print/title = 'ERROR: A linear or near linear dependency (singularity) exists in the data.'.
end if.
do if (errcode(i,1)=32).
print/title = 'ERROR: Models 80 and 81 require between 3 and 6 mediators.'.
end if.
do if (errcode(i,1)=33).
print/title = 'ERROR: Model 82 requires 4 mediators.'.
end if.
do if (errcode(i,1)=34).
print/title = 'ERROR: This model number requires between 2 and 6 mediators.'.
end if.
do if (errcode(i,1)=35).
print/title = 'ERROR: In a model with only one moderator, that moderator must be W.'.
end if.
do if (errcode(i,1)=36).
print/title = 'ERROR: A serial mediation model cannot have more than 6 mediators.'.
end if.
do if (errcode(i,1)=37).
print/title = 'ERROR: No more than 10 mediators are allowed in a PROCESS command.'.
end if.
do if (errcode(i,1)=38).
print/title = 'ERROR: XCATCODE is not provided, not the correct length, or is otherwise invalid.'.
end if.
do if (errcode(i,1)=39).
print/title = 'ERROR: WCATCODE is not provided, not the correct length, or is otherwise invalid.'.
end if.
do if (errcode(i,1)=40).
print/title = 'ERROR: ZCATCODE is not provided, not the correct length, or is otherwise invalid.'.
end if.
do if (errcode(i,1)=41).
print/title = 'ERROR: Models 1, 2, and 3 cannot be customized.'.
end if.
do if (errcode(i,1)=42).
print/title = 'ERROR: WS option available only in PROCESS v2. Or use the MEMORE macro instead.'.
print/title = '       MEMORE can be downloaded from www.akmontoya.com.'/space=0.
end if.
do if (errcode(i,1)=43).
print/title = 'ERROR: PROCESS does not allow dichotomous mediators.'.
end if.
do if (errcode(i,1)=60).
print/title = 'PROCESS is now ready for use.'.
print/title='Copyright 2017-2022 by Andrew F. Hayes. ALL RIGHTS RESERVED.'/space=0.
print/title='Workshop schedule available at http://haskayne.ucalgary.ca/CCRAM'/space=0.
end if.
do if (errcode(i,1)=50).
print/title = 'ERROR: A multicategorical moderator cannot be specified as a covariate.'.
end if.
do if (errcode(i,1)=51).
print/title = 'ERROR: A variable you specified as a covariate is a moderator in all equations.'.
end if.
do if (errcode(i,1)=52 and mcerpt=0).
compute mcerpt=1.
print/title = 'ERROR: A variable specified as multicategorical must have at least three categories'.
end if.
do if (errcode(i,1) = 47).
do if (iterrmod=1).
print/title = 'ERROR: Iteration for Y model didn''t converge to a solution. Interpret results with caution.'.
print/title = '       Try increasing the number of iterations, though this will slow down computation.'/space=0.
end if.
do if (bootiter=1).
print/title = 'ERROR: Nonconvergence during bootstrapping. Interpet bootstrap results with caution.'.
end if.
end if.
do if (errcode(i,1) = 61).
print/title = 'ERROR: Variable names should be no more than eight characters in length.'.
end if.
do if (errcode(i,1) = 62).
print/title = 'ERROR: After listwise deletion of cases with missing data, too few cases remain.'.
end if.
do if (errcode(i,1) = 63).
print/title = 'ERROR: The XMINT option is available only for model 4.'.
end if.
do if (errcode(i,1) = 64).
print/title = 'ERROR: Incorrect number of values specified in CDEVAL option.'.
end if.
do if (errcode(i,1) = 65).
print/title = 'ERROR: Only indicator or sequential coding of X is allowed with the XMINT option.'.
end if.
do if (errcode(i,1) = 66).
print/title = 'ERROR: XMINT option not available with continuous X using PROCESS GUI. Use syntax.'.
end if.
do if (errcode(i,1) = 67).
print/title = 'ERROR: Too many elements provided in XREFVAL option.'.
end if.
do if (errcode(i,1)=68).
print/title = 'ERROR: Covariate assignment is not allowed with XMINT option.'.
end if.
do if (errcode(i,1) = 69).
print/title = 'ERROR: Incorrect number of values specified in COVAL option.'.
end if.
do if (errcode(i,1) = 70).
print/title = 'ERROR: Incorrect value(s) in XREFVAL for this dichotomous X variable.'.
end if.
do if (errcode(i,1) = 71).
print/title = 'ERROR: The CENTER option is not available when using the XMINT option.'.
end if.
do if (errcode(i,1) = 72).
print/title = 'ERROR: The XMINT option is not available for models with a dichotomous Y.'.
end if.
end loop.
END MATRIX.	
Ressources	Temps de processeur	00:00:06,75	
	Temps écoulé	00:00:06,78	


Run MATRIX procedure:

***************** PROCESS Procedure for SPSS Version 4.2 *****************

          Written by Andrew F. Hayes, Ph.D.       www.afhayes.com
    Documentation available in Hayes (2022). www.guilford.com/p/hayes3

**************************************************************************
Model  : 4
    Y  : HOMAIR
    X  : GDR
   M1  : TG
   M2  : HDL
   M3  : LDL
   M4  : TC

Sample
Size:  169

**************************************************************************
OUTCOME VARIABLE:
 TG

Model Summary
          R       R-sq        MSE          F        df1        df2          p
      ,1182      ,0140  3995,0140     2,3676     1,0000   167,0000      ,1258

Model
              coeff         se          t          p       LLCI       ULCI
constant   136,0731    31,5501     4,3129      ,0000    73,7847   198,3616
GDR         -4,4861     2,9155    -1,5387      ,1258   -10,2420     1,2699

Standardized coefficients
         coeff
GDR     -,1182

**************************************************************************
OUTCOME VARIABLE:
 HDL

Model Summary
          R       R-sq        MSE          F        df1        df2          p
      ,1041      ,0108   176,2049     1,8301     1,0000   167,0000      ,1779

Model
              coeff         se          t          p       LLCI       ULCI
constant    32,3032     6,6260     4,8752      ,0000    19,2217    45,3847
GDR           ,8283      ,6123     1,3528      ,1779     -,3805     2,0371

Standardized coefficients
         coeff
GDR      ,1041

**************************************************************************
OUTCOME VARIABLE:
 LDL

Model Summary
          R       R-sq        MSE          F        df1        df2          p
      ,0365      ,0013  1188,4688      ,2222     1,0000   167,0000      ,6380

Model
              coeff         se          t          p       LLCI       ULCI
constant    68,2988    17,2082     3,9690      ,0001    34,3251   102,2724
GDR           ,7496     1,5902      ,4714      ,6380    -2,3898     3,8890

Standardized coefficients
         coeff
GDR      ,0365

**************************************************************************
OUTCOME VARIABLE:
 TC

Model Summary
          R       R-sq        MSE          F        df1        df2          p
      ,0549      ,0030  1447,5609      ,5042     1,0000   167,0000      ,4787

Model
              coeff         se          t          p       LLCI       ULCI
constant   122,4391    18,9915     6,4470      ,0000    84,9446   159,9335
GDR          1,2462     1,7550      ,7101      ,4787    -2,2186     4,7109

Standardized coefficients
         coeff
GDR      ,0549

**************************************************************************
OUTCOME VARIABLE:
 HOMAIR

Model Summary
          R       R-sq        MSE          F        df1        df2          p
      ,3830      ,1467    22,8789     5,6041     5,0000   163,0000      ,0001

Model
              coeff         se          t          p       LLCI       ULCI
constant    -3,6467     2,8239    -1,2913      ,1984    -9,2229     1,9296
GDR           ,3626      ,2250     1,6119      ,1089     -,0816      ,8069
TG            ,0462      ,0098     4,7208      ,0000      ,0269      ,0656
HDL           ,1267      ,0429     2,9553      ,0036      ,0421      ,2114
LDL           ,0988      ,0357     2,7705      ,0062      ,0284      ,1692
TC           -,0948      ,0355    -2,6693      ,0084     -,1649     -,0247

Standardized coefficients
         coeff
GDR      ,1189
TG       ,5752
HDL      ,3307
LDL      ,6662
TC      -,7058

************************** TOTAL EFFECT MODEL ****************************
OUTCOME VARIABLE:
 HOMAIR

Model Summary
          R       R-sq        MSE          F        df1        df2          p
      ,0709      ,0050    26,0381      ,8437     1,0000   167,0000      ,3597

Model
              coeff         se          t          p       LLCI       ULCI
constant     1,8841     2,5471      ,7397      ,4605    -3,1445     6,9128
GDR           ,2162      ,2354      ,9185      ,3597     -,2485      ,6809

Standardized coefficients
         coeff
GDR      ,0709


************** TOTAL, DIRECT, AND INDIRECT EFFECTS OF X ON Y **************

Total effect of X on Y
     Effect         se          t          p       LLCI       ULCI       c_cs
      ,2162      ,2354      ,9185      ,3597     -,2485      ,6809      ,0709

Direct effect of X on Y
     Effect         se          t          p       LLCI       ULCI      c'_cs
      ,3626      ,2250     1,6119      ,1089     -,0816      ,8069      ,1189

Indirect effect(s) of X on Y:
          Effect     BootSE   BootLLCI   BootULCI
TOTAL     -,1464      ,1359     -,4665      ,0610
TG        -,2074      ,2721     -,9461      ,0967
HDL        ,1050      ,1634     -,0518      ,5854
LDL        ,0741      ,2890     -,3876      ,7741
TC        -,1181      ,3064     -,8189      ,4243

Completely standardized indirect effect(s) of X on Y:
          Effect     BootSE   BootLLCI   BootULCI
TOTAL     -,0480      ,0430     -,1469      ,0207
TG        -,0680      ,0844     -,2895      ,0326
HDL        ,0344      ,0515     -,0167      ,1801
LDL        ,0243      ,0932     -,1166      ,2588
TC        -,0387      ,0987     -,2632      ,1318

*********************** ANALYSIS NOTES AND ERRORS ************************

Level of confidence for all confidence intervals in output:
  95,0000

Number of bootstrap samples for percentile bootstrap confidence intervals:
  5000

------ END MATRIX -----
